# Genomic and Genotypic Characterization of *Cylindrospermopsis raciborskii*: Toward an Intraspecific Phylogenetic Evaluation by Comparative Genomics

**DOI:** 10.3389/fmicb.2018.00306

**Published:** 2018-02-26

**Authors:** Vinicius A. C. Abreu, Rafael V. Popin, Danillo O. Alvarenga, Patricia D. C. Schaker, Caroline Hoff-Risseti, Alessandro M. Varani, Marli F. Fiore

**Affiliations:** ^1^Center for Nuclear Energy in Agriculture, University of São Paulo, Piracicaba, Brazil; ^2^School of Agricultural and Veterinarian Sciences, São Paulo State University, Jaboticabal, Brazil

**Keywords:** cyanobacteria, genome assembly, pan-genome, bioinformatics, natural products, cyanotoxins, nitrogen fixation

## Abstract

*Cylindrospermopsis raciborskii* is a freshwater cyanobacterial species with increasing bloom reports worldwide that are likely due to factors related to climate change. In addition to the deleterious effects of blooms on aquatic ecosystems, the majority of ecotypes can synthesize toxic secondary metabolites causing public health issues. To overcome the harmful effects of *C. raciborskii* blooms, it is important to advance knowledge of diversity, genetic variation, and evolutionary processes within populations. An efficient approach to exploring this diversity and understanding the evolution of *C. raciborskii* is to use comparative genomics. Here, we report two new draft genomes of *C. raciborskii* (strains CENA302 and CENA303) from Brazilian isolates of different origins and explore their molecular diversity, phylogeny, and evolutionary diversification by comparing their genomes with sequences from other strains available in public databases. The results obtained by comparing seven *C. raciborskii* and the *Raphidiopsis brookii* D9 genomes revealed a set of conserved core genes and a variable set of accessory genes, such as those involved in the biosynthesis of natural products, heterocyte glycolipid formation, and nitrogen fixation. Gene cluster arrangements related to the biosynthesis of the antifungal cyclic glycosylated lipopeptide hassallidin were identified in four *C. raciborskii* genomes, including the non-nitrogen fixing strain CENA303. Shifts in gene clusters involved in toxin production according to geographic origins were observed, as well as a lack of nitrogen fixation (*nif*) and heterocyte glycolipid *(hgl*) gene clusters in some strains. Single gene phylogeny (16S rRNA sequences) was congruent with phylogeny based on 31 concatenated housekeeping protein sequences, and both analyses have shown, with high support values, that the species *C. raciborskii* is monophyletic. This comparative genomics study allowed a species-wide view of the biological diversity of *C. raciborskii* and in some cases linked genome differences to phenotype.

## Introduction

In recent decades, the cyanobacterial species *Cylindrospermopsis raciborskii* has received growing attention because of its increasing geographical distribution and the frequency of toxic blooms (Padisák, 1997; Briand et al., 2002; Kokociński and Soininen, 2012; Sinha et al., 2012; Sukenik et al., 2012). The invasive potential of *C. raciborskii* is constantly reported and mostly attributed to its ability to tolerate a wide range of climatic conditions and global warming (Neilan et al., 2003; Wiedner et al., 2007; Antunes et al., 2015; Kokociński et al., 2017). Toxic bloom episodes are considered harmful to ecosystems due to their impact on water quality, which decreases the efficiency of energy transfer from primary producers to primary consumers (Lürling and Roessink, 2006) and poses potential health risks to both animals and humans (Humpage et al., 2000; Murphy and Thomas, 2001; Chellappa et al., 2008). In addition to the production of cyanotoxins, *C. raciborskii* strains may also synthesize other biologically active natural compounds with different activities. An important step in understanding the opportunistic behavior of *C. raciborskii* is advancing our knowledge of morphological and genetic variation in this cyanobacterial species.

The high phenotypic plasticity of the species *C. raciborskii* (Hawkins et al., 2001; Komárek and Komárková, 2003; Chonudomkul et al., 2004; Moore et al., 2004; Piccini et al., 2011; Bonilla et al., 2012) affects identification accuracy. The major problems in identifying species arise because the morphology of the trichome varies during population growth, and under certain environmental conditions the differentiating akinete and heterocyte cells are absent. The morphological variability of *C. raciborskii* strains has also been documented under laboratory culture conditions. This morphological plasticity often leads to misidentification as the morphologically, phylogenetically, and ecologically related genus *Raphidiopsis*. The genera *Cylindrospermopsis* and *Raphidiopsis* belong to the same order Nostocales, and family Aphanizomenonaceae (Komárek et al., 2014), and members of both genera are morphologically very similar and can co-occur in nature. The presence or absence of heterocytes is commonly used as a diacritical morphological feature differentiating these genera. Heterocytes are morphologically distinct cells that develop from vegetative cells and are specialized for nitrogen fixation. In *Cylindrospermopsis*, heterocytes are always terminal at one or both ends of trichomes, and the absence of this specialized cell is not unusual (Komárek and Anagnostidis, 1989). Several attempts were made to solve the taxonomic relationship between *Cylindrospermopsis* and *Raphidiopsis* genera using molecular and phylogenetic approaches (Gugger et al., 2005; Stucken et al., 2009; Alster et al., 2010; Wu et al., 2011; Li et al., 2016). Recently, a proposal to unify *Cylindrospermopsis* and *Raphidiopsis* genera under the single genus *Rhaphidiopsis* was published (Aguillera et al., 2018). The name *Rhaphidiopsis* was retained as the genus name since it has nomenclatural priority over the name *Cylindrospermopsis*.

Although the morphological variability in *C. raciborskii* has been well-documented, only few comparative genomics studies of this species are available (Stucken et al., 2010; Sinha et al., 2014). These few genomic studies are concentrated in Australian *C. raciborskii* strains, and focused mainly on the toxigenic aspects of the strains. Currently, five draft genomes of *C. raciborskii* and one of *Raphidiopsis brookii* are available in public databases. This number of genomes is the minimum recommended to perform a pan-genome analysis (Rouli et al., 2015). A microbial pan-genome is the combination of genes present in all strains (the core genome) and genes that are present in only one or a few strains (known as a dispensable or flexible/accessory genome) (Tettelin et al., 2005). Pan-genome analyses provide a framework for estimating the genomic diversity of a bacterial species but are also helpful to predict, via extrapolation, how many additional genome sequences would be necessary to characterize the entire pan-genome or gene repertoire (Rouli et al., 2015). To shed some light on the knowledge of genetic variation among *C. raciborskii* strains, we combined all available *C. raciborskii* genomes with two new genome sequences to perform the first large-scale comparative genomics study for this species, with representatives from different world regions. More specifically, the goals were (1) to define the core genome and accessory genome of *C. raciborskii*, and (2) to identify toxin, heterocyte differentiation and nitrogen fixation genes, as well as other genomic features associated with the synthesis of natural products.

## Materials and Methods

### Cyanobacterial Strains

This study was performed using eight cyanobacterial genomes. Two genomes were from the *C. raciborskii* strains CENA302 and CENA303 isolated from water bloom samples collected in two different Brazilian localities (Supplementary Table S1). The origin of the other six cyanobacteria whose genomes were used in this comparative study is presented in Supplementary Table S1. The genome of the strain *R. brookii* D9 was included in this study because it showed identity above 90% to the *C. raciborskii* CS-505 genome (Stucken et al., 2010).

### Evaluation of Heterocyte Differentiation under Different Nutritional Conditions

The formation of heterocyte cells in the *C. raciborskii* strains CENA303 and CENA302 was evaluated in cultured cells exposed to different nutritional conditions through the use of the liquid media ASM-1 (Gorham et al., 1964), Z8 (Kotai, 1972), and Jaworski (Thompson et al., 1988). These culture media were also used without a combined source of nitrogen. Inocula (5 mL) from the exponential growth phase were placed into 125-mL Erlenmeyer flasks containing 50 mL of each culture medium, and the flasks were incubated for 20 days at 25 ± 1°C, under 14/10 light/dark cycle with white fluorescent light (40 μmol photon m^-2^ s^-1^). This procedure was performed four times to establish satisfactory growth conditions and better enable the observation of phenotypic characteristics. The morphological features developed by the strains under different nutritional conditions were evaluated with images obtained using an optical microscope GmbH (Carl Zeiss, Göttingen, Germany) equipped with AxionVisionLE 4.6 digital imaging system (Carl Zeiss).

### Genome Sequencing, Assembly, and Annotation

Total genomic DNA from the cyanobacterial cells was extracted following a phenol:chloroform protocol (Lin et al., 2010). Genome sequencing of the CENA302 and CENA303 strains was carried out on HiScan SQ and MiSeq platforms (Illumina, San Diego, CA, United States). For sequencing in the HiScan SQ, the extracted genomic DNA was initially fragmented by sonication (CPX-600, Cole Parmer, Vernon Hills, IL, United States). Genomic libraries were constructed with the Illumina TruSeq DNA Sample Preparation v2 kit, while clustering and sequencing were performed with the Illumina TruSeq PE Cluster Generation v3 and TruSeq SBS v3 kits, respectively. For sequencing in the MiSeq, genomic libraries were constructed with the Nextera XT DNA Sample Prep Kit (Illumina) and sequencing was performed with the MiSeq Reagent Kit v2 (Illumina).

Bases with quality scores below a Phred score of 20, sequences shorter than 250 bp and adapters were removed with PRINSEQ 0.20.4 (Schmieder and Edwards, 2011). *De novo* genome assembly was performed with SPAdes3.5 (Bankevich et al., 2012) and Geneious 9.1 (Kearse et al., 2012). Assembly statistics were obtained with Assemblathon 2 (Bradnam et al., 2013).

The automatic annotation of genes and functional classification in subsystems were performed using the Rapid Annotation using Subsystems Technology (RAST) Server (Aziz et al., 2008) and the SEED viewer (Overbeek et al., 2014). Strain-specific proteins were annotated with Blast2GO v.4.0 (Conesa et al., 2005). The antiSMASH Server was used for automatic annotation of secondary metabolite gene clusters (Weber et al., 2015). Manual annotation and curation were performed with Artemis (v. 16) and BLASTP (Altschul et al., 1990; Rutherford et al., 2000).

### Comparative Analyses

Genome-wide analysis of orthologous clusters was carried out using the OrthoVenn Server (Wang et al., 2015). Genome maps were generated in the GView Server (v.3) (Petkau et al., 2010) and constructed using the Inkscape program (v. 0.91)^1^. To compare the gene annotations among genomes, we performed Gene Ortholog (GO) enrichment analysis using Blast2GO 4.0.

The pan genomes and core genomes at the gene level were estimated using OrthoMCL implemented in GET_HOMOLOGUES with e-values of 1e^-05^ and 75% coverage as a default set (Chen, 2006; Fischer et al., 2011; Contreras-Moreira and Vinuesa, 2013).

### Phylogenetic and Phylogenomic Analyses

The phylogenomic tree was generated with 31 conserved protein sequences (Wu and Eisen, 2008). BLASTP searches were used for the identification of homologs of each protein in 39 cyanobacteria genomes. Sequence alignment for each protein was conducted in Geneious 9.1 using the Muscle option with default settings (Edgar, 2004). A Maximum Likelihood (ML) tree was constructed with RAxML v. 7.7.8 (Stamatakis, 2014) using the PROTGAMMAGTR amino-acid substitution model and a bootstrap resampling value of 1,000.

The 16S rRNA gene phylogenetic tree was inferred using a Bayesian algorithm and GTR+I+G evolution model with 36 cyanobacteria nucleotide sequences implemented in MrBayes v.3.2.5 with 5,000,000 generations (Ronquist and Huelsenbeck, 2003).

## Results

### General Features of *C. raciborskii* Genomes

The genome sequencing and assembly of the *C. raciborskii* strains CENA302 and CENA303 resulted in 49 and 73 scaffolds, respectively. General draft genome features of both *C. raciborskii* strains sequenced in this study and the genome sequences available in the NCBI GenBank are shown in **Table 1**. The draft genomes of the Brazilian *C. raciborskii* strains CENA302 and CENA303 obtained were 3.47 and 3.39 Mb in size, respectively, while the nearly complete genome (six scaffolds) of the Australian *C. raciborskii* CS-505 strain was 4.16 Mb. The two Brazilian strains showed the highest scaffold N50 among all currently available *C. raciborskii* genomes sequenced through Illumina platforms.

**Table 1:** Comparison of the genomic features and subsystem annotation of the studied strains.

	CENA302	CENA303	D9	ITEP-A1	MVCC14	CS-508	CS-505	CR12
Genomic statistics
No. of contigs	58	77	47	195	99	162	6	136
Total size (bp)	3,476,418	3,398,605	3,186,511	3,605,836	3,594,524	3,556,598	4,159,260	3,723,955
Longest contig length (bp)	324,204	403,228	526,794	266,816	299,478	193,915	4,011,384	279,631
Shortest contig length (bp)	580	511	3,501	1,008	1,035	1,027	2,519	1,058
Mean contigs size (bp)	59,938	43,581	67,798	18,491	36,308	21,954	693,210	27,383
Median contigs size (bp)	18,631	7,321	29,593	2,823	4,917	5,661	14,642	6,201
GC content (%)	40.08	40.26	40.06	40.15	40.08	40.15	40.28	40.03
N50	162,402	135,818	127,752	91,008	150,437	62,252	4,011,384	79,912
**Subsystem statistics – SEED**
No. of subsystems	350	354	347	349	351	346	360	356
No. of coding sequences	3,392	3,360	3,120	3,391	3,533	3,346	4,073	3,475
Coding sequences in subsystems	1,363 or 41%	1,342 or 40%	1,307 or 42%	1,364 or 41%	1,384 or 40%	1,355 or 41%	1,590 or 40%	1,366 or 40%
Coding sequences not in subsystems	2,029 or 59%	2,018 or 60%	1,813 or 58%	2,027 or 59%	2,149 or 60%	1,991 or 59%	2,483 or 60%	2,109 or 60%

Although the majority of the cyanobacterial strains investigated in this study were isolated from different locations all over the world (Supplementary Table S1), the results of the RAST Server and SEED viewer subsystem annotation showed that their genomes are considerably similar (Supplementary Figure S1). An average of 60% of genes in the studied genomes were identified with unknown functions, and those with annotated functions were mainly involved in: (I) Protein Metabolism; (II) Cofactors, Vitamins, Prosthetic Groups, and Pigments; (III) Amino Acids and Derivatives; and (IV) Carbohydrates.

**FIGURE 1:**
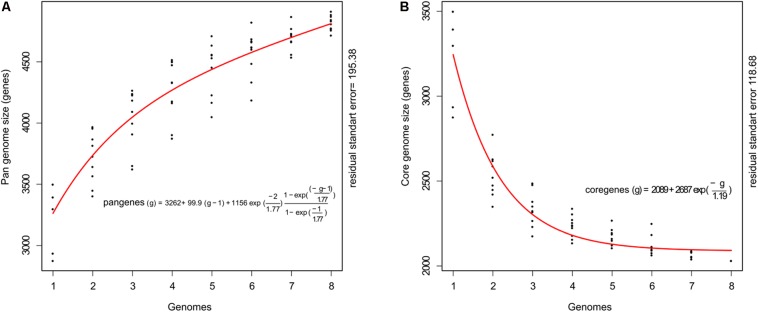
Pan- and core-genome plot of eight cyanobacterial strains (CS-505, CS-508, CR12, CENA303, CENA302, ITEP-A1, D9, and MVCC14). **(A)** Shows the progression of the pan-genome: the y axis indicates the number of non-redundant genes and the x axis indicates the number of genomes. **(B)** Shows the progression of core genome: the y axis indicates the number of genes presents in all strains and the x axis indicates the number of genomes.

The genome sequences were used to calculate the total gene repertoire encountered in these strains. The pan-genome size plot showed a power trend line that had not reached a plateau (**Figure 1A**). This open pan-genome within *C. raciborskii* suggests that full knowledge of the genetic diversity of these strains has not yet been reached. The estimated pan-genome contains a total of 4,716 genes, which is 1.77-fold higher than the average number of genes in each cyanobacterium. Moreover, the core genome size indicated that only a few more genomes are necessary to stabilize the curve (**Figure 1B**). The estimated core genome was 2,031 genes and represented 62% of the average number of genes per genome.

The Venn diagrams were constructed using genome-based protein translations separated according to two aspects: strain origin due to their proximity in phylogenetic analysis (Supplementary Figure S2A), and the presence or absence of gene clusters for the toxins cylindrospermopsin (CYN) and saxitoxin (STX) (Supplementary Figure S2B). The diagram of strain origin showed that South American and non-South American strains shared 75.7% of the total proteins (19,207 proteins), while 11.2% (2,846 proteins) and 3.7% (957 proteins) of the total proteins were exclusive to South American and non-South American genomes, respectively. In the Venn diagram of the presence or absence of toxin gene clusters, 56.4% of the total proteins (14,320 proteins) were shared among toxic and non-toxic strains, while 1.2% (295 proteins), 6.6% (1684 proteins), and 0.18% (47 proteins) of the total proteins were exclusive of CYN-producing, STX-producing and non-toxic strains, respectively. Furthermore, OrthoVenn identified 1,783 proteins in the South American group, 566 in the non-South American group, 1093 in the CYN-producing group, 1876 in the STX-producing group and 704 in the non-toxic group with no orthology (singletons) in both analyses (Data Sheet S1). Consequently, they did not appear in the diagrams.

To elucidate and compare the function of the singletons present in each of the five groups, we performed GO enrichment analysis using the Blast2GO platform. The non-toxic group was not included in the analysis, since few sequences were successfully annotated. Only 37.67% (1071) of the singletons from the South American genomes (Supplementary Figure S3) and 30.09% (288) of the singletons from the non-South American genomes (Supplementary Figure S4) showed known functions. Therefore, most of the singletons from both groups remain unidentified. Nevertheless, within the annotated singletons in the South American genomes, it was observed that the majority were involved in responses to chemicals and other stimuli, biosynthesis of vitamins, biosynthesis and modification of macromolecules, and regulation of gene expression (Supplementary Figure S2A). The known protein singletons from non-South American genomes were involved mainly in the biosynthesis of polyketides, antibiotics and other compounds, transport, and association with chromatin. In the CYN-producing strains, only 21.36% (63 proteins) were annotated (Supplementary Figure S5), and these appeared to be involved in the biosynthesis of drugs, antibiotics, polyketides and other compounds, transport, and membrane components. In the genomes of the STX-producing strains, 50.89% (624 proteins) were annotated (Supplementary Figure S6) and appeared to be involved in the biosynthesis of vitamins, cofactors and other compounds, the cell envelope, and DNA modification and acclimation. In the genomes of strains within the non-toxic group, only 8.51% (4 proteins) were annotated, but without enough information to infer their function.

**FIGURE 2:**
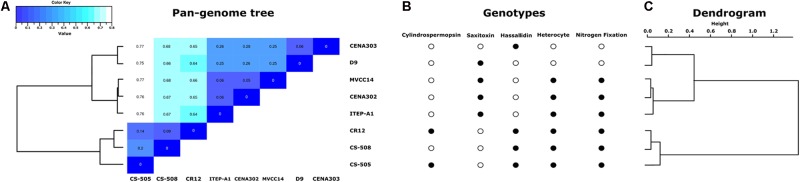
**(A)** Pan-genome tree and matrix of similarity among strains. **(B)** Comparison of presence (filled circles) or absence (empty circles) of the genotypes evaluated in this study (hassallidin, cylindrospermopsin, saxitoxin, heterocyte glycolipids, and nitrogen fixation gene clusters). **(C)** Dendrogram based on the results of OrthoMCL clustering.

To analyze the correlations among the genomes of the strains, a heatmap from an average nucleotide identity matrix derived from the pan-genome tree was performed (**Figure 2A**). In addition, a comparison between the presence or absence of some specific genotype traits (**Figure 2B**) and a dendrogram based on clustering from OrthoMCL (**Figure 2C**) were included. The two Brazilian non-heterocyte strains CENA303 and D9 were grouped together. The strain CENA302 from São Paulo state (Southeast region of Brazil) clustered with the Uruguayan strain MVCC14, and these two strains formed a major clade with the Brazilian strain ITEP-A1 (isolated from the Northeast region of Brazil). All South American strains were separated from the non-South American strains CS-505, CS-508, and CR12.

**FIGURE 3:**
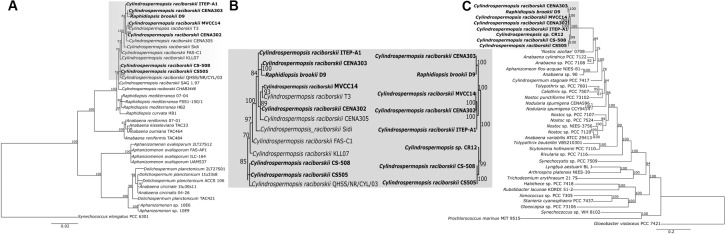
**(A)** Bayesian inference tree based on the 16S rRNA nucleotide sequences from 36 cyanobacterial strains. **(B)** Representation of the clades containing the strains involved in this study. **(C)** ML phylogenetic tree based on 31 conserved proteins from 39 cyanobacterial strains. The strains evaluated in this study are represented in bold.

To evaluate the evolutionary relationships among *C. raciborskii* strains, a phylogenetic reconstruction analysis was performed based on 16S rRNA gene nucleotide sequences and Bayesian inference, which revealed a highly supported clade (posterior probabilities of 1.0) containing nucleotide sequences of *Cylindrospermopsis* and *Raphidiopsis* strains (**Figure 3A**). Within this major clade, two distinct clades were formed that separated the *Cylindrospermopsis* and *Raphidiopsis* strains. However, the sequence of the strain identified as *R. brookii* D9 clustered within the *C. raciborskii* clade. Furthermore, the *R. brookii* D9 16S rRNA gene sequence retrieved from its genome showed 100% identity with the sequence of *C. raciborskii* T2 (also known as SPC388, GenBank accession number MF671763). The cyanobacteria strains originating from South America (Brazilian strains ITEP-A1, CENA303, T3, CENA302, CENA305 and D9, and Uruguayan strain MVCC14) clustered together in an internal clade. Unfortunately, the available *Cylindrospermopsis* sp. strain CR12 draft genome sequence only contained a short fragment (86 bp) of the 16S rRNA gene, which prevented its use in the phylogenetic analysis. CS-505, CS-508 from Australia was grouped with QHSS/NR/CYL/03 from the United States.

The phylogenomic reconstruction tree based on 31 highly conserved protein sequences (**Figure 3B**) from the eight cyanobacteria genomes showed a topology similar that observed in the phylogenetic tree based on 16S rRNA gene sequences (**Figure 3C**). Furthermore, for the majority of strains, both phylogenetic tree topologies corroborated those observed in the pan-genome tree and dendrogram (**Figures 2A, 3C**). An exception was found for the Australian strains CS-505 and CS-508, which were grouped together in both the 16S rRNA and phylogenomic tree but were separated in the pan-genome tree and dendrogram.

### Nitrogen Fixation Gene Cluster in *Cylindrospermopsis raciborskii*

**FIGURE 4:**
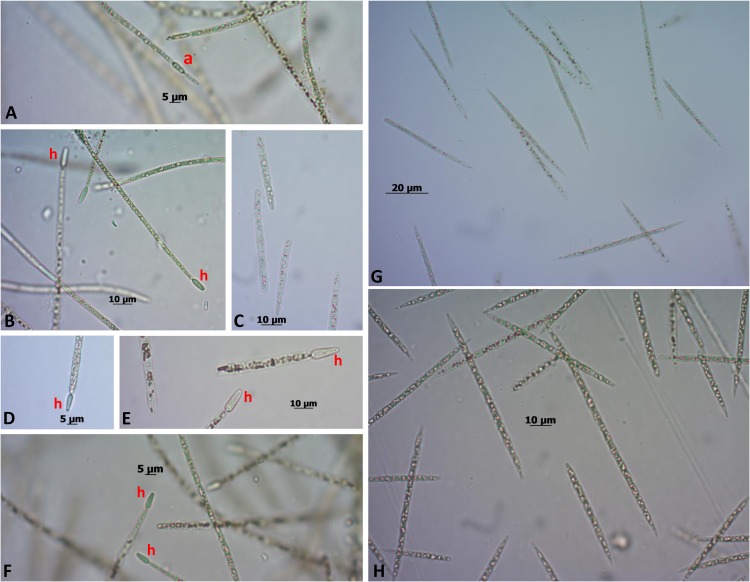
CENA 302 growth in **(A)** ASM-1, scale bar = 5 μm; **(B)** ASM-1 without combined nitrogen, scale bar = 10 μm; **(C)** Jaworski, scale bar = 10 μm; **(D)** Jaworski without combined nitrogen, scale bar = 5 μm; **(E)** Z8, scale bar = 10 μm; and **(F)** Z8 without combined nitrogen, scale bar = 5 μm. CENA303 growth in **(G)** ASM-1, scale bar = 20 μm; and **(H)** Z8 medium, scale bar = 10 μm. “a”: akinete; “h”: heterocyte.

In culture, the *C. raciborskii* strain CENA303 never showed heterocytes, while the *C. raciborskii* strain CENA302 always showed differentiated heterocytes. Before genome sequencing of both strains, growth tests were conducted under different nitrogen concentrations in an attempt to induce heterocyte differentiation in the CENA303 strain. Exposure of cells of the *C. raciborskii* strains CENA303 and CENA302 to various nutritional conditions affected their development in several ways. The lack of a combined nitrogen source prevented the growth of the CENA303 strain, as well as the Jaworski medium with nitrogen. This medium has a low nitrogen concentration, which is likely quickly exhausted in the first cycles of cell growth, inhibiting the development of a non-diazotrophic cyanobacterium. The CENA303 strain did not grow in the absence of combined nitrogen and did not differentiate heterocytes in any tested culture medium (**Figure 4**). However, the CENA302 strain showed growth in all media and differentiated heterocytes under all conditions, especially in media without combined nitrogen. Both strains showed akinete differentiation in the culture media where they were able to grew.

**FIGURE 5:**
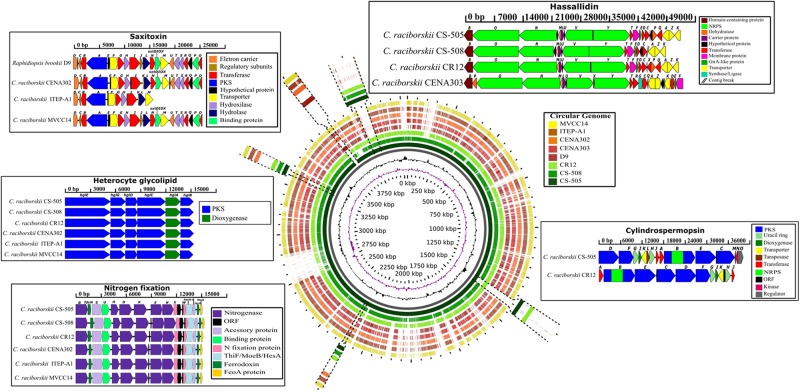
BLAST atlas analysis of seven *Cylindrospermopsis* strains (CS-505, CS-508, CR12, CENA303, CENA302, ITEP-A1, and MVCC14) and *Raphidiopsis brookii* D9, using the strains CS-505 as reference. The hassallidin (*hass*), cylindrospermopsin (*cyr*), saxitoxin (*sxt*), heterocyte glycolipid (*hgl*), and nitrogen fixation (*nif; fdxN; hesA and B; feoaA*) gene clusters are shown inside the boxes and their location in the genomes are indicated. The *Cylindrospermopsis raciborskii* ITEP-A1 genome showed an incomplete *sxt* gene cluster. The length in base pairs (bp) of the gene cluster and the function of the gene are also shown. ORF, open reading frame; PKS, polyketide synthase; NRPS,non-ribossomal peptide synthase.

All genomes harbored two genes (*hetR* and *hetN*) involved in the regulation of heterocyte differentiation (Supplementary Table S2). However, while the *nif* and *hgl* gene clusters involved in nitrogen fixation and the formation of a thick heterocyte glycolipid envelope, respectively, were identified in six *C. raciborskii* genomes (CENA302, ITEP-A1, MVCC14, CS-505, CS-508, and CR12), they were absent in the genomes of the CENA303 and D9 strains (**Figure 5** and Supplementary Tables S2, S3). The *nif* gene cluster of the Brazilian strain CENA302 comprised the nitrogenase structural genes *nifH*, *nifD*, and *nifK* flanked on the left by the *nifU*, *nifS*, *fdxN*, and *nifB* genes and on the right by *nifE*, *nifN*, *nifX*, *nifW*, *hesA*, *hesB*, *fdxH*, and *feoA*. The heterocyte glycolipid gene cluster of the CENA302 strain contained the six conserved genes for *hgl* encoding enzymes related to envelope formation. The general organization of the *nif* and *hgl* gene clusters was almost identical in the genomes of the six nitrogen-fixing *C. raciborskii* strains.

### Genetic Potential for the Synthesis of Natural Products in *C. raciborskii*

A BLAST atlas analysis was performed in the GView Server to compare the genome of the Australian strain CS-505 used as a reference with the other seven genomes and verified the overall conservation among them, and the exact genomic location and gene organization of hassallidin, cylindrospermopsin, saxitoxin, nitrogen fixation, and heterocyte glycolipid clusters (**Figure 5**). Based on genome conservation analysis, the Australian CS-508 and Singaporean CR12 strains showed considerably similar genomes to the Australian CS-505 strain, while the Brazilian strains CENA302, CENA303, ITEP-A1 and D9, and the Uruguayan strain MVCC14 showed genomes more divergent from the reference. Furthermore, two conserved regions were identified in the first three genomes (CS-505, CS-508, and CR12), which were considerably divergent in the other five genomes (Supplementary Figure S7). The genes in these two regions were automatically annotated in the RAST Server, and although most were not divided into subsystems by the SEED tools, many were shown to be involved in protein metabolism.

The anti-SMASH software and manual curation identified gene clusters involved in non-ribosomal peptides synthesis (cylindrospermopsin, saxitoxin, and hassallidin), but also ribosomally synthesized natural products (terpene and phytoene). The survey of NRPS (non-ribosomal peptide synthase) and PKS (polyketide synthase) genes in the investigated genomes demonstrated a low frequency of these enzymes, as well as terpene synthase and phytoene synthase (Supplementary Table S4).

#### Hassallidin Gene Cluster

The *has* gene cluster coding for the biosynthesis of hassallidins was identified in four *C. raciborskii* genomes: CENA303, CS-505, CS-508, and CR12 (**Figure 5** and Supplementary Table S5). A putative *has* gene cluster ∼48-kb-long was found in the Brazilian non-nitrogen-fixing strain CENA303, containing four genes encoding NRPS (*hasO*, *hasN*, *hasV*, and *hasY*) and 15 genes encoding putative tailoring enzymes (*hasA*, *hasB*, *hasC*, *hasD*, *hasE*, *hasF*, *hasG*, *hasK*, *hasL*, *hasM*, *hasP*, *hasR*, *hasT*, *hasU*, and *hasZ*). Furthermore, the *hasQ* and *hasX* genes are also present, but with partial sequences. An insertion sequence (IS) was identified inside the *hasR* gene, and manual curation allowed reconstruction of the cluster into a single contig. The *has* gene cluster encoding 18 genes was also identified in a single contig in the Australian strain *C. raciborskii* CS-505, whose topology is very similar to that found in the Singaporean strain CR12. The *has* gene cluster of the Australian strain CS-508 is split into two contigs between the *hasC* and *hasA* genes, and some *has* genes encoding putative tailoring enzymes are absent. The content and architecture of the Brazilian *C. raciborskii* CENA303 *has* gene cluster are the most divergent from the *C. raciborskii* strains.

#### Cylindrospermopsin Gene Cluster

The *cyr* gene cluster encoding the biosynthesis of the cylindrospermopsins was identified in the Australian toxic strain *C. raciborskii* CS-505 and the Singaporean *C. raciborskii* CR12 genomes in a single contig containing 15 and 11 ORFs, respectively (**Figure 5** and Supplementary Table S6). The *cyr* gene cluster in the CR12 strain showed rearrangements in the order of some genes and lacked the *cyrL* and *cyrM* genes coding for transposases, and *cyrN* and *cyrO* coding for a kinase and a regulator, respectively, compared with the *cyr* gene cluster of the CS-505 strain. Vestiges of *cyr* genes (*cyr* L, M, and N) were found in the six remaining genomes.

#### Saxitoxin Gene Cluster

The *sxt* gene cluster encoding the biosynthesis of saxitoxins was identified in the genomes of the Brazilian *C. raciborskii* CENA302 and *R. brookii* D9, and in the Uruguayan *C. raciborskii* MVCC14 (**Figure 5** and Supplementary Table S7). Considerably conserved saxitoxin gene clusters containing 21 genes were found in the genomes of CENA302, D9, and MVCC14. The only difference was the presence of *sxtK* gene (unknown protein) in the genome of CENA302, while D9 and MVCC14 showed the *sxtJ* gene (regulatory protein). An incomplete *sxt* gene cluster containing 12 ORFs was found in the *C. raciborskii* ITEP-A1 draft genome.

## Discussion

In this study, we used comparative genome analysis to investigate the *C. raciborskii* pan-genome based on eight genome sequences to comprehend the genetic landscape of this species. The genome dynamics of the *C. raciborskii* strains showed that the pan-genome size increased steadily without reaching a plateau, while the core genome appeared near saturation, suggesting that further sampling of *C. raciborskii* genomes is likely to cause a closed state in the core genome. The *Cylindrospermopsis* genomes from different origins generally showed a significant resemblance, with similar patterns in gene functions. Notably, in all eight genomes, it was only possible to assign functions to approximately 40% of protein sequences, which emphasizes the lack of knowledge of cyanobacterial proteins and/or a more cursory annotation of proteins in this phylum.

Subsequently, increasingly refined examinations showed that specific and recurrent genomic variations occur among the seven strains of *C. raciborskii* and the single *R. brookii* D9 strain. The genomic features of these strains appear to be influenced by their geographic origin, since evolutionary analysis differentiated the South American strains (CENA302, CENA303, ITEP-A1, MVCC14, and D9) from the Australian and Singaporean strains (CS-505, CS-508, and CR12). However, the lack of European and African genome sequences of this species did not allow a more precise conclusion about the influence of the environment on their genomic plasticity. Previous studies on phylogeny of several *C. raciborskii* genes already observed biogeographic variation correlated to genetic variability (Neilan et al., 2003; Gugger et al., 2005; Moreira et al., 2013, 2015; Antunes et al., 2015).

Despite the separation between South American and non-South American genomes, neither the genotypes, subsystem annotation nor enrichment analysis appeared sufficient to explain this separation. By examining the subsystem annotations of the two largest regions of genomic divergence in the BLAST atlas, we once again found that most gene functions could not be assigned (Supplementary Figure S7). While little can be inferred from region (B), region (A) showed that almost half of the genes are involved in N-linked glycosylation, which is a process of protein modification. These processes are not yet fully understood but appear to be frequently present in the three domains of life and control a wide range of cellular processes, such as signal transduction, protein folding, targeting, stability, cell–cell and virus–cell interactions, and host immune responses (Nothaft and Szymanski, 2013). Therefore, although more information is necessary before we can fully determine the differences between these groups (South American and non-South American), the results of our analyses indicate that they have a common primary metabolism and minor differences in very important cell processes, such as cell regulation, biosynthesis, and transport, which allows the exploration of a wide range of environmental conditions.

The identification of significant associations of GO terms between South American and non-South American genomes was not deeply accessed through the GO enrichment analysis. Low resolution may occur in this type of analysis, since functional enrichment analysis is directly associated with sequence annotation level depth (Wang et al., 2011; Glass and Girvan, 2015). The sequence set analyzed in this study gave insufficient functional categories, which influenced the results of this type of functional enrichment analysis. In other words there was an enrichment bias.

Since the group containing CYN-producing strains (Australian CS-505 and Singaporean CR12) was also within the non-South American group (CS-505, CS-508, and CR12), it is possible to evaluate the results of the enrichment analyses of these two groups together. The percentage of genes annotated as involved in the biosynthesis of polyketides might be influenced by the *cyr* gene cluster, which contains polyketide synthase genes and is present only in Australian CS-505 and CR12 strains. Polyketides boast a wealth of medicinally important activities, including antibiotic, anticancer, antifungal, antiparasitic, and immunosuppressive properties (Staunton and Weissman, 2001). In addition, the Cellular Component ontology showed that almost half of the singletons successfully annotated in the non-South American genomes are involved in the cell membrane. Therefore, these singletons could be directly associated with the adaptation of cellular machinery to particular environmental conditions. The strains not only synthesize secondary metabolites and antibiotics found in the South American genomes, but also show a high percentage of genes involved in cell transport, membrane, and regulation of gene expression.

All genomes of STX-producing strains are from South America, and consequently, these two groups were also analyzed together. Similar to the singletons from the non-South American genomes, the South American singletons were also related to adaptation to a particular environment. However, whereas the first were mainly associated with changes in cellular machinery, the latter were also linked to differences in cellular metabolism and recognition of environmental stimuli and chemicals. This hypothesis is sustained by the fact that the South American genomes presented singletons that participate in water-soluble vitamin biosynthetic processes, similar to the pattern found in cyanobacteria typical of lakes and rivers (Wetzel, 2001), as well as genes related to post-translational/co-translational cofactors, cell movement, and cellular cycle.

The strains CENA303 and CS-508 did not show either the *cyr* or *sxt* gene clusters, and therefore they formed their own group (non-toxic strains). The enrichment analyzes of this group resulted in only four annotated genes, and thus not much information can be inferred. This result illustrates the current lack of information on cyanobacteria and the difficulty of functional annotation in this phylum.

This comparative genomic analysis showed that the five South American (Brazil and Uruguay) genomes are slightly smaller and more conserved than the non-South American (Australia and Asia) genomes, suggesting that genomes from South America underwent gene loss events. For example, the Brazilian strains CENA303 and D9 have smaller numbers of coding genes than the other strains and have substantial differences in their genomes compared with the Australian CS-505 and Singaporean CR12. Signs of genome reduction have already been reported for the *R. brookii* D9 strain (Stucken et al., 2010). Genome reduction is an evolutionary strategy strongly related to genome efficiency and selective pressure, and can distinctly affect various cellular processes, directing selection for the maintenance of specific genes. Usually, events of gene loss and genome shrinkage are related to the accessory genome, i.e., genes that are not conserved among genomes, and do not involve crucial housekeeping functions (Bolotin and Hershberg, 2016; Paul et al., 2016). Housekeeping genes are conserved, fitted in the core genome, and they are considered good molecular markers for phylogeny (Wu and Eisen, 2008; Calteau et al., 2014; Uyeda et al., 2016). In this study, the similarity matrix indicated that the presence of additional genotype traits results in more distantly related genomes. Accessory genes contribute to increase the distinction among genomes, however, they are not conserved and they are not good molecular markers for phylogeny (Sinha et al., 2014).

Evolutionary relationships among the eight strains elucidated by this comparative genomic study demonstrated that the strain deposited in the GenBank as *R. brookii* D9 is highly related to *C. raciborskii* strains, as already evidenced in previous reports (Stucken et al., 2009, 2010; Hoff-Risseti et al., 2013; Aguillera et al., 2018). Previous studies identified a different morphotype within the culture of *C. raciborskii* T2 (also known as SPC338) as *Raphidiopsis* likely due to the absence of heterocytes in their filaments (Castro et al., 2004; Stucken et al., 2009). However, the lack of this specialized cell is not unusual in *Cylindrospermopsis* strains (Komárek and Anagnostidis, 1989). Misidentification between *Cylindrospermopsis* and *Raphidiopsis* strains is common, since they are morphologically and genetically close; specifically, both genera are included in the order Nostocales and the family Aphanizomenonaceae (Komárek et al., 2014). Indeed, this study also showed that *C. raciborskii* CENA303 filaments do not contain heterocytes. Even after complete depletion of inorganic nitrogen sources in culture media, vegetative cells of CENA303 were incapable of initiating heterocyte differentiation. The CENA303 and D9 strains are phylogenetically close and do not possess *nif* genes, although they contain regulatory genes (*hetR* and *hetN*) involved in heterocyte differentiation (Herrero et al., 2013). The presence of these genes suggests that selective losses of the *nif* and *hgl* clusters have occurred in the CENA303 and D9 strains, which lost the capacity to differentiate heterocytes.

Given the metabolic potential for the production of natural products, this comparative genome study emphasized gene clusters involved in the synthesis of the antifungal hassallidin and the toxins cylindrospermopsin and saxitoxin. Much of the natural products synthesized by cyanobacteria are attributed to the versatility of NRPS and PKS enzymes that catalyze chain elongation from simple building blocks to create a diverse array of molecules (Welker and von Döhren, 2006). The extent and distribution of secondary metabolite synthesis in cyanobacteria appears to be correlated to genome size (Shih et al., 2013). The *C. raciborskii* genome is the smallest known genome in multicellular cyanobacteria (Stucken et al., 2010) and therefore a low frequency of NRPS and PKS enzyme systems for the production of secondary metabolites is expected, which was observed in this study.

Hassallidins are antifungal cyclic glycosylated lipopeptides that were first identified as being produced by cyanobacteria of the genus *Hassalia* (Neuhof et al., 2005, 2006). Other cyanobacteria were also shown to be producers of this class of peptides and the gene cluster in the *Anabaena* sp. SYKE748A strain was characterized as spanning 59 kbp and consisting of 26 ORFS (*hasA*-*hasZ*) (Vestola et al., 2014). The biosynthesis of hassallidins involves many genes, and the role of some of these in the biosynthetic pathway is not yet clear. While NRPS genes (*hasO*, *hasN*, *hasV*, and *hasY*) encode the nine modules responsible for the incorporation of amino acids into the hassallidin peptide backbone, the glycosylation genes (*hasD*, *hasQ*, *hasT*, and *hasX*) are then responsible for the incorporation of sugars, and correspondingly, the lipidation genes (*hasG*, *hasH*, and *hasL*) for the addition of lipids (Vestola et al., 2014). The Australian CS-505 strain is a known producer of hassallidins (Vestola et al., 2014) and the *has* gene cluster was recently described (Pancrace et al., 2017). Clusters showing similar genetic organization to that found in the CS-505 strain were identified in the genomes of the CENA303, CS-508, and CR12 strains. Predictions of the A-domains of NRPS genes indicate that compared with the *Anabaena* sp. SYKE748A strain, which incorporates a methylthreonine at position nine in the molecule, the *Cylindrospermopsis* strains incorporate a threonine. In addition, the number of glycosylation and lipidation genes varies among strains and may act in more than one step in hassallidin assembly. Therefore, *in silico* analyses indicated that the CENA303, CS-508, and CR12 strains are potential producers of new hassallidin variants. The IS insertion found inside the *hasR* gene in the CENA303 strain may irreversibly inactivate this gene. Nevertheless, HasR has been described as an acyltransferase that acts on the acetylation of hassallidin sugars (Vestola et al., 2014). Thus, its inactivation cannot affect the production of the molecule but merely the variants produced. As half of the strains included in the present study are potentially hassallidin producers, and the two others contain fragments of the gene cluster, this molecule appears to be widespread among *C. raciborskii*.

The shift in toxin production according to geographic origins had already been reported in *C. raciborskii* strains (Lagos et al., 1999; Piccini et al., 2011; Hoff-Risseti et al., 2013). In South America, *C. raciborskii* strains produce the neurotoxin saxitoxin and some of its derivatives, while in Australia and Asia, the isolates synthesize the alkaloid cylindrospermopsin. Both toxins are synthesized by the NRPS and PKS enzyme complexes (Kellmann et al., 2008; Mihali et al., 2008). The results of this study showed that the genomes of South American strains contain the *sxt* gene cluster, while Australian and Singaporean strains possessed the *cyr* gene cluster, thus corroborating information available in the literature. Furthermore, among the 26 genes proposed to be involved in STX production (Kellmann et al., 2008), 21 were found in the genomes of the STX-producing strains CENA302, D9, and MVCC14. Since the Brazilian strains CENA302 and D9 are known as STX producers (Stucken et al., 2010; Hoff-Risseti et al., 2013), the genes missing (*sxtJ* or *sxtK*, *sxtV*, *sxtZ*, *sxtY*, and *sxtW*) should be not essential for STX production or may be involved in generating toxin variants.

Vestiges of *cyr* genes were found in the genomes of South American STX-producing *C. raciborski* strains. However, the annotated gene fragments were different from the gene fragments amplified by PCR using specific primers in previous studies (Hoff-Risseti et al., 2013; Piccini et al., 2013). No vestiges of *sxt* genes were found in the genomes of the CYN-producing strains. The finding that STX-producing *C. raciborskii* strains also carry fragments of *cyr* genes suggests either a remnant or an otherwise ancestral intermediate of a functional CYN gene cluster (Hoff-Risseti et al., 2013).

This study provides new insights into the genotypic and phenotypic plasticity of the species *C. raciborskii*, including the presence of nitrogen-fixing and non-nitrogen-fixing strains. Both strains may co-occur in nature in large populations, such as those formed during bloom events. Environmental conditions usually found during blooms likely favor gene loss events found in the smaller genomes of non-nitrogen-fixing *C. raciborskii* strains, without compromising their survival. This comparative genomic analysis also successfully estimated the genetic diversity among *C. raciborskii* strains and the *R. brookii* D9 strain revealing a conserved core genome with major differences at the accessory genome levels. The biosynthetic capacity for producing the secondary metabolites of the investigated genomes was low, which is expected due to its small genome size.

### Nucleotide Sequence Accession Number

The genome sequence data of the *C. raciborskii* strains CENA302 and CENA303 sequenced under this study have been deposited at the GenBank/EMBL/DDBJ under the accession numbers NZ_MTPU00000000.1 and NZ_NBYN00000000.1. Sequence of 16S rRNA gene from the *C. raciborskii* T2 (SPC-338) have been deposited in NCBI’s sequence read archive under accession number MF671763.

## Author Contributions

MF conceived the study. CH-R, PS, and DA performed the experiments. VA, RP, DA, CH-R, PS, AV, and MF analyzed the data. All authors were involved in writing the paper and had final approval of the manuscript.

## Conflict of Interest Statement

The authors declare that the research was conducted in the absence of any commercial or financial relationships that could be construed as a potential conflict of interest.

## Footnotes

1 https://inkscape.org/
