# Corrigendum: Genomic and Genotypic Characterization of *Cylindrospermopsis raciborskii*: Toward an Intraspecific Phylogenetic Evaluation by Comparative Genomics

**DOI:** 10.3389/fmicb.2018.00979

**Published:** 2018-05-15

**Authors:** Vinicius A. C. Abreu, Rafael V. Popin, Danillo O. Alvarenga, Patricia D. C. Schaker, Caroline Hoff-Risseti, Alessandro M. Varani, Marli F. Fiore

**Affiliations:** ^1^Center for Nuclear Energy in Agriculture, University of São Paulo, Piracicaba, Brazil; ^2^School of Agricultural and Veterinarian Sciences, São Paulo State University, Jaboticabal, Brazil

**Keywords:** cyanobacteria, genome assembly, pan-genome, bioinformatics, natural products, cyanotoxins, nitrogen fixation

There was an error in the placement of some of the decimal points and the thousands separators of Table 1 as published. The correct version of Table 1 appears below. The authors apologize for the mistake. This error does not change scientific conclusions of the article in any way.

**Table 1:** Comparison of the genomic features and subsystem annotation of the studied strains.

	**CENA302**	**CENA303**	**D9**	**ITEP-A1**	**MVCC14**	**CS-508**	**CS-505**	**CR12**
**Genomic Statistics**								
No. of contigs	58	77	47	195	99	162	6	136
Total size (bp)	3,476,418	3,398,605	3,186,511	3,605,836	3,594,524	3,556,598	4,159,260	3,723,955
Longest contig length (bp)	324,204	403,228	526,794	266,816	299,478	193,915	4,011,384	279,631
Shortest contig length (bp)	580	511	3,501	1,008	1,035	1,027	2,519	1,058
Mean contigs size (bp)	59,938	43,581	67,798	18,491	36,308	21,954	693,210	27,383
Median contigs size (bp)	18,631	7,321	29,593	2,823	4,917	5,661	14,642	6,201
GC content (%)	40.08	40.26	40.06	40.15	40.08	40.15	40.28	40.03
N50	162,402	135,818	127,752	91,008	150,437	62,252	4,011,384	79,912
**Subsystem Statistics–SEED**								
No. of Subsystems	350	354	347	349	351	346	360	356
No. of coding sequences	3,392	3,360	3,120	3,391	3,533	3,346	4,073	3,475
Coding sequences in Subsystems	1,363 or 41%	1,342 or 40%	1,307 or 42%	1,364 or 41%	1,384 or 40%	1,355 or 41%	1,590 or 40%	1,366 or 40%
Coding sequences not in Subsystems	2,029 or 59%	2,018 or 60%	1,813 or 58%	2,027 or 59%	2,149 or 60%	1,991 or 59%	2,483 or 60%	2,109 or 60%

The original article has been updated.

## Conflict of interest statement

The authors declare that the research was conducted in the absence of any commercial or financial relationships that could be construed as a potential conflict of interest.

