# Supplementary material for: Genomic and Genotypic Characterization of Cylindrospermopsis raciborskii: Toward an Intraspecific Phylogenetic Evaluation by Comparative Genomics
Source: Front Microbiol. 2018 Feb 26;9:306. doi: 10.3389/fmicb.2018.00306 (PMC5834425; doi:10.3389/fmicb.2018.00306)
Supplement: Supplementary file 1 [file Image_1.pdf]

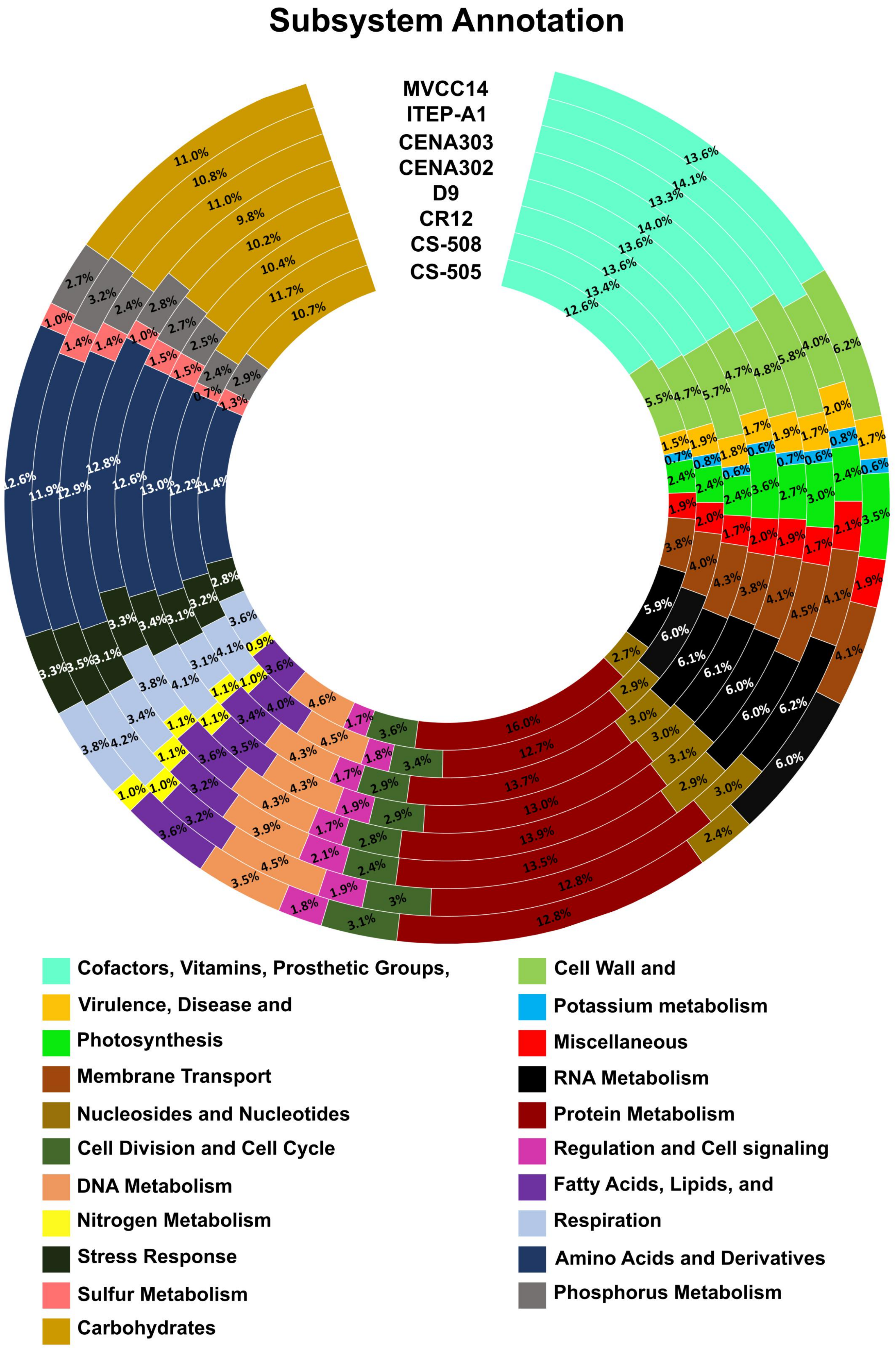

Supplementary Figure S1. Comparison of the automatic annotation of *Cylindrospermopsis raciborskii* strains and *Raphidiopsis brookii* D9 strain. Each layer of the circle represents a genome and each piece of the layer represents a subsystem. The values represent the percentage of the subsystem in the genome of the strain.

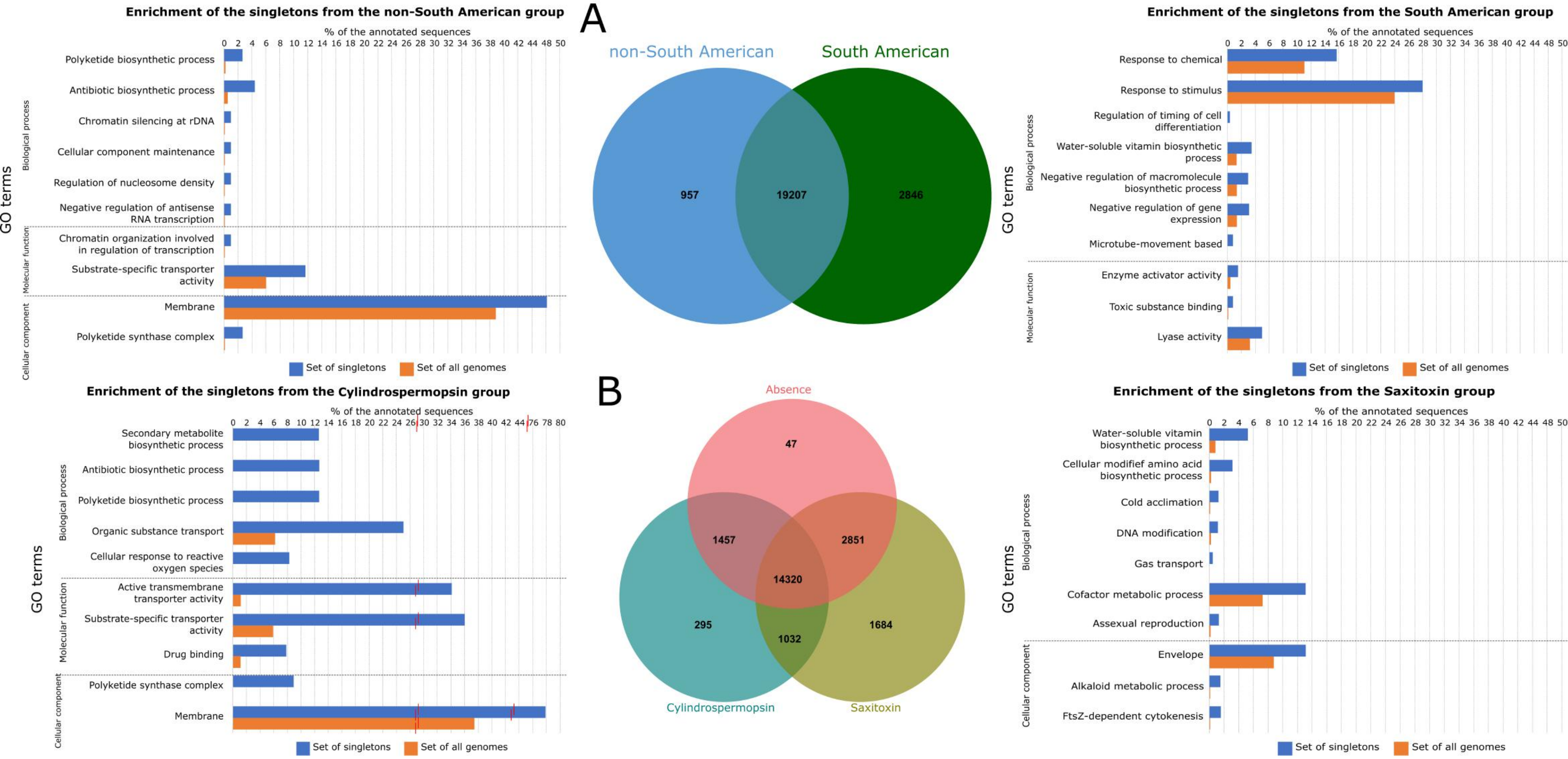

Supplementary Figure S2. Venn diagrams of the strains that were separated in five groups depending on the origin **(A)** South American (CENA302, CENA303, D9, ITEP-A1 and MVCC14) or non-South American (CS-505, CS-508 and CR12); and **(B)** presence of gene clusters for the toxins cylindrospermopsin (CS-505 and CR12) or saxitoxin (CENA302, D9, ITEP-A1 and MVCC14) or absence of both gene clusters (CENA303 and CS-508). The diagrams show the numbers of orthologous proteins. The result of the enrichment analysis of the singletons from the South American, non-South American, Cylindrospermopsin, Saxitoxin and Absence groups are shown in graphs next to their respective circles; the y axis indicates the GO term divided accordingly to the Biological process, Molecular function and Cellular component ontologies and the x axis indicates the % of the annotated sequences.

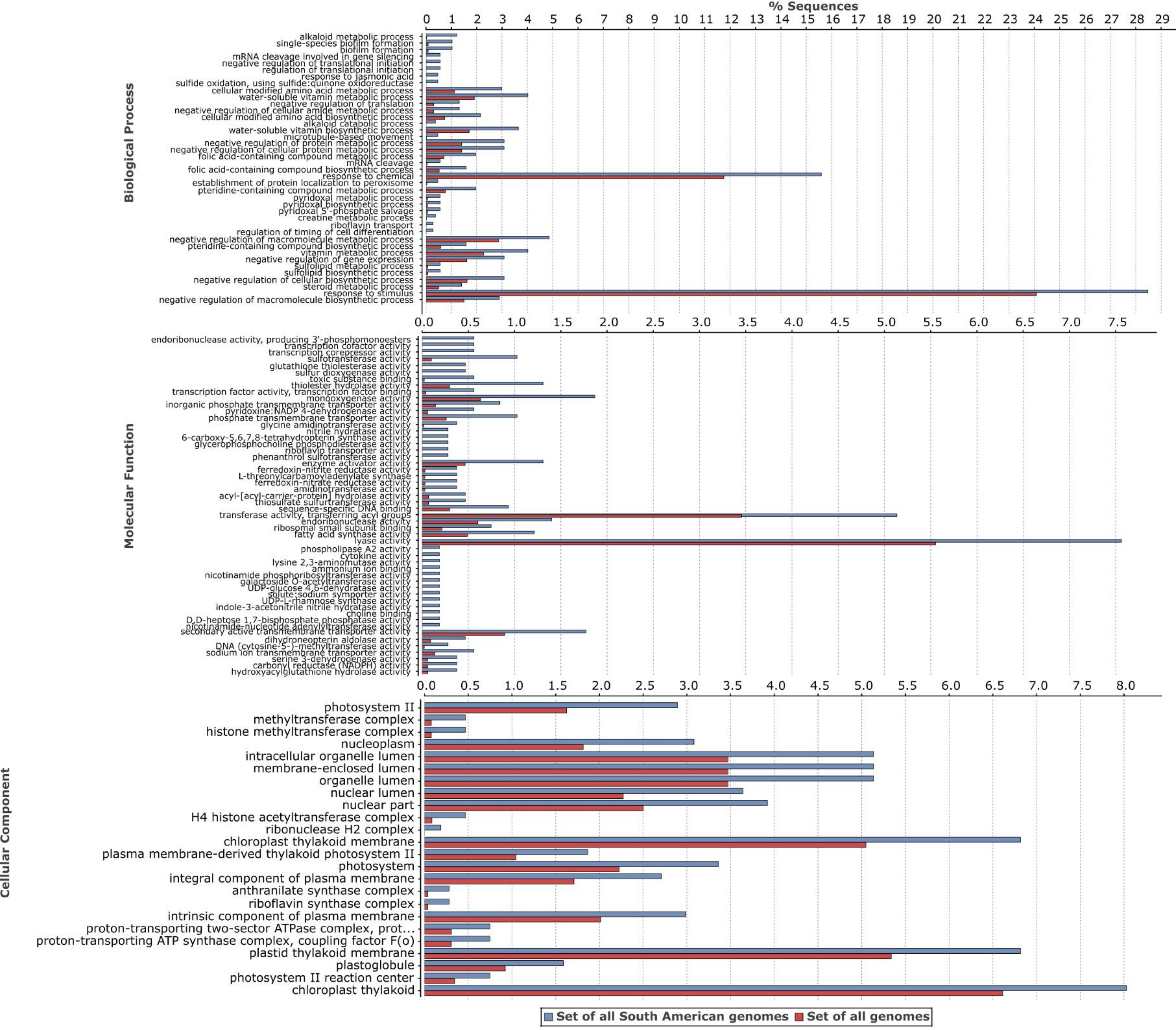

Supplementary Figure S3. GO enrichment analysis based of 1,071 singletons from the South American genome set.

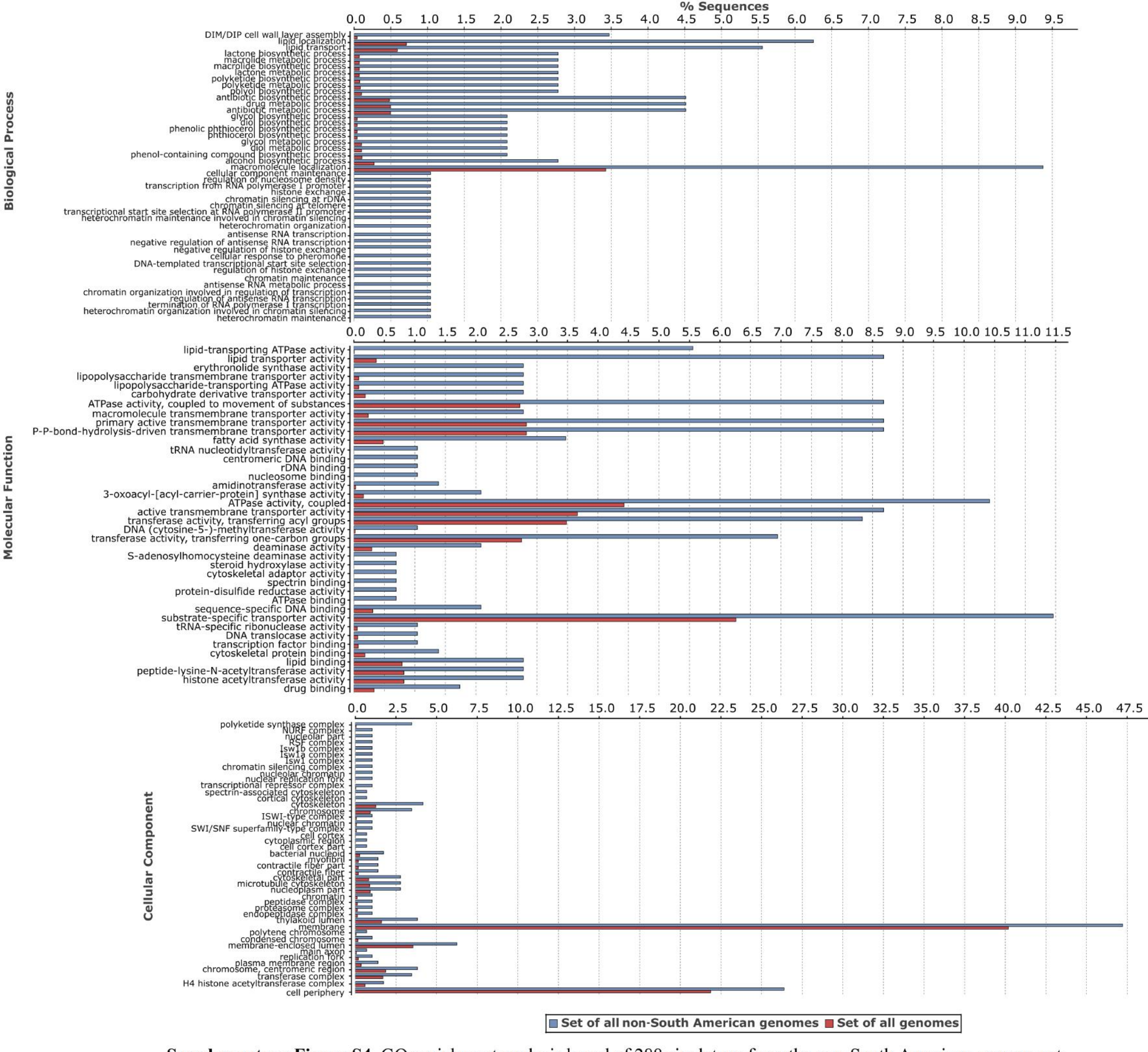

Supplementary Figure S4. GO enrichment analysis based of 288 singletons from the non-South American genome set.

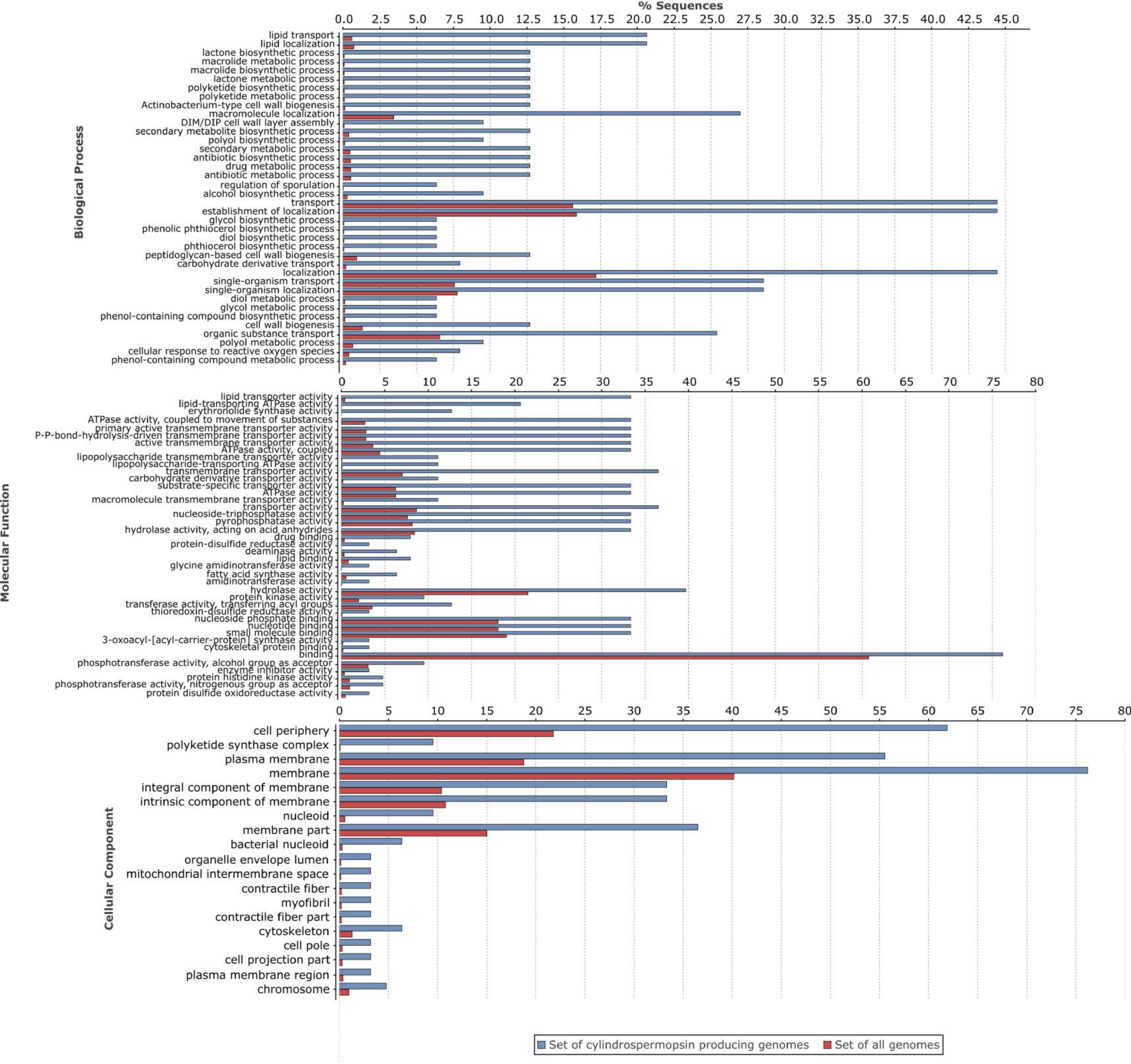

Supplementary Figure S5. GO enrichment analysis based of 63 singletons from the Cylindrospermopsin set.

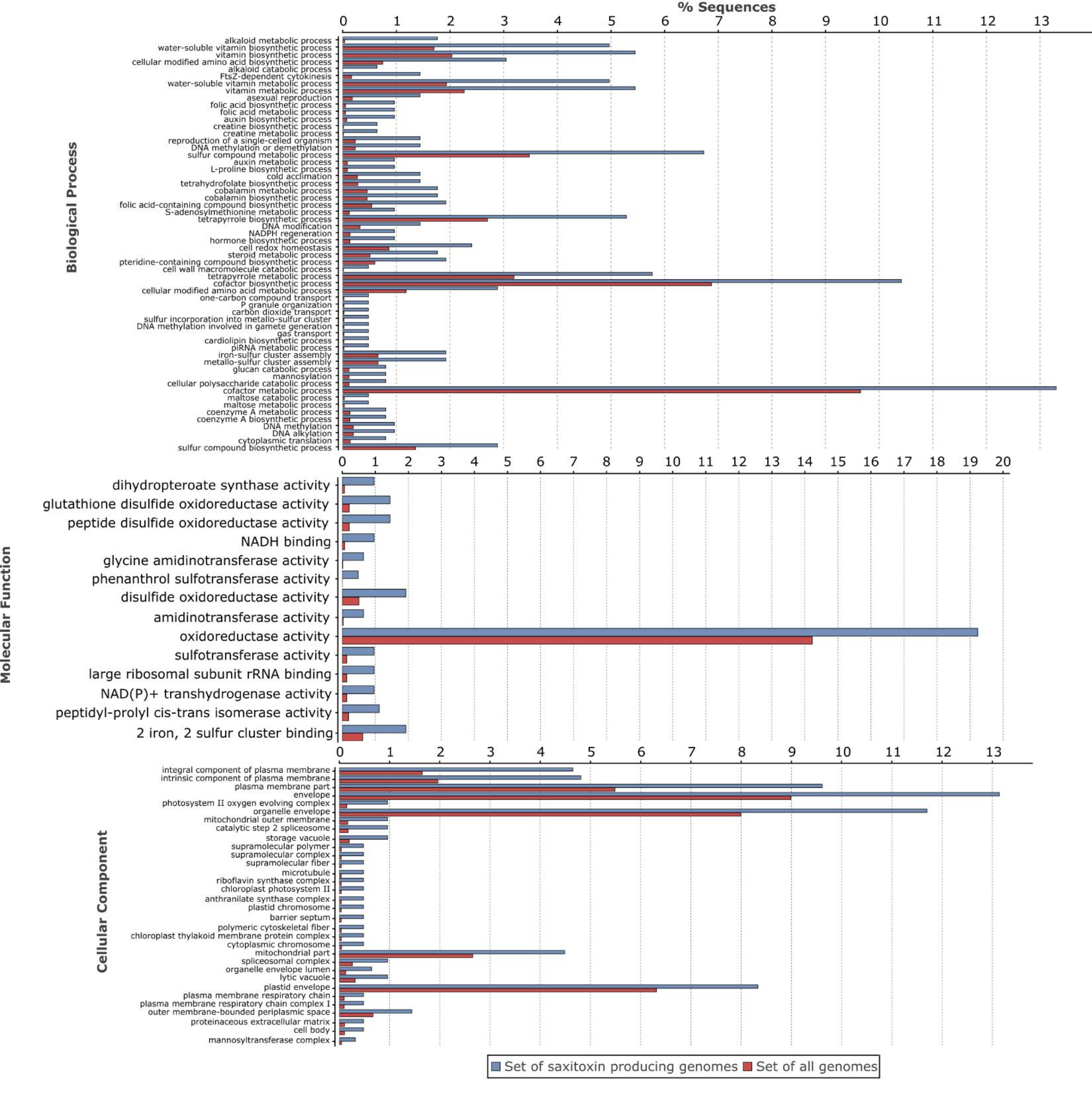

Supplementary Figure S6. GO enrichment analysis based of 624 singletons from the Saxitoxin set.

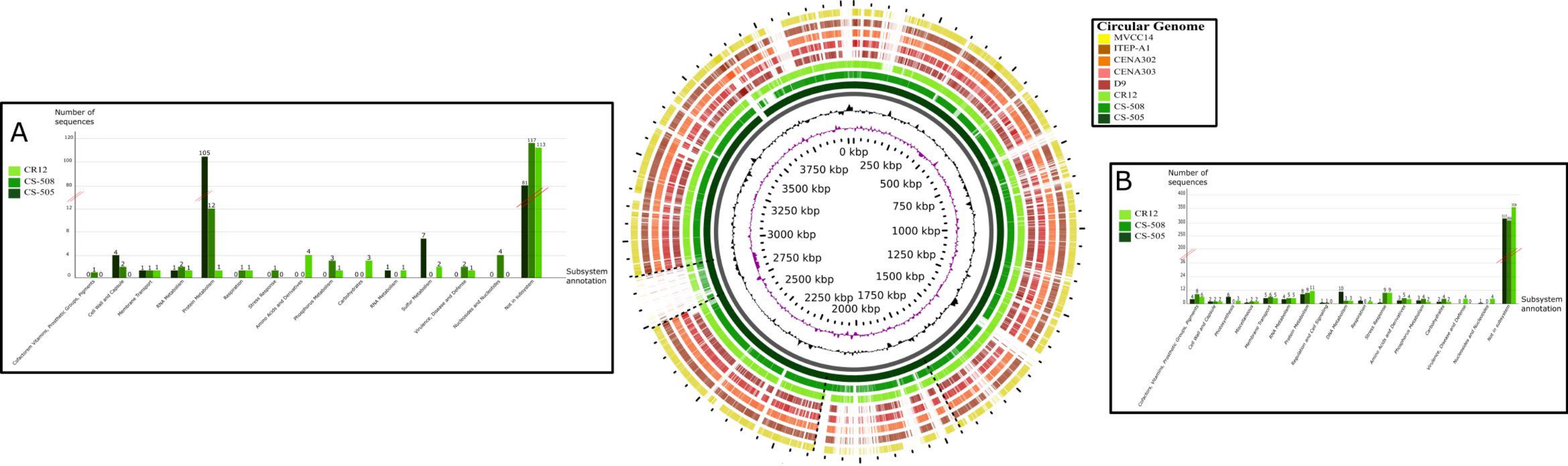

Supplementary Figure S7. Two conserved specific regions (**A** and **B**) which are divergent from the South American five genomes were identified on the non-South American CS-505, CS-508 and CR12 genomes.
