# Supplementary material for: Genomic and Genotypic Characterization of Cylindrospermopsis raciborskii: Toward an Intraspecific Phylogenetic Evaluation by Comparative Genomics
Source: Front Microbiol. 2018 Feb 26;9:306. doi: 10.3389/fmicb.2018.00306 (PMC5834425; doi:10.3389/fmicb.2018.00306)
Supplement: Supplementary file 2 [file Data_Sheet_1.PDF]

Data Sheet 1- Protein sequences of the South American group which were not included in the Veen diagram.

>1170769.6.peg.1
MCNGDLESTVKAVKRLFAGIPWRNFTNNDLANFEGYYASVLYAFLSSLNARIIPEDITNY
GQADITAILGDHIYVMEIKVIDGDKVKENLALKQIRKCNYAQKYRGEPGKTVHEVGLVFS
SSKRNLIQADWE
>1170769.6.peg.7
MSNQNDNSQNDNSRDKSQKPQSSGWFKIKRLRPPKGIGKPKTE
>1170769.6.peg.23
MLLGRRSPWLPKKIANYQFPRVFAQTILHNLGRVTRLLEKIARPRLTKLANHDITWRCNG
FCISWLAILLISPVPLTNPIPTIGILLFAAASIESDGLLICICYVLTLLITLIFYLIVYG
VLQLPGLIT
>1170769.6.peg.24
MSPGGIGNKNGKTNKAMTRMNPRWEVVSSTSPRVRGWCARRCSKDFISSEKVKRIVQGLG
I
>1170769.6.peg.43
MESNNYREIMNSKNFTFSGLTLKSLILLSSLLICNGLTIRGLELSANAQLLSNAEKEELS
RLRTETKIQKQLQSYLSIWLTLLSLFAVGLIATLWFLRKAIIRDIVERAMRQIGNIENLQ
TELIAANQKTTGLIEYSQDLALELEQKVNHLKTTIEGEGGKLSVLLSDLPKSKQEFLTAL
EREVIAAQENISSLEFKLNTQLEQVTLAAQQQRVTIENIKKLESELFAQFSEIKLSIENH
RDTSVSDINKYRSELMEQFEILALETLESKTQVVQSITEHASQFTSNLSEFQTHAQEQMD
GFTSNLSGFQTHAQEQMDGFTSNLSEFQTHAQNQMDGFTSNLSEFQTHAQNQMDGFTSNL
SEFQTYAQEQMDGFTSNLSEFQTHAQNQIDSFTSSLSDLNQTSALQLIDLQTDTQKQKDH
IFKDLAKSQSEFSHYLLELRNTTESRQREIIENWQKSVDEVEQMLSNLHNDVEEQKTDLL
ENLQVLGLGFQKQIGDLQNSIEQRHRKFFQTSQNSVEQLISEFMSELSVMKSDIKTDVDH
QKATLIARLAKLEKLESQFVEQLKKLQLDAENRQYETLKELSQMTPQTPVKHISPLSSVE
TDTNTTNTIDVQTVNNLDNHLDHEIQEAEELLSQNRYEDALSIYEKITSIQPDHGEYWLK
RGFILNKLKRYKEAIGAYNQVIRINPAHYQGWFDIGITCGKLGKHQEAFNCFNKATEIKP
EESVAWLNRGLSLVELENYEEAISSFDKALEIQPSSFKIWDKRGYTLVRLGRDEEAITNF
NKALELNPDYGSALYHKSACYALQKNVELALVNLQQAIKHKPSYREDAAGDIDFDDINND
TRFQQLIHGE
>1170769.6.peg.57
MDIVKTVLADVIELQIETSVIDESSYNDNASSTKPENKMVTRINLVDGDIRNEIGTSFIG
SGPYTELREFHLSQVQEGREIIQKNIESVQKLGEILMSMVKQSQNSQSSQLAKLP
>1170769.6.peg.68
MPKLVIHEYQRTAQYFREDLGNDIQLDIDVDSWWHFCHGFTGRREG
>1170769.6.peg.71
MLGVAGVFGGSLFSAMHGSLVTSSLLDSKNTFNTVFYKFI
>1170769.6.peg.72
MYHLEKGRYKQVKANRRNHYAIPELGVELGILLDQERPPIPWLRWWDSRGNLLLTGNERA
EEESQRRELAEAIAIQERQQKELAEALAIQERQQKEMAEALAIQERGKKEKLAAYLRSLG
INPDEI
>1170769.6.peg.73
MQVTTTSTPIPPGELSPSNWPDHTQLPDSDDNFVKNFQEHPQSVILTTSIEPLLDKIHPD
KESGEVLGL
>1170769.6.peg.76
MELIQFFDSLIGRSRIICGVIFLELTSNLSNLLLILD
>1170769.6.peg.78
MPGIRNAWNGKVWWKSGSLQMFTDAGNLLCVTLCMSG
>1170769.6.peg.79

MQYNPFSDFYSTLTNNPGYFPGPQGVGNFVAISAIAALLEWVRVGNAPSIPDIFGYLV
>1170769.6.peg.80
MNLSITTKTPRKTTIKTARINLFLLASALITAITSTTSLATDTISAQDIAQIAKKTSVQI
NTEGDITPGGSGVIIAQQGNRYSVLTANHVVCDIIDRPGKITCAKDITYSVRTNDGKEYP
IQSQDIIVLQSTKNDPDLALVSFVATEEYPAANLGDSDQMTEASDVFVGGFPAVFGKVGS
ARDFSFSKGIVLSRGRTSINGYSLIYDAKTLTGNSGGPVFDIKGRVVGIHGLADASNKSK
TETGELVSQKTGFNAGIPINTFLNFNNPLVKDLPIKRNTIATGEAPQERLNSPQSARDFY
ARGITKLEQFNYKESLADLDQAIKIDPKYAEAYFKRGYALSWLRRYEEALLDFNQVIALD
PNYLDGYLNRGWTYIWLQNDQAALEDFNRVIRINPNYAEAYAHQGMAYIKLGKYQAALES
SKQAIRLDPNKSYGYTIQGDVFNYLKDYPAAINVSTFAIKIDPDDFNAYINRALAYTLTG
NYSDALVDYQKSAEIFERRYTRKPSN
>1170769.6.peg.99
MVMERLKQDLKNDLIAGLLVVIPLATTIWLTITIANWVINFLTQIPKQLNPFDGLNPILV
NLLNFLVGLAVPLISILFIGLMARNIFGKWLLDFGERILHAIPLAGQVYKTLKQLLETIL
KDSNGKFRRVVLLEYPRRGIWSIGFVTGVIASDIQAKLSRPMLSIFIPTTPNPTTGWYAV
VPEDEAINLTMSIEDAFKIIVSGGIVAPSNGIVMSQLPLTTPALTTESKSHLVGVEPDF
>1170769.6.peg.105
MQIGVPKETKDQEFRVGLTPASVRVITENNHSVFIETMAGYPAGFTDEDYLAAGAQIVAS
AESAWHQDLVVKVKEPLRSEYQFLRKDLVLFTYLHLAADRELTEHLIDSGTCAIAYETVE
QVGTNRLPLLTPMSIIAGRLAVQFGARFLESQQGGRGVLLGGVPGVKPGKVVILGGGVVG
TEAAKIAVGMGAGVQILDVNVERLSYLETLFGSRVELLYSNSAQIEAVVREADLLIGAVL
IPGKKAPILVPRKLVKQMRPGSVIVDVAVDQGGCVETLYPTSHSNPVYVDEGIVHYGVPN
MPGAVPWTSTQALNNSTLPYVLQLANLGLEALEVNSALAKGLNIKSHHLLHPAVQEVFPD
L
>1170769.6.peg.107
MGIVDALIGGLDDSWSLFAFGLGTTGVALGLKLASKLSRAQQRSPREEGRTFQYYLPPTS
SSSSLPIIKATKNKSYY
>1170769.6.peg.110
MGNLHSVCKALEKAGAIARVTACGKELARADAIVLPGVGAFDPAMQNLRSRNLEQPIKDV
VTSGKPFLGICLGLQILFESSAEGNEPGLGIIRGKVRRFRPEAGIAIPQMGWNQLQLTQP
NSILWEHLPSQPWVYFVHSYYVDPLEPEVQSATVTHGSQTVTAAIARENLLAVQFHPEKS
SNIGLQVLSNFVAQVREKIPA
>1170769.6.peg.118
MKQNKPQVRPVALGCRKYRRCPAWYFGGVMELRAAVVC
>1170769.6.peg.121
MGANITPIAKIKPVNHPETIVYIQGRIEKHAPLIGEQAYQIADSTGKIWVVINQNSGQNQ
NNLQLGQEVVIKGKVKYKGITLHQQEYGEVYLEEE
>1170769.6.peg.128
MTIEFTKYHGLGNDFILIDNRCDLTPLITPEMAIKMCDRHFGIGADGVIFALPGQKGTDY
TMRIFNSDGSEPEMCGNGIRCLAAFLTELEGISRSKDYYLIHTLAGIIRPQLTDDGQVKV
DMGSPRLLAQEIPTTLVPGDSKVINLPLEIGKQTWHITCVSMGNPHCITFVEDVASIPVE
IIGPQFEHHPVFPQRTNTEFIQVVNPSHLKMRVWERGAGITLACGTGACASLVAGVLNNL
CDRMATIELPGGNLEIQWSETDQRIYMTGPAERVFTGKI
>1170769.6.peg.130
MVTSIVFMSGIGFINVYEFFVLTFLYHFPHIFYLWFQIRSLFQFYTG
>1170769.6.peg.131
MVVAAVLLSLVVIYLASKVGGELSNKLGFPPVLGELVGGVVIGTSVLHLLVFPEGGTDSS
SSLIMSFLQITAGLTPEATPAVFAAQSEVISVLAELGVIILLFEIGLESNLKDLMEVGIQ
AFVVAVVGVAVPFGAGTAGLMIIFGIAPVPAIFAGAALTATSIGITSRVLSEIGRLNSKE
GQIILGAAVIDDILGIIVLAVVASLAKDGAVDVGNVIYLIASATGFIIGAVILGNVFNKS
FVAIADVLKTRGGVVIPAFIFAFIMSYLADIINLEAILGAFAAGLVLEETEKRKELQKQV
IPIADMLVPIFFVAVGAKTDLGVLNPAIPTNREGLVMATFLITIAIIGKVITGLAVFGQP

GINRLAIGVGMIPRGEVGLVFAGVGAASGVLSKPLGAAIIMMVIITTFLAPPLLRVVFPQ
GESSVAIDSTSEV
>1170769.6.peg.136
MSKVLVSDPIDQAGIDILSQVATVDVKTGLKPAELVAIIGEYDALMIRSGTRVTEEIIEA
GTQLKIIGRAGVGVDNVDVPTATRKGIVVVNSPEGNTIAAAEHTLAMMLSLSRHIPDANT
SLKKGEWDRKTFVGAEIYKKILGVVGLGKIGSHVAHVAKAMGMKLLAYDPFISTERAEQI
GCQLVDLDLLFQQADYITLHIPKTPETTNLINAKTLGKMKPTTRIINCARGGIIDELALA
DAIKNGKIAGAALDVFQSEPLGDSPLRSLGKEIILTPHLGASTTEAQVNVSIDVAEQIRD
VLLGLPARSAVNIPGLGPDIIEELKPYMQLAETLGNLVGQLAGGRIETLNVKLQGDLATN
KSQPLVVAALKGLLYQALRERVNYVNATIEAKERGIRVIETRDASARDYAGSLHLEATGT
LGTHSVTGALLGEKEIHLTDVDGFPINVPPSKYMLFTLHRDMPGIIGKLGSLLGSFNVNI
ASMQVGRKIVRGDAVMALSIDDPLPDGILEEIKQVSGIRDAYTVTL
>1170769.6.peg.144
MVRALVGDSTITRVDMEISKKRIALKLNSQVEIILAESQG
>1170769.6.peg.145
MPSKSIFIFNLCLTYGGCAWRIKLGNSDSSLILPFTWVHDLVHNSIISSYRS
>1170769.6.peg.158
MTVHWGIFRLQYFTVLRWLAIAFLRLLSSNFSNILFYGIKNTKQ
>1170769.6.peg.168
MKANPVNLPNFRSAVDGDSQDSPVASSKTLVQLLSEEIESQVKASSRCIQAVVSRITKEV
ERICDKSSRIQTSGQIKSWQNNLAKHRLQKCLRYYQLGSRQGRIELHSSLGSIIYRHVTT
SGSELGFEARYNLIEDFLQAFYMESIKAFRRENELAQEYTPRTQLELAEYMAFTEQYAKR
RINLPSGVNQQLIVLRAQGFARRQPQETTVDIEMAVDSAKTEETESYQRNLAVQQIRSQM
VAKSTFDPSEDSERDRVITELMKYLQSQGQGDCMNYLSLKLQDLSAPEIDQILGLTSRQR
DYLQQRFKYHVEKFAKQHHWQLVHQWLGAGLEHKLGLSSQQWDLFWNQLTQQQQQIFQLK
TTMENDQAIAKAVQCTPKQLQKRWTQMLELAWAIRNGNVEVKTC
>1170769.6.peg.171
MRKGIVSRQQPIYTLCQYIPAREWVLVEYELEKCDFLLRDRIGDLIGREQWQND
>1170769.6.peg.185
MVKLQRNVQSLVESNIIKPSDSIWKIAFLYADEWKYWKQELLDFGFSMQDPIGDLLAVET
WDED
>1170769.6.peg.191
MIADYGQLCQLYCILACQIWTLGSLKMTRKLTAKAQPA
>1170769.6.peg.196
MIVHVPGSRADDPTHPLKVVGWGNLAKDIHQNYHAGDRVILEGRLGMNTFDRPEGFKEKR
AELTVQKIHPVTKNTGTSQPAGQLPQETPNYQASRPTPTPVVEETARSVTTLDPSPQAVI
QPTPNPDEIPF
>1170769.6.peg.201
MSSLNARIIPEDITNYGQADITAILGDHIYVMEIKVVDGEKVKENLALKQIQECNYAQKY
RGEPGKTVHEVGLIFSRSKRNLIRADWE
>1170769.6.peg.203
MPTSIAELLPTISELSRADKLQLVQIVLQQLAAEEGIKKPKKSLRGTLKHYANPSLIEQE
QTAWLNAVGDDYEPS
>1170769.6.peg.204
MPQVFSKLNSLLQAQNFKQADRETRKIMLAIAKREEEGWLRIEDAEKFPCKELRSIDQLW
LKYSGGKFGISVQQQIYQSLGGTKEYNYDVAKHSPCDRS
>1170769.6.peg.217
MSDGGNVEVVELDGPIVKLRLQGACGSCPSSTMTLRMGIERRLKEMIPEIGEVEQVF
>1170769.6.peg.219
MNLQKPILVGGLGLSFSLWMLNTWHDSIIQMAELGLLGALAVGGGLWFLPKSQPSEQLHD
NPPDRSRVEAVISQAQSIIHQIANEVVNHPSLSILQEQLAKLTLELDRKEVQIAVTGGKS
VGKSTIIRLLKTVSNLSANPWELVETLPLFTRLSQDDDPAILLTLQKSDLVLFITNGDLT

ASQLDVLQKLRSTQQLVLLVFNKQDQYMTEERIIILQSLQQTFPGHVLATSASPLPIKVR
KHLADGSIQEGMEQPTPNIQQLVEQLTVILAQQIPQLVCATTWRKSLFLKTQARSCLNSI
RRDRSLPIVEQNQWIAGAAAFANPLPALDILATVAITGQMVMDLSNIYLQKISLDQAQKV
ARTLANLMFKLGLVELSTKAVTGVLKTNAVTFFAGGMVEGVSAAYLTRIAGLSLIEYFEQ
QDIALTSDRQLNMETLGGVLQRVFQENQTLLLLETFVKQGVKRLSRETKPSTTSVHF
>1170769.6.peg.229
MIERSGRNLIPWVICYGFLILCPLPLTIRLTDFQILE
>1170769.6.peg.234
MVEETNQKPVGKKEKPPAVEDKPFQEFMQEHYLPALQKAIAQEGISDVKLTFVKQKYAIV
GFNSSQECWQITGSWQNGAKQFNVYFPDQDIQGKKGFSCHEGKKPSTLESFLIDERKITL
DLLVSRLIYRLNGQKWLGRN
>1170769.6.peg.243
MMIIANMAGVVPDVYPFLSHALWNGCTPTDLVYPFFLFIVGVAMSFSLSKYSLESKLDKF
VYFNLCRRAVILFTLGLLLNGFWNQGVGSFDLQSLRVMGVLQRISLAYLVASLIVLKFPE
KTQWALAGILLIFYWLTMMYIPVPDYGAGMLTREGNFGAFIDRLIIAKPHLYAGDGFNFR
GDPEGLFSTIPAIVNVLFGYFAGQWMRKSTINSHTSMDLVLWGLCSLVVGMIWDGLFPIN
KKLWTSSYVLFSTGWGLVFLAACYDLIEVRKIKRWSKGFEIIGLNAIALFVASVFLIKVT
VKLKIGEGENAISVYNWIYRNLFASWVGNTNGSFLFALATLLLWYGLAFFMYRQRWFIKV
>1170769.6.peg.252
MTQSETTTAKSFPQLLAGLSLISLSLVISTWIGSRAILDFKKANDALIVTGSAKRPIRSD
YIVLRLSLVSQRPTIEEAFKDLKNQTIRVQAYLKENGVPDAAITSNPVETMTIPEITENG
RETGRILAHKLTQNLQIRSQDVDKYSQLSQKSTELINEGINLTVQPPEYLYTQLSKLRVE
MVAEATKDAQARAKAIADSAGGQVGSVRSAQTGVFQITARNSTDVSDSGLYDTSSIDKDI
TAVVSITFSMK
>1170769.6.peg.265
MYHLEQGRYKQVKANRRNHYPIPELGVELGMLLDQERPPIPWLRWWDNGGNLLLTGNERA
EQECQRRELAEAIAIQERFEKEQERQQKELAESLAIQEREKKEMAEALAIQERTEKEQER
QQKELAEALAIQEREKKEKLAAYLRSLGINPDEI
>1170769.6.peg.271
MPKPYSVDLRNRVIVVWATQEGSQRQLAERFKVSLSFVTNLVRRYRETGQVEPKQCGGYE
KPIIEGQYLNMIKAWIDEKNDLLLSELSDRLREKTGIKLMLVSQPCIEHYKSWVYIVKKM
VWESTICWCSI
>1170769.6.peg.272
MLTHNIQTKIKNSPLLFLLPLALAVRLYNINSPIIGIHSWRQADTAAIARNFYESRFNIF
YPQIDWGGNTPGYCETEFPIYSFIVAIFYKIFGVHEIFGRLTSVIFSLLTIYFLYKLIAK
FLDPKIAIWSCLFFAILPLTVYYSRTFQPEAILLTCSVAGIYYFSNWLESEKIHFLCISG
IFVSLACLIKVLPIIYVGLPLLYLAYCKFKHKIFTNIYLWIYTLFIGISFGMWYYHAHQL
YLEYGNTFGFWGSSANGRYQYNIIFTLKFWLDIIFRTVVRHFAVFMFPIFIAGLFIPRTN
KQEYLFDIWLISVIITWVLVPTVSLVHEYYQLPLMLPGVVFIGKFLAKYLDHNSLKINIK
KISITCICLSIITGSLIYTIDYMFKEKIHKSATFQLAQIVQKKTDAKSLMIFTTGGDPTL
MYLSHRKGWLIDPSDLTRQYLETKIKLGANYLISSFQFVESYNFNINENQKQNIIDTLVK
YPNILDSKELIIAFLNLRKN
>1170769.6.peg.273
METVWDAQAEEVILSGTTSQLGILTGHAPLLSALDTGVMRVRATRNSNWQAIALLQAIAL
LGGFAEVENNEVTILVNGAETGDKINLEEAHAAYNEAQQRLSQVPAGESQAQIKATQAFK
RARLQAAGDMV
>1170769.6.peg.274
MLLNNPHFHLPYKNQQSQGKNTLLSRGILSIIGRVRVRCRSYPKE
>1170769.6.peg.294
MSAGNHFPLAFTLVYVVGFVAAVTIGSIAWYNSKRPPGWESKDRPDFVPKIDKE
>1170769.6.peg.308
MDFSCSSIVYQLSAQITSIDVSRTISAKGSSRKSVKEVLAVWN

>1170769.6.peg.331
MNLSNSNLNSNLEKIYAFDYLRAISCIFVIALHSNIICFVKENQIIHDLIIFNLFDLAVP
LFFQISLILFFLKREKQPDYFFKKRFFKLLKMYIFWGLFYQLFSWGLHFKNIDFTNIQNL
IHIEANMKNIIIFIITDGKSPTFYFLFSLFFITSLAELFVYCLEKIKTRYQINPELISYT
ALCLTVWYNS
>1170769.6.peg.335
MNQLLPLSINQQEIYIDQRMWTNGSHLNIGAIVIVRGVFNREILNCAMSKIIDCHPGLRT
RVYEVDGKPLQTVTEYQHRDICFIDFSNYESSEAKAQEYISQEFAKPFDFNIDAPLADFK
LIKLSDDSHIIYAKYHHVITDGWGTAIFFREVIKTYTQIINDGKDSQIPRDWVIQEYIEE
EKKYLESSIFLRDRDYWQQRLQNVSPKIFSLIQPQGLEGKRHSIYIPREEYNRVNRLCQD
MQSNVFHFILSLISIYLTRRYLKDDVVVGLSLLNRSKKNFKDAIGLFVSTIPFRLTINRQ
QTIHQLLDNIRSLLRQDYRHQRFPLGEMKRFSGLQNKIKENLFEVFLSYERHDYSESFPD
TQTSCIPLYSGQQKIPLIVYVREYEETSDVKIDFDYNLSYLDEKTVEQIVTGFQNLFIQA
TDNLEIFIGDLEDSLADSRDISKNISVSQPEIPFVNYQETLISAFEEIATQYPGNPAVQF
NNKILGYAQLNAHANRLANYLIGQGIKPQSKVGICLERSDQMIIAILGIIKTGSSYVPLD
PNSPIARRQLILQDSGMTALITQSSLLTELNTENIIAFTLESIDSELNKEPNTSPQIAIS
TDFPAYVIYTSGSTGTPKGCIVTHRNAVRLMRATEAWFRFNETDIWTLFHSFAFDFSVWE
LWGALLYGGKVIIVPFGLSRNPEKFREFLTTEKVTVLNQTPSAFYQLIRADESAVGDLSL
RYIIFGGEALDLQSLQAWLEKYGDKKPRLINMYGITETTVHVTYRPITRQDLKTKGSFIG
REIPDLAIYLLDDQLIPVADGVSGEIYVSGAGVTNGYLNRPTLTAERFLPNPFGTGRIYR
TGDLARRLPNGDLEYLGRADQQVKIRGFRIELGEIQAALTSHGEVQEAVVVTDEWQEEKR
LVAYYVPGESSPTVNELRQFLKNTLPDYMIPAAYVSLKAFPLNVNGKIDIQALPAPDWNS
LRVEEDYIGPRNIDEEILCTIVAKILGLEKVGIDDNFFEIGGDSILALQVIAKAKKEGFA
ISARELYELTTVRNLATKKAAVATLEDIAETSILSLVSDTDKAVLPKDIEDAYPLSSLQG
GMLYHSELHTGSAIFHQIFTFNLEISYSELAWRQAIADVCLANPVLRTSFHWTGYSQPLQ
IVHRQVESPLSIVDLRSSPNANQHVQEWVELEKTRGFDIGNPPLFRFQIHRISNENLCFS
FSFHHVILDGWSVATLLTQLLRRYVQYLDGKNLPPLVFPETIYRQFIAQEQNAIANEEIR
EFWSQHLSNLQVTFLPRLNTTGIKITTTDYHNRQLKRLSLTISDELTDRLRKISQNIGVP
LKTCLLALHLRVVSFVTGQKEVVTGNVTNARSETSDSENALGLFVNTIPLRLELPSGSWI
DLIKAVFHAETAVLPYRMFPLAEIQRLLGKRPLFEVGFNYVHFHVYDSLLSLPQVQVGNV
DIFEETDFPFLAEFCLVPGSQTLQLNLIYDTQQFADAQVEQYGRYYQTAMVEMTTAPQMA
YHRRSLISISERQQLLQAANLDPQNFPSPDTLVSTFTQAVVKYTNKTALVYQQTTLSFAE
LETRANCLAHYLRTKKVNTEKLVGVCLERSEQLIITILGILKAGGCYVPIDPSYPSDRLE
FIFQDSQMMLLITEKSLISQLPECGGEIVTIEDITTEIDTRNCHPPVVQILPENPAYVIY
TSGSTGKPKGCIVTHSNVIRLLKATQSWFNFNSEDVWTLFHSYAFDFSVWEMWGALLYGG
QIVIVPYWTSRSPKDLLQLLTKNRVTVLNQTPSAFKQLIPAVQEQSEKLSLRYVIFGGEA
LEPATLQPWFDLYGDEKPKLINMYGITETTVHVTYKPITQTDIISNRGSVIGQPIPDLEL
YILDENLEPTPIGVTGEIYIGGAGVTRGYLQQPRLSAERFIPHPYSEKPGSRLYRSGDLA
KRLSDGEIQYLGRSDQQIKIRGFRIELGEISSVISSYPQVKQALVMVQKAPNGENRIIAY
VIFDSTAQAIDPLKEFLKTKLPDYMIPSALMAIETIPLTINGKIDYESLPIHDWHWKNKD
YVSPRNEREATICSLMASLLKLEQVGVQDDFFEIGGDSLLVTQLAIRLRQTYNIEFPLPQ
LFTHRTPEAIALLLPEVSQSSVETQIPKLRRTSRSVTLSDDGVLSKN
>1170769.6.peg.339
MEVYVLPLSLSQKQFWYREKLSSGNTAYNIPLALSLLGNLDQVVLETCFQKIINRHEILR
TTFALENGEPVQLVHDKKEFYLECQTLPLHLEKRPIEVSTIKEILERESRQPFDLINGPL
MRVKLYQISSEEHILLINLHHIISDGWSLGIFIQELTKLYATSGKINLPELPIQYGDYAQ
WEETYLQADKIQEQLTYWQDKLTLPLPILDLPLDKNRPILQTFNGAVLRQSLPGDVISSL
EILAAKEGVTFFMLTLAVYQVLLFRYSRQTDIIVGSPCANRTRSEVQNLIGCFINTLPIV
CSLEGEISFRQILQQISITSVEAFANQDVPLELIIDQLRIKRDPSRSQVFQTLFALQNAP
IGTIEMAGITVQPIHLDNGGAKFDISLMLEPDFEHGWTAALEYNTDLFTAETAQEILTRY
QQLLVAVVDNFDTRIDALPWLSKQEIKELLTLGSTEQTAEKIEPISLIDIFTQTVNNYKN
KVAVIDSSQKITYQELDHISNQLAASLIQKGVGRETRVGIFQERNIELIASILAVLKAGA

TCVPLDPQYPGERLNFIARDSGIELVLTTELLQSKIPVGIPEVLLVESGKFTEKLEQLNL
PAKIFPQQAAYIIYTSGSTGQPKGCVVTHQNVVRLMRNTQKWFEFNEKDVWTMFHSFAFD
FSVWEIWGALLYGGKLVIVPYFESRSPQAFRELLLAHEVTILNQTPSAFRQLIRADEEFS
HPLNNLRAIIFGGEALELQSLRPWIERYGDSHPRLVNMYGITETTVHVTYHQILAQDLLE
QRPSVIGIPIPDLCLYILNDSFEPVPYGVAGEIYVGGMGVSRGYLNLRSLTAQRFIPDPI
SQKPGARLYRTGDLARKLRNGDIEYLGRCDFQVKIRGFRIELGEIEAALIKLLQVSEAVV
TVHSFTEEEQRLVAYIVVSSVGNKGITTSELRTELKQKLPDYMIPAAFVFLDTMPLTSQG
KINRSALPAPDWNQSATKKDFTPPATDAEKILCHAWQLTLGVDNIGIEDNFFDLGGDSIL
ALRMVTQMRNQGWIVTPKQIFQEQTVKKLALVAQKQTNLQTLTVENHIARNEVPLSPIQQ
WFFDLTLSNPNHWNQSLLLQVHPSLEPKEVLAAMKVVCAHHDSFRLRFEKQAKGWRQFYA
SESQDGFSWEEKVDLDFKTEIEQNVIISQVRERVEKSLDLTHGPLCRLVWFNLGESLPPQ
LLIVIHHLIIDGVSWRILLQDLVEVISGAEISPTTGSWQNWCQFLQDSVKLQNIQDERQF
WQSTLAKETAKLPLDFSEGVKTNLECHVKTVSCQLTAQETQILLTTANKSYRTNPQELLL
AALGKTLANVTQSSDVRIMLEGHGREELSSGLDITRTLGWFTTLYPFTLKLPETDCHSEI
IKSVKEQLRAVPQKGFSYGILRYYNQETKTSLTTTVEVSFNYLGQVRNEGGNKHQLFSLL
NSQGSPARDPQGVRPHLIDINAIVLEGKLQVDWLYSSNLHRTTTIEQWVSDFKKNLLEIL
ELCLDLGVSEYTPTDFPLIKIPQSKLDIIQNQYPWIEDIYPLSPLQEGMLFHAIYEPEEG
IYFEQVIGKIIGKLDADNFAHAWQVVVDRHSILRTCFVWEEQEEPLQIVNKQTTFSVTCM
DWRNLSQELQLERVQEYLIADKQEGFSLSQNPLMRVTLVRLNDDTWQWLWSHHHIILDGW
SLPVIFKEVLTIYQWINQNSQRVTGERATNTLPPAVPYRHYIQWLRSRDQQKAQQFWRKY
LAGISTVTRLAWNTHDLETNPGLPAYQEIELKLTESEFALVQKMAQSWRVTINTITQGAW
AICLQKHGAGEDVVFGVTVSGRPPELPEMENMVGLFINTLPMRVKIDPTLSIANWLQNIQ
QHHLEMRDYEYSKLADIQKDCNLAGSALFESLLVFENYPVDQSLRGKLGDFQVEDIQFYE
RTNYPLTVGVIPNQGILLKLNYQTSFLSRGAAEKLISRFRNIIVNMAVEADETLDRIQTL
SVSEQKELITSSRGNIIKWQGFKSAHQLFENSADLYPDVTALVCGDRNISYGELEKRANN
LAFKLLAKGIRYESLVGLYFEPSIDYIVALLAVLKVGAAFLPLDRNYPDNRLEFIFLDSQ
IPLVLTNNSSVVPSKLPEKVEVLDISQIDWRENHPRLNLQVSPENIAYVIYTSGSTGKPKG
VLVPHSGIENLVRAQIDSFGVNTESRVYQFASLNFDAAISEIFMALGSGAMLYMQPQAER
SPSSELWEKLTSWKITHLTLPPSLVAAISTTALPQLKTLIMAGEAVSGDLLRLWGGEQRK
CFNAYGPTEATVCSSLIDCTNLLGDASIGRTIPNVEIYLLDSFLDPVAPGVVGEIYIGGV
GLARGYLQRPGMTASVFIPHPFSEKPGQRLYKTGDRGAYDFHGNICFMGRYDDQVKVNGY
RIELSEIESALTKHKAVDSAVVILRQDLIGRNRILGYALVKPAEGKDELTGQELKDYLTN
ILPAYMVPGAIILMREWPLTPNGKIDRRLLPTPEFANAELIPKTYTEQILGQIWMELLGI
ETVNPQDNFFELGGDSIISLQVVARARTAGWEISPKDIFEAQTLSRVATRAKLISQQAEI
VEPLTGLIPLSPIQNWFFAQNLLHPHHWNQSVALTCREPLNTEALIVALDALVAYHDIFR
IGFSQDKGKWQQFYVSETKSPSLKIIDFASDSPETQLASLDTALEAEHASFQLDAPPLLR
VLYATNLTEYGDILFVFAHHLITDGVSWRILLEDLSKAYQQTIDQQTISLSLKTGSYRQW
TTYLQTLANTDKVIQDIPFWQNILDASVTRLPIDKSGKNSVDSTNAICTQLSLQETLLLV
KQATATYHASVQEIMLAALLSTLVNVYKSDHWLIDLEGHGREQIGDALDTSRTVGWFTCL
YPILLKLPANQDNHEVLLKEVKTQLRSIPHYGISFGLLRYLNQNQPLHRQGNSDISFNYL
GKIDNLSKKNGIFGLSNVPTGTGLFYLQERTHFLAINAKIQNEILQIEWSYSTNIHHDHT
IENIAKTYLQYLALYVTGCESPDSLFYTASDFHLADISESELGSILEDLE
>1170769.6.peg.341
MKKLLVTGSSGLIGSEVCAYFAQKGWSIHGIDNNQRAVFFGQQGDTRWNQKRLESQIKGF
VHHEVDIRNRKSILELIERIIPDAIVHTAAQPSHDLAAKIPFDDFDINAVGTLNLLEATR
QFAASIPFVYLSTNKVYGDAPNEIPLIELETRWDYNVTKYQNGIAESFRIDQSKHSLFGA
SKLAADIMVQEYGRYFGLNTCCLRGGCLTGPNHSGVELHGFLSYLVKCNLEGRTYKVFGY
KGKQVRDNIHSYDVARFIEEFIAAPRTGEVYNLGGGKENTCSILEAFKIVTELTNKPMVH
EYIATNREGDHICYYSNLQKIREHYPNWSITKPLNSIFVELVAAWSKKMYTT
>1170769.6.peg.346
MLSETANRAPANPDSIAIPPIPMTNNNQRPDFDFLGFLPLDLLLTGLLPAADVL
>1170769.6.peg.351

MKFDITNPGMQFSEYLPWNETLGLAYQLGVDGLSILMLILNSLLTWIAIYSSSENTERPK
LFYSLVLLVSGGVAGAFLAQNLLLFFLFYEMELIPFYLLISIWGGNKRAYAGMKFLIYTA
VSGALILATFLGIVWLSGSHSFALDAVNTQNLSTTMQIVLLAGIILGFGIKIPLIPFHTW
LPDAYVEASAPIAILLGGILAKLGTYGLLRFGFGLFPQAWNVVAPTLAIWGAISAIYGAV
VAISQKDIKRMVAYSSIGHMGYILLATASGTKLALVGAVAQMFSHGLILAILFHLVGIIE
GKVGTRELDKLNGLMSPIRGLPIVSALLVLGGMASAGIPGLTGFIAEFIVFQGSFSTFPI
PTLLCVASSGLTAVYFVILLNRTCFGKLNNDLAYYPKVVWAEKIPALVLAGLIIFLGVQP
TWLVRWNETTTSAMVGAISTAGTIIISQVETNQHQQ
>1170769.6.peg.371
MVILLSTFKYLKVFYRKIDPPDIPSDIKVAGKLFDGGWEVLVGLRCNHL
>1170769.6.peg.379
MLKLKLHVQQLTNGKTGIFLDILGIPNFVKLGLFASYSNSNTK
>1170769.6.peg.395
MKLEDRIDNAIDSIDKYLTIVMIWTKSPRFACQISKQQMPTL
>1170769.6.peg.397
MFLRCLSTFCILSPIESPGLFILIYSQSALMRLRLLRLKTNPTSIGNDRE
>1170769.6.peg.432
MVIFRRLITSGLIGFLASSCANAAQSSTEYTKQIVQENNVARLMAQVQDSQKVSNLLSQG
NGFLDSGRYEEALQLYNRAIEIEKDSVPSWVNRGNALLSLKRHQEALESYNQAIALRPNK
NEAWYNRGNALSALGRYEEAIRSYNESIVIDPNKFEAWINKGIALTKLQRYQEGLASYNQ
AISINPNFPTAYYNKACNYALQKQVNLAVESLAKAIKIDGQKYTQLARVDKDFAKISDNR
DFQELLK
>1170769.6.peg.434
MEWNNTQTDYPHQCIHQLFEQQVERTPNAIAVQWENQQVTYRELNNRANQLAHYLQFLGV
SAQTLVGIYLERSPKIIIAMLGILKAGGAYLPLDPTYPSDRLAFMLQDAKAFLILTEQQL
GGKLTDVEQICLDRDWSVIAQKNQQNLNCDTTPDNLAYVIYTSGSTGQPKGVMVPHCGVV
RLVVNTDYITLQPTDVIAQVSNNSFDVATCEIWGALLNGAKVAIFDRETILSPRDFATSL
QNEGITILMITTALFNQMVQQVPMAFRHLHYLLFGGEAVDTQWVRHLLAVDGPQKLLHAY
GPTENTTISTCYYIQQIEPKAKTVPIGRAIANTQVYILDRHLQPVSIGVSGELYLGGTGL
AIGYLNRPELTSERFLPNPFVANDRLYKTGDLARFLPDGNIEFIGRVDHQVKIRGFRIEL
GEIETVLTQHPQVKQVVVIVREDNPGNKYLTAYIVSESLTLSSSELRQFLKERLPEYMIP
LAFVILNAFPLNPNGKIDRRALPIPIVDNPQTVFVAPRNPTEETIAHIIANVLGLEKVGI
YDNFFELGGHSLLATSVISRIRESLSLELPLRSLFKAPTIVQLSQVINVHQLEVEQQSAG
SMTFDTLPPLLPQVRNTYIPLSFAQESIWHSQQLAPDNYAYNSFVTLRFTGSLSATVLES
SFNEIIRRHEILRTAFTLIEGQPVQVITPLLTIPLEIIDLQNLPNTKRTSEAERLAALEY
EHHFDLGSLPLIKTKLLQVNQKEYWLTINMHHIITDGWSLGLLLEELGILYTAFSNGLSS
PLPELPVQYADFTLWQHQCFNEKVIEKQLAYWVEKLTNTSPISHNVSNIPPQVSSSASVY
SIVLPVSMVRSIQAFSLEQKVTIFVIILTAVNILLFNYSGKDDILVITTVGNRSSVKTET
MLGCFINDVILHSHFSSEETGLTLLQQVQQTLMEAINNKEIACQTVIDTVTSKQPLNIST
SLTMLPPQNWHNRMLDFEFVSIKRDRSLWDEEIPLEIYVSSPSVNNPTMEIKVFYSRELF
TDNAIEFMFSYYQEILQKLVQHPNSPIG
>1170769.6.peg.445
MKSGFLLGSLVAVCVVGFPLALHHHKAIRPVLVELGLVLQ
>1170769.6.peg.446
MSGNTPTTSTTATCARNLSKPSSTSWSTGVLRKPTSPDPRYSVRQKGRSSGPFHFKNHGC
MTQPPLNSGPVNRPPALMPFLANQPWFISSTNRTGPDEQWESVVWGFMSARLTMVPSSLT
KAAVRGSSVFIIQKHCTDGCSKTKIMPSCWGISLRNMRPVLRCSGVDATCASIWCIPAES
LTRGRSICGDWAPTKGPVRARRAAKAYWTSGFMGRHFARSP
>1170769.6.peg.448
MQVPVYIIFDPDGGLLEYYELKNERYELKQPDENGRHWIESMELFLGTWQGAKEGRTGYW
LRWWEETGNLLPWALELIEQERQRAEQEHQRAEQEHQRAEQERQLAEQERQLAEQERQLA
EQERQRAEREHQLAEQERQEKERLIAYLRSQGIDPNNLPNHAE

>1170769.6.peg.449
MQVPVYIIFDPDGGLLEYYELKNERYELKQPDENGRHWIESMELFLGTWQGTKEGRTGYW
LRWWGETGNLLPWALELIEQERQQVEQERQRAEREHQRAEQERQEKEREHQRAEQERQRA
EQERQEKEMLIAYLRSQGIDPNNLPNHTE
>1170769.6.peg.454
MTIVSQQSDRYLPYLLLQHRRILRVEPRYEHDVPMKRSLFTLSPNYSTAGFLEIEY
>1170769.6.peg.456
MHHDSLPVLIAEIEEGQKVSKDLEKLYNTYKPFFTDESKRDIRDAVLLADIFCNTYTCLE
TIFLRISREFENHLDDSQWHKELLRKMRIEIPGIRQALLSHHSYELKGLR
>1170769.6.peg.463
MQTLPKERRYETLSYLPPLSDVQIEKQVQYILSQGYIPAVEFNETSEPTEFFWTLWKLPL
FNAKTTREVLGEVQSCRSQYSNCYIRVVGFDNIKQCQVLSFIVHKPTRY
>1170769.6.peg.473
MDVMAVFIFNIQVKNVTMNRELASVSEFSQVPNSSSQLSGISLQQQKLP
>1170769.6.peg.491
MQVKAVIITHSETSTGVINDLAAINSHVKAHGEALIIVDAVTSLGAYNVAVDELGLDVVA
SGSQKGYMIPPGLGFVSVSTKAWEAYKTAKLPKFYLDLGKYRKSTAKNTTPFTPPVNLIV
ALHTTLGMMKKEGLESIFTRHERQKNATRAAMKALNLPLFAADECASPAITAVSVPGMEA
DKIRSLMKKRFDIALAGGQDHLTNKIFRIGHLGFVSDRDILSCVSSLEVVLGELGYENFT
PGTGVAAGVKVFAAH
>1170769.6.peg.492
MSLSSDGGIILARQVEEKVKICQDMADCLTDNRDQTKVKHSLSQLISQRIYQIIAGYEDS
NDSNKLRQDPIFKLVCNQVPTVGENLLASQPTMSRLENQVTQKDIKQIRRLFVDKFLESY
PRESKEIVLDIDAWDALTHGHQQLSLFNGYHRHDIYFPVLINEASSGYPLVLQLRAGNSH
SGKGVAGILKWLFLRIKRALPEIRIVLRGDGGFSLPEIIEVCEKSGVGYVFGFSNNDVLK
RKINYLLDRARLEYCRTGEKVRLFDDVYYAARSWSEPRRVIMKAEWLEKGPNPRFIITSL
ETEAQDLYDKFYVQRGATSEHRIKELKLGIKSDRLSCEKFIVNQFRLFLSQAAYILMLGI
RQAAQGTKLAKAQVPRLRETIIKIAAKVTVSARRVLVELPYYCPFSSEINLIMERLASEF
EIIFS
>1170769.6.peg.493
MSRGDRILVGNNGKFGERWVEVGEAFGLTWIIHQSRIKIAERVILQ
>1170769.6.peg.501
MQLEQAQQDLEQKLDLDFEGFTVEHLVINQQQALAIENLPGYHLEGVYDLKVKLPTRQIA
QSHNHFNIYLQIQKEGKSWRLLIPEKSGEKQPLVWRGYLII
>1170769.6.peg.507
MISNLLKFSLLPILTAVSLIFTLALTSKANGQNPAITSTEITNYAQTVMIMEPKRQQAFK
EIKTLINTRSIPIIICNDVNSIKLLPRDAQKIAINYCNEYEETVSENNLTVDRFNQITVE
VNNNSVLRERVQKLMMEKMGL
>1170769.6.peg.508
MEYVVQVLLSTIPSLTQPQAVTIMMEAHSNGLALVISCALEHAEFYCESLNNHGLTSTIE
PDD
>1170769.6.peg.509
MFRPSQIALWISQRPAPQRLGCFVLCLLVLWLPFVIPIYLLVEDGNLESIFSMVLLYGEF
IVLVRLWGKKIYQRDKILTDYGLELSSLNGVDFCQGLSIGVLSIILLFSIQGLLGWILWQ
PPKAFIVQIIWEGLLVASGVGFAEELLFRGWLLDELNRDYGSRLSTAINAILFAVAHFIR
PISAIISTLPQFPALVLLGLTQVWGKHKKRGRLGLPMGLHSGLVWGYYIINVGGLVQPSG
IVPDWVTGVNNNPFQGIVGMLGMALLAYQMRVK
>1170769.6.peg.512
MNIETLKSEKTKQLPGANLEDQDLSEFDLTAANLAGANLMGAHLVSVNLEGSHLEGANLM
GASLQGADLRANLLGANLMQADLTGADLRGSNLRGANLMGATVAGASLTAAFLSGANLMS
VNLQGVDLRDADLRGANLTGANLKGADLSRADLQGALLNQANLEESDLRGANLAGANLAG
ANLLCAELEAASLNGANLYQACLLGTILETYHD

>1170769.6.peg.523
MVITSCFYSYLVSQLLVSYLYLQRRQFVFGGSPKTMPTASI
>1170769.6.peg.531
MSSSLNLSSNIKSPHEENYQLWLESTIDNLKQGNFDRIDIDKLIEEISEMGGSLKDALEN
NLIVILAHLLKWQYQPQKRSGSWKASIKEHRRRINKSIQKHPYLKKYYEKIFSESYLPAV
DWAMEETGLSSETFPSQSPFTPEQVLNDQFLPGD
>1170769.6.peg.544
MNDLPCQELKEIIIQHGRSLCDNPEYCEAFLRDWLMNDLPRQKLKEIIIEHARSLCNNPQ
RCEAFLNNYHGEYKRDLVLIISALKQAAAKDLLNFNYINIELLVGRLIKKMHNSLSLTQK
AAHCADKSLDQVLDKMVQLITFVENVLSNEESIALDHKKAALDREKETLLLEGKISSLSV
KSQQIEEIKGQTREINERTGKILASLDNLLTEVNLAAPENGDEMTIIMDSDLVKDRYA
>1170769.6.peg.554
MRGGKSFWENFAPNHLTGKKNLVIICGVGENNENLLGL
>1170769.6.peg.558
MFSFFMVWHVYTWFSIGVIMPPTYILLSLGTTCLLTNLGAIVWRVNFRHGTSSPRSKLID
EINSSF
>1170769.6.peg.567
MADLILKLLPTNKKAKEAFVYYRDGMSAQAEGEYAEALEYYEEALSLEEDTNDKSFIVYN
MGLIYASNGDHDRALDFYHKAIDLNPRLPQALNNIAVIYHYKGEKAKEEGDNDGGEALFD
QAADYWIRAIRLAPNNYIEAQNWLKTTGRSQIDVFF
>1170769.6.peg.577
MSFYAVGDRHFHLFSKRILEEVSNLYDLWYNGICSQDLCIS
>1170769.6.peg.578
MPPRSFSWQTFIYLSVFSWGISSLATGIIKDIIAFTGWIFLFAGTAWYTTDSPVRIPGTF
MPVGALLTGFIFSVFVFGHGENTITVRTIVLWPTIAAIITALPQFFTGNGISPKATLPKL
EVRQKIILLLSWSMLISCWLQFNFVTDKWLKEYPSLSAQSFQRSNFVIKFEPKANKPEPG
NVILNRIEPLILQQITNRPWSEAERWLLEANQQVDNLGKIIINKNLQKFEEKGLWKIEPR
VVNIKSGYRLDLLSIWQGPTDSKKGFYLRKSCYIEPIASEYTTDNSNVKAKIKCDPKTKF
FRGSPPAQQ
>1170769.6.peg.580
MTRNGIGILTAQARSERLTGQIHVYDGAGKGKSQAALGVVLRSIGLGINTKSNCNRVLLL
RFLKGPGRDYDEDGAIAALQRGFPHLIDQVRTGRAEYFGHDEITPFDRAEAARGWDVAKG
ALASDLYSVVVLDEINPVLDLGLLPVQEVVETLKSKPQELEIITTGRAAPQQLLDIADLH
SEMKPHHHPQAAELLLDGIEIYTGSGKGKSTSALGKALKSIGRGINHPGSARVLIMQWLK
GGTGYTEDAAIAALQQSYPDVVDHLRCGRDAIVWRNSRQHLDYVEAERGWEIAKTAIASG
VYKTIILDELNPTVDLELLSVEPILQALLRKPKGTEIIITGRCQNQPAYFDLASIHSEVY
CHKHYANQGVELKRGVDF
>1170769.6.peg.584
MGDCYPLPPVHTGGTVLPTIIDGYGGGWGFPVSPTYDYLIFYYTI
>1170769.6.peg.604
MNVWLLSLLFHYFQHPITILSIAAILAFLLNYPVQLLEKARITRTYSVIIVLVITLTLLV
ILGFTLVPMLVEQTTQLLRNIPDWVTSSQENLSKLQVLARQKRLHIDFSVVSSQINASVQ
NILQQIASGAVGFAGTLLSALLNIVLVVVLAFYMLIYGDRLWSGLINQLPSYIGLPLSKS
LQLNFHNFFLSQLLLALFMVIALTPIFLFLRVPFALLFAIIIGISELVPVIGATLGIGLV
TLLVSLQTWWLAFPVAMVAIIIQQIRDNILAPKLLGNFTGLNPLWIFIAILMGFEIGGLL
GTLVAVPIAGTIKSTIDAIKNNKSGIT
>1170769.6.peg.606
MKAMILAAGKGTRVRPITYTIPKPMIPILQKPVMEFLLELLRKHGFDQIMVNVSHLAEEI
ENYFRDGQRFGVQIGYSFEGKIDDQGKLVGEAIGSAGGMRRIQDFSPFFDDTFVVLCGDA
LIDLDLTAAVKWHRSRGAMATIITKSVPQEEVSSYGVVVTDNENRVRAFQEKPSVEEALS
TNINTGIYIFEPEVFKYIPSGVQYDIGSQLFPHLVEINAPFYAIPMDFEWVDIGKVPDYW
RAIRGVLLGEIKNVQIPGYQVAPSIYTGLNVAVNWDRVDITGPVYIGAMTRIEDGAKIVG

PSMIGPNCWICGGVTVDNSVIFEWSRLGPGVRLIDKLVFGRYCVDKMGTAIDVQAAALDW
LITDARQTPPAEIPPEHQAIAEFLGGITV
>1170769.6.peg.609
MSAKVKLATFPFQEFGLVDAEVLQISPITRRFNEAFSVR
>1170769.6.peg.618
MHHFSEQYAKRTGTYFCSDPSVTAVVIEGLAKHKDDLGAPLCPCRHYEDKEAEVSAAYWN
CPCVPMRERKECHCMLFLTPDNEFAGKNLQEIPVETIKEVRDSMG
>1170769.6.peg.623
MITASVNFQFLQSHDLQLLRLSVLAEGYVWNQPKTCLVKLCYFGQLLTQLIAARTGNFHS
TVENQTDLLRQLELKGILPQKVALLFHQVRVVSDRATYEHTSDPSQALTILKIARELAIW
FHRTFGGNTTFTPNPFISPPDPVDYRTELETLQHVETEFEQALATFSLHLAQLQVISSTL
SPRQTDTIISLGNQAVLEIDLEENAHTYLCRGIARSDLGDNRGAINDFTQSISINSNLAQ
PYMERGIARTNLGDGQGAIDDFNQALDINPNLALAAYSRGVAHRDMGYLQKAIEDFNQTL
HLNSAFFDAYTKRGLARYDLGDKQGAIDDFNQVITINPHFADGYAARGLVYCDLRNYQEA
INDFNQAIRINPNYAQAYHNRGVARSQLGDKQGAIDDYTHSLNLNPKFASAYYNRGIIRS
DLGSQKAAMDDYTQAIRIDPNYAQAYNNRGAIRTYLGNYQGAIDDYIQALRVDSNYAEVY
HNWGTTRINLEDNQGAIDDYTQALNLNPDYAQAYYGRGIARFNLGDKQGAIDDYTQALNI
NPNYDQAYYNRGIARTSLGDKQGAVDDYTQALNLNPNYDQAYYAWGMVCSELGDKPGAVN
NYTQALNINPDDPETYIARGLTRSELGDNQGAIDDYTQSLNLNPDYAYIYNNRGVVRSDI
ADYQRAIDDYTQALNISPDYADAYYNRGIAYYDLGNYQSAIDDYTRSIEIKPNCADTYVG
RGTALYKLGDSQGAINDFHHALDIDASYADAYNNRGIVRYELGDYQGAINDFNHALNINP
NYAQAYNNRGIVRYELRDNQGAMEDFNHAVNINSNYAQAYNNRGVVRICLGERQLAIEDF
SQAIIIAYNYTESYINRGYARYELGNRQKAIEDFNQALNINPNYAQAYNNRGVAYTDLGD
REWAKDDFSQAIQINPYYAEAYNNRAIVCYELGDHQGAIEDFNQALNINSNYVEAYNKRG
NIRYELGDRQGAIEDFNRALNLGSLDLGENLKFWERGGLTLTIKLRNYLNLKILSFEVVS
KKNFGEIIKTDASVIYFVEDLGDDITLEMVEIPGGTFTINSAENRGNPEESAEHLVTVPS
FFMGKYEITQEQYQAVTGDNPSYFKGDKRPVERVKWNQAVDFCEKLSQITGRIYTLPSEA
QWEYACRGGTSTEFHFGDNITTDLVNYHGDHKQSLAPQGQYRKQTTEVGSFPPNPFGLYD
MHGNVWEWCLDHWHDHYQGIPKDSSPWVEKGIFGVFRGHVLRGGSWCDSAQHCGCGSRNR
HLSDKNNLGFRVVCSNHNR
>1170769.6.peg.624
MNFGPLNKKGGERRLNVAITRAKVKIVLISSIRAGDIDLAGTESEGIRMLRDYLQYVATG
GERLQGDSYTNTLKFDSPFEEDVYETISRHSSLQEYIIRTQVGCSGYRIDLAIAHKDSPG
EFLLGIECDGASYHSSHTARDRDRIRQKVLEGLGWTIHRIWSTEWFRNKGQQVDLLIQKI
NKLR
>1170769.6.peg.630
MEPFGGAWIQFNIRYYMFALVFVVFDVETVFLYPWAVAFHRLGLLAFIEALIFIAILVIA
LVYAWRKGALEWS
>1170769.6.peg.636
MVYVNLFEMKMRVRMSMRSVITSAYCQGLPGLYQTYENSDRSQ
>1170769.6.peg.640
MLIVLVLLYLIRAIAAIALQKTINKYKQIFATGVNYFSYK
>1170769.6.peg.660
MGIKPWSTKELLVSFIGFRTLEVITVATFRGMEGGLSKPDLYLGFYNPKDSKNFMNQNSS
FSHI
>1170769.6.peg.684
MRVLRYFSNQEVLDLRENLGRGGEAFIYTVSSDENLVAKIYHRPSSDHIKKLQAMIANPP
ANPAASFGHISIAWPQELLTAVDGSDTIIGFLMPRIRDMVPIIDFYNPGNRRQNCPLFNY
QYLLRTARNLAAAVAALHASNYCIGDVNESNILVSNTALVSLVDTDSFQVPDLSQSRVYR
CLVGKPEYTPPELQNKTFADYNRETYHDLFGLGVLIFQLLMEGNHPFSGVFQGLGDPPSY
EYRILAGHFTYSQKQKVPYLPTPITPSWQTLHPAVRDLFVSCFEDGHHSPHLRPSAQTWL
SVLSTAEASLVSCAVNPQHVYHFDLNTCPWCERTVKLGGRDPFPSLQTISAREYHQPRPK

SRKRYRYASHVRKSATPVLVTYTQSSLKSSSAIYKPIQISNRSKFYALMFGFLGLGVLGY
LDIMIKFTRIFISPSPYTKQSLLSSRSENVHSPLSLSFHDYYQRGNQAYQQQDYQQAIED
FTQGIKQNTNFSKLYMHRGNARYNLNDYQGALSDYSIALKINPQEVKAFINSGNAYFKLA
DYSNDPDYEYKKAIDSFNNAININKQDDDAYVRRGVVRSQIAKYSHNSQQEYKKSISDFT
QAIKLNPFKAEAYFQRGLSRYQFGQYSSNYAEIYKQAIADFDQALNINPQMAEVFLKRGT
IYYELAQYGERTTKNNQQKALEDLEKSAQLYLNKKDFNNYQQAMSNICVIAEKKCDYFLQ
NSSIIYSVNP
>1170769.6.peg.693
MLLLLVANKVTSELIITLGTASEEIFRGDRLPPLKFPQESCAEDRITS
>1170769.6.peg.694
MVFSQGLKRNSFVRTQSTPYIIASVISNVTSDSKTTNPQNKSCLLRFPIPLYYK
>1170769.6.peg.695
MEGFPSSFVKKSWIHHIEQFFFDIFVTFVAIEMSQDSLDG
>1170769.6.peg.706
MTITLEEIRKKLKQVWGYENFRPPQEEIVSSLLSQKDALIIMPTGAGKSICFQLPALLSN
GLTLVVSPLIALIENQVEELKQRNQKADLLHSELPASQRYKVLESISKQQLRLLYLSPET
LLSSAVWEKISHPHIGITSLILDEAHCLVQWGETFRPVYRRLAAVRPSLLNTKPPGTKIS
VAAFTATADPSTQNIIKDVLQLQQPDIYRLNPYRQNLQPTVKTVWTPKARKQQLLKFLQL
HPHQTGLIYVRTRKDSEELAQWLMNLGYDTASYHGGLSGEERRAIEKSWLHGKKSFVVCT
CAFGMGINKADVRWIVHFHAPYLLSEYVQEIGRAGRDGIVAEVLTFISEPTGFFDGEDQR
RKLFFQQQILQQYKKAQELIKKLPLQGEVKSVVKEFQHGATALAILHSSGRLLWNDPFHY
QILGKDIHQSRTYFNPAQQMVEYLRTKNCRWQFLLQSFGFNKDRENWRCGHCDNCRFKSG
GFQGIS
>1170769.6.peg.710
MTTPQEVLKMIQDQNIQMIDLKFIDAPGTWQHLTVYHNQIDESSFTSGVPFDGSSIRGWK
GIEESDMTMVLDASTAWIDPFMKEPTLSIICSIKEPRTGEWYNRCPRVIAQKAIDYLGTT
GIGDTAFFGPEAEFFIFDDVRYDQTANEGYYHVDSVEGRWNTGRKGKNGEADGPNLGYKT
RFKEGYFPVPPTDTFHDMRTEMLLTMAKCGVPVEKQHHEVATGGQCELGFRFGKLIEAAD
WLMTYKYVIKNVARKYGKTVTFMPKPIFGDNGSGMHCHQSIWKGGQPLFAGDKYANMSDM
GLYYIGGILKHAPALLAITNPTTNSYKRLVPGYEAPVNLAYSQGNRSASVRIPLSGDNPK
AKRLEFRCPDATSNPYLAFAAMLCAGIDGIKNKIHPGEPLDRNIYELSPEELAKIPSTPG
SLELALEALENDHAFLTETGVFSEDFIQNWIDYKLANEVKQLQLRPHPYEFFLYYDC
>1170769.6.peg.711
MRDAVTNLINSYDLAGKYLDRNALDSLKSYFDSGTSRVQAATAINANAAAIVKQAGSKLF
EELPELIRPGGNAYTTRRYAACLRDMDYYLRYATYALIAANMNVLDERVLQGLKETYNSL
DVPIGSTVRGIQIMKDLAKEQAIAAGVANAAFVDEPFDYITRELSEQNI
>1170769.6.peg.718
MIYGTAALMAYVTGVDSQLALYVAIAYLTARVLFSVFYILNIPILRSLMFGVGSACIISL
FVLSILKGT
>1170769.6.peg.720
MSIFNTAILYDIENLTKGYSFSKDFIKELSLKQIYRQILEVDIVNKICLQRAYANWSDHR
LSLLRGEINELGIDPIQIFGFARYHKKNAADIQLVVDTMDITIRFPHIEVYVIVSGDGGF
ASLAKKLHEYGKQVIGCAYENAANDIFKSVCDYFIKLELPEEYSPEDINTDPKNTTFGNN
KGLGIGITHPLVVRMANNIQPIYQADKKTIFSHGQKIISWFGQDPESRKQMYGHGIPLST
VREAFKYAIPEFKPEMVGFMRFAEFLQFICANTEFCVGTLPPSNTLLVFRNSIPNGVVIL
LDILNQDLHTPERYQSLLASGKPRITIEDKYSLETFVDTLMSKRDILMNISEILDIFSQE
LPDFESNKLNNLCLSLIHCNILKGYPEDENISEQKFHISQDFKDTAQILEHVKQTSLNKL
ISILVDDFKSDVFKEVIPF
>1170769.6.peg.728
MERKKQRSNLHRDRNLGLPGGIMIPQKYFNKFLPFTNSHAELL
>1170769.6.peg.732
MHPDFEIMVFRDGELRNHRYVDSLYAGFINVGNQYAPS

>1170769.6.peg.735
MVFPFTAIVGQEEMKLALLLNVIDPKIGGVMIMGDRGTGKSTTIRALADLLPEIPVVAND
PFNSDPEDPDLMSDEVRQQVAQGLEIAIGHKKVQMVDLPLGATEDRVCGTIDIEKALSEG
VKAFEPGLLAKANRGILYVDEVNLLDDHLVDVLLDSAASGWNTVEREGISIRHPARFVLV
GSGNPEEGELRPQLLDRFGMHAEIHTVKEPALRVQIVEQRSEFDQNPAQFLENYQFQQES
LQQQIVSSQELLPKVNIDYDMRVKISEVCSQLDVDGLRGDIVTNRAAKALTAFEGRTEVT
VEDIRRVITLCLRHRLRKDPLESIDSGYKVEKAFARVFGVELAEDVAGKNGTGMRSGVR
>1170769.6.peg.745
MGYNPLHYIPQRTYSRIAKENNPVVTTAIPIPQEALETINSHS
>1170769.6.peg.759
MFKTQAESEEFLRGFLPELEETPSIREVVLCPPFTNLNVMSKYLHGSRVGLGAQNVHWAE
NGAYTGEIAAPMLLEIGVRYVIIGHSERRQYFGETDETVNLRLKAAQNYGLTPILCVGET
KQQRDAGETEKLISYQLEHDLVDIDQTKLVIAYEPIWAIGTGDTCESAEANRVIGLIRSQ
LTNDKVPIQYGGSVKPNNIDEIMAQSEIDGVLVGGASLEPDSFARIVNYR
>1170769.6.peg.762
MGDKQFFPRRFAIPIEKYGDVASLNQLRSLVQPFKQSEPIMKHLQNIYRDVTQKACLLAM
NMG
>1170769.6.peg.776
MVTIGIIPNNHQSNLHRAVAAPSLKRLFSANAHQSNVETRQAKNMARAALV
>1170769.6.peg.777
MVTIAIAVFYINRSSEAHFSLMAFFLLDAILLYWLPTATSSPEAVKYVPPLAFWLRHKLM
GD
>1170769.6.peg.788
MIGKIDGRTRFLIDLQQKLPSFKTISTVLKPNTKSINQLKNLLFL
>1170769.6.peg.810
MGFIAYGLDNATLNLVGFFYGFPLLLGGLALKANELQPIPFSEPTTESVLELRKQQATIT
QNKIRKDITRFCYGQQAHLDRALDYLGLNPTDEERPVVVGLRETSLDGSYCLILEFDSPL
IPLDTWLEKQEKMTKYFAPNVHVKITQPDEDKIELELITTNHSN
>1170769.6.peg.811
MYEEENFVVLETNQEEQFLTKLELLEKLQNTLSQMPIEDIPLDVRKIGSLIEQVNHLIDT
TCELDLGPGRYLQWYAVRLEK
>1170769.6.peg.815
MSSIVKLGMWGKEKGDRLLRTFADKEIGAIAMETYRSP
>1170769.6.peg.819
MSYIIPVFGRSLPLVNRLVMVTSILFILIVGGIFPFRWWGEHLRESRVVGFLGNS
>1170769.6.peg.822
MKILVLNAGSSSQKSCLYEIGEFLPDSPIPPIWEGSIDWGTTQAKITIKVSGHIKTALLP
SSNRFHALKNLILTVIQGEHSVVESLKEINRVGHRVVHGGRRYQQATLITPEVEAEIERL
IPLAPNHNPCHLEGIAAIRQILEDVPQIAVFDTAFHAQIPQSIAAYPIPYKWYEQGIRRY
GFHGISHQYCAKRAAHLLGCELDKLKIITCHLGNGASLAAIRNGISINTTMGFTPLEGLM
MGTRCGSIDPSILIYLLKNQGLSTDELNHILNRESGLKGIFGKSGDMRDVLASWTAGDEQ
AVLALDMYIERLKSAIGAMTATLGGVDCLVFTAGIGENSAVVRQLTCNGLGFLGISVDHN
LNEGVSSDIDIATPDSRVRIFVIHTQEDWEIAAECWWLTLHHGNSYN
>1170769.6.peg.823
MIYLRANPLLREPLEEAHIKHRLLGHWGASPALSFSYIHLNRLINKYDLNVLFMAGPGHG
APGVIGPVYLEGTYSEVYPDKSEDIEGMEKLFRWFSFPGGIGSHCTPELPGSIHEGGELG
YSLAHAYGAAYDHPDLLVACVVGDGEAETGPLATAWHSNKFLNPIRDGAVLPILNLNGYK
IANPSILARISHSELESLFRGYGYNPYWVEGDDPEIMHQTMATTLERCFQEIRHYQKEAR
TTGVVSRPHWPMIILKSPKGWTGPKTVDGHITEGFWRSHQVPMGAMHSNPQHLKMLEDWL
GSYRPNELFDENGTLFPEFKAIAPQGHRRMSANPIANGGIIRKELRMPDFRDFAVSFTKP
GTIEVENTYILGNFLREVMRKNMTNFRIFSPDETASNRFQSVYEVTEKAWVGDYLPEDKD
GGHLAVDGRVMEMLSEHTLQGWLEGYLLSGRHGFFHTYEAFAHVVDSMFNQHAKWLDICK

TKVPWRSPISSLNILLSSLVWRQDHNGFSHQDPGYIDLVMNKSPDVIRVYFPPDANCLLS
VADHCLRSRDYVNVIVSDKQMHLQYLTMDEAIAHCTKGLGIWKWASNDDCGTAPDEPDVV
MACCGDIPTMESLAATAILREEFPDLKVRFINVVDLLTLNDEREHPHGLSHRDFDTLFTP
DKPIIFNFHGYPWLIHKLVYRRSNQERIHVRGYKEKGNINTPLDLAIKNQIDRFNLVIDV
IDRVTQLGSRAAYVKERMKNRIIECVHYAYTEGIDEPDITNWKWPY
>1170769.6.peg.826
MYQPVNVAVVDCGIEYQEPSKFNRIIILDKFGISMTIPANYRAMAKDNGTVEILDGGTYD
AFVCHAQNPGATGGSGYYSIEIYKSKASYLYENVWDKVPGKENMYIVWEKTYAGQELNYH
YIKLRIKTKKGLVEIDAGSEHSTQTEDDVKAALSTHAGLTQSALLYSAQTSPNSPG
>1170769.6.peg.827
MNPKKLTQLITIGILVTFITIFHPFIAIALTSKKIGAIATENDLIRNKNNRFTLLPSSSL
LRFLPNVL
>1170769.6.peg.828
MGQFFGVHPLFLVDDKKVPAIAILENEKALLFKGVRGNR
>1170769.6.peg.837
MSTSSTSAQTTTGIEDIARKTTVQINSNANPGGSGVIIKKEGTIYTVLTANHVVCDNLGT
IKIRCRTDSNYTVVTYDGKEYPMKYRQSLQINVQDPDLAIIRFESRENYQIAPLGNSDNV
KIQSDILVAGFPTIFGRVGKQRTFTITNGKVVTFIPNSDRGYGLVYNATTFIGNSGGPVF
DIYGRVIGIHGLADTDDGETNNNNQSETVNGVKPTQKTGFNAGIPINIFFSLSNFNQQVN
PTVSINRQPNPINPNNVNLSNGAIAYNDRGVNRYQLGDKQGAISDFTQAINLNPNFAQPY
YNRGATRNDLGDKQGAINDFSQFINFYPRNSLAYFNRGIARHELGDKQRAISDFTQVIKL
NPNNVAAYYNRGASRSDLGDKQGAINDFSTVINLNPNFAQAYNNRGLARHNLGDKQGAIS
DFTQSLRLDYRDPTAYNNRGIARHDMGDRQGAINDFTQAIQISPNFAQAYNNRGLARHNM
GDKQGAISDFTQAIQINPNFAQAYNNRGATRNDLGDKEGAIRDFNKASQLFDSRR
>1170769.6.peg.838
MYIGGDINPDLPASNYSYGSLSGGFGSQSDVEDITGNRKVGDIRFNSAFSFNAAIGRQLE
QFRVEIEFGNQFLSAKEFKFNGDLIPPTTSLSGNINASTILLNGYYDIPTGSKFRPYVGG
GLGVARISGKVTDNEGFYDDDDVSLNGTSFAYQLKGGVQYEVTKKGNVFGEVKYSSISSY
KAEDYTNVDFGPYNSFSFAVGYRQGF
>1170769.6.peg.870
MQRIVARFRSKSDMDGYIQHLRQLVPNDSFKMFFDSQILLSAI
>1170769.6.peg.873
MFLQEVIMEQAIAQISCTPEEERLAYEQLTQEYQGQKEQGISQEQLQNMATRQLKLERFK
EVTWAKDIDSYFYQRKPQLDRVIYSLITTNDRGVAQEIYFRVQEGEQSFAQLAQEYSQGP
EAKTSGLVGPVELQSLHPLLVRILSTSQPQQLSLPTAIEDWIVIVRLEKMLPAQLDSGMR
QRLINERFQSWLKAQVSPQNWQIKESEN
>1170769.6.peg.894
MLVYILSLVVAIASLTIYASAFFFPEVHRKDDFIWSGVGLFYALALWVFASRITGGLLLG
HVASVSLLLWFGGQTLSLRGQLVPKGKPISVSTAQPEIKEIQQPVSKISLPEKLQQLLSL
IIRPFNDLIVKVQQVVFKNPSVNTQISPSPAPEPSTPEPSTPEPSTPEPSTPEPSTPEPS
TPEPSTPEPSTPES
>1170769.6.peg.897
MDYLPDVDDRFDQFMMWYILFQLTAKHCKATTLEDAIAIVYLLLGMS
>1170769.6.peg.911
MPMAVGVIETLGFPAVLAAADAMVKSAAVTIVYYGIAESGRLLVAVRGQVAEVKTAVAAG
IASEETVYGGQVITHYIVPNPPENVETILPIHFTSESEPFRIF
>1170769.6.peg.920
MNLTSCLPILFLVPTLMLSSSPQSLAKTTLKQESSCELEREGEFYSPGQLKTIAQRITVR
VIADNSGGSGTLIAREGNSYLVLTTNDVISGTTPTALRITTHDGRTHQGRMLYNYKLDDK
QKLDKANLAILEFTSSRKYCLTKQIVNTESRQDTAVMASGYSINSSKIIFSPGRIKRIVS
QPTFSQGYEIGYDSTIQQGMGGGPIINSTGDLIGIHGKSAFPILNNGYVYADGKKPPLSE
IAEFRKLSWGIPVSSVLAQLKPEILTRYGLPIPRINRSVPEIPKLPEWLGNIESKVRQFT

VRIDGGGNNGSGVIIAREGNTYTVLTSAHVVCKRPQKISRSKEDKNKCIEENYTVIAASG
QKYPLDNSSIKLGGGGGRFGHSKI
>1170769.6.peg.921
MATVKFNSGENYPVATLADYPVANHQYIFTVGYPKLGQTPSWRFTIGQIFSRENGLLALT
STGQDLKSIDNTIQDANLGKEYELVYTSITLGGMSGGPVVDSQGRVIGIHGKAEGQVLME
DTTRVQLGYSLGIPISTFLRIAPELNTRVNRVENTPAPQLKSWEIESIRKAILSVNVSGG
NASAIEWIERGNQLWLLGRYQEAATAFENAIEQRPAFIHLAYYGKGLSLWWNGNDTAAVE
AFKQVVQAKPDFVPALYFLSLTNQNLGNLDQALFNVRQAIFHQNQAKYQPISPNLYALKG
NLLSDLKRYKEAIEAIDQAILVDPRAIFYVIRGDIHNNLGDKQEAMNDYTQALDLNPNVA
YTYTARGSVRKELGDNKGAIDDYTQALRLNSYGVVIWDIRNGK
>1170769.6.peg.922
MFSSSPQSLAKTTPKREPSCELQREGEFYSSGQLKTIAQRITVRVIGDNSGGSGTLIARE
GGSYLVLTSSDVIRGITPSALRIQTHDGRIHQGRTLYNYKLADQQQLDKINLVVLEFTSN
QKYCLTKQIVNTETRQDTAVLSSGYSVNSSKIVFSPGRIKRIVSQPTFAQGYEIGYDSTI
QQGMGGGPIINSTGELIGIHGRSAFPILNNGYVYADGKKPLVSEIKEFRQLSWGIPVRSI
LVQLRPEILARYRLPVPRRNRSVPETPILPEWLGNIESKVRQFTVRIDGGVDNGSGVIIT
REGNTYTVLTSAHLLCKISQKISSNRQNQYKCGNKNYTLVTASGKKYPLDNIKLGEGVDI
ATVKFNSGENYPVAKLANYPVENNQYVFTIGDPRVGQTPGFTVGQIFSRENGLLALTITG
QDLNGIDYTRIENANSGKEYELVYTSITPGGMSGAPVVDSQGRVIAIHGKSEGQVVMEER
TEDEGISIDNRVQLGYGTGIPISRFLSIVPQLNTQVDEVENTAAPALKLGEIESIRKIIV
LVNASMGKASAIEWIERGNQLWLLGRYQEAAMAFENAIERKPAFIHLAYYGKGLSLESNG
NDTEAIGAFEQAVKAKFDFSVAWNRLTALNIKFGRLRMALAATNQAIKLKPRDTSLYSQK
FYILISLTMYQQAVEVMDQAILLNPHQDFYINRGFARRELGDYKEAIDDYTQAIRVSPEF
ASAYYERANVRRELRDYKGAVDDYTQAIRISPEFALAYYERANVHRELRDYKKAVDDYTQ
AIRISPEFALAYYERANVHRELRDYKGAIDDYTQVIKIDPESVPSVYYERANVRRQLGDY
KGAIDDYTQVIRISPEFASAYYERANVRRELGDHQGAGADFQKASDLQVSSPKIPPKD
>1170769.6.peg.925
MTASIGGILGYSNPDSPVRHTPISGLTPPGFDSDMGFAVVLGGDGTVLAASRQVAPSGVP
LLTVNTGHMGFLTETYLNQLPTAMEQVIEGHYEIEDRAMLNVQVWRRDSVLWEALCLNEM
VLHREPLTSMCHFEIEIGRHAAVDIAADGVIVSTPTGSTAYSLSAGGPVIAPGVPVLQLV
PICPHSLASRALVFPDHEPVSIYPVNIPRLVMVVDGNGGCFVLSEDRVYLRRSEYKARFI
RLQPPEFFRILREKLGWGLPHIAKPSSVELP
>1170769.6.peg.933
MRRLFALILVIGLWFNFAPQAHALGANLVPCKDSPAFQDLALNARNTTADPESGKKRFER
YSQALCGPEGYPHLIVDGRLDRAGDFLIPSILFLYIAGWIGWVGRAYLQAIKKEADSEQK
EIQIDLGLALPIITTGFAWPAAAIKEFLSGELTAKDSEITVSPR
>1170769.6.peg.935
MAQAANKSKNLPNGPRNQQTVRAAGGNAQDGNLETPINSSPLVKWFVGNLPAYRPGLTPF
RRGLEVGMAHGYLLFGPFDKLGPLRDSANANLAGLLGSVGLVIILTACLSLYANSNPAPA
LASVTVTKVPGDAFNSKESWNNFTSAFLIGGIGGAVVAFFLTLNSGIIQGLIG
>1170769.6.peg.940
MVKIHLCSFFFPQALNLKFTEPGKLKMATLQQQKIRIRLQAFDRRLLDTSCEKIVDTANR
TNATAIGPIPLPTKRKIYCVLRSPHVDKDSREHFETRTHRRIIDIHQPSSKTIDALMKLD
LPSGVDIEVKL
>1170769.6.peg.943
MRRVMRHGKKSLAARIVYDAFKTIEDRTGGSPLEVFERAVRNATPLVEVKARRVGGATYQ
VPMEVRADRGTALALRWLVQFSRSRPGRTMASRLANELMDASNETGSAIRKREETHRMAE
ANKAFAHYRY
>1170769.6.peg.948
MANSLTKHISSYIYSPILIHTCINSYLVFVGGWGQRVPFLLKDKTWAGIWFYLTLGYGFY
RLIVNPY
>1170769.6.peg.975

MTKITNQALNSALNQLWVFSLSSDFWAVFDAVFGTEYNRKNAEILRSQWQIGDFSQLPEI
EILDSSILGSANGAYSSSENRIYLSSNLMENGTSSKIREVLIEEIGHFVDSRINQIDTPG
DEGEYFAGLVTDKKLNKDEIDRLKAEDDSNWISVDGERLLIEQSSPGTVTRTPIAPASPG
RTRYEVGNYNAFAALKSNGSVVTWGDSSYGGDSSSVASQLTSGVTQIFSTYYAFAALKSD
GSVVTWGDSSGGGDSSSVASQLTSGITQIFSNGSAFAALKSNGSVVTWGDSSGGGDSSSV
ASQLTSGVTQIFSTGFAFAALKSDGSVVTWGSGSGGDSSSVASQLTSGVTQIFSNWFAFA
ALKSDGSVVTWGSDWSGGDSSSVASQLTSGVTQIFSNGLAFAALKSDGSVVTWGSDWSGG
DSSIVTYNYNTGSSSYVSVASQLTSGVTQIFSNWRAFAALKSDGSVVTWGFSDQGGNSSI
ATYNYNTNSYSYVSVASQLTSGVTQIFSTPYAFAALKSDGSVVTWGDSSYGGDSSSVASQ
LTSGVTQIFSTGWAVPFKNASNEGISISGEGAFAALKSDGSVVTWGSGSGGDSSSVATQL
TSGVTQIFSNGSAFAALKSDGSVVTWGSYNYGGDSSSVASQLTSGVVSFADPFNDDRLVP
GSSVTLAVSPSTVAEDGTSNLIYTFTRTGVISNELTVNYTAGGTATNGTDYSNIGTSVTF
AANSATATVTVDPTADTTVESDETVSLTLASGTGYTIGTTSAVTGTITNDDNPLNSNVDT
PKVNISTGLIFTAQSNGNITLDTNRGSATPDEVTSVQNGTKSNFNHIIGLYEVLNSQGEI
KDNQGNTLKPGDANYALHALTTARVKNFAVRAGGNDTPSTATQLGSGVSVLAGKLYAPFA
IANGGTYFPGNQGIEDFVAAEQGDINRFSSAPQYVRNLVDIEGKNGDVFNNAPRFVQEPV
AYFSFGAANPDGSPHFRSHGNGVYGFEDLPVSYTQYSNNDFNDGVFALTLSI
>1170769.6.peg.977
MSAFAKHSPDGIINKTLIVNPNLVFGKGDLFNNETLLYHMSWR
>1170769.6.peg.987
MEVFLRDRLWVNPRTTIPDFTRLQPFSSQQFFNTSHN
>1170769.6.peg.1022
MSATNSRRIIIGDVHGHYQGLMLLMEKIAPNSGDQVYFLGDLIDRGPQSAQVVKFVKENN
YPCLLGNHEEMIMNVMVHHYTSSKAVQSWLYSGGQATMASYRSARIPQEDLDWFGSLPTY
LDLGDILLAHAGVNPNKSLSEQTEGDLCWIREEFHSMKTPYFTNKLIVTGHTITFTLPGV
KPGDLAQGQGWLDIDTGAYHPRSGWLTGLDITNSLVYQVNVFKNSTRCLPLEKALSKINP
QEIKLSGRYKQVS
>1170769.6.peg.1025
MSFPFHVHHKVQSFNLDTTYYFFLPNVDYGMGNPDSDHQTV
>1170769.6.peg.1027
MLLSIKTKLKLNKTQETLMAVQPLIASAKAHSKAIHSKRTQL
>1170769.6.peg.1034
MTTKSDPNRILRLLPLVVGSLGTVLLLVNRVLTPQLTESQARGDVLGVILSAVLILTGLI
WQQVQPKSPDTVELIGKEGFILASDLPETIKTELAWASRLLLTNTVTRSLVVYYKGQVLL
RRGILGSKAEVTPGPILERVLGTQKPIYLVALRVYPGKIEFDYLPDNTQGVICQPIGKEG
VLILGANAPRSYTKQDENWIEGIADKLAVTLENRTE
>1170769.6.peg.1036
MRTIDPGTTPPPSTLSNSEYPDSHRAIWRSLIWSKVSDALGLEGLVDLADGWALCQPPTP
KELAFCSTRES
>1170769.6.peg.1037
MVLIGGDPGIGKSTLLLQVSNELSQRYRILYVTGEESGQQVKLRASRLGMSKPLQVVSME
DSNTSSESSHPLPETDPGNNVGDAVEDNHRVTVEHHTISSDLYVLPETDLEEILREIDSL
KPNLAVIDSIQTVFFPALTSAPGSVAQVRECTAALMKVAKHEDITMLIVGHVTKEGTIAG
PRVLEHLVDTVLYFEGDRFASHRLLRTVKNRFGATHEIGIFEMVSNGLREVPNPSELFLG
NRDEPSPGTAIVVACEGTRPIVVELQALVSPTSYPSPRRAGTGVDFNRLVQILAVLEKRV
GVPMSKLDSYVASAGGLSVEEPAVDLGIAVAIVASFRDRIVDPQTVLIGEVGLGGQVRSV
SQMELRLKEAAKLGFKRAIIPKGQKFPDLKIEILEVSKVIDAIIAAIPHQSLENGDLDLE
EEDED
>1170769.6.peg.1047
MPSLSFGNPILVKSYSTLGRLQKAKHRTYKFQEKSDKL
>1170769.6.peg.1050
MIPVKKNLLVIYILNLRFPIFWTITGQGEKNQSSLDSLTNS

>1170769.6.peg.1068
MLHDLCCPQTVLITHPIKLMKLIAIQNVEGFDITGVLS
>1170769.6.peg.1081
MANCATPVREFQQFSFLKLGLKIRKTTSFSYCLCYHLE
>1170769.6.peg.1087
MGAGVFGFVGFFAAGGFLLSGFLVASGFTSVVFCTSGFLVASGFTSVVFCPLLIVGPACA
ENGATPKRSIMLASKDPATL
>1170769.6.peg.1103
MFEHFTSEAIRVIMLAQEEARRLGHNFVGTEQILLGLIGEGTGVAAKVLTDLGVTLKDAR
REVEKIIGRGSGFVPPEIPFTPKVKSLFEQSFREAHGLGHNYINTEHLLLGLTDAGEGVA
AKVLKSLGIELQTVRSRVMSILGEDNRVVAGRQDNPRRNQNLSIEEFGRNLTKLAQQGRL
DPVVGRQTEIERTVQILGRRTKNNPVLIGEPGVGKTAIAEGLAQRIVNQDVPEILLNKQV
ISLDMGLLVAGTRFRGDFEERLKKVMEEIRSVGNIILVIDEIHTIVGAGGTEGGLDAANI
LKPALARGELQCIGATTLDEYRKYIERDAALERRFQPILVGEPSVAETIEILRGLRSAYE
QHHKVNISDDAVIAAAELSDRYISDRFLPDKAIDLIDEAGSRVRLRHSRIIDNKELKQQL
KNTSQEKAEAVRVQDFGKASKLRQEELDLQTQLAIAQNLPKITIPQVDEEDIAEIVSSWT
GVPVNKLTESESELLLHLEDTLHKRLIGQEQAVTAVSRSLRRARVGLKSPNRPIASFIFS
GPTGVGKTELAKALAAYFFGAEDSMIRLDMSEYMESHNVSKLIGSPPGYVGYDEGGQLTE
AVRRKPYTLLLFDEIEKAHPDVFNMLLQILDDGHLTDAKGRKVDFKNTLIILTSNIGSKL
IEKGGMSLGFEFDNQANASYNRIRNLVNEELKSYFRPEFLNRVDEIIVFSQLNKDEVKEI
SQIMLEEVAKRLQEKGIKLEVTEAFKDLVVTEGYDPSYGARPLRRAIMRLLEDSLAEAIL
SGEIGEGDQAIVDVDDDGLVKVKKAETRDLVLVSAPA
>1170769.6.peg.1124
MTTPQEKLFGPDYPNQDLRDNQESRVGYWQILTISLC
>1170769.6.peg.1128
MAENKPNLWGIIPQVTQMQSEGGAAAAVHGALQTGSLSTTFTASQGLLLMIPNLFKIAGE
LTSFVLHVSARSLATHALSIFGDHSDVMAARTTGFDLLCSASVQESHDFDLIAQVATLQA
RVPFLHFFDGFRTSHNDKGGKPPPLILFLRRNEHHHISPFFLRSLLNLTKGFQVCSHTI
>1170769.6.peg.1129
MAKLFAAIDGNEAVARVAYKLNEVIAIYPITPSSNMGGWRKINLIYGG
>1170769.6.peg.1138
MSDLQNIANHHYEPIKTILRVIDFSQGHFSLVFLHCNDAQLRQQVAAQLRESSPNKIEEI
TLPQSAISLYDNISLSLDRHPEVLMVFGLENVNNLDSILQFSNQIREEFRKNFAFPLLIW
IDDRILRKILRIAPDLETWGSIIGTPNYYNGENHVKGLAQIHRFLAEAALENQQWSEAKK
QAQYAQEILTSADDGLYYLLTARSQIGLGETGAAIKTLEFAKTHTMPPQDPQLHIEILKT
LGSLYFDHGNYLQAFYVRQEQLQVEQQYRLRAFLGSSYLNPQRPIINKTNGNGRNSRPMV
FGREEDIKSLWQRISDNEHPLIVIHGQFAVGKTSIIQGGLVPILEQELVDDKQILPIILQ
KYTNWLEELSEQLLNKLEKKLQSTIAIETFNGLSPQERIVKLLNISGDKNLLTVLIFDQF
EEFFLVTSNLEQKTRFYQLLRSCLDIPFVKIILTLREEYLHYLLEIDRLVDLRVTNNNIL
DKNIRYYLGNFSKAVAKRIIQTLMVKDQFELQMELIDQLVEELGDNLGEVRPLELQIVGA
QLEKEGIDTLEQYKQFGGKQKLLEKFLQDVIKSCGPENESTAQLVLYLLTEENGTRPLKT
QSEIITQFSVESDKLDLVLKIFVGSGLVWLLRESLRDRYQLIYDCLVPFVRQQYVQLYYA
HLPEQLEKIQAELRQEREAEEQAQLVTKLEEDALIALDQFQADPLLSLVNAVSSANLLKS
IVQNNPLDKYPTVSPIYALNTILDAISDRNIIKGHEGGITSVCFSPDGQSIATGSWDKTV
RLWNLRGENIQQFRGHEGGITSVCFSPDGQSIGTGSEDGTARLWNLQGKNIQQFRGHEGG
ITSVCFSPDGQSIGTGSEDGTARLWNLQGKNIQQFRGHEGGITSVCFSPDGQNIGTGSED
RTARLWNLQGENIQQFHGHEDWVTSVSFSPDGQTLATTSVDKTARLWNLQGETIQQFHGH
ENWVTSVSFSPDGQTLATTSVDKTARLWGLHRHKIQEIRGHEDWVTSVSFSPDGQTIATG
SRDNTARLWNREGHLVQEFKGHQSRVTSVNFSPDGQTIGTGSADKTARLWNLQGDVLGEF
PGHQDWVTSVSFSPNGQTIATGSRDKTARLWNLQGDVLREFPGHEDWVTSVSFSPNGQTL
VTGGADKIARLWNLQGDLLGEFPGHEGGVTSVSFSPNGETLVTGSVDKIARLWNLKGYLI
REFKGHDSGITNVSFSPDGQTLATASVDKTVRLWNLKGQLIQEFKGYDDTFTSVSFSPDG

QTLATGSLDKIARLWPVRYLDRALEDGNTWLIDTNF
>1170769.6.peg.1161
MIDELTILPFIAAGAIAMHQFVSPARIWFGQKSDSQRIFGYHRSGYVWIGAALFTSISFV
ALQVVWQLGTSLQNTGWGLQTYSWSALLALTFGMYGLALSASSTPFTALLVDITDEDDRP
KLIAVVWSMLMMGIVIGASISSRLLERPEICGTALLAYDPSQMNKLVDISKLQTTINPVF
IILPGAVFVLTLLATLGIEKKYSRYGIRGNMVEREDQITLGKALKILTANRQTGIFFGFL
MVLTLSIFMQDSILEPYGGEVFGMCISQTTRLNIPFGIGTLLGIGSTGFLLIPRLGKKQT
TKTGCIGAAMSFCLMIMAGVSQNSGLLMGSLFFFGLASGVITTGATNLMLDLTATETAGT
FVGAWGLSQAMARGMATVLGGTVLNIGKLVFTSPTLAYGMVFALQAMGMLLAIWLLRRVN
VVEFQQNSKQVLASVLESDLD
>1170769.6.peg.1162
MQDKALANVFRQMATGAFPPVVETFERNKTIFFPGDPAERVYFLLRGAVKLSRVYEAGEE
ITVALLRENSVFGVLSLLTGNKSDRFYHAVAFTSVELLSSPIEQVEQSLKENPELSMLML
RGLSSRILQTEMMIETLAHRDMGSRLVSFLLILCRDFGVPCADGVTVDLKLSHQAIAEAI
GSTRVTVTRLLGDLREKKIISIYKKKITVHKPVALSKQFT
>1170769.6.peg.1170
MSDLFKGFEQFLELVKTLEEKIESGEVKTDIQVNSRPLSSIPRTGNIPRSAAKNSNINDI
GTSRIRTPSSPAAPQPSNGSSDSATPPENSGISLKDIGGLSQVVKELKELIAIPLKRPDL
LAKLGLEPTHGVLLVGPPGTGKTLTARALAEELGVNYIALVGPEVISKYYGEAEQKLRGI
FEKAAKNAPCIIFIDEIDSLAPDRSAVEGEVEKRLVAQLLGLMDGFSHTPGVIVLAATNR
PDHLDPALRRPGRFDREIQFRIPDANGRKEILQVLTRAMPLDETVDLEFISDRTVGFVGA
DLKAVCQKAAYMALRRQVPSMETEIPETMTVNQSDFLQALKEIKPAVLRSMEVEVPHVEW
EDIGGLEAIKQTLRESVEGALLYPELYRQTKAVAPRGILLWGPPGTGKTLLAKAVASQAR
ANFIGVNGPELLTRWVGASEQAVRELFAKARQADPCVIFIDEIDTLAPARGTYTGDSGVS
NRVVGQLLTELDGLETGTNILVIGATNRPDALDPALLRAGRLDLQLKVDLPNLASRLEIL
RVYTEGRPLLDVDLEYWAQTTEGWNGADLVLLCNQASVEAIRRFRAKGETDPAAIRITID
DFQHSYRILSQQRNS
>1170769.6.peg.1178
MTPAIEKVALRALLYLRSGFPVHLRGPAGTGKTTLALHLAHCLDRPVMLLFGDDEFKSSD
LIGSESGYTHKKLLDNYIHSVVKIEDEFRQNWMDSRLTLACREGFTLVYDEFNRSRPEVN
NVLLSALEEKILSLPPSSNQPEYLHVNPQFRVIFTSNPEEYCGVHSTQDALMDRLVTISM
PEPEHLTQTEILAQKTNIDKESAGFIVDLVRSFRLATHAEKTSGLRAGLMIAKVCADNDI
LVAPEDPTFREIAMDILFNRSSLPVAECTDIFMDLLNLDEPELPIEKEVNGHINEEENNH
TAIPIEEIEGLVTAKVVPFEKEVYNYLLQKRSESVNGIKKFLNSEYHTALNALRSLEQKG
LVSKNKRIYTI
>1170769.6.peg.1183
MIAQTNKQSSRIYPNSMIFGSQVQKGSFFTNWLNRLGISIFILNKCSFL
>1170769.6.peg.1191
MIDQEADIAVDSRNITGGGTSDWLGLATKRVLPFILPPLLV
>1170769.6.peg.1197
MGIGSAGMVQASKLPFTHGITPAVVWGFSQTVGTVAVTRLSVTGTFPNNPVLIAIAAMIL
GNLFGYLSEAWGKAMTTSNVATVGE
>1170769.6.peg.1210
MAKKSMIEREKKRAGLVAKYAAKREALLEEFRTTESPLEKLEVHRKIQQLPRNSAPTRRQ
NRCWLTGRPRGVYRDFGLSRNVLREWAHQGLLPGVVKSSW
>1170769.6.peg.1214
MVTTLKPVSTLPTQDEILRQLRSGFPHSVGLIGMRDVRDYKVKSGGSERLNTSSPFNIKA
ESLTLSNFSLPEVEELYLQHTQATGQVFTTEAIQRAYYLTDGQLWLVNALARQATQVLVK
DVTQPITTEVINRAKEILIQRQDTHLDSLAERLREDRVKAIIQPMLSGSDLPDTPEDDRR
FLLDLGLVKRSPLGGLTIANPIYQEVIPRVLSHGSQDSLPQTYGREN
>1170769.6.peg.1219
MLKHSDLFEELPEKFHDSAFLDRIHFYIPGWEVDIIRGEMFSDNYGFVVDYLAEILRAMR

NHDYSDRYKQYFSLSPDISTRDRDGIHKTFSGLMKILFPHGGATEREVEELLQMAMEGRK
RVKDQLLRIDSTYAKVNFAYENKEGISKTVVTLEEEEYPKYYHQTLTEEREGEEVVKLEG
ASAPESKSGVHPGEAHLTFQENQRGVSFDSLFGPYLKGAKKITITDPYIRVFYQARNLMD
LIETVIKLKPQEDEVKINLITTLDDFKGEQQVEYLGRIQESCATVGIDFTWEFDGTCTIH
GRHIVTDTGWKILLDRGLDIFQHYDIKDAFSIANRLQEFRGCKAFEVTFLRRETVEGN
>1170769.6.peg.1220
MKFLRELLEKVRVFEHVRHCVLGVSYEYHRCFRPQGFYSPGKGLVGHVVFHNVHKRLVHG
LLFASKLIEGYNIPVAHQADLTSRVIDKELRHSYLAARYQNAMGRELREDMGFPSALGTK
FDQVVIALAERYEANELEQLAPSTEHLRIEADTLNKQVNPLLSG
>1170769.6.peg.1238
MELIQGLDSLIGNPRIISGVILLELTNNLSILLLILD
>1170769.6.peg.1239
MRKYKEIDDYNQDIKINPNDPEYYYNRGNTRRELGDKQGAIDDYTQAITLDHNFAYAYIP
QGNVRTASGDNQDAIDDYIQVITLVNPDPKNRIIFIEQQ
>1170769.6.peg.1243
MLHKLFTEIKEYELAAYFYQISQSKISSKILKLLVNSNNITPEIILGLPIKESRPWINSI
SKFFNKDIDEQEKIFKKRREKYLELLRKYKEIDDYNQDIKINPNDPEYYYNRGNTRSDLG
DKQGAIADYNQAITLDPNYANSYNNRGNARRDLGDKQGAIADYNQAITLNPNYGNAYYNR
GLARYELGDKQGAIADFQKAADLYQKQGRDEWYRDALDRIREIQQR
>1170769.6.peg.1244
MNNNIIAGLGKAKQFAFWGAKLAVHTKIKFGAVKGEIR
>1170769.6.peg.1247
MPVGLPEFVDNPENRCPVILLLDTSASMSGKPIEQLNEALIDFKQDITRDDQACLSVEIA
LVTFGPVKLVHDFVTVENFIPPSLKADGMTPMGEAIEYALDLLEQRKQNYKTHGIVYYRP
WIFLITDGAPTDDWQTAAHRIRIAEEEHRLLFFTVAVQGADIHTLKQIAPLERPPVTLRG
LDFRSLFLWLSTSMKRISGSKIGQAVSLPPMGWGQINT
>1170769.6.peg.1248
MGWKAITRSVIGTSHKENNTPCQDYGACKIFDNIIVGAVADGAGSAQYSHIGAKKAVDTI
INSFQNIINKPTQKLNPNLELSQEVVKKVFIKITDNIIKELEKEADKHKYEIKDLACTLI
AFIATPQWLAAMQIGDGFILIDTPSQKCRMLFKPDKGEFFNETTFITSTHALEEMQIEVV
FDKIEFICVSTDGIEKVAIHLATWQPHPPFFSPLRQYLEETNNPEKEDSYIQNFLESEKL
NARTDDDKTLMLCLQI
>1170769.6.peg.1251
MIRKSICLSTWRNPRSEALIWVEVWIPISERIEQFIEPDKNLLLVWNY
>1170769.6.peg.1256
MVLTLYEKPRKCLVVGGAGYIGSHVVLTLNEAGYHVTVFDNLSTGLESSVLSPAKLIVGD
LNNLDHLEEVIRNGEFDAILHFAASIVVSESITDPLSYYTNNVVNTINLLTLAHRYRIPR
FIFSSSAAVYGITEQVPVPETAGLFPISPYGRTKLVTEWAIQDLARSAPWFSYGILRYFN
VAGCQFTSGLGGNNSRATHVIKLACQTALGKRPVFQIYGSDYPTADGTGVRDFIHVTDLS
QAHLSVLNYLEDSAESSIFNCGYGQGYSVLDVIKTVQEVSGVKFPIEIVPRRIGDPPEVV
ADVSSILQRTSWQPQHNDLKLIIQSAWNWEKGLMNNDSVS
>1170769.6.peg.1257
MPRRIFAEAMDCYLAMGFVPGNLCILQGYNKLPPAHALSLDAA
>1170769.6.peg.1258
MCGIIGLASTTSQRDRAWLSIARDTLIHRGPDDAGEWWSEDQRVEMAHRRLSILDLSPAG
HQPMELPSHGLTIVFNGEIYNYRELKQQLSTIGFTFHSTSDT
>1170769.6.peg.1259
MSINHSKLILGFDGWTLGSHHYQRLVQSLKDSGYRIKLIHFGSYGHDIRQQTSEFLGPLE
VCDISHYKGKSVREILLEEQPKAVVFLSTQSFLHQAVNRYCQALKIPTLHLFHGFVTVQA
VETSQPNKYRFWPQSKLVSQRAFKNLTKIFPIYLKSLIETKADILSWLYFFRDVVSKIFG
LFQKVASPNCSTTFCAVYGLSDVTYAHNTYRIPIHNIKVVGNPDFIKFRLSDELILSCVS
PCSSKTKIIYIDDGSPTCGLTFASQNDFLNFLVKTKLKLAEQGYELLVKLHPSQAQFDTA

QELIRLGVLLSTDASFTSDLLDCRAAITGPSSAAVIPASLGLTLLLAQYDQFEGQKYGIV
YRDYPRSLYLRNLMELKRLLNQETKPDVNRMKMWVQQYLYPLPAEDMPKRVVEIIDQMVH
KHERPCAE
>1170769.6.peg.1260
MVLKLFTPILDRYRLVLKRVHDLEIVQNTLLFDETWVNDPTKFLNGQLQRQSIVLQLRQA
FDFSIIIETGSFIGNTTGWFAELWGGEKIHSCEIDPRFHALSQIRCANMNISFFMGDSRS
FLENLRNSDISQIAFFYLDAHWGNDLPLKQELMIIKEKWPCSVVMIDDFEVPGDIGYSYD
NYGPGKALTFQEFSPFFLENKFYVYSPTVKSQDESGFRRGMVLLTLDPAISKVIDDIPSL
YRHYHH
>1170769.6.peg.1261
MQRNKIFIAHSNSGQQLLDWHARRENCARNLGYKIETMAMMDYHPYTIFPYLDKKWKKRD
TVLMRFYEKLGEKIADSDIFIHYNGALIHPKFLQQFTQLKIYHCADDPEASDVISRPVVH
AYDIHAISNPTCLDLYRSWGCKHVFFWPLGAFHYDFAVENRSQTAVRDISLSFVGTKYGV
TRWRYVHRIPILKYWSGLYTKKAFFDRLEQAFPSMVAYGGGWRMGRIEDSDIPDLYRRTL
VGINVHNNMGVHNGRLFDLAAYGVCQICDNKQHLHHVFVPGKEIIGYESTNEAIDLIRYY
LAHPNEANAIGIAGRERYYRDYTMDAIWNKFFEDLGRLSITADLSGV
>1170769.6.peg.1262
MGTYFLDALIFMPRLHAITHKTSNIDLFTTPYLIIGNALMVRVHEVAKQGRSALRQLFEQ
ATNIAASSALILAVVWILLDPISRWLFPDTENVEKFFAPLLFLIPAIITNALYVPLSDYY
GGLTKRNLFLSVMAIIQLGVLWGCSRWGDGYSAVILVVVIYLITVRGYAMIASRVLLGDF
KPPIPKEAKAFILINLMSLATSSFISETIRQSSNLHGLPPFILHLAIFFVMTCAMTGSRS
DLRRSFLQLGILRL
>1170769.6.peg.1263
MRSWLLSESVRMLKSRTLTPGFGVFFDPSKTTSGQRFFAGLCQELNQSAVPFEQRPRVVL
FNISVPWREVVKAKFRGQKIVVRVDGLYFDRLSPDFLASFLPPLRWLFGLGVRYPQFHNL
LAHLANLLDQNYKAFFRILLADYVIYQSEFSHRVHETYFPNKPSCVIVNGARYVNGSNEP
ARTTKACIRLVSIYDAWKPAKRVYDVLRFVCWLNERQQPATLTVLGYTGTVPEGSPQEMR
QMLEDSSFVSTLPRFSTFEGCFADALVGSDCYITFSYRDPCPNAVVEAMAHGVPVLALTS
GGIPDIVSDAGRLIPTDDFAQGFFSDHRFGSDFPPIDFEAVSTALNDILVNLPMYRQRVA
RRFAEQLDTTVTAKKYLQVLNYLSNS
>1170769.6.peg.1264
MQCPICKSTNYSIDPVASRLIGLCEPFKVMTCSGCGLGQLMPHLDLKEMKNLYDQAYFDS
PNADKAGLDNISLVDDYANAVESRIPKFNNTLNDLTNRFPESRTFLDIGAATGEMVMMAR
RAGYQAEGVEFSDFAVNKAREKWGIILKQTLLSEMESESFDIVHLNHVFEHFTDPVAELK
NVHRILTAGGGLYIEIPYQFHVVERLKHRFASRSVPFSLHSIHHPFFYTPKTIQRLLRDH
GFHILKLNVFAADRYPALTPSQQVKRLFWRAASWVSVGNYIEIMAIKRVSPNAKK
>1170769.6.peg.1265
MCGLTGFIETKDFSSDEARNVIVRMAQALVHRGPDDWGVWLDDEHGLALGHRRLAIVDLS
DAGHQPMVSGSGRFVLVFNGEIYNHRELRQCLPDRSWRGHSDTETLLAGIEYWGLERTLK
AAVGMFALALWDKEENTLSLARDRMGEKPLYYGWMHGTFLFASELKALRRHPAFTGEING
QALAHYVRCGDVPAPFSIFQGISKLPPGTIAILRNADRLARQEPVLTNYWSLRTVVAQRA
QSTFQGCAGEAVEQLEDLLTQSVAGQCLADVPVGAFLSGGIDSSSVVALLQSVSKKAVRT
FSIGFDESGYDESQHARNVAAHLKTEHTEFRVTAADALKIIPELPVIYDEPFADASQIPT
LLVSRLARQYVTVAITGDGGDELFCGYGRYPHTRDRWQRLARLPSVMRGVGSRVLPASPL
QECLRANSLDEFYHFTNRQWKGFPDLVCGSQEAPHALKIPDELTAANERMMFADALDYLP
NDILVKVDRAAMSCSLETRVPLLDHRIVEFAWSLPDAIKYHQGIGKWPLKQLLYRHVPRS
LVERTKMGFGVPIDHWLRGKLRDWAEDLLNEKRLQREGFFNPAPIRQEWNRHLSGKYDRH
YGLWTILIFQCWLRDWKETS
>1170769.6.peg.1266
MDGVAYLRDDLRFSPKLSRVSSYFLPLSSQLLYQTVRQSAVISNWYEIQRNLFTLVVREH
IFANLNNDRMI
>1170769.6.peg.1271

MRPELIALGTAQFGLSYGVANQKGQVPAREAYQILKYAASVGINTLDTAIAYGNSEECLG
SIGVKDWQVISKIPEFPLETYDIQGWVRNSVQGSLERLKTPQLYGLLLHNPQQLLEPQGV
ELYDALNLLKTEGLVKKIGISVYSPEELTLLFNHFSFDLVQAPFNVVDRSLDQSGWLNRL
SGLGVEVHVRSIFLQGLLLMQAEQRPAYFQSWAALWKAWEEWLLVTGITPLQACLAFVLH
YPGINRVVVGVDSLEQLQEILSATRIKSVTLPDYLYSNDTDLINPAQWRLT
>1170769.6.peg.1272
MSWWVGLTNSSSVARKPSICFAFQKDFLLIHNLCSGLELN
>1170769.6.peg.1273
MSKQKNIFLESEGDAWFERNHYAIQNREFGDQDQIIHALLRCQLSVARGVNCWKLVAGKV
SDWNGFRKALI
>1170769.6.peg.1274
MGFREGDFPVAEAYYGRAISLPMYPELTDADQLRVVEILGHVLGFGNYEQAKKHISRK
>1170769.6.peg.1275
MDRSKVAIIVPAFNEAQSIAGVISELLPYGIVIVIDDASTDGTSSTALESSAVVVKHPYN
QGYDAALNSGFSKAADLGCDYAITFDADGQHDASLIPKFIDLFSHGVDMVIGVRPQPARL
GEYVFSLYARWKFGVRDPLCGMKGYRMSIYQARGWFDSYQSIGTELMLFGLRKQYTYEQM
EVPIAARQGSGPRFGNRIKANWRILRAITMSLWHYPVG
>1170769.6.peg.1276
MKAITIQDRKVGLHHPTYFVADIAANHDGDIGRSKDLIYLCAEAGANAAKFQHFSAKTIV
SDYGFKSLGTQISHQAEWKKSVFEVYEDASLNIDWTSILKETCDDAGIAFFTSPYSLELV
DAVDPFVPAYKVGSGDITWLEIIQYMATKKKPLLLATGAATIDEVDRAVATALTRTDDVV
VMQCNTNYTGSVDNFYHINLNVLKLYREMYPDLVLGLSDHTPGPTTVLGAVALGARVIEK
HFTDDANREGPDHGFSMTPSTWREMVDRTRELEAALGKAIKKVETNEQETVILQRRGIRA
KETLTPGVTLTRDLLEVLRPCPSDALPPYRLDEILGKTVVNQIQEGDHLRWTDLK
>1170769.6.peg.1277
MKVLITGASGLLGTALVDHLADDFEVVGVSRKPGFCPKHVSWVLADLLDLSETSKLLQRI
QPQAVIHCAALVNVDLCEKDGYVADQLHRRTTEVIVKTLGKWNGRLIYISTDSVFNGRKD
DPYTEKDLPDPPNSYARTKLGGELAALSYSESVVLRTNIFGWSRAEKLSFAEWVLKGLVL
GIPLTMFTDVAYTPIHVSHLANIILQVLQCISLKGVYHATGSQVLTKYDFAMTMASLFNL
ENDHIKPISVDDLNLVADRPKNMALLNQALASSLECTIPGAQSGIELMKYQYDTGWVSRI
KNRPMKTGYQFWETL
>1170769.6.peg.1280
MATALRILQSRPHLRLVLLEKECTLAKHQTGNNSGVIHSGLYYRPGSLKALNCIAGYRQL
LAFCQDEGIPHEICGKVVVATKEKELPQLEMLYHRGIANDLDGIHY
>1170769.6.peg.1281
MGKVGVKGDRLVLYKNVNRHSYPLCTAQPLISELRGRN
>1170769.6.peg.1282
MHTTTNNTKLGKMQHLILKILLVLSLLFKSTHTWRLSMGEDRE
>1170769.6.peg.1285
MCWIFPYLSVLKGNLSRSLSAIALGLLRKCSGYYHDLPILKGNL
>1170769.6.peg.1289
MRSQFPPIPPYIDYKLLNAIAQFWLATCNNVQFPAVH
>1170769.6.peg.1294
MGTTANTTTSHPPPKVSIQLMSPASGNWEDSQEEMGKHAVAFPFN
>1170769.6.peg.1295
MNGNAFTPWRRFSLLSLGSHSLGTLIEWKLIIPYPESLLVWVSFNVPTRWGH
>1170769.6.peg.1319
METILQQEKEQLIYQGSSHSLGTLIEWKLVVKATGLISSGSSHSLGTLIEWKQELTRTYQ
RLQRVLQFPLAGDIN
>1170769.6.peg.1335
MVFPTAKKWEVALIIETTLLKDQQVTESDIKSGGVLHTLT
>1170769.6.peg.1336

MFLKTKISYYGAIVQRCKTEKGLTQITATVTKDLPKSTQH
>1170769.6.peg.1337
MMEHEAQESEKLGRIRVIVQVTYQLSILLTLFNPKLISRFGT
>1170769.6.peg.1340
MRPARQHVGFGAREFGGRAAAAIERLSDRHRRRDRRARALVIDRLLVARTVGAVDGGGRI
VEVARLAGIARQRDRRAPLRQRLRHGFVGRAQSGTLGEQFGIAAIGVRERLVDRLGAGHR
RQRHHGRQGRARHRRLQCHPSRRHRITLFQISNAPCPDRPARPIDRLRPPS
>1170769.6.peg.1341
MEPMVFARLVQALALEPGQRVLIVGDFTGYAAAVLKDMGVTLASDADDSAVDAVLFAGAI
GELLDTYTRRLNEGGRIVGVLTAPGEPGRATLWRKFAGDVTSITMFDAATPVLPGFEKQP
GFVF
>1170769.6.peg.1342
MHQPREHHRLHEILSAAQRQILVHVSDASELGGHEFFARDRGHRRDQALVDHLVGAQLAF
HHVAAAFGEIHVFGLPWCRAIRLADLYMVRRHKEQLKARKCR
>1170769.6.peg.1343
MAEQPERARRRRAYGRRRRGRDRFRSPPSRASCRQRRRPFLFRRLERTGIAVDLDAPDER
DRDDRCVRTARFGAKPRPCGNDRCGRHLGRHLSGRRRARRTICSRRRLHDRGRRRPDPER
RLRQFLEEIRHRGRLAAGGGDRRRRWQGAHRQRSPRPRAVLRAARRRRRQFRRRYEADAA
HARSARNVRRRVRADRREIGHGLRRTS
>1170769.6.peg.1344
MKRDVPMPARVEQRAGEVGLARLFQRRGGQPGGPVGVQELAGFFAIARPENAVFRRAGLV
VL
>1170769.6.peg.1345
MFDASRHWNVSLHMNKGLAGAPDAVRAQAAQTAINPAVNTAFALAICGAAGPPAYPGIAG
HEPDLARARANADKVAAAMQALETRVPSTGTYLAESDYFDANWRSGYWGGANAARLEAIK
RHYDPDNLFRVHHGIGA
>1170769.6.peg.1346
MRAIIGLTGLLLASTAEAEPKQERCLPPAVFAPRVLYMPCRDQRAEEPKSNERGPFDRRH
CLEPPWRRDKSC
>1170769.6.peg.1347
MAPRQIMLILRPLLALAALLALSACGPPMRWEHPALSDAQAQAEMGDCRQQAWSEAQSRA
FYNRFAYGPSYVRGRDGRLYMADPWMRPGFNNTWFEEQRLRDFCLRNKGFRLVPGE
>1170769.6.peg.1348
MGPQSYAEQTQKEYHKLIGARAPMGWNNQLKNEPGIALFYERKWRRMRTPLPDFPLFELD
MTPHVGASVGNVFTYGAAGATFRIGRDLGVDYGPPRIRPGLAGSLHIDPPLDRYAYYAFF
GFEGRAVARDITLDGNTFARSHSVNRRPLVGDLQMGFAVVVERIRGTFSYVMRTREFEDQ
KNPDRFGALSISYRF
>1170769.6.peg.1349
MVLLLRLLGVALRAHDAELQFQRVEPAAILPLAQIGGQRRREIRPARIGPVVGDQVAPRN
ILGRVHVLPEAEPRARLARGVEHRDPAREFAQGAPAQAGLRHPVEPNPVGEIAVGLLGLI
EGIELVVFDQHRIGAAPRPFRRILGGDGTGESCKNDGNKGAIHRNALNRTRRLRLVCRS
>1170769.6.peg.1364
MDEFLIAGLAEINGAFNLSPSWYVEALKYIKANHGLGGQAANEANTYIDYAINALS
>1170769.6.peg.1365
MSHIIHPILLCGGSGTRLWPLSRKSYPKQFARIVGNESLFQASARRLSGADFAAPTIITG
SDYRFIVVEQLAGIEIAADAILIEPSARNTAAAVAAAAVALQAQSPGALMLVAPSDHVIP
DAAAFRAAVKAAVPVAKAGGLVTFGIRPDRAETGYGWLELSADPGADFAPVPQPLRRFVE
KPNAATAADMLAKGTFLWNAGIFLFSTDGILAAFRAHAPTLLEQVEAAVAAAKRDLGFTR
LDAAAWGKIEDISIDYAVMEKASNLSVVPYAGVWSDLGGWEAVWRDGAQDGTGVVTSGPA
TALDCHDTLLRSESSAQQIVGIGLTDIVAVAMPDAVLIAHKDRAQDVKLAVAAMKKAGVS
QAETLPRDYRPWGWYESLVIGSRFQVKRIVVHPGAALSLQSHHHRAEHWIVVEGTAKVTI
DAEVRIISENQSVYIPLGAVHRMENPGKIPMVLIEVQTGSYLGEDDIIRYEDVYARS

>1170769.6.peg.1366
MARYYIAGHRGMVGGAILRRLDQRRAAGEAVEIVTRTHAELDLTDQAQVRAFMQAERPDV
VILAAAKVGGIHANNTYPADFIYENLMIECNVIHQAFAAGVTRLLQLGSSCIYPRAVAQP
MAESALLTGVLEPTNEPYAVAKIAGIKLCESYNRQHGVDYRSVMPTNLYGPGDNFHPQNS
HVLPALIRRFHEAALAGAEVVTIWGTGTPMREFLHVDDMAEASLFVLDLPKAIYDANTEP
MLSHINVGSGSDVSILNLARMVAEVTGFTGRIETDPTKPDGTKRKLMDVGRLAAMGWRAK
IGLRDGIADAYRWFLAHQADLRV
>1170769.6.peg.1367
MKKALITGITGQDGSYLAEFLLAKGYEVHGIKRRASLFNTQRIDHIYEDPHSNHQRLKLH
YGDLTDTSNLTRILQEVQPDEVYNLGAQSHVAVSFEAPEYTADVDGVGTLRLLEAIRFLG
LEKKSRFYQASTSELYGLVQEIPQRETTPFHPRSPYAVAKMYAYWITVNYREAYGIYACN
GILFNHESPRRGETFVTRKITRGLSNIAQGLEPCLYMGNIDSLRDWGHAKDYVRMQWMML
QQDAPDDFVIATGVQYSVREFISWSAAELGITLDFRGEGVEEQAIVADVTGDKAPAVKPG
DVIVRIDPRYFRPAEVETLLGDPSKAKQKLGWVPEITAQEMCAEMVAEDLKTARRHALLK
QHGYGLPVSLEG
>1170769.6.peg.1369
MVRFSIDLDPRAVHGRAGGMRAVYGLPERFFYLPNQFWSHKNHATVVEALGLLAQAGRLD
ALPPVVMTGRTEDARDPGLFGQVMARAKALGVQDHFRHLGLIPYADVFALNAAAHRLINP
SLFEGWSTTVEEAKALGTPMILSDIPLHREQAPEATFFARSSAQALAEALVAAAAAGPRP
AVDLEALDQAQTARRNAHADAFLAAVAAARTGGRT
>1170769.6.peg.1370
MREIPTEEVLKQYSLEEVLKQYSSEEVLKQYSPEEVLKQYSPEEVLKQYSSEEVLKQYSS
EEVLKQYSPEEVLKQYSPEEVLKQYSPEEVLKQYSPEEIKQYSPKEVLKQYSPQEFLEGL
SPETLEHLAIILSQLGVNQIKNQEQ
>1170769.6.peg.1408
MVKRVQLVLTKDVSKLGKSGDLVEVAPGYARNYLIPQSLATQVTPGILKQVERRREIERQ
RQLELKQQAEEQKSALEKLIKVAIAKQVGENEAIFGTVTTQDVVDAIQAATGQIIDRRGI
TIPDINHLGTYKADIKLHSEVTAKIDIEVVAS
>1170769.6.peg.1453
MAQIDEKFLEEAALLGSYRKNIGWTELQRNTSNQDSTYLIIN
>1170769.6.peg.1469
MPGMEKARNSKPSSKSGNLQVFTDAGNPYVLHLKSMEGKLSVIKIVWYVLILSEKNIRRG
K
>1170769.6.peg.1470
MGEFYLVQEYIEGKNLSQVGQIKPEQATVILSSLLNTL
>1170769.6.peg.1471
MVREIAPKTQLGVIKSNVNQGGKTIAPDIVYPESDGKPMADNTEQFKWIVKIKENLEILF
KSNPEVFVAGDLFWYPVKGSNRIKLAPDTMVVFGRPKGQRGSYRQWEEDNIPPQVVFEIL
SPCNSKGEMTRKKLFYLKHGVEEYYVYDPDEISLEVSIRENNSFKEIEDFATWTSPRLNI
RFDMTGDELVIYYPDGSRFLSPVELSNYAEQERFLKEQANQRAEQERLLREQERFLKEQA
NQRAEQERFLKEQSNQRAEQERFLKEQANQRAEQERFLKEQANQRAEQERFLKEQSNQRA
EQERLLKEQERFLKEQEQLKYQILLSQLKAKGIDITALE
>1170769.6.peg.1473
MGGIDMVREIAPKTQLGVIKSNVNQGGKTIAPDIVYPESDGKPMADNTKQFAWIVKIKEN
LEILFKSNPDVFVAGDLFWYPVKGSNRIKLAPDTMVVFGRPKGQRGSYRQWEEDNIPPQV
VFEILSPCNSKGEMTRKKLFYLKHGVEEYYVYDPDEISLEVFIREDNSFREVEDFATWTS
PRLNIRFDMTGDELVIYYPDGSRFLSPVELSNYAEQERLLKEQERFLKEQANQRAEQERL
LREQERFLKEQANQRAEQERLLKEQEQLKYQTLLAQLKAKGIDITTLE
>1170769.6.peg.1474
MVREIAPKTQLGVIKSNVNQGGETIAPEIVYPESDGKPMADNTKQFTWIVKIKENLEILF
KFNPEVFVAGDLFWYPVKGSNRIKLAPDTMVVFGRPKGQRGSYRQWEEDNIPPQVVFEIL
SPCNSKGEMTRKKLFYLKHGVEEYYVYDPDEISLEVSIREDNSFREVEDFATWTSPRLNI

RFDMTGDELVIYYPDGSRFLSPVELSNYAEQERFLKEQANQRAEQERFLKEQANQRAEQE
RLLKEQERFLKEQANQRAEQERLLKEQSNQRAEQERLLKEQERFLKEQEQLKYQTLLSQL
KANGIDVTGLE
>1170769.6.peg.1481
MDLSRGNYFIKYSPREYQRRSHLLETLPNVRHFWQLFQS
>1170769.6.peg.1496
MFIIGEGNFSEMLEGVGHEGKLSAIAQIIKFVLFITVMIYHWVNR
>1170769.6.peg.1497
MELMETNLKFQLQGRTLEEVRFLLGSGINGNITPSIISV
>1170769.6.peg.1498
METAVLLERVPFIILMVRFLLGSGINGNTLDLSNQETQVDTSAFF
>1170769.6.peg.1500
MTVPKLRFPEFRDVREWEEKKLADVTQNIGDGIHSTPIYNENGEYYFINGNNLSKLD
>1170769.6.peg.1501
MSEIEREKSYLSLVDEIAARVTEGRKKAVTQLNTTLLETNWHIGRHIVEFEQQGAERAEY
GTQLLTNLARDITARYGKGFSRSSLFLCRQLYLRIPKIQTLSGKLSWSHYCEILKADDDL
EVNFYMKQCEAENWSVRELKRQMKSMLFHRLALSSDKAKVLELAEKGQVVSTPLDIVKDP
YVLEFLNLPQRQVYLESDLEEALISNLQSFLLELGKGFAFIGRQYRINIGGRQFKVDLVF
YHRILKCFVLIDLKQGEIEHGDIGQMNLYLNYFKQEESVDDDNEPIGIVLGAYKDKILME
YATQNISNQLFVSRYQLYLPDRKQLEQELQRLLESRESQDGGEL
>1170769.6.peg.1502
MTEQIIEQELIEKLGNLKYIYREDIRDRITLEKNFREKFETLNRVHLTDAEFTRLLDQII
TADIFAAARHLRDRNSFERDDGTPLFYTLVNIKDWCKNTFEVINQLWINTTNSYHRYDVI
LLINGIPVVQIELKTLTISPRRAIQQIIDYKNDLGNGYTKTLLCFLQLFIVSNRSDTWYF
ANNNNRHFKFDAEERFLPIYQFAAEDNKKITHLDSFAEKFLTKCTLGQMISRYMVLVASE
QKLMIMRPYQIYAVKSIVECIHQNCGNGYIWHTTGSGKTLTSFKASTLLKEVQRLKTQLD
QYTDLTTENINEIGEVISQDQLQGFRGVYLETAKRLKAEQGKDKPIREVEQLDFEFVLFA
STVIDYDYIMGLIARYTQEESGRQEMTREQLIGLIQSDAKFMDEDITAYIDTLPTGEGLN
ETAIREGYERFKSEKNARELATIAEKHGLEAAALQGFVDGIVRRMIFDGEQLSNLLSPLE
LGWKARARAESALMADLIPFLHRLAQGREITGLKAYE
>1170769.6.peg.1503
MKIDRHSKAKILSQEEIQRLFTTGLTTARDRTLCAVMLYTGCRVNEAVTLKISDVYDKKG
RIRTELILRKGNTKGHLATRTIPVLEDLKHFLEQYQPPATKDGFLFPGRWGRGHLHSDSA
SIIFREGCKQVDIEGASTHSFRRTALTLMSNAGIPLRVIQEISVHRNLEQLQRYLEVESS
QVRGAIASLSMLTPVAPSPEIDDITITKITVKTDS
>1170769.6.peg.1514
MSISVRVYLLKCQLRTSSDYSTTGFLGVEPRCDQISQSSDRYLPYLLLQRHGIFFEGG
>1170769.6.peg.1520
MAGDRKALRAIARRRYCDRLHNCSLAIEYKRIYGYDEVRKYSWEGMISQCTYEASDLTFG
LNSNSGNKKRIKTLIEEARSRYFSDCY
>1170769.6.peg.1529
MYQSEAPPLKTIATDLPSQDIDEELNSTIHPRPPWETLPTMYDLPSENPEEPGLPDEFHN
FQPQLLRETCQSSVYPRAEREKQRAEQERLAKEQAEAMTIQERQQRELAEAIVIQEREKK
EKLAARLRSLGINPDDI
>1170769.6.peg.1534
MKVNLQPGVNNTNVDASKSSSQRLIAISVSAIGETIDSRVPLNLCLILDHSGSMKGQPVE
NVKRAAWLLVDKLRDQDRLSIVVFNHRAEVLLSNQNVVDRDHIKQQINRLSANGGTSIDE
GLRLGIEELAKGRRDTISQAFLLTDGENEHGDNNRCLKFAQLAADYNLTVNTLGFGNNWN
QHILEKISDAGLGSLSHIEHPDQAVDKFNSLLMRMQTVGLTNAYLLFSLAPNIRLAEFKP
VAQVSPDTIELPVQVEPDGRFGVKLGDLMKDVERVILTNIYLEQLPEGQQAIANVQVRYD
DPSVDKTGLYSPNLPVYVNVERTYQENISPKVQNYILALAKYRQTQLAEDRLQQGDRLGA
VTMLQAAAKTALQMGDANAGTVLQNSATRLQAGEDLSASDRSKTKIVSKTVLQDPTLT

>1170769.6.peg.1535
MPQRRGRGGRGQPVQRGQCPRDRQRVGRQPGRPAFDGLDQQRAQRQAGRGRERIEQARGV
AAGGGALEAGALVGQVVHGRQAHLALLGHTGQPGRASQPHMAVRQPRVGQRLQRRRRQRG
VGGAQRPAQGVGQVGNGLGHGGGRNDRSEGRCNNAPRSNDHRANHSGHCREHPQIAVAAG
LGRCAGAVLGARPRSLPEPGTTQGQPGCLHRALPAPPRPGGRGLRRPVRAGHRALAARGH
AHHPGRRCDLRPVLGHGDRVVRLQHRRHAGLSGGALPVARRGAGALRPAAGRHPARPRPR
RRVLPVHAAADPGRALLPHQFAHGPHAHEGAHLLRGEPVGHARGHRGVRERGHTTRAARF
AARHPEPGTDRLVRPAGRVPAAGALAGERRAAPAGVRALARPAAAPLRPQPDRDRRRRGR
SGHRLHRRRGESARDAGRGAQDGRRLPELRLRAEQGADPQRQAGAADAPGLAAGAAGHPA
HVPLQGRDAARARRDPRHRAARQRRALHRPGRGGAARPCAAGEPVDGVDRAGRRRHADPH
RAQHRARHRGAALRAAAARPRRGPRADQRHGVGPPGRARTRARAAGGARRWPHRLRTGAG
LRASGLAGDADRNGTAPAGARRRRRERLRPRLARGRWRAGVHRAQGPARGTRGRAAMAGG
RTRWA
>1170769.6.peg.1536
MAEHGGREERFAFDELLCAVGRVARLDGFGLQELGIPTGRVIETNAWLQTRYPHIYAVGD
AAGPHQFTHVAAHQAWYAAVNALFGEWRRFRVDYRVIPWTTFIDPEVARVGLNERDAREQ
GVPFEVTRFEIGELDRAIADGETRGFVKVLTVPGKDRILGVTIAGAHAGELIAEYVLAMK
HGLGLNRLLGTIHAYPTLAEANKYAAGVWKRAHAPEGLLRWVQRYHDWKR
>1170769.6.peg.1537
MARVPHVADGGGGQGLAGGAARQPQHLALDEGHARQRVVEHCADLERGVVAGLVQQGGAV
GGQVQRAVQPPTPAQRRGRGVAQGKRQRAVRPVGDDLHAAHAVALARQEARGRDLAVGRG
VAHHAPGRGDGAVGGGQRRQGLGAGDGGDQRGQGQAGHADHRGHTGEGEGESGGHHRFQS
W
>1170769.6.peg.1538
MTALAQRQAAVLQPRGRVGADGEHRHVAAQQRAVAQVHAAVVDGGAGFAQHLDAGAPQAP
QQPLAHRGRVARTHLRPAGDQGEGGAGHTVAAQPFGGRKHGFDAGRTTADHGHRQGACAG
QRDHVGPEPHQAFDGPQRERVLARAGQFGVEPLGPQHRAGVDRQQVVVLGVQVRAVGRHP
LHARVGGVDGGAAPGVPAHPPGLHQRGQRNLHLGLAVAPGQQARHHAAVGLGRVRRDQRD
LPVGGVVLGEAGQQVQVRMARAQQKESTDAAVHGRRWYVGLQRAHCGSATQPSLPLAVLS
ASAPMTICVSLGGGSPAGCSSANRRRKSAPGSTRSLSQRPVGSPWPRCTTKRTRRLWGLA
PSVPVGWRASHT
>1170769.6.peg.1539
MVGGHVYVMEIKVVEGNQVQGNAALDQILQRNYAEKYRGEPGKSVHEIGLIFSRNQRNLI
QADWR
>1170769.6.peg.1553
MWGGAAIAACVIGSLSGILPGNTSRLQLAQQKIDSVQTQAPISPLMVALNNPIVEIPKTA
VVRPAKIEEIDYIQEEMEFEGN
>1170769.6.peg.1562
MNIFGIGLPEMIVIGVVALLIFGPKKLPEIGRSLAKTIRSFQQASSEFQNEFKKEVQQLE
ETIKTTAEIEPKQIESSKEQKHS
>1170769.6.peg.1571
MIAEATAVTGELAEGAEHSGIGLNTNIFETNLINLAIIIITVLFVFGRKVLGNTLKTRREN
IETAIKSAEERAANAAKQLKVAEEKLTQAQVEANRIKADAETSAKAAGEAILVQAAADVE
KMQAAGAADLNAELERVISQLRQKVVAKALQKAEAELKAGIAEDAQIRIIDRSIAQLGG
>1170769.6.peg.1572
MTHWITLLAVEEVAKKGGLFDLDATLPLMAIQFLVLALILNATLYKPLGQAIDGRNDYIR
NNQLDAQQRLSQAEKLAAQYEQELAGARRQAQAVIAQAQAEAQKVAAQKIAAAQQEAQAQ
REKAASEIEQQKQQALASLEAQVDALSRQILEKLLGADLISQR
>1170769.6.peg.1577
MHQLLLAVQQRCYEHHHQQIVSISLAIDFIDPLFVLDKLGQRNTLSFYFENKSKGEAIVA
IDAIKKLDINGQNRFNKTEDFIKDCLKNIINFGNLNEPFTGSHFFCSFSFFEQHQNSNYP
FAAATIFLPKLQVAVKNSSCTLVINKVVDADVDVNHILQDIQNKIRTLQSLTNGLPIAVT

TCKGSQTSQINNPDVFKNSVLSALQKIEAKQLRKIVLADTLDVYSYSSFNLLKSLNNLRN
LHPNCYVFCISNGRGQNFLGASPERLISIQDHQLITDALAGSAPRGKTPKEDAVNAHRLV
NSSKEKHEHNLVIDFISHRLSQLGLFPQPLTPRLRQLANIQHLWTPITAVVPNNVHPIKI
VAQLHPTPAVAGATREIACAEIRHYEKFDRGLYAAPLGWLDSRGNCEFIVGIRSALINNN
CARLYAGAGIVAGSDPEREFAEVQLKLQALLKALV
>1170769.6.peg.1592
MMLKAKIGLPLAYIPAERPVFGREAYKKLVPWREHCTLMKSVVRTGVRKILN
>1170769.6.peg.1609
MNSIYWWNGEVQMEKEAILIMKSRQDLFAQVKDKIDSVHSYDTPCIVAMPIDYISETYLS
WLIKETETLN
>1170769.6.peg.1613
MAGFFGLFQGRTIFTPSGDIPQELTLEIVALCPIKPGCQN
>1170769.6.peg.1623
MSPELKNYLRLYAIEPFVLKCSFGFIGRFLIEQTDVSLLNQLAYI
>1170769.6.peg.1625
MVVKNRQICYIIAPNILKDGINLRYNLDLFNHIEQTKNSQLYCF
>1170769.6.peg.1632
MRKYWIERSPAVIKVYFLPEKVREKNFLELVDIPEIIG
>1170769.6.peg.1643
MIKAAGQIYHTYSQVHPEIIGQVSGVAISRTTYRGKVIFNQQPILLPQECFIPLQQIESC
VY
>1170769.6.peg.1644
MGWTLKLLRSGCERYTGTLWLLYRWSYIWFWKPLANNNAKFFLLDYLNL
>1170769.6.peg.1678
MSVGTYSTQNREIFPLVIPQGVVLLGNESKKGEGIIITGGGEYQSPSFGLQNITLLFSGD
GSILGITVTNSVSKGTGIWIESAAPTLANNTLCKCGREGILITGQAKPAILDNVFIQNIA
SGLMMARSSKGEVLRNVFENNPVGIAITDLAAPLIANNKLGKNQIGMAISRDASPVLRGN
LIYQNSQCGLSINGNAIPDLGKPQDPAGNIFREQENFDVQNSSSQSLISVGNQLNTAQIK
GLLELVAATNDTIIPVISSSFSDLYAHWSTAFITALAAKGFIGGFPDGTFKPNTPITRAQ
YAALIKKTFQLPDSQNLNRFKDVRTDFWANSPIASAADGGFLGGFPDGTFRPGQNLTRVQ
AIVSIVNGLKLTGSNPNGLLVYSDRAQIPSYAINATTIATQKLLVLNYPQVDLLEPLREI
TRAEVATLIYQALVVKGEAEPIVSPYIVKPDKEQPSFSDLVGHWAEAYIRALVSMNLTSG
FADGSYQPDKPMTRSQYAALIAAAFNPVAKRPTVDFIDIPSDFWAVKAIQIASRGGFIAG
FSDRTFRPDQNIQRIQVIVSLVNGLGLVPNQNHSSLSYTDKNSIPEYAKVAVIAASQQNI
VINYPDPKILAPTKEATRAEVAAMTYQALVAIKRVKPIAES
>1170769.6.peg.1687
MVRIKSGFNADDQHPIQATSADIILRQQLEHSISRYFYHGCDRNIQNLLSYCRWYMKTDT
KALTLVIECPDQVSNWRILQKIVPMATLLYSVVSSAKIRVCPPEPRAIPFEMRVDELYVY
RDWA
>1170769.6.peg.1688
MIPTLLAATTCFIIAFIAAPPVDIDGIREPVAGSLIYGNNIISGAVVPSSNAIGLHFYPI
WEAASLDEWLYNGGPYQLVIFHFLIGCACYLGRQWELSYRLGMRPWICVAYSAPLASATA
VFLIYPIGQGSFSDGMPLGISGTFNFMIVFQAEHNILMHPFHMLGVAGVFGGSLFSAMHG
SLVTSSLVRETTETESQNYGYKFGQEEETYNIVAAHGYFGRLIFQYASFNNSRSLHFFLA
AWPVVGIWFTALGVSTMAFNLNGFNFNQSIIDSQGRVIGTWADVINRANLGMEVMHERNA
HNFPLDLAAVEVSPVALTAPAING
>1170769.6.peg.1719
MRLNDVKPQKGSKKRRKRVGRGISAGQGASAGLGMRGQKSRSGSSTRPGFEGGQQPLYRR
IPKLKGFPVVNRRVYTTINVEKLASLPANSEVNLASLKEAGILTSAKGPLKVLGNGELGV
PLKVQAAAFTGQARSKIEAAGGSCEVLS
>1170769.6.peg.1729
MEGLAHRGSTLNFGDFALQAQEPCWITSRQIEASRRAMTRYIRRGGKIWIRIFPDKPVTM

RPAETRMGSGKGNPEFWVAVVKPGRILFEIAGVSEEIAREAMRLAAYKLPIKTKFIVRSQ
PQEQE
>1170769.6.peg.1740
MLRLEHISKIYPTGEVLKDINWEVKPGDRIGLVGVNGAGKSTQLKIISGEIEPTAGQIIR
PASLHIAYLNQEFEVDPTRTVQEEFWTVFKEANAVQLALYEVQRDMETATVEQLDKLINK
LDKLQRQFESLDGYNLDARIGKILPEMGFQVEDGDRLVSAFSGGWQMRMSLGKILLQKPD
LLLLDEPTNHLDLETIEWLENYLRSLTTPMVIISHDREFLDRLCTQIVETERGVSTTYLG
NYSAYLQQKAENEASQLSAFERQQKEIEKQQAFVDRFRASATRSTQAKSREKQLEKVERI
EAPTTGVRTLNFRFPPAPRSGREVVEIKDLTHTYGDKILFLGANLLIERGDRIAFLGPNG
AGKSTLLKMITGMEIPTEGTVKLGDHNVIPGYFEQNQAEALDLNKTVMETIHDEVPDWKN
EEVRTLLGRFLFAGDTVFKQVGDLSGGEKARLALAKMLLCPVNLIILDEPTNHLDIPAKE
MLEEALQNYDGTAILVSHDRYFISQVANKIVEIRDGEFRVYLGDYHYYLTKIEEEKEQAK
LAAKKATKTAKAAAKKK
>1170769.6.peg.1749
MYPSNVAKSLDIQPFVFRGSDGHDILADIINQAHSQNLLAIPWFEFGFMTPNTGELALNK
PEWLTKMRDGSTVSMSAAGEVSWLNPFHPQVQKFIIDLLVELTNNYDIDGIQFDDHTSLP
HQFGYDDYTVNLYKQETGKNPPANSQDSEWVAWRANKITEFMVRLNHTVKQIKPKVIFSV
SPNYYDHAYKFQLQDWLNWVRLNIVDELVMQVYRDDLESFTSKIARNEIQEVRQIIPTGI
GIMAGLRTSPVPMQQITKQVRTVQREELGIVFFYYETMWNRSPETLEQRIQGFKNFFPYP
AVRVAAE
>1170769.6.peg.1752
MAENTPLYERKLLSEPPEYVKLAWEWTSGKLPTCDVRGIEVHGEFLSWQDILLKLLNTPS
IASKSWVYRQYDHQVQNNTVLLPGGADAAVLRLRPLSGSTVTPWESGVAATVDCNSRYVY
LDPYEGAKAVVAEAARNLSCVGAQPLAVTDNLNFGSPEKEIGYWQLAYACKGISEGCKEL
GTPVTGGNVSLYNETFDAQGNPQAIYPTPVVGMVGLIEDLQKICGQGWQNVGDGIYLLGL
PVSVKLELGGSEYLAVIHHTVAGKPPKIDFALERDVQQVCRYGISHKWISSAHDSAEGGL
VVALAECCLSGNLGASINLGISSNGECRFDEVLFGEGGARILVSIPGTYQQVWESYLQAH
LGNNWQKLGSVVNLSSGLTVSTLDDYEVMRMDIPQMGNVYHQAIAQRLAFYE
>1170769.6.peg.1755
MPFLLLGVLFSSLLLFFVEERKLVNIMPKNPLLGALFGSMIGFLFPVCECGNVPVARRLL
MQGVPTSVAIGFLLAAPTINPIVIWATWTAFRDQPEIVVLRVVLSLLIATIIGFVFSFQK
DITPYLQPQIARYLKFNPPAQTEPKTPTRQLQEQVITPSPLLQSGTYILGGKAGISTRLS
GNSSQTVAKTPNKTLMVKLGLVLDNAIQELRELGAIMVLGSAMAAAIQVLAPRDVILSLG
AGPISSILTMLLLATVVSICSTVDSFFALSFASTFTSGSLLAFLVFGPMIDIKGVGLMLS
VFKPKALFYLFALAGQLTLLSTLFLNLHIM
>1170769.6.peg.1762
MGNCHDLMFFMAPSTVPCKERNFSNRTSLTAWVTRALLTIWKLPWAFWACLANCTRAPKP
ELSIKSICDRSRIVLFGPSPILASSTLRNVGSEKASNNPVRRKSWQLSRTSRVPLRLTVK
FSTSVFSAITPSCSY
>1170769.6.peg.1767
MSSVPLLRLSDKFDPYISGEVNIDPSAVIAPGVILQAALNSKIIIGPGVCIGMGSILQVS
HGILEIEMGANLGAGFLMVGEGKIGANACIGAGTTVFNDSVAAQQVIPAGSILGDGSRQA
NSQESGESVDEGDTSSQSGEQVVSKTQFTATFVNFQTQSTSVPPLSPTPKSQSPPETESS
TESQEISDGKPRSRDPTEPYPLGTQIYGQGSINRLLSTLFPHRQSLSDQDANNGAE
>1170769.6.peg.1769
MVVRSTAAPPTPWSKSLAEPDVHQTAYVHPSSNLIGDVHLGQNVIIAPGTSIRADEGTPF
YIGENTNIQDGVVIHGLEQGRVVGDDGEKYSVWIGKNASMTHMALIHGPAYVGDSCFIGF
RSTVFNARVGAGCIVMMHALIQDVEIPPGKYIASGSIITTQQQADRLPDVQAQDQQFAHH
VVGINQALRAGYRCAEDLKCITPIRDELKDHEEKTYTSITVEELDRSSEVAGKLSADTVE
QLRYLLEQGYKIGTEHVDQRRFRTGSWQSCQPIETRSLGQAISALETCLVDHAGEYVRLF
GIDSGKKRVLETIIQRPDGVVVASSSFKGPSPSSYNGNGNGNGKFAGIDAQIINQINQLL
SNGYKIGTEHVDERRFRTGTWQSCEPIHSTSTQEVVAALESCLNSHQGEYVRLIGIDTKA

KRRVLESIVQRPNGQVVTSGNGKVSLSPAGGTPAVSNHLSGEVVDHLRQLVSGGLKISLE
HVDQRRFRMGTWSSAGPIEARNEREAIATVESYLSEYPGEYVRLIGIDPQVKRRVLEVII
QRP
>1170769.6.peg.1772
MPIAVGMIETKGFPAVVEAADAMVKAARVTLVGYEKIGSARVTVIVRGDVSEVQASVAAG
IEAARRVNGGEVLSTHIIARPHENLEYVLPIRYTEAVEQFRTxISS
>1170769.6.peg.1774
MKKILKNLIVVRLTYSKPRFVLSLFKKEKLEPDDLDE
>1170769.6.peg.1791
MKNTTQSPDFLEMPMTTSRQPKQPQSTLVFECHLLHDDSKELSPHIHLVGRKPKDVEILF
MR
>1170769.6.peg.1795
MNSFNQVSYPVDFRHLLFLEDINRIVGSEDFHPLIFP
>1170769.6.peg.1809
MVIPFPHSVLSLVYRLVYGVIGQNGLRLRNIFGYPVTRK
>1170769.6.peg.1815
MCNPITQLQKAMGFLVCEAVSVRKIFITSSSPRATSGARGIK
>1170769.6.peg.1832
MLASKIAATYGFRIDGFVPAFLGSIVLSIASTLINYFMRLIV
>1170769.6.peg.1840
MSVELVESFAQSVPDVEIIKLNNVGHYPQEHYHEVILQDLLAFVRLTST
>1170769.6.peg.1843
MKYNYKVEEQTLEKYYQQAVQLIETQALDQIQKEIKESLDIFIERIETDKSLTQVIITTL
LKKIIKPQQDIRLHMAKFPNGYSARVLDTKVTTPFFKRKFLKYANKETAFLTKATRAEII
WNFEEGIKLPLKSKILVEPFLKLIDKIENQTIDIEQCLVYILSQLYFLCQSHKVVFSETL
KVANSVNIININRVVKMVEKHFEEPYSSRLPVIVIFAIYKQLFKTVRRFENKILLPLNVH
TSADKHGYGDIEIRDNDNNPFEILEIKHNIPIDRNIILDIVKKSTDTKIQRYYILTTYKE
CFISKDDEEYINELILNIKRESGLEIIANGIVNTLKYYLRFVEDYSEFINTYTEELVRES
LTSTEVRDSHIRTWQRILQE
>1170769.6.peg.1845
MEKICSNLIEDRVNSNSSFEKVREYQILAMLETSVGYGSPL
>1170769.6.peg.1846
MNAVRSRKFDCEWGCQGLIFGKSAFWVIWAEIIKVLFLTKALSK
>1170769.6.peg.1853
MHLFHMLGVAGVFGGSLFSAMHGSLVTSSLVRETTETDPWI
>1170769.6.peg.1860
MNYLTYSYLLTPLHTGASTQAGNLLGIAREAQTELPYIPSSSLRGKLRSSLESMAEIRSE
AGSFFGERIKDGQQPTEGEVWFADATLLFFPVGSFSHQFLWITCPLWLSRWGRWLRNDQL
NKLIEQWQSDLLSNGKKAITSASGKQIYLQGAILNEADIKTINNIDWNLFKDIPDGNGIL
DLKNKLVILSNEDCGALVEIGLQREVRIALDENEKIVAGGSFRSEEAIPSETIMFFPWGM
KLEKEANKTHKVRESLINILNDRLQFGGLEGLGRGWTENKTIAVNKKEE
>1170769.6.peg.1861
MQKLDTRDFSRHAYNGLIEIKKNVRSVDHKKASGIVQGLSAYISTWGLHRLSGDGLKYIN
SRSDDTKYKGQIYQEFLKTLQKLSKVPFAYDDASSLINLELEKYTGLNRLAIELAKEWSF
WAVPILGEAEQS
>1170769.6.peg.1862
MRNQKSEADIWQDFVQTEIKSKSKLGKILESSQYGGYEKRELTIYFPDENFRKGAQGQIE
AIKKKLREPYGLLCDRITVKTGSVSANVMNTSVTRGNSKSVSGTPKNHNPLQALYWVEPN
LPENDASQRMSILKETVAAEQGCNQIYTKLRQRTLQLVNGEENTVSVSFNWRIRIGGTRG
FRELLLPVLHPIFGIPYIPASTLKGAARAWARKNDAPARVQELLGMLNGKDAKAAKIEFL
DAFPTKHCLSIDVATPQWVWKDKKVMYEPVPHPLLSLEQPQFLIGLRPTSRQNSDCQDDL
KTVKSWLENALNSGIGSRVSSGYGKALGTIPITNTRKSYDFELWTQGMYGSNPPSKENNY

QGTPEFRPSAVRGILRYWFRAVALRLYDVPTCQKLEEQIFGNLGKQGKISLSTKINPSTK
KDPYFYDGKIYLEATEIKYLNLAEKLLFLALQLGGVGRGSRRPLHLLNGRMRGCHWQIVG
KELTLDYDSEQWREFFTEIEQAFQAIEATKATIGSYIVSPGKRGARQQDVLDKNAQIWLL
KSPSQIHPAKITNWQTDGSSSKVRGTALDLLYGDNRFKGQSKGQGNANVGGALETPSFVW
IKSIFTDSLPYQVITIFGSDDQDRKEFAKELKNQGAILVFPEMSPSKSTTSSPIKRK
>1170769.6.peg.1863
MESVLIATIGTRDLMFQIASGEWFNIGNDRVQNGEIISEQLEVISDLGLKDNTTFRDLTK
YLLDHIQKHIDHIQPPIIGKMFIEQASNIEKVYLIATDQEKNVKQREKDTLYSAELIKQW
LMHKFSHLHNHNVHIILLGQDGTNPSIFEDMFNWWRKIWKNTITVNKSQSVWVCLKGGVG
QTSEAARISGLSFYGDRIQFFEFKENTPANRNGIPSDYSGPFLGTNYLWDRTQKQALKLL
ESYDYTEAYELLEPYFQQPSANFGAIPNLLKAGKLWNQGQFERFLSLARSSTQISGVQGR
LWMAYEQAYLGVIRLEQMNTTEAMLHSYRAIEGLLYWWAADSFPDHIEERKNQYPSIKDS
ILQKYPSLKNYFNRPEPKREVNLQGYLLEDLLNLAIPETANSIDFKAFWGRSKDTRNYFS
HRLGGLAEQDIFTAWGEDITDSPQWQKRILNCINLVTGKSFKTLSQASIFSQIHTQVLEA
IKKQEIINYDNNK
>1170769.6.peg.1864
MVLITNFIRFIFVSLINGYQKYLSPYKGYSCAHHILHQGESCSQYVKRSLLQQDLQTAIK
LSQQRFVDCGKAAQVLSHQRSPYSPINKSSNQPRFYRPISRRIFILVILPSLFTFGLISP
ALAGRIPNRGFQKAGQCFGEAGMDEDRRDGDYGDPNMFYGLCCLSLIGAGILTEER
>1170769.6.peg.1873
MKKYFAAVEAYASNPTSESKQLVDERMSQAYSKIDKAVKRGVLHPNSGARKKSRLARKLK
PLTQPV
>1170769.6.peg.1874
MFATVSSQKNYQLICTHTTRNTRIARNLQVLQFHRKILGKILQLLTKVL
>1170769.6.peg.1878
MGLNTGEDSSIIIIYYIIVNADFHLKIGDLNGSYRSY
>1170769.6.peg.1904
MMSGLIPLDKGEIRIHGKPIHRTRIGYVFQNYRDSLFPWMSAYDNIAYPLRVKGISEREC
RHSVEHLIETFNICLDLKRYPYSFSGGQQQLVSILRALVAQPEVLFLDEPFSALDFETTL
FVRDKLQEIFMASTIPMLMVVHNLEEAIFLADKILLLSKRPTQVVEMVSFDAPRPRTPET
LTSRNFVEVSRYCLDIFRQEMQK
>1170769.6.peg.1927
MDICLRYGQVVLAMELKVWRKGSKDPLTAGLKQLDKYLSGLNLKTGWLVIFDRRPNLPPV
SDRTTTEMAISPQGRSITVIRG
>1170769.6.peg.1951
MDWLARFSPTYKERIIYHEAGHFLVAHLLGITVTGYTLSAWEAWKVGQPGQGGIILGDDE
IAKQLERGKIGVSMVEKYCNIWMAGIAAELLVFNSAEGGGDDKAKLNQFLTVLGFQETLF
EQKQRFHLLQAKNLLEQNWHTYQHLVQAMRNRLDVEECKKLIA
>1170769.6.peg.1952
MVPPLPSNRKLSKVAIPKNPNMVSAGMVGDRWISGPNRVDRVIKKTQMAIRLMAV
>1170769.6.peg.1959
METGDREFLKMIQVYNEDDCYATRRVKDWLVSFLVKKNLIKNEDADSESASTQ
>1170769.6.peg.1964
MALTCKDPLATEIFYTKNFGFRRVRVAKLPDGDQIVFIKMADSAFYFELFKAKEELPIPR
PTLDGPQYPGLRHLAFKVDNVDAKLAEIGSDAVITLGPINFDDYIPGWRTVWIADPDGRI
VEISQGFQDEIDVPPLKFI
>1170769.6.peg.1965
MKNVPDSPLGKLYRDHIELIMNKDIEAILDQYTDDALLISSFMKTPKYYQGRDQLREHMQ
GILGIVDLETEINFWAETENPQTLMITEIIHMKVGGEKLTMRFADSWVLQDGKIAIHFAG
MVQHPDGTLA
>1170769.6.peg.1975
MAQYFLPRPNRKFLFSHLLLILPILGGNIHQQELFSP

>1170769.6.peg.1981
MGGDRQTIIAQRQYSREVSAPKILAKNFFEKKVEKVVDILKEVRYIE
>1170769.6.peg.1991
MLSGLLKFVLGFLLAIAVLLGSGMTIAIYFINRTAITPQKPMFPNDNPDPKPNLPRVTRK
KVVKVKPKPIATPDLPRESPTPLPSGSYTAVVTWSQGLTMRDKPAFEGQAIGGVAGNQKV
IILETSQDGKWEKIRIPDTNQEGWVKSGNTEKSN
>1170769.6.peg.2001
MCGGNLNQLATPIVHSLFYLKVVFSQPHIHSTHGDFLLQWRGLV
>1170769.6.peg.2007
MVIFENLPILVHQLRIYHQFFNLCWIYLPNWLINKQGMVYNG
>1170769.6.peg.2016
MSQDKKPRSKQQLPETSPSGAYHKKPLFWKILIIQVLRGTIGILETMVTRLETSPASTTP
EKGGLLLWVVTKWDEFLRGFRLFLPSKVANNVSDGFLTMIFAFLAVLAIGVTATSLISQI
GSPSVPQSSQSSTDSTVDDKTETQLGQPPTDSTVEGKTEPQLGQPLTDSTVDDKTEAQLG
QPPTDSTVEGKTETQLGQPPTDSILKSQAELIPQQEVTGFIEKQLKEITATNIITKDKQR
IEVEIVQSIKTNFRISEIVIKTTEAWYKLESSQQEKLAAKILKSCQEMDLIHVKILNARN
QLIARSPVIGTKMVFFQFPNS
>1170769.6.peg.2024
MPTAVAFFNLVVEEKTIVHNNSISVAEPNLYSQRTQPRAIRMGVIGVGNMGQHHARLLSS
MKDVELVGVSDINVERGIETASRYKVRFFEEYCDLLPHVDAVCIVVPTRLHYAVGINCLL
AGIHVLIEKPIAASIPEAESLVNAAAQSQCILQVGHIERFNPAFRELSQVLKTEEVLALE
SHRMSPYSSRANDVSVVLDLMIHDIDLLLELAGSSVVKLTANGTRSLDSGYLDYVTATLG
FANGIVATLTASKVTHRKIRRIVAHCKNSLTEADFLNNEIFIHRQNTNPQNDRQTLYRQD
GIIEKVYTSNIQPLSAELEHFVNCVRGGNQPSVGGEQALKALRLASLIEQMALEERVWNP
LEWQSESVSQSLTSSV
>1170769.6.peg.2027
MTGSVSAFLGFSMGSSALKGVTSPDGRPTSKLISSGNNNLQSVPISFLKEEDIINQVKKR
IEQNKTKTNRTKKVEEEEQTVSTKDKSQQKAQELPEEPPQPGFPVVAESEGVNMSVQSAS
YSGGQLILKVKMHNQSNESVRFLYSFLDVTDNRGRVLTATTEGLPAELPGNGSVFMGNIS
IPTALLNDVSSLTLSLTDYPAQKLKLQLSDIPVEK
>1170769.6.peg.2028
MRARRTVTIANSAATKKALIVIKISIDNNFNPISVQINWGKVITSQARVE
>1170769.6.peg.2029
MVTVRRARIHQLVQSGDAPAIAVEETKFPPGCVLLIEITTNSAKSSEST
>1170769.6.peg.2039
MDFIRVSLPSLFQEAAKILQEKEEIILSAEEYAQQVIEAAQVKRSQILADNDIIHQVERE
TVQLRREAQQKCDAIMQDTLAEIERKRLDCDQEMEETRQNAIAHAREIENGADEYADRVL
ENIEEDLQKMLRIVTNGRLQLGGETRKQRGSSDIPKE
>1170769.6.peg.2044
MLVLTVGLFSLSLVPLSRTVIPGASRYTLVYDNGSNQAVIATSPDITPTQLEATLRQAAS
NLYSYGRMGSGRNNSLTIRARTVLHPESGISTPVYLGKVERTLVTREDPQMLVEVFLDKF
PQLPPS
>1170769.6.peg.2046
MQLIAMKVPIINSSRGFLYKSQILSYSIGYQQLQFYV
>1170769.6.peg.2047
MLAANLKQIQQFFVHPHSSLQLPWNSLLLGLLILPINPFFGAIAIGWASYKTWRKKYSSI
QRKTLNHLLVILSFWFLITTGFTVFARDQPDTLLGLFNFLPYFVVFAGLTPLITTVSQLR
QLTWIIVWSSLPVVIIGLGQLFLGWHARWQFLSVVVNLTIDPGGEPLAIYLGGEPLVRMS
SVFMNPNTLAAYLITVLILGLGLWLENYHKIRKKANPLGFIFLSVVIIANFLALILTGSR
NGWGIAVMACIAYALYQGWRLIIAGVIGLTTSIILASFATERIASVFRSFVPRFIWARLN
SDTPLALMRKTQWQFAWDLTLQQPLSGWGLRSFPHLYEQKMGVSVNHPHNLFLMLSAETG
LVTTCLFLVFLAWILIAAIKFWYKCSLPKENRLVFLSYLLAFIGWIMFNMADVTTFDLIL

SALFWVILAALYGAAHRYEGSHH
>1170769.6.peg.2055
MLRVENDTFISKLPGDKTRNYPTFLAFLGCIGVKLSKP
>1170769.6.peg.2065
MQRSEDLINAASNRYRITVQVANRAKRRRYEDFENNEDSIMKPVLRAIIEMSDELTQPEI
IGELPSDWL
>1170769.6.peg.2090
MKVLVIGGDGYCGWATALYLSNRGHEVGILDSLVRRHWDNTLGVETLTPIAPIQQRLQRW
QDLTGKSIDLFIGDITDYGFLHKALHEFEPEAIVHFGEQRSAPFSMIDREHAVLTQVNNV
VGTLNLLYIMREDFPDCHLVKLGTMGEYGTPNIDIEEGYITIEHNGRKDTLPYPKQPGSM
YHLSKVHDSHNIHFACRIWGLRATDLNQGIVYGVITEETGLDELLINRLDYDGVFGTALN
RFCIQAAVGHPLTVYGKGGQTRGFLDIRDTVRCIELAIVNPAQPGEFRVFNQFTELFSVG
DLALMVKKASYAMGLNVEIDHIDNPRIEKEEHYFNARNTKLLDLGLQPHYLSDSLLDSLL
NFAVKYKGRVDNNQILPKVSWHRK
>1170769.6.peg.2092
MAISDFPQPGTPKINIPFGCGNPYSLALSVKALDLCFSHFFNSSNPPTASIVSSSSMYSK
MPLRRISCFFSDKTISTSSSVSLPLVTWAFRYTFSASSLVSPKAALSSFSLASVVKRRPL
FCSWWAVRTLLNSVMSGSWKSITTTSFSNSIGICCIGDIKIMFFCIPLKLAIASRRDLVV
AGASIKGCKSLKINIPGSFCLIAHSIAASGDTVLCWFVLLVCPYSTMPWVTVHT
>1170769.6.peg.2093
MLILGVPGCGKSLIAKTTSRLWGLPILRLDMGRVYDGSMVGRSEANLRNALKTAESISPT
ILFIDELDKSFAGSTGSSDSDGGTSSRIFGSFLTWMQEKKSPVFVMATANRVERLPGEFL
RKGRFDEIFFVDLPTPEERQDIFRIHLTKRREEIARFDLEQLAKMSDGFSGAEIEQAIIA
AMYEAFAQDREFTQLDIIAALKSTLPLSRTMQEQVTALRDWARQRARPAASSVAEYQRLE
F
>1170769.6.peg.2124
MILKSPGQLKFTVITFTLAMLGLLGGCLGLTVSFDSQPSDSMSQAPEKSPFLENHTATPS
PQLQRVEPKITSNPTLEKSKFGNLRISNKTYQPIRLALLLRHSPSSSSGKKGLIPAHWDF
APQEGSQGGLILSLPEGSLKLEKGDILVAFAQDGSRRYWGPYIVGETSGPLWDPKTGEWQ
LTLVP
>1170769.6.peg.2127
MILVILSLYPALEKFLHSLFTHYYNLINTLISLAPDSFISVFTSNLFVKNISGLELPNTY
LLSYIKEKRYLGLDQKEGYTLVLACENSHTNQQFQDMTDGERQEILKKLKLDYGKILLNY
FSVDENLKTKIDQFISTLFCANIPVPQVIEIHMELIDEFSKQLKLEGRSDETLLDYRLTL
IDVLANLCEVYRCSTSKIN
>1170769.6.peg.2130
MKGQQLFHSFLPGVTAAVLTTQSAWAGTFKANDLKLVSSPVVSTATNPKVSVVENNWHLV
ANTVDHALGFDYQLDFGQSILPELPSSSSRTNVPSPTKFKTVLSLPPVNPKVRSGKTYNQ
VTQITLPTDKSSETEPVKPNSQSVNPTSDSPQMILERLKPNPNLLDVPQDSQGVKVQTTE
AISLEQALDLAKQNNNDLQVAVLQLQRSQSSLKEAQAALLPSLNLVGGVTRSRSSSATLR
ARQENAPENPEATSVFDSQAELRYDLYTSGRRTAAIKEAEEQVRLQQFEVEKQSEEIRLN
VATEYYSLQQSDESVRIARSAVENSQASLRDAQALERAGVGTKFDVLRSQVNLANSQQEL
TDALSQQAIARRRLALRLNLPQSVSITASDPVQLAGLWKSSLEDSIVLAYQNRAELQQKL
AERNIREQQRKQALATLGPQISFIARYDLLDRFNDGVAINDGYSVGLRASMNLYDGGASQ
ARAARAKTEIAIAEAEFAERRNQVRFQVEEAYSSHLANLENVQTAATALDQAKESLRLAR
LRFQAGVGTQTDVINAQSELTRSEANRVRAILNYNLALTRLQRYVTSRAVQKS
>1170769.6.peg.2145
MRNYHPCPKNPLTIHTSNGQIKNSSLCDFCWNEYCFCWSNYCIDEWLELQNKYMQQRLQQ
KTSNIKT
>1170769.6.peg.2150
MFKTLAISKQQLYNVQVVVVEQAPPAKYTNSQNGQNHRVAVT
>1170769.6.peg.2152

MARDYYEILGVSRDADKEQIKQAYRRQARKYHPDVNKEPGAEEKFKEINRAYEVLSEAET
RERYNRFGEAGVSGAAGFQDMGDAGGFADIFESIFSGFAGGMGGPTQQQRRRGGPVRGDD
LRLDLKLEFREAVFGGEKEIRIAHQETCEVCSGTGAKPGTRPRTCSTCSGSGQVRRVTRT
PFGSFTQVSTCPTCNGTGSVIEDKCESCDGKGMKQVTKKLKVTIPPGVDNGTRLRISQEG
DSGQRGGPAGDLYVYLFVNDDEEFQRDGINILSELKISYLQAILGCRIDVNTVDGPVELT
IPPGTQPNTVIKLENRGVPRLGNAVSRGDHLLTVLIDIPTKVTLEERELLEKLAKIKGDR
TGKGGLEGFLGNLFKA
>1170769.6.peg.2155
MPPSTIPSILLVDGYNIIGSWPCLKKTRDDSSLEAARYHLVELITNYSAFEGYESHIVFD
AHYQNTPSNREMITDFLTVHYTEFGQTADTYIEKVCAGLRHQVAQCLVSRVIVATSDRAQ
QLVVQGYGAEWLSARQLCNQVEAKVCQMRNQHQSRKKSKGRFLSHSINDEARQKLIRLRM
GL
>1170769.6.peg.2158
MPKRINNAGPLYLLYDHDLRIGLLTGCKSVNWQLGRSSLVGNLLPGNPCYIIAKSLIGKF
IKFFVETPLQN
>1170769.6.peg.2170
MKGRVGLLRLGMPWAKLSGDTAIEEIRRPVETNISAFTGVY
>1170769.6.peg.2174
MLIWNEPDEQISYGQLSAMIDLALLIFIFAGAENGQKI
>1170769.6.peg.2178
MLLIGEKTQKNAIALDLPSCDRPLITFNISPEKNEGNGDYRSSRFLKTLMKDFN
>1170769.6.peg.2180
MQFGITQVSIAQVATAQVGTVQVSTAQVGMRQVGTAQVSIA
>1170769.6.peg.2182
METYPNFTLPPTPIHRSHSLGTLIEWKRARASGLGAYQEIPKGVPTRWGH
>1170769.6.peg.2183
MYSSHSLGTLIEWKQEIRELNADLKELIGSHSLGTLIEWKHIPKITI
>1170769.6.peg.2184
MSAAVAPVKIVSIQLMSPASGNPKPNLQSRAWGCFHSINVPSEWEPKK
>1170769.6.peg.2186
MICSKISKIIRDRTSPVLLKGKTEGGKQAIAWRLFPNPTSAILLKGNFERGEKAIAWQLI
QCDRL
>1170769.6.peg.2195
MEINHRISRTNRYKLTMIYSGISTITDNLLNVGFLLSLNLSKKGK
>1170769.6.peg.2198
MIKQNFNYTSVALIALSILGLAGGFYVTQQMLQAQERAAELEREKKEAEEKKEIAEKERL
IAQEKAMEAENLRQTAEQQRLEIQQRQAQEERRRLAAESRQARLERQRLENFSSQHDISR
QDALHLVQKWYAAKPQIFAPPFNTGLVDQLATGKLHTFTTRSNGPVEWLRQNDAYYEYNY
SEIKRVLDFSTSGRYPYIKIRVSEELYLHGKNGIDKNNSGASTNNLIYFFEKENGIWKIY
DYRKVR
>1170769.6.peg.2201
MVKKGDRIASKVRDRPFISKNQLKASSFFSFRHKFGGVCNQI
>1170769.6.peg.2206
MRLIGSIIVVYLHENCCNMISLVSRSASLRMLLFLAPWLFI
>1170769.6.peg.2214
MELSNYAEQEKLLKEQAHQRAEQERLLKEQANQRAEQERLLREQERFLKEQAHQRAEQER
LLKEQAHQRAEQERLLKEQANQRAEQERLLKEQEQLKYQTLLSQLKAKGIDITTLE
>1170769.6.peg.2220
MIENYIQQLIRKQRPYYLIQGTPIKGVNNQYWVVFKHRDSHNLLHKVITFLGSGKKQATH
KLFRIFSAKYLLSCAESIYNEVVQLKQQLSGEIEQPETLENITITSEKISKIQRRFSKMD
SLPNCLNKASVNSTLKKLNSWKETKLVQKLSKDYTDGDKAKLDDEQFAIQLVEDTDKLNL
VLEEGIKSTSLEISLAALLLRA

>1170769.6.peg.2235
MFTYHINGVGKIKKKLKKLGESKNQPGIAPWIVRTEQGA
>1170769.6.peg.2238
MFATLVNWAWFLVTKCMKPIAIVACSVGKPECPPGLLSIKAVYCL
>1170769.6.peg.2263
MFTSAATGLAVGIAFIKGLTGRKLGNFYIDLIRAITRILLPISIIGAIALVALGVPETLE
GTLVVKTLEGRTQYITRGPVASFEMIKMIGQNGGGFFAANSAHPFENPNSVSNLIETIAM
IIIPASLIHSYGVFANNLKQSWLLFWMVFLIFVVFIWVTVAGEMQGNYLANQIIGIEIPN
LEGKEMRFGVGETALWAVITATTMTGAVNGMLDSFMPQGIFCNLSSLFLQMVWGGQGTGT
ANLFIYLILTVFITGLMVGKTPEFLGRKIEQREIFLASVILLIHPIIVLVPSAIALAYPN
SLSGISNPGFHGISQVVYEYASASANNGSGLEGLQDNTLWWNLSTSLTILLGRYVPIIAM
LLLANSMSSKSTVPQTRSTLRTDSVMFITITACLTMILTLLTFFPVLVLGPVAEGLNLVS
GN
>1170769.6.peg.2267
MNQPIQLSLEQQFNVYSFASQVKEMSREQAQEFLVKLYEQMVVREATYKELLKHQWGLDL
GSMA
>1170769.6.peg.2284
MNSYASTTTFDPPTASSTPIILENLPDPPLVNGVCPRRTATDIDLILLAIESIELGGSEA
ILTFAQELELNQVIRNRVNLWRMRAANPLRKAHTRRPLTTMEAKCLVIITSYMAKRLTVV
IRQLLMIYQQLSEKQIPLSHNLRLANYLERFRTHFKSRMNPRRSVLLTPNNSDEKLDQLA
IDLLAKLLFCTGTAGMQRFWISLFDGEVE
>1170769.6.peg.2297
MVFTYRSLNSGISVMTEFDVLITGGSGFIGSAIARYLVSVGKSVVCMDAVDQGRLDDIDQ
SQVNRIHKISGNVLDAEFVDQWISRCGRVIHLAAVVGVDEYVTRPHDVLDVNILGTRNVL
MACLQHNRPVLIASSSETYGLNNGILEEDSDRIYGTSRNHRWSYAISKTAGEHYAYALGR
LGLTVTSVRYFNVYGPQLDAPGQGRVISKFLGRIRDSLPLMLVDGGHAVRTLCYIDDAAE
ATARLALELSPDCGYNHSAVNIGRPEPTTMRELADIMIRLSGHKAGTQEISGKEFFGEGF
EEIPVRVPDVSKLERVINFKARIDLEEGLRRTLDYWGLLNPETNQDTSLSSSPAAVVPMV
RPHFAPNGVLLQTLHRSLATGQVTNGGLHLRSFEEELAEYLGVPDVVVLSNGADALTLGL
QVLGRKGKAILPSYTFIATLNSVESAGLEPIFCDIDPETFTMSPTALAQILDQERDVAAV
IPVNVFGVTPDLPAIADLCRQGGVEIIYDNCHGFGTETHGRRVPQEARLQMFSFHATKVL
PAVEGGALVGADEELLDLVRKLRNHGIDSHNPSASGLGMNAKMDELRAATGRHVLRQFPE
QLEQRRYYAQQLRTFFQESCHGALIPQRIPDGVVSNFQNLGVLIPSASQFGLKAAITALH
DRGVECRSYFNPALHTLTRARSYARYPLPVTDQVWNSLLCFPIHSQMDPQDIDQVQLAAR
SVVDALVMQQV
>1170769.6.peg.2299
MTIEPLPITPWSSMQWYNPPAVWQNRGDRLWVKTGLKTDFWRTTHYGFIRDSGHCYYREV
LGNFRARVCFSGQYQDLYDQAGLMVRVSKTHWLKCGIEYVDGVQYASTVVTRGSSDWAVA
PLAGQPDRLWFEVRRRNEAIAVLYSVDGEKFVLLRLTDFPEDPMVWVGPMCASPEREGFE
VTFESFTVEPSTAPVLNHV
>1170769.6.peg.2300
MTTETPDQSPDLDRSPLAFLWLILGLIALSSTAIFIKLSIQEISAEATVFNRLWIATLAF
TGLNWIRPVNTQSSETEPDQSGEVDKSRKGPLGLYWSWEIIGLLLTLGFVHLIGRYLWTW
SLTSTTAANGAMLANMPPLFTALGGWLFLGQRFDRRFLLGLAIAVVGAITLALGDWIQPK
EVLFGTGALLGDGAALLSSVFYAASFLLVEKLRRNLSTSTILVWRCALGLMLATPLVWLI
DDTIFPISTLGWVAVFGLGLISELTGHGLIVYSLKYFSSAFVTIVLLLEPAPVAVVAWLW
FGEFLDPLNIAGFCLITVGIYLAKTGSGSTQDSPNRNILKLEPSDISSDIPSES
>1170769.6.peg.2308
MIQFDTCHNFLLENLFLLVNMGAFEICSTDTPIFMGENLKNQGFELC
>1170769.6.peg.2316
MNIQAQAPTANDYFNSGLDKQNQGDNSGAIADYTEAISIDPLHTFSYYNRGLARYDLGDK
QGAIEDYNQAIDVDPEYVNAYINRGLARSDLGDKQGAIEDYNQAIKISPNNDLAYYNRGL

ANYELGNKQEAIDDYKESIRINPNSVVAYYNMGLARYDLGDKQGAIEDYDKAIDVDPGYV
NAYINRGLARYDLGDKQGAIKDYNQAIELNPSNFLAYYDRGLARYDLGNIIGAIEDYNQS
IKFKSDYPNSYINRGLARYDLGDKQGAIRDYNQAIELNPNDVLPHYNRGIARHDLGDKQG
AIEDYNQAIELNPNLAQAHYNRGVIRSEIGDTYGALRDLKNAAQIYREQGKTQDYQETLD
RISRLEKL
>1170769.6.peg.2322
MLDLTKLAGQMQGLSQHLCSEVAESNRKLELAKENLKKACQCQDEIVKRQEKWRDRITFA
NATPLEPLDTCIQISTPPKVHTIIATDGSQIAPNHHEIAYCYLLNIGRVVLYYGQNRHPL
LDSLPEIFYREEDLYIARQWGLKTEEWMSHCRTASEITVLADLACSVKNDHPSLAMVDGS
LIYWFLEQLPLEARDQILPPILEAWGKLRQAGIPIVGYLSAARNNEAKNLLRLLNCPYPV
PDCINYCPDQLDYVPCKKFEGLRDTTLWATQLPQGQRSALWRSNSRILQLYDDQVIYFCY
LHVGTEIARIEFPAWVVNDPQMIDQALGLVLAQIQKGYGYPVAIAEAHNQAVVRSGDRNQ
FFALLEREMIKAGVKNVSISYKEARKRGSIA
>1170769.6.peg.2324
MNKIESDQKQATAKKLLVITEFFPPDYAATEQLIEELVRQLNQEKLKVEVFTGQPGYVYS
VDKAPSREYLEGVHIISTERGLQHKRK
>1170769.6.peg.2326
MIKSKLAAKVTLCQVLSVGVDLGAALATSMGHTKSGRADKTLCDRSLREWIKKKVINPLF
SRVLYISLILSLML
>1170769.6.peg.2332
MTQNLLKIQQRMSYILNLEKNCGCRENLLISTRLLPKSSINWY
>1170769.6.peg.2336
MLTERINRLSQHLQANKKDHSSRRGLLKMIGERKRLLNYIQKNNSDKYQSLISRLGIRG
>1170769.6.peg.2343
MGNLGYGIDPEVVKGIAQELAEVVATGVQVAIVVGGGNIFRGVKAASAGMDRATADYIGM
IATVMNAMTLQDSLERIGVQTRVQTAIAMQEVAEPYIRRRAIRHLEKGRVVIFGAGSGNP
FFTTDTTAALRAAEIEANVIFKATKVDGIYNADPKVHSNAKRYKTLTYGHVLAEDLRVMD
STAIALCKENNIPILVFDLTVRGNIRRAVMGESIGTLVGGSCEIS
>1170769.6.peg.2348
MSGLLAGCFGYPRILNYPLDSGGRGLNSLASEFNPHIGGRYIVFVTDRRGSQDVYLFDTI
TRSLVDLPELNTFDSMADHPAVSQDGRYIVFAASRQGKSGIFLYDRETRQSRNLTTNLAA
QFRNPTISADGNRIAFEFTNSGQWDILVYDQFGQKLNVP
>1170769.6.peg.2362
MSKKSAEIPYVTTVEIIEDFKPFLPHLQTSVREYSQYINDKDLVWSYTGIANYYNGQGLY
AIAETYYQDCLTVNRIRLGDDHPHVASSLNNLAVLYESQGRYTEAEPLYLQALDLRKRLL
GDDHPDVANSLNNLAVLYAAQGRYKEAEPLYLQALDLRKRLLGDDHPHLATSLNNLALLY
QSQGRYTEAEPLYLQALDLTKRLLGDDHPHVATSLNNLALLYQSQGRYTEAEPLYLQALD
LTKRLLGDDHPHVASSLNNLGGFYQYQGRYTEAEPLYLQALDLRKRLLGDDHPDVATSLN
SLAVLYESQGRYTEAEHLFIQALDLRKRLLGDDHPDVATFLNNLGELYAAQGRYTEAEPL
YLQVLDLTKRFLGDDHPHVAISLNKIGGLYESQGRYTEAEPLYLQALDLRKRLLGDDHPH
VAISLNKIGGLYESQGRYTEAEPLYLQALDLRKRLLGDDHPDVAISLNSLGGLYESQGRY
TEAEPLYLQALDLTKRLLGDDHLHVATFLNNLGGLYESQRRYTEAEPLYLQALDLTKRLL
GDDHPHVATFLNNLGELYRSQGRYTEAEPLYLQALDLRKRLLGDNHPHVASSLNNLALLY
AAQGRYKEAEPLFIQALDLTKRLLGDNHPNTQTVYHNYLSMLSQYPEA
>1170769.6.peg.2363
MFNLKAPFLRGLGGILKMLNRTGHFWEKRYHSTGFLTTETMMAFTVLC
>1170769.6.peg.2379
MTYTLEQFKKDFVINHLRDIPTEEVLKQYSPEEIKQYSPKDLLKQYSPEEVLKQYSPKEV
LKQYSLQDLLEGLSPETLERLAIILSQLGVNQIKNQEQ
>1170769.6.peg.2380
MLLNVSLLSDVNGKPLHFIWQIQDISEQHEVEQLKNEFVSVVSHELRTPLTAIKGALEIL
ATGILVDEPREAEQMLKIAVSNSERLLRLVNNILNLEILDSGKMRLNLELCDINIMVKKA

MEIVQPIAQQKNMEISYSTLDVHNSKILADEETIIQVLTNLLSNSVKFSHAGTKVEIYTK
MGDKEIIIAVKDYGKGIPADKLKTIFERFEQVDVSDARQNGGTGLGLAICKNIVEKHQGK
IWIESTLDSGSTFYFTLPQ
>1170769.6.peg.2382
MYEPQSQPGRFFAWAIPQSLGGGWNPQNRCQAIASRLELYRPDGLQELQIARQNNENIIC
VTTEVVSTCRIVFTVPRTRDPYSVRSSIFSSLTAADQGQQTVGVNTYISSPRRSGNNPHS
RRGINLKPFLSMEDGGTGTNLNNGLLIRSRTPGKTILNPRLFR
>1170769.6.peg.2393
MYGNISCYFFFRQIRQFIPTWSLICIIHEAKSWQICQ
>1170769.6.peg.2394
MSEFVLEQQNEAVENHPQELIRRLVWKICIATLILMAIGSATRVMNAGLACPDWPLCYGE
LVPTKQMNLQVFLEWFHRLDAALIGLSAIALTGLCWWHRRVLPLWLPGAATFALLLIVFQ
GILGGLTVTELLRFDIVTAHLATALLFFTTLLVIGTFLIPYKGTGAVGKLPWVSLTAAVL
VYLQSLLGALVGSRWALHQCLAGEELCGVMYSHIFGLLPPTVATLSMVFISWRTPALHPV
LRKLANLAGGLLISQLLLGLATFRLHLQVEPLTITHQAVGATLLGTLVVFTVLALRDRSI
SNPSYLNN
>1170769.6.peg.2395
MIETNVSRHHDSFLQVIQSYYQLTKPRIIPLLLITTSGSMWIAAKGQVDPWLLLVTLLGG
TLAAASAQTINCIYDRDIDYEMERTRHRPMPSGKVQFRDALIFAIALAIASFTLLTVFAN
LLAALLAFSGIVFYVLVYTHWLKRHSTQNIVIGGAAGAIPALVGWAAVTDTLSWAAWLLF
AIVFLWTPPHFWALAMMIRDDYAKVGIPMLPVVAGNQATVRQIWFYTVITVTSTVLLFYP
LHASGIVYVVIAMTLGGIFLHKSWRLLQNPEDKTIAKELFLYSISYMMLLCLGMVIDSLP
FTHYLVNTILHSFHLVG
>1170769.6.peg.2397
MEDPNILLTIGLPNSANFANTISRTNGDENFDQLVARSGAAVVVNGTFAYTNPQKTVMGN
LVAGGRSLKYSPWENFGTTLGLGVGNKPEMITARVEGRPEWNKHWFSITSGPRLLRNGEV
SVNPRLEGFKDPAVLGTSLRTAIGFSEDGKKLFLANFDEKLYLEEEAEAMKAIGCYEAMN
LDGGPSRALASDNVILVPPARKLTNVILVYDGKNPPPEELKLSWERFQTRWRPDR
>1170769.6.peg.2416
MIISESSASPRTVPTLTENKQTTVNSDENQIIYLPGTNCVIKSNISLSGFTSYKVGGEAQ
WYSAPRDLMAIRATVKYAQDLDLPITILGAGSNLLVSDEGIPGMVIATRHFRHKHFDNQT
GRLTVAAGEPIPSLAWEAASLGWEGLEWSVGIPGTVGGAVVMNAGAHNKCMGEMLVSAEL
LSPDGTLETVNRSQLGYTYRSSLLQRSKRIVTQATFQLQPGADPAKVTARTKEHKQHRLS
TQPYNFPSCGSVFRNPLPQTAGWLIEQSGLKGYQIGRAQVAQLHANFIVNRGGARANDIF
RLIRHIQHQIQDQWSILLEPEVKMIGQFPVLDGYS
>1170769.6.peg.2418
MYFFWDLRFHSYKQSPPLRYDRGQRYQFIGINTEGKTLWLELQSMVSGALAETLPVAG
>1170769.6.peg.2419
MLGRLQNADISADDNSITVNGKTIKCVSDRNPENLPWKEWQIDLIIEATGVFISKEGALK
HVNAGAKKVLITAPGKNEDGTFVMGVNHHDYNHDEHHIISNASCTTNCLAPIAKVLNEKF
GIIKGTMTTTHSYTGDQRILDASHRDLRRARAAGINIVPTSTGAAKAVALVIPALKGKLN
GVALRVPTPNVSMVDFVVQVEKPTITEEVNQALKEASENSLKGILGYSELPLVSSDYQGT
NESSIVDSNLTMVMGNDMVKVMAWYDNEWGYSQRVLDLAELVAQKWV
>1170769.6.peg.2431
MPAVAVDWTHPLSFSNAELGRHNFSGQNLQAAEFSNANLEMANFANADLRGAVFSASVMT
QANLHGADLTNAMLDQVKLTGADLSDAIFLEAILLRSIFTEANIDGADFTEAILDRGQVG
ELCKSARGVNSQTHVQTRDSLGCR
>1170769.6.peg.2438
MKLELHLKTFNFDGYWGVHTVQTTPSSQIRKFWEIDHKVE
>1170769.6.peg.2450
MGFYVWISKYKEILVEYLYRYYKDDTVGWEMIIFGSKCM
>1170769.6.peg.2451

MTLEQAEKILLSLKGQGICEILILTGEVHPLSSRRQEWLELIYDLCLLALSMGFLPHTNA
GILSFSEMQKLKSVNASMGLMLEQLTPKLLHTVHRHAPSKIPELRLQHLQWAGELQIPFT
TGLLLGIGENEDDRRQTLMAIADLHLKYQNIQEVILQPHSPGSQQTFDEVAFDPYQLPGV
IAQAREILPSDITIQIPPNLVPEENWLLTCLDAGARDLGGIGPKDEVNPDYPHREVNRLR
EVLLSGGWTLLPRLPVYQRTHSVLGTMVFS
>1170769.6.peg.2467
MCIRHGAFEADPKTEELVHLQSGRHWRKWGNDWYRQHTHPDGIRFEIHEALDKLYTQGYR
ATRVIIARRYQELMSGYLERSSSWRSEQTETGNARLYGLPVEFGPDPLDEPCWDVINFDL
DKEPGAPVRYPYFRLFE
>1170769.6.peg.2470
MEGGIEGWGEASPFSVGSYRETTDVIFQSLQEMSAVLESYSPWQRDDISNILTQHQIPSA
AKTAIDMALHDWMGKNVGLPLWQLWGLNINTIVPTSVTIGINSPQGAATRAREWLDYMDV
QLLKVKLGAKEGIDADKKMILAVKDAAPKVDLFVDANGGWSLADAIAMSHWLADLGVKYL
EQPLPKGEEEKLASLKKQSPLPIFVDESCFTSTDIPPLADYVDGINIKLMKSGGLREAWR
MLNTAKAHNLQVMFGCYSDSSLANTAASHIAPLADYLDLDSHLNLINDPFVGASVTEGRI
IPNSLPGLGVQYSALTT
>1170769.6.peg.2498
MVDFSLQDLTFPLVSEIEANKNRWLFSKDNLPIGFIDL
>1170769.6.peg.2507
MYAYTNKYTILKNISFKLKTGDRVALIGATGSGKSTLLENLIGLKYPQSGTITINGIPVE
PNTVAKIRKQIGFVFQDANDQLFMPTVLEDITFGPLNYGVAPAVAKENARQLLADFGLEK
YAHRSHHELSGGQRRLAAIASVLALEPEILILDEPTTGLDPAWRRHLAQVLLKLPVQVLL
IASHDLNWLGKVTQRALVLTDGKIQIDHPIQPLLADGKTLDNLGLPLGW
>1170769.6.peg.2523
MNITQSTFINKISIGWTSLLIFTLISVPTSAQTIDRIRDILEKTTVQINSNANPGGSGVI
IKKEGNTYTVLTANHVVCENLGVETIRCRTDFTYSIRTYDGREYPIKKRQILQTKLQDPD
LAIVTFESQQNYKIASLTPPSPSISTPQTSGIPKPTPSFNSATGP
>1170769.6.peg.2525
MQRVYEVLATGDREALAMRDRPFYHSPKNPWGEECFFCGSV
>1170769.6.peg.2531
MVTAQEREYFRELVRQDQKLQSKRTTTNHAFRRFKLSLKQQK
>1170769.6.peg.2533
MVSGKDGLFSQQQKMTKTAMIEKTTFKNSPDEKHQYFLT
>1170769.6.peg.2539
MRKYKEIDDYNQDIKINPNDPEYYYNRGNTRRELGDKQGAIDDYTQVIKLNPNSAYAYIL
RGNVRSALGDKQGATDDYIQTIKLDHNFAYAYIPQGNARTASGDNQDAIDDYIQVITLVN
PDPKNRIIFIEQVLNCKYHGTEFLLEVLKKDDSWEVKDAAYSLLSSKDSELAKSSGYIHT
TNIPQIFLKLDSLLKAQDFRAADEETQKVMLAVANREREGWLRIEDAEKFPCKKLRSIDQ
LWLKYSGGKFGISVQQQIYQSLGGTKQYNQDVWSSMGDCVGWRQRGKWLSYNDLSFSQTA
PSGHLPRFVGLVGVGVISLLSRYAECNT
>1170769.6.peg.2547
MNAVRSHSLGTLIEWKPIVLGSKPKALFCSHSLGTLIEWKLLVLGYTSH
>1170769.6.peg.2548
METKMPKYAGQLLALSGSHSLGTLIEWKLGVQVIAVVTDTDLFPLAGDIN
>1170769.6.peg.2553
MKVKSKASKTQQGKGCTMSNCTSGGMELELPLFAIAELVPQNNEMTLIGKLPTCNPSIQE
QRSSRMSGQDLTLKGKVCSPYWTDFCAEINSSLLLPVGIDSQDSDLISYNTWSSRMVDKS
WFSIKLYTAHNQNLPRIYSQFFTSFPTEYRDSGNTLKKSKKIYLKMTSAKKQKIKYWFGV
SRFVYNETIGYLQNMVEP
>1170769.6.peg.2587
MLILVEVGKICSLPLYCKKHTWQKWQSFFDLTIAFFKILVYS
>1170769.6.peg.2589

MLLLVGCQSRDKIKDSIVQARVVRVVSGQTVEVVKIGEPTSLVSSVRLIGLEAPDLRQYP
WGEDARKLLEKLIEDANSDNTSNNNTNSAQIKLEFDLQTQDKFGRNLAYVWKDQVLVNEQ
IIKQGYALFAGRSPNHKYDLRLENAQHWARLMGEGIWNPENPLRLAPGQFRRING
>1170769.6.peg.2598
MFPPNHTKKTLQGKYYNNYHYHAIGNGKSRKREWRRSNILMGAMDTTTIKWEINQGLLT
>1170769.6.peg.2633
MASSLLISCEQVCKTWGFQDIYLHVLEKNDQARKLYLKLGYEIYRVESSWQDFFFPSRQF
FLHKRLR
>1170769.6.peg.2643
MYQPMHEDKFLTHDSNPGYTGKTVNLDLRVLNFLNTTLVLGIRLRKKYASSLAIVGTSWQ
VDN
>1170769.6.peg.2655
MFLAKCNYAQKYREEPRKTIHEVGLVFSSSKRNLIQADWE
>1170769.6.peg.2656
MGEVGFGVTTDGGVSSINGDVRELVDPAKKADFGKFGHSGHEDELQVGILLLEDGV
>1170769.6.peg.2664
MELFLGTWQGAKEGRTGYWLRWWEETGNLLPWALELIEQERQRAEQEHQRAEQERQLAEQ
ERQEKEREHQRAEQERQLAEREHQLAEQERQEKERLIAYLRSQGIDPNNLPNHTE
>1170769.6.peg.2665
MTGDELVIYYPDGSRFLSPVELSNYAEQERFLKEQANQRAEQERLLKEQERFLKEQANER
AEQERFLKEQANQRAEQERLLKEQERFLKEQEQLKYQTLLAQLKANGIDVTGLE
>1170769.6.peg.2666
MLLDLNKMLPASAYLGRGFLKTLLLVESALNRLSQFFKSINFK
>1170769.6.peg.2668
MRTLTWDEEGTAITLGYWGPGDITGHVLSKVTPYQIQCLTSVEATIIPPDLWHEDLKALL
SHIQQTEQILHILHCKPTSLRLWHFLLWLGNKFGRDLERGKLIDLNLTHQDISEVLNTTR
VTITRLLQKFETQGKISRHKRSIILRLTQDLQQD
>1170769.6.peg.2673
MWKLAKFGCTNSQKLGTPVLLLIQGFLDYISITIFPIWK
>1170769.6.peg.2674
MQLVKTHPIYKTNKYWRGCDLICFKAKNLYNLCTYYIRQSFFKTGKILSSRKLYGLVFTS
RAYQEMAVTHRGFSVIRQVLQRWKGYIRSWQDWQANPSKYVDAPKIPNYKHKIKGRFPIV
VYNVYQGCPTMDQPSLVQGMCQLFESLFEGDVNDSIGKVRKLVEAMIIPRNNGSYLLKFT
YEVQEPTTRSTQVIAGIDLGVNNLVALTTNSSTIRPLLVNGKPLKSLNRLFNRKRDLIGS
YQSESQPSRRLGGITSKYHNRVDNYLHQTSSIVINYLKSNGIKTLVVGKNDEWEKETRMG
ISKRHNFMPIPYSRLIGMLEHKCRLAQIELITVNEAYTSKCSAWDFEPIAKHQKYLGERV
HRGLFRSANGQVINADLNSSLNIIRMYSSEALTAERIGSCGVQPLKVNPLARVKQI
>1170769.6.peg.2680
MFYHRPVNLHVHINLQLTKVEGQPDLTHNQDSSINNVPKTVKLGTKLEWFGQIKQSFYPS
VHRCTGENENTKINSLSYC
>1170769.6.peg.2684
MRDRAGVKQLSTALTLARFRFEDINAKPVAKPKRKMEGQGKFMSSPKIADYILTIIYIY
>1170769.6.peg.2685
MPATILQMNPLINNGQVIPGTQLQIPPFDGMVVQVPNGQGWQQIAKKYGVRPDTLFELNG
CQQNPRVVFVPSSSKIKPIYGSAPSITTIMGSPVSNSTGVSFPYGWQIHPITNQVFFHSG
IDLLAEVGTPVRATASGVVVFAKEQGSYGNLVIINHQGGMQTRYAQLESIKVKLGQQVKV
NQVLGTVGATGEPSSREPHLHFEVRAREDLGWTAKDPVEYLK
>1170769.6.peg.2691
MLIIVGWGYAAVAADPPKLEDLAKQSADLKVGIDTMWVMFAGMLVFFMNAGFGMLETGLC
RQKNAVNVLAKNLIVFALSTVAFWAIGFGLMFSDGNPFIGLGGLFLQGADNSPAMGDAYK
GIFSSLNWTGVPLGAKFFFQLVFAGTAATIVSGAVAERVKFLSFLVFSLLLVGLAYPITG
HWIWGGGWLYKLGFWDFAGCTVVHSVGGWSALVGAFLLGPRIGRYNPDGSANAMPGHNMS

IATLGCLILWLGWFGFNPGSTMSVSDGTAIAHIALTTNTAGAFGGIAATIAAWAFLGKPD
LSMIINGILAGLVGVTASCAYISVPSSAIVGAIAGVLVVKAVPFFDKLKIDDPVGAVSVH
LCCGVWGTLAVGLFSQGNVYYQGGPTGLFFGGGIGQLWTQFVGVLTVGLFTVLISGIFFL
ALKYTMGIRVEESEELEGLDVGEHGMEAYPGFVKEASSPDLLGFGNR
>1170769.6.peg.2708
MALASIREWIITQLNRWFNFAERFLYTSNTEFEKTRTARESQNAFYASIMSILPFLVFGA
LSYWSLEISLGSSWGISTGILTTVGASIYELGRINGKNSD
>1170769.6.peg.2716
MASILEKIAQKYGLTTWNEASYNAYLSCPYFWIFLNQ
>1170769.6.peg.2717
MFADYGEVKRVVLPTDRETGKMRGFAFVEMIEDAQEDAAISELDGAEWMGRQLRVNKAKP
KEENRPRKGHSKG
>1170769.6.peg.2720
MLHLDPYLYLYPKINILGKGKHILFWTGLCKMSIFLGILFSALGKTREN
>1170769.6.peg.2728
MSTKIIALAISTLAMSGCGLGNNSLGEGQKPPAIAQFTDPVVPAKINSVVNPVIESNKNQ
VIPSASQNRSVRSLVKARRDPFAQIIRVPQTIPTLPSVVKSNNTSRLNSLKKPLAIRQAD
LEIRGKNPIKPSPPKPPDTKLAQSILVSGVILVNQQAQAIIKLPDDSNSRYVHAGETLTN
GILVKRIEVNQCDNPVVVLEQFGVEVRRVISGQEDGIC
>1170769.6.peg.2740
MAENVDRGWDEELNTIIDDNYFVFNAKLAQKIKFSVA
>1170769.6.peg.2762
MIKEVQWLENQLGIQQVTALEHDGKMQQLMSVETQPVSNPLPGLGTEVKAEHVNSQRTVI
KSMGEIPYQLFEKLGLSMPGWLLWVLTFAISITLSGLLMSAVALWTPLWSNLEQAEDDGY
TPTNRENLKVSDGLWNKLSLYQLSKPMNILVMGVETIKGTLDGSPESFAGSSDTMLLVRL
NPSDKSIRVLSIPKGTMVSLPEDGLSKISEANAKGGPVLAARVISRTFSDAPIDRYIRIS
TSGLRELVDQLGGVDIFVPQSMTSQDQSGRTPINLVTGWQTLNGEQAELFARFRESSVGD
MARVQRQQALIGGLVQRLNNPVVLPRLPQLTRMMRKYFDTNLRMEEMMALANFAVNVERD
KFEMTMLPGTFSKFSKDPESYWLNLTGQQSLLKNYVGVDIYQVRSDSRSVFQLKIAIQNA
SSQPQVTAKVINHLKSKGFANIYTVPDWAENQRQTQIFVRRGTRHPGVELRKILGVGQIE
VSAQGDLDADLTIRIGEDWK
>1170769.6.peg.2764
MTIGNGKKIDLEVAKTPEQLMMGLMYRPALPDNRGMLFVFSSPQPVGFWMKNVPVSLDMV
FINRGVVKYIKTAPPCENEPCPTYGPRVLIDQVVELRAERARELGLKIGDRVKIEVFKSL
R
>1170769.6.peg.2767
MELVIFLHLPEQTLFVASPGQHHYRWWEFLSKEALSFSSDISQCFCDVVVNVPKLT
>1170769.6.peg.2768
MHWMSPRANAGLNIFEASKEPDAPPAPTMVCSSSMNKITSGDFSNSFITAFIRSSNWPRY
LVPATSEARSSVTTRLPNNTRDTFFCTILRAKPSAIAVLPTPGSPMRTGLFFLRRLSTWA
TRSISFSRPTIGSNLPNSAIFVRSRPKLSNTGVRDFSALLGSFPCCEPPLNILRSSSSSV
SGPAWGLVPDD
>1170769.6.peg.2769
MQDSPSSKNLSILSGSPCTAQYATVVYAWFFAGIIHGAIA
>1170769.6.peg.2771
MELPIGTTIGQIFAITFLPIAIGMSIRQFVPKLSTKLEKVTSISATILLAVIILLLIIKE
WSRLPNFIVQVGIGVLLLNTLSMGAGFYLSKLFNLNYKQQICISIEVGMQNGTLAIAITA
GLLNNPDMAIPGAIYSLLMYLTGCMVIIYGRNLSASRV
>1170769.6.peg.2772
MGIVNTVIKLLKAVSVTLKATSPIAKNVTKFEETPPGHKAKIIKPVAIAGCMGKSFAIAN
PMRGSMSNWLINPIATAFGYLPTR
>1170769.6.peg.2773

MLQGQFLKTLINGIQYLYIPDITYRLRSNDAWYGFIPCARRLSASG
>1170769.6.peg.2776
MFLKTSSQFVGLFILSYLDGLVFECDRIFLSGEPSHLCS
>1170769.6.peg.2784
MGFFIGFQPYIWKIGKGINYGMYYLIAIVLIAIVIRLN
>1170769.6.peg.2785
MFWFFPWSLVLTVIYQYCCQNSKTVLINTFAKKEYEKVGTM
>1170769.6.peg.2786
MKLPNFCIRTIIALATLGINVQPGFSQTEKGANRPNQVTFFCQQLYDPTSGEKVPMTVVW
VPEREAHVRLIAWKSQAFGKNWTPQKRCDVVSSKFQKFYQSGQLNYLTVGRIKTRSQKGN
ITEKQVICAVQEAQGICDDQNQLFTIKDEEKNNIEKADDVLNSLVDLLYQGGNAGPIWQ
>1170769.6.peg.2787
MKNDQGHGSVFLISQDRLVITNISNAYVNKLNFFNYCGFFT
>1170769.6.peg.2788
MGVSSVNTGTESSNKQDVMVKSGFISGSFLLSGFIERVIRT
>1170769.6.peg.2789
MKPDFTITSCLLLLSVPVLTLLTPTPSFPKSTTPRSCQLQSENKEGYSPEQLKTIAERIT
VRIRGNNTAASGTLIAKQGNSYFLVLTNQHVTRRIAPGNIKIQTVDGKIHQGRVLDKFNL
LNQQEFAKYDLAILEFTTSENYCLPTRVFNNQVPDQIEVLASGYSVQTGRITFAEGQIKK
IVTEPALARGYEIGYDSRVEQGMSGGPIISSKGELLGINGKSAFPILNGGYIYADGTKPT
STQIQELRKLSWGIPITSILAQMKPEFLTAYELPVPNIAPKVPVQTLTLPQWIGKIEEKS
KQFTVRIDDSKGENGSGVIIAKQENTTASNWLETGNQLWRLGRYPEAITAFDNAIKQKPE
FIHLAYYGKGLALSRSGKVMEAVIALEEAVKAKSDSVLAWTILSLANTKLGRSDQALLAI
NQAIKLQPNNPNLYNEKLVVLSNLKRYQEAIDAIDQAIKLSPHADFYYNRGAVRSRLGDN
QGAIADLQKAADLLQKQGLSAE
>1170769.6.peg.2796
MDQLIWLRKNLFNNWYNGVLTIVCVIVLFSLGKGILFWIFNQAKWEVVTANIHLFLVGRF
PQALYWRIWLALAINSIIGLITWYGLMQKCHLPENQLLQKAYQLIVPLLAPIWFLTFIIT
IWLLGGGLGLQSVPTNLWNGLLLTLLMAFVSIVLSFPLGVLLALGRNSELPVIRWFSILY
IELVRGLPLIGILFIAQVMLPLFLPDNWRLDRLVRGVAGLVLFSAAYMAENVRGGLQSIP
RGQFEAARALGLNTFLLLILVILPQALRAVIPTIVGQFIGLFKDTSLLSLVGLVELTGIA
RSILAQPQFLGRYMEVYIFIGVIYWVFCYSMSLVARQLEI
>1170769.6.peg.2804
MILVHLKQFATFTNDNLVLYKPLYLHLLDTMALNLNL
>1170769.6.peg.2824
MDLPEPLGPEMTVHPGSKGTEVVPPKDLKLVSSTRLICTII
>1170769.6.peg.2855
MAIRFNLSDNLANLPNLTSFSWNNNKDIWINNHIFGLYLLKLKNSHFCLELK
>1170769.6.peg.2862
MDVPDMGRRQFMNLLTFGTVTGVAAGVLYPVVNYFIPPASGGAGGGSVAKDELGNDVSVS
KFLSSHNVGDRTLVQGLKGDPTYIVVESKEAIADYGINAICTHLGCVVPWNVAENKFKCP
CHGSQYDATGKVVRGPAPKSLALAHANVNDDTIVLTPWTETDFRTNENPWWA
>1170769.6.peg.2869
MNQICCLNPTYECDNPQVPDNNSYCPTCSIPLVILKNRYQPVKRLGGGGFAKTYLALDTH
KLNEPCVIKQLAPSLGNQTTQALIKATELFLQEAQQLQKLAEHTQIPSLFAYFEENRQLY
LVEQFVDGKNLLEELQTEGVFNEAKIREFLQDLLLILQEVHKQGIIHRDIKPENIMRRHK
DGKLVLIDFGASKELQGGATSGTRIGTDGYAPWEQRVDGVASTAGDLYSLGVTCFYLLTS
KNPYELWLKDGYNWVANWRNYLNQPLSQKLQQILDKLLVASSDNRYGLAEKVLEELRQPY
SIIPSNSPKTIISHTEKPQRFSYILALISVFILGIGYLVITKSPQFQPRTEPNVIPETDR
GL
>1170769.6.peg.2872
MGTWNNEPDYQWDEVSIQLMSPASGNEGRRYVSVARVDPNLGVQSFHSINVPSEWEQSLR

C
>1170769.6.peg.2873
MNGNSKILIFLTPKNCSHSLGTLIEWKPEGCGVAVALTDGSTGSHSLGTLIEWKLFVESF
SFIIVMFPLAGDIN
>1170769.6.peg.2877
MCLSSYTIYGDWRLTRIIPLRRIAQVRLGSTRHARSPPFAWEDKTPNAIALQSVWEIVLF
YHLQIFP
>1170769.6.peg.2878
MVSIQLMSPASGNIEVQPATIFNGSFHSINVPSEWELTLV
>1170769.6.peg.2879
MNKTELLNQLLGFHSINVPSEWEQERGGLREDKKEEFPFN
>1170769.6.peg.2884
MQQVKKAPDAVAIIFENKQLTYGELNCKANQLAHYLQSIGVGPEVFVGLCVTRSIEIVIG
IMGILKAGGAYVPLDPAYPQERLAFMLEDASPKVVLTESQWLEALPVINATVLCLDAEWE
KIEQQSQDSPSCSVTSENLAYLIYTSGSTGKPKGVQMPHASIVNYLQGITNIIPVDNQDI
YLHIASFSFTASVRQLFLPLSQGAAIVIATREQTRDPLRLFELIETQEITICDGVPSVWR
YGLMALESLDKKYTRAIGESKLKYLIFGGELLPYQLIKKLRNLFQTPPQFFNILGQTESI
GNAFYPIPENCDTEQGYVPVGNPLQDMQQVYVLNSQLEPVKNGESGELHIAGGTLARGYL
NRAQANAEKFINNPFNPQQKLFKTGDIARHSQDGNLEILGRIDFQANIRGMRVELEEIEA
ILKLHPSVKEGAISLREDIPGDQRLVAYIVPNTQTLDLAEIRNFIEHKLPDYMIPNAFVL
IEKLPVLPNGKLDRNNLPAPNLSATIGNFVAPRNPQEELIANIWAEVLGLEKVGIYDNFL
ELGGHSLLASLVISRLREALSLELSISILFEAPTIASLSEKVTTFRDDFHPTNSLSVLQP
VSRTAESPLSLIQQRFWIVDQMEGANAAYNITRALRLVGSLNLMALQQAVQSIIQRHETL
RTSFGISEGKPVQFIAETLPFTLPLVDLQTLAEVAREAELQRLITTEYTEAFDLSQAPLL
RVKLIRLESNSHILLVTMHHIISDAWSVAIFFQELSSLYANSPLANLPVQYADYAYWQRQ
GLQNDVINTQISYWKQQLADAPPITELPTDYPRSTIETFHGSIHRFRLGNHLTNKLKILS
QKSGTSLFMTLQAAFVTFLYRYTGQEDIIIGSPITNRNRQALESLIGFFVNTLVLRTRLE
NNPTFKQLLSQVRQVSLDGYVHQELPFDILVEALQPKRQKNLSPLFQVMFVLQNSPREKF
NLPGLNVTQIELNRPTAGATFDLTLSMQEANLELIGAFEYNANLFDATTIARMVDTAVFM
>1170769.6.peg.2885
MRIYHNSNTLAKKGYQKAGMTYLTLQTNSSFISVPNK
>1170769.6.peg.2886
MAVVIIITPTSNSTGWVRYTVNDVITSAEWCTFKMQRQKYTQLARVDKDFAKISDNREFQ
ELLIYSD
>1170769.6.peg.2902
MSITQNYKLNIIQWYPGHIAKAEKNLKEQLKRVDVVLEVRDARIPLSTHHPQVKEWIVNK
SRVLVINRLDMILPQVKSIWSEWLKKQGEVPYFANAQQGQGITAIAKAAQIAGTELNERR
KQRGMLPRPVRAVVIGFPNVGKSALINRLLGKRVVESAARPGVTRQLRWVKISEHLELLD
APGIIPSRLEDQQAAVKLAICDDIGEASYDNQLIAAAFVDMVNQFQETSPHLLPPHPLLS
RYGVDSIIHTGEAYLEVLAASRYQGDVERTARTIISDFRKGLLGAIVLEVPKINNS
>1170769.6.peg.2903
MQLLESPPIKFIPSIYVEWQVDHLRLGIFRPQKDHESEFVVHGKNPLSLCIEVSNLENAI
SDLKSLGYLPTGEISITSHGQEIYAQDPDGNRLILYSSSNIDKQCP
>1170769.6.peg.2908
MFYTSIPLPHLPDLKFQRVALFAPAIGLLIGAILGLLDMLFDYLGISALTQSVLIVIIWI
GITGGLHLDGAMDTADGLAVTDPQRRLEVMTDSATGAFGVMAALAILLIKMAALTDISQN
RFFLLMVACGWGRWGQQLAIFQYPYLKSTGKGAFHKQAIRHQMDLFPSWVLLLGFTLLIW
AFNQGNFVLVIFTLIIGNIISFIVPAWFNHKLGGHTGDTYGAVVEWTEALFLCCMSSLT
>1170769.6.peg.2934
MKPTPKRSGKMTGSNPKVKTLRVPAHPKKYAHPSRKARLKPAANIILYALRLLIVGVGLG
AIVGTLLSVLDPANRITTNSINPPVTPSSSPQSPINSSGLVISREITPLKTTIENLSAAN
PNLIPGVFIVDIDSGAYVDVSGNKNFPAASTIKIPVLVAFLEDVDRGKIRLDEILTMEQE

MVAGGSGNLRTMPVGTKLKSIEVATKMMTISDNTATNILISKLGGKELLNARFRSWGLVN
TAIQSPLPDLEGTNTTSPKELASLIAKVNQGELISMRSRDLMLDIMRRTQRDDLLPAGLG
EGATAYHKTGDIGTMLADAGLIDVPTGKRYIASIMVKRPHNEPAAAKLINSISQATYSYL
SQSNFPPDGSTNNQPSSNQQLNNSSTPVPQLQPFTQPFLQPQGGNSNIRNATINNAPLGN
YQSPLNNPPYYPPQSHPN
>1170769.6.peg.2955
MITSGLQIYNANPVFGGRGGLHIPPIFTLGAWLAGGRHWHFAAMWLFSVNLFSYGIYILL
TRRWQHRFVGNNDIKALQKTDNIKRLTYSWHRIVYTAIIPILLLAICTGIGMYKPAQFPW
LVDIFGNWQGLRIVHFASVPLIIIFVVIHWQLGKRAGGDKLLESMFW
>1170769.6.peg.2967
MIVVFLTSFSSASESLGLTGKSWTWANYLESWERGRFLVVFANSTLVAIAVYK
>1170769.6.peg.2968
MFDLTIPDSDATTLFMTQKSLVFPSITMEIRVGGDRVLSLKFQSQYNIIE
>1170769.6.peg.2977
MLSFTPQPTILCFGWEILYPQPVPCSLLVYTQRKIPVIMGILVIFT
>1170769.6.peg.2984
MLRNIKRVRRRILGKIHGDCYSQSYISRDIWGIGSSEPNPIWASNS
>1170769.6.peg.2989
MALVEASWLRSVFSVSLVSKLHPSSLATASKRKWMSLSLS
>1170769.6.peg.2990
MTTAIQQRQSANLWDRFCEFITSTNNRLYIGWFGVLMIPTLLAATTCFIIAFIAAPPVDI
DGIREPVAGSLMYGNNIISGAVVPSSNAIGLHFYPIWEAASLDEWLYNGGPYQLVIFHFL
IGVACYLGREWELSFRLGMRPWICVAFSAPLAAATAVFLIYPIGQGSFSDGMPLGISGTF
NFMIVFQAEHNILMHPFHMLGVAGVFGGSLFSAMHGSLVTSSLVRETTETESQNYGYKFG
QEEETYNIVAAHGYFGRLIFQYASFNNSRSLHFFLAAWPVVGIWFTALGVSTMAFNLNGF
NFNQSIIDSQGRVIGTWADVINRANLGMEVMHERNAHNFPLDLAAGEVAPVALTAPAING
>1170769.6.peg.2992
MYHLEKGRYKQVKANKRKHYPIPELGVELGMLLERERPPIPWLRWWDNRGGCFMISAIMG
FTRDWGMME
>1170769.6.peg.2993
MGDDGITTWHPAFLALGTSLEECAIKYKGFCQKYRVQEKPVKKNRWG
>1170769.6.peg.2995
MVGHFPTVPETAFSGRSHSLGTLIEWKLAKSKLASINSV
>1170769.6.peg.2996
MSKKFEYKNIRFDFKGRGITQEINLLDIDGKRVKGWFANTEEVPTLPELLSAAGADGWDL
VSHSVNQDNQANGVTFHYLYFKRELV
>1170769.6.peg.2998
MTYTLEQFKKDFVINHLREIPTEEVLKQYSLEEVLKQYSPEEVLKQYSPEEVLKQYSPEE
IKQYSPKEVLKQYSPQEFLEGLSPETLEHLAIILSQLGVNQIKNQEQ
>1170769.6.peg.2999
MLGIGCFWSVLEEAHITILAVHPEYQGQGLGQALLYSLIKDAVDMGLERATLEVRVSNTP
AISLYKKFGWKTAGIRPRYYQDNQEDGLILWISQLQHPHFLQTLDKWHVLVQERLGQFSW
LLIQEEN
>1170769.6.peg.3001
MGNMTLPSSRFNWAEDIPRHLRKAWDFYQERFGQVPRGLWPSEQSVSPEILPYVIKQGFK
WICSDEAVLGWTTRHFFHRDGAGNVQEPELLYRPYRLQTPEGEVSIVFRDHRLSDLIGFT
YSSMQPRQAVANLVGHLEAISRQQKERSTEQPWLVTIALDGENCWEFYPEDGKPFLETLY
QTLSQEPNIQLVTVSEFLDKYPPTATINGDRLHSGSWVDGSFTTWIGDPVKNRAWDYLVQ
ARQTLARHPEATEENNPAAWEALYAAEGSDWFWWFGEGHSSNQDAIFDQLFREHLYGIYR
ALNEPIPAYLNSPLEVHEVKADRRPESFIHPVIDGKGDEQDWDKAGRVEVGGARGTMHQS
SLIQRLWYGVDHLNFYLRVDFKNGLTPGKELPPELNLLWYYPDRPMVNSSIPLAEVPDMA
PVNYLFHHHLEINLISQAVQFREAVENYQWLPRASRAQVALNTCLEVAVPWVDLQVPPDY

PLRLILVLADDGCFHSYLPENALIPIEVP
>1170769.6.peg.3007
MTTASAYSLNVPGHPTGQISKEELRSLLKEVESQLYHSHAYRAVVDKAQKLFDISHEQLT
NFNKLIQAISREAIALTFERFVTKQSATSTDSSKNDSADSTNSNEIKELSNQTVEVCEQV
LENRSQPNDPEPTAKKQQKSDRVKGENEPDPRKKLKSWPWKNQKPSKSRLAAKKAEEERL
ESLRQIGEQLKKARESQGLTLHKLTMYTYISPNQMAAVENGDMEKLPEDVLLRGFIRIMG
NALGLNGADLANSLPVRNECKSILPSWYKNQRPSRSLSIELSPTHLYLGYTALVAGAVSG
LSMMYEQANNQGIKNFQSTTPCLSSLCGSDEKATNVNDQSTMQHGRVGNSFGPQISPPEA
L
>1170769.6.peg.3008
MLLIFDENSNFIYFAYSEIYCYIRIFIHKFIIGTINTLGGI
>1170769.6.peg.3026
MSGELDEYGMVLNLSDVKQVIKSEVTSELDFSYLNEVWEEFQATLPTTENIARVIWNRLT
PHLPLVRVQLFQHPQLWTEYEGKGEKVSLTARSHFSAAHRLAPNLSAEKYGKCTRTHGHN
YHLEVTVEGEMDGRTGMIVDLGCLHETVEREILELFDHSCINEDIPYFSTSHIVPTTENI
ARYMSDLLQFPISELGVKLSRVKLFESDHLWVEYEGKDSEIFFSVATGFSAAHRLADPTL
SLEKNQTIYGKCSRINGHGHNYHLEVTVRGEIDSVTGMSVDLVGLNQIIQHYVIEPMDHS
FLNQDLPYFTEVVPTAENIAVYISDVVRSPIEELGAKLHKVKLIESPNNSCEIYAKDIEE
SRVDRVDRELAVV
>1170769.6.peg.3031
MLSSAIFVVETYNISPDARTELSILNTCILAIFTGEYLLRLWSAKQKSKYFFSVYSIIDL
MAIIPYFIGFVDIRFIRLLRWLRILRLLRFIDKKFLFFSISEDSVIFARILFTLFAIIFV
YSGLIYQVEHPINPERFSTFLDAFYFSVVTMTTVGFGDVTPVSEWGRLLTVFMILTGVAL
IPWQVGDLIKRFLKTSNQVENTCSSCGLAFHDGDALFCKRCGAKLPQVSPSPQ
>1170769.6.peg.3040
MEGGITPNQIMSGIVLLAIQNYELEGTMFSANCLHFLMKAIPVDTTATGVTEFILSLANE
SINIGMLLDAFAFACQKQGSRNIASLVSLTYQRLEADRVISQLINDQL
>1170769.6.peg.3041
MIVLISSLTLRAEIVVSQEKRLGKMGISESRGVWLTNIDSDVLFARERLKRSLKTLGKLN
FNTVYPTVWNWGYTLYPSQIASRVIGKSLDPTPGLKNRDILREITTQGHKQGLTVIPWFE
FGFMAPADSLLAQARPEWITTRRDGTKIVKEGIHDRVWLNPFHPEVQKFMENLIVEIVRN
YDIDGIQFDDHFGLPSELGYDSYTVGLYKQEHQGKAPSENFQDPEWVKWRADKITNLMKR
VFFAIKANKKNCLVSVAPNPQRFSYEYYLADWQKWEQMGLIEELVLQVYRDDLKVFISEL
EYPEVKAAQKHIPVSIGILTGLKNRSVPMEQIATQVKKTRDRNFAGVAFFFYETLWNMSE
ETVAKRQASFQKLFPRTVQRNT
>1170769.6.peg.3050
MNQICCLNPTYECDNPPIPDNNSYCPTCSRRLVRLKTRYQPVKRLGGGGFAKTYLALDTH
KLNELCVIKQLAPSLGNQTTQALIKATELFLQEAQQLQKLAEHTQIPSLFAYFEEDRQLY
LVEQFVDGKNLLEELQTEGVFNEAKIREFLQDLY
>1170769.6.peg.3054
MIPGEIDQKAEELLCVEGSSDFYRKIIFIMFLYFCHISI
>1170769.6.peg.3066
MGRSGVTPESGGWDFHPYPTESSTFIYVLLIVEVGEVITD
>1170769.6.peg.3069
MDVLQWYLTFRLLPLQKLLLILSKKGHSPHYIWKGGAIKAP
>1170769.6.peg.3077
MKKRVTLTFPKRAIQIPVTYRLARDFNVAANIIRAQVAPNQIGKLVVELSGDIDQLDAAI
DWMRSQHIAVSHNLGEIFIDDQLCVDCGLCTGVCPTEALSLNRETYKLTFTRSRCIVCEQ
CISSCPLQAISTNL
>1170769.6.peg.3081
MLSIPGGRQYCAHPFGYSENYDDVFAEPGNILIRYYVPTRMGIKMIFQGLFFQLNNPQNW
SLTFPKTISKE

>1170769.6.peg.3096
MNKPILLSTPHISVEELDFVQEAFDTNWIAPVGPHITLFEQEFCQTVGIAHAAALVSGTA
ALHIALRLVGVQPEDEVFCSTLTFIASAAPITYLGAKPVFIDSDRTSWNMDPALLTVEID
RRSKSGNLPKALVLVHLYGQSSDIDPILTCCNQYNIPIIEDAAEALGATYKGKTPGTFGK
VGIYSFNGNKIITTSGGGMLVSDDPEIVNQAKFLATQARDPAPHYQHTTIGYNYRLSNVL
AGIGRGQLRVLGDRVQARRRNFTIYQEALSDIPGIEFMPEASFGTCTRWLTCITVEAEKF
GCSREQLRLALAAKEIESRPVWKPMHLQPIFSQCDRIGGEVAEDIFDRGLCLPSGSNLTD
SDLNRVINVIRETYATTKHA
>1170769.6.peg.3097
MILDILHHQGRLIKAFVDDNPPEMLHSIHGTPIIHSQVLSQITVDSSLWIVGIGNNRNRK
TIVEKLKNQGHSFTSAIHPSAQIGLGVEIGVGTVVMANAVVNIDTVLGDHVIINTGATID
HDCNIGDYCHVAPGSNVSGHVKLGNSVLLGVGTHVSSCVEIGDNTTCGAGSVVIRSIDGN
CLAYGCPAKVVEVLPP
>1170769.6.peg.3098
MTQIFTENHTKNFLDWFVAILFLTLTSPLLLVITILVRIKLGTPVLFSQQRPGLKGRPFT
ILKFRTMIDKRDAHGNLLPDSARLTPFGRWLRSTSLDELPELLNVIRGEMSFVGPRPLLM
KYLNLYTPEQMRRHNMKPGITGWAQVNGRNALEWEEKFKLDLWYIDHWSLMLDLRILFIT
VVKVIKRQGISSSNHATMGEFTGANLKSTR
>1170769.6.peg.3099
MLIRIAVVASYAPSLVNFRGSLIVTLEKMGAKLLLIAPELTSKTIEPLELERAETIDLPM
NRNGVNPFEDLSTCYFLWKSLRKFSPDIVLTYTAKPVIYGTLASYFARVPKSYALVTGLG
STFTDNSDISKYTAFLTRYLYKIAMRCVTASIFQNPDDEETFREKKILPASVTSYVVNGS
GVDLKYFSPSPLPESASFLMMARLLVDKGVREYVAAARQIKLQHPQTRFLLAGPLDNNPT
SISADELQSWIDDSTVEYLGFLNDVRPAISRCSVYVLPSYREGTPRSVLEAMSMGRAIIT
TNTPGCRETVQHGVNGYLIPVRDVNALVSSMKELFDPALRCQLGLASRSIAEEKYDVHKV
NQQMLTAMGL
>1170769.6.peg.3102
MRNESSFTPLISIIIPALKADIELRRCIDSVRVACPDHRKCEVIVVLPVSEIDKASLLLP
EEYVITEGNPGIYSAMNNGVGASSGRYLYFLGKDDIVLPRFSKALDLIESNSPDSLFFDV
YWGVIGLVKGNPSRWRILRGNICHQGIIYSREVLLIHGPYLTKMHVRADHLLNIRLLWDR
VHSFQVEYINLPLVWYSGTGFSSVNTDNTFWHLYPLILQKYVGKWAACLLIAYRKLRILV
PLLPDR
>1170769.6.peg.3106
MLTLYENLGASSRLRFFQYVPWLERCGIQVTISSLLSNEYIRHLQDGGKNKLEVLKGYFS
RLKIIPSLEKYDILWIEKECYRWLPAWFERILVLNRIPYVLDYDDAVFHNYDQHPNFLIR
YLLANKHPQLMRSSALVIAGNYYLADFARKAGAKNIETIPTAIDLNRYQSYPSPSVKSQL
PCIGWIGQRSTASYLHPFAPLFKRLLAEGKSQFLAIGINTQQLGIPMESVPWSEETEVEQ
ISRINIGIMPLDNSPFIHGKCGYKLIQYMACGLPVVASPVGVNTKIIEHGVNGFLAGTLM
EWETALERLLSDPDLSFRMGQAGRQKVEREYCVQVTAPRLIESLRNVMYSRHDSDK
>1170769.6.peg.3108
MKLGIKLVKKYCGSYLCKQAIAFNLPLRKTIKHNLNPSPSAIARH
>1170769.6.peg.3112
MSPNNWKVRIANLKKHYTVREAIRKVFFYPPPYLPYMEHISNSLQSFFATTHRILNFRKE
KYYLDLPLAVYDLRFSPASYDLAYFLYEADCYFRGNGYEKFNLVIVPEIAESTPSGINKD
WKQVISEDSRRQRIFNMLLPMASMYESCSSVSLINDVELIVHICRSHFCVFPQNYDGVFI
RTHMSYLNVYDFERKKTYFSGFSSFSSDLDKVKSWISSQGINLPFITFTLRHYVHQPERN
SNIADYLKFANYLCEVGISSVFIPDTDDLSYISELSEYPVFFTGAFNLYQRQAIYELALT
NIFACSGCHSLCALNKRCSYIMSGIINENYNMENLYKPRGLKYGDQPFCDNRGVWEWGGE
TFDSLKNSFDLLLKLKSKQLYIQHFV
>1170769.6.peg.3115
MVNEFSSCPVLIVGSSGTIGRSLHRFFAQREVPVWLTTRNVDQANKQFDQRTLLLDLSQP
ASIWKLDDLSVCVAIICAAVTSQKACQEDYAATYAINVTATVELAKRLMDAGVFVVFLST

NLVFDGSIPHVPPDQPVNPKTAYGQQKAESEQMLLTMGCDSVAIVRLTKVVDQGFPLFHH
WVDSLGSGQSIHPFEDLYFAPVSLDFTTQLLFEISQRRINGIIQVSATKDISYADAAYYL
CQKLQLNINLIRPVSSRTAGISHSPRFTTLDTKRLESFGMFPPEPWQFIDGIFASIILQN
SNNPDTTDNN
>1170769.6.peg.3116
MQTKEPQYNLIFDVIEKHGITQLGLMINESWNQDPKRTLFTLARYKFVAKILADQNRVLE
IGCADAFGTRLVQQTVQHITAVDFDPVFVQDVQNRLDPNWPLDCFVHDLLECPVPGEFDA
VYSLDVLEHIPPEREAEFLKNALASLKETGVMILGMPSLESQIYASPQSKAGHINCKSGN
DFRVLMRQYFHQVFLFSMNDEVVHTGFYPMAHYLMALCCGKR
>1170769.6.peg.3117
MTRLVFLVPVWGRSYVQVMIEGLLPSLLTPRNFPLLCQHRRSTLKIISNSEGEMLARDIE
VIEKCREFLDVDFVVDDNTIDSNKYASMSRMYLHGLSTLNKENRLSKEDNFIVYLTPDMF
CSDGTIETLLECSKSSFKKVMVLGIRVIKDKFLSGIKNYSPGDSSNSLDFLIQLMLSTLH
PISEVLNVNAGTFNNQWPSHLYWIEKNILIAHGFHWHPLMQSHSEIWIKTLLEQYSTSTP
PTIDDHRFMRESDGKFSYFEHCCVNTDPKRIMLVELSDLPKQRQNNRKTLVSIPSILLWC
HSNVDEFQWYLFTHEVIFSLSSSILNEKVINESRVFTEKILAFNKKMPRFFIKLIIKFRH
LIGFVKLETKRLLRKITQFVT
>1170769.6.peg.3118
MENEYFESNCHICGGNLSHIASFSNFLQVTSDCRPWKTGGSLVLCQSCGTVQKPVTEIYL
KEAEIIYAQYEIYSQSGGVEQSAFDSITGAAKSRSTKLVEWLTTNWNLPSTGKLLDIGCG
NGAFLRAFGSTYPKWLMTGLELNDRNREVVEAISGVQSLHVGSIESLQDKFNVISLIHAL
EHIPDPSSFLHGLKEKLLPGGILLIEVPNLRTSPFDILIADHCTHFTSTILSKVVDRANF
SVIRLDQDYIPKELTVAIEVKNGSGFSLEPQSDDNQEYTQNTHTLLHQHINYLQSLLDLA
KSVPGNVGILGTSIAGTWLAESLHNKVKFFLDEDPSRIGRYHLKRPIISPDAVEDGYPVL
VPLPHAIAVKVAKRLSHLNCNFLTVR
>1170769.6.peg.3119
MSPESLLKLTSQYLRKERIIIVKGWFKDTVPNIPESKKFALLHIDGDLYESAIDVLDSLF
SRNMISKGACLFFDDWNCNAADPKFGERRAWQEMVEKYNVKFSDLGSYGIVSHRFIVHEY
AREY
>1170769.6.peg.3120
MLISKLNTLNKIYKSRGVTGLLDATLTRIFRRTYNFNRVAQEVTNHLSNPTSPEVYNRLS
EGVYYVFGMGVEGDIAEFGTMSGRTAVALAASLNYCNNTLSDSDLLHGFKEPRKLWLFDS
FEGLPQARTNVDQSSLHVASGIWGG
>1170769.6.peg.3122
MALVDFMSVLHKSTTRDYLARVNDPEYPKDKAAKLAKQWDYDYWDGDRRINYGGYRYIEG
RWEKVARAMVDHYDLPRRPRILDIGCGKGFLLFDFLKVLPDAEVYGLDVSNYAISHSKEE
IRDQLTVGSATQLPWTDNYFDLVISINTFHNLHNYDLDVALREMERVGKTHKYLCVESYR
TEEEKTNLLYWQVTCEAFCTPKEWEWWFQQTGYTGDYSFIYFE
>1170769.6.peg.3123
MNVLTTGVEDFLLGFGLINPQLERIRAGRNSRVWRVKCYDEVYILKEYFRHPSDPRDRLS
TEYNFLTFLGSQGITHVPQPLNRDPDRGIALYSCLPGVSINTIRQDYIQQAAKFIHEINQ
KRNVIVAQSLPQASEACFSLLEHIERVKFRMELLQAALVETTNQWQRKAAQLVYKRLCPT
YNKVESEIRSYYSFAQLSKKLNRDHWILSPSDFGFHNMLESRGELYFLDFEYAGWDDPVK
LICDFACQPQVPVSSLQSDMFREHLNLLLELDQEQVHLLLPLYRLKWCCILLNEFRSQDL
ERRQHAGDGHGDILEQQLQKAQNYFYQHLGDG
>1170769.6.peg.3124
MLLGLDFDNTLISYDTLFHLVAVERGLIPVDLPAQKNCVRNYLRQQNQEDEWTSMQGEVY
GNRITEAVSFPGVLPTLQKLTELGVPMCLVSHKTRTPYQGPSYDLHQAARNWLTQQGFFS
PVGLNWGTGQVFFELTKQEKVNRIVELGCSHYVDDLPEILEMLPKTVQPILFAPTGEVST
PTDWYQFQSWMDLPQLIA
>1170769.6.peg.3125
MRNAFAKAVTELADRNPNLVLLAGDIGNRMFDSFKEKYPTRFYNCGVAEAGMTGIAAGLA

ASGLQPITYTITPFNTLRCLEQIRDDICYPDLPVILVGTGSGLSYASLGATHHSMDDIAA
LRILPNMHVICPGDPVEVELAVGAALDLGRPTYMRIGKKGEPVVHHQPPVFRLGKGITLQ
DGTDIALVSVGNVLPLALECSQVLNDKGFSTGVVSLHTVKPLDDSLLADLFNRYRLIAVL
EEHGLAGGAGSAILEWGCTQKVDLRKLRCFAGPDRFLSACGNQDQARAAIGLDVATIVQK
LVQESMP
>1170769.6.peg.3126
MTTTPNFFTVSAQLRSQIVQMSHQAKAAHLASALSCVDILTVLYGQVLNLDPSNPRHADR
DRFILSKGHAAAALYATLAWKGIISPEQLPTYGKQHSLLEEHPSPKLPGVEAATGSLGHG
LPIANGMTLAARIQNRSYRVFVLMSDGECNEGSVWEAAMFASAQKLDNLTAIVDFNKWQA
TGRSQEVLQLDPLPDKWASFGWEVTELDGHDHGQLLAALTQRPTGRPHAIIAHTIKGKGI
SFMEDDNNWHYRVPTAQEVLDAKLELGVI
>1170769.6.peg.3127
MGKFKILRISQLAYPKALIVFESKHPEVQILPYEEQKRLFFSEKLVYSDSFSRAMRQLGH
EADEIVSDVDWIQKTWAKENSVNYSDSSWQEEILLSQISKIRPEILYFQHNPPLPYGVWK
NLKHTCPSIKKILVHRAFPGNFNTLGAADLLMVGTRRLVSQYADHDIKAKLLYHYFDEAV
PDLLDRPQIKYPLTFLGSSGFSYGISHATRYWLLRTVLEQTQAKMWLEEPLSISPNANPT
QQVKKLLSSIIKGFVNFLPRTSQKSLGNAAWLPLKARNLIQSEYEQIELRPTGLKVPEKR
LLDLFPQRCHSPVYGLDYYEIIANSLISFNCHTDAAVDQVGNMRMFQATGMGSCLLTDTG
DNMSDLFEEDKEVVTYSSKEEALEKIDYLLQNETKCREIASAGQNRTLRDHTALKRCEQI
DEWLQEIMMC
>1170769.6.peg.3128
MSFKPVPMVDLHAQYLSIKTEIDEAIASVIAESAFIRGKYVDNFESAFAQLLGVNHCVSC
ANGTDALYIAMKSLGVKPGDEVITTAHSWISTSETITQAGGKVVFCDTDSSTFTIDPSQI
TDKITARTVGIIPVHLYGQPADMDYIMNIAQKYGLWVIEDCAQSHLARYKGQLVGTIGQF
GTFSFYPGKNLGAMGDAGCLVTQDAKLADWCSLYARHGGKGNHQMEGVNSRMDGLQAAIL
LAKLPHLPNWTEKRQRVAELYNRKFNNFPFVMTPFVAPGRDHVYHLYVICVSNRDRVREY
LSMQGILTGINYPKLLPFYPAYGYLNHQPSDFPHAVSNQERILSLPMYPEMTENQVEYVV
SSIQKFFFQ
>1170769.6.peg.3129
MPVTICDDVVIGAGSVVTRNITSPGIYAGSPARLLRKIDNTIEKV
>1170769.6.peg.3130
MGLNSRTIANLYTSLNFNKWSDKYIVTKDASVDIYGFDQYNPFAKHRI
>1170769.6.peg.3131
MALSLEDADSMIKACDYYGINLFVVKQNRFNLPILKLREAVQSQRFGKMVLGTVRVRWCR
TQSYYDQDSWRGTWSMDGGVISNQASHHIDMLLWMMGDVDTVYALGATQLVNIEAEDTAV
VSLRFRNGALGVIEATTATRPQDQEGSISILGEEGMVEVGGFAMNVIKTWQFSHSTHEDQ
EVATKYSVNPPNVYGFGHQEYYAHVVRSILSEEPNMIDGLAGRKSLELIIAIYESMEKEQ
PIKLLLSPNHTRLGKSYP
>1170769.6.peg.3132
MCGISGWWSFNRPLGKEFNIVGLTSGLSHRGPDGKSIISLDNDMLQLGHTRLSILDLSDS
GKQPMSYAQGKFYITFNGEIYNFLELRQELVSLGHQFRSDSDTEVILASYVEWGEDCLFK
FNGMWAFALWDSCKQRLFLSRDRFGVKPLFYLFDGQNFIFASELKAFMALHKEVKPDLDP
EIISLFSNLESTSLTLLKGVKNLNASHSLILARYGQPHLKQWWRTSEHLIEVPSNYADQR
EQYRELFFDACKVRMRSDVPIGTALSGGMDSSSVICAMAEIQNSNGDLFHSRQASNWRKA
FVLDYTGSTHSERHYAQEVINYVQAEPNFRELGLEKIEPEDLNRSIFALEAIQEPGLGPW
LIYQQMRSQGIVVSLDGHGGDEQLAGYHFYCQAAFQDALWRWRGIGSFGDIQNVFNGLYD
VADLPEGMSSQLPPSQLKVLFDFFRQGLREKVFSKPTLYQLLRQIKYLLNNRAFSFSKIA
TARTLPVGNRQSASIATTFLKKPKIKGYDYLNQVLYDDFHGGTLPVILRNFDRLSMSHGV
EIRAPFLDYRLVTYAFSLPSSTKLGNGFTKRILRDAMHGFMPESIRTRKSKIGFASPMIK
WIEAPLKDFVLDQVHSQIFLDCPIWDGKQVRERVVQAYRNFQPENVYKDWKYIQAHTLMQ
AFYQAY
>1170769.6.peg.3139

MIIFTSGSTGKSKAAIHDFILMQEKFQIIRYAQRTLLFLLFDHIGGINTLLHVLANGGCA
VIPSDHSSETVASAIAQYGVQVLPTSPTFLTLLLLSGVYQRYDLSSLEIITYGTEVMPES
VLTSLNQVFPHIRFHQTYGLTELGIMRSKSQSSDSLWFKVGGEGYETRIVNGMLEIKAKS
AMLGYLNAPAPFTTYGWYMTGDVVEIDGEWLKILGRKSEIINVGGQKVYPGEVENVLQTM
PGVAEVSVKGEANPITGNIVTAKVRLKTDESLRDFRVRLRAFCQDKLESYKVPVRVTLVQ
ERLHSDRFKKSR
>1170769.6.peg.3153
MVFLGLLALSGVYSISYLRGQNQVKSSFNPPSVQVAKVVTNRPSSIKVARPVVKINKQKA
VKAVWNLRQVQRKAKEIKTLSQGAINVGVVVSSYPKISQPFYTVKVLENHPDSTTSPVYW
FRVSSSNGAIQPLDLVSNKYTTLANWNPDGI
>1170769.6.peg.3166
MINPPEIEEELLLIEEEEDLLLVDDGDIDDFLEPQTDEDDAKSGKAAKSRRRTQTKKKPF
TEDSIRLYLQEIGRIRLLRADEEIELARKIADLLELERVRDDLYEQLEREPEFREWAEAV
KLPLPTFRYRLHVGRRAKDKMVQSNLRLVVSIAKKYMNRGLSFQDLIQEGSLGLIRAAEK
FDHEKGYKFSTYATWWIRQAITRAIADQSRTIRLPVHLYETISRIKKTTKLLSQEMGRKP
TEEEIATRMEMTIEKLRFIAKSAQLPISLETPIGKEEDSRLGDFIESDGETPEDQVSKSL
LREDLERVLDSLGPRERDVLRLRYGLDDGRMKTLEEIGQIFNVTRERIRQIEAKALRKLR
HPNRNSVLKEYIR
>1170769.6.peg.3202
MAIDPVTVTMKLGQVPSKLEGLNLVKVAIQLDYDSLALEA
>1170769.6.peg.3206
MIDEWQKYKYSVNGKSILEPMKLPNTKVSFNANDLLFVLTNTRKYFQDYSQKDQNISRNG
LLVSQGVKVEDVLKTLDFMISTLREDVGKNRPPRLQDPNFINANFRVIKWKAYNPDKPEQ
KQLRITKYAVFTHPGSHTKTNTYNIPIYSLKDNSATDKFYTKYTKQDVLSGIYEPGGKEF
GKVETLAYLTRQGLEEALMQGTILINFTDGSKGFFNVDRNNGISYIRGVKDTLQKRYWYF
RPVDQIKGYGYKIDAKISIRPGVTFAGDVLNIGLGRVVLIEYNQGKNKRLQMGVVADTGG
AFLPNLHQLDYLAGVFKNQTEFRQYIRQLPEYASAYILVKK
>1170769.6.peg.3208
MGGCALIWKYIPKPRQVEFSANSLSHSRSDQVDTKGW
>1170769.6.peg.3225
MHGEGNCATVERLYRHLISRIPQLNQEYRKPIQNPYLKAEPPYKMYYILLPKSANSRDIL
PLQNEAMNAEIHNDLELAEKLWIRILAVDGTDNQALSGIKRIAIKTDKVDRGENPQIEKT
GESKKIDGQDKSAGNTSSETTQPAVERWAVVLDKILPQKIQQPRIQETKDIDKFNQPIVN
PPIGTDLERVKKEPRVVEAVPAYSPHKYITDKDKKELIKTPRRGFLKIAGLATGGLILVL
MTQQIFENQEATIPTDKLPQPEPTKTTQIDYTNLESLLKVRNFKQADEETYKLMLAVANR
ESEGYLITYQDAEKFPCKELRTIDQLWLKYSQGKFGISVQQQIYQSLGGTKESRLDVYDA
LGDRLGWRRKGQWLDYNNLNFSQTAPSGHLPAAAWDTGNFDGGLNFIAGGSDYNCF
>1170769.6.peg.3227
MLVELRDYLSQLFGLKVDIVTKNGLKPLIRERFLAEAIYI
>1170769.6.peg.3249
MQWLGRWFGVLEFLNCFPNPWQHRFRQFVAERGRKLLGIIQVSPFNRTRSTWHVDQVILD
PCADKLGIGSQLLRHCFESILEARMWILEVNINDVNALALYRQNGFQRLAETTYWEIKPE
LLQELAQAEPDLPNLLPVSNADAPLLYQLDTASMPPLVRQVFDRHTHDFKTSLFDIIGDA
IKQWVEQTEVVSAYVFEPQRKAAIGYFELQLDRKGTSPHVATLTVHPAYTWLYPELLSQL
ARISQDFPQQSLKLASSDYQPEREQYLENIGAQRIEHTLIMSRSVWHKIRESKFVSLEGI
QWTEVLQGLQPSRKPIPGGMSWVPKSPPVPEVTMGKSEMVALTIEPSEPPQENN
>1170769.6.peg.3262
MQFCISVVKDSVIIIIIIKTPDLFGGQFQKFILAKVWRSNWPLWQ
>1170769.6.peg.3288
MPSDNGELQVNLFTVNRPQEEAKYTVAYIDYPAQYIQLLRSKNLVEQAIEQGKSTALQRV
RGTIVSEEKKTLGDNVGIEVNYTTADAKVVKQRVFLVGNRFYQITAETTQKRQRFLTRSM
QGFCDSFKLLP

>1170769.6.peg.3292
MSVELVLPRDEINHHKSPHKSPHKSPSLPTIYLYEFRNR
>1170769.6.peg.3298
MRRLGELPGLTRKSARRAYAPGQHGQNRKKRSEYAIRLEEKQKLRLNYGLTEKQMLRYVR
KARRVTGSTGQVLLQLLEMRLDNTVFRLGMAPTISAARQLVSHGHVTINGRVVNIASYQC
RPGEEIGVKDKEASRKLVENNLQYPGLANLPNHLEFDKNKLTGKVNGVIEREWVALQVNE
LLVVEYYSRQA
>1170769.6.peg.3299
MDQIMIYLAFSAMRTSGHRHGAFLDAAATAAKCAIYMTYLEQGQNLRMTGHLHHLEPKRV
KIIVEEIREALTEGKLLKMLGSQEPRYLIQLPHVWMEKYSWQPGKSRIPSSNLTTEEKKQ
IERKLPANLPDAQLVTSFEFLELIEFLHKRSQEELPHHHQMPLSEALAEHIKRRLIYSGT
VTRIDSPWGMPFYVLTRQFYAPADDQERTYTMIEDTARYFRMMKNWAERKSHAMRAVEEL
DIAPEKIQAAMDELDEIIRVWADRYHQEGGKPVVLQMAFGEQDD
>1170769.6.peg.3300
MEVSQDQPIHFEAPLQLLLFVDGRPKSRQQVQRICAYLQDLEVDYSFDLQIIDVREEPYL
AEHFRLVATPALVKIHPKPQQTLAGSNIIAQLQNWWPRWQTVIDTSLALQKDLHELPEPD
ISMTHPPSTIHSVALSAERIKLSDQIFYLNQEKAKLQEQLQFKERIIAMLAHDLRNPLTA
AAIAIDTLQSDYNPDLGQFQRLKPNMTENLLKQARHQTRIIDRMIADLLEIGRENDNDFN
IAPQRLELGKLSFEVLEELRDRYVGKSQTVETDLPSDLPCVYADPERIRQVLINLLDNAI
KYTPKEGKISLAGLHRTTQKVQFSVGDTGPGIPHENRDHIFENHFRLERDQAADGYGIGL
SLCQRIIRAHYGQIWVDSTPNGGAWFHFTLPVYPS
>1170769.6.peg.3302
MIITPSALALTSIKLFDLSYQDCPSDLAQGAVVSGSSRFANCFIITGKAENGTYKTVYDA
DVYGRIYDANNDPILQNRSRLGSIAQVPPGISNFELRISVPANQPTPLKLKQFKASGFST
MIRK
>1170769.6.peg.3303
MLIVRVLLTWFPQINWYNQPFAALSQVSDPYLNLFRNIIPSLGGIDISPILAFLVLNIVS
SLLENLSRVTSLGGF
>1170769.6.peg.3315
MTGFGGPVAHIAMIEDEVVKRRQWLTQEHFLDLLGATNLIPGPNSTEMAIHIGYVYAGWL
GLIVSGISFILPAVLITGVFAFIYVSYGSVPEFSPLLHGIKPVVLAIIILNAVFGLGKKAL
KNKQLLIIAVLVALVNYFGKVNEVITLLLGGILGMIWLHNATHNNKPTNLLITALTLGTT
LPKVALTPTVSIWQLGLFFLKVGSVLFGGGYLLIAFLQGELVDQYHWLTQQQLLDAIAIG
QFTPGPILSTATFIGYIISGLPGAIVATLGIFLPSFLFVILLNPVIPWLRKSPWTRGFLD
AVNASAVALMIVTTLQIAVKTLDLAKFPLLDLFSVFMFLIAAVLITRFRINAPWLVLGGG
LISIGLGLLGYL
>1170769.6.peg.3327
MRARRDRRRHARHRRRDRQRRAARPARHCDGVPILRPLSAYECRAEHGLRAALGRYAARR
NRQARGRGRRNPAPHAVARPQAAPIVGRSASARGDRPRYRARAAHFPVRRAAVQSRRLAA
RRYARGNRGAQTASRHDDDLRDPRSDGSDDARRQDRGDERGPPRTSRPAARSLSQTGQPF
RRALHRLAAHESAAGRFVGRSRGRDRPAPGGFEAG
>1170769.6.peg.3328
MIHQWRVYSFPASKRVSARPRCCTASISKSATANSPCSSVRRVAANPRCCA
>1170769.6.peg.3329
MRDDLRCAVPSSCFLGEGPVWSAADKRLLFVDILAPSVILADPESGQFVSKPMPELIGAA
VPRARGGFLAATQNGFKTFDWSSDTLTPIAHPEAGKPGNRFNDGKCDRRGRFWAGTLAIT
TAPGEGALYRLDPDGSVATMGKGFHISNGLGWSPDDRRFYFTDSGARRIYVYDFDLDGGE
IANRRVFVQLPENAGIPDGLTVDAEGFVWSAHWDGWCVTRYDPDGTVDRVVTLPVRPTS
CCFGGPDLSTLYITTARIRMSQRQLAEAPLSGGVFALRAGVRGQADTPFAG
>1170769.6.peg.3330
MEYLECDPVVFCPHPDGKYFLAHKSLEKTCAEIARYNPKDAKKYAEFTQYWQRVINAMMP
IFNAPPKSIIDIFGNYNLEKFQDLFSVVGSTQKSLDFIRTMLTSAEDILNEWFDEEFLKA

PLSRLASELGAPPSQKNLAIGVMMMSMRHHPGMSRPRGGTGALIKALVNLVTAKQGKILT
DQLVEKVLIDNGEAVGVRVAGGREYRAKYGVISNIDAQRLFLQLVDPTEVDDADPQLRER
LARRIVNNNETILKIDLALDEPLRFPYHEHKDEYLVGSILIADSMHHVEQAHSKCTLGEI
PDSNPSMYVVVPSFLDPSLAPPGKHTVWIEFFAPYQIAGAEGRGLKGTGWTDELKNRVAD
KVVEKLATYAPNVKTATIARRVESPAELGERLGAYKGNYYHIDMTLDQMIFFRPLPELAN
YKTPINNLFLTGAGTHPGGSISGMPGRNCAKVFLQSKHPITQTLKDAGNSIKSTMGSVFG
IS
>1170769.6.peg.3332
MFIILRFSRFLKTLIKDFDSINVPSKWEPDLIIFCHL
>1170769.6.peg.3334
MGNSRHAIACFPPSVYPLRRTGEVRSRIILLILERIIISSYILILEIL
>1170769.6.peg.3336
MQRPQNRERKKEYYSGKKKRHTCKQITVSTRKKRVIILTKTRADKIHDKRPLQESDGEPI
IVSETPGANMSDLLTDFTIPSNIVTFELELKNMEVRKRLIYDLLEGRNKKFIVPVYQRDY
AWKISNCKKLWEDILSLEESDHTHFLGTLVTIYIQGDKSMIIDGQQRLITVSLLLLAISN
YLKSKTNKTSDEEALQEDILNDYLINKRCKYDAERIRLKPSKRDKTYFENLFDVPAHELP
IAKQDSNIIQNYSFFYEKISGSEFLAQNIFDLFKKLEIVHIELDSKIDKPQLIFESINST
GVELTDGDLIRNYILMDLESEEQEYLYSKYWIEIERLCGNIAKFTRVFLIYTLGKEISEK
QRAVYNEFKLYAERNLKRNNVKILEEMVKYAQIYSYLIVLSEHPNKKIRQHLNRIDYLEF
TVCHSFLMDVFDLFEHKTINDETVLKVLNLIESYAFRKKLVDSSTQSLNKFFFGLAKEIK
KEDNWQKNYFDIMAFTIKSKSGNLKFPTDDEFIQTLTYKDIYKLNKKNKYFLLENLENYN
SPYQTDVQELTIEHIMPQKLTQEWKSALGNNYKEIHDKYLHTLGNLVLTAKNSNLSNKDF
KDKQKIDFENSKLRLGYDLKDVKEWNEDSIVKRAKSLAEEAVEIWKYPKSNFTKPLEEER
NLIYLSDSCKVTNTTPRTLIVEENESKVKTWKEVLIKICRILFKQSPTDFKTIINNSKIG
KYFSNGEEDEKSKKLIRPLDFIPSYYVETNLSAEKIIGLCSQLCDELDFDKEKIGIQIEL
LENKG
>1170769.6.peg.3340
MCGGAITRSEGSPINFPASSQGKIMEWSTGLSPIVTAVRSHFP
>1170769.6.peg.3341
MILPLFFLNRTVLTGDRTSPRFHSPELRSPFNPLQIFP
>1170769.6.peg.3343
MLLDPLSIDALLPAITGESNKISPYRSGPDLISWFNPYGFQDEYLSGLPENKSRSKYALA
RVQEINNDDEKMRRFIESIVDPRRFIDSELDVAKTVEFVNSIIKMDGLELRESLRGYRLY
PLRDSSEPAGITPVFEEIQSQILDSIEAAEFCIWVAVAWFTDPVLYNALAKKRREGISVR
VILIDDEINRNRSNFKEFPVKWISPEGTHNNLMHCKFCVIDLKKVIHGSYNWTNKARFNN
EQITVIEDKICAEDFAKEFVKLAAD
>1170769.6.peg.3347
MYCNFELGSREQDGRSRVGSTVRSPKLINNTLAKKGYEKVGPNLQKA
>1170769.6.peg.3348
MTYTLEQFKKDFVINHLREIPTEEVLKQYSLEEVLKQYSSEEVLKQYSPKEVLKQYSPQE
FLEGLSPETLEHLAIILSQLGVNQIKNQEQ
>1170769.6.peg.3353
MDRALSLVAALHGDRRDERARAACGDARQLPSVVGSRALRRGGGHRAHLRASAPSVELVI
TLRSTLDRAQAPAHQSIPRPPVSATCDHSFEVRPVVSTVGERVTLFSRMGKAKCPTAPPT
RFPACTPKAGAAESSSHEKGRTATGTEAMPTAVAASPPYGVPSLAKEMDALLPPSEKKPV
PST
>1170769.6.peg.3354
MVAWIGRVVRTGLERLCGVGGGYVPQHEPCGHEKPDQRRRPRGARGARGARGARGARGAR
AEKARGPWRAGVVRTRGRQGVFGHESRLKEGAVAAMTTA
>1170769.6.peg.3355
MLRVRKGDTEEPAVVPLGQSRILDHAVSGSERVAVGEILEGHHRRDVDRAAGDESAEELG
TPERIRREQGENRRRRKRVVLEQHGAADQEAHHERGHRRGPRRTISGAPKLTFDQREGGE

HHRRFERVAREHEGVPEEVRTETERCGGAQTNTCIPHVRAQPSLEKHEEGEAGKLHGQET
QPPGGVTEPPGSRLGWICRRRATVSEELGALLEGQVSGHAEEAVGRSLHRDMLVGAVLEG
HLGRVVGPAEGGDLVVHREGAVIDQRHGHVRAHVLVRVVHRGEVREHEKQRAHHEGEEEP
RDRGRFPGGRPPPGANVAVHLEGHGLSTPRRVSPPLPTSPTRPTLLIFRDFLPRLGDASG
RGAHVSHDARENGLLPESGDVARGVRVYCSSMGILVDGKWHTDMVGATSKTGAFERKPVT
FRERIEPKSEANQAARFVAEAGRYHLYVSLACPWAHRTLVLRALKGLEEVVSVSVVDPLM
LDQGWVFSDGPGCVPDSLFGSSALHEIYTRAMPDYSGRVTVPVLWDKRHGTIVNNESSEI
VRMFNTAFDALATKELPDLYPEALRAEIDALNARIYDTLNNGVYRSGFATTQSAYETAVT
ELFATLDMLEARLEKGPYLFGERMTEADWRLFPTLVRFDAVYHGHFKCNLRKLVEYPNLW
AYTKRLYHVPGVAPTVSIDHIKRHYYGSHLHLNPKGIVPVGPALTFD
>1170769.6.peg.3356
MSTAVLTRVRDAEGVHGRGGEACIPTESEIEPSQEPRAHEADGPVEHVPNDDRGGRVRER
HGERDGRAHHTLRVVPEWQTATLVRVPQGELVVAPKAYFARVEHLLEVLAVATSRVRIEK
DRGRPRDGGEHEPGDGDHPQLAARGGGREQTLELLFGADDSAHGQTAPGGTAR
>1170769.6.peg.3357
MYAFGVSHACQYGGGHTPLAPFIFLPLCLYLWRKAETSYEHAVGLGLVYALTFYNGGVYP
LGFVSLVVACETLTRMWPVPRAGRVVRAGVVAGLVFVTVSAARLVPVVDQLAHHKRALEP
EIDFISWQTLKDMYLDRTHAWHPVGQTYVWPEYSTYTGILGVSLALLGILHLRRKEAWLF
AVAAVTFLLMLGHFASWAPWSFLKANVFPYKSMRVPSRFRLLESAFVAFFVGYAIDRAPA
TFAKILRKPTALRQARLAVSCLALIGIGDIFGTASSVVASKFDGPPETRVVASTRLYVDN
ESANFEDQPRQNRARLACWDEWNFTMGAPLWYGDVPQARAVDPGAVVEVANRTQNSFTLD
VDVKREGAEVLVNGAWDRGWRTSVGRIFERNKQIVLSLPQGRHRVRVHYWPVGLTAGLVT
TGLSLVAVALFFARGRLFGKPVAEEAAGASASTASTTPEDTSDESASNDEPSEATPREAS
SDAKVEAHEPSTETSSEPLEIPKPEPSKPRSPSTLADSPDAKKRES
>1170769.6.peg.3358
MRIVIAYVHAFRHHLRDQDTKAGLDEVLTKDELARVSLQENVPLAILALLGDRLASARRR
GWLHVMHAPVLEASLVSLTDVQGACERIKTTPIPYSYTVLMHRIVGGYCILLPFGIGEAM
GWVSVPVVVVVSYALFGLDAVGDELEQPFGTDTHDLALFAISRTIEINLLRRIGATDVPA
PAKPHQGVLA
>1170769.6.peg.3359
MEAPGHRRSERRDHGHAPPDVRQGPSPHDHVVKPGIFRFDPHRTAELNSLRHRRAARVSR
SERSLLDRSTPGSNPGNPGESKETREKEAM
>1170769.6.peg.3360
MVELEPAPLEAIDLEVGRGPGVARERDPGLVALHGAVGVVRARGEDRVLSSNRGARTARR
HVVGLELLAREQREALHVEGAERLAIDHVRSRGDARERLFGRLLRGSVGRLGRAPGERAG
REHRDGEEGEGTSESEHAPP
>1170769.6.peg.3361
MKKDPSFKPVPTVTKTLQCTGPRVDARLTLDKSENRRGTLKITRKSGATDDDPKSATIGV
SITLDEATRDWVHYEGTNGEQDFFFGIVKADLDRATGAAKVNLSWAEFGQEFRVNVDCSF
VR
>1170769.6.peg.3362
MLGGAEREVHVDLDLARIDALKLSPLAILQQLKAQNLNVPAGHFEEGTKEISVRTVGELK
TVEAIRDTIVATTKDGSSVRLSDVATVEDGHEELRTRIRTNGQPAVSFDVVKQSGKNTVA
IADAVKAKLATIEATFPKGYKTSVIVDQSKFIRENAHEVEIAIVFGGAMAILVILVFMLD
LRSTLISSVALPTSVVSTFFVMYALNFTLNMMTLLALSLAIGLLIDDAVVVRENISKHLE
RGVDPKTAALEGTKEISLSVLATTLTIVAVFVPVAFMSGIVGQFFRQFGLTITAAVLVSL
FVAFTLDPMLSSRFSKAHVKGAVDRFMIVKRPFLAVFEGIEALYRRVLGFALNHKILVGV
VAFLALFSIGPVAGLMGNEFVNQEDRGQFVLEAELAAGTKLDETSRLSLPAEQKVLEDKR
FLTILSTLGPSGEVNKVRWRVVTVPKSERDVPLSELKERARTIALAALPGAKVTVTDPAF
VEGAATEAPIMLQVRGASYEELAPLAREFEQAMKAIPGIADLQVKYSPGQPELRVGVDRD
KAARAQVPVAQIALALRAAVEGDEAGKMRQGKDEVPIKVRLRQGDRSTVDDVLRMTVQTP
QGPMALADLATVERGEGPSVIEREDRERQIVVWASTKGRSLGEVVPEMTAAFAKIKMPPG

ATYHFDGQIRQMNETNGSMGAAMILGIIFIYLILASQFESFIHPLTIMLTLPLGFVGAFY
ALFMAERTMAMGAMIGIILLMGLVTKNAILLLDRALVRVREHGETPLQAILEAGPERLRP
ILMTSAAMILGMLPTATSNGEGSEFRSPMAIAVIGGVISSTILSLVVVPVVYLTIENAKG
FLGRLFGITPKTPEPTAPPPAPAE
>1170769.6.peg.3363
MHLAFGAAEHVHRRYAVDLFEARFDDVTGEFRSLRERASSALEGVGDGRSRAHVDAGDDG
LFGLGGQLVAGLRHALAHLGGGLLDVDVELEEHDDLRGALAGVRLDAVDAVERDDRVFER
LRDQLLHLARRRARIRHGDGDDREGHVREEVGAEVLVAHDPEDHEGRRDHHGEDRALDRD
L
>1170769.6.peg.3364
MDLDSNRPYQGPVDAPPPATKAGGKAVIVVGAVIFLGFLGVMGSRVGKALDKRKATAVER
ENAVVELAKKLPVEVASPSAMRWKPRIELTGTLRPWREADIGFELSGRLSKLNVQTGDKV
KSGSLLAVLDASRAGAQVNQAVAQSKAAEANLALAEDNLKRTEALVLTKSIPEAQAEQAR
QQVALAKAQLDGARASTSLAQQGAGMHAIVAPFEGVITKAPTAIGAVVNPGVPLIRVEDV
SKFRLSASVGEDDAALVKVDSPVTITYQGRTVTGKVIAVIPSLDQATRRAPVEVEVPNDP
KSPLLGYGFVRAHIDGKNEVDALRVPAAARRPGSQNEVVRLVGGKAQRTRVSHTVDTDGS
WIVTDGLTAGDTLVLNPSDDVKDGDALEVAAPKADKGQAPAPAEKK
>1170768.4.peg.1
MKFYQQKITFLFFFTTDFENALLFKGGWGGSNAMKQPKFPLTLS
>1170768.4.peg.8
MDGPNGFPKTGPSGCEKLFFDPFGRMTFQFQHKLAQNDSSPAH
>1170768.4.peg.9
MFSLKAPLFKGGWGDQVLKLSKIHPFLRGLGGLNAVK
>1170768.4.peg.10
MLLNNPHFHPQVNVESDRTSASQSLSLMRLQTSQYKFTHRV
>1170768.4.peg.17
MVYIDDDDNIDADLEEGDLDYFWLNSLSDSYQRYQEELEDAEMEIAYEEEELG
>1170768.4.peg.18
METILKNINCCDILSCSHSLGTLIEWKLLVGCKPHPGSA
>1170768.4.peg.19
MEDVQRGSHSLGTLIEWKQLMLNIENSEVAVPTRWGH
>1170768.4.peg.20
MSPASGNGKADPGDSLSGIIIGFHSINVPSEWEREINHSSPIVIGGFHSINVPSEWEQLR
>1170768.4.peg.21
MWGKKRQIGVSIQLMSPASGNSSAALRKIGLTFSFHSINVPSEWEQVGWTWT
>1170768.4.peg.22
METPGENTFKIPTGVRSHSLGTLIEWKLVPCYLAINQELLNVPTRWGH
>1170768.4.peg.23
MIQTWYGSHSLGTLIEWKPEKFFNTSWDLTAIISVPTRWGH
>1170768.4.peg.24
METLMAVPPLSCFLNSSHSLGTLIEWKRYTYYSLLRL
>1170768.4.peg.30
MVGRNNTVTDYQCIHEWVEEQTQQTPDHIAVVFGSEQLTYQQLNQRANQLAHYLQA
>1170768.4.peg.31
MIGRGDQFLDLSINQHSKLSLKLSCRSLGEGRSFFVDGVLEDFGLLRSLLSAQSG
>1170768.4.peg.44
MPWIYELVRSYNLYGPKILGDLRRNNKGTARLLNNQQLQLLQQTLQLPPEDGGLWNGPKV
ANWMSKLLNRKVYPQRGWEYLQKLGYVVPSSKSHSCHPSKSYTNL
>1170768.4.peg.45
MAYPNIFSKSCSFYRILKTAGGDLGEIWWRNWGEYHSFLTIMSLGF
>1170768.4.peg.47
MLVFPQVSNTGGNVTSIHSLVAQGVKADTSLDRISKSRESPGP

>1170768.4.peg.53
MSISDVVILRTPAGTRVALSSVEVAFRLYPLGRAIALLLKYDSA
>1170768.4.peg.56
MPLQLRQVISYDLRIDKNRIIYVPDQELLLCGRIRNRID
>1170768.4.peg.63
MILFPIITISNTYWQAVDKNQPLKSLQIAVDSKPYLSRYQQP
>1170768.4.peg.65
MLKDTWDFIKDIAGFIKEQKNYLLIPLIITLVSLGALIVFAQSSAIAPFIYTLF
>1170768.4.peg.70
MSDFAKIVVEDLNVSGMLSNHKLAQSIADCGFYESMSIDRN
>1170768.4.peg.84
MSTSNSGRGLLLADSSKPSPNQPEEPAPHRGSGRKESIEFLGNSFLA
>1170768.4.peg.98
MGFTQTAKGSAPIKQASTKGDAVKVGEYQSPTREPELEGVTRIQPHSLQGRQAATLYIRN
TPVLTFLGSRGETKVGDRSSKVVKYGSVNTGKSPQITSGGGIRGAKESPRSILVNDPVYR
ASLVAAKINQLVLDSADGRQVTVSWNPKGKYSANNRPSGNESAPTSVSSGNYTIRFDGKE
LVEINESTRLADGTNNLAKDALQATNHLRKILGEAAEVKEIANLPKPGNISIPKLPQQVA
IGGIKINFRGVASWYGYDWSGRKTANGERFNPEAMTAAHRSLPMGTRVRVTNTHNGRSVV
VRINDRGPYIGSRVIDLSVGAARILGMVSSGVAAVRIEVLGR
>1170768.4.peg.117
MVGSDVFQFSVSTFPLVALMTLLNAKSMVGVAALTLPFFSLILVLL
>1170768.4.peg.118
MSQENKNKLNRARVLVMEQLQTFASRGDFWTVFAVPFGQWYDPNRGEALRSQWLSGEGVL
PTEIVSQQTLGNAVGAYGKGKIFLSEQFIANGTSSELVRVLLEEYGHHVDAQVNVTDSPG
DEGAIFAAVVQGEILEGAALAQLKAEDDSNWISVDGERELVEESGSVTRTPIAPALPGRT
RYEVGNYYAFAALKSNGSVVTWGIDWSGGDSSIATWNSTGNYSYVSVASQLTSGVTQIFS
TYYAFAALKSNGSVVTWGDSGSGGNSSSVASQLTSGVTQIFSNVYAFAALKSNGSVVTWG
NSSSGGDSSSVASQLTSGVTQIFSTDRAFAALKSNGSVVTWGNSSSGGDSSSVASQLTSG
VTQIFSTGSAFAALKSNGSVVTWGDSSYGGDSSSVASQLTSGVTQIFSTYFAFAALKSNG
SVVTWESTGGNSSSVASQLTSGVTQIFSTGNAFAALKSNGSVVTWGDSRYGGDSSSVASQ
LTSGVTQIFSNVYAFAALKSNGSVVTWGWGSSGGDSSIATWNSNTGNYSYVSVASQLTSG
VVSFADPFNDDRLVPLSTITLAVNPTSVTEDGGSNLVYTFTRAEDTANTLTVNYTVGGTA
TNGSDYNNIGTSVTFAAGSSTATVTVDPTADNRVEANETVSLTLVSGTGYTVTTTSAVTG
TITNDDLISLINQTLNLPGNQTATISQSQLSVTSTISPTNIRYTITDLPDYGKIMFLGAE
IGVGDSFTQSAINNNRISYQDGNEDNATDSITLTVTDGVSILENVIFNIVIIPTITLAVS
PNNVTEDGTSNFIYTFTRTGSTTNSATINYTIGGTATNGVDYNSVGTSVTFAAGSSTATV
IVDPTVDTTPEADETVDLTLATGTSYNIGTSGAVRGTITNDDLPSITLTVSPSNVTEDGM
SNLMYTFTHNGITTNTFTVNYTVGGTATNGSDYNNIGTSVTFAAGSSTATVTVDPTADNR
VEANETVSLTLTTGTGYITGTSGAVTGTITNDDFISLTNQTLNLLENQTATISQSQLSVT
STISPANIRYTITDLPDYGKILFLGAEIGVGDSFTQSGVNNNRVSYQHGNHENNTADSIT
LTVTDGVTILENVTFNISVTLVDDPPILSKNQILSVNQGQVVTITKEILAATDVDTPVSQ
LVFTINNIPQNGILKKGTLFLDDGDRFTQQDIDNGNISYTQTNRNNLRDSFSFTLSDSTN
NISGLVFEINSQSNGILGITSTNGVQTEGNSGTKAFTFTVTRTGNTSNSSSANWGVTGSG
TNQADASDFGGTLPAGTVNFAVSESSKTITVNVSGDTTVEPDEEFTVTLSSPTNATITTG
TAAGTITNDDLPSITLAVSPTSVTEDGTSNLVYTFTRTGYITNTLAVNYTIGGTATSGSD
YGTIGTSVTFAANSSTATVTVDPTSDTTVESDDTVSLTLASGTGYTIGTTNAVTGTITND
DLSDTQQITSTSTRLTAIPAANLTIPLFYNTSTGDNTLAGIGLRLHYDSTDLTYQQVSNL
FQTNLFGSVTDNSDTQNFDSDNSTNRYIQFQYFATNGNWPNQNLPLKMADFAFNTSANFQ
ETQLNITGIDVAPGYTLEAAPLQVYRQNWTLDVDGNGSFGALSDGIIIMRYLFGFAGDSL
TKDVIGPGATRSTSEIRSYLAEGVNSSILDIDGNGSVTALSDGIIAVRYLFGGTFSGNAL
INGAVAPNATRGLSAIESYLASIRGNQSSLSVATVARSSSVPQQFTTFNTASNTTPKQIV
DLVNNSSSVSAGNSVTISVNYSVDNGDSTLTGIGLRLHYDSSKLSYETVTNLLQTNSFGN

VTDNVDNEDLDGDTSTNRYIQFQYVDFEGNWPNQSLPVKLANFTFNTSANFQKSKINVTA
VDVASGYTMEARPLDLVVPGVSETNVLSGTGGADTIDGSNGKYTIMPGKGDDTIIVGSAG
SVIIELPNEGNDTIVSSVNYNLAALNQIENLRLTGAGDINGIGNRKDNVITGNSGQNRLT
GLEGNDTFVFNFGDSQVEKPDQITDFQIGKDKIKINTVVGVVSTLTRAGNGSGNLRSLVD
SVFLDANGAVSGNQSLGVNSAALVVSGGGTYMIVNDGTAGFNAGTDLVINITGYSGVLPG
LGSVVASNWFV
>1170768.4.peg.119
MSQENKNKLNRARVLVMEQLQTFASRGDFWTVFAVPFGQSYDPNRGEALRSQWLSGEGVL
PTEIVSQQTLGNAVGAYGKGKIFLSEQFIANGTSSELVRVLLEEYGHHVDAQVNVTDSLG
DEGAIFAAVVQGEILEGAALAQLRAEDDSNWISVDGERVLVEESGSVTRTPIAPASSGRT
RYEVGNGSAFAALKSDGSVVTWGFSDRGGDSSSVASQLTSGVTQIFSNSLAFAALKSNGS
VVTWGYSSSGGDSSSVASQLTSGVTQIFSTRFAFAALKSNGSVVTWGYSDWGGNSSSVAS
QLTSGVTQIFSTRFAFAALKSNGSVVTWGDSSCGGNSSIATWNSTGNYSYVSVASQLTSG
VTQIFSTDAAFAALKSNGSVVTWGDSRYGGDSSIATWKSNTGNYSYVSVASQLTSGVVSF
ADPFNDDRLVLSTSESSVTLAVSPSSVTEDGSSNLVYTFTRTGVISNALTVNYTLGGTAT
SGTDYGTIGTSVTFAANSSTATVTVDPTADTTVESDETVSLTLASGTGYTIDTTSAVTGT
ITNDDTQVTLAVSPSSVTEDGSSNLVYTFTRTGDTANTLTVNYTVGGTATNGSDYGTIGT
SVTFAANSSTATVTVDPTADTTVESDETVSLTLASSTSYTIGTTSAVTGTITNDDILLPV
ITLTVFPSSVTEDGPQNLFYTFIRSGDTANALTVNYTVGGTATFNSDYTQREAASFTNTS
GSITFAPGASTATFIIDPASDTVVEGNESVSISLTSGTGYTIGTISAVSGSILDNDVTPG
TVVRTPIAPALPDRTGYEVRNRVAFAALKSDGSVVTWGYSYWGGDSSSVASQLTSGVTQI
FSNDGAFAALKSNGSVVTWGIDWIGGQYCSVKEKKG
>1170768.4.peg.121
MTKLKIFVEDLDRLFVPENLPVTYTKNKNFWGILGLMVIIY
>1170768.4.peg.122
MEVSGTSKTVPSTAIILNSPKKAPGVNIVAKGFATSSNNLLMTAAPVLWRASVIDEADGG
>1170768.4.peg.124
MTEDGTSNLVYTFTRTGVISNTLTVNYTVGGTATNGSDYGTIGTSVIFAANSSTATVTVD
PTFDTTVESDETVSLTLASGTGYTIDTTSAVTGTITNDDTQVTLAVSPSSVTEDGTSNLV
YTFTRTGVISNALTVNYTVGGTATNSTDYGTIGTSVTFAANSSTATVTVDPTSDTTVESD
ETVSLTLASGTGYTIGTTSAVTGTITNDDTQVTLAVSPSSVTEDGSSNLVYTFTRTGDTA
NTLAVSYTVSGTATFNSDYTQNGATSFSTTSGTITFASGSSTVTLSIDPTADTRVENNEI
VSLTLPSGTGYTIGTTSAVTGTIANDDTSSITIADVKQNPSVFMDKIRDYDGNNLGGSSS
WKLIGDADAQGDGDLESIFVNPLIGRWATVGVVNNFVDFSNHGQGGDTRVVGIYIDPTLK
DRPENIGGPFDSQRRFQNDLRIDNLRLVAAADYNKDGFQDMYFKLVDGTAVLRAFMHKDG
NIQYANYQSKGDLATFMIENNVDSAIWINWL
>1170768.4.peg.125
MGQQGTGDKTERAIHLTIEELQLLVRPFLRELEEFYSGVKD
>1170768.4.peg.126
MTTINYDYADGKTREIAEGFELKLLQKEKINEQEISWEERQLVVHSYAIAQTEEKYLRER
IQKTVHDLEQLKIPKRGKKKLTSFPEWELSVAEILKRYRKRILVLLGFPPTIYTQLSGQS
FTPE
>1170768.4.peg.128
MMHADGNIQYANYQSKADLTAFMTANNVSSSIWGSWL
>1170768.4.peg.129
MINLNPVAGETAVLNFIKLFSEKEVGKAINNTHDLRCSNMMC
>1170768.4.peg.143
METMYTLMDARNNLSKVRFHLGSGINGNISEARLWRITLA
>1170768.4.peg.160
MDISQNKITAMIGPSGCGKSTFLKSLNRMSELEGEVKLEGTVEFFGQNIHGKRVNLNRLR
RQISIIYAIPNLFPMSIYDNVVYGVKLIGWRPKAELDEIVEIALKRANIWEELKNKLHKS
ALELSMGQQQRLCIARSLAVKPQVLLIDEFCLGLDPIAIRKMEELIECLRSELTIVFVSH

NIQQVLRLSDFTAIFQYNQNHIGKLADFGPSKRIIANSFHHRIRDSIGSYWR
>1170768.4.peg.162
MSPQNLLAILLAVEQKFGRVRSERWGARTLDLDLLMYDNLILNQSNLQIPHPRMCERGFV
LVPLEEIAGDWQDPISGMTIRNLLRNVDHSDVHLCSGSAVKIIIQRP
>1170768.4.peg.164
MSDSDKEKLLLPVGNDQKNENPTDEKPRLTIPPKLRLLVGYIQQLSLMVREKIGRSEKPF
YRHVWFWAGLGVGTTIIGFNYGIRTVDSSLPDKSELNAMIRQQTLTIKAGDNTVLMQQGE
ATREQLKIQQIPDKLQLAFIASEDRRFKQHDGVDFQGIVRAVISNFQSQNVVQGGSTITQ
QLARILFLSQERTLWRKLKEVRLAQKIEGELSKEEILERYLNLVYLGSGAYGVADASWVY
FGKTVDQLNLSEIATIAGLAPAPNIYAPDKNLPGAIARRNTVLQRMEEDGVITPQEKQSA
LQQTLTVKTNLPRRFQVTAPYFCAYVQQQLPKYLKPDVLAAGGLTVETSLNSNWQRLAEK
VLVRTLKNEGTWGRFREGALVSINPRNGEIQVMIGGKDFTKTQFNRVTQAQRQPGSTFKT
FVYAAAIASGKNPGDTYIDQPISFRGYEPKNYSEKFRGLMTMQDALALSINTISLQVLID
VGYEPTIKIAHNMGIKSPLEHNYSLALGSNEVNLLELTSAYGSFANQGLHTEAHTIRRIL
NRQGDVIWSANFQPKRALDADSSGIVTKMLTNVVTNGTGRAAQLPDRPVAGKTGTSDESR
DLWFIGYIPQMVTGIWLGNDNNRPTHGSSSTAAYTWSQFMEGAVKDLKVEKFPRPSRVAK
RKATIKAQPISKKNSGFKISNRDDNSDSQDGNSSTRQGRRRRSNYEQLDQDQDTSTRRRR
RRRNDVQTVPSSTEENTTSTRRRRRRRNDVQTVPSSTEENTTSTRRRRRRRNDFQTVPSS
LPTPNSPAGQPSWRERLKPE
>1170768.4.peg.173
MPGTSLTQAQSGGILVSNKNLKDNTTLAQAGAAFSVNINKLSAAITAYNNIVIESKPPIL
KRLSQDVSFVGIGNTLKQLRKAIP
>1170768.4.peg.174
MEGENKPLRAFTNATANCGLIPAVVNANVILAAASVTPGRASTRVTRSVVLAVVPST
>1170768.4.peg.175
MVVPLPPPGVLLPIGGDGCEVGDGDGDGDGDGMMVGGVGVGNGIDGGEGCTAPLPAPGGV
LPPLVVNCKDANKKGTRLKAKMRLLGFNMMVSTNLFI
>1170768.4.peg.180
MQVTTTSTPLPPGELSPSSWPDHTQLPDSDDNFVKNFQEHPQSVILTTSIEPLLDKIHPD
KDYCIGQDSGIYWRFTEPVEKGVEAPDWFYVAGVPSRLEGKLRRSYVMWKEKVPPLIVIE
FVSGDGEEEKDNSPPPEGDEVDPKTKKVKKAGKFWVYEQAVKIPYYAIFDGFEGTLEMYH
LEKGRYKQVKANRRNHYAIPELGVELGILLDQERPPIPWLRWWDNRGNLLLTGNERAEEE
CQRRELAEAIAIQERQQKELAEALAIQERTEKERERQQKELAEALAIQERSEKEQEREKK
EKLAAYLRSIGINPDEI
>1170768.4.peg.181
MAIAWQLTQWDRLLKTLGIKTNDLNLIRVNANRAQVSS
>1170768.4.peg.182
MLKSIYSCYTMINDQNIINKHQVKTRIAELMAITVFSFVVQKLPDVHLSLA
>1170768.4.peg.183
MTKITNQALNSALNQLWVFSLSSDFWAVFDAVFGTEYNRKNAEILRSQWQIGDFSQLPEI
EILDSSILGSANGAYSSSENRIYLSSNLMENGTSSKIREVLIEEIGHFVDSRINQIDTPG
DEGEYFAGLVTDQKLNKDEIDRLKAENDSNWISVDGERLLIEQSSPGTVTRTPIAPASPG
RTRYEVGNAYAFAALKSNGSVVTWGQSWAGGNSSVATYNPVTNNYNPVTNNYSYVSVASQ
LTSGVTQIFSNLEAFAALKSDGSVVTWGQSWAGGNSSVATYNPVTNNYSYVSVASQLTSG
VSRIFSTGYAFAALKSDGSVVTWGDSSDGGNSSIATWNSTGNYSYVSVASQLTSGVTQIF
SNWGAFAALKSDGSVVTWGQSWAGGNSSIATYNPVTNNYNPVTNNYSYVNVASQLTSGVT
QIFSNWGAFAALKSDGSVVTWGDSSDGGNSSIATWNSTGNYSYVSVASQLTSGVTQIFSN
GGAFAALKSDGSVVTWGDSLGGGNSSIATWNSTGNYSYVSVASQLTSGVTQIFSNGGGAF
AALKSDGSVVTWGSSTSGGDSSIATYNPVTNNYSYVSVASQLTSGVTQIFSNGGAFAASG
VPVPHPQIGGAFAALKSDGSVVTWGSSTSGGDSSIATYNPVTNNYSYVSVASQLTSGVTQ
IFSNGGGAFAALKSDGSVVTWGSSTSGGDSSVATYNPVTNNYSYVSVANQLTSGVVSFAD
PFNDDRLVLSTSESSVTLAVSPSSVTEDGSSNLVYTFTRTGVISNALTVNYTVGGTATNG

TDYGNIGTSVTFAANSATATVTVDPTADTTVESDETVSLTLASGNGYTIGTITAVTGIVI
NDDKINTPPVLENLTFTGVEDKILNFVISDNNSQYRDDDNNDLAAVKVVSLPSFGSLTFV
NGQPISLNQRVSAVELLNVRYNPNANVNGQDTFNITAIDNGTPEAESGQAKVTINVTPVN
DAPEFTLTGNIQALTGAVDQTVTGFAQNISAGPNESSQKFEFITTVTSGNEIFTKLPSID
VTGNLTYSLSKTPGTAAVKVLLKDDGGVANSGLDTTEKEFTVQSAIPLNPSESSVTLAVS
PSSVTEDGSSNLVYTFTRTGVISNALTVNYTVGGTATNGTDYGNIGTSVTFAANSATATV
TVDPTADTTVESDETVSLTLASGNGYTIGTITAVTGIVINDDKINTPPVLENLTFTGVED
KILNFVISDNNSQYRDDDNNDLAAVKVVSLPSFGSLTFVNGQPISLNQRVSAVELLNVRY
NPNANVNGQDTFNITAIDNGTPEAESGQAKVTINVTPVNDAPEFTLTGNIQALTGAVDQT
VTGFAQNISAGPNESSQKFEFITTVTSGNEIFTKLPSIDVTGNLTYSLSKTPGTAAVKVL
LKDDGGVANSGLDTTEKEFTVQSAIPLNPSVFEPNVDISAGLTFKPQTNGTVNLDTSRGS
ASLDQVQSVQSGTKSNFNHIIGLYEVMNNQGEIKDNQGNTLKPEDANYTLHALTTARVKN
FTLQAGGNDTPSTATQLGSGVSVLGGKFYAPFAIANGGTYFPGNQGIEDFVAAEQGDINR
FSTALPYVRDLVAREGKNGDVFNNAPRFVQEPVAYFTFGAANPDKSAHFRSYGNGVYGFE
DLPADLTQYSNNDFNDAVFALKLTT
>1170768.4.peg.184
MIAVKDKVPKLTPEEYFIWEEKQLLRHEYINGEVYAMTGGTQNHGRIASNIIFIVKGHLR
GSGCQVGNSDCGVNILETKNYVYPDVSVTCDERDRTAIQAIQYPCLIVEVLSPSTAMPYL
>1170768.4.peg.185
MIINSFPIYASLIAYLILQLVSVRQEWGNKMLDKFRYLQACMC
>1170768.4.peg.188
MLGDDILRSCSMVIFRRLITSGLIGFLVSSCADAAKSSTEYTKQIVQENNVARLMAQVQD
SQKVSNLLSQGNAFLDSERYEEALQLYNRAIEIEKDSVPSWVNRGNALLSLKRYQEALES
YNQAIALRPNKNEAWYNRGNALSALGRYEEAIKSYNESIVIDPNKFEAWINKGIALTKLQ
RYQEGLASYNQAISINPNFAAAYYNQACNYALQKQVDLAVNSLAKAIKMERQKYSELAKA
DKDFVQVKYNREFQDLLR
>1170768.4.peg.192
MNQGQNLASFSFFMSNQERMDNMENQLIDIRLAVSALLETSVIYQRNFEVMQRNFDSVVI
EIREMQSEIREMQSDIREMQSEIREMQSEIREIQSEVREIQLDVRGLQTENRRILDVLQN
VPPDKYE
>1170768.4.peg.197
MQQEAKQLFEDYQALIQLGNNYGKFDREGKKLFIEQMENMMDRYRVFMKRFELSEDFMAQ
MTVEQLKTQLNQFGVTPQQMFEQMHLTLERMKTELEKQV
>1170768.4.peg.201
MIAIEDFLQSFYLESLNAFRRENQQPPDYCPQTLLELAEYMAFTERYAKRRIPLPGRQQQ
LIILRAQTFSQQQPLETSVDIEQATQASGGESDRSWDEPILTQLRAAIAMQPEPEIEEGS
LRSVVITELMNYLEQKQQLDCAEYFSLRLQDFSAQEIESVLGLTPRQRDYLQQRFKYHLM
RFSLLHRWELVHEWLEASLPTNLGLTPHEWELYIRNLDEKDRYLLDLKHQGESDQIIAKN
LGLSVAQLQKRWFKILEKAWEIRNSFSGSGLSAYE
>1170768.4.peg.213
MSDLQNIANHHYEPIKTILRVIDFSQGHFSLIFLHCNDAELRQQVVAQLRESSPNKIEEI
TLPQSAISLYDNISLSLDRHPEVLMVFGLETVNDLDSVLQFSNQIREEFRKNFAFPLLIW
IDDQILRKILRIAPDLETWGSIIGTPNYYNGENQVKGLAQIHRFLAEAALENQQWSEAKK
QAQYAQEILTSVDDGLYHLLTARSQIGMGETSAAIKTLEFAKTHTVLPQDPQLHIEILKT
LGSLYFDHGNYLQAFYVRQEQLQVEQQYRLRAFLGSSYLNPQRPIINKTNGNGRNSRPMV
FGREEDIKSLWQRISDNEHPLIVIHGQFAVGKTSIIQGGLVPILEQELVDDKQMLPIIFQ
KYTNWLEELSEQLLNKLEKKLQSTIAIETFNGLSPQERIVKLLNISGDKNLLTVLIFDQF
EEFFLVTSNLEQKTRFYQLLRSCLDIPFVKIILTLREEYLHYLLEIDRLVDLRVTNNNIL
DKNIRYYLGNFSKAVAKRIIQTLMVKDQFELQMELIDQLVEELGDNLGEVRPLELQIVGA
QLEKEGIDTLEQYEQFGGKQKLLEKFLQDVIKSCGPENESTAQLVLYLLTEENGTRPLKT
QSEIITQFSVESDKLDLVLKIFVGSGLVWLLRESLRDRYQLIYDCLVPFVRQQYVRLYYA
HLPEQLEKIQAELRQEREAEEQAQLVTKLEKDALIALDQFQADPLLSLVTAVSSANLLKS

IVQNNPLDKYPTVSPIYALNTILDAISDRNIIKGHEGGITSVCFSPDGQSIATGSWDKTV
RLWNLRGENIQQFRGHEGGITRVCFSPDGQSIGTGSEDGTARLWNLQGKNIQQFRGHEGG
ITSVCFSPDGQSIGTGSEDGTARLWNLQGENIQQFHGHEDWVTSVSFSPDGQTLATTSVD
KTARLWNLQGETIQQFHGHENWVTSVSFSPDGQTLATTSVDKTARLWNLQGETIQQFHGH
ENWVTSVSFSPDGQTLATTSVDKTARLWNLQGETIQQFHGHENWVTSVSFSPDGQTLATT
SVDKTARLWGLHRHKIQEIRGHEDWVTSVSFSPDGQTLATGSRDNTARLWNREGHLVQEF
KGHQSRVTSVSFSPDGQTIGTGSADKTAK
>1170768.4.peg.221
MVVQVSNRLSVNEAICFKETCRDLIESNLDSKVLLIDFNNTIFMDSSGLGALVSIFKFAQ
EKGIEFILDNVTPQVMAVLNLTGLDQVFIIKSCVTNKTSELTNSGNSRVSNCRPEDLPAT
HPCVKSRMKRIIDIVGSLVGLVITICLLIPIGIAISINDPGPIFFRQIRCGWMGKRFYIW
KFRSMCVDAEAKKSQVKNQVAGAFFKNDHDPRITKVGRFLRRTSLDELPQFWNVLKGEMS
LVGTRPPTPDEVELYEVPEWQRLDVKPGMTGEWQVNGRSKVRNFEDVIRLDLQYQKNWSL
VYDLQLIVKTISILFNKNSGAV
>1170768.4.peg.223
MGLTGKPHPPPYPSIMVGSTVPPVCTGGTQSPKNIVQQSANSGWLQL
>1170768.4.peg.225
MTSRIAVPYLNKPVPGALWNFYRYRHSSMDRNRQEAIAVRSKEM
>1170768.4.peg.251
MTLFLTFVNRPRRQVFQSAGIAAVFSFFMVWHVYTWFSIGVIMPPTYILLSLGTTCLLTN
LGAIVWRVSFRHSTPSPPSKLIDEINSSF
>1170768.4.peg.258
MGVMLMVSSLLVAPSQAQSIPAATAAIGKSSFVTTAVNRVGSAVVRIDTEKTISRPVDPI
MEDPFFRRFFGDTFPPMSPTEQLRGLGSGFIIDKSGLVLTNAHVVDQADKVTVRLKDGRT
FEGKVQGIDEVTDLAVVKVNAGKDLPVAALGSSNNVQVGDWAIAVGNPLGFDNTVTLGIV
STLKRSSAQVGISDKRLDFIQTDAAINPGNSGGPLLNAEGEVIGINTAIRADAMGIGFAI
PIDKAKVIAAQLQKNGKVAHPYLGVQMITLTPQLARQNNTDPNSTFELPEVNGVLVMRVV
PNSPAAEGGVRRGDVITTIDDQPISNAEQLQQVVEDSRLGQVLRVKVQRGNKIQNLLVTT
AELQNIS
>1170768.4.peg.289
MFTNCLRGYIAPFYGISKSHKIIANYPFPGNISHHRAVFIT
>1170768.4.peg.295
MYKLPCQVIILWGTADHCPQHEAPELVNSLLQEWIHSYLI
>1170768.4.peg.301
MQSVREGRFPEPKWGLKLFAHGLNLSLPVNNVWELIRFRIGVNRNF
>1170768.4.peg.306
MHPFHMLGVAGVFGGSLFSAMHGSLYSLEPKSQNQIAHPIINGVSDYVIINLASSYFDIG
QPPYYRRRNSVSPLSSGRDQCGSTAP
>1170768.4.peg.315
MGCGGAIACYTAQGIPVRVIFLADGISSTDSFIMKLLSPQPWINFLSLLPGTVLTFLTIT
VAFLRFYDEQDFSVLGYITQPRV
>1170768.4.peg.318
MAEKYGWKLVDVEPTGNNILPVDCIFEGETEFPQSHYDTEEEKNA
>1170768.4.peg.320
MGLTYYSKFGDGREDPKERSLALSINEQLKLAAITSFGH
>1170768.4.peg.333
MGGRNRDPLDHQSSASQYKMSYYFSIQFFDLPNPMLFYGLLHLLT
>1170768.4.peg.334
MPKVLITTVPFAERNSFPLEMLESNGIDYVINPLGKKLTEAELADMIAEFDAVIAGTEPI
TDHVMACAPNLKHISRVGIGLDSVDLMAAERRGIKVSYTPDAPAPAVAELTIGLMLTLLR
SIHTANNQMHQGKWQRHFGRRLAEITVGIIGVGRIGTRVLRRIRGFGTPRLLVNDTLPSL
ELNREFKLEWAAKTDIYQQADLISLHVPLTAQTKNMICKEQLLMMKPDALIVNTSRGGII

NEDDLYHVLSTGHLGGAAIDVFTQEPYKGRLGEIERCVLTCHMGSMSVDCRTRMEIEATE
EAVKFLTGKSLSSPVPETEYNVQRQKL
>1170768.4.peg.335
MVQQVCVIGGAGFLGSHVCDQLSAIGHRVRVYDRVSSPWLQSHQEMVVGDILDLASLESA
IAGCDAVYNFAALADLNQALDKPIETVKVNVLGNAYALEASHRHGVKRFIYASTVYVYSR
EGGFYRCSKQSAEHYVEEYQRVYGLDYTILRYGSLYGPRSDQTNGLYRILKRVLETGVIR
YEGSAEALREYIHVEDAARASVVALGDEFRNESVVLTGQDPMRVLDLLNMIREILGFDQP
VEFVESHQPGHYVRTPYAYQPKLGRKYTPPLHVDLGQGLIQLIDEVRNDTGT
>1170768.4.peg.336
MPNPLKEAMKAILSGTVSVLGRTGISQYIFQLAVNNAMDSTLQVLHRGIKLSFSTPNSLN
YWRVSTFSSKEPETLEWIDSFPENVVLWDIGANVGLYSVYASVTKNCTVFAFEPSVFNLE
LLARNIFLNGLSQKVTIIPLPLSDRISLSSMRMTSTEWGGALSTFGKEFGFDGEPIKQTF
EFSTLGMTMDEAVTLLKIPTPDFIKMDVDGLEHFILQGGANVLKNTKGVLIEVNDDFEEQ
AKSCHDLLVNAGLSLKEKRHSDIIASSTSGFANCYNQIWVRS
>1170768.4.peg.337
MKIINSAKLIFWDFDGVIKDSVDVKTQAFKSLFLPYGAEVAARICSHHESNGGVSRFEKI
PLYLTWSGLDATEQNVSEFCDRFSASVLEAVVNSPWVPGVLDYLNENYQNQYFVLVTATP
HSEIKIILNRLNIAHLFQEVWGAPTSKSDAIAIVMGKVEFNQEESLMIGDAESDMIAAQS
NGIPFLLRRTPINASLQVHYQGPQLDNFNP
>1170768.4.peg.338
MRRQIAIQQVRKNLQSGGISVGSWMQIPHPSVAEIMGQSGYDWVAIDMEHGSIAHHQLPD
LCRALELGNTLPLARIVEGTSKECKQSLDAGCGGVIVPMVETGSQLSAVRNACRWPPSGT
RGVGFSRANLFGQHFGSYQDEAQSPLLIAMIEHCRAVQNLDEILVVEGLDAILIGPYDLS
ASMGLTAQFEHPDFHGAMIQILEKAKQRKVAAGIHVVQPSKQELSQRIAEGYQFLAYSID
AVMLSTTAKQN
>1170768.4.peg.339
MTQNPVPSCGQSDQRNQEYIESFSPEKYYRSIVATDSPVIFDVGAHRGESVRFFKGIFPG
SCIYSFEPDPANFEYLEVVCQSYYSPLGGGRECLPINMGIAEKEGTMPFYRQGISHLSSL
LPINNASTDSLGYAVKALNQPIQISVTTIDTFCSKFAISHIDIMKIDVQGYEVGVLVGAK
KMLMSTSCCTVEVSFYDFYERSTSLLDVEQIMQEAGHRVWDISKISKNPKNFRTDWVELV
YRNSYLKQS
>1170768.4.peg.340
MKEEEIRPKKIFDEYLRLAAQDTHTYFDQTERRKIACPACGNLGSIAFEKSGFVYEKCSR
CQTLYVSPRPLAEAFSRYYLESPSSRYWATTFYKETAEARREKLWKPKARQIQKIIWEFF
SKSDTYPTSMPSIETNSNYDLLQDTLVVDIGGGYGLFAEEIRRLLPREPIVIEPAPHLAE
VCRKKGIRVVEKFLEDIQPDDLPKTPKAFVSFELFEHLHDPMSFMSGLHSLMQSGDLFIF
TTLSGNGLDIQVLWENSKSIMPPHHLNFFNPQSVKLLLERTGFKTLRVTTPGKLDIDILS
NNQSPIGDRFWSTFLSVATDAEKQIWQSWIAEQGWSSHMWVVCCKP
>1170768.4.peg.342
MSILIWDNCDPPNPRGDDQTIVLWCSYNTTSLHGAVSIPQWVEEHSDSIRHKYLSWVYQI
GETPICGKRVVDHLQLHPGFSLWWMSLLAEKCNFAKSTHIDDAIKLIAFVDWMDSQNSKR
IVLLSSKNRLADCLRQWCDQKGVEFEWQRSLPEGTSPFSRHLYNNLPHVLQAIIWLIRRI
LYRWKLRRVGLREWRESQGGVTFISYLFNLVPDTAKQGIFESRYWGTLPKKLHQERIKTN
WLHIYFEHEFLPSAKHAAQQIADFNRMADTLQNHVTLDSFLSPQLIARTLLDWFYLLGKV
ITIPMQNYMPRLNGINLWPLFKDDWKSSAIGKTSIANTLHLNLFEAAFGVLPKQSLGIYL
QENMDWEYASIHTWRSHMHGQLIGCAHATVRFWDLRYFFDTHIYDAAGQNSMPMPDRVAV
NGPISRQIYLEGGYPEDQLIEVEALRYLYLNREPEGDSKITTETTPAIGKLVTSPLRLLV
LGGYNEVDNQLQMQLLSEIAPDLPAETIITVKPHPAKPIKPQDYPLIEFSVKELPLAELL
QDTDVAYSSPVTSAAADAYCYGLPVISVLDPKTLNMSPLRGCKGVTFVSTPLDLINAIKS
AATHIPRNDQANDFFHLDKDLPRWKALITNNII
>1170768.4.peg.343
MTQTPIVSVVIPTYNRSLEIARCLSSLVSQTFREFEVLVCDDGSTDNTKQVVSTFTETLS

IKYFWLDNFGGPARPRNIGIQNARGKYIAFLDSDDWWTPEKLEKSVEALESGADFVYHDL
YIVSSNGVMSPFSRKAKTRQVKVPIFNDLLSNGNAINNSSVVVRREFIDSVNGFSEEKAL
IAAEDYDAWLRVAKYTDKFLKIDGCFGYYSLSNNSISLPDRLINATSRLLNLYNAELQTL
TCCPDWASLALADAYYAKGNKYLCISYCLSGISSKLSISSVIYKLKFLVRLILAFSSL
>1170768.4.peg.346
MNKPKVSSISYNSFLSFANHILFFGTVLFYSFYNPPKLVNWTTLLLYGMLYVQTILILRY
EAKRPNPFILILVYLLTAYYFPRIITLYFESQLSISEINNFSVLNRLRPSTVNDLNYTLT
FIFAANLSMFLGWLFADKKVNKLNFAQKTNAINKPRVNPVVIIILTVVSIFSSFYFSFFA
KSYSVKYITSYINLILNPLILIQMILVYLFYVYYSGRGNESSFFSWTKRRYSVLLVGLLI
LVFLLRIIVGSRSSIISVVQGIFLVSLTLGIYRVPRKILLLCLLLLALSFPLFIISTTSR
QLKSVTSDTTNLEEQFIQTIELSGSAFRSQSLVSILAPSFSRASFLDTSLDLIKNCNNYR
QIINPIFYSKSIIDNALTPGAVDFFDTPKAANALISIYGNISNISLRSTRISYQSDQMNI
YGEFFVLFYGWFSMPAFFLFTYFFQVIFFHNLNREKFDYGRSVLDIFWMNVFFSVLISFG
IDWIIGETIQSYITTRMMLFFIKTRIRL
>1170768.4.peg.347
MYLNGMGFRAIERVMGVHHTTVIDWVKQKGKKLPDAIAPENIPDVGELDELETFVGSKKT
RFGCGQQ
>1170768.4.peg.348
MTRVEGENSRLRHYLARLHRKTFCYSKSLDMLRYSVRLLIHYLHDPSVLFSS
>1170768.4.peg.350
MMKNSHPNNDKSYHFNTSLNRSIGLVYVNTVFFLLLNPFLRFLQLPIGEIDPLGSILSLP
VAMYLVLSGKIKARLFCVYVFIFLVLVYALSEIIFNIGDLNFYRFLFSLCLVILPLSSFI
FLDTYKNFFSNKLFNLNLYLWTLVAIVQVLFPWIRIPILDQLLSRGVLSAENILSGRGVQ
SMAVEPAAATYFIVFSIFYSIHLFKSDKITKSNLYVNLTALLALILLTRSATLLTNLLII
ILIYYFTKLKRSTFLTSFSLLTVLPLVIFIILPLLGVLGGRFNDFIDFFSNYDFSSSTVD
YFLIDFTQNLDGGRLASSLTSYNTLRISPLGLGISNYEENFRNSWVDLYGSDLYLRDSAS
RASTYAGHVMTIMGIPGLASLILVHCFFCLSSLNSCENIPTYKIISFSIAIFWIYFSSIV
TLPIPWVILALVY
>1170768.4.peg.353
MYNTVGAIAKHSHLRDGLLTHVKFNRFCTMGFSFSLAFTPSITT
>1170768.4.peg.356
MVKLQRNVQSLVESNIIKPSDSIWKIAFLYADEWKYWKQELLDFGFSMQDPIGDLLAVET
WDED
>1170768.4.peg.362
MVLGLIITLFFVLLAFMAPVWQNLGWLSDPKELLTNPIHQPPSGKYWFGTSRLGYDVFSR
TIFGAQAALQVVILATGLSMVVGVPLGMISGYLGGKLDKTLLFLMDSIYTLPGLLLSVTL
AFVVGRGILNAAIAISIAYIPQYYRVVRNHTVSVKTEVYIEAAQAMGASTWTVLSRYLFL
NVIQSVPVLFTLNAADAILVLGGLGFLGLGLPEEVPEWGYDLRQALEALPTGIWWTTLFP
GLAMTIMVVGLSLLGEGLNELIHPRMQKRF
>1170768.4.peg.369
MAVFSSIAAWWEKVKEIEPWSRLRVSNGEDIEKFCKTLVTAMTIITRITSQLIAP
>1170768.4.peg.370
MDKLMLSLKAVTVKSNPGGKITPEPSLEYRHTGDVFFEVFCNPNIWPTPFAAKVLLTVRN
LGIRLTTEADLTRLVDDINQYLQNTELTS
>1170768.4.peg.371
MRKGIVSRQQPIYTLCQYIPAREWVFVEYELEKCDFLLRDRIGDLIGREQWEND
>1170768.4.peg.386
MTQDIPNDILNFLATFGTGSPLHGLLIIGLTFGFVGGLFDVYVYYRHQVLSFNSEGLIQN
STITVFCESLENEEVYLKPLTRTPGAIS
>1170768.4.peg.387
MSPNKHRQDNKKPTNNIGAIKLNTMASKTERRKLVKTIISGHLQD
>1170768.4.peg.409

MVAATTGLTLRTFQNSFVMFLPFLTPKENQEDLNPYHQTP
>1170768.4.peg.411
MTGDELVIYYPDGSRFLSPVELSNYAEQERFLKEQERFLKEQANQRAEQERSLKEQANQR
AEQERLLKEQAHQRAEQERFLKEQANQRAEQERFLKEQANERAEQERLLKEQEQLKYQTL
LSQLKAKGIDITALE
>1170768.4.peg.412
MGSFFVGEKIPVFKNYMLLVGLLEGRSLFGIFRPCHSQGKI
>1170768.4.peg.413
MIAGAVNATGATSPAAKSKGKFWVYEQAVKIPYYAIFNGFKGILEFHFAQKNRSQ
>1170768.4.peg.416
MGVTSINPIKLEPIPLPRINHASNPYLNSHPTGEAIAL
>1170768.4.peg.417
MPKSDRPINLLNKFPLRRGNSGDLKCDRASASALHSPFNHPQKFP
>1170768.4.peg.420
MLNHIDSLIEPNKRSLKLLANDYKINIRRSSTSVETLN
>1170768.4.peg.423
MTYTLEQFKKDFVINHLREIPTEEVLKQYSPEEVLKQYSPQEFLEGLSPETLEHLAIFKN
SLLKNHCCS
>1170768.4.peg.424
MFEGLYIHDKWDWGRKFPVIKIDFADGVLKNREELGRRILDLLRKNAERLGVSYESNDIP
GKFGTLIGEAVAKYGTRAVVLVDE
>1170768.4.peg.428
MEGLVSTIKRLFASIPWRNFTNNDLADFEGYYASVIYAFLSSLDARVIPEDITNHGQSDL
TVMVGGHVYVMEIKVVEGNQVEGNAALDQILGRNYAEKYRGEPGKYVHEIGLIFSRSQRN
LIQADWH
>1170768.4.peg.429
MNLKNLPLGINTLSVLRENNCVYVDKTKLAYHLIRIAGRFFLSRPRRFGKSLFVDTLKEI
FEGNEKLFEGLYIHDKWDWSRKFPVIKIDFADGVLKNREELDEKIRDILWTNGDRLGVGA
KKNSISGIFGEIITGAREQFGERVVVLVDQYDKPILDNRVIPEDITNHGQSDLTVMVGVH
IYVMEIKVIEGNQVQGNAALDQILGRNYAEKYRGEPGKYVHEIGLIFSRNQRNLIQADWR
>1170768.4.peg.431
MVKVATSKLLEGGSFHSINVPSEWELHPNGLSRLKETQIVSIQLMSPASGNTAQKNPTQT
GGYIETSFHSINVPSEWEQ
>1170768.4.peg.432
MNGNSSLRDLNHSDEFCSHSLGTLIEWKRARMREAIEKLFVEVPTRWGH
>1170768.4.peg.433
METPTHQMGDVFLHFGSSHSLGTLIEWKHRGVTGVTFFQTNWVPTRWGH
>1170768.4.peg.434
METQNRLAGFGHLISGSHSLGTLIEWKRWSYWQHPVYGETSNEVPTRWGH
>1170768.4.peg.440
MGGMKPDKKGKKPPPGQLRLFWDEVDMGNRENLEVVKRAIAWQLI
>1170768.4.peg.441
MLNRTGHFWEKRYHSTGFLTTDRKRALNTLRYIHANRKAAGIQRGMFYDFSNYGVHERLG
DDGITTWHPAFLALGRSLEECAIKYKGFCQKYRVQEKPVKKNR
>1170768.4.peg.455
MEPFGGAWIQFNIRYYMFALVFVVFDVETVFLYPWAVAFHRLGLLAFIEALIFIAILVVA
LVYAWRKGALEWS
>1170768.4.peg.476
MGANCNKLLLKVHSQSIIAWTLLAAEAASHIRWIGIISQPSEWHEFKAIVSGLAMTKPVE
FIPGGTTRQESVFNGLQALPSEAKEVLIHDGARCLATPELFNACAIAILNCPGLIAAVPV
KDTIKVVGESGIIESTPQREKLWAAQTPQGFNVQLLKQCHAEGVRQGWEVTDDAALFEKC
GIQVRIVPGEETNLKLTTPQDLAIAELILSLR

>1170768.4.peg.492
MNTPMVETEDSSGKLALTIAQAASERKAGEILLLKVTDVSYLSDYFLVMTGYSRVQVRAI
SSAIEEKVQTELQRRPLRTEGKGEGSWVLQDYGDVIVHIMMPKEREFYNLEAFWSHAEHI
PLENP
>1170768.4.peg.519
MVNSPNPKSFVYTRKTLSRAERALICSPFRLKLFRDMQSQSIPQGAIATVNGVQQGYTQV
SLSGLRCDNALGWLIEVGVLRREVDGQGITDRFRLTPLGHQLVEKFAHQPWTTASWGDRL
QDAITRWLRLPSI
>1170768.4.peg.523
MSIILACQVWILGSLKMTHQGSQPVCTPETYKGDKQDG
>1170768.4.peg.532
MMLDVKFSQPNLHLYHFTNHDVQYLDIGYRTGKRCDRHLGSQICYN
>1170768.4.peg.534
MSFWAGLWVESNTRNLRGVHISDTSDLMRRMVKSGSRAISRESLILGLVSE
>1170768.4.peg.538
MAVNVWLLSLLFHYFQHPITILSIAAILAFLLNYPVRLLEKARITRTYSVIIVLVITLTL
LVILGFTLVPMLVEQTTQLLKNIPDWVTSSQENLSRLQVLARQKRLHIDFSVVSSQINAS
VQNILQQIASGAVGFAGTLLSGLLNMVLVVVLAFYMLIYGDRLWSGLINQLPSYIGLPLS
KSLQLNFHNFFLSQLLLALFMVIALTPIFLFLRVPFALLFAIIIGISELVPVIGATLGIG
LVTLLVSLQTWWLAFPVAMVAIIIQQIRDNILAPKLLGNFTGLNPLWIFIAILMGFEIGG
LLGTLVAVPIAGTIKSTVDVIRNNKAGIT
>1170768.4.peg.578
MGGLGGAYFIPLAPTGSPRTATGNKNFPHGHSFTNQEAYGPL
>1170768.4.peg.584
MGLPWYRVHTVVLNDPGRLISVHLMHTALVAGWAGSMALYELAVYDPSDPVLNPMWRQGM
FVLPFMARLGVTESWGGWSVTGGTAVDPGFWSFEGVAAAHIVLSGLLFLAAVWHWVYWDL
ELFRDPRTGESALDLPRMFGIHLFLSGLLCFGFGAFHLTGLFGPGMWVSDAYGLTGSIQP
VAPEWGPAGFNPFNPGGVVAHHIAAGVVGIIAGLFHLSVRPPERLYKALRMGNIETVLSS
SIAAVFFAAFVVAGTMWYGNATTPIELFGPTRYQWDQGYFRQEIQRRVQSSVAGGATLVE
AWSQIPEKLAFYDYVGNSPAKGGLFRTGPMVKGDGIAQSWQGHAVFTDSEGRELTVRRLP
NFFETFPVILTDKDGVVRADIPFRRAESRYSFEQTGVTVSFYGGNLNGQTFTEPADVKKY
ARKAQGGEIFEFDRETLGSDGVFRTSPRGWFTFGHAVFALLFFFGHIWHGARTIYRDVFA
GVEADLEEQVEWGLFQKLGDKTTRVRKEA
>1170768.4.peg.592
MELLLPTAFVQLLMGISALVVAVLSLLGLGNLLLQVVVWLTLSSFLTIFSRRFFTVRQTK
SKILTSTVGETMTEILPGKVGRVLYEGNSWRGRCDDDKLTIPPGQKVYVVAREGTTLIVM
PETVLDH
>1170768.4.peg.600
MAFCLFKSRTLQWDVGNHCVHRKNGDRTLTKPSTGIFF
>1170768.4.peg.604
MAILAVGAGQTYTTIQTAINAANNDDIIVVRPGIYQEDLTINKSVTLIGPYGTFEGIDGF
ENRLGVKPLGPDINAALGVGGLPTANEDFRRYQDDNGTIDNAFGNTQEAWIKGTVTVTED
NVTIDGFRLRNENGPLQWNDTPDNFKLLNNYLTGYTANNSPSFGDANINNPTGVVTGWQI
DSNYIGGLLGGGGTGGSIYLAGLQDSNIDNNTFWRPRAAHLYLASLTNVTIEGNKFYHGL
HTGGANFDGFGEFFSGSGYGYGYGGYGDGFFGRNYWLELKGDNDQVLIKNNEGEYNSGGI
QLFGETDSPFAFDNITIEGNSFPDNNFINAYNEAPDNGKSGLIPAVMATARLGGPSGSNL
VIRDNNITMDLAQVKFITDHKSSLEVRGNFNGVTIEGNTLTPKNINGGVDIITGLSLYGI
LPGETLIKGNQLLGQDGDPLEASYYGIDLIPTFADYGTYAGDLAVEDNTINSWQVGVNLR
DTNEITGDINISGNTFENNAYGVVLDATATTNSINIAGNTFSNNFSNVFDNIDPVITMDQ
VLSYEENQDLGSVLGTVSATDNLPNTDNVGITQYFISSGNDDGFFTINSSGEITLTETGL
AAANDFETSPSSFDLGITVTDGGGLQDTETITLSIINVNEGLGQLPPITTEGAGFIVGAT
LIAAIPFDDPDGTPTDISYQWQRFIEGVWTNIPDATEQNYIATEDDNNNQLRVEVTYIAG

GFEKVIYSNNVSISLVSVSGTFDSITGDNNLDISEKEAGVTLTGSVSETGTTVTILFGGQ
TRVAQVDGLSWSYVLKPNDYSFFAPGSNLFTAIFIRTDGGETGSFTTFQTLNIPDGILPP
NTSNAFDPTQPKGLKSEVIDAAQTLEIDGVSILEISKTVGILGEGESFNDPNIAVLPIGT
RDIFDQGANAGAANYAAFKTEPGTSIQGIYIVPAVEEEGVKKLQVVLADGTVVEAEIPLD
LISPIGDPLAVTISGVQPGGTTTFVLYLSQNVINQLPDDLNLGRYAKFNYESEQFELYDD
SNYDYIFNDVDGDGARDFGEVYLTVNLTDGDKWDGDGLENGIIVDPGQLGIATGSDSDNN
PPIAIELKGIVAENDPGAFIGSLTVTDDPGDSHTFTVNDSRFEVINVDGNNILKLRDGES
LDYEAASNVVLTITAIDNGGLEFTRDFTITVTDVNEAPVAIELNQITVIENDPGAIIGTL
TVSDPDGNDGHTLKVNNDTRFEIVDFDGNQTLKLKAGESLDYEAGSVELSITATDNGGLE
FTQDLTISVNDVNEPPVVSFSFFVPESTTLVTNLTVEDPENDPITLSLAGVDASLFSISP
TGELAFNTAPDFEEPLNADKNNLYKLQVVARDGENNKSIQDISILVTNVNEAPIAIDDVL
AIIPGSSFGTLNPLDNDSDPDLDDTLTIINKTDGNYGRVEIRDNELIYTLLDATYIGDDV
FSYTIVDEQGLAATANVNVTITGTDIITYPVEILDPEDSLIPDEAGSLSDIVNDISFNFL
TDYDKVQAKLALQQALSKTEAAFTNLFGLYEVDNALTGSVNGILPEDKSAYAKAALSKVV
PNFVVRAGGLGGGVNGDVIVTEGKIYAPFVIAHGGNFPGSVQDAVNAFFQVNPDNSPATA
QNYTTLPVAYFSFGSANPDGAAHIKSFGNNVFGFEDLPAGVGVSDYDFNDTVFSFG
>1170768.4.peg.607
MHHPPGLQAPAFEAHGHGLVDRLPLAVGRQFVHGAHDVDGGAALAQPGAANVVDGCAAVF
VVGGQRRSAGADVYADALVEGPTHAAGRVDDDGEADAGVALLLGGLGVVVVTPRGVARGD
IGNRQRDALGGVVGIGVVGDPLGGGDGRVVGVSLAGRGRDQGGPGEQGEGQASGEVREIH
ARVLHGASFRPWYIGAGSVYPRGFRV
>1170768.4.peg.608
MVYRRGISVPARVSGVTGGLEARLWMARRSPPVFARWPAGPAGTAGPPKRLVAEHEQVIP
RLGGGRGFARARGRAELDQGRGGDCLTPLGQVVTAQAVRRVDVFFFGLFFGRPAPPPAHG
EVAHHPLGPRGVALGAAGGLVLHDLRDADAAPCAGPDVLGWGFQAIGGQAEPDDIHAPGL
QHAGDGAQVALQRGVGGQVADGVDDVEGDVDTQARANRVEVFHAGLKDAGASAGGQFAGA
GLGVGNHGGFDVESDGAAAPAGEVAKQAARAAGGLEVGGRGSGGAAEALGDAGDAAGFGG
GAWSVEDVVEVGDGVEVGEHGARVPPRTGRGCTDAKLAPWTTPRSSRSNQANAAASPASV
ACGSRCTTCSTTWPRG
>1170768.4.peg.609
MSDQSSASSRTPFEILQFRVAEAYWIWKVWKQLYMVGQGGIQEAEERMDVLNRFGVNFFR
MLKGSLLQDVILRVCKWTDPEATQVRGVERPNLSLANALANAKSSLTPEQACKAEALLKK
FKSVTPGLVQRRHWLYAHDDFNVATGKEDVPKVSDTDVDIALQCAVDLMTVLDPKSADVE
FGYGETIAIGDGNSIVEAMRYAKRWVKHCRRYGRALDWDGVPGTDEKPL
>1170768.4.peg.610
MKFARGSYFCLAIAAFAGGCQFVPFVPLEDDYARRVSESRLRQIDQAGLERFRKAQAAAT
VPAADPTKASEQQADAARSRFAGLAKVDLSLEQCRADALINNLDLQVALIDPTIAAETVS
QEEAKFNSAFTLQAGYQSLDEPTASTLVSAEAKNLQVTPGVRIPTRTGGTVTVDLPFTRN
ENNNQFSTLNPSYTSDLEFSISQPLLRGAGRRTTTAALRIAGYDQQAAASRTKLEVIRQL
AAVDRSYWRLYQTKRELEVRQQQYELAVEQLARAQRRNNAGAVAEIEVIRAQAGVSDSLQ
AIITSQNNVLLQQRELKRIVNQPGLEVDTASMIDPTTQPDPVAFEFDSQTLLAEAMHNRM
ELLELELQLASDAVRIGLARNGTLPLFSLDYTYRINGLGDSFEDTFHNLKRNRFEDWTVG
LSAEIPLDNEEAESRVRSAILTRLQRLASRASREQSIRQEVLNAIDTLTADWQRILAARQ
SVILNTRALQAEQRQFDVGNSTSTDVLDAAARLADAQSTEVAALTDYQIAQVDLAFATGT
LLGASKVDWTPAATPSLETPDPKEELPRELQPQPEPQPEGMGAGAMPHAGEK
>1170768.4.peg.612
MVCPGDITGNFKEVNIKDVREVEDNYTPIGRPGTGEDIARVIAFLCHRDSDFITGSVIIV
TGGKDVLTKYRYQS
>1170768.4.peg.613
MERHTALITGSATGLGKKTAIELAKQGVNVILNSVTSLGKANELKDFIENNYPVQAIVIQ
GDISTYSECERIVSSANDRIGKINILINNAGAYIVERKDMVNYDLHEWEKVIQVNLTSVF
YLAKLVIPTMRQNRWGRIINMGFDRAETAPGWRYRSAFAAAKTGLVSLTKTLAIEESENR

ITVNMVCPGDITGNFKEVNIKIE
>1170768.4.peg.616
MFKTQDIKQPETYIYTRAKESLTRSLLEEMSPQKITKYQASEI
>1170768.4.peg.617
MPSIQFDPKPEISVVLTTYNRSRYLKNCINSVTRQTFEDWELVIVDDGSTDDTFEIVSLY
LAEHRNIRYLKHQNRQVGYARNAGIQASFGKYITFIDSDDTYKASHLESRLEFMRTHPEI
DIIRRRVFCA
>1170768.4.peg.618
MRFFQTVVPWASVFWIFFRHDTYLLAGDEVVISKAGKKTHGLAIP
>1170768.4.peg.641
MDAAATAAKCAIYMTYLEQGQNLRMTGHLHHLEPKRVKIIVEEIREALTEGKLLKMLGSQ
EPRYLIQLPHVWMEKYSWQPGKSRIPGSSLTTEEKKQIERKLPANLPDAQLVTSFEFLEL
IEFLHKRSQEELPQQHQMPLSEALAEHIKRRLIYSGTVTRIDSPWGMPFYVLTRHFYAPA
DDQERTYTMIEDTARYFRMMKDWAERKSHAMRAVEELDIAPEKIQAAMDELDEIIRVWAD
RYHQEGGKPVVLQMAFGQQDD
>1170768.4.peg.661
MSNLENAISDLKSLGYPPTGEISITSHGQEIYAQDPDGNRLILYSSSNIDKQCP
>1170768.4.peg.691
MQFSEYLPWNETLGLAYQLGVDGLSILMLILNSLLTWIAIYSSSENTERPKLFYSLVLLV
SGGVAGAFLAQNLLLFFLFYELELIPFYLLISIWGGNKRAYAGMKFLIYTAVSGALILAT
FLGIVWLSGSHSFALDAVNTQNLSTTMQIVLLAGIILGFGIKIPLIPFHTWLPDAYVEAS
APIAILLGGILAKLGTYGLLRFGFGLFPQAWNVVAPTLAIWGAISAIYGAVVAISQKDIK
RMVAYSSIGHMGYILLAAASGTKLALVGAVAQMFSHGLILAILFHLVGIIEGKVGTRELD
KLNGLMSPIRGLPIVSALLVLGGMASAGIPGLTGFIAEFIVFQGSFSTFPIPTLLCVASS
GLTAVYFVILLNRTCFGKLNNDLAYYPKVVWAEKIPALVLAGLIIFLGVQPTWLVRWNET
TTSAMVGAIPTAGTIIISQVETNQQ
>1170768.4.peg.697
MSSAVDIVSVPPELFAKFQTSFYTDPVDAVFKASAVD
>1170768.4.peg.728
MATLLSESKSQTGPVEALPDDFQLEDEPVDNTGQLLLAGALCESLEISGFIQPQMLIAVN
FGLCATLNEQFVAKAPDWLYIPSVY
>1170768.4.peg.730
MLIIILIFAYLRSRAIAWNPIPIPLRRLNTKCDRIGLAPGRSPFLTTQGVAPARDDV
>1170768.4.peg.740
MKYDYFSLQTCDRQVLYSFEVCLKKKAPHLVRSFSFNSIDY
>1170768.4.peg.741
MGVTSRIKVFKNWNQGKFNSLEFSQLGEEYFLCGIVAFFMGEKIPVFKN
>1170768.4.peg.742
MLLVSLLEGQSQIIRYGSNNSRFLINPFINSKNSEFPSNSNNRALF
>1170768.4.peg.752
MKANRRNHYPIPELGVELGMLLDQEKPPVPWLRWWDNRGNLLLTGNERAEEECQRRELAE
AIAIQERQQKELAEALAIQERTEKEQERQQKELAEALAIQERTEKEQERQQKELAEALAI
QERSEKEQEREKREKLAAYLRSLGINPDEI
>1170768.4.peg.760
MNDLPRQKLKEIIIQHGHALCDDPKRCEAFLRDYCGEYGREIFILISVLKQGIVKDLLNS
NNIPIELLLGRLTKQMQNNLGLTEEAARYGVESWAVVLDKMTLQPIETKSATLRNKMRTF
SFEVVSSNKEGKILNKKSVSANYFVEDLGNGIILEMIEIPGGIFMMGSQENEKDRNINEG
PLHQVKISSFFMGKYLITQEQYYAIIGNNPSNFRGNKNPVEEVSWQNAVKFCQKLSQRTG
KIYTLPSEAQWEYACRSKTITPFYFGDNITTDLVNYNGNYPYGLASKEQWRKKTTEVGTF
PPNAFGLYDMSGNLWEWCLDTWHNNYQGAPSDGSPWFSNSMIPRHVLRGGSWFNSAKDCR
SASRNCNFDHGNDYGFRVVTAPLLWN
>1170768.4.peg.765

MLTEIAPNHVHKFPLRTEGLGDAEFVYEVLATGDRTSRHFHSPELRSPF
>1170768.4.peg.783
MADEYGRQLLAALKVEANVKRNPEAIKRVKDQLTGRNREN
>1170768.4.peg.784
MAGPSSQNCAQAAEALAAGRSSLIWRRRIADTETPVSAALKLIEPERGDFLLESVEGGET
RARHSFVGLAPDLVFRASGDAAEINPSWATDRGAFAPCERGSLDALRGLAEACRMDVPPE
LPRALAFLVGYFGYETIGLVEKLPRAPQSDLTLPDMLFVRPTVLLIFDRLKDELFLIAPI
WAGSGDPEQAIAAAEERLDAAERALHAPLPASVRDASLDPETLKPQPVLAPGRYGEMVLQ
AKDYIEAGDIFQVVLAQRFTAPFALPPIELYRALRRVNPSPFLYFLDLPGLALIGSSPEI
LVRVRDGEVTIRPIAGTRPRGKTPAEDLENRQSLLADPKELAEHQMLLDLGRNDVGRVAA
AGTVRVTDSFTVEQYSHVMHIVSNVVGALEPSRDAIDALFAGFPAGTVSGAPKVRACEII
ASLEPETRGAYAGGVGYFAPDGSVDSCIVLRTAIVRDGTMHVQAGAGIVADSVPDYEQRE
CEAKAGALFAAAREALRLASEPGYGQ
>1170768.4.peg.788
MQVPVYIIFDPDGGLIEYYELRNGRYELKQPDENGRHWIGSMELFLGTWQGAKEGRTGYW
LRWWEETGNLLPWALELIEQERQQAEQERQRAEREHQRAEQERQEKEREHQRAEQERQRA
EQERQRAEQERQEKEREHQRAEQERQEKERLIAYLRSQGIDPNNLPNHAE
>1170768.4.peg.794
MKIYSLLLNNPPQTLQDKYAGIHYTESKSPFFQGELNAVKSELG
>1170768.4.peg.797
MGLEVKIATPSVKQQSQQEIIGVFLRLIVIRLTRNHFSARYGQG
>1170768.4.peg.804
MLHKLFTEIKEYELAAYFYQISQSKISSKILKLLVNSNNITPEIILGLPIKESRPWINSI
SKFFNKDIDEQEKIFKKRREKYLELLRKYKEIDDYNQDIKINPNDPEYYYNRGNTRSDLG
DNQGAIDDYTQVIKLNPNSAYAYILRGDVRSDLGDKQGAIVESPVQTCRV
>1170768.4.peg.826
MKVSVKIAIPSSNTEIILFICFTAKLDFGPDGNFLVYFIGAIFH
>1170768.4.peg.831
MFTELVLIRNILIDTLQIFGVTVDSPNLPKYPLGESRLYLYFSLRKFTDCKVL
>1170768.4.peg.835
MVGLATAAPDFGPAANVSAGRTAKPLSVTILGILVKKN
>1170768.4.peg.841
MAQSLKSQRRVYLWTVTHGIVEYGQARSTNQHNTVSPEAAIEWAIRQKEPSIFIFKDLHP
FIDAPATTRSLRDAIASFKGMQKNIILMSPMQQVPIELEKEVVVIDFQLPDMTELSKVLT
VHQEQNRGRRLTTEAREKLLRAALGLTKDEAEKVYRKAQVTSGRLTEDEVDIVLSEKKQL
IRRNGILEYIEEDETIEAVGGLEELKKWLKQRSNAFTERAREYGLPQPKGMLILGVPGCG
KSLIAKTTSRLWGLPILRLDMGRVYDGSMVGRSEANLRNALKTAESISPTILFIDELDKS
FAGSTGSSDSDGGTSSRIFGSFLTWMQEKKSPVFVMATANRVERLPGEFLRKGRFDEIFF
VDLPTPEERQDIFRIHLTKRREEIARFDLEQLAKMSDGFSGAEIEQAIIAAMYEAFAQDR
EFTQLDIIAALKSTLPLSRTMQEQVTALRDWARQRARPAASSVAEYQRLEF
>1170768.4.peg.851
MIVRYKLDTCGIKLKLAHWHQISLSERDTLVTLPCFTTGEIQNYQDYLKKLVFKYTGTYP
SLLPMDPHPPWLDSTTLPKQLIEKLETLGARIILEQWQDLTPLQRFVLVKLSLPSHENKN
FPRAMAEFHLDPPTGSLITNE
>1170768.4.peg.852
MHLIENIVVIVSKVLSILMVVVILVAIADLTVFLLKELFDAPYGKFNTILFKIFGLFLNI
LIALEILENITAYLKKHVFQVELVIVTSLIAVARKIIILDLEKVTGIDIIGLGIAILSLS
ISYLIIQSRLKS
>1170768.4.peg.860
MLDQCDFPTTNLANRKSPGVTVSSVLILFCMGGNSITLPPTERFDYQKSYKK
>1170768.4.peg.890
MYEPQSQPGRFFAWAIPQSLGGGWNPQNRCQAIASRLELYRPDGLQELQIARQNNENIIC

VTTEAVSTCRIVFTVPRNRDPYSVRSSIFSNLTAADQGQQTTGVNTYINSPRRSGNNSHF
RGGINLKPFLSMEDGGTGTNLNNGLLIPSRTPGKTILNPRLFR
>1170768.4.peg.900
MQGRVQMLYIAKNDNLGGYLPIPPNKPMVACTFVVESTG
>1170768.4.peg.902
MGLLPWRLRDTSINETTISSKQQATKNTTEIMTKKFVDLSTLDGKNGFTIINSNDKNDNL
GYSISNAGDINGDGIHDIIIGAPLSDNNDQSNAGNSYIVFGSNNGFANIIDISTLDGING
FTVNGSEIGDQSGRSVSAAGDINGDGIDDLIIGAPFADSNGDDSGAAYVIFGRSSFSSLP
TINPSNLGDNGFIINGLNPQDQLGYRVSSAGDLNQDGFDDVIIAAPPNAYVYPPVTGDQA
GKVYVIFGSEKFNPSNPNFDTSNFDLNVINGSRADDYLGVGLNRGGDFNGDGIDDLIIGS
PFNDFNGFRSGQAYVIFGSKESFSSSLNVSQLDGVNGFVINGQEGDQLGFSVSTAGDINH
DGIGDIIVSAHDADPQGVDAAGAAYVVFGARTQFNSELDLSSLNGNNGFVINGIGELDKT
SWAVTGLGDVNGDGIDDLLVSAIHADANGDNSGQGYVIFGSKESFSSSLNVSQLDGVNGF
IINGKSENHNLGYSASGAGDINGDGISDILISAPFAGSGEVYVVFGNNGSTDTGEITDEV
TEGDGGESEEFTIDGTEDEITGGVIEDNNSESEITNIDGNPDEILDGVMGEGDSDNNSTE
GLGSDITDSPDIESTPTGSVDNILGNNNVLSSSTTKVEFQLMDKIPASIRELGVFTVDDA
SGKINGIAPGQVGYSEAALARSKVVFSVLSKTPNEFNNANVTRILGFQEANPNLRFYVID
NGTTDAVKNDLLPINQVTFLDSSNLQVTQLPDNSFSLQSNDLVFKARPTTQPLPMGTNLQ
EKSQGESIDLRGVTGPVNAQFTVYREASFDNYVGFYKVTDEKGGIDTNSDGTADLLPGDA
GYIQSAVNQHLSGLGLNVANGNKSTFNNTLSGGGLYVPFIIVNGRPDTVLNSDLSANSNP
NIYFTYLGANSDQVDHVRLLGDNTFGFEDLRGGGDMDYNDLVVQVNMSARV
>1170768.4.peg.903
MTLPDSAAKEAWEEAGVIGQVNTQKLGDYKYRKRGNIYQVHLFWLTVEKILEDWPEASQR
ERIWLDINHAAIIVRENSLKKILQNSQEQVRVFTS
>1170768.4.peg.910
MGRLSFENNHRFLFTSISDTRGKVKSCNEKSTTISNEVQFPTNDKF
>1170768.4.peg.919
MIDQSKGSQVSTSLKLAQDLGNTSKSIKKLHLTDPQLQKFQSDLGQNFAGLSHYISKAAT
SLSEAKKTSKSPSGQEKMRYAKRGIESSLTTAEAAGKQLDILGNKLNKYCNPNK
>1170768.4.peg.924
MTHTGKGKININNAILSELDKLEGKLGVPALSNKIQASRPYGSTADLVSKKVITQEQFEQ
IKNLVTVEEVVLTGEAKDIDYMTKLALMKGHLLVAEELLKENQPKQAQPHIGHPVEEIYV
DIEEQLNERKVKQFKEDLVKLTELVRFRPQDKKVATNLTVAMSDIDTAITALPNEQRLQP
KFILQVIRGLLDAATAEYQAAIAKNKITAPIEYQDSRGFVIYSHELYQSVSPQMATVNPE
AQKAIDIALTELLQVWPSAIPPNQIVKTPEQVNSLVKTIEENAKFFTVM
>1170768.4.peg.925
MEFELAGVVRYTHYSLMVTGPNRLPIVAFFKAQASESLLHAQQVGEILTGLDGHPTLRIA
QMEETFKHSVKDILQESLIHEGKALDMYKSLLENVADASIYLEEFARNMIGQEEMHNLEL
KKMLRDFS
>1170768.4.peg.931
MQPKLGKNQFPMVLAMVLLFAIIGLPLAFRFTNGSGMIIWATIVVLLATLPAIIAWFNYR
P
>1170768.4.peg.934
MSELERYYTLLDLEPGATIEEINQAYKDLVFVWHPDRLPKDNHRLQKKAHDKIKALNQAR
EKLRSFQYQSQPGHNSEPSTSKRSPYQPNQPPAQNPDLSGRDFSHANLSNKDLSGRNLSY
ANLSGSNLSDTFMHKVNLRGANLSEANLFRANLLLADLREANLRSANLIGADLSGADLRG
ADLTGARMRSGERLLVKLVGANLTGAIMPDGAIYG
>1170768.4.peg.938
MTFIHQEIAATPVVVADVWVRAGATSESDPLFGMAHFLEHMIFKGTASLGPGEFDYNIER
IGGISNAATSHDYTHYYLATANHYLADTLPHLGELLLNAAIFEDEFMRERDVVLEEIRSC
ADDPDTMGFEALLKTVYENHPYGRPILGTKKELMENSPEAMRCFHRRHYQPENMTVVIVG
GIERDTAWEIVNKTFKNFKNQDDFPTSNPLAPPQIRDVKRQELILPRIEQARLIMAWNLP

GMDELAIANALEILSVILGQGRTSRLVNDLREEKQLVRGIYTNFSVQKDSSLLTITAYLE
PEYLDRVENLILEHLHRLQIHGVTEQELKRTQRSLCNDYAFNTETPNQLASLYGYYNTVA
KAQLSVAYPEQIQSFNTKKLQKVAQNYLSLQDYAVTIMKPY
>1170768.4.peg.956
MYEEENFVVLETNQEEQFLTKLELLEKLQNTLSQMPIEDIPLDVRKIGSLIEQVNHLIDT
TCELDLGPGRYLQWYAVRLEK
>1170768.4.peg.970
MTDDSVFDHGTWGIFDAWDSVHYRAIATSGYEFYPDGQQYNLAFFPMFPVTIWVLMKIGL
PFEIAGLLINNLSFLGAIYLLYFWVKKHCSLRIAQWTTIVICCCPMSMFTGVIYTEGLYL
LFSIGCLRAFDEEKYSLTAFWGAMATATRPTGMALIPALLLTTWRQRRGKIAYFTSLLTA
TGLILFSIYCAINFHDPLAFIAAQKGWRPSLGFDWQGWLNMLMQIILGRNWNFGWVWNDD
GGIRDPWYPVFFGFMVYGTVTLWLRQKYWHPFITYLVYTTLAVLLILVDQRVINNLLNVL
MVVGGSYLVWHFRRKLTPVMVIYGLCGVGLLLASGGTISLSRLAYGIVPLSIAIGAWLSR
FPRQAYLAVGLFVVLLFRLAIGFAQHHWVG
>1170768.4.peg.972
MNTSQEKVALMSLTGWGNNGAIPSIDTQTIMRLDSHIHKGK
>1170768.4.peg.974
MANLKINKDYIILCLIWLIGLVIDRIWFFLDDSIPAYDQSAHLTTALHHYRIFQNLNLLS
SDWWSSLWELTPSYRAPFVYICTVPFFILFGKGYDQASLVNLLYTGIIILSVHVKKI
>1170768.4.peg.982
MPLSAELSWAFWGWLYIPWYTLLYVEDIASEQGIWFSDALVRAHYVLAAVLLAWVTAH
>1170768.4.peg.1030
MIQPISNLRTKMNIETIKSEKTKQLPGANLEDQDLSEFDLTAANLAGANLMGAHLVSVNL
EGSHLEGANLMGASLQGADLRANLLGANLMQADLTGADLRGSNLRGANLMGATVAGASLT
AAFLSGANLMSGNFQGVDLRGADLRGANLTGANLKGADLSRADLQGALLNQANLEESDLR
GANLAGANLAGANLLCAELEAASLNGANLYQACLLGTILETYHD
>1170768.4.peg.1034
MMEAHSNGLALVISCALEHAEFYCESLNNHGLTSTIEPDD
>1170768.4.peg.1035
MLICLDLNQAMLDLAEKFLEHNYRNRIIEYNGWWDQTQTSKC
>1170768.4.peg.1046
MVVRITIQTDDFLTNHHIAALKRHHYCDRSPLEYCLNI
>1170768.4.peg.1047
MNGRILDHPLATNQIDVTPLIHGTYPLSQGLIAFEKAQSKGVLKILLEMN
>1170768.4.peg.1051
MSNLFKTKPKHTRIQRQLLILMLIIFGGSVVLGFIMGLLTGIQPVT
>1170768.4.peg.1058
MGYVDKSEVNGPGRRAVVWMQGCSSHCSSCFNPQSWSFEINKLVAIDNLAEDILRNSENT
GVTFSGGEPFLQAKSLAILARKVKAKGLNVMSFSGFTLQELQSESAPPGSRELLAELDIL
IDGPFVDSLAINSPTSPVSSRNQKVHVFNPDFNDKITWASDQIEVHILKDGARIVTGYQG
RAF
>1170768.4.peg.1059
MSTYPMMFRYPLGISFMGDTISSSFEAVVILLLSFPKYKML
>1170768.4.peg.1068
MIVLISSLTLRAEMVVSQEKRLGTMGISESRGVWLTNIDSDVLFTRERLKKSVKTLRKLN
FNTVYPTVWNWGYTLYPSQIASKVIGKSLDPTPGLKNRDILKEITTQGHKQGLTIIPWFE
FGFMAPADSLLAQARPEWITTRRDGTKIVKEGIHDRVWLNPFHPEVQKFVENLIVEIVRN
YDIDGIQFDDHFGLPSELGYDSYTVGLYKQEHQGKAPSENFQDPEWVKWRADKITNLMKR
VFFAIKANKKNCLVSVAPNPQRFSYEYYLADWQKWERMGLIEELVLQVYRDDLKVFISEL
EYPEVKAAQKHIPVSIGILTGLKNRSVPMEQIATQVEKTRDRNFAGVAFFFYETLWNMSQ
ETVAKRQASFQKLFPRTVQRNA
>1170768.4.peg.1071

MPTESQAPASMFTWSDIWQQIQQRLKGGDRLRQPNSVLHLVAKDRLLDSRQLQQMKDTLN
EMQLELKLVVTSRRQTAIAACTIGCSVEQTKIQTSFGNDSQENPKALADALYLETTIRSG
GEIRHPGTVVILGDINPGGIVIAEGDIMVWGRLRGVAHAGAGGNRESLIMALQMEPTQIR
IADALARSPEKSLTSFFPEVAYITNNGIRIARATSFSRNQLSKI
>1170768.4.peg.1072
MTTSGKGGVGKTTVSANLGMALAKTGRKVALVDADFGLRNLDLLLGLENRIVYTALEVLG
GECRLEQALVKDKRQPNLVLLPAAQNRTKDAVTPEQMKLLVDELAQKYEYVLIDSPAGIE
MGFKNAINAAREALIVTTPEISSVRDADRVVGLLEAQDIKKIHLIVNRIRPAMVRANDMM
SVEDVQEILAIPLIGVVPDDERVIVSTNRGEPLVLSETPSLAAVAFENIARRLQGGTVEF
LDLDSANDNILLRLRKLLWK
>1170768.4.peg.1078
MLSSAIFVVETYNISPDARTELSILNTCILAIFSGEYLLRLWSAKQKSKYFFSVYSIIDL
MAIIPYFIGFVDIRFIRLLRWLRILRLLRFIDKKFLFFSISEDTVIFARILFTLFAIIFV
YSGLIYQVEHAINPEGFNTFLDAFYFSVVTMTTVGFGDVTPVSEWGRLLTVFMILTGVAL
IPWQVGDLIKRFLKTSNQVENTCSRCGLAFHDSDALFCKRCGATLPQVSPSSQYQ
>1170768.4.peg.1083
MSGELDEYGMVLNLSDVKQVIKSEVTSELDFSYLNEVWEEFQATLPTTENIARVIWNRLT
PHLPLVRVQLFEHPQLWTEYEGKGEKISLTARSHFSAAHRLAPNLSAEKYGKFIRTHGHN
YHLEVTVEGEMDGRTGMIVDLGCLHETVEREILELFDHSCINEDIPYFSTSHIVPTTENI
ARYMSDLLQFPISELGVKLSRVKLFESDHLWVEYEGKDSEIFFSVATGFSAAHRLADPTL
SLEKNQTIYGKCSRINGHGHNYHLEVTVRGEIDSVTGMSVDLVGLNQIIQHYVIEPMDHS
FLNQDLPYFTEVVPTAENIAVYISDVVRSPIEELGAKLHKVKLIESPNNSCEIYAKDIEE
SRVDRVDRELAVV
>1170768.4.peg.1090
MNHAKFESDLAQLQDEVSNCERDVLNLIKGKKTMEYSQNHNGGNQSNQIAIIGMASLFPQ
SRNLQEYWQVIMDKIDCITDVPASRWSVEDYYDPNPKTPDKTYCKRGGFIPDIDFNPMEF
GLPPNILEVTDVSQLLGLVVAKAAMEDAGYGESQQFDRDRTGVILGVAIGRQLAVPLGSR
LQYPIWKKVFKNCGLSDDETEKVIEKLKSAYIQWEENAFPGMLANVISGRIANRLDLGGT
NCVVDAACASSLGALNMAISELLAHRADMMITGGVDTDNSIFAYMCFSKTPAVSPSENVR
PFDVNSDGMMLGEGVGMLVLKRLEDAVKDGDRIYAVIKGIGSSSDGKYKSIYAPHSQGQV
KAIRRAYENAGFAPQTVGLIEAHGTGTMVGDPTEFISINQVFGDNNSLKQHIALGTVKSQ
IGHTKAAAGAASLIKTALALHHKVLPPTINVTQPHPKLNIENSPFYLNTETRPWISNQPR
RAGVSAFGFGGTNYHVVLEEYESEHHQSYRLHNCAKSIFLSAPTTPELLSQCQHLYQQLE
STHKEQHYQRIIAESEQLIIPVDHARVGFTTLSLSQAIAHLAIIIDLLKNQPSVDFWEHP
KGIYYRQQGMETTGKVVALFSGQGSQYLEMGRELVINFPWLRQTYSHLDDLFSREGLESL
SQVVFPAPVFSPQERQEQLEKLQKTEYAQPAIGAFSAGLYKILQQAGLKVDFVAGHSFGE
LTALWSAGVLTEEDYFFLVKARGKAMSTSPEVDAGGMLAVKGNISQVTEFIKDFPQVAIA
NYNSPQQIVLAGNKSEITQLQNVLQAQGFSCFLLGVSAAFHTPLVSHAQKPFAHAIAQVN
FQPPRIPVYSNVTGKLYPNEPGSMQKILQEHLLNQVLFQQQIENIYQAGGNCFIEFGPKN
ILTNLVKEILVDKPHVAVALNANYRQDSDLLVREAVTKLRVFGVPLKNLDPYQIPAKISS
ASQKNQQKTLNIRLNATNINDRSQKAFAQALATGSVIKMPAVSENNYQTQPEKIRETLVE
TSTKINPEISSSSMLTSQIIPAVKNHSSVEPKMEISLDNYDRVLNSLEQSLVELTRQQSE
VNQVHEQSLQNQIEYNKTFYELMRQQSLFLAQEEINEYQSQTQQLAISSTERSMMRLHDH
QAETIRIHEKYLNYQQEYTNNYFQLLEQHYSLLEVGSPNAYSHLPSSHVSQSPAQKLMDS
LETENNSQNNLPDITVGFPIATYLDKEKLRETLINIVSDKTGYPVEMLDLSMDIEADLGI
DSIKRVEILGGLLELYPDLPRPNPEELAQLATLEQIAEYINNLITQLGQNQPLEETVSEH
HNHPQFLVLPHEELHEDLSDRSTTITIPDDLSQILLTIVSDKTGYPVEMLDLSMDMEADL
GIDSIKRVEILGGLLELYPDLPRPNPEELAQLRTLGEIADYMRNQAETVGRSNLSTSEKP
DTSTTEVADKILRLPVQLKTLPQPDSLDLTIPENHFVLITNDGSEVTHRLVAKLTDKGCK
TVVLTFPCLESNLSEEIAQIRLNDWHEETLQEHLTELTTKFGCVGGFIHLHPYSNNNLGI
DQAIVQHVFLIAKHLKEDLNHLAKKERACFFAVVRLDGELGTAKTHNFSPISGGLFGLTK
SLNQEWPEVFCRTLDLSPDLDGDTTVKHILAELQDPNLLVTEVGYNKTDRFTLVAEPTKS

SIIPDSLNITKDQVFLVSGGAKGITAKCVIKLAEEYQCKFILLGRSSAEIEPVWSEGYED
ENELKRRIMEDFLSKGEKPTPIMVQKKYQTIASQREIHNTLKAINEAGGKAEYICVDITD
GMMLKEKLRPIIDQFGTITGIIHGAGNLADKRIEKKTVQDFETVYAAKVHGLENLLNIVE
TNQLEYLILFSSVVGFYGNVGQTDYAIANEILNKSAHVIKHKHPNCHVVSINWGPWDSGM
VSPELQTAFAQRGIETIPQELGSSILVDQLRNSDSTMTQVVIGSPLVYIPSTLSSELKTH
QITRQLKLNYNPFLQDHVIAGNPVLPATCGLSWISSSCEQLYPGFQTFHCPNFKVLKGIV
FDQNSPHEYILEIQEVAKIDNQEIHLVGKISSVTNHGKIRYHFSSNLILKRQIPLADNYE
LFNLTQDGQFLASNSLLYQTGVGSLFHGNTFQGVKSVLNISPGKLTMKCELPEPTLNQQG
QFRVQTLNPYIADVQVHSLWIWTQHFHQVGCLPSEIENFEQFAPVPFGETFYVTCEIKSK
TESYVVADVITHNQKGQVYNRMKGAKATILPNS
>1170768.4.peg.1091
MREELLKLPKGYSFYEDELVGFLSKRRWPDTERRIDRTTFWRWRNDNGIEHQKVFSRLDV
LKLCQICDHYRVDGTRNEYLALMKKKKELMLNK
>1170768.4.peg.1116
MDLINEAKEILIQRQDTHLDSLAERLRENRVQAIIEPILSGEELPNTPEDDRRYLLDLGL
VIRSKEGGLKIANPIYQEVIPRVLSQGTQDSLPMIQPSWLMPTGELNPQALLEAFLEFWC
QHGEPLFKSTPYPEIAPHLVLMAFLHRVVNGGGTLEREYAIGSGRMDICLRYGQVVLAME
LKVWRKGSKDPLTAGLKQLDKYLSGLNLKTGWLVIFDRRPNLPPVSDRTTTEMAISPQGR
SITVIRG
>1170768.4.peg.1130
MNFNRLGKFDQLSASVWKIGEVLQILMGNYCFFFLKTADCDTIRVNLAG
>1170768.4.peg.1138
MVIANNRFDSNPTNRAIAVPGNDIEILLTPDQYRRIEETGQERHEYADGRIMIMPGGSEV
HSRITVDITTFLNLALRDSSFET
>1170768.4.peg.1139
MRLNNSDLRIWLPSLNHGTYADIFVIDGSPNRRDEVLNPLLIIEVLSPSTEKYDRGDKFR
KYRSLPSFIEYVLVAQDEPYVELYYKQHGEKNNLWQLEIYDQIEQSVIFHSINVEVPIRE
IYRRTKLP
>1170768.4.peg.1147
MSLFSWRFFPMQLVIFSESVYSVEVVGTPRRHDLKQIGSDRKTT
>1170768.4.peg.1148
MMKNLKELPSIQSAKVTVLHVVPSQSTAAAMTEKWEEGGKILANAIQSLNFDPSQVSSIL
RQGDPKDVVCQVADEIGVDLIVMGSRGLKRLESILSNSVSQYVFQLSSRPMLLVKDDIYV
KRIKRVMVAVDGSPSSSQCLQLALFLLSGVESGQLILTNVNTDLGGKISGITDIKPERNS
VLGNAVAIAESRGIPVRCVTSSGKPGEEICRLAQELNADLLLLGSPDRRPSIAKSFVDLD
RLLGSSLSDYVRVNATCPVLLARTVS
>1170768.4.peg.1165
MGLKSIYNQGNLEPRGPWFWSRNWPWFRNWPETSTHQVNF
>1170768.4.peg.1173
MGSQIVITYYKPIQNNQPPFLRGVGGIPNSNNLLQTNPKELAPLFKGGWGDPM
>1170768.4.peg.1176
MSPCNSKGEMTRKKLFYLKHGVEEYYVYDPDEISLEVSIRENNSFREVEDFATWTSPRLN
IRFDMTGDELVIYYPDGSRFLSPVELSNYAEQERFLKEQERFLKEQANERAEQERFLKEQ
ERLLKEQEQLKYQTLLSQLKAKGIDITALE
>1170768.4.peg.1180
MLPRIGQDSGIYWRFTEQGVFILQNCDRQNLFTINVWAGF
>1170768.4.peg.1186
MVFSFYGLNLSALNFGDFSCDHKRDARAKIANQRAHKLWESHQRVRKITKEIPVTVEPRI
PMDLLGVYILHPS
>1170768.4.peg.1188
MFNLKAPLSKQDVFKVGGKLQNDFLKTLFHTQDRPFPIQHNQQELDLGMLEYGVTSLNRI
GNKLV

>1170768.4.peg.1194
MSDRTLWIISGIIAIITFIVPIITTGLKPLWAKVVTVISLFIILVCVIFPLTISKLEITY
PVNKGTVTKEGYTIKSYDGKELVTIQVKITGKDLPKGKYVQLITSQAGGDESWVNGGSIP
SDVIGETDFSIDNVTFGEKTDQQENYYLYLIMTNKQLRSGDVFNNNEIPDYIDKSNVVEI
RVQKK
>1170768.4.peg.1209
MAFPSTGFANDYIDPGGKIDGQVFEDCEVFQSQICDHI
>1170768.4.peg.1211
MKLRSKQIPPHKIHVALQLIFLKLYILQHTNIRLHWLAQKPPILFPRLYHQRKRSQLPRP
LINLNAIEVVGENLPWNLGRLIPLLFVNGIEQIEGIHQHVPTTTGRVAYLDLLRLTDPQE
IRLRFFRLDVKLHSLCQPRVRPIQHPQPPQGIFHQIPNNPVWGKKLRRCWNILWRNLLIL
LQPLEYLVLLLRDVVLVQPPNHLNILTDIGSHRLTGIGKNRVPRQQVIRQQELGVIVDAL
EQEGHGLVQKVAGRHHQQTVGLALRVSAGYFAVEQSEDLLPDLVVDHLVVDVPHLGFGQN
LGLELSGVGWRDDIHPVIAVHIHEAQGAKAVEPDVGDPLGNLFLAVLLNRLFELLDGLST
FTPCCAISGQHQFQLGEDVFHQITSSILSEAL
>1170768.4.peg.1219
MLSLKRQQREIAETLAIQEREKKEKLAAHLRALGINPDEI
>1170768.4.peg.1228
MFSPGVLSVANFVAILSMAFIAFLSVTLQYHKFLRKGDRTGILILCNLCFPSSPKCDCEA
PVPWE
>1170768.4.peg.1229
MGPQTPSIYQGTEQEFDGALGWHEDENKPDYMGHDCYVREDMFDNPACTWKFGPINRKTF
QPYNKNHPTFILIHKLAETRWQYKLIKNGRRTLLYENHKIRCVLIHDPAEKYSFLVAMNM
GKDIFDFCFKPPHGYGDFTQLKILVVTSGEATKLSTGEIHLQLPAFAFVLAQVF
>1170768.4.peg.1230
MHYFDEYCEVHLDPDIAPYNWVFRSQEKISITFGVHVSKHPDFGQVVYELNDGKGYCDID
RITTQTEGIIENGFKMFSVILPPIGNGGGYQYRVGYIDIDGVEHISEISHFLFVCDEAPR
SMREIPSVFLGMVNNHHVYGPKPSVPMTSSPKNSNSRLFYSIMIDRFARSKHPIRSRLSL
VNYDLSCPHSSHGGTIAGVTEHLDYLKSLGIRVIILSPVYVNAPDGYHGYHPIHLLMVDP
RLGTIHSLRQLVKKAHELDIAIVLDVIVNHIADSINWPEYGGPPQGEFKYIQGDESAVMP
FPIEARNTLLFHGPEYTDMVNQRLFGFLV
>1170768.4.peg.1244
MGGTQTAGVTGGISEALVSTATGLIVAIFTLLFANSFRGLYQRQIALIQEYGGQLELLYR
RRYERGDKSYASTR
>1170768.4.peg.1250
MVALLQKIRLENQSIFQEVNDFLSTRKSNRLAQAQKQAEDLSQFRQQLEQEIDQFLTKTA
QSRQAQAQAQAQELHQFRSQLEQETSEFLTKTTQARHAQAQEQAAELSAFHKQLEQKTEL
FLADTAKARIAQAQKQAAELSAFHKELEQKTSAFLADTSKTRIEEAQKQAAELSAFHKEL
EQKTSAFLTATTSDRAAKAAAQKEALRQFRQDLFLSVIGV
>1170768.4.peg.1264
MVSKNFWKPIIHSSTALVLLTTLNTAWPLVSLAQSKPQPKANSAASSLFTDYLLGGGDRI
RVNVFEAPEYTGEYQIPPGGEINMPLIGSIPVSGLTTQQAADEIARRYARFLKRPLISVN
LLAPRPINVFVAGEVTRPGSYSLSLQGTGGNNPGVQYPTVLAALTTAEGVTLAADVTKVQ
LRRQVGRSGEQVVSLDLKQITQTGRIPIDITLRDGDTIFVPTATDFNVAEARNLFAASYA
ASRTAPRRVAITGQVYRPGSYLVAAGGGGGNDSGGLPTVMRAIQLSGGITSQADVRNIKV
RRPTRTDKEQTLNINLWELLQSGDLNQDVVVQDGDTIIVPTATQVNTAEVTQLATTTLSP
ATIKVGVVGEVKRPGVTELQPNSSLNQALLAAGGFNDARASSSSVDLVRLNPNGTVTKRA
VKVDLSKGINEETNPILRNNDVIVVNRSVLARTGDTLGAVTAPLAPVFSIISLFRLLGF
>1170768.4.peg.1265
MNRLEAAEFAFGWGFDWAKLTNGQAVLRVVSKTRAVLEWIIGFQKFLLTIFT
>1170768.4.peg.1266
MGPNGAGKTTLLKILLGISRPSSGKGLLLGKPLGDRSVKQFIGYLPENPYFYDYLTGYEL

LELTGGVFRISSRVQRQRIVHLLELVGLSQNQARKQRIGSYSKGMVQRIGLAQALINDPE
VLFLDEPMSGLDPLAQYQMREIILSLKSAGKTIFLNSHMLGDVEQICDRVGILNQGEIIC
TGSLSELLGEGKSYHIRGQGGDKEILQKRLGEIQFEREAIWHGILKEDLYDFLASLRLMG
GKVIKISLCRQSLEDFFIQQLSQLPTSSKL
>1170768.4.peg.1269
MQDKALANVFRQMATGAFPPVVETFERNKTIFFPGDPAERVYFLLRGAVKLSRVYEAGEE
ITVALLRENSVFGVLSLLTGNKSDRFYHAVAFTGVELLSSPIEQVEQALKENPELSMLML
RGLSSRILQTEMMIETLAHRDMGSRLVSFLLILCRDFGVPCADGVTVDLKLSHQAIAEAI
GSTRVTVTRLLGDLREKKMISIHKKKITVHKPVALSKQFT
>1170768.4.peg.1270
MAIKQYLNFAPQTMVKSSKVYMEAFLAVGLRWPLGTSS
>1170768.4.peg.1272
MGNQTTAKTWKQQLEQELNPSLDIILVDERYTTLAARDRYWQMFPPRGLTNLVPKGLRQP
PRVIDDIVAILLIERYLEKIGG
>1170768.4.peg.1300
MIKIIITLLEFFASFIKLLLWILFLKTNLVVLYVAIYFFL
>1170768.4.peg.1310
MVTSLVVSIFWEKIHGQCQEFPHSLTFNTKKSFFASLI
>1170768.4.peg.1317
MNIQCGYNSLNPSPSSIVPMVVEQSGMGERAFDIYSRLLRERIIFLGTAIDDNVANSIVA
QLLFLDAEDPEKDVQLYINSPGGSVYAGMAIYDTIQQIRPDVVTICFGLAASMGAFLLTA
GTAGKRMSLPDSRIMIHQPLGGAQGQAIDIEIQAREILYIKANLNQLMSKHTGQPLERIE
ADTERDFFMSPQEAKDYGLIDQVISRQNLPSSGAPVTIVK
>1170768.4.peg.1319
MQFGEVCGGNLNRHCPLRYKPYFIEKLFLANLISILLMVIFCYNRGD
>1170768.4.peg.1323
MVEEEDRVLPLWPHRDWEGWKSTNCHLSPGMLRFVTLVIKPM
>1170768.4.peg.1326
MLEREYHEVWYKQVTSTSHYRPYLAYLLANLAEKFNTDGKIDDKFSVDGLK
>1170768.4.peg.1335
MVTRLETSPASRTPEQGGLLLWVVTKWDGFLRGFRLFLPSKVANNVSDGFLTMFFAFLAV
LAIGVTATSLISQIGSPSVPQLSQPPTDSTVEGKTQTQLSQPPTDSTVEGKTQTQLGQPP
TDSTVEGKTQTQLSQPPTDSTVEDKTETQLGQPPTDSTVEGKTETQLGQPPTDSILKSQA
ELIPQQEVTGFIEKQLKEITATNIITKDKQRIEVEIVQSIKTNFRISEIVIKTTEAWYKL
ESSQQEKLAAKILKSCQEMDLIHVKILNARNQLIARSPVVGTKMVFFQFPNS
>1170768.4.peg.1343
MGVIGVGNMGQHHARLLSSMKDVELVGVSDINVERGIETASRYKVRFFEEYCDLLPHVDA
VCIVVPTRLHYAVGINCLLAGIHVLIEKPIAASIPEAESLVNAAAQSQCILQVGHIERFN
PAFRELSQVLKTEEVLALESHRMSPYSSRANDVSVVLDLMIHDIDLLLELAGSSVVKLTA
NGTRSLDSGYLDYVTATLGFANGIVATLTASKVTHRKIRHIVAHCKNSFTEADFLNNEIF
IHRQNTNPQNDRQTLYRQDGIIEKVYTSNIQPLSAELEHFVNCVRGGNQPSVGGEQALKA
LRLASLIEQMALEERVWNPLEWQSESVSQSLTSSV
>1170768.4.peg.1346
MLMTGSVSAFLGFSMGSSALKGVTSPDGRPTSKLISSKNNDLQSVPISFLKEEDIINQVK
KRIEQNKTKNNQTKKVEEEEETVYTKDQSQQKAQEISEEPPQSGFPVVAESEGVNMSVRS
ASYSGGQLILKVKMHNQSNESVRFLYSFLDVTDDKGRVLTATTDGLPAELPGNGPIFTGK
ISIPTALLNDVSSLTLSLTDYPAQKLKLQLSDIPVEK
>1170768.4.peg.1380
MGINPIPYFSELPGIKPTKKFSGMGIIKNCCYKNSVTDK
>1170768.4.peg.1384
MDLHDLAEGLPTDYEKLMTVAAETYEIYKELDRLKQQLKTMETS
>1170768.4.peg.1399

MHFIDSSGLTCLVAGMRNVDKLKGTFRICNIHPDAKLVFEVTMMDTVFEICETEEEAFAI
PF
>1170768.4.peg.1408
MGSFAVGVLAFRPQVGKLADHQGRKLVLLIGMVVATIAPLGYLAVKSLVGLMLIRAFHGI
SIAAFATAYIALVSDLAPDDRRGEIIGYMSLVNPIGVAVGPALGGYLQAIAGYTPLFIFS
SLLAGLGLICVIPITNPPRWKNNKQEKQEKQETGDDFWGILISPRVRVPAIILLMIGFSI
GTIHTFIALYIKSIGVDLNPGLFFAAAAISSFVIRLFVGRASDKYGRGLFVTLSLISYTV
AMLIIWQANSSPTLLLGAIMEGAASGIAIPMISAMMTDRALPHERGRIFSVSLTGFDLGL
GIAGPVVGYIAQSTSYRHVFGLSFGLTLLAILIFISQSNHGVFQSLRFALGRSKDVYQQS
ATDSPNLSTNT
>1170768.4.peg.1409
MYNGNVGNRDAKADQNNKPHVKITTKFFLKSGSKLGEFLTLNTT
>1170768.4.peg.1423
MPIVGWLWLRGKCADCQSKISYSYPLVELLTGIIFLIVFWVFQFSLLTIGYWVFCSWLLA
LSLIDWETMTLPGSLTKSGLVLGLIFQTTLGYVAHNTWSSAIGQLIWGIGGMVLGLWLFD
IITSLGFLFYGKPVMGGGDGKLAAMMGAWLGWQYLLVASFIACFSGVLIGCGAIIVCDGQ
IEQKMPFGPFLAWGSVISIFGGEIILDHYLRFVLSHS
>1170768.4.peg.1425
MGSATAVELDEATRTVKLNEAGDTIVISPKQLQQGKRLFQDTCAQCHAGGVTKTNQNVGL
EPEALAGAVPARDNIEGLVDFLKEPKTYDGELDISEIHPGLKSADIFTEMKNLTEDDLKE
ISAYILIQPKVVGTRWGGGKIYY
>1170768.4.peg.1438
MPIDQGVWNGFLLILPIYQVFGDQMSSIYNIYPWFGDDFTRKR
>1170768.4.peg.1455
MDWFLQPSHPHRARWLKSFVGDSLCVHDTRLKQNLFGLNFPNPLGLAAGFDKDAMGSHFW
SMFGFGLAELGTVTYHGQPGNPQPRLFRLPMDLAALNRMGFNNSGAAAMAARLTLLYQQQ
LPAIPIGINLGKSKVTPLEAAATDYLQSFRLLRNLGDYFVVNVSSPNTPGLRSLQDRPML
SQILEVIQTENSIPNQPKPLFVKIAPDLEWEAIVDIINLAKTYKLAGIIATNTTISREGL
KTQVIEKTGNPPQAEPGGISGLPLRQPSTEIIRFIYKQSQGQIPIIGVGGIFTAEDAWEK
ITAGASLIQVYTGWIYEGPMMASRILTGLLAKLQNHGLGSIGDAVGINN
>1170768.4.peg.1478
MQMAQAAGLRGRLLVTVGILILVRLGIFLPVPGIDRERFAQAISGNNAIFGLLDIFSGRG
LSTLGVFALGILPFINASIIIQLLTAAIPSLENLQKNEGEAGRRKISQITRYVSLGWAIL
QSTAFSALFLQQFALNPGPIFVAETAIALTAGSMFVMWASELITERGIGNGASLLIFVNI
VASLPKSLGDTIDLVQVGGREIVGRVIVLVLVFVATIIGIVFVQEGIRRIPIISARRQVG
RRVLAEQRSFLPLRLNQGGVMPIIFAAAILSLPLLIANFTKNVELANIVNTYLSPSGSSS
WVYALVYMVSIIFFSYFYSSLILNPVDVAQNLKKMGSSIPGIRPGKATSEYIERVSNRLT
FLGAMFLGLVAIIPTAVESTLNVPTFRGLGATSLLILVGVAIDTARQIQTYVISQRYEGM
VKQ
>1170768.4.peg.1489
MEGLAHRGSTLNFGDFALQAQEPCWITSRQIEASRRAMTRYIRRGGKIWIRIFPDKPVTM
RPAETRMGSGKGNPEFWVAVVKPGRILFEIAGVSEEIAREAMRLAAYKLPIKTKFIVRSQ
PQEQE
>1170768.4.peg.1498
MALITTGSGFIRDLEKFGSLGVFIPLEGGYEGRYRRRLRAAGYVNLHITARGLGDVAAYL
MGVHGIRPPHLGKKTTSNGAAVGEVQYLPPLISSHLAQLPPNSKGLLLWVIEGNILSDQE
VEYFTNLPRLEPRVKVVIERGGDRYFRWTPLEKTLLAS
>1170768.4.peg.1501
MSVIWGCCSFYIIQILQPVIWSILTLRIKPINMLLTFVKIESDLKVSLF
>1170768.4.peg.1510
MSREEIRGVWVTSNDLNVFKDRAQVKDAVTKLRRLNFNTIYPVVWNSGYVMYPSNVAKSL
DIQPFVFRGSDGHDILADIINQAHSQNLLAIPWFEFGFMTPNTGELALNKPEWLTKMGDG

STVSMSAAGEVSWLNPFHPQVQKFIIDLLVELTNNYDIDGIQFDDHTSLPHQFGYDNYTV
NLYKQETGKNPPANSQDPEWVAWRANKITEFMVRLNHTIKQIKPKVIFSVSPNYYDHAYK
FQLQDWLNWVRLNIVDELVMQVYRDDLESFTSKIARNEIQEVRQIIPTGIGIMAGLRTSP
VPMQQITKQVRTVQREELGIVFFYYETMWNRSPETLEQRIQGFKNFFPYPAVRVAAE
>1170768.4.peg.1526
MVKFTRQLPAVINLQWVPSNTLYNVPQFPQVDRQTTV
>1170768.4.peg.1527
MFQIKCQYMKSRLESPGEAASVAYKDESFANSLFLRKLTTIL
>1170768.4.peg.1546
MGATLQEISHSYYCQHYNSWVHHNNIIIIILRSPILL
>1170768.4.peg.1563
MILRGELVTPESDLIDRTVYNGMFTMHGTVMLFLWTFPSLVGLANYLVPIMIGARDMAFP
RLNAAAFWMVPVVGILLMSSFAVPGGPAQAGWWAYPPVSLQNPTGNLINGQVLWLLAVAI
SGVSSIMGAVNFVTTIVKMRAPGMGFFKMPLFVWAVFSAQIIQLFGLPALTAGAVMLLFD
LTVGTGFFNPINGGNPVMFQHYFWFYSHPAVYVIILPVFGIFSEIFPVYSRKPLFGYKVV
AISSMLIAVVSAIVWVHHMYVSGTPGWMRLLFMLTTMFVSVPTGIKVFAWVGTIWGGKIK
LTTPMLFALGGLIMFVFAGITGIMLSSVPIDVHVNNTYFVVGHFHYVLYGTVTMGMYAAI
YHWFPKMTGRMFNEGWGRVHFWLAFIGTNLNFLPMHPLGLQGMLRRVASYAPEYTFWNIL
ASLGAFLLGMSTLPFIFNILVSWLDGEKAPPNPWRAIGLEWLISSPPSVENFEEIPIIIS
EPYGYGKSGPLTANLENSGMEMVE
>1170768.4.peg.1573
MMGWYLNENIWVYLGKYSGIPIDEEVDDLPVSIGITG
>1170768.4.peg.1574
MTPIDKIPAHFLPNYVILSHSMSSYVLNTLPKYHPMTINIILRVLNGNFNEGFIIDVITD
GKTEQLSIIQNRNIPKLYSKWQYILHRNTIRKISDIEEQETHISTEKLRNAKTELEKEYK
QWLNNQNIGMLISNMIELSTSSPSQSPQNPNLIRIQLILSDTLNPNAQQDNDILRRLPWN
AYLSSYIRFNNYDTTFLLSKDHTSTPSHPNLFSSNPPKILAIFGGHQGELACSQADEANR
LKEICEPNGATVIFRNITDRNEFHQLLINDNYDILFYSGHSTSDQGGTIQFEQETSIIRD
FKNDLIRAVKKGLKIAIFNSCDGLKIADFLIEEVGMPAVIVMKEPVPDEFAREFLERFLI
HFVEECNFLSVAVEKAAKDIRYKEDQEFPGATWLPITYYSNAKSEEFTWSKKNQIYKNRI
DIKIHNYLYQRQEENSLCPYQGLFHFGPEEAEYFFGRQIFIEQLYEATQNRKFIPILGAS
GSGKSSVVLAGLVPKLKNQGNWEFTYFRPGKEPFPALATALIPLLYTSELDTIEKMAQGR
ILATHLQTGTIPLSDVFTQIQQKHPTEKVLIIADQFEEIYTMCPEPETRQQFLDCLIASL
SIPNSPIVLVTTMRADFLSDALSYRPFGDTLQNADLKILAMNREELTEVIIKPAEKLGVS
FEQGLAARILNDLEQQPGNLPLLEFALTQLWNQHHEWQLTHNAYEKIGQVQGALARYADE
KYSNLTKTDQEKMQRVFIQLIKPGEGAEDTRRIAFKTEIGEENWLLVRQLADARLVVTSR
NATEQEIVEVVHEALIRDWGELRKWINNNREFRNWQERLRVAREQWRITQNDRESLMRGA
ALAQAEEKLRERPEELIDEREFIEESIKERKRTEEIENERKKIQVMTAWAIAAGSLVAVV
ISTTLWLIAIQQRNQAEVSQAESLSRYSSSLFTEDKQLEASINAIKARKLVQKKPGTHPL
VITALQNIFSTNQRHSQKHESSVRSVSISPDGKTLASGSLDKTIKIWDLATGNLINTLKG
HESWVLSVSISPDGKTLASGSLDKTIKIWDLATGNLINTLKGHESSVLSVSISPDGKTLA
SGSYDKTIKIWDLATGNLINTLKGHESWVLSVSISPDGKTLASGSDDKTIKIWDLATGNL
INTLKGHESSVLSVSISPDGKTLASGSDDKTIKIWDLATGNLINTLKGHESSVRSVSISP
DGKTLASGSDDKTIKIWDLATGNLINTLKGHESWVLSVSISPDGKTLASGSDDKTIKIWD
LATGNLINTLKGHESSVRSVSISPDGKTLASGSDDKTIKIWDLATGNLINTLKGHESWVL
SVSISPDGKTLASGSDDKTIKIWNINMDLDYLIGVNCNKIGNYLLYSNEVKEQDKSLCNN
>1170768.4.peg.1578
MSPCNSKGEMTRKKLFYLKHGVEEYYVYDPDEISLEVSIRENNSFREVEDFATWTSPRLN
IRFDMTGDELVIYYPDGSRFLSPVELSNYAEQERFLKEQERFLKEQANQRAEQERFLKEQ
ANERAEQERFLREQERLLKEQANERAEQERFLREQERFLKEQANQRAEQERLLKEQEQLK
YQTLLSQLKANGIDVTGLE
>1170768.4.peg.1598

MLTLTKVFIKFSSPVDIAQLFTYHINGAGKIKKKLKK
>1170768.4.peg.1601
MGGYFFANWFTFSHRHPASTPEEKFLSLIISLVTTVFWPLIIPISCVKILKTRQLRSDTV
IPLLLVMLGLIVSYISCQLLKLVYLPS
>1170768.4.peg.1614
MLPALKRRGFTENPKHNFCVELALIKHKPFPEKSKLKR
>1170768.4.peg.1618
MVTNADEILAQMMETCPEMRDEFNSTFKLKDDPRITKVGHFLRITSLDEFPQFWNVLKGD
MSVVGPRPLVVKELQKYGSHIDQVLTIRPGITGLWQVSGRNDIPYPKRVQIDLHYVRSKG
FLLDLWIILKTINIVIIPKNNGAY
>1170768.4.peg.1633
MVFRSAVRQQLLYDCGLDSFTLYNPKKLDNSGFYRLEVWK
>1170768.4.peg.1640
MYSILLKRQISIYIDNYLTVHKNMLKNYTKLSVNIRDQFYKGKIGNRGEGVGS
>1170768.4.peg.1644
MLVLTAGLFSLSLVPLSRTVIPGAARYTLVYDNGSNQAVIATSPDITPTQLEATLRQAAS
NLYSYGRMGAGGNNSLTVRARTILHPESGISTPVYLGKVERTLVTREDPQMLVEVFLDKF
PQLPPS
>1170768.4.peg.1647
MAAATIYLPYLLVAYARVKVGYDLSAPRSMFDKLPPYARRATWAHQNCFESFMIYAPAAL
MAYVTGVDSQLALYAAIAYVTARVLFSVFYILNVPILRSLMFGVGSACIINLFVLSILRA
TT
>1170768.4.peg.1649
MNLAELGRMEILDNAVEQEEPSLDNLEAVVFEDSSILESLESDERDGDEMGAARPSGYNK
TEHDDAVGAFFKEMARYPLLKPDEEVELARRVRFLEEVKDLQAALEEQLGHSASKSEVAS
KFDLNEKQLESRLYQGRVAKRKMIRSNLRLVVSIAKRYLNRGVPFLDLIQEGAMGLNRAT
EKFDPDKGYKFSTYAYWWIRQAITRAIANDARTIRLPIHIVEKLNKLKKAQRELKQKFAR
NPTEVEMAEFLEITVQQLRQLQQLRRQALSLNHRVGKEEDTELMDLLEDEDNQSPEAKMN
ENMMRQEIWEVLGDVLTPREKDVISLRYGLTTSEPCTLEEVGNMFNLSRERVRQIQSKAM
RKLRRPHIAKRLKGWLI
>1170768.4.peg.1650
MSHKKNLHIYQEVEKPYYLLGKYKDKQKVNKNNKIEDLIFNILIFM
>1170768.4.peg.1659
MQAFFVGRAIAEVINERLEVTLTDALSELGKFDAEMKEQLHQFTEEVMARASRAAEASQT
GVTPSSTNANSVTDLQAEIDDLRAEVALLRTELQKYRSGAK
>1170768.4.peg.1672
MSGYNQPISGGREFFESLVRAISGYAQEFLSNVPNPQAHSKGSELVEVERINNDRHQLII
YSDSTDNRDNPLRIDMNTVGLFDLVEAVDQFFADSQTLPELILELQPVSRSYGMSGQLAL
RQAVPATVGVSSLAIAALALGIIPPPEWNPTTQNTQTSSGVPESSAASTTSPTPDSTVAS
NIQDLEALLNSVPEITDPSQLKSLNRQLYNRINSEWQNRGQIGENLVYRIGVATDGRIIG
YKSLNQSSSDQIEKTPLPRLLYNPVSGTSNKEPIAQFRVVFTNKAQLQVSPWIGYSGTPD
VIGDKITDPSSIRQLNRTLYQTIRQNWSIKPSFPRELRYRVAINKNGVIADYEPLNQVAF
DYYTETPLPEIFKNLYGSNLAAPKNREPLAHFRVLFKPNGVLEVSPWEGYR
>1170768.4.peg.1694
MFCGNFPIQTAIALQGNQTSIKPYLDRVVKQLTEFTLDNGLKFIILERHQAPVVSFLTYA
NVGGIDEPDGQTGVAHFLEHLAFKGTRRIGTTNYKAEKPLLDKLEQLDSQIRAAKSENRT
EELEKLQKEFKTVEAEAGKLVKQNEMGQIVEQAGGVGLNANTSSEATRYFYSFPANKLEL
WMSLESERFLEPVFREFYKERDVILEERRMRVENSPVGLMVEKFTDVAFKVHPYRRPVIG
YDEDIRNLSPANVREFFNKYYVPSNLTIAVVGDVNPNQVKRLAKIYFGRYPAKPKAQAKI
APEPKQTSTREITVKLPSQPWYLEGYHRPSITDPDNAVYDIISSLLSNGRTSRLYKSLIE
TQRVALVAEGISGFPGDKYPNLMLFYALTAPGHTVDKLAIALGQEINKLQTQLVSEKELE
RVKTQARAGLLRSLDSNMGMAQQLLEYEVKTGSWRNLFKQLDDITKVTPADIQRVAQSTF

TAENRTIGKLLSQKS
>1170768.4.peg.1699
MENRVRTLREEFTRNFDPSQAQSIMREHSYRLQPNQRQIILVNPDRKTVEPSDSTQ
>1170768.4.peg.1701
MRGYFECENLKKSNYPYISAQANCPMKSNDKIFLAGSLGVLLTIYYYAICVPFRD
>1170768.4.peg.1721
MNYRSPETILQVANLSKSFILHQQGGVSLPVIQGVSFQLNQGESLVLSGASGSGKSTLIR
CIYGNYRPNNGEVWVKHEGNWIDLCQLAPHELLAVRQKTIGYVSQFLRVIPRVPAIEVAA
ELLLDLGEERKVALAKVKELFHRLHLPERLWQLSPTTFSGGEKQRVNIARALVVNFPILL
LDEPTSALDTHNCQIVMELLEERKIKGCALIGIFHNIDQGKNLSTNTAKKLFDRELFVHE
>1170768.4.peg.1723
MILTKTIVCKLSPCPFKIDFPQPPQEKNVRRSIKLRSPSVVLVVLSN
>1170768.4.peg.1729
MIGQNGCGKTTLLLLANGLYKPNRGVVSWCGEPLTYNRNYLGRLRQKVGLVFQDPEQQLV
ATTVEEDISYGLCNLGLPVVEIQQRVEEALMEFELTNLAQTPTHHLSLGQKKRVSIADVM
VLRPELLLLDEPTAYLDIKQTRNLIAMLNKIHEYGTTLVMATHDLDLVYRWADWVFVMDR
GKLVLEGSPQDVFSQRRLLEELELGLPLICEMLFYGLSDEWLEK
>1170768.4.peg.1733
MLLLLIANKVTSEVITTLGTASEEIFRGDRLPPLKFPETSCAEDRITG
>1170768.4.peg.1739
MLIIILLKEIAKKHITIYNQGTTEGNFTENQGFLLSDNL
>1170768.4.peg.1741
MVLVGPSGCGKSTLLRLIAGLEVMTGGNIWIGNDLINDLPPKARDIAMVFQNYALYPHLT
VYENIAFGLRRRLPINHTSTKGTVGRWGENLLMGLTQKLPKQLRYQTQTEKIVEQQVLKV
AHLLQIDTMLHRLPKQLSGGQRQRVALGRAIARDPQVFLMDEPLSNLDAKLRAETRVQIV
KLQRQLGTTTIYVTHDQTEAMTMGDRIAIMNMGQIQQVARPLEIYNYPKNRFVAEFIGSP
PMNFISVEFYAPLLITHSNFRFTLPNEWGKALQKYSGQTVILGIRPEHLMLSAPATKNIP
VKVDLVENLGNDSFLSVRVINPDLPKLDGQILQVRVPSDRLINMGDQIWLSPIVQKLHFF
DPETELAIFTDNKTINF
>1170768.4.peg.1769
MFSRMLGKEENSFFPLSSKYYWFFEDFVNTEKLRFQNSIFLF
>1170768.4.peg.1772
MVVYSVPENSGMGRPQHIYHLSPGGKEYLQRSTTKVSGGYGEFAVSLLDTLAETVGTEQV
KTILQKQWERKAQEYQEKVGQGCLEERVATLVELRKAEGFMAEFRPVEEKDDGHEGNHGF
LFMEHTCAISNVAESFPSVCGHELEMFAAILPDCSVQRTHWLIDGQHRCVYLIKKN
>1170768.4.peg.1815
MFYLNWRWYSATSVERGFMYFLVFFFFPGLLLLSPIANFRPRPRQIV
>1170768.4.peg.1829
MWGSYCEEVLTKREPYRQAHLEGLAKQKDAGILITIGPTKDITKVFGIYEAESESAVSQL
IEADPYWKNGIWTEYIVKEWIQAF
>1170768.4.peg.1843
MIWVVTAKLLLPKDSILGKFQPLRFWQKTFLKKSRKSS
>1170768.4.peg.1854
MRRQRQNRTDMCQDITECQHDINKLLDRVRAGQVLKLPKISISKIVDTKGNRIDRKINSG
QINQVWRYGAEVIKIEEVDRIQVATDGDIENRLLKISEWIQTELKD
>1170768.4.peg.1908
MGENIALNVERNGFPIAVYNRSREKTDAFMSNRAGGRNVKAAFTLEEFVASLERPRKILV
MVQAGKPVDAVIQQLKPLLQEGDIIIDGGNSWFEDTERRTQELEPTGLRYIGMGVSGGEE
GALNGPSLMPGGTKSSYEYLSPIFNRIAAQVDDGPCVTYIGPGGSGHYVKMVHNGIEYGD
MQLIAEAYDLLKNVGGLDATQLHQVFTEWNQTDELNSFLIEITANIFPYVDPGTKQPLVD
LIVDAAGQKGTGRWTVQTALELGVAIPTITAAVNSRIISSIREERIAASKQLTGPVPTQF
KDTRTFVNMVRDALYCSKICSYAQGMALISTASKTYNWGLNLGEMARIWKGGCIIRAGFL

NKIKKAFDENPALPNLLLAPEFKQTILDRQSAWREIIVTAAKTGIPVPAFSASLDYFDSY
RRERLPQNLTQAQRDYFGAHTYKRVDIEGTFHTEWVPIAEAKK
>1170768.4.peg.1936
MDDEASIRRILETRLSMIGYDVVTAGDGEEALETFRKADPDLVVLDVMMPKLDGYGVCQE
LRKESDVPIIMLTALGDVADRITGLELGADDYVVKPFSPKELEARIRSVLRRVDKNGTTG
IPSSGVIHVGNIKIDTNKRQVYKGDERIRLTGMEFSLLELLVSRSGEAFSRSEILQEVWG
YTPERHVDTRVVDVHISRLRAKLEDDPSNPELILTARGTGYLFQRILEAGED
>1170768.4.peg.1937
MEQNANSFGVGGWHSAQPSAKSTKPSKPIASLTFDQISDRQIARWESGYSELDRVLGGGV
VPGSMVLIGGDPGIGKSTLLLQVSNELSQRYRILYVTGEESGQQVKLRASRLGMSKPLQV
VSVEDSNTSSESSHPLPEQDLGNNVGDAVEDDHRVTVEHHTISSDLYVLPETDLEEILRE
IDSLKPNLAVIDSIQTVFFPALTSAPGSVAQVRECTAALMKVAKHEDITMLIVGHVTKEG
TIAGPRVLEHLVDTVLYFEGDRFASHRLLRTVKNRFGATHEIGIFEMVSNGLREVPNPSE
LFLGNRDEPSPGTAIVVACEGTRPIVVELQALVSPTSYPSPRRAGTGVDFNRLVQILAVL
EKRVGVPMSKLDSYVASAGGLSVEEPAVDLGIAVAIVASFRDRIVDPQTVLIGEVGLGGQ
VRSVSQMELRLKEAAKLGFKRAIIPKGQKFPDLKIEILEVSKVIDAIIAAIPHQSLENGD
LDLEEEDED
>1170768.4.peg.1952
MATCLHLEKIQCQDFGLIEQFLIALDFFFLNESHNWIVDRQNACPKTYLGAICERVVFGR
NTHQM
>1170768.4.peg.1957
MLIRVYISHNFYLLITLITRYEKGVHPVDVPLLSLPTWQI
>1170768.4.peg.1967
MQKRGFGRRTASLVISQYTAVTIQGEWLEAFWCPECEEKKWYYVRQSNNGIYEISLAPMD
LWKQVSGVIDPYGNPSVSEFTRLSAKQITYRGLSAFYSL
>1170768.4.peg.1980
MVKLLDDLQLHRSQHGTKIVYSTGRSLHLYQELQESQKRKQRDLIKPDILVCAVGTEIYH
YDGKEELVLDREWSKHLSCNWDRELVARTAANFPSLKPQPESEQRPFKVSYFVREEKAAQ
IALELENLLVKEGKVEIQIICSHSDHKEYNRNLDILPSSANKGMAMTFVREKLAIDVEKT
VACGDSGNDIALFANRQEKGIIVGNAQGELLDWHHNNPNPNRYLAKNNFADGIAEGLRHF
SLL
>1170768.4.peg.1982
MDCKKALNETQGNIDEAIDWLRKKGIASAGKKSDRVTAEGLVEIYTTPDRSGGVLIEVNC
QTDFVARNDAFKLLVKNLAKQAVNADNVTSLLAQPYIENTAVTVDEYIKETIATLGENIQ
VRRFVNFSLEGSPGAIDGYIHTGGRVGVLISIGVDTETAAKNEEVQSLAKNTAMQVAACP
NVEYVSVDKIPDQVVQKEKEIEMGKEDLGNKPENIKEKIVQGRIDKRLKEMTLLDQPYIR
DQSISVAELIKQVESKVSEPIKVHQFVRYILGEGIEKQESNFAQEVAAQIGAK
>1170768.4.peg.2003
MFEHFTSEAIRVIMLAQEEARRLGHNFVGTEQILLGLIGEGTGVAAKVLTDLGVTLKDAR
REVEKIIGRGSGFVPPEIPFTPKVKSLFEQSFREAHGLGHNYINTEHLLLGLTDAGEGVA
AKVLKSLGIELQTVRSRVMSILGEDNRVVAGRQDNPRRNQNLSIEEFGRNLTKLAQQGRL
DPVVGRQTEIERTVQILGRRTKNNPVLIGEPGVGKTAIAEGLAQRIVNQDVPEILLNKQV
ISLDMGLLVAGTRFRGDFEERLKKVMEEIRSVGNIILVIDEIHTIVGAGGTEGGLDAANI
LKPALARGELQCIGATTLDEYRKYIERDAALERRFQPILVGEPSVAETIEILLGLRSAYE
QHHKVTISNDAVIAAAELSDRYISDRFLPDKAIDLIDEAGSRVKLRHSRIIDNKELKQQL
KNTSQEKVEAVRVQDFGKASKLRQEELDLQTQLAIAQNLPKITIPQVDEEDIAEIVSSWT
GVPVNKLTESESELLLHLEDTLHKRLIGQEQAVTAVSRSLRRARVGLKSPNRPIASFIFS
GPTGVGKTELAKALAAYFFGAEDSMIRLDMSEYMESHNVSKLIGSPPGYVGYDEGGQLTE
AVRRKPYTLLLFDEIEKAHPDVFNMLLQILDDGHLTDAKGRKVDFKNTLIILTSNIGSKV
IEKGGMSLGFEFDNQANASYNRIRNLVNEELKSYFRPEFLNRVDEIIVFSQLNKDEVKEI
SQIMLEEVAKRLQEKGIKLEVTEAFKDLVVTEGYDPSYGARPLRRAIMRLLEDSLAEAIL
SGEIREGDQAIVDVDDDGLVKVKKAETRDLVLVSAPA

>1170768.4.peg.2026
MNDIEWSTTEKELARKAFDRAYEREISALIETVREQASTITKLDEIWQLHDFLSARRHQI
DGKYDYRYSVLIFVFAQLVREGWLHIKDLEGLEVDKIAKVSALTRM
>1170768.4.peg.2036
MDRYVVYVESDSQYVLQQVRRVDSNAYIRPFGGRSIIQSGVFSESYYAAERVRDLELNGV
SDIRVMVVSPPGQISSSVSTTTSNLGYDSGAANSSDNSTNIDPTKQAHYYAESYYVVVPT
SAENLRTIWQQIRDQVKGRVHVLMRNEPRGFHVALGKFKDRYQAEQWSNYLKRLGYGNAR
VYYGR
>1170768.4.peg.2038
MQGDVLGEFPGHQDWVTSVSFSPDGQTLATGSRDKTARLWNLQGDLLREFPGHEDWVTSV
SFSPNGQTLVTGGADKIARLWNLQGDLLGEFPGHEGGVTSVSFSPDGETLVTGSVDKIAR
LWKLKGYLIREFKGHDSGITNVSFSPDGQTLATASVDKTVRLWDLKGQLIQEFKGYGDTF
TSVSFSPDGQTLATGSLDKIARLWPVRYLDRALEDGNTWLIDTNF
>1170768.4.peg.2042
MQVIEKNSLFAQVSGEQSAVVSGGSISEAQTLIIAYQLGQSSVLNDTQLFNVFLVSLST
>1170768.4.peg.2047
MIDQVINVAFDVAFSTLKNDEPLSNKLYGFCLVGDEVFDPS
>1170768.4.peg.2056
MWGKEKGDRYGDLPFALVILTKGQVLFSTGMVTHTLY
>1170768.4.peg.2067
MVIVVMRTGTSFPVVLCERTTTHRFDGNTRRLILQPAR
>1170768.4.peg.2078
MDELPLLLHYYYLTIASECYQDAVKNISKEYDLNYLTISPYSL
>1170768.4.peg.2079
MTNIQSPKLTSPKLTAYEFKNLPLLGTTTTNQNILLGGFSGLYFQGVADNGNLQFVTHTD
RGPNSEPTGQNRPFLLPNFQPEIVSFELNKASGEINITNRTKLFRADGKTPLTGIPNVQA
GAGNTAYTDEIGVNLNGTVLNNDPLGADLEGIVIADNGDYWMVDEYRPAIYHFNRNGILV
SRFIPQGTASKSNQPVGTFGIEVLPEVYAQRRNNRGFEAVALAGNKLYAFIQTPIDNPDN
TGDTTSRSSRNLRILEFDIVSEKVTGEYLYLLDDITGTGNAKTDKIGDAVFLGGSQFAVV
ERDDRSDTTSNKLIYQIDLIGATNINNTSKLSLPQGKTIEQLTPVELGPAGITPVNKSLI
VNAAQIGYTGVEKLEGLALVSASTLAIVNDNDFVTTIPEKLGIIELPNNLVEVTGTANKD
ELFVKKGEYVLGLDGDDILDASDGLGNNVLDGGAGNDQLYGGKSDTLIGGAGNDQLYVVE
GNNNILYGGAGNDRLFVTEGNNNILYGEDGDDNLYIIEGGNNTLSGGGGADKFYIVNGVI
PVVPGQVLDFTRGDDKVIISGISQIQGFDNLILEQNANNTTIKTKDLIGGSQKVLGILKG
VVANTLTADDFRINDVTITNPQVSGTFNPIATDNILTQEEKKGGLTLSGTVSDPNAQVTL
QFGGQFRVAKVQSSNSSWSYTVTDNDYNFFREGQNQLTAIFTLTNNNRTGTFRASQTLTI
NSGVLPQPTSPTLDPTKPKGISEAVTQKAQSANIGQGILGSGEKLTDPNIVVIPFGGINT
FNQGANAAIEDFGLIKTGSNTTIESVRVFPKSPTGEVSVTLSDGTSVLASIPPGISSIGD
PLGLTMSGLVPGGSSTLDFYLPRNILNQLPSDLQSATYAKFNFEAKKFEQYADEQGKPLY
QYKFTDKNSNGIRDFGELYLSLNLTDGDKWDSDRIQNGIIVDPGQLGIAVVVTGTGVTYS
SPGNSLIPREAPALSNIADNVFYKFSNTYKPAEARQALQNALGQTDAAFKNIFGLYEIDN
ATGSVNGIEPGKPGYAKAALDKSKVISNFTVRAGGSVTPNINGDLIGIGDKTYAPFVIAN
GGNYSGSIQEAINEFFKVNPNNSAATAQNYMGLPVAYFSFGAANPDGAAHIKSFGNNIFG
FEDLPAGVGVSDYDFNDMVFSFG
>1170768.4.peg.2101
MINFCFSVYFCLVYLYYISGEIYLNPFPQVDVPTPDNCYL
>1170768.4.peg.2106
MVCRITAFYGGLAPLNLFYIISGPSVLLIVIIFPQTVRWLIDIYAV
>1170768.4.peg.2113
MNQQTSWHRVILGNGNFSATVISIAGILIKQSCFLKGIVDP
>1170768.4.peg.2118
MLTERINRLSQHLQANKKDHSSRRGLLKMIGERKRLLNYIQKNNSDKYQSLISRLGIRG

>1170768.4.peg.2122
MKTSKVRKMIFLVDIILVVSWLVSIVTQLSYNFIYAM
>1170768.4.peg.2129
MLNLKTVIFVLSFTPFIVLSTQLTGRAQVSSPSLGTAGSVSGGGATGSDNNPISAPSLGT
AGSMSGGGATGSNSAPSLGTAGSMSGGGSTGSDNITVIVSTNNLRELRNAIGQLVSSGSD
SLSPITFSSLRASLISDGVSPGHTDQLIVSFSRVLSQLGVPNTNFSANNLNLEKLVASTK
IFKPTVTIVQDSPAEGEIALILDSNNLTEAINIYNKIVLESDDPTIIKLSRNQDFLKISR
ILKTLRSGII
>1170768.4.peg.2134
MGKININDTTLRDGEQAARVTFTLKEKVAIAQFLDAIGIPELEVGIPAMGEGQINAIRAI
NDLDLNAKLLAWNRAVISDIKASISCGIKRVHIAIPVSGIQIASKFHGQWRVSLQRLQDC
IHFALDHNLWVGVGGEDCSRADESFLEDVALYAQEWGASRFRFCDTVGVLEPFRTHLKIK
RLVSRLSIPIEIHTHNDFGLATANAIAGIRGGALSVNTTVNGLGERAGNAALEEVVMALK
YIYGVDLGINTDNFSQLSQLVANAVGSNVPPWKAIVGQNTHDRWLGLDDHTIVKNPEIYR
RFALPGEW
>1170768.4.peg.2135
MITTLIAGLTILVITGMTNSYISHKLFIKDSDQPTNKPS
>1170768.4.peg.2138
MKISMGVFKNSMGVISKHEKHLETYRQFPEDDGCYHQR
>1170768.4.peg.2153
MKSYARQTEAVWDDVLVPILKNFIPVLTYIIGFSLFFTVLGVDLSGIGLALGSITLVLGL
AVRDILSNFFSGLVLLIDTPFEFGDVIVFDGSLAIIKEIGIRVTKLYLIEEHCEKYVPNA
TLSNQSITNLSRPTTHYAYKIPVAVRIDADSALATNILKEIVIGHPDTIANFDDKLRYLD
SFYGLKEAQDNKPSKKEAGRNRIELDREISLQLKKIAATSETLLGEIKVLERGGLDGQEL
MVLQKTYMDILELVGMVIVTERKGKRQRSRLEEESSQKTNLISLVRIWYRTWLEDPDLVM
EDRQILPDEWERKIDLLKLKLTKLFQIISNPGVKETRLDNYVENFAEWLESSFKESSTAW
KEPQVQITDIQGSSMEFVVRFYVDNIQLEHWRRGERVKNEVRREMIRRLRLAHIYTG
>1170768.4.peg.2155
MILSITIPVFIWLVVSNSGLVKPLFLPTPQAVLTALQKLWATGDLQTDIGFSLLRVLGGF
LLAAVISIPLGVLMGSFPVVRALFEPAISIFRYMPAPAFIPLLILYFGLGETPKILLIFI
GTVFFNTLMIMDAVKFVSRELIETSYTLGGKNHQIILQVILPFIVPNIIDACRVNMAASW
NLVIVAELVAATEGLGRRISVAQRFLKTDEIFAGLIVIGLIGVVIDILLVMLHRIVCRWA
HN
>1170768.4.peg.2156
MIDKIMNNQVELSPIPQETAGELFFLINPLIVYEFYEFLVY
>1170768.4.peg.2162
MGLNYQQHVKVLPTGASNKHERNETDKVRKSQRLVVSGNVKPTPKLSSKMTGSNPKVKTL
RVPAHPKKYAHPSRKARLKPAANIILYALRLLIVGVGLGAIVGTLLSVLDPANRITTNSI
NPPVTPSSSPQSPINSSGLVISREITPLKTTIENLSAANPNLIPGVFIVDIDSGAYVDVS
GNNNFPAASTIKIPVLVAFLEDVDRGKIRLDEILTMEQEMVAGGSGNLRTMPVGTKLKSI
EVATKMMTISDNTATNMLISKLGGKELLNSRFRSWGLVNTAIQSPLPDLEGTNTTSPKEL
ASLIAKVNQGELISMRSRDLMLDIMRRTQRDDLLPAGLGEGATAYHKTGDIGTMLADAGL
IDVPTGKRYIASIMVKRPHNEPAAAKLINSISQATYSYLSQSNFSPDGSTNNQPSSNQRL
NNSSTPFTQPFLQPQGGNSNIPNATINNAPLGNYQSPLNNPPYYPPQSHPN
>1170768.4.peg.2178
MHLQIATGQPWRPYTAFGQFSVPDYPIPGGSKGWATYQKLLKAGWTLVPSARAEEFSRDL
VESSIHK
>1170768.4.peg.2179
MQDFLTITTKFRDNQHICRIIANSFEYCTQTWKSWCQNAFS
>1170768.4.peg.2185
MVTVSGVLLAILGLAIWMKLTPIFWILELLKGLLALLTDILPHYVSGPLVLLLGGLLVLW
GQSRTVGSITQALRPQGSQEELIDVILAHHRLYRGPKIVVVGGGTGLSTLLRGLKTYSAN

ITAIVTVADDGGSSGRLRQEFGVLPPGDIRNCLAALADEEKLLTELFQYRFRAGDGLTGH
SFGNLFLTAMSDITGDLERAVAASSKVLAVRGQVLPATLSDVRLWAEMADGRRIEGESSI
PKAGGKIVKIGCLPANPPAIPAAIKAIRSADYIIVGPGSLYTSLIPNLLVPEITQAIACA
NVPRIYICNIMTQPGETEGYSVSDHIRAIDHACGDRKLFDAVVVHRRSPSAQALIRYAQQ
NSHPVFLDTEAVSQLGRRIVPANVLYEDETGFVRHDPEKLARVLLKWYGAASKK
>1170768.4.peg.2190
MREAIPAQLLHPRYRRGEKNKILICSHFLIHSHQRESCGIIVLKWSKF
>1170768.4.peg.2197
MFTPSLYYIETEILRIELCAPHPYSYGMVRLGKTNDF
>1170768.4.peg.2202
MLIGVGEDPEREGLLKTPKRVAEAMKFLTSGYNQSLEELVNGAIFDEGHEEMVLVRDINF
FSLCEHHMLPFMGRAHVAYIPNKKVVGLSKLARIVEMYARRLQVQERLTRQIAEAVQTIL
EPKGVAVVMEATHMCMVMRGVQKPGSWTVTSSMIGVFQEEQKTREEFLNLIRHQAAFF
>1170768.4.peg.2217
MTSDNGELQVNLFTVNRPQEEAKYTVAYIDYPAQYIQLLRSRNLVEQAIEQGKSTALQRV
RGTVVSEEKKTLGDNVGIEVNYTTADGKVVKQRVFLVDNRFYQITAETTQKRQRFLTRSM
QGFCDSFKLLP
>1170768.4.peg.2225
MQKGKCGVDLATVTFEAEENYSVAILGNSDQVKRGTDVIVAGFPTISGNKGKDRTFTITY
GKLTSDILPKALWGYRLVYDATTKVGNSGGPVLDASGRVVGIHGLADGGEDEKDCATGQR
ETGSPSVGKTGFNFGIPINTFFSLKGQKPPTSTVQPVLPPPPPVAPGNSPRRPTRYKAPT
GPAVCPGTVC
>1170768.4.peg.2228
MGTVIALLTLIMPVLIISHYSSAEVQNQPQPVIYNLTRDKP
>1170768.4.peg.2238
MQSAGLDFSAFLDARNRQLQIYDASGKAKGLLVRAQDVPVYVEYGQAQLGIVGFDVLKEK
KPQVGQLVDLKFGYCRMSVAVKSTSSYKSPLDLPAHGRVASKYVNCAREYFESLDLPVEI
VPLYGSVELGPITGMSEAIVDIVSTGKTLRENGLVEIATLYESTARLIVHPLSYRLDLGG
IYNLAQSVKSSVSS
>1170768.4.peg.2243
MRLLSRKKEQKIVSKIAQVRVMATLTGLTFGGGTWTPKFAQGIDKDKCIGCGRCMKVCGY
SVLGLMALNEEGEFVEDEDDEEIERKVMVVTQPENCIGCQACSRICPKNCYSHTVLEK
>1170768.4.peg.2282
MANNQVDVATNNNESLERLEKTNPSARQKIEIIWTSPIIPSDPIAYRKDLPADVKKKLQN
FFYNFKDKKILEPLQWSALVPANDKTWNPIRELDLAKQVLDLQSKTDLTAEDKQKLNDLN
SRLRKLQGR
>1170768.4.peg.2301
MAHSFADVYKSLCWEAIYVSPMKRTIATAQPFCDATGLNMQLRTGIREGSYGEWETKSKS
FVQENYTENYIKWLTESAWNAPQGGETAVEIANRSIPVIREIKEKHHQGNVLLVSHKATI
RIILCSLLGIDLGCYRYRINILVASLSMVRFDVNGPMLEMLGDRNHIPEHLRLRPGT
>1170768.4.peg.2314
MLNTLLIAIASVTFFLASDFVQPQSAAAYPFWAQETAPETPREATGRIVCANCHLAAKNT
EVEVPQSVLPDTVFKAVVKIPYDTNVQQVGADGSKVGLNVGAVLMLPEGFKIAPEDRIPE
ELKEEVGDLPFQTYKEDQENVIIVGPLPGEEYQEIVFPVLSPNPATDKNIHFGKYSIHVG
GNRGRGQVYPTGEKSNNNIYNASAAGTISKIAQTEDEDGNVKYIVSITTASGNVVEDAVP
AGPELIVSEGQTVASGDALTNNPNVGGFGQKDAEIVLQDSSRVVWLIAFICLVMMAQVML
VLKKKQVEKVQAAEMNF
>1170768.4.peg.2327
MLVLSSCGYWIWEWPHWLCFGINTLSLHFSGTIIHDACHQSAHRNRIANAILGHASALVL
AFAFPVFTRVHLQHHGNVNHPKDDPDHYVSTGGPLWLIAVRFLYHEVFFFQRQLWRKYEL
LEWFLSRLLIFTIVYISVQYHFLGYILNFWFVPAFVVGITLGFFFDYLPHRPFVERDRWK
NARVYPGKILNMLILGQNYHLIHHLWPSITWYNYQPAYYLMKPLLDEKGSPQTSGLLQKK

DFLEFLYDIFIGLHFHHHE
>1170768.4.peg.2328
MINKVAIAGSCLWSLALYLSLASIREWIITQLNRWFNFAERFLYTSNTEFEKTRTARESQ
NAFYASIMSILPFLVFGALSYWSLEISLGSSWGISTGILTTVGASIYELGRINGKNSN
>1170768.4.peg.2329
MLNLTLYDQEGEVFGRPEQVELDIIIKNGLTIVCELKSSIDKAGMYIFGRKADFYAKNQN
RVVNRKIVISPMVDERAVPVAKSLGIEIYSYADVVVP
>1170768.4.peg.2330
MIEAEIKALIQKELPRAIAEEPGVRDFVLRTVSEYYTPRTEFDEKFDRVLNELQRDREEQ
ARKWDEQNRKFDAFQAEQAHKWDEQNRKFDAFQAEQARKWDEQNRKFDAFQAEQARKWDE
QNRKWDEQNRKWEENNQRLDRIEAQNSATLEEIQKANRRYESAIGAIGSRWGLYSEASFR
NGLQAILGQSFGVEVLNLTLYDQEGEVFGRPEQVELDIIIKNGLTIVCELKSSIDKAGMY
IFGRKADFYAKNQNRVVDRKIVISPMVDERAVPVAKSLGIEIYSYADVVVP
>1170768.4.peg.2346
MVVKTVVNIITIPPSKMRTASPPVIKSKVLVLSIIYAFLSLIARLECQG
>1170768.4.peg.2354
MFADYGEVKRVVLPTDRETGKMRGFAFVEMNEDAHEDAAISELDGAEWMGRQLRVNKAKP
REENNRENRPRKGHSKG
>1170768.4.peg.2355
MVSKKMQPGNFSELVYGQVGSQLDALIFATPPNTAIH
>1170768.4.peg.2379
MGNLGYGIDPEVVKGIAQELAEVVATGVQVAIVVGGGNIFRGVKAASAGMDRATADYIGM
IATVMNAMTLQDSLERIGVQTRVQTAIAMQELAEPYIRRRAIRHLEKGRVVIFGAGSGNP
FFTTDTTAALRAAEIEADVIFKATKVDGIYNADPKVHSNAKRYKTLTYGHVLAEDLRVMD
STAIALCKENNIPILVFDLTVRGNIRRAVMGESIGTLVGGSCEIS
>1170768.4.peg.2380
MNKTVEATQRAFNSIRTGRANASLLDKVQVDYYGSPTPLKSLTNITTPDASTILIQPYDR
SSLNIVEKAISLSDVGLTPSNDGSVIRLNIPPLTSDRRKELVKLAAKYAEEGRVGIRNIR
RDALDSIRKQEKAGEISEDESRDQQDKLQKITGKYTAKIDELLTEKEKDITTV
>1170768.4.peg.2410
MSQGRKNQSAIIPVCNFGEIVLCMLKSPLRYPVNKRQFLLDEIAIF
>1170768.4.peg.2412
MPRRQDIHKILLLGSGPIVIGQACEFDYSGTQACKALREEGFEVVLVNSNPATIMTDPET
ADRTYIEPLTPEMVAKVIAKERPDALLPTMGGQTALNIAVALAKNGVLEEYNVELIGAKL
PAIEKAEDRKLFNDAMEKIGVNVCPSGTASSLEESKAIAQRIGSYPLIIRPAFTMGGTGG
GIAYNKEEFEVMAQVGIDASPVSQILIDQSLLGWKEYELEVMRDLADNVVIICSIENLDP
MGIHTGDSITVAPAQTLTDKEYQRLRDMAIKIIREIGVETGGSNIQFAINPVNGDVVVIE
MNPRVSRSSALASKATGFPIAKMAAKLAVGYTLDEIKNDITKKTPASFEPTIDYVVTKIP
RFAFEKFPGSDPVLTTQMKSVGEAMAIGRTFNESFQKALRSLETGRAGWGADKAEKLPSG
EQVRAQLRTPNPERIFALRHAMQLGMSNEEIYELTAIDPWFLDKLHQILETEKFLKRTPL
QQLTKVQMYEVKRNGFSDRQIAFCTKTKEDEVRVYRKQLGVIPVYKTVDTCAAEFEAFTP
YYYSTYEEETEILPTDKPKVMILGGGPNRIGQGIEFDYCCCHAAYALKSANYETIMVNSN
PETVSTDYDTSDRLYFEPLTKEDVLNIIEAENPVGIIVQFGGQTPLKLAVPLQEYLQQSP
STVTRIWGTSPDSIDMAENRERFEKILEELKIAQPANGIARSYEDALIVAKRIGYPVVVR
PSYVLGGRAMEIVYSDSELERYMSFAVQVEPEHPILIDKFLENAIEVDVDAIADHQGRVV
IGGIMEHIEQAGIHSGDSACSLPSISLSPAVLNQIRTWTVELAKALSVVGLMNIQFAVVG
ASSYSPQVYILEANPRASRTVPFVSKATGVPLARLASLIMSGKTLEELNFTQEVIPQHIA
VKEAVLPFNKFPGTDTLLGPEMRSTGEVMGIDVDFGRAFAKAEMGAGEKLPLQGTVFVSM
SDRDKSLVVEVIKEFIQLGFKVIATQGTSEFLREQGLKIETILKLHEGRPHVLDAIKNRQ
IQLIINTPSGQEARTDGQLIRRTALGYKIPIITTIAGAKATVAAIRSLQNINLDVKAIQE
YSF
>1170768.4.peg.2413

MTTEKPEPFWFYRVRLIYSRSHPKEEEGEPERFLNNHF
>1170768.4.peg.2417
MPKGSVVFLPVTRFPIDNIVSEKQYQEELKTTDYFPMNAGMRWCGDYFLYCSQIF
>1170768.4.peg.2418
MIKQKVALVTGASRGIGKTVALGLIKDGFRLALVARSAENLQAVASEIVELNLDADSQPL
LFPIDITQRDSVQTIVAEIDKIWGRIDLFFNNAGTWIEGTLDVEIDELDRIFSVNLKAGF
YFLQAVVPIMKRQGFGQIVNLVCITAKGAEPDWGIYSASKYAFFGLTEAIHFDLIKYGIK
VTAICPSWVDTNMAQQAATPYPSDQMIQKEDILNTIRWLIKMSPKAAIKEVVIDCISEYV
REK
>1170768.4.peg.2419
MHEQNILPHEWYCEILNRHVGTFNIIICSHVKGPLTQEIIRQALDMAQSRHPRLNWRIVG
EQNCLRFETEGTQKIPLQVVDKLHNKYWQEVVTAQANEGIDSSKCLMRAVLVRALNEEDT
NYLITTIHHAIADGLSFVQLQSEILSYAQKIASGEALTQVTPLPTLPSLEKLLPYSATTS
QLYESYPETDTLGFEKWVPIENRKTAWIYRQMDEKLTQQLKKIAKQEKTTVHGAFCAAML
LETARIIRANQNIEVVRACCSTPFNLRSYLQVPVSSEHLNFMVWDVRLWHTLEKNTSFWD
LAREARQQIRSVLKSGAFISEITSEKLEDFIPLLDKEVEITVEITNLGKLNIPTIYGIFE
IEEIHVICGTAAFAGIPTLVVTEFRGKTLLSFLFSEPSLSQTTMEILANNVVSNLMDACY
>1170768.4.peg.2420
MAEVIQKVVEEEADFFKALGLELPKEVIIYEEFTAEKCEWQGLGTVKKTAVKEKPFSNLI
NDFPEHFENTACVFLLDDSRCGLQILSEAKGLHPWYYKPFTCWLFPIFIAPGESQREIFL
PSPETEPWYLPDDDYDGFYTQVLCGKHSDCGQLGYILLQEELNFLAKIVGRNFVQEIQDA
VVADSAERS
>1170768.4.peg.2422
MVGQFLGGGGQGEVYRADLGGKSVAVKWYYSHSIQADPYQRDRLEAAIQSGSPSDRCHNW
LFGFL
>1170768.4.peg.2426
MEISYLEWNQGHQPLLLLHGMADNALVWLSLGDYLSPNYHIIAPDMRGHGNSSKPETDYS
FTSAIADLEALMNSLGWFSANVVSHSWTGKLAAIWARQNPGRLKTMTLVDPIFIWKMPSV
LKLIFPLLYNVLPSLQTMGPFSSYEAAEEKIKKLVHFREWNDLQQQVFQGGIEQKPDGTW
GSKFTVAARDGIFDAVLEVAGFIHPVETPTLFVQPEKGVNRQDWQIKSYKDNLKNLTWKK
IPGTHWPFLSNPEEFNLSIAEFLAQAI
>1170768.4.peg.2435
MGWDVPRGVNHNNYLPYITAAVTILNIFVSKLSQTFLLIVNF
>1170768.4.peg.2450
MTVIKPVEYRTQSIDSSFEAEQVQFKLWRNLSASEKELLLKRVTKQGTSRL
>1170768.4.peg.2459
MGVSLSSNPPEFSQGNLDSSASDTFNSAINNAMVITSTKMSRSIFRECALNLQ
>1170768.4.peg.2462
MGTVAQGFLPLPGDTSIWIEISPGIEINKITDVALKSASVRPGWQIVERLYGMLELHSGS
QGETRAAGQAVLDLLGVKKEDCLKPRVISSQIIRNIDAYQTQLINRSRRGQLLLAGQTLY
VLEVEPAAYAALAANEAEKAAAINILEVLPVGSFGRLYLGGTERDILAGAAGALAAIENV
PGRAPQGHRKE
>1170768.4.peg.2468
MFTVSLIGNHRHLFGLSELARGGDFLQKHQEAVRTKLTASRKLCG
>1170768.4.peg.2493
MISITVKKNGARMAIAMLFTNPGSEDIIYLDSLPGILKYRKSIIYILLTFILFIN
>1170768.4.peg.2503
MESAVSVSSPTRLVAQATQSIALDWLNKGLKAIAEGKVQDAIIAFRQAAQLDPTLAPAHY
NLGLALRQTGKLQPAADAFYRATQADTQFASAFANLGAALLEGNNLPLAIDYLKTALQLD
PKLGFAHYNLGLARQLQKDWQQAIASFQKAIIYSPNSAEPVYHLGNCYLQQGKLELAKST
FIKAINLNSNYTEAHYNLGLILFEQGQLEDSLSAFRKAAQTNHNYPNAYYGAGLVFVQLK
QYEQAIKVIKYARDIYQKQGNMAWANNAEKLLTQIRNLRR

>1170768.4.peg.2504
MILTFWWYLASMLLTNTLFLKTSLFFILYSIYNLSFDIRVNNG
>1170768.4.peg.2512
MRNSGLEFAMVGNLHGVCFGTASSVPLKLEEIHEITKY
>1170768.4.peg.2514
MSTCRHHNEWLSLLDVSGPFLSLPVLLKVFPNGLDAHNPENWKLLRLAYQEWQDALNNRR
FNTNDIHHAWINFVLQRILELPRGQLLPGESNPSLAFADVPQYGETLHPDYILINPQTQL
FPESESNREYQIRLLIQILPPEQNLEKPIVNKRWKASPATRMMELLHSNNIRLGLITNGE
HWMLLNAPKGEATGYISWYSSLWLEENITLRAFSSLLGIRRFFGVDDTQILEQLLAESIT
DQQEVTNQLGYQVRKAVEVLVHALDRIDQDKNRTLLQGISETQLYEAVLTIMMRLVFMLS
AEERGLMSIGNPIYDQYYAVSTIWEQLQKKADKEGEEILERRCDAWCRLLATFRAVYHGV
NHEKLKLPGYGGSLFDPDRFAFLEGRKQGSDGKTPPSEPIPVNNRIVLHLLQALQILQVR
FAGNVESRRLSFRALDIEQIGHVYEGLLDHTAVRASSPVLGLVGTKYQEPEINLADLGIS
NNISNSNNNNSDVESLLTPDRVKTLKKLTGKSDSALKKALETEFNHYEEQKLMTACNNDK
ELFQQVRPWMGLIRLDTLNYPMIIPAGSVYVTQVSDRRETGTHYTPRSLTEELVKHSLDP
LVYEGAVEGKPQREWKLKPAAELLKLKICDPTMGSGAFLVQTCRYLGEKLVEAWDNAETA
HPGKVVITPEGTLSSKSSSKLSLQSSLESPLQQSIIPQEADERLIVARRIIADRCLYGVD
KNPLAVEMAKLSLWLITLQKNRPFTFLDHALKCGDSLIGVGIEQLGYWNLNLRKTAEIFA
NEIRYEVDKIIKLRKEIIQLPVLTSQDQNRKEYLLARVKANSLDLVQRCNLLVGSYLNNW
HEKEQEGLRQTLLINIREGTDIPQEKAMALPDFEKLRPFHWELEFPEVFIDHSVSLFANA
SSTKKNQSANYSQGFDAIVGNPPFMGGQKITGNVGTEYRDFLVKWLANGKKGSADLCAYF
FLRAKHLLQPNGVLGMIATNTIAQGDTREVGLDQLIADCTIYRAVPSRTWPGTASLEVAY
VWLKKGGWKGNFILDEKPVGGITAFLTVPGKVFGNPERLVANQNKSFQGSIVLGMGFVLT
PEEAEALIEKDPRNKDVLFPYLNGEDLNTSPDQSPSRWVINFRDWPLDAEHDNPKNPQGA
PYASDYPDCLAIVREKVKPERDKNKDKQRREIWWRFTRPTVELYRAIANMENVLVLSIVN
NHLGFAFASKNTVFANRLVVFPLYDYGLFTVLQSNLHYHWAWNYSSTMRRDINYSPTDCF
ETFPFPPSIKELEEIGEKYYNHRQKIMSNCQQGLTKTYNRFHLPEETTSDIKELRNLHIE
MDKKVATAYGWQDIELDHDFHVTKQGIRFTICENARREILDRLLKLNHQIYEEEVAKGLH
HKGNKKAKTGGKEKVKENVNNDIQQMDLFGEQ
>1170768.4.peg.2515
MVQILAKIEILRHDPKPQGNLKKKLHGYLGNVYRLRSGDYRIIYTYDDGWVALLGVDSRK
DIYKGNKLFAEGADFDVKSLPDVESLLTAESGYSAVNSQSNSQSILPNQQIQNTENFLPL
LLTEDLLERLLVPKELRTNLINCRTFDDLIKVDIPENLRDRLFDCIYSPNFDLVLSQPSY
RTDSTSDLLKFVEGSLVGFLLKLNPEQEKYVNWAIDASGPTLLKGGPGTGKSTVALYRVR
ALLQVLKKHGVVEPKILFTTYTNALITFSEQLLNNLLGEDMRYVKVKTADWIAYSLYYQY
QNSPYTLAKNNELQNIMKRAISNAIDSLEGNLLKKQAQAQTLQRLTSQYLIDEICTVIEG
RMIKTLELYQNTPRHGRMVSLNRTQRQAIWHLRRHFYQLLEEHKLQTWHQLRSRALAILE
TMQNPPIYDAVIVDETQDLDANSLRLLTKLCRHPNRIFITADANQSIYGSGFTWSDIHED
LKFVGRTGILKINHRTTREINEAAHSYLGTPGLQNATLDKELCNSQISPLNASREYIHTG
PLPAVRAVKNINEENQLLITFFQQATREFRLGMNACAIFTPNEKVGRRIAEQLTALGSIA
NFMSGKNLDLNRRGVKVITLKSAKGLEFPVVAIAGFVDSNYPIAPKNTSIEGMTEILNRE
RRTLFVGMTRAMRGLLIIIPAYGRSSLLNSFDPQLWNIAT
>1170768.4.peg.2519
MINPPEIEEELLLIEEEEDLLLVDDGDIDDFLEPQTDEDDAKSGKAAKSRRRTQTKKKPF
TEDSIRLYLQEIGRIRLLRADEEIELARKIADLLELERVRDDLYEQLEREPEFREWAEAV
KLPLPTFRYRLHVGRRAKDKMVQSNLRLVVSIAKKYMNRGLSFQDLIQEGSLGLIRAAEK
FDHEKGYKFSTYATWWIRQAITRAIADQSRTIRLPVHLYETISRIKKTTKLLSQEMGRKP
TEEEIATRMEMTIEKLRFIAKSAQLPISLETPIGKEEDSRLGDFIESDGETPEDQVSKSL
LREDLERVLDSLGPRERDVLRLRYGLDDGRMKTLEEIGQIFNVTRERIRQIEAKALRKLR
HPNRNSVLKEYIR
>1170768.4.peg.2525
MDIKQLFIAGGVVMWPLLLSSIIAVGLIIERIWFWSKITKRQEKVVKYVLNLYRQNNLVN

AIDTLQKNADLPIARIFLTALELEEPNPEEFRLALETEAQAEIPLLKRFTTVFDTIIALA
PLLGLLGTVLGLINSFASLNIRAVGK
>1170768.4.peg.2570
MSTWIFLPQGGLIIHLKILLIIQVTYTSMVIKNRNLGWIIGD
>1170768.4.peg.2575
MGKLICTLIFRIKLIKFFKPLSIFPRLEEDGGESLLSDNG
>1170768.4.peg.2587
MLQRLAHQPLTLGDVLETTSQRGFILVIALLVLPFLFPMPPGLTGPLGSACLLLSLQMLL
GRRSPWLPKKIANYQFPRVFAQTILHNLGRVTRLLEKIARPRLTKLANHDITWRCNGFCI
SWLAILLISPVPLTNPIPTIGILLFAAASMESDGLLICICYVLTLLITLIFYLIVYGVLQ
LPGLIT
>1170768.4.peg.2589
MQRSLIVAVLIGLLCAVVGTYLMVQRLALLGDAISHSVLPGLAIAFIIGANIFVGALIAA
MVSTVAIAVIKNRSPIKEDAAMGIVFSGFFALGVTLITGVQKTNKIDLNHFLFGNILGVT
PNEVRDTAILAAVALIMVFLLYKELLFYTFDPIGARVAGLPVNQLNISLMLLISLTIVAS
MKAVGVILVLSMLITPGATAYLLVNRLHQVMILGAAIAIISSIVGIYLSYFYNLPSGPAI
VLVVCTAFVLAFLFSPKSRVLGKHGR
>1170768.4.peg.2592
MRPQKFFLHSTLKKFNRLTLVKLIKLWFTTQVTRKNKTHPNLS
>1170768.4.peg.2596
MAHYDHKSIQLGSSIIEPPIAPYNLLYGYFTQTLSSR
>1170768.4.peg.2620
MSFPWLSAGKLAPIGRACITVAMLIIVGWGYAAVAADPHKLEDLAKQSADLKVGIDTMWV
MFAGMLVFFMNAGFGMLETGLCRQKNAVNVLAKNLI
>1170768.4.peg.2621
MLGPRIGRYNPDGSANAMPGHNMSIATLGCLILWLGWFGFNPGSTMSVSDGTAIAHIALT
TNTAGAFGGIAATIAAWAFLGKPDLSMIINGILAGLVGVTASCAYISVPSSAIVGAIAGI
LVVKAVPFFDKLKIDDPVGAVSVHLGCGVWGTLAVGLFSQGNVYYQGGPTGLFFGGGIGQ
LWTQFVGVLTVGLFTVLISGIFFLALKYTMGIRVKESEELEGLDVGEHGMEAYPGFVKEA
SSPDLLGFGNR
>1170768.4.peg.2623
MASEIPGKVAFSKLVAIEVLVDKSKAENETWINLVVKSEELPLRLDNHCRQVFDQIRQEV
EDNRKWQLIESVVNF
>1170768.4.peg.2634
MPILTTIVSVICNRQKFAQRLSLYGEGRRCTVLYLLMII
>1170768.4.peg.2685
MELKVWRKGSKDPLELLYFKKHYFVSHPNILPNILNIPTQNSTLIYLSFKSDRS
>1170768.4.peg.2686
MTGKGGVGKTSVAAATGLRCAELGYKTLVLSTDPAHSLADSFDLELGHDAKQVRPNLWGA
ELDALQELEGNWGAVKRYITQVLQARGLEGIQAEELAILPGMDEIFGLVRMKRHYDEGVF
DVLIIDSAPTGTALRLLSLPEVGGWYMRRFYKPFQNISVALRPLVEPIFKPIAGFSLPDK
EVMDAPYEFYEQIEALEKVLTDNTQTSVRLVTNPEKMVIKESLRAHAYLSLYNVATDLVV
ANRIIPEEVADPFFLRWKQSQEQYRREIHDNFLPLPVKEVPLFSEEMCGLAALERLKDTL
YPGEDPTQVYYRETTIRVVQENNQYSLELYLPTIPKSQIQLSKSGDELNITIGNHRRNLV
LPQALAALQPAGAKMEGDYLKIRFADNVRV
>1170768.4.peg.2700
MGYSVFGFPIPLGGRGLFILNHLLDSGFWLLSRELSFWELSF
>1170768.4.peg.2703
MRANSTRRRVFKNPLPERSHNLWYTRIVGGKLTGDRSFHLRDTP
>1170768.4.peg.2704
MVREIAPKTQLGVIKSKVNQGGETIAPDIVYPESDGKPMADNTKQFAWIVKIKENLEVLF
KSNPDVFVAGDLFWYPVKGSNRIKLAPDTMVVFGRPKGQRGSYRQWEEDNIPPQVVFEIL

SPSNSKGEMTRKKLFYLKHGVEEYYVYDPDEISLEVSIRENNSFREVEDFATWTSPRLNI
RFDMTGDELVIYYPDGSRFLSPVELSNYAEQERFLKEQERFLKEQANQRAEQERLLREQE
RFLKEQANERAEQERLLKEQEQLKYQTLLAQLKAKGIDITTLE
>1170768.4.peg.2706
MVREIAPKTQLGVIKSKVNQGGETIAPDIVYPESDGKPMADNTKQFAWIVKIKENLEVLF
KSNPDVFVAGDLFWYPVKGSNRIKLAPDTMVVFGRPKGQRGSYRQWEEDNIPPQVVFEIL
SPCNSKGEMTRKKLFYLKHGVEEYYVYDPDEISLEVSIRENNSFREVEDFATWTSPRLNI
RFDMTGDELVIYYPDGSRFLSPVELSNYAEQERFLKEQANERAEQERFLKEQANQRAEQE
RFLKEQANQRAEQERFLREQERFLKEQANERAEQERFLKEQERLLKEQEQLKYQTLLSQL
KAKGIDITALE
>1170768.4.peg.2712
MDGVNAREERKNYNFNGGKGRSVLGVLRKRISLVLEVLNKYSHKKV
>1170768.4.peg.2718
MKLWDSSFQLIRFEIVDLIGFLGFCSCFFTSQFLNCLLDKDLGDCQLYDSFAV
>1170768.4.peg.2719
MEIKRNWNTLFDVDENGDFFSVSISYGDKEKLEQMGHKNSWHFLPLR
>1170768.4.peg.2720
MEIKRNWNTPMLDRFKVLEAIVSISYGDKEKLELILVV
>1170768.4.peg.2727
MTHPNSGTNVTFLTSVIAEFSGDQTAIAENLKLFLNALLGEYQISYIQPNAERGTLHKVE
VQVKDGNKKVNSKSVSYIMPVFGRSLPLVNRLVMVTSTCLSLVGCQTIIPNLDNR
>1170768.4.peg.2728
MASLWQIIQSSLRDIPPQVVFEILSPCNSKGEMTRKKLFYLKHGVEEYYVYDPDEMSLEV
SIRENNSFREVEDFATWTSPRLNIRFDMTGDELVIYYPD
>1170768.4.peg.2737
MKRRSFIESSALFGASLLGTQAIASIGNRGIEPVQAQQIKELNFGIISTESQANQRPLWE
PFIAALSSSIGIPVRAFYATQYAGVIEAMRFGQVQIAWYGGKSYIEAAKIANAEAFAQTV
SSDGKKGYYAHLIANKSNPITAAAKKQGGDKYVIRNASKLTFAFNDPNSTSGFLVPSYYV
FGRNKVDPKKVFKRLIFSGSHEAK
>1170768.4.peg.2740
MVSQTRSSFIGEITQETLALTRRLFIQLQRRPSTLIAGIIQPVMWLVLFGALFQNAPQGL
FGSTANYGQFLAAGVIVFTAFAGALNAGLPVMFDREFGFLNRLLVAPLASRFSIVFASAI
FIISQSLLQAAVIVGAAAILGAGIPDVNGLVAIALIVFLLALGVTAISLGLAFALPGHIE
LIAVIFVTNLPLLFASTALAPLSFMPEWLKIVVALNPLSYAIEPIRHLYLNSNWGLNDVV
MEVFWGNVSLGGSLLVLLGFAIVALLSIQPQLRKTLA
>1170768.4.peg.2741
MLVCGAGIAFIGLFAPQPSFSNTASTSQPLNSPDNTNPLANDSGLDMFNIIHRLNFGPLN
WDPNQQNQQLEDAAAAFKARQNQILQNQQPSKVKGEENRSGANTDGSNTERK
>1170768.4.peg.2752
MLIPNGRFLGFIVMNVCLLSYPSLSLIQSPELFMGIM
>1170768.4.peg.2765
MAGYDVSQAEDGIKGQALALQLQPDLILLDLMLPRVDGFTICQRLRRDERTAEIPVLMLT
ALNQTQYKVEGFNAGADDYLTKPFEVEEMLARVRALLRRADRIPQAAKHSEILNYGSLTL
VPERFEAIWFHETVKLTHLEFELLHCLLQRHGQTVSPSEILREVWGYDPDDDIETIRVHI
RHLRTKLEPDPRRPRYIKTVYGAGYCLELPAVPPSGEASNMTLVE
>1170768.4.peg.2777
MLTLSAAWTVVHIVKYCVQAIALNNPVNMVAFLWFFLVRAFFIMVFSYG
>1170768.4.peg.2778
MLSIPGGHQYCARPFGYSENYDDVFAEPGNILIRYYVPTRMGIKMIFQGLFFELNNPQNW
SLTFPTTIPKE
>1170768.4.peg.2787
MKKYFAAVEAYASNPTSESKQLVDERMSQAYSKIDKAVKRGVLHPNSGARKKSRLARKLK

PLTQPV
>1170768.4.peg.2788
MAVLAVLIVSFLLLGPIITYFSKSLTLSYCQFELELFPSLPTLDRTL
>1170768.4.peg.2793
MTDQDISGGFSPVETTKPEQNPRLSPDLEVQVNQSSGGNPIQHSDRNFGSLMQQQNNNDL
YEKGRKNNWYRPVDFILSGKIWELAMWKKSWIWWFFVLAFIPSSIGIISVSILLKLPSAP
NCPRIFWPLASASMRVHCATLAASKQTVSDLLQAIALVKDLPQDHPLRGQINDLLQEWSR
DIINLAEKSFQLGNLEEAIATAKQIPENLEDRQFVEEKILKWQSTWSTAEEIYQSSIGEL
ENRRWQSAFMLSSKLLRINNRFWSTTKYDQLNQIIVTAREDGDKLDKADSLADRNSVNDI
LSAIKLVKSVKPESYLYKKAQELVPQFGRKMLKLAQAQMERRDADKGLEIAGKIPPIPSL
QSEIDDFIDLGEAQRNAWLGTILGLENAISQAQQIDPSRGIYGRAQELISVWQLEIEDVT
KLEQARDLASGGTIEDLRSAISQAQQIPSQNPRAQEAQTQINRWNDQIETIQDKPYLDRA
EQIANAGDINSLQSAIAEASQISSGRALYSKARRRIRSWNASIQRIEDQPYLDRAIVLAE
SGDLNSAVQEARKIAISGRALAGEAQAVIDTWQEQIRARENWRKARELASIGTADALSQA
IRIANRVSRRNVLRMDINIAIDRWGEQILEMARSQSQVDLVKSIETAKLIPRSSSAYADA
QLQIRTWKEQLVPVPTTPFLSPGEPSP
>1170768.4.peg.2812
MAITTAASRLGTEPFSEARRVELRPSASREEVELVIRTAYRQVLGNDYILASDRLVSAES
LLRDGNLTVREFVRAIAKSELYKAKFFYNSFQTRLIELNYKHLLGRAPLDESEVVYHLDL
YNNKGYDAEIDSYIDSVEYQNNFGENIVPYYRGFDFQPGQTSVGFTRMFRLYRGYANSDT
AQVEGSKSRLARELAGKKASSIVGPSGSNDNWSFRPSADNAPKQNLGNAVGQSDRVYRIE
VAGIRSPGYPSVRRSSTAFIVPYERLSDKIQQIHKQGGKIVSIIAT
>1170768.4.peg.2863
MKWQLLTHNKQVLGKIFTILVFTGLTGVLCVSCNRNQDLLVTEIGVNPPKRPTRKTSGAG
EFYLQGQNQHSRGNFQAAIAAYSKSISLNSDYAPAFKARGLAYFDLNNKERAINDYNQSL
QINPNDPETYNYRGNARASLGNQKGAIEDYNEAIRLSPNYAEAFNNRGNSHAAQGNKNAA
LEDYTQAIRIDQNYPVAYNNRGNAYSSLGNTSKAIADYNQAIRLNPQFAPAYNNRGNAFA
SSGDKRRALQDLQKAATIFDQEGNRGLYQQTMKNIEELEN
>1170768.4.peg.2864
MPVSAQMTIANGKKIDLEVAKTPEQQMMGLMYRSALPDNRGMLFAFLSPQPVGFWMKNVP
VSLDMVFIHSGVVQYIKTAPPCQNEPCPTYGPKVLIDQVVELRAERARELGLKIGDKVKI
ELFKPLR
>1170768.4.peg.2877
MLDLGLVKRSPLGGLTIANPIYQEVIPRVLSQGSQDSLPQTYGREN
>1170768.4.peg.2887
MRISNFRGPQQTAILTTSDRRHADTSDSPVLFSWGVLWDFS
>1170768.4.peg.2890
MYKIVKNKIYVANLFLFVLISMSTSSASAQTTTGIEDIARKTTVQINSNANPGGSGVIIK
KEGTIYTVLTANHVVCDNLGTIRIRCRTDLTYTVVTYDGKEYPMKYRQSLQINVQDPDLA
IITFESRENYQIAPLGNSDNVKIQSDILVAGFPTIFGRVGKQRTFTITNGKVVTFIPNSD
RGYGLVYNATTFIGNSGGPVFDIYGRVIGIHGLADTDDGETNNNDQSETVNGVKPTQKTG
FNAGIPINIFFSLSNFNRQLDPAVSINRQPNSTNPNNVSLNNRAIAYHDRGVNHYQSGDR
QGAISDFTQAIQINPNFAQAYNNRGIARHNMGDKQGAISDFKKAAQLFDLKNSRSRKIMR
IP
>1170768.4.peg.2891
MSQKLNALCFDGKWNHVVLPPDNSDYSQGITVEAWVWYGSFAQNWSRIVDFGNGQGRNNI
VLAHAGTSNSLSFHTFTSTGGYAVEVPNALEIGKWAHVAATIDKSGEAKLYKNGKLIQLI
QTKPFRLPDNVERKLNYIGKSNWTNNDGFFQGKMAEVRLWNVARTPEEIEQNMNRRLSGN
EAGLVAYYPLNGDANDKTKNARHGIIIGATWQQEELPIQEPNVNQNQTTTQTTTFITPDR
LLPKYDAVIVGAGIAGAIRVVGK
>1170768.4.peg.2903
MSCTNLEVICWELAIMANRKMKTLSLPKIITPQCQVSTLPNLIVEQNCGKR

>1170768.4.peg.2919
MGKKRKFHLGAFIQATGHHVSAWRHPEAQIDAGSNFQHYRKITQTAERGLFDAVFLADSP
GIWGGTPETQKRNGKLVHFEPVTLFAALSSVTKSIGFIATASTTYEEPYNLARKFASLDY
LSNGRAGWNVVTTGNENAAANFGLEYHPEHGERYERAEEFVEVVKGLWDSWEDDAFLRNR
ESGIYFDPDKLHVLNHKGKYFSVKGPLNVGRPPQGYPVIVQAGASEAGRELKVMTEYSRL
KSSRSAVIKSKLNYPVIDTDVHTNDFTPALEDYIANYGGSRLVDALRKAESSRLNSKVRG
KDWYQQTPEERQYYRTIRSPWWARVTRNTLDLATYTLPELFYERQAEQGSDYSVLFPNNV
LAPTGASNEDRQALQRAINHYHADLYRKYSVFLN
>1170768.4.peg.2920
MLEGGADWGSHVYIHLVDRFSKRNLKGLQNYNPDLTNGHELYELFIRFGTELLEGYSLSQ
EELTQTVLGSSFNRYSRSPVGSELEDFAAAGIESIEDIRDRWVNSFFFGSESDDRTIATA
FNDRANPLNVKINAIYSSDVGHWDVPDLTDPLAESWDLVQEGVISEDEFQSYVFRNPYKF
YTQANPDFFRGTVIESKVNNIASHQVQKVEQNLVSV
>1170768.4.peg.2922
MLFQHQLELYRSLVGKAHLIYSSEFLGKLGADNSFDYLQRGIDG
>1170768.4.peg.2923
MNFQEVFGVGSSFGNIAFIIRARVEGLRDFGPSLSPFNAFLLLQGLETLSLRVDRHLSNA
LELAHWLEKQPQVEWVNYPGLPHHPYYERAKKYLRHGFGGVLNFGIKGGLEAGKNFIDRL
KLASHLANVGDAKTLVIHPASTTHQQLTDSEQLSAGVTPDLVRVSVGIEHIEDIQEDFVQ
AFG
>1170768.4.peg.2926
MKFIDPRPADLFQGKENIWVRNGHIPGAKNIPWPTFTDPQNPHKLKSLEEIKKILADKKI
APNDDIIVSCSTGREATLQYVVLKHLLGYPKVRVYEGSWTEYSTYSDLPVATGPEEVS
>1170768.4.peg.2930
MQKITSPVISEQQRIADLFYNEGVIPTKIAVRESFLTPQQYTTITPARIGNR
>1170768.4.peg.2931
MGLGLTFLITACSPSSTNNAGNNQTSSPNEAANSSSSSRLTNSSTVIRIGYQKAATVLYA
LKAKGELEKSLQKSGVSVTWTEFPAGPPMLEALNAGSIDFGYTGEAPPVFAQAAGTPLLY
VAYDPLATKAEAIIVHKDSPIKSLADLKGKKVAFAKGSNTNYLIVKALEKVGLKYSDIKP
TFLHSDCKK
>1170768.4.peg.2942
MAVMVGIINHGVTIAVPPPKEDIPEEILRTEIITIGRSPIDGKILTASEYAELQEQLRTI
PPRKLSPSLRHTVFLLRMRRILLQIFPFLDI
>1170768.4.peg.2947
MNQGRTYDYIDLIELLIGTIFESKGDSQVIYPLLENNLHLLDATFIDQLQSWGGHQVNKQ
ATPEQNAQLGASLYLLARIFYHFPGGDPLINLTIAICGYEFCAAIERQLGLEKELAQVLN
HLGAAYQTQAQMGTDSHSNLEKAIAAYNEAIAIRRQPGLERELAQVLNNLGAAYQIQAQM
SKEANLERAVTAYAEAISILRELDLERDLAQTLYNLGSAYQTQAQLGKDSQANLQKAIPP
YTEAISILRELGLERDLAKTLTSVGSVYISQGKLERAITPFREAITMFRQLGLERDLAET
LNQLGTAYLSEVERGKDPEANLQRAIIVFTKALTILRQPGLEPDLAQTLHNLGKAYQTQA
ELGTESQSNLEMAIAHYREALAIATPELSPADYLKFASHLGDLGFQQGWWDIALEGYERG
IKALETIQFWQPGDKSPDIQYPHIYQSAVRACIELHQYGKALELVERSKSRNLVELLENK
HLYPKGNIPSRIYAELDRLRQAVRNEVRLLEIQGEKRSHAQLNGLIEELDQFITSQVKPL
DPHFSLTQRVEPITISKILKLVGADPRRVILEWYFTDDVLYTFIITANPDVPIFPHKVVI
SPSVIRQFSQKYLDIYRDRHNLGNWKQALPELLGEMKEILQVETLLTQLWKLSPPCEELV
LIPYGCLHIFPLHALFPWERFPLGFSYFHSCQLLSSVEVTEREEVVKSLAIQNPTQNLQY
ASFEVETIITRLLIGTEVITEKEATRETIFSRSIPEKNYLHFSCTGYSNFQKPLHSAILL
ANCILPAPPKGGEYLPLPNGAAVDLSQCLTLGDICGLDMRRCHLVSLSAYEMGIGDMVDT
KNDDYISLPTGFLLAGASTIISSLWAVSDYTTALLMVRFYQFYTDTENLLTYGRPRLALY
HAQQWLRDAPAGKLLTWAAKLPFSGTRLTLLADFDTMPESQRPFQDPYYWAAFTCVGI
>1170768.4.peg.2952
MISAKMGNLNQAKAIIKEMQSINNQEDFGANILEQILTPSPEEDG

>1170768.4.peg.2970
MTAVASAINSKKVPMFFYPQLRSQIQIFLSVVILQVDDL
>1170768.4.peg.2973
MGKHLYWQIGNLKLHGQVFLTSWFVIGVLTLASILASSNVKRIPRGIQNLMEFALEFIRD
LAKNQIGEKEYRPWVPFVGTLFLFIFVSNWSGALVPFKLIHLPEGELAAPTSDINTTVAL
ALLTSLAYFYAGFSKKGLGYFGNYVQPVSFMLPFKIIEDFTKPLSLSFRLFGNILADELV
VGVLVLLVPLFVPLPVMALGLFTSAIQALIFATLAAAYIGEAMEDHHGEEHEGSH
>1170768.4.peg.2984
MVLNSLYPKIREMEQLKYRIVREIEQLEQHRVLAYTESKQLENQILDCRNKRDTIHREIG
NYAAEKRQLELEIGTMEGTMGELQNSRQELHKSCIDLTAEKRRLDINCQVLRSEIGQLQS
QIAIYKQEKEELESNLVLSQRLKPQLEEKLHELRTESQDLENQVLTQKNLLQEAIETEQG
LQAKMVDLQHHKQKQETEVSQLQNRISLLRDEHDLLQNQVWELLQNLETLAPQSTLADDE
DNNGENNVELFPFDDLL
>1170768.4.peg.3006
MEREAILIMKSRQDLFPQIKDKINSVHSYDTPCIVAMPIDYISETYLSWLIKETKILKQI
PLL
>1170768.4.peg.3016
MQIEQAQEELEQKLDLDFEGFTVEHLVINQQQALTIENLPGYHLEGVYDLKVKLPTRQIT
QLHNHFNIYLQIQKEGKSWRLLIPEKSGEKQPLVWRGYLII
>1170768.4.peg.3019
MLPATNKCAEIASNFPLRMGKSEYVKGKTKLSDLPSGH
>1170768.4.peg.3032
MLRFSYLKMKLKVKEFLTTQVQGIKGQIIWQVTNCTFTH
>1170768.4.peg.3036
MSAEYSEDILVQQTTADFFEHTLKWHCVYAYLPATTPL
>1170768.4.peg.3038
MKITNIPIITGIFLSVYTNREQGTGWGYKISHPKHKIVGCGVKLNTGVE
>1170768.4.peg.3045
MIFPPKITQQGLKLNFSFAQFLRSLFSFSEIQYIEPEEKDEHFFINLYT
>1170768.4.peg.3055
MKDSDPVNLFRYVVGGIRNVGLAYIHLIEPRSTSAGGSDKTDESAPSTARKLRRT
>1170768.4.peg.3064
MFEGLYIHDKWDWGRKFPVIKIDFADGVLKNREELDRRILDLLRKNAERLGVSYESNDIP
GKFGTLIGEAVAKYGIRAVVLVDEYDKPILDNIDNPNIAAEMREGLKNLYSVLKGQDANL
QFVFMTGVTKFSKVSLFSGINQLTDITIDTQYSSICGYTETDLRESFGDHLEGVDWDAVR
HWYNGYNWTGSQTVYNPYDILLFIGKGQVFRNYWFETGSPSFLLKLFQKEQYFLPNLEGI
QVTEEILDSFDVEQINPVTLLFQSGYLTIKDIFTDINQMVFCLGIPNMEVKIALNNQFIN
AYSNLVNEKLGIQRLIHTQLRSGDVEGLVSTIKRLFASIPWRNFTNNDLADFEGYYASVI
YAFLSSLDARVIPEDITNHGQSDLTVMVGGHVYVMEIKVVEGNQVEGNAALDQILGRNYA
EKYRGEPGKSVHEIGLIFSRSQRNLIQADWR
>1170768.4.peg.3090
MGLCQVCYHLTAEPVCEICRNPHRDSQTICVVADSRDVIALEKTREYRGKYHVLGGVISP
MDGIGPEQLTLQALVRRVSQQKPQEVIMAISPSVEGETTTLYIAGLLKPFTRVTRIAFGL
PMGGDLEYADEITLAKALEGRRELD
>1170768.4.peg.3107
MTDTYLCIRHGAFEADPKTEELVHLQSGRHWRKWGNDWYRQHTHPDGIRFEIHEALDKLY
TQGYRATRVIIARRYQELMSGYLERSSSWRSEQPETGNARLYGLPVEFGPDPLDEPCWDV
INFDLDKEPGAPVRYPYFRLFE
>1170768.4.peg.3110
MLPTMERLPRLNRLIDQQNYFVIHAPRQTGKTTAMLNLAQELTASGEYTAAMVSVEIGSA
FNHQPDVAEQAILGAWQQSARFWLPQELEPPTWPAALPGQRINAALTTWAEISPRPLVVF
IDEIDSLQDETLISVLRQLRDGFPRRPKGFPQCVALIGMRDVRDYKVVSGGSDRLNTASP

FNIKVESLTLNNFSLKDVTNLYQQHTQATGQIFTPEAVHRAYYLTQGQPWLVNALARQTT
EYLTTDPQIPITIDLIDQAKEILIQRQDTHLDSLAERLREDWVQAIIEPILAGEELPNTP
EDDRRYLIDLGLVIRSQEGGLKIANPIYQEVIPRMLSRGTQDSLPMIQPTWLTTTGLLNI
PALLEAFLDFWRQHGEPLQKSAAYPEIAPHLVLMAFLHRVVNGGGTLNREYAIGSGRMDI
CLRYGKEVLAMELKVWRKGSSDPLKKGLQQLDKYLSGLNLPTGWLVIFDRRPNLPPISER
TSTEMAISPQGRNITVIRG
>1170768.4.peg.3156
MIDEWQKYKYSVNGKSILEPMKLPNTKVSFNANDLFFVLTNTRKYFQDYYQKDQNISRNG
LLVSQGVKVEDVLKTLDFMISTLREDMKKNRPPRLQDPNFINANFQVIKWKAYNPDKPEQ
KQLRITKYAVFTHPGSHTKTSTYNIPIYSLKDNLSTDKFYTKYTKQDVLSGIYEPGGKEF
GKVETLAYLTRQGLEEALMQGTILINFTDGSKGFFNVDRNNGISYIRGVRDTLQKRYWYF
RPVDQIKGYGYKIDTKISIRPGVTFAGDVLNIGLGRVVLIEYNQGQNKRLQMGVVADTGG
AFLPNLHQLDYLAGVFKNQTEFRQYIRQLPEYASAYILVKK
>1170768.4.peg.3161
MGILLVIPNAELIFEEVLNLSEVGQTAMQWSMAGGGVAYMILGAVGVFLYACRNLGWARA
LYFLIPSLSISLTSELLGTSTGFPFGHYSYLSGLGYKIAGLVPFTIPLSWFYVGIVSYLL
GRTGFDVDRKPTLSRHLGAIALGALLLTSWDFVLDPAMSQTSLPFWYWHKPGSFFGMPYQ
NFAGWFGTGSLFMTVSALLWRNNPINLERSQLNVALIVYLANFGFATVMSVAAGFPIPII
LGLFLGVAPAILLWSKAPSKSISAGLESGNQSVMSNVNNVNVVLK
>1170768.4.peg.3167
MSFLNAEDPTACDLIADFGIKILWHPSLGLEMGTNLQTLLWECILGKTPLDILVFEGTVV
NAPNGTGEWNRFADRPMKQWLEDLAKAANFVVAIGDCATWGGIPAMSPNPSESQGLQFLK
RKKGGFLGENFISKAGLPVINIPGCPAHPDWISQILVAIATGRISDIALDELHRPQTFFN
TFAETGCTRNIHFAYRATTTEFGDRKGCLFYDLGCRGPMTHSSCNRILWNGVSSKTRAGM
PCIGCTEPEFPFHDLKPGTVFKTQTIMGVPKEIPPGVNPEDYAVFTVVAKDTAPKWTDED
FFLV
>1170768.4.peg.3173
MSLITLQSVKKDFGIKEILKEATFSIDGTDKVGLIGTNGSGKSTLLKIIAGIEPIDGGQI
LTNYGAKIIYLPQQPDIDENLTVLEQIFMDSGEHTKLVKEYEELSDKLAHYPEDTLLMSR
LSEVMQRMDATGAWELETNAKIILTKLGIGDFEVKVGTLSGGYRKRIALATALLAQPDVL
LMDEPTNHLDALSVEWLQSYLNRFRGALLLITHDRYFLDKVTNRIIEIDRGDIYTYSGNY
SYYLEKKALAEESAVSSQRKHQGILRRELEWLKRGPKARSTKQKARIQRVESMRETQFKQ
AQGKVDISTIGRRIGKKVIELSGICKSYNGKTLISNFSYEFSPEDRIGIIGGNGTGKSTL
MNMITGRTSPDAGTVEIGSTIHIAYFDQHSEELISAVNDNQRVIDYIKEEGEFIKIADGT
KITASQMLERFLFPGSQQYAPIHKLSGGEKRRLFLLRLLIGAPNVLILDEPTNDLDVQTL
SVLEEYLEDFLGSVIVVSHDRYFLDRTVDTIFALEEGGNLRQYPGNYSVYLDYKKSEELT
QQETINGRDNRKSKNLTQPKSGEQEVQNKKRRRLSNWEKREFEQLEAKIVDLEAQRTLVE
TSLLAVAPENYTQVQNLYEQMEVLKQAIDIATERWLELAEMDV
>1170768.4.peg.3185
MAGNHVIFIHPDGTSPSHFAFARFVDKGPDGRLNWDNLEKTGVYLGHMKDQLGGTSNSGA
VTHATGTKAYAESFGFEAGNTPIVSLDGSNKTIVEAARDAGKVTALVQSGAIFEPGTAAF
VAKTKEIVNPGASRTVPRSQAAEIAKQVILSGVDFIMSGGELNLLPVGTNGIHGTAAQLD
ALTCIIHEGKNRKNKT
>1170768.4.peg.3187
MNRYFQAKDFWDKKDIEERAEYFADIALGTWGYLINQLPRNPVFPVKFLPEIPDENVAIN
ILYYRNNLKLTVRNL
>1170768.4.peg.3188
MSNDIILINEKATNFLNKLCKSRNIIGNLKITFPIFVKVFESYIASIFVYVNIGMGYGIT
>1170768.4.peg.3194
MLDAFSEPTFRKVLEGKIGEGPNNIILDLSKIDFIDSSGLGALVQLAKQAQNQGSFQIVS
NARVTQTVKLVRLEKFLSLQGTVEGALENIKS
>1170768.4.peg.3199

MSSVPLLRLSDKFDPYISGEVNIDPSAVIAPGVILQAALNSKIIIGPGVCIGMGSILQVS
HGILEIEMGANLGAGFLMVGEGKIGANACIGAGTTVFNDSVAAQQVIPAGSILGDGSRQA
NSQESGESVDEGDTSSQSGEQVVSKTQFTATFVNFQTQSTSVPPLSPTPKSQSPPETESS
TESQEISDGKPRSRDPTEPHPLGTQIYGQGSINRLLSTLFPHRQSLSEQDANNGAE
>1170768.4.peg.3201
MVVRSTAAPPTPWSKSLAEPDVHQTAYVHPSSNLIGDVHLGQNVIIAPGTSIRADEGTPF
HIGENTNVQDGVVIHGLEQGRVVGDDGEKYSVWIGKNASITHMALIHGPAYVGDSCFIGF
RSTVFNARVGAGCIVMMHALIQDVEIPPGKYIASGSIITTQQQADRLPEVQAQDQQFAHH
VVGINQALRAGYRCAEDLKCITPIRDELKDHEEKTYTSITVEELDRSSEVAGKLSADTVE
QLRYLLEQGYKIGTEHVDQRRFRTGSWQSCQPIETRSLGQAISALETCLVDHAGEYVRLF
GIDSGKKRVLETIIQRPDGVVVATSPSFKSPSPSSYNGNGNGKFAGIDAQIINQINQLLS
NGYKIGTEHVDERRFRTGTWQSCEPIHSTSTQEVVAALESCLNSHQGEYVRLIGIDTKAK
RRVLESIVQRPNGQVVTSGNGKVSLSPAGSTPAVSNHLSGEVVDHLRQLVNGGLKISLEH
VDQRRFRMGTWSSAGPIEARNEREAIATVESYLSEYPGEYVRLIGIDPQVKRRVLEVIIQ
RP
>1170768.4.peg.3219
MGPHRILLDCGAADISSITKENSPADLVLISHAHPDHCRGLLSLNRAFPLLPIYTSEVTS
KLLPLNWTELGAAENITFCQALPLRSPVEVEENLVVELFPAGHLPGAVAILLTYQGPERD
YKLLYTGDFFLSNSRLVDGLRLEELRGLSLDVLLIEGSYGTSRHPHRRHQENQIAERINR
AIIDRSSVLLPTPALGLGQELLMLLRSHHNFTGQDIDIWVDGEVGTGCDAYLELLSHLPA
SVQNFARHQPLFWDERVRPRVRRLKVEDLHTLGSSPCIVLTDYKSDLNKYIKHGDGASWV
VLFPEKISSDLENPLVQKETYLLAQHSDGPGTTQLIHNLRPQHVVFIHGDPTYLADLTTL
EELQNRYHVHSPPGGTLVELPIGETLIQSPVPEANYQGELTELETIITIILPREISEDPR
WQDFADTGLVEARWQGEDLVLKGLSQRYLLNRNSLDTAKSKYHYNWSEIECCGTCKHQRG
QRCWNAAAPLYNFKVTLEGYCPAFERLSENES
>1170768.4.peg.3237
MSDGKIVLIRRSDDECWALPGGMVDWGEDIPSVVRRELREETGLELVKIRRLVGVYSSPE
RDPRIHSICVVVEAEVEGEMNIQDHLEVLEIEAFPPTSLPLTPMSHDHYQQLQDYLSGLT
TLA
>1170768.4.peg.3249
MKPNYSNWDDDLPPDPQEIYQDLISTLERKVGFGLYFVQCTPIEADNFVQQISRDLANKK
IALLNLYEPMEKFYEYVKDYVQGQNIDILLVKGLEYSLYKYEKRNFGEVTEGQFTNLTKV
PPILNHLNQQRERFRDDFSFCFVFLLRSFSLNYLIHRAPDFFDWRSGVYELPTTAELVDE
ESRRLIVEGDYKKYLELTPQQKIETMLEIQELLTEKYQNDSNRARLLFEMGNLLYSANEY
ETAITFYEQELKLQPDDHSAWCNHGHALFSLSRYEAAIVSYRQALKLRPDDPFCWYALGN
SQRKLHRDQEAIISYNQAIKIKTDDHYFWYNRGNALRNIGCNEEAILSYGQAIKIKPDES
NVWNNRGIALRNLGRYQEAIFCYDQVLKLQPDDYYAWYNRGVALKKLKQNEAAVLSYDQA
LKLKPDDHYTWNNRGNALDDLGRIAEAIFSYDQALKIKPDDQYAFYNKACCYAVQGKIQE
ALENLENAVSLKPEEFTQRAKADPDFDRIREDTRFQALINKTFHD
>1170768.4.peg.3259
MVTPQADLEIRDEKPIKPSLPKLPDTKLAHSILVSGVILVDQQAQAIIKLPDDLNSRYVQ
AGERLTNGILVKRIEVNPCNNPVVVLEQFGVEVRKVISAQDDERELC
>1170768.4.peg.3267
MDNSSSFPQFYPERKTKFPKKIDILHSPVQNSVCLPLPKMLIFG
>1170768.4.peg.3273
MVNKTEIAQISHFSHFARFDLYYLYFVAKNPEVFTNLIRR
>1170768.4.peg.3283
MKTLPGKNTRPTSGRVREAVFNIWQGKIDQCCWLDLCAGSGSMGAEALCRGAKLVVGIEK
SSHACAIIQENWQHLVTEQQVFHILRGDVIQQLKKLSGQTFDRIYFDPPYASDLYDQVLN
AIAGFKLLHKDGEIAVEHSSDFKVPIIPVWQVIRQRNYGNTSLTFYSCREECESDGALYY
DISPSLGTHN
>1170768.4.peg.3287

MGIMDALIGGLDDSWSLFAFGLGTTGVGLGLKLASKLSRSPREEGRTFQYYLPPTSSSCS
LPIIKATKNKPQY
>1170768.4.peg.3314
MNLSITTKTTIKTARINLFLLASALVTAITSNTSLAADTISAQDIAQIAKKTSVQINTEG
DITPGGSGVIIAQQGNRYSVLTANHVVCDIIDRPGKITCAKDIIYSVRTNDGKKYPIKSQ
DIIVLQSTKNDPDLALVSFVATQEYPTANLGDSDQMTEASDVFVGGFPAVFGKVGSARDF
SFSKGIVLSRGRTSINGYSLIYDAKTLTGNSGGPVFDIKGRVVGIHGLADASNKSKTETG
ELVSQKTGFNAGIPINTFLNFNNPLVKDLPIKRNTIATGEAPQERLNSPQSARDFYARGI
TKLEQFNYKESLADLDQAIKIDPKYAEAYFKRGYALSWLRRYEEALLDFNQVIALDPNYL
DGYLNRGWTYIWLQNDQAALEDFNRVIRINPNYAEAYAHQGMAYIKLGKYQAALESSKQA
IRLDPNKSYGYTIQGDVFNYLKDYPAAINVSTFAIKIDPDDFNAYINRALAYTLTGNYSD
ALVDYQKSAEIFERRYTRKPSN
>1170768.4.peg.3318
MRLKIKNQNLHLKTKDHLPEDREETIDGLANKGITIS
>1170768.4.peg.3326
MRLLHILASLDPALVDYVKLETGDRPPDHAQNFPTAIAAFFRNFGLINIKNQPKH
>1170768.4.peg.3328
MFNSIGSPPTPLKRGAFRLDVLVSPVKVLLVSPQENITYLVIGCDRL
>1170768.4.peg.3347
MTPQPGVPPEEAGAAVAAESSTGTWTTVWTDLLTDLDRYKGRCYDIEPVAGEDNQYIAYV
AYPLDLFEEGSVTNMFTSIVGNVFGFKALRALRLEDLRIPVAYLKTFQGPPHGIQVERDK
LNKYGRPLLGCTIKPKLGLSAKNYGRAVYECLRGGLDFTKDDENINSAPFQRWRDRFLFV
AEAIKKSQAETGEIKGHYLNVTAPTCEEMLKRAEFAKELEMPIIMHDYLTAGFTANTTLA
RWCRDNGLLLHIHRAMHAVIDRQKNHGIHFRVLAKALRMSGGDHIHTGTVVGKLEGDRAI
TMGFVDLLRENYVEQDKSRGIYFTQDWASMPGVMAVASGGIHVWHMPALVEIFGDDSVLQ
FGGGTLGHPWGNAPGATANRVALEACVQARNEGRNLAREGNDIIREAAKWSPELATACEL
WKEIKFEFETVDTV
>1170768.4.peg.3353
MTEFCSLFVSLFVTFFAISWLPRYIPVIFDHVTLARGEVNTF
>1170768.4.peg.3356
MDNLATSKSKSQSKSPSGGGLPVVQYWVEQTLTPQGFKLWQTLNHKSACLSCAWGTGGQK
GGFVNEAGEYLQRCAKSVEAIAAELQPGIKLEFFRKHSIGELQKLTS
>1170768.4.peg.3366
MIKGRSHEGKSSAIAFFWVFSPISSTALYGKLLTRNRVW
>1170768.4.peg.3368
MATASMTLSVSRSAALKSCAFNRVSNFAYLLGVGAVVVEALDIDVAFK
>1170768.4.peg.3377
MLGHSIEQEELVQLSIIVALANIIKLTPIREAVTIFTI
>1170768.4.peg.3378
MIGSGTIIRDGSPDNYSYRVITNSHVLRSAKGPYTIHTPDGKVYIGKVSLLHNRFKEDDI
AILNFDANKTVYEGGKINRESLQLDEKVFVGGFITEREKRYRFIFTSGKISLLLKKPLLG
GYQIGYTNKVRKGMSGAPVLNRKGEVVGINGLQSEPLWQAQELYQDGEKPDSETEKLIVS
SSMAVPMRDQWGK
>1170768.4.peg.3388
MLLFPNSSNLTYQTAPGQAQIPTGAWEIITGSVAYDAPFGPTVAGLFLTPQQKAIYNSLP
ATNDVDSTVNDKDDFIKQSVNSSLQAFGYDPIGLNNNLSQADGLINSKLIQGDYIATHTY
GWTEFNIDPVTQKLSVTTYGVKPYTEAELVANPTAVINLEPQIVSQFEVNPDANNVPQAS
QLVFGTTGDDVVLAPSQTDGIKDLIFTGGGKDVVDISLDTPLAGFPRGENTVYTGSSKDV
IYAGNGDRIFGGSGDDEMYATDAKNYRLSGGSGNDVFHLGVNGRALGGDGDDKFFVTEGG
GNLISGGAGADQFWIATGDIPRVENKNVANSIVDFQMGIDVLGINGQGQSFGFKDLTLTN
NDIIINGNTIATLIGVNTSTLTVSNFSFV
>EFA71501.1

MFFSVIIPTYNRLPILQKCLTALENQSYTSQVTGYEVVLVDDGSTDGTLDWLAKHQDLFP
HVRCFQQDHAGPAAARNLGVQNSMGDTIIFIDSDLVVLSNFLDAHSDALTQGRKRLGGDR
LFTYGAVINTCNFEDPTAEPYKITDFSAAFFATGNVAIPKHWLEKAGLFDTGFQLYGWED
LELGVRLKKLGLQLIKCPQAVGYHWHPPFNLQQIHNLIDKEIQRGKMGVLFYQKHPTWEV
KMMIQMTFFHRLLWGLLSLNGLLNEKTNGSPVAVVN
>EFA71511.1
MSAEIICVGTELLLGDILNSNAQFIAQELAQLGVPHYYQTVVGDNPERLKRVIEIAASRV
EILIFTGGLGPTPDDLTCETIADYFGVPLIEDPEIIEDIREKFSQRGRVMSPSNRKQALI
PQGAKVLPNLTGTAPGIIWQPRSNLTILTFPGVPSEMHQMWQDTAVPFLKSQGWGKEIIY
SRSLKFWGVGESVLAEKVSDYLSLSNPTVAPYAGKGEVRLRICAKAPDTVSAQELIAPVE
KKLREIGGLDYYGVDGETLASVVGNLLRLSGTSLSVAESCTGGGVGQMLTEIPGSSDYFW
GGVIAYDNSVKVRLLGVDPGDLDQFGAVSPVVAQKMAIGVKIV
>EFA71517.1
MKSLKQIFVINQIRNLTTNNPDITWGSFAILYRTNAQSRPFEELLVKYQIHYTVVGGMKF
YDRKEIKDVISYLRAINNPADTVSLLRVINTPRRGIGKSTVDALINASQQLGTTLWEILS
DETSVNTLTGRAAKSVTGFTSIMKKWQEKTTQVPASEVLEGILEDSGYLRDLMDQGTDEA
DNRISNVKELYNAVSQFQDENRGNDISLQAFLQSAALSSDLDNLSESRDAVSLMTLHASK
GLEFPIVFLVGLEQGLFPGYRSLQDPKALEEERRLCYVGITRTQERLYLSHARERRLYGS
REPALPSQFLGELPQDLLTTKSKIHAARSTKSTTIMPETPGETNNWRVGEQVLHKAFGKG
VITHVFASDDKISLAIKFTSLGQKIIDPKVAQLQKIN
>EFA71518.1
MKDRIQRLFADQFAINQYGQKLDLLPAQQQTEVISSVYRTYIKNIWCGTFHSLFSRILRF
DVEKYQDEKGRHWQKNFSIFDESDVQTLIKEIVTKQLNLDSKKFDPKSVRYAISNAKNQG
LSPREFEEQQPNYRGRVIGEVYNLYQDQLAVNNSLDFDDLILVPTRLFGQNEQVLAYWHN
KFRHILVDEYQDTNRTQYELLRLLVTNGECRKNQWNWDNRSVFVVGDADQSIYSFRMADF
TILLEFQQDFGDGLDDDDTRTMVKLEENYRSCENILQAANELIENNTERIDKILKPTRDV
GESIYFHKADDEVAEADFCYQSNSEFNN
>EFA71533.1
MGRVGAVGFMSEADVAKVRGCDGSVTDGSVILASSMPLTLSDEANCPLVATDASRAFEVV
FLNILGLRISKICC
>EFA71545.1
MTTKSDPNRILRLLPLVVGSLGTVLLLVNRVLTPQLTESQARGDVLGVILSAVLILTGLI
WQQVQPKSPDTVELIGKEGFILASDLPETIKTELAWASRLLLTNTVTRSLVVYYKGQVLL
RRGILGSKAEVTPGPILERVLGTQKPIYLVALRVYPGKIEFDYLPDNTQGVICQPIGKEG
Y
>EFA71551.1
MTNPEGKEIEQQLEQLENELMLLKQRYSQIERDQQKKS
>EFA71555.1
MEKIAPNSGDQVYFLGDLIDRGPQSAQVVKFVKENNYPCLLGNHEEMIMNVMVHHYTSSK
AVQSWLYSGGQATMASYRSARIPQEDLDWFGSLPTYLDLGDILLAHAGVNPNKSLSEQTE
GDLCWIREEFHSMKTPYFTNKLIVTGHTITFTLPGVKPGDLAQGQGWLDIDTGAYHPRSG
WLTGLDITNSLVYQVNVFKNSTRCLPLEKALSKINPQEIKLSGRYKQVS
>EFA71567.1
MEWKEIRGNWVIVPRHPVGIIHFLGGAFVASAPHLTYRWLLEQFANKGYVIIATPFVNGL
DHQAIAQSVLLKFERTLERLHYYGELHKLYLPIYGVGHSMGCKLQLLIGSLFPVERAGNI
LISFNNYTAKDAIPLVEQLNSTFMSDLMIEFSPTPTETNQIIKESYQIRRNLLIKFHNDN
LDQSVELTKILKNRFSQMVTVKLSPETIQLL
>EFA71573.1
MTQKVIIFDFDGTIADTLDALVTIANRLAREFGYMQISANELKLLRNLTARQIIKYSGIS
LFKIPFLVKRVKGELKNKIKNVQPIPEIPEALRELSNQGYKLGIITSNAQENVHEFLKCH
QLDNLFEFVHSGVTIFGKNTIMSNVIKQRQIKPETVIYVGDEIRDIEAAKKAHVQVIAVT
WGFNSPEALARENPDFLIDHPWELLEVINRY

>EFA71580.1
MAEDGNSALVKAIIGNHPETFQLLLTKGANVNLQDPVGITPLMYATKQGYIRAVDMLIKA
GAKVNTRNHGGYTALMIAKSNNYAETSNLLIQAGAKE
>EFA71581.1
MIDLLTAAKNGNVQQVRQLLGSGFPVDAGDRHGTTALMFAANFGYTEIVRCLLEFGADID
LPRKLHGLTALMLAAAHNQVEVVKLLTSQGANTNAVNEDGSTALMIAVEKGHIETVQNLL
NFGADPKIVDEHNEDAFKLAIRQNNKVILNILLKHSQVKGETESLLIMGADNGHLDIVKT
LLLYGVNPNLENSDGTTALLAAAAGGHTEIIQVLLDGGAEINHQDQDGETAMHFSVVENH
LETVKTLINRGANLEIRNNLGDTPLILAVFQGYQEIVRVLLAAGADGSKKEFGGIPLDLS
SIPRTY
>EFA71582.1
MSLELTPDVKFGLQFFHPAIMWILLALSLYAAYLGLQVQRTRNAQGEEKKQLIKGKYSDK
HHKIGSVLLALMVGGSIGGMAVTYINNGKLFVGPHLLAGLGMTGLIAFSAALSPFMQKGA
NWARATHILLNFGILGLFIWQAVSGVEIVLKIISQA
>EFA71596.1
MNIFKDSGNLVLLFGICGNVIKSGGSNMTSKTKETKPSYVFRASWAILLLAINFLVAAYY
FHIIE
>EFA71599.1
MGHALIAYSGGVDSTLVAKVAHDVLGDRAVAVTAVSPSLLPEELTEASAQAATIGITHKI
VHTQEMANPNYTSNPVNRCYFCKSELHDTLKPLALEWGYSYVIDGVNADDLKDYRPGIQA
AKERGARSPLAEIGITKMEVRQLSQQLALPWWDKPAQPCLSSRFPYGEEITIEKLQRVGR
AEIYLKTLGWQDLRVRSEGETARIELPPEKIKDFVLKTDLPTLVTTLENLGFIYVTLDLE
GYQSGKLNRVLTRQDPILTKKES
>EFA71601.1
MIGYFTNDIAIKMLFRPYRPIYVLGRKLPFTPGLIPSNQERLGNNIAHAIMGSLLTPDEL
HKLAGRLLATDRVKAAILWLLQLALAQIKQEKNQKTAQILAGILRDLLGDSLPRLLRVLA
RKEDFLEVQINQVFDRLILEFRLNEEQAIWLADWLMKIAFPPDVLRQAIINFLTDKTIQT
IDDSFREKTSGTYWVVANLFGLRNTLTRLRSFCLDEKELTNQRLQELVQDLQLGDRLKKL
LQDLSLQNLPIGTVRELRKSVKESIRYYLQDSGSNLIQEVTESADWEKISILLLNRLRDS
VIVNSSLESVAQELALILDKYLEKDLETIVAQTIPILSLDKVIVEKVKDTSPADLEAAIE
GIVKNELQAIVNLGGILGLGVGLLQVGFLLLNQV
>EFA71604.1
MFRELIVNACVHRNYSITGSRIRIFMFHNRIEFMSPGKLPNTVTIDKLRFGVSYSINPVI
VKFMENLRYIDKLGRGLPMVYQEAKKLGKDVLFEEIGEEFKVTLLT
>EFA71606.1
MTESANTENHQENTHKSNTAEELLVKLRQKKGNWVEWGNAIAYLQKNGYNPQDIFEATGF
EPIQQNQVVVGAQVYSSLEKFGASEATKAYYGTRASDILYELRLLTQGDRATAAEFNFCP
QAGCG
>EFA71607.1
MDEAREIAKAIKDFSRFSTPPEGFSTHPGDAVGYQCWKLARQNSDLQERSRLIAKGLRFV
QSSTARKQIEQLLTDFTIVPQRNAPLLPYFRLESDEELPRLVPVVGELPLTPEDVKSVPL
IIEEAPFNIVKFAGEQAWVALPGWQVLRSAEDPIVIIGESNIFPQSKSSKTEQILIVIDR
DQRDWDEGGYFAFDNDGEVDFQWFETQPEQTILGRIIVILRPKKVLDEDFTKDSWQIDE
>EFA71615.1
MKTPIGADFNLAGSSNAMDRNHIHQHILFLQGVVTIGAMIFKNCHQVEIDEYLP
>EFA71626.1
MRLVAAARVRRAQEQVIATRPFADRLAQVLYGLQTRLKFEDVDLPLLKKREVKSVGLLVV
SGDRGLCGGYNSNVIRRAENRAKELKAEGLDVTFVIVGRKANQYFQRRGYKIDATYSGLE
QIPTATEATNIADELLSLFLSEKVDRIELVYTRFVSLVSSRPVVQTLLPLDTQGFETSDD
EIFRLTTRGGKFEVEREKVTSTVAPLPRDMIFEQDPVQILDSLLPLYLSNQLLRALQESA
ASELAARMTAMSNASDNAGQLISSLTLSYNKARQAAITQELLEVVGGAEALG
>EFA71629.1

MGTFLLLIAEATAVTGELAEGAEHSGIGLNTNIFETNLINLAIIITVLFVFGRKVLGNTL
KTRRENIETAIKSAEERAANAAKQLKVAEEKLTQAQVEANRIKADAETSAKAAGEAILVQ
AAADVEKMQAAGAADLNAELERVISQLRQKVVAKALQKAEAELKAGIAEDAQIRIIDRSI
AQLGG
>EFA71640.1
MPAFPFSFEVLYNPISYRRYDTATLQFNCEDYRAIHKILEPTYLETEIYFQPQIPLFTKY
LAPGLSLAEEPNQKFSPEESFGMNRCQIIANALLEAWQKGKNAIEERIQIIQQHFASELI
DLHYPYLNPSSQDIYIP
>EFA71641.1
MLSSYQVNNSPVNSQISQISSSQSSPVSRLDNYHLSVIESLFDIARNIEISADFSIQHPQ
YQPFAIPSTVAQRFRHNSLDLQRKYLNLLLRNFLHGIYYNGALQPILSTNNHQCSHLLTN
GLEPNYKLGVDRNFYQQLHENNHGIGNYDADWEVLRIEPDGTMAVMKNNLTLYVEPECYL
KSDRSPTPGDLIGIWMPKNRLQNGCYIAIGNCNQQHPSHSHNHHINYHSKKIAKIYFNIT
PSGAIALMNILTKRLNDASIPF
>EFA71646.1
MSITSSDAKIAVEFRDVSFEINHRLLVSKLNLQINQGEALILLGRSGSGKTTTLKLINHL
LKPTQGEVSVQGLSTNDWDVIKLRRSIGYVIQETGLFPHFNVAENIGIVPSLEKWPSKKN
>EFA71647.1
MLDLVGLEPTKFAERYPHQLSGGQKQRIGVARALAADPPILLMDEPFGALDPITRLELQQ
QFQYLQRQLGKTVIFVTHDIQEAFFLASRIGLMYEGSLVALGTKREFIQSQHGEAKAFFG
LFDCCAPLGT
>EFA71666.1
MPYQEDLFVADISETHVCILNKFNVVDYHLLIITRAFVEQESLLTGEDFAAMWACLAEFD
GLAFYNGGKVAGASQRHKHLQIIPLAGNEIPIAPLLTTADLENGITTIPAFPFLHAFTTF
SPDQTPQDILSKYYALLDIVSITANDDRKQSAAYNFLATRNWMLVVPRLKEDFVSIPVNS
LGFAGSLLVKNSAQMQLVKDIGPMNILKNVAISNI
>EFA71678.1
MLPIPDEFSFLTSGFAQCISVFIVFLILFTYLPILGSGGVFFLMKY
>EFA71690.1
MIFIIVETEAETGMDKPQTLEPKSKLTSKVNQRANYKQLTLFDLV
>EFA71696.1
MLNLVPSNIIVLVLGACVGSFINVVVYRLPRGLSLLWPSSHCPHCLNPLKVYHNLPIVGW
LWLRGKCADCQSKISYSYPLVELLTGIIFLIVFWVFQFSLLTIGYWVFCSWLLALSLIDW
ETMTLPGSLTKSGLVLGLIFQTTLGYVAHNTWSSAIGQLIWGIGGMVLGLWLFDIITSLG
FLFYGKPVMGGGDGKLAAMMGAWLGWQYLLVASFIACFSGVLIGCGQLLSVMVK
>EFA71709.1
MQRQKKSTVFISSEGDVSESINPSIQEFSLASGNAINSLPIPEKFLPDGKNQRGVRNNLA
FESLTITPNNQMLFTATENALVQDGEAAKPNFGTPCRILQYNLSDNQPQKEFLYPMEPVT
PLFNFTGRFDSGLSDLVALDNRGNFLSLERTFTGLGFAVSLFEVSLNNADDIHNIPNLST
VDMSKIKPVEKKLLLDLQTLPLPLDNIEGLTIGPKLSDGNISLILVSDNNFNRLQTTQIL
AFKLKRESPLKKLLRQLGLT
>EFA71710.1
MKESKKAKWKNPRMVIYGIITMIIVSIMGLFLGQIANATVRITDIEFLGVATLPTGYTFQ
NTKVGGLSGITYDVDNDLYYIVSDDRGQKGPPRFYNFKIDLSKGKIDQSKALPVGVTTLL
DGKNQKFALGGIDPEGIAATKKIHRLYFFRGGCQ
>EFA71713.1
MLSWLNNPRFEIIRHDITEPIRLEVDQVYHLACPASPVHYQYNPIKTVKTNVMGTLNMLG
LAKRVKARFLLASTSEVYGDPEIHPQTEDYHGSVNPIGIRSCYDEGKRVAETLTFDYYRE
NKVDVRVARIFNTYGPRMLENDGRVVSNFVVQALRGNPLTVYGEGQQTRSFCYVSDLVEG
LIKLMNGDYTGPINLGNPEEYTILELAQTIQNMINPEVQIKFTPLPADDPRRRRPDITKA
KTWLNWEPKISLQTGLKLTVEDFHSRIHAND
>EFA71720.1

MDECTNKSRRVFVNLEGVNFIDSSGLTALVAGMRNVDKLKGTFRICNIHPDAKLVFEVTM
MDTVFEICETQEEAFAIPF
>EFA71729.1
MQSTNIRAIAQMFRLAGWISFWIQLVLGVISGIIVLLYAFFSQRPGSPNNNPGTGFGIFL
AVCGLIVLGAGIYLGYRYTRIGKQLDSSNPSNRPRKLESVQVIRLGVWTNLGGILVTLLG
AQAIVGTLVARSISPQAVTTQLFDPNRVISGLDMLVVQANTNTISAHFAGLVLFAIFTKS
NHKIVD
>EFA71733.1
MLLGNESKKGEGIIITGGGEYQSPSFGLQNITLLFSGDGSILGITVTNSVSKGTGIWIES
AAPTLANNTLCKCGREGILITGQAKPAILDNVFIQNIASGLMMARSSKGEVLRNVFENNP
VGIAITDLAAPLIANNKLGKNQIGMAISRDASPVLRGNLIYQNSQCGLSINGNAIPDLGK
PQDPAGNIFREQENFDVQNSSSQSLISVGNQLNTAQIKGLLELVAATNDTIIPVISSSFS
DLYAHWSTAFITALAAKGFIGGFPDGTFKPNTPITRAQYAALIKKTFQLPDSQNLNRFKD
VRTDFWANSPIASAADGGFLGGFPDGTFRPGQNLTRVQAIVSIVNGLKLTGSNPNGLLVY
SDRAQIPSYAINATTIATQKLLVLNYPQVDLLEPLREITRAEVATLIYQALVVKGEAEPI
ASPYIVKPDKEQPSFSDLVGHWAEAYIRALVSMNLTSGFADGSYQPDKPMTRSQYAALIA
AAFNPVAKRPTVDFIDIPSDFWAVKAIQIASRGGFIAGFSDRTFRPDQNIQRIQVIVSLV
NGLGLVPNQNHSSLSYTDKNSIPEYAKVAVIAASQQNIVINYPDPKILAPTKEATRAEVA
AMTYQALVAIKRVKPIAES
>EFA71734.1
MIEVDHLSKIYGSTLAITDVTFKVEPGEILGFLGPNGAGKTTTMRILAGYLPLVRARLKL
LDMMSRIIP
>EFA71735.1
MPETPPLYPEMTVEGFLYFVAQIKGIPAGDRPTKVKAAMDRCNITEKRQVIIRKLSKGFR
QRVGIAQAIVHDPPAIILDEPTVGLDPRQIIEVRNLIKSLAGTHTIILSTHILPEVSMTC
NRVAIINHGKIVATNSPENLMTNLIEGSGYELTISGEVDLAKQVLQNIPGVSLVESMPNL
STCQNVILRVLSEPGSNSGKYIASTLIHGGFELHEMRRIGASLEDVFLRLTTEEKTLSQF
VEAKDLVETSGDNI
>EFA71743.1
MATLLYSVVSSAKIRVCPPEPRAIPFEMRVDELYVYRDWA
>EFA71744.1
MTTTEMVRIKSGFNADDQHPIQATSADIILRQQLEHSISRYFYHGCDRNIQNLLSYCRWY
MKTDTKALTFSY
>EFA71745.1
MTTTLQQRSNASVWDRFCEFITSTENRVYVGWFGVLMIPTLLAATTCFIIAFIAAPPVDI
DGIREPVAGSLIYGNNIISGAVVPSSNAIGLHFYPIWEAASLDEWLYNGGPYQLVIFHFL
IGCACYLGRQWELSYRLGMRPWICVAYSAPLASATAVFLIYPIGQGSFSDGMPLGISGTF
NFMIVFQAEHNILMHPFHMLGVAGVFGGSLFSAMHGSLVTSSLVRETTETESQNYGYKFG
QEEETYNIVAAHGYFGRLIFQYASFNNSRSLHFFLAAWPVVGIWFTALGVSTMAFNLNGF
NFNQSIIDSQGRVIGTWADVINRANLGMEVMHERNAHNFPLDLAAGEVSPVALTAPAING
>EFA71751.1
MDIYKAVILPLLFTLVKADPEWLHYVLISGMDWFLQPSHPHRARWLKSFVGDSLCVHDTR
LKQNLFGLNFPNPLGLAAGFDKDAMGSHFWSMFGFGLAELGTVTYHGQPGNPQPRLFRLP
MDLAALNRMGFNNSGAAAMAARLTLLYQQQLPAIPIGINLGKSKITPLEAAATDYLQSFR
LLKNLGDYFVVNVSSPNTPGLRSLQDRPMLSQILEVIQTENSIQNQPKPLFVKIAPDLEW
EAIVDIINLAKAYKLAGIIATNTTISREGLKTQVIEKTGNPPQAEPGGISGLPLRQPSTE
IIRFIYKQSQGQIPIIGVGGIFTAEDAWEKINSRCKFNPGLHRLDL
>EFA71757.1
MKSYQDNHQQRSVKGFSMEKTQTSDSPKSPPTNSDLPLKVNPINGSLETTHEKEKAVLEE
TQSQIDLEDKDGSKSYLKAGGKHGKEGFSQY
>EFA71758.1
MIALINSEFQQRGQSISGWVSALMMIFTIPAVLFGSVAGVFVDRWSKKAVLVASNAWRGI

LVITIPFLIWLTHDWHPIGALPVGFLMILLVTFLVSTFTQFFAPAEQATIPLVVEEQHLL
SANSLYTTTMMASVIIGFAVGEPVLVLADSLWAKFGGAGGLGKEILVGGSYGIAGIILLF
IQTQEKPHPPETEFPHLLSDLWDGLNYLKSNRSIKNAMLQLTILFSVFAALTVLAVRMAE
IIPNLKASQFGFLLAAGGVGIAAGATIVGQFGRGFTYGQLSLWGSLGMSGSLIGLAIFTN
QLWPVLLFIASLGVFGALLGIPMQTTIQTETSAEMRGKVFGLQNNVINIALTLPLALAGI
AENFIGLSSVFLLLSIIVFGGGIITWYNSSSH
>EFA71776.1
MLSLKRALKNDRKRVGRGISAGQGASAGLGMRGQKSRSGSSTRPGFEGGQQPLYRRIPKL
KGFPVVNRRVYTTINVEKLASLPANSEVNLASLKEAGILTSAKGPLKVLGNGELGVPLKV
QAAAFTGQARSKIEAAGGSCEVLS
>EFA71780.1
MLTWQGIKQRKCQQQKMTRSIAKVLQQEGFISEYTEAGEGVKRNLVISLKYKGKSRQPLI
TTLKRVSKPGLRVYSNKKELPRVLGGIGIAIISTSSGIMTDREARRQNLGGEVLCYVW
>EFA71786.1
MEGLAHRGSTLNFGDFALQAQEPCWITSRQIEASRRAMTRYIRRGGKIWIRIFPDKPVTM
RPAETRMGSGKGNPEFWVAVVKPGRILFEIAGVSEEIAREAMRLAAYKLPIKTKFIVRSQ
PQEQE
>EFA71794.1
MALITTGSGFIRDLEKFGSLGVFVPLEGGYEGRYRRRLRAAGYVNLHITARGLGDVAAYL
MGIHGIRPPHLGKKSTSNGAAVGEVQYLPP
>EFA71795.1
MWVIEGNILSDQEVEYFTNLPRLEPRVKVVIERGGDRYFRWTPLEKTLLAS
>EFA71797.1
MLRLEHISKIYPTGEVLKDINWEVKPGDRIGLVGVNGAGKSTQLKIISGEIEPTAGQIIR
PASLHIAYLNQEFEVDPTRTVQEEFWTVFKEANAVQLALYEVQRDMETATVEQLDKLINK
LDKLQRQFESLDGYNLDARIGKILPEMGFQVEDGDRLVSAFSGGWQMRMSLGKILLQKPD
LLLLDEPTNHLDLETIEWLENYLRSLTTPMVIISHDREFLDRLCTQIVETERGVSTTYLG
NYSAYLQQKAENEASQLSAFERQQKEIEKQQAFVDRFRASATRSTQAKSREKQLEKVERI
EAPTTGVRTLNFRFPPAPRSGREVVEIKDLTHTYGDKILFLGANLLIERGDRIAFLGPNG
AGKSTLLKMITGMEIPTEGTVKLGDHNVIPGYFEQNQAEALDLNKTVMETIHDEVPDWKN
EEVRTLLGRFLFAGDTVFKQVGDLSGGEKARLALAKMLLCPVNLIILDEPTNHLDIPAKE
MLEEALQNYDGTAILVSHDRYFISQVANKIVEIRDGEFRVYLGDYHYYLTKIEEEKEQAK
LAAKKATKTAKAAAKKK
>EFA71804.1
MRVFISTGEVSGDLQGAMLITALKNQAATLGLPLEIVALGGSQMAKAGARVLGDTSGIGS
MGIVEALPYIIPTIMMQRQAIAYLKKNPPDIIVLIDYMTPNMGIGSYMQQHFPQVPVVYY
IAPQEWVWSMSLDRTRKIVNFTDKLLAIFPEEARYYQAKGANVNWVGHPLVDKMVNTPSR
ESARKILGIQEQELAIALLPASRHQELKYLLPGIFQAAKNIQSQLPKANFLIPLSLERFR
GKVTRAIKDYGLKARIFSVNQQEIFAAADLAITKSGTANLELALANVPQVVVYSLSPFTA
WVGRKILKGSIPFASPVNLVLMREIVPELLQEKATADNITKAAMELLLNREKRTKNSIGL
SRDASVFGRSRSLRSRS
>EFA71806.1
MQVYRDDLESFTSKIARNEIQEVRQIIPTGIGIMAGLRTSPVPMQQITKQVRTVQREELG
IVFFYYETMWNRSPETLEQRIQGFKNFFPYPAVRVAAE
>EFA71807.1
MKLHLFSFNYHPKDRTNWPGKIWKQFSPWLCLISLLTVVLIHSVPSVTAQMSREEIRGVW
VTSNDLNVFKDRDQVKDAVTKLRRLNFNTIYPVVWNSGYVMYPSNVAKSLDIQPFVFRGS
DGHDILADIINQAHSQNLLAIPWFEFGFMTPNTGELALNKPEWLTKMRDGSTVSMSAAGE
VSWLNPFHPQVQKFIIDLLVELTNNYDIDGIQFDDHTSLPHQFGYDDYTVNLYKQETGKN
PPANSQDSEWVAWRANKITEFMVRLNHTVKQIKTKSHLFCFAKLLRPCL
>EFA71811.1
MNLVVLQNWLDNASFAILFCTMLIYWVGTAFPGLSITAALGTAGMAIANLSIAVLLTARW

IEAGYFPLSNLYESLFFLTWGITAIHLIAEYTSRSRLVGVVTAPVAMGITAFATLTLPSQ
MQNSEPLVPALKSNWLMMHVSVMMLSYSALMVGSLLAIAFLIVTQGQNIELQGSSVGNGG
YRANGYKVIKSQELIMEGSGGEKNGFARVESNQNGNGYSTAVLEVVKADHISPVTTLSPQ
RLTLAETLDNISYRVIGLGFPLLTIGIIAGGVWANEAWGSYWSWDPKRNLGPNYLVGFCR
LSSR
>EFA71812.1
MVFAAYLHARITRGWQGRKPAILAAGGFLVVWTCYLGVNLLGKGLHSYGWFF
>EFA71815.1
MTKIHKYILSVSQLDRAAIALILVLGLLIGITIFKGDGVKPNVRNFSWQDRQIGAEDRAF
SLVFSRPMDAKSVEDNLQINPPLAGKISWAGRRMVYTLLTPAPYGTNYQVSLTKAKDKFS
RSKDNNRLIKAFTGKFSTRNRALVYIGVSPQEYGRLVLYNLTKEEKTILTPRDLIVMDFE
PFPLGDKILFSARASNNPDLLSAQIYTVTTGVGNGQEEKSVGKLGLILDNQEFQNLKFDL
SPDGGTLVVQRGKKDNPGDFGLWYSSLDDLGSNGQLILKRLEGQPAGDFVITPDSKAVAV
AQGQGTAILSLAGDTSKPLDFLPQFGLVQAFSKDGSQAAMVKFNSDYTRDLFLVTNQGVQ
KPLLKTSGSILYCSFDPSSPTLYCLLTSLVSQDKYVEQPYLMAINLDTKKYKPILMLPVG
QRNVQMSLAPDGLAVLFDQIVPVSNNSLSLPPSILKTDDGEAIAKSSLWFMPLLPIAEDQ
AKSMIKPEALPIDGFNPVWLP
>EFA71816.1
MAAINFMLFHNYTKEKQIMVDSEIQQLVNLNQELKNS
>EFA71823.1
MTAKVINHLKSKGFANIYTVPDWAENQRQTQIFVRRGTRHPGVELRKILGVGQIEVSAQG
DLDADLTIRIGEDWK
>EFA71824.1
MIKEVQWLENQLGIQQVTALEHDGKLQQLMSVETQPVSNPLPGLGTEVKAEHVNSQRTVI
KSMGEIPYQLFEKLGLSMPGWLLWVLTFAISITLSGLLMSAVALWTPLWSNLEQAEDDGY
TPTNRENLKVSDGLWNKLSLYQLSKPMNILVMGVETIKGTLDGSPESFAGSSDTMLLVRL
NPSDKSIRVLSIPKGTMVSLPEDGLSKISEANAKGGPVLAARVISRTFSDAPIDRYIRIS
TSGLRELVDQLGGVDIFVPQSMTSQDQSGRTPINLVTGWQTLNGEQAELFARFRESSVGD
MARVQRQQALIGGLVQRLNNPVVLPRLPQLTRMMRKYFDTNLRMEEMMALANFAVNVERD
KFEMTMLPGTFSKFSKDPESYWLNLTGQQSLLKN
>EFA71830.1
MGGFFSAVSKVPITAIVIVFELTTDFNLVLPLMIVAVTAYLVADYFVPGSLYDELLKLNG
ITIEKNSPIEGVLTKLKAEDVMQKRVETLDAQMSIKEAIQAFSRSHHRGFPVVDQGQLVG
IITQSDIKNIYPFQYTTLREIMTPGPVTVQPDQGLSEVLYLLDRHQISRLPVVEKQKILG
IITRGDIIRAEADSINCKNIASGPQPEPSYLVYQTRSPSTGRGRLLVTLSNPDTAETLLE
MAATIAQERHYELECLQIILVSRHSSPSETKVRTTKSKRILRQAESLAKRWQVPIHTQIR
VAHDVAQAILETIKERHIDLIFMGWKGNTSTPGRIFGTVVDTIIRQATCDVVLVKLSSLH
NKSYLANFHRWLVPMAGGPNAPLAIKLLPALVSLVENPQIRLTQVVKPWEKEPDMHVLEE
STRQLIRNRNIHGEVIAASLQGESVSQAVIELVNTEGFDVVVLGASREGLLQQAIHGNIP
ETIASGVNSTVILVRGEI
>EFA71831.1
MLIVDRLAKQFRQIWQPRKGLAIAEASIIGIVAAVSAVLLKQSSGWLGTWRIHSTHILPA
WLVLPLIGMSFGFFAGWLIQRFAPEAAGSGIPQVKASLGNVPIRLSWQVVGIKLISTIFA
LGSGLILGRQGPTIQVGAGLAAGMSRLVPTSPEHRRQMIAAGAGAGLSAAFNAPITGVLF
IIEELLKDLSELTLGTAIIACFIGGVISRLLGGGTLQLNRELVNYSAQFYVPEIPIFLLL
GVIIGLFSAVFRHGLRLSLKIYQRLHITLPLRVALAGLISGLIVALLPEYYRDNAGLREY
MIASEPNLLLATITFISQFILTLIAFGSGAPGGLFAPSLILGSCLGHIIGVFEVQFFGFL
VPLILTL
>EFA71851.1
MEVTPIIAKIKQALPKGGVIYNTLNGDSNVAFFKQLKGAGLTPDKYPSMSVSIAEEEVKA
IGVEYLKGHYAAWNYFQTVDTPANKKFVEAFKKEYGADRVLNDPMEAAYIAVYLWKQAVE
KAGSTDLAKVRQAAYGQTIDAPEGKVTMNGNHHISKVVRIGEVRDDGLFKIIYATPAPVE

PIPWNQYVKETKGFACDWSDPAKGGKYKQS
>EFA71854.1
MNVKILETENVTVSFDGFKAINRLNFTMEKGELRVVIGPNGAGKTTFLDVITGKVKPTQG
RVLFKGRNLRNFSEHEIARLGIGRKFQTPRIYLNLTPRENLELTSNRQKNCFFYPI
>EFA71855.1
MGTIGLTSKADIPAGLLSHGEKQRLEIGMLVAQSPDLLLVDEPVAGLTDEETYNIGELLL
TLSQDHSILVIEHDMEFVRQIARKVTVLHEGSVLCEGDFKQVQNDPRVIEVYLGQQQE
>EFA71858.1
MLTSSVTVAQNSLLVVFPPKNHQTSTEKIFFIGTAPPQGEVVINGRKVKRSQSGHFSPSF
PLQLGENLFKIRYQNQEQEIRVTRVSTQPELPTGLGFAPNSLQPGVDLARLPGELICFSA
IAPPQATVFVKLGEQMVSLTPQPLQANLPANSSVLTGRNQPTGYIPNKYQGCTTVTSVAD
LGQPQFTLTLNNQTISQTAPGKIQILHPAQLSVAEVTSESCVTRTGPSTDYSRMTPLPKG
TRVMITGQEGDWFRLDYGAWVNRKEIQIIPGAAPPKTIIRSVGYSQLPKVTEIRFPLQVP
VPLKVQQGDRTFTLTLYNTTAQTDTIRLDDDPLISRLDWQQVTPDQVKYTFNLKKLQQWG
YKLKYESSTLVLTLRHQPTIALARRLPLLGMKIVLDPGHGGKESGAIGPTGYAEKDVNLV
VSKLLRDELVKRGAVVVMTREDDREVSLVERQEIISKEEPAIALSIHYNSLPDNGDAENT
RGFGSFWYHPQSHSLAVFLHNYVVSQLKKPSYGVFWNNLALTRPSYAPAVLLELGFYEQP
SRL
>EFA71861.1
MKFYLGTKNGYSFVRFHLGSGINGNDLSKRGTENTGEVRFHLGSGINGNIHGTFCFWTAE
KYVRFHLGSGINGNS
>EFA71862.1
MEEKLKELRLVRFHLGSGINGNFVDTFSLVSVTSVRFHLGSGINGNSHA
>EFA71864.1
MRYLVEMLDNLIDEVGEDETHPLASLMEIIGVLIEQYENQNIPETFKANDRHTTNMPQVF
SKLNSLLQAQNFKQADRETRKIMLAIAKREEEGWLRIEDAEKFPCKELRSIDQLWLKYSG
GKFGISVQQQIYQRTLNMT
>EFA71865.1
MNSKFPIKHISPLCTLFLLLTGTNLIRVTPALGIDQHQIIAKQLIQSKKSQVNQVFQPIL
SKIKKNHPNQDSITHPYSHWEK
>EFA71866.1
METVTKNKYQILLGFTPDCGGGTACGFGAISAELVSSNTPKPVGKEVNLNNNKKAYFEDF
KCGANCSNANLIWREKGVQYTIGLKAGSLSDLVKMANSVVSPK
>EFA71879.1
MEAVISQAQSIIHQIANEVVNHPSLSILQEQLAKLTLELDRKEVQIAVTGGKSVGKSTII
RLLKTVSNLSANPWELVETLPLFTRLSQDDDPAILLTLQKSDLVLFITNGDLTASQLDVL
QKLRSTQQLVLLVFNKQDQYMTEERIIILQSLQQTFPGHVLATSASPLPIKVRKHLADGS
IQEGMEQPTPNIQQLVEQLTVILAQQIPQLVCATTWRKSLFLKTQARSCLNSIRRDRSLP
IVEQNQWIAGAAAFANPLPALDILATVAITGQMVMDLSNIYLQKISLDQAQKVARTLANL
MFKLGLVELSTKAVTGVLKTNAVTFFAGGMVEGVSAAYLTRIAGLSLIEYFEQQDIALTS
DRQLNMETLGGVLQRVFQENQTLLLLETFVKQGVKRLSRETKPSTTYVHF
>EFA71886.1
MGDFGIDLTSTHVFKPLMPLGVEHIKSIKIDKTMLTRENFFEAESFLPGA
>EFA71887.1
MEVMDLESEGVKKTSPQDISKTIRVSALHALAYCRRLFYLEEVEELYTQDAAIFAGRRLH
TELEKQEDEVWEELFLESEELGLRGRLDALRTRDGQTIPYEHKRGYCHRDENKQPQAWES
DKLQILAYALLLEFALGITVKEGRIRYHTDNILIHVPLDHSGRESVHQAIKEARNLRESA
YRPPVTENERLCARCSLAPVCLPEEARLTHDKEWQPIRLFPEDDERLIIHILEPGALVGK
TGSQIKINRRNQPIENIPASQIGQLVLHSFSQISTQALYFCAHQDIGIHFISGGGRYVGS
FDNRRGSIQRRIRQYTALSDPAFCLELARKLVLCRGQGQRKFLMRGTRGRTQVPENLHKS
IAQIKTMLKQVPQAKSLESLLGLEGNIAASYFSALPHLIVADVVDELYFNGRNRRPPKDR
FNALLSFGYALVLKDVMNAILTVGLEGALGFYHQPRSQAAPLALDLLEIFRVPLVDMTVM

SSINRGQWHPKEDFEIRREQVWLSEPGKRKFVELYERRKQETWKHPVTGYSLTYRRLFEL
EVRLLEKEWSGEPGLFGNLILR
>EFA71889.1
MFQRLGAEDYTHYDPKDPGYFEFEGSGYVYKGGNHFSGTHIMGTTPKNSVVDQNLKCWDH
PNLYLVGSGSMPTIGTSNTTVTIAALTFKAVEQMLKEL
>EFA71899.1
MIQCPECKSSKINKNGHKKANKIIFVKTVVDNLLIITKPTEDIQKRQEMD
>EFA71911.1
MGFWLRNSQFNNNSHRESAQWKKDIFLGNHPGNEHNPRDAGHFWGSDPNQKYLWGNHTRR
PVPVYYQGAYSGNLTIYAGQNVKFTDSTGSTYTIPGGRGLVDQTHIFQTMKRAITGSNL
>EFA71917.1
MVLYYGQNRHPLLDSLPEIFYREEDLYIARQWGLKTEEWMSHCRTASEITVLADLACSVK
NDHPSLAMVDGSLIYWFLEQLPLEARDQILLPILEAWGKLRQAGIPIVGYLSAARNNEAK
NLLRLLNCPYPVPDCINYCPDQLDYVPCKKFEGLRDTTLWATQLQPGQRSALWRSNSRIL
QLYDDQVIYFCYLHVGTEIARIEFPAWVVNDPQMIDQALGLVLAQIQKGYGYPVAIAEAH
NQAVVRSGDRNQFFALLEREMIKAGVKNVSISYKEARKRGSIA
>EFA71922.1
MKGNNYCAKIFEFNNNKSGFTDFWLPIILVNLVLTVSTPISPANATEQSPDQIGREENQP
HRDQSTREVNLDVKLPQINAQNNSQNADNSEEIDKIRRRLLLEPVIKQKPAPKPQPTNAP
GLSFAGVSAFGANMGDVFLGTSVATAGNRGNSLKDIDGSMSAGFGLGDARGLIGLEFTFN
NGSIKNFGSNGTFDLKVHRTIQTDNSSSIGVAVGWKTFGQYKTKASGEAVRPSSLYGVVT
SHSLLRANDPVNKMPISFSLGVGGGDFRQGNDSVGVFGGAGLQVHPQIGVGLGWSGIGLN
VGASFLPVPTLPLTVTTSVSDLTSNSPGSTVFSLSVGYGFNFLPK
>EFA71924.1
MVPTPQETAKSEMFKANQAMQIARRSNERAGGIWIYQFIRSPIWFISVLFT
>EFA71925.1
MSDLSKLSILIIGSYNGQDSFGDKCLARCVAQQIRETCNFSGHQSPNILSHIDGNLETSQ
GEIPEVEFSTGISLLYWAWNNKLRHLHLPTALQRFMGVITLPVYLMATQVNRRKLQRIKR
EIRESPLVYFYGGTQLSEQWFWYNFIPLMITAFLCKLYQVPLYFGPQQYGPENNWQRWFL
RTTVKYLVRDIRVRNKNCLELLNLSPEKLCYDEVFSCAIRYPVVKHHVPPKNFILVNMRG
TNFLRDGESREFEVFSNLLVAVKNQLGLPFKLFQMSGSSFCDDTKLLGFLQSRPEFSDIH
LEVLPLVVEEAELIKVAIQAYGTISMSFHGCILSMIGGCPSVPVTSGDYYDYKYIDFDKY
SGEQGTPLITLGNLDVKQASATISDYFSKYSPVKTAIARERAAQQMKYWYKTIV
>EFA71928.1
MAKFLRSKLIHPKLSYKIWQLLCKLNHGQSFFFPLRSSGNFPVLMYGGICGYSDSAIFFE
SLQYEQENIAVYRSIIIYYQILF
>EFA71930.1
MSKLSFLIFILDKYLRNQSRDCPHCGSENTILKQRKKLLLELRECQDCFLMYRYPKDTMV
DNFNFYQLNYDELLVIATPIESKARLVYL
>EFA71931.1
MKVLVSCGTKFHSDHLAYELQQRNSLYKLITSHPSVAYKRKPIDRKKILFLPPIFVISLV
LSRLLGKFYVFRSRLEWVLAIIYDWMASLFVQSPDISISWAWASLRTIREVKKRGGIAIV
EECGSCNLYQEKLLREEYQNLNVHYKPQTFHKIILRELEECQEADYILCPSQHVANSLNQ
CGIAQEKLLIIPYGVELSLFNPQPKKDDTFRVLFVGTVGIRKGLIYLFKALEQLATKNNL
ENFECLIIGRLDYDFKHIFSKYKDYFKYIPRVPHDQLKEYYSNSSLFVFPSLDEGMAYVQ
LEAMACGLPVICTPNSGGDSVIRNGEEGFIIPIRDSEAIQQKIEYLYFHPQELQKMSQQA
LERAKEFTWENYGNSLSKKLQELKLRREKC
>EFA71932.1
MYSRIKLATKELDLSSYESCLSISYSKPLCNLIGFLDSQILEANYPQYNICNLDLPSETF
DAVVTDQVLEHIECDPCEAINECYRVLKPGGIMVCTTCFMMPFHGSPDFSVSGGGDYWRY
TPQGLQFLCRNFSKIIQSDGWGNPLLPILGGLGLVHKPIPDAAWHPLHKIATYDRKSYAC
VVWIIAQK

>EFA71933.1
MKRVTFITPAHISCNPRLFKEANTLHSAGFQVRVVAGDYSPEARILDNSISPEPPWSWVK
VPLGSKPIYLATRFLQKLAQKLAAHKYILNLSIATFSNSPISYQLAKAAAAEPADLYIAH
CLAALPAAVMAAEKHHGKVGFDAEDFHIGELPDIPENQAEIAVRDVIERSLLPRCDYLTA
ASPLIAEAYGQRYGVEMTTILNVFPLSEAPPSLSQKQEINSSPSLYWFPKPLVVVGGLSL
>EFA71935.1
MSLPYFKEFGWEPHILTVKPEFVIGVNDPLLVKTIPSHTSVTYAQALPFQKTKLIGIGSL
GLRCFPFLLKTGNQLLKQNKI
>EFA71936.1
MALGRIWHDKFNIPYVLDFQDPWLSDYYEKSGVTPPGGN
>EFA71937.1
MSKVSHIVSVSPEYPNSLRQPYPYLKPEQFTVLPFGAPTVDFEQLPDLNIHNRIFNCQDG
KRHWVYVGVVGNIMSLALRILFLGIQSHRRQHPETWQSVKLHFVGTSYATDNRAVKTVEP
MAREFGVDDLVEEYTQRIPYFEALQTLVDSDGVLLIGSDDPSYTASKLYPCVLARKPILA
IFHEKSSVVNILKQCQGGECVTFNSTIQPQNLLEDILKKLKLANFATKSL
>EFA71939.1
MQNPHVSVVEKLQWCDQHREELAAMVHRVYQDFQPRDWAEVAKDFTKIVRDCLNQNSDHQ
EK
>EFA71944.1
MKNLALAISGKMDKHFTALWDGGHIKFFSVVTMKSLLISEGYEDIKFKFAGRIPYLWKSM
LCSAKLIK
>EFA71946.1
MVGLISTYGGSSYPTAVGVEAGMKAAVDFYWGKKNLQGLKVAIQGVGNVGKNLCEILSHQ
EVEIFVSDISNHKLAEVERLYPVNIVDVEEIYELDVDIFAPCALGGIINSRTIPKLQANI
IAGAANNQLENEELDSQLLADRNIVYCPDYVINAGGLINVYDGMIGLSEESSLARVRNIY
NTLKQVFAISKEYNVPPLTASKQLAENRFLQHQTQN
>EFA71947.1
MEIFLKIAEMGHKQVTFCYDQESGLKAIVAIHNTNLGSGVALGGTRLLPYSTEEDALKDA
LRLSYSMTYKAACANIPMGGGKAVIIADPEQKNNKLFSAYGSFINSFRGSFITGQDVNIS
WEDAHKIGEKHPTWLA
>EFA71954.1
MIRRVATFSGIPTGLGISTLVVSYLLVSYAHIQLPPIAVLLVNMGLFGLGVLGITYGVLS
ASWDEDNPGTLLGVGEFGTNWSRMVEVWRETRKK
>EFA71955.1
MSAQESEPSRLPFEPNKKRQKPSKVSAKAVVKTEESPQKLPNQPPLLKRKWRFPK
>EFA71956.1
MALTQLRKQELISGFQVHETDTGSADIQIAMLTERINRLSQHLQANKKDHSSRRGLLKMI
GERKRLLNYIQKNNSDKYQSLISRLGIRG
>EFA71971.1
MTDEPSSVIDITRKRAMINFGINGTGLLAQVNFGANSASVLGIFLAVAGAALYFLRTVRP
ELSRDQDIFFAAVGLLCGFILIFQGWRLDPILQFGQLLLVGSTVFFAVESIRLRSIATQQ
AKRNTPIVDDEREVSKKYSYSQRRNYQAEVDEDLDPLPYYEEEEPEPPNVLGFAVVGMIV
LVKRATKTLPSAHHVVVVRNNQIHELKNAVLVMEAPPTVLIVMILMKIGALLLDQLMTGK
LPLQKPENHLVLVNLNLQNFPRMNHPLDQEGVVPLWSLLSLEMMNQLMLIMYPIIP
>EFA71973.1
MTSVMSGVDLISWTVGPLLGVMTFLFYFPDHFDLVSPGES
>EFA71974.1
MPPLGGVDITPIIWVGIFSLVREFLLGQQGLLTMAARLNG
>EFA71975.1
MGDKSTAKETMQKAGVPTVPGSDGLVETETQGLAIAHQIGYPVMIKATAGGGGRGMRLVR
SEDEFIKLFHAAQGEAGAAFGNAGVYIEKFIERPRHIEFQILGDNYGNVIHLGERDCSIQ
RRNQKLLEEAPSPALDEDLREKMGHAAVKAAQFINYTGAGTVEFLLDKSGHFYFMEMNTR

IQVEHPVTEMVTGVDLLVEQIRIAQGERLKFDQDKVTLQGHAIECRINAEDPDHDFRPSP
GKISGYLPPGGPGVRIDSHVYTDYQIPPYYDSLIGKLIVWGPDRHTAINRMKRALRECAI
TGLPTTIAFHQRIMEHPQFLQGQVYTSFIQDMKLP
>EFA71976.1
MKFDKILIANRGEIALRILRACEEMGISTVAIHSTVDRNALHVQLADEAVCIGEPASAKS
YLNIPTLLQQP
>EFA71978.1
MSESAPVNENSQSSAIDTENSGERTSKTRQLLGMKGAASGETSIWKIRLQLMNLLPGFLN
LGVVCGAASSGNYSWSLENVLKAALCMLLSGPLLTGYTQTINDYYDREIDAINEPYRPIP
SGAISEKQVVSQFVILLLLGYGVAYILDIWAGHTFPNVLMLSVFGSLVAYIYSAPPLKLK
QNGWLGNYALGASYIALPWWAGHALFGELNWKIVVLTLFYSLAGLGIAIVNDFKSVEGDR
QLGLNSLPVMFGIETAALICVVMIDLFQGLVAGYLVSIHENLYAAILALLIIPQITFQDM
YFLRDPIANDVKYQASAQPFLVLGMLVTGLALGHGGI
>EFA71979.1
MALILTFLGKNGTGRSKIAIAAAQFLANQGKRVLLAGLADPTLPILLGTSLSADPEQIAP
NLEAVQFQVSVMLERNWEEVKKLEAQYLRTPIIQDVYGQELVVLPGMDNALALNAIREYD
ASGKYDVIIYDGTGDIFTLRMLGMPESLSWYIRRFRQLLANSDLGKAISESPLIQPVITS
LFNVNWTAENFAQPANQINNFLDQGKAALANPTRACGFLVTTADSIDVANARYLWGDCPT
SWFNCWGIILLSADTSNNLSTEFTPLPVTMVPDVSSGNWQPMIDALPNFVEQAAKAPKPI
EIDVHNKQVRLFLPGFDKKQVKLTQSGPEVTIEAGDHRRNIFLPPALSGKPITGAKFQNS
YLIISF
>EFA71987.1
MPPSTIPSILLVDGYNIIGSWPCLKKTRDDSSLEAARYHLVELITNYSAFEGYESHIVFD
AHYQNTPSNREMITDFLTVHYTEFGQTADTYIEKVCAGLRHQVAQCLVSRVIVATSDRAQ
QLVVQGYGAEWLSARQLCNQVEAKVCQMRNQHQSTKKIQG
>EFA71990.1
MCSGTGAKPGTRPRTCSTCSGSGQVRRVTRTPFGSFTQVSTCPTCNGTGSVIEDKCESCD
GKGMKQVTKKLKVTIPPGVDNGTRLRISQEGDSGQRGGPAGDLYVYLFVNDDEEFQRDGI
NILSELKISYLQAILGCRIDVNTVDGPVELTIPPGTQPNTVIKLENRGVPRLGNAVSRGD
HLLTVLIDIPTKVTLEERELLEKLAKIKGDRTGKGGLEGFLGNLFKA
>EFA71991.1
MARDYYEILGVSRDADKEQIKQAYRRQARKYHPDVNKEPGAEEKFKEINRAYEVLSEAET
RERYNRFGEAGVSGAAGFQDMGDAGGFADIFESIFSWFCWGKWGDLLNSKGDGVDRFVVT
ISD
>EFA71992.1
MLRHAMVKVAAAIEDTSTEEKDQSDQDQI
>EFA71993.1
MTSDYPTGINSQESDSTVSDHQPPDANLSEQSTVGEENGVAAVEEVVDRDLITQLTQQNQ
SLKAQLEERNSQYMRIAADFDNYRRRVSKEKEDTETQVKRNTIMELLPVVDNFERARAHL
KPQDDGEMTIHKSYQGVYKQLVDSLKKMGVSPMRPEGQEFDPNLHEAVMREQTSEHPEGT
VLEELVRGYF
>EFA71995.1
MITLLGYSLNLVRQGITTLEEVERVVYTDKELIRELKVSRQKSLTCQCCDATLKPEWLDC
PYCLTPRLID
>EFA71998.1
MKKELEMAQITYMTGCGATPGLLTAAAALAAQSYAEIHKVEITFGVGIANWEAYRATIRE
DIAHIPGYNVEIARAMTDEEVEALLDKTNGVLTLENMEHADDVMLEIAGICPRDRVTVGG
VVDTRNPKKPLSTNVKITGRTFEGRISTHTFTLGDETSMAANVCGPAFGYLKAGYQFHKK
GIYGLFTAAEVMPQFVR
>EFA71999.1
MKHKLTMTQDTKTTMNTEITSQPLRIGVLGFGGLGQAAAKVLSGKREMLLVAVADQKGYA
YSPVGLKTNIAITTYQDRGSVGYLQSHGNLSNHSIQDLIANAPGVDAYFWLYLTFLMTLS

PKLPSNLSLLVGKVCWWMLLNAPVQ
>EFA72009.1
MSVQFSRGCPFQCEFCDIIVLYGRKPRTKTPEQLLAELDCLYRLGWRRGIFMVDDNFIGN
KRNVKLLLKELKVWMVEHEYPFRFDTEASVDLAQDPELMELMVESGFAAVFLGIETPDED
SLQLTKKFQNTRSSLAESVETIIKTGLRPMAGFIIGFDGEQKGAGDRIVRFAEQAAIPST
TFAMLQALPNTALWHRLQKEGRLRENQDGNINQTTLMNFIPTRPLEDIAKEYVEAFCALY
DPIKYLDRTYRCFLIMGSPKWKAPFKMPDWVIVKALLIVIWRQGIKRETRWKFWHHLFSI
IKHNPQVAEHYLATCAHNEHFLEYRQIVRDQIEGQLAAYLAQGTEKPYVVPQKEKVGV
>EFA72010.1
MRVLLVYPIFPKTFWSYEKILDLVDRKVLLPPLGLITVAAILPQEWEFKLVDRNIRPATE
EEWAWADVVIFSAMIVQKQDLLDQVREAKRRGKLVALGGPYPTSTPHEVQEAGADFLILD
EGKLPYPCLSPPYKVVRNPAFSGPRKNQMSQAHQFLDLIC
>EFA72015.1
MAERNIREQQRKQALATLGPQISFIARYDLLDEFNDGVAINDGYSVGLRASMNLYDGGAS
RARAARAKTEIAIAEAEFAERRNQVRFQVEEAYSSHLANLENVQTAATALDQAKESLRLA
RLRFQAGVGTQTDVINAQSELTRSEANRVRAILNYNLALTRLQRYVTSRAVQKS
>EFA72016.1
MKGQQLFHSFLPGVTAAVLTTQSAWAGTFKANDLKLVSSPVVSTATNPKVSVVENNWHLV
ANTVDHALGFDYQLDFGQSILPELPSSSSRANVPSPTKFKTVLSLPPVNPKVRSGKTYNQ
VTQITLPTDKSSETEPVKPNSQSVNPTSDSPQMILERLKPNPNLLDVPQDSQGVKVQTTE
AISLEQALDLAKQNNNDLQVAVLQLQRSQSSLKEAQAALLPSLNLLGGVTRSRSSSTTLR
ARQTDKFLNRPTENPDATSVFDSQAELRYDLYTSGRRTAAIKEAEEQVRLQQLEVERQSE
EIRLNVATEYYSLQQSDESVRIARSAVENSQASLRDAQALERAGVGTKFDVLRSQVNLAN
SQQELTDALSQQAIARRRLALRLNLPQSVSITASDPVQLAGLWKSSLEDSIVLAYQNRAE
LQQKIGREEY
>EFA72019.1
MTDGERQEILKKLKLDYGKILLNYFSVDENLKTKIDQFISTLFCANIPVPQVIEIHMELI
DEFSKQLKLEGRSDETLLDYRLTLIDVLANLCEVYRCSTSKIN
>EFA72022.1
MILKSPGQLKFTVITFTLAMLGLLGGCLGLTVSFDSQPSDSMSQAPEKSPFLENHTATPS
PQLQRVEPKITSNRTLEKSKFGNLRISNKTYQPIRLALLLRHSPSSSSGKKGLIPAHWDF
APQEGSQGGLILSLPEGSLKLEKGIF
>EFA72045.1
MFPMMAENEESKALDQGFVLKPMNCPFHIQIYKSELRSYRELPMRLAEFGTVYRYEQSGE
LGGLTRVRGFTVDDSHLFVTPEQLDGEFLNVVDLILTVFRSLQLKNFKARLSFRDPTSDK
YIGGDEVWDKAEGAIRRAVQQLGMDHFEGIGEAAFYGPKLDFIFSDVLEREWQLGTVQVD
YNLPERFDLEYVAEDGSRKRPVMIHRAPFGSLERLIGILIEEYAGDFPLWLAPVQIRLLP
VGEQQIEFTKEIARKMTELGIRAQADLSGDRLAKLIRNGEKDKIPVMAVVGAKEVETNSL
SIRTRASGELGVIPVEKVVEKMQESIINYSNF
>EFA72046.1
MAMAVQKLFPKAQVTIGPWIENGFYYDFDNPEPFSDKDLKAIQKEMVKIINRKLPLIREE
VSREEAKKRIEKIQEPYKLEILADIKQEPITIYHLGDEWWDLCAGPHVENTKDINPKAIE
LESVAGAYWRGDETKAQLQRIYATAWETPEQLGEYKRRKEEALRRDHRKIGKELGLFIFS
DPVGPGLPLWTPKGTLLRSILEDFLKKEQLKRGYLPVVTPILPE
>EFA72060.1
MRGYDEGKLAAGRTYVQASAEYRFPIFSVVGGSLFFDCGSDLGSATRAAEILNKNGSGYG
YGLGLRIQSPLGPIRIDYGISDKGDSRINFGIGERF
>EFA72066.1
MVEGWVFHISFNFQDSSEVYREFVQLTLHRPCDMSGLSHEFLTTNGPTHWPFSQDNYQEV
KRLYTDLHFYTHDGKAQFAACFSHGLAEPPDPEYPFILTSGRLYGHWHTQTRTGRIDKIC
QMHPEPFLEIHPRDALKLQLADHQLVMVRSRRGAAEFPAKITLTISPGTVFVPIHWGKLW
AENSEANALTHSASCPDSFQPELKACAVQLIPIIPIQS

>EFA72067.1
MTEFIKTLCPYCGVGCGLEVSSLTQPPSFTTDNWRVRGDKSHPSSQGMVCVKGATVAEAI
CKNRLQYPMFRESLTQDFRLISWEEAFTIITNHIQQTLVSYGPESICMYGSGQFQTEDYY
IAQKLLKGCLGTNNFDANSRLCMSSAVSGYLQSFGADGPPCCYDDLELTDCVFLIGTNTA
ECHPIIFNRLTKYHKKNPHVKMVVVDPRSTPTAKTADLHLAIVPGTDIDLLNGIAHLLVK
WNSWDVNFVDNYTSNFPDYIQVINYYSPDLVTSRCGISLADLETAAKYWGQAQSVLSLWS
MGVNQSSEGTAKVRSIIDLHLLTGQVGKPGAGPFSLTGQPNAMGGREAGGLAHLLPGYRL
IKNPQHRLEVEEFWGLPPGTISPIPGLNVWEMIMALESGNVQLLWIAATNPAVSMPDLER
TKKALLQSPFTIYQDAYYPTETANYAHLLLPAAQWGEKTGVMTNSERRITLCSAFKNPPQ
LAKSDWEIFAEVGRRLGFSHQF
>EFA72069.1
MVAGMPLAMTLGAMGKTPTPMVTAMTLSRNGSAITLSQELWANGVRNAADLEKTVNTDLD
KTYTLGVVHPASMQNLLLRYWLAASGIEPDKDVRLIVIPPDEMVDFIQERKVDGYCVSEP
WNTCAVEQNLGFIMLPTSDIWQGHVDKILGVTENWSQQYPQTHLSLVKSLLAACEYCDDI
RNESEIISLISQPQYIGDHHVNLRSELLESYQTNYHRHSQTSHQFYLHQANYPNRHEMLW
ILTQLARWGFIAFPKNWVEIIDRVCRPDIFGVAAREVGILDIGREESIQLFDGKTFNPSQ
PLEYLTGLAIKSQVRVEEALV
>EFA72070.1
MNTFVEIDHVDKIFNLPYGGKYIALKNIELKVSQGEFISLIGHSGCGKSTLLNIIAGLDQ
ATIGGVTLEGREVREPGPDRMVIFQNYSLLPWLTVEGNIALAVDEVHQDLSVGERQKIIE
EHIDLVGLRRAAKKLPGQLSGGMKQRVAIARALATRPKLLLLDEPFGALDALTRGNLQEQ
LMKICNEHKITCVMVTHDVDEALLLSDRIVMLTNGPEAHIGQILEVSIPRPRQRLEVVEH
PSYYNLRNQMIYFLNQQKLAKQRQKQTISPILISTNQPEKVHLQIGYLPITQAAPLIIAQ
EKGFFAECGLQVNLIPEKIGKILPKV
>EFA72071.1
MLVGGVGIGSFIWDAYNTTTDTNLSEIIVALIYVGIVGLMLDRTVAFIAEKIVQKEQK
>EFA72072.1
MSVGVTNQKISTKRQKAIRTINKFLVHKVLPPLCGLVIFLMLWQLLCSIPGFQLPGPLET
FVETWDPFIIKPFFDNGDSDKGLGWQILTSLGRVGLGFSLATIVGIFLGILIGANRFLYN
AVDPIFQVLRTIPPLAWLPISLAAFQQANPSAIFVIFITSIWPILINTTVGVQNIPQDYV
NVARVLRLRQGKYFLKISFPSHSSLYLYRTENWYWVILVGHCSCRNVGRWCGYRFIYLGC
L
>EFA72086.1
MSKKLIQPLPIKKSKQWSSLQEKWQQLVKRDDQGNWDSQGKVWGAPYRWGNTVLVYNQKQ
LQNFNWQPIDWSDLWRSDLRSRISLPNHPREVIGLVLKKLGRSYNTVNPVEIPNLKIELE
SLNQQVKFYESTNYLEPLITGDTWLAVGWSNDIIPILSRYPQLRAVIPQSGTAIWADIWV
QPANTLITETDKTDNLILQWIDFCWQPDIARQIVMLGKTNTPTFVNTSKVQGISDDTQNL
LVIEHELLEKCEFLKPLSLESINQYEELFLKMKAS
>EFA72087.1
MKRRNFLFNVGGLLLGQTLMGCNGKNQSEFQVLLLKDSIPGYVVNQFNKRLQSDKTLSHV
KLSFVPKSQIHDLFKQLQIWRETRDKSPGGKKSPHCDLVTLGDYWLKPAIEQKINSTLTN
KKK
>EFA72088.1
MALRQIFDLYACVRPCRYYAGTPSPHKSPDKLDVIVYRENTEDIYLGIEWKQGSEIGERL
INILNKDLIPATPEHGKKQIPLDAGIGIKPISRSGSQRLVRRALKHALQLPKEKQQVTLV
HKGNIMKYTEGAFRDWGYELVTQEFRAECITERESWILGNKEKNPQISPEENARMIDPGF
DNLTPEKKGQIVKEVETVINSIWSTHGDGQWKDKVMVNDRIADSIFQQIQTRPDEYSILA
TMNLNGDYLSDAAAAIVGGLGMGPGANIGDSCAIFEATHGTAPKHAGLDKINPGSVILSG
VMMLEYMGWQEAADLIKRGLGASIADGKVTYDLARLMEPPVEPLKCSEFADAIIQQFNC
>EFA72089.1
MLENSTKAELISLFWNVLTMYEKITPPPTGEKITFQNGEPIVPDNPIIPFIRGDGTGIDI
WPATEKVLDTAVAKAYSGKRKISWFKVYAGDEACDLYGTYQYLPQDTLTAIKEYGIAIKG

PLTTP
>EFA72094.1
MRDIRNLDRISFSEWFYSHGGSKGSIKRMWNPIAYALGFIDCDHISARCMLTIFQFFAAK
TEASILRMLEGSPQEYLHQPIVNYLTNRGTKIHTRRQVREIKFTESDSQSEVTGILVAQG
EQEELITADAYVFACDVPGIQRVLPPSWRKWPEFDNIYKLDAVPVATVQLRFDGWVTELQ
DSEKRHQLHQAVGIDNLLYTADADFSCFADLALTSPADYYRPGQGSLMQLVLTPGDPFIK
QSNEAIAHHVLKQVHELFPSSRELNMTWYSVVKLAQSLYREAPGMDPYRPDQKTPVGNFF
LAGSYTQQDYIDSMEGATISGKRAAKAILATGGIFK
>EFA72095.1
MRVVIVGAGLAGLATAVDLADAGWDVEIFEARPFVGGKVSSWVDGDGNHIEMGLHVFFGC
YYNLFELMAKVGAGNNLRLKEHTHVFVNKGGNTGALDFRFITGAPFNGLKAFLPLPNFHY
KTSYKMLLLWALVLSFEV
>EFA72104.1
MLAANLKQIQQFFVHPHSSLQLPWNSLLLGLLILPINPFFGAIAIGWASYKTWRKKYSSI
QRKTLNHLLVILSFWFLITTGFTVFARDQPDAFLGLFNFLPYFVVFAGLTPLITTVSELR
QLTWIIVCSSLPVVMIGLGQLFLGWHARWQFLSVVVNLTIDPGGEPLVRMSSVFMNANTL
AAYLITVLILGLGLWLENYHKIRKKANPLGFIFLSVVIIANFLALILTGSRNGWGIAVMA
CIAYALYQGWRLIIAGVIGLTTSIILASFATERIASVFRSFVPRFIWARLNSDTPLALMR
KTQWQFAWDLTLQQPLSGWGLRSFPHLYEQKMGVSVNHPHNLFLMLSAETGLVTTCLFFG
FFWLGY
>EFA72105.1
MAWILIAAIKFWYKCSLPKENRLVFLSYLLAFIGWIMFNMADVTTFDLILSALFWVILAA
LYGAAHRYEGSHH
>EFA72106.1
MPTTADFLQYSQWSGITTIALAALTILALLFKWGLRFRLVGATGFMLVLTVGLFSLSLVP
LSRTVIPGASRYTLVYDNGSNQAVIATSPDITPTQLEATLRQAASNLYSYGRMGSGRNNS
LTIRARTVLHPESGISTPVYLGKVERTLVTREDPQMLVEVFLDKFPQLPPS
>EFA72108.1
MSLSWITPADRIKKLPPYVFARLDELKAKAREQGLDLIDLGMGNPDGATPQPIIEAAKTA
LANPANHGYPPFEGTANFRKAITEWYYRRYHVLLDPDSEALPLLGSKEGLAHLAIAYINP
GDTVLVPSPSYPVHFRGPIIAGGVIHNIILQEKDDWLIDLGSISDEVAQKAKILYFNYPS
NPTGATAPREFFEDIVAFAKKHEILLVHDLCYAELAFDGYQPTSLLEIPGAKDIGVEFHT
LSKTYNMAGWRVGFVVGNRHVIQGLRTLKTNLDYGIFSALQTAAETALRLPDSYLHTVQQ
RYSIRRDFLIAGLSKLGWNINPTKATMYLWVKCPAGMGSTDFALNILQKLVW
>EFA72109.1
MVVTPGNAFGVGGEGYVRISLIADCDRLGEALERFRQAGICYQSDVVPVCF
>EFA72116.1
MEKSHIQLPGYKILEVIHVSSRTSVYRGEQETTKRSVIIKTANAQYPPLNELIALKNQFA
ITQKIDHPHIIKSYALEPYGNSYALIGEDIGGISVYDYAQSQPLGINMFLKIAIAVVKAL
EYLYNYKVIHKDIKPKTLLLIQKPIKLS
>EFA72117.1
MTDFSISCLLPQEIAEIKKFNPLEGTLSYMSPEQTGRMNCGIDYRSDFYSLGVTFYELLT
GRLPFISKNPLKLVHSHLAKMPPLPQEINPEIPDLLAEITIKLMSKSPENRYQTARGIRY
DLERFQEALLNHGQIKSWQLGSRDIADRFIICDKVYGRDKEIAILLDAFARVSQGSKELM
LVLGSSGIGKTAIVYEINQPILEQQGYFTAGKYEQLQNNKNNIPVSALLKALGNLLQQVL
TQSPEELVAWKNKILLTLGEQAQVIIDVIPELEKIIGSQPAAPKLTGNAAQNRFNVLFAK
FIQIFANEEHPLVIFLDDLQWADSTSLKLIQFLIKEPDTKYLLLIGAYRDHEVYSRHPLT
VTLESLRKIEPKIAINEIDLQPLDKNQLNLLISGNLACEETLSLSLTEFVFNQARGNPFF
SNQLIKSMHEDGLLRFDFQLGCWQCDIYKAQALYGNDILQLLATQIHKLPIATQEVLKIA
ACIGNEFDLLTLAIVCEKSQLEIVSNLQNALTAELIIPQDEKHNLLYPETHQNDQSKKYK
FFHDRIQQAAYLLIPDNEKESTHWHLGQLIYEYSKNRDKKELEEKIFIIANQLNVAQTII
DTAREVERYQLAELNLIAGHKAKLSTAYEAAINYLRFALELLPANSWQTHYHLTLNLYLE

AVEVEFLNINFDQAEIYIKLVQQKAVTLLDQVPVYEIQIQIYMAKVQIKLAIETGIHIIN
MLGIQLVEESPKILNDQNQNYVDELINLPVMTAPDKIAAMGILGNITTATYCFDLELFDR
IVFTMIYLSLQYGNCSTSASGYAHYGLLLCKLAGNIDNGYRYGQLALNLANRFNAQEVKC
VVLLTCNSNINFWKNHLQQTIGSLSECMNYGMETGDLEHVGYASAIYNQNKFLIGENLTC
LLQELEPI
>EFA72118.1
MYRFKQQGAVLVHLIWKQLVLELLNYDPSSGSFHNSFDNIELLSILIQSKASTLIFTFYL
AKTIFFYLFHNYQKSLEAVLQMTQHLNYVSTQIGCSQYNFYYSLVLLGEYSQKSNANQRK
FKKNTKNTYKLLNSIKNLWQYGPVNVQKIISISMI
>EFA72119.1
MAIWASKCPENYQHKYDLIAAEMARVLGNHWQAGELYDQAIMGARKNRYIQEEALANELA
ARFYLNCGRNKIAQTYLIDAYYCYLNWGARSKVIDLEISYPELLAPVIKPRTIIAEETSF
MTKIMDSNKNVSAILDLETVTKASLAISSEIKIDKLLGTLLNVIIENTGAKKAALVLKKE
SSLFTVVQENQNTGWELIPIKDSQDIPINIINYVNNTQNEILIDSNSIDKKFAVDPYIIK
YQPKSMLCNPILKHGQIIGIIYLENRLTTGAFTPERLKVLKLLSSQAAISLENAELYENL
EEKVAIRTKELNDNNLYLEKTLQELKSTQMQLIHTEKMSSLGQMVASIAHEINNPVNFIY
ANVDHAIDYIQYLLDLLNIYQQEYPTPHQIIVEKSKSINLDFLVSDLPKVLNSMSVGAER
IRDLVVSLRNFSRVDESEIKPLDVHQSIDSTLILLQPQFKEKLGSAKNIIFKNYSNLPLI
TCYASQLNQVFMNMISNAIDAIYEHRKNLSGAAKETFTGQIIISTLVINRDWVGVCIKDN
GKGMTNEVKNRIFEPFFTTKPVGEGTGLGLSISYQIIVNQHRGKIDCLSQPGMGTEFVIQ
IPTNCSQYD
>EFA72125.1
MITFPQLIWTDIGLKLLSILILITINAFFVAAEFAMVTVRRARIHQLVQSGDAPAIAVEE
TKFPPGCVLLIEITTNSAKSSEST
>EFA72126.1
MTTNSTAVLTLILVALMLMTGSVSAFLGFSMGSSALKGVTSPDGRPTSKLISSGNNNLQS
VPISFLKEEDIINQVKKRIEQNKTKTNRTKKVEEEEQTVSTKDKSQQKAQELPEEPPQPG
FPVVAESEGVNMSVQSASYSGGQLILKVKMHNQSNESVRFLYSFLDVTDNRGRVLTATTE
GLPAELPGNGSVFMGNISIPTALLNDVSSLTLSLTDYPAQKLKLQLSDIPVEK
>EFA72129.1
MHNNSISVAEPNLYSQRTQPRAIRMGVIGVGNMGQHHARLLSSMKDVELVGVSDINVERG
IETASRYKVRFFEEYCDLLPHVDAVCIVVPTRLHYAVGINCLLAGIHVLIENPLPRAFPK
QSL
>EFA72130.1
MLKTEEVLALESHRMSPYSSRANDVSVVLDLMIHDIDLLLELAGSSVVKLTANGTRSLDS
GYLDYVTATLGFANGIVATLTASKVTHRKIRRIVAHCKNSLTEADFLNNEIFIHRQNTNP
QNDRQTLYRQDGIIEKVYTSNIQPLSAELEHFVNCVRGGNQPSVGGEQALKALRLASLIE
QMALEERVWNPLEWQSESVSQSLTSSV
>EFA72131.1
MLEWITNTINSLGYIGIAMMMFLENLFPPIPSELIMPLAGFTASPYQPGGAKLNIIGVFF
SGLVGSTLGALIWYYPGKLLQEQQLKKLARKYGKWLAISSDDIDKAKTWFNRQGGKAVLM
GRLVPGIRTLISIPAGMANMPMLPFLLYTTLGSAVWVGFLTYSGYLLGTQYKLVEEYIDP
ISKIVLVGILVLFFFGYLSAKVNRSVDKF
>EFA72138.1
MSQDKKPRSKQQLPETSPSGAYHKKPLFWKILIIQVLRGTIGILETMVTRLETSPASTTP
EKGGLLLWVVTKWDEFLRGFRLFLPSKVANNVSDGFLTMIFAFLAVLAIGVTATSLISQI
GSPSVPQSSQSSTDSTVDDKTETQLGQPPTDSTVEGKTEPQLGQPLTDSTVDDKTEAQLG
QPPTDSTVEGKTETQLGQPPTDSILKSQAELIPQQEVTGFIEKQLKEITATNIITKDKQR
IEVEIVQSIKTNFRISEIVIKTTEAWYKLESSQKEKLAAKILKSCQEMDLIHVKILNAQN
QLIARSPVVGTKMVFFQFPNS
>EFA72145.1
MISIASIDPNTITTYLQTQREFFATGKTKDVNFRLAQLQKLRTLVTDNKESIIAALKGDL

NKPEFESYAMEIGAIKEIDYAIKHIKKWTKPKKTGVPLEFFNYSAKILPEPLGMVLIISP
WNYPLQLVISPLVGSIAAGNCTIIKPSELAPHTATLLTQLISEYFPPEYIRVVEGGVETS
KQLLEQKFDHIFFTGGTAIGKIVMTAAAKHLTPVTLELGGKSPCIVDKEINLDHTSKRII
WGKFINAGQTCIAPDYLLVNKKIKISFNQFSPANSSRILWR
>EFA72146.1
MGGEINPEQLYIAPTLIDNISLTDKIMEEEIFGPILPIIEYTDIQEAIEIINSQPKPLAL
YLFSENKKLQEQVLTNTSSGTVCINDTIIQVAVSSLPFGGVGDSGMGSYHGKVGFDTFSH
HKSVLYNGFRLDLNWRYAPYLGKMSTLKKIIGA
>EFA72149.1
MATIILQNHASFRLHKLSTSHDTSSTSEFSQNLTSPLLDRAKSFIRPDQASSYGDAIKMA
KKYTRDNRSLIWPKLLLMTGATKFYNLLKTMLIRENLAKL
>EFA72152.1
MAGKLASHLVDNGTDTVLVCGTTGESPTLSWDEEYQLFVEVLQAVSGKAQVIAGCGSNST
TEAIAATQKAAKLGLHGSLQVVPYYNKPPQSGLYNHFQAIAKSCPDMPVLLYNVPSRTGQ
NLIPETVMRLAEIDNIVGIKEASGNLDQTSEIRRLTPREFQIYSGDDSLTLPLLAIGAKG
VVSVASHLVGNQIKQMIESFSLGQVKTATEIHLQLFPLFKALFLTANPIPVKQALKIIGW
DVGSLRLPLLEADMEVCQKLKTVLCEMKLV
>EFA72156.1
MRKLIAGPGVYICDECVELCNEILDEELLDAANNPANSSPPKPEQTPKRRTNSANLSLNQ
IPKPREIKNYLDDHVIGQDEAKKVLSVAVYNHYKRLAVAQGKAEDSVELQKSNILLIGPT
GCGKTLLAQTLAKILDVPFAVADATTLTEAGYVGEDVENILLRLLQVANLDVEEAQRGII
YIDEIDKIARKSENTSITRDVSGEGVQQALLKMLEGTVANVPPQGDVNIPIRTAFRLTLA
TSCLFAAELLSV
>EFA72157.1
MGFVQSGEGQSKEKRVATTLKQLEPDDLVKFGMIPEFIGRMPMVAVVEPLDEDALMAILT
QPRSALVKQYQKLLKMDNVNLEFKEEALSAIAQEAYRRKTGARALRGIVEELMLDVMYEL
PSRKDVTTCTVTREMVEKRSTAELLVHPSSLPKPESA
>EFA72159.1
MIQYWQEEHEINSHHWQSELLGFRYQMQDRLTTNLQLFLAQEMENTYQRALKFVQIKTKF
KVQFPAECPYTLEQLLNIDYTG
>EFA72163.1
MLSGLLKFVLGFLLAIAVLLGSGMTIAIYFINRTAITPQKPMFPNDNPDPKPNLPRVTRK
KVVKVKPKPIATPDLPRESPTPLPSGSYTAVVTWSQGLTMRDKPAFEGQAIGGVAGNQKV
IILETSQDGKWEKLGFQILIKKVGLKVAIRKNQINSDKWEPSWFLFTSIRA
>EFA72168.1
MDASIKQSLKGVNLYLIGMMGSGKTTIGSLLAQAVNYSFIDTDEVIVKAAGKPISDIFTT
EGETAFRQLESNVLAQVCAYTKLTIATGGGIVLRRENWSYLHHGLIIWLDVSVEILLERL
KEDQTRPLLQHPDPQSKLRSLLDERYSLYSQADLHIEITQQEHQDKL
>EFA72174.1
MNLETSTKLVLLSILTVLTPLNDVIAQNTTKLSQNPTSRYTRLESLLKAQNFKGADTETS
KVMLALANRQKEGFLTAEDVEKFPCRELRIIDKLWLKYSRGKFGISVRQKIYESLGGVEG
INSSELDWSFAKRVGWVSRTGSWIGYDRLNFSQKAPSGHLPRRIFFSDRMRTYQTIFITD
NFPSMLFRCNT
>EFA72175.1
MIAVTEQDRFFTPEEIYNIVKQRMFSSICSNAAGEYMEVYKKSRLVLPSGCKDSSCAQSY
PFHPELFNLLTKKIASIPNFQRTRGALRLLALLAIVIRYLWQKLEYLAQNSPTTSENASF
WIPMIHPHHIPLGIEEEITGDLTSRLDRQLMRIPIQADIYNSDGREAHSQIQHREWSAAG
KPPFTKVNQVRICFILTIR
>EFA72215.1
MTKLTATPISPVTAERTPPSVVLQTLELRRCFDKFVAVDSLNISVSTGEIFGLLGPNGAG
KSTVIKTLTTLLPLTSGRAYLAGYDVIHSPNSVRRAIGYVPQALSADGTLTGYENLLISA
KIYDIPSRRRKDRINQMLEFMGLENVTHRLVRNYSGGMIRKLEIAQAILHQPKILFLDEP

TVGLDPVARSQVWQLMQQLRNECGTTNFFNNSLLRRSGFSLRSSGNHESRQGNYHGFPVR
VKDLYWQTRGQFR
>EFA72219.1
MKHNPYNWIEESLNTIHRADWYRSVQTINSPPGAVVILSGQKMINFASNDYLGLAADDNL
KMSAIEAIRQFGTGSTGSRLLTGDRQLHRELEQAIASTKQTEDAVVFSSGYLANVGTITA
LVGKRDIIFSDQYNHSSLKSGGILSGGTVIEYPHCDMIVLEKKLKEERQKYRRCLIVTDS
VFSMDGDLCPLPNLLDLAEEFNSMLLIDEAHATGVMGKSGAGCVEHFNCTGRELIQIGTL
SKALGSLGGYATGSSALIDFLRNRAPSWIYSTALSPADTAAALAGINVVQKEPERRKRLW
ENVNYLQKVVKENLGKLKILPTQSPYYVLSYLMLQQHCRWENT
>EFA72221.1
MFSVHAFEHYLVKLYEYLQVLENRLFSSGLHTLGEPPNEEELTGYLNAYFGEGTEVQKES
QKSQENLIVDLLNQSTDELTNLLRGLNGEYIPPAPGGDLLRDGPGVLPTGRNIHALDPYR
MPSPAAYERGREIARKILDQHLQEHHSYPETVAVLLWGLDAIKTKGESLGILLELVGAEP
VKEGTGRIVRYDLKPLETVGHPRIDVLGNLSGIFRDSFVNIIELLDDLFLRAAQIDEPQE
WNFIRKHALLLQAQGIENSSARLFSNPSGDFGSLVNDRVVDGNWDSGDELGKTWESRNVF
SYGRQDKGQARPEVLQTLLKTSDRIVQEIDSVEYGLTDIQEYYANTGGLKKAAEQQSGKR
VTASFVESFSKDTTPRNLDDLLRMEYRTKLLNPKWANSMANQGSGGAFEISQRMTALIGW
GGTVDFRDHWVYEQAADTYALDQEMAEKLRQANPEAFRNIVSRMLEAHGRGLWNADENRL
NQLRQLYELTDEQLEGVGV
>EFA72223.1
MNKKYVLIGVEGNHDQAFISKILRKLLDFCPSNEVDKIDKIWRKFIPVYPAKNGKLHFRL
NIPTILCKDNISVAIYAGEGGNLITNLSDKLSDIDCSYLFAFGIIADADKKTPDKIVEKY
HNDLREHFPNFPNKVNSTGNVIDSSPKLGIYILPDNNRQGVLDTLICDCGDLAYPEYMQR
AREYIDKFSEEERNKPPLKWKPFDKEKAIIATVVSVLKPGKTNQTSISDDAWISDKTAEF
PAIQNLTQFLINLIC
>EFA72224.1
MKSLDSVIIHQFRGIRDLELKDLGRINLLVGINNSGKTSVLEALQIYSNPLNITTWLITA
CQRELQNRLPRDLLIDALRWLFSQNPVSTIESPELIIILISSTGLFSVKKLIASYEVIEEI
WLKKSENDQIQENEEEDDLIQENEEIPGVRKGMNLKIELFTEDGQVSLFDTTPTITKNFP
LWQNKGLYLHLYKPSGTREPSLNTATVTSSSHRSADQIRLLSQARFRNFKSDVVRLLQQI
DSNISDIEILSPPQSTSSVSTSSVYIYIQHKKLGLVPLSSFGDGIRRLLHIALKLSSVKE
GGILLIDELDSTIHTEALQNYFQWLVKWCAEMDVQLFATTHSLEAVDALLEVTESDSDLV
LYRLEPKEEKQE
>EFA72226.1
MSQLWGLLVILITCILLGALPIIPWITRIIKGSQLEQVGTGNLSVAAAFYHGGRVVGVLA
VISEALKGIAAVLITRIFFPQGSFWELIALIALVIGRYTFTRGAGTTNVAWGFLLHDPLI
AGSVTLFAAIGFLLLRSRQVIQFGVLILFPLLVAFLHGQDFSKIIAAFTLAGLMGWIYQQ
IPDDLELPPQGAQLPVKPIMEYLSGNKPTIITLDDFLDPEVFGVKSATLSQIKHRGYSVP
KGWILAPFDDPSQLINFLQPSPLSPLVVRSSAIGEDSQQASAAGQYTTVLNVTSKQGLSL
AIAEVKGSYNSENAVKYRQDLGVKDVGMAVLIQPQVQSVYSGVAFSRDPISQQGDAVVIE
AVVGSPEQVVSGKVTPEQYRLFVLGKINYLRCNLKEREKIPQSLIKQVAYLARRLENNYY
GIPQDIEWSYDGQTLWVLQARPITTLVPIWTRKIAAEVIPGVIRPLTWSINLPLTCGVWG
KLFTIVLGERASGLDFTKMATLHYSRAYFNASLLGEVFLSMGLPQKV
>EFA72227.1
MQREIGLEKQFKLDYSRLFLPGMTQLTNESLGELSPSQLLNRVDQILDLLEKVTYYSILS
PLSAAIRQKLFRVQDEEIDHSNAPEISSLHSLQRLALAAKNLLPNLEPQRVFDQLAETTS
GQNILEEFYEVLEEYGYLSQVGTDIAVSRWKEQPDLFKQMFIQILETHEVISNQGSSKRQ
QGNVQNRVNLKGTVTEVYSRLLAELRWTFLALEKTWLTSHLLTAPGDIFYLNLGEIRRLI
ADADPQLTEQLVKLLASRRSQFEQDSEISQIPAVVYGNNPPYPMTNSKPMNLSDRVLVGI
PASRGQAIGKVKIVRNFQEAGEINKQTILVVPYTDSGWATILVRAGGVIAETGGKLSHGA
IVAREYGIPAVMDIHGATDLLRDGQKVRIDGSRGTVEMGRFPELGSN
>EFA72234.1

MRICLSFFLFVSYRNKNANSFLLILPLFFLNRTVLGLDPAWRRHLAQVLLKLPVQVLLIA
SHDLNWLGKVTQRALVLTDGKIQIDHPIQPLLADGKTLDNLGLPLGW
>EFA72245.1
MFLQVKGKVDLVKIVDFQELLDPNSRIVHGKDQEGEEEQDMDIYEKQDFGFSLGGRIAQM
LAGS
>EFA72265.1
MIICPNCNHPNPDAAVQCEACYTPLPNTITCPSCGANVQADAAFCGQCGYNLVSTPIEVP
PLVTPDPLEISTQSPCSVPPTLVSTAPDPGPPVKVLKTQLQQVVGRLFHLQGNQEVELPQ
NLSVIHIGKPNDRIPPDIDVAGFSNSEIVSRIHADIRVEGDAYYIEDVGSSNGTYINNLP
LLPGNRHRLRPGDRISLGKGDLVTFLFQLS
>EFA72268.1
MFIDHLHSQHLEELVHGSSVNLHLAILNFHSLQGVNAYQHILISNNLPRTNMGIIKSGWL
ERYEHITAGGWWCSGVDPLNNWQKMEWGCFKPTQPRRNKNGKSIKYEHPPSTATRVFCLR
VTEEIWHQISQRYHVTMPKDITINDDGEAQGFWPWVIESNIPIIICEGVKKAAALLTQGY
VAIGIPGITSGYRVIKNEFGKVTRRCLIPDLEVFANRQPSFYICFDFENQPRKVSAINNA
ISKLGYLLKRKNCSVKVIKLPGKEKGVDDFITAKGAKEFEQIYHQSMDLEVYIAQTKPHI
DLTVTPSVTINQSYLDRICLPSNGLVGVKSAKGTGKTTRLQAVVEEAKNRCQPILLITHR
ILLGKFLCEKLVLGGESTLKTSHHHLAAHLSNPLGCVLIPCGSSIPKIGKEL
>EFA72280.1
MYNLEINFLQHRSSSKLKVEDGNEVNSINHPVNRRSFIPIFVGLGLGLCFPALAWSSLWW
LQDQNSSLEGEIAQLNNHSQNLDQQLTQMQKIREQTTTIEQQTENLVTVFEQMRSWSAIL
QELGNRIPSKVQIESLEHKQITTLSTKTEQLEITGYALNFKTVNQFLLNIGKSKLFNGKN
SRITMAELVDAPPVTGAIVTQQSPMEFKPPQVVKYNMTISLTDVPVSNLVRELEKKGSHG
VSGKNSSFRKNRSY
>EFA72287.1
MFGGLMFGPDLDIYSRQFQRWGWFRWIWLPYQQSLRHRSFLSHGPIIGTTLRVVYLMVFI
AATTGLLLLILAIFSNISVNWGDIWAKVRQNISTYAREFFAWFVGCELGAMSHYLSDWTN
STHKRFQKKGVHALLPTSKIRKRKIHSRRSPSSKPKPDKKQ
>EFA72288.1
MPSGQTHDRITIWSIPVVASVTLVATGSGNITLVVLGGLCLVV
>EFA72289.1
MLPKISCLLIITVMQIFIAENLRVNPVLAQSRQSASSNTVNNGDANNVFNSPNQNFNEFT
VPNIYPLDNSINTPVNTENDFGLNLSMGVNTLDSRNVTVYLGLIFQPGRTYSHTVRMNRI
NKETELLEVQRKIAEAKLQLLQKQVSEAEMKLQKLQEPGSEPPPTGN
>EFA72290.1
MNVKKVVSCISYFALAIAIIPLAPNWVLAVTISVLSYNFNAAQANSTIGNITRGNIGVGV
TKLKESKQETIIDLSMDELKQDCFLKIIADEGVNLTGYIQVNDKFVQKITENKTEILISK
LLQKGKNTIEIIGNYDPANSNIKIEFIATNTELTQEVSGGGHIQGALTIYVE
>EFA72291.1
MSHIGIQANMSRNPATQTSNVEMQGPQSCTGNTSSSTSVQVNTDNNGPVRQNQRVRQEIR
DRQSNGNQAGGPTVRNSVIVPVNVKTPENFNP
>EFA72292.1
MSIFQGSQHLLIELPELTISMAQGELTILFRDYQKAKKQLSELTAENQALLLVKRQHKQP
LLGGVIQKGVKPLCLVVSIQW
>EFA72293.1
MNRFIVNLLAAGFLTLGLSQVIPAHAQSGNTGVSQESDQTTVITGDGNTVVNEARQNNQR
VRRGRGTDGDDATIQRVNQTSDVQGNNNTSINRAEQNNQGVNESGRRRRTNININNGN
>EFA72294.1
MTLEQAEKILLSLKGQGVCEILILSGEVHSSSSRRQEWLELIYDLCLLALSMGFLPHTNA
GILSFSEMQKLKSVNASMGLMLEQLTPKLLHTVHRHAPSKIPELRLQHLQWAGELQIPFT
TGLLLGIGENEDDRRQTLMAIADLHLKYQNIQEVILQPHSPGSQQTFDEVAFDPYQLPGV
IAQAREILPSDITIQIPPNLVPEENWLLACLDAGARDLGGIGPKDEVNPDYPHREVNRLR

EVLLSGGWTLLPRLPVYQKNWYKTPLGAPLTELTK
>EFA72296.1
MTESLLNIQNLCVAYPQDYDQAPIWAVNDVSFSLKPGEKIGLVGESGCGKSTIGRAIMRL
LPDHSRVQGQVNFRESLVLDLTPAQMRQFRGEAVALVFQDPMTRLDPLMTISEHCLETLA
AHSPQLTKKQAKERVLATLEKVKIPGSRWSQYPHEFSGGMRQRVAIALALLLNPKLIIAD
EPTTSLDVTVSAQILQELTRLCAEDNMGLLLISHDLAMVAEYCDRIGVMYQGKIVEMGKT
ESVFKQPQHEYTRSLLRAALHIQQEPVGVGEEEKKENPILKITELKQYYTIEPNFLERLF
QSQQQTIKAVDDINLELYPGEILGLVGESGCGKSTLSRTILQLIPPTGEKWNF
>EFA72297.1
MEFLGQELTRLSREEVRGFRREIQMIFQDPHACLNPAMTVGESIADPLLIHKIARGKQAE
EQVLWMLEKVGLTPWQTYYGRYPADLSGGQQQRVAIARALITRPKLVICDEPVSMLDASV
QTQVLDLMLELKAEFDLTYLFITHDLWLARFLCDRIAVMNSGKIVELGKTKEIFSHPQHP
YTQTLLGAAPLLARV
>EFA72345.1
MLLIFAIALAIASFTLLTVFANLLAALLAFSGIVFYVLVYTHWLKRHSTQNIVIGGAAGA
IPALVGWAAVTDTLSWAAWFTFCHCIFMDTPHFWALAMMIRDDYAKVGIPMLPVVAGNQA
TVRQIWFYTVITVTSTVLLFYPLHASGIVYVVIAMTLGGIFLHKSWRLLQNPEDKTIAKE
LFLYSISYMMLLCLGMVIDSLPFTHYLVNTILHSFHLVG
>EFA72346.1
MLLLIGLSAIALTGLCWWHRRVLPLWLPGAATFALLLIVFQGILGGLTVTELLRFDIVTA
HLATALLFFTTLLVIGTFLIPYKGTGAVGKLPWVSLTAAVLVYLQSLLGALVGSRWALHQ
CLAGEELCGVMYSHIFGLLPPTVATLSMVFISWRTPALHPVLRKLANLAGGLLISQLLLG
LATFRLHLQVEPLTITHQAVGATLLGTLVVFTVLALRDRSISNPSYLNN
>EFA72352.1
MTLDDFQVLDGDLDHPTLSEYLRSDALAVDTETMGLVPQRDRLCLVQLCNPEGKVTAVRI
AKGQTHAPNLQQLLESTHVVKVFHFARFDLATLRHNLKIYVQPVFCTKIASKLARTYTNR
HGLKELVQELEQLELDKSSQSSDWGNPVSLSDAQLSYAANDVRYLLSLKQKLSQMLQREE
RWQLVQECFSFLPTLVSLDLLQFKDLFEH
>EFA72353.1
MFRQTAFGIGLTTLVLTSGLVTGAISTSNVTKKISSISSFNGSTRAVASHQNNEGNKHDN
NRLEKLVFEQINQYRVAQGLSKLTLNLNITKQARIHSQNMANGTVKFSHHGFEQRVKSIH
LKYNNAAENVAFNIGYNEPAKQAIIGWLNSPGHLRNIRGKFQLTGVGVAKNDKGEVYLTQ
IFINTTLRYSRKISPRSPAF
>EFA72356.1
MNPNHITNFDNLWLELFSLYTYPYLLLLIGNSIAFGQTGVVVPTVPSDSTTPSPSNPPSS
TPTQIPSPNNLSTTRFSCQYYDGKYTVMYEPQSQPGRFFAWAIPQSLGGGWNPQNRCQAI
ASRLELYRPDGLQELQIARQNNENIICVTTEVVSTCRIVFTVPRTRDPYSVRSSIFSSLT
AADQGQQTVGVNTYISSPRRSGNNPHSRRGINLKPFLSMEDGGTGTNLNNGLLIRSRTPG
KTILNPRLFR
>EFA72361.1
MESEYRQRREQVMAKISTGAAIFRSAPTAVMHNDVEYVYRQDSDFYYLTGFNEPEAVAVL
APHHGEHRFILFVQPKDREKEVWSGYRCGVEGAKEIYGADMAYPITELDDKLPQYLQKAE
RIYYHLGRDSHFNDRVIRHYQNLLVTRPRRGTGPIAIEDTGPILHGLRLHKTNFELDLMR
QAADIAVSAHNHAMSIARPGSYEYEIQAEIEHIFRLQGGMGPAYPSIVAAGKNACVLHYI
ENNYQMQEQELLLIDAGCAYRYYNSDITRTFPVNGKFTPEQKALYEIVLEAQKQAIQEVK
PGNGFDAPHKKAVQVLTEGLIEVGLLKGEVNQLIQEGKYKQFYMHRTSHWLGLDVHDVGV
YQHGEVPQVLQPGQVLTIEPGLYVVPHTPSAEDQPPIDDRWVGIGIRIEDDVLVTPKEMR
S
>EFA72374.1
MSITHSAVINLGKEVSKIIVGQSGLIKQCLIAFLAGGHIILEGVPGTGKTLLVKVLAQLI
QGEFKRIQLTPDVLPSDITGTNIFDLNTRNFYLRKGPIFTEILLADEINRTPPKTQAALL
EAMEELQVTLDGESLPLPDLFWVVATQNPLEFEGTYPLPEAQLDRFLFKLVVGYPDQVAE

KQMLFNRQSGFTGRRIDISNLNPVTTVNNILQARQAVKQVNVAEAIVDYILEIVSKTRKH
PDLALGASPRAAGAWLQTSQACAWLAGRDFVTPDDVKAVAAPLLRHRLILNPEAMLDGSK
IDSVITTVINQVPVPR
>EFA72381.1
MQDLLLILQEVHKQGIIHRDIKPENIMRRHKDGKLVLIDFGASKELQGGATSGTRIGTDG
YAPWEQRVDGVASTAGDLYSLGVTCFYLLTSKNPYELWLKDGYNWVANWRNYLNQPLSQK
LQQILDKLLVASSDKRYGLAEKVLEELRQPYSIIPSNSPKTIISHTEKPQRFSYILALIS
VFILGIGYLVITKSPQFQPRTEPNVIPETDRGL
>EFA72384.1
MDGELYDFGTKGSGRTPYSRGGDGMLTLKGGVREVLAGEALHRLGVRTSRCLSMIETGLS
LWRGDEPSPTRSSVMVRVSKSHIRFGTFERLHYLKRPDLIQKLLDHVIDQYYQDLNNQQD
KYALFYAELVKRVAELVAQWMCAGFCHGVLNTDNMSITGESFDYGPYAFIPTYDLNFTAA
YFDYYRRYCYGQQPSICYLNLELLQEPLKAVIDPVDLDYGLAKFQEYYHAEYGNLMLKRL
GFAQTKFPEANDLLDLTIGFLKESQINYHQFFADMANTFSPRWCEEPSLIMEESQILPPV
RSSSVFKKWCALYHQVLNNLVSQEMANVGTTLVQYNPQSNLLRPVIEETWQPIVEQDNWQ
PFYDLIQNFHT
>EFA72392.1
MSRSKALQYYIASRLLFAPLQLLTILTIVFLLLRATPGDPADAILGGRAPESAKEELRKQ
LGLDLPIWLQYINYLGNILRFDLGTSLTSRGQNVWQIISQHFPATVELAVCSMLVALIVG
ILVGTLSASRPGTPLDLGGRLFGIITYALPMFWAGMLLQLVFSVQLQWFPNSNRFPPNIS
PPTTITGLYTIDSLLGGNLNLFFPGLASFSSTQFNPRNFIKWHFERIVRVNLKETLKADY
VEAARARGIPENKILVSHALKNALIPVITVLGLTFASLLGGAILTEVTFSWPGLANRLYQ
AIADRDYTTVQGVLVFFGAIVVSASIVIDILNAYIDPRIRY
>EFA72410.1
MSVVNISELEAKRIQTFPDNYRFIGCWTESMRQIGNAVPVELGYFLASSLSKALC
>EFA72414.1
MTICEDLWNDEEFWGKKCYAVNPIADLSVVGVDLIVNLSASPYTVGKQKTREAMLRHTAV
NFQQPIIYTNQVGGNDDLIFDGYSFAVNSQGEILYRGRGFTPDFLIVEFNQHTQEVELAS
NPDQNPIAPIYESEDEEIWHALVLGVKDYVKKCRFSQVILGLSGGIDSALVAAIATAALG
KENVLGVLMPSPYSSQHSVSDALKLGQNLGIKTQILPIGELMKSFDHTLFELFTGTEFGI
AEENIQSRIRGILLMAISNKFGYLLLSTGNKSEIAVGYCTLYGDMNGGLAVIADVPKTRV
YSICNWLNGQNQQEVIPQNILTKPPSAELKPGQTDQDSLPPYNILDDILQRLINQHQSVE
EIIAGGHDLGIVNRVIKLVAGSEFKRRQAAPGLKITDRAFGTGWRMPIAAIRSL
>EFA72415.1
MKIAIAQLNPIIGDVKGNCQKILEIAQQANDVRLLLTPELSLCGYPPRDLLLNPGFVEAM
DMSLQELAQNLPPHLAVLVGTVVRNGEHHIRGGKIYLIVLLG
>EFA72420.1
MAPGVGKTYKMLEEAHQLKQEGIDVIIGILETHGRKETAQKATGLEMIPKRVMVKENITL
AEMDTDAILDRSPQLVLIDELAHTNIPGSPREKRYQDVEVILESGIDVYSTVNIQHIESL
NDIVARITGIVVRERIPDRLLDEADAVVVIDITPETLEERLREGKIYPHSQVEQSLKNFF
QRRNLIALRELSLREVADTVEEEANTSSLEPSSCNIHERVLVCVSTYPNSVQLLRRGARI
ANYMNARLYGVLVADPERFLSKKEAGHIDTCEKLCREFGGEFLHVKSQNVAREIAQIAAK
YHITQIVIGESQQPRWQRWFKGSFTQRLVDLIRNQNIDLHIIAE
>EFA72423.1
MATCLAKHHGKKVLVLDLDSQISATLSLMSPGDFAKRRKQRKTLRYLLDEVINPDPQPEY
KIHDVIEPELCKLSSLSLLPGDIDLYDEFVVSEMLHNQAVALEERDFETIWNRFERVLVR
DILKPVRDQYDFILLDCAPGYNLMTRSALATSDFYLLPAKPEPLSVVGIQLLERRIAKLK
DSHEHEAKINIQMLGIVFSMCNTNMLTGRYYKQVMHRIVEDFGVETICQAQIPVDVNVAK
AVDSFMPVTLLNPNSSGSKAFIQLTEELVRRL
>EFA72440.1
MNITDLSSPHVLLDSLLNLRQAVERDGKTIFDQWRSHIQRSQFLPSALNLAQYLALRRHD
LRPLQAALMPWGLSSLGRIEARVMANLDAVIATLALICEVPIPTSVTRPVITSFFEGENR

LREQTECLFGPALPHRRVRIMVTLPTEAASEYEMVREIIERGATCLRINCAHDNPSIWEK
MIQNIRQAEQELSCQCTVMMDLGGPKIRTEMVLSPVGKNRVFRGDLIVLCRSLSNQAIAD
PIDNIQISCTVPEILDLLKVGTLVYIDDGKLRTRVVDQDYPLPDGGSGFLLEVTHAKPKG
VKLSPEKGLNFPNIVLPLIPLTPKDLTDLDFLATHADIIGYSFVQQTTDIQLLQSELARR
CLHGEHPAIIAKIETAVAVTNLPELIVHAAGKHPFGVMIARGDLAVEIGYQRLAEIQEEI
LWLCEAAHVPVIWATQVLESLVKEGAPSRGEMTDAAMAERAECVMLNKGPFITQAVTILD
DVLTRMEAHQLKKTPNCGPCVPGRTFLLRGATFN
>EFA72447.1
MLIMDQLIWLRKNLFNNWYNGVLTIVCVIVLFSLGKGILFWIFNQAKWEVVTANIHFIFS
WQIPPGFILANLASFGNQFYYRVDNLVWVDAKYHLPENQLLQKAYQLIVPLLAPIWFLTF
IITIWLLGGGLGLQSVPTNLWNGLLLTLLMAFVSIVLSFPLGVLLALGRNSELPVIRWFS
ILYIELVRGLPLIGILFIAQVMLPLFLPDNWRLDRLVRGVAGLVLFSAAYMAENVRGGLQ
SIPRGQFEAARALGLNTFLLLILVILPQALRAVIPTIVGQFIGLFKDTSLLSLVGLVELT
GIARSILAQPQFLGRYMEVYIFIGVIYWVFCYSMSLIARELEI
>EFA72450.1
MSELRRLKSLLPPELQSWVIVEGTTEVSPPLIRSEEIGKDQVEIQIDLIKWDALAMDQRN
LLFWHEVGRIQNDTIPKDGWEMAALAIGLGGAVGELWVQDGLLLVLALALCGVSGWRLYQ
KNNGTKQTKELIDADEKAITLATRFGYTLPNAYKSLGSALKTLIDTTPGKRQRSRYEARL
SALKRSANKAKSRSNNSNNVDENY
>EFA72454.1
MGRYPEAITAFDNAIKQKPEFIHLAYYGKGLALSRSGKVMEAVIALEEAVKAKSDSVLAW
TILSLANTKLGRSDQALLAINQAIKLQPNNPNLYNEKLVVLSNLKRYQEAIDAIDQAIKL
SPHADFYYNRGVVRSRLGDNQGAIADLQKAADLLQKQGLSAE
>EFA72455.1
MEDGKKFIADLLAGAKVKLEGTAPWLSASNLPIDDRGELTIQVTEKHLIPPPDCLVYHPQ
RVAITLSSLEVETINHIYELLAQAEISSQALAVIIAPLSLANNSILQEISKTFSIPARFL
TTNQIQELTDKGYSFHEAVVAAGMTGNNTYHTSFESPFSPITLSIATDIIDPETIGKGRG
KLAIIGTGPGGVQWMSPEVQEILRNATDLVGYKTYINLIGELADGKNIHESDNREEESRA
KKALDLAVQGKYVAVVSSGDPGIYAMATAVFEVLEKGQYNQPEWQTIDIQVAPGISAMQA
TAATIGAPLGHDFCVISLSDILKPWEVIVQRITNAAQGDFVIVFYNPISQSRTWQLTAAK
NILLEYRKPDTPVILGSNIGRPGQRLKVTNLEELNPEDADMRTLIIVGSSHTRKIQSLGG
NIWVYTSRHYSV
>EFA72456.1
MRNLKSPVVVMEGLTFKPAPICRASSFTPVNPPNRGTTALLSSATANRGGS
>EFA72457.1
MVIILSQNSLTVAKKIVGVIPGAKIYGLANRTCDVDVSFTDFGDTLRQLFVTGTP
>EFA72463.1
MASLARWPNASPDSPVQTVVPVDEGGEDLVNKAQNILEQGLQLEFTTLENPDQIRDCTLQ
ITIKQKNWVPNY
>EFA72464.1
MGSQLLNLNISHKDYSGEFFRDLLHRNQDPLLQEYLEDCVQANGIMFLVDGNSRRKDPEY
ANGLDKLLIALDRHDIGGMKRRIALVLNKCEQSDLWVNRDKPAFLASARFPQVSQKLKVW
QQMGGGEVEFFTASAFGMLGTKYPEPNVNLVQKDRGGVSAVIKNPKLWRPFGLVAPIYWL
CKGSRHPDLDKS
>EFA72467.1
MIFELILKNLDVNQYKSIKYGDFNTDKIQQTHKRRIAQSIYKFQESQKNPQPTGYLDYKS
AEFGELKRKISEQLPISNTPL
>EFA72468.1
MESNIFTSLILPIALGAMMLGMGLSLVPEDFQRVGKYPKAVAIGLISQLLILPLIGLAIA
KFLPMQPAIATGLMILALCPGGVSSNLVTFLAMGDVALSVTLTALSSLITVFTIPIFANL
ASQHFFWARCSSGVANWNDYWANFCNYLLTNSDWYEYSAICAKTVYKTRKVTSISATILL
AVIILLLIIKEWSRLPNFIVQVGIGVLLLNTLSMGAGFYLSKLFNLNYKQQICISIEVGM

QNGTLAIAITAGLLNNPDMAIPGAIYSLLMYLTGCMVIIYGRNLSASRV
>EFA72470.1
MGLLHPDYPGFFLSARAYLEWYGGRGDGHIQEKPVVAILLYRKHVITKQNYIPQLIKKFE
NSGLIPLPIFINGVEGHVAVRDWMTTDYERKERQKGNIETPSLSTEAVKVDILVSTIGFP
LVGGPAGSMEAGRQVEVAKRILMAKNIPYIVAAPLLIQDIYSWTRQGIGGLQSVVLYALP
ELDGAIDTVPLGGLVGENIYLVPERVQRLIDRVSNWVALSRKPTSARKIAIILYGFPPGY
GAVGTAALLNVPRSLIKLLHALKEQGYTVGEIPQDGEELIRQVKVVDEELETWEKNKPFP
TKTNTVNVKTLEKWLGYLRTSRIEKQWQSLTGAGIKTYGDELHIGGVQLGNIWIGIQPPL
GIQGDPMRLMFERDLTPHPQYAAYYKWLQNEFQADAVVHFGMHGTVEWLPGSPLGNTGYS
WSDILLGNLPNLYIYAANNPSESILAKRRGYGVLISHNVPPYGRAGLYKELVSLRDLIAE
YREDPQKNYVLKEGICKKIVDTGLDVDCPFDDDQKIGNSLLPPRKMYKNVL
>EFA72471.1
MKRIVLIAGFESFNADLYRKAALLASSRCSDLDIHVFSDRNISTNQEEIKNALKNADVFF
GSLIFDYDQVLWLREHVSHIPIRLVFESALELMSLTQIGKFSIGDKPAGMPKPIKFILDK
FSNGKEEDKLAGYISFLKLGPKLLKFVPVQKVQDLRNWLIIYGYWNAGGKENVAAFILDP
GGKIFRFESREYSRTYRNT
>EFA72476.1
MLLLGERGFADGDLGQKITRTITKLRSCGFEIKSAPNQPYKLVTSDFPVIITEEQRQALA
MACELLASLGFSAEAGHIYRIANSENRISHKLTTDFHPPTNYSEDKIQEVVQDLQNRIGK
KRRFVVWYRSRNGNDKQWDLDKSELRLHNGVLYLFSIVPSFIGQHIHKTPNPEQNCTLRV
DRIARVGSSSQIPWTYTKFPTLKIQYRLTGNLASYKPRRPHEKIISPPDKTEHVDIETQE
DCIFWFHQRILQYGANARVLQPDWLVKWWWKV
>EFA72480.1
MNGNQVTVWSRHGSQHLGDLINNVDLVLCAVSMKGVREVASLVRSFPVSPHTIFVTATKG
LDPETTCTPSQIWQREFPDHSIVVLSGPNLSQEIAQELPAATVVASKNNMAAQVVQQVFS
SSRFRVYTNPDPVGVELGGTLKNVIAIAAGICDGLHLGTNAKAALVTRGLAEIIRIGAIF
GAKTETFYGLSGLGDLLATCNSPLSRNYQVGYQLACGKTLVQILANLPGTAEGVNTCYVL
MHLSRQQSIPMPITQQVYRLLESLITPQQALEELMLRGMKSEYHE
>EFA72486.1
MNNLLSKLPELSGLIVSCQAPVTSPLHNPTIIAAMAQASANNGAKAVRIDSPNHIKAVKQ
KVSVPIIGLWKQIIAESDVYITPQFHHALAVAEAGADIIAMDATPRKRAGGEKLVDIIRG
IHQQIGKPVMADVDTFASAKLAIDSGADIVGTTLFGYTEETKNLIPPGWELLKQIVENLK
VEHPEILVICEGGFPPRKKQKKH
>EFA72487.1
MGLVLKEDLKVFMMDPNTEIRRLLDIVPASGRMTIKIISKPEQNQVISAEFPLPWIQSKP
VYINFELWRQLKKPQRDLLILYHTSWLIGIKWIQPDIYQGVVLVGLLGG
>EFA72488.1
MDTTGYLSGSSPSWTIRGVIETLQGDIVGLVIAGGLTVISGVKLWQNNKSQELQLRADKT
AIAIAQKRGYSESEAAGHLLTAIETVAKLEGRLGLNFNELIRCQNLRAIAS
>EFA72505.1
MNIAVVGLSHKTAPVEVREKLSIPEPQTESAIAHLLNYPHIDEVAILSTCNRLEIYVVSS
DIPQGIREVTQFLAEHSKLPVGSLRQHLFTLIHTDAVTHILRVAAGLDSLVLGEGQILAQ
VKNTHKLGQQYNGVKTILNRLFKQALTAGKRVRSETSIGTGAVSISSAAVELAQIKVENL
ELYRVLIVGAGKMSRLLVQHLISKGASHISIVNRSRERAAELAKQFPEVTIHIHLLPEMM
SVISHSDLVFTSTSSTEPIINRSKLEMALDPNHSLMLFDISVPRNVDSDVSHLNNVQVFN
VDDLKAVVAQNYESRRKMAQEAEKILDEEVESFDIWWRSLETVSTISSLRNKIEHIREQE
LQKALSRLGSEFGEKHQEVIEALTRGIVNKILHDPMVQLRSQQDIETRRNCMRTLQMLFN
LDTEEQFS
>EFA72509.1
MLTTDQIGKAGYNPGNFTNNFGGTSSATPVVAGVAALMLSANPDLTAQQVNRILQETTDK
IVDPQPDPQLGLQGGTYNSNGHCLWFGYGKVNAAKAVRAAQKLRGNGLVANNANKLIRGE
NKNQVTIPDGEKQGVRTTIVVPQNIIINDIRIGVNISHDFLGDLEVYLIPPNSQSLLLQS

RTLGRQTRLEKTYTVSSQPVLKQLLNSSAKGNWQLHVVDCSPQDIGKLNGWELLIGYRD
>EFA72510.1
MINDQPVVLQRGGEELILIKSTERFTLRLYADLNREELSRISWGKWQRTIPRAKLELFTV
TPDQIEAAMSQARSDQKVAFASHVYQLQDNPSSYVYLSDQITVQFAAGVDNDKIGIITSS
LNLTENQPIADLPNTFTFLVSKQSPHNPLKIANQLQTLPEVLAAEPNVFIQQETHYRPSD
SLYPQQWYLNHSGGNQLAIASHISVEQAWDITRGVRSVVVAVVDDSFDLNHPDLQGVGKI
VAPRDLKEKDFLPLPGEQETSHGTACAGLAVAEENGQGIVGVAPGCALMPIRTTGYLDDE
SIEDIFNWALKYNASVISCSWGASAVYFPLSLRQKAAITRAATKGRNGKGCVILFAAGNA
NRPISGTIDEQNWPKDLLKGKTKWLSGFAIHPDVIAVSASTSLNKKAAYSNWGENISVCA
PSNNAPPGMWFKKVVLCTPNQLFQQL
>EFA72522.1
MISRSKILGGIENQYQRATVCFNCGYIYNIDTKDWCENCERDIKAYSDKNERAKVNQILS
MDTVYTQKKQRITCDEEERLKYGYNITTHFCYSEGNKKTVIIKTKEGKNLLKLTYGATAS
ISRINRGSAKKKDQGFCLDTQTGKWISNENEENKEEIGKNSTTTKSDIFLMVNITCNILI
IELIKEAEESKIDTIDQGKSENREDTETIMTSLQYALERGIQEHYNLEPNELDSEKMGNG
KYLLFWEAAEGGAGVLSQILQDKQHLNKIAEKALDICHFYEPKEDCVYACYQCLLSYRNQ
NEHNLINRHLIKELLEEIKESYLEREREMDREKEFETLYLQTDRRSELEREVIKEIYERG
YRMPDAAQFLIPGTDCEADFVYIDKIAIFCDGSVHDTEEQKREDKRKREKVRRETKYQVL
VLKYNEEWRENIKKLAHL
>EFA72525.1
MGDVNTYSIFAETARNLIKEDGRVGIIVPTGIATDDTCKMFLVIWLNNSIWRVFMTLKIE
KQYL
>EFA72527.1
MEIGSIVNCRQRQWVSIPSEDPEISLLKPLSGNEQEIIGIYKKLNLEEPTPDKFPQPNPE
YIKDHQRACLLMDATRHLLRSGAGPFRCFGRISFRPRPYQIVPLLMALRQETVRLLIADD
VGIGKTMEAGLIARELLDRGEIERIAVLCPPHLCDQWQRELLTKFHIKATVIRSGTANKL
ERENPTTKSIFEFHRHIIVSLDYAKMEKRKASFILHCPDLVIIDEAHTCTIANAQNRREQ
QRYQIVSEIAKNP
>EFA72529.1
MATAALINIILLPNHLSVSFNQPVKFISLFLLLLSLHPVVLNPLLRFLFKIKSQIKSQIK
SQLKSEDDIPNSMNSMNNLTLEKYPLLPLVGELIFVCLRGTGFILTIFALGDVNLNQIPI
LIGAFSLAWVLGLVIPGAPGGLGVFESTAILLLQNTFPPSLLIGAIALYRLMSILAEAIG
AGLSYIP
>EFA72530.1
MTMGKFLRWVVFGVTLFFLVTTLKAHWLEVKSLNLGGIRWRILPMATMVTLLAHTWAGLV
WTWILNHLNQSVPTGLFIRVY
>EFA72538.1
MQTLPSLETGKIITSQPTFDTTIKRRKTRAVKVGDVIIGGGYPVVVQSMINEDTLDIDGS
VSAVRRLHEIGCEIVRVTVPSMAHAKALAEIKQKLQQTYRDVPIVADVHHNGMKIALEVA
KHIEKVRINPGLYVFEKPNSDRTEYTKTEFDEIGEKIRETLAPLVVSLRDQGKAMRIGVN
HGSLAERMLFTYGDTPEGMVESALEFIRICESLNFYNIVISMKASRVPVMVAAYRLMAKR
MDDLGMDYPLHLGVTEAGDGEYGRIKSTAGIATLLADGIGDTIRVSLTEAPEKEIPVCYS
ILQALGLRKTMVEYVACPSCGRTLFNLEEVLHKVREATKHLTGLDIAVMGCIVNGPGEMA
DADYGYVGKIPVLSLCIGVGRRLKRFQKTKG
>EFA72554.1
MARLLFGGILTLLVLSITACSSLVLIPTYELVVKAVNMQLEQAQQDLEQKLDLDFEGFTV
EHLVINQQQALAIENLPGYHLEGVYDLKVKLPTRQIAQSHNHFNIYLQIQKEGKSWRLLI
PEKSGEKQPLVWRGYLII
>EFA72560.1
MEPKRQQAFKEIKTLINTRSIPIIICNDVNSIKLLPRDAQKIAINYCNEYEETVSENNLT
VDRFNQITVEVNNNSVLRERVQKLMMEKMGL
>EFA72562.1

MFRPSQIALWISQRPAPQRVGCFVLCLLVLWLPFVIPIYLLVEDGNLESIFSMVLLYGEF
IVLVRLWGKKIYQRDKILTDYGLELSSLNGVDFCQGLSIGVLSIILLFSIQGLLGWILWQ
PPKAFIVQIIWEGLLVASGVGFAEELLFRGWLLDELNRDYGSRLSTAINAILFAVAHFIR
PISAIISTLPQFPALVLLGLTQVWGKHKKRGRLGLPMGLHSGLVWGYYIINVGGLVQPSG
IVPDWVTGVNNNPFKVLWEC
>EFA72563.1
MKELKTLYFLSMQNLIHVIFAAFCIIINLTLIVITTCLPAEAISLQTKPDTIESNVTSIH
LLLGNPSNATSSLDNPDNYLMIKPQYALSYNRSHGSANWVTWQLDKSWLGDAKRQDDFRP
DDTLPNGWTRVKPSVYNSSGYDRGHIARSADRTQSVEDNSATFLMTNIIPQTPDNNRNTW
GNLEDYSMKLAGEGKQLYIIAGGFGDKGKLKNLVTIPQYTWKIIVVLDRPGLGLKDVNVN
TRVIAVNIPNDEQLDNNWRVFRTSVDKLEELTGYDFLSTVSPDIQKVIESQVDNL
>EFA72565.1
MIQPISNLRTKMNIETLKSEKTKQLPGANLEDQDLSEFDLTAANLAGANLMGAHLVSVNL
EGSHLEGANLMGASLQGADLRANLLGANLMQADLTGADLRGSNLRGANLMGATVAGASLT
AAFLSGANLMSVNLQGVDLRDADLRGANLTGANLKGADLSRADLQGALLNQANLEESDLR
GANLAGANLAGANLLCAELEAASLNGANLYQACLLGTILETYHD
>EFA72570.1
MTESTTFFLSPLIRITLLTLYIALTLPLPFLAEVTQAPVTPLVLWLGIIIGLVALYGVLT
QRVIVNEQEIQVTYPIWVPKLLRQDWSLPWSEIKSLKCRTTGQGGLVYYFLTDEGKAYLL
PMRVTGFNRLVKFVQLKTGMDITDVRPLSQPWMYIILLTCTFILLLVDAWTINMANSMI
>EFA72575.1
MTPFTIRIGGVVLYTGTLFFSYLASRKLFGDRVATLTLGILTTIPIFQIAFGILTLPDNA
LMFFWSICLWLCATEFFPSGESCDPIYDTRPYRPTSKLAFVGLVVGLSFLGKYHGALLGS
GLVLFCLISNRHRCALFSIWTLAAVVLFLIAISPVLYWNSQHEWASFRFQSGRAVPSVGY
DLERLLVTILVGIGYLFPTFGFPIWWTSFRTLWEWLPFNKSHTNKIEQKAIMPNQLAHDL
VDQKRLLILCVSMPIFFGFTFMGGFIQILPSWHMPGFFGATLLLGERAALVQVKHPKFIR
NWLWGSGMVILPLLLISLLHIHWGIAQKGGNVAIAGGFWEAKDDPSTQMIDIEQLRQAFV
DSPVLKTELEKADFVFSNNFFVAGQVGMAIEPLGKKVTCFDEDLRGFAYWSQGTDFVGKT
SLYVTSKQFMEDERFPQPLDKYKDYFQSLEKIADIAIKRGGEAVQIFPVYRASPMLKPYP
RPYG
>EFA72582.1
MSSSLNLSSNIKSPHEENYQLWLESTIDNLKQGNFDRIDIDKLIEEISEMGGSLKDALEN
NLIVILAHLLKWQYQPQKRSGSWKASIKEHRRRINKSIQKHPYLKNITRRFFQNPTYLL
>EFA72595.1
MNDLPCQELKEIIIQHGRSLCDNPEYCEAFLRDWLMNDLPRQKLKEIIIEHARSLCNNPQ
RCEAFLNNYHGEYKRDLVLIISALKQAAAKDLLNFNYINIELLVGRLIKKIAQ
>EFA72596.1
MVQLITFVENVLSNEESIALDHKKAALDREKETLLLEGKISSLSVKSQQIEEIKGQTREI
NERTGKILASLDNLLTEVNLAAPENGDEMTIIMDSDLVKDRYA
>EFA72616.1
MPRTQKNDNFVDKSFTVMADLILKLLPTNKKAKEAFVYYRDGMSAQAEGEYAEALEYYEE
ALSLEEDTNDKSFIVYNMGLIYASNGDHDRALDFYHKAIDLNPRLPQALNNIAVIYHYKG
EKAKEEGDNDEGEALFDQAADYWIRAIRLAPNNYIEAQNWLKTTGRSQIDVFF
>EFA72621.1
MLLFLKLIDYLLAAVFIGAALIIYFDSNNQPYLLAGIGASAIAILLFLINRNSVGAAEKQ
AKKNEFTKKAELYTSLLQNSNSLEHNTIVPVRANALEYCQDLINDYKKTRNIARSLYYVL
QISTVILSGVTPILVLVDKLETGQPWLKWLPVICPAVASIVASIVTSFPFQKNWVTANTI
VELLEAEQEKFILGITPAYRCYDVVGDLEQQQKASQAVELFISQVNSIHLQQVQQATEQQ
SDKRKEETKTQDPALN
>EFA72622.1
MQAYLSDSGWELTPKPPDLDVNETGTTFAENACLKAVEVAKYTSQWAIADDSGLWVDWLN
GAPGVYSARYGNTDEERIGRLLSELANTENRQAKFICAIAVANPQGEIIFQSEGSCEGEI

LYEVRGEGGFGYDPIFYVPEKKLTFAQMSPELKKSISHRGHALRKIIPQLLEVD
>EFA72625.1
MNDMDFKQLLKQQLPVLVNEDVEMRNLLVRAISSYFAGSLETESHFDLVLGEIRRDREEQ
SRKWDENKQEFDRVITQMELDRQEQARKWDENKQEFDRVITQMELDRQEQARKWDENKQE
FDRVITQMELDRQEQARKWDENKREMELDRQEQARKWDENKREMELDRQEQARKWDENKR
EMELDRQEQARKWDENKQEFDRVITQMELDRQEQARKWDENKREMELDRQEQARKWDENK
REMELDRQEQARKWDENKREMELDRQEQARKWDENKQEMELDRQEQARKWDENKREMELD
RQEQARKWDENKREMELDRQEQACKWDENKREFDQVIAAIERMSRKHEVGISGLGARWGL
CSESSFRNGLKAILEESFGVEVLNIVEYDDQGIVFGRPDQVELDLIIRNGQLIICEIKSS
LSKGDLYTFYRKVDFYQQQHQRQATRKIVISPMVHPSANSIASKLGIEIYSYGEDVPPEL
ASSPSL
>EFA72626.1
MEANQQVDNLGKIIINKNLQKFEEKGLWKIEPRVVNIKSGYRLDLLSIWQGPTDSKKGFY
LRKSCYIEPIASEYTTDNSNVKAKIKCDPKTKFFRGSPPAQQ
>EFA72627.1
MNFPLLKEIIQLFTFIKDLFLGIQKLLMPPRSFSWQTFIYLSVFSWGISSLATGIIKDII
AFTGWIFLFAGTAWYTTDSPVRIPGTFMPVGALLTGFIFSVFVFGHGENTITVRTIVLWP
TIAAIITALPQFFTGNGISPKATLPKLEVRQKIILLLSWSMLISCWLQFNFVTDKWLKEY
PSLSAQSFQRSNFVIKI
>EFA72629.1
MTRNGIGILTAQARSERLTGQIHVYDGAGKGKSQAALGVVLRSIGLGINTKSNCNRVLLL
RFLKGPGRDYDEDGAIAALQRGFPHLIDQVRTGRAEYFGHDEITPFDRAEAARGWDVAKG
ALASDLYSVVVLDEINPVLDLGLLPVQEVVETLKSKPQELEIITTGRAAPQQLLDIADLH
SEMKPHHHPQAAELLLDGIEIYTGSGKGKSTSALGKALKSIGRGINHPGSARVLIMQWLK
GGTGYTEDAAIAALQQSYPDVVDHLRCGRDAIVWRNSRQHLDYVEAERGWEIAKTAIASG
VYKTIILDELNPTVDLELLSVEPILQALLRKPKGTEIIITGRCQNQPAYFDLASIHSEVY
CHKHYANQGVELKRGVDF
>EFA72639.1
MPIPNPDQRGGVDCRINGIMPFVIKLKPTIKDTIKRIIKGIGTGKIIMVETDIVVTIIPS
IIIGRLPNLLAQNPAGKDAKASAKPNDSIVMVILSGDNWVLSVLPKVSISKRDPQDMVKP
VRKMKDRL
>EFA72640.1
MVAMLALSSLRSFIFYFGRKIHILLTEKARAALIPATKGLIAGEKSEPKRLMAKENPPK
>EFA72641.1
MATNTADTGKQKALSMVLNQIERSFGKGAIMRLGDATRMRVETISTGALTLDLALGGGLP
KGRVIEIYGPESSGKTTVALHAIAEVQRNGGIAAFVDAEHALDPTYAGALGVDIENLLVS
QPDTGEAGLEIVDQLVRSAAVDIVVIDSVAALVPRAEIEGDMGDAHVGLQARLMSQALRK
ITGNIGKSGCTVIFINQLRQKIGVTYGSPETTTGGNALKFYASVRLDIRRIQTLKKGSDE
FGNRVKVKVAKNKSCTTL
>EFA72642.1
MLIRKGAWYSHNGENISQGRDNAIKYLEEKSDFAEKIKEQVRAKLDQGALVSANSVAKST
EEEVEEEIEEEE
>EFA72655.1
MNVWLLSLLFHYFQHPITILSIAAILAFLLNYPVQLLEKARITRTYSVIIVLVITLTLLV
ILGFTLVPMLVEQTTQLLRNIPDWVTSSQENLSKLQVLARQKRLHIDFSVVSSQINASVQ
NILQQIASGAVGFAGTLLSALLNIVLVVVLAFYMLIYGDRLWSGLINQLPSYIGLPLSKS
LQLNFHNFFFESTVVSIIHGNCFNTNFFIFKSSLCSIICDYYRNLRTSSCDWCYFGYRVS
DLTSILTDLVVSISSSNGGNYHTTDQR
>EFA72656.1
MVAIIIQQIRDNILAPKLLGNFTGLNPLWIFIAILMGFEIGGLLGTLVAVPIAGTIKSTI
DAIKNNKSGIT
>EFA72658.1

MKAMILAAGKGTRVRPITYTIPKPMIPILQKPVMEFLLELLRKHGFDQIMVNVSHLAEEI
ENYFRDGQRFGVQIGYSFEGKIDDQGKLVGKLLVQLGECDVSRISHLFLMTHLWFCVVML
>EFA72659.1
MVLCGDALIDLDLTAAVKWHRSRGAMATIITKSVPQEEVSSYGVVVTDNENRVRAFQEKP
SVEEALSTNINTGIYIFEPEVFKYIPSGVQYDIGSQLFPHLVEINAPFYAIPMDFEWVDI
GKVPDYWRAIRGVLLGEIKNVQIPGYQVAPSIYTGLNVAVNWDRVDITGPVYIGAMTRIE
DGAKIVGPSMIGPNCWICGGVTVDNSVIFEWSRLGPGVRLIDKLVFGRYCVDKMGTAIDV
QAAALDWLITDARQTPPAEIPPEHQAIAEFLGGITV
>EFA72661.1
MGVESITNEIVKVHINGHKSELKNQKHKKLVPPALNRVIYPAAGK
>EFA72663.1
MLKFFADRGGTFTDIVALTDNQDIINRLLNYPQRFLIVPLPNQQWVIVYKLLSENPDQYT
DAVIQGIRHIMGVPPNRVFLVKP
>EFA72666.1
MKIATWNVNSIRTRLEQVISWLGENNVDVLCLQETKVIDRDFPLTVFHDVGYHTYIYGQK
AYNGVALISRQPMKSVSTGFCQVLENLEPEWDDQKRVITGIVDEVRIINLYVPNGSAVGS
EKYQYKLQWLAVLKTYLEVLLKSNSHIIMCGDFNIALEDIDIYKQVDTKNQIMASLPERE
ALREILNLGFGDGFRKFNSQGVIIVGGTIVRELLKEIRDGE
>EFA72670.1
MTKLNSELNTKSSDKSLEAMHHFSEQYAKRTGTYFCSDPSVTAVVIEGLAKHKDDLGAPL
CPCRHYEDKEAEVSAAYWNCPCVPMRERKECHCMLFLTPDNEFAGKNLQEIPVETIKEVR
DSMG
>EFA72672.1
MLPVTIITVTLTGKLSTAQSSGGNRPLTIRADIQEYDAKTQVITARGNVQMLYPARQIQA
TAAQAQYFSKERRIDFSGNVYILQQGNNSIRAEKVTYLIDEGRFIALPQSNRQVESIYMI
GDDDSIQPTSRPTTKPTPKRGSKTRSPL
>EFA72675.1
MITASVNFQFLQSHDLQLLRLSVLAEGYVWNQPKTCLVKLCYFGQLLTQLIAARTGNFHS
TVENQTDLLRQLELKGILPQKVALLFHQVRVVSDRATYEHTSDPSQALTILKIARELAIW
FHRTFGGNTTFTPNPFISPPDPVDYRTELETLQHVETEFEQALATFSLHLAQLQVISSTL
SPRQTDTIISLGNQAVLEIDLEENAHTYLCRGIARSDLGDNRGAINDFTQSISINSNLPQ
PYMERGIARTNLGDGQGAIDDFNQALDINPNLALAAYSRGVAHRDMGYLQKAIEDFNQTL
HLNSAFFDAYTKRGLARYDLGDKQGAIDDFNQVITINPHFADGYAARGLVYCDLRNYQEA
INDFNQTIRINPNYAQAYHNRGVARSQLGDKQGAIDDYTHSLNLNPKFASAYYNRGIIRS
DLGSQKAAMDDYTQAIKIDPNYAQAYNNRGAIRTYLGNYQGAIDDYIQALRVDSNYAEVY
HNWGTTRINLEDNQGAIDDYTQALNINPNYAQAYYGRGIARFNLGDKQGAIDDYTQALNI
NPNYAQAYYNRGIARTSLGDKQGAVDDYTQALNINPNYDQAYYAWGMVCSELGDKPGAVN
NYTQALNINPDDPETYIARGLTRSELGDNQGAIDDYTQALNLNPDYAYIYNNRGVVRSDI
ADYQRAIDDYTQALNISPDYADAYYNRGIAYYDLGNYQSAIDDYTRSIEIKPNCADTYVG
RGTALYKLGDSQGAINDFHHALDIDASYADAYNNRGIVRYELGDYQGAINDFNHALNINP
NYAQAYNNRGIVRYELRDNQGAMEDFNHAVNINSNYAQAYNNRGIVRICLGERQLAIEDF
SQAIIIAYNYTESYINRGYARYELGNRQKAIEDFNQALNINPNYAQAYNNRGVAYTDLGD
REWAKDDFSQAIQINPYYAEAYNNRAIVCYELGDHQGAIEDFNQALNINSNYVEAYNKRG
NIRYELGDRQGAIEDFNRALNLGSLDLGENLKFWERGGLTLTIKLRNYLNLKILSFEVVS
KKNFGEIIKTDASVIYFVEDLGDDITLEMVEIPGGTFTINSAENRGNPEESAEHLVTVPS
FFMGKYEITQEQYQAVTGDNPSYFKGDKRPVERVKWNQAVDFCEKLSQITGRIYTLPSEA
QWEYACRGGTSTEFHFGDNITTDLVNYHGDHKQSLAPQGQYRKQTTEVGSFPPNPFGLYD
MHGNVWEWCLDHWHDHYQDIPKDSSPWVEKGIFGVFRGHVLRGGSWCDSAQHCGCGSRNR
HLSDKNNLGFRVVCSNHNR
>EFA72680.1
MFVLSGYEYLLGFLILCSLVPALALSASKILRPSSYSPERRTTYESGMEPFGGAWIQFNI
RYYMFALVFVVFDVETVFLYPWAVAFHRLGLLAFIEALIFIAILVIALVYAWRKGALEWS

>EFA72690.1
MLELIELTETIDHQLLQNKINNHLRYLESCGNEGEFADFSNLKIENFDFSNCKLSGINAH
NSVFIKCCFYQCDLYGSVFKDSVFMNINFEKANLGKTEFYRIKNEDVTFDNANLGSAEFY
DCQLNGTSFKNANLGGSSFDECDLTNVVFEGAVLENASLTKNIEKNTNWSNIKLIS
>EFA72691.1
MDLVLLDLVDVISSDYVPQSLLQAMFIIAKKTDKPLYRSMELFTSNPAQAIDLFHDRGSL
EVGKRADFITVCDDGIVPRLISTICAGCRIS
>EFA72692.1
MNEQIYTNYRLLLPNEEILGTLVVRNGKIADIQPGITKKGENGEGKYLIPGLIELHTDNL
ERCMSPRPGIRWPLEAAAIYHDRDLASAGITTVCDAIAIGDVNPKSPRLKNYSQMINVLC
QGKADRRFLVDHYIHLRCELAYPEVDQITQEYVHNSLLLMISLMDHTPGQRQFIKLEKFK
EYYMGKHGVTAQEMEEFITTRQERHKLYSKKNRHSLVELARERKIALASHDDATVDHVQE
AVEDGVVLAEFPTTVEAASKAHSLGLKVLMGAPT
>EFA72717.1
MRHPILYLGFTWWIWFITPLLARLIDYRVGWDPTRQILVAPYLVVLITTASVIKNLPRTL
KDGGLPFILALIGVIYGLLVGLLYNQPISVIRGFLDWFTPIIFAFHLFINWRDYPSYGRN
TQRVFVWAVLLIGAYGIYQFIFAPEWDKFWLIESKMFTSAGSPEAFGMRVWSILHSPGTF
GAVMQTGLILLFTSYGPLIFPASLVGYLSFLLSQVRTSWGCWLLGIIIMLGSVKTKIRMR
LGMIILVIVLTIIPLVTIKPISEVVTTRLESFSNLEKDSSFQDRSRTYNESLNVALSTAF
GHGLGNIWEVDEKTGQIKVIVIDSGILDMFLTLGWFGAIPYTSGLFLLLVSVIKYTEAKR
DVFISAARAIGISACAQLIIYSGMLGVAGMIMWGFLAVSMAAHKYYKHHY
>EFA72718.1
MTSKQLLFNSFLESSYSPEDRSAQSWVIIFPLLYLQVPVILVVLLTY
>EFA72735.1
MRVLRYFSNQEVLDLRENLGRGGEAFIYTVSSDENLVAKIYHRPSSDHIKKLQAMIANPP
ANPAASFGHISIAWPQELLTAVDGSDTIIGFLMPRIRDMVPIIDFYNPGNRRQNCPLFNY
QYLLRTARNLAAAVAALHASNYCIGDVNESNILVSNTALVSLVDTDSFQVPDLSQSRVYR
CLVGKPEYTPPELQNKTFADYNRETYHDLFGLGVLIFQLLMEGNHPFSGVFQGLGDPPSY
EYRILAGHFTYSQKQKVPYLPTPITPSWQTLHPAVRDLFVSCFEDGHHSPHLRPSAQTWL
SVLSTAEASLVSCAVNPQHVYHFDLNTCPWCERTVKLGGRDPFPSLQTISAREYHQPRPK
SRKRYRYASHVRKSATPVLVTYTQSSLKSSSAIYKPIQMSNRSKFYALMFGFLGLGVLGY
LDIMIKFTRIFISPSPYTKQSLLSSRSENVHSPLSLSFHDYYQRGNQAYQQQDYQQAIED
FTQGIKQNTNFSKLYMHRGNARYNLNDYQGALSDYSIALKINPQEVKAFINSGNAYFKLA
DYSNDPDYEYKKAIDSFNNAININKQDDDAYVRRGVVRSQIAKYSHNSQQEYKKSISDFT
QAIKLNPFKAEAYFQRGLSRYQFGQYSSNYAEIYKQAIADFDQALNINPEMAEVFLKRGM
IYYELAQYGEKTARNNQQRALEDLEKSAQLYLNKKDVNNYQQAISNICVIAEEKCDYFLQ
NSSIIYSVNP
>EFA72743.1
MSSTISHSPLLQIGTASMLLLLVANKVTSELIITLGTASEEIFRGDRLPPLKFPQESCAE
DRITS
>EFA72754.1
MTITLEEMRKKLKQVWGYENFRPPQEEIVSSLLSQKDALIIMPTGAGKSICFQLPALLSN
GLTLVVSPLIALIENQVEELKQRNQKADLLHSELPASQRYKVLESISKQQLRLLYLSPET
LLSSAVWEKISHPHIGITSLILDEAHCLVQWGETFRPVYRRLAAVRPSLLNTKPPGTKIS
VAAFTATADPSTQNIIKDVLQLQQPDIYRLNPYRQNLQPTVKTVWTPKARKQQLLKFLQL
HPHQTGLIYVRTRKDSEELAQWLMNLGYHTASYHGGLSGEERRAIEKSWLHGKKSFVVCT
CAFGMGINKADVRWIVHFHAPYLLSEYVQEIGRAGRDGIVAEVLTFISEPNRFF
>EFA72755.1
MNLTGFFDGEDQRRKLFFQQQILQQYKKAQELIKKLPLQGEVKSVVKEFQHGATALAILH
SSGRLLWNDPFHYQILGRDIHQSRTYFNPAQQMVEYLRTKNCRWQFLLQSFGFNKDRENW
RCGHCDNCRFKSGGFQGIS
>EFA72756.1

MSLDKIYKTDVLVVGGGTGGTTAAIQAARRGCKTILVSEFSWLGGMLTSAGVCAPDGNEL
NSFQTGIWGDFIRELQKRQSGGLNNSWVSFFSYQPQVGAGIFADWIKELPNLQWITHRIP
LEVLRQGDRIVGVSFADCTIYAQIILDGTELGDVLALGEVPYRWGWELQSEWGETSAPSS
FNSLTKTYPIQAPTWVVIMEDFGEVLAPEIPPAPNYDPALFAGAWENYGKEKFLNYGRLP
QNWFMINWPICGNDYGQGTSRLLESKARNEFYQECFWHSQNFARYIQTSFGRRYGLAQGA
FPTVSPAFALHPYFREGRRLQGVTTICEQDLLPLSNGSVAPLMKDTVAIGNYANDHHYPG
IKFPLQPKSLLWGGRWTGTPFTIPYSSLIPKSIDGLLVCEKNISVSHIANGATRLQPVVM
GIGQAAGMAAALCCELNCQPRDLPVKTLQMALLTDKHSPAAVIPLLNLSMNHPDWLRWQI
DILNHPEIYPPSGNILTCGGNIHQSTFHHF
>EFA72757.1
MRATISILVSSMILGSLAFNRETLFTNPSRLSTSNSGTGLLFADSSKPSPNRPGEPVTYR
GSGRKESINL
>EFA72762.1
MIVGYESQMVKGALSKNSDKHSDLEFVEQSVQLGTGHAIQQLLPYLSDYDGNLLILNGDV
PLLKTETLQQLLQTHQEHDNACTILTANLAQPDGYGRVFCDDNQIVHQMVEHKDCTHIQR
KNNRVNAGIYCFNWQKLATILPHLDNNNVQKEYYLTDAVVQVGKVMAVDVSDEREIWGIN
DRRQLADAYQLLQQRIKEKWLLAGVTITDPSSVTIDETVEMEPDVIIEPQTHLRGKTLIG
SGSRIGPGSLIENSQIGENVTALYSVITDSFVEQGTKIGPFAHLRGHVEAGENCRIGNFV
ELKNTQLGDRSNVAHLSYLGDTSAGTQVNIGAGTITANYDGVKKHRTRIGDRTKTGSNSV
LVAPITIGSDVYIAAGSTVTEDVENDALVIARSRQVVKPGWKIKRELAE
>EFA72784.1
MAEKTIANSIPIINYGYLNNDRVQAALSKSQPQKEDILKMRVTAIFSVHST
>EFA72786.1
MKQQGVLVLDAPANQISEQLVERYLQIKAKNQI
>EFA72791.1
MLNLPVPSTLLYSIPLAAVTIYLPYLLVAYARVKVGYDMSAPRSMFDKLPPYARRATWAH
QNCFESFMIYGTAALMAYVTGVDSQLALYVAIAYLTARVLFSVFYILNIPILRSLMFGVA
SACIISLFVLSILKGT
>EFA72793.1
MSIFNTAILYDIENLTKGYSFSKDFIKELSLKQIYRQILEVDIVNKICLQRAYANWSDHR
LSLLRGEINELGIDPIQIFGFARYHKKNAADIQLVVDTMDITIRFPHIEVYVIVSGDGGF
ASLAKKLT
>EFA72794.1
MANNIQPIYQADKKTIFSHGQKIISWFGQDPESRKQMYGHGIPLSTVREAFKYAIPEFKP
EMVGFMRFAEFLQFICANTEFCVGTLPPSNTLLVFRNSIPNGVVILLDILNQDLHTPERY
QSLLASGKPRITIEDKYSLETFVDTLMSKRDILMNISEILDIFSQELPDFESNKLNNLCL
SLIHCNILKGYPEDENISEQKFHISQDFKDTAQILEHVKQTSLNKLISILVDDFKSDVFK
EVIPF
>EFA72811.1
MNLEQKDPCFERLLTYIRQNRGFDFTGYKRSTLVRRVTKRIQALNIDNFIDYMDYLEVDP
EEFHNLFNTILINVTDFFRDVSAWDHLGNQVIPNIIKRQKKRMNKFVYGVLVVLPGKKLI
P
>EFA72812.1
MAENLGAEDFRQRVKIYATDIDEEALNQARQALFSVKSVESVPRKFRDKYFDLTGNNYIF
RQDLRRSVIFGRHNLFIDAPISRLDLLVCRNTLMYFNSEIQGRIMARFHFALNDTGYLFL
GKAEMLLMYSSLFMPVDLKNRVFTKLSSTNIRDRLLVMANSVDDESSRQLSQNIRLRDLA
FESAPVAEIVIDINGLLIIINEAARNLFGLSKMDLERQFYELELSYRPIELRSLIERAYN
ERRPITLNSIERYVPNSEQQYFDVLITPLQEDDLSLLGVSIAFNDVTRYVELQEALQRSR
QDLETTNEELQSTNEELETTNEELQSTNEELETTNEELQSTNQELETMNEELQSANEELQ
TINHELSERTLELNRRNNVFINCILKSLQKGIVVIDRNFSILNWNELVEDLWGLRYDEVIN
KSLFSLDISLPVEELRSPILDIISGKTDFQEVSIESTNRRGRIIQCYIALTPLIDKKIEG
VVLIMSDSQN

>EFA72815.1
MKRIGILTSGGDCPGLNCVIRAVVSHAILTYNWEVVGIPYATQGLQQRQTIPLSIHGWNL
RGIDPLLNMGGTILGSINEGDTLAAAGEILSGYEALGLDALIAVGGDGSLKIIHELATMG
NWHLVGIPKTIDNDVALTERSIGFDTAVNTIVDAINRLTFTAASHDRVMIVEVMGRTAGH
LALHSGIAGGADVILIPEIPYTIQGICEHLKELRDTWKRKFAIVVVAEGATLCSEDLNND
HGVNGEHIPDTNIGSAKCGRGQYVAGQISNFAHGFDTRVSVLGHIQRGGIPSALDRLVAT
AFGKTAVDLIAQGKNHEMLAWQKWQGYKYFHRKFFT
>EFA72825.1
MCIQVELKQNRDAWVYNFSQESLIDNMTRMIDFYNQQVEGFRKFLEGQTLSNAEQRKQKV
ERFIDTDPKKIKLVWRTKGRLW
>EFA72829.1
MKVKILDLPGLSYVGDETSMEVIRQCREALCLVTYNSQETDPQKVRSLLLQVVEEVKGLG
GSPDRMLFVFNKIDVFRDDKNWEESERRFIEKTTKNIKDELRIHLGEYTQAIKNLKLIKL
STLPALLSLQILSDNYNHSNTACRDADRKFNKLIEESILEDLPRNAEKWSGQDRRRIAQD
LWRKSYAQEFQECLNEHIAEHFPKLVIPQAIERFNTSAGNSITQWTVQTTTAILNSSEES
YQQECHRISQVKSDLDHFIQVSSNSLRNPFEKISQEIGEYFDKENKDVQHLSKTLTSVVI
ELQNTEPCRNNPEIQEKLVPLYDWRDSLSREVSTILEAVTSSLEKGTVTLDHPNFKKVNS
YHVKLLEGNIIRLIKLGYSHDIAKHGKTIEAKTELEKENLRQTNNEINELSIHLSLIIPQ
VLEKTSEQEIQRIYEAVNGLFAFYLEFLEEECNEIAGNKLGINFSKSELVQVKKDLKFIQ
PKFKSDFAIETREYTEETRSWDYWFWLVPKQVVCSSDNAKIPKMRDILTTWELEIKKIDP
ELLKPFIEWLLEQIDNLKKKVDKTQSEIIKSYRDRLEKARKEVNFDYEQRQSIWQPMQKR
AKELEWEFSQLVNFEKPQVNNNDK
>EFA72832.1
MIAAQCGIFANNHNFAERSLPIKKQGVSCQGIVIEDDCWLGHGVTVLDGVTIGKGSVIGA
GAVVTKNIPPYSVALGVPAKVVKSRL
>EFA72838.1
MAEIDKSISFDGRDIRLKVGLLAPQAGGSVLIESGDTAVLVTATRSEAREGIDFLPLTVD
YEERLYAAGRIPGGIMRREGRPPERAILTSRLIDRPLRPLFPSWLRDDLQVIALTLSMDE
LVPPDVLAVTGASIATLIAQIPFYGPMAAVRVGLVGDDFIINPTYAEIEAGDLDLVVAGS
PDGVIMVEAGANQLPERDIIEAIDFGYEAVRDLIQAQRDLIAELGLTIVLQEPPIPDQSL
ENYIRDRASEEIKKITRSV
>EFA72839.1
MTKTERDAALDVVKSAIASSIESLGEEDPIRTAATANSQVLTNTFKDITKYFMRRQIVED
NVRVDGRKLDEVRPVSSQVGLLPRRVHGSGLFNRGLTQVLSACTLGTPGDAQSLSDDLQV
DQSKRYLHHYNFPPFSVGETKPLRAPGRREIGHGALAERAILPVLPTKEQFPYVIRVVSE
VLSSNGSTSMGSVCGSTLALMDAGVPITKPVSGAAMGLIKEGSEVRVLTDIQGIEDFLGD
MDFKVAGTDTGITALQMDMKISGLSLDVIKQALEQAKDARLHILDKMLQTIDKPRTETSP
FAPRLVTIKIDPDMIGLVIGPGGKTIKGITEETGAKIDIEDSGIVTISAIDESRAKRAKS
IIQGMTRKLHEGDVYLGRVTRIIPIGAFVEFLPGKEGMIHISQLADYRVGKVEDEVAVGE
EVIVKVREIDSKGRINLTRLGIHPDQAAAAREAAATNR
>EFA72850.1
MTLISLIAAISQDRILADSKNEHIRGGIPWDIPSDGRYFKEITWRHPVIMGRKTYATFNH
PLPNRTNLIVTKNTDYQAPGCVVFHSLEEAIKWSKMCETEEIFIAGGEQIYTQTMELAHK
LYLTIVEGSFEGDIYFPEFSNFGKLTKRGKIRRKWF
>EFA72853.1
MSIYVIGIGGTGAKSIEAIIQLTAIGLFGEQSVKLLFVDTDETNGNVSRALNSLDTYNKC
YELGLNGKYPWMRTEIEPFPLWSPFADNLTNKNLAVIFNYNLVKENDAALGNLFDVLYTS
KEREANLDVGFRGRPNIGAAVMSQVKMDTIDNDFWGSLMGKIQQDTGGGKRPKIFLCGSI
FGGTGASGLPTIGRLIHNRLETLKIREKVDLGCLFVLPYFSFTPDENSEEVYARSEQFLL
NTEAALRYYGSQPQEIFDVVYLLGNQQLSTVKKFSIGKNTQCNDPHFIELYAALAARQFS
LKTTPGQKRWY
>EFA72854.1

MNRENSRNLTWKDIPDMQEVYPEIVNATRFALVWLSNIAPELEQAKKMGEKVFRTGAGRS
WFVKFFPASRGILGGGSQPIVDFNNIKEQEAIQIISNWCNDYLRWLWSIHQCQEESIQLF
NGDYLNTGNLNAENLANLIIEQTDSRDRNKKSLDTIQKLKERLDPQSIPAPNFGVIGLAK
SLYHVCRLTD
>EFA72855.1
MKIGELKKQGKLYMTSLLLPRLKDDCDVKSGVAGVWDAQAPNTFNQVASSLDYKAPGEIK
NISSVPTIWARPLSVEMALHNDKYPIREQIIVQWQGMLTVLALAEVRGFPIKAQLLELGA
KKDTDNFARSLYELLPDDGNSIYKFSNNKNPWEDLYILPGIILP
>EFA72860.1
MGVGTPGPVDATGRVAKVAINLEGWDNVPLAEWLENRTGIPTILANDANCAGLGEAWLGA
GRDFQNLILLTLGTGVGGAIILHGKLFVGHQGAAGELGLITLNPNGPSCNSGNQGSLEQY
TSIGAIHRRTGKDPARLGELAKQGDIQSLTFWQEYGRDLGIGLTSLIYVLTPQAIIIGGG
ISASFEYFLPAMQEEIERRVLPSSRMGLQILPAQLGNSAGMVGAAKLAWDND
>EFA72861.1
MEVVIGIDIGGTSIKLGVFDPNGNCLQTIILQTPKPATPEAVLSTMLGG
>EFA72864.1
MLLVFGFKTVDIVLKLGSFCCKSIKNLVLPSIFPIMAELSNLILAEMGYYGANCLFIALG
KTL
>EFA72881.1
MMAKPKKNNPPIVSLDEPINLNDPEYYINRELSWLQFNSRVLHEGCDERTPLLERLKFLA
IFSSNLDEFFMVRVAGLKQQVEAKVNLLTPDGRTPQQQLDDIRLHLSPLVAKQNQQFEEI
LQPLLSNQGIYIIKYMDMNEQQKLYLDEYFKEQIFPVLTPLAVDPSHPFPYISNLSLNLA
VVVKNPETEEEYFARVKVPKVLPRFIPLPSELASDNGRPIHWIGVPLEQAIAHNLDRLFP
GMNIQECHPFRITRDADLELEEDEADDLLLAIEQELRKRRMGGTPVRLEIQSQTPENLRS
RLLEDLELTANDVYQVNGLLGLRDLMYFMGLSLPELKDPPRQSVIPSRLQRLKEPCLDPD
VLETEDGKDFFSVIREKDLLVHHPYQSFSGTVEQFITHAAWDPQVLAIKMTLYRTSGDSP
IVNALIAAAENGKQVSVLVELKARFDEENNIYWARRLEKVGVHVVYGLAGLKTHSKIVLV
VRREKEKICRYVHIGTGNYNPKTARLYTDLGLFSCREELGADLTDVFNFLTGYSLQKN
>EFA72882.1
MRNRFLELIYREITNVQNGFSGRIVAKMNALVDPEIIATLYTASCAGVQIDLIVRGICCL
RPGMKNISDNIRIMSIIGRFLEHSRIFYFHNNGQEEIYIGSADWMRRNLDRRVEVITPIR
DQEIAKDLQEILGIMLADNRQAWDLQPDGNYTQRTPKDNSSEANSQTILMSMNQNNYPKP
LMQN
>EFA72886.1
MYEEENFVVLETNQEEQFLTKLELLEKLQNTLSQMPIEDIPLDVRKIGSLIEQVNHLIDT
TCELDLGPGRYLQWYAVRLEK
>EFA72908.1
MAKIIAFDEESRRALEKGINALADAVKITLGPRGRNVLLEKKIWYPQIVNDGITVAKEIE
LEDPLENTGARLIQEVASKTKDVAGDGTTTATVLAQALVKEGLKNVAAGTNPIALKRGID
KTVEALVKEIAKIAKPVEDSELDSPAASIAQVATVSAGNDEEVGQMLAEAMAKVTKDGVI
TVEESKSLTTELEVVEGMQIDRGYISPYFITNNDRMTVEFDNPRILIADKKISSIQDLVP
ILEKVARLGQPLLIIAEDVEGDALATLVVNKARGVLAVAAIKSPGFGERRKALLQDIAIL
TDGQMISEEIGLSLDTATLEMLGKARKVTIDKENTTIVSGTENKPEIQKRIAQIRKQLEE
TDSDYDAEKLQERIAKLAGGVAVIKVGAATETELKDRKLRIEDALNATKAAVEEGIVPGG
GTTLIHLVAKVDAIKDTLDGEEKIGAEIVQRSLEAPLRQIANNAGVEGSVIVSQVRNSDF
NIGYNAATGEFEDLIAAGIIDPAKVVRSSLQNAASIAGMVLTTEVLVVEKPEKKAPAAPD
PGMGGMGGMGGMGGMGGMGMF
>EFA72918.1
MGFFDSEIVQQEAKQLFEDYQALIQLGNNYGKFDREGKKLFIEQMENMMDRYRIFMKRFE
LSEDFMAQMTVEQLKTQLNQFGVTPQQMFEQMHLTLERMKTELEKQV
>EFA72923.1
MDNMENQLIDIRLAVSALLETSVIYQRNFEVMQRNFDSVVIEIREMQSEIREMRSDIREM

QSEIREMQSEIREIQSEVREIQLDVRGLQTENRRILDVLQNVPPDKYE
>EFA72927.1
MLGDDILRSCSMVIFRRLITSGLIGFLVSSCADAAKSSTEYTKQIVQENNVARLMAQVQD
SQKVSNLLSQGNGFLDSGRYEEALQLYNRAIEIEKDSVPSWVNRGNALLSLKRYQEALES
YNQAIALRPNKNEAWYNRGNALSALGRYEEAIRSYNESIVIDPNKFEAWINKGIALTKLQ
RYQEGLASYNQAISINPNFPTAYYNKACNYALQKQVNLAVESLAKAIKIDGQKYTQLARV
DKDFAKISDNRDFQELLK
>EFA72947.1
MLYGLYIPTQGAKWICLIIFLIAALTDWLDGYLARKLNQVTDLGKFLDPLVDKLLVLAPF
LVFVELGKIPGWGVFIILARELAIAGWRINQTTISGANIWGKLKTITQILSIALLIAPLP
TNWQIYAKSAFWLSVLLTVISGLIYLLPVLSPKHGN
>EFA72948.1
METPRFMPVGTLANVKTVTPAQLEDTGAQMILSNTYHLHLQPGEKIVAGGGGLHKFMAWN
GPMLTDSGGFQVFSLSEMRKITEEGVVFRSPHDGQMIKLTPEKSIEIQNILGADVIMAFD
ECPPYPATYQEVTAATDRTYRWLERCIMSHQRLDQALFPIVQGGVYLDMRIRAATDLAKL
DMPGYAIGGVSVGEPPELMAQIVRTTAPLLPRNKPRYLMGVGTYREMVIAIAAGVDLFDC
VIPTRWARHGTAMVQGERWNLKNSKFREDFIPIDETCHCYTCSNFSRAYVSHLVRSQEIL
AYTLLSIHNITELIRFTQAIRESILQNRFLTDFGHWMNDPEKDLE
>EFA72964.1
MAKKLKIHGLDGSIDAYLAQPVATGIYPGIVVLQEIFGVNEHIRDVTERIARLGYVAIAP
ALFQRQAPGFEAGYSPDEIEIGRKYAWSQTKATELLGDIGSSINYLKTLENVQPANFGCI
GFCFGGHVAYLAATLPDIKATASFYGAGIPTRTPGGNHPTLLRTSEISGTIYLFFGMEDA
SIPQEQVDEIEDTLKKHQVPHRIFRYDGANHGFFCDHRASYNVKAAGEAWEQVQQLFNLL
VKTND
>EFA72966.1
MRVYPQTDFPERFEEPGTRWLLLPGEPSPQPVQLLHGRYVEGKNLYVIKLHGIGDRNAAE
NLREGRLFVPISDRVELGPGEFHVIDLIGLPVFMQESGERIGDVTDILPSGHDLLEVKCD
PSWNRNLGGKTVLIPFVMEIVPVVDLANRRIEITPPPGLLSINE
>EFA72967.1
MKRENKKRKQATGEQKKRGNKSESPIPQPR
>EFA72989.1
MIPERYQGSDLLTSLDNWLQFVIEETADFVCAYKPTLGFYQALGVRGIELLIKIMASIPK
EVPIILDAKHGDLNTTSMFAHSIFVDWGVDAVTLNPYAGQDHIAPFLVYPDKAVFILCCT
SNPSAAILQQYPATSPFYLQVVQESKTWGTPEQLCLEVGTTNPEILKSIRSAAPERIIMV
RSIWSQENSIQPILAAGLDSHGDGLLIPVPQDMLISANLSQEIGSLRTELNQIRSDLINS
TSSCDVWFPDVNMKDKHPHQDLILQLFDIGCIMFGEFVQASGAIFPYYIDLRKIISNPQV
FSQVIGAYEKILTGLTFDRLAGIPYGSLPTATGLSLRLGYPMIFPRKEVKAHGTRKAIEG
NFLPGETIAVVDDILISGKSVMEGAEKIKSVGLTVNDIVVFIDHEKGVKDKLKENGYCGH
SVLTISEIVNTLYEAGRINQQQLLAFQQE
>EFA72999.1
MVSYRIKQFLFLLLVLSKPRYLSASHHALTMFWVCFIDNPGNNLV
>EFA73003.1
MAQVQLFPVNPRLSLAEEKEVRKTTIKGGKMMSDPEKLQKCLTLPFQIQMQQHCL
>EFA73011.1
MDLRRDALQILKETSRTFYIPISIMPSGLQEAVASAYLCMRAIDEIEDHATLENHTKGIL
LQSISQTLQAGVDGFAVDAFSIGFKGYEDSLPEVSLRIREWAILAPESIAPRIWDATAAM
ADRMAYWSQINWKITNEYDLDRYTFGVAGAVGLLLSDLWSWYDGTTTNRMEAIAFGRGLQ
AVNILRNNSEDLTRGVNFSRRVGITRTFNSMLVVI
>EFA73013.1
MKATLIKTTQPGIKRKRFFVPIVNVLLLMELSVKEFVWLTVTTDNHN
>EFA73025.1
MLKVANRQEEGWLRTEDAEKFACKELRSIDQWWFVPFFNWLHPRQ

>EFA73043.1
MNTSQIQTLLQNLFQQDSRWPHTQRRIIFWYDPDGQFVSIFEELEINDVKKIQLGDTPFT
LKYRLLIEEPEQNFLLYAPFLNRNPKKTGS
>EFA73046.1
MVFNRYTVEILKYTRFVYNSDRLSEMLGGKLSPDQKVPLIHKFLLFSEILAK
>EFA73047.1
MLKEAKPECEVEGNIMSIFVGETGKKVRDKRNELFHKLLGLKETDVFEAWDTTDKDSWKT
KVLDCLNFVSDQSFKSLEEVSVMYKVHQLLNEMLNDYKINAFVSHQD
>EFA73069.1
MNSLLPPNLELIIRPIQYRDLDGLERLRQEVPPKCGLFGMQWLGRWFGVLEFLNCFPNPW
QHRFRQFVAERGRKLLGIIQVSPFNRTRSTWHVDQVILDPCADKLGIGSQLLRHCFESIL
EARMWILEVNINDVNALALYRQNGFQRLAETTYWEIKPELLQELAQAEPDLPNLLPVSNA
DAPLLYQLDTASMPPLVRQVFDRHTHDFKTSLFDIIGDAIKQWVEQTEVVSAYVFEPQRK
AAIGYFELQLDRKGTSPHVATLTVHPAYTWLYPELLSQLARISQDFPQQSLKLASSDYQP
EREQYLENIGAQRIEHTLIMSRSVWHKIRESKFVSLEGIQWTEVLQGLQPSRKPIPGGMS
WVPKSPPVPEVTMGKSEMVALTIEPSEPPQENN
>EFA73086.1
MLTVALPKGELLKNSIQLMQSAGLDFSAFLDAGNRQLQIYDASGKAKGLLVRAQDVPVYI
EYGQAQLGIVGFDVLKRKKPQVGQLVDLKFGYCRMSVAVKSTSSYKSPLDLPAHGRVASK
YVNCAREYFESLDLPVEIVPLYGSVELGPITGMSEAIVDIVSTGKTLRENGLVEIATLYE
STARLIVHPLSYRLDLGGIYNLAQSVKSSVNS
>EFA73095.1
MDLSLIPAQPKPGVINVLIEIAGGSQNKYEFDKELNAFALDRVLYSSVKYPYDYGFIPNT
LAEDGDPLDGMVIMDEPTSPGCVIPARPIGFLEMIDGGERDEKILCVPVKDPRYAHVNSL
KDIPPHRLEEIAEFFRSYKNLEKKVTQILGWHDADKVAPLVQKCVAAAK
>EFA73098.1
MNRGLLNQIPVMPVKNYMVSGFALLVVILTGMRVSLGSGGVGEIAEKPQLKSIAMCIQAD
QV
>EFA73105.1
MITKTTKLISFISVISLPLVSVFIYDDYTTGQTTTTSQLAQVKSVWQQFSSQEGKFTVLF
PGTPRFSQQKMPSDNGELQVNLFTVNRPQEEAKYTVAYIDYPAQYIQLLRSKNLVEQAIE
QGKSTALQRVRGTIVSEEKKTLGDNVGIEVNYTTADAKVVKQRVFLVGNRFYQITAETTQ
KRTKVFD
>EFA73113.1
MNQINYLRISLIDRCNFRCLYCMPNDTELNHILKKQLLTNDELLTLIQDVFIPVGFNRFR
LTGGEPLLRPGVVDLVNKIAHLPPNPRFIHDHQWFLLAPLAQDLYNAGLTRINISLDSLN
PNTFNLIIGDHSPHGWEQVWHGIQSAYKVGFNPLKLNVVVIPGINDHEILNLAALTLDKN
WHVRFIEFMPIGNTSLFSDRGWVSSAQLRDQIGQRWGLTESQVRGNGPADVFQIPGAKGT
LGFISQMSECFCDRCNRLRLSADGWLRPCLLNESGQIDLKTALRSGQTMGELQQQVGELL
HLKSEINFKERNPGTIGTYSRTMSQIGG
>EFA73115.1
MNNDIDLIKRLGPSAMDQIMIYLAFSAMRTSGHRHGAFLDAAATAAKCAIYMTYLEQGQN
LRMTGHLHHLEPKRVKIIVEEIREALTEGKLLKMLGSQEPRYLIQLPHVWMEKYSWQPGK
SRIPGSNLTTEEKKQIERKLPANLPDAQLVTSFEFLELIEFLHKRSQEELPHHHQMPLSE
ALAEHIKRRLIYSGTVTRIDSPWGMPFYVLTRQFYAPADDQERTYTMIEDTARYFRMMKN
WAERKSHAMRAVEELDIAPEKIQAAMDELDEIIRVWADRYHQEGGKPVVLQMAFGEQDD
>EFA73116.1
MTENLLKQARHQTRIIDRMIADLLEIGRENDNDFNIAPQRLELGKLSFEVLEELRDRYVG
KSQTVETDLPSDLPCVYADPERIRQVLINLLDNAIKYTPKEGKISLAGLHRTTQKVQFSV
GDTGPGIPHENRDHIFENHFRLERDQAADGYGIGLSLCQRIIRAHYGQIWVDSTPNGGAW
FHFTLPVYPS
>EFA73117.1

MLPVLKQDYMEVSQDQPIHFEAPLQLLLFVDGRPKSRQQVQRICAYLQDLEVDYSFDLQI
IDVREEPYLAEHFRLVATPALVKIHPEPQQTLAGSNIIAQLQNWWPRWQTVIDTSLALQK
DLHELPEPDISMTHPPSTIHSVALSAERIKLSDQIFYLNQEKQNFKNSYNLKSVS
>EFA73120.1
MYLLFQTLASFVEIYSYVLIVRVLLTWFPQINWYNQPFAALSQVSDPYLNLFRNIIPSLG
GIDISPILAFLVLNIVSSLLENLSRVTSLGGF
>EFA73126.1
MNEIEFPFLLASLTGKWQRFCLQFLLFLVCLLIIATSINIDTALAGSKDDRYEGNIFVVF
AGNGSLVPPKPNPGTEFSRTQTHSTNVLY
>EFA73127.1
MRIIPVSVDAIPPKKVYNPNEPGYYYSGSVPQIVIFDQSGKVVLNKNGQVPFEEIDDKFR
QVFNLAPRDQSIKYQQRSFNEYSSELSP
>EFA73130.1
MGTTSAVTGTITNDDIQDTTPPLATSLTPTDNATGVAVNANLVMNFNEAIQKGTGNIFIK
KVSDNSTVETIAVTNSNVTISGTQLTINPTNDLASGTNYYVQIANTAIRDIAGNNYAGFP
NSTTTWRFTTNKAPTDLNLTKNVVEENVNSVTSVGYFGTKDPDIDNTFTYSLVSGTDSND
NNSFTISSNELKTKTKAQFDFETKNSYNIRVRTTDQGGLFYEKQFTINVTDVNEAPTGIN
LSKTSVLENQNIGTPVGDFDTVDPDTGNTFTYSLVPVQNSDNHNLFSISGNQLKTEAQFD
FETKNSYNIRVKTTDKSGLSYEKQFLITVTDVLEPNITLEVSPTPSNVQEDRTTNLVYTF
TRTNVNIANALTVNYSIGGTAYYNDNNTQFNDYTQKGAATFTGTTGTITFAANSNKATLT
IDPTADTTVEENETVILTLAAGSGYTIGTPSAVMGTITDDEPPLVSLKLSSPNIVRESGG
TSLKYEFTRIGSNSDSLTVYFKIGGTLGRELDPNDFSYKTSTNTVLRNRTTRGGPGKYNE
GQIEFKEGQKTAFFEITPHLDKENERIEDISLQLEKPRRDQNQYTIQTEDPVKGMVSDTR
FYLYSGDMFKELNSSNIGNYLNKNSKTVLFIHGYEPISSNQLNSFENIPIEYRKLHPDTN
VILVDWSEDASYSFQVYEEGVYEEAKISTIPIGDELSDFLISLQIDPKKIELIGHSLGSH
VAGIAGSTYYNKTGEKLGLIIGLDAAGVKYEKTGPIDRLDSSDAKRVVGIHTDPEGFGDP
KRYGHLDVYVNNQKGESIGDHSYVKDIYRSLINGTRHVPVQNSKNLFKDMFDISDLYDTE
VQGEGHIIVQEDRDALEINGTNTTGFGSEDYLFGNERDNILTGFQGIDYLYGGPGKDIFV
FRFGMAIEVLGNKTIISADSLVTKPDYIYDFTIGEDMFRIHSSPSYSSSDVVKLNRFTRA
ANTNVNTLEEMVRDVFTDSNGGVAG
>EFA73140.1
MAEALAIQERTEKEQERQQKELAEALAIQEREKKEKLAAYLRSLGINPDEI
>EFA73146.1
MGTQGQMGGIYTDYFSYVLGWLFLVFLVFTALRLAWQGWRSLKSARECPTINLIGQSARL
LPTPALFAGQIGFWSPELVVSQGLLKHLSPEQLESVLAHEQGHYSYRDTFCFFWLGWIRS
CSACLPNTEPLWQELLMLRELRADSYAAARVDPLVLAESLLLVVSSQPLAWDICCAALSS
SKVDRLEQRIDALLAFSEPNWPEWDLKSKLHYWRVFALALLPLVTVFSTVRRLAIMNSIA
VDAY
>EFA73166.1
MQLQPPFLSTFTDREKKAIQDLTSYSGVHSLPEIWPLAAQHFRDITALYNPHSKPEVKIT
YSQLWDQIQRFAIGLQVLGINKNNDSYPPRIALIADNSPRWFIADQGIMTAGAVNAVRSS
QAEKNELLYIISHSGSTVVVVEDVKTLNKLEPDLGELPIKLVILLSDETDMPEWNTLPII
ETYSVMNFTQLLNLANNYTLTPLTIGGDALATLIYTSGTTGKPKGVMLSHNNLLHQVQTL
GTIVQPQPGDVALSILPTWHSYERSGEYFLLSQGCTQVYTNLRMIKEDFKKFKPNFMIAV
PRLWESIYEGVQKQFRGQPAKKQKLIQFLLKMGEEYIFARRIAQGLSLEHIGASEWIKWA
AKIKQLVLLPLQILGKS
>EFA73167.1
MREATGGKIKQVISGGGALPRHIDNFFEIIGVEILQGYGLTETSPVTNARRIWRNLRGSS
GQPIAGTQVKIVHPETKDPLPAGKIGLVLLKGPQVMGGYYQNLEATKQVIDNHGWFNSGD
LGWVTPENDLVLTGRAKDTIVLSNGENIEPQPIEDACLRSPYIDQIMLVGQDQRSLGALI
VPNLDALEKWAEVENINLPTQDDSTENEDQKIGQKIDLENKIIQDLYRKELNREVQNRPG
YRADDRIGPFKLILEPFSMENGMMTQTMKIRRHVVAQNYVNTIDSMFVK

>EFA73172.1
MDTTEDSVMVEETNQKPVGKKEKPPAVEDKPFQEFMQEHYLPALQKAIAQEGISDVKLTF
VKQKYAIVGFNSSQECWQITGSWQNGAKQFNVYFPDQDIQGKKGFSCHEGKKPSTLESFL
IDERKITLDLLVSRLIYRLNGQKWLGRN
>EFA73177.1
MRLLLKIKKAYRRLVKMFHPDTNQDTANHEEIIKINAAYEVLSDVEQRVKYDEQIGNWDT
SHPAVGNRENPRQTKKPGGKEADEKVEEWLKLVYQPINRWLCSIVSSLEQQLEDLAADPF
DEQLIDEFQEYLDTCRDKLKKAQTAFRSLPNPPSLATSAAHLYYALNQVSDGLEELAYFP
ASYDDRYLHTGKEMFRIAKQLLDFSPGSNTREK
>EFA73179.1
MTTQFNRRKFLIYGSAALGSSVLLKACANNTQTNTPADSSAGGNTIKVGILHSLSGTMAI
SEKSVVDAENLAIKEINAQGGVLGKQIEAITEDGASNWDTFREKATKLIEQDKVAVVFGC
WTSASRKNVKPVFEDKDHMLWYPVQYEGQECSKNIFYTGAAPNQQIEPSVDWLLKNKGKE
FFLVGSDYVFPRTANTIIK
>EFA73189.1
MVKARRDPFAQIIRVPQTIPTLPSVVKSNNTSRLNSLKKPLAIRQADLEIRGKNPIKPSP
PKPPDTKLAQSILVSGVILVNQQAQAIIKLPDDSNSRYVHAGETLTNGILVKRIEVNQCD
NPVVVLEQFGVEVRRVISGQEDGIC
>EFA73192.1
MQVFPGDNFLAPGKLTDHKLDLVSLIASHFSTSNSHIFNVQVPATGSIHIQPTELTIASW
LHRLTIKDHNWQPILDWSVKSQIRSYSSLYNSVSIFAIQHAYSRCVSLISLAKREGWIKI
VENDTDSQPLLSVSSIPWLDDCQKLRFYQPDEIHLIHLLVRTVDDLAFPHIQRSINWLAV
AEKLSSAFEKFWSSCPIWGYVKIHDLELAKARIGMLILTQLVIKYILEVKLGVTAISQL
>EFA73199.1
MTIYIGNLSYRATEADLKSVFADYGEVKRVVLPTDRETGKMRGFAFVEMIEDAQEDAAIS
ELDGAEWMGRQLRVNKAKPKEENRPRKGHSKG
>EFA73221.1
MSKGTLFDKVWEIHTVGTLPSGLTQLFIGLHLIHEVTSPQAFAMLKERGLKVLFPHRTVA
TVDHIVPTDNQVRPFADNMAEEMIRALEKNCQENDITFYNIGSGNQGIVHVIAPELGLTQ
PGMTIACGDSHTSSHGAFGAIAFGIGTSQVRDVLASQTLALSKLKVRKIEVNGNLPPGVY
AKDVILHIIRTLGVKGGVGYAYEYAGTTFEQMNMEERMTVCNMAIEGGARCGYVNPDEIT
YEYLKNKDFAPKEAAWEEALKWWQSLRSDEDAEYDDVVVFRAEDIPPTVTWGITPGQGIG
VDEKVPAAQDLPEEDRFVAEEAYRYMDLYPGQPIQGTKIDVCFIGSCTNGRISDLREAAK
NCPRSSSCTGYESLCGSWIGKGEKNKLKLKGWIKSFIQAGFEWREPGCSMCLAMNPDKLQ
GRQISASSSNRNFKGRQGSASGRTLLMSPAMVATAAIKGEVADVRELLSANS
>EFA73229.1
MATGLALISSNLNLASVSAVESCLTPALSRIKKHQVNPGENLVTIAERYKLMPATILQMN
PLINNGQVIPGTQLQIPPFDGMVVQVPNGQGWQQIAKKYGVRPDTLFELNGCQQNPRVVF
VPSSSKIKPIYGSAPSITTIMGSPVSNSTGVSFPYGWQIHPITNQVFFHSGIDLLAEVGT
PVRATASGVVVFAKEQGSYGNLVIINHQGGMQTRYAQLESIKVKLGQQVKVNQVLGTVGA
TGEPSSREPHLHFEVRAREDLGWTAKDPVEYLK
>EFA73235.1
MNNFELDTYLNRLSQKLSEKLNGDSHKRFPGWLAVDFGTSNSTVTMFDPIEVPIAETLPR
EQEVRLRQRLGEWLNSPPHLALPDIGVNEWEKFLVNLGRNLEIPPEAIGEIFENDHKDKF
LEALRQIELCLGNSERFRRAVSKKLYQIYHEVFRVPTLESQNLIPVVLDFNRRQTEIPSE
IEICKIQPLKLQMGRTARDNRKKAIAQGTITAVKDIISRFHHSPKRYFGQNRTFPVVINE
GEKNDLENNNIEVHQLIQAAWGHLIELTEDYRQRAGRRFSQGDLLTAVVTYPTVAPPVVR
KEIKALVEELGLDDVQTAYDEAVSVAIFFLWREFGGNLNIGIESFKTRCRQEKNNWSQNV
LVLDIGGGTTDLALIKLTLEDKTPVFTNNEDRGLGGRYYKLTPKLLGSSGHLQLGGELIT
LRVFRLLKIALADFLLTAVTDGNITSDKLEDLINSELNERFLQDGKFKSGSLLKCVDKEN
PEGDVAFKDALDTAEKVLPTRWQQAPQRLQTFYTLWEHAESAKLKLGRKDLKMVY
>EFA73236.1

MLLQSSVKFQLVSADSIYLTINAQQFERCAISSIREAIGIAKGLMESRLNEDQKVDWLIL
SGKTCNLDLVKTQIYQEFSKSPYFIWNPERITFVLEFTKLATSAGACYAEKLRRLRFDPE
ASKNLLRKGANQLEIDVKNLFYYLPCNFKRKTQTQELLSIFNAGQELYQLIPWESVAKVR
TTWQGIQLTNIIYRQDYQDGELRLWGSFDGKTLMENLRMEEAEFLKKIQVQFEIDQTLQF
TVLLCQGSPHYLIDVPGIDINSVIDPHATGSDIFVDGKLKWNIALIGDYENLKDGDIAIN
VLESATVDQPDAYHLVFAVDNSQNQILETFHYLQDGTKETGKGLISSPLPPFPHNGQHNF
YICQTDSLTKTKKWIRIGSLSKPEISTDYPCQYYVTLDSKGILRMHPGAVPYWTSHSLES
LQQPGCVYCTELELQPNEIDRERDPFCGVH
>EFA73239.1
MAVTHRGFSVIRQVLQRWKGYIRSWQDWQANPSKYVDAPKIPNYKHKIKGRFPIVVYNVY
QGCPTMDQPSLVQGMCQLFESLFEGDVNDSIGKVRKLVEAMIIPRNNGSYLLKFTYEVQE
PTTRSTQVIAGIDLGVNNLVALTTNSSTIRPLLVNGKPLKSLNRLFNRKRDLIGSYQSES
QPSRRLGGITSKYHNRVDNYLHQTSSIVINYLKSNGIKTLVVGKNDEWEKETRMGISKRH
NFMPIPYSRLISMLEHKCRLAQIELITVNEAYTSKCSAWDFEPIAKHQKYLGERVHRGLF
RSANGQVINADLNSSLNIIRMYSSEALTAERIGSCGVQPLKVNPLARVKQI
>EFA73242.1
MFPNGTTQTISANSRTITRTETITRGSDTGQILTDAAIGAGAATLIALVTGNKKVEVLEP
IGGAAAGALASVLLRKNTADVFVLRPEQDLAITLTSNLVLSR
>EFA73243.1
MSGLITWKSTAAALMTIAINTVAVIPWLYPNPAQAQFNFNQPRTITIPANVTLPVTYEKE
KIILQPGERIPLTLRIANDIMDSNRNILIPVNSEVTGELTPVNLGVIIQEG
>EFA73245.1
MTAKIISIQVGNLAGLFSWVAGTDHYYKKIKIYKFCVPEQK
>EFA73246.1
MNPTENIIHLPRKHLAQQILTQHSIIPIKNDVLWRIERGVVRTLTWDEEGTAITLGYWGP
GDITGHVLSKVTPYQIQCLTSVEATIIPPDLWHEDLKALLSHIQQTEQILHILHCKPTSL
RLWHFLLWLGNKFGRDLERGKLIDLNLTHQDISEVLNTTRVTITRLLQKFETQGKISRHK
RSIILRLTQDLQQD
>EFA73247.1
MVTTLKPVSTLPTQDEILRQLRSGFPRRPQGFPHSVG
>EFA73250.1
MNLQRSQVTWVQALRSLIQLSHFDFDGYVEARKALTTEEWIDLLVQTIGFNPEMFGRRSK
LLQLIRLIPFCERNYNLIELGPKGTGKSHIFSEFSPHGILISGGEVTVAKLFVNNSTGKI
GLVGYWDVVAFDEFAGKQKTVNKSLVDIMKNYMANKSFSRGVETLGAEASMVFVGNTKHT
VPYMLKHSDLFEELPEKFHDSAFLDRIHFYIPGWEVDIIRGEMFSDNYGFVVDYLAEILR
AMRNHDYSDRYKQYFSLSSDISTRDRDGIHKTFSGLMKILFPHGGATEREVEELLQMAME
GRKRVKDQLLRIDSTYAKVNFAYENKEGISKTVVTLEEEEYPKYYHQTLTEEREGEEVVK
LEGASAPESKSGVHPGEAHLTFQENQRGVSFDSLFGPYLKGAKKITITDPYIRVFYQARN
LMDLIETVIKLKPQEDEVKINLITTLDDFKGEQQVEYLGRIQESCATVGIDFTWEFDGTC
TIHGRHIVTDTGWKILLDRGLDIFQHYDIKDAFSIANRLQEFRGCKAFEVRFLRRETVEG
N
>EFA73258.1
MQAKDIKEQLKSLSQTAMTIDPQLAKRLDEVNRWVKCIRPGSLTAKPYVLAFLLEVITDS
RIWLVIQSTSDPVAKKTKFEQMTANERYWYGYLFPKWINSSDTKFYIWKKKIMAGEFNQL
DNDIIRLMAQEIERREGQVWQRYIADLSMATDLIVSNHQHKPLCIQVTSVSKELHAKK
>EFA73267.1
MGLWLENGNSQGTIGIDLQPQPHFVYCPQEAVEKLNERVEKRLQELLGIIDNNNPETEVL
MIGIGQGEIKLIQFAPELSPRTCFERVNQDVDTLLELLEQKMIEQIAV
>EFA73275.1
MALVPMRLLLDHAAENGYGIPAFNVNNLEQIQSIMKAAAETDSPVILQASRGARNYAGEN
FLRHLILAAVETYPQIPIVMHQDHGNAPSTCYSAIKNNFTSVMMDGSLEADAKTPASFEY
NVAVTREVVNVAHSLGVSVEGELGCLGSLETGAGEAEDGHGFEGTLDHSQLLTDPDQAVD

FVEATQVDALAVAIGTSHGAYKFTRKPTGEILAISRIEEIHRRLPNTHLVMHGSSSVPED
LIALINEFGGAIPETYGVPVEEIQKGIKSGVRKVNIDTDNRLAITAAVREALAKNPKEFD
PRHFLKPSIKYMQKVCSDRYEQFGTAGNASKIKQVSLEDFAAKYAKGELAVKAAAAV
>EFA73296.1
METFFIIPAHSYGLGVALEKLSMPANVTAICLGKSTYARVGLVANVTPAEASWNGSLTLE
FSNSSSADCRIYANEGVVQLLFLEGEPCQTTYADRGGKYQDQPERVTLARV
>EFA73316.1
MKSLYQYQGDSQDQIENGYDDPNFEHLHPVEVNEFLPQIHRLTTIGAGIMLTIFLVRDSF
SNCTDL
>EFA73317.1
MGEIKLVESAISGEVQRILVQNHQVVRQGDSMAFVDDSQVNTQKKQLEDSIEKSQLQLLG
IESQLNEIRNQILDQANLNEQTVFAAQSEFMGTQRNFDDQRIKGNAELLQAEIAFHFAKS
QLQRLEKQGILKSTIQEAETALKLAILQRDRLQAIARSGAIPANLLEEKQQAIKSARAKL
ESAKNNAQTLWDDKQQAFKIAQTNLYKARTAMNPSNSAVIIASQRIKQERARGEMTIAAL
NKELGNLLQQRLEIQKQLTRYRQDLLQTEINLQKTVIRAPITGTLLQLKLRNIGQVVQSG
QAIAQIAPLNVPLEIKAYIPSQEINKVQVGRKFQMRVSACPYPDYGTLRGRVKNISPDVL
SSNVITNNITDNITNTTGINGYEVTMEPETLYLGKGENKCYLQPGMEGGTDIISREENVM
QFILRKARLISN
>EFA73318.1
MFNPFQSRKKYECVLQASEEDCGAACLVSLCKYYGRFLTINKSREAVGTGQLGTTLLGLK
RGSDSLGFNSRAVKAAPEILDRITEIPLPAIIHWRGHHWVILYGTRGKKYVIADPAVGVR
YIERQELLAAWNGVTLLLEPDPEQFSTQSQEQPHSGLLRFFLRILPYRGLLAQVIIINII
LGVLALGTPILIQLLTDDVLVRGDIQLLSVVVYAVVVMTIFSSSLQLIQSTMIAHFGQRL
QLGLVLDFGRKLLQLPLSYYESRRSGEITSRLSDINEINQLVSQLVILLPSQFFVALISL
GLMVFYSWQLTIAVIIFAAVMTLVALPFLPILQQKTRSLLVLGAENQGVLVETFKGAQVV
KTTNAAPQFWDEFQSRFGRFANLNFSTIQLDIINGTIAKLLSSLGGVILLGLGSILVINH
QLSIGQMLAFNTLQINVLALIVFVVGLVDEYFRSQTAISRLLEVIDATPEVVGGGQKPIA
QIAGDADYFFFSLDISPSRKGRPIR
>EFA73319.1
MTFHHLGRVDLLDDFSLKLPGGKVIAIIGKSGCGKSTLAKLLGGLYQPSSGNIRIGVFNI
HDLALDCLRQQVVYVPQDPHFWSRSILENFRLGIPNLSFDQIVQACEIADADGFISQLPN
KYQTVLGEFGANLSGGQRQRLAIARGILNNPPVLILDEATAGLDPVSETHVLDRLLAFRK
GKTTILITHRPSVMHRADWIVLLEQGKIQLQGSLETFIAQPGEHLHFLTI
>EFA73332.1
MDFIAPWERVIDLKFARRPGEAPLYHLYVEIMGKYSNVILTDAKNEIITAAHQVSQQQSS
VRPIQTGQPYEIPPKLTGTIPTLTETEARWQERVSLVPGSIKKQILKSYSGVSSALLDSL
LLVAHISPETSTTSLTNQDWYRLFARWQEWLRRLEEGDFKPAWTKQGYTVMGWGEIQSVD
NIQELINQYYTQQLNQQVFYHLRHQLTQKLQNILGKLNTKAQAFKDRLEQSEDADKYKQQ
ADLLMAHLHQLQPGMTEIILPDFETDQPQRIVLEADKNAVQNAQRFYKQHQKLKRARNAV
YPLLLEVNREIEYLEQVEAATNQIDNYQTKQDLEAIEEIRDELIEQKYLEELEYRRPSQS
ETVSHNFHHYCTPSGLEILIGRNNHQNDYLTFRVAGDYDLWFHAQEIPGSHVLLRLPPGT
VPEEADLQYTANIAAYFSRARHSEQVPVVYTRPNRVYKPKGAKPGLVIYKQEKIIWGNPG
KISQVINHLQQGV
>EFA73336.1
MLVSLNACDWQVRVATSSDLSGIAQIITESFYSQNGLWGWAFPLFRIGIYEDLKYRLQTL
MPHQTCLVAVHTSQSGNCKVLGTVELGVRSIHSWTSYSRLLPYVSNLAVAPRYRRYGLAS
SLLISCEQVCKTWGFQDIYLHVLEKNDQARKLYLKLGYEIYRVESSWQDFFFPSRQFFLH
KRLR
>EFA73337.1
MSQLKYCQQLFVSSQWLFGNITNSDNNTQDDSCIVVVDCRFSLADPLWGRNQYQMGHIPG
AYYLDLNQDLSGVVGQHGGRHPLPDVSILADKLAQMGINHGQSLVVAYDDSRLAFASRLW
WLLRYLGHENVAVLDGGFSGWQSLGYPVSNIPSTIPGKREILLPTYKQQKLWILIMLNIM

>EFA73338.1
MDFNYVKHYVEQINQSTLTENKPVLIDAREGDRYRGEREPIDAIAGHIPGAINYPWQGVT
NAQGYLLSDQGERWQAIANSPEIIVYCGSGVTACVNLLSLEIAGISTAKLYPGSWSDWIS
YQTNNMFSFIRPDLSEFTSYKPHPSSDTLEPVNTELDRLDTNESPYDLPQEIKKKLAETF
QHTIESNRYPDGGHEALKAEIAQYVSESAGLSSDSFSAVNISVGNGSDELIRSLLISTCL
GGAGSILVANPTFSMYAILAQTLGIPVVSVGRNHGHFEMELDAAQTALKEANPPVRAVFV
VHPNSPTANCLTVSELNWLQGLPLDILVVIDEAYFEFSQNTLVERLIKHPNWVILRTFSK
AFRLAAMRVGYCVGHPQVISILEKVRLPYNLPSFSLVGALVALQNRQVLLSSIAETLTER
DKLIQDLSTHPILEVVPSNTNFIYVRLKHNHSHDGVLKNISQKLRSEGTLIRLLPGGLRI
TIGTPEENARTLKRLGQI
>EFA73339.1
MDFLPQVTKDGSLTFFSEEFQESFHSLYGAKQESFLKFVIPTQVPLFAQTGILKF
>EFA73340.1
MKEIAFKHCYQDDNLSVRLIINDARKSIQEILSSGFLADVIFLDPFSPPHCPQLWTVEFI
EQIALCLQPGGLLATYSCAAAVRTALISAGLIIGSTTPVGRRTPGTIAGYAPHQHKYITE
TYPLGAAEIEHLKTRAAIPYRDPQLQDEAPNIMKRREQEVLTSTLESTSQWRKRWLTNNT
PTQ
>EFA73350.1
MAAMGSPDDRQIDGIGGATTLTSKVVVLSLSNHHWADIDFRD
>EFA73360.1
MPKPYSVDLRNRVIVAWAAQEGSQRQLADRFKVSLSFVRNLVRRYRETGQVEPKQCGGYE
KPIIEGQYLNMIKAWIDEKNDLLLSELCDRLS
>EFA73365.1
MHYFDEYCEVHLDPDIAPYNWVFRNQEKISITFGVHVSKHPDFGQVVYELNDGKGYCDID
RITTQTEGIIENGFKMFSVILPLLVMVGDISIE
>EFA73369.1
MMLLIAEWQLSLKRITFIYECLIELPVEIRRGDVAQEVIRFARENNTGLVITTKSPSPKF
AQICDLIRPHLDLEVLAVEPFFDYDGFLDLKRFSRYWKVAEKYVFRPNSDQDG
>EFA73372.1
MHPNQKLLFSTALFSFWLSLDIVSCAYAQTQTSTPGNAPPQLKKLIAEIDAASSRGDIKG
VMGFYSSKFTHGDGLTRQAMEQSLKSFWQRYPKLSYQTKLQSWKSNGKTIIAETVTRITG
LSSDKTSPLAINAAITSRQHIRGTQITHQEILSERTQMTSGKKPPQVEFRLPQQVKVGEK
YAFDAIVKEPLGNNFLLGTAMEETIKPNKYLNPTPINLELLSTGGYLKLALLPYNQ
>EFA73389.1
MGRLKWKSVSQAVYHIESVGMKVRLIVLSKIPKSANNELWRLFSVRSEVVEEAISHYQES
YRNSEYAV
>EFA73391.1
MDPLNPFLKGEINALCLVYEKLGLLDPPKPLKKGANCFVFSL
>EFA73402.1
MVRLGLFQMGLGIMSLLTLGVLNRVMIDELTILPFIAAGAIAMHQFVSPARIWFGQKSDS
QRIFGYHRSGYVWIGAALFTSISFVALQVVWQLGTSLQNTGWGLQTYSWSALLALTFGMY
GLALSASSTPFTALLVDITDEDDRPKLIAVVWSMLMMGIVIGASISSRLLERPEICGTAL
LAYDPSQMNKLVDISKLQTTINPVFIILPGAVFVLTLLATLGIEKKYSRYGIRGNMVERE
DQITLGKALKILTANRQTGIFFGFLMVLTLSIFMQDSILEPYGGEVFGMCISQTTRLNIP
FGIGTLLGIGSTGFLLIPRLGKKQTTKTGCIGAAMSFCLMIMAGVSQNSGLLMGSLFFFG
LASGVITTGATNLMLDLTATETAGTFVGAWGLSQAMARGMATVLGGTVLNIGKLVFTSPT
LAYGMVFALQAMGMLLAIWLLRRVNVVEFQQNSKQVLASVLESDLD
>EFA73403.1
MQDKALANVFRQMATGAFPPVVETFERNKTIFFPGDPAERVYFLLRGAVKLSRVYEAGEE
ITVALLRENSVFGVLSLLTGNKSDRFYHAVAFTSVELLSSPIEQVEQSLKENPELSMLML
RGLSSRILQTEMMIETLAHRDMGSRLVSFLLILCRDFGVPCADGVTVDLKLSHQAIAEAI
GSTRVTVTRLLGDLREKKLFLSTRKKSLCINQLH

>EFA73411.1
MSDLFKGFEQFLELVKTLEEKIESGEVKTDIQVNSRPLSSIPRTGNIPRSAAKNSNINDI
GTSRIRTPSSPAAPQPSNGSSDSATPPENSGISLKDIGGLSQVVKELKELIAIPLKRPDL
LAKLGLEPTHGVLLVGPPGTGKTLTARALAEELGVNYIALVGPEVISKYYGEAEQKLRGI
FEKAAKNAPCIIFIDEIDSLAPDRSAVEGEVEKRLVAQLLGLMDGFSHTPGVIVLAATNR
PDHLDPALRRPGRFDREIQFRIPDANGRKEILQVLTRAMPLDETVDLEFISDRTVGFVGA
DLKAVCQKAAYMALRRQVPSMETEIPETMTVNQSDFLQALKEIKPAVLRSMEVEVPHVEW
EDIGGLEAIKQTLRESVEGALLYPELYRQTKAVAPRGILLWGPPGTGKTLLAKAVASQAR
ANFIGVNGPELLTRWVGASEQAVRELFAKARQADPCVIFIDEIDTLAPARGTYTGDSGVS
NRVVGQLLTELDGLETGTNILVIGATNRPDALDPALLRAGRLDLQLKVDLPNLASRLEIL
RVYTEGRPLLDVDLEYWAQTTEGWNGADLVLLCNQASVEAIRRFRAKGETDPAAIRITID
DFQHSYRILSQQRNS
>EFA73412.1
MIDFKFQLSESRRLLQDSQFTGFLIVGIAEPMCLAETERFLEQLNTLEVPFGGILINRIL
TDPIIDQDRYAEQQNYVQKFLNLSPGQPVFIIPNNPPPPLGSQALDKLAGEIQHISHVEG
VAPPIIHWPQRIPPSFTDFLAQGCKLIIVGGKGGVGKTTVSAAMSWAFASHYPQKNISVI
SIDPAHSLGDAFGQKLGHDSQAITPNLCGQEIDADKILDQFRTDYLWELADMISGEGTTK
DTDVNIAYLPEAWRQIMSQALPGIDEMLSLITINNLLETNQQDLIILDTAPTGHLLQFLS
MPSALGDWLSWIFKLWMKYQDVLGRVDFIGRLRQLRQQVVKAQKKLKDPRHTQFVGVIQA
ESAITSEHVRLTASLKNMGIEQRYVVQNRYTQAVEVDHNLFPEQTIIHLPLLPRSVEPME
RIKGAANLLFAFEK
>EFA73413.1
MLSGKGGVGKTTLSCCLARYWARKFPEEKILLLSTDPAHSLGDVLLTKVTNEPESAVDLP
NLSIQALDAQNLLLEFKAKYSQYLEILVERGSLADGEDLAPVWDLDWPGLNELMGLLEIQ
RLLSEKNVDRIVVDMAPSGHTVSLLKLKDFLDVILNSLELFQKK
>EFA73421.1
MVALLQKIRLENQSIFQEVNDFLSTRKSNRLAQAQKQAEDLSQFRQQLEQEIDQFLAKTA
QARQAQAQAQAQELNQFRSQLEQETSKFLTKTTQARYAQAQEQAAELNAFHKQLEQKTEV
FLADTAKARIAQAQKQAAELSAFHKELEQKTSAFLADTAKARIDEAQKQAAELSAFHKRT
RAKTSAFLTATTSDRAAKAAAQKEALRQFRQDLFLSVIGV
>EFA73427.1
MPEGTILVFPLAIREDKSDQSKNQKFQPFASGDKADIYLGGLESIGFGHCELTLKGQVNI
N
>EFA73429.1
MLTDKPVPYRFRSQGTLGRGRYAVPAGSVYILKEPINQSWWEWNEKWFPEEGYSLKKVGC
GLCLPITIQGVN
>EFA73430.1
MFKYLISISPLGFMYGSAGAFLSPENLVGRSGAKFPPDASTLAGLMFSINKVKNKLSQSE
LRDNLYVAGPFWAKSQDKNNFFVPIPKHKIITQKEVREWSVEKEEWYLEKKQRKTRI
>EFA73444.1
MGVSPDSSKSHCKFITKHNLTITLLTDPEHQLIETYGAWRMKKFMGKEYMGVARSTFLIA
PDKIIAYSWPNAKSKGHAQAVLKKLQELKN
>EFA73445.1
MSDIPQLGKPAPDFAKLDQDGNLVSLYQINQWVVLYFYPKDDTPGCTTEAKEFTELAPQF
Y
>EFA73448.1
MGEDDKQEDSIRPRRFADYIGQQDLKDVLDIAIKAAKSRGDVMDHLLLYGPAGLGKTTMA
MILASEMGVNYKITSAPALERPRDIVGLLVNLKPGDVLFIDEIHRLSRMTEEILYPAMED
YRLDVTIGKGSGAKVRTIPLSKFTLVGATTRVGALTSPLRDRFGLVQKLRFYQVEELSQI
VLRSAEVLQTIVNLEGAREIAKRSRGTPRIANRLLKRVRDYAIVKSRPQIDQPTAAEALT
LFQVDPCGLDWTDRKMLTVIIENFHGGPVGLETLAAATGEDTQTIEEVYEPYLMQIGYLS
RTPRGRVVTSAAYQHLGFQLNSEQLSLL

>EFA73453.1
MTNNQSTIPVIVNGAGGKMGREVIKAISQATDMTLMGAIDTSPEHQGKDAGELAGLNIAL
EVPITNQLDPILGYVAGERQLQPAVMVDFTHPDAVYDNVRSAIAYGIRPVVGTTGLSPQQ
LENLADFAEKASTGCLIIPNFSIGIVLLQQAAITASQYFDHVEIIELHHNQKADAPSGTA
IQTAQLLADMGKPSTHPQVTETEKIAGARGCLADEGIRIHSVRLPGLIAHQEVIFGAAGQ
IYTLRHDTSDRSCYMPGVLLAIRKINQLKSLVYGLEKIL
>EFA73464.1
MMKNLPIVSRPLNFNQRYPCPSCRVGKIETMPLMEAMFCDFCQEIFTVNLQSQQVKMPSR
QPPLIWHWNGDNWDQGQIEGMELGWGYAVAGIAFVMLPTFLIGISAYYLPSSPMLFWIPC
IWTLLTFILHLAIVIWLFMEFIRSL
>EFA73472.1
MDTSKETSTKWFSTRLLPPQAIRQALQPCSQEKVEEVKEEYPQFKTWVEKTLPSLPEKKI
PFDVNHLKADQNTIRVLEEIGVIYEDKDKDEATRFYIPEIFRAGLGFSGTTGRPRTLALK
QKIFGKGN
>EFA73476.1
MAITLVSTIVSVVGYFAARIVPGIGIQMNPLLAPLTAVISAMIIAPHAGAPVAFAGGILG
TVIGADLLHLKDIQFMSEGVLSIGGAGVFDGIALCGLFALLLS
>EFA73477.1
MIYLPVSILLFLVLLLLLPFIWFALAVDIVEIAVAKLGFSPQIAFFLFLLIIITSTINIP
LYRLENTIEVVDEFATLWLREFWGIPLRRLERSTVIALNVGGGLIPVLLALYQIPEETCW
QSPW
>EFA73479.1
MLLGMIFLGISTTGWTSYAALPSGNAITDGRALLRYALPIENQPVRKLQASLEDISNQLR
ANKRWGAISRDLSQASRILDRPSQILTSIPPERQPQAESWIGELKSGIGELQEVVKTRQK
TPILEGRAKLLNLVSLLEESMVQGFPFEVPAEFSNLPQLKGRATIAIKTNKGDLTVVVDG
YSAPVTAGNFVDLVQRGFYNGLKFTRSEESYVLQTGDPEGKEVGFIDPVTGKYRAIPLEI
LAEGDKQPTYGITLEDAGRYLDMPVLPFSSFGALAMARPEGDPDGGSSQIFFFLFEPELT
PAGRNLLDGRYAVFGYLVEGKEILDKLKAGDIIESAQVIQGIENLVEPPA
>EFA73489.1
MTVTATLSQLAAVLSASLLNIPSSSLSQVIVGIQTDTRVLQPGEIFLALGGEKFDGHQFV
EMAIARGAVAAIVDYTYDYPNLPVIKVSNTLNAYQELAKWWRGQFGIPVIGVTGSVGKTT
TKELIASVLGTRGKVHKTYGNFNNEIGVPKTLLELSEGDDFAVIEMAMRGRGQIGELTHI
AKPNIGVITNVGTAHIELLGSEQAIAEAKCELLAEMPNDSVAVLNYDNPLLMTTAKQVWS
GEVISYGFSGGDIQGKLVDYQKIEVGGMTLPLPLTGVHNGSNFLAALAVAKVLDVDWKLL
EAGINVTMPQGRSQSWKLPKDIMILDETYNAAPEAMLAALELLAQTPGRRKIAVLGAMKE
LGERSAKLHQQVGETVRKLNLDGLLVLVDGPDAEIIATSAQGIPCERCHNHSDLVSLLKP
TYNQAIAYYLKLLIPLD
>EFA73508.1
MVIFGGDAFPDATPPPYVQEAFASQFRRLVDAHIPTVLLVGNHDLHSQGIGGASLNIYRT
LGVPGFVVGDSLTTHHIQTPNGKVQVITLPWLTRSMLMTRKETTGMSMAEVNQLLTDKLQ
VVLEGEVRNLDPNLPTILLAHLMADNAMLGAERLLAVGKGFTLPASLLIRPCFDYVALGH
VHCHQNLNKSNDPPVVYPGSIERVDFSEEKEQKGYVMVELEKGRANWQFCPLPARVFRTI
EVDLSPSDNPQQTLLQAIAKHDLTGTVVRLVYKLRSEQLDVIENAVIHKALGDAHHYTIH
PELVSQLARPRIPELNASSSIDPIEALKTYLNNREDLKDIATYMLEAAQELLGDEETKIL
VDR
>EFA73513.1
MKKSVSTVKIDTLDLTITAPQTKKSDSVWVVFATTFITIFWRK
>EFA73514.1
MSAQSHAPWLVFLGAGAALVTTSLLGVLLGGFIASRLSPKTVEKSAGLVLLLVSSMLFWD
VIHG
>EFA73527.1
MTAENTALSFECETGNYHTFCPISCVAWLYQKIEDSFFLVIGTKTCGYFLQNAMGVMIFA

EPRYAMAELEEGDISAQLNDFNELKRLCEQIKRDRNPSVIVWIGTCTTEIIKMDLEGLAP
KLEAEIGIPIVVARANGLDYAFTQGEDTVLAAMANRCPAQTPVTETEKNERNAIQKLLHF
GKKKEEIIEEESEYVNHPPLVLFGSLPDPVVTQLTLELKKQGIKVSGWLPAKRFTELPVI
EEGYYVAGVNPFLSRTATTLMRRRKCKLISAPFPIGPDGTRAWVEKICSVFGITPQGLEA
REAQIWENIEDYVKLIRGKSVFFMGDNLLEISLARFLVHCGMTVPEIGIPYMDKRYQAAE
LSLLEKTCQEMGVSLPKIIEKPDNYNQVQRIYELKPDLVITGMSHANPLEARGINTKWSV
EFTFAQIHGFGNARDILELVTRPLRRNNNLKDLGWDKLVREATTA
>EFA73533.1
MSRIGAIALFAPLLVINGWALSSIFNYFHSLIVILVGASVLAFLLNYPITWMQKQGARRE
QVAVLVFLIALSILLALGVTLFPLALTQARQLVNRLPEWIDSGRSQLMILNEKAEMMGLP
INLDAIVVQINDRVKSQLQAITAQVLNLAVVTVTSLLDFLLTMVLTFYLLQHGDELWQSL
VEWLPNKFREPFSRTVRLSFQNFFITQLILSTCMASALIPSFLWLKVPYGLLFGLTIGIM
ALIPFGGSVGIAITTLLVSLQDISMGVRVLAAAVIVQQILENILAPRILGNFTGLNPVWV
LISVLTGARVGGLLGVIVAVPCAVVIKTVIGVIRSPMVIDQTNHQLDETGEQDLAPAENN
LSINTLVNHPHIQPKN
>EFA73546.1
MKEFNTAGICLPKKHYMCDITAKFNRCRHLIESGKYFAINFPRQYGKTTMQHLLEKSFQG
VEEYLVISTSFEGIGDLPFQSEEQMASVVPAVLAKGLFFNNPELADWLEVKSKQINSFKQ
LSRFVSDWVRESKLKAGSG
>EFA73547.1
MDVDIDFTFSQEEIEPMLADYASEHGFQMDCPVLARLLFYYTSGNPFLVSKMCQVIDEMH
QNQRPWSVSDIEDAYKHLVDVSYSNTNFNDVYKNIENNPDFSQLVRAIAIDGEDLVFDRG
NPLIDLGATYGIIKSNSVGRCDIANKIYEFRIISYFISKRETAHQHLDRYRDSGFISNGK
LQISLILERFQWFMREHHSSRDNDFLEKNGRMLLMSFFRRIINGNGYMFKENVVAENRRM
DLVVTYKNQRYVIELKIWYGEKRLQDGIEQLCSYLDSYGLNEGHILIFNFNSNKTYDIRE
INHQGKHLTAFFV
>EFA73607.1
MVVRSTAAPPTPWSKSLAEPDVHQTAYVHPSSNLIGDVHLGQNVIIAPGTSIRADEGTPF
YIGENTNIQDGVVIHGLEQGRVVGDDGEKYSVWIGKNASMTHMALIHGPAYVGDSCFIGF
RSTVFNARVGAGCIVMMHALIQDVEIPPGKYIASGSIITTQQQADRLPDVQAQDQQFAHH
VVGINQALRAGYRCAEDLKCITPIRDELKDHEEKLNTSITVEELDRSSEVAGKLSADTVE
QLRYLLEQGYKIGTEHVDQRRFRTGSWQSCQPIETRSLGQAISALETCLVDHAGEYVRLF
GIDSGKKRVLETIIQRPDGVVVASSSFKGPSPSSYNGNGNGNGKFAGIDAQIINQINQLL
SNGYKIGTEHVDERRFRTGTWQSCEPIHSTSTQEVVAALESCLNSHQGEYVRLIGIDTKA
KRRVLESIVQRPNGQVVTSGNGKVSLSPAGGTPSVSNHLSGEVVDHLRQLVSGGLKISLE
HVDQRRFRMGTWSSAGPIEARNEREAIATVESYLSEYPGEYVRLIGIDPQVKRRVLEVII
QRP
>EFA73608.1
MGSILQVSHGILEIEMGANLGAGFLMVGEGKIGANACIGAGTTVFNDSVAAQQVIPAGSI
LGDGSRQANSQESGESVDEGDTSSQSGEQVVSKTQFTATFVNFQTQSTSVPPLSPTPKSQ
SPPETESSTESQEISDGKPRSRDPTEPYPLGTQIYGQGSINRLLSTLFPHRQSLSDQDAN
NGAE
>EFA73612.1
MLDSILPRTSVIVMTSTLATLIVACSGHKQTNQGINRATDVGNLAAVQPPKRLPHSQEVS
VLELALDKGVSAVRISQWAQSAEDWQLVESNFNRAIALLKQIRPNSPNFAFARKKIIEYE
GQLQLARQQGNPSKGSSLSPDSPVVTRSNPGRKILYSPPIPVRETPTITDFQSPSPDIAT
KKIPTEKTNDQVYIVPIKRRIGGTPIVEVTFNGKSRFEMIVDTGASGSVITQEMARSLGV
VPVGVARANTVSSQGIEFPIAYVDSMEVGGVMAKRVPVAIAGLALTTGLLGHDFFGNYDI
TIKRNVVEFRPQNTASTSVKIPRAVPTFPKDYQLPEFP
>EFA73618.1
MSQVFKNWIVRISSILLSTLLWSALILPTYAQTQIDLKLEQQVLEIIRRNPITIIEAVQA
YQEEQQQRVITRRQEFIKNFSQNPQDVIANSPTIGSSKLQTVLLEFSDFECPYCSEAHKT

LKNLLNKYPNRFTLVYKHFPLFQIHSQALPAARAAWAAHQQGKFWQYHDTLFTKQNQLGE
SLYIETAKSLKLDLGKFNQDRQLADKAIQKDLDLVNNLNLSGTPLLLSLVQILPDLSSYQ
SWKHFWQLLEIK
>EFA73630.1
MEPTPPTPPTETLSTQETVEGKLEPTPPTPPTESLSTPETVEGKSEPTPPTPPTESLSTP
ETLT
>EFA73643.1
MNNYLYQYNQLYCGKLLKRYKRFFVDVELESGEIVTAHCANTGPMTGVSQIGSAVQLSKT
DNPQRKLAYTLELIQVDNSGPTWVGVNTNLPNNIIKLALEKQLFPQLGEYKQIKGEVVYG
KEKRSRVDFYLTGNEMPIYLEIKNTTWSQGSLALFPDTETTRGQKHLRELMEVLPKNRAV
MLYFINRGDCVKFAPGDSADPLYGQLLRQAIKVGLEVLPCRFDVSPQGVSYLGLAELKI
>EFA73656.1
MRLGLKVQILAGLITFLSVLSVNQYQFIHAQLTNKPLHTQQRVEQHQSQESSVLYSVIDE
WQKYKYSVNGKSILEPMKLPNTKVSFNANDLLFVLTNTRKYFQDYSQKDQNISRNGLLVN
QGVKVEDVLKTLDFMISTLREDVGKNRPPRLQDPNFINANFRVIKWKAYNPDKPEQKQLR
ITKYAVFTHPGSHTKTNTYNIPIYSLKDNSATDKFYTKYTKQDVLSGIYEPGGKEFGKVE
TLAYLTRQGLEEALMQGTILINFTDGSKGFFNVDRNNGISYIRGVKDTLQKRYWYFRPVD
QIKGYGYKIDAKISIRPGVTFAGDVLNIGLGRVVLIEYNQGKNKRLQMGVVADTGGAFLP
NLHQLDYLAGVFKNQTEFRQYIRQLPEYASAYILVKK
>EFA73671.1
MKIFPRLLYIDGLGEPGSDGETYQKMMDANTRSIVEGLGVDI
>EFA73672.1
MNRLLKCNFCNICLPIGLPIILAILGCNHRTPNNNYTQDQPGQQNQKISQNLPQVVVTTT
ILCDITNQIARESINLICLVPPGKEPPRYQPTLGDIKAIKQADLILYHGYNFEPRLIKSF
KNSRKNIAKISVGQRAVKQPQKLQQNGKIINEPHIWHDVRNAIKMVEVVNFQLGKLSAEN
RQKYNSNTRQLTEELKKLNQWIKSTLSTIPDKNRKLLTTHRAMIYYVKAYGLDYKGTLPD
ISNEDKLTSKKAKSLAEYIKTTQAPTIFADRAVNLMLLAPIAKKNKSENFSSTTLY
>EFA73680.1
MTIQQTELLYLPPDLKPELLPKHVAVIMDGNGRWAKGQGLPRIMGHKRGVDALKNLLRCC
KDWGIQALTAYAFSTENWNRPHEEVEFLMTLFQGVLRQELREMVEENVEIQFVGNLQALP
VALQREISHSMAETRGNQSIKFTVATNYGGRQEIVQACQAIAQKVKEGLLNPEEISEELF
AGHLYTAGIADPDLLIRTSGEMRLSNFLLWQMAYAEIYITDTLWPDFGRREFHLALLAYQ
QRERRFGVV
>EFA73687.1
MQNYILALAKYRQTQLAEDRLQQGDRLGAATMLQAAAKTALQMGDANAGTVLQNSATRLQ
AGEDLSASDRSKTKIVSKTVLQDPTLT
>EFA73688.1
MKVNLQSGVNNTNVDASKSSSQRLIAISVSAIGETIDSRVPLNLCLILDHSGSMKGQPVE
NVKRAAWLLVDKLRDQDRLSIVVFNHRAEVLLSNQNVVDRDHIKQQINRLSANGGTSIDE
GLRLGIEELAKGRRDTISQAFLLTDGENEHGDNNRCLKFAQLAADYNLTVNTLGFGNNWN
QHILEKISDAGLGSLSHIEHPDQAVDKFNSLLMRMQTVGLTNAYLLFSLAPNIRLAEFKP
VAQVSPDTIELPVQVEPDGRFGVKLGDLMKDVERVILTNIYLEQLPEGQQAIANVQVRYN
DPSVDKTGLYSQIYQSMSMWSALTKKILVQKCKITF
>EFA73694.1
MSIYVGNLSYDVTLEDLKSAFSKYGNVSKVQLPTDRETGRPRGFGFVEMSNEAEENAAID
ALNGKEFKGRQLKVNKARPREERESSRGGWGGGRSGSAGHDHRY
>EFA73699.1
MKQSFFNRQLTQFVGILLGMGVALFILRGLRIITFLPSGIIGLLFLGAIAIGVLSYLQTK
WWRF
>EFA73700.1
MLLSQDKHKSLIEGEILVQTRPHNAWGGAVTASIYLPLVRSYVWEQITEYPRWVNYFPDL
TKSELISPGNSSQGNVKFLYQRAQKAFLFFTAQVEIYLTVIEVLGKQIQFRMERGTFEDF

YANLQFQDMGDGTLLIYTVEATPNIPIPSMLIEQGMSRGLPDNLRKMRQFLCSRA
>EFA73707.1
MILGGGIGSEAKAFGKAKGYADRATITQNRSH
>EFA73736.1
MSYSSQELSEFLHKIPGFERLSATEINHLLSKQQPLRYRLGQKIVGKEKLPERIVIVYQG
KVRLLAYHLQSQLPITLQMLEPGAIIGEISYLRQIACETAIASTEVVCLTWNPVDYLSVF
SQNSDFAKERQEKNHIIEVFDVISHHVAQKAYGNLNLKDITRDILPESKIQYLPPGKTPL
DQLGSDRTWFVSSGQITNFPVNSQIVSSNNREVLQVKGKIPARLLGISCEALLLLEQQEN
REPEKIEDPRKKTDVDVIDIPFAITTEFSPQENRKAKSSKNRQKYPFFAGWGELNSTLAC
FQMLAKHLQIPLRKEVVRRILSDNLKRQGNVSFQLCAYVGKLLG
>EFA73737.1
MNPVLQFRNFSGRESYHTLTAPEVLKLLNKYQLIPMFLQEVIMEQAIAQISCTPEEERLA
YEQLTQEYQGQKEQGISQEQLQNMATRQLKLERFKEVTWAKDIDSYFYQRKPQLDRVIYS
LITTNDRGVAQEIYFRVQEGEQSFAQLAQEYSQGPEAKTSGLVGPVELQSLHPLLVRILS
TSQPQQLSLPTAIEDWIVIVRLEKMLPAQLDSGMRQRLINERFQSWLKAQVSPQNWQIKE
SEN
>EFA73738.1
MVQPEDFLAVVDREWQMLSSDGLPLSLIFCAIDNFDPNQETTKNTSNLRQIALVIRKCLY
PPSCFAYYKQGNFGVLLPNMSLDAAVRLAHDLHETVVSTVEISFSLGITGTFPRSSSTVE
KLLNTANQALTGAQRHGGNTFCLYPG
>EFA73739.1
MKILVVDDDTSLCQLVKTSLVAHRYVVDVANDGEMGLEMGYQFNYDLILLDILMPKLDGL
SLCHTLRNKGYQGQIIMVTAKTTQEDIIIGLDAGADDYLIKPYYIHELLARIRACSRRIG
SQLTLNTLVYNSLSLDPELIEVKYDEASINLSRREFQLLELFLRYPQKIFTRGQIIDKLW
SIDDSPTDGAVTNLVKDLRHKLKKAGMQEELIETVHGLGYRLTSKRELEREVVETIPTQA
SSPITDSSVSEGLVLMEQIKLEFEMSLPKKIADIRAGLVKLETEITNGQQQQYLMRMTHS
LLGSLGTFGYPQCSQVAAKMENILVKESLTNVDIQAIDSLIHALEWEVNPKKRKNRSQFQ
HFF
>EFA73755.1
MQGTLAGISRLGKPTPYVFYFGKESGDTVIYFFETQSPVGKKITRVCKQNTLCRGKAQLQ
VVKSIPKSIPESTSGTYKIISVSQVNGKR
>EFA73762.1
MTEQPQKQKSSRSFAGIAGIVAAATLISKIFGLIRQQAIAAAFGVGAAATAYSYAYIIPG
FLLILLGGVNGPLHSALVSVLAKRKQEEAAPIVETVTTLVSGLLLIVTVAQIFLAEPLID
LVGYGLDVKTREIAVRQLQIMSPMALFSGLIGLGFGTLNVANQYWLLSISPLLSSITVVI
GIGILALEYGKQIIQPEFAFIGGMVLAWGTLIGAILQWSVQLIFQWRLGLGKLKLRFDFK
SPAVQEVIKIMIPATISSGMMPINVATDLFFASPIKGAAAAFNYANLLVQTPLGIISNII
FTSLLPMFAKLAEPQSWPDLKLRIRQGIILTAVTMLPLGGLMIGLSTPIVQVVYQRGAFN
QQATELVASLLIAYGIGMFAYLGRDVLVRVFYALGDGQTPFKISAFNIFLNVVLDFVLVK
PFGAPGLVLATVGVNCSSMLMLLWLLNRKLNGLPLREWTWPILGFNFWQCGCWC
>EFA73766.1
MAKLFAAIDGNEAVARVAYKLNEVIAIYPITPSSNMGGWRKINLIYGGIIPQVTQMQSEG
GAAAAVHGALQTGSLSTTFTASQGLLLMIPNLFKIAGELTSFVLHVSARSLATHALSIFG
DHSDVMAARTTGFDLLCSASVQESHDFDLIAQVATLQARVPFLHFFDGFRTSHNDKGGET
PTPYLIS
>EFA73767.1
MPVIEKKRTRDLPQINERIRFPKIRVIDNEGAQLGIMAPLEALRLAEEKELDLVLLSDKA
DPPVCRIMDYGKYKFEQEKKAREARKKQHTADVKEVKMRYKIEEHDYNVRVKQAERFLKD
GDKVKATVMFRGREIQHSDLAETLLNRMATDLEPFGEIQQAPKKEGRNMMMLISPKK
>EFA73772.1
MAARGIDRQLPFLICFIQGKLEIVRSIFNHSAYPPV
>EFA73777.1

MQTLEKKKDRANELIFECVGKQKCLFYIRRQGNVRDTEEEILDLTIATDESEFTLPKRLK
ELDKTLGLRAALQKNGNGGLKILSTYLLPIARGKKDAYTVPLNLQLVPNHYEPVHIPPKT
LAQIVKMPICGNHVPTEEQLRAWKAFLKVEERVAKARQFCVNFLNSYYDLKQREIVLKIN
INSATLDGDEENYINEDNFWERVRQAKNQEIKFSDVIPTEKSVAPVVNWEQLKR
>EFA73782.1
MNTRKSPIQIFGVYALLLAIALLTLFPLLWLISTALKSPTENLLETPPKLLPLEPTLDNF
LRVWESLPFGQYLYNSFLVAILTVALNLLFCSLAAYPLARLSFPGRNTIFIAIVSTIMIP
FQIVMIPLYIITVQLGLTNSYLGMIFPSLASAFGIFLLRQAFMGVPKEIEEAARIDGSSE
LGLWWFIMLPAIKPALITLAIFVFIGAWSDFLWPLIVIQDESLYTLPLGVAKLAGTFSLD
WRLVAAGSIISVAPVLLLFLFLQKFIVPTDTGSGIKG
>EFA73792.1
MFEHFTSEAIRVIMLAQEEARRLGHNFVGTEQILLGLIGEGTGVAAKVLTDLGVTLKDAR
REVEKLLVGVLVSSHPKSLSPRKSRVYLSSLLEKLTVLVTTT
>EFA73793.1
MLGLTDAGEGVAAKVLKSLGIELQTVRSRVMSILGEDNRVVAGRQDNPRRNQNLSIEEFG
RNLTKLAQQGRLDPVVGRQTEIERTVQILGRRTKNNPVLIGEPGVGKTAIAEGLAQRIVN
QDVPEILLNKQVISLDMGLLVAGTRFRGDFEERLKKVMEDIRSVGNIILVIDEIHTIVGA
GGTEGGLDAANILKPALARGELQCIGATTLDEYRKYIERDAALERRFQPILVGEPSVAET
IEILRGLRSAYEQHHKVNISDDAVIAAAELSDRYISDRFLPDKAIDLIDEAGSRVRLRHS
RIIDNKELKQQLKNTSQEKAEAVRVQDFGKASKLRQEELDLQTQLAIAQNLPRITIPQVD
EEDIAEIVSSWTGVPVNKLTESESELLLHLEDTLHKRLIGQEQAVTAVSRSLRRARVGLK
SPNRPIASFIFSGPTGVGKTELAKALAAYFFGAEDSMIRLDMSEYMESHNVSKLIGSPPG
YVGYDEGGQLTEAVRRKPYTLLLFDEIEKAHPDVFNMLLQILDDGHLTDAKGRKVDFKNT
LIILTSNIGSKLIEKGGMSLGFEFDNQANASYNRIRNLVNEELKSYFRPEFLNRVDEIIV
FSQLNKDEVKEISQIMLEEVAKRLQEKGIKLEVTEAFKDLVVTEGYDPSYGARPLRRAIM
RLLEDSLAEAILSGEIGEDDQAIVDVDDDGLVKVKKAETRDLVLVSAPA
>EFA73799.1
MTPQLLFNSSTMKNLPSLFTLFISVVLATWILAIALISVQNTASISLRFLVFQSIQVPFG
LMLAFWVVVGLITVSFGQLLGNMGGSGNSADEEGDFFVDEDFR
>EFA73806.1
MKIQIELSGKFARSLLNATEVIPRTLNHLIHEVQAIGTRYHRRTPQLLRQSFGLDFIPEP
VLINLCKNSRGVTKESDIYQVPPLKLLKPLKSQLDNLDIYQYAYNFICEQGNLNLTPRDP
QLPVFSSDVCEQMGSYIITISEPNYPDLNLDFIQIIWNNSWQYWQKLSDIEKIDYALKSH
ELS
>EFA73807.1
MSPAYVYKLREQFRLHPKIAETVYPVICDHWVYTHYPNIDYNVSNLSQIARGVTWKNMSE
RLTVSQSVGEKIYELVENLTQTLSSEIISEIGIITFSETQRDNLREVLTANHPQFSPIFI
GTAKEWSGAERRIIIVNCMTACSGGPNNIALEDINIALTRAKDQLFLVGDHVLWQESRSP
IRSLLYQSSLSIETQVVI
>EFA73809.1
MGDWMLTLEIHQLDSMELNSNKLQFPIWVFWYLPSRDVINQMGYYYLHNLRNQELENNGL
FAGAIVLYLQDHTQNYSLDDSPSYEELFPNLRLVKLPENFTEQGFSQTVEIIFNTLIEVV
GGFTL
>EFA73810.1
MADAIYIYLIVGLLRLQGVNIDPTVGVSCKLIPDQGRYLSPDQNIKVRVFYKPGFVYLG
>EFA73817.1
MHKLRSMAEFTQRFPDVKEVIVNGTEPPVQRPQNRERQKEYYSCKKKQHTCKQITVSTRK
KRVIILTKTRAGKVHDKRLLQESEIVQYIPDEVAIEGDLGFQGL
>EFA73818.1
MFKSLYFLSSVLLTSIVISSMPHSTSYAQVGRSGFRSISRGSYGSIRGSLRLPQQIKVSR
YGYASFYFNLGNDCGAGISYGKKGGMDGQWYTFMNCGNNRGGSGRPIGLQPINRIELSYD
GISQVTLLVNGRRVQSAMTTLSPSSVSMVHAYYDEPGGTEKTYYDSAAFEEVECNYSRSS

CNFSSPEFFGTARRLFYRSTPNTLNTYMR
>EFA73825.1
MPAVIVMKEPVPDEFAREFLERFLIHFVKECNFLSVAVEKAAKDILYKEDQEFPGATWLP
ITYYSNAKSEEFTWSKKNQIYKNRIDIKIHNYLYQRQEENSLCPYQGLFHFGPEEAEYFF
GRQIFIEQLYEATQNRKFIPILGASGSGKSSVVLAGLVPKLKNQGNWEFTYFRPGKEPFP
ALATALIPLLYTSELDTIEKMAQGRILATHLQTGTIPLSDVFTQIQQKHPTEKVLIIADQ
FEEIYTMCPEPETRQQFLDCLIASLSIPNSPIVLVTTMRADFLSDALSYRPFGDTLQNAD
LKILAMNREELTEVIIKPAEKLGVSFEQGLAARILNDLEQQPGNLPLLEFALTQLWNQHH
EWQLTHNAYEKIGQVQGALARYADEKYSNLTKTEQETVEVLHEALIRDWGERIEYHSQAY
YKEPKTSTLRK
>EFA73826.1
MNITQSTFINKISIGWTSLLIFTLTSVPTSAQTIDRIRDILEKTTVQINSNANPGGSGVI
IKKEGNTYTVLTANHVVCENLGVETIRCRTDFTYSIRTYDGREYPIKKRQILQTKLQDPD
LAIVTFESQQNYKIASLGNSSQVKIQSDIFVAGFPTIFGFTGRDRTFSITNGKVVAFIPQ
QKAPKGYGLIYDATTRIGNSGGPVFDSNGNVIGIHGLADADALENNNTNQSETRNNLRPN
LSTIRKLINPQTESGVSFQVKTGFNAGIPIDILYGVLPQTYFNTRSITSPSDSMNSGESG
TSNENIDDSIKSQSIAYFDKAAEQYNGGDKQGAIANYNQAIKLDPDYTDAYINRGLVRSE
LGDRQGAIADYNQAIKLDPNYALAYYNRGIVHSELGDRQGAIADYNQAIKLDSNYTDAYY
NRGIVRSELGDKQGAIVDYNQAIKLNPNYTNAYINRGLVRSELGDNQVAIADYNQAIKLN
PNYALAYYNRGIVHSELGDKRGAIADYNQAIRLDHNYTDAYYNRGILRSELGDKQGAIVD
YNQAIKLNPNYTNAYINRGLVRSELGDNQVAIADYNQAIKLNPNYALAYYNRGTVHSELG
DKRGAIADYNQAIRLDPNYTDAYYNRGILRSELGDKQGAIDDYNQAIKLDPNYANAYYNR
GIIRSELGYNQGAIADFQKAVNIYQQQGKENNYRDALDRIREIQQR
>EFA73834.1
MTLVFQFALVSLVLFSFVLVVGVPVAYATPQNWVDSKKLLWLGSGIWIALVLLVGVLNFF
VV
>EFA73842.1
MTSLTSYSSLTNFFPETLVLEVRNLMYAYTNKYTILKNISFKLKTGDRVALIGATGSGKS
TLLENLIGLKYPQSGTITINGIPVEPNTVAKIRKQIGFVFQDANDQLFMPTVLEDITFGP
LNYGVAPAVAKENARQLLADFGLEKYAHRSHHELSGGQRRLAAIASVLALEPEILILDEP
TTGLDPILFG
>EFA73851.1
MKMKFVYILAGIALFLKMLFLSDGTSNLSGMSTIKDTMSTIVPAGISITEKVVAESGVVN
AVSGIIFRNRLYDTIFEVIVFTIAILGCNFLLASENPSCAIYQFKDRASITLARLGATIA
ALVGIELAIRGHLSPGGGFAAGVAGGTAIGLIAITSSYQWMQDIYQRYHAAVWEKISVLI
FYCVITGHFIGT
>EFA73858.1
MYYDEDANLDFLAGKTVAIIGYGSQGHAHALNLKDSGVNVIVGLYPGSKSIAKAEAAGLT
VKNVADAAKAADLIMILLPDEVQKTIYTNEILPNLEAGNILAFAHGFNIHFGQIVPPGDV
DVIMVAPKGPGHLVRRTYEQGQGVPALFAVYQDATGKARDRAMAYAKGIGGTRAGVLETT
FREETETDLFGEQAVLCGGLSALIKAGFETLIEAGYQPELAYFECLHEVKLIVDLVVEGG
LATMRDSISNTAEYGDYTRGPRVVTAQTKAEMKKILGEIQSGQFAREFVLENQAGKPGFT
AMRRQEAEHPIEEVGKDLRAMFSWLKKA
>EFA73862.1
MSSIWEENQVKSKFFRTFFIFLSLVGLGGCIYFSLFDQQPWLIIMSVVLMISMLITSQLI
SQHNRQFIPILFGGMYLVLFFLMISKSWVWELNEKFPVQPVAALIRQHVPPGTKIYTSFP
DARPSLDFYSDCQIIPTSLTDLSSRFYHQSYLLINNKQLVKINLKNSKLLGEYQEFTLIA
PI
>EFA73864.1
MSLQEFTLLLFSVLISVTGQFLLKTGALKLGKVDAGNFITHILNMITVPELLLGLACYGV
GAIAYILLLTRVNLSIAGPAVSVGYIFQS
>EFA73870.1

MYQGIYNHPKKPNSALRKVARVRLTSGFEVTAYIPGIGHNLQEHSVVMIRGGRVKDLPGV
RYHIIRGTLDTAGVKDRKQGRSKYGTKRPKQTKK
>EFA73871.1
MIMRRVMRHGKKSLAARIVYDAFKTIEDRTGGSPLEVFERAVRNATPLVEVKARRVGGAT
YQVPMEVRADRGTALALRWLVQFSRSRPGRTMASRLANELMDASNETGSAIRKREETHRM
AEANKAFAHYRY
>EFA73876.1
MMNTILESDRRFGVLMVNPINGAIANVGCCAEIIHYQRLEDGRMEILTLGQQRFRVLEYV
REKPYRVGLVEWMEENPPALDLRPLAGEVEQLLRDVVRLSSKLTDRDIELPEDLPDLPRE
LSYWVASNLYGVADEQQALLELQDTQARLNREAEILTSTRNHLAARSVLKDTFDDIK
>EFA73888.1
MPKAGIIYNDVKPIAVRVAIELKDQLTAAGWDVCMTASIGGILGYSNPDSPVRHTPISGL
TPPGFDSDMGFAVVLGGDGTVLAASRQVAPSGVPLLTVNTGHMGFLTETYLNQLPTAMEQ
VIEGHYEIEDRAMLNVQVWRGDSVLWEALCLNEMVLHREPLTSMCHFEIEIGRHAAVDIA
ADGVIVSTPTGSTAYSLSAGGPVIAPGVPVMQLVPICPHSLASRALVFPDHEPVSIYPVN
IPRLVMVVDGNGGCFVLSEDRVYLRRSEYKARFIRLQPPEFFRILREKLGWGLPHIAKPS
SVELP
>EFA73893.1
MDWIFYCWFSRILNGVRRGDYYSPLLTSVFGVDIRYAIGASLVSVIATSLGSASTYIKKG
FTNIRLGMFLEVATTTGAIIGALMATFISVKFLTIILAFVLIYSAYLSQSPKSEYQEVAT
PDTLAEYLELNGTYPISDGVIPYYVNCLPAGFSIMLLAGILSGLLGIGSGAFKVLAMDQV
MRLPFKVSTTTSNFMIGVTAATSAGVYLSRGYIDPGLSMPVMLGVLPGAFLGARILIGAK
TQTIRIIFSIILIVMALKMVYNGLTGGV
>EFA73898.1
MPMAVGVIETLGFPAVLAAADAMVKSAAVTIVYYGIAESGRLLVAVRGQVAEVKTAVAAG
IASEETVYGGQVITHYIVPNPPENVETILPIHFTSESEPFRYFLTCTGNFIYTGD
>EFA73905.1
MLYTHRRKFISSVDNPQFLPPVSRWTSLAGIFLVGTVATAIALASWVKYNVTVKADAVVR
PMGEIRVVQPEIEGTIKSILVKPNQTVKIGDVIAYLNTDDLLIQKKSVTRKYPTG
>EFA73906.1
MICSYKKSQLQENIQQGNLQILQIHAQVRILDRQILAETQVAQNAINSARVDLLRNQREY
QQQQVSTQGEFLSAQANWQKAKASLDKARADLNFAKMDKERYQELSQIGAVGRRELEQKA
LTVQQTQSILDTERKSLEMAKIKVQSAQVAINPTPAAVMIAQERIAQETARGVSNIASLN
KEKQALIERRVQLETQIKQSIKELEQVENQMRKSMILATSNGIILKLNLRNPGQVIRPSE
SVAEIVPDSSELLIRALIPTEEIQKVNIGQQVKLRVGACPYPDYGTLHGVVQTISPDVIT
NQPNNPGTTTNRMGYFEAMVKPASLQFGQGRHQCYLQSGMEVKADIISKQETALEFMFRK
ARLITDL
>EFA73931.1
MTDKGEISLHQKVLGSRRLSNYSWATIVTLGASGFSLASISSYLKVNLLIVTDATELIFV
PQGLVMGIYGIAGMLLALYLWLVILWDVGGGYNEFNQETGKFKIFRWGFPGKDRQIAIES
SIGDIQSVRISIKEGLNPQRALYLKVKGRRDIPLTRVGQPLSLSELETQGAQLARFLGVP
LEGL
>EFA73934.1
MKNPSSYLILVMTLLLGNPALLIGTGTSAIAQFSDCQKAVTQAQLNQCAAINAKTADQKL
NDAYNKVLAIYKGKR
>EFA73936.1
MQVTTTSTPIPPGELSPSSWPDHTQLPDSDDNFVKNFQEHPQSVILTTSIEPLLDKIHPD
KDYCIGQDSGIYWRFTEPVEKGVEAPDWFYVPGVPSRLKGKLRRSYVMWKEKVPPLIVIE
FVSGDGKEEKDNSPPPERDEVDPKTKKVKKAGKFWVYEQAVKIPYYAIFDGFEGTLEMYH
LEKGRYEQVKANRRNHYPIPELGVELGMLLDQEKPPVPWLRWWDNRGNLLLTGNERAEEE
CQRRELAEAIAIQERQQKELAEALAIQERTEKERERQQKELAEALAIQERSEKEQEREKK
EKLAAYLRSLGINPDEI

>EFA73942.1
MKLWVDLAQAAKTDAIKDTVDYRSVISLVQNLLTTSKFDLLERLAGAIADAILQESSLTT
QVQVTLIKPAAPIPDFDGNIRIELTRSKSNL
>EFA73965.1
MTITVKDVSTCKWDFFVWNADIMTQPNYGWQWKIGTEEFAIVFHMFCFAFNQQNDGTSPS
GNVEWFVGGV
>EFA73981.1
MKFLNALIEAEGIQLDGAPGANGRGGRSASYRVSVQSNNNLLIGSAYTKQMNLKPGDEFV
ISLGKKHIRLRQVEPEERDDDDQLEEVTA
>EFA73984.1
MNLPVVLDIALGLVFTYLILSLLASEIQELLATVLQWRAVHLKKSIEIFLAGDIKNSDAK
DVLDLVNRIYNNPIIRSINQETKGFLSTLPRKMTWKVADIVSSVKKLVSRSSRENKIFAN
QNSGPSYISSDSFVSGLLEELKLPKIIHSLVEVRLENFKIQHLRAIKLILTRSLRQIASN
ELSTNVTHDINDDFVNLDSEYRQIVDDFKNQKFDVDTSMNRMQDSLNKYINNFQANIENN
HPALMETHKRLQTLQNHIFPSLEEAITVAGLKPSISEIIQLMETGKAGYNEIQTTSQLRD
GERYVTIRDLIDSLPPGMKQNIATMAKRAQYKAKTTEEGIRVLRQEIENSFDSSMQRAGG
VYKRNAKGVAILIGIVIAFGANADTFYIIDRLSKDTALREAIVYKAQQTIDQQVLDPNLK
NIDTNQILEDISLPIGWSEKNLNRATGY
>EFA73985.1
MIMGWLVSGFAIAMGAPFWFDLLGKVMNVRNTGKGNKGTGN
>EFA73991.1
MLQRLAHQPLTLGDVLETTSQRGFILVIALLVLPFLFPMPPGLTGPLGSACLLLSLQMLL
GRRSPWLPKKIANYQFPRVFAQTILQNLSRVTRLLEKIARPRLTKLANHDITWRCNGFCI
SWLAILLISPVPLTNPIPTIGILLFAAASIESDGLLICICYVLTLLITLIFYLIVYGVLQ
FPGLIT
>EFA73997.1
MKYWRETLAIAQRILTELLRRRRSLIFWTIFPVSILILNGFVLSERGELAMEVALEKAAP
SSLVGAALFFSCLGGTVATVVAEREQQTLKRLFISPLSGTSYFLGIFLAHSCIGIGQTLL
VYIVAGFWGATFQGSILLGIVIIFLSIIGYVGLGFVLGTQLARRTEDVNALVATFGVPLL
ILGGTFFPSSLFPKTLLDIAKYNPIYHMNEALLGVSANGEKFSDVSLHLRFLFIFCYTHP
RLWLDIL
>EFA73999.1
MLSPNMATTYLPPMGERISMEEVISPTGRTITLIRS
>EFA74000.1
MFAASHQLLWSMIGLLLTIGGTFLEAYGTTLPWSWSQQGIKTFPLGASYQIAAVLFVGCL
GVKVLVPFPKLLI
>EFA74001.1
MFRGKSAGALSQIAYLVMGLTLLPVFAQGGGIGYVKLPNLVTYWGLFQEHGFADILPLKP
DLD
>EFA74003.1
MNGIYSLSDVTKKGLGFWGGCWELMVWDLVKLIYIKINWEFYA
>EFA74024.1
MTLHLIRGKIHRRNTVEQLAVVGPDSLFIALLTAIFVGAVFTIQVAREFINFGAGNLVGG
VLAVALTRELSPVLTAVILAGRVGSAFAAEIGTMRVTEQIDALLMLKTDPIDFFSHTSSF
GLFINATNFDLAIPGYWNVGRLIIATNLYNLSDTVFLDSARNFLDLRDIISAMIKAACFG
ILIAIIGCSWGLTTTGGAKGVGQSTTTAVVTALLVIFISNFFLSWLMFQGAGNPSIRP
>EFA74028.1
MSRCVNNWGNLFTLINGEYVNTPTIIDSLNPSDFSQVVGKIGLISVEQAEEAMQAARAAF
PHWQKTPVPQRADILRRAANLMEIRRAELAAWIVLEVGKPVKEADAEVSEAIDFCRYYAQ
EMERLDGGVVYDVAGETNRYIYQPKGIAVVISPWNFPLAIACGMTVAALVAGNCTLLKPA
ETSSVITAKFTQILLEAGIPKGVFQYVPGKGSQVGAYLVNHPQTHVIAFTGSQEVGCRIY
AEAAIVKPGQKHLKKVIAEMGGKNAIIVDESADLDQAVVGVVHSAFGYSGQKCSACSRVI

VLQPIYETFLSRLIEATKSLNIGAAELPSTQVGPVIDSQAKNRILEYIEMGKKEAKLVLQ
LESPSQGYFVGPVIFAEVPPQGAIAQQEIFGPVLAVIPAPDFHQAVEIANSTNYALTGGI
YSRTPSHIEQAKRELEVGNLYINRNITGAIVARQPFGGFKLSGVGSKAGGPDYLLQFLEP
RTITENIQRQGFAPIEGAE
>EFA74029.1
MALKTQNSNYEVKTQEIAQQILTHTQEGRSFFAALRDQMRWDDKLLGWTMDNPGLRVQLF
RFIDTLPALHSKVEIASHLQEYLGDDSVELPPALKSLLNFAYPDSMPGQVAATTVETAVQ
TLAHKYISGENIQQVIKTVEKLRKEKMAFTIDLLGEAVITEVEAQSYLEKYLELMQQLVE
ASKKWHHVPIIDEADGEIIPKVQVSVKLTAFYSQFDPLNAEGSEAKVSERIRILLRRSRE
LGAAVHFDMEQYAYKDITLKILKKLLLEEEFRQRTDIGITIQAYLRDSKEDVQGVIDWLK
QRGYPLTIRLVKGAYWDQETIKAAQKHWHQPVYNDKVATDANFEAITQLLLENHQYVYSA
LGSHNVRSQARAIAIAETLKVPRRCFEMQVLYGMGDKIAKALVDKGYRVRVYCPYGDLLP
GMAYLIRRLLENTANSSFLRQNLENRPVAELLAPPIVEETPSPENPTQDFRGAADIDYAE
EQKREDSRLAFEQVRQQLGKPIYPN
>EFA74037.1
MGERLISNLNIIATYNSSEFNLQFKQKHKKRLIIITIL
>EFA74048.1
MVLGLIITLFFVLLAFMAPVWQNWGWLSDPKELLTNPIHQPPSGKYWFGTSRLGYDVFSR
TIFGAQAALQVVILATGLSMVVGVPLGMISGYLGGKLDKTLLFLMDSIYTLPGLLLSVTL
AFVVGRGILNAAIAISIAYIPQYYRVVRNHTVSVKTEVYIEAAQAMGASTWTVLSRYLFL
NVIQSVPVLFTLNAADAILVLGGLGFLGLGLPEEVPEWGYDLRQALEALPTGIWWTTLFP
GLAMTIMVVGLSLLGEGLNELIHPRMQKRF
>EFA74050.1
MEESFQGVNPEYFLKQLNTFPLLGKQIKLWLKLGFLNHQQFSLPSLLLGAALGELERRIN
WSLTSLIADSNQAGITTSSCDVIPHFVKHRGGFVLVHQELEVIILCEHIITQWLSEVGLE
LKGEGIRVSHSLYDYQGNVGCNFLGFYIRQFVAQDKSHDKSSQNSDLGSPHYNTSYNTLI
SISKESLEHHTQSLGAIIDQHRSAKQSVLISRLNPLIERWTKYYSPLISDRIFGKVDFEL
HSKLRAWSRRRHNQKGKRWISKKYWSICKEGWRFCHQNSEGKTYELMKHQYIHQDLKLRK
RERRSE
>EFA74051.1
MVVFAIYNQALTLLISMAMSPEWDAKFNLGLHEANSDISCYNAVRSIRREVVKNLSMF
>EFA74052.1
MAITEISSAKTSFCSGEKDLAPWFGGNTPFTAIHLADQDLINSFWSL
>EFA74054.1
MKTKLQVKLLQYILIRDKTAQGFTLLELLVVIVIIGILSAIALPSFLNQANKAKESEAKT
YISSFNKAQTLYRLENTGFATTLNQLSIIIPPSTEFYNYTIGGVALLPTLQQVLRTLTPS
KGLVVE
>EFA74055.1
MSATGQTQSVACQTEQVQQIHPVVVPILDSTQAKCDDTKNMTTMK
>EFA74056.1
MVKLQRNVQSLVESNIIKPSDSIWKIAFLYADEWKYWKQELLDFGFSMQDPIGDLLAVET
WDED
>EFA74067.1
MAELVAFCQINGIPGDSIGDFVAALGEYAGESLIRMVALICVDGILPLGPDFVQKSLSFL
SSMNPQELDQNSTFRSIKDAIPGNGTGGKIDFIGQSLDSVQGWMNGIVSANALTAQKVLN
NISGFLEFADDKLDYVAAFLDVSTNYYEHTGTQTLARRLIERALAEI
>EFA74068.1
MSKPIFELVDELPTSGLTVSLLNALDFVAPGEWQNTVGFVNTIKTVTGETDEDLIQAIGE
RAVYLFNDKSQGYQTALWLYQTVDGTDKALGAAALANKVGEPYPYLVS
>EFA74072.1
MLNEKYAQEIKGFHSINVPSEWELNNIGNLILLGGHKGSVSIQLMSPASGNNTI
>EFA74076.1

MNTNELKFMLKLLGYQTYRGNWSLFKDKEKTKVCQELERREYVDYSREIVSAQILPAGKA
LLQIETSQLPITAEELKVLEKIAKAGKKIAPSAIKITKLKVRSKRGNPKNPR
>EFA74079.1
MYNNRGEAKTLLPRGIQMHISDGERFMISMVSDLARRMAIANPKIRKSIERQGDYFN
>EFA74081.1
MPKTEKIVINTSPLIALVAAWGDLSILSC
>EFA74082.1
MHYLTDVTEIYRQISQLTRFRTLWLDTEIANWNTPYPRLSLIQVLAYPSDLTGEFAYIFD
VLDKPDLTAYFIQHIMVNSNIQKVFHNADFDLKYLGKNQAQNVVCTFKLAKKINRKVLQT
TNLKLKNLSC
>EFA74083.1
MGTSDWGERPLSSRQLKYAAMDVIYLAAVHHRLLEISDPNAVSTIFSISFDSSFNGNFDN
SNDSNLNNWDNRVNLERENSPFSPTKLRLAFECPRLFYLNYKFNCKSIFIPADSTPGIGN
IFHQLADNLIDVLLTDPSFTNLFIPPSDQLDVEKLAAQIQRLFYQISFFPYLQTTISQEK
SKGVLLYKVWARLQELIKKITKLLVDNRRYCTAKALMANTFINEDRNLEYDFNLPDGTKE
LIRGKYDCLVFSFASQRLSVIEFKTYQPMDISGQLAQTAIYSYMLSQRKKMPVDSAVYCF
LPEFQEYTYTWEQLENTTHQIIHHKLLQMRQWSGWESPQPNPPPMTTQTHLCQICPEQQR
CQTFFAAEVTTPQSTQAIQSIQLPKINEIPEPKIVDANAIEVKLVNTLASFGIGVEYQGT
MVGPAFIRVKLKPHLGVKVNSFVEIV
>EFA74084.1
MLKLSKDLQVQLELENPPLIASQAGYVSVDLPRKDRQIARFEDYIQRNFYLQPLS
>EFA74085.1
MNIDGHLLEADLSDSNTCHFLVGGTTGSGKSEFLRSLLLSLLYRHSPQHLKIVLVDPKRV
TFPEFERIPWLYSPVVKDSDRAVEIMGELVAEMDSRYQKFELVKCPNITTYNQNSGKILP
RLVCIFDEYADFMAEKEIRSVLEQSIKRLGAMARAAGIHLIISTQRPEAGVVTPIIRSNL
PGRIALRTSSAADSQIYFRCQDITSSRFIG
>EFA74092.1
MLERGIYLAPSQFEAGFTSLAHTEEDINHTLEVVKEVLSNL
>EFA74093.1
MVDTTIQTTKSQEIFAAAQNLMPGGVSSPVRAFKSVGGQPIVFDKVKGAHIWDVDGNKYI
DYVGTWGPAICGHANPEVISALHQALEKGTSFGAPCVLENILAEMVIDAVPSIEMVRFVN
SGTEACMAVLRLMRAYTNREKIIKFEGCYHGHADMFLVKAGSGVATLGLPDSPGVPKSAT
KGTLTAPFNDLEAVKALFQQNPGEIAGVILEPVVGNAGFIAPDAGFLEGLREITQENGAL
LVFDEVMTGFRIAYGGAQAKFGITPDLTTLGKVIGGGLPVGAYGGRREIMSMIAPAGPVY
QAGTLSGNPLAMTAGIKTLELLQKPGTYDYLERITQKLVNGMLTIAKEAGHSVCGGSISA
MFGLFFTSGPVHNYEDAKKIRHSQIW
>EFA74096.1
MMRQAGRYMKAYRDLREKYPSFRERSEIPEVAIEVSLQPWRAFQPDGVILFSDIVTPLPG
MGIDMDIAEGKGPIIFSPIRTQKQVDGLHPLDPETALPFIRQILGALRQEVGSASTVLGF
VGAPWTLAAYAVEGKGSKTYSIIKNMAFSDSNILHQLLDKLAESIAVYIRYQIDCGAQVV
QMFDSWAGQLSPQDYDIFALPYQKKVFELVKRTHPQTPLILLVTGSAGLLERMATSGADI
LTIDWTVDMADARRRLGNNVKVQGNLDPGVLFGSKQFIRERILDTVRKAGNWGHILNLGH
GVLPETPEENVAFFFETAKNLHVSV
>EFA74101.1
MIEREKKRAGLVAKYAAKREALLEEFRTTESPLEKLEVHRKIQQLPRNSAPTRRQNRCWL
TGRPRGVYRDFGLSRNVLREWAHQGLLPGVVKSSW
>EFA74105.1
MDSLAERLREDRVKAIIQPMLSGSDLPDTPEDDRRFLLDLGLVKRSPLGGLTIANPIYQE
VIPRVLSQGSQDSLPQTYGREN
>EFA74106.1
DVKNLYEQHTQATGQIFTPDAINHAYYLTQGQPWLVNALARQATEFLNTDPSVPITVDLI
NEAKEILIQRQDTHLDSLAERLRENRVQAIIEPILSGEQLPDVPQDDIRFVLDLGLCRQD

TGRGLEIANPIYKEVLPRVLAYTPTMSLGVIEPEWLSPTGELVPEQLLAAFLEFWRQHGE
PLFKSTPYPEIAPHLVLMAFLHRVVNGGGTLEREYAIGSGRMDICLRYGQVVLAMELKVW
RKGSKDPLTAGLKQLDKYLSGLNLKTGWLVIFDRRPNLPPVSDRTTTEMAISPQGRSITV
IRG
>EFA74118.1
MIVPTGAGAAMSAGRKILALTAAKATKNTKTTSRGAMY
>EFA74122.1
MENLRKTLCQLADGVLPLQLISNLLSQVQLQGQKELRLKQIQLQNQVARDILTTRDKRLL
SFLNQLNLELETITSIENFLVQDIDSLYVDLSTKLYDSEPVKANNYQECFLHADEETLTS
LDRLVYSLPTVQNNAKNLLLELRNCLELIVSLDRQIQAAAPPEAYIKLQKIRELAEKEWS
RANTNLEILNRQFINLTTTIDKTKKELNSYTNKNLKYRSNQHLIDSVHRVQENLKLFKEK
LTLRKLNKLEEEVKNCFLYLLHKSDLVYRVGIDSKSFQLSLYDLHGKLVPKHRLSAGEKQ
LLAIAFLWGLAKVSGKQLPVAIDTPLGRLDSSHRNNLLERYFPTASHQVILLSTDTEIAR
KEIAILREHQAIAREYVLEYDSGKRETSIKEGYFW
>EFA74123.1
MIFLELVLQNFGPYAGKQVINLDTRIDRNNIRPIILLGGMNGGGKTTLMDAIRLALYGAR
AQCSTRGNLSYGDFLTQCVNNKADPINKTRIELVFEHIEDDKPVRYRVVRTWEKNPKDGK
DSLGILGEDETWPQSLANIWDEYIENILPLGISNLFLFDGEQVKELAEQEVPTPIVVDAI
NGLLGLELVDKLSLDLEILVNRKKKENADDRDLAKLEELENRLHEQMEQKNSQKAQLQDL
EEQGKKFKV
>EFA74125.1
MAKIPQDNRNIFPQYPVGQPPQLPSQIMVQQTKMGNVTAYLGSVTLEWFAKKVKFASTLP
ILQNKYNPVTDNIEIDADSIEQIQERSLDWSRQSSLLQYLAIHRNHKFPPVLVVISQPWV
NDEKSDLWDGEGRAKKATTDFIPSDANSHIGLLNVAEEDTSIYVLDGQHRLMGVQGLLEL
LENGRIARYNRDKIPTDSYIDLSELVDQYRIDVDYLKQLPQEKLALNLFAQSIVEKPTQK
PNRESDQFLCM
>EFA74126.1
MNLMATPLTKGQLIQLNEDNGFAIVARKIAVSHPLLAHRENRKPRINWNSATVAANSTVL
TTLQALQDMSTKYLGYKFPHWQPQIKGLIPMRPDPDQLGEGMREFQQLFDHLATLPSYKI
LDHEDTPVLRRFSFEKGGGEGNILFRPVAQVALAEALGILVFKNGLLLENIFKKLQKFDL
QGGFSQMEFPNSVWYGVLYDPNKKRVQVSKKELAVKLLIYLLGGIKDRMEIAQLRKALAS
ARTREDRAMDFDGNWVSDQEVGLPSI
>EFA74143.1
MAAGGEAVQVFSDLNELIEVSEQWPAQKRLRAGERGGGKVAGFELGNSPLDCTAELVEGR
RLFISTTNGTRALKRIEDAPIVLAAALINRSAVVEFLLEKQPQTIWIVGSGWEGSFSLED
TVCAGAIAHSVWQKTGLELDEIAGNDEVTSAIALYEQWQDNLIGLFHQASHGKRLLRLEC
VEDLKYCSQVDILDILPLQKQPGILTSHNAH
>EFA74150.1
MLFSKASTRTRVSFTVAMYQLGGQVIDLHPNVTQVSRGEPVQDTARVLDRYLDVLAIRTF
AQQELVTFAHYAKIPVINALTDLEHPCQILADLLTIREVFSDLTGLTLTYVGDGNNVANS
LMLGCALAGMNVRIAFPQGYAPNQDIVEKSRVIAGDKTEVMLTHDPVIASKNAHILYTDV
WASMGQESEADNRLPIFQPYQISQDLLSLADPQAIVLHCLPAHRGEEITDEVIEGSQSKV
WDQAENRLHAQKALLASILGAS
>EFA74156.1
MTDQDISGGFSPVETTKPEQNPRLSPDSEVQVNQSSGRNAIQHSDRNKIWELGMWKKSWI
WWFLVLAFIPSTMGIISVAILLKLPSAPNCPRIFWPLASASMRVHCANLAASKQTVNDLL
QAIALVKDLPQDHPLRGQINDLLQEWSRDIINLAEKSFQSGNLEEAIATDQKNSRKFGRS
SVCGRENTQMAIYLVNS
>EFA74166.1
MNKDISLLLVEDGQFVEASTEVVKDIFCQTSGVVEITQKNDILREIVIKPGELLMVDDPE
AVLGRDNTFIHPQEELLGTTVTEMRYIQYVESPEGPALLSRPVVEFQVSSHPNLPSTTSV
SQQTGRSIQLRAVQRLPYKDGERVKAVDGVELLRTQLVLEIEQEGEQDHASPLAADIELV

PDSENSEIQRLQLVILESLSLRRDMAADATQGSTHTTLEVEDGITIPPGSVVARTQILSK
EGGIIRGVRPGAEAVRRCLILRDTDMIVVKTNLPPTVKKGDLLVQGTEIAPGITAPVSGQ
LVDISHQLPPNNNQQEQLVEAAYSLKIRTGRPYRVSPGAVLQIEDGGLVQRGDNLVLLVF
ERAKTGDIIQGLPRIEELLEARKPKEACILSRRSGEVKVVYGEGDENLVSREAYSVKVVE
SDGVVIDYPLGPGQNLIVPDGAMVEAGQPLTDGPSNPHEILEIFFSLGSEDGLYACASHA
LQKVQSFLVNEVQMVYQSQGIDIADKHIEVIVRQMTNKVRIDDGGDTIMLPGELVELKQV
EQVNEAMSITGGARAQYTPVLLGITKASLNTDSFISAASFQETTRVLTEAAIEGKSDWLR
GLKENVIIGRLIPAGTGYNTYEEVGAIEDYGTDVATGVLDEVDDPLDIVLDDRTAKLYSL
DNSGLDGTYDDSYDHDNTYGSNHSTVLEDEDEDLITDEVITGVDEDEDEDDYEDEDEDDF
DIDVDL
>EFA74176.1
MWSQHPAPSLEAIVPWTRLFCDRRDQFQNDQDATKDFQQKLAEFLKALFITTEPKELDNE
IQNWLKLAAFVKRKRDIKVGGDN
>EFA74179.1
MGFEGTGAAQYFSVFSECLTNPDFVFSGRSRRPPGNPVNAMLSFGYQVLWNHLLALIEVQ
GLDPYYACLHQANDGHAALASDLIEEFRAPLVDSLVMWLINRNIMDVKGDFQFKNGGCYL
NDSGRKKFLRGFLQRMTEEIQTNDGKQPKWDLLTQQVRNYKQFVYNPSHHYKPYQID
>EFA74180.1
MQTLYISEQNCYVCLQKETLIVKQGDTVHVEVQLPLLEQILIFGRSQITTQVIRACLWRD
IPIAYLSRMGYCYGRILPISRGYRQLSRYQQELSPVDRLITARAIVKGKLKIVEFY
>EFA74197.1
MSFANNKKTNEYKELKEKPHWQGTTVTPATKTTTRSRDTKSQEPVQGADMSDDLNRGRVA
IFIDGLNLFHAALQIGIEIDYVKLLCRLTQTSRLLRAFFYTGVDTSKEKQQGFLLWMRRN
GYRVVTKDIIALTENGKKPKSQCGNCCGYDYLSSLL
>EFA74198.1
MVKNPNLNVEIAVDMITLAPYYDTAVLVSGDGDLAYAVNAVTSLGSRVEVIGLQTMTSDS
LIDVADYFIDFDSIKQYIQKDSQFGYNYRSSPTSHL
>EFA74206.1
MEGWSWEDAAYMTVITLATVGYGETHPLGSHGRLFTIALILMGVINIGYIVNRFTEAIIG
GYFQQGIKVRQQRRLMESLIDHYIICGFSRTGKQIAKEFQSESVSFVVIDSKIESVQQAE
SQGYIVYQGDATLDDTLSKVGIQRAVCIVAALPSDAENLYTVLSAKTLNPQIRAIARAST
EESLQKLQRGGADTVISPYITGGKRMAAAALRPQVLDFVDGILSGTDRQLYMEEFLLDGD
CPFLGQTLQKAKLRSQTGALVLAIRRSDGNLIGGPTGDTILISGDTLICMGTAEQLRGLN
KVLAPINSRQLRRPKHI
>EFA74210.1
MLVKGVNQNVNALGYFGYAYYKKNQDKLKLVAVDNKKGPILPSPETVGKSKYQPLSRPLF
IYVNLWSGKHRADLYKFVDFYLKKAPAIVNSVGSVPLPKEAYNIGYVHLHNGKAGTVFGG
KSEFNLTIGELLRKQKEF
>EFA74211.1
MKPTAVRLLITVGMLTLAASCTADVNKNSSVEATKDVNSQTASTIRIDGSSTVYPITQAI
VKEFSKDGKNPTSVQVSFSGTGGGFKKFCRNETEINNASRPISAKEMAECNKNGVRYIEI
PVAFDALTVVVHPQNNWVKDITVDELRKIWEPPIPGENFPVESSTCFLARPPIKIIRTR
>EFA74219.1
MFKRLKDHQKKVLINGRWVVGDASCKYNAHSEIIRCAIKPDGPCNSCGFKE
>EFA74228.1
MNQPIQLSLEQQFNVYSFASQVKEMSHEQAQEFLVKLYEQMVVREATYKELLKHQWGLDL
GSMA
>EFA74229.1
MGGHAGGEEASRIAIQQIQAYLTNQWDSPENTQSLLRLAISQANNSILQDQLSHPERSDM
GTTVVVVVFRSPEPPICAHLGDSRLYLLTNSQLCQITQDHTWIAKAMEIGEISPEEARSH
PYRHVLSRCLGRADLSDPEVQSFKLNPGDRLLLCSDGLTEELLDEEIFQLYQNLDLGQTA
SSLVQAAKAKGGKDNITVILVAAEKQE

>EFA74233.1
MGPVLRVLFSIPIALVYLRWGNRASWMAAITSGLLLSVLTGPVRSLLFIMPFAFLGVLLG
ASWHRRVPWFVSITLGTILCTLGVFFRLWLLSILSGEDLWIYLTNQVTEILQWLFLNLRI
LTPPSTLGVKLAALLLIVINNLIYMFIVHLAAWLLLERLGNSIPNPPHWIQTLMNYED
>EFA74239.1
MASVYDWFEERLELEAIAEDVTSKYVPPHVNIFYCLGGITLVCFLIQFATGFAMTFYYKP
TVAEAFSSVQYIMNEVNFGWLIRSIHRWSASMMVLMMILHVFRVYLTGGFKNQRELTWVS
GVILAVITVSFGVTGYSLPWDQVGYWAVKIVSGVPEAIPVVGVLISDLLRGGSSVGQATL
TRYYSAHTFVLPWLIAVFMLFHFLMIRKQGISGPL
>EFA74243.1
MIIRNATELDLPAIVAIYNAAVPTRMATADLEPVTLESRMTWFQERVPSRSPLWVVEVND
NIGGWLSFQSFYGRPAYHATAEISIYISPSFHRRGLGKTLLLKAIHESPNLGIKTLLSFY
FCP
>EFA74245.1
MRAANPLRKAHTRRPLTTMEAKCLVIITSYMAKRLTVVIRQLLMIYQQLSEKQIPLSHNL
RLANYLERFRTHFKSRMNPRRSVLLTPNNSDEKLDQLAIDLLAKLLFCTGTAGMQRFWIS
LFDGEVE
>EFA74249.1
MVSSLAVIITLIWWWNRRSSVSDMPNIASSRETVEAINLQTAKTEIVSSQAITELSQGNL
ERGLVAVEKLLDRGDFKLADTALSSIPSKHWKNPAVNFLRGRLAWQSAQMRDKKYSIDDA
RRYWEVAVKSQPSSIMYNNALGFAYYAEGKLNYANDFWFRSVNLSLKSNKVSLDSLTAHG
GLALGLYKSANDQPPNRRQKYIDEAIKLRQMIIEKEPEQFTVERLTRNWLWTEQAISDWQ
SLLEENPKR
>EFA74250.1
MTQEFHISVTPVGQSDYLVRTEQVAPGVPLAEELVTWPVAEWLAAAEHLMNDPLKSVLQG
EGLTQPAAGTARSSVNLVALGQKLYNALFQGTLRDSWITAQGVAQNQQQLLRLRLGLKDN
KLARLPWEVMHAGDRPVATGPYIAFSRYQNGVLSVSSPTRLPTRQEENLIKVLMVISCPV
DQAKLDLLKQEAFNLKSELNRNDWVQKESNKDLPEIELTVLEQPGREELTRSLEQGRYHV
LHYSGHSNVGSQGGEIYLVSRRTGLTESLTGDDLAGLLVNNNIQMAVFNSCLGAYRAKSD
SEGDTGEQNLTESLVKRGISSVLAMSERIPDEVALTLTQLFYRNLRQGYPLDLCVSRVRQ
GLISAYGSHQVYWALPTLYLHREFEGFVRPQLGPSGSSEFFGDYQSTISGFESEVPEHKI
DDLEANPGLEEIMDYGLTKETSDLDWLEDSQWDLMGGVNHDQENDDQDAAIVADLLRRID
NKPVASEQLIVKAEPGILIKGQESTGGENEFDGWNDSKTAKSSNQLHRDPRRRVSPDVPL
PPITKVAIDRKPKLIWECWWSVV
>EFA74257.1
MAVILCGTILLMMPFSTSNGTWNDPIVALFTSTSAVCVTGLSVVDPGTYFSFWGQLLILL
LVQIGGLGYMTTTTFLILLIGKRFDLRQKVAIQQALDRPGMSGSSQIIRSIIATTIIFEI
TGIFLLLPAFVPDHGWSYGMWLAIFHSINSFNNAGFSLFKDNLIGYQTSLLVVFTVTGLI
IFGGIGYQVILDMYLWLRDRLKRKTTFMAFSLDFKVAVSTTLLLLVVGTVAFFLIEIRNP
ETFGKFRFSDQLLLAWFQSVTPRTAGFNTIDIGKMSDAGLFITIALMFIGASPGGTGGGI
KTTTLRVLTSCTKAILQGKEEVWLYERKIAINLILKAIGVVFGSLATVLSATVLISLTDP
KLEFIQILFEVVSAFGTVGLSTGITGSISTAAKIVIIVTMYIGRVGVLLSMSAILGDPRP
SRVHYPEGNLLVG
>EFA74259.1
MQNSDLDISLSGYDYELPPELIAQNPVVPRDNSRLMVINPPAGTLTPPLDHIFRDLPELL
KPGDLLVMNNTRVIPARLYGRKSTGARVEVLLLEEKQHNCWLALVKPGKYFNIGTEIIFD
DQGLSSVNKLIPQLTATVIERDENTGGRLLRFDLPINVSLLHFLHKLGKIPLPPYINNSH
ALEEQYQTVYAKHDGAVAAPTAGLHFTPELLVKLRESGIQQEFLTLHVGVGTFRPVEVED
VTTHQMHEEWIEVSGDLVEKIYATKNAGGRIIAVGTTVVRALEGAAQSGSLQPYTGKVNL
FYLSRLSMACRGRSYY
>EFA74260.1
MSQKNVLCFDGKWNHVVLPPDNSDYSQGITVEAWVWYGSFGQNWSRIVDFGNGQGRNNIV

LAHAGTSNSLAFHTFTSTGGYAVEVPNALEIGKWAHVAATIDKSGEAKLYKNGKLIQTKP
FRLPDNVERKLNYIGKSNWPNNDGFFQGKMAEVRLWNVARTPEEIEQNMNRRLSGNEAGL
VAYYPLNGDANDKTKNARHGIIIGATWQQEELPIQEPNVNQNQTTTQTTTFITPDRLLPK
YDAVIVGAGIAGAIVAKTLSQQGKTVLILEAGEAKELTLSGFQHYLDTFYGSTEKHPNSP
YPENHYVQSPMDDNGYFVEKGPMTLSGSYTRVLGGTTMHWEAKTPRMLPNDFKTKSLYGQ
GLDWPISYDDLMPYYQQAEYEIGVSGDVEEQKSLGLKFEDGYVFPMEKMPPSFLDQKVIE
KVDGTKVQVCGETVELKFSTFPQGRNGIPNPKYNQGNLFVPQGVSSVTPVQYGERCQGNA
NCVPICPAQAKYDARRTLARALETGRVHILPKAVAFHINYNRENGRIQSIEYKYYGNEKT
GSVDSPLKVEGTLFVLGANAVENARLMLSCNLPNTSGMIGRHLMDHPFVLAWALMPEVTG
TMRGPLVTSGIGTLRDGTFRAKQSGFAMDIHNDGWGWATGSPDTELFDAVDHKNKYGAEL
RETLINRISRQLLLAFMCEMPPDISNRVTIDPKYKDKLGNYRPVINYNLP
>EFA74263.1
MSKVLVSDPIDQAGIDILSQVATVDVKTGLKPAELVAIIGEYDALMIRSGTRVTEEIIEA
GTQLKIIGRAGVGVDNVDVPTATRKGIVVVNSPEGNTIAAAEHTLAMMLSLSRHIPDANT
SLKKGEWDRKTFVGAEIYKKILGVVGLGKIGSHVAHVAKAMGMKLLAYDPFISTERAEQI
GCQLVDLDLLFQQADYITLHIPKHRKTTNLINAKTLGKMKPTTRIINCARGGIIDELALA
DAIKNGKIAGAALDVFQSEPLGDSPLRSLGKEIILTPHLGASTTEAQVNVSIDVAEQIRD
VLLGLPARSAVNIPGLGPDIIEELKPYMQLAETLGNLVGQLAGGRIETLNVKLQGDLATN
KSQPLVVAALKGLLYQALRERVNYVNATIEAKERGIRVIETRDASARDYAGSLHLEATGT
LGTHSVTGALLGEKEIHLTDVDGFPINVPPSKYMLFTLHRDMPGIIGKLGSLLGSFNVNI
ASMQVGRKIVRGDAVMALSIDDPLPDGILEEIKQVSGIRDAYTVTL
>EFA74264.1
MNISNDKNIMTNTWWEIQILCTPDLEDSIFWRLETLGCRGVAVEKKDRLLLIRAYLSTLQ
TQLSDLTNLSTLLHEDSTAIGLPTPELNWHQINEEDWSTSWKQYWQPQEIGNQFLINPAW
LPIPLSTSRLVIRLDPGVAFGTGNHATTQLCLESLEKYLTQNSDPQVIADIGCGSGILGI
GALLLGAKKVYGVDNDPLAVESTNSNCILNHLNPEKLTCALGSVHTLTEILTEPLDGIVC
NILADVIIELIPQMTDLVKPGSWGIFSGILVEQSPSVITILEKTVGY
>EFA74268.1
MPLINTIDVTIFTRFTIPLLGSSTNEPVDNTMVVAAVLLSLVVIYLASKVGGELSNKLGF
PPVLGELVGGVVIGTSVLHLLVFPEGGTDSSSSLIMSFLQITAGLTPEATPAVFAAQSEV
ISVLAELGVIILLFEIGLESNLKDLMEVGIQAFVVAVVGVAVPFAAGTAGLMIIFGIAPI
PAIFAGAALTATSIGITSRVLSEIGRLNSKEGQIILGAAVIDDILGIIVLAVVASLAKDG
AVDVGNVIYLIASATGFIIGAVILGNIFNKSFVAIADVLKTRGSGNSRFYFCVHHVLPGG
HY
>EFA74270.1
MCDRHFGIGADGVIFALPGQKGTDYTMRIFNSDGSEPEMCGNGIRCLAAFLTELEGISRS
KDYYLIHTLAGIIRPQLTDDGQVKVDMGSPRLLAQEIPTTLVPGDSKVINLPLEIGKQTW
HITCVSMGNPHCITFVEDVASIPVEIIGPQFEHHPVFPQRTNTEFIQVVNPSHLKMRVWE
RGAGITLACGTGACASLVAGVLNNLCDRMATIELPGGNLEIQWSETDQRIYMTGPAERVF
TGKI
>EFA74275.1
MEMTNKLPRWLTFALAFPIIILNGWLLIQVIKYFQPLVSVVVLAVLLSFVLNYPIKFFHS
LGVPRILAIVGVLLLTIVMLGAIGVILLPLIFQQLNELINILPAWISSGTQQLQAFLDWA
ATQQDLPVNIIGIATQLLEKISNQIQSFTGKILSFAFDTIGFLLNLILTIVLTIYLILNG
EKLWDGLYGWLPTYLGVKARALLKEDFQNYFIGQATLGAVLGVTVTLTFIALKIPPSLII
WNYHWFFSLFPFGTGIGIGIVSLLIALENFWQAVEVALIAVTIDQINSNIVAPRLLGNLT
GLNPVWVVISLLVGAKLGGVLGLLIAIPIASFIKDFADIWRQGQLQTDSMEGKITTPS
>EFA74285.1
MREAVFNIWQGKIDQCRWLDLCAGSGSMGAEALCRGAKLVVGIEKSSHACTIIQENWQHL
ITEQQVFHILRGDVIQQLKKLSGQTFDRIYFDPPYTSDLYDQVLNAIAGFKLLHKDGEIA
VEHSSDFKVPIIPVWQVIRQRKYGNTSLTFYSCREECESDGALYYDTSPSLGTHN
>EFA74286.1

MPVIAVVDYDMGNLHSVCKALEKAGAIARVTACGKELARADAIVLPGVGAFDPAMQNLRS
RNLEQPIKDVVTSGKPFLGICLGLQILFESSAEGNEPGLGIIRGKVRRFRPEAGIAIPQM
GWNQLQLTQPNSILWEHLPSQPWVYFVHSYYVDPLEPEVQSATVTHGSQTVTAAIARENL
LAVQFHPEKSSNIGLQVLSNFVAQVREKIPA
>EFA74289.1
MNAILPQFLKSFYRKDPIISVLITMGIVDALIGGLDDSWSLFAFGLGTTGVALGLKLASK
LSRAQQRSPREEGRTFQYYLPPTSSSSSLPIIKATKNKSYY
>EFA74291.1
MQIGVPKETKDQEFRVGLTPASVRVITENNHSVFIETMAGYPAGFTDEDYLAAGAQIVAS
AESAWHQDLVVKVKEPLRSEYQFLRKDLVLFTYLHLAADRELTEHLIDSGTCAIAYETVE
QVGTNRLPLLTPMSIIAGRLAVQFGARFLESQQGGRGVLLGVFPELSPVKW
>EFA74292.1
MVILGGGVVGTEAAKIAVGMGAGVQILDVNVERLSYLETLFGSRVELLYSNSAQIEAVVR
EADLLIGAVLIPGKKAPILVPRKLVKQMRPGSVIVDVAVDQGGCVETLYPTSHSNPVYVD
EGIVHYGVPNMPGAVPWTSTQALNNSTLPYVLQLANLGLEALEVNSALAKGLNIKSHHLL
HPAVQEVFPDL
>EFA74304.1
MENWSPNTTPIDLTINGAFIQMKRSNYATGTYSTSINSQPQDFQYKIAVNFGRSSGHLPP
RRQWHYDVGLLSQPLDLFAQKIPIKSQKLSKDYFRQVGRDDEWIQTLLCAKQTSDATYAV
DPDQRFNCF
>EFA74312.1
MTWGNVGDKTVICALSLKDEGGCDGNNTLFALKPENARNPNRVLETLTQISIRGSSAGVI
TETRGRRPNRASVNLGEVISQAAKRLPKQTLRTRPNPGVKPTVRPTRGGL
>EFA74316.1
MNLSITTKTPRKTTIKTARINLFLLASALVTAITSTTSLATDTISAQDIAQIAKKTSVQI
NTEGDITPGGSGVIIAQQGNRYSVLTANHVVCDIIDRPGKITCAKDITYSVRTNDGKEYP
IQSQDIIVLQSTKNDPDLALVSFVATEEYPVANLGDSDQMTEASDVFVGGFPAVFGKVGS
ARDFSFSKGIVLSRGRTSINGYSLIYDAKTLTGNSGGPVFDIKGRVVGIHGLADASNKSK
TETGELVSQKTGFNAGIPINTFLNFNNPLVKDLPIKRNTIATGEAPQERLNSPQSARDFY
ARGITKLEQFNYKESLADLDQAIKIDPKYAEAYFKRGYALSWLRRYEEALLDFNQVIALD
PNYLDGYLNRGWTYIWLQNDQAALEDFNRVIRINPNYAEAYAHQGMAYIKLGKYQAALES
SKQAIRLDPNKSYGYTIQGDVFNYLKDYPAAINVSTFAIKN
>EFA74317.1
MFRLLQLKIDPDDFNAYINRALAYTLTGNYSDALVDYQKSAEIFERRYTRKPSN
>EFA74323.1
MSTSQQTVSNPLKTPLLPPLLGASVDELTTWVQQQGQPGYRGKQLHNWIYHHGVHRISDI
SVFPKTWREQVTDVSIGRSSVNYQCSATDGTVKYLLNLADGEIIETVGIPSDKRLTVCVS
TQVGCPMACDFCATGKGGFKRNLNRGEIVDQVLTVQEDFQQRVSHVVFMGMGEPLLNTEN
VILALKCLNQDLGIGQRSLTVSTVGIRDRIRQLAEHHLQVTLAVSLHAPNQILREQIIPS
AKTYPIEQLLAECRQYVEITGRRVTFEYILLSGVNDLPEQALELSQRLRGFQSHVNLIPL
>EFA74331.1
MTLTFPKRAIQIPVTYRLARDFNVAANIIRAQVAPNQIGKLVVELSGDIDQLDAAIDWMR
SQHIAVSHNLGEIFIDDQLCVDCGLCTGVCPTEALSLNRETYKLTFTRSRCIVCEQCISS
CPLQAISTNL
>EFA74359.1
MGSDSDLPTMKEAIAICEEFSVSYEVAIVSAHRTPLRMVEYAQTAHIRGLKVIIAGAGGA
AHLPGMVASLTPLPVIGVPVPTRHLQGVDSLYSIVQMPAGIPVATVAIGGAKNAGLLAVQ
ILATYKPDLLVKVQAYRQSLSDMVMDKQNRLNS
>EFA74363.1
MGLIEELVLQVYRDDLKVFISELEYPEVKAAQKHIPVSIGILTGLKNRSVPMEQIATQVK
KTRDRNFAGVAFFFYETLWNMSEETVAKRQASFQKLFPRTVQRNT
>EFA74364.1

MAPADSLLAQARPEWITTRRDGTKIVKEGIHDRVWLNPFHPEVQKFMENLIVEIVRNYDI
DGIQFDDHFGLPSELGYDSYTVGLYKQEHQGKAPSENFQDPEWVKWRADKITNLMKRVFF
AIKANKKELSSFCCTQSPKIFLRILFSRLAKMGTDGINRRTGITSI
>EFA74365.1
MFPKEKRLGKMGISESRGVWLTNIDSDVLFARERLKRSLKTLGKLNFNTVYPTVWNWGYT
LYPSQIASRVIGKSLDPTPGLKTEIY
>EFA74366.1
MNIKLDKYTPSSLASLFILLMEGGITPNQIMSGIVLLAIQNYELEGTMFSANCLHFLMKA
IPVDTTATGVTEFILSLANESINIGMLLDAFAFACQKQGSRNIASLVSLTYQRLEADRVI
SQLINDQL
>EFA74367.1
MPLFSFVLTNTRSLTPEAADRTTREVCHNLKIALAQENIEDFLVVSRSDSTLRGHYPIET
DVIAEELGPFDAHFLIPAFFEGGRITRDAIHYLIIDGIPTPVHETEFARDSVFGYNYSYL
PKYVEEKTQGRIPESAVTKFTLEDIRQGSLNRLLTLHNNQCGVVDGESQEDLNMFARDIL
TAATGGKRFLFRSAASILTALANLPPQPIAPENMAKCVRGGKPGVVIVGSHVKKTTQQLE
SLLKIPQTVGIEVNVAKLLYESVNESGELLEILQQVEQVHHAGKIPVVYTSRQELTFPDV
KTRLDFGIRVSALLMDIVRGLPKDIGFLISKGGITSNDVLSTGLSLTSARLLGQILPGCS
MVTTPSDHPHFPLLPVVLFPGNVGNTDALATICQRLTISNPVK
>EFA74373.1
MGPKPPSSFKITGKYPQGWELISDEQGQWGKIE
>EFA74375.1
MTDLTTPVGQIINFVIALLVLLSSAIFVVETYNISPDARTELSILNTCILAIFTGEYLLR
LWSAKQKSKYFFSVYSIIDLMAIIPYFIGFVDIRFIRLLRWLRILRLLRFIDKKFLFFSI
SEDSVIFARILFTLFAIIFVYSGLIYQVEHPINPERFSTFLDAFYFSVVTMTTVGFGDVT
PVSEWGRLLTVFMILTGVALIPWQVGDLIKRFLKTSNQVENTCSSCGLAFHDGDALFCKR
CGAKLPQVSPSPQ
>EFA74380.1
MECIINRRAQFSASHRYWLPELGEAQNVGKFGLCAKFPGHGHNYVLFISMSGELDEYGMV
LNLSDVKQVIKSEVTSELDFSYLNEVWEEFQATLPTTENIARVIWNRLTPHLPLVRVQLF
EHPQLWTEYEGKGEKISLTARSHFSAAHRLAPNLRAEKYGKCTRTHGHNYHLEVTVEGEM
DGRTGTIVDLGCLHETVEREILELFDHSCINEDIPYFSTSHIVPTTENIARYMSDLLQFP
ISELGVKLSRVKLFESDHLWVEYEGKDSEIFFSVATGFSAAHRLADPTLSLEKNQTIYGK
CSRINGHGHNYHLEVTVRGEIDSVTGMSVDLVGLNQIIQHYVIEPMDHSFLNQDLPYFTE
VVPTAENIAVYISDVVRSPIEELGAKLHKVKLIESPNNSCEIYAKDIEESRVDRVDRELA
VV
>EFA74386.1
MEVIGETYEQEDIELILPTHSSPLFDINFDINPI
>EFA74388.1
MNHAKFESDLAQLQDEVSNCERDVLNLIKGKKTMEYSQNYNGGNQSNQIAIIGMASLFPE
SRNLQEYWQVIMDKIDCITDVPASRWSVEDYYDPNPKAPDKTYCKRGGFIPDIDFNPMEF
GLPPNILEVTDISQLLGLVVAKAAMEDAGYSESQQFDRDRTGVILGVAIGRQLAVPLGSR
LQYPIWKKVFKNCGLSDDETEKVIEKLKSAYIQWEENAFPGMLANVISGRIANRLDLGGT
NCVVDAACASSLGALNMAISELLAHRADMMITGGVDTDNSIFAYMCFSKTPAVSPSENVR
PFDVNSDGMMLGEGVGMLVLKRLEDAVKDGDRIYAVIKGIGSSSDGKYKSIYAPHSQGQV
KAIRRAYENAGFAPQTVGLIEAHGTGTMVGDPTEFISINQVFGDNNSLKQHIALGTVKSQ
IGHTKAAAGAASLIKTALALHHKVLPPTINITQPHPKLNIENSPFYLNTETRPWISNQPR
RAGVSAFGFGGTNYHVVLEEYESEHHQSYRLHNCAKSIFLSAPTTPELLSQCQHLYQQLE
STDKEQHYQRIIAESEQLIIPVDHARVGFTTLSLSQAIAHLAIIIDLLKNQPSVDFWEHP
KGIYYRQQGMETTGKVVALFSGQGSQYLEMGRELVINFPCLRQTYSHLDDLFSREGLESL
SQVVFPTPVFSPQERQEQLEKLQKTEYAQPAIGVLSAGLYKILQQAGLKVDFVAGHSFGE
LTALWSAGVLTEEDYFFLVKARGKAMSTSPEVDAGGMLAVKGNISQVTEFIKDFPQVAIA
NYNSPQQIVLAGNKSEITQLQNVLQAQGFSCFLLGVSAAFHTPLVSHAQKPFAHAIAQVN

FQPPRIPVYSNVTGKLYPNKPGSMQKILQEHLLNQVLFQQQIENIYQAGGNCFIEFGPKN
ILTNLVKEILVDKPHIAVALNANYRQDSDLLLREAVTKLRVFGVPLKNLDPYQIPTKISS
ASQKNEQKTLNIRLNATNINDRSQKAFAQALATGSVIPITRVSEENYQTQPEKTLVEINP
EITSSSILNSKTNSEVKNYSMVEQKIEIPVENYDRILDSLEQSLAEFTRQQSEINHAHQQ
SLQNQIEYNKTFYELMQQQCLFLGKEETNEYQAQTQQLAISSTERSMMQLHDHQAETIRI
HEKYLNYQQEYTNNYFQLLEQHYSLLEVGSPNGYSHLPSSHVAQSDPPAQKLIYPLEPEN
NSQNNLPDITVDFPIATHIDKGKLRETLINIVSDKTGYPVEMLDLSMDIEADLGIDSIKR
VEILGGLLELYPDLPRPNPEELAQLATLEQIAEYINNLITQLGQNQPLEETLSEHHNHPQ
FLVLPHDDLHEDLSNRSTTITIPDNLSQILLTIVSDKTGYPVEMLDPSMDMEADLGIDSI
KRVEILGGLLELYPDLPRPNPEELAQLATLEQIADYMRNQAETVERSNLSTSEKPDTSTT
EVADKILRLPVQLKALPQPDSLDLTIPENHFVLITNDGSEVTHRLVAKLADKGCKTVVLT
FPYLESNLSEEIAQIRLNDWHEETLQEHLTELTSKFGCVGGFIHLHPYSNNNLGIDQAIV
QHVFLIAKHLKEDLNQLTKKERACFFAVVRLDGELGTAKTHNFSPISGGLFGLTKSLNQE
WPEVFCRTLDLSPDLDGDTTVKHILAELQDPNLLVTEVGYNKTDRFTLVAEPTKSSIIPD
SLNITKDQVFLVSGGAKGITAKCVIKLAEEYQCKFILLGRSSAEIEPVWSEGYEDENELK
RRIMEDFLSKGEKPTPIMVQKKYQTIASQREIHNTLKAINEAGGKAEYICVDITDGMMLK
EKLRPIIDQFGTITGIIHGAGNLADKRIEKKTVQDFETVYAAKVQGLENLLNIVETNQLE
YLILFSSVVGFYGNVGQTDYAIANEILNKSAHVIKHKYPNCHVVSINWGPWDSGMVSPEL
QTAFAQRGIETIPQELGSSILVDQLRTSDSSMTQVVIGSPLVYIPSTLSSELKTHQITRQ
LKLNYNPFLQDHVIAGNPVLPATCGLSWISSSCEQLYPGFQIFHCPNFKVLKGIVFDQNS
PHEYILEIQEIAKIDNQEIHLVGKISSVTNHGKIRYHFSSNLILKRQVPPADNYELFNLT
QDGQFLASNSLLYQTGVGSLFHGNTFQGVKSVLNISPGKLTMKCELPEPTLHQQGQFRVQ
TFNPYIADVQVHSLWIWTQHFHQVGCLPSEIENFEQFAPVPFGETFYVTCEIKSKTESYV
VADVITHSQKGQVYNRMKGAKATILPNSY
>EFA74395.1
MWQSQEKFAVLLLDVKNRFLGTKIITIGTATETLASPRDIFREVIRHGATRMIIAHNHPS
GNLDPSQADLDLTRQLLSGAQLLNIPILDHLILGNGNHQSLREITSLWDDFPQEN
>EFA74396.1
MTYCLRISDLPENERPRERLITHGAKVLATAELIAILLGTGQGPGKLSAVGLGQHILQEL
GRDQGDPLAALRDATPAQLTEIHGVGSAKATSILAAVELGKEFFISSCGGSDY
>EFA74400.1
MTTASAYSLNVPGHPTGQISKEELRSLLKEVESQLYHSHAYRAVVDKAQKLFDISHEQLT
NFNKLIQAISREAIALTFERFVTKQSATSTDSSKNDSADSTNSNEIKELSNQTVEVCEQV
LENRSQPNDPEPTAKKQQKSDRVKGENEPDPTKKTQKLAMEKSKTF
>EFA74401.1
MKTNQIQRKKLKSWPWKNQKPSKSRLAAKKAEEERLESLRQIGEQLKKARESQGLTLHKL
TMYTYISPNQMAAVENGDMEKLPEDVLLRGFIRIMGNALGLNGADLANSLPVRNECKSIL
PSWYKNQRPSRSLSIELSPTHLYLGYTALVAGAVSGLSMMYEQANNQGIKNFQSTTPCLS
SLCGSDEKATNVNDQSTMQHGRVGNSFGPQISPPEAL
>EFA74407.1
MSKPILEGFDAKLEEKNPVEGGSDAIEQPTFPLIL
>EFA74410.1
MTTKILVTGGAGYIGSHVVLQLAESGYDIVVYDNCSTGTPESVLHGELVVGDLADVDRLY
QIFSQHKFSAVLHFAASLVVPESVAHPLDYYANNTRNTLNLLRCCSVMGVNQFVFSALHN
KV
>EFA74412.1
MLNAYSLGVSRRTVVSLALQIANERVNKDRLVRRRTAQVQIAFDIYLRALYQNRPDISFF
FTNHVASSLHRYWPSLFPNDFKSLKYDQFWVERWSDEILFAMREAAYQIGQLMAFVERYP
GSSRWSQ
>EFA74416.1
MQCLFMTKNQNPSLSSHVNVLSTNLLDASPINQRLNVLQGLAQFSLCFLIVGCQQLGMQV
VCYPLVIVISLVFSKRIKLLSYKYTVAPLALISLAALSLNLRDFPSGDVLNLIRFFTGFL

LISFFFANSTVKLRYFSYSFIAWSLIELCFLIFLNDQPPYIKSYLSNSDIESFSRAEFTY
GNLRLLGPFINSSINGTYAGVLTLMLLLDPDLIFGKFELRSKKREILLLSILSGVTLLYS
GSATGFITTGFLLFLRLSPYLFILRSKYVKILLTLSLIAFASFSLGGIYFSKLDSNYLSF
IFDLKLNQFFDRVNNVYDLVLGSPYETGFIYGGDFLVLSMIDNFGIILAMLVFFTAWNQC
GRKYRPYFLALSFSSLHYGTIFSLTGQVLFASLVLSPNKSYDLK
>EFA74419.1
MSVLDREFLGMGEEVKLFEEELTAFLGRQVACVVNGTAALHLGLQACGIGQGDEVLVQSL
TYVASFQAISAVGATPIPCDIDPKTLTIDWKDAEKRITPRTRAIMPVHYSGGVGELDQIY
AFAQSHEIRVIEDAAHAFGTKYQNQLVGSFGDIACFSFDGIKNITSGEGGCIVSNSPDIM
SRVRDARLLGVEKDTEKRFQGQRSWDFDVRSQGWRYHMSNIMAAIGRVQLRRFPELSFRR
QQLAHCYDKRLSGISFITLLKHNYEEVVPHIYPIYLTEGVSRDYVRQRLLEHGIQTGLHY
QPNHHLSFFSHGLDNELPITDRTYSGLITLPLHPDLSEEQIDFICSILIQVINNIDNATL
N
>EFA74420.1
MKVIILAGGFGTRLAEYTDIIPKPMVSVGGLPVLLHIMQLFDNYGHKNFYIACGYKAEVI
KQYFLNYPTLNADFTIDLATGQVQTVKKKEIDWQVTLIQTGLKSMTGGRVKRLQSLIGNE
TCFLTYGDGLANINLDDLLSYHYNHGRMVTITAVRPSARFGELEICDGQVISFEEKPQLR
QGWINGGFFVIEPSFFDLIAGDDTVLEAEPLEKATALGELMAYQHDGFWQCMDTKRDLDL
LESLWKSGKPPWIKE
>EFA74421.1
MLDGVILTPIRRIPSDAGDVLHGIKASDPGYVKFGEAYFSTIHYGTIKPWKRHNQMTLNL
VVPCGCIRFVLYDDRPESTSYGRVESFILSPEDQDYRRLTIPPGIWMAFQGVSQNLNLVL
NIADIEHDPKESDRADIDFITFDWSLKP
>EFA74423.1
MYYVLLLIGRIFGKLSRIVFNLAKDFEPEDIQSKLVQKSTQYDMVTAPDEPYYFQQYWKF
ILPHIEKLLEPNQVLELGCSQGRFTLPLARLFPNTQVIACDFSATAISTAKKYASEALVT
NIDYRVQSISECLKSLKAGTCDIVFMTEVTFFYPKWKLDLPKIVEVLKPGGVVVMSFKTK
YFYALTIARHRFWDNVDMLITQTQGRIGVGSPVEFTWQTSTEIREILSKFGLVLLDLVGI
GVLSGMKGHDPHDGIVQPSLLDDSEREALMKLELNLASEVPDGGRYILAVARKT
>EFA74424.1
MKTWEKQLVGNRISLKEFIQIESIRQRPLILDWIRRQREANNDSIHWWMTDLAGRNNVSC
RFFDDVIHISALKSWITTNASYPDEILVVCENSYVLAAAKTNLESTVQLRCQIGWQITMA
FETSYLILRACVNFARQILQFWKHHRAAQISRPFNLQPPQGEVYLINQCLDDKAFNKDGP
LACRYFTVLPEWLEKQGKQVYRLPWLYNVSLPLEEVYQKLRDSSCFLPEDWLTWRDYVRA
FYDGIKTVSTIRKSIPYKDNFHLEFLLIRERFRQLTTGISVSRFLLYLPALRRWGNNLTK
LVSITHFESMQPERVQPYFCRFVLGNKSKSIGYYHSLVASDFLGYHIPLGESESKFFPDI
IVTNGGLGHSVLISRGLDEKKLLSGPGLRQNFVQTATENSGNCLGIPLPLDLQGAVEMLD
GIYAHFDWVRNYLKSSVILKPHPMMSRREINILLSHMGWTDLPQDWQWCEGEMTDLLKIS
RCCLVMSTASIIDVLLSGRVAISLGRELTSSWNYGDFLENEFPILRSVSPKNLCQRLEEV
FLTDTQWYDDEFRKIRQKFLAGLNQTSDQTLSVFL
>EFA74425.1
MKLEPQVCPLCNSSKPHQRIRSTDVYGGVSEQAFWQCNDCNAIYLYPVTSREDDKAFYEN
EFDKWMAKRSGDESWSDPESQFKSLSSREMPLRKPWLDKLCPAGSRVLEIGSSSGFMLKP
LQDSGCEVIGVELSHQYRDFANSKGVKTVASMDALNRDHTGYFDILLHYYVLEHVTDPLS
FLKSLLPFLKPGGKMMFEVPNGNDPLISFYKVPEFEKFYWWRAHHWYFTPESLKYVLAGL
DRPFEIFPGQRYDLSNHIHWLMTGKPGGFRKYSHIFSQETERAYAEDLKRSGWCDYLIAI
VS
>EFA74426.1
MKAIVTGGAGFIGSHLVVRLIDDGWDVIVVDNLSSGHERNIPGGAHFIWMDLTTEDSFSL
LPDNGVDAIFHLASHVGQELSFENPTYDLKANALSTIFLLKWALAKRVPRFIFASTMNIY
GDPLNLPVSEDSEIKPPSPYSVGKVASEYLCKIYQGFGIHTTCLRLFNVYGPLQDMKNMK
QGMVSIFMSYVAKNVPIHVKGSKDRFRDFIYVDDVVDAFVKSLDNRASGKIYNVSTGTKT

YVWELIDYILDAFGKKADEYPITFGDGTPKDQFGIYGDNSSLVGDLDWVPRTDLKSGLKV
MADWVRTLPSEMLPNL
>EFA74427.1
MYDFFLGSCPKNLDEEINYLISVKRLLPRWINSIPDSEFATICTIIHDLGNRATITGEKL
CLIETGVGASSIAIVFYAIKHHGLALTWDFNSKKGSEIRTACVETICTLFSANINSVWKL
IGYNSRSPYLGIGITSEWTDTVHFTMHDSEHVWENVEGELNLVDPFLKDGSVVAVDDAYY
TFLHTDTAYINLTRKKLGLKAINSIEGNVCQSHSIEAENFLKKRWGKVKSLAGDYQKICQ
DDVSIAYFNNELKVRSSLGMEQVQQLETRFGVWEVKSRLRQKEEK
>EFA74428.1
MANKVLNTTSRVAVTSRSFSRHFDLRQELLASYAQVTFNESGQVLAGNDLVEFLQGHDKA
ITALEKIDENLLAQLPQLKVISKYGVGLDTIDLEAMDRHGVQLGWKGGVNRRSVAEMVIA
AAISLLHRTSESHAEVRAGQWRQLQGRQLTGKTVGIVGCGHIGKEVAILLSGFGCRILAH
DIKNYAEFYIAKDIVPVSLEDLLSDADVVTIHLPLDISTRKMFSLERLKLMKKGACLINF
ARGGIVDESGLKQLLKDGYLAGAALDVFNEEPPLDLELLNLPNLIATGHIGGSTGEAVLA
MGRAAIIGLDSAKPARNYTEIF
>EFA74429.1
MCEALVIQSHRGSYTVRYAQNALHLLALESHEKRHFIIDANVVAIYHEVLNPIISGSSAL
VIEATESAKTLDRFTGYVEALVSQSMRRDHRLIAIGGGITQDITAFLATTLLRGVAWEFY
PTTLLAQADSCIGSKSSINVGPVKNILGTYCPPNQITIDPDILKTLKEVDFRSGIGEILK
VHAIAGPSHYDEIAAVQDQLKTDHNLLRYFINRSLEIKKTFIESDEFDTGPRNVMNYGHS
FGHAIEAATDFFVPHGIAVTIGMDLANHVAMQLGHVSDDHFNRMHPTLMKNSEGFHTVEV
PRDRFFAALSKDKKNIGTQLSLILLDAAGLPKRTLIDNSEEFCTICNNYFLDVLPYG
>EFA74430.1
MSFYPVDFADTHATESFVDKIRSSKFDILVNNAGINKVAPFANIAPKDFDLIHLVNVRAP
FRLCQAVIPYMLEQRWGRIVNVSSIFGIVSREHRASYSTSKFAIDGMTAALAAEVAQFGI
LANCVAPGFIDTELTRSILGTQGIADLADRIPARRLGQAEEVASLVCWLCSPENTYISGQ
RLVIDGGFVRV
>EFA74433.1
MKIVRYVTHNPPGGVHNGVVKDGNVFELEGDILSGNTHAGKMVGSIEDLRLVSPCQPAKI
ICVAINFPGILHFSPTMSEPLVFVKPPSCICGPGDTVENPFPGLSWWGEAELAVVIGKRL
RNISDCEAREGILGFTIANDTTVENVDHRDHHLARSKCADKFCAVGPWIDTEFDASDCVI
EAIQNGEVIRRGRSSDQFWQWQRIISWLSTWMTLNPWDLVLTGNPPDTVGMRFLGDDDIY
TARVSGLGELTNRFVRTLPIRRSF
>EFA74450.1
MGDKQGAISDFTQAINLNPNFAQPYYNRGATRNDLGDKQGAINDFSQFINFYPRNSLAYF
NRGIAWHELGDKQRAISDFTQVIKLNPNNVAAYYNRGASRSDLGDKHGAINDFTTVINLN
PNFAQPYYNRGLARHNLGDKQGAISDFTQSLRLDYRDPTAYNNRGIARYDMGDRQGAIND
FTQAIQISPNFAQAYNNRGLARHNMGDKQGAISDFTQAIQINPNFAQAYNNRGATRNDLG
DKEGAIRDFNKASQLFDSRR
>EFA74451.1
MYKILKNKIYVANLSLFVLISVSTSSTSAQTTTGIEDIARKTTVQINSNANPGGSGVIIK
KEGTIYTVLTANHVVCDNLGTIKIRCRTDSNYTVVTYDGKEYPMKYRQSLQINVQDPDLA
IIRFESRENYQIAPLGNSDNVKIQSDILVAGFPTIFGRVGKQRTFTITNGKVVTFIPNSD
RGYGLVYNATTFIGNSGGPVFDIYGRVIGIHGLADTDDGETNNNNQSETVNGVKPTQKTG
FNAGIPINIFFSLSNFNQQVNPTVP
>EFA74455.1
MSRKGGDFQVDFNRYGGPDLVIIDEAHEGIPNYRNRIHQTCLQIQQSDRLYSIQDNQQEL
DLGILEYGVTSLVALMRWK
>EFA74456.1
MEVEKDLTIRKQLLDVIIIKRYKDSGFNLYPAGLENMSKHNLISYKSIHESFTVWSLMEL
IGHYVNYRKQTSKSLSNLLPEKDYKLYGLTTHEPTQLMGKLKWKSVSQGVYDIECVGMNV
RLIVLSKIPKSANNELWRLFSAKAEVVEEAISHYQESYRNSEYSVLMQQLYEFYLKEKLP

MTYTLKQFKKDFVISHLREIPTEEVLKQYSPEEVLKQYSLQDLLEGLSPETLERLAIILS
QLGVNQIKNQEQ
>EFA74460.1
MGTAIVIGYGNELFGDDGIGPLIAKVIQRWRLPCVQSLAVHQLTPELAEPIANSRLAIFV
DTCINSYIIRCKYNYYCHYH
>EFA74468.1
MLEPLFRCNLACSGCGKIQHPTEVLKQNLSPEQCFAAVEECGAPVVSIPGGEPLLHPQIN
QIVQGLVDRKKYVYLCTNGLLLEKSLHKFKPSPYLTFSVHLDGMKEWHDHCVDRKGVFDV
AVAAIRAAKSQGFRVTTNTTIFTGCNVEEMQGFFDFLETLKVDGMMISPGYSYEWAPDQD
HFLQREQTRALFREILAPYQSGKKNWNFNHNPLFLDFLIGEKDYECTPWGSPSYSVLGWQ
KPCYLLNEGYYQTFQELLDQTDWSKYGHASGNPKCADCMVHCGYEPTAAIDAMQPQNIAR
SLSTVFGR
>EFA74470.1
MDICLRYGKVVMGIELKVRREKLDPLTKGLTQLDKYLDGLGLDTGWLVIFDRRPGLPPMG
ERISTEEAISPGGRTIMVIRS
>OHY31408.1
MSQEVTLKNLPLGINTLDKLRGSNCVYVDKTPFALKLIKQPGAFFLSRPRRFGKSLFVDT
LKEIFEGNEKLFEGLYIHDKWDWSRKFPVIKIDFADGVLKNREELDEKIRDLLWNNGDRL
GVGAKKNSISGIFGEIITGAREQFGERVVVLVDEYDKPILDNIDNPNIAAEMREGLKNLY
SVLKSQDANLQFVFMTGVTKFSKVSLFSGVNQLKDITISEAYSSICGYTETDLGESFGDH
LEGVDWDTVRHWYNGYNWTGSETVYNPYDILLFISEGMKFRNYWFETGSPTFLVKLFQTN
RYFLPNLEHLEVTEEILESFEVERINPVTLLFQSGYLTIERTFTRRQRYMFALKIPNLEV
RLALNDQFINAYTEIVNEKSGIQDSLYEFMNRGDVESMIMAIRRLFAGIPWRNFTNNDLA
DFEGYYASVIYAFLSSLDARVIPEDISNYGQADITAMLGVHIYVMEIKVVEGNQVQGNAA
LDQILQRNYAEKYRG
>OHY31503.1
MSLITLQSVKKDFGIKEILKEATFSIDGTDKVGLIGTNGSGKSTLLKIIAGIEPIDGGQI
LTNYGAKIIYLPQQPDIDENLTVLEQIFMDSGEHTKLVKEYEELSDKLAHYPEDTLLMSR
LSEVMQRMDATGAWELETNAKIILTKLGIGDFEVKVGTLSGGYRKRIALATALLAQPDVL
LMDEPTNHLDALSVEWLQSYLNRFRGALLLITHDRYFLDKVTNRIIEIDRGDIYTYSGNY
SYYLEKKALAEESAVSSQRKHQGILRRELEWLKRGPKARSTKQKARIQRVESMRETQFKQ
AQGKVDISTIGRRIGKKVIELSGICKSYNGKTLISNFSYEFSPEDRIGIIGGNGTGKSTL
MNMITGRTSPDAGTVEIGSTIHIAYFDQHSEELISAVNDNQRVIDYIKEEGEFIKIADGT
KITASQMLERFLFPGSQQYAPIHKLSGGEKRRLFLLRLLIGAPNVLILDEPTNDLDVQTL
SVLEEYLEDFLGSVIVVSHDRYFLDRTVDTIFALEEGGNLRQYPGNYSVYLDYKKSEELT
QQETINGRDNRKSKNLTQPKSGEQEVQNKKRRRLSNWEKREFEQLEAKIVDLEAQRTLVE
TSLLAVAPENYTQVQNLYEQMEVLKQAIDIATERWLELAEMDV
>OHY31513.1
MAGNHVIFIHPDGTSPSHFAFARFVDKGPDGRLNWDNLEKTGVYLGHMKDQLGGTSNSGA
VTHATGTKAYAESFGFEAGNTPIVSLDGSNKTIVEAARDAGKVTALVQSGAIFEPGTAAF
VAKTKEIVNPGASRTVPRSQAAEIAKQVILSGVDFIMSGGELNLLPVGTNGIHGTAAQLD
ALTCIIHEGKNRKNKT
>OHY31528.1
LAGALSESLEIGGFIQPQMLIAVNFGLCATLNEQFVAKAPDWLYIPSVKEILPGRKSYTP
HLEGDVPAVVMEFLSDKDGGEYSFKRTYPPGKWFFYEQILQVPVYIIFDPDGGLIEYYEL
RNGRYELKQPDENGRHWIGSMELFLGTWQGTKEGRTGYWLRWWEQTGNLLPWALELIEQE
RQLAEQERQRAERERQRAERERQEKERLIAYLRSQGIDPNNLPNHAE
>OHY31532.1
MEIGQLTNIYERWKFLYVTLCMSGQEGRDPKPHDNQPPPSPHPNPPSRQPARAGALTVLF
L
>OHY31540.1
MAKNWAIVIGINNYLNLQRLDFAQSDAEAMKEWFLQEAKFDQVFLFTENSPPIPTNTQPI

PTQPTFGILMSFLGRQFKKPLLTTGDNLWFFFSGHGQRSRDKDYLMLKDTNPGDIERTAI
SVEYVTERLRGSGADNVILFLDACRSQGAKDGLGYGEEQYQGVITFYSCKAEQTAWEIPK
LKKGAFTHTLLEGLRLGVKGNCAAVHTLHRYLENEVPKLNQKHGKPIQNPYLKADPPYKM
YYILLPQSANSSDILNLKNEVRNAESRNDSELADKLRIRILAVDDKDNEEVDSRYAKLET
LLKAKNFRAADEETYKVMLAVANRESEGWLRREDAQRFPCRELRSIDKLWLKYSRGKFGI
SVQQQIYQRLGGTEDFNLDVWRSMGDLVGWRSGGNWLSNLRSWMRGGENWLFYSDLNFSQ
TAPLGHLPFAFALFREKPAFFVFEKERFTISSSGRTVIIGPERWVRKRWEDLVYRRVIGG
GDFELRMFESWTRSHMTEAQRWFPWVVPFFPSLLI
>OHY31647.1
MEEYYVYDPDEISLEVSIRENNSFREVEDFATWTSPRLNIRFDMTGDELVIYYPDGSRFL
SPVELSNYAEQERFLKEQANQRAEQERLLKEQANQRAEQERLLREQERFLKEQANQRAEQ
ERLLREQERFLKEQANQRAEQERLLKEQEQLKYQTLLSQLKAKGIDITTLE
>OHY31751.1
MLTVALPKGELLKNSIQLMQSAGLDFSAFLDARNRQLQIYDASGKAKGLLVRAQDVPVYV
EYGQAQLGIVGFDVLKEKKPQVGQLVDLKFGYCRMSVAVKSTSSYKSPLDLPAHGRVASK
YVNCAREYFESLDLPVEIVPLYGSVELGPITGMSEAIVDIVSTGKTLRENGLVEIATLYE
STARLIVHPLSYRLDLGGIYNLAQSVKSSVSS
>OHY31839.1
MLNLKTVIFVLSFTPFIVLSTQLTGRAQVSSPSLGTAGSVSGGGATGSDNNPISAPSLGT
AGSMSGGGATGSNSAPSLGTAGSMSGGGSTGSDNITVIVSTNNLRELRNAIGQLVSSGSD
SLSPITFSSLRASLISDGVSPGHTDQLIVSFSRVLSQLGVPNTNFSANNLNLEKLVASTK
IFKPTVTIAQDSPAEGEIALILDSNNLTEAINIYNKIVLESDDPTIIKLSRNQDFLKISR
ILKTLRSGII
>OHY31884.1
MTNIQSPKLTSPKLTAYEFKNLPLLGTTTTNQNILLGGFSGLYFQGVADNGNLQFVTHTD
RGPNSEPTGQNRPFLLPNFQPEIVSFELNKASGEINITNRTKLFRADGKTPLTGIPNVQA
GAGNTAYTDEIGVNLNGTVLNNDPLGADLEGIVIADNGDYWMVDEYRPAIYHFNRNGILV
SRFIPQGTASKSNQPVGTFGIEVLPEVYAQRRNNRGFEAVALAGNKLYAFIQTPIDNPDN
TGDTTSRSSRNLRILEFDIVSEKVTGEYLYLLDDITGTGNAKTDKIGDAVFLGGSQFAVV
ERDDRSDTTSNKLIYQIDLIGATNINNTSKLSLPQGKTIEQLTPVELGPAGITPVNKSLI
VNAAQIGYTGVEKLEGLALVSASTLAIVNDNDFVTTIPEKLGIIELPNNLVEVTGTANKD
ELFVKKGEYVLGLDGDDILDASDGLGNNVLDGGAGNDQLYGGKSDTLIGGAGNDQLYVVE
GNNNILYGGAGNDRLFVTEGNNNILYGEDGDDNLYIIEGGNNTLSGGGGADKFYIVNGVI
PVVPGQVLDFTRGDDKVIISGISQIQGFDNLILEQNANNTTIKTKDLIGGSQKVLGILKG
VVANTLTADDFRINDVTITNPQVSGTFNPIATDNILTQEEKKGGLTLSGTVSDPNAQVTL
QFGGQFRVAKVQSSNSSWSYTVTDNDYNFFREGQNQLTAIFTLTNNNRTGTFRASQTLTI
NSGVLPQPTSPTLDPTKPKGISEAVTQKAQSANIGQGILGSGEKLTDPNIVVIPFGGINT
FNQGANAAIEDFGLIKTGSNTTIESVRVFPKSPTGEVSVTLSDGTSVLASIPPGISSIGD
PLGLTMSGLVPGGSSTLDFYLPRNILNQLPSDLQSATYAKFNFEAKKFEQYADEQGKPLY
QYKFTDKNSNGIRDFGELYLSLNLTDGDKWDSDRIQNGIIVDPGQLGIAVVVTGTGVTYS
STTVNPGNSLIPREAPALSNIADNVFYKFSNTYKPAEARQALQNALGQTDAAFKNIFGLY
EIDNATGSVNGIAPGQPGYAKAALDKSKVISNFTVRAGGSVTPNINGDLIGIGDKTYAPF
VIANGGNYSGSIQEAINEFFKVNPNNSAATAQNYMGLPVAYFSFGAANPDGAAHIKSVGN
NIFGFEDLPAGVGVSDYDFNDMVFSFG
>OHY31900.1
MVIIGQGLRLFIILSSLDARVIPEDITNHGQSDLTVMVGVHIYVMEIKVIEGNQVQGNAA
LDQILGRNYAEKYRGEPGKYVHEIGLIFSRSQRNLIQADWR
>OHY31901.1
MLKSQDANLQFVFMTGVTKFSKVSLFSGINQLTDITIDTQYSSICGYTETDLRESFGDHL
EGVDWDAVRHWYNGYNWTGSETVYNP
>OHY31933.1
MLIWEDNNILLKAQDFRAADRETDKVILAVANREREGYLEIEDAEKFPCKELRSIDQLWL

KYSRGKFGISVQQQIYQSLGGTKEYNQDVLRSMGERVGWRREGNWLYYSDLNFSQQAPSG
HLPVFAYGWVWGGWVGRYGGDTSLLSRHAECNT
>OHY31937.1
MLFFLFGGFKYFMVTKMVTKVDSPLFYYDTLGYSYHIYYIFFILGIVVTYFNCYDVTDY
>OHY31945.1
YNGGPYQLVIFHFLIGVACYLGREWELSFRLGMRPWICVAFSAPLAAATAVFLIYPIGQG
SFSDGMPLGISGTFNFMIVFQAEHNILMHPFHMLGVAGVFGGSLFSAMHGSLVTSSLVRE
TTETESQNYGYKFGQEEETYNIVAAHGYFGRLIFQYASFNNSRSLHFFLAAWPVVGIWFT
ALGVSTMAFNLNGFNFNQSIIDSQGRVIGTWADVINRANLGMEVMHERNAHNFPLDLAAG
EVAPVALTAPAING
>OHY32019.1
MLGFVFPVFAFMNNAGYGNLATVLCSRNILLQMPKPLPIQEKSRVLGCQTLHFADAAFAS
PLTWGKLDLSR
>OHY32020.1
MANYNKLRTGMTYEQVVKILGEEGKEISSNDIAGYKNVMYMWKAGGFSVGNMNAMFQNGA
LVQKAQFELPE
>OHY32037.1
MVTLKGFAVLPADTFAAGPKSGAAVANPTNGRTTPFVGQPIQGFSGVQFAPNTNGSRFWF
LADNGFGAKNNSADFLLRIYQLDPNFTGVENGNAKVEVEKFIQLSDPNRLIPFSIVNQNT
SERQLTGADFDVESFVIDAKGDIWVGDEFGTYLLHFDSNGVLLDAPISTPNLFKLNTLNG
QKPIVIGHRGASGELPEHTIEAYRLAILRGADFIEPDLVSTKDGVLIARHEPNLINTTDV
ANRPEFANRKKKVVVDGVEEEGFFASDFTLAEIKTLRAVMPQGYRDQVFNGLLEIPTLGD
IIDLVKEVEAQTGKKIGIYPETKHPTYHDNLNLSLEEKLIDTLKSKSFTDPTRIFIQSFE
VSNLQDLNNNIMPARGVNIPLVQLIDAYDVADDGKLIYKDAYARPYDFTVKGDTRTYGDL
LTPAGLQEIAKYADGIGPWKRQIISVKTVDKNNDGKPDDLNNDGVINDSDKVTLPPTSVV
SDAHKVGLLVHPYTFRNESRFLASDYNNNPELEYRQFISLGVDGYFTDFPGTGDLVRDQI
TTNQVRSPQNPTVLSKPNFDTLNGQKPIVIGHRGSSGERPEHTLASYKLAIAQGADFVEP
DLVVTKDNILIARHEPMLAVVNLNTDGTIKLVSGKPEINFTDTSTDVYLRDKFQDRLKVK
NLDGRNVGGWFAEDFTLAEIKELNAIERLPSLRSTAFDKDGLKVPTLKEVIDLVKQVELE
TGRKIGIYPETKHPTFFQQQGFNTSQLLVNTLKTENFTDASRVFIQSFEVSNLKELKSTI
MPGASIDIPLVQLFGGSGKPYDFVVNGDSRTYDNLSTPTGLKEIAQYAKGIGPNKQRIVP
MTTVDNNKDGQPDDLNGDGQISDADRALGASTTLIQDAHQAGLLVHLYTLRNDGFFLSAD
YKGDPGAEVRKFVNLGVDGFFTDFPKTGTSVIVNNYLAGTGYANPNNNLNSPYFADSPVY
FNPNQPYYGDLVTANLNRSQGFEGMAFSPDRQTVYPMLEGTVVGDPAGSVRIYKFDVATE
TYTGLVGLYQLASPSNAIGDFTPINDKEFLVIERDNNQGTSAAFKKIFKVDFSQINAQGF
VPKEEVANLLDIQDPNDLNSDGNKTYNMPFQTIEDVVVWDNKTIVVANDNNYPFSIGRPP
LIDNNEIVVLELDKALSLDARLGLAATIAESSQLVFGTPGVDNVSVPQATDGINDAIFTG
AGDDKVDTLGVTNPYAGNNTVYSGSGKDVIYVNNGDRIFGGSGNDEILATDAKDYRISGG
SGNDVFYLGTNGRALGGDGEDKFFVTEGGGNLISGGAGGDQFWITTGDIPSVGNKNFANT
IVDFQIGVDVLGISGQGSNFGFNNLTLTNNDIIINGNKVATLTGINTSTLTASNFAFA
>OHY32042.1
MSHFSTLRTKITDAEILKASLSDLGIAVKTEADVRGYNGQRVRSDIVAVLEGEYDLGWSR
NSDGSFDLIADLWGVAKKHNQTELINSINQKYAVNKTLAEVKQRGFKNANVKLVLQ
>OHY32043.1
MKEELNILIQAQYPLIYLVTSEEERAEQAIYTMAQSLKPQRRVYVWTVTHGIVEYGQARS
TNQHNTVSSEAAIEWAIRQKEPGIFIFKDLHPFIDAPAITRSLRDAIASFKGMQKNIILM
SPMQQIPIELEKEVVVIDFQLPDMTELSKVLTAHQEQNRGRRLTTEAREKLLRAALGLTK
DEAEKVYRKAQVTSGRLTEDEVDIVLSEKKQLIRRNGILEYIEEDETIEAVGGLEELKKW
LKQRSNAFTERAREYGLPQPKGMLILGVPGCGKSLIAKTTSRLWGLPILRLDMGRVYDGS
MVGRSEANLRNALKTAESISPTILFIDELDKSFAGSTGSSDSDGGTSSRIFGSFLTWMQE
KKSPVFVMATANRVERLPGEFLRKGRFDEIFFVDLPTPEERQDIFRIHLTKRREEIARFD
LEQLAKMSDGFSGAEIEQAIIAAMYEAFAQDREFTQLDIIAALKSTLPLSRTMQEQVTAL

RDWARQRARPAASSVAEYQRLEF
>OHY32090.1
MGLLPWRLRDTSINETTISSKQQATKNTTEIMTKKFVDLSTLDGKNGFTIINSNDKNDNL
GYSISNAGDINGDGIHDIIIGAPLSDNNDQSNAGNSYIVFGSNNGFANIIDISTLDGING
FTVNGSEIGDQSGRSVSAAGDINGDGIDDLIIGAPFADSNGDDSGAAYVIFGRSSFSSLP
TINPSNLGDNGFIINGLNPQDQLGYRVSSAGDLNQDGFDDVIIAAPPNAYVYPPVTGDQA
GKVYVIFGSEKFNPSNPNFDTSNFDLNVINGSRADDYLGVGLNRGGDFNGDGIDDLIIGS
PFNDFNGFRSGQAYVIFGSKESFSSSLNVSQLDGVNGFVINGQEGDQLGFSVSTAGDINH
DGIGDIIVSAHDADPQGVDAAGAAYVVFGARTQFNSELDLSSLNGNNGFVINGIGELDKT
SWAVTGLGDVNGDGIDDLLVSAIHADANGDNSGQGYVIFGSKESFSSSLNVSQLDGVNGF
IINGKSENHNLGYSASGAGDINGDGISDILISAPFAGSGEVYVVFGNNGSTDTGEITDEV
TEGDGGESEEFTIDGTEDEITGGVIEDNNSESEITNIDGNPDEILDGVMGEGDSDNNSTE
GLGSDITDSPDIESTPTGSVDNILGNNNVLSSSTTKVEFQLMDKIPASIRELGVFTVDDA
SGKINGIAPGQVGYSEAALARSKVVFSVLSKTPNEFNNANVTRILGFQEANPNLRFYVID
NGTTDAVKNDLLPINQVTFLDSSNLQVTQLPDNSFSLQSNDLVFKARPTTQPLPMGTNLQ
EKSQGESIDLRGVTGPVNAQFTVYREASFDNYVGFYKVTDEKGGIDTNSDGTADLLPGDA
GYIQSAVNQHLSGLGLNVANGNKSTFNNTLSGGGLYVPFIIVNGRPDTVLNSDLSANSNP
NIYFTYLGANSDQVDHVRLLGDNTFGFEDLRGGGDMDYNDLVVQVNMSARV
>OHY32115.1
MSELERYYTLLDLEPGATIEEINQAYKDLVFVWHPDRLPKDNHRLQKKAHDKIKSLNQAR
EKLRSFQYQSQPGHHSEPSTSKRSPYQPNQPPPQNPDLSGRDFSHANLSNKDLSGRNLSY
ANLSGSNLSDTFMHKVNLRGANLSEANLFRANLLLADLREANLRSANLIGADLSGADLRG
ADLTGARMRSGERLLVKLVGANLTGAIMPDGAIYG
>OHY32182.1
MVALLQKIRLENQSIFQEVNDFLSTRKSNRLAQAQKQAEDLSQFRQQLEQEIDQFLTKTA
QSRQAQAQAQAQELHQFRSQLEQETSEFLTKTTQARHAQAQEQAAELSAFHKQLEQKTEL
FLADTAKARIAQAQKQAAELSAFHKELEQKTSAFLADTSKTRIEEAQKQAAELSAFHKEL
EQKTSAFLTATTSDRAAKAAAQKEALRQFRQDLFLSVIGV
>OHY32195.1
MVSKNFWKPIIHSSTALVLLTTLNTAWPLVSLAQSKPQPKANSAASSLFTDYLLGGGDRI
RVNVFEAPEYTGEYQIPPGGEINMPLIGSIPVSGLTTQQAADEIARRYARFLKRPLISVN
LLAPRPINVFVAGEVTRPGSYSLSLQGTGGNNPGVQYPTVLAALTTAEGVTLAADVTKVQ
LRRQVGRSGEQVVSLDLKQITQTGRIPIDITLRDGDTIFVPTATDFNVAEARNLFAASYA
ASRTAPRRVAITGQVYRPGSYLVAAGGGGGNDSGGLPTVMRAIQLSGGITSQADVRNIKV
RRPTRTDKEQTLNINLWELLQSGDLNQDVVVQDGDTIIVPTATQVNTAEVTQLATTTLSP
ATIKVGVVGEVKRPGVTELQPNSSLNQALLAAGGFNDARASSSSVDLVRLNPNGTVTKRA
VKVDLSKGINEETNPILRNNDVIVVNRSVLARTGDTLGAVTAPLAPVFSIISLFRLLGF
>OHY32339.1
MQVLRSFSNQEVLDLQENLGRGGEACVYTVPSNENLVAKIYHHPTPNHIQKLRAMIANPP
ANPAASFGHISIAWPQELLTAAESSDTIIGFLMPRIRNMRPIMDFYNPGNRRQNCPLFNY
QYLLRTARNLAAAFAALHASNYCIGDVNESNILVSNTALVSLVDTDSFQVPDLSQNTVYR
CLVGKPEYTPPELQNKTFADYNRETYHDLFGLGVLIFQLLMEGTHPFSGVFQGLGDPPTY
ESRILAGHFTYSQKQKVPYLPTPITPSWQTLHPDLRDLFISCFEDGYHAPYLRPSAQTWL
SVLSTAEASLVSCAVNPQHVYHPHLDKCPWCERTIKLGGRDPFPSLQAISAREHLQPRRK
SRKRPRYQPRVRKPAVPLLAPYTQSSLRSTLPGYPTVQTSSRSKFYTFMFGILGLGVLGY
LDIMVKFTRPFLSPNPYTQQSLLSSSSENVHSPLSLSFNDYYQRGNQAYQQDYQQAIED
FSQGIKQNTNFSKLYMHRGNARYNLNDYEGALTDYNLALKINPQEVKALINRGNAYIKLA
EYSNDPDYEYKKAIDSFNNAININQQDDEAYVRRGIVRSQIARYSNNSQEEYERSIGDFT
QAIKLNRFKAEAYFQRGLARYQFAQYSSNYAQIYKQAIADFDQALNINPEMAEVFLKRGM
IYYELAQYGEKTARNNQQRALEDLEKSAQLYLNKKDVNNYQQAISNICVIAEEKCDYFLQ
NSSIIYSVNP
>OHY32645.1

MRSVDDKIGGKITLNNNIIAGLGKAKQFAFWGAGGGAIGALIAEILYPIIIQDNSKSLGN
TVIQVAIWFGIIGATIAIALLSASFQYLKRGIQLGQATQNGIGVGLLAGAIAFLTALKIS
LENN
>OHY32646.1
MRKYKEIDDHNQDIKINPNSAYAYILRGIARFQSGDKQGAIADYNQAIKLDPNYALAYYN
RGIARRQLGDKQGAIADYTQAITLNPKLALAYIGRGNARDDLGDKQGAIADYTQAITLDP
NDADAYNNRGVVRDDLGDKQGAIDDYNQAIKLDPKNADAYYNRGLARRDLGNKQGAIADF
QKAADLYQKQGRNEWYRYALDRIREIQQR
>OHY32647.1
MNTQKLHIIMKRINLTFISSTLIALTQSPVFGAGPIGIGELKLGMTQEQVQALKGSVELS
SELSQWKPSESQNYTPEPGKKILKGMLNNPVTGKSEVTLTFTNDRLSSLSLVLDNESEMA
AAKNMISSKYGSPKTDNRQRDEQCIYRNGNSFTLKNGTLFYQWEQNHGGGVVSTTISEIL
INTCPYNLRYGTTDGLVFRTLSIKYGPKKPDVNNLF
>OHY32651.1
LARELTDSGEYTAVMLSVEVGSVFPDEPERAERAILGSWQDAIDIWLPEELHPPFDPERR
ESIGAFLKSWAKSSTRPLVVFIDEIDSLENQTLISVLRQLRDGFPRRPQGFPHSVGLIGM
RDVRDYKVKSGGSERLNTSSPFNIKAESLTLSNFSFTDVKNLYEQHTTETGQIFTPEAIQ
RAYYLTDGQPWLVNALARQATQVLVKDVNQPITAEVINQAKEILIQRQDTHLDSLAERLR
EERVKTIIEPILAGEDLPDTPEDDRRFLLDLGLVKRSPLGGLTIANPIYQEVIPRVLSQG
SQDSLPQIQPTWLNTDNSLNPQALLNSFLEFWRQHGEPLLKSAPYHEIAPHLVLMAFLHR
VVNGGGTLEREYAIGSGRMDICLRYGKVVM
>OHY32680.1
MSVNTVPSINYYSLDVIQYEASQLVRKGIVSRQQPIYTLCQYIPAREWVFVEYELEKCDF
LLRDRIGDLIGREQWEND
>OHY32714.1
MVVFGRPKGQRGSYRQWEEDNIPPQVVFEILSPCNSKGEMTRKKLFYLKHGVEEYYVYDP
DEISLEVSIRENNSFREVEDFATWTSPRLNIRFDMTGDELVIYYPDGSRFLSPVELSNYA
EQERFLKEQERFLKEQANQRAEQERSLKEQANQRAEQERLLKEQAHQRAEQERFLKEQAN
QRAEQERFLKEQANERAEQERLLKEQEQLKYQTLLSQLKAKGIDITALE
>OHY32803.1
APRQVGKTTAMLALARELTDSGEYTAVMLSVEVGSVFPDEPERAERAILGSWQDAIDIWL
PEELHPPFDPERTLDIQTVLKTWAMASPRPLVVFIDEIDSLENQTLISILRQLRAGYPNR
PQGFPHSVGLIGMRDVRDYKVKSGGSERLNTSSPFNIKAESLTLSNFSFTDVKNLYEQHT
TETGQIFTPEAIQRAYYLTDGQPWLVNALARQATQVLVKDVNQPITAEVINQAKEILIQR
QDTHLDSLAERLREERVKTIIEPMLAGEDLPDTPEDDRRFLLDLGLCRRDRGGALEIANP
IYREILPKTLAAVAIASLTSVEPNWLNSDGTLNPQILLNSFLEFWRQHGEPLLKSAPYHE
IAPHLVLMAFLHRVVNGGGTLEREYAIGSGRMDICLRYGKVVMGIELKVRREKLDPLTKG
LTQLDK
>OHY33028.1
GGPYQLVIFHFLIGVACYLGREWELSFRLGMRPWICVAFSAPLAAATAVFLIYPIGQGSF
SDGMPLGISGTFNFMIVFQAEHNILMHPFHMLGVAGVFGGSLFSAMHGSLVTSSLVRETT
ETESQNYGYKFGQEEETYNIVAAHGYFGRLIFQYASFNNSRSLHFFLAAWPVVGIWFTAL
GVSTMAFNLNGFNFNQSIIDSQGRVIGTWADVINRANLGMEVMHERNAHNFPLDLAAGEV
APVALTAPAING
>OHY33033.1
GFPRRPQGFPHSVGLIGMRDVRDYKVKSGGSERLNTSSPFNIKAESLTLSNFSFTDVKNL
YEQHTTETGQIFTPEAIQRAYYLTDGQPWLVNALARQATQVLVKDVNQPITAEVINQAKE
ILIQRQDTHLDSLAERLREERVKTIIEPILAGEDLPDTPEDDRRFLLDLGLVKRSPLGGL
TIANPIYQEVIPRVLSQGSQDSLPQIQPTWLNTDNSLNPQALLNSFLEFWRQHGEPLLKS
APYHEIAPHLVLMAFLHRVVNGGGTLEREYAIGSGRMDICLRYGKVVMGIELKVRREKLD
PLTKGLTQLDKYLDGLGLDTGWLVIFDRRPGLPPMGERISTEQVISPSGRTITLIRS
>OHY33034.1

MPKNWLNTDNTLNPQALLNSFLEFWRQHGEPLLKSAPYHEIAPHLVLMAFLHRVVNGGGT
LEREYAIGSGRMDICLRYGKVVMGIELKVRREKLDPLTKGLTQLDKYLDGLGLDTGWLVI
FDRRPGLPPMGERISTEQVISPSGRTITLIRS
>OHY33131.1
MNSKNYTFSGLTLKSLILLSSLLICNGLTIGGLELSANAQLLSNAEREELSRLRTETKIQ
KQLQSYLSIWLTLLSLFAVGLIATLWFLRKAIIRDIVERAMRQIGNIENLQTELIAANQK
TTGLIEYSQDLALELEEKVNHLKTKIEGEGGKLSVLLSDLPKSKQEFLTALEREVIAAQE
NISSLEFKLNTQLEQVTLAAQQQRVTIENIKKLESELFAQFSEIKLSIENHRDTSVSDIN
KYRSELMEQFEILALETLESKTQVVQSITEHASQFTSNLSEFQTHAQNQIDGFTSNLSEF
QTHAQEQMDGFTSNLSEFQTHAQNQIDGFTSNLSEFQTHAQNQMDGFTSNLSEFQTHAQN
QIDGFTSNLSEFQTHAQNQIDSFTSSLSDLNQTSVSQLVDLQTDTQKQKDHIFKDLAKSQ
SEFSHYLLELKNTTESRQREIIYSWQKSVDEVEQMLSNLHNNVEEQKTDLLENLQVLGLG
FQKQIGDLQNSIEQRHRKFFQTSQNSVEQLISEFMSELSAMKSDIKTDVDHQKANLIARL
AKLEKLESQFVEQLKKLQLDAENRQYETLKELSQMTPQTPDISPLSPVGTDTNTTNTIDV
QSVNNLDNHLDHEIQEAEELLSQNRYEDALSIYEKITSIQSDHEEYWLKRGFILNKIKRH
KEAIGVYNQVIRINPAHYQAWFDIGITCGKLGKHQEAFNCFNKATEIKPEESVAWLNRGL
SLVELENYEEAISSFDKALEIQPSSFKIWDKRGYTLVRLGRDEEAITNFNKALELNPEYG
SALYHKAACYALQKNVESALVNLQQAIKHNPSYREDAAGDIDFDDINNDTRFQQLIHRE
>OHY33271.1
MNPKNLPLGINTLSMLRENNCVYVDKTEIAHRLIRIPGRFFLSRPRRFGKSLFVDTLKEI
FEGNQKLFEGLYIHDQWDWSRKFPVIKIDFAGGVLKNRQELDQKINGILLKTAQSLGVDY
ELKDIQGRFGEIIAGAYQRFGERTVVLVDEYDKPILDNIDNPPIAAEMREGLKNLYSVLK
EQDANIQFIFMTGVTKFSKVSLFSGVNQLTDITIDTRYSSICGYTETDLTQSFGEHLAGA
DREAVRSWYNGYNWTGSESVYNPYDILMFIDKGKIFHNYWFETGNPTFLVKLFQANSYFL
PNLEHLEVTEEILESFEVERINPVTLLFQSGYLTIDHTFIRRHRSMFALKIPNMEVRLTL
NDHFINAYTEIVNEKSAIQDSLYEYMCNGDLESTVKAVKRLFAGIPWRNFTNNDLANFEG
YYASVLYAFLSSLNA
>OHY33273.1
MKEKKFIDELNRRNSTYNNPDLALTMANLLNTISKDINTDSQRFIYEILQNADDASNQNN
MLDVQISFVGNYAIISHQGDTFKESDIESISSAGDGTKREDSTKTGFKGIGFKSVFSHSK
YVIIKSKNFCFRYDENHWKNYWDDSNWGPKTNWQNQRLANRKDPDFKMPWQIIPIWTDFP
KELESIDVWEKFNVSIIIKYDEIHNLQTTFTQLLSNTQILLFLRSQEIRITLNYKQNSTI
TKSKNGEVTTIKTRNKNGTIISEWIINKYEFPLPDDVKQKIKQDEKLPEKLRDSSYTEIA
FAIKLEEGKLKAVHKDHRLIFTYLPTSINYNFPFLVNAPFLADAGREHLHQDLFWNKFIF
EQIPFKLLAFASELASKSSKYNKQFLKIIPHKSQGITVLEKEFYRSYKSALDTIAFIPNQ
NGKLLKVSEAIFDKPSISNVIGSDILISYINKKDKICFTLESLVSQLEDVNILKEIGVRI
FDVDELQHFLISDTFTNKHELTNNFALISFLYEEVQKLEISHKNIWKDKLHNIPFIFDQY
EKLKSPQNIYFDSVEFSENLSLNVSIIHNHLTTQIDKNSDIRNWLGDLGVKEPSVLSFIE
KTIIAECETYITTNNALKIGRFLFDAHKKGQLKEEHYEGLKNSKFLTQNQNLIAAKSAYL
SDFYEPELCLESVYSYDFYVFNQYVEDVNIKGEWKNFLLKIGVNQNITLQNITINRSDDL
DKIEMDYFLKVEEEAKKEHKYPWLINESNPIKITKISYSEIARENCDFAKQFWQQVFKKI
SLNSINKTALMRWGYYGSCESVENYFYWSLHNSTFIPTNQRTCLKASDVFNNSIPKIREI
AGKYLAIFDYDEFIPPNWLDYLKFKPHLEVSDYLQILKAIYEDVNVSEEEKEDNKKRICL
IYQMLSDLCKTNLDEEEIRLWGSTNKLLSKNSWEFLLPQDLCLIRVEGFQSGNSVYTEKI
NLQIIELLQLFGVSIIDSIQLSVPQDNTFEIFHLKNKLIEIAPFIALIYTSEFKVIESWE
REYKLIKDKIDNVHIFQTSEISLYYGNENDKQKRISWGENSNFYYVGDWKSPRVLDGLVE
PLCRFLNIQYAERLLIVLLLESYTSGVDYLKEKGYNTDIIHKSATNNLISETNEITTNHE
NRPYNQYDEDLGRKGEEFVYGELQRIYIQKYNSSIKETEDGFKIVNIQKNNKINRLEVFW
RNKNQNTTENHDFKIVENGQETYIDSKATPYGKNEEKIPFYISCREFALLEKVERYLIAR
VFNVTSNPELVFIKLDIENL
>OHY33284.1
MSSVPLLRLSDKFDPYISGEVNIDPSAVIAPGVILQAALNSKIIIGPGVCIGMGSILQVS

HGILEIEMGANLGAGFLMVGEGKIGANACIGAGTTVFNDSVAAQQVIPAGSILGDGSRQA
NSQESGESVDEGDTSSQSGEQVVSKTQFTATFVNFQTQSTSVPPLSPTPKSQSPPETESS
TESQEISDGKPRSRDPTEPHPLGTQIYGQGSINRLLSTLFPHRQSLSEQDANNGAE
>OHY33285.1
MVVRSTAAPPTPWSKSLAEPDVHQTAYVHPSSNLIGDVHLGQNVIIAPGTSIRADEGTPF
HIGENTNVQDGVVIHGLEQGRVVGDDGEKYSVWIGKNASITHMALIHGPAYVGDSCFIGF
RSTVFNARVGAGCIVMMHALIQDVEIPPGKYIASGSIITTQQQADRLPEVQAQDQQFAHH
VVGINQALRAGYRCAEDLKCITPIRDELKDHEEKTYTSITVEELDRSSEVAGKLSADTVE
QLRYLLEQGYKIGTEHVDQRRFRTGSWQSCQPIETRSLGQAISALETCLVDHAGEYVRLF
GIDSGKKRVLETIIQRPDGVVVATSPSFKSPSPSSYNGNGNGKFAGIDAQIINQINQLLS
NGYKIGTEHVDERRFRTGTWQSCEPIHSTSTQEVVAALESCLNSHQGEYVRLIGIDTKAK
RRVLESIVQRPNGQVVTSGNGKVSLSPAGSTPAVSNHLSGEVVDHLRQLVNGGLKISLEH
VDQRRFRMGTWSSAGPIEARNEREAIATVESYLSEYPGEYVRLIGIDPQVKRRVLEVIIQ
RP
>OHY33310.1
MLRVGNLDKSLEFYCDVLGMKLLRRKDYPTGEFTLAFVGYGDETDNAVIELTYNWGVEKY
ELGTGYGHIALGVDDIYSTCEAIGTRGGKVVRQPGPMKHGSTVIAFVEDPDGYKVELIQM
AKS
>OHY33326.1
MKPNYSNWDDDLPPDPQEIYQDLISTLERKVGFGLYFVQCTPIEADNFVQQISRDLANKK
IALLNLYEPMEKFYEYVKDYVQGQNIDILLVKGLEYSLYKYEKRNFGEVTEGQFTNLTKV
PPILNHLNQQRERFRDDFSFCFVFLLRSFSLNYLIHRAPDFFDWRSGVYELPTTAELVDE
ESRRLIVEGDYKKYLELTPQQKIETMLEIQELLTEKYQNDSNRARLLFEMGNLLYSANEY
ETAITFYEQELKLQPDDHSAWCNHGHALFSLSRYEAAIVSYRQALKLRPDDPFCWYALGN
SQRKLHRDQEAIISYNQAIKIKTDDHYFWYNRGNALRNIGCNEEAILSYGQAIKIKPDES
NVWNNRGIALRNLGRYQEAIFCYDQVLKLQPDDYYAWYNRGVALKKLKQNEAAVLSYDQA
LKLKPDDHYTWNNRGNALDDLGRIAEAIFSYDQALKIKPDDQYAFYNKACCYAVQGKIQE
ALENLENAVSLKPEEFTQRAKADPDFDRIREDTRFQALINKTFHD
>OHY33380.1
MNLSITTKTTIKTARINLFLLASALVTAITSNTSLAADTISAQDIAQIAKKTSVQINTEG
DITPGGSGVIIAQQGNRYSVLTANHVVCDIIDRPGKITCAKDIIYSVRTNDGKKYPIKSQ
DIIVLQSTKNDPDLALVSFVATQEYPTANLGDSDQMTEASDVFVGGFPAVFGKVGSARDF
SFSKGIVLSRGRTSINGYSLIYDAKTLTGNSGGPVFDIKGRVVGIHGLADASNKSKTETG
ELVSQKTGFNAGIPINTFLNFNNPLVKDLPIKRNTIATGEAPQERLNSPQSARDFYARGI
TKLEQFNYKESLADLDQAIKIDPKYAEAYFKRGYALSWLRRYEEALLDFNQVIALDPNYL
DGYLNRGWTYIWLQNDQAALEDFNRVIRINPNYAEAYAHQGMAYIKLGKYQAALESSKQA
IRLDPNKSYGYTIQGDVFNYLKDYPAAINVSTFAIKIDPDDFNAYINRALAYTLTGNYSD
ALVDYQKSAEIFERRYTRKPSN
>OHY33513.1
MKKFDICFINFSFKPGGGNRVILELSESLAKKNFSCRLISLVNNNNRGFSKISIIREESI
FSFIKSDYKLAPVINLFLTLSHLVADRNNYRVIIINNPLIAPIFGMLGFSNVYYYIQADD
YRIFDDRFLIKSSLLLHFYRFITKSITYNVYGERYLFNSHFSFEKFSEISKQNIPNPFLI
LPGVDLSIFTPSSEKYSRLEKDSSRPIVISTILRKHPWKGSLDFLRAVRIIGETNELDSY
RFVAITNEDISSISIPKQVKILYPKNDRELSGCLNQSDIFIVTSWWEGFGLPGLEAMACG
CAVITTRNGGCNEYAVDKVNCLMYEPKDVQQLTKLILELSHNEPLRKEISKSGIKTSQDH
SWDNSASALVSLCNI
>OHY33514.1
MKTFFKKILPFLDVLLVVFVYPAGYLLKYLRMAGVQRMPLSKMALVSMGVFPIRDHYYEP
QFDYRDPKLDLSKDRNLCGINWNISGQIKFLEKLAFSHEILDIPAKKSASISFHFNNESF
ESGDAEYWYQIIRFLKPKRIIEVGSGYSTLMAIKATAKNQKEDQYYTCDHVCIEPYEMPW
LEMSGVSVIRKKVEDLELSFFDILQENDILFIDSSHIIRPYGDVLFEYLQLLPSLKKGVV
VHIHDIFSPKNYPSRFLEDEVKFWNEQYLLEAFLTHNNSWEVIGSLNYLHHNHYQNLKLV

APFLSPEREPGSFYIRKIV
>OHY33515.1
MTLSLLEATWFLLPFTVLGTLCEILYKDKKYSLITFFLSSLGVVLFSITHVSICSDYSSY
EQIYSSALNGSINFSDVIGLDASYRTLASIWGSLIPLDLDFVLSYHLFTAVYQTTSILIL
VVFLFFFCPRWKNTLLFFILFGSLQFQHIILCGIRHGLSSSITVLLFFAFINFITKREGY
LSIVFLALLSVFTHWQAVIVILIIIILLQIVTSKSFTSVFRSKTLKLFRSRIILVPVFTLI
FIVLYAAPQIFTNLFLGFSQIVLSGQAGRISIYLDSNLDLGYGTKSSLLFYIDMILIYFS
LFRGEIIKSRLSQGNDKTDFNYCTQCKLLDWIGYLSIVNLIVKLLLAVGLGVLLRVSVTI
HLLQIFCIPVLLDSLKMKSRVVLVLLMSLPYLYFLFFISAEKFLMFV
>OHY33592.1
MTLPDSFRTQVQHIQESSKFQSVDGVLRATPFPGYTLITPPASEDPQNADFYQQIETFGR
SLLELPIASDLIVPLPVSSFHLTLADLIWDHAFIHACEKKHNFEEELNSYLGDLFGQYQK
SRSSLQGPISWQVLGLIVMPRAIALGLIPKDERSYEEIIQLRRLIYQNRKLMGLGIEQHY
HFTAHITLGYFSEIPANLDRVNLSNLLSELNQHWLLNFSEIVVSQAEVRKFDDMTHYYRQ
LNWASFRF
>OHY33644.1
METADNIESLIRKAQDLLDNKKTQAAIDTIHKVLEFNPNSIEGIKVYGQCLVKLNQIEDA
LAIFKKGLEIAPNNVSLMRNYGITLYQAKEIKRAVNVLDSASKIEPNNIKVLNSYARVLS
DSGNYEKAREIFERSLQIHPNNTIALTSYGKALADSGNYEKAREIFERSLQIHPNDTIAL
TSYGKALADSGNYEKAREIFERSLQMDPNDIIALTSYGKALADSGNYEKAREIFERSLQI
HPNNTIALTSYGKALADSGNYEKAREIFERSLQMDPNNTIALTSYGKALADSGNYEKARE
IFERSLQIHPNNIYILRMVNVWKS
>OHY33650.1
MNQGRTYDYIDLIELLIGTIFESKGDSQVIYPLLENNLHLLDATFIDQLQSWGGHQVNKQ
ATPEQNAQLGASLYLLARIFYHFPGGDPLINLTIAICGYEFCAAIERQLGLEKELAQVLN
HLGAAYQTQAQMGTDSHSNLEKAIAAYNEAIAIRRQPGLERELAQVLNNLGAAYQIQAQM
SKEANLERAVTAYAEAISILRELDLERDLAQTLYNLGSAYQTQAQLGKDSQANLQKAIPP
YTEAISILRELGLERDLAKTLTSVGSVYISQGKLERAITPFREAITMFRQLGLERDLAET
LNQLGTAYLSEVERGKDPEANLQRAIIVFTKALTILRQPGLEPDLAQTLHNLGKAYQTQA
ELGTESQSNLEMAIAHYREALAIATPELSPADYLKFASHLGDLGFQQGWWDIALEGYERG
IKALETIQFWQPGDKSPDIQYPHIYQSAVRACIELHQYGKALELVERSKSRNLVELLENK
HLYPKGNIPSRIYAELDRLRQAVRNEVRLLEIQGEKRSHAQLNGLIEELDQFITSQVKPL
DPHFSLTQRVEPITISKILKLVGADPRRVILEWYFTDDVLYTFIITANPDVPIFPHKVVI
SPSVIRQFSQKYLDIYRDRHNLGNWKQALPELLGELKEILQVETLLTQLWKLSPPCEELV
LIPYGCLHIFPLHALFPWERFPLGFSYFHSCQLLSSVEVTEREEVVKSLAIQNPTQNLQY
ASFEVETIITRLLIGTEVITEKEATRETIFSRSIPEKNYLHFSCTGYSNFQKPLHSAILL
ANCILPAPPKGGEYLPLPNGAAVDLSQCLTLGDICGLDMRRCHLVSLSAYEMGIGDMVDT
KNDDYISLPTGFLLAGASTIISSLWAVSDYTTALLMVRFYQFYTDTENLLTYGRPRLALY
HAQQWLRDAPAGKLLTWAAKLPFSGTRLTLLADFDTMPESQRPFQDPYYWAAFTCVGI
>OHY33699.1
GFPRRPQGFPHSVGLIGMRDVRDYKVKSGGSERLNTSSPFNIKAESLTLSNFSFTDVKNL
YEQHTTETGQIFTPEAIQRAYYLTDGQPWLVNALARQATQVLVKDVNQPITAEVINQAKE
ILIQRQDTHLDSLAERLREERVRDIIQPMLAGEDLGDTPEDNLRYVLDLGLCRRDRGGAL
EIANPIYREILPKTLAAVAIASLTSVEPNWLNSDGTLNPQILLNSFLEFWRQHGEPLLKS
APYHEIAPHLVLMAFLHRVVNGGGTLEREYAIGSGRMDICLRYGKVVMGIELKVRREKLD
PLTKGLTQLDKYLDGLGLDTGWLVIFDRRPGLPPMGERISTEQVISPSGRTITLIRS
>OHY33720.1
MNPKNLPLGINTLDKLRGNNCVYVDKTRIAFQLIKQPGAFFLSRPRRFGKSLFVDTLKEI
FEGNQKLFEGLYIHDQWDWSRKFPVIKIDFAGGVLKNRQELDQKINGILLKTAQSLGVDY
ELEDIQGRFGEIIAGAYQRFGERTVVLVDEYDKPILDNIDNPSIAVEMREGLKNLYSVLK
EQDANIQFIFMTGVTKFSKVSLFSGLNQLTDITISRDFSTICGYTQEDLEQTFAQHLQGV
DWDELRLWYNGYSWRGESVYNPYDILLFIREGMEYGNYWFETGNPTFLIKLFGTNRYFLP

NLEHLEVTEEILKSFEIEQINPVTLLFQSGYLTIERNFIRHRRSMFALKIPNMEVRLTLN
DQFINVYTGMVNEKSAIQDILYECMWSGDLESIVKAVKRLFAGIPWRNFTNNHLADFEGY
YASVLYAFLSSLNARIIPEDISNYGQADITAILGDHIYVMEIKVVDGENVKENL
>OHY33732.1
MQCAIGTMVIIGQDPRLFIIPTTSCCLLERAKFRNYWFETGSPSFLLKLFQKEQYFLPNL
EGIQVTEEILDSFDVEQINPVTLLFQSGYLTLLFQSGYLTIKDTFTDINQMVFCLGIPNM
EVKIALNNQFINAYSNLINEKLGIQRLIHTQLRSGDVEGLVSTIKRLFASIPWRNFTNND
LANFEGYYASVIYAFLSSLDARVIPEDISNYGQADITAMLGVHIYVMEIKVVEGNQVQGN
AALDQILQRNYAEKYRGEPGKYVHEIGLIFSRSQRNLIQADWH
>OHY33733.1
MIMAIKRLFAGIPWRNFTNNDLADFEGYYASVIYAFLSSLDARVIPEDISNYGQADITAM
LGVHIYVMEIKVVEGNQVEGNAALDQILQRNYAEKYRGEPGKSVHEIGLIFSRSQRNLIQ
ALAIIKNLPLGINCVKMCLCFLSRPRRFGKSLFVDTLKEIFEGNEKLFEGLYIHDKWNWG
RKFPVIKIDFADGVLKNREELDKYDFETDAWGLCEKEYTRDFGEIITGAREQFGERVVVL
VDEYDKPILDNIDNPNIAAEMREGLKIFTQC
>OHY33744.1
MTTAIQQRQSANIWDRFCEFITSTNNRLYIGWFGVLMIPTLLAATTCFIIAFIAAPPVDI
DGIREPVAGSLMYGNNIISGAVVPSSNAIGLHFYPIWEAASLDEWLYNGGPYQLVIFHFL
IGVACYLGREWELSFRLGMRPWICVAFSAPLAAATAVFLIYPIGQGSFSDGMPLGISGTF
NFMIVFQAEHNILMHPFHMLGVAGVFGGSLFSAMHGSLVTSSLVRETTETESQNYGYKFG
QEEETYNIVAAHGYFGRLIFQYASFNNSRSLHFFLAAWPVVGIWFTALGVSTMAFNLNGF
NFNQSIIDSQGRVIGTWADVINRANLGMEVMH
>OHY33747.1
MIPTLLAATTCFIIAFIAAPPVDIDGIREPVAGSLMYGNNIISGAVVPSSNAIGLHFYPI
WEAASLDEWLYNGGPYQLVIFHFLIGVACYLGREWELSFRLGMRPWICVAFSAPLAAATA
VFLIYPIGQGSFSDGMPLGISGTFNFMIVFQAEHNILMHPFHMLGVAGVFGGSLFSAMHG
SLVTSSLVRETTETESQNYGYKFGQEEETYNIVAAHGYFGRLIFQYASFNNSRSLHFFLA
AWPVVGIWFTALGVSTMAFNLNGFNFNQSIIDSQGRVIGTWADVINRANLGMEVMHERNA
HNFPLDLAAGEVAPVALTAPAING
>OHY33789.1
MENSESAKPKPKPWQPGSVKAQFGSTIITGGDASMKPEHLSFLIIWRHRQHHQGQPKKQ
>OHY33943.1
MKWQLLTHNKQVLGKIFTILVFTGLTGVLCVSCNRNQDLLVTEIGVNPPKRPTRKTSDAG
EFYLQGQNQHSRGNFQAAIAAYSKSISLNSDYAPAFKARGLAYFDLNNKERAINDYNQSL
QINPNDPETYNYRGNARASLGNQKGAIEDYNEAIRLSPNYAEAFNNRGNSHAAQGNKNAA
LEDYTQAIRIDQNYPVAYNNRGNAYSSLGNTSKAIADYNQAIRLNPQFAPAYNNRGNAFA
SSGDKRRALQDLQKAATIFDQEGNRGLYQQTMKNIEELEN
>OHY34004.1
MTDQDISGGFSPVETTKPEQNPRLSPDLEVQVNQSSGGNPIQHSDRNFGSLMQQQNNNDL
YEKGRKNNWYRPVDFILSGKIWELAMWKKSWIWWFFVLAFIPSSIGIISVSILLKLPSAP
NCPRIFWPLASASMRVHCATLAASKQTVSDLLQAIALVKDLPQDHPLRGQINDLLQEWSR
DIINLAEKSFQLGNLEEAIATAKQIPENLEDRQFVEEKILKWQSTWSTAEEIYQSSIGEL
ENRRWQSAFMLSSKLLRINNRFWSTTKYDQLNQIIVTAREDGDKLDKADSLADRNSVNDI
LSAIKLVKSVKPESYLYKKAQELVPQFGRKMLKLAQAQMERRDADKGLEIAGKIPPIPSL
QSEIDDFIDLGEAQRNAWLGTILGLENAISQAQQIDPSRGIYGRAQELISVWQLEIEDVT
KLEQARDLASGGTIEDLRSAISQAQQIPSQNPRAQEAQTQINRWNDQIETIQDKPYLDRA
EQIANAGDINSLQSAIAEASQISSGRALYSKARRRIRSWNASIQRIEDQPYLDRAIVLAE
SGDLNSAVQEARKIAISGRALAGEAQAVIDTWQEQIRARENWRKARELASIGTADALSQA
IRIANRVSRRNVLRMDINIAIDRWGEQILEMARSQSQVDLVKSIETAKLIPRSSSAYADA
QLQIRTWKEQLVPVPTTPFLSPGEPSP
>OHY34083.1
MNSSDNKIHEIHEFSTGIQFERRGHSWIWTGFTTKYMNSTMGEEIPKVVERSIANEEFAL

AEGSWTEKPAIIARILGNGNNIWSVIAVVTRAKDEAGRSATLHRYFLCQGEHKLRVILAW
WEQNNRPTFNPFDFQVLGSPHSFAGEVPPPSDLEQIEQILSLFKEQAQPQEDKEQILPLS
REESVFTGQLGEQPQPRFAQGFDQQQILATSTGGTKPQTDTEEVWQFHAIYN
>OHY34084.1
MLSTTERIPQLKQLIEQRNYFVIHAPRQTGKTTAMLNLAQELTASGKYTAVMVSVEVGSA
FNDQPQIAEESILTAWQDAIEFWLPPELQPTFFTQGNTPKKIGSFLKMWAETSSRPLVIL
IDEIDSLQNQTLINVLRQIIRSYQ
>OHY34096.1
NLYSVLKSQDANLQFVFMTGVTKFSKVSLFSGVNQLKDITISEAYSSICGYTETDLRESF
GDHLEGVDWDTVRHWYNGYNWTGSETVYNPYDILLFISEGMKFRNYWFETGSPTFLVKLF
QTNRYFLPNLEHLEVTEEILESFEVERINPVTLLFQSGYLTIERTFIRRQRYMFALKIPN
LEVRLALNDQFINAYTEIINEKSGIQDSLYEFMNRGDVESMIMAIKRLFAGIPWRNFTNN
DLADFEGYYASVIYAFLSSLDARVIPEDISNYGQADITAMLGVHIYVMEIKVVEGNQVEG
NAALDQILQRNYAEKYRGEPGKSVHEIGLIFSRSQRNLIQANWQ
>OHY34126.1
MNHPAKILTFIFLWFQPILTIIHHKQTLAVTSEEKTNLICTQASKAVLTIKTDYSKHGSG
FLVNNKGLIITNAHVVSDGPTVATVVFSDGQELSADLIGFANNGVDLAALKIHNRNNLPY
LKIARNKKVKVGFSVFAIGSPINPNYQNTCTQGIISNIHPNGTIQHTATTNPGNSGGPLL
NNKSEVIGVNTWGILGGTAINLSQSIDKVHTFLADIQKQTTSPVSTLPTPPKSVFKTILP
DGKPIKGKIYGDPDVYTFAGVVGQKILIQMNSDEINSHLTLSLYKKTESDENEQIIAQND
DKVAGDFNAEIITTLPENGIYQVLVKTSRTGETGIYNLRVITQP
>OHY34127.1
MKVVSSIAISITLIGLTIQPKPVYSQQNASFRCENIFDQASRKKVPMTVAWVPERKTHVG
VIGWKSTYFEVGGWTSEKRCQEVSKKFQDFQNRGTINYLMTGKNNGYPIICAVAEVRDEC
NSQSQLFTIKLGSNPELVLRKLIASLEASGQEQEKPIWQSSKDKIYLNLSNYLQQARSIT
ISP
>OHY34128.1
MYKKGKGDLILTTNTLTKINNMKHYITIIFCLFLLAPSENTLANPITKNLTAKTNSCEIR
PEDQENAQYSDLQQQEIARRITVRIIANNNIGSGTILSRNNNSYLVATNSHVLLEANSDN
ISIQTVSGKTYTGKIIPQTNLKKLDLSILEFYSAEYYCSAPEIVDTTINLEMPIMAAGYT
KGKIIFRKGNVQRIAEPSLKEGYGIGYSVNIIPGMSGGPIINSRGYLIGINGKRPYPILN
SGYIDINGKQPSAAEIQEFRKLSWGIPVTTLLSQVRTDLLQKYSLPLPNIATKVAVQPLP
PWIAKIEEKVKQFTVRIDNRDGENGSGVIIAKEGSTYTILTAAHVLCEKASNNRCIEQEY
SILTGDDKKHPIDRNSIKKEEGVDLAVVKFTSSENYPIATLADYPTQDNQYIFTAGYPKL
GENRSPWRFTLGQTFSKEQGLLATRSSDFKSSGSGLQAISVSLTEGYELVYTNITLGGMS
GGPVLDTEGRVIGIHGRADGQVAIDEKTGDIGPNNGQVQLGYSLGIPIRTFLGIAPRLNS
QAQQIEKTTARELQSGKIDSIKQVLLSVDISKGSTTASNWLERGNQLLRLGRPREAITAF
DNAIQQKPAFIHLAYYGKGAGLALLSLALFDSGKVEEAAIALEQAIKAKSDFVPAWDYLS
LAHGRLGKFNEALVAINQAIKLQPDNSNLYNTKWQVLSSLKRYQEAIDAIDQAIKLSPRV
AFYNNRGVARFQSGDKQGAIADYNQAITLDPKFALAYNNRGLARFQSGDKEGAIADYNQA
ITLNPKNALAYINRGLARFQSGDKEGAIADYNQAITLDPKNALAYIGRGFARFQSGDKQG
AIADFNQAITLDPKNALAYIGRGFARFQSGDKQGAIA
>OHY34132.1
MKRVVPFTRLSKFQSRDSILADKYTALEKNVFASKSAPIIFPPETTLATLIASLAVMDNK
KGFLACLGKAKTIEKAISNSINAPNIRTDFLLKKWLGVAPARDDV
>OHY34136.1
MCGLTGFFYPGGFHLEDATNLAQQMAKTLSHRGPDDGGLRSLVKYFYHQAHHGRCTCTG
>OHY34137.1
MSIRIAFIITGLNTGGAEMMLLKLLERLSPEFVPKVISLTDIGVIGQSIQSLGIPDGFFH
TKTIRQKWEEHLSGGRN
>OHY34138.1
MEAMECGCAIIATDVGETRQLLDDSCALLISPNSTSLANAVMDLLEDPVRLNTIAKAAQR

RVKENQSLEKFSEYFSKRILKLSESEITSDVN
>OHY34139.1
MKLAYLNVIYSILPGVTSKIESQALALKSIDSQSKIVCFYCDNDNSLDTTNKCLTYVKLP
KPVRSAQLFRKLYNFQLTQFIFDYTTNNRFDVVILRSIALSPLFWYYFRSKKYKLITEHH
TKIVPEMMIKKSYLGAASTILSRGIIDSVIDGKICVTREIANYEAFNKPIRVIPNGHNAV
QKTSFLPYNGKTVRLVMLCSVSQPWHGITRLFASMISWQRDRSDIQFYVDIIGNIQTSEF
VSQNLPNNVVFHGYKDANDIAIIMSHANIGVSSIALYLKNMQEACSLKSREYIGRGMPFI
CGYYDPDILETDSYILRVPNDGSIIEVSVLLEFLQYLNRHREKVTFELDAAARRISWESK
MKDYYDFAKSIYLE
>OHY34140.1
MKVVTIIGARPQFIKTAVVSRAFRDHFPDLQEIIVHTGQHYDANMSDVFFRELDIPYPDH
YLGIGGGTHGQNTGRMIESIETVLTATKPDYVLVYGDTDSTLAGAIAASKLHIPVAHIEA
GLRSFNRFMPEEINRVLTDHVSQLLFTPTKTADQNLAREGIQENSVHRVGDVMYDASLYY
SKCMQGNGSILDQSSLDTKNYILVTIHRAENTDYPDKLREIIRGLSGVTLTVVWPLHPRT
RKCLTELQLSMPDNIKLIEPVGYLDMLMLEKHAKFIVTDSGGVQKEAYFHQVPCITVREE
TEWIETVEAGWNCLVGANAEKIVTALRMCYNPNKQITALYGNGQAGLAIAKVIACGFD
>OHY34141.1
MPDRILVISHAPFDNPYGASTSIREQYQAFEQLDKYEFIHVSKASPKEGILKGFQIYYPK
KSCIKPTNVSNIFSACLPWSDNYDTAAMHHSAIDVRFICKQLILDTNWRLRKKKAINTFL
MFYPKIIHLNSLVLAEVIPTLRINYNRPIISHVRELLKHKISEKNRQAILQLDAVITIDS
AVTKRFIEVVPEYPVEKIHQILNPFSARPFDDSLSSLFPDGGKFVFAIIGTVSRDKGVDL
VCECFYKADLEDSVLIVFGSTDSEYASSIKMKWLNRTSKIIWVGEHEYLAKRGAYSRIDV
VVRADPSFRTGRTVYEGLYAESDIVIQGDINDLITDSSLLKWRDNITMYKPRDRISLVNA
LRIVHKKLKFSRSVSPERKLSSNYDDYRERMDMVYSQLLKSFDFINC
>OHY34142.1
MKIPVLDLKPQYEQIKDQVQGAINKVLESGQFIMGPDVKLFEQEVADYLGVKHTIAVNSG
TDALVIGLRALGIGSGDEVITTPFSFFATAESISNVGAIPIFADIDQLSFNINPEAIKKH
ITPKTKAIMPVHLYGNPAVMGQIMDIAQQYGLKVIEDCAQSFGARYNGGCVGCEENCQAS
TRESIQGKFTGTIGDVGAYSFFPSKNLGCYGDGGMLVTNDDQVAELAQMLRVHGSKKKYH
NEILGYNSRLDTLQAAILRVKLRYIDQWNRGRRRVAELYNTLLSGVNDIVAPTINDSHVF
HQYTVRIVNGQRDKVQQYLTSEGIGSMIYYPVPQDKLPIYNGQYGVNPVSDLLAREVLSL
PIWPELELEKIEVVVKVIGQSVKENHL
>OHY34143.1
MSNYFAHESAYVDEGAQIGEGTKIWHFCHIFGKAKIGNNCNLGQNVLVANNVVIGNNCKI
QNNVSLYEGVVLEDYVFCGPSMVFTNIKTPRCEYPRNTSDDYRQTLVKRGASIGANATIV
CGVTLHECAFVAAGAVVTKDVPAYAMVAGVPARIIGWMSSYGDVLDFDTDGYATDSQGMK
YQRISESTIVKL
>OHY34144.1
MNREKNSVQVAVVGCGYWGKNLVRNFAQLGALTWICDKSETALKAQSQLYPQIGITNDFR
EIISNPEIQGVVLATPAAMHFSQAREVLLHGKDVFVEKPLSLRYKEGRELVEIAETRGAV
LMVGHILEYHPAVTLLKKLVYSGELGKLLYIYSNRLNLGKVRQEENILWSFAPHDIAVIS
SLLHLEPTEVISVGGTYLQSGIADVTVTNLVFPQNIRAHIFVSWLHPSKEQKLVVIGDRK
MAVFNDTVKEGKLKLYDKGIEWQGGLPIARQTSETILFTDEKEPLLLECQHFLECIKKRD
TPLTDGSSALKVLKILEASELSLQKGGLPVSLNEIEE
>OHY34145.1
MKNLDELREKITSRTAIIGVIGLGYVELPFAVEKAKVGFQVIGIEQNPVRAERVNSADNY
ISDVKDDDLKHVVATGNLRAITGFDCLPEIDVLVIWEVLDAANTKPFGIMPFYPGPGVSG
HCIPIDPHYLER
>OHY34146.1
MKTEKPYQRCKRCVMDTSALDITFDVNGVCNYCTEFLERSAHLINEDPVAKKARLDELVL
RVKRSGHGKPYDCVVGVSGGVDSSWTLVQTVKLGLRPLAVHMDNGWNSELAQNNIANLVR
SLGVDLYTHVIDWEEYRNLMQAFFDANVIDVELLYDNAMLAVNYQQASRYDIKFILAGTN

QATEGMRMPKEWNWFKHDKRNIQALGRNAGTRLKTFPSIGTLEYIYYEFLRKIKWTSFLD
FVPYNKFEALDNLERDFGYKRYPFKHYESIFTRFYQGYLLPRKFNVDKRKLHLGTLVASG
QMTRDDALIGLEGIPYPSLAALEEDVEYFLKKMHWNGEQLNNYLIQPEIPHSYYPTEKPL
WDFLTKVYKIFKIG
>OHY34147.1
MLKHRVIPALLLQNAGLVKTLQFKNPKYVGDPINAIRIFNDKEVDELMVLDILASKQGLE
PNYAIIEQIAGECFMPLCYGGGIRTVEQAGRIFALGVEKICIQTAALANPQLINDLASKF
GSQSVVISVDVKRDWLGHYRLYRSENAKTAALPWYKFLQEAVAAGCGEVLLNAVDRDGTM
AGPDLELIHQANHNLNVPLIAVGGVGSLEDIKAAVDAGASAVAVGAFFVFHGPHRAVLIT
YPRYQDLEILFASEVGR
>OHY34148.1
MQYKKLTFLFKTYLEILKSLAVKDTYRSLSKCDVLLCCHDVDLGETLEGLPYSKLVDSVF
EDLSNKGWVCRKFAHPWSVLTGRKAWGNPPSANQRIFFVRIQRKICKFFPFLPNSKKDKK
YSSLDPVENFYHELFDITLPRCVITIGSPPEMCRSAKSRGIPVFELLHGIGYDPVPWGWD
QALACNLPTGILSLDNVSTKTFTSLHSKGVHVKQIPHPFFRRFLDPEARQKLPPQWQDYP
AWLPTDKIIILVSLSCGLVPDEVIDAVDQTHDSIFWLFRLHPVQLRQNQYNHHRQFLERL
TKNHPNCEWRESSVLPLPLILSYCSGHLTMSSMTTYDAAFMGVKSLLLCPTLKDNSMFSD
LRSSGFAELGNFDVASIIKWVTTIKRCSHKFTTSTNSDQDWDSAVQWMLGRAYKADGEL
>OHY34149.1
MGWRSIEFKELYQSACEYLDVDRVHKIEIDREQAYLPQVKQALNKLQPTHYVYDPRTGSE
NWLRGLWQAFQISFLLASRHITPVVLLTDLAIRTWRAQSAVVTATSGVVVTFMAPSRIQP
IFPHRRLVGPSLMPFSQATLAWLDHLPRTQPDNNPPRAIFTGSLYEPRTSLLNAIKVGLE
ARGLTLDIQGRQLGSPRVSDEEYWLRLANADIVLTTANQMIQPGADWTWVLHLLYRYLEV
VACGTLLLAPEVPGIGRFFTSGEHFVSFKKSDDAVDAIEYYLNHPVEREKIAKQGYARAR
SLIESRIFWLTIDVALGHESLA
>OHY34150.1
MRAIIVGHTGQDGTLLCNSLKHKGYKVLGFSRSSVYTNSDANFNLRPNLTDEQSIYQLVQ
GFQPSEIYYLAAYHTSSERQQSESLSQNFALSQSTHVTGLLYFLCAIKDLLPSCKLFYAS
SSLVFSGEDGEVQTELTPLSPQGFYGITKAQAMYLCQEFRSKFGIFVSVGILYNHESCLR
SHHFLSQKIIQAALCIASGSQEKLMLGDLSVRVDWSYAPDFIKAFQNIIQLPSSDDFIIS
RGEAHSVEEFVDIVFDYFNLDYTHHVMQDRSLLQRRLLTRVGDCSKLKRVSGWTTSLNFY
DFVRQLVIDSESLCKTKRL
>OHY34152.1
MIKDAESLRKIGNYETYSQAHQDLFVRIMLGFKQNGIYVEIGAAEPKQSNNTYILERDLQ
WIGLSLEIDAALVGEYNSIRNNKCLLADATKFDFDGYFRENRFPTVIDYLSLDIDPAHIT
YQALENLPLSDYRFSVITYEHDRYASGPEMMVKSRQLLENLGYIRVVSNVRCCGRDFEDW
WVDPLIVSPQIYEPFISENIECRDIFKSV
>OHY34153.1
MQCRILLHAIAWEPDPENFKLLRCNIILNDLEEKVTLYNLALGRDSGKTLKFELSEDNFG
DHRIRVSDDSGKYHEESRKIIEVKSETLDSYIDIFRQENLSGNQLILWIDVQGYEGEVLA
GARTLIEQMKPAIGMEFWPYGLDRAGGIDSLIEAINHYEIYFDLTQSNPSGQPVNKLFDI
YDYHKNNDYFMMDILLF
>OHY34156.1
MLRKIGYTSYLSTTPDTNTIADLIIILPGVGAYDTGMTMLEEHGWKDYLQEVAHHSTTKIL
GICLGMQLLCEGSEEGNRSGLGFIPGYFKRFKPDVSSLKVPHMGWNTVEFRDSRYLPFPI
HGENPRFYFVHSYYYSAVNDNFVSGWCNYGKKFGAVIDNKSVTGVQFHPEKSHRFGMQFF
RNYINSICLNTE
>OHY34168.1
MLHKLFTEIKEYELAAYFYQISQSKISSKILKLLVNSNNITPEIILGLPIKESRPWINSI
SKFFNKDIDEQEKIFKKRREKYLELLRKYKEIDDYNQDIKINPNDPEYYYNRGNTRSDLG
DNQGAIDDYTQVIKLNPNSAYAYILRGDVRSDLGDKQGAIVESPVQTCRV
>OHY34169.1

MKRFFTWVPTLVLVIICSLFLFNSPSYAFNQADLDQLLQNNICEKCDLRNADLSDANLIR
AKLSRANLSYTDLRRVNLSRANLSGADLTYAKLTYAILIGADLSGANLSYTDLSRANLSG
ADLSGANLSYTDLSRANLSGANLSGVNLSDAKLHGANLSGVDRSGAQY
>OHY34205.1
MISKERKFTIIIPTRDRADTLLYTLASAISQNYPNLEIIVSDNASQDVTHEVVKSFCDSR
IKYINTGHRVSMSHNWEFALSHVDEGWITVLGDDDAIIPGAISQVNQIIDATGTKAVRSN
GCGYMWPTMTSNCHGHLHLSTKKGYKKVGSEKALSRVMSGNLYYTNLPVLYNGGFVDISL
VKEAKAITDSFFLSMTPDVYAGIVFSLLTDEYVYSHEPLAINGASHHSGGTAAFEAVKRN
RSYNPAEKFWSEENIPFHPSLPLTKEGRPVKSIQAIVYEAYLQAAVFHERKRIVYNLVSP
KQQLEIILRDCTPVTKPEVMSWSSEFAEKNNIIPDQSLNLIAGKKSVLFLLKKITVKIVN
LCLSLSLSGRQVVGLDNVYEASILVALIKRGYLASFQLAFSNLMCRAASIRELDFRRKLM
QYFFG
>OHY34206.1
MSNFEIEVLERLSLVAKNEEIARSSHTFLKATIQSKYSYNFYWLGRPIIQYPQDIVAMQE
LIWSIQPDLIIETGIAHGGSLIFSASMLELNAACGGPQSAEVLGIDIDIRQHNREAIESH
PMYKRISMIQGSSIAPEIIEQVKLKAVNKQKILVCLDSNHTHDHVLAELEAYAPLTSVNS
YCVVFDTMIEDFSDDMFADRPWGKGNNPKTAVWEYLKTHPEFEIDKSVQHKLLITVAPDG
YLRRVC
>OHY34207.1
MKVAVTGATGFIGRHITSALLKKGYDVLLVGRSNNVQQSGLPFVQLDLLEERNHNWISEY
KPSHLLHLAWYTEHGDYWESPLNINWCHSTINLIHAFAVQGGKRIVVSGSCAEYDWSFGY
CNEIKTPSYPSSLYGTGKDCARRMSERICNTYKVSLAWGRIFLPFGSGENPKRLIPSITR
AIIGSDPPFPIRVCQWRDFLPVEMVADGFLFLLEQDYPGIFNICSGKPVQLSEIVKRIGF
ILNKSADGLMLEAEFSTGYDSFLVGDNRCITMHGWHPDYDLWHHLGVYVNSLVRAI
>OHY34209.1
MKIVTTPIAGVWIVKTTPFVDQRGYLYRGFCNQELEPILKDRSISQVNVSKTEDVGAIRG
IHFQYPPYSEMKFIRCIRGRIWDVAVDLRQGSATFLQWFYAELSADNGDMIVIPEGCGHG
FQVLEPNTEMLYLNTSPYEPNHQSGLLYNDPLLNISWPIDVTTISERDQSHPLLSENFQG
LLL
>OHY34210.1
MKAVILAGGYGTRLSEETHLKPKPMIEIGDRPILWHIMKLYSAHGVNDFIICCGYKGYVI
KEYFANYFLHMSDVTFDMQINEMQVHQKHAEPWRVTLVDTGENTLTGGRLKRIKKYINGE
TFCFTYGDGVSSVNIQKLINYHQKSGLWATVTAVQPPGRYGALNINDKGIVLNFQEKPQG
EGGWINGGFFILEPQVFDLIPGDNTSWESETLPLIASKSQLAAFQHHGFWQPMDTLRDKN
MLEELWRSDKAPWRVW
>OHY34211.1
MNKYKNLLKKAIGYFPFGLGGKFLSLCRVVTNTILLPVSLPLSLMTRFLGFRVLPVSSTR
IGHLVCETDCLIKEITMQRIKPKKWLIFYDGANVSNSAYLELLPDYFFPVNTKNFILKIV
QNLLLRRVVNTEINHYVTAMYQSAQIYSIQAAWEGKGATIHCPADWLEAKNQVLKRHGIS
SGQPYVCIHARESGYSPIDEIWHCKRNVNIMSYSLAVEYLIACGYAVIRMGDHTMTPLVQ
EFSGVFDYARSSDKSPWLDLAISSECSFFLGSASGAYTMANVFLRPVVCVGMSLPFNFSP
SGYSFDIGIPKLFRERYSDTFLSIKDIFKLGLAELRLAEDVNRRGYELVENTAEEILDTV
KEMVGRLKGNWVETAEDKQLQSIMRSYIGIGSYSYGSSARCGTAFLRKYKFLL
>OHY34214.1
MVDELPVKLSDYVADFIAAQGTKCVFAVSGGASLHLIHSVANHAKLHYVCTHHEQSAAMS
ADAYARVTGNLGVAIATSGPGATNLITGICCSFYDSVPVLLVTGQVSTFRMVGDTGVRQI
GFQETPITDICQSITKYACQIRDSSQIRYELEKAVYISRSGRPGPVIIDIPDNLQRSQIS
VATLEKYTPDNFLLRKFPCFGDVKHKFVELITSSKRPVLIAGWGIHLSRTKSEFVKLAEL
LNVPVALTWGAADILPADHPLYVGTFGTHGMRHANFAVQNADLIISLGSRLDTKSTGSPI
NTFSRGAKKIVVDIDPHELGKFSYFGLDIDLLIQEDLVDFFREIQKGDIPVLSDNHRDWI
TIISRWKQDFLSFDRRNVLPTGLDPYEFMDKLSIHTPDQSQVFVDTGCAIAWTMQNFRFS
RGQRLFHDFNNTAMGWALPAAIGGFFARPDLPLVCIVGDGSLMMSLHELATVKHHRIPIK

LILLNNSGYSMIRQTQDQWLDSKYYASSHEGGLSFPDYKALATAFGFDYVEIACIEDCSE
KMGTVFLSSDPLICNVIIPPDARVIPQVKFGRPNEDMEPLLPRDLFLRNMIVNPLDVSKS
Y
>OHY34215.1
MELVCDIGLALSNYFLDKGFSVLGSFRNSSFQLEQLKTKFTALIQSDFSDSSSVDQLVQS
IKAIPFTWDFIIVCPATMQPIGKFESCQIDDWERNIKINFLSQLRLLHGILPLKQNFGEQ
LPTVIFFAGGGTNSAPVNFSAYTISKIALIKAVEVLDAESEDVCFTILGPGWVKTKIHSE
TLESKDTSGKAYDETSRRFATGEFTPMSKVIDCCEWLLNSPKNVIGGRNFSLVYDQWGND
NLNKLLSENSSMYKLRRYGNDLTI
>OHY34216.1
MTETTFAQAINLALHEAMFLDPAVICYGLGVTDPKAVFGTTAGLEQRFGKARVFDMPTSE
NAMTGVAIGAALNGIKPVMTHQRLDFFLLALDQLVNGAAKWYYMFGSQVSVPITIRLILG
RGWGQGPTHSQNLQAWFAHIPGLKVVMPSTPEDAKGLLLSAIFDPNPVVFLEHRWLHNTV
GSIPDGDVRTPLGKGKIMREGEDITLVSMSYMTIEALHAADYLVTQGISCEVIDLRTIKP
LDWEMVFTSVVSTGRLLALDSGFTTGSVAGEIVARVAMDLFGRLKVAPGRLAMPDVPEPT
SFALTKGFYVRAADIAVKVMEMMGRSAEGVREALPEPCPHDVPGDWFKGPF
>OHY34217.1
MNTDLQLALLRQMWLIRTVEETIAQHYHEGKMRCPTHLSIGQEAVPAAVSACLKTTDFAV
STHRGHAHYLAKGGNLKAMIAEIYGKANGCSKGKGGSMHLIDLGVGFMGTSAIVGNSIPV
GVGLALSAQLQGTDQVSCVFIGDGAVEEGVFYESVNFAVLRNLPVLFICENNLYSVYSPL
SVRQPQGRQISSMVGSMGIKVVTGDGNRVLDCYHIFHSALDSLRSGGGPQFLEFFTYRWR
EHCGPSFDNDLGYRTPEEFSYWQTRDPIVQLTQELTQDLASHPERIIKEVKQEVADAFAF
AEASPFPDPSSAYEGVYK
>OHY34218.1
MGKEIDLLVNYPKTKRNLDERAASKTEADRAIARRFGKEFFDGDRNHGYGGFNYHPRFWQ
PVIPTFKDYWGLDATSSVLDVGCAKGFMLHDLAELIPGITVKGVDISDYATANAIEDMLP
HVSVANATCLPFDDNSFDVVISINTVHNLNREECGQSLQEIERVSRGKSFITVDAYRNVE
EQTRMEAWNLTAKTIMSVDNWVAFFAEVGYTGDYYWFIP
>OHY34225.1
MTSIPALRDFHSPSTQVYRFLESVSKVSINNLFGKHGVQKHAVGKFGTIDFPYFSMGSIN
STHLFGLDELIIFAFYESNKQRYKNVADLGANIGLHSIMMTRLGWNVTAYEPDPTHFNQL
QNNVIRNDCMHNNVKLLNKAVSVEVGEVEFIRVVGNTTGSHIAGSKNNLYGDLDRFMVSV
DSFRDICNHFDLLKIDVEGHESDILLSTVGQDWQGTDAIVEIGSASNAEKVFHHFSKLAV
NLFSQKRAWQKVNSVEEMPFSYKDGSLFISSGDVMPW
>OHY34226.1
MCDVLVVGGGIVGLATALRILQSRPQLRLVLLEKESHLACHQTGRNSGVIHSGLYYRPGS
LKARNCRAGYDQLVAFCREEGIAHDICGKVVVATSTEELSPLATLYDRGVANGLTGLRWL
SSEEIKEVEPYCVGLRGLRVPQTGIVDYKAVALKYAEKLRDGGAEIILGSKVEHVLTQDS
SVEVVTADRIWQAKFLVVCGGLQSDRLALKTEPDLPLRIIPFRGEYYELKSEVHHLVRHL
IYPVPNPAFPFLGVHFTRMIGGGVECGPNAVLAWGRECYKKSDFNVRDVWETVTWGGFHK
VAIRYWRSGLDEMYRSWSKAAFVRALQKLIPSIQGEDLVSGGVGIRAQACHINGQLLDDF
ELRSHGRVIHVCNAPSPAATASLAIGQTIAQEVLSRLS
>OHY34297.1
MNHAKFESDLAQLQDEVSNCERDVLNLIKGKKTMEYSQNHNGGNQSNQIAIIGMASLFPQ
SRNLQEYWQVIMDKIDCITDVPASRWSVEDYYDPNPKTPDKTYCKRGGFIPDIDFNPMEF
GLPPNILEVTDVSQLLGLVVAKAAMEDAGYGESQQFDRDRTGVILGVAIGRQLAVPLGSR
LQYPIWKKVFKNCGLSDDETEKVIEKLKSAYIQWEENAFPGMLANVISGRIANRLDLGGT
NCVVDAACASSLGALNMAISELLAHRADMMITGGVDTDNSIFAYMCFSKTPAVSPSENVR
PFDVNSDGMMLGEGVGMLVLKRLEDAVKDGDRIYAVIKGIGSSSDGKYKSIYAPHSQGQV
KAIRRAYENAGFAPQTVGLIEAHGTGTMVGDPTEFISINQVFGDNNSLKQHIALGTVKSQ
IGHTKAAAGAASLIKTALALHHKVLPPTINVTQPHPKLNIENSPFYLNTETRPWISNQPR
RAGVSAFGFGGTNYHVVLEEYESEHHQSYRLHNCAKSIFLSAPTTPELLSQCQHLYQQLE

STHKEQHYQRIIAESEQLIIPVDHARVGFTTLSLSQAIAHLAIIIDLLKNQPSVDFWEHP
KGIYYRQQGMETTGKVVALFSGQGSQYLEMGRELVINFPWLRQTYSHLDDLFSREGLESL
SQVVFPAPVFSPQERQEQLEKLQKTEYAQPAIGAFSAGLYKILQQAGLKVDFVAGHSFGE
LTALWSAGVLTEEDYFFLVKARGKAMSTSPEVDAGGMLAVKGNISQVTEFIKDFPQVAIA
NYNSPQQIVLAGNKSEITQLQNVLQAQGFSCFLLGVSAAFHTPLVSHAQKPFAHAIAQVN
FQPPRIPVYSNVTGKLYPNEPGSMQKILQEHLLNQVLFQQQIENIYQAGGNCFIEFGPKN
ILTNLVKEILVDKPHVAVALNANYRQDSDLLVREAVTKLRVFGVPLKNLDPYQIPAKISS
ASQKNQQKTLNIRLNATNINDRSQKAFAQALATGSVIKMPAVSENNYQTQPEKIRETLVE
TSTKINPEISSSSMLTSQIIPAVKNHSSVEPKMEISLDNYDRVLNSLEQSLVELTRQQSE
VNQVHEQSLQNQIEYNKTFYELMRQQSLFLAQEEINEYQSQTQQLAISSTERSMMRLHDH
QAETIRIHEKYLNYQQEYSNNYFQLLEQHYSLLEVGSPNGYSHLPSSHVAKSDPPAQKLI
YPLEPENNSQNNLPDITVDFPIATYIDKEKLRETLINIVSDKTGYPVEMLDLSMDIEADL
GIDSIKRVEILGGLLELYPDLPRPNPEELAQLATLEQIAEYINNLITQLGQNQPLEETVS
EHHNHPQFLVLPHEELHEDLSDRSTTITIPDDLSQILLTIVSDKTGYPVEMLDLSMDMEA
DLGIDSIKRVEILGGLLELYPDLPRPNPEELAQLRTLGEIADYMRNQAETVGRSNLSTSE
KPDTSTTEVADKILRLPVQLKTLPQPDSLDLTIPENHFVLITNDGSEVTHRLVAKLTDKG
CKTVVLTFPCLESNLSEEIAQIRLNDWHEETLQEHLTELTTKFGCVGGFIHLHPYSNNNL
GIDQAIVQHVFLIAKHLKEDLNHLAKKERACFFAVVRLDGELGTAKTHNFSPISGGLFGL
TKSLNQEWPEVFCRTLDLSPDLDGDTTVKHILAELQDPNLLVTEVGYNKTDRFTLVAEPT
KSSIIPDSLNITKDQVFLVSGGAKGITAKCVIKLAEEYQCKFILLGRSSAEIEPVWSEGY
EDENELKRRIMEDFLSKGEKPTPIMVQKKYQTIASQREIHNTLKAINEAGGKAEYICVDI
TDGMMLKEKLRPIIDQFGTITGIIHGAGNLADKRIEKKTVQDFETVYAAKVHGLENLLNI
VETNQLEYLILFSSVVGFYGNVGQTDYAIANEILNKSAHVIKHKHPNCHVVSINWGPWDS
GMVSPELQTAFAQRGIETIPQELGSSILVDQLRNSDSTMTQVVIGSPLVYIPSTLSSELK
THQITRQLKLNYNPFLQDHVIAGNPVLPATCGLSWISSSCEQLYPGFQTFHCPNFKVLKG
IVFDQNSPHEYILEIQEVAKIDNQEIHLVGKISSVTNHGKIRYHFSSNLILKRQIPLADN
YELFNLTQDGQFLASNSLLYQTGVGSLFHGNTFQGVKSVLNISPGKLTMKCELPEPTLNQ
QGQFRVQTLNPYIADVQVHSLWIWTQHFHQVGCLPSEIENFEQFAPVPFGETFYVTCEIK
SKTESYVVADVITHNQKGQVYNRMKGAKATILPNS
>OHY34306.1
MNPFNREKIQFYLTDLTTPVGQIINFVIALLVLLSSAIFVVETYNISPDARTELSILNTC
ILAIFSGEYLLRLWSAKQKSKYFFSVYSIIDLMAIIPYFIGFVDIRFIRLLRWLRILRLL
RFIDKKFLFFSISEDTVIFARILFTLFAIIFVYSGLIYQVEHAINPEGFNTFLDAFYFSV
VTMTTVGFGDVTPVSEWGRLLTVFMILTGVALIPWQVGDLIKRFLKTSNQVENTCSRCGL
AFHDSDALFCKRCGATLPQVSPSSQYQ
>OHY34422.1
MSLTARSHFSAAHRLAPNLSAEKYGKFIRTHGHNYHLEVTVEGEMDGRTGMIVDLGCLHE
TVEREILELFDHSCINEDIPYFSTSHIVPTTENIARYMSDLLQFPISELGVKLSRVKLFE
SDHLWVEYEGKDSEIFFSVATGFSAAHRLADPTLSLEKNQTIYGKCSRINGHGHNYHLEV
TVRGEIDSVTGMSVDLVGLNQIIQHYVIEPMDHSFLNQDLPYFTEVVPTAENIAVYISDV
VRSPIEELGAKLHKVKLIESPNNSCEIYAKDIEESRVDRVDRELAVV
>OHY34427.1
MNIETIKSEKTKQLPGANLEDQDLSEFDLTAANLAGANLMGAHLVSVNLEGSHLEGANLM
GASLQGADLRANLLGANLMQADLTGADLRGSNLRGANLMGATVAGASLTAAFLSGANLMS
GNFQGVDLRGADLRGANLTGANLKGADLSRADLQGALLNQANLEESDLRGANLAGANLAG
ANLLCAELEAASLNGANLYQACLLGTILETYHD
>OHY34521.1
MPVGNDQKNENPTDEKPRLTIPPKLRLLVGYIQQLSLMVREKIGRSEKPFYRHVWFWAGL
GVGTTIIGFNYGIRTVDSSLPDKSELNAMIRQQTLTIKAGDNTVLMQQGEATREQLKIQQ
IPDKLQLAFIASEDRRFKQHDGVDFQGIVRAVISNFQSQNVVQGGSTITQQLARILFLSQ
ERTLWRKLKEVRLAQKIEGELSKEEILERYLNLVYLGSGAYGVADASWVYFGKTVDQLNL
SEIATIAGLAPAPNIYAPDKNLPGAIARRNTVLQRMEEDGVITPQEKQSALQQTLTVKTN

LPRRFQVTAPYFCAYVQQQLPKYLKPDVLAAGGLTVETSLNSNWQRLAEKVLVRTLKNEG
TWGRFREGALVSINPRNGEIQVMIGGKDFTKTQFNRVTQAQRQPGSTFKTFVYAAAIASG
KNPGDTYIDQPISFRGYEPKNYSEKFRGLMTMQDALALSINTISLQVLIDVGYEPTIKIA
HNMGIKSPLEHNYSLALGSNEVNLLELTSAYGSFANQGLHTEAHTIRRILNRQGDVIWSA
NFQPKRALDADSSGIVTKMLTNVVTNGTGRAAQLPDRPVAGKTGTSDESRDLWFIGYIPQ
MVTGIWLGNDNNRPTHGSSSTAAYTWSQFMEGAVKDLKVEKFPRPSRVAKRKATIKAQPI
SKKNSGFKISNRDDNSDSQDGNSSTRQGRRRRSNYEQLDQDQDTSTRRRRRRRNDVQTVP
SSTEENTTSTRRRRRRRNDVQTVPSSTEENTTSTRRRRRRRNDFQTVPSSLPTPNSPAGQ
PSWRERLKPE
>OHY34537.1
MANLQIAARNSIQAILISLIRSLKNILNTKIVLFSRFMANADGNSYLVKKAKISGSTYIL
HSNDYGVSRRIYIGLDDEHLKAIKAINLINDSSRFAKRGGRITHLLDIGANIGHISIPLL
EQGILGVAG
>OHY34538.1
MTSFAQLMSLRGRRALITGAAGGLGRIMSETLAELGADLVLVDRPGAVFEPLLQRLQALT
ETTPVGFQTIACDLERQDHRHDLIQTLGADGQGISVLINNAAFVGSSDLQGWAVPFEAQT
LETWRRALEVNLTAAFHLCQGLAPLLRSAPGGNIINVISIYGHHGPDWSLYEGTTMGNPA
AYGASKGGLIQLTRWLATTLAPRVRVNGISPGGIYRNQPEVFVQRYNQRTPLGRMATEDD
FRGVVGFLASDLSGYMTGQILQVDGGWSAW
>OHY34539.1
MIDRVLIVGHGSIGQRHLRIVRHNLPQADIRVLRHRPGSGVPELANGVYHHLADACAFAP
QVAIIANPAPFHLHTAAALVAVGSHLLIEKPLSHDLEGVGALLAQAHSQGVVLQVGYNLR
FLPTLSRFRDLVHQGEIGRVLSVRCEIGQYLPTWRPDRDYRQGVSAQASLGGGVLLELSH
ELDYLRWIFGEVDWVRAYLGRHSQLEIDVEDLAHLTLGFSPGSAFESASGPVAALSLDFL
RHDPTRLCTALGEKGSLRWNGLTGTVDLYPAGGDHWQLVYHHPHQRDDSYQAQWAHFVAC
VQSNRPPLVSGEDGYAVLKIVAAARSALPPSALAS
>OHY34540.1
MLKKRIIFTLLYDSGSFMLSRNFRLQRVGNLDWLQRHYNFAHVSFFIDELIVLDVTRKER
NLEDFCHTLQALTTGCFVPIAAGGGVRSLDQARSLLRSGADKIVVNSPLFDQPDLVQALA
SSFGQQCVVGSVDMKRKANGTYQIYTQSGSHPLEQSPQTALQWLSQDWVGELYLNSIDRD
GTGQGYDFDLLDQLPVDCSVPVILAGGVGNGQQLAVGFADSRVDAVATAHLFNFVGDGLQ
RARQTLMDQGANLPVWPTLEQLGLGNAPRQTGA
>OHY34541.1
MSPEIVLIDYGMGNLWSVRSALDFLGKKPVISGDPAQVVRANALVLPGVGSFRQATEALR
SSGLDEAITEAVLGRGRKILGICLGMQLFAEYGSEDGVSQGLGFIPGRVERFSSEELGSL
KVPHIGFNRVISSEQSRLFRGLNAPTDFYFVHSYRLLPAGLPGQSSTCHYGVDFLAAYEH
ENIFATQFHPEKSQTNGLFLLKNFLQG
>OHY34543.1
MKPNIHTQNWDRSIISCTATIRDVIQNLNDVAIQIALITDLEGALIGTISDGDIRRGLLR
GLGLDSPIESIIHRNPLVVPESMECNHVFQLMKANKIRQIPVVSEDNRVVGIHLWDNLVD
GDVLSDSMGATNLMVIMAGGKGTRLRPHTENCPKPMLKIGGRPMLEHIIERARSQGFRDF
VISIHYLGHMVEEYFGNGEKHGVKINYLREEYPMGTAGALSLLTPLPKQPFIVTNGDIIT
EINYNDLLSFHQQHRASATMAVRLYEWQHPFGVVQMEGLKIIGFNEKPVVRTHVNTGVYA
LNSETLSHLKSNEPCDMPSLFDRLQDKGDLTIAYPIHEIWLDVGRPDDLTKANGIIG
>OHY34682.1
GGPYQLVIFHFLIGVACYLGREWELSFRLGMRPWICVAFSAPLAAATAVFLIYPIGQGSF
SDGMPLGISGTFNFMIVFQAEHNILMHPFHMLGVAGVFGGSLFSAMHGSLVTSSLVRETT
ETESQNYGYKFGQEEETYNIVAAHGYFGRLIFQYASFNNSRSLHFFLAAWPVVGIWFTAL
GVSTMAFNLNGFNFNQSIIDSQGRVIGTWADVINRANLGMEVMHERNAHNFPLDLAAGEV
APVALTAPAING
>OHY34728.1
MGTRKTVSFTANSSKATVTVNPTPDTRQEGNETVSLTLASGNGYTVGTTSAVTGTITNDD

FIGNASNNTLTGTAGNDHVNGGIGQDTLTGGAGNDVFVFQFGQSGVSGADRISDFAIGSD
KIDLLSLSGAGVSDPTAFTRASNSTASSLANMVNNVFTDADGSLVGNQGLGINSAALVGV
TTLGIAGTYLVINDGVADFQSGNDLLVNITGYSGALPSFGTIPVTSFFV
>OHY34761.1
MPSIQFDPKPEISVVLTTYNRSRYLKNCINSVTRQTFEDWELVIVDDGSTDDTFEIVSLY
LAEHRNIRYLKHQNRQVGYARNAGIQASFGKYITFIDSDDTYKATHLESRLEFMRTHPEI
DIIQGGFEMEGEFFVRDYFRPDKTISIRECVSCATFFGKRNVYFQLSGFKNLACSEDTEF
WQRAKKMFKTQDIKQPETYIYTRAKESLTRSLLEEMSPQKITKYQASEI
>OHY34762.1
MLGSFFSASYVKAEEILKTDVNISPLTSSNYQRPIIIAEKINLDVCQQAPKSILCEIRRS
QENRRIQLEKEEREREELRQRKLEEKAMEIPGVPEIPTDQDWKSPNSKIPWSRIVKLKSD
FDGNVDYAIFDRDWTKRDGEEVGFVTKWTSDSIQGLIYKTDCGASFCIGTRIKSIVDMPS
PIVLRLGEQEYKIYGDEGTFNIPSSFLKKIVESSGKPSLKIKIGESGMILPIGQNTVESL
KTLYSRISLRQKAPDFNLQAVPIVDGNSFQKLVAASLNKVVMIRAGGSLGTGFIAENSGL
IMTNRHVVRSYRTVDISYADGTKTIAHVIFRDREKDLAIIRPSKPSVTGSLPLCYAIYPK
PGEEVFALGSPLGLANTVSKGIASSVRISEADSKSIIPEGSTLIQTDAAVSPGNSGGPLL
NNRGEVIGMITFKRTAGEGLNFAVSIIDLLNAIEAQRPTIPDGQAATECGNLIPIANKDT
R
>OHY34783.1
MSTTAEEIWELLGELIKVQKETDRLLREQSQETNRKFQETDRKFQETDRKFQETDKKFQE
TDRLLREQSQETDRKFQETDKKFQETDRFLREQSQETDRKFQETEYLLREQSERANLRFQ
ETERLIKEESIRLDKQLGQIGNSLGQFVEFQVRPAAVRLFQEMGIAVKEIATNVSVQGSE
GTEIDILVVNSHEAIAIEVKSKLSDDDVKEHIARLSEFKKLLPRYENLNIMGAVAGMVVP
ENV
>OHY34784.1
MEHLVLYLSIPPNPFKKQGFFILQNCDLHNLIEEMFRDFKKGGYNLEETNVTGERFISLV
LLIAIAYSSATIQGQQIKRKGIQKYVSRIKEYGRTERRHSSFYIGLYGQTWVNFKEICMD
MVMELMRLNRNKCKYYQQGLRAMRLIESVL
>OHY34788.1
MVIFTSRLETLNFLCEQLARDLRLGKDAICYLKGEGMTDMEQMKVVEKFGQEDDPVRVLV
ATEVAAEGINLHYLSHRLIHFDIPWSLMTVQQRNGRVDRYGQRQRPEIRYLITRCSLPEM
DESNRIIKVLLKKNDQAIKNIGDPSIFLGKYDANQEDDYVSSHIEMGTSAERFEEILDGN
AQKGGDLGIFSWMEESHFDEVFSEDDNPEFVGESTGTILSLFNSTFDFVVSALESNIVNI
PNRQINDKSRFIELEMPEELKKRYERLPRELAPDNKTLLFLTDDRQVIMNEIAIARNQKG
GWNKKQYLWELHPLVEWLKDRCLFHFPRHQAPVIQLHYGVDADESIFICFGSFSNRRGYP
IINCQVSVIFKGNKFSRIEVFAETVKRLRLGEKISNTGSVELESLLPLREEAIKRTKRYL
SKMRQEEQEKLNVKLGEEKRRLEGLRDNHLNQLQLWLEKTEGEKIKQQGEKRRGEIDKMF
ESYEEWVKSSMTTESEPYIKLVAVLRG
>OHY34802.1
MFNLKAPLFKQGVFILQILPYKKATIPPNVLSYIVFNLKAPLFKQGVFILQILAEKRKNL
SLLDIQ
>OHY34815.1
MAQTQNLINKEHRVKDISNDTIDNLISLQSLDDEISLREILAILRRRALTIISVASLAMV
GCVGYLLTAEKTYQGNFQILVEPLTNDRPNLLSLTDNLPSSSGVNGLDYGSQIKVLKSSE
MINQVLPKLKVAYPNLTYKILSKNLDIERLQQEKILKISYKSPDATQINTILSKLSDFYL
KYSLERRKTTLNQGLQFIDNQLPVVERRVNNLQIQLQRFRQKYQFVNPDEQSTSVVAQIQ
DLEKQRVAVEQRLGTTRLSYSTLLTAEGQEAILNQAPIYSGLITQLRQLEAQLSAELARF
QSDNPLIETLQEKIQNLLPLIENEKNRFVGLRLAEVANEIKLLEEQNSQLLKAEQQYRAR
FQLLPILAKQHTDLQRKLQLANESLNRFLEAREKLAIEVAQTEIPWELIQSPTTSKDPIS
PKLYETLLIGLLASISTGIVFGLILEKLDNSYHDPTLLQEKTKLPLLGTVPMIKNIGASY
QSRHDADNNKTETLAKVPLKVPLFDISNLLSPKKKRRKGYGYGYGYGYYGEGHFWQSLQVLY
ANIELLNSDQIVKSIAISSSVKGEGKSTMSIHLAQIAASVGKKVLLVDSDLRLPQIHKRL

DLPNIMGLTNAITSNIPVEEVIQRVPDLDMLSVITSGILPPDPMRLLSSEKMKQMMRHFR
EEYDLVIYDCPPISGLVDTRLLASQVDGLLLVVKMHNTDIDIIKQVQESLRQSSISILGV
VANQYKRSSHQNYDYYYSYYSYRASQSAN
>OHY34857.1
MLATFKLSIGLMDYKVIECTNAYDDEKFYTGYEHVVSLEIEGEKSPLLINHTPYDDQYIE
ECSLAEIIQECEEWDFESILESITEKEEEIQQLGREIAILVVYSISLFTLKPTVSVFEYN
SLIPEKYLSWFAPPKISDDC
>OHY34905.1
MRDAVTTLINSYDLAGKYLDRNALDSLRSYFDSGTSRVQAATAINANAAAIVKQAGSKLF
EELPELIRPGGNAYTTRRYAACLRDMDYYLRYATYALIAANMKVLDERVLQGLRETYNSL
DVPIGSTVRGIQIMKNLAKEQAIAAGVANAAFVDEPFDYITRELSEQNI
>OHY34970.1
MRADSLLDKIHPDKDYCIGQDSGIYWRFTEPVEKGVEAPDWFYVPGVPSRLKGKLRRSYV
MWKEKVPPLIVIEFVSGDGEEEKDNSPPPEGDEVDPKTKKVKKAGKFWVYEQAVKIPYYA
IFDGFEGTLEMYHLEKGRYKQVKANRRNHYAIPELGVELGILLDQERPPIPWLRWWDNRG
NLLLTGNERAEEECQRRELAEAIAIQERQQKELAEALAIQERTEKERERQQKELAEALAI
QERSEKEQEREKKEKLAAYLRSIGINPDEI
>OHY34972.1
MTKITNQALNSALNQLWVFSLSSDFWAVFDAAFGTEYNRKNAEILRSQWQIGDFSQLPEI
EILDSSILGSANGAYSSSENRIYLSSNLMENGTSSKIREVLIEEIGHFVDSRINQIDTPG
DEG
>OHY35012.1
MGKLKWKIVSQGVYDIECIGINVRLIVLSKIPKSANNELWRLFSARAEVVQEAISHYQES
YRNSEYSVLMQQLYEFYLKEKLPMTYTLEQFKKDFVINHLREIPTEEVLKQYSPEEVLKQ
YSPKEVLKQYSPQEFLEGLSPETLEHLAIFKNSLLKNHCCS
>OHY35051.1
MSPKNLPLGINTLSVLRENNCVYVDKTEIAHRLIRIPGRFFLSRPRRFGKSLFVDTLKEI
FEGNQKLFEGLYIHDQWDWSRKFPVIKIDFAGGVLKNRQELDLRILDILQENAEHLGVSY
ESTDIPGKLGTLIRKAMAKYGERAVVLVDEYDKPILDNIDNPSIAGEMREGLKNLYSVLK
QQDANIQFIFMTGVTKFSKVSLFSGLNQLTDITISRDFSTICGYTQEDLEQTFAQHLQGV
DWDELRLWYNGYSWRGDSVYNPYDILLFIREGMEYGNYWFETGNPTFLIKLFQTNRYFLP
NLEHLEVTEEILKSFEIEQINPVTLLFQSGYLTIERNFIRHRRSMFALKIPNMEVRLTLN
DQFINVYTGMVNEKSAIQDILYECMWSGDLESIVKAVKRLFAGIPWRNFTNNHLADFEGY
YASVLYAFLSSLNARIIPEDISNYGQADITAILGDHIYVMEIKVVDGENVKENL
>OHY35102.1
MFVAGDLFWYPVKGSNRIKLAPDTMVVFGRPKGQRGSYRQWEEDNIPPQVVFEILSPCNS
KGEMTRKKLFYLKHGVEEYYVYDPDKISLEVSIRENNSFREVEDFATWTSPRLNIRFDMT
GDELVIYYPDGSRFLSPVELSNYAEQEKLLKEQANERAEQERFLKEQANQRAKQERLLKE
QEQLKYQTLLSQLKAKGIDITTLE
>OHY35242.1
MRDIPPQVVFEILSPCNSKGEMTRKKLFYLKHGVEEYYVYDPDEMSLEVSIRENNSFREV
EDFATWTSPRLNIRFDMTGDELVIYYPDGSRFLSPVELSNYAEQERFLKEQERFLKEQEI
LVCLDLYYYNNLYSYDLAYSTTTTLYSTSFGNS
>OHY35248.1
MADNTKQFAWIVKIKENLEVLFKSNPDVFVAGDLFWYPVKGSNRIKLAPDTMVVFGRPKG
QRGSYRQWEEDNIPPQVVFEILSPCNSKGEMTRKKLFYLKHGVEEYYVYDPDEISLEVSI
RENNSFREVEDFATWTSPRLNIRFDMTGDELVIYYPDGSRFLSPVELSNYAEQEKLLKEQ
ANERAEQERLLKEQANQRAEQERLLKEQANEGAEQERLLRTGKISQRAGK
>OHY35377.1
MTVLAENDLEFDFSSAMEAIIFDDDTLHNPSTIKRVDFIAEFNDRFIFLEVKDPDMPGVA
NPEAFKKKLLTGNLIPDLAGKYRDSSWFRTLSGKATKPIHYVVLISMATLEPALLLAKQD
ELKRSMPITHKDWSAPCVASCAILNIEQYKKQFGANSVRRLSAGG

>OHY35399.1
MTKFILKILWLDDNVALAVDQVVGKGTSPLTKYFFWPRNDAWEELKKELESKHWISDPDR
MELLNKATEVINYWQEEGKNRPMSEAQLKFPEVAFTGST
>OHY35438.1
MNQAEISTLIRQELEQLIVEDPVIKNLILGSMSQYYIGRQDADSKFDQTLAQLQSYQEEQ
NRKWEEQNRHNREIMAQLQSYQEEQNRKWEEQNRHNREIMAQLKSYQEEQNRKWEEQNRH
NLQVLEEIKQMNRKHESTVGSLGSRWGLSSEASFRNGLKGILKDSFGVEVLNFLDFDNEG
EVFGRPDQVEIDVIIKNGLIILCEIKSSIDKAGMYIFDRKVAFYEKHHQRRVDRKLVISP
MVDPRALPVAQNLGIEIYSYAEDVNSI
>OHY35484.1
MPRRQDIHKILLLGSGPIVIGQACEFDYSGTQACKALREEGFEVVLVNSNPATIMTDPET
ADRTYIEPLTPEMVAKVIAKERPDALLPTMGGQTALNIAVALAKNGVLEEYNVELIGAKL
PAIEKAEDRKLFNDAMEKIGVNVCPSGTASSLEESKAIAQRIGSYPLIIRPAFTMGGTGG
GIAYNKEEFEVMAQVGIDASPVSQILIDQSLLGWKEYELEVMRDLADNVVIICSIENLDP
MGIHTGDSITVAPAQTLTDKEYQRLRDMAIKIIREIGVETGGSNIQFAINPVNGDVVVIE
MNPRVSRSSALASKATGFPIAKMAAKLAVGYTLDEIKNDITKKTPASFEPTIDYVVTKIP
RFAFEKFPGSDPVLTTQMKSVGEAMAIGRTFNESFQKALRSLETGRAGWGADKAEKLPSG
EQVRAQLRTPNPERIFALRHAMQLGMSNEEIYELTAIDPWFLDKLHQILETEKFLKRTPL
QQLTKVQMYEVKRNGFSDRQIAFCTKTKEDEVRVYRKQLGVIPVYKTVDTCAAEFEAFTP
YYYSTYEEETEILPTDKPKVMILGGGPNRIGQGIEFDYCCCHAAYALKSANYETIMVNSN
PETVSTDYDTSDRLYFEPLTKEDVLNIIEAENPVGIIVQFGGQTPLKLAVPLQEYLQQSP
STVTRIWGTSPDSIDMAENRERFEKILEELKIAQPANGIARSYEDALIVAKRIGYPVVVR
PSYVLGGRAMEIVYSDSELERYMSFAVQVEPEHPILIDKFLENAIEVDVDAIADHQGRVV
IGGIMEHIEQAGIHSGDSACSLPSISLSPAVLNQIRTWTVELAKALSVVGLMNIQFAVVG
ASSYSPQVYILEANPRASRTVPFVSKATGVPLARLASLIMSGKTLEELNFTQEVIPQHIA
VKEAVLPFNKFPGTDTLLGPEMRSTGEVMGIDVDFGRAFAKAEMGAGEKLPLQGTVFVSM
SDRDKSLVVEVIKEFIQLGFKVIATQGTSEFLREQGLKIETILKLHEGRPHVLDAIKNRQ
IQLIINTPSGQEARTDGQLIRRTALGYKIPIITTIAGAKATVAAIRSLQNINLDVKAIQE
YSF
>OHY35545.1
EQNRKFDAFQAEQAHKWDEQNRKFDAFQAEQAHKWDEQNRKWEENNQRLDRIEAQNSATL
EEIQKANRRYESAIGAIGSRWGLYSEASFRNGLQAILGQSFGVEVLNLTLYDQEGEVFGR
PEQVELDIIIKNGLTIVCELKSSIDKAGMYIFGRKADFYAKNQNRVVNRKIVISPMVDER
AVPVAKSLGIEIYSYADVVVP
>OHY35582.1
MATGILVDEPQETEQMLKIAVSNSERLLRLVNNILNLEMLDSGKMRLNLELCDINIMVKK
AMETVQPIAQQKNMEISYSTLDVHNSKILADEETIIQVLTNLLTNSVKFSRAGTKVEIYT
KMGDKEIIIAVKDYGKGIPPDKLETIFERFEQVDVSDSRQKGGTGLGLAICKNIVEKHQG
KIWVESILDSGSTFYFTLPQQ
>OHY35583.1
MLDVNPSFLRFSGYSELELIGYSFDNLPLIYNPEQGLLLKEQLKNQGYVRNLEFDWLIKS
GEVKTSLLSCEPIVIDNKDCVISIIKDITDRKKTEENLRQSEERFRTAFENAGSGMALVS
LNILFGRFRILVNDTKLSN
>OHY35587.1
VYPESDGKPMADNTEQFRWIVKIKENLEILFKSNPDVFVAGDLFWYPVKGSNRIKLAPDT
MVVFGRPKGQRGSYRQWEEDNIPPQVVFEILSPCNSKGEMTRKKLFYLKHGVEEYYVYDP
DEISLEVSIRENNSFREVEDFATWTSPRLNIRFDMTGDELVIYYPDGSRFLSPVELSNYA
EQERFLKEQERFLKEQANQRAEQERFLKEQANERAEQERFLREQERLLKEQANERAEQER
FLREQERFLKEQANQRAEQERLLKEQEQLKYQTLLSQLKANGIDVTGLE
>OHY35596.1
MALTNFVATPIISSLPISTSSQNNLNCPGGPEKDYCIGQDSGIYWRFTEPVEKGVEAPDW
FYVPGVPSRLEGKLRRSYVMWKEKVPPLIVIEFVSGDGEEEKDNSPPPERDEVDPKTKKV

KKAGKFWVYEQAVKIPYYAIFDGFEGKLEMYHLEKGRYKQVKANRRNHYPIPELGVELGM
LLDQEKPPVPWLRWWDNRGNLLLTGNERAEEECQRRELAEAIAIQERQQKELAEALAIQE
RQQKELAEALAIQERSEKEQEREKREKLAAYLRSLGINPDEI
>OHY35608.1
MVSKKPKPRKKNSSHNPNRDWHRLLGMFITVHFYGSPYEVEVEKDLTIRKQLLDIIIIKR
YKDSGFNLYPAGLENMSKHNLISYKSIHESFTVWSLMELIGHYVNYRKQTSKSLSNLLPE
KDYKLYGLTTHEPTQLMGKLKWKSVSQGVYDIECVGMNVRLIVLSKIPKSANNELWRLFS
ARAEVVEEAISHYQESYRNSEYSVLMQQLYEFYLKEKLPMTYTLKQFKKDFVISHLREIP
TEEVLKQYSPEEIKQYSPKDLLKQYSPEEVLKQYSPEEVLKQYSPEEVLKQYSPKDLLKQ
YSLQDLLEGLSPETLERLAIILSQLGVNQIKNQEQ
>OHY35609.1
MTIHFYGSPYEVEVEKDLTIRKQLLDVIIIKRYKDSGFNLYPAGLENMSKHNLISYKSIH
ESFTVWSLMELIGHYVNYRKQTSKSLSNLLPEKDYKLYGLTTHEPTQLMGKLKWKSVSQG
VYDIECVGMNVRLIVLSKIPKSANNELWRLFSAKAEVVEEAISHYQEIYLNSEHSVLMQQ
LYEFYLKEKLPMTYTLEQFKKDFVINHLRDIPTEEVLKQYSPEEVLKQYSPEEVLKQYSP
EEVLKQYSLQDFLEGLSPETLERLAIILSQLGVNQIKNQEQ
>OHY35611.1
MKKFISSAIFTSVITLSGLIIFSGYVNNKYAQAQPSVCTDVLNGQCTPKHYSNNIIDKAK
TFTGLTYKQIDCSHLVHKTFNLYFFGKPELDKNQDPIIPYLTATDGLYDSKNAKKYFDEI
QPENVKVGDLVLFDRPPQGKYRHVGFIESYNVDQQVGKFFGSQTSTGPATAIFTTNQKKT
GYYWGVKQKFIKFLRPKETILRRT
>OHY35612.1
MKKFISSAIFTSVIGISSIFVSSVAKADFNVSQYAQSQADGCTAFPDRIRFAYDFTGACN
SHDWCYSYSEVSRKNCDDSLLREAEASCNGKRVCLKAAQVLYKGVRRFGQPYFLRARQAS
GRPQNDSR
>OHY35614.1
MVIFRRLITSGLIGFLVSSCADAAKSSTEYTKQIVQENNVARLMAQVQDSQKVSNLLSQG
NAFLDSERYEEALQLYNRAIEIEKDSVPSWVNRGNALLSLKRYQEALESYNQAIALRPNK
NEAWYNRGNALSALGRYEEAIKSYNESIVIDPNKFEAWINKGIALTKLQRYQEGLASYNQ
AISINPNFAAAYYNQACNYALQKQVDLAVNSLAKAIKMERQKYSELAKADKDFVQVKYNR
EFQDLLR
>OHY35657.1
MFEHFTSEAIRVIMLAQEEARRLGHNFVGTEQILLGLIGEGTGVAAKVLTDLGVTLKDAR
REVEKIIGRGSGFVPPEIPFTPKVKSLFEQSFREAHGLGHNYINTEHLLLGLTDAGEGVA
AKVLKSLGIELQTVRSRVMSILGEDNRVVAGRQDNPRRNQNLSIEEFGRNLTKLAEQGRL
DPVVGRQTEIERTVQILGRRTKNNPVLIGEPGVGKTAIAEGLAQRIVNQDVPEILLNKQV
ISLDMGLLVAGTRFRGDFEERLKKVMEEIRSVGNIILVIDEIHTIVGAGGTEGGLDAANI
LKPALARGELQCIGATTLDEYRKYIERDAALERRFQPILVGEPSVAETIEILLGLRSAYE
QHHKVTISNDAVIAAAELSDRYISDRFLPDKAIDLIDEAGSRVKLRHSRIIDNKELKQQL
KNTSQEKVEAVRVQDFGKASKLRQEELDLQTQLAIAQNLPKITIPQVDEEDIAEIVSSWT
GVPVNKLTESESELLLHLEDTLHKRLIGQEQAVTAVSRSLRRARVGLKSPNRPIASFIFS
GPTGVGKTELAKALAAYFFGAEDSMIRLDMSEYMESHNVSKLIGSPPGYVGYDEGGQLTE
AVRRKPYTLLLFDEIEKAHPDVFNMLLQILDDGHLTDAKGRKVDFKNTLIILTSNIGSKV
IEKGGMSLGFEFDNQANASYNRIRNLVNEELKSYFRPEFLNRVDEIIVFSQLNKDEVKEI
SQIMLEEVAKRLQEKGIKLEVTEAFKDLVVTEGYDPSYGARPLRRAIMRLLEDSLAEAIL
SGEIREGDQAIVDVDDDGLVKVKKAETRDLVLVSAPA
>OHY35672.1
MSNQERMDNMENQLIDIRLAVSALLETSVIYQRNFEVMQRNFDSVVIEIREMQSEIREMQ
SDIREMQSEIREMQSEIREIQSEVREIQLDVRGLQTENRRILDVLQNVPPDKYE
>OHY35924.1
MLLDQEKPPVPWLRWWDNRGNLLLTGNERAEEECQRRELAEAIAIQERQQKELAEALAIQ
ERTEKEQERQQKELAEALAIQERTEKEQERQQKELAEALAIQERSEKEQEREKREKLAAY

LRSLGINPDEI
>OHY35925.1
MHGEGNCATVERLYRHLTIETPKLNQEHGKPIQNPYLKAEPPYKMYYILLPKIANIRDIV
PLKNEALHAEVNNDLELAEKLWIRVLAVDGTDNDALSGIKRITIRREQNPQIKPPGEGNT
IDGQDKSPGNNKGATQPADENTVPPSKEPRIQETKHGSGFLKIWLKIALLASSGLILVYT
TLWQIFQQQWNQITNPEPTETSYNPNPSKSSPINVDSRYAKLETLLKAQNFRAADLETDK
VMLAVANRESEGWLRIEDAEKFPCKELRSIDQLWLKYSRGKFGISVQQQIYQSLGGTKAF
RQNVWDKMGDRVGWRREGNWLSYSDLNFSQQAPSGHLPAWGWLGVDYIGGGWGEYGKYFP
PVQTCRV
>OHY36257.1
MALITTGSGFIRDLEKFGSLGVFIPLEGGYEGRYRRRLRAAGYVNLHITARGLGDVAAYL
MGVHGIRPPHLGKKTTSNGAAVGEVQYLPPLISSHLAQQPPNSKGLLLWVIEGNILSDQE
VEYFTNLPRLEPRVKVVIERGGDRYFRWTPLEKTLLAS
>OHY36404.1
MSQDKKPRSKQQLPGTPPPGVYHKKPLFWKILIIQVLRGTIGVLERMVTRLETSPASRTP
EQGGLLLWVVTKWDGFLRGFRLFLPSKVANNVSDGFLTMFFAFLAVLAIGVTATSLISQI
GSPSVPQLSQPPTDSTVEGKTQTQLSQPPTDSTVEGKTQTQLGQPPTDSTVEGKTQTQLS
QPPTDSTVEDKTETQLGQPPTDSTVEGKTETQLGQPPTDSILKSQAELIPQQEVTGFIEK
QLKEITATNIITKDKQRIEVEIVQSIKTNFRISEIVIKTTEAWYKLESSQQEKLAAKILK
SCQEMDLIHVKILNARNQLIARSPVVGTKMVFFQFPNS
>OHY36453.1
MASYKVELKTPSGLQLIDCPAYDYILDAAESAGLDLPYSCRNGSCSSCVGKLAFGTVDQD
EQSFLTDDQIKAGYILTCVAYATSNCTIETNKEADLLKR
>OHY36458.1
MTQTTEKLKKFTILHSNDMHGDFLAEAKSGEGNLIGGLSLLSGYINKVRQTEKNTLYVIS
GDMLQGSMIDTEFKGLSTMEIMNYLAPDVVTLGNHELDYGFPHLLFLEKMANFPIVNANL
YIKKYSKRLMNPYIILNVDGFDIMFIGIVTEEVLSALKLDTSIGTFVGLEDAAAEVGKIC
NTYKNEDIDLTILLTHIGFEEDKKLAAMLDPEWGVDIIIGGHSHTVLEQPAQVNNILISQ
AAVGTDQIGRFDITVDDDTNSIVEWKWQLVPISNQVATPDVELQNFINAYKEQVDRKYNR
IVSRLSRKLIHPVREQETELGNLIADIFSEIDMIDVVLAGSSAIRGKELGPVVTLEDLKK
TYPYDGALYKIRVNGTQLTKVFAHILRPENRNPVESQCYQISKGVQVVYNDTKKSIESFN
INGQPVEAGRQYIMCVENYHYQNSLKNLNLTPEEVANAKVVATSAQSVLEEYWALASLLW
SQLKSPKFNADKFKP
>WP_006276885.1
MTRNGIGILTAQARSERLTGQIHVYDGAGKGKSQAALGVVLRSIGLGINTKSNCNRVLLL
RFLKGPGRDYDEDGAIAALQRGFPHLIDQVRTGRAEYFGHDEITPFDRAEAARGWDVAKG
ALASDLYSVVVLDEINPVLDLGLLPVQEVVETLKSKPQELEIITTGRAAPQQLLDIADLH
SEMKPHHHPQAAELLLDGIEIYTGSGKGKSTSALGKALKSIGRGINHPGSARVLIMQWLK
GGTGYTEDAAIAALQQSYPDVVDHLRCGRDAIVWRNSRQHLDYVEAERGWEIAKTAIASG
VYKTIILDELNPTVDLELLSVEPILQALLRKPKGTEIIITGRCQNQPAYFDLASIHSEVY
CHKHYANQGVELKRGVDF
>WP_061544881.1
MGIREEVYYEGGPHIGDLILNLLIGLTIVGLPLTIGAIIRALWLRFKITDLRVAVMGGWM
GQNRTDIIYSEVVKVVKVPRGVGLWGDMVLTMKNGSRLEIRAIPNFREVYEYINDRVAAK
NPQYTSNPS
>WP_061544934.1
MALITTGIGFIRDLEKFGSLGVFVPLEGGYEGRYRRRLRAAGYVNLHITARGLGDVAAYL
MGVHGIRPPHLGKKTTSNGAAVGEVQYLPPLISSHLAQLPPNSKGLLLWVIEGNILSDQE
VEYFTNLPRLEPRVKVVIERGGDRYFRWTPLEKTLLAS
>WP_061545005.1
MFFRAGARISVIYYPDGSRFLSPVELSNYAEQERLLKEQEQLKYQTLLAQLKAKGIDITT
LE

>WP_061545032.1
MFKTWQIALVAEGPTDFEIIQAVLKAVLSTPFILTLIQPEVTHPQRGSGWGGVLKWCHQA
QERWSGPLMEDPTLSNFDIIIIHLDADVAYFEYNDVSYSKTEVQAHRWASIPCAQPCPPI
LDTIHSLRNVLSSWLDKIQQDVYTVLCLPAQSSGTWLAVALLSADHPLLTNEIECEPLEE
KLAQLPKSQRVKKTIKEYRLHAPQITAQWSQVKKICSQATRFEKDVLTAIRQAIAEK
>WP_061545097.1
MPKQKKIDPLVGEELLQRVKELGNETREEKAKQCGYYTITKNGIERVNIMKFLNALIEAE
GIQLDGAPGANGRGGRSASYRVSVQSNNNLLIGSAYTKQMNLKPGDEFVITLGKKHIRLR
QVESEERDDDDQLEEVTA
>WP_061545103.1
MNSKNYTFSGLTLKSLILLSSLLICNGLTIGGLELSANAQLLSNAEREELSRLRTETKIQ
KQLQSYLSIWLTLLSLFAVGLIATLWFLRKAIIRDIVERAMRQIGNIENLQTELIAANQK
TTGLIEYSQDLALELEEKVNHLKTKIEGEGGKLSVLLSDLPKSKQEFLTALEREVIAAQE
NISSLEFKLNTQLEQVTLAAQQQRVTIENIKKLESELFAQFSEIKLSIENHRDTSVSDIN
KYRSELMEQFEILALETLESKTQVVQSITEHASQFTSNLSEFQTHAQSQIDGFTSNLSEF
QTHAQSQMDGFTSNLSEFQTHAQSQMDGFTSNLSEFQTHAQEQMDGFTSNLSEFQTHAQN
QIDSFTSSLSDLNQTSVSQLVDLQTDTQKQKDHIFKDLAKSQSEFSHYLLELKNTTESRQ
REIIDSWQKSVDEVEQMLSNLHNNVEEQKTDLLENLQVLGLGFQKQIGDLQNSIEQRHRK
LFQTSQNSVEQLISEFMSELSAMKSDIKTDVDHQKANLIARLAKLEKLESQFVEQLKKLQ
LDAENRQYETLKELSQMTPQTPDISPLSPVGTDTNTTNTIDVQSVNNLDNHLDHEIQEAE
ELLSQNRYEDALSIYEKITSIQSDHEEYWLKRGFILNKIKRHKEAIGVYNQVIRINPAHY
QAWFDIGITCGKLGKHQEAFNCFNKATEIKPEESVAWLNRGLSLVELENYEEAISSFDKA
LEIQPSSFKIWDKRGYTLVRLGRDEEAITNFNKALELNPEYGSALYHKAACYALQKNVES
ALVNLQQAIKHNPSYREDAAGDIDFDDINNDTRFQQLIHRE
>WP_061545156.1
MPHPLMYEEENFVVLETNQEEQFLTKLELLEKLQNTLSQMPIEDIPLDLRKIGSLIEQVN
HLIDTTCELDLGPGRYLQWYAVRLEK
>WP_061545172.1
MSELERYYTLLDLEPGATIEEINQAYKDLVFVWHPDRLPKDNHRLQKKAHDKIKALNQAR
EKLRSFQYQSQPGHNSEPSTSKRSPYQPNQPPPQNPDLSGRDFSHANLSNKDLSGRNLSY
ANLSGSNLSDTFMHKVNLRGANLSEANLFRANLLLADLREANLRSANLIGADLSGADLRG
ADLTGARMRSGERLLVKLVGANLTGAIMPDGAIYG
>WP_061545245.1
MVTLKGFAVLPADTFAAGPKSGAAVANPTNGRTTPFVGQPIQGFSGVQFAPNTNGSRFWF
LADNGFGAKNNSADFLLRIYQLDPNFTGVENGNAKVEVEKFIQLSDPNRLIPFSIVNQNT
SERQLTGADFDVESFVIDAKGDIWVGDEFGTYLLHFDSNGVLLDAPINTPNLFKLNTLNG
QKPIVIGHRGASGELPEHTIEAYRLAILRGADFIEPDLVSTKDGVLIARHEPNLINTTDV
ANRPEFANRKKKVVVDGVEEEGFFASDFTLAEIKTLRAVMPQGYRDQVFNGLLEIPTLGD
IIDLVKEVEAQTGKKIGIYPQTKHPTYHDNLNLSLEEKLIDTLKSKSFTDPTRIFIQSFE
VSNLQDLNNNIMPARGVNIPLVQLIDAYDVADDGKLIYKDAYARPYDFTVKGDTRTYGDL
LTPAGLQEIAKYADGIGPWKRQIISVKTVDKNNDGKPDDLNNDGVINDSDKVTLPPTSVV
SDAHKVGLLVHPYTFRNESRFLASDYNNNPELEYRQFISLGVDGYFTDFPGTGDLVRDQI
TTNQVRSPQNPTVLSKPNFDTLNGQKPIVIGHRGSSGERPEHTLASYKLAIAQGADFIEP
DLVVTKDNVLIARHEPMLAVVNLNTDGTIKLVSGKPEINFTDTSTDVYLRDKFQDRLKVK
NLDGRNVGGWFAEDFTLAEIKELNAIERLPSLRSTAFDKDGLKVPTLKEVIDLVKQVELE
TGRKIGIYPETKHPTFFQQQGFNTSQLLVNTLKTENFTDASRVFIQSFEVSNLKELKSTI
MPGASIDIPLVQLFGGSGKPYDFVVNGDSRTYDNLSTPTGLKEIAQYAKGIGPNKQRIVP
MTTVDNNKDGQPDDLNGDGQISDADRTLGASTTLIQDAHQAGLLVHLYTLRNDGFFLSAD
YKGDPGAEVRKFVNLGVDGFFTDFPKTGTSVIVNNYLAGTGYANPNNNLNSPYFADSPVY
FNPNQPYYGDLVTANLNRSQGFEGMAFSPDRQTVYPMLEGTVVGDPAGSVRIYKFDVATE
TYTGLVGLYQLASPSNAIGDFTPINDKEFLVIERDNNQGTSAAFKKIFKVDFSQINAQGF
VPKEEVANLLDIQDPNDLNSDGNKTYNMPFQTIEDVVVWDNKTIVVANDNNYPFSIGRPP

LIDNNEIVVLELDKALSLDARLGLAATIAESSQLVFGTPGVDNVSVPQATDGINDAIFTG
AGDDKVDTLGVTNPYAGNNTVYSGSGKDVIYVNNGDRIFGGSGNDEILATDAKDYRISGG
SGNDVFYLGTNGRALGGDGEDKFFVTEGGGNLISGGAGGDQFWITTGDIPSVGNKNFANT
IVDFQIGVDVLGISGQGSNFGFNNLTLTNNDIIINGNKVATLTGINTSTLTASNFAFA
>WP_061545300.1
MNASEQATNFELASKIATVVNLFKLQFPDAKSDLKPWQNDRETRQLVDPDSIDIGFHFPG
ISRSWRSRSVLIQIRFHQDPINKQSKAIGVEVSGFDYRGQVWRLSTIEQWSFVGEFTPSA
VVGEKLKHICKQILEIFNI
>WP_061545333.1
MLNLKTVIFVLSFTPFIVLSTQLTGRAQVSSPSLGTAGSVSGGGATGSDNNPISAPSLGT
AGSMSGGGATGSNSAPSLGTAGSMSGGGSTGSDNITVIVSTNNLRELRNAIGQLVSSGSD
SLSPITFSSLRASLISDGVSPGHTDQLIVSFSRVLSQLGVPNTNFSANNLNLEKLVASTK
IFKPTVTIAQDSPAEGEIALILDSNNLTEAINIYNKIVLESDDPTIIKLSRNQDFLKISR
ILKTLRSGII
>WP_061545445.1
MQVLRSFSNQEVLDLQEKLGRGGEACVYTVPSNENLVAKIYHHPTPNHIQKLRAMIANPP
ANPAASFGHISIAWPQELLTAAESSDTIIGFLMPRIRNMRPIMDFYNPGNRRQNCPLFNY
QYLLRTARNLAAAFAALHASNYCIGDVNESNILVSNTALVSLVDTDSFQVPDLSQNTVYR
CLVGKPEYTPPELQNKTFADYNRETYHDLFGLGVLIFQLLMEGTHPFSGVFQGLGDPPTY
ESRILAGHFTYSQKQKVPYLPTPITPSWQTLHPDLRDLFISCFEDGYHAPYLRPSAQTWL
SVLSTAEASLVSCAVNPQHVYHPHLDKCPWCERTIKLGGRDPFPSLQAISAREHLQPRRK
SRKRPRYQPRVRKPAVPVLAPYTQSSLRSTLPGYPTVQTSSRSKFYTFMFGILGLGVLGY
LDIMVKFTRPFLSPNPYTQQSLLSSSSENVHSPLSLSFNDYYQRGNQAYQQQDYQQAIED
FSQGIKQNTNFSKLYMHRGNARYNLNDYEGALTDYNLALKINPQEVKALINRGNAYIKLA
EYSNDPDYEYKKAIDSFNNAININQQDDEAYVRRGIVRSQIARYSNNSQEEYERSIGDFT
QAIKLNRFKAEAYFQRGLARYQFAQYSSNYAQIYKQAIADFDQALNINPEMAEVFLKRGM
IYYELAQYGEKTARNNQQRALEDLEKSAQLYLNKKDVNNYQQAISNICVIAEEKCDYFLQ
NSSIIYSVNP
>WP_061545646.1
MSLITLQSVKKDFGIKEILKEATFSIDGTDKVGLIGTNGSGKSTLLKIIAGIEPIDGGQI
LTNYGAKIIYLPQQPDIDENLTVLEQIFMDSGEHTKLVKEYEELSDKLAHYPEDTLLMSR
LSEVMQRMDATGAWELETNAKIILTKLGIGDFEVKVGTLSGGYRKRIALATALLAQPDVL
LMDEPTNHLDALSVEWLQSYLNRFRGALLLITHDRYFLDKVTNRIIEIDRGDIYTYSGNY
SYYLEKKALAEESAVSSQRKHQGILRRELEWLKRGPKARSTKQKARIQRVESMRETQFKQ
AQGKVDISTIGRRIGKKVIELSGICKSYNGKTLISNFSYEFSPEDRIGIIGGNGTGKSTL
MNMITGRTSPDAGTVEIGSTIHIAYFDQHSEELISAVNDNQRVIDYIKEEGEFIKIADGT
KITASQMLERFLFPGSQQYAPIHKLSGGEKRRLFLLRLLIGAPNVLILDEPTNDLDVQTL
SVLEEYLEDFLGSVIVVSHDRYFLDRTVDTIFALEEGGNLRQYPGNYSVYLDYKKSEELT
QQETINGRDNRKSKNLTQPKSGEQEVQNKKRRRLSNWEKREFEQLEAKIVDLEAQRTLVE
TSLLAVAPENYTQVQNLYEQMEVLKQAIDIATERWLELAEMDV
>WP_061545658.1
MAHKKGTGSTRNGRDSNAQRLGVKRFGGQVVRAGNILVRQRGTKFHPGNNVGIGNDDTLF
ALIDGVVTFERKGKSRKKVSVYPPVTEESVAS
>WP_061545707.1
MPKHFNTAGPCQSDIHYMLSPTARLPDLKALIHGRNYFIIHAPRQVGKTTAMIALAEELT
DSGEYTAIMLSLEVGAPFSAPYHEIAPHLVLMAFLHRVVNDGGTLEREYAIGSGRMDICL
RYGKVVMGIELKVRREKLDPLTKGLTQLDKYLDGLGLDTGWLVIFDYRPGLPPMGERISK
EEAISPRGRTITVIRS
>WP_061545746.1
MQQLYEFYLKEKLPMTYTLKQFKKDFVISHLREIPTEEVLKQYSPEEIKQYSPKDLLKQY
SPEEVLKQYSPEEVLKQYSPEEVLKQYSPEEVLKQYSPEEVLKQYSLEEVLKQYSLQDFL
EGLSPETLERLAIILSQLGVNQIKNQEQ

>WP_061545749.1
MVESNLFSKLENLLRSQNFREADTETLMVVLKVANREKEGRLTLEDAKTFSYENLLIIDN
LWCRWSNGKFGISVQQKIWCECGGARKYDADMVLNTSISFGDRVGWRCKGKWLPYDQLNF
SLNAPVGHLPCGGSPHTYIWTMCMDMNDLP
>WP_061545776.1
MQQLKAKTLEMRTPQATKTAVLVIGGAEDKVHGREILRTFVARAGASKAYITIVPSASRE
PAIIGGRYIRLFEEMGAQKVEILDIREREQCENSQIKASLENCTGVFLTGGDQLRLCGVL
ADTPAMDLIRQRVRAGQLTLAGTSAGAAVMGHHMIAGGGSGETPNRSLVDMATGLGFIPE
VIVDQHFHNRNRMGRLISAIAAHPDRLGIGIDEDTCAVFERDGWLQVVGKGSVTIVDPTE
LTHTNEPHVSANEPLNVHNLRLHILSYGDRFHLYQRTVLPAVHRLPSYGDNVS
>WP_061545872.1
MTYCLRISDLPENERPRERLITHGAKVLATAELIAILLGTGQGPGKLSAVGLGQHILQEL
GRDQGDPLAALRDATPARLTEIHGVGSAKATSILAAVELGKRVFLSRPAEGAIIDSPIAA
AATLSQDLMWQSQEKFAVLLLDVKNRFLGTKIITIGTATETLASPRDIFREVIRHSATRM
IIAHNHPSGNLDPSQADLDLTRQLLSGAQLLNIPILDHLILGNGNHQSLREITSLWDDFP
QEN
>WP_061545944.1
MNIETIKSEKTKQLPGANLEDQDLSEFDLTAANLAGANLMGAHLVSVNLEGSHLEGANLM
GASLQGADLRANLLGANLMQADLTGADLRGSNLRGANLMGATVAGASLTAAFLSGANLMS
GNFQGVDLRGADLRGANLTGANLKGADLSRADLQGALLNQANLEESDLRGANLAGANLAG
ANLLCAELEAASLNGANLYQACLLGTILETYHD
>WP_061546085.1
MDKIKLATVWLGGCSGCHMSFLDLDEWLIDLATQVDVVYSPIADIKEYPEGVDVVLVEGA
IANEEHLELIHKIRQRTKTIISFGDCAVTGNVTAMRNLTGTANVSLQLAYIEGADVNQKI
PHSLGIVPPLIDRVVPVHNIVDVDIYLPGCPPSATRIRATLEPLLRGETPPMVGREMIKF
G
>WP_061546113.1
MFEHFTSEAIRVIMLAQEEARRLGHNFVGTEQILLGLIGEGTGVAAKVLTDLGVTLKDAR
REVEKIIGRGSGFVPPEIPFTPKVKSLFEQSFREAHGLGHNYINTEHLLLGLTDAGEGVA
AKVLKSLGIELQTVRSRVMSILGEDNRVVAGRQDNPRRNQNLSIEEFGRNLTKLAEQGRL
DPVVGRQTEIERTVQILGRRTKNNPVLIGEPGVGKTAIAEGLAQRIVNQDVPEILLNKQV
ISLDMGLLVAGTRFRGDFEERLKKVMEEIRSVGNIILVIDEIHTIVGAGGTEGGLDAANI
LKPALARGELQCIGATTLDEYRKYIERDAALERRFQPILVGEPSVAETIEILLGLRSAYE
QHHKVTISNDAVIAAAELSDRYISDRFLPDKAIDLIDEAGSRVKLRHSRIIDNKELKQQL
KNTSQEKVEAVRVQDFGKASKLRQEELDLQTQLAIAQNLPKITIPQVDEEDIAEIVSSWT
GVPVNKLTESESELLLHLEDTLHKRLIGQEQAVTAVSRSLRRARVGLKSPNRPIASFIFS
GPTGVGKTELAKALAAYFFGAEDSMIRLDMSEYMESHNVSKLIGSPPGYVGYDEGGQLTE
AVRRKPYTLLLFDEIEKAHPDVFNMLLQILDDGHLTDAKGRKVDFKNTLIIILTSNIGSKV
IEKGGMSLGFEFDNQANASYNRIRNLVNEELKSYFRPEFLNRVDEIIVFSQLNKDEVKEI
SQIMLEEVAKRLQEKGIKLEVTEAFKDLVVTEGYDPSYGARPLRRAIMRLLEDSLAEAIL
SGEIREGDQAIVDVDDDGLVKVKKAETRDLVLVSAPA
>WP_061546126.1
MPQVVLINPQIPPNTGNIARTCAATATELHLVGPLGFEITDRYLKRAGLDYWPHVKLNYH
ESYQTFLKVHQQRGGRLLGFSVRGNINYIQHEFHPDDWLLFGSETTGIPENILDICDSTL
YIPMGQPGVRSLNLSVSVAVALFESRRQLGYLK
>WP_061546157.1
MPPVPVNPGNPLFPRDAGSLSGIVDQVSFNFLPGYNPTQARQTLQNTLGNTSAAFTNLFG
LYEVDSVTGAVNGLTPDKPGYAKAALNRMVPNFVVRAGGSGNGTTGDVIVSGGKIYAPFV
IANGGNYSGTIQEAINEFFKVNPNNSPATAQNYTTLPVAYFSFGAANPDGAAHIKSLANN
IFGFEDLPSNVGVSDYDFNDMVFSFG
>WP_061546170.1
MSNQERMDNMENQLIDIRLAVSALLETSVIYQRNFEVMQRNFDSVVIEIREMQSEIREMR

SDIREMQSEIREIQSEIREIQSEVREIQLDVRGLQTENRRILDVLQNVPPDKYE
>WP_061546173.1
MPRRQDIHKILLLGSGPIVIGQACEFDYSGTQACKALREEGFEVVLVNSNPATIMTDPET
ADRTYIEPLTPEMVAKVIAKERPDALLPTMGGQTALNIAVALAKNGVLEEYNVELIGAKL
PAIEKAEDRKLFNDAMEKIGVNVCPSGTASSLEESKAIAQRIGSYPLIIRPAFTMGGTGG
GIAYNKEEFEVMAQVGIDASPVSQILIDQSLLGWKEYELEVMRDLADNVVIICSIENLDP
MGIHTGDSITVAPAQTLTDKEYQRLRDMAIKIIREIGVETGGSNIQFAINPVNGDVVVIE
MNPRVSRSSALASKATGFPIAKMAAKLAVGYTLDEIKNDITKKTPASFEPTIDYVVTKIP
RFAFEKFPGSDPVLTTQMKSVGEAMAIGRTFNESFQKALRSLETGRAGWGADKAEKLPSG
EQVRAQLRTPNPERIFALRHAMQLGMSNEEIYELTAIDPWFLDKLHQILETEKFLKRTPL
QQLTKVQMYEVKRNGFSDRQIAFCTKTKEDEVRVYRKQLGVIPVYKTVDTCAAEFEAFTP
YYYSTYEEETEILPTDKPKVMILGGGPNRIGQGIEFDYCCCHAAYALKSANYETIMVNSN
PETVSTDYDTSDRLYFEPLTKEDVLNIIEAENPVGIIVQFGGQTPLKLAVPLQEYLQQSP
STVTRIWGTSPDSIDMAENRERFEKILEELKIAQPANGIARSYEDALIVAKRIGYPVVVR
PSYVLGGRAMEIVYSDSELERYMSFAVQVEPEHPILIDKFLENAIEVDVDAIADHQGRVV
IGGIMEHIEQAGIHSGDSACSLPSISLSPAVLNQIRTWTVELAKALSVVGLMNIQFAVVG
ASSYSPQVYILEANPRASRTVPFVSKATGVPLARLASLIMSGKTLEELNFTQEVIPQHIA
VKEAVLPFNKFPGTDTLLGPEMRSTGEVMGIDVDFGRAFAKAEMGAGEKLPLQGTVFVSM
SDRDKSLVVEVIKEFIQLGFKVIATQGTSEFLREQGLKIETILKLHEGRPHVLDAIKNRQ
IQLIINTPSGQEARTDGQLIRRTALGYKIPIITTIAGAKATVAAIRSLQNINLDVKAIQE
YSF
>WP_061546233.1
QNRKFDAFQAEQAHKWDEQNRKFDAFQAEQARKWDEQNRKWDEQNRKFDAFQAEQARKWD
EQNRKWDEQNRKWEENNQRLDRIEAQNSATLEEIQKANRRYESAIGAIGSRWGLYSEASF
RNGLQAILGQSFGVEVLNLTLYDQEGEVFGRPEQVELDIIIKNGLTIVCELKSSIDKAGM
YIFGRKADFYAKNQNRVVNRKIVISPMVDERAVPVAKSLGIEIYSYADVVVP
>WP_061546401.1
MDLSLIPAQPKPGVINVLIEIAGGSQNKYEFDKELNAFALDRVLYSSVKYPYDYGFIPNT
LAEDGDPLDGMVIMDEPTFPGCVIPARPIGFLEMIDGGERDEKILCVPVKDPRYAHVNSL
KDIPPHRLEEIAEFFRSYKNLEKKVTQILGWHDADKVAPLVQKCVAATK
>WP_061546407.1
MLTVALPKGELLKNSIQLMQSAGLDFSAFLDAGNRQLQIYDASGKAKGLLVRAQDVPVYV
EYGQAQLGIVGFDVLKEKKPQVGQLVDLKFGYCRMSVAVKSTSSYKSPLDLPAHGRVASK
YVNCAREYFESLDLPVEIVPLYGSVELGPITGMSEAIVDIVSTGKTLRENGLVEIATLYE
STARLIVHPLSYRLDLGGIYNLAQSVKSSVSS
>WP_061546453.1
MQSLVIRSFTTLADSGNYEKVRENFERSLQIHPNDTIALNSYGKALADSGNYEKASEIFE
RSLQIQPDNYIYFTYGKCLEELGKYENAISQLKLITVDKLPQYHKNVVHISLGRVYYFLK
LYIEGDKHFNIAIANSDDREKSILSSARSILAHSPHNQTAVKMLQQIAEESPRYAQAMEM
LTLNLTEEEYFKTVPDDSNNLKDAQILNRAIYHKIANEITILKSIAYKILRVSSIKDALL
ESIISNIEEILQEINHRRSLEKSQIEQIEIIANNEYGKILEIIAKTAHDISDFVNNELAI
VESKTRRAIKKFTGNDPQFSQFNKLLTQLEFTQDALNDLKAINEGIRIKNRQFKVKKIFE
TWEFNTQIDNAQISLNIENGDSDFNGDEEKIKSILNELVENSLKHNPDNSDLQIKINSQD
VTNPPGIRGANIPGSKKYLYIEFIDNGLGIAPDKKDWVFQPLNTTATGTYSSGLGLFIIR
KTLTQMGGYIREVGVNGVKFEIYIPYPDQNI
>WP_061546699.1
MFFKLGPNRQFPPGHLDLIPSWFVVYGNLDEINKSRENEVNNLLKRDRIVRNLEVFQDII
VISLCVALFCVILLRFISLYITLIK
>WP_061546709.1
MTIQQTELLYLPPDLKPELLPRHVAIIMDGNGRWAKQRGLHRIKGHEAGAKQVKDLVRCC
VQWGIETLTVYAFSTENWNRPLREVNSLMALMRRFLREELREMHEKNIKIRFMGDLTKLP
QSLQREISHSTLLTCDNTGLNFYIATNYGGRQEILQACQAIAGRVQTGDLKADQISQELF

ESHLYTSGISDPDLLIRTSGEMRLSNFLLWQMAYGEIYIADTLWPDFDRGQFHLALLAYQ
QRERRFGAV
>WP_061546747.1
MINIEQFRQEIEDWIINVVSIPNPLTGNFPPCPYAKAAWLNNRVSVRWFHGPELPELLME
QIRTWNNDFEMVIFGCDPQNLDAQRLERYITKANYVLPEYDLVALGSHPDKQYVGDDAEN
VNNVIITHPKYVLASVQSFSQLQEASDELLRLGYFQYWSAEKLAEMKSERASHNLSSIQR
KNSYRIIPTNH
>WP_061546750.1
MRDAVTTLINSYDLAGKYLDRNALDSLRSYFDSGTSRVQAATAINANAAAIVKQAGSKLF
EELPELIRPGGNAYTTRRYAACLRDMDYYLRYATYALIAANMNVLDERVLQGLRETYNSL
DVPIGSTVRGIQIMKDLAKEQAIAAGVANATFVDEPFDYITRELSEQNI
>WP_061546821.1
MPLGRELPQLLKQRLFHKGRKFDFEVSRLRLPNKSEGEWECIRHPGGALAIPVTDDGKLI
LVRQYRFAVQGRLLEFPAGTVEPNEKPLITVQREIQEEIGYKAHQWDKLGEFFLAPGYSD
EIIYAFLARDLEKLETPPQQDEDEDIETVFLSPAQLEAAIDRGEPVDAKTVTSFFLARSF
LMLKP
>WP_061546834.1
MPVGNDQKNENPTDEKPRLTIPPKLRLLVGYIQQLSLMVREKIGRSEKPFYRHVWFWAGL
GVGTTIIGFNYGIRTVDSSLPDKSELNAMIRQQTLTIKAGDSTVLMQQGEATREQLKIQQ
IPDKLQLAFIASEDRRFKQHDGVDFQGIVRAVISNFQSQNVVQGGSTITQQLARILFLSQ
ERTLWRKLKEVRLAQKIEGELSKEEILERYLNLVYLGSGAYGVADASWVYFGKTVDQLNL
SEIATIAGLAPAPNIYAPDKNLPGAIARRNTVLQRMEEDGVITPQEKESALQQPLTVKTN
LPRRFQVTAPYFCAYVQQQLPKYLKPDVLAAGGLTVETSLNSNWQRLAEKVLVRTLKNEG
TWGRFREGALVSINPRNGEIQVMIGGKDFTKTQFNRVTQAQRQPGSTFKTFVYAAAIASG
KNPGDTYIDQPISFRGYEPKNYSEKFRGLMTMQDALALSINTISLQVLIDVGYEPTIKIA
HNMGIKSPLEHNYSLALGSNEVNLLELTSAYGSFATEGLHTEAHAIRRILNRQGDVIWSA
NFQPKRALDADSSGIVTKMLTNVVTNGTGRSAQLPDRPVAGKTGTSDESRDLWFIGYIPQ
MVTGIWLGNDNNRPTHGSSSTAAYTWSQFMEGAVKDLKVEKFPRPSRVAKRKATIKAQPI
SKKNAGFKFSKRDDNSDSQDGNSSTRQGRRRRSNYEQLDQDQDTSTRRRRRRRNDVQTVP
SSTEENTTSTRRRRRRRNDFQIVPSSTEENTTSTRRRRRRRNDFQTVPSSLPTPNSPAGQ
PSWRERLKPE
>WP_061546874.1
MIMPSPINLGTILQNRYHIIRLLGQGGFGRTYLAEDQGRFNELCAIKELVILEPDSYEGK
KAQELFDREASILYQIDHPQIPKFREKFAQDQRLFLVQDFVGGKTYHTILNERRTQGQSF
TQAEVLYLLQSLLPVLEYIHKAKIIHRDISPDNLILRSTDQKPVLIDFGVVKEVATRLSN
SSIYQATTVGKPGYSPIEQVQTGKAYPNSDLYALAVTAIVLLTAKEPADLFDETTFVWKW
QHWVQVSPKFAQVINRMLNRIPGDRYQTAKEVLLDLNNLEVSSPLNPRDPNLSYLPTVAI
SHPSPTPTNSPEPVISPHTNSSILDNTLSMVAIGAFVVILTGFSSWSLVNYFRGQRLSPL
LSTTPQNFDSPVIPRSTPNSTLTPTPSKMEPRNLAWDLSNNANEEGIIKFGEVIEYSFRG
IPGQKLTVAVNEESGVLLTILTADGQPLSTDAQQVTSYEGVLTSRGRLTIQLTLSTTVSE
STYSLSVALENPIKRAPIPPRNT
>WP_061546902.1
MAILAVGAGQTYTTIQTAINAANNDDIIVVRPGIYQEDLTINKSVTLIGPYGTFEGIDGF
ENRLGVKPLGPDINAALGVGGLPTANEDFRRYQDDNGTIDNAFGNTQEAWIKGTITVTED
NVTIDGFRLRNENGPLQWNDTPDNFKLLNNYLTGYTANNSPSFGDASINNPTGVVTGWQI
DSNYIGGLLGGGGTGGSIYLAGLQDSNIDNNTFWRPRAAHLYLASLTNVTIEGNKFYHGL
HTGGANFDGFGEFFSGSGYGYGYGGYGDGFFGRNYWLELKGDNDQVLIKNNEGEYNSGGI
QLFGETDSPFAFDNITIEGNSFPDNNFINAYNEAPDNGKSGLIPAVMATARLGGPSGSNL
VIRDNNITMDLAQVKFITDHKSSLEVRGNFNGVTIEGNTLTPKNINGGVDIITGLSLYGI
LPGETLIKGNQLLGQDGDPLEASYYGIDLIPTFADYGTYAGDLAVEDNTINSWQVGVNLR
DTNEITGDINISGNTFENNAYGVVLDATATTNSINIAGNTFSNNFSNVFDNIDPVITMDQ
VLSYEENQDLGSVLGTVSATDNLPNTDNVGITQYFISSGNDDGFFTINSSGEITLTETGL

AAANDFETSPSSFDLGITVTDGGGLQDTETITLSIINVNEGLGQLPPITTEGAGFIVGAT
LIAAIPFDDPDGTPTDISYQWQRFIEGVWTNIPDATEQNYIATEDDNNNQLRVEVTYIAG
GFEKVIYSNNVSISLVPVSGTFDSITGDNNLDISEKEAGVTLTGSVSETGTTVTILFGGQ
TRVAQVDGLSWSYVLKPNDYSFFAPGSNLFTAIFIRTDGGETGSFTTFQTLNIPDGILPP
NTSNAFDPTQPKGLKSEVIDAAQTLEIDGVSILEISKTVGILGEGESFNDPNIAVLPIGT
RDIFDQGANAGAANYAAFKTEPGTSIKGIYIVPAVEEEGVKKLQVVLADGTVVEAEIPLD
LISPIGDPLAVTISGVQPGGTTTFVLYLSQNVINQLPDDLNLGRYAKFNYESEQFELYDD
SNYDYIFNDVDGDGARDFGEVYLTVNLTDGDKWDGDGLENGIIVDPGQLGIATGSDSDNN
PPIAIELKGIVAENDPGAFIGSLTVTDDPGDSHTFTVNDSRFEVINVDGNNILKLRDGES
LDYEVASNVVLTITAIDNGGLEITRDFTITVTDVNEAPVAIELNQITVIENDPGAIIGTL
TVSDPDGNDGHTLKVNNDTRFEIVDFDGNQTLKLKAGESLDYEAGSVELSITATDNGGLE
FTQDLTISVNDVNEPPVVSFSFFVPESTTLVTNLTVEDPENDPITLSLAGVDASLFSISP
TGELAFNTAPDFEEPLNADKNNLYKLQVVARDGENNKSIQDISILVTNVNEAPIAIDDVL
AIIPGSSFGTLNPLDNDSDPDLDHTLTIINKTDGNYGRVEIRDNELIYTLLDATYIGDDV
FSYTIVDEQGLAATANVNVTIIGTDIITYPVEILDPEDSLIPDEAGSLSDIVNDISFNFL
TDYDKVQAKLALQQALSKTEAAFTNLFGLYEVDNALTGSVNGILPEDKSAYAKAALSKVV
PNFVVRAGGLGGGVNGDVIVTEGKIYAPFVIAHGGNFPGSVQDAVNAFFQVNPDNSPATA
QNYTTLPVAYFSFGSANPDGAAHIKSFGNNVFGFEDLPAGVGVSDYDFNDTVFSFG
>WP_061546918.1
MLNPNLDEIQLTKDDYERYSRHLILPEVGLEGQKRLKAASVLCIGTGGLGSPLLLYLAAA
GVGRIGIVDFDVVDFSNLQRQVIHGTSWVGKPKIASAKDRIHEINPYCQVDLYETRLSSE
NAIDILSPYDVIVDGTDNFPTRYLVNDACVLLNKPNVYGSIFRFEGQATVFNYQGGPNYR
DLYPEPPPPGMVPSCAEGGVLGILPGIIGVIQATETVKIIIGKGTTLSGRLVLYNALDMK
FRELKLRPNPVRPVIDKLVDYEEFCGIPQARAAEAQQQIETQEMTVKELKTLIDSGSQDF
ILLDVRNPHEYEIARIPGSVLIPLPEIENGDGVARVKQLLNGHSLIAHCKMGGRSAKALA
ILKAAGISGTNVKGGINAWSQEIDPSVPQY
>WP_061546994.1
MQYMHSEQNAEIISQINYFIGVVFNYENLIQNGQIENEEPLLIVVNDIKPPHSSDSLSKY
HPSYSYLINCINYFVNQSGLGHMLTPPVTLMVNNSDKYNSIRLGTLLDVKHFDVEFKQQF
FDPKEAITYLFDKLEVNSPEDKLEAINEIINRSFYPSVDVLFKFVESYNKSWLPQQNNKS
INQLYTEFMNKFDYHYPPATYEDIYGEYEDDLA
>WP_061547005.1
MPLKAVLFDFNGVIIKDESIHLKLIDEILVEENLQPQKPDERLRCLGRSDRACFEELLKR
RGRVVSQDYLTHLLRNKANKYIKELESLEQLPLYSGIEDLIIQARSQNLPVGLVSGALGR
EIELVLERANIREYFQVIIAGDDIATSKPQPEGYLLAVDRLNQLYSDINVDLSLQPKDCL
ALEDTLAGIEAAKRAGMKVVGIANTYPFHILQRQANWTVDYVTDLEWERIWETFEISLS
>WP_061547055.1
MVSKNFWKPIIHSSTALVLLTTLNTAWPLVSLAQSKPQPKANSAASSLFTDYLLGGGDRI
RVNVFEAPEYTGEYQIPPGGEINMPLIGSIPVSGLTTQQAADEIARRYARFLKRPLISVN
LLAPRPINVFVAGEVTRPGSYSLSLQGTGGNNPGVQYPTVLAALTTAEGVTLAADVTKVQ
LRRQVGRSGEQVVSLDLKQITQTGRIPIDITLRDGDTIFVPTATDFNVAEARNLFAASYA
ASRTAPRRVAITGQVYRPGSYLVAAGGGGGNDSGGLPTVMRAIQLSGGITSQADVRNIKV
RRPTRTDKEQTLNINLWELLQSGDLNQDVVVQDGDTIIVPTATQVNTAEVTQLATTTLSP
ATIKVGVVGEVKRPGVTELQPNSSLNQALLAAGGFNDARASSSSVDLVRLNPNGTVTKRA
VKVDLSKGINEETNPILRNNDVIVVNRSVLARTGDTLGAVTAPLAPVFSIISLFRLLGF
>WP_061547056.1
MNIRINNKLTGLATSVLTLTSGLAIVTSTSQTVLAQVVSQLATPQPPQPTIETTQNGPTT
SGWGVTPEISTLGVGATISKSITPNLNAKLGVRGFGTSANISESGIDYDAKLNLFSVSTL
VDYHPWKNSGFRLTGGLVFQDNNIEGNGKSNSDQKIQIGDQEYTSDQLGSVKAKVSFPNS
VAPYLGIGWGNAVKPGNRWSFSANLGVVFTGSPKVNLTPQFGAAATPEIQNQIQADVEKE
RQQLENDLKWLNIYPVLSLGISYQF
>WP_061547067.1

MVALLQKIRLENQSIFQEVNDFLSTRKSNRLAQAQKQAEDLSQFRQQLEQEIDQFLTKTA
QSRQAQAQAQAQELHQFRSQLEQETSEFLTKTTQARHAQAQEQAAELSAFHKQLEQKTEL
FLADTAKARIAQAQKQASELSAFHKELEQKTSAFLADTSKTRIEEAQKQAAELSAFHKEL
EQKTSAFLTATTSDRAAKAAAQKEALRQFRQDLFLSVIGV
>WP_061547103.1
QFIAKAPDWLYIPSVKEILPGRKSYTPHLEGDVPAVVMEFLSDKEGGEYSFKRTYPPGKW
FFYEQILQVPVYIIFDPDGGLLEYYELRNGRYELKQPDENGRHWIGSMELFLGTWQGTKE
GRTGYWLRWWEQTGNLLPWALELIEQERQQAEQERQRAEKEHQRAEQERQEKEREHQRAE
QERQEKERLIAYLRSQGIDPNNLPNHAE
>WP_061547114.1
MVQSRYTRLINFLQEDLAISAASLAVALRHPEHDTGSLTMILWQYGLITLDQLEQIYDWL
ETI
>WP_061547116.1
MKWQLLTHNKQVLGKIFTILVFTGLTGVLCVSCNRNQDLLVTEIGVNPPKRPTRKTSGAG
EFYLQGQNQHSRGNFQAAIAAYSKSISLNSDYAPAFKARGLAYFDLNNKERAINDYNQSL
QINPNDPETYNYRGNARASLGNQKGAIEDYNEAIRLSPNYAEAFNNRGNSHAAQGNKNAA
LEDYTQAIRIDQNYPVAYNNRGNAYSSLGNTSKAIADYNQAIRLNPQFAPAYNNRGNAFA
SSGDKRRALQDLQKAATIFDQEGNRGLYQQTMKNIEELEN
>WP_061547132.1
MTTDLPSTLDLLNPTLKALQLLGGSGSILEIANIICEMQNFSEEQQSIPHKNGPQTEISY
RLGWARTYLGKCGLIENKSHGVWSLTNKGKETESVNPKEVNRIVSSKYKKNNQVKSNQES
LNDLELHNTINEIDEISQSEQLNISDESGTINLLKTLLEGAIPLKSELWSEQLLDVLLDM
PPDSFERLCQRLLRESGFIKVNVTGRKGDGGIDGIGVLKIALLSFQVLFQCKRYRGIVGP
GEIRDFRGAMVGRTDKGLFITTGRFSRSAEQEATRDGAPAIELIDGQELCLLLANLQIGI
TRKTIEVVEINNEYFKNV
>WP_061547147.1
MIMNLKTKKAIEQLEKFLGETIQTLADFQKLPVICKEKYISVYSLPELCKDGKLGGCDMI
TASSCSVEKPTFWPRFFPLSQF
>WP_061547189.1
MSTSSTSAQTTKRIEDIARKTTVQINSNANPGGSGVIIKKEGIIYTVLTANHVVCDNLGR
IKIRCRADLTYSVVTYDGKEYLMKSRQSLQTNVQDPDLAMVTFESRENYEIAPLGNSDNV
SIQSDVLVAGFPTIFGRVGKQRTFTITNGKVVTFIPNSDRGYGLVYNATTFIGNSGGPVF
DIYGRVIGIHGLADTDDGETNNNNQSETVNGVKPTQKTGFNAGIPINIFFSLSNFNRQLD
PAVSINRQPNSTNPNNVNLSNDAIAYHDRGVNRYQSGDRQGAISDFTQAIQINPNFAQAY
YNRGATRNDLGDKQGAINDFSHFINFYPRNSLAYFNRGIAWHELGDKQRAISDFTQVIKL
NPNNVAAYYNRGASRSDSGDKQGAINDFTTVINLNPNFAQAYNNRGLARHNLGDKQGAIS
DFTQSLRLNYRDPTAYNNRGIARHDMGDTQGAINDFTQAIQISPNFANAYDNRGLARHNM
GDKQGAISDFTQAIQINPNFAQAYNNRGATRNDLEDRQGAISDFTQAIQINPNFAQAYNN
RGLARHNMGDKQGAISDFTQAIQINPNFAQAYNNRGATRNDLGDKEGAISDFKKAAQLFD
LKNSRSRKIMRIP
>WP_061547248.1
DAITFALSGVDANLLSLDPQTGELTFKQPPDFEKPGDQNKDNTYEVKITVTDSSNLSVTR
DIPVTVTNVNEAPIAKEDFLTIFSATSGSIEPLKNDEDPDQNDQLKIIAKTDGKYGKVEI
NSDQLKYTLLDAAYIGDDVFTYTISDKEGLTATANVRVNVTATNVIPAITPVPVNPGDPL
IPKEAGSLAGIVNNVYFNFLAGYNPTQARQTLQNTLVQTNAAFNNLFGLYEIDGKDATGS
VTGSVNGIAPGDNGYAKAALSRAVSSFAVRAGGSGNGITGDVIVSGGKIYAPFVIANGGN
LFGSMQDAINTFFQLNAENSPATAENYTSLPVAYFSFGAANPDGAAHIRSFGNNIFGFED
LPSNVGVSDYDFNDMVFSFG
>WP_061547262.1
MSFIYYGESQDIYGGAGLEPTPINREEYGIPIKTYQTCASQVIDSRYTRLETLLKARSPA
LSWRSLVDWGLGVGMGFGGDYFPPVQTCRL
>WP_061547263.1

MKNADNPPGQLVLLVVLGVNCVFGLLGLKSGSLKFGSGIVGVILIGLKSGSLKFGSGIVG
GILIG
>WP_061547274.1
MPDFALILKDFVTQIFPRLRTELRFHRKLAKGGLKPDGTPFINKRTGKPYVATTQLTHVL
VGLSALTRLLNYLEKNSLLTSQINITEADFRRVCALFCLHDLHKDDDPSRDVQQRDTSIR
PSLMLEIANKASLTDWLNNHDLNGYEYREAMIHLSDTTHGDCKHCRGEIGYEKLYSLVRL
ADAMASIQNLDEGTNSLKNRIKDFARSLKNLHFSYHKIDDYRGVTTNLYHQSVAGVLQQE
YNVYPLLFFDHGTIYIGQNELQSFDKSSFINQVHQRFNDSLKNLGSISSDELQYNSKTQR
FEKYIFGFSGIKEQLEYLKNHAIPRVKAKKEALHICGMI
>WP_061547278.1
MVGVHIYVMEIKVIEGNQVQGNAALDQILQRNYAEKYRGEPGKYVHEIGLIFSRTQRNLI
QANWQ
>WP_061547286.1
MLLMLPMVVLFFPSINFGLIEAETFPWAIALTIATSFIFFEWIDRAVAIFLAILSVSALI
TIAEFGSELVFESIRSVFAYINAISGFYVLMRFRVKVGETAIFRIAEYAFCIMLIVGIGQ
HLSLIGNENNAIIQFLALHICGMI
>WP_061547290.1
MRQPENIGAPLNFRFVLDEAVGEYFMWAAADDVWESNWIQTLLPVTETYRCLAYGMVVVI
DERGKQMANPSSGRVFNYHGLKFVRRIKYMFAMGALGKANPIYGLIPRSTLNRGNFSIFD
RVVYGSDMVFLFDLLRITEIRSPSENTRLYKRVWTKSIIQGKISGNGQFDSLLRKFFVVF
LSWSGITYSSYCTPIESCVILLTIPLMFLRAGTFYASIFIYKTLGGVAPARDDV
>WP_061547303.1
ICFIDFSPYESSEAKAQDYITQEFAKPFDFNIDAPLADFKLIKLSDDSHIIYAKYHHVIT
DGWGTAIFFREVIKTYTQIINDGKDSQIPRDWVIQEYIEEEKKYLESSIFLRDRDYWQQR
LQNVSPKIFSLIQPQGLEGKRHSIYIPREEYNRVNRLCQDMQSNVFHFILSLISIYLTRR
YLKDDVVVGLSLLNRSKKNFKDAIGLFVSTIPFRLTINRQQTIHQLLDNIRSLLRQDYRH
QRFPLGEMKRFSGLQNKIKENLFEVFLSYERHDYSESFPDTQTSCIPLYSGQQKIPLIVY
VREYEETSDVKIDFDYNLSYLDEKTVEQIVTGFQNLFIQATDNLEIFIGDLEDSLADSRD
ISKNISVSQPEIPFVNYQETLISAFEEIATQYPGNPAVQFNNKILGYAQLNAHANRLANY
LIGQGIKPQSKVGICLERSDQMIIAILGIIKTGSSYVPLDPNSPIARRQLILQDSGMTAL
ITQSSLLTELNTENIIAFTLESIDSELNKEPNTSPQIAISPDFPAYVIYTSGSTGTPKGC
IVTHRNAVRLMRATEAWFRFNETDIWTLFHSFAFDFSVWELWGALLYGGKVIIVPFGLSR
NPEKFREFLTTEKVTVLNQTPSAFYQLIRADESAVGDLSLRYIIFGGEALDLQSLQPWLE
KYGDKKPRLINMYGITETTVHVTYRPITRQDLKTKGSFIGREIPDLAIYLLDDQLIPVAD
GVSGEIYVSGAGVTNGYLNRPTLTAERFLPNPFGTGRIYRTGDLARRLPNGDLEYLGRAD
QQVKIRGFRIELGEIQAALTSHGEVQEAVVVTDEWQEEKRLVAYYVPGESSPTVNELRQF
LKNTLPDYMIPAAYVSLKAFPLNVNGKIDIQALPAPDWNSLRVEEDYIGPRNIDEEILCT
IVAKILGLEKVGIDDNFFEIGGDSILALQVIAKAKKEGFAISARELYELTTVRNLATKKA
AVATLEDIAETSILSLVSDTDKAVLPKDIEDAYPLSSLQGGMLYHSELHTRSAIFHQIFT
FNLEISYSELAWRQAIADVCLANPVLRTSFHWTGYSQPLQIVHRQVESPLSIVDLRSSPN
ANQQVQEWVELEKTRGFDIGNPPLFRFQIHRISNEKLCFSFSFHHVILDGWSVATLLTQL
LRRYVQYLDGKNLPPLVFPETIYRQFIAQEQNAIANEEIREFWSQHLSNLQVTFLPRLNT
TGIKITTTDYHNRQLKRLSLTISDELTDRLRKISQNIGVPLKTCLLALHLRVVSFVTGQK
EVVTGNVTNARSETSDSENALGLFVNTVPLRLELPSGSWIDLIKAVFHAETAVLPYRMFP
LAEIQRLLGKRPLFEVGFNYVHFHVYDSLLNLPQVQVGNVDIFEETDFPFLAEFCLVPGS
QTLQLNLIYDTQQFADAQVEQYGRYYQTAMVEMTTAP
>WP_061547306.1
KTRLAFQLIKQPGAFFLSRPRRFGKSLFVDTLKEIFEGNQKLFEGLYIHDQWDWSRKFPV
IKIDFAGGVLKNRQELDQKINGILLKTAHSLGVDYELEDIQGRFGEIIAGAYQRFGERTV
VLVDEYDKPILDNIDNPSIAGEMREGLKNLYSVLKEQDANIQFIFMTGVTKFSKVSLFSG
LNQLTDITISRDFSTICGYTQEDLEQTFAQHLQGVDWDELRLWYNGYSWRGDSVYNPYDI
LLFIREGMEYGNYWFETGNPTFLIKLFQANRYFLPNLEHLEVTEEILKSFEIEQINPVTL

LFQSGYLTIERNFTRHRRSMFALKIPNMEVRLTLNDQFINVYTGMVNEKSAIQDILYECM
WNGDLESIVKAVKRLFAGIPWRNFTNNHLADFEGYYASVLYAFLSSLNARIIPEDITNYG
QADITAILGDHIYVMEIKVVDGENVKENLALKQIRECNYAQKYRGEPGKTVHEVGLVFSR
SKRNLIQADWE
>WP_061547314.1
MTTAIQQRQSTNIWDRFCEFITSTNNRLYIGWFGVLMIPTLLAATTCFIIAFIAAPPVDI
DGIREPVAGSLMYGNNIISGAVVPSSNAIGLHFYPIWEAASLDEWLYNGGPYQLVIFHFL
IGVACYLGREWELSFRLGMRPWICVAFSAPLAAATAVFLIYPIGQGSFSDGMPLGISGTF
NFMIVFQAEHNILMHPFHMLGVAGVFGGSLFSAMHGSLVTSSLVRETTETESQNYGYKFG
QEEETYNIVAAHGYFGRLIFQYASFNNSRSLHFFLAAWPVVGIWFTALGVSTMAFNLNGF
NFNQSIIDSQGRVIGTWADVINRANLGMEVMHERNAHNFPLDLAAGEVAPVALTAPAING
>WP_061547329.1
MIEAEIKALIQKELPRAIAEEPGVRDFVLRTVSEYYTPRTEFDEKFDRVLNELQRDREEQ
ARKWDEQNRKFDAFQAEQARKWNEQNRKWDEQNRKFDAFQAEQARKWDEQNRKWDEQNRK
WEENNQRLDRIEAQNSATLEEIQKANRRYESAIGAIGSRWGLYSEASFRNGLQAILGQSF
GVEVLNLTLYDQEGEVFGRPEQVELDIIIKNGLTIVCELKSSIDKAGMYIFGRKADFYAK
NQNRVVNRKIVISPMVDERAVPVAKSLGIEIYSYADVVVP
>WP_061547330.1
MIEAEIKALIQKELPRAIAEEPGVRDFVLRTVSEYYTPRTEFDEKFDRVLNELQRDREEQ
ARKWDEQNRKFDAFQAEQARKWNEQNRKFDAFQAEQAHKWDEQNRKFDAFQAEQARKWDE
QNRKWEENNQRLDRIEAQNSATLEEIQKANRRYESAIGAIGSRWGLYSEASFRNGLQAIL
GQSFGVEVLNLTLYDQEGEVFGRPEQVELDIIIKNGLTIVCELKSSIDKAGMYIFGRKAD
FYAKNQNRVVNRKIVISPMVDERAIPVAKSLGIEIYSYADVVVP
>WP_061547331.1
MIEAEIKALIQKELPRAIAEEPGVRDFVLRTVSEYYTPRTEFDEKFDRVLNELQRDREEQ
ARKWDEQNRKFDAFQAEQAHKWDEQNRKFDAFQAEQARKWDEQNRKFDAFQAEQARKWEE
NNQRLDRIEAQNSATLEEIQKANRRYESAIGAIGSRWGLYSEASFRNGLQAILGQSFGVE
VLNLTLYDQEGEVFGRPEQVELDIIIKNGLTIVCELKSSIDKAGMYIFGRKADFYAKNQN
RVVNRKIVISPMVDERAVPVAKSLGIEIYSYADVVVP
>WP_061547342.1
MVNSGSEQLPDHSSQIVTVRPDSETATLQKLPYFVGISGKTAGTKGISMNLVIIPAGGKA
EPHFHRDYETAIYLVKGRVETRYGKGLSQSVINEAGDFIFIPAGVPHQPYNLSDTEAAHA
IVSRNDPNEQENVVLYDPSVYS
>WP_061547343.1
MKERIIALLFLIIIFEIVFRACGGTTLETPQSIPETTAEETTKECIDRVSNELIERDRGE
VLTEEKMVLYYAEFITRCNLTPNR
>WP_061547355.1
MEIYFSNELQIDVNAITIGDNTGTIPTGGRTAGTDTNNADGDSSTGNFVLLSFQNLTTSA
TNKSFITIPFVTNSTFDGQATVNFIARSSNPSLTVDPITPVAISNQVPAIIEVGPGQTYT
TIQAAISAASNGDVIRVLSGVYNENLTINKSVTLEGPNKGIKPITPDINPTSGININQGY
RTNEEAWIKGTITVAADGVTIDGFRLRNENGPLQWSGTPDNFKLLNNYVTGYNANQGPRF
GDANSNNPTNVVTGWQIDGNYIGGLLGGGGTGGSMYLAGLKDSSINNNTFWRPRAAHLYL
ASLTNVTIEGNKFYHGLHAGGANYDDLGKFFSGAGYGYGGYGGYGGSYGGGYGRNYWLEL
KGTNNTVNIKSNTGEYNSGGIQLYGEVNDPFQFNNITIEGNTFPANNFINAYTADPTNNL
SGLIPAVMATAKVVNNGPAGSNLVIRNNTITKDISQLKFSKDHTSAIDVRGNFNGVTIDN
NSLTSVGTNGGVSLITGLSLYGGLSGQVLIQNDVLSGAGGNWGTANYYGIDFIPNPAL
>WP_061547363.1
MAQIRNFIGNVNHNTVVDSTVVPESDRREPLKHLLIGSKKTVLSTIHYLHVLGYAHATDW
SDLIPTNNPGLVCE
>WP_061547367.1
MSTLTRAGNGSGNLRSLVDSVFLDANGAVSGNQSLGVNSAVLVVSGGGTYMIVNDGTAGF
NAGTDLVINITGYSGVLPGLGNVVASNWFV

>WP_061547368.1
MLIIFDITVFFYSLFIIKSATSLPYLVIWVFSFYRKNLIFVLAFALTFVFVVVIGYQSEL
SQSRAIIFLSQLLTAEDLNIDDLMYIVFDNSGFRDPSVVASYFYGATNIVGGGVGNWKQS
SLDALNASSIDVSRISYFGGGYYSSVRPTSYLSSLFLDTGLIGVLVFMVLLWGEIKKYNA
NYSLVNKTAVIACIFSITLLGQIGDPVVWACLALSLAPRSLDISGEEENCSN
>WP_061547379.1
AELRFMTRLWGQPSITFKGHEYHAPFECNESFEDMDEETLKSFAAIIVPSGMVSDRLRYT
EDVTQVPPATEFLKRAFAQPSILKGIICHGLWLVAPTPELVKGRTLTCHNNLHGDAIAYG
AHYRNEDVVIDGDLVTGRSGAHAHLFARAIIDRLS
>WP_061547386.1
MNSVGTARKLKQALSQMFGGENLGEAHGIVQASEKKDALKKVHTTGTSGIEVGIDFTDEY
FKYLLLFEGRTSSQFLQR
>WP_061547399.1
MVVSNEVGRLTPLAAPTGTEPAKLGIIRHVLKVYPMPESMINKTTGFMATNLQSYHSLHT
HRAEATVIFDLSPTRVLDLSFRIKIVA
>WP_061547402.1
MTKKYIIWGSSGHSKVLHSLITLCQGKVIALFDNNPNSSVSIKGLPLYIGLDGLHQWMLD
YEDFQNIYGIAAIGGSRGKDRIATHQLFCSFGIKVETLKHPNSTICQTATIGKGSQLLCQ
SLIAAGSTIGDACIINHQASIDHECLIGNGVHLAPSSTLCGCVNLGDNVMIGAGAVVLPR
ISIGE
>WP_061547416.1
GVLKNREELDEKIRDLLWNNGDRLGVDAKKNSISGIFGEIITGAREQFGERVVVLVDEYD
KPILDNIDNPNIAAEMREGLKNLYSVLKSQDANLQFVFMTGVTKFSKVSLFSGVNQLKDI
TISEAYSSICGYTETDLRESFGDHLEGVDWDTVRHWYNGYNWTGSETVYNPYDILLFISE
GMKFRNYWFETGSPSFLLKLFQKERYFLPNLEGIQVTEEILDSFDVEQINPVTLLFQSGY
LTIKDTFTDINQMVFCLGIPNIEVKIALNNQFINAYSNLVNEKLGIQRLIYTQLRSGNVE
GLISTIKRLFASIPWRNFTNNDLADFEGYYASVIYAFLSSLDARVIPEDITNHGHSDLTV
MVGVHIYVMEIKVIEGNQVQGNAALDQILQRNYAEKY
>WP_071985094.1
MAKSKGARIIVTLECTECRTNPDKRSPGVSRYTSTKNRRNTTSRLELKKFCPHCNKHTVH
KEIK
>WP_071985099.1
MSHTVKIYDTCIGCTQCVRACPTDVLEMVPWDGCKAAQVASSPRIQDCVGCKRCETACPT
DFLSIRVYLGAETTRSMGLAY
>WP_071985113.1
MNNQLFFLVKLLLLSTLISGLIKYVGPIFLFPPTAVNALMMVLLPTIFMISFLVLPMVLK
RQS
>WP_071985120.1
MQVNDLGFVASILFVLVPAVFLIILYIQTASREGGKN
>WP_071985140.1
MGRKLSKSHLPEKICPVCQLPFTWRKKWQDCWDDVKYCSERCRRRRSQTT
>WP_071985141.1
MKVRASVKKICDKCNVIKRRGRVMVICENPKHKQRQG
>WP_072301454.1
MKEQANQRAEQERLLKEQANEGAEQERLLKEQERFLKEQANERAEQERFLKEQANERAEQ
ERLLKEQEQLKYQTLLSQLKAKGIDITALE
>WP_072301459.1
MVGGLLASVISAVICLILPKFLSLFSSGKNNHLERDRNRGITSFPYCTVYKLTGSEWCKF
RPQFCGRCSSYRKSEKLKNLWKW
>WP_072301474.1
MLYRRKSTVYTRPLARLIEQLQRLPGVGPKSAQRLALYILKRPESEIEALAQTLIDAKKQ
VGLCQVCYHLTAEPVCEICRNPHRDSQTICVVADSRDVIALEKTREYRGKYHVLGGVISP

MDGIGPEQLTLQALVRRVSQQKPQEVIMAISPSVEGETTTLYIAGLLKPFTRVTRIAFGL
PMGGDLEYADEITLAKALEGRRELD
>WP_072301475.1
MEVSETEDSKSHRWSGGKDPAIFEANHKSRGDYWIIDNQYLVPKYGQKINQHSYETISTL
FECLNYHYNDSIGFRSMILVKPAKVSPIHDQEKWKLQDTGTLQF
>WP_072301492.1
MNKQLTHTELENIYQGAFRYVRQKTRFEVSFPQDCPYGLEQLLNQSYF
>WP_072301502.1
MLKRNLVSNFVGQGWNAVMAIAFIPVYIHYLGIESYGLIGLFALVTAWTTLLDIGLTPTI
SREMARFTAGAYTPELVRDLLHSIETITFGIAAIIAVTLTFGSSWLATYWIQSNTISVGV
VSQAITIMGFVSAFRFTETIYHGSLVGLQRQTPLNLIKSSIATLAHCCT
>WP_072301503.1
MVFEILSPCNSKGEMTRKKLFYLKHGVEEYYVYDPDEMSLEVSIRENNSFKEVEDFAIWT
SPRLNIRFDMTGDELVIYYPDGSRFLSPVELSNYAEQERFLKEQANQRAEQERFLKEQAN
ERAEQERFLKEQANERAEQERFLKEQERLLKEQEQLKYQTLLSQLKAKGIDITALE
>WP_072301513.1
MSKSVIDLLKQTTEGLFMPSESEYPFNVVYWEFFNLNETTIQQKTGITGNVRTVTVDDFF
QGVTKQEDWYEEEERNVAKRFESLVLVLKNNLAEAKVYEIGNKEVHAYILGTKDGEIIGI
STVVIRT
>WP_072301527.1
MNDRITQSLTKLFEKHRIVFWYDAKQELRQEFEAIALDGIEKIELKNNEFGVKYRILRQQ
PRGKFLLYHEGPQPADLDNWLLDTQLAHTDFRADQFAMWLAELELGREFTDVVQSHIAFF
QAVERRKALKRLITSHDTPSQLRLKMVALCAGCGSEARLEAILEQLLGELSGDSVQYMEG
YAGDRYRLIERCELKQYFWEQMKRRYGYSSPEPSIKDFVLQLFADCYFKNFTAARKQRTE
TLSPDALVFLKRWKDSIKCKESFETLSQQCAEDLDIKRDLEERDIRDLIELDYFRLVDQK
ILSDLVRCVERKTVSSGDVGQWIWQRKQSHWYPEFEHLYEAVATAAQFIHELDGAYLTMD
SLAEGIQRYASSWFRIDQFYRKFIYHVRRSGESSLMERLVDLIENLYDNNYLLKLNDRWE
MLVDKAKSWSAPSIYLQNKFFDQWVQPFLRTEKKIFVIISDALRYEIGDELMSLIRKEDR
Y
>WP_072301528.1
MNISHQDWDSYETSWDFTNLPLLNPDFRQPTLKATYQKLRSHWQQMTLETQRLEEENNRI
FISTYGLQDELTPEVPLNEITLTCNPYYRYDHTKPEAELETLLLADTMKEYISYAVGCMF
GRYSLEKPGLILANQGETVEDYLKQIPNPTFPPDADNVLPILDGEWFIDDITERFRQFLR
VTFAEEHYDENLKFIEAALGKDIRKYFLKDFYKDHIKRYKKRPIYWLFCSPKGSFNALIY
MHRYRPDTVSVVLNDYLREFRAKLEARRDHLKRVEVSADASQSEKTKAVKEIAKLTATIE
ELNDYERQVLYPLAIQQIQIDLDDGVKANYQKFGLALKKIPGLEAKEED
>WP_072301529.1
MKWFFTWVPTLVLVIICSLFLFNSPSYAFNQADLDQLLQNKICEKCDLSDADLSDADLRD
ANLRDANLSFANLSDANLRNADLRDADLSGAYLSFAYLSFADLSGAVLDGAELSGAVLDG
AIFCDDYGRKKGWCN
>WP_072301531.1
MVREIAPKTQLGVIKSKVNQGRETIAPDIVYPESDGKPMADNTKQFAWIVKIKENLEILF
KSNPDVFVAGDLFWYPVKGSNRIKLAPDTMVVFGRPKGQRGSYRQWEEDNIPPQVVFEIL
SPCNSKGEMTRKKLFYLKHGVEEYYVYDPDEISLEVSIRENNSFREVEDFATWTSPRLNI
RFDMTGDELVIYYPDGSRFLSPVELSNYAEQERFLKEQANQRAEQERLLREQERFLKEQA
NQRAEQERLLKEQANERAEQERLLKEQEQLKYQTLLSQLKAKGIDITALE
>WP_072301540.1
MVFEILSPCNSKGEMTRKKLFYLKHGVEEYYVYDPDEISLEVSIRENNSFREVEDFATWT
SPRLNIRFDMTGDELVIYYPDGSRFLSPVELSNYAEQERFLKEQANERAEQERLLREQER
FLKEQANQRAEQERLLREQERFLKEQANQRAEQERLLKEQANEGAEQERLLKEQERFLKE
QANERAEQERFLKEQANERAEQERLLKEQEQLKYQTLLSQLKAKGIDITALE
>WP_072301541.1

MTGDELVIYYPDGSRFLSPVELSNYAEQERFLKEQANERAEQERFLKEQERLLKEQEQLK
YQTLLSQLKAKGIDINALE
>WP_072301544.1
MLFQSGYLTIESTFTAMERYMFRLKIPNREVKVALGDQLVNAYTDFVEEKLGIQRPLYEK
LFQGDVNGFIDTVRRLFASIPWRNFTNNDLANFEGYYASVLYAFLSSLNARIIPEDITNH
GQADITAILGDHIYVMEIKVVDGEKVKENLALKQIRKCNYAQKYREEPGKTVHEVGLVFS
SSKRNLIQADWE
>WP_072301546.1
MFLLIEAPFAAFRPFQSGSYRSTTSVPSPSTVYGILLNLAGIEQRVGTDQDITLIKDDLP
TIEIAIGIPHLPNKPKTEIATLSQQLHSYLVGNSERGKELAKKTYGNKYWIAPVRREVII
NFRLIVGVKANQDLCNRIIEGLNGELKETRYGIPFAGDNNFFFDNIEVINRPPCARWYCP
LDKSTYPERGICRLTTWINRSDNTKTKIGLFYPTDFVLDPPESAWSKLPSNT
>WP_072301547.1
MYRIGGIKGMSGGPILDENGKLVGIHGQTYSQSLGDGRGTPEEYGIPIKTYQTWASQLST
PSVVNTRYKRLESLLRTQNFREADEETNKVILKVARRERQGWLRKEDEENFPCKELRTID
QLWLKHSKGKFGISVQQEIYESLDKDPSLFGDRVGWRRTGRWLSYKNINFSQTAPSGHLP
FVLPFLFREGGGWLERFGRWVTFPSVQTCRV
>WP_072301548.1
MLFQSGYLTIERTFTRRQRYMFALKIPNLEVRLALNDQFINGYTEIINEKSGIQDSLYEF
MNRGDVESMIMAIKRLFAGIPWRNFTNNDLADFEGYYASVIYAFLSSLDARVIPEDISNY
GQADITAMLGVHIYVMEIKVVEGNQVQGNAALDQILQRNYAEKYRGEPGKYVHEIGLIFS
RSQRNLIQADWH
>WP_072301549.1
MAGDLFWYPVKGSNRIKLAPDTMVVFGRPKGQRGSYRQWEEDNIPPQVVFEILSPCNSKG
EMTRKKLFYLKHGVEEYYVYDPDEISLEVSIRENNSFREVEDFATWTSPRLNIRFDMTGD
ELVIYYPDGSRFLSPVELSNYAEQERLLKEQAHQRAEQERFLKEQANQRAEQERLLKEQA
NQRAEQERFLKEQANERAEQERFLKEQERLLREQEQLKYQTLLSQLKAKGIDITALE
>WP_072301550.1
MPPQVVFEILSPCNSKGEMTRKKLFYLKHGVEEYYVYDPDKISLEVSIRENNSFREVEDF
ATWTSPRLNIRFDMTGDELVIYYPDGSRFLSPVELSNYAEQERFLKEQERFLKEQANQRA
EQERLLKEQERFLKEQANERAEQERFLKEQERLLKEQEQLKYQTLLAQLKAKGIDITTLE
>WP_072301553.1
EFWRQHGEPLLKSAPYHEIAPHLVLMAFLHRVVNGGGTLEREYAIGSGRMDICLRYGKVV
MGIELKVRREKLDPLTKGLTQLDKYLDGLGLDTGWLVIFDRRPGLPPMGERISTEQVISP
SGRTITLIRS
>WP_072301555.1
MAGDLFWYPVKGSNRIKLAPDTMVVFGRPKGQRGSYRQWEEDNIPPQVVFEILSPCNSKG
EMTRKKLFYLKHGVEEYYVYDPDEISLEVSIRENNSFKEVEYFATWTSPRLNIRFDMTGD
ELVIYYPDGSRFLSPVELSNYAEQERLLKEQANQRAEQERLLKEQANQRAEQERLLKEQE
RFLKEQANQRAEQERSLKEQAHQRAEQERLLKEQEQLKYQTLLAQLKAKGIDITTLE

-Protein sequences of the non-South American group which were not included in the Veen diagram.

>OBU74745.1
MATTADEVWKLLGELIESQKETERKFQETERFLREQSQETDRKFQETERFLREQSQETDR
KFQETERLLREQSQETDRLLREQSQETDRKFQETDRKFQETDRLLREESKRVNNQIGQLG
NRLGEFVESQVRPAAVKLFQERGIAVKEIASNTYIQTGKEGLEIDLLVINSSDIILIEAK
SKVSEDDVNEHLERLSKFKRFFPRYESYRVLGAVAGMVIPLDVSRYAYRKGLFVIGQSGD
NLVILNDDKFRPRGW
>OBU74749.1
MATTADEVWKLLGELIESQKETERKFQETERFLREQSQETDRKFQETERLLREQSQETER

FLREQSQETDRKFQETERLLREQSQETDRKFQETDRKFQETDRLLREESKRVNNQIGKLG
NRLGEFVESQVRPAAVKLFQERGIAVKEIASNTYIQTGKEGLEIDLLVINSSDIILIEAK
SKVSEDDVNEHLERLSKFKRFFPRYESYRVLGAVAGMVIPLDVSRYAYRKGLFVIGQSGD
NLVILNDDKFRPRGW
>OBU74752.1
MATTADEVWKLLGELIESQKETERKFQETERFLREQSQETDRKFQETERLLREQSQETDR
LLREQSQETDRKFQETDRKFQETDRLLREESKRVNNQIGQLGNRLGEFVESQVRPAAVKL
FQERGIAVKEIASNTYIQTGKEGLEIDLLVINSSDIILIEAKSKVSEDDVNEHLERLSKF
KRFFPRYESYRVLGAVAGMVIPLDVSRYAYRKGLFVIGQSGDNLVILNDDKFRPRGW
>OBU74755.1
MATTADEVWKLLGELIESQKETERKFQETERFLREQSQETDRKFQETDRKFQETDRKFQE
TERLLREQSQETDRKFQETDRLLREESKRVNNQIGQLGNRLGEFVESQVRPAAVKLFQER
GIAVKEIASNTYIQTGKEGLEIDLLVINSSDIILIEAKSKVSEDDVNEHLERLSKFKRFF
PRYESYRVLGAVAGMVIPLDVSRYAYRKGLFVIGQSGDNLVILNDDKFRPRGW
>OBU74760.1
MKKRQQPKAAELAKTLQRYFSEQEQEQILQVLASTERHERFNTLLWVNGFQDIIEQRKQL
IERITAEQQGKSIGKA
>OBU74761.1
MTITFRTAKKQGISIRIDGTPVTPRTKDKRLFDLVNNQLNEFNKTLTLVIFPNKSFNTVQ
YELMLNGFEESLSGTETKSPKEDLTEMRRQITLEIEYTFMKKENDSLKRENNLLRSKINE
VEILVHNAQSKRRFCN
>OBU74762.1
MKIELIQQLINQKMRLRNYAGFSVLFREFRFKANEALTFKAPGQFWFLEDNIPPQFLITS
EMGIYDVLDNRINELQHEHSGTVKMINRSSGVLLLRVVILIPVKRKGHATTRK
>OBU74763.1
MKTRFFVHTEQIDFNGQIRCFVIDFPPNTAKIISLVATAEPLAKDYEIRHLEVGLLTLYN
AAGVWYESQIRFSGQLTWFPKIPPYSYLDHIRQMPVSGAKLTGQTLDIPALEPYLNARFE
TSPSLSGFRLKLTFTYLENEN
>OBU74772.1
MKINKLHLSITSIAIFALINILWIPQYTIASKKNISQRSNSSRMANKATYLTTPFNNSTK
IPPNFLGHDLEAISNALIKRQESLKKDEFETTQAFNQRVIKEGLKPILGTINVDSTLAFV
LSDKFESSYNADLSLFRIEIPNSDYTEFTWKSKTFNYRTYKASNAFGATIDVSSWDSESF
SIKPDRRSNEQTITLSISSNLAKKLKENFGIIFISKIEGYSPVKRQIGRSNATFDNPYSI
SYSDTSWFLRRFYGEKVN
>OBU74774.1
MSIKQAQEPTSLNVKILEFKIFDKTTGKVYYSGTLYPPINYTKLETLLKAQMFKEADEET
DRVMLAVANRQSEGWLREEDAEKFPCKELRTIDNLWLKYSQGKFGISVQQEIYKNLGGTK
QFDWNVWRSFGDRVGWRKDDSWLIYSDLNFSLSAPTGHLPVGMGVSGDGIRDSSLLSRHV
ECNP
>OBU74824.1
MTIVHNFSYSCQPTSQKFSQAKDNIDDRCERTDNLDKTELKEQTFSFLTRLGFTVGKELW
IKTSKRQVFRAVVGKGKLEVFPQRKVSKVEDPKGGSVWRDVGRSYGWEFLYQLSQETSLF
FLPNHPQGGIGKGHCTNFSNLFFEVDDLPLDQQFQNIENLKSLGLLPSAIVFSGGKSFHT
FLSLTEDPGPDLWVILQKKLICLTQSDPAIKNLNREMRLPGFFRPDKGKYQSLVSTGDRR
YSIKEIEDILQPFFPHGLSDERWNDWRLAEKAEKNGILSMPEESLPTVVRKMEKQKQLAA
RKQLVCEEENNLIELVNKTAEQATIEDFEKLHPLKVKWFGKKAKSDCPFHQSQSGTAGWF
GNIGGKLGWACPIDTDNRLLDHFRFFSKLRYGKENPTGKEWVEIAKAYLTEMGIPFSDWN
RFKNGKLAKFGEEIRAKDLEDNRVEEAQDLLVLLKKVGRSLCKTVKGFGKEKYVVSNPSH
TVTRKKINVKEIKKEILDAYRSGYKNIIVKTPPGSGKTHTAGTFMPDELSVNKILYVTNG
VQNPTVSSLVDWSPATARHGGLKWDTSQKTADGRYYLRRAKKGEKPDIEPNCIANEVFEG
LRQQEVNVDNSSVICDGCPVAGTCKSFQGFLIDRKTALEQDRVRIHPESIDPTIFDSDLQ
IGAIWDENIIKFKKDVTFTETDLNATLGFLARNIEVLVKFLPLLNGIREFFKEKSSPYNG

YEREEILARLPEVTREHLDLATNLVPDLSFLENFFNGVHGNRSANLSLRKESIKETAETI
NNMGKQWLLPFVRAITEKGFLYYKKGVFTISVAEEKHTKIMEAAKVNIILDATADPEYIV
TALKMEKEKTIVLTSEVETPNLKIKILTGMGNLRPQSKERTSSKQKRVDAIREKFLTLFP
DGVVFEYKSVAREGDRVHFRDSRANNTDQEKKACLLLGVPIPNLADARAAWEIGLGGGKG
FDDYLGRLTVAEICQTIGRLRASRRPKEEITVYIAGDTKFDLSKALKENFNGAEITTQDV
GEICPEAGDASHRILALVSKGVRQLIEEGKKVTQQNLAKVAGIGQSTIAESIPWGKFKKI
SEILLGRFNTESDKKEPELLPDESWFIDQYLPILIEEEVGPQEVIETLERVAQTPERIGE
MLSRLPKYLKQGMLWLLYKDLEVPWQFSTS
>OBU74827.1
MLNILTSLLVAKKSIKPIVKKQYSAYGDFRYFTYSDILEAVEPVLLENGLLITFTSTGTT
LTATLWHVASGEMLESAYDLTVFMETSHKKMNKAQLVGSATTYAKKIALCGLLNLDTQDA
DPDSLGDETPTVNTSTTTHKKVPAGQPLVTPPRKPQKLPAVGQEQKATKTEVTVSTETPV
TTLQEPVPDAPPKPMDVISKEQLATLSTTVQQLGIPKGKALAIARNVCGEHLYNASYIRQ
PFFNLVMTALTKVAT
>OBU74840.1
MNRKKLTQLITIAILVTFITIFHPFITIALTTKEIGAIAERVTVRLSGPDQGSGVIINKN
GNTYTVLTNSHVFQYTGAFEIITYDGRKYQSNNVTENT
>OBU74841.1
MLAVANRQSEGWLREEDAEKIPCKELRTIDNLWLKYSQGKFGISVPTRNIQKPGRNKTI
>OBU74851.1
MATTADEVWKLLGELIESQKETERKFQETERFLREQSQETDRKFQETERFLREQSQETDR
RFQETERFLREQSQETERLLREQSQETDRKFQETDRLLREESKRVNNQIGQLGNRLGEFV
ESQVRPAAVKLFQERGIAVKEIASNTYIQTGKEGLEIDLLVINSSDIILIEAKSKVSEDD
VNEHLERLSKFKRFFPRYESYRVLGAVAGMVIPLDVSRYAYRKGLFVIGQSGDNLVILND
DKFRPRGW
>OBU74853.1
MALLQPDFEPELQFAYSSFHLFKPDFDYLLPLRETKTVVHLPEIRCKEYGDRGKNTSNKV
YN
>OBU74857.1
MPRVAFFFGISIYMYMDDHGIPHCHAMYGDFAGSFSLEDGEPLAGEMPPAQAKKIKIFIL
NNQVELLEKWHELSD
>OBU74860.1
MVRQIPSKTQLRVIESNISQGVETPKIVYPESDNKPMADNTRQFTWIVKIKENLEILFKY
NTDVFVAGDLFWYPVEGSNKIKLAPDTMVVFGRPKGHRGSYRQWEENNIPPQVVFEILSP
GNNNTEMDRKKLFYLEHGVEEYYVVNPDKISLEVSIRENNSFKEIENFTTWTSPRLKITF
DMSQDELVIYYPDGSKFLSPVELSNYAEQETQRAEREKLLKEQETQRAEQERLIKEQETQ
RAERERLLKEQETQRAEQERFLKEQETQRAERERLLKEQETQRAEQERFLKEQETQRAER
ERLLKEQEQIKYQTLLSQLKAKGIDITALE
>OBU74861.1
MVRQIPSKTQLRVIESNISQGVETPKIVYPESDNKPMADNTRQFAWIVKIKENLEILFKS
NADVFVAGDLFWYPVEGSNKIKLAPDTMVVFGRPKGHRGSYRQWEENNIPPQVVFEILSP
GNNNTEMHRKKLFYLEHGVEEYYVVNPDKISLEVSIRENNSFKEIENFTTWTSPRLKITF
DMSQDELVIYYPDGSKFLSPVELSNYAEQERFLKEQETQRAERERLLKEQETQRAEQERL
IKEQETQRAERERLLKEQETQRAEQERFLKEQETQRAERERLLKEQETQRAEQERFLKEQ
ETQRAERERLLKEQEQIKYQTLLSQLKAKGIDITALE
>OBU74862.1
MDRKKLFYLEHGVEEYYVVYDPDKISLEVSIRENNSFKEIENFTTWTSPRLKITFDMSQDE
LVIYYPDGSKFLSPVELSNYAEQERFLKEQETQRAERERLIKEQETQRAEREKLLKEQET
QRAERERLIKEQETQRAERERLLKEQETQRAERERLLKEQETQRAERERFLKEQETQRAE
RERFLKEQETQRAERERLLKEQEQIKYQTLLSQLKAKGIDITALE
>OBU74863.1
MVRQIPSKTQLRVIESNISQGVETPKIVYPESDNKPMADNTRQFTWIVKIKENLEILFKY

NADVFVAGDLFWYPVEGSNKIKLAPDTMVVFGRPKGHRGSYRQWEENNIPPRWCLKFYLL
VIIILRWTEKSSFI
>OBU74866.1
MVRQIPSKTQLRVIESNISQGVETPKIVYPESDNKPMADNTRQFTWIVKIKENLEILFKY
NADVFVAGDLFWYPVEGSNKIKLAPDTMVVFGRPKGHRGSYRQWEENNIPPQVVFEILSP
GNNNTEMDRKKLFYLEHGVEEYYVYDPDKISLEVSIRENNSFKEIENFTTWTSPRLKIRF
DMSQDELVIYYPDGSKFLSPVELSNYAERERFLKEQETQRAEREKLLKEQETQRAERERL
LKEQETQRAERERLLKEQETQRAERERLLKEQETQRAERERLLKEQETQRAEQETQRAEQ
ERFLKEQETQRAERERLLKEQEQIKYQTLLSQLKAKGIDITALE
>OBU74875.1
MLGSISTSAEATSRIEDIARKTTVQINSNANPGGSGVIIKKEGKTYTVLTANHVVCENLG
TIKIRCRTDLTYTIRTHDGREYPVKKHQSMQVNVQDPDLAIVTFESEENYQIAPVGNSDS
VKIQSDVLVAGFPSIFNRVGKQRTFTITNGKVVTFIPDSDRGYGLIYNATTFIGNSGGPV
FDIDGRVIGIHGLADTDDGGDQQETGSPQQKTGFNAGIPINIFLSLSNLNLPSPPVPVTP
TNPTNPNNNATVYAIAYNDRGVNRYQSGDKQGAINDFTAAINVNPNFAKSYYNRAAIRNE
LGDKQGAISDFTQFLTFHPYNALAYYNRGILHHELGNKQGAINDFTQVIKLNPGNIRAYY
NRGASRSDLGDKQGAISDFTKVIEINPNFAEAYNNRGLARRDSGDKQGAITDFTQSINLN
PRDPIAYNNRGIARDELGDKPGAISDFTQAVTINPNFAQAYNNRGLARHNLGDKPGAITD
FTQAIKIIPNFAQAYNNRGLSRSDLGDKPGAISDFTQAIKIIPNFAQAYNNRGLALHSLG
DKQGAVSDFTQAIKINPNFAQAYNNRGLSRSDLGDKEGAISDFKNAAVLFNRNAR
>OBU74876.1
MTQNNLGKFAAPIKAIILHYFSDLGFVLPGLDKNWKSWSQITEATKEYIQNRQSLQVNLN
HR
>OBU74911.1
MSVKASGGGSVARPQLYQTLPVATISQAEQQDRFLGRGELTELESYFASGAKRLEIAQVL
TENSETIVSRAANRIFVGGSPMAFLEKPQEPEMALVGAGTMNVQEGMKLGTITYVESRGG
FFESLRSIFNTSAGGPTPPGFRPINIARYGPSNMSKSLRDLSWFLRYATYAIVAGDPNII
SVNTRGLREIIENACSGEATIVALQEIKVASLSFFRKDPVATEIVTQYMDVLLTEFQAPT
PSTKVRQRPSADQQGLQLPQIYFNAAERRPKYVMKPSLSATEKTEVVKAAYRQIFERDIT
RAYSLSISDLESKVKNGSISMKEFVRRLAKSPLYQKQFYQPFINSRVIELAFRHILGRGP
SSREEVQKYFAIISNGGLPALVDTLVDSREYSDYFGEETVPYIRGLGQEAQECRNWGPQQ
DLFKYSAPFRKVPQFITTFAAYEQPLPDQHPYGSGNDPLEIQFGAIFPKETRNPSSSPAP
FGKDTRRILIHQGPGINNQLSNPKARGLAPSSLGAKVFKLDQLPGTIGKKAAKGASVKFS
ESSTQAVIKACYLQVFGRDVYEGQRLKVAEIKLENGEITVREFIRILAKSDLFRKMYWTS
LYVCKAIEYIHRRLLGRPTYGREENNKYFDIASKKGFYAVVDAILDTVEYSESFGEDTVP
YERYLTPAGLSLRQLRVGTIREDINKVEKEETPRFVELGAVKELRTQPSVDFRINQGVSK
QREQTKVFTLFATDKVAVQTLIGAAYRQVFERDIPPYIIQNEFTDLESKLGNGEITVREF
IQSLGGSGLYIKEFYTPYPNTKVIELGTKHFLGRAPLDQAEIRKYNQILATQGIKAFINA
MVNTAEYAESFGEDTVPYRRFPTLPAANFPNTEKLYNQLTKQNDDLVVPSFETLQPRIKS
ENTPLLGNAIADLAIKAKQMDKSKPLFIELGRSFNDGRGQSVEVGVGTSRRKPARIYRMT
VGTNKPEINQVINAIYVQVMDVFSGQIPDYIRRTDLDSRLRNGEITVREFVIELASSEIY
RKRFYTPYPNTKVIEFLFRHILGRAPATQSEIRQYNKILADSGLRTAVETIVNTGEYSRY
FGEDVVPYNRFPSLPAGNYLGSVKAEADLVKQSWSSLSPSVLTGRGTNR
>OBU74914.1
MSESLPLRDRYLALIDEIVSNTLKGKISSVYQIYQMLLNGISLDTGEVFELALSDRTYDY
IPELLEGL
>OBU74971.1
MSGWFGYTKAHQESGDKNNATIINGALATTLLDLGKKGNIGGFVIGVPPKAISNTIANRQ
DNSTSLHLEAFYTHRINNNVSITPAIYVIDNPDHKSSNGAIWVGSVRTGITF
>OBU75018.1
MPRRQDIHKILLLGSGPIVIGQACEFDYSGTQACKALREEGFEVVLVNSNPATIMTDPET
ADRTYIEPLTPEMVAKVIAKERPDALLPTMGGQTALNIAVALAKNGVLEEYNVELIGAKL

PAIEKAEDRKLFNDAMEKIGVNVCPSGTASSLEESKAIAQRIGSYPLIIRPAFTMGGTGG
GIAYNKEEFELMAQVGIDASPVSQILIDQSLLGWKEYELEVMRDLADNVVIICSIENLDP
MGIHTGDSITVAPAQTLTDKEYQRLRDMAIKIIREIGVETGGSNIQFAVNPVNGDVVVIE
MNPRVSRSSALASKATGFPIAKMAAKLAVGYTLDEIKNDITKKTPASFEPTIDYVVTKIP
RFAFEKFPGSDPVLTTQMKSVGEAMAIGRTFNESFQKALRSLETGRAGWGADKAEKLPSG
EQVRAQLRTPNPERIFALRHAMQLGLSNEEIYELTAIDPWFLDKLHQILETEKFLKRTPL
QQLTKVQMYEVKRNGFSDRQIAFCTKTKEDEVRAYRKQLGVIPVYKTVDTCAAEFEAFTP
YYYSTYEEETEILPTDKPKVMILGGGPNRIGQGIEFDYCCCHAAYALKSANYETIMVNSN
PETVSTDYDTSDRLYFEPLTKEDVLNIIEAENPVGIIVQFGGQTPLKLAVPLQEYLQKSP
SSVTRIWGTSPDSIDMAENRERFEKILQELKIAQPANGIARSYEDALIVAKRIGYPVVVR
PSYVLGGRAMEIVYSDSELERYMSFAVQVEPEHPILIDKFLENAIEVDVDAIADHQGRVV
IGGIMEHIEQAGIHSGDSACSLPSISLSPAVLNQIRTWTVELAKALSVVGLMNIQFAVVG
ASSYSPQVYILEANPRASRTVPFVSKATGVPLARLASLIMSGKTLEELNFTQEVIPQHIA
VKEAVLPFNKFPGTDTLLGPEMRSTGEVMGIDVDFGRAFAKAEMGAGEKLPLQGTVFVSM
SDRDKSLVVEVIKEFIQLGFKVIATQGTSEFLREQGLQIETILKLHEGRPHVLDAIKNRQ
IQLIINTPSGQEARTDGQLIRRTALGYKIPIVTTIAGAKATVAAIRSLQNISLDVKTIQE
YSF
>OBU75231.1
MNLQNLPLGINTLSVLRENNCVYVDKTKLAYHLIRIAGRFFLSRPRRFGKSLFVDTLKEI
FEGNEKLFEGLYIYDKWEWSRKFPVIKIDFADGVLKNREELDEKIRDLLWNNGDRLGVGS
KKKSISGIFGEIIAGAREQFGERVVVLVDEYDKPILDNIDNPNIAAEMREGLKNFYSVLK
SQDANLQFVFMTGVTKFSKVSLFSGINQLTDITISEAYSSICGYTETDLRESFGDHLEGV
DWDALRHWYNGYNWTGSETVYNPYDILLFISEGMRFRNYWFETGSPSFLLKLFQKERYFL
PNLEGIEVTEEILDSFDVEQINPVTLLFQSGYLTIKDTFTDINQIVFCLGIPNMEVKIAL
NNQFINAYTKLVNEKLGVQRLIHTQLSSGDVEGLVSTIKRLFASIPWRNFTNNDLADFEG
YYASVIYAFLCSLDARVIPEDITNHGQSDLTVMVEGHIYVMEIKVVEGNEVQGNPALDQI
LQRNYAEKYRGERGKSVHEIGLIFSRSQRNLIQANWQ
>OBU75253.1
MSLITLQLVKKDFGIKEILKEATFSIDGTDKVGLIGTNGSGKSTLLKMIAGIEPVDGGQI
LTNSGAKIIYLPQQPDMDENLTVLEQIFMDSGEHTKLVKEYEELSDKLAHYPEDTLLMSR
LSEVMQRMDATGAWELETNAKIILTKLGIGDFEVKVGTLSGGYRKRIALATALLAQPDVL
LMDEPTNHLDALSVEWLQSYLNRFRGALLLITHDRYFLDKVTNRIIEIDRGDIYTYSGNY
SYYLEKKALAEESAVSSQRKHQGILRRELEWLKRGPKARSTKQKARIQRVESMRETQFKQ
AQGKVDISTIGRRIGKKVIELSNIYKSYDGKILINNFSYEFSPEDRIGIIGGNGTGKSTL
MNMITGRTSPDAGRVEVGSTIHIAYFDQHSEELILAVNDNQRVIDYIKEEGEFIKIADGT
KITASQMLERFLFPGSQQYAPIHKLSGGEKRRLFLLRLLISAPNVLILDEPTNDLDVQTL
SVLEEYLEDFVGSVIVVSHDRYFLDRTVDTIFALEEGGNLRQYPGNYSVYLDYKKSEELT
QQETINGKDNRKSKNLTQPKPGDQEVQNKKRRRLSNWEKREFEQLEAKIADLEAQITLVE
TSLLAVTSENYTQVQNLYEQMEALKQAIDVATERWLELAEIDV
>OBU75324.1
MQQVEKSPDAVAIIFENQQLTYGELNCKANQLAHYLQSIGVGPEVFVGLCVTRSIEIVIG
IMGILRAGGAYVPLDPAYPQERLAFMLEDAKPKVVLTENQCLEALPIINATVLCLDADWQ
KIEQQSEDNPSCDVTPDNLAYLIYTSGSTGKPKGVQMPHSSIVNYLQGITKIIPVDNQDI
YLHTASFSFTASVRQLFLPLSQGAAVVIATREKTRDPLRLFELIETQEVTICDGVPSVWR
YGLMALESLDKKYTVAIGESKLKYLLKHLSQATRQSAKAF
>OBU75336.1
MQADGPAVNTIGQIIGIGHGRGIGLMFVIMGLLSILFTFICYFYPRLRFVEDELPDTIVD
S
>OBU75348.1
MKWQLLTHNKQVLGKIFTILVFTGLTGILCVSCNRNQDLLVTEIGVNPPKRPTRKTSGAG
EFYLQGQNQHSRGNFQAAIAAYSKSISLNSDYAPAFKARGLAYFDLNNKERAINDYNQSL
QINPNDPETYNYRGNARASLGDQKGAIEDYNEAIRLSPNYAEAFNNRGNSHAAQGNKNAA

LEDYTQAIRIDQNYSVAYNNRGNAYSSLGNTSKAIADYNQAIRLNPQFAPAYNNRGNAFA
SSGDKRRALQDLQKAATIFDQEGNRGLYQQTMKNIEELGN
>OBU75427.1
MQSIQFMLRVDNLCKSFGVIPACDGVSFDLYPGQVLGIVGESGSGKSTLLKAIAHYITVD
EGSIIYRNRQEQYLKIQELAEYQRRWLMRTEWGFVQQNPRDGLRMQVSAGANIGERLLDI
GMRNYGQIRQEAIRWLQEVEIDPDRLDDLPTTFSGGMQQRLQLARVLVTRPRLILMDEPT
GGLDVSVQARLLDLLRSLVRNFNLSVILVTHDIGVVRLLAHRLLVMQQGKVVESGLTDQV
LDDPQHPYTQLLVSAALTP
>OBU75439.1
MRNVGYIGWFGVLMIPTLLAATTCFIIAFIAAPPVDIDGIREPVAGSLMYGNNIISGAVV
PSSNAIGLHFYPIWEAASLDEWLYNGGPYQLVIFHFLIGVACYLGREWELSFRLGMRPWI
CVAFSAPLAAATAVFLIYPIGQGSFSDGMPLGISGTFNFMIVFQAEHNILMHPFHMLGVA
GVFGGSLFSAMHGSLVTSSLVRETTETESQNYGYKFGQEEETYNIVAAHGYFGRLIFQYA
SFNNSRSLHFFLAAWPVVGIWFTALGVSTMAFNLNGFNFNQSIIDSQGRVIGTWADVINR
ANLGMEVMHERNAHNFPLDLAAGEVAPVALTAPAING
>OBU75440.1
MRQPLTTPLAGESLNLPDHTQLPDSDDNFVKNFQEHPQSIILTTSIEPLLKEIHPNGDYC
IGQDSGIYWRFTEPPEKGVEAPDWFYVPGVPSRLNGQLRRSYVLWKEKVPPFIVIEFASK
NGKEEKDSSPPPEGDEIDPETNKPKKAGKFWVYEQAVKVPYYAIFNGFKGTLEVYHLQGK
RYKEIKANRRGHYAIPEMGIELGILYDNQNPPTPWLRWWDEKGSLLLTGNERAEQAEAIA
IRERLAKEQEREAKEQAEAVAIRERLAKEQAEAIAIRERLAKEQEQEAKEQAEAIATRER
QQKEKLVAYLRSLGIDPEKI
>OBU75442.1
MKKFLTLALILTLFLVSSFSLGTSPSYAYSQSDLYRLLETHECAECDLSDADLSGANLED
ADLLIANLSGANLDHADLKGADLSDADLSGANLEDADLLIANLSGANLDHADLKGADLSE
ANLFAADLSDADLSYANLKYANLKYADLNGARLPGAIQ
>OBU75519.1
MPKKNKIKKTELQNQSPSKIKPTVTENWSKGISFSYKYFQSDHRDFSVVGRNSDYLLGLL
ERLRDISSWTAQELISNRSKALRCHPITWEDTTESGFGIPGEEQIVDTPYQFSICSNEHG
RVHGFFIEEIFYIVWLDPDHLLYRR
>OBU75534.1
MTTTIQQRQSANVWDRFCEFITSTNNRLYIGWFGVLMIPTLLAATTCFIIAFIAAPPVDI
DGIREPVAGSLMYGNNIISGAVVPSSNAIGLHFYPIWEAASLDEWLYNGGPYQLVIFHFL
IGVACYLGREWELSFRLGMRPWICVAFSAPLAAATAVFLIYPIGQGSFSDGMPLGISGTF
NFMIVFQAEHNILMHPFHMLGVAGVFGGSLFSAMHGSLVTSSLVRETTETESQNYGYKFG
QEEETYNIVAAHGYFGRLIFQYASFNNSRSLHFFLAAWPVVGIWFTALGVSTMAFNLNGF
NFNQSIIDSQGRVIGTWADVINRANLGMEVMHERNAHNFPLDLAAGEVAPVALTAPAING
>OBU75541.1
MLAGLDLPDTPEDDRRFSLDLGLVKRIPLGRMTIANPIYQEVIPRVLSQGSQDSLTQIQP
TWLNTDK
>OBU75542.1
MAVLVLDDVLIGLDMSNPLPIIDIIDEYFIDKYQIFLMTYDLEWFEILCEHFVERNGKYW
KAFEFYCADNTELELPIFAERGKGRDEYIKRAEQYYATNDYKAAAVYTRSAYEATLKFFC
ARHRVPVPYVSKPKDLKTNQLWEAVKTYIKTHPKVTNKKTGYEEDYLDSKTINHVEKANG
RILNPLSHSRAVSIYRREVQYAIAVVKKLQDRLQ
>OBU75552.1
MDSYSFLNSHTHNNPLYKRNNWQSSYNSYNQKELADSDIGTLKDEKYMRSYALKLAHQGE
YRKAIALLDRIIDSHPENAIDYNNRGLIYFQSGHAQKALLDYNTAMQLNPRLASVYNNRA
NYYAARGDLVKALSDYDQALDLNPRYVRAWINRGITLRELGEYKDAIDDLEIALLFGQLE
IHIWSERGRSYHLWGDWNCAIADYRRVLSHTMSLDKIEDIITYRLRLQVENWLGELGFST
YK
>OBU75554.1

MKDLIIVSGLSAGVGIGVILTGISNGQASIGVLSSTAGAIMGASVVRKLEDDRNRVTLVK
LEELKNRELTRQESTNLIQQIETLRPTVKQLQQDREREEENLVVLRGKLGNQQAELEFIR
REWEQNQSRLEDITQTVANLTAQKQELENRISVINQENPRLLELEELRRAIEQSRIEKSA
LEGKISGLTSQLESLDSRRQETVSIELELRAKEIDLEKLTTQVENLKNQSQELEQRAADL
ELLRFTYDNIIKQNQDYEVKVNQLRPEIQRLELEKQQILHSIQENQNEYQKVQELREKLH
SLRLSIADESSRLREVETETQTWQGKKAALEESNGHLQEQKQELERTISRYKQEIVEIEN
SAELALQPLKEKLWNNLPQIIKPRKQESEFIADFITRIRSQGLTFSTRTVNAFHTSLKVQ
DISALVILAGISGTGKSELPQRYANYLGAQLLTLAVQPRWDSPQDLQGFYNYVEKKFKPT
DLMRGLYQYGQPDMQDRIVIVLLDEMNLARVEYYFSEFLSKLESRRNHATYLELDVGSLR
IRENERRLQIPNSFLFVGTMNEDETTQTLSDKVLDRANVLTFGRPQNLKLRQENQGAAVQ
HHDSSYLPYSHFQSWVKLPNPNSEIVRSVKYFLNQANEVMEKMGHPFAHRVYQAITQYVV
NYPQVESIESPAFKFALADQFGQKLLPKLRGVMVDEAHEQLEEMGRIIDDINDQPLITAF
DKARKGRYGQFTWQGLVYEDEEV
>OBU75556.1
MDFSMNFNEPNETEENKTEELDRGGFKRQLKKLIYTAAEISPVSQAQWEKLLEIYNFASL
SIEVIIQTICRNNARNVECRYKTLHS
>OBU75564.1
MNSLLINRYEIINPLGSGGFGETFLARDTQMPSQRLVVIKHLKPALQNSHSSTELIENLF
QKEAAVLEELGNHCSQIPQLYSYFSEEGEFYLVQEYIEGKNLSQVGQIKPEHATVILSSL
LNTLKYVHSKNIIHRDIKPENIILRDSDRLPVLIDFGAVKETMGVVTLGSGSTVSSVVVG
TRGFMAPEQTAGRPVFSTDLYALGLTIIYALTQRLPIEFSISQLTGEIDWTSYVPNLDPK
LVQVLNKSIKIDLGSRYLTAEAMYSDLHTSSGIPLSTVLAPKSQEDTLVVSPGGESKNLI
SSLTSVVFSKVKSQNKDTKVPVNYTRVAVIALSILGLAGGFFVTQQMLEAQERAAQLERE
KKEAEEKKEIAEKERLQAQQKALEAENLRQTAEQERLAVEKRQAQEERRRLVAESRQARL
ERRRLAAERRLSINSSQTSQTDAIVVGQPGYKNIRSGPGTTYKVLGTADTGDPVKILGSS
YDQDNYQWYQVYHPNSGTTGWIAAQLINLN
>OBU75565.1
MKLFLSFLSFFRHFIPTMILIIGVGIYMLLRPSDVPFPTTFTNSSPGSSSYQIPNPPPHQ
STESLTNTPQYSLSPPASQVANDTDIIPQPPGKSFEPTLPNSKSIQSQPPAHFRYPENKQ
NLVEVGTYYNRTAYLNLEAATAFKKMKLAANQVGIKLAPISGFRSFVEQENLFQKQIERR
GSAQAAQLLSAPPRFSEHHTGYAIDISDDRHPETDLKFAFESTEAYRWLEVNASQYGFEL
SFPRNNFQGVSHEPWHWRFVGSLTAKEIFKAARIQQNSRKF
>OBU75595.1
MICCLNPDCQNPQNPHGSKFCQSCDTPLVPLLRNRFRIIRVLSDEGGFGRTYLAEDTDKL
NDNCVVKQLAPKFQGTWAQKKAMELFSQEAKRLQELGEHPQIPTLLAYFEQDKCLYLVQQ
FIDGNNLLQELQLRKHYKDSDIQSLLLDLLPILKFLHSRGIIHRDIKPENIIRRKRDGRL
ILIDFGSAKQLTVAVQKKYGTSIGSHGYSAIEQIRDGKAYPASDLFSLGATCFHLLTGLS
PFQLWIEHGYSWVKNWQDCLNNSRSAELIIIILDKLLQLDLKNRYQSADEVIKDLSKKNVY
GSKKFGIYLKKGKHEKHKHTILRNIFLILVTISVVGGLGYRNLGQIQTAIFSQFNPLLIP
SNKSSTSPEYGQISGRTSSIRTNKVSLLKTITQVDKSLAVVAITPDGNTIVSAGHKEIKL
WNSKTGKQIISLPGHTQNINALAISPDGNNLVSAGDDKTIKVWNLQTKKLTFNLVGHQDS
IQALAISQDSKILVSAGDDKTIKVWSLLTGKFLKTLLGHNYWVRSLALSPDGFTLASGSF
DKTIKIWNINQTSGQKPTTLLDTTSQTVTSLAFSPDTSTLVSTSRDRQIKFWDIKNKEII
FASKKQNVTSVIFSPDGKTLISKAKSCPDCSEKISSVIKLWDVSTKEEIYALPGNTKIVT
SLVLSADGKTLVGGTEDNKINIWEISP
>OBU75600.1
MLAIYGGTVYCQQIWSHSYRKLESLQRQERQLTAANAIIISKMAQEAEAMGLGLSAPGKM
VFLPAVSNNSQPPSRAKIPPSPGKRTSIPPLGY
>OBU75748.1
MSKKHLSPVFLVTVAIAASLESSLSASGQTPSQSQQLLDGVNTKASQLLSSNLYSVKGET
FIGQTAPVDPNSNGNAVDNNQPDNQPNGQPTNPNNTEQPTETAEPRVLISEVVVKSAVGQ
LPSEIETQVYQVIRTKAGQPTTRSQLQEDINGIFAIGFFTNVQAVPEDTPSGVRITFVVS

LNPVLSKVELDANPGAGVASVIPADTVDKIFSQQYGKVINLRDLRAGIAQLTKEYQDKGY
VLANLIAAPKVSETGVITLQVAEGIVENVQVAFRNKEGQDVDEKGRPIRGRTKDYIIKRE
MQLKPGQVFNRNIVQKDLQRVFGLGLFDDINVSLNPGTDPSKVVVVVVNVTERNSGSIAVG
GGISSASGLFGTISYQEQNLSGRNQKLGSELQIGQRELLFDLRFSDTWIDGDPYRTSYTT
NVFRRRSISLVFEGKDSNIKTFNLDNTTDVNSQATPRIARLGGGVSFGRPLSTNLYENPE
WFASLGLQYQRISSRDGNGNIRPQGAIFDDSGNRLSPTIPLTQSPTGEDDLFLLQLSAQS
DRRNNAVQPTKGSYLRVGLDQSVPIGQGSILMSRVKANYSQYVPIKLLGSKNPETLAFNL
QGGTILGDLPPYEAFSLGGSNSVRGYDEGKLAAGRTYVQASAEYRFPIFSVVGGSLFFDC
GTDLGSATRAAEILNKNGSGYGYGLGLRIQSPLGPIRIDYGISDRGDSRINFGIGERF
>OBU75794.1
MTLQSFGVIGLAVMGENIALNVERNGFPIAVYNRSREKTDAFMSNRAGGRNVKAAFTLEE
FVASLERPRKILVMVQAGKPVDAVIQQLKPLLQEGDIIIDGGNSWFEDTERRTQELEPTG
LRYIGMGVSGGEEGALNGPSLMPGGTKSSYEYLSPIFNRIAAQVDDGPCVTYIGPGGSGH
YVKMVHNGIEYGDMQLIAEAYDLLKNVGGLDATQLHQVFTEWNQTDELNSFLIEITANIF
PYVDPETKQPLVDLIVDAAGQKGTGRWTVQTALELGVAIPTITAAVNSRIISSIREERIA
ASKQLTGPVPTQFKDTRTFVNMVRDALYCSKICSYAQGMALISTASKTYNWGLNLGEMAR
IWKGGCIIRAGFLNKIKKAFDENPALPNLLLAPEFKQTILDRQSAWREIIVTAAKTGIPV
PAFSASLDYFDSYRRERLPQNLTQAQRDYFGAHTYKRVDIEGTFHTEWVPIAEAKK
>OBU75905.1
MRAFVTGGTGFVGAHVVRCLLQSGYKVTALVRKNSNLANVKGLEIEVVTGDLNDPGIWEQ
MGGCDYLFHLAAHYSLWQKDRQLLYHHNVEGTRNLLRSAQKAGIERTVYTSSVAAIGVGK
SGEIVDETHQSPLNKLVGDYKKSKFLAEQVAKTAVQEGQDIVIVNPSSPIGPLDIKPTPT
GDIILRFLRQQMPAYVNTGLNFIDVRDVAQGHLLALEKGKTGDRYILGNENLSLKQLLDT
LSEITGIKAPQLSLPSFIPLTVAWIEEKVLAPLGKTPTVPIDGVRMAQQPMYYNASKAIR
ILGLPQSSVRVALQDAVRWFVSNGYVKY
>OBU75953.1
MLNLDRILNQERLLREMTGLNRQAFNELLSQFADTYERTVFNSLANRKRAPGGGRKPTLR
SIEEKLFYILLYCKFYPTYRNRVTDFDDQLMLVSAGLWNFYLDAA
>OBU75961.1
MLNLDRILNQERLLREMTGLNRQAFNELLSQFADTYERTVFNSLANRKRAPGGGRKPTLR
SIEEKLFYILLYCKCYPTFDLLSVLFNFDRSCAHDWVHRLLSVLETTLGEKQVLPARKLR
SMEEFTKRFPDVKEVIVDGTERPVQRPQNRERQKEYYSGKKKRHTCKQITVSTREKRVII
RTETRAGKVHDKRLLHESEIVQYIPDEVAIEGDLGFHGLEKEFVNVHLPHKKPKGIEARR
HGGGMGQFL
>OBU75968.1
MENFLVRNTGLMEYLRVVGKCDRLYEWGCLSEYQILVVEKRPYMGTQIPMMKIYHCQVK
>OBU76008.1
MNMGLLPWRLRDTSINKTTINSKQQATKNTTEIMTKKFVDLSTLDGKNGLTIINGNDKDD
NLGYSISNAGDINGDGINDIIIGAPLSDPNDQSNAGNSYIVFGSDNGFANIIDISTLDGI
NGFTVNGGGIGDQSGRSVSAAGDINGDGIDDLIIGAPFADSNGDDSGAAYLIFGRRSFSS
LPTVNPSNLGDNGFIINGLNPQDQLGYRVSGAGDINQDGFDDVIIAAPPNAYVYPPVTGD
QAGKVYVIFGSEKFNPSNLNFNPNFDLNSLNGNNGFVINGSRADDYLGVGLNRGGDFNGD
GIDDLIIGSPFNDFNGFRSGQAYVIFGRKESFSSSLDVSQLDGVNGVVINGQEGDQLGFS
VSSAGDINHDGIGDIIVSAHDADPNGIDAAGVAYVVFGARTQFSSQLDLSNLNGNNGFVI
NGIGELDKASWAVTGLGDVNGDGIDDLLVSASHADANGDNSGQGYVIFGGDKFSSAINLA
EIDDTKGFIINGKSENHNLGYSASGVGDINGDGVSDILISAPFAGSGEVYVVFGKNSPTD
TGEITDEVTEGNGGESDIINEAPSGLFLKTNNRVDENGLESNVIGTFTTVDPNADDSFTY
SLSDDENYPDNGLFSIQDNQLISKESLDFENQPIYTISVITTDRGGLSLTAEFTIDGTED
EITGGVIEDNNSESEGTPDEILDGVMGEGDSDNNSTEGSGSDLTDSSDIPSTSTGSADNI
LSSNNNVLSSSTTRVEFQLMDKIPASIRELGVFTVDDPSGKINGIAPGQVGYSEAALARS
KVVFSILSKTPNEFNNANVTRILGFQEANPNLRFYVIDNGTTDSVKNGLFPIDQVTFLDS
SNLQVTQLPDNSFSLQSNDLVFKVRPTTKPLPMGANLQEKSQGESIDLRGVTGPVNAQFT

VYREASFDNYVGFYKVTDEKGGIDTNNDGTADLLPGDAGYIQAAVNQHLIGLGLNVPDGN
KSNFNSTLSGGGLYVPFIIVNGRPDAFLNSDLSANSNPDIYFTYLGANPDRVDHVRLLGD
NTFGFEDLRGGGDRDYNDLVVQVNMSANV
>OBU76074.1
MNPVPPLTVPQSSTTTQNVTLPEEPLDLPDHTQLPDSNDDFVKNFQEHPQSIILTTSIEP
LLKKIHPNGDYCIGQDSGIYWRFTEPPEKGVEAPDWFYVPGVPSRLNGQLRRSYVLWKEK
VPPFIVIEFASKNGKEEKDSSPPPEGDEIDPETGKLKKAGKFWVYEQAVKIPYYAIFNGF
KGTLEVYHLERKRYKEIKANRRGHYAIPEMGIELGILYDNQKPPTPWLRWWDNKGNLLLT
GNELAEQAEAIAIRERLAKEQAETIASQERLAKEQEREAKEQAETIASQERLAKEQEREA
KERAEEIASQERMAKERAETIASQERLAKERAETIASQERLAKERAETIASQERMAKEQE
RQQKEKLAAYLRSLGIDPEKI
>OBU76075.1
MNPVPPLTVPQSSTTTQNVTLPEEPLDLPDHTQLPDSNDDFVKNFQEHPQSIILTTSIEP
LLKKIHPNGDYCIGQDSGIYWRFTEPPEKGVEAPDWFYVPGVPSRLNGQLRRSYVLWKEK
VPPFIVIEFASKNGKEEKDSSPPPEGDEIDPETGKPKKAGKFWVYEQAVKVPYYAIFNGF
KGTLEVYHLERKRYKEIKANRRGHYAIPEMGIELGILYDNQKPPTPWLRWWDNKGNLLLT
GNELAEQAEAIAIRERLAKERAETIASQERLAREQAENIASQERLAKEQEREAKERAEEI
ISQERMAKEQEREAKEQAENIASQERLAKEQEREAKERAEAVASQERMAKEQERQQKEKL
AAYLRSLGIDPEKI
>OBU76077.1
MPQSSTTTQNVTLPEEPLDLPDHTQLPDSNDDFVKNFQEHPQSIILTTSIEPLLKKIHPN
GDYCIGQDSGIYWRFTEPPEKGVEAPDWFYVPGVPSRLNGQLRRSYVLWKEKVPPFIVIE
FASKNGKEEKDSSPPPEGDEIDPETGKLKKAGKFWVYEQAVKIPYYAIFNGFKGTLEVYH
LERKRYKEIKANRRGHYAIPEMGIELGILYDNQKPPTPWLRWWDNKGNLLLTGNELAEQA
EASAIRERLAKERAETIASQERLAREQAENIASQERMAKEQEREAKERAEAIASQERMAK
EQERQQKEKLAAYLLSLGIDPEKI
>OBU76080.1
MSQPSTQNVTPPREPFNLPDHTQLPDSDDNFVKNFQEHPQSIILTTSIEPLLKKIHPNGD
YCIGQDSGIYWRFTEPPEKGVEAPDWFYVPGVPSRLNGQLRRSYVLWKEKVPPFIVIEFA
SKNGKEEKDSSPPPEGDEIDPETGKPKKAGKFWVYEQAVKVPYYAIFNGFKGTLEVYHLE
RKRYKEIKANRRGHYAIPEMGIELGILYDNQKPPTPWLRWWDNKGDLLLTGNERAEQAEV
IAIRERLAKEQEREAKEQERQQKEKLAAYLRSLGIDPEKI
>OBU76107.1
MHFEDQAFDRLGTAVEGNTGPVWYYLLEIIKYGFPWLLFFPGGLYLSWKNRQTPWGSLVF
IGTVIYFTIVSLMSTKLPWYIMPIYPFLALAVGVNLGYIWEEHSLGKFLKTPLTIKFLIG
FFIFLSVAGVGGCIYFSLFDRQVPLILMSVVLTISMVITSWFISQHSRQFIPVLFGGMYL
VLTLLMISQSWVWELNEKFPVQPVAGLIRQHIPAGQKIYTSFPDSRPSLDFYSDCQIIPA
SLGDLEDKFSSHSFLLVNEEDLGKINLKNSRVLGKSQGFRLIAPTSPSSPTNKL
>OBU76150.1
MLQDTQTIRYYQRLTDAFVELWNRGYHRDDMRMYLDGYLAALRQSNAIEPYLIHRLEEEA
IRYLHDGSNFAVVQPEPERYHGY
>OBU76191.1
MNSTTASLSPTLNFEVGQISNFPILNNTFDKITINSLENIEQAINIAREDWDNFETSWDF
QTHPLLRENSPNISTSFTNWQNRTETAFRQLQLLEQENNRYWIKSYGLETELTPEVPLNE
ITLTCNPHYRYDHTKPQTELETLLLADTIKEYISYAVGCMFGRYSLDQPGLILANQGETL
PDYLKQIPNPTFTPTKNNVIPILDGQWFTDDMSDRFRQFLRLTFGEEHYEENLKFIEKAL
GKDIRKYFLKDFYNDHIKRYKKRPIYWLFSSPKSNFNALIYLHRYRPDTVSIVLNDYLRE
FRAKLEARQNHLKRVEVSADASQSEKTKAVKEINKLATIIEELNDYEREILYPLAIEQIH
IDLDDGVKVNYPKFGLALKKIPGLETKEED
>OBU76235.1
MSILIANIGTSDLAIKINVNGQQRYFNVLIILDTAISETVKSHLSPILVEILLWIKTYLI
QKGTGLKVSWYMVL

>OBU76237.1
MSNLLERITVNLRQCDGRPCIRGMRIRVTDVLDLFVAGLSAEQILEELPDLEMDDLKAAL
TYQVATPNQQ
>OBU76242.1
MATTADEVWKLLGELIESQKETERKFQETDRKFQETDRLLREQSQETERFLREQSQETDR
KFQETERLLREQSQETERLLREQSQETDRKFQETDRLLREESKRVNNQIGQLGNRLGEFV
ESQVRPAAVKLFQERGIAVKEIASNTYIQTGKEGLEIDLLVINSSDIILIEAKSKLSEDD
VNEHLERLSKFKRFFPRYESYRVLGAVAGMVIPLDVSRYAYRKGLFVIGQSGDNLVILND
DKFRPRGW
>OBU76256.1
MIMNLKTKKAIEQLEQFVSIPLEETLRQHEKTTHQEIATNLFKNVASTVPAYQSFLATHN
IKRETIQTLADFQKLPVICKENYISVYSLPELCKDGILGGCDMIAASSGSTGKPTFWPRF
FTDELQIATRFEQIFHDSFGANIYPENVSVGLEQPIIREWVTGKFVLQVKEDQDQNRFLS
VVVELAPGLEGSEEKIIEITNSILAQLLRLNSEFANYVPGEYQRPQVELKATGDGEYFPT
GVKHKYTRNL
>OBU76278.1
MFGLCLLGQKVQKTSIDYSSLKNRLKTDINKVMKLTFINEAIEERYWTKTKYTINEIIIT
TIKEKEESDVENNKLANSDPKPSPEEKTINLPKKPTLTKQLIFQSILKSLGMNEYKNIDY
VTFQTDKIQQTHKRKIAQSIYKFQESQKNIQPTGYLDHKSQESHELKRKISQQFLSQVL
>OBU76285.1
MLKVTNTNANVNIKTNNHITITDRTKQFAIRIIKACYFLDEKSGVYRTISKQLLRSGTSI
GANVRESQSAQSDKDFIHKLEIALKEARETQYWLEILIESELVSKPKFTSLLQEANEIGK
ILVASTKKLKEK
>OBU76315.1
MDKQNLLGLVIFLILLLIPGSLFSPLATPIDGWRAMLAAVTSATFATLLEGISPRGTDNL
SVPLITAIVVWLIIGR
>OBU76356.1
MITLETNTRNRLILQLLFFCGLRVGELTILLWADIKDNGSTAYVHITGKGNKQRTLIIPP
LLWASLKSHKTTNESPVFKSRKGGKNLTQKAVWDIVRTASQRIGISASPHWLRHTHASLA
LHNGADINQVSTSLGHSSVATTTKYLHARPNDCSSLYL
>OBU76359.1
MDTGTDKGIPDWLNPDRLIELIELLREAGYKIGISQHIAAQDIILFLITQGQTLDNPKQL
RNLLGPIFSKSLIEQENFQYHFDNWLKLIKQIGLDTEKVSLPVARRRIPWRQLQWILIFI
AVLMGTPTTQPKPLSTNPPSSPTTTPTPKQTLPSINPTPSTTGTPTPEQPSSVNWQITLI
YFLLSLCFLYIGSQLLWLWRANLFLHRSSTKVIPDLQTISIPDLEQNLFPRYLFISIAKN
FRQRILVPTNILDVEKTIDAFFQRGRWVEPIYRNSAVIPEYLFLVERTSYRDHQSKFVAE
MIEQLKNDGVYVETYFYDEDPRICFSSDEHNSSLKLHQIATKYSQHYLIVVSDTEKLFSS
ITGELEPWVNQLLDWQNRVILTPTPVENYSYEKFVLAQDFLILPATSTGLQTLSQKLQQQ
IATNHYLITETPSPLPESLRLRPLHWIERNSPPTKDIDAMLVSLEEYLGKNNFYWLGACA
VFPQLHWNITVYLGNTLKTEMGHSLLEVGSLTKLARLPWLRYGYMPDWLRSLLIAKLTNE
QKHTIRTVLKDLLVTAVQGSKGGLQLEVAKKHHSFLSKLLANPIMFYLLSRRVFEGSELR
DHLFVSFMTRQSKLTIEVSDTFIRLLQRTPQPLITRGILKKTGFALGLLTLTIIAQEVSV
RVTYPPTVIRTESLLSKLEALLKAKNFKEADLETDRVMLAVANRQSEGWLRIEDAENFPC
KELRTIDNLWLKYSQGKFGISVQQEIYKNLGGTKQFDENVWRSFGRMVGWRDHYSDYNFS
LSAPTGHLPSLWSVDGRRRQRERLQLVIELRLWVGGLFPPVKTCRV
>OBU76361.1
MTRSPQLTASLNSSVVRIYNPTSNNVVGAGFLVEDRLVLTCAHVVADALGIHIRTVEIPN
GTVELDFPLVAPKQRLRARIVFWRPVNPDMFGEDIARLELEPPLPDKAQAARLVRSENLY
NHPFRVLGFPLGEDNGAWAHGKIKGRRANGWLQLEGTRQTGYSVDHGFSGAPIWDEQLQG
IVGMAVAADINRPKIKVASMIPTEVLDTALTPSDLPKRKIPSLLPYLVNRIEQEYELGRV
IVRNSPSPLICIIHGDEFQSHDKFLERLHKVSLPRFLGEESIKKYHLPSPPKLNNWDEFS
GHLHRTLADIVIGRNSASETEIKTEINDFFHRYPCPILIHTHLLTEQLKEKFETLDNLVK

FWCDEWTINSNQNLIICIFIKYQIKRKMNTENSGIIKLFNFLNLNYLVKKYRYEKANQKI
SQHIKSLCNSERSNPLVLVPELTGVSRSDAENWVRDHTQNFIGEAMAENLMKRIRNLFDN
WEEKNSSNKIPMDHLAQELIKVLKSNC
>OBU76362.1
MKSIIEFPLENGESILVEVDEPGFTDSRISRRDDIAKKAEKTFESALEKVRPIADIIMTR
VNSLSQPADEVEVKFGIKMSGTLGVLIASGNSEVNYEITLKWKRNP
>OBU76366.1
MGTGDRIALPWGDREAIQETAYSQLRRTVTLSAIAPISLVVRAIVINGWNIVIKVTRIAI
VISWVSFLRFISWDG
>OBU76377.1
MKRFLTLALILTLFLVSSFSFSTSPSYAYSQSDLEKLLETKICIKCDLSHANLERADLKY
AYLNGANLSGANLKYADLSGANLKYADLSGANLEDTNLEGTDLEGANLKDADLLGANLED
ANLEHADLKGAYIRSANIEHTHTLDANLTNTQL
>OBU76379.1
MKSQDFRKADIETAIVMLAVANRQSEGWLRTEDAENFTCKELRTIDNLWLKYSQGKFGIS
VQQEIYKNLGGTKQFDVNVWRSFGDRVGWRKDGSWLKYSDLNNFSLSAPTGHLPWVGVVG
GGREGFFWFLWVVVEGRFPS
>OBU76404.1
MKSQDFREADLETDRVILAVANRQSEGWLRVEDAENFPCKELRTIDNLWLKYSQGKFGIS
VQQEIYKNLGGTKQFDVNVWISFGDRVGWRKQGSWLDYSDLNFSLSAPKGHLPNEGRKWY
ILDGWKVHLPRGFSRPSLLSRHVECNP
>OBU76405.1
MKTQKFTQLVIIGIIVAFITIPSHITLALRPKEIADIATQVTIRITGISNGSGVIISRNG
NTYTVLTNSHIFENHPNGKFEIITPDGRKHQLNNLRRIANLDLATLEFNSIQEYRVVEPG
DSSRITRGEDIYVSGFPANQDLNFSSDRITRIITKARPGGYALVYRIGAFPGMSGGPILD
SDGKLVGIHGETQSVSLGPGRSTPEEYGIPLQTFLNATSISSPPPTRAGTQN
>OBU76411.1
MVRQIAPKTQLGAIKSNKNQLGETIVPEIVYPESDGEPMADNTKQFTWIVKIKENLEILF
KSNPDVFVAGDLFWYPVEGSNKIKLAPDTMVVFGRPKAHRGSYRQWEEDNIPPQVVFEIL
SPGNTQDEMDKKSCFI
>OBU76412.1
MFYLKHGVEEYYVYDPDRISLEVSIRENNSFKEIENFTTWTSRRLKIRFDMTQDELVIYY
PDGSKFLSPVELSNYAEQETQRAEREKLLKEQERFLKEQEQIKYQTLLSQLKAKGIDITA
LE
>OBU76414.1
MRQPLTTPLAGESLNLPDHTQLPDSDDNFVKNFQEHPQSIILTTSIEPLLKEIHPNGDYC
IGQDSGIYWRFTEPPEKGVEAPDWFYVPGVPSRLNGQLRRSYVLWKEKVPPFIVIEFASK
NGKEEKDSSPPPEGDEIDPETNKPKKAGKFWVYEQVVKVPYYAIFNGFKGTLEVYHLQGK
RYKEIKANRRGHYAIPEMGIELGILYDNQNPPTPWLRWWDEKGSLLLTGNERAEQAEAIA
IRERLAKEQEQEAKEQAEAIAIRERLAKEQAEAVAIRERLAKEQAEAVAIRERLAKEQAE
AIAIRERLAKEQEQEAKEQAEAIATRERQQKEKLVAYLRSLGIDPEKI
>OBU76435.1
MRITKSALTAGVLAGVLGIFPPMITLALTSEQVASIAEKFTVRISGVAPGSGVIFNKNGN
TYTVLTNFHIFDRGRTLRDGAYRVTTADGKTYPMINTVRIPDLDLARFNFRSNEEYRVVK
IGSSDKIVRGKRIYVNGFPEQQEVNFLPGQVNRILAKPRRQGYVLVYRIGAFTEMSGSPI
LDEDGNLVGIHGLTEDVDVEGGGTTPEEYGIPINAYKSSSVYSNNAEFYFNRAYNLYESG
DKQGAIVDYTQAIQINPNYALAYIGRGNARSDLGDKQGAIVDYNQAIQINPNYALAYYNR
GNARSELGDKQGAIVDYTQAIQINPNYAYAYNNRGLARSELGDKQGAIVDYTQAIQINPN
YADSYYNRGIARSELGDKQGAIVDYTQAIQINPNYAYAYNNRGLARSELGDKQGAIVDYN
QAIQINPNYAYAYNNRGLARSELGDKQGAIVDYTQAIQINPNYADSYYNRGIARSELGDK
QGAIVDYNQAIQINPNYADSYYNRGIARSELGDKQGAIVDYNQAIQINPNYAYAYNNRGL
ARSELGDKQGAIVDYNQAIQINPNNAYAYNNRGLARSELGDKQGAIVDYNQAIQINPNYA

DAYIGRGNARSELGDKQGAIVDYNQAIQINPNYADAYYNRGLARSELGDKQGAIVDYNQA
IQINPNYADAYIGRGNARSELGDKQGAIVDYNQAIQINPNYADAYNNRGIARSELGDKQG
AIVDYNQAIQINPNDADAYYNRGLARSELGDKQGARGDFQTAARLYQQQGKQNDYQDALN
RISQLR
>OBU76441.1
MNDLPRQKLKEIIIQHGRSLCDDPHRCEAFLRDYCGQYKREIFLLISALKQGVVKDLLNS
NNTPIEVLLGRLINQMQNDLGLTKEGSCACFMDN
>OBU76487.1
MIQSYSMNSTSSLISPDVSGYQSHDIKPVKLGVMASGNGSNFEVVAQAIKSGDLNAQIQV
LIYNNHLAKAAERALNHGVEAILLNHRHYQKREDLDREIVSTLRQYQVELVVMAGWMRLV
TQELIDAFPNHIINIHPSLLPSFKGVRAVEQALEAGVKITGCTVHLLRLEMDSGPILMQA
AVPVLPNDTAETLHARIQVQEHRILPLAIAQVADKI
>OBU76645.1
MSQNNSVNGNGTVSPFLKTLVQQIRASDSYGFYRNWSDELILKPFVVTKQNKKQISVEGE
IDPATIARINAFFRAVAASIEQETGMISNVVIQLGHEGFGWALVFSGRLLLAIKTLRDAH
RFGFESLEKLNEEGQNFVQKGIDLAQCFPQVGNL
>OBU76690.1
MSQDKKPRSKPQLPDTPPLGGYHKKPLFWKILIIQVLRGTIGILERMVTRLETSSSTTSE
KGGLLLWVVRKWDGFLRGFRLFLPSKVANNVSDSFLTLIFVFPALLAIGVTAISLISQIH
SQPVPQLGESIADSIVEDKSQPQLGESIADFTVENKSELQLGESTSDSTVEDKSEPQLGE
PTADSIVEGKSKPELSQPATDFILRSQSEIEPVELIPQPEVTVFIEKQLKEITGISIITK
DKQRIELELVQSIKTNFRISEIVIKVNENWYKLESSQQEKLAAKILKSCQEMDLIHVKLV
NARNQVIARSPVIGTKMLFFQFPTS
>OBU76735.1
MTKESVNFQFLQLQDLQQLVRLGILAEGYVWNQPNTCLVKLCYFGQLLAQLIAARTGNFH
STVENQADLLNQLELKGILPQKVALLFHQVRVVSDRAAYEYTSDSSQALTILKIARELAI
WFHRTFGGNTTFTPNPFISPSSSVDYGAELEILQHIETEFEQALPKFSVHLAQMQAVCST
LSPRQTDTIISLGNQAVLEIDLEEDAHTYLCRGIARSDLGDHRGAINDFTQSISINSNLA
QAYMERGIARSNLGDGQEAIEDFNQALDINPNLALAAYSRGVTHSDMGYLEKAIDDFNQT
LHLNSAFFDAYTRRGLARYDLGDKQGAIDDFNQVIRINSHFADGYAARGLVYCDLGNHQE
AINDFSQAIRINPNYAQAYHNRGVARSQLGDKQGAIDDYTQSLNLNPKFASAYYNRGIIR
SDLGSNKAAMDDCTQAIRINPNYAEAYNNRGAIRTYLGNYQGAIDDYIQALRVDSNYVEA
YYNWGTTRINLEDNEGAIDDYTQAININPNYAQAYYGRGIARFNLGDKQGAIDDYTQAIN
TNPNYAQAYYNRGIARFNLEDKQGSVDDYTQAININPNYAQAYYAWGMLRSELGDKPEAV
NNYTQALNINPDDTETYVARGLTRSELGDNQGAIDDYTQALNLNPDYACIYNNRGIVRSD
IADYQRAIDDYTEAINISPDYADAYYNRAIVYYDLGNYQRAIDDYTQSLEIKSNCADAYI
GRGTALYKLGDSQGAINDFHHALDIDPSYADAYNNRGIVRYELGDHQGAIGDFHHALDID
PSYADAYNNRGIVRYELRDNRGAIEDFNHALNINSNYAQAYNNRGIVRICLGERQLAIED
FTQATIIASNYTESYINRGYARYELGNRQKAIEDFNQALNINPNYAQAYNNRGVAYTDLG
DSEWAKDDFSQALQINPYYAEAYNNRGIVCYKLGDRQGAIEDFNQALKINSNYVEAYNIR
GNIRYELGDRQGAIEDFNRALSLGSLDLTENLKFWEKGGLTLTIKLRNYLNLKILSFEVV
SKKNSGEIIKRDASAIYFVEDLGDDITLEMVEIPGGTFTINPAENRGNAEGIAEHLVTIP
SFFMGKYEITQEQYQAVTGDNPSYFKGDKRPVERVKWSQALDFCEELSQITGRIYTLPSE
AQWEYACRGGTSTEFHFGDTITTDLVNYHGDHGDSLAPQGQYRKQTTEVGSFPPNPFGLY
DMHGNVWEWCLDHWRDHYQDIPKDGSPWVEKGIFGVFRGHLLRGGSWCDSAKHCRSGSRN
RHLSDKNNLGFRVVCSNPNR
>OBU76739.1
MADVESQAESKLVPAGQVSTWLTENGFDHESLSPDVNGVEIIKVAPDFLLPTATALYAYG
FNYLQFQGGIDLGPGQDLVSVYHLIKVSSDADKPAEVRLKVFLPRENPVVPSVYWIWRTA
DWQERESYDMFGIIYDGHPNLKRILMPEDWVGWPLRKDYISPDLYELQDAY
>OBU76748.1
MLSYTTRVSIGVIEPRWLNEQGELLPDELLEAFLEFWRQHGEPLLKSAPYHEIAPHLVLM

AFLHRVVNGGGTLEREYAIGSGRMDICLRYGKVVMGIELKVRKEKLDPLTQGLIQLDKYL
DGLGLDTGWLVIFDRRPGLPPMGERISTEEAISPSGLTITVIRS
>OBU76750.1
MKDRTATIILCLFVGGLGIHRFYLGQTGLGLLYLLFCWTLIPSFIAFFELFIFIFTSDDE
FNRKFNSASK
>OBU76752.1
MALGDSNTVTIGQEVYVSGFPSKQDFTFRGVVGGGVWVGVFGFSPPGELLEVGVGTFPPV
KTCRV
>OBU76792.1
MKIIPKTFNTTNFIQFHILEYFLIDVQIFARSANFIIVKFATEIWQFLCLYYEKLSLKSK
FIPDRIHF
>OBU76944.1
MKKTVVLLKGGLGNQMFQYAFARSISLKNSSKLVIDNWSGFTFDYKYHRQYELGTFSIVG
PPRQPNRKVSFLVLRTKV
>OBU76945.1
MPPEPMEKHFLELGKLLRETESVALGIRLYEESKNPGSHSSSGELKSHFEINQAILKLRE
LCNGAKFFVFCTHRSPLLQELALPENTIFVTHDDGYVGSMERMWLLTQCKHHIFTNSTFY
WWGAWLSQKFYIQGSQIVFAADNFINSDAIPKHWKPF
>OBU76946.1
MNLLKRLNINEYNRQQWLARVLTELPDGYRILDAGAGELKNRVYCNHLNYVSQDFCEFQG
RGECNEGLQFNGWDTSRIDIVSDISDIPEPDASFDAILCSEVLEHIPEPTHALDEFARLL
KPGGVLILTAPFSSNVHMSPYYFCTGFSKYWYEYHLVQRNFNITELVANGDWYTLLRQEI
MRLGGVDRQGGIWVWPIGYAYALLGLLYFGLRGKYLREDIACFGWHVLAVKRTV
>OBU76947.1
MNFSVLISVYYRDDHLCLDQAIRSVITQTCQPSEIVIVKDGSLTPILDSVLETFVATSPI
PVKLVPLPKNVGLGLALQTGLQSCSYNWVARMDSDDLSLPQRFEKQTQLIKEKQLDAVSA
WIEEFDVTPGDLKRVRKLPTEHQQLVQFSQRRNPLNHPCAFFNKNAVMSVGSYESMPLFE
DYYLWLKLIQNGYRIGNLPEVLLYFRVGNGMIRRRHGWEYLKKELKFYYRVMLNGLIPSQ
TLVVSVLLRLPLRLLPIRLLQFLYEKVVRY
>OBU76960.1
MLSITNEKSQKNYAGPKKIILIEHLNEIGGASRVSISLVHGLSDRFDFHVIIPSGQYLQE
FEDGKTKVYKMSYSSFRPTLIPNKWVDQISLVLEIRSCVRKILKDKETAVIHINGLPNIL
PAIATRFLGLPTVWHVHETAFNPNWAFRLLTCFACSISNSVIAVSNSTAEDLIKNILFKS
QANKIKVVYNSAFSSIKSKETDQKNIIKSDNTFIVAMAARIVPQKGIIEFLKIAQKTTEQ
SKNIEFWLAGPKVIKHNRYFQSVISVIEDDSSKQIKYMGELSSCEYFFKMSNIVVNTSLF
VEPFGLTLVEAMSLGKPVIGPPYGGPGEIISNNENGLLIDPKNTLLFSNTIIELSKDSER
LNRLGQAGFMKYQNSFSQENFFNSFSSEYFTLLE
>OBU76963.1
MLFKVPRIIRPVYLFLTALFPGWKDSWLLVARKKQDWSVKKTLSQSELAAILGNYSSYDY
TQ
>OBU76964.1
MNINKSKVPKPRILVFIDYYLPGYKAGGPLRTLHNMVEQLSDCFQFLIVTRDHDINDSHP
YDGLETGVWTSQGKAQVFYIPKNQWGVCSIRRILRETSYDILYFNSFFSPWMAGLPLLLR
YLKQFDPVSIILAPRGEFSPGALQLKAGKKKLYMAVLFRLGLCRNLIWQASSDKEGVDIY
NVFHKAGKGSDIKIAPDLPSLLYANKYIPNDNQISPTRLNSLKILFISRISPMKNLDFLL
RVLRNVSSPVDLSIYGPIDDSNYWSICQALINDLPRQIKFEFGGEIEHERVGEIFKQHDL
FAFPTRGENFGHVIIESLCAGTPVIVSDQTPWIAAPDGALEVLPLDAEKWQAAIERWANM
QDSVLLKMRRGAFAYARSYLESNNALEQNKELFFAAMKNGVK
>OBU76965.1
MPTIAAITTCFNRKKKTISALTSLYTSLLNTQDVNLSVYLVDDGSTDGTSEEVSKQFPTV
KIIKGSGNLYWSGGMRLGFTEAMKTVHDFYLWLNDDVILNSYAIENLLKCYLRNLSISGV
ESVVVGALSNLDNDQVIYGGHRLVGSKFLRKNQFVYSPDEDLECDSFHGNCVLIPNNIAS

KVGGIDPVFIHAMGDSDYGYRCSLQGFKIFCCKEIVGRCDNDQGNYKLYSTLAQGNWKFL
PLFLRLKVVTNPKNFPFKPWFIYSYRHLGWLWLLRFLRPYLLAIFPQLIRTQ
>OBU76966.1
MIQRQNCFHELFAYLGKLKYTILLLGLVIWLLSPMLGLIPLLIFSQIDVSQSVIKNTRKK
NYILNYFCILAVLFTVTITASTYSVVGDLKQYTIIYDQLGGTNFFDFFKKINMEPVTFII
PDLVKRYFYLNRFDFILLQAITMNCAYFALARVFMPNYYPTVIMLNICSIAYFQQLFLMR
QYYSFIFLVPFIYLVNWKYKLVLGLIALFTHSSTVIFTIPQIIVTMTITDQRVKHGKEDS
ISILKLVQSFQRWLKKLLLDRFVLYVALLTITLSFILLTKSGFISNTQVLLGSNAFVQEV
SPRLATTLSSYSNTEYLLGLSRDLWKVAVIDIMFLPLLLVQIRFNREPLVCYSWVLAFAS
SAISLLLFYVGIPAFGRLVYFLSGLSGFFYTFVLKSSNLFHKRNFFSFAIFGAISAKIVY
FYYFLFSFLRSDANPYLWGGHVIQANIYDYIEYLLNINRNL
>OBU76967.1
MLRDRLSAHNGEKKGKFREIFERSTNWGDSIIDLLDWMHDAKSFFPKTIGTMIRWFGEIV
GYFDGRTTSGIVEGINNKLKLIKRLGYGFRNFNNLRLCSLLHRHFSINCP
>OBU76969.1
MLNSEIKSPLTNESKVEYVRSLSPQEIANKWQSSMDIDVGSVFRNLPAIEHWRCVQTGIV
>OBU76970.1
MKKVNLACGSVYIKQSDWINLDYQPMGDGVIKSDLLGTLPFKQGSVSLVYSSHFLEHIPR
SKVAHFLSECFRVLEPGGTIRLVLPDLEEICSQYLYNRQRSDHKKADFCVVEMIDQCVRL
ESGGELGSLYKLYSQSYDRDLEMVEYIRSFNGELLCKAENHNNSPLSKIASLLEEPKLLW
GKIAYHLEQYYIRMIVSLLPKAFRTQNVSLATVGERHHWLWDFRQIQQALESVGFIATSR
CSFNTSMFPDFPFQSLDMDANGFPRKGLESMYVEAKKPK
>OBU76972.1
MLSFSTPVIFLIFRRPDLTAQVFERIRAAKPPKLLVVADGPRNTQEELLCQQARKVTEQI
DWNCEVLRNYSDVNLGCRDRVSSGLTWAFEHVEEAIILEDDCLPHPSFFSYCENLLNYYR
HDERVMVVSGDNFQDGKHRTPYSYYFSRYNHCWGWATWRRAWQHWEFNPHKWIDFRDSNL
MRFIFDDPDEENYWISIFNTLFLEGKPDTWDYVWTFACWSQGGLTALPSVNLVSNIGFRS
DGTHIVGDGRLANMITQDIGEIHHPPFVARHKDADTYTFKYVFFNNTSRLTAFLKSLYQK
AKKFLTFTK
>OBU76984.1
MLKVRVIPTLLWKQFGLVKGNRFNSWRRVGSVLPAIKVYNQRDVDELILVDITANTTGES
PDFESIADFSQECFVPFAVGGGVTNIDHVQNLLRSGADKIVVNTAAYSSPSLITDIARRY
GTQCVVSSIDVKRKSRSDWICFSHSGSKDTGREVVSWARELEDRGAGEILITSIDNDGMM
QGYDLSLIETISQSVKIPVIASGGAGNYNHMIEAIKQSGASAVAAASMFHFTEQTPAGAK
EAMKKAGIPVRLNFTRR
>OBU76985.1
MKVGIIDYGVGNLGSIAMAVEQLRSKPVLIDRAIDLHAVDALILPGVGNFTDCMQILVKG
GWVDAIKEEVTNYHRPLLGICLGMQLLANIGLEGALDSTTGTQGLGLIPGRVVSLKSQGC
SLRVPHVGWNNITKLDSKPWLLNGISDGTDFYFVHSYVFMPDEQSTVLARAEYDISFTAA
IGLGRVWGTQFHPEKSSRAGIQLLRNFLDG
>OBU76987.1
MNYKTQQEQFWAGDFGTDYIQRNQSEQLLASNLAFFSRTLYAAKGVRSCIEFGANIGMNL
KALKLLYPGIDLHGIEINQQAARELTNVIPADHVYSESILEFCELRHWDLVLIKGVLIHI
NPEYLPVVYTKLNNATSRYLLIAEYYNPSPVAIPYRGHLDRLFKRDFAGEILDRYPEFSL
VDYGFFYRRDPNFPQDDITWFLLEK
>OBU76996.1
MATTADEVWKLLGELIESQKETERKFQETERFLREQSQETERLLREQSQETDRKFQETER
LLREQSQETDRKFQETDRLLREESKRVNNQIGQLGNRLGEFVESQVRPAAVKLFQERGIA
VKEIASNTYIQTGKEGLEIDLLVINSSDIILIEAKSKVSEDDVNEHLERLSKFKRFFPRY
ESYRVLGAVAGIVIPLDVSRYAYRKGLFVIGQSGDNLVILNDDKFRPRGW
>OBU76999.1
MATTADEVWKLLGELIESQKETERKFQETERFLREQSQETDRKFQETERLLREQSQETDR

KFQETERLLREQSQETERLLREQSQETDRLLREESKRVNNQIGQLGNRLGEFVESQVRPA
AVKLFQERGIAVKEIASNTYIQTGKEGLEIDLLVINSSDIILIEAKSKVSEDDVNEHLER
LSKFKRFFPRYESYRVLGAVAGMVIPLDVSRYAYRKGLFVIGQSGDNLVILNDDKFRPRG
W
>OBU77006.1
MATTADEVWKLLGELIESQKETERKFQETERFLREQSQETDRKFQETERLLREQSQETER
FLREQSQETDRKFQETDRLLREQSQETDRKFQETERLLREQSQETDRKFQETERLLREQS
QETERLLREESKRVNNQIGQLGNRLGEFVESQVRPAAVKLFQERGIAVKEIASNTYIQTG
KEGLEIDLLVINSSDIILIEAKSKVSEDDVNEHLERLSKFKRFFPRYESYRVLGAVAGMV
IPLDVSRYAYRKGLFVIGQSGDNLVILNDDKFRPRGW
>OBU77008.1
MATTADEVWKLLGELIESQKETERKFQETERFLREQSQETERLLREQSQETERLLREQSQ
ETDRKFQETERLLREQSQETERFLREQSQETDRKFQETERLLREQSQETDRKFQETDRLL
REESKRVNNQIGKLGNRLGEFVESQVRPAAVKLFQERGIAVKEIASNTYIQTGKEGLEID
LLVINSSDIILIEAKSKVSEDDVNEHLERLSKFKRFFPRYESYRVLGAVAGMVIPLDVSR
YAYRKGLFVIGQSGDNLVILNDDKFRPRGW
>OBU77014.1
MATTADEVWKLLGELIESQKETERKFQETERFLREQSQETERLLREQSQETDRKFQETER
LLREQSQETERFLREQSQETDRKFQETDRLLREQSQETERLLREQSQETDRLLREESKRV
NNQIGQLGNRLGEFVESQVRPAAVKLFQERGIAVKEIASNTYIQTGKEGLEIDLLVINSS
DIILIEAKSKVSEDDVNEHLERLSKFKRFFPRYESYRVLGAVAGMVIPLDVSRYAYRKGL
FVIGQSGDNLVILNDDKFRPRGW
>OBU77020.1
MATTADEVWKLLGELIESQKETERKFQETERFLREQSQETDRKFQETDRLLREQSQETDR
KFQETDRKFQETDRLLREQSQETERFLREQSQETDRKFQETERLLREQSQETERLLREES
KRVNNQIGQLGNRLGEFVESQVRPAAVKLFQERGIAVKEIASNTYIQTGKEGLEIDLLVI
NSSDIILIEAKSKVSEDDVNEHLERLSKFKRFFPRYESYRVLGAVAGMVIPLDVSRYAYR
KGLFVIGQSGDNLVILNDDKFRPRGW
>OBU77026.1
MATTADEVWKLLGELIESQKETERKFQETERFLREQSQETDRKFQETERFLREQSQETDR
KFQETERLLREQSQETDRLLREQSQETDRKFQETDRKFQETDRKFQETDRLLREESKRVN
NQIGQLGNRLGEFVESQVRPAAVKLFQERGIAVKEIASNTYIQTGKEGLEIDLLVINSSD
IILIEAKSKVSEDDVNEHLERLSKFKRFFPRYESYRVLGAVAGMVIPLDVSRYAYRKGLF
VIGQSGDNLVILNDDKFRPRGW
>OBU77028.1
MATTADEVWKLLGELIESQKETERKFQETERFLREQSQETDRKFQETERFLREQSQETDR
KFQETERLLREQSQETDRLLREQSQETDRKFQETDRKFQETDRLLREESKRVNNQIGQLG
NRLGEFVESQVRPAAVKLFQERGIAVKEIASNTYIQTGKEGLEIDLLVINSSDIILIEAK
SKVSEDDVNEHLERLSKFKRFFPRYESYRVLGAVAGMVIPLDVSRYAYRKGLFVIGQSGD
NLVILNDDKFRPRGW
>OBU77034.1
MATTADEVWKLLGELIESQKETERKFQETERFLREQSQETERFLREQSQETDRKFQETER
LLREQSQETDRKFQETERLLREQSQETDRKFQETDRLLREESKRVNNQIGQLGNRLGEFV
ESQVRPAAVKLFQERGIAVKEIASNTYIQTGKEGLEIDLLVINSSDIILIEAKSKVSEDD
VNEHLERLSKFKRFFPRYESYRVLGAVAGMVIPLDVSRYAYRKGLFVIGQSGDNLVILND
DKFRPRGW
>OBU77037.1
MATTADEVWKLLGELIESQKETERKFQETERFLREQSQETDRKFQETERLLREQSQETER
FLREQSQETDRKFQETDRLLREQSQETDRKFQETERLLREQSQETDRKFQETERLLREQS
QETERLLREESKRVNNQIGQLGNRLGEFVESQVRPAAVKLFQERGIAVKEIASNTYIQTG
KEGLEIDLLVINSSDIILIEAKSKVSEDDVNEHLERLSKFKRFFPRYESYRVLGAVAGMV
IPLDVSRYAYRKGLFVIGQSGDNLVILNDDKFRPRGW

>OBU77039.1
MATTADEVWKLLGELIESQKETERKFQETERFLREQSQETERLLREQSQETERLLREQSQ
ETDRKFQETERLLREQSQETERFLREQSQETDRKFQETERLLREQSQETDRKFQETDRLL
REESKRVNNQIGKLGNRLGEFVESQVRPAAVKLFQERGIAVKEIASNTYIQTGKEGLEID
LLVINSSDIILIEAKSKVSEDDVNEHLERLSKFKRFFPRYESYRVLGAVAGMVIPLDVSR
YAYRKGLFVIGQSGDNLVILNDDKFRPRGW
>OBU77041.1
MATTADEVWKLLGELIESQKETERKFQETERFLREQSQETDRKFQETERLLREQSQETDR
KFQETERLLREQSQETERFLREQSQETDRKFQETERLLREQSQETDRKFQETDRLLREES
KRVNNQIGQLGNRLGEFVESQVRPAAVKLFQERGIAVKEIASNTYIQTGKEGLEIDLLVI
NSSDIILIEAKSKVSEDDVNEHLERLSKFKRFFPRYESYRVLGAVAGMVIPLDVSRYAYR
KGLFVIGQSGDNLVILNDDKFRPRGW
>OBU77043.1
MSNFPTVKAKEFIKVIEKLGFYLDRQKGSHAIYKNINGSRVVVPIHSGQDIKQGTLMGMI
QDIGIDKEMFFELLQK
>OBU77047.1
MDTFLDGKAGNARLDFNLQGAFTRLLCCNQLQDSSQLQVTHSNFDYSQHITDFVDSNTAD
ATAYMVIPPSIENTSCEAIVYPDRSPGDYLAGITTMQPIEDIFSLAVGQNSSIIKFTRSD
NKLPSRIVTGLRLYQKGLVAECSLGVIHSKRPGKHFHWMLVSGLYETYVLFTAITEIYGE
LPSNLSLCLTLYGENSSESVQTNYGIELKSVGFITMF
>OBU77048.1
MAIIKQTDNFSANFGEVLRSSSIFWITQNSTTKTTICLSNYWRFKNNLDVTILVSWRAMS
GELLKRHKVEFVNTAVLNLAPPFDEFEGSCEIETFCLNNLRIPYSAIMAVYETKDSISMV
HSYTRTYSHHEIEEGRTITDGHESCWTIYGGGAKFRALLLVTTVIMLCNHIIANSGSGVM
MVEK
>OBU77052.1
MATTADEVWKLLGELIESQKETERKFQETERFLREQSQETDRKFQETERLLREQSQETDR
KFQETERLLREQSQETDRLLREESKRVNNQIGQLGNRLGEFVESQVRPAAVKLFQERGIA
VKEIASNTYIQTGKEGLEIDLLVINSSDIILIEAKSKVSEDDVNEHLERLSKFKRFFPRY
ESYRVLGAVAGMVIPLDVSRYAYRKGLFVIGQSGDNLVILNDDKFRPRGW
>OBU77056.1
MATTADEVWKLLGELIESQKETERKFQETERLLREQSQETDRKFQETERLLREQSQETDR
KFQETERLLREQSQETDRKFQETDRLLREESKRVNNQIGQLGNRLGEFVESQVRPAAVKL
FQERGIAVKEIASNTYIQTGKEGLEIDLLVINSSDIILIEAKSKVSEDDVNEHLERLSKF
KRFFPRYESYRVLGAVAGMVIPLDVSRYAYRKGLFVIGQSGDNLVILNDDKFRPRGW
>OBU77061.1
MNNNSKQIYNYTVILEKEEQRSKFRKLLTVINLWEIEAETVFEQKSRVVGATSNAVLADG
FEGKARPKS
>OBU77066.1
MFRLKIPNQEVKVALGDQLVNAYTDFVEEKLGIQRPLYENLFQGYVSRFIDTVRSLFASI
PWRNFTNNNLANFEGYYASVLYAFLSSLNARIIPEDITNYGQADITAILGNHIYVIEIKV
VDGENVKENLALKQIRECNYAQKYRGEPGKTVHEVGLVFSRSKRNLIQADWE
>OBU77067.1
MNPKNLPLGINTLSMLRENNCVYVDKTEIAHGLIRIPGRFFLSRPRRFGKSLFIDTLKEI
FEGNQKLFEGLYIHDQWDWSRKFPLSVREWSMATTGLRQETPPS
>OBU77100.1
MANYHLPIFCEIIVPASEVVKVMSSVPLLRLSDKFDPYISGEVNIDPSAVIAPGVILQAG
LNSKIIIGPGVCIGMGSILQVSHGILEIEMGANLGAGFLMVGEGKIGANACIGAGTTVFN
DSVAAQQVIPAGSILGDSSRQQSTSPPSPTPKSQSPPETESSSTESQETSDGKPHSRDPT
EPHPLGTQIYGQGSINRLLSTLFPHRQSLSDQDANNGEE
>OBU77142.1
MRNVGSIETEIFDSPDTLEKLCLMSGGHVRNLLLLTQDAIGRTQDLPISERAVRRAITQA

RDTYRRAVENHQWYLLAEVSISKRILNDDQYRNLMFNRCLLEYRYLDKEGEIQRWYDIHP
LIQGIQEFREALATLL
>OBU77205.1
MPYSHKSTKKQRQYNQDLTAKRFASTVPAQTLSWLSSISLLSGGFVFAQTESPVDNIVST
VEVSQPTTLGDKGQNGQVSLPVESKVTELKTGVQTQGKNSEVNSNLESVATEGNSESRSE
SSPTSVILKVPALPVLKSQPETLPTSTQPIPETSTLVIPVTSQPNNSDNGNTGKDYNSTQ
IDPTEYNGNSTVKYDAPSSVEVTGRTQDCKAVISQPGKSVGTCGGKNPTNPSVANSTRKS
APTWLKKSGAVNLGKSQLKTVPNENALSTNNSSPRSQGQKSEEVVNSLAATVNNSQNWHS
LGSNSSSRHTSAGGYMGRISTKTSYNRPRDPREFSSNTTVTPITPSFGTLPPPMIEGKVA
PRPSKVAYDFDLASVLPQVPYISSFADNGTNSGSVSGITYPLSFAAPITSLFGWRTHPIT
GDRRFHAGMDIAAPTGTPILAAEKGQVEMADWMGGYGLAVTINHDQRQQTLYGHMSEILV
RPGQWVEPGMIIGRVGSTGNSTGPHLHFEVRHLTANGWVAVDPSMQLQAGMNSLYNRVAY
NYNSQSKQLR
>OBU77212.1
MGRFYRTVLIINHGNAELNASSLGMAKAALEAINGLNLFGVYGSQASVIHVLPDEIARGR
ITLKSLLPRESASKEVDAALLSIISYPAFAVEDPGLCDRTFQKIINELSGNYVCKRFCVK
PNARMGLFPRSIL
>OBU77215.1
MKFTPVKFCLTFLWLTLGVGLGSFDPNNPNNIVVAQLPPEPPEIFPLPDNQPVPVFKSEF
GVRIVDSQGKVNFFPTTRVPLRRGDIYGWRITLENYRGQVKWREVLRLPKAPETWITQDK
KHNFSLGADGTTAITRRTQMAKDGVIENYWQISPGDPLGEHKIEVYVDDSLITTFQFETV
EF
>OBU77216.1
MATNTADTGKQKALSMVLNQIERSFGKGAIMRLGDATRMRVETISTGALTLDLALGGGLP
KGRVIEIYGPESSGKTTVALHAIAEVQRNGGIAAFVDAEHALDPTYAGALGVDIENLLVS
QPDTGEAGLEIVDQLVRSAAVDIVVIDSVAALVPRAEIEGDMGDAHVGLQARLMSQALRK
ITGNIGKSGCTVIFINQLRQKIGVTYGSPETTTGGNALKFYASVRLDIRRIQTLKKGSDE
FGNRVKVKVAKNKVAPPFRVAEFDIIFGKGVSTLGCLVDLAEETGVLIRKGAWYSHNGEN
ISQGRDNAIKYLEEKSDFAEKIKEQVRAKLDQGAVVSANSVAKSTEEEVEEEMEGEE
>OBU77234.1
MAILAVGAGQTYTTIQAAINAANNDDIIVVRPGIYQEDLTINKSVTLIGPYGTFEGIDGF
ENRLGVKPLDPDINAALGVGGLPTANEDFRRYQDDNGTIDNAFGNTQEAWIKGTITVTED
NVTIDGFRLRNENGPLKWNDTPDNFKLLNNYLTGYTANNSPSFGDASINNPTGVVTGWQI
AGNYIGGLLGGGGTGGSIYLAGLQDSNIDDNTFWRPRAAHLYLASLTNVTIEDNKFYHGL
HTGGANFDGFGEFFSGSGYGYGGYGDGYGGYGDGFFGRNYWLELKGDNDQVLIKNNEGEY
NSGGIQLFGETDSPFAFDNITIEGNTFPDNNFINAYSEAPSNGKSGLIPAVMATARLSGP
SGSNLVIRDNDITMDLAQVKFITDHKSSLEVRGNFNGVTVEGNTLTPKNINGGVDIITGL
SLYGSLPGETLIRGNQLLGQDGDPLEASYYGIDLIPTFADYGTYTGDLTVEDNTINSWQV
GVNLRNTNEITGDININGNTFENNAYGVVLDATATTNSINIAGNTFSNNFSNVFDGIDPV
ITMDQVLSYEENQDLGSVLGTVSATDNLPNTDNVGVTQYFISSGNDDGFFTINSSGEITL
TEAGLAAANDFETSLSSFNLGIIVTDGGGLQDTETITLSIINVNEGLGQLPPITTEGAGF
TVGATLIAAIPFDDPDGIPTDISYQWQRLIEGVWTNIPDATEQNYVATEDDNNNQLRVEV
TYIAGGFEKVIYSNNVSISLVLVSGTFDSITGDNNLDISEKEAGVTLTGSVSEIGTTVTI
LFGGQTRVAQVDGLSWSYVLKPNDYNFFAAGSNLFTAIFTRTDGGETGSFTTFQTLTIPD
GILPPNTSNAFDPTQPKGLKSEVIDAAQTLEIDGVSILEISKTVGILGEGESFNDPNIAV
LPIGTRDIFDQGANAGAANYAAFKTEPGTSIQSIYIVPAVEEEGVKKLQVVLADGTVVEA
EIPPDLISPIGDPLAVTISGVQPGGTTTFVLYLSQNVINQLPEDLNLARYVKFNYESQQF
ELYDDFNYDYIFNDVDGDGVRDFGEVYLTVNLTDGDIWDGDGLANGIIVDPGQLGIATDS
GTDNNPPIAIELRGIVAENDPGAFIGSLTVTDDPGDSHTFTVNDSRFEVINVDGNNILKL
REGESLDYEAASNIVLTITAIDNGGLEITQDFTITVTDVNEAPVAIELNQITVIENDPGA
IIGTLTVSDPDGNDGHTLKVNNDTRFEIVDFDGNQTLKLKAGESLDYEAGSVKLSITATD
NGGLEVTQDLTVSITDVNEPPVVSFSFFVPESTTLVSNLTVEDPENDPITLSLAGVDASL

FSISPTGELTFNTAPDFEEPLNADKNNLYKLQVVARDEQNNKSIQDISILVTNVNEAPIA
IDDVLAIIPGSSFGTLNPLDNDSDPDLNDPLTIINKTDGNYGRVEIRDNELIYTLLDATY
IGDDVFSYTIIDEQGLAATANVNVTITGTDIITYPVEILDPEDSLIPDEAGPLSDIVNDI
SFNFLTDYDKVQAKLALQEALSKTEASFTNLFGLYEVDNALTGSVNGVLPEDKSAYAKAA
LSRVVPNFVVRAGGSGDGVNGDVIVSEGKIYAPFVIAHGGNFSGSVQDAVNAFFHVNPDN
SPATAQNYTTLPVAYFSFGSANPDGAAHIKSFGNNVFGFEDLPAGVGVSDYDFNDTVFSF
G
>OBU77256.1
MPKQFNTAGPCKANIHYMLSPTGRLPQLKALIDGENYFIIHAPRQVGKTTAMMALAQELT
DSGQYLAMLLTVETGAPFPDAPEQAQQSILRRWQNEIRFRKLPLPTLTQIQRETETSPRL
VNALARQATQVLVQDVNEPITAEVINQAKEMLIQRQDTHLDSLAERLREERVKTIIEPIL
AGEDLPDVPPDDIRYVLDLGLCRDQGQGLEIANPIYKEVLPLVLSYTTRVSIGAIEPLRL
NEQGELLPDKLLHAFLEFWRQHGEPLLKSAPYHEIAPHLVLMAFLHRVVNGGGTLEREYA
IGSGRMDICLRYGKVVMGMELKVWRERKSDPLIKGLTQLDKYLDGLGLDTGWLVIFDRRP
GLPPMGERISTEEVISPNGRTITLIRS
>OBU77263.1
MPKHFNTAGPCQSDIHYMLSPTGRLPQLKALIDGRNYFIIHAPRQVGKTTAMIALAQELT
DSGEYTAVMLSVEVGSVFPDEPERAERAILGSWQEDFCLDLQL
>OBU77287.1
MTTPKLTSYEFKNLPSLGTTTTNQNILLGGFSGLYFQGVANNGNLKFVTHTDRGPNGEPT
GQNRPFLLPNFQPEIVSFELDKASGEINITKRTKLFRADGKTPLTGLPNVQAGVGNTAYT
DEIGVDLNGTVLTNDPLGADLEGIVVTDNGDYWMVDEYRPAIYHFNGNGILVSRFIPQGT
ASKSNQPVGTFGTEVLPEVYAQRRNNRGFEAVALAGNKLYAFIQTPIDNPDSTGDTTSRG
SRNLRILEFDIVAQKVTGEYLYLLDDITGTGNAKTDKIGDAVFLGGSRFAVVERDDRSDT
TSNKLIYQIDLTGATNINDTSKLTLPQGKTIEQLTPVELGPAGIIPVNKSLIVNAAQIGY
TGVEKLEGLALVSANTLAIVNDNDFITTIPEKLGIIELPNNLVEVTGTANKDELFVKKGE
YVLGLDGEDILDASDGLGNNVLDGGAGNDQLYAGRSDTLIGGAGNDQLYVVEGNNNTLYG
GAGNDQLYVVEGNNNTLYGEDGDDNLYIIEGGNNTLSGGGGADKFYIVNGAIPAAPSQVL
DFTRGNDKVIISGISQIKSFDNLILDQNGDDTIIKTKDLIDGSQKILGILKAVLANTLTA
DDFRIDDNRAPSFVTTTATFSTSENTTAVGTITPATDPDAGDVLSYTLGGADASKFDFNP
TTRVLSFKTAPNFESPGSAAGTNTYTLVVTATDSKNAIATQNITVNVTDVDDTAPVVNAN
QTFSYAEGKTANFQVGTVTATDAVGVTSFAIASGNNSGFFAISNSGVITLTAAGAATSAA
SNDFETNPNTFTLGITASDAANNTSSPVNVTINVNDVDDNQTPNVSGTFNIIATDGILTI
EEKNAGVTLSGRVSDQKAQVTVQFGGQIRVANVIGTNWSYKLTSNDYNFFKEGSNQLTAI
FTLNNVTPQFKSSQTLTIGTGVLPSLGSPTLDPTQPKGISAEVTQKAQSANIRQGILGTQ
EKLTDPNIVVIPFGAIDTFNKGANAAIGDFGLIKTGANTTIESVRVLPKNTTGEVSVTLN
DGTVVLASIPPGISSIADPLGLIMSGVLLGGSSTLDFYLPKNILNQLPNDLQSATYAKFN
FEAKKFERYADEQGKPLYQYKFTDKNSNGIRDFGELYLSLNLTDGDKWDGDRIQNGIIVD
PGQLGIAVAVTGTGVTYSSTTVNPGNSLIPREAPPLSNIADNVFYNFPSTYRPAEARQAL
QNALGQTDAAFKNIFGLYEVDNATGAVNGIAPGQSGYAKAALDKSKVISNFTVRAGGSVT
PNINGDLIGIGGKTYAPFVIANGGNYSGSIQEAINEFFKVNPNNSAATAQNYMGLPVAYF
SFGAANPDGAAHIKSFGNNIFGFEDLPAGVGVSDYDFNDMVFSFG
>OBU77379.1
MSADSINTALAELENLINSCENDLIRFIEARKMNSGNEISTKKVNRQLQQNPIAIVGMAS
LLPQSRNLRQYWQNIVSKADCITDVPESHWSVKDYYDPNPRTPEDKTYCKRGGFIPEVDF
NPMEFGIPPSILEVTDVSQLLSLVVAKEAMEDAGYGEAREFNRENVGVILGVAMAKQLGM
PLSARLEYPVWEKVLISSGLSPEDTQKIVEKIKSAYIKWDENAFPGMLANVVAGRIANRL
NFGGMNCVVDAACASSFGALKMAISELVEYRSDMMLTGGVDTDNTIMAYISFSKTPAVSP
GENVKPFDAKSDGMMLGEGIAMLVLKRLEDAQKDGDRIYAVIKGIGTSSDGRYKSIYAPR
KEGQVKALERAYNDAGFSPTTLGLMEAHGTGTMAGDPTEFASLQSFFSKHDERKQYIALG
SVKSQIGHTKAAAGAASLVKTALALYHKILPPTINITEPNPKLDIENSCFYLNTETRPWI
RGESESPRRAGVSSFGFGGTNYHLVLEEYQQEQQQPYRLHDVASQILFSAPNPSELIKNL

ETSLENLQAGDGNRYYSQLVEECRNIQIPQNAARIGFVAGNKADACKLLALSIDLLKNKQ
STLNWEHPQGIYYRASGIKLRGKVVALFSGQGSQYLEMGREAVMNFPALRRLYGLMDSLL
IEDNLQPISQVVFPHPTFNQTEKADQIATLQRTEYAQPAIGVFSAGLYSIFQQAGFKSDF
TAGHSFGELTALLAAGVLSESDYLYLVKARGKAMAAPKDPDHDAGSMLAVKEEISKVELV
LKNFPKITIANFNSPSQVVLAGPSHEIQKIHQKFQDLGYGAVLLPVAAAFHTPLIAFAQK
SFAIATKSVKLLNPKIPVFSNVTAQQYPQESDKIQRILESHLASSVNFTQQIENIYAAGG
YCFVEFGPKRILTNLVKDILGERPHITISLNPSTHKSSDISLREAAVQLQVLGMELGNID
PYQLPEIFSKEISQKSLNVKLKGINYVSEKTKNAFEKALNDGFKIQGVPQIQPVSVIDKP
SELIPEKEVVEIVHTTNGNGNRDQQTSIISSLSTDSQMNSANPINSSLRAEEQLPILTYP
MVQVLGGKMTISDKNTEFQQVLASLENLLSKFQNSQSDNLQIHDTYLQHQIEYAKTFFQL
VQQQNTLFMNNKSPETGETKKSIMDSFERSMMQFHHQQAETLRIHEQYLREQLEHTKNFF
HLIQQEYSLLIDGAEELTPVIPLQLTDIHTENLREISPQNSQLTVSEMVVSSTSKTLEVE
IPQPTIIETQPVITPIPETRVEQPPVPVTEIAISSQTKKSAIDIQDIGKSLLSITSEKTG
YPIEMLEFDMDMEADLGIDSIKRVEILGGLQELYPDLPKPNLEELAEKRTIGQIVEYLEK
QVLTNPTTNQVNHSEHIVKNVDPKVTSELIIPSPVDIPHSPPTSDEYSSIAETLLKITSE
KTGYPVEMLELDMDMEADLGIDSIKRVEILGGMQEVYPDLPKPNLEELGDLRTIRQIVNY
LQSLVVVEKKNLDFEQIKATVVDLTPPPTIDPNLPRRPVKLKILPEPDFWECQLPGGHIG
LITDDGSLTTTKLVHGLIDKGWKVVVLSFPQSIVPERSPLPVGVTRINLANMSEEHLQLL
LQSISVQYGKIGAFIHLHPYFSTTNLAYLEAEKVIVKHIFLIAKHLKQSLNNAANLEGRV
SFCTVVHLDGTFGLDHTENFGIIGGGLFGLTKSLRWEWPKVFLKAIDLSPKLEPHQSAEY
IVAELYDSNRYIGEVGYGSKGRVTLVATAD
>OBU77498.1
MPKPYSIDLRNRVIVAWVAQEGSQRQLAERFKVSLSFVRNLVRRYRETGQVEPKQCGGYE
KPIKGSLCRAREIVN
>OBU77511.1
MALIVQKYGGTSVGSVERIQAVARRIHGTAQVGNSVVVVVSAMGKTTDGLVKLAHEISPS
PTRREMDMLLSTGEQVTIALLSMALQEIGQPAISLTGAQVGIVTEAEHTRARILHIETER
LISHLNAGQVVVVAGFQGISNTSAMEITTLGRGGSDTSAVALAAALKADFCEIYTDVPGI
LTTDPRLVPEAQLMKEITCDEMLELASLGAKVLHPRAVEIAKNYGVPLVVRSSWTDQPGT
WVTSNKVQERAMVNLELARPVDAVEFDVDQAKVSLLRVPDQPGVAARLFNEIADQQVDVD
LIIQSIYEGNSNDIAFTVNTLILKRAEAVASAIAPALRNNDGSDEDEVEVLVERNTAKVS
ISGAGMIGRPGVAAKMFATLAKAGVNIQMISTSEVKVSCLVAAVDCDRAILSLCQEFEVN
ASTGNVSSSNSIYSTVCGVALDMNQSRLAIRHVPDQPGIAGKLFGLLAESNISVDMIIQS
QRCRVIDGVACRDIAFTTNRTDGENAQAKINQVAAQLGWGEVILDDAIAKVSVVGSGMVG
QPGVAAKMFTALAQNQINIQMITTSEIKISCVVSEKEGVKALQIIHTAFGLAGTHKFVVP
A
>OBU77514.1
MVSKNFWKPIIHSSTALILLTTLNTAWPLVSLAQSKPQPKVNSASSSLFTDYLLGGGDRI
RVNVFEAPEYTGEYQIPPGGEINMPLIGSIPVSGLTTQQAADEIARRYARFLKRPLISVN
LLAPRPINVFVAGEVTRPGSYSLSLQGTGGNNPGVQYPTVLAALTTAEGVTLAADVTKVQ
LRRQVGRSGEQVISLDLKQITQTGRIPIDITLRDGDTIFVPTATDFNVAEARNLFAASYA
ASRTAPRRVAITGQVYRPGSYLVAAGGGNDSGGLPTVMRAIQLSGGITSQADVRNIKVRR
PTRTDKEQTLNINLWELLQSGDLNQDVVVQDGDTIIVPTATQVNTAEVTQLATTTLSPAT
IKVGVVGEVKRPGVTELQPNSSLNQALLAAGGFNDARASSSSVDLVRLNPNGTVTKRAVK
VDLSKGINEETNPILRNNDVIVVNRSVLARTGDTLGAVTAPLAPVFSIISLFRLLGF
>OBU77608.1
MQEKGVKWVKTLAPNYIEMYRKQKQQDTTPEDFELSLEGKIAPDNRWVILAKIIPWSQFE
AEYAAIFSEGKGAPAKTFRMA
>OBU77618.1
MTGKGQGFGFGLGKVKEIADAFKKAQEMQQGAKRLQEELEQMEILGESGGGLVKVIISGN
QEPKRVEISPNALNEGPDVLSDLVTAAMKDAYLKSTQTMRERMEELTGGLELPGF
>OBU77647.1

MTESLLNIQNLCVAYPQDYDQTPIWAVNDVSFSLKPGEKIGLVGESGCGKSTIGRAIMRL
LPDHSRVQGQVNFRESSVLNLTPAQMRQFRGEAVALVFQDPMTRLDPLMTISEHCLETLA
AHSPQLTKKQAKERVLATLEKVKIPGSRWSQYPHEFSGGMRQRVAIALALLLNPKLIIAD
EPTTSLDVTVSAQILQELTKLCAEDNMGLLLISHDLAMVAEYCDRIGVMYQGKIVEMGKT
ESVFKQPQHEYTQSLLRAALHIQQEPVGVGEEEKKENPILKITELQQYYSIEPNFLERLF
QSQQQTIKAVDGINLELYPGEILGLVGESGCGKSTLSRTILQLIPPTGGKVEFLGQELTR
LSREEVRSFRREIQMIFQDPHACLNPAMTVGESIADPLLIHKIARGKQAEEEVVWMLEKV
GLTPWQTYYRRYPADLSGGQQQRVAIARALITRPKLVICDEPVSMLDASVQTQVLDLMLQ
LKAEFDLTYLFITHDLWLARFLCDRIAVMNSGKIVELGKTKEIFSHPQHPYTQTLLGAAP
LLARV
>OBU77661.1
MIEAEIKALIQKELPRAIAEEPGVRDFVLRTVSEYYTPRTEFDEKFDRVLNELQRDREEQ
ARKWDEQNRKFDAFQAEQAQKWDEQNRKFDAFQAEQAQKWDEQNRKFDAFQAEQAQKWDE
QNRKWDEQNRKWDEQNRKWEENTQRLDRIEAQNSATLEEIQKANRRYESAIGAIGSRWGL
YSEASFRNGLKAILGQSFGVEVLNLTLYDQEGEVFGRPEQVELDIIIKNGLTIVCELKSS
IDKAGMYVFGRKAEFYAKNQNRVVDRKIVISPMVDERAIPVAKSLGIETYSYADMVVS
>OBU77662.1
MIEAEIKALIQKELPRAIAEEPGVRDFVLRTVSEYYTPRTEFDEKFDRVLNELQRDREEQ
ARKWDEQNRKFDAFQAEQAQKWDEQNRKFDAFQAEQAQKWDEQNRKFDAFQAEQAQKWDE
QNRKWEENTQRLDRIEAQNSATLEEIQKANRRYESAIGAIGSRWGLYSEASFRNGLKAIL
GQSFGVEVLNLTLYDQEGEVFGRPEQVELDIIIKNGLTIVCELKSSIDKAGMYVFGRKAE
FYAKNQNRVVDRKIVISPMVDERAIPVAKSLGIETYSYADMVVS
>OBU77674.1
MEDSQMTISIGDTAKELGVSVKTVRRWADSGKLRFERSPSGHRRFYLADIKRITPRDFNQ
LEDRVTINYARVSSSDQKEELTRQIQVLEAFSGANGWQFETISDLGSGLNYNKKGLQKLL
KRIMQGDVGRLVLTHKDRLLRFGSELVFAMCEEYETEVVIINKSTRHLRK
>OBU77675.1
MPRDRPLFSSPTLKDRPENIGGPFDSQRRFQNDLRIDNLRLLSAADYNKDGFQDMYFKVA
DGTAVLRALMHADGNIQYANYQSKADLTAFMTANNVSPSIWSGWI
>OBU77711.1
MNQILPLSINQKEIYVDQIMSPNSCHMHIGATVTVRGIFDREILNYAMSKTIDCHPGLKT
RIYEVDGEPFQTIASHTSNHIPFIDFSGHDNSDEQAENYINQEFIKPLTFGENATLADFQ
LIKVCDDKHIVYAKYHHVITDGWGAAIFFREVIKTYNQILQEGREDQETRDWVITEYIEE
ETKYLASDIFMRDRHYWQQRLNNLSPMIFHPIKQPQELDGKRHSIYIPRHQYDQVDELCK
NVKSNVFHFILSLIAIYVSKHYLKNDVVVGLSLLNRSKNIFKDAIGMFVSTIPFRLEVEQ
ENTIHQLLDKIRYSLRQDYRHQKFPLAEMKQLSGLKATSKQHLFEVFLSYERHDYADSLP
NTKTTCVPLYSQQQKVPLIIYVREYEKTDDVKIDFDYNLSYLDGEAVGEIVRSFETLFTQ
AATNLEISIGDLAICDSETINISQPDSPSTKFFADDTETLVSAFEKVVSQYPQNLAVQFD
GELYKKFLSYTELNDQANRLANYLISQGVKPGSRVGICLERSEQIIVAILAIIKTGSAYV
PIDPHAPSVRRQFIVQDSGMTTLITETSLIAELVTENISTLTIESINLALAKQANTLPRI
SIKPDFPAYIIYTSGSTGTPKGCVVTHKNAIRLMRATEPWLGFNEKDIWTLFHSFAFDFS
VWELWGALLYGGKVIVVPFWLSRNPEKFREFLSTEKVTVLNQTPSAFYQLIHADESSVGD
IYLRYIIFGGEALNIQSLQPWLERYGDKKPYLINMYGITETTVHVTYRPITRQDLKVRRS
FIGKEIPDLHIYLLDEKLSPVADGIPGEIYVGGAGVTNGYLNRPALTAERFLPNPFGSGR
MYRSGDLAKRLPNGDLEYLGRIDQQVKIRGFRIELGEIQAALISHYQVREAVVITDEWEE
EKRLVAYYVPDESSPTAHELRQYLKSKLPDYMIPAAYVKLEVLPLNVNGKVDIKALPMPD
WNLLRVEEDYIAPRNLDEETLCTIMAEILGLQKVGIDDNFFEIGGDSILALQVVARAKKE
GFLISAGELYELATVRYLATKKAIITSGDGVKKLPNIGSALVCDADKLLLPQDVADAYPL
SSLQSGMLYHSELDPDSAIFHQIFTFDLQIGYSELAWKQAIADICSANPVLRTSFHWTGY
SQPLQIVHEQVELPLNVVDLRGCDNANEQIGKWIELEKNHNFDITQAPIFRLQIHRVSDL
KLSFSFSFHHVILDGWSVATLLTQLLRRYVEYLATENLPPLPVTQISYKDFIAQEQNVIT
NHTAREFWLQHLRNLQVTFLPRLSTNVAKTTATSHQKRQLKRLSVTIDTKLAEKLRQITK

NLGVPLKTSLLALHLRVISFITGQKQVVTGNVINARPETSGSENLLGLFVNTIPFRLELP
QGNWLDLVRAVFRLEREILPHRTFPLAEIQRVVDQRPLFDLGFNYVHFHVYEGLLNLPQI
QVENVDIFEETDFPFLTEFCLVPGSGALQLNLIYDTQQFADTQVEQYANYYQAAMFDMVT
NPQTPYHRRSLISSQERQQLIQSANQNQKDFVSSQTLVSAFNQTVAKHANKTALVYQQTS
LSFGELEIQANRLAHYLQAKGIGPETLVGVCLERSEQLIISILAVLKAGGAYVPIDPAYP
SDRLEFLFQDSGIVLLITQRKVISQLPECGAEIIILEDIAKQIEGNNSQTPAVNILPENA
AYVIYTSGSTGKPKGCIVTHANVIRLFNSTTTWFNFHSEDIWTLFHSYAFDFSVWEMWGG
LLYGGQVVVVPHWTVRSPKEFLQMLATHRVTVLNQTPSAFKQLISVVRQKPEKLSLRYVI
FGGEALELADLQPWIDLYGHTQPELINMYGITETTVHVTYRPITQKDIINNLNHRPSVIG
QSIPDLELYILDENLDPTPTGVTGEIYIGGAGVTRGYLQQPGLTAQRFIPHAHRPGSRLY
RSGDLARYLPDGEIQYLGRADQQIKIRGFRIELEEIQLVITSHPDVKQALVVCQKSPTGE
NRIVAYVIFSGVAQPQNDLGKFLKTKLPDYMVPSVFVPIETIPLTINGKVDYAALPVHNW
NSIKKDYIAPRNDREATICSLMASLLKLERVGVDDDFFEIGGDSLLVTQLAISLRQTYDT
EFPLPELFTHRTPGEIALLVGDESPALPEIEIPKASRTRRSVTLTDDGILSKY
>OBU77715.1
MEVYVLPLSVSQKQFWYREIMFTGNTAYHIPIGLNLLGDLDFTLLEKCFRYIINRHEILR
TTFALENGEPVQLVRSEQQFHLQHRNLELPPEKWSDYDRLLTIKKILEDESRQLFDLIKG
PLLRATLYKISNKEHILLVNLHHIISDGWSLGIFIQELTQLYVSRAEISLPELPIQYGDY
AEWQETYLQTQRIQDQLAYWQKKLALPLPILDLPSDKNRPALQTFSGAVLRKTLPRDLID
SLEAVAAKEGVTFFMVTLAVYQILLFRYSGQTDIIIGTPCANRNRGELKNLIGCFINTLP
IVCSLTGQLSFKQVLQQVASTCVEAFANQEVPLEIIIDKLNITRDPAHSQVFQSLFALQN
APIEEIKLPGLTVQPVYLDNGGAKFDLILMLEPTFEKGWIAALEYNTDLFTAHTAEDILR
HYEQLLSAVIGNLDTKIDTLPWLNTEERRELLSFGSSASDQEVEATNLVDVFSHIVNTYG
EKVALIESERTLTYQKLNQISNQLASHLIEKGVGAETRVGIFQERSLELVASILAVLKCG
ATYVPLDPQYPAERLNFIAQDSGIRLVITTEPLRAKIPGEIPELLLIDTMKPTNQLVHPI
SKIFPEQAAYIIYTSGSTGKPKGCLVTHKNVLRLMRNTQKWFEFNEKDVWTMFHSFAFDF
SVWEMWGALLYGGKLVIVPYLESRSPHDFRQLLATQQVTILNQTPSAFRQLIRADQEFTH
PLNNLRAIIFGGEALELQSLKPWIERYGDSHPRLINMYGITETTVHVTYRQILAEDILEN
RGSVIGIPIPDLCLYILDSYFEPVPYGVAGEIYVGGMGVSRGYLNRPSLTAERFIPNPIS
QKPGARLYRTGDLARRLRNGDIEYLGRCDSQVKIRGFRIELAEIEAAINKLTQVSESVVT
VHSTSDQDRRLVAYIVLRSINNNEIDRNELRKALKESLPDYMVPAAFVFLDAIPLTNQGK
INRGALPLPDWDQSTTKRSFIPPETDAQKALCHIWQRVLGIEEIGIEDNFFDLGGDSILA
LRVITEMRSQGWILTPKQIFEQQTVQRLALAIQEQTDLQSFAAAENNNIPTGEVPLSPIQ
QWFFDLKLSNPHHWNQTLLLQVDPSLQVTQVATAIKVVFAYHDNFRLRFQKESQGWRQFY
VEKDQDDGFPWEVVDLGLKSEIEQNSIMKEVREKSEKSLNLTEGPVYMVKWFNLGANRPS
QLLIVIHHLIIDGVSWRILLQDLVDVIGGNKLSFKTTSWQKWCEFIHGYVNSYNIQSEKL
FWQNTLARKTAKLPLDFPESLAENLEYSVSTVSCQLTAKETLTLLTTANKTYRTNPQELL
IAALGKTLADITQNSYVQIMLEGHGREELSSDLDVTRTLGWFTTLYPVSLDLPKGDSQTE
AIREIIKSVKEQLRAVPQRGFGYGILKYLDQETKPLLASAVEISFNYLGQVRNEGGARHK
FFTLLNSQGLPTRDPQATRPHIIDVNAIVVEGELRVDWLYSSNLHQASTINRWVSDFKSN
LLKLLNFCTEVGVGEYTPTDFPLAQLTQSKLDILQNQHPDLEDIYPLSPLQQGMLFHAIY
EPDQGIYFEQVTGKIIGKLDVDKFAYAWQVIVNRHPVLRTCFVWEDQGQPLQIVRKELPF
SLIYKDWRDLSPAQQTEQMGKYLVKDKQQGFNLNDSPLMRFTLICLDDSTWQWLWSHHHI
ILDGWSLPVIFKEVLSVYQSATERVPHSLLPVPPYRHYIQWLTGRDQLKAKQFWQQYLAG
ISTGTRLAWQIPDLENDSSLPPYREVELRLTESEFDLVQKMAQSQRLTLNTIIQGAWAMC
LQKHGAGEDVIFGVTVAGRPPEISEMENMVGLFINTLPMRVKIDPTLSVSDWLQNIQQHH
LEMREYEYSKLADIQKDLNLAGLPLFESLLVFENYPVDQNLKKQLPDFRVDDIQFYERTN
YPLTVGVIPDQGLLLKLNYQTKFLSGNAAEKMISRFRHIIVNMAFKPEQTLAKVPALSIS
DREELTTCNQGNTIIEGGFKTAHQLFEYYADLQPDSIAVVCDEESVTYGELENRANNLAG
QLLDTGIGYESIVGLYFDPGIEYIISLLAVLKVGAAFLPLDRIYPEYRLKFIIKNSQVSV
ILTNNIIGLPLQENLQVLDISNLESKGNHPRLNLKVRAENLAYVIYTSGSTGNPKGVLVT
HSGIQNLVQCQTMSFGVTGKSRVYQFAALNFDSAIAEIFMSLGSGAVLYIQSQSHRSPGS

ELWRKLTNWEITHVTLPPSLVASIDTQDLPGLQTLILAGEAVSGDLLRRWSRGQRRCFNA
YGPTEATVCASLMDCTHLLGEPSIGKGIPNVEIYLLDSFLEPVVPGVIGEIYIGGIGLAR
GYLQRTGLTAALFIPHPFTKTPGSRLYKTGDRAVYDPEGNIRFIGRYDNQVKINGYRIEL
GEIEAALTKHEAVESALAVLRTNLIGRNRILGYALIKPVEGREQPNDLKEHLAKILPDYM
IPGAVIVVDEWPLTPNGKIDRQRLATPDFSSTEVIPKTDIEQIFAQIWIELLGLETVNPQ
DNFFEMGGDSIISLQMVTRARAAGWEISPKDIFEGQTLSRIATRAKLMETQTEIVEPLTG
FIPLSPIQNWFFAQNFLHPHHWNQSVALTCKQPVNTDALVVALHTLVSHHDIFRIGFAEN
EGEWQQFYVGEAKSPNVKIIDFSHYLPTTHLAFLNSALETEHSSFKLDRPPLIRILCGRN
LNNYGDVLFIFAHHLIIDAVSWRILLEDLNQAYQQTIDQLDQLQQPNSPLSLPVKTASYR
SWTTHLHSLASSKMTQDTPFWKDILNAVITPLPVDKSGNNSVDSTAIISTYLTAEQTVTL
IKQATVTYHASVQEIMLAALLNTLISIYKSDQWLIDLEGHGREDIGHNLDLSRTVGWFTC
LYPILLKQPTNPDNHEILLKEIKNQLRTIPHHGISFGLLRYLNLNQPLKESKNADISFNY
LGSLDSPLGDNHNFSVSDAPVGAGVFALQQRTHILAINAKIQDKILQVEWSYSRNIHYDQ
TIENIAKTYLQSLGLYLTNSDSANSSFYSASDFQLVDLSESELGELLEDLE
>OBU77716.1
MAGLKDKIEDIFPLTPLQKGLLFHTIYEPESGVYFEQLHCRLEGDVSTVAVRQAWQTLVD
RHAILRTAIVTKGQSEPVQVVFRHLKFNIEEEDWRGLSDDVQKHRLQDFLEADKRKGFVL
NRPPLMRVTLIRFREDVWYLVWSHHHIILDGWSWPILLKEFLTLHKAAKENIEVALPNRR
PYGDFIAWLKRKDLQESKIFWQQYMSGFESDTPLLMTSKSRVNPKFKGGEITKQLSPEIT
DLLGRLARNCSVTLNTVIQGAWAILLNRYSRSNDVVYGITVAGRPPEIPGVEQMIGPFIN
TLPFRVAISGDQTLNTWLQNLQFQAASMRQFEHTSLSDIRAWSDIPAGSEMFETLLAFEN
FPVDKLLKISDFGLNVAESHFLETTHYPITLVVIPSDTIAIKLSYNTNRFSDTTMKLLLD
QFSNLLLNMANHPQSLLQDISLLSHQEKQQIKNKREQTNSSYTTINEMFAETASKYPERI
ALTYENQTLTYRELEERSNAIARHLKSLGIGSEKRVVICLDRTPELIISMLGVVKAGGIY
VPVDPSYPRDRIEFTITDCGAEVIITTTNTSQELPNGITRVCLDVEDAAYLKTSAEPIDT
DNVTPNSGAYIIYTSGSTGKPKGVLVTHKNLTRLFKSTEHWFKFNQQDVWTFFHSFAFDF
SVWEIWGALLYGGRLVIVPYCVSRDPQTFLRLLEEQKVTVLNQTPSAFAQLLAVADSNCN
LDNLRYVIFGGEALNLGSLSPWFEKYGDNQTRLVNMYGITETTVHVTYRPISKQDIDNLS
GSLIGEPIPDLDIYILDADGNLLPTGVIGEMYIGGAGVAHSYLNRPELTAERFIEARFRP
NCRLYRTGDLGRFLPNGDLEYLGRIDYQVKIRGFRIEIGEIENVLAEISDVHENIVLVTT
DIETGENRLIAYLVCSPEKQPTIKDIRDHLQRSLPDYMIPSQVVYLDKFPLTANGKIDRK
SLPIPEMKRENIGIEFAAPETEIERKLASIWQQVLGVERVGRFDNYFVLGGDSIRSIRVC
SLAQAAGFNLKIEHIFTHPILADLATDLETTLQPLSGGKKQDYYQEPFGLIATEDREKLT
NIADDAYPLAQLQAGMWFHTEYSQTSTTYQDLFSFRIRIPFNLQVWEQAYSQMFTRHPVL
RTAFFLGEFSQPLQAIIKDVSGRVIFKNLTNLSPSAQDDYLRVFIEGEQQSRFNYKNPPL
IRFFIHQLSEDVVEATLVFHHAIMDGWSLAQFLTDLTGLYLHLMGRGVPSLSPQTGLEYS
QFIALEQEALQDKKQREFWQQQLEDIPLTKLPRLPRKNLPKMHPTIGKVDITLSQTTSQG
LKDVAHQLGVPLRTVLLAIHLHILSVFSGDEEIVTGLVSNGRPDTTDGDQVLGLFLNTLP
LRLRLPHGSWVDLIKATWLAEQALVANRRFPLPEVQRLNGGLPLYETSLNFIHFHVYQGL
LNWREVELLKSTSFEETNIPFAVTWNQEVASANISLNIAYSHAEFTQDQVDNIANYYQLC
SELVSENCVGNREFSLAPQEFIYEKQLSTSRPTITPVHQIVAQQAAVRPKEIAVICEGEG
WTYEQLNQKANQLAHFLQSQGITQEKPVGICLERSLDMVCAMLGVMKAGGCYVPIDPHYP
SARIQSMLEDGNLDLLLTRSDLKVNYHQNSQKTIFVDTQHREIREQSKANLNVSVFPDNI
AYIIFTSGSTGKAKGVAISHGALVSHQTWFVDKFSVTNADIVLQKTPFSFDASVWEFWTP
LMVGGKLVMAKPGGHQDPAYLVKTIQQEKVTLFQLVPSLLEVVLGDPELEHCSSLRLVFS
GGEVLKNRIWQKFQEKLPIPLINLYGPTETTIDITFHNCQENDNIANLDQIPLGQPVTNV
KLYILNSLLQPVPIGTPGKIFVSGCQVARGYWHAPGMTAERFLPDPFVPGQRMYDTGDHA
RYLPGGNIEFLGRIDQQIKVRGFRIETSEIVAALEAQSWVNSALVKVISDSQISRSDRSN
YLVAYVQPRLNTPNNWQKILRLELGQILPEYMIPNLFISIDSWPLLPNGKIDINSLPDPQ
VVETTVSQEYVPPESETEKILTKLWQQVLRLPRLGTRENFFELGGDSIIGLQIIAKARDL
GIYFTAQDLFKYSTVADLANHLRKEDHFSHLPTVEIGEIPLTPIQEWFFKQSLPHPHYWN
QAVFLDVKAQITTGDLEKAVNQIMGKHPAFKLKFCETEKGWIQELDNNSQHFNLDIVDLT

DTPETELSTHLQTWATQFQGQLNLETGKLFRVVYFQTAPTTADKILLIIHHLIVDGVSWR
VILQDLVGSMRNVTQDQSLSSFQAKPSVSFPQWSRYLRNMSHITWEVDVEFWQKQTVHDF
TLPLDFPEKIADNKENSAAQIEFTLTEAETNKMLLEISRTRKVSIQEILLTALATTLREW
TGRSEIVIALETHGRGSSLDLDISDSVGWFTSLFPFRITTVNSNVLDNLSGIKGQLNGIP
NNGLSYGILSQKLEHGEILPPIPQGIIFNYLGQFDEQIPLDAPFAPAVQDSGISRHLENQ
RTFQLEITGLIVNRKLEIRFGFSTNLHREQTIRNLLHTYQQCLRNLLMGSEEKLVRWNPE
DFPLANLNQEQLNIAVSGITDLQDIYPLAPVQQGIIFHANYEIEKDIYLQQVTGQITGDL
NVEIFKSAWENCINHYSILRTSYIWESLPRPLARVHSTVNLPFVYQDWCGTDDWEQKWSD
LLMEDRQQGFSMERPPLMRLILVKTDKAKWRFCWTHHHVLIDGWSLPLVFQHVISFYQAE
QKHQFLKWPPVPNYRDFIVWLNQKPIQQAEVFWRDQLAGLDSVTKIGLTSDPLDNGADYQ
VLQTTLQPEVYAQLKNYANKHLVTVSTLINAAWAILISKYSGSNEVIFGVTVSGRPTELS
GFEQMVGLFINTLPLRLKFQPDKPVATWLKEVSDRILAISEHSYSSLVDIQGWSNINRGE
SLFESIVVYENYPVGEKIRNHNDDLIISDVESLEKNHYPITLYALPGEDLTLKIAFQKIG
TTKERQQLLQHITDILNTLVTKSPRFLGEICLPTPSLIPIKHQPPVVTLADLFNQRVSEN
ANSPAILQGEKWLTYGELNDQANKIAQALVDLGVTQETLVGVCVDRHAGLVTALLGIIKS
GAAYLPIDPAFPINRLEWIINDSQAAIILTESSVVSRLPKSSARVLVLEAILQEEIAVSF
QPRPLDLGSIAYIIYTSGSTGKPKGVQIEHQSLVNCLLSFKNQLQLDITDTLVAVTTISF
DIAGLELFLPLISGAKLVIANQETAQDGFKLNDLLERSQATIMQATPTTWRLLLTAGWQP
KNQFCALCGGEAIPRELATVLLQLNLNLWNVYGPTETTIWSTVKQLEKSKDVISIGREIA
NTSLYILDDGLNLLPEGIVGELYIGGLGLARGYRNNPTLTSTTFIPDPFAQDPGSRLYRT
GDLARRLSNGEIEFLGRIDYQVKIRGYRIELGEIETILSSHPAITQAIVQAIGDTPAEQR
LVAYVVSKTQAPTIEDLRLYLYKYLPEYMIPSAWVFIDKIPLTGNNKINRRALPIPTYKG
ENKDWLPPRTSIEEVLVLMWQELLKVEKVGVQDNFFHLGGHSLLAGQFHGYIKKVFAIDL
ALRELFDAVTIEKIALLLIEKETKLGNTEKIAKAFLRLKSMTPEEKANLLQNSRLKKLN
>OBU77769.1
MVETEFSQVRFHGDREKAKKVYEGIKPLTAQDVADVIFFCATRPAHVNINQVILMPVDQA
SATLVNRQN
>OBU77800.1
MVRQIAPKTELRAIESNTNQGVETPKIVYPESDNKPMADNTRQFTWIVKIKENLEILFKY
NADVFVAGDLFWYPVEGSNKIKLAPDTMVVFGRPKGHRGSYRQWEEDNIPPQVVFEILSP
GNNNTEMDRKKLFYLKHGVEEYYVYDPDRISLEVSIRENNSFKEIENFTTWTSPRLKIRF
DMTQDELVIYYPDGSKFLSPVELSNYAEQETQRAEREKLLKEQETQRAEQERFLKEQETQ
RAERERLIKEQETQRAEQERFLKEQETQRAEREKLLKEQERFLKEQEQIKYQTLLSQLKA
KGIDITALE
>OBU77810.1
MLDLDRIPNQERLLRAYKVFFQPPIFDIWIDNPLHKSDRISQPSEYSTNPVLRARKFSIG
GLTT
>OBU77833.1
MNHAKLESDLAQLQDEVSNCERDLLNLIKGKKTMEYPQNHNGGNQSNQIAIIGMASLFPQ
SKNLQEYWQVIVDKIDCIMDVPASRWSVEDYYDPNPKAPDKTYCKRGGFIPDIDFNPMEF
GLPPNILEVTDISQLLGLVVAKAAMEDAGYGESQQFDRDRTGVILGVAIGRQLAVPLGSR
LQYPVWKKVFKNCGLSDDETEKVIEKLKSAYIQWEENAFPGMLANVISGRIANRLDLGGT
NCVVDAACASSLGALNMAISELLAHRADMMITGGVDTDNSIFAYMCFSKTPAVSPSEKVR
PFDVNSDGMMLGEGVGMLVLKRLEDAVKDGDRIYAVVKGIGSSSDGKYKSIYAPHSQGQV
KAIRRAYENAGFAPQTVGLIEAHGTGTMVGDPTEFTSINQVFGDNNSLKQHIALGTVKSQ
IGHTKAAAGAASLIKTALALYHKVLPPTINITQPHPKLNIENSPFYLNTETRPWISNQPR
RAGVSAFGFGGTNYHVVLEEYESEHHQSYRLHNCAKSIFLSAPTTPELLSQCQHLYQQLE
STDKEQHYQRIIAESEQLIIPADHARVGFTILSLSQAIAHLAIIIDLLINQPSVEFWEHP
KGIYYRQQGMETTGKVVGLFSGQGSQYLEMGRELVINFPCLRQTYSHLDDLFSREGLEPL
SQVVFPTPVFSPQERQEQLEKLQKTEYAQPAIGALSAGLYKILQQAGLKVDFVAGHSFGE
LTALWSAGVLTEEDYFFLVKARGKAMSTSPAVDAGGMLAVKGNISQVTELIKDFPQVAIA
NYNSQQQIVLAGHKSEITQLQNVLQSKGFSCFLLGVSAAFHTPLVSHAQKPFAHAIAQVN

FQPAHIPVYSNVTGKLYPNEPASMQKILQEHLLNQVLFQQQIENIYQAGGNCFIEFGPKN
VLTNLVKEILVDKPHFAVALNANYRQDGDLLLREAVTKLRVFGVPLKNLDPYQIPAKISS
SSQKNEQKTLNIRLNATNINDRSQKAFAQALATGPVIKKSTVSEDNYQIQPQKILVEKNP
QINPEIISSSILTSKNSSQVKNHSMVEPKMEIPVENYDRLLDSLEQSLAEFTRQQSEINQ
VHQQSLQNQMEYNKTFYELMQQQCLFLAKEETNEYQAQTQQLAISSTERSMMRLHDHQAE
TIRIHEKYLNYQQEYTNNYFQLLEQHYSLFEVGSPNGYPHLPSSHVAQSDPPAQKLIYPL
EPENNSQNNLPDITVGFPIATYLDKEKLRDTLINIVSDKTGYPVEMLDLSMDIEADLGID
SIKRVEILGGLLELYPDLPRPNPEELAQLATLEQIAEYINNLITQVYENQPVEETVGSGK
SPLSQEIPVKAINEQTATIETPSPVTELDNHPQFLVLSHEDSLDRSHQPTITIPDNLSQI
LLTIVSDKTGYPAEMLDLSMDMEADLGIDSIKRVEILGGLLELYPDLPRPNPEELAQLAT
LEQIAEYMRNQAETVERSNLSPLEKPDMSTTEVGDKILRLPVQLKPLPQPDSLDLTIPEN
HFVLITNDGSEVTHRLVAKLADKGCKTVVLTFPCLESNLSEEIAQIRLNDWHEETLQEHL
TELTTKFGCVGGFIHLHPNSNNNLGMDKAIVQHVFLIAKHLKEDLNQLAKKERACFFAVV
RLDGELGTAKTHNFSPISGGLFGLTKSLNQEWPEVFCRTLDLSPDLDADTTVKHILAELQ
DPNLLVTEVGYNKIDRFTLVAEPGKSSIIPDSLNITKNQVFLVSGGAKGITAKCVIKLAE
EYQCKFILLGRSSAEIEPVWSEGYEDENELKRRIMEDFLAKGEKPTPIMVQKKYQTISSQ
REIHNTLKAITEAGGKAEYVCVDITDGMMLREKLTPIIDQFGTITGIIHGAGNLADKRIE
KKTVQDFETVYAAKVQGLENLLNIVETNQLEYLILFSSVVGFYGNVGQTDYAIANEILNK
SAHVIKHKHPNCHVVSINWGPWDSGMVSPELQTAFAQRGIETIPQELGSSILVDQLRNSN
STMTQVVIGSPLVYIPSTLSSELKTHQITRQLKLNYNPFLQDHVIAGNPVLPATCGLSWI
SSSCEQLYPGFQTFHCPNFKVLKGIVFDQNSPHEYILEIQEVAKIDNQEVQLAGKISSVT
NHGKIRYHFSSNLILKRQIPPADNYEFFNLTQDGQFLASNSLLYQTGGGSLFHGNTFQGV
KSVLNISPGKLTMKCELPEPTLYQQGQFRVQTLNPYIADVQIHSLWIWTQHFHQVGCLPS
EIENFEQFAPVPFGETFYVTCEIKSKTESYVVADVITHNQKGQVYNQIKASKATILPNSY
>OBU77856.1
MSQQNQVKNKLNGALTSVIQTLQEFAEQTNFWQILDIAFGRTYNHLRVKELRTQWRQRDK
GALPLIEIVNQEVLGSSLGAYSIDTDKIYMSEQFVVNAKLADLVLVLLEEYGHHVDAQVN
AKDTPGDEGEIFAALVLGKTLDDESLRNLRAEDDSAVIALGGEVIKI
>OBU77857.1
MVRQIAPKTQLGAIKSNKNQLGETIVPEIVYPESDGEPMADNTKQFTLNSDREALPAGAR
PFTLRA
>OBU77859.1
MSNSAFRLIGLDALRNDPRYANIIDGNAPDRNSDGQPDQRLTVVVLDTGVDTGHQLLSPN
ILAYVDFINGSRFY
>OBU77860.1
MVVDFINGTPVVTNRPPAFSSGNFTVPENTTGVGSVMATDEDEGTTLTFSIVGGADRDKF
SINRVTGALSFNTAPDFEAPGDVGTNNVYDLRIQVRDGQNSVEQDITITVTDVNELPANQ
APTFTSAPSFTVPENRTTVGNVTASDPENDPLSFSITGGSDQARFAINASTGSLSFVTAS
DYETPTDVGRNNIYDLQISVFDGQNTVNQNIAVTVTDVNDRTLLGGILSDNNDPDGHGTH
VSGIIGATDPNIGVANNVDLIGLRVLGEGQDGSEVAQALQWVLDHRSEYNIVAVNMSLGI
PSAFYTQPQKVRVIQFTIHGDV
>OBU77864.1
MVVFGRPKGHRGSYRQWEENNIPPQVVFEILSPGNNNTEMDRKKLFYLEHGVEEYYVYDP
DKISLEVSIRENNSFKEIENFTTWTSPRLKIRFDMSQDELVIYYPDGSKFLSPVELSNYA
EQERFLKEQETQRAEREKLLKEQETQRAEQERLIKEQETQRAERERLLKEQETQRAEQER
FLKEQETQRAERERLLKEQEQIKYQTLLSQLKAKGIDITALE
>OBU77865.1
MSQGVETPKIVYPESDNKPMADNTRQFTWIVKIKENLEILFKYNTDVFVAGDLFWYPVEG
SNKIKLAPDTMVVFGRPKGHRGSYRQWEENNIPPQVVFEILSPGNNNTEMDRKKLFYLEH
GVEEYYVYNPDKISLEVSIRENNSFKEIENFTTWTSPRLKITFDMSQDELVIYYPDGSKF
LSPVELSNYAEQETQRAERERFLKEQETQRAERERLLKEQEQIKYQTLLSQLKAKGIDIT
ALE

>OBU77888.1
MADNTRQFTWIVKIKENLEILFKSKADVFVAGDLFWYPVKGSNKIKLAPDTMVVFGRPKG
HRGSYQQWEEDNIPPQVVFEILSPSNNNTEMDRKKLFYLEHGVEEYYVYDPDRISLEVSI
RENNSFKKLENFTTWTSPRLKIRFDMSQDELVIYYPDGSKFLSPVELSNYAEQERFLKEQ
ETQRAEREKLLKEQERFLKEQETQRAEREKLLKEQERFLKEQETQRAEREKLLKEQERFL
KEQEQIKYQTLLSQLKAKGIDINALE
>OBU77896.1
MSQEVDLKNLPLGINTLDKMRGSNCVYVDKTSFALKLIKQPGAFFLSRPRRFGKSLFVDT
LKEIFEGNEKLFEGLYIHDKWDWSRKFPVIKIDFADGVLKNREELDEKIRDLLWNNGDRL
GVGSKKKSISGIFGEIIAGAREQFGERVVVLVDEYDKPILDNIDNPNIAAEMREGLKNLY
SVLKSQDSNLQFVFMTGVTKFSKVSLFSGINQLTDITIDKQYSSICGYTETDLQEFFGDH
LEGVDWDTLRHWYNGYNWTGSETVYNPYDILLFIGKGQVFRNYWFETGSPTFLVKLFQTN
RYFLPNLEHLEVTEEILESFEVERINPVTLLFQSGYLTIERTFTRRQRYMFALKIPNLEV
RLALNDQFINAYTETVNEKSGIQDSLYEFMNRGDVESMIMAIKRLFAGIPWRNFTNNDLA
DFEGYYASVIYAFLSSLDARVIPEDISNYGQADITTMLGSHIYVMEIKVVEGNQVQGNPA
LDQILQRNYAEKYRGEPGKSVHEIGLIFSRSQRNLIQANWQ
>OBU77907.1
MSQGVETPEIVYPESDNKPMADNTRQFTWIVKIKENLEILFKSNADVFVAGDLFWYPVEG
SNKIKLAPDTMVVFGRPKGYRGSYRQWEENNIPPQVVFEILSPGNNNTEMDRKKLFYLEH
GVEEYYVYNPDKISLEVSIRENNSFKEIENFTTWTSPRLKIRFDITQDELVIYYPDGSKF
LSPVELSNYAEQETQRAEREKLLKEQETQRAEREKLLKEQETQRAEREKLLKEQETQRAE
RERLLKEQEQIKYQTLLSQLKAKGIDITALE
>OBU77981.1
MNIETLKSEKTKQLPGANLEDQDLSEFDLTAVNLAGANLMGAHLVSANLEGSHLEGANLM
GASLQGADLRANLLGANLMQADLTGADLRGSNLRGANLMGATVAGASLTAAFLSGANLMS
VNFQGVDLRGADLRGANLIGANLKGADLSRADLQGALLNQANLEESDLRGANLAGANLAG
ANLLCAELEAASLNGANLYQACLLGTILETYHD
>OBU78003.1
MADNTKQFTWIVKIKENLEILFKSNPDVFVAGDLFWYPVEGSNKIKLAPDTMVVFGRPKA
HRGSYRQWEEDNIPPQVVFEILSPGNTQDEMDKKKLFYLKHGVEEYYVYDPDRISLEVSI
RENNSFKEIKDFSVWTSPRLDVRFDMTGDELIIYYPDGGRFLSPVELSNYAEQETQRAER
EKLLKEQERFLKEQETQRAERERLLKEQERLLKEKETQRAERERLLKEQERLLKEKETQR
AERERLLKEQEQIKYQTLLSQLKAKGIDITALE
>OBU78005.1
MILILQHLRTHRVLTKKLRLVPYALVGIGVFAFNANTDTNTYLQIYLILLEAKVGIVLVY
FFTKNLEKFYK
>OBU78008.1
MTGLNRQAFNELLSQLIVAWVAQEGSQRQLAERFKVSLSFVKNLVRRYRETGQVEPKQCG
GYEKPIIAGQYLNMIKSWLDEKNDLLLSELCDRLRETTGTSVSITTMHRALEKLGLRHKK
KSKCQ
>OBU78082.1
MADNTRQFTWIVKIKENLEILFKCNADVFVAGDLFWYPVEGSNKIKLAPDTMVVFGRPKG
HRGSYRQWEEDNIPPQVVFEILSPGNSQDEMDKKKLFYLKHGVEEYYVYDPDRSSLEVSI
RENNSFKEIKDVSVWTSPRMNVRLDMTGDELVIYYPDGGRFLSPVELSNYAEQENQRAER
EKLLKEQETQRAERERLLKEQERFLKEQETQRAEREKLLKEQETQRAEQERLLKEQEQLK
YQTLLAQLKAKGIDISTLE
>OBU78089.1
MATTADEVWKLLGELIESQKETERKFQETERFLREQSQETERFLREQSQETDRKFQETER
LLREQSQETERLLREQSQETDRKFQETDRLLREESKRVNNQIGQLGNRLGEFVESQVRPA
AVKLFQERGIAVKEIASNTYIQTGKEGLEIDLLVINSSDIILIEAKSKVSEDDVNEHLER
LSKFKRFFPRYESYRVLGAVAGMVIPLDVSRYAYRKGLFVIGQSGDNLVILNDDKFRPRG
W

>OBU78090.1
MATTADEVWKLLGELIESQKETERKFQETERFLREQSQETDRKFQETERLLREQSQETDR
KFQETERLLREQSQETDRLLREQSQETDRKFQETDRLLREESKRVNNQIGQLGNRLGEFV
ESQVRPAAVKLFQERGIAVKEIASNTYIQTGKEGLEIDLLVINSSDIILIEAKSKVSEDD
VNEHLERLSKFKRFFPRYESYRVLGAVAGMVIPLDVSRYAYRKGLFVIGQSGDNLVILND
DKFRPRGW
>OBU78091.1
MATTADEVWKLLGELIESQKETERKFQETERFLREQSQETDRKFQETERLLREQSQETER
LLREQSQETDRKFQETDRKFQETDRLLREESKRVNNQIGQLGNRLGEFVESQVRPAAVKL
FQERGIAVKEIASNTYIQTGKEGLEIDLLVINSSDIILIEAKSKVSEDDVNEHLERLSKF
KRFFPRYESYRVLGAVAGMVIPLDVSRYAYRKGLFVIGQSGDNLVILNDDKFRPRGW
>OBU78092.1
MATTADEVWKLLGELIESQKETERKFQETERFLREQSQETDRKFQETERLLREQSQETDR
KFQETERLLREQSQETDRKFQETDRKFQETDRLLREESKRVNNQIGQLGNRLGEFVESQV
RPAAVKLFQERGIAVKEIASNTYIQTGKEGLEIDLLVINSSDIILIEAKSKVSEDDVNEH
LERLSKFKRFFPRYESYRVLGAVAGMVIPLDVSRYAYRKGLFVIGQSGDNLVILNDDKFR
PRGW
>OBU78099.1
MIAHRLSTLEKCDRIFQLDQGQVCQEGDRHGDSPTNSATGGAESEPAKLMCNSILERENM
ATTADEVWKLLGELIESQKETERKFQETERFLREQSQETERFLREQSQETDRKFQETERL
LREQSQETDRKFQETDRLLREESKRVNNQIGQLGNRLGEFVESQVRPAAVKLFQERGIAV
KEIASNTYIQTGKEGLEIDLLVINSSDIILIEAKSKVSEDDVNEHLERLSKFKRFFPRYE
SYRVLGAVAGMVIPLDVSRYAYRKGLFVIGQSGDNLVILNDDKFRPRGW
>OBU78101.1
MATTADEVWKLLGELIESQKETERKFQETERLLREQSQETDRKFQETERLLREQSQETDR
KFQETERLLREQSQETDRKFQETDRLLREESKRVNNQIGQLGNRLGEFVESQVRPAAVKL
FQERGIAVKEIASNTYIQTGKEGLEIDLLVINSSDIILIEAKSKVSEDDVNEHLERLSKF
KRFFPRYESYRVLGAVAGMVIPLDVSRYAYRKGLFVIGQSGDNLVILNDDKFRPRGW
>OBU78104.1
MATTADEVWKLLGELIESQKETERKFQETERFLREQSQETDRKFQETERLLREQSQETER
FLREQSQETDRKFQETERLLREQSQETDRKFQETERLLREQSQETERLLREESKRVNNQI
GQLGNRLGEFVESQVRPAAVKLFQERGIAVKEIASNTYIQTGKEGLEIDLLVINSSDIIL
IEAKSKVSEDDVNEHLERLSKFKRFFPRYESYRVLGAVAGMVIPLDVSRYAYRKGLFVIG
QSGDNLVILNDDKFRPRGW
>OBU78169.1
MGETIVPEIVYPESDGEPMADNTKQFTWIVKIKENLEILFKSNPDVFVAGDLFWYPVEGS
NKIKLAPDTMVVFGRPKAHRGSYRQWEEDNIPPQVVFEILSPGNNNTEMDKKKLFYLKHG
VEEYYVYDPDRISLEVSIRENNSFKEIENFTTWTSPRLKIRFDMTQDELVIYYPDGSKFL
SPVELSNYAEQETQRAEREKLLKEQERFLKEQEQIKYQTLLSQLKAKGIDITALE
>OBU78172.1
MTNFSQADDIDLLGLLLEEEGIELEIKEVVPRVGSTEAPTSFQQRRLWFLYELEPTSSAY
NICSIFDLKGTLNITALRVAFKQLQQRHESLRTTFMDVDGEPWQKIHANSATELRLEDWS
NDRSEDKIPEIIAEIARHESDHQFNLQTGPLIRAQLFKIESKQHILSINLHHIIADAWSV
GVILQEIAMLYQAEISKTPIGLPELKFQYTDYALWQKENFQNSNILEKSLTYWEKQLAQL
PTLQFPLDFPRPRLQTFRGGLIKFEISKETTNRIHNFIVKEGATLFMFLMAVFQTLLSRY
TGQEDIAVGTSIANRPSDSENLIGFFVNMLVIRTNLADEPNFNSLLKTVKKTILSAFEHK
EIPFETLVEKLNLPRDTSRNPLFQIAFTLLNAPKPQFGTGDLEVSILATQEAARFDLELF
ITETEDTLNGAVSYNIDLLKRETVERVARHFCQLLESVLAQPEIPVSRLPFLLSEEIAVL
APSQPAQTFPVHFCLHDIFTEQAKLRPQQTALIFGQERLTYSEVNYRANQLAHYLMRVGV
KPEARVGLWLSRSLDLVIGIIAILKAGGVYVPFDPNYPKDRITYMLEDSQIRVLLTHSEF
EAQIPFEFTNNTNNVSNTNNNILTIFIDKYKSEFTQVVTTEPEVLILPDNAAYIIYTSGS
TGKPKGVVVTHRHVVRLMLSTEKWFKFNAKDVWTLFHSCAFDFSVWEIWGALFYGGVLVI

VPYLISRSPEEFYNLLCEEKVTVLNQTPSAFQQLIQAESTLCREGELELRYVIFGGEALD
LASLEPWFERHDDQFPLLVNMYGITETTVHVTYLPLTFKDVKKGSGSLIGKQIPDLSLYI
LDRHLQPVPIGVVGEMYVGGAGVTRGYFHRPQLTAERMIPNPFATNDLTSVRLYKTGDLA
RFLDNGNIEYIGRNDHQVKIRGFRIELGEIEALIKSHPEVRDALVIAREESKEDVRLDAY
IIPINQIANTETLTQEQTQEWQYTFNDTYNITSGETEEDFNIIGWNSSYSNQPIPGVEMR
QWLNNTLLRIQSLKPRKVLEIGCGTGMILLNIAPQVESYWGTDFSQAAINRLDTIVKNRS
LKNVNLLTREAIDFSEIPTGYFDTVVINSVAQYFPSIEYLQQVIKSVWQLLKTGGSLFIG
DNRNLSLSNYFYASVAYFQANDNTDCETFKTQVRRIAKKENELIIAPHFFTDLRKSFPDL
TAVEIQIKSENNENELTKYRYDVILHKLGISTEQPPEIIWRDWETDNLQLTDLKQQVIEM
RSIGWHSVPNGRLSKDAAIYQWMLKNSHENEQKTIGELRTVLNNIHEPKGFNPADFYAIA
EEIGLEVSISYSPGKVDCFDVCFYPAGSGKSMAPSMPIVNDLLGRDNHPSWIDPLKNRLT
KLLISQLKQRLEEKLPEYMCPSAFMILENFPLTPSGKLDRRALPIPDRDLIINQQSLVPP
KTPTEYKLSQLWMDVLGIDKIGVTEDFFHLGGHSLLATKLVSRIREEFNVALPLRSIFEY
STIARLGDEIDCLIDVNTTKTGPEDIIPVSNRENLPLSFSQSRLWFLDLLEKENAAYNIS
VAFRLEGDLNVDALRESWQNIIQRHEVLRTTFDNVQGSPIQIVHDWSELKLTIRNLSCLD
FQTQQETLRKSIQEVVITPFNLNQLPLLRIHLYQLSADVSVLLLVIHHIIADGWSLGVMV
KELSLFYTAICQRNIPSIPPLSIQYGDFANWQREVFQKTQLPIQLAYWKQKLTGANQILE
LPTDYPRSPIPSYQGSAVNFAINPQTTQEFKKLCESQGATLFMGLLAVFSILLMRYSGQE
DLLIGTPIANRNRKQTEDLIGFFVNTLVIRNNLSGNPNFINLLSITKEETLQAYAHQDVP
FEKIVEEINPQRNLSQHPLFQVMFVWQNAPMNKLELPNLQLSPWRLEQRLAKFDLTLLMT
ETEQGIDGTWEYRTDLFAPETINRMIGHFETLLKGIIAEPQKPITHLPILTSHEKNQLLF
QWNQTQFEYPLYQQNKCLHQLFELQVEKTPNNVAVVFKNQSLTYFQLNQRANQLAHYLQA
KGIGPETLVGVCLERSEQLIISILAVLKAGGAYVPIDPAYPSDRLEFLFQDSGIVLLITQ
RKVISQLPECGAEIIILEDIAKRIEGNNSQTPAVNILPENAAYVIYTSGSTGKPKGCIVT
HANVIRLFNSTTTWFNFHSEDIWTLFHSYAFDFSVWEMWGGLLYGGQVVVVPHWTVRSPK
DFLQMLATHKVTVLNQTPSAFKQIISVVQQKPEKLSLRYVIFGGEALELADLQPWIELYG
DMQPELINMYGITETTVHVTYRPITQKDIINNLNYRASVIGQSIPDLELYILDENLDPTP
IGVAGEIYIGGAGVTRGYLQQPGLTAQRFIPHVHSKNPGSRLYRSGDLARHLPDGEIQYL
GRADQQIKIRGFRIELGEIEAVLAQHPNVLNAVVVVSGDSSATNSLIAYYVSTEKQFTSS
GVLRNFLKEKLPDYMIPNSFIVLDHLPMTPNGKIDRKLLAGLNINRTFDAHQHVSPRTLL
EYKLVEIWEEILQVRPIGVTENFFDLGGHSLLAIRLIAAIEQKLKCNLPVVSLFREGTIE
KIALLSDQDHQKASNHSDILIPLQTQGDLLPLFLVHQAGGYGLSYSVIAEKLAVGMGKKL
PIYAIQSPGLDGKQSPLESIEEMANTYINTIREIQPHGPYLLGGHSLGGLIAFAMASQLE
AMGEQIERVLIIDTHPPMPTDETIASLEDNAGIICFMVEQIALFFNKNVTINYQTISSLD
QDSQLDYVAQTLEQHNLIPPNSGNSLIARLIKVYKANLRASVVYQPPVNRSNITLFITPS
LAAKFPNDPTVGWQKLTTQKVQVCRVMGEHQTMLKEPEVENLVTEIMATLVNTP
>OBU78181.1
MRSHFSAAHRLAPNLSAEKYGKCTRTHGHNYHLEVTVEGEMDGRTGMIVDLGRLHEIVER
EILELFDHSCINEDIPYFSTSHIVPTTENIARYMSDLLQFPISQLGVKLSRVKLFESDHL
WVEYEGKDSEIFFSVATGFSAAHRLADPTLSLEKNQTIYGKCSRINGHGHNYYLEVTVQG
EIDSVTGMSVDLVGLNQIIQHYVIEPMDHSFLNQDLPYFTEVVPTAENIAVYITNVVRSP
IEELGAKLHKVKLIESPNNSCEIYARDIEESKVDRIYRELAAV
>OBU78182.1
MPGKGDDIIRVNSASVVIIELPNEGNDTVFSSINYNLASLPQIENLTLWGTEDINGIGNR
RDNVITGNSGQNVLTGLQGNDTFVFNLGDSVVGKPDRIGDFQFGKDKIKVNGVLPSVLTR
AGNNSASTLNSLVDSVFIDGNGATSGNQGLGTNSAALVVSTAQGIGGTYLIVNDGVGGFN
PATDLVINLTGYSSNLPGLGNIAVGSLFV
>OBU78183.1
MRVIESNISQGVETPKIVYPESDNKPMADNTRQFTWIVKIKENLEILFKYNADVFVAGDL
FWYPVEGSNKIKLAPDTMVVFGRPKGHRGSYRQWEENNIPPQVVFEILSPGNNNTEMDRK
KLFYLEHGVEEYYVYNPDKISLEVSIRENNSFKEIENFTTWTSPRLKIRFDMSQDELVIY
YPDGSKFLSPVELSNYAEQETQRAEREKLLKEQETQRAERERLLKEQETQRAEQERLLKE

QETQRAEQERFLKEQETQRAEQERFLKEQETQRAERERLLKEQEQIKYQTLLSQLKAKGI
DITALE
>OHY31526.1
MILLQNPCFNYEQETKKPGVKITVNKFNQLLIGGTAMVSMLGWNINMAQAQSLPAYCTGS
AIGALLGLAIGANPRAAANAIPAECTTQNQNSPSSSPKLIAAYNSTKSDSDVGIKQDCIS
FFYDSGDFLEKCSSFINSDYTRPRTRITNIEGIWKRTGDLVYVKVRSSAGLSGERKYQII
PDGLLNLDTGTRLTKE
>OHY31530.1
MTMNKFNQLLMGTTTVVLTAVMLTSCGGGTSSNTGGGTTQGGGGTTQGGGSVTPVQPTPT
TPQTLTVTVPSLGYVQTIDKYTTLADITTAARDGAAECINRFGLSSASNTCLGEVRAAQS
QAVNFIN
>OHY31549.1
MKWQLLTHNKQVLGKIFTILVFTGLTGILCVSCNRNQDLLVTEIGVNPPKRPTRKTSGAG
EFYLQGQNQHSRGNFQAAIAAYSKSISLNSDYAPAFKARGLAYFDLNNKERAINDYNQSL
QINPNDPETYNYRGNARASLGDQKGAIEDYNEAIRLSPNYAEAFNNRGNSHAAQGNKNAA
LEDYTQAIRIDQNYSVAYNNRGNAYSSLGNTSKAIADYNQAIRLNPQFAPAYNNRGNAFA
SSGDKRRALQDLQKAATIFDQEGNRGLYQQTMKNIEELGN
>OHY31590.1
MAITTAASRLGTEPFSEARRVELRPSASREEVELVIRTAYRQVLGNDYILASDRLVSAES
LLRDGNLTVREFVRAIAKSELYKVKFFYNSFQTRLIELNYKHLLGRAPLDESEVVYHLDL
YDNKGYDAEIDSYIDSVEYQNNFGENIVPYYRGFDFQPGQTSVGFTRMFRLYRGYANSDT
AQVEGSKSRLARELAGKKASSIVGPSGSNDNWSFRPSADNAPKQNLGNAVGQSDRVYRIE
VAGIRSPGYPSVRRSSTAFIVPYERLSDKIQQIHKQGGKIVSITAT
>OHY31928.1
MAERLREERVKAIIQPMLAGLDLPDTPEDDRRFSLDLGLVKRIPLGRMTIANPIYQEVIP
RVLSQGSQDSLTQIQPTWLNTDK
>OHY31951.1
MPRRQDIHKILLLGSGPIVIGQACEFDYSGTQACKALREEGFEVVLVNSNPATIMTDPET
ADRTYIEPLTPEMVAKVIAKERPDALLPTMGGQTALNIAVALAKNGVLEEYNVELIGAKL
PAIEKAEDRKLFNDAMEKIGVNVCPSGTASSLEESKAIAQRIGSYPLIIRPAFTMGGTGG
GIAYNKEEFELMAQVGIDASPVSQILIDQSLLGWKEYELEVMRDLADNVVIICSIENLDP
MGIHTGDSITVAPAQTLTDKEYQRLRDMAIKIIREIGVETGGSNIQFAVNPVNGDVVVIE
MNPRVSRSSALASKATGFPIAKMAAKLAVGYTLDEIKNDITKKTPASFEPTIDYVVTKIP
RFAFEKFPGSDPVLTTQMKSVGEAMAIGRTFNESFQKALRSLETGRAGWGADKAEKLPSG
EQVRAQLRTPNPERIFALRHAMQLGLSNEEIYELTAIDPWFLDKLHQILETEKFLKRTPL
QQLTKVQMYEVKRNGFSDRQIAFCTKTKEDEVRAYRKQLGVIPVYKTVDTCAAEFEAFTP
YYYSTYEEETEILPTDKPKVMILGGGPNRIGQGIEFDYCCCHAAYALKSANYETIMVNSN
PETVSTDYDTSDRLYFEPLTKEDVLNIIEAENPVGIIVQFGGQTPLKLAVPLQEYLQKSP
SSVTRIWGTSPDSIDMAENRERFEKILQELKIAQPANGIARSYEDALIVAKRIGYPVVVR
PSYVLGGRAMEIVYSDSELERYMSFAVQVEPEHPILIDKFLENAIEVDVDAIADHQGRVV
IGGIMEHIEQAGIHSGDSACSLPSISLSPAVLNQIRTWTVELAKALSVVGLMNIQFAVVG
ASSYSPQVYILEANPRASRTVPFVSKATGVPLARLASLIMSGKTLEELNFTQEVIPQHIA
VKEAVLPFNKFPGTDTLLGPEMRSTGEVMGIDVDFGRAFAKAEMGAGEKLPLQGTVFVSM
SDRDKSLVVEVIKEFIQLGFKVIATQGTSEFLREQGLQIETILKLHEGRPHVLDAIKNRQ
IQLIINTPSGQEARTDGQLIRRTALGYKIPIVTTIAGAKATVAAIRSLQNISLDVKTIQE
YSF
>OHY31980.1
MTSHNNNYSESSGLGVFSISKSGDLTTDLNTSFPINVSPEPLAEQPILPTTPPLGELPKI
PISANPNPNPYLTSAAIVPDFNGDGKTDKMWVNVQTGEIFVRLMDGTRIIEQASLGQYDL
RTWSYKTADFNSDNKTDFLLRNETTGENLVVLMDGTKVASFLMLDRVDPGWKAEIGDFNG
DRKTDIFWRNTTTGQNAIWEMNGTKVVNATMLETKDVALTATIVDFDGNGKSDIFWRDSL
TGANSAWFMNGTEVTNYDLQAQDGAWTSTLGDFNGDLKTDILWRNNTTGENKIWTMNGVF

ITEGVVNTLGQGWTANIGDFNGDGRTDIFWHNSSTGENTAWLMNGTSIQSEAFLPSNPPG
LTASLGDFNGDGKTDIYWRDQQTGADKIWNMNGTIASETPVSEVDRLTPEWYTA
>OHY31986.1
MGIELRSYVFLDNLQPQHAAYMGTVAQGFLPLPGDTSIWIEISPGIEINKITDVALKSAS
VRPGWQIVERLYGMLELHSGSQGETRAAGQAVLDLLGVKKEDCLKPRVISSQIIRNIDAY
QTQLINRSRRGQLLLAGQTLYVLEVEPAAYAALAANEAEKAAAINILEVLPVGSFGRLYL
GGTERDILAGAAGALAAIENVPGRDPQGHRKE
>OHY32018.1
MEQLIVEDPVIKNLILGSMSQYYIGRQDADSKFDQTLAQLQSYQEEQNRKWEEQNRHNRE
IMSQLQSYQEEQNRKWEEQNRHNREIMAQLQSYQEEQNRKWEEQNRHNLGILEEIKQMNR
KHESTVGSLGSRWGLSSEASFRNGLKGILKDSFGVEVLNFLDFDNEGEVFGRPDQVEIDV
IIKNGLVILCEIKSSIDKAGMYIFDRKVAFYEKHHQRRVDRKLVISPMVDPRALSVAQNL
GIEIYSYAEDVNRI
>OHY32218.1
MGLFDESTLKCKAEVWRSLINRWRDIRGGYNKCEEYRPLAQLFERFKEYDLAGYFYQVSD
GVVKKDLFYEIPKAQNRPSPVIYGLGIEREVTLEDIIIGLADGVVKFFNQDVDMKLVFVI
PVSLLILGSGWFIGSKTWQYVYANEAEKFLCQKSGGGENCPVIVLDGKNHYSFDEIKQVI
PKVVNQVIDQQTKAPTAEPTQSGSNTSGQNYGQQDIKKKVIENLIQILGDKNLKYQDLNS
TDKIEEAVKTQWVIAVYNYQLKSKIDQKVTTQKSNKEECLLQIFFCLKKNIKTEETYSLE
AKLTNDINKKINPPKKNQPGQNSPVK
>OHY32222.1
MTTALSWQKRVGNQRDWVWRGWQIRYTFIRPVNHHQTATPLILLHGFGASIGHWRHNLEV
LGKHHTVYALDMLGFGGSEKVPANYSVNLWVEQLYDFWQTFIHHPVILIGNSLGSLVTLV
AAAVHPDMVQGMVMMSLPDPNLEQEVLPPFLHPLVRGIKGIFASPLLLKPLFNFIRQPAV
LRRWAGLAYAHPQAITDELIDILAGPPQDRGSTRAFIALFKA
>OHY32223.1
MGNIIANDADGNTLTYSISGGADQSLFTINANTGVLSFVTAPNFEAPGDVGADNRYNLQI
QVTDGNNRVTQVLIIDVTNLNEAPTDLTLSATTIEENQASGTVVGNFSTTDPDAGNTFTY
SLVTGAGSTDNSFFTIDGGKLKTAAAFDFETKNSYSIRVRSTDQGGLFFEKQLTIGVKGV
NEPPVFSAVSFSVRENSKSIGRISVQDPEGDNITFALAGVDAKLLSIDPTTGELTFNQAP
DFEKPEDADNNKIYQVQVTVRDGNTPVTRNIDIKVEDVNEAPAAIGDFLAIVGDTSGSIE
PLRNDTDPDSGDKLKIIGVTDGKQGKVEIIGDQLKYTLLDAAYTGDDVFSYTISDQGNLT
ATANVKVNVTGTKVVVNSGVITDVQPGDPLIPSEAGSLSGIVNNVSFNFRAGYNPTQARD
ILQRTLVRTDAAFNNLFGLYEIDDATGTVNGVAPGQPGYARAALNRAVSSFAVRAGGSGN
GITGNVVVGGDKFYAPFVIANGGNLFGSMQDAINTFFQLNADNSRATAENYTSFPVAYFS
FGAANPDGAAHIKSFGNNIFGFEDLPAGVGVNDYDFNDTVFSFG
>OHY32224.1
MSPASGSVAGAMVPTVVLFSAVEKVAVALAKLGASLIPVTLTVTVWVAFKPLTSVAVMVR
L
>OHY32225.1
MGNITATDADGNILTYSISGGADRSLFTINANANTGVLSFVTAPNFEVPTDVGTNNVYNL
QIQVTDGNNPVTQDLIINVTNVNETPTDLTLSATTIAENQAIGTVVGNLSTIDPDAGNTF
TYSLVTGAGATDNSSFTIDGVQLKNRCCF
>OHY32226.1
MSTPKLVITPKIQSVSPRSGKINLDYRLENFTDQAVSSASIEVYFSNELQIDVNAITIGD
NTGTVPLGGRITGADTNNADGDGTTGNFVVLTFQNLTTSATNKSFINIPFVTTSTFDGQA
AVNFIARSTNSNLTVDTIAPVAINNQVAAILEVGPDQTYKTIQAAIDAASNGDVVRVLSG
VYNENVTINKSVTLEGPNKGIRPTTPDINLTGGININQGYRTNPEAWIKGTVTVTADNVT
IDGFRLRNENGPLQWTGTPDNFKLLNNYVTGYNANKGPRFGDANSNNPTNVVTGWQIDAN
YIGGLLGGGGTGGSMYLAGLSNSSINNNTFWRPRAAHLYLASLTNVTIDGNKFYHGLHAG
GADFDGFGKFFSGTGYGYGGYGGYGGSYGGGYGRNYWLELKGTNNTVNIKNNSGEYNSGG
IQLYGEVNDPFLFNKVTIENNTFPANNFINAYTQASNNNLSGLIPAVMATARVVNGGPSG

SDLVIRDNKITMDLAQVKYDKDHKSSLEVRGNFNGVTIENNTLTPTGTNGGVNLITGLNL
YGSLPGQVSVKNNEFFGEGGTRQNASYYGIDVNPTFTGYGTYNSNLNIQNNTIRNWEVGV
VLRDAVQITANSINIAGNNFSNNSSNVFDGINPTITASQVLSYPENQQQGATLGTVSASD
NLPNTDNVGIIQYSISSGNESGFFSINSSSGQITLTSAGISAAANNFESLPNTFTLGITV
TDGGGLTATNAVTLSVTNVNEAPSFANATATFSRAENSTTVGTISAAIDPDAGDTLTYTL
SGADVAKFNIDNTTRSLTFKTAPDFEAPGSAAGTNTYSVTVIATDGGGLTATQAVTVNVT
DVDDTPPDAPQIINFIDNVAPVTGTFGNGTTTDDLTPTLNIKAEAGSSVQVFRNSLTYGD
ATAANTPGDYTFTTANLAPGNTYSFTARATDAAGNVSPLSNPFTLTVGLPGYQRYNFTYR
YGNGDSYSGYVYAPVGTYTQGQNIPVSNTNETGQTGSYTIDSFGEITTDSSFNNLVYLTS
YNDADTGFGTTTNIWPPQGTVSGSSGLGSEYGFAYDANFFSSDPYFSNFFEADIRSNNVF
FEFTYYYGEDTNNDYYKGYGYASRDYINAPGRYLAINSKPNDTGKTGYYQVTSVQNSFDF
GLRNINTYIWVNEYFDIQTDQDGIGTGGYGKANYIWSYGGNRGLGSEEGYAYNLGFEGGD
NQFNHINSADIATTKTFLSIRNPGNAWLQTRFEGNQGTSTNYTFEVVRQGNLNSALSVNW
NTQSFFFPNADANDFVGSTFPSGTVNFTPGQSTAPLTISVQGDIDIEFPEWFQAVIDNPD
PTSIVLAQNYAWSLILNDDGWFWGWGFGDPHLVTLDGLAYDFMAVGEFVLVETTPGSANP
FQVQVRYEPYPGSEVVSVTTRMAVKLGERRIELQLGPDPLLVDGSIVSIAPTEAGVDING
DGTLDVERNGNVYIITLNDLGEQVRVEIYDAFMDVNVLIVESPSGVNRGFRGLLGNRNND
RTDDLTGRDGTLYSQPVSFENLYGAFANSWRLDAVGTNNGKASLFSYGVGERFGGFDRSN
FPQGVIDLDQVPADLLTAARTAAAGITDPILKDAAIYDYLLTGERSFIAAAEVFPDKPKD
DTDPTLARVITSVGVAATPLSITEGNSASQDVTFRVWRTNPSGNLTVDYRLEGSINADDL
SPGTPFSGLINFADGETEKLVKVTVLGDTLIETDEQLVMRIETPNIGSVMVAAGQAATTI
ISDDLPPVTIGVIAGNDILNEAEKAAGAVITGTATGLAQVQVTIAGQRKTVNVIDGNWTA
NFTPQELPGDGSYTVEAIGIAQSGSQTIPASRTLLLDTIPPNAPVINPVTGDDIINPAER
SSGITITGTAEANSRVRLTFGNVTRTVTAINGQWSVNISASELPSEGILSLLATATDTAG
NTSAAIAREVRFNRAPSFANATATFSTAENSTRVEIITAATDPDAGDTLIYTLSGADGDK
FNIDSSTRLLSFKTAPNFEAPGSAAGTNAYSVTVTATDRGGLTATQAVTVNVTDVVEIGN
PPVITSGSTFSIAENSTTVATIIATDVESSTLNYSISGGVDQNLFAIDPTTGVLRFVTAP
NFEAPTDVGADNRYNLQIQVKDSDNNTVTKDLIITVTDVNEAPSFNTPTATFSTGENTIL
VGSVVATDPDRGDTLTYTLSGADAGKFDIDSTTQFLTFKTAPNFEAPGSAGGNNTYNVTV
TARDGAGLTTTQAVTISVTNVNEAPSFANPTATFSTAENTTTVEIITATDPDGGDTLTYS
LSGADEGKFNIDSSTRLLSFKTPPDFEAPGSAAGTNTYRVTVTAKDAAGLTATQEVTVNV
TGVVENGNPPLITSPSTFSIAENSTAVETIIATDADSNTLIYSISGGVDRSLFTINANTG
ALSFVNAPNFEAPGTDNIYNVQIQVTDGNNPVTQDLIINVTNVNEAPSFTNTTATFPVAE
NSTTVGTIAPATDPDAGDTLTYTLSGADAGKFNIDSSTRLLSFKTPPDFEAKGSAAGSNT
YSVTVTATDGGGLTTTQASYC
>OHY32502.1
MSQDKKPRSKPQLPDTPPLGGYHKKPLFWKILIIQVLRGTIGILERMVTRLETSSSTTSE
KGGLLLWVVRKWDGFLRGFRLFLPSKVANNVSDSFLTLIFVFPALLAIGVTAISLISQIH
SQPVPQLGESIADSIVEDKSQPQLGESIADSTVENKSELQLGESTSDSTVEDKSEPQLGE
PTADSIVEGKSKPELNQPATNFILRSQSEIEPVELIPQPEVTVFIEKQLKEITAISIITK
DKQRIELELVQSIKTNFRISEIVIKINENWYKLESSQQEKLAAKILKSCQEMDLIHVKLV
NARNQVIARSPVIGTKMLFFQFPTS
>OHY32545.1
MTIEDLRNTIKRLNSKAGQMKMDLHDLAEGLPTDYEKLMAVAAETYEIYKELDRLKQQLK
TMETS
>OHY32795.1
MCGPLAVAFSFSYPEKTQSWQRQLQFHILLNLGRVFSYALVGTAIGTLGSALVQGGQLGG
VGSDLRRWIAIITGMMLIWLGLAQVKPHLIPKIPIFHPLLQNSLHNRLSAVMVNLSLHTK
WWTPALLGMTWGLMPCGFLYAAQIKAAATGNGWHGAITMLAFGLGTLPTMLGVSISTSLM
SKDQRSQLFRMGGWVSMIIGIITISRTGETMVDYSGYAALICLILALIARPTSVLLPALI
RYRRGLGVGAFLLSLVHTVHKLEHFLAWNVSAIWFLPVEFQWGMGAGILALIFMTPAAFT
SFDFMQKSLGPNWRRIHLLTVPALILTTIHAVLIGSTYLGALKLTIFNQMATLLLVFIIL

AVLMMRSPLVWSIFKLKEFYTPLKQKE
>OHY32901.1
MGIELKVRKEKLDPLTQGLIQLDKYLDGLGLDTGWLVIFDRRPGLPPMGERISTEEAISP
GGRTITVIRS
>OHY33030.1
MQRPELIVIIVKLILKRLGLPKIPKTSLTKDLSLQSALSRITTKDIKIKTVIDIGASDGQ
WTKVVKAYFPWAFYYLIEANPIHCSISK
>OHY33031.1
MSIKQAQEPEKDNLEFPLVLVGSDQGNVSYVKGKVKELNLSKQVYFLGFVPQKDMASLYI
NAFALTFVSFFGPDNLPPLEAMALNCPVIASKVSGSEEQLGNSALLVNPKEPQEIANAIK
SLWHDSTLRQNLIRKGKDRAFQWTARDYVQSLFCLLDQFEPIRRCWQ
>OHY33032.1
MIPIQKILLRSLPRYNTPAYRTITTTMNRNRSLVVFTFVSNLLSATLETATLGIIFLALG
VLQDNQLPQLPDTIKSALPWLADRWKGENQEVFLLLIGLAVLSQVVRSLMTYISLVSSGD
LTARVQAQMTEKVFARIMSFTFSCASRYKIGDLSTYVGQAGSTVDMQMRLWSQFLTGIMM
FFAYSITVLTISLPLSAVALLLFVLLIWLQRYLIPRIQSTARELSQAQVDVAKDMVENIQ
GLRVVHTFGYQHSTINRVVYLQKQVLVFLQRQARLLSITSPLNNALTILVIAALLLTGSF
LLQRGQGNVLPALATFILALNRLSMQVQSLAGTMNGLAERSGMMDRLDAILGGEGQELSR
VGGEIFEGLKSAITFNHVSLKYEGTSLPALSDICFKLPRNRVVALIGSSGAGKSSVADLL
IGLYAPTTGEILVDGLNLQSYSWESWRSKLGVVSQDTFIFNQSILENIRYGMTNATDEQV
LEAARVAQADQFIQLLPRGYETVVGERGYRLSGGQRQRVALARAILKQPEILILDEATSA
LDSESERLVQQALGQFQAERTVLVIAHRLSTIVNADEILVMEQGCIVERGTHQELLELGA
KYANYWQMQSAH
>OHY33044.1
MDSYSFLNSHTHNNPLYKRNNWQSSYNSYNQKELADSDIGTLKDEKYMRSYALKLAHQGE
YRKAIALLDRIIDSHPENAIDYNNRGLIYFQSGHAQKALLDYNTAMQLNPRLASVYNNRA
NYYAARGDLVKALSDYDQALDLNPRYVRAWINRGITLRELGEYKDAIDDLEIALLFGQLE
IHIWSERGRSYHLWGDWNCAIADYRRVLSHTMSLDKIEDIITYRLRLQVENWLGELGFST
YK
>OHY33074.1
MNHAKLESDLAQLQDEVSNCERDLLNLIKGKKTMEYPQNHNGGNQSNQIAIIGMASLFPQ
SKNLQEYWQVIVDKIDCIMDVPASRWSVEDYYDPNPKAPDKTYCKRGGFIPDIDFNPMEF
GLPPNILEVTDISQLLGLVVAKAAMEDAGYGESQQFDRDRTGVILGVAIGRQLAVPLGSR
LQYPVWKKVFKNCGLSDDETEKVIEKLKSAYIQWEENAFPGMLANVISGRIANRLDLGGT
NCVVDAACASSLGALNMAISELLAHRADMMITGGVDTDNSIFAYMCFSKTPAVSPSEKVR
PFDVNSDGMMLGEGVGMLVLKRLEDAVKDGDRIYAVIKGIGSSSDGKYKSIYAPHSQGQV
KAIRRAYENAGFAPQTVGLIEAHGTGTMVGDPTEFTSINQVFGDNNSLKQHIALGTVKSQ
IGHTKAAAGAASLIKTALALYHKVLPPTINITQPHPKLNIENSPFYLNTETRPWISNQPR
RAGVSAFGFGGTNYHVVLEEYESEHHQSYRLHNCAKSIFLSAPTTPELLSQCQHLYQQLE
STDKEQHYQRIIAESEQLIIPADHARVGFTILSLSQAIAHLAIIIDLLINQPSVEFWEHP
KGIYYRQQGMETTGKVVGLFSGQGSQYLEMGRELVINFPCLRQTYSHLDDLFSREGLEPL
SQVVFPTPVFSPQERQEQLEKLQKTEYAQPAIGALSAGLYKILQQAGLKVDFVAGHSFGE
LTALWSAGVLTEEDYFFLVKARGKAMSTSPAVDAGGMLAVKGNISQVTELIKDFPQVAIA
NYNSQQQIVLAGHKSEITQLQNVLQSKGFSCFLLGVSAAFHTPLVSHAQKPFAHAIAQVN
FQPAHIPVYSNVTGKLYPNEPASMQKILQEHLLNQVLFQQQIENIYQAGGNCFIEFGPKN
VLTNLVKEILVDKPHFAVALNANYRQDGDLLLREAVTKLRVFGVPLKNLDPYQIPAKISS
SSQKNEQKTLNIRLNATNINDRSQKAFAQALATGPVIKKSTVSEDNYQIQPQKILVEKNP
QINPEIISSSILTSKNSSQVKNHSMVEPKMEIPVENYDRLLDSLEQSLAEFTRQQSEINQ
VHQQSLQNQMEYNKTFYELMQQQCLFLAKEETNEYQAQTQQLAISSTERSMMRLHDHQAE
TIRIHEKYLNYQQEYTNNYFQLLEQHYSLFEVGSPNGYPHLPSSHVAQSDPPAQKLIYPL
EPENNSQNNLPDITVGFPIATYLDKEKLRDTLINIVSDKTGYPVEMLDLSMDIEADLGID
SIKRVEILGGLLELYPDLPRPNPEELAQLATLEQIAEYINNLITQVYENEPVEETVGSGK

SPLSQEIPVKAINEQTATIETPSPVTELDNHPQFLVLSHEDSLDRSHQPTITIPDNLSQI
LLTIVSDKTGYPAEMLDLSMDMEADLGIDSIKRVEILGGLLELYPDLPRPNPEELAQLAT
LEQIAEYMRNQAETVERSNLSPLEKPDMSTTEVGDKILRLPVQLKPLPQPDSLDLTIPEN
HFVLITNDGSEVTHRLVAKLADKGCKTVVLTFPCLESNLSEEIAQIRLNDWHEETLQEHL
TELTTKFGCVGGFIHLHPNSNNNLGMDKAIVQHVFLIAKHLKEDLNQLAKKERACFFAVV
RLDGELGTAKTHNFSPISGGLFGLTKSLNQEWPEVFCRTLDLSPDLDADTTVKHILAELQ
DPNLLVTEVGYNKIDRFTLVAEPGKSSIIPDSLNITKNQVFLVSGGAKGITAKCVIKLAE
EYQCKFILLGRSSAEIEPVWSEGYEDENELKRRIMEDFLAKGEKPTPIMVQKKYQTISSQ
REIHNTLKAITEAGGKAEYVCVDITDGMMLREKLTPIIDQFGTITGIIHGAGNLADKRIE
KKTVQDFETVYAAKVQGLENLLNIVETNQLEYLILFSSVVGFYGNVGQTDYAIANEILNK
SAHVIKHKHPNCHVVSINWGPWDSGMVSPELQTAFAQRGIETIPQELGSSILVDQLRNSN
STMTQVVIGSPLVYIPSTLSSELKTHQITRQLKLNYNPFLQDHVIAGNPVLPATCGLSWI
SSSCEQLYPGFQTFHCPNFKVLKGIVFDQNSPHEYILEIQEVAKIDNQEVQLAGKISSVT
NHGKIRYHFSSNLILKRQIPPADNYEFFNLTQDGQFLASNSLLYQTGGGSLFHGNTFQGV
KSVLNISPGKLTMKCELPEPTLYQQGQFRVQTLNPYIADVQIHSLWIWTQHFHQVGCLPS
EIENFEQFAPVPFGETFYVTCEIKSKTESYVVADVITHNQKGQVYNQIKASKATILPNSY
>OHY33097.1
MRSHFSAAHRLAPNLSAEKYGKCTRTHGHNYHLEVTVEGEMDGRTGMIVDLGCLHEIVER
EILELFDHSCINEDIPYFSTSHIVPTTENIARYMSDLLQFPISQLGVKLSRVKLFESDHL
WVEYEGKDSEIFFSVATGFSAAHRLADPTLSLEKNQTIYGKCSRINGHGHNYYLEVTVQG
EIDSVTGMSVDLVGLNQIIQHYVIEPMDHSFLNQDLPYFTEVVPTAENIAVYITNVVRSP
IEELGAKLHKVKLIESPNNSCEIYARDIEESKVDRIYRELAAV
>OHY33260.1
MWGFTGDGGAMYTIQSLWTAARHNVDAKFVICNNSSYRLLQLNIQAYWNERNIPLHDFPL
SFDLSKPSIQFAQLAQAMGVEAIRVEDPNQIEPAIQKALEHSGPFLIDLVLEGDVHPELV
GVRCG
>OHY33391.1
MGQNWAISIGINHYDNLQPLKYAKRDAEAMKEWFRTSGFNQVFLFTEDSPGIPTSPPIVT
RPTYGTLRRFLNVQFERPLLQSGDNLWFFFAGHGQRHQDKDYLILNDTDPHDIEHTAISV
EYATQRLRRCGADNVILLLDACRNEGRRDGLGFGDKKYQGVITFYSCSANQQAWEIDALQ
HGTFTHTLLEGLKIEGEGNCATVERLYRHLTSEIPNLNTRYGKQPQNPYLSADPPYKMNY
ILLPRFANERDIDPLVMEAQNAEIRKDLDLAEKLWERILAANGTNQRALEGIRRIAAVRA
TTVPGTSDGIQSETETQSLHTPNRYTKLETLLKDKNFREADSETDSVMLAVANRQSEGYL
RVEDAENFPCQELRTIDNLWLKYSQGKFGISVQQEIY
>OHY33416.1
MQKNNIKNILIQALKPNRFSVMIGKIFKRFNDKQGMHSSNENLSWIESHSCEFQQLAMKL
DMELWQEAECFSQKLQINSEEKLKNINYPLGGGGLYPLLYFITRYVKPSSILETGVAAGW
SSCAFLEAIKINGKGKLYSSDFPYFRLPNPERYIGILVDESLKSNWDLYIDGDEKNLPMI
LNKLDAIDMFHYDSDKSYSGREMVMFGIETKLSQNSIILMDDIQDNSFFYDYIERNNIQE
WYVFEFQGKYVGMIGSLH
>OHY33418.1
MKILVTGAAGFIGFHLSQRLLSRGDMVVGLDNLNNYYDASLKQDRLSQLESQRTFTFAKL
DLEDQEGVNSLFKKHNFDTVVNLAAQAGVRYSLENPHAYINSNILGFTNILEACRYKQVK
HLVFASSSSVYGANTKTPFSVHDNVDHPISLYAATKKANELMAHTYSHLYGIPSTGLRFF
TVYGPWGRPDMALFLFTKAILSGKPIDVFNFGKMKRDFTYIDDIVEGLVRVIDRIPQGNS
NWSGYNPDPGTSKAPYKIYNIGNNNPVELLHFINVIEECLGIKAQKNMLPLQLGDVTMTY
ADVDDLVADVGFKPSTSIELGVQRFIEWYKKYYIS
>OHY33430.1
MNQAHKAVFLDRDGVINRSLVKQGKPYPPATIDELEILPGVDEALISLKKEGFLLIVVTN
QPDVARGKTKKEFVNAINSRLASSLPIDDFFTCFHDDSDNCDCRKPKPGSLFSAATRHDI
CLPSSFMVGDRWRDIEAGYGAGCRTIFIDYGYDEKQPDHFDFRVSSLFEAARIILKTPEK
FDEKD

>OHY33431.1
MTYAQQHLEEATRIIEKIDFDTVEQVADILACVKAEEGRIFFLGVGGSAGNCSHAVNDFR
KIVGIESYAPTDNVSELTARVNDEGWATIFVEWLKTSKLNSKDCVFVFSVGGGNLEKNIS
PNLVEALKYTKTVSAKITGVVGRDGGYTAKVADACVIIPTVNPDTITPHSEAFQAVIWHL
LVSHPKLKANQTKWESTVK
>OHY33433.1
MIITRSPLRITLGGGGTDLPSYYRDHEGFLIAAAIDKYVYVTVMRPFTEGIFLKYSQLEH
VNEIAEVKHPIIRECLHILDLKTPQVEITTLADIPAGTGLGSSGSFTTALLKALYTHRRC
HLHQEELAELACHIEIDRLGEPIGKQDQYAAAIGGITCFTFHKDDQVTANPLAISMDTMF
DLEDNLLLFFTGFSRSASGILKDQKERTQKSDGDMLANLHYVKDLGYRSKAALESGDTYL
FGQLMHEHWEHKKKRSGGMSNPQIDEWYQLAMNNDAIGGKLVGAGGGGFLMFMASDRNKL
RHAMTNAGLEEVRFGFDFEGTKVVLTS
>OHY33434.1
MIFNLLYKSAYILEKLSAFAQGKGYGSRSIRQEIAVAKKLMQSLQPELLMIDIGGNIGDY
TYQLRKGFKQAEVHIFEPSIVNVNKLSQRFKGDPLVILNPVGVSNCEGSFLLYSNEQGSG
IASLSKRRLDHFDISFDFSEQIQTICFENYWINQLNRKRINLVKLDIEGHELDALRGFGS
AIWATELIQFEFGGSNLDTHTTFQDFFYFFKEHNYEIYSRKNK
>OHY33436.1
MLLYQFQGQSKKYSFYTKNNAGYKLMRRLGIKLSKGYPKTTMEKLFYSHIFKASEVAFFR
RRFDWAIK
>OHY33437.1
MNSKVALITGASRGLGKVLAHRFWESNYSLYLIARSYEELQKVRSSLPPRPSQNCDIYGC
DLGISESIERLRSEIYNNLSRLNVLINNAGTHGPIGQSWINNTSDWQKTIQVNLFAPVAL
CQIAVPLMEQTGGGVIINLSGGGATGPRPNFSAYATAKAALVRFSETLAEETRGISIRVN
CIAPGAMKTALLAEILEKGTQLSGEREFDLASKVLVEGGASMDRVADLALFLASEDSKGI
TGKLISAVWDRWEDWPLYLDELSKTDVYTLRRIVGRDRGMTWGDK
>OHY33438.1
MKKVFITGCAGFIGSNLTDRLLSLGTKVTGYDNFSTGQERFLALASKNANFNLVRGDLLD
QTVLTNAMEGCEMVFHLAANADVRFGTNHPRRDLEQNTIATYNVLEAMRQNGIQHIAFSS
TGSVYGEAPVIPTPEDAPFPIQTSLYGASKLAGEGLIAAYCEGFGFQSWIFRFVSILGER
YTHGHVFDFYKQLKADPTRLAVLGNGTQRKSYLYIQDCLDAILLALERASNRVNIFNLGV
DDYCQVNNSIGWICQELGVNPQLEYSGGDRGWIGDNPFIHLDVSKIQALGWEPKLTIREG
VIKTVQYLRANEWVFEVRK
>OHY33563.1
MKVREGDLEIDFTDAIEALIFDQMKDKTLPDYHGVAEMYRVDFVVEFETRIVFVEIKDPG
NPKAQVKGLEKFWEELKNGSLSRTFANKFVDSFLYRWAEEKIHKPVDYLNIITLDDAVLL
DNFSDEIRKVIPPMGKTVPRWRRQLVENCQVFNLETWNETFPGWPVTRLNQSTERREN
>OHY33721.1
MCYGTKQINAALSTTLNPKVMTSFRKFAQNCQVGDVNPCFDKYSDLDSGAAAFVNNEVDA
FFGYSERLNYILKNSSNSDVQLSSLPLSEGSNPLLFADALVLRKDCDQTCENAANTFAAY
LDNPDTQEWILSSKDAGENAVPRYLIPATYSAFTTNSLAKDSYYQTLEKVVKNADAYPNS
GFAEIRKTLKKAILQELQSSS
>OHY33730.1
MKKLQNLLFSFLIFTLVFLGITAFFIPNIAYAQTTSKTILKVALFPYIPDSAEDQYQTLL
NRIESEFETQNPNIDLVLKPLNPEEEGFYDIDTLKQWLTNPSKQDGYDLVEVDTLLLGDL
VKANVVKTWNKPENIKDWYPAGLQGVTVNGDIYGVPHLLCGHFIISRNDKVAKTKSVEKL
LNILTAITPDTPNLAGDLTGSWNLPALYLDGWADTYGTRSVEC
>OHY33754.1
MNDLPRQKLKEIIIQHGRSLCDNPQRCEAFLRDYCGGYRREIFILISALKQDAAKDLLNS
NNVPLELLVSRLIKKMQNELGLTEEAAHYAVESWAQALDKMPQQQIQQPRFDVINKANKK
LNHPVSSQQTTVVSPLFTTNQQQQKLVLGRGLLKKAGVSVGLFILVVIVQQIFTANSTEP
EINGYPTTEITELPTPEITRSSTPKVTRSLRRKITEPTEPEITEPTEPEITEPTEPEITE

PTEPEITEPTEPEITESTEVEVDESSKPENAEFLEREIREVNESPTPEVTESPTLEVTES
PTPEVTESPTPEKTNPI
>OHY33900.1
MALQLKLQLFTNNNGLIGDPINEVPFENSFFLQILAGDFRSDAEGLIGFVTDLQWQPGQI
QALDDPFGPKTLVTSSFPLFVGGTLDKTLGLINDLTGGSLPEFEIGEAIGIKKWEPFATL
LFQAIGTKIINVTDFTLTPDLSNLSFADGYINNAPIISDPGIVLENSNPTILTVSDPNPS
DILIFKITGGADQQWFTLNTNGELLFNLGENKSPNYEDPLDSDQNNSYQVEITAYDNFGE
LKFGDLEKTVLGVTTVRMLIIEVINVNETPTNISLNATTVDENIPTNTVIGTFSTTDPDA
GNTFTYSLVGGDTDNSVFSIVDNQLQINNSPDFETKSSYSIRVKTRDQGGLGFEKTLTIT
VNNLNEAPTFPTDTATFSSVENSTSVGTITGATDPDGHTLTYSIEGADANKFNFDTSNRV
LSFKTAPDFEASGSAVGTNVYTVTVTATDGAGLTATQAVTVNVTDVVEVGNPPLITSSST
FFVAENSTVVEAITATDANLEDILTYIISGGLDQSLFTIDANTGVLGFVTAPDFEAPGDT
GTDNFYNLQIQVTDSKNPVTQDLIVAVNNLNEAPTDIILININLYENVPINTVIGAFSTT
DPDAGNTFTYSLVGGGADNPVFSIVGNQLQINNSPDFETKSSYSIRVKTTDQDGLEFEKT
LGITVNNLNEAPTDIILDNFSIDENVPINTVIGAFSTTDPDAGNTFTYSLVGGGADNPVF
SIVGNQLQINNSPDFETKSSYSIRVKTTDQDGLEFEKTLGITVNNLNEVPSRVISGTDQA
DNINATTGQTTVMPGKGDDIIRVNSASVVIIELPNEGNDTVFSSINYNLASLPQIENLTL
TGTGDINGIGNRKDNVITGNSGQNVLTGLQGNDTFVFNFGDSVVGKLDRIGDFQFGKDKI
KVNAVSPSVLTRASNSGASTLSSLVDSVFIDANGAESGNQGLGTNSAALVVSIAQGIAGT
YLIVNDGVGEFNPTTDLVINLTGYSGTLPGVGNIGIANLFV
>OHY33901.1
MIEILLLRLSSLTIFSRSRFTCSVVAIKVEAGNFANSVLKTFSICSKNERAEQAINNRPS
LLSIFGGMP
>OHY34129.1
MGEGGGNIISGGAGADQFWILTDDPTRLQTPNRIVDYTVGTDVIGITNQFASSVGDLTFS
GSDISLNGVLIATLNGVNAAGATFVFANPPANLP
>OHY34199.1
MYWQNFINKSTNRKIFGAALVVAAMTALVYLSRTANELIIAWKFGTGDDIEAFLIALLIP
SLLITVIGGSFNAAFIPTYIQVKQLQGSPASQKLLSGVNGWSSVLLVLATIIMLLTAPVY
LRFVAGGFGEEKLHLTFQLLCIISTKVVFSGTLTIWRAVLNAGERFAYAALTPTITPILS
IFLLLALPSWGIYAVAGGLVGGSILELTAMGIALKRQRISLIPKFNSFDANLRQVAAQYV
PMVAGAFLMSSTTLVDQSMAAMLSPGSVAALNYGNKLVGLPMVLATTALSTAVTPYFSQM
VAAQDWSSVRNTLKKYLFCIFLISIPLTGIFILLSHPITAIFLQRGSFTSEDTNLVSQIQ
SCYALQIPFYIGGIFVVRLISALKCNNILMLAAAINLLLNISFNFILMNIIGAAGIALST
SLVYMFSFLFVLFSLHQKIQCFDS
>OHY34201.1
MQQEKIWQYYQNEGLSSFKNNWGRLEFLARQIRKKLSGKPRGLNIGVGNGSLEKIAISLG
LDIYSLDPDNCAIERLIQELGMDGKAKVGYLQNLPFPDNFFDFIVVSEVLEHLSDDILDQ
SLHEFNRVLKSKGMIIGTVPARENLQDKIIICPSCGEKFHRWGHVQSFDSVRLFNLLSKH
FQVEKIKEKWFFTIPNWKAKIVDCIKITIKITMEKCGISVPGKTIYFLISKK
>OHY34232.1
MAILAVGAGQTYTTIQAAINAANNDDIIVVRPGIYQEDLTINKSVTLIGPYGTFEGIDGF
ENRLGVKPLDPDINAALGVGGLPTANEDFRRYQDDNGTIDNAFGNTQEAWIKGTITVTED
NVTIDGFRLRNENGPLKWNDTPDNFKLLNNYLTGYTANNSPSFGDASINNPTGVVTGWQI
AGNYIGGLLGGGGTGGSIYLAGLQDSNIDDNTFWRPRAAHLYLASLTNVTIEDNKFYHGL
HTGGANFDGFGEFFSGSGYGYGGYGDGYGGYGDGFFGRNYWLELKGDNDQVLIKNNEGEY
NSGGIQLFGETDSPFAFDNITIEGNTFPDNNFINAYSEAPSNGKSGLIPAVMATARLSGP
SGSNLVIRDNDITMDLAQVKFITDHKSSLEVRGNFNGVTVEGNTLTPKNINGGVDIITGL
SLYGSLPGETLIRGNQLLGQDGDPLEASYYGIDLIPTFADYGTYTGDLTVEDNTINSWQV
GVNLRNTNEITGDININGNTFENNAYGVVLDATATTNSINIAGNTFSNNFSNVFDGIDPV
ITMDQVLSYEENQDLGSVLGTVSATDNLPNTDNVGVTQYFISSGNDDGFFTINSSGEITL
TEAGLAAANDFETSLSSFNLGIIVTDGGGLQDTETITLSIINVNEGLGQLPPITTEGAGF

TVGATLIAAIPFDDPDGIPTDISYQWQRLIEGVWTNIPDATEQNYVATEDDNNNQLRVEV
TYIAGGFEKVIYSNNVSISLVLVSGTFDSITGDNNLDISEKEAGVTLTGSVSEIGTTVTI
LFGGQTRVAQVDGLSWSYVLKPNDYNFFAAGSNLFTAIFTRTDGGETGSFTTFQTLTIPD
GILPPNTSNAFDPTQPKGLKSEVIDAAQTLEIDGVSILEISKTVGILGEGESFNDPNIAV
LPIGTRDIFDQGANAGAANYAAFKTEPGTSIQSIYIVPAVEEEGVKKLQVVLADGTVVEA
EIPPDLISPIGDPLAVTISGVQPGGTTTFVLYLSQNVINQLPEDLNLARYVKFNYESQQF
ELYDDFNYDYIFNDVDGDGVRDFGEVYLTVNLTDGDIWDGDGLANGIIVDPGQLGIATDS
GTDNNPPIAIELRGIVAENDPGAFIGSLTVTDDPGDSHTFTVNDSRFEVINVDGNNILKL
REGESLDYEAASNIVLTITAIDNGGLEITQDFTITVTDVNEAPVAIELNQITVIENDPGA
IIGTLTVSDPDGNDGHTLKVNNDTRFEIVDFDGNQTLKLKAGESLDYEAGSVKLSITATD
NGGLEVTQDLTVSITDVNEPPVVSFSFFVPESTTLVSNLTVEDPENDPITLSLAGVDASL
FSISPTGELTFNTAPDFEEPLNADKNNLYKLQVVARDEQNNKSIQDISILVTNVNEAPIA
IDDVLAIIPGSSFGTLNPLDNDSDPDLNDPLTIINKTDGNYGRVEIRDNELIYTLLDATY
IGDDVFSYTIIDEQGLAATANVNVTITGTDIITYPVEILDPEDSLIPDEAGPLSDIVNDI
SFNFLTDYDKVQAKLALQEALSKTEASFTNLFGLYEVDNALTGSVNGVLPEDKSAYAKAA
LSRVVPNFVVRAGGSGDGVNGDVIVSEGKIYAPFVIAHGGNFSGSVQDAVNAFFQVNPDN
SPATAQNYTTLPVAYFSFGSANPDGAAHIKSFGNNVFGFEDLPAGVGVSDYDFNDTVFSF
G
>OHY34262.1
MPYSHKSTKKQRQYNQDLTAKRFASTVPAQTLSWLSSISLLSGGFVFAQTESPVDNIVST
VEVSQPTTLGDKGQNGQVSLPVESKVTELKTGVQTQGKNSEVNSNLESVATEGNSESRSE
SSPTSVILKVPALPVLKSQPETLPTSTQPIPETSTLVIPVTSQPNNSDNGNTGKDYNSTQ
IDPTEYNGNSTVKYDAPSSVEVTGRTQDCKAVISQPGKSVGTCGGKNPTNPSVANSTRKS
APTWLKKSGAVNLGKSQLKTVPNENALSTNNSSPRSQGQKSEEVVNSLAATVNNSQNWHS
LGSNSSSRHTSAGGYMGRISTKTSYNRPRDPREFSSNTTVTPITPSFGTLPPPMIEGKVA
PRPSKVAYDFDLASVLPQVPYISSFADNGTNSGSVSGITYPLSFAAPITSLFGWRTHPIT
GDRRFHAGMDIAAPTGTPILAAEKGQVEMADWMGGYGLAVTINHDQRQQTLYGHMSEILV
RPGQWVEPGMIIGRVGSTGNSTGPHLHFEVRHLTANGWVAVDPSMQLQAGMNSLYNRVAY
NYNSQSKQLR
>OHY34268.1
MAFNLNGFNFNQSIIDSQGRVIGTWADVINRANLGMEVMHERNAHNFPLDLAAGEVAPVA
LTAPAING
>OHY34437.1
MYCISALDSKNCQLLISVNLLSIHKIYARAAKVKFLPPYSPDLSPIELCWSKLKQFLRSR
EARTLEALNEAMTSAVNYITAEDALNWFNHCGLFT
>OHY34438.1
MSNTKVDVSFILGDIFAAGDQNIHIDLNAYEIGKNHPVDIGLVADPKLTLARMAEILRES
MTPAQQEAAELRVKMLKNAKQNTIRDQRVKDREDWDNRPLHIVQFAEALGRLLPDNVVVF
DEALTNSPPLSRYLRVTEPGSYFLTRGGFKLFGNFYP
>OHY34474.1
MKRELLATIALVAPLLFVSSVNAGNNRDLQKLLSTGECQKCQLSRVNLSGSHLIGADLRG
ANLQGADLTGVNLEGADLTGANLSGANLTSAFVTNVNMKRANLNGANFTSAVINDSNVYQ
ASMNNLTIADAQIYNTGIGVGGEDGQIPDWD
>OHY34530.1
MLQATTIQISCKNNLHNILPDIKELLDTCYPRPPRNVFYLLIEKYCVGFPVYIAIDNFSR
IVGFTYLAINSKGGTLESLAVHPDFRNQNLGSQLVNTLLKENKGVIQITTRIPKFFEKLG
FEYVKTLPDQSHYMININFS
>OHY34532.1
MIILIPTYYKNEKYCNLLLDLLEVNWPNHPELYFLTDGGNINYPNVIKVDNKNWLIVLYK
GLKYLINKYPDLDYIYLVLEDLIPLWSLSVEELTKIENVVINNKLKCVCFPTYPAYWGQE
NEVKLDGITLYKTPEEFDFYSQLQPSIWQVGHLMKICEHALENNLLDAWSFEWIKSEEQH
YVSSYQWSTVFNGFLVRGRVNLAAINKIKLPEGKKLKNQLLKSFIFDLPSLIKYRINRKL

KLIG
>OHY34559.1
MYLLFQTLASFVEIYSYVLIVRVLLTWFPQINWYNQPFAALSQVSDPYLNLFRNIIPSLG
GIDISPILAFFVLNIVSSLLENLSRATSLGGF
>OHY34602.1
MGDNTGGSGTLIAQEGGSYLVLTSGDVISGITPAALRIQTHDGRVHQGRALYNYKLADQQ
QLDKINLVILEFTPNRKYCLTKQILNTAIKQDTAVMASGYSVNSSKIIFSPGTIKQIVSQ
PTFVQGYEIGYDSASATQQGMSGGPIINSTGDLIGIHGKSAFPILNNGYVYADGKKPLVS
EIKEFRKLSWGIPVGSILAQLKPEILARYGLPVPRRNRSVPEIPILPEWLNNIESKVREF
TVRIDGDGNNGSGVIIAREGNTYTVLTSAHVLCKIPHKTSNNHCATKNHTLVTASGQKYP
LDNSSIKLVQGVDIATVKFNSGENYPVATLANYAVENHQYVFTVGEPKLGQTPRLTVGQI
FSKENGLLALKSAGQELKDIDYTTIEDANLGKEYELVYTSVSQRGMSGGPVVDSQGRVIG
IHGKSEGQVVMEETTEDGGVDDRVQLGYNLGIPISTFLRIAPQLNTRPERVENTPAPQLK
SWEIESIRKTIVLVNASMGKASAIEWIERGNQLWLLGRYQEAVTAFENAIERKPAFIHLA
YYGKGLSLESNGNDTEATGAFEQAVKAKFDFSVAWNRLAALNIKFGRLSMALAATNQAIK
LQSMDTSLYSQKFYILLSLTMYQEAIQVMDQAILLNPHHGFYINRGAARRELGDYKGAID
DYTQAIEISPELASVYYERGGARRELGDYKGAIDDYTQAIKISPELASVYYERGGARREL
GDYKGAIDDYTQAIEISPEFASAYYDRGGVRRKLRDYKGAIDDYTQTIKTDPESAFLAYY
ERGGARRELGDYKGAIDDYTQAIEISPEFASAYYERGFARRELGDENGAGFDFQRASDLQ
VSRPRILPKD
>OHY34603.1
MNLTSCLPILFLVPTLVLSSSPQSLAKTTPKQESSCELEREGEFYSPEQLKTIAQRITVR
VIADNSGGSGTLIAQEGNSYLVLTSNDVISGTTPSALRIQTHDGRIHQGRMLYNYNNAEQ
QQLDKINLVILEFTSNRKYCLTKQIVNTEIRQDTAVLASGYSVNSSKIIFSPGTIKQIVS
QPTFAQGYEIGYDSTIQQGMGGGPIINSTGDLIGIHGKSAFPILNNGYVYADGKTPPLSE
IEEFRKLSWGIPVSSILAQLKPEVLSRYGLPIPRRNRSVPEIPILPEWLGNIESKVRQFT
VRIDGGGNNGSGVIIAREGNTYTVLTSAHVVCKIPQKISRSKEDKNKCVEENYTVIAASG
REYPLDNGSIKLGKGVDLATVKFNSGENYPVATLADYPVANHQYIFTVGYPKLGRTPSWR
FTIGQIFSRENGLLALTSTRQNLKSIDYTIQDANVGKEYELVYTSITLGGMSGGAVVDSQ
GRVIGIHGKAEGQVVMEETTEDEAVSIGNRVQLGYSLGIPISTFLRIAPELNTRVDRVEN
TPAPQLKSWEVESIRKAILSVNVSRGNASAIEWVERGNQLWLLGRYQEAATAFENAIERR
PAFIHLAYYGKALSLWWNGNDTETGRGILLSRTIKNHSSKNYSQSYGRQHWWFRNSNCPG
RWLLFGADKW
>OHY34620.1
MLQDTQTIRYYQRLTDAFVELWNRGYHRDDMGMYLDGYLAALRQSNAIEPYLIHRLEEEA
IRYLHDGSNFAVVQPEPERYHGY
>OHY34663.1
MNIETLKSEKTKQLPGANLEDQDLSEFDLTAVNLAGANLMGAHLVSANLEGSHLEGANLM
GASLQGADLRANLLGANLMQADLTGADLRGSNLRGANLMGATVAGASLTAAFLSGANLMS
VNFQGVDLRGADLRGANLIGANLKGADLSRADLQGALLNQANLEESDLRGANLAGANLAG
ANLLCAELEAASLNGANLYQACLLGTILETYHD
>OHY34808.1
MNRTTIKNFAIWARNHLKEQVSTRATQLTITEKTITEKTITDQRTFAGGLLSGEQTLNSE
EAKQYQKLHSHIEYLLKQQASKNLDKKFNSNNRRKQLSRVVEKNKRGSDFDGGFNIHTVI
SLGDQIDQRLPQHQPRECFAIALKFL
>OHY34812.1
MQQVEKSPDAVAIIFENQQLTYGELNCKANQLAHYLQSIGVGPEVFVGLCVTRSIEIVIG
IMGILRAGGVYVPLDPAYPQERLAFMLEDAKPKVVLTENQCLEALPIINATVLCLDADWQ
KIEQQSEDNPSCDVTPDNLAYLIYTSGSTGKPKGVQMPHSSIVNYLQGITKIIPVDNQDI
YLHTASFSFTASVRQLFLPLSQGAAVVIATREKTRDPLRLFELIETQEVTICDGVPSVWR
YGLMALESLDKKYTVAIGESKLKYLIFGGELLPYQLIKKLRNLFQTPPQFFNILGQTETI
GNAFYPIPENCDIEQGYVPVGNPVL

>OHY34821.1
MNPKNLPLGINTLSMLRENNCVYVDKTEIAHGLIRIPGRFFLSRPRRFGKSLFIDTLKEI
FEGNQKLFEGLYIHDQWDWSRKFPVIKIDFAGGVLKNRQELDLRILDILHENAEYLGVSY
ESTDIPGKLGTLIRKAMAKYGERAVVLVDEYDKPILDNIDNPPIAAEMREGLKNLLPGDK
VVKQLVS
>OHY34844.1
MIPACDGVSFDLYPGQVLGIVGESGSGKSTLLKAIAHYITVDEGSIIYRNRQEQYLKIQE
LAEYQRRWLMRTEWGFVQQNPRDGLRMQVSAGANIGERLLDIGMRNYGQIRQEAIRWLQE
VEIDPDRLDDLPTTFSGGMQQRLQLARVLVTRPRLILMDEPTGGLDVSVQARLLDLLRSL
VRNFNLSVILVTHDIGVVRLLAHRLLVMQQGKVVESGLTDQVLDDPQHPYTQLLVSAALT
P
>OHY35001.1
MDSYSFLNSHTHNNPLYKRNNWQSSYNSYNQKELADSDIGTLKDEKYMRSYALKLAHQGE
YRKAIALLDRIIDSHPENAIDYNNRGLIYFQSGHAQKALLDYNTAMQLNPRLASVYNNRA
NYYAARGDLVKALSDYDQALDLNPRYVRAWINRGITLRELGEYKDAIDDLEIALLFGQLE
IHIWSERGRSYHLWGDWNCAIADYRRVLSHTMSLDKIEDIITYRLRLQVENWLGELGFST
YK
>OHY35003.1
MKDLIIVSGLSAGVGIGVILTGIANGQASIGVLSSTAGAIMGASVVRKLEDDRNRVTLVK
LEELKNRELTRQESTNLIQQIETLRPTVKQLQQDREREEENLVVLRGKLGNQQAELEFIR
REWEQNQSRLEDITQTVANLTAQKQELENRISVINQENPRLLELEELRRAIEQSRIEKSA
LEGKISGLTSQLESLDSRRQETVSIELELRAKEIDLEKLTTQVENLKNQSQELEQRAADL
ELLRFTYDNIIKQNQDYEVKVNQLRPEIQRLELEKQQILHSIQENQNEYQKVQELREKLH
SLRLSIADESSRLREVETETQTWQGKKAALEESNGHLQEQKQELERTISRYKQEIVEIEN
SAELALQPLKEKLWNNLPQIIKPRKQESEFIADFITRIRSQGLTFSTRTVNAFHTSLKVQ
DISALVILAGISGTGKSELPQRYANYLGAQLLTLAVQPRWDSPQDLQGFYNYVEKKFKPT
DLMRGLYQYGQPDMQDRIVIVLLDEMNLARVEYYFSEFLSKLESRRNHATYLELDVGSLR
IRENERRLQIPNSFLFVGTMNEDETTQTLSDKVLDRANVLTFGRPQNLKLRQENQGAAVQ
HHDSSYLPYSHFQSWVKLPNPNSEIVRSVKYFLNQANEVMEKMGHPFAHRVYQAITQYVV
NYPQVESIESPAFKFALADQFGQKLLPKLRGVMVDEAHEQLEEMGRIIDDINDQPLITAF
DKARKGRYGQFTWQGLVYEDEEV
>OHY35045.1
MKTRPSPVYAMASAPTVTPDQGTQVVRKPYPNYKVIVLNDDFNTFEHVAKSLMKYIPGMT
TEQAWELTNQVHYEGQAIVWVGPQEQAELYHQQLHRAGLTMAPLEAA
>OHY35058.1
MLDQISQYLQFNISIEAPIVLTVLVFLEAVLSADNAIALAVIAQGLEDKTLERRALNVGL
IIAYILRISLLLTATWVQQFWQFELLGALYLLWLVFQHFTSQEKADHHHHGPRFKSLWQA
IPVIAFTDLAFSLDSVTTAIAVSQETWLVLIGTTIGVITLRFMAELFIRWLDEYENLADA
GYITVALVGLRLLLKVLNEAFVPPQWLMVGAIAIILVWGFSKRNIVGEVEIEQIEETEKT
QV
>OHY35093.1
MPKTNSFCIEVLVEEPSAEEALKTLLPKLLKGRSRHKIINFGSKSKLLKLLSQRLSAYGD
RISKGENVRIIVLVDQDNDDCKKLKNQLEAIAQQTGLSTKSKPDSEGRFVVLNRIVIKEL
ESWFIGDTEALRKAFSSLPSISPKSGIFRSPDNGGSWEALHRFLKKHGIYKNGYPKIEAA
RRISLHMNPDQNYSPSFNCFKAGIEAFLSQ
>OHY35094.1
MKRLLTVALLTTGTITGSFFLNVYAEPSLTAFQSDSNSYFSIPSAEAANRKGSRYVGGTS
SDGTSRSASGRCNSPSGRAASVRPGGR
>OHY35099.1
MMNQNKTPPRIQYLKIENFRALRKVELKNLTPLTVLLGPNGSGKSTLFDVFAFLSECFEL
GLRRAWDKRGRAKELKTRGSDEAIVIEIQYKEPDYPTITYHLAVDERNGAPFVKEEWLKW
RRGKHGQPFNFLDYKEGNGKAISGELPDEKDKRIEIPLKSPDLLAVNALGQFAEHPRVAA

LRDFITGWHVSYLSADSIRTQPEAGPQEHLNRSGDNLANVIQYLNEQHRDRLEAIFTTLR
RRIPRIERVLTESMPDGRLLLQIKDTPFTHPIIARFASDGSLKMLAYLVLLQDPAPPSFI
GIEEPENYLHPRLLPELAEECQQATARTQMLVTTHSPFFINQLKPKQVRIIYRGTDGYTR
TQLIEDIPGIKEFIQHGANLGDLWMEGHFEVGDPLNQEGI
>OHY35592.1
MGIELGILYDNQKPPTPWLRWWDEKGSLLLTGNERAEQAEAIAIRERLAKEQEQEAKEQA
EAIAIRERLAKEQAEAVAIRERLAKEQAEAIAIRERLAKEQEQEAKEQAEAIATRERQQK
EKLVAYLRSLGIDPEKI
>OHY35704.1
MVSKNFWKPIIHSSTALILLTTLNTAWPLVSLAQSKPQPKVNSASSSLFTDYLLGGGDRI
RVNVFEAPEYTGEYQIPPGGEINMPLIGSIPVSGLTTQQAADEIARRYARFLKRPLISVN
LLAPRPINVFVAGEVTRPGSYSLSLQGTGGNNPGVQYPTVLAALTTAEGVTLAADITKVQ
LRRQVGRSGEQVISLDLKQITQTGRIPIDITLRDGDTIFVPTATDFNVAEARNLFAASYA
ASRTAPRRVAITGQVYRPGSYLVAAGGGNDSGGLPTVMRAIQLSGGITSQADVRNIKVRR
PTRTDKEQTLNINLWELLQSGDLNQDVVVQDGDTIIVPTATQVNTAEVTQLATTTLSPAT
IKVGVVGEVKRPGVTELQPNSSLNQALLAAGGFNDARASSSSVDLVRLNPNGTVTKRAVK
VDLSKGINEETNPILRNNDVIVVNRSVLARTGDTLGAVTAPLAPVFSIISLFRLLGF
>OHY35795.1
MWFQYVFKNWITLFPTPRIFPIRVTSIDPGMVETEFSQVRFHGDREKAKKVYEGIKPLTA
QM
>OHY35854.1
MNERLPLSINQKEIYVDQIMSPDSCHMHIGATVTVRGIFDRQILNYAMSKTIDCHPGLKT
RIYELDGQPFQTIASDTSNHIPFIDFSGHDNSDEQAENYINQEFIKPLTFGENVPLADFQ
LIKVCDDKHIVYAKYHHVITDGWGAAIFFREVIKTYNQILQEGREGQETRDWVITEYIEE
ERKYLASDIFMRDQHYWQQRLNNVSPMIFSPIKQPQELDGKRHSIYIPRHQYDQVDELCK
NVKSNVFHFILSLIAIYVSKHYLKNDVVLGLSLLNRSKNIFKDAIGMFVSTIPFRLEVEQ
EDTIHQLLDKIRYTLRQDYRHQKFPVAEMKQLSGLKATSKQHLFEVFLSYERHDYADNFL
GTKTTCVPLYSQQQKVPLIIYVREYEKTDDVKIDFDYNLSYLDGEAVGEMVRSFETLFTQ
AATNLEISIGDLAICDSETINISQPDSPSTKFFADDTETLVSAFEKVVSQYPQNLAVQFD
GELYKKFLSYTELNDQANRLANYLISQGVKPGSRVGICLERSEQIIVAILAIIKTGSAYV
PIDPHAPSVRRQFIVQDSGMTTLITETSLIAELVTENISTLTIESINLALAKQANTLPRI
SIKPDFPAYIIYTSGSTGTPKGCVVTHKNAIRLMRATEPWFGFNEKDIWTLFHSFAFDFS
VWELWGALLYGGKVIVVPFWLSRNPEKFREFLSTEKVTVLNQTPSAFYQLIHADQSSVQD
IYLRYIMFGGEALNIQSLQPWLERYGDKKPYLINMYGITETTVHVTYRPITRQDLKVRGS
FIGKEIADLHIYLLDEKLSPVADGIPGEIYVGGAGVTNGYLNRPALTAERFLPNPFGSGR
MYRSGDLAKRLPNGDLEYLGRIDQQVKIRGFRIELGEIQAALISHYQVREAVVITDEWEE
EKRLVAYYVPDESSPTAHELRQYLKNKLPDYMIPAAYVKLEVLPLNVNGKVDIKALPMPD
WNLLRVEEDYIAPRNLDEEILCTIVAEILGLQKVGIDDNFFEIGGDSILALQVVARAKNA
GFLISAGELYELATVRYLATKKAIITSGDGVKKLPNIGSALVSDADKLLLPQDVGDAYPL
SSLQSGMLYHSELDPDSAIFHQIFTFDLQIGYSELAWKQAIADICSANPVLRTSFHWTGY
SQPLQMVHEQVELPLSVVDLRGCDNANEQIREWIELEKNHNFDITQAPLFRLQIHRVSDL
KLSFSFSFHHVILDGWSVATLLTQLLRRYVEYLATENLPPLPVTQISYKDFIAQEQNVIT
NHTVREFWLQHLRNLQVTFLPRLSTNTAKTTAASHQKRQLKRMSVLVDTKLAEKLRQITK
NLGVPLKTSLLALHLRVLSFITGQKQVVTGNVINARPETSGSENLLGLFVNTIPFRLELP
QGNWLDLVRAVFRLETEILPHRTFPLAEIQRVLDQRPLFDVGFNYVHFHVYEGLLNLPQI
QVENVDIFEETDFPFLTEFCLVPGSAALQLNLIYDTQQFADTQVDQYANYYQAAMFDMVT
NPQTPYHRRSLISSQERQHLIQSANQNLKDFVSSQTLVSAFNQTVAKHANKTALVYQQTS
LSFGELEIQANRLAHYLQAKGIGPETLVGVCLERSEQLIISILAVLKAGGAYVPIDPAYP
SDRLEFLFRDSGIMLLITQRSVISQLPECGAEIIILEDIAKEIEGNNSQTPAVNILPENA
AYVIYTSGSTGKPKGCIVTHANVIRLFNSTTTWFNFHSEDIWTLFHSYAFDFSVWEMWGG
LLYGGQVVVVPHWTVRSPKEFLQMLATHRVTVLNQTPSAFKQLISVVRQKPEKLSLRYVI
FGGEALELADLQPWIDLYGHTQPELINMYGITETTVHVTYRPITQKDIINNLNHRPSVIG

QSIPDLELYILDENLDPTPTGVTGEIYIGGAGVTRGYLQQPGLTAQRFIPHAHRPGSRLY
RSGDLARYLPDGEIQYLGRADQQIKIRGFRIELEEIQLVITSHPDVKQALVVCQKSPTGE
NRIVAYVIFSGVAQPQNDLGKFLKTKLPDYMVPSVFVPIETIPLTINGKVDYAALPVHNW
NSIKKDYIAPRNDREATICSLMASLLKLERVGVDDDFFEIGGDSLLVTQLAISLRQTYDT
EFPLPELFTHRTPGEIALLVGDESPALPEIEIPKASRTRRSVTLTDDGILSKY
>OHY35864.1
MTNFSQADDIDLLGLLLEEEGIELEIKEVVPRVGSTEAPTSFQQRRLWFLYELEPTSSAY
NICSIFDLKGTLNITALRVAFKQLQQRHESLRTTFMDVDGEPWQKIHANSATELRLEDWS
NDRSEDKIPEIIAEIARHESDHQFNLQTGPLIRAQLFKIESKQHILSINLHHIIADAWSV
GIILEEIAMLYQAEISKTPMALPELKFQYTDYALWEKGNFQNSNILEKSLTYWEKQLAQL
PTLQFPLDFPRPRLQTFRGGLIKFEISKETTNRIRNFIVKEGATLFMFLMAVFQTLLSRY
TGQEDIAVGTSIANRPSDSENLIGFFVNMLVIRTNLADEPNFNSLLKTVKKTILSAFEHK
EIPFETLVEKLNLPRDTSRNPLFQIAFTLLNAPKPQFGTGDLEVCILATQEAARFDLELF
ITETEDTLNGAVSYNIDLLKRETVERVARHFCQLLDSVLAQPEIPVSRLPFLLSEEIAVL
APSQPTQTFPVHFCLHDIFTQQAKLRPQQTALIFGQERLTYLEVNERANQLAHYLMHVGV
KPEARVGLWLSRSLDLVIGIIAILKAGGVYVPFDPNYPKDRITYMLEDSQIRVLLTHSEF
EAQIPFEFTNNTNNVSNTNNNILTIFIDKYKSEFTQVVTTEPEVLILPDNAAYIIYTSGS
TGKPKGVVVTHRHVVRLMLSTEKWFKFNAKDVWTLFHSCAFDFSVWEIWGALFYGGVLVI
VPYLISRSPEEFYNLLCEEKVTVLNQTPSAFQQLIQAESTLCREGELELRYVIFGGEALD
LASLEPWFERHDDQFPLLVNMYGITETTVHVTYLPLTFKDVKKGSGSLIGKQIPDLSLYI
LDRHLQPVPIGVVGEMYVGGAGVTRGYFHRPQLTAERMIPNPFATNDLTSVRLYKTGDLA
RFLDNGNIEYIGRNDHQVKIRGFRIELGEIEALIKSHPEVRDALVIAREESKEDVRLDAY
IIPINQIANTETLTQEQTQEWQYTFNDTYNITSGETEEDFNIIGWNSSYSNQPIPGVEMR
QWLNNTLLRIQSLKPRKVLEIGCGTGMILLNIAPQVESYWGTDFSQAAINRLDTIVKNRS
LKNVNLLTREAIDFSEIPTGYFDTVVINSVAQYFPSIEYLQQVIKSVWQLLKTGGSLFIG
DNRNLSLSNYFYASVAYFQANDNTDCETFKTQVRRIAKKENELILAPHFFTDLRKSFPDL
TAVEIQIKSENNENELTKYRYDVILHKLGISTEQPPEIIWRDWETDNLQLTDLKQQVIEM
RSIGWHSIPNGRLSKDAAIYQWMLKNSHENEQKTIGELRTVLNNIHEPKGFNPADFYAIA
EEIGLEVSISYSPGKVDCFDVCFYPAGSGKSMAPSMPIVNDLLGRDNHPSWIDPLKNRLT
KLLISQLKQRLEEKLPEYMCPSSFMILENFPLTPSGKLDRRALPIPDRDLIINQQSLVPP
KTPTEYKLSQLWMDVLGIDKIGVTEDFFHLGGHSLLATKLVSRIREEFNVALPLRSIFEY
STIARLGDEIDCLIDVNTTKTGPEDIIPVSNRENLPLSFSQSRLWFLDLLEKENAAYNIS
VAFRLEGDLNVDALQESWQNIIQRHEVLRTTFDNVQGSPIQIVHDWSELKLTIRNLSCLD
FQTQQETLRKSIQEVVITPFNLNQLPLLRIHLYQLSADVSVLLLVIHHIIADGWSLGVMV
KELSLFYTAICQRNIPSIPPLSIQYGDFANWQREVFQKTQLPIQLAYWKQKLTGANQILE
LPTDYPRSPIPSYQGSAVNFAINPQTTQEFKKLCESQGATLFMGLLAVFSILLMRYSGQE
DLLIGTPIANRNRKQTEDLIGFFVNTLVIRNNLSGNPNFINLLSITKEETLQAYAHQDVP
FEKIVEEINPQRNLSQHPLFQVMFVWQNAPMNKLELPNLQLSPWRLEQRLAKFDLTLLMT
ETEQGIDGTWEYRTDLFAPETINRMIGHFETLLKGIIAEPQKPITHLPILTSHEKNQLLF
QWNQTQFEYPLYQQNKCLHQLFELQVEKTPNNVAVVFKNQSLTYFQLNQRANQLAHYLQS
RGVRPDVLVGICMERSLEMVIGLLGILKAGGAYVPMDSNYPRERLDFMLVDAGISLLLTQ
ENQVTTLDILPPHQIICLDKEWQVIAQEDTHNPSTNLVVENLAYLIYTSGSTGQPKGVMI
SHSAICNHMLWMQKTFSFGEREKVLQKTPFSFDASVWEFYAPLLTGGQLIIAEKDGHKDV
SYLLKLICEQQVTVLQMVPSLLQMFLEYGEIENCHSLTHIFCGGEALPVAMVENLLSKLN
VNFHNLYGPTEACIDATFLSFTKENNHYIKQNMLPIGRPIANTQTYVLDAHLQPVPIGVP
GELYIGGMGLARGYCQLPQLTRDKFIAHPFSDNPDSRLYKTGDLVRYLPDGNIEFIGRID
HQVKIRGFRIELGEIEAVLTQHPNVLNAVVVISGDSSATNSLIAYCLSTEKQFTSSGVLR
DFLKEKLPDYMIPNSFIVLDHLPMTPNGKIDRKLLAGLNINRNFDAHQHVSPRTLLEYKL
VEIWEEILQVSPISVTENFFDLGGHSLLAIRLIAAIEQKLKCNLPVVSLFREGTIEKIAL
LLDQDHQKASNHSDILIPLQTQGDLLPLFLVHQAGGYGLSYSVLAEKLAVGMGKKLPIYA
IQSPGLDGKQSPLESIEEMANTYIHTIREIQPHGPYLLGGHSLGGLIAFAMASQLEAMGE
QIERVLIIDTHPPMPTDETIASLEDNAGIICFMVEQIALFFNKNVTINYQTISSLDQDSQ

LDYVAQTLEQHNLIPPNSGNSLIARLIKVYKANLRASVVYQPPVNRSNITLFITPSLAAK
FPNDPTVGWQKLTTQKVQVCRVMGEHQTMLKEPEVENLVTEIMATLVNTP
>OHY37328.1
MTTISFPSGAVCIEQNNSNNYGYTITAHCSAATVSGESLSLKEFRGQLVSVPGSGGDKCE
GQIELTLRDESNGVDVKYDVQLAENFFMRLITSNGSYIKSGAVEINRSSKEGGSFGRPRV
FTMDMTINLSNLSFADAEDM
>OHY37831.1
MLRLLIYSVTSGFLVLLNTGIVDLANAQETSGRYVKTFVGNQSVTIDCIDETYDKKGDGS
VWISVYDYHQRYSTLRKIIQSEYVRLCGRMLNSDSIRAGLNQERQRKAWERRFQKERQRE
REFQREQQRQQKREQRFQKEQQRERRRKRKF
>OHY37848.1
MDICLRYGVVVMGMELKMWKPGKKDPLPQGLQQLDKYLAGLGLDTGWLVIFDRRPDLPPI
EERTTTEEVVSPGGRAIIVIRG
>OHY38788.1
MVQELKRPRQIASFPETAPAANPVFFRTYSRRTQTGLRESWSNVCDRTLKGLVELGKLNL
EETALLEKMQLQMKALPSGRWLWVGGV
>OHY38887.1
MKIISVAAIQKREYWIEEIRKLSGNFGDDSERLEKELSYEIQNEGLETLLNHLRLSGDIP
ESYGHDTSEEKQYSKYTDALLSELYKALGLKSFVLKERADAADVEVVAKDYSFVADAKAF
RLSRTAKNQKDFKVQAMDGWKRGKPFAMVVCPIYQLPARSSQIYEQASTRNVCIFTYSHL
AMLVSYSMIEGKSKAEELIHEVFKVIPALNPSKEASAYWLAVNKTMLGFSPQIEKLWKIE
KMAAIESIAAAKEEALKFLANEREKIMRMSHDEAIKELINVHKIESRIRTIKTVSDNGIL
GIR
>OHY38888.1
MEDYINKILNEDSIKGIKKLSDNSIHLILSDIPYGIGVEDWDVLHDNTNSAYLGTSPGQE
KAGAVFKKRGKPINGWSEADREIPKQYYEWCSSWASEWLRVMKPGGSVFIFAGRRYAHRC
ISALEDAGFSFKDMFAWMRQRAPHRAQRISVVYDRRGDTDSSQIWEGWRVGNLRPTFEPV
LWFTKPYKIGTTIADNVLSHGVGAFNESAFMKYEKSPDNILTSGFMSGETGYHPTQKPVR
LMQSLIELATQEEQIVLDPFCGSGSTLIAAKLLNRKFIGYELNKEYYSIAEERLKEKKEV
QLSLL
>OHY39335.1
MQRGATSFGTSTGSVTFATGSSVVILPIDPSSDVVSDGNETVALTLAAGTGYAVGSSGAV
TGIILDNDVAPGTVVRGSVAIRLVCWVSCLNPTYDYLRQN
>OHY39387.1
MICCLNPDCQNPQNPHGSKFCQSCDTPLVPLLRNRFRIIRVLSDEGGFGRTYLAEDTDKL
NDNCVVKQLAPKFQGTWAQKKAMELFSQEAKRLQELGEHPQIPTLLAYFEQDKCLYLVQQ
FIDGNNLLQELQLRKHYKDSDIQSLLLDLLPILKFLHSRGIIHRDIKPENIIRRKRDGRL
ILIDFGSAKQLTVAVQKKYGTSIGSHGYSAIEQIRDGKAYPASDLFSLGATCFHLLTGLS
PFQLWIEHGYSWVKNWQDCLNNSRSAELIIIILDKLLQLDLKNRYQSADEVIKDLSKKNVY
GSKKFGIYLKKGKHEKHKHTILRNIFLILVTISVVGGLGYRNLGQIQTAIFSQFNPLLIP
SNKSSTSPEYEQISGRTSSIRTNKVSLLKTITQVDKSLAVVAITPDGNIIVSAGHKEIKL
WNSKTGKQIISLPGHTQNINALAISPDGNNLVSAGDDKTIKVWNLQTKKLTFNLVGHQDS
IQALAISQDSKILVSAGDDKTIKVWSLLTGKFLKTLLGHNYWVRSLALSPDGFTLASGSF
DKTIKIWNINQTSGQKPTTLLDTTSQTVTSLAFSPDTSTLVSTSRDRQIKFWDIKNKEII
FASKKQNVTSVIFSPDGKTLISKAKSCPDCSEKISSVIKLWDVSTKEEIYALPGNTKIVT
SLVLSADGKTLVGGTEDNKINIWEISP
>OHY39512.1
MIKIKTKKTVINEEIAKVKKKRWNAYYFVGNLAALWCKGFSHYFEGSGGEKQRNFADLRK
VGLDTLTG
>OHY39754.1
MPESNLSVSQLLQSLLEPLLEDFEYWFARSQQLLENEKISFMTEQEQSDLLDRVKQAQLE
VNTSRMLFNATGKQVGLDMATLAPWHQLLGECWRVGMRYRQIK

>OHY39803.1
MVALLLDLLIRDRHQNTVSLNDVMVEMWRKFGTQETGYTTQDLKEVIEQVAQIDLSDFFK
QYIDGLEHLPFLKYLEPFGLALVEQSESAPYLGIRVEPENGQETIKFVEAHSPASTAGLD
VGDELLAIDGIKVGINQLGHRLQDYQPKDTIEITVFHQDELRNHKVTLGKPRPHKYQLRP
VENPSTTQKNNFEGWLEVPITTIQ
>OHY39892.1
MDAYQHPEALEALQKEQLVQIARHIGLETSLRGKAKTAPALKAEIRNYLNYCEQLTIPEV
MPLSG
>OHY39966.1
MGLLPWRLRDTSINKTTINSKQQATKNTTEIMTKKFVDLSTLDGKNGLTIINGNDKDDNL
GYSISNAGDINGDGINDIIIGAPLSDPNDQSNAGNSYIVFGSDNGFANIIDISTLDGING
FTVNGGGIGDQSGRSVSAAGDINGDGIDDLIIGAPFADSNGDDSGAAYLIFGRRSFSSLP
TVNPSNLGDNGFIINGLNPQDQLGYRVSGAGDINQDGFDDVIIAAPPNAYVYPPVTGDQA
GKVYVIFGSEKFNPSNLNFNPNFDLNSLNGNNGFVINGSRADDYLGVGLNRGGDFNGDGI
DDLIIGSPFNDFNGFRSGQAYVIFGRKESFSSSLDVSQLDGVNGVVINGQEGDQLGFSVS
SAGDINHDGIGDIIVSAHDADPNGIDAAGVAYVVFGARTQFSSQLDLSNLNGNNGFVING
IGELDKASWAVTGLGDVNGDGIDDLLVSASHADANGDNSGQGYVIFGGDKFSSAINLAEI
DDTKGFIINGKSENHNLGYSASGVGDINGDGVSDILISAPFAGSGEVYVVFGKNSPTDTG
EITDEVTEGNGGESDIINEAPSGLFLKTNNRVDENGLESNVIGTFTTVDPNADDSFTYSL
SDDENYPDNGLFSIQDNQLISKESLDFENQPIYTISVITTDRGGLSLTAEFTIDGTEDEI
TGGVIEDNNSESEGTPDEILDGVMGEGDSDNNSTEGSGSDLTDSSDIPSTSTGSADNILS
SNNNVLSSSTTRVEFQLMDKIPASIRELGVFTVDDPSGKINGIAPGQVGYSEAALARSKV
VFSILSKTPNEFNNANVTRILGFQEANPNLRFYVIDNGTTDSVKNGLFPIDQVTFLDSSN
LQVTQLPDNSFSLQSNDLVFKVRPTTKPLPMGANLQEKSQGESIDLRGVTGPVNAQFTVY
REASFDNYVGFYKVTDEKGGIDTNNDGTADLLPGDAGYIQAAVNQHLIGLGLNVPDGNKS
NFNSTLSGGGLYVPFIIVNGRPDAFLNSDLSANSNPDIYFTYLGANPDRVDHVRLLGDNT
FGFEDLRGGGDRDYNDLVVQVNMSANV
>OHY40959.1
MGLFDESTLKCKAEVWRSLINRWRDIRGGYNKCEEYRPLAQLFERFKEYDLAVYFYQVSD
GVVKKDLFYEIPPRSRNLRLSEKDVLGLKIKTKLNLVDNFGLRIGDMINSFINFSRYDMK
IPQVMIVSLVFFVSGLSLGNIFPIRNSLNEQTTVNQSKPSTNPTEEPDVENVNNDWNKTT
KAFQEIISDLSNNIKKENTPKIIKDLYPNLHTHIFPLISPAQPNKQLKEELIFSLILNEM
NITQYQKFSYAKLGNITDEQKIKIYQAIKKFQTSNQSKVKDGYFDFENPKDTSLNKLKER
VRQKI
>OHY41050.1
MPTLIVGSGSQYTTIQAAINAASDGDIIEVQAGTYAEQLTIDKQLTIRGPNSEKTGNASD
RIGEAVVTFPAGITVTAPSLITVNVSGVTIEGLELRSNDYLINKFPYLIETNKVNNLTVR
NNHMYGGEVGIYVLTSNDKTVNRSGLLIEGNYIDGGPYVNSKFNRGIYVQSTAGTIQNNT
VINTNIGIQYLPYANPVGGTIRGNTVSAGSIGLYHNYQDKGAAPVTWENNVVSVAQNDRA
GLKSQVFDPWTTPVIFRGIELITFGDQGSGNAPQVTFTNNSVNADISGTGYNSTVSEGLR
FSNPYGNGQAIFNGNTLTGWTVRAVNNQVNQTSRDFSFSFITGFTDDVAPVTGTNATNGS
STNDTTPTINITAEVNSTVEIFNGNTKLGNATATGAGTYTFTPTDLSPGTYNFTARATNA
AGNVSVNSGVFTLTVDTAAPSAPIITGFTDDVVPVTGVITAGGSSTNDPTPILNITAEAG
STVEVFRNGVSLGNAIATGAGTYTFTPPTLNPGAYSITARSTDGAGNISANSTAFSLTVD
TGVAAPTILSRTGSDNAPVLTISAEANSTVEVFRNGVSLGNATDITEGGTTYTITSSVLT
DGNYSFTARATDRAGNVSVDSAPFNLSVDTTPPSAPIIVSAADNVDPIQSSFLVDGNITN
DTTPSISISAEPGAIVEVFINGVSAGNAIATGPGTYTFTPAVPLTQGNYTFTARATDAAG
NLSASLSNDFRLTIDAVASTPVITAITDNEAPFTGNVANNGITNDTTPTINITAEANSTV
EVFNGPTRLGNATATAPGIYTFTPVTALSPGSYAFTARSTDVAGNISSNSTPFNLTIDTN
AVQITFNAIVDDTGNSASDFLTNATSMLLQGRATPGATITVTVNGIAQTTTVTSGNIAGE
DGLAAWTYVLPTLNPGTYTVGVRASTPAAGNSQTVTRSLTIDTTAPAVAITGINDGASSG
NILTNDTTLIFNGTAESGSLVTLTVTNAQNNTQVFQQTVNAVAGTWRIDRTNSNLPGGSY

NITATATDAAGNVSTTVTRSLEIDTTAPNAPTITSVTDDVAPITGTISSTTGDTTPTLNI
TAEAGSTVEVFNGNTKLGNATATGLGIYTFTTTALTPGTYSLTARATDAAGNTSGSSAAF
SLIIDTTAPSAPTITGFTDDVAPVTGVINTSSTNDLTPTLNITAEAGSTVEVFRDGVFVG
NATANAPGIYTLTPPALTPGTYSFTARATDAAGNTSGSSAAFSLTLDTTAPSAPTITGFT
DDVAPVTGVVNTSSTNDPTPTLNITAEAGSTVEVFVGNTKLGDAAATTTPGIYTFTPATA
LSAGAYSFTARATDGVGNISQSSSIFSLILITTSPQTPIITAVSDNVGSQQSNVPSGGST
DDTTPTLTIAAAAGTTVRIYNGDPGVQTNLLGTATESSQLGVFTFTPSTPFLNNQTVNLQ
VIATDTAGNLSTASTVYPITIVTGPPAAPTITAVADDAGSIVGNLTPGQSSDDVLPVLTI
TAAQGSTIKVFNGTDEIPGSALITGTASGISTYTFTPARLLASGSVSLTATATNAAGLTS
IASAPFNYTLDRQPPAAPTITNIGTNNVFAAVGGTISGTGEAGATLALQFSSGRNLGGGT
SPTTVTVGPDGTWSVNVSAADVNAFGEGAERVTVRQRDAAGNQSESTSQNFNVFTSAVQV
SIEAIQEDRGLSNSNFITNDRTLTLLGKATAGSTVTITVGTTTLGGTVSVPGTGGLADWS
YVIPGSLPANQTTQITARATSGVDSSPLITQEVIVDTASPTLAISKSFNGSTANIGTGPI
TYTFSFSEPVVDFTTSKVLVVGGSKGPLLNPSGDLRNYTMVVTPTQVFEGNMTVIVGVNE
VRDRAGNSNTQDTEITQPVDILAPTITSGSVSPPISENSGSNQLVYRTTALGTGNSLSLS
TNGDGSLFTIDSTGQVRLTANPNFETKPNYTFTVVATDEASNVSEQTVTLGIINLDEVAP
TITSGTGATAIPENSSLNQVVYTVTSTDSGDISGGVTYSLKPNTGDGAMFTINPTTGRVR
LNASANFEAKNSYAFTVIATDAAGTASEQAVTLPITDVNESPTAIALSNNSIDENGVLNV
GTLSTIDPDSNDTFQYTLVGGTGSGDNNAFTLTTAGQLSFNTPPDFETKSNYSIRVRSTD
AGGLFTDTAFTINVIDTNDAPSTLSLSSTTVNENVGVGTLVGLLSSTDPDASPQSFTYTL
VSGTGGVDDVDNSSFVIVGEQLRLSVSPDFETKSNYRINVRSTDQGGLSRDQAFTINIRD
QRESLNDQITQEESSINPFILPVVGSSGAVSYQLLSLNSTPRPDWISFNPSTRQVSGTPP
TNFAEAVQLRLNELDSGNNLLGSYNFKLVVQNVNDAPTTASTSRTGQEDTDITLQLSDFA
FTDVDSGDSLKAVQIVAPPTAGSLLLNGNPVTYPLQVTREQISQGALRFRPAANANGLYA
TFQFRVLDQGDGVSSPATFTINLTAVNDPATGQPGITGTLEEGQTLTVNTGGIGDVDGLP
ASGFSYQWFRASVDGSGNPGLFQEITGITTPTHQLAATDINKVIRVQVSFTDQGGNLESV
ISNRTNLIQNRPDTPTWSGQQITGTPTQGQTLTVNTGGIGDLDGLGSFSYQWQQSSDNSV
WTAISGATGTSLRLGQDQVGKLVRNLVSFTDGYGNRETLTSNPTTQVANINDPASGTPQI
IGTPAKGQVLSVSTQGLRDADGIDTSTFSYQWQQSSDGNTWSDISGGTGSNLTLGDSQVN
NRVRVRVSYRDYPGGPETQRTLETRESGATALVSNSNLNPVEGAVVLTLNGNPLTGSPSQ
NQIVVANTSGIRDGDGIGALSYEWLADGQVISGATGDRLTLGQPQVGKAIAVRVRYIDAG
GTNEEISSPATPPVLNVNDPALALILTGAIVQNQTLTAQLDSITDLDGAPAIQRDSQGNI
TNYTFQWQQSSDGTTWSNIPNATTARLNLTETLVSQRLRVTMSFTDNGGFSETVTSVATN
PILNVNDAPLGDPTIGVTVPNQAQTLTASTNGISDIDGLGTFNYQWQRLNDTTWEAITGA
NAATYNPSQRDVNRSLRVRVTYTDGRGTQETLFSTATGLVIDLPDGPTGQPTISGTLTQG
ELLTADPSSIADLDGIGTFSYQWQQSTDGTTWNNIPNATGNTYRLTQNQVGRQVRVRVNY
IDGLGTRETVTSTATTPILNVNDAPTGSVTVRGTPAIGVTLSADTRSLVDPDDEVNSVRA
FSYQWFANGVSITGATNATYTPIDDDLNKTLTVQVTYTDQRGTVERVLSPNSLAVISADQ
LLTGDVSILSTNALEGVDASGNLIAQENVTLVADPWTTQITFNGIRRVQFSYQWYADGVA
IIGANSDLFTPGDDHVGKVITVAVTPTGLSSSLLSKGTAPVVNVNDQPEGIPTFSGLVNG
AAQEDSTLTVNVSGISDADGIDATTPFSFQWQQSTDSNNWNNITNATGRSFTPGDAQSGQ
RLRLVTTYTDLRGTTEIVPTPVGSLNNIPGQVRPVNDLPTGSIGINGFLVVGQVLTAAST
LVDADGIPPSGVTYTWQRQNPATTSVRGTVGEWSDIATGATYTIADTVSGSPLRVAARYT
DQQGFSETITSSPTTNVVRNVREGSLITWSSSQAATSQWQVFSSSTGWQDLAGETRASLQ
TKNDLGGRQVRLLLNGTPTPALFIQTVDNGVGTVSPITIAPESSQPSFVLGATLKTGSVV
GDLDGLDPNSTNVIYQWQRSNNDSSWNNILKDGDKAIYIVTDEDLNQNLRVQITYTDLQG
FTSTIYSAATSVIQTAPPPVASGSFDAMTANGDDIASVEEFPVNNDTSSLAGGLTLTGTV
TSPNTSVTLTFGGQTRVARLAVDTDGNRISDASTSTTSPVAWSYTLTAADLTWLVGNEDN
QVTARFTRTEGNQIGIGKTSRTLFISQGVLADRTAPTVIDASQPGGLTADVLRNLQALGV
EFQGVDISQPNALVFTYGTADEFNAGADTADKGTGAGLNTSTGTIQGIEQFETGQDGTYT
IPGSDGSSTPISAPPRSSFTPVADPLAMTVSEVRPGATVTTQFYLPANVASRLAANDTPL
TRWTYYKLDSTDNQFKAFTDNNGNDLYAFRVEGGNNDNLWQRGEQVVLTLTLTDGDKWDR

DGLANGIIVDPGLAGLSVNQAPTITSPATTNFAENGTGTAYTVLATDPDAGATLTYSLLG
TDAPLFNINSSTGAVTFKTAPNFEVPGDGGGNNIYDITVSASDGSLSTSQAVAITVTNVN
EAPTDLTLSATTIAENQAIGTVVGNFSTTDPDAGNSFTYSLVTGAGATDNSFFTIDGGQL
KTAAAFDFETKNSYSIRVRTADQGGLSFEKQLTINVTNVNEAPSFTNTTATFPVAENGTT
VGTIDPATDPDGGDILNYTLSGADATKFNFNTITRALSFKTPPNFEAPGSAAGTNAYSVT
VTATDVRGLNATQTVTVNVTDVVEIGNPPVITSSSNFSVAENSTTVGNITATDADGDPLT
YSISGGTYQSLFTINANTGNLSFVAAPNFEAPGDGGTNNVYNLRIGVTDGKNTVNQELII
NVTNVNEAPTITSPATANFAENGTDTAYTVLATDPDAGTTLTYSLSGTDAPLFNINSTTG
AVTFQTPPNFEAPGDGGGNNVYDITVSASDGSLSASQAVAINVTNANEAPTITSPATANF
AENGTGTAYTVLATDPDAGTTLTYSLSGTDAPLFNINSSTGAVTFKTAPNFEAPTDAGGN
NVYDITVSASDGSLSASQAVAITVTNEAPVFRSVSFKVPENRKLVGKIDVQDPEGDNITF
ALAGVDAKLLSIDSQGNLTFNKEPDLEKPEDADRNNIYQVQVTVKAGSDTVTQDIPITVE
NVNEAPIAIGDFLAIVGNNSGSIEPLKNDSDPDSGDKLEISGVTNGKHGTVEIIGEQLKY
TLFNAAYTGDDVFSYTISDRENLTATANIKVNVTGTKVVVNPDLIKVEPGDRLIPSEAGS
LSDIVNNVSFTFRAGYSQPQAEQTLRNVLSRTDAAFTNLFGLYEIDNATGTVNGIAPGQN
GYAKAALNRAVSSFAVRAGGSGNGVTGSVIVGGDKFYAPFVIANGGNLSGSMQDAINQFF
QANPINSPATAQNYTTFPVAYFSFGAANPDGAAHIKSFGNNIFGFEDLPAGVGVNDYDFN
DTVFSFG
>OHY41055.1
MVGGLETLNNQTLSLIVIEGVSHAVHDKLTQAGFIKVFYNPYTREILSNPVASLKSGNNI
YIRNQKFVSDKLAQSPAYFIHNMGLRI
>OHY41057.1
MTINKIIDDWYLENLICPQDNTKLNLVGNDLVSQSGNTYPIVNGIPIMLIDDVPQTIDLA
NTSLADSKLKNDSDPYFINTLGISEYEKNQVKKYTENLQVQERIIGIINTSVDPVIEFLI
GATNGILYSQNIGNLKRLPIPDINLPVATRKQFNL
>OHY41059.1
MSNSTDNTHGILIIGFTTHPHDVGSALVLAAQSLNLPLAVANISDYESSLNHLWGKVFYR
IAQRRTLEWFDFQRKILDLIGKFQPQLVIVTGIIPLGQDIFHTIHNYQGKIVNYLTDNPW
NPALGSPIFRDNLRLYDCVFSTKTAIIPQLLRVGVRNLFSHF
>OHY41062.1
MKTVVIVAPHFPPSNLTAGHRCRYFATHLPKFGWNVKVISIQPQYYEEKLEPQLTELLPP
ELEVIRTTALPTRPLRLIGDVGIRAFWWHYQALCKLIESGTINPKTDLIYIPIPSNYSSL
LGYLIYKRYGIAYGIDYIDPWVNTWPGCEVWLSKAWFSYNLGKILEPIALRHVRIITAVA
PGYYEGVLKSYPWINPDCCFAMPYGVEPEDFKYIESHPRATYLFDPLDGDYHIIYAGAML
PKAYSTLEALFTAINQIKSVNPQLGKRLKFHFVGTGQNPTDPESYSIKPYAEKYQLLDTV
TEHPARIPYLDVLNHLQQAQGVLIVGSPERHYTPSKVFQSVLSRRPVIALLHSESTAVSI
LNQVNTGYLVTFDERKPAHVCIDDMATAIEKAVTNPDNTEQINWDAFYTYSTVAMTEKLA
QAFDLTLSANVR
>OHY41063.1
MRILITVDPEIPVPPQLYGGIERIVDILVRGLRKRGHDVGLVANGKSTTSASELFPWWGR
RSQNQLDTVKNTILLWSIVQRFRPDIIHSFSRIFYLLPWLGSPLPKIMSYQRDPSYRTTS
WAVKLSGNSLKFTGCSRYICDLGKRSGGNWYPIHNCVELEKYTFNPTVSQDSPLVFLSRV
EKIKGAHTAIQIALKTGHPLIIAGNHNPDDPYWQREILPHINNNGQIQYIGPVNDQQKNH
LLGQAKAMIVPIEWEEPFGIVFAEALACGTPVISCPRGALPEIIRPGVDGYLINSIEEGC
QAVQKLNHIHRVNCRQRVEEYFASDVIVEQYEQLYKVHLS
>OHY41064.1
MLTDTIYQSLKSVPSLQRQFKRVVLKLPHRQRLVNHFQCKLWVDPAELHGFYLYYEREYD
DYIFEFLLTQVRNLQSKYHRAIDIGANIGIYTTFLAQISNHVDAFEPEKQVLARLRKNLS
LNGINNVAIHEKCVGQFSGNVGFTSPDKHNQGVGSISLESGIGQVPCITLDDFLGGVLSE
SCLIKMDIEGGEWLALQGAREALTQRKAPVSILLEVHPEEIERLGGTVKELKQLLESMRL
EVSALTPQGLKPLPENGNRAQVLNPAIKLYNLCVLPR
>OHY41065.1

MPSLKQKVGAWLIPKLPINRHVFDHIRLELNALRVRGLHSFHPGIRRKVKQLQSQNNLLV
NIGCGPFGEDGWVNLDLFTHPNLTLVADTRRRLPLADSSCVGIHVEHFFEHLNPEDECPA
FLSECRRCLQPDGVLRIIVPDAELYIKAYLSPGWDMFNAIGCGGDQPETAFNSKMQALNH
VFIQGWEHYGGYDTQSLTLMLQQAGFTKINRCGWRSGDFPVTPIDREQHRPYSLYLEAIL
>OHY41066.1
MVLYLLNRHVNPITAALLGLSFLLDPIFVASSRLRVDCWAIALCLGSCYLLRVSLKLIQN
NQRCFVNIGVAGSLSAAAFFVWPSVLIIYPLILLELWYVLVAVIQGKKIWKDACQLLLVF
GTSFLLICLLLIIPIIGNLDMIISDFSRATRAVSKIGIGEFINFATSFIRGRSLVLPILA
SFALVYVTEKSLAITTLLALSIVISTGVTPDRCLYAFPYLILLVSNLYSQPTKSIKSRDL
NPRIKAAFLLVLVGYAVTISLIIRPVIGLSNSSGRNTNLLVNAGKTHIGEGEYKIWDSTW
QFYETGRLLKWKMYQPFWAFWGIEDNDNSHRFIDNLDHVILQHKSEYKIIEKRGFRLQKT
IRLDSGNQSNEGVFLNHLTKSLNSQTAYGPYDLYSR
>OHY41067.1
MLLTNKLNQLRHNFWLIWFLILLLVALIHSLTLTVSPPIMGDEVQIIEYGRAFLSPNTDW
SMNWDVANNRPFTSLFYLGCLLQEIAFSVANFSIFGSRFMGLKAIRIKYSSNHIGIILIK
SPC
>OHY41069.1
MQLLLSSPGVGPFIQQTAKALYEASILHSYATTFVSYPESTWQKSLCGMAKIFKFNLERE
LQRRAITEIPLTYVHNYPWREILRTISSKLDQDGRLTDKLWEWSTKGFDHWVANHHLGTV
GGVYGYETACLATFRAAKKQGLATIYELPAPEHDFVANILEQELTLYPQLRTSYYQYTQQ
LQQQRTEHGRQEWELADVIIVNSQFTKNSYAAAGLDMDKVRLIPLGAPPVREKLPNNSIN
SDKTMQFLWAGTFSIRKGAHYLVSAWQKLQPQEARLKVFGAMGLPENLLINLPKSIEFFP
TVPRTELVKIYQVCDVLVFPTLCDGFGMVITEALAQGLPVITTNCAGASDLIQDGVNGLI
IPPRDTEALAAAINWCLTHPSQVKEMSTAALKTAAQWQWSDYRQSLVANIQAGLKSAGYQ
>OHY41070.1
MTDPDKIQSDYYTQTASSYDDMHGDPEHDVALSYISSLITGLNISNILDVGCGTGRGIKY
FLSKHKNLTIKGVEPVEALIEIAVNKNHISHQLISKGNGENLPFTDQSFDAVFELAMLHH
VPHPNLVVSEMIRVARKAIFISDSNRFGQGSYLARWVKLILYKLGLWKWADLIRTQGKGY
TITPGDGLAYSYSVFDSYDLLAQWADRIILIPTVPFSSKTWFHPLLTSGHILMCAIRE
>OHY41613.1
MSESLPLRDRYLALIDEIVSNTLKGKISSVYQIYQMLLNGISLDTGEVFELALSDRTRDI
QLQVDNEKDETKKGKANRSLRAIKTVQSQWQRWEEQNQATEVITSAVREIISASPEENLT
EFLRYLDPNQKHALNLSQLQQIAKSLDQFSTVNRDIKEICDWC
>OHY41660.1
MGLDLNLSGKTAIVTGGSAGIGLATAKGLYKEGVNVVIAARSPSTLEEAIQEIKNAPSPN
SQNEVISINADLTKVEDIEKVVLGTSEKFGRIDILINNAGSARAGSFLDLEDEVFLDAWH
LKLLGYIRFVRLVVPYFKQQGDGRIVNIIGGAGRTPRPNFLPGGTTNAALLNFTRGISKE
LAQNNIRINAISPGLTDTRRAKTLAQQNAQSLGISVEEYNLQAVKGIPLGKIVQPDEIAA
LALFLVSDLASSITGTEIQVDGGATPGV
>OHY42057.1
MINLEQTLNEQQNLSELCSDTPPKLLNTCEVFAPNAYYGNDLIYKLYADLPVKYPLKAVL
PHAPDFYVNSRDKVWESELVNSLPEIWCYGNRSTQIYSQALKNIKIDKKVVPSASPFLYL
LKLIQPDSIIPERRGTIFFPTHSTHHIIDNTSFEILASKLDCLGEEYRPISVCIYWRDFN
LGRHLPFEKRGMKIVSAGHMYDPEFLFRFYHLCSLHKYSCANDYGTAILYSIKSGCSYFH
LDADDLYSNTVKKINSNVCLPDDPASYVSTEVTSLEEKINEVKTFRDLFAVPRQELISNQ
IEFVNELLGNQSLKTPTELRDMIIAAEIKYVATAEIAIRDNNLLPRLLRRLRGYPLYKLL
RKN
>OHY42059.1
MKTRIHIISASFNAGNCIGKLIQSLESQTDKDFVWILVDGGSNDDTLGKAEKIQGIAKKS
CWKIRDNNQADK
>OHY42232.1
MQYRNLGDSDLLVSELCLGTMNYGKQNTLEEAKNQLSYAFDRGINFIDTAEMYPAPTCSE

TQGKTEEYIGKWLVTQPRDQVIIATKIAGPSGGQTLPITWIREGKNRIDRKNIQEAVEGS
LRRLQTDYIDLYQIHWPDRYVPLFGAPDYDPNYERETVAIAEQLEAFAELVKAGKIRYLG
VSNETPWGVCEFCHLAQQLGLPKIVSIQNAFNLTNRVFQINLAETCRFHNVGLIAYSTLG
FGHLTGKYLSQTPPRSRIDLFPKFDRRYRKPHFQEAVQAYVDLADKNGLTPVQLALGFVR
SRWFVTSTIIAASTLEQLQENISCVDVVLTPEILTEIDQIHARYPNPVP
>OHY42491.1
MGCPNFNIFWATFITTFLTFNTSAPIPLPQELTPDDWFTSSQISSYNLQTTTVYFGNFKQ
KGFNGKASYEIYGDDNIKKTANILSHFAFYCGTGYKTTIGMGQTNITSKSLDFSKDNNQP
TNSNT
>OHY42509.1
MNDLENIPNHHYEPIKTILRVIDFSQGQFSLIFLHCNDAELREQVAAQLRERSPNKIEEI
TLPHSAISLYDNISLTLDRHPEVLMVFGLETVNNLDSILQFSNQIREEFRKNFTFPLLIW
IDDQILRKILRVAPDLETWGSIIGTPNYYNGENQVKSLAQIHRFLAEAALENQQWSEAKK
QAQYAQEILTSVDHIILPDDGLYHLLIARSQIGLGETSTAIKSLEIAKTHSAPPQDPQLY
IEILKTLASLYFDHGNYLEAFYVRQEQLQVEQQYGLRAFLGSSYLNPQRPIMNGTNGSNG
RSMAFGREEDIKRLWQRISDNEHPLVVIHGQFAVGKTSIIQGGLVPILEQELIDDRQLLP
IILREYTNWLEELWEQLLNKLENKLQSTIAIETFNGLSPQERIIKLLNISGDKNLLTVLI
FDQFEEFFFVANNLEQKRTFYQLLRSCLDIPFVKIILTLREEYLHYLLEIDRLVDLRVTN
NNILDKTIRYYLGNFSKAVAKRIIQTLMVKDQFELQMELTDQLVEELGDNLGEVRPLELQ
IVGVQLEKEGIDTLEQYEQFGGKQKLLEKFLQDVIKSCGPENESTAQLVLYLLTGENGTK
PLKTQAEIITQLSVESDKLDLVLKIFVGSGLVWLVRESLRDRYQLVHDCLVQFIRYQYAR
SYYTQLSAQLERIQAELRQEREAEEQAQLVTKLEEDALIALDQFQTDPLLSLVTAVGNAN
LLKSIVQNNPLDKYPTVRPIYTLNTILDTISDRNIIKGHEGGITSVCFSPDGQSIGTGSW
DKTIRLWNLRGENIQQFRGHEGGITSVCFSPDGQSIGTGSEDGTARLWNLQGKNIQQFRG
HEGGITSVCFSPDGQSIGTGSEDGTARLWNLQGKNIQQFRSHEGGVTSICFSPDGQSIGT
GSEDGTARLWNLQGENIQQFHGHEDWVTSVSFSPDGQILATTSVDKTVRLWNLQGETIQQ
FHGHENWVTSVSFSPDGKTLATTSVDKTARLWGLHRQKIQEIRGHEDWVTSVSFSPDGQN
IATGSRDNTARLWNWEGRLIQEFKGHQSRVTSVNFSPDGQTIGTGSADKTARLWNLQGDI
LGEFQGHEDWVTSVSFSPNGQILATGSRDKIARLWSLQGDLLGEFPGHEDWVTSVSFSPN
GQTLATGSADKIARLWNLQGDLLGKFPGHEGGVTSVSFSPDGQTLVTGSVDKIARLWNLN
GYLIREFKGHDSGITNVSFSPDGQTLATASVDKTVRLWDLKGQLIQEFKGYDDTVTSVSF
SPDGQTLATGSLDKIARLWPVRYLDRALKDGNTWLIDTNF
>OHY42533.1
MATDITKILIGETTALIPQAGASTAPEHLLDGGSGDTDFPYGKFKAMATVGEIDPKTGHV
LTGWPDGQAAWLLDEDTIRVAYQSESYATFVKETYGWKMDSGVSFTGSHVHVIDYNRAAF
ADFLNNNSPASKMFEGAGHLFNTVYNVFGEIVDGKNADPKDLSAKWGNQTGADGTLYEFD
ERYRLTQGDWFFHSFCGAYYEEANKYGNGIGFADDVWLMGEEWNIGQMYSSRGGDKFFTD
NTMGLASMVVDIANKTAYTVPVLGQSGYEKILPINSGHKDYVVLVMSGYNLEVEPAPLKI
YIGKKNVDAAGKAMNYNTASARDAFLGRNGLLFGQLYGMAATNDTYADLGIANVDADTEM
LNAYTADADAPDTFKVRYYPTKYRWDGFDTPENAGKTEVYRWLQDGDTVGGVKEANEQPE
GYTFFNGDSKVEHPAVDPDITQSRYVINLTDARSILGIDFNNIVTDLTNDADGNGLPDYL
SADVTRVLAGVDGALVLETNGKGAAPTGPNNPASSLTHAIHVEQGKAYADQPDGLQWVKT
SDGDYLILDEDSGNDYGERKYVLPIDSETLQLTDPGTGYFLASAGGSLNPRAKAKVAAIP
GTFSRATGAEFSGTWNVTHLVAKKEDGSFYTQEELDGTGAQRIIGSLPLEEQTFIGVVQQ
GGESGGILAERKADQGGQIFMFNITEPLEFVKPLITGTPNADTIEAGVGEFTGVNSLVFT
GAGKDEVDIPIGGAKLYLGSNSIFTGSGADTISVTDKDRAFGGSGDDKFDATEATGYRIS
GGVGNDIFYLGVNGRAIGGDGDDRFFVGEGGGNIISGGAGADQFWILTDDPTKLKASNTI
VDYTIGTDVIGIANQVADSVDDLTLSGSNISLNGVLIATLNGVNVASATFVFGNPLAS
>OHY42534.1
MGEGGGNIISGGAGADQFWILTDDPTKLKASNTIVDYTISTDVIGIANQVADSVDDLTLS
GSNISLKGVLIATLNGVNAASATFVFGSPLAS
>OHY42587.1

MEPNWLNPDGTLNPQILLDSFLEFWRQHGEPLLRSAPYHEIAPHLVLMAFLHRVVNGGGT
LEREYAIGSGRMDICLRYGQVVMGIELKVRREKLDPLTKGLTQLDKYLDGLGLDTGWLVI
FDRRPGLPPMGERISTEEAISPGGRTITVIRS
>OHY42712.1
MEKFLATYECAGCDLSGARLFSAYLSGARLSRANLSRADLGDADLSRANLSGANLSGANL
EAADLRNARLSYANLSGANLYYAKLSGADLSGANLEGADLSELDPFD
>OHY42751.1
MRLVSTLLANIALAAQTHCDVILFNIDSKADANNKNDKETITKVFQKVFDDHLGYFGTTP
EIARFERQLENKGKYQPFKEAFYRQTNQSWEETREAWAFYQDDIVAALTVSTGMTGEQAN
RLLDFNEKYSLSPEEFAKTVKEHLHTKGPKHHLVFMVDEVGQYIGEDTKLMLNLQTVVED
LGIHCQGRAWVLVTSQEAMDEITKNKIKGFKLFGNFYP
>OHY42758.1
MKLKKLTELVIISILITVITILQPVVTLALTSAQIAAIATQITVRISGADKGSGVIISRS
GNTYTVLTNNHVFKNPGNYEVTTPDRRKYQVTNIRRIENLDLATFQFNSTETYNIVKQGN
SKQMTIGKAVYVSGFPADKGLNFLRSEISRIDPPGKGGYSLVYRIGAFPAMSGGPILNED
GKLVGIHGKTESIPISIYDSTQEEYGIPLQTFLNATSISSPPPTPSRYTKLETLLKSQDF
READIETAKVMLAVANRQSEGWLRIEDAENFPCKELRTIDNLWLKYSQGKFGISVQQEI
>OHY43326.1
MNMVWSRNLRVFRPEKLNLEGTVVPTPQEAVRIEVDKGIAMVERERLKAIQAQQSVEQER
LRALQAEQDAKQSKAELRELQDKVKTLGISID
>OHY43339.1
MANYHLPIFCEIIVPASEVVKVMSSVPLLRLSDKFDPYISGEVNIDPSAVIAPGVILQAG
LNSKIIIGPGVCIGMGSILQVSHGILEIEMGANLGAGFLMVGEGKIGANACIGAGTTVFN
DSVAAQQVIPAGSILGDSSRQQSTSPPSPTPKSQSPPETESSSTESQETSDGKPHSRDPT
EPHPLGTQIYGQGSINRLLSTLFPHRQSLSDQDANNGEE
>OHY43451.1
MPIIVWTKQGAQSFGENYNPKSRCEIVTANLNQAVEDSAGNLYDVVLTWGKLDDGKTVIC
ALSLKDEGGCDGSNTLFALKPENAKNPNRVLETLTQISIKGSSAGVVSETKPRGRLSVNL
GRVVSQAAKRLPRETPKTRKTPRSTKGSV
>KRH95363.1
MTIIVFGSINMDLVATTPRLPIPGETLLGESFFTAPGGKGANQAVALAKLGIPTQMIGRV
GNDNY
>KRH95378.1
MNASEQATNFEIASKIATVVNLFKLQFPDAKSDLKPWQNDRETRQLVDPDSIDIGFHFPG
ISRSWRSRSVLIQIRFHQDPINQQSKAIGVEVSGFDHRGQVWRLSTIEQWSFVGEPTPSS
VVGEKLKHICRQILEIFNI
>KRH95387.1
MLKKMIFYSTYLSLLGCQTVNPNGSISSNLLSSPGCPDKPTVSLSSKNVEQIMLDQNIIT
KSSQVSSNKHIGYTFAAKAGEKLNYSTDTNVCVWVFAPNNEIVKGINLPKKGKYTIQVAA
PQGVKTFSLQMSLGSTLKSDLVTMEDPVEKSTVIATTVAPIPNTKSQIPARADSVQKSYT
ATQNTSRISPEQVIRDYYDKINNQQYDTAWDIYPSTVKEDLNLHPNGYDSFLGWWTQVES
VRVIRVSVKSEDNDSAIVNFRGQYSMKNGRLLPVRLRFYLDWNQDNATWYVTQIKVRTPN
>KRH95395.1
MISGQKAIWKRLRSSKLFVIGLAIFSCLLITPLLHLPAWGNVTNSRIQFVSPNWVAENIK
LYR
>KRH95396.1
MKTYTQPEKLIVAIAPIGVSGVIGAFIFGIGMQLGGACGCGTLYTISSGNYTMIITRKSP
KGTEATWL
>KRH95397.1
MASSGVFMGIPATTGCLVRSRSHLNPLTYQVDALRGIMLVNGSSIYGLGLDCAILLLTLI
GLTFLCGRLYPLVVM
>KRH95405.1

MEVIYIFFIVIVIVVLIIQVAIVRWVFRINHIVMILERIVAHLEDIKKQNNILIRHQNEM
LELLDTDLERIVAHLENIKKQNNILIRHQNEMLELLDTDAES
>KRH95408.1
MLTRQHEFTEKIVEILNYHFADQGIIVLSSSELLQYLNIKTQAANRGSKSRAGLANHYAV
CVLVEDYLNHKFHINGGYEDYQGARFVNLFRRQRELPFGNKLQNHALNHRLNQEFKKYFP
TIPYVPIIRDTKTSRYWINENLIKILVNGNQINIAQSVKDIIDAYVHARVNSFTDFIMYC
KRMINMQLQTQPEYLEFIQSLLKPNIDARIFEIVSYAILKQYYGDQKIYWGWSYDNINAE
YLILYKTGRTNANDGGIDFVMKPLGRFFQITETVDVGKYFLDIDKVQRYPITFVVKTEQT
PKEILYKIQQQAVAKYSVKAIVKRYMESIEEVINIPELIRRFNLILQQDKGNLVIEEIVL
QSHIEFNMETEDVLK
>KRH95409.1
MMNIERYEHGGHILFHGDALSTLSNQVASQSVDLIFVDPPYNIGKRFSNFYDKWESEDKY
ATWVYNVLEECTRVLKPNGSMYVMASTQAMPYFDLYLRKTMTILSRIVWHYDSSGVQATK
YFGSMYEPILYCVKDKGNYIFNSDDIKIEAKTGAKRKLIDYRKTVPRQYNSEKVPGNVWY
FPRVRYRMEEYENHPSQKPESLLERIILASSDPSNIVLDPFAGTFTSACVAKRLGRNSIS
IEFQEEYLRIGLRRILGWKEYKGEKLLPPSKNYVSKKTQNKCYPERSL
>KRH95410.1
MVKSPSKQIQEKLKPILFYIVPLSSGILMGVTVAPINAWFLAWIALAPLWVIVVKYTRKS
LPSASWWAIAYHQ
>KRH95458.1
MYLLFQTLASFVEIYSYVLIVRVLLTWFPQINWYNQPFAALSQVSDPYLNLFRNIIPSLG
GIDISPILAFLVLNIVSSLLENLSSATSLGGF
>KRH95471.1
MSNTTSGQGTLPPASAIDKLTKNEVDTWFDYPIRVQPHHTDYAGLVWHGTYLTWMEEARV
ECLRSFGVDYADLVALGCDLPVVELSIRYHISLQLGNMALVKTRMLEVTGVRINWDYKIV
SLDNQQLCVTAQVSLVALDRERGKIMRQLPATFQEVIAKIAASYK
>KRH95501.1
MHHRNCLEVRNLRVEFSGDGAQVQAIDGISFDVLQGQTLGIVGESGSGKSVTALAIMGLL
PYPGRVTGGQILFRNQKNAQPLDLLALPSREIQLYRGGDMAMIFQEPMTSLNPVYNIGFQ
LTEAIERHQNVTLAEAKRIAIARLQEVKLLPKDENIKQQYLENWHQTHPKTPLPNDYQLA
QLVKEYKMAMLERYPHELSGGQLQRVMIAMGICCNPSLLIADEPTTALDVTVQATIMELM
RELQKSRNMAMIFISHDLGLVAEIADQVAVMYKGKIVECGLASQIFTHPQHPYTKGLVAC
RPTLNQRPQKLLTVSDYMIVQRYPNGELIIKSKEPNIPRQITPAEIETRIDDLTRKSPLL
QIHNLKVGFPVRGLLGGNKRYNIAVNGVSFDVYPGETLGLVGESGCGKTTLGRTLIRLIQ
PMAGKIIFEGQNITNFAGTQLQTLRKQMQIIFQNPFSSLDPRIKIGEAIMEPLLIHSLGK
TKQQRQDIVVQLLERVGLSANDMNRYPHQFSGGQRQRICIARSLALNPKFIICDESVSAL
DVSVQAQVLNLLKELQQEFQLTYIFISHDLSVVKFMSDRILVMNEGKIVEVGTSESIYHQ
PREEYTKKLIAAIPQGNINY
>KRH95522.1
MAHTRNFISNVDDSRVVSQAGHREPLKHLLIGSKKAVISTIHYLHVLGYANATDWSDLTP
TGNPGEVMSILVRHIVIN
>KRH95525.1
METRINAQIQSQNLWDGATLIDIMRKQAIEYDFNKGRMVINSILLADKTEVNNRGFLLDK
IRDYGCAYQGWNLYAPYEGVGFRSSTQPTFMFNSTHPLILFIFT
>KRH95532.1
MNHAKLESDLAQLQDEVSNCERDLLNLIKGKKTMEYSQNHNGGNQSNQIAIIGMASLFPQ
SKNLQEYWQVIVDKIDCITDVPASRWSVEDYYDPNPKAPDKTYCKRGGFIPDIDFNPMEF
GLPPNILEVTDISQLLGLVVAKAAMEDAGYGESQQFDRDRTGVILGVAIGRQLAVPLGSR
LQYPVWKKVFKNCGLSDDETEKVIEKLKSAYIQWEENAFPGMLANVISGRIANRLDLGGT
NCVVDAACASSLGALNMAISELLAHRADMMITGGVDTDNSIFAYMCFSKTPAVSPSEKVR
PFDVNSDGMMLGEGVGMLVLKRLEDAVKDGDRIYAVVKGIGSSSDGKYKSIYAPHSQGQV
KAIRRAYENAGFAPQTVGLIEAHGTGTMVGDPTEFTSINQVFGDNNSLKQHIALGTVKSQ

IGHTKAAAGAASLIKTALALHHKVLPPTINITQPHPKLNIENSPFYLNTETRPWISNQPR
RAGVSAFGFGGTNYHVVLEEYESEHHQSYRLHNCAKSIFLSAPTTPELLSQCQHLYQQLE
STDKEQHYQRIIAESEQLIIPADHARVGFTILSLSQAIAHLAIIIDLLKNQPSVEFWEHP
KGIYYRQQGMETTGKVVALFSGQGSQYLEMGRELVINFPCLRQTYSHLDDLFSREGLEPL
SQVVFPTPVFTPQERQEQLEKLQRTEYAQPAIGVLSAGLYKILQQAGLKVDFVAGHSFGE
LTALWSAGVLTEEDYFFLVKARGKAMSTPPAVDAGGMLAVKGNISQVTELIKDFPQVAIA
NYNSQQQIVLAGNKSEITQVQNVLQSKGFSCFLLGVSAAFHTPLVSHAQKPFAHAIAQVN
FQPARIPVYSNVTGKLYPNEPASMQKILQEHLLNQVLFQQQIENIYQAGGNCFIEVGPKN
VLTNLVKEILINKPHIAVALNANYRQDSDLLLREAVTKLRVFGVPLKNLDPYQIPAKISS
SSQKNEQKTLNIRLNATNINDRSQKAFAQALATGPVIKKSTVSEDNYQIQPQKILVEKNP
QINPEIISSSILTSKNSSQVKNHSIVEPKMEIPVENYDRLLDSLEQSLAEFTRQQSEINQ
VHQQSLQNQMEYNKTFYELMQQQCLFLAKEETNEYQAQTQQLAISSTERSMMRLHDHQAE
TIRIHEKYLNYQQEYTNNYFQLLEQHYSLFEVGSPNGYPHLPSSHVAQSDPPAQKLIYPL
EPENNSQNNLPDITVGFPIATYLDKEKLRDTLINIVSDKTGYPVEMLDLSMDIEADLGID
SIKRVEILGGLLELYPDLPRPNPEELAQLATLEQIAEYINNLITQVYENQPVEETVGSGK
SPLSQEIPVKAINEQTATIETPSPVTELDNHPQFLVLSHEDSLDRSHQPTITIPDNLSQI
LLTIVSDKTGYPAEMLDLSMDMEADLGIDSIKRVEILGGLLELYPDLPRPNPEELAQLRT
LGEIAEYMRNQAETVERSNLSPLEKPDMSTTEVGDKILRLPVQLKPLPQPDSLDLTIPEN
HFVLITNDGSEVTHRLVAKLADKGCKTVVLTFPCLESNLSEEIAQIRLNDWHEETLQEHL
TELTTKFGPVGGFIHLHPNSNNNLGMDKAIVQHVFLIAKHLKEDLNQLAKKERACFFAVV
RLDGELGTAKTHNFSPISGGLFGLTKSLNQEWPEVFCRTLDLSPDLDADTTVKHILAELQ
DPNLLVTEVGYNQIDRFTLVAEPGKSSIIPDSLNITKNQVFLVSGGAKGITAKCVIKLAE
EYQCKFILLGRSSAEIEPVWSEGYEDENELKRRIMEDFLAKGEKPSPIMVQKKYQTISSQ
REIHNTLKAITEAGGKAEYVCVDITDGMMLREKLTPIIDQFGTITGIIHGAGNLADKRIE
KKTVQDFETVYAAKVQGLENLLHIVETNQLEYLILFSSVVGFYGNVGQTDYAIANEILNK
SAHVIKHKHPNCHVVSINWGPWDSGMVSPELQTAFAQRGIKTIPQELGSSILVDQLRNSD
STMTQVVIGSPLVYIPSTLSSELKTHQITRQLKLNYNPFLQDHVIAGNPVLPATCGLSWI
SSSCEQLYPGFQTFHCPNFKVLKGIVFDQNSPHEYILEIQEVAKIDNQEIQLAGKISSVT
NHGKIRYHFSSNLILKRQITPADNYEFFNLTQDGQFLASNSLLYQTGGGSLFHGNTFQGV
KSVLNISPGKLTMKCELPEPTLYQQGQFTVQTLNPYIADVQIHSLWIWTQHFHQVGCLPS
EIENFEQFVPVPFGETFYVTCEIKSKTESYVVADVITHNQKGQVYNRMKGAKGTILPNSY
>KRH95577.1
MIQFRIQPDSEIPASSQLFNQIRFAIACGQYGSGYKLPSTRALAIQTGLHRNTISKVYRQ
LEEEGFVESLAGSGIYVRVQGHETGSRLQLPILQQYPQAFKSIQQTLDELLNQGCSLNQA
RELFLAEVDWRLRCSARVLVAVPLQDIGVGQLMVDELEESLEIPVELVAMEELAAVLEET
TSATLVTSRYFIGDVEAIAAPKAVRVIPLDIHDYNKELNVVKNLPRSSCVGIVSLSPGIL
RATEVILHGLRGDDLLVMTAQPKDAYKLNAIAKRCEVIFCTDATSHSGVQTVVQGIAEDI
IRPPKLIRCANYIALQSVNLLQRELGLI
>KRH95587.1
MPEITRFYGIIIKLFFAGHPPAHFHAICGEYNAIFNLETLEFIEGEYEIWTNGQHCSQNL
EP
>KRH95596.1
MPKHFNTAGPCQSDIHYMLSPTARLPELKALIDGRNYFIIHAPRQVGKTTAMIALAQELT
ASGHYTSVMLSLEVGAPFSQDPHKAEQSILAEWRQSLRFRLPPDLQPTEWPATESSSQLS
TFLSNWAATADRPLAVFLDEIDALSDETLIFVLRQLRSGFPNRPHGFPHSLGLIGMRDVR
DYKVKSGGSERLNSASPFNIKAESLTLSNFSFDEVQELYLQHTEATGQIFTPEAIHHAFY
LTDGQPWLVNALARQATTVLVKDVNQPITIDVIKQAKEILIQRQDTHLDSLAERLREDRV
KAIIQPILAGSDLPDTPEDDRRFVLDLGLLKRSSLGGLTIANPIYQEVIPRVLSQGSQDS
LPQIQPSWLNTDHSLNPEKLLNAFLDFWRQHGEPLLKSVPYHEIAPHLVLMAFLHRIVNG
GGTLEREYAIGSGRMDICLRYGVVVMGMELKVWKPGKKDPLPQGLQQLDKYLAGLGLDTG
WLVIFDRRPDLPPIEERTTTEEVVSPGGRAIIVIRG
>KRH95609.1

MLKQVKDLKLLSLTQLIVMLLLRLLMSLEGYISIFALRVIINNIIPSQHVEFEYIQTSKS
NSEFDSELSLTSTTNFTLQVLHGSDFEGGIPAVTDVVGFSAVVNKLKDDPKYKTNTLILS
SGDNYIPGAFFNASSDTKLNNVGGLGSSTTPVIGRGDIGILNGIGIQASALGNHEFDLGV
RQVRDILRTGSGNPGTNFPYLSTNLDFSPEITAGNLSATDLAPNQNTAHASSIKGKIAKS
TIINVAGIDGITGTADDQRIGIVGATTPTLANISSSGSTIVKPANPIDYDALAAEIQTSV
DILKAQGINKIILLAHMQQLTIERDELAKRLRDKVDTALTLAPGKFTFALPGTEQDVLAE
YLGDRFSTNAYGVADVSPALDRRIQNLTFQGEREKYKCFDKRSLWSRVIVKRLR
>KRH95659.1
MHITEFDEKFDRVLNELQRDREEQARKWDEQNRKLDAFQAEQNRNRVVDRKIVISPMVDE
RAIPVAKSLGIETYSYADMVVS
>KRH95660.1
LFGLAWDLLYDFLQKYLWDHDWPGVFQFYAGIVEGVFLGLILGTIGLPKIPRAEFQLVTF
IQHYGLVWLGIYLSAWIVMRLLFPRWRFRGGEWLGNWPKGS
>KRH95661.1
MPMNFSPNYTITPKIAKFLMRIETVRERVCYLPITAMVLASLRETARLYTTHYSTMIEGN
RLAPDEIENVVKYEGHFPGRHRDEREVKGYYAALAKLEQWVAAGVQVSEKIIQTLHAVVM
SDGRTTAQPSAYRDGQNVIWDASNGTIVYMPPEAKDVPFLMKAMVKWIHQSQEVPCPVVA
GIAHYQFATIHPYYDGNGRTARLLTTLILHLGGYDLKGLYSLEEYYARNLTAYYEAISVG
ESHNYYLGRVEADITKWVEYFVEGMAIAFENVLKRMAEAELQGSVDQDPILRQLDPRQRK
ALSLFQQFEVVTSKQIGELFGFKPRTSAQICKDWVESGFLEMVDPSNRGRKYKLSTQYKD
LLN
>KRH95693.1
MALIVQKYGGTSVGSVERIQAVARRIHGTAQVGNSVVVVVSAMGKTTDGLVKLAHEISPS
PTRREMDMLLSTGEQVTIALLSMALQEIGQPAISLTGAQVGIVTEAEHTRARILHIETER
LISHLNAGQVVVVAGFQGISNTSAMEITTLGRGGSDTSAVALAAALKADFCEIYTDVPGI
LTTDPRLVPEAQLMKEITCDEMLELASLGAKVLHPRAVEIAKNYGVPLVVRSSWTDQPGT
WVTSNKVQERAMVNLELARPVDAVEFDIDQAKVSLLRVPDRPGVAARLFNEIADQQVDVD
LIIQSIHEGNSNDIAFTVNTPILNRAEAVASAIAPALRNNDGSNEAEVLVERNTAKVSIS
GAGMIGRPGVAAKMFATLAKAGVNIQMISTSEVKVSCLVAAVDCDRAILSLCQEFEVNAS
TRNVSSSNSIYSTVCGVALDMNQSRLAIRHVPDQPGIAGKLFGLLAESNISVDMIIQSER
CRVIDGVACRDIAFTTNRTDGENAQAKINQVAAQLGWGEVILDDAIAKVSVVGSGMVGQP
GVAAKMFTALAQNQINIQMITTSEIKISCVVSEKEGVKALQIIHTAFGLAGTHKFVVPA
>KRH95735.1
MNHDVFLVPPGKKISLGDYDPSYKAEFHQKVDAVKKLRAGIKELARYQDVLYAQNTYALL
IIFQAMDAAGKDSTIKHVMSGVNPQGCQVFSFKAPSDEELDHDYLWRSTRCLPERGRIGI
FNRSYYEELLVVRVHPETLARQQLHHFPQGNQLWKQRFEEINNFEKYLVNNGIVILKFFL
HISPQEQKKRFLQRIESPAKHWKFSASDVRERAFWHDYMIAYEDVFNHTSTKHAPWYIIP
ADRKWFTRLVVSEIICDKLKELDLQYPIVSEEHQQQLLQAKKLLESEDITNFHPRTSDRI
S
>KRH95741.1
MTSIDKILLKYKVLVETHANRFRPQLDALYHFVDESMKEIQNTEREILESQNVELKKIID
ALQVDPRILLSTDEFKQFVEILGIAECWWEWEELEDLPAIDKDPTNWLLAKLQLPLIIRD
YQEFEDPYAYDDTSTYTLYGYKISLKLGNRICTMEVERRRVYENRCKEFSPEKQIAYYIL
SPIRDLLRSMNYSEQEIDQLGGEMGILVFYVAKLFELKPTVSVFEYNSMKRIY
>KRH95745.1
MTWWQEELLDIVLTVSGEAERLFEGIDEIVDTLFDLTEEIDEQVGSAGTCLSELLEPLVG
FYWDLEDPNLPYEVEADSLKNSACVGCSNYHGEVYGGNLLVCAMYPYGWDGEKCPDWEE
>KRH95746.1
MSHSVSNILPSEDPVKEVKYGEREIEEGKLITFPNPRVGREYTIDITLPEFTCKCPFSGY
PDFATIHIAYIPDQRVVELKALKLYINSYRDKYISHEEVTNQILDDLVIACAPLEMTVKA
DFSPRGNVHMVVEVKHKKGAAS
>KRH95753.1

MGNQFKTLALLAALSGLLIAISYWIIGGSGGLVLGIGLAAITNLFSWYQSDKIALAVYNA
QPVSEAQLPVLHRMVAKLSSRANMPMPKLYIVPTYTANAFATGRDPEHAAVAVTEGILNI
LPEEELEGVIAHELTHIVNRDTLTQAVAATVAGAISFLAQMLSYSLWFGGGSRDNNRGGN
PLGVLLTVMLAPLAATIIQLAISRTREFSADAGAAKITGNPRALARALQRLEAAARETPL
DANPAFEPLLIINPISGQFLANLFSSHPSTQSRVQALLQLEN
>KRH95773.1
MDKLTGLATSVLTLTSGLAIVTSTSQTVLAQVVTLQPGTFSYQAADLLAETPINLEEFCK
VYPLNSRCVPTQPVTPQPPQPTQETTQTGPTTSGWGVTPEISTLGIGATVTKSITPNLNA
KLGVRGFGTNANISESGIDYDAKLNLFSVSTLVDYHPWKNSGFRLTGGLVFQDNNIEGTG
KSNSNQKIQIGDQEYTSDQLGSVKAKVSFPNSVAPYLGIGWGNAVKPGNRWSFSANLGVM
FTGSPKVNLAPQFGGAATPEIRNQIQADVEKERQQLENDLKWLNIYPVLSLGLSYQF
>KRH95784.1
MTTETTAQNPTTGADAVDIAIAQGIDFDGSPIPTVKLELYNYVMGLEAGRQRSGVSNTMR
SRIVRIGAKHIPQAELDEKLVAAGFAPLKEKEIAFFYGSK
>KRH95797.1
MIFTQTSLAGAFIIELEDKPDHRGFFARTFCAQEFAEHGLKPTVAQCNLSFNHQKGTLRG
MHYQITPATETKLIRCTQGAIYDVIVDMRPESPTYLSYIGVELTATNRRALYVPEMFAHG
YQALTDGAEVVYQVGEFYTPGYERGLRYDDPILDIVWPLNVTEISQKDLSWPLLESVLIG
V
>KRH95809.1
MKKFLRLALILTLFLVSSFSFSTSPSYAYSQSDLDKLLETGDCRNCDLSGADLRKANLSG
ADLSSADLSYAILTSANLSTANLKKANLSGADLSGADLVDAYLPDADVRDADLSYAFVLH
ADLSYANFIGANFEGANVRGANFWGARLDRGWESLRYEK
>KRH95828.1
MANVEVNKITFVLIVVVNLSMSIVHLKAIQKKLNKVACALILTVWDLEQLNAIKAFIIQL
LELLENKG
>KRH95848.1
MENRLFVFSPCGTSLLTNQAAQEERGLVSKHANAKHIEEIPSEDSLKLRSLAKRVEEKLA
SADLELAGKMSAELNGIIKLYRRP
>KRH95857.1
MLNSLNLENFKPFENQSFVLRPLTFCVIPADVHPGNSGHQIPHSLVSGSGSSMTRHFIVL
DIHRTILKL
>KRH95865.1
MAILEQVWQLCQRQFLCVIASVLFYTSIPLPHLPGLKFQRVALFAPAIGLLIGAILGLLD
MLFDYLGISALTQSVLIVIIWIGITGGLHLDGAMDTADGLAVTDPQRRLEVMTDSATGAF
GVMAALAILLMKMAALTDISQNRFFLLMVACGWGRWGQQLAIFQYPYLKSTGKGAFHKQA
IRHQMDLLPSWVLLLGFNLLTLAFNQGNFVLVLFTLIIGNIISFIVPAWFNHKLGGHTGD
TYGAVVEWTEALFLCCMSSLT
>KRH95896.1
MYKIYTTSGKRIIIRSKLYLIFRRVTAVFIYLNQILITFSLCAKQKPWFIYLKFAVKDMQ
NFHTLGDRYFCSFDAIIDFMIHLYGIIKVHEHLHSLQEMYLSVLKEHNYIVLESDNKLHV
FYGD
>KRH95897.1
MKFTIDEICHLFDHKGSKMYGAEAVTQLEHALQTANLALQAGETRELITACLLHDLGHLI
HNLGDDPAAQGVDDKHEYRAIPFLGQIFSLEVTEPIRLHVVAKRYLCAVDSQYWQGLSPA
SQRSLQLQGGIFSPQEAEEFIRLPFAPDAVKLRIYDDQAKVPHLSTPELSYFVELVPRS
>KRH95900.1
MSVSTKHPDFNSGFNFAYLDEQTKRSIRRALLKAVAIPGHQIPFSSREMPMSYGWGTGGI
QVTAAIIGQNDVLKVIDQGADDTTNAVNIRRFFRKVCAVETTEETGRATLIQTRHRIPET
PLREGQILVYQVPIPEPLRWLEPSTVESRKMHALQEYGPIYIKLYEDITRYGYIATGYDY
PVIVEDRYLMSPSPIPRFDNPKMHLNPALQLFGAGREKRIYAVPPYSRVKSLDFEDYPFG
VERWNHACELCGSTESFLDEVLVDDRGGRMWICSDTDYCQQQRRKK

>KRH95906.1
MSTAIAKKADKVCEVKIVRVKRGLFNPTLIAIISFCILVSVPKPAYAMHIMEGFLPVGWA
VFWWVVALPFFILGLRSLTRTTQANPQLKLLLGLAGAFAFVLSALKIPSVTGSSSHPTGT
GLGAVLFGPLTMSVLGSLVLLFQSLLLAHGGLTTLGANAFSMAIAGPFAAYWIYNLTIKI
SGKEKIAIFLSAAIADLLTYVITSIQLALAFPAPVGGFMASFIKFTGIFAITQVPLAISE
GLLTLLVWNWLQSYNPQELELLQLIKGGNGNES
>KRH95908.1
MRDVRDYKVKSGGSERLNTSSPFNIKAESLTLSNFTLSEVEELYLQHTQATGQVFTPEAI
HQAFYLTDGQPWLVNALARQATQVLVKDITQPITAEVINQAKEILIRRQDTHLDSLAERL
REERVRDIIQPMLAGEDLADTPEDNLRYVLDLGLCRRDRGGGLEIANPIYREILPKALAS
VAIASLTSVEPNWLNPDGTLNPQILLDSFLEFWRQHGEPLLKSAPYHEIAPHLVLMAFLH
RVVNGGGTLEREYAIGSGRMDICLRYGKVVMGIELKVRKEKLDPLTQGLIQLDKYLNGLG
LDTGWLVIFDRRAGLPPMGERISTEQAISPGGRTITVIRS
>KRH95909.1
LFGLAWDLVYDFLQKYLWDHDWPGVFQFYAGIVEGVFLGVILGTIGLPKIPRAEFQVVTF
IQHYGLVWLGVYLSAWIVMRLVFPRWRFRGGEWLGNWPRGS
>KRH95924.1
MQKFLLYLLTSSVLGYAISMSGSALAEDVSKTESSVQVSEPNYISQMTSVSQLSDVQPGD
WAFQAIQSLIERYGCVGGYPDGKFRGNRTLTRFEFAAALSACLDRVNELISSATADQVTK
QDIANIERLQKQFGPELETFKRRVDTLEAKTTRLEATQFSTTTKLQGQVVAVVSDISARK
VNVDTRTVTDKNATLGVRTRLELVTSFTGKDTLFTRLQSNNIRTPKLVDPAKSPTASDYA
VDFKEAGFYFGGGVDSTALSVTALSYKFPIGDKTQVIAVANDGAAEDLTTTITSFNGDGA
FGALSTFGTRNPIYSQLGASGLGINHEFNKNLTLSLGYLGGDMSTGSSSAASPASGKGLF
EGPYGALAQLTVKPSDRVTLGLTYINSYNLALAAGSNNATGGLGGNFSSNSYGVQASLGV
TPKLRLEGWAGYTKSQLLTGIKGDVDIWNYAVNLAFPDLWKKGNLGGLIVGMQPKVTNAS
TTLSGLKDKDTSYHLEGFYQFKVNDNITVTPGLIWLTAPNHNKQNDSVIIGALRTTFSF
>KRH95925.1
MKNFGKTTKTTKITRRILGWSSSILFLSLIYGCERSQVNIPKEESTSTIVATFLPVYLFT
KAIVGDVAKVDVLVKPGTDIHEYQSTPDNVKAIATGSILVKNGLGLEEFLEGTVKNAKNP
KLVEIDASKGIKVMDDGSPVEKIKGEKHDHKHDHALGNPHVWLDPVLAKQQVINIRDGLI
AADAQNKQTYQTNAATYIQELDNLNNEFEQTLSKTPNCTFITFHDAFPYMAKRYNLKQVA
VVELPEDKLAPGDIQAVINTVRKYKVKVLFSEPGIDNKLLTSLAQDLNLTLYSLNSLETG
ERNPQYYFQAMRDNLKSLATGCQK
>KRH95928.1
MLVEFSPPNNNGTLYPAGVSTLLVFDITPLRERAIATPCGEAQLFVGWVGGVGFHPSTQP
TLSYI
>KRH95968.1
MYRRNPSLQDYLLVDAEKIAIDLYRKNDRGNWEIFNYQSGDNIELQSIDLSFPIQSVYED
IVFEELA
>KRH95969.1
MKRRSFIESSALFGASLLGTQAIATIGNRGIEPVQAQQIKELNFGIISTESQANQRPLWE
PFIAALSSSIGIPVRAFYATQYAGVIEAMRFGQVQIAWYGGKSYIEAAKIANAEAFAQTV
SSDGKKGYYAHLIANKSNPITAAAKKQGGDKYVIRNASKLTFAFNDPNSTSGFLVPSYYV
FGKNKVDPKKVFKRLIFSGSHEATALAVANNQVDVATNNNESLERLEKTNPSARQNIEII
WTSPIIPSDPIAYRKDLPADVKKKLQNFFYNFKDKKILEPLQWSALVPANDKTWNPIREL
DLAKQVLDLEAKTDLTPADKQKLNNLNSQLRKLQGR
>KRH95979.1
MKLVGEKVFSIITDNLRAFSLSDKFWQSMDSAFGTSYNSTIAELLRGKWQKGDFSDLPPI
EMVDSAVLRGGQGAYSQQENRIYLSGDLIGNVEAISRVIIEEIGHYIDAQINQVDSPGDE
GSIFVALVQGEELSPDVLAVLKTEDDTAWINLNGRSIVIEQATFTSTGRVNDTINGTVEN
DTINAGLGRDTVNGGGGTDLLIVDYSSNTYTGNQAGITSSISGSGGFNGSYTAYTYYNYS
WDNNYDQVSFSNIERFQITGTVAGDNITTGDGNDIIDGGDGNDIINAGNGDDTINGGDGN

DTINGGGGINIIDGGDGIDTVDVNLSSITSTQTIEDSDIAKNFTLADGTNIFNVENFRDL
TLGSGADVVNFTRRYNNTINTGTGNDTINAGLGRDTVNGGGGTDLLIVDYSSNTYTGNQA
GITSSISGSGGFNGSYTAYTYYNYSWDNNYDQVSFSNIERFQITGTVAGDNITTGSGNDI
INGGDGNDTINGGGGDDTINGGDGSDTINGGDGNDIINGVNWNSSTPGSSEVDTLTGGQG
NDRFILGDLNWVGYDNGDTSSAGTTDYALITDFTVNDIIQLQGKSSNYSLVVSGSDTHLY
LNKLGSEPDELIATLRSTSTLNLSLTGSYFNYVNAAVAPELAIASTNATQTEGNSSTKSF
TFTVTRTGDANNSSSANWAVTGSGTNQADATDFGGTMATGTVNFAANETSKTITVNVSGD
TTVEPDEEFTVTLSNPTNATIATATATGIIQNEDVAAPLPTITLAVSPTSVTEDGTTNLV
YTFARTGPTNDVLSVNYNIYGTADETDYTGASPGIFPASGKTITFTTGASTAMLTIDPTA
DTTVESDETVILTLASGTGYTIGTTGAVTGTITNDDLGNTQQITSTSNRLRATPGANLTV
PLFYNTSNSNNAVNGIGIRLHYDSTDLSYQQVTNLFSTNLFGSITNGLDTENFDKDNTTD
RYIQLQYFATTGNWPNQTLPVKLGDFGFTTSSIFQGTQLNITGVDLAPGYTLEAAPIEIY
KQNWTLDVDGNGTISALSDGIIIMRYLFGNFSGDALTRNAIAPNATRTPSEIRTYLGEAG
SILDIDGDGAVRPLSDGIMAVRYLFGNFPGNALINGAISPNATRNLSQIESYLASISGTS
SASPLVPQQTTFLPLFAQTFNATSSSKQIIDLTTSNSSLTPGAPVSIGVTYNVDSGDNTL
TGIGIRLHYNSNQISYQGASNLLSTDLFGDVTDNLDEQDLDGDTSTNRYIQIQYADFSGN
WPNQNLPVKIGDFAFNTVSSFQQSKVNVTAVDVAPGYTLEARPLTLAGDGQPKVISGTDQ
GDDINATRGQTTVMPGKGDDIIRVNSASVVIIELPNEGNDTVFSSINYNLASLPQIENLT
LWGTEDINGIGNRKDNVITGNSGQNVLTGLQGNDTFVFNLGDSVVGKPDRIGDFQFGKDK
IRVNGVSPSVLTRASNNSASTLNSLVDSVFIDGNGATSGNQGLGTNSAALVVSTAQGIGG
TYLIVNDGVGGFNPATDLVINLTGYSSNLPGLGNIAVGSLFV
>KRH95980.1
MSQEVNLKNLPLGINTLDKLRGSDCVYVDKTHLALKLIKQPGAFFLSRPRRFGKSLFKDI
TISEAYSSICGYTETDLRESFGDHLEGVDWDALRHWYNGYNWTGSETVYNPRGIKSGIMQ
HLTKSSSETMLKNTVGNRENLSMKLV
>KRH95992.1
MSLITLQLVKKDFGIKEILKEATFSIDGTDKVGLIGTNGSGKSTLLKMIAGIEPVDGGQI
LTNSGAKIIYLPQQPDMDENLTVLEQIFMDSGEHTKLVKEYEELSDKLAHYPEDTLLMSR
LSEVMQRMDATGAWELETNAKIILTKLGIGDFEVKVGTLSGGYRKRIALATALLAQPDVL
LMDEPTNHLDALSVEWLQSYLNRFRGALLLITHDRYFLDKVTNRIIEIDRGDIYTYSGNY
SYYLEKKALAEESAVSSQRKHQGILRRELEWLKRGPKARSTKQKARIQRVESMRETQFKQ
AQGKVDISTIGRRIGKKVIELSNIYKSYDGKILINNFSYEFSPEDRIGIIGGNGTGKSTL
MNMITGRTSPDAGRVEVGSTIHIAYFDQHSEELISAVNDNQRVIDYIKEEGEFIKIADGT
KITASQMLERFLFPGSQQYAPIHKLSGGEKRRLFLLRLLISAPNVLILDEPTNDLDVQTL
SVLEEYLEDFVGSVIVVSHDRYFLDRTVDTIFALEEGGNLRQYPGNYSVYLDYKKSEELT
QQETINGKDNRKSKNLTQPKPGDQEVQNKKRRRLSNWEKREFEQLEAKIADLEAQRTLVE
TSLLAVTSENYTQVQNLYEQMEALKQAIDFATERWLELAEMDV
>KRH96005.1
MTTPQEVLKMIRDQNIQMIDLKFIDTPGTWQHLTVYHNQIDESSFTSGVPFDGSSIRGWK
GIEESDMTMVLDASTAWIDPFMQEPTLSIICSIKEPRTDEWYNRCPRVIAQKAIDYLGTT
GIGDTAFFGPEAEFFIFDDVRYDQTANEGYYHVDSVEGRWNTGRKGKNGEADGPNLGYKT
RFKEGYFPVPPTDTFHDMRTEMLLTMAKCGVPIEKQHHEVATGGQCELGFRFGKLIEAAD
WLMTYKYVIKNVARKYGKTVTFMPKPIFGDNGSGMHCHQSIWKDGKPLFAGDKYAGMSDM
GLYYIGGILKHAPALLAITNPTTNSYKRLVPGYEAPVNLAYSQGNRSASVRIPLSGANPK
AKRLEFRCPDATSNPYLAFAAMLCAGIDGIKNKIHPGEPLDKNIYELSPEELAKIPSTPG
SLELALEALENDHAFLTETGVFSEDFIQNWIDYKLVNEVKQLQLRPHPYEFFLYYDC
>KRH96006.1
MRDAVTTLINSYDLAGKYLDRNALDSLRSYFDSGTSRVQAATAINANAAAIVKQAGSKLF
EELPELIRPGGNAYTTRRYAACLRDMDYYLRYVTYALIAANMNVLDERVLQGLRETYNSL
DVPIGSTVRGIQIMKDLAREQAIAAGVANAAFVDEPFDYITRELSEQNI
>KRH96053.1
MSTKIYQQIQEFYDASSGLWEQIWGEHMHHGYYGADGRERKERRQAQIDLIEAVLNWSGV

KHADDILDVGCGIGGSSLYLAQKFHAMSTGITLSPIQSARAKERALEANLQSRSSFLVAN
AQEMPFGDNSFDLVWSLESGEHMPDKTKFLQECYRVLKPGGTLIMVTWCHRPTDVLPLTE
DEQKHLQDIYRAYCLPYVISLPEYYAIASQLGLKNIKTADWSTGVAPFWNVVIDSAFTPQ
AFLGLLFSGWTTIQAALSLSLMRRGYERGLIKFGLLCGIK
>KRH96089.1
MAWQKILATSDLLPGGREVVKVGKRNILVLNHDNQYYAVENSCPHLKVPMKSAKIESGTI
VCSFHRSAFDLATGEVKTWCPWPPAVGKLMGMVSQQRSLPVFPLRVENDHVLIDIPE
>KRH96106.1
MEVKELLNRYARGERNFNGICLRAVNLRGVNLGGIDFARADLSWSDMTGISLSGANLSQA
NLRGAKLENAHLSEVILCGADLTQAILINAHLNESDLSGALLVDANLCDADLHQASITAA
NLQSAKLNGAKMGGVRMWKADLQGADLTGADLSEANMCGVNLSMANLSATDMSETFLTGA
IMPDGSLHS
>KRH96121.1
MSGSWSQTSRRKFLTTAGIATGAVFIKGCLGNPPEKGGGTSSQSQQVEAVNLTPEITPET
TKVKLGYLPIVEAAPLIIAQELGFFKRWGMTEVELAKQASWGSMRDNTEIGSAGGGVDGG
QYQMPMPHLITEGLITKGNAKIPMYILAQLNTQGNGIAIASKHAGKQISLDLSKGGKAVF
DKLKSTPSPFTAAFTFAKVNQEFWLRYWLAAGGVNPDTDVKLIPVPTAQTVANMRTGTVD
AFSTGDPWPYRIVKDKIGFISALTAQIWKNHPEEYLAIRGEWVDANPKATKAILKAVMEA
QQWLDKFENRETAANILARPNYYNLSPTFLTDPFQGNYDMGDGQQVKDKSMAVLYWKDER
GSVSYPYKSHDLWFLTESIRWGFLPPEYLEKSEDIINRVNKENIWREAAKEAGIPDADIP
TSTSRGVEKFFDGTTFDPSDPRGYLKSLKIKKVNI
>KRH96143.1
MITRQNRHAKLPENTDTKSLNQFPSLKDELYLRFYLSSPEEFALPLISIKEVIEVTPNQI
IPIPNTSPLVLGVVNWRSRLIWVVDLGKFMGEMIPLNLERRSQVSVITTEYEDTIIGLAV
DQICATFWLDMESVVAPTDVPDDTVPFVHGEWLDSENNKSVKLINQKIILQSDKWTSMVK
FNQEEK
>KRH96151.1
MQIASGSSNDMKNLQGFDSNHPPDPKLIDSCVHCGFCLSTCPSYRVIGKEMDSPRGRIYL
MDAINEGEIALNTATVQHFDSCLGCLACVSTCPSGVQYDKLISATRHQVERNYQRSFADK
LIRQLIFSLFPNPDILRILLFPLFLYQKLGISQLLRATRLIQKISPRLAAMESILPKITI
QSFGNNLPDIIPAQGAKRYRVGMILGCVQRLFFSPVNEATVRVLTANGCEVIIPKSQGCC
AALPEHQGQTEQAKALARQMIDSFADSNVDFIIINAAGCGHTLKEYGHILADDPEYAAKA
QIFAAKVKDAQEFLVSVGLTAELSSLSDKPLTLVYQDACHLLHGQKISLQPRQLLKQIPG
VTLKEPLDAALCCGSAGVYNLLQPEIAQELGEQKAQNLLNTGADIIASPNPGCSLQISKY
LGKVSVMHPMELLDYSIRHQKLEREISR
>KRH96154.1
MIFDLKVPGEWDSVLSDEFRKPYWKQLEEFLVQERSSQTIYPPESEVFSAFELTPYQQVN
VLLLGQDPYHQRNQAHGLCFSVKPGIKPPPSLKNIYRELQQDIGCPIANHGYLANWAKQG
ILMLNAVLTVREGQANSHKNQGWELFTDEVIKKVNEKTKPVIFVLWGGYARKKVKLIDTN
RHLVIESAHPSPLSAYHGFFGSKPFSAINSALKIWDKPEIDWGL
>KRH96173.1
MNQGRTYDYIDLIELLIGTIFESKGDSQVIYPLLENNLHLLDATFIDQLQSWGGHQVNKQ
ATPEQNAQLGASLYLLARIFYHFPGGDPLINLAIAICGYEFCAAIERQLGLEKELAQVLN
HLGAAYQTQAQMGTDSHSNLEKAIAAYNEAITIRRQPGLERELAQVLNNLGAAYQIQAQM
SKEANLERAVTAYAEAISILRELDLERDLAQTLYNLGSAYQTQAQLGKDSQANLQKAIPP
YTEAISILRELGLERDLAKTLTSVGSVYISQGKLERAITPFREAITMFRQLGLERDLAET
LNQLGIAYLNEVERGKDPDANLQRAIIVFTKALTILRQPGLEPDLAQTLHNLGKAYQTQA
ELGTESQSNLEMAIAHYREALAIATPELSPADYLKFASHLGDLGFEQGWWDIALEGYEQG
IKALETIQFWRDKSPGEIQHSHIYQSAVRACIELHQYGKAVELVERSKSRNLVELLENKH
LYPKGNIPSRIYGELDRLRQAIRNEVRLLEIQGEKRSHGQLNTLIEELDQFITSQVKPLD
PHFSLTQRVEPITISKILKLVGADPRRVILEWYFTDDVLYTFIITANPDVPIFPHKVVIS
PSVIRQFSQKYLDIYRDRHNLGNWKQALPELLGELKEILQVETLLTQLWKLSPPCEELVL

IPYGCLHIFPLHALFPWERFPLGFSYFPSCQLLSSVGVTEREEVVQSLAIQNPTQNLQYA
SFEVETIITRLLIGTEVITEKEATRETIFSRSIPEKNYLHFSCTGYSNFQKPLHSAILLA
NCILPAPPKGGEYLPLPNGAAVDLSQCLTLGDICGLDMRRCHLVSLSAYEMGIGDMVDTK
NDDYISLPTGFLLAGASTIISSLWAVSDYTSALLMIRFYQFYTDTENLLTYGRPRLALYH
AQQWLRDAPAGKLLTWAAKLPFSGTRLTLLADFDTMPESQRPFQEPYYWAAFTCVGI
>KRH96179.1
MICCLNPDCQNPQNPHGSKFCQSCDTPLVPLLRNRFRIIRVLSDEGGFGRTYLAEDTDKL
NDNCVVKQLAPKFEGTWAQKKAMELFSQEAKRLQELGEHPQIPTLLAYFEQDKCLYLVQQ
FIDGNNLLQELQLRKHYKDSDIQSLLLDLLPILKFLHSRGIIHRDIKPENIIRRKRDGRL
ILIDFGSAKQLTVAVQKKYGTSIGSHGYSAIEQIRDGKAYPASDLFSLGATCFHLLTGLS
PFQLWIEHGYSWVKNWQDCLNNSRSAELIIILDKLLQLDLKNRYQSADEVIKDLSKKNVY
GSKKSGIYLKKGKHEKHKHTILRNIFLILVTISVVGGLGYRNLGQIQTAIFSQFNPLLIP
SNKSSTSPEYGQISGRTSSIRTNKVSLLKTITQVDKSLAVVAITPDGNTIVSAGHKEIKL
WNSKTGKQIISLPGHTQNINALAISPDGNNLVSAGDDKTIKVWNLQTKKLTFNLVGHQDS
IQALAISQDSKILVSAGDDKTIKVWSLLTGKFLKTLLGHNYWVRSLALSPDGFTLASGSF
DKTIKIWNINQTSGQKPTTLLDTTSQTVTSLAFSPDTSTLVSTSRDRQIKFWDIKNKEII
FASKKQNVTSVIFSPDGKTLISKAKSCPDCSEKISSVIKLWDVSTKEEIYALPGNTKIVT
SLVLSADGKTLVGGTEDNKINIWEISP
>KRH96199.1
MTTLYEQDFALWSEKMADLIANGHFDKLDIINLVEEIRDLSKRERDQLLSSMRLILHHKK
IAERHGSHLALDSGGSANGEFIRLDI
>KRH96200.1
MKRGIQGHYVTISTAGERAHAFIPKPLPPHPAIEWTQTLSSKFDQALVSLGRLSSISTLL
PDTSFLIYMYIRKEAVLSSMIEGTQSSLSDLLIFELEEQPGVPLDDVREVSNYLAALEHG
LRLLRGGLPISLRLFKEIHGVLLNKGRGSSQTPGEFRRTQNWIGGTRPGNAAFVPPPANE
VLECMSKLELFLHDEPEPTPILLKAALAHVQFETIHPFLDGNGRLGRLLITLLLCEHQVL
QEPMLYLSLHFKTHRQYYYELLNQVRLTGDWEAWLDFFAESVIITATQAVETAQQLLELG
NQDRDKIKALGRRTASMLQIHRALTERPIATSPWLVERTGLSPATVNKALAQLQKLGIVR
ELTSQKRNRLFSYADHVEILSRG
>KRH96210.1
MTTFTAKRRTSNNSSENFIVAFITKQTPEILPPLVAIVAFLVVWQIFSLTGGTLPGPIQV
VQDTWQLIIYPFYDRGGIDKGLFWQVFASLQRVAISYTLAAVVGIGLGILIGVNSTMSKA
LDPLFQLLRTVPPLAWVPISLAALRQNEPAALFVIFITSLWPILINTAVGVKEIPTDYNN
VAKVLQLSQKEYFLNILIPAALPYIFTGLRISIGLAWLAIIAAEIVMSGIVGIGFFIWDA
YQANKVSEVILALVYIGVVGLLLDKLMAWLQTRILPEQK
>KRH96231.1
MEVELKERKPSGRNYRNLIKKAEIQLKSTISVFIENKNSEKFVTKRAYIANAKKPEFHQS
HKERMQRFKDDTGFILKIENKIII
>KRH96232.1
MPLYLPQSEIDRIEVINLDKWVDNFWREQNIDKKIIFLDSDTKHLWEQAYQLLSVPIGIE
FFDTPQP
>KRH96250.1
MLAYQPINREISRVIIAGGIHGNELIGVYLVKTFEKYGNLIERETFETICLLGNTPAINA
GRRYVDKDLNRCFTKDILFNANTSIYEESRAKEIWQLLQPHHPQKLDAIIDIHTTTANMG
LCIIISNIHPILVTLAVQLTSISPLIKVCFRPVTPDGGFLRSLSEVGFTLEVGPVPQGVL
NAQLFQQTAKIVYAILDFFETYNQGKICINQKSNEKKLKVYEFIGTIDYPRNELGQLQGM
IHPQLEYKDYQPLNFGEPIFLTFDNQEVFYQGESTVYPVFINEAAYYEKGIAMHLTQKKL
VDI
>KRH96256.1
MQITINIPDNLTDKFRDKLGHLSEKVMNKLALEAFLEGLINFNEFRQMLSFQDENAFKAF
LSANFPLHSGGLLNLAGSCADIDFTIDEEGIYDHKNDETILNWPIQ
>KRH96274.1

MTVYTTGSLKAELNDRGWRLTPQRETILHIFQELPQGEHLSAEDLHYRLENEGESISLST
VYRTLKLMARLGILRELELGEGHKHYELNQPYPHHHHHLICVKCNATIEFKNESILKIGT
KTAQKEGYQLLDCQLTIHAVCPRCQRALMPL
>KRH96295.1
MESNIFTSLILPMALGTMMLGMGLSLVPEDFQRVGKYPKAVAIGLVSQLFILPLIGLAIA
KLVPMQPAIATGLMILALCPGGVSSNLVTFLAMGDVALSVTLTALSSLITVFTIPIFANL
ASQHFFGQGAVVELPIWTTIGQIFAITFLPIIIGMSIRQFVPKLSTKIEKITSISATVLL
AVIILLLIIKEWSRLPNFIFQVGIGVLLLNTLSMAAGFYLSKLFNLNYKQQICISIEVGM
QNGTLAIAITAGLLNNPDMAIPGAIYSLLMYLTGCIAIVYGRKMKSEMRYRR
>KRH96304.1
MARNSKDFKDLMRQKQSSRGKHKNVEALIEKMQRAGFGESSANMLREPKGHPKMAEIRVS
SPRDNSQPPRWRKKWARR
>KRH96313.1
METYSQPTLSTIQSPRRHNNLKDTALGLVSTLSFPAIVGTADMMLKSAGVHLVGYEKIGS
GHCTAIVRGNIADVRLAVEAGVQTAEQFGQLVSSLVIPRPYPNLDIVLPINRLTQIMADG
TYSRLSNQAIGLVETRGFPAMVGACDAMLKSAEVHLASYEKIGAGLCTAIIRGTVANVAV
AVEAGMYEAERIGELNAVMVIPRPLDELEQTLPIASCWVEEHRPLDIPLHVKEKIVDAQA
VELPDLAKLPVKIKEELLVDE
>KRH96356.1
MKPNYSNWDDDLPPDPQEIYQDLISTLERKVGFGLYFVQCTPIEADNFAQHISRDLANKK
IALLNLWEPIEKFYEHVKNYVQGQTIDILLVKGLEYSLYKYEKRNFGEVTEGQFTNLTKV
PPILNHLNQQRERFRDDFSFCFVFLLRSFSLNYLIHRAPDFFDWRSGVYDLPTTAELVDE
ESRRLIMEGDYKKYLELTPQQKIETMLEIQELLTEKYQNDSNKARLLFEMGNLLYSANEY
ETAITFYEQELKLQPDDHGAWCNHGHALFSLSRYEAAIVSYRQALKLRRDDPFCWYALGN
CQRKLHRDQEAILSYNQAIKIKTDDHYFWYNRGNALRNIGCNEEAILSYGQAIKIKPDES
NVWNNRGIALRSLGRYQEAVFCYDQALSLKPDDYYAWYNRGVALKKLKQNEAALLSYDQA
LKLKPDDHYSWNNRGNALEDLGHIEEAIFSYDQALKIKPDDQYAFYNKACCYAVQGKIQE
ALENLENAVSLKPEQFTQMAKADPDFDRIREDARFQALINKTFHD
>KRH96380.1
MGKYHYVLASQKFLLEEEPLEEVLRERIRNYHEREKEIDFWLVKNPAFLEAPEMSDIKKK
CPQLPVAIISTDSQFITWLKLRLEHVIVGEFTTPLGTDPLASLTNV
>KRH96396.1
MNYQSSNEGFTLWENLIVLAIIGILSAILTPSWLSFFTNYRLNVAQEQVYQALRQAQSQA
KKEKSTWQASFKQENGIVKWAVHPILVHPPNAVWHSLDSAIQLDRETTLRESHGIKYIQF
DDLGTFRNPPWGTVTLSMKSGGTAKRCVIVSTILGSLRTARENTVLRNNAYCY
>KRH96399.1
MEPIIREIIWHKKLEITQIQQEMSLASLQRQLTAAPSVRDFFTALQQNIYKPSLIAEVKR
IFSSENILSSDFDALSIAKSYERTGAACISVVTDQKFFHGGFDQLRIVRHKVTLPILCKD
FVLDPCQIYLARAAGADAILLIAAILTDQQINNLLRVIHYLGMNAVVEVHNLMELDRVIR
LEDVRIIAINNRSLEDLTVNINTTLELMAARKSHLHNLGILVVSESGIETSQDLSLMANV
GVNGVLIGDCLLREENLEDAVKELLKSQIYGFGGPSYKS
>KRH96408.1
MLESLVAISTLSLLLMVITPIWIMSTAIRMQSRRVEMAAQAATSFVNGVKIGSIVTPQVI
SEITPSQQASRNISTSPQDYLITSSKMPAPTSANGLYCYNQNGIIGFTECQNNSNNLFYI
QAGTIATSSKNESYLLSIRVFRADIDFTQQIPINKSTPSPITHNLGDKQVPLIQMTTEIT
NNKTSFYSLCQRLGAIAPTTYSSSTLCQ
>KRH96416.1
MDSYSFLNSHTHNNPLYKRNNWQSSYNSYNQKELADSDIGTLKDEKYMRSYALKLAHQGE
YTKAIALLDRIIDSHPENAIDYNNRGLIYFQSGHAQKALLDYNTAMQLNPRLASVYNNRA
NYYAARGDLVKALSDYDQALDLNPRYVRAWINRGITLRELGEYKDAIDDLEVALLFGQLE
IHIWSERGRSYHLWGDWNCAIADYRRVLSHTMSLDKIEDIITYRLRLQVENWLGELGFST
YK

>KRH96421.1
MSMDQISPAATHEINAYLPYIQGNKRNLLPWAITLYQKGCIDGERKIEGSDNIPFTAKWN
ISTLPTDLTCCSVHFHAPGEFAYEVTMTGFEFVDFLIQVIENYKRNRIVDFSKAFYRKLL
CPE
>KRH96434.1
MALTEDDLQTLLEEGIDTSNPGIYEELLLDLQGNILKGHGRDHTVHLFLQFKPGQVEPLK
DWIREFAKNITSAHQQAAEAELYRTEKIAGKPFINFFLSHAGYEYLQFRSFQIPGNQPFR
FGMKNDTIRNLLGDPVIEEWEPGLQEEIHALLLIADDNLVSLLQTVNQITRTLYLMAQVV
HREDGFILKNEKGDHIEHFGFIDGISQPLFTKSDIQKAKTSDDFSKWDPRAPLSLVLAKD
PNGKSEDSYGSYLVYRKLEQDVIALLDAEKDLAEKLGVDKELAGALMMGRFRDGTPLTLA
DKPGGDYGNDFDYSEDEKAYKCPFHTHVRKTNPRGDTGRVISSGESFEDALKTERNHRIA
RRAVSYGETDYTNSTSTGSGLLFLCFQADLENQFNFMQAAWANASNFVEWNVGVDPIIGQ
TGPDADRNQKWPVAWGKPEKSDQSNTFNFGLWVHMKGGEYFFAPSISSLTSI
>KRH96435.1
MNKKVRLLIILLTCLGIIINPNIVSADTLIAANLESINGGEWQTVPLPEKREDWMQAVHT
SLLPNGKVLVVNGSSNRNTLVQDNTGNQFIDGVNGRDYNVVNNSTLFDPKTNTFERIASP
PSIANGKSNDPFCSGNVHLADGNVVFVSGSHRYYPGEKFEGSKQTNVYNWQTNSWGTLGQ
LKEGRWYPSPVTLADGKLVIFSGLKYDKPNQITPSVEIFDPITKKFQFIDLTYVENSPFN
TKITYQDNYIYNGKTVSRTIDAYDSIDIYPRIFPTPDGKLLITGDGAGKSPLEIHESNKT
YLMSIKQDSQGKFSVSFEIGPNRKDVSKVYGTGILDPNKEGDVLLMGGIIGTNDINFGRP
YLGKYNDGLAAKGVRIASSLERWSAPENSGKPNGEWEAYPDFFAKPRSMVQAVILPSKQI
LAVNGGEYGEYKPIQEPVLMTADPLSPGGYKTETMNPGKFPRLYHNNALLLPDARVLVIG
GNPSRAGRAMDGTVHVDVLPNPKTYYTIPEFKNASGEVETFDLAKYYESPKSYFVDGDPE
PFVPAEIWQAEIFSPPYLFKSGLRPEIVDAPTTLKYGELQTVSLKNATSNSSLVLIKLSS
GTHSFDYGQRLADLEIENVSADNSTINFKAPTNANLYPPGYYMMFYVNDIGKPSEAKFVK
LES
>KRH96439.1
MTASGSRGLSFPKAKHILVEIPKFEIEKNLMEIIQVIYRGRGEDDIDNQDKYINFYLAER
SFYYGDQMEIAVRENCLSLLNLLLILKASIMTRIQGYGSLGKKNYILIPIGGKSVFTAGD
SFASKIENLITQLKREYSYNKSHTCLRDAYMKLESLMSRAKFTASKSEQESYLKIQESFN
SKFADSTKTLDQLLDFTPLELAYISGGLLIVPCSQIRQSYSMMFLEIQKYANQELLNKLK
NISSNPSYPENLTSAVKDGIELVEKLINAPKKSQKYDQYQENIQADQYYAFPLFAFISGD
VMKQYFETHPQEPEDMRFKDILSMYIRSLYPVTNILPIGHTYEEFPFIVFNSYSLDEMRY
KLFTEKYLLNSQELNVLSLILSSKKKFLCWTFSKSCDIL
>KRH96455.1
MAILAVGAGQTYTTIQAAINAANNDDIIVVRPGIYQEDLTINKSVTLIGPYGTFEGIDGF
ENRLGVKPLDPDINAALGVGGLPTANEDFRRYQDDNGTIDNAFGNTQEAWIKGTITVTED
NVTIDGFRLRNENGPLKWNDTPDNFKLLNNYLTGYTANNSPSFGDASINNPTGVVTGWQI
AGNYIGGLLGGGGTGGSIYLAGLQDSNIDDNTFWRPRAAHLYLASLTNVTIEDNKFYHGL
HTGGANFDGFGEFFSGSGYGYGGYGDGYGDGYGGYGDGFFGRNYWLELKGDNDQVLIKNN
EGEYNSGGIQLFGETDSPFAFDNITIEGNTFPDNNFINAYSEAPSNGKSGLIPAVMATAR
LSGPSGSNLVIRDNDITMDLAQVKFITDHKSSLEVRGNFNGVTVEGNTLTPKNINGGVDI
ITGLSLYGSLPGETLIRGNQLLGQDGDPLEASYYGIDLIPTFADYGTYTGDLTVEDNTIN
SWQVGVNLRNTNEITGDININGNTFENNSYGVVLDATATTNSINIAGNTFSNNFSNVFDG
IDPVITMDQVLSYEENQDLGSVLGTVSATDNLPNTDNVGVTQYFISSGNDDGFFTINSSG
EITLTEAGLAAANDFETSPSSFNLGIIVTDGGGLQDTETITLSIINVNEGLGQLPPITTE
GAGFTVGATLIAAIPFDDPDGIPTDISYQWQRLIEGVWTNIPDATEQNYIATEDDNNNQL
RVEVTYIAGGFEKVIYSNNVSISLVPVSGTFDSITGDNNLDISEKEAGVTLTGTVSETGT
TVTILFGGQTRVAQVDGLSWSYVLKPNDYNFFAAGSNLFTAIFTRTDGGETGSFTTFQTL
TIPDGILPPNTSNAFDPTQPKGLKSEVIDAAQTLEIDGVSILEISQTVGILGEGESFNDP
NIAVLPIGTRDIFDQGANAGAANYAAVKTEPGTSIQSIYIVPVVEEEGVKKLQVVLADGT
VVEAEIPPDLISPIGDPLAITISGVQLGGTTTFVLYLSQNFINQLPEDLDLARYVKFNYE

SQQFELYDDFNYDYIFNDVDGDGVRDFGEVYLTVNLTDGDIWDGDGLANGIIVDPGQLGI
ATDSGTDNNPPIAIELRGIVAENDPGAFIGSLTVTDDPGDSHTFTVNDSRFEVINVDGNN
ILKLREGESLDYEAASNIVLTITAIDNGGLEITQDFTITVTDVNEAPVAIELNQITVIEN
DPGAIIGTLTVSDPDGNDGHTLKVNNDTRFEIVDFDGNQTLKLKAGESLDYEAGSVELSI
TATDNGGLEVTQDLTVSITDVNEPPVVSFSFFVPESTTLVSNLTVEDPENDPITLSLAGV
DASLFSISPTGELTFNTAPDFEEPLNADKNNLYKLQVVARDEQNNKSIQDISILVTNVNE
APIAIDDVLAIIPGSSFGTLNPLDNDSDPDLNDPLTIINKTDGNYGRVEIRDNELIYTLL
DATYIGDDVFSYTIIDEQGLAATANVNVTITGTDIITYPVEILDPEDSLIPDEAGSLSDI
VNDISFNFLTDYDKVQAKLALQEALSKTEASFTNLFGLYEVDNALTGSVNGVLPEDKSAY
AKAALSRVVPNFVVRAGGSGDGVNGDVIVSEGKIYAPFVIAHGGNFSGSVQDAVNAFFQV
NPDNSPATAQNYTTLPVAYFSFGSANPDGAAHIKSFGNNVFGFEDLPAGVGVSDYDFNDT
VFSFG
>KRH96459.1
MGLDYKLPMPTPIIIAHRGASGYRPEHTLAAYQLAIDMGADYIEPDLVISQDGVLIARHE
NEISMTTDVENHPEFAHLRTTKMIDGEIRTGWFTEDFTLKQLKTLTVKERIGQIRPQNTV
YDGLETIPTLEEIIDLAENQSSQKGYAIGIYPETKHPSYFQSIGLPLEPALLRSLANTQL
PIFIQSFEVGNLQNLNKNTDFPLVQLINDLGQPNDFRVDGKSCTYQDMIKPGGLKKIAQY
AQAIGVNKNLLIPRNSQGKLLSPTSLVKDAHQQNLLIHTWTFRNENCFLPLDYQNHPQGE
YELFFNLGVDGVFTDFPDTAVYARGKV
>KRH96472.1
MLNPNLDEIQLTKDDYERYSRHLILPEVGLEGQKRLKAASVLCIGTGGLGSPLLLYLSAA
GVGRIGIVDFDVVDFSNLQRQVIHGTSWVGKPKIASAKDRIHEINPYCQVDLYETRLSSE
NAIDILSPYDVIVDGTDNFPTRYLVNDACVLLNKPNVYGSIFRFEGQATVFNYQGGPNYR
DLYPEPPPPGMVPSCAEGGVLGILPGIIGVIQATETVKIIIGKGTTLSGRLVLYNALDMK
FRELKLRPNPVRPVIDKLVDYEEFCGIPQARAAEAQQQMETQEMTVKELKTLIDSDSQDF
ILLDVRNPNEYEIARIPGSVLIPLPEIENGDGVARVKELLNGHSLIAHCKMGGRSAKALA
ILKAAGISGTNVKGGINAWSQEVDPSVPQY
>KRH96475.1
MKFTSVKFCLAFLWLTLGVDLGGLEFNNRVVAQLPPEPPEIFPLPNNQSIPVLKSEFGVR
IVDGQGKVNFFPTTRIPLKRGDVYGWRITLENYRGQVKWREVLRLPKAPETWITQDKKHN
FSLGADGTTAITRRTQMAKDGVIENYWQISPGDPLGQHKIEVYVDDSLITTFEFETVQF
>KRH96496.1
MNDLPRQKLKEIIIQQGRSLCDNPQRCEAFLRDYCGGYRREIFILISALKQGVAKDLLNS
NNVPLELLVSRLIKKMQNELGLTEEAAHYAVESWAQALDKMPQQQIQQPRFDVINKANKK
LNHPVSSQQTTVVSPLFTTNQQQQKLVLGRGLLKKAGVSVGLFILVVIVQQIFTANSTEP
EINGYPTTEITELPTPEITRSSTPKVTRSLRRKITEPTEPEITEPTEPEITEPTEPEITE
PTEPEITESTEPEITESTEPEITESTEVEVDESSKPEIAEFLEREIREVNESPTPEATES
PTPEVTESPTPEITESPTPEITESPTPEKTNPI
>KRH96500.1
MNELPRQKLCEIITQYSKDICNNPQRCEGLLRDFCGQYSKEVFLLINALKKGVATELVKS
QAQIPESVILAKLTKRLQDELGIAEEAAYWAVDSWGLALGIISEPRSKNDLESREQLILQ
REQEVEKQQKQKEEYEKELHKSDREIEIWVYISALLSGSYTFLLIGLAILSVIIYVYQQS
EKTQEISNLSEEMNNLTSQYNQKVAKLTEQSNNLKGQIDNLTDKKKSLYNEVQNFQNSLK
DLENNLENNLNFSGDTYLNLCNTTSNDTISTTFMYQEGEVWKSKGWWVIKKGECTKLFVR
LNYRGYIYLHGQTNNLTWGSKDFSFCVKNSAFEFEKADEIECSGENYKANAIQFFVFPGV
NNYNFKDYKLYTE
>KRH96502.1
MKKVEDLTKIFLEIDEELIRKGVEPYTRPFAACSEIAQHLFPGTNLPMDDPIFQSINQIY
RDFYGSAYLHLPPAYIGCFMFRDVFLPIRLPVIYGEFLINPIDFLTDIPETQNLKRWLFK
DHKAVLTFFDQVIDVMDFVYGIDDLEKGDKLPPKAIEFFYLAKQQMQAAAATLLGSFDKY
AVIQNCCLATELLLKGALIAKKIDIETLKKKYGHDLKCLLKKNFDLLPGLNQDMLSDIIH
KLPDYTKSRYELKDFSRLKLGEVFMKTQFISGDILRQFSDRNSRANFNMGQELDVTIRTF

PKKQLE
>KRH96503.1
MSEETIWIITREYTPSDEDAKGKNTYSNPWGDDKQNITEVATGLVKVSVEKLETELSHFL
QLIGKVFSHAQKQINQQTGFKLDEVELCVEITGEGEVKLLGTGVKTGTKGGLTLKFKQEA
NNTQNPN
>KRH96505.1
MQVSLKSSILGLSMVGAAVLVFGEPGVAGENKFFCTREGGVPVTKVRTARGNETFIIWER
DFHKYPATKRCGIISNKLQRFYENGEVHFKTGIVNQYPVVCISNRQNTRCSGDNLLVTLP
QGEDSVSVLE
>KRH96508.1
MNSETKLIKSRIGLLNLAEQLNNVTRACKLMGTSRDSFYRIRELYNTGGEEALREISRRK
PIPKNRVEPDVEEAVVQMAFDYPAYGQSRACNELRKKGIFISAAGVRCVWQRHNLEVFEK
RLKALEERVAQDGLILTEAQVIAMERKKEKLEAHGEIETEHPGYLVAQDTYYVGTIKGVG
RIYQQTVIDTYTRVAFAKLYTTKHAITSADVLNDRVIPFFEGQEIPILRMLTDRGTEFNG
RPENHEYELYLQLENIDHSKTKVRHPQSNGICERLHRTMQDEFYAVAFRKKIYQTLESLQ
TDLDDWINYYNQERPHSGRYCFGKTPMQTFLESITLAKQKLLNNLCPAA
>KRH96532.1
MTQNRSQKPIVIAPSILSANFSRLGEDIRAVDAAGADWIHVDVMDGRFVPNITIGPLVVE
AIRPVTTKPLDVHLMIVEPEKYVEGFAKAGADIISVHAEHNASPHLHRTLGQIKELGKQA
GVVLNPGTPLQLIEYVLELCDLVLIMSVNPGFGGQSFIPSVVPKIRQLRQMCDEKGLDPW
IEVDGGLKANNTWQVLEAGANAIVAGSAVFNVPDYAQAIENIRHSKRPVKELAVSL
>KRH96544.1
MPTSLHTTPYLMAQTFHALSDPIRISVIELLRHRELCVCDLSNALGISQSKLSFHLKILK
QTGLVVNRQEGRWIYYSLNLYQFQILEQYLQDIRVNSPVLPLTSFCE
>KRH96551.1
MKIWVNEQIDPSGMIHACIACCDEDQAKECHNSFSHSLTEGQKAAGWIAKLRTVETWDEV
PVNALKLN
>KRH96577.1
MNQILPLSINQKEIYVDQIMSPASCHMHIGATVTVRGIFDRQILNYAMSKTIDCHPGLKT
RIYELDGEPFQTIAIHTSNHIPFIDFSGHDNSDEQAENYINQEFIKPLTFGENAPLADFQ
LIKVCDDKHIVYAKYHHVITDGWGAAIFFREVIKTYNQILQEGREDQETRDWVITEYIEE
ETKYLASDIFMRDRHYWQQRLNNLSPMIFHPIKQPQELDGKRHSIYIPRHQYDQVDELCK
NVKSNVFHFILSLIAIYVSKHYLKNDVVVGLSLLNRSKNIFKDAIGMFVSTIPFRLEVEQ
ENTIHQLLDKIRYSLRQDYRHQKFPVAEMKQLSGLKATSKQHLFEVFLSYERHDYADNFL
GTKTTCVPLYSQQQKVPLIIYVREYEKTDDVKIDFDYNLSYLDGEAVGEIVRSFETLFTQ
AATNLEISIGDLAICDSETINISQPDSPSTKFFADDTETLVSAFEKVVSQYPQNLAVQFD
GELYKKFLSYTELNDQANRLANYLISQGVKPGSRVGICLERSEQIIVAILAIIKTGSAYV
PIDPHAPSVRRQFIVQDSGMTTLITETSLIAELVTENISTLTIESINLALAKQANTLPRI
SIKPDFPAYIIYTSGSTGTPKGCVVTHKNAIRLMRATEPWFGFNEKDIWTLFHSFAFDFS
VWELWGALLYGGKVIVVPFWLSRNPEKFREFLSTEKVTVLNQTPSAFYQLIHADQSSVQD
IYLRYIMFGGEALNIQSLQPWLERYGDKKPYLINMYGITETTVHVTYRPITRQDLKVRGS
FIGKEIADLHIYLLDEKLSPVADGIPGEIYVGGAGVTNGYLNRPALTAERFLPNPFGSGR
MYRSGDLAKRLPNGDLEYLGRIDQQVKIRGFRIELGEIQAALISHYQVREAVVITDEWEE
EKRLVAYYVPDESSPTAHELRQYLKNKLPDYMIPAAYVKLEVLPLNVNGKVDIKALPMPD
WNLLRVEEDYIAPRNLDEEILCTIVAEILGLQKVGIDDNFFEIGGDSILALQVVARAKNA
GFLISAGELYELATVRYLATKKAIITSGDGVKKLPNIGSALVSDADKLLLPQDVGDAYPL
SSLQSGMLYHSELDPDSAIFHQIFTFDLQIGYSELAWKQAIADICSANPVLRTSFHWTGY
SQPLQMVHEQVELPLSVVDLRGCDNANEQIREWIELEKNHNFDITQAPLFRLQIHRVSDL
KLSFSFSFHHVILDGWSVATLLTQLLRRYVEYLATENLPPLPVTQISYKDFIAQEQNVIT
NHTVREFWLQHLRNLQVTFLPRLSTNTAKTTAASHQKRQLKRMSVLVDTKLAEKLRQITK
NLGVPLKTSLLALHLRVLSFITGQKQVVTGNVINARPETSGSENLLGLFVNTIPFRLELP
QGNWLDLVRAVFRLETEILPHRTFPLAEIQRVLDQRPLFDVGFNYVHFHVYEGLLNLPQI

QVENVDIFEETDFPFLTEFCLVPGSAALQLNLIYDTQQFADTQVDQYANYYQAAMFDMVT
NPQTPYHRRSLISSQERQHLIQSANQNLKDFVSSQTLVSAFNQTVAKHANKTALVYQQTS
LSFGELEIQANRLAHYLQAKGIGPETLVGLCLERSEQLIISILGILKAGGAYVPIDPAYP
SDRLEFLFRDSGIMLLITQRSVISQLPECGAEMIILEDIAEEIEQENSQAPEVNILPQNA
AYVIYTSGSTGQPKGCIVTHANVIRLFNSTETWFNFDSEDIWTLFHSYAFDFSVWEMWGG
LLYGGQVVVVPHWTVRSPKEFLQMLATHRVTVLNQTPSAFKQLISVVRQKPEKLSLRYVI
FGGEALELADLQPWIDLYGHTQPELINMYGITETTVHVTYRPITQKDIINNLNHRPSVIG
QSIPDLELYILDENLDPTPTGVTGEIYIGGAGVTRGYLQQPGLTAQRFIPHAHRPGSRLY
RSGDLARYLPDGEIQYLGRADQQIKIRGFRIELEEIQLVITSHPDVKQALVVCQKSPTGE
NRIVAYVIFSGVAQPQNDLGKFLKTKLPDYMVPSVFVPIETIPLTINGKVDYAALPVHNW
NSIKKDYIAPRNDREATICSLMASLLKLERVGVDDDFFEIGGDSLLVTQLAISLRQTYDT
EFPLPELFTHRTPGEIALLVGDESPALPEIEIPKASRTRRSVTLTDDGILSKY
>KRH96590.1
MIIVFDIDGVIRDVSGSYRRAMADTVEYFTNYAYRPTVTDIDDLKSEGIWNNDWEGSQEL
IYRYFVSQGQQREDLQLDYESIVAYFQSRYRGTDTENWNGYICHEPLLLEPSYLESLTQA
NISWGFFSGATRGSASYVLERRLGLKSPVLIAMEDAPGKPDPTGLFATINLLEIAKQDQN
KKQPVIYVGDTVADMHTVAKAKILDSSRTWVAVGVLPPHVQETPSHRDAYQKIMLQAGAN
ILLTNVQELTPSKIAELVNIYI
>KRH96596.1
MVTTAEKTNIGYITQVIGPVVDVKYPGGKLPQIYNALTINGTNEAGQEINLTVEVQQLLG
DNQVRAVAMSTTDGLVRGLEVVDTGAPISVPVGKATLGRIFNVLGEPVDNQGPVNNEETL
PIHRESPKLTDLETKPSVFETGIKVVDLLTPYRRGGKIGLFGGAGVGKTVIMMELINNIA
TQHGGVSVFAGVGERTREGNDLYNEMIESGVINKENLNESKIALVYGQMNEPPGARMRVG
LSGLTMAEYFRDVNKQDVLLFVDNIFRFVQAGSEVSALLGRMPSAVGYQPTLGTDVGALQ
ERITSTTEGSITSIQAVYVPADDLTDPAPATTFAHLDGTTVLSRGLAAKGIYPAVDPLGS
TSTMLQPSIVGKEHYDIARSVQSTLQRYKELQDIIAILGLDELSEDDRLIVARARKIERF
LSQPFFVAEVFTGSPGKYVKLEETIKGFQKILAGELDDLPEQAFYMVGDINEAIAKAQKL
KG
>KRH96607.1
MTISIREKQPKPNLSLQLQRFWEILYVLVVRTLKVRYRGSILGVYWSLLNPLIMTSLYTA
IFGATFSSYYNNSITNYVLAAFTGLVVINFFSASTTQCLFSVVENSSLLNKIRLPVSVFP
VSMVASNIFQFSVGTFPLLAVMTLLNTRNVVNVLAMVFPLFALVLVCMGVGFLVSTLYVF
FRDLHYFYELVVFVLWISSPVFYPAAIVPPQVRPFLGLNPLSPIIESLRQITLSGSPPDL
GLIWGALLSGMIILSFGWTCFHLWRHQFMDLL
>KRH96608.1
MLSSMFDFLIVGAGFAGCTLANCIATHLDKKVLVIDTRSHIGGNAYDCYDKAGILIHRYG
SHIFHTNSKKIIDYLSRFTEWRVYQHEVLARVDGELYPIPINLNTINKLYGFNFNSQQLS
DFYEQIRERYERIENSEQAVVGKVGTDLYERFFKNYTYKQWNLWPHELDASVCARIPVRT
NKDNRYFADKYQMMPVDGYTKMFERMLDNPNIKFMLNTSFQEVEKWLKFDHLIYTGPIDQ
FFDYKFGRLPYRSLKFQFETHDIEYFQPVAVVNYPNDYDFTRIVESKHITGQKHSKTTIY
YEFPQSEGDPYYPVPRPENRELFQKYKDEADKLQTVTFVGRLAQYQYYNMDQVVAAALTV
FENRISKLY
>KRH96609.1
MSIKQVAIIVVTWNKIRDVSFLIEDIKKLDLNEISINVFVVDNNSSDGTETHLNKYYSFV
KVLQTGDNLGGSGGFSYGMDYVKELEYDYVWLLDNDVRLDTQSLLNLVDTLNNYPDIGLV
GSQIRKLDQPNIIQDLGMFIYYKKAHVKGNFSDNTIESAINNLSHLNSQTGYIDVDFCSA
ASLLVRKEVIQQIGVFEDYFLHFDDTEWCLRAKKVGWSVVVNPSSIVWHSSPDFKQRPWI
SYYDERNLCYCWQKYFPDLLLKRIRVLLPKLIYYSITGRFFWVSTHLMGLDDFISGIKGR
MPAPLPYKQLSIEEVLDNNLRVTVQASLYQDQFESQILDQINSLIDKERFTVFNKNKSLL
YTSYMWLLSCFHKSFDLALVSCYQTEVWQFNLARKVYFFTGSGYVLVNTSIINLARAAIT
TFLRLLKIYWQIHKLTSKKSIMLDLNRIM
>KRH96610.1

MSITVSIILVNYNGADVLVDCLKSINKFVPGYNCEVIVVDNASQDNSIDIIENKFPNVQL
IKLTKNVGFGAGNNAGAKVAKGEFLFLLNTDTIITSNILPHLVDLMEKNPTVGIIGPKLL
FPDGRFQISFSPKISIKGELEARKLHRYAESRTKLALLENNFQEIQEVDIVVGAAFFIRG
DLFRTLGGFDEKFFMYFEESDLCQRAKNIGYQILYTPKISLIHIRGYSTSKLANPMAVEY
RRSQLYYYQKHRPLWERLTLRIYLLISFLYKFTITSNPYCWQIIILVLKPK
>KRH96616.1
MFDLIKGPLLRATLYKISNKEHILLVNLHHIISDGWSLGIFIQELTQLYVSRAEISLPEL
PIQYGDYAEWQETYLQTQRIQDQLAYWQKKLALPLPILDLPSDKNRPALQTFSGAVLRKT
LPRDLIDSLEAVAAKEGVTFFMVTLAVYQILLFRYSGQTDIIIGTPCANRNRGELKNLIG
CFINTLPIVCSLTGQLSFKQVLQQVASTCVEAFANQEVPLEIIIDKLNITRDPAHSQVFQ
SLFALQNAPIEEIKLPGLTVQPVYLDNGGAKFDLSLMLEPTFEKGWIAALEYNTDLFTAH
TAEDILRHYEQLLSAVIGNLDTKIDTLPWLNTEERRELLSFGSSASDQEVEATNLVDVFS
HIVNTYGEKVALIESERTLTYQKLNQISNQLASHLIEKGVGAETRVGIFQERSLELVASI
LAVLKCGATYVPLDPQYPAERLNFIAQDSGIRLVITTEPLRAKIPGEIPELLLIDTMKPT
NQLVHPISKIFPEQAAYIIYTSGSTGKPKGCLVTHKNVLRLMRNTQKWFEFNEKDVWTMF
HSFAFDFSVWEMWGALLYGGKLVIVPYLESRSPHDFRQLLATQQVTILNQTPSAFRQLIR
ADQEFTHPLNNLRAIIFGGEALELQSLKPWIERYGDSHPRLINMYGITETTVHVTYRQIL
AEDILENRGSVIGIPIPDLCLYILDSYFEPVPYGVAGEIYVGGMGVSRGYLNRPSLTAER
FIPNPISQKPGARLYRTGDLARRLRNGDIEYLGRCDSQVKIRGFRIELAEIEAAINKLTQ
VSESVVTVHSTSDQDRRLVAYIVLRSINNNEIDRNELRKALKESLPDYMVPAAFVFLDAI
PLTNQGKINRGALPLPDWDQSTTKRSFIPPETDAQKALCHIWQRVLGIEEIGIEDNFFDL
GGDSILALRVITEMRSQGWILTPKQIFEQQTVQRLALAIQEQTDLQSFAAAENNNIPTGE
VPLSPIQQWFFDLKLSNPHHWNQTLLLQVDPSLQVTQVATAIKVVFAYHDNFRLRFQKES
QGWRQFYVEKDQDDGFPWEVVDLDLKSEIEQNSIMKEVREKSEKSLNLTEGPVYMVKWFN
LGANRPSQLLIVIHHLIIDGVSWRILLQDLVDVIGGNKLSFKTTSWQKWCEFIHGYVNSH
NIQSEKLFWQNTLARKSAKLPLDFPESLAENLEYSVSTVSCQLTAKETLTLLTTANKTYR
TNPQELLIAALGKTLADITQNSYVQIMLEGHGREELSSDLDVTRTLGWFTTLYPVSLDLP
KGDSQTEAIREIIKSVKEQLRAVPQRGFGYGILKYLDQETKPLLASAVEISFNYLGQVRN
EGGARHKFFTLLNSQGLPTRDPQATRPHIIDVNAIVVEGELRIDWLYSSNLHQASTINRW
VSDFKSNLLKVVNFCTEVGVGEYTPTDFTLAQLNQSKLDILQNQYPHLEDIYPLSPLQQG
MLFHAIYEPDQGIYFEQVTGKIIGKLDVDKFAYAWQVIVDRHPVLRTCFVWEEQDQPLQI
VNKGTGFSLIYKEWRDLSPAQQIEKMGEYLVKDKEQGFNLNDSPLMRFTLICLDDSTWQW
LWSHHHIILDGWSLPVIFKEVLSVYQSATERVPHSLLPVPPYRHYIQWLTGRDQLRAKQF
WQKYLVGISTGTRLAWEIPDLENNSSAYREVELRLDDLEFDLVQKMAQSQRLTLNTIVQG
AWAMCLQKHGAGEDVMFGVTVAGRPPELSNMENMVGLFINTLPMRVKIDPSLSLTDWLQN
IQQQHLEMREYEYSKLADIQKDLNLAGLALFESLLVFENYPVDQNLKKQLPDFRVDDIQF
YERTNYPLTVGVIPDQGLLLKLNYQTKFLSGNAAEKMISRFRHIIVNMAFKPEQTLAKVP
ALSISDREELTTCNQGNTIIEGGFKTAHQLFEYYADLQPDSIAVVCDEYSITYGELENRA
NNLATQLVDTGIAHESIVGLYFDPGIEYIISLLAVLKVGGAFLPLDRSYPDHRLRFIIEN
SQVSVILTNNIIGLPLQENLQVLDIRNLESKGNHPRLNLKVRPENLAYVIYTSGSTGNPK
GVLVTHSGIQNLVQCQTMSFGVTAKSRVYQFAALNFDSAIAEIFMSLGSGAVLYIQSQAH
RSPGSELWQKLTNWEITHVTLPPSLVASIDTQDLPRLQTLILAGEAVSGDLLRRWGRGQR
RCFNAYGPTEATVCASLIDCTHLLGEPSIGKGIPNVEIYLLDSFLEPVVPGVIGEIYIGG
IGLARGYLQRPGLTAALFIPHPFTKTPGSRLYKTGDRAVYDPEGNIRFIGRYDNQVKING
YRIELGEIEAALTKHEAVESALAVLRTDLIGRNRILGYALIKPVEGREQPNDLKEHLAKI
LPDYMIPGAVIVVDEWPLTPNGKIDRQRLATPDFSSTEVIPKTDIEQIFAQIWIELLGLE
TVNPQDNFFEMGGDSIISLQMVTRARAAGWEISPKDIFEGQTLSRIATRAKLMETQTEIV
EPLTGFIPLSPIQNWFFAQNFLHPHHWNQSVALTCKQPVNTDALVVALHTLVSHHDIFRI
GFAENEGEWQQFYVGEAKSPNVKIIDFSHYLPTTHLAFLNSALETEHSSFKLDRPPLIRI
LCGRNLNNYGDVLFIFAHHLIIDAVSWRILLEDLNQAYQQTIDQLDQLQQPNSPLSLPVK
TASYRSWTTHLHSLASSKMTQDTPFWKDILNAVITPLPVDKSGNNSVDSTAIISTYLTAE
QTVTLIKQATVTYHASVQEIMLAALLNTLISIYKSDQWLIDLEGHGREDIGHNLDLSRTV

GWFTCLYPILLKQPTNPDNHEILLKEIKNQLRTIPHHGISFGLLRYLNLNQPLKESKNAD
ISFNYLGSLDSPLGDNHNFSVSDAPVGAGVFALQQRTHILAINAKIQDKILQVEWSYSRN
IHYDQTIENIAKTYLQSLGLYLTNSDSANSSFYSASDFQLVDLSESELGELLEDLE
>KRH96619.1
MTKQVESKESNFYTASSEKKKINIQNIIFPKLDICTVEELFFRGDDGVLFDYQNRTLGIK
KDTVVSFNTYFNGFFANKWKSYTSITNIGVCLSLQGSFNIHIYSVDSFVQSRTIVMQKTV
KNAHFDSPIVLENFDIYPYNGMIYIEVQALSNNCQMEGGFFYSYVQEIKDIRLGIVICTY
KRETYVRKNIDLLEKNLLSRDEYRDKLKIFVIDNGKTLSSFDNPLIEIIPNKNAGGSGGF
ARGMIEVANHHRGFSHILLMDDDVLFDPEVILRLSNFLSVINQDDICVGGDMLRLDKKHI
QHERGGYWNKLRGCTPVKYNLDLTVLENILFNEIEEYCEYNAWWLYCFPVDSIKKIGLPY
PFFIRLDDVEFSKRINNKIIALNGICVWHEQFENKHSPVTEYYNIRNGLIFNSLYYEKNA
SIFSHLSWFLLPTIRHLFCYRYETAEYVLQAASDFLCGPENLFSQNPQQNHSKLSLCAEK
TRRNKNGVSPFIMKKYMESINENENLLHRIWRVFTLNGHILPRSFFWDDRNLTDKGYKVV
SSYGSKPLNVFRAKTIIYYNIETQESFAVQFSRTRFFRILFHSIYLGILMLLKYGRLAKL
YKTTLGKFTSQSFWEEYLELKKQF
>KRH96629.1
MVVAQSSNQGKMYSLLMMKSFLIWTFTLAVCLLVVGFPLVVVMATVGCLLSIVLQSVMPA
SAVLLVAGTLVIFNVMSVLIVAGVLTAKSVHPKEVKWLSWLYGDAERVQTTVYASCPLTC
EIK
>KRH96656.1
MNFKLGITNNSLLVTTLITAATAVLSTTSIAADTISIQDIAQIAKKTSVQINTEGDVTPG
GSGVIIAKQGNRYSVLTANHVVCDIIDRPGKILCAKDITYSVRTNDGKEYPIKSKDIVVL
QSTKNDPDLALVSFVATQEYPTANVGDSDQMTEASDVFVGGFPAVFNKIGSARDFTFTKG
IVLSRGRTSINGYSLIYDAKTLTGNSGGPVFDIKGRVVGIHGLADTSAKSKTETGEIVSQ
KTGFNAGIPINTFLNFNNPLLKELPIQRNTTATGEVPQQRLNSPQSARDFYARGITKLDQ
FSYKESLDDFDQAIKIDPKYAEAYFKRGYALSWLRRYEEALSDFNQVIALDPNYLDGYLN
RGWTYIWLQNDQAALEDFNRAIRLNPSYSIAYAHQGMAYIKLGKYQAALESSKQAIRLDP
NNSYGYTIQSDVFNYLKDYAASIKVSTLAIIIDPDDFNAYINRATAYTLTGNFSAALVDY
QKSAEIFERRYTKKPSPATDNSRTTPKP
>KRH96661.1
MTPLINQALSLVYTQLSYFSESSNYYQVLSTAFGNNYDRDIAEKLKLHWQQVNFTQIPQI
EILDGGILGSAYGAYARETNKIYLSRNFVTNNTAEVIGRVILEEVGHFVDGQLNVEDSEG
DEGAIFAELVVGNSLDDQALRKLRTESDFGIITLNGQQVHVEQSSSLSLPGVRFSSVAWA
DYDGDKKQDFLLTGYSSSGPIAKLYINTVNGFQEDTTVSLPGVEYSSVAWADYDKDGDQD
FLLTGFSSSGPIAKLYRNTGNGFEEDTNVSLPGVAASSVAWADYDGDGKQDFLLTGFSSS
GPIAKLYRNTGNGFEEDTTVSLPGVEYSSVAWADYDGDGNQDFLLTGYSSSGPIAKLYRN
TGNGFEEDTTVSLPGVEYSSVAWADYDKDGDQDFLLTGFSSGFTPIAKLYINTGNGFKED
TNVSLPGVVFISVAWADYDKDGDQDFLLTGSSSDGRIAKLYRNTGNGFEEDTNVSLPGVV
FSSVVWADYDKDGDQDFLLTGDSSDGPIAKLYRNTGNGFTETLSEPNEINDVTLAVSPTI
VNEDGRENLVYTFTRRGDLAQELTVTYNIGNIGGTANIGGTADARDYIGATPGEGKTIKF
TANSPTVKLEINPTADNLSEFNETVRLTLVEGDGYTVGTTTPVTGTIENDDLPGVEYSSV
AWADYDGDGKQDFLLTGFDGSNPIAKLYRNTGNGFEDKTPKGLPGVEYSSVAWADYDKDG
DQDFLLTGSSSDGRIAKLYRNTGDGFEEDTNVSLPGVAASSVAWADYDGDGKQDFLLTGF
SSDFTPIAKLYRNTGNGFEEDTNVSLPGVGYSSVAWADYDGDGKQDFLLTGDSSDFTPIA
KLYRNTGNGFEEKTPKDLPGVRDSSVAWADYDGDGKQDFLLTGYSSDFTPIAKLYRNTGN
GFEEDTNVSLPGVGYSSVAWADYDGDGKQDFLLTGYSSDGQIAKLYRNTGNGFEEDTTVP
LPGVSDSSVAWADYDGDGRQDFLLTGYSSDFTPIAKLYRNTGNGFKDATDIGIGKIDKFP
GVEDSSVAWSKDGKYFLLTGFDGSKPIAKLYTSEGNGLFKESQDLPGVIGGSVAWSKDGK
YFLLTGWDGSNPIAKLYENTGNGFEDKTPKGLPGVRNSSVAWADYDKDGDQDFVLTGWDG
SRSVAKLYENTGNGFKEPSNVSLTGVYNSSVAWSENGEYLLLTGQSASGLVSRVYKNSVE
NGSRRLLVTDNPVTGVENGSVAWSKDGEYFLLTGWDGSKSVAKLYKKNKDGKFEEDNSIS
LPGVRDSSVAWADYDGDRKQDFILTGLSSDGRISKLYKKNKDGKFEEDNSISLPGLALGS

VNWGDYDGDGRQDLLLTGESDYGRISQVYRNTSSGDKTFFTATLSPTGLPDITLAVSNTI
VKEGEKLVYTFTRTGDLTQELTVTYDRGGTADFSDYIGIISGSAVFKKHKNKVELEITTR
QDLSSEGDETLELTLLQSQEYTVGTNTPVKGIIRDAKQERVPLITSIPKNMEMLPKNIKP
GEELSKLSYQFLDYNSPTVRAKLDSALNLTNSVFTNFLGLYEVDDPNTGAVNGFFPGDPG
YAEAALSRRVQNFYVKAGGSATNITTNGVFAQNQTPVGDPSKINDVRYLAPFLIANIGSK
DITAEIGEIIKRSNSSAEYQDKSVAYFSFGAANPGGMSQIKHFGNGIFGFEDLPLNVSDR
DFNDTVFSFG
>KRH96713.1
MIDGVPQLVDGGKHYGHLEVNIEPFTPIAGITAKIEFTPVYSFPILDATYNLVATERRVY
DDPVVMLVTDEGSVINIPYSEGTKS
>KRH96718.1
ALLETVAIHQRNFEVMQRNFDSMVTEMREIQLEIREMRSDTREMQSEIREMQSEVREIQL
DVRGLQTENRRILDILQNLPPGGSYE
>KRH96727.1
MQRDLQQRIESISNYNTRLRQYITELEICVEQIKQEKEDQQKQIAELLKEQTKLNEMVAK
LQTVQDVRQMQQELENKEAQIRNLQEKNEAIQAENLKLQGEITTLQAARNSEDEQQTTPS
IPTPSRYTNLETLLKAENFKLADQETSSVMLALAKRQKEGYLRVEDAENFPYEELRTIDN
LWLKYSQGKFGISVQQEIYKNLGGTKQFNLNVWRSFGDRVGWRKQGSWLNYSDLNFSTLA
PVGHLPTVRLAPGSSHSGWKNGVGVWVSRSVLPSCQDM
>KRH96729.1
MQIKLVSENKITQEQVEQVFNLPIAIGRDLSELPPNLNNEPVSPIVLLDNNRQISRFHAQ
IRLNNNRVYLEDKSSNGTKVNGKLLVKQGQVLNTGDTIIIGGYTITVVILEGGGQDEGTV
IVGEAATVFNFDPQSQVLPASRLEPIPNVASSISFNPHTGILEQQVSSSPSISRSEFPYN
LSFWHRPQISLREIESSGFLVRETEYLACGGGMGSFVWVDMLRIAGVKTENIKILSNQEK
PYQRYKTLLKNCQISEDKRIRSGSDSCPDNIWGWPGYGLRDAKKALFSGQIGAAIGFLWQ
VFAEPVHADTYTPIAGDVFDSMDRESERINWQKMLEPGAILSLRQTEDGRYCIAYCSDPQ
NRRHYQFLLARYVHICTGYPGLKLLPDLEKYRQEYPQETGNSRTVVQGYEEHDHIYTHLE
KHGGTLILRGSGIVASQVLDRIYQARKRNKNIDVIHLNRNPRSGN
>KRH96730.1
IDCTGLISDPLQSPFLKDLINHYDLDLNPDRRLYVKNNFEIRQLRHPRDSQSRVYAAGII
TLGGPYAPVDTFLGLQYAAHRSVEALAAIKAPGVRYIQGIYSVWQWFKWALNLKP
>KRH96731.1
MYTEDQIKNLFGNKTVEYIKNKNTGGVSNSKGNTYENIFAIYKISSLSKSIIEDEREIYL
LSQCLSFIDDLVIELTSENTLQHYQLKNSSNITWGTGEKSINDDFKKQYELNKSISKESK
LALVVSSLELRDKLQANIPDDIKNYSQVIYFYFADSLPKIIAQEPEFRRSLEYLCAFENP
EPDKLECLATVLLGAWVASEKSKLPIMDILQKAQDFIPSYIRSFKTELQLNPEIKEIFDK
IDGFTYNLTRGFLQWEYFDGLNAGTLPYSIETARFQKFQELIKKNRPTSFEDLEVFLI
>KRH96746.1
MSTIYVLSNPSTTITKEDSITNAINQWLYGKSNHSKRSYNRVIRSYISYLDDIHIANSTA
SHFRDYQ
>KRH96748.1
MLKISLEKIRQSHLTKKSSVTHALRCDRLIYDVSKSMLNSDGKRFPSGNKAAISRFFASV
EFLVTIFLLEVSQKLRIGATQ
>KRH96749.1
MTDGATETLSTIEKIKIRKNINIPNFYRKWQRILHSYTTRKISDVPNQETSINTEEWKLA
KANIQDEYEQWLETQNISTLIKNLIKNLIDKNLIERRISSQNRELVRIHLNFSDNLNESS
QQDNDILRRLPWGHYFNTHISNHNYDITFILSKDHTNKPTHANLFTSKAPKILAIFGGHQ
GELASSQSHEANELLKICEPNGATVILKNVTEQREFHQLLINDSYDILFYSGHSSSKDGG
TIQFEQENSIIRDFKSDLIQATEKGLKIALFNSCDGLKIADFLTKEVGIPAVIVMKEPVP
DEFAREFFERFLCRVTTATEFTAIFM
>KRH96754.1
MESLRLAILSIVYGIVASTIPEPELVCAKIEDNLGIKQSVAKIPHAPRAQAIKIRVIWLF

IRITPLF
>KRH96757.1
MSIMQKITIDNLESPAGYDLFLDSESFLDEVHESEYHRINGGATPTFTITSTAWCWAGGV
ALGAAISGGIVWGAKKLFG
>KRH96768.1
MKWQLLTHNKQVLGKIFTILVFTGLTGILCVSCNRNQDLLVTEIGVNPPKRPTRKTSGAG
EFYLQGQNQHSRGNFQAAIAAYSKSISLNSDYAPAFKARGLAYFDLNNKERAINDYNQSL
QINPNDPETYNYRGNARASLGDQKGAIEDYNEAIRLSPNYDEAFNNRGNSHAAQGNKNAA
LEDYTQAIRIDQNYSVAYNNRGNAYSSLGNTSKAIADYNQAIRLNPQFAPAYNNRGNAFA
SSGDKRRALQDLQKAATIFDQEGNRGLYQQTMKNIEELGN
>KRH96776.1
MRNWWKQWLTTNLEWSQSLLIGTLDILLVVGLTYMILVIISERRTLWMVRGFIFLMLASA
ISGALNLILLNFVLEKLVIGCAVAMAVSLQLEFRRFLEQLGRGEFRQLFQPHRLTVTKSD
SVIDEIVDSIKELSKNRIGALLILETTEPIDERDFSVPGVKLNAQVSKELLQTIFQPKTL
LHDGATLIRGSRIVSSGIILPLSGRTASRQLGTRHRAAMGITERVENCICVVVSEETGSI
SLAERGTLYRPLTIRKLKESLEARFSTTVDREVVAPGVFGLVSQIASQIFRFVFRLFRLP
LSFAKKNQTEK
>KRH96819.1
MRFTASVLQKIATEIGAKILIEPEYELIGHITFRNGKRTVFRNTRFNINGFGSANLAQDK
AFSNYFLGTLGYKVTEGKTFFSDKMCQKVANPRNIDEGWQYAEKLGLPVIVKPLNLSSGI
LVTKVYNKNEYYEVAKKIFNVQSVLIVERFYLGNDFRILVLDNEVMAAYQRIPLSIIGNG
KSTILELLTIKQEELVKKVEKISIDLADFRIFKNLQKQNLTFESIVPQGMIVYLLDNANL
SAGGQAVDMTDNIHPDFQQLAINITKDMGLRLAGVDILTQNITQPLVDYTLLEVNSAPGL
THYASLGEKQRQQVEHLYRKILQELEKG
>KRH96823.1
MNIKRVFKNQIFLVILSLSLLVIIPLTCLVGITSTALALEYNKEILIGANFSQRDLRDSS
FTKANLRQSDFSGSNLSGVSFFAANLESANFNGADLTNATLDSARFIRANLTNAILEGAF
AASAKFDGAIIVGADFTNVLLRRDEQNKLCEVAKGINPTTGRDTRETLLCR
>KRH96853.1
MKSVLKWYRSQYLAPSPLFVKRQVLVRNAIVNGIYVETGTYLGDTTNFLSQRFPKVISIE
PEATLFEKAKKRFSDKKDVHILHGCSEDIFPTLLTELNGDINFWLDGHYSGGDFWKTYKG
KSDTPIISELRHIRDNIHKFNQVAILVDDIRCFQEGSRLPDYPTLDYLVDWARELSLNWH
IEHDIFVARSAGP
>KRH96858.1
MLKPKNKSDRNKIVIGIDATNIRGGGGITHLIELLNAAEPIKQGISRVIIWGGSKTLASL
SERPWLKKINPPQLDQGVFSRILWQNLQLSKSSQDLGCDLLFVPGGSYFGNFHPVVAMSQ
NLLPFDLPEMRRYGWSLMQLRILLLRETQRQTFENADAVIFLTKYAEECVTKTTGFLKGK
TTIIPHGLNTRFRHSIKPQISILEYSRENPYRIVYVSIVDVYKHQWNVVEAVSRLRHSGL
PVELDLVGPAYTPSLVRLQKTLDKCDPHGDWCRYHGSIPYQDIHEFYNKADLGVFASSCE
NMPNILLENMSAGLPVACSNRGPMPEILEDAGVYFDPESADQIASALEELIYNPSLRKDL
AERAYLKSQDFDWMRCAEETFKFLRRIATTFKYA
>KRH96863.1
MCGIAGFVNGGHESTLWKMVNNQIHRGPDDTGIYLDSQSGVGLAHCRLAILDLSPLGHQP
MASLDGSITLVFNGEIYNFLELRKELIDKGFHFRGNSDTEVLLNLYLSQGHSMLSRLNGI
FAFALWDTSKQCLFIARDALGVKPLYYTNTPDYFAFASEIKALSPFLPSTPSLDYEALHR
YLTFLWCPGAGTPFNSVRKLLPGEAMTVTGRVTRQWTWYKLPIFRYPQIISDQKTVLEQT
VTHLKDAVHRQMIADVPVGAFLSGGLDSSSIVAFAREVNPHIRCFTIDVNGQEKGMTDDL
PYSRLVAAHLKVPLDVVKIDSAQMARDLPKMVEQLDEPLADPAPLNVLYISQLAQQQGIK
VLLSGSGGDDLFTGYRRHLALGWESYWRWLPLSIRSWLEHISGGFNQKISFLRRVSKFFN
GATLTRDQRLVNYFAWIRQPDLLKLYTPEFRAALSDTVAATPMLEFLSPLCDGVPPLEKM
LALEQRFFLSDHNLTYTDKMAMAVGVEVRVPFLDLELVDFAAQIPPSLKQRRGVGKWILK
KAMEPYLPREVIYRPKSGFGAPLRRWMRFELRELLGDILSAESLKQRGLFDPRAVQQLIS

DNDSGRVDATYTLLSLISIELWCRRFIDTDKQSQLP
>KRH96865.1
MQANPHQVCILGLGYIGLPTAAILSQHGYQIKGVDINPKVVETINQGKIHIVEPDLDLVV
SEAVHNQKLSASTTPSAADIFIICVPTPFHKNDNGIPQPNIDYVLAAAQSIIPVLKPGNI
VILESTSPVGTTEKVGEIFNLAGLSPDHIHLAYCPERVLPGKILQELIHNDRVVGGLTPA
ATTLVQTFYQTFCQGQILTTDARTAELCKLTENAYRDVNLAFANQLSMLCPHLGIDVREL
IHLANHHPRVNILQPGCGVGGHCIAVDPWFIAAADPENTSLIQTARHINDSKPQWVVQQI
ITLAQEFQQKYHRAPTIGCFGLAFKPNVNDLRGSPALEIATTLISSGYSVLVVEPNLEYH
VSLELTPWKLALENADILVFLVGHREFIGLDLANKPYLDFCGTH
>KRH96866.1
MLQQHLAIVTFNFPPDFGAASFRMLSLVEAIQKQASTRKIDLKITVVCAKPFRYKYNNKN
EPGSSSPKHNLIRGLENIDIVRLDVPMFGRGFVAESFSYLFFLIQALPVLIWNRPHLIFA
TSAKLLTSYLGALASLITGAILCVDIRDTFSENFLSFFRRQRKVLVYLILLYIENFVANR
ATSINLVSPGFSQLYDELLDSSKVSYFTNGVDEQFVEFYANSQHPSLKTFLNKTFNHTTN
NTLVVRKTLDNKINSARLLEIDTVKQTNHTDKHKTILYAGNLGIGQDLLKLLEPLIEEKE
IVEELIELKWSIKIMGDGAQAPALRELAKFPHLREIITVLRPIPRQELIREYGQVDALFL
QVGSYRSLDMVIPSKIFEYAATGLPILAGVRGYTRDFIGQIPGVQFFTQKDIKSFLNQLK
QIKTGWHNRDEFIEQYDRRNIMRKYADHLLSYIQASK
>KRH96877.1
MATRERLAKEQEREAKEQAEAIATRERLAKEQEREAKEQAEAIATRERQQKEKLVAYLRS
LGIDPEKI
>KRH96881.1
MKKFLTLALILILFLVSSFSLGTSPSYAYSEFDLDRLLKTDMCLGCDLSSANLEDAILIN
ANLQGANLSGANLKDAFLIDANLSDANLQGANLEGTNLKYADLEDAFLIDANLSDANLSD
ANLQGANLQGANLEGASLSGAIR
>KRH96882.1
MNNMMQYKDYFGSIHYSDDDKIFYGQVEYIRSLISFEGEDVASLRASFEEAIDDYLALCE
EKGIEPEEPFKGSFNVRVGSQLHRQAALFAQQRGVNLNNLVTDALERYLKEESLENA
>KRH96883.1
MTRREKLIKRFLSIPKDFTWEELLSLLSGFGFEEVSTGKTGGSRRRFLNDAGVIITLHKP
HPQNILKRYQIEQIIEILQEEELL
>KRH96930.1
MNIETLKSEKTKQLPGANLEDQDLSEFDLTAVNLAGANLIGAHLVSVNLEGSHLEGANLM
GASLQGADLRANLLGANLMQADLTGADLRGSNLRGANLMGATVAGASLTAAFLSGANLMS
VNFQGVDLRGADLRGANLTGANLKGADLSRADLQGALLNQANLEESDLRGANLAGANLAG
ANLLCAELEAASLNGANLYQACLLGTILETYHD
>KRH96934.1
MVKSVEIRNSDLRSFYQSYQRDPFPPSSFLRRLLPRREIDSLLEAARMSNDLLRVHFERI
IKTYRIPIQYHSRILDEVRIKQEENPESLKDVIKQINANIIATKDSIQNLKF
>KRH96937.1
MFEDYEGIERNSLNILNNFTTNPQAEVLVFDKIDPCFIDKVCFNSVQDMKQWDNLDTSNY
PQRFSVNLYYFKPRNDYKIWQAKKTDV
>KRH96939.1
MLATSKSEASVDENLNQLLFVQNLKGHESKVSAVAFSPDGRNLVSGSDDKTIKIWDLITQ
THRTLPAHQDSPWNGGINSVAVSPDGNIVASASKDKTIRLWNFTSGEKIITLTGHQEQVN
SLVFSPLGKILASGSNDKTIKLWNLESGEEIYSFQGHSDGVLCVAFSPDGQLLASGSRDG
TIMILKLAEKQVRTIVVNSNWFNGGINSLAFTPNNQILVSGGNDTIIKLWNVETGEEIRT
LNGHSQAVYTIAISPDGNIVASGSKDKTAKLWNLESGEEISTVKCAEDAIYTITFSPNGK
LLATGSGDKTITVFPYNYPKT
>KRH96986.1
MQSSFEGFHGIIPEILVGKAFQGYSGLLPNNSIESVGAKVKFLPPYSPDLSPIELCWSKL
KQFLRSREARTLEALNESMTSAVNYITAEDALNWFNHCGLFT

>KRH96987.1
MNSHYSMIIFWSQEDNCYVVHLPDFPFQDIHTHGNTYEEAAKHGQQVIDSYLQLYQENNQ
PLTRA
>KRH96996.1
MASQERLAKEQEREAKERAEAIASQERLAKEQEREAKEQERQQKEKLAAYLRSLGIDPEK
I
>KRH96997.1
MNQYPVILIPERIQELKLALPPVPPAPIEPIRPGNPPVRPQEPRPAPRSNMSIQIFLIVA
TLLSALFIYLTSQSWWLLFIPILFFISYQTCFYYLRQEYKRNLESYGRQLQIYNKRMQLH
RQNINFYPLRLEQYRREQQLYEETVQVARTPERIADYYFNCLLQLLRETKSHDGDNSDAR
IGKREREFGKHLIHYFPGKIHTQLKVQNPRWQGFPYTPDFAYIDLDIQMYIDIELDEPYA
VDGTPIHFIGLRTELDRNNHFVNERGWIVIRFAEEQVARYPHSCCKKVATVISEITNNYS
ILNHFVNVPDLQFVSRWTEKESKQMYLQNFRDAY
>KRH96999.1
MTEIGMLIMGEFNYKQTNLPHLLDEQLLKAENAQYKTKNSHFSQLPHGITSKITAPEFMT
VGVTEKIAISRTIRTGLMDKNEGRVAIPDRYRWQQASKRTKKRQLPPQFQLADRSNLPKR
RPTPTDYLAYNRPQMPTLRFGDSGLSIRVLQRLLISNGYNVRVDGVFGALTETAIKAFQS
QRNLSVDGVVGPKTWSQLCSI
>KRH97012.1
MSKIFRISHLLVTTLLSLSFPHPVGATPGTILQINPPVSTPSTTELTVIVNGILNKTGDI
CLRVYNSEVGFLTNGSSEVKSGCTKITGSSVKTVFSGLKPGTYAVAVMDDQNGDRKLNKD
FFGIPTEGFGISRDPIVSMRTGMPKFRRASFKMTQNTTIDITMKYSLDP
>KRH97034.1
MSADSINTALAELENLINSCENDLIRFIEARKMNSGNEISTKKVNRQLQQNPIAIVGMAS
LLPQSRNLRQYWQNIVSKADCITDVPESHWSVKDYYDPNPRTPEDKTYCKRGGFIPEVDF
NPMEFGIPPSILEVTDVSQLLSLVVAKEAMEDAGYGEAREFNRENVGVILGVAMAKQLGM
PLSARLEYPVWEKVLISSGLSPEDTQKIVEKIKSAYIKWDENAFPGMLANVVAGRIANRL
NFGGMNCVVDAACASSFGALKMAISELVEYRSDMMLTGGVDTDNTIMAYISFSKTPAVSP
GENVKPFDAKSDGMMLGEGIAMLVLKRLEDAQKDGDRIYAVIKGIGTSSDGRYKSIYAPR
KEGQVKALERAYNDAGFSPTTLGLMEAHGTGTMAGDPTEFASLQSFFSKHDERKQYIALG
SVKSQIGHTKAAAGAASLVKTALALYHKILPPTINITEPNPKLDIENSCFYLNTETRPWI
RGESESPRRAGVSSFGFGGTNYHLVLEEYQQEQQQPYRLHDVASQILFSAPNPSELIKNL
ETSLENLQAGDSNRYYSQLVEECRNIQIPQNAARIGFVAGNKADACKLLALSIDLLKNKQ
STLNWEHPQGIYYRASGIKLRGKVVALFSGQGSQYLEMGREAVMNFPALRRLYGLMDSLL
IEDNLQPISQVVFPHPTFNQTEKADQIATLQRTEYAQPAIGVFSAGLYSIFQQAGFKSDF
TAGHSFGELTALLAAGVLSESDYLYLVKARGKAMAAPKDPDHDAGSMLAVKEEISKVELV
LKNFPKITIANFNSPSQVVLAGPSHEIQKIHQKFQDLGYGAVLLPVAAAFHTPLIAFAQK
SFAIATKSVKLLNPKIPVFSNVTAQQYPQESDKIQRILESHLASSVNFTQQIENIYAAGG
YCFVEFGPKRILTNLVKDILGERPHITISLNPSTQKSSDISLREAAVQLQVLGMELGNID
PYQLPEIFSKEISQKSLNVKLKGINYVSEKTKNAFEKALNDGFKIQGVPQIQPVSVIDKP
SELIPEKEVVEIVHTTNGNGNRDQQTSIISSLSTDPQMNSANPINSSLRAEEQLPILTYP
MVQVLGGKMTISDKNTEFQQVLASLENLLSQFQNSQSDNLQIHDTYLQHQIEYAKTFFQL
VQQQNTLFMNNKSPETGETKKSIMDSFERSMMQFHHQQAETLRIHEQYLREQLEHTKNFF
HLIQQEYSLLIDGAEELTPVIPLQLTDIHTENLREISPQNSQLTVSEMVVSSTSKTLEVE
IPQPTIIETQPVITPIPETRVEQPPVPVTEIAISSQTKKSAIDIQDIGKSLLSITSEKTG
YPIEMLEFDMDMEADLGIDSIKRVEILGGLQELYPDLPKPNLEELAEKRTIGQIVEYLEK
QVLTNPTTNQVNHSEHIVKNVDPKVTSELIIPSPVDIPHSPPTSDEYSSIAETLLKITSE
KTGYPVEMLELDMDMEADLGIDSIKRVEILGGMQEVYPDLPKPNLEELGDLRTIRQIVNY
LQSLVVVEKKNLDFEQIKATVVDLTPPPTIDPNLPRRPVKLKILPEPDFWECQLPGGHIG
LITDDGSLTTTKLVHGLIDKGWKVVVLSFPQSIVPERSPLPVGVTRINLANMSEEHLQLL
LQSISVQYGKIGAFIHLHPYFSTTNLAYLETEKVIVKHIFLIAKHLKQSLNNAANLEGRV
SFCTVVHLDGTFGLDHTENFGIIGGGLFGLTKSLRWEWPKVFLKAIDLSPKLEPHQSAEY

IVAELYDSNRYIGEVGYGSKGRVTLVATAD
>KRH97043.1
FLLSTISVLSNPSTTITKEDSITNAINQWLYGKSTHSKRSYNRVIRSYISYLDDIHIANS
TASHFRDYQEYLYQSGKTINTINTYTNIIKSFFTFLRDESVLPTNITHRVKSPKPVSALR
ERILTRAEVDAMITLETNTRNRLILQLLFFCGLRVSELTILLWADIKDNGSTAYVHITGK
GNKQRTLIIPPLLWTSLKSHKTTNESPVFKSRKGGKNLTQKAVWDIKHARSWLHRRALCG
NFACQIGFRDLISGQTPAVGHALEFLVSCRWHD
>KRH97044.1
MARLGGDTNHRRAAFGRYVAEAVFWTPAALLPGSGVVWDMIDGNSARVTVSRGALSQSVD
VYVNASGQPVVVQFMRWNNANPEKIYGYQPFGGYLSDFREVQGFTIPFRVEAGNQFGTDT
YFPFFRAQLKAVNFPANTGQSP
>KRH97046.1
MLLFPNSSIDEIDSLQNQTLISVLRQLRDGFPHSLGLIGMRDVRDYKVKSSGSERKSDPL
TKGLTQLDKYLDGLGLHTGWLVIFDRRPGLPPMGERISTEEVISPSGRTITVIRS
>KRH97047.1
MGWRKNLVHLPHKKPKGKELTQQQKEENRELSRQRVVCEHAHSGIKRYNCVHSVYRNRVT
DFDDQLMLVSAGLWNFYLDAA
>KRH97080.1
MYSSSENLHLLYDIDENLWLEKTIVLLKEKRFYDLDLQHLVEELEALSKRDKNAVASLLE
QIIRHLLLLQYWHEESEINRNHWQTEIVGFRNQLERLLTTNLHNYLYTELEKIYQGAFRY
VRQKTRFEVSFPQDCPYCLEQLLNQSYF
>KRH97117.1
MTQSSNRENHQGNIQNTDNTAEELLVKLRQKKGNWVEWGNAIAYLQKNGYNPQDIFEATG
FEPIQQNQVVVGAQVYSSLEKFGASEATKAYYGTRASDILYELRLLTQGDRATAADLIFA
HKLDVDEAREIAKAIKDFSRFSTPPEGFSTHPGDAVAYQCWKLARQNSDLQERSRLIAKG
LRFVQSSTARKQIEQLLTDFTIVPQRNAPLLPYFRLESDEELPRLVPVVGELPLTPKDVK
SVPLIIEEAPFNIVKFAGEQAWVALPGWQVLRSAEDPIVIIGESNIFPQSKSGKTEQILI
VIDRDERDWDDGSYFAFDNDGEVDFQWFETQPEQTILGRIIVILRPKKVLDEDFTKDSWQ
IDE
>KRH97167.1
MIVAKDQTPYLTPEEYFTWESTQPEKYEYIDGQVYAMGGGSINHGRISIRFTSMLDSYLE
DTGCITGNSDIKVNILGSNNYTYPDISVTCDDRDKANTQYITYPCLIIEVLSPSTEAYDR
GGKFRMYRKNPALIDYLLVSSTSMEVDLYHKNDRGDWLIINYKPGEVIELQSINFNFPID
QVYRGLDLTKQCYF
>KRH97224.1
MYTVERQLYESLLGKRVSDSHWSNLKKTAQEADHVITLDEPPELSQNKEITLAEKQTIST
VTKDLGFILPQDELKAIAQHSLREIASSLREDSEITSITEIKEAILRFIAKKGERTRREV
DTALSEIATAYAAEQLISSRLFASLLANLSKEVKVAKGWNGVRHIVAVELRNNMDLAQAV
KVVKVIPSDDVLAPYFALGGTALLITAATISHKLTQYQAVQLLSSLRGIKGAAGRYAGGI
CSGLT
>KRH97227.1
MNLDTIKQDIASLPPYAQQIIIELVEVLKKRYPLNQQRTSENSLQDWSDFIGCIEAETDL
SKNYKNYLDTELNQKYDHS
>KRH97228.1
MKPKKLTQLLIIGILVTCITILQPLITLALTRQEIDAIATQVTVRITGTRNGSGVIIKQD
NNTYTVLTNFHTFQEKGRFEIITPDGVTHQLNKISRVGSSLDLATLEFNSNRRYRVVELG
DSTKITRGRDIYVSGFPADKGLNFLRAEISRIDPPEKGGYSLVYRIGAFPGMSGGPILDE
NGKLVGIHGKTDYILVPPHDSTSEEYGIPLHTYLNVSKRYDKLETLLKAQNFREADLETD
RVMLAVANKQNQGYLTVEDAKKFPCQELRTIDNLWLKYSQGKFGISVQQEIYKNLGGTQQ
YDDKVMESFSYRVGWKYGEEWLNYNSLNFTLSAPKGHLPTPVISWEFLVSYVVVSLLSRH
VECNL
>KRH97233.1

MTQKDLFLIWTKEADAALKVNDSGVAVDLWKCVGSHRLIAIVDVPTTDALDQILFDLPIM
RKVGQHVHVDVTSLKVYEDFTTYVTSQL
>KRH97245.1
MENIHNLNITDEEYLHLISKGYDPKLESQFIELGETEDQARKLAKVVGMFKDGPPQSDEE
WEHFLEVWEN
>KRH97247.1
MQSSFEGFHGIIPEILVGKAFQGYSGLLPNNSNGNFSIMRRAWTALIRVRFPLGSGINGN
GRSLKYYKIWFFVRFPLGSGINGNEEEKLLLGRGDYLPRRRDNGLRPIFLFFFLRKGKRR
GI
>KRH97260.1
MKLGQWIGLIALVVSLYILWQLREVLLLIFAAVVLATTLNRLARTCQNLGIKRGLAVFLS
VMFFLVGIVAFFWVIVPPFVHQFQELTLRVPQGFERVNTWVDEQRSHIPQELEPVIPDLN
RLIAEAQPLINRVLGNSFAFVSGSLVLVLNILLVLVLTAMFLTNPAAYQKLFVRLFPSFY
RRRVEGILNQCEDSLERWLTGAFIAVCLVGLMSLIGLSILGVKAALALGVLAGLMNLIPN
LGPTMSVVPAMAIALLDSPWKPIFVLILYFFIQQLESSFITPMVMAHQVSLLPAVTLISQ
LFFVTFFGFLGLFLALPLTVVAKIWIQEVLVKDVLDCWEHHGHDEVDLVILTDDDSQGE
>KRH97264.1
MQNYARDINSLKSHATMWWPQNLRDKNATTSIIPRLLETQDDFISILQLSKNNPTQVFEL
AEAANFPANLFLKHLVVISDYGGELMKRLGESFTTIFTKRDKTNKLVMDYVWKGKNHQYV
FESMPIQSKLDNKKLNIDGKGLQFEEPFDGLKRDITMILLYASTSDVSHCAALDLCTLGS
ILGDKVALEHHIKQKYILVSRITGGANANSLGQLAQSYILKYLKEKLGTEFYISSNERIP
LQGTEISFDIVVTKADKKVGIEISFQVTTNSTIERKAAQASERQALMTKAGYKTAYVIDG
AGNFERFAAISKICQHSDCTVAFSDSEFNILVDFLRENL
>KRH97285.1
MLTICPHLFVVLIKLMSDLIGPLGFSDFYLLVLPKLVRVLTSPWELIFDKWVSIYNHSS
>KRH97292.1
MLIPILIFDVALVAWSLHLMEKAIENKEFSLMLAGALVAVAAAAMLVVYFLMGNCMSYLL
KIG
>KRH97293.1
MLSKRILPCLDVKAGRVVKGVNFVDLKDAGDPVELARIYDEAGADELVFLDITATHEDRD
TIIDVVYRTADQVFIPLTVGGGVQTLENVKDLLRAGADKVSINSAAVRNPHLINEASDRF
GNQCIVVAIDARRRVEPNSTGWDVYVRGGRENTGIDALSWAQEVEKRGAGELLVTSMDAD
GTQAGYDLELTRAIAQAVEIPVIASGGAGNCQHIHQALTLGQAEAALLASILHYGHLSIA
QIKNYLQEQSVPIRLPC
>KRH97319.1
MVVSPAKNKQHHVVIIGGGFGGLYAAKTLANTNVNVTLIDKRNFHLFQPLLYQVATGTLS
PADISAPLRSVFRNIKNTQVLLGEVTDIDPKGQKVFLGGEVVQYDTLVLATGANHSYFGK
DHWKDLAPGLKTVEDAIEMRRRIFSAFEAAEKESDPAKRRALLTFVIVGGGPTGVELAGA
IAELAYQTMKDEFRSINTSETKILLLQGGDRLLPHIAPELSEEAKLSLQKLGVEIQTQTR
VTNLENDIVTFKTGERIQQIASKTILWAAGVQGSPIGKILAERADIERDFSGRVIVEPNL
TIPGFKNIFVIGDLASFSHQNGKPLPGVAPVAKQQGEYVGTLILLRLQGQTLPEFNYNDV
GSLAMIGQNLAVVDLGFIKLKGFIAWVFWLVVHIYFLIEFDTKLVVVFQWAWNYITRNRR
SRLITGKAAFLETQTINNNNPYQAAETAQHTVKV
>KRH97327.1
MTIDQVDIRKSEVIQLLKQINVPILNNNLVSLGMVRNLRIIDDYVYLRLYLGSCELDLKE
EVRTKLSQLGWCKKTYIEIRTISQVRRTIGISSGKGGVGKSTVAVNLAAALSLSGAKVGL
LDADVYGPNIPQMMGLGHSEIIVTDTADGQRFIPLEAHGIKLMSVGLLAEPDHPLAWRGP
VLHKIINQFIHQVEWGEMDYLLIDLPPGTGDAQITIVQESPICGVILVTTPQQVAIADVR
RSVYMFRQVGVPVLGIIENMSYLLNENVDSFQAPQYIFGKDGGKLLSEELEAPLLGQIPI
HQRICESGDRGLPIVLGDRHFLPSRILEQIAQGLRKTFNNL
>KRH97334.1
MKPNTPTGYPTTKWASGIKHKMNVGWVEERNPTPPQVTLPLTYPTNNCASLLNKLSLKDF

IKVQRNWK
>KRH97336.1
MQVIEKNSLFAQVSGEQSAVVSGGNAVVGSWAFVTFLANDGALSYAEAVASIILTSPYF
>KRH97341.1
MFLRIDASGYFTNRKQFRLFAPMINFPILTKSILLTFFGNVDKLSFFPLEQHKTLYLEIG
S
>KRH97350.1
MTTAIKPDLTSNLVNRVLSIKPLFDFAKHQAREMMIKRAQKIGVNWHQEVEKLQARDWSN
DLAQVQDPQLTYPDYYLTSFHAYETGNMSWQAAFEVESAAYAVHAKVWPDFLPDGDAKLR
QSYHHILKTLIPDTPKDILDLGCSVGMSTFALQAIYPQSQITGLDLSPYFLAVANYRSQK
SPQYQNSINWLHAAAESTGMPDNSYDLVSIFLMCHELPQSATQKIFVEARRVLRPGGHLT
IMDMNPQSEIYKKMPTYVFTLLKSTEPYLDDYFTLDMEKSLIDAGFKTPTMTSNSPRHRT
VIAQVIK
>KRH97384.1
MNSKALPCQVNNLEVGVYECEIHLKFRLIEEKSLLGDREQLLQVFLDALTEGSDEFLEML
QASVKAQEISEFKASPQMRRQLMRLRNSLDNTQQ
>KRH97420.1
MVGIHQFPTDSLPKYGLLHLYPVFGQNSPPLSLVTLDTQEKIRQVLSQGYKITVEYVDKR
RFSMGSWQTCGNLHISDYAQAVFALESCLLEHAGEYVRLVVIDPKLKRRVIETIIQRP
>KRH97443.1
MSNSSVLCLGEILFDCLADQIGLKLEEVNSWTPYPGGAPANVACALVKLGTKAGFIGAVG
QDEPGDTLVKLLGDVGVDTRGVQRHPTAPTRQVYVVRDLNGDRTFAGFGKYHTREFADTC
LKASDLPEELFNEADFLVLGTLELAYPESEAATHQALKLAERYDLKIILDVNWRPVFWQD
SELAKQKIHQILPNCDFIKLTKEEGEWLFNTSDAGAITYRINSLEGVLVTDGENGCSYCL
AENEGKLPAFSVPVVDTTGAGDSFLAGFVHQLNQYGIQALSDPQIAKSVITYASAVGALT
TINPGAIASQPTAQEVETFLHRFNPE
>KRH97444.1
MPKHFNTAGPCQSDIHYMLSSLDRLPTLGNLIDQRGYFVIHAPRQTGKTTAMMTLAQQLT
ESGNYTAIVLSVETGSAFKHDPILAEKYIIRSWIGTTKVTLPGELQPPKLRDIQEEMGLL
DPSDLDIKTYLQAWTLASPRPLVVFLDEIDSLEDETLITVLRQLRAGFPLRPHGFPHSLA
LIGVRDVRDYKVASGGSFRLNTASPFNIKLESFTLSNFTLSEVTTLYQQHTDATGQVFLP
EAVALVFHLTQGQPWLVNAIARQLVEVLVTDPTQPITVAEVAQAKELIIQRQETHLDSLV
KRLREPEIQAIFEPMLSGDELGNIPEDDVQLLLDLGLCRLQNGSGLQVANPIYKEIIPRV
LAYVTTASLPAPSLNPHWLNPDRSLNPEALLGSFLDFWRQHGEPLLKSAPYHEIAPHLVL
MAFLHRVLNGGGRLEREYAIGSGRMDICLRYGAVVMGMELKVWKPGKKDPLPQGLQQLDK
YLAGLGLHTGWLVIFDRRPGLLPIEERTTTEEVVSPGGRAIVVIRG
>KRH97455.1
MYLSQPDLNNFSVQPNQTALIDPAVIKAAGQIYHTYSQVHPEIIGQVSGVAISRTTYRGK
VIFTQQPVLLPQECFIPLQQIESYVY
>KRH97463.1
MDFGIGFLSNNVMLPIIDFFYGVFPSYGLAIVALTLIIRFALYPLSAGSIRSMRRMRIVQ
PLMQKRMAEIKERYKDNPQKQQEEMVNVQKEFGNPLAGCLPLLLQMPVLLALFATLRGSP
FAGANYSVNLQIVPSEQIERIQPQAFATSPQNIYVADGVHTKITAILPGGNKLAVGEKTK
IQYQTMEGKPFDALLLEYPQTKLTPEWKIIKGEDRIKIDSEGNVEALQPGDVTIQGTIPG
LAANSGFLFIDALGRVGAIDPDGKVHWDIVGMIIFFGISLYFSQMLSGQNSSGGNPQQET
VNKITPVIFSGMFLFFPLPAGVLMYMVIGNVFQTLQTYILSREPLPEELQKIVAIQEKEK
QAATVDVKTLPFEPKGSKKKQPNNKESKT
>KRH97465.1
MDAIFLPQLTKAPQCTEEIQVDEFLPGLETLTPVRGVVRLQHHGNYLEVSGKAESIITCS
CNRCLQQYNQRLAIKTKEIIWFDTNSSPVEDLPLEREVAMEDLVETIAPDGYFDPGEWVY
EQMCLAIPQRQLCNSNCPGIIATGVNESSVDRRWSALEKLKNQLS
>KRH97480.1

MDTAIIPSTLLLTLLLLVGLFFFIRASTKDRTEQAKIASEDDETILMGQLKDYFQSRAYR
VVSADPEKKEVIFEGFVQPSWFLAVFLTVLAALGLACLGLVLAQLFSSQNPFFLVLVAPL
SGIFYWRQSGRIEKVLLKMEFCQNEQHPSSIITVTAHRDELAELKRALQVKALQVKIVDV
IT
>KRH97494.1
MTPQDNKKIVEERKELIKEVLQAYPEKAAKKREKHLNVHEEGKTDCGVKSNIKSLPGVMT
ARGCAYAGSKGVVWGPIKDMIHISHGPVGCGYWSWSGRRNYYLGTTGVDTFGTMHFTSDF
QERDIVFGGDKKLLKLIQELEELFPLNRGVSVQSECPIGLIGDDIEAVSKTAAKELGKPV
VPVRCEGFRGVSQSLGHHIANDQIRDWVFPRADQAKKDGSLKFESTPYDVAIIGDYNIGG
DAWASRILLEELGLRVVAQWSGDGTINEMLLTPNVKINLIHCYRSMNYISRHMEEAYGIP
WMEYNFFGPTKIAESLRAIAARFDSKIQENAEKVIAKYQPSMDAIIAKYGPRLEGKTVAM
MVGGLRPRHVVPAFNDLGMKLVGTGYEFAHSDDYKRTTHYIDNGTIVYDDVTAFEFEEFI
KALKPDLIASGVKEKYVFQKMGLPFRQMHSWDYSGPYHGYDGFAIFARDMDLALNSPTWG
LIGAPWNKSAKKALRKATAAV
>KRH97540.1
MKVFENTTKKIFLTSWVSPHQMDANPGSVVNEEQHSQTGWDMLGPVRNWLDNIKVQDRDF
AHILCKLIPAQCPFERDVKLLGKVLFHIPPLCKLNPFYDQLTYLRFRALCYLADECGEA
>KRH97558.1
MRLSASLQTILTYGLACPLIAVNVWLLSLLFHYFQHPITILSIAAILAFLLNYPVQLLEK
ARITRTYSVIIVLVITLTLLVILGFTLVPMLVEQTTQLLKNIPDWVTSSQENLSRLQVLA
RQKRLHIDFSVVSSQINASVQNILQQIASGAVGFAGTLLSGLLNIVLVVVLAFYMLIYGD
RLWSGLINQLPSYIGLPLSKSLQLNFHNFFLSQLLLALFMVIALTPIFLFLRVPFALLFA
IIIGISELVPVIGATLGIGLVTLLVSLQTWWLAFPVAMVAIIIQQIRDNILAPKLLGNFT
GLNPLWIFIAILMGFEIGGLLGTLVAVPIAGTIKSTVDTIRNNGIT
>KRH97592.1
MLTLVTSSVVVLLLGACIGSFINVVVYRLPRGLSLLWPPSRCPHCLNQLKVYHNLPILGW
LWLRGKCAYCQGEISHRYPLVELLTGIIFLIVFWVFQFSLPTIGYWVFCSWLLALSLIDW
ETMILPSSLTKSGLVLGLIFQTTLGYVTHNTWSGTIGQLIWGIGGMVLGIWLFDIIANLG
FVFYGQPVMGGGDGKLAAMMGAWLGWQYLLIASFIACFSGVLVGFGAIIVSDGQIGQKMP
FGPFLAWGALISIFGGQVILDHYLRFVLSHS
>KRH97593.1
MKEHKKAKWKNPRMVIYGIITMVIVSIIGLFLGQIATASVIITDIEFLGVATLPTGYTFQ
NTEIGGLSGITYDVDHDLYYVVSDNRGQKGPPRFYNFKIDLSKGKLDQSKVLPVGVTTLL
DENNRKFALGGIDPEGIAVTKKSTVFISSEGDVSQSINPFIREFSLASGNAINSLPIPEK
FLPDGKNQRGVHNSLTFESLTITPNNQMLFTATENALVQDGEAAKPKFGTLCRILQYNLT
YNQPQKEFLYPMEPVTPLFNFMDRFYSGLSDLVALDNRGNFLSLERSFTGVGFAVSLFEV
SLNNADDIQNIPSLKAIDIGKIKPVEKKLLLDLQTLPLPLDNIEGLTIGPKLSDGNISLI
LVGDNNFNRLQSTQILALKLKRESPLKRLLHQLGLTW
>KRH97605.1
MILIIFMSLILSGCVEYNLGINFHNSNNAEWVQHIRIAENLTSFSSDYLQEWFNVLSRRV
SNIGGSTKYISPGELVVKIPFTHGQELEEKLATFYPHSLQTSLHEPINITSNITLKENNF
LFLSRNNLIYQLDLRSLAGVVAQKNSSDKTQISLFNIDFTLKTPWGNIKNINSTVKPLPP
ETNNQQITWQMQLGELNQIEVVFWLPNMLSIGTLIIIIIVLFGYYLKYSSLNIPNQN
>KRH97609.1
MVCENLGTTEIRCRTDFNYTIRTYDGSEHAIKHRQSLQKTVNDPDLAIVTFESQQDYEVA
PIRNFDDVQIQSDILVAGFPTIFGRVGKNRIFTVTNGKVVGFIPDPPKGYGLVYNATTFI
GNSGGPVFDSSGNVIGIHGLADTDGVEIDNKNQSETRNGLKPNVSIIRKLINPQIESGTS
FIQKTGFNAGIPINIFLSVTADAGLQLPNVKSPVVIAPGKTDQVAISPLVPSDREATIPF
SSSPSSDSTDKVNSNVAIAPKIVPNVNDALSYIARGIALYELGDKQGAIVDYNQAIKIDP
NYANAYIAKGVARSDLGDKQGGIDDYNQAIQINHNYAKAYYNRGNARSNLGDKKRAISDF
QTAARLYQQQGKQNDYQDALNRICRILSICHDNKLNSNILSICHDNKLNSNLVF
>KRH97610.1

MNNQHNIQPVPEFNPYLALIVKERLRQARRSFDFALIATALSFGISLVGAGCLLTNKVSE
GAVTTAVGLVASMGYMRIAKDANNRLDKIADDLDVAIPR
>KRH97640.1
MTVSIIEQKKAEDKPKKVSTPEEYLSLEEKAEYKHEYRNGEIVAMVGGTTNHNEIVTNLC
TTLKVALKGQHYRVFIGDVRLWIPRYNQYTYPDVMVISGDPVYYQGKTTITNPLLIIEVL
SQSTQDYDRGTKFTHYRSIPELQEYILVDQYSVNIEQFTKTSQGQWLLTEYEKGAETFSM
QSLKLQLKITDIYEQVEFA
>KRH97642.1
MPTSSYSQLYELFIDQVIGLSQKTDFSLTALISNYISMNYQIKLEQIDKLKAWLDEFRPF
DQTIIAEIKKLYDVRFTYNSNAIEGNTLTQSETELVLTKGITIGGKTLDEHLEVVGHKEA
IDYIESLAQKDTVINEWEIKQVHNLILRKIHPDEAGCYRQLDVMAAGTNYIYPPHYLLSQ
LMTDFVIWLNSDAALTLHPVEYATMAHYRFVSIHPFRDGNGRTARLLMNLLLIRAGYPIV
VINNQIRNDYIHALSYGQRNQDDLNQLFDLVCDATISSLVETLRLLVTASSSREKGRVFY
QEITNFIDNFLIK
>KRH97646.1
MEQLKKERLESKGWKVGTVSDFLELTPEEAFLLEIKLVLSRSLKERRQKLMTQVELASKI
GSSQPRIANAGNGSDSVELLIRAMLATGATPKDIGQIIASVG
>KRH97652.1
MVGDLDGLDPNSTNVIYQWQRSNNDSSWNNILKDGDKAIYVVTDEDLNQNLRVQITYTDL
QGFSS
>KRH97654.1
MSQPVLQYIATKNYKNLYLPDPVEFNNLNILLGANGSGKSNYINSLKFLRDCLYPTKYAL
EINGFEDAVNKIGGAGILDNTIEDPASVYFVYCFAGLSSDSTSKNHILELSLLVNRKEDR
KQAVVIDQESLKVGDKVIIDFPHLYLYEPASNVIEDARKLLDFVSQWQFYNANSFDLEKI
KTSETKITSPDKYVSASGDNLALVFENLIREDIFFEETINKAMTLVLPRTYRIRCVRSQE
LSLVMEWYSLDTKKPLCLKELSDGTIRMLCWAIILHSPVLPSLLVIDEPELGLHVAWMRI
LSEWIKMAAHKTQIIIATHSPDLLDHFTDCLEKVYCFDSYGKSHFSIKKLTQEMLADKLE
EGWQLGDLYRVGDPTIGGWPW
>KRH97663.1
MSFENVSLEMQRRFEVLDEMIEKGYIEPIKPLVDWLPTLCYSPARTSTFDNAPENLKRKF
TFMFSWYAFLFAIFAFVQTRLARDYLICISTINLITLIYPFSELYLYSSALVINIFAAQC
FVFSRYYQYKTFGRCPHSRNAFSTICLSLIYLFGLIIIDVIIQP
>KRH97664.1
MRMNIKRLVSVGMIVATIGINSKAFAQNQEYGTQRVSEIPNAFPEFTIPRNIKLVECFNN
LNFDSDKATLRYLNRTLKGSPNNPCLYLARGLTYMNLGRIQAAKTDFDTFIGMDSNFPYV
YVFRGFCFMLLNSPVTAISDLRKASGLFEAQGNLSFMELLNGLIKNLELLSNP
>KRH97666.1
MKIKETLVSLILVGGAMTIGSQAIAEDFSSIKNSSPLNASSQLWQTNNPGVNNFNGYGQF
TQLGLSRYTQDGIPYKIQNVQVSSSDVCEYINMAISAFGSGDFQKAKMILDVVVDLDPNT
SYAYVLRGLSFLMLESPKSALTNLEKGVSLLIREDDSESQYLAEMVNNLIPMVKLLYSL
>KRH97667.1
MTLEEFIQILAEHQLSKYIRSQEALIRRAKQTGRMSYQERLIYIRQAQAAIQDLKQILSN
RNMCERLLIYTQKQSSADQVETIDWILRMIELSDGIYGKISFSSDSSDIYQEGLQRTRIW
FFDRFVENYNPQVSSILTWFNKNLDYKFRDLRREREREPQSLDDTTRIGPPHIPSPENRN
FVEEIEAMLESLENCLNSPNSNCPRSCIRNAPHANCNSVIRSILNLLLAGEVEKLGPVWD
TLSRKYEVSSYSMKQFIRKECFTCFKRLTE
>KRH97669.1
MSLEVNISFRVEGSFTEGFTMTPMIPQQGSQEFRELGSRIRLPACPVLPNLYNECRNFTD
EYTRRIEGVPDQTTTVNERERWTRTQMNLNRSFLDWIRNANFEVIINEIITRIFNKRLTD
TSVRIHFVSNNSNNNDYDIFRRMPWDEFTRSTRSRMKFVLSQYSLTYNSCPTRLSELRIL
VIRGGRDLENSQNREINALRKIPGVRLIDKTRTIDNIDCLHKVLTENVYDILFYTGHSSS
NNGGEIRVGSLTVSINVFVEDLRMAVSRGLKIALFNSCDGLGIADFLVTQVRVPVVLIMK

EPVPDIFAAKFFIEFITRFAADRMSLSIALEKARSSSHFESVNFPGATSLPILFYLYPVK
KDFRISRFLYIKVVIKIIKEGVWKKLNNMFGIIQRLSRRQYMYMLIFLLPLFSVFLLKIG
ILGPENYPLEGVIDNILPANIVDIDISDDEQYVAYVHNRGILLQKMDDLTAKNNIQLCSK
NFEEIPPNSLPSTKLVFKKNKTLAIATYLDSARSNIRYLNVDNCKFEEKVIESSNIIFTS
LDFSTHKNVIVATRFDIEKDAITAGLYDSDSGENINIKGKDSVFEGVVDAKFWNSQKIIL
LKNNGQPRIEIYNIDVSNEDNSYKLERVNSLVLKELKFEYAEKIVLSQDRKYISILGSGR
IHIWEIDDKFKLKEIPKNIQNQLEGRTDVRDAIFYKKQILIPNTTLVD
>KRH97671.1
MMNIQSLKSQPQKTAWILLLPAVLLLVIVFAYPIGRTFWLSFFTENLGTKLRPVYSGLDN
YVRLLGDSRFWESFKSTTIFTVVSVSLELILGLNIALLLNQQFLGRDIVRTIAILPWALP
TAIIGLVWNWIFNDQFGVFNDLLLKWHFIDTGINWLGEPIPAMIAVIIADVWKTTPFISI
ILLAGLQAIPRDLYEAHSVDGANPWQSFYLITLPLLIPQIVIALLFRLAQAFGIFDLISV
MTGGGPGGATEVVSLYTYSTIMRYLDFGYGASLIVITFLLLIIAVIIFTIALKNKGDQNF
LF
>KRH97672.1
MTQFQLKTRVKNLTKLLVIITGIILLAVFCLAPIIWQLLTSFKSNEDILKIPIIYFPSKI
TLNHYIQLFITHPFWRYIINSIFISSSSTILSLVFGAPAAYALARLNPWGGKIIIGTLLI
ITLIPGILLLSGLLEIVRVFHLGNNYLSIIIPYVAINLPLTVLVLRNFFQQLPIEIEDAA
KIDGYNTIQMLLKIILPISMPALVTTGLLNFIFAWNEFIFALTFITREEMKTIPIAVAQI
GGATEFEIPYGPIAAATMISTLPLMLIVLVFQRKIIQGLTSGAVKA
>KRH97673.1
MAKLQLKDLTKSYSSQITSVKNINLTVEDNEFLTLLGPSGCGKSTILRMIAGLETPTKGQ
ILLAGEDITLKSPGSRNIAMVFQSYALYPHLTVFENLASGLKLRKIPHGEIYRRVKKVSE
SLELEELLLRKPAQLSGGERQRVALGRALVRNAKVYLLDEPLSNLDATLREKVRADIKQI
FTQETSPVVYVTHDQTEAMTLSSKVAVINYGSIQQLDLPAVIYKQPANLFVAGFVGSPQM
NLLPLSCNSNYAILGNNKLFLPNMNKIPPKIVLGIRPEDIYIAQSGDEHTISGKIYLVEN
LGMHNLVTLHIDNHHPIPLTIRILLPTNQTWENQQITINLPPKNLHWFDYQSGNRL
>KRH97676.1
MASFTEKCYKLRYLLAEQNWKQANQETKNIIFGIINETKEIPFYLQNISQISSRNLRLID
RLWLKYSQGKFGFSVQQKIYREIGGTQDYDSEKWAIFIGKVGWNESLENENNENNNITLE
NVPEGHFPMLIPQNDYRPFFFIVIILSSPLLSFLVMKIFSNLWFFLTEFGIWGLLLWVIL
FILLKIIWITGWGYLQLRLSLHYVPKFNYTCSQIMSSIFERLKSSNL
>KRH97695.1
MIRQFTDKSYLKSALKNSSIRVPKHLIFNQSQYRHEGISYLQFVIKELGNDIFVKPVIGA
GSEKTRRIHTIDELKAWCNSNVDSDDEFEFNEFIRGELYNTSVVMKNGLPCYFAACKHYR
PNDDFIYGHAIGNIVVREEDPKFEKLRQFSSDTLQSLEHDYPKNGVLNIDFFLQEGSEEP
ILMEVAARTPGGLVSKMFYTYQGVRLNELHLQLQMGDDPEIILKNRSEWKYSAYSIHPKQ
EGVVTEIEKPILDSDVEVYWQIYLGETLKVSESMRDVAIAMLLSNHDFSTLEQDYKLANS
LSLYKTKKDSFQELKAVFVALTV
>KRH97696.1
MPNQHNDVNILVIGAGVSGLTTAICLREAGFNVIIIADRFAPDLTSVVAGALWEWPPAVC
GRHGTPRSLERSKEWCMTAYNKFKEIHAEFGSQETGVYLRDSYFYFRDILENRPADFRKM
NELKDKVDGFERGLHIVKETIDLTFQ
>KRH97697.1
MLESNPSVSISIPTYNESSNIERVIKGFLATEYQNLIEVIIADGGSNDNTQEIVKRLSTQ
DSRIKLIHNPLKVQSAGLNLILQECRGDIFLRVDAHSDYAPDYIERCVEALLESKALNVG
GAQRFVAKTPFQAGIALSSKSILGSGGAKYRDPNYNGYAETVYLGCFWKKALTEVSGYST
EATTNEDAELNQKLLKKDQNAIYISSKIRVWYYPRKTFKSLYIQYFKYGRGRYLTSIKHS
IKSQIRGIVPFLVISSITLLLLIDLLFPRLGLPMVILVILGLLFPFLESLRVTLKSRTSF
DSEIWRGDKGKIPSFLSRWFFCGIVLLTMPIAHSLGYAYQLIKHKILGLNGWL
>KRH97700.1
TTSIEPLLKKIHPNGDYCIGQDSGIYWRFTEPPEKGVEAPDWFYVPGVPSRLNGQLRRSY

VLWKEKVPPFIVIEFASKNGKEEKDSSPPPEGDEIDPETGKLKKAGKFWVYEQAVKVPYY
AIFNGFKGTLEVYHLQGKRYKEIKANRRGHYAIQEMGIELGILYDNQNPPTPWLRWWDEK
GSLLLTGNERAEQAEAIAIRERLAKEQERGAKEQAEAIAIRERLAKEQERGAKEQAEAIA
IRERLAKEQAEAIAIRERQQKEKLVAYLRSLGVDPEKI
>KRH97702.1
MRQPLTTTQNGTTPLAGESLNLPDHTQLRDSDDNFVKNFQEHPQSIILTTSIEPLLKEIH
PNGDYCIGQDRDGHRIGNTV
>KRH97713.1
MDEEFIIPQAPANFKSGFIGIIGRPNVGKSTLMNQLIGQKIAITSPVAQTTRNRLRGILT
REKAQLIFVDTPGIHKPHHPLGEVLVQNAKIAITSVDVVLFVVDGTAVCGGGDRFIVDLL
TKCEIPVIMGINKIDQQPAEAEKIDESYRELAQGNGWEIVKFSALENQGILELETLLIGY
LETGPLYYPPDLVTDQPERFIMGELIREQILLLTREEVPHSVAIAIDLVEENPTITRVVA
TINVERDSQKGILIGKGGTMLKSIGTVARQQIQKLIAGKVHLELFVKVQPKWRHSRLRLA
ELGYRVEE
>KRH97721.1
MLYRRFGRTNLQMPVFSCGGMRYQFNWQDVPLQEIPVDSQQNLANIIHRAVDLGINHLET
ARFYGTSEMQLGQVLPNLDREKLIVQTKVTPCSDPQEFRQIFDKSLAYLELDYVDLLGLH
GINNQELLDYSIGGCLQVAKELQAQGKVRFIGFSTHAPLEIILQAVNSNKFDYINLHWYY
INQWNWPAIEAANRLDMGVFIISPANKGGILYQPSPKLVELCQPLSPMVFNDLFCLSHSQ
VHTLSIGAAQPSDFDEHLKTLELLDRADEILPPIIAKLEKAAIDTLGEDWVKTWDTNLPV
WENTPGNINIKLILWLLNLALAYDMVEYGKMRYNLLGNADHWFPGNRADKLEELDLQECL
GNSPHREKIPLMLSKAHTIFKGEEVKRLSQS
>KRH97723.1
MKRELFERIYEEELTPIQRRILHRILQGKSNTAIRGDVTVWFDHKVYSGSERDKLPRSKR
TILEGLESGKKLMDQSNLSHHLRKICEQFELIDLQEVIKQFIKYRRQLVSYETLEQFELL
QTTQFPSGATNSLYYEYRHPIEEKCESYIKKVGEEAILLRIKAPAQMGKTSLINRLLHHE
YIKIIVEPRCQLYREYFTTFI
>KRH97724.1
MSIVPRIFYYIYLNLLLGDKKMATSFYKVGGTLPINSSSYVIRTADTELFNLLKQGEYCY
VFNSRQMGKSSIKDQTQYKLKKEGFFCIDIDLQGIGTQVTQQEWYVSFIYNLLNAIALEE
KIEIDLGSWIEPRSYLSPSQLLNNFVSHELLTSVQKKIVIFIDEIDCSFGLSFRDNFFAL
IRFFYNQRNQNINYQRLTFCLLGVATPSDLIKNPVESPFNIGKAVELNGFEFHESLVLAE
GLKGLASNQNETLKQVLYWTGGQPFLTQTVCDLLVKLDIKVSEGKEKETIRQLVYSQLIN
NYHDYQHFDQIKTRLIANENRVGKLLEIYQYILKHGIFLSKDLGKEKEFESELRLSGLVV
RRNGGLEVFNPIYQEIFNQDWVQEQLDSIRPYFRVV
>KRH97725.1
MILGDFGSDGEIFFEIDLITADDLELPVTAMLDTGFTELLAVNSQDLEALGWRFLRERPL
KTAQGIKTFRIYLGKVRIDRREFEIPVYGGEEITEILLGSQWLKIMRLVADMSADVLSLE
IV
>KRH97726.1
MTETKSHKVVRRGRLFPEIQWTEDRINLYRSELETHHQRCRVVFEKLQPDLIKNHYNWFM
AVDSESGDYFLDKDEEVVTQMFLQKHPTAIPFIFVINETGVAGRI
>KRH97729.1
MRYGEGKLSCTVLKTNGIGDNLVEFNTKPNKPTGYKRDIYKCDCIAFSTGSREAFPCHLY
YILLNKTTDTFKNQHKTKNKC
>KRH97783.1
MIKKSCKKYHLTLVLTGHIHEDERIAAPKFLRQAERKLKSAQRRVSRRKRGSNRRKKAIK
KLGILHKKVVDTRKDFQFKTANNLLKKYDVVAVEKLNIKGLAKTRLAKSINDGGTVHHHT
FKQSRECWLEEESLRSPLSPVRECVEYVTIEPSSSGFCISVVGSMSQMYTICISSQNY
>KRH97812.1
MLTQDVRNALDVADLNKLKYDLNSLQPVDVGEYISELPEQQRAIAFRLLNKNQAIDVFEY
LPTDVQEELINSLHDVQVVHLVEEMSPDERAYLFDELPAGVVKRLLQQLSPEQRQATATI

LGYPEGTAGRVMTTEYVRLRQGLTVGEALSKIRLQDEDKETIYYAYVTDDNRKLVSVVSL
RQLLFTFPEVFIRDIASSQVVKVTTETSQEEVARIMQRYDLIAIPVVDREDRLVGIITID
DVVDILQEEATEDIQKLAAVSGDEEALSPPHLTIRKRLPWLLGIMALYIGAASAIAPFQR
VIAAVPVLAVIMPIFSNTGGTVGIQALTVTIRGLGVGEVTTKDAGKILRKELIAGLGTAL
ALGSTMILLSLIWAKPDEKWVALIAGVVMATNTMVAVSLGTLLPMGLQRLKLDPALMSGP
LVTTMLDTIGFLTFLSMISLALKVFNLSY
>KRH97817.1
MARLGYTPDHYTMIILLRIQLDWDSLCSHSKFLKIFVLLLLTMAQTGGNMDSMNPYYQYR
AVRDVKR
>KRH97824.1
MPDYSLRRGSILDKALLVKFIQRTYQEIFPSRDFSHLSRTVENYLSNDTPLWWVYKNQQP
SPIACLWAGNAIDQVTGSRHTHIFLLYVEPTHRRQGIAKTLMQHVENWAKQKGDPQIGLQ
VFTTNTPALELYKQLGYQTQSLWMIKSLEG
>KRH97888.1
MSIVTKSIVSADAEARYLSPGELDRIKSFVSGGVARLRIAQVLTDNRERIVKQAGDQLFQ
KRPDVVSPGGNAYGQEMTATCLRDLDYYLRLVTYGIVSGDVTPIEEIGIVGVREMYKSLG
TPIDAVAGGVAAMKSVAATLLSAEDSAEAGGYFDYVVGAMS
>KRH97889.1
MSVKASGGGSVARPQLYQTLPVATISQAEQQDRFLGRGELTELESYFASGAKRLEIAQVL
TENSETIVSRAANRIFVGGSPMAFLEKPQEPEMALVGGSTMNVQEGMKLGTITYVESRGG
FFESLRSIFNTSAGGPTPPGFRPINIARYGPSNMSKSLRDLSWFLRYATYAIVAGDPNII
SVNTRGLREIIENACSGEATIVALQEIKVASLSFFRKDPVATEIVTQYMDVLLTEFQAPT
PSTKVRQRPSADQQGLQLPQIYFNAAERRPKYVMKPGLSATEKTEVVKAAYRQIFERDIT
RAYSLSISDLESKVKNGSISMKEFVRRLAKSPLYQKQFYQPFINSRVIELAFRHILGRGP
SSREEVQKYFAIISNGGLPALVDTLVDSREYSDYFGEETVPYIRGLGQEAQECRNWGPQQ
DLFKYSAPFRKVPQFITTFAAYEQPLPDQHPYGSGNDPLEIQFGAIFPKETRNPSSSPAP
FGKDTRRILIHQGPGINNQLSNPKARGLAPSSLGAKVFKLDQLPGTIGKKAAKGASVKFS
ESSTQAVIKACYLQVFGRDVYEGQRLKVAEIKLENGEITVREFIRILAKSDLFRKMYWTS
LYVCKAIEYIHRRLLGRPTYGREENNKYFDIASKKGFYAVVDAILDTVEYSESFGEDTVP
YERYLTPAGLSLRQLRVGTIREDINKVEKEETPRFVELGAVKELRTQPSVDFRINQGVSK
QREQTKVFKLIATDKLAVQTLIGAAYRQVFERDIPPYIIQNEFTDLESKLGNGEITVREF
IQSLGGSGLYIKEFYTPYPNTKVIELGTKHFLGRAPLDQAEIRKYNQILATQGIKAFINA
MVNTAEYAESFGEDTVPYRRFPTLPAANFPNTEKLYNQLTKQNDDLVVPSFETLQPRIKS
ENTPLLGNAIADLAIKAKQMDKSKPLFIELGRSFNDGRGQSVEVGVGTSRRKPARIYRMT
VGTNKPEINQVINAIYVQVMDVFSGQIPDYIRRSDLDSRLRNGEITVREFVIELASSEIY
RKRFYTPYPNTKVIEFLFRHILGRAPATQGEIRQYNKILADSGLRTAVETIVNTGEYSRY
FGEDVVPYNRFPSLPAGNYLGSVKAEADLVKQSWSSLSPSVLTGRGTNR
>KRH97900.1
MTLPDSFRTQVQHIQESSKFQSVDGVLRATPFPGYTLITPPASEDPQNADFYKQIETFGQ
LLLELPIASDLIVPLPVSSFHVTLADLIWDHAFIHACEKKPDFEEELNSYLGDLFGQYQK
LRSSSQGPISWQVLGVIVMPRAIALGLIPKDERSYEEIIQLRRLIYQNRKLMGLGIEQHY
HFTAHITLGYFSEIPANLDRVNLSNLLSELNQYWLLNFSEIVVSQAEVRKFDDMTHYYRQ
PNWSSFRF
>KRH97916.1
MVNYDLPWNPNRLEQRFGRIHRIGQTEVCHLWNLVAHETREGDVYRTLLEKLEAEQKALG
GQVFDVLGKAIAGRETTGTSVSITTMHRALEKLGLRHKKKSKCQ
>KRH97918.1
METIYSSSRPSIPIFHSALIMIKATLVTTLITFALGFGTAKAQTYTKQQLQEIYSSHLVN
EGFRPKVDNDGDISFRSEGRTFWILLDEDDPTFFRMFMGFIQEDKSPQMRLRRLEGCNIA
NHKITANFR
>KRH97920.1
MAISNRERVGRALDSLKEGLYPFVDREMKQTYGKQWTTMAINCLPESYTTRKTADTIFQE

DVSALLIVMWEYWNDVFKKTLGRSDRSLVSELRETRNSWAHNSSFSLDDTYRAFDSITRL
LNAVSGDTEEVEKQKQEILRTRYEEQAKRETRRKAEAPTEGQPSSWLKPWREIATPHPDV
ASGRYQQAEFAADLWQVYQDEGSDEYRLPTEFFRRTYLTEGLKKLLSNALIRLTGKGGDP
VIELQTNFGGGKTHAILALYHIFFGLKRQELPGLDSVFEETSIKELPQNVNTVVIVGNKI
SPGTIYKKSDGTQIRTLWGEIAWQLGGKEGYEMVRDSDQTATNPGDTLKHLFNRYAPCMI
LIDEWVAYARQLHEQPDLPGGSFDTHFTFAQTLSESAKNADRTLLVVSIPSSSDNEIGGD
RGKQALDRLKNAMGRVESPWRPSSAEESFEIVRRRLFETTTNPDLFVERDRVIRAFYDMY
RQQKQEFPSECAEAKYQNRLKESYPIHPEIFDRLYSEWSTLDKFQRTRGVLRLMAKVIHS
LWEREDKGLLIMPGQIPMDDAQVQSELTRYLDDNWVPIIEKDVDGINSLPLEIDRQNTPI
GRYSACRRVARTIYLGSAPIQQAANIGLEEQRIKLGCVQPGEVVATFVDALRRLTDRATY
LYIDGNRYWISNQPNVTRTAQDRTNQFLEELYKVTEEIIRRLKSDKERGEFTAIHTAPDS
SSDIPDDPNLGVRLVVLSPELQHNKAKKNSSAIEWIKDVLNHRGTSPRYYKNTLVFIAAE
EDNIENLNKNVAQYLAWDSILNDKDTLNLNVSQTKQATAQKEQSEKYVKTILNQTYQWLI
SPEQPNPHEPIEINCERIPGESSPILRASRQLVNDGQLITEYSSNTLRMEALDKYLWRDT
NHIDLKQLWEYLAQYVYLPRLKNPEVLLEAVKNGVQKIDIQNHFGYAQGWEESKQKYKNL
VVLHNINPSISSENLIVKPEIATKQLKEEQVKELTSPSRSESTSKKPIQPTQEPTPTERT
RETKPQLKRFHGTVEIDPLRVNRDAPAIANEIIQHLTSLKDAKVRIVLEIEADIPDGVPD
DVVRTVTENCRTLKFNSQAFEQE
>KRH97930.1
MNPVDIGIIGGSGLYKMSALQDVEELDIKTPFGSPSDTIIIGTLAGARVAFLARHGRNHG
LLPSELPFRANIYAMKQLGVKYLLSASAVGSLRAEVKPLDMVIPDQFIDRTKNRVSTFFG
EGIVAHIAFGNPICQNLAAVLADAIASLNLPDVTLHREGTYLCMEGPAFSTKAESNMYRS
WGATVIGMTNLTEAKLAREAEIAYATLALVTDYDCWHPDHDHVTVDMVISNLQHNGINAQ
EVIIETVKRLSENSPPSEAHSALKYAILTNLADVPTETKQKLGLLLEKYLGV
>KRH97933.1
MAVVYTKCQHLGVKYLLSASAVGSLRAEVKPLDMVIPDQFIDRTKNRVSTFFGEGIVAHI
AFGNPICQNLAAVLADAIASLNLPDVTLHREGTYLCMEGPAFSTKIENGSDFC
>KRH97945.1
MTTTSISKKEIAAMTPKDVKDLAARLELDNYSNAFDGLNDWHLLRAIAFQRPELVEQYVY
LLDLEPYDEG
>KRH97953.1
MAIANFSRGQGIHSPYLAKLLPLIWRSGDLATRDRLPISQFTSYTLEQNGVAKMNRYTKL
ENLLKAQDFKEADLETNRVIVEVANREREGYLRE
>KRH98013.1
MSVIESLFDIARNIEISADFSIQHPQYQPFAIPSTVAQRFRHNSPDLQQKYLNLLLRNFL
HGIYYNGALQPILSTNNQQCGHLLTNGLEPNYKLGVDRNFYQQLHENNHGIGNYDAEWEV
LRTEPDGTIAVMKNNLTLYVEPECYLKSNRSPNPGDLIGIWMPKNRLQNGCYIAVGNCNQ
QHLSYNHNINYYSKKIVKIYFNITPSGAIALMNILTKRLNDASIPFSFEVLYNPISYRRY
DTATLHFNCQDYPAIRKILEPTYLETEIYFQPQIPLFTKYLAPGLSLAEEPNQKFSPEES
FGMNRCQIIANALLEAWQKGKNAIEERIQIIQQHFASQLIDLHYPYLNPSSQDVY
>KRH98025.1
MRFIRDLTFDTAKLLNRIYKESRHYQVRQRAHCILLSYKGVTIPELIEFFQVSRRTIYNW
MNDWEERRLLGLYNRKGRGRKAIFNEGQKQKIKEWVKLYPKDLKKVLNEIQEEWGITVSK
DTIKRVLRSMSMTWRRFQRGLAGEPDPLEYKEKELALTKLKEQEKCGEIDLRYLDESGFC
LTPYVPYGWQKKGENIPIKSGRSRRLNILGLMNRYQELDAYTFEGTITSEVVISCLDKFA
ENLPIKTFVVMDKASFHRSKKIQDKINEWQQKNLEIFWLPSYSPQLNLIEILWRFMKYEW
IEMDAYSSWQNLVNYVEKVIRDFGKEYVINFA
>KRH98026.1
MQIDHVHFYVENAHRWRDWFVNNLGFQQVPLWGTFFDLINHQKLRDTDTQVVRSLPGDKG
VEDEMWQ
>KRH98039.1
MTINDMDFKQLLKQQLPALIQEDVEVRDLLVRAISSYFAGSLETESHFDLVLGEIRRDRE

EQSRKWDENKRELELDRQEQARKWDENKRELELDRQEQARKWDEQTGKWNENRQEFDRVI
AAIDRMSRKHDIAISGLGSRWGLCSESSFRNGLKAILEESFGVEVLNIVEYDDQGIVFGR
PDQVELDLIIRNGELIICEIKSSVSKADLYTFYRKVDFYQQQHQRQATRKIVISPMVHPS
ANSIASKLGIEIYSSSEDVSAELPPSPSL
>KRH98054.1
MRSPKGSAAQSPIGIASRSPSKYPQNFEITDLSVDGKRFPSTFYQMGTEHEMSLDMNSAS
WSKVIAHRECFAIARRECFAIAHRGKTNPRKFAREMLPNQGARAESNPVSTRSQYLFLLT
KVKM
>KRH98058.1
MTSKKREQIRNYFTKRKPSPSEIMIEEMLTRFAIFFILGSFLIRFILVFTNSGAISIFLI
LLIFGVSLLKLKKFYFSKELIFTTRYADIPEEQTVDAWLIDDIEDLKERSIRRLNINKAE
LIRDSIVIRGPILWSTNGIPSQDLLWKKGKDQHIRFSINTITVIHLTDYGISSYQCDFNF
LKGVPLNERDDEFHYRDVVAVSTRDDSTNYRLPNNVLIRHAQLFKLSVSSGDSIQVVINS
SELLKFTGGTILDTGLDSAIRTLRKVLNQKKS
>KRH98059.1
MNLEQKDPCFERLLTYIRQNRGFDFTGYKRSTLVRRVTKRIQALNIDNFIDYMDYLEVDP
EEFHSLFNTILINVTDFFRDVSAWDHLGNQVIPNIIKSKKKNEQIRIWCAGCASGEEAYT
LAILMAENLGAEDFRQRVKIYATDIDEEALNQARQALFPVKSVESVPRKFRDKYFDLTGN
NYIFRQDLRRSVIFGRHNLFIDAPISRLDLLVCRNTLMYFNSEIQGRIMARFHFALNDTG
YLFLGKAEMLLMYSSLFMPVDLKNRVFTKLSSTNIRDRLLVMANSVDDESSRQLSQNIRL
RDLAFESAPVAEIVIDINGLLIIINEAARNLFGLSKMDLERQFYELELSYRPIELRSLIE
RAYNERRPITLNSIERYVPNSEQQYFDVLITPLQEDDLSLLGVSIAFNDVTRYVELQEAL
QRSRQDLETTNEELQSTNEELETTNEELQSTNEELETTNEELQSTNQELETMNEELQSAN
EELQTINHELSERTLELNRNNVFISCILKSLQKGIVVIDKNFSILNWNELVEDLWGLRYD
EVINKSLFSLDISLPVEELRSPILDILSGKTDFQEVRVESTNRRGKIIECYVGLTPLIDK
KIEGVVLIMTGSQG
>KRH98065.1
MVQERTLPKFDTATVQITREEGLGLYEDMVLGRYFEDKCAEMYYRGKMFGFVHLYNGQEA
VSSGIIRGAMRPGEDFVSSTYRDHVHALSAGVPAREVMAELFGKATGCSKGRGGSMHMFS
AEHRLLGGYAFVAEGIPVASGAAFQSKYRREVLGDERSDQVTACFFGDGAANNGQFFETL
NMAALWKLPILFVVENNKWAIGMAHERATSDPEIYKKASVFNMVGVEVDGMDVLAVRQVA
QEAVARARAGEGPTLIEALTYRFRGHSLADPDELRSKEEKEFWFSRDPIKKLGAYLVEHN
LAVESDLKQIEKKIQSLIEDAVRFAQESPEPDSSELYRFIFAEDE
>KRH98069.1
MINDAGVRVFISSLNTDINWATISTWLVIAVILSMVGGALGGMMIAGKDLGFKFAAIIGS
LFAPAGVIPTLILGLLLLNFLGNY
>KRH98085.1
MNSINNLIDTDSMCSSNLLNVPQLDKVWLKHRSNADQVSQYYIGTEFQDYAFRINECSQL
LNFHIVDLGTDQISTQFKLNTARFCRVRYCPVCQWRRSLMWKAKAYSVLPRIVADYPTSR
WLFITLTVRNCLITELRVTLDWMHQSFKRLTKLKDFPAFGWLKSTEVTRSKDNSAHPHFH
CLLMVKSSYFGKNYLKQSDWLELWRHSLRTNYSPVLHVQALKKDKCPSEIIPELLKYCTK
ESDLIIDKDWFLELTRQMQKARTISTGGVVKEYLKHLMIERENLISDDNDTSRFEDKEHL
YFKWNRISKKYNLFSE
>KRH98086.1
MLTIALLIAFIIKVKIWFIYIDFTLFVNPRFYFTVIKIKEEIKQIDFHAYSGRFNFTYSL
DK
>KRH98092.1
MKIISFLGFNNYIETNYIHPTTSQAVRTKFFQEALVEFYQPDTLYVLLTPTVATKIPRNG
TISNWQGLQEQLANKSVKLEPVFDIPESNSLDDSWLIFDKITNCLNEGDRVIFDLTHSFR
SIPVIALLAISYLRTVKQVQIEGVLYGAFDPNAQGADTPTYDLLPMLSLLDWLAAADRFV
KVGDGSPLAELLENEISENETRYDQTVRPLARKFKHTADIINRISLAIALVRPVEILEET
TKLEEIIKKAESSFDKRAKPFGLISQKLTQEYGQFALENPTNSGNLAQGLRLQFQLIDWY

IKRHQVVQAMTLAREWLVSVLAYRLGEQDPLNKDQRKQLENALNNGQSKLCGKEITLISP
WDDEFEKLPDFDLFSTIWNQLRDIRNDIAHVGMRKGAEKAKTLKEVSAKIVLKLKTIADT
LS
>KRH98095.1
MNGKNPHELLEAFLEFWRQHGEPLLKSAPYHEIAPHLVLMAFLHRVVNGGGTLEREYAIG
SGRMDICLRYGKVVMGIELKVRREKLDPLTKGLTQLDKYLDGLGLD
>KRH98108.1
MLCGRVYPHEKACFYFFAWIIRDAPQQRLAPLIARMRKMEDIDRIVAESDTIVELINEYR
TCVKSFSWLTVREVVIDRLEGSRRSIKGHHLEASVRTALITAFQNYFSIHGNYGQYKKIK
IADKQIKIGNHTIDVSAELTPTGNGLNETLLLPIKTRETEGGGHSHIFTRDIIAAVRELK
EDGNRYHMIAVIVALNWSVTELDNIDDQIDKVFHFNMNPNKFVGFDEASQIQLNRYIQGV
LDNG
>KRH98109.1
MDNLSRIINKVLCGDIRDVASSIPDNYIQAIVTSLPYFGHRNYSGNEASVREIGREGNLL
NYVKNVIDCFEALKPKLRNDGLLWLNIGDTYRNKELQGVPWRVAFALKDTGWILRSDIIW
KKPNAMPSSVKNRPTTDHEYVFLFSKSADYYYDADSIREPHVTFSENSKMRGGRNHLGKR
NGTPENGKNAGNQNLHDGRWDQAFHPMGRNKRTVWEIPLGKFRDAHFAVFPEALVETCVL
ASTKKGDVVFDPFTGSGTTGVVALRNDRKFIGCDLVKEYQEMAQKRIDEIVAQPSLFQ
>KRH98110.1
MEIIRTQQTIARTLENIGNLYKDEFNEAILNFEDKFINDSHQALSMRRITDESTIEMQRL
LTRKGFSLIRQGNNHEIWKHPNLKKVTSLPRHKKLSTFVIASIQKDIEDAIQ
>KRH98117.1
MITTENLPSIEKQIWTWRNYKIQYTVMGVGQPLVLVHGFGASIGHWRKNIPILAKAGYQV
FALDLLGFGGSEKADIKYSMEVWVELLRDFYHEHIQSPTIFIGNSIGALLSLMVVTQHPE
IASGAVLINSAGGLNHRPRELNPITRFFMATFRQLVTNPITGTIVFNRVRTKSQIRRTLY
QVYCDRNAVTDELVDILYEPSCDRGARKVFASIVTAPPGPAPITLLPKLTHPLLVLWGEK
DPWIPITGTNIYAEAASGKDIEIVPIPNAGHCPHDEVPDLVNRVIIDWLGKKNL
>KRH98134.1
MLILELVRVQRCGKSAPAVSRGTGSVNPGWEQGQRNYGWSFTSFGKLEPLEVVGNNYPRQ
ITTLAREQNPAYVLLSLF
>KRH98141.1
MTALTTPQVVAPKEISNYQVKPGDTLEEIAGSYGTSVTELIQANGLSDPHDLRVNQKLII
PQVKSGTGIQELPTPTSPVVIPPKRIAQVAISPLVPPDREVNLPNAAELPLPTSASNEGV
AIAVPQIPSPSSGSAISVPVAGKNLPQVPEAGSITPNTRGIGGSTDGFTTSVAITPKTVP
TQLEKVTPNNDRIRSLQAEIERLRQKYRDQRSAITLANSGKVENTPSPLGGGNIDPVKVS
PGVQIKAVTITVPRLGENGNVSVNSQLGVDAIAPNDEPVNPQFVPGNTRRNPSGIRLNVP
PRRMNARDSLGKLRGTTVSPALPPLAAVDIYLPRNTEEENNSPALTGHIWPAKGVLTSGY
GWRWGRMHRGIDIANGVGTPIYASAPGRVERAGWNNGGYGLLVEIRHEDGSMTRYGHNSR
ILVRVGQEVQQGETIAAMGSTGFSTGPHTHFEIHPTGKGAVNPIAFLPSQARL
>KRH98147.1
IFAVKPLTFSCAGTVNSSFVKMSDFIKDYKPAGIQEIEISVTEPMDYRKLFTAIPLVAKL
PMYINHIATISLEEQFLRLEYQGPEKGFKVFQGVLNSFLNNPQVKADLLLKLEFKFLSPI
MVEGGEIRDLKKALERNPVDNLNLVAKVTY
>KRH98171.1
MQNTRLNNLLDTIATNVAQWFMNPWRRLCLLLISWLFGFFLGSVVSTTAGQQAQLDIWAA
AVLVLLTEVASRLFYSRGFFSQRAIWVESLNWLKIGLIYSLFLEAFKLGS
>KRH98177.1
MKGQQLFHSFLPGVTAAVLTTQPAWAGTFKANDLKLVSSPVVSTATNPKVSVVENNWQLA
ATTVDHAPLFDYQLDFGQAVLPELPSSSPLPVVNGANVPSPTKLKTVLSLSPVNPKVISG
KTYNQVAQITLPQDNPSGIAQTNSQSVNPTSDSPQTILERFKPNPNFLDVPQDSQGVKVQ
TTEAISLEQALELAKQNNNDLQVAVLQLQRSQSSLREAQAALLPSLNVLGGVTRSRSSSA
TLQERQTAKRLNIPSENPDATSVFDSQAELRYDLYTSGRRTAAIKEAEEQVRLQQFEVER

QSEEIRLNVATEYYGLQQSDESVRIARSAVENSQASLRDAQALERAGVGTKFDVLRSQVN
LANSQQELTDALSQQAIARRRLSLRLNLPQSVSITASDPVQLAGLWKSSLEDSIVLAYEN
RAELQQKLAERNIREQQRKQALATLGPQISFIARYDLLDQFNDGVAINDGYSVGLRASMN
LYDGGASRARAARAKTEIAIAETEFAERRNQVRFQVEEAYSSHLANLENVQTAATALDQA
KESLRLARLRFQAGVGTQTDVINAQSELTRSEANRVRAILNYNLALTRLQRYVTSRAVQK
S
>KRH98225.1
MIDLLTAAKNGNVQQVRQLLASGFPVDTGDRHGTTALMFAANFGYTEIVRCLLDFGADID
LPRKLHGLTALMLAAAHNQVDVVKLLTSQGANTNAVNEDGSTALMIAVEKGYIETVQNLL
DFGADPKIVDQHNEDAFKLAIRQNNRVILNILVKNSQTKGETESLLIMGADNGNLEIVKT
SLLYGVNPNLENSDGTTALLAAAAGGHTEIIQVLLDRGAEINHQDQEGETAMHFSVVENH
LETVQTLVNRGANLEIRNNLGDTPLILAAFQGYQEIVRVLLDAGADGGKKNLGEFPLTLA
AFQGHTETVKVLLESGVNIDVIAEDGKSALVKAIIGNHPEIFQLLLTKGANVNLQDPVGV
TPLMYATAQGYTQAVDMLIQAGANVNIKNQGGYTALMIAKSNNYTKTSNLLIQAGAKE
>KRH98226.1
MSLELTPDVKFGLEFFHPAIMWILLALSLYAAYLGLQVQRTRNAQGEEKKQLIKGKYSDK
HHKIGSVLLALMVGGSIGGMAVTYINNGKLFVGPHLLAGLGMTGLIAFSAALAPFMQKGA
NWARATHILLNFGILGLFIWQAVSGVEIVLKIIGQA
>KRH98232.1
MAIETHLTLLKAGAVTWLDWRARNPELQVDLSTSNLRGENFRGANFQNVNLNQVDFSHAL
LVRADFQDANLSAANLNSAKLVQANLRKANLSVANLQNANLMRANLDGAVLIGADLKNSN
LQDAVVTSANLIGTDFYYANLNGVDFAYSKLIRSNLSFANLIGANLIGSNLQDCNLYEAE
IINSYLYNTNLSRANLSRSHLGSSYLCRANFMEANLTSADLTGANLKDANLAGANLQGAN
LRCANLTGANLTGANLQNAILPPVFICN
>KRH98247.1
PLSPGKMSNIVPAGISITEKVVAESGVVNAVSGIIFRNRLYDTIFEVIVFTIAILGCNFL
LASENPSCTIYQFKDRASITLARLGATIAALVGIELAIRGHLSPGGGFAAGVAGGTAIGL
VAITSSYQWMQDIYQRYHAAIWEKISVLIFIVLSVVTLSGFELPHGELGKLFSGGILPIL
NIIVAVKVALGSWAAVLIFIRHRGLL
>KRH98250.1
MTDGERQELLKKLKLDYGRILLNYFSVDQNLKTTIDQFISTLFCANIPVPQVIEIHMELI
DEFSKQLKLEGRSDETLLDYRLTLIDVLANLCEVYRCSTSRIT
>KRH98257.1
MEQLIVEDPVIKNLILGSMSQYYIGRQDADSKFDQTLAQLQSYQEEQNRKWEEQNRHNRE
IMAQLQSYQEEQNRKWEEQNRHNREIMAQLQSYQEEQNRKWEEQNRHNLGILEEIKQMNR
KHESTVGSLGSRWGLSSEASFRNGLKGILKDSFGVEVLNFLDFDNEGEVFGRPDQVEIDV
IIKNGLVILCEIKSSIDKAGMYIFDRKVAFYEKHHQRRVDRKLVISPMVDPRALSVAQNL
GIEIYSYAEDVNRI
>KRH98260.1
MTASVRERYQSVGEVIKELNFAESNQSETKSDVNPVNYWQTLVSRAVEKGKQSVQQWFSI
NDSQVAEILATVTSQLPTTEALLLGKPQTGKSSIVRGFTGVSPEIIGQGFRPHTQNTERY
IYPNNDLPLIIFTDTVGLGDTDKDTEVIIQEIIKDLNTGTKRARVFILTVKINDFATDTL
RNIAQKLRQQYTHIPCLLAVTCLHEIYPPDMKNHPDYPPNFAEINRAFDEIKANFSGLYD
RATLVDFTLEEDGYSPVFYGLEAFRDSLTSLLPEAEAKTIYQLLDEQAGDKLGNIYRDTA
RRYILPFSIMATTLAAVPLPFTTMPVLTALQVSMVGLLGKLYGQTLTPSQAGGIVSTIAG
GFLAQAIGRELIKFIPGFGTVIAASWAGAYTWSLGEAACVYFGDLMGGKKPDLQTIQNVM
EQSFQSKQNTQKEE
>KRH98261.1
MKKFLTLALILTLFLVSSFSLGTSPSYAYSRSDLERLLETRKCPECELRNADLSDADLKG
AKLKDADLSYAYLTGANLRGADLKGADLSDADLKGADLKGADLSDADLKGAKLKGADLTN
ADLSRAEVAGSSFKNAKLDRKWQFLQLYDR
>KRH98317.1

MNFTIKNALSPVKEGYTTLDIQVMGGIITQVGQNLNVIGTLIDGQDKLVLPGFFNAHTHS
VEKWQRGIIPPLPLELWLAHLCDFSPLDMERVYLSALGTGVETLLSGGTSVVDHLVLIPG
QELETIATVYQPSSGMRIFYII
>KRH98327.1
MNPVLQFGNVSGQESYHTLTAPEVLKLLNKYQLIPPLLKEVIIEQAIAQISSTPEEEQLA
YEKLNQQYQGQKEQGISQEQLQSMATRQLKLEKFKEVTWGKEIDSYFYQRKPQLDKVIYS
LITTADIGIAQEIYFRIQEGEQSFNQLAQEYSQGPEAQTGGLVGPVELQSLHPLLVRILS
TSQPQQLSLPTPIGDWIVIVRLEKLLPAQLDNGMRQRLLNERFQSWLQAQVSPQNWQIKE
SEN
>KRH98352.1
MIRRRSTPWIHYWSRPLLGAIATLGILNTGYLTYEKLTGGTPICTAGEQVKGCVDVLSSP
WGTVFNQPLALFGLLAYTSMLLLAVFPIVLISKDNDSKKSSQNKSTRQLENLSWWLLWIG
AIAMTVFSGYLMYVLAFKLQAVCWYCIASAVFALSMLTLTVLGREWEDIGQVLFIGLIVV
VVTLIGTLGIYSGIDTSKNENIDTIASSEQRINFSPKEEPNPNFGWKITTKSGESEIALA
QHLVKIGAKEYVAYWCPHCHEQKLLFGKEAYEIINDNNVTVECAADSPKGKPELCRAAKI
QGFPSWIINGKIYSGVQNLSDLARLSGYTGPQNFKYFR
>KRH98375.1
MTHIDKSCDILIVGGGIVGLATAFRIFQSRPDLRLVLLEKESTLAKHQTGNNSGVIHSGL
YLSW
>KRH98376.1
MTRCVIEICNVKPSYQSFNTPISIIRPFNTYGPRQSAQLVGWEPLYGGREGFKQGLAETA
EWFMNPTNLAGYKSDRYNI
>KRH98377.1
MPLHEPEFMGNEWELVKNCLDSTFVSSVGKYVDRFEVMLAEYTGAKYAVAVVNGTAALHI
ALLLAGVKPVL
>KRH98380.1
MWWITESKNLSVQATEAINKADIIGIAAISCWELAMLVAKNRIGLSMDVQIWINLALQHP
KIQLLALTPEIAVLSTRLPGNFHGDPADRLIVASSLVHQKFP
>KRH98381.1
MVTLIEKLTKFLQFTMHNNALKISICIPTYNAEKFIRTTISSCLEQTQAPYEILLSDDGS
SDRCWQSS
>KRH98382.1
MATTADEVWKLLGELIESQKETERKFQETDRKFQETDRLLREQSQETERFLREQSQETDR
KFQETDRLLREQSQETERLLREQSQETDRKFQETDRLLREESKRVNNQIGQLGNRLGEFV
ESQVRPAAVKLFQERGIAVKEIASNTYIQTGKEGLEIDLLVINSSDIILIEAKSKLSEDD
VNEHLERLSKFKRFFPRYESYRVLGAVAGMVIPLDVSRYAYRKGLFVIGQSGDNLVILND
DKFRPRGW
>KRH98386.1
MYQSDVYSKPSYEPLPPQETLPTMYELPSEYPEEPGLPDEFHLLQPELLRSTFCPHSYPK
DNVFIGSELNLYYDSKHTQWYKRP
>KRH98387.1
MAYSDFTLERITKIFGINIEERTNVFTAFDQLRVDDFFIKYLQNNIPLAQAIGTEKAKSE
MIIAPVLIEVRRLLNNKISLFSGIDFNVNIEQGLNGFCDFLIGLSSQQLYVTAPVIALVE
AKNDNLKQGFAQCIAEMIAAAQLNQSEGNNVENIYGCVTNGNQWVFLQLTGNLVVVDLDE
YYINQPEKIISVFVSLIKSEKDF
>KRH98388.1
MPYSQFTIPKVVEDFGLTLIESGAFLNATQTVTLSPYLEEFITKNLQLAIALNTEKARSE
LIICPVLLAIKETLPSISFFSGEEFNVDADLGLNGVCDYILSQSAEQLYVTAPVTMVVEA
KKENLKGGLGQCIAEMVAAWKFNTERNSTISCIYGVVTTGTVWRFLKLQEQTVTIDLNEY
PLPPINSILAKLTQMMFPQSMDTVR
>KRH98405.1
MEEKLMTIKQVCTALNVSIQTLRLWDESGKLSAIRTVGNHRRYKQSDIYKLIGIDKPSEE

VEESVALYSRVSSGEQKTKGDLDRQNTRLTEYAAKKKYKVNYIFTEVGSGMNDSRAELHQ
LMKLAIERRITKVVIEHKDRLIRFNFNILKMFFESHNVEVEYVEEVLPKSYEAELIEDML
SLMASFSAKTYGERSAERRRNK
>KRH98432.1
MAEKSKKDIQSTLSPVEYSQLLLEVKERVRTAQYAALKAVNTQLVGLYWDIGKMIVERQK
DSGWGKSVVERLSADLQEKFPGIRGFSVQNLWYMRQYYLEFSGDEKLQPLVGEIAWELEK
AIIPRIKELKGQLPSSEAISNLLEGIYD
>KRH98434.1
MNTREVVVNKLQQLPDPLISRVDEFIDFLIAQKQNQVVNVQSSESLTDRWKRWFEKVDQL
PILNHEPQNEYQQRLLQKYRQQSLDL

-Protein sequences of the Cylindrospermopsin-producing group which were not included in the Veen diagram.

>OBU74745.1
MATTADEVWKLLGELIESQKETERKFQETERFLREQSQETDRKFQETERFLREQSQETDR
KFQETERLLREQSQETDRLLREQSQETDRKFQETDRKFQETDRLLREESKRVNNQIGQLG
NRLGEFVESQVRPAAVKLFQERGIAVKEIASNTYIQTGKEGLEIDLLVINSSDIILIEAK
SKVSEDDVNEHLERLSKFKRFFPRYESYRVLGAVAGMVIPLDVSRYAYRKGLFVIGQSGD
NLVILNDDKFRPRGW
>OBU74749.1
MATTADEVWKLLGELIESQKETERKFQETERFLREQSQETDRKFQETERLLREQSQETER
FLREQSQETDRKFQETERLLREQSQETDRKFQETDRKFQETDRLLREESKRVNNQIGKLG
NRLGEFVESQVRPAAVKLFQERGIAVKEIASNTYIQTGKEGLEIDLLVINSSDIILIEAK
SKVSEDDVNEHLERLSKFKRFFPRYESYRVLGAVAGMVIPLDVSRYAYRKGLFVIGQSGD
NLVILNDDKFRPRGW
>OBU74752.1
MATTADEVWKLLGELIESQKETERKFQETERFLREQSQETDRKFQETERLLREQSQETDR
LLREQSQETDRKFQETDRKFQETDRLLREESKRVNNQIGQLGNRLGEFVESQVRPAAVKL
FQERGIAVKEIASNTYIQTGKEGLEIDLLVINSSDIILIEAKSKVSEDDVNEHLERLSKF
KRFFPRYESYRVLGAVAGMVIPLDVSRYAYRKGLFVIGQSGDNLVILNDDKFRPRGW
>OBU74755.1
MATTADEVWKLLGELIESQKETERKFQETERFLREQSQETDRKFQETDRKFQETDRKFQE
TERLLREQSQETDRKFQETDRLLREESKRVNNQIGQLGNRLGEFVESQVRPAAVKLFQER
GIAVKEIASNTYIQTGKEGLEIDLLVINSSDIILIEAKSKVSEDDVNEHLERLSKFKRFF
PRYESYRVLGAVAGMVIPLDVSRYAYRKGLFVIGQSGDNLVILNDDKFRPRGW
>OBU74760.1
MKKRQQPKAAELAKTLQRYFSEQEQEQILQVLASTERHERFNTLLWVNGFQDIIEQRKQL
IERITAEQQGKSIGKA
>OBU74761.1
MTITFRTAKKQGISIRIDGTPVTPRTKDKRLFDLVNNQLNEFNKTLTLVIFPNKSFNTVQ
YELMLNGFEESLSGTETKSPKEDLTEMRRQITLEIEYTFMKKENDSLKRENNLLRSKINE
VEILVHNAQSKRRFCN
>OBU74762.1
MKIELIQQLINQKMRLRNYAGFSVLFREFRFKANEALTFKAPGQFWFLEDNIPPQFLITS
EMGIYDVLDNRINELQHEHSGTVKMINRSSGVLLLRVVILIPVKRKGHATTRK
>OBU74763.1
MKTRFFVHTEQIDFNGQIRCFVIDFPPNTAKIISLVATAEPLAKDYEIRHLEVGLLTLYN
AAGVWYESQIRFSGQLTWFPKIPPYSYLDHIRQMPVSGAKLTGQTLDIPALEPYLNARFE
TSPSLSGFRLKLTFTYLENEN
>OBU74772.1

MKINKLHLSITSIAIFALINILWIPQYTIASKKNISQRSNSSRMANKATYLTTPFNNSTK
IPPNFLGHDLEAISNALIKRQESLKKDEFETTQAFNQRVIKEGLKPILGTINVDSTLAFV
LSDKFESSYNADLSLFRIEIPNSDYTEFTWKSKTFNYRTYKASNAFGATIDVSSWDSESF
SIKPDRRSNEQTITLSISSNLAKKLKENFGIIFISKIEGYSPVKRQIGRSNATFDNPYSI
SYSDTSWFLRRFYGEKVN
>OBU74774.1
MSIKQAQEPTSLNVKILEFKIFDKTTGKVYYSGTLYPPINYTKLETLLKAQMFKEADEET
DRVMLAVANRQSEGWLREEDAEKFPCKELRTIDNLWLKYSQGKFGISVQQEIYKNLGGTK
QFDWNVWRSFGDRVGWRKDDSWLIYSDLNFSLSAPTGHLPVGMGVSGDGIRDSSLLSRHV
ECNP
>OBU74793.1
MKRFLTLALILTLFLVSSFSFSTSPSYAYSQSDLEKLLETSICIKCDLSHANLERADLKY
AYLNGANLSGANLKYADLSGANLEDTNLEGADLEGANLEGADLEGANLKDADLLGANLKD
ANLEHADLKGAYIRSANIEDTHTLDANLTNTQL
>OBU74814.1
MLRGGHLGTFPRILLKGKKGFCQKYRPQPKKVEKNRWGSKLLAGMKPNQKKKGTSPGQLK
LFWDELDITNEEIVKAATIFVKANCYTPNHAKTRN
>OBU74819.1
MELQGIEHQSSESAIAPIGGKLPYSLEITSGKVLDVLLALFPGKATRKQLNGFLYSRTRP
GFYLELNDALHLAYADQRDGHGTAHWWRLFFGDRLRSQIEELAKSNALVVAKTPSNQANS
GVRADYEWGGMYFRSKVEMKVAQELDKRGVTFFANVRGRYSLEGSPVSKDLLNGRVELDF
LVFHRGKCAILQVDGPQHKGQPERDYAGDRMMLREGISTVRFTAKECHEKTADVVTEFLG
VLGV
>OBU74822.1
MLGFKLLSIRKLKLADGESLRIYKVVSSWENGDGEYTLTLAELTDDLFRYWEQDSEIMAL
SHFLGSKVTKEEICYWEQQITDPRIVEYLRLKFS
>OBU74824.1
MTIVHNFSYSCQPTSQKFSQAKDNIDDRCERTDNLDKTELKEQTFSFLTRLGFTVGKELW
IKTSKRQVFRAVVGKGKLEVFPQRKVSKVEDPKGGSVWRDVGRSYGWEFLYQLSQETSLF
FLPNHPQGGIGKGHCTNFSNLFFEVDDLPLDQQFQNIENLKSLGLLPSAIVFSGGKSFHT
FLSLTEDPGPDLWVILQKKLICLTQSDPAIKNLNREMRLPGFFRPDKGKYQSLVSTGDRR
YSIKEIEDILQPFFPHGLSDERWNDWRLAEKAEKNGILSMPEESLPTVVRKMEKQKQLAA
RKQLVCEEENNLIELVNKTAEQATIEDFEKLHPLKVKWFGKKAKSDCPFHQSQSGTAGWF
GNIGGKLGWACPIDTDNRLLDHFRFFSKLRYGKENPTGKEWVEIAKAYLTEMGIPFSDWN
RFKNGKLAKFGEEIRAKDLEDNRVEEAQDLLVLLKKVGRSLCKTVKGFGKEKYVVSNPSH
TVTRKKINVKEIKKEILDAYRSGYKNIIVKTPPGSGKTHTAGTFMPDELSVNKILYVTNG
VQNPTVSSLVDWSPATARHGGLKWDTSQKTADGRYYLRRAKKGEKPDIEPNCIANEVFEG
LRQQEVNVDNSSVICDGCPVAGTCKSFQGFLIDRKTALEQDRVRIHPESIDPTIFDSDLQ
IGAIWDENIIKFKKDVTFTETDLNATLGFLARNIEVLVKFLPLLNGIREFFKEKSSPYNG
YEREEILARLPEVTREHLDLATNLVPDLSFLENFFNGVHGNRSANLSLRKESIKETAETI
NNMGKQWLLPFVRAITEKGFLYYKKGVFTISVAEEKHTKIMEAAKVNIILDATADPEYIV
TALKMEKEKTIVLTSEVETPNLKIKILTGMGNLRPQSKERTSSKQKRVDAIREKFLTLFP
DGVVFEYKSVAREGDRVHFRDSRANNTDQEKKACLLLGVPIPNLADARAAWEIGLGGGKG
FDDYLGRLTVAEICQTIGRLRASRRPKEEITVYIAGDTKFDLSKALKENFNGAEITTQDV
GEICPEAGDASHRILALVSKGVRQLIEEGKKVTQQNLAKVAGIGQSTIAESIPWGKFKKI
SEILLGRFNTESDKKEPELLPDESWFIDQYLPILIEEEVGPQEVIETLERVAQTPERIGE
MLSRLPKYLKQGMLWLLYKDLEVPWQFSTS
>OBU74827.1
MLNILTSLLVAKKSIKPIVKKQYSAYGDFRYFTYSDILEAVEPVLLENGLLITFTSTGTT
LTATLWHVASGEMLESAYDLTVFMETSHKKMNKAQLVGSATTYAKKIALCGLLNLDTQDA
DPDSLGDETPTVNTSTTTHKKVPAGQPLVTPPRKPQKLPAVGQEQKATKTEVTVSTETPV
TTLQEPVPDAPPKPMDVISKEQLATLSTTVQQLGIPKGKALAIARNVCGEHLYNASYIRQ

PFFNLVMTALTKVAT
>OBU74840.1
MNRKKLTQLITIAILVTFITIFHPFITIALTTKEIGAIAERVTVRLSGPDQGSGVIINKN
GNTYTVLTNSHVFQYTGAFEIITYDGRKYQSNNVTENT
>OBU74841.1
MLAVANRQSEGWLREEDAEKIPCKELRTIDNLWLKYSQGKFGISVPTRNIQKPGRNKTI
>OBU74850.1
MDEATSALDNSTEKEVMAAIEGLSHQLTVILIAHRLSTLEKCDRIFQLDQGRVYQEGDR
>OBU74851.1
MATTADEVWKLLGELIESQKETERKFQETERFLREQSQETDRKFQETERFLREQSQETDR
RFQETERFLREQSQETERLLREQSQETDRKFQETDRLLREESKRVNNQIGQLGNRLGEFV
ESQVRPAAVKLFQERGIAVKEIASNTYIQTGKEGLEIDLLVINSSDIILIEAKSKVSEDD
VNEHLERLSKFKRFFPRYESYRVLGAVAGMVIPLDVSRYAYRKGLFVIGQSGDNLVILND
DKFRPRGW
>OBU74853.1
MALLQPDFEPELQFAYSSFHLFKPDFDYLLPLRETKTVVHLPEIRCKEYGDRGKNTSNKV
YN
>OBU74857.1
MPRVAFFFGISIYMYMDDHGIPHCHAMYGDFAGSFSLEDGEPLAGEMPPAQAKKIKIFIL
NNQVELLEKWHELSD
>OBU74860.1
MVRQIPSKTQLRVIESNISQGVETPKIVYPESDNKPMADNTRQFTWIVKIKENLEILFKY
NTDVFVAGDLFWYPVEGSNKIKLAPDTMVVFGRPKGHRGSYRQWEENNIPPQVVFEILSP
GNNNTEMDRKKLFYLEHGVEEYYVYNPDKISLEVSIRENNSFKEIENFTTWTSPRLKITF
DMSQDELVIYYPDGSKFLSPVELSNYAEQETQRAEREKLLKEQETQRAEQERLIKEQETQ
RAERERLLKEQETQRAEQERFLKEQETQRAERERLLKEQETQRAEQERFLKEQETQRAER
ERLLKEQEQIKYQTLLSQLKAKGIDITALE
>OBU74861.1
MVRQIPSKTQLRVIESNISQGVETPKIVYPESDNKPMADNTRQFAWIVKIKENLEILFKS
NADVFVAGDLFWYPVEGSNKIKLAPDTMVVFGRPKGHRGSYRQWEENNIPPQVVFEILSP
GNNNTEMHRKKLFYLEHGVEEYYVYNPDKISLEVSIRENNSFKEIENFTTWTSPRLKITF
DMSQDELVIYYPDGSKFLSPVELSNYAEQERFLKEQETQRAERERLLKEQETQRAEQERL
IKEQETQRAERERLLKEQETQRAEQERFLKEQETQRAERERLLKEQETQRAEQERFLKEQ
ETQRAERERLLKEQEQIKYQTLLSQLKAKGIDITALE
>OBU74862.1
MDRKKLFYLEHGVEEYYVYDPDKISLEVSIRENNSFKEIENFTTWTSPRLKITFDMSQDE
LVIYYPDGSKFLSPVELSNYAEQERFLKEQETQRAERERLIKEQETQRAEREKLLKEQET
QRAERERLIKEQETQRAERERLLKEQETQRAERERLLKEQETQRAERERFLKEQETQRAE
RERFLKEQETQRAERERLLKEQEQIKYQTLLSQLKAKGIDITALE
>OBU74863.1
MVRQIPSKTQLRVIESNISQGVETPKIVYPESDNKPMADNTRQFTWIVKIKENLEILFKY
NADVFVAGDLFWYPVEGSNKIKLAPDTMVVFGRPKGHRGSYRQWEENNIPPRWCLKFYLL
VIIILRWTEKSSFI
>OBU74868.1
MQIRRRSPSPAIDVSIVRYQVVLKDASPNNILEEIVWSKEIEIEQRRDKVPLRELQKQVL
VAPPTRDFLAALRQSKTKPALIAEVKKASPSKGVLREDFNPLEIAQSYVAGGASCLSVLT
DGKFFQGSFENLSLIRSAVYLPLLCKEFIIYPYQMYLARVNGADAILLIAAILSDQDLQY
FIKIAKSLKMTALIEVHSLEELDRVLKLDGVNLIGINNRNLEDFSVNLQTTGELLKQRGS
QIQERNILVVSESGIHTPEDISIVEKAGASAILVGESLVKQPDPELAIKQLFGK
>OBU74875.1
MLGSISTSAEATSRIEDIARKTTVQINSNANPGGSGVIIKKEGKTYTVLTANHVVCENLG
TIKIRCRTDLTYTIRTHDGREYPVKKHQSMQVNVQDPDLAIVTFESEENYQIAPVGNSDS

VKIQSDVLVAGFPSIFNRVGKQRTFTITNGKVVTFIPDSDRGYGLIYNATTFIGNSGGPV
FDIDGRVIGIHGLADTDDGGDQQETGSPQQKTGFNAGIPINIFLSLSNLNLPSPPVPVTP
TNPTNPNNNATVYAIAYNDRGVNRYQSGDKQGAINDFTAAINVNPNFAKSYYNRAAIRNE
LGDKQGAISDFTQFLTFHPYNALAYYNRGILHHELGNKQGAINDFTQVIKLNPGNIRAYY
NRGASRSDLGDKQGAISDFTKVIEINPNFAEAYNNRGLARRDSGDKQGAITDFTQSINLN
PRDPIAYNNRGIARDELGDKPGAISDFTQAVTINPNFAQAYNNRGLARHNLGDKPGAITD
FTQAIKIIPNFAQAYNNRGLSRSDLGDKPGAISDFTQAIKIIPNFAQAYNNRGLALHSLG
DKQGAVSDFTQAIKINPNFAQAYNNRGLSRSDLGDKEGAISDFKNAAVLFNRNAR
>OBU74876.1
MTQNNLGKFAAPIKAIILHYFSDLGFVLPGLDKNWKSWSQITEATKEYIQNRQSLQVNLN
HR
>OBU74891.1
MEYQFSFRHFSQKFTHAVITNHGVWDMREGVMIRLVDRTGKVGWGEISPIAWFGSETLEQ
AREFCSQLPKIITLEMILGIPAHLPACQFAFESARENFSNSMPVFKNQHLGDESLISKKM
KYSALLPRGEAAVQGWKNLWQKGYETFKLKIAVDDITQELEILHLLVGQLPESAKIRLDA
NGGLNYQQAKLWLEICDQFSEKIEFIEQPLGMDRLEEMLELSQVYLTEIALDESVATWQK
LESCYQMGWRGVFVVKPAILGSPSRLREFCQNHTIDLVFSSVFETPIGREAALKLAGELS
YGISNLSGNCRSLGFGIDHYFALQSTNWPEVLWNPIY
>OBU74911.1
MSVKASGGGSVARPQLYQTLPVATISQAEQQDRFLGRGELTELESYFASGAKRLEIAQVL
TENSETIVSRAANRIFVGGSPMAFLEKPQEPEMALVGAGTMNVQEGMKLGTITYVESRGG
FFESLRSIFNTSAGGPTPPGFRPINIARYGPSNMSKSLRDLSWFLRYATYAIVAGDPNII
SVNTRGLREIIENACSGEATIVALQEIKVASLSFFRKDPVATEIVTQYMDVLLTEFQAPT
PSTKVRQRPSADQQGLQLPQIYFNAAERRPKYVMKPSLSATEKTEVVKAAYRQIFERDIT
RAYSLSISDLESKVKNGSISMKEFVRRLAKSPLYQKQFYQPFINSRVIELAFRHILGRGP
SSREEVQKYFAIISNGGLPALVDTLVDSREYSDYFGEETVPYIRGLGQEAQECRNWGPQQ
DLFKYSAPFRKVPQFITTFAAYEQPLPDQHPYGSGNDPLEIQFGAIFPKETRNPSSSPAP
FGKDTRRILIHQGPGINNQLSNPKARGLAPSSLGAKVFKLDQLPGTIGKKAAKGASVKFS
ESSTQAVIKACYLQVFGRDVYEGQRLKVAEIKLENGEITVREFIRILAKSDLFRKMYWTS
LYVCKAIEYIHRRLLGRPTYGREENNKYFDIASKKGFYAVVDAILDTVEYSESFGEDTVP
YERYLTPAGLSLRQLRVGTIREDINKVEKEETPRFVELGAVKELRTQPSVDFRINQGVSK
QREQTKVFTLFATDKVAVQTLIGAAYRQVFERDIPPYIIQNEFTDLESKLGNGEITVREF
IQSLGGSGLYIKEFYTPYPNTKVIELGTKHFLGRAPLDQAEIRKYNQILATQGIKAFINA
MVNTAEYAESFGEDTVPYRRFPTLPAANFPNTEKLYNQLTKQNDDLVVPSFETLQPRIKS
ENTPLLGNAIADLAIKAKQMDKSKPLFIELGRSFNDGRGQSVEVGVGTSRRKPARIYRMT
VGTNKPEINQVINAIYVQVMDVFSGQIPDYIRRTDLDSRLRNGEITVREFVIELASSEIY
RKRFYTPYPNTKVIEFLFRHILGRAPATQSEIRQYNKILADSGLRTAVETIVNTGEYSRY
FGEDVVPYNRFPSLPAGNYLGSVKAEADLVKQSWSSLSPSVLTGRGTNR
>OBU74914.1
MSESLPLRDRYLALIDEIVSNTLKGKISSVYQIYQMLLNGISLDTGEVFELALSDRTYDY
IPELLEGL
>OBU74937.1
MKVAISNLGNIQKAEIELSPFTVFIGRSGTGKSWTAYTIASIVSNYGFKSYLQIYEDKIK
REQYNLEYDVLEESIQEFLESGSCQINLVNFAQKFAEKYVNDLAKMSPNWLSKFLGTSRL
NFNNLEVKFSINELKQDLVTRIRQISFQQERNYGNQGLKVFKQQDEEEIYFYQVYEGNRD
SLKDSLKLHPLLIRDLFLKTILGIIHECFYADVHIFPTERTAYIGFPFSVSKLMELEMEK
TISPEEQKPIEEQKPIMENLMKFMKFKRSTRSGIIDSLTSVIASAVIKTDQERDEEAEKD
PQIKKYIDLANFLQENILSGHVKFDSSPLVNEIIFEPIPDVKLEMHITSSMIKELTPLYL
CLRYLAKPNELLVIDEPEMNLHPAAQVEITEFLAMLVNAGIKVLITTHSPYIVNHISNLI
KAANYENKDTIKEKFYLERTDAFIAQKNVSIYLFEDGTAKNILHENGQIDWDTFADVSDD
VGHILF
>OBU74971.1

MSGWFGYTKAHQESGDKNNATIINGALATTLLDLGKKGNIGGFVIGVPPKAISNTIANRQ
DNSTSLHLEAFYTHRINNNVSITPAIYVIDNPDHKSSNGAIWVGSVRTGITF
>OBU75018.1
MPRRQDIHKILLLGSGPIVIGQACEFDYSGTQACKALREEGFEVVLVNSNPATIMTDPET
ADRTYIEPLTPEMVAKVIAKERPDALLPTMGGQTALNIAVALAKNGVLEEYNVELIGAKL
PAIEKAEDRKLFNDAMEKIGVNVCPSGTASSLEESKAIAQRIGSYPLIIRPAFTMGGTGG
GIAYNKEEFELMAQVGIDASPVSQILIDQSLLGWKEYELEVMRDLADNVVIICSIENLDP
MGIHTGDSITVAPAQTLTDKEYQRLRDMAIKIIREIGVETGGSNIQFAVNPVNGDVVVIE
MNPRVSRSSALASKATGFPIAKMAAKLAVGYTLDEIKNDITKKTPASFEPTIDYVVTKIP
RFAFEKFPGSDPVLTTQMKSVGEAMAIGRTFNESFQKALRSLETGRAGWGADKAEKLPSG
EQVRAQLRTPNPERIFALRHAMQLGLSNEEIYELTAIDPWFLDKLHQILETEKFLKRTPL
QQLTKVQMYEVKRNGFSDRQIAFCTKTKEDEVRAYRKQLGVIPVYKTVDTCAAEFEAFTP
YYYSTYEEETEILPTDKPKVMILGGGPNRIGQGIEFDYCCCHAAYALKSANYETIMVNSN
PETVSTDYDTSDRLYFEPLTKEDVLNIIEAENPVGIIVQFGGQTPLKLAVPLQEYLQKSP
SSVTRIWGTSPDSIDMAENRERFEKILQELKIAQPANGIARSYEDALIVAKRIGYPVVVR
PSYVLGGRAMEIVYSDSELERYMSFAVQVEPEHPILIDKFLENAIEVDVDAIADHQGRVV
IGGIMEHIEQAGIHSGDSACSLPSISLSPAVLNQIRTWTVELAKALSVVGLMNIQFAVVG
ASSYSPQVYILEANPRASRTVPFVSKATGVPLARLASLIMSGKTLEELNFTQEVIPQHIA
VKEAVLPFNKFPGTDTLLGPEMRSTGEVMGIDVDFGRAFAKAEMGAGEKLPLQGTVFVSM
SDRDKSLVVEVIKEFIQLGFKVIATQGTSEFLREQGLQIETILKLHEGRPHVLDAIKNRQ
IQLIINTPSGQEARTDGQLIRRTALGYKIPIVTTIAGAKATVAAIRSLQNISLDVKTIQE
YSF
>OBU75023.1
MSQELLNSLFINRVAVLATMHHKENVIAPLLKKQLGLEVVVPPNFNTDQFGTFTRDIKRP
DTQIVTAKLKAKKAMEMTGETIGIGSEGSFVPHPNFPYIYANREIVLLLDQQNDLQIVGE
VFSTETNFNHLVINSFEAAQKFAAKVGFPDHGLVVWFESSNGQDPEIIKGITTQEQLYES
VNFALNNSPHGNLHIETDMRALYNPTRMNNIAKATQDLVEKIYSCCPECGTPGFSITDRT
QGLPCESCGQPTSLTMGVIYQCQKCNFIEEKLYPDGKFFADPGLCPYCNP
>OBU75027.1
MSRNTSDQLGEDQEIGRLIDEVMSSSLSDEQYRQKMQRRKQIQDQRIADAIPQKGLIIVN
TGHGKGKTTAALGMIVRALGHGYKVAIVQFIKGAWEPSEKRVFSSWSDQLEFHAMGEGFT
WETQDRDRDLDKANVAWEKSLEFIRHPEFHLVLLDEINIALKLGYLKVDQVLAGLAEKPP
DKHVILTGRSAPPALIEKADLVTEMTLIKHPFKDQGVKAQPGIEY
>OBU75032.1
MVDSSSNTPPHHVVIVGGGFGGLYAAKALAKTNVNVTLIDRRNFHLFQPLLYQVATGALS
PADISSPLRSILSKNKNTTVLLGEVNDINPEDQTVMVGAEAVPYHTLIVATGAKHSYFGK
DNWEEFAPGLKTLEDAIEMRRRIFTAFEAAEKETDAAKRRALLTFVVVGGGPTGVELAGA
IAELARKTLKEDFRNIDTSETRVLLLEGLDRVLPPFAPELSQTAADSLAELGVEVQTKTL
VTNIEDDIVTVKQGEEVKTIATRTVLWAAGVSASPMGKVLMARTQAECDRAGRIMVEPDL
SIKGYPNIFVVGDLANFCHQSGKPLPGVAPVAMQEGEYVAKLIKNRLLGEVTPSFNYVDR
GSLAMIGQHSAVVDLGFIKIRGFLAWLFWLFIHIYFLIEFDNKLVVMIQWMWSYFTRSRR
ARLITGKEIIKYVSDEEQANYKPLHAKQPINV
>OBU75049.1
MIIVIDNYDSFTYNLVQYLGELAVEFSAAADLKVFRNDKITVEGIRELKPHGLVISPGPG
RPEDAGISLDVIEKLGSSLPILGVCLGHQSIGKVFGGKVVAATELMHGKTSPVLHNSVGV
FQGLENPITATRYHSLVIERETCPDVLEITAWVEDGTIMGVRHRNYPHIQGVQFHPESVL
TIAGKQLLSNFLRELET
>OBU75057.1
MKPKPINSNYTRKPRIFNQPERFIEGWYWVLPSHKLLVGEVKPITILGKELVIYRGEDGL
VVIFDAYCPHMGAHLGEGKVKGNELRCFLHHWQYDQEGFCTKIPCLDESIDTTATKSIKA
RNWPTEEKYGLIWVWTGENPQQSLPFIPELEVAELDIAFDGKFLIHCHPHIVMVNPIDAQ
HFYNIHRQVWESSFQKQELNENAIIFRYNQQCNNYNSNSPIKILRFLLNPPISYHLCYWY

GSTMIVRVEIRFLHFQFNCHILYALRLIESGKTTGIRIFIAKKRPGIIGHIYGKFLLWLS
KIVINAINYMSNQHGRLFPKPGKNNRNNIEFNLQHPLELDQPIIEFISHLERQKPLKWGT
WSWERFTDNQQMESQKPQNVQKWRDDLAND
>OBU75101.1
MHHVSIRTGNIHRAIAFYQELGFVVGTRFTTGYTLACWLEGLDSRIELIQIPQLKPAPDA
FTDENYVGYYHLSFDLTSRVSDLSAWLLELQQKMSLVLELSPLKVLLEPTQQQIGDRILE
VTFIADMDNLPLEFIRFLPNSLSVDNKG
>OBU75168.1
MALPIVAIIGRPNVGKSTFVNRLAGDQTAIVHDEPGVTRDRTYRPAFWQNREFLVVDTGG
LVFNDDTEFLPLIRQQAITALSEACAAIFVVDGQTGLTPADEEIAEWLRQQPVPTLLAVN
KCESPDQGIIQASEFWELGLGEPYPISAIHGSGTGDLLDDLIQYIPQVEELQENTEVKVA
IVGRPNVGKSSLLNAFVGEERAIVSPISGTTRDTIDTLIEREGQAYRLIDTAGIRKKKHV
EYGTEFFSINRAFKAIRRADVVLLVLDALDGVTEQDQKLAGRIVEDGRACIIVVNKWDVV
EKDSYTIYDHEKSLEARLHFTEWADTIFVSAITGQRVEKILDLVNKAAESHKRRVSTSVV
NEVLTDAVSWHSPPASRSGKQGKIYYGTQVSSQPPTFALFVNDAKRFNENYRRYIERQFR
KQLGFEGTPIRILWRSKKVREMEVGTLNRATRV
>OBU75181.1
MLRIITQQAHVVSELQRICERTQDEQVFNKEATVREVLLSVKNQGDKAVLHYTAEFDHQT
LEPGELRVKGSEMDVAYQQISKDLLASIRLACERIEAFHRQRVPKTWVHFGEDEVVLGKR
YTPVDRAGLYIPGGRACYPSTVLMNAIPAKVAGVPRIVMVTPPGPDKVINPAVLVAAQEA
GIGEIYRIGGSQAIAALAYGTETIPKVDVITGPGNIYVTLAKKLVYGTVGIDSLAGPSEV
LIIADETANPKHLAADMLAQAEHDPMAAAILLTTDTGLAKKVQLEVDRQLVDHPRRIDTE
KAIAHYGLVVIVESLEAAAEFSNIFAPEHLELEVKDPWSLIQHIRHAGAVFLGDSTPEAV
GDYLAGPNHTLPTSGAARYASALGVETFLKHSSIIQYSRGALDKVASAITTLARAEGLPS
HADSVRIRFDTDS
>OBU75190.1
MKYYQIQNNIFPSSYLKDLGGEIQASPYFTTNNLNRDFIQTKGFSVVFQRQGIKIVHEKF
PFFKPYLDLALQPNCNAFYLNPLLLQEGSQVDPHIDRSLRSYCKTIEPPSLVTVLYVRVP
ENMEGGELVLKSNNRQIQKIKPQTNTLVCFQGNLTHSVNPMKTSGNRLSLVCEQYDLTDR
EIVHIPIYTLESRAGQPTKGKMKSRSSFIPGM
>OBU75216.1
MAGHSKWANIKRQKAVVDAKKGSVFTQLSRAIIIAAKNGIPDPTGNFQLRTAIDKAKAAG
IPNDNIERAIAKGAGTLGSDSNNLEEIRYEGYGPGGVAILVEALTDNRNRTAADLRVAFS
KNGGNLGETGCVSWMFSQKGVCIVTGVEDEENLLEASLVGNAESYEMIDQQVAEVFTQVS
DLEKLSQTLKAKDFKLTEVEIRWIPQTEVEVTHVDQAKSLLKLIYTLEGLDDVQSVTANF
DMAENIIQAFAR
>OBU75218.1
MITKTTKLISLVSVISLPLVSLFIHNQYTSGQTPTISQLAQVKSIWQRFSSQEGKFSVLF
PGTPRLSQQKMTSDNGELQVNLFTVNRPQEEAKYTVAYIDYPAQYIQLLRSRNLVEQAIE
QGKSTALERVRGTIVSEEKKTLGDNVGIEVNYTTPAGKVVKQRVFLIDNRFYQITAETTQ
KRQRFLTRSMQGFCDSFKLLP
>OBU75225.1
MKPINPLASACRYCRHYQPEGRRGGVCSQLSAPVEGCWKACSLAVPAFAPTWESLEESWN
LPLTKPMLPILSVPGYLDTRTENLPVPEVQEITSIEETAFSTLPGSR
>OBU75231.1
MNLQNLPLGINTLSVLRENNCVYVDKTKLAYHLIRIAGRFFLSRPRRFGKSLFVDTLKEI
FEGNEKLFEGLYIYDKWEWSRKFPVIKIDFADGVLKNREELDEKIRDLLWNNGDRLGVGS
KKKSISGIFGEIIAGAREQFGERVVVLVDEYDKPILDNIDNPNIAAEMREGLKNFYSVLK
SQDANLQFVFMTGVTKFSKVSLFSGINQLTDITISEAYSSICGYTETDLRESFGDHLEGV
DWDALRHWYNGYNWTGSETVYNPYDILLFISEGMRFRNYWFETGSPSFLLKLFQKERYFL
PNLEGIEVTEEILDSFDVEQINPVTLLFQSGYLTIKDTFTDINQIVFCLGIPNMEVKIAL
NNQFINAYTKLVNEKLGVQRLIHTQLSSGDVEGLVSTIKRLFASIPWRNFTNNDLADFEG

YYASVIYAFLCSLDARVIPEDITNHGQSDLTVMVEGHIYVMEIKVVEGNEVQGNPALDQI
LQRNYAEKYRGERGKSVHEIGLIFSRSQRNLIQANWQ
>OBU75257.1
MSTEDTKFMIDKPDEEWKSILTPEQFQVLRKHGTERSHTSPLDKNYEPGTYVCAGCGQPL
FTSDTKYNSGTGWPSFFQPIEGAISTTVDRSLFMTRTEVHCSKCGGHLGHVFNDGPQPTG
LRYCMNGVSLNFQPV
>OBU75336.1
MQADGPAVNTIGQIIGIGHGRGIGLMFVIMGLLSILFTFICYFYPRLRFVEDELPDTIVD
S
>OBU75345.1
MIKQVQWLENQLAIQQVTALEHEGQLEQFMSVETQTVSNPVLRAEVTAGGVNSQRTVAEL
MGSMPYQISDKLGLSMPRWLLWVLTFSISITLSGLFMSAVALWTPLWSNLEKAEDDGFTP
SNRDTLKVSDGLWNKLSLYQLSKPMNILVMGVEPIKGTLDGSPESFAGSSDTMLLVRLNP
SDKSIRVLSIPKGTMVSLPEDGLSKISEANTKGGPVLAARAISRAFSNAPIDRYIRISTS
GLRELVDQLGGVDIFVPQSMSSQEQTGGTPTNLVSGWQTLDGEQAELFARFRESSLGDIA
RVQRQQALIGGLVQRLNNPVVLPRLPQLTRMMRKYFDTNLRMEEMMALANFAVNVERDNF
QMTMLPGTFSKFSKDPESYWLNLTGQQSLLKNYVGVDIYQVKSDSRSVSQLKIAIQNASS
QPQVTAKVINQLKSKGFANIYTVPDWAENQRQTQIFVRRGTRQPGVELQTILGRGQIEVS
AQGDLDADLTIRIGEDWK
>OBU75348.1
MKWQLLTHNKQVLGKIFTILVFTGLTGILCVSCNRNQDLLVTEIGVNPPKRPTRKTSGAG
EFYLQGQNQHSRGNFQAAIAAYSKSISLNSDYAPAFKARGLAYFDLNNKERAINDYNQSL
QINPNDPETYNYRGNARASLGDQKGAIEDYNEAIRLSPNYAEAFNNRGNSHAAQGNKNAA
LEDYTQAIRIDQNYSVAYNNRGNAYSSLGNTSKAIADYNQAIRLNPQFAPAYNNRGNAFA
SSGDKRRALQDLQKAATIFDQEGNRGLYQQTMKNIEELGN
>OBU75380.1
MFTIKRWWEHKTLQLALLGLILSGAWTLKETNGELLMELYQSFIFPLTTLQSSNHAENKI
RDAKLMELQTRIVELQTQNQRLQNLLGYVEKGGTSNRPVVARVVGRSADHWWQEVTVNRG
AQVGIKPGFVVKAEGGLVGLVRTVTPHTSRILLISDATSQIGVTISRTAAKGILRGDYSG
EGVLEFYEKVPNIKIGDLVSTSTYSRKFPPGEPVGKIKSLDLKKLPAPLAKVELFPPIRY
LDWVTIYPHVPDNLEGIPNNSNDNQSNK
>OBU75381.1
MGIDLGTANTLVYVSGKGIVLQEPSVVAIDHNEKIALAVGEDAKRMLGRTPGNITAVRPL
RDGVIADFDTAELMLKSFIQRVNDGKPLLLPRIVIGIPSGVTGVERRAVMDAAAQAGARE
VYLIDEPVAAAIGAGLPVAEPTGNMIIDIGGGTTEVAVLSLQGTVVSESVRIAGDELTDS
ITQYMKKVHNLVIGERTAEEIKIRIGSAYPTSDEDQGTMEVRGLHLLSGLPRTVTVQGQE
VRESMLEPLAIIVEAVKRTLEKTPPELAADIIDRGIMLAGGGALLKGLDTLISHETGIVT
HIAADPLSCVVLGTGRVLENFKQLERVFSGRSRNT
>OBU75384.1
MSSAYLLVSHGSKDSRPNLAIEELARLLFEKLNAGEDPEQHLVGVGTLEFNSQPLSGQIK
DFAQKAVVCNCKSIQVIPLFLLPGVHLMSDLPMEVKLAQDSVGNDLPIKIKPYLGSHAMM
WKLLAVGMAQISAEAFILLAHGSRRANSHHPVETIANNLGAFSAYWSVSPSLEARVIDLV
STGYQRIGILPYFLFSGGITDEIVIMVERLTLKFPEIKLELASPLGVTSQLVDIIWDLAI
>OBU75385.1
MGKVYLVGAGPGDPGLMTLKGKDLLERANVVVYDALVSPQVLDMISPQAEKIDAGKRMGR
HSLLQEETTQLLIEKAQEHEIVVRLKGGDPFIFGRGGEEMAELIAGGISVEVVPGITAGI
AAAAYAGIPLTHRLYSSSVTFVTGHEAMGKYRPAVNWQAIAQSSETIVIYMGMHNLPYIL
EQLMGVGMSSQTPIALVRWGTRLEQEELIGELGTIIQQVAKTGFSAPAIAVIGSVVKMHG
VLSVHQGVLKPSN
>OBU75418.1
MIGVAIVGSGFGQKVHIPAFSSHHKTKIFSIYHRDIKQAHSLARAYDIPHHSDNLEEILA
LPEVQAVSISTPPFLHYPMGKLVLAAGKHLLLEKPTTLNVTQAKELYQLAKQKNIIATVD

FEFRFVPAWQYLHQLLNDSYVGNIRLIKIDWLGSSRANTDRPWNWYSQQEQGGGVLGSLG
SHAFDYINWLFGEVTRLNAYLTTAITQRRDPVDGELKVVNSDDNCLISLELEERIPCQVC
ISAVVQAKRTHAIEVYGDKGTLIIASENQKDYIYGFRVWGCEIGGSFTELEIPKQLLFPQ
DYTDGRISAFLRVVDQWVNGIETQKEIVPSLKQGVYSQLLMDLCHQSSHSRTWVDVPSW
>OBU75427.1
MQSIQFMLRVDNLCKSFGVIPACDGVSFDLYPGQVLGIVGESGSGKSTLLKAIAHYITVD
EGSIIYRNRQEQYLKIQELAEYQRRWLMRTEWGFVQQNPRDGLRMQVSAGANIGERLLDI
GMRNYGQIRQEAIRWLQEVEIDPDRLDDLPTTFSGGMQQRLQLARVLVTRPRLILMDEPT
GGLDVSVQARLLDLLRSLVRNFNLSVILVTHDIGVVRLLAHRLLVMQQGKVVESGLTDQV
LDDPQHPYTQLLVSAALTP
>OBU75431.1
MDITKSLYQACNPNKTLNSNNPEDKQRYIDFSPVRGLEIINELKRTITRLSPDSPTCQLF
TGHIGVGKSTELMRLKSELETEFFHVVYFESSQSFDLGDVDVTDILLAIAKEVSHSLEVT
QINVKSGFFQNLFKDVIEFLQTPLDIDFEAELSVGIAKITAKTKESPKLRDQLRQYLEPR
TASILQSINRELLVPAQEELKKQGKSGLVVIIDNLDRIDNSPKANGQYQPEYLFVERGEQ
LKALSCHIVYTIPLVLIFSNALGRLINRFGVDPKVLPMISVKQRNGEYYSAAMTLLQQMI
MVRAFPSVNWQQSPEKLALTTKIFDSPRTFDRLCQVSGGHLRSLLKLLFRCLQSQDVPIS
RLCLENTIKEQCNLLSLAITSDEWELLRKVWKTKTYRGEEQYDILLRSLFVFEYRDDQGS
WFDINPILLESTEFPRDYDYKD
>OBU75439.1
MRNVGYIGWFGVLMIPTLLAATTCFIIAFIAAPPVDIDGIREPVAGSLMYGNNIISGAVV
PSSNAIGLHFYPIWEAASLDEWLYNGGPYQLVIFHFLIGVACYLGREWELSFRLGMRPWI
CVAFSAPLAAATAVFLIYPIGQGSFSDGMPLGISGTFNFMIVFQAEHNILMHPFHMLGVA
GVFGGSLFSAMHGSLVTSSLVRETTETESQNYGYKFGQEEETYNIVAAHGYFGRLIFQYA
SFNNSRSLHFFLAAWPVVGIWFTALGVSTMAFNLNGFNFNQSIIDSQGRVIGTWADVINR
ANLGMEVMHERNAHNFPLDLAAGEVAPVALTAPAING
>OBU75440.1
MRQPLTTPLAGESLNLPDHTQLPDSDDNFVKNFQEHPQSIILTTSIEPLLKEIHPNGDYC
IGQDSGIYWRFTEPPEKGVEAPDWFYVPGVPSRLNGQLRRSYVLWKEKVPPFIVIEFASK
NGKEEKDSSPPPEGDEIDPETNKPKKAGKFWVYEQAVKVPYYAIFNGFKGTLEVYHLQGK
RYKEIKANRRGHYAIPEMGIELGILYDNQNPPTPWLRWWDEKGSLLLTGNERAEQAEAIA
IRERLAKEQEREAKEQAEAVAIRERLAKEQAEAIAIRERLAKEQEQEAKEQAEAIATRER
QQKEKLVAYLRSLGIDPEKI
>OBU75442.1
MKKFLTLALILTLFLVSSFSLGTSPSYAYSQSDLYRLLETHECAECDLSDADLSGANLED
ADLLIANLSGANLDHADLKGADLSDADLSGANLEDADLLIANLSGANLDHADLKGADLSE
ANLFAADLSDADLSYANLKYANLKYADLNGARLPGAIQ
>OBU75461.1
MKKFLTLALILILFLVSSFSLGTSPSYAYSQSDLDRLLETRECPECDLSDADLSDADLSR
DDLRRANLRGAKLKDADLSDADLSDADLRRAKLRHANLRGAKLKDADLSSAYLSGADLTG
ANLSGADLRDAKLKNADLSGAFLTSADLMRADLTGANLTCAVGADFIVAEIVAERWKEKE
RQLLVPSGSKARNSSRAIFFEKEREKLLGKDCKNAIEEEE
>OBU75484.1
MKIALVHDYLTQRGGAERVFELLCKYYPEADIFTSVYDAQKTIDLGDRIVKTTFLQSIPG
AKKYFRLIAPLYFPAFRSLDLQDYDLIISSSTSFAKAVRKKKEAQHICFCHNVTRFLWDT
QTYLREYGDYRYFAPVIEKIFALMRNVDLKYSQEPDLYIANSSVVAKRIQQIYGKEAMVV
NYPIDTNNFVFSEIKDDYYLASARMISYKRFDIIVEAFNWLGWPLLISGDGPELQRLKSK
ALDNIQFLGHVSDVKRKDLFSRAKSIIVAALEDYGLVPVEANASGTPVIAYGAGGVLDTQ
IPGKTGVFFNRQSADSLQTGLCRAKEIAWNYQSIRNHAVNNFSESVFFQKIEEIVMKNSG
IHH
>OBU75519.1
MPKKNKIKKTELQNQSPSKIKPTVTENWSKGISFSYKYFQSDHRDFSVVGRNSDYLLGLL

ERLRDISSWTAQELISNRSKALRCHPITWEDTTESGFGIPGEEQIVDTPYQFSICSNEHG
RVHGFFIEEIFYIVWLDPDHLLYRR
>OBU75527.1
MLTSLIDSEDRAAVYAAKTKTAVCDRSHWGRIEVTGEDRLRFLHNQSTNNLESLQPGSGC
DTVMVTSTARTIDLVTGYVLEDRVLLLVSPNRREFLLSWLDRYIFFADQVTLTDITEQTA
TFTLLGPESDTIISKLGVASLLSQPDGHHISINGIIFAVGTGLAIPGYTLILPRAEKQQI
WQQLLDWGAVKLSDRHWEMLRISQGRPAPDAELTDDYNPLEVGLWQTVSFNKGCYIGQET
IARLNTYKGVKQHLWGIKLKNCAQPGTIITISEEKVGKLTSYIETPEGHFGLGYIRAKAG
GVGLTVEVGETQGEIVPVPFVSWEYPQ
>OBU75534.1
MTTTIQQRQSANVWDRFCEFITSTNNRLYIGWFGVLMIPTLLAATTCFIIAFIAAPPVDI
DGIREPVAGSLMYGNNIISGAVVPSSNAIGLHFYPIWEAASLDEWLYNGGPYQLVIFHFL
IGVACYLGREWELSFRLGMRPWICVAFSAPLAAATAVFLIYPIGQGSFSDGMPLGISGTF
NFMIVFQAEHNILMHPFHMLGVAGVFGGSLFSAMHGSLVTSSLVRETTETESQNYGYKFG
QEEETYNIVAAHGYFGRLIFQYASFNNSRSLHFFLAAWPVVGIWFTALGVSTMAFNLNGF
NFNQSIIDSQGRVIGTWADVINRANLGMEVMHERNAHNFPLDLAAGEVAPVALTAPAING
>OBU75541.1
MLAGLDLPDTPEDDRRFSLDLGLVKRIPLGRMTIANPIYQEVIPRVLSQGSQDSLTQIQP
TWLNTDK
>OBU75542.1
MAVLVLDDVLIGLDMSNPLPIIDIIDEYFIDKYQIFLMTYDLEWFEILCEHFVERNGKYW
KAFEFYCADNTELELPIFAERGKGRDEYIKRAEQYYATNDYKAAAVYTRSAYEATLKFFC
ARHRVPVPYVSKPKDLKTNQLWEAVKTYIKTHPKVTNKKTGYEEDYLDSKTINHVEKANG
RILNPLSHSRAVSIYRREVQYAIAVVKKLQDRLQ
>OBU75556.1
MDFSMNFNEPNETEENKTEELDRGGFKRQLKKLIYTAAEISPVSQAQWEKLLEIYNFASL
SIEVIIQTICRNNARNVECRYKTLHS
>OBU75558.1
MGNFYHNFLNIQHFWNNAFYSQERLSLNFPSISPRIPLSANQQIALEMALSNSPITVISG
TGATGKTSIAKNLAQVAIDSSHKVLILAHNLASLSPYYNLTNYPLVLSQQGDYYQKVINR
LRYDNLAQAKMDYLPLHLLPDVELSKLRTPAKLERWLPIIENNSYQQLADILKLESEFQN
LTQARLHLLAYRLKQLLPFLQEQLRLNQIYNHLSQAGIEEIARQLLANPQITITGTVAEF
MQVENRLLWETSFDIIIVEEANRLHWIQLLFLAGLCKKLVLFGEDIPPRYGQPKKSIPYP
YNCLQFNCFQWLQSHLSPAYVYTLKEQFRLHSAMAKIVYPSICNHWVYIQSHYTDNSLSG
VNLSQINRRVVWHTMVGDKPVAENMIRFLEKLIRNHDGQVMSEIGMITFSPTQTDAIKEI
LPTISSIYQNQLFVGTAGEWSGKERKIIMVNCSGNPKDISVEDINIALTRAKDYLFLFGD
CDVWLRYNSPLKALLYHPAVSQERQVVI
>OBU75564.1
MNSLLINRYEIINPLGSGGFGETFLARDTQMPSQRLVVIKHLKPALQNSHSSTELIENLF
QKEAAVLEELGNHCSQIPQLYSYFSEEGEFYLVQEYIEGKNLSQVGQIKPEHATVILSSL
LNTLKYVHSKNIIHRDIKPENIILRDSDRLPVLIDFGAVKETMGVVTLGSGSTVSSVVVG
TRGFMAPEQTAGRPVFSTDLYALGLTIIYALTQRLPIEFSISQLTGEIDWTSYVPNLDPK
LVQVLNKSIKIDLGSRYLTAEAMYSDLHTSSGIPLSTVLAPKSQEDTLVVSPGGESKNLI
SSLTSVVFSKVKSQNKDTKVPVNYTRVAVIALSILGLAGGFFVTQQMLEAQERAAQLERE
KKEAEEKKEIAEKERLQAQQKALEAENLRQTAEQERLAVEKRQAQEERRRLVAESRQARL
ERRRLAAERRLSINSSQTSQTDAIVVGQPGYKNIRSGPGTTYKVLGTADTGDPVKILGSS
YDQDNYQWYQVYHPNSGTTGWIAAQLINLN
>OBU75565.1
MKLFLSFLSFFRHFIPTMILIIGVGIYMLLRPSDVPFPTTFTNSSPGSSSYQIPNPPPHQ
STESLTNTPQYSLSPPASQVANDTDIIPQPPGKSFEPTLPNSKSIQSQPPAHFRYPENKQ
NLVEVGTYYNRTAYLNLEAATAFKKMKLAANQVGIKLAPISGFRSFVEQENLFQKQIERR
GSAQAAQLLSAPPRFSEHHTGYAIDISDDRHPETDLKFAFESTEAYRWLEVNASQYGFEL

SFPRNNFQGVSHEPWHWRFVGSLTAKEIFKAARIQQNSRKF
>OBU75592.1
MKKLINQPENFVKESLMGMARAHSGLIKVNFDPTFVYRAETPNKSKVAIISGGGSGHEPM
HTGFVGKGMLDAACPGEIFTSPTPDQMLAAAEKVDSGLGTLYIVKNYSGDIMNFEMATEL
ARSQGIRTLNMIIDDDVAVKNSSYTQGRRGVGTTVLAEKICGAAAEQGYDLPQVANLCKK
VNLLGRSIGVALSSCTVPARGTPTFDLGDDQIELGIGIHGEPGRERVPLMRGDEVTEILA
RSLFDDTDYCRTMREWDEKQEEWKEVELLNKPWEKGDRLLAFVNSMGGTPISELYLVYRK
LVELCEQEGLEIVRNLIGPYMTSLEMQGCSITLLKLDEEMLDLWDAPVKTASLRWGV
>OBU75600.1
MLAIYGGTVYCQQIWSHSYRKLESLQRQERQLTAANAIIISKMAQEAEAMGLGLSAPGKM
VFLPAVSNNSQPPSRAKIPPSPGKRTSIPPLGY
>OBU75604.1
MQGKDWFIPESGKHELCKAAREWDLLRENYRFYRFLTEVEDVLNDVEEESDFLPHLRMLV
RRLIVNSYWVKSRVLKPHSPTGTSVLLLYDELGFPLTVQTVTFAPGITSNIHNHGTWGIV
AILQGQEKNTFWQPDPANPDRIQQVGSLDLFPGDLISFTSQTIHQVYSVGPEPTFTFNIY
GETNSQNRREFDIVNHTSKNF
>OBU75662.1
MRLTFSEEIKSLLQRLAHQPLTLGDVLETTSQRGFILVIALLVLPFLFPMPPGLTGPLGS
ACLLLSLQMLLGRRSPWLPKKIANYQFPRVFAQTILHNLGRVTRLLEKIARPRLTKLANH
DITWKCNGLCISWLAILLISPVPLTNPIPTIGILLFAAASMESDGLLVCICYILTLLITL
IFYLIVYGVLQLPGLIT
>OBU75668.1
MDTIEDNVMVEETNQKPVAKKEKPPAVEDKPFEEFMQEHYLPALQKAIVQEGISDVKLTF
AKQKYAIVGFDSSQECWQIIGSWQNGSKQFNVYFPDQDIQGKKGFSCHEGKKPSTLESFL
IDERKITLDLLVSRLVYRLNGQKWLGRN
>OBU75677.1
MSDNSPISPHRCLLYNEGLKPYLDVLVWQRQLLQERIENSDLEDVIILVEHPPVYTLGQG
ADPNFLKFDLDKSEYNVHRVERGGEVTYHCPGQLVGYPILNLRRYSQDLHWYLRQLEQVL
IDVLASYGLVGERISGLTGVWVEGKKVAAIGIKVSRWVTMHGFALNVCPDMTGFEKIVPC
GIKDKKVASLAEWIPDIDCLAVRLRVAECFAKVFGVKLQK
>OBU75735.1
MNMPNFPWLTTIILFPIAASLLIPIIPDKEGKTVRWFALIVGLIDFALIVAAFYTGYDFS
NPDLQLVESYSWIPQIDLRWSVGVDGLSMPLIILTGFITTLAALAAWPVTFKPRLFYFLL
LAMYGGQIAVFAVQDMLLFFLVWELELIPVYLLLAIWGGKKRHYAATKFILYTAGASLFI
LLGSLTMAFYGDNITFDMSSLALKDYALNLQLLLYAGFLIAYAVKLPIIPLHTWLPDAHG
EATAPVHMLLAGILLKMGGYALIRMNAQMLPDAHAYFAPLLVILGVVNIIYAALTSFAQR
NLKRKIAYSSISHMGFVTIGIASFTDLGLSGAVLQMVSHGLIGASLFFLVGATYDRTHTL
MLDEMGGVGKRMKKIFAMFTTCSMASLALPGMSGFVAELMVFVGFATSDAYNPTFKVIVV
LLMAVGVILTPIYLLSMLREIFYGQENDELVSHQALIDAEPREVFIIACLLVPIIGIGFY
PKLLTQIYDATTVQLTQRLRDSVPTLTAEKEIQHVSVNTPVIAQ
>OBU75794.1
MTLQSFGVIGLAVMGENIALNVERNGFPIAVYNRSREKTDAFMSNRAGGRNVKAAFTLEE
FVASLERPRKILVMVQAGKPVDAVIQQLKPLLQEGDIIIDGGNSWFEDTERRTQELEPTG
LRYIGMGVSGGEEGALNGPSLMPGGTKSSYEYLSPIFNRIAAQVDDGPCVTYIGPGGSGH
YVKMVHNGIEYGDMQLIAEAYDLLKNVGGLDATQLHQVFTEWNQTDELNSFLIEITANIF
PYVDPETKQPLVDLIVDAAGQKGTGRWTVQTALELGVAIPTITAAVNSRIISSIREERIA
ASKQLTGPVPTQFKDTRTFVNMVRDALYCSKICSYAQGMALISTASKTYNWGLNLGEMAR
IWKGGCIIRAGFLNKIKKAFDENPALPNLLLAPEFKQTILDRQSAWREIIVTAAKTGIPV
PAFSASLDYFDSYRRERLPQNLTQAQRDYFGAHTYKRVDIEGTFHTEWVPIAEAKK
>OBU75825.1
MIIPLTRQKLEQIIPLIGSGDQYKYYWGKFNNFLQRLLISVVTVVVILLIKNLLQLDFGL
VFVFGVFGGFFWLWYPVYQASIRNFKYRSYKYGGFFRGRIVDWWITEKVIGKQETVNNRG

ELVIIENREKRINLEIGDNTGFSIEFDAPLRSTHKAIARGQVAEMLVMSNSADLSTIEDF
SNIYIPSRNLWVSDYPYVREDLFNQVSRRIRSSPRGRSQTPKYRAKPQDDYYDDES
>OBU75870.1
MPLKAVLFDFNGVIIKDESIHLKLIDEILVEENLQPQKPDERLRYLGRSDRACFEELLKR
RGRVVSQDHLTNLLRNKANKYIKELESLEQLPLYSGIEDLIIQARSQNLPVGLVSGALVR
EIELVLERANIREYFQVIIAGDDIATSKPQPEGYLLAVDRLNQVYSDINVDLSLQPEDCL
ALEDTLAGIEAAKRARMKVVGIANTYPFHILQRQSNWTVDHVRDLEWERIWETFEQKDWK
NGERVTIG
>OBU75878.1
MDWINLLRSLQSDFIKRLNSGCLLNCEIPGSHSELTIISGQRLKSLREFCWSMAEKYKRT
STVRDVFISNLKGKLGEEVVKERLADFITEVDYEKRLGGDGKSDFTVTAHPWIGIEVKSR
HGDFDKVRWSVSSQEVGKNAVIVCILIREEVTEAQSAYHLFLAGFLPTKMIKLKTGRISF
GIDQLLYGGGLLAYLEDLPAMISNGISQEVISSNTNGLYNSHNLPAPDMGRDVVEFYVKL
GDKHFDDGDYIVAISNYSQALQNNKKNSYYQGELQAELNLSHKYTDNVGNIDIYAYYKLG
LAYYKLGDYDMAIFNYNQVINANVNHSNAYNKRGLAHYKSRNYHSAIEDFSQAISINPEL
AINYKNRAEARYLIGDYQGATEDYSQAVSIHPDLLDQPILVEDLGELFNIKCHDEVIYKN
RADHLYQLGAYEEALENYNQAIALNINYVDAYYQRGKIYFNKGIYEAAVDDFSMVIKTQP
NYGDAYYYRGNCWLIMGNKQTASGDFKKAADIYWQDGRLAEYKETQAMILDVEIEQSLDI
LDF
>OBU75893.1
MSAALEVTDASFEQEVIDSPIPVLVDFWAPWCGPCRMVAPVVEEIATQYAGKLKVVKINT
DENPGVASKYGIRSIPTLIVFKEGVKVGLHVGAVPKSTLSKTVEEILTPTQG
>OBU75905.1
MRAFVTGGTGFVGAHVVRCLLQSGYKVTALVRKNSNLANVKGLEIEVVTGDLNDPGIWEQ
MGGCDYLFHLAAHYSLWQKDRQLLYHHNVEGTRNLLRSAQKAGIERTVYTSSVAAIGVGK
SGEIVDETHQSPLNKLVGDYKKSKFLAEQVAKTAVQEGQDIVIVNPSSPIGPLDIKPTPT
GDIILRFLRQQMPAYVNTGLNFIDVRDVAQGHLLALEKGKTGDRYILGNENLSLKQLLDT
LSEITGIKAPQLSLPSFIPLTVAWIEEKVLAPLGKTPTVPIDGVRMAQQPMYYNASKAIR
ILGLPQSSVRVALQDAVRWFVSNGYVKY
>OBU75906.1
MEPTINCAEACVNGCILGDKCPHTEYRAATTKFIEDTPLEKMLEIAEERLRKKMMEPPKW
VFPEDP
>OBU75942.1
MQKYRSVLALIIILLIAAIAVIVKIPVPLGLDLQGGSQLTIQVKPTEQIKEITDRELEAV
KKVVEGRINGLGVSEPIIQTVGKDKILVQLPGVNDPQQAENVLGGTAQLEFRLQKPDTET
LLQSLRVSQLDIKSKQETLRKGKDENAIAANQAELKKNYQAIAELFASTDPPLTGKYLKD
AYGEPTQGKNWNVALRFDNEGGALFAELTKQLAGTGRGIGIFLDNELISSPSVGVEFAAT
GITGGSAIITGRFQAEEANNLGVQLRGGALPVPVEIAERRTVGATLGQDSIQSSIYAGIG
GLALVLVFMVLYYRLPGLIADISLIIYAILTWATFSLLGVTLTLPGIAGFILSIGMAVDA
NVLIFERTREELRSGKTLYRSVESGFYRAFSSILDSNVTTWIACAALFWLGSGLVKGFAL
TLALGVAVSMFTAITCSRTLMFLAISFPSWRKPALYCPKLADRTEIIK
>OBU75953.1
MLNLDRILNQERLLREMTGLNRQAFNELLSQFADTYERTVFNSLANRKRAPGGGRKPTLR
SIEEKLFYILLYCKFYPTYRNRVTDFDDQLMLVSAGLWNFYLDAA
>OBU75957.1
MQKRESPQILFDGNGTQSEFPDSCIHHLFEDQAAKRPDAIALIDGEQSLTYGELNVRANH
LAQHLLSLGCQPDDLLAICIERSAELFIGLLGILKAGCAYVPLDVGYPGDRIEYMLRDSD
ARILLTSTDVAKKLALTIPALQECQTVYLDQEIFEYDFHFLAIAKLLHNQYLRLLHFYFY
TLIQQCQATSVSQGIQTQVLPNNLAYCIYTSGSTGNPKGILMEHRSLVNMLWWHQQTRPS
VQGVRTLQFCAVSFDFSCHEIFSTLCLGGILVLVPEAVRQNPFALAEFISQQKIEKLFLP
VIALLQLAEAVNGNKSTSLALCEVITTGEQMQITPAVANLFQKTGAMLHNHYGATEFQDA
TTHTLKGNPEGWPTLVPVGRPLHNVQVYILDEAQQPVPLGGEGEFCIGGIGLARGYHNLP

DLTNEKFIPNPFGANENAKKLYRTGDLARYLPDGTIEHLGRIDHQVKIRGFRVELGEIES
VLASHQAVRECAVVAREIAGHTQLVGYIIAKDTLNLSFDKLEPILRQYSEAVLPEYMIPT
RFINISNMPLTPSGKLDRRALPDPKGDRPALSTPLVKPRTQTEKRLAEIWGSYLAVDIVG
THDNFFDLGGTSLLLTQAHKFLCETFNINLSAVSLFQYPTIQTLAQYIDCQGDTTSSDTA
SRHKKVRKKQSGDSNDIAIISVAGRFPGAETIEQFWHNLCNGVESITLFSDDELEQTLPE
LFNNPAYVKAGAVLEGVELFDATFFGYSPKEAAVTDPQQRILLECAWEAFERAGYNPETY
PEPVGVYAGSSLSTYLLNNIGSALGIITEQPFIETDMEQFQAKIGNDRSYLATRISYKLN
LKGPSVNVQTACSTSLVAVHMACQSLISGECQMALAGGISVVVPQKGGYLYEEGMVRSQD
GHCRAFDAEAQGTIFGNGGGLVLLKRLQDALDDNDNIMAVIKATAINNDGALKMGYTAPS
VDGQADVISEAIAIADIDASTIGYVEAHGTATQLGDPIEVAGLARAFQRSTDSVLGKQQC
AIGSVKTNIGHLDEAAGIAGLIKAALALQYGQIPPSLHYANPNPRIDFDATPFFVNTELR
EWSRNGYPRRAGVSSFGVGGTNSHIVLEESPVKQPTLFSSLPERSHHLLTLSAHTQEALH
ELVQRYIQHNETHLDINLGDLCFTANTGRKHFEHRLAVVAESIPGLQAQLETAQTAISAQ
KKNAPPTIAFLFTGQGSQYINMGRTLYDTESTFRAALDRCETILQNLGIESILSVIFGSS
EHGLSLDDTAYTQPALFAIEYALYQLWKSWGIQPSVVIGHSVGEYVSACVAGVFSLEDGL
KLIAERGRLIQALPRDGSMVSVMASEKRIADIILPYGGQVGIAAINGPQSVVISGQQQAI
DAICAILETEGIKSKKLNVSHAFHSPLVEAMLDSFLQVAQEVTYSQPQIKLISNVTGTLA
SHESCPDELPITTAEYWVRHVRQPVRFAAGMESLEGQGVNVFIEIGPKPVLLGMGRDCLP
EQEGLWLPSLRPKQDDWQQVLSSLRDLYLAGVTVDWSSFDQGYARRRVPLPTYPWQRERH
WVEPIIRQRQSVLQATNTTKLTRNASVAQHPLLGQRLHLSRTQEIYFQTFIHSDFPIWVA
DHKVFGNVIIPGVAYFEMALAAGKALKPDSIFWLEDVSIAQALIIPDEGQTVQIVLSPQE
ESAYFFEILSLEKENSWVLHASGKLVAQEQVLETEPIDLIALQAHCSEEVSVDVLYQEEM
ARRLDMGPMMRGVKQLWRYPLSFAKSHDAIALAKVSLPEILLHESNAYQFHPVILDAGLQ
MITVSYPEANQGQTYVPVGIEGLQVYGRPSSELWCRAQYRPPLDTDQRQGIDLLPKKLIA
DLHLFDTQGRVVAIMFGVQSVLVGREAMLRSQDTWRNWLYQVLWKPQACFGLLPNYLPTP
DKIRKRLETKLATLIIEANLATYAIAYTQLERLSLAYVVAAFRQMGWLFQPGERFSTAQK
VSALGIVDQHRQLFARLLDILAEADILRSENLMTIWEVISYPETIDIQVLLDDLEAKEAE
AEVTLVSRCSAKLAEVLQGKCDPIQLLFPAGDTTTLSKLYREAPVLGVTNTLVQEALLSA
LEQLPPERGWRILEIGAGTGGTTAYLLPHLPGDQTKYVFTDISAFFLAKAEERFKDYPFV
RYQVLDIEQAPQAQGFEPQIYDLIVAADVLHATSDLRQTLVHIRQLLAPGGMLILMEDSE
PARWADLTFGLTEGWWKFTDHDLRPNHPLLSPEQWQILLSEMGFSQTTALWPKIDSPHKL
PREAVIVARNEPAIRKPRRWLILADEEIGGLLAKQLREEGEDCILLLPGEKYTERDSQTF
TINPGDIEEWQQLLNRVPNIQEIVHCWSMVSTDLDRATIFSCSSTLHLVQALANYPKNPR
LSLVTLGAQAVNEHHVQNVVGAALWGMGKVIALEHPELQVAQMDLDPNGKVKAQVEVLRD
ELLARKDPASAMSVPDLQTRPHEKQIAFREQTRYVARLSPLDRPNPGEKGTQEALTFRDD
GSYLIAGGLGGLGLVVARFLVTNGAKYLVLVGRRGAREEQQAQLSELEQLGASVKVLQAD
IADAEQLAQALSAVTYPPLRGVIHAAGTLNDGILQQQSWQAFKEVMNPKVAGAWNLHILT
KNQPLDFFVLFSSATSLLGNAGQANHAAANAFLDGLASYRRHLGLPSLSINWGTWSEVGI
AARLELDKLSSKQGEGTITLGQGLQILEQLLKDENGVYQVGVMPINWTQFLARQLTPQPF
FSDAMKSIDTSVGKLTLQERDSCPQGYGHNIREQLENAPPKEGLTLLQAHVREQVSQVLG
IDTKTLLAEQDVGFFTLGMDSLTSVELRNRLQASLGCSLSSTLAFDYPTQQALVNYLANE
LLGTPEQLQEPESDEEDQISSMDDIVQLLSAKLEMEI
>OBU75961.1
MLNLDRILNQERLLREMTGLNRQAFNELLSQFADTYERTVFNSLANRKRAPGGGRKPTLR
SIEEKLFYILLYCKCYPTFDLLSVLFNFDRSCAHDWVHRLLSVLETTLGEKQVLPARKLR
SMEEFTKRFPDVKEVIVDGTERPVQRPQNRERQKEYYSGKKKRHTCKQITVSTREKRVII
RTETRAGKVHDKRLLHESEIVQYIPDEVAIEGDLGFHGLEKEFVNVHLPHKKPKGIEARR
HGGGMGQFL
>OBU75968.1
MENFLVRNTGLMEYLRVVGKCDRLYEWGCLSEYQILVVEKRPYMGTQIPMMKIYHCQVK
>OBU76008.1
MNMGLLPWRLRDTSINKTTINSKQQATKNTTEIMTKKFVDLSTLDGKNGLTIINGNDKDD

NLGYSISNAGDINGDGINDIIIGAPLSDPNDQSNAGNSYIVFGSDNGFANIIDISTLDGI
NGFTVNGGGIGDQSGRSVSAAGDINGDGIDDLIIGAPFADSNGDDSGAAYLIFGRRSFSS
LPTVNPSNLGDNGFIINGLNPQDQLGYRVSGAGDINQDGFDDVIIAAPPNAYVYPPVTGD
QAGKVYVIFGSEKFNPSNLNFNPNFDLNSLNGNNGFVINGSRADDYLGVGLNRGGDFNGD
GIDDLIIGSPFNDFNGFRSGQAYVIFGRKESFSSSLDVSQLDGVNGVVINGQEGDQLGFS
VSSAGDINHDGIGDIIVSAHDADPNGIDAAGVAYVVFGARTQFSSQLDLSNLNGNNGFVI
NGIGELDKASWAVTGLGDVNGDGIDDLLVSASHADANGDNSGQGYVIFGGDKFSSAINLA
EIDDTKGFIINGKSENHNLGYSASGVGDINGDGVSDILISAPFAGSGEVYVVFGKNSPTD
TGEITDEVTEGNGGESDIINEAPSGLFLKTNNRVDENGLESNVIGTFTTVDPNADDSFTY
SLSDDENYPDNGLFSIQDNQLISKESLDFENQPIYTISVITTDRGGLSLTAEFTIDGTED
EITGGVIEDNNSESEGTPDEILDGVMGEGDSDNNSTEGSGSDLTDSSDIPSTSTGSADNI
LSSNNNVLSSSTTRVEFQLMDKIPASIRELGVFTVDDPSGKINGIAPGQVGYSEAALARS
KVVFSILSKTPNEFNNANVTRILGFQEANPNLRFYVIDNGTTDSVKNGLFPIDQVTFLDS
SNLQVTQLPDNSFSLQSNDLVFKVRPTTKPLPMGANLQEKSQGESIDLRGVTGPVNAQFT
VYREASFDNYVGFYKVTDEKGGIDTNNDGTADLLPGDAGYIQAAVNQHLIGLGLNVPDGN
KSNFNSTLSGGGLYVPFIIVNGRPDAFLNSDLSANSNPDIYFTYLGANPDRVDHVRLLGD
NTFGFEDLRGGGDRDYNDLVVQVNMSANV
>OBU76032.1
MTKFKVPRRSFLLFGGAFLTQIWRLDLPVRPTTPSQPSVTPTIIPTVRAYRSQINGIPFY
QTIIDLKDPNIFVTIGLPNDANFANTISRTNGDENFDQLVARSGAAVVVNGTFASTNPQK
TVMGNLVAGGRFLKYSLWENFGTTLGLGVGNRPEMITARVDGRPVWNKHWFSITSGPRLL
RNGEVSLNPRLEGFKDPTVLGASLRTAIGFSQDGKRLFLASFDEKLYLDEEAEAMKAMGC
YEAMNLDGGSSRALASDNVILVPPTRKLTNVILVYDGKNPPPEELKLSWERFQTRR
>OBU76034.1
MTEHILSISQDPINVPKISFLTMLRLGLFQMGLGIMSLLTLGVLNRVMIDELTVLPFIAA
GAIAMHQFVSPARIWFGQKSDSQKIFGYHRSGYVWIGAALFTSISFVALQVVWQLGTSLQ
NTDWSLQTYGWSALLALIFGMYGLALSASSTPFTALLVDITDEDDRPKLIAVVWSMLMMG
IVIGASISSRLLERPEICGTALLAYDPSQMNKLVDISKLQTTINPVFIILPGAVFVLTLL
ATLGIEKKYSRYGIRGNMVEREDQITLGKALKILTANRQTGIFFGFLMVLTLSIFMQDSI
LEPYGGEVFGMCISETTRLNIPFGIGTLLGIGSTGFLLIPRLGKKQTTKTGCIGATMSFC
LIIMAGFSQNSGLLMGSLFFFGLASGVITTGATNLMLDLTAAETAGTFVGAWGLSQAMAR
GMATVLGGTVLNLGKLVFTSPMLAYGMVFALQAIGMLLAIWLLRRVNVVEFQQNSKQVLA
SVLESELD
>OBU76068.1
MTWYSITPTGTITLGNLTPVGQNSGLVGCRWPPNGNQLAAALNLPKTTQMWGPFWLHEKN
LYLSVPQGVYTHRLPLRQNTKVLDLYRMYWQEKWQLHHQTTPDIEIEQLGGKYLIKSQDF
RQLWEQGFLNQVEVQPLPWQTLTLSHNRREDFQVVEEGGLYAEKTILMASKWSLVIKVIG
KGEPQKYGTLGAGATPIVTVPLENLETENWEFLGAEIPDADGAVLLTSALWSNGRTKTSH
PYPDQHPPLAYLAQQGEPWQTWKTVSHKLTPERKLTPGEWLTPAGAIYRWHKAPITKSGP
LLDPFNRHVWGYGHLWLFKENLI
>OBU76074.1
MNPVPPLTVPQSSTTTQNVTLPEEPLDLPDHTQLPDSNDDFVKNFQEHPQSIILTTSIEP
LLKKIHPNGDYCIGQDSGIYWRFTEPPEKGVEAPDWFYVPGVPSRLNGQLRRSYVLWKEK
VPPFIVIEFASKNGKEEKDSSPPPEGDEIDPETGKLKKAGKFWVYEQAVKIPYYAIFNGF
KGTLEVYHLERKRYKEIKANRRGHYAIPEMGIELGILYDNQKPPTPWLRWWDNKGNLLLT
GNELAEQAEAIAIRERLAKEQAETIASQERLAKEQEREAKEQAETIASQERLAKEQEREA
KERAEEIASQERMAKERAETIASQERLAKERAETIASQERLAKERAETIASQERMAKEQE
RQQKEKLAAYLRSLGIDPEKI
>OBU76075.1
MNPVPPLTVPQSSTTTQNVTLPEEPLDLPDHTQLPDSNDDFVKNFQEHPQSIILTTSIEP
LLKKIHPNGDYCIGQDSGIYWRFTEPPEKGVEAPDWFYVPGVPSRLNGQLRRSYVLWKEK
VPPFIVIEFASKNGKEEKDSSPPPEGDEIDPETGKPKKAGKFWVYEQAVKVPYYAIFNGF

KGTLEVYHLERKRYKEIKANRRGHYAIPEMGIELGILYDNQKPPTPWLRWWDNKGNLLLT
GNELAEQAEAIAIRERLAKERAETIASQERLAREQAENIASQERLAKEQEREAKERAEEI
ISQERMAKEQEREAKEQAENIASQERLAKEQEREAKERAEAVASQERMAKEQERQQKEKL
AAYLRSLGIDPEKI
>OBU76077.1
MPQSSTTTQNVTLPEEPLDLPDHTQLPDSNDDFVKNFQEHPQSIILTTSIEPLLKKIHPN
GDYCIGQDSGIYWRFTEPPEKGVEAPDWFYVPGVPSRLNGQLRRSYVLWKEKVPPFIVIE
FASKNGKEEKDSSPPPEGDEIDPETGKLKKAGKFWVYEQAVKIPYYAIFNGFKGTLEVYH
LERKRYKEIKANRRGHYAIPEMGIELGILYDNQKPPTPWLRWWDNKGNLLLTGNELAEQA
EASAIRERLAKERAETIASQERLAREQAENIASQERMAKEQEREAKERAEAIASQERMAK
EQERQQKEKLAAYLLSLGIDPEKI
>OBU76080.1
MSQPSTQNVTPPREPFNLPDHTQLPDSDDNFVKNFQEHPQSIILTTSIEPLLKKIHPNGD
YCIGQDSGIYWRFTEPPEKGVEAPDWFYVPGVPSRLNGQLRRSYVLWKEKVPPFIVIEFA
SKNGKEEKDSSPPPEGDEIDPETGKPKKAGKFWVYEQAVKVPYYAIFNGFKGTLEVYHLE
RKRYKEIKANRRGHYAIPEMGIELGILYDNQKPPTPWLRWWDNKGDLLLTGNERAEQAEV
IAIRERLAKEQEREAKEQERQQKEKLAAYLRSLGIDPEKI
>OBU76101.1
MGEAKRRKNTIGENYGQETPILPWVPITKSQAELFVKITTRGAWIGIGTMVAIWVTIRFI
GPAFGWWQVVD
>OBU76107.1
MHFEDQAFDRLGTAVEGNTGPVWYYLLEIIKYGFPWLLFFPGGLYLSWKNRQTPWGSLVF
IGTVIYFTIVSLMSTKLPWYIMPIYPFLALAVGVNLGYIWEEHSLGKFLKTPLTIKFLIG
FFIFLSVAGVGGCIYFSLFDRQVPLILMSVVLTISMVITSWFISQHSRQFIPVLFGGMYL
VLTLLMISQSWVWELNEKFPVQPVAGLIRQHIPAGQKIYTSFPDSRPSLDFYSDCQIIPA
SLGDLEDKFSSHSFLLVNEEDLGKINLKNSRVLGKSQGFRLIAPTSPSSPTNKL
>OBU76136.1
MYKLNSGFRWTSSAEIEIELMGLVLIPEETDSDIEQLEQQQLDQNNSINNTNFSSEITSF
CHPNKSQSGQQLENILSNLLGHGVLIASTVVFAGGMLYLIRHGFEPAEYHRFRGTPSQFC
SPMGVINAIFSGSGRGIIQLGLLILISVPILRVIISFLVFMFQRKFTYVVMTLLVIATIS
YSLVSASL
>OBU76155.1
MHLPSFLWLWRIAAWSMGLAIFVYTILAITAYWLWQVRTNGRSPLGLVAPKVNNLVKAFH
YLLGITLIFLVLLLLLIGIVGTLGHFGSLGHSSHLFAGLTVVILVLTSALSATQISQGKF
WARPLHITLNAILFFGFAWVCLTGWNVVQKYL
>OBU76171.1
MSRSKALQYYIASRLLFAPLQLLTIVTIVFLLLRATPGDPADAILGGRAPESAKEELRKQ
LGLDLPIWLQYINYLGNILRFDLGSSLTSRGQNVWQIISQHFPATMELAVCSMLVALIVG
ILVGTLSASRPGTPLDLGGRLFGIITYALPMFWAGMLLQLVFSVQLQWFPNSNRFPPNII
PPTTITGLYTIDSLLSGNLNYFFLALHHLALPSLTLGILLSGIFERIVRVNLKETLKADY
VEAARARGIPENKILVSHALKNALIPVITVLGLTFASLLGGAILTEVTFSWPGLANRLYQ
AIADRDYTTVQGVLVFFGGIVVSASIVIDILNAYIDPRIRY
>OBU76174.1
MIKSDSPVNHSVPSPGGKTPLYQTGVEMKARFTNFGGWEMPLQYTSIIEEHQAVRNGAGM
FDISHMGKFNLQGKNLMAQLEKLVPSDLRRLQPGQSQYTVLLNPQGGIIDDIIIYRQSGK
NTDNEKVVIIVNASTTDKDRNWLSQNLDLNQIQFEDLSRDKILIALQGPKATAILQSFVA
DDLTPIKAFGHLETEILGGVAFLARTGYTGEDGFEIMVDSQTGLEFWQRLHGAGVTPCGL
GCRDTLRLEAAMSLYGQDIDDNTTPLEAGLAWLVHLDRKGDFIGRDILERQKIQGVERKL
VGLQTQGRNIPRHGYSLLSSGKIIGQVTSGTLSPTLNYPIALAYVTAELANIKQQIEVDI
RGKTYPAQVVKRPFYKSQNRVHK
>OBU76179.1
MVVLIALFVIGWVAAGLIGSLAYFMGEQSKPIHERNWRSESFARLAKSITGQEINYETRT

PAYGMDAYISQGLSE
>OBU76191.1
MNSTTASLSPTLNFEVGQISNFPILNNTFDKITINSLENIEQAINIAREDWDNFETSWDF
QTHPLLRENSPNISTSFTNWQNRTETAFRQLQLLEQENNRYWIKSYGLETELTPEVPLNE
ITLTCNPHYRYDHTKPQTELETLLLADTIKEYISYAVGCMFGRYSLDQPGLILANQGETL
PDYLKQIPNPTFTPTKNNVIPILDGQWFTDDMSDRFRQFLRLTFGEEHYEENLKFIEKAL
GKDIRKYFLKDFYNDHIKRYKKRPIYWLFSSPKSNFNALIYLHRYRPDTVSIVLNDYLRE
FRAKLEARQNHLKRVEVSADASQSEKTKAVKEINKLATIIEELNDYEREILYPLAIEQIH
IDLDDGVKVNYPKFGLALKKIPGLETKEED
>OBU76208.1
MNNISNLQFKRSGWQTTVIFTLGFWLSATMVLDGLIMPSLYITGMMKEANFTMAGYSIFW
NFNRVELVAAAVVLTAILAMGKAKSKWNLSSICWSVLLLMVALLDTYLLTPQMSALGSNL
SLMVTPGTVPETMNLLHGGYFILEVIKLVGAGCLFNRCWNDREIMAI
>OBU76230.1
MNFHQRKLYALLSTITENSPNTEFICQHLEVFQADIDALSQWWETQGHIAAAISSSSDRV
NLKTVENNGDTIELRHPLSGQEQIINYPQNLSLEAIAAEIQTVWQRISPQTDIDQEDKIA
KLYWWCWRFYPHLLASYNVALLTPAHSILPDCSHHSYKSTVAALVGAMYPQTWQQGEAET
HPYLLIFTFSPVQEFIKASRKFLDFWGGSYLLHYLSAIICWEVAKLYGPDAVITPSLWSQ
EIIDALLLKRYPDFKADFHKYEGTDPISKFWNQNNPPQSLCTAGFPNVITVLVGSKDEAI
KLGKHLEATLKNHWQEMARKVREDVKSKFRDKIADNDKLKEVVNAIAKDLLGENSEAKVK
DKNKEEPVEKAKKTPREELETELTKLRQAGCWEWNGLWDAQINHTWETYFVAVPLGNPEE
DLQIQINSNIQKKWIEGQNQIADIRITQQNNDTENPNHQKSIPTAAELNAYDSLNVGTWW
GSFQRRLAKAIQAIKNTRSWQIPIASGERSTLSGQYTALHPRFLYQNFHNGLGLPLESLG
LFWKVMAFVYPGVFNGSEKLNAIELTKRLAWRHGGVAENLGIELNSDDDYEGLIRFPNLC
SIAAANFATSHPRIIQSFWSDLRQKIEQNSELQSKHDDFCSRTNRPFQVRCADAALKKAL
PNYKKGLNAVMFSSKWLADDMNLKATEISALSTVVDTVQRKHFGDGSPADWWVLVLGDGD
GMGGYVDGSSLKNYSDYIVANLVDTQNIEWEKWDNLLNTKKRMGPATHVGLNRALLDFSN
RLVPYLAEQRHCGKVIYSGGDDVMAALPLADLPGFLRSLRAAWRGEDDPENEFISQGGYW
FWKNEETQPLEIPRRPLFTMGKDATMSLGVVIAHKSVPLPTVLESIWDAEKERAKKLSGV
KEKRQDSLGDEIIYPNKDGLCFRVIYGSGNTLEALMKGHLLPLWWNFLQAYQEVDFSPVL
YRLSEELPRHAEVTKDSFMFRKAAQAILVSRDQQLAEEVENALLGWLDAWEQWAWGAKET
ARESGEGEVLGTKSEDLANLLRFSAFLVSRRQQEVSWQDVGGKKV
>OBU76231.1
MEWYVIEPIDILLFRETKPFSPGEGAWAKSIFPPVPITVFQALRSLVDKAQKLEFFGAFL
LHHEPGRTPEIFLPTPKDLLSVSTKQIYEEETVSKDKTAKEWQRLTYLEPLNRQDPKWQH
LGFDPEHFPDGGISPMVTPVLSATPNNPESQNSPTEEYISGHPNPWIKAQALIKYLKGAY
LTYKHNGKDFHSDPWQKQVLSHIQMAANQRQVKSENGYFTEVAVRLHKHWKLIAAVNTKL
PSSIVRLGGEGHQALVYPLKDLFNNADKNNKKDNNKKHNFDDFQESVLQELESFRKPTNT
SNKAYLLTPGLAQTQPEKMIYGVYPYYWQEILAGCVSDRPLLWGGKSRYVKTPMSPQRAY
VAPGAVYRFKDNYEKLSETDKKSLQQLLPLPQAEVKEKWLQTLCSLNYGTLLWNR
>OBU76235.1
MSILIANIGTSDLAIKINVNGQQRYFNVLIIILDTAISETVKSHLSPILVEILLWIKTYLI
QKGTGLKVSWYMVL
>OBU76236.1
MENRLVVFSPCGTSLLTNQAAQEERALVTKYANTKHIEEIPPEDSLKLQSLAKRVEEKLA
FADLELAGKMSAELNGIIKLYNGKLEKKADTHYLLCTDTWLGEQTATLVEQWLRERGFTV
NRIRQTDLQTKDIESFQIALSDIVKFFENTIPGYRDSQHKIIFNLTGGFKSVQGFLQTLA
TFYADETIYIFETAKDLLRIPRLPVKMVVEDSVRDHLKVFRRLANGMKTAEVSGVPETLL
MRIGDEVSLSPWGGLVWQRTKKEIYRERLHPSPSEKVSYGPKFENSLRGISADRLILINE
RIDQLAKNLESGGEYNVSSLDLKALKGNPRPPSTLEIDAWSDQDAKRIFVHKSGNHFVLD
KLDSGLHV
>OBU76237.1

MSNLLERITVNLRQCDGRPCIRGMRIRVTDVLDLFVAGLSAEQILEELPDLEMDDLKAAL
TYQVATPNQQ
>OBU76242.1
MATTADEVWKLLGELIESQKETERKFQETDRKFQETDRLLREQSQETERFLREQSQETDR
KFQETERLLREQSQETERLLREQSQETDRKFQETDRLLREESKRVNNQIGQLGNRLGEFV
ESQVRPAAVKLFQERGIAVKEIASNTYIQTGKEGLEIDLLVINSSDIILIEAKSKLSEDD
VNEHLERLSKFKRFFPRYESYRVLGAVAGMVIPLDVSRYAYRKGLFVIGQSGDNLVILND
DKFRPRGW
>OBU76256.1
MIMNLKTKKAIEQLEQFVSIPLEETLRQHEKTTHQEIATNLFKNVASTVPAYQSFLATHN
IKRETIQTLADFQKLPVICKENYISVYSLPELCKDGILGGCDMIAASSGSTGKPTFWPRF
FTDELQIATRFEQIFHDSFGANIYPENVSVGLEQPIIREWVTGKFVLQVKEDQDQNRFLS
VVVELAPGLEGSEEKIIEITNSILAQLLRLNSEFANYVPGEYQRPQVELKATGDGEYFPT
GVKHKYTRNL
>OBU76278.1
MFGLCLLGQKVQKTSIDYSSLKNRLKTDINKVMKLTFINEAIEERYWTKTKYTINEIIIT
TIKEKEESDVENNKLANSDPKPSPEEKTINLPKKPTLTKQLIFQSILKSLGMNEYKNIDY
VTFQTDKIQQTHKRKIAQSIYKFQESQKNIQPTGYLDHKSQESHELKRKISQQFLSQVL
>OBU76280.1
MTRLYTDICNQDVRKLLETLANKGVNPEQYKETMTKIGDNLGNFLLTKINDKTSDIYLAC
TVEDADFLAKGILSELENHYHNIAFACFWNKRFSPFEIEDLKVAPILKKYQEPSHGKVQY
LIVIKSIISGACVVRTNLVNLIQKIEPEKIFIVAPVIYKNAEQKLKDEFDENIYSKFEFL
YFAKDDERTNEGEVIPGIGGNVYLRLGFDGQDNKNEYIPEIVKKRRSQFMRQKQEIHLSK
NISN
>OBU76285.1
MLKVTNTNANVNIKTNNHITITDRTKQFAIRIIKACYFLDEKSGVYRTISKQLLRSGTSI
GANVRESQSAQSDKDFIHKLEIALKEARETQYWLEILIESELVSKPKFTSLLQEANEIGK
ILVASTKKLKEK
>OBU76289.1
MKPYFTIISSLLLLFSPGLNLAGLTQVPSPIDQSQSVNCNPEKEGEDGAYSLQQQEAILR
QITVKVIGDNNGGSGTLLGRQGNHYLVASNSHTLVGVNLNNIRVETIDGQVYPAQIIPNV
NFQENNLDLTILKFTSNKTYCLPKIANNEINKQLPIIAAGYSGGDNSFISKEGMVEKITE
LVFKKGYEIGYTSDIEQGMSGGPIINRDGELVGINAMAPYPVLNHAYVYADGKLPTSSEL
RELRKLSWGITLKTLLAQIEPELMVAYRLTIPRIKQIVPESKLVGWLQEVEAKAKKITVK
IDSSNESNGSGVIIAKEGDVYTVLTANHVVCERQLVAQPCGNFNYKLMTHDGNVYPIDKS
TIQFQPGVDLAVVKFNSRENYPVATLANYNPTTDDYVFTTGYPKLKEKSDWRFSLGQVYS
KERGLLTTTSQLDLPNSSAGGLNSVTQSAASLAGGYELVYTTITFGGMSGGPVLDSQGRV
IGIHGKSEGEHAYDAITGDSGTSDGNKVQIGNSLGIPTSTFLAVASRINVQPQILETTPP
PQLTATEVESIRNAILSVDVTKTNTSASQWLERGNQLWRLRRYNDSIQAFEQAIEQKPDF
VYLGYYGKGLGHYGNKQYEEAITAFKQVLESRPDFVPARFYLSVIYRELKQLDLALTEVD
QAIKLQPNDSRLYNQKFLILMDAKRYAQAEESISKAIEINPIAPFYNNRGVVYSRNKKWE
LALVDFKRALEGNPQYAHAYANGAFVYYNKGIWNSVISSYTKAIEIDPQYPLYYNNRGVA
YYNNEQWQSALNDFSKVVTLEPRYARGYKHLGEVYAQFGDIQKARENLQQAARLFQDQRR
NAEYRETVSILQGF
>OBU76300.1
MLVSLNACDWQVRVATSSDLSGIAQIITESFYSQNGLWGWAFPLFKIGIYEDLRYRLQTL
MPHQTCLVAIHTSQSGNHQVLGTVELGVRFVHSWTNYNRLSPYVSNLAVDPRYRKYGLGS
SLLTSCEQVCKSWGFQDIYLHVLEKNHQARKLYLKMGYEIYRVESSWQDFFLPSRQFFLH
KRLK
>OBU76313.1
MTISIQVSRDSINNLDLSPALKVIESILQKESIISQEQQLRFDIDYPRQDDDPREISEIP
EIRLWFVRLDAQYPWLPFLLDWKSGELGRYTAMLVPHEFHKKEGIQYNPESLEIFLMHKI

FILSNWLKQNQIPARFRLKSLAQMLGYELEDGFFEMIDS
>OBU76315.1
MDKQNLLGLVIFLILLLIPGSLFSPLATPIDGWRAMLAAVTSATFATLLEGISPRGTDNL
SVPLITAIVVWLIIGR
>OBU76317.1
MDAKELWQRYQDWLYYHEGLGLYLDISRMRFDNTFVESLQSKFEQAFREMVDLEKGAIAN
PDENRMVGHYWLRNPDLAPNSQLRAEIVRTLEEIEVFADQVHTGSIHPPKENRFTDIISI
GIGGSALGPQFVAEALAPDLPPLNIHFIDNSDPAGIDRVLSRVGDRLSSTLVLVISKSGG
TPEPRNGMIEVKQAYSRRNLDFAQYAIAITSMGSNLDKVAKSENWLGTFPMYDWVGGRTS
EMSAVGLVPAALQGINIRAMLDGAKQMDDATRIANIKNNPAALLALSWYFSGNGKGEKDM
VVLPYKDSLLLFSRYLQQLVMESLGKERDLDGKTVYQGIAVYGNKGSTDQHAYVQQLREG
VPNFFATLIEVLEDRQGASSEIDPGVTAGDYLSGFLLGTRQALYENHRDSITVTIPQVNA
QTVGALIALYERAVGLYASLVNVNAYHQPGVEAGKKAAAVILDLQNKVIKVLQSEKKGLT
IGEIADKAGASEQVEPIYKILRHLHANNRGVVLTGDLSKPGTLTVSLT
>OBU76320.1
MFPLWLKIAIAAGWVFLIIILIAWLVSRFTPSQPEIIRKIVHIGSGNVILLAWWFHIPAYV
GISAAILAGLISVISYFVPILPVINSVGRQSLGTFFYAVSIGILVGYFWYLQKPEYAALG
ILIMTWGDGLAALIGQRFGKHKYYLFGANKSWEGSLTMTVVSYLVSVIILLATRGSSWQI
WLVSALVSVTATLLESVSFLGIDNLTVPIGSAILAYGLTSQ
>OBU76345.1
METITLTNSRFLSILQLASPSLPVGAYSYSEGLEALVENRTIHNRVGLKNWLDSELRYGS
IRIDGAVMIRAIQSTNLADLEGLRKWNYWLSAFRDTQELRAASWQMGRSLLQLLGKLSPP
ITSISNTVGYPCNYAIAFGIGCGHWQIDARVGLLVYLHSWASNLITAGIKLIPLGQTSGQ
ELLWELQGLLDITVEEILSLEDDHLSCCSWGLSLASMQHETQYTRLFRS
>OBU76356.1
MITLETNTRNRLILQLLFFCGLRVGELTILLWADIKDNGSTAYVHITGKGNKQRTLIIPP
LLWASLKSHKTTNESPVFKSRKGGKNLTQKAVWDIVRTASQRIGISASPHWLRHTHASLA
LHNGADINQVSTSLGHSSVATTTKYLHARPNDCSSLYL
>OBU76359.1
MDTGTDKGIPDWLNPDRLIELIELLREAGYKIGISQHIAAQDIILFLITQGQTLDNPKQL
RNLLGPIFSKSLIEQENFQYHFDNWLKLIKQIGLDTEKVSLPVARRRIPWRQLQWILIFI
AVLMGTPTTQPKPLSTNPPSSPTTTPTPKQTLPSINPTPSTTGTPTPEQPSSVNWQITLI
YFLLSLCFLYIGSQLLWLWRANLFLHRSSTKVIPDLQTISIPDLEQNLFPRYLFISIAKN
FRQRILVPTNILDVEKTIDAFFQRGRWVEPIYRNSAVIPEYLFLVERTSYRDHQSKFVAE
MIEQLKNDGVYVETYFYDEDPRICFSSDEHNSSLKLHQIATKYSQHYLIVVSDTEKLFSS
ITGELEPWVNQLLDWQNRVILTPTPVENYSYEKFVLAQDFLILPATSTGLQTLSQKLQQQ
IATNHYLITETPSPLPESLRLRPLHWIERNSPPTKDIDAMLVSLEEYLGKNNFYWLGACA
VFPQLHWNITVYLGNTLKTEMGHSLLEVGSLTKLARLPWLRYGYMPDWLRSLLIAKLTNE
QKHTIRTVLKDLLVTAVQGSKGGLQLEVAKKHHSFLSKLLANPIMFYLLSRRVFEGSELR
DHLFVSFMTRQSKLTIEVSDTFIRLLQRTPQPLITRGILKKTGFALGLLTLTIIAQEVSV
RVTYPPTVIRTESLLSKLEALLKAKNFKEADLETDRVMLAVANRQSEGWLRIEDAENFPC
KELRTIDNLWLKYSQGKFGISVQQEIYKNLGGTKQFDENVWRSFGRMVGWRDHYSDYNFS
LSAPTGHLPSLWSVDGRRRQRERLQLVIELRLWVGGLFPPVKTCRV
>OBU76361.1
MTRSPQLTASLNSSVVRIYNPTSNNVVGAGFLVEDRLVLTCAHVVADALGIHIRTVEIPN
GTVELDFPLVAPKQRLRARIVFWRPVNPDMFGEDIARLELEPPLPDKAQAARLVRSENLY
NHPFRVLGFPLGEDNGAWAHGKIKGRRANGWLQLEGTRQTGYSVDHGFSGAPIWDEQLQG
IVGMAVAADINRPKIKVASMIPTEVLDTALTPSDLPKRKIPSLLPYLVNRIEQEYELGRV
IVRNSPSPLICIIHGDEFQSHDKFLERLHKVSLPRFLGEESIKKYHLPSPPKLNNWDEFS
GHLHRTLADIVIGRNSASETEIKTEINDFFHRYPCPILIHTHLLTEQLKEKFETLDNLVK
FWCDEWTINSNQNLIICIFIKYQIKRKMNTENSGIIKLFNFLNLNYLVKKYRYEKANQKI
SQHIKSLCNSERSNPLVLVPELTGVSRSDAENWVRDHTQNFIGEAMAENLMKRIRNLFDN

WEEKNSSNKIPMDHLAQELIKVLKSNC
>OBU76362.1
MKSIIEFPLENGESILVEVDEPGFTDSRISRRDDIAKKAEKTFESALEKVRPIADIIMTR
VNSLSQPADEVEVKFGIKMSGTLGVLIASGNSEVNYEITLKWKRNP
>OBU76366.1
MGTGDRIALPWGDREAIQETAYSQLRRTVTLSAIAPISLVVRAIVINGWNIVIKVTRIAI
VISWVSFLRFISWDG
>OBU76373.1
MAQTRNFISNVDDTGVVNSRVVSPGGHREPLKHLLIGSKKAVISTIHYLHVFGYANATDW
SDLTPTGNSGEVMSILVRHIVIN
>OBU76377.1
MKRFLTLALILTLFLVSSFSFSTSPSYAYSQSDLEKLLETKICIKCDLSHANLERADLKY
AYLNGANLSGANLKYADLSGANLKYADLSGANLEDTNLEGTDLEGANLKDADLLGANLED
ANLEHADLKGAYIRSANIEHTHTLDANLTNTQL
>OBU76379.1
MKSQDFRKADIETAIVMLAVANRQSEGWLRTEDAENFTCKELRTIDNLWLKYSQGKFGIS
VQQEIYKNLGGTKQFDVNVWRSFGDRVGWRKDGSWLKYSDLNNFSLSAPTGHLPWVGVVG
GGREGFFWFLWVVVEGRFPS
>OBU76404.1
MKSQDFREADLETDRVILAVANRQSEGWLRVEDAENFPCKELRTIDNLWLKYSQGKFGIS
VQQEIYKNLGGTKQFDVNVWISFGDRVGWRKQGSWLDYSDLNFSLSAPKGHLPNEGRKWY
ILDGWKVHLPRGFSRPSLLSRHVECNP
>OBU76405.1
MKTQKFTQLVIIGIIVAFITIPSHITLALRPKEIADIATQVTIRITGISNGSGVIISRNG
NTYTVLTNSHIFENHPNGKFEIITPDGRKHQLNNLRRIANLDLATLEFNSIQEYRVVEPG
DSSRITRGEDIYVSGFPANQDLNFSSDRITRIITKARPGGYALVYRIGAFPGMSGGPILD
SDGKLVGIHGETQSVSLGPGRSTPEEYGIPLQTFLNATSISSPPPTRAGTQN
>OBU76411.1
MVRQIAPKTQLGAIKSNKNQLGETIVPEIVYPESDGEPMADNTKQFTWIVKIKENLEILF
KSNPDVFVAGDLFWYPVEGSNKIKLAPDTMVVFGRPKAHRGSYRQWEEDNIPPQVVFEIL
SPGNTQDEMDKKSCFI
>OBU76412.1
MFYLKHGVEEYYVYDPDRISLEVSIRENNSFKEIENFTTWTSRRLKIRFDMTQDELVIYY
PDGSKFLSPVELSNYAEQETQRAEREKLLKEQERFLKEQEQIKYQTLLSQLKAKGIDITA
LE
>OBU76414.1
MRQPLTTPLAGESLNLPDHTQLPDSDDNFVKNFQEHPQSIILTTSIEPLLKEIHPNGDYC
IGQDSGIYWRFTEPPEKGVEAPDWFYVPGVPSRLNGQLRRSYVLWKEKVPPFIVIEFASK
NGKEEKDSSPPPEGDEIDPETNKPKKAGKFWVYEQVVKVPYYAIFNGFKGTLEVYHLQGK
RYKEIKANRRGHYAIPEMGIELGILYDNQNPPTPWLRWWDEKGSLLLTGNERAEQAEAIA
IRERLAKEQEQEAKEQAEAIAIRERLAKEQAEAVAIRERLAKEQAEAVAIRERLAKEQAE
AIAIRERLAKEQEQEAKEQAEAIATRERQQKEKLVAYLRSLGIDPEKI
>OBU76435.1
MRITKSALTAGVLAGVLGIFPPMITLALTSEQVASIAEKFTVRISGVAPGSGVIFNKNGN
TYTVLTNFHIFDRGRTLRDGAYRVTTADGKTYPMINTVRIPDLDLARFNFRSNEEYRVVK
IGSSDKIVRGKRIYVNGFPEQQEVNFLPGQVNRILAKPRRQGYVLVYRIGAFTEMSGSPI
LDEDGNLVGIHGLTEDVDVEGGGTTPEEYGIPINAYKSSSVYSNNAEFYFNRAYNLYESG
DKQGAIVDYTQAIQINPNYALAYIGRGNARSDLGDKQGAIVDYNQAIQINPNYALAYYNR
GNARSELGDKQGAIVDYTQAIQINPNYAYAYNNRGLARSELGDKQGAIVDYTQAIQINPN
YADSYYNRGIARSELGDKQGAIVDYTQAIQINPNYAYAYNNRGLARSELGDKQGAIVDYN
QAIQINPNYAYAYNNRGLARSELGDKQGAIVDYTQAIQINPNYADSYYNRGIARSELGDK
QGAIVDYNQAIQINPNYADSYYNRGIARSELGDKQGAIVDYNQAIQINPNYAYAYNNRGL

ARSELGDKQGAIVDYNQAIQINPNNAYAYNNRGLARSELGDKQGAIVDYNQAIQINPNYA
DAYIGRGNARSELGDKQGAIVDYNQAIQINPNYADAYYNRGLARSELGDKQGAIVDYNQA
IQINPNYADAYIGRGNARSELGDKQGAIVDYNQAIQINPNYADAYNNRGIARSELGDKQG
AIVDYNQAIQINPNDADAYYNRGLARSELGDKQGARGDFQTAARLYQQQGKQNDYQDALN
RISQLR
>OBU76444.1
MLNFFSVLIYIINNLQPLLIPICFVMAWSIITLLVVNLWTATKDTVQIAQKMHQIPCPNC
QFFTNNYRLKCTVNPYTASTEAAIGCKDYIGS
>OBU76487.1
MIQSYSMNSTSSLISPDVSGYQSHDIKPVKLGVMASGNGSNFEVVAQAIKSGDLNAQIQV
LIYNNHLAKAAERALNHGVEAILLNHRHYQKREDLDREIVSTLRQYQVELVVMAGWMRLV
TQELIDAFPNHIINIHPSLLPSFKGVRAVEQALEAGVKITGCTVHLLRLEMDSGPILMQA
AVPVLPNDTAETLHARIQVQEHRILPLAIAQVADKI
>OBU76495.1
MNVQHKTDLDVARDIKELCDVINEAIEDARDVLKDARDILQNAQVVKSDLEEIETRSLAN
IESNLGQINQILLDIGGMENLRQLLDQSITAQTSLDGTKQEAIALHDQISQILLETGGVE
NLRQLLDQSTTVQTSLDRTRQEAIAFQNQIIELGITSDFWANIQQFLTKIQQVDSSSQLT
YQDIQNIQQNLQSIEAVVQSRSQELASQYNQLQTYLSQSETKLENLIQTYGRISTEIETN
YGYINQANENIESNLGQINQILLETGGTENLRQLLDQSRNVQTSLEITKQGAIAVHNQIS
QILLETGGTENLRQLLDQSRNIQTSLDRIKQEAIAVYNQISQLGISPNLWADIQQVLTNI
QQAESSSQLTYRNIQHIEQNLQSIEAVAQTRSQEVASQYNQLQTYISESETRLENLVQTY
GRISTEIATHYRYINQSNKSIESNLGQVNQILLETGGTENLRQLLDQCRMVRNSLDRSKQ
EAIAVHNQINQIILETGGLENLRQLLDQYTTVQTLLDRNRQEAIAVRSLDAYLMEFRKPR
NHRELRKFLWNELGFVGLIIYFLRLITSRK
>OBU76503.1
MAGNHVIFIHPDGTSPSHFAFARFVDKGPDGRLNWDNLKKTGVYLGHMEDQLGGTSNSGA
VTHATGAKAYAESFGFEAGNTPIVSLDGSNKTIVQAARDAGKVTALVQSGAIYEPGTAAF
VAKTKEIVNPDGSRIVPRAQAAEIAKQVIDSGVDFIMGGGELNLLPVGTNGFHGTAAQLD
AVSTNSLQRPTENLIEKAKTLGYTVVYNKDQLNDLLTLPTPPTKVLGVFAIVHTFNDRAE
EQLAASNLPLYSPAAPTIGEMLDVTQKLMERHPNFSKGSITILEEEGTDNFGNNNNASGT
LEALRRTDAAIGVAMDFVKKYENTLIITAADSDAGGLQIRDPLGTGNVGNINNNPIDSTS
ARNVPMDGRTGVASPPFVSAPDADGDRFNFGVAWAGLPDFSGSIISKAHGLNADRLPPTL
DNTKIYELMYETLFEKELTARNPADNNLAAPAATKPTGNVIFIHPDGTSPSHFMALRNVD
LGPDGRLNWDKMSHAGVYLGHMENQLTGTSNAGAVTHANGVKVFNESFGLNEDNSIVTPA
SGKVGYTILEEAIASGKATALIQSGHIGEPGTAAFAAATTNRDGNNIRARDKTAEIAEQV
IRSGTQIIMAGGEVYLLPKGTTGFHVTAAIDAEYTDAADRPTINLINLAKSLGYTVVYTE
EQMNAAVATATANTKLLGVFAANHTFDDRREESLGLNTNSPLPLYVSTAPTVAEMLTASL
KILRQDPQGFFVVIEEEGTDNFANNNNAVGTIEAIRRADAAIGVAMDYVKQDPNTLVITA
ADSDAGGMQVFQFAPYNRPSGNSTSSPDLADSEPSAPFINVNRTTTNNSRVFLDGFEGST
GTTADPWIPFAAKNSIDGPMGNFGIAWVGTPDFPGSIVSKAYGMNADKLPGTVDNTQIYD
LMYQTLFGVTPEFAATQQEVKLVSGTSGNDVLFAGANSSPSSFDGINDLVFTGAGADQVD
ASTAKSAIAGNNSVFTGSGADEIYLNNGDRTFGGSGNDIFYATDASSYRISGGTGNDTFY
LGANGRALGGDGDDRFFVGEGGGNVISGGAGVDQFWILTDNPTLLKTPNTIVDYTIGTDV
IGITNQVATSVSDLTFSGSNISINGVLVATLNGVNAASATFVFANPSI
>OBU76576.1
MTTDYPKIDIAPFIDHSLLLPTTTEPQVEKWCEDAYRLNFAAVCIHPSHVKQAAELLHNK
KPQVCTVIGFPTGLTTSTVKLHEAQEAVENGATELDLMINLGWLKIGKTEQLHREIATIC
EETGKPVKVILETSLLTDTEKKVAAEIAMDAGAAFLKTSTGWNGGATVKDVEFLKQITKE
RVGIKASGGIRTHQDAIDLIMAGATRLGTSRGIDLLHQRLDTEKSQG
>OBU76581.1
MSVLAAIAVLATLILVHELGHFIAARSQGIYANRFSLGFGPILLKYQGSQTEYTIRAFPL
GGFVGFPDDDPDSTIPPNDSNLLRNRPILDRAIVISAGVMANLVFAYLVLALQLGVVGIP

KEFQYQPGVLIKPINEQSIAYQSGIREGDIVISVNGRELVGGKDSTLYLTREIQNHPRQP
IDLQLQRQDQEIALQITPGENPEGKGLVGVELAPNGKAIYERPQNPIQIFTVAGERFQQL
FVGTIKGFGQLITNFQQTASQVSGPVNIVKIGAKLAADNSANLLSFAAIISINLAVINIL
PLPALDGGQLFFLLIEGLFGKPLPMKIQEGVMQTGLVVLLGLGIFLIFKETLQLSFIQQI
FQKM
>OBU76641.1
MTITLTEKAEFRLRAFLKTSATQENQSGVRISVKNGGCSGYEYGIEITSQPQPDDIVTKQ
GNVLLYIDAKSAPLLEGVEIDFIEGVMDSGFKFSNPNATDTCGCGKSFKSEDCSPNGVPY
S
>OBU76645.1
MSQNNSVNGNGTVSPFLKTLVQQIRASDSYGFYRNWSDELILKPFVVTKQNKKQISVEGE
IDPATIARINAFFRAVAASIEQETGMISNVVIQLGHEGFGWALVFSGRLLLAIKTLRDAH
RFGFESLEKLNEEGQNFVQKGIDLAQCFPQVGNL
>OBU76660.1
MRQVLKINFIKPLIVFGFLLGPFFLALGLTIGTTSQWTILPLGFITAGVVICLFWVLVQA
HKSQFWQQRSTQSNTNAVIAILAVLTILGLINFLGNRYHIRLDLTETQLFTLAPQSQEIL
RTLPEPAKLWLFTREKNPEDQELLKRYGQQNPQFSFEYVDPQTRPGLAEKFGVNDFGKVY
LEYNNKHQLVQNVNENERLSEVKLTSQLQKIISNDSPKVYLLQGHGELEIANSKNPENSI
SQAIQGLTDNNFTPLALSLTQQTTVPADAGVVVIASPKRELLPGEITALENYLNIGGNLM
LMIDPNHDPKLDTLLKSWGVQLDNRLAIDITGGNLGLGPASPLITEYGQHPITQDFRNGI
SFYPIARPILVEPTPGIQSTPLLRTKAYPDSWAESDQENEKLEFNEGKDLKGPLTLGVAL
TKKLASPMQENSPTKTRESRLVIFGNSQFARDGLFQQQLNGDVFLNSVSWLSQKEEQPLS
IRPKEQTNRRIIMSNMKANLLGVSSLLILPLIGFLSSVVIWWFRR
>OBU76665.1
MPIYIYWGEDDFAIEKAVTLLRDRILDPLWTSFNYTTFLPDQGDSVIQALNQVMTPSFGA
GGRLVWLINSNLCQNCPENIFSELKRTLPVIPESSFLLVTSPNKPDERLKSTKLLKQFAD
FREFSLIPPWKTELLVQAVNQAAQDIGIKLTPQVAETLAESIGNDSRLLYTEIEKLRLYL
ASSNIPLTSTIVTQLVRNTTHNTLQLAAAIKAGDSAKALGILADLIGAAEPGLRIVATLT
GQFRTWLWVKMMMESGERNPQAIAQGAEVSNPKRIYFLQQEVKSLSVEQLVFSLPLMLEL
EVSLKQGASEILTLQTKVIELCQLYQKSGTFQPENKSNLLT
>OBU76707.1
MNTMIFAKHLFCLSSLLTLVVLGSGSSATAQVIPSDNAAPPGQSAQSQMDAALTRPIQEG
GSYIGVGGNIGISGGSSPLADGNFGIVSKLSVFNSFSIRPGAVLGSDTTFLVPITYDFAY
PQIKQVLNGSALPIAPYAGIGVAIKDSKDTNVRVQNDKYPKIALLLSGGVDMPLSDRFTA
TASINAGFFRSVDIGLLFGVGYRFSGI
>OBU76710.1
MNFREEFKLLLRARYPLIYIPTQEEERVEATIREEATNQGNRPVYTWDFVDGYQGNPNDQ
GFGKRNPLQALEFVEKIPSSVCAIIILRDYHRFLDDVAIARKLRNLSRLLKSQPKNIVLL
SPRVLIPDDLTEVMTVVEFPLPTVPEIKTEVEKLLQVTGNSLSGKLVDDLVRSCQGLSME
RIRRVLSRALASHGELLPEDVDLVLEEKRQTIRQTQILDFYPATEQISDIGGLDNLKDWL
IRRGGSFTQRARQYGLPHPRGLMLVGIQGTGKSLTAKAIAHYWHLPLLRLDVGRLFGGLV
GESESRTRQMIQVAEALAPCVLWIDEIDKAFSGLGSRGDAGTTSRVFGTFITWLAEKTSP
VFVVSTANDIQSLPPEMLRKGRFDEIFFVGLPTQEERKAIFHVHLSRLRPHNVKGYDIDR
LAYETPDFSGAEIEQTLIEAMHIAFSQNRDFITDDVLEAASQIIPLARTAVEQIQQLQEW
AASGRARLASKHSSLTERIQRQL
>OBU76739.1
MADVESQAESKLVPAGQVSTWLTENGFDHESLSPDVNGVEIIKVAPDFLLPTATALYAYG
FNYLQFQGGIDLGPGQDLVSVYHLIKVSSDADKPAEVRLKVFLPRENPVVPSVYWIWRTA
DWQERESYDMFGIIYDGHPNLKRILMPEDWVGWPLRKDYISPDLYELQDAY
>OBU76748.1
MLSYTTRVSIGVIEPRWLNEQGELLPDELLEAFLEFWRQHGEPLLKSAPYHEIAPHLVLM
AFLHRVVNGGGTLEREYAIGSGRMDICLRYGKVVMGIELKVRKEKLDPLTQGLIQLDKYL

DGLGLDTGWLVIFDRRPGLPPMGERISTEEAISPSGLTITVIRS
>OBU76750.1
MKDRTATIILCLFVGGLGIHRFYLGQTGLGLLYLLFCWTLIPSFIAFFELFIFIFTSDDE
FNRKFNSASK
>OBU76752.1
MALGDSNTVTIGQEVYVSGFPSKQDFTFRGVVGGGVWVGVFGFSPPGELLEVGVGTFPPV
KTCRV
>OBU76791.1
MSLELFSLEKIQEFAQTYGYWAVFVGILLENLGIPLPGETLTLVGGFLAGSDELNYWLVL
GDAVTGAVIGGICGYWIGRLGGWSLLVKAGKIFRISEERLVSIKDQFSENAGKTVFFGRF
FALLRIFASPLAGIAEMPFGKFLVYNLAGASAWASIMVTLAFFAGKIVSLEQLVAWVSQF
ALLALLILVLVIVLPIWWESRQVKHSGE
>OBU76792.1
MKIIPKTFNTTNFIQFHILEYFLIDVQIFARSANFIIVKFATEIWQFLCLYYEKLSLKSK
FIPDRIHF
>OBU76852.1
MTIEFTKYHGLGNDFILIDNRCDLTPLITPEMAIRMCDRHFGIGADGVIFALPGQKGTDY
TMRIFNSDGSEPEMCGNGIRCLAAFLTELEGISRSKDYYLIHTLAGVIRPQLTDDGQVRV
DMGSPRLLAQEIPTTLAPGNSKIINLPLEIGNQTWHITCVSMGNPHCITFVEDVASIPLE
IIGPEFEHHPVFPQRTNTEFIQVVNPHHLKMRVWERGAGITLACGTGACASLVAGVLNNL
CDRMAIIELPGGNLEIEWSETDQRIYMTGPAERVFTGKM
>OBU76858.1
MLTNWRNFLKNAGEWRGSFTRISGQGEILDSTLSILNLEATNNNETVLFRLRRFQGHDYD
SLVIQDYQQEYTSLAKENIFFETGAFSKGTVQLAPFAEFGAEYGFVHENRRSRLVQLYNK
DGELSGLTLIREFRSFTDAQERPQLTVEQLIGKWQGISHTVYSDLRPSNKETTYLEIKKL
DNGHLEKQQLFAGEEMISLGKIVDNRLIFEPSSRQQSNQKLNNEEILLPDGVSSNVPPKL
ERRKEFFIEAGWLVKDTERQRLIRSYNAQGEWISSSHIVEIKMA
>OBU76889.1
MSDWAKIQSDYEDFHQQFSSIIIATVSDQGIPNASYAPFVMDELKNIYIYVSGLSIHTIN
IESRPDVSVLFIEDEDESPNIFARRRLNFDCRASLLERESSKWQEIVNQFQARFGTIIEV
LKGLADFRVFQLTPTGGRFVIGFGAAYHIHGDNLHQLVSISRD
>OBU76908.1
MNSQQLATYLELTDSISKPWLLVQLRLKKLQERRHDISTEVYIEELADIHQDMMNLGEWW
KGMEDEVF
>OBU76944.1
MKKTVVLLKGGLGNQMFQYAFARSISLKNSSKLVIDNWSGFTFDYKYHRQYELGTFSIVG
PPRQPNRKVSFLVLRTKV
>OBU76946.1
MNLLKRLNINEYNRQQWLARVLTELPDGYRILDAGAGELKNRVYCNHLNYVSQDFCEFQG
RGECNEGLQFNGWDTSRIDIVSDISDIPEPDASFDAILCSEVLEHIPEPTHALDEFARLL
KPGGVLILTAPFSSNVHMSPYYFCTGFSKYWYEYHLVQRNFNITELVANGDWYTLLRQEI
MRLGGVDRQGGIWVWPIGYAYALLGLLYFGLRGKYLREDIACFGWHVLAVKRTV
>OBU76947.1
MNFSVLISVYYRDDHLCLDQAIRSVITQTCQPSEIVIVKDGSLTPILDSVLETFVATSPI
PVKLVPLPKNVGLGLALQTGLQSCSYNWVARMDSDDLSLPQRFEKQTQLIKEKQLDAVSA
WIEEFDVTPGDLKRVRKLPTEHQQLVQFSQRRNPLNHPCAFFNKNAVMSVGSYESMPLFE
DYYLWLKLIQNGYRIGNLPEVLLYFRVGNGMIRRRHGWEYLKKELKFYYRVMLNGLIPSQ
TLVVSVLLRLPLRLLPIRLLQFLYEKVVRY
>OBU76960.1
MLSITNEKSQKNYAGPKKIILIEHLNEIGGASRVSISLVHGLSDRFDFHVIIPSGQYLQE
FEDGKTKVYKMSYSSFRPTLIPNKWVDQISLVLEIRSCVRKILKDKETAVIHINGLPNIL
PAIATRFLGLPTVWHVHETAFNPNWAFRLLTCFACSISNSVIAVSNSTAEDLIKNILFKS

QANKIKVVYNSAFSSIKSKETDQKNIIKSDNTFIVAMAARIVPQKGIIEFLKIAQKTTEQ
SKNIEFWLAGPKVIKHNRYFQSVISVIEDDSSKQIKYMGELSSCEYFFKMSNIVVNTSLF
VEPFGLTLVEAMSLGKPVIGPPYGGPGEIISNNENGLLIDPKNTLLFSNTIIELSKDSER
LNRLGQAGFMKYQNSFSQENFFNSFSSEYFTLLE
>OBU76963.1
MLFKVPRIIRPVYLFLTALFPGWKDSWLLVARKKQDWSVKKTLSQSELAAILGNYSSYDY
TQ
>OBU76964.1
MNINKSKVPKPRILVFIDYYLPGYKAGGPLRTLHNMVEQLSDCFQFLIVTRDHDINDSHP
YDGLETGVWTSQGKAQVFYIPKNQWGVCSIRRILRETSYDILYFNSFFSPWMAGLPLLLR
YLKQFDPVSIILAPRGEFSPGALQLKAGKKKLYMAVLFRLGLCRNLIWQASSDKEGVDIY
NVFHKAGKGSDIKIAPDLPSLLYANKYIPNDNQISPTRLNSLKILFISRISPMKNLDFLL
RVLRNVSSPVDLSIYGPIDDSNYWSICQALINDLPRQIKFEFGGEIEHERVGEIFKQHDL
FAFPTRGENFGHVIIESLCAGTPVIVSDQTPWIAAPDGALEVLPLDAEKWQAAIERWANM
QDSVLLKMRRGAFAYARSYLESNNALEQNKELFFAAMKNGVK
>OBU76965.1
MPTIAAITTCFNRKKKTISALTSLYTSLLNTQDVNLSVYLVDDGSTDGTSEEVSKQFPTV
KIIKGSGNLYWSGGMRLGFTEAMKTVHDFYLWLNDDVILNSYAIENLLKCYLRNLSISGV
ESVVVGALSNLDNDQVIYGGHRLVGSKFLRKNQFVYSPDEDLECDSFHGNCVLIPNNIAS
KVGGIDPVFIHAMGDSDYGYRCSLQGFKIFCCKEIVGRCDNDQGNYKLYSTLAQGNWKFL
PLFLRLKVVTNPKNFPFKPWFIYSYRHLGWLWLLRFLRPYLLAIFPQLIRTQ
>OBU76966.1
MIQRQNCFHELFAYLGKLKYTILLLGLVIWLLSPMLGLIPLLIFSQIDVSQSVIKNTRKK
NYILNYFCILAVLFTVTITASTYSVVGDLKQYTIIYDQLGGTNFFDFFKKINMEPVTFII
PDLVKRYFYLNRFDFILLQAITMNCAYFALARVFMPNYYPTVIMLNICSIAYFQQLFLMR
QYYSFIFLVPFIYLVNWKYKLVLGLIALFTHSSTVIFTIPQIIVTMTITDQRVKHGKEDS
ISILKLVQSFQRWLKKLLLDRFVLYVALLTITLSFILLTKSGFISNTQVLLGSNAFVQEV
SPRLATTLSSYSNTEYLLGLSRDLWKVAVIDIMFLPLLLVQIRFNREPLVCYSWVLAFAS
SAISLLLFYVGIPAFGRLVYFLSGLSGFFYTFVLKSSNLFHKRNFFSFAIFGAISAKIVY
FYYFLFSFLRSDANPYLWGGHVIQANIYDYIEYLLNINRNL
>OBU76967.1
MLRDRLSAHNGEKKGKFREIFERSTNWGDSIIDLLDWMHDAKSFFPKTIGTMIRWFGEIV
GYFDGRTTSGIVEGINNKLKLIKRLGYGFRNFNNLRLCSLLHRHFSINCP
>OBU76969.1
MLNSEIKSPLTNESKVEYVRSLSPQEIANKWQSSMDIDVGSVFRNLPAIEHWRCVQTGIV
>OBU76970.1
MKKVNLACGSVYIKQSDWINLDYQPMGDGVIKSDLLGTLPFKQGSVSLVYSSHFLEHIPR
SKVAHFLSECFRVLEPGGTIRLVLPDLEEICSQYLYNRQRSDHKKADFCVVEMIDQCVRL
ESGGELGSLYKLYSQSYDRDLEMVEYIRSFNGELLCKAENHNNSPLSKIASLLEEPKLLW
GKIAYHLEQYYIRMIVSLLPKAFRTQNVSLATVGERHHWLWDFRQIQQALESVGFIATSR
CSFNTSMFPDFPFQSLDMDANGFPRKGLESMYVEAKKPK
>OBU76972.1
MLSFSTPVIFLIFRRPDLTAQVFERIRAAKPPKLLVVADGPRNTQEELLCQQARKVTEQI
DWNCEVLRNYSDVNLGCRDRVSSGLTWAFEHVEEAIILEDDCLPHPSFFSYCENLLNYYR
HDERVMVVSGDNFQDGKHRTPYSYYFSRYNHCWGWATWRRAWQHWEFNPHKWIDFRDSNL
MRFIFDDPDEENYWISIFNTLFLEGKPDTWDYVWTFACWSQGGLTALPSVNLVSNIGFRS
DGTHIVGDGRLANMITQDIGEIHHPPFVARHKDADTYTFKYVFFNNTSRLTAFLKSLYQK
AKKFLTFTK
>OBU76977.1
MSIAIASKILGKSHPPFIIAEMSGNHNQSLERALEIVEAAAKAGVDALKLQTYTRECFAI
ALIYSGFHVNKPQYNSIKPEKSD
>OBU76984.1

MLKVRVIPTLLWKQFGLVKGNRFNSWRRVGSVLPAIKVYNQRDVDELILVDITANTTGES
PDFESIADFSQECFVPFAVGGGVTNIDHVQNLLRSGADKIVVNTAAYSSPSLITDIARRY
GTQCVVSSIDVKRKSRSDWICFSHSGSKDTGREVVSWARELEDRGAGEILITSIDNDGMM
QGYDLSLIETISQSVKIPVIASGGAGNYNHMIEAIKQSGASAVAAASMFHFTEQTPAGAK
EAMKKAGIPVRLNFTRR
>OBU76985.1
MKVGIIDYGVGNLGSIAMAVEQLRSKPVLIDRAIDLHAVDALILPGVGNFTDCMQILVKG
GWVDAIKEEVTNYHRPLLGICLGMQLLANIGLEGALDSTTGTQGLGLIPGRVVSLKSQGC
SLRVPHVGWNNITKLDSKPWLLNGISDGTDFYFVHSYVFMPDEQSTVLARAEYDISFTAA
IGLGRVWGTQFHPEKSSRAGIQLLRNFLDG
>OBU76987.1
MNYKTQQEQFWAGDFGTDYIQRNQSEQLLASNLAFFSRTLYAAKGVRSCIEFGANIGMNL
KALKLLYPGIDLHGIEINQQAARELTNVIPADHVYSESILEFCELRHWDLVLIKGVLIHI
NPEYLPVVYTKLNNATSRYLLIAEYYNPSPVAIPYRGHLDRLFKRDFAGEILDRYPEFSL
VDYGFFYRRDPNFPQDDITWFLLEK
>OBU76996.1
MATTADEVWKLLGELIESQKETERKFQETERFLREQSQETERLLREQSQETDRKFQETER
LLREQSQETDRKFQETDRLLREESKRVNNQIGQLGNRLGEFVESQVRPAAVKLFQERGIA
VKEIASNTYIQTGKEGLEIDLLVINSSDIILIEAKSKVSEDDVNEHLERLSKFKRFFPRY
ESYRVLGAVAGIVIPLDVSRYAYRKGLFVIGQSGDNLVILNDDKFRPRGW
>OBU76999.1
MATTADEVWKLLGELIESQKETERKFQETERFLREQSQETDRKFQETERLLREQSQETDR
KFQETERLLREQSQETERLLREQSQETDRLLREESKRVNNQIGQLGNRLGEFVESQVRPA
AVKLFQERGIAVKEIASNTYIQTGKEGLEIDLLVINSSDIILIEAKSKVSEDDVNEHLER
LSKFKRFFPRYESYRVLGAVAGMVIPLDVSRYAYRKGLFVIGQSGDNLVILNDDKFRPRG
W
>OBU77006.1
MATTADEVWKLLGELIESQKETERKFQETERFLREQSQETDRKFQETERLLREQSQETER
FLREQSQETDRKFQETDRLLREQSQETDRKFQETERLLREQSQETDRKFQETERLLREQS
QETERLLREESKRVNNQIGQLGNRLGEFVESQVRPAAVKLFQERGIAVKEIASNTYIQTG
KEGLEIDLLVINSSDIILIEAKSKVSEDDVNEHLERLSKFKRFFPRYESYRVLGAVAGMV
IPLDVSRYAYRKGLFVIGQSGDNLVILNDDKFRPRGW
>OBU77008.1
MATTADEVWKLLGELIESQKETERKFQETERFLREQSQETERLLREQSQETERLLREQSQ
ETDRKFQETERLLREQSQETERFLREQSQETDRKFQETERLLREQSQETDRKFQETDRLL
REESKRVNNQIGKLGNRLGEFVESQVRPAAVKLFQERGIAVKEIASNTYIQTGKEGLEID
LLVINSSDIILIEAKSKVSEDDVNEHLERLSKFKRFFPRYESYRVLGAVAGMVIPLDVSR
YAYRKGLFVIGQSGDNLVILNDDKFRPRGW
>OBU77014.1
MATTADEVWKLLGELIESQKETERKFQETERFLREQSQETERLLREQSQETDRKFQETER
LLREQSQETERFLREQSQETDRKFQETDRLLREQSQETERLLREQSQETDRLLREESKRV
NNQIGQLGNRLGEFVESQVRPAAVKLFQERGIAVKEIASNTYIQTGKEGLEIDLLVINSS
DIILIEAKSKVSEDDVNEHLERLSKFKRFFPRYESYRVLGAVAGMVIPLDVSRYAYRKGL
FVIGQSGDNLVILNDDKFRPRGW
>OBU77020.1
MATTADEVWKLLGELIESQKETERKFQETERFLREQSQETDRKFQETDRLLREQSQETDR
KFQETDRKFQETDRLLREQSQETERFLREQSQETDRKFQETERLLREQSQETERLLREES
KRVNNQIGQLGNRLGEFVESQVRPAAVKLFQERGIAVKEIASNTYIQTGKEGLEIDLLVI
NSSDIILIEAKSKVSEDDVNEHLERLSKFKRFFPRYESYRVLGAVAGMVIPLDVSRYAYR
KGLFVIGQSGDNLVILNDDKFRPRGW
>OBU77022.1
MSIAIASKILGKSHPPFIIAEMSGNHNQSLERALEIVEAAAKAGVDALKLQTYTADTMTL

GNGLTCGGVEKSLASFRSSSVWIHRRGEKIVNI
>OBU77026.1
MATTADEVWKLLGELIESQKETERKFQETERFLREQSQETDRKFQETERFLREQSQETDR
KFQETERLLREQSQETDRLLREQSQETDRKFQETDRKFQETDRKFQETDRLLREESKRVN
NQIGQLGNRLGEFVESQVRPAAVKLFQERGIAVKEIASNTYIQTGKEGLEIDLLVINSSD
IILIEAKSKVSEDDVNEHLERLSKFKRFFPRYESYRVLGAVAGMVIPLDVSRYAYRKGLF
VIGQSGDNLVILNDDKFRPRGW
>OBU77028.1
MATTADEVWKLLGELIESQKETERKFQETERFLREQSQETDRKFQETERFLREQSQETDR
KFQETERLLREQSQETDRLLREQSQETDRKFQETDRKFQETDRLLREESKRVNNQIGQLG
NRLGEFVESQVRPAAVKLFQERGIAVKEIASNTYIQTGKEGLEIDLLVINSSDIILIEAK
SKVSEDDVNEHLERLSKFKRFFPRYESYRVLGAVAGMVIPLDVSRYAYRKGLFVIGQSGD
NLVILNDDKFRPRGW
>OBU77034.1
MATTADEVWKLLGELIESQKETERKFQETERFLREQSQETERFLREQSQETDRKFQETER
LLREQSQETDRKFQETERLLREQSQETDRKFQETDRLLREESKRVNNQIGQLGNRLGEFV
ESQVRPAAVKLFQERGIAVKEIASNTYIQTGKEGLEIDLLVINSSDIILIEAKSKVSEDD
VNEHLERLSKFKRFFPRYESYRVLGAVAGMVIPLDVSRYAYRKGLFVIGQSGDNLVILND
DKFRPRGW
>OBU77037.1
MATTADEVWKLLGELIESQKETERKFQETERFLREQSQETDRKFQETERLLREQSQETER
FLREQSQETDRKFQETDRLLREQSQETDRKFQETERLLREQSQETDRKFQETERLLREQS
QETERLLREESKRVNNQIGQLGNRLGEFVESQVRPAAVKLFQERGIAVKEIASNTYIQTG
KEGLEIDLLVINSSDIILIEAKSKVSEDDVNEHLERLSKFKRFFPRYESYRVLGAVAGMV
IPLDVSRYAYRKGLFVIGQSGDNLVILNDDKFRPRGW
>OBU77039.1
MATTADEVWKLLGELIESQKETERKFQETERFLREQSQETERLLREQSQETERLLREQSQ
ETDRKFQETERLLREQSQETERFLREQSQETDRKFQETERLLREQSQETDRKFQETDRLL
REESKRVNNQIGKLGNRLGEFVESQVRPAAVKLFQERGIAVKEIASNTYIQTGKEGLEID
LLVINSSDIILIEAKSKVSEDDVNEHLERLSKFKRFFPRYESYRVLGAVAGMVIPLDVSR
YAYRKGLFVIGQSGDNLVILNDDKFRPRGW
>OBU77041.1
MATTADEVWKLLGELIESQKETERKFQETERFLREQSQETDRKFQETERLLREQSQETDR
KFQETERLLREQSQETERFLREQSQETDRKFQETERLLREQSQETDRKFQETDRLLREES
KRVNNQIGQLGNRLGEFVESQVRPAAVKLFQERGIAVKEIASNTYIQTGKEGLEIDLLVI
NSSDIILIEAKSKVSEDDVNEHLERLSKFKRFFPRYESYRVLGAVAGMVIPLDVSRYAYR
KGLFVIGQSGDNLVILNDDKFRPRGW
>OBU77043.1
MSNFPTVKAKEFIKVIEKLGFYLDRQKGSHAIYKNINGSRVVVPIHSGQDIKQGTLMGMI
QDIGIDKEMFFELLQK
>OBU77047.1
MDTFLDGKAGNARLDFNLQGAFTRLLCCNQLQDSSQLQVTHSNFDYSQHITDFVDSNTAD
ATAYMVIPPSIENTSCEAIVYPDRSPGDYLAGITTMQPIEDIFSLAVGQNSSIIKFTRSD
NKLPSRIVTGLRLYQKGLVAECSLGVIHSKRPGKHFHWMLVSGLYETYVLFTAITEIYGE
LPSNLSLCLTLYGENSSESVQTNYGIELKSVGFITMF
>OBU77048.1
MAIIKQTDNFSANFGEVLRSSSIFWITQNSTTKTTICLSNYWRFKNNLDVTILVSWRAMS
GELLKRHKVEFVNTAVLNLAPPFDEFEGSCEIETFCLNNLRIPYSAIMAVYETKDSISMV
HSYTRTYSHHEIEEGRTITDGHESCWTIYGGGAKFRALLLVTTVIMLCNHIIANSGSGVM
MVEK
>OBU77052.1
MATTADEVWKLLGELIESQKETERKFQETERFLREQSQETDRKFQETERLLREQSQETDR

KFQETERLLREQSQETDRLLREESKRVNNQIGQLGNRLGEFVESQVRPAAVKLFQERGIA
VKEIASNTYIQTGKEGLEIDLLVINSSDIILIEAKSKVSEDDVNEHLERLSKFKRFFPRY
ESYRVLGAVAGMVIPLDVSRYAYRKGLFVIGQSGDNLVILNDDKFRPRGW
>OBU77055.1
MERLQGAARLAQIADFIEGREGGYEEIVGERGIRLSGGQRQRIGIARALYKGASVIVLDE
ATSALDNTTEKEVMAAIEGLSHQLTVILIAHRLSTLEKCDRIFQLDQGQVCQEGKG
>OBU77056.1
MATTADEVWKLLGELIESQKETERKFQETERLLREQSQETDRKFQETERLLREQSQETDR
KFQETERLLREQSQETDRKFQETDRLLREESKRVNNQIGQLGNRLGEFVESQVRPAAVKL
FQERGIAVKEIASNTYIQTGKEGLEIDLLVINSSDIILIEAKSKVSEDDVNEHLERLSKF
KRFFPRYESYRVLGAVAGMVIPLDVSRYAYRKGLFVIGQSGDNLVILNDDKFRPRGW
>OBU77061.1
MNNNSKQIYNYTVILEKEEQRSKFRKLLTVINLWEIEAETVFEQKSRVVGATSNAVLADG
FEGKARPKS
>OBU77066.1
MFRLKIPNQEVKVALGDQLVNAYTDFVEEKLGIQRPLYENLFQGYVSRFIDTVRSLFASI
PWRNFTNNNLANFEGYYASVLYAFLSSLNARIIPEDITNYGQADITAILGNHIYVIEIKV
VDGENVKENLALKQIRECNYAQKYRGEPGKTVHEVGLVFSRSKRNLIQADWE
>OBU77067.1
MNPKNLPLGINTLSMLRENNCVYVDKTEIAHGLIRIPGRFFLSRPRRFGKSLFIDTLKEI
FEGNQKLFEGLYIHDQWDWSRKFPLSVREWSMATTGLRQETPPS
>OBU77100.1
MANYHLPIFCEIIVPASEVVKVMSSVPLLRLSDKFDPYISGEVNIDPSAVIAPGVILQAG
LNSKIIIGPGVCIGMGSILQVSHGILEIEMGANLGAGFLMVGEGKIGANACIGAGTTVFN
DSVAAQQVIPAGSILGDSSRQQSTSPPSPTPKSQSPPETESSSTESQETSDGKPHSRDPT
EPHPLGTQIYGQGSINRLLSTLFPHRQSLSDQDANNGEE
>OBU77123.1
MTAKQTWSQRFESALHPTIARFNASITFDIELLEYDITGSQAHAKMLGHSQIITPEEAEQ
LVTGLEQVRQEYRQGKFQPGIDAEDVHFAVEKRLTEIVGDVGKKLHTARSRNDQVGTDTR
LYLREQIQQIRQHLREFQQVLLDLAEKNIETLIPGYTHLQRAQPLSLAHHLLAYFQMAQR
DWERLGDVYKRVNICPLGSGALAGTTFPIDRNYTAQLLNFDSIYANSLDGVSDRDFAIEF
LAAASTIMVHLSRLSEEVILWACEEFRFIHLKDSCATGSSIMPQKKNPDVPELVRGKTGR
VFGHLQSMLVIMKGLPLAYNKDLQEDKEGIFDSVNTVKSCLQAMTILLREGMEFRQERLA
AAVTEDFANATDVADYLAARGVPFREAYNIVGKVVKTSISAGKLLKDLTLEEWQQVHPSF
ESDIYEAISPRQVVAARNSYGGTGFEQVRQAIASAHIQILQS
>OBU77135.1
MTSAVAIESPKMPVSKQGLPVTIITGFLGSGKTTLLNHILTNQQGVKTAVLVNEFGEIGI
DNELIVTTDDNMVELSNGCICCTINNDLVDAVYQVLEREEKIDYLVVETTGLADPLPVAM
TFLGSELRDLTRLDSIITVVDAANYSLDLFNSQAAHSQIVYGDVILLNKVDLVDEATLTV
LERKIHDVKEGTRIIRTKNSQVPLALILSVGLFESDKYFDTSTDKHEHHDHSSCDHDHHE
HEHHHHSHHLENDGFVSISFQSDKPFAIRKFQYFLDNQLPTNVFRAKGIMWFEESPNRHI
FHLCGKRFTIDDDQWHGEKKNQLVLIGQNLDERELLQQIEHCLCLPSVNKDKGFKGG
>OBU77142.1
MRNVGSIETEIFDSPDTLEKLCLMSGGHVRNLLLLTQDAIGRTQDLPISERAVRRAITQA
RDTYRRAVENHQWYLLAEVSISKRILNDDQYRNLMFNRCLLEYRYLDKEGEIQRWYDIHP
LIQGIQEFREALATLL
>OBU77188.1
MDPISGVNIVSTGNSVTNPYKTITFALEQAQPGTIIQLAPGSYTNESFPIILKQGITLRG
DESTKGKTVVISGGGDYNSRSFARQNVTILAEQDSTINGVTVTNRNARGTGVWVESTNPV
IKNSTFAQSLREGIFVTAGGDPTVENNQFTANNGNGISITKSSKGKISNNVIEKSGFGLS
INHDSKPVLTKNQITNNRDGIVITDSAQPLLRSNVVKDNERDGIVITLNSSPDLGTRSNP
GGNVIVNNGRSNIYNVATTGVAISALGNTIDDSGILGEVEIDGIQITGNRKPVSSDDPNL

ALLIRKWQLTAVSCSSTPVIVIFIGNKQYCFSPQPDLTAKAYEYNPTTGTLRALRTGPRP
TKPGNL
>OBU77205.1
MPYSHKSTKKQRQYNQDLTAKRFASTVPAQTLSWLSSISLLSGGFVFAQTESPVDNIVST
VEVSQPTTLGDKGQNGQVSLPVESKVTELKTGVQTQGKNSEVNSNLESVATEGNSESRSE
SSPTSVILKVPALPVLKSQPETLPTSTQPIPETSTLVIPVTSQPNNSDNGNTGKDYNSTQ
IDPTEYNGNSTVKYDAPSSVEVTGRTQDCKAVISQPGKSVGTCGGKNPTNPSVANSTRKS
APTWLKKSGAVNLGKSQLKTVPNENALSTNNSSPRSQGQKSEEVVNSLAATVNNSQNWHS
LGSNSSSRHTSAGGYMGRISTKTSYNRPRDPREFSSNTTVTPITPSFGTLPPPMIEGKVA
PRPSKVAYDFDLASVLPQVPYISSFADNGTNSGSVSGITYPLSFAAPITSLFGWRTHPIT
GDRRFHAGMDIAAPTGTPILAAEKGQVEMADWMGGYGLAVTINHDQRQQTLYGHMSEILV
RPGQWVEPGMIIGRVGSTGNSTGPHLHFEVRHLTANGWVAVDPSMQLQAGMNSLYNRVAY
NYNSQSKQLR
>OBU77212.1
MGRFYRTVLIINHGNAELNASSLGMAKAALEAINGLNLFGVYGSQASVIHVLPDEIARGR
ITLKSLLPRESASKEVDAALLSIISYPAFAVEDPGLCDRTFQKIINELSGNYVCKRFCVK
PNARMGLFPRSIL
>OBU77215.1
MKFTPVKFCLTFLWLTLGVGLGSFDPNNPNNIVVAQLPPEPPEIFPLPDNQPVPVFKSEF
GVRIVDSQGKVNFFPTTRVPLRRGDIYGWRITLENYRGQVKWREVLRLPKAPETWITQDK
KHNFSLGADGTTAITRRTQMAKDGVIENYWQISPGDPLGEHKIEVYVDDSLITTFQFETV
EF
>OBU77216.1
MATNTADTGKQKALSMVLNQIERSFGKGAIMRLGDATRMRVETISTGALTLDLALGGGLP
KGRVIEIYGPESSGKTTVALHAIAEVQRNGGIAAFVDAEHALDPTYAGALGVDIENLLVS
QPDTGEAGLEIVDQLVRSAAVDIVVIDSVAALVPRAEIEGDMGDAHVGLQARLMSQALRK
ITGNIGKSGCTVIFINQLRQKIGVTYGSPETTTGGNALKFYASVRLDIRRIQTLKKGSDE
FGNRVKVKVAKNKVAPPFRVAEFDIIFGKGVSTLGCLVDLAEETGVLIRKGAWYSHNGEN
ISQGRDNAIKYLEEKSDFAEKIKEQVRAKLDQGAVVSANSVAKSTEEEVEEEMEGEE
>OBU77223.1
MENTTEITKLFRAHVFITGQVQGVGYRYATVDTATQLGLTGWVRNLVDGRVEAVFEGSRD
IVEEMVRWCHTGPTAAIVKQVVINYEQAEGLRTFELCLSHNVG
>OBU77233.1
MEELLELKTLLTQGKVLDALVLVEEMTEMSKDDKINKIYSFAVILILHLIKQQVEQRTTR
SWDISISNAVRQINRTNKRRKVNGYYLSNSELKVALADAYYSALDGASLEAFEGRYSSEE
LAERIDYSRLMKDAWDLISTQQN
>OBU77234.1
MAILAVGAGQTYTTIQAAINAANNDDIIVVRPGIYQEDLTINKSVTLIGPYGTFEGIDGF
ENRLGVKPLDPDINAALGVGGLPTANEDFRRYQDDNGTIDNAFGNTQEAWIKGTITVTED
NVTIDGFRLRNENGPLKWNDTPDNFKLLNNYLTGYTANNSPSFGDASINNPTGVVTGWQI
AGNYIGGLLGGGGTGGSIYLAGLQDSNIDDNTFWRPRAAHLYLASLTNVTIEDNKFYHGL
HTGGANFDGFGEFFSGSGYGYGGYGDGYGGYGDGFFGRNYWLELKGDNDQVLIKNNEGEY
NSGGIQLFGETDSPFAFDNITIEGNTFPDNNFINAYSEAPSNGKSGLIPAVMATARLSGP
SGSNLVIRDNDITMDLAQVKFITDHKSSLEVRGNFNGVTVEGNTLTPKNINGGVDIITGL
SLYGSLPGETLIRGNQLLGQDGDPLEASYYGIDLIPTFADYGTYTGDLTVEDNTINSWQV
GVNLRNTNEITGDININGNTFENNAYGVVLDATATTNSINIAGNTFSNNFSNVFDGIDPV
ITMDQVLSYEENQDLGSVLGTVSATDNLPNTDNVGVTQYFISSGNDDGFFTINSSGEITL
TEAGLAAANDFETSLSSFNLGIIVTDGGGLQDTETITLSIINVNEGLGQLPPITTEGAGF
TVGATLIAAIPFDDPDGIPTDISYQWQRLIEGVWTNIPDATEQNYVATEDDNNNQLRVEV
TYIAGGFEKVIYSNNVSISLVLVSGTFDSITGDNNLDISEKEAGVTLTGSVSEIGTTVTI
LFGGQTRVAQVDGLSWSYVLKPNDYNFFAAGSNLFTAIFTRTDGGETGSFTTFQTLTIPD
GILPPNTSNAFDPTQPKGLKSEVIDAAQTLEIDGVSILEISKTVGILGEGESFNDPNIAV

LPIGTRDIFDQGANAGAANYAAFKTEPGTSIQSIYIVPAVEEEGVKKLQVVLADGTVVEA
EIPPDLISPIGDPLAVTISGVQPGGTTTFVLYLSQNVINQLPEDLNLARYVKFNYESQQF
ELYDDFNYDYIFNDVDGDGVRDFGEVYLTVNLTDGDIWDGDGLANGIIVDPGQLGIATDS
GTDNNPPIAIELRGIVAENDPGAFIGSLTVTDDPGDSHTFTVNDSRFEVINVDGNNILKL
REGESLDYEAASNIVLTITAIDNGGLEITQDFTITVTDVNEAPVAIELNQITVIENDPGA
IIGTLTVSDPDGNDGHTLKVNNDTRFEIVDFDGNQTLKLKAGESLDYEAGSVKLSITATD
NGGLEVTQDLTVSITDVNEPPVVSFSFFVPESTTLVSNLTVEDPENDPITLSLAGVDASL
FSISPTGELTFNTAPDFEEPLNADKNNLYKLQVVARDEQNNKSIQDISILVTNVNEAPIA
IDDVLAIIPGSSFGTLNPLDNDSDPDLNDPLTIINKTDGNYGRVEIRDNELIYTLLDATY
IGDDVFSYTIIDEQGLAATANVNVTITGTDIITYPVEILDPEDSLIPDEAGPLSDIVNDI
SFNFLTDYDKVQAKLALQEALSKTEASFTNLFGLYEVDNALTGSVNGVLPEDKSAYAKAA
LSRVVPNFVVRAGGSGDGVNGDVIVSEGKIYAPFVIAHGGNFSGSVQDAVNAFFHVNPDN
SPATAQNYTTLPVAYFSFGSANPDGAAHIKSFGNNVFGFEDLPAGVGVSDYDFNDTVFSF
G
>OBU77256.1
MPKQFNTAGPCKANIHYMLSPTGRLPQLKALIDGENYFIIHAPRQVGKTTAMMALAQELT
DSGQYLAMLLTVETGAPFPDAPEQAQQSILRRWQNEIRFRKLPLPTLTQIQRETETSPRL
VNALARQATQVLVQDVNEPITAEVINQAKEMLIQRQDTHLDSLAERLREERVKTIIEPIL
AGEDLPDVPPDDIRYVLDLGLCRDQGQGLEIANPIYKEVLPLVLSYTTRVSIGAIEPLRL
NEQGELLPDKLLHAFLEFWRQHGEPLLKSAPYHEIAPHLVLMAFLHRVVNGGGTLEREYA
IGSGRMDICLRYGKVVMGMELKVWRERKSDPLIKGLTQLDKYLDGLGLDTGWLVIFDRRP
GLPPMGERISTEEVISPNGRTITLIRS
>OBU77263.1
MPKHFNTAGPCQSDIHYMLSPTGRLPQLKALIDGRNYFIIHAPRQVGKTTAMIALAQELT
DSGEYTAVMLSVEVGSVFPDEPERAERAILGSWQEDFCLDLQL
>OBU77282.1
MKFGIDIGHNCPPDTGAAGIRSEDKLTMEVGNKVISKLRGLGHQVIPCKPDSASSVSQSL
GRRCDIANRNRVDVFASIHFNAFNGKANGTEVLAGSDAGRKIAQSIVNEIVSLGFFNRGV
KNGSHLYVLRNTNMTSVLIECCFVDSAKDMQLYDGEAMANAIVKGLTGKLPTAPVKPVED
VTGDKDTSILRLQKALNQLKITDRNNRPLTEDNFTGPATSSAVEKFQRVVGIIPTGMATQ
TTWDAINQILAKRTVQGNQTSGPIMRYLQYRVGTTPDGIYGRQTEAAIKRFQQQNGLTAD
GIIGPATWGKLLG
>OBU77287.1
MTTPKLTSYEFKNLPSLGTTTTNQNILLGGFSGLYFQGVANNGNLKFVTHTDRGPNGEPT
GQNRPFLLPNFQPEIVSFELDKASGEINITKRTKLFRADGKTPLTGLPNVQAGVGNTAYT
DEIGVDLNGTVLTNDPLGADLEGIVVTDNGDYWMVDEYRPAIYHFNGNGILVSRFIPQGT
ASKSNQPVGTFGTEVLPEVYAQRRNNRGFEAVALAGNKLYAFIQTPIDNPDSTGDTTSRG
SRNLRILEFDIVAQKVTGEYLYLLDDITGTGNAKTDKIGDAVFLGGSRFAVVERDDRSDT
TSNKLIYQIDLTGATNINDTSKLTLPQGKTIEQLTPVELGPAGIIPVNKSLIVNAAQIGY
TGVEKLEGLALVSANTLAIVNDNDFITTIPEKLGIIELPNNLVEVTGTANKDELFVKKGE
YVLGLDGEDILDASDGLGNNVLDGGAGNDQLYAGRSDTLIGGAGNDQLYVVEGNNNTLYG
GAGNDQLYVVEGNNNTLYGEDGDDNLYIIEGGNNTLSGGGGADKFYIVNGAIPAAPSQVL
DFTRGNDKVIISGISQIKSFDNLILDQNGDDTIIKTKDLIDGSQKILGILKAVLANTLTA
DDFRIDDNRAPSFVTTTATFSTSENTTAVGTITPATDPDAGDVLSYTLGGADASKFDFNP
TTRVLSFKTAPNFESPGSAAGTNTYTLVVTATDSKNAIATQNITVNVTDVDDTAPVVNAN
QTFSYAEGKTANFQVGTVTATDAVGVTSFAIASGNNSGFFAISNSGVITLTAAGAATSAA
SNDFETNPNTFTLGITASDAANNTSSPVNVTINVNDVDDNQTPNVSGTFNIIATDGILTI
EEKNAGVTLSGRVSDQKAQVTVQFGGQIRVANVIGTNWSYKLTSNDYNFFKEGSNQLTAI
FTLNNVTPQFKSSQTLTIGTGVLPSLGSPTLDPTQPKGISAEVTQKAQSANIRQGILGTQ
EKLTDPNIVVIPFGAIDTFNKGANAAIGDFGLIKTGANTTIESVRVLPKNTTGEVSVTLN
DGTVVLASIPPGISSIADPLGLIMSGVLLGGSSTLDFYLPKNILNQLPNDLQSATYAKFN
FEAKKFERYADEQGKPLYQYKFTDKNSNGIRDFGELYLSLNLTDGDKWDGDRIQNGIIVD

PGQLGIAVAVTGTGVTYSSTTVNPGNSLIPREAPPLSNIADNVFYNFPSTYRPAEARQAL
QNALGQTDAAFKNIFGLYEVDNATGAVNGIAPGQSGYAKAALDKSKVISNFTVRAGGSVT
PNINGDLIGIGGKTYAPFVIANGGNYSGSIQEAINEFFKVNPNNSAATAQNYMGLPVAYF
SFGAANPDGAAHIKSFGNNIFGFEDLPAGVGVSDYDFNDMVFSFG
>OBU77384.1
MEKKPLRIALFTGLYAPFLTGVSVAVHQRVHWLLEQGHEVFLIHPQINKQYSQQVGNRPM
LGLSELQIFSNFSSYAFPTQPLIFYKSLPQPLNYRHWNDTKLLENFQPDIIVVEEAAQMR
GLYSIFLQGYGRAVGVDYAKRTKTPIISVFHTDIVAYIKYYLGDILFRLMSQIIPLLVRQ
FSDQYTLNIFPSREQLKKYQKLQCQRCEYVPYQGINCEKFHPRNICHDPIPNDKRPTILF
VGRITAEKNVTQILEAYPLIAAKIPDVHLVIVGSGPLDQEIRHRAQKFADGVTIWGESHG
TELLGWFARADVFVNPSVTENFCTTNNEALASGTPVVAVMAPSTAEQVIIGYNGLLAQPN
NPKDFADKIVTILQNPDLKNQLSRQARPSILQFDWSNCSQKFEDKLYELVNVNEMEFF
>OBU77402.1
MEQLEPLQHFLPQVWFFILGLFLFLYILLDGFDLGVGILSLTASSEERRSILMTSLGNVW
DANETWLILMGGSLFGAFPLAYATILNALYLPAVVMVVGLILRAVSFEFRENADNKLVWN
IAFGVGSFLAALGQGFALGSVFEGIKVDQAGHFAGGVWDWLTWRSVVVALTLIQGYVLIG
STYLILKTTGDLQKTYYKTATIATWTTLVGALFITISTPIISGEARTQLFAAPLIYIFGL
IPVLGILFIGLLLRSLYQRQENTPLVYTFLVFCLSFIGLGFVIFPNIIPPSVTIYQAAAA
PSSLVFMLTFIGFLIPVVLFYNVYNYVVFRGKIINQE
>OBU77413.1
MTNSKLTVLKGLVFASLINIINLWPIENWAIQSLATAATHGQLLAQESVPIEISTPQPVQ
EKKSGFTLYIWQPNGTISPVIARISLKAKYGEKYLQERFLGDYQYKIKQKAKFENGFKWG
DRIVIRLYNTQNQLVGYTEFACLPNHTTVNLILPANSPPNPLVRVFYGVDSDEDGIVDLD
TTMSYDYFTEIRNQEVTFLSSPQNIDPTPYQAAGFAKVAQPSTYPASFTQGQFALVGKTI
SINDSQLAKAILSPPGSLVQLTQLNKSAYQLNNLLTKYREVGVAKGIRVRFSDLPQNYWA
KDYIEELAAMEVIDGYPDGTFRPNAPITRAQLATLLPKIFFKDTIRGKVAFRDIPKNHWA
HNAIQKTYQMGILTPSANRKFRPDQKLTRLDVLTTIAKALNYKFTGSTRDILSIYQDAST
IRSEHRELIAALTQNGVIVNYPNIRLLNTRGSCACFMDN
>OBU77417.1
MPQVVLINPQIPPNTGNIARTCAATATELHLVGPLGFEITDRYLKRAGLDYWPHVKLNYH
ESYHTFLKVHQQRGGRLLGFSVRGNIDYIQYEFRPDDWLLFGSETTGIPENILDICDSTL
YIPMDQPGVRSLNLSVSVAVALFESRRQLGYLK
>OBU77435.1
MAKIIAFDEESRRALEKGINALADAVKITLGPRGRNVLLEKKFGIPQIVNDGITVAKEIE
LEDPLENTGARLIQEVASKTKDVAGDGTTTATVLAQALVKEGLKNVAAGTNPIALKRGID
KTVEALVKEIAKIAKPVEDSELDSPTASIAQVATVSAGNDEEVGQMLAQAMAKVTKDGVI
TVEESKSLTTELEVVEGMQIDRGYISPYFITNNDRMTVEFDNPRILIADKKISSIQDLMP
ILEKVARLGQPLLIIAEDVEGDALATLVVNKARGVLAVAAIKSPGFGERRKALLQDIAIL
TDGQMISEEIGLSLDTATLEMLGKARKITIDKENTTIVSGSENKPEIQKRIAQIRKQLEE
TDSDYDAEKLQERIAKLAGGVAVIKVGAATETELKDRKLRIEDALNATKAAVEEGIVPGG
GTTLIHLVAKVDAIKDTLDGEEKIGAEIVQRSLEAPLRQIANNAGVEGSVIVSQVRNSDF
NIGYNAATGEFEDLIAAGIIDPAKVVRSSLQNAASIAGMVLTTEVLVVEKPEKKAPAAPD
PGMGGMGGMGGMGGMGGMGMF
>OBU77454.1
MFIFRRLITSGLIGFLVSSCADAAQSSTEYTKQIVQENNVARLMAQVQDSQKVSNLLSQG
NGFLDRGRYEEALQLYNRAIEIEKYSVPSWVNRGNALLSLKQYEEALRSYNQAIALRPNK
NEAWYNRGNALSALGRYEEAIRSYNESIVIEPNKFEAWINKGIALTKLRRYQEGLASYNQ
AISINPNFAAAYYNKACNYALQKQTDLAVKSLAKAIKMERQKYTELAKADNDFIQVKYNR
EFQNLLK
>OBU77459.1
MIEQMLNRVIKWLKQILKRVFQALSGQRRRSSQAPQNGEKKTIPPLSDTDLEFLFTELLA
GVRQAKGRTWARNWLDQIEHRVTTEQWLDWLRKFGNKLLKSKNPHNEIASRLVQLGELEV

GEVGDLAYEIGMELLTRTPIEPIWEYDGPDDEIVGSPAGENLSPEEQIVTQDDLLAILRE
NEPLRRQISQDLQIDSDDPQVILEGIDQAKENL
>OBU77474.1
MAAKKQGLQGNYAQTRLLLALWDLGAGEQKVTKGQLGKRIVSKGQRMIDYQQIIEHLEAD
GAITITKKGATVSYTITIPVGINTLHNGLSNKEFKFDKNVGAWVANALLLLLRQSHSTPS
TPEIKASESTNSKVIASYEEFKSVLLEVYSLLDNQYNFDNLVPIYRLRREIGERVERGKF
NQWLVEMQADDILQLISGEIPDFTADKQEDSILIPGTQLRSYAKRLV
>OBU77498.1
MPKPYSIDLRNRVIVAWVAQEGSQRQLAERFKVSLSFVRNLVRRYRETGQVEPKQCGGYE
KPIKGSLCRAREIVN
>OBU77514.1
MVSKNFWKPIIHSSTALILLTTLNTAWPLVSLAQSKPQPKVNSASSSLFTDYLLGGGDRI
RVNVFEAPEYTGEYQIPPGGEINMPLIGSIPVSGLTTQQAADEIARRYARFLKRPLISVN
LLAPRPINVFVAGEVTRPGSYSLSLQGTGGNNPGVQYPTVLAALTTAEGVTLAADVTKVQ
LRRQVGRSGEQVISLDLKQITQTGRIPIDITLRDGDTIFVPTATDFNVAEARNLFAASYA
ASRTAPRRVAITGQVYRPGSYLVAAGGGNDSGGLPTVMRAIQLSGGITSQADVRNIKVRR
PTRTDKEQTLNINLWELLQSGDLNQDVVVQDGDTIIVPTATQVNTAEVTQLATTTLSPAT
IKVGVVGEVKRPGVTELQPNSSLNQALLAAGGFNDARASSSSVDLVRLNPNGTVTKRAVK
VDLSKGINEETNPILRNNDVIVVNRSVLARTGDTLGAVTAPLAPVFSIISLFRLLGF
>OBU77517.1
MLNLSGKNALVTGIANSRSIAWGITQQLHAAGANLGVTYLPDDKGKFEKKVLELVEPLEP
SLFLPCNVENEEQIQATFDAIQQKWGKLDILIHCLAFANKEDLSGDFSQTSRLGFNKALE
VSTYSLIQLSGAAKPLMTEGGSILTLSYLGAVRAIPNYNVMGVAKAGLEASVRYLAAELG
PQNIRVNGISAGPIRTLASSAVGGILDMIHHVEQVAPLKRTVTQLEVGSAATFLCSDLSS
GITGQILYVDAGYEIMGM
>OBU77579.1
MSIIALRAWYIRDYEPITELEKRPPDIRLSKKSLLKSGLRADFLEDADQVRTATWFKHYL
EGDSIEFYIEGSGGYSVANVDLISHEIYLTKQSLLSQLEPTIFFSFQTEYARSSDLFREN
LQETITTLNSKSRVPLTLIESSRSRNKPLRLNRTIMRKIRKSLLFIADTTPITSIEGKDF
SELIPSPNVCIEIGYAFEHKRTEQIMFVHVHRPEVEGQFPFDLSAEHIAEFADADEIKQF
LTVAIESKLSRFRLFG
>OBU77581.1
MSIKNTIVMGNNTWEKTTWEWELYLFNQRIQQWLEYQYSQLFKNIPENSRFSISEQILQI
LQVLFWLILVLLLGWVIWRLWQEFYPEIYTWWKNLSSTFVHPRVPDSPPQLSSNFLLAKS
QEFYHQGDYQKACQFLYFSMLQQLHERAIALQQPSRTDGEYLQLLLGTVASIQPYETLIT
THEQLCFSNHKVSSENYQHCLQSYQELFNSP
>OBU77585.1
MSLFNPAFAQNPKPFLKWVGGKTQLINELDKLISYHISKYNTYTYIEPFVGGGAVLFYIL
SNFPQINNIIINDINSNLISTYNLIKEDYRKLVLVLAEIEKAYYNLTSLENKQQFYLAKR
DEFNQEDSEFSPVNKTALMLFLNKTCFNGLYRVNKKGKFNVPFGKYKEPKICNVDNIISV
HYHLQNVKIFQGDFIETIRYAQAPTLFYLDPPYKPINDTSAFTSYSLENFNDQDQLRLKN
FCDQIHINGHYFILSNSDVKNFNHENTFFDDLYQGYHIKRVKARRNINSKGDCRGELFEL
LISNF
>OBU77608.1
MQEKGVKWVKTLAPNYIEMYRKQKQQDTTPEDFELSLEGKIAPDNRWVILAKIIPWSQFE
AEYAAIFSEGKGAPAKTFRMA
>OBU77618.1
MTGKGQGFGFGLGKVKEIADAFKKAQEMQQGAKRLQEELEQMEILGESGGGLVKVIISGN
QEPKRVEISPNALNEGPDVLSDLVTAAMKDAYLKSTQTMRERMEELTGGLELPGF
>OBU77647.1
MTESLLNIQNLCVAYPQDYDQTPIWAVNDVSFSLKPGEKIGLVGESGCGKSTIGRAIMRL
LPDHSRVQGQVNFRESSVLNLTPAQMRQFRGEAVALVFQDPMTRLDPLMTISEHCLETLA

AHSPQLTKKQAKERVLATLEKVKIPGSRWSQYPHEFSGGMRQRVAIALALLLNPKLIIAD
EPTTSLDVTVSAQILQELTKLCAEDNMGLLLISHDLAMVAEYCDRIGVMYQGKIVEMGKT
ESVFKQPQHEYTQSLLRAALHIQQEPVGVGEEEKKENPILKITELQQYYSIEPNFLERLF
QSQQQTIKAVDGINLELYPGEILGLVGESGCGKSTLSRTILQLIPPTGGKVEFLGQELTR
LSREEVRSFRREIQMIFQDPHACLNPAMTVGESIADPLLIHKIARGKQAEEEVVWMLEKV
GLTPWQTYYRRYPADLSGGQQQRVAIARALITRPKLVICDEPVSMLDASVQTQVLDLMLQ
LKAEFDLTYLFITHDLWLARFLCDRIAVMNSGKIVELGKTKEIFSHPQHPYTQTLLGAAP
LLARV
>OBU77661.1
MIEAEIKALIQKELPRAIAEEPGVRDFVLRTVSEYYTPRTEFDEKFDRVLNELQRDREEQ
ARKWDEQNRKFDAFQAEQAQKWDEQNRKFDAFQAEQAQKWDEQNRKFDAFQAEQAQKWDE
QNRKWDEQNRKWDEQNRKWEENTQRLDRIEAQNSATLEEIQKANRRYESAIGAIGSRWGL
YSEASFRNGLKAILGQSFGVEVLNLTLYDQEGEVFGRPEQVELDIIIKNGLTIVCELKSS
IDKAGMYVFGRKAEFYAKNQNRVVDRKIVISPMVDERAIPVAKSLGIETYSYADMVVS
>OBU77662.1
MIEAEIKALIQKELPRAIAEEPGVRDFVLRTVSEYYTPRTEFDEKFDRVLNELQRDREEQ
ARKWDEQNRKFDAFQAEQAQKWDEQNRKFDAFQAEQAQKWDEQNRKFDAFQAEQAQKWDE
QNRKWEENTQRLDRIEAQNSATLEEIQKANRRYESAIGAIGSRWGLYSEASFRNGLKAIL
GQSFGVEVLNLTLYDQEGEVFGRPEQVELDIIIKNGLTIVCELKSSIDKAGMYVFGRKAE
FYAKNQNRVVDRKIVISPMVDERAIPVAKSLGIETYSYADMVVS
>OBU77672.1
MSNETITIADIFALFKESERQREAEQKRLSQEIEREREERQRENEARQQEYEKRQQEYEA
RQQREREERQRENEVRQREYEERQREYEERQRKHEEEMNQFRISMEETRKIVAETNKNMG
SITSRWGEFVENLVRPAAVRLFKEQGINVHYTSLQVKADDYAGSIEIDIWAENDGEIVAI
EVKSHLKVRDIKRFITVLDRFKDVFPKYKNYKLYGAVAGIKVDEKADQYALEQGLFLIRP
AGDSVAIDMKKDFQAKVW
>OBU77673.1
MSNETITIADIFALFKESERQREAEQKRLSQEIEREREERQQEYEKRQQEYEARQQEYEA
RQQREREERQRENEARQQEYEARQQRENEARQREYEERQRKHEEEMNQFRISMEETRKIV
AETNKNMGSITSRWGEFVENLVRPAAVRLFKEQGINVHYTSLQVKADDYAGSIEIDIWAE
NDGEIVAIEVKSHLKVRDIKRFITVLDRFKDVFPKYKNYRLYGAVAGIKVDEKADQYALE
QGLFLIRPAGDSVAIDMKKDFQAKVW
>OBU77674.1
MEDSQMTISIGDTAKELGVSVKTVRRWADSGKLRFERSPSGHRRFYLADIKRITPRDFNQ
LEDRVTINYARVSSSDQKEELTRQIQVLEAFSGANGWQFETISDLGSGLNYNKKGLQKLL
KRIMQGDVGRLVLTHKDRLLRFGSELVFAMCEEYETEVVIINKSTRHLRK
>OBU77675.1
MPRDRPLFSSPTLKDRPENIGGPFDSQRRFQNDLRIDNLRLLSAADYNKDGFQDMYFKVA
DGTAVLRALMHADGNIQYANYQSKADLTAFMTANNVSPSIWSGWI
>OBU77692.1
MTLTIRVISPDKTVWDGPADEVILPSTTGQLGVLSGHAPMLTALDTGVMRVRAEKNASWQ
SIALLGGFAEVEEDEVTILVNGAQRGDTINLEEARSEFNAAQSKINQVVAGHRQAQIQAT
AALKRARARFQAAGGMV
>OBU77711.1
MNQILPLSINQKEIYVDQIMSPNSCHMHIGATVTVRGIFDREILNYAMSKTIDCHPGLKT
RIYEVDGEPFQTIASHTSNHIPFIDFSGHDNSDEQAENYINQEFIKPLTFGENATLADFQ
LIKVCDDKHIVYAKYHHVITDGWGAAIFFREVIKTYNQILQEGREDQETRDWVITEYIEE
ETKYLASDIFMRDRHYWQQRLNNLSPMIFHPIKQPQELDGKRHSIYIPRHQYDQVDELCK
NVKSNVFHFILSLIAIYVSKHYLKNDVVVGLSLLNRSKNIFKDAIGMFVSTIPFRLEVEQ
ENTIHQLLDKIRYSLRQDYRHQKFPLAEMKQLSGLKATSKQHLFEVFLSYERHDYADSLP
NTKTTCVPLYSQQQKVPLIIYVREYEKTDDVKIDFDYNLSYLDGEAVGEIVRSFETLFTQ
AATNLEISIGDLAICDSETINISQPDSPSTKFFADDTETLVSAFEKVVSQYPQNLAVQFD

GELYKKFLSYTELNDQANRLANYLISQGVKPGSRVGICLERSEQIIVAILAIIKTGSAYV
PIDPHAPSVRRQFIVQDSGMTTLITETSLIAELVTENISTLTIESINLALAKQANTLPRI
SIKPDFPAYIIYTSGSTGTPKGCVVTHKNAIRLMRATEPWLGFNEKDIWTLFHSFAFDFS
VWELWGALLYGGKVIVVPFWLSRNPEKFREFLSTEKVTVLNQTPSAFYQLIHADESSVGD
IYLRYIIFGGEALNIQSLQPWLERYGDKKPYLINMYGITETTVHVTYRPITRQDLKVRRS
FIGKEIPDLHIYLLDEKLSPVADGIPGEIYVGGAGVTNGYLNRPALTAERFLPNPFGSGR
MYRSGDLAKRLPNGDLEYLGRIDQQVKIRGFRIELGEIQAALISHYQVREAVVITDEWEE
EKRLVAYYVPDESSPTAHELRQYLKSKLPDYMIPAAYVKLEVLPLNVNGKVDIKALPMPD
WNLLRVEEDYIAPRNLDEETLCTIMAEILGLQKVGIDDNFFEIGGDSILALQVVARAKKE
GFLISAGELYELATVRYLATKKAIITSGDGVKKLPNIGSALVCDADKLLLPQDVADAYPL
SSLQSGMLYHSELDPDSAIFHQIFTFDLQIGYSELAWKQAIADICSANPVLRTSFHWTGY
SQPLQIVHEQVELPLNVVDLRGCDNANEQIGKWIELEKNHNFDITQAPIFRLQIHRVSDL
KLSFSFSFHHVILDGWSVATLLTQLLRRYVEYLATENLPPLPVTQISYKDFIAQEQNVIT
NHTAREFWLQHLRNLQVTFLPRLSTNVAKTTATSHQKRQLKRLSVTIDTKLAEKLRQITK
NLGVPLKTSLLALHLRVISFITGQKQVVTGNVINARPETSGSENLLGLFVNTIPFRLELP
QGNWLDLVRAVFRLEREILPHRTFPLAEIQRVVDQRPLFDLGFNYVHFHVYEGLLNLPQI
QVENVDIFEETDFPFLTEFCLVPGSGALQLNLIYDTQQFADTQVEQYANYYQAAMFDMVT
NPQTPYHRRSLISSQERQQLIQSANQNQKDFVSSQTLVSAFNQTVAKHANKTALVYQQTS
LSFGELEIQANRLAHYLQAKGIGPETLVGVCLERSEQLIISILAVLKAGGAYVPIDPAYP
SDRLEFLFQDSGIVLLITQRKVISQLPECGAEIIILEDIAKQIEGNNSQTPAVNILPENA
AYVIYTSGSTGKPKGCIVTHANVIRLFNSTTTWFNFHSEDIWTLFHSYAFDFSVWEMWGG
LLYGGQVVVVPHWTVRSPKEFLQMLATHRVTVLNQTPSAFKQLISVVRQKPEKLSLRYVI
FGGEALELADLQPWIDLYGHTQPELINMYGITETTVHVTYRPITQKDIINNLNHRPSVIG
QSIPDLELYILDENLDPTPTGVTGEIYIGGAGVTRGYLQQPGLTAQRFIPHAHRPGSRLY
RSGDLARYLPDGEIQYLGRADQQIKIRGFRIELEEIQLVITSHPDVKQALVVCQKSPTGE
NRIVAYVIFSGVAQPQNDLGKFLKTKLPDYMVPSVFVPIETIPLTINGKVDYAALPVHNW
NSIKKDYIAPRNDREATICSLMASLLKLERVGVDDDFFEIGGDSLLVTQLAISLRQTYDT
EFPLPELFTHRTPGEIALLVGDESPALPEIEIPKASRTRRSVTLTDDGILSKY
>OBU77715.1
MEVYVLPLSVSQKQFWYREIMFTGNTAYHIPIGLNLLGDLDFTLLEKCFRYIINRHEILR
TTFALENGEPVQLVRSEQQFHLQHRNLELPPEKWSDYDRLLTIKKILEDESRQLFDLIKG
PLLRATLYKISNKEHILLVNLHHIISDGWSLGIFIQELTQLYVSRAEISLPELPIQYGDY
AEWQETYLQTQRIQDQLAYWQKKLALPLPILDLPSDKNRPALQTFSGAVLRKTLPRDLID
SLEAVAAKEGVTFFMVTLAVYQILLFRYSGQTDIIIGTPCANRNRGELKNLIGCFINTLP
IVCSLTGQLSFKQVLQQVASTCVEAFANQEVPLEIIIDKLNITRDPAHSQVFQSLFALQN
APIEEIKLPGLTVQPVYLDNGGAKFDLILMLEPTFEKGWIAALEYNTDLFTAHTAEDILR
HYEQLLSAVIGNLDTKIDTLPWLNTEERRELLSFGSSASDQEVEATNLVDVFSHIVNTYG
EKVALIESERTLTYQKLNQISNQLASHLIEKGVGAETRVGIFQERSLELVASILAVLKCG
ATYVPLDPQYPAERLNFIAQDSGIRLVITTEPLRAKIPGEIPELLLIDTMKPTNQLVHPI
SKIFPEQAAYIIYTSGSTGKPKGCLVTHKNVLRLMRNTQKWFEFNEKDVWTMFHSFAFDF
SVWEMWGALLYGGKLVIVPYLESRSPHDFRQLLATQQVTILNQTPSAFRQLIRADQEFTH
PLNNLRAIIFGGEALELQSLKPWIERYGDSHPRLINMYGITETTVHVTYRQILAEDILEN
RGSVIGIPIPDLCLYILDSYFEPVPYGVAGEIYVGGMGVSRGYLNRPSLTAERFIPNPIS
QKPGARLYRTGDLARRLRNGDIEYLGRCDSQVKIRGFRIELAEIEAAINKLTQVSESVVT
VHSTSDQDRRLVAYIVLRSINNNEIDRNELRKALKESLPDYMVPAAFVFLDAIPLTNQGK
INRGALPLPDWDQSTTKRSFIPPETDAQKALCHIWQRVLGIEEIGIEDNFFDLGGDSILA
LRVITEMRSQGWILTPKQIFEQQTVQRLALAIQEQTDLQSFAAAENNNIPTGEVPLSPIQ
QWFFDLKLSNPHHWNQTLLLQVDPSLQVTQVATAIKVVFAYHDNFRLRFQKESQGWRQFY
VEKDQDDGFPWEVVDLGLKSEIEQNSIMKEVREKSEKSLNLTEGPVYMVKWFNLGANRPS
QLLIVIHHLIIDGVSWRILLQDLVDVIGGNKLSFKTTSWQKWCEFIHGYVNSYNIQSEKL
FWQNTLARKTAKLPLDFPESLAENLEYSVSTVSCQLTAKETLTLLTTANKTYRTNPQELL
IAALGKTLADITQNSYVQIMLEGHGREELSSDLDVTRTLGWFTTLYPVSLDLPKGDSQTE

AIREIIKSVKEQLRAVPQRGFGYGILKYLDQETKPLLASAVEISFNYLGQVRNEGGARHK
FFTLLNSQGLPTRDPQATRPHIIDVNAIVVEGELRVDWLYSSNLHQASTINRWVSDFKSN
LLKLLNFCTEVGVGEYTPTDFPLAQLTQSKLDILQNQHPDLEDIYPLSPLQQGMLFHAIY
EPDQGIYFEQVTGKIIGKLDVDKFAYAWQVIVNRHPVLRTCFVWEDQGQPLQIVRKELPF
SLIYKDWRDLSPAQQTEQMGKYLVKDKQQGFNLNDSPLMRFTLICLDDSTWQWLWSHHHI
ILDGWSLPVIFKEVLSVYQSATERVPHSLLPVPPYRHYIQWLTGRDQLKAKQFWQQYLAG
ISTGTRLAWQIPDLENDSSLPPYREVELRLTESEFDLVQKMAQSQRLTLNTIIQGAWAMC
LQKHGAGEDVIFGVTVAGRPPEISEMENMVGLFINTLPMRVKIDPTLSVSDWLQNIQQHH
LEMREYEYSKLADIQKDLNLAGLPLFESLLVFENYPVDQNLKKQLPDFRVDDIQFYERTN
YPLTVGVIPDQGLLLKLNYQTKFLSGNAAEKMISRFRHIIVNMAFKPEQTLAKVPALSIS
DREELTTCNQGNTIIEGGFKTAHQLFEYYADLQPDSIAVVCDEESVTYGELENRANNLAG
QLLDTGIGYESIVGLYFDPGIEYIISLLAVLKVGAAFLPLDRIYPEYRLKFIIKNSQVSV
ILTNNIIGLPLQENLQVLDISNLESKGNHPRLNLKVRAENLAYVIYTSGSTGNPKGVLVT
HSGIQNLVQCQTMSFGVTGKSRVYQFAALNFDSAIAEIFMSLGSGAVLYIQSQSHRSPGS
ELWRKLTNWEITHVTLPPSLVASIDTQDLPGLQTLILAGEAVSGDLLRRWSRGQRRCFNA
YGPTEATVCASLMDCTHLLGEPSIGKGIPNVEIYLLDSFLEPVVPGVIGEIYIGGIGLAR
GYLQRTGLTAALFIPHPFTKTPGSRLYKTGDRAVYDPEGNIRFIGRYDNQVKINGYRIEL
GEIEAALTKHEAVESALAVLRTNLIGRNRILGYALIKPVEGREQPNDLKEHLAKILPDYM
IPGAVIVVDEWPLTPNGKIDRQRLATPDFSSTEVIPKTDIEQIFAQIWIELLGLETVNPQ
DNFFEMGGDSIISLQMVTRARAAGWEISPKDIFEGQTLSRIATRAKLMETQTEIVEPLTG
FIPLSPIQNWFFAQNFLHPHHWNQSVALTCKQPVNTDALVVALHTLVSHHDIFRIGFAEN
EGEWQQFYVGEAKSPNVKIIDFSHYLPTTHLAFLNSALETEHSSFKLDRPPLIRILCGRN
LNNYGDVLFIFAHHLIIDAVSWRILLEDLNQAYQQTIDQLDQLQQPNSPLSLPVKTASYR
SWTTHLHSLASSKMTQDTPFWKDILNAVITPLPVDKSGNNSVDSTAIISTYLTAEQTVTL
IKQATVTYHASVQEIMLAALLNTLISIYKSDQWLIDLEGHGREDIGHNLDLSRTVGWFTC
LYPILLKQPTNPDNHEILLKEIKNQLRTIPHHGISFGLLRYLNLNQPLKESKNADISFNY
LGSLDSPLGDNHNFSVSDAPVGAGVFALQQRTHILAINAKIQDKILQVEWSYSRNIHYDQ
TIENIAKTYLQSLGLYLTNSDSANSSFYSASDFQLVDLSESELGELLEDLE
>OBU77716.1
MAGLKDKIEDIFPLTPLQKGLLFHTIYEPESGVYFEQLHCRLEGDVSTVAVRQAWQTLVD
RHAILRTAIVTKGQSEPVQVVFRHLKFNIEEEDWRGLSDDVQKHRLQDFLEADKRKGFVL
NRPPLMRVTLIRFREDVWYLVWSHHHIILDGWSWPILLKEFLTLHKAAKENIEVALPNRR
PYGDFIAWLKRKDLQESKIFWQQYMSGFESDTPLLMTSKSRVNPKFKGGEITKQLSPEIT
DLLGRLARNCSVTLNTVIQGAWAILLNRYSRSNDVVYGITVAGRPPEIPGVEQMIGPFIN
TLPFRVAISGDQTLNTWLQNLQFQAASMRQFEHTSLSDIRAWSDIPAGSEMFETLLAFEN
FPVDKLLKISDFGLNVAESHFLETTHYPITLVVIPSDTIAIKLSYNTNRFSDTTMKLLLD
QFSNLLLNMANHPQSLLQDISLLSHQEKQQIKNKREQTNSSYTTINEMFAETASKYPERI
ALTYENQTLTYRELEERSNAIARHLKSLGIGSEKRVVICLDRTPELIISMLGVVKAGGIY
VPVDPSYPRDRIEFTITDCGAEVIITTTNTSQELPNGITRVCLDVEDAAYLKTSAEPIDT
DNVTPNSGAYIIYTSGSTGKPKGVLVTHKNLTRLFKSTEHWFKFNQQDVWTFFHSFAFDF
SVWEIWGALLYGGRLVIVPYCVSRDPQTFLRLLEEQKVTVLNQTPSAFAQLLAVADSNCN
LDNLRYVIFGGEALNLGSLSPWFEKYGDNQTRLVNMYGITETTVHVTYRPISKQDIDNLS
GSLIGEPIPDLDIYILDADGNLLPTGVIGEMYIGGAGVAHSYLNRPELTAERFIEARFRP
NCRLYRTGDLGRFLPNGDLEYLGRIDYQVKIRGFRIEIGEIENVLAEISDVHENIVLVTT
DIETGENRLIAYLVCSPEKQPTIKDIRDHLQRSLPDYMIPSQVVYLDKFPLTANGKIDRK
SLPIPEMKRENIGIEFAAPETEIERKLASIWQQVLGVERVGRFDNYFVLGGDSIRSIRVC
SLAQAAGFNLKIEHIFTHPILADLATDLETTLQPLSGGKKQDYYQEPFGLIATEDREKLT
NIADDAYPLAQLQAGMWFHTEYSQTSTTYQDLFSFRIRIPFNLQVWEQAYSQMFTRHPVL
RTAFFLGEFSQPLQAIIKDVSGRVIFKNLTNLSPSAQDDYLRVFIEGEQQSRFNYKNPPL
IRFFIHQLSEDVVEATLVFHHAIMDGWSLAQFLTDLTGLYLHLMGRGVPSLSPQTGLEYS
QFIALEQEALQDKKQREFWQQQLEDIPLTKLPRLPRKNLPKMHPTIGKVDITLSQTTSQG
LKDVAHQLGVPLRTVLLAIHLHILSVFSGDEEIVTGLVSNGRPDTTDGDQVLGLFLNTLP

LRLRLPHGSWVDLIKATWLAEQALVANRRFPLPEVQRLNGGLPLYETSLNFIHFHVYQGL
LNWREVELLKSTSFEETNIPFAVTWNQEVASANISLNIAYSHAEFTQDQVDNIANYYQLC
SELVSENCVGNREFSLAPQEFIYEKQLSTSRPTITPVHQIVAQQAAVRPKEIAVICEGEG
WTYEQLNQKANQLAHFLQSQGITQEKPVGICLERSLDMVCAMLGVMKAGGCYVPIDPHYP
SARIQSMLEDGNLDLLLTRSDLKVNYHQNSQKTIFVDTQHREIREQSKANLNVSVFPDNI
AYIIFTSGSTGKAKGVAISHGALVSHQTWFVDKFSVTNADIVLQKTPFSFDASVWEFWTP
LMVGGKLVMAKPGGHQDPAYLVKTIQQEKVTLFQLVPSLLEVVLGDPELEHCSSLRLVFS
GGEVLKNRIWQKFQEKLPIPLINLYGPTETTIDITFHNCQENDNIANLDQIPLGQPVTNV
KLYILNSLLQPVPIGTPGKIFVSGCQVARGYWHAPGMTAERFLPDPFVPGQRMYDTGDHA
RYLPGGNIEFLGRIDQQIKVRGFRIETSEIVAALEAQSWVNSALVKVISDSQISRSDRSN
YLVAYVQPRLNTPNNWQKILRLELGQILPEYMIPNLFISIDSWPLLPNGKIDINSLPDPQ
VVETTVSQEYVPPESETEKILTKLWQQVLRLPRLGTRENFFELGGDSIIGLQIIAKARDL
GIYFTAQDLFKYSTVADLANHLRKEDHFSHLPTVEIGEIPLTPIQEWFFKQSLPHPHYWN
QAVFLDVKAQITTGDLEKAVNQIMGKHPAFKLKFCETEKGWIQELDNNSQHFNLDIVDLT
DTPETELSTHLQTWATQFQGQLNLETGKLFRVVYFQTAPTTADKILLIIHHLIVDGVSWR
VILQDLVGSMRNVTQDQSLSSFQAKPSVSFPQWSRYLRNMSHITWEVDVEFWQKQTVHDF
TLPLDFPEKIADNKENSAAQIEFTLTEAETNKMLLEISRTRKVSIQEILLTALATTLREW
TGRSEIVIALETHGRGSSLDLDISDSVGWFTSLFPFRITTVNSNVLDNLSGIKGQLNGIP
NNGLSYGILSQKLEHGEILPPIPQGIIFNYLGQFDEQIPLDAPFAPAVQDSGISRHLENQ
RTFQLEITGLIVNRKLEIRFGFSTNLHREQTIRNLLHTYQQCLRNLLMGSEEKLVRWNPE
DFPLANLNQEQLNIAVSGITDLQDIYPLAPVQQGIIFHANYEIEKDIYLQQVTGQITGDL
NVEIFKSAWENCINHYSILRTSYIWESLPRPLARVHSTVNLPFVYQDWCGTDDWEQKWSD
LLMEDRQQGFSMERPPLMRLILVKTDKAKWRFCWTHHHVLIDGWSLPLVFQHVISFYQAE
QKHQFLKWPPVPNYRDFIVWLNQKPIQQAEVFWRDQLAGLDSVTKIGLTSDPLDNGADYQ
VLQTTLQPEVYAQLKNYANKHLVTVSTLINAAWAILISKYSGSNEVIFGVTVSGRPTELS
GFEQMVGLFINTLPLRLKFQPDKPVATWLKEVSDRILAISEHSYSSLVDIQGWSNINRGE
SLFESIVVYENYPVGEKIRNHNDDLIISDVESLEKNHYPITLYALPGEDLTLKIAFQKIG
TTKERQQLLQHITDILNTLVTKSPRFLGEICLPTPSLIPIKHQPPVVTLADLFNQRVSEN
ANSPAILQGEKWLTYGELNDQANKIAQALVDLGVTQETLVGVCVDRHAGLVTALLGIIKS
GAAYLPIDPAFPINRLEWIINDSQAAIILTESSVVSRLPKSSARVLVLEAILQEEIAVSF
QPRPLDLGSIAYIIYTSGSTGKPKGVQIEHQSLVNCLLSFKNQLQLDITDTLVAVTTISF
DIAGLELFLPLISGAKLVIANQETAQDGFKLNDLLERSQATIMQATPTTWRLLLTAGWQP
KNQFCALCGGEAIPRELATVLLQLNLNLWNVYGPTETTIWSTVKQLEKSKDVISIGREIA
NTSLYILDDGLNLLPEGIVGELYIGGLGLARGYRNNPTLTSTTFIPDPFAQDPGSRLYRT
GDLARRLSNGEIEFLGRIDYQVKIRGYRIELGEIETILSSHPAITQAIVQAIGDTPAEQR
LVAYVVSKTQAPTIEDLRLYLYKYLPEYMIPSAWVFIDKIPLTGNNKINRRALPIPTYKG
ENKDWLPPRTSIEEVLVLMWQELLKVEKVGVQDNFFHLGGHSLLAGQFHGYIKKVFAIDL
ALRELFDAVTIEKIALLLIEKETKLGNTEKIAKAFLRLKSMTPEEKANLLQNSRLKKLN
>OBU77732.1
MSKGKQQADNTLTMNSNNSLWKYWKQGLTSLSLILLGSGITVTGSYLTNNQEALTKSASK
LGVESVHAAPLPGNMDSNFVTEVVQKVGPSVVRINSSRTVRTQAPDEFEDLRRFFGSRIP
RRENRVERGAGSGFIISNDGRILTNAHVVEGADRVTVTLKDGRTFEGRVLGADQLTDVAV
VKIDAKNLPTVTLGNSEQLQPGQWAIAIGNPLGLDNTVTTGIISATGRSSNQVGVPDKRV
EFIQTDAAINPGNSGGPLLNARGEVIGINTAIIQGAQGLGFSIPINTAQRISNQIITTGK
AQHPYLGIQMVSITPDLRQRINSDPNRGLTVSENQGVLIIRVVPNSPAAKAGIRIGDVIV
RLNGEVITDSSAVQKAVEVAQVGGNLRLDLRRNGQTINIAVKPGIFPTGMQ
>OBU77769.1
MVETEFSQVRFHGDREKAKKVYEGIKPLTAQDVADVIFFCATRPAHVNINQVILMPVDQA
SATLVNRQN
>OBU77800.1
MVRQIAPKTELRAIESNTNQGVETPKIVYPESDNKPMADNTRQFTWIVKIKENLEILFKY
NADVFVAGDLFWYPVEGSNKIKLAPDTMVVFGRPKGHRGSYRQWEEDNIPPQVVFEILSP

GNNNTEMDRKKLFYLKHGVEEYYVYDPDRISLEVSIRENNSFKEIENFTTWTSPRLKIRF
DMTQDELVIYYPDGSKFLSPVELSNYAEQETQRAEREKLLKEQETQRAEQERFLKEQETQ
RAERERLIKEQETQRAEQERFLKEQETQRAEREKLLKEQERFLKEQEQIKYQTLLSQLKA
KGIDITALE
>OBU77810.1
MLDLDRIPNQERLLRAYKVFFQPPIFDIWIDNPLHKSDRISQPSEYSTNPVLRARKFSIG
GLTT
>OBU77849.1
MSYYRRRLSPIKPGEPIDYKDVELLRKFMTERGKILPRRITGLTSQQQRQLTLAIKRARI
LAMLPFINAEG
>OBU77856.1
MSQQNQVKNKLNGALTSVIQTLQEFAEQTNFWQILDIAFGRTYNHLRVKELRTQWRQRDK
GALPLIEIVNQEVLGSSLGAYSIDTDKIYMSEQFVVNAKLADLVLVLLEEYGHHVDAQVN
AKDTPGDEGEIFAALVLGKTLDDESLRNLRAEDDSAVIALGGEVIKI
>OBU77859.1
MSNSAFRLIGLDALRNDPRYANIIDGNAPDRNSDGQPDQRLTVVVLDTGVDTGHQLLSPN
ILAYVDFINGSRFY
>OBU77860.1
MVVDFINGTPVVTNRPPAFSSGNFTVPENTTGVGSVMATDEDEGTTLTFSIVGGADRDKF
SINRVTGALSFNTAPDFEAPGDVGTNNVYDLRIQVRDGQNSVEQDITITVTDVNELPANQ
APTFTSAPSFTVPENRTTVGNVTASDPENDPLSFSITGGSDQARFAINASTGSLSFVTAS
DYETPTDVGRNNIYDLQISVFDGQNTVNQNIAVTVTDVNDRTLLGGILSDNNDPDGHGTH
VSGIIGATDPNIGVANNVDLIGLRVLGEGQDGSEVAQALQWVLDHRSEYNIVAVNMSLGI
PSAFYTQPQKVRVIQFTIHGDV
>OBU77864.1
MVVFGRPKGHRGSYRQWEENNIPPQVVFEILSPGNNNTEMDRKKLFYLEHGVEEYYVYDP
DKISLEVSIRENNSFKEIENFTTWTSPRLKIRFDMSQDELVIYYPDGSKFLSPVELSNYA
EQERFLKEQETQRAEREKLLKEQETQRAEQERLIKEQETQRAERERLLKEQETQRAEQER
FLKEQETQRAERERLLKEQEQIKYQTLLSQLKAKGIDITALE
>OBU77865.1
MSQGVETPKIVYPESDNKPMADNTRQFTWIVKIKENLEILFKYNTDVFVAGDLFWYPVEG
SNKIKLAPDTMVVFGRPKGHRGSYRQWEENNIPPQVVFEILSPGNNNTEMDRKKLFYLEH
GVEEYYVYNPDKISLEVSIRENNSFKEIENFTTWTSPRLKITFDMSQDELVIYYPDGSKF
LSPVELSNYAEQETQRAERERFLKEQETQRAERERLLKEQEQIKYQTLLSQLKAKGIDIT
ALE
>OBU77888.1
MADNTRQFTWIVKIKENLEILFKSKADVFVAGDLFWYPVKGSNKIKLAPDTMVVFGRPKG
HRGSYQQWEEDNIPPQVVFEILSPSNNNTEMDRKKLFYLEHGVEEYYVYDPDRISLEVSI
RENNSFKKLENFTTWTSPRLKIRFDMSQDELVIYYPDGSKFLSPVELSNYAEQERFLKEQ
ETQRAEREKLLKEQERFLKEQETQRAEREKLLKEQERFLKEQETQRAEREKLLKEQERFL
KEQEQIKYQTLLSQLKAKGIDINALE
>OBU77896.1
MSQEVDLKNLPLGINTLDKMRGSNCVYVDKTSFALKLIKQPGAFFLSRPRRFGKSLFVDT
LKEIFEGNEKLFEGLYIHDKWDWSRKFPVIKIDFADGVLKNREELDEKIRDLLWNNGDRL
GVGSKKKSISGIFGEIIAGAREQFGERVVVLVDEYDKPILDNIDNPNIAAEMREGLKNLY
SVLKSQDSNLQFVFMTGVTKFSKVSLFSGINQLTDITIDKQYSSICGYTETDLQEFFGDH
LEGVDWDTLRHWYNGYNWTGSETVYNPYDILLFIGKGQVFRNYWFETGSPTFLVKLFQTN
RYFLPNLEHLEVTEEILESFEVERINPVTLLFQSGYLTIERTFTRRQRYMFALKIPNLEV
RLALNDQFINAYTETVNEKSGIQDSLYEFMNRGDVESMIMAIKRLFAGIPWRNFTNNDLA
DFEGYYASVIYAFLSSLDARVIPEDISNYGQADITTMLGSHIYVMEIKVVEGNQVQGNPA
LDQILQRNYAEKYRGEPGKSVHEIGLIFSRSQRNLIQANWQ
>OBU77907.1

MSQGVETPEIVYPESDNKPMADNTRQFTWIVKIKENLEILFKSNADVFVAGDLFWYPVEG
SNKIKLAPDTMVVFGRPKGYRGSYRQWEENNIPPQVVFEILSPGNNNTEMDRKKLFYLEH
GVEEYYVVNPDKISLEVSIRENNSFKEIENFTTWTSPRLKIRFDITQDELVIYYPDGSKF
LSPVELSNYAEQETQRAEREKLLKEQETQRAEREKLLKEQETQRAEREKLLKEQETQRAE
RERLLKEQEQIKYQTLLSQLKAKGIDITALE
>OBU77925.1
MKTKIFGLAIVLSLATMLGACEGGTEPSGDATTTPTATTTPAEPTDSGTATPATTTEPTA
TPTTTP
>OBU77971.1
MNALSIPTWIIHISSVIEWVVAISLIWKYGELTQNHSWRGFALAMIPALISALSACTWHY
FDNPQSLEWLVTLQATTTLIGNFTLWAAAVWVWHSTRASGVLNISNKE
>OBU77976.1
MFSYNFISNYLGRDNIYFPVVVWLLSRLMIWAGMLLIAPSISITDNSVFEHGTWSIFDAW
DSVHYRAIATSGYEFYPDGQQYNLAFFPMFPLTIWVLMKLGLPFEIAGLLINNLSFLGAV
YLLYFWVKKHCSLRIAQWTIIVICCCPMSMFTGVIYTEGLYLLFSIGCLRAFDEEKYSLT
AFWGAMATATRPTGMALIPALLLTAWRQRRGKIAYFTSLLTATGLILFSIYCAINFHDPL
AFIAAQKGWRPSLGFDWQGWLNMLMQIILGRNWNFGWVWNDDGGIRDPWYPVFFGFMVYG
TVTLWLRQKYWHPFMIYLVYTTLAVLLILVDQRVINNLLNVLMVVGGSYLVWHFRRKLTP
VMVIYSLCGVSLLLASGGTISLSRLAYGIVPLSIAIGAWLSRFPRQAYLSVGLFVVLLFR
LAIGFAQHHWVG
>OBU77981.1
MNIETLKSEKTKQLPGANLEDQDLSEFDLTAVNLAGANLMGAHLVSANLEGSHLEGANLM
GASLQGADLRANLLGANLMQADLTGADLRGSNLRGANLMGATVAGASLTAAFLSGANLMS
VNFQGVDLRGADLRGANLIGANLKGADLSRADLQGALLNQANLEESDLRGANLAGANLAG
ANLLCAELEAASLNGANLYQACLLGTILETYHD
>OBU77985.1
MITRTSDTELLNWTGDTLGIGLFEDAVELKDDLATLNQKYGGVFSEVIAEEEFTGKANTT
VVIRVGANSPIRKVIFIGLGKVDTLKLETLRIAAATLARTAKKQKTKTLAISFPIYNNQP
AATAQAITEGAQLALYQDIRFKSEPEDKNPPIETIDLLNLSGQQPAITRAEQIVSGVILA
RELVAAPANAVTPITMAQTAQAIAQENGLELKILEQEDCEKLGMGAYLGVAQASDLPPKF
IHLIYKPATTPKRKLAIIGKGLTFDSGGLNIKGAGSGIETMKIDMGGAAATLGAAKAIGQ
IKPDVEVHFISAVTENMISGKAMHPGDILKASNGKTIEVNNTDAEGRLTLADALVYTDQL
GLDAMVDLATLTGACVVALGDNIAGLFTPNDNIASQLQTAAESSGEKIWRMPMEEKYFEG
LKSGIADMKNTGPRYGGSITAALFLKQFVKTTPWAHLDIAGPVWADQESGYNSAGATGFG
VRTLVSWVESD
>OBU78001.1
MAIRGNSWNYLSLLPIVLCCSLMLLLVGCQSKDRTKDSIVQARVVRVVSGQTLEVAKIGD
PTNSVSSVRLIGLDAPDLRQYPWGEDARKLLEKLIQDANSDNTTNNNTNSAQIKLAQIKL
AQIKLAQIKLEFDLQTQDKFGRNLAYVWKDQVLVNEQIIKQGYALFAGRSPNHKYDLRLE
NAQHWARLMGEGIWNPENPLRLTPGQFRRING
>OBU78003.1
MADNTKQFTWIVKIKENLEILFKSNPDVFVAGDLFWYPVEGSNKIKLAPDTMVVFGRPKA
HRGSYRQWEEDNIPPQVVFEILSPGNTQDEMDKKKLFYLKHGVEEYYVYDPDRISLEVSI
RENNSFKEIKDFSVWTSPRLDVRFDMTGDELIIYYPDGGRFLSPVELSNYAEQETQRAER
EKLLKEQERFLKEQETQRAERERLLKEQERLLKEKETQRAERERLLKEQERLLKEKETQR
AERERLLKEQEQIKYQTLLSQLKAKGIDITALE
>OBU78005.1
MILILQHLRTHRVLTKKLRLVPYALVGIGVFAFNANTDTNTYLQIYLILLEAKVGIVLVY
FFTKNLEKFYK
>OBU78008.1
MTGLNRQAFNELLSQLIVAWVAQEGSQRQLAERFKVSLSFVKNLVRRYRETGQVEPKQCG
GYEKPIIAGQYLNMIKSWLDEKNDLLLSELCDRLRETTGTSVSITTMHRALEKLGLRHKK

KSKCQ
>OBU78069.1
MLYNLHKLSRSSAILLLSLVLISGLLAGCFGYPRILSYPFDSGGRGLNSLASEFNPQISG
RYIVFVTDRGGSQDVYLFDTITRSLVDLPGLNTFDTMADHPAVSQDGRYVVFAASRQGKS
GIFLYDRETRQSRNLTTNLVAQFRNPTISADGTRIAFEFTNDGQWDILVCDRFGQKLNIS
>OBU78082.1
MADNTRQFTWIVKIKENLEILFKCNADVFVAGDLFWYPVEGSNKIKLAPDTMVVFGRPKG
HRGSYRQWEEDNIPPQVVFEILSPGNSQDEMDKKKLFYLKHGVEEYYVYDPDRSSLEVSI
RENNSFKEIKDVSVWTSPRMNVRLDMTGDELVIYYPDGGRFLSPVELSNYAEQENQRAER
EKLLKEQETQRAERERLLKEQERFLKEQETQRAEREKLLKEQETQRAEQERLLKEQEQLK
YQTLLAQLKAKGIDISTLE
>OBU78089.1
MATTADEVWKLLGELIESQKETERKFQETERFLREQSQETERFLREQSQETDRKFQETER
LLREQSQETERLLREQSQETDRKFQETDRLLREESKRVNNQIGQLGNRLGEFVESQVRPA
AVKLFQERGIAVKEIASNTYIQTGKEGLEIDLLVINSSDIILIEAKSKVSEDDVNEHLER
LSKFKRFFPRYESYRVLGAVAGMVIPLDVSRYAYRKGLFVIGQSGDNLVILNDDKFRPRG
W
>OBU78090.1
MATTADEVWKLLGELIESQKETERKFQETERFLREQSQETDRKFQETERLLREQSQETDR
KFQETERLLREQSQETDRLLREQSQETDRKFQETDRLLREESKRVNNQIGQLGNRLGEFV
ESQVRPAAVKLFQERGIAVKEIASNTYIQTGKEGLEIDLLVINSSDIILIEAKSKVSEDD
VNEHLERLSKFKRFFPRYESYRVLGAVAGMVIPLDVSRYAYRKGLFVIGQSGDNLVILND
DKFRPRGW
>OBU78091.1
MATTADEVWKLLGELIESQKETERKFQETERFLREQSQETDRKFQETERLLREQSQETER
LLREQSQETDRKFQETDRKFQETDRLLREESKRVNNQIGQLGNRLGEFVESQVRPAAVKL
FQERGIAVKEIASNTYIQTGKEGLEIDLLVINSSDIILIEAKSKVSEDDVNEHLERLSKF
KRFFPRYESYRVLGAVAGMVIPLDVSRYAYRKGLFVIGQSGDNLVILNDDKFRPRGW
>OBU78092.1
MATTADEVWKLLGELIESQKETERKFQETERFLREQSQETDRKFQETERLLREQSQETDR
KFQETERLLREQSQETDRKFQETDRKFQETDRLLREESKRVNNQIGQLGNRLGEFVESQV
RPAAVKLFQERGIAVKEIASNTYIQTGKEGLEIDLLVINSSDIILIEAKSKVSEDDVNEH
LERLSKFKRFFPRYESYRVLGAVAGMVIPLDVSRYAYRKGLFVIGQSGDNLVILNDDKFR
PRGW
>OBU78099.1
MIAHRLSTLEKCDRIFQLDQGQVCQEGDRHGDSPTNSATGGAESEPAKLMCNSILERENM
ATTADEVWKLLGELIESQKETERKFQETERFLREQSQETERFLREQSQETDRKFQETERL
LREQSQETDRKFQETDRLLREESKRVNNQIGQLGNRLGEFVESQVRPAAVKLFQERGIAV
KEIASNTYIQTGKEGLEIDLLVINSSDIILIEAKSKVSEDDVNEHLERLSKFKRFFPRYE
SYRVLGAVAGMVIPLDVSRYAYRKGLFVIGQSGDNLVILNDDKFRPRGW
>OBU78101.1
MATTADEVWKLLGELIESQKETERKFQETERLLREQSQETDRKFQETERLLREQSQETDR
KFQETERLLREQSQETDRKFQETDRLLREESKRVNNQIGQLGNRLGEFVESQVRPAAVKL
FQERGIAVKEIASNTYIQTGKEGLEIDLLVINSSDIILIEAKSKVSEDDVNEHLERLSKF
KRFFPRYESYRVLGAVAGMVIPLDVSRYAYRKGLFVIGQSGDNLVILNDDKFRPRGW
>OBU78104.1
MATTADEVWKLLGELIESQKETERKFQETERFLREQSQETDRKFQETERLLREQSQETER
FLREQSQETDRKFQETERLLREQSQETDRKFQETERLLREQSQETERLLREESKRVNNQI
GQLGNRLGEFVESQVRPAAVKLFQERGIAVKEIASNTYIQTGKEGLEIDLLVINSSDIIL
IEAKSKVSEDDVNEHLERLSKFKRFFPRYESYRVLGAVAGMVIPLDVSRYAYRKGLFVIG
QSGDNLVILNDDKFRPRGW
>OBU78112.1

MLLHYCLDLNSDRQVVMFLHGFMGNIYEFDNVIKLLNNNFSYLTVDLPGHGKTEVLGGSD
YYGMENTAQAIINLLDELKIEKCFLVGYSMGGRIALYLTINFPERFIKVVLESSSPGLST
DFQRIMRIKSDAGIIRKLTRISTRNEFGVFLNNWYSQPIFGQIKNHPAYTKMIETRLANS
PLKIAKSLQFMGTGYQPSLWHKLEYNQIPLLLLVGEYDQKFIDINTVIYNLIPGSKLVII
NRSAHNTHLENPLMFVEYIMEFFRVF
>OBU78155.1
MIERTLEISREWWSQRWLDLLDSYRFKKRLERARNYSRQGNVLSIEFQKSKVLARVKGSE
VEPYQVSLSLDIFSDEEWSYVIENMSHKSLFAAKLLAGEMPQNIEDVFTSNGLSLFPFTL
NDVHSQCSCPDPAVPCKHIGAVYYQLSDRLSEDPFVLFELRGRSRERIIQDLRKLRGQKK
LVNLPIHRKNNQGENSPQLNPTPNQINYFWQYNDPLDSSLVVISPNMGETVLDILGNIPL
PKDEENTVHVLGSSSDSTMKHLQNIYRDVSQKACLLAMNMG
>OBU78167.1
MEETRKIVAETNKNMGSITSRWGEFVENLVRPAAVRLFKEQGINVHYTSLQVKADDYAGS
IEIDIWAENDGEIVAIEVKSHLKVRDIKRFITVLDRFKDVFPKYKNYRLYGAVAGIKVDE
KADQYALEQGLFLIRPAGDSVAIDMKKDFQAKVW
>OBU78169.1
MGETIVPEIVYPESDGEPMADNTKQFTWIVKIKENLEILFKSNPDVFVAGDLFWYPVEGS
NKIKLAPDTMVVFGRPKAHRGSYRQWEEDNIPPQVVFEILSPGNNNTEMDKKKLFYLKHG
VEEYYVYDPDRISLEVSIRENNSFKEIENFTTWTSPRLKIRFDMTQDELVIYYPDGSKFL
SPVELSNYAEQETQRAEREKLLKEQERFLKEQEQIKYQTLLSQLKAKGIDITALE
>OBU78172.1
MTNFSQADDIDLLGLLLEEEGIELEIKEVVPRVGSTEAPTSFQQRRLWFLYELEPTSSAY
NICSIFDLKGTLNITALRVAFKQLQQRHESLRTTFMDVDGEPWQKIHANSATELRLEDWS
NDRSEDKIPEIIAEIARHESDHQFNLQTGPLIRAQLFKIESKQHILSINLHHIIADAWSV
GVILQEIAMLYQAEISKTPIGLPELKFQYTDYALWQKENFQNSNILEKSLTYWEKQLAQL
PTLQFPLDFPRPRLQTFRGGLIKFEISKETTNRIHNFIVKEGATLFMFLMAVFQTLLSRY
TGQEDIAVGTSIANRPSDSENLIGFFVNMLVIRTNLADEPNFNSLLKTVKKTILSAFEHK
EIPFETLVEKLNLPRDTSRNPLFQIAFTLLNAPKPQFGTGDLEVSILATQEAARFDLELF
ITETEDTLNGAVSYNIDLLKRETVERVARHFCQLLESVLAQPEIPVSRLPFLLSEEIAVL
APSQPAQTFPVHFCLHDIFTEQAKLRPQQTALIFGQERLTYSEVNYRANQLAHYLMRVGV
KPEARVGLWLSRSLDLVIGIIAILKAGGVYVPFDPNYPKDRITYMLEDSQIRVLLTHSEF
EAQIPFEFTNNTNNVSNTNNNILTIFIDKYKSEFTQVVTTEPEVLILPDNAAYIIYTSGS
TGKPKGVVVTHRHVVRLMLSTEKWFKFNAKDVWTLFHSCAFDFSVWEIWGALFYGGVLVI
VPYLISRSPEEFYNLLCEEKVTVLNQTPSAFQQLIQAESTLCREGELELRYVIFGGEALD
LASLEPWFERHDDQFPLLVNMYGITETTVHVTYLPLTFKDVKKGSGSLIGKQIPDLSLYI
LDRHLQPVPIGVVGEMYVGGAGVTRGYFHRPQLTAERMIPNPFATNDLTSVRLYKTGDLA
RFLDNGNIEYIGRNDHQVKIRGFRIELGEIEALIKSHPEVRDALVIAREESKEDVRLDAY
IIPINQIANTETLTQEQTQEWQYTFNDTYNITSGETEEDFNIIGWNSSYSNQPIPGVEMR
QWLNNTLLRIQSLKPRKVLEIGCGTGMILLNIAPQVESYWGTDFSQAAINRLDTIVKNRS
LKNVNLLTREAIDFSEIPTGYFDTVVINSVAQYFPSIEYLQQVIKSVWQLLKTGGSLFIG
DNRNLSLSNYFYASVAYFQANDNTDCETFKTQVRRIAKKENELIIAPHFFTDLRKSFPDL
TAVEIQIKSENNENELTKYRYDVILHKLGISTEQPPEIIWRDWETDNLQLTDLKQQVIEM
RSIGWHSVPNGRLSKDAAIYQWMLKNSHENEQKTIGELRTVLNNIHEPKGFNPADFYAIA
EEIGLEVSISYSPGKVDCFDVCFYPAGSGKSMAPSMPIVNDLLGRDNHPSWIDPLKNRLT
KLLISQLKQRLEEKLPEYMCPSAFMILENFPLTPSGKLDRRALPIPDRDLIINQQSLVPP
KTPTEYKLSQLWMDVLGIDKIGVTEDFFHLGGHSLLATKLVSRIREEFNVALPLRSIFEY
STIARLGDEIDCLIDVNTTKTGPEDIIPVSNRENLPLSFSQSRLWFLDLLEKENAAYNIS
VAFRLEGDLNVDALRESWQNIIQRHEVLRTTFDNVQGSPIQIVHDWSELKLTIRNLSCLD
FQTQQETLRKSIQEVVITPFNLNQLPLLRIHLYQLSADVSVLLLVIHHIIADGWSLGVMV
KELSLFYTAICQRNIPSIPPLSIQYGDFANWQREVFQKTQLPIQLAYWKQKLTGANQILE
LPTDYPRSPIPSYQGSAVNFAINPQTTQEFKKLCESQGATLFMGLLAVFSILLMRYSGQE
DLLIGTPIANRNRKQTEDLIGFFVNTLVIRNNLSGNPNFINLLSITKEETLQAYAHQDVP

FEKIVEEINPQRNLSQHPLFQVMFVWQNAPMNKLELPNLQLSPWRLEQRLAKFDLTLLMT
ETEQGIDGTWEYRTDLFAPETINRMIGHFETLLKGIIAEPQKPITHLPILTSHEKNQLLF
QWNQTQFEYPLYQQNKCLHQLFELQVEKTPNNVAVVFKNQSLTYFQLNQRANQLAHYLQA
KGIGPETLVGVCLERSEQLIISILAVLKAGGAYVPIDPAYPSDRLEFLFQDSGIVLLITQ
RKVISQLPECGAEIIILEDIAKRIEGNNSQTPAVNILPENAAYVIYTSGSTGKPKGCIVT
HANVIRLFNSTTTWFNFHSEDIWTLFHSYAFDFSVWEMWGGLLYGGQVVVVPHWTVRSPK
DFLQMLATHKVTVLNQTPSAFKQIISVVQQKPEKLSLRYVIFGGEALELADLQPWIELYG
DMQPELINMYGITETTVHVTYRPITQKDIINNLNYRASVIGQSIPDLELYILDENLDPTP
IGVAGEIYIGGAGVTRGYLQQPGLTAQRFIPHVHSKNPGSRLYRSGDLARHLPDGEIQYL
GRADQQIKIRGFRIELGEIEAVLAQHPNVLNAVVVVSGDSSATNSLIAYYVSTEKQFTSS
GVLRNFLKEKLPDYMIPNSFIVLDHLPMTPNGKIDRKLLAGLNINRTFDAHQHVSPRTLL
EYKLVEIWEEILQVRPIGVTENFFDLGGHSLLAIRLIAAIEQKLKCNLPVVSLFREGTIE
KIALLSDQDHQKASNHSDILIPLQTQGDLLPLFLVHQAGGYGLSYSVIAEKLAVGMGKKL
PIYAIQSPGLDGKQSPLESIEEMANTYINTIREIQPHGPYLLGGHSLGGLIAFAMASQLE
AMGEQIERVLIIDTHPPMPTDETIASLEDNAGIICFMVEQIALFFNKNVTINYQTISSLD
QDSQLDYVAQTLEQHNLIPPNSGNSLIARLIKVYKANLRASVVYQPPVNRSNITLFITPS
LAAKFPNDPTVGWQKLTTQKVQVCRVMGEHQTMLKEPEVENLVTEIMATLVNTP
>OBU78181.1
MRSHFSAAHRLAPNLSAEKYGKCTRTHGHNYHLEVTVEGEMDGRTGMIVDLGRLHEIVER
EILELFDHSCINEDIPYFSTSHIVPTTENIARYMSDLLQFPISQLGVKLSRVKLFESDHL
WVEYEGKDSEIFFSVATGFSAAHRLADPTLSLEKNQTIYGKCSRINGHGHNYYLEVTVQG
EIDSVTGMSVDLVGLNQIIQHYVIEPMDHSFLNQDLPYFTEVVPTAENIAVYITNVVRSP
IEELGAKLHKVKLIESPNNSCEIYARDIEESKVDRIYRELAAV
>OBU78182.1
MPGKGDDIIRVNSASVVIIELPNEGNDTVFSSINYNLASLPQIENLTLWGTEDINGIGNR
RDNVITGNSGQNVLTGLQGNDTFVFNLGDSVVGKPDRIGDFQFGKDKIKVNGVLPSVLTR
AGNNSASTLNSLVDSVFIDGNGATSGNQGLGTNSAALVVSTAQGIGGTYLIVNDGVGGFN
PATDLVINLTGYSSNLPGLGNIAVGSLFV
>OBU78183.1
MRVIESNISQGVETPKIVYPESDNKPMADNTRQFTWIVKIKENLEILFKYNADVFVAGDL
FWYPVEGSNKIKLAPDTMVVFGRPKGHRGSYRQWEENNIPPQVVFEILSPGNNNTEMDRK
KLFYLEHGVEEYYVYNPDKISLEVSIRENNSFKEIENFTTWTSPRLKIRFDMSQDELVIY
YPDGSKFLSPVELSNYAEQETQRAEREKLLKEQETQRAERERLLKEQETQRAEQERLLKE
QETQRAEQERFLKEQETQRAEQERFLKEQETQRAERERLLKEQEQIKYQTLLSQLKAKGI
DITALE
>KRH95354.1
MSEIEKVPRGKKPIYILKKSRKMLLYIALGLVALILVITLGSVISEPLERIISVAENLYQ
KWFEKQNTANPFVLLPLAFIGGLLASISPCILALLPVNLSYIGTLKITSRWDAFSQAGLF
VLGSVTILSLFGLVSSFAGMVIIEYRGYINIVVGLIMAVMGLWLIGVIKIYLPQIDLKLP
NTGPYSVGLTFALVSSPCASPVLFAVLAAAAATGSQVLGTLTMVSYALGYTILIFLASLF
TGLVKQSRKLLQHSETIIQLGSVALMLTGIYYLYTGTVWFLGG
>KRH95358.1
MKSVLNHRLITIVLTGWISLFVSGLVLVLATPSITVIIDRSYCPSDQWQTQVVIPYRDLY
QKHQDKQLKIESITLLSDLGQESSKKIPTPEEIAILNTYGKLSSDRQKSLEKLSQKAKLL
RCL
>KRH95362.1
MVDNKNILLRNLWYYALPSSQLKTGKMVSRVLLKEPILFTRDKNGQVFAIEDICPHRAVP
LSCGRFDGEQVECCYHGWRFNSQGKCTEIPSLLPEQSIDLNRFNVKSYPVYETQGNIWIY
MYDKEGPPINAEIGDIPQVPGFSNREPDLVEVMKFPCFIDHAVTGLMDPAHSPYVHQVWW
WRSGKLHEEVKQFDPSEYGFTVRRHTLSDNTENMSRLYWLVGGGIPEVEISFRLPGVRIE
EITFGKHRVCNLTAVTPISDTETEVNFVLYGIPAWLKIFTPLIQILTRKFLDQDRNVVEK
QQIGLQYNPILRLIKDSDMQAQWYFQLKREFSRAAAENREFVNPVKSVLLRWRA

>KRH95363.1
MTIIVFGSINMDLVATTPRLPIPGETLLGESFFTAPGGKGANQAVALAKLGIPTQMIGRV
GNDNY
>KRH95365.1
MKLQVSTFTVNKRFALTISRGTTAQTTNIWVKIMEGGIEGWGEASPFGVGSYRETTDVIF
QSLQEISSMLESYSPWQRDDISKILTQHQIPSAAKTAIDMALHDWMGKNVGLPLWQIWGL
NINTIVPTSVTIGINSPQVAAARARDWLQYMDVQLLKVKLGAKEGIDADKKMILAVKEAA
PKVDLFVDANGGWSLPDAIAMSHWLADLGVKYLEQPLPKGEEQKLPSLKKQSPLPIFVDE
SCFTSTDIPPLADYVDGINIKLMKSGGLEEAWRMVNTAKAHNLQVMFGCYSDSSLANTAA
SHIAPLADYLDLDSHLNLIDDPFVGASVTEGRIIPNSLPGLGVQYSALTT
>KRH95373.1
MVWQRLDGRQPDQLRTLNFYPHFTRFAPGSVLAQCGETQVLCTVSITEGVPKFLMGSGKG
WLTAEYRMLPSATQQRQERELLKLSGRTQEIQRLIGRSLRAALNFEILGEKTLTVDADVL
QADAGTRTISITGGFIALAHAITNLLQQGVLERSPLCGQIAAVSVGLLKGEAFLDLDFTE
DVNAEVDFNVVMNSRLNIIEVQGTAEAGSFSRQQLNQLLDVAETGIRELLIAQRSVIPDF
NTLLNV
>KRH95374.1
MIFTQIDSPNINPAPSLPNSVAGSLQVFTSSERYFFTNVISQSLRIASHGTPVLIIQFLK
GGINQGINNPIQIGNKLDWIRCDLARSPDTPNFNEEEIGSLHSLWEYTQKVVYEGKYSLV
VLDELSLAVDFGLIPEKEVLQFLIDRPTHLDMILTGPQMPKSFLDLADQITEIRRLQP
>KRH95375.1
MKKLFIWLIQGYRLFISPLFPPSCRFQPTCSMYAMEAIERFGVFHGSWMALGRILRCHPF
HPGGYDPVPEKTEKSP
>KRH95376.1
MRPYHQIPIIESGEPLVEIPLELFAVENPHPYAKLGARYGQYSPYFLRKTVVKNLIHAQN
CLNLLSPGWHIQIFDAYRPVGVQQFMVDYSFIQLVKAGGILEQNLSDDQREKIWQQVYEI
WAPPSSNPHTPPPHSTGAAVDITLVNERGEIINMGSPIDEISERSHPDYYLNKHSQYHQG
REMLNNIMCQAGFQRHPREWWHFSFGDQMWAWLSHQSTAIYGFCE
>KRH95378.1
MNASEQATNFEIASKIATVVNLFKLQFPDAKSDLKPWQNDRETRQLVDPDSIDIGFHFPG
ISRSWRSRSVLIQIRFHQDPINQQSKAIGVEVSGFDHRGQVWRLSTIEQWSFVGEPTPSS
VVGEKLKHICRQILEIFNI
>KRH95385.1
MVKYTFHLKPEGSPSESYSYSLDLNNMEEDAPEKVFTPLVREKIRKTLQDLSLSAIRDYQ
LNRIIQTWTEDIKHGYRFSSLSLNLRLLIDENIDQLQEDGNQEIPQMIAPDISDIQPQSG
VLPALNFV
>KRH95387.1
MLKKMIFYSTYLSLLGCQTVNPNGSISSNLLSSPGCPDKPTVSLSSKNVEQIMLDQNIIT
KSSQVSSNKHIGYTFAAKAGEKLNYSTDTNVCVWVFAPNNEIVKGINLPKKGKYTIQVAA
PQGVKTFSLQMSLGSTLKSDLVTMEDPVEKSTVIATTVAPIPNTKSQIPARADSVQKSYT
ATQNTSRISPEQVIRDYYDKINNQQYDTAWDIYPSTVKEDLNLHPNGYDSFLGWWTQVES
VRVIRVSVKSEDNDSAIVNFRGQYSMKNGRLLPVRLRFYLDWNQDNATWYVTQIKVRTPN
>KRH95389.1
MLKKMIFYSTCLSLLGCQGLTLPTPKGTGILNLGSPGCSDKPTVSLNAKNVEKITFNQGR
ISKSNQVSVEQHVGYTFAAIAGDKLSYSTDSDVCIWVFTPDTEIVKGKDLLKTGQYTIQV
GAPQGVKNFNLEVGLGDLQTNISPTTESTPTINLDAKENNLQPEHDISQEDAVELVKQWY
AAKPQIFASPFDTNLVDQLATGKMHNFTTKADGPVEWLRQNGAYYEYISSEIKRVVNFSN
SGKRPYIRVRVREERYLHSRYGIDRAKSGKFTRNLTYFFEKENTRWKISEVYPAWQ
>KRH95390.1
MNTKQIILLSAREIEKMRRAGSLAAQLLQHLEPLVKPGVTTQELNDEAEAWTQAHGAKSA
PLGYMGFPKSICTSLNEVICHGIPSSQRVLKKGDIINIDVTPVLDGYHGDTSKTFIVGEA
RPIAKKLVEVTQKCLYLGIAEIRPGGRIGDIGAAIQEHAEGHGFSVVRDFVGHGINKIFH

TAPDIPHFGTRGTGRLLRPGMVFTIEPMINEGSYEFEMLADGWTAITRDRKLSAQFEHTI
AVTENGVEILTLP
>KRH95391.1
MYNYGLFTEKSIGVGLIGTGYAAKHRSQAIKQDERANLIAIAGHSPESITALAKSYETQV
SNSWEELVEREDVDLIVISTINSDHGKIARAALNNNKHLVVEYPLSLNLKDAEELIALAK
DKKRLLHVEHIELLSGWHQVLKGNLPMLGQLFYVRYSTMKAEHPARRKWTYNHQLFGFPL
MGALSRLHRLIDVFGQVVTVNCHQRYWETEEEYYQGCLCTTQLCFADGLLAQVIYGKGET
IWQSERKLEVSAENGGLILDGDKGIFIGSEDTRSIEIANRQGLFARDTKIVLDHIFHGSP
LYVTAEQSLYTLKIADAARRAAETGLTIFLDQD
>KRH95394.1
MADVLAIAGSPAHPSRTYGILEYATQLISEQGLETTIISVRDLPAEDLVYGRYNSPALEK
PKELIQQASGIIIATPIYKAAYTGVLKSFLDLLPQKALSGKILLPFATGGTIAHLLAIEY
ALKPVLSELGARHILSTVYAVDKQIQFSQEGSIQLEEELAQRLHENVGELVRLVNSVKVN
EQKLSVS
>KRH95395.1
MISGQKAIWKRLRSSKLFVIGLAIFSCLLITPLLHLPAWGNVTNSRIQFVSPNWVAENIK
LYR
>KRH95396.1
MKTYTQPEKLIVAIAPIGVSGVIGAFIFGIGMQLGGACGCGTLYTISSGNYTMIITRKSP
KGTEATWL
>KRH95397.1
MASSGVFMGIPATTGCLVRSRSHLNPLTYQVDALRGIMLVNGSSIYGLGLDCAILLLTLI
GLTFLCGRLYPLVVM
>KRH95398.1
MSLDKPPQQCAARVMETIPLLMRFIRSDMCTHSADSLTIPQLRSLAFLKRNPGTSLSAVA
EHLGVTCATASTTIERLVQRHLVQRTDHPQERRKIVLNLTTQGKSLLEESQEKTRLHIAE
IIESLTSEELLQIETSLTLLKNVFEKTESNH
>KRH95399.1
MSLKKPNLTTDHSSIHDPFAALRFRDYRLFTIGRILLFTGNQMQTVALGWELYERTNSPL
ALGIVGLAQVLPVILLTLIAGHVADKYNRQRTTVVASLLLALCSLGLGIISYTQAPVFLI
YICLVLTGIARAFLKPASDAMMWQLIPPQVFTNAATWVSGSFQLASVIGPALGGFVIAIF
NSATQVYILTSIASLSFLIAVVAMNPPEGNLSKEPISLRSLAAGAEFIWKNQIILAAITL
DLFAVLFGGAVALLPIYAKDILKVGPVELGYLQAAPAIGALIMGAVLIQLPPITKTWSTL
LWSVFGFGVTTIIFGLSKWMWLSLLMLALGGGLDTISVVIRHTLVQLKTPEELRGRVAAI
NTVFITASNELGAFESGLVAALVGPILCVVGGGIGTILVVLATMVIWPELNKT
>KRH95400.1
MTQVTISWVIYDLTKSSWLLGLTGFLQFLPTVLLTPFSGVLCDRWNQKKLLILVQILGLM
VSTTLTTLTFIGGSGMLQVSCGNTIIQTLVEDDKRGRVMSLYSLAIIGTLPLGNLIVGSL
AQNIGAPNTVIGCGIFCLLESIWFNQQLPLLRGKIRETLILSTSTLETGA
>KRH95405.1
MEVIYIFFIVIVIVVLIIQVAIVRWVFRINHIVMILERIVAHLEDIKKQNNILIRHQNEM
LELLDTDLERIVAHLENIKKQNNILIRHQNEMLELLDTDAES
>KRH95408.1
MLTRQHEFTEKIVEILNYHFADQGIIVLSSSELLQYLNIKTQAANRGSKSRAGLANHYAV
CVLVEDYLNHKFHINGGYEDYQGARFVNLFRRQRELPFGNKLQNHALNHRLNQEFKKYFP
TIPYVPIIRDTKTSRYWINENLIKILVNGNQINIAQSVKDIIDAYVHARVNSFTDFIMYC
KRMINMQLQTQPEYLEFIQSLLKPNIDARIFEIVSYAILKQYYGDQKIYWGWSYDNINAE
YLILYKTGRTNANDGGIDFVMKPLGRFFQITETVDVGKYFLDIDKVQRYPITFVVKTEQT
PKEILYKIQQQAVAKYSVKAIVKRYMESIEEVINIPELIRRFNLILQQDKGNLVIEEIVL
QSHIEFNMETEDVLK
>KRH95409.1
MMNIERYEHGGHILFHGDALSTLSNQVASQSVDLIFVDPPYNIGKRFSNFYDKWESEDKY

ATWVYNVLEECTRVLKPNGSMYVMASTQAMPYFDLYLRKTMTILSRIVWHYDSSGVQATK
YFGSMYEPILYCVKDKGNYIFNSDDIKIEAKTGAKRKLIDYRKTVPRQYNSEKVPGNVWY
FPRVRYRMEEYENHPSQKPESLLERIILASSDPSNIVLDPFAGTFTSACVAKRLGRNSIS
IEFQEEYLRIGLRRILGWKEYKGEKLLPPSKNYVSKKTQNKCYPERSL
>KRH95410.1
MVKSPSKQIQEKLKPILFYIVPLSSGILMGVTVAPINAWFLAWIALAPLWVIVVKYTRKS
LPSASWWAIAYHQ
>KRH95414.1
MTVTATLSQLAAALSASLVNIPSSSLSQVIVGIQTDTRVLQPGEIFLALGGEKFDGHQFV
EMAIARGAVAAIVDYTYNYPDLPVIKVSNTLNAYQELAKWWRGQFGIPVIGVTGSVGKTT
TKELIAAVLGTRGKVHKTHGNFNNEIGVPKTLLELSQGDDFAVIEMAMRGRGQIAELTHI
AKPNIGVITNVGTAHIELLGSEQAIAEAKCELLEEMSNDSVAVLNYDNPLLMATAKQVWS
GEVITYGFSGGDIQGKLVDYQKIEVGGMTLPLPLPGVHNGSNFLAALAVAKVLDVDWKLL
EAGINVTMPQGRSQSWKLPRDIMILDETYNAAPEAMLAALELLAQTPGTRKIAVLGAMKE
LGERSTKLHQQVGETVKKLNLDGLLVLVDGPDAETIVTSAQGIPCERCHNHSDLVSLLKT
YVQPGDRLLFKAAHSVGLEKVVQQFCGEINS
>KRH95417.1
MLNISNLLLFKTQTPHSGYHWDGTSRRFFEGWYNRVTLPEIGQTFAFMYSIEDPISNQPH
SGGAAQVLGPDDSYLLRTFPDIKAFWASRDVLALGHWGKTDLQITPQYLTPHKFTTHIQQ
GYQATATLNQGIITDPGTGNYCRWQYEIKPIYGWGQPNYPQQSTAGWLSFLQIFEPGWQI
LMAHGLASGYIDWNGKIYQFTNAPAYGEKNWGGAFPQKWFWINCNCFDYETDLALTAGGG
KRGVLCWMESVGMIGIHYQGHFYEFVPWNSQVTWQIEPWGSWQMQARNYKYEVTVTGTTD
LPGTLLRAPTNQGLAICCRDTMQGKVSLELRRLHQNHSTVIVKASSDVCGLEVGGDSWEN
SWQFQ
>KRH95425.1
MSDLPFTLDQLRILKAIAQEGSFKRAAESLYVSQPAVSLQVQNLERQLDVPLFDRGGRRA
QLTEAGHLLLNYGEKILGLCQETCRAIEDLQNLQGGTLIVGASQTTGTYLLPRMIGMFRQ
KYPDVAVQLHVHSTRRTAWSVANGQVDLAIIGGEIPTELAEYLEVLPYAEDELALILPTF
HPFTKLHTIQKEDLYKLQFIALDSQSTIRKVIDHVLIRCGLDTRRFKFEMELNSIEAIKN
AVQSGLGAAFVSTSAIVKELQMGMLHRSTIEGVIVKRTLWLIFNPNRYRSKAAEAFSEEI
LPRFANPGWNASALKTSPPTTNLLDIDTDNDSLFA
>KRH95435.1
MIRQMCWLSKFSGDGEKILHLQIAAGQPWRPYTAFPQFSVPDYPIPGGSKGWATYQKLLK
AGWTLVPSARAEEFSRDLVESSIQK
>KRH95441.1
MGPTPILTFDRGTLILHPPPRSKSWIYFATWDDRIEKFRIPAIKYRCLVETLEADDTDFI
DEAKKFYPLELLPSVQMTPYPHQTEALVAWKLAGRQGVVVLPTAAGKTYLAQMAMEATPR
STLIIVPTLDLMHQWYAHLKAAFPDGELGLLGGGSRDKTAILVATYDSAAIYAESLGDKY
GLIIFDECHHLPTDFNRVIAEYAIAPYRLGLSATPERTDGKHVDLEILIGKEVYRQYVEE
LAGKVLADHEIIQIKVKLSPTEREKYNRLIQTRNDFLKHSRISLGSLQGWQTFVQVSGRS
QDGRRAMLAHRQAKEIALGTDGKMRVLADLLADHYPEMVLIFTADNATVYRISRELLIPA
ITHQTPVKERHEILTKFKQGIYNTLVASHVLNEGVDVPAAAVAIILSGTGSIREYTQRLG
RILRKGNQENKRAILYEVIAEDTSEEGTSIRRKGLRGMETPSGNKPEEKSKKGELSIVYG
DNQEPELKVAQPVEINYLIKKLTKSKHQKDVTD
>KRH95442.1
MLPTELLIYRQTGEEIIPKRLKLDDKHLALANDLINFFQDTLGKTQGYLERQLIDFEGET
TDYRMKRGLGYILKSSFCTFEIISPLEPQMLRERVFTLAAQSVASPQNTQIIFSQIAAKL
SQELEREILPIQIAEGLYADLLENRILTAFDAPKPEDLLHRYNLSQVQGIFYKATQLILN
AHRNVPREYKLLFRYLKLFQLMAYIEGNANHGFTITIDGPTSLFTSSTRYGLSIAKMIPA
LLHVTKWSLSTTLQIKDLYTQEWKTSRFTLNSECGLISHYPQGKPYDSMLDQSFADKWDA
MKSGWFLETEVDLIPIPGSVIIPDFRLVHPDGRSFLLEIVGYWRPEYLKKKFAQVKRANC
HNLILAISEKLNLEKSGVNLDNVPAKIIWFKEKLLPKAVLSSYAK

>KRH95443.1
MTELPKTLEEAIAQSRAAVRSALSDGKTRIQVELLFPELQFMPVAEQFLPLFTQYESRLK
VFFADAGAAALARRDWGDIPFKITDIGTGRAASSESKIQPEDEIFLFIAPTPVEVAQLEK
LCQIIGERPFVMLNPRLEDSSVVGIGYAARETRRRFISTIESCYYLRPIDEQSALMRSYP
GNWEIWLETDGEYQKIAELPQKPSGDEIDSILIKGQPETGITASKKPTVFKSLQRFIKAL
SS
>KRH95445.1
MRSLVGPGINLSLIASTLGLSLSHSGTVFALPPQEIKGKLDAVPVYLITNDQGTPLSRMI
SSPDRKQERAMTDVYMSRQEALNFVQKFRQILGKDKNPKTQEMLKTLQVTTVPLGLIYQK
QQQQQNQPNQLLFSFNPVSQEMTGAAQLMKASGQRVEQLKSVPIFMVISGKDKSHITIQV
GGGKPQTIIPLFFSKQDAQNLLTKVKGKFPQAYIQVVGVDGLINTLTQKNDDWLKQLVLI
PSPESRQHLNNLRSNSAQPKTIPPKPRS
>KRH95458.1
MYLLFQTLASFVEIYSYVLIVRVLLTWFPQINWYNQPFAALSQVSDPYLNLFRNIIPSLG
GIDISPILAFLVLNIVSSLLENLSSATSLGGF
>KRH95471.1
MSNTTSGQGTLPPASAIDKLTKNEVDTWFDYPIRVQPHHTDYAGLVWHGTYLTWMEEARV
ECLRSFGVDYADLVALGCDLPVVELSIRYHISLQLGNMALVKTRMLEVTGVRINWDYKIV
SLDNQQLCVTAQVSLVALDRERGKIMRQLPATFQEVIAKIAASYK
>KRH95478.1
MSPQEFTLLLFSVLISVTGQFLLKTGALKLGKVHAGNFITHILNMITVPELLLGLACYGV
GAIAYILLLTRVNLSIAGPAVSVGYIFSVMIGLLVFKESMPLTRILGLGFIVTGVILLVR
PN
>KRH95483.1
MISLSSAATNEIGRLKSKHQPHSLFRVRVQPGGCSGWLYHISFDQLINPEDQVFDIDNLQ
LVMDQETIKYINGLTIDYSEDLMGGGFRFRNPLATSTCSCGNSFSMKS
>KRH95495.1
MAKIEIHGVPHAYELTVPTSHPETLVFIHGWLNSRSYWQPLISRISLDFQCLSYDLRGFG
ESQCQSCDQPDNSHEHSPYSTGAYSQDLITLLELLSISRVWLIGHSWGGTIALRTALQLP
NLVQGVICINAGGGIYLKEAFERFRATGQKFLQIRPRWLYQLPLIDLFFTRTNVARPLDR
HWARQRIMDFIIADPQSAIGTLMSSTTEEEVNSLPQLVSQIKQPVYFLTGANDKVIEPKY
VRHLASFHWLFQYVGDNVIEIPNCGHLAMLEQPDAVSQHIISIITG
>KRH95501.1
MHHRNCLEVRNLRVEFSGDGAQVQAIDGISFDVLQGQTLGIVGESGSGKSVTALAIMGLL
PYPGRVTGGQILFRNQKNAQPLDLLALPSREIQLYRGGDMAMIFQEPMTSLNPVYNIGFQ
LTEAIERHQNVTLAEAKRIAIARLQEVKLLPKDENIKQQYLENWHQTHPKTPLPNDYQLA
QLVKEYKMAMLERYPHELSGGQLQRVMIAMGICCNPSLLIADEPTTALDVTVQATIMELM
RELQKSRNMAMIFISHDLGLVAEIADQVAVMYKGKIVECGLASQIFTHPQHPYTKGLVAC
RPTLNQRPQKLLTVSDYMIVQRYPNGELIIKSKEPNIPRQITPAEIETRIDDLTRKSPLL
QIHNLKVGFPVRGLLGGNKRYNIAVNGVSFDVYPGETLGLVGESGCGKTTLGRTLIRLIQ
PMAGKIIFEGQNITNFAGTQLQTLRKQMQIIFQNPFSSLDPRIKIGEAIMEPLLIHSLGK
TKQQRQDIVVQLLERVGLSANDMNRYPHQFSGGQRQRICIARSLALNPKFIICDESVSAL
DVSVQAQVLNLLKELQQEFQLTYIFISHDLSVVKFMSDRILVMNEGKIVEVGTSESIYHQ
PREEYTKKLIAAIPQGNINY
>KRH95505.1
MIEAEVHLSLHNFLRSQVGFPTWPHHLTMARLVARAFRLGRSALIQVGALCGYQGRYRTS
FIASALMWPGPVIIVALPEIQQRLLKIEIPRLEQWLGTTKSIRVGDVWPDPDFKGLLLTS
PQAWLKEQLSNEHVGFPPHIPTIIDGVDDLEDWVRNQLTQNLDPQSWDQLIWACPHQADL
IREVRVKLTHELFQHPENPYHCYLICQREIEILQTLFSALNPDHLPAIWRSFQACFYNAI
DNSVTPVGLTSSLFWATVARRQGLFYLHCAPVELATKLAPIWQRQPVVLIGSALEPETEA
PLFRQGLGLVDVTCLKFSSDNHGEAIQLYVPHKLPLPNTPEFQSSFIHQVRTLICLSTTA
LGLTVVLVGDVPLKSRVAAILAADFGSRVQVEKTCLDDNGILVSGWEFWRSHQSVLPAPQ

LLIIATLPLPSLENPLVAGRVAYYKRSHQDWFRLYLLPTALSELQRALAPVRESQGIVAL
LDSRVVNRSYGSQILACLSPRARLDYLDPSLFSSHTPENY
>KRH95508.1
MHSPIYSLVVPIYNEEENIEEMYRRLSNVMAQLQGEVELILIDDGSRDQSLTMIRKLHHH
DSRVRYLSLARNFGHQIAVTAGLNFVQGQAIIVMDADLQDPPELILSMIDKWHEGYQVVY
AQRISRHKETWFKRLTAYLFYRLLQRLAKVTIPVDTGDFCLMDKQVVDILNGMPERNRYI
RGLRAWVGFKQTSVMFERSPRYAGKVKYTFSKSLSLAIDGIISFSTVPLRLATYLGIISA
TVALIMIFLVLYWRIFAPVSQLIGYSLITIAIFFLGSVQLICIGILGEYIGRIYEEVKAR
PLYTLKETGGFDPLNI
>KRH95511.1
MSILAFSVLVWVGSFSAGLVGSLTGLGGGVVIVPLLTSVFGVDIRYAIGASLVSVIATSL
GSASTYIRKGFTNIRLGMFLEVASTIGAVVGASIATIVSVKFLTIVLAFVLIYSAYLSQR
PKTECVEVVKSDPLAEYLQLNGTYPINDGVMSYQVNSLPAGFSIMLLAGILSGLLGIGSG
AFKVLAMDQAMGLPFKVSTTTSNFMIGVTAAASAGIYLSRGYIEPGLSMPVLLGVLPGAF
LGARILIGAKTQTLRIIFSIILVLMALKMVYNGLTGGV
>KRH95522.1
MAHTRNFISNVDDSRVVSQAGHREPLKHLLIGSKKAVISTIHYLHVLGYANATDWSDLTP
TGNPGEVMSILVRHIVIN
>KRH95524.1
MYDRMFDFLIVGAGFAGCTLANCIATQLDRKVLVIDTRHHIGGNAFDCYDNAGVLIHKYG
AHIFHTNSKKIIDYLSQFTQWRVYQHQVLARVDGDLYPIPINLNTINKMYGLNLNNEEVA
DFYEQIKQKYDRIENSEQAVISKVGNDLYEKFFKNYTYKQWNLWPHQLDASVCARIPVRT
NKDNRYFGDKYQLMPLHGYTKMFENMLAHPNIKIMLNTSFQDVEKWLKFDHLIYTGPIDQ
FFDYKFGQLPYRSLRFEFETHDVEYFQPVAVVNYPNDYDFTRIVESKHITGQKHPKTTIY
YEYPQSEGDPYYPIPRPENRDLFEQYKTAADKLASVTFVGRLAQYQYYNMDQVVAAALTV
FENRISQLSYYPLITGKISHGN
>KRH95525.1
METRINAQIQSQNLWDGATLIDIMRKQAIEYDFNKGRMVINSILLADKTEVNNRGFLLDK
IRDYGCAYQGWNLYAPYEGVGFRSSTQPTFMFNSTHPLILFIFT
>KRH95526.1
MKITKVAAIIVTWNKRKDVCAVIKDIESLDLQNISLDIFVVDNASQDGTQNYLELHYPHI
KVLQTGKNLGGSGGFSQGMNFVSNLDYQYIWLLDNDVRLAPYALVPLVETLNTYAEVGLV
GSQIRKLDNPDIVQEIGSYIHEPKAHLQTYLGNSPVQSPAEILNSKNYLTVDICAAASLL
FRREIIHQIGVFENYFLHFDDVEWCLRAKQFGWVIAAHPASIIWHCSPDFKQRPWISYYD
ERNLLYCWHKHRPDLLLRRLRILFPKLVYYSLTGRHFWALIHLQAMTDFLNLIQGEMPNL
LSSFTLGEILPHNGQVLIQDSIYQHICDFLDVKQEQKFTLWYPPNKYNKLWAWVYLSFIS
CFSKPVDLAIVSYWNPNFYYLFLSKQLYFFTGNGYVKSQICFTQIILDSLGVVYGFLKIY
LRMTHLVKKSDKSFNKLVISNLLHPFWVNKVKKLLLIISTIYS
>KRH95528.1
MNKWFNTAGPCKSDIHYMLPPTERLPKVRTLIDRQNYFVIHAPRQVGKTTAMLALAQELT
EGGKYTAVMLSAEVGAAFPHDPVAAQGAILTEWQQSICFRLPSELQPTRWPAVDNSSQLS
IFLSNWAVTSPRPLVFFLDEIDALSDETLISILRQLRSGYPNRPQGFPHSLGLIGMRDIR
DYKVKSGGGDRLNTASPFNIKVESLTMGNFTFTDVKTLYEQHTTATGQVFTPEALEYSFY
LTDGQPWLVNALARQATEDVVKDINQPITVEVIEEAKEILIQRQDTHLDSLAERLREPRV
RTIIEPILAGEELPDVPSDDIRYVLDLGLCQDKNDQGLEIANPIYREVIPRVLTYTTRSS
IGVIEPNWLSDQGELLPDKLLQVFLEFWRQHGEPLLKSTPYPEIAPHLVLMAFLHRVVNG
GGRLEREYAIGSGRMDICLRYGKVVMGIELKVWHPKRKDPLSQGLKQLDKYLAGLNLETG
WLVIFDRRPNLPPIGDRTTTQEVVSPAGRCITVIRG
>KRH95530.1
MILNIPLAWLQLAKQKVRFLVALAGISFVAVLMFMQIGFQDALYASATQLHKNIEGDLFL
ISAQYKSLTSNQSFPRSRLYQTLGFKDVESVDPLYVQFAKLKNPLTGRKYPIYVLGFDPV
KSIFKLPEIQQDFKLLQIPDQVFFDRAARPEFGPIAQHYSQNKPVSMEIFSYLGLIGYKV

RVSGLFSLGPSFGVDGNLIVSDSTFMRVFRERNANQIDIGLIHLRPGADSQRVLGELSAN
LPEDVMVMNRHDFIEFEKNYWTLRTPIGFVFNLMVIMGFVVGVIVVYQILYSNISSHLIE
FATLKAMGFKNKYLLRVVFQQALILAGLGYIPGFAISLGLYDIAKNATQLPIVMDGDKAF
IVFISAVVMCLTSGFFSTNKLRKLDPADIF
>KRH95532.1
MNHAKLESDLAQLQDEVSNCERDLLNLIKGKKTMEYSQNHNGGNQSNQIAIIGMASLFPQ
SKNLQEYWQVIVDKIDCITDVPASRWSVEDYYDPNPKAPDKTYCKRGGFIPDIDFNPMEF
GLPPNILEVTDISQLLGLVVAKAAMEDAGYGESQQFDRDRTGVILGVAIGRQLAVPLGSR
LQYPVWKKVFKNCGLSDDETEKVIEKLKSAYIQWEENAFPGMLANVISGRIANRLDLGGT
NCVVDAACASSLGALNMAISELLAHRADMMITGGVDTDNSIFAYMCFSKTPAVSPSEKVR
PFDVNSDGMMLGEGVGMLVLKRLEDAVKDGDRIYAVVKGIGSSSDGKYKSIYAPHSQGQV
KAIRRAYENAGFAPQTVGLIEAHGTGTMVGDPTEFTSINQVFGDNNSLKQHIALGTVKSQ
IGHTKAAAGAASLIKTALALHHKVLPPTINITQPHPKLNIENSPFYLNTETRPWISNQPR
RAGVSAFGFGGTNYHVVLEEYESEHHQSYRLHNCAKSIFLSAPTTPELLSQCQHLYQQLE
STDKEQHYQRIIAESEQLIIPADHARVGFTILSLSQAIAHLAIIIDLLKNQPSVEFWEHP
KGIYYRQQGMETTGKVVALFSGQGSQYLEMGRELVINFPCLRQTYSHLDDLFSREGLEPL
SQVVFPTPVFTPQERQEQLEKLQRTEYAQPAIGVLSAGLYKILQQAGLKVDFVAGHSFGE
LTALWSAGVLTEEDYFFLVKARGKAMSTPPAVDAGGMLAVKGNISQVTELIKDFPQVAIA
NYNSQQQIVLAGNKSEITQVQNVLQSKGFSCFLLGVSAAFHTPLVSHAQKPFAHAIAQVN
FQPARIPVYSNVTGKLYPNEPASMQKILQEHLLNQVLFQQQIENIYQAGGNCFIEVGPKN
VLTNLVKEILINKPHIAVALNANYRQDSDLLLREAVTKLRVFGVPLKNLDPYQIPAKISS
SSQKNEQKTLNIRLNATNINDRSQKAFAQALATGPVIKKSTVSEDNYQIQPQKILVEKNP
QINPEIISSSILTSKNSSQVKNHSIVEPKMEIPVENYDRLLDSLEQSLAEFTRQQSEINQ
VHQQSLQNQMEYNKTFYELMQQQCLFLAKEETNEYQAQTQQLAISSTERSMMRLHDHQAE
TIRIHEKYLNYQQEYTNNYFQLLEQHYSLFEVGSPNGYPHLPSSHVAQSDPPAQKLIYPL
EPENNSQNNLPDITVGFPIATYLDKEKLRDTLINIVSDKTGYPVEMLDLSMDIEADLGID
SIKRVEILGGLLELYPDLPRPNPEELAQLATLEQIAEYINNLITQVYENQPVEETVGSGK
SPLSQEIPVKAINEQTATIETPSPVTELDNHPQFLVLSHEDSLDRSHQPTITIPDNLSQI
LLTIVSDKTGYPAEMLDLSMDMEADLGIDSIKRVEILGGLLELYPDLPRPNPEELAQLRT
LGEIAEYMRNQAETVERSNLSPLEKPDMSTTEVGDKILRLPVQLKPLPQPDSLDLTIPEN
HFVLITNDGSEVTHRLVAKLADKGCKTVVLTFPCLESNLSEEIAQIRLNDWHEETLQEHL
TELTTKFGPVGGFIHLHPNSNNNLGMDKAIVQHVFLIAKHLKEDLNQLAKKERACFFAVV
RLDGELGTAKTHNFSPISGGLFGLTKSLNQEWPEVFCRTLDLSPDLDADTTVKHILAELQ
DPNLLVTEVGYNQIDRFTLVAEPGKSSIIPDSLNITKNQVFLVSGGAKGITAKCVIKLAE
EYQCKFILLGRSSAEIEPVWSEGYEDENELKRRIMEDFLAKGEKPSPIMVQKKYQTISSQ
REIHNTLKAITEAGGKAEYVCVDITDGMMLREKLTPIIDQFGTITGIIHGAGNLADKRIE
KKTVQDFETVYAAKVQGLENLLHIVETNQLEYLILFSSVVGFYGNVGQTDYAIANEILNK
SAHVIKHKHPNCHVVSINWGPWDSGMVSPELQTAFAQRGIKTIPQELGSSILVDQLRNSD
STMTQVVIGSPLVYIPSTLSSELKTHQITRQLKLNYNPFLQDHVIAGNPVLPATCGLSWI
SSSCEQLYPGFQTFHCPNFKVLKGIVFDQNSPHEYILEIQEVAKIDNQEIQLAGKISSVT
NHGKIRYHFSSNLILKRQITPADNYEFFNLTQDGQFLASNSLLYQTGGGSLFHGNTFQGV
KSVLNISPGKLTMKCELPEPTLYQQGQFTVQTLNPYIADVQIHSLWIWTQHFHQVGCLPS
EIENFEQFVPVPFGETFYVTCEIKSKTESYVVADVITHNQKGQVYNRMKGAKGTILPNSY
>KRH95535.1
MSILNIFQKNSKYFFNPVNKRQLSSLETTMELLNQRAKTWNLVTISRIRGHIQKPVLREA
LDLLQYGHLVLNSHIVTHQHNFQNRFYLQAGTTEEIPLRMVINSEEKQWQEVVNQEMNQP
IDSGRYLMRVVLIINRENPKVNYLVTTLHHAIADGLSSVNLHSEIFTYYEQITSGNLLNP
VSTLPPLPPPEKLLGHLQGAKLNGWILLLKIAWEKLTNPPQTLKVEKYVPISQRNSQIIH
RQILSDTAEKFFAQCRAENATVQSVLSAAMLLTVAKKILNQQRKSIRLNCLSYFDLRRRL
QPPINEQNIGLLATSQMSFHTVTTNTYFWDLARRIKRTLAASIHRGDIFKMVFLAKHLIN
FCFLFPHQIAASVSVSNIGKVNIPGIYGELQLEEISFAGSHALYAGMFILHVATFQGKML
LNFVFSQPSLSQDTMENLVNEFMKVIEEISLLPSQIS

>KRH95542.1
MSANEIFDKTFQRVKKIVVDQLSAEAKTVIPEASFANDLSADSLDTVELVMALEEEFGVE
IPDEAAEKITTVQEAVDYIINNAPESAL
>KRH95545.1
MRVNSKKLFSFGLITLLVVGGVSQSAFASERTLLTGTPVNQTREKRNSFQRLNLTSEQQA
KIRDIRRDTHSKIEGVLTPEQKIKFQAAVKQRETEYPNPSESKPRYYGRRGHMGDVLRSL
GLTDGQKNQIREIRASSRQQIQSVLTPEQRAQWQQFQQRNRRQYR
>KRH95547.1
MSSNLASKLRVGTKKAHTMAENVGFVKCFLKGVVEKNSYRKLVANFYFIYSAMEEEMEKH
KTHPVVGKIYFPELNRKRSLEQDLAYYYGYNWREQIQLSSAGEAYVKRIREISATAPELL
VAHSYTRYLGDLSGGQILKNIAVTAMNLTAGEGTSFYEFADISDEKSFKTQYRQNLDAMP
IDNQTGDRIVEEANAAFGVNMKMFQELEGNLIKAIGIMVYNALTRKRSKGSTELVIAE
>KRH95548.1
MIVELLEKLFVRTTDTSRNQVKRRLQVVIAHDRSSIDPQTLDKMRQEILEVVCRYVEIDT
NGSEFSLESDQRTTALIANLPIRRVKGVTSELADSSAGS
>KRH95554.1
MLTSSETPIIAAVILVALGILAWGFYRSRPFGKLGILAWLQSVVLMAPWLVFFGLFAVGI
YINIAGVLLLLVISTSIYIFLGWQLRKAGQDVILKTKATERIAKETSPTVSQTENGGPPV
VKLEPITIPEEDLNTIKGIFSIDTFFATETIPYQEGVVFKGNLRGDAQEVHKRLTKNLAG
QLGDKYRLFLVENTDGKPVVIILPSRSDPRPMQLGQKVFAVILLLATIATSLETGGLLLN
FDLFTTPSRITEALPIALGILAILVAHELGHWLFAKKHQVQLTWPFFLPAVQIGSFGAIT
RFQSLLPDRKALFDIALAGPGFGGLVSLVMLVTGLLLSHPGSLFQIPNKFFQGSILVGSL
ARVILGSTLQAPVVNIHPLVIIGWLGLVITALNLMPAGQLDGGRIVQAIYGRKTARTTTI
ATLAVLALVSLGNTTAFYWAIVIFFLQRDGERPSLNEITEPDDARAALGLLALFLMISTL
LPLTPALAGKLGIG
>KRH95563.1
MSHFSQIKTQIRNLESLQDALSDLGIDWKSGSREVRGYRGQTHTAEVTIEQDNGYDIGFK
WNGQEYELVADFQYWQQNLSVDGFLRQITQRYAYQTVMKETAKVGFQISQEQKNADGSIS
LVLQRWSA
>KRH95569.1
MHQTHSAALEINLLHANQEGADHNRSHFNSWGITCVNIMSSPGAGKTTLLERTLSVLTSE
LKIAVIEGDMNTELDANRLRQYGVPVIAINTGRSCHLDSQMVAGGIHQLENKHNPTELDL
VLVENVGNLVCPAEFEVGEHFKVALLSITEGEDKPLKYPIMFQAADCLLITKIDLAPYLD
TDINQIVTNVQQMNPGVKIIPFSAKTDEGLDTWCDWVKTQVKSNYHLDIKGTK
>KRH95571.1
MTKNNSPAVSWGIGDNSTWLFMILSITIPVFIWLVVSNSGLVKPLFLPTPQAVLSALQKL
WATGDLQTDIGFSLLRVLGGFLLAAVISIPLGVLMGSFPVVRALLEPAISIFRYMPAPAF
IPLLILYFGLGETPKILLIFIGTVFFNTLMIMDAVKFVSRELIETSYTLGGQHHQILLQV
ILPFIVPNIIDACRVNMAASWNLVIVAELVAATEGLGRRISVAQRFLKTDEIFAGLIVIG
LIGVVIDILLVMLHRMVCKWAHN
>KRH95572.1
MHLQVSQLHKQFKTRRGTLNALENINLHIDQGEFVCAVGASGSGKTTLLRLISGLDTPTA
GEILVDGVPVQGPGKERGLVFQSYTLYPWMNVADNVGFGLKLQGVPPIKRKQSISYYLEV
VGLSEFAQALPRQLSGGMKQRVAIARALASQPKILLMDEPFGALDVQTKESMQKFLRQIW
QQTGTTILMITHDVEEAIFLSQRIYVLTSRPGKIRQEINITLPQNEYDQVKQSWEFQNYK
RLIFNLLSGY
>KRH95574.1
MKRENKNRKQEIGDQKKGETNQSFPFPNLDDWIEIGKIVAPQGLTGEMRVYPQTDFPERF
EEPGTRWLLLPGEPSPQPVQLLHGRYVEGKNLYVIKLHGIGDRTAAENLREGRLFVPIGD
RPELAPGEFHVIDLIGLPVFMQESGERIGDVTDILPSGHDLLEVKCDPSWNKNLGGKTVL
IPFVMEIVPVVDLGNRRIEITPPPGLLSINE
>KRH95577.1

MIQFRIQPDSEIPASSQLFNQIRFAIACGQYGSGYKLPSTRALAIQTGLHRNTISKVYRQ
LEEEGFVESLAGSGIYVRVQGHETGSRLQLPILQQYPQAFKSIQQTLDELLNQGCSLNQA
RELFLAEVDWRLRCSARVLVAVPLQDIGVGQLMVDELEESLEIPVELVAMEELAAVLEET
TSATLVTSRYFIGDVEAIAAPKAVRVIPLDIHDYNKELNVVKNLPRSSCVGIVSLSPGIL
RATEVILHGLRGDDLLVMTAQPKDAYKLNAIAKRCEVIFCTDATSHSGVQTVVQGIAEDI
IRPPKLIRCANYIALQSVNLLQRELGLI
>KRH95578.1
MQLKQVIIAYKARDSQSKRWAELCAKQLEKRHCQVLVGPSGPKDNPYPVFLASATQPIDL
ALVLGGDGTVLTGARHLAPAGIPILGVNVGGHLGFLTESMDEFQEPERVWDRLLEDRYAI
QRRMMLQAAVYEGPRVNLEPITENFLALNEFCIKPASADRMITSILEMEIDGEVVDQYVG
DGLIVSTPTGSTGYTVSANGPIMHDGMEAITVTPICPMSLSSRPLVLPPGSVVSVWPLGD
YDLSTKLWMDGVLSTSIWPGHRVDVRMTDCRAKFIILRANNSYYQTLREKLLWAGTRVHY
ANNHHN
>KRH95584.1
MYLYLIRHGIAQEHQLDIKDEERSLTIEGKKKMEKIAQRLIQLKFKFDLILTSPLVRARQ
TAEILMQAGLGSQLETSLHLSWEGNVHNWIQEWLEPRRHDQKYDQQTHLALVGHEPCLSS
WAEILVWGEAKEKLILKKAGMIGIEVPDNGSIVGHGQMFWLTPPRYLL
>KRH95587.1
MPEITRFYGIIIKLFFAGHPPAHFHAICGEYNAIFNLETLEFIEGEYEIWTNGQHCSQNL
EP
>KRH95588.1
MINCKTDKKLLHFLYHELELSHADVAVALRQRKFDEAPIPMLLWQYGLIDLQQLEKIFDW
LAENV
>KRH95590.1
MKVMLRVNDAGTLVVYVAKKDLEEEVVKQTDSEAGKVLTLANGWQLEFSQIPPRENLPQT
VKAKLLH
>KRH95596.1
MPKHFNTAGPCQSDIHYMLSPTARLPELKALIDGRNYFIIHAPRQVGKTTAMIALAQELT
ASGHYTSVMLSLEVGAPFSQDPHKAEQSILAEWRQSLRFRLPPDLQPTEWPATESSSQLS
TFLSNWAATADRPLAVFLDEIDALSDETLIFVLRQLRSGFPNRPHGFPHSLGLIGMRDVR
DYKVKSGGSERLNSASPFNIKAESLTLSNFSFDEVQELYLQHTEATGQIFTPEAIHHAFY
LTDGQPWLVNALARQATTVLVKDVNQPITIDVIKQAKEILIQRQDTHLDSLAERLREDRV
KAIIQPILAGSDLPDTPEDDRRFVLDLGLLKRSSLGGLTIANPIYQEVIPRVLSQGSQDS
LPQIQPSWLNTDHSLNPEKLLNAFLDFWRQHGEPLLKSVPYHEIAPHLVLMAFLHRIVNG
GGTLEREYAIGSGRMDICLRYGVVVMGMELKVWKPGKKDPLPQGLQQLDKYLAGLGLDTG
WLVIFDRRPDLPPIEERTTTEEVVSPGGRAIIVIRG
>KRH95598.1
MLQLAPHIFHILHQHGIEHAFGIPGDFALTLYDALKESKIKPVIMTHEPSVGFAADVYAR
IRGLGLAVVTYGVGGLNMVNAVAGAYAEKSPLVILSGSPGIKERRQDSLLHHKVKTFDSQ
RRVYEEVTVYASAITDVETAERKIHRAIDYAKTFKRPVYLEIPRDMVYAELPEAEYERVP
IKHTDRDTLKEAVSETLEILEKAKSPVIIAGVEVHRFGLQVELLALAEKLGVPICATMLG
KSVFPETHPQYLGIYNGEAGDENINKIVEESDCLLMLGVFMTDINLGMFTAHINQKHTIS
ATSERIAIKHHEYQNILFTDFIAGLLKNPHLPHFQFPNTYRMHPRVEEKIDNISMGGLIY
EINQFIDHKTIIITDVGDSLFAADDIQTKQGTSYLAPAFYASMGFAIPGIIGAQLADPFR
RVLALVGDGAFQMTGMELLTAKRLGMNPIVIIINNGSFASLRAMGHEDADFVNISTIDYA
DLAKVLGGNGFVIHTGLELRRALSVAKDSENFSILDVRISADDISPALQRLKTLFTQTLK
>KRH95600.1
MQILGLEIKIQNSQSNDIMNEFQPPIPESEAQQALELERQLPLTGWQQEVSRSLELGLEA
AASINDRTISTFARGELPHYAGINTFLKAPYLEDIWKVGEYDVAIVGVPHDSGTTYRPGT
RFGPQGIRRISALYTPYNFELGIDLREQITLCDVGDIFTIPANNEKSFDQISKGIAHIFS
SGAFPIIMGGDHSIGYPTVRGICRHLGDKKMGIIHFDRHVDTQETDLDERMHTCPWFHAT
NMKNAPAQNLVQLGIGGWQVPRQGVKICRQRSTNILTITDIVEKGLDYAVEFALERALDG

TDCVYISFDIDCIDAGFVPGTGWPEPGGFMPREALYILGKIVQRAPICGLEVVEVSPPYD
ISDITSLMATRVICDTMGHLIKSGQLPRKDKPAYIEMEARQESIVDWS
>KRH95601.1
MKPALTKIGAQMSNLTGVRAIMKDINETLRANQGQVLYNLSAGNPLILPEVEQLWRDCTA
DLLSSGEYGEVVCRYGSSQGYAPFIAAIVKDFNQRYGLQLTERNILVTAGSQTIYFYAAN
AYGGYTEECKLKKIVLPLSPDYTGYGGVSICPESLIAYKPALDVDGVNHRFKYRPDFTQL
SITQETGCVIFSRPCNPTGNVLTNEEVEKIAALATPYDVPVFIDSAYAPPFPALNFTEMK
PVFGENIIHCISLSKAGLPGERIGVAIGEEKLLQVLECFQTNAGIHSSRYGQAIATRAIE
SGALANIAETVIRPFYQHKFDVLESTLDAVMPKDLPWFLHRGEGAIFAWLWLQELPISDW
EFYQQLKKVGVIVVPGSSFFPGLEENWEHKHQCLRISLTGTDEEISIGMQRLAKIAQEVY
HQ
>KRH95609.1
MLKQVKDLKLLSLTQLIVMLLLRLLMSLEGYISIFALRVIINNIIPSQHVEFEYIQTSKS
NSEFDSELSLTSTTNFTLQVLHGSDFEGGIPAVTDVVGFSAVVNKLKDDPKYKTNTLILS
SGDNYIPGAFFNASSDTKLNNVGGLGSSTTPVIGRGDIGILNGIGIQASALGNHEFDLGV
RQVRDILRTGSGNPGTNFPYLSTNLDFSPEITAGNLSATDLAPNQNTAHASSIKGKIAKS
TIINVAGIDGITGTADDQRIGIVGATTPTLANISSSGSTIVKPANPIDYDALAAEIQTSV
DILKAQGINKIILLAHMQQLTIERDELAKRLRDKVDTALTLAPGKFTFALPGTEQDVLAE
YLGDRFSTNAYGVADVSPALDRRIQNLTFQGEREKYKCFDKRSLWSRVIVKRLR
>KRH95659.1
MHITEFDEKFDRVLNELQRDREEQARKWDEQNRKLDAFQAEQNRNRVVDRKIVISPMVDE
RAIPVAKSLGIETYSYADMVVS
>KRH95660.1
LFGLAWDLLYDFLQKYLWDHDWPGVFQFYAGIVEGVFLGLILGTIGLPKIPRAEFQLVTF
IQHYGLVWLGIYLSAWIVMRLLFPRWRFRGGEWLGNWPKGS
>KRH95661.1
MPMNFSPNYTITPKIAKFLMRIETVRERVCYLPITAMVLASLRETARLYTTHYSTMIEGN
RLAPDEIENVVKYEGHFPGRHRDEREVKGYYAALAKLEQWVAAGVQVSEKIIQTLHAVVM
SDGRTTAQPSAYRDGQNVIWDASNGTIVYMPPEAKDVPFLMKAMVKWIHQSQEVPCPVVA
GIAHYQFATIHPYYDGNGRTARLLTTLILHLGGYDLKGLYSLEEYYARNLTAYYEAISVG
ESHNYYLGRVEADITKWVEYFVEGMAIAFENVLKRMAEAELQGSVDQDPILRQLDPRQRK
ALSLFQQFEVVTSKQIGELFGFKPRTSAQICKDWVESGFLEMVDPSNRGRKYKLSTQYKD
LLN
>KRH95666.1
MNTKDPYQRITTYWIGLKSWIINTPHRAVLAAYRAAWEIRNIEIQQFNGQKISPQSANYS
GNLMEFWQGDLNRNLTTIKIRLAEFNLGMTFTDRSTDTNNQYVYDNDLLEKLKFIDEVIG
NYTQKRNDSKYMEITQPLKTSDKEVQFNNKSTDVDSNDNQKKGVFPGSIARTLSKIAKDF
TPTAESDFINNYRISRNKTRIGIRFLLLIIIIPLIVQNFSKNLLFTPLFTQWMSQDNQRV
FLNREMEEKAMNELKNFERKLQFQNLLHTAPPLSVEEIELEVKDKALDLAEEFRRDSSLA
IGNIFADVISLISFAIIIGWRKKDIEVVQSLLDKIAYGLSDSAKAFLIILITDIFVGFHS
PHGWEIILESLAEHLGLPANRNVIFLFIATFPVILNTIFKYWIFRYLSRLSPSALATLKE
MDE
>KRH95671.1
MIREASISLELPLILLVFQVPAVLILISRLLKGPTRHPPIEPQQPTPDMLGSVSVIVPTL
NEALRITPLLSGLTRQSYEVREVIVVDSRSKDGTPDLVKAVQQKDPRFRVMTDDPLPTGW
VGRPWALHNGFLFSCEESKWFLGMDADIQPHPGLVASLVRTAEAEGYDLVSLSPQFILKY
PGECWLQPSLLLTLLYRFDPAGTRTSQPERVMANGQCFLCRRSVLATMNGYTSARSSFCD
DVTLARNIAAAGFKVGFLDGARVFQVRMYEGAMETWKEWGRSLDLKDASPPGQVWGDLWL
LFCVQGLPLPVILAFLLVSPSPYLPGSLLLALNVFLLSIRFALLLAISPSYDRTNAYGGW
LFWLSPFSDPLAVIRIFLSALRTPKEWRGRKY
>KRH95679.1
MPRKRLTDLLQEEVQKFTPDESVVETTAKKIPDLELPEDGSNSVTIVSSNSTSDNSNGLH

WGVTVQELQESLNRYHVRETAMQQEICELKNALSEQRSLSEKLGKELYETKKTALKLAES
NSTLIEENNQLKQQPAQQVIPNSTGESVQSTQPTKDVAKSLVINPRKSYSPPGIFIDKSK
ADTKKDDFSNNTWLYD
>KRH95693.1
MALIVQKYGGTSVGSVERIQAVARRIHGTAQVGNSVVVVVSAMGKTTDGLVKLAHEISPS
PTRREMDMLLSTGEQVTIALLSMALQEIGQPAISLTGAQVGIVTEAEHTRARILHIETER
LISHLNAGQVVVVAGFQGISNTSAMEITTLGRGGSDTSAVALAAALKADFCEIYTDVPGI
LTTDPRLVPEAQLMKEITCDEMLELASLGAKVLHPRAVEIAKNYGVPLVVRSSWTDQPGT
WVTSNKVQERAMVNLELARPVDAVEFDIDQAKVSLLRVPDRPGVAARLFNEIADQQVDVD
LIIQSIHEGNSNDIAFTVNTPILNRAEAVASAIAPALRNNDGSNEAEVLVERNTAKVSIS
GAGMIGRPGVAAKMFATLAKAGVNIQMISTSEVKVSCLVAAVDCDRAILSLCQEFEVNAS
TRNVSSSNSIYSTVCGVALDMNQSRLAIRHVPDQPGIAGKLFGLLAESNISVDMIIQSER
CRVIDGVACRDIAFTTNRTDGENAQAKINQVAAQLGWGEVILDDAIAKVSVVGSGMVGQP
GVAAKMFTALAQNQINIQMITTSEIKISCVVSEKEGVKALQIIHTAFGLAGTHKFVVPA
>KRH95697.1
MFNNSPAILTSQVSKVYRTGFWSHHKIISLHNCSLEVYPGETFGLLGPNGAGKTTLLKIL
LGISRPSSGKGFLLGKPLGDRSVKQFIGYLPENPYFYDYLTGYELLELTGEIFRISSRVQ
RQRIVHLLELVGLSQNQARKQRIGSYSKGMVQRIGLAQALINDPKVLFLDEPMSGLDPLA
QNQMREIILSLKSAGKTIFFNSHMLGYVEQICDRIGILNQGEIICSGSLSELLGEEKGYY
IRGKGGDKEILQKRLGEIQFEREAIWHGILKEDLYDFLASLRLMGGKVIKISLCRQSLED
FFIQQLSPLPTQSKL
>KRH95708.1
MRKIVIAGNWKMFKTQAESEEFLRGFLPELEETPSIREVVLCPPFTNLNVMSKYLHGSRV
GLGAQNVHWSENGAYTGEIAAPMLLEIGVRYVIIGHSERRQYFGETDETVNLRLKAAQNY
GLTPILCVGETKQQRDAGETEKLISYQLKHDLVDVDQTKLVIAYEPIWAIGTGDTCESAE
ANRVIGLIRSQLTNDKVPIQYGGSVKPNNIDEIMAQSEIDGVLVGGASLEPESFARIVNY
Q
>KRH95713.1
MSNLSLSCLELFAGAGGLAKGLEMAKIKHKALIELDYNACLTLANNYNHQLIYNVDVRKF
KFEEVGEVDIIAGGPPCQPFSLGGKHKGHMDQRDMFPHACKAISICKPKAFIFENVKGML
RPAFSNYFEYLILSLTYPEVCLKDLQSWKEHLILLKTISNSNNYPGVKYNVVFGLLDAAN
YGIPQRRERVFIVGIRQDLNINWSFPQPSHSYDSLLWSQFVSYDYWERHQIKPISIEWVD
QRTKQKINQLIQKPTLFPPVLKPWKTIRDQIGELPKPDSQGSFDREHILRQGARSYPGHN
GSYIDMPSKTIKAGDHGVPGGENMIRYPDETVRYYTTLEAKRIQTFPDDYRFIGCWTESM
RQIGNAVPVELGYFLASSLSRALC
>KRH95721.1
MLLSNQTTCTNSISQGSLANFKVGVDNVIFSVDTARNRLLVLLVMRQQEPFLNFWSLPGT
LVRQGESLEDAAYRIMAEKIRVSNLYLDQLYTFGGPHRDPREKSNSYGVRYLSVSYFALV
RFEEAELITNKVAGTAWHPVKNIPELAFDHDKIINYGHKRLKNKLEYSPVAFDVLPETFT
LNELYQLYTTVLGDNFSDYSNFRARLLKLGFLLDTGSKVCRGAGRPASLYKFDAQAFAPF
KDKPLVFI
>KRH95729.1
MGYVIATANMKGGVGKTTVTVNLATCLAKHHGKKVLVLDLDSQISATLSLMSPGDFAKRR
KQRKTLRYLLDEVINPDPQPEYKIHDVIEPELCKLPSLSLLPGDIDLYDEFVVSEMLHNQ
AVALEERDFETIWNRFERVLVRDILQPVRDQYDFILLDCAPGYNLMTRSALATSDFYLLP
AKPEPLSVVGIQLLERRIAKLKDSHEHEAKINIQMLGIVFSMCNTNMLTGRYYKQVMHRV
VEDFGVETICQAQIPVDVNVAKAVDSFMPVTLLNPSSSGSKAFIHLTEELLRRL
>KRH95732.1
MPYTTEEGGRLNNFAQEPKVYEAEPPTGKQKLNYVILGGLGALLIVGVVFIAFAVSNPS
>KRH95735.1
MNHDVFLVPPGKKISLGDYDPSYKAEFHQKVDAVKKLRAGIKELARYQDVLYAQNTYALL
IIFQAMDAAGKDSTIKHVMSGVNPQGCQVFSFKAPSDEELDHDYLWRSTRCLPERGRIGI

FNRSYYEELLVVRVHPETLARQQLHHFPQGNQLWKQRFEEINNFEKYLVNNGIVILKFFL
HISPQEQKKRFLQRIESPAKHWKFSASDVRERAFWHDYMIAYEDVFNHTSTKHAPWYIIP
ADRKWFTRLVVSEIICDKLKELDLQYPIVSEEHQQQLLQAKKLLESEDITNFHPRTSDRI
S
>KRH95736.1
MDNSSILFPNQIFGLLTNHTEVNQDINQYTIWLLPICIVTGMTFVLEGYFIGLREGGTLR
NVVLLSFIVSFIPLVIAAWYFHSNHLLWSSLLAYMTSNMLLLSASIPQTLKDESSQNVLA
>KRH95738.1
MPKAIVMGLGKSGIAAARLLKQEGWEVEIWDSNTSPFLLEQQQKLAQEQITVKLGNNPEL
KENQLPKLIVVSPGVPWDIPLLVKAREMGIETMGEMELAWRQLQNIPWVGITGTNGKTTT
TALTAAIFQAAGLHAPACGNIGYAVAEVALEVALGAKPPDWVIGELSSYQIESSWSLFPH
IGIWTTFTPDHLARHKTLENYYDIKAKLLRNSRLQIINGDDAYLSKVGLQHWPYAYWTSV
KGLGHLIGGKGFYIENGWVTEKLSSDSEPQPIVAVTDLKMVGEHNQQNLLMSVGAARLAG
IEIAAINHGVQQFPGVPHRLQHVCTWEGIQFINDSKATNYDAAEVGLKSVKSPTILIAGG
EAKAGDDTGWLKRIQEKTAAVLLIGSAAETFSKRLETVGYGNFEIVETMEKAIPRAAQLA
KQYQATTILLSPACASFDQYANFEVRGDHFQQLSLEWASSAITNNS
>KRH95741.1
MTSIDKILLKYKVLVETHANRFRPQLDALYHFVDESMKEIQNTEREILESQNVELKKIID
ALQVDPRILLSTDEFKQFVEILGIAECWWEWEELEDLPAIDKDPTNWLLAKLQLPLIIRD
YQEFEDPYAYDDTSTYTLYGYKISLKLGNRICTMEVERRRVYENRCKEFSPEKQIAYYIL
SPIRDLLRSMNYSEQEIDQLGGEMGILVFYVAKLFELKPTVSVFEYNSMKRIY
>KRH95745.1
MTWWQEELLDIVLTVSGEAERLFEGIDEIVDTLFDLTEEIDEQVGSAGTCLSELLEPLVG
FYWDLEDPNLPYEVEADSLKNSACVGCSNYHGEVYGGNLLVCAMYPYGWDGEKCPDWEE
>KRH95746.1
MSHSVSNILPSEDPVKEVKYGEREIEEGKLITFPNPRVGREYTIDITLPEFTCKCPFSGY
PDFATIHIAYIPDQRVVELKALKLYINSYRDKYISHEEVTNQILDDLVIACAPLEMTVKA
DFSPRGNVHMVVEVKHKKGAAS
>KRH95753.1
MGNQFKTLALLAALSGLLIAISYWIIGGSGGLVLGIGLAAITNLFSWYQSDKIALAVYNA
QPVSEAQLPVLHRMVAKLSSRANMPMPKLYIVPTYTANAFATGRDPEHAAVAVTEGILNI
LPEEELEGVIAHELTHIVNRDTLTQAVAATVAGAISFLAQMLSYSLWFGGGSRDNNRGGN
PLGVLLTVMLAPLAATIIQLAISRTREFSADAGAAKITGNPRALARALQRLEAAARETPL
DANPAFEPLLIINPISGQFLANLFSSHPSTQSRVQALLQLEN
>KRH95757.1
MFGFIKNLIAGILSFISGIFGKKDEYYLELKEENEPTTVVQVAPVVKVESVVTPKVESIP
APVEKPVVAKETTFAPQYLLTLTSRARRRPGANMSGFMDMAREAKVPS
>KRH95758.1
MKTIFHLAFPIGDISQTKGFYIDGLGCIAGRENPHALILNLYGHQLVAHITKEPLTPQKA
IYPRHFGLVFIQEEDWQELLERAKTKELNFREEPKNRFVGSPLEHRTFFLEDPFYNIMEF
KHYRYPEAIFGSSQYTGIGDT
>KRH95762.1
MPKCIVIEPHLTVQELENRYQQSQNKIESTHYQTIWLLASGKTTAEVSAITGYGVPWIYE
LVRSYNLYGTEILGDLRRNNKGTARLLNNQQLQLLQQTLQLPPEDGGLWNGPKVANWMSK
LLNRKVYPQRGWEYLKKLRNIPSFRSLTQL
>KRH95779.1
MKFTSVLYGLFLVIVLITYWNIKQSKFRLWTIIISSIVFYASWDVQYIPLMVVLTFINFR
LGLEIAENSASNNYSKKNNNNQDSDQDIKNIEADWNPNRLKILALGICLNVGLLLVFKYS
TILSQWILRWDITLENASFKLGIPLGISFFTFENIAYLIDVYRGVPPADNLLKFASYKLF
FPKLISGPITRYHQISWQFDQTKINHIHIRTEGLWLIARGAVKKGVLADNLGIFVELCFN
NIQRAGSTDLWLATFAYGFQLYLDFNGYVDIARGSALLLGLVLPENFDFPYLSSSIADFW
RRWHITLGDWLRNYLYFPLGGSRRGLLRTCVNLLIVMLIAGIWHGSAWGFLVWGGSHGIA

LCIHRVTDVVSNNFKILRLFWHHPLGIILAWLLTQLTVFISWIWFRLPRIEDSALVMGNL
WGHTADVQFIKKVYMEALNTSPNQVLWGLILIFVVMIVAYVFKRKLHLELRWQLKLVFVP
LCFYAVWLLAPQGSLPYIYFDF
>KRH95784.1
MTTETTAQNPTTGADAVDIAIAQGIDFDGSPIPTVKLELYNYVMGLEAGRQRSGVSNTMR
SRIVRIGAKHIPQAELDEKLVAAGFAPLKEKEIAFFYGSK
>KRH95790.1
MNSSKQVAQWRVAAASVCGTSHIKNDQLCQDAHYWHILPNNVLLIAVADGAGCANLGKVG
AVIATQTAIEYISQRKDIATVITDDILLRELLHEAMINAKTALENEAQVGKYELSDLATT
LIIVVATPKLAAVAQIGDGLAVTRDSTGKLQALTIPYRGEYVNETIFLTSSEAVTTTQLQ
ILRHNIVNIGVLTDGLQMLALNMLVQEPHQPFFLPLFDFVAKVKDHRLAKEQLTSFLSSR
KIIERTDDDLTLVLAAFSN
>KRH95795.1
MSKLLTIAIPTYNRADLLDQQLEWLSHAIKGYEQDCEILVSDNCSSDYTPQVIKKWQKIL
SSVTFTSNRNSSNLGVMRNIIYCLNSATTKYVWTIGDDDPIQDRTVGYVIDKLQKHQDLS
LMFLNFSGRNKITGEAVHPPTISGNRWFDIDVEDGAGNGKAIFEHCLAKSVGAVIFLTAS
IYRADLVKQALQIWPDAINNWISLAYFAGYCAAHGKVIVTKENFLECIVGVSYWQKEPKS
ALLMQYKHIPEVISKLHENGYSKQFYARMMIQNWRGVNPKVFLGALRRWPISAIQTILPF
FAVVTVSAVEVMAAPELKIADCNQQISNSSLRRNKDS
>KRH95797.1
MIFTQTSLAGAFIIELEDKPDHRGFFARTFCAQEFAEHGLKPTVAQCNLSFNHQKGTLRG
MHYQITPATETKLIRCTQGAIYDVIVDMRPESPTYLSYIGVELTATNNRRALYVPEMFAHG
YQALTDGAEVVYQVGEFYTPGYERGLRYDDPILDIVWPLNVTEISQKDLSWPLLESVLIG
V
>KRH95801.1
MTTSIFPSLQQSGVVSDLSENFAFPSYSLKWRRDQLLVTFSRNSSQIHLPSLNNEQQLIN
CLKHSTVNLVTIDSKLGTSTLKFWANACEKANKPIFIRPVNKNQPLTIGDDILVVLERTI
NIFLALFFLSLFSPLIGFIVLLMLLKSPGSIFKYEWCIGKKGKLFRLVNFDHNLQQNLPI
SSLGMTKLRLHRLPELFNILRGETSLFNSKHSKL
>KRH95802.1
MKTAICTVFEKDYHYGVGALINSLHHHGFKGICWAGYRGKLPSWAKYLKTGAGYEEFTVS
PDCAIRFIEVKTNKHLGFYKPDFMSLLWQKYCPDMEALFYFDPDIVNKCKWNFYENWVSR
GIAICGDSWYLVPANHPRRLAWKEFAESNGFICERQLDYHYNSGFIGLPKQYQSVLTTWQ
NLQEIGEKLGYSDLTDLYGSRTFNFPPYLYGDQTYLNLALMLNNYPLSTVGPDGMDFIPG
GTIMSHATVPNVKPWRKKLMLSALLGSAPSITDKLYWKHSHTPIKLYSNAKLIQQNLEIL
LGSAIGRFIRRAPM
>KRH95804.1
MTIIAQLKHKFNGKVYFLCDPRELDDVGDQFQHLLVCLAEGFQELGISFFANVNYWLESS
QEEKYLFNYNPNITFDDCDLVILTNIWLSVNYAWVDNLFKPNRGYLTVYLDGEDSDKTYR
FRPEFNQFDFIFRTHYNRKLQYGNSKSRNNFYPWAFGLSSRILRELNTVPDFDDRQKQIL
VNFRHWKKGHPVRNISSRMFIPQISKIFSIDHTIDSPHNLTSDPYHQLQWLQTGKRHYPS
FYQRLKNSIACACFGGFFVPSWPDDPASLLNRIGKQMLNHLQLKSHQIVQWDSWRLWESL
AAGCVTFHVDFEKYGVCLPVMPENWAHYIGVDLDNVKATIERIADQPEILPAIATEGRKW
AMKNYSPAPTALRFLETIYQKVS
>KRH95805.1
MDGGGYSGLGSEYGYANDGNGFDPYFTQYYEADIIGQQYTFTYFYGNGDNYSGYGFAPLG
TYTVGQLPDYYDNETGTQKGYYVINSVEDGATGTKDYVQVTSYTDADTGFGETTSIYSGS
GYYGLGSEYGYAFNANPWTGDTYFSRYNEADLPAALKVQVTAIFM
>KRH95809.1
MKKFLRLALILTLFLVSSFSFSTSPSYAYSQSDLDKLLETGDCRNCDLSGADLRKANLSG
ADLSSADLSYAILTSANLSTANLKKANLSGADLSGADLVDAYLPDADVRDADLSYAFVLH
ADLSYANFIGANFEGANVRGANFWGARLDRGWESLRYEK

>KRH95813.1
MSNENDPMQLKRELSWLSAAIMGLASVVGAGIFVSIGVVAEISGSTAIAALVVAGILGAC
NSVNLAQLAVSHPVSGGIYEYGYKYLTPWLGFTGGWIYLLSKTAVAATAALGFSGYLLNN
IGLADAGILIPVAEIAVVIITLIVFGGMRSSQISTIVVVLITMASLLGLITAGLFFCFSH
GWGKLTFSGINSYQGATSFLQSVALMFVSYNGAARISMVSEEIVDPKKSIPRAIIFTVVT
TMVLYIGVSLVSLGSIGAESFAAATRTNAAPLKAVADSFGIPLVSNLLALGAITSMLSIL
LTTVLGVSRLLLAMGRRGDMPKLFAKLNTAGTTPEFAVIFVGIAIAVLVLIGDVRVTWSF
GTFGALYRSFITSLSALQIGDQERLYPKWISWFSLCSSVVLAFCIEWYYWTIGLGLVIVG
LIWRFIFRQLYTSENIQLITKD
>KRH95817.1
MLDFSGSYFSSRIKYLIYYGFINTREKVKSMTAKDLFNNHEDITDPRHKKTHQFTISAKA
ENGELYTDLDHGQWKASMEAMYQHKNTKIVFEMGEKSMTATIQGSWEW
>KRH95822.1
MTSSGIEVEHLNFFWPNGEQAIKSCSLTVPKGEFWMLLGTNGSGKSTLLRLIAGLLVPQS
GRIDILSPLGFVFQNPDHQLVMPTVGADVAFGLVPEKLSTSMVKARVEEALQAVNLLSVQ
RRPIYALSGGQKQRVAIAGAIARHCEVLLLDEPTALLDPDSQLELVTTVRQLVKTRSIAA
LWVTHRLEELNYCDGAFLLEQGVLVAAGDPELLKQRLTGSQ
>KRH95825.1
MSLSTPDDQLDLRGTPCPINFVRTKLCLEQMSDGSLLEVWLDPGEPIEQVPDSLTMAGFH
VENITDKSEYFSLLVRRPLV
>KRH95828.1
MANVEVNKITFVLIVVVNLSMSIVHLKAIQKKLNKVACALILTVWDLEQLNAIKAFIIQL
LELLENKG
>KRH95832.1
MQKRESPQILFDGNGTQSEFPDSCIHHLFEDQAAKRPDAIALIDGEQSLTYGELNVRANH
LAQHLLSLGCQPDDLLAICIERSAELFIGLLGILKAGCAYVPLDVGYPGDRIEYMLRDSD
ARILLTSTDVAKKLALTIPALQECQTVYLDQEIFEYDFHFLAIAKLLHNQYLRLLHFYFY
TLIQQCQANSVSQGIQTQVLPNNLAYCIYTSGSTGNPKGILMEHRSLVNMLWWHQQTRPS
VQGVRTLQFCAVSFDFSCHEIFSTLCLGGILVLVPEAVRQNPFALAEFISQQKIEKLFLP
VIALLQLAEAVNGNKSTSLALCEVITTGEQMQITPAVANLFQKTGAMLHNHYGATEFQDA
TTHTLKGNPEGWPTLVPVGRPLHNVQVYILDEAQQPVPLGGEGEFCIGGIGLARGYHNLP
DLTNEKFIPNPFGANENAKKLYRTGDLARYLPDGTIEHLGRIDHQVKIRGFRVELGEIES
VLASHQAVRECAVVARKIAGHTQLVGYIIAKDTLNLSFDKLEPILRQYSEEVLPEYMIPT
RFINISNMPLTPSGKLDRRALPDPKGDRPALSTPLVKPRTQTEKRLAEIWGSYLAVDIVG
THDNFFDLGGTSLLLTQAHKFLCETFNINLSAVSLFQYPTIQTLAQYIDCQGDTTSSDTA
SRHKKVRKKQSGDSNDIAIISVAGRFPGAETIEQFWHNLCNGVESITLFSDDELEQTLPE
LFNNPAYVKAGAVLEGVELFDATFFGYSPKEAAVTDPQQRILLECAWEAFERAGYNPETY
PEPVGVYAGSSLSTYLLNNIGSALGIITEQPFIETDMEQFQAKIGNDRSYLATRISYKLN
LKGPSVNVQTACSTSLVAVHMACQSLISGECQMALAGGISVVVPQKGGYLYEEGMVRSQD
GHCRAFDAEAQGTIFGNGGGLVLLKRLQDALDDNDNIMAVIKATAINNDGALKMGYTAPS
VDGQADVISEAIAIADIDASTIGYVEAHGTATQLGDPIEVAGLARAFQRSTDSVLGKQQC
AIGSVKTNIGHLDEAAGIAGLIKAALALQYGQIPPSLHYANPNPRIDFDATPFFVNTELR
EWSRNGYPRRAGVSSFGVGGTNSHIVLEESPVKQPTLFSSLPERSHHLLTLSAHTQEALH
ELVQRYIQHNETHLDINLGDLCFTANTGRKHFEHRLAVVAESIPGLQAQLETAQTAISAQ
KKNAPPTIAFLFTGQGSQYINMGRTLYDTESTFRAALDRCETILQNLGIESILSVIFGSS
EHGLSLDDTAYTQPALFAIEYALYQLWKSWGIQPSVVIGHSVGEYVSACVAGVFSLEDGL
KLIAERGRLIQALPRDGSMVSVMASEKRIADIILPYGGQVGIAAINGPQSVVISGQQQAI
DAICAILETEGIKSKKLNVSHAFHSPLVEAMLDSFLQVAQEVTYSQPQIKLISNVTGTLA
SHESCPDELPITTAEYWVRHVRQPVRFAAGMESLEGQGVNVFIEIGPKPVLLGMGRDCLP
EQEGLWLPSLRPKQDDWQQVLSSLRDLYLAGVTVDWSSFDQGYARRRVPLPTYPWQRERH
WVEPIIRQRQSVLQATNTTKLTRNASVAQHPLLGQRLHLSRTQEIYFQTFIHSDFPIWVA
DHKVFGNVIIPGVAYFEMALAAGKALKPDSIFWLEDVSIAQALIIPDEGQTVQIVLSPQE

ESAYFFEILSLEKENSWVLHASGKLVAQEQVLETEPIDLIALQAHCSEEVSVDVLYQEEM
ARRLDMGPMMRGVKQLWRYPLSFAKSHDAIALAKVSLPEILLHESNAYQFHPVILDAGLQ
MITVSYPEANQGQTYVPVGIEGLQVYGRPSSELWCRAQYRPPLDTDQRQGIDLLPKKLIA
DLHLFDTQGRVVAIMFGVQSVLVGREAMLRSQDTWRNWLYQVLWKPQACFGLLPNYLPTP
DKIRKRLETKLATLIIEANLATYAIAYTQLERLSLAYVVAAFRQMGWLFQPGERFSTAQK
VSALGIVDQHRQLFARLLDILAEADILRSENLMTIWEVISYPETIDIQVLLDDLEAKEAE
AEVTLVSRCSAKLAEVLQGKCDPIQLLFPVGDTTTLSKLYREAPVLGVTNTLVQEALLSA
LEQLPPERGWRILEIGAGTGGTTAYLLPHLPGDQTKYVFTDISAFFLAKAEERFKDYPFV
RYQVLDIEQAPQAQGFEPQIYDLIVAADVLHATSDLRQTLVHIRQLLAPGGMLILMEDSE
PARWADLTFGLTEGWWKFTDHDLRPNHPLLSPEQWQILLSEMGFSQTTALWPKIDSPHKL
PREAVIVARNEPAIRTPRRWLLLADEEIGGLLAKQLREEGEDCILLLPGEKYTERDSQTF
TINPGDIEEWQQLLNRVPNIQEIVHCWSMVSTDLDRATIFSCSSTLHLVQALANYPKNPR
LSLVTLGAQAVNEHHVQNVVGAALWGMGKVIALEHPELQVAQMDLDPNGKVKAQVEVLRD
ELLARKDPASAMSVPDLQTRPHEKQIAFREQTRYVARLSPLDRPNPGEKGTQEALTFRDD
GSYLIAGGLGGLGLVVARFLVTNGAKYLVLVGRRGAREEQQAQLSELEQLGASVKVLQAD
IADAEQLAQALSAVTYPPLRGVIHAAGTLNDGILQQQSWQAFKEVMNPKVAGAWNLHILT
KNQPLDFFVLFSSATSLLGNAGQANHAAANAFLDGLASYRRHLGLPSLSINWGTWSEVGI
AARLGLDKLSSKQGEGTITLGQGLQILEQLLKDENGVYQVGVMPINWTQFLARQLTPQPF
FSDAMKSIDTSVGKLTLQERDSCPQGYGHNIREQLENAPPKEGLTLLQAHVREQVSQVLG
IDTKTLLAEQDVGFFTLGMDSLTSVELRNRLQASLGCSLSSTLAFDYPTQQALVNYLANE
LLGTPEQLQEPESDEEDQISSMDDIVQLLSAKLEMEI
>KRH95845.1
MTSEYPTGINSQESDSTVSDHQPADVNLSEQSTVSEENGVPTVEEVVNRDLITQLTQQNQ
SLKVQLEERSSQYMRIAADFDNYRRRISKEKEDTETQVKRNTIMELLPVVDNFERARAHL
KPQDDGEMTIHKSYQGVYKQLVDSLKKMGVSPMRPEGQEFDPNLHEAVMREQTSEHPEGT
VLEELVRGYFLGDRVLRHAMVKVAAAIEDTVTEEKDQSDQD
>KRH95848.1
MENRLFVFSPCGTSLLTNQAAQEERGLVSKHANAKHIEEIPSEDSLKLRSLAKRVEEKLA
SADLELAGKMSAELNGIIKLYRRP
>KRH95856.1
MTLDFIKISAQTTKTPLGLIVTLHGWGANAEDVASLLPHVNLHDYEFLLPNAPYPYPYSD
NGRAWYDLRDDKMYEGLVVSRQLLIDWLQSLPTTTGIPLSKTILTGFSQGGAMTLDVGLN
LPLAGLAVMSGYLHPQVSTIEKRQFPPTLIMHGTQDQIVPLSAAIKSQQVAQSLGVPVEY
HHFDAGHEVNLAMLKVLRNFVIKLTNPEKG
>KRH95857.1
MLNSLNLENFKPFENQSFVLRPLTFCVIPADVHPGNSGHQIPHSLVSGSGSSMTRHFIVL
DIHRTILKL
>KRH95859.1
MSLVKTPVQAIAKKPLIGLKADSFRHPLDLEATKTLRQIPGIDIMVRNLLGPVAEQVFYA
ENIASSILVSEKQLPDLHYLLIDACKNLDIELPQLYIRQHPSPNAYTFAMRGKKPFIVLH
TSLVDMLTPEEIQAVIGHELGHLKCDHSVYLTPANLLILATSILPNIGVVLAQSLQNQLL
EWVRCAEFTCDRAALLATQNPKVVMSVLMKLAGGSPTLAPRLNLDAFVAQARAYDAISKT
ELGMMVKDAHTAQLSHPVPVLRAREIDRWSSSVEYHNLLGNKGLENRDQTQGGWRNW
>KRH95863.1
MSNFSFQLVVNCSQTQARAGVFSTPHGIVETPRFMPVGTLANVKTVTPAQLADTGAQMIL
SNTYHLHLQPGEKIVAGGGGLHKFMAWDGPMLTDSGGFQVFSLSEMRKITEEGVVFRSPH
DGQMIKLTPEKSIEIQNILGADVIMAFDECPPYPATYQEVTAATDRTYRWLERCIKSHQR
LDQALFPIVQGGVYLDLRTRAATDLAKLDMPGYAIGGVSVGEPPELMAQIVRTTAPLLPR
NKPRYLMGVGTYREMVIAIAAGVDLFDCVIPTRWARHGTAMVQGERWNLKNAKFREDFAP
IDETCHCYACSNFSRAYVSHLVRSQEILAYTLLSIHNITELIRFTQAIRESILQNRFLTD
FGHWMNDPEKDLE
>KRH95865.1

MAILEQVWQLCQRQFLCVIASVLFYTSIPLPHLPGLKFQRVALFAPAIGLLIGAILGLLD
MLFDYLGISALTQSVLIVIIWIGITGGLHLDGAMDTADGLAVTDPQRRLEVMTDSATGAF
GVMAALAILLMKMAALTDISQNRFFLLMVACGWGRWGQQLAIFQYPYLKSTGKGAFHKQA
IRHQMDLLPSWVLLLGFNLLTLAFNQGNFVLVLFTLIIGNIISFIVPAWFNHKLGGHTGD
TYGAVVEWTEALFLCCMSSLT
>KRH95869.1
MLDLTKLAGQMQGLSQHLCSEVAESNRKLELAKENLKKACECQEEIVKRQEKWRDRITFA
NATPIEPLDTCIQISTPPKVHTIIATDGSQIAPNHHEIAYCYLLNIGRVVLYYGQNRHPL
LDSLPEIFYREEDLYIARQWGLKTEEWMSHCRTTSEITVLSDLACSIKNEHPSLAMVDGS
LIYWFLEQLPLEARDQILPPILEAWGKLRQAGIPIVGYLSAARNNEAKNLLRLLNCPYPV
PDCINYCPDQLDYVPCKKFEGLRDTTLWATQLPQGQRSALWRSNSRILQLYDDQVIYFCY
LHVGTEIARIEFPAWVVNDPGMIDQALGLVLAQIQKGYGYPVAIAEAHNQAVVRSGDKNH
FFALLEREMIKAGIKNVSISYKEARKRGSIA
>KRH95871.1
MISTLPNSGTQNLPKVRLQIRALLPQLIEWRRKIHQKPELGFQEKLTAQFISHQLQSWGI
EHQTGIAQTGIVATITGAKSATGKVLAIRADMDALPVQEENKVSYCSQRDGIMHACGHDG
HTAIALGTAYYLQKHRQDFSGQVKIIFQPAEEGPGGAKPMIDAGVLKNPDVDAIIGLHLW
NDLLVGTVGVRPGPFMAAVDFFNCTILGRGGHGALPHQTIDSVVVGAQIVSALQTIVARN
VNPLDSAVVTIGELHAGTRMNVIADTARMSGSVRYFNGQLAEFFKQRITEIIRGICESHG
ANYELEYTHLYPPVINDGGMAQLVRKVAEQVVETPGNIIPECQIMGSEDMSFFLQEVPGC
YFFLGSANPEKQLNYPHHHPRFDFDEIALAIGVEIFVRCVENFLIPQN
>KRH95873.1
MFTTTLTRTQSKPPLDLFTAIENLKTELNAVILAHYYQEPDIQDIADFIGDSLQLAKAAA
KTSADVIVFAGVHFMAETAKILNPDKLVLLPDLNAGCSLADSCPPEKFAAFKAAHPDHIV
ISYINCSAEIKAMSDIICTSSNAVKIVQQIPKDQGIIFAPDRNLGKYVSQQTGRDMLLWR
GSCIVHETFSEKKIVELKIAHPHAQAIAHPECESSVLRHADYIGSTAALLNYCQKSPAVE
FIVATEPGIIHQMQKLAPGKHFIPAPAENNCNCNECPFMRLNTLEKLYLAMNNRTPEITM
SEKIRVQALKPIQRMLEMSN
>KRH95874.1
MTAHKLNEMEIAQALKSLPGWQVQGGKLSKEYQFRDFATALGWMVKVGVYADTLGHHPEW
FNVYNKVVVNLATHDLGNVISNLDVELARKMEESF
>KRH95876.1
MNQINYLRISLIDRCNFRCVYCMPDDTELNYILKKQLLTNDELLTLIKDVFIPVGFNRFR
LTGGEPLLRPGVVDLVNKIAHFPQTQDLSMTTNGFLLAPLAQDLYNAGLTRINISLDSLN
PHTFNLIIGHHSSHGWEQVWCGIQSADKVGFNPLKLNVVVIPGVNDHEILNLAALTLDKN
WHVRFIEFMPIGNNTLFSDRGWVSSAQLRDQIRQRWGLIESQVCGNGPADVFQIPGAKGT
LGFISQMSECFCDRCNRVRLSADGWLRPCLLNESGQIDLKTALRSSQTMEQLQQQVRELL
NLKSEINFKERNPGTLGTYSRTMSQIGG
>KRH95880.1
MNQLIIASLLSFYLAFNLGANDIANAMGTSVGSKAVTLKQAMIIAGVLEFAGAVLFGRGV
TETLGTKIAHPELFITTPRTLLLGMMAVLISSGLWLQLATALSLPVSSSHAVVGAIAGFT
WVATGVDNIDWQAIRTITLIWVLTPIVSASIAGIFYSIIQNYILSPTNSQQRLQEWIPWL
SVIVLSVFGAIVLVPLAEPITRFFDQQVGLQIPPHSIAIFTIVLGIIVLTIYSWKQVENT
TGQISASPIHNLIEGLFAKFQLLSACFVAFAHGANDIGNAIAPLAVISYIDQTQKVPIHG
ITVPGWVIILGGVGIVSGLGIWGRKVITTIGQHIIPLQPSAGFCAELATATTVLLASRLG
LPVSTSHGIIGSIIGVGLVQSPRLINFSTIRGITAAWLITVPVSAFISALIFIIIRIV
>KRH95888.1
MYNYIRDAGEIYKKSFDIIRSEAQLDSLPEDVAKIAVRLIHACGMTDIVKDLAYSLDAVN
LGKKALASGAPILCDCQMVAQGITKKRLPTTNAIICSLNDPEVPTIAKRLGNTRSAAALE
LWRFHLDGAVVAIGNAPTALFRLLEMLDEGVPRPALILGFPVGFVGAAESKAALAENSRG
VPFLTLHGRRGGSAIAASAINALACEEET
>KRH95894.1

MLKAGIVGLPNVGKSTLFNAVVANAKAEAANFPFCTIEPNVGVVAVPDDRLNVLAKLASS
EQIIPARVEFVDIAGLVKGASQGEGLGNQFLSHIREVDAIVHVVRCFENDDIIHVSGSVD
PVRDIDIINLELGLSDLAQIERRIERSRKLARTSKDAQFEITVLDKLVAALNEGKLVRQV
SLTPEEAGVIKNLGLLTSKPIIYAANVAEDDLATGNDFVEKVRAVAAQENAQVVIVSAQV
EAELVELPDADKFDFLASLGVQEGGLKSLIRATYALLGLRTYFTSGPKETRAWTIHAGMS
APQAAGVIHSDFERGFIRAETVAYDDLVTYGSINAAKEKGLVRSEGKEYIVQEGDVMLFR
FNV
>KRH95896.1
MYKIYTTSGKRIIIRSKLYLIFRRVTAVFIYLNQILITFSLCAKQKPWFIYLKFAVKDMQ
NFHTLGDRYFCSFDAIIDFMIHLYGIIKVHEHLHSLQEMYLSVLKEHNYIVLESDNKLHV
FYGD
>KRH95897.1
MKFTIDEICHLFDHKGSKMYGAEAVTQLEHALQTANLALQAGETRELITACLLHDLGHLI
HNLGDDPAAQGVDDKHEYRAIPFLGQIFSLEVTEPIRLHVVAKRYLCAVDSQYWQGLSPA
SQRSLQLQGGIFSPQEAEEFIRLPFAPDAVKLRIYDDQAKVPHLSTPELSYFVELVPRS
>KRH95900.1
MSVSTKHPDFNSGFNFAYLDEQTKRSIRRALLKAVAIPGHQIPFSSREMPMSYGWGTGGI
QVTAAIIGQNDVLKVIDQGADDTTNAVNIRRFFRKVCAVETTEETGRATLIQTRHRIPET
PLREGQILVYQVPIPEPLRWLEPSTVESRKMHALQEYGPIYIKLYEDITRYGYIATGYDY
PVIVEDRYLMSPSPIPRFDNPKMHLNPALQLFGAGREKRIYAVPPYSRVKSLDFEDYPFG
VERWNHACELCGSTESFLDEVLVDDRGGRMWICSDTDYCQQQRRKK
>KRH95906.1
MSTAIAKKADKVCEVKIVRVKRGLFNPTLIAIISFCILVSVPKPAYAMHIMEGFLPVGWA
VFWWVVALPFFILGLRSLTRTTQANPQLKLLLGLAGAFAFVLSALKIPSVTGSSSHPTGT
GLGAVLFGPLTMSVLGSLVLLFQSLLLAHGGLTTLGANAFSMAIAGPFAAYWIYNLTIKI
SGKEKIAIFLSAAIADLLTYVITSIQLALAFPAPVGGFMASFIKFTGIFAITQVPLAISE
GLLTLLVWNWLQSYNPQELELLQLIKGGNGNES
>KRH95908.1
MRDVRDYKVKSGGSERLNTSSPFNIKAESLTLSNFTLSEVEELYLQHTQATGQVFTPEAI
HQAFYLTDGQPWLVNALARQATQVLVKDITQPITAEVINQAKEILIRRQDTHLDSLAERL
REERVRDIIQPMLAGEDLADTPEDNLRYVLDLGLCRRDRGGGLEIANPIYREILPKALAS
VAIASLTSVEPNWLNPDGTLNPQILLDSFLEFWRQHGEPLLKSAPYHEIAPHLVLMAFLH
RVVNGGGTLEREYAIGSGRMDICLRYGKVVMGIELKVRKEKLDPLTQGLIQLDKYLNGLG
LDTGWLVIFDRRAGLPPMGERISTEQAISPGGRTITVIRS
>KRH95909.1
LFGLAWDLVYDFLQKYLWDHDWPGVFQFYAGIVEGVFLGVILGTIGLPKIPRAEFQVVTF
IQHYGLVWLGVYLSAWIVMRLVFPRWRFRGGEWLGNWPRGS
>KRH95911.1
MGRIFISAAHGGKEDGKIDPGTIAGGTTESREMILLRDLIVTELRGRNLEVLSVPDDLSA
VQTISWINSRSRAGDVALEIHTDGANNPTVRGAGVFYIANNTERKQNAEILLTGLLRRVN
QLPSRGVRPDTDSGLGRLQFCRQTNLPALLMQVGFITSPEDRNLLQTRRRDFALGIVDGL
VGWSKIIAPQPETPVDANYLAININININGQNYGEQGILINGNSYIPVDLVDRLRIDLSRGS
NINRVTYRRIVYVKAIELREFNISIAWDSSTKTVKLRSVLAICAGHIDQIISNGSTTEVQ
LQLFLRNNNENALNQFGDIPKLYREEATLEGVNHDIAFCQMCLETGFLRFGGDIKPLQNN
FAGLGGISGGAEAASFSSARIGVRAHIQHLKAYASLEPLVQEEVDPRFRFVTRGVAPSVD
QLSGRWSADLDYGTKIKALFKRLYESAGLI
>KRH95915.1
MLILTKLKSDHGHTAFDLVLPLTAEERRRSRYRLILENGQEICWRLSRGRVIHHGDMLAD
ESETTWIKIVAKPEPVLTVVGTTPIQLLRAAYHLGNRHIPVELTTDYLRLSPDSVLQSML
QKLQLEVTEEVAPFYPELGAYGDHHSHE
>KRH95916.1
MTNLQVFLDIATEAALAAGVILQDYLGKVEDAVTEKGRPGDLVTAADQTAEKAILAVINR

HFPEHSILAEESGKVGNQTSQYLWVIDPLDGTTNYTHQYPCFCTSIGLLVEGEPKVGVIY
DPLRGELFQAAAGLGATRNRRPIRVSSTTQLSKSLLTTGFAYDRRETSDTNYPEFCHFTH
LTQGVRRGGSAALDLAYISCGRVDGYWERGLAPWDMVAGIILVREAGGKVTAYNGSDLQI
DSGRILATNSYLHQVISEELMQVNR
>KRH95917.1
MVYQPAAGARDLLPLDVAQKRWIEDRLQQVFHRWGYHRIITSTLERMDTIMAGEAIDRHK
VIQLQTGQDEELGLRPELTASIARTVVTRMAMAHYPQRLYYNANVFRRNWEKRHNHQQEY
YQAGVELIGAEGLLANAEVLLLTSNCLTALGLNQWHLILGEAGITKSLLNAFPPEIRTQV
RMAIANLNRVAIDTLPLGEDLHKQARIMMDLRGKSGDVLQKVSNFPLDSSQQAAVNHLKS
LVELLEAEGKFSLILDLSLIQTIDYYTGIVFEVVGDLNGQAQVLGRGGRYDQLLGLYHPR
GENIPGIGFELSIDDLYQVLSSTQQLPQTIPSSNWLVVPEGDNAQVATFAYAQKLRDSAD
LIRVEMELGGKDPQGVREYAKERSIAQIAWIKSDGTGTIETVS
>KRH95918.1
MPHTIVTETCEGVADCVDACPVACIHEGPGKNIKGTDWYWIDFTTCIDCGICLQVCPVEG
AIVPQERPELQKTPQ
>KRH95920.1
MVQVAPKLITVNEFITQYGDNNHYELIDGELIEMEPTGPHEQISAFTSRKLNVEIDHQDL
PYFTPSRCWVKLLGTNTAFRPDVIVLDQTQLINEPLWQQEPVITSGKLIKLIAEVVSTNW
QNDYARKVEDYALLGVPEYWIIDYLGLGGREYIGKVKQPTITICKLIEDEYQKQLFQHDD
LLVSAILPSLQLTAKQLLTAGGSVNMVGRL
>KRH95922.1
MRKLTDSDKTEILKLYRETAETTSTLAERYGVSNSTISRLLKGILPEEDYEYLVSLKRAA
RTPEGRALVSYEQLPLLGQSQEDEIAATQTPVEDTQTPVEELEPPKVEVTRVDTPQMEVS
KAVIKEIAGVVDIQAIPIPAPIIRRVKTRSSATDKPPFPVSSDLEPVKNQVTILEETDSQ
ASIIANMFGDDLLEDSEDLDDLDDDLYEDEEDFEEEEFERPTPFVTRPRAGDSSVQILPL
SVAPLPKTCYLVIDRCAELITRPLKDFGDLGAIPIVETQQKTLPVFDNHRVAKRFSTKRD
RVIRIPDSKVLHKTSNHLQAKGITRLLIDGQVYSLT
>KRH95924.1
MQKFLLYLLTSSVLGYAISMSGSALAEDVSKTESSVQVSEPNYISQMTSVSQLSDVQPGD
WAFQAIQSLIERYGCVGGYPDGKFRGNRTLTRFEFAAALSACLDRVNELISSATADQVTK
QDIANIERLQKQFGPELETFKRRVDTLEAKTTRLEATQFSTTTKLQGQVVAVVSDISARK
VNVDTRTVTDKNATLGVRTRLELVTSFTGKDTLFTRLQSNNIRTPKLVDPAKSPTASDYA
VDFKEAGFYFGGGVDSTALSVTALSYKFPIGDKTQVIAVANDGAAEDLTTTITSFNGDGA
FGALSTFGTRNPIYSQLGASGLGINHEFNKNLTLSLGYLGGDMSTGSSSAASPASGKGLF
EGPYGALAQLTVKPSDRVTLGLTYINSYNLALAAGSNNATGGLGGNFSSNSYGVQASLGV
TPKLRLEGWAGYTKSQLLTGIKGDVDIWNYAVNLAFPDLWKKGNLGGLIVGMQPKVTNAS
TTLSGLKDKDTSYHLEGFYQFKVNDNITVTPGLIWLTAPNHNKQNDSVIIGALRTTFSF
>KRH95925.1
MKNFGKTTKTTKITRRILGWSSSILFLSLIYGCERSQVNIPKEESTSTIVATFLPVYLFT
KAIVGDVAKVDVLVKPGTDIHEYQSTPDNVKAIATGSILVKNGLGLEEFLEGTVKNAKNP
KLVEIDASKGIKVMDDGSPVEKIKGEKHDHKHDHALGNPHVWLDPVLAKQQVINIRDGLI
AADAQNKQTYQTNAATYIQELDNLNNEFEQTLSKTPNCTFITFHDAFPYMAKRYNLKQVA
VVELPEDKLAPGDIQAVINTVRKYKVKVLFSEPGIDNKLLTSLAQDLNLTLYSLNSLETG
ERNPQYYFQAMRDNLKSLATGCQK
>KRH95928.1
MLVEFSPPNNNGTLYPAGVSTLLVFDITPLRERAIATPCGEAQLFVGWVGGVGFHPSTQP
TLSYI
>KRH95960.1
MSKAQNNLRMEWKNNGPVPGEIESDVNWQEIPWKKLERHVFKLQKRIYKASQRGDVKTVH
KLQKLLIKSWSARCIAVKGVFPQDQGAKSFSPEQKLVLARELSVIAKSLLVRKNLSLVVF
TIYNQALTLLISMAMSPEWDAKLNLGLHEANSDISCYDAIRSIRREVVKKPKYVLVGELE
ESFQGVNPEYFLKQLNTFPLLGKQIKLWLKLGFLNHPHSLCSLLLGAALGELQRRINWVG

LIADTTSFSCDVIPHFVKHKGRFVLVHQELEVIILCQNIITQWLSEVGLQLKAGGSRVSH
SLYDYQGNVGCNFLGFYIRQFVTHDKLSQNSDSGSNHYNTSYSTLISISKESLEHHTKSL
GAIIDQHRSAKQSVLISKLNPLIESWTKYYSPLISDRIFSKVDFELQSKLRAWSRRRHNQ
KGKRWISKKYWSMGESWRFCHQNSGGKTYELMKHQHIHQDLKLRRSGSTQNIHPQAKAVK
DGMLGRQDKSHPIEEPCELKNSCTVLKTSQEGDLLA
>KRH95964.1
MQEFQSRVVSQETHLNRARASIKQTLSWYAYLRKSGHLSSRPDLASLIKPQMEILNATLH
KLDSNLIKIAAFGLVSRGKSAVLNSLLGEKILETGPLNGVTQWPRSLRWQSGTKVCVELI
DTPGLDEINGEVRGEMARTVAQQADLILFVVSGDITRTEYQALLELRQAQKPLILVFNKI
DLYPDTDKSTIYHNLQVLGAATGDQESDPLLPDEIVMVSAEPAPMEVRVEYADGRISYEW
ETPKPQVQELKQTILNILNREGRSLLALNALVQARESEAIIAEKTLYFRQQEAEALIWRF
CKYKALAIALNPIGFLDVIGGLITDLALIRSLARLYSLPMTSYEATRLLKTILLSSGGLL
LAEIASNLVLGMGKSAAAVAGEGSINITTFAGGALAQSGVASYGAYAVGKAAQVYLENGC
TWGRLGANTVIEEILTQVEKNTILYRLKQKIK
>KRH95966.1
MVILTLLDPQYQTPVQEWRFENATLIRIGRAVDNHVVLHSNLVSRYHLELKKLPETGDVD
SWQIVSRGTNGTFVNGVLTSKCRLMDNYLFQLAQGGPIVRFQIQSIPNLTNPSWMTPQVT
GQVSPLACKHEGNLPNNMFCVHCGQPISFIKQIRHYQVLRVLGQGGMGTTYLAWDSAGIT
TGVPQLLVLKQMNRDMSVIAKAQELFEREANTLKCLHHPGIPKYYDFFVEGGKKFLAMEL
IQGQDLEKVVSYGGSVTVTQAIAWMVQTCEILDYLHSQNPPLIHRDIKPANLMVRNGNHR
IVVLDFGAVKEIGTGSGTRIGAEGYCAPEQERGQPLTQSDLYAVGPTLIFLLTGQNPLKF
FRPRGKGFCFDVQNVPTITPKIQKVIERVTEPLPGDRFQSAQDLAQALLEC
>KRH95968.1
MYRRNPSLQDYLLVDAEKIAIDLYRKNDRGNWEIFNYQSGDNIELQSIDLSFPIQSVYED
IVFEELA
>KRH95969.1
MKRRSFIESSALFGASLLGTQAIATIGNRGIEPVQAQQIKELNFGIISTESQANQRPLWE
PFIAALSSSIGIPVRAFYATQYAGVIEAMRFGQVQIAWYGGKSYIEAAKIANAEAFAQTV
SSDGKKGYYAHLIANKSNPITAAAKKQGGDKYVIRNASKLTFAFNDPNSTSGFLVPSYYV
FGKNKVDPKKVFKRLIFSGSHEATALAVANNQVDVATNNNESLERLEKTNPSARQNIEII
WTSPIIPSDPIAYRKDLPADVKKKLQNFFYNFKDKKILEPLQWSALVPANDKTWNPIREL
DLAKQVLDLEAKTDLTPADKQKLNNLNSQLRKLQGR
>KRH95970.1
MVALIGASGSGKSTLLRHINGLQTADAGEVTVYGTTLQSQGKLHSKVRLLRSRIGCIFQQ
FNLVNRLTVIENVLVGNLARVSTLRSTLHLFTKEEKIQALAALEQVGIIEHAYKRASTIS
GGQQQRVAIARCLVQRAKIILADEPIASLDPESARKVMELLVQLNRDNGISVVASLHQIQ
MVRNYFSRAIALKDGEVKFDGATVELDDRKLNQIYGAAVEELVMRGHGEVLL
>KRH95972.1
MKTDINLPSVTSPSLMVSDTGSSFIGEITQETLALTRRLFIQLQRRPSTLIAGIIQPVMW
LVLFGALFQNAPQGLFGSTANYGQFLAAGVIVFTAFAGALNAGLPVMFDREFGFLNRLLV
APLASRFSIVFASAIFIISQSLLQAAVIVGAAALLGAGLPDINGLVAIALIVFLLALGVT
AISLGLAFALPGHIELIAVIFVSNLPLLFASTALAPLSFMPEWLKIVVALNPLSYAIEPI
RHLYLNSDWELNDVVMEVFWGNVSFGGSLLVLLGFAIVALLSIQSQLRKTLA
>KRH95979.1
MKLVGEKVFSIITDNLRAFSLSDKFWQSMDSAFGTSYNSTIAELLRGKWQKGDFSDLPPI
EMVDSAVLRGGQGAYSQQENRIYLSGDLIGNVEAISRVIIEEIGHYIDAQINQVDSPGDE
GSIFVALVQGEELSPDVLAVLKTEDDTAWINLNGRSIVIEQATFTSTGRVNDTINGTVEN
DTINAGLGRDTVNGGGGTDLLIVDYSSNTYTGNQAGITSSISGSGGFNGSYTAYTYYNYS
WDNNYDQVSFSNIERFQITGTVAGDNITTGDGNDIIDGGDGNDIINAGNGDDTINGGDGN
DTINGGGGINIIDGGDGIDTVDVNLSSITSTQTIEDSDIAKNFTLADGTNIFNVENFRDL
TLGSGADVVNFTRRYNNTINTGTGNDTINAGLGRDTVNGGGGTDLLIVDYSSNTYTGNQA
GITSSISGSGGFNGSYTAYTYYNYSWDNNYDQVSFSNIERFQITGTVAGDNITTGSGNDI

INGGDGNDTINGGGGDDTINGGDGSDTINGGDGNDIINGVNWNSSTPGSSEVDTLTGGQG
NDRFILGDLNWVGYDNGDTSSAGTTDYALITDFTVNDIIQLQGKSSNYSLVVSGSDTHLY
LNKLGSEPDELIATLRSTSTLNLSLTGSYFNYVNAAVAPELAIASTNATQTEGNSSTKSF
TFTVTRTGDANNSSSANWAVTGSGTNQADATDFGGTMATGTVNFAANETSKTITVNVSGD
TTVEPDEEFTVTLSNPTNATIATATATGIIQNEDVAAPLPTITLAVSPTSVTEDGTTNLV
YTFARTGPTNDVLSVNYNIYGTADETDYTGASPGIFPASGKTITFTTGASTAMLTIDPTA
DTTVESDETVILTLASGTGYTIGTTGAVTGTITNDDLGNTQQITSTSNRLRATPGANLTV
PLFYNTSNSNNAVNGIGIRLHYDSTDLSYQQVTNLFSTNLFGSITNGLDTENFDKDNTTD
RYIQLQYFATTGNWPNQTLPVKLGDFGFTTSSIFQGTQLNITGVDLAPGYTLEAAPIEIY
KQNWTLDVDGNGTISALSDGIIIMRYLFGNFSGDALTRNAIAPNATRTPSEIRTYLGEAG
SILDIDGDGAVRPLSDGIMAVRYLFGNFPGNALINGAISPNATRNLSQIESYLASISGTS
SASPLVPQQTTFLPLFAQTFNATSSSKQIIDLTTSNSSLTPGAPVSIGVTYNVDSGDNTL
TGIGIRLHYNSNQISYQGASNLLSTDLFGDVTDNLDEQDLDGDTSTNRYIQIQYADFSGN
WPNQNLPVKIGDFAFNTVSSFQQSKVNVTAVDVAPGYTLEARPLTLAGDGQPKVISGTDQ
GDDINATRGQTTVMPGKGDDIIRVNSASVVIIELPNEGNDTVFSSINYNLASLPQIENLT
LWGTEDINGIGNRKDNVITGNSGQNVLTGLQGNDTFVFNLGDSVVGKPDRIGDFQFGKDK
IRVNGVSPSVLTRASNNSASTLNSLVDSVFIDGNGATSGNQGLGTNSAALVVSTAQGIGG
TYLIVNDGVGGFNPATDLVINLTGYSSNLPGLGNIAVGSLFV
>KRH95980.1
MSQEVNLKNLPLGINTLDKLRGSDCVYVDKTHLALKLIKQPGAFFLSRPRRFGKSLFKDI
TISEAYSSICGYTETDLRESFGDHLEGVDWDALRHWYNGYNWTGSETVYNPRGIKSGIMQ
HLTKSSSETMLKNTVGNRENLSMKLV
>KRH95992.1
MSLITLQLVKKDFGIKEILKEATFSIDGTDKVGLIGTNGSGKSTLLKMIAGIEPVDGGQI
LTNSGAKIIYLPQQPDMDENLTVLEQIFMDSGEHTKLVKEYEELSDKLAHYPEDTLLMSR
LSEVMQRMDATGAWELETNAKIILTKLGIGDFEVKVGTLSGGYRKRIALATALLAQPDVL
LMDEPTNHLDALSVEWLQSYLNRFRGALLLITHDRYFLDKVTNRIIEIDRGDIYTYSGNY
SYYLEKKALAEESAVSSQRKHQGILRRELEWLKRGPKARSTKQKARIQRVESMRETQFKQ
AQGKVDISTIGRRIGKKVIELSNIYKSYDGKILINNFSYEFSPEDRIGIIGGNGTGKSTL
MNMITGRTSPDAGRVEVGSTIHIAYFDQHSEELISAVNDNQRVIDYIKEEGEFIKIADGT
KITASQMLERFLFPGSQQYAPIHKLSGGEKRRLFLLRLLISAPNVLILDEPTNDLDVQTL
SVLEEYLEDFVGSVIVVSHDRYFLDRTVDTIFALEEGGNLRQYPGNYSVYLDYKKSEELT
QQETINGKDNRKSKNLTQPKPGDQEVQNKKRRRLSNWEKREFEQLEAKIADLEAQRTLVE
TSLLAVTSENYTQVQNLYEQMEALKQAIDFATERWLELAEMDV
>KRH95994.1
MNTTLKDFLPACENLGTLRLIVTSSAAVLEARGKIEKLFYAELAKGKYANMHNDGFEFHL
NMDKIVEVKFETGEAKRGNFSTYAIRFLDEKKEVALSLFLQWGKPGEYEPGQVENWLQLR
EQYGEVWEPLPVTSL
>KRH96000.1
MKITSEEIRKNLKQIWGYENFRPPQEEIVNSLLSQKDALIIMPTGAGKSICFQLPALLNN
GLTLVVSPLIALIENQVEELKQRNQKADLLHSELPASQRYKVLKSITKQELRLLYLSPET
LLSSAVWEKISHPDIGITSLILDEAHCLVQWGETFRPVYRRLAAVRPALLNTKPPGTKIS
VAAFTATADHSTQNIIRDVLQLQQPDIYRLNPYRQNLQPTVKTVWTPKARKQQLLKFLQL
HPHQTGLIYVRTRKDSEELAQWLINLGYDTASYHGGLSGEERRAIEKSWLHGKKSFVVCT
CAFGMGINKADVRWIIHFHAPYLLSEYVQEIGRAGRDGMVAEVLTFISEPTGFFDGEDQR
RQQFFQQQILQQYKKAQELIKKLPLQGEVKSVVKEFQHGATALSLLHSSGKLLWNDPFHY
QILDKDIHQNRTQFNAAQQMVEFLKTKNCRWQFLLQSFGFNKGRENWRCGHCDNCR
>KRH96005.1
MTTPQEVLKMIRDQNIQMIDLKFIDTPGTWQHLTVYHNQIDESSFTSGVPFDGSSIRGWK
GIEESDMTMVLDASTAWIDPFMQEPTLSIICSIKEPRTDEWYNRCPRVIAQKAIDYLGTT
GIGDTAFFGPEAEFFIFDDVRYDQTANEGYYHVDSVEGRWNTGRKGKNGEADGPNLGYKT
RFKEGYFPVPPTDTFHDMRTEMLLTMAKCGVPIEKQHHEVATGGQCELGFRFGKLIEAAD

WLMTYKYVIKNVARKYGKTVTFMPKPIFGDNGSGMHCHQSIWKDGKPLFAGDKYAGMSDM
GLYYIGGILKHAPALLAITNPTTNSYKRLVPGYEAPVNLAYSQGNRSASVRIPLSGANPK
AKRLEFRCPDATSNPYLAFAAMLCAGIDGIKNKIHPGEPLDKNIYELSPEELAKIPSTPG
SLELALEALENDHAFLTETGVFSEDFIQNWIDYKLVNEVKQLQLRPHPYEFFLYYDC
>KRH96006.1
MRDAVTTLINSYDLAGKYLDRNALDSLRSYFDSGTSRVQAATAINANAAAIVKQAGSKLF
EELPELIRPGGNAYTTRRYAACLRDMDYYLRYVTYALIAANMNVLDERVLQGLRETYNSL
DVPIGSTVRGIQIMKDLAREQAIAAGVANAAFVDEPFDYITRELSEQNI
>KRH96029.1
MVDSWLKPKEQLVMVVWAVNQLFGWFGFGLSLVKKDNSYLTNCPVLYISGFRSTIGIVGW
GIGCISYIKGLTFRSNKPVG
>KRH96040.1
MLTKVQQDRTTVKSELKLQNQVQEWFETFCLRYLVQQGWSESHIYRMNLALAEGFTNAVR
HAHRNLPPETNIEIELGLWVDRLEIRIWDYGQPFNPDQIPEPKPGTLQEHGYGWFLIRRL
ADQVVYERIDNQRNCLLIVKNRFEGIPA
>KRH96043.1
MNIQRKYSLPNCTILLEGFSTAPSTTFSQARPEMSILINAECYLSGYNQPISGGREFFES
LVRAVSGYAQEFLSNVPNPQAHSKGSELVEVERINNDRHQLIIYSDSTDTRDNPLRIDMN
TVGLFDLVEAVDQFFADSQTLPELILELQPVSRSYGMSGQVALRQAVPATVGVSSLAIAA
LALGIIPPPQWNPTTQNTQTSSGVPESSPASTTSPTPDSTLASDIQDLEALLNTVPEITD
PSQLKSLNRQVYNQINSEWQSRGKIGENLIYRIGVAADGRIVGYKSLNQSSNDQIEKTPL
PRLLYNPVSGTGNKEPIAQFRVVFTNKAQLQVSPWIGYSRTPDVIGDKITDPTSIRELNQ
TLYQTIRQNWSIKPSFPRELRYRVAINKNGVIADYEPLNQVAFDYYTETPLPEIFKTLYG
SNLAAPKNREPLAHFRVLFKPNGVLEVSPWEGYR
>KRH96044.1
MNSYASTTTFEPPTDSSTPMILETLPDPAIGTRACPRRTAIDIDLILLAIESIELGGSEA
ILTFAQELELNQVIRNRVNLWRMRAANPLRKAHTRRPLTTMEAKCLVIITCCIAKRLTVV
IRQLLMIYQQLSEKQIPLSHNLRLANYLERFRTHFKSRMNPRRSLLLTPNSNSDEKLDQL
AIDLLAKLLFCTGTAGMQRFWISLFDGEVE
>KRH96049.1
MERGLFWLPLLGVFFWLAWQGAREYQKVETYRVWAESFQRSKYDIYGVIGQKDNYITWGK
PNPQAIVQVQTFSLVDVQEIRLLIDGQSVRLDQPPQKGRLIELEFIFYPGAAFAPSLNIP
FTEIPLAAQWGHFLQNRLQNLPS
>KRH96050.1
MSINISITPLSGKTILVTRAVGQSSELSQGLTSLGARVIELPALEIGPPSSWMFLDQSIL
ELSTFDWLVFTSTNGVEYFFERMALQNKNQQALIKQAINRIKIAVVGEKTAQSLKTHHLE
PDFIPPDFIADSLVNNFPENLAGKKILFPRVESGGREILVKELTQKGAQVVEVPAYESLC
PQSIPTTAQQVLINQSVDVITFASSKTVKFFCVLLENNLPQEIEHYLKKTAIASIGPQTS
KTCIDLLGRVDITTQQYTIDGLIKAIVEWSLRR
>KRH96053.1
MSTKIYQQIQEFYDASSGLWEQIWGEHMHHGYYGADGRERKERRQAQIDLIEAVLNWSGV
KHADDILDVGCGIGGSSLYLAQKFHAMSTGITLSPIQSARAKERALEANLQSRSSFLVAN
AQEMPFGDNSFDLVWSLESGEHMPDKTKFLQECYRVLKPGGTLIMVTWCHRPTDVLPLTE
DEQKHLQDIYRAYCLPYVISLPEYYAIASQLGLKNIKTADWSTGVAPFWNVVIDSAFTPQ
AFLGLLFSGWTTIQAALSLSLMRRGYERGLIKFGLLCGIK
>KRH96063.1
MANSPPDQPDRESKTSQLPDQPLRLVETAFFASTSALVWFINFYFPLGPVLRVFFSIPIA
LVYLRWGNRASWMAAITSGLLLSVLTGPVRSLLFIMPFAFLGVLLGVSWHRRVPWVVSIS
LGTILCTLGVFFRLWLLSILSGEDLWVYLTNQVTEIIEWLLLNLQILTTPSALGVKLAAL
LLIMINNLIYMFIVHLAAWLLLDRLGNLIPNPPHWIQVLMNYED
>KRH96064.1
MDLLEYQVKEWFNKIGIPVLPSQRIDHPTDLKRLKIPYPIVLKSQVYAKDRSQVGGVKIV

ETTIDAIAAAQSIFSLPIGGELPKVLLAESKYNAQEEFYLAVVLDTVISRPVLLGCRYID
WQSPGENMQYVVVEQEFSPFYARRLAYLMGLRGGLMQSVSDIVTKMYHLFLHKDLDLVEI
NPLGVSSSCQVMTLNGKIRVNERAINRHPDIADIASKIARDPGSKRINGMLVDMLGRNNQ
GEIGILANGTGSALTTWDAVVAAGGKPAVSLNLRHSWLNHTEPTKFSQRMETGLRVLAAD
RNIQVILINLLGTIPELSEVPRVMTDFMALQLEELRWFLNTSQKTLPQRLPKIVMRLAGN
DFQVARKSLLSILQVQSENFILVENLDVAVKEAVSLTKSQVYRK
>KRH96067.1
MKIFVYHTPELTPTGEIPECAIAVDVLRATSTIATVLAAGGEAVQVFSDLNELTEVSEQW
PAQKRLRAGERGGGKVAGFELGNSPLDCTPELVEGRRLFISTTNGTRALKRIEDAPIVLA
AALINRSAVVNFLLEKQPQSIWIVGSGWEGSFSLEDTVCAGAIAHSIWQKTGLELDQIAG
NDEVTSAIALYEQWEDNLIGLFHQASHGKRLLRLECAEDLKYCAQTDILNVLPLQKQPGV
LTSHNAH
>KRH96071.1
MATINDNYLKLKAGYLFPEIARRVNAFAQANPDAQIIRLGIGDVTEPLPDACRHAMIQAV
HDLGDRSTFKGYGPEQGYVWLREKIAVHDFQARGAAIEADEIFISDGSKCDTGNILDIFG
RNNIIAVTDPVYPVYVDTNVMAGNTGEANDKGEYGGLVYLPVTAENNFTAEIPQQKVDLI
YLCFPNNPTGATATKEHLKDWVNYARSQGSIIFFDAAYEAFITDPDLPHSIYEIEGAREC
AIEFRSFSKNAGFTGTRCALTVVPKTLTAKAADGSNIELWKLWNRRQSTKFNGVSYIVQK
GAEAVYSEEGQAQIKALVSFYLENAQIIRQQLTSAGLKVYGGVNAPYVWVKTPAGLTSWE
FFDKLLQTVNVVGTPGSGFGAAGEGYFRISAFNSRENVEVAMQRITEKFSINLP
>KRH96076.1
MVNIVPANWNVVPVPSVKPNPIIASIDIGTNSLHIVVVRIEPTLPTFTVIAKEKETVRLG
DRNLETGDLKPEVMKKAIACLGRFQELAKSFETDSIIAVATSAVREAPNGRIFLHQIETE
LGLDVDLISGQEEARRIYLGVLSGMEFNQQPHIIIDIGGGSTELILGDSQEPRSLTSTKI
GAVRLTGELITTDPIVNTEFQYLQAYAKGMLERSVEDILSKLQPGEIPKLVGTSGTIETI
ATIHAKEKMGIVPSTLNGYCFSLHDLQTWVTRLKRMSNVERSAISGMPDRRSEVILAGAV
ILQEAMTLLGLETITVCERSLREGVIVDWMLTHGFIADRLQYQSSIRERNVLKLAQKYHI
NLECSQRVAEFALTLFSQTQGKLHNWGEDTRELLWAGAVLHNCGHHISHSAHHKHSYYLI
RNGELLGYNETEIEIIANLARYHRKSPPKKKHENFQNLTHKGHRQMVSQLSAILRVAVAL
DRRQIGAVRGIKCEYLPTIRQLEMVVSPSRYDDDCDLEMWSLDLKKQVFEQEFGVKLVAH
LARFNR
>KRH96079.1
MNSLLPPNLELIIRPVQYRDLDALEQLRRPREVPTKYGIFGMQWLGHWFGVLKFLNCFPN
PWQHRFRQFVAERGRKLLGAIQVSPFNRTRSTWRVDQVMLDTCADKLGIGSQLLRHCFES
ILEARTWILEVNINDVSALALYRQNGFQRLAETTYWEIKPDLLQELAQAEPDLPNLLPVS
NADAPLLYQLDTASMPPLVRQVFDRHTHDFKTSLFDIIGNAIKQWVEETEVVSAYVFEPQ
RKAAIGYFELQLDRQGTSPHVATLTVHPAYTWLYPELLSQLARITQDFPQQNLKLASSDY
QPEREQYLENIGAQRIEHTLIMSRSVWHKIRESKFVSLEGIQWTEVLQGLQPARKPIPGG
MSWIPKSPPVPEVTMGKSEMVALTIEPSESPQENN
>KRH96080.1
MVEFQSKSFISALGLDVGSKRVGVAGCDGTGLIATGITTIQRKSFVEDLQEIRGLVEKRR
VQILVVGIPYSGDGSMGEQARRIQKFANRLAKALGLPLEHVDERLTSFQAEQILLAENRS
PSYNKGLVDRKAASIILQQWLDDRRLRTPQNIEL
>KRH96081.1
MYSSDFSEENDRTDADLITLTDEKGRSLDCYIENSFEIEGQEYILLLPVDSPIQIFAWEG
YGDEEEATLVDDDQTIDRIFSTAQAVLSEQNLTLKNTAYALTVTGELPPVEESEILTLEV
EGEEEQETEPEQLQLLTNFYYEDQEYEIYTPIDPLLFFARKTQSGKPQLLSPEEFNQVQP
LLEEYLFDTVE
>KRH96089.1
MAWQKILATSDLLPGGREVVKVGKRNILVLNHDNQYYAVENSCPHLKVPMKSAKIESGTI
VCSFHRSAFDLATGEVKTWCPWPPAVGKLMGMVSQQRSLPVFPLRVENDHVLIDIPE
>KRH96092.1

MTNLQERGHLLTEQINPHSLNLDQINALELVELFNNEDQKALSAIAGAKMELAAAIEQIA
PRLHQGGRLFYIGAGTSGRLGVLDAAECPPTFCTSPDLVQGIIAGGAGALLRSSEGLEDL
AEDGENAIIQHEVNCLDVVVGITAGGTTPYVHGALHGARQRGALTIFIACVPVQQVPVEA
DVDIRLLTGPEILAGSTRLKAGTATKLALNILSTGVMVKLGKVYGNRMVDVAVTNQKLHD
RALRILGDLTDLNREAAETLLERSGKWVKLALLMHWKDLDREEAQQLLAAHKGNLRAAVN
SF
>KRH96094.1
MLNQINKISDRALPLVGDDIDTDRIIPARYLKAITFDGLGEGAFIDDRKALNGQHPFDLP
QYQGAKVLIVNRNFGCGSSREHAPQALAKWGIRGIIGESFAEIFFGNCVAMGIPCVTAES
AIVRQLQELVTADPQALLTIDVENLQVQIHDLTLPVFMGQGTKTAFVSGTWDACGQLVAN
AVQVTAVASQLPYIAWGFAN
>KRH96113.1
MKNKNIRIWIVIFLPLYLLGCSVSQVETSKNGSRGKLSKIRVVATSTIITDLVAQIGGEE
ISLTGILQPGTDPHVYEPVPGDGKVLETADLIVYNGYNLEPGIIKLMNSTGVMARKLAVG
EVVKPLKLKTHRGEIVPDPHVWGSAKNTITMVNAIRDTLVELSDKDREKFTRNAAQLTKE
LEKLDNWIKQEIETIPPPNRKIVTSHDAFQYYADAYGMEVIGTLIGISTEEKPSAKTVKE
LVDAIIRADIPTIFTETTINPDLMKTVAQEAGVKMATNQLYSDSLGARGSDADTYIKMMT
VNTKTIVLGLTETVK
>KRH96121.1
MSGSWSQTSRRKFLTTAGIATGAVFIKGCLGNPPEKGGGTSSQSQQVEAVNLTPEITPET
TKVKLGYLPIVEAAPLIIAQELGFFKRWGMTEVELAKQASWGSMRDNTEIGSAGGGVDGG
QYQMPMPHLITEGLITKGNAKIPMYILAQLNTQGNGIAIASKHAGKQISLDLSKGGKAVF
DKLKSTPSPFTAAFTFAKVNQEFWLRYWLAAGGVNPDTDVKLIPVPTAQTVANMRTGTVD
AFSTGDPWPYRIVKDKIGFISALTAQIWKNHPEEYLAIRGEWVDANPKATKAILKAVMEA
QQWLDKFENRETAANILARPNYYNLSPTFLTDPFQGNYDMGDGQQVKDKSMAVLYWKDER
GSVSYPYKSHDLWFLTESIRWGFLPPEYLEKSEDIINRVNKENIWREAAKEAGIPDADIP
TSTSRGVEKFFDGTTFDPSDPRGYLKSLKIKKVNI
>KRH96123.1
MQEGSYIWIWPQQQYGAVNNRTDRIFLTILVLAAILLFIIDLGEAPLTSQEISVSVVASK
ISQIGTIGYSTSDSVLTPHLIHWLVALAYFLGGNNEWTTRLPAAILTSFSVPLLYCIARE
TFRIRAIAIYSSFIYLTILPVVCYGRLAISDGIFTTMLMVLILSVLRSRRDLRYCLGIGI
CLGMICLTKGLLAILLTFIMVMVFLFWDTPRILISSYLWLGIFIGLLPSLTWYICQLVEH
NHWSGDWGNQTQPPWYYCVELIKWTWPWLVFLPQTIRATWENRNFSWAKLTMVWGGVYLL
MISLVKFKLTWYLFPVYPSLALALGFYLTTIEDWTSFFSYPRIWVISFAILSLVASGASI
YLGLTIPGTTELQVVLGTAAITMILAAILAQKQDRQFLPILVWGSYISLLLLVRSHHWIW
>KRH96125.1
MPKQKKIDPLVGEELLQRVKELGSETREEKAKQCGYYTITKNGIERVNIMKFLNALIEAE
GIQLDGAPGANGRGGRSASYRVSVQSNNNLLIGSAYTKQMNLKPGDEFVITLGKKHIRLR
QVEPEEREDDDQLEEVTA
>KRH96127.1
MSYIIILIMAAFLDYIIGDPWGWPHPVRVMGWFISGWTQFSWQHFYGPLSQRLAGIGLALT
TIGAAAVSGWLIVHIAQWLHPWLGITVASILLASCFALHSLTKAALDVIKPLSDGQLSQA
REILSQYVGRDTADLSESEILRAVLETVTENATDGVMAPLFYAIIGILIPAVGPIPLALG
YKASSTLDSMVGYREPPYTYIGWFSAKLEDCLTWVPCRLTVITLAILSLKPLRVWRLCRR
DAIQDPSPNSGWSECAYAAILGVQMGGLNWYKGVAKYKPLLGDPIYPITSTSIYRALQLT
RYSFLLWLGLTIIAYKIITTYYINYHVY
>KRH96143.1
MITRQNRHAKLPENTDTKSLNQFPSLKDELYLRFYLSSPEEFALPLISIKEVIEVTPNQI
IPIPNTSPLVLGVVNWRSRLIWVVDLGKFMGEMIPLNLERRSQVSVITTEYEDTIIGLAV
DQICATFWLDMESVVAPTDVPDDTVPFVHGEWLDSENNKSVKLINQKIILQSDKWTSMVK
FNQEEK
>KRH96144.1

MISTDEPNSKYQQALTAYIRGDYQSAATLIDQVVSILGEDPNSHLLRGNIYYALGKFKVA
KAEYDRVLGLTNNQEILGSARQKLQVIEQELVTDSSALKSGSDGAFIGDPSSVEQLFSDI
EENDPSHLLESDKKYSYNDMDKIEFLVNFDEFDDLKSISSWEPVEMDENIDTHDELAGNH
NQRLLEDKSVSTQDPLPENVPVRQNSSLNAVLKKQQWSTALVVGFTSALTAAVVGFGTSS
WVSQREWMRNANWGVPLGAGIIGGITAASMGGLAHQSVRRAFHEQNSGQAKELAVTLNSV
VTMTQSLEEVRKIGKAAQTSVHRVNDINSQVSEALENMVAEILVVGETLTKTEPQLKHLV
ESCQEINSLATLASQLASRTNLLTLNVSIEATKYGVGGRGLAIMANEISQLADKTAKSIK
QIQDMFGNIDKETNHILLTMEGSKQRVINSTKLAQQARQSLEDITPIIADIDSSIEKATL
NSP
>KRH96145.1
MHDANSFWEDTGNKPEKKPVDENLEDLIKELEDSLLAGSKQVSSQIVREKGAGPTPTERK
SQVAKIPEPSSLAGKEGVNISFDHLNQMASLVGDLLTSHHVLAHNHQYLCQSLDHLLSQI
QHLSQIGIRIQELNDPSSPFYSLHQEIIECTSTMVESAVDIDFVQEEIQTLSEQCDQVTE
KLRDSCVSIQRIPFARASERLRNQVTIDGVKYGKKVDLVIQGQETLVDKLILPHLTDCLI
YILHHAMSQETKTPSVRIASGKSPAGKITIQVSVKGKYNLLSITFISVANNTLGINYHRL
IQNLLPILHQVYGTISTDSSRTLTIKLPISLSGLTACGAICCVFHNYTIAFHQEYVMETM
DILVKDLARDAKGKPFIRWGERILPLRTLSEVLGFHRQIFNTPSTDNGQNTSVVILHPGS
SDIVVALQVDQVLGEREIFVKQFPGILPKPTGLAGVTIDSDGTIVFVADPWEIITQLNLV
SPT
>KRH96148.1
MLSARKLIIAPLVFSLSILAVTSNQYLRLQELVTAPENRSGESVEKDDQKNALRLKLLDN
MPSFGYNNILSKWVYIEFLQYFGDDQQRQRTGYGLSPEFLKIVLKHDPRFLDAYLSLSVS
TSLYAGMPKTAVNLMTTNLQLLSPKLPPKSYYVWSYKGIDELLFLGDHQAARQSFIKAAT
WAREYDDPEAQKVVFFSEKTANFLLRNPDSKFARIATWSMVLQNNMDGKTRQIAIGEIEN
LGGKVVVNNLGLPIVIFPERD
>KRH96149.1
MSETNLSANLEIQTVQIEKVESAKVNKFQSQLNNLDFKSNAVNLSSVMDIVKTVLADVIE
LQIETSVIDESSYNNNASSAKPENKMVTRINLVDGDIRNEIGTSFIGSGPYVELREFHLS
QVQEGREIIQKNIESVQKLGEILMSMVKQSQNSQSSQLAKLP
>KRH96150.1
MNIFQENYQKPDLNHSLNYGFLFPWQFPQNNGQDNCCLEVKAEEGAYSFYLNRTILEKIA
QLKLQGISLDIPKGLLLNLWYYSIFDSLVDRDENYDKSVDIYQHPQLSVLIYFFLVKTWK
ILTSQNRNKTIKFQSGFTFNSYYQSTENQHFDNLNIMMQSTILFDGDIIQKISQDLINNG
DASGIVNSHYWLTEQVTKCLRSNLNSVYWFVSAIFPAAVITWKISSGMSLWLSILISTLG
WIIIFLVLGILSLLIISELKKLVVRIKNKSVDKFIEPVISWLVWVGTNLVISINVFSRDW
VLLIANSLFMLLLPNLLKYFLPKAGKYLFKFFMK
>KRH96151.1
MQIASGSSNDMKNLQGFDSNHPPDPKLIDSCVHCGFCLSTCPSYRVIGKEMDSPRGRIYL
MDAINEGEIALNTATVQHFDSCLGCLACVSTCPSGVQYDKLISATRHQVERNYQRSFADK
LIRQLIFSLFPNPDILRILLFPLFLYQKLGISQLLRATRLIQKISPRLAAMESILPKITI
QSFGNNLPDIIPAQGAKRYRVGMILGCVQRLFFSPVNEATVRVLTANGCEVIIPKSQGCC
AALPEHQGQTEQAKALARQMIDSFADSNVDFIIINAAGCGHTLKEYGHILADDPEYAAKA
QIFAAKVKDAQEFLVSVGLTAELSSLSDKPLTLVYQDACHLLHGQKISLQPRQLLKQIPG
VTLKEPLDAALCCGSAGVYNLLQPEIAQELGEQKAQNLLNTGADIIASPNPGCSLQISKY
LGKVSVMHPMELLDYSIRHQKLEREISR
>KRH96154.1
MIFDLKVPGEWDSVLSDEFRKPYWKQLEEFLVQERSSQTIYPPESEVFSAFELTPYQQVN
VLLLGQDPYHQRNQAHGLCFSVKPGIKPPPSLKNIYRELQQDIGCPIANHGYLANWAKQG
ILMLNAVLTVREGQANSHKNQGWELFTDEVIKKVNEKTKPVIFVLWGGYARKKVKLIDTN
RHLVIESAHPSPLSAYHGFFGSKPFSAINSALKIWDKPEIDWGL
>KRH96157.1
MTDLAFTPALELARLIRKREISPLELTELYLDRISRFNPQLGSYFTVMAESAIADSRYKT

EILANTRETALPPFFGVPISIKDLNAVAGVTCTYGNPTLVNNIPNYDDGVVTKIKQAGFV
ILGKTATSELGSYPYTEPPGFPPARNPWNLEYTPGGSSGGAASAVAAGLCAIAQGSDGGG
SIRVPAACCGLVGIKPARGRVTNAPGTSIIATSGPLARTVADAAAMLDVISGYFPGDPFW
LNDPEPCFLESRTKVGLNIAFSTSIPSVGEADGNGQQGVLKAVRLLEELGHNVVEKCPDI
SGLVQPFQVVWQAGVAAARIPPQILQPLNQFLLERTGSAGEYLRAVTQMQILSRQIVSFF
DPIDILVLPVYLHSPIKIGEWSQLNPDDTLERIIKWIAPCPIANATGLPAISIPMGFDAN
GLPLSVQLIGKPAAESTLISIASQLEMVNPWVQYCELPKSSKNY
>KRH96159.1
MYQSEAPPLKTIATNLPSEDIDEELNSTIHPRPPWETLPTMYDLPSENPEEPGLPDEFHD
FQPQLLRETCQSSVYPREKMFIGTDLNLYYDVHHFSWYKRPDWFLVLGAAASETQQDMRL
SYVIWQEGFAPFLIVELLSPGTEAEDLGKTLRSANKPPTKWQTYEQYLRSPYYIIFDRYE
NQLRVFQLSGIKYQAVELTEPKFWFPELKLGVGVWLGKYQGTEGLWLRWYNEDGDWIATL
AETAEQEKQRAEQEKLRADKLAEKLAALGVNLDE
>KRH96162.1
MGNIWELDFYSRPILDANQKKVWEVLICESPTDVLTKVDSLFRYAQYCPSTQVNSVWLRQ
ALQEAIEKAGVAPIKIRFFRRQMNNMITKACQDMGIPALPSRKTLVLNQWIQQRMEEVYP
QEPGYEQVTNSSVRLERPLPQRLPDALEGKQWTFVSLGSSDITDMPEWEIAFGEAFPLEL
AGLSPEIPIPGILIFSPRALPIAGWMSGLELAYLRLDSNRNNQGDRLVLETGGTESWILA
NLRTPQLLAEAKGFEEAKQKADGVHFIGVQSDPQSQSFAGFWLLKEINL
>KRH96167.1
MTAITTDKYLSGDHHEHPDLRVWGLLTFLVSESLMFGGFFATYLFFKGTTPVWPPEGTDV
ELLIPTINTIILVSSSFVIHLGDAAIKRNRVGWMRFWYFITAIMGAVFLVGQIYEYMNLG
YGLTSNVFANCFYLMTGFHGLHVFVGLLLILGVLWRSLKRNHYSAIKHTGIEMAEIYWHF
VDIIWIILFTLVYLMNAF
>KRH96168.1
MNINCDQFSWFDASAEVKELLVLATKTWENPEESLKYMEQALAETKENNHMDVLVAAYRY
FYYQNNYALAEKTANTIIARIRKTHQLPDNWEKLRSVLLARKDESQIRIFLNAYTALGMI
SAKLGKLDQAKAIIKEIQNINDKEDFGANILWQILNPSLEEDG
>KRH96173.1
MNQGRTYDYIDLIELLIGTIFESKGDSQVIYPLLENNLHLLDATFIDQLQSWGGHQVNKQ
ATPEQNAQLGASLYLLARIFYHFPGGDPLINLAIAICGYEFCAAIERQLGLEKELAQVLN
HLGAAYQTQAQMGTDSHSNLEKAIAAYNEAITIRRQPGLERELAQVLNNLGAAYQIQAQM
SKEANLERAVTAYAEAISILRELDLERDLAQTLYNLGSAYQTQAQLGKDSQANLQKAIPP
YTEAISILRELGLERDLAKTLTSVGSVYISQGKLERAITPFREAITMFRQLGLERDLAET
LNQLGIAYLNEVERGKDPDANLQRAIIVFTKALTILRQPGLEPDLAQTLHNLGKAYQTQA
ELGTESQSNLEMAIAHYREALAIATPELSPADYLKFASHLGDLGFEQGWWDIALEGYEQG
IKALETIQFWRDKSPGEIQHSHIYQSAVRACIELHQYGKAVELVERSKSRNLVELLENKH
LYPKGNIPSRIYGELDRLRQAIRNEVRLLEIQGEKRSHGQLNTLIEELDQFITSQVKPLD
PHFSLTQRVEPITISKILKLVGADPRRVILEWYFTDDVLYTFIITANPDVPIFPHKVVIS
PSVIRQFSQKYLDIYRDRHNLGNWKQALPELLGELKEILQVETLLTQLWKLSPPCEELVL
IPYGCLHIFPLHALFPWERFPLGFSYFPSCQLLSSVGVTEREEVVQSLAIQNPTQNLQYA
SFEVETIITRLLIGTEVITEKEATRETIFSRSIPEKNYLHFSCTGYSNFQKPLHSAILLA
NCILPAPPKGGEYLPLPNGAAVDLSQCLTLGDICGLDMRRCHLVSLSAYEMGIGDMVDTK
NDDYISLPTGFLLAGASTIISSLWAVSDYTSALLMIRFYQFYTDTENLLTYGRPRLALYH
AQQWLRDAPAGKLLTWAAKLPFSGTRLTLLADFDTMPESQRPFQEPYYWAAFTCVGI
>KRH96175.1
MAFCTGALGNIDLELGSSFLSAGLVEDTGERSPYGIGEESGLNHELFVETADLGNADIMV
MNAKFSPEQVAC
>KRH96182.1
MVTQVQIVEWLQVFSSVIEHHKQELTELDAAIGDADHGINMDRGFKRVSSILPSIEGKDI
GSILKTVSMTLISSVGGASGPLYGTWFLRGSDITMGKTQLSTKDILAFLKAGLDGVIERG
KAQLGDKTMVDVIYPAVLAFEKAVAEDKETILALKIAVDAAEQGLKDTIPMIAKKGRASY

LGERSIGHQDPGGTSAYLMLRSLLEVVEGARRRIYPQSSIDIY
>KRH96185.1
MTNYPLPATNEKLSSSQYKALDISVVLPIKDELESLPLLLEGICHALQVNRLNYEIVCVD
DGSKDGSAEFLKTQAQMRNDLKVIILRRNYGQTAAMSAGFNYATGETIVTLDADLQNDPM
DIPALLAKLDEGYDLVTGWRQNRQDGAINRLIPSKIANWLIRRATGVYVHDYGCSLKAYR
AELVADMNLYGELHRFLPALAYIEGARIAEIPVRHHARRFGKSKYGIYRTFRVLMDLITI
LFMKKFLTSPMHVFGLLGLGSIIIGGGLSIYLTILKLFFYLNIGSRPLLILAVLLLVTGV
QLFCFGLLAEMLMRTYHESQGRPIYRVREVVGKIPN
>KRH96188.1
MQLLNSRKFQLTQLPEQLQDSLQDMVSHIQIESYYSIKHTRYKHSILPDSVTSQFERLPL
EIQQQHLKLQLRNFIYNNYYNVNIKNKSSTDENIDSSKLNKQLENNSLFGVDLEFYDRLH
NGNKGKGYWSHNWEIVKEETDGTLAVYKNGLTLHIDPNLHLSPTTQGLSAGKTISVKMPK
NLVQNGFYMAVANAGTQNNQEITRIYFNIAAEGAATVMESVTEYLNDLDISFSFKALYNP
GDYQRYDSAVLYFNKSQHQIVWPVLQKVYAENELYFQQQVPMFTKILAPGLGCAEEPEQK
FGDQESFGTHRCQIIANGLIEAWLAGDHTPEDRISAIFKQFALQNIQLQHPYLNPHSQDI
YTPFF
>KRH96196.1
MTHWIFTCMRRFLVITLAFFLFCGNFPIQTAIALQGNQSSIKPYLDRVVKQLTEFTLDNG
LKFIVLERHQAPVVSFLTYANVGGIDEPDGQTGVAHFLEHLAFKGTKRIGTTNYKEEKPL
LDKLEQLDSQIRAAKSENRTEELEKLKKEFKTVEAQAGKLVKQNEMGQIVEQAGGVGLNA
NTSSEATRYFYSFPANKLELWMSLESERFLEPVFREFYKERDVILEERRMRVENSPVGLM
VEKFTDVAFKVHPYRRPVIGYDEDIRNLSPANVREFFNKYYVPSNLTIAVVGDVNPNQVK
RLAKIYFGRYPAKPKAQAKINPEPKQTSTREITVELPSQPWYLEGYHRPSITDPDNAVYD
IISSLLSNGRTSRLYKSLIETQRVALVAEGISGFPGDKYPNLMLFYALTAPGHTVDKLAI
ALGQEITKLQTQLVSEKELERVKTQARAGLLRSLDSNMGMAQQLLEYEVKTGSWRNLFKQ
LDDITKVTPADIQRVAQSTFTAENRTIGKLLSQKS
>KRH96199.1
MTTLYEQDFALWSEKMADLIANGHFDKLDIINLVEEIRDLSKRERDQLLSSMRLILHHKK
IAERHGSHLALDSGGSANGEFIRLDI
>KRH96200.1
MKRGIQGHYVTISTAGERAHAFIPKPLPPHPAIEWTQTLSSKFDQALVSLGRLSSISTLL
PDTSFLIYMYIRKEAVLSSMIEGTQSSLSDLLIFELEEQPGVPLDDVREVSNYLAALEHG
LRLLRGGLPISLRLFKEIHGVLLNKGRGSSQTPGEFRRTQNWIGGTRPGNAAFVPPPANE
VLECMSKLELFLHDEPEPTPILLKAALAHVQFETIHPFLDGNGRLGRLLITLLLCEHQVL
QEPMLYLSLHFKTHRQYYYELLNQVRLTGDWEAWLDFFAESVIITATQAVETAQQLLELG
NQDRDKIKALGRRTASMLQIHRALTERPIATSPWLVERTGLSPATVNKALAQLQKLGIVR
ELTSQKRNRLFSYADHVEILSRG
>KRH96202.1
MRYSLRSRVRGIFLGMLLGETLTNQESVSLGEIAVSGSQSLIKWGRLEIEEWWENYQNTT
STAGIIVIAALPLAIFYHEDPDKFKENLQQFLKLGNADPEERDNALAWGYVLIKCLTETL
NTVTLIPETIDFLGNTTSPLPESLLKLNNLLKQQAGIVRMGAEFNLQENISHNMALSLYC
FLSSVEDFKLSLLRSNKQAQIFNCYCSVLTGVMSGAYNSVQGIPVNWKILLSLKPGMDNL
SQIEELTDNLVRLWSGAYNVNIQLAPHSIFAAPHLIRAR
>KRH96208.1
MSIHEVFMPALSSTMTEGKIVSWVKSPGDKVEKGETVVVVESDKADMDVESFYEGFLAHI
LVQAGETAPVGAAIAYVAETQEEITSAKILGGGASAVTPTSPVAPVSASVVPVPITVSQN
GSNHQQGRLVVSPRARKLAKELKVDLNNLQGSGPYGRIIAGDIEAAVGKQPTSPVISTIP
TIPSTPPAIPTKSVPSVVNSGQVVPLTTLQNAVVRNMMSSLSVPTFHVGYTITTDGLDKL
YKQIKSKGVTMTALLAKAVAVTLQKHPLLNASYSEQGIVHHPQINVSVAVAMDDGGLITP
VLQNADQIDIYSLSRNWKSLVDRARAKQLQPEEYSTGTFTISNLGMFGVDTFDAILPPGQ
GAILAVGAGRSQVVATGDGSFALRQQMKVNITCDHRIIYGAHAAAFLQDLAKLIETDPQS
LTV

>KRH96210.1
MTTFTAKRRTSNNSSENFIVAFITKQTPEILPPLVAIVAFLVVWQIFSLTGGTLPGPIQV
VQDTWQLIIYPFYDRGGIDKGLFWQVFASLQRVAISYTLAAVVGIGLGILIGVNSTMSKA
LDPLFQLLRTVPPLAWVPISLAALRQNEPAALFVIFITSLWPILINTAVGVKEIPTDYNN
VAKVLQLSQKEYFLNILIPAALPYIFTGLRISIGLAWLAIIAAEIVMSGIVGIGFFIWDA
YQANKVSEVILALVYIGVVGLLLDKLMAWLQTRILPEQK
>KRH96214.1
MLAKDSNSLESQPSQGTAGRAKDLLGKFVDTVGVIVRDITSLEVNTIVVSSISGSKFNPW
QSYYNIYAISQPNYFASKNIVSELEDRYINIFLQLEREYIYTLLIKELESPSPKARVVQE
YRSRLQYIDENRLNSDGTKVSQIVLPSPFDQESQVENYQKIAELVTDDKFVLTLRKVSEM
KAALDGGDVTSENVDTIYAQTIIQLDGDIITRYHRDLFSLKESDKDLIMKVHNDGVVSGE
KQWREVIDFLINFIKGIAS
>KRH96222.1
MEETRKMVAETNKHMGSITSRWGEFVENLVRPAAVRLFKEQGINVHYTSLQVKAHDYKGS
IEIDIWAENDGEIVAIEVKSHLKVRDIKRFIKVLDRFKDIFPKYKNYRLYGAVAGIKVDE
KADQYALEQGLFLIRPAGDSVAIDMKEDFQAKVW
>KRH96223.1
MFVHSSVSVLLTAAGAAASAASNDFETTPNTFTLGITASDAAGNTSTSTNITINVTDIPN
DVPTVTIAATDPYAAEIQTPRVNNGKFTFTLSEAAPVGGIAVNYTVSGTAIGGEDYTLLP
GTVTIAGGQTTAVVDVLPINDAIIEGNQSVILSLTDGVTYDLGATTGATVTIADGAIGDI
DGNGVFTGSDAFLINQFLAERNNPNRNSILETTFARFPSETVGSTNTTGATLANGIEAQL
SLFDIDGNSTTSPGDIFLMNQYLLLGSNPNRNQIFQLVASAFGSELNGPNNTGDELNQAL
SNLIGTNI
>KRH96229.1
MNQILTEISTQVHIQTPKTICILGGGFGGLYTALRLSQLDWGNTEKPEIVLVDQGDRFIF
SPLLYELLTNELQTWEIAPSYQEILENTGVHFHQAKVSEIDTDNQQVKLCDGKIFPYHRL
VLALGSETNLDLVPGAVKHAYPFRTIYDVHRLEEKLRVLTATNPEKIRVAIVGAGYSGVE
LACKLADRLGEKGRLRLIETGDEILRTSSEFNRQQAKKALEQKSVFIDLETKVVSIGENT
ISLEYKNQIDEIPVDLVIWTVGTRISSLVQNLPLAHNQRGQITCTPTLQVIEHPEIFALG
DLADCKDIEEQQLPGTAQVAFQQADYAAWNIWASLTDRPLLPFRYQALGEVMALGVDNAT
LTALGITLDGYLGYLVRRLVYLYRLPTLEHQLKVGFSWLLTPIIKTLSQESRE
>KRH96231.1
MEVELKERKPSGRNYRNLIKKAEIQLKSTISVFIENKNSEKFVTKRAYIANAKKPEFHQS
HKERMQRFKDDTGFILKIENKIII
>KRH96232.1
MPLYLPQSEIDRIEVINLDKWVDNFWREQNIDKKIIFLDSDTKHLWEQAYQLLSVPIGIE
FFDTPQP
>KRH96239.1
MQRYLKVIRLFWSAAIAAEMEYRLNFIIAALSSLGNLLGGIFGLFLFYRTGYTFSGWSWD
SALVVLGVFTLLQGFWATFLAPNLNRIVRHVQEGTLDFVLLKPIRSQFWLSTHILSLWGL
PDLLFGLIIIGYAGKNLGLGLDDYFWGILPLGCSLVILYSLWFMLGSTSIWFTKVYNTTE
VLRGLLEAGRYPMSAYPMGYRVFFTFVVPVSFLTTVPAQAILGQIHVVWLISAVFLALFL
FFLSTWFWRFALRFYTSASS
>KRH96244.1
MEIAQLVEKSKLITPTGPLKIAEYPAFELGWNDDVVLLSLVIPTYQERENIHKMINIINS
ILEESIPNNYELIVVDDNSPDLTWQVAESLIPEYPQLRVMRRQQERGLSSAVIRGWQAAK
GQVLGVIDGDLQHPPEILLQLLSQIKRGADLAVASRHVDGGGVSSWNLVRRFLSRGAQIL
GLVILPHILGKVSDPMSGYFMVRRNAIAGVEMNPIGYKIILEVIARGKVKEIGEVGYVFC
EREEGESKVTWKQYLEYIQHLIRLRLSTGRLGKISQKFNLQITLPLGRFMRFGLVGLSGV
FVDTVVLYLLSDTLALPLIGSKILAGELAIFNNFLWNDAWTFGDISIQQRSWKQRIKRFY
KFNLICLAGLMINVLILKFLVQFLISNRYIANLIAIGVTTIWNFWLNLKLSWRVTDIK
>KRH96246.1

MKSYWSRLLALILVITIGLMGCSGTPDSLTGDYRQDTLAVVKVMRQAVELSPNDPNKAAI
QAEARQKINDFSARYQRSSSVSSLSSFTTMRTALNALAGHYSSYPNRPVPQKLKDRLEFE
FNRVELALKRGY
>KRH96248.1
MQKTTLSKLQRLIPIHFLSTIQYIHACLLSHWILRWGSQPIALTTAPVMVFSPHQDDETF
GCGGMIARKRQQAIRVGVVFLTDGRGSHGLEPNIQNQVMQIRQQESLQALEILGVPQTEI
KFLNREDGSLLDLNISQKRQLISEIVTLLRDYQPGEVYVPHFKDCHRDHEATYSLVKEAI
AESGISVELLQYPIWIFWRSPLFILLKLRDIAPAYRLSVAQVQSQKQQAIAAYSSQISTL
PRGFIKRFLQAEEIFFKTEY
>KRH96249.1
MRILHLTNHIQNVGNGIVNVAVDLACLQATDGLEVAIASLGGEYEILLEKYGISHFELDQ
SRRVTKLIKAVYSYRDIIKKFQPDIVHCHMMTGVLLAGIFRNNHEYGLVATVHNEFQRSA
ILMGLADRVIAVSHAVANSMIRRGIPSRKLRVVANGTLGSPRHKKIQEYQPMKLHHPAIT
TVAGMYTRKGISELIQAFQIIAQDFTQAHLYIVGEGPDRTMFEAMVQKCGELKHRIHFEG
FQPEPQRYMLSTDIFVLASHCESFGLVLTEAREAGCAIVATDVDGIPETLDYGQAGILVP
PQDSQSLAYALIQLLRDRHKLQAWKLRGQQNLQRFSTTRVSEETLSIYRELSNKYNVFSP
IDTRQLVTGK
>KRH96250.1
MLAYQPINREISRVIIAGGIHGNELIGVYLVKTFEKYGNLIERETFETICLLGNTPAINA
GRRYVDKDLNRCFTKDILFNANTSIYEESRAKEIWQLLQPHHPQKLDAIIDIHTTTANMG
LCIIISNIHPILVTLAVQLTSISPLIKVCFRPVTPDGGFLRSLSEVGFTLEVGPVPQGVL
NAQLFQQTAKIVYAILDFFETYNQGKICINQKSNEKKLKVYEFIGTIDYPRNELGQLQGM
IHPQLEYKDYQPLNFGEPIFLTFDNQEVFYQGESTVYPVFINEAAYYEKGIAMHLTQKKL
VDI
>KRH96251.1
MTSKQLLFNSFLQSSYSPEDRSAQSWVIIFSFILFISTCYFVGAANLLRIIFPVSALLVG
IFLYLRHSILYLGFTWWIWFITPLLARLIDYRVGWDPTRQILVAPYLVVLITTASIIKNL
PRALKEGGLPFILALIAVIYGLLVGLLYNQPISVIRGFLDWFTPIIFGFHLFINWRDYPS
YRQNIQRVFIWAVLLIGAYGIYQFIFAPEWDRFWLVESKMFTSAGSPKPFGMRVWSILHS
PGTFGAVMQTGLLLLFTSYGPLIFPASVVGYLSFLLSQVRTSWGCWLLGILIMLGSVKAK
VQMRLAVIILIMVLSIIPLVTIKPISEVVTTRLESFSKLEEDSSFQDRSKTYDRNLNLAL
STPLGNGVGNIWKVNEKTGQIEVVVIDSGILDMFFTLGWFGAIPYTSGLVLILVAVINCT
EAKRDVFISAARAIGISSCAQLIIYSGMLGVAGMIMWGFLAVSMAAHKYYKHHY
>KRH96252.1
MRNTVLIPTYRRPHDLWRCLVALQAQTRPANQIIIVVRDVDTQTWEFLNQLPSELSSDNL
PLQIVTVTKPGVVQALNTGLKVVNGDVLSITDDDAAPHPDWLEKITAHFTKDADIGAVGG
RDWVHRGDKVEGDSRAVVGRVQWFGRVIGNHHLGTGKAREVDVLKGVNMSFRTQSIGKLT
FDERMRGTGAQVHFEMAFTLALKRAGWKIIYDPHIAVDHYPATRFDEDQRENFNEIALTN
LVHNETLVLLEYLPPFRQIVFLLWAIFIGTRDALGIVQWLRFLPSQGRIVTKKLHASLRG
RWQAWQTNKTNQLIISNS
>KRH96254.1
MGLPVIDIQNLDYYFGSGYLKKQILFNINLTINAGEIVIMTGPSGSGKTTLLTLVGGLRS
VQSGSLKVLGKDLSKASLKQLTQSRISNGYIFQSHGLHGSLTAVQNVRMGLEVHPRIPAS
EMMIRSRQILEAVGLGDRVNYYPDNLSGGQKQRVAIARALVSDPKIVLADEPTAALDKQS
GRDVVELMQKLAKEHNSTILLVTHDHRILDIADRIIYMEDGHLSAGG
>KRH96256.1
MQITINIPDNLTDKFRDKLGHLSEKVMNKLALEAFLEGLINFNEFRQMLSFQDENAFKAF
LSANFPLHSGGLLNLAGSCADIDFTIDEEGIYDHKNDETILNWPIQ
>KRH96262.1
MKNNPYSWIEESLNTIHRADWYRSVQTINSPPGAVVLLSGQKMINFASNDYLGLAADDNL
KMSAIEAIRQFGTGSTGSRLLTGDRQLHRELEQAIASTKQTEDALVFSSGYLANIGTITA
LVGKRDIIFSDQYNHSSLKNGGILSGGTVIEYPHCDMTVLGKKLQEERQKYRRCLIVTDS

VFSMDGDLCPLPNLLDLAEQFNSMLLIDEAHATGVMGKSGAGCVEHFNCTGRELIQIGTL
SKALGSLGGYAAGSSALIDFLRNRAPSWIYSTALSPADTAAALAGINIVQKEPERRKRLW
ENVNYLQTVVKENLGKLKILPTQSPILCFELPDAATALQVGKYLREEGIFAPAIRPPTVP
TSRIRITIMATHNREQIDKLVDSLQRIS
>KRH96270.1
MKIAPEITYRNLDKSQVIDKLVREKIAKLENICNYINSCHIAIEKSHDRPRSGSPYRVRI
DLTVPPGHELVAEKNPGESIRYEPLDAVIRQTFDAMVHQLSKLTQMQRASEQYDRDEETR
ESRGFITKLFKEDGYGFLQALDGREIYFHKNSVLHQDFNQLKLGACVHFSQEEGEQGPQA
TTIQLVDKR
>KRH96274.1
MTVYTTGSLKAELNDRGWRLTPQRETILHIFQELPQGEHLSAEDLHYRLENEGESISLST
VYRTLKLMARLGILRELELGEGHKHYELNQPYPHHHHHLICVKCNATIEFKNESILKIGT
KTAQKEGYQLLDCQLTIHAVCPRCQRALMPL
>KRH96277.1
MNNLLSKLPELSGLIVSCQAPITSPLHNPTIIAAMAQASANNGAKAVRIDTPNHIKAVKE
KVPVPIIGLWKQIVAESDVYITPQFHHALAVAEAGADIIAIDATQRKRPGGEKLVDMIRG
IHQQIGKPVMADVDTFTSAKLAIDSGADIVGTTLFGYTEETKNLIPPGWELLKHIVENLK
VEHPDILVICEGGISSPEEAKKALELGADAVVVGTAITGIDLLVKAYIKRI
>KRH96280.1
MLLIMTNVHTSDYEGHYDADFALWVEETVAKLKSQNFQQVDWKNLIQEVESLGKSQRSSV
RSYLVRLLEHLLKRCYVQTPDCNRSWEIEIRNFRQRLMFELEDSPSLKNFILEILPKCYR
IGLDNVKDSYPSVIFPEDFPFSPELERLLNEKFWEKG
>KRH96291.1
MNVGDVNTDGGDLSEELSLDDLQDIEQEEDIEIAAVDPQNLVGNNRRSTDLVRVYLQEIG
KVRLLGRDEEVGEAQKVQRHLKIRLLLANAAKQGDALVVPYLQLIQVQERLVSELGHRPS
LERWASTAGIKLADLKPTLSEGKRRWAEITQLTVEELDQIQSQGIQAKEHMIKANLRLVV
SVAKKYQNRGLELLDLVQEGTLGLERAVEKFDPTKGYRFSTYAYWWIRQGITRAIATSSR
TIRLPVHITEKLNKIKKAQRKIAQEKGRTPTLEDLALELDMTPNQVREVLLRVPRSVSLE
TKVGKDKDTELGELLETDTITPEETLMRESLQKDIHHLLSDLTTREREVILMRFGLADGH
SYSLAEIGRALELSRERVRQIESKALQKLRQPKRRNLVRDYLESLS
>KRH96298.1
MTSPEQLKPPSEAFINPLLELRNYYASLVQKYQKLFIEARSQLDHVEALLSSWSYSDDHE
PISELEEDQVSSQLFLASNHQSTLESFASECLEHKNQKEVCPETPEPITDAIDATVLPPS
EETHKSFGKGEEVPMIAQYKSLNRMEALRLFFDEHAGTACHIDFILRSLYGNLEPAVLRI
VKGRLQSSLTQGREKGDWYAVPNEPGCYTLALNLLDYSRRSSSSASHTKKKKSVTASASP
STIIPVLEQFEGQFLIDAISLFLEQHRGQIFTVNEVIQGIYGNLDDQQIARIKNKTLNEL
SRGHRTGRFSRVPNQVGFYTWNANLLPRDI
>KRH96302.1
MVNSLKKPTFEEIRPGVKVPAKETLLTPRFYTTDFDEMARMDISVNEDELRAILEEFRVD
YNRHHFVRDAEFEQSWDHIDGQTRQLFIEFLERSCTAEFSGFLLYKELGRRLKDKSPLLA
ECFNLMSRDEARHAGFLNKAMSDFNLSLDLGFLTKSRSYTFFKPKFIFYATYLSEKIGYW
RYITIYRHLEKHPEDRIYPIFRFFENWCQDENRHGDFFDAIMKAQPQMLRGWQGKLWSRF
FLLSVFATMYLNDIQRKDFYAAIGLDAREYDIHVIKKTNETAGRVFPIILDVESPEFYQR
LDICIKNNERLTAISNGNSPKFLQFLQKIPIYISHGWQFLKLYLMKPIDVLATQGEPK
>KRH96304.1
MARNSKDFKDLMRQKQSSRGKHKNVEALIEKMQRAGFGESSANMLREPKGHPKMAEIRVS
SPRDNSQPPRWRKKWARR
>KRH96308.1
MSLRGTLEVRDKDNYQLFRLTGLLDAFSEPTFRKVLEGKIGEGPNNIILDLSQIDFIDSS
GLGALVQLAKIAKLGENQGSFQIVTNARVTQTVKLVRLEKFLSLQTSVERALENIKS
>KRH96313.1
METYSQPTLSTIQSPRRHNNLKDTALGLVSTLSFPAIVGTADMMLKSAGVHLVGYEKIGS

GHCTAIVRGNIADVRLAVEAGVQTAEQFGQLVSSLVIPRPYPNLDIVLPINRLTQIMADG
TYSRLSNQAIGLVETRGFPAMVGACDAMLKSAEVHLASYEKIGAGLCTAIIRGTVANVAV
AVEAGMYEAERIGELNAVMVIPRPLDELEQTLPIASCWVEEHRPLDIPLHVKEKIVDAQA
VELPDLAKLPVKIKEELLVDE
>KRH96322.1
MSKTVAQVMTHNPIMVNPQTPLKQAIQILAEKQISGLPVVDDMGKLVGIISETDLMWQET
GITPPAYIMFLDSVIYLQNPATYERDLHKALGQTVGEVMSNNPITISPDQSLKTAAKIIQ
DHKVRRLPVVDDAGTVIGILTRGDIIRTMACD
>KRH96324.1
MLNQSQTPLIDALKSSISKNHTPFYTPGHKRGAGVSPILTDLLGKDVFRADLTELSELDN
LFAPESAILAAQELAAMAFGAQRTWFLVNGSSCGIIAAIMTVCGPNQHILLPRNIHLSVV
SGLIIAGAIPIFINPQYDQDVDITCSITSKDLEIALAQHPQAKAVLVVYPTYNGVCGNLK
AICQVTHEHNIPLIVDEAHGAHLNFHDHLPISALTAGADLTIQSIHKTLGSMTQTAMLHV
QGNRINIDRLNQALQLVQSTSPSFILLASLDAARQQMAIDGQNLMQQTLELANIARNKIR
EIPGLSVLELPKIRQPGFFDLDKTRLTVNVKELGITGFAAENFLIEMGIVPEVSSFENVT
FIISLGNNESDINALVKVFKKINDIPRSRKYEMITSNIISKIIFNYQPDNRLGISPREAF
FATSEILPLEKTVDRICAENICPYPPGIPILMPGERITKSALDYLQQVQDLGGVITGCLD
ASCHTLKVVK
>KRH96325.1
MKGQLDDYNRQINKKINTDQIEQIIKAIIAGKYSWACVLLLRFSGLNPIDYIPYRTYIRL
LKNNYLLGGSGQNQPSKKEVEIMC
>KRH96327.1
MTAESSKKILVIEDDHLMQNLLLESLKAEGFATIAAETGELGLYKAKENLPDIVICELIM
PDMDGYTVLTKLRQNSQLAIMPFIFLSTNNSKAAVRKAMELGADDYLTKPTTIDELLKAI
HIRLEKQSLMKSWYVNNNSPITNKPENPALVFPNIPHFQKVFDYIESHYQLGITLSDVAQ
AVGYSPAYLTNQVSQQTGNSVNSWIVKRRITQACFLLKNTALTIEEIATKIGYQNCCHFS
RQFSQYQSLSPKMWRKQHQLSHVCTQKGISSTNLPKSLTIFPLD
>KRH96330.1
MVRPQKQFAQHWLRSEKALNSIVKAAECQVNDRILEIGPGTGILTKRLLPLVDSLLAVEI
DRDLCELLAKKLGARENFLLLQGDFLTLGIASQLTAFPKFQRQNKVVANIPYNITGPIIE
KLLGTIADPNPQPFDSIVLLIQKEVAQRLYAKPGSRNFGALSVRVQYLAECEFICTVPAS
AFYPPPKVDSAVVRLLPRNIEIGANDPKLLENMVKLGFGCKRKMLRNNLQSVIDRESLTK
LLEGLNINPHVRAEEISVSQWVSLVNSLNTTSLMSVHTQPK
>KRH96348.1
MEIQPHHDMTAIIANCAVITVSDTRNIQTDRSGQLIQQLLLTANHVVADYVIIPDDPATI
QSHLNYLSNKTEINVVICNGGTGVAPRDTTYDAIASLLEKTLPGFGELFRWLSYQEIGSR
AIASRAVAGTYRNKLVFSLPGSSNAVRLGMEALILPEIAHLVKQMEQ
>KRH96351.1
MTLSIYNTLTRRQEEFKPVEPGQVRMYYCGVTVYDYCHLGHARACIVWDVVRRYLEFIGY
RVRYIQNFTDIDDKILNRARKENSTMETVAERYIQAYFEDMGRLGVREADEYPRATHTMN
GIKRLIQDLEVRGYAYPADGDVYYAVRKFNEYGKLSGRKLADLQAGASERVNIEDPEYQK
KKDPFDFALWKAAKPCEPAWESPWGKGRPGWHIECSAMVRDRLGDTIDIHAGGADLIFPH
HENEIAQSEAVTGKPLANYWLHNGMVKVDGEKMSKSLGNFTTIRDLLDRGVDPMALRLFV
LMAQYRKPLDFTEEAILAATNGWHTLKEGLLFGYHHGGKLDWDLQSAKDNTNIDQVEIEK
FTTTVDDDFNFPGGLAVIFEIAKELRKEGNIIVHEGKTQTPCAKLLTKWQTLVTLTSVLG
LIAKPEEQKTIDESLSDKVIENLVQKRQEARKAKDFAESDRIREELKAKGITLIDSKEGT
RWQREQDF
>KRH96356.1
MKPNYSNWDDDLPPDPQEIYQDLISTLERKVGFGLYFVQCTPIEADNFAQHISRDLANKK
IALLNLWEPIEKFYEHVKNYVQGQTIDILLVKGLEYSLYKYEKRNFGEVTEGQFTNLTKV
PPILNHLNQQRERFRDDFSFCFVFLLRSFSLNYLIHRAPDFFDWRSGVYDLPTTAELVDE
ESRRLIMEGDYKKYLELTPQQKIETMLEIQELLTEKYQNDSNKARLLFEMGNLLYSANEY

ETAITFYEQELKLQPDDHGAWCNHGHALFSLSRYEAAIVSYRQALKLRRDDPFCWYALGN
CQRKLHRDQEAILSYNQAIKIKTDDHYFWYNRGNALRNIGCNEEAILSYGQAIKIKPDES
NVWNNRGIALRSLGRYQEAVFCYDQALSLKPDDYYAWYNRGVALKKLKQNEAALLSYDQA
LKLKPDDHYSWNNRGNALEDLGHIEEAIFSYDQALKIKPDDQYAFYNKACCYAVQGKIQE
ALENLENAVSLKPEQFTQMAKADPDFDRIREDARFQALINKTFHD
>KRH96366.1
MLTITQTETKIEIEFPCLPLAVYKEIAAHLCQVKGVHVELVTQTSPEFDYHQSQIKSLCI
SWQADSDSQRIQQILGYYQKRCHNP
>KRH96375.1
MPQFSPLTNSRLLTNLSNLGFIYQVLTWALLLMIGSCARGNLINFHTLGTNITPIAKIKP
MGQPETTVYIQGKIEKHAPLIGQHAYQIADSTGRIWVVINQNKLQLEQEVVIGGKIKHKS
IKLNEQEYGEVYLEEE
>KRH96378.1
MQDIGDIFYNRVLLVALVACFISQGLKLIFEVIKHRKLDLRVLVTTGGMPSAHSALVTAL
AAGVGQTIGWSSPDFALAAVVAIIVMYDATGVRQAAGKQARILNQMVDQLFHEKPEFFQD
RLKELLGHTPLQVVAGSILGATISCLASYWLTKAPS
>KRH96379.1
MQIRLPKTKLKLNLPNNLSLALVSLGAFLVPTVIYPNYVLSQPTLAACQPPNAEEYLLLF
LTPTANNQEQLRTVLPSELKSVTCKYLDQVVTRVGGFKKVEDANRWAKYVSNVIGLASII
TQVTQPMGSEPPKPNYSYNPTVLGDGFAVLVDYFRRPELANNIQKIVGGNVGLVSYGERP
YLLAVYTTNQTEAYRTLQKLNENGLFAILADGKKVMLLRSVVTLK
>KRH96380.1
MGKYHYVLASQKFLLEEEPLEEVLRERIRNYHEREKEIDFWLVKNPAFLEAPEMSDIKKK
CPQLPVAIISTDSQFITWLKLRLEHVIVGEFTTPLGTDPLASLTNV
>KRH96383.1
MSLRIYGNRLLKTLPGKNTRPTSGRVREAVFNIWQGKIDQCCWLDLCAGSGSMGAEALCR
GAKLVVGIEKSSHACAVIQENWQHLVAEQQVFHILRGDVLQQLKKLSGQTFDRIYFDPPY
ASNLYDQVLNAIAGFKLLHKHGEIAVEHSSDFKVPIIPIWQVIRQRNYGNTSLTFYSCRE
ESKSDGVVYYDISPSLGTHISCEGLTT
>KRH96386.1
MLLVLPDLILAQANPIASPEVIQNQQVRSLPGSLDDVPVFNSNSPELVLREGILLSTFPP
QDKKNPKAHLNFPFRGRFDVFSHHVAKADPPENLRSLYHAIILHNPSQKIATVNILHAAS
YLSQPDAPFIPLESLLPNYSGMVFAGPGSRVMSDILRGKRQEKFPPQIIIPPGESRILLN
LPIPTRGLTPPLNGRSTYMRLWSNETVYAASLAMFAKLNADGSERAPNQAEWEDLLNNED
LSTPRDKVPTPIEGTAKPTIYGRVAGVARGSQWRGFLADTPQSAYLTIPEPGRAFSYVLS
TLHGGTLGTGQIQSAPMLVRYADTAYFAHGNYGIQYSLRLPLWNNSTTQQNVTLALQTPI
KENQLTNPGLRFFTTPQRQVFFRGTVRIRYQNEKGQPATKFVHLVQTRGQVGVPLISVNI
KPGASSLVDVDFLYPPDASPPQVLTLSTQN
>KRH96391.1
MSQFPSQSTEINSSATRDVTPMVALKEIVARLHREQNKIQELLGSLGFALRSFNNLNQFL
ELIPFMAAKVTDADGSALFLYKPNGQVTLEQLHWQDSQQRKSIRKALETASSQITLFPSS
APVTTTGILDDQMQMYLGPDVQVFGTAIIVKHTERGWLYVLSRDPDYSWTETRQKLVRLV
ADQTGVAIGNDELSVELRKKELLDQELEIAAEIQRRLLPRLCPNIPGAVLAARFKPANRV
GGDYYDFIATNHTTNYPPHHLSQENTCWALVIADVMGKGVPAGLIMTMLRGMLRGEVLHG
NSAAGILQNLNRVMYADLENSNRFVTMFYSEYDPKTRVLSYSNAAHNPPLWWHARSKTVT
RLDTMGMLIGLDANSQYENGQALLEPGDTVIYYTDGLTDAAAASGDRFSEDNFITSFSAA
CRYCNGPQEIADYLFDKVQEFIGTDKQNTDDMTLVVLQISQDEEP
>KRH96395.1
MVNRPTNSNTSLTLKVVGIVCILSFFIDFLILMLGFNFTDKEAQVGLTTALVDRGVVPMV
GLAMILIAHWLDTNDQGKNPGMDFKFPSLILSSIFGLMFLLIFPLHLTNVDQVSKEKVNQ
IAQDAQQAESQLNTQLAQFQGQLNNDQGRAQLQQAQLQAKAQITELLKDPQKYKQALENP
QLPPEQKELLKKLQANPQDIDKFIAQQTDPNEIAKQKIEQIRQRQTEAEKQARENAWKSG

LRIGIGSLLLSIGYIIIGWTGLKTTSGTRRV
>KRH96396.1
MNYQSSNEGFTLWENLIVLAIIGILSAILTPSWLSFFTNYRLNVAQEQVYQALRQAQSQA
KKEKSTWQASFKQENGIVKWAVHPILVHPPNAVWHSLDSAIQLDRETTLRESHGIKYIQF
DDLGTFRNPPWGTVTLSMKSGGTAKRCVIVSTILGSLRTARENTVLRNNAYCY
>KRH96397.1
MQLLVGLTIGVISITLLLRLLINVMEINQQEQAKITTGAEIQTALDYIANDLKQAIYIYD
AQGINAIRSQLPSTPTGIDRVPVLVFWRQETIENVLPVPGSLIKDDAFVYSLVVYYLIRD
TTASVSEWSKSARIARWKIRDGVLANTRDFDQIVLCNGYTGRDYIKGPNNNKREQNPEFC
PEPGFAAFNLNNSGSLEQIMNSWRKHSAKYTNDPIVLVDYIDSRINNIPPAVCPPNSTNP
KITWSRVTSSIFSHTTTGRMTSFYACVDRLNVTAQVFIRGDALARIPNNLNTNSNQLSHY
FPTVSTQVQGKGFFYK
>KRH96398.1
MATEQSSQILALPGQSPQFLCEAQVKKISPAALAYLGDAIYELYVRMFYLWPQQRPEIYH
SLVVAQVRAEKQASHLRSLIPELRNHELEIVRRGRNAATGRPKRLDPEIYQQATSLETLV
GYLYLTDYPRLTELLQKLPLEK
>KRH96399.1
MEPIIREIIWHKKLEITQIQQEMSLASLQRQLTAAPSVRDFFTALQQNIYKPSLIAEVKR
IFSSENILSSDFDALSIAKSYERTGAACISVVTDQKFFHGGFDQLRIVRHKVTLPILCKD
FVLDPCQIYLARAAGADAILLIAAILTDQQINNLLRVIHYLGMNAVVEVHNLMELDRVIR
LEDVRIIAINNRSLEDLTVNINTTLELMAARKSHLHNLGILVVSESGIETSQDLSLMANV
GVNGVLIGDCLLREENLEDAVKELLKSQIYGFGGPSYKS
>KRH96408.1
MLESLVAISTLSLLLMVITPIWIMSTAIRMQSRRVEMAAQAATSFVNGVKIGSIVTPQVI
SEITPSQQASRNISTSPQDYLITSSKMPAPTSANGLYCYNQNGIIGFTECQNNSNNLFYI
QAGTIATSSKNESYLLSIRVFRADIDFTQQIPINKSTPSPITHNLGDKQVPLIQMTTEIT
NNKTSFYSLCQRLGAIAPTTYSSSTLCQ
>KRH96415.1
MKSFEYIPPLQPEKKSSTVRRQLKRHLRQRSHLIMGLEVFGKIIVNLLIISVSISALTRL
FPHYLLQQDKLENIGTQVKIMKNRVGTLREEFTRNFDPSQAQSIMREHSYRLQPNQRQII
LVNPDRKTVEPPDSTQ
>KRH96416.1
MDSYSFLNSHTHNNPLYKRNNWQSSYNSYNQKELADSDIGTLKDEKYMRSYALKLAHQGE
YTKAIALLDRIIDSHPENAIDYNNRGLIYFQSGHAQKALLDYNTAMQLNPRLASVYNNRA
NYYAARGDLVKALSDYDQALDLNPRYVRAWINRGITLRELGEYKDAIDDLEVALLFGQLE
IHIWSERGRSYHLWGDWNCAIADYRRVLSHTMSLDKIEDIITYRLRLQVENWLGELGFST
YK
>KRH96421.1
MSMDQISPAATHEINAYLPYIQGNKRNLLPWAITLYQKGCIDGERKIEGSDNIPFTAKWN
ISTLPTDLTCCSVHFHAPGEFAYEVTMTGFEFVDFLIQVIENYKRNRIVDFSKAFYRKLL
CPE
>KRH96423.1
MNELLAPLRSLRQSNWFKLICGASFQHLPSVRSLTLAYTLAGADCIDVAADPAVIDVVKQ
AFSVAKTLVKETQAKGFNWQGNLPLLMVSLNDGEDPHFRKAKFNPRDCPDNCSKPCEKIC
PAQAIVFNSQINNQKDSSSGIIGEKCYGCGRCVPICPYGIIDTVSHLSPLGAILPLIMST
GIDAIEIHTKVGRLPEFEDLWSKIAPWANKLKLLAISCNDGEGLIDYLKSVYNLIVPLPQ
VLIWQTDGRSMSGDIGDGTTIAAIKLGQKVLAANLPGHVQLAGGTNNYTASKLKALGILG
NGKLENSPGQLAGVAYGSYARVLLSPVIDQLESWEVNTSLRKSQRLEDEPELLWQAVKLA
SSLVSQIKSQSDL
>KRH96434.1
MALTEDDLQTLLEEGIDTSNPGIYEELLLDLQGNILKGHGRDHTVHLFLQFKPGQVEPLK
DWIREFAKNITSAHQQAAEAELYRTEKIAGKPFINFFLSHAGYEYLQFRSFQIPGNQPFR

FGMKNDTIRNLLGDPVIEEWEPGLQEEIHALLLIADDNLVSLLQTVNQITRTLYLMAQVV
HREDGFILKNEKGDHIEHFGFIDGISQPLFTKSDIQKAKTSDDFSKWDPRAPLSLVLAKD
PNGKSEDSYGSYLVYRKLEQDVIALLDAEKDLAEKLGVDKELAGALMMGRFRDGTPLTLA
DKPGGDYGNDFDYSEDEKAYKCPFHTHVRKTNPRGDTGRVISSGESFEDALKTERNHRIA
RRAVSYGETDYTNSTSTGSGLLFLCFQADLENQFNFMQAAWANASNFVEWNVGVDPIIGQ
TGPDADRNQKWPVAWGKPEKSDQSNTFNFGLWVHMKGGEYFFAPSISSLTSI
>KRH96435.1
MNKKVRLLIILLTCLGIIINPNIVSADTLIAANLESINGGEWQTVPLPEKREDWMQAVHT
SLLPNGKVLVVNGSSNRNTLVQDNTGNQFIDGVNGRDYNVVNNSTLFDPKTNTFERIASP
PSIANGKSNDPFCSGNVHLADGNVVFVSGSHRYYPGEKFEGSKQTNVYNWQTNSWGTLGQ
LKEGRWYPSPVTLADGKLVIFSGLKYDKPNQITPSVEIFDPITKKFQFIDLTYVENSPFN
TKITYQDNYIYNGKTVSRTIDAYDSIDIYPRIFPTPDGKLLITGDGAGKSPLEIHESNKT
YLMSIKQDSQGKFSVSFEIGPNRKDVSKVYGTGILDPNKEGDVLLMGGIIGTNDINFGRP
YLGKYNDGLAAKGVRIASSLERWSAPENSGKPNGEWEAYPDFFAKPRSMVQAVILPSKQI
LAVNGGEYGEYKPIQEPVLMTADPLSPGGYKTETMNPGKFPRLYHNNALLLPDARVLVIG
GNPSRAGRAMDGTVHVDVLPNPKTYYTIPEFKNASGEVETFDLAKYYESPKSYFVDGDPE
PFVPAEIWQAEIFSPPYLFKSGLRPEIVDAPTTLKYGELQTVSLKNATSNSSLVLIKLSS
GTHSFDYGQRLADLEIENVSADNSTINFKAPTNANLYPPGYYMMFYVNDIGKPSEAKFVK
LES
>KRH96437.1
MSRRYLFTSESVTEGHPDKICDQISDTILDALLTEDPTSRVAAEVVVNTGLVLITGEITT
KANVNFVHLARKKIAEIGYTDAVNGFSANSTSVLLAIDEQSPDIAQGVNTAHETRTQDSE
EQFDKIGAGDQGLMFGFACNETPELMPLPISLAHRIARRLAAVRKTGDLSYLRPDGKTQV
TVVYEDGKPVGIDTILISTQHTPNIGDITDEAGVQAKIKEDLWALVVEPVFGDITVKPDQ
TTRFLVNPTGKFVIGGPQGDSGLTGRKIIVDTYGGYSRHGGGAFSGKDPTKVDRSAAYAA
RYAAKNIVAAGLAEKCEVQLSYAIGVARPVSIFVDTFGTSKVDDEVLLQLVKDNFELRPA
GIIHAFNLRNLPSERGGRFYQDVAAYGHLGRSDLDLPWERTDKAAVLKQAASEYVNSPVS
SLR
>KRH96439.1
MTASGSRGLSFPKAKHILVEIPKFEIEKNLMEIIQVIYRGRGEDDIDNQDKYINFYLAER
SFYYGDQMEIAVRENCLSLLNLLLILKASIMTRIQGYGSLGKKNYILIPIGGKSVFTAGD
SFASKIENLITQLKREYSYNKSHTCLRDAYMKLESLMSRAKFTASKSEQESYLKIQESFN
SKFADSTKTLDQLLDFTPLELAYISGGLLIVPCSQIRQSYSMMFLEIQKYANQELLNKLK
NISSNPSYPENLTSAVKDGIELVEKLINAPKKSQKYDQYQENIQADQYYAFPLFAFISGD
VMKQYFETHPQEPEDMRFKDILSMYIRSLYPVTNILPIGHTYEEFPFIVFNSYSLDEMRY
KLFTEKYLLNSQELNVLSLILSSKKKFLCWTFSKSCDIL
>KRH96444.1
MKAIRTRSQDRILTLLGSIKQGISAQDMYIELRNQNQSMGLATVYRALESLKLEGKVQVR
TLSNGESLYSLTQQDKHHLTCLQCGISIPIHQCPVHELENELQVSHNFKVFYHTLEFFGL
CNKCQEI
>KRH96448.1
MHLLMLITAVTIAYTLRYSAYIPQGNWHLRWSRTLFFFLFPPLLILMTVMAVVCMGTQGQ
MVGIYTDYFSYVLGWLFLGFLVFVALKLAWQGWQSLKSARECPTINLTGQSARLLPTSAL
FAGQIGFWSPELVVSQGLLKHLSPEQLESVLAHEQGHHSYRDTFCFFWLGWIRSCSACLP
NTEPLWQELLMLRELRADSYAAARVDPLVLAESLLLVVSSQPLAWDICCAALSSSKVDRL
EQRIDALLSPLEPNWPEWDLKSKLHYWRVFALALLPLVTVFFHS
>KRH96452.1
MRLTSSIFSTIVLLNIGAVNLVLDTFVSSVIFPLQAQAKPTTILSDLSEQKSQPKEVDPT
KLRQKMRVRLMRMVNGKRVYAPGTSAGIPSAFGANWGDFYVAIAGATADRVRPVIDGGIS
TSLGLGNATDIFGLELNYNNLSMRRFATNGSFNAKIHRSIYSTPSTYIAGAVGWNNFAKY
GSDTAHTSSSVYGIVSAYHFLKTNLANPLPINLSLGVGGAPLFSNAGVGLIAGAGIQVHP
NIGVSTAWSGRGLNLAASYLPMRTLPFTLNVVYGDVLNNTEAGSVLSFSIGYGFDFTP

>KRH96454.1
MVLNQTATQTLTELQIIAPTVAGTIISNVSTPPVTLTSPVSQITITPSAAFTLPSLATAT
ESVPKDLPTGILSSPTGEVTLAVTPLQAGQTIATQVVSGRGAEMTINATTLRIAPINQNA
DNKEVSTTATLRIANGQNLDLVLTGTSERVANAAGFIATATSAGLSLTQIQTGTSIALTG
ANFVQVTSLINSLSGLMVTGTNQTMKPDLSITLVASGITKDPVTTTTKSEVKHGPVKDVT
IEPLQLGRAIVVFNQILDTSSDDTVIALSENTEFLTIGKMLQRLRDAFDD
>KRH96455.1
MAILAVGAGQTYTTIQAAINAANNDDIIVVRPGIYQEDLTINKSVTLIGPYGTFEGIDGF
ENRLGVKPLDPDINAALGVGGLPTANEDFRRYQDDNGTIDNAFGNTQEAWIKGTITVTED
NVTIDGFRLRNENGPLKWNDTPDNFKLLNNYLTGYTANNSPSFGDASINNPTGVVTGWQI
AGNYIGGLLGGGGTGGSIYLAGLQDSNIDDNTFWRPRAAHLYLASLTNVTIEDNKFYHGL
HTGGANFDGFGEFFSGSGYGYGGYGDGYGDGYGGYGDGFFGRNYWLELKGDNDQVLIKNN
EGEYNSGGIQLFGETDSPFAFDNITIEGNTFPDNNFINAYSEAPSNGKSGLIPAVMATAR
LSGPSGSNLVIRDNDITMDLAQVKFITDHKSSLEVRGNFNGVTVEGNTLTPKNINGGVDI
ITGLSLYGSLPGETLIRGNQLLGQDGDPLEASYYGIDLIPTFADYGTYTGDLTVEDNTIN
SWQVGVNLRNTNEITGDININGNTFENNSYGVVLDATATTNSINIAGNTFSNNFSNVFDG
IDPVITMDQVLSYEENQDLGSVLGTVSATDNLPNTDNVGVTQYFISSGNDDGFFTINSSG
EITLTEAGLAAANDFETSPSSFNLGIIVTDGGGLQDTETITLSIINVNEGLGQLPPITTE
GAGFTVGATLIAAIPFDDPDGIPTDISYQWQRLIEGVWTNIPDATEQNYIATEDDNNNQL
RVEVTYIAGGFEKVIYSNNVSISLVPVSGTFDSITGDNNLDISEKEAGVTLTGTVSETGT
TVTILFGGQTRVAQVDGLSWSYVLKPNDYNFFAAGSNLFTAIFTRTDGGETGSFTTFQTL
TIPDGILPPNTSNAFDPTQPKGLKSEVIDAAQTLEIDGVSILEISQTVGILGEGESFNDP
NIAVLPIGTRDIFDQGANAGAANYAAVKTEPGTSIQSIYIVPVVEEEGVKKLQVVLADGT
VVEAEIPPDLISPIGDPLAITISGVQLGGTTTFVLYLSQNFINQLPEDLDLARYVKFNYE
SQQFELYDDFNYDYIFNDVDGDGVRDFGEVYLTVNLTDGDIWDGDGLANGIIVDPGQLGI
ATDSGTDNNPPIAIELRGIVAENDPGAFIGSLTVTDDPGDSHTFTVNDSRFEVINVDGNN
ILKLREGESLDYEAASNIVLTITAIDNGGLEITQDFTITVTDVNEAPVAIELNQITVIEN
DPGAIIGTLTVSDPDGNDGHTLKVNNDTRFEIVDFDGNQTLKLKAGESLDYEAGSVELSI
TATDNGGLEVTQDLTVSITDVNEPPVVSFSFFVPESTTLVSNLTVEDPENDPITLSLAGV
DASLFSISPTGELTFNTAPDFEEPLNADKNNLYKLQVVARDEQNNKSIQDISILVTNVNE
APIAIDDVLAIIPGSSFGTLNPLDNDSDPDLNDPLTIINKTDGNYGRVEIRDNELIYTLL
DATYIGDDVFSYTIIDEQGLAATANVNVTITGTDIITYPVEILDPEDSLIPDEAGSLSDI
VNDISFNFLTDYDKVQAKLALQEALSKTEASFTNLFGLYEVDNALTGSVNGVLPEDKSAY
AKAALSRVVPNFVVRAGGSGDGVNGDVIVSEGKIYAPFVIAHGGNFSGSVQDAVNAFFQV
NPDNSPATAQNYTTLPVAYFSFGSANPDGAAHIKSFGNNVFGFEDLPAGVGVSDYDFNDT
VFSFG
>KRH96459.1
MGLDYKLPMPTPIIIAHRGASGYRPEHTLAAYQLAIDMGADYIEPDLVISQDGVLIARHE
NEISMTTDVENHPEFAHLRTTKMIDGEIRTGWFTEDFTLKQLKTLTVKERIGQIRPQNTV
YDGLETIPTLEEIIDLAENQSSQKGYAIGIYPETKHPSYFQSIGLPLEPALLRSLANTQL
PIFIQSFEVGNLQNLNKNTDFPLVQLINDLGQPNDFRVDGKSCTYQDMIKPGGLKKIAQY
AQAIGVNKNLLIPRNSQGKLLSPTSLVKDAHQQNLLIHTWTFRNENCFLPLDYQNHPQGE
YELFFNLGVDGVFTDFPDTAVYARGKV
>KRH96470.1
MIQKILLAVSGLGHAEEMMKNLKELPSIQSAKVTVLHVVPSQSTAAAMTEKWEEGGKILA
NAIQSLNFDPSQVSSILRQGDPKDVVCQVADEIGVDLIVMGSRGLKRLESILSNSVSQYV
FQLSSRPMLLVKDDIYVKRIKRVMVAMDGSPSSSQCLQLALFLLSGVESGQLILTNVITD
LGGKVSGITDIKPERNSVLGNAVATAESRGIPVRCVTSSGKPGEEICRLAQELNADLLLL
GSPDRRPSIAKSFVDLDRLLGSSLSDYVRVNATCPVLLARTVS
>KRH96471.1
MGNIRFIKENKEVIAADGANLRLKALENGIDIYKLWGKMTNCGGYGQCATCIVEVTEGLE
NLSSRTDVEKQKFKNSPDTYRLACQTLVNGPVSVITKP

>KRH96472.1
MLNPNLDEIQLTKDDYERYSRHLILPEVGLEGQKRLKAASVLCIGTGGLGSPLLLYLSAA
GVGRIGIVDFDVVDFSNLQRQVIHGTSWVGKPKIASAKDRIHEINPYCQVDLYETRLSSE
NAIDILSPYDVIVDGTDNFPTRYLVNDACVLLNKPNVYGSIFRFEGQATVFNYQGGPNYR
DLYPEPPPPGMVPSCAEGGVLGILPGIIGVIQATETVKIIIGKGTTLSGRLVLYNALDMK
FRELKLRPNPVRPVIDKLVDYEEFCGIPQARAAEAQQQMETQEMTVKELKTLIDSDSQDF
ILLDVRNPNEYEIARIPGSVLIPLPEIENGDGVARVKELLNGHSLIAHCKMGGRSAKALA
ILKAAGISGTNVKGGINAWSQEVDPSVPQY
>KRH96480.1
MTKIYTKDFKINCVPKRRTWQNIAQEIMKLPLPGIPIRVILTSAQDDTLSFECSFIAIEK
KLTWPSLLEINVRKRVSNQPFVAVSIVPTGVRAEIGGFAGDATPSTNLLAAACDYVITNP
NAVTASDVYFAKDNVLYLEGNLICQLLLGNIGIIPEKRTNIAAIIEKPRDERFLNNVLNA
LNGLRAVGGINIDPVIVTGGAIETKCTYSQYGNASGEFKGIEELIKALDVIENSSARAVA
LISTLLVDDQVRQAYYKGESIPNPWGGAEAILTHTVTNFYPFTAAHAPLLLEWEHTGFGK
LVDPRDGAELISSAYVCSPLSGLINSPRPVSFDTPIAPGETRISVENISALVMPETTVGN
IPFFAGLDQGVPIILVKDNTTQYKITPELLQIPETENRKIYRVRSYMEAAGLLLALRNGI
LPESTTRPIPQLKPIFI
>KRH96482.1
MIQLQEQQKKSPWLPIGSISLAVLTFAFTPILIRTCQEEIGPVSTIFNRYWIAAMILLLW
NILKNPRQLLMKNQNLFSQMSQQKLCSICNKTVFLLLLSGFFLATTTVLWSWSLVHTGVA
NSALLHNLSVFFTGIVEWFFFKKYFNKHFIIGGVIAGGGIIILGLNDLKFEVGQIQGDLV
SLFSSLAFCGFLMTVERVRIKVSSVTTMLWCYGLGIILTLPIALINGEQLFPLSWHGWYG
PVVLGINAVVVNFLEIYSLQQLSASFVTLVFLIDPILTGILSWWIFGETLSWLNFVAFTV
ILFGLYLAISFDIGQLTISEHAVMANEISDA
>KRH96483.1
MQSLNIRKSYDVVVAGGGHNGLIAACYIAKAGRSVLVLEQQDKVGGTAVSEQLFPGIEAQ
LSVYAYLVGMLPQKIVSDLNLDFQFRQRDPASFSPFEKNGKHQGLLISNSSQSITKNSFE
QLTGNLNEYDSYCKFHRLQKYLGKHIWPSLLEPMQTREEIKNRFQTEEEKLAWKLFIEQP
LGVAIEEFFDSDLVRGLVLTDGVIGTFAHPHDPSLQQNRTFLYHLIANGSGDWCVPVGGM
GKLTQQLVTQCQKFGVDILNCAEVIKINPDPEQAIVQFQYNNQESYVRGNYILANIAPKK
LSQLLPGYEVVNDPKDVGSFVKINMVLSKLPRPRANYDSVQAFTGTFHINEGYQNIIHSY
NMAIQGVIPEQPPGEFYCHSLTDNSILSPLLKAKGYQTLTLFGLEMPYTLFIPDNETNKK
KVLALYLKAVNQYLLDPIEDCIARDENGDFCVDITTPVDVERKMGIPTGHVFHQALSWPF
VESEEERGMWGVEIGFDNIFLCGSGAKRGGCVSGIPGHNAAMKILGY
>KRH96485.1
MEFNQQLLKHYWKRHLESEHQQLHLFSSVHIFESLPSTNQMAWQLLSQGEKRGCVVIAQQ
QTAGKGQWGRQWLSPQGGLYISLGIPLQLEVANGYQLTLASAWGIASQLKNCGVEARIKW
PNDLVLNRRKLGGILTETKVKQGQITQVVIGVGVNWKNPVPETGINLHSWQNENLWPISG
LEMLAAQVLLGIQSGLDCLQDEGISIVLSRYLDLLINLGDQVCVNIPFSGKNEDNLVETS
VLATVVGVTPQGHLRLQTSGNREELIQGEISLEPGTISLGYTQFSV
>KRH96489.1
MKYRRFGKTNLDLSVFSLGTMRCITDVEIFHHTIEKALEWGINHLETARGYGKSEEYLGR
AIKTGLSVPRGQFYITTKIPPCADNSTMERHIDESLQKLNVDYLDALGIHGLNTEQHWKW
VKSNNGCMKAIQKAIEDGRVKHVGFSTHGSLKIILDAINTDYFEFVNLHYYYFFQRNNLA
VTVAAEKDMGVFIISPADKGGRLYTPSDKLKELCQPFTPLELNYRFLLSDSRITTLSVGA
GNPEELTLPLAVSDNTSNLTPAEVKVFKNLENYQTQILGTDKCSQCYDCLPCPENINIPE
ILRLRNLSVAYNMIDYGKYRYGMLENAGHWFSGMRGNRCTECGDCLPKCPESLDIPHLLQ
NAHETLNGKSSRRLWD
>KRH96491.1
MLFGVMVNQWQAINKMSKSLTDLLKQTTEGLFMPSESEYPFNVVCWEFFNLNETTIQQKT
GIIGNVRTVTIDDFFQGVTKQEDWYEEEERNLAKRFEGLVLVLKNNLAEAKVYEIGDKEV
HAYILGTKDDEIIGISTVVIRT

>KRH96493.1
MHIHTLEEWQHSHDFSSNQHQAEKSTKIVMVLTALTMIAEIAAGTIFGSMALLADGWHMA
THVAAFGITVFAYQYARNNANNPKYTFGTGKVSVLAGFTSAVVLGIIALFMGIESLQRFF
TPTGIQFNEAITIAVIGLFVNIVSAFLLQDHHDHEHHHEHQQDHNLRAAYFHVLADALTS
VFAIIALFAGKFLGWVWMDAAMGLVGAGVISQWSYGLLQDTGLILLDGSGDKQIRLAIVN
AIEENSDNRVTDIHIWYVGQHHLAAMISLVTHDPKTPEYYKMLLRDIPTISHVSIEVNPC
HGESCQETRTC
>KRH96496.1
MNDLPRQKLKEIIIQQGRSLCDNPQRCEAFLRDYCGGYRREIFILISALKQGVAKDLLNS
NNVPLELLVSRLIKKMQNELGLTEEAAHYAVESWAQALDKMPQQQIQQPRFDVINKANKK
LNHPVSSQQTTVVSPLFTTNQQQQKLVLGRGLLKKAGVSVGLFILVVIVQQIFTANSTEP
EINGYPTTEITELPTPEITRSSTPKVTRSLRRKITEPTEPEITEPTEPEITEPTEPEITE
PTEPEITESTEPEITESTEPEITESTEVEVDESSKPEIAEFLEREIREVNESPTPEATES
PTPEVTESPTPEITESPTPEITESPTPEKTNPI
>KRH96500.1
MNELPRQKLCEIITQYSKDICNNPQRCEGLLRDFCGQYSKEVFLLINALKKGVATELVKS
QAQIPESVILAKLTKRLQDELGIAEEAAYWAVDSWGLALGIISEPRSKNDLESREQLILQ
REQEVEKQQKQKEEYEKELHKSDREIEIWVYISALLSGSYTFLLIGLAILSVIIYVYQQS
EKTQEISNLSEEMNNLTSQYNQKVAKLTEQSNNLKGQIDNLTDKKKSLYNEVQNFQNSLK
DLENNLENNLNFSGDTYLNLCNTTSNDTISTTFMYQEGEVWKSKGWWVIKKGECTKLFVR
LNYRGYIYLHGQTNNLTWGSKDFSFCVKNSAFEFEKADEIECSGENYKANAIQFFVFPGV
NNYNFKDYKLYTE
>KRH96502.1
MKKVEDLTKIFLEIDEELIRKGVEPYTRPFAACSEIAQHLFPGTNLPMDDPIFQSINQIY
RDFYGSAYLHLPPAYIGCFMFRDVFLPIRLPVIYGEFLINPIDFLTDIPETQNLKRWLFK
DHKAVLTFFDQVIDVMDFVYGIDDLEKGDKLPPKAIEFFYLAKQQMQAAAATLLGSFDKY
AVIQNCCLATELLLKGALIAKKIDIETLKKKYGHDLKCLLKKNFDLLPGLNQDMLSDIIH
KLPDYTKSRYELKDFSRLKLGEVFMKTQFISGDILRQFSDRNSRANFNMGQELDVTIRTF
PKKQLE
>KRH96503.1
MSEETIWIITREYTPSDEDAKGKNTYSNPWGDDKQNITEVATGLVKVSVEKLETELSHFL
QLIGKVFSHAQKQINQQTGFKLDEVELCVEITGEGEVKLLGTGVKTGTKGGLTLKFKQEA
NNTQNPN
>KRH96505.1
MQVSLKSSILGLSMVGAAVLVFGEPGVAGENKFFCTREGGVPVTKVRTARGNETFIIWER
DFHKYPATKRCGIISNKLQRFYENGEVHFKTGIVNQYPVVCISNRQNTRCSGDNLLVTLP
QGEDSVSVLE
>KRH96508.1
MNSETKLIKSRIGLLNLAEQLNNVTRACKLMGTSRDSFYRIRELYNTGGEEALREISRRK
PIPKNRVEPDVEEAVVQMAFDYPAYGQSRACNELRKKGIFISAAGVRCVWQRHNLEVFEK
RLKALEERVAQDGLILTEAQVIAMERKKEKLEAHGEIETEHPGYLVAQDTYYVGTIKGVG
RIYQQTVIDTYTRVAFAKLYTTKHAITSADVLNDRVIPFFEGQEIPILRMLTDRGTEFNG
RPENHEYELYLQLENIDHSKTKVRHPQSNGICERLHRTMQDEFYAVAFRKKIYQTLESLQ
TDLDDWINYYNQERPHSGRYCFGKTPMQTFLESITLAKQKLLNNLCPAA
>KRH96516.1
MTNLTPNSSFSVTLRLKIPNRVGMLASVTQAIALTGGNIGQIDLIEQNRHNSIRDITVDA
ASGEHGETIVNSVKELTDIQVINVYDRTFNLHEGGKISIASRIPIKTVSDLAMAYTPGVG
RICQAIAQKPEEVYRLTIKQNTVAIVTDGSAVLGLGNLGPEAALPVMEGKAMLFKEFANI
DAFPICLDTQDTEEIIRAVKNIAPVFGGVNLEDIAAPRCFEIEQRLQSELDIPIFHDDQN
GTAIVTLAALLNALKLVNKSINQIRIVINGAGAAGIAVARLLRKAGAEQIWMCDSQGIIS
TNRTDLNPEKLEFAVKAQGTLVGATQGADVFIGLSKPGVLTPEMVKSMTKDAIVFAMANP
IPEIQPELAPKNVAVMATGRSDYPNQINNVLAFPGVFRGALDCRSKVITSNMCLEAAKAI

ASLVNTSDLNREYIIPSVFDNRVASAVAAAVKQAARAEGNAQA
>KRH96524.1
MKVAVTGATGFVGSRLVQCLHDRGEQILVLSRNPRSAARTFPSEVFPNVEILAYTPGVSG
VWQNRIAGCDAVVNLAGEPIAQQRWTPEHKLEILNSRKLGTQKIVEAIAEANPKPSVLIN
ASAIGFYGTSQTATFDENSPTGTDFLAQVCQQWEEQAQMVKNYDVRLVILRFGIVLGNGG
ALGKMITPFKLFAGGPIGSGNQWFSWIHLNDIVNLIIQSLVDPNAQGVYNATAPHPVQMK
DLSNTMAQVMNRPAWLPVPEIALEALLGEGAMVVLEGQRVLPQRTQALGFEYQYPLLPSA
LRQILT
>KRH96525.1
MKEKILNWVNIILVADFFLVILGFGWFTIAIVGNASGVNLGLDLWHRLWIPLFNPAIGLL
MGGALLSGISSWVSKKFSN
>KRH96530.1
MRVVIVGAGLAGLATAVDLADAGWEVEIFEARPFVGGKVSSWVDADGNHIEMGLHVFFGC
YYNLFELMAKVGAGNNLRLKEHTHVFVNKGGNTGALDFRFITGAPFNGLKAFFTTSQLSL
QDKLQNALALGTSPIVRGLIDFEGAMKDIRNLDKISFSEWFYSHGGSKGSIKRMWNPIAY
ALGFIDCDHISARCMLTIFQFFAAKTEASILRMLEGSPQEYLHQPIVNYLTDRGTKIHTR
RQVREIKFTESDSQSEVTGILVAQGEQEELITADAYVFACDVPGIQRVLPPSWRKWSEFD
NIYKLDAVPVATVQLRFDGWVTELQDSQKRHQLHQAVGIDNLLYTADADFSCFADLALTS
PADYYRPGQGSLMQLVLTPGDPFIKQSNEAIAHHVLKQVHELFPSSRELNMTWYSVVKLA
QSLYREAPGMDPYRPDQKTPVGNFFLAGSYTQQDYIDSMEGATISGKRAAKAILASGQSK
I
>KRH96532.1
MTQNRSQKPIVIAPSILSANFSRLGEDIRAVDAAGADWIHVDVMDGRFVPNITIGPLVVE
AIRPVTTKPLDVHLMIVEPEKYVEGFAKAGADIISVHAEHNASPHLHRTLGQIKELGKQA
GVVLNPGTPLQLIEYVLELCDLVLIMSVNPGFGGQSFIPSVVPKIRQLRQMCDEKGLDPW
IEVDGGLKANNTWQVLEAGANAIVAGSAVFNVPDYAQAIENIRHSKRPVKELAVSL
>KRH96533.1
MKKTLIVCVLGIYSLGIPALASFQSSSFLGTNGINALILHQPPYKLTGRKIALGQVEIGR
PGLFGLDKAVSKNRAVTPTAVFSLNAPAQSNFGVDTHAYNVAGVMTSQDKSLPGVAPQAR
LYSSAVGFSRNLGQSEECLSAQHVALQNGGDVRAINFSFGEPLNRDPRPEPVLDGKALLT
LCVDWSSRVHDVTYVIAGNQGKGGISIPTDNFNAINVAFSSARRGIFDKLHVSNLAGNNQ
EVSDRLLGKEFNIDGRSSVGLVAPGTNIPLINPDGKLNKSTGSSFAAPQVTAAIGLLQEF
ADRQIRKQAVNWRIDARRHQVMKAVLLNSAEKILDNGNGLRLGMTRTLVDKQNRDWLVSQ
AYQNPGIPLDPQMGSGHLNVFRAYQQFMWGQWGPGNEVPTIGWDYHRISANSSIDYSLGK
PLKKDSFVAITLTWDRLVELEDTNQNQLYDTDETFIDKGINNLDIYLLKDDEKNIKISNC
SSTSQIDNVEHIFCSIREGGKYKVRVDFKNQVNRSKQDYALAWWTVGEN
>KRH96535.1
MKRRNFLFNVGGILLGQTLIGCNGKNQSEFQVLLLKDSIPGYVVNQFNKRLQSDKTLPHV
KLSFVPKNQIYDLFKQLQTWRETRDKSPGEKKSPHCDLVTLGDYWLKPAIEQKLIRPLPI
KKSKQWSSLQEKWQQLVKRDDQGNWDSQGKVWGAPYRWGNTVLVYNQKQLQTLNWQPRDW
SDLWRSDLRSRISLPNHPREVIGLVLKKLGRSYNTVNLLEIPNLNIELESLNQQVKFYDS
TNYLEPLITGDTWLAVGWSNDIIPILSRYPQLRAVIPQSGTAIWADLWVQPANTLMTEET
DNLISQWIDFCWQPDIAGQIVMLGKTNTPPFINTSKVQGISDSIQNLLLIEQELLEKCEF
LKPLSLESINQYEELFLKMKTG
>KRH96536.1
MNELTKSDLRERLGNIDQIRDILIGPHLREYNNRLEQLERGLASVQYELRNRGEETRQTI
LVELNATADLLEKKIRNLIIKDEEEKFNLNQQIDNINKRILTATQELQDSVTAELDELAE
NADKRFKSIQTKDEEEKLEIRQQIELVNKRLGGNIETVKETFQQQTKTLQENLQATRSKL
QDEITELRAQTIEELERYSSILTQEKVSKDDMAELLFELGLRLQGKEFVPELQQVANFTE
ELQTIITESPDQSATDLIPENPPEVTSESKPTVSSSSPRPKVEGSTPRRTRTSRLKGGN
>KRH96541.1
MNRIKKALITGITGQDGSYLSEFLLEKGYEVHGIIRRTSTFNTDRIDHIYEDPHQQGARF

FLHYGDLTDGTTLRRILEEVKPVEIYNLGAQSHVRVSFDSPEYTVDSVAMGTLRLLEAVR
DYQNRTGIQVRFYQAGSSEMYGLVQAVPQSETTPFYPRSPYACAKVYAHWQTINYRESYG
LFACNGILFNHESPRRGETFVTRKITRAVSRIVAGKQNKLYMGNLDAKRDWGYAKDYVRA
MWLMLQKEQPDDYVIATGETHSVREFLDLAFSYVNLKWEDYVEFDQRYLRPAEVDLLIGD
STKARQKLSWQPSVTFEQLVALMVEADLQALGLNSPHSSSLQIPWDMAIIRHEIGALHF
>KRH96543.1
MRILVIGGTRFIGVYLTQLLIKAGHEVVLFNRGNHPAPDGVGQIIGDRTDPSQLSKLSQE
SFDVIFDNNGRELTDTEPLAKMFQGRVKQFVYMSSAGVYLKSDQLPHVEGDTIDPKSRHR
GKHETESFLQQLGIPFTSIRPTYIYGPKNYNPLESWFFDRIVRDRPIPIPGNGLHITQLG
HVQDLAQAMLQVIGNETAIGKIYNVSGDRFVTFDGLARACAIAAGKSADSVKIVHYDPKK
FDFGKRKAFPMRAQHFFASVNRAITELNWQPQYDLISGLQDSFQNDYLTGGAAQGEIDFS
VDDEILA
>KRH96544.1
MPTSLHTTPYLMAQTFHALSDPIRISVIELLRHRELCVCDLSNALGISQSKLSFHLKILK
QTGLVVNRQEGRWIYYSLNLYQFQILEQYLQDIRVNSPVLPLTSFCE
>KRH96546.1
MKATAVRLLITVGMLTLATSCTADVNKNSSVEATKDVNSQIARKIKIDGSSTVYPITQAI
VKEFTKDGKNSSSVEVSVSGTGGGFKKFCSNETDINNASRPISAKEMAECNKNGVRYIEI
PIAFDALTVVVNPQNNWVEDMTVDELKKTWEPQSQGKITKWNQIRASWPDRPLKLYGPGR
DSGTYDYFVEAIIGREKASRNDYTASEDDNVLVEGVNKDINALGYFGYAYYEKNQDKLKL
VAIDNKKGPVLPSQETVGKSRYQPLSRPLFIYVNLWSGEKRSKIYQFVDFYLKKAPAIVN
SVGSVPLPEEAYKIGYVHLHNGKAGTVFGGKSEFDLTIGQLLRKQKEF
>KRH96548.1
MNHTADSGANLRNYAVVTLAYWGFTITDGALRMLVLLYFNQIGYTPLQIAFLFLFYEIFG
IVTNFLGGWVGSQLGLKVTLYSGIGLQVFSLFMLSWLNTDWVQWLAVLYVMVAQAFSGIA
KDLTKMSSKSAIRLVVPQDAQSSLFKWVAILTGSKNALKGIGFFVGSALLSLFGFVNALL
IMAGALFLIMFTGLLLPKGMGKIKKKVKFSQLFSKSEEINILSAARFFLFGSRDVWFVVG
LPVFLREVLGWSFYQVGGFLATWVIGYGIVQFFAPNLMKKFGSGRAPTAKNIQFWTFALT
AVPCVIALALQSGLPANIVMISGLMLFGVVFAFNSAVHSYLVLAFTDDDKVALNVGFYYM
ANSGGRLAGTVLSGLVYQLFGLVGCLWTSMIFVLGAGLVSLKLPNPNPSKDIVWKGGEGD
>KRH96551.1
MKIWVNEQIDPSGMIHACIACCDEDQAKECHNSFSHSLTEGQKAAGWIAKLRTVETWDEV
PVNALKLN
>KRH96556.1
METVIHDLTNQLQLTLVITYTLMGGYFFANWFTFSHRHPASTPEEKFLSLIISLVTTVFW
PMIIPISCVKIFKTRQLRSDTVIPLLLVLLGLTISYISCQLLKLIYLPT
>KRH96562.1
MKRRILVVDNEQYIQEVAKICLETVAGWEVLTASSGKEGILQAENHQPDAILLDVMMPDM
DGLSAFKKLQANPATQTIPVILLTAKIQAADRRRYSQLGIKSAIAKPFNPLELAHQVAAE
LDWNLECNKTK
>KRH96564.1
MLSKILVGLDLSSTGEEVFQQALNLAKLTSAELMLIHVLSPEEDGIPDTMMFSQIDYYPA
WTDESMGIYLKKLEAYKEEGLEMLQGFCARANTENIKTEFSQNVGNPGKVICQVAGAWGA
DLIIIGRRGVSKITEFFMGSVSNYVLHHAPCSVHIVHHPDKKS
>KRH96567.1
MTVKTLTIDGQMVSAHEHNTLLEAAQESGIHIPTLCHLDGVGDVGACRLCLVEIAGMNKL
LPACVTKVSEGMEVTTKSDRLQRYRRTITEMLFAEGNHTCSVCVANGNCELQDLAIEMGM
DHVRLAYQFPQRSVDTSHERFGIDHNRCVLCTRCVRVCDEIEGAHTWDMAGRGTNSHLIT
DLNQPWGTSLTCTSCGKCVNACPTGALFTKGVSVEEMKHDRGKIEFLVTAREKQQWNF
>KRH96570.1
MKKIVIDPVTRIEGHAKISIYLDDEGHVDDARFHVTEFRGFEKFCVGRPFPEMPGITARI
CGICPVSHLLASAKTGDKILSVTIPEVATKLRRLMNLGQIIQSHALSFFHLSAPDLLLGM

DSEPAKRNIFGLIGTDPELARGGIRLRQFGQEIIELLGGKKIHPAWAVPGGVSDPLTSEN
RTHIQNRIPEAKATVLNAIGLFKNLLDNYQKEAQTFGNFPSLFMGLVTAQGLWETYDGHL
RFVDSGGNIVADKLDVDNYHEFIGEAVQEDSYLKSPYYRPLGYPDGKDHCRLDSGMYRVG
PLARLNICSQIGTPLADQELREFRSYGSGTVKSSFFYHYARLIEILASIEHIEILLDDPD
ILSTRLRAAAGINRLEAVGVSEAPRGTLFHHYRIDENGLMQKVNLIIATGQNNLAMNRTV
AQIARHFIQGSQVQEGMLNRVEAGIRAFDPCLSCSTHAFGKMPLVIDLVDRNGNVVNQVC
RN
>KRH96571.1
MTKRLIGLTGGIATGKSTVANYLASVYGLPILDADIYARDAVSESSVILSQITQRYGKEI
LLTDGNLNRKKLAEIIFNQSPERSWVENLIHPYVRNCFLKTIEESPHDTLVLVIPLLFEA
GLENLVNEIWVVYCQGEIQKQRLMSRNDLTGVQAMARINSQLPLEEKVARADVVLDNSSD
LESLLRQVDLSIARFH
>KRH96574.1
MFFNPAIFPFTSALEASWPDIRKELEQLQPADFIDWPERNIYNHGWGVLGLYAFGQRLEE
NCRLCPKTAGVVENIPGMITAGFSSLAPGTYIGPHFGVSKAVLRCHLGVVVPDNNCAIRV
DKETKNWQEGKCLVFDDTYEHEAWNRSNKTRIVLLVDFMRSNPTSEELVTGDQNVDHDYW
INILRQKDVNLK
>KRH96575.1
MKLEDTKSLVTGAASGIGRCIALELASAGATVVGGDVDMEGLKSLELEAKELPGKIYVLQ
LDVANESNVKEFIFAACEKIGHPNTLINNAGILRDGLLVTQDEDGWLRKLPTAQWKRVID
VNLTGAFFMAREFAAVAIEQNVSPALIVNISSVTRSGNPGQSNYSASKAGLDADTRTWAL
ELAPFGFRVAGVAPGLTNTPILSRVSPTALADMTGSIPLKRIAEPYEIWQAVRFIIECDY
FTASVIDVDGGARF
>KRH96577.1
MNQILPLSINQKEIYVDQIMSPASCHMHIGATVTVRGIFDRQILNYAMSKTIDCHPGLKT
RIYELDGEPFQTIAIHTSNHIPFIDFSGHDNSDEQAENYINQEFIKPLTFGENAPLADFQ
LIKVCDDKHIVYAKYHHVITDGWGAAIFFREVIKTYNQILQEGREDQETRDWVITEYIEE
ETKYLASDIFMRDRHYWQQRLNNLSPMIFHPIKQPQELDGKRHSIYIPRHQYDQVDELCK
NVKSNVFHFILSLIAIYVSKHYLKNDVVVGLSLLNRSKNIFKDAIGMFVSTIPFRLEVEQ
ENTIHQLLDKIRYSLRQDYRHQKFPVAEMKQLSGLKATSKQHLFEVFLSYERHDYADNFL
GTKTTCVPLYSQQQKVPLIIYVREYEKTDDVKIDFDYNLSYLDGEAVGEIVRSFETLFTQ
AATNLEISIGDLAICDSETINISQPDSPSTKFFADDTETLVSAFEKVVSQYPQNLAVQFD
GELYKKFLSYTELNDQANRLANYLISQGVKPGSRVGICLERSEQIIVAILAIIKTGSAYV
PIDPHAPSVRRQFIVQDSGMTTLITETSLIAELVTENISTLTIESINLALAKQANTLPRI
SIKPDFPAYIIYTSGSTGTPKGCVVTHKNAIRLMRATEPWFGFNEKDIWTLFHSFAFDFS
VWELWGALLYGGKVIVVPFWLSRNPEKFREFLSTEKVTVLNQTPSAFYQLIHADQSSVQD
IYLRYIMFGGEALNIQSLQPWLERYGDKKPYLINMYGITETTVHVTYRPITRQDLKVRGS
FIGKEIADLHIYLLDEKLSPVADGIPGEIYVGGAGVTNGYLNRPALTAERFLPNPFGSGR
MYRSGDLAKRLPNGDLEYLGRIDQQVKIRGFRIELGEIQAALISHYQVREAVVITDEWEE
EKRLVAYYVPDESSPTAHELRQYLKNKLPDYMIPAAYVKLEVLPLNVNGKVDIKALPMPD
WNLLRVEEDYIAPRNLDEEILCTIVAEILGLQKVGIDDNFFEIGGDSILALQVVARAKNA
GFLISAGELYELATVRYLATKKAIITSGDGVKKLPNIGSALVSDADKLLLPQDVGDAYPL
SSLQSGMLYHSELDPDSAIFHQIFTFDLQIGYSELAWKQAIADICSANPVLRTSFHWTGY
SQPLQMVHEQVELPLSVVDLRGCDNANEQIREWIELEKNHNFDITQAPLFRLQIHRVSDL
KLSFSFSFHHVILDGWSVATLLTQLLRRYVEYLATENLPPLPVTQISYKDFIAQEQNVIT
NHTVREFWLQHLRNLQVTFLPRLSTNTAKTTAASHQKRQLKRMSVLVDTKLAEKLRQITK
NLGVPLKTSLLLALHLRVLSFITGQKQVVTGNVINARPETSGSENLLGLFVNTIPFRLELP
QGNWLDLVRAVFRLETEILPHRTFPLAEIQRVLDQRPLFDVGFNYVHFHVYEGLLNLPQI
QVENVDIFEETDFPFLTEFCLVPGSAALQLNLIYDTQQFADTQVDQYANYYQAAMFDMVT
NPQTPYHRRSLISSQERQHLIQSANQNLKDFVSSQTLVSAFNQTVAKHANKTALVYQQTS
LSFGELEIQANRLAHYLQAKGIGPETLVGLCLERSEQLIISILGILKAGGAYVPIDPAYP
SDRLEFLFRDSGIMLLITQRSVISQLPECGAEMIILEDIAEEIEQENSQAPEVNILPQNA

AYVIYTSGSTGQPKGCIVTHANVIRLFNSTETWFNFDSEDIWTLFHSYAFDFSVWEMWGG
LLYGGQVVVVPHWTVRSPKEFLQMLATHRVTVLNQTPSAFKQLISVVRQKPEKLSLRYVI
FGGEALELADLQPWIDLYGHTQPELINMYGITETTVHVTYRPITQKDIINNLNHRPSVIG
QSIPDLELYILDENLDPTPTGVTGEIYIGGAGVTRGYLQQPGLTAQRFIPHAHRPGSRLY
RSGDLARYLPDGEIQYLGRADQQIKIRGFRIELEEIQLVITSHPDVKQALVVCQKSPTGE
NRIVAYVIFSGVAQPQNDLGKFLKTKLPDYMVPSVFVPIETIPLTINGKVDYAALPVHNW
NSIKKDYIAPRNDREATICSLMASLLKLERVGVDDDFFEIGGDSLLVTQLAISLRQTYDT
EFPLPELFTHRTPGEIALLVGDESPALPEIEIPKASRTRRSVTLTDDGILSKY
>KRH96578.1
MSKIPQIIHQIFFLGAAAVPEKYRRYQQTVLQNHPHWQYQFWDEQKARGFMTDNYPWFLP
VFDAYPHDIQRRDAIRYFILYHYGGFYLDMDVESIKPLDNLLADFELILSKLVGFSNAIM
GSIPKHPLWLKVFEELKNRQHNSDHKIMPLYVGHSTGPIMLNDCVVGGKFDQNSNVLVCP
GYIFEPGAPMELNGKIFKSRVNPETYTIHHMTTSWLPKKHQIARFLFGLMLEPYWFFRSL
FKGKI
>KRH96587.1
MSHITKLLQTVNELRNFIIFWLGQSVSEIGNRLTGFGLGIWVYQNTHEVAGLGLVVFFTT
LPGVLITPFVGALVDRWNRKWTIIFSDLAAATVTLTLIVLLLTNNLQIWHTYITAFCTSV
CGSFQMIAKGAALPMMVKKHQIGRANGLIHFSTALGQLTAPILAGILLSSIQIQGLLMVD
LSSYFVGLLTLLIIDIPQPEPIIQSNQSVRVNTIIHDIAYGWNAVVENNTLLILLAFMSI
HFLVDGMTTVLINPLVLSFSSTKSFSVIMSIAACGMVGGSLSMTLWGGGKKHTSTLFTFT
ALNGVGLVIAGFSPSIPIIALGLFISFFSLPITLSTNSTIWQSSVNGNIQGRVLSLFNTV
IGLALAIGNLTASPLSDDLFEPMLLEGGLLSTSMGSIIGTGEGRGIGLLIIMAGMILFCL
SIIFYTHFNWKDKNLETNDEFSISAKEVVKDS
>KRH96590.1
MIIVFDIDGVIRDVSGSYRRAMADTVEYFTNYAYRPTVTDIDDLKSEGIWNNDWEGSQEL
IYRYFVSQGQQREDLQLDYESIVAYFQSRYRGTDTENWNGYICHEPLLLEPSYLESLTQA
NISWGFFSGATRGSASYVLERRLGLKSPVLIAMEDAPGKPDPTGLFATINLLEIAKQDQN
KKQPVIYVGDTVADMHTVAKAKILDSSRTWVAVGVLPPHVQETPSHRDAYQKIMLQAGAN
ILLTNVQELTPSKIAELVNIYI
>KRH96591.1
MFKVYPKQLLTYYLQEQFRYYHLVFLGLLALSGAYSITYLMGQNQVKSSLNPPSVQVAKV
VTNRPSSIKVAVVKINKQKAVKSVWNLPQVQRKAKEIKTLSQGTIDVGVVVSSYPKTSQP
FYTVKVLENHPDSTTSPVYWFRVSSSNGAIQPLDLVSNKYTNLTNWNPDGI
>KRH96596.1
MVTTAEKTNIGYITQVIGPVVDVKYPGGKLPQIYNALTINGTNEAGQEINLTVEVQQLLG
DNQVRAVAMSTTDGLVRGLEVVDTGAPISVPVGKATLGRIFNVLGEPVDNQGPVNNEETL
PIHRESPKLTDLETKPSVFETGIKVVDLLTPYRRGGKIGLFGGAGVGKTVIMMELINNIA
TQHGGVSVFAGVGERTREGNDLYNEMIESGVINKENLNESKIALVYGQMNEPPGARMRVG
LSGLTMAEYFRDVNKQDVLLFVDNIFRFVQAGSEVSALLGRMPSAVGYQPTLGTDVGALQ
ERITSTTEGSITSIQAVYVPADDLTDPAPATTFAHLDGTTVLSRGLAAKGIYPAVDPLGS
TSTMLQPSIVGKEHYDIARSVQSTLQRYKELQDIIAILGLDELSEDDRLIVARARKIERF
LSQPFFVAEVFTGSPGKYVKLEETIKGFQKILAGELDDLPEQAFYMVGDINEAIAKAQKL
KG
>KRH96603.1
MKLDYDIIIIGDSIAGYEAALYAAQLHAKVALVKSQPTYQLNYVYPLRELSRISYQYLEM
MGLGICADQPPSTQVLKQLQQPERCLISSYQRAIFYAQGMESQFNLINSLDNLATQGVDI
IIGRGEFVNSRKPSFRVQGRTLRGSRYLLACGSVTKIPHIENLATTGYVTLTNIWHYLVS
TNLPKHWVIIGGLPQSIEIAQTLAHLGCHIDLVLNHPTVLSHLEPEIVELLIAQLEAEGV
SIFREQPVTQTTKIKGQKWVQLKDQAIETDEILIANHQQPKIENLNLASPRIKSSSDRLI
TNDKLQTTNPNIYACGDIIGGYDMENIAKYEAKIAVKNALFFPRHRVNYSAIPWIISTQP
IVARVGITELQAKKTYNKNRVLVFKNYFKTTTAGQIKNETTGICKLIVLENGKILGCSIF
GQEAEEIINLIGLAIAQNLKIDNLENLAVVYPSCSEILTQTAREWSTNKLHKNHLLQEFL

QSFLHFRREWKF
>KRH96607.1
MTISIREKQPKPNLSLQLQRFWEILYVLVVRTLKVRYRGSILGVYWSLLNPLIMTSLYTA
IFGATFSSYYNNSITNYVLAAFTGLVVINFFSASTTQCLFSVVENSSLLNKIRLPVSVFP
VSMVASNIFQFSVGTFPLLAVMTLLNTRNVVNVLAMVFPLFALVLVCMGVGFLVSTLYVF
FRDLHYFYELVVFVLWISSPVFYPAAIVPPQVRPFLGLNPLSPIIESLRQITLSGSPPDL
GLIWGALLSGMIILSFGWTCFHLWRHQFMDLL
>KRH96608.1
MLSSMFDFLIVGAGFAGCTLANCIATHLDKKVLVIDTRSHIGGNAYDCYDKAGILIHRYG
SHIFHTNSKKIIDYLSRFTEWRVYQHEVLARVDGELYPIPINLNTINKLYGFNFNSQQLS
DFYEQIRERYERIENSEQAVVGKVGTDLYERFFKNYTYKQWNLWPHELDASVCARIPVRT
NKDNRYFADKYQMMPVDGYTKMFERMLDNPNIKFMLNTSFQEVEKWLKFDHLIYTGPIDQ
FFDYKFGRLPYRSLKFQFETHDIEYFQPVAVVNYPNDYDFTRIVESKHITGQKHSKTTIY
YEFPQSEGDPYYPVPRPENRELFQKYKDEADKLQTVTFVGRLAQYQYYNMDQVVAAALTV
FENRISKLY
>KRH96609.1
MSIKQVAIIVVTWNKIRDVSFLIEDIKKLDLNEISINVFVVDNNSSDGTETHLNKYYSFV
KVLQTGDNLGGSGGFSYGMDYVKELEYDYVWLLDNDVRLDTQSLLNLVDTLNNYPDIGLV
GSQIRKLDQPNIIQDLGMFIYYKKAHVKGNFSDNTIESAINNLSHLNSQTGYIDVDFCSA
ASLLVRKEVIQQIGVFEDYFLHFDDTEWCLRAKKVGWSVVVNPSSIVWHSSPDFKQRPWI
SYYDERNLCYCWQKYFPDLLLKRIRVLLPKLIYYSITGRFFWVSTHLMGLDDFISGIKGR
MPAPLPYKQLSIEEVLDNNLRVTVQASLYQDQFESQILDQINSLIDKERFTVFNKNKSLL
YTSYMWLLSCFHKSFDLALVSCYQTEVWQFNLARKVYFFTGSGYVLVNTSIINLARAAIT
TFLRLLKIYWQIHKLTSKKSIMLDLNRIM
>KRH96610.1
MSITVSIILVNYNGADVLVDCLKSINKFVPGYNCEVIVVDNASQDNSIDIIENKFPNVQL
IKLTKNVGFGAGNNAGAKVAKGEFLFLLNTDTIITSNILPHLVDLMEKNPTVGIIGPKLL
FPDGRFQISFSPKISIKGELEARKLHRYAESRTKLALLENNFQEIQEVDIVVGAAFFIRG
DLFRTLGGFDEKFFMYFEESDLCQRAKNIGYQILYTPKISLIHIRGYSTSKLANPMAVEY
RRSQLYYYQKHRPLWERLTLRIYLLISFLYKFTITSNPYCWQIIILVLKPK
>KRH96615.1
MVGALALVLVFFVGTCWYCLVEGWSWEDAAYMTVITLATVGYGETNPLGSRGRLFTIALI
LMGVINIGYIVNRFTQAIIEGYFQQGIKLRQRRRLMESLVDHYIICGFSRTGRQIAKEFQ
AESVSFVVVDSDMESVQQAESQGYMVYQGDATLDDTLSKVGIQRAVCIVAALPSDAENLY
TVLSAKTLNPQIRAIARASTEESLQKLQRGGADTVISPYITGGKRMAAAALRPQVLDFVD
GILSGTDRQLYMEEFLLDGDCPFLGQTLQKAKLRSQTGALVLAIRRSDGNLIGGPTGDTI
LISGDTLICMGTAEQLRGLNQVLAPMNSRELRRPKHI
>KRH96616.1
MFDLIKGPLLRATLYKISNKEHILLVNLHHIISDGWSLGIFIQELTQLYVSRAEISLPEL
PIQYGDYAEWQETYLQTQRIQDQLAYWQKKLALPLPILDLPSDKNRPALQTFSGAVLRKT
LPRDLIDSLEAVAAKEGVTFFMVTLAVYQILLFRYSGQTDIIIGTPCANRNRGELKNLIG
CFINTLPIVCSLTGQLSFKQVLQQVASTCVEAFANQEVPLEIIIDKLNITRDPAHSQVFQ
SLFALQNAPIEEIKLPGLTVQPVYLDNGGAKFDLSLMLEPTFEKGWIAALEYNTDLFTAH
TAEDILRHYEQLLSAVIGNLDTKIDTLPWLNTEERRELLSFGSSASDQEVEATNLVDVFS
HIVNTYGEKVALIESERTLTYQKLNQISNQLASHLIEKGVGAETRVGIFQERSLELVASI
LAVLKCGATYVPLDPQYPAERLNFIAQDSGIRLVITTEPLRAKIPGEIPELLLIDTMKPT
NQLVHPISKIFPEQAAYIIYTSGSTGKPKGCLVTHKNVLRLMRNTQKWFEFNEKDVWTMF
HSFAFDFSVWEMWGALLYGGKLVIVPYLESRSPHDFRQLLATQQVTILNQTPSAFRQLIR
ADQEFTHPLNNLRAIIFGGEALELQSLKPWIERYGDSHPRLINMYGITETTVHVTYRQIL
AEDILENRGSVIGIPIPDLCLYILDSYFEPVPYGVAGEIYVGGMGVSRGYLNRPSLTAER
FIPNPISQKPGARLYRTGDLARRLRNGDIEYLGRCDSQVKIRGFRIELAEIEAAINKLTQ
VSESVVTVHSTSDQDRRLVAYIVLRSINNNEIDRNELRKALKESLPDYMVPAAFVFLDAI

PLTNQGKINRGALPLPDWDQSTTKRSFIPPETDAQKALCHIWQRVLGIEEIGIEDNFFDL
GGDSILALRVITEMRSQGWILTPKQIFEQQTVQRLALAIQEQTDLQSFAAAENNNIPTGE
VPLSPIQQWFFDLKLSNPHHWNQTLLLQVDPSLQVTQVATAIKVVFAYHDNFRLRFQKES
QGWRQFYVEKDQDDGFPWEVVDLDLKSEIEQNSIMKEVREKSEKSLNLTEGPVYMVKWFN
LGANRPSQLLIVIHHLIIDGVSWRILLQDLVDVIGGNKLSFKTTSWQKWCEFIHGYVNSH
NIQSEKLFWQNTLARKSAKLPLDFPESLAENLEYSVSTVSCQLTAKETLTLLTTANKTYR
TNPQELLIAALGKTLADITQNSYVQIMLEGHGREELSSDLDVTRTLGWFTTLYPVSLDLP
KGDSQTEAIREIIKSVKEQLRAVPQRGFGYGILKYLDQETKPLLASAVEISFNYLGQVRN
EGGARHKFFTLLNSQGLPTRDPQATRPHIIDVNAIVVEGELRIDWLYSSNLHQASTINRW
VSDFKSNLLKVVNFCTEVGVGEYTPTDFTLAQLNQSKLDILQNQYPHLEDIYPLSPLQQG
MLFHAIYEPDQGIYFEQVTGKIIGKLDVDKFAYAWQVIVDRHPVLRTCFVWEEQDQPLQI
VNKGTGFSLIYKEWRDLSPAQQIEKMGEYLVKDKEQGFNLNDSPLMRFTLICLDDSTWQW
LWSHHHIILDGWSLPVIFKEVLSVYQSATERVPHSLLPVPPYRHYIQWLTGRDQLRAKQF
WQKYLVGISTGTRLAWEIPDLENNSSAYREVELRLDDLEFDLVQKMAQSQRLTLNTIVQG
AWAMCLQKHGAGEDVMFGVTVAGRPPELSNMENMVGLFINTLPMRVKIDPSLSLTDWLQN
IQQQHLEMREYEYSKLADIQKDLNLAGLALFESLLVFENYPVDQNLKKQLPDFRVDDIQF
YERTNYPLTVGVIPDQGLLLKLNYQTKFLSGNAAEKMISRFRHIIVNMAFKPEQTLAKVP
ALSISDREELTTCNQGNTIIEGGFKTAHQLFEYYADLQPDSIAVVCDEYSITYGELENRA
NNLATQLVDTGIAHESIVGLYFDPGIEYIISLLAVLKVGGAFLPLDRSYPDHRLRFIIEN
SQVSVILTNNIIGLPLQENLQVLDIRNLESKGNHPRLNLKVRPENLAYVIYTSGSTGNPK
GVLVTHSGIQNLVQCQTMSFGVTAKSRVYQFAALNFDSAIAEIFMSLGSGAVLYIQSQAH
RSPGSELWQKLTNWEITHVTLPPSLVASIDTQDLPRLQTLILAGEAVSGDLLRRWGRGQR
RCFNAYGPTEATVCASLIDCTHLLGEPSIGKGIPNVEIYLLDSFLEPVVPGVIGEIYIGG
IGLARGYLQRPGLTAALFIPHPFTKTPGSRLYKTGDRAVYDPEGNIRFIGRYDNQVKING
YRIELGEIEAALTKHEAVESALAVLRTDLIGRNRILGYALIKPVEGREQPNDLKEHLAKI
LPDYMIPGAVIVVDEWPLTPNGKIDRQRLATPDFSSTEVIPKTDIEQIFAQIWIELLGLE
TVNPQDNFFEMGGDSIISLQMVTRARAAGWEISPKDIFEGQTLSRIATRAKLMETQTEIV
EPLTGFIPLSPIQNWFFAQNFLHPHHWNQSVALTCKQPVNTDALVVALHTLVSHHDIFRI
GFAENEGEWQQFYVGEAKSPNVKIIDFSHYLPTTHLAFLNSALETEHSSFKLDRPPLIRI
LCGRNLNNYGDVLFIFAHHLIIDAVSWRILLEDLNQAYQQTIDQLDQLQQPNSPLSLPVK
TASYRSWTTHLHSLASSKMTQDTPFWKDILNAVITPLPVDKSGNNSVDSTAIISTYLTAE
QTVTLIKQATVTYHASVQEIMLAALLNTLISIYKSDQWLIDLEGHGREDIGHNLDLSRTV
GWFTCLYPILLKQPTNPDNHEILLKEIKNQLRTIPHHGISFGLLRYLNLNQPLKESKNAD
ISFNYLGSLDSPLGDNHNFSVSDAPVGAGVFALQQRTHILAINAKIQDKILQVEWSYSRN
IHYDQTIENIAKTYLQSLGLYLTNSDSANSSFYSASDFQLVDLSESELGELLEDLE
>KRH96619.1
MTKQVESKESNFYTASSEKKKINIQNIIFPKLDICTVEELFFRGDDGVLFDYQNRTLGIK
KDTVVSFNTYFNGFFANKWKSYTSITNIGVCLSLQGSFNIHIYSVDSFVQSRTIVMQKTV
KNAHFDSPIVLENFDIYPYNGMIYIEVQALSNNCQMEGGFFYSYVQEIKDIRLGIVICTY
KRETYVRKNIDLLEKNLLSRDEYRDKLKIFVIDNGKTLSSFDNPLIEIIPNKNAGGSGGF
ARGMIEVANHHRGFSHILLMDDDVLFDPEVILRLSNFLSVINQDDICVGGDMLRLDKKHI
QHERGGYWNKLRGCTPVKYNLDLTVLENILFNEIEEYCEYNAWWLYCFPVDSIKKIGLPY
PFFIRLDDVEFSKRINNKIIALNGICVWHEQFENKHSPVTEYYNIRNGLIFNSLYYEKNA
SIFSHLSWFLLPTIRHLFCYRYETAEYVLQAASDFLCGPENLFSQNPQQNHSKLSLCAEK
TRRNKNGVSPFIMKKYMESINENENLLHRIWRVFTLNGHILPRSFFWDDRNLTDKGYKVV
SSYGSKPLNVFRAKTIIYYNIETQESFAVQFSRTRFFRILFHSIYLGILMLLKYGRLAKL
YKTTLGKFTSQSFWEEYLELKKQF
>KRH96622.1
MQLVKTHPIYKNDKYWQGCDLICFRAKNLYNLCTYYLRQSFFKTGKILSSGKLYSLVFSS
RAYQEMAVTHRGLSVIRQVLQRWRNYIRSWQDWQANPSKYVDPPKIPNYKHKIKGRFPIV
VYNVYQGCPTMDQPSLVQGMCQLFEGLFEGDVNDSIGKARKLVEAMIIPRNNGLYLLKFI
YEVQEPTTRSTRVIAGIDLGVNNLVALTTNSSTIRPLLVNGKPLKSLNRLFNRKLDLIGS

YQSESQPSQRLGGITTKHHNRLDNYLHQTSSIVINYLKSNGIKTLVVGKNDEWEKETRMG
TSKRHNFMPIPHSRLIDILKHKCRLAQIELITVNEAYTSKCSAWDFEPIAKHQKYLGERL
HRGLFRSANGQVINADLNSSLNIIRMYSSEALTAERIGSCGVQPLKVNPLARVKQI
>KRH96624.1
MIPHLQQLLATQDSVISQLLRFNLYGLKETLGQAKTAYPADPGYDLCSQVIGEIDGLLQL
NSVGEFEDVELAIHDSTSGQLLTELGEIFNSDLEVRFYLGDQPLHSDNDCDLWNEIHHKL
LRLPEDLATIWKNRSLEIAKKLGALEDYVHLRYLPFVRDEIIYPGLNGTIVSPGLSLSKK
ALLKSDLAQLHNSDNWHFLTSFLSLYTSFIDIDPDLHHALKSVFSFDIISFQSHPEQKQL
YLESFSDRLQRHQKLNDDSSSIINLRSWLDLDEAIHSLVFVPPAERYSWWGKLQQESRRI
LKKMADQATKAGNNIKIKQLSGLYADICQYTKDDLQLEDGGTPGEVLSCLRLYAKINEEE
YPGRVIYRAC
>KRH96626.1
MYFTWLDSNSWLLEIGGWRILLDPWLVGDLTFNNVDWLFKSYRLQDRPIPNNIDLILLSQ
GLEDHAHPPTLKQLDRHIPVLGSPQAAKVVEKLGYYQVKTLHHGESFTLEDTLKNSTLKD
QLEIKALPGSPVGPNVRENGYVIRNISNNVGLYYEPHGYHSSALEELSPVDVVITPIINL
SLPLLGPVIKGMNSALEVAKLLKPQIMLPTAAGGDVFFDGILSKVLQAKGNVAEFKELLE
LNSLSTQVIEPKPGERISV
>KRH96628.1
MLSKLEDRFWVRSMGIAIPKVIYPGIYGELGVVNQTRFGHQIHDYEKLSVKINKEMLIFF
FRFYILNFTE
>KRH96629.1
MVVAQSSNQGKMYSLLMMKSFLIWTFTLAVCLLVVGFPLVVVMATVGCLLSIVLQSVMPA
SAVLLVAGTLVIFNVMSVLIVAGVLTAKSVHPKEVKWLSWLYGDAERVQTTVYASCPLTC
EIK
>KRH96634.1
MARGEIIRKLFKSFSRNEREEFLAAAQELIEEERNKNHILLARDLEKLLHNGYTKPLASN
LAPLTKFPQPPKDKDTGLALLDVKQFDLTWDHIVLSEKIFDILQEIVLENRKQDILAAYN
LKPKNKLLFCGPPGCGKTQTAKILSSVLSLPLVYVNLTAVFSSYLGETATNLQKIFTYIE
QGEWLVLFDEFDAIARDRDNLNEHGEVKRLVNSLLQLIDNANNQSVFVAATNHEKLLDSA
VWRRFDEVIFFDNPTVELRTALLNRYLSGIRYTVVNLSTFAERLENATGADIERICSDAI
KAVILRGERTLTADDLEVAIGRFLERQSIIANSK
>KRH96640.1
MTTKKQFNSFEEMLSGSDVPVLVDFYAEWCGPCQMMAPILEQVNTHFQGQLRVVKIDTEK
YSQLATQYRIEALPTLILFKNGQPVNKIEGVVQAPQLIERLKTLI
>KRH96644.1
MTNTWWEIQILCTPDLEDSIFWRLETFGCRGVAVEKKDRFILIKAYLSTLQAQLSDLTNL
STLLHEDSIAIGLPTPELNWHQINEEDWSTSWKQYWHPQEIGNLFLINPAWLPIPPSTSR
LVIRLDPGVAFGTGNHATTQLCLESLEKYLTQNSIHTSITAPQVIADIGCGSGILGIGAL
LLGAKKVYGVDNDPLAVESTNSNCILNHLNPEKLTCALGSVHTLTEILTESLDGIVCNIL
ADVIIELIPQMTDLVKPGSWGIFSGILVEQSPSVITTLEKNNWVVDKVWQRQEWCCLNAR
R
>KRH96654.1
MKFSATVLATFIAVSAGWHPSMVRAVPVESGDNPPNPPTIPASDNPSDSEPTGDESYNGT
KFACVSQGNGKWATVGQRPGGQPIPVIIWTQSASKYFGSQFAPENRCQIVTRKFNTAVQE
SGGRLQDVLLISGNVNGQVVICVISDRDNRCQDKNTLFTLKPENAKNPDKVLSQLLEISK
KGSEGGVITETRSSRQRVTVKLSDVLAARRSSSSPVKRPKVQNNRRNQPGL
>KRH96656.1
MNFKLGITNNSLLVTTLITAATAVLSTTSIAADTISIQDIAQIAKKTSVQINTEGDVTPG
GSGVIIAKQGNRYSVLTANHVVCDIIDRPGKILCAKDITYSVRTNDGKEYPIKSKDIVVL
QSTKNDPDLALVSFVATQEYPTANVGDSDQMTEASDVFVGGFPAVFNKIGSARDFTFTKG
IVLSRGRTSINGYSLIYDAKTLTGNSGGPVFDIKGRVVGIHGLADTSAKSKTETGEIVSQ
KTGFNAGIPINTFLNFNNPLLKELPIQRNTTATGEVPQQRLNSPQSARDFYARGITKLDQ

FSYKESLDDFDQAIKIDPKYAEAYFKRGYALSWLRRYEEALSDFNQVIALDPNYLDGYLN
RGWTYIWLQNDQAALEDFNRAIRLNPSYSIAYAHQGMAYIKLGKYQAALESSKQAIRLDP
NNSYGYTIQSDVFNYLKDYAASIKVSTLAIIIDPDDFNAYINRATAYTLTGNFSAALVDY
QKSAEIFERRYTKKPSPATDNSRTTPKP
>KRH96657.1
MVECPVRSNILNSTQPYFTSGYNHRGLVFSAISLTLPSILLTLVLSPDLVFAEEGVTNQN
GYETRSVPPQISQAENISPEEVRRIRQELLIEPLVKTKPREVQKPTYPPGLTFAGVSAFG
ANTGDVFIGITGTTPGKLRDGQYDGGINLGAGIGESNKLAALELGFSNNSIRKFGANGSF
DLKVHRLVYAQGNNQVAVAAGWNYFAQYGTDGVTPSGVYGVVTSYSLLQPDDEVNKMPIS
VSFGAGGGPFRQEFASTGVFGSVGMQVPQLGVGFGWSGVGFNLGASYVPIPTLPLTITA
QGADITNNSPGGTILSLSVSYGFNFLPK
>KRH96658.1
MLKPKSLIFTLSLVPFLLASLQLTSEGGSTTPGGPSGNVLPSPPSIPQDPVQPTQPPPPN
PQDPVQRPQPERPNPQDPVQPPQPERPTGKTTPGGSSGGSGVTFIPNAESIIVTRQGQSA
TLAVTQEVQSALNQLVPNIVQVLGTSANTTDLVTLVLARPGVTEAAARITAALTSAGISP
QLAIALVNALNGLFSPSTASSPGAMLVSTKDWKGNNTVAQAGAAFSVNIDKLSAAITAYN
GIVIESKPPTLKRLSQDASFVGIGRTLKQLRNAIP
>KRH96661.1
MTPLINQALSLVYTQLSYFSESSNYYQVLSTAFGNNYDRDIAEKLKLHWQQVNFTQIPQI
EILDGGILGSAYGAYARETNKIYLSRNFVTNNTAEVIGRVILEEVGHFVDGQLNVEDSEG
DEGAIFAELVVGNSLDDQALRKLRTESDFGIITLNGQQVHVEQSSSLSLPGVRFSSVAWA
DYDGDKKQDFLLTGYSSSGPIAKLYINTVNGFQEDTTVSLPGVEYSSVAWADYDKDGDQD
FLLTGFSSSGPIAKLYRNTGNGFEEDTNVSLPGVAASSVAWADYDGDGKQDFLLTGFSSS
GPIAKLYRNTGNGFEEDTTVSLPGVEYSSVAWADYDGDGNQDFLLTGYSSSGPIAKLYRN
TGNGFEEDTTVSLPGVEYSSVAWADYDKDGDQDFLLTGFSSGFTPIAKLYINTGNGFKED
TNVSLPGVVFISVAWADYDKDGDQDFLLTGSSSDGRIAKLYRNTGNGFEEDTNVSLPGVV
FSSVVWADYDKDGDQDFLLTGDSSDGPIAKLYRNTGNGFTETLSEPNEINDVTLAVSPTI
VNEDGRENLVYTFTRRGDLAQELTVTYNIGNIGGTANIGGTADARDYIGATPGEGKTIKF
TANSPTVKLEINPTADNLSEFNETVRLTLVEGDGYTVGTTTPVTGTIENDDLPGVEYSSV
AWADYDGDGKQDFLLTGFDGSNPIAKLYRNTGNGFEDKTPKGLPGVEYSSVAWADYDKDG
DQDFLLTGSSSDGRIAKLYRNTGDGFEEDTNVSLPGVAASSVAWADYDGDGKQDFLLTGF
SSDFTPIAKLYRNTGNGFEEDTNVSLPGVGYSSVAWADYDGDGKQDFLLTGDSSDFTPIA
KLYRNTGNGFEEKTPKDLPGVRDSSVAWADYDGDGKQDFLLTGYSSDFTPIAKLYRNTGN
GFEEDTNVSLPGVGYSSVAWADYDGDGKQDFLLTGYSSDGQIAKLYRNTGNGFEEDTTVP
LPGVSDSSVAWADYDGDGRQDFLLTGYSSDFTPIAKLYRNTGNGFKDATDIGIGKIDKFP
GVEDSSVAWSKDGKYFLLTGFDGSKPIAKLYTSEGNGLFKESQDLPGVIGGSVAWSKDGK
YFLLTGWDGSNPIAKLYENTGNGFEDKTPKGLPGVRNSSVAWADYDKDGDQDFVLTGWDG
SRSVAKLYENTGNGFKEPSNVSLTGVYNSSVAWSENGEYLLLTGQSASGLVSRVYKNSVE
NGSRRLLVTDNPVTGVENGSVAWSKDGEYFLLTGWDGSKSVAKLYKKNKDGKFEEDNSIS
LPGVRDSSVAWADYDGDRKQDFILTGLSSDGRISKLYKKNKDGKFEEDNSISLPGLALGS
VNWGDYDGDGRQDLLLTGESDYGRISQVYRNTSSGDKTFFTATLSPTGLPDITLAVSNTI
VKEGEKLVYTFTRTGDLTQELTVTYDRGGTADFSDYIGIISGSAVFKKHKNKVELEITTR
QDLSSEGDETLELTLLQSQEYTVGTNTPVKGIIRDAKQERVPLITSIPKNMEMLPKNIKP
GEELSKLSYQFLDYNSPTVRAKLDSALNLTNSVFTNFLGLYEVDDPNTGAVNGFFPGDPG
YAEAALSRRVQNFYVKAGGSATNITTNGVFAQNQTPVGDPSKINDVRYLAPFLIANIGSK
DITAEIGEIIKRSNSSAEYQDKSVAYFSFGAANPGGMSQIKHFGNGIFGFEDLPLNVSDR
DFNDTVFSFG
>KRH96664.1
MLTIGKPSVFFKDFSPEYTIIPKTIFQTQSTKLLTQTMKNSLYLPMILATLCVATPAWGE
EICDHHTQDSNGQGKQVSGKAADLIITQSPGVQPDGSSLAQTNESSTQEIRLDQDAVIDP
SQSYRLPVNNYWYVSGSSGLFSSQSDIPIKVTRNITGQVPGDLSLVSPFRFNVAGGYQWK
LFRTELELGYTSTVINGIKYKVNNSSQPLEGNIDRVTVLLNGYYDVFTGSRLRPYLGAGV

GVGFNGGNIKQPQSGDSFTVSGTSLSYQLKLGVQYEIAKKRSVFVDVKYLNVGGYNSKPN
VNDVSDIELGSANSYGLSIGYKQGF
>KRH96667.1
MNQSAKPYSPLRVALVGTMVATSATLSLFGQAWNKQVKAALQDSPKALVDQVWQLVNRDY
VDGKFNQQNWQAIRQSLLSKNYTSKQEAYVAIRSALQKLEDPYTRFMDPKQFEALTNQTS
GEVTGIGIRMEINEQTKRLTVVEPIQNSPADKAGIKAGDEIIAINGKSTSKMKIDEASSL
IRGPAGTAITLKISRPGNSFLDIKLTRATIEVPTVRYILKRDNGRRIGYIRLQEFSSHAA
EQMDRAIRDLNNQKVEFYVLDLRGNPGGLLQASIEIARMWLDKGGIVKTVDRVGGSEETK
ANGTALTNRPLAILVDGNSASASEILTGALKDNNRAVVVGSQTYGKALVQSVHELIDGSG
LAITIAHYYTPKGTDINKKGITPDIQLDLTQAQERELAANPNLIGTLSDPQYARAISAIL
DSKFAQPPQKVENF
>KRH96671.1
MPGSNSVVRRYTPPTCTLEILAESSPLSRWMSQTVVDQLRFQLHFDDPTLPQDLRIPIEG
NREQLEVLCNAVSSYVQQLLQESSDRFCVIFRESTPSKPPTLPSEAEDIASSASPASASL
PHHRDNTGVLEATIYLEFQENLTHKLYLGSLATQRSGPVIELTLLQLFDLSSALDEYSAD
IIALPTMGAERFISSKLPRWAPVAAVLTLALGLTPFTWQYASNMRSVRVSEQQKPQEPMS
TGAPTMNPDAGVKSPDLADLPSGLPNFNLPSPNQENLDVLRSGTVFPTPTPGTTGKNPLK
TPEIAGDIIPNSPNSTQVPPKILGYPTDPRIQGRMPNSNIPPGDVNRLPDSLPNPSLPQT
QPLNRSNRTGENDTAGTAKQDLTTITSQENKSYDTPQVAEAGKYLQGKWRPPADFTQTLE
YSVTLEVDGTIQRILPLNQAAREYIDNTGMPEIGKPFVSSNKAGKTLRIRVVYSPDGKVK
TFTETP
>KRH96689.1
MSDRLLTFSHLGALLIDSDPIFRLGLRIALEASSSIKVVGDVSNDSAALQLLGDTASSQE
QTDENENSQEPKKDHREINLIVLELGRNSQEAELGLQFCEQLKALYPHIPVLLLTAVSNS
ETLLTAKNLGVNGYCPKGISISLLVGIMEEIAKGGYYWWEKNPSKSLDSPLTRWTHKIHS
SGISYITRDLTKITLRLRIPGIPLLERVILAGHRRELLAAGWLVHQLFGSSEPQPVPPLR
NPGESESVPIARSPLQTKKALQSIIFTSCLDKLQFPLPNIGEVPLEIDILREEKKKELLY
LILQKLSQRLDKLRDCQFEISQLSNLKKEILYDLWEGVVREFYGQLPQVNVGKSTIETVG
FLLDNARGVETEILDKIPLVEELVSYLIWQTDLYVDNRLYSPGSESANYQILMILENLLI
QIANGVLQPLINLLADVETVKQHFYDRHFISTREIERFRNNLSWKYWLSRYFGEPQAIFE
SRYELFVIAPRGITKTSIYSPRNHQLANLSGIPLAVTLALEFRDAIAPRLQSLLSFVGNV
IVFILTKIVGRGLGLIARGVLQGLGSVNLGDKPRK
>KRH96693.1
MKFLYQYQRDSQSGIENGYDDPNFEHLHPVEVNEFLPQIHRLTSIGAGIMLTIFLVGIAL
TTILTYKTTVKVSGTIRPVGEIKLVESAISGEVQRILVQNHQVVRQGDSMAFVDDSQLQT
QKKQLEDSIQKSQLQLLEIDSQLNEIRNQILDQTNLNEQTIFAAQSEFMGTQRNFDDQRI
KGNAELLQAEIAFQFAKSQLQRLEKQGILKSTIQEAETALNLAIIQRNRLQAIASSGAIP
ASLLEEKQQAIKSARAKLESAKNNAQSLWDDKQQAFKTAQTNLYKARTTMNPSNSAVIIA
SQRIKQEQVRGEMTIAALNKELGNLLQQRLEIQKQLTRYRQDLLQTEINLQKTVIRAPIT
GTLLQLKLRNLGQVVQSGQAIAQIAPLNVPLEIKAYIPSQEINKVQVGQKIQMRVSACPY
PDYGTLRGRVKNISPDVLPGNIITNNITNNINNNITNSNGINGYEVTMQPETLYLGKGQN
KCYLQPGMEGGADIISRQENVMQFILRKARLISN
>KRH96698.1
MFLYLSKLLPLFFYPLGLASISLVIALITLWKRPKIAAGWIGISLAIVIISGNGWVSKSL
VASLEWQNIPLEKIPQAEAIVVLGGATKPAAWPRSTVDLNESGDRVIYAAQLYGQKKAPL
IILSGGRIDWRGGGTPESADMATILISLGIPPEVIIEEPNSLNTYENAVNVKKILESRGI
KKVLLVTSAMHTPRSLKIFQRQGVEVIPAPTDFIVSRSDLEELIATPKSTILNLFPNADN
LNDFTNALKEYIGYLVYALRGWL
>KRH96700.1
MIRSSVDNCPVPIEQQPLHEYEELKNSWFFGESTLGSRGYLTRILWIWGWSWLIAGPVSA
SSFPVEKHIFHFILCGTAIASLVVVLVLIRLYLGWFYIKDRLYSATVLYEESGWYDGQIW
HKPREIIDRDRLIVAYEIKPILGRLQMTFGVVAILYFTGILVWNLF

>KRH96703.1
MQIVICPGIHQRELTQRFIEDLWSVGENNLNNLQMDNMLVFPEEGILTLSTFHILLFLGD
RLGNRLELPVIFIGFSAGVVGAMGAAIKWQMRGGNVKALIAIDGWGVPVGGNFPIHRLSH
DYFTHWSSAILGSKQDNFYADPPVEHLSMWGSPGKVQGYWQNLSTGFFGCPTYLSATEFL
HLLLKSYDSKL
>KRH96704.1
MVVTPEKLQHTHDHLPNHDSIAVLLMGYGEVESYEDFANYNEQALNLLTAKFAPVPTWIY
PPLAKLLALFDRHEWGHTHHDFISPHNAIFEKQRAGIEHELQHKWGNKVQVFKAFNFCAP
FLPGQVLAEIKNQGFEKILIYPLLVVDSIFTSGIAIEQVNNALAELSDGEQHWLKGTRYI
PSFYNEPEYISLMARLVEEKVSSDLATNYLPSQIGIVLLNHGCPHKAKGFTSGIDESQIL
YELVREKLIHRYPLISVGWLNHDTPLIEWTQPNATQAAKNLIQLGAKVIIFMPIGFATEN
HETLLDVHHIIHDLEKQYSGLDYVQMPCVNDHPQFLKMAAGWANTQISELMKEAGQEVNL
QLSTHHHHHH
>KRH96711.1
MASNKNILSEILSTSPNFFSQFVFGFLLAAISRFAGASLILCVFLGIVGGLALGWFTIAN
ENNETIPNVAANDGIDAALKYCLVFMFSFLFIGYSAPISILFGCLAGLGGGWIIAWWRSK
ELTVTQIQDDLVEDDDLEQSDTRVTKRKKRLPVRRLRRPPGSFTFRFWEK
>KRH96713.1
MIDGVPQLVDGGKHYGHLEVNIEPFTPIAGITAKIEFTPVYSFPILDATYNLVATERRVY
DDPVVMLVTDEGSVINIPYSEGTKS
>KRH96718.1
ALLETVAIHQRNFEVMQRNFDSMVTEMREIQLEIREMRSDTREMQSEIREMQSEVREIQL
DVRGLQTENRRILDILQNLPPGGSYE
>KRH96727.1
MQRDLQQRIESISNYNTRLRQYITELEICVEQIKQEKEDQQKQIAELLKEQTKLNEMVAK
LQTVQDVRQMQQELENKEAQIRNLQEKNEAIQAENLKLQGEITTLQAARNSEDEQQTTPS
IPTPSRYTNLETLLKAENFKLADQETSSVMLALAKRQKEGYLRVEDAENFPYEELRTIDN
LWLKYSQGKFGISVQQEIYKNLGGTKQFNLNVWRSFGDRVGWRKQGSWLNYSDLNFSTLA
PVGHLPTVRLAPGSSHSGWKNGVGVWVSRSVLPSCQDM
>KRH96729.1
MQIKLVSENKITQEQVEQVFNLPIAIGRDLSELPPNLNNEPVSPIVLLDNNRQISRFHAQ
IRLNNNRVYLEDKSSNGTKVNGKLLVKQGQVLNTGDTIIIGGYTITVVILEGGGQDEGTV
IVGEAATVFNFDPQSQVLPASRLEPIPNVASSISFNPHTGILEQQVSSSPSISRSEFPYN
LSFWHRPQISLREIESSGFLVRETEYLACGGGMGSFVWVDMLRIAGVKTENIKILSNQEK
PYQRYKTLLKNCQISEDKRIRSGSDSCPDNIWGWPGYGLRDAKKALFSGQIGAAIGFLWQ
VFAEPVHADTYTPIAGDVFDSMDRESERINWQKMLEPGAILSLRQTEDGRYCIAYCSDPQ
NRRHYQFLLARYVHICTGYPGLKLLPDLEKYRQEYPQETGNSRTVVQGYEEHDHIYTHLE
KHGGTLILRGSGIVASQVLDRIYQARKRNKNIDVIHLNRNPRSGN
>KRH96730.1
IDCTGLISDPLQSPFLKDLINHYDLDLNPDRRLYVKNNFEIRQLRHPRDSQSRVYAAGII
TLGGPYAPVDTFLGLQYAAHRSVEALAAIKAPGVRYIQGIYSVWQWFKWALNLKP
>KRH96731.1
MYTEDQIKNLFGNKTVEYIKNKNTGGVSNSKGNTYENIFAIYKISSLSKSIIEDEREIYL
LSQCLSFIDDLVIELTSENTLQHYQLKNSSNITWGTGEKSINDDFKKQYELNKSISKESK
LALVVSSLELRDKLQANIPDDIKNYSQVIYFYFADSLPKIIAQEPEFRRSLEYLCAFENP
EPDKLECLATVLLGAWVASEKSKLPIMDILQKAQDFIPSYIRSFKTELQLNPEIKEIFDK
IDGFTYNLTRGFLQWEYFDGLNAGTLPYSIETARFQKFQELIKKNRPTSFEDLEVFLI
>KRH96733.1
MCSHELTNSHIISYYVRGQEKCNYLTTFFIRSGSIAEAAIKTGRKAIGIEINPNYVNIAV
QKVKNIRYEEENQVKQGSLFDLVTDIF
>KRH96743.1
MSNQERIDNMENQLIDIRLAVSALLETSVIYQRNFEVMQRNFDNVVVEMRQMQSEMREMQ

SEMREIQLEIREMQSEVREIQLDVRGLQTENRRILDILQNLPPGGNYE
>KRH96746.1
MSTIYVLSNPSTTITKEDSITNAINQWLYGKSNHSKRSYNRVIRSYISYLDDIHIANSTA
SHFRDYQ
>KRH96747.1
MVKLKTLFLVSFAFILVLHGARQIELKAQQKTKQESPHLLLGNPSSAKNSIDSSNNYLMV
KKQYALSYNRSHGTANWVAWELNQSWLGNAERQDNFRPDPTLPKQWKRIKPSIYKSSGYD
RGHLVPSGDRTANIEDNSSTFLMTNIIPQTPDNNRNTWGNLEDYSRDLVEQGKTLYIIAG
TWGSQGKINNLVNIPKYTWKIIVVLDRPSRISDVTPNTQVIAVNIPNQEELDNDWKKFLT
TVDQIEKLTKY
>KRH96748.1
MLKISLEKIRQSHLTKKSSVTHALRCDRLIYDVSKSMLNSDGKRFPSGNKAAISRFFASV
EFLVTIFLLEVSQKLRIGATQ
>KRH96749.1
MTDGATETLSTIEKIKIRKNINIPNFYRKWQRILHSYTTRKISDVPNQETSINTEEWKLA
KANIQDEYEQWLETQNISTLIKNLIKNLIDKNLIERRISSQNRELVRIHLNFSDNLNESS
QQDNDILRRLPWGHYFNTHISNHNYDITFILSKDHTNKPTHANLFTSKAPKILAIFGGHQ
GELASSQSHEANELLKICEPNGATVILKNVTEQREFHQLLINDSYDILFYSGHSSSKDGG
TIQFEQENSIIRDFKSDLIQATEKGLKIALFNSCDGLKIADFLTKEVGIPAVIVMKEPVP
DEFAREFFERFLCRVTTATEFTAIFM
>KRH96754.1
MESLRLAILSIVYGIVASTIPEPELVCAKIEDNLGIKQSVAKIPHAPRAQAIKIRVIWLF
IRITPLF
>KRH96757.1
MSIMQKITIDNLESPAGYDLFLDSESFLDEVHESEYHRINGGATPTFTITSTAWCWAGGV
ALGAAISGGIVWGAKKLFG
>KRH96763.1
MSIIQAQNLSKSYSVAIKQPGFVGTINHFFNRQYRHIQAVKDVTFTIEPGEIVGFLGPNG
AGKTTTLKMLTGLIHPSSGSLKVGGFSPFRRQEAFLQRITLVMGQKQQLLWDLPAIDSLK
INAAVYNISDREFQQRVGELTEMLSLGSKLNQPVRKLSLGERMKAEILAALLHRPQVLFL
DEPTLGLDINAQVNVRDFLREYNQLYQSTILLTSHYMADITALCERVLVIHQGELMYDGR
LEELIQKFAPYRQIYIELSQSLPIEKLTYYGEVEYLEGRRVSLLVQREGLTRTVGQILAD
LDVVDLTVTEPPVEEVIGKVFQSVLISPTHRK
>KRH96768.1
MKWQLLTHNKQVLGKIFTILVFTGLTGILCVSCNRNQDLLVTEIGVNPPKRPTRKTSGAG
EFYLQGQNQHSRGNFQAAIAAYSKSISLNSDYAPAFKARGLAYFDLNNKERAINDYNQSL
QINPNDPETYNYRGNARASLGDQKGAIEDYNEAIRLSPNYDEAFNNRGNSHAAQGNKNAA
LEDYTQAIRIDQNYSVAYNNRGNAYSSLGNTSKAIADYNQAIRLNPQFAPAYNNRGNAFA
SSGDKRRALQDLQKAATIFDQEGNRGLYQQTMKNIEELGN
>KRH96769.1
MNKLLNCKFCNICLPIGLPIILAILGCNHRNLNNNYTQDHQNQKISQNLPQVVVTTTILC
DITNQIAKESINLICLVPPGLEPPIYQPTPEDIKAIKKADLILYHGYNFEPNLIKSFKNS
RKNIAKIPVGQRAVKQPQKLRQNGKIINEPHIWHDVRNAIKMVEVVNFQLGKISPENQQR
YNSNTRQLTRELKELNQWIKSTLSTIPDKKRKLLTTHGAMIYYVKAYGLDYKGTLPDISN
EDKLTAKKAKSLAEYIKTTQAPIIFADRAVNLMLLAPIAKKTKVKIFPRPLYIDGLGEPG
SDGETYQKMMDANTRSIVEGLGGTYLRFVPNIGR
>KRH96776.1
MRNWWKQWLTTNLEWSQSLLIGTLDILLVVGLTYMILVIISERRTLWMVRGFIFLMLASA
ISGALNLILLNFVLEKLVIGCAVAMAVSLQLEFRRFLEQLGRGEFRQLFQPHRLTVTKSD
SVIDEIVDSIKELSKNRIGALLILETTEPIDERDFSVPGVKLNAQVSKELLQTIFQPKTL
LHDGATLIRGSRIVSSGIILPLSGRTASRQLGTRHRAAMGITERVENCICVVVSEETGSI
SLAERGTLYRPLTIRKLKESLEARFSTTVDREVVAPGVFGLVSQIASQIFRFVFRLFRLP

LSFAKKNQTEK
>KRH96780.1
MQNLQNFRDYYEILGVTKDASNEEIKKVYRRLARQYHPDLNPGNKESEEKFKMIGEAYEI
LSDSARRSQYDQFSRYWQQRGFTGAKTAAKSKSWGTNRPSESSNQGVNPADFPDFESFIN
QVIGVSSRKEAKKSPNTTTQDPFGNPRTKVAYTVNNRTPPKDIEARLTLPLEKAYQGGNE
RIRLEDGRSLEVTMPPAMLTGQTIRLRNQGISGGDLYLKITVEPHPLFKMEGFNISCQVP
VTPSEAVLGGQVEAPTLDGPVKMTIPPAVRSGQRFRLANKGYPMEDGKRGDQLVEIQIIT
PKNISDQERQLYEKLREIESLKLKP
>KRH96784.1
MKIQLLSALNNTNIDASQDNSQRQLEVSISAIADELDTSLPLNLCLILDKSGSMHGESMS
MVINAVEQLIDQLQSGDRIAIVAFAGSGEVIIPNQIIKDPKTIKSQLHNKLKAGGGTIIG
EGLSLGITELLKGSKGACSHAFLLTDGYGDNGFKIWRLQIGPNDNQRCLELAQKAAKLNL
TINSFGFGDEWNQDLLEKIADAGGGTLAYIETPQNAIEQFNRIFKRIQSVGLTNAHLLLS
LVPGVRLADLKPVAQVAPETIELPISTELNGTFVFRLGDLMKDGTRRVLANIYLGSLPEG
EQIIGHIQVRYDNPAVNKEAILSPLIPVYANITKTYQPANNPDVLNSVLALAKYRQTQVA
ETKLESGDRTGAITMLQTAAKTALQIGDSRGATVLQSSATRLQNGEELSDAERKKTRIAS
KTILMD
>KRH96793.1
MKKSALFIGLITLDLIYLAESPPKNNQKLVAMDYTVAAGGPATNASVTFSYLDNNSTILG
VLGSHHLTKLISTDLANYEVKTIDLDPHKNTPPPVSSIIVTQQTGERAVISLNAVKSPGE
ISSIPSNILEGIDLILIDGHQMVASKTLVMKAKNQNIPIVMDGGSWKEGLEEILPYIDYA
ICSANFQPPPCETRQDIFTYLMGFNIPHIAITQGEKPIEYYSIGQTGSIHVPQIKTVDTL
GAGDIFHGAFCHYILQSSFTMALALASQIAAKSCELFGTRRWMEEQKP
>KRH96803.1
MKLSVIIISLSALATAGLGVTMAHTNPREVTYQEYAVKKITTVLKTDGCQKVPIFLRNLV
KFDCNQLVDSAKSQIKDVVVATTKRQNYVLLSIYITNLKIHNSLPGYTFETLGAFNSFYT
YRVKQE
>KRH96807.1
MSTTIPINRYRFFQKIQPLSILVKIVNDSTTGCLQVFSPSGTWSIYLQEGHLVYASRSEN
IWEPIYRNLQRLSWQNSNLMRINEQLENFVEQCVQNQTISHADYLAICWLVDEQYISLVE
AGILIEQLVLEFLESFFEIDQGSYEFIPQSFLDPLPKFCHLNVNSLVQKYKSEIHVSREE
SGLDPQNLSPTYTIACASQNIVLLNYIRKLLNQTIFNIIDITDPDVMGVSVIRPDMIILD
VTMPKLSSYGIGLLLCKQFSSKGIPIILVTKKNNLINKIITRLVGATACLGKPFNQDELM
KVIFENIN
>KRH96813.1
MTQELIGALGGVEQLNQLIDAVNQAETPLEMVTAVRNLAAAKSPAAIATLIAVFGYNNPP
AAGVALAALTDLGTVAVPSLLAQIDDYNYGARAYSIRTLAAIADPRALDVLITAGVTDFA
PSVRRAAAKGLGNLNWSQLGDNERDLAIERALEALLLIYQDTDWSIRYAAIVGLEHLGKT
RRETILNKFREMIANEEEKSIRIRIQLATSKVTRDN
>KRH96817.1
MINIIILVPQGAEHQVVCKGINGAHNAKIQVIGIPIGISSLKPFLKNNYPHWNPYLNRQVL
LMGLCGSLHPDYGVGNIVLYETCIYETNQVNQINCDVNLTKDIYKHLRDQAFLVRGVKGI
TSDTVVSKSVEKGGLHRKFGVDVVDMEGFGFLEFFQGTGISTAILRVVSDDSTHNIPDLS
KTINPNGSLEILPLLIALTQQPIAASHLISGSLRGLKQLEKLARCLATWQQ
>KRH96818.1
MPQDTGYKNTKLEKKAISYKNRLNGWTVARVAEDDQRVVVARFRSKSDADGYIQHLRQLV
PDGYFEMFFDSQVLIASM
>KRH96819.1
MRFTASVLQKIATEIGAKILIEPEYELIGHITFRNGKRTVFRNTRFNINGFGSANLAQDK
AFSNYFLGTLGYKVTEGKTFFSDKMCQKVANPRNIDEGWQYAEKLGLPVIVKPLNLSSGI
LVTKVYNKNEYYEVAKKIFNVQSVLIVERFYLGNDFRILVLDNEVMAAYQRIPLSIIGNG
KSTILELLTIKQEELVKKVEKISIDLADFRIFKNLQKQNLTFESIVPQGMIVYLLDNANL

SAGGQAVDMTDNIHPDFQQLAINITKDMGLRLAGVDILTQNITQPLVDYTLLEVNSAPGL
THYASLGEKQRQQVEHLYRKILQELEKG
>KRH96822.1
MKQLQTRLVFIIFSIFFTGWLICGLSIWSLEFNKAKKDILRTAGILLDTAAAVRDYTSNQ
VKPQFDLIETSLKAPAADQAIQGGNNQAQGYQQTETEVAEFNRVTVPSYAAQQVLNQLEK
NKNGYSYRETAINPTNRKDLAAPWELEIINYFAENPKAPPKMGERFDTLTKQKTYYIAKP
IQITKESCLVCHSTPERAPASLIKTYGSENGFGWKLNEVVGARIISVPSIVQYNEARRSI
GSYLLAIASIFLVAYTAVIVIIYRWVTKPLDFITHLLEEVSLHQAEGAQLPEDKSNPLHH
LNRSINRLLISLNKALKSPQQQ
>KRH96823.1
MNIKRVFKNQIFLVILSLSLLVIIPLTCLVGITSTALALEYNKEILIGANFSQRDLRDSS
FTKANLRQSDFSGSNLSGVSFFAANLESANFNGADLTNATLDSARFIRANLTNAILEGAF
AASAKFDGAIIVGADFTNVLLRRDEQNKLCEVAKGINPTTGRDTRETLLCR
>KRH96824.1
MSKLPSSNYGKIGLTGEDLVTKWLKSKGWQILHSRFSCRWGEVDIIAQYDNKTLVPTSVN
RKTPLLAFVEVKTRSAGNWDAGGKDSVTTNKQRKILIAAEMFLAKHPDKADYSCRFDVAS
VFYKKSIPKKQVIQPKALASLSTQEYEFILWDYIESAFDISARLSSI
>KRH96830.1
MNSNDIVWQQGKIIELEISDLNDNGEGVGRFEGRVVFVPDTVVGDRLLVRLLRVKNKYAH
GAVTAIFQPSLQRTRPQCIVADKCGGCQWQHINYEYQLQVKQNQVIQALERIGNFVNPPV
ASVLTTKSPLGYRNKATYPIKTSSTGQVQAGYYQKGSHLLINLNQCPVQDPRLNPLLTEI
KQDIQKQGWSVYDENRHQGEIRHLGLRIGRYTGEILLTLVVKNGQLPGIETQAQQWLHRY
PQLVGICLNHNGDRTNAIFGKVTTCIAGVPYLRERFAGLEFKVRPDTFFQVYTETAEALL
EVIQSQLNLQGDEILVDAYCGIGTLTLPLAKQARQTIGLEIQPAAVEQAIYNAQHNGITN
AKFQIGAVEDILPKLDTLPDIVLLDPPRKGCEPIVLQSLLKLKPKKIAYISCKASTLARD
LSLLCASGEYKLKKVQPADFFPQTPHVETVAFLES
>KRH96833.1
MSLSPDSLPPALGKIVQRFQRASDPKRRYEQLIWYAQKLPEFAEANKIPENKVPGCVSQV
YVTARLNDSKVMFAGDSDSQLTKGLLALLIEGLNGLTPKEIIQLTPEFIQETGLNVSLTP
SRANGFYNIFRTMQKKALECDPPTGGG
>KRH96840.1
MIPDAPLDPNTPTQSTLGNRVRNFLIVIVAIALTGLLFLGLRNQTSAVSLQELDQASTPL
EMAITNNLPSLVEFYADWCTVCQKMAPDIAKLKDKYGDKMNFVMLNVDNSKWLPEMLKYR
VDGIPHFVFLGNQGETIAEAIGDTPRTIMDSNLDALIKGSPLPYAQMNGRASGFSAPAST
NQDDPRSHGGQVVN
>KRH96844.1
MKQFLKLNDYQAAYTLKGKGEPLVLLHGFFGDASSLNCLVDQLQSQFQCFSLELLGFGDC
SKSNKPKINYLIEDQVAFLKQFVDNLQLQKFYLAGYSYGAWIASAYAVHYSFSLHGLGLI
GPAGIRDDGFVGRYDHLKPLLWQTKLVDLAIALYKPFAYFSGNRNGYQKIAQARHMLMTQ
PSAAAMLNSILKATNALNTVEKHIHQISTPTIVIAAENDTTIPQWHSETYAKTIPQTAFY
VIPGAEHDFVNTHAPDICRLVYSFFTSVAIDV
>KRH96846.1
MQAEPKPGTLYIVGTPIGNLEDMTFRAVRILQAVDIIAAEDTRHTGKLLQHFQVHTPQIS
YHEHNRTGRIPEILTYLHYGKAIALVSDAGMPGISDPGHELITACVAAGIDVVPIPGATA
AITALSVSGLATSKFVFDGFLPAKRQHRREYLATLLTETRTLLFYESPHRLRETLEDLGE
ILGGSRTMVMARELTKLYEEIWRGDIKEAIAYYREKDPQGEYTLVVGGASPSQPEITEAQ
LRAELLEIIKQGVSRSQASRQLAQETSISRRYLYQLALSIDQEGV
>KRH96847.1
MVVKIHLSITQKTQRLDRYLSSVIPDLSRSRIQDLIEQGYVQVNGKVCYSKNITLDGGEY
LEVEIPPVQAAQLVAQDIPLDILYEDEQLLILNKPAGLVVHPAPGHLEGTLVHALLAHCP
NLPGIGGVQRPGIVHRLDKDTTGAIAIAKTDIAYKQLQLQLQSKIARREYLGVVYGAPKS
ESGTIDLPIGRHPQQRKKMAIVPVEQGGRNAITNWHIKERLGNYTLIYFQLETGRTHQIR

VHSAKMGHPIVGDPVYSSGRSVGVNLPGQALHAWQLKLQHPTSGEWVEVTAPLPDSLTKL
IEVLRRR
>KRH96848.1
MGTSLLSHLKANFKRYLEKIDRDVTPSAGKSYPYNKCAQSQLAGPVHNLGGGGVDVDDGI
QWMINQVRGGDNSDQKVNVLVVRASGNDNYNELIYRMRGVNYVKTLIVKNRQEANRNDIF
DQVRNAGVIFFAGGDQCEYIRHWKNTKLEAAIKSVYDKGGAVGGTSAGAMIQSEFVYDSC
ACEESIETWEALSDPYRNITFTYNFFQWKYLKRTVIDTHFDERKRMGRIMVFIARQIQDG
ISTDALGIAISEQTSLLVDKNGMAKVVGKGAVYFVLGDHPPQVCQPGQPLTYHEYKIWRL
IRGETFNLKHPPSTGYYFRSVKRGKFDRDPY
>KRH96849.1
MEETRKMVAETNKHMGSITSRWGEFVENLVRPAAVRLFKEQGIDIHYTSLQVKAHDYAGS
IEIDIWAENDGQIVAIEVKSHLKVRDIKRFIKVLDRFKDVFPKYKKYKLYGAVAGIKVDE
KADQYALEQGLFLIRPAGDSVAIDVKKDFQAKVW
>KRH96851.1
MVHATKPIMLKARLLYTAKILFHVLGEFGYNPIKMVVSILELPLYIYQLTSFSIRSKSPV
KIRLFPYLTDRRDFAGSISRHYFHQDLWAARKVYENKPENHIDVGSRIDGFVAHLLTFRT
VEVLDVRKMTSSVKGMTFRQVNLMEFQKVPGSVCDSLSCLHALEHFGLGRYGDPIDPEGH
IKGLKSLTKMLKSGGTLLLSVPTGKERIEFNGHRIFSVTTILNLTTSDYDLVSFSYIDDT
NTLYENVDTSFVPTMLYGCGLYELKKK
>KRH96853.1
MKSVLKWYRSQYLAPSPLFVKRQVLVRNAIVNGIYVETGTYLGDTTNFLSQRFPKVISIE
PEATLFEKAKKRFSDKKDVHILHGCSEDIFPTLLTELNGDINFWLDGHYSGGDFWKTYKG
KSDTPIISELRHIRDNIHKFNQVAILVDDIRCFQEGSRLPDYPTLDYLVDWARELSLNWH
IEHDIFVARSAGP
>KRH96854.1
MSTFKFFRKSDTVWYFLLFALLLLTLYIQYLSKYGYIPLTGIYVPDGYSYELRALENREL
EEFSAFSYNLFNQFIYNSFLGAFGFFLFNSSLLFLSVWLCRPVFASISENSINYARFAIV
FNPYLLIGAIGPNKESILLFINLLFWYLCFSGYRDHESRSNNQGNLVAKIIDPVAVFTLA
SVPLFIRPIVSIILYISLIYKIVGFKKTRLVATWLLVCYFLSVSIPVINEIYAQQVDQGL
SSFSGSTVYDAAETLSRYSLDPIMQYPAFIAKTILLFFGFVIRPFSFSALLDVGYTFLAL
AYFPINLSLILTFLKNTKRNYDNLSRPDNDNTMNTFLFFTLLSMFIVIVSPGFTFRYFIP
VTPFSFGLFIFQNSSTRKILVTISVLIGVLVIVGNLIFYQKSYYQDVSLPVFMDWL
>KRH96858.1
MLKPKNKSDRNKIVIGIDATNIRGGGGITHLIELLNAAEPIKQGISRVIIWGGSKTLASL
SERPWLKKINPPQLDQGVFSRILWQNLQLSKSSQDLGCDLLFVPGGSYFGNFHPVVAMSQ
NLLPFDLPEMRRYGWSLMQLRILLLRETQRQTFENADAVIFLTKYAEECVTKTTGFLKGK
TTIIPHGLNTRFRHSIKPQISILEYSRENPYRIVYVSIVDVYKHQWNVVEAVSRLRHSGL
PVELDLVGPAYTPSLVRLQKTLDKCDPHGDWCRYHGSIPYQDIHEFYNKADLGVFASSCE
NMPNILLENMSAGLPVACSNRGPMPEILEDAGVYFDPESADQIASALEELIYNPSLRKDL
AERAYLKSQDFDWMRCAEETFKFLRRIATTFKYA
>KRH96866.1
MLQQHLAIVTFNFPPDFGAASFRMLSLVEAIQKQASTRKIDLKITVVCAKPFRYKYNNKN
EPGSSSPKHNLIRGLENIDIVRLDVPMFGRGFVAESFSYLFFLIQALPVLIWNRPHLIFA
TSAKLLTSYLGALASLITGAILCVDIRDTFSENFLSFFRRQRKVLVYLILLYIENFVANR
ATSINLVSPGFSQLYDELLDSSKVSYFTNGVDEQFVEFYANSQHPSLKTFLNKTFNHTTN
NTLVVRKTLDNKINSARLLEIDTVKQTNHTDKHKTILYAGNLGIGQDLLKLLEPLIEEKE
IVEELIELKWSIKIMGDGAQAPALRELAKFPHLREIITVLRPIPRQELIREYGQVDALFL
QVGSYRSLDMVIPSKIFEYAATGLPILAGVRGYTRDFIGQIPGVQFFTQKDIKSFLNQLK
QIKTGWHNRDEFIEQYDRRNIMRKYADHLLSYIQASK
>KRH96871.1
MKSIMVVGTTSHAGKSLITTALCRIFSQNGWRVAPFKGQNMALNAYVTANGGEIGYAQAV
QAWAAKIPPTVEMNPILLKPQGDMTSQVIIKGKVVGKVSARDYYEQYFEIGWQTIQECLK

QLSSEFDLIVCEGAGSPAEINLKHRDLTNMRVAKYLGASTILVVDIDRGGAFAHVVGTLE
LLEPEERALIKGIIINKFRGMRSLLDPGITWLEERTGIPVIGVIPYISEIFPAEDSLDLL
DRKSTKFQSDLNIAVIRLPRIANFTDFDPLESEPTISVKYISPKQELGYPDAVIIPGTKT
TIADLIVLQKSGMAEAIQQYAASGGTVLGICGGFQMLGQVVADPEGIEGPPGKYQGLNLL
PIKTIITGQKTARQRQVISNYPQMGLPIQGFEIHQGRSRVEEPTDKSSHESPCHPLFDDA
NLGLVDNCQSIWGTYLHGIFDNGAWRRSWLNRLRQQRGLKSLPTGVANYGEHREKILDSL
AAEIESHLNLSPLTNIDTTRL
>KRH96877.1
MATRERLAKEQEREAKEQAEAIATRERLAKEQEREAKEQAEAIATRERQQKEKLVAYLRS
LGIDPEKI
>KRH96881.1
MKKFLTLALILILFLVSSFSLGTSPSYAYSEFDLDRLLKTDMCLGCDLSSANLEDAILIN
ANLQGANLSGANLKDAFLIDANLSDANLQGANLEGTNLKYADLEDAFLIDANLSDANLSD
ANLQGANLQGANLEGASLSGAIR
>KRH96882.1
MNNMMQYKDYFGSIHYSDDDKIFYGQVEYIRSLISFEGEDVASLRASFEEAIDDYLALCE
EKGIEPEEPFKGSFNVRVGSQLHRQAALFAQQRGVNLNNLVTDALERYLKEESLENA
>KRH96883.1
MTRREKLIKRFLSIPKDFTWEELLSLLSGFGFEEVSTGKTGGSRRRFLNDAGVIITLHKP
HPQNILKRYQIEQIIEILQEEELL
>KRH96886.1
MNQICCLNPTYECDNPQVPEGTSYCPTCGTPLVILKNRYQPVKRLGGGGFAKTYLALDTH
KLNELCVIKQLAPSLGNQTTQALIKATELFLQEAQQLQKLAEHTQIPSLFAYFEENRQLY
LVEQFVDGKNLLEELQTEGVLNEAKIRQFLQDLLLILQEVHKQGIIHRDIKPENVMRRHK
DGKLVLIDFGASKELQGGATSGTRIGTDGYAPWEQRVDGVASTAGDLYSLGVTCFYLLTS
KNPYELWLKDGYNWVANWRNYLNQPLSQKLQQILDKLLVANSENRYSLAEKVLEELRQPY
SIIPSNSPKTIISHTKKPRTFGYILALISVVLLGIGYLLITKTPQLQPKTEPNGIQETDR
GL
>KRH96891.1
MRNALTPARLTRAAKVMLNTLLIAIASVTFSLVSDFVQPQSAAAYPFWAQETAPETPREA
TGRIVCANCHLAAKNTEVEVPQSVLPDTVFKAVVKIPYDTSVQQVGADGSKVGLNVGAVL
MLPEGFKIAPEDRIPEELKEEVGDLPFQTYKEDQENVIIVGPLPGEEYQEIVFPVLSPNP
ATDKNIHFGKYSIHVGGNRGRGQVYPTGEKSNNNIYNASAGGTISKIAQTEDEDGNVKYI
VSITTASGDVVEDTVPAGPELIVSEGQTVASGDALTNNPNVGGFGQKDAEIVLQDSSRVV
WLIAFICLVMMAQVMLVLKKKQIEKVQAAEMNF
>KRH96911.1
MALIDGLKNDDKRQMLVADCMNLLETRVANIGGISGIAIKAGYATIKGISPKYCAGAVER
LLPESFAALEPLWNEGLEAGDAVTHLTQNRSRTADAIFSVTDIRIEKSTNSTIKGVYGKL
RVPVKKHVEEVVPDLAQILDKYAKN
>KRH96912.1
MLKGSILQQLETVCRHSNRPIRYGVYYKNTLVSLCHALEDHILDKREQPIVITCFQRGKW
YLQEADRYRDIAACSQDVVIMATDDAGFAAHSTSQLPNVNLVELNTTDPLSQEWHLIILA
PSYASMVICQELSDADYGMQGLPTSDLERKFYGMWTFEPDLVLKTTELAIAHIENYNQEL
AHKLNSHKQIIETQMASSEEVEVIVSRVIEYLKSSENTVTSATGKNSLSRNLVSNELQAL
LRMAELIEMRDIENPMAAVQVAGMAEVMAQLLDLPPWQIKRLRLAALLHRIYNLKQNKTN
GEVQVLDRMPELQDIAQIIIHQNEWWNGNGVPEGLSGEKIPLESRILALLMEFQREINQQ
HKNPQGMEDKDIFALALSKCKQQEQTRFDPELINTLNLLVLGLQQGLDLPCMTPKFSNSM
WLIDSRQS
>KRH96915.1
MAQILDSLPPEQSGKILCCYINATSKIQVARISNVPNWYFERVIFPGQRLIFEAPKYAQM
EIHTGMMASAILSDTIPCDRLRLTETDEDENDGNQTLAVDVDNNRDISSEIHKQFALLEA
RTLASYSTSYSKD

>KRH96920.1
MIKILDSTLREGEQTPGVYFSPEKKLVIAQLLDEIGVDVIEVGNPSVDNEIALAIQQIVK
EGLKAKIGAHSLCKIDAVKKAIDCGVDFLGVFFSVSQKRLKQDYNICLSAAIEKIVEVIE
YAKTQKPDLLIRYTPEDTVRSSMENVVQAATAAIKAGADIVSIADTTGYTTPFDPSRSIY
VQVKTLKEELANQGLYPQIEVHCHNDKGLALTNALDAYRAGVDMIDTAVMGLGERAGIVD
LAELLVNLVDMGEAKKWQLHFLRDLYDFVSKYAHIVIPPNRPITGKNAFTHYAGVHVKAV
VKDESLYQSLDPQTLGMKSGIALGMQSGSTAVEQALIHIGRQDLAQNKDLVSKILQAVKE
IAKRGVPIDIQEDLPSIIDRFQIQGSEKFLNIP
>KRH96930.1
MNIETLKSEKTKQLPGANLEDQDLSEFDLTAVNLAGANLIGAHLVSVNLEGSHLEGANLM
GASLQGADLRANLLGANLMQADLTGADLRGSNLRGANLMGATVAGASLTAAFLSGANLMS
VNFQGVDLRGADLRGANLTGANLKGADLSRADLQGALLNQANLEESDLRGANLAGANLAG
ANLLCAELEAASLNGANLYQACLLGTILETYHD
>KRH96934.1
MVKSVEIRNSDLRSFYQSYQRDPFPPSSFLRRLLPRREIDSLLEAARMSNDLLRVHFERI
IKTYRIPIQYHSRILDEVRIKQEENPESLKDVIKQINANIIATKDSIQNLKF
>KRH96937.1
MFEDYEGIERNSLNILNNFTTNPQAEVLVFDKIDPCFIDKVCFNSVQDMKQWDNLDTSNY
PQRFSVNLYYFKPRNDYKIWQAKKTDV
>KRH96939.1
MLATSKSEASVDENLNQLLFVQNLKGHESKVSAVAFSPDGRNLVSGSDDKTIKIWDLITQ
THRTLPAHQDSPWNGGINSVAVSPDGNIVASASKDKTIRLWNFTSGEKIITLTGHQEQVN
SLVFSPLGKILASGSNDKTIKLWNLESGEEIYSFQGHSDGVLCVAFSPDGQLLASGSRDG
TIMILKLAEKQVRTIVVNSNWFNGGINSLAFTPNNQILVSGGNDTIIKLWNVETGEEIRT
LNGHSQAVYTIAISPDGNIVASGSKDKTAKLWNLESGEEISTVKCAEDAIYTITFSPNGK
LLATGSGDKTITVFPYNYPKT
>KRH96942.1
MNPTENIIHLPRKSLPQQILTQHSIIPVRNDVLWRIERGVVRTLTWNEEGTGITLGYWGP
GDITGHALSKVTPYQIQCLTSVEATIIPPHLWHEHIECLLSHIQQTEQILHILHCKPTSL
RLWHFLLWLGDKFGRDLEQGKLIDLNLTHQDISEVLNTTRVTITRLLQKFEVQGKISRHK
RSIILRLSQVE
>KRH96948.1
MFWADKIAADAQGYQVVNDSKTPSGRVHVGSLRGVVIHDVIYRALKHAGKPVKFTYGVDD
YDALDTVPKYLDREKFKPYLGFPLCNVPSPGEGAPDYAKYFIGEFFEIFEYLGIQPETYF
LRDLYRSGKLNSHINIFLKNAHLVRQVYKQVSKADRPENWYPFQVICENCGKIATTVVTD
YNGSEVFYTCQPDSTNYVQGCGHSAWVSPLNGNGKLPWKVEWVAKWDVLGVTIEMAGKDH
SQKGGSRDVANAICRQVLEKQPPFHSPYEFILVNGTKMSSSKGVGSSAREIANLLPPELL
RFLMLRTQPRTVINFAPNYETITRLFRDYDTLISKYEVNPELTEELMSLFYAQLGDEVKV
FQPFDYSTLISLLQIPRLNIQDEVVQRSANSLTEYDQFIVNQRIASAQRWLEDYADEEEK
LVLYLEQVPEKARGLSSEQVTYIQKLAENLECVTPWEAEALQTVIFSTTKELNIPPADAF
KALYLGFLNKEKGPKAGGLFSYLEKSFVISRLQEITRKSPKGTEATWL
>KRH96949.1
MLELLGSGLISIWLDLAGIKIQPVSALETLAGQTSPGFVIAPDPSLVGAMTTRQYLQGLV
SSKLIEPNLVGHQGIWLQSGPILMANHQGTVPLPAASLTKVATSLASFKTLGPNYQFQTL
VGITSPIVNRVVNGDLVVSGGGDPMFVGEEAIAIGNTLNKMGIQQIKGNLVVSGKFAMNF
SSDPNVAGQIFKQALNHKTWNRNLTYQHSIMPKGTLKPQLVINGAIKVAPPSRHNQLLPQ
TLLIRHLSLPLHQIIKEMNVYSNNEIAEMLSQYIGGADVVRSISSQLAMVPQSEIQLING
SGLGRENRISPRAVAAMFMALQREAMASNLSLADLFPTSGLDNRGTMQHRNMPNATVMKT
GTLSDVSALAGVLPTRDRGLVWFTIINRGNQVSSFRAEQDKLLQQLAKQLATYNIFPKTL
TPHSGNKSVLSLGSPNRNEVVYKVHYTCQNRTLRKLC
>KRH96951.1
MEIQLGRGKKARRAYGIDEIALVPGKRTVDPSLADTRWKIGKIERE IPIIASAMDGVVDV

DMAVKLSELGALGVLNLEGIQTRYDDPNPILDKIASVGKEEFVSLMQDLYAEPIKPELIE
KRIQEIKQQGAIAAVSATPAGAGKYGEVVSRSGADLFFIQATVVSTDHISPESITPLDLV
QFCHSMPIPVILGNCVTYEVTLNLMKAGAAAVLVGIGPGAACTSRGVLGVGVPQATAVAD
CAAAREDFYQETGKYVPIIADGGLITGGDICKCIACGADGVMIGSPFARAAEAPGRGYHW
GMATPSPVLPRGTRIRVGTTGTLEQILKGPAGLDDGTHNLLGALKTSMGTLGAKNLKEMQ
QVEVIIAPSLLTEGKVYQKAQQLGMGK
>KRH96953.1
MGWGCRLPVSILRVKPTKSFISPCLIGIFLGVVVSGCGDRGTNSSSKKEVEITLVSFAVT
KTAHEAIIPKFVEKWQQEHGQKVIFKQSYGGSGSQTRAVIDGLPADVVHLALGLDTKKIE
KAGLIQPGWEKEAPNNAIVSQSVAALVTRPGNPKNINTWADLSRDDVKLITADPKTSGVA
RWNFLTLWNSVIRIGGDEAKAKEFVSQAYKNVPILTKDAREATDTFFKQGQGDALINYEN
EIFLAQENGMKVNYTIPDVNISIDNPIAIVDRNVDKHNNREVVEAFVQFLFTPQAQEEFA
KVGFRPVNTTVAKSKIFVDKYPPVKTLSTVKDLGGWGTVQKKFFDDGALFDQIQARGK
>KRH96970.1
MTQKNQTLINLLSLVATTTLIFGGSWFLMERWGQIVNHQTNNQNSAQKFNIFKVFNTCDI
SNSLEGTYSYGGSTTWAPVRTIADSILQRNCPQFILRYTQPVNQSPGSGTGIRMLIDNQL
TFSQSSRPLKPEENVKAQQKGFSLREIPVGIDGIAIAVNHSLNIPGLTIVQIKDIYTGKI
TNWQQVGGPNLGIKPISRDKQAGGTVEFFVENILNKENFGAQVIYIGTTTEALRKIDATP
GGIYYGSAPEVVPQCGVKSLPVGRVSGKFIAPYQKPEVPRFNCPKERNRLNIEDFRNGNY
PITRNLFVITKQNNQIDQQVGEAYANWLLTNEGQELIEKSGFVRIR
>KRH96971.1
MEVYCTRPHCPRPQNYFPDLDNITTLQAAQQKYCVSCGMPLLLDGRYLPVKLLGIGGFGA
AFLARDRRIPGIPNCVVKQFQPSTSLNAAQLDLAQELFEREATVLADVGMNHDQIPYLFA
FFPVVVPSLQPGRQDQFFYLVQEYIDGKNLEEELQHRGNFSETEVLVILKEILPVLQFIH
DRGIIHRDIKPSNIMRRQDGRLFLLDFGSVKQVTSAKVSSASTAIYTPGFAAPEQTTRGQ
VFPSTDIYALGVTILTLLTGKEATELFDPQINQWRWRQEVKISPHLSGILDKMLMPAINE
RFSSATEVLSALIPSITIPDQPQPSNLPFKGLMVKKFSFGELLLRGAVTGFEGALIVIAV
GLLVETPIIRLAISLVMVSSLVVVQSQGWLAVKDLLLILLISFLVIFLIALLPGGLDIQI
VVVLGIASALICIGAITLFSLIHKILSTIL
>KRH96986.1
MQSSFEGFHGIIPEILVGKAFQGYSGLLPNNSIESVGAKVKFLPPYSPDLSPIELCWSKL
KQFLRSREARTLEALNESMTSAVNYITAEDALNWFNHCGLFT
>KRH96987.1
MNSHYSMIIFWSQEDNCYVVHLPDFPFQDIHTHGNTYEEAAKHGQQVIDSYLQLYQENNQ
PLTRA
>KRH96989.1
MNKKVLVTGGAGYIGSHVVLQLAESGYDIVVYDNCSTGTPDSVLHGELVVGDLSDIDRLY
QIFSQHRFSAVLHFAASLVVPESVAHPLDYYTNNTRNTLNLLRCCSVMGVNQFVFSSTAA
VYGQPQENPVTEESPTSPINPYGRSKLMSEWIIQDHGLASDFRYVILRYFNVAGADSRGR
LGSNSPHANHLIANACNVALKRQPELKIFGVDFPTVDGTGVRDYIHVEDLASAHVDALKY
LENNGTSQILNCGYGKGYSVLQVVERIRAISGMDIPITIASRRPGDPACVTAHAQKIKQV
LNWEPKYDNLDDIISTTLDWEKSKATR
>KRH96994.1
MTLISLIAAISQDRILSDSKNEHIRGGIPWDIPSDGRYFKETTWRHPVIMGRKTYATFNH
PLPNRTNIIVTKNTDYQAPGCVVFHSLEEAIKWSKMSETEEIFIAGGEQIYTQAMEFANK
LYLTIVEGNFEGDIHFPEFSNFGKLTKEEKLEENGFKFKFIEIERQ
>KRH96995.1
MVATTAKKMTFEEFLNYDDGTDYLYELENGEIILMPFESEINRRIAVFLLIYFSQLGIPY
YRLSMKTEIVVNSRMVGVRVPDLVVFSEELAQVMQNATRSLILMDMPPPLLVVEVVSPNQ
EKRDYRYKRSEYAARGINEYWIVDPIGQKVTVLEWVEGLYEERVFMDDEVICSPLFAEVK
LTVNEILR
>KRH96996.1

MASQERLAKEQEREAKERAEAIASQERLAKEQEREAKEQERQQKEKLAAYLRSLGIDPEK
I
>KRH96997.1
MNQYPVILIPERIQELKLALPPVPPAPIEPIRPGNPPVRPQEPRPAPRSNMSIQIFLIVA
TLLSALFIYLTSQSWWLLFIPILFFISYQTCFYYLRQEYKRNLESYGRQLQIYNKRMQLH
RQNINFYPLRLEQYRREQQLYEETVQVARTPERIADYYFNCLLQLLRETKSHDGDNSDAR
IGKREREFGKHLIHYFPGKIHTQLKVQNPRWQGFPYTPDFAYIDLDIQMYIDIELDEPYA
VDGTPIHFIGLRTELDRNNHFVNERGWIVIRFAEEQVARYPHSCCKKVATVISEITNNYS
ILNHFVNVPDLQFVSRWTEKESKQMYLQNFRDAY
>KRH97012.1
MSKIFRISHLLVTTLLSLSFPHPVGATPGTILQINPPVSTPSTTELTVIVNGILNKTGDI
CLRVYNSEVGFLTNGSSEVKSGCTKITGSSVKTVFSGLKPGTYAVAVMDDQNGDRKLNKD
FFGIPTEGFGISRDPIVSMRTGMPKFRRASFKMTQNTTIDITMKYSLDP
>KRH97020.1
MQIIQLSALSDNYIFLLYDSYHNIAAVVDPAQPEPVMEKLTELQCNLVAIFNTHHHHDHV
GGNQKLIEKFPQVTIYAGIQDRGRIPGQQVFLQANDIVKFGDRQAIVLFIPGHTRAHIAY
YFPPVTPGDTGELFCGDTLFAGGCGRLFEGTPAQMVESLTQLRALPDNTRVWCAHEYTWN
NLRFALTVDSENQELQKRLTEVTALRQLQQPTVPSLLGTEKLTNPFLRWDQPSLQLAVNS
SDPIQTFAKIRGMKDKY
>KRH97023.1
MAKFSRWVVFGVTLLFLAKTLQDHWLEVNSLSLVTIKWKTLILATIVTLLAHTWAGWVWT
WILKDLNQSVPTGLFIRVYLKTNVAKYLPGNVWHYYGRILTAKNTNIPTLVATLSVLLEP
LLMATAALFNIILLPNQLFLTFRYIPQILQFISLCLLLSAMHPAVLNPGLDILYQIKSKV
KFPGKTSPTVNSPIQPINNLILDHYPLLPLVGELIFVYLRGTGFILTISAVSNVDINQIP
ILMGAFSLAWVLGLVIPGAPGGIGVFEATAILVLDDVFPPAILISAIALYRLISILAETI
GAAIGYLL
>KRH97025.1
MPLKILVVDDDIGTSLSISDYLELCGYSVLTADDGENALSIIQQEHLDLMVTDIIMPRMN
GYELVRRVRQNIEFRLLPVILLTARTKTQERILGYQSGCDLYLPKPFELEELSAAVRNLL
ERSQIIQSEYCLSSQISGSFPPDDKTRPVSPSIPRTETLNHPDSHILMSISTREREVLEL
LTHGLSNAEIGSKLYLSPRTVEKYVSSLLRKTSSNNRAELVRFAIKHGIVD
>KRH97027.1
MNSEVPPKYEQDFYGWIQWQLRAISQRQVSQLDWENLQTELEGLGRQEYRELVSRLTVLL
GHLLKWEYQPENRCRSWFLTIREQRRAIHRHFQRNPTLESRIPYALEDAFEGGVDLALRE
TNLPLRTFPQVCPYQFEQAISHGFMCDTSQDWQ
>KRH97031.1
MKLYLYLLAGISSALLGWNIGQFFITDLGLFPQYPEIILFPCIAVSLSCSMVMNEIFISN
PTRVKRSFETAKQPLLIATGLGIISGLVAGIISQILFWPVIRVPTPIVRTLGWLLIGVSV
GLAEGLTWRWHSMEASGKKRLQQRLKISVIAASGASLMAAVLFEILRLTIGKMPPDLKSI
EDIIGFSILGLLLGAVFSVTNSPSYIAALRAGKGFEYKDFKEDTQDVPSYPLINSHQLSF
VNNVEETNEIQEGLSIQLPARGKIRIGSDSINTDITIPGLPPHIADIEIQKRAANLIPDS
EFFHAIAVNGVTLRHNRQVALRHNYLLTLYTFNKAGVKEEKYYRFVYYNRFLDPQA
>KRH97035.1
MNLNNKTILITGTDEFIGSRAAELAVGQGLKVRALQADPFLDKTSGESLEKLGVEIIIGS
INDPGIATKVCQGVDIVLHTSQLTEEGGDIKKFREINVGGTCNIAQAAKQAKVKTFIHLS
SALVYGFNYTPNVAETETLSGDNNPYCQTKIEAEIEILKLNSPPDFGVTIIRAGDVYGPG
SVPWIVRPVLMMRQKLFAYPNDGKGVMNHLYVDNLIDAIFLAMAQQTYGEVFNITDGENT
SWKEYFTHLAAMEGLPIPMSLPKEEMRLFLKVRNQGQKLFRKKVDILPESVDFMSRPYSY
SIAKARSILNYQPKISLEVGMNNTHQWLQTTDIQKLIK
>KRH97040.1
MIHQIDTLSYTNRLRKLSPWHKLIFAFTTLAISLASDPRVQILTVIWMSIWTVIYAKIPA
KVYLQLLMFTIVFGLMSLPALIVNGVSIFDIESVKLDSWYGITLGNFYVVISHNGTIQAW

NILTRSLSSASCVYFIILTIPFTEILGILRYLGVPILITDLLLLMYRFIFILLSTANELW
TAQTSRGGYRTAGVGMKSLAILIGQLLQRSLQQYNQFSLGLEARGFVSEFRVWRQYRYSL
ELRYIIESIVGCVVLIGLNFW
>KRH97041.1
MREYLLEFKEVYYSYMEFQQLALNSLTLRIPSGKKCALIGQNGCGKTTLLLLANGLYKPN
RGVVSWCGEPLTYNRNYLGKLRQKVGLVFQNPEQQLVAATVEEDISYGLCNLGLPVVEIQ
QRVEEVLIEFELTNLAQTPTHHLSLGQKKRVSIADVMVLRPELLLLDEPTAYLDIKQTRN
LIAMLNKIHQHGTTLVMVTHDLDLVYRWADWVFVMDRGKLMLEGSPQDVFGQRRLLEELE
LGVPLISEM
>KRH97043.1
FLLSTISVLSNPSTTITKEDSITNAINQWLYGKSTHSKRSYNRVIRSYISYLDDIHIANS
TASHFRDYQEYLYQSGKTINTINTYTNIIKSFFTFLRDESVLPTNITHRVKSPKPVSALR
ERILTRAEVDAMITLETNTRNRLILQLLFFCGLRVSELTILLWADIKDNGSTAYVHITGK
GNKQRTLIIPPLLWTSLKSHKTTNESPVFKSRKGGKNLTQKAVWDIKHARSWLHRRALCG
NFACQIGFRDLISGQTPAVGHALEFLVSCRWHD
>KRH97046.1
MLLFPNSSIDEIDSLQNQTLISVLRQLRDGFPHSLGLIGMRDVRDYKVKSSGSERKSDPL
TKGLTQLDKYLDGLGLHTGWLVIFDRRPGLPPMGERISTEEVISPSGRTITVIRS
>KRH97047.1
MGWRKNLVHLPHKKPKGKELTQQQKEENRELSRQRVVCEHAHSGIKRYNCVHSVYRNRVT
DFDDQLMLVSAGLWNFYLDAA
>KRH97048.1
MIRVLILGGTGDAIEITNKINQIPEIQAIASWAGRTKNPVMPPNVRIGGFGGVPGLVEYL
REQKIDIVIDGSHPFADRISWNADTAAREVGIPRLLFHRPGWEKESGDNWIEVENNREAA
VILDKQRVSHQVFKNQINPIKRVFLTIGRQEIGAFAHLEEIWFLMRMIDPPEVDSKIPRG
EILFNRGPFDLRDEREICTRYNIDAIVSKNSGGSATYPKIIAAREMGIPVVMVKRPPLPP
GEQVTDVEGVINWLIDKIR
>KRH97055.1
MNSKFPIKHISPLCTVFVLLTGTNLITVTPALGIYQHQIIAKQPIQGKKSQVNPVFQPIL
SKLKKTTQINILLPTHIPIGKNEPPLYSIVETVTKNKYQILLGFTPDCSGGTACRFGAIS
AELVSSNTPKPVGKELNLNNNKKAYFEDFKCGANCSDANLTWREKGVQYTIGLKAGSLSD
LVKMANSVVSANKRIRP
>KRH97062.1
MSKGVITITDAEFASEVLAADQPVLVYFWAPWCGPCKLMTGLMNLAAEKYSDRLKIVKME
VDPNPDSVKTYQVEGVPALRLIASKKLKASTEGVIGKEKLLGWLNENLDN
>KRH97064.1
MSQNTRTNSHNPFPKTSRLNPILATALKNLEVRLDEELVRYRRTRYSARPVDDKYITSNS
VELKVPSGEEGDPLSNRFSPDSTLVYSQGGSYTDTTTSFPPEQEIFDSPSLEEESAFQAP
PKKLNKKNFNKNNKKKSNRSKGGLLSPLGVFSIFLMLLTSLGFGYVLFNLRSLSKLSLSK
FNPFNTNPEISSSNTSENTNSPAVNNQSSIGSQSLTSVPQLPRVSQRKSANNGQLPSPSQ
PDSPVNQVSGVKTPPKSTNLPVSLQSRSIRNSTRQNTSSRSGLGVSSSNPSTPRSNTQFA
SIGEGRIKRRIKQEVPRQNLQLGEGSNAAAQAVNNPYYYLITDNESDTILSAAKEVVPDA
YLSPNQKYIYLGAFLTPEEAKQRLQQLEAKGIKARLRN
>KRH97065.1
MSLKSVNQILGILQIESRCQEEPLLKLLNFWPEVVGVKVSRETRPLSIRRHVLWVATSSA
AWSQNLTFGRYAILLKLNQRLSQLQIPALTDIRFSTAQWNEIAQNQTQETVCGQEHPSYL
AQSNNINYTSQIAQPVVSVKMVFENWFRTKQVQDSHLPLCPQCQCPSPPGELERWTICSP
CAAKVMERL
>KRH97066.1
MVFVLPGVKFDLDLIQKYDTRAPRYTSYPPATELSETFTTQDWEMAISHSNHRHTPLSLY
FHIPFCQTPCYFCGCNTVISHNKKIAEPYVNNLAQEIKNTSRLIDTSRPVLQIHWGGGTP
NYLEIRQVEFLWETINRYFSVDANAEVSIEINPRYINRDYINSLRSIGFNRISFGIQDFD

PQVQLAVNRIQPEKMLFDAMNWIKQANFSSVNVDLIYGLPYQNLQGFQETLTKTIALDPD
RIVVFNFAYVPWMKPVQKKIDPTTLPTAQEKLDIMKMTIEQLTSHQYLFIGMDHFAKHND
ELAIAQVNGTLKRNFQGYTTHAETELFGFGITAISMLDNAYSQNSKDLKHYYQSVEMGAV
PISKGIQLTPDDMIRRDVIMSIMSHFCLRKLEIEAKYHINFDQYFHEELLELGRLERDGL
VNLFADEIQVTDIGRLLVRNIACHFDAYSKMKESKFSRTI
>KRH97072.1
MQVLILALAILLVSCGTGEVRMDIFSVNTRYTEQDPALSGDGKFLAFVSNRSGSQQLLVY
DLQNQGFAPISGLNRRETIVENPSLSYTGRYICYLTSDRAKPVIALYDRVTKRSQVLTPT
YGGWIKNPDISPDGRYIVFQSAGRGQWDIEVLDRGPTVELDIPNGRAVGN
>KRH97080.1
MYSSSENLHLLYDIDENLWLEKTIVLLKEKRFYDLDLQHLVEELEALSKRDKNAVASLLE
QIIRHLLLLQYWHEESEINRNHWQTEIVGFRNQLERLLTTNLHNYLYTELEKIYQGAFRY
VRQKTRFEVSFPQDCPYCLEQLLNQSYF
>KRH97081.1
MPYSQFTFSKVKEQFDLTVTEGVRFFPPDIDPIVPSQKLLAILEDIPWAIAVDTEKARSE
VIINPVLLELRRIFDRQISIFSGEEFSVDPGIGLTGFCDFLISKSAEQLAIEAPAMVIIE
AKKADLKVGIGQCIAEMVAAQRFNQACNRQVSTVYGCISSGTQWRFLQLEGSVVTIDLTD
YPLPPVEVILGFLVWMIKNC
>KRH97082.1
MQINEKTDIKKSQPSQDESFSAKEFFNRQWEVYQKVLNNNYMGHQEIYDVLHKLLAEWSK
PFTMLDLGCGDASFTSGALLNTQITEYTGVDVSTAALVDAEQNIALIGCERKLVSADCWQ
FTNDLVQDGTHKFDVVLISFALHHLQPEEKERIINNIRTLLNPHGVFILIDIIRREKEDR
ESYIQRYLGNVKRDWSLIDPQEYTMVENHISSSDFPETQSWFQTISQKLGFSNFTPVYCD
NLDTTELLCFYR
>KRH97085.1
MTNQSFKEIRVQELQQRLIQDGSNLQLLDVREPQEIAIAQISGFVNLPLSEYNQWQGEIS
TRFDTSKETLVLCHHGSRSAQMCQWLLSQGFRDVSNIVGGIDAYSLLVDNSIPRY
>KRH97100.1
MGSTFGHLFRITTFGESHGGGVGVIIDGCPPRLEISPAEIQFELDRRRPGQSKITTPRKE
ADSCEILSGVFEGKTLGTPIAILVRNKDTRSQDYDEMSEKYRPSHADATYDAKYGFRNWQ
GGGRSSARETIGRVAAGAIAKKILLQVAGVEIIAYVKRIKDLEGTVDTNTVTLADVESNI
VRCPDAEIAPQMIELIEKTGRDGNSIGGVVECVVRNVPKGLGEPVFDKLEADLAKAVMSL
PASKGFEIGSGFAGTLLTGFEHNDEFYIGPHGDIRTVTNRSGGIQGGISNGENIILRVAF
KPTATIRKEQKTVTKEGEETVLAGKGRHDPCVLPRAVPMVDGMVALVLCDHLLRHYAQCK
LL
>KRH97108.1
MVDTTIRTTKSQEIFAAAQNLMPGGVSSPVRAFKSVGGQPIVFDRVKGAHIWDVDGNKYI
DYVGSWGPAICGHANPEVISALHQALEKGTSFGAPCVLENILAEMVIDAVPSIEMVRFVN
SGTEACMAVLRLMRAYTNREKIIKFEGCYHGHADMFLVKAGSGVATLGLPDSPGVPKSAT
KGTLTAPFNDLEAVKALFEQNPGEIAGVILEPVVGNAGFIAPDAGFLEGLREITQENGAL
LVFDEVMTGFRIAYGGAQAKFGITPDLTTLGKVIGGGLPVGAYGGRREIMSMIAPAGPVY
QAGTLSGNPLAMTAGIKTLELLQKPGTYDYLERITQKLVNGMLTIAKEAGHSVCGGSISA
MFGLFFTSGPVHNYEDAKKSDTVKFGRFYRAMLERGIYLAPSQFEAGFTSLAHTEEDIDQ
TLAVVKEVLRGVNSPT
>KRH97111.1
MTLTEKLEKLKTLFAEMGHALIAYSGGVDSTLVAKVAHEVLGDRAVAVTAVSPSLLPEEL
TEATAQAATIGITHKIVHTQEMANPNYTSNPVNRCYFCKSELHDTLKPLALEWGYSYVID
GVNADDLKDYRPGIQAAKERGARSPLAEIGITKMEVRQLSQQLALPWWNKPAQPCLSSRF
PYGEEITIEKLQRVGRAEIYLKTLGWEDLRVRSEGETARIELPPEKIKDFVLKTDLPTLV
TTLENLGFIYVTLDLEGYQSGKLNRVLTKQGPVLPKK
>KRH97114.1
MKQGIRDIFDRIAPVYDQLNDRLSLGQHRIWKEMTVKWSGVKPGDTCLDLCCGSGDLTFR

LARRAGIAGRVYGVDFSNNLLNAAKNRQKLSQNPYSITWTEADVLSLPFVDDQFDVVTMG
YGLRNVTDITRSLQEIYRVLKPGGRAAILDFHRPDDHIWRTFQQWYLDYLVVPLATNLGV
REEYAYISPSLQRFPRGKEQVALARQVGFVNAVHYPISNAMMGVLVIVVP
>KRH97116.1
MTTLNLVKLPITTIEIAPGSHLLIHDVTWEQYEALYKDWGDERQVPRMNYCNGTLEIVSP
LPAHERPHRIISDIVKTLLDAENRPWEDFGSTTFKKPEQAGLEPDTCFYIENADRVRSLM
RMNMETDPPPDLAIESDLTSQTTLDTYLTLQVPEIWIYENDRLTIYLLEKNNYQKSTTSR
VFPSLSITDLIPELVQQAIKRGTSSMLRNLRHQLST
>KRH97117.1
MTQSSNRENHQGNIQNTDNTAEELLVKLRQKKGNWVEWGNAIAYLQKNGYNPQDIFEATG
FEPIQQNQVVVGAQVYSSLEKFGASEATKAYYGTRASDILYELRLLTQGDRATAADLIFA
HKLDVDEAREIAKAIKDFSRFSTPPEGFSTHPGDAVAYQCWKLARQNSDLQERSRLIAKG
LRFVQSSTARKQIEQLLTDFTIVPQRNAPLLPYFRLESDEELPRLVPVVGELPLTPKDVK
SVPLIIEEAPFNIVKFAGEQAWVALPGWQVLRSAEDPIVIIGESNIFPQSKSGKTEQILI
VIDRDERDWDDGSYFAFDNDGEVDFQWFETQPEQTILGRIIVILRPKKVLDEDFTKDSWQ
IDE
>KRH97128.1
MIEVITPPTLVIPQLIVGLGNPEPKYNQTRHNIGFAAIDAISRNWHIPVTENRKFQGQFG
EGLALGSKKIRILKPLTYMNRSGESIQAVTSWYKLPPESVLVIYDDMDLPLGKIRLRLSG
SAGGHNGMKSTISHLNSQNFPRLRIGIGKPKNLPNNDGDTVSHVLGKFSPQEAKLIDPIL
AFVVECVELTLKQGVEKAMNRCNSWNGVL
>KRH97129.1
MKLSSLKIVVVGGGAAGFFGAIACTKTNSQAQVTLLEAGLEPLAKVLISGGGRCNVTNAC
FVPQDLVQNYPRGSKALRGAFSRFQPQDTIAWFEEHGVKLKTEADGRVFPITDRSETIAE
CLIKFAAHGGVRLKTRTSVVSVERQNGQFKIDCKSAGDVYSLYCDRLLLATGSGLVGYKI
ARALGHHIESPVPSLFSFKITDPKLQSLSGISVNSVSLTLSLQEKGVLKQTGSLLVTHWG
VSGPAILKLSAYGARLLYENRYQGKLYINWLPDLSLEEVKQKLLDVKQEWGKKAIALHRG
VDLPHRLWQYLIDRVNISVEERWAEISSKVLNQLAQEVHRGKYVITGKGEFKEEFVTCGG
VDLKEVDFKTMESKIVPGLYFAGEILDIDGITGGFNFQSAWTTAYLAGCAMGM
>KRH97130.1
MTTKSDPNRILRLLPLVVGSLGAVLLLVNRVLTPQLTESQARGDVLGVILSAVLILTGLI
WQQVQPKSPDTVELIGKEGFILASDLPETIKTELAWASRLLLTNTVTRSLVVYYKGEVLL
RRGILGSKAEVIPGPILERVLGTQKPIYLVALRVYPAKIEFDYLPENTQGVICQPIGKEG
VLILGANAPRSYTKQDENWIEGIADKLAVTLENRMEQQSS
>KRH97133.1
MKILLTGISGQVGWELQRSLMTVGDVICLGRNELDLSQYETITSTIREIKPDLIVNPAAY
TAVDRAELEPDLAMSINGVAPGILAEEAKRLGAAIVHYSTDYVFGGNQNTPYQENDPTFP
QNVYGKTKLAGEKSIQAVGTNHLIFRTSWVYGLRGKNFLLTMQRLAKEREEIRVVDDQIG
SPTWSRMIAEVTAQIIAQIRGQMVISGTTYLADFMAEKGGIYHLSCGGKTSWYGFAKAIL
IDKFADDQYGQYKLQRLVPITTPEYPTPAPRPSYSLLDNQKLLDTFGLKISNWQQVLDLA
LDS
>KRH97134.1
MKVSKTEIPDVLLIEPQVFGDERGFFYESFNEKLFTEKTGISPHFVQDNHSRSGKNVLRG
LHYQIQQPQGKLVRVAVGEVFDIVVDLRKSSPTFSQWVCVYLSSANKHQLWIPPGFAHGF
LVLSEYADFLYKTTDYYAPEYDRCILWNDPDLAISWPIGDEPIVSSKDKSGKLFSQAEVY
P
>KRH97135.1
MKGIILAGGSGTRLYPLTQVVSKQLMSVYDKPMIYYPLSVLMLAGIKEILIISTPRDLPL
FEKLLKDGSQWGLKFSYVEQPKPEGLAQAFILGKDFIKNQPVCLILGDNIFYGHGLTEIL
TRAVQLEHGGLVFGYKVTQPENYGVIEFNHLGKAISIEEKPKIPKSKYAVPGIYFYDAQV
VEIAASLKPSARGELEITDINLTYLRQEQLRVEILGRGYAWLDTGTHDSLRKASNFIYTL
EERQGVKIACIEEIAYNQGYIDRPQLQFLVDSMGKSSYGMYLRRILQDEITFK

>KRH97136.1
MSNYQRNDLPNLLVTGGAGFIGSNFVLHARRLGYGNIINLDKLTYASNLQNLTEIERNEK
YQEGYRFIQGDIGNSELVSYLLEEYEVDAVINFAAETHVDRSIFSPGNFIETNVVGTFKL
LEASKTYWQKLSSKKQESFRFLHISTDEVYGSLNAEDTAFREDSQYAPNSPYAASKASAD
HLVRSYHHTYGLPTLTTNCSNNYGPLQFPEKLIPLMIINAINGKPLPIYGDGQNIRDWLY
VTDHCDAIYLVLQEGRIGENYNIGGMNEQTNLVVVNKICEILERLAPKPNCEYSSLITFI
KDRPGHDRRYAIDCSKIAKELGWQPKENFESGLIKTVRWYLDNAAWVESILSGAYQNWIK
QNYESRETSQLKG
>KRH97146.1
MTTSLIKDQENIVYPTLGEDEVKFKHIIKSLPKECFQKNRRKAWTTAIISLTTVGLGYYF
LAISPWFLLPIAWIFTGTALTGFFVIGHDCGHRSFANRRWVNDLVGHLFMMPLIYPFHSW
RIKHNYHHKHTNKLEEDNAWHPIRVEVFANWGKVRQSAFELFIRKRLWWIGSIGHWAVVH
FNPGKFQRKDRASVKLSVGVVIAFAIIVFPTLIFTTGLWGFIKFWFIPWMVYHFWMSTFT
IVHHTTADVPFKTADKWNEALAQLFGTIHCDYPRWVEILCHDINVHVPHHISTAIPSYNL
RSAYKSIKQNWRPFLHKECKFSWNLMKKITNECQLYQTDIGYITFDEYYAQKQPTRK
>KRH97148.1
MNNLEQLRAAERVLLEIFYGIDAQVKHNLQRVLNAFRDHHLGSHHFAGVSGYGHDDLGRD
TLDQVFAQVMGAESALVRVQIVSGTHAIACALYGVLRPGDEMLAVVGSPYDTLEEVIGLR
GQNQGSLIDFGIKYRQLELTDQGKINWQKLSTAIQQNTRLVLIQRSCGYSWRSSLSIDEI
KRVVEIVKQQNPRTICFVDNCYGEFIDTREPTHVGVDLMAGSLIKNPGGTIVTAGGYIAG
KADLVEAAACRLTAPGIGSAGGATFDQNRLLFQGLFLAPQMVGEAMKGTYLTGYVFDKLG
YPVNPPPLAPRGDVIQAVKLGSAKKLLAFCKAIQQSSPVGSYLDPVPDAMPGYESQVVMA
GGTFIEGSTLELSADGPLREPYIVYCQGGTHWTHVSLALQAAMEAVGEF
>KRH97149.1
MTNINSNKHKKSKALKPGSRRPAKELCSECGLCDTYYIHYVKEACAFITQRIEELEVNTH
QRCRDLDKENELYFGVHQEMMAARKQLPIVGAQWTGIVSTIAIEMLNRGLVEGVVCVQNS
QEDRFQPMPIIARTPEAILAAKVNKPTLSPNLSVLEEIEQSGMKKLLVIGVGCQIQALRA
VEKQLGLEKLYVLGTPCVDNVTRAGLQKFLETTSRSPGTVVSYEFMQDFRVHFKHEDGSE
EKVPFFGLKTNILKDIFAPSCMSCFDYVNSLADLVVGYMGAPYPWQWIVVRNDTGKQMLD
LVKEQLEIQPVMSQGNRQPAVQQGIKAYDEAVTLPMWVAQLMGIVIDRIGPKGLEYGKFS
IDSHFARNYLYVKRHHPQKLEAHVPEFAKRIVAQYNLPETD
>KRH97153.1
MLQLPDSPPRMKTLVSVHESYKLCQELTAKYAKTFYLGTLLMSPTKRQSVWAIYAWCRRT
DELVDGPAATKTTPETLAQWETQLDSIFAGCPLDNYDVALVDTLQHFPLEIQPFRDMIAG
QRMDLYRSRYETFEDLYLYCYRVAGTVGLMSTAIMGVDTNVYSAPWYRDIQPYLPVEEAI
ALGIANQLTNILRDVGEDARRGRIYIPLEDLKKFSYSPEELLQGVLDDRWRSLMRFQIKR
AREFYTKADRGISYLAQDARWPVWAASMLYGKILDVIERNDYQVFNQRAYVPQLQKISTL
PLAWMRSQVL
>KRH97154.1
MRVVIAGAGLAGLSCAKYLVDAGYIPIVLERRDVLGGLVAAWKDSDGDWYETGLHAFFGA
YPNMLQLLKELGIEDRLQWKQHTLIFNQPDKPGTLSRFDVPDIPSPFNIIVSILRNNDML
TWEQKLRFAVGLLPAIVRGQEYVEEMDKYSFAEWLKRQGIGERVVSDVFIAASKALTFIN
PDEVSSTILLTALNRFLQERYGSKIAFLDGSPTERLCQPIVDYITKKGGEVRLNAPLKEI
LLNPDGTVGGFLLRGLDGKPDEVITADFYVSAMSVDPLKVMLPEPWRQMEFFQKLEGLEG
VPVINLHLWFDQKLTDIDHLLFSRSPLLSVYADMSNTCREYSNPDRSMLELVLAPAQDWI
DKSDEEIVSATMTELQKLFPHHFGGEEPAKLLKSHVVKTPRSVYKATPGRQKYRPPQKTP
ITNFFLSGSYTMQRYLGSMEGAVLSGKLTAQAISHIPTLNRPPAKNAATA
>KRH97155.1
MSQHLQQFGNHLILGVSGTTLTDDDKRVLSDLKPVGVIFFAKNFLDGTPYPIWLEEFKKL
LIDIREYTERELLFTALDHEGGKVIRTPLPITRFPVAHLVRSRAYQVAKATGLELRSLGI
NLSFAPVADVFSNPQNPVIGLRAFGQTADLAGENAKEYYRGLKESGILGCAKHFPGHGDT
SQDSHLELPILNLSLEDLKNRELIPFEILIQEQIPLMMTAHILFPQIDPDLPATISTTIL

HQILRQELNFSGVVVSDDLDMKAVADMFVYPETVARAFNAGCDLFLVSRNISSSSIEKTY
KVAENFISCLHNGSLTPSVIENSHNRIAQLLTKTPQYEVFPLEKQILVDHAELAINCCFD
LN
>KRH97162.1
MTTVYETTYILRPDLGDDQVDQAIVKYQNLLNEQGAENLEIQNRGKRRLAYEINRHRDGV
YVQFNYTAPAGAIAVFERSMRLNEDVIRYLTVKQEVKTTAPDSEALPA
>KRH97165.1
MGRAKKVVLAYSGGVDTSVCIPYLQHEWGVEEVITLAADLGQGDELEPVKEKALKSGASE
SLVADVKESFVQDYAFPAIQANALYENRYPLGTALARPLIAKVLVETAEKYGADAIAHGC
TGKGNDQVRFDVSCTALNPKLKILAPAREWGMSREQTIAYGEKFGIPAPVKKSSPYSIDK
NLLGRSIEAGILEDPANEPPEEIYEMTKAVADAPNEPEYLEIGFEKGIPVAINGTVKTPV
QLIQEINTIVGNHGIGRIDMIENRLVGIKSREIYESPAMVVLINAHRDLESLTLTADVTQ
YKRGLEETYTKLVYNGLWYSPLKNAIDAFIQQTQIRVSGVVRLKLFKGNATIVGRCSENT
LYTPDLATYGAEDQFDHKAAEGFIYVWGLPTRIWAQYNRD
>KRH97167.1
MIVAKDQTPYLTPEEYFTWESTQPEKYEYIDGQVYAMGGGSINHGRISIRFTSMLDSYLE
DTGCITGNSDIKVNILGSNNYTYPDISVTCDDRDKANTQYITYPCLIIEVLSPSTEAYDR
GGKFRMYRKNPALIDYLLVSSTSMEVDLYHKNDRGDWLIINYKPGEVIELQSINFNFPID
QVYRGLDLTKQCYF
>KRH97175.1
MEINQLIHTTILVTDLEKSEQFYGTILGLAKIERPLKYPGVWYQIGHHQIHLILAPSVPT
QNQNQKWSLNPHMAFSVLDLTTAQLELQNQNVTFQTSSSGRRAIFIQDPDGNIVELAQAG
>KRH97179.1
MSNPSNSGKIKFGTDGWRGIIADDFTFSNVRKVTRAIAAYLETAYDKSRPVLIAYDTRFL
ADEFARTSGAVLADLGWNVKITVRDCPTPVIAYNAKHLNSAGALMFTASHNPAPYCGIKY
IPDYAGPATPEITDTIVANIETASDELPGSNPSGTISTFDPKPAYLDFIYKLLDVEKIRS
ANLKVKYDALYSTSRGYLDEVLQYCGTQLESFHTWRDVLFGGGMPEPKGDQLVELVEAVK
NEKADLGLATDGDSDRFGIVDELGNVLTPNTVLLVLARHLIKNKGKSGAIVRTVATTHLL
DNFAAKYGLPIYETAVGFKYIGEKMRETTVLIGGEESGGLSVIGHIPEKDGVLADMLVAE
AIAYEGKPLSQLVQEAIAEADGPLYNNRLDLHLTEAHKNAVIKSYTQNPPSQVAGISVKE
VGRKDGIKLYLAEGSWILLRPSGTEPLVRVYIETNSPEKLGEIAKTMEAEIAQLA
>KRH97180.1
MKIIPDKDNINFDSHGSNPYLWKFWGVLPIYPYNQRRTVRQEVVKDTIWTFDQIQGVFYV
VVPIRMTVIRLAQGGLLVYAPVAPTRECIKLVNELVSLHGDVKYIILPTVSGLEHKVFVG
PFAREFSRARVFVAPEQWSFPLNLPLSWLGLPGRRTQILPEDPYETPFGDEFDYAILGPI
NLGLGKFGEVALFHKQSRTLMVTDTIVSVSVDPPAIIQLDPFPLLYHAQEKGGDNVPDSL
QNRRKGWQRTALFAFYFRPSVVDIPPWKDIWQDAKNLSQRNLRNYFGFFPFRWKPDWEES
YELLTRGGEIFVAPVLRTLILNRAPQVTINWANRVAKWDFARIIPCHFDAPVTATPQEFR
RAFNFLEKGSGDSPDSNNKVEDDLQVLRDIDQGLNNWKIVPPAQDYSKP
>KRH97187.1
MTDASPSKEQNHPRYHSDRLTINSLLSEDKTDYNLAELARLKIRYQGFPGARDLQNDLDR
ILQLWGLTAEELFVKTRAIHHVGGIYKSRAKREEEDWN
>KRH97190.1
MSQFLIRTFTSLSMLVGFMVISGCNMSLFENTNIKDVVEKVSDGDTLILRDGSGEKHKVR
LGCIDAPEIPHSHKQKRSKKRRDMNQFNWGVRAKNRLAQLIKNSGRRVKINVVDQDEYGR
KVIELRLRDDTLVQEVLLTEGLAKVHPEYMKLCSSKDIMLQAQTQAQRQKIGIWGDDEFI
NPWEYRKL
>KRH97202.1
MIFLPSAIEQLSEPTSVALAQSIQIEEMITPLSPQPIRTSYVVQGSGNTPILLIHGFDSS
VLEFRRLLPLLAPTHPTWAVDLLGFGFTERQRDIGYSPAAIKTHLYHFWKTLIGQPVILL
GASMGGAAAIDFALTYPELVQKLILIDSAGLKGGSALSKLMFPQLYSLAAEFLRNSQVRD
RICRSAYKNPNLINDDTLCCRDLHIEMANWKESLITFTQSGGYQAFKLEELGKIGQPTLI

LWGDSDRILGTKDGDKFRQAIPQSQLIWIPDCGHIPHVEKPEITAQHILDFTGKIL
>KRH97209.1
MIKPLLQLNRWQLSRQISILLCLLSLCSLAILSGCQNNIPQKNHITHLTLWQGINPPANR
DVFQKLVTKFNQTHPNIQVDSLFILGSDMALPKILTSVVGNATPDLLVYNPGITGQIVEL
DAVTPLDQWWNQFPHKSEVFPNLLEQLKLNGKLWSLPLWNSNVGIFYRPDLFEAAGITQT
PKTWEELIAVAQKLTLDKNRDGHPEQYGILLPLGKGGWTVFTWFPFVLSAGGGIMTDNYP
NLNNPAAITAIKFWQQLLQTGVATLSPPERGYEEDAFFAGRVAMQITGPWSYITKSPIPF
NAFPIPTYTTPATVTGTGNIFMMKTTSEKQQAALKFLEFIVSQEFQTPWSIGTGFLPVNI
KSLNSPEYQDYLQTRPWLKVFLDQIPLAGSLPTIAGFSRISENLGRAIEQTLLGKSSAEV
ALKQAQERLDVIWGDMSSP
>KRH97211.1
MDRFNYQEYHEGDQFELLSAYLDGEVTATERQRIEQWLATDESGKRLYMKLLRLRHGVRS
MSVPSCYAASSELFDLVWKRIIFRRRINWMWGGAAIAACVIGSLSGILPGNTSRLQLAQQ
KIDSVQTQAPISPLMVALNNPIVEIPKTAVVRPAKIEEIDYIQEEMEFEGN
>KRH97215.1
MTHPLVPQIIELATPLAAELGLEVVGMVFHTNQRPPILRVDIRNPQQDTSLNDCEKMSRA
LESSLDAAEIIPDTYVLEVSSPGISRELATDREFISFKGFPVVVSTCDPDDGEKEWRGQL
IRRDETKIYLNQKGRVVEILRSLVTKVQLDDHP
>KRH97221.1
MTPQLLFNSSTMKNLPSLFTLFLSVVLATWILAIALISVQNATPISLRFLVFQSIQVPFG
LMLAFWVVVGLITVSFGQLLGNMGGSGNSADEEGDFFVDEDFR
>KRH97222.1
MNTRKSPIQIFGVYALLLAIALLTLFPLLWLISTALKSPTENLLETPPKLLPLQPTLDNF
VRVWESLPFGQYLYNSFLVAILTVVLNLLFCSLAAYPLARLSFLGRNTIFIAIVSTIMIP
FQIVMIPLYIITVQLGLTNSYLGMIFPSLASAFGIFLLRQAFMGVPKEIEEAARIDGSSE
LGLWWFIMLPAIKPALITLAIFVFIGAWSDFLWPLIVIQDESLYTLPLGVAKLAGTFSLD
WRLVAAGSIISVAPVLLLFLFLQKFIVPTDTGSGIKG
>KRH97224.1
MYTVERQLYESLLGKRVSDSHWSNLKKTAQEADHVITLDEPPELSQNKEITLAEKQTIST
VTKDLGFILPQDELKAIAQHSLREIASSLREDSEITSITEIKEAILRFIAKKGERTRREV
DTALSEIATAYAAEQLISSRLFASLLANLSKEVKVAKGWNGVRHIVAVELRNNMDLAQAV
KVVKVIPSDDVLAPYFALGGTALLITAATISHKLTQYQAVQLLSSLRGIKGAAGRYAGGI
CSGLT
>KRH97227.1
MNLDTIKQDIASLPPYAQQIIIELVEVLKKRYPLNQQRTSENSLQDWSDFIGCIEAETDL
SKNYKNYLDTELNQKYDHS
>KRH97233.1
MTQKDLFLIWTKEADAALKVNDSGVAVDLWKCVGSHRLIAIVDVPTTDALDQILFDLPIM
RKVGQHVHVDVTSLKVYEDFTTYVTSQL
>KRH97245.1
MENIHNLNITDEEYLHLISKGYDPKLESQFIELGETEDQARKLAKVVGMFKDGPPQSDEE
WEHFLEVWEN
>KRH97247.1
MQSSFEGFHGIIPEILVGKAFQGYSGLLPNNSNGNFSIMRRAWTALIRVRFPLGSGINGN
GRSLKYYKIWFFVRFPLGSGINGNEEEKLLLGRGDYLPRRRDNGLRPIFLFFFLRKGKRR
GI
>KRH97252.1
MAKHFNTAGPCQSDIHYMLSPTARLPDLKALIDGRNYFIIHAPRQVGKTTAMIALAQELT
DSGQYTAVMLSVEVGSGFSHNPQQAEQVILQEWKQAIRFYLPKELQPSYWPEGETDSGIG
KTLSEWSTQSPRPLVILLDEIDSLTDEALIFILRQLRSGFPRRPQGFPHSVGLIGMRDVR
DYKVKSGGSERLNTSSPFNIKAESLTLSNFTLPEVEELYLQHTQATGQVFTIEAIQQAFY
LTDGQPWLVNALARQATQVLVKDITQPITVEVINRAKEILIQRQDTHLDSLAERLREDRV

KAIIQPMLSGSDLPDTPEDDRRFLLDLGLVKRSPLGGLTIANPIYQEVIPRVLSQGSQDS
LPQIQPTWLNPDNTLNPDKLLHAFLEFWRQHGEPLLRSAPYHEIAPHLVLMAFLHRVVNG
GGTLEREYAIGSGKMDICLRYGQVVMGIELKVRREKLDPLSKGLTQLDKYLDGLGLDTGW
LVIFDRRPGLPPMGERISTEEAISPRGRTITVIRS
>KRH97253.1
MNFQSVIAALHQFWGQRGCLIAQPYDMEKGAGTKNPHTFLRALGPEPWSVAYVEPCRRPT
DGRYGENPNRFQHYYQYQVLIKPSPDNIQEIYLDSLRVLGILPEDHDIRFVEDNWEDATV
GAWGTGWEVWLDGMEITQFTYFQQCGGIDCRPVSIEITYGLERLAMYLQQVEAITKIHWT
DHITYGDVHLQGEIEQCVYNFEASNPEMLLNLFHIYEQEASQLSEKGLVLPTLDYVIKCS
HTFNLLDARGVISVTERTRYIARIRHLARKVAHLYVEQRQKLGFPLLKNNGGREG
>KRH97260.1
MKLGQWIGLIALVVSLYILWQLREVLLLIFAAVVLATTLNRLARTCQNLGIKRGLAVFLS
VMFFLVGIVAFFWVIVPPFVHQFQELTLRVPQGFERVNTWVDEQRSHIPQELEPVIPDLN
RLIAEAQPLINRVLGNSFAFVSGSLVLVLNILLVLVLTAMFLTNPAAYQKLFVRLFPSFY
RRRVEGILNQCEDSLERWLTGAFIAVCLVGLMSLIGLSILGVKAALALGVLAGLMNLIPN
LGPTMSVVPAMAIALLDSPWKPIFVLILYFFIQQLESSFITPMVMAHQVSLLPAVTLISQ
LFFVTFFGFLGLFLALPLTVVAKIWIQEVLVKDVLDCWEHHGHDEVDLVILTDDDSQGE
>KRH97263.1
MIRFIDLFAGIGGMRLGFQQACDVLGVEYECVLSSEIDKKAVETYKINFDDQPRGDIREI
DIMPEFDFMLAGFPCQPFSYAGKQQGFGDTRGTLFFEIERLLTSYQPEAFLLENVRGLTT
HDHGRTFNTIINSLEKLGYGIHYLLLNSSNFGVPQNRVRIYILGLLGKNPRLTINSDKGA
TDSHSFKEQFYQLSLFPDSYSVVKVKNILEQKVSENYYLSKRFQQQIFDAVDGKFEKLHG
VRLIDFRGGNSIHSWDLGIKGKCSKNERDFMNALISHRRHKEFGNHQDGKSLTIEQIKTF
FDHEDMENIIYSLIQKKYLKEINGRYNPVCGNMSFEVFKFLDPESISITLTSSDANKLGI
VQNGKPRRITPRECARLQGFPDSYIVHQDDNAAYKQFGNAVTVPVIQAIVEDLIKYNNLH
ST
>KRH97264.1
MQNYARDINSLKSHATMWWPQNLRDKNATTSIIPRLLETQDDFISILQLSKNNPTQVFEL
AEAANFPANLFLKHLVVISDYGGELMKRLGESFTTIFTKRDKTNKLVMDYVWKGKNHQYV
FESMPIQSKLDNKKLNIDGKGLQFEEPFDGLKRDITMILLYASTSDVSHCAALDLCTLGS
ILGDKVALEHHIKQKYILVSRITGGANANSLGQLAQSYILKYLKEKLGTEFYISSNERIP
LQGTEISFDIVVTKADKKVGIEISFQVTTNSTIERKAAQASERQALMTKAGYKTAYVIDG
AGNFERFAAISKICQHSDCTVAFSDSEFNILVDFLRENL
>KRH97273.1
MNQTAINLIAICVFLMTLSTLLGPLIHLSPTIPALTILGFLGIATLDNFGFEGRGGTIFL
DWLARFSPTYQERIIYHEAGHFLVAHLSGITVTGYTLSAWEAWKIGQPGQGGIILGDDEI
APQLERGKIGVSMVEKYCTIWMAGIAAELLVFKSAEGGGDDKAKLNQFLTVLGFQETLFE
QKQRFHLLQAKNLLEQNWQTYQNLVQAMRNRLDVEECKKLIA
>KRH97277.1
MLELFNTINPWLAGGALNAALLTLVDRIPNKLLTPAGIRHAWLLGVIIWGTLGWPGYLVV
GFYFIVGSGVTRIGIKQKQVQGIAEKRSGARGPENVWGSALIGALCSLGVLLLPAWSYLL
CLGYVASFSTKLSDTTASEIGKAYGKRTFLITTLQPVPRGTEGAISLEGTLAGMVGSILV
AIVGWSVNLIDMPGIFWCAIASFIATNLESVIGATLQSKYTWLTNEVVNIFNTLIGAIVA
MILAVVFQRVLI
>KRH97278.1
MESSSFFTINDEQIELSQVVNYLQVSGRLNQFINDVVRQYILEKEIKNRNDVDISTALIE
QAIIDFRLKNQLTDQEQFQNWLTNNGTDYGTFYESVSFSFKLEKLKVVITEPKIPEYFIE
RKIYLDRVVLSRIMVSTRELAEELHTQIQEGGSFEQLAKEYSLADEKTFNGMMGPISRGS
LPDILRASVDGANMGELIGPIEIEGTFNLFRLENILLSSLENVQLKQSLQNELFEKWLGE
KIQNVTVKLQVS
>KRH97284.1
MPKYLGLISVLVVCGLSSLPDPVQAQALLPYAIQLNRANLEKQGLRLAQEAAQLARLDQF

ELAIPRAKMAVQLAPTNDKVWFLLSQLHVQRKDFPEAIASLTQAQKLNPNNPDILFALGS
ANFQQKNYQLSVKYYQSGLKLRPNDPEGLFNLGNTYYILGRLPEAIAQLNQAANQDKKFW
PAINNIGLIKYEQGDVKGAIKEWEKAIAIDQRAAEALLALAVALYTRGESQQGLTKGEQA
LKIDQRYADLDFLKENLWGTKLLLDAKKFLELPEIRSALEPKEETPSPTDNQ
>KRH97285.1
MLTICPHLFVVLIKLMSDLIGPLGFSDFYLLVLPKLVRVLTSPWELIFDKWVSIYNHSS
>KRH97286.1
MVSISKHVEIPLIGKKGQYSWRWIIGIVASGVLILGATTIIKAGNSENRKPDITKLTVPV
EAKDLTVRITASGKVQPIQSVNVSPKNAGLLAELNVEQGEKVEEGQIIARMDDSEIRMGI
LQFQANLEQAKAQLADAEAGSRVEDIAQAKARVNQAKAQLEIISSGSRSQEIEQAKASVE
GARSQLELTQARLNRYQKLAKEGAISQDTLDQYIAENKRAKSNLREAEKRLSLQEAGNRE
QEIRRQQAVVTQETEGLRKLQNGSRPQEIARLTAVVEAAKAQLKRQQVQLEDTIIRAPFA
GIITQKYANIGAFVTPTTSASSSTSATSSSIVALARGLEVLALIPEADIARIKQGQQVEI
ISDAYPNQVFTGRVRLIAPEAVIEQGVTSFQVRIFIVNGGDKLRSGLNVDVTFLGDRLED
AITIPTVAILTEDGKTGVLVPDSDNKPEFREISIGAQIKNETQVLTGIQTGDLIFINPPK
DYKSKKPN
>KRH97288.1
MSTTLMISDLVNESLDGNSCSQPEIIRLENIFKVYGTGETEVKALNNINLVINQGEYCAI
MGPSGSGKSTAMNIIGCLDRPSGGNYYLDNVNVADMNDQQLAQIRNLKLGFVFQQFHLLT
QLTALENVMLPMVYAGVKPGERKARAIEALIRVGLEKRLHNKPNQLSGGQQQRVAIARAI
VNAPVVLLADEPTGALDSRTTQEVLDIFTELNSTGITVIMVTHETEVASQTKRIVWFRDG
EVVHSHLTPEELHHLVSS
>KRH97292.1
MLIPILIFDVALVAWSLHLMEKAIENKEFSLMLAGALVAVAAAAMLVVYFLMGNCMSYLL
KIG
>KRH97293.1
MLSKRILPCLDVKAGRVVKGVNFVDLKDAGDPVELARIYDEAGADELVFLDITATHEDRD
TIIDVVYRTADQVFIPLTVGGGVQTLENVKDLLRAGADKVSINSAAVRNPHLINEASDRF
GNQCIVVAIDARRRVEPNSTGWDVYVRGGRENTGIDALSWAQEVEKRGAGELLVTSMDAD
GTQAGYDLELTRAIAQAVEIPVIASGGAGNCQHIHQALTLGQAEAALLASILHYGHLSIA
QIKNYLQEQSVPIRLPC
>KRH97295.1
MLRDFANREELVTYIREQFPQAAQTGDYISGTSGGRKAAVEQLEKVNAVLYAKTRNYLTG
AVTRLSPYIRHGVLSLREVREYILNTVENSEDGSKLINELGWRDYWQRLYMKLGKGIWEN
QEEYKTGYHRGDYSHTLPEDIQEGKTGLVCIDSFSQELRVTGYLHNHIRMWLASYIIHWR
KIQWQAGAKWFLQHLLDGDPASNNMSWQWVASTFSHKPYFFNRENLERYTKGVYCSRCPL
YGKCVFEGSYEDLQSTLFPHSPLT
>KRH97296.1
MSDIPQIGKPAPDFAKLDQDENLVSLYQINQWVVLYFYPKDDTPGCTMEAKEFTELASEF
IDLDGKVIGVSPDSSKSHCKFITKHNLAITLLTDPEHQLIETYGAWRMKKFMGKEYMGVA
RSTFLIAPDKIIAYSWPNAKSKGHAQTVLKKLQELKN
>KRH97297.1
MSIRPIYLDSHATTPLDERVLNAMIPYFTEKFGNPASNSHVYGWEGQAAVKRTREVLSTA
INCTPEEIVFTSGATEANNLAIKGVAEAYFSKGQHIVTVATEHKAVLDTCEYLKTIGFEL
TILPVEKDGLIDLEKLEKSLRADTILVSVMAANNEIGVLQPLSEIGRICHQQGVIFHTDA
AQAIGKISLDVEALNIDLMSLTAHKVYGPKGIGALYVRRKNPRVKLAPQQHGGGHERGMR
SGTLYTPQIVGFGKAIEIALEEQETENFRLETLRERLWKQISKVGGIHLNGHPSKRLAGN
LNISVEGIDGAALSLGLQPVVAVSSGSACSSHHVAPSYVLTALGYPEELAHASVRFGIGR
FNTAEQIDEVAAHFLKTVESLRFNSVVTC
>KRH97301.1
MTSSTVTDKGEISLHQNVLGSRRLSNYSWATIVTLGASGFSLASISSYLKVNLLIVTDAT
ELIFVPQGLVMGIYGIAGILLALYLWLVILWDVGGGYNEFNQETGKFKIFRWGFPGKDRQ

IAIESSIRDIQSVRISIKEGLNPQRALYLKVKARRDIPLTRVGQPLSLSELETQGAQLAR
FLGVPLEGL
>KRH97306.1
MSVYVGNLSYEVTQDALTAVFAEYGSVKRVQIPTDRETGRVRGFAFVEMNSEAEETAAIE
ALDGAEWMGRDLKVNKAKPKEDRGGSGGRGGGYGGGRNRY
>KRH97308.1
MVLVPNESRTTVKVLSIIPSGSWVSQISHHRHKILLVSVLFLGILATGCVSTVKESAEAQ
SQNSSGKRSAKPISVDVAIARTDSLNQELIYTGSTVPRKIISVRSQVEGRLIGLDLEIGD
RVSKGQRVGRLDDILLKTGLEQQEAELGNRESEVERARIQVGNIEAEVEKVRLELMQAKS
DSDRQQKLLQEGAISQQAAQQAVTKVKTYQQILKSTIEKQRTENKAVAAAQNRVLAQRAV
VKAARERLSYTDLISPITGVVTEKITEPGNLLQSGNEVIKIADLSQIKVVVKVSELELGK
VEIGQRVEVNLDAFPDEKIIGRIERISPVADSTARVVPVEIVIPNSQGKIRSGMLARVNF
SRQESSRVVVFKTAINNQDQETSSPNNNSTIFVIERNEKRVKVKEQPVVLGKEADGKIEI
ISGIQPGDSYVVRSSKPLQDGQRVKLSALSELPN
>KRH97312.1
MSYATDEPRDAFQSEDTLETTVNEARWYAVQVASGCEKRVKTTLEQRIQTFDVADKIIQV
EIPHTPAVKIRKDGTRQQTEEKVFPGYVLVRMTMSDDTWQIVKNTSHVINFVGAEQKHGS
GRSRGHVKPVPLSNSEVERIFKQTTEQEPVVKIDMATGDKIMVLSGPFKDFEGEVIEVSP
ERSKLKALLSIFGRDTPVELEFNQVQKQS
>KRH97317.1
MTTQNQLGSPNIIRSKDKNALEQNSDGVSIQVEKLSDRQRQITARVQIHQPVQKVWKILT
DYESLVEFIPNLTKSSLIEHPDGGIRLEQIGSQCLLNFKFCARVVLDLEEIFPKLIKFAM
VEGDFKGFSGFWSLKPYKLGTGEGTDLCYTIRVWPKLTMPIGIIENRLANDLQCNLLAIR
QRASW
>KRH97319.1
MVVSPAKNKQHHVVIIGGGFGGLYAAKTLANTNVNVTLIDKRNFHLFQPLLYQVATGTLS
PADISAPLRSVFRNIKNTQVLLGEVTDIDPKGQKVFLGGEVVQYDTLVLATGANHSYFGK
DHWKDLAPGLKTVEDAIEMRRRIFSAFEAAEKESDPAKRRALLTFVIVGGGPTGVELAGA
IAELAYQTMKDEFRSINTSETKILLLQGGDRLLPHIAPELSEEAKLSLQKLGVEIQTQTR
VTNLENDIVTFKTGERIQQIASKTILWAAGVQGSPIGKILAERADIERDFSGRVIVEPNL
TIPGFKNIFVIGDLASFSHQNGKPLPGVAPVAKQQGEYVGTLILLRLQGQTLPEFNYNDV
GSLAMIGQNLAVVDLGFIKLKGFIAWVFWLVVHIYFLIEFDTKLVVVFQWAWNYITRNRR
SRLITGKAAFLETQTINNNNPYQAAETAQHTVKV
>KRH97320.1
MLVYILSLVVAIASLTIYASAFFFPEIHRKNDFIWSGVGLFYALVLWVFASRITGGLLLG
HVASVSLLLWFGGQTLSLRGQLVSKGKPTSVPTAQPEIKGIQQPVSKISLLEKLQQLPSL
IIRPFNGLIVKVQQVVFKNPSVNTKISPSPTPEPPAPESPAPEPPAPESPTSEPPAPEPP
APESSNPQS
>KRH97327.1
MTIDQVDIRKSEVIQLLKQINVPILNNNLVSLGMVRNLRIIDDYVYLRLYLGSCELDLKE
EVRTKLSQLGWCKKTYIEIRTISQVRRTIGISSGKGGVGKSTVAVNLAAALSLSGAKVGL
LDADVYGPNIPQMMGLGHSEIIVTDTADGQRFIPLEAHGIKLMSVGLLAEPDHPLAWRGP
VLHKIINQFIHQVEWGEMDYLLIDLPPGTGDAQITIVQESPICGVILVTTPQQVAIADVR
RSVYMFRQVGVPVLGIIENMSYLLNENVDSFQAPQYIFGKDGGKLLSEELEAPLLGQIPI
HQRICESGDRGLPIVLGDRHFLPSRILEQIAQGLRKTFNNL
>KRH97329.1
MQITFLGTSSGVPTRSRNVSSVALRLPQRAELWLFDCGEGTQHQILRSDLKISQLSRIFI
THLHGDHIFGLMGLLASCGLAGNVERVDIYGPAGLNEYLQGASRYSHTHFSYPIKVHTVQ
PGVIYEDEEFTVTCGMLHHRITAFGYRVMEKNRYGRFDVEQAKALQIPSGPIYGKLKRGE
TVKLEDGRIINGKELCGPTEIGRKFAYCTDTVYCDGAVKLAEDADVLIHEATFAHQDAEM
AFQRLHSTSTMAAQTAYVAGVNQLIMTHFSPRYTPGNDIELKDLLKEARAIFPNTIMAHD
FMVYDIPRRREKGSGDPD

>KRH97330.1
MQSKPWKKIRAFLPINHLSNYISNIYQKQILSYLAIFNLLSITYLIVNWHYLNSPVLAVY
IIIANLLELIILQHKDKNLKSEVKSRQTIIEIIFETIHNGPLQTLDRILRILNQVNQQEF
SPHTLMKTTIPELTTELEKLNQELRGIYEFWHRETISPHTNLYLDKNLVINLEAPLPEIL
YQVYTHTLERNFSCFQTIKLKVRSFDPLDETGLTLEHKRGICRFLEESLCNVGKYANGVT
CLKVTGLSSMGWYTLSIVDDGLGVNSCKEGEGTKQFKYLAQQIQGKFQRIPQHPQGTICE
LSWPTSSVVGSHQT
>KRH97334.1
MKPNTPTGYPTTKWASGIKHKMNVGWVEERNPTPPQVTLPLTYPTNNCASLLNKLSLKDF
IKVQRNWK
>KRH97336.1
MQVIEKNSLFAQVSGEQSAVVSGGNAVVGSWAFVTFLANDGALSYAEAVASIILTSPYF
>KRH97337.1
MTQTKNFIGNVNDTTAVGSTVLPESDRREPLKHLLIGSKKTVISTIHYLHVLGYAHATDW
SDPIPTSNPGEVMSILVRQILIN
>KRH97341.1
MFLRIDASGYFTNRKQFRLFAPMINFPILTKSILLTFFGNVDKLSFFPLEQHKTLYLEIG
S
>KRH97342.1
MTSYTTSSAKAEMSELRRLKSLLPPELQSWVIVEGTTEVSPPLIRSEEIGKDQVEIQIDL
IKWDALAMDQRNLLFWHEVGRIQNDTIPKDGWEMAALAIGLGGAVGELWVQDGLLLVLAL
ALCGVSGWRLYQKNNGAKQTKELIDADEKAINLATRFGYTLPNAYKSLGSALKTLIDTTP
GKRQRYRYEARLSALKRSANKAKSRSNNVDENY
>KRH97350.1
MTTAIKPDLTSNLVNRVLSIKPLFDFAKHQAREMMIKRAQKIGVNWHQEVEKLQARDWSN
DLAQVQDPQLTYPDYYLTSFHAYETGNMSWQAAFEVESAAYAVHAKVWPDFLPDGDAKLR
QSYHHILKTLIPDTPKDILDLGCSVGMSTFALQAIYPQSQITGLDLSPYFLAVANYRSQK
SPQYQNSINWLHAAAESTGMPDNSYDLVSIFLMCHELPQSATQKIFVEARRVLRPGGHLT
IMDMNPQSEIYKKMPTYVFTLLKSTEPYLDDYFTLDMEKSLIDAGFKTPTMTSNSPRHRT
VIAQVIK
>KRH97365.1
MSIFPISKPTVLVTGGAGYIGSHTVKALLQDGYHVLILDNLVYGHRDLVEQVLQVELIQG
DIQDIPLLNSIFQRYQVEVVMHFSAYAYVGESVTDPAKYYRNNVVATLSLLEAMLGAGIY
KFVFSSTCATYGVPQFIPLTEEHPQHPINPYGATKLMVERILSDFDIAYGLKYVSFRYFN
AAGADPSGILGEDHNPETHLIPLVLQTALGKRSSISIFGTDYPTPDGTCIRDYIHVTDLA
IAHILGLEYLLQGGTSTVFNLGNGNGFSVKEVIAAAKEVTGNNIPITECDRRPGDPPILI
GSSEKARKILGWQPVYPHINEIVSHAWKWHQKRHG
>KRH97367.1
MLPTKSYLQLTPDLRICRILNGMWQVSGGHGRIVPKNALSAMFKYVDAGFTTWDLADHYG
PAEDLIGEFRRQLIAQRGELAANNIQTFTKWVPRPVNMTKGIVEENINISLKRMDVPSLD
LMQFHWWEYGNSNYLNALKYMVELQAQGKIKHLALTNFDTEHLQIITQAGIRIVSNQVQY
SLVDRRPEVNMIKFCEAHNIKLLTYGTICGGFLSEKYLGQSEPRSFDLNTISLKKYKNMI
DAWGGWQLFQSLLTTLEQIADKHQASIANVAINYILKQPAVAGVIVGARLGISEHIADNQ
RVFEFDLDDEDIKIINRVCQQSRDLYRVIGDCGDEYRR
>KRH97370.1
MTKTYKSILSVSQLDRAAIALILVLGLLIGITIAQGDGVKPTVRSFSWQDRQIGAEDTAF
SLVFSRPMDAKSVEDNLQITPPLAGKISWAGKRMVYTLLTPAPYGTNYQVSLNKAKDKFS
RSQNSNRLIKSFMGSFSTRNRALVYIGASPQEYGRLVLYNLTQEQKTILTPRDLIVMDFE
PFPIGDKILFSARASNNPDLLSAKIYTVTTGIVNGQEQKSEPAGKLGLMLDSQEYQNLKF
DLSPDGETIVVQRGKKDNPGDFGLWYSSLDNLDSNGKVTLKRLEGKPAGDFIITPDSKAV
AVAQGQGTAILSLAGDTSKPLDFLPQFGLVQAFSKDGSQAAMVKFNNDYTRDLFLVTNQG
VQKPLLKTSGSILYCSFDPSSPTLYCLLTSLVSQDKYVEQPYLVAINLDTKKYKPILMLP

LGQRNVQMSLAPDGLAVLFDQIVPVSNNSLSLPPSILKTNDGEAIAKSSLWFMPLVPIAE
DQGKSLIKPETLPMDGFNPVWLP
>KRH97384.1
MNSKALPCQVNNLEVGVYECEIHLKFRLIEEKSLLGDREQLLQVFLDALTEGSDEFLEML
QASVKAQEISEFKASPQMRRQLMRLRNSLDNTQQ
>KRH97407.1
MTRLIFLGPPGAGKGTQAKVLADFLQVPHISTGDILRQAITDQTALGVKAQEYMDKGDLV
PDQLVQDMVEERLQKSDAQKGWILDGFPRTVSQAVFLGNLLDQIQGDSERVVNLDAPDEI
VVSRLLGRGRKDDSEDVIRHRLNVYRRDTAPLIQYYGDRQKLLTVNGNQSQEEVTSALKM
AITVLRK
>KRH97413.1
MMSDSRQSTPTYRVALVIQYLGTNFHGWQRQKAQRTVQEEIETAIASVLGYHVTLHGAGR
TDAGVHAAAQVAHFNATGHIPAHKWATVLNSYLPPDILIRASAGVKESWHARFSATYRRY
RYTIYTEALPNLFVSPFSWHYYYAPLEEKLMQAALTPLVGKHHLAAFHRAGSARSHSWVE
IQAVECGRQGSLVHIEIQANGFLYGMVRLLVGMLVQVGSKQKSLEDFTNIWQTEDREKVK
YAAPPQGLCLLRVGYSDFPFSQEIWYETQPYLVFGHRTDDESIKPDKGKNNE
>KRH97420.1
MVGIHQFPTDSLPKYGLLHLYPVFGQNSPPLSLVTLDTQEKIRQVLSQGYKITVEYVDKR
RFSMGSWQTCGNLHISDYAQAVFALESCLLEHAGEYVRLVVIDPKLKRRVIETIIQRP
>KRH97427.1
MDIYKAVILPLLFNLVKADPEWLHHGLISGIDWFLQPSHPHRARWLKSFVGDSLCVHDNR
LKQNLFGLNFPNPLGLAAGFDKDGMGSHLWSMFGFGLAELGTVTYHGQAGNPQPRLFRLP
MDLAALNRMGFNNSGAAAMAARLTLLYQQQLPAIPIGINLGKSKITPLEAAATDYLQSFR
LLNSLGDYFVVNVSSPNTPGLRSLQDRPMLSQILEVIQTENSTQNQPKPLFVKIAPDLEW
EAIVDIINLAKTYKLAGIIATNTTISREGLKTQVIEKTGNPPQAEPGGISGLPLRQPSTE
IIRFIYKQSQGQIPIIGVGGIFTAEDAWEKITAGASLIQVYTGWIYEGPMMASRVLTGLL
AKLQNHGLGSIGDAVGINS
>KRH97431.1
MLSRVWSASIVGIDAVKVGVEVDISGGGLPGIIILGLPDSAIQESKERVKATLRNAGFNV
PVRKIVINLTPADLRKEGPAFDVPISIGILAASEQVNLDLLGEFLFLGEVSLDGTLLPVT
GVLPIAAAAEKLGISSLVVPMENTQEAAVVEGLNVYGCTNILQVVDLLNNIKNHKKVSLK
PTQESLLSTSSNSADLQDVKGQSHARRALEIAAAGGHNLIFVGPPGSGKTMLARSLPGIL
PPLEFSESLEVTRIHSVAGLLKNRGSLVYERPFRSPHHSASGPSLVGGGSFPRPGEISLS
HRGVLFLDELTEFKRDVLEFLRQPLEDGYVTISRTRQSVVFPALFTLVASTNPCPCGYYG
DAVQPCTCSPRQREQYWAKLSGPLMDRIDLQVAVNRLKPEEITRETRGESSKIVRERVQK
VRAIANLRFQSEPHVKSNAHMQSRHLQKWCKLDDTSRKLLESAITRLGLSARASDRILKV
ARTIADLADEGNLKSQHVAEAIQYRTIDRMQ
>KRH97433.1
MVRIKSGFNADDQHPIQATSADIILRQQLEYSISRYFYHGCDRNIQNLLSYCRWYMITDT
KALTLVIECPDQVSNWRILQKIVPMASLLYSVVSSAKIRICPPDAGAIPFEMRVDELSVY
RDWA
>KRH97434.1
MLTIIGCGNLNRNDDGVGVIIAQKLQQYVAENPHPQVRIFDCGTGGIEVMFQARGSKKLI
IVDASCTNSQPGAIFRVPGKELEGLPQVGYNLHGFRWDHALAAGRKIFADDFPQDVTVYL
IEAENLDFGLDLSPAVNHSAQLVFAELITTIENANFT
>KRH97435.1
MLNRIKEISNNIAPRLREIYRHLHAHPELSGQEHQTAAFVAGVLSSSGLHVLEEVGKTGV
VGELLTNHPREEILAIRTDMDALPIQERTGLDHSSSRDGVMHACGHDVHTTVGLGTAMVL
SEIANHVEGRIRFLFQPAEEIAQGAGWMVQEGAMNDVCAILGVHVFPSISAGSVGIRYGA
LTAAADDLEIIILGESGHGARPHEAVDAIWIASQVITALQQAISRTQNPLRPVVLSIGKI
SGGRAPNVIADRVQLLGTVRSLHPETRSQMPAWIDKIVANVCNCYNAKYQVNYRHGISSV
QNDYSLTQLLQSAAEEAWGSDYVQVLPEPSLGAEDFSVYLDYAPGSMFRLGVGYKDRIIN

HPLHHPQFEVDDSAIITGVVTLAYAAYKYWQK
>KRH97440.1
MAKERPPMEEMTLRQLRKVASEYGVSRYSRMRKSQLLASIQEVQTRKFSISPSQSLEAQE
NVEATKFELGQEDRNGGSLSDVDAGLGDLPGGYGESRIVLLPRDPQWAYTYWDIPNEHKQ
ELRRQGGQQLALRIYDVTDVDLDHQSPHSLQEYPADELAREWYLPIPVSDRDYVIDIGYR
TPDGRWLVLARSARVHIPPVYPSDWIEDVFITVDFEEDLRGKTKYELIPPAKKAAIGVST
AIPGGDPVRDKIYDLAESGEAQRVAGSLFGSMQHVPGSVRSEQAISSYVFPSGVGMWAVP
TASGINVNMSGVGMSGVGFSASAIPVRPRQFWLVADAELIVYGATEPDATVTIGGRPIKL
NPDGTFRFQMSFQDGLIDYPILAVAADGEQTRSIHMKFERETPSRHTNTKEEAVLEWLS
>KRH97441.1
MRKLYFLLPGTNGKFACGGLWAELKTISLVRNICNAEVVTYRQREKNLLFLDDLLPNPNL
QDVIFVVSWGFDIPKLVRKLQKYNVVYHAHSAGYKFHLPSSIPIIAVSRNTMGYWGQKAP
NNLIYYLPNQISDEFTNLHLDRDIDVLVQSRKSSEYLLQQLIPALQQKCRVLVADSYVPD
LPGLFNRSKIYLYDSAEYWAQQSVTEGFGLQPMEALACGCQVFSSINGGLSDYLDPAFNC
YKIAGYSIEYDLQRIIKILDSSVHLTLSNQVLSEHRTESITARLLVILSEINHFFDHQSH
QLSTIPQLTKFRLATLLMKRVYGKFIQSCLGRMS
>KRH97443.1
MSNSSVLCLGEILFDCLADQIGLKLEEVNSWTPYPGGAPANVACALVKLGTKAGFIGAVG
QDEPGDTLVKLLGDVGVDTRGVQRHPTAPTRQVYVVRDLNGDRTFAGFGKYHTREFADTC
LKASDLPEELFNEADFLVLGTLELAYPESEAATHQALKLAERYDLKIILDVNWRPVFWQD
SELAKQKIHQILPNCDFIKLTKEEGEWLFNTSDAGAITYRINSLEGVLVTDGENGCSYCL
AENEGKLPAFSVPVVDTTGAGDSFLAGFVHQLNQYGIQALSDPQIAKSVITYASAVGALT
TINPGAIASQPTAQEVETFLHRFNPE
>KRH97444.1
MPKHFNTAGPCQSDIHYMLSSLDRLPTLGNLIDQRGYFVIHAPRQTGKTTAMMTLAQQLT
ESGNYTAIVLSVETGSAFKHDPILAEKYIIRSWIGTTKVTLPGELQPPKLRDIQEEMGLL
DPSDLDIKTYLQAWTLASPRPLVVFLDEIDSLEDETLITVLRQLRAGFPLRPHGFPHSLA
LIGVRDVRDYKVASGGSFRLNTASPFNIKLESFTLSNFTLSEVTTLYQQHTDATGQVFLP
EAVALVFHLTQGQPWLVNAIARQLVEVLVTDPTQPITVAEVAQAKELIIQRQETHLDSLV
KRLREPEIQAIFEPMLSGDELGNIPEDDVQLLLDLGLCRLQNGSGLQVANPIYKEIIPRV
LAYVTTASLPAPSLNPHWLNPDRSLNPEALLGSFLDFWRQHGEPLLKSAPYHEIAPHLVL
MAFLHRVLNGGGRLEREYAIGSGRMDICLRYGAVVMGMELKVWKPGKKDPLPQGLQQLDK
YLAGLGLHTGWLVIFDRRPGLLPIEERTTTEEVVSPGGRAIVVIRG
>KRH97445.1
MSADIITLETQETGSHNLIIHRSDSDLSLQGRIQVPGDKSISHRALMLGAIAEGETEIQG
LLLGEDPRSTSSCFRSLGAEISDLNTQLVRVKGIGLGNFQEPLDVLNAGNSGTTIRLMLG
LLASHPGRFFAVTGDDSLRSRPMSRVVKPLQQMSAQIWGRKGNTLAPLAIQGQSLKPIHY
HSPIASAQVKSCILLAGLNTEGKTTVTEPALSRDHSERMLKAFGADLTIDPGSNSVTITG
NAKLYGQKVIVPGDISSAAFWLVAGSIVPGSDLVVENVGVNPTRTGILEALTIMGANIQL
ENQREVAGEPVADLHVRSSQLQSCTIAGDIIPRLIDEIPILSVAATFAKGTTIIRDAAEL
RVKESDRITVMAQQLNKMGARVTELPDGMEITGGTNLVGTEVDSYTDHRIGMSLAIAALN
ALGTTTIHRAEAAAISYPNFTSTLVEVCHRHC
>KRH97446.1
MLTHHHKPVCLSIISTDLPVWSVVETPATLYQKDSERFHLLLTAPPLISCEADNMSTTEN
AIAPIHKHLQTPPSPRVLWLEISPYRITMTMQGNTQISYRHFWEKGVYGISRYWLPLESL
QPCQPIRLRNFTTNLKLTGKILPENLRIEYELWSQKLQLGRYILNLEIHH
>KRH97447.1
MTPEVKMDRFNYIAKYKSNQLIYGHGNTAVVTGWTVKEALCKRLQPSEYAVIGQLYSPTR
GINILVRNLLLNPHVHYLIVINATKEDKNAGAIECLLDFFAHGVEENFSASGRRSWVICS
SITGYIDIDIDLNALEKLRHSIEVATAKSISLAVEKIKYYAQKPMIAPWGMALEFPISQF
ETTILPGVRYGHRIEGKTIAETWVKIIHRIKTTGIIRPSAYDSQWQELIDLMAIITDEPD
DFYFPEPNYLPIERSYLPEYISQVLDDAHDQEGVKYTYGKRLRSWFGKDQIQQVIDKLIN

DPDSARAVMSLWDAVKDEHDSPPCLNHIWVRIVDDQLSLTATFRSNDMFSAWPANGMGLR
ALQKYIYDGIVRGSDIHQNLQLGPLITVSQSAHIYDECWEHAENVIASHYHKICQKKDYY
DPAGNFIIYLQNGSIIVEHTTPNSGEVVNCYSGKSSTQLSREITLNSPGLQVDHAMYLGS
ELQKAEIALSSDYHLIYQQDKPLKFSLNPA
>KRH97455.1
MYLSQPDLNNFSVQPNQTALIDPAVIKAAGQIYHTYSQVHPEIIGQVSGVAISRTTYRGK
VIFTQQPVLLPQECFIPLQQIESYVY
>KRH97459.1
MKIAVAKEIEVSERRVSLVPDMVAKLVKQGLEISVETGAGEKAYFSDGDYEAAGAKIITD
AAVLWGEADILLKVSPPQEREDGRHEIDLLKPGAVLLSFLNPLGNPEVARKLAQRQITAL
SMELIPRTTRAQSMDALSSQASIAGYKTVLLAAAALPKYFPMLTTAAGTIAPAKVFVMGA
GVAGLQAIATARRLGALVEAFDIRPAVKEEVQSLGAKFVEIKLTEETTAAGGYAKEISED
SKKRTQEVVAEHVKHSDIVITTAQVPGRKAPILVTEDMVKGMKPGSVIVDLAAEQGGNCA
CTAPGKDIVYHGVTIIGPINLPSSMPVHASQLYAKNVTALMQLVVKDKALNINFADDIVD
AACITHNGEIRNQRIKDALQTVTV
>KRH97460.1
MKRLLPALALSSCLLTGISTTTLAQSLPGLTLFSGVKSENQLSFFLDFGGQTNSTDRYRL
RVPANKMKLPVSQFNITYPEHYKGSFDTKEIEVRVKGKSVGLKEVKWDKETRVIEIVTQE
PVPARSKVELILSNVQNPSFGGMFYFNCQVLSPGGVQIPRYLGTWIISIS
>KRH97461.1
MPLPKAYRLKSRGDFQAVFREGVRCHSSHFTLRALKPLSPTHLHSSSINIPANTCENLPN
TKIGISISTKVSKRAVVRNRIKRQITGVLYQLLPKLSKGWRLVVIVKPKTGEWQCISQQF
LRELEQLLVKAEVINGHS
>KRH97463.1
MDFGIGFLSNNVMLPIIDFFYGVFPSYGLAIVALTLIIRFALYPLSAGSIRSMRRMRIVQ
PLMQKRMAEIKERYKDNPQKQQEEMVNVQKEFGNPLAGCLPLLLQMPVLLALFATLRGSP
FAGANYSVNLQIVPSEQIERIQPQAFATSPQNIYVADGVHTKITAILPGGNKLAVGEKTK
IQYQTMEGKPFDALLLEYPQTKLTPEWKIIKGEDRIKIDSEGNVEALQPGDVTIQGTIPG
LAANSGFLFIDALGRVGAIDPDGKVHWDIVGMIIFFGISLYFSQMLSGQNSSGGNPQQET
VNKITPVIFSGMFLFFPLPAGVLMYMVIGNVFQTLQTYILSREPLPEELQKIVAIQEKEK
QAATVDVKTLPFEPKGSKKKQPNNKESKT
>KRH97465.1
MDAIFLPQLTKAPQCTEEIQVDEFLPGLETLTPVRGVVRLQHHGNYLEVSGKAESIITCS
CNRCLQQYNQRLAIKTKEIIWFDTNSSPVEDLPLEREVAMEDLVETIAPDGYFDPGEWVY
EQMCLAIPQRQLCNSNCPGIIATGVNESSVDRRWSALEKLKNQLS
>KRH97466.1
MRNSPSLDPDLRKNLVVIFTCGLLFWSALASLLPTLPLYIESLGSTKQQIGIVMGSFAVG
VLVFRPQVGKLADRQGRKLVLLIGMVVATIAPLGYLAVKSLVGLMLIRAFHGISIAAFAT
AYIALVSDLAPDNRRGEVIGYMSLVNPIGVAVGPALGGYLQAIAGYTPLFIFSSLLAGLG
LICVIPITNPPTWKNNKQETGDDFWGILISPRVRVPAIILLIIGFSIGTIHTFIALFIKS
IGIDLNAGLFFAAAAISSFVIRLFVGRASDKYGRGLFVTLSLIGYGIAMLTIWQANSSPI
LLLGAIVEGAASGIAIPMISAMMTDRALPHERGRIFSVSLVGFDLGLGIAGPVVGYIAQS
TSYRHVFGLSFGLTLLAILIFMSQSNHGVFQSLRFALGRSKDVYQRSASDSPTLSTNT
>KRH97468.1
MRILVTGGAGFIGSHLIDRLMSNNHEVICLDNFYTGSKQNLLSWLNNPRFEIIRHDITEP
IRLEVDQVYHLACPASPVHYQYNPIKTVKTNVMGTLNMLGLAKRVKARFLLASTSEVYGD
PEIHPQTEDYRGSVNPIGIRSCYDEGKRVAETLTFDYHRENKVDVRVARIFNTYGPRMLE
NDGRVVSNFVVQALRGNPLTVYGEGQQTRSFCYVSDLVEGLIKLMNGDYTGPVNLGNPEE
YTILELAQTIQNMINPEVQIKFTPLPADDPRRRRPDITRAKTWLNWEPTISLQTGLKMTV
EDFYSRIHAND
>KRH97472.1
MLTNSQTPTLSAELSKFLPPPDSQTRASQFMKQLQDKITTELELLDGGSKFTEDSWERIE

GGGGRSRVLREGTVFEQAGVNFSQVWGDQLPPSILVQRPEAAGHGFYATGTSLVLHPRNP
YIPTVHLNYRYFEAGPVWWFGGGADLTPYYPFAEDPIHFHQTLKNACDKHHVDYYPVFKR
WCDEYFYLKHRGETRGVGGIFLDYQDGQGDIYRGPDSQGEAANYSRNLTPLPRRTWEQVF
SLIQDCGQAFIPAYAPIVQRRRNTEYGDRQRSFQLYRRGRYVEFNLVYDRGTIFGLQTNG
RTESILMSLPPLVRWEYGYQPQPNSPESELYETFLKPQDWLNWKLQSSH
>KRH97473.1
MLSIQEKSYQTKSDETVIVLAPAGRLDITTAWEFRLKLQECISKKNCHLVVNLGGVDFID
SSGLTSLVAGMRDANKLNRTFRICNIHPDAKLVFEVTMMDTVFEICETEEEAFAIPF
>KRH97474.1
MNNFRTVSDTKRTFYSRHTRPINTIYRRVVEELMVEMHLLSVNVDFSYNSIYALGVVTTF
DRFMQGYQPSKDLVSIFNAIICAVEQDPQVYRQDAAKLKAIANSFSVKDLIAWCSQTTPL
DQDANLQAELQAIAQNPNFKYSRLLAIGLFSLLELSDPEFVKDETQRNQTIAVIAQGLKL
SEDKLNKDLDLYRSNLDKMEQALTVMADMLAADRKKRDQRQQNSGNPPLAPTNE
>KRH97476.1
MDILDLFKKGGPAMWPLLVLSVLSLSVIFERLWFWLRILSQEKQVVERVLDAAIDSWEIA
TEIAQKATDQPIGRFLYAPLRLQKSDAETFKLALESTAAEEIAGMRRGEKLLESVIALSP
LLGLLGTVLGLIQSLRAIKIGDLGTESTAGVTTGIGESLISTASGLIVAIVTLVFYRLFQ
SFAVNQVKVFNKAGNDLELLYRQSPPESKKREFVFITEESPVEEELTHPIENPVTSSESE
PENES
>KRH97478.1
MLMSASSDFIALCQEQVSLLTQGLGASSSVVYLTQELVDHPSGEGLLIPVLVYPEASRLN
YSQDVGRIKVNNLKFGNLENLSDSSYVLSLPDQKLLAPRLDYIPSSPQSTGAYTSDYNKS
LNLEDKYLFGQYQVVLPLVYEGLMVGLLLTAREDRQWKQVEEEEVRRIATTLAIACILDQ
RQVWLQHQLQQEKALQKEQGDLLDNLLHQFRNPLTAIRTFGKLLLKRLRSNDTNREVAIN
IINQSDRLQELLQNFDQVLDSKNRDTTAIPTLGLALTVEASPQKTAPLLLPGTGEEPTSC
HLKDILLPLLASAQVLAQEKTIQLLIDIPNHLPPVKANIKALTEVFSNIIDNAIKYTPVG
GKISIQSLQKNTDFQGIAISDTGPGIPKEDLDRLGERNYRGVQANTDIPGTGLGMAIAKQ
LIAQMQGEIEVFSPAVEFDSASSSLPGTSFIVWLPQLPHLLNQHPL
>KRH97480.1
MDTAIIPSTLLLTLLLLVGLFFFIRASTKDRTEQAKIASEDDETILMGQLKDYFQSRAYR
VVSADPEKKEVIFEGFVQPSWFLAVFLTVLAALGLACLGLVLAQLFSSQNPFFLVLVAPL
SGIFYWRQSGRIEKVLLKMEFCQNEQHPSSIITVTAHRDELAELKRALQVKALQVKIVDV
IT
>KRH97492.1
MKMTQGKINELLNESGCEHNQKKQAQKKNKSCTQQAQPGAAQGGCAFDGAMIALVPITDA
AHLVHGPIACAGNSWGSRGSLSSGPMLYKTGFTTDLTENDVIFGGEKKLYQAILEIHKRY
QPSAVFVYATCVTALIGDDMEAVCKVAAEKIGIPVIPVISPGFIGSKNLGNRFAGESLLE
YVVGTGEPEHTTPYDINLIGEYNIAGEMWGVLPLFEKLGIRVLAKITGDARYQEITYAHR
AKLNVMICSRALLNMARKMEERYGIPYIEESFYGIDDINHCLINVAAKLGDGELQARTKK
LIAEETAALDIALAPYRERLKGKRVVLYTGGVKSWSIISAAKDLGIEVVATSTRKSTEED
KAKIKKLLGNDGIMLEKGNAQELLKLVRDTKADMLIAGGRNQYTALKARIPFLDINQERH
HPYAGYMGMVEMARELYEALYSPIWEQIRKPAPWDNGETF
>KRH97494.1
MTPQDNKKIVEERKELIKEVLQAYPEKAAKKREKHLNVHEEGKTDCGVKSNIKSLPGVMT
ARGCAYAGSKGVVWGPIKDMIHISHGPVGCGYWSWSGRRNYYLGTTGVDTFGTMHFTSDF
QERDIVFGGDKKLLKLIQELEELFPLNRGVSVQSECPIGLIGDDIEAVSKTAAKELGKPV
VPVRCEGFRGVSQSLGHHIANDQIRDWVFPRADQAKKDGSLKFESTPYDVAIIGDYNIGG
DAWASRILLEELGLRVVAQWSGDGTINEMLLTPNVKINLIHCYRSMNYISRHMEEAYGIP
WMEYNFFGPTKIAESLRAIAARFDSKIQENAEKVIAKYQPSMDAIIAKYGPRLEGKTVAM
MVGGLRPRHVVPAFNDLGMKLVGTGYEFAHSDDYKRTTHYIDNGTIVYDDVTAFEFEEFI
KALKPDLIASGVKEKYVFQKMGLPFRQMHSWDYSGPYHGYDGFAIFARDMDLALNSPTWG
LIGAPWNKSAKKALRKATAAV

>KRH97496.1
MWDYTDKVLELFYEPKNQGTIEDNREPGVKIATGEVGSIACGDALRLHLKVEEATDKILD
ARFQTFGCTSAIASSSALTEMVKGLTLDEALRVTNKEIAAYLGGLPEAKMHCSVMGQEAL
EAAIYNYRGIPLASHEDDDEGVLICSCFGITDAKIKKAVRQNNLFSAEQVTNYVKAGGGC
GSCLAKIDDIIKQVQQDFALEIGSGNGTVSHQQFSALGDNLREQLEQQKPLTNVQKIALI
QKVLDEEVRPVLIADGGDVELYDIEGNKVKVILKGACGSCSSSTATLKIAIESRLRERVN
KEIIVEAV
>KRH97501.1
MLTKFPSPYDELAVNLLVHYGFDLNGHTAIDLVTYWGKEYPHDWLHIGIIEALYQGRYKA
ISVQQILTFWQRRGCVYYHFNTEFERMICNKFPQILKQDSGTVLSVGTSLNLARPRNGVK
KVSSHSDNYHFQNNDLSEDLFMETYLASPPILPQTRNFPYNSSAPLKDVINHESLSAPEP
LSEVIPVNINHPPIGQFTPESTNNSELFTSKLIAMTIN
>KRH97510.1
MQVNESRTNANLKANIEAEAWHSLEQSILYYQKQPVGTLAAVDQSVEALNYDQCFVRDFV
SSALVFLIKGRTDIVKNFLEATLKLQPKQKDLNPYKPGRGLIPASFKVVTNHGEEHLEAD
FGEHAIARVTPVDSCFWWLILLRAYVVSTNDYDLAYRPDFQTGIRLIMDICLANRFDMYP
TILVPDGACMIDRRMGIYGHPLEIQVLFFAALRAARELLVCEGNEDIVEAIDHRLPLLGG
HIREHYWIDINRLSDIYRFKSEEYGKTAVNLFNIYADSLPYYNLDKWLPRKGGYFAGNVG
PSQLDTRFFTLGNLMAVICDLATKTQAQAVMNLIEKRWEDLVGDMPIKICFPALENEEYR
VVTGCDPKNIPWSYHNAGNWPVLMWMLAAAAVKTGRVSMAQEAIEIAQSRLSEDQWPEYY
DGKKGRLIGKQARKYQTWTIAGYLLSQEMIENPDCLSLVSFEKLPPQGAFKACKLEFST
>KRH97515.1
MGKLAEVSYFGCNMINSLKPAVYIVGAGPGDPDLLTVKAQRLLAAADLVLFADSLVPQQI
LDICRPDAQVIGTATKTLEEIVGTIIAAVQSNKFVVRLHSGDVSLYSAIHEQIELLNASN
IPFEIVPGVSVFQAAAAKLKVELTVPDLVQSIILTRVSGRTKVPDQEQLASLAAHQASLC
LYLSARHVASAQNQLLQHYPPQTPVAICFRVGWPDEKIFIVPLEKMAESTDEQKLLRTTL
YIISPALSTVSGRSHLYDAKYNHLFRVSTQ
>KRH97522.1
MANESEPASELRLNKAVKWTDTLRTKVQCGKVTPMLSDRNSDLTDHTVESRSLSEHNNGW
>KRH97524.1
MKAVILLSGGLDSSTVLYQARADGYECYAISFDYHQRHRRELQSAMSIAQSLGVVEHQLV
TFDLRSWGGSALTDNSIPVPHNRPLDQMSESIPITYVPARNSIFLSFGLAYAEAIRAERV
YVGVNALDYSGYPDCRPDYIHAMQEVFRLGTKQGREGKPIDIIAPLINLTKTQIIQLGNR
LGVPWELTWSCYSGGEHPCGVCDSCQLRWVAFHELGLKDPGV
>KRH97528.1
MFQWSKKTVKSVTFNPGISDESLLAQVESYLQANPSKTFSDLCKETLWQYLCGLESSPPT
RGTIPVSPDQKISELQRQVAELERRFLSRESHILQLSQQVAQLANMVNQGQSVVSSVSLG
VFNPSVSQPVPVEKTDPVIDRLSSLIDDF
>KRH97529.1
MVEQQKQEPDIPELSRTQVLIAMGITAIILWTVAKLWLGFGDVILFRLYWQPTHLVWGLG
LGFSITIFSSVAYRISSDYRESADYYLRMVLKPLALPDLIWLGLLPGLSEELLFRGVMLP
ALGADNVAVIISSLCFGVLHLTGAKQWPYVVWATIIGIVLSYGALWSGNLLLPIVAHTST
NWLASYLWKRWQMAT
>KRH97531.1
MTQTFHVEINHQGKKYNLEVPSDETILSAAEQARLDLPSSCHAGVCTTCAALIIEGTVDQ
SDGMGVGMELQAQGYALLCVAKPLSNLKIETEKEDVVYQKQFGTAN
>KRH97540.1
MKVFENTTKKIFLTSWVSPHQMDANPGSVVNEEQHSQTGWDMLGPVRNWLDNIKVQDRDF
AHILCKLIPAQCPFERDVKLLGKVLFHIPPLCKLNPFYDQLTYLRFRALCYLADECGEA
>KRH97543.1
MSKSYSVAILGATGAVGTELLELLESRKFPVDNLKLLASERSAGKVIQFQGENLVIESVN
DRCFDQIDVVLASAGGGTSKIWAPKAVDRGAVVIDNSSTFRMHPDVPLIVPEVNPQEAAN

HKGIIANPNCTTILMALVVWPLHQVKPVKRIVAATYQSASGAGAKAMEEVKVQSQFILEG
KQPVAQVLPYPLAFNLFPHNSPMTSMGYCEEELKMVNETRKIFGNQDIRITATCVRVPVL
RAHSEAINLEFDTPFAPDTARQILSGSPGVQLLEDWHKNYFPMPMEASGKDEVLVGRIRQ
DISHPCGLELWLCGDQIRKGAALNAVQIAELLVKENLLG
>KRH97544.1
MKITQEKLEKSQIGLEIEITQESTREKYEQVIKDLMRNVNIPGFRKGKVNRQILLQRIGT
NRVKASVLEELIPEAIEQAVKQENIKAIGQPKLVSSFDDLIGKYQPGETLTFSAAVDVEP
EVNIKQYTGMQIKAEEAKYDSTKVDRAIEEEREKIATLVPVEGRAAQLGDVVLVDFSGSL
AETPEQEPALIPGAQGEDFSVDLNEDRFIPGFVTGIVGMIPGETREIPAQFPHNYPDENL
SNKAALFTVTLKEIKEKELPELDDNFAQDVSDFKTLAELRASLEKQFQQKVEEKNTENKH
EALLQELLQHVEIDLPETMIEQQVDHRLEKTAFNLAQQGLDVKRLFTKETVVQLRENIRP
SAIEDLKRDLAIREIAKRESITVDKEEVKKKADEVLQQYSGEDIDVKNLKLVLGIELERE
KVLEWLIANSSLELVPEGSLSGGDQEPETVTPE
>KRH97545.1
MLISQSENPPITSLSTMNIQCGYNNLNPISLSSIVPMVVEQSGMGERAFDIYSRLLRERI
IFLGTAIDDNVANSIVAQLLFLDAEDPEKDVQLYINSPGGSVYAGMAIYDTIQQIRPDVV
TICFGLAASMGAFLLTAGTAGKRMSLPDSRIMIHQPLGGAQGQAIDIEIQAREILYIKAN
LNQLMSKHTGQPLERIEADTERDFFMSPQEAKDYGLIDQVISRQNLPSSGAPVTIVK
>KRH97548.1
MSIGNLEDLGLGDLQQLHETDDYQWLLTNIKLLRNKQFDQLDLENLIEELTDLGNEKKNA
VESLLQQVIRHLLLYQYWHVEIERNSGHWQAEIYNFRDQVNSKLTSNLRSHLVAEMPKIY
QRALGYVQRKTNYQIEIDFPRESPYSLEQLLDINYLG
>KRH97551.1
MLSGLLKFVLGFLLAIAVLLGSGMTIAIYFINRTAITPEKPMFANDNPDPKPNLPKVTPK
KVVKVKPKPTATPELNRESPTALPPGSYTAVVTWSQGLSVRDKPAFEGQAIGGVAGNQKV
IILETSQDGKWQKIRIPDTDQEGWVKAGNTEKSN
>KRH97554.1
MDGSIKQLLKGVNLYLIGMMGSGKTTIGNLLAQAVNYSFIDTDEVIVKAAGKPISDIFIT
EGEPAFRQLESNVLAQVCAYTKLTIATGGGIVLRRENWSYLHHGLIIWLDVPVEILLERL
KEDQTRPLLQDPDTQNKLRSLLDKRYSLYSQADLHIEITQQETPEQTVTNILQAIPGVLK
TPEIPKLTG
>KRH97556.1
MGTENLETAKNLYSQGKIAFENGEYQQSVDNLEKATSLLFQNSRFAGEVNIWLVNAYEAT
GRSQEAIALCQELSHHPHYEVKSQAKRLVYILKAPKLKRPKEWMTEIPDFATISERQTKT
LIAPQKSTSPQKSPDSEYVDLSQVNTQDNLFIWVALIIIAACTISYLVWLSF
>KRH97557.1
MDRTIADLRQDYSLQELDEKSINGNPLVQFRIWFDQAIAAELPEPNAMTLATCTCDGKPS
ARMVLLKDFDDRGFVLFTNYNSHKGQELGINPHAALVFWWAQLERQVRIVGGVEKISPQE
SDGYFEVRPHGSRLGAWASNQSEVIPHREFLQLKLAELEQKYENQSIPRPPHWGGFRVIP
QEIEFWQGRSSRLHDRLLYTRLHNHEWRIERLSP
>KRH97558.1
MRLSASLQTILTYGLACPLIAVNVWLLSLLFHYFQHPITILSIAAILAFLLNYPVQLLEK
ARITRTYSVIIVLVITLTLLVILGFTLVPMLVEQTTQLLKNIPDWVTSSQENLSRLQVLA
RQKRLHIDFSVVSSQINASVQNILQQIASGAVGFAGTLLSGLLNIVLVVVLAFYMLIYGD
RLWSGLINQLPSYIGLPLSKSLQLNFHNFFLSQLLLALFMVIALTPIFLFLRVPFALLFA
IIIGISELVPVIGATLGIGLVTLLVSLQTWWLAFPVAMVAIIIQQIRDNILAPKLLGNFT
GLNPLWIFIAILMGFEIGGLLGTLVAVPIAGTIKSTVDTIRNNGIT
>KRH97559.1
MDAKDLLATINNLIEQAKRGEIDPWNVEVVEVIDRYLEFMSPQGTTQGYESDLHQSGQAF
LSASMLVLFKANTLMELSRIDSEIDNSSEDVVTDDNNTESHSIERLQLERQLRRRTAAMP
PPKRRVTLVELIEQLQIMASQLKEVEKPNKSERPRRQPTMKTMKAALELAHQENLTQVAV
EVEQVLLLAAKEENSSEPSWGLEELVELWSGTQQANQVDKKSSSEHNSEHGNLVSVFWAL

LLLCAQSKVELFQEEFYQEVKVRLLRQK
>KRH97566.1
MKIATWNVNSIRTRLGQVISWLGENNVDVLCLQETKVIDTDFPLTVFHDMGYHTYIYGQK
AYNGVALISRQPMKSVTTGFCQVLENLEPEWDDQKRVITGIVDEVRIINLYVPNGSAVGS
EKYQYKLQWLAVLKTYLEVLLKCNSDIIMCGDFNIALEDIDIYKQVDTENQIMASLPERQ
ALREILKLGFGDGFRKFNSQGGNYSWWDYRTGAFKRNSGWRIDHHYLTDVLYKQAKSCFI
DISPRKLEQPSDHAPVIVEV
>KRH97569.1
MNSTTNKRIALISVHGDPAIEIGKEEAGGQNVYVRQVGEALSQLGWQVDMFSRKVSPDQE
NIVQHNSHCRTIRLTAGPVEFVSRDHGFQYLREFVEQLLKFQKESGFKYELVHTNYWLSS
WVGLQLRQIQGTKQIHTYHSLGVVKYNTIENIPPIANQRLAVEKEVLEKAEVIIATSPQE
EEQMRTLISRKGNITVIPCGTNVRRFNCVDRGVARDTLGIDKKAKVVLYVGRFDPRKGIE
TLVRAVRESRFFGDQNLKLIIGGGSTPGNSDGKERDRIEGIVRELGMVEYTLFPGLLKRD
ILPYYYSAADVCVIPSHYEPFGLVALESMACGTPVIASDVGGLQFTVVSENTGLLAPVQD
VTAFSYAIDRIIGNPQWRDKLGLAGSKRVVEKFSWEGVASQLNTVYTQVLDRMIIGSLTH
PL
>KRH97575.1
MAAKSFPISSLSIKGYKSIKNLQDFPMRSLNILIGANGAGKSNFVTFFAMLSELVEQRLQ
VWVQKQGGAERVLSFGIRETPELYSRIYFARNQYSFTLGPTASSGFVFLEEEEYFDGPRF
GPKKKTKSGQTESCLRDWKAAAKQDSMEDYIYSSMSSWKVFHFHDTSDMAGVKRPCSVHD
NKHLRPEASNLAAYLYLLQQKETAIYNQIRKTVALAIPFFDDFVLEPTTLPTEEQQIRLL
WKQKDSDYAFWPSQLSDGSLRFICLVTVLMQPNPPSTIIIDEPELGLHPHAIVLLGSLIR
ANSNRTQVIVSTQSVPLLNEFSIDDLIVVEREEGASVFKRYNGEDFSSWLENYSIGELWE
KNILGGRPRIGS
>KRH97580.1
MSEQAQEALETQAPDRYECRACGYVYEPEKGDDKYNIPAGTAFADLPTNWKCPVCSAKKV
AFANIGPTGTASGFKENLGYGFGVNQLTPTQKNILIFGALALGFLFFISLYGLQ
>KRH97582.1
MNQNKERQKRRPIAIDLFSGAGGLSLGIESAGFEVVISIEIDPVHSAIHNYNFPNCANIC
RDISNVSSEELWNILNDKDINEVDLLAGGPPCQGFSQMGYRHIEDPRNKLVFEYVRVVRD
IKPRYFIFENVPGIVSGKHKGFIEDLCHEFIQIGYNTIVPVLILNAADFGVAQNRSRLIL
LGWRKDMPKPVYPGVLFEKPTANSKSRELSSLPPWLGASDVLSDLEGVSVFIGKDEGLSP
DKLEYCGPRSNFSFHPNGVFDLCHRRTFTQKLIYGHLSSTHTEQSIVRFKNVPQGVVEPT
TRLFKLHPYRPSNTLRAGTDSKRGAHTAPRPIHYSHPRCISIREAARLHSFPDWFQFHRT
IWHGHRQIGNSVPPLLAKEIAQSLIGLLGYDLSKIPVDNLPPSDPGLLSLTMHQACNLLG
VCKDVIGKRNRNKLNT
>KRH97583.1
MSHSILQQNLTQKKISPVSFFADILLAGMVLGPSLAPFLAASNVFVLQIISNIIYFIGDH
VCPQPETGLELAPPYIMTVCMRCYGTVTGLLITRVLYGLTQGKGVFWLHQYGWMGASVAT
ILMTAYPWELAAEVFGLWEFNNYVVTPFGLVTGLAWGLFAMPLLHSSKRTFLINQ
>KRH97585.1
MRTTNQKIIQVDAFTNKPFQGNPAAVCVLTNSQSDEWMQQVAQEMNLSETAFLLAENDGF
NLRWFTPTTEVPLCGHATLASAHVLWSEGYLSPEQTARFYTKSGLLIAKKQDDWIELDFP
VNHSQIVEPLPLLNEVLGVGYKSVSLNSLGYLVELASPQLVRQIQPDLQQMRLLPVRNVI
VTSTGDLEYDFVSRFFAPGFGIDEDPVTGAAHCCLAPFWRHKLQKDSFLAYQASQRGGVV
KVTYSGGNRVFLSGQAITIIRGELVNY
>KRH97589.1
MVDSEIQQLVTLNQELKNVNNDLCRKLEKLNKELGEAEKILQWQKKRSSVSESMLNQQNQ
EITAAQERIQSLSQQLETALQNMESQEMFIETHKAESQINQQRIAQLERECTLLQTKCSE
QSQQLLQSENTCRELGMRLLRQQRQTLQFKAALEKCLDNPASSDQEIDDNACYLDVNTED
SRFYKKVQSLLSHREPIRPWSAMDYVDSEFNQPTENTMDVPEETSHVDQRLEDILSVFFA
SNPDVSDISNTNRTNNTKETNIIDSNPEENEPRPTMTSESSKDINNLPKVNLLSDCPINT

INMNTSDQQNNYDLQWSINKINNSSNNFSPVEPLEQDSEEKSPSPLIYPGRTVKVRKTLA
AVELPNFPPQSQNQDAR
>KRH97590.1
MISQLNNGFTLFLSLLVEAMPFLLLGVLFSSLLLFFVEERKLVDIMPKNPFLGALFGSMI
GFLFPVCECGNVPVARRLLMQGVPTSVAIGFLLAAPTINPIVIWATWTAFRDQPEIVVLR
VVLSLSIATIIGFVFSFQKDITPYLQPQIARYLKFNPPAQTEPKTPQLQEQVTTPSPLLQ
SGTYILGGKAGISTRLSGNFSQPVVKTPNRTLIGKLGLVLDNAIQELRELGAIMVLGSAI
AAAIQVLAPRDVILSLGAGPISSILTMLLLATVVSICSTVDSFFALSFASTFTSGSLLAF
LVFGPMIDIKGVGLMLSVFKPKALFYLFALAGQLTLLFTLFLNLHIM
>KRH97592.1
MLTLVTSSVVVLLLGACIGSFINVVVYRLPRGLSLLWPPSRCPHCLNQLKVYHNLPILGW
LWLRGKCAYCQGEISHRYPLVELLTGIIFLIVFWVFQFSLPTIGYWVFCSWLLALSLIDW
ETMILPSSLTKSGLVLGLIFQTTLGYVTHNTWSGTIGQLIWGIGGMVLGIWLFDIIANLG
FVFYGQPVMGGGDGKLAAMMGAWLGWQYLLIASFIACFSGVLVGFGAIIVSDGQIGQKMP
FGPFLAWGALISIFGGQVILDHYLRFVLSHS
>KRH97593.1
MKEHKKAKWKNPRMVIYGIITMVIVSIIGLFLGQIATASVIITDIEFLGVATLPTGYTFQ
NTEIGGLSGITYDVDHDLYYVVSDNRGQKGPPRFYNFKIDLSKGKLDQSKVLPVGVTTLL
DENNRKFALGGIDPEGIAVTKKSTVFISSEGDVSQSINPFIREFSLASGNAINSLPIPEK
FLPDGKNQRGVHNSLTFESLTITPNNQMLFTATENALVQDGEAAKPKFGTLCRILQYNLT
YNQPQKEFLYPMEPVTPLFNFMDRFYSGLSDLVALDNRGNFLSLERSFTGVGFAVSLFEV
SLNNADDIQNIPSLKAIDIGKIKPVEKKLLLDLQTLPLPLDNIEGLTIGPKLSDGNISLI
LVGDNNFNRLQSTQILALKLKRESPLKRLLHQLGLTW
>KRH97597.1
MKRTTLVLVILALALGGFVYFDEMKRKSQLEQVAQNTQKQINQPNNSQESNQREKKQIFS
FNTGDIQSITIKTQDYTLDLEQRSKSEPPNWLVKLPPSTTPKPAQKAIVSYLTDLLTKGK
TENIIPVPVSRLSEFGLDKPFAVIDIKLQSQEKHQLVLGKPNFNNTLLYAKVNCTDSNSQ
SIEVLLVSKDFANAVNRQLSEWEEPTLSSSDNLPN
>KRH97600.1
MFPTHRPRRLRTHPQLRRMVRETVLTTSDLIYPLFAVPGESIANEVKSMPGVYQLSIDKI
VEEAKEVYDLGIPAIILFGIPEDKDIDATGAWHDCGIVQKATTAVKQAIPDLIVVTDTCL
CEYTSHGHCGYLQTGDLTGRVLNDPTLELLKKTAISQAQAGADIIAPSGMMDGFVQAIRA
GLDEAGFENIPIMSYAAKYASAYYGPFRDAADSTPQFGDRRTYQMDPANSREAIKEIELD
IIEGADMLMVKPALAYMDIIWQVKKASNLPVAAYNVSGEYSMVKAAALNGWIDEQRVVME
TLTGFKRAGADMILTYHAKEAAKWL
>KRH97603.1
MELEADEGLLQEAETTINQMHRDLDQWELQQLLSGPYDSQGAVLTINAGAGGTDAQDWAF
MLMRMYTRWAEAHGYKVTLAEESEGDEAGIKSATLEITGRYAYGYLRAEMGTHRLVRISP
FNANGKRQTSFAGVEVMPQIDNSVTLEIPEKDLEITTSRAGGKGGQNVNKVETAVRIVHI
PTGVAVRCTEERSQLQNKEKALARLKAKLLVIAREQRAQEIAEIRGDMVEASWGNQIRNY
VFHPYQIVKDLRTNLETTAIGDVMNGDLDSFIQAYLRQENQLVTSGE
>KRH97605.1
MILIIFMSLILSGCVEYNLGINFHNSNNAEWVQHIRIAENLTSFSSDYLQEWFNVLSRRV
SNIGGSTKYISPGELVVKIPFTHGQELEEKLATFYPHSLQTSLHEPINITSNITLKENNF
LFLSRNNLIYQLDLRSLAGVVAQKNSSDKTQISLFNIDFTLKTPWGNIKNINSTVKPLPP
ETNNQQITWQMQLGELNQIEVVFWLPNMLSIGTLIIIIIVLFGYYLKYSSLNIPNQN
>KRH97609.1
MVCENLGTTEIRCRTDFNYTIRTYDGSEHAIKHRQSLQKTVNDPDLAIVTFESQQDYEVA
PIRNFDDVQIQSDILVAGFPTIFGRVGKNRIFTVTNGKVVGFIPDPPKGYGLVYNATTFI
GNSGGPVFDSSGNVIGIHGLADTDGVEIDNKNQSETRNGLKPNVSIIRKLINPQIESGTS
FIQKTGFNAGIPINIFLSVTADAGLQLPNVKSPVVIAPGKTDQVAISPLVPSDREATIPF
SSSPSSDSTDKVNSNVAIAPKIVPNVNDALSYIARGIALYELGDKQGAIVDYNQAIKIDP

NYANAYIAKGVARSDLGDKQGGIDDYNQAIQINHNYAKAYYNRGNARSNLGDKKRAISDF
QTAARLYQQQGKQNDYQDALNRICRILSICHDNKLNSNILSICHDNKLNSNLVF
>KRH97610.1
MNNQHNIQPVPEFNPYLALIVKERLRQARRSFDFALIATALSFGISLVGAGCLLTNKVSE
GAVTTAVGLVASMGYMRIAKDANNRLDKIADDLDVAIPR
>KRH97612.1
MSNIIELSKYDAALTSINNVPNNPAGSGIGELIINYYSSPLNRKTEIMQQALNYGKEGTK
FLLCILNDKQENPEVKDVVYSLLFKCSGLISSLNNNKTLITDTSHTSQGSSLKADEPAVN
IYTQLNKLLKEKNWKEADLETEKIMLTVAKRTEQGYLQLQDIENFPCFDLHTIDQLWLEH
SGGKFGFSVQRRIYQSLGGGKNYDLLIWRAFGDQVGWRREGKWLDYSHINFAITAPVGHL
PLWGDVDDGL
>KRH97620.1
MGGTHKTAIALGSNLGDSANILLACLDTLSQTPGLIIKAISSFYKTKAVGPSQPDYLNAC
AILEVTMSPQNLLAILLAVEQKFGRVRLERWGARTLDLDLLMYDNLILNQSDLEIPHPRM
YERGFVLVPLEEIAGDWQDPISGMTIRNLLRNVDHSDVHWVSGPAVKIIIQRP
>KRH97622.1
MVNHPDVKNLVKILIGAAWIDGRIQPEERQYLREIAHSKGLSADPEIKPWLYELVQVKPD
QCYQWVREYLGDRPSLEQCQDLIESISGLIYSDGEVATEEAKLLTRIQDLAKNSDHNQTI
YTSLLRKIQKLYRRWVDVQN
>KRH97628.1
MQILPGPKTLSTIQVLNWIFRPMPYMVECAKKYGDLFALKLQSDLPPLIFVHSPEAIQQM
LSNDQKELEAPGELNSIFEYLLGKNSVITLSGKAHQRERQLIMPPFHGERMRTYSKLIEN
ITTKVFAQQEKNQFFNVRNITQDITLQVMIEAVFGIYEGERALKLKYLLCELLEQASSPL
RVSFLYLPKLKEIFGVSEIWKRHIQKKEQADQLIYQEIKERRENFDPQRTDILNLLMSAR
DENNQPMTDVELKDELMTLLVAGHETTATAISWAFYWIHKLPEVREKLLAELDSLEENYD
SSAVFKLPYLTAVCNETLRIHPVGMLTFPRMVKEPISLGGYQLEPGTILLGSIYLVHHRE
DIYPEPEKFKPERFLEKQFSPYQFLPFGGGSRRCVGLAFAQMEMKLILAKVLKTWSMKLV
NTQEVKPKRRGLVTGPNAPINLTIEQYSYP
>KRH97629.1
MALFGNRFSQLRNENQTVMSPAEALAGIALISITADGYCSDEEVLGLMASLNRMKLFRSY
STDVIRKLFDRLLFLIKRDGFSNLLQSAVSSLPRDLHETAFAVVSDLILADGEVTQEEED
LLGDLYKALDLSEEVASKIVDVMLIKNKG
>KRH97633.1
MIASAQTADRSAPVPTLQLNITTGGDDLREGSVAYAEIRLRDGRTLPKTNLNGGRGLGGN
SRNSFSVSLPSGIQLGDLDGSLLTISHDGAPRRFPDGYDNWNVDAMSVTTARVCSGGLSV
ASATGSPWVRFTGGKTFEAVPFRAPDTSREASPSSLGLKIVTGGDDLREGSVAYAEIRLR
DGRTLPRVNLTGGRGLTGNSVRNFSVSLPAGTRLGDLATLTLSHDGAPRRPFDGYDNWNV
DSLNVTTPEVCSSVSLANLSGRPWVRFTGGKTFETVTLRVR
>KRH97636.1
MENWQAVVSVITFVAVILAITVELLDLTVAALLGALVLVFTNIMSLGEAVGYIGKSHSTL
GLFFGVMVLVRSFEPTNIFSYIATQIVILAQGSGRKLLLGIVLLVTPICAFLPNATTVML
LAPLIPPMAEEIGVNFVPLLILMVFVANSSGLLTLVGDPATFIVGDAVNISFIDYLTQLS
LGGAIAVITVVVTLPILFRKIWRTNLENLAELPHPQINHPRVLMLGAVIVAFVLIFFIIG
DYLPIPISPAAVALLGATLALLLAHKTRIDSVHNILQDVDWGTLIFFMSIFVLIGGLDKT
GVINSLSGILSIILGKNILFASLLVLFVVGALSSVIPNIPLVVAMVPMIKQYIVNVGLAP
AAVLATDFQGKFPPEVLPLFYAMMFGATLGGNGTLVGASSNIVAAGIAEQHGKRISFKTF
LHYGIPITILQLITAALYVLVRFLL
>KRH97637.1
MSEIEKVPRGKKPIYILKKSRKMLLYIALGLVALILVITLGSVISEPLERIISVAENLYQ
KWFEKQNTANPFVLLPLAFIGGLLASISPCILALLPVNLSYIGTLKIKSRWDAFSQAGLF
VLGTVTTLSLFGLVSSFAGMVIIEYRGYINIIVGLIMAVMGLWLIGVIKIYLPQIDLKLP
NTGPYSVGLTFALVSSPCASPVLFAVLAAAAATGSQVLGTLTMVSYALGYTILIFLASLF

TGLVKQSRNLLQHSETIIQLGSAALMLTGIYYLYTGTVWFFGG
>KRH97640.1
MTVSIIEQKKAEDKPKKVSTPEEYLSLEEKAEYKHEYRNGEIVAMVGGTTNHNEIVTNLC
TTLKVALKGQHYRVFIGDVRLWIPRYNQYTYPDVMVISGDPVYYQGKTTITNPLLIIEVL
SQSTQDYDRGTKFTHYRSIPELQEYILVDQYSVNIEQFTKTSQGQWLLTEYEKGAETFSM
QSLKLQLKITDIYEQVEFA
>KRH97642.1
MPTSSYSQLYELFIDQVIGLSQKTDFSLTALISNYISMNYQIKLEQIDKLKAWLDEFRPF
DQTIIAEIKKLYDVRFTYNSNAIEGNTLTQSETELVLTKGITIGGKTLDEHLEVVGHKEA
IDYIESLAQKDTVINEWEIKQVHNLILRKIHPDEAGCYRQLDVMAAGTNYIYPPHYLLSQ
LMTDFVIWLNSDAALTLHPVEYATMAHYRFVSIHPFRDGNGRTARLLMNLLLIRAGYPIV
VINNQIRNDYIHALSYGQRNQDDLNQLFDLVCDATISSLVETLRLLVTASSSREKGRVFY
QEITNFIDNFLIK
>KRH97645.1
MPTISMFYGLIIRMFFTDIQQHNLPYLHVEYQGAEAVVSIPDGEIVQGVLPPKKLRMLQA
WIVIHEEELMADWSLAVKGEPIFKIEPLR
>KRH97646.1
MEQLKKERLESKGWKVGTVSDFLELTPEEAFLLEIKLVLSRSLKERRQKLMTQVELASKI
GSSQPRIANAGNGSDSVELLIRAMLATGATPKDIGQIIASVG
>KRH97647.1
MEYRRAKTPGATYFFTLVTYCRRPILGKSENIDLLREAFRYVMKNYQFKIDAIVILPEHL
HCLWTLPENDGDFSTRWRLIKSYFSRKCQVSCQGTMTLSQEQKGEKPIWQRRFWEHQIRD
DRDFINHVEYIHYNPVRHGLVNAPKDWQYSSFHRYVQAGFYDVMWGAEERHVFGGHIGHE
>KRH97652.1
MVGDLDGLDPNSTNVIYQWQRSNNDSSWNNILKDGDKAIYVVTDEDLNQNLRVQITYTDL
QGFSS
>KRH97654.1
MSQPVLQYIATKNYKNLYLPDPVEFNNLNILLGANGSGKSNYINSLKFLRDCLYPTKYAL
EINGFEDAVNKIGGAGILDNTIEDPASVYFVYCFAGLSSDSTSKNHILELSLLVNRKEDR
KQAVVIDQESLKVGDKVIIDFPHLYLYEPASNVIEDARKLLDFVSQWQFYNANSFDLEKI
KTSETKITSPDKYVSASGDNLALVFENLIREDIFFEETINKAMTLVLPRTYRIRCVRSQE
LSLVMEWYSLDTKKPLCLKELSDGTIRMLCWAIILHSPVLPSLLVIDEPELGLHVAWMRI
LSEWIKMAAHKTQIIIATHSPDLLDHFTDCLEKVYCFDSYGKSHFSIKKLTQEMLADKLE
EGWQLGDLYRVGDPTIGGWPW
>KRH97655.1
MVVWVFAGGGETEVQSLIPFLEKNFNCKFQRKTPARRKPGPKPGVKTTAYGRTGQGLINQ
ISQELPIALKNEPKVCSLILVFDDLDCRDPTEEKEKFLDAISKFPECADIPRIVGFAAPE
IESWIIGDWHSTMAKHKDFRAKSDAILWRLSHKMKEMGFSFDAPESFSKYDSQRNCCEDK
LSELLMECSQESSVLFRKGYHTPSLLLEIQPSQLMGKCPLFRRFYTDLQSFCK
>KRH97658.1
MYHNHHLTEKNLFEHKVIIATDGNVILYPDFFSVEQSNQLFCELYGNIKWKQEIIHLFGK
KMPIPRLTAWYGDEGKSYTYSGIEQHPESWNPTLKFIKSKIEEIVPVRFNSVLINLYRDG
KDSMGWHSDDEPELGKNPLIASLSFGATRRFYLRHKYDKSQKTVIDLENGSLLLMQDQTQ
HFWQHQVGKTAKKVQSRINLTFRIVN
>KRH97661.1
MAQTRNFIGNVSDTTVVGTKIVPEGDRREPLKHLLIGSKKTVISTIHYLHVLGYAHATDW
SDLMPTSNPGEVMSILVREMVIK
>KRH97662.1
MKRFLTLALILTLFLVSSLSLSTSASYAYSQTDLDRLWVRSKFRKNCIECDLTGANLRNA
DLDSANLDRADLTGADLRNAALRNADLRLANLDDADLEGANLEAANLEAAHLWHARLRCA
DLKGANLVDADLEGADLEGADLEGANLTGTNITGTNITGANITCDRTF
>KRH97663.1

MSFENVSLEMQRRFEVLDEMIEKGYIEPIKPLVDWLPTLCYSPARTSTFDNAPENLKRKF
TFMFSWYAFLFAIFAFVQTRLARDYLICISTINLITLIYPFSELYLYSSALVINIFAAQC
FVFSRYYQYKTFGRCPHSRNAFSTICLSLIYLFGLIIIDVIIQP
>KRH97664.1
MRMNIKRLVSVGMIVATIGINSKAFAQNQEYGTQRVSEIPNAFPEFTIPRNIKLVECFNN
LNFDSDKATLRYLNRTLKGSPNNPCLYLARGLTYMNLGRIQAAKTDFDTFIGMDSNFPYV
YVFRGFCFMLLNSPVTAISDLRKASGLFEAQGNLSFMELLNGLIKNLELLSNP
>KRH97666.1
MKIKETLVSLILVGGAMTIGSQAIAEDFSSIKNSSPLNASSQLWQTNNPGVNNFNGYGQF
TQLGLSRYTQDGIPYKIQNVQVSSSDVCEYINMAISAFGSGDFQKAKMILDVVVDLDPNT
SYAYVLRGLSFLMLESPKSALTNLEKGVSLLIREDDSESQYLAEMVNNLIPMVKLLYSL
>KRH97667.1
MTLEEFIQILAEHQLSKYIRSQEALIRRAKQTGRMSYQERLIYIRQAQAAIQDLKQILSN
RNMCERLLIYTQKQSSADQVETIDWILRMIELSDGIYGKISFSSDSSDIYQEGLQRTRIW
FFDRFVENYNPQVSSILTWFNKNLDYKFRDLRREREREPQSLDDTTRIGPPHIPSPENRN
FVEEIEAMLESLENCLNSPNSNCPRSCIRNAPHANCNSVIRSILNLLLAGEVEKLGPVWD
TLSRKYEVSSYSMKQFIRKECFTCFKRLTE
>KRH97669.1
MSLEVNISFRVEGSFTEGFTMTPMIPQQGSQEFRELGSRIRLPACPVLPNLYNECRNFTD
EYTRRIEGVPDQTTTVNERERWTRTQMNLNRSFLDWIRNANFEVIINEIITRIFNKRLTD
TSVRIHFVSNNSNNNDYDIFRRMPWDEFTRSTRSRMKFVLSQYSLTYNSCPTRLSELRIL
VIRGGRDLENSQNREINALRKIPGVRLIDKTRTIDNIDCLHKVLTENVYDILFYTGHSSS
NNGGEIRVGSLTVSINVFVEDLRMAVSRGLKIALFNSCDGLGIADFLVTQVRVPVVLIMK
EPVPDIFAAKFFIEFITRFAADRMSLSIALEKARSSSHFESVNFPGATSLPILFYLYPVK
KDFRISRFLYIKVVIKIIKEGVWKKLNNMFGIIQRLSRRQYMYMLIFLLPLFSVFLLKIG
ILGPENYPLEGVIDNILPANIVDIDISDDEQYVAYVHNRGILLQKMDDLTAKNNIQLCSK
NFEEIPPNSLPSTKLVFKKNKTLAIATYLDSARSNIRYLNVDNCKFEEKVIESSNIIFTS
LDFSTHKNVIVATRFDIEKDAITAGLYDSDSGENINIKGKDSVFEGVVDAKFWNSQKIIL
LKNNGQPRIEIYNIDVSNEDNSYKLERVNSLVLKELKFEYAEKIVLSQDRKYISILGSGR
IHIWEIDDKFKLKEIPKNIQNQLEGRTDVRDAIFYKKQILIPNTTLVD
>KRH97671.1
MMNIQSLKSQPQKTAWILLLPAVLLLVIVFAYPIGRTFWLSFFTENLGTKLRPVYSGLDN
YVRLLGDSRFWESFKSTTIFTVVSVSLELILGLNIALLLNQQFLGRDIVRTIAILPWALP
TAIIGLVWNWIFNDQFGVFNDLLLKWHFIDTGINWLGEPIPAMIAVIIADVWKTTPFISI
ILLAGLQAIPRDLYEAHSVDGANPWQSFYLITLPLLIPQIVIALLFRLAQAFGIFDLISV
MTGGGPGGATEVVSLYTYSTIMRYLDFGYGASLIVITFLLLIIAVIIFTIALKNKGDQNF
LF
>KRH97672.1
MTQFQLKTRVKNLTKLLVIITGIILLAVFCLAPIIWQLLTSFKSNEDILKIPIIYFPSKI
TLNHYIQLFITHPFWRYIINSIFISSSSTILSLVFGAPAAYALARLNPWGGKIIIGTLLI
ITLIPGILLLSGLLEIVRVFHLGNNYLSIIIPYVAINLPLTVLVLRNFFQQLPIEIEDAA
KIDGYNTIQMLLKIILPISMPALVTTGLLNFIFAWNEFIFALTFITREEMKTIPIAVAQI
GGATEFEIPYGPIAAATMISTLPLMLIVLVFQRKIIQGLTSGAVKA
>KRH97673.1
MAKLQLKDLTKSYSSQITSVKNINLTVEDNEFLTLLGPSGCGKSTILRMIAGLETPTKGQ
ILLAGEDITLKSPGSRNIAMVFQSYALYPHLTVFENLASGLKLRKIPHGEIYRRVKKVSE
SLELEELLLRKPAQLSGGERQRVALGRALVRNAKVYLLDEPLSNLDATLREKVRADIKQI
FTQETSPVVYVTHDQTEAMTLSSKVAVINYGSIQQLDLPAVIYKQPANLFVAGFVGSPQM
NLLPLSCNSNYAILGNNKLFLPNMNKIPPKIVLGIRPEDIYIAQSGDEHTISGKIYLVEN
LGMHNLVTLHIDNHHPIPLTIRILLPTNQTWENQQITINLPPKNLHWFDYQSGNRL
>KRH97676.1
MASFTEKCYKLRYLLAEQNWKQANQETKNIIFGIINETKEIPFYLQNISQISSRNLRLID

RLWLKYSQGKFGFSVQQKIYREIGGTQDYDSEKWAIFIGKVGWNESLENENNENNNITLE
NVPEGHFPMLIPQNDYRPFFFIVIILSSPLLSFLVMKIFSNLWFFLTEFGIWGLLLWVIL
FILLKIIWITGWGYLQLRLSLHYVPKFNYTCSQIMSSIFERLKSSNL
>KRH97680.1
MAIRVLVPTMLQKETNNQAVVECSGSSVNELLDTLEKTFPGIKGRLRDETGTPRRFLNLY
VNSEDIRFLRGTETVLQDGDEISIVPAVAGG
>KRH97682.1
MSSNLVLVKIKLFAAYQEAYGVTELEWEFPPHTPVKSICDRMIWEHPKLSQWRDITRFGI
NLMFVDPDTILNHGDEVVLIPPVNGG
>KRH97684.1
MSRNCRTAANPLYQIIADYIDTSDQRRITFAEYMDLVLYHSEYGYYSSHSGQIGFAGGDF
FTSPSLGDDFGELLAKQFLQMWENLDQPRPFHLVEMGGGTGVLAFQILKFLKNHHPDFWE
IIEYIIIEKSPKLKWEQQQRLEGFSVQWLDLPEILPGSMVGCFFSNELVDAFPVHEFILE
KGKLQEIYVTYRASNPIEFMEVVGEPSTPKLAEYLQLVEIDISQNAYPENYRSEINLASL
DWLSIVANCLQRGYVLTIDYGYPATRYYHPRRSQGTLQCYYQHRYHHNPYIKVGEQDITT
YIDFTALENWGKRCGLNPVGWTQQGLFLMALGLGDRISALSYQQHPLSQLLKRREALHQL
ISPEGLGNFGVLVQSKGLTTTQSQLPLQGFITPVVF
>KRH97686.1
MPINTQQLKQWKQQGRSIVALTAWDYAIAQLLDAAGVDLILVGDSLSVILGYETTLPVTL
EEIIHHAKAVKRGIKRALLVVDLPFLTYQESIAQAMQSAGRVLKETGAQGVKLEGGYPAM
IDIISRLVQAGIPVLGHLGLTPQSIHQLGLKQQGKTPQTADLIFHQAIALEQAGVFALVL
EHIPSQLAVKITKELSIPTIGIGAGQECNGQILVTSDVLGLSEKQPPFAKVYTDLRQEIT
KAVEKYALEVRNQDF
>KRH97695.1
MIRQFTDKSYLKSALKNSSIRVPKHLIFNQSQYRHEGISYLQFVIKELGNDIFVKPVIGA
GSEKTRRIHTIDELKAWCNSNVDSDDEFEFNEFIRGELYNTSVVMKNGLPCYFAACKHYR
PNDDFIYGHAIGNIVVREEDPKFEKLRQFSSDTLQSLEHDYPKNGVLNIDFFLQEGSEEP
ILMEVAARTPGGLVSKMFYTYQGVRLNELHLQLQMGDDPEIILKNRSEWKYSAYSIHPKQ
EGVVTEIEKPILDSDVEVYWQIYLGETLKVSESMRDVAIAMLLSNHDFSTLEQDYKLANS
LSLYKTKKDSFQELKAVFVALTV
>KRH97696.1
MPNQHNDVNILVIGAGVSGLTTAICLREAGFNVIIIADRFAPDLTSVVAGALWEWPPAVC
GRHGTPRSLERSKEWCMTAYNKFKEIHAEFGSQETGVYLRDSYFYFRDILENRPADFRKM
NELKDKVDGFERGLHIVKETIDLTFQ
>KRH97697.1
MLESNPSVSISIPTYNESSNIERVIKGFLATEYQNLIEVIIADGGSNDNTQEIVKRLSTQ
DSRIKLIHNPLKVQSAGLNLILQECRGDIFLRVDAHSDYAPDYIERCVEALLESKALNVG
GAQRFVAKTPFQAGIALSSKSILGSGGAKYRDPNYNGYAETVYLGCFWKKALTEVSGYST
EATTNEDAELNQKLLKKDQNAIYISSKIRVWYYPRKTFKSLYIQYFKYGRGRYLTSIKHS
IKSQIRGIVPFLVISSITLLLLIDLLFPRLGLPMVILVILGLLFPFLESLRVTLKSRTSF
DSEIWRGDKGKIPSFLSRWFFCGIVLLTMPIAHSLGYAYQLIKHKILGLNGWL
>KRH97700.1
TTSIEPLLKKIHPNGDYCIGQDSGIYWRFTEPPEKGVEAPDWFYVPGVPSRLNGQLRRSY
VLWKEKVPPFIVIEFASKNGKEEKDSSPPPEGDEIDPETGKLKKAGKFWVYEQAVKVPYY
AIFNGFKGTLEVYHLQGKRYKEIKANRRGHYAIQEMGIELGILYDNQNPPTPWLRWWDEK
GSLLLTGNERAEQAEAIAIRERLAKEQERGAKEQAEAIAIRERLAKEQERGAKEQAEAIA
IRERLAKEQAEAIAIRERQQKEKLVAYLRSLGVDPEKI
>KRH97702.1
MRQPLTTTQNGTTPLAGESLNLPDHTQLRDSDDNFVKNFQEHPQSIILTTSIEPLLKEIH
PNGDYCIGQDRDGHRIGNTV
>KRH97704.1
MYGCQQVLIKSDKSITAILEYVIPKFGEKPEGWEASGKRVSRGVYQTSDGFKINADCNGA

ANILKKVAVMLGIDLSGISRGCLSQPQKVRLWTLQKSPCL
>KRH97713.1
MDEEFIIPQAPANFKSGFIGIIGRPNVGKSTLMNQLIGQKIAITSPVAQTTRNRLRGILT
REKAQLIFVDTPGIHKPHHPLGEVLVQNAKIAITSVDVVLFVVDGTAVCGGGDRFIVDLL
TKCEIPVIMGINKIDQQPAEAEKIDESYRELAQGNGWEIVKFSALENQGILELETLLIGY
LETGPLYYPPDLVTDQPERFIMGELIREQILLLTREEVPHSVAIAIDLVEENPTITRVVA
TINVERDSQKGILIGKGGTMLKSIGTVARQQIQKLIAGKVHLELFVKVQPKWRHSRLRLA
ELGYRVEE
>KRH97714.1
MWQRMINALTKWLTKIKNLLFKNKPPAEKKERPKVSEYTATKGPESSSEPPSSKPPNKKK
VRDPFANARGQNILFTKGVLFEDPQPEINSLQKKVLEILFSVFNLSIKEDDIYVAYFPSR
LLYYIEKQDSFSAFRRFLNDIGRTPDEVFKNPKTATFIKDIILGLYEADINCWDTGISTE
PLYQHLRETAANVDYNLESFINLLSRIEKLAILIHQLQRIAGQIPFDSFRKFSQTVNSSL
KSWHTLSDTTFDQWTNSLNDYKSQFRDYQYFSEVIDQNCFRLRSTMAVIPQEIINVIENL
LEEVEQLKQKLKIGSISINEGLGELGTLASEIKGFVDEVLHRNQETNSSRKEDFAYTSNT
LSLDEAFRLLNLTRETISMKLLKTARNKCARDYHPDMGGDVNMMKRINEAYEILKDYLEN
RHT
>KRH97721.1
MLYRRFGRTNLQMPVFSCGGMRYQFNWQDVPLQEIPVDSQQNLANIIHRAVDLGINHLET
ARFYGTSEMQLGQVLPNLDREKLIVQTKVTPCSDPQEFRQIFDKSLAYLELDYVDLLGLH
GINNQELLDYSIGGCLQVAKELQAQGKVRFIGFSTHAPLEIILQAVNSNKFDYINLHWYY
INQWNWPAIEAANRLDMGVFIISPANKGGILYQPSPKLVELCQPLSPMVFNDLFCLSHSQ
VHTLSIGAAQPSDFDEHLKTLELLDRADEILPPIIAKLEKAAIDTLGEDWVKTWDTNLPV
WENTPGNINIKLILWLLNLALAYDMVEYGKMRYNLLGNADHWFPGNRADKLEELDLQECL
GNSPHREKIPLMLSKAHTIFKGEEVKRLSQS
>KRH97723.1
MKRELFERIYEEELTPIQRRILHRILQGKSNTAIRGDVTVWFDHKVYSGSERDKLPRSKR
TILEGLESGKKLMDQSNLSHHLRKICEQFELIDLQEVIKQFIKYRRQLVSYETLEQFELL
QTTQFPSGATNSLYYEYRHPIEEKCESYIKKVGEEAILLRIKAPAQMGKTSLINRLLHHE
YIKIIVEPRCQLYREYFTTFI
>KRH97724.1
MSIVPRIFYYIYLNLLLGDKKMATSFYKVGGTLPINSSSYVIRTADTELFNLLKQGEYCY
VFNSRQMGKSSIKDQTQYKLKKEGFFCIDIDLQGIGTQVTQQEWYVSFIYNLLNAIALEE
KIEIDLGSWIEPRSYLSPSQLLNNFVSHELLTSVQKKIVIFIDEIDCSFGLSFRDNFFAL
IRFFYNQRNQNINYQRLTFCLLGVATPSDLIKNPVESPFNIGKAVELNGFEFHESLVLAE
GLKGLASNQNETLKQVLYWTGGQPFLTQTVCDLLVKLDIKVSEGKEKETIRQLVYSQLIN
NYHDYQHFDQIKTRLIANENRVGKLLEIYQYILKHGIFLSKDLGKEKEFESELRLSGLVV
RRNGGLEVFNPIYQEIFNQDWVQEQLDSIRPYFRVV
>KRH97725.1
MILGDFGSDGEIFFEIDLITADDLELPVTAMLDTGFTELLAVNSQDLEALGWRFLRERPL
KTAQGIKTFRIYLGKVRIDRREFEIPVYGGEEITEILLGSQWLKIMRLVADMSADVLSLE
IV
>KRH97726.1
MTETKSHKVVRRGRLFPEIQWTEDRINLYRSELETHHQRCRVVFEKLQPDLIKNHYNWFM
AVDSESGDYFLDKDEEVVTQMFLQKHPTAIPFIFVINETGVAGRI
>KRH97729.1
MRYGEGKLSCTVLKTNGIGDNLVEFNTKPNKPTGYKRDIYKCDCIAFSTGSREAFPCHLY
YILLNKTTDTFKNQHKTKNKC
>KRH97731.1
MDILSYGLMAIGILFWFWGTSHLVSNKSVLFKLHGLSVADTLGSMLIIILAILLIIPSKWP
LLVLAIISLALWNTMLGYVIAHCSTEDITPEVENE
>KRH97734.1

MNTITTIWVGIPFFLGFIVFLLPPLNRHLAFLGTLVSAVYSMELLIKQPEIQLNLLDSFG
VTLVADQLSGYFILTNALVTMAVVLYCWKSDKSAFFYAQVLLVHGSLNAAFICADFISLY
VALEVSGIAAFLLIAYSRSDRSIWVGLRYLFVSNISMLFYLVGAVLIYQKSSSFSFAGLE
NAPPEAIALIFLGLLVKAGIFVSGLWLPLTHSESETPVSAMLSGIVVKASVLPLLRCAAV
GENIGHIVVIFGVATALMGVSYAILEKDTKRMLAFHTISQLGFILAAPGVGGFYALTHGL
VKSSLFLIAGSLPTRNFKELQSKPINTAIWIPLFTASLSISGFPLLAGFAAKVLTLKNIT
SWQFVAMNIAAVGTAISFAKFIFLPHKLGDKQETVTAVKPGFWVAIAVLITGLLIANIAY
LGAYNLEDIIKAIVTMIVGWLAYHLIVQKLAPKLVAYLPRAVEKFEHLIGVMSLTLILLF
WMALS
>KRH97741.1
MIERYTLPEMGNLWSESYKLQTWLQVEIAVCEAQGELDYIPLAAVDEIKAKAKFDPKRVL
EIEAEVRHDMIAFLTNVNEYVGDAGRYIHLGLTSSDVLDTALALQLVASLDLLCQKVENL
IQAIRIKAHEHRYTVMIGRSHGIHAEPITFGFKLAGWLAEVLRHQERLQLLRKNVAVGKI
SGAVGTYANIEPRVEAIACAKLGLKPDTASTQVISRDIHAEFVQQLALLAASIERFAVEI
RNLQKTDVLEVEEYFSKGQKGSSAMPHKRNPIRSERLTGMARLVRSHAGAALENVALWHE
RDISHSSVERVMLPDACILTDFMLHEIIDLITNLLVYPQNMARNLNCYGGVVFSQRVLLA
LIDKGTRREEAYAIVQQNAHIAWNNPEGNFRHLITQDLRVREKLSDSEIDACFDAKQHLK
YLDQVYERLGI
>KRH97744.1
MKKKQKRFQHTGIPIAAYIILIIVIISISTLIKQPIVLGQTPRLDTEEELKHTCASQIPS
SIEKIINSPTFERMRWGILIKNLSSDQILYSRDAQKYFIPASTTKILTAAAAWQKLGKDF
RIRTSIHQGDEGNFYLVGRGDPSFNNAQLTALAQKLQQRGIRSINKLIVDDSYFQGEYID
ASWQWEDIQADYGAPVNSLMVNENTGILTLSPTKIGEKLNITWSNDMELYSGIENNSITV
AENESRFVQVTRDLKGQVLKIDGKLPINSPSQTIGLSVIDPIDNFLKNLRLTWEKVGINV
KEIQPIFEYQHYKIAEQKPEIAVVESLTLPDLLKEVNRNSNNLYAESLLRHLGNSESTNK
NETTVNQGLKILKTTLSEWGIEPNTYMIVDGSGLSRKNLISPEALVKILQVMAKSPDGNL
FRASLATGGMNGTLKNRFLKTPAWGIVQGKTGSMTGVISLSGYINVPNYDDLVFSMIVNQ
SQHPSAVRKAMDEIIILLAKLHKC
>KRH97746.1
MSHTRKTLSIPNMEISYLEWSQGHEPLLLLHGMADNALVWLSLGDYLSPNYHVIAPDMRG
HGNSSKPETDYSFTSAIADLEALMDSLGWLSANVVSHSWTGKLAAIWARQNPGRLKTMTL
VDPIFIWKMPRVLKLIFPLLYNVLPSLQTMGPFSSYEAAEEKIKKLVHFREWNDLQQQVF
QGGIEQKPDGTWGSKFTVAARDGIFDAVLEVAGFIHPVEIPTLFVQPEKGVNRQDWQIKP
YKDNLKNLTWKKIPGTHWPFLSNPEEFNLTIAEFLAQSI
>KRH97755.1
MKRIISVLLLGIAIFTLAFSNSALAVDAEAGASVFKANCAQCHLGGKNLVNAAKTLKKEA
LEKYDMYSQEAIVSQVTKGKGAMPAFGKRLKSAQIENVAAYVLEQADEGWKK
>KRH97757.1
MVITKSRLVLSATAVAVSTIVVGGLGIHCRGQALFQSSPKELIDEVWQIVNRQYVDGSFN
QVNWQAVRQEYLGKSYGNKQIAYKSIREMLKKLGDPYTRFMDPDEFKNMQVDTSGELTGI
GITISQDEKTKQLVVIAPIEDTPAFKVGILARDVILEINGKTTQGMDTNRAVSLIRGEPG
TQVKLKISRNGKTKTFDITRARIEIHPVKFSQKQTPAGNIGYIRLNQFSANASKEMRGAI
ENLEANRVDGYILDLRGNPGGLLYTSIDIARMWLDKGTIVSTIDRQGEKQRESARDRALT
SKPLVVLVDKGSASASEILSGALQDNRRAMVVGTQTFGKGLVQSVRPLRDGSGLAVTIAK
YHTPAGRDINKHGIDPDVVVDLTDAQRQNLWVKEREKLATLEDPQFAKALDILGKEIAKN
P
>KRH97768.1
MNFPFSIKNKNSNQGLNLDFILNRCLPLTGVLLFFGLWWFIAVSGFVNPVLLPTPLATVQ
TLIDGLFGGSMFSDLATTVLRTFSAFFLATLFGIPLGVGLGSSEKIYRSVEFLIEFFRST
PASALIPLFILFFGISDFSKVVIASFSAFLLIVFNSAYGVIHAKQSRILAARVMGANRWQ
IFKDVLLLESLPQTFIGLRSGISIALVIVIVSEMFIGSQQGLGKRIIDAQQILNVRDMYA
SILITGSLGYLLNMLFLGLEKRLIHWSGK

>KRH97769.1
MDSLLQTKTAKSEEFITVKGLCKSFAGQPVYKNFDLNLANHQLVSIFGPNGCGKSTLINM
MSGLIPVDQGEIKIHGKPIQRTRIGYVFQNYRDSLFPWMSAYDNIAYPLRVKGISERECR
HSVEHLIETFNICLDLKRYPYSFSGGQQQLVSILRALVAQPEVLFLDEPFSALDFETTLF
VRDKLQEIFMATTIPMLMVVHNLEEAIFLADKILLLSKRPTRVVEMVTFDAPRPRTPETL
TSHKFVEVSRYCLDIFRQEMQK
>KRH97774.1
MAKIPQDNRSIFPQYPVGQPPQLPSQIIVQQTKMGDITAYLGSVTLEWFAKKVEFASTLP
ILQNKYNPVTDNIEIDADSIEQIQQRSLDWSRQASLLQYLTIHRNHKFPPVLVVINQPWA
NDAKSELWGGEGRAKKATTDFTPLDANGHIGLLNVAEKDVNIYALDGQHRLMGVQGLLEL
LENGRITKYNRDKIPTDSYIDLSELVDQYRIDIDYLKQLPQEKIGIEFICAVNSGETYSE
AKQRVRSIFVHVNLMATPLTKGQLIQLNEDNGFAIVARKIAVSHPLLAHRENRKPRINWN
SATVAANSTVLTTLQALQDMSTKYLGYKFPHWQTQIKGLIPMRPDPDQLAEGIREFQQLF
DHLATLPSYKILDHEDTTVLRRFSFEKVGGEGNILFRPVAQVALAEALGILVFKNGLLLE
NIFKKLQKFDLQGGFSQMEFPNSLWYGVLYDPNKKRVQVSKKELAVKLLIYLLGEIKDQM
EIAELRKAIASARTTEDRAIDFDGNWVSAQEVGLPSI
>KRH97782.1
MSLEISLQHTSTLKEIAQKTRLAALNLSLLNSNQKDYALDTIALALESAQEEILQANIAD
CQTAMSQGISKSLYKRLYLDSNKLQEAIAGVRDLVKLKDPVGQVQIHREIDRGLILKRIT
CPLGVLGVIFEARPEAAIQIVSLAIKSGNGVILKGGKEAIGSCEAIVSAIKQGLSKTDVN
PDVVQLLTTREEILELLNLDKYVDLIIPRGSNSFVRFVQENTRIPVLGHADGICHLYVDK
AADIHKAIDITVDAKTQYTAACNAIETLLIHSSIAQEFLPQVASALESENVELRGDNRTL
DILPKIELATEKDWQTEYSDLILSIKIVDSLEEAIAHISEYGSRHTEAIVTEDLIAANTF
QALVNAAGVYHNCSTRFADGFRYGFGAEVGISTQQMPPRGPVGLEGLVTYKYHLIGDGHV
VKTYTGDRRKGFTHKDLGV
>KRH97783.1
MIKKSCKKYHLTLVLTGHIHEDERIAAPKFLRQAERKLKSAQRRVSRRKRGSNRRKKAIK
KLGILHKKVVDTRKDFQFKTANNLLKKYDVVAVEKLNIKGLAKTRLAKSINDGGTVHHHT
FKQSRECWLEEESLRSPLSPVRECVEYVTIEPSSSGFCISVVGSMSQMYTICISSQNY
>KRH97797.1
MLRILTVSPTYFMKIALFTETFLPKVDGIVTRLSHTVDHLQRDGHQVMVFCPEGGIKEYK
GAKVYGVSGFPLPLYPELKLALPRPAIGHVLQQFAPDLIHVVNPAVLGLSGILHSKLHKI
PLVASYHTHLPQYLQHYGLSVLEGLLWELLKITHNQAALNLCTSTAMIEELAAHGIERLD
LWQPGVDTELFHPDLASQEMRSYLSQGHPNSTLLLYVGRLSAEKEIEQIKPILEAIPHGR
LALVGDGPHRQNLQNHFAHTNTHFVGYLKGQQLASAFASADVFVFPSRTETLGLVLLEAM
AAGCPVIAARSGGIPDIVTDGVDGYLFDPNAPIQQAIDLTIKLLREKQEIAAMRSNARKK
AEQMGWSAAVRQLQEYYQRTLN
>KRH97803.1
MIFPDFQQFIELAQQGNFVPVYQEWVADLDTPVSAWYKVCAGQPYSFLLESVEGGEKVGR
YSLLGCDPLWILEARGNQTTQIYRDGRRQVFQGDPFRVLADCLAPYHPVKLPELPSGIGG
LFGFWGYELINWIEPTVPIHSQDDRYIPDGLWMQVDHLLIFDQVKRKIWAIAYADVREGV
EESYQKACNCIKQMVEKLSLPLTKENTQLTWKSPQNRPKVDIKEYSSNFTREQFCASVEK
AKEHIRAGDIFQVVISQRLGTEYRGNPFELYRSLRQINPSPYMAFFNFDDWQIIGSSPEV
MVKADRDDEEGGIIATVRPLAGTRPRGKTTKEDGELAADLLADPKEVAEHIMLVDLGRND
LGRVCQNGTVKVDELMIVERYSHVMHIVSNVVGKLAEDKTAWDLLKACFPAGTVSGAPKI
RAMQIINQLEPTRRGVYSGVYGYYDFEGQLNTAISIRTMVLKDGTVSVQAGAGLVADSQP
EKEYEETLNKARGLLEAIRCLKEIT
>KRH97809.1
MMVDNFPWLTTIIVFPLLAACLIPLIPDKDGKTVRWYALGVGIADFALICYAFWTHYNSL
NTGFQLVESYNWMPILGLKWAVAVDGISAPLVLLAGFVTTLSMFSAWQVDRRPRLFYSLM
LVLYAAQIGVFVAKDLLLFFIMWEVELIPVYLLVSIWGGQRRRYAATKFIIYTAAASIFI
LIAALAMGLYGGDNLSFDVSDLATKNYPLTLQLLLYAGLFIAFGVKLAIFPLHTWLPDAH

GEASSPVSMILAGVLLKMGGYGLIRLNMELLPDAHIYFAPVIAVLGVVNIIYGALNSFAQ
TNMKRRLAFSSISHMGFVLLGLASFTDLGMNGAMLQMLSHGLIASVLFFLAGVTYDRTHT
MVMKDMGGVGQAMPVVFALFTMGAMASLALPGMSGFVGELSVFVGVTTSDVYTSTFCTVT
VFLAAVGVILTPIYLLSMLRQVFYGKDAALTCDITSAGVENQEDEGTACFGTDCLLPNQS
IYSDAKPREVFIAGCFLVLIIGIGLYPKVFMQMYDAKTVAVNAHIRQSYTVISQSSPSIY
ALANLR
>KRH97810.1
MPIPVSLILMIALFSFLGMFPLAIASGAGNEILQPLAIVVLGGLFTSTALTLLVIPALYA
KFGKWLRPKMEQQLGLTD
>KRH97812.1
MLTQDVRNALDVADLNKLKYDLNSLQPVDVGEYISELPEQQRAIAFRLLNKNQAIDVFEY
LPTDVQEELINSLHDVQVVHLVEEMSPDERAYLFDELPAGVVKRLLQQLSPEQRQATATI
LGYPEGTAGRVMTTEYVRLRQGLTVGEALSKIRLQDEDKETIYYAYVTDDNRKLVSVVSL
RQLLFTFPEVFIRDIASSQVVKVTTETSQEEVARIMQRYDLIAIPVVDREDRLVGIITID
DVVDILQEEATEDIQKLAAVSGDEEALSPPHLTIRKRLPWLLGIMALYIGAASAIAPFQR
VIAAVPVLAVIMPIFSNTGGTVGIQALTVTIRGLGVGEVTTKDAGKILRKELIAGLGTAL
ALGSTMILLSLIWAKPDEKWVALIAGVVMATNTMVAVSLGTLLPMGLQRLKLDPALMSGP
LVTTMLDTIGFLTFLSMISLALKVFNLSY
>KRH97813.1
MSTPASNWNRHHILSLADFTVNEYNAVLQTAASFKEVLSRRTKKVPTLQGQVVANLFFEP
STRTRSSFEIAAKRLSADTLNFSSSSSSMTKGETILDTAKTYLAMGTDIMVIRHKEAGVP
QAIAREMDRLGVKVSVLNAGDGQHEHPSQGLLDLFTICSLIDPEQPKIASLAGKKIAIVG
DILHSRVARSNIWSLTTSGAQVHLAAPPTLLPKYFSEYVGSRENSQNLFIHWQLDPALED
ADFVMTLRLQKERMTGNLLPSLREYHQLFGITRQRLKACKSQVKVLHPGPVNRGVEISSD
LMDDPKFSLIQDQVTSGVAVRMALLYFIGGAKG
>KRH97817.1
MARLGYTPDHYTMIILLRIQLDWDSLCSHSKFLKIFVLLLLTMAQTGGNMDSMNPYYQYR
AVRDVKR
>KRH97819.1
MSSSLDANSTNNIYETYDKHESKFLTPFERKTLLKNLQTNSHPEYRRRIEIILLADMGKS
QSQICEIIDCSQEMARYWMGIAEAGMAHKWKERKIGRPKTVNNQYLERLKDLVNHSPREY
GYAFSNWTAQWLSKHLAKELGITISDRHINRLLKQMGLSTRAKNPCAIKDPANQDKSISI
CDLPSNLEPSFNWSFSLLPNHQ
>KRH97821.1
MPQSTYNSSSAILISEQPPIAEIITQENPNQIETSVKDWFYGTEELLDALPKVWTRSMLY
LLVTFTAVALPWIMFSQIDETGSASGRMEPKGATHKLDSSVMGSVVAVNVKEGAKVKAGQ
PLIEIDSQLLRTQLQEAQAKLEGLVNRQTQVELLYNQVLLAINIQKQQNQSQSLEKLAQL
NQARENLDAKQSAYQLQKLERLTQVEQVKQNIQSTQIAYGLAENRLRRDSLEVARYNQLL
EEGIIPQTRFVEIEKTAEDSQRLRQEASSNLQRASLQLREELSRYNSVMNQLRSEIQQAK
LRLQEQESSYQSIIQAGKLTVLKNQEQLKDLQTQISGIKSEISQTKSQIKSLQLQLQQRT
LRSPVDGIVFELPIKKPGAVVQVGQMVAQIAPKDANLILKAEIPSQQSGFIKVGMPVKIK
FDAYPFQEYGVTQGRVLWISPDAKVSPNRPTSVETYQLEIALEKPYIKSGDKPIPLIPGQ
TATAELIVRQRRVMDLILDPFKRLQKNGLDL
>KRH97823.1
MQEIQALKQAFYSRKMGSLLLLGFASGLPLFLTTRTLQLWMKDADVEVAKITLFSLVSLP
YSLKFIWSPLIDRFSPSFLGGRRGWLFLTQLGLVLAIVIMALQQPTQNTQVLITLAIISF
IIAFFSATQDIAGDAYRTEILKPLELETGASVWVLGYRVALFIAFSLAAWLAGFLSWNMV
YLLMAGFMTVGLITTLLAPPETQEKNNSPKTNNRFLKTKDLIFLLLIITITAALVGGVIS
QVIPLQVFYWILGGLLITWVLASIILPKPQLNEHSQDLTPHTLQEAVILPLQIFLEKYGI
SKALIIILIFIILYKLGDSLVGITANLFAKEINFNNQELATVYIIGLIATTGVILGGIIM
SKIGINRALWIFGILQLLSNLGYYTLAIVGKDYPLLAIAIMIENSSAGLVTVATVAYLMS
LCSHNFTTTQFALFSSLMALSRDILSAPAGDWAKATGWPNFFLLSILAALPGLILLPIAA

PWNNKSLTVNRPGLEKEDLWNHKQ
>KRH97824.1
MPDYSLRRGSILDKALLVKFIQRTYQEIFPSRDFSHLSRTVENYLSNDTPLWWVYKNQQP
SPIACLWAGNAIDQVTGSRHTHIFLLYVEPTHRRQGIAKTLMQHVENWAKQKGDPQIGLQ
VFTTNTPALELYKQLGYQTQSLWMIKSLEG
>KRH97826.1
MTETGSEKLNKFEKLKAEKDGLLVKQEISEFARQGWEAMEENDLNHRLKWLGVFFRPVTP
GKFMMRMRIPNGILTSTQMCVLAEVIRRYDREGSADITTRQNIQLRGIDFSDIPDIFNKF
HKVGLTSIQSGMDNVRNITGDPLAGLDANELYDTRELVQQIQDLLTNGGEGNPEFTNLPR
KFNIAVTGGKDNSIHAEINDLAFVPAFKEGFGFNVLVGGFFSGKRCEAAIPLNVWVKPEE
VVSLSKAIVEIFRDHGLRLNRQKARLMWLIDEWGLEKFRDEVEKQWGKSLQTAAPKDEID
WEKRDHIGVYPQKQLGLNYVGLNIPVGRLSADDMFELARLAEVYGSGEIRFTVEQNAIIP
NIHDSALNTLLTEPILKKFSVSPGLLMRSVVSCTGAQFCNFALIETKKRAIAMTEALEEE
LILSKPVRIHWTGCPNSCGQPQVADIGLMGTKVRKNGQTLEGVDIYMGGKVGKDARLGTC
IQKSVACEDLQPILKDILLKQFGAKVR
>KRH97829.1
MYQSDVYSKPSYEPLPPQETLPTMYDLPSEYPEEPGLPDEFHLLQPELLRSTFCPPSYPK
DNVFIGSDLNLYYDSKHTQWYKRPDWFAVLGVSRFYEQTELRLSYVTWCEGTYPFVVVEL
ISPGTEKEDLGKNLQEVNQPPNKWTVYEQILRIPYYFVYNRYTNEFHCFGLVMNRYQPLS
INGLGIWLEEAELGLGLWVGEYQGLTRQWLRWYDKDNNWLPTPEERATQLAKQEKQRADL
AEAELAKLRQLIAEQGINL
>KRH97836.1
MLRLITDFDGPIMDVSERYYQVYLLCLEKTRYPSQVIHKLTKEEFWQFKRSHTAEKQIAF
KSGLDAEQAQEFAKIRKETVHTEPYFDYDIMVPGALEALVKVKEAGIDLVVMTMRRVREL
NYAFDKFNLDKFFPEDRRYCLSNDYIKTRDIEDKPLLMAKAIKELPPATEIWMVGDTEAD
ITAAKKHAVKMIGVESGIRDRTQLELYQPDFILEDLKAAVNFINQS
>KRH97838.1
MSVRVRIAPSPTGNLHIGTARTAVFNYLFAHHQGGKFIVRIEDTDLERSRSEYTDNILQG
LSWLGLTWDEGPFFQSQRLELYQLAVKKLLDQGLAYRCYTTTEELEALRELQKARNEAPR
YDNRHRELSQEQEAEFVKQGRSYVIRFKIDDQRQILWNDLVRGEVSWRGSDLGGDMVIAR
ASDDGIGQPLYNFVVVVDDIDMEITHVIRGEDHIANTAKQILLYEALGANIPEFAHTPLI
LNQEGRKLSKRDGVTSISDFQKMGFTAPALVNYMTLLGWSAPDSTQEIFTLEEAAKEFTF
DRVNKAGAKFDWDKLDWINSQYIHNMPIEKLTDLLIPFWQDAGYSLTDGRDRPWLEQLVS
LIAPSLTRLVDALPMTKIFFTNGVELTEEGRQQLEQEGVKALLKAILVALDINTITGDTA
QNILKQVVKEQGVKKGLVMRSLRAALTGDVHGPDLITSWVLLNKIGLDKTRLSQALI
>KRH97843.1
MAKVVLDDVKRKFDKVTAIENISFEVPDGEFWVLVGPSGCGKSTILRTIAGLETITSGSL
YIGDRLVNNIPARSRDVAMVFQNYALYPHKTVAENIAFGLEMRQVDRKTIQSRIMSVAKA
LSLEHLLDRKPKQLSGGQQQRVALGRAIARQPQVFLLDEPLSNLDTQLRDDTKTELKQLH
QKLKITTIYVTHDQVEAMTLADKIVVLNRGKIQQIGDPQSIYGLPANQMVATFLGNPPMN
IIPATVKEDMFDVGGQLLAIPNQIAVVSAVYPGQNYHLGIRPEHIRIICQETDSQNILKE
PGELWVEVKVVEPLGKEILIRVELPSCPGLINMQVERDRHLHPGEKLKICLDLNNLFVFE
TSDGVRIFPL
>KRH97845.1
MNMEGLFQRTGVMRQHLLNLYQTTIALPWIPSDLLPQVFKELHTTLKMLLGAIDELHQQN
EEFVQTRNLVEIERQHYQELFEYLPVGYLHTNLQGIIEDANQEAGRLLNISQKFLVGKPL
ISFIVQECQQYFCRELIELSKSDQVRQLFLVLKPRYVSSFDACLIVRSCFNLHSNQRNLY
WLIQKSSTSRTVEMTAIDMHQEILQDRQVHKYSKRDNIPLSNTYFGYVLQGLVKISTLSQ
TGTEIMIGLATSGMIFGSTMTNLPLYEATAISDVELVLIYVSEMRAIPNLNQMLSPKIKQ
RLQQAESFLFIISHYNVEDRLSSLLEMLKLEFGEPVVEGTRLLFYLTHEDIATACNSTRV
TITRLLNRLQKQGKIKYDTNKHIIICGPQ
>KRH97850.1

MEYTIQELLQNLVDMGGSDLHLSASLRPYFRIGGKLTPIGNEILTPNECERLIFSMLNND
QRHRLEKTWELDCSYEIKALARFRVNVYKERGNYAACLRVLSYKIPNFQELGLPNIVREI
AEKPRGLVLVTGPTGSGKTTTLAAMIDLINQNRAEHILTIEDPIEFVHEPIKSLIHQRQL
GEDTRSFANALKAALREDPDIILVGEMRDLETISLAISAAETGHLVLATLHTNSAAQTID
RIIDVFPAERQMQIRVQLSNSLLAVFSQILVPRKTVRPGGSSQVMAQEIMIVTPAIANLI
REGKIAQIYSFIQTGSKLGMQTLEKALYELFKAGLISQELALSKTSRPDEVQRLIGIL
>KRH97856.1
MNHQQQNNNFQVNEPVTTQEAIDLLLGEGWLDEATRIEDEANCTISAGLDWGNVLPPLML
NLPLFSRLSTLRVSLNREIRLIIETWDLGVGTSSAIETARRRIKERLLSPPPELLPYLEA
TLLQDNLYSEEFISNREALKSLLAVLLTDSDRAEIAETAANSIRAQVMSFEIQKQKW
>KRH97861.1
MSISIKPDEISSIIQQQIEQYDQEVKVANVGTVLQVGDGIARIYGLEQVMSGELLEFEDG
TVGIAQNLEEDNVGAVLMGEGRNIQEGSTVTATAKIAQVGVGEALIGRVVDALGRAIDGK
GEIKATTSRLIESPAPGIIARRSVHEPMQTGITAIDSMIPIGRGQRELIIGDRQTGKTSI
AIDTIINQKGEDVVCVYVAVGQKASTVANVVQTLQEKGALEYTVVVAANASDPATLQFLA
PYTGATIAEYFMYQGKATLVIYDDLSKQAQAYRQMSLLLRRPPGREAYPGDVFYIHSRLL
ERAAKLSDELGKGSMTALPIIETQAGDVSAYIPTNVISITDGQIFLSSDLFNSGVRPAVN
PGISVSRVGSAAQTKAMKKVAGKIKLELAQFDDLQAFAQFASDLDKTTQDQLARGQRLRE
LLKQPQNDPLSVAEQVAILYAGINGYLDDIAVNQVTSFVKGFRDYLKSGNNSYYQGVQTS
KVLGEAEEVALKEALADYKKTFLATV
>KRH97872.1
MESYLLKSLSDLEDKNISRENWLIVKNGEQFEPLVKELYGEIRHQLDRQIDHNHEPVYKP
LKIILAQRDPVKFLAGFIAGCAAECAVFLCNPDWEKNEWEQVWNLVKPDIIWGDEIKFPD
FTTTNIPEEPMISGIMIPTGGSSGKIKFAIHTWETLTASVRGFTEYFNLSSVNSFCVLPL
YHVSGLMQFIRSFTTGGKLVITSFKQLTSESNQTSDIYKIKNSDFFISLVPTQLQKLLDN
QKLTQWLSEFETVLLGGGPSWNDLLIKARANGIRLAPTYGMTETASQIATLKPDEFLKIA
PDEFLKTKNSGRTLPHAEIVMEHFLENEHYEGNHLGAIRINAKSLALGYYPTIWQDDQGF
LTDDMGFLDQNGYLHIMGRRSNKIISGGENIYPPEVEAAIRATNLIMDVCVIGIPDHNWG
EAVTAIYVPQSPPTPIPEIKNQLQQTLTRFKIPKYWIPVVSLPRNPQGKINISQLQQIVL
ESLDQEMII
>KRH97873.1
MKISLSSENVIQYLYDTGLFSSQDSPSPESDLPKTTIDNHIFLVKLSGNRQILIKQETPN
GEYPSNYQFSNEWVFHQLLVQFPLLGNIPAIASLLLHFDAENSILVCTFLSEYMELEEFY
KNRSIFPLEIATSIGSALACLHRSTFQKQEYRDFIDTAPEGELRYNYYNPVQGISSLTQQ
VWGKIPTAALEFHALYQHYEEIESAIADLSYHWKPCCLTHNDLQLDNILVHSRWQNLDDC
LVRFKQWGVYAWGDPAFDLGTLLASYLQIWLSSLVVDSSLDLEESLDLALVSLDTVQPSL
IAIMRTYLQIFPMVLEYFPQFIIRVVQFTGLALIHYIQKCINYYKYFDKTHLSMLEVGKK
LLTMPENSLLNIFGTSPQEILPSVIGVKIGVKTYSKKNGRETDRQIAPIYYPKTRLRGC
>KRH97874.1
MVKYTYTDQLMSTINTSFHTKVQFLQRQAASVLLYQSVLQTEVGIAFLELLQAIRYTDGD
GRNCLQAYGNYFHSLASSQQTWEEYLIRQILLSDNPFTKLAQKREFTDLPSALIAAAQHD
LQILQNLYECNCALLSEWVQTVCHLPVSPVVWYQEPQPQPIQTVILQDLMSTWQQLENWG
DIIGDLVTYYQHQGSGLFAEYRAFRWENGEFFGIPYPDRVKLETLVGYEEQKQILLKNTR
FLLSGQPALHVLLYGSRGSGKSSLVKSLLNQDGYHNLRLVEVRKSELYSLPQIVEYLRDL
PQKFIIFVDDLSFEEDDDVFKSLKVVLEGGLTPRPNNVVVYATSNRRHLIREYFTDRPNP
KDHQEVHSWDTMEEKLSFADRFGLTLTFEAADQRTYLQIIHHLAAETGMDLGGEDLEFQA
LQWATRHNGRSGRTARQFIDFLQADSNDNNNY
>KRH97877.1
MSSDGKIAVEFRDVSFEINHRILLSKLNLEINQGEALILLGRSGSGKTTTLKLINHLLVP
TQGEVFVQGLSTNKWDVIKLRRSMGYVIQETGLFPHFNVAENIGLIPSLEKWSGKKIKTR
VYEMLDLVGLEPTKFVHRYPHQLSGGQRQRIGVARALAADPPILLMDEPFAALDPITRWE
LQQQFQHLQQQLGKTVVFVTHDIQEAFFLASRIGLMCEGNLVALGTTEQFLQSSHPEAKA

FLACLNAGKRR
>KRH97882.1
MLLAPITVLEQQYQHLHDDYKDVIEGLLASPKTLPPKYFYDDRGSLLFEEICDLPEYYPT
RTEASILRRSAEEIAQITNCCELIELGSGSSTKTQLLLNAYHKVRDLFSYIPVDVSGGIL
KDSVLQLKDKYPQIDIHGLLGTYEQALFHLESNYLQSRLLFFLGNSLGNFSQQECDRFLT
QVSKTLKTSDFFLLGVDLHKDKGILEPAYNDAQGVTAAFNLNMLTHLNWRFNGNFDTNLF
KHQAKYNLKERQIEMRLHCQESHWATLASLDLQVFFEAGESILTEISRKFDLLQMSQQLE
SKGLKTVRTWKDDNGWFGLILCQKSL
>KRH97884.1
MNLRQLIPFFDHSAGNWAPEARLLRWLTLLWLILGLIMLFSASYPVAAERQDDGLYYFKR
QIIWVFVGLILFNWIVNTPLSKILAASHWFLFLFLLLIFLILVPGVGKKAFDAARWIAIG
PIPIQPSELIKPFLVLQSARLFAQWEKLSPQVRLFWLGVFCLVLLGILAQPNLSTTALCG
MTIWFIALASGIPYRYLVGTALGGFSLALLSMSIKEYQRRRVMSFLNPWADPTGDGYQLV
QSLLAVGTGQTWGVGFGMSQQKLFYLPIQDTDFIFAVFAEEFGFVGGIVLLLVLGMFATL
GLIIALKAKNPIHKLVATGVTVLIIGQSLLHIAVTTGAIPTTGLPLPMFSYGGNSMVASL
MACSLLIRVARESSEAEILSLKKSD
>KRH97888.1
MSIVTKSIVSADAEARYLSPGELDRIKSFVSGGVARLRIAQVLTDNRERIVKQAGDQLFQ
KRPDVVSPGGNAYGQEMTATCLRDLDYYLRLVTYGIVSGDVTPIEEIGIVGVREMYKSLG
TPIDAVAGGVAAMKSVAATLLSAEDSAEAGGYFDYVVGAMS
>KRH97893.1
MTYSQILLPPGTLWKKLQETTHHALQCGALIPISTKSEFVEQDGVIFLVRVLSNLNRKQS
AQKKQDQIIKVSGTDFNPFLPYQEDLFVADISETHVCILNKFNVVNYHLLIITRAFVEQE
SLLTGEDLAAMWACLAEFDGLAFYNGGKVAGASQRHKHLQIIPLAETEIPIAPLLTTAKL
ENGITTIPAFPFLHAFTAFSPDETPQDILSKYYALLDMVSITATDNLQQSAAYNFLATRN
WMLVVPRSKEDFISIPINSLGFAGSLLVKNSAQMQLVKDIGPMNILKNVAISNI
>KRH97896.1
MIQGVNSVPAFDIKQQYLAIESKISRAVLEVLSSGRYIGGPGVEGFEQQFAAYHGVNECV
ACNSGTDALYLSLRALDIGAGDEVITTPFSFFATTEVISAVGAIPVFVDIDPTTFNLDVS
KISAAITKKTKAIMPVHLFGLPVDMTALMEIAHSHNLAVIEDCAQATGASWEGQKVGSIG
NIGCFSFYPTKNLGGCGDGGAITTNDREIADKIRLLKEHGSKTRYIHEEVGVNSRLDALQ
AAILEIKLPYLDIWNEQRQKIAAYYYQHLGQISGITPPQELLGGIGVWNQYTIRVSSQER
NGASSKYRDWVRTALQERGVSTMVYYPVPLHLQPVYQCLGYECGRLPVSEAACHQVISLP
MFPELTQEQQDRVIYSLKDCLS
>KRH97897.1
MIMLPQLQNAFLRRNVLKVISGLNNFNLDSVAAIVKAADLGGATFVDIAADQRLIHLVKS
LTSLPVCVSAVEPEQFVLAVNAGADLIEIGNFDSFYAQGRKFEADEVLSLTHKTRALLPE
ITLSVTVPHILTLDQQVQLAEALVQAGADIIQTEGGTSSKATHAGTLGLIEKAAPTLAAT
FEISRAVSIPVLCASGISNVTAPLAVAAGAAGVGVGSAINQLNSEIAMIASVRSLVESLS
NSNISSPV
>KRH97899.1
MVYRILDANLNRSREGLRIIEEWCRFGLNDASLAETCKNLRQEVARWHTPQIRAARDTVG
DPGTVLSHPQEEQRSSITSLLQANFCRIQEAFRVLEEYGKLHHEEMGKTFKQMRYQVYTL
ESTLMGHQRHHLLWQSRLYLVTSPADNLLAIVESCLQGGLTILQYREKTADDMVRWDRAK
KLRELCRTYGALFIVNDRIDLALAVDADGVHLGQQDLPVAVARELLGSQRILGRSTTNPQ
EMQAAITEGADYIGVGPVYETPTKPGKAAAGFEYVSYAARNCPIPWFAIGGVDMGNIHDV
IKAGAQRVAVVRSLMEAEQPTLATQYFISQLLRK
>KRH97900.1
MTLPDSFRTQVQHIQESSKFQSVDGVLRATPFPGYTLITPPASEDPQNADFYKQIETFGQ
LLLELPIASDLIVPLPVSSFHVTLADLIWDHAFIHACEKKPDFEEELNSYLGDLFGQYQK
LRSSSQGPISWQVLGVIVMPRAIALGLIPKDERSYEEIIQLRRLIYQNRKLMGLGIEQHY
HFTAHITLGYFSEIPANLDRVNLSNLLSELNQYWLLNFSEIVVSQAEVRKFDDMTHYYRQ

PNWSSFRF
>KRH97901.1
MHLFNKVKFNTPESVELEFTLAGIGSRAWAFLIDYLVLTTTLILVIIAWVFIFSQLVIST
ILDSAFGLWIWAIAFLTVFTIYTGYFVFFETLWQGQTPGKRMAKIRVIRDDGRLVGLQQS
VLRALLRPVDEFFFIGAFLIILAKNEKRLGDMVAGTIVIQSQIENKVVNLTISPEAQLLC
VKLQQNCDLSQLLPDDFAVIREYLKTRGGMSSKAKASLSLELSQQVQSIINLSVLPGDMS
ADVFLEAVYLGYQKLEFV
>KRH97902.1
MAENRGFYTTGQVLTVGNAVSASVRLYRSHFKDYFLLSLKAWLWIFVPVYGWAKFCALSA
LISRLGFGDLVNQPESVHSGERFVNSRLWQFLGTLILMFFIYVGIFVSFSILGFLLVLIP
TAILGGLDIQNPTNIGTWWVLIFLLVLLLAIVAIVGSIWLEIRFSLVTLPLAIEENVDAT
STIGRSWELTKGHVWRIFLILFVASLITLPMQILLQIISFIVQGILEFTSDNNPLLFIAI
FIGGSALILPFWQSLRAVIYYDLRSRREGLGLKLREREI
>KRH97903.1
MNIKRWISTREENWQRLDSLLSKIERKRLKSLKSSEIRELASLYRSITADLARARTYNVS
HTLFQSLQLLATRAYTQIYQGSRKQEWEGVIQFYRWRFPAIVQQTFPYIIAATGLFILGM
LVGWWYSWQDPSFMSLLIPESLISQVRDQGELWMGSIVGIEPLASSNIMINNISVSFSAV
AGGITGGIFTIYILVFNGLLIGSIATLVAENNLAYPFWAFVFPHGALELPAIFFAGGAGL
LLGRGILFPGKYLRLEAIKYYSSLAAQLVFGIVPLLIIAGIIEGFFSPNPIIPDPVKYLV
GLAIFILLVMYCNRK
>KRH97908.1
MSTSISRIRGKEALVRLGIITSIFTGIGSIIASNIHWVTHGGVGFHTSTQPTFILYLIPP
TQLFGLILTDPNGKNIISYSGKNSSTPISWRAALNPEELKNPPYDLLLDPPAIFPQSVYA
NPRATERTANPFSTSGWIESYAVAIIATIVMIILMTLEHTLARKREKKLQENNRKLQIDL
AEKIQERELQQVQIDSQRSEFEQEIKQLHNKIGLLNQSIAQLQSQCQNESFELQNKLREA
QLQSQQNLNQQQEYENRIELLTQQLIEEKHNQSQKPGRQILIKIFCIQLNLNRKKN
>KRH97911.1
MRTNIAHLYLLSPLHTGGASQEGNLVGIARETHTNLPYLPSSTIRGRLRAETDSNLRNQL
WGNTLEDVRNPECADENLTQGQLWVGDGSILWFPVPSLSHGVVWVTSPFLLRRWSRLTQP
NVQIPEPGSFSGGNNQNLYLKDAIFKRGDLIPWGNEWKEYLPPGSTETDIISQALVLTDR
DCQVLVQLSLWQQVKIKLNENKNVDGGFRYEEAIPPETLMYFPWGTTTNANRNAAQAQEC
LKTLLRSHETFQIGGQESLGRGFVEIWTYNDK
>KRH97915.1
MIKLEELKQGSIINGILPGQGVTVIDAKWFGTDTVELTYKDIHGSPYTELLFRDREESLQ
IVNEGKPWSFDGDGETLCLVSEAHRINLAYLFDPLLAVHTSLVEPLPHQITAVYSEMLNR
QPLRFLLADDPGAGKTIMAGLLIRELLIRGDLARCLIVCPGSLAVQKSSSDG
>KRH97916.1
MVNYDLPWNPNRLEQRFGRIHRIGQTEVCHLWNLVAHETREGDVYRTLLEKLEAEQKALG
GQVFDVLGKAIAGRETTGTSVSITTMHRALEKLGLRHKKKSKCQ
>KRH97917.1
MTEITRPFTPDWVSPPGDTILDLLEERDWTQGQLSERLGYTTKHISQLINGKAPINEETA
LKLERVLGSTAGFWLNREAQYRAQLAKIEEQERLQTWTPWLDELPVKELMQQGVIPKRRI
DAKSKPGIVRELLHFFGVASPDDWQTFYVGMECAFRRTREAQSDVGAIAAWIRQGEILAE
RLNCPKYSKPKFEKAVREIRTLTMLEPEEFDPEMRKLCWEAGVVFVIVPSIPRAHVSGMA
RWLNPHKALIQLSLYGKQNDRFWFTFFHEAAHILLHDKKDIFLDEWDGGAVVQSKQEEEA
NCWSREFLIPLHHELELPNLKSKEAVIDFAKRIGIHPGIVIGRLQHDKVIAPTWMNGLKV
SFRFKGNCASLLNSSQKSLSW
>KRH97918.1
METIYSSSRPSIPIFHSALIMIKATLVTTLITFALGFGTAKAQTYTKQQLQEIYSSHLVN
EGFRPKVDNDGDISFRSEGRTFWILLDEDDPTFFRMFMGFIQEDKSPQMRLRRLEGCNIA
NHKITANFR
>KRH97920.1

MAISNRERVGRALDSLKEGLYPFVDREMKQTYGKQWTTMAINCLPESYTTRKTADTIFQE
DVSALLIVMWEYWNDVFKKTLGRSDRSLVSELRETRNSWAHNSSFSLDDTYRAFDSITRL
LNAVSGDTEEVEKQKQEILRTRYEEQAKRETRRKAEAPTEGQPSSWLKPWREIATPHPDV
ASGRYQQAEFAADLWQVYQDEGSDEYRLPTEFFRRTYLTEGLKKLLSNALIRLTGKGGDP
VIELQTNFGGGKTHAILALYHIFFGLKRQELPGLDSVFEETSIKELPQNVNTVVIVGNKI
SPGTIYKKSDGTQIRTLWGEIAWQLGGKEGYEMVRDSDQTATNPGDTLKHLFNRYAPCMI
LIDEWVAYARQLHEQPDLPGGSFDTHFTFAQTLSESAKNADRTLLVVSIPSSSDNEIGGD
RGKQALDRLKNAMGRVESPWRPSSAEESFEIVRRRLFETTTNPDLFVERDRVIRAFYDMY
RQQKQEFPSECAEAKYQNRLKESYPIHPEIFDRLYSEWSTLDKFQRTRGVLRLMAKVIHS
LWEREDKGLLIMPGQIPMDDAQVQSELTRYLDDNWVPIIEKDVDGINSLPLEIDRQNTPI
GRYSACRRVARTIYLGSAPIQQAANIGLEEQRIKLGCVQPGEVVATFVDALRRLTDRATY
LYIDGNRYWISNQPNVTRTAQDRTNQFLEELYKVTEEIIRRLKSDKERGEFTAIHTAPDS
SSDIPDDPNLGVRLVVLSPELQHNKAKKNSSAIEWIKDVLNHRGTSPRYYKNTLVFIAAE
EDNIENLNKNVAQYLAWDSILNDKDTLNLNVSQTKQATAQKEQSEKYVKTILNQTYQWLI
SPEQPNPHEPIEINCERIPGESSPILRASRQLVNDGQLITEYSSNTLRMEALDKYLWRDT
NHIDLKQLWEYLAQYVYLPRLKNPEVLLEAVKNGVQKIDIQNHFGYAQGWEESKQKYKNL
VVLHNINPSISSENLIVKPEIATKQLKEEQVKELTSPSRSESTSKKPIQPTQEPTPTERT
RETKPQLKRFHGTVEIDPLRVNRDAPAIANEIIQHLTSLKDAKVRIVLEIEADIPDGVPD
DVVRTVTENCRTLKFNSQAFEQE
>KRH97930.1
MNPVDIGIIGGSGLYKMSALQDVEELDIKTPFGSPSDTIIIGTLAGARVAFLARHGRNHG
LLPSELPFRANIYAMKQLGVKYLLSASAVGSLRAEVKPLDMVIPDQFIDRTKNRVSTFFG
EGIVAHIAFGNPICQNLAAVLADAIASLNLPDVTLHREGTYLCMEGPAFSTKAESNMYRS
WGATVIGMTNLTEAKLAREAEIAYATLALVTDYDCWHPDHDHVTVDMVISNLQHNGINAQ
EVIIETVKRLSENSPPSEAHSALKYAILTNLADVPTETKQKLGLLLEKYLGV
>KRH97933.1
MAVVYTKCQHLGVKYLLSASAVGSLRAEVKPLDMVIPDQFIDRTKNRVSTFFGEGIVAHI
AFGNPICQNLAAVLADAIASLNLPDVTLHREGTYLCMEGPAFSTKIENGSDFC
>KRH97941.1
MATTQQSSTKQDILQYLLKHSQATAGQLSEILEVSPQAVRRHLKDLETDEMVVYSIPENS
GMGRPQHIYHLSPGGKEYLQRSATKVSGGYGEFAVSLLDTLAETVGREQVKTILQKQWER
KAQEYQEKVGQGCLEERVATLVELRKAEGFMAEFRPVEENDDGHEGAHGFLFMEHTCAIS
NVAESFPSVCGHELEMFAAILPDCSVQRTHWLIDGQHRCVYLIKRN
>KRH97944.1
MQYKQTSFFLATLILSVISNVSPVMTTQSQAGEVLLVQADNRKLRELLEQGRKLVDSGDY
SGAIAVYQDAAKLAPKNAKIYSGIGYLYAQQGNFSQSLSAYRQAISINPNNSDFYYAVGY
IKGNLGDAVGAKEAYRRAIQINRNNFHAYLGLGASQTRLGDYDAAQWAFEQATKIDRNNP
RVYELIGAMFKQRRQMQEAGNALRQALRLYRTGRDADGMVRVEEMLKEMGG
>KRH97945.1
MTTTSISKKEIAAMTPKDVKDLAARLELDNYSNAFDGLNDWHLLRAIAFQRPELVEQYVY
LLDLEPYDEG
>KRH97949.1
MIKNALTRKNHKKAIIFTGLLSAIAWTQYLTMCATVHAADRVTIRYGFLEESTSVAELKK
ATETGQLPGSLQIYTSALSQEQRNWLVQGLKTRIPINVVTLDWLLNTQLGQTVINDIATV
FDRRQDQSGVQAVRSGLILAASSPEGLSMLSFIAAYPSQTLRLNLPQVLTVARFLNIDFL
QTQQFLLMHSPQSDRQKPSINIPFDPTQPGTQKVEISKLNLSDDKRKRNIIVDIYWSTPT
NGENNIEKPLIVFSHSSSSAGTDLQYLAQHLASYGYVVAALQNPGSNFFTNKGKVGLNPQ
EFLALPQDVSFVLDELAKVNQNPNNSLQGKLTTNKVMFVGYSLGGTTALALAGGELQIAS
LKSSCEKNAGKLSDVQSFMCLARQLPQNNYQLQDQRVKQIIALKPASSLLFGETGLTKVK
VPTLVFTASADHVTPSLTEQINGFNRIASPKWLAAAVGASHYSVVDPLVVNASLNTNNTP
ITSREVVGEKSADVRSYVKAITLAMAAQLTPDANKYNVFLTPEYAQFASTPLFPFRLIAP
LHSINTTSKN

>KRH97953.1
MAIANFSRGQGIHSPYLAKLLPLIWRSGDLATRDRLPISQFTSYTLEQNGVAKMNRYTKL
ENLLKAQDFKEADLETNRVIVEVANREREGYLRE
>KRH97966.1
MSSSSLNIKQSRVIVVGGGIGGLTAAALLARRGYQVLIVDQALVPGGCASTFKRQGFIFD
VGATQVAGLEPGGIHYRIFQELDMDLPPATPCDPACAVFLPGETTPINVWRDPQKWQQER
QQQFPGSEPFWQLLATLFRASWEFQGRDPILPPGNLWDLLQLIKAVTPSTFITAPFTFMN
VGDALRLYGLDHDIRLRTFLDLQLKLYSQVSAQETALLYAATALSISQLPQGLYHLQGSM
QVLSDRLVKSLEKNGGQLLMRHTVEGINVEDGKAQGVTIRNQRTGQVFTETADHVIANVT
VQNLMELLGEKVPSGYKTRIEKLPPASGAFVVYLGVEQSAIPQNCPPHLQFMYDINGPVG
ENNSLFVSVSHEGDGRAPAGKATIIASSFVDFAPWWNSDNYPQLKAKFTQEAISRLAEYF
DLNPETIILVEAATPKTFADYTARHKGIVGGIGQRISTFGPFGFANRTPVRNLWLVGDST
HPGEGTAGVSYSALTVVRQIDDLQRRLSR
>KRH97968.1
MWQEIDRQISELTGQNFQTNQRYSVGGGCINQGYAISNGQITYFVKINQPSQGEMFAAEM
LGLQQMYNTKTIRVPQPLCWGTADNGSYIVLEWLEMSAGNNKSWQQMGRNLAAMHKTTSD
QGFGWHINNTIGSTPQINSWMLNWDEFFFKNRLGYQFQLARRRGGNFPGEQKLLDVIPSL
LADHKPETSLVHGDLWGGNVGFTINGEPVIFDPATYFGDREVDIAMTELFGGFPPSFYQG
YEEEFPLAPGYEKRKVLYNLYHILNHFNLFGGGYSNQANGMIGRILESIR
>KRH97971.1
MLDFTIIISFIIASAGIGYFSTDLLPPGSLKGVTNLDALRLVVAVFAALIGGAVGLSFQT
SYRRLETQVREMPIEVILTRAIGLVIGLLLANLMLAPLFLLPIPVDFSFIKPLVAVVGSV
ILSVTGMNLADTHGRGLLRLINPNTVETLVVEGTLKPANTKVLDTSCIIDGRIELLLETG
FLEGLIIVPQFILQELQQVADATKDVKRVRGRRGLEILNRIRENYPERIVINSVEYDDLS
TVDTKLVKFAQEINGTLLTNDYNLSKVASVQKVPVLNVNDLVNAVRPSYLPGDNIDIKIL
KEGKEPSQGIGYLDDGTMVVVEEGSGYVGGEVRVVVTSALQTSAGRMIFAKPQASALA
>KRH97973.1
MISWFPWNLKLQKLRDKLLHDPYYRLQSGEEIHLAAQLGIRIDVNQANVDDWLRLPGLSI
HQGRSLVALSRSGVKFYCIEDIAAALSLPIHRLEPLKPLLSFTYYDEHSLVHHPSININT
ASIETLLEIPTIDIVLAEAVVKNRLARGAYRNLVDFQNRLGLSGDLISKLMYYLHFQ
>KRH97977.1
MYQWILPSLAEILAQSQSHTVPCSSEKAEQQWRVGLAAAEHLLLTTLTPTTLHYTTPGLV
LTAPTPLFGQPNLTQNLQTVTFATKPFNPLALMPFDVPPKVLADQGNLLFDQDTTIRNNT
YYSHLYNNNNSILPGDSILTLLAPDPLGNEQFCLVFTEKFTLALVLSAPTGSKKEFLFSF
EPNVVKQAWQALGTRLVLTNPELFSQLDARVENYPLTAPNYQTIIQFSQFLLAELPESTD
RSTHSFPIFSFPQDPVLSSAKPPSRSDVELLQAFSHEVRTPLATIRTMTRLLLKRQDLPG
AVVKRLEVIDHECTEQIDRMELLFKAAELETSETSSSQTTQIGSQLTPMSLNQILEQIIP
RWQQAATRRNLTLDVALPQQLPTVVSNPAMLDRVLTGLMESFTRSLPPGSSIQVQVIPAG
HQLKLQLLPRLGCHNSTKDVHIPIRKALGQLLIFQPETGTISLNVAATKHLFQSIGGKLI
VRQNSQYGEVMTIFLPLEIDHKHLY
>KRH98000.1
MSFSSSEVAQTPNSRLATTESHDYDLMIVGGGIVGLTLAAALKDSGLTVLLVEAQVTSTA
VAKGQAYAIHMLSARIFQGIGIWGKILPHIAKYRQVFLSDAEYPNVVKFQTSDLGGETPE
LGYVAEHFALLEPLQEFVRTCANVTYLCPATVVSTKTSGDIVTVNIQINGADQVFRTKLM
VAADGSKSPLRQAAGIKTKGWKYWQSCIVAFVRPEKSHNYTAYEKFWQSGPFAILPLTGN
RCRIVWTAPHEEAKTLCTLSDEEFLAELTRRYGHQMGKLELLGERFVFQVQLMQSDRYVL
PRLALVGDAAHNCHPVGGQGLNLGIRDAAALAEVIQTAHQQGKDIGNIQILQQYERWRKN
ENLAILGFTDLLDRIFSNNILPMVIIRRLGLWLMQRIPILKVFALKLMIGFQGRTPQLAQ
LSLNTNFHGLNNQL
>KRH98005.1
MKLNRRQFLTVSLASLASLSPLGYSFPTLGTTTNSTIKPRRLKTGNGVGLISPAGATFLP
EEVEIVQDAVKALGMIPYLAPHLLARYGYLGGTDVQRAADINQFFADSTISMLLPMRGGW

GCARILPYLDYQVIRKNAKVLVGFSDLTALNLAIYAKTGLITFHGPNGLTSWRAEQVNWF
RRVLFAGEKLNFANEADPDDRGRLMRVKNRIQTITHGKARGRLIGGNLSVISAMVGSAYL
PNFAGAILFVEDVGESIYRIDRMLTHLKLAGILQNLRGFIFGQCTNCAPDGGYGSLTLEE
VVRDHIQSLAVPACMGLQIGHLENIVTLPIGIEVEMDATDGTIVMLDSAVN
>KRH98012.1
MIFTYQRTIRFADTDAAGVVYFANILNICHEAYEDSLIHVNINMRNFFTHPSIAYPIVHA
SADYLRPIYCGDKLIINLIPKKITEDKFDINYEISVNNVIVAKAITRHVCIDVATRNKRQ
MSGEIISWLETNRRDVEDIERKKSREEII
>KRH98018.1
MSYHRDAIAPHGGELINRVASPEQKEFFCAKADFLPRVILDERAVSDLEMIAIGGFSPLT
GFMNQVDYNRVVEEMRLANGVVWSIPITLSVTEEVASPLQVGGLVRLDNSQGEYIGVLEL
SEKYTYNKKREAANVYRTDEAQHPGVQVVYAQGSVNLAGDIWLLQRDAHPHFPTYQIDPA
ASRQMFREKGWKTIVGFQTRNPIHRAHEYIQKCALETVDGLFLHPLVGATKEDDIPADVR
MRCYEILIEHYYPLDRVILAINPAAMRYAGPREAIFHAIVRKNYGCTHFIVGRDHAGVGD
YYGTYDAQYIFDEFQPSELGIVPMKFEHAFYCTRTKQMATTKTSPSTPGERVHLSGTKVR
EMLRRGELPPPEFSRPEVAAELARAMRIGQTALI
>KRH98021.1
MKETSTSLNNLNKQGGWKENLTLIGVALILALLIRVFIAEPRLIPSASMYPTLQIGDRLV
VEKISYRLHPPQAGDIVVFQTPPELQERGYDDNQAFIKRIIGLPGDIVGIVNGQVYVNGK
QLEETYIAEPANQPFPLIKIPENKFFVMGDNRNDSNDSRYWGFLPRRNLIGHAAFRFWPL
NRLGLIG
>KRH98024.1
MDSNGPSTNLSNTYSQRVADIVGTVIALLTLIMPVLIISHYSSAEVQNPPQPVIYNLTTD
KP
>KRH98025.1
MRFIRDLTFDTAKLLNRIYKESRHYQVRQRAHCILLSYKGVTIPELIEFFQVSRRTIYNW
MNDWEERRLLGLYNRKGRGRKAIFNEGQKQKIKEWVKLYPKDLKKVLNEIQEEWGITVSK
DTIKRVLRSMSMTWRRFQRGLAGEPDPLEYKEKELALTKLKEQEKCGEIDLRYLDESGFC
LTPYVPYGWQKKGENIPIKSGRSRRLNILGLMNRYQELDAYTFEGTITSEVVISCLDKFA
ENLPIKTFVVMDKASFHRSKKIQDKINEWQQKNLEIFWLPSYSPQLNLIEILWRFMKYEW
IEMDAYSSWQNLVNYVEKVIRDFGKEYVINFA
>KRH98026.1
MQIDHVHFYVENAHRWRDWFVNNLGFQQVPLWGTFFDLINHQKLRDTDTQVVRSLPGDKG
VEDEMWQ
>KRH98033.1
MQIILYGKPGCHLCEGLIEKLEQITHHNSKSSNKDANISFTLEVRDITTREDWFAAYQYE
IPVLFLWRGETEYLQSVPRPSPRASVQQLENLLLTTASKLLILADQER
>KRH98035.1
MLLFLKLIDYLLAAVFIGAALIIYFDSNNQPYLLAGIGASAVAILLFLINRNSVGAAEKQ
AKKNEFTKKAELYTSLLQNSNSLEHNTIVPARAKALEYCQDLINDYKKTRNIARSLYYVL
QISTVILSGVTPILVLVDKLETGQPWLKWLPVICPAVASIVASIVTSFPFQKNWVTANTI
VELLEAEQEKFILGITPAYRCYDVVGDLEQQQKASQAVELFISQVNSIHLQQVQQSTEQQ
SDKRKEETKTQDPALN
>KRH98037.1
MPILTNMLLSPIKFGTDGWRGVIADEFTFERLTLVASVAAKVLYNTYYSLTGSRTIVVGY
DRRFMAENFARVVADAIKALGFDVLFSQDYAPTPAFSWAAKDFNALGALVITASHNPGSY
LGLKVKSAFGGSVPPEVTQEIERLLTVEFSPVGNLGKEETFDPWISYCQALAKKVNIEAI
QEAISSDKLTLFVDVMHGAAASGLGRLLGQKVKEINGNRDPLFGGKPPEPLPKYISEISA
EIKNYTEIHHQQNRSTLTVGLVFDGDGDRIAALDRNGNFLSSQILIPILIDHLKTRRNFT
GEIVKTVSGSDLIPSVARSLGLSIFETAVGYKYIADRMLMTEVLLGGEESGGIGYGSHIP
ERDALLSALYVLEAVVDSGLDLGEYYQQLQEKNNFFSAYDRIDLPLASMQIRDRLLQQLK
TQKLTEIAGKKVIDCQTIDGYKFRLADESWLMIRFSGTEPVLRLYCEASTIEQVHQTLNW

AKTWAESN
>KRH98039.1
MTINDMDFKQLLKQQLPALIQEDVEVRDLLVRAISSYFAGSLETESHFDLVLGEIRRDRE
EQSRKWDENKRELELDRQEQARKWDENKRELELDRQEQARKWDEQTGKWNENRQEFDRVI
AAIDRMSRKHDIAISGLGSRWGLCSESSFRNGLKAILEESFGVEVLNIVEYDDQGIVFGR
PDQVELDLIIRNGELIICEIKSSVSKADLYTFYRKVDFYQQQHQRQATRKIVISPMVHPS
ANSIASKLGIEIYSSSEDVSAELPPSPSL
>KRH98040.1
MNIITTLVIAKSLLQQVVRDRLLYILGFYAVILIPANQVIFQFAPATQDKIFLDAGLGIM
NIIGLIVAVFIGTSTMNQEIEKRTILTILAKPISRGQLIISKYLGLCGVLLLLLTCMTII
YLGFLQFQKITYNPYTIILAVIFLFLQLSLITTVAITLGVFTSTLLATFLSIAIYLIGNT
TTDLVNLVRAGENPFIVNIVTILYLTLPDLSRLDLKNDVVYGWEAIPDTITLFSNAGYGL
TYSLMLLAIAIFIFSRKEF
>KRH98045.1
MKVLVIGNGGREHALAWKLLQSERVNQVVCVPGNGGTANLKGCQNLPLSVNDFPAIGKFA
LEHNISLVVVGPEVPLANGITDYLQELGLMVFGPNRNGAQIEASKSRAKSLMQEAGVPTA
KAAVFDQAHLAKIYVQQQGIPIVIKADGLAAGKGVTVAETLTQAEMAIDAIFQGQFGSAG
NFVVIEEYLLGQEVSVLAITDGVTIRPLLPAQDHKRIGEGDTGENTGGMGAYAPTPIATP
ELMSRVQTEVLENTISALNQRGIDYRGILYAGLMVADNGDFRVLEFNCRFGDPETQVILP
LLETPLDELILACIHQRLAQIPPLVWKSGAAATVVAASAGYPGNYQKGQVITGIPDAEIS
GAKVFHAGTKLNAQGQIISDGGRVLNITGWGENFHQAIAQAYHGIQQIEFSGMYYRRDIG
HRVLKFIG
>KRH98046.1
MPDPSLYITDFLTWLNHQVSALKHRNGDVLDWDNLAEELTLTGISEKNELKSRLIVLLSH
LLKWQYQADKRSISWFTTIANQRDDLQDLLEENPGLGKYIPDLVSKAYRNARREAAAETG
MELAIFPDVCCYKIEQILNPQFICNTTKDFQKAIIEAQQ
>KRH98048.1
MAENTVKVDVLFPALLKAISSLGIDEKHQLWELLEAELFPDDEDSPEDLDDIQAARSDYQ
AGNFMTFDEYQIYRNNLPN
>KRH98054.1
MRSPKGSAAQSPIGIASRSPSKYPQNFEITDLSVDGKRFPSTFYQMGTEHEMSLDMNSAS
WSKVIAHRECFAIARRECFAIAHRGKTNPRKFAREMLPNQGARAESNPVSTRSQYLFLLT
KVKM
>KRH98058.1
MTSKKREQIRNYFTKRKPSPSEIMIEEMLTRFAIFFILGSFLIRFILVFTNSGAISIFLI
LLIFGVSLLKLKKFYFSKELIFTTRYADIPEEQTVDAWLIDDIEDLKERSIRRLNINKAE
LIRDSIVIRGPILWSTNGIPSQDLLWKKGKDQHIRFSINTITVIHLTDYGISSYQCDFNF
LKGVPLNERDDEFHYRDVVAVSTRDDSTNYRLPNNVLIRHAQLFKLSVSSGDSIQVVINS
SELLKFTGGTILDTGLDSAIRTLRKVLNQKKS
>KRH98059.1
MNLEQKDPCFERLLTYIRQNRGFDFTGYKRSTLVRRVTKRIQALNIDNFIDYMDYLEVDP
EEFHSLFNTILINVTDFFRDVSAWDHLGNQVIPNIIKSKKKNEQIRIWCAGCASGEEAYT
LAILMAENLGAEDFRQRVKIYATDIDEEALNQARQALFPVKSVESVPRKFRDKYFDLTGN
NYIFRQDLRRSVIFGRHNLFIDAPISRLDLLVCRNTLMYFNSEIQGRIMARFHFALNDTG
YLFLGKAEMLLMYSSLFMPVDLKNRVFTKLSSTNIRDRLLVMANSVDDESSRQLSQNIRL
RDLAFESAPVAEIVIDINGLLIIINEAARNLFGLSKMDLERQFYELELSYRPIELRSLIE
RAYNERRPITLNSIERYVPNSEQQYFDVLITPLQEDDLSLLGVSIAFNDVTRYVELQEAL
QRSRQDLETTNEELQSTNEELETTNEELQSTNEELETTNEELQSTNQELETMNEELQSAN
EELQTINHELSERTLELNRNNVFISCILKSLQKGIVVIDKNFSILNWNELVEDLWGLRYD
EVINKSLFSLDISLPVEELRSPILDILSGKTDFQEVRVESTNRRGKIIECYVGLTPLIDK
KIEGVVLIMTGSQG
>KRH98064.1

MTSTAQSKTSTRRVVFPFAAIVGQEEMKLALLLNVIDPKIGGVMIMGDRGTGKSTTIRAL
ADLLPEIPVVANDPFNSDPSDPDLMSDEVRQQVAEGLEIAIGHKKVQMVDLPLGATEDRV
CGTIDIEKALSEGVKAFEPGLLAKANRGILYVDEVNLLDDHLVDVLLDSAASGWNTVERE
GISIRHPARFVLVGSGNPEEGELRPQLLDRFGMHAEIHTVKEPALRVQIVEQRSEFDQNP
AQFLDNYQFQQELLQQQIVSAQELLPSVNIDYDMRVKISEVCSQLDVDGLRGDIVTNRAA
KALTAFEGRTEVTVEDIRRVITLCLRHRLRKDPLESIDSGYKVEKAFARVFGVELPEDVA
GKNGTKTGVR
>KRH98065.1
MVQERTLPKFDTATVQITREEGLGLYEDMVLGRYFEDKCAEMYYRGKMFGFVHLYNGQEA
VSSGIIRGAMRPGEDFVSSTYRDHVHALSAGVPAREVMAELFGKATGCSKGRGGSMHMFS
AEHRLLGGYAFVAEGIPVASGAAFQSKYRREVLGDERSDQVTACFFGDGAANNGQFFETL
NMAALWKLPILFVVENNKWAIGMAHERATSDPEIYKKASVFNMVGVEVDGMDVLAVRQVA
QEAVARARAGEGPTLIEALTYRFRGHSLADPDELRSKEEKEFWFSRDPIKKLGAYLVEHN
LAVESDLKQIEKKIQSLIEDAVRFAQESPEPDSSELYRFIFAEDE
>KRH98067.1
MPANSWTDQDSYTANSDGLDSLLSDLSAQGKSKNGIKDICSRFQGRRPKAILVLTMVWGG
TIALHLVSWGFAFILGLTTILGVHALRIIFVRPRHHHKQIQGDLPSVSVLVSAKNEQAVI
ARLVHNLCSLEYPHGEYEVWLIDDHSTDKTSEILAQLQQDYKQLNVFRRDANATGGKSGA
LNQVLPMTKGEIIAVFDADAQISPDLLLQVIPTFQREKVGAVQVRKAIANAKENFWTKGQ
MAEMALDTWFQQQRTAIGGLGELRGNGQFVRREALNGCGGWNEETITDDLDLTIRLNLTG
WDIECMFYPPVLEEGVTNVVALWHQRNRWAEGGYQRYLDYWDLILKGRMRAGKTVDLLIF
MLIMYIIPTAAVPDLLMSLIRHRPPILAPITSLSVTMSFIGMFSGLKRTRQDQKNSNYFM
LLLQTIRGSIYMLHWLVVMSSTTARVSLRPKRLKWVKTVHTGSQH
>KRH98069.1
MINDAGVRVFISSLNTDINWATISTWLVIAVILSMVGGALGGMMIAGKDLGFKFAAIIGS
LFAPAGVIPTLILGLLLLNFLGNY
>KRH98074.1
MSKLRVGLLFGGRSGEHEVSIVSAGAIAKALTTGENSQKYEVYPFYIQKDGVWQGEEIAR
KVLESGSAQENTLTTVNLWQFPQKSPQIDLWFPILHGPNGEDGTIQGLLTLMQVPFVGSG
VLGSAMGMDKIAMKTAFAQAGLPQVQYQTVTRAQIWSNPCVFPKLCDQIETTLGYPCFVK
PANLGSSVGIAKVRSRQELETALDNAATYDRRIIVEAGVVAREVECAVLGNDHPQASVVG
EISFNSDFYDYETKYTQGKADLLIPAPLSQTVVEQIQEMSLKAFAAVDAAGLARVDFFYV
ESTGEILINEINTLPGFTSTSMYPQLWANTGISFPQLVDQLIQLAIERQPTSDRLTN
>KRH98075.1
MIKQTLGLGTDLYNYLLRNSLREVEILSELRQETAKLPMSIMQISPEQGQFMALLIKILG
AKKTLDIGVFTGYSSLVVALSLPDDGKIIACDISEEYTSMARIYWQRAGVADKIDLQLAP
ALETLDKLLVAGEAGTFDFAFIDADKANYENYYERSLELIRPGGLIAVDNVLWSGRVADP
EIQDNQTSKIRAFNQKVHQDSRITLSLVPIADGLTLARKN
>KRH98076.1
MSRPIILGIVGDSAAGKTTLTKGIAQVLGPENVTLICTDDYHRYDRQQRAETGITALHPD
CNHLDIMQQHLALLRTGQPILKPVYSHKTGTFEPPQYIKPNKFVIIEGLLGYSTRAARDA
YDVKVYLAPPESLRADWKIKRDTQKRGYTKEQVLAELEKREPDSEQFIRPQRQWSDIVVT
FYPPNDNETENGGHLNVRLILRPTIPHPDFTQILHPDNDDSESPVRLGLDRDMGKPVDVL
EVDGHATLEQVNKIEHVMCNDMPYLRNVCDREINPELGKIAGTTGETLQSYPLAITQLIV
TYHMLKATQGHS
>KRH98079.1
MKYVDEFRNPEKAQGLQKEIAQLSLQISRNSHKNKHLKIMEVCGGHTHAIFKYGIEEILP
DNIELIHGPGCPVCVMPKGRVDDAIALCQNQQIIFTTFGDAMRVPGSKTSLLQAKAEGAD
IRMVYSPLDSLKIAKENPEKEIVFFGLGFETTAPSTAFTILQAAAENIRNFSLFSNHVLV
IPALEALLANPDLQLDGFVGPGHVSMVIGTDPYEFIPERYHKPIVISGFEPLDIFQSIWM
LLKQIEENQCEVENQYNRLVEKGGNQNAIQAMNQVFTVREKFEWRGLGEIPNSGLKIRPE
YAQFDAEAKFTIPNLKVPDHKACQCGEILKGVLKPWECKVFGTACTPETPIGTCMVSSEG

ACAAYYKYGRLSALI
>KRH98085.1
MNSINNLIDTDSMCSSNLLNVPQLDKVWLKHRSNADQVSQYYIGTEFQDYAFRINECSQL
LNFHIVDLGTDQISTQFKLNTARFCRVRYCPVCQWRRSLMWKAKAYSVLPRIVADYPTSR
WLFITLTVRNCLITELRVTLDWMHQSFKRLTKLKDFPAFGWLKSTEVTRSKDNSAHPHFH
CLLMVKSSYFGKNYLKQSDWLELWRHSLRTNYSPVLHVQALKKDKCPSEIIPELLKYCTK
ESDLIIDKDWFLELTRQMQKARTISTGGVVKEYLKHLMIERENLISDDNDTSRFEDKEHL
YFKWNRISKKYNLFSE
>KRH98086.1
MLTIALLIAFIIKVKIWFIYIDFTLFVNPRFYFTVIKIKEEIKQIDFHAYSGRFNFTYSL
DK
>KRH98092.1
MKIISFLGFNNYIETNYIHPTTSQAVRTKFFQEALVEFYQPDTLYVLLTPTVATKIPRNG
TISNWQGLQEQLANKSVKLEPVFDIPESNSLDDSWLIFDKITNCLNEGDRVIFDLTHSFR
SIPVIALLAISYLRTVKQVQIEGVLYGAFDPNAQGADTPTYDLLPMLSLLDWLAAADRFV
KVGDGSPLAELLENEISENETRYDQTVRPLARKFKHTADIINRISLAIALVRPVEILEET
TKLEEIIKKAESSFDKRAKPFGLISQKLTQEYGQFALENPTNSGNLAQGLRLQFQLIDWY
IKRHQVVQAMTLAREWLVSVLAYRLGEQDPLNKDQRKQLENALNNGQSKLCGKEITLISP
WDDEFEKLPDFDLFSTIWNQLRDIRNDIAHVGMRKGAEKAKTLKEVSAKIVLKLKTIADT
LS
>KRH98093.1
MENPLFVFSPCGTSLLTNQAAQEERGLVSKHANAKHIEDIPLEDSLKLRSLAKRVEEKLA
FADLELAGKMSAELNGIIKLYNGKLEKKADTHYLLCTDTWLGEQTATLVEQWLREKGFIV
DKNRQTDLQTKDIDSFQIALSDIVEFFEKTIPGYRKSQYKIIFNLTGGFKSVQGFLQTLA
TFYADETIYIFETAKDLLRIPRLPVKMVVEDSVRDHLLVFRRLANEMKIADVIGVPETLL
MRVEGKVFLSPWGDLVWGRTKQEIYGEKLHPSPSEKVSYGPKFEDSLRGISADRLILINE
RIDQLAKNLESGGKYNVSSLDLKALKGNPRPPSTHEIDAWSDQDAKRIFGHFESGNHFVL
DKLDSGLH
>KRH98094.1
MFFQYFGINYEHELANESRNITTELVNQRGLTWNTKINTGDRDYLRLKLGMQTKFVNNLS
VSLGYETLLGKQNVSDNYINGQIRWQF
>KRH98095.1
MNGKNPHELLEAFLEFWRQHGEPLLKSAPYHEIAPHLVLMAFLHRVVNGGGTLEREYAIG
SGRMDICLRYGKVVMGIELKVRREKLDPLTKGLTQLDKYLDGLGLD
>KRH98096.1
MKAVVKYTIFEKPQPIRAIKRLERDVLRMGALVEQSFRLSHQALFNRDLTAAEQIRRLDK
KIDRFYRQIEVDCATIMSNQAPTDQESRCLSSFMQLVRDLERIGDYAKDLAEIAMKIFPY
PPHPTLGEVAIMSDHAQSMLATSLVALADLDEISGRRIKLLDDTVDDAYKRLYRNLAQQK
DVPGVVEPILLLTLAIQCLERMADHATNIGQRVAYIVTGQR
>KRH98097.1
MFLLGFLLGLAIGFGFWLWQQFQLNSHLEQLTQPSNPHAEKILLPLLAGLHRKICTVRDE
QQNLRLSLKAYEQLLNAAPLGYLQVDEENQLLWCNQCAREMLHLQRWQPGQVRLLLELVR
SYELDQLIEQTRDWQKPQMQEWIFHPSRDHGQGTLELKPLSLAANSFPLPGGQVGVFLES
HQQFVDINQQRDRSFSDLAHELRTPLTSIRLVAETLQTRLDPPLNRWVIRLMQEVDRLIN
LVQNWLDLTQMEITSSIQLNLEMLEVRSLIFSVWENLEPLAANQHLSISYSGPEKVYICA
DKSRIYQVFLNLLDNCIKYSNLNGTIFIEMNTVCGEKSINGVAPETGTISNQILEINIID
SGVGFAPVDLPHVFQRFYRGDKARHRESRSENETVEITGSGLGLSIVRQIIIAHGGKIRA
MNHPDTGGAWIQIHLPQVVQHDSGYF
>KRH98099.1
MEVIPAIDLLEGRCVRLYKGDYAQSQVYSHNPVETAKMWADQGATRLHLVDLDGAKAGRV
VNLSAIEAITNAVSIPIEVGGGIRDRSSVIQLFSLGVQWAILGTVAVEQPDLVQGLCEQF
PHQIMVGIDARNGLVAIRGWLETSQILAPQLATQMQELGAAAIIYTDINRDGTLQGPNLE

ALRGLTSAISIPVIASGGVGSVTDLLTLLSLEHQGVTGVIVGKALYTGDISLPEALRAIG
PGRIQDIPPTLDFSSFA
>KRH98108.1
MLCGRVYPHEKACFYFFAWIIRDAPQQRLAPLIARMRKMEDIDRIVAESDTIVELINEYR
TCVKSFSWLTVREVVIDRLEGSRRSIKGHHLEASVRTALITAFQNYFSIHGNYGQYKKIK
IADKQIKIGNHTIDVSAELTPTGNGLNETLLLPIKTRETEGGGHSHIFTRDIIAAVRELK
EDGNRYHMIAVIVALNWSVTELDNIDDQIDKVFHFNMNPNKFVGFDEASQIQLNRYIQGV
LDNG
>KRH98109.1
MDNLSRIINKVLCGDIRDVASSIPDNYIQAIVTSLPYFGHRNYSGNEASVREIGREGNLL
NYVKNVIDCFEALKPKLRNDGLLWLNIGDTYRNKELQGVPWRVAFALKDTGWILRSDIIW
KKPNAMPSSVKNRPTTDHEYVFLFSKSADYYYDADSIREPHVTFSENSKMRGGRNHLGKR
NGTPENGKNAGNQNLHDGRWDQAFHPMGRNKRTVWEIPLGKFRDAHFAVFPEALVETCVL
ASTKKGDVVFDPFTGSGTTGVVALRNDRKFIGCDLVKEYQEMAQKRIDEIVAQPSLFQ
>KRH98110.1
MEIIRTQQTIARTLENIGNLYKDEFNEAILNFEDKFINDSHQALSMRRITDESTIEMQRL
LTRKGFSLIRQGNNHEIWKHPNLKKVTSLPRHKKLSTFVIASIQKDIEDAIQ
>KRH98117.1
MITTENLPSIEKQIWTWRNYKIQYTVMGVGQPLVLVHGFGASIGHWRKNIPILAKAGYQV
FALDLLGFGGSEKADIKYSMEVWVELLRDFYHEHIQSPTIFIGNSIGALLSLMVVTQHPE
IASGAVLINSAGGLNHRPRELNPITRFFMATFRQLVTNPITGTIVFNRVRTKSQIRRTLY
QVYCDRNAVTDELVDILYEPSCDRGARKVFASIVTAPPGPAPITLLPKLTHPLLVLWGEK
DPWIPITGTNIYAEAASGKDIEIVPIPNAGHCPHDEVPDLVNRVIIDWLGKKNL
>KRH98123.1
MAITIDSARGIFPQTLSADAVPATIARFKQLSAEDQLAWIWFAYLEMGKTVTIAAPGAAS
MQFAEPTLNQIRQMSFPEQTQAMCDLANNADTPICRTYAAWSPNIKLGFWNQLGEWMEQG
IVAPIPAGYQLSANAAAVLQALREMDQGQQITTLRSTVVDMGFDPNKLGDYTRVSEPVVV
PTEMSKRTQVTIEGLTNSTVLSYVNNLNANDFSALIDLFAPDGALQPPFQKPIVGRDAIL
RFFNEECQNLTLVPERGVSEPVEDGYTQIKVTGKVQTPWFGSSVGMNIAWRFLLNPQGKI
FFVAIDLLASPKELLNLMR
>KRH98129.1
MKLISEPSVPTKIQRMKQRVRWRHPAILEHNIDQTVMKFDDQGREKNQEFSFMVMGDSGT
KSNYGSHPQRQVTEMMLNHLDDCRFILHTGDVVYVVGSREYYPANFIQPYREFLLGGENP
ENIAYNHMTFKLPILPVLGNHDYYDVPLLYRLITGSTLPLRQMLRYKDIEIGWHGSNQGD
TYARAFIDYLAAIGSSDLVAHLNKYYTGKVNTGRCLNYQPGEFTRLPNRYYSFNYGGIDF
FALDSNTFNTPEPLPKSRAQDIRQKLWESRRSLEREELEILAVYEKLDSKNPRESDLLAD
LAGKLDQLNEVKLDIEKQLESHTNADTDFEQLEWLRDRLIASWQDLNVRGRVLFFHHPPY
VTEATKWQQGQTLAVRHRLREVLEQVRVALGNMGREITGGRPLVDLVFNGHAHCLEFLKT
TDTGYGDSGINYIVCGGSGRRPRRQREEGTELLEDFSEHDSAHTRKVADSLLYVGRTGYD
LETRKPYSCVRVDVKAGIPPQFVITPLVTELIEGKWCNKQLDAIIV
>KRH98130.1
MNNQDLKHLKEIFVVTAAQMRDVESRVFAAGMPIPALMEKVGRLICDRLLFMKLKGSRVG
ILAGPGHNGGDALVVGRELHFRGYNVWIYQPFGQLKELTNQHFHYAQSLGIPCFSQLAEL
PDCDFLIDGLFGFGLEREITGNIAEAINHLNLWNKPIFSIDLPSGIHTDTGAVLGTAICA
SHTFCLGLWKQGLLQEQAIQYTGKTELIDFDIPLADIQAVLGEVPTIKRITQSLVINTLP
LPLSPIVHKYKSGHLLLICGSKRYAGGAILTGLGARASGIGMLSIAVPESLKPLLVSHLP
EALIIGCPETNNGAISHLQLPGETTLNSFTVIACGPGLTTEATPIVEQVIKSEVPIILDA
DALNILAQLGTITTLKNRHQPTILTPHGGEFHRLFPEIDITNRVKGVQTAASQTRAIVLL
KGARTAIAKHQGVTWINSESTPALARGGSGDVLTGLMGGILGQVVNRQVNMEDVVATAAW
WHSQAGILAAKERTELGVDAFTLTQYLNRVLAQIQTDEISKWVELNIR
>KRH98134.1
MLILELVRVQRCGKSAPAVSRGTGSVNPGWEQGQRNYGWSFTSFGKLEPLEVVGNNYPRQ

ITTLAREQNPAYVLLSLF
>KRH98136.1
MNIDSSLKALGIANPSGTGWLAVVFTFILAWLITWGSIPTVRQFALRVGWADQPNARRLN
REPLPNAGGLAIYAGVIAAVVFASLFRPIELQGVLAQVLTILLGGSILVLVGFIDDQFGL
PPFIRLWTQIITALLLVANGMSIKVMFGTPIDSFLSTALTVLWIVGITNAVNLMDGMDGL
AGGISFITAMSLLGVSAQFDNRAAATLVLAALSGAALGFLRHNFYPSRIIMGDAGAYFLG
YVLAATSILGKLQESTIYSLIPTVLFLMLPVLDTTQVFIKRLLAGKNPLSTPGKDHLHHR
LLAWGFSQRRASLILWSLTLFFNILAMSIQAMTLPVIFASASAIVLFMSSTLIPRVHSNS
>KRH98137.1
MTSVKYQVAIVTGASRGIGRAIALQLAEKGAKIVVNYASSSTAAEKVVSEIIALGGEAIA
LQADVSQAGQVEDMVNKTLETFNRIDLLVNNAGITRDTLLLRMKLEDWQAVIDTNLTGVF
LCTKAVSKIMLKQRSGRIINISSVAGQMGNPGQANYSAAKAGVIGFTKTVAKELASRGIT
VNAVAPGFIQTDMTSEIKAEGILQYIPLGRFGKPEEIAGMVSFLATDPAAAYITGQVFNV
DGGMVI
>KRH98139.1
MTNLEKQIQLLIQNAPKDGITPKLVATIAPVLRVAAKNLRHPRYYILQNSEGSWVSTTLC
NKANPELEKQVIYAFPRLNDAIRSSHIEIKPQVVAKPIPIIDILFQLLAITPVDSIVFME
TPGTNTNAVEVRRADLENMIQQTLQQHLPPPTNIA
>KRH98141.1
MTALTTPQVVAPKEISNYQVKPGDTLEEIAGSYGTSVTELIQANGLSDPHDLRVNQKLII
PQVKSGTGIQELPTPTSPVVIPPKRIAQVAISPLVPPDREVNLPNAAELPLPTSASNEGV
AIAVPQIPSPSSGSAISVPVAGKNLPQVPEAGSITPNTRGIGGSTDGFTTSVAITPKTVP
TQLEKVTPNNDRIRSLQAEIERLRQKYRDQRSAITLANSGKVENTPSPLGGGNIDPVKVS
PGVQIKAVTITVPRLGENGNVSVNSQLGVDAIAPNDEPVNPQFVPGNTRRNPSGIRLNVP
PRRMNARDSLGKLRGTTVSPALPPLAAVDIYLPRNTEEENNSPALTGHIWPAKGVLTSGY
GWRWGRMHRGIDIANGVGTPIYASAPGRVERAGWNNGGYGLLVEIRHEDGSMTRYGHNSR
ILVRVGQEVQQGETIAAMGSTGFSTGPHTHFEIHPTGKGAVNPIAFLPSQARL
>KRH98146.1
MDGAKTGKSFTQEYYHQPGTIPGTIIIHEDAQQPQIVLMDYNSTDLVERRIINPEECSDY
LRTQSVSWVDVQGLGNRDVIHRLGQTFDLHPLILEDVVNMAERPKIEDYEEQLVIIARMV
VPNTNNNRSFYSEQVSLVLGTHYVLTIQEESEHDCFDSVRARINKNKGIIRGEKSDYLAYS
LLDAIIDGFFPVLELYGERIAELEEEVITNPTSETLKQIYQVKRELLQLRRGIWPQRDAI
SSLIRDGSHLISQEVSIYLRDCYDHAVQVLDMVENYRELVSGLMDVYMSAVSNKMNEIMK
LLTVISSIFIPLTFVVGVYGMNFSTEKSPYNMPELNWYWGYPFCLGLMAVIASSLMLFFW
RRGWLTNSFEINQK
>KRH98147.1
IFAVKPLTFSCAGTVNSSFVKMSDFIKDYKPAGIQEIEISVTEPMDYRKLFTAIPLVAKL
PMYINHIATISLEEQFLRLEYQGPEKGFKVFQGVLNSFLNNPQVKADLLLKLEFKFLSPI
MVEGGEIRDLKKALERNPVDNLNLVAKVTY
>KRH98152.1
MSNNFVWKSMVVSPAVLGVALLASGTAIASTTEAPQSSSELAQFTPVSQFSDVQPTDWAF
QALQSLVERYGCVSGYPNGTFRGKRALSRYEFAAALNSCLGRVNELIATATADMVTKQDL
VTIQRLQEEFSAELATLRSRVDTVEERTAQLEANQFSTTTKLKGEAIFVLTDSSGNDSVP
GETVLQNRVRLNLQSSFTGKDVLDTRLTAGNAQGFGNTGETKQTFEIGKTNPGNNVELDQ
LTYQTPLGRARIYLAATGGQHSDYVKVNNPYFSDKNDDGSLSTFTAENPIYRIGGGSGVA
VNIPLGEGGSGVKSSSVSIGYLASGNRTSGAENPASGGGLFDGNYAALGQLNFNLSNRLA
LAATYVHGYHGVGSTLFDSGRDNGAVVGTPQANSLGRSALTGSQISASSSNSYGLSAAFH
PTNKLAITGFISYHDITGFGVNDDYEAWSYGMGVALPDMGKKGNVLGIFGGAQPYSFSND
GGVGNRNIPYQIEGFYRYQVSDHISVTPGLVWLPAIGQNDNQPDTFIGTLRTKFSF
>KRH98155.1
MHDSNHYQTLQINNKATAAEIKKAYRRLVKMFHPDSNQDTADHEEIIKINAAYEVLSDVE
QRVKYDEQIGNTSHPRTGNRENPRQTKKPGGKEADEKVEEWLKLVYQPINRWLCSIVSSL

EQQLEDLAADPFDEQLIDEFQAYLDTCRDKLKKAQTAFRSLPNPPSLATSAAHLYYALNQ
VSDGLEELAYFPLSYDDRYLHTGKEMFRIAKHLLDFSPGSNTREK
>KRH98160.1
MLQISNLNVYYGESYILRNVDLKIASGEMVCIIGRNGVGKTTLLKTIMGLLNPRDGQIHF
AGNMINNQSPDQRAKMGIGYVPQGREIIPRLTVKENLLLGLEARRKRPKKMEISDEIFSL
FPVLATMLHRQGGDLSGGQQQQLAIARALMGDPQLLLLDEPTEGIQPSIVLEIESAVRRI
VETRGVSVLLVEQHLHFVRQADYYYAMQKGGIVASGPTNELSQDVIQSFLAV
>KRH98166.1
MTKTHSIPRLAITQGDPAGIGSEVILKALANPQLHKNCHLVVVGNRDLLTRTYENLTSLT
EKTDPLLPNPRQLDIINLDLPSLTDIIPGVGNQASGAASFAYMERAIAQTLAGEFDGIVT
APIAKSAWKAAGHHYPGQTELLAQRSGIDRFGMLFVGRSPFTGWTLRTLLATTHIPINQV
CQTLTPELLTGKLDLLEECLATDFGIKTGKIVVAGLNPHSGEMGQLGTEEIDWLIPWLQR
EREKRPHLQLEGPIPPDTMWVKPGQAWYGHSLVTNPADGYLALYHDQGLIPVKLMAFDRA
VNTTIGLPFVRTSPDHGTAFDIVGKGIADGSSMQAAIELAIELVNQRLGSLRQIE
>KRH98168.1
MLQKIPLSWLQLTREKTRLAVALSGIAFADILMFMQLGFRDALYYSNVRMHSSLKGDIVI
INNQSNAVLAMKPFSSRRLYKALDAPSVSSVHPIYLDYTSWKNPVTGRSRSILTFGFNPE
YNVFDLPGVSNNIDKLKLPDVVLFDRSSRVEYGPIASDFDQGKTVTAEIKRRRVQVVGLF
TLGASFGADGNLITSDVNFLRIFRNRQLGLIDIGLVRIKPGADVNQVATYLRGYLPPDVN
VLTKQEFIDFERNYWATSTAIGFIFTLGTIMGFIVGTVIVYQILYTEVTDHLSEYATLKA
IGYTQNYLLSVILQEAFMLAILGYVPGFFSVLFLYQVAKNATLLPVMMSYGRAIMVLILT
IIMCFISGAIAIRKLRSADPADIF
>KRH98169.1
MNYKKPVISVKNLNHYYGKGILKRQILFDINLDVYSGEIVIMTGPSGSGKTTLLSLIGGL
RSVQEGSLKFLGKELLAARQDQLVQIRRNIGYIFQAHNLLGFLTARQNVQMAVELNDNIS
PLQAMKKSTTMLEAVGLGSRINYYPDNLSGGQKQRIAIARALVNHPPLVLADEPTAALDK
QSGRDVVEIMQRLAKEQGTAILLVTHDNRILDIADRILEMEDGLLTRDSSTIT
>KRH98170.1
MSNSWLSQVSLASLLAGEREHWQVLAQEVRTWEAGAKLFGITPENVNQVVEERLSLLRCV
FPDFKQLCENNLQIPTQTMLQALWDLWLPLGVKIASSRKSLGRPMIQGILGPQGTGKTTM
CQILNLVLRHLGYSSLSLSLDDLYKTHSDRVKLREQDPRLIWRGPPGTHDIHLGLSLLDQ
ILQNKFPVTVPRFDKSALCGIGDRTTPEIIDQVDIVLFEGWFVGVLPIDPETFTNAPAPI
ITPEDQAFARSRNQQLGAYVPLWEKIDSLIIVKPTDYRFSLKWRKEAEHKMIAAGFSGMN
DAQIEEFVKYFWRSLHPELFMELLIKSPPKSLPVDLVIEVKEDHSLSYGGGVAGQ
>KRH98171.1
MQNTRLNNLLDTIATNVAQWFMNPWRRLCLLLISWLFGFFLGSVVSTTAGQQAQLDIWAA
AVLVLLTEVASRLFYSRGFFSQRAIWVESLNWLKIGLIYSLFLEAFKLGS
>KRH98183.1
MLSVRDAESTILNAVKPLDNQRDIEYIDLLTANGRILASPVVSSRGFPHWDHALMDGYAV
RYHDVQHARADKPVILQVVAEIRTGDEPSVTLESGQAVRIFTGAMIPKGADTVIMQEKTH
RQENRVLVFIAPQSHEFVIHQGEFYQAGNDLLPSGIVLSATEIALLAAAGREKVSVFRNI
RVAIFSTGNELVTLEELPKPGQIFDSNQYALATLMRQLGAEVLLLGIVPDDPIALEQTID
YAITSADIVISTGGVSVGDYDYIYRTLASLGATIHFNRVKMRPGKPLTFATFPNEGLNYA
GIDKLQKLYFGLPGNPGSVLVTCWRFVQPAVRKMSGMARGWEGKLLKVKSSSELQSNGKM
ETYIQGELKLRNGECEFRQAEGKHSSANLVNLAQTNALAILPVGKTIVHPGEEVWTLMVK
EF
>KRH98190.1
MSGAFNYLTVNLPSTPYLVPYYAVDSLLQPFKHFGVNLGLTRIVKLLETLNSPHRQVPII
HVAGTNGKGSVCAYLSSILTESGYKTGRYTSPHLIDWNERICINEKPIASEELTKLIERV
KAVINSNEEQPTQFEIITAASWLYFAQEKVDIAVVEVGLGGRLDATNVCDQPLVTVITSI
SRDHWQQLGSSISDIAREKAGIIKAGCPVVIGKLPEDAKKAVICRGVELESPIMVVSPAR
EISPGWAEYQTVEPGGHIKAIKYPLPLKGEIQLTNSALALASLGMLQKQGWEISGQAIIK

GMEKTKWPGRMQWFNWKNHQLLIDGAHNPESAEVLRNYVDSIGDRNKNITWVMGMLTTKD
HKDIFRELLKTKDRLYLVPVPESDYANLDYLKALALETCPDLDFCAIYEDIFLALNAAFG
LLKTKDNTNNNRNNSTNINGPVVLCGSLYLIGHFFSQVSPAK
>KRH98192.1
MVKNLGFSDFLSYWGNFLGKSTRGVGVELTGDRLNIAQLHRVRQGLRIESLSTFPIPEGI
MVNGEICDCPRMGEIISQAIVESKIKTTQIRTCIPEKHAIVRIIPAPLELEGQELYDLFI
NQEAGLYLPYPREEADIDYQKLGYFIDQDGIEKAQVLLVATRREITDSYIHTFALAGLKI
EVLEIKSFATIRTIQKHLLILRPEEAVVLVNIEFDHTEVAMIINGVPQFSRTIAIGTFQL
ETAISREISLPAREMMLMTIPYRAEPELDMLEQLAAEIGRTIDFCINQIESMEVSQIFLT
GAGGGMEQLDEFLTDRLGFTTSRINPCEDLLVDRDKFPSSQDPSLAVVLGLGMRQI
>KRH98198.1
MNISLDRLIRGAKEGKIISFPTDTVPGMATIPTKAELIYIAKQRSLDKPLILMGAEPESL
WEYVKGSDQEYEIWQEVVNRYWPGALTLVLPASDKVPRVMNPQDPTTIGIRVPNHPIARA
ILAQTGPMATTSVNLSGKPPLETQAEIAGEFPDVLTEELIEYKGLGVPSTVAKWTENHWQ
ILRQGSTIIG
>KRH98201.1
MTVELPSFTAELLAAKKAKGLSFADLERLIGRDEVWIASLFYGQNSTSLEEATKIADILG
LGQDIVAALTSFPSKGLGPVVPTDPLIYRFYEIIQVYGYPIKEIIHEKFGDGIMSAIDFT
LDIEKVEDEKGDRVKVTMNGKFLPYKKW
>KRH98202.1
MSATNSRRIIIGDVHGHYQGLMILMEKIAPNSGDQVYFLGDLIDRGPQSAQVVKFVKENN
YPCLLGNHEEMLLNVMIHHYTSNKAVQSWLYSGGQATMASYRSAIIPQEDLDWFSSLPTY
LDLGDILLAHAGVNPNKSLSEQTGGDLCWIREEFHSMETPYFTNKLIITGHTITFTLPGM
RPGELAQGQGWLDIDTGVYHPRSGWLTGLDITNKLVYQVNVFKNTTRCLPLEKAVSRINP
QEIKLSGRYKQVG
>KRH98207.1
MTEPLQLKSHKFADLCFISIWSFTTLASFLLSLLVVEVGEKSDLEVVDAAIGGLAIAIPQ
SYLLRKIIPPLNWIISTVLGWVLITVIGIGTMGWFVLSTHTLYNRVLISMIDSGIGGLVI
GLSQSCLAIPPSVPQKWSWVFVNAINCILGFTIGSLIGILTGKSSFLSEVFGLSIGWLIV
GILTSISANRFLKIDEAKY
>KRH98208.1
MLSGLAGLTDPKGSDWGERMLNTVASQTIRHLFTQSESVEVLVRCYPSSKLLQGSIDSFK
MSGQGLVIRRDFAIEEMCFETDAVSIDFSSVLSGKLKLKQPTQAVAQVVLLESGINHAFK
AELVKKRLINLSEPALMEISNGEPVSFPEIKIQLLPENRLHLIAKADINNGELVPLSMTI
SISIEKRRRVSFKDARFQLDEVPYQQREISQRLGAALVTILDGMVDLDRFDLDGVKMRLN
RLETQGEKLIFSGYAEIDHIPQSGY
>KRH98219.1
MTQKVIIFDFDGTIADTLDALVTIANRLAREFGYMQISAKELKLLRNLTARQIIKYSGVS
LFKIPFLVKRVKGELKNKIKDLQPIPEIPEALRELSNQGYKLGIITSNAQENVHEFLKCH
QLDNLFEFVHSGVTIFGKNTIMSSVIKQRQIKPQTVIYVGDETRDIEAAKKANVQVIAVT
WGFNSPEALARENPDFLIDHPRELLEAINHSFAEPSPN
>KRH98226.1
MSLELTPDVKFGLEFFHPAIMWILLALSLYAAYLGLQVQRTRNAQGEEKKQLIKGKYSDK
HHKIGSVLLALMVGGSIGGMAVTYINNGKLFVGPHLLAGLGMTGLIAFSAALAPFMQKGA
NWARATHILLNFGILGLFIWQAVSGVEIVLKIIGQA
>KRH98227.1
MKAFVAGATGQTGQRIVEELVSRNIPVRALVRDEQKARNLLPSQVELIVGDILQPETLVA
ALGDSTVVLCATGARPSFDPTGPYQVDFQGTKNLVKAAQDRKIQHFVLVSSLCVSQLFHP
LNLFWLILVWKKQAEEFIRKSGITYTIVRPGGLKNDDNSDEVIMQGHDTLFEGSISRKKV
ARVCVESLFEKARWNQIVEIIAKPLSSS
>KRH98232.1
MAIETHLTLLKAGAVTWLDWRARNPELQVDLSTSNLRGENFRGANFQNVNLNQVDFSHAL

LVRADFQDANLSAANLNSAKLVQANLRKANLSVANLQNANLMRANLDGAVLIGADLKNSN
LQDAVVTSANLIGTDFYYANLNGVDFAYSKLIRSNLSFANLIGANLIGSNLQDCNLYEAE
IINSYLYNTNLSRANLSRSHLGSSYLCRANFMEANLTSADLTGANLKDANLAGANLQGAN
LRCANLTGANLTGANLQNAILPPVFICN
>KRH98236.1
MSNHIDKSNNDRNHSKPLIIGVSGASGLIYAVRALKFLLESDYSIELVASKSTYTVWQAE
QNIRMPPEAIAQEQFWRSQAGVTSMGKLRCHPWSDVGAGIASGSFRSLGMIIIPCSMSTV
AKLAVGLSSDLLERAADVQVKEGRKLVIVPRETPFSLIHLRNLTTLAETGVRVVPAIPAW
YHNPKSIDDLVDFVVARALDQLDIDCIPIKRWQGHI
>KRH98237.1
MSSQTKVQTQLQVELFIENSYDLNLTNQVSEEKWYMWFSQWLTLLAAELPRALSYEIGLQ
LTNNAQIQELNAQYRQKDQPTDVLAFAALENNFPYTEEMLASQPLYLGDIVISIDTAISQ
AEEREHSLTTELAWLSAHGLLHLLGWDHPDEKSLIEMLNKQSMLLKSVGIVSNI
>KRH98241.1
MTVSKPVNYRTQSMDTSFAAEQVQFRLWRNMSSGEKESLFKRITKRGSILALAGIKSQFP
NASQDIIREYYIRKRLGDKWADLLSGLKYERDLMIEDPIWLALELASILSSLDIIYYVGG
SVASSLQGEVRLTQDLDVIANIENSQIQPLIRSMTDQFHISYTAVEEAVNGKTLSFNVIH
LTTTEKADIFVMKEDEFSLSQMSRRVLHLPDGDRTKSFYICTPEDTILQKLLWFRMDNSE
SQKQWRDILGVLKLQKELLDFDYLGEWGKKLNLTDLLLQALRESGNG
>KRH98247.1
PLSPGKMSNIVPAGISITEKVVAESGVVNAVSGIIFRNRLYDTIFEVIVFTIAILGCNFL
LASENPSCTIYQFKDRASITLARLGATIAALVGIELAIRGHLSPGGGFAAGVAGGTAIGL
VAITSSYQWMQDIYQRYHAAIWEKISVLIFIVLSVVTLSGFELPHGELGKLFSGGILPIL
NIIVAVKVALGSWAAVLIFIRHRGLL
>KRH98250.1
MTDGERQELLKKLKLDYGRILLNYFSVDQNLKTTIDQFISTLFCANIPVPQVIEIHMELI
DEFSKQLKLEGRSDETLLDYRLTLIDVLANLCEVYRCSTSRIT
>KRH98257.1
MEQLIVEDPVIKNLILGSMSQYYIGRQDADSKFDQTLAQLQSYQEEQNRKWEEQNRHNRE
IMAQLQSYQEEQNRKWEEQNRHNREIMAQLQSYQEEQNRKWEEQNRHNLGILEEIKQMNR
KHESTVGSLGSRWGLSSEASFRNGLKGILKDSFGVEVLNFLDFDNEGEVFGRPDQVEIDV
IIKNGLVILCEIKSSIDKAGMYIFDRKVAFYEKHHQRRVDRKLVISPMVDPRALSVAQNL
GIEIYSYAEDVNRI
>KRH98258.1
MVANNWEFQSLELGVDDLDSSLSSPISHLISEDALTENYEAFPSKFYSHHREFMKMQASS
EQVMEYFNCHSAWFIRCAQPMVVHPLGENGYTLVIGRFGAFGYEVEPKIGLELLVPGKNQ
CQIRSIPIPNYQSPGYHINYNAHMQLIENTPGITRVEWQLDLTVCLQFPKFIRRLPSSLV
QSTGDRLLNQVVRQVSRRLTRKVQEDFHGKSES
>KRH98259.1
MTKTAWVFPGQGSQCLNMGIDLLSIESAKAKFDQARNILGWSVDEICQGDEAKLSQTVYT
QPCLYVVESIIADLLRERGQKPDLVAGHSLGEYIALYVAGVFDWSTGLQLVKRRGEIMDN
AAGGMMAALLNFDRQQLEEEIAKTPGVVLANDNSPAQVVISGIPTAVEAVMSQVKAKRAV
ALKVSGAFHSPLMKGASEEFQAILKDIAFETAIIPVSSNVDPIPSTNPQVLKERLIEQMT
GSVRWREISLQLPQSGIEKVIEIGPGNVLTGLIKRTVQGIELKNIQNLEQISSSPVKEKN
HTKTGHS
>KRH98260.1
MTASVRERYQSVGEVIKELNFAESNQSETKSDVNPVNYWQTLVSRAVEKGKQSVQQWFSI
NDSQVAEILATVTSQLPTTEALLLGKPQTGKSSIVRGFTGVSPEIIGQGFRPHTQNTERY
IYPNNDLPLIIFTDTVGLGDTDKDTEVIIQEIIKDLNTGTKRARVFILTVKINDFATDTL
RNIAQKLRQQYTHIPCLLAVTCLHEIYPPDMKNHPDYPPNFAEINRAFDEIKANFSGLYD
RATLVDFTLEEDGYSPVFYGLEAFRDSLTSLLPEAEAKTIYQLLDEQAGDKLGNIYRDTA
RRYILPFSIMATTLAAVPLPFTTMPVLTALQVSMVGLLGKLYGQTLTPSQAGGIVSTIAG

GFLAQAIGRELIKFIPGFGTVIAASWAGAYTWSLGEAACVYFGDLMGGKKPDLQTIQNVM
EQSFQSKQNTQKEE
>KRH98261.1
MKKFLTLALILTLFLVSSFSLGTSPSYAYSRSDLERLLETRKCPECELRNADLSDADLKG
AKLKDADLSYAYLTGANLRGADLKGADLSDADLKGADLKGADLSDADLKGAKLKGADLTN
ADLSRAEVAGSSFKNAKLDRKWQFLQLYDR
>KRH98277.1
MSILEIKLPIHETFQSTVQGEGYWTGSLVDFIRLSGCPLSCPWCDTGYADGGANLRRFER
TIGELLAELKSPRIVISGGEPFIHKHLPELVEALLDAGKQVNIETSGSFWKEVPLSAWIT
LSPKEHINPKYPVQNQFWSRANEAKIVIETGQEIDFYQEHLSAHPDLCVYLQPEWNSSSK
SLALILQLLQQKPDYKLSLQTHKYIGLQ
>KRH98279.1
ESLLSSFLQYILPKEVYTHPSSVTQYKYFFAIALFEIVFVEPIAIYLDNIADITNKALTE
ITNITAPWAISNAHIWHNLIFTLFIGLLADFANFFYHYLSHKNQTLWQFHKVHHSAEVIT
PLTVYRTHPVELLVGIIIITTATGLGTGIWSYLFGTEIKRLLVCGVSLDLFIFYLAGYNL
RHSHIWLAYPKWVSHIFISPAQHQIHHSVDPKHYDKNFGYIFGIWDWIFGSLYIPTSYEK
LNFGLANGESNLFNSVSNIFLQPFKSILQQIKQINQTTV
>KRH98281.1
MSLEVGIIMGSDSDLPTIKEAIAICEEFSVSYEVAIVSAHRTPRRMVEYAETAHIRGLKV
IIAGAGGAAHLPGMVASLTPLPVIGVPVPTRNLQGVDSLYSIVQMPAGIPVATVAIGGAK
NAGLLAVQILATYKPELLVKVQAYRQSLSDMVMDKQNRLTQLGYQQYF
>KRH98283.1
MTITEKILSQLPGNILENLRRTDKVLTSLKTDNQTFTEVVKQESTPLESIDWDVIICGGT
LGILIGSALAVRGVRVALLEKGVLQGREQEWNISRQELSVLIKLDLLTQEELATTIVTTY
NPARVGFASGGELWVEDVLNIGVDPICLLKTLKNRFLLAGGTLLENTPFNNAVVHSNGVM
VNNQFSGRLLLDAMGHLSPISKQARGGKKPDAICLVVGSCAEGFTSNDSGDLLLSFTTLQ
NQCQYFWEAFPARDGRTTYLFTYIDPASQRLSLEELIGEYFRLLPEYQKVEIDKLTFKRV
LFGFFPSYRQSPLQTPWNYILPVGDSSGNQSPLSFGGFGAMIRHLERLTMGIEEALQTNE
LWASSLKLLQPYQPSLSVTWLFQKAMSVRINQQIPPDQINQLLSVVFTEMAKLGTRVLKP
FLQDIVQFSALSQTLWRTGINHPLLIAKIIPQVGLFSLIDWIGHYINLLIFTLLFSFSSI
PALGKIINNLPKSQQYYIHRWIDGWKFGSGRDYHLNP
>KRH98284.1
MILIQSIEIDWILVNTGLQFITWGVFSLLLAEILRDIYHALCHQVTWLSKWHNKHHAAYR
RDLTIASQKAYIDSQLYHDIVESVILVVLLIFVTLLTKQWGLWLGVAYSITFLYGASLRY
FQGTIDTDYNHLPGPLATIPSVWFVNRTYHWRHHFDDVNAYYSGVFSLVDKVLGTGLSLK
GKTIAITGASGSLGKALTTQLTKQNAKVVALTTNPENIPNHSGIKVIPWQLGNEIELKAN
LEKVDILIINHGINVYGDRTSAAMNNSYEINTFSALRLIDIFSETVTGPHAKATKEIWVN
TSEAEVSPALSPLYELSKRALGDLVTLKRLNGVCVIRKLILGPFKSQLNPYGVMSSTQVA
KGIVFLAQRDFRNIIVTVNPLTYLLFPIKEFTTWLYYRIFTNSYSLRRL
>KRH98290.1
MFEELIGQQQAIELLTASVKQNRIAPAYLFAGKDGVGRSLAATCFIQLLFAGSRDGEELS
VLKNRVSQRNHPDLFWVEPTYQYQGERLTAAQAAEKKVKRKAPPVIRLEQIREISQFLSR
PPLESVRSVVVIEQAEAMAESAANALLKTLEEPGRATLILIAPSPESILPTLVSRCQKIP
FYRLDSSSLTQVLTRTGNLEILEHPEILNLAAGSPGSAILAHQQLQSINSELLQEVKKLP
TSYLHALELGRRIDRELDTEGQLWLIEYLQQYYWQKTHKPQIIQELEKTRKNLLCYAQPR
LVWECTFLAILRV
>KRH98293.1
MSELERYYTLLDLEPGATIEEINQAYKDLVFVWHPDRLPKDNHRLQKKAHDKIKALNQAR
EKLRSFQDQSQPGHNSEPPTSKKSPYPPPQTNYYRPNQHPPQNPDLSGRDFSHANLSNKD
LSGRNLSYANLSGSNLSDTFMHKVNLRGANLSGANLFRANLLLADLREANLRSANLIGAD
LSGADLRGADLTGARMRSGERLLVKLVGANLTGAIMPDGAIYG
>KRH98299.1

MTTLSSIILAGGKSSRMGKDKALISIGGVPLLEKVYHVARSCTNNIYIVTPWVERYQDLH
LPGCEFIQEDPNHTQGPLVGFARGMEKTVTEWVLLLACDLPNLQIPVFQDWVQELDNTQP
QNIARLVKNYYGWEPLCGFYRTSCLPLLVDFINQGGLSFQGWLKLYPVAALPLSIPDMLF
NCNTPDDLRMVTKEDEPHGYS
>KRH98300.1
MNFSVALPTFVITLREGVEAALVVGIVLALLNKSKQSQLNVWVYAGVVVGVIVSGLIGIL
FTGIIKFLGSVNPEYTSTVEPILEGIFSILAIIMLSWMLIWMTQQAKFLKLQVEGAVRQA
LTKNRHGGLGIFSLVLIAVVREGFETVLFVAANFQQGLLPTLGAIGGLATAAGIGVLLFK
LGVRINIGKFFQVMGILLVLIVAGLLVSGMGHFDDAIASLAITSRASENLCFYYEHFTKI
HSCILGPLVWNFSSILPDEKFPGIIFKSLFGYRDKLYLVQGIGYLLLLTTVGGLYFRSLN
SRNNPPKKNVLTTQE
>KRH98305.1
MTSLTSYSSITHLFPETLVLEVKSLVYAYTNKCTILKNISFKLKTGDRVALIGATGSGKS
TLLENLIGLKYPQGGTITINGVPVEPNTVAKIRKQIGFVFQDANDQLFMPTVLEDITFGP
LNYGVAPVVAKEKARQLLADFGLEKYAHRSHHELSGGQRRLAAIASVLALEPEILILDEP
TTGLDPAWRRHLAQVLLKLPIQVLLIASHDLNWLGKVTQRALVLTDGKIQIDRPIQPLLA
DGKTLDDLGLPVGW
>KRH98314.1
MAKINSTELLESLASEIGDNVYIDIAKWHLYLTDAKLHHLLAERCYSLLIEHSVTEERVL
GLLSSIKIQIGGGRKEVPLIDLLPLQCQVHLVDILEKYQQEF
>KRH98317.1
MNFTIKNALSPVKEGYTTLDIQVMGGIITQVGQNLNVIGTLIDGQDKLVLPGFFNAHTHS
VEKWQRGIIPPLPLELWLAHLCDFSPLDMERVYLSALGTGVETLLSGGTSVVDHLVLIPG
QELETIATVYQPSSGMRIFYII
>KRH98319.1
MRKKNNKSCKQSGVIPYRLCDGKVEVLLITSRKRQSLVIPKGGICKGMTPPDSAAKEAWE
EAGVVGQVNTQKLGAYKYRKRGNIYQVHLFWLPVEKILEDWPEASQRQRIWLDINHAAII
VKENSLKKILQNSQEQVRVFTSERLETHSF
>KRH98321.1
MLKSLPQSGWFHPQPRFNRVVVVILIVVPILSTSAYFSLARQSQVKIDKSTSQSGESKDL
SGFNSPSVKLGPTLPVTQTPNKSSSVSSRSKISNKKQNAVKLERENMLGVKEMEESTSTR
TNRSAIYDKGVNHPKSKNYPNQSIATGVIPSDARSSQIVPTEEQVSISNGVPADPINSPH
PIPWKWIVMTQEAIGGQGRSGVRQYRSTPVVSPDGRYAVYSRVQLEVEPEMHNSQVSSLL
FIEDRQTKRLNILAKTSDAVDMLSKNGKFTPETNNQGKIGILVPVSWSEKGDQFLARKFV
GTFNTADVTDNAVIWNRQQNATTTVVPPQGEGEHEKIAILLGWSKKQPNYVLFRSGELGE
ENWPLVQVSGDGKNINMNVSDDQPITFGNKGQNIWLDPEVAAR
>KRH98325.1
MSQFNDHPTNGNGNGKPASIGVLQTPSKAKAGNLSHAYQDSFEQSIVLRQSPIWSRTIMI
TLMLVACFGVGWAYYAKIEQVVPATGQLKPEGTVKDVQAPISGVVKSVHVKDGQEVKPGD
LLLTFESVATLAELGALNKVRAALIKENDIYRRLMNASGAISSELNFLGGSLPAESAFLL
KSRISLVEENQLLRSQLGNSRPETGVGIDEQKRLIVARKELESRSNAAKFEVEKIRKQLS
QTIVKRKDTQNSLSIQQGILDRVEVLAKEGGISQLQYLNQQQQVQNLKAEIAQLEEEEKR
LGFDIQRAEQEVTNTIAVTDKNVLEEIATNKKRIAEIDSQFMRIILDNEQKLADISSKIS
QTKLNVKYQELRAPVSGIIFDMQAKNPGFVANPTQKLLQIVPNDKYIAEVFITNKDIGFV
KEGMKVDVRIDSFPFSEFGDIKGKVTNIGSDALPPDQTHQFYRFPARVSLDKQAMESQGR
SIPLQSGMAITANIKVREERSVMSLFTEMFTKQVESLQEVR
>KRH98327.1
MNPVLQFGNVSGQESYHTLTAPEVLKLLNKYQLIPPLLKEVIIEQAIAQISSTPEEEQLA
YEKLNQQYQGQKEQGISQEQLQSMATRQLKLEKFKEVTWGKEIDSYFYQRKPQLDKVIYS
LITTADIGIAQEIYFRIQEGEQSFNQLAQEYSQGPEAQTGGLVGPVELQSLHPLLVRILS
TSQPQQLSLPTPIGDWIVIVRLEKLLPAQLDNGMRQRLLNERFQSWLQAQVSPQNWQIKE
SEN

>KRH98333.1
MFRQTAFGIGLTTLVLTSGLVTDAISASNVTKKISSAPSFNGSTRAVASHQNNESNKHEN
NKLEKLVFEEINQYRVAQGLSKLTLNLNITKQARIHSQNMANGTVKFSHHGFEQRVKSIH
LQYNNAAENVAFNIGYNEPAKQAIIGWLNSPGHLRNIRGKFELTGVGVAKNDKGEVYLTQ
IFINTPHRYSRKISSRSPGF
>KRH98334.1
MKLDDFQVVDGDLDYPTLSEYLRSDALAVDTETMGLLPQRDRLCLVQLCNPEGKVTAVRI
AKGQTHAPNLQQLLESTDVVKVFHFARFDLATLRHNLKIHVQPVFCTKIASKLARTYTNR
HGLKELVQELEQVELDKSSQSSDWGNPLGLSDAQLSYAANDVRYLISLQQKLSHMLQREE
RWQLAQECFSFLPTLVSLDLLQFKELFEH
>KRH98343.1
MFNGEIAGIFSMLICPTCQFENPHDHKFCQSCGTPLNLGSVNSQSSLGESPTLLQDDGEQ
NPLLSDTAPTSLLSKHLKTVEYVAGTNVGRQRQKNEDYFGITSEQQKVELPHGQYLQVRG
LYIVCDGMGGHAGGEVASELAVNTIKQYFDQHWVEGELPTLEMIRQSILVTNQAIYDINQ
EASRSGIGRMGTTLVMLIMSNNKLAVSHVGDSRIYSVTITKGLEQITVDHEVAQREISKG
VDAKIAYSRPDAYQLTQALGPRDGVYPDISFLEVCEDTLFLLVSDGLSDHELLENHWQTH
LLPLLTSNGNLESGLSNLIDLANEYNGHDNITAILILVRVFPR
>KRH98352.1
MIRRRSTPWIHYWSRPLLGAIATLGILNTGYLTYEKLTGGTPICTAGEQVKGCVDVLSSP
WGTVFNQPLALFGLLAYTSMLLLAVFPIVLISKDNDSKKSSQNKSTRQLENLSWWLLWIG
AIAMTVFSGYLMYVLAFKLQAVCWYCIASAVFALSMLTLTVLGREWEDIGQVLFIGLIVV
VVTLIGTLGIYSGIDTSKNENIDTIASSEQRINFSPKEEPNPNFGWKITTKSGESEIALA
QHLVKIGAKEYVAYWCPHCHEQKLLFGKEAYEIINDNNVTVECAADSPKGKPELCRAAKI
QGFPSWIINGKIYSGVQNLSDLARLSGYTGPQNFKYFR
>KRH98357.1
MAKSSQKSEFGDFQTPDNLAKCATHLLKDKYGVRPDLIIEPTCGKGAFIRASLTEFKHTK
IVGFDINEEHIQYAKTSSLRYPNSENVTLCVQDFFSMNWDAFLADFVGSILVIGNPPWVT
TSELTILNSKNLPSKSNFQNRQGIHAITGSSNFDISEWMLLKHVQWLTKKEGTLAVLCKY
SVARKIINQVITKFGNRFSADIYLIDAKTLFGASVDACFFVLSNSGNTNCDFKIYQDLQS
DQTIYSIGNRDGRMVRDTTKYEKWKHLSGQDLRYTWRSGIKHDCSKIMEFQCVDDGLFIN
GVGEKYFLEREYLYPLLKGSDIANSRIDSHHKFVLVTQKSVGEDTIIIRDKAPKIWQYLL
EHDQFLKTRKSSIYKNKPPYSIFGIGEYSFKNWKIAISGLYKKLNFCLVEPIGNQPVMLD
DTVNFLSFDTQREAEFIFSLITSDPSIEFLNSMIFWDEKRPITIDILRRVSLKAVAREID
VLEIYLALAQVVRSNPNGQLELAIV
>KRH98375.1
MTHIDKSCDILIVGGGIVGLATAFRIFQSRPDLRLVLLEKESTLAKHQTGNNSGVIHSGL
YLSW
>KRH98376.1
MTRCVIEICNVKPSYQSFNTPISIIRPFNTYGPRQSAQLVGWEPLYGGREGFKQGLAETA
EWFMNPTNLAGYKSDRYNI
>KRH98377.1
MPLHEPEFMGNEWELVKNCLDSTFVSSVGKYVDRFEVMLAEYTGAKYAVAVVNGTAALHI
ALLLAGVKPVL
>KRH98380.1
MWWITESKNLSVQATEAINKADIIGIAAISCWELAMLVAKNRIGLSMDVQIWINLALQHP
KIQLLALTPEIAVLSTRLPGNFHGDPADRLIVASSLVHQKFP
>KRH98381.1
MVTLIEKLTKFLQFTMHNNALKISICIPTYNAEKFIRTTISSCLEQTQAPYEILLSDDGS
SDRCWQSS
>KRH98382.1
MATTADEVWKLLGELIESQKETERKFQETDRKFQETDRLLREQSQETERFLREQSQETDR
KFQETDRLLREQSQETERLLREQSQETDRKFQETDRLLREESKRVNNQIGQLGNRLGEFV

ESQVRPAAVKLFQERGIAVKEIASNTYIQTGKEGLEIDLLVINSSDIILIEAKSKLSEDD
VNEHLERLSKFKRFFPRYESYRVLGAVAGMVIPLDVSRYAYRKGLFVIGQSGDNLVILND
DKFRPRGW
>KRH98384.1
MSFNNYKSIADVLNEFPLIYQEETFIKENVSEISPYFIERLQLILKEGVVFNSEYAICEN
IISPILVEIWLKYKDKLLLWSHQALNYDEKLSGTPDYIPDYIIAQRSPRGKVILYQPYLI
LVEAKKDNFDEGWGQCLAELIAAQKLNNNQQHKIFGVVSNGKLWEFGQLQNDIFTKNIKY
YVLENLLELMGVIDFIFSESVNQVLSN
>KRH98385.1
MRIVDYEADFYAWANQQAELLRQQQGNHLDWMNLAEEIEAMGRSEKRQLASRLEVLIMHL
LKWQYQPNFRSRSWQLTIQEQRLRLGKLLQENPSLKPMVAEIILSAYPLAVISAERETGL
SNYPEDCPYSPEQLLSDLFLP
>KRH98386.1
MYQSDVYSKPSYEPLPPQETLPTMYELPSEYPEEPGLPDEFHLLQPELLRSTFCPHSYPK
DNVFIGSELNLYYDSKHTQWYKRP
>KRH98387.1
MAYSDFTLERITKIFGINIEERTNVFTAFDQLRVDDFFIKYLQNNIPLAQAIGTEKAKSE
MIIAPVLIEVRRLLNNKISLFSGIDFNVNIEQGLNGFCDFLIGLSSQQLYVTAPVIALVE
AKNDNLKQGFAQCIAEMIAAAQLNQSEGNNVENIYGCVTNGNQWVFLQLTGNLVVVDLDE
YYINQPEKIISVFVSLIKSEKDF
>KRH98388.1
MPYSQFTIPKVVEDFGLTLIESGAFLNATQTVTLSPYLEEFITKNLQLAIALNTEKARSE
LIICPVLLAIKETLPSISFFSGEEFNVDADLGLNGVCDYILSQSAEQLYVTAPVTMVVEA
KKENLKGGLGQCIAEMVAAWKFNTERNSTISCIYGVVTTGTVWRFLKLQEQTVTIDLNEY
PLPPINSILAKLTQMMFPQSMDTVR
>KRH98389.1
MYQSEAPPIKTISTDLPSQDIDKDIDEELDSTIHPRPPWETLPTMYDLPSENPEEPGLPD
EFHNFQPQLLRETCQSSVYPREEMFIGTDLNLYYDVHHFSWYKRPDWFLVLGAPASETQQ
DMRLSYVIWQEGFAPFLIVELLSPGTEGEDLGKTLRSANKPPTKWQTYEQYLRSPYYIIF
DRYENQLRVFQLLGIKYQAVELTESKFWFPELKLGVGVWSGKYQGIEGLWLRWYNEDGDW
IATLAETAEQEKLRAEQEK
>KRH98400.1
MTKTPKHPLFNHYSQLVFWLRNIIILLVVVGVYVWALQGLKVDFELVKNSWPYVIDFIVR
LFPPDWKVLDIAVRALIETVQMSLWGTSIGALLSLPIAVASANNIAPLWLQWLANLLQNA
VRSVPSIILGLIFVAATGLGSPAGTLALAIYTIGYLGKFYQQAIEAVDSRSLESLRVIGA
SKLQMVQYGILPQVLPLGLGYTLWMFEYNIRAASVLGVVGAGGIGFQLKSYIDGFEYNKA
TTMMLVLLVVVTVIDIFSSKLRRYLDSI
>KRH98405.1
MEEKLMTIKQVCTALNVSIQTLRLWDESGKLSAIRTVGNHRRYKQSDIYKLIGIDKPSEE
VEESVALYSRVSSGEQKTKGDLDRQNTRLTEYAAKKKYKVNYIFTEVGSGMNDSRAELHQ
LMKLAIERRITKVVIEHKDRLIRFNFNILKMFFESHNVEVEYVEEVLPKSYEAELIEDML
SLMASFSAKTYGERSAERRRNK
>KRH98406.1
MTVATAFDLESLNEQFETATPTEILAWSVENVPTGLVQTSAFNVDDLIITHILYEVLDNP
TPVIFLDTLHHFEQTLDLVAKAKTVYGLDLKTYKVPNVYSREEFAAKYGEALWDTDISKF
HQITKIEPLQRGLDELNTVAWITGRRRDQAVTRANMPVFELDNQGRLKINPLATWTRKES
WEYVAEYKVIYNPLHDQGYPSIGDEPITTRVGDGEDERAGRWRGTGKTECGIHI
>KRH98426.1
MALDIETQPIQERFQHLLKVISGDRFLKKQGLGNEVPFFICPYRPEESVEMEYIQQQLTK
SLNQAGLGVLEINLYNLSIELLQKRGVWEQILDIEPSVSKEQLKELLQGVLDPEKHLVPA
IAEKMAQREFAVMFISGVGEVFPYIRSHNVLNNLQSTAKDRPTVMFFPGAYTHSLETGPS
LDLFGRLRDDKYYRAFNIFHYEP

>KRH98429.1
MNFLVDHNLRGHSVFLAGSFTASGWLDLISIRFILFEEVGLAVTSDDRVVWRYAQANQMI
LITGNRSMKGKDSLEQVMREENTPTSLPVVTIGNIDRLLSEPDYRNRCVNRLVDIVVDIE
EYQGARRIFIP
>KRH98432.1
MAEKSKKDIQSTLSPVEYSQLLLEVKERVRTAQYAALKAVNTQLVGLYWDIGKMIVERQK
DSGWGKSVVERLSADLQEKFPGIRGFSVQNLWYMRQYYLEFSGDEKLQPLVGEIAWELEK
AIIPRIKELKGQLPSSEAISNLLEGIYD
>KRH98434.1
MNTREVVVNKLQQLPDPLISRVDEFIDFLIAQKQNQVVNVQSSESLTDRWKRWFEKVDQL
PILNHEPQNEYQQRLLQKYRQQSLDL

-Protein sequences of the Saxitoxin-producing group which were not included in the Veen diagram.

>1170768.4.peg.1
MKFYQQKITFLFFFTTDFENALLFKGGWGGSNAMKQPKFPLTLS
>1170768.4.peg.2
MRVPEYFWYDPFDPKDWRGFKLMNGVYEPLSLLEGGYISEQMNLKLVLWEGNYKGLSIVW
LRWATLEGKLLPTQEEQTQWERVQKEWWHAQREWELAQREWWRVQRETVDTFILYC
>1170768.4.peg.8
MDGPNGFPKTGPSGCEKLFFDPFGRMTFQFQHKLAQNDSSPAH
>1170768.4.peg.9
MFSLKAPLFKGGWGDQVLKLSKIHPFLRGLGGLNAVK
>1170768.4.peg.10
MLLNNPHFHPQVNVESDRTSASQSLSLMRLQTSQYKFTHRV
>1170768.4.peg.17
MVYIDDDDNIDADLEEGDLDYFWLNSLSDSYQRYQEELEDAEMEIAYEEEELG
>1170768.4.peg.18
METILKNINCCDILSCSHSLGTLIEWKLLVGCKPHPGSA
>1170768.4.peg.19
MEDVQRGSHSLGTLIEWKQLMLNIENSEVAVPTRWGH
>1170768.4.peg.20
MSPASGNGKADPGDSLSGIIIGFHSINVPSEWEREINHSSPIVIGGFHSINVPSEWEQLR
>1170768.4.peg.21
MWGKKRQIGVSIQLMSPASGNSSAALRKIGLTFSFHSINVPSEWEQVGWTWT
>1170768.4.peg.22
METPGENTFKIPTGVRSHSLGTLIEWKLVPCYLAINQELLNVPTRWGH
>1170768.4.peg.23
MIQTWYGSHSLGTLIEWKPEKFFNTSWDLTAIISVPTRWGH
>1170768.4.peg.24
METLMAVPPLSCFLNSSHSLGTLIEWKRYTYYSLLRL
>1170768.4.peg.30
MVGRNNTVTDYQCIHEWVEEQTQQTPDHIAVVFGSEQLTYQQLNQRANQLAHYLQA
>1170768.4.peg.31
MIGRGDQFLDLSINQHSKLSLKLSCRSLGEGRSFFVDGVLEDFGLLRSLLSAQSG
>1170768.4.peg.32
MGGFLGRKGDGSPGVKVLWRGLSRLHDLVQGWLICQSLVVN
>1170768.4.peg.33
MMALATYSIVAWRLLWLTYLARCSPDASCEQVLETHE
>1170768.4.peg.44
MPWIYELVRSYNLYGPKILGDLRRNNKGTARLLNNQQLQLLQQTLQLPPEDGGLWNGPKV

ANWMSKLLNRKVYPQRGWEYLQKLGYVVPSSKSHSCHPSKSYTNL
>1170768.4.peg.45
MAYPNIFSKSCSFYRILKTAGGDLGEIWWRNWGEYHSFLTIMSLGF
>1170768.4.peg.47
MLVFPQVSNTGGNVTSIHSLVAQGVKADTSLDRISKSRESPGP
>1170768.4.peg.51
MGQSGLIKQCLIAFLAGGHIILEGVPGTGKTLLVKVLAQLIQGEFKRIQLTPDVLPSDIT
GTNIFDLNTRNFYLRKGPIFTEILLADEINRTPPKTQAALLEAMEELQVTLDGESLPLPG
LFWVVATQNPLEFEGTYPLPEAQLDRFLFKLVVGYPDQTAEKQMLFNRQSGFTGKRLDIS
HLNPVTTVDNILQARQAVKQVNVAEAIVDYILELVTKTRKHPDLALGASPRAAGAWLQTS
QACAWLAGRDFVTPDDVKAVAAPLLRHRLILNPEAMLDGSKIDSVITTVINQVPVPR
>1170768.4.peg.53
MSISDVVILRTPAGTRVALSSVEVAFRLYPLGRAIALLLKYDSA
>1170768.4.peg.56
MPLQLRQVISYDLRIDKNRIIYVPDQELLLCGRIRNRID
>1170768.4.peg.63
MILFPIITISNTYWQAVDKNQPLKSLQIAVDSKPYLSRYQQP
>1170768.4.peg.65
MLKDTWDFIKDIAGFIKEQKNYLLIPLIITLVSLGALIVFAQSSAIAPFIYTLF
>1170768.4.peg.70
MSDFAKIVVEDLNVSGMLSNHKLAQSIADCGFYESMSIDRN
>1170768.4.peg.84
MSTSNSGRGLLLADSSKPSPNQPEEPAPHRGSGRKESIEFLGNSFLA
>1170768.4.peg.98
MGFTQTAKGSAPIKQASTKGDAVKVGEYQSPTREPELEGVTRIQPHSLQGRQAATLYIRN
TPVLTFLGSRGETKVGDRSSKVVKYGSVNTGKSPQITSGGGIRGAKESPRSILVNDPVYR
ASLVAAKINQLVLDSADGRQVTVSWNPKGKYSANNRPSGNESAPTSVSSGNYTIRFDGKE
LVEINESTRLADGTNNLAKDALQATNHLRKILGEAAEVKEIANLPKPGNISIPKLPQQVA
IGGIKINFRGVASWYGYDWSGRKTANGERFNPEAMTAAHRSLPMGTRVRVTNTHNGRSVV
VRINDRGPYIGSRVIDLSVGAARILGMVSSGVAAVRIEVLGR
>1170768.4.peg.117
MVGSDVFQFSVSTFPLVALMTLLNAKSMVGVAALTLPFFSLILVLL
>1170768.4.peg.118
MSQENKNKLNRARVLVMEQLQTFASRGDFWTVFAVPFGQWYDPNRGEALRSQWLSGEGVL
PTEIVSQQTLGNAVGAYGKGKIFLSEQFIANGTSSELVRVLLEEYGHHVDAQVNVTDSPG
DEGAIFAAVVQGEILEGAALAQLKAEDDSNWISVDGERELVEESGSVTRTPIAPALPGRT
RYEVGNYYAFAALKSNGSVVTWGIDWSGGDSSIATWNSTGNYSYVSVASQLTSGVTQIFS
TYYAFAALKSNGSVVTWGDSGSGGNSSSVASQLTSGVTQIFSNVYAFAALKSNGSVVTWG
NSSSGGDSSSVASQLTSGVTQIFSTDRAFAALKSNGSVVTWGNSSSGGDSSSVASQLTSG
VTQIFSTGSAFAALKSNGSVVTWGDSSYGGDSSSVASQLTSGVTQIFSTYFAFAALKSNG
SVVTWESTGGNSSSVASQLTSGVTQIFSTGNAFAALKSNGSVVTWGDSRYGGDSSSVASQ
LTSGVTQIFSNVYAFAALKSNGSVVTWGWGSSGGDSSIATWNSNTGNYSYVSVASQLTSG
VVSFADPFNDDRLVPLSTITLAVNPTSVTEDGGSNLVYTFTRAEDTANTLTVNYTVGGTA
TNGSDYNNIGTSVTFAAGSSTATVTVDPTADNRVEANETVSLTLVSGTGYTVTTTSAVTG
TITNDDLISLINQTLNLPGNQTATISQSQLSVTSTISPTNIRYTITDLPDYGKIMFLGAE
IGVGDSFTQSAINNNNRISYQDGNEDNATDSITLTVTDGVSILENVIFNIVIIPTITLAVS
PNNVTEDGTSNFIYTFTRTGSTTNSATINYTIGGTATNGVDYNSVGTSVTFAAGSSTATV
IVDPTVDTTPEADETVDLTLATGTSYNIGTSGAVRGTITNDDLPSITLTVSPSNVTEDGM
SNLMYTFTHNGITTNTFTVNYTVGGTATNGSDYNNIGTSVTFAAGSSTATVTVDPTADNR
VEANETVSLTLTTGTGYITGTSGAVTGTITNDDFISLTNQTLNLLENQTATISQSQLSVT
STISPANIRYTITDLPDYGKILFLGAEIGVGDSFTQSGVNNNRVSYQHGNHENNTADSIT
LTVTDGVTILENVTFNISVTLVDDPPILSKNQILSVNQGQVVTITKEILAATDVDTPVSQ

LVFTINNIPQNGILKKGTLFLDDGDRFTQQDIDNGNISYTQTNRNNLRDSFSFTLSDSTN
NISGLVFEINSQSNGILGITSTNGVQTEGNSGTKAFTFTVTRTGNTSNSSSANWGVTGSG
TNQADASDFGGTLPAGTVNFAVSESSKTITVNVSGDTTVEPDEEFTVTLSSPTNATITTG
TAAGTITNDDLPSITLAVSPTSVTEDGTSNLVYTFTRTGYITNTLAVNYTIGGTATSGSD
YGTIGTSVTFAANSSTATVTVDPTSDTTVESDDTVSLTLASGTGYTIGTTNAVTGTITND
DLSDTQQITSTSTRLTAIPAANLTIPLFYNTSTGDNTLAGIGLRLHYDSTDLTYQQVSNL
FQTNLFGSVTDNSDTQNFDSDNSTNRYIQFQYFATNGNWPNQNLPLKMADFAFNTSANFQ
ETQLNITGIDVAPGYTLEAAPLQVYRQNWTLDVDGNGSFGALSDGIIIMRYLFGFAGDSL
TKDVIGPGATRSTSEIRSYLAEGVNSSILDIDGNGSVTALSDGIIAVRYLFGGTFSGNAL
INGAVAPNATRGLSAIESYLASIRGNQSSLSVATVARSSSVPQQFTTFNTASNTTPKQIV
DLVNNSSSVSAGNSVTISVNYSVDNGDSTLTGIGLRLHYDSSKLSYETVTNLLQTNSFGN
VTDNVDNEDLDGDTSTNRYIQFQYVDFEGNWPNQSLPVKLANFTFNTSANFQKSKINVTA
VDVASGYTMEARPLDLVVPGVSETNVLSGTGGADTIDGSNGKYTIMPGKGDDTIIVGSAG
SVIIELPNEGNDTIVSSVNYNLAALNQIENLRLTGAGDINGIGNRKDNVITGNSGQNRLT
GLEGNDTFVFNFGDSQVEKPDQITDFQIGKDKIKINTVVGVVSTLTRAGNGSGNLRSLVD
SVFLDANGAVSGNQSLGVNSAALVVSGGGTYMIVNDGTAGFNAGTDLVINITGYSGVLPG
LGSVVASNWFV
>1170768.4.peg.119
MSQENKNKLNRARVLVMEQLQTFASRGDFWTVFAVPFGQSYDPNRGEALRSQWLSGEGVL
PTEIVSQQTLGNAVGAYGKGKIFLSEQFIANGTSSELVRVLLEEYGHHVDAQVNVTDSLG
DEGAIFAAVVQGEILEGAALAQLRAEDDSNWISVDGERVLVEESGSVTRTPIAPASSGRT
RYEVGNGSAFAALKSDGSVVTWGFSDRGGDSSSVASQLTSGVTQIFSNSLAFAALKSNGS
VVTWGYSSSGGDSSSVASQLTSGVTQIFSTRFAFAALKSNGSVVTWGYSDWGGNSSSVAS
QLTSGVTQIFSTRFAFAALKSNGSVVTWGDSSCGGNSSIATWNSTGNYSYVSVASQLTSG
VTQIFSTDAAFAALKSNGSVVTWGDSRYGGDSSIATWKSNTGNYSYVSVASQLTSGVVSF
ADPFNDDRLVLSTSESSVTLAVSPSSVTEDGSSNLVYTFTRTGVISNALTVNYTLGGTAT
SGTDYGTIGTSVTFAANSSTATVTVDPTADTTVESDETVSLTLASGTGYTIDTTSAVTGT
ITNDDTQVTLAVSPSSVTEDGSSNLVYTFTRTGDTANTLTVNYTVGGTATNGSDYGTIGT
SVTFAANSSTATVTVDPTADTTVESDETVSLTLASSTSYTIGTTSAVTGTITNDDILLPV
ITLTVFPSSVTEDGPQNLFYTFIRSGDTANALTVNYTVGGTATFNSDYTQREAASFTNTS
GSITFAPGASTATFIIDPASDTVVEGNESVSISLTSGTGYTIGTISAVSGSILDNDVTPG
TVVRTPIAPALPDRTGYEVRNRVAFAALKSDGSVVTWGYSYWGGDSSSVASQLTSGVTQI
FSNDGAFAALKSNGSVVTWGIDWIGGQYCSVKEKKG
>1170768.4.peg.121
MTKLKIFVEDLDRLFVPENLPVTYTKNKNFWGILGLMVIIY
>1170768.4.peg.122
MEVSGTSKTVPSTAIILNSPKKAPGVNIVAKGFATSSNNLLMTAAPVLWRASVIDEADGG
>1170768.4.peg.123
MAVDGTVFDVPDTSTNARVFGYPGSPKGTYPGFPKVRLVFLVEAGTHLIIDAFCYPYRMG
ERRGALKLLRSINSSMLLMWDRGLHSFKMVHTVIKQQGNFLGRVPGNVKFQVVKTLADGS
YLSWIAPDGQSRKKGAKRMEVRIIEYVIEEDGTLKTYRLITNLMDVDKFPALLLAQEYHK
RWEAENTLDELKVHLLARKIPIRSKNPREVVQELYGWLLAHYCLRCLMFQSATLKNISPL
RLSFVGSLRVIRRAIPEFQRQVNTNVDINLYYSWLMAEISDLEIPLRQQRSNPRVVKKAR
SKFKSKKRSHRNNCTPRQQLSFQIIKRAS
>1170768.4.peg.124
MTEDGTSNLVYTFTRTGVISNTLTVNYTVGGTATNGSDYGTIGTSVIFAANSSTATVTVD
PTFDTTVESDETVSLTLASGTGYTIDTTSAVTGTITNDDTQVTLAVSPSSVTEDGTSNLV
YTFTRTGVISNALTVNYTVGGTATNSTDYGTIGTSVTFAANSSTATVTVDPTSDTTVESD
ETVSLTLASGTGYTIGTTSAVTGTITNDDTQVTLAVSPSSVTEDGSSNLVYTFTRTGDTA
NTLAVSYTVSGTATFNSDYTQNGATSFSTTSGTITFASGSSTVTLSIDPTADTRVENNEI
VSLTLPSGTGYTIGTTSAVTGTIANDDTSSITIADVKQNPSVFMDKIRDYDGNNLGGSSS
WKLIGDADAQGDGDLESIFVNPLIGRWATVGVVNNFVDFSNHGQGGDTRVVGIYIDPTLK

DRPENIGGPFDSQRRFQNDLRIDNLRLVAAADYNKDGFQDMYFKLVDGTAVLRAFMHKDG
NIQYANYQSKGDLATFMIENNVDSAIWINWL
>1170768.4.peg.125
MGQQGTGDKTERAIHLTIEELQLLVRPFLRELEEFYSGVKD
>1170768.4.peg.126
MTTINYDYADGKTREIAEGFELKLLQKEKINEQEISWEERQLVVHSYAIAQTEEKYLRER
IQKTVHDLEQLKIPKRGKKKLTSFPEWELSVAEILKRYRKRILVLLGFPPTIYTQLSGQS
FTPE
>1170768.4.peg.128
MMHADGNIQYANYQSKADLTAFMTANNVSSSIWGSWL
>1170768.4.peg.129
MINLNPVAGETAVLNFIKLFSEKEVGKAINNTHDLRCSNMMC
>1170768.4.peg.143
METMYTLMDARNNLSKVRFHLGSGINGNISEARLWRITLA
>1170768.4.peg.148
MSKLSIQERLNQLLPKLQDSRLLENRGIGNEIGFYVFDYAPEDELYVQEYTKVLVSQLTK
DPINLIVKEFNLYKIILEILQEKGILNKAFMVEAKEGCKSLGNKIQSIVRPEKVIAQIQN
HLQGNEQLVFLTGVGASWPLIRSHSILNGLQPYLDHIPLVLFFPGSYDGHELCLFNTFKS
DNYYRAFALIPHHGAVYEHY
>1170768.4.peg.160
MDISQNKITAMIGPSGCGKSTFLKSLNRMSELEGEVKLEGTVEFFGQNIHGKRVNLNRLR
RQISIIYAIPNLFPMSIYDNVVYGVKLIGWRPKAELDEIVEIALKRANIWEELKNKLHKS
ALELSMGQQQRLCIARSLAVKPQVLLIDEFCLGLDPIAIRKMEELIECLRSELTIVFVSH
NIQQVLRLSDFTAIFQYNQNHIGKLADFGPSKRIIANSFHHRIRDSIGSYWR
>1170768.4.peg.162
MSPQNLLAILLAVEQKFGRVRSERWGARTLDLDLLMYDNLILNQSNLQIPHPRMCERGFV
LVPLEEIAGDWQDPISGMTIRNLLRNVDHSDVHLCSGSAVKIIIQRP
>1170768.4.peg.167
MLTLSYWQSKITPWKNQIIQMVKTIMRKLTIVCLALTLCLTTIACGGGSQNTTYSNGSQS
ATPTKLNDGQYQVQQVTYNDANGEYTLFLLGNQPPTFATEKLQMARLTDEEVKEGKKSYL
KVENGQPALYITEDFKIEYVHNVTETRSNPQTGQQETVFVRQESSFWTPFAAAVAGNIAG
QAIGSMLFRPQYYVPPVYQPGGIYGHGGYGSSYNAAVSSYQSRYNQPPAAVRNRAVFRTT
GSLRSNSGLTNRTNTGGRATGSGFGGSNLRSSGRSSSLRRNSGSSFGSGSRSSGVRRSGG
FGSRRR
>1170768.4.peg.173
MPGTSLTQAQSGGILVSNKNLKDNTTLAQAGAAFSVNINKLSAAITAYNNIVIESKPPIL
KRLSQDVSFVGIGNTLKQLRKAIP
>1170768.4.peg.174
MEGENKPLRAFTNATANCGLIPAVVNANVILAAASVTPGRASTRVTRSVVLAVVPST
>1170768.4.peg.175
MVVPLPPPGVLLPIGGDGCEVGDGDGDGDGDGMMVGGVGVGNGIDGGEGCTAPLPAPGGV
LPPLVVNCKDANKKGTRLKAKMRLLGFNMMVSTNLFI
>1170768.4.peg.177
MTKITNQALNSALNQLWVFSLSSDFWEVFDAVFGTEYNRKNAEILRSQWQIGDFSQLPEI
EILDSSILGSANGAYSSSENRIYLSSNLMENGTSSRIREVLIEEIGHFVDSRINQIDTPG
DEGEYFAHLLTDQKLNKDEIDRLKAEDDLVVVTIDGKGVEVEQNNATLAIAPAIASQTEG
NSGTKPFTFTVTRTGDTSGTSSANWAVTGSGINPANAADFGGILPSGTVNFAVGETSQTI
TINVSGDTTIENDEGFTVTLSDPIGAVLGTSSSGNTINESASGGFGVTEKYYNISSPGGT
FQLNYDMYGIPDRAEIFVNNVLQNQTSGFVSGSGSLNLNSIQLKAGDQIKVTITGNVQGT
AWDYNVNYTGGLSSVNYIASGVITDDDNRFTPNVVTPKLNISTGLIFTAQSNGNITLDTN
RGSATPDEVTSVQNGTKSNFNHIIGLYEVLNSQGEIKDNQGNTLKPEDANYALHALTTAR
VKNFTVQAGGNDTPSTATQLGSGVSVFAGKSYAPFVIANGGTYFGPGDQGIENFVAAEQG

DINRFISKDQYVRNLVADEGKDGDIFNNAPRFVQEPVAYFSFGVANPDKAPHFRSYGNGV
YGFEDLPANFTQYSNNDFNDGVFALTLSI
>1170768.4.peg.178
MTLLLGNSALLIGTGTSAIAQFSDCQKAVTQAQLNQCAAINAKTADQKLNDAYNKVLAIY
KGKRQAKLLIAAEEAWIKYRDASCAFSRSRVEGGSIMPMVYLNCLERLTKERTQELEIYQ
KEGSF
>1170768.4.peg.179
MGSSFIGLINSRLFNFPWFGDGRIRVVLFLLGVVILYQWIFF
>1170768.4.peg.180
MQVTTTSTPLPPGELSPSSWPDHTQLPDSDDNFVKNFQEHPQSVILTTSIEPLLDKIHPD
KDYCIGQDSGIYWRFTEPVEKGVEAPDWFYVAGVPSRLEGKLRRSYVMWKEKVPPLIVIE
FVSGDGEEEKDNSPPPEGDEVDPKTKKVKKAGKFWVYEQAVKIPYYAIFDGFEGTLEMYH
LEKGRYKQVKANRRNHYAIPELGVELGILLDQERPPIPWLRWWDNRGNLLLTGNERAEEE
CQRRELAEAIAIQERQQKELAEALAIQERTEKERERQQKELAEALAIQERSEKEQEREKK
EKLAAYLRSIGINPDEI
>1170768.4.peg.181
MAIAWQLTQWDRLLKTLGIKTNDLNLIRVNANRAQVSS
>1170768.4.peg.182
MLKSIYSCYTMINDQNIINKHQVKTRIAELMAITVFSFVVQKLPDVHLSLA
>1170768.4.peg.183
MTKITNQALNSALNQLWVFSLSSDFWAVFDAVFGTEYNRKNAEILRSQWQIGDFSQLPEI
EILDSSILGSANGAYSSSENRIYLSSNLMENGTSSKIREVLIEEIGHFVDSRINQIDTPG
DEGEYFAGLVTDQKLNKDEIDRLKAENDSNWISVDGERLLIEQSSPGTVTRTPIAPASPG
RTRYEVGNAYAFAALKSNGSVVTWGQSWAGGNSSVATYNPVTNNYNPVTNNYSYVSVASQ
LTSGVTQIFSNLEAFAALKSDGSVVTWGQSWAGGNSSVATYNPVTNNYSYVSVASQLTSG
VSRIFSTGYAFAALKSDGSVVTWGDSSDGGNSSIATWNSTGNYSYVSVASQLTSGVTQIF
SNWGAFAALKSDGSVVTWGQSWAGGNSSIATYNPVTNNYNPVTNNYSYVNVASQLTSGVT
QIFSNWGAFAALKSDGSVVTWGDSSDGGNSSIATWNSTGNYSYVSVASQLTSGVTQIFSN
GGAFAALKSDGSVVTWGDSLGGGNSSIATWNSTGNYSYVSVASQLTSGVTQIFSNGGGAF
AALKSDGSVVTWGSSTSGGDSSIATYNPVTNNYSYVSVASQLTSGVTQIFSNGGAFAASG
VPVPHPQIGGAFAALKSDGSVVTWGSSTSGGDSSIATYNPVTNNYSYVSVASQLTSGVTQ
IFSNGGGAFAALKSDGSVVTWGSSTSGGDSSVATYNPVTNNYSYVSVANQLTSGVVSFAD
PFNDDRLVLSTSESSVTLAVSPSSVTEDGSSNLVYTFTRTGVISNALTVNYTVGGTATNG
TDYGNIGTSVTFAANSATATVTVDPTADTTVESDETVSLTLASGNGYTIGTITAVTGIVI
NDDKINTPPVLENLTFTGVEDKILNFVISDNNSQYRDDDNNDLAAVKVVSLPSFGSLTFV
NGQPISLNQRVSAVELLNVRYNPNANVNGQDTFNITAIDNGTPEAESGQAKVTINVTPVN
DAPEFTLTGNIQALTGAVDQTVTGFAQNISAGPNESSQKFEFITTVTSGNEIFTKLPSID
VTGNLTYSLSKTPGTAAVKVLLKDDGGVANSGLDTTEKEFTVQSAIPLNPSESSVTLAVS
PSSVTEDGSSNLVYTFTRTGVISNALTVNYTVGGTATNGTDYGNIGTSVTFAANSATATV
TVDPTADTTVESDETVSLTLASGNGYTIGTITAVTGIVINDDKINTPPVLENLTFTGVED
KILNFVISDNNSQYRDDDNNDLAAVKVVSLPSFGSLTFVNGQPISLNQRVSAVELLNVRY
NPNANVNGQDTFNITAIDNGTPEAESGQAKVTINVTPVNDAPEFTLTGNIQALTGAVDQT
VTGFAQNISAGPNESSQKFEFITTVTSGNEIFTKLPSIDVTGNLTYSLSKTPGTAAVKVL
LKDDGGVANSGLDTTEKEFTVQSAIPLNPSVFEPNVDISAGLTFKPQTNGTVNLDTSRGS
ASLDQVQSVQSGTKSNFNHIIGLYEVMNNQGEIKDNQGNTLKPEDANYTLHALTTARVKN
FTLQAGGNDTPSTATQLGSGVSVLGGKFYAPFAIANGGTYFPGNQGIEDFVAAEQGDINR
FSTALPYVRDLVAREGKNGDVFNNAPRFVQEPVAYFTFGAANPDKSAHFRSYGNGVYGFE
DLPADLTQYSNNDFNDAVFALKLTT
>1170768.4.peg.184
MIAVKDKVPKLTPEEYFIWEEKQLLRHEYINGEVYAMTGGTQNHGRIASNIIFIVKGHLR
GSGCQVGNSDCGVNILETKNYVYPDVSVTCDERDRTAIQAIQYPCLIVEVLSPSTAMPYL
>1170768.4.peg.185

MIINSFPIYASLIAYLILQLVSVRQEWGNKMLDKFRYLQACMC
>1170768.4.peg.188
MLGDDILRSCSMVIFRRLITSGLIGFLVSSCADAAKSSTEYTKQIVQENNVARLMAQVQD
SQKVSNLLSQGNAFLDSERYEEALQLYNRAIEIEKDSVPSWVNRGNALLSLKRYQEALES
YNQAIALRPNKNEAWYNRGNALSALGRYEEAIKSYNESIVIDPNKFEAWINKGIALTKLQ
RYQEGLASYNQAISINPNFAAAYYNQACNYALQKQVDLAVNSLAKAIKMERQKYSELAKA
DKDFVQVKYNREFQDLLR
>1170768.4.peg.192
MNQGQNLASFSFFMSNQERMDNMENQLIDIRLAVSALLETSVIYQRNFEVMQRNFDSVVI
EIREMQSEIREMQSDIREMQSEIREMQSEIREIQSEVREIQLDVRGLQTENRRILDVLQN
VPPDKYE
>1170768.4.peg.197
MQQEAKQLFEDYQALIQLGNNYGKFDREGKKLFIEQMENMMDRYRVFMKRFELSEDFMAQ
MTVEQLKTQLNQFGVTPQQMFEQMHLTLERMKTELEKQV
>1170768.4.peg.201
MIAIEDFLQSFYLESLNAFRRENQQPPDYCPQTLLELAEYMAFTERYAKRRIPLPGRQQQ
LIILRAQTFSQQQPLETSVDIEQATQASGGESDRSWDEPILTQLRAAIAMQPEPEIEEGS
LRSVVITELMNYLEQKQQLDCAEYFSLRLQDFSAQEIESVLGLTPRQRDYLQQRFKYHLM
RFSLLHRWELVHEWLEASLPTNLGLTPHEWELYIRNLDEKDRYLLDLKHQGESDQIIAKN
LGLSVAQLQKRWFKILEKAWEIRNSFSGSGLSAYE
>1170768.4.peg.208
MNKKHARSGNNRSNRVIDGAKSGKSFTREYYHQPGTIPGTIIIHEDAQQPQIVLMDYNPT
DLVEKRIINPEECSDYLRTQSVSWVDVQGLGNRDVIHRLGQTFDLHPLILEDVVNMAERP
KIEDYEEQLVIIARMVVPNTNNRSFYSEQVSLVLGTHYVLTIQEESEHDCFDSVRSRINK
NKGIIRREKSDYLAYSLLDAIIDGFFPVLELYGERIAQLEEEVITNPTSETLKQIYQVKR
ELLQLRRGIWPQRDAISSLIRDGSHLISQEVSIYLRDCYDHAVQVLDMVENYRELVSGLM
DVYMSAVSNKMNEIMKILTVISSIFIPLTFVVGVYGMNFSTEKSPYNMPELNWYWGYPFC
LGLMAVIAGSLMLFFWRRGWLTNSFEINSK
>1170768.4.peg.213
MSDLQNIANHHYEPIKTILRVIDFSQGHFSLIFLHCNDAELRQQVVAQLRESSPNKIEEI
TLPQSAISLYDNISLSLDRHPEVLMVFGLETVNDLDSVLQFSNQIREEFRKNFAFPLLIW
IDDQILRKILRIAPDLETWGSIIGTPNYYNGENQVKGLAQIHRFLAEAALENQQWSEAKK
QAQYAQEILTSVDDGLYHLLTARSQIGMGETSAAIKTLEFAKTHTVLPQDPQLHIEILKT
LGSLYFDHGNYLQAFYVRQEQLQVEQQYRLRAFLGSSYLNPQRPIINKTNGNGRNSRPMV
FGREEDIKSLWQRISDNEHPLIVIHGQFAVGKTSIIQGGLVPILEQELVDDKQMLPIIFQ
KYTNWLEELSEQLLNKLEKKLQSTIAIETFNGLSPQERIVKLLNISGDKNLLTVLIFDQF
EEFFLVTSNLEQKTRFYQLLRSCLDIPFVKIILTLREEYLHYLLEIDRLVDLRVTNNNIL
DKNIRYYLGNFSKAVAKRIIQTLMVKDQFELQMELIDQLVEELGDNLGEVRPLELQIVGA
QLEKEGIDTLEQYEQFGGKQKLLEKFLQDVIKSCGPENESTAQLVLYLLTEENGTRPLKT
QSEIITQFSVESDKLDLVLKIFVGSGLVWLLRESLRDRYQLIYDCLVPFVRQQYVRLYYA
HLPEQLEKIQAELRQEREAEEQAQLVTKLEKDALIALDQFQADPLLSLVTAVSSANLLKS
IVQNNPLDKYPTVSPIYALNTILDAISDRNIIKGHEGGITSVCFSPDGQSIATGSWDKTV
RLWNLRGENIQQFRGHEGGITRVCFSPDGQSIGTGSEDGTARLWNLQGKNIQQFRGHEGG
ITSVCFSPDGQSIGTGSEDGTARLWNLQGENIQQFHGHEDWVTSVSFSPDGQTLATTSVD
KTARLWNLQGETIQQFHGHENWVTSVSFSPDGQTLATTSVDKTARLWNLQGETIQQFHGH
ENWVTSVSFSPDGQTLATTSVDKTARLWNLQGETIQQFHGHENWVTSVSFSPDGQTLATT
SVDKTARLWGLHRHKIQEIRGHEDWVTSVSFSPDGQTLATGSRDNTARLWNREGHLVQEF
KGHQSRVTSVSFSPDGQTIGTGSADKTAK
>1170768.4.peg.220
MRTSTPWVGDKQGERFTLEMFIFTQNQTLRVGKRGGILA
>1170768.4.peg.221
MVVQVSNRLSVNEAICFKETCRDLIESNLDSKVLLIDFNNTIFMDSSGLGALVSIFKFAQ

EKGIEFILDNVTPQVMAVLNLTGLDQVFIIKSCVTNKTSELTNSGNSRVSNCRPEDLPAT
HPCVKSRMKRIIDIVGSLVGLVITICLLIPIGIAISINDPGPIFFRQIRCGWMGKRFYIW
KFRSMCVDAEAKKSQVKNQVAGAFFKNDHDPRITKVGRFLRRTSLDELPQFWNVLKGEMS
LVGTRPPTPDEVELYEVPEWQRLDVKPGMTGEWQVNGRSKVRNFEDVIRLDLQYQKNWSL
VYDLQLIVKTISILFNKNSGAV
>1170768.4.peg.223
MGLTGKPHPPPYPSIMVGSTVPPVCTGGTQSPKNIVQQSANSGWLQL
>1170768.4.peg.225
MTSRIAVPYLNKPVPGALWNFYRYRHSSMDRNRQEAIAVRSKEM
>1170768.4.peg.244
MINSRSQAAKKAEQVHRQNILKNLEHRLEAARTKGDESLIRQLEAEQASYQ
>1170768.4.peg.251
MTLFLTFVNRPRRQVFQSAGIAAVFSFFMVWHVYTWFSIGVIMPPTYILLSLGTTCLLTN
LGAIVWRVSFRHSTPSPPSKLIDEINSSF
>1170768.4.peg.258
MGVMLMVSSLLVAPSQAQSIPAATAAIGKSSFVTTAVNRVGSAVVRIDTEKTISRPVDPI
MEDPFFRRFFGDTFPPMSPTEQLRGLGSGFIIDKSGLVLTNAHVVDQADKVTVRLKDGRT
FEGKVQGIDEVTDLAVVKVNAGKDLPVAALGSSNNVQVGDWAIAVGNPLGFDNTVTLGIV
STLKRSSAQVGISDKRLDFIQTDAAINPGNSGGPLLNAEGEVIGINTAIRADAMGIGFAI
PIDKAKVIAAQLQKNGKVAHPYLGVQMITLTPQLARQNNTDPNSTFELPEVNGVLVMRVV
PNSPAAEGGVRRGDVITTIDDQPISNAEQLQQVVEDSRLGQVLRVKVQRGNKIQNLLVTT
AELQNIS
>1170768.4.peg.275
MLLGTNGSGKSTLLRLIAGLLVPQSGRIDVLSPLGFVFQNPDHQLVMPTVGADVAFGLVP
EKLSTPMVKARVEEALQAVNLLCVQRRPIYALSGGQKQRVAIAGAIARHCEVLLLDEPTA
LLDPDSQLELVTTVRQLVKTRSIAALWVTHRLEELNYCDGAFLLEQGVLVAAGDPELLKQ
RLTGSQ
>1170768.4.peg.276
MSVQRKSAEDAEAVYQLILAAMGSGSPAIVELKCEGKTEKKIAVLASEISGIQIVQKDGS
ASSSGKLPGFAAFTVKEETQV
>1170768.4.peg.285
MNSEIPPQYEQDFYGWIQWQLRAISQRQVSQLDWENLQTELEGLGRQEYRELVSRLTVLL
AHLLKWEYQPENRCRSWFLTIREQRRAIDRHFQRNPTLKSRIPHALEDAFEGGIDLALRE
TNLPLRTFPQVCPYQFEQAVSHGFMCDTSQDWQ
>1170768.4.peg.289
MFTNCLRGYIAPFYGISKSHKIIANYPFPGNISHHRAVFIT
>1170768.4.peg.295
MYKLPCQVIILWGTADHCPQHEAPELVNSLLQEWIHSYLI
>1170768.4.peg.301
MQSVREGRFPEPKWGLKLFAHGLNLSLPVNNVWELIRFRIGVNRNF
>1170768.4.peg.302
MGGGKLIASLLEFDLVDELWLTTCALILATVLTSSFMWAVYTIDH
>1170768.4.peg.306
MHPFHMLGVAGVFGGSLFSAMHGSLYSLEPKSQNQIAHPIINGVSDYVIINLASSYFDIG
QPPYYRRRNSVSPLSSGRDQCGSTAP
>1170768.4.peg.314
MVCNKLLLFEIPPTPLKREANSFGLVCNKLLLFEIPPTPLKRGANSFRLVCNKLLLFGIP
PTPLKKGG
>1170768.4.peg.318
MAEKYGWKLVDVEPTGNNILPVDCIFEGETEFPQSHYDTEEEKNA
>1170768.4.peg.320
MGLTYYSKFGDGREDPKERSLALSINEQLKLAAITSFGH

>1170768.4.peg.333
MGGRNRDPLDHQSSASQYKMSYYFSIQFFDLPNPMLFYGLLHLLT
>1170768.4.peg.334
MPKVLITTVPFAERNSFPLEMLESNGIDYVINPLGKKLTEAELADMIAEFDAVIAGTEPI
TDHVMACAPNLKHISRVGIGLDSVDLMAAERRGIKVSYTPDAPAPAVAELTIGLMLTLLR
SIHTANNQMHQGKWQRHFGRRLAEITVGIIGVGRIGTRVLRRIRGFGTPRLLVNDTLPSL
ELNREFKLEWAAKTDIYQQADLISLHVPLTAQTKNMICKEQLLMMKPDALIVNTSRGGII
NEDDLYHVLSTGHLGGAAIDVFTQEPYKGRLGEIERCVLTCHMGSMSVDCRTRMEIEATE
EAVKFLTGKSLSSPVPETEYNVQRQKL
>1170768.4.peg.335
MVQQVCVIGGAGFLGSHVCDQLSAIGHRVRVYDRVSSPWLQSHQEMVVGDILDLASLESA
IAGCDAVYNFAALADLNQALDKPIETVKVNVLGNAYALEASHRHGVKRFIYASTVYVYSR
EGGFYRCSKQSAEHYVEEYQRVYGLDYTILRYGSLYGPRSDQTNGLYRILKRVLETGVIR
YEGSAEALREYIHVEDAARASVVALGDEFRNESVVLTGQDPMRVLDLLNMIREILGFDQP
VEFVESHQPGHYVRTPYAYQPKLGRKYTPPLHVDLGQGLIQLIDEVRNDTGT
>1170768.4.peg.336
MPNPLKEAMKAILSGTVSVLGRTGISQYIFQLAVNNAMDSTLQVLHRGIKLSFSTPNSLN
YWRVSTFSSKEPETLEWIDSFPENVVLWDIGANVGLYSVYASVTKNCTVFAFEPSVFNLE
LLARNIFLNGLSQKVTIIPLPLSDRISLSSMRMTSTEWGGALSTFGKEFGFDGEPIKQTF
EFSTLGMTMDEAVTLLKIPTPDFIKMDVDGLEHFILQGGANVLKNTKGVLIEVNDDFEEQ
AKSCHDLLVNAGLSLKEKRHSDIIASSTSGFANCYNQIWVRS
>1170768.4.peg.337
MKIINSAKLIFWDFDGVIKDSVDVKTQAFKSLFLPYGAEVAARICSHHESNGGVSRFEKI
PLYLTWSGLDATEQNVSEFCDRFSASVLEAVVNSPWVPGVLDYLNENYQNQYFVLVTATP
HSEIKIILNRLNIAHLFQEVWGAPTSKSDAIAIVMGKVEFNQEESLMIGDAESDMIAAQS
NGIPFLLRRTPINASLQVHYQGPQLDNFNP
>1170768.4.peg.338
MRRQIAIQQVRKNLQSGGISVGSWMQIPHPSVAEIMGQSGYDWVAIDMEHGSIAHHQLPD
LCRALELGNTLPLARIVEGTSKECKQSLDAGCGGVIVPMVETGSQLSAVRNACRWPPSGT
RGVGFSRANLFGQHFGSYQDEAQSPLLIAMIEHCRAVQNLDEILVVEGLDAILIGPYDLS
ASMGLTAQFEHPDFHGAMIQILEKAKQRKVAAGIHVVQPSKQELSQRIAEGYQFLAYSID
AVMLSTTAKQN
>1170768.4.peg.339
MTQNPVPSCGQSDQRNQEYIESFSPEKYYRSIVATDSPVIFDVGAHRGESVRFFKGIFPG
SCIYSFEPDPANFEYLEVVCQSYYSPLGGGRECLPINMGIAEKEGTMPFYRQGISHLSSL
LPINNASTDSLGYAVKALNQPIQISVTTIDTFCSKFAISHIDIMKIDVQGYEVGVLVGAK
KMLMSTSCCTVEVSFYDFYERSTSLLDVEQIMQEAGHRVWDISKISKNPKNFRTDWVELV
YRNSYLKQS
>1170768.4.peg.340
MKEEEIRPKKIFDEYLRLAAQDTHTYFDQTERRKIACPACGNLGSIAFEKSGFVYEKCSR
CQTLYVSPRPLAEAFSRYYLESPSSRYWATTFYKETAEARREKLWKPKARQIQKIIWEFF
SKSDTYPTSMPSIETNSNYDLLQDTLVVDIGGGYGLFAEEIRRLLPREPIVIEPAPHLAE
VCRKKGIRVVEKFLEDIQPDDLPKTPKAFVSFELFEHLHDPMSFMSGLHSLMQSGDLFIF
TTLSGNGLDIQVLWENSKSIMPPHHLNFFNPQSVKLLLERTGFKTLRVTTPGKLDIDILS
NNQSPIGDRFWSTFLSVATDAEKQIWQSWIAEQGWSSHMWVVCCKP
>1170768.4.peg.342
MSILIWDNCDPPNPRGDDQTIVLWCSYNTTSLHGAVSIPQWVEEHSDSIRHKYLSWVYQI
GETPICGKRVVDHLQLHPGFSLWWMSLLAEKCNFAKSTHIDDAIKLIAFVDWMDSQNSKR
IVLLSSKNRLADCLRQWCDQKGVEFEWQRSLPEGTSPFSRHLYNNLPHVLQAIIWLIRRI
LYRWKLRRVGLREWRESQGGVTFISYLFNLVPDTAKQGIFESRYWGTLPKKLHQERIKTN
WLHIYFEHEFLPSAKHAAQQIADFNRMADTLQNHVTLDSFLSPQLIARTLLDWFYLLGKV
ITIPMQNYMPRLNGINLWPLFKDDWKSSAIGKTSIANTLHLNLFEAAFGVLPKQSLGIYL

QENMDWEYASIHTWRSHMHGQLIGCAHATVRFWDLRYFFDTHIYDAAGQNSMPMPDRVAV
NGPISRQIYLEGGYPEDQLIEVEALRYLYLNREPEGDSKITTETTPAIGKLVTSPLRLLV
LGGYNEVDNQLQMQLLSEIAPDLPAETIITVKPHPAKPIKPQDYPLIEFSVKELPLAELL
QDTDVAYSSPVTSAAADAYCYGLPVISVLDPKTLNMSPLRGCKGVTFVSTPLDLINAIKS
AATHIPRNDQANDFFHLDKDLPRWKALITNNII
>1170768.4.peg.343
MTQTPIVSVVIPTYNRSLEIARCLSSLVSQTFREFEVLVCDDGSTDNTKQVVSTFTETLS
IKYFWLDNFGGPARPRNIGIQNARGKYIAFLDSDDWWTPEKLEKSVEALESGADFVYHDL
YIVSSNGVMSPFSRKAKTRQVKVPIFNDLLSNGNAINNSSVVVRREFIDSVNGFSEEKAL
IAAEDYDAWLRVAKYTDKFLKIDGCFGYYSLSNNSISLPDRLINATSRLLNLYNAELQTL
TCCPDWASLALADAYYAKGNKYLCISYCLSGISSKLSISSVIYKLKFLVRLILAFSSL
>1170768.4.peg.346
MNKPKVSSISYNSFLSFANHILFFGTVLFYSFYNPPKLVNWTTLLLYGMLYVQTILILRY
EAKRPNPFILILVYLLTAYYFPRIITLYFESQLSISEINNFSVLNRLRPSTVNDLNYTLT
FIFAANLSMFLGWLFADKKVNKLNFAQKTNAINKPRVNPVVIIILTVVSIFSSFYFSFFA
KSYSVKYITSYINLILNPLILIQMILVYLFYVYYSGRGNESSFFSWTKRRYSVLLVGLLI
LVFLLRIIVGSRSSIISVVQGIFLVSLTLGIYRVPRKILLLCLLLLALSFPLFIISTTSR
QLKSVTSDTTNLEEQFIQTIELSGSAFRSQSLVSILAPSFSRASFLDTSLDLIKNCNNYR
QIINPIFYSKSIIDNALTPGAVDFFDTPKAANALISIYGNISNISLRSTRISYQSDQMNI
YGEFFVLFYGWFSMPAFFLFTYFFQVIFFHNLNREKFDYGRSVLDIFWMNVFFSVLISFG
IDWIIGETIQSYITTRMMLFFIKTRIRL
>1170768.4.peg.347
MYLNGMGFRAIERVMGVHHTTVIDWVKQKGKKLPDAIAPENIPDVGELDELETFVGSKKT
RFGCGQQ
>1170768.4.peg.350
MMKNSHPNNDKSYHFNTSLNRSIGLVYVNTVFFLLLNPFLRFLQLPIGEIDPLGSILSLP
VAMYLVLSGKIKARLFCVYVFIFLVLVYALSEIIFNIGDLNFYRFLFSLCLVILPLSSFI
FLDTYKNFFSNKLFNLNLYLWTLVAIVQVLFPWIRIPILDQLLSRGVLSAENILSGRGVQ
SMAVEPAAATYFIVFSIFYSIHLFKSDKITKSNLYVNLTALLALILLTRSATLLTNLLII
ILIYYFTKLKRSTFLTSFSLLTVLPLVIFIILPLLGVLGGRFNDFIDFFSNYDFSSSTVD
YFLIDFTQNLDGGRLASSLTSYNTLRISPLGLGISNYEENFRNSWVDLYGSDLYLRDSAS
RASTYAGHVMTIMGIPGLASLILVHCFFCLSSLNSCENIPTYKIISFSIAIFWIYFSSIV
TLPIPWVILALVY
>1170768.4.peg.353
MYNTVGAIAKHSHLRDGLLTHVKFNRFCTMGFSFSLAFTPSITT
>1170768.4.peg.362
MVLGLIITLFFVLLAFMAPVWQNLGWLSDPKELLTNPIHQPPSGKYWFGTSRLGYDVFSR
TIFGAQAALQVVILATGLSMVVGVPLGMISGYLGGKLDKTLLFLMDSIYTLPGLLLSVTL
AFVVGRGILNAAIAISIAYIPQYYRVVRNHTVSVKTEVYIEAAQAMGASTWTVLSRYLFL
NVIQSVPVLFTLNAADAILVLGGLGFLGLGLPEEVPEWGYDLRQALEALPTGIWWTTLFP
GLAMTIMVVGLSLLGEGLNELIHPRMQKRF
>1170768.4.peg.369
MAVFSSIAAWWEKVKEIEPWSRLRVSNGEDIEKFCKTLVTAMTIITRITSQLIAP
>1170768.4.peg.370
MDKLMLSLKAVTVKSNPGGKITPEPSLEYRHTGDVFFEVFCNPNIWPTPFAAKVLLTVRN
LGIRLTTEADLTRLVDDINQYLQNTELTS
>1170768.4.peg.371
MRKGIVSRQQPIYTLCQYIPAREWVFVEYELEKCDFLLRDRIGDLIGREQWEND
>1170768.4.peg.386
MTQDIPNDILNFLATFGTGSPLHGLLIIGLTFGFVGGLFDVYVYYRHQVLSFNSEGLIQN
STITVFCESLENEEVYLKPLTRTPGAIS
>1170768.4.peg.387

MSPNKHRQDNKKPTNNIGAIKLNTMASKTERRKLVKTIISGHLQD
>1170768.4.peg.397
MDNQSSLSPQPQKTKSPSLPARRLLAWLAEITLVTASGVIPFSIGAYINSQTDLQRTPLN
PVLAGIERAIAQPLALPVNYGIRNVASPTNFLWTIGLLTPITLGGWQLYLLATTGSTLVK
KKLGIKVVNQEGKPPGFKAIIIREGIGRWGIPMSMAYVLWRYSFAFPNLQLFTFLVLVMV
IAEGIGINSRRNSQAIHDQLAHTYTLDVNIAIPGKQLTESSPSRANFTFNFPPNLTLIVV
GATGMIALLSTLVGTQIYIQNQANQRQTQLVNNQNFLELSKQINPQSGTSIEERQRAILA
MGGVNDLQSIKYLVDLLTKETDSSILNSIQQALSNIGITSIPELKRVNQFIGKEIKSGAI
SGSVGEQLLNINQQTINKILAVYSGKTHNLDLTEIQLGPQNADENSQSQLVLENVDLSGV
ILKSANLNQASFKGSRFRSVGEDGRWDTYDDIIADLSKAQLKRSNLSNANLSRVLMSRVD
LSRSVLNRANLANSKLIGANLSSAQLVGSDLQQATLQDATLTGADISGAQLQEADLYAAQ
LARVSAIGSQLSHSNLTKTNWQGADLSESYLNHANLNSANLSAANLSGAILRSANMTNAN
LSNADISRADLRGANLEGTDFQGAILFPGKQDPKDRFVETSDLGSQAAIVQGVDFSNAKN
LDVQQLAFICTNGGIHSRCP
>1170768.4.peg.409
MVAATTGLTLRTFQNSFVMFLPFLTPKENQEDLNPYHQTP
>1170768.4.peg.411
MTGDELVIYYPDGSRFLSPVELSNYAEQERFLKEQERFLKEQANQRAEQERSLKEQANQR
AEQERLLKEQAHQRAEQERFLKEQANQRAEQERFLKEQANERAEQERLLKEQEQLKYQTL
LSQLKAKGIDITALE
>1170768.4.peg.412
MGSFFVGEKIPVFKNYMLLVGLLEGRSLFGIFRPCHSQGKI
>1170768.4.peg.413
MIAGAVNATGATSPAAKSKGKFWVYEQAVKIPYYAIFNGFKGILEFHFAQKNRSQ
>1170768.4.peg.416
MGVTSINPIKLEPIPLPRINHASNPYLNSHPTGEAIAL
>1170768.4.peg.417
MPKSDRPINLLNKFPLRRGNSGDLKCDRASASALHSPFNHPQKFP
>1170768.4.peg.420
MLNHIDSLIEPNKRSLKLLANDYKINIRRSSTSVETLN
>1170768.4.peg.423
MTYTLEQFKKDFVINHLREIPTEEVLKQYSPEEVLKQYSPQEFLEGLSPETLEHLAIFKN
SLLKNHCCS
>1170768.4.peg.424
MFEGLYIHDKWDWGRKFPVIKIDFADGVLKNREELGRRILDLLRKNAERLGVSYESNDIP
GKFGTLIGEAVAKYGTRAVVLVDE
>1170768.4.peg.428
MEGLVSTIKRLFASIPWRNFTNNDLADFEGYYASVIYAFLSSLDARVIPEDITNHGQSDL
TVMVGGHVYVMEIKVVEGNQVEGNAALDQILGRNYAEKYRGEPGKYVHEIGLIFSRSQRN
LIQADWH
>1170768.4.peg.429
MNLKNLPLGINTLSVLRENNCVYVDKTKLAYHLIRIAGRFFLSRPRRFGKSLFVDTLKEI
FEGNEKLFEGLYIHDKWDWSRKFPVIKIDFADGVLKNREELDEKIRDILWTNGDRLGVGA
KKNSISGIFGEIITGAREQFGERVVVLVDQYDKPILDNRVIPEDITNHGQSDLTVMVGVH
IYVMEIKVIEGNQVQGNAALDQILGRNYAEKYRGEPGKYVHEIGLIFSRNQRNLIQADWR
>1170768.4.peg.431
MVKVATSKLLEGGSFHSINVPSEWELHPNGLSRLKETQIVSIQLMSPASGNTAQKNPTQT
GGYIETSFHSINVPSEWEQ
>1170768.4.peg.432
MNGNSSLRDLNHSDEFCSHSLGTLIEWKRARMREAIEKLFVEVPTRWGH
>1170768.4.peg.433
METPTHQMGDVFLHFGSSHSLGTLIEWKHRGVTGVTFFQTNWVPTRWGH

>1170768.4.peg.434
METQNRLAGFGHLISGSHSLGTLIEWKRWSYWQHPVYGETSNEVPTRWGH
>1170768.4.peg.439
MQVTTTSTPPGELSPSSWPDHTQLPDSDDNFVKNFQEHPQSVILTTSIEPLLDKIHPEKD
YCIGQDSGIYWRFTEPVEKGVEAPDWFYVPGVPSRLKGKLRRSYVMWKEKVPPLIVIEFV
SGDGEEEKDNSPPPERDEVDPKTKKVKKAGKFWVYEQAVKIPYYAIFDGFEGTLEMYHLE
KGRYKQVKANRRNHYPIPELGVELGMLLDQEKPPVPWLRWWDNRGNLLLTGNERAEEECQ
RRELAEAIAIQERQQKELAEALAIQERQQKELAEALAIQERSEKEQEREKREKLAAYLRS
LGINPDEI
>1170768.4.peg.440
MGGMKPDKKGKKPPPGQLRLFWDEVDMGNRENLEVVKRAIAWQLI
>1170768.4.peg.441
MLNRTGHFWEKRYHSTGFLTTDRKRALNTLRYIHANRKAAGIQRGMFYDFSNYGVHERLG
DDGITTWHPAFLALGRSLEECAIKYKGFCQKYRVQEKPVKKNR
>1170768.4.peg.455
MEPFGGAWIQFNIRYYMFALVFVVFDVETVFLYPWAVAFHRLGLLAFIEALIFIAILVVA
LVYAWRKGALEWS
>1170768.4.peg.462
MQAIVKIFLENIHKSYGKRVIVNRVSLSVSQGEIVGLLGPNGAGKTTTFYIATGLEKPDQ
GKVWLDELDVTSMSMHKRARLGLGYLAQEASIFRQLSVKENILLVLEQTNVPRREWSSRL
GELLTEFRLQKVVNSKGIQLSGGERRRTELARALAAGREGPKFLFLDEPFAGVDPIAVAE
IQQIVARLRERGMGILITDHNVRETLAITDRAYIMREGQILAFGIADELYNNPLVRQYYL
GNHFLV
>1170768.4.peg.466
MLLSIKTKLKLNKTQEILMAKHAGIARFTYNWGLTTWQDLYKDGLSPKLN
>1170768.4.peg.476
MGANCNKLLLKVHSQSIIAWTLLAAEAASHIRWIGIISQPSEWHEFKAIVSGLAMTKPVE
FIPGGTTRQESVFNGLQALPSEAKEVLIHDGARCLATPELFNACAIAILNCPGLIAAVPV
KDTIKVVGESGIIESTPQREKLWAAQTPQGFNVQLLKQCHAEGVRQGWEVTDDAALFEKC
GIQVRIVPGEETNLKLTTPQDLAIAELILSLR
>1170768.4.peg.492
MNTPMVETEDSSGKLALTIAQAASERKAGEILLLKVTDVSYLSDYFLVMTGYSRVQVRAI
SSAIEEKVQTELQRRPLRTEGKGEGSWVLQDYGDVIVHIMMPKEREFYNLEAFWSHAEHI
PLENP
>1170768.4.peg.501
MTIYQGKYLAIRDVSFELFPGTDTAIVGPNGAGKSTLVKGILDLIPRTAGKIEVFGFPVS
RLGNLRQLLGYVPQNFIFDRSFPISTSELVRLGISNNSSVNSWLCKLGKFRQIRQQESTA
IKVALQRTNSYHLRHKPLGTLSGGELKRVLLAYCLVSPRKLLVLDEAFAGVDIQGAADFY
ALLNQLKLEEGWTILQVSHDIDMVNRHCDRVLCLNQTLVCSGKPEIALSPQNLLATYGPG
FSRYQHHH
>1170768.4.peg.503
MQKFLLYLLTSSVLGYAISMGGIALAEDVSKTESSLQVSDPNYISQMTSVSQLSDVQPGD
WAFQAIQSLIERYGCVGGYPDGKFRGNRTLTRFEFAAALSACLDRINELISSATADQVTK
QDIANIERLQKQFGPELETFKSRVGTLEAKTTRLEATQFSTTTKLEGQVVAVISDISAKK
VGEVTPTDTNATLGAWTILDLVTSFTGKDTLFTRLESNNIRTPNVGTTEAGFYFGGDGDS
TALSVTALSYKFPIGDRTQVIAVANGGAAEDLTSTITSFNGDGAFGALSTFGTRNPIYSQ
LGASGLGINHEFNKNVTLSLGYLGGSGLSAASPASDSGLFKGHYGALAQLTVKPSERITL
GLTYINSYNLALDTGSNNATFTNTNLPDESKFSSNSYGLQASLGVTPQLRLEGWLGYTKS
QLLTGEKGDVDIWNYAVNLAFPDLWKKGNLGGLIVGMQPKVTSASTTLSNPKEINLKDKD
TSYHLEGFYQFTVNDNITITPGLIWLTAPNHNKQNDSVIIGALRTTFSF
>1170768.4.peg.519
MVNSPNPKSFVYTRKTLSRAERALICSPFRLKLFRDMQSQSIPQGAIATVNGVQQGYTQV

SLSGLRCDNALGWLIEVGVLRREVDGQGITDRFRLTPLGHQLVEKFAHQPWTTASWGDRL
QDAITRWLRLPSI
>1170768.4.peg.523
MSIILACQVWILGSLKMTHQGSQPVCTPETYKGDKQDG
>1170768.4.peg.532
MMLDVKFSQPNLHLYHFTNHDVQYLDIGYRTGKRCDRHLGSQICYN
>1170768.4.peg.534
MSFWAGLWVESNTRNLRGVHISDTSDLMRRMVKSGSRAISRESLILGLVSE
>1170768.4.peg.538
MAVNVWLLSLLFHYFQHPITILSIAAILAFLLNYPVRLLEKARITRTYSVIIVLVITLTL
LVILGFTLVPMLVEQTTQLLKNIPDWVTSSQENLSRLQVLARQKRLHIDFSVVSSQINAS
VQNILQQIASGAVGFAGTLLSGLLNMVLVVVLAFYMLIYGDRLWSGLINQLPSYIGLPLS
KSLQLNFHNFFLSQLLLALFMVIALTPIFLFLRVPFALLFAIIIGISELVPVIGATLGIG
LVTLLVSLQTWWLAFPVAMVAIIIQQIRDNILAPKLLGNFTGLNPLWIFIAILMGFEIGG
LLGTLVAVPIAGTIKSTVDVIRNNKAGIT
>1170768.4.peg.569
MRSPERFYHQCLGWFLILINPKLRKKAAIAVWKRVFKKSFCNLPPTLKTPCLRRGLNALC
LVYEKLELLGSPQPPLKRGANCFMFSL
>1170768.4.peg.578
MGGLGGAYFIPLAPTGSPRTATGNKNFPHGHSFTNQEAYGPL
>1170768.4.peg.584
MGLPWYRVHTVVLNDPGRLISVHLMHTALVAGWAGSMALYELAVYDPSDPVLNPMWRQGM
FVLPFMARLGVTESWGGWSVTGGTAVDPGFWSFEGVAAAHIVLSGLLFLAAVWHWVYWDL
ELFRDPRTGESALDLPRMFGIHLFLSGLLCFGFGAFHLTGLFGPGMWVSDAYGLTGSIQP
VAPEWGPAGFNPFNPGGVVAHHIAAGVVGIIAGLFHLSVRPPERLYKALRMGNIETVLSS
SIAAVFFAAFVVAGTMWYGNATTPIELFGPTRYQWDQGYFRQEIQRRVQSSVAGGATLVE
AWSQIPEKLAFYDYVGNSPAKGGLFRTGPMVKGDGIAQSWQGHAVFTDSEGRELTVRRLP
NFFETFPVILTDKDGVVRADIPFRRAESRYSFEQTGVTVSFYGGNLNGQTFTEPADVKKY
ARKAQGGEIFEFDRETLGSDGVFRTSPRGWFTFGHAVFALLFFFGHIWHGARTIYRDVFA
GVEADLEEQVEWGLFQKLGDKTTRVRKEA
>1170768.4.peg.588
MKFGVLVFPGSNCDRDAAYVTRDILGHTTRMVWHQERDISDIDVIIIPGGFSYGDYLRCG
AIARFSPVMQEVVNHAEKGKFIIGICNGFQVLTEVKLLPGALTRNQNLHFICDRISVKVE
RNNLPWTSAYGDKEVITLPIAHGEGRFYADKDTLAAIEDNQQVLFRYDGENPNGSLNNIA
GICNRGGNVLGMMPHPERAADRSLGGTDGLRLFQGLIGENLTRSILF
>1170768.4.peg.592
MELLLPTAFVQLLMGISALVVAVLSLLGLGNLLLQVVVWLTLSSFLTIFSRRFFTVRQTK
SKILTSTVGETMTEILPGKVGRVLYEGNSWRGRCDDDKLTIPPGQKVYVVAREGTTLIVM
PETVLDH
>1170768.4.peg.600
MAFCLFKSRTLQWDVGNHCVHRKNGDRTLTKPSTGIFF
>1170768.4.peg.604
MAILAVGAGQTYTTIQTAINAANNDDIIVVRPGIYQEDLTINKSVTLIGPYGTFEGIDGF
ENRLGVKPLGPDINAALGVGGLPTANEDFRRYQDDNGTIDNAFGNTQEAWIKGTVTVTED
NVTIDGFRLRNENGPLQWNDTPDNFKLLNNYLTGYTANNSPSFGDANINNPTGVVTGWQI
DSNYIGGLLGGGGTGGSIYLAGLQDSNIDNNTFWRPRAAHLYLASLTNVTIEGNKFYHGL
HTGGANFDGFGEFFSGSGYGYGYGGYGDGFFGRNYWLELKGDNDQVLIKNNEGEYNSGGI
QLFGETDSPFAFDNITIEGNSFPDNNFINAYNEAPDNGKSGLIPAVMATARLGGPSGSNL
VIRDNNITMDLAQVKFITDHKSSLEVRGNFNGVTIEGNTLTPKNINGGVDIITGLSLYGI
LPGETLIKGNQLLGQDGDPLEASYYGIDLIPTFADYGTYAGDLAVEDNTINSWQVGVNLR
DTNEITGDINISGNTFENNAYGVVLDATATTNSINIAGNTFSNNFSNVFDNIDPVITMDQ
VLSYEENQDLGSVLGTVSATDNLPNTDNVGITQYFISSGNDDGFFTINSSGEITLTETGL

AAANDFETSPSSFDLGITVTDGGGLQDTETITLSIINVNEGLGQLPPITTEGAGFIVGAT
LIAAIPFDDPDGTPTDISYQWQRFIEGVWTNIPDATEQNYIATEDDNNNQLRVEVTYIAG
GFEKVIYSNNVSISLVSVSGTFDSITGDNNLDISEKEAGVTLTGSVSETGTTVTILFGGQ
TRVAQVDGLSWSYVLKPNDYSFFAPGSNLFTAIFIRTDGGETGSFTTFQTLNIPDGILPP
NTSNAFDPTQPKGLKSEVIDAAQTLEIDGVSILEISKTVGILGEGESFNDPNIAVLPIGT
RDIFDQGANAGAANYAAFKTEPGTSIQGIYIVPAVEEEGVKKLQVVLADGTVVEAEIPLD
LISPIGDPLAVTISGVQPGGTTTFVLYLSQNVINQLPDDLNLGRYAKFNYESEQFELYDD
SNYDYIFNDVDGDGARDFGEVYLTVNLTDGDKWDGDGLENGIIVDPGQLGIATGSDSDNN
PPIAIELKGIVAENDPGAFIGSLTVTDDPGDSHTFTVNDSRFEVINVDGNNILKLRDGES
LDYEAASNVVLTITAIDNGGLEFTRDFTITVTDVNEAPVAIELNQITVIENDPGAIIGTL
TVSDPDGNDGHTLKVNNDTRFEIVDFDGNQTLKLKAGESLDYEAGSVELSITATDNGGLE
FTQDLTISVNDVNEPPVVSFSFFVPESTTLVTNLTVEDPENDPITLSLAGVDASLFSISP
TGELAFNTAPDFEEPLNADKNNLYKLQVVARDGENNKSIQDISILVTNVNEAPIAIDDVL
AIIPGSSFGTLNPLDNDSDPDLDDTLTIINKTDGNYGRVEIRDNELIYTLLDATYIGDDV
FSYTIVDEQGLAATANVNVTITGTDIITYPVEILDPEDSLIPDEAGSLSDIVNDISFNFL
TDYDKVQAKLALQQALSKTEAAFTNLFGLYEVDNALTGSVNGILPEDKSAYAKAALSKVV
PNFVVRAGGLGGGVNGDVIVTEGKIYAPFVIAHGGNFPGSVQDAVNAFFQVNPDNSPATA
QNYTTLPVAYFSFGSANPDGAAHIKSFGNNVFGFEDLPAGVGVSDYDFNDTVFSFG
>1170768.4.peg.606
MRWWLLAWLMRGSGIGSSFIGLINSRLFNFPYSDRMAVNPVGSPVKNSRDKD
>1170768.4.peg.607
MHHPPGLQAPAFEAHGHGLVDRLPLAVGRQFVHGAHDVDGGAALAQPGAANVVDGCAAVF
VVGGQRRSAGADVYADALVEGPTHAAGRVDDDGEADAGVALLLGGLGVVVVTPRGVARGD
IGNRQRDALGGVVGIGVVGDPLGGGDGRVVGVSLAGRGRDQGGPGEQGEGQASGEVREIH
ARVLHGASFRPWYIGAGSVYPRGFRV
>1170768.4.peg.608
MVYRRGISVPARVSGVTGGLEARLWMARRSPPVFARWPAGPAGTAGPPKRLVAEHEQVIP
RLGGGRGFARARGRAELDQGRGGDCLTPLGQVVTAQAVRRVDVFFFGLFFGRPAPPPAHG
EVAHHPLGPRGVALGAAGGLVLHDLRDADAAPCAGPDVLGWGFQAIGGQAEPDDIHAPGL
QHAGDGAQVALQRGVGGQVADGVDDVEGDVDTQARANRVEVFHAGLKDAGASAGGQFAGA
GLGVGNHGGFDVESDGAAAPAGEVAKQAARAAGGLEVGGRGSGGAAEALGDAGDAAGFGG
GAWSVEDVVEVGDGVEVGEHGARVPPRTGRGCTDAKLAPWTTPRSSRSNQANAAASPASV
ACGSRCTTCSTTWPRG
>1170768.4.peg.609
MSDQSSASSRTPFEILQFRVAEAYWIWKVWKQLYMVGQGGIQEAEERMDVLNRFGVNFFR
MLKGSLLQDVILRVCKWTDPEATQVRGVERPNLSLANALANAKSSLTPEQACKAEALLKK
FKSVTPGLVQRRHWLYAHDDFNVATGKEDVPKVSDTDVDIALQCAVDLMTVLDPKSADVE
FGYGETIAIGDGNSIVEAMRYAKRWVKHCRRYGRALDWDGVPGTDEKPL
>1170768.4.peg.610
MKFARGSYFCLAIAAFAGGCQFVPFVPLEDDYARRVSESRLRQIDQAGLERFRKAQAAAT
VPAADPTKASEQQADAARSRFAGLAKVDLSLEQCRADALINNLDLQVALIDPTIAAETVS
QEEAKFNSAFTLQAGYQSLDEPTASTLVSAEAKNLQVTPGVRIPTRTGGTVTVDLPFTRN
ENNNQFSTLNPSYTSDLEFSISQPLLRGAGRRTTTAALRIAGYDQQAAASRTKLEVIRQL
AAVDRSYWRLYQTKRELEVRQQQYELAVEQLARAQRRNNAGAVAEIEVIRAQAGVSDSLQ
AIITSQNNVLLQQRELKRIVNQPGLEVDTASMIDPTTQPDPVAFEFDSQTLLAEAMHNRM
ELLELELQLASDAVRIGLARNGTLPLFSLDYTYRINGLGDSFEDTFHNLKRNRFEDWTVG
LSAEIPLDNEEAESRVRSAILTRLQRLASRASREQSIRQEVLNAIDTLTADWQRILAARQ
SVILNTRALQAEQRQFDVGNSTSTDVLDAAARLADAQSTEVAALTDYQIAQVDLAFATGT
LLGASKVDWTPAATPSLETPDPKEELPRELQPQPEPQPEGMGAGAMPHAGEK
>1170768.4.peg.612
MVCPGDITGNFKEVNIKDVREVEDNYTPIGRPGTGEDIARVIAFLCHRDSDFITGSVIIV
TGGKDVLTKYRYQS

>1170768.4.peg.613
MERHTALITGSATGLGKKTAIELAKQGVNVILNSVTSLGKANELKDFIENNYPVQAIVIQ
GDISTYSECERIVSSANDRIGKINILINNAGAYIVERKDMVNYDLHEWEKVIQVNLTSVF
YLAKLVIPTMRQNRWGRIINMGFDRAETAPGWRYRSAFAAAKTGLVSLTKTLAIEESENR
ITVNMVCPGDITGNFKEVNIKIE
>1170768.4.peg.616
MFKTQDIKQPETYIYTRAKESLTRSLLEEMSPQKITKYQASEI
>1170768.4.peg.617
MPSIQFDPKPEISVVLTTYNRSRYLKNCINSVTRQTFEDWELVIVDDGSTDDTFEIVSLY
LAEHRNIRYLKHQNRQVGYARNAGIQASFGKYITFIDSDDTYKASHLESRLEFMRTHPEI
DIIRRRVFCA
>1170768.4.peg.618
MRFFQTVVPWASVFWIFFRHDTYLLAGDEVVISKAGKKTHGLAIP
>1170768.4.peg.635
MGLVCESKYMGISWQIFGYSHQTNWQSLHFALHQKKRRVHYLQKQIEHLKTAPIHSYSIG
QIRTPIRISR
>1170768.4.peg.641
MDAAATAAKCAIYMTYLEQGQNLRMTGHLHHLEPKRVKIIVEEIREALTEGKLLKMLGSQ
EPRYLIQLPHVWMEKYSWQPGKSRIPGSSLTTEEKKQIERKLPANLPDAQLVTSFEFLEL
IEFLHKRSQEELPQQHQMPLSEALAEHIKRRLIYSGTVTRIDSPWGMPFYVLTRHFYAPA
DDQERTYTMIEDTARYFRMMKDWAERKSHAMRAVEELDIAPEKIQAAMDELDEIIRVWAD
RYHQEGGKPVVLQMAFGQQDD
>1170768.4.peg.647
MNLKTKKAIEQLEKFVSTPLEETLRQHEKTTHQEIATNLFKTVVSTVPAYQSFLATHKIK
GETIQTLADFQKLPVICKENYISVYSLPELCEDGTLGGCDMIAASSGSTGKPTFWPRFFT
DELQIATRFEQIFHDSFGADNKSTLAIVCFSLGTWVGGMFTTNCCRYLATKGYPITVITP
GNNKTEILRVVQELGSNFEQVVLLGYPPFLKDVIDTGIANSLEWKQYKIKLVMAGEVFSE
EWRSLVGERIGTKNLCYDFASMYGTADAGVLGNETPLSICIRRFLAANPAAAKALFGESR
LPTLVQYDPCSRFFEVEDSKLIFSGNNGIPLIRYNILDHGGLITYEGMVKFLAEWGFDPV
AELGKNRGIHQLPFVYVFGRSNFAVSYFGANIYPENVSVGLEQPIIREWVTGKFVLQVKE
DQDQNRFLSVVVELASGLEGSEEKIIQITNSILAQLLRLNSEFANYVPGEYQRPQVELKP
TGDGEYFPTGVKHKYTR
>1170768.4.peg.650
MLAGDIVTKGTHKSSQKAKILQLPVVGIFTLIIYKSTMLKAGIVGLPNVGKSTLFNAVVA
NAKAEAANFPFCTIEPNVGVVAVPDDRLNVLAKLASSEQIIPARVEFVDIAGLVQGASQG
EGLGNQFLSHIREVDAIVHVVRCFENDDIIHVSGSVDPVRDIDIINLELGLSDLAQVERR
IERSRKLARTSKDAQFEVTVLDKLVAALNEGKSVRQVSLTPEEAGVIKNLGLLTSKPIIY
AANVAEDDLATGNDFVEKVRAVAAQENAQVVIVSAQVEAELVELPDADKVDFLASLGVQE
GGLKSLIRATYALLGLRTYFTSGPKETRAWTIHAGMSAPQAAGVIHSDFERGFIRAETVA
YEDLVTHGSINAAKEKGLVRSEGKEYIVQEGDVMLFRFNV
>1170768.4.peg.658
MNKVDIHKLLSDLGRVHYIYTQGDKLLSEGDGDVMEVFAHPQRSTLVANNTLYLNIYSFD
YLELKQSDAQETYLDLIQEEYCLRLIPLFVPPEERCDDQFNNGVIEAMMDQVLSTRWDVE
IDDDCSDCL
>1170768.4.peg.661
MSNLENAISDLKSLGYPPTGEISITSHGQEIYAQDPDGNRLILYSSSNIDKQCP
>1170768.4.peg.691
MQFSEYLPWNETLGLAYQLGVDGLSILMLILNSLLTWIAIYSSSENTERPKLFYSLVLLV
SGGVAGAFLAQNLLLFFLFYELELIPFYLLISIWGGNKRAYAGMKFLIYTAVSGALILAT
FLGIVWLSGSHSFALDAVNTQNLSTTMQIVLLAGIILGFGIKIPLIPFHTWLPDAYVEAS
APIAILLGGILAKLGTYGLLRFGFGLFPQAWNVVAPTLAIWGAISAIYGAVVAISQKDIK
RMVAYSSIGHMGYILLAAASGTKLALVGAVAQMFSHGLILAILFHLVGIIEGKVGTRELD

KLNGLMSPIRGLPIVSALLVLGGMASAGIPGLTGFIAEFIVFQGSFSTFPIPTLLCVASS
GLTAVYFVILLNRTCFGKLNNDLAYYPKVVWAEKIPALVLAGLIIFLGVQPTWLVRWNET
TTSAMVGAIPTAGTIIISQVETNQQ
>1170768.4.peg.697
MSSAVDIVSVPPELFAKFQTSFYTDPVDAVFKASAVD
>1170768.4.peg.698
MQITTSWMRQGLEQGITQGLEQGIAQGLEQGIAQGLEQGIAQGLEQGIAQGLEREKVLVI
RLLKRKFGDEAINVDLEARIMALNIDVIERLGEEILDFSTVEDLVEWLKKLKPSK
>1170768.4.peg.701
MDHRQILTMAKHFNTAGPCQSDIHYILPPTARLPDLKALIHGRNYLEGDVPAVVMEFLSD
KEGGEYSFKRTYPPGKWFFYEQILQVPVYIIFDPDGGLIEYYELKNERYELKQPDENGRH
WIESMELFLGTWQGTKEGRTGYWLRWWEQTGNLLPWALELIEQERQQVEQERQRAEREHQ
RAEREHQRAEQERQEKEMLIAYLRSQGIDPNNLPNHAE
>1170768.4.peg.704
MTIEVAPGARILCRDAEWLVKSVSLTSDGDKVIEAVGVSEFLRGKRVQFLEELEEQLDIL
QPEETRLVQDPSPNYSNALLFVEANLKQVIPQDDKIYIANRGAMDTLKYQLYPTSLALKM
PRQRILIADAVGLGKTLECGMLVSELILRERGRRILVVTTKSMISQFQKEFWLRFTIPLV
PLDSFEIQRIKSIIPTNYNPFFFYDRTIISIDTLKQDREYSRFLDHSHWDIIIIDEAHNV
AERGNNVSKRSKLADKLSILSDTLILLSATPHDGKSESFASLMNMLDPTAIANESEYTKD
DIRNLYVRRFKKDVWQDLRENMLPEPNIKQVESMASREEERVFEVLNNLRLVGIDKNQRA
GHLFKTTLLKSFLSSPQACKKTVDNRLRLLEKKREQLQGVSLSEVMEDIGELTVLASYFF
GFINSNCLLFRNNSRSTNQT
>1170768.4.peg.728
MATLLSESKSQTGPVEALPDDFQLEDEPVDNTGQLLLAGALCESLEISGFIQPQMLIAVN
FGLCATLNEQFVAKAPDWLYIPSVY
>1170768.4.peg.730
MLIIILIFAYLRSRAIAWNPIPIPLRRLNTKCDRIGLAPGRSPFLTTQGVAPARDDV
>1170768.4.peg.733
MGGSKELKPYYGCIYLKPPEQKSVFYLWFWNMGCDRMVVTPVRLHFLPFSKKSLRRRVFF
L
>1170768.4.peg.740
MKYDYFSLQTCDRQVLYSFEVCLKKKAPHLVRSFSFNSIDY
>1170768.4.peg.741
MGVTSRIKVFKNWNQGKFNSLEFSQLGEEYFLCGIVAFFMGEKIPVFKN
>1170768.4.peg.742
MLLVSLLEGQSQIIRYGSNNSRFLINPFINSKNSEFPSNSNNRALF
>1170768.4.peg.745
MWFWNIGCDRMAVTPVHLRSTRYVRLRSTHHVRSHVYHL
>1170768.4.peg.750
MFSLGEVGDAYTIMDGVSAREERKNSHKNGRTGSYNSDWLWN
>1170768.4.peg.752
MKANRRNHYPIPELGVELGMLLDQEKPPVPWLRWWDNRGNLLLTGNERAEEECQRRELAE
AIAIQERQQKELAEALAIQERTEKEQERQQKELAEALAIQERTEKEQERQQKELAEALAI
QERSEKEQEREKREKLAAYLRSLGINPDEI
>1170768.4.peg.756
MNDLLKPGQIVHTESSKTACVVGQFLGGGGQGEVYQANLGGKSVALKWYYSHSIQADPYQ
RDRLEAAIQCGSPSDRFLWPIDIVSESGIPGFGYVMPLRELRYKGFVDLMKRRIQPDPTF
RTLATAGFELANGFFKLHSKGLCYRDISFGNVFFDPKTGDILICDNDNVTINGDQEGGVM
GTPRFIAPEIIANQARPSTQTDLYSLAVLLFYMLIIHHPLEGRRETEIKCLDAPAMNKLY
GTDAVFIFDPNDNSNAPVLGYHDNALVFWKIYPQFLRDLFIKAFTNGIKDPQNGRIRESE
WRGAMVNLRDSIIYCPHCGSENFYDPDVLKVSGGNPNPCWSCQKEILLPPRIRIDRNIIM
LNYDTKLFPHHINVNLFDFSEPIGEVIQNPKNPAIWGIKNLSSEKWVCTTTDNQVKNVEP

GYSATIAVGTKINFGKAEGEIRL
>1170768.4.peg.758
MRPDKKGKKPLPGQLRLFWDEVDMGNEEIIGVATKFVKANCYSPNPNLF
>1170768.4.peg.760
MNDLPRQKLKEIIIQHGHALCDDPKRCEAFLRDYCGEYGREIFILISVLKQGIVKDLLNS
NNIPIELLLGRLTKQMQNNLGLTEEAARYGVESWAVVLDKMTLQPIETKSATLRNKMRTF
SFEVVSSNKEGKILNKKSVSANYFVEDLGNGIILEMIEIPGGIFMMGSQENEKDRNINEG
PLHQVKISSFFMGKYLITQEQYYAIIGNNPSNFRGNKNPVEEVSWQNAVKFCQKLSQRTG
KIYTLPSEAQWEYACRSKTITPFYFGDNITTDLVNYNGNYPYGLASKEQWRKKTTEVGTF
PPNAFGLYDMSGNLWEWCLDTWHNNYQGAPSDGSPWFSNSMIPRHVLRGGSWFNSAKDCR
SASRNCNFDHGNDYGFRVVTAPLLWN
>1170768.4.peg.765
MLTEIAPNHVHKFPLRTEGLGDAEFVYEVLATGDRTSRHFHSPELRSPF
>1170768.4.peg.782
MGGSKELKPYWVSIYLRSPEQKSVFYLWFWNIGCDRMAVNPVRLRSTRYARSHFLPSTKK
KP
>1170768.4.peg.783
MADEYGRQLLAALKVEANVKRNPEAIKRVKDQLTGRNREN
>1170768.4.peg.784
MAGPSSQNCAQAAEALAAGRSSLIWRRRIADTETPVSAALKLIEPERGDFLLESVEGGET
RARHSFVGLAPDLVFRASGDAAEINPSWATDRGAFAPCERGSLDALRGLAEACRMDVPPE
LPRALAFLVGYFGYETIGLVEKLPRAPQSDLTLPDMLFVRPTVLLIFDRLKDELFLIAPI
WAGSGDPEQAIAAAEERLDAAERALHAPLPASVRDASLDPETLKPQPVLAPGRYGEMVLQ
AKDYIEAGDIFQVVLAQRFTAPFALPPIELYRALRRVNPSPFLYFLDLPGLALIGSSPEI
LVRVRDGEVTIRPIAGTRPRGKTPAEDLENRQSLLADPKELAEHQMLLDLGRNDVGRVAA
AGTVRVTDSFTVEQYSHVMHIVSNVVGALEPSRDAIDALFAGFPAGTVSGAPKVRACEII
ASLEPETRGAYAGGVGYFAPDGSVDSCIVLRTAIVRDGTMHVQAGAGIVADSVPDYEQRE
CEAKAGALFAAAREALRLASEPGYGQ
>1170768.4.peg.785
MGWFLILINPKLRKKAAIAVWKRVFKKSFCNLPPTLKMPYFLSYPLPTSFLT
>1170768.4.peg.786
MDCDRMAVDPVRLRSTRTCAIALYPLHNFPSIYPIHYSISSSNFS
>1170768.4.peg.788
MQVPVYIIFDPDGGLIEYYELRNGRYELKQPDENGRHWIGSMELFLGTWQGAKEGRTGYW
LRWWEETGNLLPWALELIEQERQQAEQERQRAEREHQRAEQERQEKEREHQRAEQERQRA
EQERQRAEQERQEKEREHQRAEQERQEKERLIAYLRSQGIDPNNLPNHAE
>1170768.4.peg.789
MATLLSESRSQTGPVISWEALPDDFQLEDEPVDNTGQPLLAGALSESLEIGGFIQPQMLI
AVNFGLCATLNEQFVAKAPDWLYIPSVKEILPGRKSYTPHLEGDVPAVVMEFLSDKEGGE
YSFKRTYPPGKWFFYEQILQVPVYIIFDPDGGLIEYYELKNERYELKQPDENGRHWIESM
ELFLGTWQGTKEGRTGYWLRWWEQTGDLLPWALELIEQERQRAEREHQRAEQERQEKERE
HQRAEQERQEKERLIAYLRSQGIDPNNLPNHAE
>1170768.4.peg.794
MKIYSLLLNNPPQTLQDKYAGIHYTESKSPFFQGELNAVKSELG
>1170768.4.peg.797
MGLEVKIATPSVKQQSQQEIIGVFLRLIVIRLTRNHFSARYGQG
>1170768.4.peg.804
MLHKLFTEIKEYELAAYFYQISQSKISSKILKLLVNSNNITPEIILGLPIKESRPWINSI
SKFFNKDIDEQEKIFKKRREKYLELLRKYKEIDDYNQDIKINPNDPEYYYNRGNTRSDLG
DNQGAIDDYTQVIKLNPNSAYAYILRGDVRSDLGDKQGAIVESPVQTCRV
>1170768.4.peg.812
MNDKKRNQWDLCRFIRTLTYFEVFPLLNWIQNILQNRPTNRQDQPTGRIQMGIILVAGAT

GGVGKRVVKKLLTQGYRVRCLVRDIEKAREVLGNEADLVVGDITKPESLNDLVMSNIQGV
VCCTAVRVQPVEGDTPDRAKYNQGVKFYQPEIVGDTPENVEYKGVKNLIVAAKRYLPTTG
EKIIFDFTQPSSDLKNIWGALDDVVMGGVSSSNFYILEKTAVFNGNVSTANSGGFASVRT
KNFSPAINLSGFTGIRLRVKGDGQRYKIFLRTETTWDGIGYSYSFDTMANTWIDVNIPFV
NLVPVFRAKTVKDCPKIDESKICSLQLMLSKFEYDGGLNPKFTPGVFTLELESIRAYGGE
GVSQFVLVSSAGVTRPGRPGINLEEEPPAVRLNDQLGGILTWKLKGEDSLRDSQIPYTII
RPCALTEDRGGKELIVDQGDNIRGKISRDDVAEICLQSLQQPQAKNITFEVKQGQNDAVS
LNWGQLLSQLQPDRPNPL
>1170768.4.peg.815
MTVTQISAQELFRAAYQNRYTWDENFPGYTADITYKYEGQEFTAKIRIDANFKWEVTQVE
DEAAKKAIDSQTWEIAVHRVRRTFAQTHGENTFTYGEKDSTGAVEIIVGGKSAGDKYKVR
NDVVTLVHRHIHGVVVTINTFSIHETEAGYLSHTYDSVYHDPKTGEQKGGRSEFTDEYEK
VGNYYILNRREIRTETPAGMSIQEFVFANLELLG
>1170768.4.peg.822
MRGSGIGSSFIRLINSRLFINFPWFGDGHIRVVLFLLGVVILY
>1170768.4.peg.826
MKVSVKIAIPSSNTEIILFICFTAKLDFGPDGNFLVYFIGAIFH
>1170768.4.peg.831
MFTELVLIRNILIDTLQIFGVTVDSPNLPKYPLGESRLYLYFSLRKFTDCKVL
>1170768.4.peg.835
MVGLATAAPDFGPAANVSAGRTAKPLSVTILGILVKKN
>1170768.4.peg.841
MAQSLKSQRRVYLWTVTHGIVEYGQARSTNQHNTVSPEAAIEWAIRQKEPSIFIFKDLHP
FIDAPATTRSLRDAIASFKGMQKNIILMSPMQQVPIELEKEVVVIDFQLPDMTELSKVLT
VHQEQNRGRRLTTEAREKLLRAALGLTKDEAEKVYRKAQVTSGRLTEDEVDIVLSEKKQL
IRRNGILEYIEEDETIEAVGGLEELKKWLKQRSNAFTERAREYGLPQPKGMLILGVPGCG
KSLIAKTTSRLWGLPILRLDMGRVYDGSMVGRSEANLRNALKTAESISPTILFIDELDKS
FAGSTGSSDSDGGTSSRIFGSFLTWMQEKKSPVFVMATANRVERLPGEFLRKGRFDEIFF
VDLPTPEERQDIFRIHLTKRREEIARFDLEQLAKMSDGFSGAEIEQAIIAAMYEAFAQDR
EFTQLDIIAALKSTLPLSRTMQEQVTALRDWARQRARPAASSVAEYQRLEF
>1170768.4.peg.851
MIVRYKLDTCGIKLKLAHWHQISLSERDTLVTLPCFTTGEIQNYQDYLKKLVFKYTGTYP
SLLPMDPHPPWLDSTTLPKQLIEKLETLGARIILEQWQDLTPLQRFVLVKLSLPSHENKN
FPRAMAEFHLDPPTGSLITNE
>1170768.4.peg.852
MHLIENIVVIVSKVLSILMVVVILVAIADLTVFLLKELFDAPYGKFNTILFKIFGLFLNI
LIALEILENITAYLKKHVFQVELVIVTSLIAVARKIIILDLEKVTGIDIIGLGIAILSLS
ISYLIIQSRLKS
>1170768.4.peg.860
MLDQCDFPTTNLANRKSPGVTVSSVLILFCMGGNSITLPPTERFDYQKSYKK
>1170768.4.peg.864
MGVDFCLMERGKKIVKGLVRLLTVFSLLLGCWGWLGTTPTAQAWDFSHVNLPQTALLAIA
RQNKADQKLATDFGKKIDLNNTNISRFQEIRGFYPVLAKKIIANAPYAKVEDVLEIKDLS
DRQKQLLENNLSQFTVTEYDAEFNEGDDRINNGIYR
>1170768.4.peg.868
MNLYKLFKTRKPIIGVVHLLPLPTSARWGGSLKAVIDRAEQEATALASGGVDALIVENFF
DAPFTKNHVDPAVVSAMTLAVQRIQNLVTLPLGLNVLRNDAESAMAIASCVKAEFIRVNV
LTGVMDTDQGLIEGRAHQLLRYRRELGSDVKILADVLVKHARPLGHPHLSVAIKDTIERG
LADGVILSGLETGSPPSLEDLKLASRVAASTPVFIGSGADWENIGTLMPVVDGVIVSSSL
KRHGQISQPIDPTRVSQFVEAAHRSITTKI
>1170768.4.peg.873
MHYARQGVITEEMHHVAKRENLPPELIKEEVARGRMIIPANINHTNLEPMAIGIASRCKV

NANIGASPTSSNVEEELDKLRLSIKYGADTVMDLSTGGGNLDQIRTAIINASSVPIGTVP
VYQALESVHGKMENFTADDFLHVIEKHAQQGVDYQTIHAGLLIEHLPLVKTRLTGIVSRG
GGILAKWMLLHRKQNPLYTRFQDIIEIFKKYDVSFSLGDSLRPGCTHDASDPAQLAELKT
LGQLTRKAWEHDVQVMVEGPGHVPMDQIEFNVRKQMEECSEAPFYVLGPLVTDIAPGYDH
ITSAIGAAMAGWYGTAMLCYVTPKEHLGLPNAEDVRNGLIAYKIAAHAADIGRHRPGARD
RDDELSQARYNFDWNRQFELALDPDRAREYHDETLPEEVYKQAEFCSMCGPKFCPMQTKV
DADAIAELEQFLAKEPVGQV
>1170768.4.peg.886
MFRQTAFGIGLTILVLTSGLVTDAISTSNVTKKISSASSLNGSTQAVAFHQNNESNKHEN
NRLEKLVFEQINQYRVAQGLSKLTLNLNITKQARIHSQNMANGTVKFSHHGFEQRVKSIH
LQYNNAAENVAFNIGYNEPAKQAIIGWLNSPGHLRNIRGKFELTGVGVAKNDKGEVYLTQ
IFINTPHRYSRKISPRSPAF
>1170768.4.peg.887
MADHGWLKAKHTFSFANYHDPSRIGFGALRVINDDHIAPGGGFATHPHRDMEIVTIPLQG
SLRHEDSMGNSQVLQKGEIQSMSAGTGILHSEFNHGDEDLKLLQIWVLPKKIGVQPRYEQ
KAFNPSDRKQKLQTVVSPDGEGLGINQDAWFFLADLEGTSVEYKLHEKGQGVYVFVISGF
ANVNGKELFPRDGLGLEEDLHIQVFQQCELLVMEVPMLSS
>1170768.4.peg.890
MYEPQSQPGRFFAWAIPQSLGGGWNPQNRCQAIASRLELYRPDGLQELQIARQNNENIIC
VTTEAVSTCRIVFTVPRNRDPYSVRSSIFSNLTAADQGQQTTGVNTYINSPRRSGNNSHF
RGGINLKPFLSMEDGGTGTNLNNGLLIPSRTPGKTILNPRLFR
>1170768.4.peg.900
MQGRVQMLYIAKNDNLGGYLPIPPNKPMVACTFVVESTG
>1170768.4.peg.902
MGLLPWRLRDTSINETTISSKQQATKNTTEIMTKKFVDLSTLDGKNGFTIINSNDKNDNL
GYSISNAGDINGDGIHDIIIGAPLSDNNDQSNAGNSYIVFGSNNGFANIIDISTLDGING
FTVNGSEIGDQSGRSVSAAGDINGDGIDDLIIGAPFADSNGDDSGAAYVIFGRSSFSSLP
TINPSNLGDNGFIINGLNPQDQLGYRVSSAGDLNQDGFDDVIIAAPPNAYVYPPVTGDQA
GKVYVIFGSEKFNPSNPNFDTSNFDLNVINGSRADDYLGVGLNRGGDFNGDGIDDLIIGS
PFNDFNGFRSGQAYVIFGSKESFSSSLNVSQLDGVNGFVINGQEGDQLGFSVSTAGDINH
DGIGDIIVSAHDADPQGVDAAGAAYVVFGARTQFNSELDLSSLNGNNGFVINGIGELDKT
SWAVTGLGDVNGDGIDDLLVSAIHADANGDNSGQGYVIFGSKESFSSSLNVSQLDGVNGF
IINGKSENHNLGYSASGAGDINGDGISDILISAPFAGSGEVYVVFGNNGSTDTGEITDEV
TEGDGGESEEFTIDGTEDEITGGVIEDNNSESEITNIDGNPDEILDGVMGEGDSDNNSTE
GLGSDITDSPDIESTPTGSVDNILGNNNVLSSSTTKVEFQLMDKIPASIRELGVFTVDDA
SGKINGIAPGQVGYSEAALARSKVVFSVLSKTPNEFNNANVTRILGFQEANPNLRFYVID
NGTTDAVKNDLLPINQVTFLDSSNLQVTQLPDNSFSLQSNDLVFKARPTTQPLPMGTNLQ
EKSQGESIDLRGVTGPVNAQFTVYREASFDNYVGFYKVTDEKGGIDTNSDGTADLLPGDA
GYIQSAVNQHLSGLGLNVANGNKSTFNNTLSGGGLYVPFIIVNGRPDTVLNSDLSANSNP
NIYFTYLGANSDQVDHVRLLGDNTFGFEDLRGGGDMDYNDLVVQVNMSARV
>1170768.4.peg.903
MTLPDSAAKEAWEEAGVIGQVNTQKLGDYKYRKRGNIYQVHLFWLTVEKILEDWPEASQR
ERIWLDINHAAIIVRENSLKKILQNSQEQVRVFTS
>1170768.4.peg.910
MGRLSFENNHRFLFTSISDTRGKVKSCNEKSTTISNEVQFPTNDKF
>1170768.4.peg.919
MIDQSKGSQVSTSLKLAQDLGNTSKSIKKLHLTDPQLQKFQSDLGQNFAGLSHYISKAAT
SLSEAKKTSKSPSGQEKMRYAKRGIESSLTTAEAAGKQLDILGNKLNKYCNPNK
>1170768.4.peg.924
MTHTGKGKININNAILSELDKLEGKLGVPALSNKIQASRPYGSTADLVSKKVITQEQFEQ
IKNLVTVEEVVLTGEAKDIDYMTKLALMKGHLLVAEELLKENQPKQAQPHIGHPVEEIYV
DIEEQLNERKVKQFKEDLVKLTELVRFRPQDKKVATNLTVAMSDIDTAITALPNEQRLQP

KFILQVIRGLLDAATAEYQAAIAKNKITAPIEYQDSRGFVIYSHELYQSVSPQMATVNPE
AQKAIDIALTELLQVWPSAIPPNQIVKTPEQVNSLVKTIEENAKFFTVM
>1170768.4.peg.925
MEFELAGVVRYTHYSLMVTGPNRLPIVAFFKAQASESLLHAQQVGEILTGLDGHPTLRIA
QMEETFKHSVKDILQESLIHEGKALDMYKSLLENVADASIYLEEFARNMIGQEEMHNLEL
KKMLRDFS
>1170768.4.peg.931
MQPKLGKNQFPMVLAMVLLFAIIGLPLAFRFTNGSGMIIWATIVVLLATLPAIIAWFNYR
P
>1170768.4.peg.934
MSELERYYTLLDLEPGATIEEINQAYKDLVFVWHPDRLPKDNHRLQKKAHDKIKALNQAR
EKLRSFQYQSQPGHNSEPSTSKRSPYQPNQPPAQNPDLSGRDFSHANLSNKDLSGRNLSY
ANLSGSNLSDTFMHKVNLRGANLSEANLFRANLLLADLREANLRSANLIGADLSGADLRG
ADLTGARMRSGERLLVKLVGANLTGAIMPDGAIYG
>1170768.4.peg.938
MTFIHQEIAATPVVVADVWVRAGATSESDPLFGMAHFLEHMIFKGTASLGPGEFDYNIER
IGGISNAATSHDYTHYYLATANHYLADTLPHLGELLLNAAIFEDEFMRERDVVLEEIRSC
ADDPDTMGFEALLKTVYENHPYGRPILGTKKELMENSPEAMRCFHRRHYQPENMTVVIVG
GIERDTAWEIVNKTFKNFKNQDDFPTSNPLAPPQIRDVKRQELILPRIEQARLIMAWNLP
GMDELAIANALEILSVILGQGRTSRLVNDLREEKQLVRGIYTNFSVQKDSSLLTITAYLE
PEYLDRVENLILEHLHRLQIHGVTEQELKRTQRSLCNDYAFNTETPNQLASLYGYYNTVA
KAQLSVAYPEQIQSFNTKKLQKVAQNYLSLQDYAVTIMKPY
>1170768.4.peg.945
MLLHLSTWPEVEKYLESYSGIIIPIGSTEQHGPTGLIGTDAVCAEFISHGVGEATQAMVG
PTINVGMALHHTAFPGTISLRPSTLILVVKDYVSSLAKAGFTKFYFINGHGGNIATLKTA
FSETYAYLEDIQAKNFQKVQCQLGNWFTCSSVYKLAQELYGDKEGSHATPSEVALTQYIY
PQAIKQSFLAPEVATGHRIYGPSDFRLRYPDGRMGSHPDLATPEHGKQFYDLAVEELSAA
YSNFLNQD
>1170768.4.peg.970
MTDDSVFDHGTWGIFDAWDSVHYRAIATSGYEFYPDGQQYNLAFFPMFPVTIWVLMKIGL
PFEIAGLLINNLSFLGAIYLLYFWVKKHCSLRIAQWTTIVICCCPMSMFTGVIYTEGLYL
LFSIGCLRAFDEEKYSLTAFWGAMATATRPTGMALIPALLLTTWRQRRGKIAYFTSLLTA
TGLILFSIYCAINFHDPLAFIAAQKGWRPSLGFDWQGWLNMLMQIILGRNWNFGWVWNDD
GGIRDPWYPVFFGFMVYGTVTLWLRQKYWHPFITYLVYTTLAVLLILVDQRVINNLLNVL
MVVGGSYLVWHFRRKLTPVMVIYGLCGVGLLLASGGTISLSRLAYGIVPLSIAIGAWLSR
FPRQAYLAVGLFVVLLFRLAIGFAQHHWVG
>1170768.4.peg.972
MNTSQEKVALMSLTGWGNNGAIPSIDTQTIMRLDSHIHKGK
>1170768.4.peg.974
MANLKINKDYIILCLIWLIGLVIDRIWFFLDDSIPAYDQSAHLTTALHHYRIFQNLNLLS
SDWWSSLWELTPSYRAPFVYICTVPFFILFGKGYDQASLVNLLYTGIIILSVHVKKI
>1170768.4.peg.982
MPLSAELSWAFWGWLYIPWYTLLYVEDIASEQGIWFSDALVRAHYVLAAVLLAWVTAH
>1170768.4.peg.989
MDIQLINIGFGNIVSANRVVAIVSPESAPIKRIIGDAKDRGQLVDATYGRRTRAVIITDS
SHVILSAIQPETVANRFVLSRDHHTADN
>1170768.4.peg.998
MGNSPPRGGGSQPQQKCEKCKVLFPYLNSVKIFITKHKNL
>1170768.4.peg.1008
MEISGKEELEVLDASQVKELLENQRVHLIDVREQEEFMGEHIPGSQLLSLSKLDPKKISL
LTGKKIVLYCHSGNRSKQAAHRLIEFGFRDFSQLQGGISAWKKSGYVTNRNAPISIMRQV
QIVAGTLVVTGTVLGVLVSPWFLILSGFVGTGLVFAGLTNTCTMAMLLKKLPYNQRG

>1170768.4.peg.1030
MIQPISNLRTKMNIETIKSEKTKQLPGANLEDQDLSEFDLTAANLAGANLMGAHLVSVNL
EGSHLEGANLMGASLQGADLRANLLGANLMQADLTGADLRGSNLRGANLMGATVAGASLT
AAFLSGANLMSGNFQGVDLRGADLRGANLTGANLKGADLSRADLQGALLNQANLEESDLR
GANLAGANLAGANLLCAELEAASLNGANLYQACLLGTILETYHD
>1170768.4.peg.1032
MDSIHLLLGNPSNATSSLDNPDNYLMIKPQYALSYNRSHGSANWVAWQLDKSWLGDAKRQ
DDFRPDDTLPDVWTRVKPSVYNSSGYDRGHIARSADRTQSVEDNSATFLMTNIIPQTPDN
NRNTWGNLEDYSMKLAEEGKQLYIIAGGFGDKGKLKNLVTIPQYTWKIIVVLDRPGLGLQ
DVNSNTRVIAVNIPNDEQLDNNWRLFRTSVDKLEELTGYDFLSTVSPNIQKVIESQIDNL
>1170768.4.peg.1034
MMEAHSNGLALVISCALEHAEFYCESLNNHGLTSTIEPDD
>1170768.4.peg.1035
MLICLDLNQAMLDLAEKFLEHNYRNRIIEYNGWWDQTQTSKC
>1170768.4.peg.1046
MVVRITIQTDDFLTNHHIAALKRHHYCDRSPLEYCLNI
>1170768.4.peg.1047
MNGRILDHPLATNQIDVTPLIHGTYPLSQGLIAFEKAQSKGVLKILLEMN
>1170768.4.peg.1051
MSNLFKTKPKHTRIQRQLLILMLIIFGGSVVLGFIMGLLTGIQPVT
>1170768.4.peg.1058
MGYVDKSEVNGPGRRAVVWMQGCSSHCSSCFNPQSWSFEINKLVAIDNLAEDILRNSENT
GVTFSGGEPFLQAKSLAILARKVKAKGLNVMSFSGFTLQELQSESAPPGSRELLAELDIL
IDGPFVDSLAINSPTSPVSSRNQKVHVFNPDFNDKITWASDQIEVHILKDGARIVTGYQG
RAF
>1170768.4.peg.1059
MSTYPMMFRYPLGISFMGDTISSSFEAVVILLLSFPKYKML
>1170768.4.peg.1071
MPTESQAPASMFTWSDIWQQIQQRLKGGDRLRQPNSVLHLVAKDRLLDSRQLQQMKDTLN
EMQLELKLVVTSRRQTAIAACTIGCSVEQTKIQTSFGNDSQENPKALADALYLETTIRSG
GEIRHPGTVVILGDINPGGIVIAEGDIMVWGRLRGVAHAGAGGNRESLIMALQMEPTQIR
IADALARSPEKSLTSFFPEVAYITNNGIRIARATSFSRNQLSKI
>1170768.4.peg.1072
MTTSGKGGVGKTTVSANLGMALAKTGRKVALVDADFGLRNLDLLLGLENRIVYTALEVLG
GECRLEQALVKDKRQPNLVLLPAAQNRTKDAVTPEQMKLLVDELAQKYEYVLIDSPAGIE
MGFKNAINAAREALIVTTPEISSVRDADRVVGLLEAQDIKKIHLIVNRIRPAMVRANDMM
SVEDVQEILAIPLIGVVPDDERVIVSTNRGEPLVLSETPSLAAVAFENIARRLQGGTVEF
LDLDSANDNILLRLRKLLWK
>1170768.4.peg.1074
MSSNLASQLRVGTKKAHTMAENVGFVKCFLKGVVEKNSYRKLVANFYFIYSAMEEEMEKH
KTHPVVGKIYFPELNRKRSLEEDLAYYYGYNWREQIQLSPAGESYVKRIREISATAPELL
VAHSYTRYLGDLSGGQILKNIAVTAMNLTAGEGTSFYEFADISDEKSFKTKYRQNLDAMP
IDNQTGDRIVEEANAAFSINMKMFQELEGNLIKAIGIMVYNALTRKRSKGSTELVIAK
>1170768.4.peg.1078
MLSSAIFVVETYNISPDARTELSILNTCILAIFSGEYLLRLWSAKQKSKYFFSVYSIIDL
MAIIPYFIGFVDIRFIRLLRWLRILRLLRFIDKKFLFFSISEDTVIFARILFTLFAIIFV
YSGLIYQVEHAINPEGFNTFLDAFYFSVVTMTTVGFGDVTPVSEWGRLLTVFMILTGVAL
IPWQVGDLIKRFLKTSNQVENTCSRCGLAFHDSDALFCKRCGATLPQVSPSSQYQ
>1170768.4.peg.1083
MSGELDEYGMVLNLSDVKQVIKSEVTSELDFSYLNEVWEEFQATLPTTENIARVIWNRLT
PHLPLVRVQLFEHPQLWTEYEGKGEKISLTARSHFSAAHRLAPNLSAEKYGKFIRTHGHN
YHLEVTVEGEMDGRTGMIVDLGCLHETVEREILELFDHSCINEDIPYFSTSHIVPTTENI

ARYMSDLLQFPISELGVKLSRVKLFESDHLWVEYEGKDSEIFFSVATGFSAAHRLADPTL
SLEKNQTIYGKCSRINGHGHNYHLEVTVRGEIDSVTGMSVDLVGLNQIIQHYVIEPMDHS
FLNQDLPYFTEVVPTAENIAVYISDVVRSPIEELGAKLHKVKLIESPNNSCEIYAKDIEE
SRVDRVDRELAVV
>1170768.4.peg.1090
MNHAKFESDLAQLQDEVSNCERDVLNLIKGKKTMEYSQNHNGGNQSNQIAIIGMASLFPQ
SRNLQEYWQVIMDKIDCITDVPASRWSVEDYYDPNPKTPDKTYCKRGGFIPDIDFNPMEF
GLPPNILEVTDVSQLLGLVVAKAAMEDAGYGESQQFDRDRTGVILGVAIGRQLAVPLGSR
LQYPIWKKVFKNCGLSDDETEKVIEKLKSAYIQWEENAFPGMLANVISGRIANRLDLGGT
NCVVDAACASSLGALNMAISELLAHRADMMITGGVDTDNSIFAYMCFSKTPAVSPSENVR
PFDVNSDGMMLGEGVGMLVLKRLEDAVKDGDRIYAVIKGIGSSSDGKYKSIYAPHSQGQV
KAIRRAYENAGFAPQTVGLIEAHGTGTMVGDPTEFISINQVFGDNNSLKQHIALGTVKSQ
IGHTKAAAGAASLIKTALALHHKVLPPTINVTQPHPKLNIENSPFYLNTETRPWISNQPR
RAGVSAFGFGGTNYHVVLEEYESEHHQSYRLHNCAKSIFLSAPTTPELLSQCQHLYQQLE
STHKEQHYQRIIAESEQLIIPVDHARVGFTTLSLSQAIAHLAIIIDLLKNQPSVDFWEHP
KGIYYRQQGMETTGKVVALFSGQGSQYLEMGRELVINFPWLRQTYSHLDDLFSREGLESL
SQVVFPAPVFSPQERQEQLEKLQKTEYAQPAIGAFSAGLYKILQQAGLKVDFVAGHSFGE
LTALWSAGVLTEEDYFFLVKARGKAMSTSPEVDAGGMLAVKGNISQVTEFIKDFPQVAIA
NYNSPQQIVLAGNKSEITQLQNVLQAQGFSCFLLGVSAAFHTPLVSHAQKPFAHAIAQVN
FQPPRIPVYSNVTGKLYPNEPGSMQKILQEHLLNQVLFQQQIENIYQAGGNCFIEFGPKN
ILTNLVKEILVDKPHVAVALNANYRQDSDLLVREAVTKLRVFGVPLKNLDPYQIPAKISS
ASQKNQQKTLNIRLNATNINDRSQKAFAQALATGSVIKMPAVSENNYQTQPEKIRETLVE
TSTKINPEISSSSMLTSQIIPAVKNHSSVEPKMEISLDNYDRVLNSLEQSLVELTRQQSE
VNQVHEQSLQNQIEYNKTFYELMRQQSLFLAQEEINEYQSQTQQLAISSTERSMMRLHDH
QAETIRIHEKYLNYQQEYTNNYFQLLEQHYSLLEVGSPNAYSHLPSSHVSQSPAQKLMDS
LETENNSQNNLPDITVGFPIATYLDKEKLRETLINIVSDKTGYPVEMLDLSMDIEADLGI
DSIKRVEILGGLLELYPDLPRPNPEELAQLATLEQIAEYINNLITQLGQNQPLEETVSEH
HNHPQFLVLPHEELHEDLSDRSTTITIPDDLSQILLTIVSDKTGYPVEMLDLSMDMEADL
GIDSIKRVEILGGLLELYPDLPRPNPEELAQLRTLGEIADYMRNQAETVGRSNLSTSEKP
DTSTTEVADKILRLPVQLKTLPQPDSLDLTIPENHFVLITNDGSEVTHRLVAKLTDKGCK
TVVLTFPCLESNLSEEIAQIRLNDWHEETLQEHLTELTTKFGCVGGFIHLHPYSNNNLGI
DQAIVQHVFLIAKHLKEDLNHLAKKERACFFAVVRLDGELGTAKTHNFSPISGGLFGLTK
SLNQEWPEVFCRTLDLSPDLDGDTTVKHILAELQDPNLLVTEVGYNKTDRFTLVAEPTKS
SIIPDSLNITKDQVFLVSGGAKGITAKCVIKLAEEYQCKFILLGRSSAEIEPVWSEGYED
ENELKRRIMEDFLSKGEKPTPIMVQKKYQTIASQREIHNTLKAINEAGGKAEYICVDITD
GMMLKEKLRPIIDQFGTITGIIHGAGNLADKRIEKKTVQDFETVYAAKVHGLENLLNIVE
TNQLEYLILFSSVVGFYGNVGQTDYAIANEILNKSAHVIKHHKHPNCHVVSINWGPWDSGM
VSPELQTAFAQRGIETIPQELGSSILVDQLRNSDSTMTQVVIGSPLVYIPSTLSSELKTH
QITRQLKLNYNPFLQDHVIAGNPVLPATCGLSWISSSCEQLYPGFQTFHCPNFKVLKGIV
FDQNSPHEYILEIQEVAKIDNQEIHLVGKISSVTNHGKIRYHFSSNLILKRQIPLADNYE
LFNLTQDGQFLASNSLLYQTGVGSLFHGNTFQGVKSVLNISPGKLTMKCELPEPTLNQQG
QFRVQTLNPYIADVQVHSLWIWTQHFHQVGCLPSEIENFEQFAPVPFGETFYVTCEIKSK
TESYVVADVITHNQKGQVYNRMKGAKATILPNS
>1170768.4.peg.1091
MREELLKLPKGYSFYEDELVGFLSKRRWPDTERRIDRTTFWRWRNDNGIEHQKVFSRLDV
LKLCQICDHYRVDGTRNEYLALMKKKKELMLNK
>1170768.4.peg.1116
MDLINEAKEILIQRQDTHLDSLAERLRENRVQAIIEPILSGEELPNTPEDDRRYLLDLGL
VIRSKEGGLKIANPIYQEVIPRVLSQGTQDSLPMIQPSWLMPTGELNPQALLEAFLEFWC
QHGEPLFKSTPYPEIAPHLVLMAFLHRVVNGGGTLEREYAIGSGRMDICLRYGQVVLAME
LKVWRKGSKDPLTAGLKQLDKYLSGLNLKTGWLVIFDRRPNLPPVSDRTTTEMAISPQGR
SITVIRG

>1170768.4.peg.1118
MPKKPSHPYTEPLAFERLLLLIATLLKYPGVGSPDFLEFSNNKNHDAIGVVKIYLQQIAK
DLNIKLPDNYPAVDTLRKDLKTLRRYGILDQRMYRWGYYLGTGALSITELKVAFNTLASQ
AQYQGDAQTRRIYETLSKRLRSLDTELKGEFFYPIRQHLNRAIIHTDPEEMAAKGENRDT
LFHQLPLLEQAISQGQAIEISRTKSFYGSGNVGRFQVFPLQLIYHDIAWYLLYELCESGH
LVMGRINRFNSYCSLVKIPARGIKKQRESLAKAYQLLENGWGLNLGEPEPQQLELAGNLK
FIEVEVRFYPPISNFIVEGERRHPNQKIVLGEPDKNSGEHKFIEYSIKLPPRSLNEFIQW
VYRYMDKAQILSPSELIEQHHQLAQNLYNRYQNV
>1170768.4.peg.1130
MNFNRLGKFDQLSASVWKIGEVLQILMGNYCFFFLKTADCDTIRVNLAG
>1170768.4.peg.1132
MPYSHRSTKKQRKYNQELTARRFASTVPAQTLSWLSSISLLSGGFVFAQTESPADNIVST
VEVSQPTTLGDRGQNGQGSLPLESKVTELKTGVQTQGKNSEVNSNLESVATERNSESKSE
SSPTSVILKVPAPPTLKSQPETLPTSTQPIPETSTLVIPVTSQPNNSDNGNTGKDYNSTQ
IDPTEYNGNSTVKYDAPSSVEVTGVTQDCKAVISPTGKSVGTCGGKNSTNPSVANFTQKS
APTWLKKSAPGNLGKSPLKTVPSETPLSTNNSNPRPQSEKSEEIVNSLAGTVNNSQNWHS
LGSNSSSGHTSAGKQDYTGRISTKTSYNRPRIPDPREFSSGTTVTPVTPSFGTLPPPMIE
GKVAPRPSKVAYDFDLASVLPQVPYISSFADNGTNSGVTFPLSFAAPITSLFGWRTHPIT
GDRRFHAGMDIAAPTGTPILAAEKGQVEAADWMGGYGLAVTINHNQRQQTLYGHMSEILV
RPGQWVEPGMIIGRVGSTGNSTGPHLHFEVRHLTDNGWVAVDPSMQLQAGMNSLYNRVAY
NYNPQSKQLR
>1170768.4.peg.1138
MVIANNRFDSNPTNRAIAVPGNDIEILLTPDQYRRIEETGQERHEYADGRIMIMPGGSEV
HSRITVDITTFLNLALRDSSFET
>1170768.4.peg.1139
MRLNNSDLRIWLPSLNHGTYADIFVIDGSPNRRDEVLNPLLIIEVLSPSTEKYDRGDKFR
KYRSLPSFIEYVLVAQDEPYVELYYKQHGEKNNLWQLEIYDQIEQSVIFHSINVEVPIRE
IYRRTKLP
>1170768.4.peg.1147
MSLFSWRFFPMQLVIFSESVYSVEVVGTPRRHDLKQIGSDRKTT
>1170768.4.peg.1148
MMKNLKELPSIQSAKVTVLHVVPSQSTAAAMTEKWEEGGKILANAIQSLNFDPSQVSSIL
RQGDPKDVVCQVADEIGVDLIVMGSRGLKRLESILSNSVSQYVFQLSSRPMLLVKDDIYV
KRIKRVMVAVDGSPSSSQCLQLALFLLSGVESGQLILTNVNTDLGGKISGITDIKPERNS
VLGNAVAIAESRGIPVRCVTSSGKPGEEICRLAQELNADLLLLGSPDRRPSIAKSFVDLD
RLLGSSLSDYVRVNATCPVLLARTVS
>1170768.4.peg.1162
MVFSFYGLNLSALNFGDFSCDHKRDARANFPWFGDGRVSRRKVVFEVCHKFN
>1170768.4.peg.1165
MGLKSIYNQGNLEPRGPWFWSRNWPWFRNWPETSTHQVNF
>1170768.4.peg.1171
MRGSGTGSSFIGLINSRLFNFPWFGDGRIRVVLFLLGVVILYQWIF
>1170768.4.peg.1172
MESPRHKLQLEILTETLTSWLEQREDGFIGGEMFVYFSANEVKTEDFKGPDFFTVLGVPK
GERKSWVVWQEGKSPDVIVEP
>1170768.4.peg.1173
MGSQIVITYYKPIQNNQPPFLRGVGGIPNSNNLLQTNPKELAPLFKGGWGDPM
>1170768.4.peg.1175
MLKQYSPEEVLKQYSPEEIKQYSPKEVLKQYSLQDLLEGLSPETLERLAIILSQLGVNQI
KNQEQ
>1170768.4.peg.1176
MSPCNSKGEMTRKKLFYLKHGVEEYYVYDPDEISLEVSIRENNSFREVEDFATWTSPRLN

IRFDMTGDELVIYYPDGSRFLSPVELSNYAEQERFLKEQERFLKEQANERAEQERFLKEQ
ERLLKEQEQLKYQTLLSQLKAKGIDITALE
>1170768.4.peg.1178
MNPIELEWKHLKKDELSGKMFEDELELAYAVMDGVNARGKRNKHSTERIKFNNSCLSQPF
VT
>1170768.4.peg.1179
MQVPVYIIFDPDGGLIEYYELRNGRYELKQPDENGRHWIGSMELFLGTWQGTKEGRTGYW
LRWWEQTGNLLPWALELIEQERQLAEQERQRAEREHQRAEQERQEKEREHQRAEQERQEK
ERLIAYLRSQGIDPNNLPNHTE
>1170768.4.peg.1180
MLPRIGQDSGIYWRFTEQGVFILQNCDRQNLFTINVWAGF
>1170768.4.peg.1181
MWFWNIGCDRMAVNSVHLRSTPYERLQSTRTCAIALYPR
>1170768.4.peg.1186
MVFSFYGLNLSALNFGDFSCDHKRDARAKIANQRAHKLWESHQRVRKITKEIPVTVEPRI
PMDLLGVYILHPS
>1170768.4.peg.1188
MFNLKAPLSKQDVFKVGGKLQNDFLKTLFHTQDRPFPIQHNQQELDLGMLEYGVTSLNRI
GNKLV
>1170768.4.peg.1194
MSDRTLWIISGIIAIITFIVPIITTGLKPLWAKVVTVISLFIILVCVIFPLTISKLEITY
PVNKGTVTKEGYTIKSYDGKELVTIQVKITGKDLPKGKYVQLITSQAGGDESWVNGGSIP
SDVIGETDFSIDNVTFGEKTDQQENYYLYLIMTNKQLRSGDVFNNNEIPDYIDKSNVVEI
RVQKK
>1170768.4.peg.1198
MVREIAPKTQLGVIKSKVNQGGETIAPDIVYPESDGKPMADNTKQFTWIVKIKENLEVLF
KSNPDVFVAGDLFWYPVKGSNRIKLAPDTMVVFGRPKGQRGSYRQWEEDNIPPQVVFEIL
SPCNSKGEMTRKKLFYLKHGVEEYYVYDPDEISLEVSIRENNSFREVEDFATWTSPRLNI
RFDMTGDELVIYYPDGSRFLSPVELSNYAEQERFLKEQANQRAEQERLLKEQANQRAEQE
RLLREQERFLKEQANQRAEQERLLREQERFLKEQANQRAEQERLLKEQEQLKYQTLLSQL
KAKGIDITTLE
>1170768.4.peg.1199
MVREIAPKTQLGVIKSKVNQGGETIAPDIVYPESDGKPMADNTKQFAWIVKIKENLEILF
KSNPDVFVAGDLFWYPVKGSNRIKLAPDTMVVFGRPKGQRGSYRQWEEDNIPPQVVFEIL
SPCNSKGEMTRKKLFYLKHGVEEYYVYDPDEMSLEVSIRENNSFREVEDFATWTSPRLNI
RFDMTGDELVIYYPDGSRFLSPVELSNYAEQERFLKEQANQRAEQERLLREQERFLKEQA
NQRAEQERLLREQERFLKEQANQRAEQERLLKEQEQLKYQTLLSQLKAKGIDITALE
>1170768.4.peg.1209
MAFPSTGFANDYIDPGGKIDGQVFEDCEVFQSQICDHI
>1170768.4.peg.1211
MKLRSKQIPPHKIHVALQLIFLKLYILQHTNIRLHWLAQKPPILFPRLYHQRKRSQLPRP
LINLNAIEVVGENLPWNLGRLIPLLFVNGIEQIEGIHQHVPTTTGRVAYLDLLRLTDPQE
IRLRFFRLDVKLHSLCQPRVRPIQHPQPPQGIFHQIPNNPVWGKKLRRCWNILWRNLLIL
LQPLEYLVLLLRDVVLVQPPNHLNILTDIGSHRLTGIGKNRVPRQQVIRQQELGVIVDAL
EQEGHGLVQKVAGRHHQQTVGLALRVSAGYFAVEQSEDLLPDLVVDHLVVDVPHLGFGQN
LGLELSGVGWRDDIHPVIAVHIHEAQGAKAVEPDVGDPLGNLFLAVLLNRLFELLDGLST
FTPCCAISGQHQFQLGEDVFHQITSSILSEAL
>1170768.4.peg.1219
MLSLKRQQREIAETLAIQEREKKEKLAAHLRALGINPDEI
>1170768.4.peg.1220
MPAGKALLQIGTSQLPITAEELKVLEKIAKAGKKIVPSAIKITKLKSDQKEVILKTLGER
GLIKTELKIKKNQAEVWLTDRALEFLRSEYIPSKTYSPVISLELLGNYIRFLRKNAIPAD

PSGAKPVVDKITDRKITDEEILQTIKNLDRELGTENYLPIFHLREKLQPPLLRDEVDQAL
YRLQKSDKIDFSSLQEVTAYTPEQIDAGIPQNIGGQLFFIMVN
>1170768.4.peg.1222
MHIHKITLTNFRGATSLSICLHEHLNVIIGENGTGKSTVLDAMAILLSWIISGIRSNNRN
SRMGHLHISEDDISNNQGLASIEITCIDDGETINWRLEKSREESGSPEPTGEGLSNYTKR
VRHKTISDNNQNNIPVFVYYRLNRAVSGTTSETANISNQMTAYDSALTKGADFKNFFKWF
RTREDLENECKIRQGYDYIDPQLKAVRNALSQILPEFTHLNIRRDPLRMEVDKEGRTLKF
DQLSDGERFMISMVSDLARRMAIANPKLENPLKGKGIILIDEIDLHLHPRWQRMIVPKLA
EIFSACQFIFSTHSPSIITHVHPENILLLKLEKGVISVSNPSNSYLSAIYQMIDNNNLAL
GSTKEQFEREVKWAMAIKLFEMKWLSSGMAASLLGVNRTTFLMKLADYNIPLIDLTEEEL
LSDLANA
>1170768.4.peg.1228
MFSPGVLSVANFVAILSMAFIAFLSVTLQYHKFLRKGDRTGILILCNLCFPSSPKCDCEA
PVPWE
>1170768.4.peg.1229
MGPQTPSIYQGTEQEFDGALGWHEDENKPDYMGHDCYVREDMFDNPACTWKFGPINRKTF
QPYNKNHPTFILIHKLAETRWQYKLIKNGRRTLLYENHKIRCVLIHDPAEKYSFLVAMNM
GKDIFDFCFKPPHGYGDFTQLKILVVTSGEATKLSTGEIHLQLPAFAFVLAQVF
>1170768.4.peg.1230
MHYFDEYCEVHLDPDIAPYNWVFRSQEKISITFGVHVSKHPDFGQVVYELNDGKGYCDID
RITTQTEGIIENGFKMFSVILPPIGNGGGYQYRVGYIDIDGVEHISEISHFLFVCDEAPR
SMREIPSVFLGMVNNHHVYGPKPSVPMTSSPKNSNSRLFYSIMIDRFARSKHPIRSRLSL
VNYDLSCPHSSHGGTIAGVTEHLDYLKSLGIRVIILSPVYVNAPDGYHGYHPIHLLMVDP
RLGTIHSLRQLVKKAHELDIAIVLDVIVNHIADSINWPEYGGPPQGEFKYIQGDESAVMP
FPIEARNTLLFHGPEYTDMVNQRLFGFLV
>1170768.4.peg.1244
MGGTQTAGVTGGISEALVSTATGLIVAIFTLLFANSFRGLYQRQIALIQEYGGQLELLYR
RRYERGDKSYASTR
>1170768.4.peg.1259
MALIVQKYGGTSVGSVERIQAVARRIHGTAQVGNSVVVVVSAMGKTTDGLVKLAHEISPS
PTRREMDMLLSTGEQVTIALLSMALQEIGQPAISLTGAQVGIVTEAEHTRARILHIETER
LISHLNAGQVVVVAGFQGISNTSAMEITTLGRGGSDTSAVALAAALKADFCEIYTDVPGI
LTTDPRLVPEAQLMKEITCDEMLELASLGAKVLHPRAVEIAKNYGVPLVVRSSWTDQPGT
WVTSNKVQERAMINLELARPVDAVEFDIDHAKISLLRVPDRPGVAARLFNEIADQQVDVD
LIIQSIHEGNSNDIAFTVNTPILNRAEAVASAIAPALRNNDGSNEAEVFVERNTAKISIS
GAGMIGRPGVAAKMFTALAKAGVNIQMISTSEVKVSCLVAAVDCDRAILSLCQEFEVNAS
TRNVSSSNSIYSTVCGVALDMNQSRLAIRHVPDQPGIAAKLFGLLAESNISVDMIIQSQR
CRVIDGVPCRDIAFTTNRTDGENAQAKISQVAAQLGWGEVILDQAIAKVSVVGSGMVGQP
GVAAKMFTALAQNQINIQMITTSEIKISCVVSEEEGVKALQIIHTAFGLAGTHKFVVPA
>1170768.4.peg.1264
MVSKNFWKPIIHSSTALVLLTTLNTAWPLVSLAQSKPQPKANSAASSLFTDYLLGGGDRI
RVNVFEAPEYTGEYQIPPGGEINMPLIGSIPVSGLTTQQAADEIARRYARFLKRPLISVN
LLAPRPINVFVAGEVTRPGSYSLSLQGTGGNNPGVQYPTVLAALTTAEGVTLAADVTKVQ
LRRQVGRSGEQVVSLDLKQITQTGRIPIDITLRDGDTIFVPTATDFNVAEARNLFAASYA
ASRTAPRRVAITGQVYRPGSYLVAAGGGGGNDSGGLPTVMRAIQLSGGITSQADVRNIKV
RRPTRTDKEQTLNINLWELLQSGDLNQDVVVQDGDTIIVPTATQVNTAEVTQLATTTLSP
ATIKVGVVGEVKRPGVTELQPNSSLNQALLAAGGFNDARASSSSVDLVRLNPNGTVTKRA
VKVDLSKGINEETNPILRNNDVIVVNRSVLARTGDTLGAVTAPLAPVFSIISLFRLLGF
>1170768.4.peg.1265
MNRLEAAEFAFGWGFDWAKLTNGQAVLRVVSKTRAVLEWIIGFQKFLLTIFT
>1170768.4.peg.1266
MGPNGAGKTTLLKILLGISRPSSGKGLLLGKPLGDRSVKQFIGYLPENPYFYDYLTGYEL

LELTGGVFRISSRVQRQRIVHLLELVGLSQNQARKQRIGSYSKGMVQRIGLAQALINDPE
VLFLDEPMSGLDPLAQYQMREIILSLKSAGKTIFLNSHMLGDVEQICDRVGILNQGEIIC
TGSLSELLGEGKSYHIRGQGGDKEILQKRLGEIQFEREAIWHGILKEDLYDFLASLRLMG
GKVIKISLCRQSLEDFFIQQLSQLPTSSKL
>1170768.4.peg.1269
MQDKALANVFRQMATGAFPPVVETFERNKTIFFPGDPAERVYFLLRGAVKLSRVYEAGEE
ITVALLRENSVFGVLSLLTGNKSDRFYHAVAFTGVELLSSPIEQVEQALKENPELSMLML
RGLSSRILQTEMMIETLAHRDMGSRLVSFLLILCRDFGVPCADGVTVDLKLSHQAIAEAI
GSTRVTVTRLLGDLREKKMISIHKKKITVHKPVALSKQFT
>1170768.4.peg.1270
MAIKQYLNFAPQTMVKSSKVYMEAFLAVGLRWPLGTSS
>1170768.4.peg.1272
MGNQTTAKTWKQQLEQELNPSLDIILVDERYTTLAARDRYWQMFPPRGLTNLVPKGLRQP
PRVIDDIVAILLIERYLEKIGG
>1170768.4.peg.1289
MGARLRVFLTSEEDKTLFNLRSADVPQKVKDRAEVIRLNAHGWYVEKIAAHFNWTSQTVR
EVLHKWEKFGLEGLWEKSGRGGKPKYKDSDIEFLEECLKKEPRTYNSVQLAQKLETERSV
KLSPDRLRRVLKKGDHLEKKQKKPQRKTRPRK
>1170768.4.peg.1293
MAYQPQGYQILIFIFLQIRKNKRKLLTAFLLFSGEGQNFNAVVGNMSTSQSDLIISQPPP
NLQ
>1170768.4.peg.1300
MIKIIITLLEFFASFIKLLLWILFLKTNLVVLYVAIYFFL
>1170768.4.peg.1301
MGTENLETARNLYSQGKIAFENGEYQQSVDNFEKATSLLLQNSRFAGEVNIWLVNAYEAT
GRSQEAIALCQQLSHHPHYEVKSQAKRLVYILKAPKLKRPKEWMTEIPDFATISEGQTKT
LIAPQKSTAPKKSPDSEYVDLSQVNTQDNLFIWVALIIAACTISYLVWLSF
>1170768.4.peg.1310
MVTSLVVSIFWEKIHGQCQEFPHSLTFNTKKSFFASLI
>1170768.4.peg.1317
MNIQCGYNSLNPSPSSIVPMVVEQSGMGERAFDIYSRLLRERIIFLGTAIDDNVANSIVA
QLLFLDAEDPEKDVQLYINSPGGSVYAGMAIYDTIQQIRPDVVTICFGLAASMGAFLLTA
GTAGKRMSLPDSRIMIHQPLGGAQGQAIDIEIQAREILYIKANLNQLMSKHTGQPLERIE
ADTERDFFMSPQEAKDYGLIDQVISRQNLPSSGAPVTIVK
>1170768.4.peg.1319
MQFGEVCGGNLNRHCPLRYKPYFIEKLFLANLISILLMVIFCYNRGD
>1170768.4.peg.1323
MVEEEDRVLPLWPHRDWEGWKSTNCHLSPGMLRFVTLVIKPM
>1170768.4.peg.1326
MLEREYHEVWYKQVTSTSHYRPYLAYLLANLAEKFNTDGKIDDKFSVDGLK
>1170768.4.peg.1335
MVTRLETSPASRTPEQGGLLLWVVTKWDGFLRGFRLFLPSKVANNVSDGFLTMFFAFLAV
LAIGVTATSLISQIGSPSVPQLSQPPTDSTVEGKTQTQLSQPPTDSTVEGKTQTQLGQPP
TDSTVEGKTQTQLSQPPTDSTVEDKTETQLGQPPTDSTVEGKTETQLGQPPTDSILKSQA
ELIPQQEVTGFIEKQLKEITATNIITKDKQRIEVEIVQSIKTNFRISEIVIKTTEAWYKL
ESSQQEKLAAKILKSCQEMDLIHVKILNARNQLIARSPVVGTKMVFFQFPNS
>1170768.4.peg.1337
MTTHFITAEIDLQENPAELQQTIEAEIKKQGQPLRWAVTSVDNTRAKATVEAVVTR
>1170768.4.peg.1343
MGVIGVGNMGQHHARLLSSMKDVELVGVSDINVERGIETASRYKVRFFEEYCDLLPHVDA
VCIVVPTRLHYAVGINCLLAGIHVLIEKPIAASIPEAESLVNAAAQSQCILQVGHIERFN
PAFRELSQVLKTEEVLALESHRMSPYSSRANDVSVVLDLMIHDIDLLLELAGSSVVKLTA

NGTRSLDSGYLDYVTATLGFANGIVATLTASKVTHRKIRHIVAHCKNSFTEADFLNNEIF
IHRQNTNPQNDRQTLYRQDGIIEKVYTSNIQPLSAELEHFVNCVRGGNQPSVGGEQALKA
LRLASLIEQMALEERVWNPLEWQSESVSQSLTSSV
>1170768.4.peg.1346
MLMTGSVSAFLGFSMGSSALKGVTSPDGRPTSKLISSKNNDLQSVPISFLKEEDIINQVK
KRIEQNKTKNNQTKKVEEEEETVYTKDQSQQKAQEISEEPPQSGFPVVAESEGVNMSVRS
ASYSGGQLILKVKMHNQSNESVRFLYSFLDVTDDKGRVLTATTDGLPAELPGNGPIFTGK
ISIPTALLNDVSSLTLSLTDYPAQKLKLQLSDIPVEK
>1170768.4.peg.1380
MGINPIPYFSELPGIKPTKKFSGMGIIKNCCYKNSVTDK
>1170768.4.peg.1384
MDLHDLAEGLPTDYEKLMTVAAETYEIYKELDRLKQQLKTMETS
>1170768.4.peg.1392
MNPTYLLLMIVDLFWNQTSQVGFLIVTKKKATRSEVS
>1170768.4.peg.1398
MHLLSVNADFSYNSIYALGVVTTFDRFMHGYQPSEDLVSIFNAIICAVEQDPQAYRQDAA
KLKAIANSFSVKDLIAWCSQTTPLDQDANLQGELQAIAQNPNFKYSRLLAIGLFSLLELS
DPEFVKDETQRNQAIAVIAQGLKLSEDKLNKDLDLYRSNLDKMEQALIVMADVLAADRKK
RDQRQQNSGNPTLPPTNEN
>1170768.4.peg.1399
MHFIDSSGLTCLVAGMRNVDKLKGTFRICNIHPDAKLVFEVTMMDTVFEICETEEEAFAI
PF
>1170768.4.peg.1405
MFLPLPQFATEDRHPDHIAEVIETSSTEFLAQCLDPEDLRFPLMPAFGSWISAFDEESDN
QIYAVVYYATTAPIDSVHRARALGLSLKNLRQEQPQIFAMLKTEFRAAIVGFEAPSDIST
QRQMYHYLPPRPPQIHQAVYRCRPESMLRFTDNMDFLRTLLLVPGAPVESLAASAIREVY
KLRKLDREWLVKACRYLSLLLKDDYDQLRFILGQIHP
>1170768.4.peg.1408
MGSFAVGVLAFRPQVGKLADHQGRKLVLLIGMVVATIAPLGYLAVKSLVGLMLIRAFHGI
SIAAFATAYIALVSDLAPDDRRGEIIGYMSLVNPIGVAVGPALGGYLQAIAGYTPLFIFS
SLLAGLGLICVIPITNPPRWKNNKQEKQEKQETGDDFWGILISPRVRVPAIILLMIGFSI
GTIHTFIALYIKSIGVDLNPGLFFAAAAISSFVIRLFVGRASDKYGRGLFVTLSLISYTV
AMLIIWQANSSPTLLLGAIMEGAASGIAIPMISAMMTDRALPHERGRIFSVSLTGFDLGL
GIAGPVVGYIAQSTSYRHVFGLSFGLTLLAILIFISQSNHGVFQSLRFALGRSKDVYQQS
ATDSPNLSTNT
>1170768.4.peg.1409
MYNGNVGNRDAKADQNNKPHVKITTKFFLKSGSKLGEFLTLNTT
>1170768.4.peg.1423
MPIVGWLWLRGKCADCQSKISYSYPLVELLTGIIFLIVFWVFQFSLLTIGYWVFCSWLLA
LSLIDWETMTLPGSLTKSGLVLGLIFQTTLGYVAHNTWSSAIGQLIWGIGGMVLGLWLFD
IITSLGFLFYGKPVMGGGDGKLAAMMGAWLGWQYLLVASFIACFSGVLIGCGAIIVCDGQ
IEQKMPFGPFLAWGSVISIFGGEIILDHYLRFVLSHS
>1170768.4.peg.1425
MGSATAVELDEATRTVKLNEAGDTIVISPKQLQQGKRLFQDTCAQCHAGGVTKTNQNVGL
EPEALAGAVPARDNIEGLVDFLKEPKTYDGELDISEIHPGLKSADIFTEMKNLTEDDLKE
ISAYILIQPKVVGTRWGGGKIYY
>1170768.4.peg.1435
MSNSSVLCLGEILFDCLADQIGLKLEEVNSWTPYPGGAPANVACALVKLGTKAGFIGAVG
QDDPGDTLVKLLGDVGVDTRGVQRHPTAPTREVYVVRDLNGDRTFAGFGKYHTSDFADTC
LKASDLPKELFNEADFLVLGTLELAYPESEAATHQALKLAERYDLKIVLDVNWRPVFWQD
SELAKQKIHQILPNCDFIKLTKEEGEWLFNTSDAGAITYRLNSLEGVLVTDGENGCSYCL
AENEGKLPAFSVPVVDTTGAGDSFLAGFVHQLNQYGIQALSDPQIAKSVITYASAVGALT

TINPGAIASQPTAQEVEDFLHSNKLSLI
>1170768.4.peg.1438
MPIDQGVWNGFLLILPIYQVFGDQMSSIYNIYPWFGDDFTRKR
>1170768.4.peg.1442
MEPFLDSPGSLKIDCEMSNALKRAWIRIAFDPTDKGKVFGRGGRNIQAIRTVVTAAAELA
GQSVYLDIYGNNSHGPDGSDEEEKIFPPKSRNTPGSFSKPRFRR
>1170768.4.peg.1455
MDWFLQPSHPHRARWLKSFVGDSLCVHDTRLKQNLFGLNFPNPLGLAAGFDKDAMGSHFW
SMFGFGLAELGTVTYHGQPGNPQPRLFRLPMDLAALNRMGFNNSGAAAMAARLTLLYQQQ
LPAIPIGINLGKSKVTPLEAAATDYLQSFRLLRNLGDYFVVNVSSPNTPGLRSLQDRPML
SQILEVIQTENSIPNQPKPLFVKIAPDLEWEAIVDIINLAKTYKLAGIIATNTTISREGL
KTQVIEKTGNPPQAEPGGISGLPLRQPSTEIIRFIYKQSQGQIPIIGVGGIFTAEDAWEK
ITAGASLIQVYTGWIYEGPMMASRILTGLLAKLQNHGLGSIGDAVGINN
>1170768.4.peg.1473
MTLIWYGEQDSPLMGFPQGEWKTLDNQLGKLNFYHWKDWFKLWHFDPPKSQIRA
>1170768.4.peg.1478
MQMAQAAGLRGRLLVTVGILILVRLGIFLPVPGIDRERFAQAISGNNAIFGLLDIFSGRG
LSTLGVFALGILPFINASIIIQLLTAAIPSLENLQKNEGEAGRRKISQITRYVSLGWAIL
QSTAFSALFLQQFALNPGPIFVAETAIALTAGSMFVMWASELITERGIGNGASLLIFVNI
VASLPKSLGDTIDLVQVGGREIVGRVIVLVLVFVATIIGIVFVQEGIRRIPIISARRQVG
RRVLAEQRSFLPLRLNQGGVMPIIFAAAILSLPLLIANFTKNVELANIVNTYLSPSGSSS
WVYALVYMVSIIFFSYFYSSLILNPVDVAQNLKKMGSSIPGIRPGKATSEYIERVSNRLT
FLGAMFLGLVAIIPTAVESTLNVPTFRGLGATSLLILVGVAIDTARQIQTYVISQRYEGM
VKQ
>1170768.4.peg.1501
MSVIWGCCSFYIIQILQPVIWSILTLRIKPINMLLTFVKIESDLKVSLF
>1170768.4.peg.1510
MSREEIRGVWVTSNDLNVFKDRAQVKDAVTKLRRLNFNTIYPVVWNSGYVMYPSNVAKSL
DIQPFVFRGSDGHDILADIINQAHSQNLLAIPWFEFGFMTPNTGELALNKPEWLTKMGDG
STVSMSAAGEVSWLNPFHPQVQKFIIDLLVELTNNYDIDGIQFDDHTSLPHQFGYDNYTV
NLYKQETGKNPPANSQDPEWVAWRANKITEFMVRLNHTIKQIKPKVIFSVSPNYYDHAYK
FQLQDWLNWVRLNIVDELVMQVYRDDLESFTSKIARNEIQEVRQIIPTGIGIMAGLRTSP
VPMQQITKQVRTVQREELGIVFFYYETMWNRSPETLEQRIQGFKNFFPYPAVRVAAE
>1170768.4.peg.1526
MVKFTRQLPAVINLQWVPSNTLYNVPQFPQVDRQTTV
>1170768.4.peg.1527
MFQIKCQYMKSRLESPGEAASVAYKDESFANSLFLRKLTTIL
>1170768.4.peg.1542
MVGEFSTDSYYIGCITGKLTNKSQEHHYMQLLAGDIGGTGTRLRLVEFSPSLGLRTLYED
NYRSADFDDLVPIVIRFLKAGQTSTGTIFDPKTACFAIAGPVVNNQVQLTNLPWFLDGER
LSRELDIPQVSLINDFAAVGYGILGLQSQDLITLQDVPPQPGAPIGVIGAGTGLGEAFLI
QQGENYQVFATEGGHGDFAPRNELEFNLLQYILNKHGIARSSIERVVSGLGIISIYQFLR
DTTGEAENPEIAQVVRNWENGQGGSDPAATIGTAALNNSDRLSIETMRIFVSCYGAEAHN
FALKLLPYGGLYIAGGIAPRNLPLMQNGNFIQNFVEGGTMTSLLQNIPVHIIVNEQVGLI
GAALFASRL
>1170768.4.peg.1546
MGATLQEISHSYYCQHYNSWVHHNNIIIIILRSPILL
>1170768.4.peg.1558
MKYVDEFRNPEKAQGLQKEIAQLSLQISRNSHKNKHLKIMEVCGGHTHALFKYGIEEILP
DNIELIHGPGCPVCIMPKGRIDDAIALCQAANIIFTTFGDAMRVPGSKTSLLQAKAEGAD
IRMVYSPLDSLKIAKENLTKEIVFFGLGFETTAPSTAFTILQAAAENITNFSLFSNHVLV
IPALEALLANPDLQLDGFVGPGHVSMVIGTEPYQFIPQRYHKPIVISGFEPLDIFQSIWM

LLKQIEENRCEVENQYNRLVEKGGNKNALQAINQVFTVREKFEWRGLGEIPNSGLKIRPE
YAQFDAEAKFTIPNLKVPDHKACQCGEILKGVLKPWECKVFGTACTPETPIGTCMVSSEG
ACAAYYKYGRLSILV
>1170768.4.peg.1559
MMLNFNYIKRGIKMCLGIPGQIIEISNPEHKLAIVKVGGVKRQVNIACIVDEEHPPEKCL
GDWVLVHVGFAMNRINEQEAAETLALLQEIANG
>1170768.4.peg.1563
MILRGELVTPESDLIDRTVYNGMFTMHGTVMLFLWTFPSLVGLANYLVPIMIGARDMAFP
RLNAAAFWMVPVVGILLMSSFAVPGGPAQAGWWAYPPVSLQNPTGNLINGQVLWLLAVAI
SGVSSIMGAVNFVTTIVKMRAPGMGFFKMPLFVWAVFSAQIIQLFGLPALTAGAVMLLFD
LTVGTGFFNPINGGNPVMFQHYFWFYSHPAVYVIILPVFGIFSEIFPVYSRKPLFGYKVV
AISSMLIAVVSAIVWVHHMYVSGTPGWMRLLFMLTTMFVSVPTGIKVFAWVGTIWGGKIK
LTTPMLFALGGLIMFVFAGITGIMLSSVPIDVHVNNTYFVVGHFHYVLYGTVTMGMYAAI
YHWFPKMTGRMFNEGWGRVHFWLAFIGTNLNFLPMHPLGLQGMLRRVASYAPEYTFWNIL
ASLGAFLLGMSTLPFIFNILVSWLDGEKAPPNPWRAIGLEWLISSPPSVENFEEIPIIIS
EPYGYGKSGPLTANLENSGMEMVE
>1170768.4.peg.1573
MMGWYLNENIWVYLGKYSGIPIDEEVDDLPVSIGITG
>1170768.4.peg.1574
MTPIDKIPAHFLPNYVILSHSMSSYVLNTLPKYHPMTINIILRVLNGNFNEGFIIDVITD
GKTEQLSIIQNRNIPKLYSKWQYILHRNTIRKISDIEEQETHISTEKLRNAKTELEKEYK
QWLNNQNIGMLISNMIELSTSSPSQSPQNPNLIRIQLILSDTLNPNAQQDNDILRRLPWN
AYLSSYIRFNNYDTTFLLSKDHTSTPSHPNLFSSNPPKILAIFGGHQGELACSQADEANR
LKEICEPNGATVIFRNITDRNEFHQLLINDNYDILFYSGHSTSDQGGTIQFEQETSIIRD
FKNDLIRAVKKGLKIAIFNSCDGLKIADFLIEEVGMPAVIVMKEPVPDEFAREFLERFLI
HFVEECNFLSVAVEKAAKDIRYKEDQEFPGATWLPITYYSNAKSEEFTWSKKNQIYKNRI
DIKIHNYLYQRQEENSLCPYQGLFHFGPEEAEYFFGRQIFIEQLYEATQNRKFIPILGAS
GSGKSSVVLAGLVPKLKNQGNWEFTYFRPGKEPFPALATALIPLLYTSELDTIEKMAQGR
ILATHLQTGTIPLSDVFTQIQQKHPTEKVLIIADQFEEIYTMCPEPETRQQFLDCLIASL
SIPNSPIVLVTTMRADFLSDALSYRPFGDTLQNADLKILAMNREELTEVIIKPAEKLGVS
FEQGLAARILNDLEQQPGNLPLLEFALTQLWNQHHEWQLTHNAYEKIGQVQGALARYADE
KYSNLTKTDQEKMQRVFIQLIKPGEGAEDTRRIAFKTEIGEENWLLVRQLADARLVVTSR
NATEQEIVEVVHEALIRDWGELRKWINNNREFRNWQERLRVAREQWRITQNDRESLMRGA
ALAQAEEKLRERPEELIDEREFIEESIKERKRTEEIENERKKIQVMTAWAIAAGSLVAVV
ISTTLWLIAIQQRNQAEVSQAESLSRYSSSLFTEDKQLEASINAIKARKLVQKKPGTHPL
VITALQNIFSTNQRHSQKHESSVRSVSISPDGKTLASGSLDKTIKIWDLATGNLINTLKG
HESWVLSVSISPDGKTLASGSLDKTIKIWDLATGNLINTLKGHESSVLSVSISPDGKTLA
SGSYDKTIKIWDLATGNLINTLKGHESWVLSVSISPDGKTLASGSDDKTIKIWDLATGNL
INTLKGHESSVLSVSISPDGKTLASGSDDKTIKIWDLATGNLINTLKGHESSVRSVSISP
DGKTLASGSDDKTIKIWDLATGNLINTLKGHESWVLSVSISPDGKTLASGSDDKTIKIWD
LATGNLINTLKGHESSVRSVSISPDGKTLASGSDDKTIKIWDLATGNLINTLKGHESWVL
SVSISPDGKTLASGSDDKTIKIWNINMDLDYLIGVNCNKIGNYLLYSNEVKEQDKSLCNN
>1170768.4.peg.1578
MSPCNSKGEMTRKKLFYLKHGVEEYYVYDPDEISLEVSIRENNSFREVEDFATWTSPRLN
IRFDMTGDELVIYYPDGSRFLSPVELSNYAEQERFLKEQERFLKEQANQRAEQERFLKEQ
ANERAEQERFLREQERLLKEQANERAEQERFLREQERFLKEQANQRAEQERLLKEQEQLK
YQTLLSQLKANGIDVTGLE
>1170768.4.peg.1598
MLTLTKVFIKFSSPVDIAQLFTYHINGAGKIKKKLKK
>1170768.4.peg.1601
MGGYFFANWFTFSHRHPASTPEEKFLSLIISLVTTVFWPLIIPISCVKILKTRQLRSDTV
IPLLLVMLGLIVSYISCQLLKLVYLPS

>1170768.4.peg.1614
MLPALKRRGFTENPKHNFCVELALIKHKPFPEKSKLKR
>1170768.4.peg.1615
MPTSLRTNHHLIAQTFHALSDPIRIGVIELLRHRELCVCDLCNTLDISQSKLSFHLKILK
QTNLVINRQEGRWIYYSLNLCQFQILEQYLQDIRIDSPIVPITSWCN
>1170768.4.peg.1618
MVTNADEILAQMMETCPEMRDEFNSTFKLKDDPRITKVGHFLRITSLDEFPQFWNVLKGD
MSVVGPRPLVVKELQKYGSHIDQVLTIRPGITGLWQVSGRNDIPYPKRVQIDLHYVRSKG
FLLDLWIILKTINIVIIPKNNGAY
>1170768.4.peg.1633
MVFRSAVRQQLLYDCGLDSFTLYNPKKLDNSGFYRLEVWK
>1170768.4.peg.1640
MYSILLKRQISIYIDNYLTVHKNMLKNYTKLSVNIRDQFYKGKIGNRGEGVGS
>1170768.4.peg.1644
MLVLTAGLFSLSLVPLSRTVIPGAARYTLVYDNGSNQAVIATSPDITPTQLEATLRQAAS
NLYSYGRMGAGGNNSLTVRARTILHPESGISTPVYLGKVERTLVTREDPQMLVEVFLDKF
PQLPPS
>1170768.4.peg.1647
MAAATIYLPYLLVAYARVKVGYDLSAPRSMFDKLPPYARRATWAHQNCFESFMIYAPAAL
MAYVTGVDSQLALYAAIAYVTARVLFSVFYILNVPILRSLMFGVGSACIINLFVLSILRA
TT
>1170768.4.peg.1649
MNLAELGRMEILDNAVEQEEPSLDNLEAVVFEDSSILESLESDERDGDEMGAARPSGYNK
TEHDDAVGAFFKEMARYPLLKPDEEVELARRVRFLEEVKDLQAALEEQLGHSASKSEVAS
KFDLNEKQLESRLYQGRVAKRKMIRSNLRLVVSIAKRYLNRGVPFLDLIQEGAMGLNRAT
EKFDPDKGYKFSTYAYWWIRQAITRAIANDARTIRLPIHIVEKLNKLKKAQRELKQKFAR
NPTEVEMAEFLEITVQQLRQLQQLRRQALSLNHRVGKEEDTELMDLLEDEDNQSPEAKMN
ENMMRQEIWEVLGDVLTPREKDVISLRYGLTTSEPCTLEEVGNMFNLSRERVRQIQSKAM
RKLRRPHIAKRLKGWLI
>1170768.4.peg.1650
MSHKKNLHIYQEVEKPYYLLGKYKDKQKVNKNNKIEDLIFNILIFM
>1170768.4.peg.1659
MQAFFVGRAIAEVINERLEVTLTDALSELGKFDAEMKEQLHQFTEEVMARASRAAEASQT
GVTPSSTNANSVTDLQAEIDDLRAEVALLRTELQKYRSGAK
>1170768.4.peg.1669
MISTDILLEPTGYQRVLNKAVKWLNNNFRLRSMLTKVQQDRTTVRSDLKLQNQVQEWFEA
FCLRYLVQEGWSESQIYRMNLALAEGFTNAVRHAHRTLPPETNIEIELGLWVDRLEIRIW
DYGQPFNPDQIPEPQPGTLQDHGYGWFLIRRLADWVVYERTDNERNCLLIVKNRF
>1170768.4.peg.1672
MSGYNQPISGGREFFESLVRAISGYAQEFLSNVPNPQAHSKGSELVEVERINNDRHQLII
YSDSTDNRDNPLRIDMNTVGLFDLVEAVDQFFADSQTLPELILELQPVSRSYGMSGQLAL
RQAVPATVGVSSLAIAALALGIIPPPEWNPTTQNTQTSSGVPESSAASTTSPTPDSTVAS
NIQDLEALLNSVPEITDPSQLKSLNRQLYNRINSEWQNRGQIGENLVYRIGVATDGRIIG
YKSLNQSSSDQIEKTPLPRLLYNPVSGTSNKEPIAQFRVVFTNKAQLQVSPWIGYSGTPD
VIGDKITDPSSIRQLNRTLYQTIRQNWSIKPSFPRELRYRVAINKNGVIADYEPLNQVAF
DYYTETPLPEIFKNLYGSNLAAPKNREPLAHFRVLFKPNGVLEVSPWEGYR
>1170768.4.peg.1682
MMPFSTSNGTWNDPIVALFTSTSAVCVTGLSVVDPGTYFSFWGQLVILLLVQIGGLGYMT
TTTFLILLIGKRFDLRQKVAIQQALDRPGMSGSSQIIRSIIATTIIFEITGIFLLLPAFV
PDHGWSYGMWLAIFHSINSFNNAGFSLFKDNLIGYQTSLLVVFTVTGLIIFGGIGYQVIL
DMYLWLRDRLKRKTTFMVFSLDFKVAISTTLLLLMVGTVAFFLIEIRNPETFGKFTFSDQ
LLLAWFQSVTPRTAGFNTIDIGKMSDAGLFITIALMFIGASPGGTGGGIKTTTLRVLTSC

TKAILQGKEEVWLYERKIAINLILKAIGVVFGSLATVLSATVLISLTDPKLEFIQILFEV
VSAFGTVGLSTGITGSISTAAKIVIIVTMYIGRVGVLLSMSAILGDPRPSRVHYPEGNLL
VG
>1170768.4.peg.1694
MFCGNFPIQTAIALQGNQTSIKPYLDRVVKQLTEFTLDNGLKFIILERHQAPVVSFLTYA
NVGGIDEPDGQTGVAHFLEHLAFKGTRRIGTTNYKAEKPLLDKLEQLDSQIRAAKSENRT
EELEKLQKEFKTVEAEAGKLVKQNEMGQIVEQAGGVGLNANTSSEATRYFYSFPANKLEL
WMSLESERFLEPVFREFYKERDVILEERRMRVENSPVGLMVEKFTDVAFKVHPYRRPVIG
YDEDIRNLSPANVREFFNKYYVPSNLTIAVVGDVNPNQVKRLAKIYFGRYPAKPKAQAKI
APEPKQTSTREITVKLPSQPWYLEGYHRPSITDPDNAVYDIISSLLSNGRTSRLYKSLIE
TQRVALVAEGISGFPGDKYPNLMLFYALTAPGHTVDKLAIALGQEINKLQTQLVSEKELE
RVKTQARAGLLRSLDSNMGMAQQLLEYEVKTGSWRNLFKQLDDITKVTPADIQRVAQSTF
TAENRTIGKLLSQKS
>1170768.4.peg.1699
MENRVRTLREEFTRNFDPSQAQSIMREHSYRLQPNQRQIILVNPDRKTVEPSDSTQ
>1170768.4.peg.1701
MRGYFECENLKKSNYPYISAQANCPMKSNDKIFLAGSLGVLLTIYYYAICVPFRD
>1170768.4.peg.1721
MNYRSPETILQVANLSKSFILHQQGGVSLPVIQGVSFQLNQGESLVLSGASGSGKSTLIR
CIYGNYRPNNGEVWVKHEGNWIDLCQLAPHELLAVRQKTIGYVSQFLRVIPRVPAIEVAA
ELLLDLGEERKVALAKVKELFHRLHLPERLWQLSPTTFSGGEKQRVNIARALVVNFPILL
LDEPTSALDTHNCQIVMELLEERKIKGCALIGIFHNIDQGKNLSTNTAKKLFDRELFVHE
>1170768.4.peg.1723
MILTKTIVCKLSPCPFKIDFPQPPQEKNVRRSIKLRSPSVVLVVLSN
>1170768.4.peg.1729
MIGQNGCGKTTLLLLANGLYKPNRGVVSWCGEPLTYNRNYLGRLRQKVGLVFQDPEQQLV
ATTVEEDISYGLCNLGLPVVEIQQRVEEALMEFELTNLAQTPTHHLSLGQKKRVSIADVM
VLRPELLLLDEPTAYLDIKQTRNLIAMLNKIHEYGTTLVMATHDLDLVYRWADWVFVMDR
GKLVLEGSPQDVFSQRRLLEELELGLPLICEMLFYGLSDEWLEK
>1170768.4.peg.1733
MLLLLIANKVTSEVITTLGTASEEIFRGDRLPPLKFPETSCAEDRITG
>1170768.4.peg.1739
MLIIIILLKEIAKKHITIYNQGTTEGNFTENQGFLLSDNL
>1170768.4.peg.1741
MVLVGPSGCGKSTLLRLIAGLEVMTGGNIWIGNDLINDLPPKARDIAMVFQNYALYPHLT
VYENIAFGLRRRLPINHTSTKGTVGRWGENLLMGLTQKLPKQLRYQTQTEKIVEQQVLKV
AHLLQIDTMLHRLPKQLSGGQRQRVALGRAIARDPQVFLMDEPLSNLDAKLRAETRVQIV
KLQRQLGTTTIYVTHDQTEAMTMGDRIAIMNMGQIQQVARPLEIYNYPKNRFVAEFIGSP
PMNFISVEFYAPLLITHSNFRFTLPNEWGKALQKYSGQTVILGIRPEHLMLSAPATKNIP
VKVDLVENLGNDSFLSVRVINPDLPKLDGQILQVRVPSDRLINMGDQIWLSPIVQKLHFF
DPETELAIFTDNKTINF
>1170768.4.peg.1761
MVRDVDTQTWEFLSQLPSKLSSDNLPLQIVTVTRSGVVQALNTGLKVVDGDLVSITDDDA
APHPDWLEKITAHFAKDSSIAAVGGRDWVHGDRIEQDSRAVVGRIQWFGRVIGNHHLGTG
KAREVDILKGVNMSFRIQSIGELTFDERMRGTGAQVHFEMAFTLALKRAGWKIIYDPQIA
VDHYPATRFDEDQRENFNEIALTNLVHNETLVLLEYLPPLRQIVFLLWAIFIGTRDALGI
VQWLRFLPSQGRIVNKKLYASLRGRWQAWQTNKTNPLIIFNS
>1170768.4.peg.1767
MTPTDVKDLAARLELDNYSNAFDGLNDWHLLRAIAFQRPELVEPYVYLLLDLEPYDEG
>1170768.4.peg.1769
MFSRMLGKEENSFFPLSSKYYWFFEDFVNTEKLRFQNSIFLF
>1170768.4.peg.1772

MVVYSVPENSGMGRPQHIYHLSPGGKEYLQRSTTKVSGGYGEFAVSLLDTLAETVGTEQV
KTILQKQWERKAQEYQEKVGQGCLEERVATLVELRKAEGFMAEFRPVEEKDDGHEGNHGF
LFMEHTCAISNVAESFPSVCGHELEMFAAILPDCSVQRTHWLIDGQHRCVYLIKKN
>1170768.4.peg.1781
MHQAFDNLLIDWQQNYKLLNYGRITLSYHRDAIAPHGGQLINRVASPEQKEFFLAKAEFL
PRVILDERAVSDLEMIAIGGFSPLTGFMNQVDYNRVVEEMRLANGIVWSIPITLSVTEEV
ASPLQVGGLVRLDNSQGEYIGVLELSEKYTYNKKREAVNVYRTDQAQHPGVQVLYAQGSV
NLAGDIWLLQRDAHPHFPTYQIDPAASRQMFREKGWKTIVGFQTRNPIHRAHEYIQKCAL
ETVDGLFLHPLVGATKEDDIPADVRMRCYEILIEHYYPLDRVILAINPAAMRYAGPREAI
FHAVVRKNYGCTHFIVGRDHAGVGDYYGTYDAQYIFDEFEPSELGIVPMKFEHAFYCTRT
KQMATTKTSPSTPAERVHLSGTKVREMLRRGELPPPEFSRPEVAAELARSMRIGQPALV
>1170768.4.peg.1782
MDSIGKWHLSPDDTYPCYNEWKGYQEQTSATKDYGIITNSLIENNQPNFNTQYKQQEQGR
LGKVSEICLTYGLLKDNLNSVRNRQVLQTINQTQEKINGLEQENANIRTQYDSTLLEKIA
RQPDGKSINQVKAEEARQNLDRNTRQIAQLKEENKKSKNQLLNQPASVKFLSVLQDKTQF
SNLVSGYKTASFWYPSIQVTLQAIFLLPLIFIASLIYGRSQRQGYGLVSLISWHLLVIFV
IPLVIKVLEFLQVGVVFKFLLDIISSLLGGLLFLVSYVYIIVIPLLGFAIIKLLQRNVVF
NSKIQAGKRVQNSQCINCGRTLRSREAHCPHCGYYQYVECHHCHNLTYKKLPYCYHCGTE
QISL
>1170768.4.peg.1795
MLHAYFGGKIYGVENVPQTGGLVIVSNHASYFDPPIVSNCVCRPVAYMAKEELFKIPVLA
QAIKLYGAYPVSRGTADRGAIKYALEYLENGWAVGVFLEGTRTRDGRVHDPKRGAALLAA
KAKVPLLPVSLWGSEKIVQPGTPIPRKVPLTIRIGKLIATPSSTSKEELEAVTHQCANAI
NTLHDLGR
>1170768.4.peg.1796
MSDLREKLTENLDEAEWEWLVPHAQRDVIIVVASGLDLLDVGEAIASDNTQSVGVWIDEQ
LISKPSPIQLGDWNGDPTKRFNALIIEPYVLIQEK
>1170768.4.peg.1807
MVLLLAVAAFIGVSIIPILGTLNAPRPQNSPENPQLADQIRGYELVLQREPENQAVLKQL
LQARLQILSQKSNTQVQPADIQGVIETLQKLSRLNPDNLEYKVLLAQATQQIGNKQEAIQ
IYRSILRTQPGNIQSLQGIVKLELDQKRPEAAIQFLKETISNAEKSNSVQPGSFDIIAIQ
LLLGSVYSYQKNTDQAISLYEQVMKQYPQDFRPVLAKAILLKEQGKINEAKPLFDSALTL
APAQYKDEIKNLTSGF
>1170768.4.peg.1815
MFYLNWRWYSATSVERGFMYFLVFFFFPGLLLLSPIANFRPRPRQIV
>1170768.4.peg.1823
MERSKDKAAAGLRVQTSGYLHTLIGFSGALFQLDPQNLLATIVVPLSYALFTSIIGWFFG
GELVNEFDGQPAGGITNEAEILIREFQSFADSLSAIHKQYIQKIKDASDSFESQLQKVGA
AYERMVDTQEVQFDRLSKKQSELLAQLEDYQKRFIDSQNSCYGEMDNAIQESINSSNKLN
ASVDALTSALSTKELFSITSNFVYLSEQTKVASDNMGQVATTSKNVAKYLEESKILIDQL
EKLLSSINSYRR
>1170768.4.peg.1829
MWGSYCEEVLTKREPYRQAHLEGLAKQKDAGILITIGPTKDITKVFGIYEAESESAVSQL
IEADPYWKNGIWTEYIVKEWIQAF
>1170768.4.peg.1841
MLIENNVLFAQVLAEESVGINGGSLLEIVNVPNEVLNYFQNLPSELENWILGYL
>1170768.4.peg.1843
MIWVVTAKLLLPKDSILGKFQPLRFWQKTFLKKSRKSS
>1170768.4.peg.1849
MWRVVEFLHVKPGKGSAFVRTKLKNVQNGNVVEKTFRAGETVPQASLEKITMQHTYKEGN
EFVFMDMETYEEDRLNATQIGDGVKYLKEGMEVNVTFWMKQEEKQVLKQVLEVELPNSVV
LEVVETDPGVKGDTATGGTKPATLETGAIVMVPLFISKGERIKIDTREDKYLGRE

>1170768.4.peg.1854
MRRQRQNRTDMCQDITECQHDINKLLDRVRAGQVLKLPKISISKIVDTKGNRIDRKINSG
QINQVWRYGAEVIKIEEVDRIQVATDGDIENRLLKISEWIQTELKD
>1170768.4.peg.1856
MDVHGDYQQRDIPHELLVKMQQDKITHRESVLEHLIYDQPDYPPRNWEMGAAATIRLMQQ
GVNMIYRGVLLATYEDKYTLLSCPDLLVKQPGKSSLGDWLYVPVDIQLGKRPKQEYQVVA
AFHAEIAGALQEVMLAECWLVLRSKETTYAVDLYKWRPQMLDILADYVQVMSSSEAPEVF
IARQKCNLCHWYNHCYSIAREQQHLSLLPGVTPVRYNYLRSKSINSLDLLAKTQPSSLEN
VTGFDYKIAWKLVIQAQSTLRQTALILPETVVRGNLISNVGIELYFDIEAQPDLNLNYLL
GVLVVDIENKQETFYSFLATKSEEEELIWQQFVDLVCQYPHSPIYHFCNYEVETVNKLGK
LYGTPDSITRMILTRFVDIYEQLIETVALPIESYALKAIANWLGFTWRDPKANGAKCIYW
YDQWLETGDREFLKMIQVYNEDDCYATRRVKDWLVTFTKDFLL
>1170768.4.peg.1877
MLYETDFYTWTQEQCQALLNRQVNSLDWQHLAEEIGDLGNRHYDQLSSRLSILIGNLLKW
KYQPANRSNSWKATIREQRRKIDRLLERNPGLNNKWNEALEEAWLDGKDLAIRETGLDDQ
DFPEECPFSSFQIQDSNYFG
>1170768.4.peg.1878
MITQPVHASPTITPGKTRLSCVRSPHSPTTGKQICIQIPDSTSPPKSESIVIASSQPDQS
ETDEFVLTDAESDEAIQLFGCDCPVCIRAVRQLHGLAPGPI
>1170768.4.peg.1895
MFLGLLATGCVSTVKESAEAQSQSSPGKKSAKPISVDVAIARTGSLNEELIYTGSTVPKK
IISVRSQVEGRLIGLDLEIGDKVSKGQRVGRLDDILLKTGLEQQEAELANRESEVERVRT
QVGNIEAEVEKVRLELMQAKSDSDRQQKLLQEGAISQQAAQQALTRVKTYQQILKATIEK
QRTEKKAVAAAQNRVLAQRAVVKAARERLSYTDLISPITGVVTEKITEPGNLLQSGNEVI
KIADLSQIKVVVKVSELELGKVEIGKSVGVNLDAFPNEKIMGRIERISPVADSTARVVPV
EIVIPNSQGKIRSGMLARVNFSRQESSRVVILKTAINNQEEETSSPNNNSTIFVIERNEE
RVKVKEQTVVLSKEADGKVEIISGIQPGDSYVVRSSKPLEDGQRVKLSALSELPN
>1170768.4.peg.1908
MGENIALNVERNGFPIAVYNRSREKTDAFMSNRAGGRNVKAAFTLEEFVASLERPRKILV
MVQAGKPVDAVIQQLKPLLQEGDIIIDGGNSWFEDTERRTQELEPTGLRYIGMGVSGGEE
GALNGPSLMPGGTKSSYEYLSPIFNRIAAQVDDGPCVTYIGPGGSGHYVKMVHNGIEYGD
MQLIAEAYDLLKNVGGLDATQLHQVFTEWNQTDELNSFLIEITANIFPYVDPGTKQPLVD
LIVDAAGQKGTGRWTVQTALELGVAIPTITAAVNSRIISSIREERIAASKQLTGPVPTQF
KDTRTFVNMVRDALYCSKICSYAQGMALISTASKTYNWGLNLGEMARIWKGGCIIRAGFL
NKIKKAFDENPALPNLLLAPEFKQTILDRQSAWREIIVTAAKTGIPVPAFSASLDYFDSY
RRERLPQNLTQAQRDYFGAHTYKRVDIEGTFHTEWVPIAEAKK
>1170768.4.peg.1923
MPVGSMSQSIAVSWSTVDANYSQASVQVDKLSNQDLILRCQSGLRPDKAAFAELLRRYQT
QVDRVLYHLAPDWSDRADLAQEVWIRVYRNIHRLQEPAKFRGWLSRIATNLFYDELRKRK
KSGSSLSLDAPRLAGDGEIDWEIASDTPSPEEQLRTREFYEQLREAIADLPEIFRTTIVL
REIEGLAYEEIAQITGASLGTVKSRIARARSRLQSQLQSYLDG
>1170768.4.peg.1936
MDDEASIRRILETRLSMIGYDVVTAGDGEEALETFRKADPDLVVLDVMMPKLDGYGVCQE
LRKESDVPIIMLTALGDVADRITGLELGADDYVVKPFSPKELEARIRSVLRRVDKNGTTG
IPSSGVIHVGNIKIDTNKRQVYKGDERIRLTGMEFSLLELLVSRSGEAFSRSEILQEVWG
YTPERHVDTRVVDVHISRLRAKLEDDPSNPELILTARGTGYLFQRILEAGED
>1170768.4.peg.1937
MEQNANSFGVGGWHSAQPSAKSTKPSKPIASLTFDQISDRQIARWESGYSELDRVLGGGV
VPGSMVLIGGDPGIGKSTLLLQVSNELSQRYRILYVTGEESGQQVKLRASRLGMSKPLQV
VSVEDSNTSSESSHPLPEQDLGNNVGDAVEDDHRVTVEHHTISSDLYVLPETDLEEILRE
IDSLKPNLAVIDSIQTVFFPALTSAPGSVAQVRECTAALMKVAKHEDITMLIVGHVTKEG
TIAGPRVLEHLVDTVLYFEGDRFASHRLLRTVKNRFGATHEIGIFEMVSNGLREVPNPSE

LFLGNRDEPSPGTAIVVACEGTRPIVVELQALVSPTSYPSPRRAGTGVDFNRLVQILAVL
EKRVGVPMSKLDSYVASAGGLSVEEPAVDLGIAVAIVASFRDRIVDPQTVLIGEVGLGGQ
VRSVSQMELRLKEAAKLGFKRAIIPKGQKFPDLKIEILEVSKVIDAIIAAIPHQSLENGD
LDLEEEDED
>1170768.4.peg.1952
MATCLHLEKIQCQDFGLIEQFLIALDFFFLNESHNWIVDRQNACPKTYLGAICERVVFGR
NTHQM
>1170768.4.peg.1957
MLIRVYISHNFYLLITLITRYEKGVHPVDVPLLSLPTWQI
>1170768.4.peg.1961
MQEPQYTETKTKETPIPDLSAQTGSITKLQSPPKSQEQWLKYGQEVSNFLGTLPEYLVGL
FDKYKQPLLTLGLIVTAGVTVKVILAVLDSLNDIPLVAPTFELIGIGYSGWFVYRYLLKA
STREELTSEIDTLKSQVFGQD
>1170768.4.peg.1963
MKTLVSVHESYKLCQELTAKYAKTFYLGTLLMSPTKRQSVWSIYAWCRRTDELVDGPAAT
KTTPETLAQWENQLDSIFAGCPLDDYDVALVDTLQHFPLEIQPFRDMIAGQRMDLYRSRY
ETFEDLYLYCYRVAGTVGLMSTTIMGVDASVYSAPWHRNIQPYLPIEEAIALGIANQLTN
ILRDVGEDAHRGRIYIPLEDLKKFSYSPEELLQGVLDDRWRSLMRFQIKRAREFYTKADR
GISYLAQDARWPVWAASMLYGKILDVIERNDYQVFSQRAYVSQLQKISTLPLAWMRSQVL
>1170768.4.peg.1967
MQKRGFGRRTASLVISQYTAVTIQGEWLEAFWCPECEEKKWYYVRQSNNGIYEISLAPMD
LWKQVSGVIDPYGNPSVSEFTRLSAKQITYRGLSAFYSL
>1170768.4.peg.1980
MVKLLDDLQLHRSQHGTKIVYSTGRSLHLYQELQESQKRKQRDLIKPDILVCAVGTEIYH
YDGKEELVLDREWSKHLSCNWDRELVARTAANFPSLKPQPESEQRPFKVSYFVREEKAAQ
IALELENLLVKEGKVEIQIICSHSDHKEYNRNLDILPSSANKGMAMTFVREKLAIDVEKT
VACGDSGNDIALFANRQEKGIIVGNAQGELLDWHHNNPNPNRYLAKNNFADGIAEGLRHF
SLL
>1170768.4.peg.1982
MDCKKALNETQGNIDEAIDWLRKKGIASAGKKSDRVTAEGLVEIYTTPDRSGGVLIEVNC
QTDFVARNDAFKLLVKNLAKQAVNADNVTSLLAQPYIENTAVTVDEYIKETIATLGENIQ
VRRFVNFSLEGSPGAIDGYIHTGGRVGVLISIGVDTETAAKNEEVQSLAKNTAMQVAACP
NVEYVSVDKIPDQVVQKEKEIEMGKEDLGNKPENIKEKIVQGRIDKRLKEMTLLDQPYIR
DQSISVAELIKQVESKVSEPIKVHQFVRYILGEGIEKQESNFAQEVAAQIGAK
>1170768.4.peg.1987
MKDWIIVGGLSCGIGICGISIAGVGGITGRIGVGVLGSTTGAIIGASLVSKLEEKKLNKT
IATLKQQEQELQNRVLVINQQNPNLSELEERKGKIEASKIERATLEARITQLNSQVESLE
SRRLELNSIELQIVSQQTELNRLNTRVQELTNQSQELEQRAAELELLRFTYDAILSQKQD
YEAKVSQLNPEIDRLELEKQRILQTIEENQNNYQKLIELREKCRSLTLTIRDQESRLREL
ENEGNNLQTRNHNLQTENHNLQTETDNLQQTRREIETTIANLRGEIREIENSGRVALEPL
REKLWTNLPRQVRQLEGETQFIQNFITSLRSEGLSFSSRTIKAFHTSLKVQDISALVILA
GISGTGKSELPQRYANYIGAQLLTLAVQPRWDSPQDLQGFYNYLEKKFKPTQLMRGLYQY
KDPEMSNRIVIVLLDEMNLARVEYYFSEFLSKLESRRNHATYLEIDVGSLPIAESERRLE
IPKAFLFVGTMNEDETTQTLSDKVLDRANVLTFGRPQNLKLRQRSGQQITVNQHSDNLVN
NRDSSYVTYSDFQNWVRTPVPESDVVKKVESYLNEANKVMEKMGHPFAHRVYQAIAQYVV
NYPEVNGIDSESFKFALADQFGQKLLPKLRGVMVDEAHQQLQEMKEIIDKINDQPLIKAF
DKAKEGRYGQFSWQGLVYEDEEA
>1170768.4.peg.2026
MNDIEWSTTEKELARKAFDRAYEREISALIETVREQASTITKLDEIWQLHDFLSARRHQI
DGKYDYRYSVLIFVFAQLVREGWLHIKDLEGLEVDKIAKVSALTRM
>1170768.4.peg.2036
MDRYVVYVESDSQYVLQQVRRVDSNAYIRPFGGRSIIQSGVFSESYYAAERVRDLELNGV

SDIRVMVVSPPGQISSSVSTTTSNLGYDSGAANSSDNSTNIDPTKQAHYYAESYYVVVPT
SAENLRTIWQQIRDQVKGRVHVLMRNEPRGFHVALGKFKDRYQAEQWSNYLKRLGYGNAR
VYYGR
>1170768.4.peg.2038
MQGDVLGEFPGHQDWVTSVSFSPDGQTLATGSRDKTARLWNLQGDLLREFPGHEDWVTSV
SFSPNGQTLVTGGADKIARLWNLQGDLLGEFPGHEGGVTSVSFSPDGETLVTGSVDKIAR
LWKLKGYLIREFKGHDSGITNVSFSPDGQTLATASVDKTVRLWDLKGQLIQEFKGYGDTF
TSVSFSPDGQTLATGSLDKIARLWPVRYLDRALEDGNTWLIDTNF
>1170768.4.peg.2042
MQVIEKNSLFAQVSGEQSAVVSGGSISEAQTLIIAYQLGQSSVLNDTQLFNVFLVSLST
>1170768.4.peg.2047
MIDQVINVAFDVAFSTLKNDEPLSNKLYGFCLVGDEVFDPS
>1170768.4.peg.2053
MSVSQHVGYTFTGTAGEKLSYSTDADVCLWVFTPDNQIVKGGDLSKDGKYVIQVAAPEGV
KTFNLHLSLGSLQIAVATPTPESSLNIKTQNDNSSNESSAQHDISQEDALQLVQKWYAAK
PQIFAPPFETGLVDQLATGKLHTFTTKANGPVEWLRQNDAYYEYNYSEIKRVLDFSTSGK
RPYIKIRVSEELYLHGKNGIDKNNSGASTNNLIYFFEKENGIWKIYDYRKVR
>1170768.4.peg.2054
MKNTPRLLQMTNSQRIYGKIYSTIYNGKVNSKIAKSYLKTYLIGKSITLSWFLIAGLFSP
TLGAIKKAEIVGLPNIKDDRVTIKIKVEGEGARPVMGLERNNFDLKVDGKKLKVKPRDWK
SPEEIIPPKAWIIVLLDFSGSMNQIDSGGSKKIAGAINAIREFTKVSSDRGGDTQISVVP
FGEAGKNCPEYTVNKDTLDKFLSASDFKLQNSLEYLSSLNPCGSTNLYQPLKKALEFLGN
PEDARFTLPENSSEPNPRLSIILLSDGYHNAMNEFQDFNELKSLLQSYENITVHTLGYGL
TPSQLGIKYNLNRPATRRDINQGKVPEEEFVDQQRLAEIANLTGGIAEFSGDQTAIAENL
KLFLNALLGEYQISYIQPNAERGTLHKVEVQVKDGNKKVNSKSVPYIMPVFGRSLPLANR
LVMVTSILFILIVGGIFPFRWWGEHLKGQAQGE
>1170768.4.peg.2056
MWGKEKGDRYGDLPFALVILTKGQVLFSTGMVTHTLY
>1170768.4.peg.2062
MKSYTEGMRYFQNAQLRGADLHNLDLKGSDLSYADLSSANLSMANLRGCDLSYADLSDAN
LQDADLRGTMLFYTNLRQANLQGSKLDNADWDKNSPLFPNTESTKDPIQ
>1170768.4.peg.2067
MVIVVMRTGTSFPVVLCERTTTHRFDGNTRRLILQPAR
>1170768.4.peg.2068
MQVPVYIIFDPDGGLIEYYELRNGRYELKQPDENGRHWIGSMELFLGTWQGTKEGRTGYW
LRWWEQTGNLLPWALELIEQERQQAEQERQRAEQERQEKERLIAYLRSQGIDPNNLPNHT
E
>1170768.4.peg.2078
MDELPLLLHYYYLTIASECYQDAVKNISKEYDLNYLTISPYSL
>1170768.4.peg.2079
MTNIQSPKLTSPKLTAYEFKNLPLLGTTTTNQNILLGGFSGLYFQGVADNGNLQFVTHTD
RGPNSEPTGQNRPFLLPNFQPEIVSFELNKASGEINITNRTKLFRADGKTPLTGIPNVQA
GAGNTAYTDEIGVNLNGTVLNNDPLGADLEGIVIADNGDYWMVDEYRPAIYHFNRNGILV
SRFIPQGTASKSNQPVGTFGIEVLPEVYAQRRNNRGFEAVALAGNKLYAFIQTPIDNPDN
TGDTTSRSSRNLRILEFDIVSEKVTGEYLYLLDDITGTGNAKTDKIGDAVFLGGSQFAVV
ERDDRSDTTSNKLIYQIDLIGATNINNTSKLSLPQGKTIEQLTPVELGPAGITPVNKSLI
VNAAQIGYTGVEKLEGLALVSASTLAIVNDNDFVTTIPEKLGIIELPNNLVEVTGTANKD
ELFVKKGEYVLGLDGDDILDASDGLGNNVLDGGAGNDQLYGGKSDTLIGGAGNDQLYVVE
GNNNILYGGAGNDRLFVTEGNNNILYGEDGDDNLYIIEGGNNTLSGGGGADKFYIVNGVI
PVVPGQVLDFTRGDDKVIISGISQIQGFDNLILEQNANNTTIKTKDLIGGSQKVLGILKG
VVANTLTADDFRINDVTITNPQVSGTFNPIATDNILTQEEKKGGLTLSGTVSDPNAQVTL
QFGGQFRVAKVQSSNSSWSYTVTDNDYNFFREGQNQLTAIFTLTNNNRTGTFRASQTLTI

NSGVLPQPTSPTLDPTKPKGISEAVTQKAQSANIGQGILGSGEKLTDPNIVVIPFGGINT
FNQGANAAIEDFGLIKTGSNTTIESVRVFPKSPTGEVSVTLSDGTSVLASIPPGISSIGD
PLGLTMSGLVPGGSSTLDFYLPRNILNQLPSDLQSATYAKFNFEAKKFEQYADEQGKPLY
QYKFTDKNSNGIRDFGELYLSLNLTDGDKWDSDRIQNGIIVDPGQLGIAVVVTGTGVTYS
SPGNSLIPREAPALSNIADNVFYKFSNTYKPAEARQALQNALGQTDAAFKNIFGLYEIDN
ATGSVNGIEPGKPGYAKAALDKSKVISNFTVRAGGSVTPNINGDLIGIGDKTYAPFVIAN
GGNYSGSIQEAINEFFKVNPNNSAATAQNYMGLPVAYFSFGAANPDGAAHIKSFGNNIFG
FEDLPAGVGVSDYDFNDMVFSFG
>1170768.4.peg.2101
MINFCFSVYFCLVYLYYISGEIYLNPFPQVDVPTPDNCYL
>1170768.4.peg.2106
MVCRITAFYGGLAPLNLFYIISGPSVLLIVIIFPQTVRWLIDIYAV
>1170768.4.peg.2113
MNQQTSWHRVILGNGNFSATVISIAGILIKQSCFLKGIVDP
>1170768.4.peg.2114
MSADSINTALAELENLINSCENDLIRFIEARKMNSGNEISTKKVNRQLQQNPIAIVGMAS
LLPQSRNLRQYWQNIVSKADCITDVPESHWSVKDYYDPNPRTPEDKTYCKRGGFIPEVDF
NPMEFGIPPSILEVTDVSQLLSLVVAKEAMEDAGYGEAREFDRENVGVILGVAMAKQLGM
PLSARLEYPVWEKVLISSGLSPEDTQKIVEKIKSAYIKWDENAFPGMLANVVAGRIANRL
NFGGMNCVVDAACASSFGALKMAISELVEYRSDMMLTGGVDTDNTIMAYISFSKTPAVSP
GENVKPFDAKSDGMMLGEGIAMLVLKRLEDAQKDGDRIYAVIKGIGTSSDGRYKSIYAPR
KEGQVKALERAYNDAGFSPTTLGLMEAHGTGTMAGDPTEFASLQSFFSKHDKRKQYIALG
SVKSQIGHTKAAAGAASLVKTALALYHKILPPTINITEPNPKLDIENSCFYLNTETRPWI
RGESESPRRAGVSSFGFGGTNYHLVLEEYQQEQQQPYRLHDVASQILFFAPNPSELIKNL
ETSLQNLQAADSNGYYSQLVEECRNIQIPQNAARIGFVAGNKEDTCKLLALSIDLLKNKQ
STLNWEHPQGIYYRASGIKLRGKVVALFSGQGSQYLEMGREAVMNFPALRRLYGLMDSLL
IEDNLQPISQVVFPHPTFNQTEKADQIATLQRTEYAQPAIGVFSAGLYSIFQQAGFKCDF
TAGHSFGELTALLAAGVLSESDYLYLVKARGKAMAAPKDPDHDAGSMLAVKEEISKVELV
LKNFPKITIANFNSPSQVVLAGPSHEIQKIHQKFQDLGYGAVLLPVAAAFHTPLIAFAQK
SFAIATKSVKLLNPKIPVFSNVTAQQYPQESDKIQRILESHLASSVNFTQQIENIYAAGG
YCFVEFGPKRILTNLVKDILGERPHITISLNPSTHKSSDISLREAAVQLRVLGMELGNID
PYQLPEIFSKEISQKSLNVKLKGINYVSEKTKNAFEQALNDGFKIQGVPQIQPVSVIDKP
SEFTPKKEVVETVYTTNSNGTKHNQTSIISSLSTDSQMNSANAINSSLRAEEQLPILTYP
MVQVLGGKMTTSDKNTEFQQILASLENLLSQFQNSQSDNLQIHDTYLQHQMEYAKTFFQL
IQQQNTLFMNNKSAETGETKKSIMDSFERSMMQFHHQQAETLRIHEQYLREQLEHTKNFF
HLIQQEYSLIIDGAEELTPVIPLQLTDIHTENLREISPQNPQLSVPEMVVTPTIKTLEVE
IPQAITETRVEKPPVSVTETAISSQTKKSAIDIQDIGKSLLSITSEKTGYPIEMLEFDMD
MEADLGIDSIKRVEILGGLQELYPDLPKPNLEELAEKRTIGQIVEYLEKQVVTNPTNNQI
NHSEHIVKNVDPKVIDIPHSSTTSDEYSSIAETLLKITSEKTGYPVEMLELDMDMEADLG
IDSIKRVEILGGMQEVYPDLPKPNLEELGDLRTIRQIVNYLQSLVVVEKKNLDFEQIKAT
VVELNPPPTVDPNLPRRPVKLKILPEPDFWECQLPGGHIGLITDDGSLTTTKLVHELIDK
GWKVVVLSFPQSIVPERSPLPAGVTRINLANMSEEHLQLLLQSISVQYGKIGAFIHLHPC
FSTTNLAYLETEKVIVKHIFLIAKHLKQSLNNAANLEGRVSFCTVVHLDGAFGLDHTENF
GIIGGGLFGLTKSLRWEWPKVFLRAIDLSPKLEPHQSAEYIVAELYDSNRYIGEVGYGPK
GRVTLVATADxxSFIGNQELxVxPENIYSPFPVSxVQxxSEKYMITTFQIPPSSVFVVSG
GAKGITAQCTIKLAQSQIGNFGSLSHSGIFILLGRSEITPEPGYAHDCLEESVLKKRIME
NLISQGEKPTPMMVQKIFNQINSVREIKKTLAAIEQTGAKAEYINVDVTDGKVLHDKLRD
VCQKFGKITGVIHGAGNLADKLIEKKTADDFEKVYNAKVQGLQNILNCCDPQHLKHLVLF
SSVSGFYGNIGQSDYAIANEILNKSAHQFKKHYPQTHVVAINWGGWDSGMVTPQLKKAFT
ERGIDIIPVETGTQMLVNELHPAFENQSQVIIGSPMNLPPTPLDSELKSYRIYRRMTLEQ
NPFLYDHTIAGVQVLPATCAMSWMVHACEEIYPGYRYLHCRQFKVLKGISFNSTLGSEYI
LEIQEISKVDGELIEFQTKILSKNSQGKTHFHFSAIVTVVGKIPKAPIYESLNLTEDNVI

TTRGKQFYQNGDSSLFHGPAFQEITRVLNVNPQKITVECCWQAISETDQGQFPVKWHNPY
TTDISTQSLWIWLNHFHEQVCLPGQLTYWEQFLTIPCNSPFYVSCEVENKTDTGVTANFI
LHSQSGEIYSRILGAKAVIWPMKMLSKK
>1170768.4.peg.2120
MIIVMKVGSPKVEIDRLSSDLTGWGLTPEKIIGKHKVVIGLVGETADLDPLQIQELSPWI
EQVLRVEVPYKRASRQFRHGEASEVVVTTPNGNVVFGEHHPIVVVAGPCSVENEAMIVET
ALRVKLSGAKFLRGGAYKPRTSPYAFQGHGESALELLAKARGVSGLGIITEVMDGADLEK
IAQVADVIQVGARNMQNFSLLKQVGAQSKPVLLKRGMAATIEDWLMAAEYILAAGNPNVI
LCERGIRTFDRQYTRNTLDLSVVPVLRKLTHLPIMIDPSHGVGWAEFVPSMAMAAIAAGT
DSLMIEVHPNPAKALSDGPQSITPDRFDQLMQELSVIGQAVGRWHQPVLSSV
>1170768.4.peg.2122
MKTSKVRKMIFLVDIILVVSWLVSIVTQLSYNFIYAM
>1170768.4.peg.2126
MLQLAPHIFHILHQHGIEHAFGIPGDFALTLYDALAESKITPVIMTHEPSVGFAADAYAR
IRGLGLAVVTYGVGGLNMVNAVAGAYAEKSPLVILSGSPGIKERRQDTLLHHKVKTFDSQ
RRVYEEITVYASAITDIETAERKIHRAIDYAKTFKRPVYLEIPRNMVYAELPEVDYERVP
VKHTDIDTLKEAVSETLEILEKAKSPVIVAGVEVHRFGLQVQLLALAEKLGVPICATMLG
KSVFPETHPQYLGIYNGEAGDKNINKIVEESDCLLMLGVFMTDINLGMFTAHINQKHTIS
ATSERIAIKHHEYQNILFTDFITGLLKNPHLPHFRSPNTYRMHPRVEENIDNISMGGLIY
EINQFIDHKTIIITDVGDSLFAADDIQTKQGTSYLAPAFYASMGFAIPAIIGAQLADPFR
RVLALVGDGAFQMTGMELLTAKRLGMNPIVIIINNSSFASLRAMGHENADFVNISTIDYG
DLAKVLGGNGFVIRTGLELRRALSIAKESENFSILDVRISADDISPALQRLKTLFTQTLK
>1170768.4.peg.2129
MLNLKTVIFVLSFTPFIVLSTQLTGRAQVSSPSLGTAGSVSGGGATGSDNNPISAPSLGT
AGSMSGGGATGSNSAPSLGTAGSMSGGGSTGSDNITVIVSTNNLRELRNAIGQLVSSGSD
SLSPITFSSLRASLISDGVSPGHTDQLIVSFSRVLSQLGVPNTNFSANNLNLEKLVASTK
IFKPTVTIVQDSPAEGEIALILDSNNLTEAINIYNKIVLESDDPTIIKLSRNQDFLKISR
ILKTLRSGII
>1170768.4.peg.2134
MGKININDTTLRDGEQAARVTFTLKEKVAIAQFLDAIGIPELEVGIPAMGEGQINAIRAI
NDLDLNAKLLAWNRAVISDIKASISCGIKRVHIAIPVSGIQIASKFHGQWRVSLQRLQDC
IHFALDHNLWVGVGGEDCSRADESFLEDVALYAQEWGASRFRFCDTVGVLEPFRTHLKIK
RLVSRLSIPIEIHTHNDFGLATANAIAGIRGGALSVNTTVNGLGERAGNAALEEVVMALK
YIYGVDLGINTDNFSQLSQLVANAVGSNVPPWKAIVGQNTHDRWLGLDDHTIVKNPEIYR
RFALPGEW
>1170768.4.peg.2135
MITTLIAGLTILVITGMTNSYISHKLFIKDSDQPTNKPS
>1170768.4.peg.2138
MKISMGVFKNSMGVISKHEKHLETYRQFPEDDGCYHQR
>1170768.4.peg.2150
MTSPSESSQIATSSFSMDDFAKALEKHDYQFQKGQTVRGKVFQLDHDGAYVDIGGKSSAF
LPRDEASLRTVTDLSEILPLNEEMEFLIIREQDAEGQVTISRRQLEIQHIWEKVAQMQED
SQSIPVKVIGVNKGGVNVEVFSLRGFIPKSHLLERENLEALKGQTLTVGFLEVNRANKKL
ILSQRLATRSSNLSLLQTDQLVTGKVTGIKPFGVFVDLDGVSALLHIKQVSQKFTESLEK
VFQIGQEIKGIVIDIDESKGRVAISTRKLENYPGEVLENLAEVMNSADARANRVSSTPE
>1170768.4.peg.2152
MVTSSYTLQLLKFVTFVTTMIKPALTKIGAQMSNLTGVRAIMKDINETLRANQGQVLYNL
SAGNPLILTEVEQLWRECTADLLASSEYGEVVCRYGSSQGYAPFIAAIVKDFNQRYGLQL
TERNILVTAGSQTIYFYAANAYGGYTEDCKLKKIVLPLSPDYTGYGGVSICPESLIAYKP
TLDVDGVNHRFKYRPDFTQLSITQETGCVIFSRPCNPTGNVLTNEEVEKIAALATPHDVP
VLIDSAYAPPFPALNFTEMKPVFGENIIHCISLSKAGLPGERIGVAIGEEKLLQVLECFQ
TNAGIHSSRYGQAIATRAIESGALANIAETVIRPFYQHKFDVLESTLDAVMPKDLPWFLH

RGEGAIFAWLWLRELPVSDWEFYQQLKKVGVIVVPGSSFFPGLEENWEHKHQCLRISLTG
TDEEISIGMQRLAKIAQEVYHQ
>1170768.4.peg.2153
MKSYARQTEAVWDDVLVPILKNFIPVLTYIIGFSLFFTVLGVDLSGIGLALGSITLVLGL
AVRDILSNFFSGLVLLIDTPFEFGDVIVFDGSLAIIKEIGIRVTKLYLIEEHCEKYVPNA
TLSNQSITNLSRPTTHYAYKIPVAVRIDADSALATNILKEIVIGHPDTIANFDDKLRYLD
SFYGLKEAQDNKPSKKEAGRNRIELDREISLQLKKIAATSETLLGEIKVLERGGLDGQEL
MVLQKTYMDILELVGMVIVTERKGKRQRSRLEEESSQKTNLISLVRIWYRTWLEDPDLVM
EDRQILPDEWERKIDLLKLKLTKLFQIISNPGVKETRLDNYVENFAEWLESSFKESSTAW
KEPQVQITDIQGSSMEFVVRFYVDNIQLEHWRRGERVKNEVRREMIRRLRLAHIYTG
>1170768.4.peg.2155
MILSITIPVFIWLVVSNSGLVKPLFLPTPQAVLTALQKLWATGDLQTDIGFSLLRVLGGF
LLAAVISIPLGVLMGSFPVVRALFEPAISIFRYMPAPAFIPLLILYFGLGETPKILLIFI
GTVFFNTLMIMDAVKFVSRELIETSYTLGGKNHQIILQVILPFIVPNIIDACRVNMAASW
NLVIVAELVAATEGLGRRISVAQRFLKTDEIFAGLIVIGLIGVVIDILLVMLHRIVCRWA
HN
>1170768.4.peg.2156
MIDKIMNNQVELSPIPQETAGELFFLINPLIVYEFYEFLVY
>1170768.4.peg.2157
MRKLLSLCTLFVFSLLLAISCTPYGGDSREIQVGFSVWPGWLPWQIAAKENLFTANNIQV
NLKWFDGYLDSINALNSGKLDANSQTLNDTISSVAAGADQVIVLVNDNSTGNDKIIVREG
INTIADLKGKKVAVEEGTVDHFLLLLGMKKAGLTSNDIILQPLETGAASAAFVAGQVDAV
GVFAPFTTKALELPGSKELFSSRDFPGAIPDHLVVNRQLIKERPQDVQALVDSWFETLDY
IKANPEKSNEIMAKRAGVTVDEYKKYAEGTKIFSVEDNLQAFSSNSNIGSLKYTAQEIAK
FLVEAKLAKKVPDLSQIFDDRFVKAYAAKQK
>1170768.4.peg.2162
MGLNYQQHVKVLPTGASNKHERNETDKVRKSQRLVVSGNVKPTPKLSSKMTGSNPKVKTL
RVPAHPKKYAHPSRKARLKPAANIILYALRLLIVGVGLGAIVGTLLSVLDPANRITTNSI
NPPVTPSSSPQSPINSSGLVISREITPLKTTIENLSAANPNLIPGVFIVDIDSGAYVDVS
GNNNFPAASTIKIPVLVAFLEDVDRGKIRLDEILTMEQEMVAGGSGNLRTMPVGTKLKSI
EVATKMMTISDNTATNMLISKLGGKELLNSRFRSWGLVNTAIQSPLPDLEGTNTTSPKEL
ASLIAKVNQGELISMRSRDLMLDIMRRTQRDDLLPAGLGEGATAYHKTGDIGTMLADAGL
IDVPTGKRYIASIMVKRPHNEPAAAKLINSISQATYSYLSQSNFSPDGSTNNQPSSNQRL
NNSSTPFTQPFLQPQGGNSNIPNATINNAPLGNYQSPLNNPPYYPPQSHPN
>1170768.4.peg.2170
MFKGKSLPIMFFILPIFIWWGYREVQNQFSIPQAILVLGGSSKRLERERFAADFAKKHPN
IPIWITGGSPADHTKKVFTKAGVNLSRLHLDYEAVDTVTNFTTIVDDLQDKKIKSVYLLT
SDFHMRRARVVGEIILGTRDISLKPISVPSKTDPEPIEKVIRDGFRAIVWVGTGYTGAEG
VNVK
>1170768.4.peg.2178
MHLQIATGQPWRPYTAFGQFSVPDYPIPGGSKGWATYQKLLKAGWTLVPSARAEEFSRDL
VESSIHK
>1170768.4.peg.2179
MQDFLTITTKFRDNQHICRIIANSFEYCTQTWKSWCQNAFS
>1170768.4.peg.2180
MVIIDDNYWTQFWNCGNLWRSFNLLLNLLYNMQPNSRKNLHTNISVTAAAIALLIASPVVN
SNVANAQTKITNTSPAAAVDKPKVVNQSITEKQVLGAQKAWEEALINISTTYEQKGIAAA
KDLAGKIIDQAYGYQFGAVLFKPTLTKAPQTFRTTRQGALAYFVGGDPAFPSDTGFALKG
WRKVEIRNVGVFITGNTAMTMGNVLLTDKSGKVTTVDKTWVFIRDSSGNLRIILHHSSLP
YGG
>1170768.4.peg.2185
MVTVSGVLLAILGLAIWMKLTPIFWILELLKGLLALLTDILPHYVSGPLVLLLGGLLVLW

GQSRTVGSITQALRPQGSQEELIDVILAHHRLYRGPKIVVVGGGTGLSTLLRGLKTYSAN
ITAIVTVADDGGSSGRLRQEFGVLPPGDIRNCLAALADEEKLLTELFQYRFRAGDGLTGH
SFGNLFLTAMSDITGDLERAVAASSKVLAVRGQVLPATLSDVRLWAEMADGRRIEGESSI
PKAGGKIVKIGCLPANPPAIPAAIKAIRSADYIIVGPGSLYTSLIPNLLVPEITQAIACA
NVPRIYICNIMTQPGETEGYSVSDHIRAIDHACGDRKLFDAVVVHRRSPSAQALIRYAQQ
NSHPVFLDTEAVSQLGRRIVPANVLYEDETGFVRHDPEKLARVLLKWYGAASKK
>1170768.4.peg.2190
MREAIPAQLLHPRYRRGEKNKILICSHFLIHSHQRESCGIIVLKWSKF
>1170768.4.peg.2197
MFTPSLYYIETEILRIELCAPHPYSYGMVRLGKTNDF
>1170768.4.peg.2202
MLIGVGEDPEREGLLKTPKRVAEAMKFLTSGYNQSLEELVNGAIFDEGHEEMVLVRDINF
FSLCEHHMLPFMGRAHVAYIPNKKVVGLSKLARIVEMYARRLQVQERLTRQIAEAVQTIL
EPKGVAVVMEATHMCMVMRGVQKPGSWTVTSSMIGVFQEEQKTREEFLNLIRHQAAFF
>1170768.4.peg.2217
MTSDNGELQVNLFTVNRPQEEAKYTVAYIDYPAQYIQLLRSRNLVEQAIEQGKSTALQRV
RGTVVSEEKKTLGDNVGIEVNYTTADGKVVKQRVFLVDNRFYQITAETTQKRQRFLTRSM
QGFCDSFKLLP
>1170768.4.peg.2221
MKPLQSGIGDKETYQSLFNLDQSEEEITPLYFQAPLAPPIAAAKENRTINLGLVWRTFLK
LQRERDFLLVESLGGLGSPVTDELTVADLAGEWRLPTVLVVPVRLGAIAQAVANAALSRQ
AKINLLGIVLNCNQPRSEEEISDLTPTHLIESLTNFPVLGCLPYLEDVNDLEALAELGSN
LTIPVWSGLQVG
>1170768.4.peg.2225
MQKGKCGVDLATVTFEAEENYSVAILGNSDQVKRGTDVIVAGFPTISGNKGKDRTFTITY
GKLTSDILPKALWGYRLVYDATTKVGNSGGPVLDASGRVVGIHGLADGGEDEKDCATGQR
ETGSPSVGKTGFNFGIPINTFFSLKGQKPPTSTVQPVLPPPPPVAPGNSPRRPTRYKAPT
GPAVCPGTVC
>1170768.4.peg.2228
MGTVIALLTLIMPVLIISHYSSAEVQNQPQPVIYNLTRDKP
>1170768.4.peg.2238
MQSAGLDFSAFLDARNRQLQIYDASGKAKGLLVRAQDVPVYVEYGQAQLGIVGFDVLKEK
KPQVGQLVDLKFGYCRMSVAVKSTSSYKSPLDLPAHGRVASKYVNCAREYFESLDLPVEI
VPLYGSVELGPITGMSEAIVDIVSTGKTLRENGLVEIATLYESTARLIVHPLSYRLDLGG
IYNLAQSVKSSVSS
>1170768.4.peg.2243
MRLLSRKKEQKIVSKIAQVRVMATLTGLTFGGGTWTPKFAQGIDKDKCIGCGRCMKVCGY
SVLGLMALNEEGEFVEDEDDEEIERKVMVVTQPENCIGCQACSRICPKNCYSHTVLEK
>1170768.4.peg.2246
MGFVQLGMLLKRKNRAIAAVLAFSGAIAISGLHKFYLGQPLWGIVYLLLSWTPIPKIASA
IEGVWYLTQDEETFNRKFNSGNLSVNSSPEVSTHVESVANALRELDALREEGLISEYEFE
QKRRQLIDRIF
>1170768.4.peg.2248
MNDPENKSIEETSTSLNNLNKQGGWKENLTLIGVALILALLIRVFIAEPRLIPSASMYPT
LQIGDRLVVEKISYRLHPPQPGDIVVFQTPPELQQRGYDDNQAFIKRIIGLPGDIVGIVN
GQVYVNGKQLEENYIAEPANQPFPPIKIPENKFFVMGDNRNDSNDSRYWGFLPRKNLIGH
AAFRFWPLNRLGLIG
>1170768.4.peg.2253
MKYYQVQNNIFPSSYLKDLGGEIQASPYFTTNNLNRDFIQTKGFSVVFQRQGIKTVQEKF
PFFKPYLDLALQSSCNAFYLNPLLLQEGSRVDPHIDRSLRSYCKTIEPPSLVTVLYVRVP
ENMEGGELILKSNNRQIGKIKPQTNLLVYFQGNLTHSINSVKTPGNRLSLVCEQYDLTDR
EIEDVPIHSVESRAIRNHHRSNKL

>1170768.4.peg.2256
MSNPEQILPVIIEILKIVVLISISVVFVNTISFLIIDFWFTHKQGKQPSKLLKFTVSVVL
YGIAAAVVLQILGKDTTQFFTTSALFAAVVGFAMQEPLGNLLSGVFLQINQPFQIGDWIE
FQALDGMMVEGVVESVDWDSTDIRQKSGEITYIPNGSIAKNLVKMAAAGNVYRTVDFTVP
PNIPPNQVMDIACKALINQPPANVNLDKPLFARMWSYGLEEINYKLFYYPKNYSEAETHT
DPEIRCRLWYALNRAGLTSEYQQSEKQHLLQLVSAVEFFRDLSLEAKSLIIEESKTLLFD
REELLDNQQLFSGTMFLLVSGSILVQNKLVRTSKGNITIPLNQNPENDYRASLALKPPVI
EQVSVQLAKYIGPVAFSLTKEAAKTAPSLYSLYSTLSNEIHNPQQREEFLLHQPPAPTEE
LLAGDFFGEMCLFLGQPLPKIKITTIEETQLLGITPNALLSALDRDGIDISVIAQQVSNY
YHSYLVSSLQEIPSEMLNGTSILEQIKQYFQSVLALKMCKPN
>1170768.4.peg.2281
MSDNSKNGDPSPSVLAMLEKEAKLVTVKKVLLALAIAVTLIASYLQSELNFLVLLQRGDN
MVEYVKSYFLPDFSDWGYYFSETVITISMGLWGTLMAAIVSVPLSILASNNMCPIWIVQP
TRRILDSMRAINEVVFALIFVVAVGLGPFAGVLALFVHTTGVLGKLFSEAVESIEPGPVD
GIRATGASQIQEVIYGVIPQVMPLWTSFTLYRFESNVRSASVLGIVGAGGIGVSLYQSFG
AFQYQKVCAILIVLVAATAVIDLLSAKIRNWLV
>1170768.4.peg.2282
MANNQVDVATNNNESLERLEKTNPSARQKIEIIWTSPIIPSDPIAYRKDLPADVKKKLQN
FFYNFKDKKILEPLQWSALVPANDKTWNPIRELDLAKQVLDLQSKTDLTAEDKQKLNDLN
SRLRKLQGR
>1170768.4.peg.2301
MAHSFADVYKSLCWEAIYVSPMKRTIATAQPFCDATGLNMQLRTGIREGSYGEWETKSKS
FVQENYTENYIKWLTESAWNAPQGGETAVEIANRSIPVIREIKEKHHQGNVLLVSHKATI
RIILCSLLGIDLGCYRYRINILVASLSMVRFDVNGPMLEMLGDRNHIPEHLRLRPGT
>1170768.4.peg.2314
MLNTLLIAIASVTFFLASDFVQPQSAAAYPFWAQETAPETPREATGRIVCANCHLAAKNT
EVEVPQSVLPDTVFKAVVKIPYDTNVQQVGADGSKVGLNVGAVLMLPEGFKIAPEDRIPE
ELKEEVGDLPFQTYKEDQENVIIVGPLPGEEYQEIVFPVLSPNPATDKNIHFGKYSIHVG
GNRGRGQVYPTGEKSNNNIYNASAAGTISKIAQTEDEDGNVKYIVSITTASGNVVEDAVP
AGPELIVSEGQTVASGDALTNNPNVGGFGQKDAEIVLQDSSRVVWLIAFICLVMMAQVML
VLKKKQVEKVQAAEMNF
>1170768.4.peg.2327
MLVLSSCGYWIWEWPHWLCFGINTLSLHFSGTIIHDACHQSAHRNRIANAILGHASALVL
AFAFPVFTRVHLQHHGNVNHPKDDPDHYVSTGGPLWLIAVRFLYHEVFFFQRQLWRKYEL
LEWFLSRLLIFTIVYISVQYHFLGYILNFWFVPAFVVGITLGFFFDYLPHRPFVERDRWK
NARVYPGKILNMLILGQNYHLIHHLWPSITWYNYQPAYYLMKPLLDEKGSPQTSGLLQKK
DFLEFLYDIFIGLHFHHHE
>1170768.4.peg.2328
MINKVAIAGSCLWSLALYLSLASIREWIITQLNRWFNFAERFLYTSNTEFEKTRTARESQ
NAFYASIMSILPFLVFGALSYWSLEISLGSSWGISTGILTTVGASIYELGRINGKNSN
>1170768.4.peg.2329
MLNLTLYDQEGEVFGRPEQVELDIIIKNGLTIVCELKSSIDKAGMYIFGRKADFYAKNQN
RVVNRKIVISPMVDERAVPVAKSLGIEIYSYADVVVP
>1170768.4.peg.2330
MIEAEIKALIQKELPRAIAEEPGVRDFVLRTVSEYYTPRTEFDEKFDRVLNELQRDREEQ
ARKWDEQNRKFDAFQAEQAHKWDEQNRKFDAFQAEQARKWDEQNRKFDAFQAEQARKWDE
QNRKWDEQNRKWEENNQRLDRIEAQNSATLEEIQKANRRYESAIGAIGSRWGLYSEASFR
NGLQAILGQSFGVEVLNLTLYDQEGEVFGRPEQVELDIIIKNGLTIVCELKSSIDKAGMY
IFGRKADFYAKNQNRVVDRKIVISPMVDERAVPVAKSLGIEIYSYADVVVP
>1170768.4.peg.2346
MVVKTVVNIITIPPSKMRTASPPVIKSKVLVLSIIYAFLSLIARLECQG
>1170768.4.peg.2354

MFADYGEVKRVVLPTDRETGKMRGFAFVEMNEDAHEDAAISELDGAEWMGRQLRVNKAKP
REENNRENRPRKGHSKG
>1170768.4.peg.2355
MVSKKMQPGNFSELVYGQVGSQLDALIFATPPNTAIH
>1170768.4.peg.2377
MNVSKDILKPNTLSHILPELSMEEQQLISSGGDPVITADVVTYAATSQIQ
>1170768.4.peg.2379
MGNLGYGIDPEVVKGIAQELAEVVATGVQVAIVVGGGNIFRGVKAASAGMDRATADYIGM
IATVMNAMTLQDSLERIGVQTRVQTAIAMQELAEPYIRRRAIRHLEKGRVVIFGAGSGNP
FFTTDTTAALRAAEIEADVIFKATKVDGIYNADPKVHSNAKRYKTLTYGHVLAEDLRVMD
STAIALCKENNIPILVFDLTVRGNIRRAVMGESIGTLVGGSCEIS
>1170768.4.peg.2380
MNKTVEATQRAFNSIRTGRANASLLDKVQVDYYGSPTPLKSLTNITTPDASTILIQPYDR
SSLNIVEKAISLSDVGLTPSNDGSVIRLNIPPLTSDRRKELVKLAAKYAEEGRVGIRNIR
RDALDSIRKQEKAGEISEDESRDQQDKLQKITGKYTAKIDELLTEKEKDITTV
>1170768.4.peg.2382
MEVILQIIRQQPNSTPVVQSYNLQVDPGNTILDCLNRIKWEQDGTLAFRKNCRNTICGSC
AMVINGRSALACKENVGSELARLRNISASVYHADHHTINSITVAPLGNMPVIKDLVVDMT
SFWDNLEAVTPYISTAARQIPEREFLQTPQERSLLDQTGNCIMCGACYSECNARQVNPDF
VGPHALAKAYRMVADSRDDNQGNRLEEYNHTTQGVWGCTRCFYCDSVCPMGVEPLAQINK
IKQKILERKQADESRSIRHRKVLVDLVKQGGWIDERQFGVQVVGNYFRDLKGLLSLAPLG
LRMLVRGKFPLSFEPSSGTQEVKSLIESIQKQELT
>1170768.4.peg.2405
MSGTTGERPFSDIVTSIRYWVIHSITIPALFIAGWLFVSTGLAYDVFGTPRPNEYFTQVR
QEVPIVSNRYEAKKQVETFIAK
>1170768.4.peg.2410
MSQGRKNQSAIIPVCNFGEIVLCMLKSPLRYPVNKRQFLLDEIAIF
>1170768.4.peg.2412
MPRRQDIHKILLLGSGPIVIGQACEFDYSGTQACKALREEGFEVVLVNSNPATIMTDPET
ADRTYIEPLTPEMVAKVIAKERPDALLPTMGGQTALNIAVALAKNGVLEEYNVELIGAKL
PAIEKAEDRKLFNDAMEKIGVNVCPSGTASSLEESKAIAQRIGSYPLIIRPAFTMGGTGG
GIAYNKEEFEVMAQVGIDASPVSQILIDQSLLGWKEYELEVMRDLADNVVIICSIENLDP
MGIHTGDSITVAPAQTLTDKEYQRLRDMAIKIIREIGVETGGSNIQFAINPVNGDVVVIE
MNPRVSRSSALASKATGFPIAKMAAKLAVGYTLDEIKNDITKKTPASFEPTIDYVVTKIP
RFAFEKFPGSDPVLTTQMKSVGEAMAIGRTFNESFQKALRSLETGRAGWGADKAEKLPSG
EQVRAQLRTPNPERIFALRHAMQLGMSNEEIYELTAIDPWFLDKLHQILETEKFLKRTPL
QQLTKVQMYEVKRNGFSDRQIAFCTKTKEDEVRVYRKQLGVIPVYKTVDTCAAEFEAFTP
YYYSTYEEETEILPTDKPKVMILGGGPNRIGQGIEFDYCCCHAAYALKSANYETIMVNSN
PETVSTDYDTSDRLYFEPLTKEDVLNIIEAENPVGIIVQFGGQTPLKLAVPLQEYLQQSP
STVTRIWGTSPDSIDMAENRERFEKILEELKIAQPANGIARSYEDALIVAKRIGYPVVVR
PSYVLGGRAMEIVYSDSELERYMSFAVQVEPEHPILIDKFLENAIEVDVDAIADHQGRVV
IGGIMEHIEQAGIHSGDSACSLPSISLSPAVLNQIRTWTVELAKALSVVGLMNIQFAVVG
ASSYSPQVYILEANPRASRTVPFVSKATGVPLARLASLIMSGKTLEELNFTQEVIPQHIA
VKEAVLPFNKFPGTDTLLGPEMRSTGEVMGIDVDFGRAFAKAEMGAGEKLPLQGTVFVSM
SDRDKSLVVEVIKEFIQLGFKVIATQGTSEFLREQGLKIETILKLHEGRPHVLDAIKNRQ
IQLIINTPSGQEARTDGQLIRRTALGYKIPIITTIAGAKATVAAIRSLQNINLDVKAIQE
YSF
>1170768.4.peg.2413
MTTEKPEPFWFYRVRLIYSRSHPKEEEGEPERFLNNHF
>1170768.4.peg.2416
MITPKPQQNTNPPHKIIGVGVIWNQEKQILIDRRLPTGSMANLWEFPGGKLEEGETIQDC
IVREIREELGIKIAVREHLITIDHTYSHLRVTLSVYHCDYLDGTPQSIECAEFRWVNLDD

LEHFEFPAANGQIIAALNS
>1170768.4.peg.2417
MPKGSVVFLPVTRFPIDNIVSEKQYQEELKTTDYFPMNAGMRWCGDYFLYCSQIF
>1170768.4.peg.2418
MIKQKVALVTGASRGIGKTVALGLIKDGFRLALVARSAENLQAVASEIVELNLDADSQPL
LFPIDITQRDSVQTIVAEIDKIWGRIDLFFNNAGTWIEGTLDVEIDELDRIFSVNLKAGF
YFLQAVVPIMKRQGFGQIVNLVCITAKGAEPDWGIYSASKYAFFGLTEAIHFDLIKYGIK
VTAICPSWVDTNMAQQAATPYPSDQMIQKEDILNTIRWLIKMSPKAAIKEVVIDCISEYV
REK
>1170768.4.peg.2419
MHEQNILPHEWYCEILNRHVGTFNIIICSHVKGPLTQEIIRQALDMAQSRHPRLNWRIVG
EQNCLRFETEGTQKIPLQVVDKLHNKYWQEVVTAQANEGIDSSKCLMRAVLVRALNEEDT
NYLITTIHHAIADGLSFVQLQSEILSYAQKIASGEALTQVTPLPTLPSLEKLLPYSATTS
QLYESYPETDTLGFEKWVPIENRKTAWIYRQMDEKLTQQLKKIAKQEKTTVHGAFCAAML
LETARIIRANQNIEVVRACCSTPFNLRSYLQVPVSSEHLNFMVWDVRLWHTLEKNTSFWD
LAREARQQIRSVLKSGAFISEITSEKLEDFIPLLDKEVEITVEITNLGKLNIPTIYGIFE
IEEIHVICGTAAFAGIPTLVVTEFRGKTLLSFLFSEPSLSQTTMEILANNVVSNLMDACY
>1170768.4.peg.2420
MAEVIQKVVEEEADFFKALGLELPKEVIIYEEFTAEKCEWQGLGTVKKTAVKEKPFSNLI
NDFPEHFENTACVFLLDDSRCGLQILSEAKGLHPWYYKPFTCWLFPIFIAPGESQREIFL
PSPETEPWYLPDDDYDGFYTQVLCGKHSDCGQLGYILLQEELNFLAKIVGRNFVQEIQDA
VVADSAERS
>1170768.4.peg.2422
MVGQFLGGGGQGEVYRADLGGKSVAVKWYYSHSIQADPYQRDRLEAAIQSGSPSDRCHNW
LFGFL
>1170768.4.peg.2426
MEISYLEWNQGHQPLLLLHGMADNALVWLSLGDYLSPNYHIIAPDMRGHGNSSKPETDYS
FTSAIADLEALMNSLGWFSANVVSHSWTGKLAAIWARQNPGRLKTMTLVDPIFIWKMPSV
LKLIFPLLYNVLPSLQTMGPFSSYEAAEEKIKKLVHFREWNDLQQQVFQGGIEQKPDGTW
GSKFTVAARDGIFDAVLEVAGFIHPVETPTLFVQPEKGVNRQDWQIKSYKDNLKNLTWKK
IPGTHWPFLSNPEEFNLSIAEFLAQAI
>1170768.4.peg.2428
MAIISINTLIKPSIVVGQTPTLNAKEEVNHTCASQIPSSIEKIINSPTFERMRWGILIKN
LSSGQIIYSRDAQKYFIPASTTKILTTAAAWQKLGKDFRIHTSIYQGDEGNFYLVGRGDP
SFNNVQLTALAQKLQQRGIRSINKLIVDDSYFQGEYIDSSWQWEDIQADYGAPVNSLMVN
ENTGILTLSPNKIGEKLNITWSNNIELYGGIENNSITVAENEPRFVQVTRDLKGQILKID
GKLRINSPSQTIGLSVIDPIDNFLKNLRFTWAKVGITVKEIQPVFEYQHYKIAEQKPEIA
VVESLVLPDLVKEANMNSNNLYAEALLRHLSNSEPTNKYETTVNQGLKILKTTLGEWGIE
PNTYMIVDGSGLSRKNLISPEALVETLQVMAKSPDGNLFRASLATGGMTGTLKNRFLKTP
AWGIVQGKTGSMTGVISLSGYINVPDYDDLVFSMIVNQSQQPSAVRKAMDEIIIILLAKLH
KC
>1170768.4.peg.2431
MGNLWSESYKLQTWLQVEIAVCEAQGELGYIPLSAVDEIKAKAKFDPKRVLEIEAEVRHD
MIAFLTNVNEYVGDAGRYIHLGLTSSDVLDTALALQLVASLDLLCQKVESLIQAIRIKAH
EHRYTVMIGRSHGIHAEPITFGFKLAGWLAEVLRHQERLQLLKNNVAVGKISGAVGTYAN
IEPRVEAIACGRLGLKPDTASTQVISRDRHAEFVQQLALLAASIERFAVEIRNLQKTDVL
EVEEYFSKGQKGSSAMPHKRNPIRSERLTGMARLVRSHAGAALENVALWHERDISHSSVE
RVMLPDSCILTDFMLHEITDLITNLLVYPQNMARNLNCYGGVVFSQKVLLALIDKGTRRE
EAYAIVQQNAHIAWNNPEGNFRHLITQDLRVRDKLSDSEIDLCFDAKQHLKYLEQVYERL
GI
>1170768.4.peg.2435
MGWDVPRGVNHNNYLPYITAAVTILNIFVSKLSQTFLLIVNF

>1170768.4.peg.2438
MIMEACVLLTVLLGFFGIIFKKNLVMKIISMDVMSTGVIAFYVFIASKAGLFTPILGEVK
KGNYADPVPQAVILTAIVIGFSIQALMLVGVMKLAGDNPTLEIDEIEKNNVP
>1170768.4.peg.2443
MNDSYLYIIIALLPLTAGLLVTQVNPYHGLILRGVLGAIAALVDAVLGAADVALTEALMG
TMLSVTLYAIAVRSSLVLRVGVIENQDGEILKLIEDFRRVFSNHYMRLEVVTYNNQQSLK
NGLISKEVHATCFPTTERQNYQTEVRVHRVYNIIEREILPNNTGLKNLEYVDILVSEEKH
P
>1170768.4.peg.2447
MYLTPKSGLFLGGSCITAIAAVGSVFELSYGQPNFGFQTTAIILAISVPVTILLFVAAVK
DARANIK
>1170768.4.peg.2450
MTVIKPVEYRTQSIDSSFEAEQVQFKLWRNLSASEKELLLKRVTKQGTSRL
>1170768.4.peg.2459
MGVSLSSNPPEFSQGNLDSSASDTFNSAINNAMVITSTKMSRSIFRECALNLQ
>1170768.4.peg.2462
MGTVAQGFLPLPGDTSIWIEISPGIEINKITDVALKSASVRPGWQIVERLYGMLELHSGS
QGETRAAGQAVLDLLGVKKEDCLKPRVISSQIIRNIDAYQTQLINRSRRGQLLLAGQTLY
VLEVEPAAYAALAANEAEKAAAINILEVLPVGSFGRLYLGGTERDILAGAAGALAAIENV
PGRAPQGHRKE
>1170768.4.peg.2468
MFTVSLIGNHRHLFGLSELARGGDFLQKHQEAVRTKLTASRKLCG
>1170768.4.peg.2478
MNQAEISTLIRQELEQLIVEDPVIKNLILGSMSQYYIGRQDADSKFDQTLAQLKSYQEEQ
NRKWEEQNRHNREIMAQLKSYQEEQNRKWEEQNRHNLQVLEEIKQMNRKHESTVGSLGSR
WGLSSEASFRNGLKGILKDSFGVEVLNFLDFDNEGEVFGRPDQVEIDVIIKNGLIILCEI
KSSIDKAGMYIFDRKVAFYEKHHQRRVDRKLVISPMVDPRALPVAQNLGIEIYSYAEDVN
SI
>1170768.4.peg.2480
MTPVENQAGANSCTANAVVGGYEYIMNRVGKDIDFSRLFVVYYNARLLGLEASGGDKIQDQ
GSNISLALVSLQDQGICHESTWSYQIAESGKVKNVNTKPSHEAYTEAAKLISTDEFQWET
PEQVNPDIYSMKHCLAEGYPFIFGLVLFKSFDRVTSQGRVPMPDLNSDEGREQHGNHAML
CVGYKDSAKVFIVRNSWGEEWGDSGYCYIPYEYMTNPDLCFECWKIKGTTDFDLTADVWV
GEDEDFDEEFYQEEEQEEEESCYLTLVEAIAVICLCGASVDNLSEEESELLMGLYETYEI
DTELLQEKIDSLVEIGGLEMLYNAAIQIILAEDAAIEAFQMSVEFALADEYFTDEEYEYW
SQLAGDLELDGDTATAFFNEVLAEYDYETIDSLF
>1170768.4.peg.2484
MLVYLAWQMWQLKHKLTIIANNLESKESASYGVLARIGDNINVEREQIYYWRQKQQMLKQ
QLQQAWQTFQLLKLLTTSGWRLLAQKVQK
>1170768.4.peg.2493
MISITVKKNGARMAIAMLFTNPGSEDIIYLDSLPGILKYRKSIIYILLTFILFIN
>1170768.4.peg.2502
MSVGLDLIPNTMIEYLYYLGNASLTLRVVQHLYNRPQIPVSFITVIHQINGWVIRIKLKE
AISTQEDGDFRAFCNELGISYEPPMRLQMAFWSLEAGQSPIEVMQRYQIAIVSHGGSEKE
EIEAFRQQFVRGLGYCPETLA
>1170768.4.peg.2503
MESAVSVSSPTRLVAQATQSIALDWLNKGLKAIAEGKVQDAIIAFRQAAQLDPTLAPAHY
NLGLALRQTGKLQPAADAFYRATQADTQFASAFANLGAALLEGNNLPLAIDYLKTALQLD
PKLGFAHYNLGLARQLQKDWQQAIASFQKAIIYSPNSAEPVYHLGNCYLQQGKLELAKST
FIKAINLNSNYTEAHYNLGLILFEQGQLEDSLSAFRKAAQTNHNYPNAYYGAGLVFVQLK
QYEQAIKVIKYARDIYQKQGNMAWANNAEKLLTQIRNLRR
>1170768.4.peg.2504

MILTFWWYLASMLLTNTLFLKTSLFFILYSIYNLSFDIRVNNG
>1170768.4.peg.2512
MRNSGLEFAMVGNLHGVCFGTASSVPLKLEEIHEITKY
>1170768.4.peg.2514
MSTCRHHNEWLSLLDVSGPFLSLPVLLKVFPNGLDAHNPENWKLLRLAYQEWQDALNNRR
FNTNDIHHAWINFVLQRILELPRGQLLPGESNPSLAFADVPQYGETLHPDYILINPQTQL
FPESESNREYQIRLLIQILPPEQNLEKPIVNKRWKASPATRMMELLHSNNIRLGLITNGE
HWMLLNAPKGEATGYISWYSSLWLEENITLRAFSSLLGIRRFFGVDDTQILEQLLAESIT
DQQEVTNQLGYQVRKAVEVLVHALDRIDQDKNRTLLQGISETQLYEAVLTIMMRLVFMLS
AEERGLMSIGNPIYDQYYAVSTIWEQLQKKADKEGEEILERRCDAWCRLLATFRAVYHGV
NHEKLKLPGYGGSLFDPDRFAFLEGRKQGSDGKTPPSEPIPVNNRIVLHLLQALQILQVR
FAGNVESRRLSFRALDIEQIGHVYEGLLDHTAVRASSPVLGLVGTKYQEPEINLADLGIS
NNISNSNNNNSDVESLLTPDRVKTLKKLTGKSDSALKKALETEFNHYEEQKLMTACNNDK
ELFQQVRPWMGLIRLDTLNYPMIIPAGSVYVTQVSDRRETGTHYTPRSLTEELVKHSLDP
LVYEGAVEGKPQREWKLKPAAELLKLKICDPTMGSGAFLVQTCRYLGEKLVEAWDNAETA
HPGKVVITPEGTLSSKSSSKLSLQSSLESPLQQSIIPQEADERLIVARRIIADRCLYGVD
KNPLAVEMAKLSLWLITLQKNRPFTFLDHALKCGDSLIGVGIEQLGYWNLNLRKTAEIFA
NEIRYEVDKIIKLRKEIIQLPVLTSQDQNRKEYLLARVKANSLDLVQRCNLLVGSYLNNW
HEKEQEGLRQTLLINIREGTDIPQEKAMALPDFEKLRPFHWELEFPEVFIDHSVSLFANA
SSTKKNQSANYSQGFDAIVGNPPFMGGQKITGNVGTEYRDFLVKWLANGKKGSADLCAYF
FLRAKHLLQPNGVLGMIATNTIAQGDTREVGLDQLIADCTIYRAVPSRTWPGTASLEVAY
VWLKKGGWKGNFILDEKPVGGITAFLTVPGKVFGNPERLVANQNKSFQGSIVLGMGFVLT
PEEAEALIEKDPRNKDVLFPYLNGEDLNTSPDQSPSRWVINFRDWPLDAEHDNPKNPQGA
PYASDYPDCLAIVREKVKPERDKNKDKQRREIWWRFTRPTVELYRAIANMENVLVLSIVN
NHLGFAFASKNTVFANRLVVFPLYDYGLFTVLQSNLHYHWAWNYSSTMRRDINYSPTDCF
ETFPFPPSIKELEEIGEKYYNHRQKIMSNCQQGLTKTYNRFHLPEETTSDIKELRNLHIE
MDKKVATAYGWQDIELDHDFHVTKQGIRFTICENARREILDRLLKLNHQIYEEEVAKGLH
HKGNKKAKTGGKEKVKENVNNDIQQMDLFGEQ
>1170768.4.peg.2515
MVQILAKIEILRHDPKPQGNLKKKLHGYLGNVYRLRSGDYRIIYTYDDGWVALLGVDSRK
DIYKGNKLFAEGADFDVKSLPDVESLLTAESGYSAVNSQSNSQSILPNQQIQNTENFLPL
LLTEDLLERLLVPKELRTNLINCRTFDDLIKVDIPENLRDRLFDCIYSPNFDLVLSQPSY
RTDSTSDLLKFVEGSLVGFLLKLNPEQEKYVNWAIDASGPTLLKGGPGTGKSTVALYRVR
ALLQVLKKHGVVEPKILFTTYTNALITFSEQLLNNLLGEDMRYVKVKTADWIAYSLYYQY
QNSPYTLAKNNELQNIMKRAISNAIDSLEGNLLKKQAQAQTLQRLTSQYLIDEICTVIEG
RMIKTLELYQNTPRHGRMVSLNRTQRQAIWHLRRHFYQLLEEHKLQTWHQLRSRALAILE
TMQNPPIYDAVIVDETQDLDANSLRLLTKLCRHPNRIFITADANQSIYGSGFTWSDIHED
LKFVGRTGILKINHRTTREINEAAHSYLGTPGLQNATLDKELCNSQISPLNASREYIHTG
PLPAVRAVKNINEENQLLITFFQQATREFRLGMNACAIFTPNEKVGRRIAEQLTALGSIA
NFMSGKNLDLNRRGVKVITLKSAKGLEFPVVAIAGFVDSNYPIAPKNTSIEGMTEILNRE
RRTLFVGMTRAMRGLLIIIPAYGRSSLLNSFDPQLWNIAT
>1170768.4.peg.2525
MDIKQLFIAGGVVMWPLLLSSIIAVGLIIERIWFWSKITKRQEKVVKYVLNLYRQNNLVN
AIDTLQKNADLPIARIFLTALELEEPNPEEFRLALETEAQAEIPLLKRFTTVFDTIIALA
PLLGLLGTVLGLINSFASLNIRAVGK
>1170768.4.peg.2530
MSKPQITIRLSPSPLQELNNYVELTSTSRTDVVVNAIAQYLGCTDNVPLN
>1170768.4.peg.2539
MSINTPPKNLSFTVPMTFSNELSVFEMNIPIDEGSWSTVKSVAYGLDLEQSLSTKNLSLL
DNTLTSARNIGTLTGTQTFSDFVGSSDTNDYYRLDLSQTSNFSLSLTGLSADADTQLLNS
SGTVIQSSQNAGKVSESINRSLNAGTYYVRVYPYSGNTNYTLTLSATPPDQAGNTLATAR
NIGTLTSTQTFSDFVGSSDTNDYYRLDLSQTSNFSLSLTGLSANADTQLLNSSGTVIQSS

QNAGKVSESINRSLNAGTYYVRVYPYSGNTNYTLTLSAVSSPPPPTAGFNSTYGYGMINA
ARSVAAALGQSSPFPDVANLGGNNWGNDLVGSPEVWSRGYTGQGVVVAVVDTGVDYTHSD
LDANIWVNTREVSGNGIDDDANGYIDDIRGWDFVGNDNDPMDGNSHGTHVAGTIAGENNG
TGVTGVAYNARIMPVRVLDNNGSGSSLGVANGIRYAADNGARVINLSLGGGYSSNIYSAI
QYATTRGATVVMAAGNSGASQPDYPAFHATEYGLAIGAVDSNSNIASFSNRAGNNSAMRY
VVAPGVSIYSTVPNNGYATYSGTSMAAPHAAGVVALMLSANSALTPVQVRNIVTGTNNVA
NISSLGTDSQLVTATRYYVEESVPEFSISYVQVPTDPVIPVDNNYNNEITTDQVEVLGSI
STQLSTAVTKDNYYVYLDDSGNYSFSLSIVLGLFGSPLVVHPRINHF
>1170768.4.peg.2543
MSIATAVKTEYEAIIGLETHCQLSTNTKIFSSSSTAFGAEPNTNIDPVCMGLPGVLPVLN
AKVLEYAVKAGLALNCQIAKYSKFDRKQYFYPDLPKNYQISQYDLPIAEHGWLEIEIVDD
HDQLIRKRIGITRLHMEEDAGKLVHAGSDRLAGSSYSLVDYNRAGVPLVEIVSEPDLRSG
EEAAEYAQELRRIMRYLGVSDGNMQEGSLRCDVNISVRPVGEKKFGTKVEIKNMNSFSAI
QRAIDYEIQRQIAAIKAGERIIQETRLWEEGSQRTSSMRVKEGSSDYRYFPEPDLAPIEV
SHSQLETWRSELPELPAQKRHRYERELGLSAYDTRVLTEDLPVTEYFESAINAGANPKSA
ANWITQDIAAYLNKQKLTISEIKLTPQTMAEVISRIEKGKISNAQAKEKLGDLLTGISVE
EVFAGQELITDPDVLGPIVDEIIAANPQQVEKYRGGNINLKGFFVGQVLKKTNKRAEPKL
TNELVEKKLHNL
>1170768.4.peg.2570
MSTWIFLPQGGLIIHLKILLIIQVTYTSMVIKNRNLGWIIGD
>1170768.4.peg.2573
MAAFLDYIIGDPWGWPHPVRVMGWFISRWTQFSWQHFSRPLSQRVAGIGLALTTIGAAAV
SGWLIVHITHWLHPWLGMTVASILLASCFALHSLTKAALDVIKPLNDGQLSQARQILSQY
VGRDTADLSESEVLRAVLETVTENATDGVMAPLFYAIIGMLIPGVGPIPLALGYKASSTL
DSMVGYREPPYTYIGWFSAKLEDSLTWIPCRLTVITLAILSLKPLGVWRLCKRDAIQDPS
PNSGWSECAYAAILGVQMGGLNWYKGVAKYKPLLGDPIYPITSTSIYRALQLTRYSFLLW
LGLTTVAYKIIIT
>1170768.4.peg.2575
MGKLICTLIFRIKLIKFFKPLSIFPRLEEDGGESLLSDNG
>1170768.4.peg.2587
MLQRLAHQPLTLGDVLETTSQRGFILVIALLVLPFLFPMPPGLTGPLGSACLLLSLQMLL
GRRSPWLPKKIANYQFPRVFAQTILHNLGRVTRLLEKIARPRLTKLANHDITWRCNGFCI
SWLAILLISPVPLTNPIPTIGILLFAAASMESDGLLICICYVLTLLITLIFYLIVYGVLQ
LPGLIT
>1170768.4.peg.2589
MQRSLIVAVLIGLLCAVVGTYLMVQRLALLGDAISHSVLPGLAIAFIIGANIFVGALIAA
MVSTVAIAVIKNRSPIKEDAAMGIVFSGFFALGVTLITGVQKTNKIDLNHFLFGNILGVT
PNEVRDTAILAAVALIMVFLLYKELLFYTFDPIGARVAGLPVNQLNISLMLLISLTIVAS
MKAVGVILVLSMLITPGATAYLLVNRLHQVMILGAAIAIISSIVGIYLSYFYNLPSGPAI
VLVVCTAFVLAFLFSPKSRVLGKHGR
>1170768.4.peg.2592
MRPQKFFLHSTLKKFNRLTLVKLIKLWFTTQVTRKNKTHPNLS
>1170768.4.peg.2596
MAHYDHKSIQLGSSIIEPPIAPYNLLYGYFTQTLSSR
>1170768.4.peg.2620
MSFPWLSAGKLAPIGRACITVAMLIIVGWGYAAVAADPHKLEDLAKQSADLKVGIDTMWV
MFAGMLVFFMNAGFGMLETGLCRQKNAVNVLAKNLI
>1170768.4.peg.2621
MLGPRIGRYNPDGSANAMPGHNMSIATLGCLILWLGWFGFNPGSTMSVSDGTAIAHIALT
TNTAGAFGGIAATIAAWAFLGKPDLSMIINGILAGLVGVTASCAYISVPSSAIVGAIAGI
LVVKAVPFFDKLKIDDPVGAVSVHLGCGVWGTLAVGLFSQGNVYYQGGPTGLFFGGGIGQ
LWTQFVGVLTVGLFTVLISGIFFLALKYTMGIRVKESEELEGLDVGEHGMEAYPGFVKEA

SSPDLLGFGNR
>1170768.4.peg.2623
MASEIPGKVAFSKLVAIEVLVDKSKAENETWINLVVKSEELPLRLDNHCRQVFDQIRQEV
EDNRKWQLIESVVNF
>1170768.4.peg.2626
MGGQEVAIATINPTTGETLKVFLPLKDEEIEAKLHLANQTFQHYRYTNFVERSHWLLETA
NILDREKTDLGKLITLEMGKTLKSAIAEVEKCALVCRYYAEHGPDFLADVNISTDASRSL
VKYQPLGVILAVMPWNFPFWQVFRFAAPALMAGNVGLLKHASNVPQCALSIEEIINRGGF
PEGAFQTLLIPAIQVGDLMADNRIKAATLTGSEPAGVSLAVASGKHIKKTVLELGGSDPF
IVLESADLELAVSTAVTARLINNGQSCIAAKRLIVAEAIADRFEEMLLAKFATLKIGDPL
ADDTDIGPLATSKILEDLDNQVQMAIASGGKILLGGYPIKEGHGNFYPPTIIVDIPPDHP
IAQEEFFGPVALLFQVPDLDAAIRLANDIPFGLGASAWTNNPQEQERLITEIEAGAVFIN
GMVKSDPRLPFGGIKRSGYGRELSIQGMHEFVNIKTVWVK
>1170768.4.peg.2630
MDYIEKVLEKLRELARKLVEALLGPEVEPEGELIPIPVHERPRGR
>1170768.4.peg.2634
MPILTTIVSVICNRQKFAQRLSLYGEGRRCTVLYLLMII
>1170768.4.peg.2680
MFNQFVQLLLPNAKYIKKIAFDRILRPFRDKQSATDLISESTETLELSTKQRDQIIEEIR
ILLKNEKTLQQTLKKEQDQRNTANEQLFLELLGIFDTLEFLVDYLSNSPEPSAKSIKRLS
KQLEVLQRKLVGILEQRKVELIEDLNHTKPDFNLCVVVDREVRNDLEDQTITKVVKKGFR
IENRVLRPIEVITSKQQ
>1170768.4.peg.2685
MELKVWRKGSKDPLELLYFKKHYFVSHPNILPNILNIPTQNSTLIYLSFKSDRS
>1170768.4.peg.2686
MTGKGGVGKTSVAAATGLRCAELGYKTLVLSTDPAHSLADSFDLELGHDAKQVRPNLWGA
ELDALQELEGNWGAVKRYITQVLQARGLEGIQAEELAILPGMDEIFGLVRMKRHYDEGVF
DVLIIDSAPTGTALRLLSLPEVGGWYMRRFYKPFQNISVALRPLVEPIFKPIAGFSLPDK
EVMDAPYEFYEQIEALEKVLTDNTQTSVRLVTNPEKMVIKESLRAHAYLSLYNVATDLVV
ANRIIPEEVADPFFLRWKQSQEQYRREIHDNFLPLPVKEVPLFSEEMCGLAALERLKDTL
YPGEDPTQVYYRETTIRVVQENNQYSLELYLPTIPKSQIQLSKSGDELNITIGNHRRNLV
LPQALAALQPAGAKMEGDYLKIRFADNVRV
>1170768.4.peg.2700
MGYSVFGFPIPLGGRGLFILNHLLDSGFWLLSRELSFWELSF
>1170768.4.peg.2703
MRANSTRRRVFKNPLPERSHNLWYTRIVGGKLTGDRSFHLRDTP
>1170768.4.peg.2704
MVREIAPKTQLGVIKSKVNQGGETIAPDIVYPESDGKPMADNTKQFAWIVKIKENLEVLF
KSNPDVFVAGDLFWYPVKGSNRIKLAPDTMVVFGRPKGQRGSYRQWEEDNIPPQVVFEIL
SPSNSKGEMTRKKLFYLKHGVEEYYVYDPDEISLEVSIRENNSFREVEDFATWTSPRLNI
RFDMTGDELVIYYPDGSRFLSPVELSNYAEQERFLKEQERFLKEQANQRAEQERLLREQE
RFLKEQANERAEQERLLKEQEQLKYQTLLAQLKAKGIDITTLE
>1170768.4.peg.2711
MEKWATQELQYADLGDTRRKKRLISIVENLSSQPSTSVP
>1170768.4.peg.2712
MDGVNAREERKNYNFNGGKGRSVLGVLRKRISLVLEVLNKYSHKKV
>1170768.4.peg.2718
MKLWDSSFQLIRFEIVDLIGFLGFCSCFFTSQFLNCLLDKDLGDCQLYDSFAV
>1170768.4.peg.2719
MEIKRNWNTLFDVDENGDFFSVSISYGDKEKLEQMGHKNSWHFLPLR
>1170768.4.peg.2720
MEIKRNWNTPMLDRFKVLEAIVSISYGDKEKLELILVV

>1170768.4.peg.2727
MTHPNSGTNVTFLTSVIAEFSGDQTAIAENLKLFLNALLGEYQISYIQPNAERGTLHKVE
VQVKDGNKKVNSKSVSYIMPVFGRSLPLVNRLVMVTSTCLSLVGCQTIIPNLDNR
>1170768.4.peg.2728
MASLWQIIQSSLRDIPPQVVFEILSPCNSKGEMTRKKLFYLKHGVEEYYVYDPDEMSLEV
SIRENNSFREVEDFATWTSPRLNIRFDMTGDELVIYYPD
>1170768.4.peg.2737
MKRRSFIESSALFGASLLGTQAIASIGNRGIEPVQAQQIKELNFGIISTESQANQRPLWE
PFIAALSSSIGIPVRAFYATQYAGVIEAMRFGQVQIAWYGGKSYIEAAKIANAEAFAQTV
SSDGKKGYYAHLIANKSNPITAAAKKQGGDKYVIRNASKLTFAFNDPNSTSGFLVPSYYV
FGRNKVDPKKVFKRLIFSGSHEAK
>1170768.4.peg.2740
MVSQTRSSFIGEITQETLALTRRLFIQLQRRPSTLIAGIIQPVMWLVLFGALFQNAPQGL
FGSTANYGQFLAAGVIVFTAFAGALNAGLPVMFDREFGFLNRLLVAPLASRFSIVFASAI
FIISQSLLQAAVIVGAAAILGAGIPDVNGLVAIALIVFLLALGVTAISLGLAFALPGHIE
LIAVIFVTNLPLLFASTALAPLSFMPEWLKIVVALNPLSYAIEPIRHLYLNSNWGLNDVV
MEVFWGNVSLGGSLLVLLGFAIVALLSIQPQLRKTLA
>1170768.4.peg.2741
MLVCGAGIAFIGLFAPQPSFSNTASTSQPLNSPDNTNPLANDSGLDMFNIIHRLNFGPLN
WDPNQQNQQLEDAAAAFKARQNQILQNQQPSKVKGEENRSGANTDGSNTERK
>1170768.4.peg.2752
MLIPNGRFLGFIVMNVCLLSYPSLSLIQSPELFMGIM
>1170768.4.peg.2765
MAGYDVSQAEDGIKGQALALQLQPDLILLDLMLPRVDGFTICQRLRRDERTAEIPVLMLT
ALNQTQYKVEGFNAGADDYLTKPFEVEEMLARVRALLRRADRIPQAAKHSEILNYGSLTL
VPERFEAIWFHETVKLTHLEFELLHCLLQRHGQTVSPSEILREVWGYDPDDDIETIRVHI
RHLRTKLEPDPRRPRYIKTVYGAGYCLELPAVPPSGEASNMTLVE
>1170768.4.peg.2777
MLTLSAAWTVVHIVKYCVQAIALNNPVNMVAFLWFFLVRAFFIMVFSYG
>1170768.4.peg.2778
MLSIPGGHQYCARPFGYSENYDDVFAEPGNILIRYYVPTRMGIKMIFQGLFFELNNPQNW
SLTFPTTIPKE
>1170768.4.peg.2788
MAVLAVLIVSFLLLGPIITYFSKSLTLSYCQFELELFPSLPTLDRTL
>1170768.4.peg.2793
MTDQDISGGFSPVETTKPEQNPRLSPDLEVQVNQSSGGNPIQHSDRNFGSLMQQQNNNDL
YEKGRKNNWYRPVDFILSGKIWELAMWKKSWIWWFFVLAFIPSSIGIISVSILLKLPSAP
NCPRIFWPLASASMRVHCATLAASKQTVSDLLQAIALVKDLPQDHPLRGQINDLLQEWSR
DIINLAEKSFQLGNLEEAIATAKQIPENLEDRQFVEEKILKWQSTWSTAEEIYQSSIGEL
ENRRWQSAFMLSSKLLRINNRFWSTTKYDQLNQIIVTAREDGDKLDKADSLADRNSVNDI
LSAIKLVKSVKPESYLYKKAQELVPQFGRKMLKLAQAQMERRDADKGLEIAGKIPPIPSL
QSEIDDFIDLGEAQRNAWLGTILGLENAISQAQQIDPSRGIYGRAQELISVWQLEIEDVT
KLEQARDLASGGTIEDLRSAISQAQQIPSQNPRAQEAQTQINRWNDQIETIQDKPYLDRA
EQIANAGDINSLQSAIAEASQISSGRALYSKARRRIRSWNASIQRIEDQPYLDRAIVLAE
SGDLNSAVQEARKIAISGRALAGEAQAVIDTWQEQIRARENWRKARELASIGTADALSQA
IRIANRVSRRNVLRMDINIAIDRWGEQILEMARSQSQVDLVKSIETAKLIPRSSSAYADA
QLQIRTWKEQLVPVPTTPFLSPGEPSP
>1170768.4.peg.2800
MLGTYGSPLYVYDSDILNQTIAHITKSFKYPETRFYFATVTNGNIALLKVFKNAHWGLHA
NTPGDIYLGLSAGFAPDSIIYSGTNLNREEIEQVLSWGVTTLNLDSLAQVQLLCEVCSSL
KNTAGKGKNLNTFSRLNQLDIGLRLNVGGESRIGVRLQDFNTAIAIAKNVGLEISGLHFY
RGTGTNATKAFTDAIDEVLVAAQNLPHWKYIDFGGGFGYPYNHQGIAFDWELFGGEIIQR

ISNLDRTIKLIIEPGRAAIASCGTLLAKVVSVKWQDNKQILGVDSTVGNIAVPSVYGGYR
EIISWKNHSPKYITDICGNTTYSRDYLGKNCQLPSLEMGDIIGILDVGAYGYAMSSHFLH
RPKPGEVLLENSQHRLIRNREDYSVLLTNQIW
>1170768.4.peg.2807
MAIPLLEYKPSSQNQRVPGYEVPNEDTPRIYRIEDYAFTGEVEELIWAAYRQLFSEHVIL
KFYRQGNLESQLKNKAITVRDFVRGLAKSQAFDDLVIKTNSNYRLVEIALKRLLGRAPYN
KEEEIAWSIKIATNGWDGFVDALVDSEEYQNSFGENIIPYQRRRYKDRPFNLVTPRYANY
WRDKIEAGRYKPGSISDFMKMAASVSIRTVIYTPVNTANIAIPNTTRETVPTGIPVSISP
SANFPVK
>1170768.4.peg.2809
MTQELIGELGGMGQLNQLIDAVNQAQTPLEMVTAVKNLAAAKSPAAIATLIAVFGYNNPP
AAGLALAALTDLGTVAVPSLLAEIDDYNYGARAYSIRTLAAIADPRALDVLITAGVTDFA
PSVRRAAAKGLGNLNWSQLGDNERDLGIERALEALLLIYQDTDWSIRYAAIVGLEYLGKT
RRETVLNKFREMIANEEEKSIRIRIQLATSKVTRDN
>1170768.4.peg.2812
MAITTAASRLGTEPFSEARRVELRPSASREEVELVIRTAYRQVLGNDYILASDRLVSAES
LLRDGNLTVREFVRAIAKSELYKAKFFYNSFQTRLIELNYKHLLGRAPLDESEVVYHLDL
YNNKGYDAEIDSYIDSVEYQNNFGENIVPYYRGFDFQPGQTSVGFTRMFRLYRGYANSDT
AQVEGSKSRLARELAGKKASSIVGPSGSNDNWSFRPSADNAPKQNLGNAVGQSDRVYRIE
VAGIRSPGYPSVRRSSTAFIVPYERLSDKIQQIHKQGGKIVSIIAT
>1170768.4.peg.2826
MYVSGKGIVLQEPSVVAIDHNEKIALAVGEDAKRMLGRTPGNITAVRPLRDGVIADFDTA
ELMLKSFIQRVNEGKPLLLPRIVIGIPSGVTGVERRAVMDAATQAGAREVYLIDEPVAAA
IGAGLPVAEPTGNMIIDIGGGTTEVAVLSLQGTVVSESVRIAGDELTESIIQYMKKVHNL
VIGERTAEEIKIRIGSAYPITEEEQGSMEVRGLHLLSGLPRTVTVQGPEVRESMLEPLAI
IVEAVKRTLERTPPELAADIIDRGIMLAGGGALLKGLDTLISHETGIVTHIAADPLSCVV
LGTGRVLENFKQLERVFSGRSRNT
>1170768.4.peg.2863
MKWQLLTHNKQVLGKIFTILVFTGLTGVLCVSCNRNQDLLVTEIGVNPPKRPTRKTSGAG
EFYLQGQNQHSRGNFQAAIAAYSKSISLNSDYAPAFKARGLAYFDLNNKERAINDYNQSL
QINPNDPETYNYRGNARASLGNQKGAIEDYNEAIRLSPNYAEAFNNRGNSHAAQGNKNAA
LEDYTQAIRIDQNYPVAYNNRGNAYSSLGNTSKAIADYNQAIRLNPQFAPAYNNRGNAFA
SSGDKRRALQDLQKAATIFDQEGNRGLYQQTMKNIEELEN
>1170768.4.peg.2864
MPVSAQMTIANGKKIDLEVAKTPEQQMMGLMYRSALPDNRGMLFAFLSPQPVGFWMKNVP
VSLDMVFIHSGVVQYIKTAPPCQNEPCPTYGPKVLIDQVVELRAERARELGLKIGDKVKI
ELFKPLR
>1170768.4.peg.2877
MLDLGLVKRSPLGGLTIANPIYQEVIPRVLSQGSQDSLPQTYGREN
>1170768.4.peg.2887
MRISNFRGPQQTAILTTSDRRHADTSDSPVLFSWGVLWDFS
>1170768.4.peg.2890
MYKIVKNKIYVANLFLFVLISMSTSSASAQTTTGIEDIARKTTVQINSNANPGGSGVIIK
KEGTIYTVLTANHVVCDNLGTIRIRCRTDLTYTVVTYDGKEYPMKYRQSLQINVQDPDLA
IITFESRENYQIAPLGNSDNVKIQSDILVAGFPTIFGRVGKQRTFTITNGKVVTFIPNSD
RGYGLVYNATTFIGNSGGPVFDIYGRVIGIHGLADTDDGETNNNDQSETVNGVKPTQKTG
FNAGIPINIFFSLSNFNRQLDPAVSINRQPNSTNPNNVSLNNRAIAYHDRGVNHYQSGDR
QGAISDFTQAIQINPNFAQAYNNRGIARHNMGDKQGAISDFKKAAQLFDLKNSRSRKIMR
IP
>1170768.4.peg.2891
MSQKLNALCFDGKWNHVVLPPDNSDYSQGITVEAWVWYGSFAQNWSRIVDFGNGQGRNNI
VLAHAGTSNSLSFHTFTSTGGYAVEVPNALEIGKWAHVAATIDKSGEAKLYKNGKLIQLI

QTKPFRLPDNVERKLNYIGKSNWTNNDGFFQGKMAEVRLWNVARTPEEIEQNMNRRLSGN
EAGLVAYYPLNGDANDKTKNARHGIIIGATWQQEELPIQEPNVNQNQTTTQTTTFITPDR
LLPKYDAVIVGAGIAGAIRVVGK
>1170768.4.peg.2895
MVKRVQLVLTKDVSKLGKSGDLVEVAPGYARNYLIPQSLATQVTPGILKQVERRREIERQ
RQLELKQQAEEQKSALEKLIKVAIAKQVGENEAIFGTVTTQDVVDAIQAATGQTIDRRGI
TIPDINHLGTYKADIKLHSEVTAKIDIEVVAS
>1170768.4.peg.2901
MTPQRETILHIFQELPQGEHLSAEDLHHRLENGGESISLSTVYRTLKLMARLGILRELEL
GEGHKHYELNQPYPHHHHHLICVKCNATIEFKNESILKIGTKTAQKEGYQLLDCQLTIHA
VCPRCQRALIPL
>1170768.4.peg.2902
MLAISLDTEVEYNPQQSHLTLPELDLSDVGDVNTDEGDLSEELSLDDLQDIEQEEDVEIA
AVDPQNLVANNRRSTDLVRVYLQEIGKVRLLGRDEEVGEAQKVQRHLKIRLLLANAAKQG
DAVIVLYLQLIEVQERLVSELGHRPSLERWASTAGIKLMDLKPTLSQGKRRWAEIAKLTV
EELDEIQSQGIQAKEHMIKANLRLVVSVAKKYQNRGLELLDLVQEGTLGLERAVEKFDPT
KGYRFSTYAYWWIRQGITRAIATSSRTIRLPVHITEKLNKIKKAQRKIAQEKGRTPTLED
LALELDMTPNQVREVLLRVPRSVSLETKVGKDKDTELGELLETDTITPEETLMRESLQKD
IHHLLSDLTTREREVILMRFGLADGHSYSLAEIGRALELSRERVRQIESKALQKLRQPKR
RNLVRDYLESLS
>1170768.4.peg.2903
MSCTNLEVICWELAIMANRKMKTLSLPKIITPQCQVSTLPNLIVEQNCGKR
>1170768.4.peg.2915
MKNNPYSWIEESLNTIHRADWYRSVQTINSPPGAVVILSGQKMINFASNDYLGLAADDNL
KMSAIEAIRQFGTGSTGSRLLTGDRQLHRELEQAIASTKQTEDAVVFSSGYLANVGTITA
LVGKRDIIFSDQYNHSSLKSGGILSGGTVIEYPHCDMIVLEKKLKEERQKYRRCLIVTDS
VFSMDGDLCPLPNLLDLAEEFNSMLLIDEAHATGVMGKSGAGCVEHFNCTGRELIQIGTL
SKALGSLGGYATGSSALIDFLRNRAPSWIYSTALSPADTAAALAGINVVQKEPERRKRLW
ENVNYLQKVVKGNLGKLKILPTQSPILCFELPDAATALQVGKYLREEGIFAPAIRPPTVP
TSRIRITIMATHNREHIDKLVDSLQRIS
>1170768.4.peg.2919
MGKKRKFHLGAFIQATGHHVSAWRHPEAQIDAGSNFQHYRKITQTAERGLFDAVFLADSP
GIWGGTPETQKRNGKLVHFEPVTLFAALSSVTKSIGFIATASTTYEEPYNLARKFASLDY
LSNGRAGWNVVTTGNENAAANFGLEYHPEHGERYERAEEFVEVVKGLWDSWEDDAFLRNR
ESGIYFDPDKLHVLNHKGKYFSVKGPLNVGRPPQGYPVIVQAGASEAGRELKVMTEYSRL
KSSRSAVIKSKLNYPVIDTDVHTNDFTPALEDYIANYGGSRLVDALRKAESSRLNSKVRG
KDWYQQTPEERQYYRTIRSPWWARVTRNTLDLATYTLPELFYERQAEQGSDYSVLFPNNV
LAPTGASNEDRQALQRAINHYHADLYRKYSVFLN
>1170768.4.peg.2920
MLEGGADWGSHVYIHLVDRFSKRNLKGLQNYNPDLTNGHELYELFIRFGTELLEGYSLSQ
EELTQTVLGSSFNRYSRSPVGSELEDFAAAGIESIEDIRDRWVNSFFFGSESDDRTIATA
FNDRANPLNVKINAIYSSDVGHWDVPDLTDPLAESWDLVQEGVISEDEFQSYVFRNPYKF
YTQANPDFFRGTVIESKVNNIASHQVQKVEQNLVSV
>1170768.4.peg.2922
MLFQHQLELYRSLVGKAHLIYSSEFLGKLGADNSFDYLQRGIDG
>1170768.4.peg.2923
MNFQEVFGVGSSFGNIAFIIRARVEGLRDFGPSLSPFNAFLLLQGLETLSLRVDRHLSNA
LELAHWLEKQPQVEWVNYPGLPHHPYYERAKKYLRHGFGGVLNFGIKGGLEAGKNFIDRL
KLASHLANVGDAKTLVIHPASTTHQQLTDSEQLSAGVTPDLVRVSVGIEHIEDIQEDFVQ
AFG
>1170768.4.peg.2924
MTSASDNISLREFHSLNPRPQKLIVTIALGVLVGAAVLLYKYSWRQSVLFIIGSLLGIAL

YNSSFGFASAYRKLLLSKDGRGMYGQLLMLSVATLLFAPLLSAGKVFGQEIEGAIAPIGI
SGIIGAFIFGIGMQLGGACGCGTLYTIGSGSYTMIITLITFCLGAFYATLTHGFWSGLPK
IPPIVLGETIGWPAAVLIQLTILLLLAVGLGTWIKNSNSIAQNTQFSPWSIFTGAIALAV
LNWLTLLISGHPWRITWGFALWTAKVAMYLGWSPSSGGFWDGDSSLSSSVFSDISSVMNL
GIILGALLAAALAGKLTPQIQITPLKTLAKVLGGLLMGYGAFLSFGCNIGAFFSGIASTS
LHGWIWIIFALLGTFVSIKLQALFDSPLDLPPMV
>1170768.4.peg.2926
MKFIDPRPADLFQGKENIWVRNGHIPGAKNIPWPTFTDPQNPHKLKSLEEIKKILADKKI
APNDDIIVSCSTGREATLQYVVLKHLLGYPKVRVYEGSWTEYSTYSDLPVATGPEEVS
>1170768.4.peg.2930
MQKITSPVISEQQRIADLFYNEGVIPTKIAVRESFLTPQQYTTITPARIGNR
>1170768.4.peg.2931
MGLGLTFLITACSPSSTNNAGNNQTSSPNEAANSSSSSRLTNSSTVIRIGYQKAATVLYA
LKAKGELEKSLQKSGVSVTWTEFPAGPPMLEALNAGSIDFGYTGEAPPVFAQAAGTPLLY
VAYDPLATKAEAIIVHKDSPIKSLADLKGKKVAFAKGSNTNYLIVKALEKVGLKYSDIKP
TFLHSDCKK
>1170768.4.peg.2939
MICCLNPDCQNPQNPDGSKFCQSCDTPLVPLLRNRFRIIRVLSDEGGFGRTYLAEDTDKL
NDSCVVKQLAPKFQGTWAQKKAMELFSQEAKRLQELGEHPQIPTLLAYFEQDNCLYLVQQ
FIDGNNLLQELRLRKHYKDSDIQSLLLDLLPILKFIHSRGVIHRDIKPENIIRRKRDGRL
ILIDFGSAKQLTAAVQKKYGTSIGSHGYSAIEQIRDGKAYPASDLFSLGATCFHLLTGLS
PFQLWIEHGYSWVKNWQDCLEDSRSAELIIILDKLLQPDLKNRYQSADEIIKDLGKKNVY
GLKKSSTYLKKGKHKKPKHTILRNIFLTLVTISVVGGLGYRNLGQIQTAIFSQFNPLLMP
SNKSSTSPEYGQICRRTSSSRANKVSLFKTITQVDKSLAVVAITPDGNTIVSAGHKEIKL
WNSKTGKQIISLPGHTQNINALAISPDGNNLVSAGDDKTIKVWNLQTKKLTFNLVGHQDS
VQALAISQDSKTLVSAGDDKTIKVWSLLTGKFLKTLLDHNYWVRSLALSPDGFTLASGSF
DKTIKIWNIHQTSGQKPTTLLDTTSQTVTSLTFSPDSSTLVSTSRDRQIKFWDIKNKEII
FASKKQNVTSVIFSPDGKTLISGAKSCPDCSEKISSVIKLWDVSTKEEIYALPVNTKIVT
SLVLSADGKTLVGGTKDNKINIWEISP
>1170768.4.peg.2942
MAVMVGIINHGVTIAVPPPKEDIPEEILRTEIITIGRSPIDGKILTASEYAELQEQLRTI
PPRKLSPSLRHTVFLLRMRRILLQIFPFLDI
>1170768.4.peg.2947
MNQGRTYDYIDLIELLIGTIFESKGDSQVIYPLLENNLHLLDATFIDQLQSWGGHQVNKQ
ATPEQNAQLGASLYLLARIFYHFPGGDPLINLTIAICGYEFCAAIERQLGLEKELAQVLN
HLGAAYQTQAQMGTDSHSNLEKAIAAYNEAIAIRRQPGLERELAQVLNNLGAAYQIQAQM
SKEANLERAVTAYAEAISILRELDLERDLAQTLYNLGSAYQTQAQLGKDSQANLQKAIPP
YTEAISILRELGLERDLAKTLTSVGSVYISQGKLERAITPFREAITMFRQLGLERDLAET
LNQLGTAYLSEVERGKDPEANLQRAIIVFTKALTILRQPGLEPDLAQTLHNLGKAYQTQA
ELGTESQSNLEMAIAHYREALAIATPELSPADYLKFASHLGDLGFQQGWWDIALEGYERG
IKALETIQFWQPGDKSPDIQYPHIYQSAVRACIELHQYGKALELVERSKSRNLVELLENK
HLYPKGNIPSRIYAELDRLRQAVRNEVRLLEIQGEKRSHAQLNGLIEELDQFITSQVKPL
DPHFSLTQRVEPITISKILKLVGADPRRVILEWYFTDDVLYTFIITANPDVPIFPHKVVI
SPSVIRQFSQKYLDIYRDRHNLGNWKQALPELLGEMKEILQVETLLTQLWKLSPPCEELV
LIPYGCLHIFPLHALFPWERFPLGFSYFHSCQLLSSVEVTEREEVVKSLAIQNPTQNLQY
ASFEVETIITRLLIGTEVITEKEATRETIFSRSIPEKNYLHFSCTGYSNFQKPLHSAILL
ANCILPAPPKGGEYLPLPNGAAVDLSQCLTLGDICGLDMRRCHLVSLSAYEMGIGDMVDT
KNDDYISLPTGFLLAGASTIISSLWAVSDYTTALLMVRFYQFYTDTENLLTYGRPRLALY
HAQQWLRDAPAGKLLTWAAKLPFSGTRLTLLADFDTMPESQRPFQDPYYWAAFTCVGI
>1170768.4.peg.2952
MISAKMGNLNQAKAIIKEMQSINNQEDFGANILEQILTPSPEEDG
>1170768.4.peg.2970

MTAVASAINSKKVPMFFYPQLRSQIQIFLSVVILQVDDL
>1170768.4.peg.2973
MGKHLYWQIGNLKLHGQVFLTSWFVIGVLTLASILASSNVKRIPRGIQNLMEFALEFIRD
LAKNQIGEKEYRPWVPFVGTLFLFIFVSNWSGALVPFKLIHLPEGELAAPTSDINTTVAL
ALLTSLAYFYAGFSKKGLGYFGNYVQPVSFMLPFKIIEDFTKPLSLSFRLFGNILADELV
VGVLVLLVPLFVPLPVMALGLFTSAIQALIFATLAAAYIGEAMEDHHGEEHEGSH
>1170768.4.peg.2979
MESYLLKSLLNLEDKNISRENWLIVENGEEFETLVEELYGEIRHQLDRQIDHNHEPIYKP
LKIILAQRDPVKFLAGFIAGCAAECAVFLCNPDWEKNEWEQVWDLVNPDMIWGDEIKFPD
FTTTNTNITSEKPIISGIMIPTGGSSGKIKFAIHTWETLTASVRGFTEYFNLSSVNSFCV
LPLYHVSGLMQFMRSFTTGGKLVITSFKQLTSESVQKSDIYKIKNSDFFISLVPTQLQRL
LENQKLTQWLSEFKTVLLGGGPSWNDLLTKARANDIRLAPTYGMTETASQIATLKPDEFL
KIPLDEFLKTKNSGRTLPHAEIVMEHLLENEHYGENHLGVIRIKAKSLALGYYPTIWQDD
QGFLTDDMGFLDQNGYLHIVGRRSNKIISGGENIYPSEVEAAIRATTLIMDVCVIGIPDH
NWGEAVTAIYVPQSPPTPILEIKNQLQQTLTRFKIPKYWIPVVSLPRNQQGKINISQLQQ
IILESLDQEEIV
>1170768.4.peg.2983
MSTINTSFHSKVQFLQRQAASVLLYQSVLQTEVGIGFLELLQAIRYTDGDGRNCLQAYGN
YFHSLASSQQTWEEYLIRQILLSDNPFTKLAQKREFTDLPSALIAAAQHDLQILQNLYEC
NCALLSEWVQNVCHLPVSPVVWYQEPQPQQSQTVILQDLMITWQQLENWGDIIRDLVTYY
QHQGGGLFAQYRAFRWETGQFVGIPYPDRVKLETLVGYEEQKQILLKNTRFLLSGRPALH
VLLYGSRGSGKSSLVKSLLNQDGYYNLRLVEVRKSQLHSLPQIVEYLRELPQKFIIFVDD
LSFEEDDDVFKSLKVVLEGGLTPRPNNVVVYATSNRRHLIREYFTDRPNPKDHQEIHSWD
TMEEKLSFADRFGLTLTFEAADQRTYLQIVHHLAAETGMNLTREDLEFQALQWATRHNGR
SGRTARQFIDFLQADSNDNNNY
>1170768.4.peg.2984
MVLNSLYPKIREMEQLKYRIVREIEQLEQHRVLAYTESKQLENQILDCRNKRDTIHREIG
NYAAEKRQLELEIGTMEGTMGELQNSRQELHKSCIDLTAEKRRLDINCQVLRSEIGQLQS
QIAIYKQEKEELESNLVLSQRLKPQLEEKLHELRTESQDLENQVLTQKNLLQEAIETEQG
LQAKMVDLQHHKQKQETEVSQLQNRISLLRDEHDLLQNQVWELLQNLETLAPQSTLADDE
DNNGENNVELFPFDDLL
>1170768.4.peg.2986
MSFEINHRLLVSKLNLQINQGEALILLGRSGSGKTTTLKLINHLLKPTQGEVSVQGLSTN
DWDVIKLRRSIGYVIQETGLFPHFNVAENIGIVPSLEKWPSKKIKIRVHEMLDLVGLEPT
KFAERYPHQLSGGQKQRIGVARALAADPPILLMDEPFGALDPITRLELQQQFQYLQRQLG
KTVIFVTHDIQEAFFLASRIGLMYEGSLVALGTKREFIQSQHGEAKAFLACLTAAHHWEH
NN
>1170768.4.peg.2991
MGIIGVNGFLIYQGKNPQEYTITLINQIPSGFWLRLGFGIAQCVGVVIAAGFVIKLLNYW
LQIAARKVKNLEQKPVNDENIDAFFTTLNRRITLGIWLWVLIVCSQFLQLSYLLTQYLYI
GLTIYLIIASGLLILKTVGVIVDTLDALSIRYSSPDNLFRFYDRLGHLVPFLKSSLEFVV
YVSTATLVVQQVQLIANLASFGIKIIKIIAIVFISRVIFEVIYLLLEEFLFKDQQLTETQ
RSRRLTMIPLFRSLLQYLIYFAVAVSILYILDINPTPILAGAGIIGLAVGLGAQTLINDI
VSGFFILFENYYLVGDYIQAGKAEEKPVEGTVESIELRTTRIRHPNGQLQIIRNGDIGSV
VNFSKQYIFACVDIGVASNCDLQPVYRLIGEIGQSLKLNDPDVLEPTFIEGVESLSESHL
LLRTLTKVKPGKHLHIQRVLRQLYIDNFPDLGILLPASIRKDED
>1170768.4.peg.3000
MSVKASGGGSVARPQLYQTLPVATISQAEQQDRFLGRGELTELESYFASGAKRLEIAQVL
TENSETIVSRAANRIFVGGSPMAFLEKPQEPEMALVGAGSMNVQEGMKLGTITYVESRGG
FFESLRSIFNTSAGGPTPPGFRPINVARYGPGNMSKSLRDLSWFLRYATYAIVAGDPNII
SVNTRGLREIIENACSGEATIVALQEIKVASLSFFRKDPVATEIVTQYMDVLLTEFQAPT
PSTKVRQRPSADQQGLQLPQIYFNAAERRPKYVMKPGLSATEKTEVVKAAYRQIFERDIT

RAYSLSISDLESKVKNGSISMKEFVRRLAKSPLYQKQFYQPFINSRVIELAFRHILGRGP
SSREEVQKYFAIISNGGLPALVDTLVDSKEYGDYFGEETVPYIRGLGQEAQECRNWGPQQ
DLFKYSAPFRKVPQFITTFAAYEQPLPDQHPYGSGNDPLEIQFGAIFPKETRNPSSSPAP
FGKDTRRILIHQGPGINNQLSNPKARGLAPSSLGAKVFKLDQLPGTIGKKAAKGASVKFS
ESSTQAVIKACYLQVFGRDVYEGQRLKVAEIKLENGEITVREFVRILAKSDLFRKMYWTS
LYVCKAIEYIHRRLLGRPTYGREENNKYFDIASKKGFYAVVDAILDTVEYSESFGEDTVP
YERYLTPAGVSLRQLRVGTIREDINKVEKEETPRFVELGAVKELRTQPSVDFRINQGVSK
QREQTKVFKLIATDKVAVQTLIGAAYRQVFERDIPPYIIQNEFTDLESKLGNGEITVREF
IQSLGGSGLYIKEFYTPYPNTKVIELGTKHFLGRAPLDQAEIRKYNQILATQGIKAFINA
MVNTAEYAESFGEDTVPYRRFPTLPAANFPNTEKLYNQLTKQNDDLVVPSFETLQPRIKS
ENTPLLGNAIADLAIKAKQMDKSKPLFIELGRSFNDGRGQSVEVGVGTSRRKPARIYRMT
VGTNKPEINQVINAIYVQVMDVFSGQIPDYIRRSDLDSRLRNGEITVRQFVIELASSEIY
RKRFYTPYPNTKVIEFLFRHILGRAPATQGEIRQYNKILADSGLRTAVETIVNTGEYSRY
FGEDVVPYNRFPSLPAGNYLGSVKAEADLVKQSWSSLSPSVLTGRGTNR
>1170768.4.peg.3006
MEREAILIMKSRQDLFPQIKDKINSVHSYDTPCIVAMPIDYISETYLSWLIKETKILKQI
PLL
>1170768.4.peg.3016
MQIEQAQEELEQKLDLDFEGFTVEHLVINQQQALTIENLPGYHLEGVYDLKVKLPTRQIT
QLHNHFNIYLQIQKEGKSWRLLIPEKSGEKQPLVWRGYLII
>1170768.4.peg.3019
MLPATNKCAEIASNFPLRMGKSEYVKGKTKLSDLPSGH
>1170768.4.peg.3029
MDQWQTQVVIPYGDLYRKYEDKQLKIESITLFSDLGQESSEKIPTPQEIAKLNTYGKLSS
NRQKSLEKLSQTSKILGCQ
>1170768.4.peg.3030
MFLGIIGAVVPAIPGSSLILISIIIWGIVNNSFVTIKIPLIVTIIVLLLSTGVDFLAGYI
GAKQAGASKWGQIGAFVGLLMGFFGLLPTLPFGGPLLGILFGPLLGAIVGEFLYQRRLWP
AVKAGIGITVGTLVGNLIQGVLAISAVIVFLLTTWPQVYGG
>1170768.4.peg.3032
MLRFSYLKMKLKVKEFLTTQVQGIKGQIIWQVTNCTFTH
>1170768.4.peg.3036
MSAEYSEDILVQQTTADFFEHTLKWHCVYAYLPATTPL
>1170768.4.peg.3038
MKITNIPIITGIFLSVYTNREQGTGWGYKISHPKHKIVGCGVKLNTGVE
>1170768.4.peg.3045
MIFPPKITQQGLKLNFSFAQFLRSLFSFSEIQYIEPEEKDEHFFINLYT
>1170768.4.peg.3055
MKDSDPVNLFRYVVGGIRNVGLAYIHLIEPRSTSAGGSDKTDESAPSTARKLRRT
>1170768.4.peg.3064
MFEGLYIHDKWDWGRKFPVIKIDFADGVLKNREELDRRILDLLRKNAERLGVSYESNDIP
GKFGTLIGEAVAKYGIRAVVLVDEYDKPILDNIDNPNIAAEMREGLKNLYSVLKGQDANL
QFVFMTGVTKFSKVSLFSGINQLTDITIDTQYSSICGYTETDLRESFGDHLEGVDWDAVR
HWYNGYNWTGSQTVYNPYDILLFIGKGQVFRNYWFETGSPSFLLKLFQKEQYFLPNLEGI
QVTEEILDSFDVEQINPVTLLFQSGYLTIKDIFTDINQMVFCLGIPNMEVKIALNNQFIN
AYSNLVNEKLGIQRLIHTQLRSGDVEGLVSTIKRLFASIPWRNFTNNDLADFEGYYASVI
YAFLSSLDARVIPEDITNHGQSDLTVMVGGHVYVMEIKVVEGNQVEGNAALDQILGRNYA
EKYRGEPGKSVHEIGLIFSRSQRNLIQADWR
>1170768.4.peg.3072
MIKDDFFMPLETALERTFIAIKPDGVQRGLAGEIIRRFETKGFTLVGLKFMKVSKELAEQ
HYDVHKERPFFASLVEFITSGPVVAMVWQGEGVVASARKIIGATNPLTSEPGTIRGDFGI
NIGRNLIHGSDAIETAQREISLWFKEEELVSWTPHLAPWLAE

>1170768.4.peg.3075
MLGYVRGGETNYVSTLKSIRNLFELSDKLPVEYYRIGSPMQKITSSEYYNYGVTTTFYDG
SSNQFPEVSSPIDAAIISPEKEQKKMTVIITDLQQNSGDVTKLNKLINNTYYNIENRDYA
VGIWAIKSEFDGKIYLEGNNPRSFAYNTGQESAKFRPFYVLFVGPYGDIKYYFSQLKKYN
TNQALLNSDNSKFMIFHPDHILDKISVLDVTPMSLPQGITEPFSLVNGGVVVSKSNQEML
KLSSSLKQSSTINYGVSFLHSEYSLLLDPSTIKAQVKGEKFDRFKRTFVQVDSNSEIISS
IELKDWQILPEENQAKFAAVIQPDKLSEPGIYNLQFDLINSSLAFPNWWKEWDWQTRTGE
EDGSKTYNLQEFFTALKVRTEAMQSEIAKSPQHSGWFIGTLCYAIQKD
>1170768.4.peg.3090
MGLCQVCYHLTAEPVCEICRNPHRDSQTICVVADSRDVIALEKTREYRGKYHVLGGVISP
MDGIGPEQLTLQALVRRVSQQKPQEVIMAISPSVEGETTTLYIAGLLKPFTRVTRIAFGL
PMGGDLEYADEITLAKALEGRRELD
>1170768.4.peg.3097
MLSPTARLPDLKALIHGRNYFIIHAPRQVGKTTAMIALAQELTDSGEYTAIMLSLEVGAP
FSQDPGMAERAILDEWQESACVYLPTNLHPPRWPPSQPGRQIGAALASWAKVATRPLVVF
LDEIDALADETLISVLRQLRSGYNRRPHSFPHSVGLIGMRDVRDYKVKSGGSERLNTSSP
FNIKAESLTLSNFTLPEVEELYLQHTQATGQVFATEAIQRAYYLTDGQPWLVNALARQAT
QVLVKDVTQPITAEVINQAKEILIQRQDTHLDSLAERLREERVKAIIQPMLSGSDLPDTP
EDDRRFLLDLGLVKRSPLGGLTIANPIYQEVIPRVLSQGSQDSLPQIQPTWLNTDNTLNP
DKLLNAFLEFWRQHGEPLLRSAPYHEIAPHLVLMAFLHRVVNGGGTLEREYAIGSGRMDI
CLRYGKVMMGIELKVRREKLDPLTKGLTQLDKYLDGLGLDTGWLVIFDHRPGLPPMGERI
SKEEAISPRGRTITVIRS
>1170768.4.peg.3103
MQLQPEFTSYFQGLISTFESQNPGIKVKWVDVPWAAMESKILTAVSAKTSPDVVNLNPDF
AAQLAGRNAWLDLDAKVSPEVRSSYLPNIWQASTLNGKSFGIPWYLTTRLTIYNTDLLKQ
ASMSKPPATYKELAQAARQIHDKTGKYAFFTTFVPQDSGEVLESLVQMGVNLVDKQEKAA
FNTPEGRAGFQYWVDLYQQGLLPKECLTQGHRHAIDLYQSGETAFLASGAEFLKTIANNA
PQIAKSSTIAPQITGNTGKKNVAVMNVVIPRSSKNPDAALKFALFVTNDDNQLAFAKAAN
VLPSTVTALSNSYFREVPANASTVEKARIISAQEMQKAQVLTPKMKDFKLLQRAIYENLQ
AAMLGQKTVDQAVSDAAKQWDSR
>1170768.4.peg.3107
MTDTYLCIRHGAFEADPKTEELVHLQSGRHWRKWGNDWYRQHTHPDGIRFEIHEALDKLY
TQGYRATRVIIARRYQELMSGYLERSSSWRSEQPETGNARLYGLPVEFGPDPLDEPCWDV
INFDLDKEPGAPVRYPYFRLFE
>1170768.4.peg.3110
MLPTMERLPRLNRLIDQQNYFVIHAPRQTGKTTAMLNLAQELTASGEYTAAMVSVEIGSA
FNHQPDVAEQAILGAWQQSARFWLPQELEPPTWPAALPGQRINAALTTWAEISPRPLVVF
IDEIDSLQDETLISVLRQLRDGFPRRPKGFPQCVALIGMRDVRDYKVVSGGSDRLNTASP
FNIKVESLTLNNFSLKDVTNLYQQHTQATGQIFTPEAVHRAYYLTQGQPWLVNALARQTT
EYLTTDPQIPITIDLIDQAKEILIQRQDTHLDSLAERLREDWVQAIIEPILAGEELPNTP
EDDRRYLIDLGLVIRSQEGGLKIANPIYQEVIPRMLSRGTQDSLPMIQPTWLTTTGLLNI
PALLEAFLDFWRQHGEPLQKSAAYPEIAPHLVLMAFLHRVVNGGGTLNREYAIGSGRMDI
CLRYGKEVLAMELKVWRKGSSDPLKKGLQQLDKYLSGLNLPTGWLVIFDRRPNLPPISER
TSTEMAISPQGRNITVIRG
>1170768.4.peg.3140
MTHEFFEDKLVLTGFLEALFNGISIGAVLLVAALGLSIIFGLMGVINMAHGELMMLGAYT
TFVVQLGCKQLGGIWFDSYIFLALIIAFLVTALVGLVLERGVISYLYGRPLETLLATWGV
SLIFQQFVRSVNSVLVIGILSFCVLFFGGVRVLNSRQRFPQFGYGVKGIILVLSLGIAMS
LGRLFSQQSSMVQPWFGAQNVDVTAPSWLQSGVSLAGVQLPFARLFIICLTAICVVAIYL
FLQRSPWGLRIRAVTQNRTMSSCLGIPTQKVDALTFALGSGLAGIAGCAISLLGSVGPNT
GQNYIIDTFMVVVVGGVGNLVGTIVAALAIGTANFIVGSGTLAFLSSGVQPLVDFFNFFA
TTSMAKVMVFALIIVFLQWKPAGIFPQKGRNIDV

>1170768.4.peg.3144
MQIAKNITELIGRTPLVQLNRIPQAEGCYAQILVKLESMNPSASVKDRIGVSMINDAEKE
GLITPGKTVLVEPTSGNTGIALAMAAAARGYKLILTMPETMSAERRAMLRAYGAQLELTP
GIEGMSGAIRKAQEIVEKTAHSYMLQQFRNPSNPKIHWQTTAQEIWQDTDGQVDIVISGV
GTGGTITGIAEFIKSKKPSFQAIAVEPANSPVLSGGKPGPHKIQGIGAGFIPQVLKVELI
DEVIAVTDEEAIAFSRRLAREEGLLSGISSGAALCAAVRVAQRSENQGRLIVMIQPSFGE
RYLSTPLFQDLEIS
>1170768.4.peg.3156
MIDEWQKYKYSVNGKSILEPMKLPNTKVSFNANDLFFVLTNTRKYFQDYYQKDQNISRNG
LLVSQGVKVEDVLKTLDFMISTLREDMKKNRPPRLQDPNFINANFQVIKWKAYNPDKPEQ
KQLRITKYAVFTHPGSHTKTSTYNIPIYSLKDNLSTDKFYTKYTKQDVLSGIYEPGGKEF
GKVETLAYLTRQGLEEALMQGTILINFTDGSKGFFNVDRNNGISYIRGVRDTLQKRYWYF
RPVDQIKGYGYKIDTKISIRPGVTFAGDVLNIGLGRVVLIEYNQGQNKRLQMGVVADTGG
AFLPNLHQLDYLAGVFKNQTEFRQYIRQLPEYASAYILVKK
>1170768.4.peg.3161
MGILLVIPNAELIFEEVLNLSEVGQTAMQWSMAGGGVAYMILGAVGVFLYACRNLGWARA
LYFLIPSLSISLTSELLGTSTGFPFGHYSYLSGLGYKIAGLVPFTIPLSWFYVGIVSYLL
GRTGFDVDRKPTLSRHLGAIALGALLLTSWDFVLDPAMSQTSLPFWYWHKPGSFFGMPYQ
NFAGWFGTGSLFMTVSALLWRNNPINLERSQLNVALIVYLANFGFATVMSVAAGFPIPII
LGLFLGVAPAILLWSKAPSKSISAGLESGNQSVMSNVNNVNVVLK
>1170768.4.peg.3167
MSFLNAEDPTACDLIADFGIKILWHPSLGLEMGTNLQTLLWECILGKTPLDILVFEGTVV
NAPNGTGEWNRFADRPMKQWLEDLAKAANFVVAIGDCATWGGIPAMSPNPSESQGLQFLK
RKKGGFLGENFISKAGLPVINIPGCPAHPDWISQILVAIATGRISDIALDELHRPQTFFN
TFAETGCTRNIHFAYRATTTEFGDRKGCLFYDLGCRGPMTHSSCNRILWNGVSSKTRAGM
PCIGCTEPEFPFHDLKPGTVFKTQTIMGVPKEIPPGVNPEDYAVFTVVAKDTAPKWTDED
FFLV
>1170768.4.peg.3173
MSLITLQSVKKDFGIKEILKEATFSIDGTDKVGLIGTNGSGKSTLLKIIAGIEPIDGGQI
LTNYGAKIIYLPQQPDIDENLTVLEQIFMDSGEHTKLVKEYEELSDKLAHYPEDTLLMSR
LSEVMQRMDATGAWELETNAKIILTKLGIGDFEVKVGTLSGGYRKRIALATALLAQPDVL
LMDEPTNHLDALSVEWLQSYLNRFRGALLLITHDRYFLDKVTNRIIEIDRGDIYTYSGNY
SYYLEKKALAEESAVSSQRKHQGILRRELEWLKRGPKARSTKQKARIQRVESMRETQFKQ
AQGKVDISTIGRRIGKKVIELSGICKSYNGKTLISNFSYEFSPEDRIGIIGGNGTGKSTL
MNMITGRTSPDAGTVEIGSTIHIAYFDQHSEELISAVNDNQRVIDYIKEEGEFIKIADGT
KITASQMLERFLFPGSQQYAPIHKLSGGEKRRLFLLRLLIGAPNVLILDEPTNDLDVQTL
SVLEEYLEDFLGSVIVVSHDRYFLDRTVDTIFALEEGGNLRQYPGNYSVYLDYKKSEELT
QQETINGRDNRKSKNLTQPKSGEQEVQNKKRRRLSNWEKREFEQLEAKIVDLEAQRTLVE
TSLLAVAPENYTQVQNLYEQMEVLKQAIDIATERWLELAEMDV
>1170768.4.peg.3185
MAGNHVIFIHPDGTSPSHFAFARFVDKGPDGRLNWDNLEKTGVYLGHMKDQLGGTSNSGA
VTHATGTKAYAESFGFEAGNTPIVSLDGSNKTIVEAARDAGKVTALVQSGAIFEPGTAAF
VAKTKEIVNPGASRTVPRSQAAEIAKQVILSGVDFIMSGGELNLLPVGTNGIHGTAAQLD
ALTCIIHEGKNRKNKT
>1170768.4.peg.3186
MLTNNNYQNVFPWELSGLGADAVNLLKKYQLKHPQTYQKLIDIYEGNPHYLQIVGHLIQD
LFAGDTNKFLQLQQPSIDIDLMAFLRQTLDGLSLLETEILKFIASQSGSIPLTDIENHYY
LHQDVVNSIQSLKRRYLLSTQLVNEQLILKISPVIKKFLSG
>1170768.4.peg.3187
MNRYFQAKDFWDKKDIEERAEYFADIALGTWGYLINQLPRNPVFPVKFLPEIPDENVAIN
ILYYRNNLKLTVRNL
>1170768.4.peg.3188

MSNDIILINEKATNFLNKLCKSRNIIGNLKITFPIFVKVFESYIASIFVYVNIGMGYGIT
>1170768.4.peg.3194
MLDAFSEPTFRKVLEGKIGEGPNNIILDLSKIDFIDSSGLGALVQLAKQAQNQGSFQIVS
NARVTQTVKLVRLEKFLSLQGTVEGALENIKS
>1170768.4.peg.3199
MSSVPLLRLSDKFDPYISGEVNIDPSAVIAPGVILQAALNSKIIIGPGVCIGMGSILQVS
HGILEIEMGANLGAGFLMVGEGKIGANACIGAGTTVFNDSVAAQQVIPAGSILGDGSRQA
NSQESGESVDEGDTSSQSGEQVVSKTQFTATFVNFQTQSTSVPPLSPTPKSQSPPETESS
TESQEISDGKPRSRDPTEPHPLGTQIYGQGSINRLLSTLFPHRQSLSEQDANNGAE
>1170768.4.peg.3202
MQIAKVRGTVVSTQKDPSLRGVKLLMVQLVDENGNLLPKYEVAADSVGAGVDEWVLFSRG
SAARQILGNEQRPLDAAVVAIIDTIHVEDRLVYSKKDQYK
>1170768.4.peg.3219
MGPHRILLDCGAADISSITKENSPADLVLISHAHPDHCRGLLSLNRAFPLLPIYTSEVTS
KLLPLNWTELGAAENITFCQALPLRSPVEVEENLVVELFPAGHLPGAVAILLTYQGPERD
YKLLYTGDFFLSNSRLVDGLRLEELRGLSLDVLLIEGSYGTSRHPHRRHQENQIAERINR
AIIDRSSVLLPTPALGLGQELLMLLRSHHNFTGQDIDIWVDGEVGTGCDAYLELLSHLPA
SVQNFARHQPLFWDERVRPRVRRLKVEDLHTLGSSPCIVLTDYKSDLNKYIKHGDGASWV
VLFPEKISSDLENPLVQKETYLLAQHSDGPGTTQLIHNLRPQHVVFIHGDPTYLADLTTL
EELQNRYHVHSPPGGTLVELPIGETLIQSPVPEANYQGELTELETIITIILPREISEDPR
WQDFADTGLVEARWQGEDLVLKGLSQRYLLNRNSLDTAKSKYHYNWSEIECCGTCKHQRG
QRCWNAAAPLYNFKVTLEGYCPAFERLSENES
>1170768.4.peg.3225
MRFDTVRFDIVTLFPDCFGSILSSGLLGKALNRSIAQVYLVNPRDFTKDKHRKVDDEPYG
GGVGMLLKPEPIFTAVESLPVLPRRDIILMSPQGQTINQPLLRELATSYDQLVVICGHYE
GVDERVLNLVTREVSLGDFILTGGEIPAMALLNGVVRLLPGTVGKVESLKAESFEEGLLD
FPQYTRPAEFRGWKVPDVLLSGNHAEISRWRFQQQIERTASRRPDLLEKWQQERSDSLDP
TEKED
>1170768.4.peg.3237
MSDGKIVLIRRSDDECWALPGGMVDWGEDIPSVVRRELREETGLELVKIRRLVGVYSSPE
RDPRIHSICVVVEAEVEGEMNIQDHLEVLEIEAFPPTSLPLTPMSHDHYQQLQDYLSGLT
TLA
>1170768.4.peg.3238
MAKRRNPKKEKALRNQAYARKFRKRTTTGKMQRRFQQRPSKGEDDETVAAAMDAAD
>1170768.4.peg.3249
MKPNYSNWDDDLPPDPQEIYQDLISTLERKVGFGLYFVQCTPIEADNFVQQISRDLANKK
IALLNLYEPMEKFYEYVKDYVQGQNIDILLVKGLEYSLYKYEKRNFGEVTEGQFTNLTKV
PPILNHLNQQRERFRDDFSFCFVFLLRSFSLNYLIHRAPDFFDWRSGVYELPTTAELVDE
ESRRLIVEGDYKKYLELTPQQKIETMLEIQELLTEKYQNDSNRARLLFEMGNLLYSANEY
ETAITFYEQELKLQPDDHSAWCNHGHALFSLSRYEAAIVSYRQALKLRPDDPFCWYALGN
SQRKLHRDQEAIISYNQAIKIKTDDHYFWYNRGNALRNIGCNEEAILSYGQAIKIKPDES
NVWNNRGIALRNLGRYQEAIFCYDQVLKLQPDDYYAWYNRGVALKKLKQNEAAVLSYDQA
LKLKPDDHYTWNNRGNALDDLGRIAEAIFSYDQALKIKPDDQYAFYNKACCYAVQGKIQE
ALENLENAVSLKPEEFTQRAKADPDFDRIREDTRFQALINKTFHD
>1170768.4.peg.3259
MVTPQADLEIRDEKPIKPSLPKLPDTKLAHSILVSGVILVDQQAQAIIKLPDDLNSRYVQ
AGERLTNGILVKRIEVNPCNNPVVVLEQFGVEVRKVISAQDDERELC
>1170768.4.peg.3265
MHGFMGNIYEFDNVIKLLNNNFSYLTVDLPGHGKTEVLGGNDYYQMENTAQAIINLLDEL
KIEKCFLVGYSMGGRIALYLTINFPERFIKVVLESSSPGLSTDSQRIMRIKSDAGIIQKL
TRISTRNEFGVFLNNWYSQPIFGQIKNHPAYPKMIETRLENSPVKISKSLQFMGTGYQPS
LWHKLEYSQIPLLLLVGEYDQKFIDINTVIYNLIPGSKLVTINRAAHNTHLENPLMFVEQ

IMEFFKP
>1170768.4.peg.3267
MDNSSSFPQFYPERKTKFPKKIDILHSPVQNSVCLPLPKMLIFG
>1170768.4.peg.3273
MVNKTEIAQISHFSHFARFDLYYLYFVAKNPEVFTNLIRR
>1170768.4.peg.3278
MLASQKFLLEEEPLEEVLRERIRNYHEREKEIDFWLVKNPAFLEAPEMSDIKKKCPQLPV
AIISTDSQFITWLKLRLEHVIVGQFTTPLGTIHDPLASLTNV
>1170768.4.peg.3283
MKTLPGKNTRPTSGRVREAVFNIWQGKIDQCCWLDLCAGSGSMGAEALCRGAKLVVGIEK
SSHACAIIQENWQHLVTEQQVFHILRGDVIQQLKKLSGQTFDRIYFDPPYASDLYDQVLN
AIAGFKLLHKDGEIAVEHSSDFKVPIIPVWQVIRQRNYGNTSLTFYSCREECESDGALYY
DISPSLGTHN
>1170768.4.peg.3287
MGIMDALIGGLDDSWSLFAFGLGTTGVGLGLKLASKLSRSPREEGRTFQYYLPPTSSSCS
LPIIKATKNKPQY
>1170768.4.peg.3288
MTEPQPTITPKLEEPRFGFNEYAERLNGRAAMTGFILMVVIEYVTNQGVLAWLGLK
>1170768.4.peg.3297
MNDGHYLIGLYQSQLSISATTPAWTDIDILDNLIDPSVFFTPDLGKYG
>1170768.4.peg.3314
MNLSITTKTTIKTARINLFLLASALVTAITSNTSLAADTISAQDIAQIAKKTSVQINTEG
DITPGGSGVIIAQQGNRYSVLTANHVVCDIIDRPGKITCAKDIIYSVRTNDGKKYPIKSQ
DIIVLQSTKNDPDLALVSFVATQEYPTANLGDSDQMTEASDVFVGGFPAVFGKVGSARDF
SFSKGIVLSRGRTSINGYSLIYDAKTLTGNSGGPVFDIKGRVVGIHGLADASNKSKTETG
ELVSQKTGFNAGIPINTFLNFNNPLVKDLPIKRNTIATGEAPQERLNSPQSARDFYARGI
TKLEQFNYKESLADLDQAIKIDPKYAEAYFKRGYALSWLRRYEEALLDFNQVIALDPNYL
DGYLNRGWTYIWLQNDQAALEDFNRVIRINPNYAEAYAHQGMAYIKLGKYQAALESSKQA
IRLDPNKSYGYTIQGDVFNYLKDYPAAINVSTFAIKIDPDDFNAYINRALAYTLTGNYSD
ALVDYQKSAEIFERRYTRKPSN
>1170768.4.peg.3317
MNPETQTSLKVAMRKLIHSSQIKPEAVEVIVSGLQNSEISAEDWEDLFNREGANIAIKQK
VYSPQLIRLITLRALVLPKTLPEFLGWLNIQASKKVNQNQMVSLQFQQAIKELFPKEKVA
EGIGYLLPSLLDGNISPDGLSWLLAKNGSNSIWSYAQKQFVNDVRNDLQLIFDQYKNSKS
LNFDGGNLKCEIGVWNQLIRGWQGIQRRYYKCEEYRPLAELFEEFREDDLAAYFYQVSDG
LVNKKLFGRLPDSQRSGYPVVFGLPIKRKETLIDVLIKFINQFVNQDIDMKILYVAPISL
LILGSGWFVGSKTWQYVYANEAEKFLCEKSGSGENCPVIVLDGKAHYSFDEIKKIIPKVV
NRVVEKQKGLLIPESTQSIRDSESNKLRQNSGRQDIEEKVIGKLIQILGDTTLKYEDLNS
TAKIEEKVKTQWVKAVYNHKAEKKIKKVQVEECKFPLFWRCFPGQEVTVNKDQIDDSPLK
KKLEKDINDAISASQNERSSSRRQRRNNRRTTQ
>1170768.4.peg.3318
MRLKIKNQNLHLKTKDHLPEDREETIDGLANKGITIS
>1170768.4.peg.3326
MRLLHILASLDPALVDYVKLETGDRPPDHAQNFPTAIAAFFRNFGLINIKNQPKH
>1170768.4.peg.3328
MFNSIGSPPTPLKRGAFRLDVLVSPVKVLLVSPQENITYLVIGCDRL
>1170768.4.peg.3347
MTPQPGVPPEEAGAAVAAESSTGTWTTVWTDLLTDLDRYKGRCYDIEPVAGEDNQYIAYV
AYPLDLFEEGSVTNMFTSIVGNVFGFKALRALRLEDLRIPVAYLKTFQGPPHGIQVERDK
LNKYGRPLLGCTIKPKLGLSAKNYGRAVYECLRGGLDFTKDDENINSAPFQRWRDRFLFV
AEAIKKSQAETGEIKGHYLNVTAPTCEEMLKRAEFAKELEMPIIMHDYLTAGFTANTTLA
RWCRDNGLLLHIHRAMHAVIDRQKNHGIHFRVLAKALRMSGGDHIHTGTVVGKLEGDRAI

TMGFVDLLRENYVEQDKSRGIYFTQDWASMPGVMAVASGGIHVWHMPALVEIFGDDSVLQ
FGGGTLGHPWGNAPGATANRVALEACVQARNEGRNLAREGNDIIREAAKWSPELATACEL
WKEIKFEFETVDTV
>1170768.4.peg.3353
MTEFCSLFVSLFVTFFAISWLPRYIPVIFDHVTLARGEVNTF
>1170768.4.peg.3366
MIKGRSHEGKSSAIAFFWVFSPISSTALYGKLLTRNRVW
>1170768.4.peg.3368
MATASMTLSVSRSAALKSCAFNRVSNFAYLLGVGAVVVEALDIDVAFK
>1170768.4.peg.3377
MLGHSIEQEELVQLSIIVALANIIKLTPIREAVTIFTI
>1170768.4.peg.3378
MIGSGTIIRDGSPDNYSYRVITNSHVLRSAKGPYTIHTPDGKVYIGKVSLLHNRFKEDDI
AILNFDANKTVYEGGKINRESLQLDEKVFVGGFITEREKRYRFIFTSGKISLLLKKPLLG
GYQIGYTNKVRKGMSGAPVLNRKGEVVGINGLQSEPLWQAQELYQDGEKPDSETEKLIVS
SSMAVPMRDQWGK
>1170768.4.peg.3379
MTSPFSREIYQGHWVRSRVASALQTRCECFANALDIPLPLRSQGKFVNMINERFQLASHD
LSILKGN
>1170768.4.peg.3381
MRSLLVYEFEEGDKSCGRGSKLLGGMKPDKKGKKPPPGQLRLFWDEVDMGNEEIIGIATK
FVKANCFSPNPKSF
>1170768.4.peg.3387
MIRATQNRCLVDGEKHLWATLESMESQGTMTVEVKRNPTRPSRIATLEIRYQTVTIQPPQ
NRAKKEQLTPITLQAILVAEVDPPTEVEPISWLLLTTLEITSLEDVKTYVQWYCYRWLIE
RYHYVLKSGCGIEKLQLETAQRLEMALATYSIVAWRLLWLTYLARCSPDASCEQVLETHE
WQILYATIHHQLYPHTSPPTLAEVVNWIARLGGFLGRKGDGSPGVKVLWRGLSRLHDLVQ
GWLICQSLVVN
>1170768.4.peg.3388
MLLFPNSSNLTYQTAPGQAQIPTGAWEIITGSVAYDAPFGPTVAGLFLTPQQKAIYNSLP
ATNDVDSTVNDKDDFIKQSVNSSLQAFGYDPIGLNNNLSQADGLINSKLIQGDYIATHTY
GWTEFNIDPVTQKLSVTTYGVKPYTEAELVANPTAVINLEPQIVSQFEVNPDANNVPQAS
QLVFGTTGDDVVLAPSQTDGIKDLIFTGGGKDVVDISLDTPLAGFPRGENTVYTGSSKDV
IYAGNGDRIFGGSGDDEMYATDAKNYRLSGGSGNDVFHLGVNGRALGGDGDDKFFVTEGG
GNLISGGAGADQFWIATGDIPRVENKNVANSIVDFQMGIDVLGINGQGQSFGFKDLTLTN
NDIIINGNTIATLIGVNTSTLTVSNFSFV
>1170768.4.peg.3396
MSIIIYSHQAIAQTGNKDSKDNIYNPILLKLEGGITDKLTLRDIPTGQGGFARDYQINLN
KGDNLVIDASSENFDTIVTLLSPNGSTVGENDDGPDGTSNSLLFARITETGKYIIRVRSF
GETGVGSFKLKVTKLLPAK
>OHY31408.1
MSQEVTLKNLPLGINTLDKLRGSNCVYVDKTPFALKLIKQPGAFFLSRPRRFGKSLFVDT
LKEIFEGNEKLFEGLYIHDKWDWSRKFPVIKIDFADGVLKNREELDEKIRDLLWNNGDRL
GVGAKKNSISGIFGEIITGAREQFGERVVVLVDEYDKPILDNIDNPNIAAEMREGLKNLY
SVLKSQDANLQFVFMTGVTKFSKVSLFSGVNQLKDITISEAYSSICGYTETDLGESFGDH
LEGVDWDTVRHWYNGYNWTGSETVYNPYDILLFISEGMKFRNYWFETGSPTFLVKLFQTN
RYFLPNLEHLEVTEEILESFEVERINPVTLLFQSGYLTIERTFTRRQRYMFALKIPNLEV
RLALNDQFINAYTEIVNEKSGIQDSLYEFMNRGDVESMIMAIRRLFAGIPWRNFTNNDLA
DFEGYYASVIYAFLSSLDARVIPEDISNYGQADITAMLGVHIYVMEIKVVEGNQVQGNAA
LDQILQRNYAEKYRG
>OHY31452.1
MNYLVAVLPDRIQAESAYLSLEKEGITSSILGRGYKTADEFGLIDPKEEAKKQVKLMASW

LIPFGFFAGFTFSLITGLDTFIWAGEIGNHIIGGLLGAASGVMGSVFVGGGVGLISGGGD
ALPYRNRLSAGKYLIVVQGSEALTRQATRILRQYDPENIQGYADNG
>OHY31476.1
MQIAKNITELIGRTPLVQLNRIPQAEGCYAQILVKLESMNPSASVKDRIGVSMINDAEKE
GLITPGKTVLVEPTSGNTGIALAMAAAARGYKLILTMPETMSAERRAMLRAYGAQLELTP
GIEGMSGAIRKAQEIVEKTAHSYMLQQFRNPSNPKIHWQTTAQEIWQDTDGQVDIVISGV
GTGGTITGIAEFIKSKKPSFQAIAVEPANSPVLSGGKPGPHKIQGIGAGFIPQVLKVELI
DEVIAVTDEEAIAFSRRLAREEGLLSGISSGAALCAAVRVAQRSENQGRLIVMIQPSFGE
RYLSTPLFQDLEIS
>OHY31503.1
MSLITLQSVKKDFGIKEILKEATFSIDGTDKVGLIGTNGSGKSTLLKIIAGIEPIDGGQI
LTNYGAKIIYLPQQPDIDENLTVLEQIFMDSGEHTKLVKEYEELSDKLAHYPEDTLLMSR
LSEVMQRMDATGAWELETNAKIILTKLGIGDFEVKVGTLSGGYRKRIALATALLAQPDVL
LMDEPTNHLDALSVEWLQSYLNRFRGALLLITHDRYFLDKVTNRIIEIDRGDIYTYSGNY
SYYLEKKALAEESAVSSQRKHQGILRRELEWLKRGPKARSTKQKARIQRVESMRETQFKQ
AQGKVDISTIGRRIGKKVIELSGICKSYNGKTLISNFSYEFSPEDRIGIIGGNGTGKSTL
MNMITGRTSPDAGTVEIGSTIHIAYFDQHSEELISAVNDNQRVIDYIKEEGEFIKIADGT
KITASQMLERFLFPGSQQYAPIHKLSGGEKRRLFLLRLLIGAPNVLILDEPTNDLDVQTL
SVLEEYLEDFLGSVIVVSHDRYFLDRTVDTIFALEEGGNLRQYPGNYSVYLDYKKSEELT
QQETINGRDNRKSKNLTQPKSGEQEVQNKKRRRLSNWEKREFEQLEAKIVDLEAQRTLVE
TSLLAVAPENYTQVQNLYEQMEVLKQAIDIATERWLELAEMDV
>OHY31513.1
MAGNHVIFIHPDGTSPSHFAFARFVDKGPDGRLNWDNLEKTGVYLGHMKDQLGGTSNSGA
VTHATGTKAYAESFGFEAGNTPIVSLDGSNKTIVEAARDAGKVTALVQSGAIFEPGTAAF
VAKTKEIVNPGASRTVPRSQAAEIAKQVILSGVDFIMSGGELNLLPVGTNGIHGTAAQLD
ALTCIIHEGKNRKNKT
>OHY31521.1
MLTGFLEALFNGISIGAVLLVAALGLSIIFGLMGVINMAHGELMMLGAYTTFVVQLGCKQ
LGGIWFDSYIFLALIIAFLVTALVGLVLERGVISYLYGRPLETLLATWGVSLIFQQFVRS
VNSVLVIGILSFCVLFFGGVRVLNSRQRFPQFGYGVKGIILVLSLGIAMSLGRLFSQQSS
MVQPWFGSQNVDVTAPSWLQSGVSLAGVQLPFARLFIICLTAICVVAIYLFLQRSPWGLR
IRAVTQNRTISSCLGIHTQKVDALTFALGSGLAGIAGCAISLLGSVGPNTGQNYIIDTFM
VVVVGGVGNLVGTIVAALAIGTANFIVGSGTLAFLSSGVPPLVDFFNFFATTSMAKVMVF
ALIIVFLQWKPAGIFPQKGRNIDV
>OHY31528.1
LAGALSESLEIGGFIQPQMLIAVNFGLCATLNEQFVAKAPDWLYIPSVKEILPGRKSYTP
HLEGDVPAVVMEFLSDKDGGEYSFKRTYPPGKWFFYEQILQVPVYIIFDPDGGLIEYYEL
RNGRYELKQPDENGRHWIGSMELFLGTWQGTKEGRTGYWLRWWEQTGNLLPWALELIEQE
RQLAEQERQRAERERQRAERERQEKERLIAYLRSQGIDPNNLPNHAE
>OHY31532.1
MEIGQLTNIYERWKFLYVTLCMSGQEGRDPKPHDNQPPPSPHPNPPSRQPARAGALTVLF
L
>OHY31540.1
MAKNWAIVIGINNYLNLQRLDFAQSDAEAMKEWFLQEAKFDQVFLFTENSPPIPTNTQPI
PTQPTFGILMSFLGRQFKKPLLTTGDNLWFFFSGHGQRSRDKDYLMLKDTNPGDIERTAI
SVEYVTERLRGSGADNVILFLDACRSQGAKDGLGYGEEQYQGVITFYSCKAEQTAWEIPK
LKKGAFTHTLLEGLRLGVKGNCAAVHTLHRYLENEVPKLNQKHGKPIQNPYLKADPPYKM
YYILLPQSANSSDILNLKNEVRNAESRNDSELADKLRIRILAVDDKDNEEVDSRYAKLET
LLKAKNFRAADEETYKVMLAVANRESEGWLRREDAQRFPCRELRSIDKLWLKYSRGKFGI
SVQQQIYQRLGGTEDFNLDVWRSMGDLVGWRSGGNWLSNLRSWMRGGENWLFYSDLNFSQ
TAPLGHLPFAFALFREKPAFFVFEKERFTISSSGRTVIIGPERWVRKRWEDLVYRRVIGG
GDFELRMFESWTRSHMTEAQRWFPWVVPFFPSLLI

>OHY31634.1
MRWINMINFNAEFNRRMIKLGLTTRATEITTPETVEGKQEPTLPTPPTETLSTQQTVRFL
LGSGINGNLLEEALEGDEYKEKAKYLEILRVQADEIERANQEMDKQLDKEFADQIASGKA
AAAKEIAKMNAYDDHMRAFREALAKGKSKKD
>OHY31647.1
MEEYYVYDPDEISLEVSIRENNSFREVEDFATWTSPRLNIRFDMTGDELVIYYPDGSRFL
SPVELSNYAEQERFLKEQANQRAEQERLLKEQANQRAEQERLLREQERFLKEQANQRAEQ
ERLLREQERFLKEQANQRAEQERLLKEQEQLKYQTLLSQLKAKGIDITTLE
>OHY31711.1
MNKYFLRGELLLVCGAGIAFIGLFAPQPSFSNTASTSQPLNSPDNTNPLANDSGLDMFNI
IHRLNFGPLNWDPNQQNQQLEDAAAAFKARQNQILQNQQPSKVKGEENRSGANTDGSNTE
RK
>OHY31751.1
MLTVALPKGELLKNSIQLMQSAGLDFSAFLDARNRQLQIYDASGKAKGLLVRAQDVPVYV
EYGQAQLGIVGFDVLKEKKPQVGQLVDLKFGYCRMSVAVKSTSSYKSPLDLPAHGRVASK
YVNCAREYFESLDLPVEIVPLYGSVELGPITGMSEAIVDIVSTGKTLRENGLVEIATLYE
STARLIVHPLSYRLDLGGIYNLAQSVKSSVSS
>OHY31758.1
MWKSCLSAVSSVILVLGCFYTSQQQAIAQAQVVIDQDSLNIAKGIPWGAGGFPFSQVANI
KDSLVDSIAGKVVIDRHGEGPSGLGGPFSNPFPGRFVIVSLWGSNLDGCFLQVVVQKSPN
DGQAELQQLVPKTLEIGLNNQIIELERNPSTKVRGFKVDYTYTEGGGTSVGNLIGKLLFG
ENNQPAQNSGKKISSNWYMANTLFAVDQGTANLLRSAQPKEIRIRLRFENGDARIVPLGK
GTVEKWPEVYGFNSTCVPQ
>OHY31814.1
MTKALIITIKQWWESQPGCPHISAIHLMIGKFTCVEPASLQFTFQVQTRNTFLDGVKLVI
KEIPLIAFCHPCQLEYLPEIGQQYSCPRCCHPMEDIRSGRELKIERIEYSLKE
>OHY31817.1
MRKNNSPAVSWGIGDNSTWLFMILSITIPVFIWLVVSNSGLVKPLFLPTPQAVLTALQKL
WATGDLQTDIGFSLLRVLGGFLLAAVISIPLGVLMGSFPVVRALFEPAISIFRYMPAPAF
IPLLILYFGLGETPKILLIFIGTVFFNTLMIMDAVKFVSRELIETSYTLGGKNHQIILQV
ILPFIVPNIIDACRVNMAASWNLVIVAELVAATEGLGRRISVAQRFLKTDEIFAGLIVIG
LIGVVIDILLVILHRIVCRWAHN
>OHY31821.1
MTSPSESSQIATSSFSMDDFAKALEKHDYQFQKGQTVRGKVFQLDHDGAYVDIGGKSSAF
LPRDEASLRTVTDLSEILPLNEEMEFLIIREQDAEGQVTISRRQLEIQHIWEKVAQMQED
SQSIPVKVIGVNKGGVNVEVFSLRGFIPKSHLLERENLEALKGQTLTVGFLEVNRANKKL
ILSQRLATRSSNLSLLQTDQLVTGKVTGIKPFGVFVDLDGVSALLHIKQVSQKFTESLEK
VFQIGQEIKGIVIDIDESKGRVAISTRKLENYPGEVLENLAEVMNSADARANRVNSTPE
>OHY31839.1
MLNLKTVIFVLSFTPFIVLSTQLTGRAQVSSPSLGTAGSVSGGGATGSDNNPISAPSLGT
AGSMSGGGATGSNSAPSLGTAGSMSGGGSTGSDNITVIVSTNNLRELRNAIGQLVSSGSD
SLSPITFSSLRASLISDGVSPGHTDQLIVSFSRVLSQLGVPNTNFSANNLNLEKLVASTK
IFKPTVTIAQDSPAEGEIALILDSNNLTEAINIYNKIVLESDDPTIIKLSRNQDFLKISR
ILKTLRSGII
>OHY31843.1
MSRISTNTSLPFRFKFKQLGKIAYFSLVISSIFTLPASAQEKAQLWRTLTVSGSGVETIA
TTLTRVSLGVEVQGKTAQEVQQEAARRSSAVVNLLKSRKVEKLETTGVRLNPVYSYTNNI
QSITGYAASNTVSFRFPTDKVGSLLDEAIKTGASEINGISFVATDEAINEAQKQALRKAT
QEAKKQAEAVLGSLGFQTKEIISIQINNASAPPPPMLQRSQMVKASASDAVTPIVGGEQQ
VEASVTLQISY
>OHY31884.1
MTNIQSPKLTSPKLTAYEFKNLPLLGTTTTNQNILLGGFSGLYFQGVADNGNLQFVTHTD

RGPNSEPTGQNRPFLLPNFQPEIVSFELNKASGEINITNRTKLFRADGKTPLTGIPNVQA
GAGNTAYTDEIGVNLNGTVLNNDPLGADLEGIVIADNGDYWMVDEYRPAIYHFNRNGILV
SRFIPQGTASKSNQPVGTFGIEVLPEVYAQRRNNRGFEAVALAGNKLYAFIQTPIDNPDN
TGDTTSRSSRNLRILEFDIVSEKVTGEYLYLLDDITGTGNAKTDKIGDAVFLGGSQFAVV
ERDDRSDTTSNKLIYQIDLIGATNINNTSKLSLPQGKTIEQLTPVELGPAGITPVNKSLI
VNAAQIGYTGVEKLEGLALVSASTLAIVNDNDFVTTIPEKLGIIELPNNLVEVTGTANKD
ELFVKKGEYVLGLDGDDILDASDGLGNNVLDGGAGNDQLYGGKSDTLIGGAGNDQLYVVE
GNNNILYGGAGNDRLFVTEGNNNILYGEDGDDNLYIIEGGNNTLSGGGGADKFYIVNGVI
PVVPGQVLDFTRGDDKVIISGISQIQGFDNLILEQNANNTTIKTKDLIGGSQKVLGILKG
VVANTLTADDFRINDVTITNPQVSGTFNPIATDNILTQEEKKGGLTLSGTVSDPNAQVTL
QFGGQFRVAKVQSSNSSWSYTVTDNDYNFFREGQNQLTAIFTLTNNNRTGTFRASQTLTI
NSGVLPQPTSPTLDPTKPKGISEAVTQKAQSANIGQGILGSGEKLTDPNIVVIPFGGINT
FNQGANAAIEDFGLIKTGSNTTIESVRVFPKSPTGEVSVTLSDGTSVLASIPPGISSIGD
PLGLTMSGLVPGGSSTLDFYLPRNILNQLPSDLQSATYAKFNFEAKKFEQYADEQGKPLY
QYKFTDKNSNGIRDFGELYLSLNLTDGDKWDSDRIQNGIIVDPGQLGIAVVVTGTGVTYS
STTVNPGNSLIPREAPALSNIADNVFYKFSNTYKPAEARQALQNALGQTDAAFKNIFGLY
EIDNATGSVNGIAPGQPGYAKAALDKSKVISNFTVRAGGSVTPNINGDLIGIGDKTYAPF
VIANGGNYSGSIQEAINEFFKVNPNNSAATAQNYMGLPVAYFSFGAANPDGAAHIKSVGN
NIFGFEDLPAGVGVSDYDFNDMVFSFG
>OHY31900.1
MVIIGQGLRLFIILSSLDARVIPEDITNHGQSDLTVMVGVHIYVMEIKVIEGNQVQGNAA
LDQILGRNYAEKYRGEPGKYVHEIGLIFSRSQRNLIQADWR
>OHY31901.1
MLKSQDANLQFVFMTGVTKFSKVSLFSGINQLTDITIDTQYSSICGYTETDLRESFGDHL
EGVDWDAVRHWYNGYNWTGSETVYNP
>OHY31913.1
MLTANHVVCDGPLWTKNKVRCATDATYTIQTNTGREYPIKDRQVLQKDECGADLATVTFE
AEENYSVAILGNSDQVKRGADVIVAGFPAIFGNKGKDRTFNTTDGKLTSDVLRKAQWGYR
LVYNAQTSIGNSGGPVFDAYGRVVGIHGLADIGGTVDGPCASKRPSIRDATGQETGVSSG
GKTGFNAGVPINTFFSLTGRQPPTGGGQVVIPPPPPVAPGNLPRRPTRYKAPTGPAVCPG
TVC
>OHY31920.1
MSADSINTALAELENLINSCENDLIRFIEARKMNSGNEISTKKVNRQLQQNPIAIVGMAS
LLPQSRNLRQYWQNIVSKADCITDVPESHWSVKDYYDPNPRTPEDKTYCKRGGFIPEVDF
NPMEFGIPPSILEVTDVSQLLSLVVAKEAMEDAGYGEAREFDRENVGVILGVAMAKQLGM
PLSARLEYPVWEKVLISSGLSPEDTQKIVEKIKSAYIKWDENAFPGMLANVVAGRIANRL
NFGGMNCVVDAACASSFGALKMAISELVEYRSDMMLTGGVDTDNTIMAYISFSKTPAVSP
GENVKPFDAKSDGMMLGEGIAMLVLKRLEDAQKDGDRIYAVIKGIGTSSDGRYKSIYAPR
KEGQVKALERAYNDAGFSPTTLGLMEAHGTGTMAGDPTEFASLQSFFSKHDKRKQYIALG
SVKSQIGHTKAAAGAASLVKTALALYHKILPPTINITEPNPKLDIENSCFYLNTETRPWI
RGESESPRRAGVSSFGFGGTNYHLVLEEYQQEQQQPYRLHDVASQILFFAPNPSELIKNL
ETSLQNLQAADSNGYYSQLVEECRNIQIPQNAARIGFVAGNKEDTCKLLALSIDLLKNKQ
STLNWEHPQGIYYRASGIKLRGKVVALFSGQGSQYLEMGREAVMNFPALRRLYGLMDSLL
IEDNLQPISQVVFPHPTFNQTEKADQIATLQRTEYAQPAIGVFSAGLYSIFQQAGFKCDF
TAGHSFGELTALLAAGVLSESDYLYLVKARGKAMAAPKDPDHDAGSMLAVKEEISKVELV
LKNFPKITIANFNSPSQVVLAGPSHEIQKIHQKFQDLGYGAVLLPVAAAFHTPLIAFAQK
SFAIATKSVKLLNPKIPVFSNVTAQQYPQESDKIQRILESHLASSVNFTQQIENIYAAGG
YCFVEFGPKRILTNLVKDILGERPHITISLNPSTHKSSDISLREAAVQLRVLGMELGNID
PYQLPEIFSKEISQKSLNVKLKGINYVSEKTKNAFEQALNDGFKIQGVPQIQPVSVIDKP
SEFTPKKEVVETVYTTNSNGTKHNQTSIISSLSTDSQMNSANAINSSLRAEEQLPILTYP
MVQVLGGKMTTSDKNTEFQQILASLENLLSQFQNSQSDNLQIHDTYLQHQMEYAKTFFQL
IQQQNTLFMNNKSAETGETKKSIMDSFERSMMQFHHQQAETLRIHEQYLREQLEHTKNFF

HLIQQEYSLIIDGAEELTPVIPLQLTDIHTENLREISPQNPQLSVPEMVVTPTIKTLEVE
IPQAITETRVEKPPVSVTETAISSQTKKSAIDIQDIGKSLLSITSEKTGYPIEMLEFDMD
MEADLGIDSIKRVEILGGLQELYPDLPKPNLEELAEKRTIGQIVEYLEKQVVTNPTNNQI
NHSEHIVKNVDPKVIDIPHSSTTSDEYSSIAETLLKITSEKTGYPVEMLELDMDMEADLG
IDSIKRVEILGGMQEVYPDLPKPNLEELGDLRTIRQIVNYLQSLVVVEKKNLDFEQIKAT
VVELNPPPTVDPNLPRRPVKLKILPEPDFWECQLPGGHIGLITDDGSLTTTKLVHELIDK
GWKVVVLSFPQSIVPERSPLPAGVTRINLANMSEEHLQLLLQSISVQYGKIGAFIHLHPC
FSTTNLAYLETEKVIVKHIFLIAKHLKQSLNNAANLEGRVSFCTVVHLDGAFGLDHTENF
GIIGGGLFGLTKSLRWEWPKVFLRAIDLSPKLEPHQSAEYIVAELYDSNRYIGEVGYGPK
GRVTLVATAD
>OHY31933.1
MLIWEDNNILLKAQDFRAADRETDKVILAVANREREGYLEIEDAEKFPCKELRSIDQLWL
KYSRGKFGISVQQQIYQSLGGTKEYNQDVLRSMGERVGWRREGNWLYYSDLNFSQQAPSG
HLPVFAYGWVWGGWVGRYGGDTSLLSRHAECNT
>OHY31937.1
MLFFLFGGFKYFMVTKMVTKVDSPLFYYDTLGYSYHIYYIFFILGIVVTYFNCYDVTDY
>OHY31945.1
YNGGPYQLVIFHFLIGVACYLGREWELSFRLGMRPWICVAFSAPLAAATAVFLIYPIGQG
SFSDGMPLGISGTFNFMIVFQAEHNILMHPFHMLGVAGVFGGSLFSAMHGSLVTSSLVRE
TTETESQNYGYKFGQEEETYNIVAAHGYFGRLIFQYASFNNSRSLHFFLAAWPVVGIWFT
ALGVSTMAFNLNGFNFNQSIIDSQGRVIGTWADVINRANLGMEVMHERNAHNFPLDLAAG
EVAPVALTAPAING
>OHY32019.1
MLGFVFPVFAFMNNAGYGNLATVLCSRNILLQMPKPLPIQEKSRVLGCQTLHFADAAFAS
PLTWGKLDLSR
>OHY32020.1
MANYNKLRTGMTYEQVVKILGEEGKEISSNDIAGYKNVMYMWKAGGFSVGNMNAMFQNGA
LVQKAQFELPE
>OHY32037.1
MVTLKGFAVLPADTFAAGPKSGAAVANPTNGRTTPFVGQPIQGFSGVQFAPNTNGSRFWF
LADNGFGAKNNSADFLLRIYQLDPNFTGVENGNAKVEVEKFIQLSDPNRLIPFSIVNQNT
SERQLTGADFDVESFVIDAKGDIWVGDEFGTYLLHFDSNGVLLDAPISTPNLFKLNTLNG
QKPIVIGHRGASGELPEHTIEAYRLAILRGADFIEPDLVSTKDGVLIARHEPNLINTTDV
ANRPEFANRKKKVVVDGVEEEGFFASDFTLAEIKTLRAVMPQGYRDQVFNGLLEIPTLGD
IIDLVKEVEAQTGKKIGIYPETKHPTYHDNLNLSLEEKLIDTLKSKSFTDPTRIFIQSFE
VSNLQDLNNNIMPARGVNIPLVQLIDAYDVADDGKLIYKDAYARPYDFTVKGDTRTYGDL
LTPAGLQEIAKYADGIGPWKRQIISVKTVDKNNDGKPDDLNNDGVINDSDKVTLPPTSVV
SDAHKVGLLVHPYTFRNESRFLASDYNNNPELEYRQFISLGVDGYFTDFPGTGDLVRDQI
TTNQVRSPQNPTVLSKPNFDTLNGQKPIVIGHRGSSGERPEHTLASYKLAIAQGADFVEP
DLVVTKDNILIARHEPMLAVVNLNTDGTIKLVSGKPEINFTDTSTDVYLRDKFQDRLKVK
NLDGRNVGGWFAEDFTLAEIKELNAIERLPSLRSTAFDKDGLKVPTLKEVIDLVKQVELE
TGRKIGIYPETKHPTFFQQQGFNTSQLLVNTLKTENFTDASRVFIQSFEVSNLKELKSTI
MPGASIDIPLVQLFGGSGKPYDFVVNGDSRTYDNLSTPTGLKEIAQYAKGIGPNKQRIVP
MTTVDNNKDGQPDDLNGDGQISDADRALGASTTLIQDAHQAGLLVHLYTLRNDGFFLSAD
YKGDPGAEVRKFVNLGVDGFFTDFPKTGTSVIVNNYLAGTGYANPNNNLNSPYFADSPVY
FNPNQPYYGDLVTANLNRSQGFEGMAFSPDRQTVYPMLEGTVVGDPAGSVRIYKFDVATE
TYTGLVGLYQLASPSNAIGDFTPINDKEFLVIERDNNQGTSAAFKKIFKVDFSQINAQGF
VPKEEVANLLDIQDPNDLNSDGNKTYNMPFQTIEDVVVWDNKTIVVANDNNYPFSIGRPP
LIDNNEIVVLELDKALSLDARLGLAATIAESSQLVFGTPGVDNVSVPQATDGINDAIFTG
AGDDKVDTLGVTNPYAGNNTVYSGSGKDVIYVNNGDRIFGGSGNDEILATDAKDYRISGG
SGNDVFYLGTNGRALGGDGEDKFFVTEGGGNLISGGAGGDQFWITTGDIPSVGNKNFANT
IVDFQIGVDVLGISGQGSNFGFNNLTLTNNDIIINGNKVATLTGINTSTLTASNFAFA

>OHY32042.1
MSHFSTLRTKITDAEILKASLSDLGIAVKTEADVRGYNGQRVRSDIVAVLEGEYDLGWSR
NSDGSFDLIADLWGVAKKHNQTELINSINQKYAVNKTLAEVKQRGFKNANVKLVLQ
>OHY32043.1
MKEELNILIQAQYPLIYLVTSEEERAEQAIYTMAQSLKPQRRVYVWTVTHGIVEYGQARS
TNQHNTVSSEAAIEWAIRQKEPGIFIFKDLHPFIDAPAITRSLRDAIASFKGMQKNIILM
SPMQQIPIELEKEVVVIDFQLPDMTELSKVLTAHQEQNRGRRLTTEAREKLLRAALGLTK
DEAEKVYRKAQVTSGRLTEDEVDIVLSEKKQLIRRNGILEYIEEDETIEAVGGLEELKKW
LKQRSNAFTERAREYGLPQPKGMLILGVPGCGKSLIAKTTSRLWGLPILRLDMGRVYDGS
MVGRSEANLRNALKTAESISPTILFIDELDKSFAGSTGSSDSDGGTSSRIFGSFLTWMQE
KKSPVFVMATANRVERLPGEFLRKGRFDEIFFVDLPTPEERQDIFRIHLTKRREEIARFD
LEQLAKMSDGFSGAEIEQAIIAAMYEAFAQDREFTQLDIIAALKSTLPLSRTMQEQVTAL
RDWARQRARPAASSVAEYQRLEF
>OHY32051.1
MKRFFRQLKTFTSDDNFMHLIENIVVIVSKVLSILMVVVILVAIADLTVFLLKELFDAPY
GKFNTILFKIFGLFLNILIALEILENITAYLKKHVFQVELVIVTSLIAVARKIIILDLEK
VTGIDIIGLGIAILSLSISYLIIQSRLKS
>OHY32090.1
MGLLPWRLRDTSINETTISSKQQATKNTTEIMTKKFVDLSTLDGKNGFTIINSNDKNDNL
GYSISNAGDINGDGIHDIIIGAPLSDNNDQSNAGNSYIVFGSNNGFANIIDISTLDGING
FTVNGSEIGDQSGRSVSAAGDINGDGIDDLIIGAPFADSNGDDSGAAYVIFGRSSFSSLP
TINPSNLGDNGFIINGLNPQDQLGYRVSSAGDLNQDGFDDVIIAAPPNAYVYPPVTGDQA
GKVYVIFGSEKFNPSNPNFDTSNFDLNVINGSRADDYLGVGLNRGGDFNGDGIDDLIIGS
PFNDFNGFRSGQAYVIFGSKESFSSSLNVSQLDGVNGFVINGQEGDQLGFSVSTAGDINH
DGIGDIIVSAHDADPQGVDAAGAAYVVFGARTQFNSELDLSSLNGNNGFVINGIGELDKT
SWAVTGLGDVNGDGIDDLLVSAIHADANGDNSGQGYVIFGSKESFSSSLNVSQLDGVNGF
IINGKSENHNLGYSASGAGDINGDGISDILISAPFAGSGEVYVVFGNNGSTDTGEITDEV
TEGDGGESEEFTIDGTEDEITGGVIEDNNSESEITNIDGNPDEILDGVMGEGDSDNNSTE
GLGSDITDSPDIESTPTGSVDNILGNNNVLSSSTTKVEFQLMDKIPASIRELGVFTVDDA
SGKINGIAPGQVGYSEAALARSKVVFSVLSKTPNEFNNANVTRILGFQEANPNLRFYVID
NGTTDAVKNDLLPINQVTFLDSSNLQVTQLPDNSFSLQSNDLVFKARPTTQPLPMGTNLQ
EKSQGESIDLRGVTGPVNAQFTVYREASFDNYVGFYKVTDEKGGIDTNSDGTADLLPGDA
GYIQSAVNQHLSGLGLNVANGNKSTFNNTLSGGGLYVPFIIVNGRPDTVLNSDLSANSNP
NIYFTYLGANSDQVDHVRLLGDNTFGFEDLRGGGDMDYNDLVVQVNMSARV
>OHY32115.1
MSELERYYTLLDLEPGATIEEINQAYKDLVFVWHPDRLPKDNHRLQKKAHDKIKSLNQAR
EKLRSFQYQSQPGHHSEPSTSKRSPYQPNQPPPQNPDLSGRDFSHANLSNKDLSGRNLSY
ANLSGSNLSDTFMHKVNLRGANLSEANLFRANLLLADLREANLRSANLIGADLSGADLRG
ADLTGARMRSGERLLVKLVGANLTGAIMPDGAIYG
>OHY32116.1
MKIALFTETFLPKVDGIVTRLSHTVDHLQRDGHQVMLFCPEGGIKEYKGAKVYGVSGFPL
PLYPELKLALPRPAIGHVLQQFAPDLIHVVNPAVLGLSGILHSKLHKIPLVASYHTHLPQ
YLQHYGLSFLEGLLWELLKIAHNQAALNLCTSTAMVEELAAHGIERLDVWQPGVDTELFH
PDLASQEMRSYLSQGHPDSPLLLYVGRLSAEKEIEQIKPILEAIPHGRLALVGDGPHRQN
LQNHFAHTNTHFVGYLKGQQLASAFASADVFVFPSRTETLGLVLLEAMAAGCPVIAARSG
GIPDIVTDGVDGYLFDPNAPIQQAIDLTIKLLREKQEIATMRSNARNKAEQMGWSAAVRQ
LQEYYQRTLN
>OHY32120.1
MKGLVRLLTVFSLLLGCWGWLGTTPTAQAWDFSHVNLPQTALLAIARQNKADQKLATDFG
KKIDLNNTNISRFQEIRGFYPVLAKKIIANAPYAKVEDVLEIKDLSDRQKQLLENNLSQF
TVTEYDAEFNEGDDRINNGIYR
>OHY32131.1

MIKHRYLILSIVACLVVCLSSCGSQPPAGNSVTQTTSDMTHTGKGKININNAILSELDKL
EGKLGVPALSNKIQASRPYGSTADLVSKKVITQEQFEQIKNLVTVEEVVLTGEAKDIDYM
TKLALMKGHLLVAEELLKENQPKQAQPHIGHPVEEIYVDIEEQLNERKVKQFKEDLVKLT
ELVRFRPQDKKVATNLTVAMSDIDTAITALPNEQRLQPKFILQVIRGLLDAATAEYQAAI
AKNKITAPIEYQDSRGFVIYSHELYQSVSPQMATVNPEAQKAIDIALTELLQVWPSAIPP
NQIVKTPEQVNSLVKTIEENAKFFTVM
>OHY32191.1
MALIVQKYGGTSVGSVERIQAVARRIHGTAQVGNSVVVVVSAMGKTTDGLVKLAHEISPS
PTRREMDMLLSTGEQVTIALLSMALQEIGQPAISLTGAQVGIVTEAEHTRARILHIETER
LISHLNAGQVVVVAGFQGISNTSAMEITTLGRGGSDTSAVALAAALKADFCEIYTDVPGI
LTTDPRLVPEAQLMKEITCDEMLELASLGAKVLHPRAVEIAKNYGVPLVVRSSWTDQPGT
WVTSNKVQERAMINLELARPVDAVEFDIDHAKISLLRVPDRPGVAARLFNEIADQQVDVD
LIIQSIHEGNSNDIAFTVNTPILNRAEAVASAIAPALRNNDGSNEAEVFVERNTAKISIS
GAGMIGRPGVAAKMFTALAKAGVNIQMISTSEVKVSCLVAAVDCDRAILSLCQEFEVNAS
TRNVSSSNSIYSTVCGVALDMNQSRLAIRHVPDQPGIAAKLFGLLAESNISVDMIIQSQR
CRVIDGVPCRDIAFTTNRTDGENAQAKISQVAAQLGWGEVILDQAIAKVSVVGSGMVGQP
GVAAKMFTALAQNQINIQMITTSEIKISCVVSEEEGVKALQIIHTAFGLAGTHKFVVPA
>OHY32195.1
MVSKNFWKPIIHSSTALVLLTTLNTAWPLVSLAQSKPQPKANSAASSLFTDYLLGGGDRI
RVNVFEAPEYTGEYQIPPGGEINMPLIGSIPVSGLTTQQAADEIARRYARFLKRPLISVN
LLAPRPINVFVAGEVTRPGSYSLSLQGTGGNNPGVQYPTVLAALTTAEGVTLAADVTKVQ
LRRQVGRSGEQVVSLDLKQITQTGRIPIDITLRDGDTIFVPTATDFNVAEARNLFAASYA
ASRTAPRRVAITGQVYRPGSYLVAAGGGGGNDSGGLPTVMRAIQLSGGITSQADVRNIKV
RRPTRTDKEQTLNINLWELLQSGDLNQDVVVQDGDTIIVPTATQVNTAEVTQLATTTLSP
ATIKVGVVGEVKRPGVTELQPNSSLNQALLAAGGFNDARASSSSVDLVRLNPNGTVTKRA
VKVDLSKGINEETNPILRNNDVIVVNRSVLARTGDTLGAVTAPLAPVFSIISLFRLLGF
>OHY32249.1
MPLIEAYISAWKRSFEYGGRSTRGDFWWFTLASFIVSLIAGVISSKIQLVYAVAAIVPSI
PLAVRRLRDIGKSWLWLFIGLIPIIGSIWLIVLYSQPSLI
>OHY32289.1
MSDLREKLTENLDEAEWEWLVSHAQRDVIIVVASGLDLLDVGEAIASDNTQSVGVWIDEQ
LISKPSPIQLGDWNGDPTKRFNALIIEPYVLIQEK
>OHY32339.1
MQVLRSFSNQEVLDLQENLGRGGEACVYTVPSNENLVAKIYHHPTPNHIQKLRAMIANPP
ANPAASFGHISIAWPQELLTAAESSDTIIGFLMPRIRNMRPIMDFYNPGNRRQNCPLFNY
QYLLRTARNLAAAFAALHASNYCIGDVNESNILVSNTALVSLVDTDSFQVPDLSQNTVYR
CLVGKPEYTPPELQNKTFADYNRETYHDLFGLGVLIFQLLMEGTHPFSGVFQGLGDPPTY
ESRILAGHFTYSQKQKVPYLPTPITPSWQTLHPDLRDLFISCFEDGYHAPYLRPSAQTWL
SVLSTAEASLVSCAVNPQHVYHPHLDKCPWCERTIKLGGRDPFPSLQAISAREHLQPRRK
SRKRPRYQPRVRKPAVPLLAPYTQSSLRSTLPGYPTVQTSSRSKFYTFMFGILGLGVLGY
LDIMVKFTRPFLSPNPYTQQSLLSSSSENVHSPLSLSFNDYYQRGNQAYQQQDYQQAIED
FSQGIKQNTNFSKLYMHRGNARYNLNDYEGALTDYNLALKINPQEVKALINRGNAYIKLA
EYSNDPDYEYKKAIDSFNNAININQQDDEAYVRRGIVRSQIARYSNNSQEEYERSIGDFT
QAIKLNRFKAEAYFQRGLARYQFAQYSSNYAQIYKQAIADFDQALNINPEMAEVFLKRGM
IYYELAQYGEKTARNNQQRALEDLEKSAQLYLNKKDVNNYQQAISNICVIAEEKCDYFLQ
NSSIIYSVNP
>OHY32394.1
MSTKIYQQIQEFYDASSGLWEQIWGEHMHHGYYGADGRERKERRQAQIDLIEAVLNWSGV
KHADDILDVGCGIGGSSLYLAQKFHAMSTGITLSPVQCARAKERALEANLQSRSSFLVAN
AQEMPFDDNSFDLVWSLESGEHMPDKTKFLQECYRVLKPGGTLIMVTWCHRPTDVLTLSG
DEQKHLQDIYRAYCLPYVISLPEYDAIASQLGLKNIRTADWSTGVAPFWNVVIDSAFTPQ
AFLGLLFSGWTTIQAALSLSLMRRGYERGLIKFGLLCGVK

>OHY32477.1
MTRKKLFYLKHGVEEYYVYDPDEMSLEVSIRENNSFREVEDFATWTSPRLNIRFDMTGDE
LVIYYPDGSRFLSPVELSNYAEQERFLKEQERFLKEQERLLKEQANQRAEQERLLKEQEQ
LKYQTLLSQLKAKGIDITALE
>OHY32478.1
MAGLFSPTLGAIKKAEIVGLPNIKDDRVTIKIKVEGEGARPVMGLERNNFDLKVDGKKLK
VKPRDWKSPEEIIPPKAWIIVLLDFSGSMNQIDSGGSKKIAGAINAIREFTKVSSDRGGD
TQISVVPFGEAGKNCPEYTVNKDTLDKFLSASDFKLQNSLEYLSSLNPCGSTNLYQPLKK
ALEFLGNPEDARFTLPENSSEPNPRLSIILLSDGYHNAMNEFQDFNELKSLLQSYENITV
HTLGYGLTPSQLGIKYNLNRPATRRDINQGKVPEEEFVDQQRLAEIANLTGGIAEFSGDQ
TAIAENLKLFLNALLGEYQISYIQPNAERGTLHKVEVQVKDGNKKVNSKSVPYIMPVFGR
SLPLANRLVMVTSILFILIVGGIFPFRWWGEHLKGQAQGE
>OHY32628.1
MLLNNSDFHLPYNQKLVAKAKKLRKKATIPEQKLWQEYLRFLKPRFLRQRPIDNFIVDFY
CASCQLVIEIDGDSHFTQEGQKYDFERTKILEGYGLTVVRFTNEQVMNHFERVCAEIERS
FFQKDRKTVFHNRRVLF
>OHY32631.1
MNGVYEPLSLLEGGYISEQMNLKLVLWEGNYKGLSIVWLRWATLEGKLLPTQEEQTQWER
VQKEWWHAQREWELAQREWWRVQRETVDTFILYC
>OHY32632.1
MVVLTNGLRLLTLRFQLRFAAAIASDLSCEVYRRTLLQPYSFHVRHSSNELISGITDDIQ
RVSSSVLPQILLLAVNALIVLALVLSVLAISPLLALGTAATLGITYFILLRVSRRVLASN
SYLITTQSRFLVKYLQEGLGGIRDILLEGSQPMFVSYYRQVDRPIRRASADIGFIGTSPR
YIIEPVAMVAICVIAVVLAYQERGLEKVVPVLGSLALAANRLLPALQGCFAAISTLRGTQ
VSLQKVLERLSMVVDEVPVVSGIGEALKSELQLQGVWFRYGESTPWVLQNLCLTIKANTT
VGFVGSTGSGKSTTADVILGLLQPQKGEILADGEVLKGERLRGWQRTIAHVPQSIFLSDA
TVAENIAFGVPISEIDIERVKRAARLAQIADFIEKRPGGYEEIVGERGIRLSGGQRQRIG
IARALYKQASVIVLDEATSALDNATERDVMAAIEGLSHQVTVILIAHRLTTVQRCDCIFQ
LDQGQVLAWGSYEELLANSPSFRAMALTVDAPLNPAKNL
>OHY32646.1
MRKYKEIDDHNQDIKINPNSAYAYILRGIARFQSGDKQGAIADYNQAIKLDPNYALAYYN
RGIARRQLGDKQGAIADYTQAITLNPKLALAYIGRGNARDDLGDKQGAIADYTQAITLDP
NDADAYNNRGVVRDDLGDKQGAIDDYNQAIKLDPKNADAYYNRGLARRDLGNKQGAIADF
QKAADLYQKQGRNEWYRYALDRIREIQQR
>OHY32647.1
MNTQKLHIIMKRINLTFISSTLIALTQSPVFGAGPIGIGELKLGMTQEQVQALKGSVELS
SELSQWKPSESQNYTPEPGKKILKGMLNNPVTGKSEVTLTFTNDRLSSLSLVLDNESEMA
AAKNMISSKYGSPKTDNRQRDEQCIYRNGNSFTLKNGTLFYQWEQNHGGGVVSTTISEIL
INTCPYNLRYGTTDGLVFRTLSIKYGPKKPDVNNLF
>OHY32651.1
LARELTDSGEYTAVMLSVEVGSVFPDEPERAERAILGSWQDAIDIWLPEELHPPFDPERR
ESIGAFLKSWAKSSTRPLVVFIDEIDSLENQTLISVLRQLRDGFPRRPQGFPHSVGLIGM
RDVRDYKVKSGGSERLNTSSPFNIKAESLTLSNFSFTDVKNLYEQHTTETGQIFTPEAIQ
RAYYLTDGQPWLVNALARQATQVLVKDVNQPITAEVINQAKEILIQRQDTHLDSLAERLR
EERVKTIIEPILAGEDLPDTPEDDRRFLLDLGLVKRSPLGGLTIANPIYQEVIPRVLSQG
SQDSLPQIQPTWLNTDNSLNPQALLNSFLEFWRQHGEPLLKSAPYHEIAPHLVLMAFLHR
VVNGGGTLEREYAIGSGRMDICLRYGKVVM
>OHY32680.1
MSVNTVPSINYYSLDVIQYEASQLVRKGIVSRQQPIYTLCQYIPAREWVFVEYELEKCDF
LLRDRIGDLIGREQWEND
>OHY32702.1
MDNQSSLSPQPQKTKSPSLPARRLLAWLAEITLVTASGVIPFSIGAYINSQTDLQRTPLN

PVLAGIERAIAQPLALPVNYGIRNVASPTNFLWTIGLLTPITLGGWQLYLLATTGSTLVK
KKLGIKVVNQEGKPPGFKAIIIREGIGRWGIPMSMAYVLWRYSFAFPNLQLFTFLVLVMV
IAEGIGINSRRNSQAIHDQLAHTYTLDVNIAIPGKQLTESSPSRANFTFNFPPNLTLIVV
GATGMIALLSTLVGTQIYIQNQANQRQTQLVNNQNFLELSKQINPQSGTSIEERQRAILA
MGGVNDLQSIKYLVDLLTKETDSSILNSIQQALSNIGITSIPELKRVNQFIGKEIKSGAI
SGSVGEQLLNINQQTINKILAVYSGKTHNLDLTEIQLGPQNADENSQSQLVLENVDLSGV
ILKSANLNQASFKGSRFRSVGEDGRWDTYDDIIADLSKAQLKRSNLSNANLSRVLMSRVD
LSRSVLNRANLANSKLIGANLSSAQLVGSDLQQATLQDATLTGADISGAQLQEADLYAAQ
LARVSAIGSQLSHSNLTKTNWQGADLSESYLNHANLNSANLSAANLSGAILRSANMTNAN
LSNADISRADLRGANLEGTDFQGAILFPGKQDPKDRFVETSDLGSQAAIVQGVDFSNAKN
LDVQQLAFICTNGGIHSRCP
>OHY32714.1
MVVFGRPKGQRGSYRQWEEDNIPPQVVFEILSPCNSKGEMTRKKLFYLKHGVEEYYVYDP
DEISLEVSIRENNSFREVEDFATWTSPRLNIRFDMTGDELVIYYPDGSRFLSPVELSNYA
EQERFLKEQERFLKEQANQRAEQERSLKEQANQRAEQERLLKEQAHQRAEQERFLKEQAN
QRAEQERFLKEQANERAEQERLLKEQEQLKYQTLLSQLKAKGIDITALE
>OHY32803.1
APRQVGKTTAMLALARELTDSGEYTAVMLSVEVGSVFPDEPERAERAILGSWQDAIDIWL
PEELHPPFDPERTLDIQTVLKTWAMASPRPLVVFIDEIDSLENQTLISILRQLRAGYPNR
PQGFPHSVGLIGMRDVRDYKVKSGGSERLNTSSPFNIKAESLTLSNFSFTDVKNLYEQHT
TETGQIFTPEAIQRAYYLTDGQPWLVNALARQATQVLVKDVNQPITAEVINQAKEILIQR
QDTHLDSLAERLREERVKTIIEPMLAGEDLPDTPEDDRRFLLDLGLCRRDRGGALEIANP
IYREILPKTLAAVAIASLTSVEPNWLNSDGTLNPQILLNSFLEFWRQHGEPLLKSAPYHE
IAPHLVLMAFLHRVVNGGGTLEREYAIGSGRMDICLRYGKVVMGIELKVRREKLDPLTKG
LTQLDK
>OHY32808.1
MPKKPSHPYTEPLAFERLLLLIATLLKYPGVGSPDFLEFSNNKNHDAIGVVKIYLQQIAK
DLNIKLPDNYPAVDTLRKDLKTLRRYGILDQRMYRWGY
>OHY32820.1
MPYSHRSTKKQRKYNQELTARRFASTVPAQTLSWLSSISLLSGGFVFAQTESPADNIVST
VEVSQPTTLGDRGQNGQGSLPLESKVTELKTGVQTQGKNSEVNSNLESVATERNSESKSE
SSPTSVILKVPAPPTLKSQPETLPTSTQPIPETSTLVIPVTSQPNNSDNGNTGKDYNSTQ
IDPTEYNGNSTVKYDAPSSVEVTGVTQDCKAVISLTGKSVGTCGGKNSTNPSVANFTQKS
APTWLKKSAPGNLGKSPLKTVPSETPLSTNNSNPRPQSEKSEEIVNSLAGTVNNSQNWHS
LGSNSSSGHTSAGKQDYTGRISTKTSYNRPRIPDPREFSSGTTVTPVTPSFGTLPPPMIE
GKVAPRPSKVAYDFDLASVLPQVPYISSFADNGTNSGVTFPLSFAAPITSLFGWRTHPIT
GDRRFHAGMDIAAPTGTPILAAEKGQVEAADWMGGYGLAVTINHNQRQQTLYGHMSEILV
RPGQWVEPGMIIGRVGSTGNSTGPHLHFEVRHLTDNGWVAVDPSMQLQAGMNSLYNRVAY
NYNPQSKQLR
>OHY32845.1
MQVTTTSTPIPPGELSPSNWPDHTQLPDSDDNFVKNFQEHPQSVILTTSIEPLLDKIHPE
KDYCIGQDSGIYWRFTEPVEKGVEAPDWFYVPGVPSRLKGKLRRSYVMWKEKVPPLIVIE
FVSGDGEEEKDNSPPPERDEVDPKTKKAKKAGKFWVYEQAVKIPYYAIFDGFEGTLEMYH
LEKGRYKQVKANRRNHYPIPELGVELGMLLDQEKPPVPWLRWWDNRGNLLLTGNERAEEE
CQRRE
>OHY32849.1
MAVPGNDIEILLTPDQYRRIEETGQERHEYADGRIMIMPGGSEVHSRITVDITTFLNLAL
RDSSFETYNSDLRIWLPSLNHGTYADIFVIDGSPNRRDEVLNPLLIIEVLSPSTEKYDRG
DKFRKYRSLPSFIEYVLVAQDEPYVELYYKQHGEKNNLWQLEIYDQIEQSVIFHSINVEV
PIREIYRRTKLP
>OHY32852.1
MGLDYKLRMPTAIIIAHRGASGYRPEHTLAAYKLAIDMGADYIEPDLVISQDGVLIARHE

NEISMTTDVEHHPEFAHLRTTKIIDGELKTGWFTEDFTLKQLKTLTAKERIGQIRPQNTI
YDGLETIPTLEEIIALAETQSSQKGYKIGIYPETKHPSYFQSIGLPLEPALLRSLSNTQL
PIFIQSFEVGNLKKLNENTDFPLVQLINDSGQPNDFSMDGKSCTYQDMIKPDGLKKIAQY
AQAIGVNKNLLIPRNSQGKLLSPTSLVEDAHQQNLLIHAWTFRNENCFLPLDYQNHPQGE
YELFFNLGVDGVFSDFTDTAVYARGKL
>OHY32954.1
MPDTIVTEICQGVADCVAACPVACIHEGPGKNIQGTDWYWIDFTTCIDCGICLQVCPVEG
AIVPQERPELQKTPQ
>OHY33028.1
GGPYQLVIFHFLIGVACYLGREWELSFRLGMRPWICVAFSAPLAAATAVFLIYPIGQGSF
SDGMPLGISGTFNFMIVFQAEHNILMHPFHMLGVAGVFGGSLFSAMHGSLVTSSLVRETT
ETESQNYGYKFGQEEETYNIVAAHGYFGRLIFQYASFNNSRSLHFFLAAWPVVGIWFTAL
GVSTMAFNLNGFNFNQSIIDSQGRVIGTWADVINRANLGMEVMHERNAHNFPLDLAAGEV
APVALTAPAING
>OHY33033.1
GFPRRPQGFPHSVGLIGMRDVRDYKVKSGGSERLNTSSPFNIKAESLTLSNFSFTDVKNL
YEQHTTETGQIFTPEAIQRAYYLTDGQPWLVNALARQATQVLVKDVNQPITAEVINQAKE
ILIQRQDTHLDSLAERLREERVKTIIEPILAGEDLPDTPEDDRRFLLDLGLVKRSPLGGL
TIANPIYQEVIPRVLSQGSQDSLPQIQPTWLNTDNSLNPQALLNSFLEFWRQHGEPLLKS
APYHEIAPHLVLMAFLHRVVNGGGTLEREYAIGSGRMDICLRYGKVVMGIELKVRREKLD
PLTKGLTQLDKYLDGLGLDTGWLVIFDRRPGLPPMGERISTEQVISPSGRTITLIRS
>OHY33034.1
MPKNWLNTDNTLNPQALLNSFLEFWRQHGEPLLKSAPYHEIAPHLVLMAFLHRVVNGGGT
LEREYAIGSGRMDICLRYGKVVMGIELKVRREKLDPLTKGLTQLDKYLDGLGLDTGWLVI
FDRRPGLPPMGERISTEQVISPSGRTITLIRS
>OHY33131.1
MNSKNYTFSGLTLKSLILLSSLLICNGLTIGGLELSANAQLLSNAEREELSRLRTETKIQ
KQLQSYLSIWLTLLSLFAVGLIATLWFLRKAIIRDIVERAMRQIGNIENLQTELIAANQK
TTGLIEYSQDLALELEEKVNHLKTKIEGEGGKLSVLLSDLPKSKQEFLTALEREVIAAQE
NISSLEFKLNTQLEQVTLAAQQQRVTIENIKKLESELFAQFSEIKLSIENHRDTSVSDIN
KYRSELMEQFEILALETLESKTQVVQSITEHASQFTSNLSEFQTHAQNQIDGFTSNLSEF
QTHAQEQMDGFTSNLSEFQTHAQNQIDGFTSNLSEFQTHAQNQMDGFTSNLSEFQTHAQN
QIDGFTSNLSEFQTHAQNQIDSFTSSLSDLNQTSVSQLVDLQTDTQKQKDHIFKDLAKSQ
SEFSHYLLELKNTTESRQREIIYSWQKSVDEVEQMLSNLHNNVEEQKTDLLENLQVLGLG
FQKQIGDLQNSIEQRHRKFFQTSQNSVEQLISEFMSELSAMKSDIKTDVDHQKANLIARL
AKLEKLESQFVEQLKKLQLDAENRQYETLKELSQMTPQTPDISPLSPVGTDTNTTNTIDV
QSVNNLDNHLDHEIQEAEELLSQNRYEDALSIYEKITSIQSDHEEYWLKRGFILNKIKRH
KEAIGVYNQVIRINPAHYQAWFDIGITCGKLGKHQEAFNCFNKATEIKPEESVAWLNRGL
SLVELENYEEAISSFDKALEIQPSSFKIWDKRGYTLVRLGRDEEAITNFNKALELNPEYG
SALYHKAACYALQKNVESALVNLQQAIKHNPSYREDAAGDIDFDDINNDTRFQQLIHRE
>OHY33165.1
MAILVLISFAPPAWAFCGFYVAKADTRLYNQASQVIIARDGTKTVLTMANDFQGDIKDFA
VVVPVPTIIQEHQVRVPDPKIIQRLDAFTAPRLVEYFDQDPCRRRYYDSPGFIPETGTRR
PSAVEKIPGDNTLGVTIEAQFNVGEYDIVILSAKESDGLETWLNLNGYKIPRGANRLLQP
YVRSGMKFFVAKVNLDKFEQSGYQFLRPLQIAYESSKFILPIRLGMINATTEQDLIVYII
SPRGQAEVTNYRTVKIPSDTNIPLFIKDEFGSFYKAMFQNSYLQEDRKVAFLEYGWDMGN
CDPCSAEPLNLEELKQAGVFWLDERGNNYNRIAPNFGFAFSETNAYITRLHVRYTRDKFP
EDLIFQTTSNQELFQGRYVLTHPFNGKLECSAGKRYKDSLPRRFEQEARNLARLTNWKID
DIRQKMKLSVGDFHTPWWEKISLFLGF
>OHY33167.1
MGKIILVTGPARSGKSEWAENLALESGKKVVYIATANENPHDLEWQQRIEKHQQRRPETW
ITMCVPRELTGSLKNQTADTCILIDSLGTWVANCLEQDDLIWEKTLGEFLADLPLIDGDL

LFVAEETGWGVVPAYPAGRKFRDRLGGLVRQLGRISHTVYLVTGGYVLNLSILGVPLPPV
KS
>OHY33172.1
MLKKINKISDRALPLVGDDIDTDRIIPARYLKAITFDGLGEGAFIDDRKALNGQHPFDLP
QYQGAKVLIVNRNFGCGSSREHAPQALAKWGIRGIIGESFAEIFFGNCVAMGIPCVTAES
GIVRQLQELVTADPQALVTIDVENLQVQIHDLILPVAMGQGTKTAFVSGTWDACGQLVAN
AAQVKSVASQLPYIAWR
>OHY33223.1
MLEDSICKNCGTNLSTILMLEGLPSRKTTSSEKAKILPDQISLVVLILLLGTGVGGFAYS
LLFRRQSPTVQVQNQPTIKATIQELKVQPPSPNVISRQPHQKGCGGFYYTVRRRNSLSLI
ASRFYGNGSLVKFIVNANPSLKNREDTLYIGEQLFIPNREESCR
>OHY33255.1
MSESTETLELSTKQRDQIIEEIRILLKNEKTLQQTLKKEQDQRNTANEQLFLELLGIFDT
LEFLVDYLSNSPEPSAKSIKRLSKQLEVLQRKLVGILEQRKVELIEDLNHTKPDFNLCVV
VDREVRNDLEDQTITKVVKKGFRIENRVLRPIEVITSKQQ
>OHY33271.1
MNPKNLPLGINTLSMLRENNCVYVDKTEIAHRLIRIPGRFFLSRPRRFGKSLFVDTLKEI
FEGNQKLFEGLYIHDQWDWSRKFPVIKIDFAGGVLKNRQELDQKINGILLKTAQSLGVDY
ELKDIQGRFGEIIAGAYQRFGERTVVLVDEYDKPILDNIDNPPIAAEMREGLKNLYSVLK
EQDANIQFIFMTGVTKFSKVSLFSGVNQLTDITIDTRYSSICGYTETDLTQSFGEHLAGA
DREAVRSWYNGYNWTGSESVYNPYDILMFIDKGKIFHNYWFETGNPTFLVKLFQANSYFL
PNLEHLEVTEEILESFEVERINPVTLLFQSGYLTIDHTFIRRHRSMFALKIPNMEVRLTL
NDHFINAYTEIVNEKSAIQDSLYEYMCNGDLESTVKAVKRLFAGIPWRNFTNNDLANFEG
YYASVLYAFLSSLNA
>OHY33273.1
MKEKKFIDELNRRNSTYNNPDLALTMANLLNTISKDINTDSQRFIYEILQNADDASNQNN
MLDVQISFVGNYAIISHQGDTFKESDIESISSAGDGTKREDSTKTGFKGIGFKSVFSHSK
YVIIKSKNFCFRYDENHWKNYWDDSNWGPKTNWQNQRLANRKDPDFKMPWQIIPIWTDFP
KELESIDVWEKFNVSIIIKYDEIHNLQTTFTQLLSNTQILLFLRSQEIRITLNYKQNSTI
TKSKNGEVTTIKTRNKNGTIISEWIINKYEFPLPDDVKQKIKQDEKLPEKLRDSSYTEIA
FAIKLEEGKLKAVHKDHRLIFTYLPTSINYNFPFLVNAPFLADAGREHLHQDLFWNKFIF
EQIPFKLLAFASELASKSSKYNKQFLKIIPHKSQGITVLEKEFYRSYKSALDTIAFIPNQ
NGKLLKVSEAIFDKPSISNVIGSDILISYINKKDKICFTLESLVSQLEDVNILKEIGVRI
FDVDELQHFLISDTFTNKHELTNNFALISFLYEEVQKLEISHKNIWKDKLHNIPFIFDQY
EKLKSPQNIYFDSVEFSENLSLNVSIIHNHLTTQIDKNSDIRNWLGDLGVKEPSVLSFIE
KTIIAECETYITTNNALKIGRFLFDAHKKGQLKEEHYEGLKNSKFLTQNQNLIAAKSAYL
SDFYEPELCLESVYSYDFYVFNQYVEDVNIKGEWKNFLLKIGVNQNITLQNITINRSDDL
DKIEMDYFLKVEEEAKKEHKYPWLINESNPIKITKISYSEIARENCDFAKQFWQQVFKKI
SLNSINKTALMRWGYYGSCESVENYFYWSLHNSTFIPTNQRTCLKASDVFNNSIPKIREI
AGKYLAIFDYDEFIPPNWLDYLKFKPHLEVSDYLQILKAIYEDVNVSEEEKEDNKKRICL
IYQMLSDLCKTNLDEEEIRLWGSTNKLLSKNSWEFLLPQDLCLIRVEGFQSGNSVYTEKI
NLQIIELLQLFGVSIIDSIQLSVPQDNTFEIFHLKNKLIEIAPFIALIYTSEFKVIESWE
REYKLIKDKIDNVHIFQTSEISLYYGNENDKQKRISWGENSNFYYVGDWKSPRVLDGLVE
PLCRFLNIQYAERLLIVLLLESYTSGVDYLKEKGYNTDIIHKSATNNLISETNEITTNHE
NRPYNQYDEDLGRKGEEFVYGELQRIYIQKYNSSIKETEDGFKIVNIQKNNKINRLEVFW
RNKNQNTTENHDFKIVENGQETYIDSKATPYGKNEEKIPFYISCREFALLEKVERYLIAR
VFNVTSNPELVFIKLDIENL
>OHY33284.1
MSSVPLLRLSDKFDPYISGEVNIDPSAVIAPGVILQAALNSKIIIGPGVCIGMGSILQVS
HGILEIEMGANLGAGFLMVGEGKIGANACIGAGTTVFNDSVAAQQVIPAGSILGDGSRQA
NSQESGESVDEGDTSSQSGEQVVSKTQFTATFVNFQTQSTSVPPLSPTPKSQSPPETESS
TESQEISDGKPRSRDPTEPHPLGTQIYGQGSINRLLSTLFPHRQSLSEQDANNGAE

>OHY33286.1
MQIAKVRGTVVSTQKDPSLRGVKLLMVQLVDENGNLLPKYEVAADSVGAGVDEWVLFSRG
SAARQILGNEQRPLDAAVVAIIDTIHVEDRLVYSKKDQYK
>OHY33310.1
MLRVGNLDKSLEFYCDVLGMKLLRRKDYPTGEFTLAFVGYGDETDNAVIELTYNWGVEKY
ELGTGYGHIALGVDDIYSTCEAIGTRGGKVVRQPGPMKHGSTVIAFVEDPDGYKVELIQM
AKS
>OHY33326.1
MKPNYSNWDDDLPPDPQEIYQDLISTLERKVGFGLYFVQCTPIEADNFVQQISRDLANKK
IALLNLYEPMEKFYEYVKDYVQGQNIDILLVKGLEYSLYKYEKRNFGEVTEGQFTNLTKV
PPILNHLNQQRERFRDDFSFCFVFLLRSFSLNYLIHRAPDFFDWRSGVYELPTTAELVDE
ESRRLIVEGDYKKYLELTPQQKIETMLEIQELLTEKYQNDSNRARLLFEMGNLLYSANEY
ETAITFYEQELKLQPDDHSAWCNHGHALFSLSRYEAAIVSYRQALKLRPDDPFCWYALGN
SQRKLHRDQEAIISYNQAIKIKTDDHYFWYNRGNALRNIGCNEEAILSYGQAIKIKPDES
NVWNNRGIALRNLGRYQEAIFCYDQVLKLQPDDYYAWYNRGVALKKLKQNEAAVLSYDQA
LKLKPDDHYTWNNRGNALDDLGRIAEAIFSYDQALKIKPDDQYAFYNKACCYAVQGKIQE
ALENLENAVSLKPEEFTQRAKADPDFDRIREDTRFQALINKTFHD
>OHY33380.1
MNLSITTKTTIKTARINLFLLASALVTAITSNTSLAADTISAQDIAQIAKKTSVQINTEG
DITPGGSGVIIAQQGNRYSVLTANHVVCDIIDRPGKITCAKDIIYSVRTNDGKKYPIKSQ
DIIVLQSTKNDPDLALVSFVATQEYPTANLGDSDQMTEASDVFVGGFPAVFGKVGSARDF
SFSKGIVLSRGRTSINGYSLIYDAKTLTGNSGGPVFDIKGRVVGIHGLADASNKSKTETG
ELVSQKTGFNAGIPINTFLNFNNPLVKDLPIKRNTIATGEAPQERLNSPQSARDFYARGI
TKLEQFNYKESLADLDQAIKIDPKYAEAYFKRGYALSWLRRYEEALLDFNQVIALDPNYL
DGYLNRGWTYIWLQNDQAALEDFNRVIRINPNYAEAYAHQGMAYIKLGKYQAALESSKQA
IRLDPNKSYGYTIQGDVFNYLKDYPAAINVSTFAIKIDPDDFNAYINRALAYTLTGNYSD
ALVDYQKSAEIFERRYTRKPSN
>OHY33392.1
MFDYTKAKQFYLDKQRSRQNERHLLWQKAKLDSERIVAMIIQEYAPKQIIQWGSVLAPEH
FSSVSDIDLAVLGIEPLTFLRLFADAEAMTNFSLDLIRWEDIHPAFQQILLLKGKVIYAS
>OHY33513.1
MKKFDICFINFSFKPGGGNRVILELSESLAKKNFSCRLISLVNNNNRGFSKISIIREESI
FSFIKSDYKLAPVINLFLTLSHLVADRNNYRVIIINNPLIAPIFGMLGFSNVYYYIQADD
YRIFDDRFLIKSSLLLHFYRFITKSITYNVYGERYLFNSHFSFEKFSEISKQNIPNPFLI
LPGVDLSIFTPSSEKYSRLEKDSSRPIVISTILRKHPWKGSLDFLRAVRIIGETNELDSY
RFVAITNEDISSISIPKQVKILYPKNDRELSGCLNQSDIFIVTSWWEGFGLPGLEAMACG
CAVITTRNGGCNEYAVDKVNCLMYEPKDVQQLTKLILELSHNEPLRKEISKSGIKTSQDH
SWDNSASALVSLCNI
>OHY33514.1
MKTFFKKILPFLDVLLVVFVYPAGYLLKYLRMAGVQRMPLSKMALVSMGVFPIRDHYYEP
QFDYRDPKLDLSKDRNLCGINWNISGQIKFLEKLAFSHEILDIPAKKSASISFHFNNESF
ESGDAEYWYQIIRFLKPKRIIEVGSGYSTLMAIKATAKNQKEDQYYTCDHVCIEPYEMPW
LEMSGVSVIRKKVEDLELSFFDILQENDILFIDSSHIIRPYGDVLFEYLQLLPSLKKGVV
VHIHDIFSPKNYPSRFLEDEVKFWNEQYLLEAFLTHNNSWEVIGSLNYLHHNHYQNLKLV
APFLSPEREPGSFYIRKIV
>OHY33515.1
MTLSLLEATWFLLPFTVLGTLCEILYKDKKYSLITFFLSSLGVVLFSITHVSICSDYSSY
EQIYSSALNGSINFSDVIGLDASYRTLASIWGSLIPLDLDFVLSYHLFTAVYQTTSILIL
VVFLFFFCPRWKNTLLFFILFGSLQFQHIILCGIRHGLSSSITVLLFFAFINFITKREGY
LSIVFLALLSVFTHWQAVIVILIIIILLQIVTSKSFTSVFRSKTLKLFRSRIILVPVFTLI
FIVLYAAPQIFTNLFLGFSQIVLSGQAGRISIYLDSNLDLGYGTKSSLLFYIDMILIYFS
LFRGEIIKSRLSQGNDKTDFNYCTQCKLLDWIGYLSIVNLIVKLLLAVGLGVLLRVSVTI

HLLQIFCIPVLLDSLKMKSRVVLVLLMSLPYLYFLFFISAEKFLMFV
>OHY33592.1
MTLPDSFRTQVQHIQESSKFQSVDGVLRATPFPGYTLITPPASEDPQNADFYQQIETFGR
SLLELPIASDLIVPLPVSSFHLTLADLIWDHAFIHACEKKHNFEEELNSYLGDLFGQYQK
SRSSLQGPISWQVLGLIVMPRAIALGLIPKDERSYEEIIQLRRLIYQNRKLMGLGIEQHY
HFTAHITLGYFSEIPANLDRVNLSNLLSELNQHWLLNFSEIVVSQAEVRKFDDMTHYYRQ
LNWASFRF
>OHY33605.1
MSIVTKSIVNADAEARYLSPGELDRIKSFVSGGAARLRIAQVLTDNRERIVKQAGDQLFQ
KRPDVVSPGGNAYGQEMTATCLRDLDYYLRLVTYGIVSGDVTPIEEIGIVGVREMYRSLG
TPIDAVAGGVAAMKSVAATLLSAEDSAEAGAYFDYVVGAMS
>OHY33643.1
MEELGRYENAISQLKLITVDKLPQYHKNVVHISLGRVYYFLKLYIEGDKHFNIAIANSDD
REKSILSSARSILAHSPHNQTAVKMLQQIAEESPRYAQAMEMLTLNLTEEEYFKTVPDDS
NNLKDAQILNRAIYHKIANEITILKSIAYRILRVSSIKDALLESIISNIEEILQEINHRR
SLEKSQIEQIEIIANNEYGKILEIIAKTAHDISDFVNNELAIVESKTRRAIKKFTGNDPQ
FSQFNKLLTQLELTQDALNDLKAINEGIRIKNRQFKVKKIFETWEFNTQIDNAQISLNIE
NGDSDFNGDEEKIKSILNELVENSLKHNPDNSDLQIKINSQDVTNPPGIRGANIPGSKKY
LYIEFIDNGLGIAPDKKDWVFQPLNTTATGTYSSGLGLFIIRKTLTQMGGYIREVGVNGV
KFEIYIPYPDQNI
>OHY33644.1
METADNIESLIRKAQDLLDNKKTQAAIDTIHKVLEFNPNSIEGIKVYGQCLVKLNQIEDA
LAIFKKGLEIAPNNVSLMRNYGITLYQAKEIKRAVNVLDSASKIEPNNIKVLNSYARVLS
DSGNYEKAREIFERSLQIHPNNTIALTSYGKALADSGNYEKAREIFERSLQIHPNDTIAL
TSYGKALADSGNYEKAREIFERSLQMDPNDIIALTSYGKALADSGNYEKAREIFERSLQI
HPNNTIALTSYGKALADSGNYEKAREIFERSLQMDPNNTIALTSYGKALADSGNYEKARE
IFERSLQIHPNNIYILRMVNVWKS
>OHY33650.1
MNQGRTYDYIDLIELLIGTIFESKGDSQVIYPLLENNLHLLDATFIDQLQSWGGHQVNKQ
ATPEQNAQLGASLYLLARIFYHFPGGDPLINLTIAICGYEFCAAIERQLGLEKELAQVLN
HLGAAYQTQAQMGTDSHSNLEKAIAAYNEAIAIRRQPGLERELAQVLNNLGAAYQIQAQM
SKEANLERAVTAYAEAISILRELDLERDLAQTLYNLGSAYQTQAQLGKDSQANLQKAIPP
YTEAISILRELGLERDLAKTLTSVGSVYISQGKLERAITPFREAITMFRQLGLERDLAET
LNQLGTAYLSEVERGKDPEANLQRAIIVFTKALTILRQPGLEPDLAQTLHNLGKAYQTQA
ELGTESQSNLEMAIAHYREALAIATPELSPADYLKFASHLGDLGFQQGWWDIALEGYERG
IKALETIQFWQPGDKSPDIQYPHIYQSAVRACIELHQYGKALELVERSKSRNLVELLENK
HLYPKGNIPSRIYAELDRLRQAVRNEVRLLEIQGEKRSHAQLNGLIEELDQFITSQVKPL
DPHFSLTQRVEPITISKILKLVGADPRRVILEWYFTDDVLYTFIITANPDVPIFPHKVVI
SPSVIRQFSQKYLDIYRDRHNLGNWKQALPELLGELKEILQVETLLTQLWKLSPPCEELV
LIPYGCLHIFPLHALFPWERFPLGFSYFHSCQLLSSVEVTEREEVVKSLAIQNPTQNLQY
ASFEVETIITRLLIGTEVITEKEATRETIFSRSIPEKNYLHFSCTGYSNFQKPLHSAILL
ANCILPAPPKGGEYLPLPNGAAVDLSQCLTLGDICGLDMRRCHLVSLSAYEMGIGDMVDT
KNDDYISLPTGFLLAGASTIISSLWAVSDYTTALLMVRFYQFYTDTENLLTYGRPRLALY
HAQQWLRDAPAGKLLTWAAKLPFSGTRLTLLADFDTMPESQRPFQDPYYWAAFTCVGI
>OHY33652.1
MKTKIFGLAIVLSLATMLGACEGGTEPSGDATTTPAATTAPAEPTDSGTTTPAATTEPTA
TPTPTTTP
>OHY33656.1
MICCLNPDCQNPQNPDGSKFCQSCDTPLVPLLRNRFRIIRVLSDEGGFGRTYLAEDTDKL
NDSCVVKQLAPKFQGTWAQKKAMELFSQEAKRLQELGEHPQIPTLLAYFEQDNCLYLVQQ
FIDGNNLLQELRLRKHYKDSDIQSLLLDLLPILKFIHSRGVIHRDIKPENIIRRKRDGRL
ILIDFGSAKQLTAAVQKKYGTSIGSHGYSAIEQIRDGKAYPASDLFSLGATCFHLLTGLS

PFQLWIEHGYSWVKNWQDCLEDSRSAELIIIILDKLLQPDLKNRYQSADEIIKDLGKKNVY
GLKKSSTYLKKGKHKKPKHTILRNIFLTLVTISVVGGLGYRNLGQIQTAIFSQFNPLLMP
SNKSSTSPEYGQICRRTSSSRANKVSLFKTITQVDKSLAVVAITPDGNTIVSAGHKEIKL
WNSKTGKQIISLPGHTQNINALAISPDGNNLVSAGDDKTIKVWNLQTKKLTFNLVGHQDS
VQALAISQDSKTLVSAGDDKTIKVWSLLTGKFLKTLLDHNYWVRSLALSPDGFTLASGSF
DKTIKIWNIHQTSGQKPTTLLDTTSQTVTSLTFSPDSSTLVSTSRDRQIKFWDIKNKEII
FASKKQNVTSVIFSPDGKTLISGAKSCPDCSEKISSVIKLWDVSTKEEIYALPVNTKIVT
SLVLSADGKTLVGGTKDNKINIWEISP
>OHY33678.1
MKIAPEITYRNLDKSQIIDKLVREKIAKLENICNYINSFHIAIEKSHDRPRSGSPYRVRI
DLTVPPGHELVAEKNPGESIRYEPLDAVIRQTFDAMLHQLSKLTQMQRASEQYDRDEETR
GSTGFITKLFKEDGYGFLQALDGREIYFHKNSVLHQDFNQLKLGACVHFSQEEGEQGPQA
TTILVVDKR
>OHY33699.1
GFPRRPQGFPHSVGLIGMRDVRDYKVKSGGSERLNTSSPFNIKAESLTLSNFSFTDVKNL
YEQHTTETGQIFTPEAIQRAYYLTDGQPWLVNALARQATQVLVKDVNQPITAEVINQAKE
ILIQRQDTHLDSLAERLREERVRDIIQPMLAGEDLGDTPEDNLRYVLDLGLCRRDRGGAL
EIANPIYREILPKTLAAVAIASLTSVEPNWLNSDGTLNPQILLNSFLEFWRQHGEPLLKS
APYHEIAPHLVLMAFLHRVVNGGGTLEREYAIGSGRMDICLRYGKVVMGIELKVRREKLD
PLTKGLTQLDKYLDGLGLDTGWLVIFDRRPGLPPMGERISTEQVISPSGRTITLIRS
>OHY33702.1
MTQSSPVSRLDNYHLSVIESLFDIARNIEISADFSIQHPQYQPFAIPSTVAQRFRHNSLD
LQRKYLNLLLRNFLHGIYYNGALQPILSTNNHQCSHLLTNGLEPNYKLGVDRNFYQQLHE
NNHGIGNYDADWEVLRIEPDGTMAVMKNNLTLYVEPECYLKSDRSPTPGDLIGIWMPKNR
LQNGCYIAIGNCNQQHPSHSHNHHTNYHSKKIAKIYFNITPSGAIALMNILTKRLNDASI
PFSFEVLYNPISYRRYDTATLQFNCEDYRAIHKILGPTYLETEIYFQPQIPLFTKYLAPG
LSLAEEPNQKFSPEESFGMNRCQIIANALLEAWQKGKNAIEERIQIIQQHFASELIDLHY
PYLNPSSHDIYIP
>OHY33720.1
MNPKNLPLGINTLDKLRGNNCVYVDKTRIAFQLIKQPGAFFLSRPRRFGKSLFVDTLKEI
FEGNQKLFEGLYIHDQWDWSRKFPVIKIDFAGGVLKNRQELDQKINGILLKTAQSLGVDY
ELEDIQGRFGEIIAGAYQRFGERTVVLVDEYDKPILDNIDNPSIAVEMREGLKNLYSVLK
EQDANIQFIFMTGVTKFSKVSLFSGLNQLTDITISRDFSTICGYTQEDLEQTFAQHLQGV
DWDELRLWYNGYSWRGESVYNPYDILLFIREGMEYGNYWFETGNPTFLIKLFGTNRYFLP
NLEHLEVTEEILKSFEIEQINPVTLLFQSGYLTIERNFIRHRRSMFALKIPNMEVRLTLN
DQFINVYTGMVNEKSAIQDILYECMWSGDLESIVKAVKRLFAGIPWRNFTNNHLADFEGY
YASVLYAFLSSLNARIIPEDISNYGQADITAILGDHIYVMEIKVVDGENVKENL
>OHY33732.1
MQCAIGTMVIIGQDPRLFIIPTTSCCLLERAKFRNYWFETGSPSFLLKLFQKEQYFLPNL
EGIQVTEEILDSFDVEQINPVTLLFQSGYLTLLFQSGYLTIKDTFTDINQMVFCLGIPNM
EVKIALNNQFINAYSNLINEKLGIQRLIHTQLRSGDVEGLVSTIKRLFASIPWRNFTNND
LANFEGYYASVIYAFLSSLDARVIPEDISNYGQADITAMLGVHIYVMEIKVVEGNQVQGN
AALDQILQRNYAEKYRGEPGKYVHEIGLIFSRSQRNLIQADWH
>OHY33733.1
MIMAIKRLFAGIPWRNFTNNDLADFEGYYASVIYAFLSSLDARVIPEDISNYGQADITAM
LGVHIYVMEIKVVEGNQVEGNAALDQILQRNYAEKYRGEPGKSVHEIGLIFSRSQRNLIQ
ALAIIKNLPLGINCVKMCLCFLSRPRRFGKSLFVDTLKEIFEGNEKLFEGLYIHDKWNWG
RKFPVIKIDFADGVLKNREELDKYDFETDAWGLCEKEYTRDFGEIITGAREQFGERVVVL
VDEYDKPILDNIDNPNIAAEMREGLKIFTQC
>OHY33744.1
MTTAIQQRQSANIWDRFCEFITSTNNRLYIGWFGVLMIPTLLAATTCFIIAFIAAPPVDI
DGIREPVAGSLMYGNNIISGAVVPSSNAIGLHFYPIWEAASLDEWLYNGGPYQLVIFHFL

IGVACYLGREWELSFRLGMRPWICVAFSAPLAAATAVFLIYPIGQGSFSDGMPLGISGTF
NFMIVFQAEHNILMHPFHMLGVAGVFGGSLFSAMHGSLVTSSLVRETTETESQNYGYKFG
QEEETYNIVAAHGYFGRLIFQYASFNNSRSLHFFLAAWPVVGIWFTALGVSTMAFNLNGF
NFNQSIIDSQGRVIGTWADVINRANLGMEVMH
>OHY33747.1
MIPTLLAATTCFIIAFIAAPPVDIDGIREPVAGSLMYGNNIISGAVVPSSNAIGLHFYPI
WEAASLDEWLYNGGPYQLVIFHFLIGVACYLGREWELSFRLGMRPWICVAFSAPLAAATA
VFLIYPIGQGSFSDGMPLGISGTFNFMIVFQAEHNILMHPFHMLGVAGVFGGSLFSAMHG
SLVTSSLVRETTETESQNYGYKFGQEEETYNIVAAHGYFGRLIFQYASFNNSRSLHFFLA
AWPVVGIWFTALGVSTMAFNLNGFNFNQSIIDSQGRVIGTWADVINRANLGMEVMHERNA
HNFPLDLAAGEVAPVALTAPAING
>OHY33789.1
MENSESAKPKPKPWQPGSVKAQFGSTIITGGDASMKPEHLSFLIIWRHRQHHQGQPKKQ
>OHY33862.1
MSNEAKTISSVPEDRNAWRWGFTPQAEIWNGRLAMIGFVAATLIELFSGQGFLHFWGIL
>OHY33871.1
MTQYQKLLRIATTGKSFQNITAKIAAIVTESGVKTGLCTLFLRHTSASLIIQENADPDVL
RDLANFMSKLVPEGNYYIHDAEGPDDMPGHIRTVLTRTSESIPINNGNLVLGTWQAIYIW
EHREYNHNRELVVHISG
>OHY33943.1
MKWQLLTHNKQVLGKIFTILVFTGLTGVLCVSCNRNQDLLVTEIGVNPPKRPTRKTSDAG
EFYLQGQNQHSRGNFQAAIAAYSKSISLNSDYAPAFKARGLAYFDLNNKERAINDYNQSL
QINPNDPETYNYRGNARASLGNQKGAIEDYNEAIRLSPNYAEAFNNRGNSHAAQGNKNAA
LEDYTQAIRIDQNYPVAYNNRGNAYSSLGNTSKAIADYNQAIRLNPQFAPAYNNRGNAFA
SSGDKRRALQDLQKAATIFDQEGNRGLYQQTMKNIEELEN
>OHY34004.1
MTDQDISGGFSPVETTKPEQNPRLSPDLEVQVNQSSGGNPIQHSDRNFGSLMQQQNNNDL
YEKGRKNNWYRPVDFILSGKIWELAMWKKSWIWWFFVLAFIPSSIGIISVSILLKLPSAP
NCPRIFWPLASASMRVHCATLAASKQTVSDLLQAIALVKDLPQDHPLRGQINDLLQEWSR
DIINLAEKSFQLGNLEEAIATAKQIPENLEDRQFVEEKILKWQSTWSTAEEIYQSSIGEL
ENRRWQSAFMLSSKLLRINNRFWSTTKYDQLNQIIVTAREDGDKLDKADSLADRNSVNDI
LSAIKLVKSVKPESYLYKKAQELVPQFGRKMLKLAQAQMERRDADKGLEIAGKIPPIPSL
QSEIDDFIDLGEAQRNAWLGTILGLENAISQAQQIDPSRGIYGRAQELISVWQLEIEDVT
KLEQARDLASGGTIEDLRSAISQAQQIPSQNPRAQEAQTQINRWNDQIETIQDKPYLDRA
EQIANAGDINSLQSAIAEASQISSGRALYSKARRRIRSWNASIQRIEDQPYLDRAIVLAE
SGDLNSAVQEARKIAISGRALAGEAQAVIDTWQEQIRARENWRKARELASIGTADALSQA
IRIANRVSRRNVLRMDINIAIDRWGEQILEMARSQSQVDLVKSIETAKLIPRSSSAYADA
QLQIRTWKEQLVPVPTTPFLSPGEPSP
>OHY34042.1
MVVKIHLSITQKTQRLDRYLSSVIPDLSRSRIQDLIEQGYVQVNGKVCYSKNITLDGGEY
LEVEVPPVQAAQLVAQDIPLDILYEDEQLLILNKPAGLVVHPAPGHLEGTLVHALLAHCP
NLPGIGGVQRPGIVHRLDKDTTGAIAIAKTDIAYKKLQLQLQSKMAKREYLGVVYGAPKS
ESGTIDLPIGRHPQQRKKMAIVPVEQGGRNAITNWYIKERLGNYTLIHFQLETGRTHQIR
VHSAKMGHPIVGDPVYSSGRSVGVNLPGQALHAWQLKLQHPTSGEWVEVTAPLPDSLTKL
IEVLRRR
>OHY34043.1
MPFSLELAEELLGTYGSPLYVYDSDILNQTIAHITKSFKYPETRFYFATVTNGNIALLKV
FKNAHWGLHANTPGDIYLGLSAGFAPDSIIYSGTNLNREEIEQVLSWGVTTLNLDSLAQV
QLLCEVCSSLKNTAGKGKNLNTFSRLNQLDIGLRLNVGGESRIGVRLQDFNTAIAIAKNV
GLEISGLHFYRGTGTNATKAFTDAIDEVLVAAQNLPHWKYIDFGGGFGYPYNHQGIAFDW
ELFGGEIIQRISNLDRTIKLIIEPGRAAIASCGTLLAKVVSVKWQDNKQILGVDSTVGNI
AVPSVYGGYREIISWKNHSPKYITDICGNTTYSRDYLGKNCQLPSLEMGDIIGILDVGAY

GYAMSSHFLHRPKPGEVLLENSQHRLIRNREDYSVLLTNQIW
>OHY34083.1
MNSSDNKIHEIHEFSTGIQFERRGHSWIWTGFTTKYMNSTMGEEIPKVVERSIANEEFAL
AEGSWTEKPAIIARILGNGNNIWSVIAVVTRAKDEAGRSATLHRYFLCQGEHKLRVILAW
WEQNNRPTFNPFDFQVLGSPHSFAGEVPPPSDLEQIEQILSLFKEQAQPQEDKEQILPLS
REESVFTGQLGEQPQPRFAQGFDQQQILATSTGGTKPQTDTEEVWQFHAIYN
>OHY34084.1
MLSTTERIPQLKQLIEQRNYFVIHAPRQTGKTTAMLNLAQELTASGKYTAVMVSVEVGSA
FNDQPQIAEESILTAWQDAIEFWLPPELQPTFFTQGNTPKKIGSFLKMWAETSSRPLVIL
IDEIDSLQNQTLINVLRQIIRSYQ
>OHY34085.1
MSKNGLPVSWAFNVEALVKPERFQIIQPASEKAVERIKRAIASTSQVKINTVNIDEAALK
SAIRSLINSSQVKPEAVGVIVNGVTNKEITPEYWEHLFNSQGANQGIRQKIYSPQMVKLV
TLRALVLPKTLPEFLGWLNIQAGKKVNQNQMVSLQFQKAIKELFPKEKVAEGIGYLLPSL
LNGKISPDGLSWLLAKNGSNSIWSYAQKQFVNDVRNDLQLIFDQYKNSKSLNFDGGNLKC
EIGVWNQLIRGWQGIQRRYYKCEEYRPLAELFEEFREYDLAAYFYQVSDGLVNKKLFGRL
AHSQKARHPIIFGLPIKRKETPIDIFINFVNQDIDMKFLYAFPLSLLILGSGWFIGSKTW
QFHVDRNEVKEYFCRELESSDNCSVMVLDSKKHYSFQDIKQIIPEVVKDLVESIKTEQGG
TADIESQLVDQVKQNLIKVLGDSTLNYTLNYDEILTQEKGKYTDSWIEKKWLMAVYNYQI
KREAQKIIQTVEVRECKLNIVVFGRCLWGGRQKVIKYQVDNSPLKTKLKEDIYRAIKEG
>OHY34092.1
MGVIYIGDRATGKTHLAVELANPKHEYVKVNSLDYDELSSLLLDRKPDKNPLGEATSLTT
HERHLDMNIQLPSGKKQVVVDCIDIGGEVWRKNWQRDNSSQWHEFLKAIRDSEGILLILP
PHRGMTFKPGVDAEEFPTQKQWCNRFERWVTFFCDQCPKIRHLALCLNKADLFCDLSTEG
QKLAYKPYNSPYNWQQRHEHVLRKYFRPVYAQIERLGQGMGSPSVKCFITSIYHRGLLEL
PWIYLGSFLGK
>OHY34096.1
NLYSVLKSQDANLQFVFMTGVTKFSKVSLFSGVNQLKDITISEAYSSICGYTETDLRESF
GDHLEGVDWDTVRHWYNGYNWTGSETVYNPYDILLFISEGMKFRNYWFETGSPTFLVKLF
QTNRYFLPNLEHLEVTEEILESFEVERINPVTLLFQSGYLTIERTFIRRQRYMFALKIPN
LEVRLALNDQFINAYTEIINEKSGIQDSLYEFMNRGDVESMIMAIKRLFAGIPWRNFTNN
DLADFEGYYASVIYAFLSSLDARVIPEDISNYGQADITAMLGVHIYVMEIKVVEGNQVEG
NAALDQILQRNYAEKYRGEPGKSVHEIGLIFSRSQRNLIQANWQ
>OHY34097.1
MLNSLIAKIKEVKDFRKTQGRRHELWVVLTIIILALLTGNVSYKEITAFSKAEEEKLIKL
LSIPAKKLPSYSTIRRVIIGINIRDIQSLLESMLQQYYREKDKEDWIAIDGKSLKNTISN
YKNKTQNMVTMVSWFSQKTKLIIKAESQESKNLSENGIVLSMIENCGLYNKVFTLDALHC
SKKITKIIIESKNDYLITVKGNQVKLHNRLKNLAEIQEPLSEYESRDQSHGRNVNRKISV
FNGEEVGHVNYPHIKSFIKVERTGFRGNKEYSQTLYYISSKKLSAEIFAKKIQEHWLIEN
QVHWVKDVIFNEDKSRIRGVEVAPKFSLLVTVTLNIYRSWGFLSIKKGQSWLGKNWEKMF
LIDHFDSS
>OHY34126.1
MNHPAKILTFIFLWFQPILTIIHHKQTLAVTSEEKTNLICTQASKAVLTIKTDYSKHGSG
FLVNNKGLIITNAHVVSDGPTVATVVFSDGQELSADLIGFANNGVDLAALKIHNRNNLPY
LKIARNKKVKVGFSVFAIGSPINPNYQNTCTQGIISNIHPNGTIQHTATTNPGNSGGPLL
NNKSEVIGVNTWGILGGTAINLSQSIDKVHTFLADIQKQTTSPVSTLPTPPKSVFKTILP
DGKPIKGKIYGDPDVYTFAGVVGQKILIQMNSDEINSHLTLSLYKKTESDENEQIIAQND
DKVAGDFNAEIITTLPENGIYQVLVKTSRTGETGIYNLRVITQP
>OHY34128.1
MYKKGKGDLILTTNTLTKINNMKHYITIIFCLFLLAPSENTLANPITKNLTAKTNSCEIR
PEDQENAQYSDLQQQEIARRITVRIIANNNIGSGTILSRNNNSYLVATNSHVLLEANSDN
ISIQTVSGKTYTGKIIPQTNLKKLDLSILEFYSAEYYCSAPEIVDTTINLEMPIMAAGYT

KGKIIFRKGNVQRIAEPSLKEGYGIGYSVNIIPGMSGGPIINSRGYLIGINGKRPYPILN
SGYIDINGKQPSAAEIQEFRKLSWGIPVTTLLSQVRTDLLQKYSLPLPNIATKVAVQPLP
PWIAKIEEKVKQFTVRIDNRDGENGSGVIIAKEGSTYTILTAAHVLCEKASNNRCIEQEY
SILTGDDKKHPIDRNSIKKEEGVDLAVVKFTSSENYPIATLADYPTQDNQYIFTAGYPKL
GENRSPWRFTLGQTFSKEQGLLATRSSDFKSSGSGLQAISVSLTEGYELVYTNITLGGMS
GGPVLDTEGRVIGIHGRADGQVAIDEKTGDIGPNNGQVQLGYSLGIPIRTFLGIAPRLNS
QAQQIEKTTARELQSGKIDSIKQVLLSVDISKGSTTASNWLERGNQLLRLGRPREAITAF
DNAIQQKPAFIHLAYYGKGAGLALLSLALFDSGKVEEAAIALEQAIKAKSDFVPAWDYLS
LAHGRLGKFNEALVAINQAIKLQPDNSNLYNTKWQVLSSLKRYQEAIDAIDQAIKLSPRV
AFYNNRGVARFQSGDKQGAIADYNQAITLDPKFALAYNNRGLARFQSGDKEGAIADYNQA
ITLNPKNALAYINRGLARFQSGDKEGAIADYNQAITLDPKNALAYIGRGFARFQSGDKQG
AIADFNQAITLDPKNALAYIGRGFARFQSGDKQGAIA
>OHY34132.1
MKRVVPFTRLSKFQSRDSILADKYTALEKNVFASKSAPIIFPPETTLATLIASLAVMDNK
KGFLACLGKAKTIEKAISNSINAPNIRTDFLLKKWLGVAPARDDV
>OHY34133.1
MNLDNNYRIAVLGLGYVGLPVALALSEKFSDVVGFDINRERLDDLMAGIDSTKEVERDRL
TETSLHFTDNSKSLEDRNFFIICVPTPVDENHQPNLTPLIKASQTIGKVIQPGSVVVYES
TVYPGVTEEICGPVIAQVSGLQQGVDFKLGYSPERINPGDKLHTLDKITKVVAGEDQNTL
ERVAAVYSQIITAGIHRAPSIKVAEMAKVIENTQRDLNIAFMNELALICDRMGIRTHDVI
QAARTKWNFLPFTPGLVGGHCIGVDPYYLTTKAEVLGYHPQVILAGRRINDSMGRYIAQR
LLKLLAQAKIPIQNSRIGILGLTFKENVPDLRNSRIPDIVKELREFGINPLVHDPLADVQ
EARREYEVELVTWEELSNLDGLILGVNHQFYLDLSLKDLIGCLKENGVLVDVKSVLDPVN
IPDQITYWSL
>OHY34136.1
MCGLTGFFYPGGFHLEDATNLAQQMAKTLSHRGPDDGGLRSLVKYFYHQAHHGRCTCTG
>OHY34137.1
MSIRIAFIITGLNTGGAEMMLLKLLERLSPEFVPKVISLTDIGVIGQSIQSLGIPDGFFH
TKTIRQKWEEHLSGGRN
>OHY34138.1
MEAMECGCAIIATDVGETRQLLDDSCALLISPNSTSLANAVMDLLEDPVRLNTIAKAAQR
RVKENQSLEKFSEYFSKRILKLSESEITSDVN
>OHY34139.1
MKLAYLNVIYSILPGVTSKIESQALALKSIDSQSKIVCFYCDNDNSLDTTNKCLTYVKLP
KPVRSAQLFRKLYNFQLTQFIFDYTTNNRFDVVILRSIALSPLFWYYFRSKKYKLITEHH
TKIVPEMMIKKSYLGAASTILSRGIIDSVIDGKICVTREIANYEAFNKPIRVIPNGHNAV
QKTSFLPYNGKTVRLVMLCSVSQPWHGITRLFASMISWQRDRSDIQFYVDIIGNIQTSEF
VSQNLPNNVVFHGYKDANDIAIIMSHANIGVSSIALYLKNMQEACSLKSREYIGRGMPFI
CGYYDPDILETDSYILRVPNDGSIIEVSVLLEFLQYLNRHREKVTFELDAAARRISWESK
MKDYYDFAKSIYLE
>OHY34140.1
MKVVTIIGARPQFIKTAVVSRAFRDHFPDLQEIIVHTGQHYDANMSDVFFRELDIPYPDH
YLGIGGGTHGQNTGRMIESIETVLTATKPDYVLVYGDTDSTLAGAIAASKLHIPVAHIEA
GLRSFNRFMPEEINRVLTDHVSQLLFTPTKTADQNLAREGIQENSVHRVGDVMYDASLYY
SKCMQGNGSILDQSSLDTKNYILVTIHRAENTDYPDKLREIIRGLSGVTLTVVWPLHPRT
RKCLTELQLSMPDNIKLIEPVGYLDMLMLEKHAKFIVTDSGGVQKEAYFHQVPCITVREE
TEWIETVEAGWNCLVGANAEKIVTALRMCYNPNKQITALYGNGQAGLAIAKVIACGFD
>OHY34141.1
MPDRILVISHAPFDNPYGASTSIREQYQAFEQLDKYEFIHVSKASPKEGILKGFQIYYPK
KSCIKPTNVSNIFSACLPWSDNYDTAAMHHSAIDVRFICKQLILDTNWRLRKKKAINTFL
MFYPKIIHLNSLVLAEVIPTLRINYNRPIISHVRELLKHKISEKNRQAILQLDAVITIDS
AVTKRFIEVVPEYPVEKIHQILNPFSARPFDDSLSSLFPDGGKFVFAIIGTVSRDKGVDL

VCECFYKADLEDSVLIVFGSTDSEYASSIKMKWLNRTSKIIWVGEHEYLAKRGAYSRIDV
VVRADPSFRTGRTVYEGLYAESDIVIQGDINDLITDSSLLKWRDNITMYKPRDRISLVNA
LRIVHKKLKFSRSVSPERKLSSNYDDYRERMDMVYSQLLKSFDFINC
>OHY34142.1
MKIPVLDLKPQYEQIKDQVQGAINKVLESGQFIMGPDVKLFEQEVADYLGVKHTIAVNSG
TDALVIGLRALGIGSGDEVITTPFSFFATAESISNVGAIPIFADIDQLSFNINPEAIKKH
ITPKTKAIMPVHLYGNPAVMGQIMDIAQQYGLKVIEDCAQSFGARYNGGCVGCEENCQAS
TRESIQGKFTGTIGDVGAYSFFPSKNLGCYGDGGMLVTNDDQVAELAQMLRVHGSKKKYH
NEILGYNSRLDTLQAAILRVKLRYIDQWNRGRRRVAELYNTLLSGVNDIVAPTINDSHVF
HQYTVRIVNGQRDKVQQYLTSEGIGSMIYYPVPQDKLPIYNGQYGVNPVSDLLAREVLSL
PIWPELELEKIEVVVKVIGQSVKENHL
>OHY34143.1
MSNYFAHESAYVDEGAQIGEGTKIWHFCHIFGKAKIGNNCNLGQNVLVANNVVIGNNCKI
QNNVSLYEGVVLEDYVFCGPSMVFTNIKTPRCEYPRNTSDDYRQTLVKRGASIGANATIV
CGVTLHECAFVAAGAVVTKDVPAYAMVAGVPARIIGWMSSYGDVLDFDTDGYATDSQGMK
YQRISESTIVKL
>OHY34144.1
MNREKNSVQVAVVGCGYWGKNLVRNFAQLGALTWICDKSETALKAQSQLYPQIGITNDFR
EIISNPEIQGVVLATPAAMHFSQAREVLLHGKDVFVEKPLSLRYKEGRELVEIAETRGAV
LMVGHILEYHPAVTLLKKLVYSGELGKLLYIYSNRLNLGKVRQEENILWSFAPHDIAVIS
SLLHLEPTEVISVGGTYLQSGIADVTVTNLVFPQNIRAHIFVSWLHPSKEQKLVVIGDRK
MAVFNDTVKEGKLKLYDKGIEWQGGLPIARQTSETILFTDEKEPLLLECQHFLECIKKRD
TPLTDGSSALKVLKILEASELSLQKGGLPVSLNEIEE
>OHY34145.1
MKNLDELREKITSRTAIIGVIGLGYVELPFAVEKAKVGFQVIGIEQNPVRAERVNSADNY
ISDVKDDDLKHVVATGNLRAITGFDCLPEIDVLVIWEVLDAANTKPFGIMPFYPGPGVSG
HCIPIDPHYLER
>OHY34148.1
MQYKKLTFLFKTYLEILKSLAVKDTYRSLSKCDVLLCCHDVDLGETLEGLPYSKLVDSVF
EDLSNKGWVCRKFAHPWSVLTGRKAWGNPPSANQRIFFVRIQRKICKFFPFLPNSKKDKK
YSSLDPVENFYHELFDITLPRCVITIGSPPEMCRSAKSRGIPVFELLHGIGYDPVPWGWD
QALACNLPTGILSLDNVSTKTFTSLHSKGVHVKQIPHPFFRRFLDPEARQKLPPQWQDYP
AWLPTDKIIILVSLSCGLVPDEVIDAVDQTHDSIFWLFRLHPVQLRQNQYNHHRQFLERL
TKNHPNCEWRESSVLPLPLILSYCSGHLTMSSMTTYDAAFMGVKSLLLCPTLKDNSMFSD
LRSSGFAELGNFDVASIIKWVTTIKRCSHKFTTSTNSDQDWDSAVQWMLGRAYKADGEL
>OHY34149.1
MGWRSIEFKELYQSACEYLDVDRVHKIEIDREQAYLPQVKQALNKLQPTHYVYDPRTGSE
NWLRGLWQAFQISFLLASRHITPVVLLTDLAIRTWRAQSAVVTATSGVVVTFMAPSRIQP
IFPHRRLVGPSLMPFSQATLAWLDHLPRTQPDNNPPRAIFTGSLYEPRTSLLNAIKVGLE
ARGLTLDIQGRQLGSPRVSDEEYWLRLANADIVLTTANQMIQPGADWTWVLHLLYRYLEV
VACGTLLLAPEVPGIGRFFTSGEHFVSFKKSDDAVDAIEYYLNHPVEREKIAKQGYARAR
SLIESRIFWLTIDVALGHESLA
>OHY34150.1
MRAIIVGHTGQDGTLLCNSLKHKGYKVLGFSRSSVYTNSDANFNLRPNLTDEQSIYQLVQ
GFQPSEIYYLAAYHTSSERQQSESLSQNFALSQSTHVTGLLYFLCAIKDLLPSCKLFYAS
SSLVFSGEDGEVQTELTPLSPQGFYGITKAQAMYLCQEFRSKFGIFVSVGILYNHESCLR
SHHFLSQKIIQAALCIASGSQEKLMLGDLSVRVDWSYAPDFIKAFQNIIQLPSSDDFIIS
RGEAHSVEEFVDIVFDYFNLDYTHHVMQDRSLLQRRLLTRVGDCSKLKRVSGWTTSLNFY
DFVRQLVIDSESLCKTKRL
>OHY34152.1
MIKDAESLRKIGNYETYSQAHQDLFVRIMLGFKQNGIYVEIGAAEPKQSNNTYILERDLQ
WIGLSLEIDAALVGEYNSIRNNKCLLADATKFDFDGYFRENRFPTVIDYLSLDIDPAHIT

YQALENLPLSDYRFSVITYEHDRYASGPEMMVKSRQLLENLGYIRVVSNVRCCGRDFEDW
WVDPLIVSPQIYEPFISENIECRDIFKSV
>OHY34153.1
MQCRILLHAIAWEPDPENFKLLRCNIILNDLEEKVTLYNLALGRDSGKTLKFELSEDNFG
DHRIRVSDDSGKYHEESRKIIEVKSETLDSYIDIFRQENLSGNQLILWIDVQGYEGEVLA
GARTLIEQMKPAIGMEFWPYGLDRAGGIDSLIEAINHYEIYFDLTQSNPSGQPVNKLFDI
YDYHKNNDYFMMDILLF
>OHY34159.1
MTVTQISAQELFRAAYQNRYTWDDNFPGYTADITYKYEGQEFTGKIRIDANFKWEVTQVE
DEAAKKAIDSQTWEIAVHRVRRTFAQTHGENTFTYGEKDSTGAVEIIVGGKSAGDKYKVR
NDVVTLVHRHIHGVVVTINTFSIHETEAGYLSHTYDSVYHDPKTGEQKGGRSEFTDEYEK
VGNYYILNRREIRTETPAGMSIQEFVFANLELLG
>OHY34162.1
MNDKKRNQWDLCRFIRTLTYFEVFPLLNWIQNILQNRPTNRQDQPTGRIQMGIILVAGAT
GGVGKRVVKKLLTQGYRVRCLVRDIEKAREVLGNEADLVVGDITKPESLNDLVMSNIQGV
VCCTAVRVQPVEGDTPDRAKYNQGVKFYQPEIVGDTPENVEYKGVKNLIVAAKRYLPTTG
EKIIFDFTQPSSDLKNIWGALDDVVMGGVSSSNFYILEKTAVFNGNVSTANSGGFASVRT
KNFSPAINLSGFTGIRLRVKGDGQRYKIFLRTETTWDGIGYSYSFDTMANTWIDVNIPFI
NLVPVFRAKTVKDCPKIDESKICSLQLMLSKFEYDGGLNPKFTPGAFTLELESIRAYGGE
GVSQFVLVSSAGVTRPGRPGINLEEEPPAVRLNDQLGGILTWKLKGEDSLRDSQIPYTII
RPCALTEDRGGKELIVDQGDNIRGKISRDDVAEICLQSLQQPQAKNITFEVKQGQNDAVS
LNWGQLLSQLQPDRPNPL
>OHY34168.1
MLHKLFTEIKEYELAAYFYQISQSKISSKILKLLVNSNNITPEIILGLPIKESRPWINSI
SKFFNKDIDEQEKIFKKRREKYLELLRKYKEIDDYNQDIKINPNDPEYYYNRGNTRSDLG
DNQGAIDDYTQVIKLNPNSAYAYILRGDVRSDLGDKQGAIVESPVQTCRV
>OHY34169.1
MKRFFTWVPTLVLVIICSLFLFNSPSYAFNQADLDQLLQNNICEKCDLRNADLSDANLIR
AKLSRANLSYTDLRRVNLSRANLSGADLTYAKLTYAILIGADLSGANLSYTDLSRANLSG
ADLSGANLSYTDLSRANLSGANLSGVNLSDAKLHGANLSGVDRSGAQY
>OHY34193.1
ERKSWVVWQEGKGPDVIVELLSESTAKTDKTTKKSIYQNQMRVPEYFWYDPFNPEDWKGF
KLINGVYKPLELVEGGYISEQINLKLVLWEGSFKGLNIVWLRWATLEGELLPTQQEKIQR
EYERREIAEALVIQERQEKEIAQALVIQERQEKEIAEALVIQERAEKEQERQKNEKLAAY
LRSLGINPDEI
>OHY34205.1
MISKERKFTIIIPTRDRADTLLYTLASAISQNYPNLEIIVSDNASQDVTHEVVKSFCDSR
IKYINTGHRVSMSHNWEFALSHVDEGWITVLGDDDAIIPGAISQVNQIIDATGTKAVRSN
GCGYMWPTMTSNCHGHLHLSTKKGYKKVGSEKALSRVMSGNLYYTNLPVLYNGGFVDISL
VKEAKAITDSFFLSMTPDVYAGIVFSLLTDEYVYSHEPLAINGASHHSGGTAAFEAVKRN
RSYNPAEKFWSEENIPFHPSLPLTKEGRPVKSIQAIVYEAYLQAAVFHERKRIVYNLVSP
KQQLEIILRDCTPVTKPEVMSWSSEFAEKNNIIPDQSLNLIAGKKSVLFLLKKITVKIVN
LCLSLSLSGRQVVGLDNVYEASILVALIKRGYLASFQLAFSNLMCRAASIRELDFRRKLM
QYFFG
>OHY34206.1
MSNFEIEVLERLSLVAKNEEIARSSHTFLKATIQSKYSYNFYWLGRPIIQYPQDIVAMQE
LIWSIQPDLIIETGIAHGGSLIFSASMLELNAACGGPQSAEVLGIDIDIRQHNREAIESH
PMYKRISMIQGSSIAPEIIEQVKLKAVNKQKILVCLDSNHTHDHVLAELEAYAPLTSVNS
YCVVFDTMIEDFSDDMFADRPWGKGNNPKTAVWEYLKTHPEFEIDKSVQHKLLITVAPDG
YLRRVC
>OHY34207.1
MKVAVTGATGFIGRHITSALLKKGYDVLLVGRSNNVQQSGLPFVQLDLLEERNHNWISEY

KPSHLLHLAWYTEHGDYWESPLNINWCHSTINLIHAFAVQGGKRIVVSGSCAEYDWSFGY
CNEIKTPSYPSSLYGTGKDCARRMSERICNTYKVSLAWGRIFLPFGSGENPKRLIPSITR
AIIGSDPPFPIRVCQWRDFLPVEMVADGFLFLLEQDYPGIFNICSGKPVQLSEIVKRIGF
ILNKSADGLMLEAEFSTGYDSFLVGDNRCITMHGWHPDYDLWHHLGVYVNSLVRAI
>OHY34209.1
MKIVTTPIAGVWIVKTTPFVDQRGYLYRGFCNQELEPILKDRSISQVNVSKTEDVGAIRG
IHFQYPPYSEMKFIRCIRGRIWDVAVDLRQGSATFLQWFYAELSADNGDMIVIPEGCGHG
FQVLEPNTEMLYLNTSPYEPNHQSGLLYNDPLLNISWPIDVTTISERDQSHPLLSENFQG
LLL
>OHY34210.1
MKAVILAGGYGTRLSEETHLKPKPMIEIGDRPILWHIMKLYSAHGVNDFIICCGYKGYVI
KEYFANYFLHMSDVTFDMQINEMQVHQKHAEPWRVTLVDTGENTLTGGRLKRIKKYINGE
TFCFTYGDGVSSVNIQKLINYHQKSGLWATVTAVQPPGRYGALNINDKGIVLNFQEKPQG
EGGWINGGFFILEPQVFDLIPGDNTSWESETLPLIASKSQLAAFQHHGFWQPMDTLRDKN
MLEELWRSDKAPWRVW
>OHY34211.1
MNKYKNLLKKAIGYFPFGLGGKFLSLCRVVTNTILLPVSLPLSLMTRFLGFRVLPVSSTR
IGHLVCETDCLIKEITMQRIKPKKWLIFYDGANVSNSAYLELLPDYFFPVNTKNFILKIV
QNLLLRRVVNTEINHYVTAMYQSAQIYSIQAAWEGKGATIHCPADWLEAKNQVLKRHGIS
SGQPYVCIHARESGYSPIDEIWHCKRNVNIMSYSLAVEYLIACGYAVIRMGDHTMTPLVQ
EFSGVFDYARSSDKSPWLDLAISSECSFFLGSASGAYTMANVFLRPVVCVGMSLPFNFSP
SGYSFDIGIPKLFRERYSDTFLSIKDIFKLGLAELRLAEDVNRRGYELVENTAEEILDTV
KEMVGRLKGNWVETAEDKQLQSIMRSYIGIGSYSYGSSARCGTAFLRKYKFLL
>OHY34214.1
MVDELPVKLSDYVADFIAAQGTKCVFAVSGGASLHLIHSVANHAKLHYVCTHHEQSAAMS
ADAYARVTGNLGVAIATSGPGATNLITGICCSFYDSVPVLLVTGQVSTFRMVGDTGVRQI
GFQETPITDICQSITKYACQIRDSSQIRYELEKAVYISRSGRPGPVIIDIPDNLQRSQIS
VATLEKYTPDNFLLRKFPCFGDVKHKFVELITSSKRPVLIAGWGIHLSRTKSEFVKLAEL
LNVPVALTWGAADILPADHPLYVGTFGTHGMRHANFAVQNADLIISLGSRLDTKSTGSPI
NTFSRGAKKIVVDIDPHELGKFSYFGLDIDLLIQEDLVDFFREIQKGDIPVLSDNHRDWI
TIISRWKQDFLSFDRRNVLPTGLDPYEFMDKLSIHTPDQSQVFVDTGCAIAWTMQNFRFS
RGQRLFHDFNNTAMGWALPAAIGGFFARPDLPLVCIVGDGSLMMSLHELATVKHHRIPIK
LILLNNSGYSMIRQTQDQWLDSKYYASSHEGGLSFPDYKALATAFGFDYVEIACIEDCSE
KMGTVFLSSDPLICNVIIPPDARVIPQVKFGRPNEDMEPLLPRDLFLRNMIVNPLDVSKS
Y
>OHY34215.1
MELVCDIGLALSNYFLDKGFSVLGSFRNSSFQLEQLKTKFTALIQSDFSDSSSVDQLVQS
IKAIPFTWDFIIVCPATMQPIGKFESCQIDDWERNIKINFLSQLRLLHGILPLKQNFGEQ
LPTVIFFAGGGTNSAPVNFSAYTISKIALIKAVEVLDAESEDVCFTILGPGWVKTKIHSE
TLESKDTSGKAYDETSRRFATGEFTPMSKVIDCCEWLLNSPKNVIGGRNFSLVYDQWGND
NLNKLLSENSSMYKLRRYGNDLTI
>OHY34216.1
MTETTFAQAINLALHEAMFLDPAVICYGLGVTDPKAVFGTTAGLEQRFGKARVFDMPTSE
NAMTGVAIGAALNGIKPVMTHQRLDFFLLALDQLVNGAAKWYYMFGSQVSVPITIRLILG
RGWGQGPTHSQNLQAWFAHIPGLKVVMPSTPEDAKGLLLSAIFDPNPVVFLEHRWLHNTV
GSIPDGDVRTPLGKGKIMREGEDITLVSMSYMTIEALHAADYLVTQGISCEVIDLRTIKP
LDWEMVFTSVVSTGRLLALDSGFTTGSVAGEIVARVAMDLFGRLKVAPGRLAMPDVPEPT
SFALTKGFYVRAADIAVKVMEMMGRSAEGVREALPEPCPHDVPGDWFKGPF
>OHY34217.1
MNTDLQLALLRQMWLIRTVEETIAQHYHEGKMRCPTHLSIGQEAVPAAVSACLKTTDFAV
STHRGHAHYLAKGGNLKAMIAEIYGKANGCSKGKGGSMHLIDLGVGFMGTSAIVGNSIPV
GVGLALSAQLQGTDQVSCVFIGDGAVEEGVFYESVNFAVLRNLPVLFICENNLYSVYSPL

SVRQPQGRQISSMVGSMGIKVVTGDGNRVLDCYHIFHSALDSLRSGGGPQFLEFFTYRWR
EHCGPSFDNDLGYRTPEEFSYWQTRDPIVQLTQELTQDLASHPERIIKEVKQEVADAFAF
AEASPFPDPSSAYEGVYK
>OHY34218.1
MGKEIDLLVNYPKTKRNLDERAASKTEADRAIARRFGKEFFDGDRNHGYGGFNYHPRFWQ
PVIPTFKDYWGLDATSSVLDVGCAKGFMLHDLAELIPGITVKGVDISDYATANAIEDMLP
HVSVANATCLPFDDNSFDVVISINTVHNLNREECGQSLQEIERVSRGKSFITVDAYRNVE
EQTRMEAWNLTAKTIMSVDNWVAFFAEVGYTGDYYWFIP
>OHY34225.1
MTSIPALRDFHSPSTQVYRFLESVSKVSINNLFGKHGVQKHAVGKFGTIDFPYFSMGSIN
STHLFGLDELIIFAFYESNKQRYKNVADLGANIGLHSIMMTRLGWNVTAYEPDPTHFNQL
QNNVIRNDCMHNNVKLLNKAVSVEVGEVEFIRVVGNTTGSHIAGSKNNLYGDLDRFMVSV
DSFRDICNHFDLLKIDVEGHESDILLSTVGQDWQGTDAIVEIGSASNAEKVFHHFSKLAV
NLFSQKRAWQKVNSVEEMPFSYKDGSLFISSGDVMPW
>OHY34226.1
MCDVLVVGGGIVGLATALRILQSRPQLRLVLLEKESHLACHQTGRNSGVIHSGLYYRPGS
LKARNCRAGYDQLVAFCREEGIAHDICGKVVVATSTEELSPLATLYDRGVANGLTGLRWL
SSEEIKEVEPYCVGLRGLRVPQTGIVDYKAVALKYAEKLRDGGAEIILGSKVEHVLTQDS
SVEVVTADRIWQAKFLVVCGGLQSDRLALKTEPDLPLRIIPFRGEYYELKSEVHHLVRHL
IYPVPNPAFPFLGVHFTRMIGGGVECGPNAVLAWGRECYKKSDFNVRDVWETVTWGGFHK
VAIRYWRSGLDEMYRSWSKAAFVRALQKLIPSIQGEDLVSGGVGIRAQACHINGQLLDDF
ELRSHGRVIHVCNAPSPAATASLAIGQTIAQEVLSRLS
>OHY34297.1
MNHAKFESDLAQLQDEVSNCERDVLNLIKGKKTMEYSQNHNGGNQSNQIAIIGMASLFPQ
SRNLQEYWQVIMDKIDCITDVPASRWSVEDYYDPNPKTPDKTYCKRGGFIPDIDFNPMEF
GLPPNILEVTDVSQLLGLVVAKAAMEDAGYGESQQFDRDRTGVILGVAIGRQLAVPLGSR
LQYPIWKKVFKNCGLSDDETEKVIEKLKSAYIQWEENAFPGMLANVISGRIANRLDLGGT
NCVVDAACASSLGALNMAISELLAHRADMMITGGVDTDNSIFAYMCFSKTPAVSPSENVR
PFDVNSDGMMLGEGVGMLVLKRLEDAVKDGDRIYAVIKGIGSSSDGKYKSIYAPHSQGQV
KAIRRAYENAGFAPQTVGLIEAHGTGTMVGDPTEFISINQVFGDNNSLKQHIALGTVKSQ
IGHTKAAAGAASLIKTALALHHKVLPPTINVTQPHPKLNIENSPFYLNTETRPWISNQPR
RAGVSAFGFGGTNYHVVLEEYESEHHQSYRLHNCAKSIFLSAPTTPELLSQCQHLYQQLE
STHKEQHYQRIIAESEQLIIPVDHARVGFTTLSLSQAIAHLAIIIDLLKNQPSVDFWEHP
KGIYYRQQGMETTGKVVALFSGQGSQYLEMGRELVINFPWLRQTYSHLDDLFSREGLESL
SQVVFPAPVFSPQERQEQLEKLQKTEYAQPAIGAFSAGLYKILQQAGLKVDFVAGHSFGE
LTALWSAGVLTEEDYFFLVKARGKAMSTSPEVDAGGMLAVKGNISQVTEFIKDFPQVAIA
NYNSPQQIVLAGNKSEITQLQNVLQAQGFSCFLLGVSAAFHTPLVSHAQKPFAHAIAQVN
FQPPRIPVYSNVTGKLYPNEPGSMQKILQEHLLNQVLFQQQIENIYQAGGNCFIEFGPKN
ILTNLVKEILVDKPHVAVALNANYRQDSDLLVREAVTKLRVFGVPLKNLDPYQIPAKISS
ASQKNQQKTLNIRLNATNINDRSQKAFAQALATGSVIKMPAVSENNYQTQPEKIRETLVE
TSTKINPEISSSSMLTSQIIPAVKNHSSVEPKMEISLDNYDRVLNSLEQSLVELTRQQSE
VNQVHEQSLQNQIEYNKTFYELMRQQSLFLAQEEINEYQSQTQQLAISSTERSMMRLHDH
QAETIRIHEKYLNYQQEYSNNYFQLLEQHYSLLEVGSPNGYSHLPSSHVAKSDPPAQKLI
YPLEPENNSQNNLPDITVDFPIATYIDKEKLRETLINIVSDKTGYPVEMLDLSMDIEADL
GIDSIKRVEILGGLLELYPDLPRPNPEELAQLATLEQIAEYINNLITQLGQNQPLEETVS
EHHNHPQFLVLPHEELHEDLSDRSTTITIPDDLSQILLTIVSDKTGYPVEMLDLSMDMEA
DLGIDSIKRVEILGGLLELYPDLPRPNPEELAQLRTLGEIADYMRNQAETVGRSNLSTSE
KPDTSTTEVADKILRLPVQLKTLPQPDSLDLTIPENHFVLITNDGSEVTHRLVAKLTDKG
CKTVVLTFPCLESNLSEEIAQIRLNDWHEETLQEHLTELTTKFGCVGGFIHLHPYSNNNL
GIDQAIVQHVFLIAKHLKEDLNHLAKKERACFFAVVRLDGELGTAKTHNFSPISGGLFGL
TKSLNQEWPEVFCRTLDLSPDLDGDTTVKHILAELQDPNLLVTEVGYNKTDRFTLVAEPT
KSSIIPDSLNITKDQVFLVSGGAKGITAKCVIKLAEEYQCKFILLGRSSAEIEPVWSEGY

EDENELKRRIMEDFLSKGEKPTPIMVQKKYQTIASQREIHNTLKAINEAGGKAEYICVDI
TDGMMLKEKLRPIIDQFGTITGIIHGAGNLADKRIEKKTVQDFETVYAAKVHGLENLLNI
VETNQLEYLILFSSVVGFYGNVGQTDYAIANEILNKSAHVIKHKHPNCHVVSINWGPWDS
GMVSPELQTAFAQRGIETIPQELGSSILVDQLRNSDSTMTQVVIGSPLVYIPSTLSSELK
THQITRQLKLNYNPFLQDHVIAGNPVLPATCGLSWISSSCEQLYPGFQTFHCPNFKVLKG
IVFDQNSPHEYILEIQEVAKIDNQEIHLVGKISSVTNHGKIRYHFSSNLILKRQIPLADN
YELFNLTQDGQFLASNSLLYQTGVGSLFHGNTFQGVKSVLNISPGKLTMKCELPEPTLNQ
QGQFRVQTLNPYIADVQVHSLWIWTQHFHQVGCLPSEIENFEQFAPVPFGETFYVTCEIK
SKTESYVVADVITHNQKGQVYNRMKGAKATILPNS
>OHY34306.1
MNPFNREKIQFYLTDLTTPVGQIINFVIALLVLLSSAIFVVETYNISPDARTELSILNTC
ILAIFSGEYLLRLWSAKQKSKYFFSVYSIIDLMAIIPYFIGFVDIRFIRLLRWLRILRLL
RFIDKKFLFFSISEDTVIFARILFTLFAIIFVYSGLIYQVEHAINPEGFNTFLDAFYFSV
VTMTTVGFGDVTPVSEWGRLLTVFMILTGVALIPWQVGDLIKRFLKTSNQVENTCSRCGL
AFHDSDALFCKRCGATLPQVSPSSQYQ
>OHY34376.1
MDIQLINIGFGNIVSANRVVAIVSPESAPIKRIIGDAKDRGQLVDATYGRRTRAVIITDS
SHVILSAIQPETVANRFVLSRDHHTADN
>OHY34413.1
MLLHLSTWPEVEKYLGSSSGIIIPIGSTEQHGPTGLIGTDAVCAEFISHGVGEATQAMVG
PTINVGMALHHTAFPGTISLRPSTLILVVKDYVSSLAKAGFTKFYFINGHGGNIATLKTA
FSETYAYLEDIQAKNFQKVQCQLGNWFTCSSVYKLAQELYGDKEGSHATPSEVALTQYIY
PQAIKQSFLAPEVATGHRIYGPSDFRLRYPDGRMGSHPDLATPEHGKQFYDLAVEELSAA
YSNFLNQD
>OHY34422.1
MSLTARSHFSAAHRLAPNLSAEKYGKFIRTHGHNYHLEVTVEGEMDGRTGMIVDLGCLHE
TVEREILELFDHSCINEDIPYFSTSHIVPTTENIARYMSDLLQFPISELGVKLSRVKLFE
SDHLWVEYEGKDSEIFFSVATGFSAAHRLADPTLSLEKNQTIYGKCSRINGHGHNYHLEV
TVRGEIDSVTGMSVDLVGLNQIIQHYVIEPMDHSFLNQDLPYFTEVVPTAENIAVYISDV
VRSPIEELGAKLHKVKLIESPNNSCEIYAKDIEESRVDRVDRELAVV
>OHY34424.1
MNNIEVSPPQRTELNRESIDENSLPSDSPNYSINHNINHNVQVQIKTEDGKILLILPTES
QAPASMFTWSDIWQQIQQRLKGGDRLRQPNSVLHLVAKDRLLDSRQLQQMKDTLNEMQLE
LKLVVTSRRQTAIAACTIGCSVEQTKIQTSFGNDSQENPKALADALYLETTIRSGGEIRH
PGTVVILGDINPGGIVIAEGDIMVWGRLRGVAHAGAGGNRESLIMALQMEPTQIRIADAL
ARSPEKSLTSFFPEVAYITNNGIRIARATSFSRNQLSKI
>OHY34426.1
MSEMAIVSARKARLQQLANQGSPNAQAALELAEAPNHFLSIVQVGITLINILNGVFGGAT
IAQRLEKYVELVPFLSPYSQTIAFGVVVLVITYLSLIVGELVPKRLALNNPEKIAAFIAI
PMRALASLASPIVYMLSISTETVLQILGIRPSEEPQVTEEEIKILIEQGTEAGTFEAAEQ
DMVERVFRLGDRPVTFFMTPRPDIVWLDLDDSPEENRQKMSASNYSRYPVCQEGMDNVLG
VIPVTDLLSRSLRNEPFDLTIGLRQPVFVPESTRGLKVLELFKQTVTHIALVVDEYGVIQ
GLVTLNDIMSEIVGDVPAQPGQEEPQAVQREDGSWLVDGMLPVEEFLELFDVEELETEAR
GNYQTLGGLVITNLGRIPTAADHFEWQGMRIEVMDMDGNRVDKVLVVPHTN
>OHY34427.1
MNIETIKSEKTKQLPGANLEDQDLSEFDLTAANLAGANLMGAHLVSVNLEGSHLEGANLM
GASLQGADLRANLLGANLMQADLTGADLRGSNLRGANLMGATVAGASLTAAFLSGANLMS
GNFQGVDLRGADLRGANLTGANLKGADLSRADLQGALLNQANLEESDLRGANLAGANLAG
ANLLCAELEAASLNGANLYQACLLGTILETYHD
>OHY34430.1
MSGKEELQVLDASQVKELLENQRVHLIDVREQEEFMGEHIPGSQLLPLSKLDPEKISLLT
GKKIVLYCHSGNRSKQAAHRLIEFGFRDFSELQGGISAWKKSGYVTNKNAPISIMRQVQI

VAGTLVVTGTVLGVLVSPWFLILSGFVGTGLVFAGLTTTCTMAMLLKKLPYNQRG
>OHY34432.1
MYSPIYSLVIPIYNEEENIQEMYRRLTHLMTQLDGDAELILVDDGSQDCSLTMIRELHDH
DGRIRYLSLARNFGHQVAVTAGLNFVQGQAIIVMDADLQDPPELILSMIDKWHEGYQVVY
AQRISRQKESLFKRLTAYLFYRLLQRLSKVKMPVDTGDFCLMDKHVVDILNAMPERNRYI
RGLRAWVGFKQTSVLFERGPRYAGKVKYTFSKSFSLAIDGIISFSTVPLRLATYLGIISA
TIALIMILLVLYWRIFAPVSQLIGYSLITIAIFFLGSVQLICIGILGEYIGRIYEEVKAR
PLYTLKETGGFDPLNI
>OHY34454.1
MRNALTPARLARAAKVMLNTLLIAIASVTFFLASDFVQPQSAAAYPFWAQETAPETPREA
TGRIVCANCHLAAKNTEVEVPQSVLPDTVFKAVVKIPYDTNVQQVGADGSKVGLNVGAVL
MLPEGFKIAPEDRIPEELKEEVGDLPFQTYKEDQENVIIVGPLPGEEYQEIVFPVLSPNP
ATDKNIHFGKYSIHVGGNRGRGQVYPTGEKSNNNIYNASAAGTISKIAQTEDEDGNVKYI
VSITTASGNVVEDAVPAGPELIVSEGQTVASGDALTNNPNVGGFGQKDAEIVLQDSSRVV
WLIAFICLVMMAQVMLVLKKKQVEKVQAAEMNF
>OHY34486.1
MANLFNKFIGRTRYVVSRIFLHLNGQEVAPILGVLNSAAREAIDAEGDLESLGEQLVTVC
ETLLRYDQCWVAAGNEGDVFWNEGEAGDYVNELFTDSAGRYGADLDTGSSPGQALSIPVT
RNVLVMITVAFEGELPEIETNLAQISSLKAGLKSLVNLHYQNKLRAIQVHFSPARLGDEL
TDDHLLEYYPELIPL
>OHY34504.1
MSKLSIQERLNQLLPKLQDSRLLENRGIGNEIGFYVFDYAPEDELYVQEYTKVLVSQLTK
DPINLIVKEFNLYKIILELLQEKGILNKAFMVEAKEGCKSLGNKIQSIVRPEKVIAQIQN
HLQGNEQLVFLTGVGASWPLIRSHSILNGLQPYLDHIPLVLFFPGSYDGHELCLFNTFKS
DNYYRAFALIPHHGAVYEHC
>OHY34522.1
MQSLRLVVACVLAFLLTNQGTAIADNCPPKGCAQKYTLALSWQPGFCETHGYKAECQEQT
ATSYDANNFTLHGLWPDKLEYCNVSASYIENDKTGNWKDLPIVEVDTETTDELDEVMPGF
GESSLERHEWIKHGTCDGRNSDNYYDLSVDLLKEFNDSQVRNLFIEHIGETVSLDQVKTA
FEGTFGSGSSSSLNLKCDSTNNLATEIRLKIKRPQSGQKLADLLLPSGGQSCNQVVVDGF
GVGNLTQ
>OHY34537.1
MANLQIAARNSIQAILISLIRSLKNILNTKIVLFSRFMANADGNSYLVKKAKISGSTYIL
HSNDYGVSRRIYIGLDDEHLKAIKAINLINDSSRFAKRGGRITHLLDIGANIGHISIPLL
EQGILGVAG
>OHY34538.1
MTSFAQLMSLRGRRALITGAAGGLGRIMSETLAELGADLVLVDRPGAVFEPLLQRLQALT
ETTPVGFQTIACDLERQDHRHDLIQTLGADGQGISVLINNAAFVGSSDLQGWAVPFEAQT
LETWRRALEVNLTAAFHLCQGLAPLLRSAPGGNIINVISIYGHHGPDWSLYEGTTMGNPA
AYGASKGGLIQLTRWLATTLAPRVRVNGISPGGIYRNQPEVFVQRYNQRTPLGRMATEDD
FRGVVGFLASDLSGYMTGQILQVDGGWSAW
>OHY34539.1
MIDRVLIVGHGSIGQRHLRIVRHNLPQADIRVLRHRPGSGVPELANGVYHHLADACAFAP
QVAIIANPAPFHLHTAAALVAVGSHLLIEKPLSHDLEGVGALLAQAHSQGVVLQVGYNLR
FLPTLSRFRDLVHQGEIGRVLSVRCEIGQYLPTWRPDRDYRQGVSAQASLGGGVLLELSH
ELDYLRWIFGEVDWVRAYLGRHSQLEIDVEDLAHLTLGFSPGSAFESASGPVAALSLDFL
RHDPTRLCTALGEKGSLRWNGLTGTVDLYPAGGDHWQLVYHHPHQRDDSYQAQWAHFVAC
VQSNRPPLVSGEDGYAVLKIVAAARSALPPSALAS
>OHY34540.1
MLKKRIIFTLLYDSGSFMLSRNFRLQRVGNLDWLQRHYNFAHVSFFIDELIVLDVTRKER
NLEDFCHTLQALTTGCFVPIAAGGGVRSLDQARSLLRSGADKIVVNSPLFDQPDLVQALA
SSFGQQCVVGSVDMKRKANGTYQIYTQSGSHPLEQSPQTALQWLSQDWVGELYLNSIDRD

GTGQGYDFDLLDQLPVDCSVPVILAGGVGNGQQLAVGFADSRVDAVATAHLFNFVGDGLQ
RARQTLMDQGANLPVWPTLEQLGLGNAPRQTGA
>OHY34543.1
MKPNIHTQNWDRSIISCTATIRDVIQNLNDVAIQIALITDLEGALIGTISDGDIRRGLLR
GLGLDSPIESIIHRNPLVVPESMECNHVFQLMKANKIRQIPVVSEDNRVVGIHLWDNLVD
GDVLSDSMGATNLMVIMAGGKGTRLRPHTENCPKPMLKIGGRPMLEHIIERARSQGFRDF
VISIHYLGHMVEEYFGNGEKHGVKINYLREEYPMGTAGALSLLTPLPKQPFIVTNGDIIT
EINYNDLLSFHQQHRASATMAVRLYEWQHPFGVVQMEGLKIIGFNEKPVVRTHVNTGVYA
LNSETLSHLKSNEPCDMPSLFDRLQDKGDLTIAYPIHEIWLDVGRPDDLTKANGIIG
>OHY34679.1
MSVYNLGTNIDKPSLPQVFKGIALFTPGGDLIYCIDPSKQKRWHLHLCGVLQEILNLSEP
PHFLVPCYTATIDHWLNPRTQKIQTFAEAYPAVMSYQPVLGAIFGTGNEVWQRSPWREDV
CDLMVIESYRSIFPELWEDHDLIVRLDSMSMPYSQKHVNQKQIIQNHEVYVLRLFVAGHN
MSTERILEKLHELLEDYLGVPYTLKVIDVLTHPEQAEIDQVSATPTLVKIWPHPVRRIVG
DLDNVEKVLHTLGKKDNF
>OHY34682.1
GGPYQLVIFHFLIGVACYLGREWELSFRLGMRPWICVAFSAPLAAATAVFLIYPIGQGSF
SDGMPLGISGTFNFMIVFQAEHNILMHPFHMLGVAGVFGGSLFSAMHGSLVTSSLVRETT
ETESQNYGYKFGQEEETYNIVAAHGYFGRLIFQYASFNNSRSLHFFLAAWPVVGIWFTAL
GVSTMAFNLNGFNFNQSIIDSQGRVIGTWADVINRANLGMEVMHERNAHNFPLDLAAGEV
APVALTAPAING
>OHY34728.1
MGTRKTVSFTANSSKATVTVNPTPDTRQEGNETVSLTLASGNGYTVGTTSAVTGTITNDD
FIGNASNNTLTGTAGNDHVNGGIGQDTLTGGAGNDVFVFQFGQSGVSGADRISDFAIGSD
KIDLLSLSGAGVSDPTAFTRASNSTASSLANMVNNVFTDADGSLVGNQGLGINSAALVGV
TTLGIAGTYLVINDGVADFQSGNDLLVNITGYSGALPSFGTIPVTSFFV
>OHY34761.1
MPSIQFDPKPEISVVLTTYNRSRYLKNCINSVTRQTFEDWELVIVDDGSTDDTFEIVSLY
LAEHRNIRYLKHQNRQVGYARNAGIQASFGKYITFIDSDDTYKATHLESRLEFMRTHPEI
DIIQGGFEMEGEFFVRDYFRPDKTISIRECVSCATFFGKRNVYFQLSGFKNLACSEDTEF
WQRAKKMFKTQDIKQPETYIYTRAKESLTRSLLEEMSPQKITKYQASEI
>OHY34762.1
MLGSFFSASYVKAEEILKTDVNISPLTSSNYQRPIIIAEKINLDVCQQAPKSILCEIRRS
QENRRIQLEKEEREREELRQRKLEEKAMEIPGVPEIPTDQDWKSPNSKIPWSRIVKLKSD
FDGNVDYAIFDRDWTKRDGEEVGFVTKWTSDSIQGLIYKTDCGASFCIGTRIKSIVDMPS
PIVLRLGEQEYKIYGDEGTFNIPSSFLKKIVESSGKPSLKIKIGESGMILPIGQNTVESL
KTLYSRISLRQKAPDFNLQAVPIVDGNSFQKLVAASLNKVVMIRAGGSLGTGFIAENSGL
IMTNRHVVRSYRTVDISYADGTKTIAHVIFRDREKDLAIIRPSKPSVTGSLPLCYAIYPK
PGEEVFALGSPLGLANTVSKGIASSVRISEADSKSIIPEGSTLIQTDAAVSPGNSGGPLL
NNRGEVIGMITFKRTAGEGLNFAVSIIDLLNAIEAQRPTIPDGQAATECGNLIPIANKDT
R
>OHY34783.1
MSTTAEEIWELLGELIKVQKETDRLLREQSQETNRKFQETDRKFQETDRKFQETDKKFQE
TDRLLREQSQETDRKFQETDKKFQETDRFLREQSQETDRKFQETEYLLREQSERANLRFQ
ETERLIKEESIRLDKQLGQIGNSLGQFVEFQVRPAAVRLFQEMGIAVKEIATNVSVQGSE
GTEIDILVVNSHEAIAIEVKSKLSDDDVKEHIARLSEFKKLLPRYENLNIMGAVAGMVVP
ENV
>OHY34784.1
MEHLVLYLSIPPNPFKKQGFFILQNCDLHNLIEEMFRDFKKGGYNLEETNVTGERFISLV
LLIAIAYSSATIQGQQIKRKGIQKYVSRIKEYGRTERRHSSFYIGLYGQTWVNFKEICMD
MVMELMRLNRNKCKYYQQGLRAMRLIESVL
>OHY34802.1

MFNLKAPLFKQGVFILQILPYKKATIPPNVLSYIVFNLKAPLFKQGVFILQILAEKRKNL
SLLDIQ
>OHY34814.1
MNLEAEYDLDFYVWINKNVELLRCGSLSEIDAEHIAQELESMGKRDRRQLRSRLQVLIMH
LLKWQYQPDKQSKSCLATIDHQRDEIQSLLLDSPSLRRDLQTALVTVYAKAVSDAQEETS
LPATTFPLSCPFALEEILSSRFLPKVS
>OHY34815.1
MAQTQNLINKEHRVKDISNDTIDNLISLQSLDDEISLREILAILRRRALTIISVASLAMV
GCVGYLLTAEKTYQGNFQILVEPLTNDRPNLLSLTDNLPSSSGVNGLDYGSQIKVLKSSE
MINQVLPKLKVAYPNLTYKILSKNLDIERLQQEKILKISYKSPDATQINTILSKLSDFYL
KYSLERRKTTLNQGLQFIDNQLPVVERRVNNLQIQLQRFRQKYQFVNPDEQSTSVVAQIQ
DLEKQRVAVEQRLGTTRLSYSTLLTAEGQEAILNQAPIYSGLITQLRQLEAQLSAELARF
QSDNPLIETLQEKIQNLLPLIENEKNRFVGLRLAEVANEIKLLEEQNSQLLKAEQQYRAR
FQLLPILAKQHTDLQRKLQLANESLNRFLEAREKLAIEVAQTEIPWELIQSPTTSKDPIS
PKLYETLLIGLLASISTGIVFGLILEKLDNSYHDPTLLQEKTKLPLLGTVPMIKNIGASY
QSRHDADNNKTETLAKVPLKVPLFDISNLLSPKKKRRKGYGYGYGYYGEGHFWQSLQVLY
ANIELLNSDQIVKSIAISSSVKGEGKSTMSIHLAQIAASVGKKVLLVDSDLRLPQIHKRL
DLPNIMGLTNAITSNIPVEEVIQRVPDLDMLSVITSGILPPDPMRLLSSEKMKQMMRHFR
EEYDLVIYDCPPISGLVDTRLLASQVDGLLLVVKMHNTDIDIIKQVQESLRQSSISILGV
VANQYKRSSHQNYDYYYSYYSYRASQSAN
>OHY34857.1
MLATFKLSIGLMDYKVIECTNAYDDEKFYTGYEHVVSLEIEGEKSPLLINHTPYDDQYIE
ECSLAEIIQECEEWDFESILESITEKEEEIQQLGREIAILVVYSISLFTLKPTVSVFEYN
SLIPEKYLSWFAPPKISDDC
>OHY34904.1
MVAVAILAAGKGTRMKSRLPKVLHPLGGKSLVERVIDSVQPLVPTRKFVIVGYESQMVKG
ALSKNSDKHSDLEFVEQSVQLGTGHAIQQLLPYLSDYDGNLLILNGDVPLLKTETLQQLL
QTHQEHDNACTILTANLAQPDGYGRVFCDDNQIVHQMVEHKDCTHIQRKNNRVNAGIYCF
NWQKLATILPHLDNNNVQKEYYLTDAVVQVGKVMAVDVSDEREIWGINDRRQLADAYQLL
QQRIKEKWLLAGVTITDPSSVTIDETVEMEPDVIIEPQTHLRGKTLIGPGSRIGPGSLIE
NSQIGENVTALYSVITDSFVEQGTKIGPFAHLRGHVEAGENCRIGNFVELKNTQLGDRSN
VAHLSYLGDTSAGTQVNIGAGTITANYDGVKKHRTRIGDRTKTGSNSVLVAPITIGSDVY
IAAGSTVTEDVENDALVIARSRQVVKPGWKIKRELAE
>OHY34905.1
MRDAVTTLINSYDLAGKYLDRNALDSLRSYFDSGTSRVQAATAINANAAAIVKQAGSKLF
EELPELIRPGGNAYTTRRYAACLRDMDYYLRYATYALIAANMKVLDERVLQGLRETYNSL
DVPIGSTVRGIQIMKNLAKEQAIAAGVANAAFVDEPFDYITRELSEQNI
>OHY34938.1
MKFSIVTPGFNSAASIAACVESVLHQSFNDYEHIIIDNLSTDDTVKIIRSLYQQHGKEAK
LRVISEADRGISDAFNKGVAKASGEYILILNSDDALYNENILELVNHFAAGESYLVIHGD
MYFTDHKFGSNIRAPLLCRVEAAMPYNHPGMFIRRDVYQLFGCYDTRYKVAMDYEFILRL
DTRMPDFTKRIGYLNWVPLTRSSAGGMSWRNEQRAIDEVVMALKEFKRYNLYARYHYCAR
TVRTTIKGVLRSVGLTSLVKLWRRWKWGN
>OHY34970.1
MRADSLLDKIHPDKDYCIGQDSGIYWRFTEPVEKGVEAPDWFYVPGVPSRLKGKLRRSYV
MWKEKVPPLIVIEFVSGDGEEEKDNSPPPEGDEVDPKTKKVKKAGKFWVYEQAVKIPYYA
IFDGFEGTLEMYHLEKGRYKQVKANRRNHYAIPELGVELGILLDQERPPIPWLRWWDNRG
NLLLTGNERAEEECQRRELAEAIAIQERQQKELAEALAIQERTEKERERQQKELAEALAI
QERSEKEQEREKKEKLAAYLRSIGINPDEI
>OHY34972.1
MTKITNQALNSALNQLWVFSLSSDFWAVFDAAFGTEYNRKNAEILRSQWQIGDFSQLPEI
EILDSSILGSANGAYSSSSENRIYLSSNLMENGTSSKIREVLIEEIGHFVDSRINQIDTPG

DEG
>OHY35012.1
MGKLKWKIVSQGVYDIECIGINVRLIVLSKIPKSANNELWRLFSARAEVVQEAISHYQES
YRNSEYSVLMQQLYEFYLKEKLPMTYTLEQFKKDFVINHLREIPTEEVLKQYSPEEVLKQ
YSPKEVLKQYSPQEFLEGLSPETLEHLAIFKNSLLKNHCCS
>OHY35051.1
MSPKNLPLGINTLSVLRENNCVYVDKTEIAHRLIRIPGRFFLSRPRRFGKSLFVDTLKEI
FEGNQKLFEGLYIHDQWDWSRKFPVIKIDFAGGVLKNRQELDLRILDILQENAEHLGVSY
ESTDIPGKLGTLIRKAMAKYGERAVVLVDEYDKPILDNIDNPSIAGEMREGLKNLYSVLK
QQDANIQFIFMTGVTKFSKVSLFSGLNQLTDITISRDFSTICGYTQEDLEQTFAQHLQGV
DWDELRLWYNGYSWRGDSVYNPYDILLFIREGMEYGNYWFETGNPTFLIKLFQTNRYFLP
NLEHLEVTEEILKSFEIEQINPVTLLFQSGYLTIERNFIRHRRSMFALKIPNMEVRLTLN
DQFINVYTGMVNEKSAIQDILYECMWSGDLESIVKAVKRLFAGIPWRNFTNNHLADFEGY
YASVLYAFLSSLNARIIPEDISNYGQADITAILGDHIYVMEIKVVDGENVKENL
>OHY35101.1
MVREIAPKTQLGVIKSKVNQGGETIAPDIVYPESDGKPMADNTKQFAWIVKIKENLEVLF
KSNPDVFVAGDLFWYPVKGSNRIKLAPDTMVVFGRPKGQRGSYRQWEEDNIPPQVVFEIL
SPSNSKGEMTRKKLFYLKHGVEEYYVYDPDEISLEVSIRENNSFREVEDFATWTSPRLNI
RFDMTGDELVIYYPDGSRFLSPVELSN
>OHY35102.1
MFVAGDLFWYPVKGSNRIKLAPDTMVVFGRPKGQRGSYRQWEEDNIPPQVVFEILSPCNS
KGEMTRKKLFYLKHGVEEYYVYDPDKISLEVSIRENNSFREVEDFATWTSPRLNIRFDMT
GDELVIYYPDGSRFLSPVELSNYAEQEKLLKEQANERAEQERFLKEQANQRAKQERLLKE
QEQLKYQTLLSQLKAKGIDITTLE
>OHY35200.1
MNSEIPPQYEQDFYGWIQWQLRAISQRQVSQLDWENLQTELEGLGRQEYRELVSRLTVLL
AHLLKWEYQPENRCRSWFLTIREQRRAIDRHFQRNPTLKSRIPHALEDAFEGGIDLALRE
TNLPLRTFPQVCPYQFEQAVSHGFMCDTSQDWQ
>OHY35218.1
MNTEITSQPLRIGVLGFGGLGQAAAKVLSGKREMLLVAVADQKGYAYSPVGLKTNVAITT
YQDRGSVGYLQSHGNLSNHSIQDLIANAPGVDAYFLALPNLPNDFIPKVTQQFITAGWQG
VLVDAIKRTSAVEELLAMKKELEMAQITYMTGCGATPGLLTAAAALAAQSYAEIHKVEIT
FGVGIANWEAYRATIREDIAHIPGYNVEIARAMTDEEVEALLDKTNGVLTLENMEHADDV
MLEIAGVCPRDRVTVGGVVDTRNQKKPLSTNVKITGRTFEGRISTHTFTLGDETSMAANV
CGPAFGYLKAGYQFHKKGIYGLFTAAEVMPQFVR
>OHY35242.1
MRDIPPQVVFEILSPCNSKGEMTRKKLFYLKHGVEEYYVYDPDEMSLEVSIRENNSFREV
EDFATWTSPRLNIRFDMTGDELVIYYPDGSRFLSPVELSNYAEQERFLKEQERFLKEQEI
LVCLDLYYYNNLYSYDLAYSTTTTLYSTSFGNS
>OHY35247.1
MVREIAPKTQLGVIKSKVNQGGETIAPDIVYPESDGKPMADNTKQFAWIVKIKENLEILF
KSNPDVFVAGDLFWYPVKGSNRIKLAPDTMVVFGRPKGQRGSYRQWEEDNIPPQVVFEIL
SPCNSKGEMTRKKLFYLKHGVEEYYVYDPDEISLEVSIRENNSFKEVEDFATWTSPRLNI
RFDMTGDELVIYYPDGSR
>OHY35248.1
MADNTKQFAWIVKIKENLEVLFKSNPDVFVAGDLFWYPVKGSNRIKLAPDTMVVFGRPKG
QRGSYRQWEEDNIPPQVVFEILSPCNSKGEMTRKKLFYLKHGVEEYYVYDPDEISLEVSI
RENNSFREVEDFATWTSPRLNIRFDMTGDELVIYYPDGSRFLSPVELSNYAEQEKLLKEQ
ANERAEQERLLKEQANQRAEQERLLKEQANEGAEQERLLRTGKISQRAGK
>OHY35252.1
MEEWITQELERTELGDKRRTKRLIKIVSNLSASPEASVPQASGTWSQTKATYDFWDSPYI
KPSMIRQGHLDATVERIAIR

>OHY35336.1
MGARLRVFLTSEEDKTLFNLRSADVPQKVKDRAEVIRLNAHGWYVEKIAAHFNWTSQTVR
EVLHKWEKFGLEGLWEKSGRGGKPKYKDSDIEFLEECLKKGRCIFAG
>OHY35377.1
MTVLAENDLEFDFSSAMEAIIFDDDTLHNPSTIKRVDFIAEFNDRFIFLEVKDPDMPGVA
NPEAFKKKLLTGNLIPDLAGKYRDSSWFRTLSGKATKPIHYVVLISMATLEPALLLAKQD
ELKRSMPITHKDWSAPCVASCAILNIEQYKKQFGANSVRRLSAGG
>OHY35393.1
MFRLTKDTHDLLNYLIARYKNQVDYLMIRLEEAEGTDILLRGDKVETLSEGVSMGAHVRA
CHKGGWGLTSFNHIADIEERIQEAISAARTVGDEQTILAPIPVIQSTCKLGITGIDPSTV
PLKKKKQLCDRYREILKGVDSRITTTSVRYNDSSQKVIIATSEGTLIEQSWVDMEMRFAA
TARNGDTVQTGRETVGSRKDYDDLVNLDTVVQGAAQRAVTALSLPPVKGNTYTVVIDPIL
TGLFVHEAFGHLSEADMAYENPDLLEVMTLGRRFGPEELQIFDGAGSPGHRGSYLYDDEG
TPATTTQLIEDGVLVGRLHSRETAGKLGEKPTGNARCLDYHFSPIVRMTNTWIERGKTPV
VDLFTDIKEGVYARNWLGGMTNGEMFTFSAGEAWMIRNGAIAEPVKDVTLSGNVFQTLAD
IEGIGDDFHWDESGGCGKGGQNGLPVGCGGPSLRIRDVVVGGES
>OHY35399.1
MTKFILKILWLDDNVALAVDQVVGKGTSPLTKYFFWPRNDAWEELKKELESKHWISDPDR
MELLNKATEVINYWQEEGKNRPMSEAQLKFPEVAFTGST
>OHY35403.1
MDIKQLFIAGGVVMWPLLLSSIIAVGLIIERIWFWSKITKRQEKVVKYVLNLYRQNNLVN
AIDTLQKNADLPIARIFLTALELEEPNPEEFRLALETEAQAEIPLLKRFTTVFDTIIALA
PLLGLLGTVLGLINSFASLNIGNVGGTQTAGVTGGISEALVSTATGLIVAIFTLLFANSF
RGLYQRQIALIQEYGGQLELLYRRRYERGDKSYASTR
>OHY35434.1
MQRSFCQKILVSIAVFVFGASIWVMNSPLALAYENPDLLPDTFTPVVDLAKTLPDPQEEK
LVKELEQFEVDTGWKLRVLTQYDRTPGRAVIKYWGLDDKSILLVADARGGNILSFSVGDA
VYELLPRTFWIELQTRFGNLYFVRENGEDQAILQALNSVKGCLAQGGCNVVPGLPKEQWI
LTLITSAIGGIICGFAAQPRNDKQVFAWQWALIFSPLWGILFIAFGIAPVITRTSDWVPL
VRNISAFLIGLLVAYLSPIFSRPVSRNES
>OHY35438.1
MNQAEISTLIRQELEQLIVEDPVIKNLILGSMSQYYIGRQDADSKFDQTLAQLQSYQEEQ
NRKWEEQNRHNREIMAQLQSYQEEQNRKWEEQNRHNREIMAQLKSYQEEQNRKWEEQNRH
NLQVLEEIKQMNRKHESTVGSLGSRWGLSSEASFRNGLKGILKDSFGVEVLNFLDFDNEG
EVFGRPDQVEIDVIIKNGLIILCEIKSSIDKAGMYIFDRKVAFYEKHHQRRVDRKLVISP
MVDPRALPVAQNLGIEIYSYAEDVNSI
>OHY35461.1
MYLTPKSGLFLGGSCITAIAAVGSVFELSYGQPNFGFQTTAIILAISVPVTILLFVAAVK
DARANIK
>OHY35463.1
MNDSYLYIIIALLPLTAGLLVTQVNPYHGLILRGVLGAIAALVDAVLGAADVALTEALMG
TMLSVTLYAIAVRSSLVLRVGVIENQDGEILKLIEDFRRVFSNHYMRLEVVTYNNQQSLK
NGLISKEVHATCFPTTERQNYQTEVRVHRVYNIIEREILPNNTGLKNLEYVDILVSEEKH
P
>OHY35482.1
MITPKPQQNTNPPHKIIGVGVIWNQEKQILIDRRLPTGSMANLWEFPGGKLEEGETIQDC
IVREIREELGIKIAVREHLMTIDHTYSHLRVTLSVYHCDYLDGTPQSIECAEFRWVNLDD
LEHFEFPAANGQIIAALNR
>OHY35484.1
MPRRQDIHKILLLGSGPIVIGQACEFDYSGTQACKALREEGFEVVLVNSNPATIMTDPET
ADRTYIEPLTPEMVAKVIAKERPDALLPTMGGQTALNIAVALAKNGVLEEYNVELIGAKL
PAIEKAEDRKLFNDAMEKIGVNVCPSGTASSLEESKAIAQRIGSYPLIIRPAFTMGGTGG

GIAYNKEEFEVMAQVGIDASPVSQILIDQSLLGWKEYELEVMRDLADNVVIICSIENLDP
MGIHTGDSITVAPAQTLTDKEYQRLRDMAIKIIREIGVETGGSNIQFAINPVNGDVVVIE
MNPRVSRSSALASKATGFPIAKMAAKLAVGYTLDEIKNDITKKTPASFEPTIDYVVTKIP
RFAFEKFPGSDPVLTTQMKSVGEAMAIGRTFNESFQKALRSLETGRAGWGADKAEKLPSG
EQVRAQLRTPNPERIFALRHAMQLGMSNEEIYELTAIDPWFLDKLHQILETEKFLKRTPL
QQLTKVQMYEVKRNGFSDRQIAFCTKTKEDEVRVYRKQLGVIPVYKTVDTCAAEFEAFTP
YYYSTYEEETEILPTDKPKVMILGGGPNRIGQGIEFDYCCCHAAYALKSANYETIMVNSN
PETVSTDYDTSDRLYFEPLTKEDVLNIIEAENPVGIIVQFGGQTPLKLAVPLQEYLQQSP
STVTRIWGTSPDSIDMAENRERFEKILEELKIAQPANGIARSYEDALIVAKRIGYPVVVR
PSYVLGGRAMEIVYSDSELERYMSFAVQVEPEHPILIDKFLENAIEVDVDAIADHQGRVV
IGGIMEHIEQAGIHSGDSACSLPSISLSPAVLNQIRTWTVELAKALSVVGLMNIQFAVVG
ASSYSPQVYILEANPRASRTVPFVSKATGVPLARLASLIMSGKTLEELNFTQEVIPQHIA
VKEAVLPFNKFPGTDTLLGPEMRSTGEVMGIDVDFGRAFAKAEMGAGEKLPLQGTVFVSM
SDRDKSLVVEVIKEFIQLGFKVIATQGTSEFLREQGLKIETILKLHEGRPHVLDAIKNRQ
IQLIINTPSGQEARTDGQLIRRTALGYKIPIITTIAGAKATVAAIRSLQNINLDVKAIQE
YSF
>OHY35488.1
MSGTTGERPFSDIVTSIRYWVIHSITIPALFIAGWLFVSTGLAYDVFGTPRPNEYFTQVR
QEVPIVSNRYEAKKQVETFIAK
>OHY35537.1
MSPIKFDLKPEISIILCTFNRAKYLVNCVESVINQTHHNWELLVVDDGSEDETFTIINEY
LLKYGNIRYLKHQNRKSPYAKNVGIQACFGKYITFIDSDDTYLPNHLTTRLKYMESNPEV
DLIAGGFVCDEDIFVADFFQPGKIINLRDCILGATFFGKRKVFFDLQGFNNLPYGEDTDF
WDRAEKLVRTHKLSQPETYIYTRAEVSITKEFTNNMLRSI
>OHY35545.1
EQNRKFDAFQAEQAHKWDEQNRKFDAFQAEQAHKWDEQNRKWEENNQRLDRIEAQNSATL
EEIQKANRRYESAIGAIGSRWGLYSEASFRNGLQAILGQSFGVEVLNLTLYDQEGEVFGR
PEQVELDIIIKNGLTIVCELKSSIDKAGMYIFGRKADFYAKNQNRVVNRKIVISPMVDER
AVPVAKSLGIEIYSYADVVVP
>OHY35554.1
MKELSLAESNHSETESDVNPVNYWQKLVSRAADKGKQSVQQWFSINDGQVAEILATVSSQ
LPTTEALLIGKPQTGKSSIVRGFTGVSPEIIGQGFRPHTQNTQRYIYPNNDLPLIIFTDT
VGLGDTDKDTEVIIQEIIKDLNTGNKRARVFILTVKINDFATDTLKNIAQKLRQQYTHIP
CLLAVTCLHEIYPPDMENHPDYPPNFPEINRAFDEIKANFSGLYDRVTLVDFTLEEDGYS
PVFYGLESFRDSLTSLLPEAEAKTVYQLLDEQMGDKLGNIYRDTARRYILPFSIMATTLA
AVPLPFTTMPVLTALQVSMVGILGKLYGQTLTPSQAGGIVSTIAGGFLAQAIGRELVKFI
PGFGTVIAASWAGAYTWSLGEAACVYFGDLMGGKKPDLQTIQNVMQQTFQSKENTQKEE
>OHY35559.1
MSQELVNSLFINRVAVLATMHHKENVIAPLLQKQLGLEVVVPANFNTDQFGTFTREIKRP
DTQIVTAKLKAKKAMEMTGETIGIASEGSFVPHPNFPYIYANREIVLLLDQQNDLQIIGE
VFSTETNFNHLVINSFEAAQKFADKVGFPDHGLVIWLESSNGQDPEIIKGITTQEQLYES
VNFALNNSPHGKLHIETDMRALYNPTRMNNIAKATQDLVEKIYSCCPQCSTPGFSITDRT
QGLPCESCGQPTSLTMGVIYQCQKCNFIEQKLYPDGKFFADPGLCPYCNP
>OHY35582.1
MATGILVDEPQETEQMLKIAVSNSERLLRLVNNILNLEMLDSGKMRLNLELCDINIMVKK
AMETVQPIAQQKNMEISYSTLDVHNSKILADEETIIQVLTNLLTNSVKFSRAGTKVEIYT
KMGDKEIIIAVKDYGKGIPPDKLETIFERFEQVDVSDSRQKGGTGLGLAICKNIVEKHQG
KIWVESILDSGSTFYFTLPQQ
>OHY35583.1
MLDVNPSFLRFSGYSELELIGYSFDNLPLIYNPEQGLLLKEQLKNQGYVRNLEFDWLIKS
GEVKTSLLSCEPIVIDNKDCVISIIKDITDRKKTEENLRQSEERFRTAFENAGSGMALVS
LNILFGRFRILVNDTKLSN

>OHY35587.1
VYPESDGKPMADNTEQFRWIVKIKENLEILFKSNPDVFVAGDLFWYPVKGSNRIKLAPDT
MVVFGRPKGQRGSYRQWEEDNIPPQVVFEILSPCNSKGEMTRKKLFYLKHGVEEYYVYDP
DEISLEVSIRENNSFREVEDFATWTSPRLNIRFDMTGDELVIYYPDGSRFLSPVELSNYA
EQERFLKEQERFLKEQANQRAEQERFLKEQANERAEQERFLREQERLLKEQANERAEQER
FLREQERFLKEQANQRAEQERLLKEQEQLKYQTLLSQLKANGIDVTGLE
>OHY35596.1
MALTNFVATPIISSLPISTSSQNNLNCPGGPEKDYCIGQDSGIYWRFTEPVEKGVEAPDW
FYVPGVPSRLEGKLRRSYVMWKEKVPPLIVIEFVSGDGEEEKDNSPPPERDEVDPKTKKV
KKAGKFWVYEQAVKIPYYAIFDGFEGKLEMYHLEKGRYKQVKANRRNHYPIPELGVELGM
LLDQEKPPVPWLRWWDNRGNLLLTGNERAEEECQRRELAEAIAIQERQQKELAEALAIQE
RQQKELAEALAIQERSEKEQEREKREKLAAYLRSLGINPDEI
>OHY35608.1
MVSKKPKPRKKNSSHNPNRDWHRLLGMFITVHFYGSPYEVEVEKDLTIRKQLLDIIIIKR
YKDSGFNLYPAGLENMSKHNLISYKSIHESFTVWSLMELIGHYVNYRKQTSKSLSNLLPE
KDYKLYGLTTHEPTQLMGKLKWKSVSQGVYDIECVGMNVRLIVLSKIPKSANNELWRLFS
ARAEVVEEAISHYQESYRNSEYSVLMQQLYEFYLKEKLPMTYTLKQFKKDFVISHLREIP
TEEVLKQYSPEEIKQYSPKDLLKQYSPEEVLKQYSPEEVLKQYSPEEVLKQYSPKDLLKQ
YSLQDLLEGLSPETLERLAIILSQLGVNQIKNQEQ
>OHY35610.1
MLSIEEIIEIADTQVFEHQGKHLNDLQRVILEETLKGKSYHDIAEKHNFTEKYIKDSASK
LWQCLTKLVGEKVNKFNVRSTLDRYSFDSTRIANTNIGIQINSGNLFEESVRSKKTKQIQ
DKKDNNIPHENLTEAPGTEDFYGRERELSLLQTKILEEKCNLIILLGIRGIGKTALALEL
VNRIKPKFDLIIYKSLHLQTTLTELITEIIEPNIDLHDDITQDNSTYQLSHLKKSLAQRR
YLIILDDVNCVFKNRSQNQQLSGEYQLYSNLFKLLSSVKTQSCVILISSERIPELSVSTN
NCHNTFSWELSGLGADAVNLLKKYQLKHPQTYQTLIDIYEGNPRYLQIIAHLIQDLFAYD
TNKFLQLQQPCIDIELTTFLKHTLEGLSSLETEILKFIANQSGSIPLTDLENHYHVHQDV
VNSIQSLKRRYLLSTQLVNERVILEISPVIKKFLSG
>OHY35611.1
MKKFISSAIFTSVITLSGLIIFSGYVNNKYAQAQPSVCTDVLNGQCTPKHYSNNIIDKAK
TFTGLTYKQIDCSHLVHKTFNLYFFGKPELDKNQDPIIPYLTATDGLYDSKNAKKYFDEI
QPENVKVGDLVLFDRPPQGKYRHVGFIESYNVDQQVGKFFGSQTSTGPATAIFTTNQKKT
GYYWGVKQKFIKFLRPKETILRRT
>OHY35612.1
MKKFISSAIFTSVIGISSIFVSSVAKADFNVSQYAQSQADGCTAFPDRIRFAYDFTGACN
SHDWCYSYSEVSRKNCDDSLLREAEASCNGKRVCLKAAQVLYKGVRRFGQPYFLRARQAS
GRPQNDSR
>OHY35614.1
MVIFRRLITSGLIGFLVSSCADAAKSSTEYTKQIVQENNVARLMAQVQDSQKVSNLLSQG
NAFLDSERYEEALQLYNRAIEIEKDSVPSWVNRGNALLSLKRYQEALESYNQAIALRPNK
NEAWYNRGNALSALGRYEEAIKSYNESIVIDPNKFEAWINKGIALTKLQRYQEGLASYNQ
AISINPNFAAAYYNQACNYALQKQVDLAVNSLAKAIKMERQKYSELAKADKDFVQVKYNR
EFQDLLR
>OHY35615.1
MEQPYSTVIITGASSGVGLYSAKSLAARGWHVIMACRDLEKTKKAAQAVGIASDSYTMIS
LDLASLDSVRKFVQDFRSTGKTLDALVCNAAIYMPLLKEPLYSPDGYELSVATNHLGHFL
LCNLMLEDLKKSSAPEPRLVILGTVTHNPNELGGKIPPRPDLGDLQGFAAGFKAPYSMID
GKKFEPVKAYKDSKVCNVLTMRELHRRYHDSTGITFSSLYPGCVATTALFRNHYPLFQKL
FPIFQKYITGGFVTEEQAGERVAQVVADPTYNQSGIYWSWGNRQKKNRQSFAQKVSNEAS
DDAKAEKMWNLSAKLVGIV
>OHY35630.1
MAKIIAFDEESRRALEKGINALADAVKITLGPRGRNVLLEKKFGTPQIVNDGITVAKEIE

LEDPLENTGARLIQEVASKTKDVAGDGTTTATVLAQALVKEGLKNVAAGTNPISLKRGID
KTVEALVKEIAKIAKPVEDSELDSPAASIAQVATVSAGNDEEVGQMLAEAMAKVTKDGVI
TVEESKSLTTELEVVEGMQIDRGYISPYFITNNDRMTVEFDNPRILIADKKISSIQDLVP
ILEKVARLGQPLLIIAEDVEGDALATLVVNKARGVLAVAAIKSPGFGERRKALLQDIAIL
TDGQMISEEIGLSLDTATLEMLGKARKVTIDKENTTIVSGTENKPEIQKRIAQIRKQLEE
TDSDYDAEKLQERIAKLAGGVAVIKVGAATETELKDRKLRIEDALNATKAAVEEGIVPGG
GTTLIHLVAKVDAIKDTLDGEEKIGAEIVQRSLEAPLRQIANNAGVEGSVIVSQVRNSDF
NIGYNAATGEFEDLIAAGIIDPAKVVRSSLQNAASIAGMVLTTEVLVVEKPEKKAPAAPD
PGMGGMGGMGGMGGMGGMGGMGMF
>OHY35659.1
MTFYALTRKFWIFDLTLNWYIPRELYSGLSIERGNRSLTPRLKSAILSQIMVNFSRLSQG
KIDSFSDNRTEFIDNGALLYHKKAKLGLNLT
>OHY35660.1
MTKRAFHLSILGIFIFAVSLRFWGLARFNTLVFDEVYFAQFGNNYLTKTPFFNAHPPLSQ
YIIGLGIWMGSHIPFWQNSVNSLTGSLLSSWSYRWANALIGSLIPVIAIFLSYQLSYRRG
FALLAGLFTALDGLFLVESRYALSNIYIVLFGLLGQCFLFLALEKRSGQKWGKLIKLLVA
GISFGASLATKWNGLWFLLGVYGIWIVGWIIRWLQPRENGSNEESRMKTEQDMVRIPLAK
ITQINIWQMGFFLGIIPLITYSLIWIPHLQLDRRYGFIEVHRQILNFHLHLGGNDSSVHP
YCAPWYKWPLMTRPMAYFYQKSQSFQDPLPVFGPPLPAASGKVIYDVHAMGNPLLWWFGV
TAIILLLQIVFIGIVLPSIQQKRLFIPRNISVDTWIGLYVVINYAANLLPWVRVSRCVFI
YHYMSALVFVFLALAWFVDCCLRSSYKSMRILGWTVTGSTIAAFIFWLPIYLGLPLSSAD
YWLRMWFRSWI
>OHY35670.1
MGGITGRIGVGVLGSTTGAIIGASLVSKLEEKKLNKTIATLKQQEQELQNRVLVINQQNP
NLSELEERKGKIEASKIERATLEARITQLNSQVESLESRRLELNSIELQIVSQQTELNRL
NTRVQELTNQSQELEQRAAELELLRFTYDAILSQKQDYEAKVSQLNPEIDRLELEKQRIL
QTIEENQNNYQKLIELREKCRSLTLTIRDQESRLRELENEGNNLQTRNHNLQTENHNLQT
ETDNLQQTRREIETTIANLRGEIREIENSGRVALEPLREKLWTNLPRQVRQLEGETQFIQ
NFITSLRSEGLSFSSRTIKAFHTSLKVQDISALVILAGISGTGKSELPQRYANYIGAQLL
TLAVQPRWDSPQDLQGFYNYLEKKFKPTQLMRGLYQYKDPEMSNRIVIVLLDEMNLARVE
YYFSEFLSKLESRRNHATYLEIDVGSLPIAESERRLEIPKAFLFVGTMNEDETTQTLSDK
VLDRANVLTFGRPQNLKLRQRSGQQITVNQHSDNLVNNRDSSYVTYSDFQNWVRTPVPES
DVVKKVESYLNEANKVMEKMGHPFAHRVYQAIAQYVVNYPEVNGIDSESFKFALADQFGQ
KLLPKLRGVMVDEAHQQLQEMKEIIDKINDQPLIKAFDKAKEGRYGQFSWQGLVYEDEEA
>OHY35671.1
MDLAVVESRRDSKSLELNSIESQIVSQQVDLNRLDARVQELTNQSLELEHRVLVINQQNP
NLPELEDLMLKIGASTIERATLEGQINGLKSQVESLDSKRLELNSIELQIVSQQVDLNRL
NTRIEELTNQSQELEQRAADLRSLRISIEESIIDRNNLEARITQLNSQVESLESRRDSKS
LELNSIESQIVSQQADLNRLGARIEELTNQSLELEHRTTELELLRFTYDAILSQKQDYEV
KVSQLKPEIDRLELDKQRILQTIEENQNNYQRLTEVREQYRSLTLAIRDQESKLRELKKE
EHNLQTRNHNLQTETDNLHQTKREIETTIANLKGEIREIENSGRVALEPLREKLWTNLPR
QVRQLEGETQFIQNFIASLTDEGLSFSSRTIKAFHTSLKVQDISALVILAGISGTGKSEL
PQRYANYIGAQLLTLAVQPRWDSPQDLQGFYNYLEKKFKPTQLMRGLYQYRQPEMRDRIV
IVLLDEMNLARVEYYFSEFLSKLESRRNHATYLEIDVGSLPIAESERVLVYVELVSLVWV
VLLEELEWEYWEVPQEQS
>OHY35684.1
MATVATHRQLLAQESVPIEISTPQPVQEKKSGFTLYIWQPNGTISPVIARISLKAKHGQK
YLQERFLGDYQYRIKQKAKFENGFKWGDRIVVRLYNTQNQLVGYTEFACLPNHTTVNLIL
PANSPPNPLVRVFYGVDGDEDGIVDPDTTMSYDYFTEIRNQQVTFLSSPPDIDLSPYQAA
GFAKVAQPSTYPGSFIQGPFALVGKPISINDSQLAKAFLYPPGEIVQVTKLSESAYQLSN
LLSKYREVGVGKGIRVRFSDLPQNYWAKDYIEELAAMEIIDGYPDGTFRPNAPITRAQLA
TLLPKIFFKDKIRGEVAFRDIPKNHWAHNAIQKTYQMGILTPSANRRFRPDQKLTRLDVL

TTIAKALNYKFTGSTRDILSIYQDASTIRSEHRGLIAAITENGVVVNYPNIRLLNTRKLV
TRAEVCALLYRAMVSKGDVPDFSSVYTVRSNGSNIK
>OHY35761.1
MREIPTEEVLKQYSLEEVLKQYSSEEVLKQYSSEEVLKQYSPEEVLKQYSPEEIKQYSPK
EVLKQYSPQEFLEGLSPETLEHLAIILSQLGVNQIKNQEQ
>OHY35770.1
MATNADQVWELLAQLVESQAQLVESQAQLTESQKETDLKFRETDLQIKELGKQIGSLGNK
FGSFTEGLALPSMQTILREQFGMEIISPSVRVKKGGENLEIDVLAYTNGDINKAMIVEVK
SHVQEKSIAQLVKILENFRTFFPEHQNKQVYGILAGVDMSEEMKNMAIEAGLYVANIQDE
VFQIDTPPSFEPKAW
>OHY35791.1
MLRKRNLLILLKGKTERGEKAIAILENENVGRIVLTGVKGDQCCECLVNPLRFYERWKFL
YVTLCMSGQEGKLVNCNLHSFFPIYLPIFRTLGSGK
>OHY35882.1
MACNKLLLFGIPPTPLKRGANSFGLACNKLLLFEIPPTPLKKGANSFMLACNKLLLFGIP
PTPLKKGANSFNSVYK
>OHY35883.1
MVTTVKLVPTLPTQDELPYDDGVPMESPRHKLQLEILTETLTPWLEQREDGFIGGDPPQP
PLKRGAFRLNMLYNITSDPPQPPLKRGAFRLNTLYNITSDPPQPPLKRGAFRLNMLYNIT
SDPPQPPLKRGAFRLNTLYNITSDPPQPPLKRGAFRLNTLYNITSDPPQPPLKRGAFRLN
MLYNITSDHPNLLKKGGF
>OHY35924.1
MLLDQEKPPVPWLRWWDNRGNLLLTGNERAEEECQRRELAEAIAIQERQQKELAEALAIQ
ERTEKEQERQQKELAEALAIQERTEKEQERQQKELAEALAIQERSEKEQEREKREKLAAY
LRSLGINPDEI
>OHY36257.1
MALITTGSGFIRDLEKFGSLGVFIPLEGGYEGRYRRRLRAAGYVNLHITARGLGDVAAYL
MGVHGIRPPHLGKKTTSNGAAVGEVQYLPPLISSHLAQQPPNSKGLLLWVIEGNILSDQE
VEYFTNLPRLEPRVKVVIERGGDRYFRWTPLEKTLLAS
>OHY36341.1
MFLPLPQFATEDRHPDHIAEVIETSSTEFLAQCLDPEDLRFPLMPAFGSWISAFDEESDN
QIYAVVYYATTAPIDSVHRARALGLSLKNLRQEQPQIFAMLKTEFRAAIVGFEAPSDIST
QRQMYHYLPPRPPQIHQAVYRCRPESMLRFTDNMDFLRTLLLVPGAPVESLAASAIREVY
KLRKLDREWLVKACRYLSLLLKDDYDQLRFILGQIHP
>OHY36416.1
MSKSYSVAILGATGAVGTELLELLESRKFPVGNLKLLASERSAGKIIQFQGENLVTESVN
ERSFDQIDIVLASAGGETSKIWAPKAVERGAVVIDNSSTFRMHPDVPLIVPEVNPQESAN
HKGIIANPNCTTILMALAVWPLHQVKPVKRIVAATYQSASGAGAKAMEEVKVQSQLILEG
KQPVAEVLPYPLAFNLFPHNSPMTSMGYCEEELKMVNETRKIFGNQDIRITATCVRVPVL
RAHSEAINLEFDTPFAPHTAREILSRSPGVQLLEDWHKNYFPMPMEASGKDEVLVGRIRQ
DISHPCGLELWLCGDQIRKGAALNAVQIAELLVEQNLLG
>OHY36453.1
MASYKVELKTPSGLQLIDCPAYDYILDAAESAGLDLPYSCRNGSCSSCVGKLAFGTVDQD
EQSFLTDDQIKAGYILTCVAYATSNCTIETNKEADLLKR
>OHY36458.1
MTQTTEKLKKFTILHSNDMHGDFLAEAKSGEGNLIGGLSLLSGYINKVRQTEKNTLYVIS
GDMLQGSMIDTEFKGLSTMEIMNYLAPDVVTLGNHELDYGFPHLLFLEKMANFPIVNANL
YIKKYSKRLMNPYIILNVDGFDIMFIGIVTEEVLSALKLDTSIGTFVGLEDAAAEVGKIC
NTYKNEDIDLTILLTHIGFEEDKKLAAMLDPEWGVDIIIGGHSHTVLEQPAQVNNILISQ
AAVGTDQIGRFDITVDDDTNSIVEWKWQLVPISNQVATPDVELQNFINAYKEQVDRKYNR
IVSRLSRKLIHPVREQETELGNLIADIFSEIDMIDVVLAGSSAIRGKELGPVVTLEDLKK
TYPYDGALYKIRVNGTQLTKVFAHILRPENRNPVESQCYQISKGVQVVYNDTKKSIESFN

INGQPVEAGRQYIMCVENYHYQNSLKNLNLTPEEVANAKVVATSAQSVLEEYWALASLLW
SQLKSPKFNADKFKP
>WP_006276885.1
MTRNGIGILTAQARSERLTGQIHVYDGAGKGKSQAALGVVLRSIGLGINTKSNCNRVLLL
RFLKGPGRDYDEDGAIAALQRGFPHLIDQVRTGRAEYFGHDEITPFDRAEAARGWDVAKG
ALASDLYSVVVLDEINPVLDLGLLPVQEVVETLKSKPQELEIITTGRAAPQQLLDIADLH
SEMKPHHHPQAAELLLDGIEIYTGSGKGKSTSALGKALKSIGRGINHPGSARVLIMQWLK
GGTGYTEDAAIAALQQSYPDVVDHLRCGRDAIVWRNSRQHLDYVEAERGWEIAKTAIASG
VYKTIILDELNPTVDLELLSVEPILQALLRKPKGTEIIITGRCQNQPAYFDLASIHSEVY
CHKHYANQGVELKRGVDF
>WP_009341847.1
MTKSTVENAENLVIIGSGPAGYTAAIYAGRANLKPIVFEGFATGGLPGGQLMSAIR
>WP_040553397.1
MLEKEAKLVTVKKVILVLAIAVTLIASYLQSELNFLVLLQRGDNMVEYVKSYFLPDFSDW
GYYFSETVITISMGLWGTLMAAIVSVPLSILASNNMCPIWIVQPTRRILDAMRAINEVVF
ALIFVVAVGLGPFAGVLALFVHTTGVLGKLFSEAVESIEPGPVDGIRATGASQIQEVIYG
VIPQVMPLWTSFTLYRFESNVRSASVLGIVGAGGIGVSLYQSFGAFQYQKVCAILIVLVA
ATAVIDLLSAKIRNWLV
>WP_040553731.1
MVAVAILAAGKGTRMKSRLPKVLHPLGGKSLVERVIDSVQPLAPTRKFVIVGYESQMVKG
ALSKNSDKHSDLEFVEQSVQLGTGHAIQQLLPYLSDYDGNLLILNGDVPLLKTETLQQLL
QTHQEHDNACTILTANLAQPDGYGRVFCDDNQIVHQMVEHKDCTHIQRKNNRVNAGIYCF
NWQKLATILPHLDNNNVQKEYYLTDAVVQVGKVMAVDVSDEREIWGINDRRQLADAYQLL
QQRIKEKWLLAGVTITDPSSVTIDETVEMEPDVIIEPQTHLRGKTLIGSGSRIGPGSLIE
NSQIGENVTALYSVITDSFVEQGTKIGPFAHLRGHVEAGENCRIGNFVELKNTQLGDRSN
VAHLSYLGDTSAGTQVNIGAGTITANYDGVKKHRTRIGDRTKTGSNSVLVAPITIGSDVY
IAAGSTVTEDVENDALVIARSRQVVKPGWKIKRELAE
>WP_040554081.1
MKDQAKKSILVEIAVVMAIALLLLVIMPLVLTEFRLNLLGRFLSLAIVGLGLDLIWGYTG
LLSLGHGIFFGLGGYAIAMYLKLQVPSGELPDFMGLYGVTELPWFWHPFYSFGFAVIAVI
LIPGLLAGVLGYLVFRNRIKGVYFSILTQAAVIIFFNFFNGQQKLFNGTNGLTAFKTLLG
VSLNDRGMQLVLYSLTIVFLILTYGLFRWLTSGRFGRLLMAIRDDETRVRFCGYDPTEYK
VLVFAVSGAIAGIAGAFYAIQSGSVSPRAMDIGFSIEMVIWVAVGGRGTLTGAILGTLLV
NYARTFLSEQFAEIWLFFQGALFLVVVTVLPDGMMGWLKTQTLGFVNRSSKVITYPSLEQ
DVEVEHERKNLGN
>WP_050773268.1
MNNIEVSPPQRTELNRESIDENSLPSDSPNYSINHNINHNVQVQIKTEDGKILLILPTES
QAPASMFTWSDIWQQIQQRLKGGDRLRQPNSVLHLVAKDRLLDSRQLQQMKDTLNEMQLE
LKLVVTSRRQTAIAACTIGCSVEQTKIQTSFGNDSQENPKALADALYLETTIRSGGEIRH
PGTVVILGDINPGGIVIAEGDIMVWGRLRGVAHAGAGGNRESLIMALQMEPTQIRIADAL
ARSPEKSLTSFFPEVAYITNNGIRIARATSFSRNQLSKI
>WP_050773314.1
MVGLSIWWICTTPLWHRDLMLVDFSLERCIVSLFQLFNSHSIFPHIFASLRRVLVGLLIA
ISLGVPMGIFLGMYRSLDLATSVLFQLVRMISPLSWMPIAVMVLGIGDLPVYFLLSISAI
WAVILNTSAGVTAVDTQWLLLSKSLCATKWETISQVIIPAIIPHLLTGIRLAMGVIWIVL
VPAEMLGVNAGLGYFILDTRDRLAYSELAAVIVIIGLIGYFLDAVLRFTHQRWTHLV
>WP_061544803.1
MSIGNLEDLGLGDLQQLHETDDYQWLLTNIKLLKNRQFDQLDLENLIEELTDLGNEKKNA
VESLLQQIIRHLLLYQYWNVEIERNSGHWQAEIYNFRDQLNSKLTSNLRSHLVGEMPKIY
QRALGYVQRKTNYQIDFPRESPYCLEQLLDINYLS
>WP_061544812.1
MDNQENSWHGKLELVYAQRQNSTQLMFSHNQAPLKVQRPFYPEGEKICHSVILHTAGGVV

AGDRLSSKIHLKSETDVLITTAAASKIYRSNGLYAKQTVSIKIDRGSCLEYLPQETIVFN
GGRYRQDVRIELGEGSSFIGWEISRLGRTARGEKFLEGEMLSHTEIWQGEVPLWIDRQHI
PGGVEAFYNPHSLKGNPVIGSFVCVGLPISEERIEKSRSGIANGWDAGVTRLEQGILCRY
RGNSTSWAKKWFTNVWQDLRQSLLNRGNCIPRVWQITRN
>WP_061544831.1
MVVDFPALLLGFEFTSPGPVVPGLEFGPFVIRWYGLLIASAVLIGVSLAQHLAKRRHLDP
DIISDLSVWLVVGAIPAARLYYVLFQWSEYSRSPGRILAIWQGGIAIHGAILGGIIAALI
FARLKRISFWQLADLVTPSLILGQAIGRWGNFFNSEAFGDPTDLPWKLYIPPEHRPPALV
NFEYFHPTFLYESLWNLMVFALLLTLFFRSLSGKPELKTGTLFLIYWVAYSIGRFAIEGL
RTDSLMLGSLRIAQLVSLTGIVVGLAGLVWLYVYKRSLPDVVSPSYNRYNHQ
>WP_061544839.1
MVKQVKSNYSIQWSDQISQIPQTAWDALAIPLKTPFLEWYWLHNMEISHSVTANTGWLPN
HLTLWRDKVLIGAAPLYIKGHSQGEFVFDHQWADLADRIGVHYYPKLLGMSPLTPAEGYR
FLIAPGEDEEEITAVMVREIDDFCVRHNISGCHFLYVDPQWQLVLEKQGFTPWLHHSYIW
QNDGFASFEDYLKMFNANQRRNIKRERKAVETAGLKLQAVTGDQIPQSLFPLMYEFYSDT
CDKFGWWGSKYLTKRFFESLYLNYRHRVVFFSAHNHQSTSQPLGMSFCLFKDDRLYGRYW
GSFQEIDCLHFDACYYTPIEWAISRKIQIFDPGAGGRHKQRRGFPATPNHSLHRFYHNHL
GQLLRRYISQANHHEAQQIIAMNADLPFNPNPS
>WP_061544873.1
MFLPLPQFATEDRHPDHIAEVIETSSTEFLAQCLDPEDLRFPLMPAFGSWISAFDEESDN
QIYAVVYYATTAPIDSVHRARALGLSLKNLRQEQPQIFAMLKTEFRAAIVGFEAPSDIST
QRQMYHYLPPRPPQIHQAVYRCRPESMLRFTDNMDFLRTLLLVPGAPVESLAASAIREVY
KLRKLDREWLVKACRYLSLLLKDDYDQLRFILGQIHP
>WP_061544881.1
MGIREEVYYEGGPHIGDLILNLLIGLTIVGLPLTIGAIIRALWLRFKITDLRVAVMGGWM
GQNRTDIIYSEVVKVVKVPRGVGLWGDMVLTMKNGSRLEIRAIPNFREVYEYINDRVAAK
NPQYTSNPS
>WP_061544909.1
MTSTPDTIFGKIIRREIPAHIVYEDELALAFTDVNPQAPTHILVIPKKPIVNLATAQAED
QELLGNLLLTVQKVAQIAGLEQGYRVVMNVGQDGGQTVHHLHIHILGGRSLSWPPG
>WP_061544913.1
MNLSTQSTQTQVQPKLIIHGGAGSSLQSKGGLATVRRSLHTIIEDVYSMLLSGVSASEAV
VRGCQLLEDDPRFNAGTGSVLQSDGQIRMSASFMNGTSGKFSGVINVSRVKNPIEMALFL
QQSPDRVLSDHGSLELARELQVPSYNALTDLRLKEWIQEREDKFKRTMAGVIAEPETPDL
EAGRGTIGVVALDTFGQLAAGTSTGGKGFERIGRVSDSAMPAGNYATKYAGISCTGIGED
IIDECLAAKIAVRVSDGMSLKEAMWRSFTEASKNQRDLGAIALDSKGNISYGKTSEILLA
AYHNGQSIGDTLEWNDGELVGIESSQPVS
>WP_061544915.1
MTQTNMENYGKSFLVIIVPISFLIIVVLNTWKFLLIGLFLIMSLNLWQKYRWEKWCDQVN
PLFYELIEENQGKISSVDLAIRGNFPGDEAKRYLEKKAKEFGTNPVDSGNDGPHYYFITG
SILGEILDGSESDVKPVGRAVTKEARSLLSPPVLVLESKEGDKEGQITNRLETKSLEKQL
AFGSLIQSELAQRLGVYSSTVYKRRNDPDFSEWTRSRDPDGIAWSYSRKNREFFPLDK
>WP_061544921.1
MAYYISTRFLDKIAVHITKNFLNLAGLRVPLILGIHGRKGEGKSFQCELAFEKMGVGVTL
ISGGELESPDAGDPARLIRLRYRETAELVKVRGKMCVLMINDLDAGAGRFDEGTQYTVNT
QLVNATLMNIADSPTDVQLPGSYDAKPIRRVPIIVTGNDFSTLYAPLIRDGRMEKFYWEP
NHDDKVGIVGGIFAEDGLSPVEIEQLVNTFPHQSIDFFSALRSRIYDEQIRDFIHEIGYD
RVSMRVVNSMEPPPQFQKPEFTLFHLIEAGNVMVGEQKHVESSHLVAEYNPRGYQAQSPV
KELPTLQSNHDHNDQNVPHKTLENSPPLSLVTLDTQEQIRQVLSQDHKITVEYVDKRRFS
MGSWQTCGNLHISDYPQAVSALESCLLEHAGEYVRLVVIDPKLKRRVLETIIQRP
>WP_061544924.1
MAESYLLEKLKSVEQTFHELTRRLADPDTAKSPDEYQKIAKSRSSLEEVVNAYETWKNSQ

EELVGARQILKESNGDLELHEMATIEVKDLESKIEHLEEQLKILLLPRDPNDEKNIMLEI
RAGTGGDEASIWAGDLLRMYSRYADTQGWKVKLVSESPGEMGGFKEVILEIQGNSVYSKL
KFEAGVHRVQRVPATEAGGRVHTSTATVAIMPEVDDVEVHIDPKDIEMTTARSGGAGGQN
VNKVETAVDLFHKPTGIRIFCTEERSQLQNKERAMQILRAKLYEIKLREQQEAVTSMRRS
QVGTGSRSEKIRTYNYKDSRATDHRLGQNFTLSPVLEGDLEILIQSCISQDQQERLAELA
TANN
>WP_061544934.1
MALITTGIGFIRDLEKFGSLGVFVPLEGGYEGRYRRRLRAAGYVNLHITARGLGDVAAYL
MGVHGIRPPHLGKKTTSNGAAVGEVQYLPPLISSHLAQLPPNSKGLLLWVIEGNILSDQE
VEYFTNLPRLEPRVKVVIERGGDRYFRWTPLEKTLLAS
>WP_061544959.1
MKRIGILTSGGDCPGLNCVIRAVVSHAILTYNWEVVGIPYATQGLQQRQTIPLSIHGWNL
RGIDPLLNMGGTILGSINEGDTLAAAREILSGYEALGLDALIAVGGDGSLKIIHELATMG
NWHLVGIPKTIDNDVALTERSIGFDTAVNTIVDAINRLTFTAASHDRVMIVEVMGRTAGH
LALHSGIAGGADVILIPEIPYTIQGICEHLKELRDTWKRKFAIVVVAEGATLCSEDLNND
HGVNGEHIPNTNIGSAKCGRGQYVAGQISNFAHGFDTRVSVLGHIQRGGIPSALDRLVAT
AFGKTAVDLIAQGKNHQMLAWQNGKVTSISIESSLLESPQLVDPKSDLVDTAHALGIYVG
LT
>WP_061544962.1
MNLEQKDPCFERLLTYIRQNRGFDFTGYKRSTLVRRVTKRIQALNIDNFIDYMDYLEVDP
EEFHNLFNTILINVTDFFRDLSAWDHLGNQVIPNIIKSKKKNEQIRIWCAGCASGEEAYT
LAILMAENLGAEDFRQRVKIYATDIDEEALNQARQALFSVKSVESVPRKFRDKYFDLTGN
NYIFRQDLRRSVIFGRHNLFIDAPISRLDLLVCRNTLMYFNSEIQGRIMARFHFALNDTG
YLFLGKAEMLLMYSSLFMPVDLKNRVFTKLSSTNIRDRLLVMANSVDDESSRQLSQNIRL
RDLAFESAPVAEIVIDINGLLIIINEAARNLFGLSKMDLERQFYELELSYRPIELRSLIE
RAYNERRPITLNSIERYVPNSEQQYFDVLITPLQEDDLSLLGVSIAFNDVTRYVELQEAL
QRSRQDLETTNEELQSTNEELETTNEELQSTNEELETTNEELQSTNQELETMNEELQSAN
EELQTINHELSERTLELNRNNVFINCILKSLQKGIVVIDRNFSILNWNELVEDLWGLRYD
EVINKSLFSLDISLPVEELRSPILDIISGKTDFQEVSIESTNRRGRIIQCYIALTPLIDK
KIEGVVLIMSDSQN
>WP_061545005.1
MFFRAGARISVIYYPDGSRFLSPVELSNYAEQERLLKEQEQLKYQTLLAQLKAKGIDITT
LE
>WP_061545021.1
MLEDSICKNCGTNLSTILMLEGLPSRKTTSSEKAKILPDQISLVVLILLLGTGVGGFAYS
LLFRRQSPTVQVQNQPTIKATIQELKVQPPSPNVISRQPHQKGCGGFYYTVRRRNSLSLI
ASRFYGNGSLVKFIVNANPSLKNREDTLYVGEQLFIPNREESCR
>WP_061545032.1
MFKTWQIALVAEGPTDFEIIQAVLKAVLSTPFILTLIQPEVTHPQRGSGWGGVLKWCHQA
QERWSGPLMEDPTLSNFDIIIIHLDADVAYFEYNDVSYSKTEVQAHRWASIPCAQPCPPI
LDTIHSLRNVLSSWLDKIQQDVYTVLCLPAQSSGTWLAVALLSADHPLLTNEIECEPLEE
KLAQLPKSQRVKKTIKEYRLHAPQITAQWSQVKKICSQATRFEKDVLTAIRQAIAEK
>WP_061545050.1
MEKLTEAQNELYEWLVEYIRVNQHSPSIRQMMHGVNLKSPAPVQSRLEHLRNKRYIDWDE
GKARTIRILHSSRYGVPILGTIAAGGLIEPFADAVECEYLDLTNLPIPPQSYALRVAGDS
MIEDLIADGDLVFLRPVSEPNLMKNGTIVAARVEGYGTTLKRFYLEGDTVTLKPANSNYQ
PIQVPAIQVEIQGSLVGVWRNYN
>WP_061545073.1
MAILVLISFAPTAWAFCGFYVAKADTRLYNQASQVIIARDGTKTVLTMANDFQGDIKDFA
VVVPVPRIIQEHQVRVPDPKIIQRLDAFTAPRLVEYFDQDPCRRRYYNSPGVIPETGTRR
PSAVEKIPGDNTLGVTIEAEFNVGEYDIVILSAKESDGLETWLNLNGYKIPRGANRLLQP
YVRSGMKFFVAKVNLDKFEQSGYQFLRPLQIAYESSKFILPIRLGMINATTEQDLIVYII

SPRGQAEVTNYRTVKIPSDTNIPLFIKDEFGSFYKAMFQNSYLQEDRKVAFLEYGWDMGN
CDPCSAEPLNLEELKQAGVFWLDERGNNYNRIAPNFGFAFSETNAYITRLHVRYTRDKFP
EDLIFQTTSNQESFQGRYVLTHPFNGELECSAGKRYKDSLPHRFEQEARNLARLTNWKID
DIRQKMRLSVGDFHTPWWEKISLFLGF
>WP_061545091.1
MTTVTTKRRSSNNGSENFILAFINKQMPEILPPLIAIVAFLVVWQIFSLTGGTLPGPIQV
VQDTWELILYPFYDRGGIDKGLFWQVFASLQRVAISYTLAAVVGIGLGILIGVNTTMSKA
LDPLFQLLRTVPPLAWVPISLAALRQNEPAALFVIFITSLWPILINTAVGVKEIPVDYNN
VAKVLQLSQKEYFFNVLIPAALPYIFTGLRISIGLAWLAIIAAEIVMSGIVGIGFFIWDA
YQANKVSEVILALVYIGVVGLLLDKFMAWLQTRILPEQK
>WP_061545097.1
MPKQKKIDPLVGEELLQRVKELGNETREEKAKQCGYYTITKNGIERVNIMKFLNALIEAE
GIQLDGAPGANGRGGRSASYRVSVQSNNNLLIGSAYTKQMNLKPGDEFVITLGKKHIRLR
QVESEERDDDDQLEEVTA
>WP_061545103.1
MNSKNYTFSGLTLKSLILLSSLLICNGLTIGGLELSANAQLLSNAEREELSRLRTETKIQ
KQLQSYLSIWLTLLSLFAVGLIATLWFLRKAIIRDIVERAMRQIGNIENLQTELIAANQK
TTGLIEYSQDLALELEEKVNHLKTKIEGEGGKLSVLLSDLPKSKQEFLTALEREVIAAQE
NISSLEFKLNTQLEQVTLAAQQQRVTIENIKKLESELFAQFSEIKLSIENHRDTSVSDIN
KYRSELMEQFEILALETLESKTQVVQSITEHASQFTSNLSEFQTHAQSQIDGFTSNLSEF
QTHAQSQMDGFTSNLSEFQTHAQSQMDGFTSNLSEFQTHAQEQMDGFTSNLSEFQTHAQN
QIDSFTSSLSDLNQTSVSQLVDLQTDTQKQKDHIFKDLAKSQSEFSHYLLELKNTTESRQ
REIIDSWQKSVDEVEQMLSNLHNNVEEQKTDLLENLQVLGLGFQKQIGDLQNSIEQRHRK
LFQTSQNSVEQLISEFMSELSAMKSDIKTDVDHQKANLIARLAKLEKLESQFVEQLKKLQ
LDAENRQYETLKELSQMTPQTPDISPLSPVGTDTNTTNTIDVQSVNNLDNHLDHEIQEAE
ELLSQNRYEDALSIYEKITSIQSDHEEYWLKRGFILNKIKRHKEAIGVYNQVIRINPAHY
QAWFDIGITCGKLGKHQEAFNCFNKATEIKPEESVAWLNRGLSLVELENYEEAISSFDKA
LEIQPSSFKIWDKRGYTLVRLGRDEEAITNFNKALELNPEYGSALYHKAACYALQKNVES
ALVNLQQAIKHNPSYREDAAGDIDFDDINNDTRFQQLIHRE
>WP_061545106.1
MKMLTPQNTYAWFSQRAWVEIDLGALSNNVKQLLGFLSPGTQLMAVVKADAYGHGAITVA
TTVLAAGATWLGVATVTEGIQLREAGIKAPILILGATYTPEQIHAITSWQLEPTLCSPGQ
ALIFSQFLNSIDHNYKLRVHIKLDTGMSRLGAEWQQAGQFAQFVQELPRLHIASVYSHFA
TADSPDTTIMEQQHGRFQQAIAQIRERGIKIPSLHLANSAATLGDKELHYDMVRAGLAIY
GLYPAAHQRNYLQLRPALQVKARITHIKTISKGTGVSYGHKFIAPREMRIGVVGIGYADG
VPRGLSNQMEVLVQGHRVQQIGMITMDQLMIDISPLVEVQEGEIVTLLGEQGEEKITADD
WANQLNTISWEIICGFKHRLPRVSIEI
>WP_061545116.1
MLAKDSNSLESQPNQGTAGRAKDLLGKFVDTVGVIVRDITSLEVNTIVVNNISGSKFNPW
QSYYNIYAISEPNYFDSKNIVSELEERYINIFLQLEREYIYTLLTKELESPSPKAQVVQG
YRSRLKYIDENRLNSDGTKASQIVLPSPFDQESQVENYQKIAALVTDDKFVLTLRKVSEM
KAALDGGDVTSENVDTIYAQTIIQLDGDIITRYHRDLFGLKESDKDLIMKVHNDGVVSGE
KQWREVIDFLINFIKGIAS
>WP_061545117.1
MNIFQENYQKPDLNYSLNYSFLFPWQFPQNNGQDNCCLEVKAEEGAYSFYLNRTILEKIA
QLKLQGISLDIPKGLLLNLWYYSIFDSAVDRDENYDKSADIYPHPQLSVLIYFFLVKTWK
ILTSQNRNKTIKFQSGFTFNSYYQSTENIMMQSTILFDGDIIQKISQNLISNGDASKIVN
SHYWLTEQVTKCLRSNLNSVYWFVSAIFPAAVITWKITSGMSLWLSILISTLGWIIIFLV
LGTLSLLIISQLKKLLVTIKNKSVDKFIEPVISWLVWVGTNLVISINVFSRDSVLLIANS
LFMLLLPNLLKYFLPKASKYLFKFFMK
>WP_061545119.1
MSATSQFLTLAGITNLENKVIPWEELGQNVQERVGLAIDQTHPSCMVYPQTQEELSGIIA

TANSNRWRVLTCGGMTKINWGGLTSTDIIVSTEHINQLIEHAVGDLTITVEAGIKFREIR
EILSKRGQTLGLDPAFPEHATIGGIVATGDTGSLRQRYGGVRDQLLGITFVRADGKIAKA
GGRVVKNVAGYDLMKLFTGAYGTLGVISQVTLRVYPLPETSGTVILTGQPEPISRVAKIL
QSSQLTPTQADLISHGLVSQLGLGDGIGLMVRFQSIESSVQQQLQRCSTMGKELGLTSTV
YLGNETKQEASLWQQLPELIYNCGHNGEITAKIGVLPTAAVSIISQIQYGLIHLNSGLGL
VRLENENPVLTLRSLCQENLGFLSILSAPVTIKKNMDVWGYNANSVEIMRRIKQQFDPNS
ILNPGRFVAGI
>WP_061545121.1
MSIATAVKTEYEAIIGLETHCQLSTNTKIFSSSSTAFGAEPNTNIDPVCMGLPGVLPVLN
AKVLEYAVKAGLALNCQIAKYSKFDRKQYFYPDLPKNYQISQYDLPIAEHGWLEIEIVDD
HDQPIRKRIGITRLHMEEDAGKLVHAGSDRLAGSSYSLVDYNRAGVPLVEIVSEPDLRSG
EEAAEYAQELRRIMRYLGVSDGNMQEGSLRCDVNISVRPVGEKKFGTKVEIKNMNSFSAI
QRAIDYEIQRQIAAIEAGERIIQETRLWEEGSQRTSSMRVKEGSSDYRYFPEPDLAPIEV
SHSQLETWRSELPELPAQKRHRYERELGLSAYDTRVLTEDLPVTEYFESAINAGANPKSA
ANWITQDIAAYLNKQKLTISEIKLTPQTMAEVISRIEKGKISNAQAKEKLGDLLTGISVE
EVFAGQELITDPDVLGPIVDEIIAANPQQVEKYRGGNINLKGFFVGQVLKKTNKRAEPKL
TNELVEKKLHNL
>WP_061545124.1
MSINTPPKNLSFTVPMTFSNELSVFEMNIPIDEGSWSTVKSVAYGLDLEQSLSTKNLSLL
DNTLTSARNIGTLTGTQTFSDFVGSSDTNDYYRLDLSQTSNFSLSLTGLSADADTQLLNS
SGTVIQSSQNAGKVSESINRSLNAGTYYVRVYPYSGNTNYTLTLSATPPDQAGNTLATAR
NIGTLTSTQTFSDFVGSSDTNDYYRLDLSQTSNFSLSLTGLIANADTQLLNSSGTVIQSS
QNAGKVSESINRSLNAGTYYVRVYPYSGNTNYTLTLSATPPDQAGNTLATARNIGTLTST
QTFSDFVGSSDTNDYYRLDLSQTSNFSLSLTGLIANADTQLLNSSGTVIQSSQNAGKVSE
SINRSLNAGTYYVRVYPYSGNTNYTLTLSAVSSPPPPTAGFNSTYGYGMINAARSVAAAL
GQSSPFPDVANLGGNNWGNDLVGSPEVWSRGYTGQGVVVAVVDTGVDYTHSDLDANIWVN
TREVSGNGIDDDANGYIDDIRGWDFVGNDNDPMDGNSHGTHVAGTIAGENNGTGVTGVAY
NARIMPVRVLDNNGSGSSLGVANGIRYAADNGARVINLSLGGGYSSNIYSAIQYATTRGA
TVVMAAGNSGASQPDYPAFHATEYGLAIGAVDSNSNIASFSNRAGNNSAMRYVVAPGVSI
YSTVPNNGYATYSGTSMAAPHAAGVVALMLSANSALTPVQVRNIVTGTNNVANISSLGTD
SQLVTATRYYVEESVPEFSISYVQVPTDPVIPVDNNYNNGITTDQVEVLGSISTQLSTDV
TKDNYYVYLDDSGNYSFSLSIVLGLFGSPLVLHPRINHF
>WP_061545150.1
MVIVASVIPFLHIPVTIASFLPSLPLDSLFSTQGIMIMLLAAYAGAMWMFLTSAPKVYTV
MVSDLEIARQLYQGLLDLPAAEVPLHYYYNYEQTIGATAIDPLYMSSVPSVPHRTISNSN
DGLWYQLKKNTQLHVITGASLGSKSQQRHVCFDRDCLDLILVRVETRGLKFKIRNQKPLN
FLVKDYEGRIIEMAEVTG
>WP_061545156.1
MPHPLMYEEENFVVLETNQEEQFLTKLELLEKLQNTLSQMPIEDIPLDLRKIGSLIEQVN
HLIDTTCELDLGPGRYLQWYAVRLEK
>WP_061545162.1
MTKIRDKTVLITGASSGIGAACATVFGQENCRLILAGRRLENLECQAQQLYELYKTETHL
LQLDVRDRAAVELAIGSLPSPWSEVDILINNAGLSRGLDKLYLGDIEDWEEMIDTNIKGL
LYLTRYVLPGMVDRGSGHIINLGSIAGHQTYPGGNVYCGSKAAVKAISEGLKLDLLGTPI
RVTSIDPGMVETEFSQVRFHGDQERAKKVYEGIKPLTAQDVADVIFFCATRPAHVNINQV
ILMPVDQASATLVNRQK
>WP_061545166.1
MQQVWVGIGCQKGVSCLLIELGLNKVCQKYRIDPRSIDQIATIESKASELGLVEFCLKNN
FRLQTFSSTTLSSISVPNPSEIITKIMGTPSIAEAAALLCAAQNHSRSQLLVPKEIFRLP
DQGAITIAVATRA
>WP_061545172.1
MSELERYYTLLDLEPGATIEEINQAYKDLVFVWHPDRLPKDNHRLQKKAHDKIKALNQAR

EKLRSFQYQSQPGHNSEPSTSKRSPYQPNQPPPQNPDLSGRDFSHANLSNKDLSGRNLSY
ANLSGSNLSDTFMHKVNLRGANLSEANLFRANLLLADLREANLRSANLIGADLSGADLRG
ADLTGARMRSGERLLVKLVGANLTGAIMPDGAIYG
>WP_061545181.1
MHIPDGFISVPVATTTSLASAAALFISFRRSQTAFGVRRAPLLGLTTAFIFAAQMVNFPV
AGGTSGHLSGAALGAIILGSPWAGTLCLATVLIIQAVLFADGGITALGANILNLGVIGVW
VGWILTQTLQRLLGGSVQRLPLAAGIAAGISMVVSAIACAIELAISGTAPVNLVLPSMTG
IHILIGIGEGLITGGVLAYLARSRPDLLPGEEEKFRGWLVPVVSILLIAGVISLFASAWP
DGLEKAAENLGFINLAQEVRIVVPTPFADYEIDGLGQIGTSITGLLGAASCFAVAFGIAK
VIKPNNA
>WP_061545182.1
MLKISLPLRLQLSLIVVIGTAFLKYHSWLQLYIYGAIALVWVVILGVEIVKLGGLLGGEL
IFLCLVALPLGWEKASFLLVRSLICLIVMNSFLLTLPPHSFGIALKGLPIPLALKENLLL
AGQYLEILLAEIQRMQRGAQLRGLSGTRGWLRYASAAMIGALYLRTLERAERVYAAMITR
GYNGQLPMDCQTNPQELLTIILVIVIASYITVSSY
>WP_061545208.1
MDNLATNIPKIMVVDDDFSVRNLIHRFLSRKYKTEAAADGKSAMTLFEKFNPSLVILDWN
LPDVNGYKLCQDMQSRTNVLVLMLTSRNDETDKIKVLAGGADDFMTKPFSLAEVEVRVDA
LLRRIRYIQPTQSQRLIFKHLAINPEGREVTLNDKPLALTALEFNILHFLACHPGQAWSR
PQLIQKIWGCDYVGDGRVVDVHIGQLRKKMEVDSSTPEFIKTVRGYGYKFESPEHNRVSI
>WP_061545229.1
MTTLNLPKNTQLQALGNPQSHQKSPDFLFIDGVNKIYPTAEGPYTVLDNISLKVQEGEFI
CFIGHSGCGKSTLLNMISGFNQPTNGMVWLQGQPITEPGPDRMMVFQNYCLLPWLSVFEN
VYLAVDSVFPHKSQAEKRSIVREHLGMVGLTEAAQNKPHQISGGMKQRVAIARALAIRPQ
VLILDEPFGALDAITKEELQEELLQIWSDHQVTVLMITHDIDEALFLADKLVMMTNGPAA
NIGEILDIPFSRPRQRRQIMESTEYYHLRNYALDFLYRRCAHVEE
>WP_061545245.1
MVTLKGFAVLPADTFAAGPKSGAAVANPTNGRTTPFVGQPIQGFSGVQFAPNTNGSRFWF
LADNGFGAKNNSADFLLRIYQLDPNFTGVENGNAKVEVEKFIQLSDPNRLIPFSIVNQNT
SERQLTGADFDVESFVIDAKGDIWVGDEFGTYLLHFDSNGVLLDAPINTPNLFKLNTLNG
QKPIVIGHRGASGELPEHTIEAYRLAILRGADFIEPDLVSTKDGVLIARHEPNLINTTDV
ANRPEFANRKKKVVVDGVEEEGFFASDFTLAEIKTLRAVMPQGYRDQVFNGLLEIPTLGD
IIDLVKEVEAQTGKKIGIYPQTKHPTYHDNLNLSLEEKLIDTLKSKSFTDPTRIFIQSFE
VSNLQDLNNNIMPARGVNIPLVQLIDAYDVADDGKLIYKDAYARPYDFTVKGDTRTYGDL
LTPAGLQEIAKYADGIGPWKRQIISVKTVDKNNDGKPDDLNNDGVINDSDKVTLPPTSVV
SDAHKVGLLVHPYTFRNESRFLASDYNNNPELEYRQFISLGVDGYFTDFPGTGDLVRDQI
TTNQVRSPQNPTVLSKPNFDTLNGQKPIVIGHRGSSGERPEHTLASYKLAIAQGADFIEP
DLVVTKDNVLIARHEPMLAVVNLNTDGTIKLVSGKPEINFTDTSTDVYLRDKFQDRLKVK
NLDGRNVGGWFAEDFTLAEIKELNAIERLPSLRSTAFDKDGLKVPTLKEVIDLVKQVELE
TGRKIGIYPETKHPTFFQQQGFNTSQLLVNTLKTENFTDASRVFIQSFEVSNLKELKSTI
MPGASIDIPLVQLFGGSGKPYDFVVNGDSRTYDNLSTPTGLKEIAQYAKGIGPNKQRIVP
MTTVDNNKDGQPDDLNGDGQISDADRTLGASTTLIQDAHQAGLLVHLYTLRNDGFFLSAD
YKGDPGAEVRKFVNLGVDGFFTDFPKTGTSVIVNNYLAGTGYANPNNNLNSPYFADSPVY
FNPNQPYYGDLVTANLNRSQGFEGMAFSPDRQTVYPMLEGTVVGDPAGSVRIYKFDVATE
TYTGLVGLYQLASPSNAIGDFTPINDKEFLVIERDNNQGTSAAFKKIFKVDFSQINAQGF
VPKEEVANLLDIQDPNDLNSDGNKTYNMPFQTIEDVVVWDNKTIVVANDNNYPFSIGRPP
LIDNNEIVVLELDKALSLDARLGLAATIAESSQLVFGTPGVDNVSVPQATDGINDAIFTG
AGDDKVDTLGVTNPYAGNNTVYSGSGKDVIYVNNGDRIFGGSGNDEILATDAKDYRISGG
SGNDVFYLGTNGRALGGDGEDKFFVTEGGGNLISGGAGGDQFWITTGDIPSVGNKNFANT
IVDFQIGVDVLGISGQGSNFGFNNLTLTNNDIIINGNKVATLTGINTSTLTASNFAFA
>WP_061545254.1
MAVTPAHLRSTHYARSLNFSLGRRVFFLWECLIALDPPQPPLKRGAFRLNTMCKNIYIVN

SVNV
>WP_061545258.1
MTSEQTIRRSDILNTQVITRDNGKRLGIVSQAWVDIDQREVVAFGLRDSLISISSLPRQM
YLSSINQIGDVILVDNEDVIEEIDVEALSNLINWEVITETGEVLGRVRSFKFNGESGKIS
SIVIASLGLPQIPEQLLSTYEISIDEVVSTGPNRLIVFEGAEERVNQLSVGVLERLGIGK
APWERDSEEEYGYSPTRTVSPANQLPSGVPLEPPKPKIRTPQPVAREQEEWTPDYVEEEI
RPQREVMQARAYESIQYEEEEDNWSETTDKDAYQAPAGPKPTTQPYNKTYAQQYEDDEDL
EGDAWLDVPKPVNIPKKVKERQVEYEEES
>WP_061545260.1
MQFTTDFENTLLMRQDSVRLIQPEDYDNLSGNKTNKIKTLSYNTKPPHFKHGDNLDQIVK
W
>WP_061545287.1
MARDLRGFIKILEERGQLKRISALVNPDMEIAEIANRMLQKGGPGLLFENVKGASFPVAV
NLMGTVERICWAMNMQHPEELESLGKKLSMLQQPKPPKNLSQAIDFGKVLFDVLKAKPGR
DFFPPCQQVVVEGGDLNLNKLPLIRPYPGDAGKIITLGLVITKDCVTGTPNVGVYRLQLQ
SAQTMTVHWLSVRGGARHLRKAAERGKKLEVAIALGVDPLIIMAAATPIPVDLSEWLFAG
LYGGSGVPLAKCKTVDLEVPADSEFVLEGTITPGEVLPDGPFGDHMGYYGGVEDSPLIRF
GCMTHRREAIYLTTFSGRPPKEEAMMAIALNRIYTPILRQQVTEIVDFFLPMEALSYKAA
IISIDKAYPGQARRAALAFWSALPQFTYTKFVIVVDKNINIRDPRQVVWAISSKVDPSRD
VFILPNTPFDTLDFASEKLGLGGRMGIDATTKIPPETEHEWGEALESDPNIAALVDRRWA
EYGLANLNLGEVNPNLFGYDISK
>WP_061545289.1
MALVRWDPIRDIERLEPFRDLERWDPFREIETLQRRMGRLFERMLPTDGGERAGLTFIPA
AELEEMEDAFKLRVELPGLTAKDVTVEVTPEAVSITGERKSETTTEREGYTRSEFRYGKF
QRVIPLPSTVKNQEVKAEYKDGILRLNLPKTEAEKQRAVKVNLG
>WP_061545295.1
MLNLINRLKNNKPLLFAIYGTSGCLTAAILLGEPFLALTKLGKSSITKPQAIVLLIDTSS
SMSDGKLAEVKTAASQFIQRRNLESDQIAVVNFGANVQTPAPLTNNINTLNNAIDQLLEI
GSTPMGEGINTAQDQLQATTLNKNIILFTDGLPDDPNFAYNSALSVRNAGIKLIAVATGG
ADTNYLTQITGDRSLVFYANSGQFDQAFSQAEAVIYKQLIESNTGENYGFGYSIFRIGGW
TAFLALGISLALIMGQNRYMRLPLLTLKKGIVTTIGSLTAGTVAGASGQLLILALSPLSS
TVFLGVLGLDVIAKVGGWVILGALVGVGTKFFVPNLTLKNSLLGGTVGGGIGASGFLVTS
SLLGDIVGRLSGVSILGFFIGLMIALIERKQLNSEPYLLVHWTPTEQTNYLLGTKPISMG
TALNVEIPLNAVDGFTPLTARIFKEGGDIIMQFDQEYAMIKKMKVTSQTLKVGDVRKLGK
ITIEVKDKT
>WP_061545300.1
MNASEQATNFELASKIATVVNLFKLQFPDAKSDLKPWQNDRETRQLVDPDSIDIGFHFPG
ISRSWRSRSVLIQIRFHQDPINKQSKAIGVEVSGFDYRGQVWRLSTIEQWSFVGEFTPSA
VVGEKLKHICKQILEIFNI
>WP_061545325.1
MSAQESEPSRLPFEPNKKRQKPSKVSAKPVVKIEESPQTLPNQPPFTKEEMAIPQVVSQR
MIRRVATFSGIPTGLGITTLVVSYLLVSYAHIQLPPIAVLLVNMGLFGLGVLGITYGVLS
ASWDEDNPGTLLGVGEFGTNWSRMVEVWRETRKKQV
>WP_061545333.1
MLNLKTVIFVLSFTPFIVLSTQLTGRAQVSSPSLGTAGSVSGGGATGSDNNPISAPSLGT
AGSMSGGGATGSNSAPSLGTAGSMSGGGSTGSDNITVIVSTNNLRELRNAIGQLVSSGSD
SLSPITFSSLRASLISDGVSPGHTDQLIVSFSRVLSQLGVPNTNFSANNLNLEKLVASTK
IFKPTVTIAQDSPAEGEIALILDSNNLTEAINIYNKIVLESDDPTIIKLSRNQDFLKISR
ILKTLRSGII
>WP_061545361.1
METLEFIIHPDGRVQEKVTGVIGISCAEITAAIEAQLGRVLSREST SEYFTPNHLQESGT
VNTQATFSEW

>WP_061545376.1
MINQQLTSGIALWKWRNLAITTALVKGISPREVDWLLQELAGMDKLTLRLESFKEYQEMT
MVLSLAELDSLWQKRLQQRVPIQYLAGRTPWRNFTLAVSDAVLIPRPETEILIDLVIESA
NQDLQSGIWVDLGTGSGAIALGLAEVLTNAKIYATDISEQALAVARTNARNLGFTQRVEF
HQGCWWEPLNHLKGKISGMVSNPPYIPSDLIGTLEPEVVKHEPHLALDGGVDGLEAIRYL
VEVSPHYLLPGGVWLIEMMAGQDEMVREMLINNGNYSHISIHTDLAGINRFALAYVSR
>WP_061545425.1
MQSIQPILQVDHLSKSFGVIPACDGISFDLYSGQVLGIVGESGSGKSTLLKAIAHYITVD
KGSIIYKNRQEQYLKIQELAEHQRRWLMRTEWGFVQQNPRDGLRMQVSAGANIGERLLDI
GMRNYGQIRQEATRWLQEVEIDPERLDDLPTTFSGGMQQRLQLARVLVTRPQLILMDEPT
GGLDVSVQARLLDLLRSLVRNFNLSVILVTHDIGVVRLLAHRLLVMQQGKVVESGLTDQV
LDDPQHPYTQLLVSAALTP
>WP_061545430.1
MSTAIAKKADKVCEVEIVRVKRGLFNPTVIAIISFCILVSVPKPAYAMHIMEGFLPLEWA
VFWWVVALPFFLLGLRSLTRTTQANPQLKLLLGLAGAFAFVLSALKIPSVTGSSSHPTGT
GLGAVLFGPLTMSVLGSLVLLFQSLLLAHGGLTTLGANAFSMAIAGPFAAYWIYNLTIKI
SGKEKIAIFLSAAIADLLTYIITSIQLALAFPAPVGGFMASFVKFTGIFAITQVPLAISE
GLLTLLVWNWLQSYNPQELELLQLIKGGKGNESVSERVN
>WP_061545445.1
MQVLRSFSNQEVLDLQEKLGRGGEACVYTVPSNENLVAKIYHHPTPNHIQKLRAMIANPP
ANPAASFGHISIAWPQELLTAAESSDTIIGFLMPRIRNMRPIMDFYNPGNRRQNCPLFNY
QYLLRTARNLAAAFAALHASNYCIGDVNESNILVSNTALVSLVDTDSFQVPDLSQNTVYR
CLVGKPEYTPPELQNKTFADYNRETYHDLFGLGVLIFQLLMEGTHPFSGVFQGLGDPPTY
ESRILAGHFTYSQKQKVPYLPTPITPSWQTLHPDLRDLFISCFEDGYHAPYLRPSAQTWL
SVLSTAEASLVSCAVNPQHVYHPHLDKCPWCERTIKLGGRDPFPSLQAISAREHLQPRRK
SRKRPRYQPRVRKPAVPVLAPYTQSSLRSTLPGYPTVQTSSRSKFYTFMFGILGLGVLGY
LDIMVKFTRPFLSPNPYTQQSLLSSSSENVHSPLSLSFNDYYQRGNQAYQQQDYQQAIED
FSQGIKQNTNFSKLYMHRGNARYNLNDYEGALTDYNLALKINPQEVKALINRGNAYIKLA
EYSNDPDYEYKKAIDSFNNAININQQDDEAYVRRGIVRSQIARYSNNSQEEYERSIGDFT
QAIKLNRFKAEAYFQRGLARYQFAQYSSNYAQIYKQAIADFDQALNINPEMAEVFLKRGM
IYYELAQYGEKTARNNQQRALEDLEKSAQLYLNKKDVNNYQQAISNICVIAEEKCDYFLQ
NSSIIYSVNP
>WP_061545450.1
MMINTWQYQLNAFTEDINYRVSVAKYKKHLPVLSPKDKLIAETLKQQGVYITSLTDLGMP
STTQMWVSATGYAGMISAPRNVESGYSLPQIYTVTDLPEFFTWGIESRLRNIIESYIELP
IAFHGVHVRKDFPNQQQLQTLLWHKDSEDRRMIKIIVYLHDVGEEHGPFEYIPLPSNIGE
WCNYYRVDYRLWKSGFLGIDDREMMNIIPKKFWKSCPGKAGTVIFVDPRNVLHHGTVRSQ
ERSTAFFVYTSHTPKRPELCTQYHDHTFTKPHGQFKTEIANKAR
>WP_061545491.1
MSDLREKLTENLDEAEWEWLVPHAQRDVIIVVASGLDLLDVGEAIASDNTQSVGVWIDEQ
LISKPSPIQLGDWNSDPTKRFNALIIEPYVLIQEK
>WP_061545511.1
MWQVIKDIIEKLQSWWGGLRDHIFGKEPDIIDDGGDGDGEESETGGSGEKIDKIIIEDFD
YGPIIDRLPPKEIFPKEILFKNPSDKIDSLQKKVLEILFSRFNLSAKEGDNYVAYFPSRL
LSYIKQKDAFFAFRQFLNDIGTTPDEVFKNREIANFVKEAILGLYEADVNCWDTPLSTEP
LYQKLKQSINRHNLDDFIHLLNKLEKFAILAHQIERLIGKIPFHEFDQFSQEVKSQLKEW
DTKWHTIPEKTVNQWKNSLNKYESQSTNYQNLCKQISQDFLRVESLSLTSEQAIVIEYLL
KEVEELKEQLQVGLIETDEGLNQLEILADEIQGFVDEVSHRSRDAESSRTEDSSNKKLSL
EEALMLLSLTLETLSLKSLKTSRNRYARIHHPDVGGDEKMMKKINQAYEILKEYLET
>WP_061545514.1
MQIRSNMEQAEIFVFIASFFLIALGFKEPDGPVKKPSEHPPIILFPESSRYNFRSGRAEL
SSEFRNHIRTKLKPKILEVVKQYKDIDAIEVIGHTDGRPINSVEQSISSHNSNLDNNLER

VNLGKLSVSRLQPGSNTDLGIMRALAVVQELKQSGGGISKLNLRAYSAGQLLLEDGQPAP
IDTTDTPERRRIEIRFTKLGRTQVFK
>WP_061545515.1
MLTQDVRNALDVADLNKLKYDLNSLQPVDVGEYISELPEQQRAIAFRLLNKNQAIDVFEY
LPTEVQEELINSLHDVQVVHLVEEMSPDERAYLFDELPAGVVKRLLQQLSPEQRQATATI
LGYPEGTAGRVMTTEYVRLRQGLTVGEALSKIRLQDEDKETIYYAYVTDDNRKLVSVVSL
RQLLFTFPEVFIRDIASSQVVKVTTETPQEEVARIMQRYDLIAIPVVDREDRLVGIITID
DVVDILQEEATEDIQKLAAVSGDEEALSPPHLTIRKRLPWLLGIMALYIGAASAIAPFQK
VIAAVPVLAVIMPIFSNTGGTVGIQALTVTIRGLGVGEVTTKDAGKILRKELIAGLGTSL
ALGSTMVLLSLIWAKPDEKWVALIAGIVMATNTMVAVSLGTLLPMGLQRLKLDPALMSGP
LVTTMLDTIGFLTFLSMISLALKVFNLSY
>WP_061545535.1
MKIVDLIAWFEEWANPAWSESWDNCGWQIQPGILEQKSRVLVCLTPTLAVVKEAIALKAN
LIFAHHPLIFHPLKSIQVGEPVGEILKLAFAHNIGIYSAHTNFDQVEDGTGDVLSKILDL
KEVLPIVHTRLGLGYGRVGKLDPAIKLEELLKIIQAKLKPPKLIFSPHGNLGEEIWRVAV
LGGSGAGFISAVVKTKAQVYLTADCKFHQFQESRDQGLILIDAGHYATERPACDCLVKKL
ESLNLEWVKLSDEDEDFRQFWN
>WP_061545538.1
MNNPQDYNPSSIAQINGNLLGLPHTYDSANLIIFPVPWEVTVSYRTGTGNGPQRILDGSY
QIDLFDLDNPDGWKQGIFLVEISQDILDKNHYYRQQAAKIIQRQAEGKDLKAEPDLTGVL
AEVNQAGRDLNKWLFDQCQQAMSQGKKVGVIGGDHSSPLGYLQALGSQYADFGILHIDAH
ADLRHAYEGFEFSHGSIMFNALEIPQISKLVQVGLRDICVDEIEIIKGSNNRIVAYYDPI
IKQQQYAGKTWNDLCEEIVANLPQHVYISFDVDGLDPKLCPHTGTPVPGGLELEQAYGLF
RKVVKTGRKIIGFDVCEVGDGEWDGNVGARIVYKLANLLPC
>WP_061545554.1
MLRDFANREELVTYIREQFPQAAQTDDYISGIPGGRKAAEAQLEKVNTVSYAKTRNYLTG
AVTRLSPYIRHGVLSLREVREYILNTVENSEDGSKLINELGWRDYWQRLYMKLGKGIWEN
QEEYKTGYHTEDYSHNLPENIQEGKTGLVCIDSFSQELRVTGYLHNHIRMWLASYIIHWR
KIQWQAGAKWFLQHLLDGDPASNNMSWQWVASTFSHKPYFFNRENLERYTKGVYCSRCPL
YGKCVFEGTYENLQSILFPHSP
>WP_061545562.1
MTILVKTTQTADEQQIADFFRDSAGTWRSERRYYTLPEGKTQELISMITIYFLPKGDQEL
LTLAQLHNLPNLDSLNCGAKVSWQSMDLENLERKLHGQTIFGALGKILYRDRGFATTKPV
TADYVISQAHTLCLRTEYNRAAFEEEIKLIGDKYRTRQTIISRDGKPLTIGQYLEKRV
>WP_061545570.1
MVVSPAKNKQHHVVIIGGGFGGLYAAKTLANTNVNVTLIDKRNFHLFQPLLYQVATGTLS
PADISAPLRSVFRNSKNTQVLLGEVTDIDPKGQKVFLGGEVVQYDTLVLATGANHSYFGK
DHWKDLAPGLKTVEDAIEMRRRIFSAFEAAEKESDQAKRRALLTFVIVGGGPTGVELAGA
IAELAYQTMKDEFRSINTSETKILLLQGGDRLLPHIAPELSEEAKLSLQKLGVEIQTQTR
VTNLENDIVTFKTGEQIQQIASKTILWAAGVQGSPIGKILAERADIERDFSGRVIVEPNL
TIPGFKNIFVIGDLASFSHQNGKPLPGVAPVAKQQGEYVGTLILLRLQGQTLPEFNYNDV
GSLAMIGQNLAVVDLGFIKLKGFIAWVFWLVVHIYFLIEFDTKLVVVFQWAWNYITRNRR
SRLITGKAAFLETQTINNNNPYQATETAQQTVKV
>WP_061545575.1
MKQEIRDIFDRIAPVYDQLNDCLSLGQHRIWKEMTVKWSGVKTGNTCLDLCCGSGDLTFR
LARRAGITGRVYGVDFSNNLLSAAKNRQELSHNPHSITWIEADVLSLPFADNQFDVATMG
YGLRNVTDITRSLQEIYRVLKPGGRAAILDFHRPDDHIWGIFQQWYLDYLVVPLATNLGV
REEYAYISPSLQRFPRGEEQVALARQVGFVNAVHYPISNAMMGVLVIVVP
>WP_061545591.1
MSKLRISKQHLWLERLMAITATLNLGLVMFDWSYVPWRDFYFRNLPQLSRIYDPIKGIEP
HRDTVNYLNNIASLQVQVSESGLGSPAVKTKLGEIRRLSMDMIDSNPFAGAGKSGTLEKI
KNRMRERVGQESSKGAFTIFWSPEYLSQGRWVEEIDFFNRQILPSIASNYYRRIGENGEL

INNFWLIDLPFVAVFGLELLWRAFYIKRQNSHLSWLEAILWRWYDLFLLFPLWRWLRIVP
VVIRLDQSNLVNLYLVRKQIHQGIVANFAEEITEIVVVRVINQIQLSIKHSDITRWVTPN
QNKREYIDINNINELEAISGILIRILIDSVMPKVQPEINAILSRSIGGALTQVPIYGNLL
QLPGLSQVQNKLSGEIADQISKNLYSIMTKVSKDPLTAQLSKDLIAKFTTTLSGEIQTKH
ILVELQSLLNDLLEEVKINYVQRLSQEDIEQIIEQTREMRSRAV
>WP_061545646.1
MSLITLQSVKKDFGIKEILKEATFSIDGTDKVGLIGTNGSGKSTLLKIIAGIEPIDGGQI
LTNYGAKIIYLPQQPDIDENLTVLEQIFMDSGEHTKLVKEYEELSDKLAHYPEDTLLMSR
LSEVMQRMDATGAWELETNAKIILTKLGIGDFEVKVGTLSGGYRKRIALATALLAQPDVL
LMDEPTNHLDALSVEWLQSYLNRFRGALLLITHDRYFLDKVTNRIIEIDRGDIYTYSGNY
SYYLEKKALAEESAVSSQRKHQGILRRELEWLKRGPKARSTKQKARIQRVESMRETQFKQ
AQGKVDISTIGRRIGKKVIELSGICKSYNGKTLISNFSYEFSPEDRIGIIGGNGTGKSTL
MNMITGRTSPDAGTVEIGSTIHIAYFDQHSEELISAVNDNQRVIDYIKEEGEFIKIADGT
KITASQMLERFLFPGSQQYAPIHKLSGGEKRRLFLLRLLIGAPNVLILDEPTNDLDVQTL
SVLEEYLEDFLGSVIVVSHDRYFLDRTVDTIFALEEGGNLRQYPGNYSVYLDYKKSEELT
QQETINGRDNRKSKNLTQPKSGEQEVQNKKRRRLSNWEKREFEQLEAKIVDLEAQRTLVE
TSLLAVAPENYTQVQNLYEQMEVLKQAIDIATERWLELAEMDV
>WP_061545658.1
MAHKKGTGSTRNGRDSNAQRLGVKRFGGQVVRAGNILVRQRGTKFHPGNNVGIGNDDTLF
ALIDGVVTFERKGKSRKKVSVYPPVTEESVAS
>WP_061545669.1
MQIAKNITELIGRTPLVQLNRIPQAEGCYAQILVKLESMNPSASVKDRIGVSMINDAEKE
GLITPGKTVLVEPTSGNTGIALAMAAAARGYKLILTMPETMSAERRAMLRAYGAQLELTP
GIEGMSGAIRKAQEIVEKTAHSYMLQQFRNPSNPKIHWQTTAQEIWQDTDGQVDIVISGV
GTGGTITGIAEFIKSKKPSFQAIAVEPANSPVLSGGKPGPHKIQGIGAGFIPQVLKVELI
DEVIAVTDEEAIAFSRRLAREEGLLSGISSGAALCAAVRVAQRSENQGRLIVMIQPSFGE
RYLSTPLFQDLEIS
>WP_061545677.1
MWKKIRTIKFILVVTLAIAFSAISTTIYPDMAVAAGFRSFVDTEDGYQFSYPNGWLQVKV
ANGPDVVFHDLIEVSENVSVVISPVLQGKSLTELGTPTEVGYKLGKSALAPEGSGRSAEL
VNVAQKEINGNNYYFLEYAVKLGNGQSRHNVASVAVSRGKLFTFNASVPERRWRKLQRTI
DEVVSSFQVY
>WP_061545678.1
MNIPEFVLASASPARHRLLQTVGIEPIIYPSDFDESQVQLSNPEELVNKLAQCKAETVSP
RFPSALVMGCDSILAMDNQIYGKPKNVEVAISRWRLMQGNWGDLYTGHVLMDNLKKKTII
KCQITRVYFAKMTDHAIINYVRTGEPLKCAGAFALEGFGSLFVEKIMGCHSNVIGLSLPL
LRQMLGELGYEVTDFWQ
>WP_061545693.1
MLPREELLKGVENRDVIAHVIDQAEQAIKTWEVVITDFLSPPELAEIERVFSRLTEVHLV
DWGGYPQAERQRVAITRSEMPLDVSQVAVTVLDIAGNFLFDTATHRDFLGAMLGTGIVRE
KTGDIIILGERGAQAIVVPELAEFLTMNLQQVRSVPVKTQVIDPSELKIREPKKKELTTV
EASLRLDAIASAGFGMSRSKMVDLIDSGDVRVNWKEITQSSYQLKMGDLIAIRGKGRLQV
GEIAVTKKDRYRIQLTRYM
>WP_061545707.1
MPKHFNTAGPCQSDIHYMLSPTARLPDLKALIHGRNYFIIHAPRQVGKTTAMIALAEELT
DSGEYTAIMLSLEVGAPFSAPYHEIAPHLVLMAFLHRVVNDGGTLEREYAIGSGRMDICL
RYGKVVMGIELKVVREKLDPLTKGLTQLDKYLDGLGLDTGWLVIFDYRPGLPPMGERISK
EEAISPRGRTITVIRS
>WP_061545715.1
MEVVIGIDIGGTSIKLGVFDPNGNRLQTIILQTPKPATPEAVLSTMLGGINSLIEQNGRI
LAIGVGTPGPVDATGRVAKVAINLEGWDNVPLAEWLENRTGIPTILANDANCAGLGEAWL
GAGRDFQNLILLTLGTGVGGAIILHGKLFVGHQGAAGELGLITLNPNGPSCNSGNQGSLE

QYTSIGAIHRRTGKYPARLGELAEQGDIQSLTFWQEYGRDLGIGLTSLIYVLTPQAIIIG
GGISASFEYFLPAMQEEIERRVLPSSRMGLQILPAQLGNSAGMVGAAKLAWDND
>WP_061545739.1
MDSLLQPFKHFGVNLGLARIVKLLETLNNPHRQVPIIHVAGTNGKGSVCAYLSSILTESG
YKTGRYTSPHLIDWNERICINEKPIASEELTKLIERVKAVINSNEEQPTQFEIITAASWL
YFAQEKVDIAVVEVGLGGRLDATNVCDHPLVTVITSIGRDHWQQLGSSISDIAREKAGII
KAGCPVVMGKLPEDAKRAVICRSVELESPIVVVSPAREISPGWAEYQTVEPGGHIKAIKY
PLPLKGQIQLTNSALALASLGMLQRQGWEISGQAIIRGMEKTKWPGRMQWFNWKKDNSDC
QLLIDGAHNPESAEVLRNYVDSIGDRNKNITWVMGMLTTKDHKDIFRELLKTKDRLYLVP
VPGSDYANLDYLKDLALETCPDLDFCAIYEDIFLALNAAFGFLKTKDKMNNNTNTNGPVV
LCGSLYLIGHFFSQVNLAK
>WP_061545741.1
MNFTNFLYLVIGILLGIAIDKLLMSKLLSNPDENTWERSQTQILQQLHQTELAYEMAKEM
SQFHAGFLARISHELRSPINGLIGLHQLILHDLCENPQEEREFINQAYERSLQFLKIIDE
MVNIARIEYGTNQLVIESVGIREVFREVEELTYMLAANRNFPLQICLPPSEIYVLVDKRW
LRQILVSLIDNTISRMESGYICLFIKSPDGNITTDIHNHLDIYLDLPSNTVISHERLDTC
LHITDNHDSEIRDISPGMKLLINQRLLEMMGGKLEIVEPDCPPINNETTNFTRLQITLPQ
ITSTSIPEVALLTQ
>WP_061545745.1
MERTFIAIKPDGVQRGLAGEIIRRFETKGFTLVGLKFMKVSKELAEHHYDVHKERPFFAS
LVEFITSGPVVAMVWQGEGVVASARKIIGATNPLTSEPGTIRGDFGINIGRNLIHGSDAI
ETAQREISLWFKEEELVSWTPHLAPWLAE
>WP_061545746.1
MQQLYEFYLKEKLPMTYTLKQFKKDFVISHLREIPTEEVLKQYSPEEIKQYSPKDLLKQY
SPEEVLKQYSPEEVLKQYSPEEVLKQYSPEEVLKQYSPEEVLKQYSLEEVLKQYSLQDFL
EGLSPETLERLAIILSQLGVNQIKNQEQ
>WP_061545749.1
MVESNLFSKLENLLRSQNFREADTETLMVVLKVANREKEGRLTLEDAKTFSYENLLIIDN
LWCRWSNGKFGISVQQKIWCECGGARKYDADMVLNTSISFGDRVGWRCKGKWLPYDQLNF
SLNAPVGHLPCGGSPHTYIWTMCMDMNDLP
>WP_061545776.1
MQQLKAKTLEMRTPQATKTAVLVIGGAEDKVHGREILRTFVARAGASKAYITIVPSASRE
PAIIGGRYIRLFEEMGAQKVEILDIREREQCENSQIKASLENCTGVFLTGGDQLRLCGVL
ADTPAMDLIRQRVRAGQLTLAGTSAGAAVMGHHMIAGGGSGETPNRSLVDMATGLGFIPE
VIVDQHFHNRNRMGRLISAIAAHPDRLGIGIDEDTCAVFERDGWLQVVGKGSVTIVDPTE
LTHTNEPHVSANEPLNVHNLRLHILSYGDRFHLYQRTVLPAVHRLPSYGDNVS
>WP_061545837.1
MFYSDFKLSQVVDDFGLTINERSGLFAEHPQTECSDLLKTILSESIDLAVAINTEKARSE
MIISPVLLEVRRKLNYQISLFSGIDFNVDPQRGLNGFCDFLISLSQEQLFVRAPVLTLVE
TKNENLKSGLGQCIAEMIAAQEFNRQKQNQQIKIVYGVVTIGTIWQFLQLENQVVSIDLS
EYYIKDVNKILGILISTI
>WP_061545839.1
MAGTDFKDYYSILGISKTASPEEIKQAFRKLARKYHPDVNPGNKQAEARFKEINEAYEVL
SDPDKRKKYDQYGQYWKQVGESGFPGGAGVDMSGFDFGQYGSFNDFLNDLFGGVGPRSST
SSRSAGRPGGFGGFGDFGFPDMGAQDSEFVITLSFAEAFAGVQKRFSLGSETIEVRIPAG
AKTGTRLRVRGKGTVNPKTGQRGDLYLKTELSPHSFFQLEGDQLICEVPIAPEEAVLGAT
IQVPTPDGHASVKLPAGVRSGQSLRLRGKGWPLPKGGRGDQLVKVAIIPPKDLTPQEREY
YEKLRAIRSYDPRAHLQQIKF
>WP_061545860.1
MENKLMLMIPGPTPVPEAALLALAKHPIGHRTGEFNNIMGEVTENLKWLHQTTNDVLMLN
VSGTGAVEAGIINFLSPGDRILVGNNGKFGERWVEVGEAFGLKVEVVTAEWGKPLDPDKF
GEILGADTNKEIKAVIITHSETSTGVINDLAAINSHVKAHGEALIIVDAVTSLGAYNVAV

DELGLDVVASGSQKGYMIPPGLGFVSVSAKAWEAYKTAKLPKFYLDLGKYRKSTAKNTTP
FTPPVNLIVALHTTLGMMKKEGLESIFTRHERQKNATRAAMKALNLPLFAADECASPAIT
AVSVPGMEADKIRSLMKKRFDIALAGGQDHLTNKIFRIGHLGFVSDRDILSCVSSLEVVL
GELGYENFTPGTGVAAAVKVFAAH
>WP_061545862.1
MSSGFDYDLVIVGAGVGGHGAALHAVHYGLKTAIVEAADMGGTCVNRGCIPSKALLAASG
KVRELRDAHHLQSLGIQLGGVSFQRDAIAQHANNLVSKIQGDLTNSLKRLGVDIIKGWGK
LAGPQKVSVVTTGSEKIITAQNIILSPGSVPFVPPGIEIDGKTVFTSDQGVKLESLPDWI
AIIGSGYIGLEFADIYTALGSEVTMIEAVDMLMPGFDRDIAKLAERVLITSRDIETKVGI
YAKKIIPGSPVVIELADFQTKEDLEVLEVDACLVATGRIPATKNLGLETVGVELDKRNFI
PVNDGMHILAGSEIVPNLYAIGDANGKMMLAHAASAQGIIAVENILGRNKKVDYRSIPAA
AFTHPEVSYVGLTETAAQELGLAQGFEIGTTKSYFKGNSKALAENEADGIAKVIYRQDTG
EVLGVHIFGVHASDLIHEASSAIAYRHSVKDLAYLVHAHPTLSEVLDEAYKRAIAS
>WP_061545872.1
MTYCLRISDLPENERPRERLITHGAKVLATAELIAILLGTGQGPGKLSAVGLGQHILQEL
GRDQGDPLAALRDATPARLTEIHGVGSAKATSILAAVELGKRVFLSRPAEGAIIDSPIAA
AATLSQDLMWQSQEKFAVLLLDVKNRFLGTKIITIGTATETLASPRDIFREVIRHSATRM
IIAHNHPSGNLDPSQADLDLTRQLLSGAQLLNIPILDHLILGNGNHQSLREITSLWDDFP
QEN
>WP_061545893.1
MRVSTRKIFSFGLVSLLMVGGISPVAFAGEKTPLMKTPVNTNIAQAQRQGNQTRQRRDSF
QRLNLTSEQQAKIRDIRRDTHSKIESILTPEQKIKFQAAVKQRETEYSNPGGSKRGRGLN
VLRSLGLTDEQKNQIREIRASSRQQMQAVLTPEQKAQWQQFQQRNRRQYR
>WP_061545910.1
MLYLHNLTYHPTACPTPILQAINLELPPQKLGLVIGPSGSGKSTLLEILSGLAEPTSGGI
FWREEELVTDHLQQLAGIVFQFPERHFCGGTILEELRLGHPELGLERVKNALKEVGLESL
SLSTSPNALSGGQQRRLALAVQLIRQPNLLLLDEPTAGLDWSMRRQLVTLLERLKKNWTI
LVVTHDAGDLLPIADCCWNLHHGILSPIAPSLESIGK
>WP_061545915.1
MDEEFIVPQAPANFKSGFIGIIGRPNVGKSTLMNQLIGQKIAITSPVAQTTRNRLRGILT
REKAQLIFVDTPGIHKPHHPLGEVLVQNAKIAITSVDVVLFVVDGTVVCGGGDRFIADLL
SKCEIPVIMGINKIDQQPVETEKIDESYRELARENQWQIVKFSALENQGILELEDLLIEQ
LETGPLYYPPDLVTDQPERFIMGELIREQILLLTREEVPHSVAIAIDLVEENPTITRVVA
TINVERDSQKGILIGKGGTMLKSIGTVARQQIQKLIAGKVHLELFVKVQPKWRHSRLRLA
ELGYRVEE
>WP_061545921.1
MKQLAIPCLLFRSGTSRGPFFLSSDLPDDNIERDKIILAAMGSPDDRQIDGIGGATTLTS
KVVVLSLSNHHWADIDFLFGQVSINQGWIDWSPFCGNMTAGVAHAAIERGLISALDPITK
VRIRNLNTNSLIETVVQTPQGEIIYDGNTSIDGVPGTAASVVMNFMEVVGSKTGQLLPTG
YCREKIQGIEVTCIDVAIPMVIARAEDLGKTGYETKAELDADRDFFTQIETIRRIAGERM
GLGDVTNRVIPKFAIIAPPRQGGTITSRYFVPDVCHAAHAVTGAICVGCCSLLKDSVADG
IAANSTLSGEEIVIVEHPSGKIQVSLVISGHGSLMMVERAGIIRTVRLLFSGNVHVSTR
>WP_061545944.1
MNIETIKSEKTKQLPGANLEDQDLSEFDLTAANLAGANLMGAHLVSVNLEGSHLEGANLM
GASLQGADLRANLLGANLMQADLTGADLRGSNLRGANLMGATVAGASLTAAFLSGANLMS
GNFQGVDLRGADLRGANLTGANLKGADLSRADLQGALLNQANLEESDLRGANLAGANLAG
ANLLCAELEAASLNGANLYQACLLGTILETYHD
>WP_061545977.1
MSPVTIPSSTIPDWEELLETAQKYTSARRIKRWGQNPSSAPIPAGWNKLPPDGQPPILLY
RDSNSWCPFCERVWLALEEKQIPFSTEFIDLTNKPKWYTDLVPTTLVPAAKIEGRLVYES
KDILLAIEEHFDETLLPEDPQENQVARQWVEKAETNELQTVAYKLLRQPPEDKNELAKLE
LELESKLDEFEEDLGQYPGPYFLSTFSLVDIMYIPHLDRLAANLPVYRDYALLGNPRFPR

INFWFAAFKERPAFSRVKSDPITNNLLLKRRFGLKPIGSPLPLNLAESELLEYRAEASER
LANNRDAAIKDIVKNSGLELLGDVVSIRESVEYYLRLLADYLVNGDNKLLLGGNAGGKEG
VDPNFAAVGAITLAYLRNRICAPRDMSSGAATALRAGVDRVLAAIY
>WP_061545980.1
MKLILPVEIAEEIEPHLPKETEIKVIRVDSDGNLDGDATDAEVYFSWFYLKPTTLHRVLE
AAPSLRWHHAPNAGVNHILTQKYLERDLILTNGAGIHGIPIAEFVITYILAHVKQLAKFY
QLHSENKWQRGVPLNELSEKTLLIIGAGGIGKEIAVRAKVFGMKVFGSSRHPKQLPNFDQ
VVGVNEWRQLLPQADFVVIATPLTPETKGMIDANTLALFRTNSYLINIARGGIVDEQELI
QALEENRIAGAALDTVLTEPLPPESPLWKLPNLLITPHNSGDSPKTKQRTFDLFLENLTR
YLEGKPLQNVVDKNAGY
>WP_061545983.1
MLLQDSTLQLSECAGYFNDGLNGFSIDKSLITLARSGYLALGLSSSLGGSGGGLSDVVEA
IASVSEQCLTSGFVFWCQRAFMEYLSASGNHYLQSEILPMVLKGELSGATGLSNAMKYLA
GIEKLRLQVEIDNENVTINGFLPWASNLRPEKFVIAVAGQTNCGKSMVIAVPADISGLNR
GEDLQLLGLQASWTSSLKFNNVQLCHSWIISDNGAEFLPKIRPAFLLMQCGLALGMARRS
LKETRQSINTNNREVLASRLGASTATLSNLENHISYLSNLSAFTLTQMRQLFEVRVKLTR
LAVEVVQMELEAKGGTGYLKPSGTARRLREVAFLPVLTPSLVQLETELYRHRLVKEKSDG
LPYNCH
>WP_061545986.1
MSTDSNYQVNQWLEMLRSPEVGDRLVAVKSLQHLGEDLAIEGLITALKDESTAVQKIAVS
ALWELGNPQAVPALLEHLGDWDPEVRIEALSALGELISSHHLSLLLDALGKENVNLQLNI
LILLRKIHHIGSLDAVLSFFNSQHPELREAAVTTLRYLNQVQICPKALSLLNDADNNVRR
ATAITLGYLKDGKIPGLLSQSISHDSDWQVRKNAAISLVNHANSVAVSTLKNGITDENWQ
VRKSIIQLLQKAPQIELLPLLIESLRSDQYSDVRKEAAVALSHLTIGHPSSLEALDALRT
GLNDEDTEVQICCQKAIEKIDSNVKQRSTASKPDVSTVDG
>WP_061545994.1
MICCLNPDCHNPQNPDGSKFCQSCDTPLVPLLRNRFRIIRVLSDEGGFGRTYLAEDTDKL
NDSCVVKQLAPKFQGTWAQKKAMELFSQEAKRLQELGEHPQIPTLLAYFEQDNCLYLVQQ
FIDGNNLLQELRLRKHYKDSDIQSLLLDLLPILKFIHSRGVIHRDIKPENIIRRKRDGRL
ILIDFGSAKQLTAAVQKKYGTSIGSHGYSAIEQIRDGKAYPASDLFSLGATCFHLLTGLS
PFQLWIEHGYSWVKNWQDCLEDSRSAELIIIILDKLLQPDLKNRYQSADEIIKDLGKKNVY
GLKKSSTYLKKGKHKKPKHTILRNIFLTLVTISVVGGLGYRNLGQIQTAIFSQFNPLLMP
SNKSSTSPEYGQISRRTSSSRANKVSLFKTITQVDKSLAVVAITPDGNTIVSAGHKEIKL
WNSKTGKQIISLPGHTQNINALAISPDGNNLVSAGDDKTIKVWNLQTKKLTFNLVGHQDS
VQALAISQDSKTLVSAGDDKTIKVWSLLTGKFLKTLLDHNYWVRSLALSPDGFTLASGSF
DKTIKIWNIHQTSGQKPTTLLDTTSQTVTSLTFSPDSSTLVSTSRDRQIKFWDIKNKEII
FASKKQNVTSVIFSPDGKTLISGAKSCPDCSEKISSVIKLWDVSTKEEIYALPVNTKIVT
SLVLSADGKTLVGGTKDNKINIWEISP
>WP_061546007.1
MKSIMNSVNGWQNEDQLTRNISVSRSLFVLGYSFLVLSLTLFGCNGSGSNTSVSNTDSKT
NGSSSNQKVIKIVRSKQLTALAVLEKQGTLEKKLQPLGYKVEWPEFAAGPQQLEALNAGG
LDIAATAESPPVFSQAADVPLVYLAANSSDGQAISLLVPTKSPVKTLKDLKGKKIAFQKA
SIGHYLTVRAVDKEGLKLSDVESVFLPPPDANVAFSQGKVDGWFIWEPFVTRNVQNKVGR
VLLDGGNGLRDTNNFYSTNRKFYQENKQAIKIFLEELQKAQVWSKNHPKEIAQLLASVTQ
LDPPTLEKMHGKYDFSLVPITESIINKQQQVADKWYSLGLIPKKVNVRDGFLTPKEYEEI
TPQEVLGSK
>WP_061546008.1
MWQKYEVKSIRTLSLLFGMGLGLTFLITACSPSSTNNAGNNQSSSPNEAANSSPSSRLTN
SSTVIRIGYQKAATVLYALKAKGELEKSLQKSGVSVTWTEFPAGPPMLEALNAGSIDFGY
TGEAPPVFAQAAGTPLLYVAYDPLATKAEAIIVHKDSPIKSVADLKGKKVAFAKGSNTNY
LIVKALEKVGLKYSDIKPTFLLPADARIAFEGKNVDAWAIWDPFLAAAQKATDARILTDA
TGLAPNLGYYLAAKSFVDKNPEGLKTVLRDVDEVSKWAKNNPSDVAKLLSPVLGIDVGVL

EISERRRDYGVLPLTEEVINNQQKVADTFYQLKLIPKQINVREVVTKIDF
>WP_061546018.1
MQNSDLDISLSGYDYELPPELIAQNPVVPRDSSRLMLINSPAGTLTPPLDHIFRDLPELL
KPGDLLVMNNTRVIPARLYGRKSTGARVEVLLLEEKQHNCWLALVKPGKYFNIGTEIIFD
DQGLSSVDKLIPQLTATVIERDENTGGRLLQFDLPINVSLLHFLHKLGEIPLPPYINNSH
ALEEQYQTVYAKHDGAVAAPTAGLHFTPELLVKLRECGIQQEFLTLHVGVGTFRPVEVED
VTTHQMHEEWIEVSGDLVEKIYATKNAGGRIIAVGTTVVRALEGAAQGGSLQPYTGKVNL
FIYPGYQWRVVEGLITNFHLPRSSLLMLVSSLIGRKRLLDVYNQAIASRYRFYSFGDAML
ILPEARICCG
>WP_061546023.1
MSINIPITPLSGKTILVTRAVGQSSELSHGLTNLGARVIELPALEIGPPSSWRSLDQSIL
ELSTFDWLVFTSTNGVESFFARMALQYKNQQALIRQAINRTKIAVVGEKTAQSLKIHYIE
PDFIPPDFIADSLVNNFPENLAGKKILFPRVESGGRDILVKELTQKGAQVVEVPAYESLC
PQSIPTTAQQALISQSVDVITFASSKTVEFFCVLLENNLRQKAEQYLNKSAIASIGPQTS
KTCIDLLGRVDITAQKHTIDGLIKAIVEWSKSLN
>WP_061546050.1
MDELRTVLELATQEELQDITAILFSRKFNPLDYVHTPEPVMVQSQNRQAWLDTLESRFRF
LAADGITVLRKRTEQVTYRQALIQVCRHLKISYYEELSTVDLEAEVFLHLLGKVWRKLPR
QQRQKISSRIKSQLITSDLPNSVLQLLPTDSLGLIFKAGSAVAVTSVIQPYLLKQIACQF
ATHLATYQVAKQATIVGTEMASQQIKNYVTAQMARRGMTLSAARYGAVRTVFSVVGPVMW
AWFLADLGWRTISTNYGRIIPTIFTLAQIRLTRTECWQPT
>WP_061546051.1
MKTVIKLTQELVIGEIESILRTYPYSPYQQVFEIIDLRQELIVFVMNRIPGISPTISQDQ
MLLVVNKEENGDNRLFNRHLSGNFLAQQLHLQNLVHQGVFAIIQAKADVISHILNEPVHP
GCEPSNWFG
>WP_061546071.1
MVNSLSVRTSDPSYPINLVGKTGQAVYISIHNPSQYICANCEQILPDWKQQQFLWVIVVL
QQSKYPLVEMTGEIETEKEKLREKFIRFGCDVTFNLRDQGYTTDLIDPRTGYPLLSHPGL
IPHDDTAVAKALLNYPVIKNKCCVLVHPQWGTAVYPSVLLSSAPPEVILSVIKSIAPLHG
WIEPDN
>WP_061546085.1
MDKIKLATVWLGGCSGCHMSFLDLDEWLIDLATQVDVVYSPIADIKEYPEGVDVVLVEGA
IANEEHLELIHKIRQRTKTIISFGDCAVTGNVTAMRNLTGTANVSLQLAYIEGADVNQKI
PHSLGIVPPLIDRVVPVHNIVDVDIYLPGCPPSATRIRATLEPLLRGETPPMVGREMIKF
G
>WP_061546094.1
MASLLSTPTTSSFLTLTVAPAKVIRGTGVLRSSAISEISTLGNRPLIVTGKHTLNIYQES
LQTIFQSGDLDALATSYSPDCCEVSLKALRKAVKEHKANVIIGLGGGKSIDTAKLLGHQL
QLPVVTIPTSAATCAAWTALSNVYSASGAFLYDVPLCRCPDLLILDYDLIKTAPRETLVA
GIGDGIAKWYEASVSSGNMQDTLIIAAVQQARVLRDILLQKSAAALDSPGSQIWQEVVDC
SVMLAGVIGGLGGARCRTVAAHAVHNGLTHICGHGSIHGEKVAYGILVQLRLEEMLQGNQ
LAETARQQLLKFYTEIGLPQKLADLGLGNISLTQLQTAAEIALAPNSDIHRLPFSLSSEQ
LMAAMISTTAPVLRQRDSSSLTTQTTN
>WP_061546095.1
MESQGIVPLKSACGISYDSLAQLLVKQDFQAADLLTIQQMCEVAGTQAVRRKWLYFTEVE
NFPIQDLQTINSLWLAHSQGKFGFSVQRELWLGVGRNWDRLWPKIGWKQGEKWTRYPHEF
TWNLAAPRGHLPLSNQLRGVRVMASLLSHPAWTTPS
>WP_061546099.1
SALDPSYTEIVLLLQTVTFTFKKVLINMKDWIIVGGLSCGIICGISIVGVGGITGRIGVG
VLGSTTGAIMGASVVKSRGEGLIALGEKLTRTRGELERINEEKIGQEQQIENIRGEYGRK
QVELEHWQNKLKYIETEYGEKQDELRELQNRVLVINQQNPNLHELEELGRRIEASKIERA
ALEGQISGLNSQVESLDSKRLELNSIELQIVSQQADLNRLGARIEELTNQSQELEQRAAD

LRSLRISIEESTIDRNNLEARITQLNSQVESLESKRAELNSIESQIVFKQPDLNRLNARI
EELTNQSQELEQRAAELRSLRISIEESTIDRSNLEARITQLNSQVESLDSRRLELNSIEL
QIVAQQTELNRLNARIEELTNQSQELEQRAAELELLRFTYDGILSQRQDYEAKVSQLKPE
IDRLELEKQRILQTIEENQNNYQRQIELREKCRSLTLTIRDQESRLRELENEEHNSQTRN
HNLQTENDKLQQTKRELETTIANLRGEIREIENSGRVALEPLREKLWTNLPKQVRQLEGE
TQFIQKFITSLRNEGLSFSSRTIKAFHTSLKVQDISALVILAGISGTGKSELPQRYANYI
GAQLLTLAVQPRWDSPQDLQGFYNYLEKKFKPTQLMRGLYQYKDPEMSNRIVIVLLDEMN
LARVEYYFSEFLSKLESRRNHATYLEIDVGSLPIAESERRLEIPKAFLFVGTMNEDETTQ
TLSDKVLDRANVLTFGRPQNLKLRQRSGQQITVNQHSDNLVNNRDSSYVTYSDFKNWVRT
PVPESDVVKGVESYLNEANKVMEKMGHPFAHRVYQAIAQYVVNYPEVNGIDSESFKFALA
DQFGQKLLPKLRGVMVDEAHQQLQEMKEIIDEINDQPLIKAFDKAKEGRYGQFSWQGLVY
EDEEA
>WP_061546100.1
MRIYPSLFAYLNQHQSPLISDIFALEASHRPILVLELDEDELLSLPSWFIPISQGIYEVG
FPYLQPTDLHYDHDHHASLRYVLSLAIPGQGYNLSITDVLDLETIKEEVEIPEWEHRLGN
RLDRTASELVRLFIGMFTHISSGTTRINSEANILLSGLSQKLDSVNIEDANLPIVISLDN
KYQLHQKLELIGSRLRHQLTRKAELIPVGKIQEMDSYCLRDYIRRPGSTPEEKAGSKQEL
MGIKRYQDFNTPENKFLVYFARIFHLNCVQYEISNANQFRSQINKIRLVIDLFQQLPTVK
TIQNRGYQFTKPNYVLQQNTIYKSFYQAYLEYIRKKHEKENLWGFRNYLLADAIYIYLIV
GLLRLKGVNIDPTVGVSCKLIPDQGRYLSPDQNIKVRVFLQTRVCVFGLEKRSNIAMGDW
MLTLEIHQLDSMELNSNKLQFPIWVFWYLPSRDVINQMGDYLHNLRNQELENNGLFAGAI
VLYLQDHTQNYSLDDSPSYEKLFPNLRLVKLPENFTEQGFSQTVEIIFNTLIEVVGGFTL
>WP_061546103.1
MYLQSLELRNFRNYQEQKVEFTAPKTILVGNNAQGKSNLLESVELLATLRSHRLGKDSDF
IQEGQDMARINAILDRTTGISNLTLNLRRNGRRTVAINGETVRRQMDFLGILNAVEFSSL
DLELVRGSPAIRRSWLDTLLVQLEPVYAHILHQYNQVLRQRNAFLKTIQQKGIKNHDSEL
AIWDAQLVTTGTKVMRRRNRAIQRLGPIATHWHSSISGKMEKLEINYMPNVPILIDEQQE
LLQFFLARVQEHSAIESYRGTTLVGPHRDEIELVVNGTPARQYASQGQQRTLVLALKLAE
LQLIEEVVNDTPLLLLDDVLAELDLSRQNQLLDAIQDRFQTLITTTHLGAFDAQWLNSSQ
ILFVKCGSISQ
>WP_061546126.1
MPQVVLINPQIPPNTGNIARTCAATATELHLVGPLGFEITDRYLKRAGLDYWPHVKLNYH
ESYQTFLKVHQQRGGRLLGFSVRGNINYIQHEFHPDDWLLFGSETTGIPENILDICDSTL
YIPMGQPGVRSLNLSVSVAVALFESRRQLGYLK
>WP_061546140.1
MAKIIAFDEESRRALEKGINALADAVKITLGPRGRNVLLEKKFGTPQIVNDGITVAKEIE
LEDPLENTGARLIQEVASKTKDVAGDGTTTATVLAQALVKEGLKNVAAGTNPISLKRGID
KTVEALVKEIAKIAKPVEDSELDSPAASIAQVATVSAGNDEEVGQMLAEAMAKVTKDGVI
TVEESKSLTTELEVVEGMQIDRGYISPYFITNNDRMTVEFDNPRILIADKKISSIQDLVP
ILEKVARLGQPLLIIAEDVEGDALATLVVNKARGVLAVAAIKSPGFGERRKALLQDIAIL
TDGQMISEEIGLSLDTATLEMLGKARKVTIDKENTTIVSGTENKPEIQKRIAQIRKQLEE
TDSDYDAEKLQERIAKLAGGVAVIKVGAATETELKDRKLRIEDALNATKAAVEEGIVPGG
GTTLIHLVAKVDAIKDTLDGEEKIGAEIVQRSLEAPLRQIANNAGVEGSVIVSQVRNSDF
NIGYNAATGEFEDLIAAGIIDPAKVVRSSLQNAASIAGMVLTTEVLVVEKPEKKAPAAPD
PGMGGMGGMGGMGGMGGMGGMGMF
>WP_061546153.1
MNRLFLLPYSFYYCLFVFLPPTYAKNTAYEPINYSSKTKFATSCSQQNWENLTTQLMLDL
PSYTNRVTQRSRRLGLSVDLYPYVIVAGRSEFKSLPLSPNINTDSDYVSKGVEQLFFTTL
ERQYSRQKSMELQQFHWLFVTKTPSGWRVVMMFSQAGDYPLKSIISPPRNSSDGAIAQAV
KLWLRDCQAGSLRS
>WP_061546157.1
MPPVPVNPGNPLFPRDAGSLSGIVDQVSFNFLPGYNPTQARQTLQNTLGNTSAAFTNLFG

LYEVDSVTGAVNGLTPDKPGYAKAALNRMVPNFVVRAGGSGNGTTGDVIVSGGKIYAPFV
IANGGNYSGTIQEAINEFFKVNPNNSPATAQNYTTLPVAYFSFGAANPDGAAHIKSLANN
IFGFEDLPSNVGVSDYDFNDMVFSFG
>WP_061546160.1
MLTSLQNPLVKQIRKLHSAKERHKQQLFVLEGTHLIAEACSVNYPLETVCCTIDWQNSHS
ELWQNVCNACHRAEIVSQEVLAAMATTIQPDGVVAMVKRCEENPIPITGLALALETIQDP
GNLGTIIRTAAAVDASGLWLSNDSVDLDHPKVLRASAGQWFRLKKQVSHNLKDTVQDCQR
GGMQVVATLPTAELTYWQVDWSLPSLILLGNEGAGLSDDLATMANIQVKIPLSPQVESLN
VAISAALILYEAQRQKACGKNTN
>WP_061546170.1
MSNQERMDNMENQLIDIRLAVSALLETSVIYQRNFEVMQRNFDSVVIEIREMQSEIREMR
SDIREMQSEIREIQSEIREIQSEVREIQLDVRGLQTENRRILDVLQNVPPDKYE
>WP_061546173.1
MPRRQDIHKILLLGSGPIVIGQACEFDYSGTQACKALREEGFEVVLVNSNPATIMTDPET
ADRTYIEPLTPEMVAKVIAKERPDALLPTMGGQTALNIAVALAKNGVLEEYNVELIGAKL
PAIEKAEDRKLFNDAMEKIGVNVCPSGTASSLEESKAIAQRIGSYPLIIRPAFTMGGTGG
GIAYNKEEFEVMAQVGIDASPVSQILIDQSLLGWKEYELEVMRDLADNVVIICSIENLDP
MGIHTGDSITVAPAQTLTDKEYQRLRDMAIKIIREIGVETGGSNIQFAINPVNGDVVVIE
MNPRVSRSSALASKATGFPIAKMAAKLAVGYTLDEIKNDITKKTPASFEPTIDYVVTKIP
RFAFEKFPGSDPVLTTQMKSVGEAMAIGRTFNESFQKALRSLETGRAGWGADKAEKLPSG
EQVRAQLRTPNPERIFALRHAMQLGMSNEEIYELTAIDPWFLDKLHQILETEKFLKRTPL
QQLTKVQMYEVKRNGFSDRQIAFCTKTKEDEVRVYRKQLGVIPVYKTVDTCAAEFEAFTP
YYYSTYEEETEILPTDKPKVMILGGGPNRIGQGIEFDYCCCHAAYALKSANYETIMVNSN
PETVSTDYDTSDRLYFEPLTKEDVLNIIEAENPVGIIVQFGGQTPLKLAVPLQEYLQQSP
STVTRIWGTSPDSIDMAENRERFEKILEELKIAQPANGIARSYEDALIVAKRIGYPVVVR
PSYVLGGRAMEIVYSDSELERYMSFAVQVEPEHPILIDKFLENAIEVDVDAIADHQGRVV
IGGIMEHIEQAGIHSGDSACSLPSISLSPAVLNQIRTWTVELAKALSVVGLMNIQFAVVG
ASSYSPQVYILEANPRASRTVPFVSKATGVPLARLASLIMSGKTLEELNFTQEVIPQHIA
VKEAVLPFNKFPGTDTLLGPEMRSTGEVMGIDVDFGRAFAKAEMGAGEKLPLQGTVFVSM
SDRDKSLVVEVIKEFIQLGFKVIATQGTSEFLREQGLKIETILKLHEGRPHVLDAIKNRQ
IQLIINTPSGQEARTDGQLIRRTALGYKIPIITTIAGAKATVAAIRSLQNINLDVKAIQE
YSF
>WP_061546176.1
MSGTTGERPFSDIVTSIRYWVIHSITIPALFIAGWLFVSTGLAYDVFGTPRPNEYFTQVR
QEVPIVSNRYEAKKQVETFIAK
>WP_061546183.1
MSTVKKENDNGKTKLTADMVRTYLREIGRVPLLNREQEIIYGKQVQQMMILVEAKEALAK
ELGYEPSLSEWAIYVNQSENDLKHKISTGKRAKQKMIEANLRLVVAIAKKYQKRNMEFLD
LIQEGTLGLERGVEKFDPTMGYKFSTYAYWWIRQAITRAIAQQGRTIRLPIHITEKLNKI
KKVQRELAQKLGRSPSPTEIAKELELEPAQIREYLNIARQPISLDVRVGDNQDTELQEML
EDDGLSPEHYTTQEFLRHDLNNLLAELTPQQREVVTLRFGLEDGNEMSLAKVGDRLNISR
ERVRQLEHQALTHLRRRRSNVKEYVT
>WP_061546222.1
MGSTFGHLFRITTFGESHGGGVGVIIDGCPPKLEISPAEIQFELDRRRPGQSKITTPRKE
ADSCEILSGVFEGKTLGTPIAILVRNKDIRSQDYDEMSEKYRPSHADATYDAKYGFRNWQ
GGGRSSARETIGRVAAGAIAKKILLQVAGVEIIAYVKRIKDLEGTVDTNIVTLADVESNI
VRCPDPEIAPQMIELIEKTGRDGNSIGGVVECVVRNVPKGLGEPVFDKLEADLAKAVMSL
PASKGFEIGSGFAGTLLTGFEHNDEFYIDPHGDIRTVTNRSGGIQGGISNGENIILRVAF
KPTATIRKEQKTVTKEGEETILAGKGRHDPCVLPRAVPMVDAMVALVLCDHLLRHYAQCK
LL
>WP_061546228.1
MNQKRILITGASGCIGHYISETLIENTNHQLYLLVRNPSKLQVNTDMRPGVTILQGDMGE

ISSLGNLLKTIDTAVLTATSWGGNNIFDINVNKTIQLMNLLNPDRCEQVIYFSTASVLNH
QNQPLKEAGEIGTDYISSKYKCLHKIEDLEIFPKITTVFPTLVLGGDENKPYSHLTSGIH
EVTKYLDIIRFLKTDGSFHFIHGKDIAKVVEHLIDHPPKKGDPRRLVLGQAPLTINQAIE
EVCEYLNKKIYFRIPLSLALADVIIKVFRIQMATWDRFCMKYRHFTYNNPINPAIFDLPN
YCPTMTDVLKTSGISKG
>WP_061546243.1
MFHSILQQNLTQKKISRVSFFADFLLAGMVLGPSLAPFLAASNVFVLQIISNIIYFIGDH
VCPQPETGLELAPPYITSVCMRCYGTVTGLLITRVLYGLTQGRGVFWLHQYGWMGASVAT
ILMTAYPWELAAEVFGLWEFNNYVVTPFGLVTGLAWGLFAMPLLHSSKKTFESKY
>WP_061546269.1
MFLYLSKLLPLFFYPLGLASVSLVIALVSLWKRPKIAAVCIGTSLAILMIAGNGWVSKAL
VESLEWQNIPLEKIPEAEAIVVLGGATKPAAWPRSTVDLNESGDRVIYAAQLYGQKKAPL
IILSGGRIDWRGGGPPESADMAAILISLGIPPEVIIEEPNSLNTYENAVNVRKILESRGI
KKVLLVTSAMHTPRSLKIFQRQGVEVIPAPTDFIVSRSDLEELIATPKSTILNLFPRADN
LNDFTNALKEYIGYLVYALRGWL
>WP_061546276.1
MRTVDLFSGCGGLSLGFQNAGFEIVAAFDQWKAAVKVYRDNFHHPIYDTDLGTQAGLEFV
KDLKPQIIIGGPPCQDFSSAGKRDETLGRANLTISYANIVVATKPEWFVMENVERIIKSP
ILQEALFIFKQAGYGISYQVLDASLCGVPQSRKRFFLVGNQHSNDNFLNPYLHKNQANKP
MTLCEYFGDSLGIEYYYRHPRSYARRGIFSIYEPSPTIRGVNRPIPKGYKKHNGDPIEIS
DQVRPLTTKERSLIQTFPKDFILNGSKTDLEQIIGNAVPIKLAEYVANCINEYIKDRNQN
KVTTVRQLELIFD
>WP_061546278.1
MTQNPSKILKVEGLTIYQGKYLAIRDVSFELFPGTDTAIVGPNGAGKSTLVKGILDLIPR
TAGKIEVFGFPVSRLGNLRQLLGYVPQNFIFDRSFPISTSELVGLGISNNSSVNSWLYKL
GKFRQIRQQESTAIKVALQRTNSYHLRHKPLGTLSGGELKRVLLAYCLVSPRKLLVLDEA
FAGVDIQGAADFYALLNQLKLEEGWTILQVSHDIDMVNRHCDRVLCLNQTLVCSGKPEIA
LSPQNLLATYGPGFSRYQHHH
>WP_061546279.1
MKNFGQPSRIAKKILGWSSFVLFLSLIYGCERSQVNLPTEKSTSTVSSPATQTKIVATFL
PVYLFTKAIVGDVAKVDVLVKPGTDIHEYQSTPDNVKAIATADVLVKNGLGLEEFLEGTV
KNAKNPKLVEIDASKGIRVMDDGSPVEKIKGEKHDHKHDHAQGNPHVWLDPVLAKQQVIN
IRDGLIAADGQNKQTYETNAATYIQELDNLNREFQQTLDKRPNCTFITFHDAFPYMAKRY
NLKQVAVVELPEDKLAPGDIQAVINTVRNYKVKVLFSEAGIDNKLLTSLAQDLNLTLYSL
NSLETGERKPQYYFQAMRDNLKSLATGCQK
>WP_061546284.1
MINNPENSPKHNSRNNYLLEVENVHAGYIKDVDILQGINFRVGYGELVTVIGPNGAGKST
LAKTIFGLLTPHTGTITFGGEKINGLKCDQIVKKGMCYVPQIANVFPSLTIEENLEMGAF
VRNIPLKPLKDKIFTMFPRLGDRRLQRAGTLSGGERQMLAMGKALMLEPRLLLLDEPSAA
LSPILVTQVFEQIKQINQGGTAIVLVEQNARKALELAHTGCVLVSGKDEISGSGQELLQD
PKVGELYLGAGEKTVDIKPELK
>WP_061546309.1
MSIALKIEAVLYLKGQSLSLSEIAEYVGCDRYTIEEGIIELMDNYARRESALEIVETEGS
YSLQLRADFQDLVQTLIPVELGVGSLRTLAAIALNSPILQTDLINLRGSSAYPHVAELVE
LGFIRKKKDPNSRSYSLQVTSKFHQYFQIDELPIDQQKIKRKEI
>WP_061546333.1
MQRSFCQKILVSIAVFVFGASIWVMNSPLALAYENPDLLPDTFTPVVDLAKTLPDPQEEK
LVKELEQFEVDTGWKLRVLTQYDRTPGRAVIKYWGLDDKSILLVADARGGNILSFSVGDA
VYELLPRTFWIELQTRFGNLYFVRENGEDQAILQALNSVKGCLAQGGCNVVPGLPKEQWI
LTLITSAIGGIICGFAAQPRNDKQVFAWQWALIFSPLWGILFIAFGIAPVITRTSDWVPL
VRNISAFLIGLLVAYLSPIFSRPVSRNES
>WP_061546360.1

MYLTPKSGLFLGGSCITAIAAVGSVFELSYGQPNFGFQTTAIILAISVPVTILLFVAAVK
DARANIK
>WP_061546362.1
MIGHLILRLTIWFLLTSDFSTVNIIIGVAIAFLLPRGYSSRARFTEWLRVFGQVLVAIVI
AFKEAFEIILFPHHREEIIRENVKHKHSYLLIFMDIFLITFTPKTIVFNHNEQGFYEVHQ
IKPGGKE
>WP_061546364.1
MFLMSKATEEKSVNLDLSTPLQLIGRSHEFERITNILLNDRDLLITGVPGSGRRTLVKIA
AQEVGALVLEIDCIRAIDGERLLQLFTETINHNWEAHKIEIWVEQNGRELFIFNVETRLK
LSHGLNDKKLWQAFVMLLDLLQNIANDLNRRVVLILQSFPHIRSWDRHNVWESTLRQEIN
SHPDVSYVILATIAETSHHQDDQKYAMEKIELAPLERDIMAVWAREVLEKQNLKFDRHSQ
ALTIFLDAVQGSIGDAMALIRRLSSLQHEQGLIKEEKVRQVLEAILRDLSITYESLLMLL
PGNQIHLLECLAIDPTDKPQSKEYIQKHGLSRGGTLQGSLTGLQTKGLIYSAQQGYKLAL
PLLALWLKQRLN
>WP_061546381.1
MKELNLAESNHSETESDVNPVNYWQKLVSRAVDKGKQSVQQWFSINDGQVAEILATVSSQ
LPTTEALLIGKPQTGKSSIVRGFTGVSPEIIGQGFRPHTQNTERYIYPNNDLPLIIFTDT
VGLGDTDKDTEVIIQEIIKDLNTGNKRARVFILTVKINDFATDTLKNIAQKLRQQYTHIP
CLLAVTCLHEIYPPDMENHPDYPPNFPEINRAFDEIKANFSGLYDRVTLVDFTLEEDGYS
PVFYGLESFRDSLTSLLPEAEAKTVYQLLDEQMGDKLGNIYRDTARRYILPFSIMATTLA
AVPLPFTTMPVLTALQVSMVGILGKLYGQTLTPSQAGGIVSTIAGGFLAQAIGRELVKFI
PGFGTVIAASWAGAYTWSLGEAACVYFGDLMGGKKPDLQTIQNVMQQTFQSKENTQKEE
>WP_061546384.1
MSQELVNSLFINRVAVLATMHHKENVIAPLLQKQLGLEVVVPANFNTDQFGTFTREIKRP
DTQIVTAKLKAKKAMEMTGETIGIASEGSFVPHPNFPYIYANREIVLLLDQQNDLQIIGE
VFSTETNFNHLVINSFEAAQKFADKVGFPDHGLVIWLESSNGQDPEIIKGITTQEQLYES
VNFALNNSPHGKLHIETDMRALYNPTRMNNIAKATQDLVEKIYSCCPQCSTPGFSITDRI
QGLPCESCGQPTSLTMGVIYQCQNCNFIEQKLYPDGKFFADPGLCPYCNP
>WP_061546396.1
MSNTLLIAGTDTGAGKTLLTCSIAAYWLKYFPQSRLGIMKPLQSGIGDKETYQSLFNLDQ
SEEEITPLYFQAPLAPPIAAAKENRTIDLGLVWRTFLKLQRERDFLLVESLGGLGSPVTD
ELTVADLAGEWRLPTVLVVPVRLGAIAQAVANAALARQAKINLLGIVLNCNQPRSEEEMS
DLTPTHLIESLTNFPVLGCLPYLEDVNDLEALAELGSNLTIPVWSRLQVD
>WP_061546398.1
MLTTKPQVSLIRATSYQQDALKESLTTLLDPLGGIGAFVKPGNRVLLKPNLLTGARPTKE
CTTRPELIRAVAQQVIEAGGKPFLGDSPAFGTAKGVARANGLLPILEELNIPIVEFRGQR
YTCPEGSGKSYEFNHLLLSKEAMETDVIINLPKVKSHMQLTVTMGVKNLFGCVPGKMKAW
WHMEAGKDAKKFGEMLVETARAINPSLTILDGIIGHEGNGPSGGEPRELGVLGASENVFA
LDRAVLEILKVDPMQVPTMVAAQNLGICPELTDIKFPHLSPDLLQIEDWRLPDNLMPIDF
AMPRVIKSTFKHLYIRFIKEPMGAYSRG
>WP_061546401.1
MDLSLIPAQPKPGVINVLIEIAGGSQNKYEFDKELNAFALDRVLYSSVKYPYDYGFIPNT
LAEDGDPLDGMVIMDEPTFPGCVIPARPIGFLEMIDGGERDEKILCVPVKDPRYAHVNSL
KDIPPHRLEEIAEFFRSYKNLEKKVTQILGWHDADKVAPLVQKCVAATK
>WP_061546436.1
MHIGLYCPVETGHLNTMLPIGEALLIKGHRVTFVGIADAQAKVEAIGIDFFPVAMESLPQ
GSIDKIFETLREREGLSAPLYTIGIFRQVSYMVLQDGAIACKDLKLDGMVIDQTSTEAVA
IAKILNIPYVTLCSALPFNQEAAIPPVFTTWQYGNSWFFKLRNQLTNYVGSLIGKIIHEP
AKQFLKNNQLPIPRTFDSSLAIICHQPKSLEYPRQTLPDNFHFTGPFHSTTSRKFVDFPW
EKLSDKPLIYASMGTLQNRRKNIFQTIAAACENLDIQLVISLGGSANPEDLPPLPGHPLV
VRYAPQIELLQRASLTITHGGMNTTLESLTHGVPLVAIPITNDQPGIAARIKWSGCGEFL
ELQQLTVQKLREKVKRVLQVPSYRDRAQQFQQEINHSGGINQAIAIIEQAILTQQPVVDW

TPVIFDNFND
>WP_061546440.1
MNKYFLRGKLLLVCGAGIAFIGLFAPQPSFSNTASTSQPLNSPDNTNPLANDSGLDMFNI
IHRLNFGPLNWDSNQQNQQLEDAAAAFKARQNQILQNQQPSKVKGEENRSGANTDGSNTE
RK
>WP_061546442.1
MDIKRGFIGTIGNTPLIRLNSFSEETGCEILGKAEFLNPGGSVKDRAALYIIEDAEKKGL
LGPGGTVVEGTAGNTGIGLAHICNAKGYKCLIIIPNTQSQEKIDALTTLGAEVRPVPAVP
YKDPNNYVRLSGRIAQEMSNAIWANQFDNLANRCAHYETTGEEIWQQTGGKIDGWVAATG
TGGTYAGVAMCLKEKNSNIRCVVADPLGSGLYSYIKTGEISIEGNSITEGIGNSRITANM
KGVPIDDAIQINDQEALRVVYQLLKRDGLLMGGSTGINVGAAVALARELGPGHTIVTILC
DSGSRYQSRIFNPEWLATKGLVMG
>WP_061546453.1
MQSLVIRSFTTLADSGNYEKVRENFERSLQIHPNDTIALNSYGKALADSGNYEKASEIFE
RSLQIQPDNYIYFTYGKCLEELGKYENAISQLKLITVDKLPQYHKNVVHISLGRVYYFLK
LYIEGDKHFNIAIANSDDREKSILSSARSILAHSPHNQTAVKMLQQIAEESPRYAQAMEM
LTLNLTEEEYFKTVPDDSNNLKDAQILNRAIYHKIANEITILKSIAYKILRVSSIKDALL
ESIISNIEEILQEINHRRSLEKSQIEQIEIIANNEYGKILEIIAKTAHDISDFVNNELAI
VESKTRRAIKKFTGNDPQFSQFNKLLTQLEFTQDALNDLKAINEGIRIKNRQFKVKKIFE
TWEFNTQIDNAQISLNIENGDSDFNGDEEKIKSILNELVENSLKHNPDNSDLQIKINSQD
VTNPPGIRGANIPGSKKYLYIEFIDNGLGIAPDKKDWVFQPLNTTATGTYSSGLGLFIIR
KTLTQMGGYIREVGVNGVKFEIYIPYPDQNI
>WP_061546454.1
MAQSQAKISQGQVIERIPLLASADPQWFAVYIYCQPNINYQLGDYQQHFPLMSVIKPFSF
LYLLEHLGKEQVLQSVGTSPSSMAFNSLEQLISDHGYPRNPMINSGAITVADKLKTIGQT
RLSSNYDGVSDDLCEHFIKWLNQLGDTQLYLDMNMLNSVRSTRSQLNLAIAQVLCQYGYI
ENIESAIDIYEQICCISGTVVDLGRLGKLLADDQGLISPQHRLLVNDVMLTCGLYQASSH
YTQKIGLPMKSGISGALLVVIPNQGAIAIYSPALDPIGNSIAGLSLVETLCSNLSS
>WP_061546459.1
MANLKAIRDRIQSVKNTKKITEAMRLVAAARVRRAQEQVIATRPFADRLAQVLYGLQTRL
KFEDVDLPLLKKREVKSVGLLVVSGDRGLCGGYNSNVIRRAENRAKELKAQGLDVTFVIV
GRKANQYFQRRGYKIDATYSGLEQIPTATEATNIADELLSLFLSEKVDRIELVYTRFVSL
VSSRPVVQTLLPLDTQGFETSDDEIFRLTTRGGKFEVEREKVTSTVAPLPRDMIFEQDPV
QILDSLLPLYLSNQLLRALQESAASELAARMTAMSNASDNAGQLISSLTLSYNKARQAAI
TQELLEVVGGAEALG
>WP_061546464.1
MTTRQTPNRKLWMAAIKPPMYSVAIMPIWVGSCVAFAQTQIFHGVPFTIFATAAILILAW
ENITNDVFDAETGIDENKHHSLVTLTGNKQLMFWLGNLCLVLGLSGIIAIAWLQRDPTVV
GLILLCCSLGYVYQGPPFRLGYKGWGEVLCFFAFGPVGMTAAYYSQTQNWSPTNLAASVI
VGIVTSLVLFCSHFHQVKDDIAAGKRSPIARLGTKRGAEVLVWFTASIYVICLLLVVLGI
FPFWTLLSWLSLPFGVKLCGHVWKNHHFPEKVSNCKFIAIGVHFWSCLLLGVGFILG
>WP_061546465.1
MEYRFSFRQFSQKFNHAVITNHGVWDMREGIIIRLVDRTGKVGWGEISPIAWFGSETLEQ
AREFCSQLPEIITPEMILDIPAHLPACQFAFESARENFSNSMPVFKNQHLGDESLIERLI
SKKMKYSALLPRGEAAVQGWKNLWQKGYETFKIKIAVDDITRELEILHLLVGQLPESAKI
RLDANGGLNYQQAKLWLEVCDQFSQKIEFIEQPLGIDRLEEMLQLSQAYLTKIALDESVA
TWQKLESCYQMGWRGVFVIKPAILGSPSRLRGFCQNHTIDLVFSSVFETPIGREAALKLA
GEISHGISNLSGNWRSLGFGIDHYFALESTNWPEILWNPIY
>WP_061546481.1
MIMLPQLQNAFLRRNVLKVISGLNNFNPESVAATVKAAGLGGATFVDIAADQRLIHLAKS
LTSLPICVSAVEPEQFVLAVSAGADLIEIGNFDSFYAQGRKFEADEVLSLTHKTRALLPE
ITLSVTVPHILTLDQQVQLAEALVEAGADIIQTEGGTSIQPTHPGTLGLIEKAAPTLAAT

FEISRAVSIPVLCASGISNVTAPLAIAAGAAGVGVGSAINQLNSEIAMIASVRSLVESLS
HVPLIPRF
>WP_061546494.1
MIINPDLPKRGDIWLVNFDPTIGAEIKKVRPAVVISSDAIGKLPIKLITPITDWKTYFSA
NFWHVKSEPNSINGLNKASAIDTLQLRGVDLQRFIRKLGSVSEIKMLEIIASIATVIEFE
V
>WP_061546495.1
MATLLSESRSQTGPVISWEALPDDFQLEDEPVDNTGQPLLAGALSESLEIGGFIQPQMLI
AVNFGLCATLNEQFIAKAPDWLYIPSVKEILPGRKSYTPHLEGDVPAVVMEFLSDKEGGE
YSFKRTYPPGKWFFYEQILQVPVYIIFDPDGGLIEYYELKNERYELKQPDENGRHWIESM
ELFLGTWQGTKEGRTGYWLRWWEETGNLLPWALELIEQERQQVEQERQLAEQERQRAEQE
RQLAEQERQEKEREHQRAEQERQRAEQERQLAEQERQEKERLIAYLRSQGIDPNNLPNHT
E
>WP_061546497.1
MTTLLSESRSQTGPVISWEALPDDFQLEDEPVDNTGQPLLAGALSESLEISGFIQPQMLI
AVNFGLCATLNEQFIAKAPDWLYIPSVKEILPGRKSYTPHLEGDVPAVVMEFLSDKDGGE
YSFKRTYPPGKWFFYEQILQVPVYIIFDPDGGLLEYYELKNRRYELKQPDENGRHWIESM
ELFLGTWQGTKEGRTGYWLRWWEETGNLLPWALELIEQERQRAEQEHQRAEQERQEKEML
IAYLRSQGIDPNNLPNHAE
>WP_061546504.1
MYCDYLVQILTAKVYDVAQETPLEYAPNLSNRIHNKLLLKREDMQSVFSFKLRGAYNKMA
NLPQDLLKQGVIAASAGNHAQGVALSASRLGAKAMIVMPVTTPLVKVNAVKSRGGEVILY
GNTYDDAYAYARKLEAEKGLTFIHPFDDPYVIAGQGTIGMEILRQYQQPIDAIFVAIGGG
GLISGIGAYVKRLRPEIKIIGVEPVDADAMYQSLKAGHRVRLPQVGLFADGVAVKEVGEE
TFRLCQEYVDEIMLVDTDATCAAIKDVFEDTRSILEPAGALSIAAAKYYAEREQIQGKTL
VSVACGANMNFDRLRFVAERAEFGERREAIFAVNIPEKPGSLKQFCECLGDRNLTEFNYR
IADDREAHIFVGVQIENRADAAKIVEKFELHGLKTIDLTDDELTKLHLRHMVGGHSSLAH
HELLYRFEFPERPGALMKFVGSMSPDWNISLFHYRNNGADYGRIVVGMQVPPGEMQEWRE
FLDSLGYCYWEETQNPAYKLFLA
>WP_061546510.1
MTGKEILPEIEQFRSLQSTLGERWKPSEIFDNSETDILIIPSLSIDQRELEKVPGCEHYE
ERLLFSLIRLRNPRNRLIYVTSMPIHPSIIDYYLQLLPGIPFSHARHRLLMLSTYDSSLK
PLSQKILERPRLIERIRQALQIDKAFISCYNSTFWEAELSLKLNIPLYAAAPDLQIWGTK
SGSREIFAQTGVHHPDGSKLVWNGKELAREVAKLWERQPHLKKIVVKLNQGISGKGNAIL
NLLPLTDFAPGKTNHVKRAESIEKSFVNLRFQAQQENWINFSQLILEMGAIAESFIEGEI
KYSPSVQGRITPDGTVEILSTHDQILGGPDGQIYLGCRFPANENYRVELQNIGLAVGKKL
SEKGALERFGVDFVVVDQGNGKWDIQAIEINLRKGGTTHPFMTLKLLTNGSYDLSTGLFY
SQQGKPKYYVATDNLQKDSYRGLLPSDLMDIIAHHRLHFDSGTETGTVFHLMGCISQFGK
LGLTSIGNSPEQAEEIHNRVIHVLDQETNNQINHYSLFSNYAFPIAEDTFGY
>WP_061546558.1
MDYRDAGVDVEAGRAFVGQIRNLVHSTFRPEVLGGLGGFSGCFQLPTGYHEPVLVSGTDG
VGTKLKIAQILNCHHTVGIDLVAMCVNDVLTSGAEPLFFLDYVATGKLEGEQLTQVVSGI
AQGCKLAGAALLGGETAEMPGFYQVGEYDLAGFCVGIVEKSKMLDGSQVQIGDLAIGLAS
SGIHSNGLSLVRKIVSDGGFAWDHTPELFHGKTLGSTFLTPTRIYVKSVLSALHQGLEIH
GMAHITGGGLPENLPRCLGANQSVKVHPASWPIPPVFKWLADFGSVGTEAMYNTFNMGLG
FVLILPPTQAQQAITHFQSQHIPAFTIGEVVGGSGELIALFDS
>WP_061546582.1
MSDLILFWHRRDLRTSDNTGLGVAREKTKKVVGVFCLDPNILGQDDIAPARITYMIGCLK
SLESLYLQAGSQLLILHDNPVRAIPNLAEALQAKAVFWNWDVEPYAQIRDRDVTLALKTR
GIETLEKNWDQLLHSPETILSGTRTPYTVYTPFWKKWHSQPKLEPIEILANCENLTAREE
EIAEKSGVIKLPTARDLGFIWDGELILEPGEVAAWERLTDFLDCAIDEYEEKRNYPAIDG
TSLLSPALKFGVIGIRTIWQKTQELLAISNSQEFSNGIITWQKELAWREFYQHAMYHFPE

LAEGAYRDVFKNFPWSDNQDHFQAWCEGKTGYPIVDAAMRQLNKTGWMHNRCRMIVASFL
TKDLIINPQWGEKYFMQKLIDGDLSANNGGWQWSASSGMDPKPLRIFNPSSQAQKFDGDG
EYIRHWVGELASVDTEYLIGGNITPLERQGLSYPPPIVDHKKQQALFKQLYQQQKVAFG
>WP_061546602.1
MEPLEPLQHFLPQVWFFILGLFLFLYILLDGFDLGVGILSLTASSEERRSILMTSLGNVW
DANETWLILMGGSLFGAFPLAYATILNALYLPAVVMVVGLILRAVSFEFRENADNKLVWN
IAFGVGSFLAALGQGFALGSVFEGIKVDQAGHFAGGVWDWLTWRSVVVALTLIQGYVLIG
STYLILKTTGDLQKTYYKTATIATWTTLVGALFITISTPIISEEVRTQLFTAPLIYIFGS
IPVLGILFIGLLLRSLYQRQENTPLVYTFLVFCLSFIGLGFVIFPNIIPPSVTIYQAAAA
PSSLVFMLTFIGFLIPVVLFYNVYNYVVFRGKIINQE
>WP_061546613.1
MHESRKWPFVSRLLKISEFIDKYTDKLGWFCNWLVLITIGVGFFNVLARYLGRFVGIQLS
SNALLELQWYLFSMTFLLGFAYILRHGDNVRVDFLYTNMSNRKRALIDFWGTVLFLIPFC
VIGIWVTFNPVLQSWGRLSDGNWGSWEVSSDANGLPRAPIKTMLPLGLLFLLLQGISQAI
KYLAILLGYEQVSEKVQLETSENIKIE
>WP_061546623.1
MGIILLFGLLLGILSGLLGSTLLIVAAIFLTRMQLTTHIQVWRVFNPTIGLLTPLFLGFA
YLFTRYRLVWDSSDGTPDYSNWFQNLYVSGFVYTVAPGLAALFAFVAVLFLSPRK
>WP_061546635.1
MKKLYDHIQPDPVRLEIFKNLYQFIAEQMGIVLQNTALSVNIKERLDFSCAIFDSSGLLV
ANAPHIPVHLGSMSESVRCLINDLGATIKPGNVYLSNNPYNGGTHLPDVTAITPIFLPPF
LNMFLKTGQELPLFFVASRGHQADIGGITPGSMPPHSTDITQEGIIFDNFLLVQEGELQE
TSVRSYLLNHPYPSRNPDQNIADFKAQIAANARGSQELIKMVEHYGLETVKVYMQFVQDN
AEESVRRTINVLKNGSFIYPMDSGAKIQVQVTIDRENRTAKIDFTGTSPQLKSNFNAPKA
VTQAAVLYVFRTLVDDNIPLNAGCLKPLEIIIPQGSMLNPTYPAAVVAGNVETSQTIVDA
LYGALGVMAASQGTMNNFTFGNEKYQYYETICGGSGAGMDFHGTDAVHTHMTNSRLTDPE
VLETRYPVIVESFSIRENSGGKGKYSGGNGVVRKIKFLESMTANILSGHRLVPPFGLHGG
APGKVGNNWIQRENGIPEVLDSTATVQMQPGDIFVIETPGGGGFQEQ
>WP_061546670.1
MQRYLKVIRLFWSAAIAAEMEYRLNFIIAALSSLGNLLGGIFGLFLFYRTGYTFSGWSWD
SALVVLGVFTLLQGLWATFLAPNLNRIVRHVQEGTLDFVLLKPIRSQFWLSTHILSLWGL
PDLLFGLIIIGYAGKNLGLGLDDYFWGILPLGCSLVILYSLWFMLGSTSIWFTKVYNTTE
VLRGLLEAGRYPMSAYPMGYRVFFTFVVPVSFLTTVPAQAMLGQIQVSWLISAVFLAMFL
FFLSTWFWRFALRFYTSASS
>WP_061546699.1
MFFKLGPNRQFPPGHLDLIPSWFVVYGNLDEINKSRENEVNNLLKRDRIVRNLEVFQDII
VISLCVALFCVILLRFISLYITLIK
>WP_061546702.1
MNFTTNKPQKFTTHYSQKLTEYVTSLNWSKSGHKLAVTSASGEVIIWENQTITNLQTSTG
KSLDCGGFSADDQYLAVGGQDGNVKIWREKELIQTLANAPAWIDKLAWNHTNNLLAFSLG
RYVQVWDVDARELVVTLNFENSSILGIDWRQDGKYLAISGYKGVKVWNRENWDEEPYILS
TDTASTGVVWSNDGKYLASANMDPSITVLEWENPDPWLMRGFPGKIRQLAWSNRTSDTGD
PILACSSVEGVVMWHKSVDESVGWESTILTNHFDIITAIAHPPHSLNYRLPNNELILASA
GADGWLCLWDENFQVWEILSGVTEGFSTLAWQPQGKFLAAGGDQGELIIWSSNNSQL
>WP_061546709.1
MTIQQTELLYLPPDLKPELLPRHVAIIMDGNGRWAKQRGLHRIKGHEAGAKQVKDLVRCC
VQWGIETLTVYAFSTENWNRPLREVNSLMALMRRFLREELREMHEKNIKIRFMGDLTKLP
QSLQREISHSTLLTCDNTGLNFYIATNYGGRQEILQACQAIAGRVQTGDLKADQISQELF
ESHLYTSGISDPDLLIRTSGEMRLSNFLLWQMAYGEIYIADTLWPDFDRGQFHLALLAYQ
QRERRFGAV
>WP_061546724.1
MQAEPKPGTLYIVGTPIGNLEDMTFRAVRILQAVDMIAAEDTRHTGKLLQHFQVHTPQIS

YHEHNRTGRIPEILTYLHYGKAIALVSDAGMPGVSDPGHELITACVAAGIDVVPIPGATA
VITALSVSGLATSKFVFDGFLPAKRQHRREYLETLLMETRTLVFYESPHRLRETLEDLGE
ILGGSRTIVMGRELTKLYEEIWRGDIKDAIAYYQGKDPQGEYTLVLGGASPSQPEITEAQ
LRAELLEIIKQGVSRSQASRQLAQETSISRRYLYQLALSIDQESVFETGNMRMDIIDKLR
TDQE
>WP_061546733.1
MQTVTLGDRGPSVSALGMGTWAWGDTLFWAYGKSFGESEVAAAFQASLAAGITFFDTAEI
YGFGESERLLGQFCQQTQQPVQIATKYFPLPWRWNRVAIVDALTASLDRLRMSRICLYQI
HWPLEFLLKTQDFMEVLAAEVKKGRIQAVGVSNYGAKQMTLAHEYLAAKGIPLATNQVPY
SLLTRQIESNGILDQARQLGVTILAYSPLAQGLLTGKYTPETAPSLQGARRLDPRFSPQG
LKKLAPLISALQQIGEKYDKTPAQVALNWLIAQGNVIPIPGAKNADQAKQNAGALGWLLS
PEEVESLGTIV
>WP_061546736.1
MGKVYLVGAGPGDPGLMTLKGKGLLERANVVVYDALVSPQVLDMISPHAEKIDAGKRMGR
HSLLQEETTQLLMEKAQEHEIVVRLKGGDPFIFGRGGEEMAELIAGGISVEVVPGITAGI
AAPAYAGIPLTHRLYSSSVTFVTGHEAMGKYRPAVNWQAIAQSSETIVIYMGIHNLPYIL
EQLMGVGMSSQTPIALVRWGTRPEQEELIGELGTIIQQVAKTGFSAPAIAVIGSVVKMHV
DFSPSA
>WP_061546737.1
MSTTIPIIPINRYRFFQKIQPLSILVKIVNNSTSGCLQVFSPSGTWSIYLQEGNLVYASR
SENIREPLYRNLQRLSWQNSNLTGINEKLGDFVEQCVQSQTISHPDYLAICWLVNEKHIS
LVEAGILIEQLALEFLESFFQIDQGSYEFIPQSFLDPLPKFCHLNVNSLVQKYKSEIHFP
PEESALDPQNLSPTYTIACASQNIVLLNHIRRLLNQTIFNIIDITDLDVMEVDAIRPDMI
ILDATMFKVSSYDIGLLLRKQFSLKDIPIILITKKNNLTNKIITRLVGATACLGKPFNQD
ELMKVIFENIN
>WP_061546747.1
MINIEQFRQEIEDWIINVVSIPNPLTGNFPPCPYAKAAWLNNRVSVRWFHGPELPELLME
QIRTWNNDFEMVIFGCDPQNLDAQRLERYITKANYVLPEYDLVALGSHPDKQYVGDDAEN
VNNVIITHPKYVLASVQSFSQLQEASDELLRLGYFQYWSAEKLAEMKSERASHNLSSIQR
KNSYRIIPTNH
>WP_061546750.1
MRDAVTTLINSYDLAGKYLDRNALDSLRSYFDSGTSRVQAATAINANAAAIVKQAGSKLF
EELPELIRPGGNAYTTRRYAACLRDMDYYLRYATYALIAANMNVLDERVLQGLRETYNSL
DVPIGSTVRGIQIMKDLAKEQAIAAGVANATFVDEPFDYITRELSEQNI
>WP_061546769.1
MGWGCISPVNILRVKPTKSFISPCLIGIFLSVVVGGCGDRGTDSSSKKDVEITLVSFAVT
KTAHEAIIPKFVEKWQQEHGQKVIFKQSYGGSGSQTRAVIDGLPADVVHLALGLDTKKIE
KAGLIQPGWEKEAPNNSIVSQSVAALVTRPGNPKNINTWADLSRDDVKLITADPKTSGVA
RWNFLSLWNSVIKTDGDEAKAKEFVSQVYKNVPILTKDAREATDAFFKQGQGDALINYEN
EIILAQENGLKVNYTIPDVNISIDNPIAVVDRNVDKHNNREVVEAFVQFLFTPQAQEEFA
KVGFRPVNNTVAKSKTFVDKYPPVKTLSTVKDLGGWGSVQKKFFDDGALFDQIQAQGK
>WP_061546770.1
MTLSTPTDVSNSNKPGFARGLLNSATRLSWTWRITIFYLLVMLFLPVLAMFLKAGTEPPT
KFWQIATSEIALATYNVTFVTSLLAAAVNGVFGTLVAWVLVRYDFPLKRIVDATVDLPFA
LPTSVAGLTLATVYSDNGWLGSLLAPWGIKVSFTRLGVGIAMVFISLPFVVRTVQPVLQE
MEKDVEEAAWSLGASPWQTFMKVILPPLLPTILTGVALGFSRAVGEYGSTVIISSNTPFQ
DLIAPVLIFQRLEQYDYSGATVIGVVLLLISLVLLVGINLLQAWARRYDNK
>WP_061546771.1
MTTNKSRQKSQYQKQQNWVPSVLIAIAVGYLFLVQYIPALNVFVQAFIKGVEPFWNNLTK
TEFLHAAWLTLLLAVIAVPINAVFGLCAAWAIARHKFPGRAIVLSIIDLPFSISPVVAGL
MIVLLYGRQGWFGGWLQSHDIQIIFAFPGMVLATCFVSMPFVAREVIPVLEEFGKDQEEA
AKTLGASEWQTFWRVTLPSIRWGLLYGLILTNARSMGEFGAVSVVSGNIANTTQSLPLFV

EDAYKQYETEAAFSAAVLLALLAVVTLVLKEILERKTRIKDDGEN
>WP_061546812.1
MTKITNQALNSALNQLWVFSLSSDFWEVFDAAFGTEYNRKNAEILRSQWQIGDFSQLPEI
EILDSSILGSANGAYSSSENRIYLSSNLMENGTSSRIREVLIEEIGHFVDSRINQIDTPG
DEGEYFAGLLVGESLNEAQLSVLKAEIDSAIILLNGVQLTVETATPNTTDNSRLIYTPSG
DGGLKVRLDKFGGFGYGGSGEWGALYDPLPVGTQNKGEAQTTYYSVVALGIINNGETTSR
KLLQNLGNNASFESSSNTTVNSTFSTDGLKFKLRQSVQDTFADQNQSRTGSRLDQTYTIT
NTTDQTINFDLIRYVDGDLDFDGSIYDGGGRIVRNAEEILFETDKGGTGQSDTTFFGITS
VGGNIPTTNRWELNEFDELNRLNDPMRKLKDKIVLNDTNADQFIDEGREYDVTLELRNEF
SLAAGGIATYTTSTLFGSGEPTQLDITPPTGKVRDLPRITTDRNITVSWDANDTQSGVKN
YDVFVSVNSGNFTEWLTDVITTSAVYPGQVGNTYAFYSLATDNQGNQQDPNTAPRTSTQV
VPIFTPNVVTPKLNISTGLIFTAQSNGNITLDTNRGSATPDEVTSVQNGTKSNFNHIIGL
YEVLNSQGEIKDDQGKTLKPEDANYALHALTTARVKNFTVQAGGNDTPSTATQLGSGVSV
FAGKSYAPFVIANGGTYFGPGDQGIENFVAAEQGDINRFSSKDQYVRNLVADEGKDGDIF
NNAPRFVQEPVAYFSFGVANPDKAPHFRSYGNGVYGFEDLPANFTQYSNNDFNDGVFALT
LSI
>WP_061546821.1
MPLGRELPQLLKQRLFHKGRKFDFEVSRLRLPNKSEGEWECIRHPGGALAIPVTDDGKLI
LVRQYRFAVQGRLLEFPAGTVEPNEKPLITVQREIQEEIGYKAHQWDKLGEFFLAPGYSD
EIIYAFLARDLEKLETPPQQDEDEDIETVFLSPAQLEAAIDRGEPVDAKTVTSFFLARSF
LMLKP
>WP_061546825.1
MVNQNLTAKEIGFTHEDFAALLDKYDYHFSPGDIVPGTVFSIEPRGALIDIGAKTAAYIP
IQEMSINRVDAPEEVLQSNETREFFILTDENEDGQLTLSIRRIEYMRAWERVRQLQKEDA
TVRSDVFATNRGGALVRIEGLRGFIPGSHISTRKPKEELVGEQLPLKFLEVDEERNRLVL
SHRRALVERKMNRLEVGEVVIGTVRGIKPYGAFIDIGGVSGLLHISEISQEHIDTPHSVF
NVNDEVKVMIIDLDAERGRISLSTKQLEPEPGDMIKNRDLVYDKAEEMAAKYREQMLAKQ
QGIAVAPVEGAETAPVEELELVAETEVPPAIEVEEEIPVAVEE
>WP_061546862.1
MTLRVAVVGSGPAGSSAAETLAKAGIETYLFERKLDNAKPCGGAIPLCMVSEFDLPADII
DRRVRNMKMISPSNREVDINLTNQEEYIGMCRREVLDGFLRNRAAKLGANLINATVHKLD
IPNNNSDPYTIHYVDHNEGGSVGVAKTLKVDLIIGADGANSRVAKEMDAGDYNYAIAFQE
RIRLPEDKMAYYNNLAEMYVGDDVSTDFYAWIFPKYDHVAVGTGTMHVNKASIKQLQAGI
RARAVEKLAGGTIIKVEAHPIPEHPRPRRVVGRIALVGDAAGYVTKSSGEGIYFAAKSGR
MCSETIVEVSNSGARIPSERELKLYIKRWDKKYGLTYKVLDLLQTVFYRSDATREAFVEM
CDDMDVQKLTFDSYLYKTVVPANPITQLKITAKTIGSLLRGNALAP
>WP_061546874.1
MIMPSPINLGTILQNRYHIIRLLGQGGFGRTYLAEDQGRFNELCAIKELVILEPDSYEGK
KAQELFDREASILYQIDHPQIPKFREKFAQDQRLFLVQDFVGGKTYHTILNERRTQGQSF
TQAEVLYLLQSLLPVLEYIHKAKIIHRDISPDNLILRSTDQKPVLIDFGVVKEVATRLSN
SSIYQATTVGKPGYSPIEQVQTGKAYPNSDLYALAVTAIVLLTAKEPADLFDETTFVWKW
QHWVQVSPKFAQVINRMLNRIPGDRYQTAKEVLLDLNNLEVSSPLNPRDPNLSYLPTVAI
SHPSPTPTNSPEPVISPHTNSSILDNTLSMVAIGAFVVILTGFSSWSLVNYFRGQRLSPL
LSTTPQNFDSPVIPRSTPNSTLTPTPSKMEPRNLAWDLSNNANEEGIIKFGEVIEYSFRG
IPGQKLTVAVNEESGVLLTILTADGQPLSTDAQQVTSYEGVLTSRGRLTIQLTLSTTVSE
STYSLSVALENPIKRAPIPPRNT
>WP_061546875.1
MGWGDPSSPNSHKLNIKHLAPLFKGGWGDPKSLNLLWMYLFKTSRKKVSFLLVVLEHRLR
SHGSYPSAFPKHSQRVCKALALARSPFLPFSKKPLRRRVFFLWKCLIALDPPQPPLKRGA
FRLNMMCKNIYIVKSVNL
>WP_061546902.1
MAILAVGAGQTYTTIQTAINAANNDDIIVVRPGIYQEDLTINKSVTLIGPYGTFEGIDGF

ENRLGVKPLGPDINAALGVGGLPTANEDFRRYQDDNGTIDNAFGNTQEAWIKGTITVTED
NVTIDGFRLRNENGPLQWNDTPDNFKLLNNYLTGYTANNSPSFGDASINNPTGVVTGWQI
DSNYIGGLLGGGGTGGSIYLAGLQDSNIDNNTFWRPRAAHLYLASLTNVTIEGNKFYHGL
HTGGANFDGFGEFFSGSGYGYGYGGYGDGFFGRNYWLELKGDNDQVLIKNNEGEYNSGGI
QLFGETDSPFAFDNITIEGNSFPDNNFINAYNEAPDNGKSGLIPAVMATARLGGPSGSNL
VIRDNNITMDLAQVKFITDHKSSLEVRGNFNGVTIEGNTLTPKNINGGVDIITGLSLYGI
LPGETLIKGNQLLGQDGDPLEASYYGIDLIPTFADYGTYAGDLAVEDNTINSWQVGVNLR
DTNEITGDINISGNTFENNAYGVVLDATATTNSINIAGNTFSNNFSNVFDNIDPVITMDQ
VLSYEENQDLGSVLGTVSATDNLPNTDNVGITQYFISSGNDDGFFTINSSGEITLTETGL
AAANDFETSPSSFDLGITVTDGGGLQDTETITLSIINVNEGLGQLPPITTEGAGFIVGAT
LIAAIPFDDPDGTPTDISYQWQRFIEGVWTNIPDATEQNYIATEDDNNNQLRVEVTYIAG
GFEKVIYSNNVSISLVPVSGTFDSITGDNNLDISEKEAGVTLTGSVSETGTTVTILFGGQ
TRVAQVDGLSWSYVLKPNDYSFFAPGSNLFTAIFIRTDGGETGSFTTFQTLNIPDGILPP
NTSNAFDPTQPKGLKSEVIDAAQTLEIDGVSILEISKTVGILGEGESFNDPNIAVLPIGT
RDIFDQGANAGAANYAAFKTEPGTSIKGIYIVPAVEEEGVKKLQVVLADGTVVEAEIPLD
LISPIGDPLAVTISGVQPGGTTTFVLYLSQNVINQLPDDLNLGRYAKFNYESEQFELYDD
SNYDYIFNDVDGDGARDFGEVYLTVNLTDGDKWDGDGLENGIIVDPGQLGIATGSDSDNN
PPIAIELKGIVAENDPGAFIGSLTVTDDPGDSHTFTVNDSRFEVINVDGNNILKLRDGES
LDYEVASNVVLTITAIDNGGLEITRDFTITVTDVNEAPVAIELNQITVIENDPGAIIGTL
TVSDPDGNDGHTLKVNNDTRFEIVDFDGNQTLKLKAGESLDYEAGSVELSITATDNGGLE
FTQDLTISVNDVNEPPVVSFSFFVPESTTLVTNLTVEDPENDPITLSLAGVDASLFSISP
TGELAFNTAPDFEEPLNADKNNLYKLQVVARDGENNKSIQDISILVTNVNEAPIAIDDVL
AIIPGSSFGTLNPLDNDSDPDLDHTLTIINKTDGNYGRVEIRDNELIYTLLDATYIGDDV
FSYTIVDEQGLAATANVNVTIIGTDIITYPVEILDPEDSLIPDEAGSLSDIVNDISFNFL
TDYDKVQAKLALQQALSKTEAAFTNLFGLYEVDNALTGSVNGILPEDKSAYAKAALSKVV
PNFVVRAGGLGGGVNGDVIVTEGKIYAPFVIAHGGNFPGSVQDAVNAFFQVNPDNSPATA
QNYTTLPVAYFSFGSANPDGAAHIKSFGNNVFGFEDLPAGVGVSDYDFNDTVFSFG
>WP_061546906.1
MNIGYLGPPGTYSEQAAWFYLNWIKGNQEFQGEVILRPYPTIAKAIQAVTVQEINLAVVP
VENSIEGSVSMTMDNLWQLENLQIKQALVLPINHCLISCATKLEDIEIVYSHPQALAQCQ
GWLGKFLPQANLSATNSTTQALEQLGKSPTTAAISSEKAAQMYDLPILNNRINDYPDNCT
RFWVVAPESGGESFSSSSTHTSLAFSVPANIPGALVRVLQVFADLGINLSRIESRPTKRS
LGEYLFFLDIEASVLSSLMTTALKNISINVEILKIFGSYTVLTVDSVDKSGTKIR
>WP_061546908.1
MKNEKLNLGTKLAYGAGDLGPAVTSNIAIFFLLVFFTNVAGIPPGLAGSILMIGKVWDAI
NDPIVGVLTDKTKSRYWGRRLPWMFYGAIPFGIFFFLQWTIPQFYLDPGQNTLALFWYYV
AIGILSQAFFTIVNLPYTAMTPELTQDYDERTSLNSFRFSFSIGGSILSLIFAQVVFSLV
KSPQAQYLVLAASCTVIAVISLYWCVYGTRERILAFEAKRTQIEEPAEIPFIDQIRIAFT
NKPFLFVIAIYLFSWLGVQITATVIPYFVIYCMKLNNSQVPTVLIAVQGTALLMLFVWSY
LSKRYGKKIVYFLGMTLWIVAGAGLFFLQPNQIPLMYVMTVMAGFGVSTAYLIPWSMIPD
VIELDELQTGQRREGVFYGFMVLLQKFGLAFGLFLVGHALQVSGFKEVTAGTANILPSQP
DSALFAIRLIIGPVPTFFLIMGLIINFFYPITREMHSEIMLKLQEKRQNLQ
>WP_061546912.1
MTHQTSGHWRLGLTLSLLTAFLWGVLPVALKMTLQVLDIYTIVWFRFSLAFVLLGAYLFT
QKKLPTQQQIFSAPIKLLIITTIFLCSNYILFMQGIALTTANNTEVLIQLAGVLFGFGGL
VVFKERYTLWQWLGISILTFGFLLFFKSQISNLITSQGQYLLGSGLVILGAISWAIYALA
QKELLQSLSSSHIMLIVYGGCALLLTPFAKITTVFTLDLASLSVLIFCGLNTLIAYGAFA
ESLEHCPASIVSAILALAPIFTLISVDLAATILPQIFTPEYINLRGIIGALLVVVGSATT
ALGKNK
>WP_061546918.1
MLNPNLDEIQLTKDDYERYSRHLILPEVGLEGQKRLKAASVLCIGTGGLGSPLLLYLAAA
GVGRIGIVDFDVVDFSNLQRQVIHGTSWVGKPKIASAKDRIHEINPYCQVDLYETRLSSE

NAIDILSPYDVIVDGTDNFPTRYLVNDACVLLNKPNVYGSIFRFEGQATVFNYQGGPNYR
DLYPEPPPPGMVPSCAEGGVLGILPGIIGVIQATETVKIIIGKGTTLSGRLVLYNALDMK
FRELKLRPNPVRPVIDKLVDYEEFCGIPQARAAEAQQQIETQEMTVKELKTLIDSGSQDF
ILLDVRNPHEYEIARIPGSVLIPLPEIENGDGVARVKQLLNGHSLIAHCKMGGRSAKALA
ILKAAGISGTNVKGGINAWSQEIDPSVPQY
>WP_061546932.1
MGLDYKLPMPTPIIIAHRGASGYRPEHTLAAYKLAIDMGADYIEPDLVISQDGVLIARHE
NEISMTTDVEHHPEFAHLRTTKIIDGELKTGWFTEDFTLKQLKTLTAKERIGQIRPQNTI
YDGLETIPTLEEIIALAETQSSQRGYKIGIYPETKHPSYFQSIGLPLEPALLRSLSNTQL
PIFIQSFEVGNLKKLNENTDFPLVQLINDSGQPNDFSMDGKSCTYQDMIKPDGLKKIAQY
AQAIGVNKNLLIPRNSQGKLLSPTSLVEDAHQQNLLIHAWTFRNENCFLPLDYQNHPQGE
YELFFNLGVDGVFSDFTDTAVYARGKL
>WP_061546934.1
MAVPGNDIEILLTPDQYRRIEETGQERHEYADGRIMIMPGGSEVHSRITVDITTFLNLAL
RDSSFETYNSDLRIWLPSLNHGTYADIFVIDGSPEFSPNRRDEVLNPLLIIEVLSPSTEK
YDRGDKFRKYRSLPSFIEYVLVAQDEPYVEIYYKQHGEKNNLWQLEIYDQIEQSVIFHSI
NVEVPIREIYRRTKLP
>WP_061546978.1
MYNYIRDAGEIYKKSFDIIRSEAELDSLPEDVAKIAVRLIHACGMTDIVKDLAYSLDAVN
LGKNALTSGAPILCDCQMVAQGITKKRLPTTNAIICSLNDPEVPTIAKRLGNTRSAAALE
LWRFHLDGAVVAIGNAPTALFRLLEMLDEGVPRPALILGFPVGFVGAAESKAALAENSRG
VPFLTLHGRRGGSAIAASAINALASEEET
>WP_061546979.1
MNLKGRLYGVGVGPGDPELLTLKALRLIRSASVIAYQSAIDRESIARKIVSPYLPGNQIE
VLYHLPRALEPEKAKDIYDREVEPIGSHLAAGRDVVVLCEGDPFFYGSFMYIFTRLADKY
ETEVVPGISSLMACPISLGVPFTYYNDILKVLPGTLPREMLINQLLSADAAAIMKIGRHF
VKIRDVLHQLGLNSRAKYIERATTTQQRIVPLDEVDPDEVPYFSMIVIPTKQRL
>WP_061546981.1
MAVTPAHLHFLPFSKKSLRRRVFFLWECLIALDPPQPPLKRGAFRLNMMCKNIYIVKSVN
V
>WP_061546985.1
MLNKETTYSQARLNLATILDQVCDQREIVVIKRRNDKNVALIAEDELSSLLECVYLLRSP
ENAKRLFRALEWTQTAMETPQTLAELKEELGIE
>WP_061546991.1
MAVSLTKGQRVSLEKVAPGLSEVFVGLGWDVKTTDTGFDFDLDSSVFLLGANEKLISDDH
FIFYNNLVSPDPGKSVQHTGDNLTGFGDGDDEVIKINLKKVPEEVKRIVITVTIHEAQQR
RQNFGQVQNAFIRIVNAQNKQEAVRYDLAEDYSIETALIMAELYRKDGQWSLNAVGAGYQ
GGLKALLDRYS
>WP_061546994.1
MQYMHSEQNAEIISQINYFIGVVFNYENLIQNGQIENEEPLLIVVNDIKPPHSSDSLSKY
HPSYSYLINCINYFVNQSGLGHMLTPPVTLMVNNSDKYNSIRLGTLLDVKHFDVEFKQQF
FDPKEAITYLFDKLEVNSPEDKLEAINEIINRSFYPSVDVLFKFVESYNKSWLPQQNNKS
INQLYTEFMNKFDYHYPPATYEDIYGEYEDDLA
>WP_061547005.1
MPLKAVLFDFNGVIIKDESIHLKLIDEILVEENLQPQKPDERLRCLGRSDRACFEELLKR
RGRVVSQDYLTHLLRNKANKYIKELESLEQLPLYSGIEDLIIQARSQNLPVGLVSGALGR
EIELVLERANIREYFQVIIAGDDIATSKPQPEGYLLAVDRLNQLYSDINVDLSLQPKDCL
ALEDTLAGIEAAKRAGMKVVGIANTYPFHILQRQANWTVDYVTDLEWERIWETFEISLS
>WP_061547008.1
MTKKIGIVLGTRPEAIKLAPVIQIFKTAVDLEVEVILTGQHKEMVAQVMVLFNLKADWDL
EIMQPKQSLNDITCRSLQGLETLYRKLNIDLVIVQGDTTTAFAAALGAFYQQIPVGHLEA
GLRTDDLFNPYPEEANRRLISQISQLHFAPTSLAVTNLQNSGVLGEIHLTGNTVIDALLN

VAKANPACEIPGLKWGEYRTILATVHRRENWGEPLQAIAQGFLEILDRFSDTALLLPLHK
NPTVREPLQEILGKHPRIFLTEPLDYAELVGAIGRSYLLLTDSGGLQEEAPSLGKPVLVL
RDTTERPEAITAGTAKLVGTQTDRIVTAASELLSDRQAYLSMANAINPFGDGHAAERILK
IVRNYLGLTVDGV
>WP_061547011.1
MYFTWLDSNSWLLEIGGWRILLDPWLVGDLTFNNVDWLFKSYRLQDRPIPNNIDLILLSQ
GLEDHAHPPTLKQLDRHIPVLGSPQAAKVVEKLGYHQVKTLHHGESFTLEDTLNNNLKDQ
LEIKALPGSPVGPNVRENGYVIRNISNKMSLYYEPHGYHSSALEELSPVDVVITPIINLS
LPLLGPVIKGMNSALEVAKLLKPQIMLPTAAGGDVFFDGILSKLLQAQGNVAEFKELLEV
NSLSTQVIEPKPGERISV
>WP_061547013.1
MTNTWWEIQILCTPDLEDSIFWRLETLGCRGVAVEKKDKLLLIRAYLSTLQAQLSDLTNL
STLLHEDSTAIGLPTPELNWHQINEEDWSTSWKQYWQPQEIGNQFLINPAWLPIPLSTSR
LVIRLDPGVAFGTGNHATTQLCLESLEKYLTQNSDPQVIADIGCGSGILGIGALLLGAKK
VYGVDNDPLAVESTNSNCILNHLNPEKLTCALGSVHTLTEILIEPLDGIVCNILADVIIE
LIPQMTDLVKPGSWGIFSGILVEQSPSVITILEKNSWVVDKVWQRQEWCCLNARR
>WP_061547023.1
MDIQLINIGFGNIVSANRVVAIVSPESAPIKRIIGDAKDRGQLVDATYGRRTRAVIITDS
SHVILSAIQPETVANRFVLSRDHHTADN
>WP_061547025.1
MAKIEILGVPHAYELTVPTSHPSTLVFIHGWLNSRSYWQPLISRLSVDFQCLSYDLRGFG
ESQCQSYDQPHKSHEHSPYSTGAYSQDLIALLELLSISRVWLIGHSWGGTIALRTALQWP
NLVQGVICINAGGGIYLKEAFERFRATGQKFLQIRPRWLYQLPLIDLFFTRTDVARPLDR
HWARQRIMDFIIADPQSAIGTLMSYTTEEEVNSLPQLVSQIKQPVYFLTGADDKVIEPKY
VRHLASFHWLFQYVGDNVIEIPNCGHLAMLEQPDVVSKHIISIITG
>WP_061547037.1
MVLLIALFVIGWVAAGLIGSLAYFMGEQTKPIHERNWRSESFARLAKSITGQEINYETRT
PAYGIDAYASQGLSE
>WP_061547055.1
MVSKNFWKPIIHSSTALVLLTTLNTAWPLVSLAQSKPQPKANSAASSLFTDYLLGGGDRI
RVNVFEAPEYTGEYQIPPGGEINMPLIGSIPVSGLTTQQAADEIARRYARFLKRPLISVN
LLAPRPINVFVAGEVTRPGSYSLSLQGTGGNNPGVQYPTVLAALTTAEGVTLAADVTKVQ
LRRQVGRSGEQVVSLDLKQITQTGRIPIDITLRDGDTIFVPTATDFNVAEARNLFAASYA
ASRTAPRRVAITGQVYRPGSYLVAAGGGGGNDSGGLPTVMRAIQLSGGITSQADVRNIKV
RRPTRTDKEQTLNINLWELLQSGDLNQDVVVQDGDTIIVPTATQVNTAEVTQLATTTLSP
ATIKVGVVGEVKRPGVTELQPNSSLNQALLAAGGFNDARASSSSVDLVRLNPNGTVTKRA
VKVDLSKGINEETNPILRNNDVIVVNRSVLARTGDTLGAVTAPLAPVFSIISLFRLLGF
>WP_061547056.1
MNIRINNKLTGLATSVLTLTSGLAIVTSTSQTVLAQVVSQLATPQPPQPTIETTQNGPTT
SGWGVTPEISTLGVGATISKSITPNLNAKLGVRGFGTSANISESGIDYDAKLNLFSVSTL
VDYHPWKNSGFRLTGGLVFQDNNIEGNGKSNSDQKIQIGDQEYTSDQLGSVKAKVSFPNS
VAPYLGIGWGNAVKPGNRWSFSANLGVVFTGSPKVNLTPQFGAAATPEIQNQIQADVEKE
RQQLENDLKWLNIYPVLSLGISYQF
>WP_061547059.1
MALIVQKYGGTSVGSVERIQAVARRIHGTAQVGNSVVVVVSAMGKTTDGLVKLAHEISPS
PTRREMDMLLSTGEQVTIALLSMALQEIGQPAISLTGAQVGIVTEAEHTRARILHIETER
LISHLNAGQVVVVAGFQGISNTSAMEITTLGRGGSDTSAVALAAALKADFCEIYTDVPGI
LTTDPRLVPEAQLMKEITCDEMLELASLGAKVLHPRAVEIAKNYGVPLVVRSSWTDQPGT
WVTSNKVQERAMINLELARPVDAVEFDIDQAKISLLRVPDRPGVAARLFNEIADQQVDVD
LIIQSIHEGNSNDIAFTVNTPILNRAEAVASAIAPALRNNDGSNEAEVFVERNTAKISIS
GAGMIGRPGVAAKTFTALAKAGVNIQMISTSEVKVSCLVAAVDCDRAILSLCQEFEVNAS
TRNVSSSDSIYSTVCGVALDMNQSRLAIRHVPDQPGIAAKLFGLLAESNISVDMIIQSQR

CRVIDGVPCRDIAFTTNRTDGENAQAKISQVAAQLGWGEVILDQAIAKVSVVGSGMVGQP
GVAAKMFTALAQNQINIQMITTSEIKISCVVSEEEGVKALQIIHTAFGLAGTHKFVVPA
>WP_061547067.1
MVALLQKIRLENQSIFQEVNDFLSTRKSNRLAQAQKQAEDLSQFRQQLEQEIDQFLTKTA
QSRQAQAQAQAQELHQFRSQLEQETSEFLTKTTQARHAQAQEQAAELSAFHKQLEQKTEL
FLADTAKARIAQAQKQASELSAFHKELEQKTSAFLADTSKTRIEEAQKQAAELSAFHKEL
EQKTSAFLTATTSDRAAKAAAQKEALRQFRQDLFLSVIGV
>WP_061547080.1
MTLTIRVVSPDKTVWDGPADEVILPSTTGQLGVLSGHAPMLTALDIGVMRVRAEKNANWQ
SIALLGGFAEVEEDEVTILVNGAQRGDTINLEEARSEFNAAQTKMNQVAGGDRQAQIQAT
AALKRARARFQAAGGMV
>WP_061547103.1
QFIAKAPDWLYIPSVKEILPGRKSYTPHLEGDVPAVVMEFLSDKEGGEYSFKRTYPPGKW
FFYEQILQVPVYIIFDPDGGLLEYYELRNGRYELKQPDENGRHWIGSMELFLGTWQGTKE
GRTGYWLRWWEQTGNLLPWALELIEQERQQAEQERQRAEKEHQRAEQERQEKEREHQRAE
QERQEKERLIAYLRSQGIDPNNLPNHAE
>WP_061547108.1
MTPTLLGRWQTRVLLLLTVGNLVSLPFAGGFLGHGSAIYFWVVFYVGLFGLAWDVVYDFL
QKYLWDHDWPGVFQFYAGIVEGVFLGAILGTIGLPKIPRAEFQLVTFIQHYGMVWLGVYL
SAWIVMRLLFPRWRFRGGEWLGNWPN
>WP_061547109.1
MGPANFSHDTQAEQLLELALKSGAEAAEVYQSRSLSRPVFFEANRLKQLETSQSEGAALR
LWKNGRPGLTVAYGPLDPPGMVERALALSQLNEPESVELGSHSPQSYLDIGISVSVEQLI
NWGKTAIAEVRDLYPDVVCNGDWECDVESIRLINSQGLNCDYSDTTLSCYMSAEWVRGDD
FLSVADGQTQRGQLDPLKVAHQILQRLTWAEQNVPTPSWRSPILFTSKAADMLWGTVQAA
LNAKQVWEGASPWSDRLGQSIMTPDLTLYQDPQAGPYSCPFDDEGTPTRSLIFVDRGVLR
NFYSDRTTGSLLGMGTTGNGFRPGLGSYPAPGMFNFLIQPGRESLLELIKSMDTGLIVDQ
MLGGSGGISGDFAINVDLGYLVEKGEIIGRVKDTMVAGNVYTALKQVVKIGGDADWNGSC
YTPSIIVEGLSTIGKNGINTSY
>WP_061547114.1
MVQSRYTRLINFLQEDLAISAASLAVALRHPEHDTGSLTMILWQYGLITLDQLEQIYDWL
ETI
>WP_061547116.1
MKWQLLTHNKQVLGKIFTILVFTGLTGVLCVSCNRNQDLLVTEIGVNPPKRPTRKTSGAG
EFYLQGQNQHSRGNFQAAIAAYSKSISLNSDYAPAFKARGLAYFDLNNKERAINDYNQSL
QINPNDPETYNYRGNARASLGNQKGAIEDYNEAIRLSPNYAEAFNNRGNSHAAQGNKNAA
LEDYTQAIRIDQNYPVAYNNRGNAYSSLGNTSKAIADYNQAIRLNPQFAPAYNNRGNAFA
SSGDKRRALQDLQKAATIFDQEGNRGLYQQTMKNIEELEN
>WP_061547130.1
MSGKEELQVLDASQVKELLENQRVHLIDVREQEEFMGEHIPGSQLLSLSKLDPEKISLLT
GKKIVLYCHSGNISKQAAYRLIEFGFRDFSQLQGGISAWKKSGYVTNRNAPISIMRQVQI
VAGTLVVTGTVLGVLVSPWFLILSGFVGTGLVFAGLTNTCTMAMLLKKLPYNQIG
>WP_061547132.1
MTTDLPSTLDLLNPTLKALQLLGGSGSILEIANIICEMQNFSEEQQSIPHKNGPQTEISY
RLGWARTYLGKCGLIENKSHGVWSLTNKGKETESVNPKEVNRIVSSKYKKNNQVKSNQES
LNDLELHNTINEIDEISQSEQLNISDESGTINLLKTLLEGAIPLKSELWSEQLLDVLLDM
PPDSFERLCQRLLRESGFIKVNVTGRKGDGGIDGIGVLKIALLSFQVLFQCKRYRGIVGP
GEIRDFRGAMVGRTDKGLFITTGRFSRSAEQEATRDGAPAIELIDGQELCLLLANLQIGI
TRKTIEVVEINNEYFKNV
>WP_061547136.1
MIRLNAHGWYSGFCMWSEPSYTYYQRREQKNLEQTKRRGRRLSIIGLLQPLISFVYGLVI
GGVNRKSYIEMMEHEAEEAEKLGRIRVIVQDNGPIHQCKEVKKLWSKWESQNLYIFFLPK

YCSEMNPIELEWKHLKKDELSGKMFEDELELAYAVMDGVNTKGKRNKHSTERIKFNNSCL
S
>WP_061547147.1
MIMNLKTKKAIEQLEKFLGETIQTLADFQKLPVICKEKYISVYSLPELCKDGKLGGCDMI
TASSCSVEKPTFWPRFFPLSQF
>WP_061547151.1
MSIFWRKVLQLPQTYLLLMAIVLGLSVFFHAVGVHSLSLGLGSIIAVGMVIAWLYSLWLQ
ISANSVNLLDTDNFRQRLQKISSDISNKSTTEWQKAQTYAIESHQLCQAIVTQESILTAE
ILETLYTVLNLSEQVIHAILALKQVKTERYRTLTKNHLQTSLDRLKTTHHNLQQLQDQVL
LSSLDKNTVNTEFPLFLKTLIQENRSVVQTIIQDSTLAKENES
>WP_061547163.1
MSLYKTDYYLWVVETVNQLQSRDFTSIDWENLIEEVWDLGRSEKRKLKSLLRNLFEDLLK
LTYWESEIDRNKFHWRGEIRNFRQQIQDELKDSPSLKNYLDEVLLECYQDAKEIFADKSQ
IPLSSLPEISMGTLEEILDAKWLPNQ
>WP_061547165.1
MDNNLIIIYLSIFVGLLLFAAISVFRQVIKTRKTENSLARLKNKLTKEKGSAQEYYELAS
IYSEKKVFSQAITLYQKAIKAAEEEQEQDIGPVYNALGYVYFCQDQYDLAIRQYKEALKL
KPDYVTALNNLAHAYEKKKLPSQALQTYQEVIKFNPNNPIAKRRIQSLQRLVSK
>WP_061547180.1
MFKFIRSGLIYSLKVMLLGMIFLGTSTTGWTSYAALPSGNAITDGRALLRYALPIENQPV
RKLQASLEDISNQLRANKRWGAISRDLSQASRILDRPSQILTSIPPERQPQAESWIGELK
SGIGELQEVVKTRQKTPILEGRAKLLNLVSLLEESMVQGFPFEVPAEFSHLPQLKGRATI
AIKTNKGDLTVVVDGYSAPVTAGNFVDLVQRGFYNGLKFTRSEESYVLQTGDPEGKEVGF
IDPVTGKYRAIPLEILAEGDKQPTYGITLEDAGRYLDMPVLPFSSFGALAMARPEGDPDG
GSSQIFFFLFEPELTPAGRNLLDGRYAVFGYLVEGKEILDKLKAGDIIESAQVIQGIENL
VEPPA
>WP_061547189.1
MSTSSTSAQTTKRIEDIARKTTVQINSNANPGGSGVIIKKEGIIYTVLTANHVVCDNLGR
IKIRCRADLTYSVVTYDGKEYLMKSRQSLQTNVQDPDLAMVTFESRENYEIAPLGNSDNV
SIQSDVLVAGFPTIFGRVGKQRTFTITNGKVVTFIPNSDRGYGLVYNATTFIGNSGGPVF
DIYGRVIGIHGLADTDDGETNNNNQSETVNGVKPTQKTGFNAGIPINIFFSLSNFNRQLD
PAVSINRQPNSTNPNNVNLSNDAIAYHDRGVNRYQSGDRQGAISDFTQAIQINPNFAQAY
YNRGATRNDLGDKQGAINDFSHFINFYPRNSLAYFNRGIAWHELGDKQRAISDFTQVIKL
NPNNVAAYYNRGASRSDSGDKQGAINDFTTVINLNPNFAQAYNNRGLARHNLGDKQGAIS
DFTQSLRLNYRDPTAYNNRGIARHDMGDTQGAINDFTQAIQISPNFANAYDNRGLARHNM
GDKQGAISDFTQAIQINPNFAQAYNNRGATRNDLEDRQGAISDFTQAIQINPNFAQAYNN
RGLARHNMGDKQGAISDFTQAIQINPNFAQAYNNRGATRNDLGDKEGAISDFKKAAQLFD
LKNSRSRKIMRIP
>WP_061547194.1
MVSDLARRMAIANPKLENPFEREVKWAMAIKLFEMKWLSSGMAASLLGVNRTTFLMKLAD
YNIPLIDLTEEELLSDLANA
>WP_061547224.1
MLIINHYYIEWIEDWCKENGWTDLFVERRGNYWAFPPGCVIPEPIPVDTLKLIKQKNGAT
NEEVLISVVALFITLLCISCAFICMSPMPLVVAFAFDAVTVAQLEIEE
>WP_061547231.1
MATLYEQDFALWSEKMADLIVRKCFDELDITNLVEEIRDLSKRERDRLFSSMRLILHHFL
KWDYQPELRSRSWLLTIQRERSNIEDYLADSPSLKKYMTDEYLYKKFQKARLDAIAETGL
EMPISCSYTLNDIISRSLTLT
>WP_061547234.1
MNGVYEPLSLLEGGYISEQMNLKLVLWEGNYKGLSIVWLRWATLEGKLLPTQEEQTQWER
VQKEWWHAQREWELAQREWWRAQREIVETLAIQ
>WP_061547237.1

MKRSYTEFLQDILHAITEIGLFVNGASYEAFESNREKTLAVVKLLEVIGEAVKKIPNERR
ELYPDIPCWG
>WP_061547246.1
MNLEAEYDLDFYGWISKNVELLRCGSLSEIDAEHIAQELESMGKRDRRQLRSRLQVLIMH
LLKWQYQPDKQRKSWLATIDHQRDEIQSLLLDSPSLRRDLETALVTVYAKAVSDAQEETS
LPATTFPLSCPFALEEILSSRFLPKVN
>WP_061547248.1
DAITFALSGVDANLLSLDPQTGELTFKQPPDFEKPGDQNKDNTYEVKITVTDSSNLSVTR
DIPVTVTNVNEAPIAKEDFLTIFSATSGSIEPLKNDEDPDQNDQLKIIAKTDGKYGKVEI
NSDQLKYTLLDAAYIGDDVFTYTISDKEGLTATANVRVNVTATNVIPAITPVPVNPGDPL
IPKEAGSLAGIVNNVYFNFLAGYNPTQARQTLQNTLVQTNAAFNNLFGLYEIDGKDATGS
VTGSVNGIAPGDNGYAKAALSRAVSSFAVRAGGSGNGITGDVIVSGGKIYAPFVIANGGN
LFGSMQDAINTFFQLNAENSPATAENYTSLPVAYFSFGAANPDGAAHIRSFGNNIFGFED
LPSNVGVSDYDFNDMVFSFG
>WP_061547250.1
MATSADDVSEILAQLAESQKETHLQFPETDRQIRELGEKIRRLGDKFGGFTERLALPSME
PILREQFAMETISPSVRVKKGGENFEIDVLAYTNGDINKAMIVEVKSEVREESITHLVKT
LKKFRTLFPEHQNKQLYGVLAGVDMSEEMKNRAMEEGLYVARIQDDVFQMDTPSGFEAKA
W
>WP_061547262.1
MSFIYYGESQDIYGGAGLEPTPINREEYGIPIKTYQTCASQVIDSRYTRLETLLKARSPA
LSWRSLVDWGLGVGMGFGGDYFPPVQTCRL
>WP_061547263.1
MKNADNPPGQLVLLVVLGVNCVFGLLGLKSGSLKFGSGIVGVILIGLKSGSLKFGSGIVG
GILIG
>WP_061547274.1
MPDFALILKDFVTQIFPRLRTELRFHRKLAKGGLKPDGTPFINKRTGKPYVATTQLTHVL
VGLSALTRLLNYLEKNSLLTSQINITEADFRRVCALFCLHDLHKDDDPSRDVQQRDTSIR
PSLMLEIANKASLTDWLNNHDLNGYEYREAMIHLSDTTHGDCKHCRGEIGYEKLYSLVRL
ADAMASIQNLDEGTNSLKNRIKDFARSLKNLHFSYHKIDDYRGVTTNLYHQSVAGVLQQE
YNVYPLLFFDHGTIYIGQNELQSFDKSSFINQVHQRFNDSLKNLGSISSDELQYNSKTQR
FEKYIFGFSGIKEQLEYLKNHAIPRVKAKKEALHICGMI
>WP_061547278.1
MVGVHIYVMEIKVIEGNQVQGNAALDQILQRNYAEKYRGEPGKYVHEIGLIFSRTQRNLI
QANWQ
>WP_061547280.1
MQTLYISEQNCYVCLQKETLIVKQGDTVHVEVQLPLLEQILIFGRSQITTQVIRACLWRD
IPIAYLSRMGYCYGRILPISRGYRQLSRYQQELSPVDRLITARAIVKGKLKNSRILLRRQ
RKKRESETLERTLQSLDYLAEQAVQADTWERLMGFEGTGAAQYFSVFSECLTNPDFVFSG
RSRRPPGNPVNAMLSFGYQVLWNHLLALIEVQGLDPYYACLHQANDGHAALASDLIEEFR
AALVDSLVMWLINRNIMDVKEDFQFKNGGCYLNDSGRKKFLRGFLQRMTEEIQTNDGKQP
KWDLLTQQVRNYKQFVYNPSHHYKPYQID
>WP_061547286.1
MLLMLPMVVLFFPSINFGLIEAETFPWAIALTIATSFIFFEWIDRAVAIFLAILSVSALI
TIAEFGSELVFESIRSVFAYINAISGFYVLMRFRVKVGETAIFRIAEYAFCIMLIVGIGQ
HLSLIGNENNAIIQFLALHICGMI
>WP_061547290.1
MRQPENIGAPLNFRFVLDEAVGEYFMWAAADDVWESNWIQTLLPVTETYRCLAYGMVVVI
DERGKQMANPSSGRVFNYHGLKFVRRIKYMFAMGALGKANPIYGLIPRSTLNRGNFSIFD
RVVYGSDMVFLFDLLRITEIRSPSENTRLYKRVWTKSIIQGKISGNGQFDSLLRKFFVVF
LSWSGITYSSYCTPIESCVILLTIPLMFLRAGTFYASIFIYKTLGGVAPARDDV
>WP_061547293.1

MNLIPAYLGSPPAPPTDRDNDGMPDAWEIARGLNPNLSNHNGYNLSSRGYTNLEVYLHEL
STKILGVRGIWGRGRNEPTAIAGYMGTSFAIANP
>WP_061547295.1
MKSNVNQGGETIAPDIVYPESDGKPMADNTKQFTWIVKIKENLEILFKSNPDVFVAGDLF
WYPVKGSNRIKLAPDTMVVFGRPKGQRGSYRQWEEDNIPPQVVFEILSPCNSKGEMTRKK
LFYLKHGVEEYYVYDPDEISLEVSIRENNSFREVEDFATWTSPRLNIRFDMTGDELVIYY
PDGSRFLSPVELSNYAEQERLLKEQEQLKYQTLLSQLKAKGIDITALE
>WP_061547300.1
MQVTTTSTPIPPGELSPSSWPDHTQLPDSDDNFVKNFQEHPQSVILTTSIEPLLDKIHPD
KDYCIGQDSGIYWRFTEPVEKGVEAPDWFYVAGVPSRLEGKLRRSYVMWKEKVPPLIVIE
FVSGDGKEEKDNSPPPERDEVDPKTKKAKKAGKFWVYEQAVKIPYYAIFDGFEGTLEMYH
LEKGRYEQVKANIRNHYPIPELGVELGMLLDQEKPPVPWLRWWDNRGNLLLTGNERAEEE
CQRRELAEAIAIQERTEKEQERQQKEIAEALAIQERQQKEMAEALAIQEREKKEKLAAYL
RSLGINPDEI
>WP_061547303.1
ICFIDFSPYESSEAKAQDYITQEFAKPFDFNIDAPLADFKLIKLSDDSHIIYAKYHHVIT
DGWGTAIFFREVIKTYTQIINDGKDSQIPRDWVIQEYIEEEKKYLESSIFLRDRDYWQQR
LQNVSPKIFSLIQPQGLEGKRHSIYIPREEYNRVNRLCQDMQSNVFHFILSLISIYLTRR
YLKDDVVVGLSLLNRSKKNFKDAIGLFVSTIPFRLTINRQQTIHQLLDNIRSLLRQDYRH
QRFPLGEMKRFSGLQNKIKENLFEVFLSYERHDYSESFPDTQTSCIPLYSGQQKIPLIVY
VREYEETSDVKIDFDYNLSYLDEKTVEQIVTGFQNLFIQATDNLEIFIGDLEDSLADSRD
ISKNISVSQPEIPFVNYQETLISAFEEIATQYPGNPAVQFNNKILGYAQLNAHANRLANY
LIGQGIKPQSKVGICLERSDQMIIAILGIIKTGSSYVPLDPNSPIARRQLILQDSGMTAL
ITQSSLLTELNTENIIAFTLESIDSELNKEPNTSPQIAISPDFPAYVIYTSGSTGTPKGC
IVTHRNAVRLMRATEAWFRFNETDIWTLFHSFAFDFSVWELWGALLYGGKVIIVPFGLSR
NPEKFREFLTTEKVTVLNQTPSAFYQLIRADESAVGDLSLRYIIFGGEALDLQSLQPWLE
KYGDKKPRLINMYGITETTVHVTYRPITRQDLKTKGSFIGREIPDLAIYLLDDQLIPVAD
GVSGEIYVSGAGVTNGYLNRPTLTAERFLPNPFGTGRIYRTGDLARRLPNGDLEYLGRAD
QQVKIRGFRIELGEIQAALTSHGEVQEAVVVTDEWQEEKRLVAYYVPGESSPTVNELRQF
LKNTLPDYMIPAAYVSLKAFPLNVNGKIDIQALPAPDWNSLRVEEDYIGPRNIDEEILCT
IVAKILGLEKVGIDDNFFEIGGDSILALQVIAKAKKEGFAISARELYELTTVRNLATKKA
AVATLEDIAETSILSLVSDTDKAVLPKDIEDAYPLSSLQGGMLYHSELHTRSAIFHQIFT
FNLEISYSELAWRQAIADVCLANPVLRTSFHWTGYSQPLQIVHRQVESPLSIVDLRSSPN
ANQQVQEWVELEKTRGFDIGNPPLFRFQIHRISNEKLCFSFSFHHVILDGWSVATLLTQL
LRRYVQYLDGKNLPPLVFPETIYRQFIAQEQNAIANEEIREFWSQHLSNLQVTFLPRLNT
TGIKITTTDYHNRQLKRLSLTISDELTDRLRKISQNIGVPLKTCLLALHLRVVSFVTGQK
EVVTGNVTNARSETSDSENALGLFVNTVPLRLELPSGSWIDLIKAVFHAETAVLPYRMFP
LAEIQRLLGKRPLFEVGFNYVHFHVYDSLLNLPQVQVGNVDIFEETDFPFLAEFCLVPGS
QTLQLNLIYDTQQFADAQVEQYGRYYQTAMVEMTTAP
>WP_061547306.1
KTRLAFQLIKQPGAFFLSRPRRFGKSLFVDTLKEIFEGNQKLFEGLYIHDQWDWSRKFPV
IKIDFAGGVLKNRQELDQKINGILLKTAHSLGVDYELEDIQGRFGEIIAGAYQRFGERTV
VLVDEYDKPILDNIDNPSIAGEMREGLKNLYSVLKEQDANIQFIFMTGVTKFSKVSLFSG
LNQLTDITISRDFSTICGYTQEDLEQTFAQHLQGVDWDELRLWYNGYSWRGDSVYNPYDI
LLFIREGMEYGNYWFETGNPTFLIKLFQANRYFLPNLEHLEVTEEILKSFEIEQINPVTL
LFQSGYLTIERNFTRHRRSMFALKIPNMEVRLTLNDQFINVYTGMVNEKSAIQDILYECM
WNGDLESIVKAVKRLFAGIPWRNFTNNHLADFEGYYASVLYAFLSSLNARIIPEDITNYG
QADITAILGDHIYVMEIKVVDGENVKENLALKQIRECNYAQKYRGEPGKTVHEVGLVFSR
SKRNLIQADWE
>WP_061547314.1
MTTAIQQRQSTNIWDRFCEFITSTNNRLYIGWFGVLMIPTLLAATTCFIIAFIAAPPVDI
DGIREPVAGSLMYGNNIISGAVVPSSNAIGLHFYPIWEAASLDEWLYNGGPYQLVIFHFL

IGVACYLGREWELSFRLGMRPWICVAFSAPLAAATAVFLIYPIGQGSFSDGMPLGISGTF
NFMIVFQAEHNILMHPFHMLGVAGVFGGSLFSAMHGSLVTSSLVRETTETESQNYGYKFG
QEEETYNIVAAHGYFGRLIFQYASFNNSRSLHFFLAAWPVVGIWFTALGVSTMAFNLNGF
NFNQSIIDSQGRVIGTWADVINRANLGMEVMHERNAHNFPLDLAAGEVAPVALTAPAING
>WP_061547330.1
MIEAEIKALIQKELPRAIAEEPGVRDFVLRTVSEYYTPRTEFDEKFDRVLNELQRDREEQ
ARKWDEQNRKFDAFQAEQARKWNEQNRKFDAFQAEQAHKWDEQNRKFDAFQAEQARKWDE
QNRKWEENNQRLDRIEAQNSATLEEIQKANRRYESAIGAIGSRWGLYSEASFRNGLQAIL
GQSFGVEVLNLTLYDQEGEVFGRPEQVELDIIIKNGLTIVCELKSSIDKAGMYIFGRKAD
FYAKNQNRVVNRKIVISPMVDERAIPVAKSLGIEIYSYADVVVP
>WP_061547331.1
MIEAEIKALIQKELPRAIAEEPGVRDFVLRTVSEYYTPRTEFDEKFDRVLNELQRDREEQ
ARKWDEQNRKFDAFQAEQAHKWDEQNRKFDAFQAEQARKWDEQNRKFDAFQAEQARKWEE
NNQRLDRIEAQNSATLEEIQKANRRYESAIGAIGSRWGLYSEASFRNGLQAILGQSFGVE
VLNLTLYDQEGEVFGRPEQVELDIIIKNGLTIVCELKSSIDKAGMYIFGRKADFYAKNQN
RVVNRKIVISPMVDERAVPVAKSLGIEIYSYADVVVP
>WP_061547342.1
MVNSGSEQLPDHSSQIVTVRPDSETATLQKLPYFVGISGKTAGTKGISMNLVIIPAGGKA
EPHFHRDYETAIYLVKGRVETRYGKGLSQSVINEAGDFIFIPAGVPHQPYNLSDTEAAHA
IVSRNDPNEQENVVLYDPSVYS
>WP_061547343.1
MKERIIALLFLIIIFEIVFRACGGTTLETPQSIPETTAEETTKECIDRVSNELIERDRGE
VLTEEKMVLYYAEFITRCNLTPNR
>WP_061547350.1
MEELLKLKGLLLQGDIPGALMIVEDLEEMSRDDKINNIRSYTVILLLHLIKQQAEKRTTR
SWDVSIRNSVREIREKNKRRKAGGYYLNTEDLLQILEVAYPMAIDQASLEVEGGRYETEE
LENLVNQGEIINHALALIDCL
>WP_061547355.1
MEIYFSNELQIDVNAITIGDNTGTIPTGGRTAGTDTNNADGDSSTGNFVLLSFQNLTTSA
TNKSFITIPFVTNSTFDGQATVNFIARSSNPSLTVDPITPVAISNQVPAIIEVGPGQTYT
TIQAAISAASNGDVIRVLSGVYNENLTINKSVTLEGPNKGIKPITPDINPTSGININQGY
RTNEEAWIKGTITVAADGVTIDGFRLRNENGPLQWSGTPDNFKLLNNYVTGYNANQGPRF
GDANSNNPTNVVTGWQIDGNYIGGLLGGGGTGGSMYLAGLKDSSINNNTFWRPRAAHLYL
ASLTNVTIEGNKFYHGLHAGGANYDDLGKFFSGAGYGYGGYGGYGGSYGGGYGRNYWLEL
KGTNNTVNIKSNTGEYNSGGIQLYGEVNDPFQFNNITIEGNTFPANNFINAYTADPTNNL
SGLIPAVMATAKVVNNGPAGSNLVIRNNTITKDISQLKFSKDHTSAIDVRGNFNGVTIDN
NSLTSVGTNGGVSLITGLSLYGGLSGQVLIQNDVLSGAGGNWGTANYYGIDFIPNPAL
>WP_061547363.1
MAQIRNFIGNVNHNTVVDSTVVPESDRREPLKHLLIGSKKTVLSTIHYLHVLGYAHATDW
SDLIPTNNPGLVCE
>WP_061547366.1
MGYVIATANMKGGVGKTTVTVNLATCLAKHHGKKVLVLDLDSQISATLSLMSPGDFAKRR
KQRKTLRYLLDEVINPDPQSEYKIHDVIEPELCKLPSLSLLPGDIDLYDEFVVSEMLHNQ
AVALEERDFETIWNRFERVLVRDILKPVRDQYDFILLDCAPGYNLMTRSALATSDFYLLP
AKPEPLSVVGIQLLERRIAKLKDSHEHEAKINIQMLGIVFSMCNTNMLTGRYYKQVMHRI
VEDFGVETICQAQIPVDVNVAKAVDSFMPVTLLNPNSSGSKAFIQLTEELVRRL
>WP_061547367.1
MSTLTRAGNGSGNLRSLVDSVFLDANGAVSGNQSLGVNSAVLVVSGGGTYMIVNDGTAGF
NAGTDLVINITGYSGVLPGLGNVVASNWFV
>WP_061547368.1
MLIIFDITVFFYSLFIIKSATSLPYLVIWVFSFYRKNLIFVLAFALTFVFVVVIGYQSEL
SQSRAIIFLSQLLTAEDLNIDDLMYIVFDNSGFRDPSVVASYFYGATNIVGGGVGNWKQS

SLDALNASSIDVSRISYFGGGYYSSVRPTSYLSSLFLDTGLIGVLVFMVLLWGEIKKYNA
NYSLVNKTAVIACIFSITLLGQIGDPVVWACLALSLAPRSLDISGEEENCSN
>WP_061547380.1
MFFIFPFFFFACIGRVHERLEDDGLTTWHPAFLALGRSLEECAVKYKGFCQKYRAQEKPV
KKNRWGSKLLGGMKPDKKGKKPLPGLLRLFWDEVDMGNEEIIGVATKFVKANCYSPNPKL
F
>WP_061547386.1
MNSVGTARKLKQALSQMFGGENLGEAHGIVQASEKKDALKKVHTTGTSGIEVGIDFTDEY
FKYLLLFEGRTSSQFLQR
>WP_061547391.1
MDILTLNIKLGNMQYLILKILLILYFTGLFKYGTAILEVISFGEQKRSPAVEIVTDYTLF
FMCHVRSLLGLE
>WP_061547394.1
MKSNVNQGGETIAPDIVYPESDGKPMADNTEQFRWIVKIKENLEILFKSNPDVFVAGDLF
WYPVKGSNRIKLAPDTMVVFGRPKGQRGSYRQWEEDNIPPQVVFEILSPCNSKGEMTRKK
LFYLKHGVEEYYVYDPDEISLEVSIRENNSFREVEDFATWTSPRLNIRFDMTGDELVIYY
PDGSRFLSPVELSNYAEQERFLKEQANERAEQERLLKEQERFLKEQANQRAEQERFLKEQ
EQLKYQTLLSQLKAKGIDINALE
>WP_061547399.1
MVVSNEVGRLTPLAAPTGTEPAKLGIIRHVLKVYPMPESMINKTTGFMATNLQSYHSLHT
HRAEATVIFDLSPTRVLDLSFRIKIVA
>WP_061547402.1
MTKKYIIWGSSGHSKVLHSLITLCQGKVIALFDNNPNSSVSIKGLPLYIGLDGLHQWMLD
YEDFQNIYGIAAIGGSRGKDRIATHQLFCSFGIKVETLKHPNSTICQTATIGKGSQLLCQ
SLIAAGSTIGDACIINHQASIDHECLIGNGVHLAPSSTLCGCVNLGDNVMIGAGAVVLPR
ISIGE
>WP_061547406.1
MATLLSESRSQTGPVISWEALPDDFQLEDEPVDNTGQPLLAGALSESLEISGFIQPQMLI
AVNFGLCATLNEQFIAKAPDWLYIPSVKEILPGRRSYTPHLEGDVPAVVMEFLSDKDGGE
YSFKRTYPPGKWFFYEQILQVPVYIIFDPDGGLLEYYELKNERYELKQPDENGRHWIGSM
ELFLGTWQGTKEGRTGYWLRWWEQTGNLLPWALELIEQERQQVEQERQLAEQERQLAEQE
RQRAEREHQRAEQERQEKEREHQRAEQERQEKERLIAYLRSQGIDPNNLPNHAE
>WP_061547415.1
MGSPQPPLKRGANSFRLACNKLLLFGIPPTPLKRGANSFRLACNKLLLLVIPLTPLKKGG
>WP_061547416.1
GVLKNREELDEKIRDLLWNNGDRLGVDAKKNSISGIFGEIITGAREQFGERVVVLVDEYD
KPILDNIDNPNIAAEMREGLKNLYSVLKSQDANLQFVFMTGVTKFSKVSLFSGVNQLKDI
TISEAYSSICGYTETDLRESFGDHLEGVDWDTVRHWYNGYNWTGSETVYNPYDILLFISE
GMKFRNYWFETGSPSFLLKLFQKERYFLPNLEGIQVTEEILDSFDVEQINPVTLLFQSGY
LTIKDTFTDINQMVFCLGIPNIEVKIALNNQFINAYSNLVNEKLGIQRLIYTQLRSGNVE
GLISTIKRLFASIPWRNFTNNDLADFEGYYASVIYAFLSSLDARVIPEDITNHGHSDLTV
MVGVHIYVMEIKVIEGNQVQGNAALDQILQRNYAEKY
>WP_071985092.1
MMERTPNPNQQPVELNRTSLYLGLLLIFVLGILFSSYFFN
>WP_071985094.1
MAKSKGARIIVTLECTECRTNPDKRSPGVSRYTSTKNRRNTTSRLELKKFCPHCNKHTVH
KEIK
>WP_071985099.1
MSHTVKIYDTCIGCTQCVRACPTDVLEMVPWDGCKAAQVASSPRIQDCVGCKRCETACPT
DFLSIRVYLGAETTRSMGLAY
>WP_071985100.1
MRPFRDKQSATDLISESTETLELSTKQRDQIIEEIRILLKNEKTLQQTLKKEQDQRNTAN

EQLFLELLGIFDTLEFLVDYLSNSPEPSAKSIKRLSKQLEVLQRKLVGILEQRKVELIED
LNHTKPDFNLCVVVDREVRNDLEEQTITKVVKKGFRIENRVLRPIEVITSKQQ
>WP_071985113.1
MNNQLFFLVKLLLLSTLISGLIKYVGPIFLFPPTAVNALMMVLLPTIFMISFLVLPMVLK
RQS
>WP_071985120.1
MQVNDLGFVASILFVLVPAVFLIILYIQTASREGGKN
>WP_071985140.1
MGRKLSKSHLPEKICPVCQLPFTWRKKWQDCWDDVKYCSERCRRRRSQTT
>WP_071985141.1
MKVRASVKKICDKCNVIKRRGRVMVICENPKHKQRQG
>WP_072301453.1
MVREIAPKTQLGVIKSKVNQGGETIAPDIVYPESDGKPMADNTKQFTWIVKIKENLEILF
KSNPDVFVAGDLFWYPVKGSNRIKLAPDTMVVFGRPKGQRGSYRQWEEDNIPPQVVFEIL
SPCNSKGEMTRKKLFYLKHGVEEYYVYDPDGISLEVSIRENNSFREVEDFATWTSPRLNI
RFDITGDELVIYYPDGSRFLSPVELSNYAEQERFLKEQAHQRAQQERLLKEQANQRAEQE
RLLREQERFLKEQANQRAEQERLLKEQERFLKEQANQRAEQERLLKEQERLLKEQEQLKY
QTLLSQLKAKGIDITTL
>WP_072301454.1
MKEQANQRAEQERLLKEQANEGAEQERLLKEQERFLKEQANERAEQERFLKEQANERAEQ
ERLLKEQEQLKYQTLLSQLKAKGIDITALE
>WP_072301457.1
MIWKRSRKSHKGKQDPESKQIKQADLDMLELSAAGGEIDLKYLDESGFCMWSELSYTYYQ
RREQKNLEQTKRRGRRLSIIGLLQPLISFVYGLVIGGVNRKSYIEMMEHEAEEAEKLGRI
TVIVQDNGPIHQCKEVRKLWSALQDKV
>WP_072301459.1
MVGGLLASVISAVICLILPKFLSLFSSGKNNHLERDRNRGITSFPYCTVYKLTGSEWCKF
RPQFCGRCSSYRKSEKLKNLWKW
>WP_072301462.1
MAKLLYEIYTNKSISKNYSLKMKKLIERDLAPTAWQNKPFNSIEGFLGEGLPENVKFYSK
MGWNSRTRNDAAIIISPDKKHKYILVVLGDDPSFFQDKKLFPEISRVVYKAMTINTSSSM
SDSECFANALPRVFSRENLKW
>WP_072301466.1
MTKLRKFVGYNLRKSSRSCVKKLFIWPIQGYRLFISPLFPPSCRFQPTCSMYAMEAIERF
GVFRGSWMALGRILRCHPFHPGGYDPVPEKTEKSP
>WP_072301471.1
LLSVSNKTNIINLARTLVEEFDFDIISSGGTAKTLKNAGIPVTKVSEYTGSPEILGGRVK
TLHPRIHGGILARRDVAQDLTDLAANEIRPIDLVVVNLYPFRETIAQPSVTLADAVEQID
IGGPAMLRAASKNFPNLTVLCDPEQYNEYLQELRQNGSTSWEFRRKCALQGFLHTASYDH
AIATYLSEIESPSLSKNDLTENYNISGRRIQTLRYGENPHQPATWYGTGSNPTGWNTAIK
LQGKELSYNNLVDLEAARQIIAEFTDLPAATIIKHNNPCGTAEADTIFAAYQNAYNADSI
SAFGGIVALNRPIDTPTAWELTKTFLECVVAPDCDREAQAILNKKGNVRVLILPDLLSGP
KETVKAIAGGLLVQTADDTIADSSKWQVVTKVKPTPEELAELLFAWKVCKHVKSNAIVIS
KQRTTLGVGAGQMNRVGSTKIALEQAGAGAKGAVLASDGFFPFDDTVKTAAAAGITAIVQ
PGGSLRDQDSIKSADELGLSMVMTGVRHFLH
>WP_072301474.1
MLYRRKSTVYTRPLARLIEQLQRLPGVGPKSAQRLALYILKRPESEIEALAQTLIDAKKQ
VGLCQVCYHLTAEPVCEICRNPHRDSQTICVVADSRDVIALEKTREYRGKYHVLGGVISP
MDGIGPEQLTLQALVRRVSQQKPQEVIMAISPSVEGETTTLYIAGLLKPFTRVTRIAFGL
PMGGDLEYADEITLAKALEGRRELD
>WP_072301475.1
MEVSETEDSKSHRWSGGKDPAIFEANHKSRGDYWIIDNQYLVPKYGQKINQHSYETISTL

FECLNYHYNDSIGFRSMILVKPAKVSPIHDQEKWKLQDTGTLQF
>WP_072301485.1
MKMNCPNCDSNNIRKNGQRRGKQNYQCKNCGRQFIESYSPRGYSQEVKEACLTMYVNGNG
FRAIERMTKVNHNTVIRWVKKLGRQLSDSNNNSQTP
>WP_072301491.1
MATVATHGQLLAQESVPIEISTPQPVQEKKSGFTLYIWQPNGTISPVIARISLKAKHGQK
YLQERFLGDYQYRIKQKAKFENGFKWGDRIVVRLYNTQNQLVGYTEFACLPNHTTVNLIL
PANSPPNPLVRVFYGVDGDEDGIVDPDTTMSYDYFTEIRNQQVTFLSSPPDIDLSPYQAA
GFAKVAQPSTYPGSFIQGPFALVGKPISINDSQLAKAFLYLPGKIVQVTKLSESAYQLSN
LLSKYREVGVGKGIRVRFSDLPQNYWAKDYIEELAAMEIIDGYPDGTFRPNAPITRAQLA
TLLPKIFFKDKIRGEVAFRDIPKNHWAHNAIQKTYQMGILTPSANRRFRPDQKLTRLDVL
TTIAKALNYKFTGSTRDILSIYQDASTIRSEHRGLIAAITENGVVVNYPNIRLLNTRKLV
TRAEVCALLYRAMVSKGDVPDFSSVYTVRSNGSNIK
>WP_072301492.1
MNKQLTHTELENIYQGAFRYVRQKTRFEVSFPQDCPYGLEQLLNQSYF
>WP_072301496.1
MSITINNVSKSFKGKLALKQVSCSIYEGDMVALIGASGSGKSTLLRHINGLQTADAGEIT
IYGTTLQSQGKLHSKVRLLRSRIGCIFQQFNLVNRLTVIENVLVGNLARVSPLRSTLHLF
TKEEKTQALAALEQVGIIEHAYKRASTISGGQQQRVAIARCLVQRAKIILADEPIASLDP
ESARKVMELLVQLNRDNGISIVASLHQIQMVRNYFSRAIALKDGEVKFDGPTVELDDRKL
NQIYGAAVEELVMRGHGEVLL
>WP_072301502.1
MLKRNLVSNFVGQGWNAVMAIAFIPVYIHYLGIESYGLIGLFALVTAWTTLLDIGLTPTI
SREMARFTAGAYTPELVRDLLHSIETITFGIAAIIAVTLTFGSSWLATYWIQSNTISVGV
VSQAITIMGFVSAFRFTETIYHGSLVGLQRQTPLNLIKSSIATLAHCCT
>WP_072301503.1
MVFEILSPCNSKGEMTRKKLFYLKHGVEEYYVYDPDEMSLEVSIRENNSFKEVEDFAIWT
SPRLNIRFDMTGDELVIYYPDGSRFLSPVELSNYAEQERFLKEQANQRAEQERFLKEQAN
ERAEQERFLKEQANERAEQERFLKEQERLLKEQEQLKYQTLLSQLKAKGIDITALE
>WP_072301504.1
MSYKLLFVCLGNICRSPSAENIMNYLIDKANLSNEIHCDSAGTSNYHIGSPPDRRMSAAA
SAKLGIDLLGKARQFQKSDFEDFDLILAMDKDNYRDILSLDSSGKYHHKVRLMCDFCSEH
SLQEVPDPYYGGPEGFNQVIDLLMDACNGLLKHIKNQ
>WP_072301509.1
MIINSFPIYASLIAYLILQLISVRQEWGNKMLDKFRYLQACMCQQISYVHWMEDIMRC
>WP_072301511.1
MQSLRLVVACVLAFLLTNQGTAIADNCPLKGCAQKYTLALSWQPGFCETHGYKAECQEQT
ATSYDANNFTLHGLWPDKLEYCNVSASYIENDKTGNWKDLPIVEVDTETTDELDEVMPGF
GESSLERHEWIKHGTCDGRNSDNYYDLSVDLLKEFNDSQVRNLFIEHIGETVSLDQVKTA
FEGTFGSGSSSSLNLKCDSTNNLATEIRLKIKRPQSGQKLADLLLPSGGQSCNQVVVDGF
GVGNLTQ
>WP_072301513.1
MSKSVIDLLKQTTEGLFMPSESEYPFNVVYWEFFNLNETTIQQKTGITGNVRTVTVDDFF
QGVTKQEDWYEEEERNVAKRFESLVLVLKNNLAEAKVYEIGNKEVHAYILGTKDGEIIGI
STVVIRT
>WP_072301517.1
MYSLVIPIYNEEENIQEMYRRLTHLMTQLDGDAELILVDDGSQDRSLSMIRELHDHDGRI
RYLSLARNFGHQVAVTAGLNFVQGQAIIVMDADLQDPPELILSMIDKWHEGYQVVYAQRI
SRQKESLFKRLTAYLFYRLLQRLSKVKMPVDTGDFCLMDKHVVDILNAMPERNRYIRGLR
AWVGFKQTSVLFERGPRYAGKVKYTFSRSFSLAIDGIISFSTVPLRLATYLGIISATIAL
IMILLVLYWRIFAPVSQLIGYSLITIAIFFLGSVQLICIGILGEYIGRIYEEVKARPLYT
LKETGGFDPLNI

>WP_072301518.1
MAGLFSPTLGAIKKAEIVGLPNIKDDRVTIKIKVEGEGARPVMGLERNNFDLKVDGKKLK
VKPRDWKSPEEIIPPKAWIIVLLDFSGSMNQIDSGGSKKIAGAINAIREFAKVSSDRGGD
TQISVVPFGEAGKNCPEYTVNKDTLDKFLSASDFKLQNSLEYLSSLNPCGSTNLYQPLKK
ALEFLGNPEDPRFTLPENSSEPNPRLSIILLSDGYHNAMNEFQDFNQLKSLLQSYENITV
HTLGYGLTPSQLGIKYNLNRPATRRDINQGKVPEEEFVDQQRLAEIANLTGGIAEFSGDQ
TAIAENLKLFLNALLGEYQISYIQPNAERGTLHKVEVQVKDGNKKVNSKSVPYIMPVFGR
SLPLVNRLVMVTSILFILIVGGIFPFRWWGEHLKEQARGE
>WP_072301521.1
MFMYFSAKELKNQDFQRPDFFAVLGVPKGERKSWVVWQEGKSPNLIIELLSENTVENDKT
TKKNIYQEQMRVPEYFWYDPFDPKDWRGFKLMNGVYEPLSLLEGGYISEQMNLKLVLWEG
NYKGLSIVWLRWATLEGKLLPTQEEKTQWERVQKDQIHFCIQLNI
>WP_072301527.1
MNDRITQSLTKLFEKHRIVFWYDAKQELRQEFEAIALDGIEKIELKNNEFGVKYRILRQQ
PRGKFLLYHEGPQPADLDNWLLDTQLAHTDFRADQFAMWLAELELGREFTDVVQSHIAFF
QAVERRKALKRLITSHDTPSQLRLKMVALCAGCGSEARLEAILEQLLGELSGDSVQYMEG
YAGDRYRLIERCELKQYFWEQMKRRYGYSSPEPSIKDFVLQLFADCYFKNFTAARKQRTE
TLSPDALVFLKRWKDSIKCKESFETLSQQCAEDLDIKRDLEERDIRDLIELDYFRLVDQK
ILSDLVRCVERKTVSSGDVGQWIWQRKQSHWYPEFEHLYEAVATAAQFIHELDGAYLTMD
SLAEGIQRYASSWFRIDQFYRKFIYHVRRSGESSLMERLVDLIENLYDNNYLLKLNDRWE
MLVDKAKSWSAPSIYLQNKFFDQWVQPFLRTEKKIFVIISDALRYEIGDELMSLIRKEDR
Y
>WP_072301528.1
MNISHQDWDSYETSWDFTNLPLLNPDFRQPTLKATYQKLRSHWQQMTLETQRLEEENNRI
FISTYGLQDELTPEVPLNEITLTCNPYYRYDHTKPEAELETLLLADTMKEYISYAVGCMF
GRYSLEKPGLILANQGETVEDYLKQIPNPTFPPDADNVLPILDGEWFIDDITERFRQFLR
VTFAEEHYDENLKFIEAALGKDIRKYFLKDFYKDHIKRYKKRPIYWLFCSPKGSFNALIY
MHRYRPDTVSVVLNDYLREFRAKLEARRDHLKRVEVSADASQSEKTKAVKEIAKLTATIE
ELNDYERQVLYPLAIQQIQIDLDDGVKANYQKFGLALKKIPGLEAKEED
>WP_072301529.1
MKWFFTWVPTLVLVIICSLFLFNSPSYAFNQADLDQLLQNKICEKCDLSDADLSDADLRD
ANLRDANLSFANLSDANLRNADLRDADLSGAYLSFAYLSFADLSGAVLDGAELSGAVLDG
AIFCDDYGRKKGWCN
>WP_072301530.1
MVFEILSPCNSKGEMTRKKLFYLKHGVEEYYVYDPDEISLEVSIREDNSFREVEDFATWT
SPRLNVRFDMTGDELVIYYPDGSRFLSPVELSNYAEQERFLKEQANQRAEKERLLKEQAH
QRAEQERFLKEQEQLKYQTLLSQLKAKGIDITALE
>WP_072301532.1
MVREIAPKTQLGVIKSKVNQGGETIAPDIVYPESDGKPMADNTEQFKWIVKIKENLEILF
KSNPDVFVAGDLFWYPVKGSNRIKLAPDTMVVFGRPKGQRGSYRQWEEDNIPPQVVFEIL
SPCNSKGEMTRKKLFYLKHGVEEYYVYDPDEMSLEVSIRENNSFREVEDFATWTSPRLNI
RFDMTGDELVIYYPDGSRFLSPVELSNYAEQERFLKEQANQRAEQERLLREQERFLKEQA
NQRAEQERLLKEQANEGAEQERLLKEQERFLKEQANERAEQERFLKEQANERAEQERLLK
EQEQLKYQTLLSQLKAKGIDITALE
>WP_072301533.1
MVREIAPKTQLGVIKSKVNQGGETIAPDIVYPESDGKPMADNTEQFKWIVKIKENLEILF
KSNPDVFVAGDLFWYPVKGSNRIKLAPDTMVVFGRPKGQRGSYRQWEEDNIPPQVVFEIL
SPCNSKGEMTRKKLFYLKHGVEEYYVYDPDEMSLEVSIRENNSFREVEDFATWTSPRLNI
RFDMTGDELVIYYPDGSRFLSPVELSNYAEQERFLKEQANQRAEQERLLREQERFLKEQA
NQRAEQERLLREQERFLKEQANERAEQERLLKEQEQLKYQTLLSQLKAKGIDITALE
>WP_072301540.1
MVFEILSPCNSKGEMTRKKLFYLKHGVEEYYVYDPDEISLEVSIRENNSFREVEDFATWT

SPRLNIRFDMTGDELVIYYPDGSRFLSPVELSNYAEQERFLKEQANERAEQERLLREQER
FLKEQANQRAEQERLLREQERFLKEQANQRAEQERLLKEQANEGAEQERLLKEQERFLKE
QANERAEQERFLKEQANERAEQERLLKEQEQLKYQTLLSQLKAKGIDITALE
>WP_072301541.1
MTGDELVIYYPDGSRFLSPVELSNYAEQERFLKEQANERAEQERFLKEQERLLKEQEQLK
YQTLLSQLKAKGIDINALE
>WP_072301542.1
MKMNCPNCDSNNIRKNGQRRGKQNYQCKNCGRQFIESYSPRGYSQEVKEACLYVNGNGFR
AIERMTKVNHNTVIRWVKKLGRQLSDSNNNSQTP
>WP_072301544.1
MLFQSGYLTIESTFTAMERYMFRLKIPNREVKVALGDQLVNAYTDFVEEKLGIQRPLYEK
LFQGDVNGFIDTVRRLFASIPWRNFTNNDLANFEGYYASVLYAFLSSLNARIIPEDITNH
GQADITAILGDHIYVMEIKVVDGEKVKENLALKQIRKCNYAQKYREEPGKTVHEVGLVFS
SSKRNLIQADWE
>WP_072301545.1
MNGTTGSLSPTLNFEVGQISNFPILNNTIDTAQIKITENIDQAINISRQDWDNFETSWDF
LTHPLLRHNSPSISQCFTQWQNQSETAFRQLQLLEEENNRYWIQTYGLETELTPEVPEDQ
ITIHRADQQRDIRSLLSYIVGCIMGRYSLDKPGIIHAGNPFDPSLHQQFPASNHAIIPIT
DQTYFTDDIITRFEEFIQIAYSADTCSENLKFIADTLTLKNGESPRERIQRYFLQEFISD
HIQTYKKRPIYWLFTSGKKRAFNALIYLHRYQEDTLARMRTDYVLELQIKLEGEITKYQK
QLEISTNNADKKIASKRLKELQDQQSELAEYQEKLQHLADARIKLDLDDGIAYNYCRFKG
LVYEGSDLKMADLEKASQWKK
>WP_072301546.1
MFLLIEAPFAAFRPFQSGSYRSTTSVPSPSTVYGILLNLAGIEQRVGTDQDITLIKDDLP
TIEIAIGIPHLPNKPKTEIATLSQQLHSYLVGNSERGKELAKKTYGNKYWIAPVRREVII
NFRLIVGVKANQDLCNRIIEGLNGELKETRYGIPFAGDNNFFFDNIEVINRPPCARWYCP
LDKSTYPERGICRLTTWINRSDNTKTKIGLFYPTDFVLDPPESAWSKLPSNT
>WP_072301547.1
MYRIGGIKGMSGGPILDENGKLVGIHGQTYSQSLGDGRGTPEEYGIPIKTYQTWASQLST
PSVVNTRYKRLESLLRTQNFREADEETNKVILKVARRERQGWLRKEDEENFPCKELRTID
QLWLKHSKGKFGISVQQEIYESLDKDPSLFGDRVGWRRTGRWLSYKNINFSQTAPSGHLP
FVLPFLFREGGGWLERFGRWVTFPSVQTCRV
>WP_072301548.1
MLFQSGYLTIERTFTRRQRYMFALKIPNLEVRLALNDQFINGYTEIINEKSGIQDSLYEF
MNRGDVESMIMAIKRLFAGIPWRNFTNNDLADFEGYYASVIYAFLSSLDARVIPEDISNY
GQADITAMLGVHIYVMEIKVVEGNQVQGNAALDQILQRNYAEKYRGEPGKYVHEIGLIFS
RSQRNLIQADWH
>WP_072301549.1
MAGDLFWYPVKGSNRIKLAPDTMVVFGRPKGQRGSYRQWEEDNIPPQVVFEILSPCNSKG
EMTRKKLFYLKHGVEEYYVYDPDEISLEVSIRENNSFREVEDFATWTSPRLNIRFDMTGD
ELVIYYPDGSRFLSPVELSNYAEQERLLKEQAHQRAEQERFLKEQANQRAEQERLLKEQA
NQRAEQERFLKEQANERAEQERFLKEQERLLREQEQLKYQTLLSQLKAKGIDITALE
>WP_072301550.1
MPPQVVFEILSPCNSKGEMTRKKLFYLKHGVEEYYVYDPDKISLEVSIRENNSFREVEDF
ATWTSPRLNIRFDMTGDELVIYYPDGSRFLSPVELSNYAEQERFLKEQERFLKEQANQRA
EQERLLKEQERFLKEQANERAEQERFLKEQERLLKEQEQLKYQTLLAQLKAKGIDITTLE
>WP_072301553.1
EFWRQHGEPLLKSAPYHEIAPHLVLMAFLHRVVNGGGTLEREYAIGSGRMDICLRYGKVV
MGIELKVRREKLDPLTKGLTQLDKYLDGLGLDTGWLVIFDRRPGLPPMGERISTEQVISP
SGRTITLIRS
>WP_072301555.1
MAGDLFWYPVKGSNRIKLAPDTMVVFGRPKGQRGSYRQWEEDNIPPQVVFEILSPCNSKG

EMTRKKLFYLKHGVEEYYVYDPDEISLEVSIRENNSFKEVEYFATWTSPRLNIRFDMTGD
ELVIYYPDGSRFLSPVELSNYAEQERLLKEQANQRAEQERLLKEQANQRAEQERLLKEQE
RFLKEQANQRAEQERSLKEQAHQRAEQERLLKEQEQLKYQTLLAQLKAKGIDITTLE
>EFA71501.1
MFFSVIIPTYNRLPILQKCLTALENQSYTSQVTGYEVVLVDDGSTDGTLDWLAKHQDLFP
HVRCFQQDHAGPAAARNLGVQNSMGDTIIFIDSDLVVLSNFLDAHSDALTQGRKRLGGDR
LFTYGAVINTCNFEDPTAEPYKITDFSAAFFATGNVAIPKHWLEKAGLFDTGFQLYGWED
LELGVRLKKLGLQLIKCPQAVGYHWHPPFNLQQIHNLIDKEIQRGKMGVLFYQKHPTWEV
KMMIQMTFFHRLLWGLLSLNGLLNEKTNGSPVAVVN
>EFA71511.1
MSAEIICVGTELLLGDILNSNAQFIAQELAQLGVPHYYQTVVGDNPERLKRVIEIAASRV
EILIFTGGLGPTPDDLTCETIADYFGVPLIEDPEIIEDIREKFSQRGRVMSPSNRKQALI
PQGAKVLPNLTGTAPGIIWQPRSNLTILTFPGVPSEMHQMWQDTAVPFLKSQGWGKEIIY
SRSLKFWGVGESVLAEKVSDYLSLSNPTVAPYAGKGEVRLRICAKAPDTVSAQELIAPVE
KKLREIGGLDYYGVDGETLASVVGNLLRLSGTSLSVAESCTGGGVGQMLTEIPGSSDYFW
GGVIAYDNSVKVRLLGVDPGDLDQFGAVSPVVAQKMAIGVKIV
>EFA71517.1
MKSLKQIFVINQIRNLTTNNPDITWGSFAILYRTNAQSRPFEELLVKYQIHYTVVGGMKF
YDRKEIKDVISYLRAINNPADTVSLLRVINTPRRGIGKSTVDALINASQQLGTTLWEILS
DETSVNTLTGRAAKSVTGFTSIMKKWQEKTTQVPASEVLEGILEDSGYLRDLMDQGTDEA
DNRISNVKELYNAVSQFQDENRGNDISLQAFLQSAALSSDLDNLSESRDAVSLMTLHASK
GLEFPIVFLVGLEQGLFPGYRSLQDPKALEEERRLCYVGITRTQERLYLSHARERRLYGS
REPALPSQFLGELPQDLLTTKSKIHAARSTKSTTIMPETPGETNNWRVGEQVLHKAFGKG
VITHVFASDDKISLAIKFTSLGQKIIDPKVAQLQKIN
>EFA71518.1
MKDRIQRLFADQFAINQYGQKLDLLPAQQQTEVISSVYRTYIKNIWCGTFHSLFSRILRF
DVEKYQDEKGRHWQKNFSIFDESDVQTLIKEIVTKQLNLDSKKFDPKSVRYAISNAKNQG
LSPREFEEQQPNYRGRVIGEVYNLYQDQLAVNNSLDFDDLILVPTRLFGQNEQVLAYWHN
KFRHILVDEYQDTNRTQYELLRLLVTNGECRKNQWNWDNRSVFVVGDADQSIYSFRMADF
TILLEFQQDFGDGLDDDDTRTMVKLEENYRSCENILQAANELIENNTERIDKILKPTRDV
GESIYFHKADDEVAEADFCYQSNSEFNN
>EFA71519.1
MEVEMQESQYTETKTKETPIPDLSTQTGSITKLQSPPKSQEQWLKYGQEVSNFLGTLPEY
LVGLFDKYKQPLLTLGLIVTAGVTVKVILAVLDSLNDIPLVAPTFELIGIGYSGWFVYRY
LLKASTREELTSEIDTLKSQVFGQD
>EFA71533.1
MGRVGAVGFMSEADVAKVRGCDGSVTDGSVILASSMPLTLSDEANCPLVATDASRAFEVV
FLNILGLRISKICC
>EFA71545.1
MTTKSDPNRILRLLPLVVGSLGTVLLLVNRVLTPQLTESQARGDVLGVILSAVLILTGLI
WQQVQPKSPDTVELIGKEGFILASDLPETIKTELAWASRLLLTNTVTRSLVVYYKGQVLL
RRGILGSKAEVTPGPILERVLGTQKPIYLVALRVYPGKIEFDYLPDNTQGVICQPIGKEG
Y
>EFA71551.1
MTNPEGKEIEQQLEQLENELMLLKQRYSQIERDQQKKS
>EFA71555.1
MEKIAPNSGDQVYFLGDLIDRGPQSAQVVKFVKENNYPCLLGNHEEMIMNVMVHHYTSSK
AVQSWLYSGGQATMASYRSARIPQEDLDWFGSLPTYLDLGDILLAHAGVNPNKSLSEQTE
GDLCWIREEFHSMKTPYFTNKLIVTGHTITFTLPGVKPGDLAQGQGWLDIDTGAYHPRSG
WLTGLDITNSLVYQVNVFKNSTRCLPLEKALSKINPQEIKLSGRYKQVS
>EFA71567.1
MEWKEIRGNWVIVPRHPVGIIHFLGGAFVASAPHLTYRWLLEQFANKGYVIIATPFVNGL

DHQAIAQSVLLKFERTLERLHYYGELHKLYLPIYGVGHSMGCKLQLLIGSLFPVERAGNI
LISFNNYTAKDAIPLVEQLNSTFMSDLMIEFSPTPTETNQIIKESYQIRRNLLIKFHNDN
LDQSVELTKILKNRFSQMVTVKLSPETIQLL
>EFA71573.1
MTQKVIIFDFDGTIADTLDALVTIANRLAREFGYMQISANELKLLRNLTARQIIKYSGIS
LFKIPFLVKRVKGELKNKIKNVQPIPEIPEALRELSNQGYKLGIITSNAQENVHEFLKCH
QLDNLFEFVHSGVTIFGKNTIMSNVIKQRQIKPETVIYVGDEIRDIEAAKKAHVQVIAVT
WGFNSPEALARENPDFLIDHPWELLEVINRY
>EFA71574.1
MSYLTSLSTHTIKEKAYKLGFHKVGVAVVLGESKEKERLRKWISLGYHADMDWMTNPKRE
DIKLVMPEVRSLVCVAINYYTPHPRPQGVEYGKISRYGWGRDYHRILHKKLKQLATWLES
KGEGIKARYYADTGPIQDKVWAEKAGIGWIGKNGNVITREYGSWVFLGEVLTNLELEGDT
PATQHCGTCNRCIEACPTEAIAQPFVVDANKCIAYHTIENRGETLPITIERNLQGWVAGC
DICQDVCPWNQRFSQITDVVDFHPYPGNLAPKLVELSEITDEQWNEKFSASALRRIKPGM
LRKNARANLTTLKLNNDSKSNHL
>EFA71580.1
MAEDGNSALVKAIIGNHPETFQLLLTKGANVNLQDPVGITPLMYATKQGYIRAVDMLIKA
GAKVNTRNHGGYTALMIAKSNNYAETSNLLIQAGAKE
>EFA71581.1
MIDLLTAAKNGNVQQVRQLLGSGFPVDAGDRHGTTALMFAANFGYTEIVRCLLEFGADID
LPRKLHGLTALMLAAAHNQVEVVKLLTSQGANTNAVNEDGSTALMIAVEKGHIETVQNLL
NFGADPKIVDEHNEDAFKLAIRQNNKVILNILLKHSQVKGETESLLIMGADNGHLDIVKT
LLLYGVNPNLENSDGTTALLAAAAGGHTEIIQVLLDGGAEINHQDQDGETAMHFSVVENH
LETVKTLINRGANLEIRNNLGDTPLILAVFQGYQEIVRVLLAAGADGSKKEFGGIPLDLS
SIPRTY
>EFA71588.1
MAIETHLTLLKAGAVTWLDWRARNPEIQVDLSTCNLRGENFRGANFQNVNLNQVDFSHAL
LVRADFQDANLSAANLNSAKLVQANLRKANLSVANLQNANLMRANLDEAVLIGADLKHSN
LQDAVVTSANLIGTDFHCANLNGVDLAYSNLVRSNLSFANLIGANLIGSNLQDCNLYEAE
IINCYLYDTNLARANLRRSHLGSSYLCRANFMEANLTNADLTGANLKDANLAGANLQGAN
LRCANLTGANLTGANLTGANLPPVFICN
>EFA71596.1
MNIFKDSGNLVLLFGICGNVIKSGGSNMTSKTKETKPSYVFRASWAILLLAINFLVAAYY
FHIIE
>EFA71599.1
MGHALIAYSGGVDSTLVAKVAHDVLGDRAVAVTAVSPSLLPEELTEASAQAATIGITHKI
VHTQEMANPNYTSNPVNRCYFCKSELHDTLKPLALEWGYSYVIDGVNADDLKDYRPGIQA
AKERGARSPLAEIGITKMEVRQLSQQLALPWWDKPAQPCLSSRFPYGEEITIEKLQRVGR
AEIYLKTLGWQDLRVRSEGETARIELPPEKIKDFVLKTDLPTLVTTLENLGFIYVTLDLE
GYQSGKLNRVLTRQDPILTKKES
>EFA71601.1
MIGYFTNDIAIKMLFRPYRPIYVLGRKLPFTPGLIPSNQERLGNNIAHAIMGSLLTPDEL
HKLAGRLLATDRVKAAILWLLQLALAQIKQEKNQKTAQILAGILRDLLGDSLPRLLRVLA
RKEDFLEVQINQVFDRLILEFRLNEEQAIWLADWLMKIAFPPDVLRQAIINFLTDKTIQT
IDDSFREKTSGTYWVVANLFGLRNTLTRLRSFCLDEKELTNQRLQELVQDLQLGDRLKKL
LQDLSLQNLPIGTVRELRKSVKESIRYYLQDSGSNLIQEVTESADWEKISILLLNRLRDS
VIVNSSLESVAQELALILDKYLEKDLETIVAQTIPILSLDKVIVEKVKDTSPADLEAAIE
GIVKNELQAIVNLGGILGLGVGLLQVGFLLLNQV
>EFA71603.1
MNINYIIELINQGENSSVEFKRSDVKLDSLCKEIIAFSNSSGGVVLIGVDDDGTILGVES
DRNYEEWVVNIARNNIIPPVNIQSGEVVWDGKKIVVVEVPKGKDRPYQDNSGRFYIRIGS
TNRIASLNELMRLFQQSGLYHFDVTAVDNTSPSYLNHNAIDRYFHSYDVHYMEMEQEEKI

TLLKNTDIIAENEQVTVGGLLVFGINPQRIFHNASISFAHFLGDTISEELIDKKNN
>EFA71604.1
MFRELIVNACVHRNYSITGSRIRIFMFHNRIEFMSPGKLPNTVTIDKLRFGVSYSINPVI
VKFMENLRYIDKLGRGLPMVYQEAKKLGKDVLFEEIGEEFKVTLLT
>EFA71606.1
MTESANTENHQENTHKSNTAEELLVKLRQKKGNWVEWGNAIAYLQKNGYNPQDIFEATGF
EPIQQNQVVVGAQVYSSLEKFGASEATKAYYGTRASDILYELRLLTQGDRATAAEFNFCP
QAGCG
>EFA71607.1
MDEAREIAKAIKDFSRFSTPPEGFSTHPGDAVGYQCWKLARQNSDLQERSRLIAKGLRFV
QSSTARKQIEQLLTDFTIVPQRNAPLLPYFRLESDEELPRLVPVVGELPLTPEDVKSVPL
IIEEAPFNIVKFAGEQAWVALPGWQVLRSAEDPIVIIGESNIFPQSKSSKTEQILIVIDR
DQRDWDEGGYFAFDNDGEVDFQWFETQPEQTILGRIIVILRPKKVLDEDFTKDSWQIDE
>EFA71613.1
MILGTSTNSNKHNPDFSSGITLGDTALGADMNSSSNSGDGEKNTRLVVPTQQNKTNIGLK
DSPSPEAGNNQTDVVVDLSDRRVYVYLYDQVVASYPIAVGKKGWETPTGTFKVIHKEHHP
IWKHPITGKIFEAGTDSPLGDRWIGFWSDGKNEIGFHGTPNKDLIGGAVSHGCLRMRNSD
VRMLYEQVDLGTFVSVRH
>EFA71615.1
MKTPIGADFNLAGSSNAMDRNHIHQHILFLQGVVTIGAMIFKNCHQVEIDEYLP
>EFA71626.1
MRLVAAARVRRAQEQVIATRPFADRLAQVLYGLQTRLKFEDVDLPLLKKREVKSVGLLVV
SGDRGLCGGYNSNVIRRAENRAKELKAEGLDVTFVIVGRKANQYFQRRGYKIDATYSGLE
QIPTATEATNIADELLSLFLSEKVDRIELVYTRFVSLVSSRPVVQTLLPLDTQGFETSDD
EIFRLTTRGGKFEVEREKVTSTVAPLPRDMIFEQDPVQILDSLLPLYLSNQLLRALQESA
ASELAARMTAMSNASDNAGQLISSLTLSYNKARQAAITQELLEVVGGAEALG
>EFA71629.1
MGTFLLLIAEATAVTGELAEGAEHSGIGLNTNIFETNLINLAIIITVLFVFGRKVLGNTL
KTRRENIETAIKSAEERAANAAKQLKVAEEKLTQAQVEANRIKADAETSAKAAGEAILVQ
AAADVEKMQAAGAADLNAELERVISQLRQKVVAKALQKAEAELKAGIAEDAQIRIIDRSI
AQLGG
>EFA71637.1
MEYQFSFRQFSQKFNHAVITNHGVWDMREGIIIRLVDRTGKVGWGEISPIAWFGSETLEQ
AREFCSQLPEIITPEMILDIPAHLPACQFAFESARENFSNSMPVFKNQHLGDESLIERLI
SKKMKYSALLPRGEAAVQGWKNLWQKGYETFKIKIAVDDITQELEILHLLVGQLPESAKI
RLDANGGLNYQQAKLWLEVCDQFSQKIEFIEQPLGINRLEEMLELSQAYLTKIALDESVA
TWQKLESCYQIGWRGVFVIKPAILGSPSRLRGFCQNHTIDLVFSSVFETPIGREAALKLA
GEISHGISNLSGNWRSLGFGIDHYFALESTNWPEILWNPIY
>EFA71640.1
MPAFPFSFEVLYNPISYRRYDTATLQFNCEDYRAIHKILEPTYLETEIYFQPQIPLFTKY
LAPGLSLAEEPNQKFSPEESFGMNRCQIIANALLEAWQKGKNAIEERIQIIQQHFASELI
DLHYPYLNPSSQDIYIP
>EFA71641.1
MLSSYQVNNSPVNSQISQISSSQSSPVSRLDNYHLSVIESLFDIARNIEISADFSIQHPQ
YQPFAIPSTVAQRFRHNSLDLQRKYLNLLLRNFLHGIYYNGALQPILSTNNHQCSHLLTN
GLEPNYKLGVDRNFYQQLHENNHGIGNYDADWEVLRIEPDGTMAVMKNNLTLYVEPECYL
KSDRSPTPGDLIGIWMPKNRLQNGCYIAIGNCNQQHPSHSHNHHINYHSKKIAKIYFNIT
PSGAIALMNILTKRLNDASIPF
>EFA71646.1
MSITSSDAKIAVEFRDVSFEINHRLLVSKLNLQINQGEALILLGRSGSGKTTTLKLINHL
LKPTQGEVSVQGLSTNDWDVIKLRRSIGYVIQETGLFPHFNVAENIGIVPSLEKWPSKKN
>EFA71647.1

MLDLVGLEPTKFAERYPHQLSGGQKQRIGVARALAADPPILLMDEPFGALDPITRLELQQ
QFQYLQRQLGKTVIFVTHDIQEAFFLASRIGLMYEGSLVALGTKREFIQSQHGEAKAFFG
LFDCCAPLGT
>EFA71650.1
MIKIDRWKIHQHHVEIIYSADGFNFSTNIFYHDVSFSRLINKYSGSYIERIIAHIVLFEG
MKFCSLFPKYYDVSSIAKYLEPRVLDLFVRIYQGVFGQHWYENNITNYQQPEIISSAVLG
GSEPQEILGDNQTILTGCGGGKDSILAMKILEEAGIPFSSLQYSHSVYGKADFQHKLISQ
VVECVKPKHKHQISIYDNFTESPFLKIYFPENSGIIAPETPVSIFQSLIIMLDHGYHSLC
LAHEKSANTGNLYWEKLGKEVNHQWGKGLLAEQILNLFIQEHLLTNFAYFSILQPIYDFR
IFQNFSKYPEFISKIHSCNIQKPWCYKCPKCAYVWLGLMASSSVNIVEKVFKNNLFDDGD
LIPIFREMMGLGQHTPFECIGEVQETWLLMRKSLERGIRGRALDIFKEEILSNPEINWEA
IEGKYNHVYGAEHTIPDWIFARVKEHL
>EFA71660.1
MTGKVTPVLQFPRPKTRKMSDCEKKYYKVLRAVINAQHNAGKYFPEG
>EFA71666.1
MPYQEDLFVADISETHVCILNKFNVVDYHLLIITRAFVEQESLLTGEDFAAMWACLAEFD
GLAFYNGGKVAGASQRHKHLQIIPLAGNEIPIAPLLTTADLENGITTIPAFPFLHAFTTF
SPDQTPQDILSKYYALLDIVSITANDDRKQSAAYNFLATRNWMLVVPRLKEDFVSIPVNS
LGFAGSLLVKNSAQMQLVKDIGPMNILKNVAISNI
>EFA71678.1
MLPIPDEFSFLTSGFAQCISVFIVFLILFTYLPILGSGGVFFLMKY
>EFA71681.1
MNNSSVLCLGEILFDCLADQIGLKLEEVNSWTPYPGGAPANVACALVKLGTKAGFIGAVG
QDDPGDTLVKLLGDVGVDTRGVQRHPTAPTREVYVVRDLNGDRTFAGFGKYHTSDFADTC
LKASDLPKELFNEADFLVLGTLELAYPESEAATHQALQLAERYDLKIVLDVNWRPVFWQD
SELAKQKIHQILPNCDFIKLTKEEGEWLFNTSDAGAITYRLNSLEGVLVTDGENGCSYCL
AENEGKLPAFSVPVVDTTGAGDSFLAGFVHQLNQYGIQALSDPQIAKSVITYASAVGALT
TINLGAIASQPTAQEVEAFLHSNKLSLI
>EFA71690.1
MIFIIVETEAETGMDKPQTLEPKSKLTSKVNQRANYKQLTLFDLV
>EFA71696.1
MLNLVPSNIIVLVLGACVGSFINVVVYRLPRGLSLLWPSSHCPHCLNPLKVYHNLPIVGW
LWLRGKCADCQSKISYSYPLVELLTGIIFLIVFWVFQFSLLTIGYWVFCSWLLALSLIDW
ETMTLPGSLTKSGLVLGLIFQTTLGYVAHNTWSSAIGQLIWGIGGMVLGLWLFDIITSLG
FLFYGKPVMGGGDGKLAAMMGAWLGWQYLLVASFIACFSGVLIGCGQLLSVMVK
>EFA71708.1
MDAIFLPQLTKAPQCTEEVQVDEFLPELETLTPVRGVVRLQHHGNYLEVSGKAESIITCS
CNRCLQQYNQRLTIKTKEIIWFDTNSDPVEDLPLEREVAMEDLVETIAPDGYFDPGEWLY
EQMCLAIPQRQLCNSNCPGIIATGVNESSVDRRWSALEKLKNQLS
>EFA71709.1
MQRQKKSTVFISSEGDVSESINPSIQEFSLASGNAINSLPIPEKFLPDGKNQRGVRNNLA
FESLTITPNNQMLFTATENALVQDGEAAKPNFGTPCRILQYNLSDNQPQKEFLYPMEPVT
PLFNFTGRFDSGLSDLVALDNRGNFLSLERTFTGLGFAVSLFEVSLNNADDIHNIPNLST
VDMSKIKPVEKKLLLDLQTLPLPLDNIEGLTIGPKLSDGNISLILVSDNNFNRLQTTQIL
AFKLKRESPLKKLLRQLGLT
>EFA71710.1
MKESKKAKWKNPRMVIYGIITMIIVSIMGLFLGQIANATVRITDIEFLGVATLPTGYTFQ
NTKVGGLSGITYDVDNDLYYIVSDDRGQKGPPRFYNFKIDLSKGKIDQSKALPVGVTTLL
DGKNQKFALGGIDPEGIAATKKIHRLYFFRGGCQ
>EFA71713.1
MLSWLNNPRFEIIRHDITEPIRLEVDQVYHLACPASPVHYQYNPIKTVKTNVMGTLNMLG
LAKRVKARFLLASTSEVYGDPEIHPQTEDYHGSVNPIGIRSCYDEGKRVAETLTFDYYRE

NKVDVRVARIFNTYGPRMLENDGRVVSNFVVQALRGNPLTVYGEGQQTRSFCYVSDLVEG
LIKLMNGDYTGPINLGNPEEYTILELAQTIQNMINPEVQIKFTPLPADDPRRRRPDITKA
KTWLNWEPKISLQTGLKLTVEDFHSRIHAND
>EFA71720.1
MDECTNKSRRVFVNLEGVNFIDSSGLTALVAGMRNVDKLKGTFRICNIHPDAKLVFEVTM
MDTVFEICETQEEAFAIPF
>EFA71729.1
MQSTNIRAIAQMFRLAGWISFWIQLVLGVISGIIVLLYAFFSQRPGSPNNNPGTGFGIFL
AVCGLIVLGAGIYLGYRYTRIGKQLDSSNPSNRPRKLESVQVIRLGVWTNLGGILVTLLG
AQAIVGTLVARSISPQAVTTQLFDPNRVISGLDMLVVQANTNTISAHFAGLVLFAIFTKS
NHKIVD
>EFA71733.1
MLLGNESKKGEGIIITGGGEYQSPSFGLQNITLLFSGDGSILGITVTNSVSKGTGIWIES
AAPTLANNTLCKCGREGILITGQAKPAILDNVFIQNIASGLMMARSSKGEVLRNVFENNP
VGIAITDLAAPLIANNKLGKNQIGMAISRDASPVLRGNLIYQNSQCGLSINGNAIPDLGK
PQDPAGNIFREQENFDVQNSSSQSLISVGNQLNTAQIKGLLELVAATNDTIIPVISSSFS
DLYAHWSTAFITALAAKGFIGGFPDGTFKPNTPITRAQYAALIKKTFQLPDSQNLNRFKD
VRTDFWANSPIASAADGGFLGGFPDGTFRPGQNLTRVQAIVSIVNGLKLTGSNPNGLLVY
SDRAQIPSYAINATTIATQKLLVLNYPQVDLLEPLREITRAEVATLIYQALVVKGEAEPI
ASPYIVKPDKEQPSFSDLVGHWAEAYIRALVSMNLTSGFADGSYQPDKPMTRSQYAALIA
AAFNPVAKRPTVDFIDIPSDFWAVKAIQIASRGGFIAGFSDRTFRPDQNIQRIQVIVSLV
NGLGLVPNQNHSSLSYTDKNSIPEYAKVAVIAASQQNIVINYPDPKILAPTKEATRAEVA
AMTYQALVAIKRVKPIAES
>EFA71734.1
MIEVDHLSKIYGSTLAITDVTFKVEPGEILGFLGPNGAGKTTTMRILAGYLPLVRARLKL
LDMMSRIIP
>EFA71735.1
MPETPPLYPEMTVEGFLYFVAQIKGIPAGDRPTKVKAAMDRCNITEKRQVIIRKLSKGFR
QRVGIAQAIVHDPPAIILDEPTVGLDPRQIIEVRNLIKSLAGTHTIILSTHILPEVSMTC
NRVAIINHGKIVATNSPENLMTNLIEGSGYELTISGEVDLAKQVLQNIPGVSLVESMPNL
STCQNVILRVLSEPGSNSGKYIASTLIHGGFELHEMRRIGASLEDVFLRLTTEEKTLSQF
VEAKDLVETSGDNI
>EFA71743.1
MATLLYSVVSSAKIRVCPPEPRAIPFEMRVDELYVYRDWA
>EFA71744.1
MTTTEMVRIKSGFNADDQHPIQATSADIILRQQLEHSISRYFYHGCDRNIQNLLSYCRWY
MKTDTKALTFSY
>EFA71745.1
MTTTLQQRSNASVWDRFCEFITSTENRVYVGWFGVLMIPTLLAATTCFIIAFIAAPPVDI
DGIREPVAGSLIYGNNIISGAVVPSSNAIGLHFYPIWEAASLDEWLYNGGPYQLVIFHFL
IGCACYLGRQWELSYRLGMRPWICVAYSAPLASATAVFLIYPIGQGSFSDGMPLGISGTF
NFMIVFQAEHNILMHPFHMLGVAGVFGGSLFSAMHGSLVTSSLVRETTETESQNYGYKFG
QEEETYNIVAAHGYFGRLIFQYASFNNSRSLHFFLAAWPVVGIWFTALGVSTMAFNLNGF
NFNQSIIDSQGRVIGTWADVINRANLGMEVMHERNAHNFPLDLAAGEVSPVALTAPAING
>EFA71749.1
MSVLAAIAVLATLILVHELGHFIAARSQGIYANRFSLGFGPILLKYRGSQTEYTIRAFLL
GGFVGFPDDDPDSAIPPNDPNLLRNRPILDRAIVISAGVMANLVFAYLVLALQLGVVGIP
KEFQYQPGVLIKPINEQSIAYQAGIREGDIVISVNGRELVAGKDSTLYLTQEIQNHPRQP
IDFQIQRQDREISLQITPGENPEGKGLVGVELAANGKAVYERPQNPIQIFTVAGERFQQL
FVGTIKGFGQLITNFQQTASQVSGPVNIVKIGAKLAADNSANLLSFAAIISINLAVINIL
PLPALDGGQLFFLLIEGLFGKPLPMKIQEGVMQTGLVVLLGLGIFLIFKETLQLSFIQQI
FQKM

>EFA71751.1
MDIYKAVILPLLFTLVKADPEWLHYVLISGMDWFLQPSHPHRARWLKSFVGDSLCVHDTR
LKQNLFGLNFPNPLGLAAGFDKDAMGSHFWSMFGFGLAELGTVTYHGQPGNPQPRLFRLP
MDLAALNRMGFNNSGAAAMAARLTLLYQQQLPAIPIGINLGKSKITPLEAAATDYLQSFR
LLKNLGDYFVVNVSSPNTPGLRSLQDRPMLSQILEVIQTENSIQNQPKPLFVKIAPDLEW
EAIVDIINLAKAYKLAGIIATNTTISREGLKTQVIEKTGNPPQAEPGGISGLPLRQPSTE
IIRFIYKQSQGQIPIIGVGGIFTAEDAWEKINSRCKFNPGLHRLDL
>EFA71752.1
MTQTNMENYGKSFLVVIVPISFLIIVVLNTWKFLLIGLFLIMSLNLWQKYRWEKWCDQVN
PLFYELIEENQGKISSVDLAIRGNFPGDDAKRYLEKKAKEFGTNPVDSGTNGPHYYFITG
SILGEILDGSESDVKPVGRAVTKEARSLLSPPVLVLESKEGDKEGQITNRLETKSLEKQL
AFGSLIQSELAQRLGVYSSTVYKRRNDPDFSEWTRSRDPDGIAWSYSRKNREFSPWINKQ
W
>EFA71753.1
MPGGLLQNNISIISILAKIDPFFVFPGKISTTGWQKPPIVGVLPFLKSYN
>EFA71756.1
MNGSYQVRGINLKSHFLGESDKILTVLTRELGLIKAIAPGARKYQSNLNGRSGIFMVNEL
LISPGKSNPTQTSGLYRINQAQTLKTHAGLSKSLGKLAASQYLAEIALHQALEEHPQIEL
YDLLNQHLDTLDSLPLIPQIAIVAHLAKGVFDMLNLAGISPQIQLCCLTQKPLIPDLSNP
KWQAGFSTTAGGTICLQTWEKFKKQENFHEPKIPENDFERVLHQQELPKISRRLGAKELM
MLQYLSQPGIIQIDSAYDDSWLSVEQILRHYTQYHLGFPIRSATLIDSYFAPNHDAIV
>EFA71757.1
MKSYQDNHQQRSVKGFSMEKTQTSDSPKSPPTNSDLPLKVNPINGSLETTHEKEKAVLEE
TQSQIDLEDKDGSKSYLKAGGKHGKEGFSQY
>EFA71758.1
MIALINSEFQQRGQSISGWVSALMMIFTIPAVLFGSVAGVFVDRWSKKAVLVASNAWRGI
LVITIPFLIWLTHDWHPIGALPVGFLMILLVTFLVSTFTQFFAPAEQATIPLVVEEQHLL
SANSLYTTTMMASVIIGFAVGEPVLVLADSLWAKFGGAGGLGKEILVGGSYGIAGIILLF
IQTQEKPHPPETEFPHLLSDLWDGLNYLKSNRSIKNAMLQLTILFSVFAALTVLAVRMAE
IIPNLKASQFGFLLAAGGVGIAAGATIVGQFGRGFTYGQLSLWGSLGMSGSLIGLAIFTN
QLWPVLLFIASLGVFGALLGIPMQTTIQTETSAEMRGKVFGLQNNVINIALTLPLALAGI
AENFIGLSSVFLLLSIIVFGGGIITWYNSSSH
>EFA71760.1
MGVGVTLISGGELESPDAGDPARLIRLRYRETAELVKVRGKMCVLMINDLDAGAGRFDEG
TQYTVNTQLVNATLMNIADSPTDVQLPGSYDAKPIRRVPIIVTGNDFSTLYAPLIRDGRM
EKFYWEPNHDDKVGIVGGIFAEDGLSPVEIEQLVNTFPHQSIDFFSALRSRIYDEQIRDF
IHEIGYDRVSMRVVNSMEPPPQFQKPEFTLFHLIEAGNVMVGEQKHVESSHLVAEYNRRG
YQAQSPVKELPTLQSNHDHNDQNVPNKTLENSPPLSLVTLDTQEQIRQVLSQGHKITVEY
VDKRRFSMGSWQTCGNLHISDYPQAVSALESCLLEHAGEYVRLVVIDPKLKRRVLETIIQ
RP
>EFA71776.1
MLSLKRALKNDRKRVGRGISAGQGASAGLGMRGQKSRSGSSTRPGFEGGQQPLYRRIPKL
KGFPVVNRRVYTTINVEKLASLPANSEVNLASLKEAGILTSAKGPLKVLGNGELGVPLKV
QAAAFTGQARSKIEAAGGSCEVLS
>EFA71780.1
MLTWQGIKQRKCQQQKMTRSIAKVLQQEGFISEYTEAGEGVKRNLVISLKYKGKSRQPLI
TTLKRVSKPGLRVYSNKKELPRVLGGIGIAIISTSSGIMTDREARRQNLGGEVLCYVW
>EFA71794.1
MALITTGSGFIRDLEKFGSLGVFVPLEGGYEGRYRRRLRAAGYVNLHITARGLGDVAAYL
MGIHGIRPPHLGKKSTSNGAAVGEVQYLPP
>EFA71795.1
MWVIEGNILSDQEVEYFTNLPRLEPRVKVVIERGGDRYFRWTPLEKTLLAS

>EFA71797.1
MLRLEHISKIYPTGEVLKDINWEVKPGDRIGLVGVNGAGKSTQLKIISGEIEPTAGQIIR
PASLHIAYLNQEFEVDPTRTVQEEFWTVFKEANAVQLALYEVQRDMETATVEQLDKLINK
LDKLQRQFESLDGYNLDARIGKILPEMGFQVEDGDRLVSAFSGGWQMRMSLGKILLQKPD
LLLLDEPTNHLDLETIEWLENYLRSLTTPMVIISHDREFLDRLCTQIVETERGVSTTYLG
NYSAYLQQKAENEASQLSAFERQQKEIEKQQAFVDRFRASATRSTQAKSREKQLEKVERI
EAPTTGVRTLNFRFPPAPRSGREVVEIKDLTHTYGDKILFLGANLLIERGDRIAFLGPNG
AGKSTLLKMITGMEIPTEGTVKLGDHNVIPGYFEQNQAEALDLNKTVMETIHDEVPDWKN
EEVRTLLGRFLFAGDTVFKQVGDLSGGEKARLALAKMLLCPVNLIILDEPTNHLDIPAKE
MLEEALQNYDGTAILVSHDRYFISQVANKIVEIRDGEFRVYLGDYHYYLTKIEEEKEQAK
LAAKKATKTAKAAAKKK
>EFA71804.1
MRVFISTGEVSGDLQGAMLITALKNQAATLGLPLEIVALGGSQMAKAGARVLGDTSGIGS
MGIVEALPYIIPTIMMQRQAIAYLKKNPPDIIVLIDYMTPNMGIGSYMQQHFPQVPVVYY
IAPQEWVWSMSLDRTRKIVNFTDKLLAIFPEEARYYQAKGANVNWVGHPLVDKMVNTPSR
ESARKILGIQEQELAIALLPASRHQELKYLLPGIFQAAKNIQSQLPKANFLIPLSLERFR
GKVTRAIKDYGLKARIFSVNQQEIFAAADLAITKSGTANLELALANVPQVVVYSLSPFTA
WVGRKILKGSIPFASPVNLVLMREIVPELLQEKATADNITKAAMELLLNREKRTKNSIGL
SRDASVFGRSRSLRSRS
>EFA71806.1
MQVYRDDLESFTSKIARNEIQEVRQIIPTGIGIMAGLRTSPVPMQQITKQVRTVQREELG
IVFFYYETMWNRSPETLEQRIQGFKNFFPYPAVRVAAE
>EFA71807.1
MKLHLFSFNYHPKDRTNWPGKIWKQFSPWLCLISLLTVVLIHSVPSVTAQMSREEIRGVW
VTSNDLNVFKDRDQVKDAVTKLRRLNFNTIYPVVWNSGYVMYPSNVAKSLDIQPFVFRGS
DGHDILADIINQAHSQNLLAIPWFEFGFMTPNTGELALNKPEWLTKMRDGSTVSMSAAGE
VSWLNPFHPQVQKFIIDLLVELTNNYDIDGIQFDDHTSLPHQFGYDDYTVNLYKQETGKN
PPANSQDSEWVAWRANKITEFMVRLNHTVKQIKTKSHLFCFAKLLRPCL
>EFA71811.1
MNLVVLQNWLDNASFAILFCTMLIYWVGTAFPGLSITAALGTAGMAIANLSIAVLLTARW
IEAGYFPLSNLYESLFFLTWGITAIHLIAEYTSRSRLVGVVTAPVAMGITAFATLTLPSQ
MQNSEPLVPALKSNWLMMHVSVMMLSYSALMVGSLLAIAFLIVTQGQNIELQGSSVGNGG
YRANGYKVIKSQELIMEGSGGEKNGFARVESNQNGNGYSTAVLEVVKADHISPVTTLSPQ
RLTLAETLDNISYRVIGLGFPLLTIGIIAGGVWANEAWGSYWSWDPKRNLGPNYLVGFCR
LSSR
>EFA71812.1
MVFAAYLHARITRGWQGRKPAILAAGGFLVVWTCYLGVNLLGKGLHSYGWFF
>EFA71815.1
MTKIHKYILSVSQLDRAAIALILVLGLLIGITIFKGDGVKPNVRNFSWQDRQIGAEDRAF
SLVFSRPMDAKSVEDNLQINPPLAGKISWAGRRMVYTLLTPAPYGTNYQVSLTKAKDKFS
RSKDNNRLIKAFTGKFSTRNRALVYIGVSPQEYGRLVLYNLTKEEKTILTPRDLIVMDFE
PFPLGDKILFSARASNNPDLLSAQIYTVTTGVGNGQEEKSVGKLGLILDNQEFQNLKFDL
SPDGGTLVVQRGKKDNPGDFGLWYSSLDDLGSNGQLILKRLEGQPAGDFVITPDSKAVAV
AQGQGTAILSLAGDTSKPLDFLPQFGLVQAFSKDGSQAAMVKFNSDYTRDLFLVTNQGVQ
KPLLKTSGSILYCSFDPSSPTLYCLLTSLVSQDKYVEQPYLMAINLDTKKYKPILMLPVG
QRNVQMSLAPDGLAVLFDQIVPVSNNSLSLPPSILKTDDGEAIAKSSLWFMPLLPIAEDQ
AKSMIKPEALPIDGFNPVWLP
>EFA71816.1
MAAINFMLFHNYTKEKQIMVDSEIQQLVNLNQELKNS
>EFA71820.1
MLPIKSYLQLTPDLRICRILNGMWQVSGGHGRIVPKNALNSMFKYVDAGFTTWDLADHYG
PAEDLIGEFRRQLIAQRGGLAANNIQTFTKWVPRPVNMTKSIVEENINISLKRMDVPCLD

LMQFHWWEYGNPNYLNALKYMAELQAQGKIKHLALTNFDTEHLQIITQAGIRIVSNQVQY
SLVDRRPEVNMIKFCEGHNIKLFTYGTVCGGFLSEKYLGQREPRGFDLNTISLKKYKNMI
DAWGGWQLFQSLLATLKQIADKHRASIANVAINYVLKQPAVAGVIVGARLGISEHIVDNQ
RVFEFELDHEDIKIINGVSQQSRDLYQVIGDCGDEYRR
>EFA71823.1
MTAKVINHLKSKGFANIYTVPDWAENQRQTQIFVRRGTRHPGVELRKILGVGQIEVSAQG
DLDADLTIRIGEDWK
>EFA71824.1
MIKEVQWLENQLGIQQVTALEHDGKLQQLMSVETQPVSNPLPGLGTEVKAEHVNSQRTVI
KSMGEIPYQLFEKLGLSMPGWLLWVLTFAISITLSGLLMSAVALWTPLWSNLEQAEDDGY
TPTNRENLKVSDGLWNKLSLYQLSKPMNILVMGVETIKGTLDGSPESFAGSSDTMLLVRL
NPSDKSIRVLSIPKGTMVSLPEDGLSKISEANAKGGPVLAARVISRTFSDAPIDRYIRIS
TSGLRELVDQLGGVDIFVPQSMTSQDQSGRTPINLVTGWQTLNGEQAELFARFRESSVGD
MARVQRQQALIGGLVQRLNNPVVLPRLPQLTRMMRKYFDTNLRMEEMMALANFAVNVERD
KFEMTMLPGTFSKFSKDPESYWLNLTGQQSLLKN
>EFA71830.1
MGGFFSAVSKVPITAIVIVFELTTDFNLVLPLMIVAVTAYLVADYFVPGSLYDELLKLNG
ITIEKNSPIEGVLTKLKAEDVMQKRVETLDAQMSIKEAIQAFSRSHHRGFPVVDQGQLVG
IITQSDIKNIYPFQYTTLREIMTPGPVTVQPDQGLSEVLYLLDRHQISRLPVVEKQKILG
IITRGDIIRAEADSINCKNIASGPQPEPSYLVYQTRSPSTGRGRLLVTLSNPDTAETLLE
MAATIAQERHYELECLQIILVSRHSSPSETKVRTTKSKRILRQAESLAKRWQVPIHTQIR
VAHDVAQAILETIKERHIDLIFMGWKGNTSTPGRIFGTVVDTIIRQATCDVVLVKLSSLH
NKSYLANFHRWLVPMAGGPNAPLAIKLLPALVSLVENPQIRLTQVVKPWEKEPDMHVLEE
STRQLIRNRNIHGEVIAASLQGESVSQAVIELVNTEGFDVVVLGASREGLLQQAIHGNIP
ETIASGVNSTVILVRGEI
>EFA71831.1
MLIVDRLAKQFRQIWQPRKGLAIAEASIIGIVAAVSAVLLKQSSGWLGTWRIHSTHILPA
WLVLPLIGMSFGFFAGWLIQRFAPEAAGSGIPQVKASLGNVPIRLSWQVVGIKLISTIFA
LGSGLILGRQGPTIQVGAGLAAGMSRLVPTSPEHRRQMIAAGAGAGLSAAFNAPITGVLF
IIEELLKDLSELTLGTAIIACFIGGVISRLLGGGTLQLNRELVNYSAQFYVPEIPIFLLL
GVIIGLFSAVFRHGLRLSLKIYQRLHITLPLRVALAGLISGLIVALLPEYYRDNAGLREY
MIASEPNLLLATITFISQFILTLIAFGSGAPGGLFAPSLILGSCLGHIIGVFEVQFFGFL
VPLILTL
>EFA71840.1
MAIDKNQKNQNPLFQKIARVSRRSHQVKDSHINLSHGSGGKAMSDLINDVFVKNFDNEIL
SQLEDQASFDLSTLGKLGDRLAFTTDSYVVDPLFFPGGNIGDLAVNGTINDLAVGGAKPL
YLSCSMILEEGLPLETLRKVVESMQHAAQKAGVQIVTGDTKVVHRGSADKLFINTAGIGI
IPQGIKISPRHIQPGDMVIINGEIGNHGAAILIARGELELETDIESDSQPLHELVAEIIK
VCPDIHAMRDITRGGLATVLNEFAQTANLGIKINEQTIPIREEVSGMCEILGLDPLYLAN
EGKIVIVVPQEQAELVLSTMQDHPLGKQAAIIGQVVDQLPGIVFLKTVFGAERIVDMLVG
EQLPRIC
>EFA71851.1
MEVTPIIAKIKQALPKGGVIYNTLNGDSNVAFFKQLKGAGLTPDKYPSMSVSIAEEEVKA
IGVEYLKGHYAAWNYFQTVDTPANKKFVEAFKKEYGADRVLNDPMEAAYIAVYLWKQAVE
KAGSTDLAKVRQAAYGQTIDAPEGKVTMNGNHHISKVVRIGEVRDDGLFKIIYATPAPVE
PIPWNQYVKETKGFACDWSDPAKGGKYKQS
>EFA71854.1
MNVKILETENVTVSFDGFKAINRLNFTMEKGELRVVIGPNGAGKTTFLDVITGKVKPTQG
RVLFKGRNLRNFSEHEIARLGIGRKFQTPRIYLNLTPRENLELTSNRQKNCFFYPI
>EFA71855.1
MGTIGLTSKADIPAGLLSHGEKQRLEIGMLVAQSPDLLLVDEPVAGLTDEETYNIGELLL
TLSQDHSILVIEHDMEFVRQIARKVTVLHEGSVLCEGDFKQVQNDPRVIEVYLGQQQE

>EFA71858.1
MLTSSVTVAQNSLLVVFPPKNHQTSTEKIFFIGTAPPQGEVVINGRKVKRSQSGHFSPSF
PLQLGENLFKIRYQNQEQEIRVTRVSTQPELPTGLGFAPNSLQPGVDLARLPGELICFSA
IAPPQATVFVKLGEQMVSLTPQPLQANLPANSSVLTGRNQPTGYIPNKYQGCTTVTSVAD
LGQPQFTLTLNNQTISQTAPGKIQILHPAQLSVAEVTSESCVTRTGPSTDYSRMTPLPKG
TRVMITGQEGDWFRLDYGAWVNRKEIQIIPGAAPPKTIIRSVGYSQLPKVTEIRFPLQVP
VPLKVQQGDRTFTLTLYNTTAQTDTIRLDDDPLISRLDWQQVTPDQVKYTFNLKKLQQWG
YKLKYESSTLVLTLRHQPTIALARRLPLLGMKIVLDPGHGGKESGAIGPTGYAEKDVNLV
VSKLLRDELVKRGAVVVMTREDDREVSLVERQEIISKEEPAIALSIHYNSLPDNGDAENT
RGFGSFWYHPQSHSLAVFLHNYVVSQLKKPSYGVFWNNLALTRPSYAPAVLLELGFYEQP
SRL
>EFA71861.1
MKFYLGTKNGYSFVRFHLGSGINGNDLSKRGTENTGEVRFHLGSGINGNIHGTFCFWTAE
KYVRFHLGSGINGNS
>EFA71862.1
MEEKLKELRLVRFHLGSGINGNFVDTFSLVSVTSVRFHLGSGINGNSHA
>EFA71864.1
MRYLVEMLDNLIDEVGEDETHPLASLMEIIGVLIEQYENQNIPETFKANDRHTTNMPQVF
SKLNSLLQAQNFKQADRETRKIMLAIAKREEEGWLRIEDAEKFPCKELRSIDQLWLKYSG
GKFGISVQQQIYQRTLNMT
>EFA71865.1
MNSKFPIKHISPLCTLFLLLTGTNLIRVTPALGIDQHQIIAKQLIQSKKSQVNQVFQPIL
SKIKKNHPNQDSITHPYSHWEK
>EFA71866.1
METVTKNKYQILLGFTPDCGGGTACGFGAISAELVSSNTPKPVGKEVNLNNNKKAYFEDF
KCGANCSNANLIWREKGVQYTIGLKAGSLSDLVKMANSVVSPK
>EFA71868.1
MNDKKRNQWDLCRFIRTLTYFEVFPLLNWIQNILQNRPTNRQDQPTGRIQMGIILVAGAT
GGVGKRVVKKLLTQGYRVRCLVRDIEKAREILGNEADLVVGDITKPESLNDLVMSNIQGV
VCCTAVRVQPVEGDTPDRAKYNQGVKFYQPEIVGDTPENVEYKGVKNLIVAAKRYLPTTG
EKIIFDFTQPSSDLKNIWGALDDVVMGGVSSSNFYIFEKTAVFNGNVSTANSGGFASVRT
KNFSPAINLSGFTGIRLRVKGDGQRYKIFLRTETTWDGIGYSYSFDTMANTWIDVNIPFV
NLVPVFRAKTVKDCPKIDESKICSLQLMLSKFEYDGRLNPKFTPGAFTLELESIRAYGGE
GVSQFVLVSSAGVTRPGRPGINLEEEPPAVRLNDQLGGILTWKLKGEDSLRDSQIPYTII
RPCALTEDRGGKELIVDQGDNIRGKISRDDVAEICLQSLQQPQAKNITFEVKQGENDAVS
LNWGQLLSQLQPDRPNPL
>EFA71879.1
MEAVISQAQSIIHQIANEVVNHPSLSILQEQLAKLTLELDRKEVQIAVTGGKSVGKSTII
RLLKTVSNLSANPWELVETLPLFTRLSQDDDPAILLTLQKSDLVLFITNGDLTASQLDVL
QKLRSTQQLVLLVFNKQDQYMTEERIIILQSLQQTFPGHVLATSASPLPIKVRKHLADGS
IQEGMEQPTPNIQQLVEQLTVILAQQIPQLVCATTWRKSLFLKTQARSCLNSIRRDRSLP
IVEQNQWIAGAAAFANPLPALDILATVAITGQMVMDLSNIYLQKISLDQAQKVARTLANL
MFKLGLVELSTKAVTGVLKTNAVTFFAGGMVEGVSAAYLTRIAGLSLIEYFEQQDIALTS
DRQLNMETLGGVLQRVFQENQTLLLLETFVKQGVKRLSRETKPSTTYVHF
>EFA71886.1
MGDFGIDLTSTHVFKPLMPLGVEHIKSIKIDKTMLTRENFFEAESFLPGA
>EFA71887.1
MEVMDLESEGVKKTSPQDISKTIRVSALHALAYCRRLFYLEEVEELYTQDAAIFAGRRLH
TELEKQEDEVWEELFLESEELGLRGRLDALRTRDGQTIPYEHKRGYCHRDENKQPQAWES
DKLQILAYALLLEFALGITVKEGRIRYHTDNILIHVPLDHSGRESVHQAIKEARNLRESA
YRPPVTENERLCARCSLAPVCLPEEARLTHDKEWQPIRLFPEDDERLIIHILEPGALVGK
TGSQIKINRRNQPIENIPASQIGQLVLHSFSQISTQALYFCAHQDIGIHFISGGGRYVGS

FDNRRGSIQRRIRQYTALSDPAFCLELARKLVLCRGQGQRKFLMRGTRGRTQVPENLHKS
IAQIKTMLKQVPQAKSLESLLGLEGNIAASYFSALPHLIVADVVDELYFNGRNRRPPKDR
FNALLSFGYALVLKDVMNAILTVGLEGALGFYHQPRSQAAPLALDLLEIFRVPLVDMTVM
SSINRGQWHPKEDFEIRREQVWLSEPGKRKFVELYERRKQETWKHPVTGYSLTYRRLFEL
EVRLLEKEWSGEPGLFGNLILR
>EFA71889.1
MFQRLGAEDYTHYDPKDPGYFEFEGSGYVYKGGNHFSGTHIMGTTPKNSVVDQNLKCWDH
PNLYLVGSGSMPTIGTSNTTVTIAALTFKAVEQMLKEL
>EFA71899.1
MIQCPECKSSKINKNGHKKANKIIFVKTVVDNLLIITKPTEDIQKRQEMD
>EFA71911.1
MGFWLRNSQFNNNSHRESAQWKKDIFLGNHPGNEHNPRDAGHFWGSDPNQKYLWGNHTRR
PVPVYYQGAYSGNLTIYAGQNVKFTDSTGSTYTIPGGRGLVDQTHIFQTMKRAITGSNL
>EFA71917.1
MVLYYGQNRHPLLDSLPEIFYREEDLYIARQWGLKTEEWMSHCRTASEITVLADLACSVK
NDHPSLAMVDGSLIYWFLEQLPLEARDQILLPILEAWGKLRQAGIPIVGYLSAARNNEAK
NLLRLLNCPYPVPDCINYCPDQLDYVPCKKFEGLRDTTLWATQLQPGQRSALWRSNSRIL
QLYDDQVIYFCYLHVGTEIARIEFPAWVVNDPQMIDQALGLVLAQIQKGYGYPVAIAEAH
NQAVVRSGDRNQFFALLEREMIKAGVKNVSISYKEARKRGSIA
>EFA71922.1
MKGNNYCAKIFEFNNNKSGFTDFWLPIILVNLVLTVSTPISPANATEQSPDQIGREENQP
HRDQSTREVNLDVKLPQINAQNNSQNADNSEEIDKIRRRLLLEPVIKQKPAPKPQPTNAP
GLSFAGVSAFGANMGDVFLGTSVATAGNRGNSLKDIDGSMSAGFGLGDARGLIGLEFTFN
NGSIKNFGSNGTFDLKVHRTIQTDNSSSIGVAVGWKTFGQYKTKASGEAVRPSSLYGVVT
SHSLLRANDPVNKMPISFSLGVGGGDFRQGNDSVGVFGGAGLQVHPQIGVGLGWSGIGLN
VGASFLPVPTLPLTVTTSVSDLTSNSPGSTVFSLSVGYGFNFLPK
>EFA71924.1
MVPTPQETAKSEMFKANQAMQIARRSNERAGGIWIYQFIRSPIWFISVLFT
>EFA71925.1
MSDLSKLSILIIGSYNGQDSFGDKCLARCVAQQIRETCNFSGHQSPNILSHIDGNLETSQ
GEIPEVEFSTGISLLYWAWNNKLRHLHLPTALQRFMGVITLPVYLMATQVNRRKLQRIKR
EIRESPLVYFYGGTQLSEQWFWYNFIPLMITAFLCKLYQVPLYFGPQQYGPENNWQRWFL
RTTVKYLVRDIRVRNKNCLELLNLSPEKLCYDEVFSCAIRYPVVKHHVPPKNFILVNMRG
TNFLRDGESREFEVFSNLLVAVKNQLGLPFKLFQMSGSSFCDDTKLLGFLQSRPEFSDIH
LEVLPLVVEEAELIKVAIQAYGTISMSFHGCILSMIGGCPSVPVTSGDYYDYKYIDFDKY
SGEQGTPLITLGNLDVKQASATISDYFSKYSPVKTAIARERAAQQMKYWYKTIV
>EFA71928.1
MAKFLRSKLIHPKLSYKIWQLLCKLNHGQSFFFPLRSSGNFPVLMYGGICGYSDSAIFFE
SLQYEQENIAVYRSIIIYYQILF
>EFA71930.1
MSKLSFLIFILDKYLRNQSRDCPHCGSENTILKQRKKLLLELRECQDCFLMYRYPKDTMV
DNFNFYQLNYDELLVIATPIESKARLVYL
>EFA71931.1
MKVLVSCGTKFHSDHLAYELQQRNSLYKLITSHPSVAYKRKPIDRKKILFLPPIFVISLV
LSRLLGKFYVFRSRLEWVLAIIYDWMASLFVQSPDISISWAWASLRTIREVKKRGGIAIV
EECGSCNLYQEKLLREEYQNLNVHYKPQTFHKIILRELEECQEADYILCPSQHVANSLNQ
CGIAQEKLLIIPYGVELSLFNPQPKKDDTFRVLFVGTVGIRKGLIYLFKALEQLATKNNL
ENFECLIIGRLDYDFKHIFSKYKDYFKYIPRVPHDQLKEYYSNSSLFVFPSLDEGMAYVQ
LEAMACGLPVICTPNSGGDSVIRNGEEGFIIPIRDSEAIQQKIEYLYFHPQELQKMSQQA
LERAKEFTWENYGNSLSKKLQELKLRREKC
>EFA71932.1
MYSRIKLATKELDLSSYESCLSISYSKPLCNLIGFLDSQILEANYPQYNICNLDLPSETF

DAVVTDQVLEHIECDPCEAINECYRVLKPGGIMVCTTCFMMPFHGSPDFSVSGGGDYWRY
TPQGLQFLCRNFSKIIQSDGWGNPLLPILGGLGLVHKPIPDAAWHPLHKIATYDRKSYAC
VVWIIAQK
>EFA71933.1
MKRVTFITPAHISCNPRLFKEANTLHSAGFQVRVVAGDYSPEARILDNSISPEPPWSWVK
VPLGSKPIYLATRFLQKLAQKLAAHKYILNLSIATFSNSPISYQLAKAAAAEPADLYIAH
CLAALPAAVMAAEKHHGKVGFDAEDFHIGELPDIPENQAEIAVRDVIERSLLPRCDYLTA
ASPLIAEAYGQRYGVEMTTILNVFPLSEAPPSLSQKQEINSSPSLYWFPKPLVVVGGLSL
>EFA71935.1
MSLPYFKEFGWEPHILTVKPEFVIGVNDPLLVKTIPSHTSVTYAQALPFQKTKLIGIGSL
GLRCFPFLLKTGNQLLKQNKI
>EFA71936.1
MALGRIWHDKFNIPYVLDFQDPWLSDYYEKSGVTPPGGN
>EFA71937.1
MSKVSHIVSVSPEYPNSLRQPYPYLKPEQFTVLPFGAPTVDFEQLPDLNIHNRIFNCQDG
KRHWVYVGVVGNIMSLALRILFLGIQSHRRQHPETWQSVKLHFVGTSYATDNRAVKTVEP
MAREFGVDDLVEEYTQRIPYFEALQTLVDSDGVLLIGSDDPSYTASKLYPCVLARKPILA
IFHEKSSVVNILKQCQGGECVTFNSTIQPQNLLEDILKKLKLANFATKSL
>EFA71939.1
MQNPHVSVVEKLQWCDQHREELAAMVHRVYQDFQPRDWAEVAKDFTKIVRDCLNQNSDHQ
EK
>EFA71944.1
MKNLALAISGKMDKHFTALWDGGHIKFFSVVTMKSLLISEGYEDIKFKFAGRIPYLWKSM
LCSAKLIK
>EFA71946.1
MVGLISTYGGSSYPTAVGVEAGMKAAVDFYWGKKNLQGLKVAIQGVGNVGKNLCEILSHQ
EVEIFVSDISNHKLAEVERLYPVNIVDVEEIYELDVDIFAPCALGGIINSRTIPKLQANI
IAGAANNQLENEELDSQLLADRNIVYCPDYVINAGGLINVYDGMIGLSEESSLARVRNIY
NTLKQVFAISKEYNVPPLTASKQLAENRFLQHQTQN
>EFA71947.1
MEIFLKIAEMGHKQVTFCYDQESGLKAIVAIHNTNLGSGVALGGTRLLPYSTEEDALKDA
LRLSYSMTYKAACANIPMGGGKAVIIADPEQKNNKLFSAYGSFINSFRGSFITGQDVNIS
WEDAHKIGEKHPTWLA
>EFA71954.1
MIRRVATFSGIPTGLGISTLVVSYLLVSYAHIQLPPIAVLLVNMGLFGLGVLGITYGVLS
ASWDEDNPGTLLGVGEFGTNWSRMVEVWRETRKK
>EFA71955.1
MSAQESEPSRLPFEPNKKRQKPSKVSAKAVVKTEESPQKLPNQPPLLKRKWRFPK
>EFA71956.1
MALTQLRKQELISGFQVHETDTGSADIQIAMLTERINRLSQHLQANKKDHSSRRGLLKMI
GERKRLLNYIQKNNSDKYQSLISRLGIRG
>EFA71971.1
MTDEPSSVIDITRKRAMINFGINGTGLLAQVNFGANSASVLGIFLAVAGAALYFLRTVRP
ELSRDQDIFFAAVGLLCGFILIFQGWRLDPILQFGQLLLVGSTVFFAVESIRLRSIATQQ
AKRNTPIVDDEREVSKKYSYSQRRNYQAEVDEDLDPLPYYEEEEPEPPNVLGFAVVGMIV
LVKRATKTLPSAHHVVVVRNNQIHELKNAVLVMEAPPTVLIVMILMKIGALLLDQLMTGK
LPLQKPENHLVLVNLNLQNFPRMNHPLDQEGVVPLWSLLSLEMMNQLMLIMYPIIP
>EFA71973.1
MTSVMSGVDLISWTVGPLLGVMTFLFYFPDHFDLVSPGES
>EFA71974.1
MPPLGGVDITPIIWVGIFSLVREFLLGQQGLLTMAARLNG
>EFA71975.1

MGDKSTAKETMQKAGVPTVPGSDGLVETETQGLAIAHQIGYPVMIKATAGGGGRGMRLVR
SEDEFIKLFHAAQGEAGAAFGNAGVYIEKFIERPRHIEFQILGDNYGNVIHLGERDCSIQ
RRNQKLLEEAPSPALDEDLREKMGHAAVKAAQFINYTGAGTVEFLLDKSGHFYFMEMNTR
IQVEHPVTEMVTGVDLLVEQIRIAQGERLKFDQDKVTLQGHAIECRINAEDPDHDFRPSP
GKISGYLPPGGPGVRIDSHVYTDYQIPPYYDSLIGKLIVWGPDRHTAINRMKRALRECAI
TGLPTTIAFHQRIMEHPQFLQGQVYTSFIQDMKLP
>EFA71976.1
MKFDKILIANRGEIALRILRACEEMGISTVAIHSTVDRNALHVQLADEAVCIGEPASAKS
YLNIPTLLQQP
>EFA71978.1
MSESAPVNENSQSSAIDTENSGERTSKTRQLLGMKGAASGETSIWKIRLQLMNLLPGFLN
LGVVCGAASSGNYSWSLENVLKAALCMLLSGPLLTGYTQTINDYYDREIDAINEPYRPIP
SGAISEKQVVSQFVILLLLGYGVAYILDIWAGHTFPNVLMLSVFGSLVAYIYSAPPLKLK
QNGWLGNYALGASYIALPWWAGHALFGELNWKIVVLTLFYSLAGLGIAIVNDFKSVEGDR
QLGLNSLPVMFGIETAALICVVMIDLFQGLVAGYLVSIHENLYAAILALLIIPQITFQDM
YFLRDPIANDVKYQASAQPFLVLGMLVTGLALGHGGI
>EFA71979.1
MALILTFLGKNGTGRSKIAIAAAQFLANQGKRVLLAGLADPTLPILLGTSLSADPEQIAP
NLEAVQFQVSVMLERNWEEVKKLEAQYLRTPIIQDVYGQELVVLPGMDNALALNAIREYD
ASGKYDVIIYDGTGDIFTLRMLGMPESLSWYIRRFRQLLANSDLGKAISESPLIQPVITS
LFNVNWTAENFAQPANQINNFLDQGKAALANPTRACGFLVTTADSIDVANARYLWGDCPT
SWFNCWGIILLSADTSNNLSTEFTPLPVTMVPDVSSGNWQPMIDALPNFVEQAAKAPKPI
EIDVHNKQVRLFLPGFDKKQVKLTQSGPEVTIEAGDHRRNIFLPPALSGKPITGAKFQNS
YLIISF
>EFA71982.1
MCYYKTTQHFPPTNNANAMQIYLDYSATTPTRPEAIAIMESVLKEQWGNPSSLHQWGNRA
ALILETARIQVAGLINAVPESIIFTSGGTESDNLAIMGVAQYYHQPQHIIISSVEHSAIS
APISMLENWGWQITRLQVDRKARVNPEDLKLAFRDNTVLVSIIYGQSEVGTVQPILDLAK
ITKSNNVLFHTDAVQVGGRLPLNVNSLPVDLLSLSSHKIYGPLGAGALYVRPGVELIPLI
GGGGQENNLRSGTQALPAIAGFGVAAKLAGQELEIERTRLIQLRDRLFSKLANIPGLIPT
GDMVERLPHHVSFCIEYADGEKISGKTLVRQLNLAGIGISAGAACNSGKLIPSPVLVSMG
YDRRLALGGIRLTIGKQTTVADIDWTAVVLEQILHRLLTY
>EFA71987.1
MPPSTIPSILLVDGYNIIGSWPCLKKTRDDSSLEAARYHLVELITNYSAFEGYESHIVFD
AHYQNTPSNREMITDFLTVHYTEFGQTADTYIEKVCAGLRHQVAQCLVSRVIVATSDRAQ
QLVVQGYGAEWLSARQLCNQVEAKVCQMRNQHQSTKKIQG
>EFA71990.1
MCSGTGAKPGTRPRTCSTCSGSGQVRRVTRTPFGSFTQVSTCPTCNGTGSVIEDKCESCD
GKGMKQVTKKLKVTIPPGVDNGTRLRISQEGDSGQRGGPAGDLYVYLFVNDDEEFQRDGI
NILSELKISYLQAILGCRIDVNTVDGPVELTIPPGTQPNTVIKLENRGVPRLGNAVSRGD
HLLTVLIDIPTKVTLEERELLEKLAKIKGDRTGKGGLEGFLGNLFKA
>EFA71991.1
MARDYYEILGVSRDADKEQIKQAYRRQARKYHPDVNKEPGAEEKFKEINRAYEVLSEAET
RERYNRFGEAGVSGAAGFQDMGDAGGFADIFESIFSWFCWGKWGDLLNSKGDGVDRFVVT
ISD
>EFA71992.1
MLRHAMVKVAAAIEDTSTEEKDQSDQDQI
>EFA71993.1
MTSDYPTGINSQESDSTVSDHQPPDANLSEQSTVGEENGVAAVEEVVDRDLITQLTQQNQ
SLKAQLEERNSQYMRIAADFDNYRRRVSKEKEDTETQVKRNTIMELLPVVDNFERARAHL
KPQDDGEMTIHKSYQGVYKQLVDSLKKMGVSPMRPEGQEFDPNLHEAVMREQTSEHPEGT
VLEELVRGYF

>EFA71995.1
MITLLGYSLNLVRQGITTLEEVERVVYTDKELIRELKVSRQKSLTCQCCDATLKPEWLDC
PYCLTPRLID
>EFA71998.1
MKKELEMAQITYMTGCGATPGLLTAAAALAAQSYAEIHKVEITFGVGIANWEAYRATIRE
DIAHIPGYNVEIARAMTDEEVEALLDKTNGVLTLENMEHADDVMLEIAGICPRDRVTVGG
VVDTRNPKKPLSTNVKITGRTFEGRISTHTFTLGDETSMAANVCGPAFGYLKAGYQFHKK
GIYGLFTAAEVMPQFVR
>EFA71999.1
MKHKLTMTQDTKTTMNTEITSQPLRIGVLGFGGLGQAAAKVLSGKREMLLVAVADQKGYA
YSPVGLKTNIAITTYQDRGSVGYLQSHGNLSNHSIQDLIANAPGVDAYFWLYLTFLMTLS
PKLPSNLSLLVGKVCWWMLLNAPVQ
>EFA72009.1
MSVQFSRGCPFQCEFCDIIVLYGRKPRTKTPEQLLAELDCLYRLGWRRGIFMVDDNFIGN
KRNVKLLLKELKVWMVEHEYPFRFDTEASVDLAQDPELMELMVESGFAAVFLGIETPDED
SLQLTKKFQNTRSSLAESVETIIKTGLRPMAGFIIGFDGEQKGAGDRIVRFAEQAAIPST
TFAMLQALPNTALWHRLQKEGRLRENQDGNINQTTLMNFIPTRPLEDIAKEYVEAFCALY
DPIKYLDRTYRCFLIMGSPKWKAPFKMPDWVIVKALLIVIWRQGIKRETRWKFWHHLFSI
IKHNPQVAEHYLATCAHNEHFLEYRQIVRDQIEGQLAAYLAQGTEKPYVVPQKEKVGV
>EFA72010.1
MRVLLVYPIFPKTFWSYEKILDLVDRKVLLPPLGLITVAAILPQEWEFKLVDRNIRPATE
EEWAWADVVIFSAMIVQKQDLLDQVREAKRRGKLVALGGPYPTSTPHEVQEAGADFLILD
EGKLPYPCLSPPYKVVRNPAFSGPRKNQMSQAHQFLDLIC
>EFA72015.1
MAERNIREQQRKQALATLGPQISFIARYDLLDEFNDGVAINDGYSVGLRASMNLYDGGAS
RARAARAKTEIAIAEAEFAERRNQVRFQVEEAYSSHLANLENVQTAATALDQAKESLRLA
RLRFQAGVGTQTDVINAQSELTRSEANRVRAILNYNLALTRLQRYVTSRAVQKS
>EFA72016.1
MKGQQLFHSFLPGVTAAVLTTQSAWAGTFKANDLKLVSSPVVSTATNPKVSVVENNWHLV
ANTVDHALGFDYQLDFGQSILPELPSSSSRANVPSPTKFKTVLSLPPVNPKVRSGKTYNQ
VTQITLPTDKSSETEPVKPNSQSVNPTSDSPQMILERLKPNPNLLDVPQDSQGVKVQTTE
AISLEQALDLAKQNNNDLQVAVLQLQRSQSSLKEAQAALLPSLNLLGGVTRSRSSSTTLR
ARQTDKFLNRPTENPDATSVFDSQAELRYDLYTSGRRTAAIKEAEEQVRLQQLEVERQSE
EIRLNVATEYYSLQQSDESVRIARSAVENSQASLRDAQALERAGVGTKFDVLRSQVNLAN
SQQELTDALSQQAIARRRLALRLNLPQSVSITASDPVQLAGLWKSSLEDSIVLAYQNRAE
LQQKIGREEY
>EFA72019.1
MTDGERQEILKKLKLDYGKILLNYFSVDENLKTKIDQFISTLFCANIPVPQVIEIHMELI
DEFSKQLKLEGRSDETLLDYRLTLIDVLANLCEVYRCSTSKIN
>EFA72022.1
MILKSPGQLKFTVITFTLAMLGLLGGCLGLTVSFDSQPSDSMSQAPEKSPFLENHTATPS
PQLQRVEPKITSNRTLEKSKFGNLRISNKTYQPIRLALLLRHSPSSSSGKKGLIPAHWDF
APQEGSQGGLILSLPEGSLKLEKGIF
>EFA72028.1
MSQLFTIQKCGKKLRKIKFILVVTLAIAFSAISTTVYPDMAVAAGFRSFVDTEDGYQFSY
PNGWLQVKVANGPDVVFHDLIEVSENVSVVISPVPQGKSLTELGTPTEVGYKLGKSALAP
EGSGRSAELVNVAQKEINGNNYYFLEYAVKLGNGQSRHNVASVAVSRGKLFTFNASVPER
RWRKLQRTIDEVVSSFKVY
>EFA72045.1
MFPMMAENEESKALDQGFVLKPMNCPFHIQIYKSELRSYRELPMRLAEFGTVYRYEQSGE
LGGLTRVRGFTVDDSHLFVTPEQLDGEFLNVVDLILTVFRSLQLKNFKARLSFRDPTSDK
YIGGDEVWDKAEGAIRRAVQQLGMDHFEGIGEAAFYGPKLDFIFSDVLEREWQLGTVQVD

YNLPERFDLEYVAEDGSRKRPVMIHRAPFGSLERLIGILIEEYAGDFPLWLAPVQIRLLP
VGEQQIEFTKEIARKMTELGIRAQADLSGDRLAKLIRNGEKDKIPVMAVVGAKEVETNSL
SIRTRASGELGVIPVEKVVEKMQESIINYSNF
>EFA72046.1
MAMAVQKLFPKAQVTIGPWIENGFYYDFDNPEPFSDKDLKAIQKEMVKIINRKLPLIREE
VSREEAKKRIEKIQEPYKLEILADIKQEPITIYHLGDEWWDLCAGPHVENTKDINPKAIE
LESVAGAYWRGDETKAQLQRIYATAWETPEQLGEYKRRKEEALRRDHRKIGKELGLFIFS
DPVGPGLPLWTPKGTLLRSILEDFLKKEQLKRGYLPVVTPILPE
>EFA72060.1
MRGYDEGKLAAGRTYVQASAEYRFPIFSVVGGSLFFDCGSDLGSATRAAEILNKNGSGYG
YGLGLRIQSPLGPIRIDYGISDKGDSRINFGIGERF
>EFA72066.1
MVEGWVFHISFNFQDSSEVYREFVQLTLHRPCDMSGLSHEFLTTNGPTHWPFSQDNYQEV
KRLYTDLHFYTHDGKAQFAACFSHGLAEPPDPEYPFILTSGRLYGHWHTQTRTGRIDKIC
QMHPEPFLEIHPRDALKLQLADHQLVMVRSRRGAAEFPAKITLTISPGTVFVPIHWGKLW
AENSEANALTHSASCPDSFQPELKACAVQLIPIIPIQS
>EFA72067.1
MTEFIKTLCPYCGVGCGLEVSSLTQPPSFTTDNWRVRGDKSHPSSQGMVCVKGATVAEAI
CKNRLQYPMFRESLTQDFRLISWEEAFTIITNHIQQTLVSYGPESICMYGSGQFQTEDYY
IAQKLLKGCLGTNNFDANSRLCMSSAVSGYLQSFGADGPPCCYDDLELTDCVFLIGTNTA
ECHPIIFNRLTKYHKKNPHVKMVVVDPRSTPTAKTADLHLAIVPGTDIDLLNGIAHLLVK
WNSWDVNFVDNYTSNFPDYIQVINYYSPDLVTSRCGISLADLETAAKYWGQAQSVLSLWS
MGVNQSSEGTAKVRSIIDLHLLTGQVGKPGAGPFSLTGQPNAMGGREAGGLAHLLPGYRL
IKNPQHRLEVEEFWGLPPGTISPIPGLNVWEMIMALESGNVQLLWIAATNPAVSMPDLER
TKKALLQSPFTIYQDAYYPTETANYAHLLLPAAQWGEKTGVMTNSERRITLCSAFKNPPQ
LAKSDWEIFAEVGRRLGFSHQF
>EFA72068.1
MTTLNLPKNTQLQALGNPQSHQKSRDFLFIDGVNKIYPTAEGPYTVLDNISLKVQEGEFI
CFIGHSGCGKSTLLNMISGFNQPTNGMVWLQGEPVTEPGPDRMMVFQNYCLLPWLSVFEN
VYLAVDSVFPHKSQAEKRSIVIEHLGMVGLTEAAQKKPHQISGGMKQRVAIARALAIRPQ
VLILDEPFGALDAITKEELQEELLQIWSDHQVTVLMITHDIDEALFLADKLVMMTNGPAA
NIGEILDIPFSRPRQRRQIMESTEYYHLRNYALDFLYRRCAHVEE
>EFA72069.1
MVAGMPLAMTLGAMGKTPTPMVTAMTLSRNGSAITLSQELWANGVRNAADLEKTVNTDLD
KTYTLGVVHPASMQNLLLRYWLAASGIEPDKDVRLIVIPPDEMVDFIQERKVDGYCVSEP
WNTCAVEQNLGFIMLPTSDIWQGHVDKILGVTENWSQQYPQTHLSLVKSLLAACEYCDDI
RNESEIISLISQPQYIGDHHVNLRSELLESYQTNYHRHSQTSHQFYLHQANYPNRHEMLW
ILTQLARWGFIAFPKNWVEIIDRVCRPDIFGVAAREVGILDIGREESIQLFDGKTFNPSQ
PLEYLTGLAIKSQVRVEEALV
>EFA72070.1
MNTFVEIDHVDKIFNLPYGGKYIALKNIELKVSQGEFISLIGHSGCGKSTLLNIIAGLDQ
ATIGGVTLEGREVREPGPDRMVIFQNYSLLPWLTVEGNIALAVDEVHQDLSVGERQKIIE
EHIDLVGLRRAAKKLPGQLSGGMKQRVAIARALATRPKLLLLDEPFGALDALTRGNLQEQ
LMKICNEHKITCVMVTHDVDEALLLSDRIVMLTNGPEAHIGQILEVSIPRPRQRLEVVEH
PSYYNLRNQMIYFLNQQKLAKQRQKQTISPILISTNQPEKVHLQIGYLPITQAAPLIIAQ
EKGFFAECGLQVNLIPEKIGKILPKV
>EFA72071.1
MLVGGVGIGSFIWDAYNTTTDTNLSEIIVALIYVGIVGLMLDRTVAFIAEKIVQKEQK
>EFA72072.1
MSVGVTNQKISTKRQKAIRTINKFLVHKVLPPLCGLVIFLMLWQLLCSIPGFQLPGPLET
FVETWDPFIIKPFFDNGDSDKGLGWQILTSLGRVGLGFSLATIVGIFLGILIGANRFLYN
AVDPIFQVLRTIPPLAWLPISLAAFQQANPSAIFVIFITSIWPILINTTVGVQNIPQDYV

NVARVLRLRQGKYFLKISFPSHSSLYLYRTENWYWVILVGHCSCRNVGRWCGYRFIYLGC
L
>EFA72073.1
MKNISRRKFLITSGVAAASTIVAHGCASDNSGSGTNSSTQISNTAKVETTKAKLGFIALT
DSAPLIIAKEKGFFAKYGMTDVEVSKQKSWPVTRDNLKIGSSGGGIDGAHILSPMPYLIT
VNDKIPMYILARLNTNGQAISVAEKFKAINLRLKSELLKEPASKAKARKKPIKFAITFPG
GNHDLWVRYWLAASGINPDQDVILEPVPPPQMVANMKVGTVDAFCVGEPWNAQLVSQKLG
YSALVTGELWKDHPEKAFTMRKDWVDQNPNAAQALLMAVMEAQQWCEKNENKEEMCQICA
DRKYFNVAVKDILERSKGNIDYGDGRKIENFPYRMKFWADNASYPYKSHDIWFLTEDIRW
GYLPRDTDVKTIVDQVNREDLWKKAATALGVPAAEIPTSSSRGVETFFDGVKFDPEKPEE
YLNSLPIKKV
>EFA72086.1
MSKKLIQPLPIKKSKQWSSLQEKWQQLVKRDDQGNWDSQGKVWGAPYRWGNTVLVYNQKQ
LQNFNWQPIDWSDLWRSDLRSRISLPNHPREVIGLVLKKLGRSYNTVNPVEIPNLKIELE
SLNQQVKFYESTNYLEPLITGDTWLAVGWSNDIIPILSRYPQLRAVIPQSGTAIWADIWV
QPANTLITETDKTDNLILQWIDFCWQPDIARQIVMLGKTNTPTFVNTSKVQGISDDTQNL
LVIEHELLEKCEFLKPLSLESINQYEELFLKMKAS
>EFA72087.1
MKRRNFLFNVGGLLLGQTLMGCNGKNQSEFQVLLLKDSIPGYVVNQFNKRLQSDKTLSHV
KLSFVPKSQIHDLFKQLQIWRETRDKSPGGKKSPHCDLVTLGDYWLKPAIEQKINSTLTN
KKK
>EFA72088.1
MALRQIFDLYACVRPCRYYAGTPSPHKSPDKLDVIVYRENTEDIYLGIEWKQGSEIGERL
INILNKDLIPATPEHGKKQIPLDAGIGIKPISRSGSQRLVRRALKHALQLPKEKQQVTLV
HKGNIMKYTEGAFRDWGYELVTQEFRAECITERESWILGNKEKNPQISPEENARMIDPGF
DNLTPEKKGQIVKEVETVINSIWSTHGDGQWKDKVMVNDRIADSIFQQIQTRPDEYSILA
TMNLNGDYLSDAAAAIVGGLGMGPGANIGDSCAIFEATHGTAPKHAGLDKINPGSVILSG
VMMLEYMGWQEAADLIKRGLGASIADGKVTYDLARLMEPPVEPLKCSEFADAIIQQFNC
>EFA72089.1
MLENSTKAELISLFWNVLTMYEKITPPPTGEKITFQNGEPIVPDNPIIPFIRGDGTGIDI
WPATEKVLDTAVAKAYSGKRKISWFKVYAGDEACDLYGTYQYLPQDTLTAIKEYGIAIKG
PLTTP
>EFA72094.1
MRDIRNLDRISFSEWFYSHGGSKGSIKRMWNPIAYALGFIDCDHISARCMLTIFQFFAAK
TEASILRMLEGSPQEYLHQPIVNYLTNRGTKIHTRRQVREIKFTESDSQSEVTGILVAQG
EQEELITADAYVFACDVPGIQRVLPPSWRKWPEFDNIYKLDAVPVATVQLRFDGWVTELQ
DSEKRHQLHQAVGIDNLLYTADADFSCFADLALTSPADYYRPGQGSLMQLVLTPGDPFIK
QSNEAIAHHVLKQVHELFPSSRELNMTWYSVVKLAQSLYREAPGMDPYRPDQKTPVGNFF
LAGSYTQQDYIDSMEGATISGKRAAKAILATGGIFK
>EFA72095.1
MRVVIVGAGLAGLATAVDLADAGWDVEIFEARPFVGGKVSSWVDGDGNHIEMGLHVFFGC
YYNLFELMAKVGAGNNLRLKEHTHVFVNKGGNTGALDFRFITGAPFNGLKAFLPLPNFHY
KTSYKMLLLWALVLSFEV
>EFA72104.1
MLAANLKQIQQFFVHPHSSLQLPWNSLLLGLLILPINPFFGAIAIGWASYKTWRKKYSSI
QRKTLNHLLVILSFWFLITTGFTVFARDQPDAFLGLFNFLPYFVVFAGLTPLITTVSELR
QLTWIIVCSSLPVVMIGLGQLFLGWHARWQFLSVVVNLTIDPGGEPLVRMSSVFMNANTL
AAYLITVLILGLGLWLENYHKIRKKANPLGFIFLSVVIIANFLALILTGSRNGWGIAVMA
CIAYALYQGWRLIIAGVIGLTTSIILASFATERIASVFRSFVPRFIWARLNSDTPLALMR
KTQWQFAWDLTLQQPLSGWGLRSFPHLYEQKMGVSVNHPHNLFLMLSAETGLVTTCLFFG
FFWLGY
>EFA72105.1

MAWILIAAIKFWYKCSLPKENRLVFLSYLLAFIGWIMFNMADVTTFDLILSALFWVILAA
LYGAAHRYEGSHH
>EFA72106.1
MPTTADFLQYSQWSGITTIALAALTILALLFKWGLRFRLVGATGFMLVLTVGLFSLSLVP
LSRTVIPGASRYTLVYDNGSNQAVIATSPDITPTQLEATLRQAASNLYSYGRMGSGRNNS
LTIRARTVLHPESGISTPVYLGKVERTLVTREDPQMLVEVFLDKFPQLPPS
>EFA72108.1
MSLSWITPADRIKKLPPYVFARLDELKAKAREQGLDLIDLGMGNPDGATPQPIIEAAKTA
LANPANHGYPPFEGTANFRKAITEWYYRRYHVLLDPDSEALPLLGSKEGLAHLAIAYINP
GDTVLVPSPSYPVHFRGPIIAGGVIHNIILQEKDDWLIDLGSISDEVAQKAKILYFNYPS
NPTGATAPREFFEDIVAFAKKHEILLVHDLCYAELAFDGYQPTSLLEIPGAKDIGVEFHT
LSKTYNMAGWRVGFVVGNRHVIQGLRTLKTNLDYGIFSALQTAAETALRLPDSYLHTVQQ
RYSIRRDFLIAGLSKLGWNINPTKATMYLWVKCPAGMGSTDFALNILQKLVW
>EFA72109.1
MVVTPGNAFGVGGEGYVRISLIADCDRLGEALERFRQAGICYQSDVVPVCF
>EFA72116.1
MEKSHIQLPGYKILEVIHVSSRTSVYRGEQETTKRSVIIKTANAQYPPLNELIALKNQFA
ITQKIDHPHIIKSYALEPYGNSYALIGEDIGGISVYDYAQSQPLGINMFLKIAIAVVKAL
EYLYNYKVIHKDIKPKTLLLIQKPIKLS
>EFA72117.1
MTDFSISCLLPQEIAEIKKFNPLEGTLSYMSPEQTGRMNCGIDYRSDFYSLGVTFYELLT
GRLPFISKNPLKLVHSHLAKMPPLPQEINPEIPDLLAEITIKLMSKSPENRYQTARGIRY
DLERFQEALLNHGQIKSWQLGSRDIADRFIICDKVYGRDKEIAILLDAFARVSQGSKELM
LVLGSSGIGKTAIVYEINQPILEQQGYFTAGKYEQLQNNKNNIPVSALLKALGNLLQQVL
TQSPEELVAWKNKILLTLGEQAQVIIDVIPELEKIIGSQPAAPKLTGNAAQNRFNVLFAK
FIQIFANEEHPLVIFLDDLQWADSTSLKLIQFLIKEPDTKYLLLIGAYRDHEVYSRHPLT
VTLESLRKIEPKIAINEIDLQPLDKNQLNLLISGNLACEETLSLSLTEFVFNQARGNPFF
SNQLIKSMHEDGLLRFDFQLGCWQCDIYKAQALYGNDILQLLATQIHKLPIATQEVLKIA
ACIGNEFDLLTLAIVCEKSQLEIVSNLQNALTAELIIPQDEKHNLLYPETHQNDQSKKYK
FFHDRIQQAAYLLIPDNEKESTHWHLGQLIYEYSKNRDKKELEEKIFIIANQLNVAQTII
DTAREVERYQLAELNLIAGHKAKLSTAYEAAINYLRFALELLPANSWQTHYHLTLNLYLE
AVEVEFLNINFDQAEIYIKLVQQKAVTLLDQVPVYEIQIQIYMAKVQIKLAIETGIHIIN
MLGIQLVEESPKILNDQNQNYVDELINLPVMTAPDKIAAMGILGNITTATYCFDLELFDR
IVFTMIYLSLQYGNCSTSASGYAHYGLLLCKLAGNIDNGYRYGQLALNLANRFNAQEVKC
VVLLTCNSNINFWKNHLQQTIGSLSECMNYGMETGDLEHVGYASAIYNQNKFLIGENLTC
LLQELEPI
>EFA72118.1
MYRFKQQGAVLVHLIWKQLVLELLNYDPSSGSFHNSFDNIELLSILIQSKASTLIFTFYL
AKTIFFYLFHNYQKSLEAVLQMTQHLNYVSTQIGCSQYNFYYSLVLLGEYSQKSNANQRK
FKKNTKNTYKLLNSIKNLWQYGPVNVQKIISISMI
>EFA72119.1
MAIWASKCPENYQHKYDLIAAEMARVLGNHWQAGELYDQAIMGARKNRYIQEEALANELA
ARFYLNCGRNKIAQTYLIDAYYCYLNWGARSKVIDLEISYPELLAPVIKPRTIIAEETSF
MTKIMDSNKNVSAILDLETVTKASLAISSEIKIDKLLGTLLNVIIENTGAKKAALVLKKE
SSLFTVVQENQNTGWELIPIKDSQDIPINIINYVNNTQNEILIDSNSIDKKFAVDPYIIK
YQPKSMLCNPILKHGQIIGIIYLENRLTTGAFTPERLKVLKLLSSQAAISLENAELYENL
EEKVAIRTKELNDNNLYLEKTLQELKSTQMQLIHTEKMSSLGQMVASIAHEINNPVNFIY
ANVDHAIDYIQYLLDLLNIYQQEYPTPHQIIVEKSKSINLDFLVSDLPKVLNSMSVGAER
IRDLVVSLRNFSRVDESEIKPLDVHQSIDSTLILLQPQFKEKLGSAKNIIFKNYSNLPLI
TCYASQLNQVFMNMISNAIDAIYEHRKNLSGAAKETFTGQIIISTLVINRDWVGVCIKDN
GKGMTNEVKNRIFEPFFTTKPVGEGTGLGLSISYQIIVNQHRGKIDCLSQPGMGTEFVIQ
IPTNCSQYD

>EFA72125.1
MITFPQLIWTDIGLKLLSILILITINAFFVAAEFAMVTVRRARIHQLVQSGDAPAIAVEE
TKFPPGCVLLIEITTNSAKSSEST
>EFA72126.1
MTTNSTAVLTLILVALMLMTGSVSAFLGFSMGSSALKGVTSPDGRPTSKLISSGNNNLQS
VPISFLKEEDIINQVKKRIEQNKTKTNRTKKVEEEEQTVSTKDKSQQKAQELPEEPPQPG
FPVVAESEGVNMSVQSASYSGGQLILKVKMHNQSNESVRFLYSFLDVTDNRGRVLTATTE
GLPAELPGNGSVFMGNISIPTALLNDVSSLTLSLTDYPAQKLKLQLSDIPVEK
>EFA72129.1
MHNNSISVAEPNLYSQRTQPRAIRMGVIGVGNMGQHHARLLSSMKDVELVGVSDINVERG
IETASRYKVRFFEEYCDLLPHVDAVCIVVPTRLHYAVGINCLLAGIHVLIENPLPRAFPK
QSL
>EFA72130.1
MLKTEEVLALESHRMSPYSSRANDVSVVLDLMIHDIDLLLELAGSSVVKLTANGTRSLDS
GYLDYVTATLGFANGIVATLTASKVTHRKIRRIVAHCKNSLTEADFLNNEIFIHRQNTNP
QNDRQTLYRQDGIIEKVYTSNIQPLSAELEHFVNCVRGGNQPSVGGEQALKALRLASLIE
QMALEERVWNPLEWQSESVSQSLTSSV
>EFA72131.1
MLEWITNTINSLGYIGIAMMMFLENLFPPIPSELIMPLAGFTASPYQPGGAKLNIIGVFF
SGLVGSTLGALIWYYPGKLLQEQQLKKLARKYGKWLAISSDDIDKAKTWFNRQGGKAVLM
GRLVPGIRTLISIPAGMANMPMLPFLLYTTLGSAVWVGFLTYSGYLLGTQYKLVEEYIDP
ISKIVLVGILVLFFFGYLSAKVNRSVDKF
>EFA72138.1
MSQDKKPRSKQQLPETSPSGAYHKKPLFWKILIIQVLRGTIGILETMVTRLETSPASTTP
EKGGLLLWVVTKWDEFLRGFRLFLPSKVANNVSDGFLTMIFAFLAVLAIGVTATSLISQI
GSPSVPQSSQSSTDSTVDDKTETQLGQPPTDSTVEGKTEPQLGQPLTDSTVDDKTEAQLG
QPPTDSTVEGKTETQLGQPPTDSILKSQAELIPQQEVTGFIEKQLKEITATNIITKDKQR
IEVEIVQSIKTNFRISEIVIKTTEAWYKLESSQKEKLAAKILKSCQEMDLIHVKILNAQN
QLIARSPVVGTKMVFFQFPNS
>EFA72140.1
MTAKVISICNLKGGVGKTTLVMTLAEYLAGDTIYDKRVLLIDLDPQTNLTSAMMSEDVWE
WEYDQKGLTLPFLLKNVDYFLEKPHSTRFIVQDQVSNVRNKNSFNCLHLIPSSPRLFDVQ
EYLPANAVAILHNILTPLKEQYDYVLIDCPPSINTVIKSAFYASDFCLIPCVPSRMSIHG
LELLLEKLRLFKKEYDHHITPMGTIISRYNGTSSQTHNLNFITVNPLLPPTFTIKIPERS
KIAEGLDFDHQLTYKQKYGDVHETMIALAKEFMYRVDNSTVFQSVIA
>EFA72145.1
MISIASIDPNTITTYLQTQREFFATGKTKDVNFRLAQLQKLRTLVTDNKESIIAALKGDL
NKPEFESYAMEIGAIKEIDYAIKHIKKWTKPKKTGVPLEFFNYSAKILPEPLGMVLIISP
WNYPLQLVISPLVGSIAAGNCTIIKPSELAPHTATLLTQLISEYFPPEYIRVVEGGVETS
KQLLEQKFDHIFFTGGTAIGKIVMTAAAKHLTPVTLELGGKSPCIVDKEINLDHTSKRII
WGKFINAGQTCIAPDYLLVNKKIKISFNQFSPANSSRILWR
>EFA72146.1
MGGEINPEQLYIAPTLIDNISLTDKIMEEEIFGPILPIIEYTDIQEAIEIINSQPKPLAL
YLFSENKKLQEQVLTNTSSGTVCINDTIIQVAVSSLPFGGVGDSGMGSYHGKVGFDTFSH
HKSVLYNGFRLDLNWRYAPYLGKMSTLKKIIGA
>EFA72147.1
MVVMAVPKKKTSKSKRDKRRATWRHKAAVEAKKALSLGKSILSGRSTFVYPTNEDEESN
>EFA72149.1
MATIIILQNHASFRLHKLSTSHDTSSTSEFSQNLTSPLLDRAKSFIRPDQASSYGDAIKMA
KKYTRDNRSLIWPKLLLMTGATKFYNLLKTMLIRENLAKL
>EFA72152.1
MAGKLASHLVDNGTDTVLVCGTTGESPTLSWDEEYQLFVEVLQAVSGKAQVIAGCGSNST

TEAIAATQKAAKLGLHGSLQVVPYYNKPPQSGLYNHFQAIAKSCPDMPVLLYNVPSRTGQ
NLIPETVMRLAEIDNIVGIKEASGNLDQTSEIRRLTPREFQIYSGDDSLTLPLLAIGAKG
VVSVASHLVGNQIKQMIESFSLGQVKTATEIHLQLFPLFKALFLTANPIPVKQALKIIGW
DVGSLRLPLLEADMEVCQKLKTVLCEMKLV
>EFA72156.1
MRKLIAGPGVYICDECVELCNEILDEELLDAANNPANSSPPKPEQTPKRRTNSANLSLNQ
IPKPREIKNYLDDHVIGQDEAKKVLSVAVYNHYKRLAVAQGKAEDSVELQKSNILLIGPT
GCGKTLLAQTLAKILDVPFAVADATTLTEAGYVGEDVENILLRLLQVANLDVEEAQRGII
YIDEIDKIARKSENTSITRDVSGEGVQQALLKMLEGTVANVPPQGDVNIPIRTAFRLTLA
TSCLFAAELLSV
>EFA72157.1
MGFVQSGEGQSKEKRVATTLKQLEPDDLVKFGMIPEFIGRMPMVAVVEPLDEDALMAILT
QPRSALVKQYQKLLKMDNVNLEFKEEALSAIAQEAYRRKTGARALRGIVEELMLDVMYEL
PSRKDVTTCTVTREMVEKRSTAELLVHPSSLPKPESA
>EFA72158.1
MAYIEVRGVNHYYEWIRKPSEIAKPVIVFLHGWAGSCRYWRSTAEVLSERFDCLLYDLRG
FGSSECQPKNNQEPIKLSYELTEYAEDLAALLNQLNLERVYINAHSMGASIATLFFNRYP
QRVVKGILTCSGIFEYDEKAFAAFYQFGGYVVKFRPQWLTKIPLADRMFMARFLHRPIPD
SERIAFLEDFVAANYDAALGTIFTSVSKEQAETMPDEFAKLTVPTLLIAGEHDIIIPADL
GKKAANLNDKIQLEIIPDTAHFPMLEDPETYLDKVQGFLVANI
>EFA72159.1
MIQYWQEEHEINSHHWQSELLGFRYQMQDRLTTNLQLFLAQEMENTYQRALKFVQIKTKF
KVQFPAECPYTLEQLLNIDYTG
>EFA72163.1
MLSGLLKFVLGFLLAIAVLLGSGMTIAIYFINRTAITPQKPMFPNDNPDPKPNLPRVTRK
KVVKVKPKPIATPDLPRESPTPLPSGSYTAVVTWSQGLTMRDKPAFEGQAIGGVAGNQKV
IILETSQDGKWEKLGFQILIKKVGLKVAIRKNQINSDKWEPSWFLFTSIRA
>EFA72167.1
MPFLVVLVVGLIILLAQNLAPTLPLMFLGMRSRPLPLALWILFSITAGGLTSLLISTFIG
TLWKITGYSTTETGSSAPPSGSKSKSFDNYSQENIPREKSRDEFDDWQMSSQEGDDWDST
QPPKSPTQRNASPSDSVYSYSSQSPKNTGVGKTESIYDADYRVIIPPYQPPSTKKSDNDT
DDDWDFLTDEE
>EFA72168.1
MDASIKQSLKGVNLYLIGMMGSGKTTIGSLLAQAVNYSFIDTDEVIVKAAGKPISDIFTT
EGETAFRQLESNVLAQVCAYTKLTIATGGGIVLRRENWSYLHHGLIIWLDVSVEILLERL
KEDQTRPLLQHPDPQSKLRSLLDERYSLYSQADLHIEITQQEHQDKL
>EFA72174.1
MNLETSTKLVLLSILTVLTPLNDVIAQNTTKLSQNPTSRYTRLESLLKAQNFKGADTETS
KVMLALANRQKEGFLTAEDVEKFPCRELRIIDKLWLKYSRGKFGISVRQKIYESLGGVEG
INSSELDWSFAKRVGWVSRTGSWIGYDRLNFSQKAPSGHLPRRIFFSDRMRTYQTIFITD
NFPSMLFRCNT
>EFA72175.1
MIAVTEQDRFFTPEEIYNIVKQRMFSSICSNAAGEYMEVYKKSRLVLPSGCKDSSCAQSY
PFHPELFNLLTKKIASIPNFQRTRGALRLLALLAIVIRYLWQKLEYLAQNSPTTSENASF
WIPMIHPHHIPLGIEEEITGDLTSRLDRQLMRIPIQADIYNSDGREAHSQIQHREWSAAG
KPPFTKVNQVRICFILTIR
>EFA72182.1
MGGCEQKSFPPNNTQQSTTKPVFSDDQPVKVGYLPITDAAPLLVAHSRKLYEAEGLTSEQ
PRLFRSWSQIVEAFLARQINVIHVLMPVTIWIRYGRKFPAKVIAWNHTNGSALTVAPEIE
SAKNLGGRTIAVPFWYSIHNVVLQQILKQQGLSVVRKAQDAPIGSNEVNLVVLPPPDMVS
ALANKSIGGYIVAEPFNAVAENLKTGKVLRFTGDVWKDHACCVVFVHEEDITQRREWTQK
VVNAIVKAQSWARNNRLEVAKILSKDGGKYTPHPLGALQRSLGYYDSKFYQQAITHPDWG

INRIDFQPYPFPSYTEKLVELLRETQVEGETNFLQTLEPKQVAADLVDDSFVKSALQQIG
GPEVFGLPKELLRSETFAI
>EFA72195.1
MVKYNQLGESNLYVSDICLGTMTYGHQNTGSEAYEQLDYAVDKGINFIDTAEMYPVPPRG
ETQGKTEVYIGEWLKNQQRDKLIIATKIAGPGRPFTWLRGGNNKVDQQNIQQAVEESLKR
LQTDYIDLYQIHWPERYVPTFGQTVYNPDLERDSVPISEQLTAFAKLIDAGKIRYLGLSN
ETPWGVTEFVRIAKELKLPKVVSIQNAYNLLNRSFDSGLAEVARHTDVGLLAYSPLAFWF
LTGKYIENEEVKNTRISLFPGFGQRYLKPNVKEAVISYIEIAKKYNLKPAQLSIAFVRSR
WFVKSTIIGATTLAQLQEDIDSVNIVLEPEIFQEIDAVNTRYPNPAP
>EFA72200.1
MFGTGLKLTLQEIWQPLANTRLVIFSLITNFLLVPVFVYLLLTVISVTEPVKDGFMIMAL
ASGPPALPKLAEIVKGNLAFATGLMMLLMLGTVFYLPITLPFFIEGVQITPWDIGKPLIF
MMIIPLVIGLSIKLIKFESEDIVIKLQAITFQISNFGLLLGLGVRLIVHFDEILLLLKTG
VIFICAVFIIFSFSVGYLLGGPGVDTQRVLGVGTAQRNFAAALLIGTSNFDDPNVVSIIM
VTSLLMMVSVLVLGKNLTELEPATTKRITNLKSYRSFDFHLLYINSIKCISKYIISFSPT
A
>EFA72203.1
MNFNFPLPRRTAIKKFVKYSALGALLFTLPITGNIVQAENPRLANKTGIQTKVVRMAYQT
SGDIVKIKGVVDKRLQSLGIQVEWSPFPAGPQLMEAMNANRVDIGTVGETPPIFAQAAGA
QLTYIAARKPSRGEGSAIVVQKDSPIKTLKDLKGKKVVFQKGSAAHYLLLRALGEVGLKY
GDIQPVSLTPAEARDAFIQKKIDAWVAWDPFIAFVQQTANARVLRNASGIATQGGFYMTR
RDFARENPELVKIILEEIDKLGQWAESNRDEVVKILAPELKIDPSILKVVVGRRTFRLQK
ITSPIISEQQRIADLFYNEG
>EFA72210.1
MSQQYRFETLQVHAGQEPAPGTNARAVPIYQTTSYLFNDTDHGARLFALREFGNIYTRIM
NPTTDVFEKRVAALEGGIAALATSSGQAAQFLAISTIAQAGDNIVSTSFLYGGTYNQFKV
SLPRLGINVKFVEGDDVENFRQAIDERTKALYVETIGNPQFNIPDFTSLAQIAHDHGIPL
IVDNTFGAGGYIARPIEYGADIVVESATKWIGGHGNSIGGVIVDSGKFDWGNGKFPIFTE
PSPGYHGLNFQEVFGVGSSFGNIAFIIRARVEGLRDFGPSLSPFNAFLLLQGLETLSLRV
DRHLSNALELAHWLEKQPQVEWVNYPGLTHHPYYERAKKYLRHGFGGVLNFGIKGGLEAG
KNFIDRLKLASHLANVGDAKTLVIHPASTTHQQLTDSEQLSAGVTPDLVRVSVGIEHIED
IQEDFVQAFG
>EFA72215.1
MTKLTATPISPVTAERTPPSVVLQTLELRRCFDKFVAVDSLNISVSTGEIFGLLGPNGAG
KSTVIKTLTTLLPLTSGRAYLAGYDVIHSPNSVRRAIGYVPQALSADGTLTGYENLLISA
KIYDIPSRRRKDRINQMLEFMGLENVTHRLVRNYSGGMIRKLEIAQAILHQPKILFLDEP
TVGLDPVARSQVWQLMQQLRNECGTTNFFNNSLLRRSGFSLRSSGNHESRQGNYHGFPVR
VKDLYWQTRGQFR
>EFA72217.1
MRFIRTDMRTHSADCLSIPQLRSLAFLKRNPGASLSAVADHLGVTCATASTTIERLVQRH
LVQRSDHPQERRRIVLNLTMEGKSLLEESQEKTRIHIAEIIDGLTSEEILQIETGLTLLK
NVFETVD
>EFA72219.1
MKHNPYNWIEESLNTIHRADWYRSVQTINSPPGAVVILSGQKMINFASNDYLGLAADDNL
KMSAIEAIRQFGTGSTGSRLLTGDRQLHRELEQAIASTKQTEDAVVFSSGYLANVGTITA
LVGKRDIIFSDQYNHSSLKSGGILSGGTVIEYPHCDMIVLEKKLKEERQKYRRCLIVTDS
VFSMDGDLCPLPNLLDLAEEFNSMLLIDEAHATGVMGKSGAGCVEHFNCTGRELIQIGTL
SKALGSLGGYATGSSALIDFLRNRAPSWIYSTALSPADTAAALAGINVVQKEPERRKRLW
ENVNYLQKVVKENLGKLKILPTQSPYYVLSYLMLQQHCRWENT
>EFA72220.1
MITPIEVRNPRTGKFDYVIVPPPAKLLSQQCHRLRRGQISWQKIGVEGRIETLKHFRESI
VREYNLLKNALVSDTGRLGTSTLEIDIFLNNIDKWCNLAPQLLQSTAKNTSIPFISLRQQ

LAPYRLVGIISPSHFPLLTSTIDAIPALLAGCAVIIKPSELTPRFFGNLMTIINKINKLK
DVLTCIEGGPETGTILIDEVDLICFRGSLQTGRLVAQNAARNFIPAFLELGGKDPAIILE
SANLDLAASAILWSAVMNRGGSSIERIYVAESIYEEFYHLLITKTHQLKFAYPDIESGEI
GPIMSENQAKIINNHLQDAIKKGAVIHCGGKVEEMEGTWWCKPTVLTQVNHSMKIMMEQT
FGPIMPIMSFSTVEEAVNLANDSIYGLNAAIFAESEELAVEIGMELDVGTISINDAGLTA
IMQEGENHPLKFSGLGGSRMGNSVFKNFLRTKAFLIKTNDLNDPWWFNIEQSI
>EFA72221.1
MFSVHAFEHYLVKLYEYLQVLENRLFSSGLHTLGEPPNEEELTGYLNAYFGEGTEVQKES
QKSQENLIVDLLNQSTDELTNLLRGLNGEYIPPAPGGDLLRDGPGVLPTGRNIHALDPYR
MPSPAAYERGREIARKILDQHLQEHHSYPETVAVLLWGLDAIKTKGESLGILLELVGAEP
VKEGTGRIVRYDLKPLETVGHPRIDVLGNLSGIFRDSFVNIIELLDDLFLRAAQIDEPQE
WNFIRKHALLLQAQGIENSSARLFSNPSGDFGSLVNDRVVDGNWDSGDELGKTWESRNVF
SYGRQDKGQARPEVLQTLLKTSDRIVQEIDSVEYGLTDIQEYYANTGGLKKAAEQQSGKR
VTASFVESFSKDTTPRNLDDLLRMEYRTKLLNPKWANSMANQGSGGAFEISQRMTALIGW
GGTVDFRDHWVYEQAADTYALDQEMAEKLRQANPEAFRNIVSRMLEAHGRGLWNADENRL
NQLRQLYELTDEQLEGVGV
>EFA72223.1
MNKKYVLIGVEGNHDQAFISKILRKLLDFCPSNEVDKIDKIWRKFIPVYPAKNGKLHFRL
NIPTILCKDNISVAIYAGEGGNLITNLSDKLSDIDCSYLFAFGIIADADKKTPDKIVEKY
HNDLREHFPNFPNKVNSTGNVIDSSPKLGIYILPDNNRQGVLDTLICDCGDLAYPEYMQR
AREYIDKFSEEERNKPPLKWKPFDKEKAIIATVVSVLKPGKTNQTSISDDAWISDKTAEF
PAIQNLTQFLINLIC
>EFA72224.1
MKSLDSVIIHQFRGIRDLELKDLGRINLLVGINNSGKTSVLEALQIYSNPLNITTWLITA
CQRELQNRLPRDLLIDALRWLFSQNPVSTIESPELIIILISSTGLFSVKKLIASYEVIEEI
WLKKSENDQIQENEEEDDLIQENEEIPGVRKGMNLKIELFTEDGQVSLFDTTPTITKNFP
LWQNKGLYLHLYKPSGTREPSLNTATVTSSSHRSADQIRLLSQARFRNFKSDVVRLLQQI
DSNISDIEILSPPQSTSSVSTSSVYIYIQHKKLGLVPLSSFGDGIRRLLHIALKLSSVKE
GGILLIDELDSTIHTEALQNYFQWLVKWCAEMDVQLFATTHSLEAVDALLEVTESDSDLV
LYRLEPKEEKQE
>EFA72226.1
MSQLWGLLVILITCILLGALPIIPWITRIIKGSQLEQVGTGNLSVAAAFYHGGRVVGVLA
VISEALKGIAAVLITRIFFPQGSFWELIALIALVIGRYTFTRGAGTTNVAWGFLLHDPLI
AGSVTLFAAIGFLLLRSRQVIQFGVLILFPLLVAFLHGQDFSKIIAAFTLAGLMGWIYQQ
IPDDLELPPQGAQLPVKPIMEYLSGNKPTIITLDDFLDPEVFGVKSATLSQIKHRGYSVP
KGWILAPFDDPSQLINFLQPSPLSPLVVRSSAIGEDSQQASAAGQYTTVLNVTSKQGLSL
AIAEVKGSYNSENAVKYRQDLGVKDVGMAVLIQPQVQSVYSGVAFSRDPISQQGDAVVIE
AVVGSPEQVVSGKVTPEQYRLFVLGKINYLRCNLKEREKIPQSLIKQVAYLARRLENNYY
GIPQDIEWSYDGQTLWVLQARPITTLVPIWTRKIAAEVIPGVIRPLTWSINLPLTCGVWG
KLFTIVLGERASGLDFTKMATLHYSRAYFNASLLGEVFLSMGLPQKV
>EFA72227.1
MQREIGLEKQFKLDYSRLFLPGMTQLTNESLGELSPSQLLNRVDQILDLLEKVTYYSILS
PLSAAIRQKLFRVQDEEIDHSNAPEISSLHSLQRLALAAKNLLPNLEPQRVFDQLAETTS
GQNILEEFYEVLEEYGYLSQVGTDIAVSRWKEQPDLFKQMFIQILETHEVISNQGSSKRQ
QGNVQNRVNLKGTVTEVYSRLLAELRWTFLALEKTWLTSHLLTAPGDIFYLNLGEIRRLI
ADADPQLTEQLVKLLASRRSQFEQDSEISQIPAVVYGNNPPYPMTNSKPMNLSDRVLVGI
PASRGQAIGKVKIVRNFQEAGEINKQTILVVPYTDSGWATILVRAGGVIAETGGKLSHGA
IVAREYGIPAVMDIHGATDLLRDGQKVRIDGSRGTVEMGRFPELGSN
>EFA72228.1
MEGEGRWGEGDRLIPPNPLKKGANSFMLACNKLLLFGIPPTPFKKGG
>EFA72234.1
MRICLSFFLFVSYRNKNANSFLLILPLFFLNRTVLGLDPAWRRHLAQVLLKLPVQVLLIA

SHDLNWLGKVTQRALVLTDGKIQIDHPIQPLLADGKTLDNLGLPLGW
>EFA72244.1
MDKINSAELLESLASEIGDNVYMDIAKWHLYLTDAKLHHLLAEGCYSLLIDHSITEERVL
GLLSTIKIQIGGGRKEVPLIDLLPLQCQVHLVDILEKYQQEF
>EFA72245.1
MFLQVKGKVDLVKIVDFQELLDPNSRIVHGKDQEGEEEQDMDIYEKQDFGFSLGGRIAQM
LAGS
>EFA72249.1
MLNLINRLKNNKPLLFAIYGTSGCLTAAILLGEPFLALTKLGKSSTIKPQAIVLLIDTSS
SMSDGKLAEVKTAASQFIQRRNLESDQIAVVNFGATVQTPAPLTNDINTLNNAIDQLLEI
GSTPMGEGINTAQDQLQATTLNKNIILFTDGLPDDPNFAYNSALSVRNAGIKLIAVATGG
ADTNYLTQITGDRSLVFYANSGQFDQAFSQAEAVIYKQLIESNTGENYGFGYSIFRIGGW
TAFLALGISLALIMGQNRYMRLPLLTLKKGIVTTIGSLTAGTVAGASGQLLILALSPLSS
TVFLGVLGLEVIAKIGGWVILGAVVGAGTKFFLSPILTLKNSLLGGTVGGGIGASGFLVT
SSLLGDIVGRLSGVSILGFFIGLMIALIERKQLNSEPYLLVHWTPTEQTNYLLGKKPISM
GTALNVEIPLNAVDGFTPLTARIFKEGGDIIMQFDQEYAMIQKNESYQSDFKSR
>EFA72268.1
MFIDHLHSQHLEELVHGSSVNLHLAILNFHSLQGVNAYQHILISNNLPRTNMGIIKSGWL
ERYEHITAGGWWCSGVDPLNNWQKMEWGCFKPTQPRRNKNGKSIKYEHPPSTATRVFCLR
VTEEIWHQISQRYHVTMPKDITINDDGEAQGFWPWVIESNIPIIICEGVKKAAALLTQGY
VAIGIPGITSGYRVIKNEFGKVTRRCLIPDLEVFANRQPSFYICFDFENQPRKVSAINNA
ISKLGYLLKRKNCSVKVIKLPGKEKGVDDFITAKGAKEFEQIYHQSMDLEVYIAQTKPHI
DLTVTPSVTINQSYLDRICLPSNGLVGVKSAKGTGKTTRLQAVVEEAKNRCQPILLITHR
ILLGKFLCEKLVLGGESTLKTSHHHLAAHLSNPLGCVLIPCGSSIPKIGKEL
>EFA72280.1
MYNLEINFLQHRSSSKLKVEDGNEVNSINHPVNRRSFIPIFVGLGLGLCFPALAWSSLWW
LQDQNSSLEGEIAQLNNHSQNLDQQLTQMQKIREQTTTIEQQTENLVTVFEQMRSWSAIL
QELGNRIPSKVQIESLEHKQITTLSTKTEQLEITGYALNFKTVNQFLLNIGKSKLFNGKN
SRITMAELVDAPPVTGAIVTQQSPMEFKPPQVVKYNMTISLTDVPVSNLVRELEKKGSHG
VSGKNSSFRKNRSY
>EFA72281.1
MIFNRDLNFAEKTNSNNSLSSYPVSFGITFTPMVTGLIIGVLGLLGSGYIIILNMLIPEFE
KHNQLKTKIIQTEQEHEQKNQQAQQINLIQKRLALSQQQKKSSPILNGDEKNLNNLLLSI
NQIIELTNSQSLGNPYIKAKLKKFTPLSEKPQVITDNSLGESLKNQLKRQIIQVEIEGNF
YQIQTIMASLEKLPPLLLIDKYKCQLLAPTIDTQQIVSTGKILTSFELVIPISLSDKEAL
EIAAKTPLQ
>EFA72282.1
MPKHFNTAGPCQSDIHYMLSPTARLPDLKALIHGRNYFIIHAPRQVGKTTAMIALAQELT
DSGEYTAIMLSLEVGAPFSQDPGMAERAILDEWQESACVYLPTNLHPPRWPPSQPGRQIG
AALASWAKVATRPLVVFLDEIHALADETLISVLRQLRSGYNRRPHSFPHSVGLIGMRDVR
DYKVKSGGSERFNTSSPFNIKAESLTLSNFTLPEVEELYLQHTQATGQVFATEAIQRAYY
LTDGQPWLVNALARQATQVLVKDVTQPITAEVINQAKEILIQRQDTHLDSLAERLREDRV
KAIIQPMLSGSDLPDTPEDDRRFLLDLGLVKRSPLGGLTIANPIYQEVIPRVLSQGSQDS
LPQIQPTWLNTDNTLNPDKLLNAFLEFWRQHGEPLLRSAPYHEIAPHLVLMAFLHRVVNG
GGTLEREYAIGSGRMDICLRYGKVMMGIELKVRREKLDPLTKGLTQLDKYLDGLGLDTGW
LVIFDHRPGLPPMGERISKEEAISPRGRTITVIRS
>EFA72286.1
MTAQNKFIKNNIADNSHLVALQELIDVVAKLRSPEGGCPWDLAQTPSSLTPYIIEEAYEV
VDAISTGEQSAICEELGDLLLQVILQAQIASEKGISVKEVAEGIAQKLIRRHPHVFGDVK
VENVEQVRQNWDEIKAAEKGQTIATQKLSDKLNRYRRSLPPLNAAMKISQKAAEVGFEWS
DIKEVWGKFHEELAEFDQALAEETLERQESELGDLLFAIIQLARWYNLDPARGLEGTSLR
FVQRLRTMEQVMDRPISDYKLAELEALWQQAKAKLIDG

>EFA72287.1
MFGGLMFGPDLDIYSRQFQRWGWFRWIWLPYQQSLRHRSFLSHGPIIGTTLRVVYLMVFI
AATTGLLLLILAIFSNISVNWGDIWAKVRQNISTYAREFFAWFVGCELGAMSHYLSDWTN
STHKRFQKKGVHALLPTSKIRKRKIHSRRSPSSKPKPDKKQ
>EFA72288.1
MPSGQTHDRITIWSIPVVASVTLVATGSGNITLVVLGGLCLVV
>EFA72289.1
MLPKISCLLIITVMQIFIAENLRVNPVLAQSRQSASSNTVNNGDANNVFNSPNQNFNEFT
VPNIYPLDNSINTPVNTENDFGLNLSMGVNTLDSRNVTVYLGLIFQPGRTYSHTVRMNRI
NKETELLEVQRKIAEAKLQLLQKQVSEAEMKLQKLQEPGSEPPPTGN
>EFA72290.1
MNVKKVVSCISYFALAIAIIPLAPNWVLAVTISVLSYNFNAAQANSTIGNITRGNIGVGV
TKLKESKQETIIDLSMDELKQDCFLKIIADEGVNLTGYIQVNDKFVQKITENKTEILISK
LLQKGKNTIEIIGNYDPANSNIKIEFIATNTELTQEVSGGGHIQGALTIYVE
>EFA72291.1
MSHIGIQANMSRNPATQTSNVEMQGPQSCTGNTSSSTSVQVNTDNNGPVRQNQRVRQEIR
DRQSNGNQAGGPTVRNSVIVPVNVKTPENFNP
>EFA72292.1
MSIFQGSQHLLIELPELTISMAQGELTILFRDYQKAKKQLSELTAENQALLLVKRQHKQP
LLGGVIQKGVKPLCLVVSIQW
>EFA72293.1
MNRFIVNLLAAGFLTLGLSQVIPAHAQSGNTGVSQESDQTTVITGDGNTVVNEARQNNQR
VRRGRGTDGDDATIQRVNQTSDVQGNNNTSINRAEQNNQGVNESGRRRRTNININNGN
>EFA72294.1
MTLEQAEKILLSLKGQGVCEILILSGEVHSSSSRRQEWLELIYDLCLLALSMGFLPHTNA
GILSFSEMQKLKSVNASMGLMLEQLTPKLLHTVHRHAPSKIPELRLQHLQWAGELQIPFT
TGLLLGIGENEDDRRQTLMAIADLHLKYQNIQEVILQPHSPGSQQTFDEVAFDPYQLPGV
IAQAREILPSDITIQIPPNLVPEENWLLACLDAGARDLGGIGPKDEVNPDYPHREVNRLR
EVLLSGGWTLLPRLPVYQKNWYKTPLGAPLTELTK
>EFA72296.1
MTESLLNIQNLCVAYPQDYDQAPIWAVNDVSFSLKPGEKIGLVGESGCGKSTIGRAIMRL
LPDHSRVQGQVNFRESLVLDLTPAQMRQFRGEAVALVFQDPMTRLDPLMTISEHCLETLA
AHSPQLTKKQAKERVLATLEKVKIPGSRWSQYPHEFSGGMRQRVAIALALLLNPKLIIAD
EPTTSLDVTVSAQILQELTRLCAEDNMGLLLISHDLAMVAEYCDRIGVMYQGKIVEMGKT
ESVFKQPQHEYTRSLLRAALHIQQEPVGVGEEEKKENPILKITELKQYYTIEPNFLERLF
QSQQQTIKAVDDINLELYPGEILGLVGESGCGKSTLSRTILQLIPPTGEKWNF
>EFA72297.1
MEFLGQELTRLSREEVRGFRREIQMIFQDPHACLNPAMTVGESIADPLLIHKIARGKQAE
EQVLWMLEKVGLTPWQTYYGRYPADLSGGQQQRVAIARALITRPKLVICDEPVSMLDASV
QTQVLDLMLELKAEFDLTYLFITHDLWLARFLCDRIAVMNSGKIVELGKTKEIFSHPQHP
YTQTLLGAAPLLARV
>EFA72333.1
MPPNIPNSTIWQQAEILMQPAFIRIVDNIRKHLDISKWKGSYHDVLIWPAGTTDGTKSIV
IQLLEDSKNATAEDLLAIKQKLALLPIPHPGYHLRLQYQEQVVNMDLWDLCYQVCFVDYS
PENVLVDIDTNLLDEFGEVDWQYLESKTKGLVQRIFDNLIES
>EFA72334.1
MWQVIKDIIEKLQSWWGGLRDHIFGKEPDIIDDGGDGDGEESETGGSGEKIPIIDDFDYG
PIIDRLPPTKIFPKEILFEHPSDKIDSLQKKVLEILFSRFNLSAKEGDNYVAYFPSRLLS
YIKQKDAFFAFRQFLNDIGTTPDEVFKNREIANFVKEAILGLYEADVNRWDTPLSTEPLY
QKLKQSINCHNLDDFINLLNKLEKFAILAHQIERLIGKIPFHEFDQFSQEVKSQLKEWHT
IPEKTVNQWKNSLNKYESQSTNYQNLCKQISQDFLRVESLSLTSEQTIVIEYLLKEVEQL
KKLLQVGPVELDDGIEQLEILADEIKGFVDEVSYRHREAESSRTEDVPPKNNKLSLDEAL

ILLSLTLETLTLQSLKTSRNRYARIHHPDIGGDEKMMKKINQAYEILKEYLEI
>EFA72336.1
MSLIKIFKIVYLVGIVLSGVLTFVSSDKNNTEISKLVWLVGVSHVFVLAMIGFRMERSKD
KAAAGLRVQTSGYLHTLIGFSGALFQLDPQNLLATIVVPLSYALFTSIIGWFFGGELVNE
FDGGGAVDIGNEGERIAQEFHIFAENLGREFQSFADSLSAIHEQYSRKIEDASNSFESQL
QKVGAAYKGMVDTQEVQFDRLSKKQLDLLAQLEDYQKRFIDSQNSCYGKMDNAIQESINS
SDKLNASVDALTSALSTKELFSITSNFVYLSEQTKVASDNMEQVATTSKNVAKYLEESKI
LIDQLEKLLSSINSYRR
>EFA72337.1
MQIRSNMEQAEIFVFIASFFLIALGFKEPDGPVKKPSEHPPIILFPESSRYNFRSGRAEL
SSEFRDHIRTKLKPKILEVVKQYKDIDAIEVIGHTDGRPINSVEQSISSHNSNLDNNLER
VNLGKLSVSRLQPGSNTDLGIMRALAVVQELKQSGGGISKLNLRAYSAGQLLLEDGQPAP
IDTTDNPERRRIEIRFTKLGRTQVFK
>EFA72345.1
MLLIFAIALAIASFTLLTVFANLLAALLAFSGIVFYVLVYTHWLKRHSTQNIVIGGAAGA
IPALVGWAAVTDTLSWAAWFTFCHCIFMDTPHFWALAMMIRDDYAKVGIPMLPVVAGNQA
TVRQIWFYTVITVTSTVLLFYPLHASGIVYVVIAMTLGGIFLHKSWRLLQNPEDKTIAKE
LFLYSISYMMLLCLGMVIDSLPFTHYLVNTILHSFHLVG
>EFA72346.1
MLLLIGLSAIALTGLCWWHRRVLPLWLPGAATFALLLIVFQGILGGLTVTELLRFDIVTA
HLATALLFFTTLLVIGTFLIPYKGTGAVGKLPWVSLTAAVLVYLQSLLGALVGSRWALHQ
CLAGEELCGVMYSHIFGLLPPTVATLSMVFISWRTPALHPVLRKLANLAGGLLISQLLLG
LATFRLHLQVEPLTITHQAVGATLLGTLVVFTVLALRDRSISNPSYLNN
>EFA72351.1
MGIKITNKQKNHLLINNLHKTMDNIATNIPKIMVVDDDFSVRNLVHRFLSRKYKTETAAD
GKSAMTLFEQFNPSLVILDWNLPDINGYKLCQDMQNRTNVLVLMLTSRNDETDKIKVLTG
GADDFMTKPFSLVEVEVRVNALLRRIRYIQPTQSQRLIFKQLAINPEGREVTLNDKPLAL
TALEFNILHFLACHPGQAWSRPQLIQKIWGCDYVGDGRVVDVHIGQLRKKMEVDSSTPEF
IKTVRGYGYKFESPEHNRV
>EFA72352.1
MTLDDFQVLDGDLDHPTLSEYLRSDALAVDTETMGLVPQRDRLCLVQLCNPEGKVTAVRI
AKGQTHAPNLQQLLESTHVVKVFHFARFDLATLRHNLKIYVQPVFCTKIASKLARTYTNR
HGLKELVQELEQLELDKSSQSSDWGNPVSLSDAQLSYAANDVRYLLSLKQKLSQMLQREE
RWQLVQECFSFLPTLVSLDLLQFKDLFEH
>EFA72353.1
MFRQTAFGIGLTTLVLTSGLVTGAISTSNVTKKISSISSFNGSTRAVASHQNNEGNKHDN
NRLEKLVFEQINQYRVAQGLSKLTLNLNITKQARIHSQNMANGTVKFSHHGFEQRVKSIH
LKYNNAAENVAFNIGYNEPAKQAIIGWLNSPGHLRNIRGKFQLTGVGVAKNDKGEVYLTQ
IFINTTLRYSRKISPRSPAF
>EFA72354.1
MIYHAANTRGVADHGWLKAKHTFSFANYHDPSRMGFGALRVINDDHIAAGRGFATHPHRD
MEIVTIPLQGSLRHKDSMGNSQVLQKGEIQSMSAGTGILHSEFNHGDEDLKLLQIWVLPK
KIGVQPRYEQKAFNPSDRKQKLQTVVSGDGEGLGINQDAWFYLADLEGTSVEYKLHGKGQ
GVYVFVISGFANVNGKELFPRDGLGLEEDLHIQVFQQCELLLIEVPMLSS
>EFA72356.1
MNPNHITNFDNLWLELFSLYTYPYLLLLIGNSIAFGQTGVVVPTVPSDSTTPSPSNPPSS
TPTQIPSPNNLSTTRFSCQYYDGKYTVMYEPQSQPGRFFAWAIPQSLGGGWNPQNRCQAI
ASRLELYRPDGLQELQIARQNNENIICVTTEVVSTCRIVFTVPRTRDPYSVRSSIFSSLT
AADQGQQTVGVNTYISSPRRSGNNPHSRRGINLKPFLSMEDGGTGTNLNNGLLIRSRTPG
KTILNPRLFR
>EFA72361.1
MESEYRQRREQVMAKISTGAAIFRSAPTAVMHNDVEYVYRQDSDFYYLTGFNEPEAVAVL

APHHGEHRFILFVQPKDREKEVWSGYRCGVEGAKEIYGADMAYPITELDDKLPQYLQKAE
RIYYHLGRDSHFNDRVIRHYQNLLVTRPRRGTGPIAIEDTGPILHGLRLHKTNFELDLMR
QAADIAVSAHNHAMSIARPGSYEYEIQAEIEHIFRLQGGMGPAYPSIVAAGKNACVLHYI
ENNYQMQEQELLLIDAGCAYRYYNSDITRTFPVNGKFTPEQKALYEIVLEAQKQAIQEVK
PGNGFDAPHKKAVQVLTEGLIEVGLLKGEVNQLIQEGKYKQFYMHRTSHWLGLDVHDVGV
YQHGEVPQVLQPGQVLTIEPGLYVVPHTPSAEDQPPIDDRWVGIGIRIEDDVLVTPKEMR
S
>EFA72374.1
MSITHSAVINLGKEVSKIIVGQSGLIKQCLIAFLAGGHIILEGVPGTGKTLLVKVLAQLI
QGEFKRIQLTPDVLPSDITGTNIFDLNTRNFYLRKGPIFTEILLADEINRTPPKTQAALL
EAMEELQVTLDGESLPLPDLFWVVATQNPLEFEGTYPLPEAQLDRFLFKLVVGYPDQVAE
KQMLFNRQSGFTGRRIDISNLNPVTTVNNILQARQAVKQVNVAEAIVDYILEIVSKTRKH
PDLALGASPRAAGAWLQTSQACAWLAGRDFVTPDDVKAVAAPLLRHRLILNPEAMLDGSK
IDSVITTVINQVPVPR
>EFA72376.1
MLNKETTYSQARLNLATILDQVCDQREIVVIKRRNEKNVALIAEDELSSLLECVYLLRSP
ENAKRLFRALEWTQTAMETPQTLAELKEELGIECKKKKPDTHNEEKELCGYFPVFSPDFK
ADLAWWYNHDRKKGDKILDLVADILDGQPFTGLGKPEPLKYIAPDTWSRRIDLEHRLVYK
VTQNKVYFLQARYHYESD
>EFA72381.1
MQDLLLILQEVHKQGIIHRDIKPENIMRRHKDGKLVLIDFGASKELQGGATSGTRIGTDG
YAPWEQRVDGVASTAGDLYSLGVTCFYLLTSKNPYELWLKDGYNWVANWRNYLNQPLSQK
LQQILDKLLVASSDKRYGLAEKVLEELRQPYSIIPSNSPKTIISHTEKPQRFSYILALIS
VFILGIGYLVITKSPQFQPRTEPNVIPETDRGL
>EFA72384.1
MDGELYDFGTKGSGRTPYSRGGDGMLTLKGGVREVLAGEALHRLGVRTSRCLSMIETGLS
LWRGDEPSPTRSSVMVRVSKSHIRFGTFERLHYLKRPDLIQKLLDHVIDQYYQDLNNQQD
KYALFYAELVKRVAELVAQWMCAGFCHGVLNTDNMSITGESFDYGPYAFIPTYDLNFTAA
YFDYYRRYCYGQQPSICYLNLELLQEPLKAVIDPVDLDYGLAKFQEYYHAEYGNLMLKRL
GFAQTKFPEANDLLDLTIGFLKESQINYHQFFADMANTFSPRWCEEPSLIMEESQILPPV
RSSSVFKKWCALYHQVLNNLVSQEMANVGTTLVQYNPQSNLLRPVIEETWQPIVEQDNWQ
PFYDLIQNFHT
>EFA72389.1
MGKTPLYHTGVEMKARFTNFGGWEMPLQYTSIIEEHQAVRNGAGMFDISHMGKFTLQGKN
LMAELEKLVPSDLSRLEPGQSQYTVLLNPQGGIIDDIIIYCQSGKNTDNEKVVIIVNAST
TDKDRNWLSQNLDLNQIQFEDLSRDKILIALQGPKATGILQSFVADDLTPIKAFGHLETA
ILGGRAFLARTGYTGEDGFEIMVDSEPGLEFWQSLHGVGVTPCGLGCRDTLRLEAAMSLY
GQDIDDNTTPLEAGLAWLVHLDRKGDFIGRDVLERQKIQGLERKLVGLQTQGRNIPRHGY
SVLSSGKIIGQVTSGTLSPTLNYPIALAYVSAELANIKQQLEVDIRGKTYPAQVVKRPFY
KSQNRAHK
>EFA72392.1
MSRSKALQYYIASRLLFAPLQLLTILTIVFLLLRATPGDPADAILGGRAPESAKEELRKQ
LGLDLPIWLQYINYLGNILRFDLGTSLTSRGQNVWQIISQHFPATVELAVCSMLVALIVG
ILVGTLSASRPGTPLDLGGRLFGIITYALPMFWAGMLLQLVFSVQLQWFPNSNRFPPNIS
PPTTITGLYTIDSLLGGNLNLFFPGLASFSSTQFNPRNFIKWHFERIVRVNLKETLKADY
VEAARARGIPENKILVSHALKNALIPVITVLGLTFASLLGGAILTEVTFSWPGLANRLYQ
AIADRDYTTVQGVLVFFGAIVVSASIVIDILNAYIDPRIRY
>EFA72396.1
MDIITSSEEINIGYLGPPGTYSEQAACFYLNWIKGNQEFQGEVILRPYPTIAKAIQAVTV
QEINLAVVPVENSIEGSVSMTMDNLWQLENLQIKQALVLPINHCLISCATKLEDIEIVYS
HPQALAQCQGWLGKFLPQANLSATNSTTQALEQLGKSPTTAAISSEKAAQMYDLPILSNR
INDYPDNCTRFWVVAPESGGESFSSSSTHTSLAFSVPANIPGALVRVLQVFADLGINLSR

IESRPTKRSLGEYLFFLDIEASVLSSLMTSALKNISINVEILKIFGSYTVLTVDSVDKSG
TKIK
>EFA72410.1
MSVVNISELEAKRIQTFPDNYRFIGCWTESMRQIGNAVPVELGYFLASSLSKALC
>EFA72414.1
MTICEDLWNDEEFWGKKCYAVNPIADLSVVGVDLIVNLSASPYTVGKQKTREAMLRHTAV
NFQQPIIYTNQVGGNDDLIFDGYSFAVNSQGEILYRGRGFTPDFLIVEFNQHTQEVELAS
NPDQNPIAPIYESEDEEIWHALVLGVKDYVKKCRFSQVILGLSGGIDSALVAAIATAALG
KENVLGVLMPSPYSSQHSVSDALKLGQNLGIKTQILPIGELMKSFDHTLFELFTGTEFGI
AEENIQSRIRGILLMAISNKFGYLLLSTGNKSEIAVGYCTLYGDMNGGLAVIADVPKTRV
YSICNWLNGQNQQEVIPQNILTKPPSAELKPGQTDQDSLPPYNILDDILQRLINQHQSVE
EIIAGGHDLGIVNRVIKLVAGSEFKRRQAAPGLKITDRAFGTGWRMPIAAIRSL
>EFA72415.1
MKIAIAQLNPIIGDVKGNCQKILEIAQQANDVRLLLTPELSLCGYPPRDLLLNPGFVEAM
DMSLQELAQNLPPHLAVLVGTVVRNGEHHIRGGKIYLIVLLG
>EFA72420.1
MAPGVGKTYKMLEEAHQLKQEGIDVIIGILETHGRKETAQKATGLEMIPKRVMVKENITL
AEMDTDAILDRSPQLVLIDELAHTNIPGSPREKRYQDVEVILESGIDVYSTVNIQHIESL
NDIVARITGIVVRERIPDRLLDEADAVVVIDITPETLEERLREGKIYPHSQVEQSLKNFF
QRRNLIALRELSLREVADTVEEEANTSSLEPSSCNIHERVLVCVSTYPNSVQLLRRGARI
ANYMNARLYGVLVADPERFLSKKEAGHIDTCEKLCREFGGEFLHVKSQNVAREIAQIAAK
YHITQIVIGESQQPRWQRWFKGSFTQRLVDLIRNQNIDLHIIAE
>EFA72423.1
MATCLAKHHGKKVLVLDLDSQISATLSLMSPGDFAKRRKQRKTLRYLLDEVINPDPQPEY
KIHDVIEPELCKLSSLSLLPGDIDLYDEFVVSEMLHNQAVALEERDFETIWNRFERVLVR
DILKPVRDQYDFILLDCAPGYNLMTRSALATSDFYLLPAKPEPLSVVGIQLLERRIAKLK
DSHEHEAKINIQMLGIVFSMCNTNMLTGRYYKQVMHRIVEDFGVETICQAQIPVDVNVAK
AVDSFMPVTLLNPNSSGSKAFIQLTEELVRRL
>EFA72440.1
MNITDLSSPHVLLDSLLNLRQAVERDGKTIFDQWRSHIQRSQFLPSALNLAQYLALRRHD
LRPLQAALMPWGLSSLGRIEARVMANLDAVIATLALICEVPIPTSVTRPVITSFFEGENR
LREQTECLFGPALPHRRVRIMVTLPTEAASEYEMVREIIERGATCLRINCAHDNPSIWEK
MIQNIRQAEQELSCQCTVMMDLGGPKIRTEMVLSPVGKNRVFRGDLIVLCRSLSNQAIAD
PIDNIQISCTVPEILDLLKVGTLVYIDDGKLRTRVVDQDYPLPDGGSGFLLEVTHAKPKG
VKLSPEKGLNFPNIVLPLIPLTPKDLTDLDFLATHADIIGYSFVQQTTDIQLLQSELARR
CLHGEHPAIIAKIETAVAVTNLPELIVHAAGKHPFGVMIARGDLAVEIGYQRLAEIQEEI
LWLCEAAHVPVIWATQVLESLVKEGAPSRGEMTDAAMAERAECVMLNKGPFITQAVTILD
DVLTRMEAHQLKKTPNCGPCVPGRTFLLRGATFN
>EFA72441.1
MTGKEILPEIEQFRILQSTLGERWKPSEIFDNSETDILIIPSLSIDQRELEKVPGCEHYE
ERLLFSLIRLRNPRNRLIYVTSMPIHPSIIDYYLQLLPGIPFSHARHRLLMLSTYDSSLK
PLSQKILERPRLIERIRQALQIDKAFISCYNSTFWEAELSLKLNIPLYAAAPDLQIWGTK
SGSREIFAQTGVHHPDGSKLVWNAKELAREVAKLWERQPHLKKIVVKLNQGISGKGNAIL
NLLPLTDFAPGKTNHVKRAESIEKSFVNLRFQAQQENWINFSQLILEMGAIAESFIEGEI
KYSPSVQGRITPDGTVEILSTHDQILGGPDGQIYLGCRFPANENYRVELQDIGLAVGKKL
SEKGALERFGVDFVVVDKGNGQWDIQAIEINLRKGGTTHPFMTLKLLTNGSYDLSTGLFY
SQQGKPKYYVATDNLQKDSYRGLLPSDLMDIIAHHRLHFDSGTETGTVFHLMGCISQFGK
LGLTSIGNSPEQAEEIHNRVIHVLDQETNNQINHYSLFSNYAFPIVEDTFGY
>EFA72447.1
MLIMDQLIWLRKNLFNNWYNGVLTIVCVIVLFSLGKGILFWIFNQAKWEVVTANIHFIFS
WQIPPGFILANLASFGNQFYYRVDNLVWVDAKYHLPENQLLQKAYQLIVPLLAPIWFLTF
IITIWLLGGGLGLQSVPTNLWNGLLLTLLMAFVSIVLSFPLGVLLALGRNSELPVIRWFS

ILYIELVRGLPLIGILFIAQVMLPLFLPDNWRLDRLVRGVAGLVLFSAAYMAENVRGGLQ
SIPRGQFEAARALGLNTFLLLILVILPQALRAVIPTIVGQFIGLFKDTSLLSLVGLVELT
GIARSILAQPQFLGRYMEVYIFIGVIYWVFCYSMSLIARELEI
>EFA72450.1
MSELRRLKSLLPPELQSWVIVEGTTEVSPPLIRSEEIGKDQVEIQIDLIKWDALAMDQRN
LLFWHEVGRIQNDTIPKDGWEMAALAIGLGGAVGELWVQDGLLLVLALALCGVSGWRLYQ
KNNGTKQTKELIDADEKAITLATRFGYTLPNAYKSLGSALKTLIDTTPGKRQRSRYEARL
SALKRSANKAKSRSNNSNNVDENY
>EFA72454.1
MGRYPEAITAFDNAIKQKPEFIHLAYYGKGLALSRSGKVMEAVIALEEAVKAKSDSVLAW
TILSLANTKLGRSDQALLAINQAIKLQPNNPNLYNEKLVVLSNLKRYQEAIDAIDQAIKL
SPHADFYYNRGVVRSRLGDNQGAIADLQKAADLLQKQGLSAE
>EFA72455.1
MEDGKKFIADLLAGAKVKLEGTAPWLSASNLPIDDRGELTIQVTEKHLIPPPDCLVYHPQ
RVAITLSSLEVETINHIYELLAQAEISSQALAVIIAPLSLANNSILQEISKTFSIPARFL
TTNQIQELTDKGYSFHEAVVAAGMTGNNTYHTSFESPFSPITLSIATDIIDPETIGKGRG
KLAIIGTGPGGVQWMSPEVQEILRNATDLVGYKTYINLIGELADGKNIHESDNREEESRA
KKALDLAVQGKYVAVVSSGDPGIYAMATAVFEVLEKGQYNQPEWQTIDIQVAPGISAMQA
TAATIGAPLGHDFCVISLSDILKPWEVIVQRITNAAQGDFVIVFYNPISQSRTWQLTAAK
NILLEYRKPDTPVILGSNIGRPGQRLKVTNLEELNPEDADMRTLIIVGSSHTRKIQSLGG
NIWVYTSRHYSV
>EFA72456.1
MRNLKSPVVVMEGLTFKPAPICRASSFTPVNPPNRGTTALLSSATANRGGS
>EFA72457.1
MVIILSQNSLTVAKKIVGVIPGAKIYGLANRTCDVDVSFTDFGDTLRQLFVTGTP
>EFA72461.1
MGVIYIGDRATGKTHLALELANPKHEYVKVNSLDYDELSSLLLDENLQTRPTENFLYNRY
LDMNIQLPSGKKQVTVDWVDIGGEVWRKSWQRDNSNQWHEFLKAIRDSEGILLILPPHRG
MTFKPGVDAEEFPTQKQWCNRFERWVTFFCDQCPKIRHLALCLNKADLFCDLSAEGQKLA
YKPYNSPYNWQQRHEHVLRKYFRPVYAQIERLGQGVGSPSVKCFITSIYHRGLLELPWIY
LGSFLGK
>EFA72463.1
MASLARWPNASPDSPVQTVVPVDEGGEDLVNKAQNILEQGLQLEFTTLENPDQIRDCTLQ
ITIKQKNWVPNY
>EFA72464.1
MGSQLLNLNISHKDYSGEFFRDLLHRNQDPLLQEYLEDCVQANGIMFLVDGNSRRKDPEY
ANGLDKLLIALDRHDIGGMKRRIALVLNKCEQSDLWVNRDKPAFLASARFPQVSQKLKVW
QQMGGGEVEFFTASAFGMLGTKYPEPNVNLVQKDRGGVSAVIKNPKLWRPFGLVAPIYWL
CKGSRHPDLDKS
>EFA72466.1
MNPETQTSLKVAMRKIIHSSQIKPEAVEVIVSGLQNSEISAEDWEDLFNREGANIAIKQK
VYSPQLIRLITLRALVLPKTLPEFLGWLEIQPGKKVNQNQIVCLQFQQTIKELFPREKVL
ESIGYLLPSLLDGKISPDGLSWLLQKGDNSIWSYAQEQFIHNIKYDLLLIGDHHPKSKEN
ISHQTSFKFGSQVWNQLITSWQDIKKGNYKCQQYRPLAQFCEQLQEYDLAAYFYQISNSK
VGKKLFVQLCLLKNCQSPVVFGLTIEKRKPPIDVIINVINQDVDMKILYVAPISLLILGS
GWFVGSRTWHSYINTSEAKEYLCKQSVNGRDCSVIVFDGKDHYSFQDIQTAITKVVDEVW
QGKQKSKLKNSNMQEKITRTLPLILADKTLKYKDLQLSTGKIEPKIERQWIIAVYNYQLK
HKLQKKITPQKGEECKGSNIFGLCLLGEKVKKISIDYSPLEKRLKADINKVMTLTAINEA
MGKRYWSATKYAINTIILTTIKNNNLDNNPEKNKIGNFRS
>EFA72467.1
MIFELILKNLDVNQYKSIKYGDFNTDKIQQTHKRRIAQSIYKFQESQKNPQPTGYLDYKS
AEFGELKRKISEQLPISNTPL

>EFA72468.1
MESNIFTSLILPIALGAMMLGMGLSLVPEDFQRVGKYPKAVAIGLISQLLILPLIGLAIA
KFLPMQPAIATGLMILALCPGGVSSNLVTFLAMGDVALSVTLTALSSLITVFTIPIFANL
ASQHFFWARCSSGVANWNDYWANFCNYLLTNSDWYEYSAICAKTVYKTRKVTSISATILL
AVIILLLIIKEWSRLPNFIVQVGIGVLLLNTLSMGAGFYLSKLFNLNYKQQICISIEVGM
QNGTLAIAITAGLLNNPDMAIPGAIYSLLMYLTGCMVIIYGRNLSASRV
>EFA72470.1
MGLLHPDYPGFFLSARAYLEWYGGRGDGHIQEKPVVAILLYRKHVITKQNYIPQLIKKFE
NSGLIPLPIFINGVEGHVAVRDWMTTDYERKERQKGNIETPSLSTEAVKVDILVSTIGFP
LVGGPAGSMEAGRQVEVAKRILMAKNIPYIVAAPLLIQDIYSWTRQGIGGLQSVVLYALP
ELDGAIDTVPLGGLVGENIYLVPERVQRLIDRVSNWVALSRKPTSARKIAIILYGFPPGY
GAVGTAALLNVPRSLIKLLHALKEQGYTVGEIPQDGEELIRQVKVVDEELETWEKNKPFP
TKTNTVNVKTLEKWLGYLRTSRIEKQWQSLTGAGIKTYGDELHIGGVQLGNIWIGIQPPL
GIQGDPMRLMFERDLTPHPQYAAYYKWLQNEFQADAVVHFGMHGTVEWLPGSPLGNTGYS
WSDILLGNLPNLYIYAANNPSESILAKRRGYGVLISHNVPPYGRAGLYKELVSLRDLIAE
YREDPQKNYVLKEGICKKIVDTGLDVDCPFDDDQKIGNSLLPPRKMYKNVL
>EFA72471.1
MKRIVLIAGFESFNADLYRKAALLASSRCSDLDIHVFSDRNISTNQEEIKNALKNADVFF
GSLIFDYDQVLWLREHVSHIPIRLVFESALELMSLTQIGKFSIGDKPAGMPKPIKFILDK
FSNGKEEDKLAGYISFLKLGPKLLKFVPVQKVQDLRNWLIIYGYWNAGGKENVAAFILDP
GGKIFRFESREYSRTYRNT
>EFA72476.1
MLLLGERGFADGDLGQKITRTITKLRSCGFEIKSAPNQPYKLVTSDFPVIITEEQRQALA
MACELLASLGFSAEAGHIYRIANSENRISHKLTTDFHPPTNYSEDKIQEVVQDLQNRIGK
KRRFVVWYRSRNGNDKQWDLDKSELRLHNGVLYLFSIVPSFIGQHIHKTPNPEQNCTLRV
DRIARVGSSSQIPWTYTKFPTLKIQYRLTGNLASYKPRRPHEKIISPPDKTEHVDIETQE
DCIFWFHQRILQYGANARVLQPDWLVKWWWKV
>EFA72480.1
MNGNQVTVWSRHGSQHLGDLINNVDLVLCAVSMKGVREVASLVRSFPVSPHTIFVTATKG
LDPETTCTPSQIWQREFPDHSIVVLSGPNLSQEIAQELPAATVVASKNNMAAQVVQQVFS
SSRFRVYTNPDPVGVELGGTLKNVIAIAAGICDGLHLGTNAKAALVTRGLAEIIRIGAIF
GAKTETFYGLSGLGDLLATCNSPLSRNYQVGYQLACGKTLVQILANLPGTAEGVNTCYVL
MHLSRQQSIPMPITQQVYRLLESLITPQQALEELMLRGMKSEYHE
>EFA72486.1
MNNLLSKLPELSGLIVSCQAPVTSPLHNPTIIAAMAQASANNGAKAVRIDSPNHIKAVKQ
KVSVPIIGLWKQIIAESDVYITPQFHHALAVAEAGADIIAMDATPRKRAGGEKLVDIIRG
IHQQIGKPVMADVDTFASAKLAIDSGADIVGTTLFGYTEETKNLIPPGWELLKQIVENLK
VEHPEILVICEGGFPPRKKQKKH
>EFA72487.1
MGLVLKEDLKVFMMDPNTEIRRLLDIVPASGRMTIKIISKPEQNQVISAEFPLPWIQSKP
VYINFELWRQLKKPQRDLLILYHTSWLIGIKWIQPDIYQGVVLVGLLGG
>EFA72488.1
MDTTGYLSGSSPSWTIRGVIETLQGDIVGLVIAGGLTVISGVKLWQNNKSQELQLRADKT
AIAIAQKRGYSESEAAGHLLTAIETVAKLEGRLGLNFNELIRCQNLRAIAS
>EFA72489.1
MVKRVQLVLTKDVSKLGKSGDLVEVAPGYARNYLIPQSLATQVTPGILKQVERRREIERQ
RQLELKQQAEEQKSALEKLIKVAIAKQVGENEAIFGTVTTQDVVDAIQAATGQTIDRRGI
TIPDINHLGTYKADIKLHSEVTAKIDIEVVAS
>EFA72503.1
MLFRQLFDRESSTYTYLIADEQTEAAVLVDPVVEQVERDLQLIEELGLTLQYCLETHIHA
DHITGTGKLRERTGCLGIVPANSDVSCADKQMEDGEILTIGAINILAIGTPGHTDSHLAF
LVNQEKLFTGDSLLIRVVGELIFKVEVRKLCLTLSPKNCLHFRTK

>EFA72504.1
MYPEALIHVAEYFKVLSEVSRLQVLCCLKTGAKNVTQVIAETGLGQANVSKHLKILTQAG
IVKRRPEG
>EFA72509.1
MLTTDQIGKAGYNPGNFTNNFGGTSSATPVVAGVAALMLSANPDLTAQQVNRILQETTDK
IVDPQPDPQLGLQGGTYNSNGHCLWFGYGKVNAAKAVRAAQKLRGNGLVANNANKLIRGE
NKNQVTIPDGEKQGVRTTIVVPQNIIINDIRIGVNISHDFLGDLEVYLIPPNSQSLLLQS
RTLGRQTRLEKTYTVSSQPVLKQLLNSSAKGNWQLHVVDCSPQDIGKLNGWELLIGYRD
>EFA72510.1
MINDQPVVLQRGGEELILIKSTERFTLRLYADLNREELSRISWGKWQRTIPRAKLELFTV
TPDQIEAAMSQARSDQKVAFASHVYQLQDNPSSYVYLSDQITVQFAAGVDNDKIGIITSS
LNLTENQPIADLPNTFTFLVSKQSPHNPLKIANQLQTLPEVLAAEPNVFIQQETHYRPSD
SLYPQQWYLNHSGGNQLAIASHISVEQAWDITRGVRSVVVAVVDDSFDLNHPDLQGVGKI
VAPRDLKEKDFLPLPGEQETSHGTACAGLAVAEENGQGIVGVAPGCALMPIRTTGYLDDE
SIEDIFNWALKYNASVISCSWGASAVYFPLSLRQKAAITRAATKGRNGKGCVILFAAGNA
NRPISGTIDEQNWPKDLLKGKTKWLSGFAIHPDVIAVSASTSLNKKAAYSNWGENISVCA
PSNNAPPGMWFKKVVLCTPNQLFQQL
>EFA72522.1
MISRSKILGGIENQYQRATVCFNCGYIYNIDTKDWCENCERDIKAYSDKNERAKVNQILS
MDTVYTQKKQRITCDEEERLKYGYNITTHFCYSEGNKKTVIIKTKEGKNLLKLTYGATAS
ISRINRGSAKKKDQGFCLDTQTGKWISNENEENKEEIGKNSTTTKSDIFLMVNITCNILI
IELIKEAEESKIDTIDQGKSENREDTETIMTSLQYALERGIQEHYNLEPNELDSEKMGNG
KYLLFWEAAEGGAGVLSQILQDKQHLNKIAEKALDICHFYEPKEDCVYACYQCLLSYRNQ
NEHNLINRHLIKELLEEIKESYLEREREMDREKEFETLYLQTDRRSELEREVIKEIYERG
YRMPDAAQFLIPGTDCEADFVYIDKIAIFCDGSVHDTEEQKREDKRKREKVRRETKYQVL
VLKYNEEWRENIKKLAHL
>EFA72524.1
MSRTKQLEEKNRVFNLKAEEIALINPNTLTCPVFRTSEDAQLTKKIYHQLPVIENEKTGI
NPWGISFMRMFDMANDSGVFKNEKGDSLVPLYEAKMFHQFDHRWGTYTSSGDMRDLTEDE
KKDPSFVCFPRYWVDKTEVDQKLAGKWDHSWFLAFRDICRATDQRTAIFSLLPRVGVGNS
APIIIILEKTSLVSCLVANLSSLVFDYVTRQKVGSTHLNFFIVKQLPIIPPERYTQKDIEY
ITPRVLELVYTTWDMRPFALEMGYEGEPFFWNIERRALIRAELDAYYGKLHNLSRDEMRY
ILDPTDIYGSDFPSESFRVLKNNELKMYGEYKTQRLILQAWDRLF
>EFA72525.1
MGDVNTYSIFAETARNLIKEDGRVGIIVPTGIATDDTCKMFLVIWLNNSIWRVFMTLKIE
KQYL
>EFA72527.1
MEIGSIVNCRQRQWVSIPSEDPEISLLKPLSGNEQEIIGIYKKLNLEEPTPDKFPQPNPE
YIKDHQRACLLMDATRHLLRSGAGPFRCFGRISFRPRPYQIVPLLMALRQETVRLLIADD
VGIGKTMEAGLIARELLDRGEIERIAVLCPPHLCDQWQRELLTKFHIKATVIRSGTANKL
ERENPTTKSIFEFHRHIIVSLDYAKMEKRKASFILHCPDLVIIDEAHTCTIANAQNRREQ
QRYQIVSEIAKNP
>EFA72529.1
MATAALINIIILLPNHLSVSFNQPVKFISLFLLLLSLHPVVLNPLLRFLFKIKSQIKSQIK
SQLKSEDDIPNSMNSMNNLTLEKYPLLPLVGELIFVCLRGTGFILTIFALGDVNLNQIPI
LIGAFSLAWVLGLVIPGAPGGLGVFESTAILLLQNTFPPSLLIGAIALYRLMSILAEAIG
AGLSYIP
>EFA72530.1
MTMGKFLRWVVFGVTLFFLVTTLKAHWLEVKSLNLGGIRWRILPMATMVTLLAHTWAGLV
WTWILNHLNQSVPTGLFIRVY
>EFA72538.1
MQTLPSLETGKIITSQPTFDTTIKRRKTRAVKVGDVIIGGGYPVVVQSMINEDTLDIDGS

VSAVRRLHEIGCEIVRVTVPSMAHAKALAEIKQKLQQTYRDVPIVADVHHNGMKIALEVA
KHIEKVRINPGLYVFEKPNSDRTEYTKTEFDEIGEKIRETLAPLVVSLRDQGKAMRIGVN
HGSLAERMLFTYGDTPEGMVESALEFIRICESLNFYNIVISMKASRVPVMVAAYRLMAKR
MDDLGMDYPLHLGVTEAGDGEYGRIKSTAGIATLLADGIGDTIRVSLTEAPEKEIPVCYS
ILQALGLRKTMVEYVACPSCGRTLFNLEEVLHKVREATKHLTGLDIAVMGCIVNGPGEMA
DADYGYVGKIPVLSLCIGVGRRLKRFQKTKG
>EFA72539.1
MISVDRESGGTLPEMAEIWQETLNWQPTIEQGNQLQKLYDLIVVANRQINLTRITQPKEF
WEKHLWDSLRGVSPEQHFLPELNAAGSIIDIGSGAGFPGLPIGIVFPHHQVTLLDATRKK
VNFINSAIEKLPLSNATTILGRAEEIGQETLYREKYDLAVIRAVSTASVCAEYTLPLIKK
GGLAIIYRGTWTQEENQNLEHTANILGARIELVDRFTTPLTKSVRHCVYLRKVLRTSVSF
PRPVGVPTQNPL
>EFA72554.1
MARLLFGGILTLLVLSITACSSLVLIPTYELVVKAVNMQLEQAQQDLEQKLDLDFEGFTV
EHLVINQQQALAIENLPGYHLEGVYDLKVKLPTRQIAQSHNHFNIYLQIQKEGKSWRLLI
PEKSGEKQPLVWRGYLII
>EFA72560.1
MEPKRQQAFKEIKTLINTRSIPIIICNDVNSIKLLPRDAQKIAINYCNEYEETVSENNLT
VDRFNQITVEVNNNSVLRERVQKLMMEKMGL
>EFA72562.1
MFRPSQIALWISQRPAPQRVGCFVLCLLVLWLPFVIPIYLLVEDGNLESIFSMVLLYGEF
IVLVRLWGKKIYQRDKILTDYGLELSSLNGVDFCQGLSIGVLSIILLFSIQGLLGWILWQ
PPKAFIVQIIWEGLLVASGVGFAEELLFRGWLLDELNRDYGSRLSTAINAILFAVAHFIR
PISAIISTLPQFPALVLLGLTQVWGKHKKRGRLGLPMGLHSGLVWGYYIINVGGLVQPSG
IVPDWVTGVNNNPFKVLWEC
>EFA72563.1
MKELKTLYFLSMQNLIHVIFAAFCIIINLTLIVITTCLPAEAISLQTKPDTIESNVTSIH
LLLGNPSNATSSLDNPDNYLMIKPQYALSYNRSHGSANWVTWQLDKSWLGDAKRQDDFRP
DDTLPNGWTRVKPSVYNSSGYDRGHIARSADRTQSVEDNSATFLMTNIIPQTPDNNRNTW
GNLEDYSMKLAGEGKQLYIIAGGFGDKGKLKNLVTIPQYTWKIIVVLDRPGLGLKDVNVN
TRVIAVNIPNDEQLDNNWRVFRTSVDKLEELTGYDFLSTVSPDIQKVIESQVDNL
>EFA72565.1
MIQPISNLRTKMNIETLKSEKTKQLPGANLEDQDLSEFDLTAANLAGANLMGAHLVSVNL
EGSHLEGANLMGASLQGADLRANLLGANLMQADLTGADLRGSNLRGANLMGATVAGASLT
AAFLSGANLMSVNLQGVDLRDADLRGANLTGANLKGADLSRADLQGALLNQANLEESDLR
GANLAGANLAGANLLCAELEAASLNGANLYQACLLGTILETYHD
>EFA72570.1
MTESTTFFLSPLIRITLLTLYIALTLPLPFLAEVTQAPVTPLVLWLGIIIGLVALYGVLT
QRVIVNEQEIQVTYPIWVPKLLRQDWSLPWSEIKSLKCRTTGQGGLVYYFLTDEGKAYLL
PMRVTGFNRLVKFVQLKTGMDITDVRPLSQPWMYIILLTCTFILLLVDAWTINMANSMI
>EFA72575.1
MTPFTIRIGGVVLYTGTLFFSYLASRKLFGDRVATLTLGILTTIPIFQIAFGILTLPDNA
LMFFWSICLWLCATEFFPSGESCDPIYDTRPYRPTSKLAFVGLVVGLSFLGKYHGALLGS
GLVLFCLISNRHRCALFSIWTLAAVVLFLIAISPVLYWNSQHEWASFRFQSGRAVPSVGY
DLERLLVTILVGIGYLFPTFGFPIWWTSFRTLWEWLPFNKSHTNKIEQKAIMPNQLAHDL
VDQKRLLILCVSMPIFFGFTFMGGFIQILPSWHMPGFFGATLLLGERAALVQVKHPKFIR
NWLWGSGMVILPLLLISLLHIHWGIAQKGGNVAIAGGFWEAKDDPSTQMIDIEQLRQAFV
DSPVLKTELEKADFVFSNNFFVAGQVGMAIEPLGKKVTCFDEDLRGFAYWSQGTDFVGKT
SLYVTSKQFMEDERFPQPLDKYKDYFQSLEKIADIAIKRGGEAVQIFPVYRASPMLKPYP
RPYG
>EFA72582.1
MSSSLNLSSNIKSPHEENYQLWLESTIDNLKQGNFDRIDIDKLIEEISEMGGSLKDALEN

NLIVILAHLLKWQYQPQKRSGSWKASIKEHRRRINKSIQKHPYLKNITRRFFQNPTYLL
>EFA72586.1
MEIQLGRGKKARRAYGIDEIALVPGKRTVDPSLADTRWKIGNIEREIPIIASAMDGVVDV
GMAIKLSELGALGVLNLEGIQTRYDDPNPILDKIASVGKEEFVSLMQDLYAEPIKPELIE
KRIQEIKQQGAIAAVSATPAGAAKYGEVVSRSGADFIFIQATVVSTDHISPESITPLDLV
EFCRSMPIPVILGNCVTYEVTLNLMKAGAAAVLVGIGPGAACTSRGVLGVGVPQATAVAD
CAAAREDFYQETGKYVPIIADGGLITGGDICKCIACGADGVMIGSPFARAAEAPGRGYHW
GMATPSPVLPRGTRIRVGTTGTLEQILKGPAGLDDGTHNLLGALKTSMGTLGAKNLKEMQ
QVEVIIAPSLLTEGKVYQKAQQLGMGK
>EFA72596.1
MVQLITFVENVLSNEESIALDHKKAALDREKETLLLEGKISSLSVKSQQIEEIKGQTREI
NERTGKILASLDNLLTEVNLAAPENGDEMTIIMDSDLVKDRYA
>EFA72606.1
MSVRVRIAPSPTGNLHIGTARTAVFNYLFAHHQGGKFILRIEDTDLERSRLEYTDNILQG
LSWLGLNWDEGPFFQSQRLELYQLAVKKLLDQGLAYRCYTTTEELEALRELQKARNEAPR
YDNRHRDLTQEQEAEFVKQGRSYVIRFKIDDQRQILWNDLVRGEVSWRGSDLGGDMVIAR
ASDDGIGQPLYNFVVVVDDIDMEITHVIRGEDHIANTAKQILLYEALGANIPEFAHTPLI
LNPEGRKLSKRDGVTSISDFQKMGFTAPALVNYMTLLGWSAPDSTQEIFTLEEAAREFSF
DRVNKAGAKFDWDKLDWINSQYIHNMPIGKLTDLLIPFWQDAGYSLTDGRDRPWLEQLVS
LIAPSLTRLVDALPMTKIFFAKGVELTEEGRQQLEQEGVKALLKAILVALDIDTVTGDTA
QNILKQVVKEQGVKKGLVMRSLRAALTGDVHGPDLIESWVLLNRIGLDKPRLSQALI
>EFA72616.1
MPRTQKNDNFVDKSFTVMADLILKLLPTNKKAKEAFVYYRDGMSAQAEGEYAEALEYYEE
ALSLEEDTNDKSFIVYNMGLIYASNGDHDRALDFYHKAIDLNPRLPQALNNIAVIYHYKG
EKAKEEGDNDEGEALFDQAADYWIRAIRLAPNNYIEAQNWLKTTGRSQIDVFF
>EFA72622.1
MQAYLSDSGWELTPKPPDLDVNETGTTFAENACLKAVEVAKYTSQWAIADDSGLWVDWLN
GAPGVYSARYGNTDEERIGRLLSELANTENRQAKFICAIAVANPQGEIIFQSEGSCEGEI
LYEVRGEGGFGYDPIFYVPEKKLTFAQMSPELKKSISHRGHALRKIIPQLLEVD
>EFA72625.1
MNDMDFKQLLKQQLPVLVNEDVEMRNLLVRAISSYFAGSLETESHFDLVLGEIRRDREEQ
SRKWDENKQEFDRVITQMELDRQEQARKWDENKQEFDRVITQMELDRQEQARKWDENKQE
FDRVITQMELDRQEQARKWDENKREMELDRQEQARKWDENKREMELDRQEQARKWDENKR
EMELDRQEQARKWDENKQEFDRVITQMELDRQEQARKWDENKREMELDRQEQARKWDENK
REMELDRQEQARKWDENKREMELDRQEQARKWDENKQEMELDRQEQARKWDENKREMELD
RQEQARKWDENKREMELDRQEQACKWDENKREFDQVIAAIERMSRKHEVGISGLGARWGL
CSESSFRNGLKAILEESFGVEVLNIVEYDDQGIVFGRPDQVELDLIIRNGQLIICEIKSS
LSKGDLYTFYRKVDFYQQQHQRQATRKIVISPMVHPSANSIASKLGIEIYSYGEDVPPEL
ASSPSL
>EFA72626.1
MEANQQVDNLGKIIINKNLQKFEEKGLWKIEPRVVNIKSGYRLDLLSIWQGPTDSKKGFY
LRKSCYIEPIASEYTTDNSNVKAKIKCDPKTKFFRGSPPAQQ
>EFA72627.1
MNFPLLKEIIQLFTFIKDLFLGIQKLLMPPRSFSWQTFIYLSVFSWGISSLATGIIKDII
AFTGWIFLFAGTAWYTTDSPVRIPGTFMPVGALLTGFIFSVFVFGHGENTITVRTIVLWP
TIAAIITALPQFFTGNGISPKATLPKLEVRQKIILLLSWSMLISCWLQFNFVTDKWLKEY
PSLSAQSFQRSNFVIKI
>EFA72629.1
MTRNGIGILTAQARSERLTGQIHVYDGAGKGKSQAALGVVLRSIGLGINTKSNCNRVLLL
RFLKGPGRDYDEDGAIAALQRGFPHLIDQVRTGRAEYFGHDEITPFDRAEAARGWDVAKG
ALASDLYSVVVLDEINPVLDLGLLPVQEVVETLKSKPQELEIITTGRAAPQQLLDIADLH
SEMKPHHHPQAAELLLDGIEIYTGSGKGKSTSALGKALKSIGRGINHPGSARVLIMQWLK

GGTGYTEDAAIAALQQSYPDVVDHLRCGRDAIVWRNSRQHLDYVEAERGWEIAKTAIASG
VYKTIILDELNPTVDLELLSVEPILQALLRKPKGTEIIITGRCQNQPAYFDLASIHSEVY
CHKHYANQGVELKRGVDF
>EFA72639.1
MPIPNPDQRGGVDCRINGIMPFVIKLKPTIKDTIKRIIKGIGTGKIIMVETDIVVTIIPS
IIIGRLPNLLAQNPAGKDAKASAKPNDSIVMVILSGDNWVLSVLPKVSISKRDPQDMVKP
VRKMKDRL
>EFA72640.1
MVAMLALSSLRSFIFYFGRKIHILLTEKARAALIPATKGLIAGEKSEPKRLMAKENPPK
>EFA72641.1
MATNTADTGKQKALSMVLNQIERSFGKGAIMRLGDATRMRVETISTGALTLDLALGGGLP
KGRVIEIYGPESSGKTTVALHAIAEVQRNGGIAAFVDAEHALDPTYAGALGVDIENLLVS
QPDTGEAGLEIVDQLVRSAAVDIVVIDSVAALVPRAEIEGDMGDAHVGLQARLMSQALRK
ITGNIGKSGCTVIFINQLRQKIGVTYGSPETTTGGNALKFYASVRLDIRRIQTLKKGSDE
FGNRVKVKVAKNKSCTTL
>EFA72642.1
MLIRKGAWYSHNGENISQGRDNAIKYLEEKSDFAEKIKEQVRAKLDQGALVSANSVAKST
EEEVEEEIEEEE
>EFA72655.1
MNVWLLSLLFHYFQHPITILSIAAILAFLLNYPVQLLEKARITRTYSVIIVLVITLTLLV
ILGFTLVPMLVEQTTQLLRNIPDWVTSSQENLSKLQVLARQKRLHIDFSVVSSQINASVQ
NILQQIASGAVGFAGTLLSALLNIVLVVVLAFYMLIYGDRLWSGLINQLPSYIGLPLSKS
LQLNFHNFFFESTVVSIIHGNCFNTNFFIFKSSLCSIICDYYRNLRTSSCDWCYFGYRVS
DLTSILTDLVVSISSSNGGNYHTTDQR
>EFA72656.1
MVAIIIQQIRDNILAPKLLGNFTGLNPLWIFIAILMGFEIGGLLGTLVAVPIAGTIKSTI
DAIKNNKSGIT
>EFA72658.1
MKAMILAAGKGTRVRPITYTIPKPMIPILQKPVMEFLLELLRKHGFDQIMVNVSHLAEEI
ENYFRDGQRFGVQIGYSFEGKIDDQGKLVGKLLVQLGECDVSRISHLFLMTHLWFCVVML
>EFA72659.1
MVLCGDALIDLDLTAAVKWHRSRGAMATIITKSVPQEEVSSYGVVVTDNENRVRAFQEKP
SVEEALSTNINTGIYIFEPEVFKYIPSGVQYDIGSQLFPHLVEINAPFYAIPMDFEWVDI
GKVPDYWRAIRGVLLGEIKNVQIPGYQVAPSIYTGLNVAVNWDRVDITGPVYIGAMTRIE
DGAKIVGPSMIGPNCWICGGVTVDNSVIFEWSRLGPGVRLIDKLVFGRYCVDKMGTAIDV
QAAALDWLITDARQTPPAEIPPEHQAIAEFLGGITV
>EFA72661.1
MGVESITNEIVKVHINGHKSELKNQKHKKLVPPALNRVIYPAAGK
>EFA72663.1
MLKFFADRGGTFTDIVALTDNQDIINRLLNYPQRFLIVPLPNQQWVIVYKLLSENPDQYT
DAVIQGIRHIMGVPPNRVFLVKP
>EFA72666.1
MKIATWNVNSIRTRLEQVISWLGENNVDVLCLQETKVIDRDFPLTVFHDVGYHTYIYGQK
AYNGVALISRQPMKSVSTGFCQVLENLEPEWDDQKRVITGIVDEVRIINLYVPNGSAVGS
EKYQYKLQWLAVLKTYLEVLLKSNSHIIMCGDFNIALEDIDIYKQVDTKNQIMASLPERE
ALREILNLGFGDGFRKFNSQGVIIVGGTIVRELLKEIRDGE
>EFA72670.1
MTKLNSELNTKSSDKSLEAMHHFSEQYAKRTGTYFCSDPSVTAVVIEGLAKHKDDLGAPL
CPCRHYEDKEAEVSAAYWNCPCVPMRERKECHCMLFLTPDNEFAGKNLQEIPVETIKEVR
DSMG
>EFA72672.1
MLPVTIITVTLTGKLSTAQSSGGNRPLTIRADIQEYDAKTQVITARGNVQMLYPARQIQA

TAAQAQYFSKERRIDFSGNVYILQQGNNSIRAEKVTYLIDEGRFIALPQSNRQVESIYMI
GDDDSIQPTSRPTTKPTPKRGSKTRSPL
>EFA72675.1
MITASVNFQFLQSHDLQLLRLSVLAEGYVWNQPKTCLVKLCYFGQLLTQLIAARTGNFHS
TVENQTDLLRQLELKGILPQKVALLFHQVRVVSDRATYEHTSDPSQALTILKIARELAIW
FHRTFGGNTTFTPNPFISPPDPVDYRTELETLQHVETEFEQALATFSLHLAQLQVISSTL
SPRQTDTIISLGNQAVLEIDLEENAHTYLCRGIARSDLGDNRGAINDFTQSISINSNLPQ
PYMERGIARTNLGDGQGAIDDFNQALDINPNLALAAYSRGVAHRDMGYLQKAIEDFNQTL
HLNSAFFDAYTKRGLARYDLGDKQGAIDDFNQVITINPHFADGYAARGLVYCDLRNYQEA
INDFNQTIRINPNYAQAYHNRGVARSQLGDKQGAIDDYTHSLNLNPKFASAYYNRGIIRS
DLGSQKAAMDDYTQAIKIDPNYAQAYNNRGAIRTYLGNYQGAIDDYIQALRVDSNYAEVY
HNWGTTRINLEDNQGAIDDYTQALNINPNYAQAYYGRGIARFNLGDKQGAIDDYTQALNI
NPNYAQAYYNRGIARTSLGDKQGAVDDYTQALNINPNYDQAYYAWGMVCSELGDKPGAVN
NYTQALNINPDDPETYIARGLTRSELGDNQGAIDDYTQALNLNPDYAYIYNNRGVVRSDI
ADYQRAIDDYTQALNISPDYADAYYNRGIAYYDLGNYQSAIDDYTRSIEIKPNCADTYVG
RGTALYKLGDSQGAINDFHHALDIDASYADAYNNRGIVRYELGDYQGAINDFNHALNINP
NYAQAYNNRGIVRYELRDNQGAMEDFNHAVNINSNYAQAYNNRGIVRICLGERQLAIEDF
SQAIIIAYNYTESYINRGYARYELGNRQKAIEDFNQALNINPNYAQAYNNRGVAYTDLGD
REWAKDDFSQAIQINPYYAEAYNNRAIVCYELGDHQGAIEDFNQALNINSNYVEAYNKRG
NIRYELGDRQGAIEDFNRALNLGSLDLGENLKFWERGGLTLTIKLRNYLNLKILSFEVVS
KKNFGEIIKTDASVIYFVEDLGDDITLEMVEIPGGTFTINSAENRGNPEESAEHLVTVPS
FFMGKYEITQEQYQAVTGDNPSYFKGDKRPVERVKWNQAVDFCEKLSQITGRIYTLPSEA
QWEYACRGGTSTEFHFGDNITTDLVNYHGDHKQSLAPQGQYRKQTTEVGSFPPNPFGLYD
MHGNVWEWCLDHWHDHYQDIPKDSSPWVEKGIFGVFRGHVLRGGSWCDSAQHCGCGSRNR
HLSDKNNLGFRVVCSNHNR
>EFA72680.1
MFVLSGYEYLLGFLILCSLVPALALSASKILRPSSYSPERRTTYESGMEPFGGAWIQFNI
RYYMFALVFVVFDVETVFLYPWAVAFHRLGLLAFIEALIFIAILVIALVYAWRKGALEWS
>EFA72690.1
MLELIELTETIDHQLLQNKINNHLRYLESCGNEGEFADFSNLKIENFDFSNCKLSGINAH
NSVFIKCCFYQCDLYGSVFKDSVFMNINFEKANLGKTEFYRIKNEDVTFDNANLGSAEFY
DCQLNGTSFKNANLGGSSFDECDLTNVVFEGAVLENASLTKNIEKNTNWSNIKLIS
>EFA72691.1
MDLVLLDLVDVISSDYVPQSLLQAMFIIAKKTDKPLYRSMELFTSNPAQAIDLFHDRGSL
EVGKRADFITVCDDGIVPRLISTICAGCRIS
>EFA72692.1
MNEQIYTNYRLLLPNEEILGTLVVRNGKIADIQPGITKKGENGEGKYLIPGLIELHTDNL
ERCMSPRPGIRWPLEAAAIYHDRDLASAGITTVCDAIAIGDVNPKSPRLKNYSQMINVLC
QGKADRRFLVDHYIHLRCELAYPEVDQITQEYVHNSLLLMISLMDHTPGQRQFIKLEKFK
EYYMGKHGVTAQEMEEFITTRQERHKLYSKKNRHSLVELARERKIALASHDDATVDHVQE
AVEDGVVLAEFPTTVEAASKAHSLGLKVLMGAPT
>EFA72696.1
MPYVAVKGGEQAIENAEKLLHSRRRGDINITEITSEQIEQQLTLAVERVMCEGSLYDREL
AALAIKQSWGDLVEAIFLVRAYRTTLARFYYTQPLNTSTMQIERRISSIFKDIPGGQNLG
PTFDYIHRLLDFKLLAENQVPPPPETIVSQESFPLVMEILDKEGLIQSEPRVTTPQTPFD
LTRQPLNFPANRDARLQNLARADEGFLLSLAYSTQRGYGKNHPFAGEIRMGEVEVIIFPE
ELGFEVSVTDITITEVQMVNQFKGSKHLPPQFTRGYGLTFGYNERKAMSMALVDRAMRSS
ELGESVDSPAQNIEFVLSHSDNVEAQGFVQHLKLPHYIDFQAELNLIRQMRQKSQDLSP
>EFA72700.1
MSNTLTEIPGFSDPVHDAQQAFRALLCADAQPGRSEKIPVQLQVPQGLTPACGAACLVLL
DLDIKLWIQPLLSSSVKNWLLFHTGCQLTLDPQQADFALIQDLASMTELSVFNPGTAEKP
EDSTTLLIQIASFNGGKPITLTGPGNLSPKTITPSIPSTFWEFWERNYPLYPRGIDVFLF

TEDSVMGLPRTAKHS
>EFA72717.1
MRHPILYLGFTWWIWFITPLLARLIDYRVGWDPTRQILVAPYLVVLITTASVIKNLPRTL
KDGGLPFILALIGVIYGLLVGLLYNQPISVIRGFLDWFTPIIFAFHLFINWRDYPSYGRN
TQRVFVWAVLLIGAYGIYQFIFAPEWDKFWLIESKMFTSAGSPEAFGMRVWSILHSPGTF
GAVMQTGLILLFTSYGPLIFPASLVGYLSFLLSQVRTSWGCWLLGIIIMLGSVKTKIRMR
LGMIILVIVLTIIPLVTIKPISEVVTTRLESFSNLEKDSSFQDRSRTYNESLNVALSTAF
GHGLGNIWEVDEKTGQIKVIVIDSGILDMFLTLGWFGAIPYTSGLFLLLVSVIKYTEAKR
DVFISAARAIGISACAQLIIYSGMLGVAGMIMWGFLAVSMAAHKYYKHHY
>EFA72718.1
MTSKQLLFNSFLESSYSPEDRSAQSWVIIFPLLYLQVPVILVVLLTY
>EFA72730.1
MMINTWQDQLNAFTEDINYRVSVAKYKKHLPVLSPKDKLIAETLKQQGVYITSLTDLGMP
STTQMWVSATGYAGMISAPRNVESGYSLPQIYTVTDLPEFFTWGIESRLRNIIESYIELP
IAFHGVHVRKDFPNEQQLQTLLWHKDSEDRRMIKIIVYLHDVGEEHGPFEYIPLPSNIGE
WCNYYRVDYRLWKSGFLGIDDQEMMNVIPKKFWKSCPGKAGTVIFVDPRNVLHHGTVRSQ
ERSTAFFVYTSHTPKRPELCTQYHDHTFTKPHGQFKTEIANKAR
>EFA72735.1
MRVLRYFSNQEVLDLRENLGRGGEAFIYTVSSDENLVAKIYHRPSSDHIKKLQAMIANPP
ANPAASFGHISIAWPQELLTAVDGSDTIIGFLMPRIRDMVPIIDFYNPGNRRQNCPLFNY
QYLLRTARNLAAAVAALHASNYCIGDVNESNILVSNTALVSLVDTDSFQVPDLSQSRVYR
CLVGKPEYTPPELQNKTFADYNRETYHDLFGLGVLIFQLLMEGNHPFSGVFQGLGDPPSY
EYRILAGHFTYSQKQKVPYLPTPITPSWQTLHPAVRDLFVSCFEDGHHSPHLRPSAQTWL
SVLSTAEASLVSCAVNPQHVYHFDLNTCPWCERTVKLGGRDPFPSLQTISAREYHQPRPK
SRKRYRYASHVRKSATPVLVTYTQSSLKSSSAIYKPIQMSNRSKFYALMFGFLGLGVLGY
LDIMIKFTRIFISPSPYTKQSLLSSRSENVHSPLSLSFHDYYQRGNQAYQQQDYQQAIED
FTQGIKQNTNFSKLYMHRGNARYNLNDYQGALSDYSIALKINPQEVKAFINSGNAYFKLA
DYSNDPDYEYKKAIDSFNNAININKQDDDAYVRRGVVRSQIAKYSHNSQQEYKKSISDFT
QAIKLNPFKAEAYFQRGLSRYQFGQYSSNYAEIYKQAIADFDQALNINPEMAEVFLKRGM
IYYELAQYGEKTARNNQQRALEDLEKSAQLYLNKKDVNNYQQAISNICVIAEEKCDYFLQ
NSSIIYSVNP
>EFA72743.1
MSSTISHSPLLQIGTASMLLLLVANKVTSELIITLGTASEEIFRGDRLPPLKFPQESCAE
DRITS
>EFA72747.1
MREYLLEFKEVYYSYMESQQLALNSLTLRIPSGKKCALIGQNGCGKTTLLLLANGLYKPN
RGVVSWCGEPLTYNRNYLGRLRQKVGLVFQDPEQQLVAATVEEDISYGLCNLGLPLVEIQ
QRVEKALMEFELTGLAQTPTHHLSLGQKRRVSKADKLVLSPELLLLDEPTAYLDIKQTRN
LIAMLNKIHEYGTTLVMATHDLDLVYRWADWVFVMDRGKLVLEGSPQDVFSQRRLLEELE
LGLPLICEMLFYGLSDEWVEK
>EFA72754.1
MTITLEEMRKKLKQVWGYENFRPPQEEIVSSLLSQKDALIIMPTGAGKSICFQLPALLSN
GLTLVVSPLIALIENQVEELKQRNQKADLLHSELPASQRYKVLESISKQQLRLLYLSPET
LLSSAVWEKISHPHIGITSLILDEAHCLVQWGETFRPVYRRLAAVRPSLLNTKPPGTKIS
VAAFTATADPSTQNIIKDVLQLQQPDIYRLNPYRQNLQPTVKTVWTPKARKQQLLKFLQL
HPHQTGLIYVRTRKDSEELAQWLMNLGYHTASYHGGLSGEERRAIEKSWLHGKKSFVVCT
CAFGMGINKADVRWIVHFHAPYLLSEYVQEIGRAGRDGIVAEVLTFISEPNRFF
>EFA72755.1
MNLTGFFDGEDQRRKLFFQQQILQQYKKAQELIKKLPLQGEVKSVVKEFQHGATALAILH
SSGRLLWNDPFHYQILGRDIHQSRTYFNPAQQMVEYLRTKNCRWQFLLQSFGFNKDRENW
RCGHCDNCRFKSGGFQGIS
>EFA72756.1

MSLDKIYKTDVLVVGGGTGGTTAAIQAARRGCKTILVSEFSWLGGMLTSAGVCAPDGNEL
NSFQTGIWGDFIRELQKRQSGGLNNSWVSFFSYQPQVGAGIFADWIKELPNLQWITHRIP
LEVLRQGDRIVGVSFADCTIYAQIILDGTELGDVLALGEVPYRWGWELQSEWGETSAPSS
FNSLTKTYPIQAPTWVVIMEDFGEVLAPEIPPAPNYDPALFAGAWENYGKEKFLNYGRLP
QNWFMINWPICGNDYGQGTSRLLESKARNEFYQECFWHSQNFARYIQTSFGRRYGLAQGA
FPTVSPAFALHPYFREGRRLQGVTTICEQDLLPLSNGSVAPLMKDTVAIGNYANDHHYPG
IKFPLQPKSLLWGGRWTGTPFTIPYSSLIPKSIDGLLVCEKNISVSHIANGATRLQPVVM
GIGQAAGMAAALCCELNCQPRDLPVKTLQMALLTDKHSPAAVIPLLNLSMNHPDWLRWQI
DILNHPEIYPPSGNILTCGGNIHQSTFHHF
>EFA72757.1
MRATISILVSSMILGSLAFNRETLFTNPSRLSTSNSGTGLLFADSSKPSPNRPGEPVTYR
GSGRKESINL
>EFA72762.1
MIVGYESQMVKGALSKNSDKHSDLEFVEQSVQLGTGHAIQQLLPYLSDYDGNLLILNGDV
PLLKTETLQQLLQTHQEHDNACTILTANLAQPDGYGRVFCDDNQIVHQMVEHKDCTHIQR
KNNRVNAGIYCFNWQKLATILPHLDNNNVQKEYYLTDAVVQVGKVMAVDVSDEREIWGIN
DRRQLADAYQLLQQRIKEKWLLAGVTITDPSSVTIDETVEMEPDVIIEPQTHLRGKTLIG
SGSRIGPGSLIENSQIGENVTALYSVITDSFVEQGTKIGPFAHLRGHVEAGENCRIGNFV
ELKNTQLGDRSNVAHLSYLGDTSAGTQVNIGAGTITANYDGVKKHRTRIGDRTKTGSNSV
LVAPITIGSDVYIAAGSTVTEDVENDALVIARSRQVVKPGWKIKRELAE
>EFA72784.1
MAEKTIANSIPIINYGYLNNDRVQAALSKSQPQKEDILKMRVTAIFSVHST
>EFA72786.1
MKQQGVLVLDAPANQISEQLVERYLQIKAKNQI
>EFA72791.1
MLNLPVPSTLLYSIPLAAVTIYLPYLLVAYARVKVGYDMSAPRSMFDKLPPYARRATWAH
QNCFESFMIYGTAALMAYVTGVDSQLALYVAIAYLTARVLFSVFYILNIPILRSLMFGVA
SACIISLFVLSILKGT
>EFA72793.1
MSIFNTAILYDIENLTKGYSFSKDFIKELSLKQIYRQILEVDIVNKICLQRAYANWSDHR
LSLLRGEINELGIDPIQIFGFARYHKKNAADIQLVVDTMDITIRFPHIEVYVIVSGDGGF
ASLAKKLT
>EFA72794.1
MANNIQPIYQADKKTIFSHGQKIISWFGQDPESRKQMYGHGIPLSTVREAFKYAIPEFKP
EMVGFMRFAEFLQFICANTEFCVGTLPPSNTLLVFRNSIPNGVVILLDILNQDLHTPERY
QSLLASGKPRITIEDKYSLETFVDTLMSKRDILMNISEILDIFSQELPDFESNKLNNLCL
SLIHCNILKGYPEDENISEQKFHISQDFKDTAQILEHVKQTSLNKLISILVDDFKSDVFK
EVIPF
>EFA72803.1
MLILPNTGAIRGGIEIFGKNVYSVGLHQKDVASIQSLHHDQQISQH
>EFA72811.1
MNLEQKDPCFERLLTYIRQNRGFDFTGYKRSTLVRRVTKRIQALNIDNFIDYMDYLEVDP
EEFHNLFNTILINVTDFFRDVSAWDHLGNQVIPNIIKRQKKRMNKFVYGVLVVLPGKKLI
P
>EFA72812.1
MAENLGAEDFRQRVKIYATDIDEEALNQARQALFSVKSVESVPRKFRDKYFDLTGNNYIF
RQDLRRSVIFGRHNLFIDAPISRLDLLVCRNTLMYFNSEIQGRIMARFHFALNDTGYLFL
GKAEMLLMYSSLFMPVDLKNRVFTKLSSTNIRDRLLVMANSVDDESSRQLSQNIRLRDLA
FESAPVAEIVIDINGLLIIINEAARNLFGLSKMDLERQFYELELSYRPIELRSLIERAYN
ERRPITLNSIERYVPNSEQQYFDVLITPLQEDDLSLLGVSIAFNDVTRYVELQEALQRSR
QDLETTNEELQSTNEELETTNEELQSTNEELETTNEELQSTNQELETMNEELQSANEELQ
TINHELSERTLELNRNNVFINCILKSLQKGIVVIDRNFSILNWNELVEDLWGLRYDEVIN

KSLFSLDISLPVEELRSPILDIISGKTDFQEVSIESTNRRGRIIQCYIALTPLIDKKIEG
VVLIMSDSQN
>EFA72815.1
MKRIGILTSGGDCPGLNCVIRAVVSHAILTYNWEVVGIPYATQGLQQRQTIPLSIHGWNL
RGIDPLLNMGGTILGSINEGDTLAAAGEILSGYEALGLDALIAVGGDGSLKIIHELATMG
NWHLVGIPKTIDNDVALTERSIGFDTAVNTIVDAINRLTFTAASHDRVMIVEVMGRTAGH
LALHSGIAGGADVILIPEIPYTIQGICEHLKELRDTWKRKFAIVVVAEGATLCSEDLNND
HGVNGEHIPDTNIGSAKCGRGQYVAGQISNFAHGFDTRVSVLGHIQRGGIPSALDRLVAT
AFGKTAVDLIAQGKNHEMLAWQKWQGYKYFHRKFFT
>EFA72816.1
MNLTTNYLGMELKSPLVPFCFSSSQDVDNIKLMEDAGAGAVVMHSLFEEQLTLEKYELHH
HLTYGTESFAEALTYFPEPADFRVGPQEYLEHIRTAKEQVNIPIIASLNGFSPGGWTEYG
QLMQQAGASALELNIYYVPTNPDLTSAEVEQNYIDILRSVKKSVTIPVSVKLSPYFTNTA
NMAKRLDRAGADGLVLFNRFYQPDINLETLEVEPQVLLSTPQAMRLPLRWIAILYGHIQG
SLAATSGIHNAQDIIKMLMVGASTTMLCSVILRNGINYLKSLQQDVIKWMETHEYESVRQ
MQGTMSQLNCPDPTTFERAQYMKVLQSYQPGSQKKIQISTHIG
>EFA72823.1
MVFGKNKNGIDKTTIIYNSKITLSQIPLESYEYIVNGKSPLEWIMERYKVTKDKDSGIVN
NPNQWSEDPRYIVDLIKRIVKVSMETVRIVKELPPLELIEDS
>EFA72824.1
MPKIFPNQNVENLVIAVTGVGSTKEFSALITNFLPDLELISKSQCFPLYTYEKQSELGEI
FATVTTEEYTKKENIPDNILKDFQKKYQDKSITKEDIFYYIYGILHSPEYKQRFAADLKK
MLPRIPYIKDFSSFSQAGAELAHYHLNYETIEPYEIKEFSVEVYLDNEDYQVEKWYLVKT
RTE
>EFA72825.1
MCIQVELKQNRDAWVYNFSQESLIDNMTRMIDFYNQQVEGFRKFLEGQTLSNAEQRKQKV
ERFIDTDPKKIKLVWRTKGRLW
>EFA72828.1
MSSFQNSYEVFQSVERKANFVSAFWHRFKTEIIQHLPHSYHDEIYQLSQNLQKALDILID
ELRNPVLVLATTGTTSSGKSTLVNFLCGADVLPTAVGEMSAGSVTIEYSEQRCLTIEETP
GALWECGRWENITDDQICRKLEQVMITYLDHQKDQAVLAYPRFFLSYPFRFFKEYKNIYR
RE
>EFA72829.1
MKVKILDLPGLSYVGDETSMEVIRQCREALCLVTYNSQETDPQKVRSLLLQVVEEVKGLG
GSPDRMLFVFNKIDVFRDDKNWEESERRFIEKTTKNIKDELRIHLGEYTQAIKNLKLIKL
STLPALLSLQILSDNYNHSNTACRDADRKFNKLIEESILEDLPRNAEKWSGQDRRRIAQD
LWRKSYAQEFQECLNEHIAEHFPKLVIPQAIERFNTSAGNSITQWTVQTTTAILNSSEES
YQQECHRISQVKSDLDHFIQVSSNSLRNPFEKISQEIGEYFDKENKDVQHLSKTLTSVVI
ELQNTEPCRNNPEIQEKLVPLYDWRDSLSREVSTILEAVTSSLEKGTVTLDHPNFKKVNS
YHVKLLEGNIIRLIKLGYSHDIAKHGKTIEAKTELEKENLRQTNNEINELSIHLSLIIPQ
VLEKTSEQEIQRIYEAVNGLFAFYLEFLEEECNEIAGNKLGINFSKSELVQVKKDLKFIQ
PKFKSDFAIETREYTEETRSWDYWFWLVPKQVVCSSDNAKIPKMRDILTTWELEIKKIDP
ELLKPFIEWLLEQIDNLKKKVDKTQSEIIKSYRDRLEKARKEVNFDYEQRQSIWQPMQKR
AKELEWEFSQLVNFEKPQVNNNDK
>EFA72832.1
MIAAQCGIFANNHNFAERSLPIKKQGVSCQGIVIEDDCWLGHGVTVLDGVTIGKGSVIGA
GAVVTKNIPPYSVALGVPAKVVKSRL
>EFA72838.1
MAEIDKSISFDGRDIRLKVGLLAPQAGGSVLIESGDTAVLVTATRSEAREGIDFLPLTVD
YEERLYAAGRIPGGIMRREGRPPERAILTSRLIDRPLRPLFPSWLRDDLQVIALTLSMDE
LVPPDVLAVTGASIATLIAQIPFYGPMAAVRVGLVGDDFIINPTYAEIEAGDLDLVVAGS
PDGVIMVEAGANQLPERDIIEAIDFGYEAVRDLIQAQRDLIAELGLTIVLQEPPIPDQSL

ENYIRDRASEEIKKITRSV
>EFA72839.1
MTKTERDAALDVVKSAIASSIESLGEEDPIRTAATANSQVLTNTFKDITKYFMRRQIVED
NVRVDGRKLDEVRPVSSQVGLLPRRVHGSGLFNRGLTQVLSACTLGTPGDAQSLSDDLQV
DQSKRYLHHYNFPPFSVGETKPLRAPGRREIGHGALAERAILPVLPTKEQFPYVIRVVSE
VLSSNGSTSMGSVCGSTLALMDAGVPITKPVSGAAMGLIKEGSEVRVLTDIQGIEDFLGD
MDFKVAGTDTGITALQMDMKISGLSLDVIKQALEQAKDARLHILDKMLQTIDKPRTETSP
FAPRLVTIKIDPDMIGLVIGPGGKTIKGITEETGAKIDIEDSGIVTISAIDESRAKRAKS
IIQGMTRKLHEGDVYLGRVTRIIPIGAFVEFLPGKEGMIHISQLADYRVGKVEDEVAVGE
EVIVKVREIDSKGRINLTRLGIHPDQAAAAREAAATNR
>EFA72850.1
MTLISLIAAISQDRILADSKNEHIRGGIPWDIPSDGRYFKEITWRHPVIMGRKTYATFNH
PLPNRTNLIVTKNTDYQAPGCVVFHSLEEAIKWSKMCETEEIFIAGGEQIYTQTMELAHK
LYLTIVEGSFEGDIYFPEFSNFGKLTKRGKIRRKWF
>EFA72853.1
MSIYVIGIGGTGAKSIEAIIQLTAIGLFGEQSVKLLFVDTDETNGNVSRALNSLDTYNKC
YELGLNGKYPWMRTEIEPFPLWSPFADNLTNKNLAVIFNYNLVKENDAALGNLFDVLYTS
KEREANLDVGFRGRPNIGAAVMSQVKMDTIDNDFWGSLMGKIQQDTGGGKRPKIFLCGSI
FGGTGASGLPTIGRLIHNRLETLKIREKVDLGCLFVLPYFSFTPDENSEEVYARSEQFLL
NTEAALRYYGSQPQEIFDVVYLLGNQQLSTVKKFSIGKNTQCNDPHFIELYAALAARQFS
LKTTPGQKRWY
>EFA72854.1
MNRENSRNLTWKDIPDMQEVYPEIVNATRFALVWLSNIAPELEQAKKMGEKVFRTGAGRS
WFVKFFPASRGILGGGSQPIVDFNNIKEQEAIQIISNWCNDYLRWLWSIHQCQEESIQLF
NGDYLNTGNLNAENLANLIIEQTDSRDRNKKSLDTIQKLKERLDPQSIPAPNFGVIGLAK
SLYHVCRLTD
>EFA72855.1
MKIGELKKQGKLYMTSLLLPRLKDDCDVKSGVAGVWDAQAPNTFNQVASSLDYKAPGEIK
NISSVPTIWARPLSVEMALHNDKYPIREQIIVQWQGMLTVLALAEVRGFPIKAQLLELGA
KKDTDNFARSLYELLPDDGNSIYKFSNNKNPWEDLYILPGIILP
>EFA72860.1
MGVGTPGPVDATGRVAKVAINLEGWDNVPLAEWLENRTGIPTILANDANCAGLGEAWLGA
GRDFQNLILLTLGTGVGGAIILHGKLFVGHQGAAGELGLITLNPNGPSCNSGNQGSLEQY
TSIGAIHRRTGKDPARLGELAKQGDIQSLTFWQEYGRDLGIGLTSLIYVLTPQAIIIGGG
ISASFEYFLPAMQEEIERRVLPSSRMGLQILPAQLGNSAGMVGAAKLAWDND
>EFA72861.1
MEVVIGIDIGGTSIKLGVFDPNGNCLQTIILQTPKPATPEAVLSTMLGG
>EFA72864.1
MLLVFGFKTVDIVLKLGSFCCKSIKNLVLPSIFPIMAELSNLILAEMGYYGANCLFIALG
KTL
>EFA72876.1
MKRNQLFVMGEAKRRKNAMGENYGQETPILPWVPITKSQAELFVKITTRGAWIGIGTMVA
IWVTIRFIGPAFGWWQVVA
>EFA72881.1
MMAKPKKNNPPIVSLDEPINLNDPEYYINRELSWLQFNSRVLHEGCDERTPLLERLKFLA
IFSSNLDEFFMVRVAGLKQQVEAKVNLLTPDGRTPQQQLDDIRLHLSPLVAKQNQQFEEI
LQPLLSNQGIYIIKYMDMNEQQKLYLDEYFKEQIFPVLTPLAVDPSHPFPYISNLSLNLA
VVVKNPETEEEYFARVKVPKVLPRFIPLPSELASDNGRPIHWIGVPLEQAIAHNLDRLFP
GMNIQECHPFRITRDADLELEEDEADDLLLAIEQELRKRRMGGTPVRLEIQSQTPENLRS
RLLEDLELTANDVYQVNGLLGLRDLMYFMGLSLPELKDPPRQSVIPSRLQRLKEPCLDPD
VLETEDGKDFFSVIREKDLLVHHPYQSFSGTVEQFITHAAWDPQVLAIKMTLYRTSGDSP
IVNALIAAAENGKQVSVLVELKARFDEENNIYWARRLEKVGVHVVYGLAGLKTHSKIVLV

VRREKEKICRYVHIGTGNYNPKTARLYTDLGLFSCREELGADLTDVFNFLTGYSLQKN
>EFA72882.1
MRNRFLELIYREITNVQNGFSGRIVAKMNALVDPEIIATLYTASCAGVQIDLIVRGICCL
RPGMKNISDNIRIMSIIGRFLEHSRIFYFHNNGQEEIYIGSADWMRRNLDRRVEVITPIR
DQEIAKDLQEILGIMLADNRQAWDLQPDGNYTQRTPKDNSSEANSQTILMSMNQNNYPKP
LMQN
>EFA72908.1
MAKIIAFDEESRRALEKGINALADAVKITLGPRGRNVLLEKKIWYPQIVNDGITVAKEIE
LEDPLENTGARLIQEVASKTKDVAGDGTTTATVLAQALVKEGLKNVAAGTNPIALKRGID
KTVEALVKEIAKIAKPVEDSELDSPAASIAQVATVSAGNDEEVGQMLAEAMAKVTKDGVI
TVEESKSLTTELEVVEGMQIDRGYISPYFITNNDRMTVEFDNPRILIADKKISSIQDLVP
ILEKVARLGQPLLIIAEDVEGDALATLVVNKARGVLAVAAIKSPGFGERRKALLQDIAIL
TDGQMISEEIGLSLDTATLEMLGKARKVTIDKENTTIVSGTENKPEIQKRIAQIRKQLEE
TDSDYDAEKLQERIAKLAGGVAVIKVGAATETELKDRKLRIEDALNATKAAVEEGIVPGG
GTTLIHLVAKVDAIKDTLDGEEKIGAEIVQRSLEAPLRQIANNAGVEGSVIVSQVRNSDF
NIGYNAATGEFEDLIAAGIIDPAKVVRSSLQNAASIAGMVLTTEVLVVEKPEKKAPAAPD
PGMGGMGGMGGMGGMGGMGMF
>EFA72918.1
MGFFDSEIVQQEAKQLFEDYQALIQLGNNYGKFDREGKKLFIEQMENMMDRYRIFMKRFE
LSEDFMAQMTVEQLKTQLNQFGVTPQQMFEQMHLTLERMKTELEKQV
>EFA72923.1
MDNMENQLIDIRLAVSALLETSVIYQRNFEVMQRNFDSVVIEIREMQSEIREMRSDIREM
QSEIREMQSEIREIQSEVREIQLDVRGLQTENRRILDVLQNVPPDKYE
>EFA72927.1
MLGDDILRSCSMVIFRRLITSGLIGFLVSSCADAAKSSTEYTKQIVQENNVARLMAQVQD
SQKVSNLLSQGNGFLDSGRYEEALQLYNRAIEIEKDSVPSWVNRGNALLSLKRYQEALES
YNQAIALRPNKNEAWYNRGNALSALGRYEEAIRSYNESIVIDPNKFEAWINKGIALTKLQ
RYQEGLASYNQAISINPNFPTAYYNKACNYALQKQVNLAVESLAKAIKIDGQKYTQLARV
DKDFAKISDNRDFQELLK
>EFA72932.1
MQNQVSDQNSGEKKNNSWIVEVGKTLLVSGILALGVRTLVAEARWIPTGSMEPTLHGVQD
QWQADKIIVDKVKYKFSPPERGDIVVFSPTDELQKQQFHDAFIKRIIGLPGERVELKNGK
VYINNEFLPEEKYLFPTVRTGIDVCTTTSQRPFLSQPQTIPPNSYLVLGDNRPSSYDGRC
WGLVPREKIIGRAVIRFWPLNKIGSIDSPPLYPTQK
>EFA72939.1
MTTAISHPNGLDLRTDDYIVIGLATCFYKEDGEVHELEIIEPIPSAALEAIIKGIPTSYK
LVCATTLGSILDGENLLLPSGFPETAQFSEEFLQRTFSATRTYKRRESAQSFIPLGTTKS
DFNYSTERKRVLNAARVVTKEDNIKQHSHTHKVL
>EFA72944.1
MSLVKTSVETIARKPLIGLKADYFRHPLDLEATKTLRQIPGIDIMVRNLLGPVAEQVFYA
ENIASSILVSEKQLPDLYYLLIDACKNLDIELPQLYIRQHPSPNAYTFAMRGKQPFIVLH
TSLVDMLTPEEIQAVIGHELGHLKCDHSVYLTPANLLILATSILPNIGVVLAQSLQNQLL
EWVRCAEFTCDRAALLATQNPKVVMSVLMKLAGGSPTLAPRLNLDAFVAQAHAYDAISKT
ELGMMVKDAHTAQLSHPVPVLRAREIDRWSSSVEYHNLLENQHQTQGGWRNW
>EFA72947.1
MLYGLYIPTQGAKWICLIIFLIAALTDWLDGYLARKLNQVTDLGKFLDPLVDKLLVLAPF
LVFVELGKIPGWGVFIILARELAIAGWRINQTTISGANIWGKLKTITQILSIALLIAPLP
TNWQIYAKSAFWLSVLLTVISGLIYLLPVLSPKHGN
>EFA72948.1
METPRFMPVGTLANVKTVTPAQLEDTGAQMILSNTYHLHLQPGEKIVAGGGGLHKFMAWN
GPMLTDSGGFQVFSLSEMRKITEEGVVFRSPHDGQMIKLTPEKSIEIQNILGADVIMAFD
ECPPYPATYQEVTAATDRTYRWLERCIMSHQRLDQALFPIVQGGVYLDMRIRAATDLAKL

DMPGYAIGGVSVGEPPELMAQIVRTTAPLLPRNKPRYLMGVGTYREMVIAIAAGVDLFDC
VIPTRWARHGTAMVQGERWNLKNSKFREDFIPIDETCHCYTCSNFSRAYVSHLVRSQEIL
AYTLLSIHNITELIRFTQAIRESILQNRFLTDFGHWMNDPEKDLE
>EFA72949.1
MNKLDIHKLLSDLGRVHYIYTQGDKLLSEGDGDVMEVFAHPQRSTLVANNTLYLNIYSFD
YLELKQSDAQETYLDLIQEEYCLRLIPLFVPPEERCDNQFNNGVIEAMMDQVLSTRWDVE
IDDDCSDCL
>EFA72954.1
MESLATNQTFYRLKQRESVQENGQKQGVSQWLSISDIAISLSPLGLVMVWAIFLLILQKV
RSALEEKMSFTVNGVSQVPCKNCKFFSNNHYLKCAVKPDIVLTEAAIDCSEYCPKKTKFT
RR
>EFA72956.1
MTVIIAGERSGVGKTTVTLSLLSSLCRQGVSVQSFKVGPDYIDPMFHQYVTNQPCRNLDA
VLTSEQYIQKCFKLHSEKSEYALIEGVMGLFDGVGASSLIIDNKSDRINYPVDFASTAHV
AKLLDVPVILVIDCSRLSGSVAAIAHGYCYLDPRIKIAGLVLNRVGSDRHLSLLKSSLKN
VGLPIVGVLKREDNISIPDRHLGLVPTGELTALDRVIDQLAKLGDNCFDWELLLPLLRAN
QFKEPKLLSNPGNLNGLSGNNFQELDDKIRIAVAWDKAFNFYYQDNLDLLQELGAELVFW
SPINDDQLPNNIQGMYFGGGFPEVFAQELEANIQVIHQVKNIILSGIPTIAECGGLMYLC
ESIIDFDNQPWSMVGIIPTSAKMDKKTNSRLSSRSSFRKQFFTPWGKNIYGHEFHRSCLN
SATTYPLFNTFRYDCEESMGWEGWNLPNVHASYIHQHWGESIEIPQNFLGECLKFLKTRS
KM
>EFA72961.1
MVICQISKGSKTVNLGEGVQSVSFGILGAMMIGAALGVVLFSNIVYSAFLLGGVFISMAG
LYLLLNGDFVAAAQVLIYVGAINVLILFAIMLVNKRQDFAAIPSAGLRKIVTGVVSLGLF
ALLSTMVLATPWGNTTTPPATQNSVVVIGEHLFSDFLLPFELASILLLIAMVGAIILARR
EYLPDQVTPSELPQTILTLPERPRELVSSTGNQE
>EFA72964.1
MAKKLKIHGLDGSIDAYLAQPVATGIYPGIVVLQEIFGVNEHIRDVTERIARLGYVAIAP
ALFQRQAPGFEAGYSPDEIEIGRKYAWSQTKATELLGDIGSSINYLKTLENVQPANFGCI
GFCFGGHVAYLAATLPDIKATASFYGAGIPTRTPGGNHPTLLRTSEISGTIYLFFGMEDA
SIPQEQVDEIEDTLKKHQVPHRIFRYDGANHGFFCDHRASYNVKAAGEAWEQVQQLFNLL
VKTND
>EFA72966.1
MRVYPQTDFPERFEEPGTRWLLLPGEPSPQPVQLLHGRYVEGKNLYVIKLHGIGDRNAAE
NLREGRLFVPISDRVELGPGEFHVIDLIGLPVFMQESGERIGDVTDILPSGHDLLEVKCD
PSWNRNLGGKTVLIPFVMEIVPVVDLANRRIEITPPPGLLSINE
>EFA72967.1
MKRENKKRKQATGEQKKRGNKSESPIPQPR
>EFA72971.1
MRKNNSPAVSWGIGDNSTWLFMILSITIPVFIWLVVSNSGLVKPLFLPTPQAVLTALQKL
WATGNLQTDIGFSLLRVLGGFLLAAVISIPLGVLMGSFPVVRALFEPAISIFRYMPAPAF
IPLLILYFGLGETPKILLIFIGTVFFNTLMIMDAVKFVSRELIETSYTLGGKNHQIILQV
ILPFIVPNIIDACRVNMAASWNLVIVAELVAATEGLGRRISVAQRFLKTDEIFAGLIVIG
LIGVVIDILLVMLHRMVCRWAHN
>EFA72982.1
MGLFGFNKKVAMPTPEQALPGRQQKMPVPAAHYVNNNPLQPPFPSEMETAMFGLGCFWGA
ERKFWQQRGVYSTAVGYAAGYTPNPTYREVCTGMTGHNEVVLVVFNPKIINYSQLLKVFW
ESHNPTQGMRQGNDVGTQYRSGIYSYSPEQRELAESSMNAYQQALTNAGYGKITTEILDA
PDFYYAEDYHQQYLAKNPGGYCGLGGTNVSCPVGVFPTSVS
>EFA72987.1
MLHQVKKTMVNIRKLVPKRKHFDTELAMIRQMCWLSKFSGDGEKILHLQIATGQPWRPYT
AFGQFSVPDYPIPGGSKGWATYQKLLKAGWTLVPSARAEEFSRDLVESSIHK

>EFA72989.1
MIPERYQGSDLLTSLDNWLQFVIEETADFVCAYKPTLGFYQALGVRGIELLIKIMASIPK
EVPIILDAKHGDLNTTSMFAHSIFVDWGVDAVTLNPYAGQDHIAPFLVYPDKAVFILCCT
SNPSAAILQQYPATSPFYLQVVQESKTWGTPEQLCLEVGTTNPEILKSIRSAAPERIIMV
RSIWSQENSIQPILAAGLDSHGDGLLIPVPQDMLISANLSQEIGSLRTELNQIRSDLINS
TSSCDVWFPDVNMKDKHPHQDLILQLFDIGCIMFGEFVQASGAIFPYYIDLRKIISNPQV
FSQVIGAYEKILTGLTFDRLAGIPYGSLPTATGLSLRLGYPMIFPRKEVKAHGTRKAIEG
NFLPGETIAVVDDILISGKSVMEGAEKIKSVGLTVNDIVVFIDHEKGVKDKLKENGYCGH
SVLTISEIVNTLYEAGRINQQQLLAFQQE
>EFA72994.1
MTRIYLQDAKATREFGINLAKTLKPGTVILLQGDLGAGKTTLVQAIGEGLGISDPIVSPT
FTLINEYTDGILPLYHLDLYRLEPQDVANLYLENYWEGIDTTLGIVAVEWPERMPYLPHS
YLKLILTYEQDNNSRYVEIISC
>EFA72999.1
MVSYRIKQFLFLLLVLSKPRYLSASHHALTMFWVCFIDNPGNNLV
>EFA73003.1
MAQVQLFPVNPRLSLAEEKEVRKTTIKGGKMMSDPEKLQKCLTLPFQIQMQQHCL
>EFA73010.1
MFWVGNFVSPTRSLFPISVHSEKNSCNNGNISYLHLVFYMPLKAVLFDFNGVIIKDESIH
LKLIDEILVEENLQPQKPDERLRCLGRSDRACFEELLKRRGRVVSQDYLTHLLRNKANKY
IKELESLEQLPLYSGIEDLIIQAQSQNLPVGLVSGALGREIELVLERANIREYFQVIIAG
DDIATSKPQPEGYLLAVDRLNRLYSDINFDLSLQPKDCLALEDTLAGIEAAKRAGMKVVG
IANTYPFHILQRQANWTVDHLTDLEWERIWETFEQKDLKNGETLC
>EFA73011.1
MDLRRDALQILKETSRTFYIPISIMPSGLQEAVASAYLCMRAIDEIEDHATLENHTKGIL
LQSISQTLQAGVDGFAVDAFSIGFKGYEDSLPEVSLRIREWAILAPESIAPRIWDATAAM
ADRMAYWSQINWKITNEYDLDRYTFGVAGAVGLLLSDLWSWYDGTTTNRMEAIAFGRGLQ
AVNILRNNSEDLTRGVNFSRRVGITRTFNSMLVVI
>EFA73013.1
MKATLIKTTQPGIKRKRFFVPIVNVLLLMELSVKEFVWLTVTTDNHN
>EFA73022.1
MTMVQKIALFNHKGGVSKTTTTFNLGWMLAEKGKRVILVDTDPQCNLTGMALKEETEEDE
ARIENIYQNSSNIKTGLAPAFESQPRAIQAVDCIPIEGQEGLFLLPGHVGFAEYEVTLGI
AQELSGSIHALKNLPGAISDLLEKTANKFNADYILIDMSPSLGAINQNLLMISDFFLVPT
TADFFSVMAIDSLARILPKWCGWAKKASINPILKEATYPFPEFRLKFLGTIVQNYRIIRG
KETRAFQTWIEKIEKTVTTKLKPILEINNLLLPNQTYAEQNMNSSFTVTKISNFNSLIAL
SQKHCTPVYALTPEQLDQEGIVLKIIKKNKKNLEKLFPI
>EFA73024.1
MKMLPLFYQKHLKSQLSLAEYLFLKILVNILQSIKNVNLERLANGVPLPIKFESRRKRIQ
RFLSLPNLTIEKIWFPIIQEWLSIYFTNEK
>EFA73025.1
MLKVANRQEEGWLRTEDAEKFACKELRSIDQWWFVPFFNWLHPRQ
>EFA73026.1
MSAMLTEFTLANFKSYRTSHLPLGSLTVLIGANAAGKSNALEGLRFLSWLAQGQKLSSIQ
YAVNSAERVVRGRVNDLCHRGESNFTIGCRLDSTEWNELNITLNVRDGELHISNERIADL
TNSVPLYDLNQPSEGINTDVSVAYNNFTRGQNKPRITCSDQMAIFVQLDSPARFDAKYEN
SQKIISETVREYQRVLQNILFLDPVPAKMREYSFKSDKRLQEDGTNLSSVLYRLWNNQPE
NQQTILNFIQSLPEQAIDGLDFLFGPRDEVMVKLAETFGNNHRYCEAALLSDGTLRVLAI
AAAMLSATEGSLVVIEEIDNGVHPNRAQHLLASIRDIAEKRKLRVLLSTHNPALMDALPD
AALRDVVFCFRDPEKENQGNSRLIRLGDIDDFPSLISQGPLGQLVTAGVVDRFVKSPHTP
EDRKQQALAWLSRLQEYSNE
>EFA73028.1

MFKPHEFAKKIGVSVKTLQRWDVQGRLPAKRTLSGHRFYTEDDLLITQGLKPVDSKRKVV
VYCRVSSSSQKPELRNQISAMETFCLSRGLAVDDWVSEIGGGLNFKRKKFLSIMLSMLKG
EISTIVVAHKDRMCRFAFDFIM
>EFA73034.1
MVAKPMKFFLFSLGFLPLTILLFSWVVLPLPGTINTSLAATMQEIEERGYITIAVKDNIP
PLAFRDKQGNLQGLEIDLAKRLTRDLLGDSPSSRVKLQVKFQPVTNMERLPVVFNHRVDL
AIARVTATPSRSRIVSLSIPYYYDGTAIVTKNLTIQKPSDLNKRRICVLNYSSTIPHVRY
AIPQGELVGVNSYLEAKKLLDNGQVDACAADSTVFQGWIEADNQYSQYKILLDKLSVEPL
SIVMPKGLQYDELRTRVNDLIADYMAQGWLKSPY
>EFA73037.1
MNLREIAKETAKTLQSYLTYQALRIVLVQLNETNPPLAVWLHNFSAGKVQDGERYIEELF
REKSDLALRIMTVREYIAAEITEFLPEMVRTNIQKANMEQRRQHLERITQLSVFEPNPDN
SLDNSAGEKGNFP
>EFA73043.1
MNTSQIQTLLQNLFQQDSRWPHTQRRIIFWYDPDGQFVSIFEELEINDVKKIQLGDTPFT
LKYRLLIEEPEQNFLLYAPFLNRNPKKTGS
>EFA73046.1
MVFNRYTVEILKYTRFVYNSDRLSEMLGGKLSPDQKVPLIHKFLLFSEILAK
>EFA73047.1
MLKEAKPECEVEGNIMSIFVGETGKKVRDKRNELFHKLLGLKETDVFEAWDTTDKDSWKT
KVLDCLNFVSDQSFKSLEEVSVMYKVHQLLNEMLNDYKINAFVSHQD
>EFA73069.1
MNSLLPPNLELIIRPIQYRDLDGLERLRQEVPPKCGLFGMQWLGRWFGVLEFLNCFPNPW
QHRFRQFVAERGRKLLGIIQVSPFNRTRSTWHVDQVILDPCADKLGIGSQLLRHCFESIL
EARMWILEVNINDVNALALYRQNGFQRLAETTYWEIKPELLQELAQAEPDLPNLLPVSNA
DAPLLYQLDTASMPPLVRQVFDRHTHDFKTSLFDIIGDAIKQWVEQTEVVSAYVFEPQRK
AAIGYFELQLDRKGTSPHVATLTVHPAYTWLYPELLSQLARISQDFPQQSLKLASSDYQP
EREQYLENIGAQRIEHTLIMSRSVWHKIRESKFVSLEGIQWTEVLQGLQPSRKPIPGGMS
WVPKSPPVPEVTMGKSEMVALTIEPSEPPQENN
>EFA73094.1
MQRTLLLAKLHNCTLTGTNVNYVGSISIDLVLLQEAGILPYEQVQVVNKNNGKRFITYAI
SAPANSGIIELNGAAARLGVTGDRLIIMTYGQFTAEELKEYSPRVVIVNEKNQILEVGNY
DHFYIRGAGVTGPV
>EFA73095.1
MDLSLIPAQPKPGVINVLIEIAGGSQNKYEFDKELNAFALDRVLYSSVKYPYDYGFIPNT
LAEDGDPLDGMVIMDEPTSPGCVIPARPIGFLEMIDGGERDEKILCVPVKDPRYAHVNSL
KDIPPHRLEEIAEFFRSYKNLEKKVTQILGWHDADKVAPLVQKCVAAAK
>EFA73098.1
MNRGLLNQIPVMPVKNYMVSGFALLVVILTGMRVSLGSGGVGEIAEKPQLKSIAMCIQAD
QV
>EFA73100.1
MSNIDFRIERDSMGDRQIPNSVYYGIQTQRALENFPISGIKPLASYVDACVYIKKATAIV
NGELNCIPLDISKAIVQATDEILGGKLRDQFVVDVYQAGAGTSHHMNINEVLANRALEIL
GDEKGNYQRVSPNDHVNYGQSTNDVIPTAIRLGGLLALTQTLHPVLEKAIATLEAKAVEF
QDIVKSGRTHLQDAVPVRLGENFRAWSHILSEHQNRIYTASGDLMVLGLGGSAAGTGMNT
HPEYRKRVVEVLSQLLEIPLEPAPHLMAVMQSMGAFVNTSGAVRNLAQDMVKISHDLRLM
DSGPKTGFKEIQLPPVQPGSSIMPGKYNPVIAEMTSMVCFQVMGYDQAIALAAQAGQLEL
NVMMPLIAYNLIHSIEILGNTIGALTDNCIQGIIANRERCLGYAEGSLALVTALNTHIGY
LNAAAVAKESLQTGKSLRQIVLEKGLMTEEQLALVLDLEQMSTIVPLS
>EFA73101.1
MKPLQSGIGDKETYQSLFNLDQSEEEITPLYFQAPLAPPIAAAKENRTIDLGLVWRTFLK
LQRERDFLLVESLGGLGSPVTDELTVADLAGEWRLPTVLVVPVRLGAIAQAVANAALARQ

AKINLLGIVLNCNQPRSEEEMSDLTPTHLIESLTNFPVLGCLPYLEDVNDLEALAELGSN
LTIPVWSGLQVG
>EFA73102.1
MAGHSKWANIKRQKAVVDAKKGSVFTQLSRAIIIAAKNGIPDPTGNFQLRTAIDKAKAAG
IPNDNIERAIAKGAGILGSDSNSLEEIRYEGYGPGGVAILVEALTDNRNRTAADLRVAFS
KNGGNLGETGCVSWMFSQKGVCIVRGIENEENLLEASLIGDAESYEMIDQQVAEVFTQVS
DLEKLSQTLKAKDFKLTEVEIRWIPQNEVEVTHIDQAKSLLKLIDTLEGLDDVQSVTANF
DMAENIIKAFSI
>EFA73105.1
MITKTTKLISFISVISLPLVSVFIYDDYTTGQTTTTSQLAQVKSVWQQFSSQEGKFTVLF
PGTPRFSQQKMPSDNGELQVNLFTVNRPQEEAKYTVAYIDYPAQYIQLLRSKNLVEQAIE
QGKSTALQRVRGTIVSEEKKTLGDNVGIEVNYTTADAKVVKQRVFLVGNRFYQITAETTQ
KRTKVFD
>EFA73113.1
MNQINYLRISLIDRCNFRCLYCMPNDTELNHILKKQLLTNDELLTLIQDVFIPVGFNRFR
LTGGEPLLRPGVVDLVNKIAHLPPNPRFIHDHQWFLLAPLAQDLYNAGLTRINISLDSLN
PNTFNLIIGDHSPHGWEQVWHGIQSAYKVGFNPLKLNVVVIPGINDHEILNLAALTLDKN
WHVRFIEFMPIGNTSLFSDRGWVSSAQLRDQIGQRWGLTESQVRGNGPADVFQIPGAKGT
LGFISQMSECFCDRCNRLRLSADGWLRPCLLNESGQIDLKTALRSGQTMGELQQQVGELL
HLKSEINFKERNPGTIGTYSRTMSQIGG
>EFA73115.1
MNNDIDLIKRLGPSAMDQIMIYLAFSAMRTSGHRHGAFLDAAATAAKCAIYMTYLEQGQN
LRMTGHLHHLEPKRVKIIVEEIREALTEGKLLKMLGSQEPRYLIQLPHVWMEKYSWQPGK
SRIPGSNLTTEEKKQIERKLPANLPDAQLVTSFEFLELIEFLHKRSQEELPHHHQMPLSE
ALAEHIKRRLIYSGTVTRIDSPWGMPFYVLTRQFYAPADDQERTYTMIEDTARYFRMMKN
WAERKSHAMRAVEELDIAPEKIQAAMDELDEIIRVWADRYHQEGGKPVVLQMAFGEQDD
>EFA73116.1
MTENLLKQARHQTRIIDRMIADLLEIGRENDNDFNIAPQRLELGKLSFEVLEELRDRYVG
KSQTVETDLPSDLPCVYADPERIRQVLINLLDNAIKYTPKEGKISLAGLHRTTQKVQFSV
GDTGPGIPHENRDHIFENHFRLERDQAADGYGIGLSLCQRIIRAHYGQIWVDSTPNGGAW
FHFTLPVYPS
>EFA73117.1
MLPVLKQDYMEVSQDQPIHFEAPLQLLLFVDGRPKSRQQVQRICAYLQDLEVDYSFDLQI
IDVREEPYLAEHFRLVATPALVKIHPEPQQTLAGSNIIAQLQNWWPRWQTVIDTSLALQK
DLHELPEPDISMTHPPSTIHSVALSAERIKLSDQIFYLNQEKQNFKNSYNLKSVS
>EFA73120.1
MYLLFQTLASFVEIYSYVLIVRVLLTWFPQINWYNQPFAALSQVSDPYLNLFRNIIPSLG
GIDISPILAFLVLNIVSSLLENLSRVTSLGGF
>EFA73126.1
MNEIEFPFLLASLTGKWQRFCLQFLLFLVCLLIIATSINIDTALAGSKDDRYEGNIFVVF
AGNGSLVPPKPNPGTEFSRTQTHSTNVLY
>EFA73127.1
MRIIPVSVDAIPPKKVYNPNEPGYYYSGSVPQIVIFDQSGKVVLNKNGQVPFEEIDDKFR
QVFNLAPRDQSIKYQQRSFNEYSSELSP
>EFA73130.1
MGTTSAVTGTITNDDIQDTTPPLATSLTPTDNATGVAVNANLVMNFNEAIQKGTGNIFIK
KVSDNSTVETIAVTNSNVTISGTQLTINPTNDLASGTNYYVQIANTAIRDIAGNNYAGFP
NSTTTWRFTTNKAPTDLNLTKNVVEENVNSVTSVGYFGTKDPDIDNTFTYSLVSGTDSND
NNSFTISSNELKTKTKAQFDFETKNSYNIRVRTTDQGGLFYEKQFTINVTDVNEAPTGIN
LSKTSVLENQNIGTPVGDFDTVDPDTGNTFTYSLVPVQNSDNHNLFSISGNQLKTEAQFD
FETKNSYNIRVKTTDKSGLSYEKQFLITVTDVLEPNITLEVSPTPSNVQEDRTTNLVYTF
TRTNVNIANALTVNYSIGGTAYYNDNNTQFNDYTQKGAATFTGTTGTITFAANSNKATLT

IDPTADTTVEENETVILTLAAGSGYTIGTPSAVMGTITDDEPPLVSLKLSSPNIVRESGG
TSLKYEFTRIGSNSDSLTVYFKIGGTLGRELDPNDFSYKTSTNTVLRNRTTRGGPGKYNE
GQIEFKEGQKTAFFEITPHLDKENERIEDISLQLEKPRRDQNQYTIQTEDPVKGMVSDTR
FYLYSGDMFKELNSSNIGNYLNKNSKTVLFIHGYEPISSNQLNSFENIPIEYRKLHPDTN
VILVDWSEDASYSFQVYEEGVYEEAKISTIPIGDELSDFLISLQIDPKKIELIGHSLGSH
VAGIAGSTYYNKTGEKLGLIIGLDAAGVKYEKTGPIDRLDSSDAKRVVGIHTDPEGFGDP
KRYGHLDVYVNNQKGESIGDHSYVKDIYRSLINGTRHVPVQNSKNLFKDMFDISDLYDTE
VQGEGHIIVQEDRDALEINGTNTTGFGSEDYLFGNERDNILTGFQGIDYLYGGPGKDIFV
FRFGMAIEVLGNKTIISADSLVTKPDYIYDFTIGEDMFRIHSSPSYSSSDVVKLNRFTRA
ANTNVNTLEEMVRDVFTDSNGGVAG
>EFA73138.1
MTHQTSGHWRLGLTLSLLTAFLWGVLPVALKMTLQVLDIYTIVWFRFSLAFVLLGAYLFT
QKKLPTQQQIFSAPIKLLIITTIFLCSNYILFMQGIALTTANNTEVLIQLAGVLFGLGGL
VVFKERYTLWQWLGISILTFGFLLFFKSQISNLITSQGQYLLGSGLVILGAISWAIYALA
QKELLQSLSSSHIMLIVYGGCALLLTPFAKITTVFTLDIASLSVLIFCGLNTLIAYGAFA
ESLEHCPASIVSAILALAPIFTLISVDLAATILPQIFTPEYINLRGIIGALLVVVGSATT
ALGKNKCVR
>EFA73139.1
MSGNQPLDTNSAALVVNTSLSSSVSGTYLVINDGIAGFQHQQDLLINITGYSGTLPGVGT
IPVTSFFV
>EFA73140.1
MAEALAIQERTEKEQERQQKELAEALAIQEREKKEKLAAYLRSLGINPDEI
>EFA73144.1
MLWWTNGKQSIKMSKSVIDLLKQTTEGLFMPSESEYPFNVVHWEFFNLNETTIQEKTGII
GNVRTVTVDDFFRGVTKQEDWYEKEEQNIAKRFESLVLVLKSNLDEAKVYEIGNREVHAY
ILGTKDGEIIGISTVVIRT
>EFA73146.1
MGTQGQMGGIYTDYFSYVLGWLFLVFLVFTALRLAWQGWRSLKSARECPTINLIGQSARL
LPTPALFAGQIGFWSPELVVSQGLLKHLSPEQLESVLAHEQGHYSYRDTFCFFWLGWIRS
CSACLPNTEPLWQELLMLRELRADSYAAARVDPLVLAESLLLVVSSQPLAWDICCAALSS
SKVDRLEQRIDALLAFSEPNWPEWDLKSKLHYWRVFALALLPLVTVFSTVRRLAIMNSIA
VDAY
>EFA73154.1
MTGSIRELVIKMGIMANKQEVKKYLAHWFQLGKKVIVGQNHHSFLPSTVLNGDRYSQEFE
NCWQKILSLETRDCYLEGTQETIAQLLTPSWEILPCGRCAMPVPIKTTGLPSNSCPCYYM
LTWPNSDLPAPRCPVNSQDHLTSIRDRLLCN
>EFA73155.1
MGKIILVTGPARSGKSEWAENLALESGKKVVYIATANENPHDLEWQQRIEKHQQRRPETW
ITMCVPRELTGSLKNQTADTCILIDSLGTWVANCLEQDDLIWEKTLEEFLADLPLIDGDL
LFVAEETGWGVIPAYPAGRKFRDRLGGLVRQLGRISHTVYLVTGGYVLNLSILGVPLPPV
KS
>EFA73159.1
MYNRGLTPYLDVLVWQRQLLQERIENSDLEDVIILVEHPPVYTLGQSANPNFLKFDLDKS
QYNVHWVERGGEVTYHCPGQLVGYPILNLRRYCQDLHWYLRQLEQVLIDVLASYGLVGER
ISGLTGVWVEGKKVAAIGIKVSRWVTMHGFALNVCPDMTGFEKIVPCGIKDKKVASLAEW
IPHIDCLAVRPRVAEYFAKVFGVKLQDMKMV
>EFA73163.1
MEVKELLNRYARGERNFNGICLRAVNLRGVNLGGIDFARADLSWSDMTGISLSGANLSQA
NLRGAKLENAHLSEVILCGADLTQAVLISAHLNDSDLSGALLVDANLCDADLHQASITAA
NLQSAKLNGAKMGGVRMWKADLQGADLTGADLSEANMCGVNFSMANLSATDMSETFLTGA
IMPDGSLHS
>EFA73166.1

MQLQPPFLSTFTDREKKAIQDLTSYSGVHSLPEIWPLAAQHFRDITALYNPHSKPEVKIT
YSQLWDQIQRFAIGLQVLGINKNNDSYPPRIALIADNSPRWFIADQGIMTAGAVNAVRSS
QAEKNELLYIISHSGSTVVVVEDVKTLNKLEPDLGELPIKLVILLSDETDMPEWNTLPII
ETYSVMNFTQLLNLANNYTLTPLTIGGDALATLIYTSGTTGKPKGVMLSHNNLLHQVQTL
GTIVQPQPGDVALSILPTWHSYERSGEYFLLSQGCTQVYTNLRMIKEDFKKFKPNFMIAV
PRLWESIYEGVQKQFRGQPAKKQKLIQFLLKMGEEYIFARRIAQGLSLEHIGASEWIKWA
AKIKQLVLLPLQILGKS
>EFA73167.1
MREATGGKIKQVISGGGALPRHIDNFFEIIGVEILQGYGLTETSPVTNARRIWRNLRGSS
GQPIAGTQVKIVHPETKDPLPAGKIGLVLLKGPQVMGGYYQNLEATKQVIDNHGWFNSGD
LGWVTPENDLVLTGRAKDTIVLSNGENIEPQPIEDACLRSPYIDQIMLVGQDQRSLGALI
VPNLDALEKWAEVENINLPTQDDSTENEDQKIGQKIDLENKIIQDLYRKELNREVQNRPG
YRADDRIGPFKLILEPFSMENGMMTQTMKIRRHVVAQNYVNTIDSMFVK
>EFA73172.1
MDTTEDSVMVEETNQKPVGKKEKPPAVEDKPFQEFMQEHYLPALQKAIAQEGISDVKLTF
VKQKYAIVGFNSSQECWQITGSWQNGAKQFNVYFPDQDIQGKKGFSCHEGKKPSTLESFL
IDERKITLDLLVSRLIYRLNGQKWLGRN
>EFA73177.1
MRLLLKIKKAYRRLVKMFHPDTNQDTANHEEIIKINAAYEVLSDVEQRVKYDEQIGNWDT
SHPAVGNRENPRQTKKPGGKEADEKVEEWLKLVYQPINRWLCSIVSSLEQQLEDLAADPF
DEQLIDEFQEYLDTCRDKLKKAQTAFRSLPNPPSLATSAAHLYYALNQVSDGLEELAYFP
ASYDDRYLHTGKEMFRIAKQLLDFSPGSNTREK
>EFA73179.1
MTTQFNRRKFLIYGSAALGSSVLLKACANNTQTNTPADSSAGGNTIKVGILHSLSGTMAI
SEKSVVDAENLAIKEINAQGGVLGKQIEAITEDGASNWDTFREKATKLIEQDKVAVVFGC
WTSASRKNVKPVFEDKDHMLWYPVQYEGQECSKNIFYTGAAPNQQIEPSVDWLLKNKGKE
FFLVGSDYVFPRTANTIIK
>EFA73189.1
MVKARRDPFAQIIRVPQTIPTLPSVVKSNNTSRLNSLKKPLAIRQADLEIRGKNPIKPSP
PKPPDTKLAQSILVSGVILVNQQAQAIIKLPDDSNSRYVHAGETLTNGILVKRIEVNQCD
NPVVVLEQFGVEVRRVISGQEDGIC
>EFA73199.1
MTIYIGNLSYRATEADLKSVFADYGEVKRVVLPTDRETGKMRGFAFVEMIEDAQEDAAIS
ELDGAEWMGRQLRVNKAKPKEENRPRKGHSKG
>EFA73207.1
MLTSEAKKPLTIPPKELLDPPGGLNPTVLLFFAVVTMLLLSSCGYWIWEWPHWLCFGINT
LSLHFSGTIIHDACHQSAHRNRIANAILGHASALVLAFAFPVFTRVHLQHHGNVNHPKDD
PDHYVSTGGPLWLIAVRFLYHEVFFFQRQLWRKYELLEWFLSRLLIFTIVYISVQYHFLG
YILNFWFVPAFVVGITLGFFFDYLPHRPFVERDRWKNARVYPGKILNMLILGQNYHLIHH
LWPSITWYNYQPAYYLMKPLLDEKGSPQTSGLLQKKDFLEFLYDIFIGIHFHHHE
>EFA73209.1
MQLQIASRNLTKMRPAILQPGHSYTFRQYFEMNYEPEDILGELGYEFQRSTLSLPQSDIN
LDRLDDLKTRIQETLPYISLTSEAARRELLIAPLLIDVLHYTHAQLRVEYSVNVSEQLKG
YLDYYLYSAGKLLIVEAKNADLTRGFTQLAVELIALDIWSTSEEPILHGAVSTGDIWQFG
ILDRGGKQIQQVLKLYRVPDELESLMRILIKILQP
>EFA73221.1
MSKGTLFDKVWEIHTVGTLPSGLTQLFIGLHLIHEVTSPQAFAMLKERGLKVLFPHRTVA
TVDHIVPTDNQVRPFADNMAEEMIRALEKNCQENDITFYNIGSGNQGIVHVIAPELGLTQ
PGMTIACGDSHTSSHGAFGAIAFGIGTSQVRDVLASQTLALSKLKVRKIEVNGNLPPGVY
AKDVILHIIRTLGVKGGVGYAYEYAGTTFEQMNMEERMTVCNMAIEGGARCGYVNPDEIT
YEYLKNKDFAPKEAAWEEALKWWQSLRSDEDAEYDDVVVFRAEDIPPTVTWGITPGQGIG
VDEKVPAAQDLPEEDRFVAEEAYRYMDLYPGQPIQGTKIDVCFIGSCTNGRISDLREAAK

NCPRSSSCTGYESLCGSWIGKGEKNKLKLKGWIKSFIQAGFEWREPGCSMCLAMNPDKLQ
GRQISASSSNRNFKGRQGSASGRTLLMSPAMVATAAIKGEVADVRELLSANS
>EFA73229.1
MATGLALISSNLNLASVSAVESCLTPALSRIKKHQVNPGENLVTIAERYKLMPATILQMN
PLINNGQVIPGTQLQIPPFDGMVVQVPNGQGWQQIAKKYGVRPDTLFELNGCQQNPRVVF
VPSSSKIKPIYGSAPSITTIMGSPVSNSTGVSFPYGWQIHPITNQVFFHSGIDLLAEVGT
PVRATASGVVVFAKEQGSYGNLVIINHQGGMQTRYAQLESIKVKLGQQVKVNQVLGTVGA
TGEPSSREPHLHFEVRAREDLGWTAKDPVEYLK
>EFA73235.1
MNNFELDTYLNRLSQKLSEKLNGDSHKRFPGWLAVDFGTSNSTVTMFDPIEVPIAETLPR
EQEVRLRQRLGEWLNSPPHLALPDIGVNEWEKFLVNLGRNLEIPPEAIGEIFENDHKDKF
LEALRQIELCLGNSERFRRAVSKKLYQIYHEVFRVPTLESQNLIPVVLDFNRRQTEIPSE
IEICKIQPLKLQMGRTARDNRKKAIAQGTITAVKDIISRFHHSPKRYFGQNRTFPVVINE
GEKNDLENNNIEVHQLIQAAWGHLIELTEDYRQRAGRRFSQGDLLTAVVTYPTVAPPVVR
KEIKALVEELGLDDVQTAYDEAVSVAIFFLWREFGGNLNIGIESFKTRCRQEKNNWSQNV
LVLDIGGGTTDLALIKLTLEDKTPVFTNNEDRGLGGRYYKLTPKLLGSSGHLQLGGELIT
LRVFRLLKIALADFLLTAVTDGNITSDKLEDLINSELNERFLQDGKFKSGSLLKCVDKEN
PEGDVAFKDALDTAEKVLPTRWQQAPQRLQTFYTLWEHAESAKLKLGRKDLKMVY
>EFA73236.1
MLLQSSVKFQLVSADSIYLTINAQQFERCAISSIREAIGIAKGLMESRLNEDQKVDWLIL
SGKTCNLDLVKTQIYQEFSKSPYFIWNPERITFVLEFTKLATSAGACYAEKLRRLRFDPE
ASKNLLRKGANQLEIDVKNLFYYLPCNFKRKTQTQELLSIFNAGQELYQLIPWESVAKVR
TTWQGIQLTNIIYRQDYQDGELRLWGSFDGKTLMENLRMEEAEFLKKIQVQFEIDQTLQF
TVLLCQGSPHYLIDVPGIDINSVIDPHATGSDIFVDGKLKWNIALIGDYENLKDGDIAIN
VLESATVDQPDAYHLVFAVDNSQNQILETFHYLQDGTKETGKGLISSPLPPFPHNGQHNF
YICQTDSLTKTKKWIRIGSLSKPEISTDYPCQYYVTLDSKGILRMHPGAVPYWTSHSLES
LQQPGCVYCTELELQPNEIDRERDPFCGVH
>EFA73239.1
MAVTHRGFSVIRQVLQRWKGYIRSWQDWQANPSKYVDAPKIPNYKHKIKGRFPIVVYNVY
QGCPTMDQPSLVQGMCQLFESLFEGDVNDSIGKVRKLVEAMIIPRNNGSYLLKFTYEVQE
PTTRSTQVIAGIDLGVNNLVALTTNSSTIRPLLVNGKPLKSLNRLFNRKRDLIGSYQSES
QPSRRLGGITSKYHNRVDNYLHQTSSIVINYLKSNGIKTLVVGKNDEWEKETRMGISKRH
NFMPIPYSRLISMLEHKCRLAQIELITVNEAYTSKCSAWDFEPIAKHQKYLGERVHRGLF
RSANGQVINADLNSSLNIIRMYSSEALTAERIGSCGVQPLKVNPLARVKQI
>EFA73242.1
MFPNGTTQTISANSRTITRTETITRGSDTGQILTDAAIGAGAATLIALVTGNKKVEVLEP
IGGAAAGALASVLLRKNTADVFVLRPEQDLAITLTSNLVLSR
>EFA73243.1
MSGLITWKSTAAALMTIAINTVAVIPWLYPNPAQAQFNFNQPRTITIPANVTLPVTYEKE
KIILQPGERIPLTLRIANDIMDSNRNILIPVNSEVTGELTPVNLGVIIQEG
>EFA73245.1
MTAKIISIQVGNLAGLFSWVAGTDHYYKKIKIYKFCVPEQK
>EFA73246.1
MNPTENIIHLPRKHLAQQILTQHSIIPIKNDVLWRIERGVVRTLTWDEEGTAITLGYWGP
GDITGHVLSKVTPYQIQCLTSVEATIIPPDLWHEDLKALLSHIQQTEQILHILHCKPTSL
RLWHFLLWLGNKFGRDLERGKLIDLNLTHQDISEVLNTTRVTITRLLQKFETQGKISRHK
RSIILRLTQDLQQD
>EFA73247.1
MVTTLKPVSTLPTQDEILRQLRSGFPRRPQGFPHSVG
>EFA73250.1
MNLQRSQVTWVQALRSLIQLSHFDFDGYVEARKALTTEEWIDLLVQTIGFNPEMFGRRSK
LLQLIRLIPFCERNYNLIELGPKGTGKSHIFSEFSPHGILISGGEVTVAKLFVNNSTGKI

GLVGYWDVVAFDEFAGKQKTVNKSLVDIMKNYMANKSFSRGVETLGAEASMVFVGNTKHT
VPYMLKHSDLFEELPEKFHDSAFLDRIHFYIPGWEVDIIRGEMFSDNYGFVVDYLAEILR
AMRNHDYSDRYKQYFSLSSDISTRDRDGIHKTFSGLMKILFPHGGATEREVEELLQMAME
GRKRVKDQLLRIDSTYAKVNFAYENKEGISKTVVTLEEEEYPKYYHQTLTEEREGEEVVK
LEGASAPESKSGVHPGEAHLTFQENQRGVSFDSLFGPYLKGAKKITITDPYIRVFYQARN
LMDLIETVIKLKPQEDEVKINLITTLDDFKGEQQVEYLGRIQESCATVGIDFTWEFDGTC
TIHGRHIVTDTGWKILLDRGLDIFQHYDIKDAFSIANRLQEFRGCKAFEVRFLRRETVEG
N
>EFA73254.1
MTIQTLDKTTTISEQRFLLPGHYTWEEFETIETLTADAAGLRITYLDGCIEFMTLGEQHE
MIKTILGFLLELYFCEKGINFIPVGSATRRAKEKGASFEPDESYYIGEKKENPDLAIEVN
ITSGSIDKLEKYKRFNITEVWFWENDKLFLYHLKNGNYEQINQSQLLPDLDIYLLVTCVL
IPSIIDGRTEFIKGIKK
>EFA73257.1
MPFLTFRYIAINFPADLLSSSKQRDDLDRQVAYMQSLYPGAEIIKDVGSGLNFKRKGLLS
LLDRLLRGDKFILVVACRDRLARFGFDLVQYMVEQNGGQILVLDQTIHCPQSELTQDLLS
ILHVFSYRIQGLRKYSKKIKEDLSENDFSKKTED
>EFA73258.1
MQAKDIKEQLKSLSQTAMTIDPQLAKRLDEVNRWVKCIRPGSLTAKPYVLAFLLEVITDS
RIWLVIQSTSDPVAKKTKFEQMTANERYWYGYLFPKWINSSDTKFYIWKKKIMAGEFNQL
DNDIIRLMAQEIERREGQVWQRYIADLSMATDLIVSNHQHKPLCIQVTSVSKELHAKK
>EFA73267.1
MGLWLENGNSQGTIGIDLQPQPHFVYCPQEAVEKLNERVEKRLQELLGIIDNNNPETEVL
MIGIGQGEIKLIQFAPELSPRTCFERVNQDVDTLLELLEQKMIEQIAV
>EFA73275.1
MALVPMRLLLDHAAENGYGIPAFNVNNLEQIQSIMKAAAETDSPVILQASRGARNYAGEN
FLRHLILAAVETYPQIPIVMHQDHGNAPSTCYSAIKNNFTSVMMDGSLEADAKTPASFEY
NVAVTREVVNVAHSLGVSVEGELGCLGSLETGAGEAEDGHGFEGTLDHSQLLTDPDQAVD
FVEATQVDALAVAIGTSHGAYKFTRKPTGEILAISRIEEIHRRLPNTHLVMHGSSSVPED
LIALINEFGGAIPETYGVPVEEIQKGIKSGVRKVNIDTDNRLAITAAVREALAKNPKEFD
PRHFLKPSIKYMQKVCSDRYEQFGTAGNASKIKQVSLEDFAAKYAKGELAVKAAAAV
>EFA73296.1
METFFIIPAHSYGLGVALEKLSMPANVTAICLGKSTYARVGLVANVTPAEASWNGSLTLE
FSNSSSADCRIYANEGVVQLLFLEGEPCQTTYADRGGKYQDQPERVTLARV
>EFA73299.1
MQKFLLYLLTSSVLGYAISMGGSALAEDVSKTESSLQVSDPNYISQMTSVSQLSDVQPGD
WAFQAIQSLIERYGCVGGYPDGKFRGNRTLTRFEFAAALSACLDRINELISSATADQVTK
QDIANIERLQKQFGPELETFKRRVDTLEAKTTRLEATQFSTTTKLQGQVVAVIRDISAKK
VVEAKTLDSNATLGARTRLELVTSFTGKDTLFTRLQSNNISNDITDPTDSSKAVPIVGTK
ESGLYSGGGNNTALSVTALSYKFPIGDRTQVIAVANDGAAEDLTSTITSFNGDGAFGALS
TFGTRNPIYSQLGASGLGINHEFNKNVTLSLGYLGGRMDPSSTAASPASDSGLFKGPYGA
LAQLTVKPSERITLGLTYINSYDLALATGSNNATFTGSQFDGSKYSSNSYGLQASLGVTP
QLRLEGWLGYTKSQLLLLTGVKGDVDIWNYAVNLAFPDLWKKGNLGGLIVGMQPKVTSAS
TTLSAFKDTNTSLHLEGFYQFKVNDNITITPGLIWLTAPNHNKQNDSVIIGALRTTFSF
>EFA73302.1
MQRAIIGALLMGALGGLLGSFVTLRQLSFFSHAVGHAALVGVALGVLLNTNPHWMLLPFT
LVLGVIVLYLIDQTDLASDSILTVVLSGALAIGIILTSMIEEYRGNLMAVLFGDILAIDQ
TDLILILIIILITSSIFILSTMRSQILLTLNEDVAKVQGSPVEMYRYIFVILLSLAVAVAI
KAVGVLLVNAFLVIPATTAKLVSDHFRGFLILSVVIGCTSSIVGMIVSGLFNQASGPSVV
IVQFLLFVFTFLWVRLRKI
>EFA73303.1
MDLVTQSRSSGQCLRPIEVKLTALPDNSTCSLTEEYYGCELVVRPNTILYLACSVAANFK

NNQKGVAEFIGNNFDNINDWKNGETVFLYVKEMILAIDKIVTSLIDKQEPLLMQTIWKTN
GKSPKLNNNCLDVFVWSNLAFTRLFIDAARDEISGGSNRITRQVRTSIMRTVDLFSGCGG
LSLGFQNVGKDFILNGSKTDLEQIIGNAVPIKLAEYVANCINEYIKDRNQNKVTTVRQLE
LIFD
>EFA73309.1
MTDYFPSNLPLKSIPHSKKTVNTPMVETEDSSGKLALTIAQAASERKAGEILLLKVTDVS
YLSDYFLVMTGYSRVQVRAISSAIEEKVQTELQRRPLRTEGKGEGSWVLQDYGDVIVHIM
MSKEREFYNLEAFWSHAEHIPLENP
>EFA73310.1
MFLYLSKLLPLFFYPLGLASVSLVIALVSLWKRPKIAAVCIGTSLAILMIAGNGWVSKAL
VGSLEWQNIPPGKIPEAEAIVVLGGATKPAAWPRSTVDLNESGDRVIYAAQLYGQKKAPL
IILSGGRIDWRGSGPPESADMAAILISLGIPPEVIIEEPNSLNTYENAVNVRKILESRGI
KKVLLVTSAMHTPRSLKIFQRQGVEVIPAPTDFIVSRSDLEELIATPKSTILNLFPSADN
LNDFTNALKEYIGYLVYALRGWL
>EFA73316.1
MKSLYQYQGDSQDQIENGYDDPNFEHLHPVEVNEFLPQIHRLTTIGAGIMLTIFLVRDSF
SNCTDL
>EFA73317.1
MGEIKLVESAISGEVQRILVQNHQVVRQGDSMAFVDDSQVNTQKKQLEDSIEKSQLQLLG
IESQLNEIRNQILDQANLNEQTVFAAQSEFMGTQRNFDDQRIKGNAELLQAEIAFHFAKS
QLQRLEKQGILKSTIQEAETALKLAILQRDRLQAIARSGAIPANLLEEKQQAIKSARAKL
ESAKNNAQTLWDDKQQAFKIAQTNLYKARTAMNPSNSAVIIASQRIKQERARGEMTIAAL
NKELGNLLQQRLEIQKQLTRYRQDLLQTEINLQKTVIRAPITGTLLQLKLRNIGQVVQSG
QAIAQIAPLNVPLEIKAYIPSQEINKVQVGRKFQMRVSACPYPDYGTLRGRVKNISPDVL
SSNVITNNITDNITNTTGINGYEVTMEPETLYLGKGENKCYLQPGMEGGTDIISREENVM
QFILRKARLISN
>EFA73318.1
MFNPFQSRKKYECVLQASEEDCGAACLVSLCKYYGRFLTINKSREAVGTGQLGTTLLGLK
RGSDSLGFNSRAVKAAPEILDRITEIPLPAIIHWRGHHWVILYGTRGKKYVIADPAVGVR
YIERQELLAAWNGVTLLLEPDPEQFSTQSQEQPHSGLLRFFLRILPYRGLLAQVIIINII
LGVLALGTPILIQLLTDDVLVRGDIQLLSVVVYAVVVMTIFSSSLQLIQSTMIAHFGQRL
QLGLVLDFGRKLLQLPLSYYESRRSGEITSRLSDINEINQLVSQLVILLPSQFFVALISL
GLMVFYSWQLTIAVIIFAAVMTLVALPFLPILQQKTRSLLVLGAENQGVLVETFKGAQVV
KTTNAAPQFWDEFQSRFGRFANLNFSTIQLDIINGTIAKLLSSLGGVILLGLGSILVINH
QLSIGQMLAFNTLQINVLALIVFVVGLVDEYFRSQTAISRLLEVIDATPEVVGGGQKPIA
QIAGDADYFFFSLDISPSRKGRPIR
>EFA73319.1
MTFHHLGRVDLLDDFSLKLPGGKVIAIIGKSGCGKSTLAKLLGGLYQPSSGNIRIGVFNI
HDLALDCLRQQVVYVPQDPHFWSRSILENFRLGIPNLSFDQIVQACEIADADGFISQLPN
KYQTVLGEFGANLSGGQRQRLAIARGILNNPPVLILDEATAGLDPVSETHVLDRLLAFRK
GKTTILITHRPSVMHRADWIVLLEQGKIQLQGSLETFIAQPGEHLHFLTI
>EFA73320.1
MDAKELWQRYQDWLYYHEGLGLYLDISRIRFDNTFVESLQSKFEQAFKEMVALEKGAIAN
PDENRMVGHYWLRNPDLAPSSQLRAEIVRTLEEIEVFAGQVHTGAIHPPKENRFTDIISI
GIGGSALGPQFVAEALAPDLPPLNIHFIDNSDPAGIDRVVSRVGDRLSSTLVLVISKSGG
TPEPRNGMIEVKQAYSRRNLDFAQYAIAITSTGSNLDKVAKSENWLGTFPMYDWVGGRTS
EMSAVGLVPAALQGINIRAMLDGAKQMDDATRIANIKNNPAALLALSWYFLVMVRVKKDM
VVLPYKDSLLLFSRYLQQLVMESLGKEKDLDGKTVHQGIAVYGNKGSTDQHAYVQQLREG
VPNFFATLIEVLEDRQGASSEIDPGVTAGDYLSGFLLGTRQALYENHRDSITVTIPQVNA
QTVGALIALYERAVGLYASLVNINAYHQPGVEAGKKAAAVILDLQQKVIKVLESERKGLT
IGEIADKAGASEQVEPIYKILRHLHANNRGVVLTGDLSRPGTLTVTLT
>EFA73332.1

MDFIAPWERVIDLKFARRPGEAPLYHLYVEIMGKYSNVILTDAKNEIITAAHQVSQQQSS
VRPIQTGQPYEIPPKLTGTIPTLTETEARWQERVSLVPGSIKKQILKSYSGVSSALLDSL
LLVAHISPETSTTSLTNQDWYRLFARWQEWLRRLEEGDFKPAWTKQGYTVMGWGEIQSVD
NIQELINQYYTQQLNQQVFYHLRHQLTQKLQNILGKLNTKAQAFKDRLEQSEDADKYKQQ
ADLLMAHLHQLQPGMTEIILPDFETDQPQRIVLEADKNAVQNAQRFYKQHQKLKRARNAV
YPLLLEVNREIEYLEQVEAATNQIDNYQTKQDLEAIEEIRDELIEQKYLEELEYRRPSQS
ETVSHNFHHYCTPSGLEILIGRNNHQNDYLTFRVAGDYDLWFHAQEIPGSHVLLRLPPGT
VPEEADLQYTANIAAYFSRARHSEQVPVVYTRPNRVYKPKGAKPGLVIYKQEKIIWGNPG
KISQVINHLQQGV
>EFA73336.1
MLVSLNACDWQVRVATSSDLSGIAQIITESFYSQNGLWGWAFPLFRIGIYEDLKYRLQTL
MPHQTCLVAVHTSQSGNCKVLGTVELGVRSIHSWTSYSRLLPYVSNLAVAPRYRRYGLAS
SLLISCEQVCKTWGFQDIYLHVLEKNDQARKLYLKLGYEIYRVESSWQDFFFPSRQFFLH
KRLR
>EFA73337.1
MSQLKYCQQLFVSSQWLFGNITNSDNNTQDDSCIVVVDCRFSLADPLWGRNQYQMGHIPG
AYYLDLNQDLSGVVGQHGGRHPLPDVSILADKLAQMGINHGQSLVVAYDDSRLAFASRLW
WLLRYLGHENVAVLDGGFSGWQSLGYPVSNIPSTIPGKREILLPTYKQQKLWILIMLNIM
>EFA73339.1
MDFLPQVTKDGSLTFFSEEFQESFHSLYGAKQESFLKFVIPTQVPLFAQTGILKF
>EFA73340.1
MKEIAFKHCYQDDNLSVRLIINDARKSIQEILSSGFLADVIFLDPFSPPHCPQLWTVEFI
EQIALCLQPGGLLATYSCAAAVRTALISAGLIIGSTTPVGRRTPGTIAGYAPHQHKYITE
TYPLGAAEIEHLKTRAAIPYRDPQLQDEAPNIMKRREQEVLTSTLESTSQWRKRWLTNNT
PTQ
>EFA73350.1
MAAMGSPDDRQIDGIGGATTLTSKVVVLSLSNHHWADIDFRD
>EFA73361.1
MDNDILPSLGEKPEGWEPSGNRSGRLFKSAAIALWKRVFKKSFCNLPLTLKTPWLRWWDN
RGNLLLTGNERAEDECRGFYHPHHR
>EFA73365.1
MHYFDEYCEVHLDPDIAPYNWVFRNQEKISITFGVHVSKHPDFGQVVYELNDGKGYCDID
RITTQTEGIIENGFKMFSVILPLLVMVGDISIE
>EFA73366.1
MDYATNLVLIVGELRRFIIFWLGQSLSEIGNRLTGFGLGIWVYQNTHEVAGLSLIVFFTT
LPGVVITPFVGALVDRSNRKWTIIFSDLAAATVTLVLIVLIATDNLQIWHTYITAFFTSV
CGSFQMISKGSALPMMVKKDDLVRANGLVHLSTAIGQLGAPILAAILIAIIQIKGLLIID
LCTYFIGLFTLLIIHIPEPERSIKSRLGFSTIIHEIVDGWQNISSQIFLIIILLMFMTLYF
FLDGMTNVLINPLILSFSSPQNFGIVMSIAGCGMILGSLFMTFWGGSKKPISTLFIFSAF
NGIGLIISGFQPSVSIIALGMFITFFTLPLVLGYNSAIWQNSVDPNVQGRVLSFLHTSVG
LGVAIGNLSASPLTDKFLEPMLHVNGILANSIGKLIATGPGRGVGFLMIIEGMIMLSISI
CLYNYFYFRDNNNEQILNDVLKMNDVRTQ
>EFA73368.1
MFRLTKDTHDLLNYLIARYKNQVDYLMIRLEEAEGTDILLRGDKVETLSEGVSMGAHVRA
CHKGGWGLTSFNHIADIEERIQEAISAARTVGDEQTILAPIPVIQSTYKLGITGIDPSTV
PLKKKKQLCDRYREILKGVDSRITTTSVRYNDSSQKVIIATSEGTLIEQSWVDMEMRFAA
TARNGDTVQTGRETVGSRKDYDDLVNLDTLVQGAAQRAVTALSLPPVKGNTYTVVIDPIL
TGLFVHEAFGHLSEADMAYENPDLLEVMTLGRRFGPEELQIFDGAGSPGHRGSYLYDDEG
TPATTTQLIEDGVLVGRLHSRETAGKLGEKPTGNARCLDYHFSPIVRMTNTWIERGKTPV
VDLFTDIKEGVYARNWLGGMTNGEMFTFSAGEAWMIRNGAIAEPVKDVTLSGNVFQTLAD
IEGIGDDFHWDESGGCGKGGQNGLPVGCGGPSLRIRDVVVGGES
>EFA73369.1

MMLLIAEWQLSLKRITFIYECLIELPVEIRRGDVAQEVIRFARENNTGLVITTKSPSPKF
AQICDLIRPHLDLEVLAVEPFFDYDGFLDLKRFSRYWKVAEKYVFRPNSDQDG
>EFA73372.1
MHPNQKLLFSTALFSFWLSLDIVSCAYAQTQTSTPGNAPPQLKKLIAEIDAASSRGDIKG
VMGFYSSKFTHGDGLTRQAMEQSLKSFWQRYPKLSYQTKLQSWKSNGKTIIAETVTRITG
LSSDKTSPLAINAAITSRQHIRGTQITHQEILSERTQMTSGKKPPQVEFRLPQQVKVGEK
YAFDAIVKEPLGNNFLLGTAMEETIKPNKYLNPTPINLELLSTGGYLKLALLPYNQ
>EFA73380.1
MEVEWQMRAIRGATTVAENSVVAITEAVTELLNELEDRNQLRPTEILSVTFSVTRDLDAI
FPAAIARSRPLWDSVAMLDVQQMYVEGSLQRCIRFLIHAYLPTSSPIHHVYLRQAAKLRP
DWGFVGVQ
>EFA73389.1
MGRLKWKSVSQAVYHIESVGMKVRLIVLSKIPKSANNELWRLFSVRSEVVEEAISHYQES
YRNSEYAV
>EFA73390.1
MTGNERAEQECQRRELAETLAIQERFEKEQEREKREKLAAYLRSLGINPDEI
>EFA73391.1
MDPLNPFLKGEINALCLVYEKLGLLDPPKPLKKGANCFVFSL
>EFA73402.1
MVRLGLFQMGLGIMSLLTLGVLNRVMIDELTILPFIAAGAIAMHQFVSPARIWFGQKSDS
QRIFGYHRSGYVWIGAALFTSISFVALQVVWQLGTSLQNTGWGLQTYSWSALLALTFGMY
GLALSASSTPFTALLVDITDEDDRPKLIAVVWSMLMMGIVIGASISSRLLERPEICGTAL
LAYDPSQMNKLVDISKLQTTINPVFIILPGAVFVLTLLATLGIEKKYSRYGIRGNMVERE
DQITLGKALKILTANRQTGIFFGFLMVLTLSIFMQDSILEPYGGEVFGMCISQTTRLNIP
FGIGTLLGIGSTGFLLIPRLGKKQTTKTGCIGAAMSFCLMIMAGVSQNSGLLMGSLFFFG
LASGVITTGATNLMLDLTATETAGTFVGAWGLSQAMARGMATVLGGTVLNIGKLVFTSPT
LAYGMVFALQAMGMLLAIWLLRRVNVVEFQQNSKQVLASVLESDLD
>EFA73403.1
MQDKALANVFRQMATGAFPPVVETFERNKTIFFPGDPAERVYFLLRGAVKLSRVYEAGEE
ITVALLRENSVFGVLSLLTGNKSDRFYHAVAFTSVELLSSPIEQVEQSLKENPELSMLML
RGLSSRILQTEMMIETLAHRDMGSRLVSFLLILCRDFGVPCADGVTVDLKLSHQAIAEAI
GSTRVTVTRLLGDLREKKLFLSTRKKSLCINQLH
>EFA73411.1
MSDLFKGFEQFLELVKTLEEKIESGEVKTDIQVNSRPLSSIPRTGNIPRSAAKNSNINDI
GTSRIRTPSSPAAPQPSNGSSDSATPPENSGISLKDIGGLSQVVKELKELIAIPLKRPDL
LAKLGLEPTHGVLLVGPPGTGKTLTARALAEELGVNYIALVGPEVISKYYGEAEQKLRGI
FEKAAKNAPCIIFIDEIDSLAPDRSAVEGEVEKRLVAQLLGLMDGFSHTPGVIVLAATNR
PDHLDPALRRPGRFDREIQFRIPDANGRKEILQVLTRAMPLDETVDLEFISDRTVGFVGA
DLKAVCQKAAYMALRRQVPSMETEIPETMTVNQSDFLQALKEIKPAVLRSMEVEVPHVEW
EDIGGLEAIKQTLRESVEGALLYPELYRQTKAVAPRGILLWGPPGTGKTLLAKAVASQAR
ANFIGVNGPELLTRWVGASEQAVRELFAKARQADPCVIFIDEIDTLAPARGTYTGDSGVS
NRVVGQLLTELDGLETGTNILVIGATNRPDALDPALLRAGRLDLQLKVDLPNLASRLEIL
RVYTEGRPLLDVDLEYWAQTTEGWNGADLVLLCNQASVEAIRRFRAKGETDPAAIRITID
DFQHSYRILSQQRNS
>EFA73412.1
MIDFKFQLSESRRLLQDSQFTGFLIVGIAEPMCLAETERFLEQLNTLEVPFGGILINRIL
TDPIIDQDRYAEQQNYVQKFLNLSPGQPVFIIPNNPPPPLGSQALDKLAGEIQHISHVEG
VAPPIIHWPQRIPPSFTDFLAQGCKLIIVGGKGGVGKTTVSAAMSWAFASHYPQKNISVI
SIDPAHSLGDAFGQKLGHDSQAITPNLCGQEIDADKILDQFRTDYLWELADMISGEGTTK
DTDVNIAYLPEAWRQIMSQALPGIDEMLSLITINNLLETNQQDLIILDTAPTGHLLQFLS
MPSALGDWLSWIFKLWMKYQDVLGRVDFIGRLRQLRQQVVKAQKKLKDPRHTQFVGVIQA
ESAITSEHVRLTASLKNMGIEQRYVVQNRYTQAVEVDHNLFPEQTIIHLPLLPRSVEPME

RIKGAANLLFAFEK
>EFA73413.1
MLSGKGGVGKTTLSCCLARYWARKFPEEKILLLSTDPAHSLGDVLLTKVTNEPESAVDLP
NLSIQALDAQNLLLEFKAKYSQYLEILVERGSLADGEDLAPVWDLDWPGLNELMGLLEIQ
RLLSEKNVDRIVVDMAPSGHTVSLLKLKDFLDVILNSLELFQKK
>EFA73421.1
MVALLQKIRLENQSIFQEVNDFLSTRKSNRLAQAQKQAEDLSQFRQQLEQEIDQFLAKTA
QARQAQAQAQAQELNQFRSQLEQETSKFLTKTTQARYAQAQEQAAELNAFHKQLEQKTEV
FLADTAKARIAQAQKQAAELSAFHKELEQKTSAFLADTAKARIDEAQKQAAELSAFHKRT
RAKTSAFLTATTSDRAAKAAAQKEALRQFRQDLFLSVIGV
>EFA73427.1
MPEGTILVFPLAIREDKSDQSKNQKFQPFASGDKADIYLGGLESIGFGHCELTLKGQVNI
N
>EFA73428.1
MYTKAYGIIETLAPLHVGATAGEESGNLNLIFRDQFTQTGIIPGSSIRGRLRAEMRNSKG
ESEANYWYGNGADDDSNNESIVKFEYASIIWIPVFCPGQPIVWVSSPRLLKRYQRIAGDS
IQVQIKEGKDKYLKDIPIPDPYTGGEKLIAKQAPGGRKTIFFNLGFLEINTTKNLSPWFP
DLQERPAVIVNDGDIGMIHDMALYRQSRVRLKSEQKVVEGGGFSILKHCQRGQF
>EFA73429.1
MLTDKPVPYRFRSQGTLGRGRYAVPAGSVYILKEPINQSWWEWNEKWFPEEGYSLKKVGC
GLCLPITIQGVN
>EFA73430.1
MFKYLISISPLGFMYGSAGAFLSPENLVGRSGAKFPPDASTLAGLMFSINKVKNKLSQSE
LRDNLYVAGPFWAKSQDKNNFFVPIPKHKIITQKEVREWSVEKEEWYLEKKQRKTRI
>EFA73438.1
MKSVEIGIIGGSGLYKMSALQDVEELDIKTPFGSPSDRIIVGTLAGARVAFLARHGRNHA
LLPSELPFRANIYAMKQLGVKYLLSASAVGSLRAEVKPLDMVIPDQFIDRTKNRVSTFFG
EGIVAHIAFGNPICQNLAAVLADAIACLNLPDVTLHREGTYLCMEGPRFSTKAESNMYRS
WGATVIGMTNLTEAKLAREAEIAYATLALVTDYDCWHPDHDHVTVDMIIANLQHNGINAQ
QVIIETVKRLNENLPPSEAHSALKYAILTNLVDVPTATKQKLGLLLEKYL
>EFA73444.1
MGVSPDSSKSHCKFITKHNLTITLLTDPEHQLIETYGAWRMKKFMGKEYMGVARSTFLIA
PDKIIAYSWPNAKSKGHAQAVLKKLQELKN
>EFA73445.1
MSDIPQLGKPAPDFAKLDQDGNLVSLYQINQWVVLYFYPKDDTPGCTTEAKEFTELAPQF
Y
>EFA73446.1
MLRDFANREELVTYIREQFPQAAQTDDYISGIPGGRKAAEAQLEKVNTVSYAKTRNYLTG
AVTRLSPYIRHGVLSLREVREYILNTVENSEDGSKLINELGWRDYWQRLYMKLGKGIWEN
QEEYKTGYHTEDYSHNLPEDIQESKTGLVCIDSFSQELRVTGYLHNHIRMWLASYIIHWR
KIQWQAGAKWFLQHLLDGDPASNNMSWQWVASTFSHKPYFFNRENLERYTKGVYCSRCPL
YGKCVFEGTYENLQSILFPHSP
>EFA73448.1
MGEDDKQEDSIRPRRFADYIGQQDLKDVLDIAIKAAKSRGDVMDHLLLYGPAGLGKTTMA
MILASEMGVNYKITSAPALERPRDIVGLLVNLKPGDVLFIDEIHRLSRMTEEILYPAMED
YRLDVTIGKGSGAKVRTIPLSKFTLVGATTRVGALTSPLRDRFGLVQKLRFYQVEELSQI
VLRSAEVLQTIVNLEGAREIAKRSRGTPRIANRLLKRVRDYAIVKSRPQIDQPTAAEALT
LFQVDPCGLDWTDRKMLTVIIENFHGGPVGLETLAAATGEDTQTIEEVYEPYLMQIGYLS
RTPRGRVVTSAAYQHLGFQLNSEQLSLL
>EFA73453.1
MTNNQSTIPVIVNGAGGKMGREVIKAISQATDMTLMGAIDTSPEHQGKDAGELAGLNIAL
EVPITNQLDPILGYVAGERQLQPAVMVDFTHPDAVYDNVRSAIAYGIRPVVGTTGLSPQQ

LENLADFAEKASTGCLIIPNFSIGIVLLQQAAITASQYFDHVEIIELHHNQKADAPSGTA
IQTAQLLADMGKPSTHPQVTETEKIAGARGCLADEGIRIHSVRLPGLIAHQEVIFGAAGQ
IYTLRHDTSDRSCYMPGVLLAIRKINQLKSLVYGLEKIL
>EFA73464.1
MMKNLPIVSRPLNFNQRYPCPSCRVGKIETMPLMEAMFCDFCQEIFTVNLQSQQVKMPSR
QPPLIWHWNGDNWDQGQIEGMELGWGYAVAGIAFVMLPTFLIGISAYYLPSSPMLFWIPC
IWTLLTFILHLAIVIWLFMEFIRSL
>EFA73468.1
MTQYQKLLRIATTGKSFQNITAKIAALVTESGVKTGLCTLFLRHTSASLIIQENADPDVL
RDLANFMSKLVPEGNYYIHDAEGPDDMPGHIKTVLTRTSESIPINNGNLVLGTWQAIYIW
EHREYNHNRELVVHISG
>EFA73472.1
MDTSKETSTKWFSTRLLPPQAIRQALQPCSQEKVEEVKEEYPQFKTWVEKTLPSLPEKKI
PFDVNHLKADQNTIRVLEEIGVIYEDKDKDEATRFYIPEIFRAGLGFSGTTGRPRTLALK
QKIFGKGN
>EFA73476.1
MAITLVSTIVSVVGYFAARIVPGIGIQMNPLLAPLTAVISAMIIAPHAGAPVAFAGGILG
TVIGADLLHLKDIQFMSEGVLSIGGAGVFDGIALCGLFALLLS
>EFA73477.1
MIYLPVSILLFLVLLLLLPFIWFALAVDIVEIAVAKLGFSPQIAFFLFLLIIITSTINIP
LYRLENTIEVVDEFATLWLREFWGIPLRRLERSTVIALNVGGGLIPVLLALYQIPEETCW
QSPW
>EFA73479.1
MLLGMIFLGISTTGWTSYAALPSGNAITDGRALLRYALPIENQPVRKLQASLEDISNQLR
ANKRWGAISRDLSQASRILDRPSQILTSIPPERQPQAESWIGELKSGIGELQEVVKTRQK
TPILEGRAKLLNLVSLLEESMVQGFPFEVPAEFSNLPQLKGRATIAIKTNKGDLTVVVDG
YSAPVTAGNFVDLVQRGFYNGLKFTRSEESYVLQTGDPEGKEVGFIDPVTGKYRAIPLEI
LAEGDKQPTYGITLEDAGRYLDMPVLPFSSFGALAMARPEGDPDGGSSQIFFFLFEPELT
PAGRNLLDGRYAVFGYLVEGKEILDKLKAGDIIESAQVIQGIENLVEPPA
>EFA73489.1
MTVTATLSQLAAVLSASLLNIPSSSLSQVIVGIQTDTRVLQPGEIFLALGGEKFDGHQFV
EMAIARGAVAAIVDYTYDYPNLPVIKVSNTLNAYQELAKWWRGQFGIPVIGVTGSVGKTT
TKELIASVLGTRGKVHKTYGNFNNEIGVPKTLLELSEGDDFAVIEMAMRGRGQIGELTHI
AKPNIGVITNVGTAHIELLGSEQAIAEAKCELLAEMPNDSVAVLNYDNPLLMTTAKQVWS
GEVISYGFSGGDIQGKLVDYQKIEVGGMTLPLPLTGVHNGSNFLAALAVAKVLDVDWKLL
EAGINVTMPQGRSQSWKLPKDIMILDETYNAAPEAMLAALELLAQTPGRRKIAVLGAMKE
LGERSAKLHQQVGETVRKLNLDGLLVLVDGPDAEIIATSAQGIPCERCHNHSDLVSLLKP
TYNQAIAYYLKLLIPLD
>EFA73492.1
MGVTVAPINAWFLAWITLAPLWVIVVKYTRKSLLSACWWAIAYHGMALSWITGIHPMNWL
NVPWWPSLLITIFCWSFITLWGALLVGLWAFLMVRLNPQKPWLRVLIGTALWCGLESWWS
TGSLWWSSLSYTQSPYNLALLHLGQLSGPNTITAVIVAVNGLIGEAWIWQNQKKREVIST
GKQLKGVTRFNKVVIGVINRYSISGLVLLIVSHILGSILYFQPYSQPLNNRQNSGLKIGI
IQGNIPNEIKLLPLGFTRAITGYTNGYINLANQGVDGVLTPEGALPLYDKNLSNTPLLDA
VKQKGVVVWIGGFGRTGTSYTNSLFNINSQGQITSRYDKSKLVPLGEFVPFAEIFGNLVK
RLSPLDEHQIHGAKQQVFDTPLGRAIVGICYESAFTEIFRYQAKNGGEFILSSSNDAHYT
AAMADQHHAQDIMRAIETDRWAVRSTNTGYSAFVDPHGRTLWKSGYNVYETHAETIYPLQ
TQTLYVRFGDWIMPLLLVLAALGLIYKSLVQIL
>EFA73497.1
MTSPEQLKPPSEAFIIPLLELRNYYASLVQKYQQLFIEARSQLDHVEALLSSWSYSEDHE
PISELGNDQVSSQLFLASNHQSTLESFTSKSLEHKNLDLAQKEVSPETPELNTDAINPTV
IPLQRNPTSSLGRGEEVPMIAQYKSLNRMEALRLFFDEHAGTACHIDFILRSLYGNLEPA

VLRIVKGRLQSSLTQGREKGDWYGVPNEPGCYTLALNLLEYSRPSSSSASHTKKKKSVTT
IASPSTIIPLLKQFEGQFVVDAISLFLEQHRGQIFTVNEVIQGIYGNLDDQQIARIKNKT
LNELSRGHRTGRFSRVPNKVGFYTWNANLLPRDIWSLP
>EFA73508.1
MVIFGGDAFPDATPPPYVQEAFASQFRRLVDAHIPTVLLVGNHDLHSQGIGGASLNIYRT
LGVPGFVVGDSLTTHHIQTPNGKVQVITLPWLTRSMLMTRKETTGMSMAEVNQLLTDKLQ
VVLEGEVRNLDPNLPTILLAHLMADNAMLGAERLLAVGKGFTLPASLLIRPCFDYVALGH
VHCHQNLNKSNDPPVVYPGSIERVDFSEEKEQKGYVMVELEKGRANWQFCPLPARVFRTI
EVDLSPSDNPQQTLLQAIAKHDLTGTVVRLVYKLRSEQLDVIENAVIHKALGDAHHYTIH
PELVSQLARPRIPELNASSSIDPIEALKTYLNNREDLKDIATYMLEAAQELLGDEETKIL
VDR
>EFA73513.1
MKKSVSTVKIDTLDLTITAPQTKKSDSVWVVFATTFITIFWRK
>EFA73514.1
MSAQSHAPWLVFLGAGAALVTTSLLGVLLGGFIASRLSPKTVEKSAGLVLLLVSSMLFWD
VIHG
>EFA73527.1
MTAENTALSFECETGNYHTFCPISCVAWLYQKIEDSFFLVIGTKTCGYFLQNAMGVMIFA
EPRYAMAELEEGDISAQLNDFNELKRLCEQIKRDRNPSVIVWIGTCTTEIIKMDLEGLAP
KLEAEIGIPIVVARANGLDYAFTQGEDTVLAAMANRCPAQTPVTETEKNERNAIQKLLHF
GKKKEEIIEEESEYVNHPPLVLFGSLPDPVVTQLTLELKKQGIKVSGWLPAKRFTELPVI
EEGYYVAGVNPFLSRTATTLMRRRKCKLISAPFPIGPDGTRAWVEKICSVFGITPQGLEA
REAQIWENIEDYVKLIRGKSVFFMGDNLLEISLARFLVHCGMTVPEIGIPYMDKRYQAAE
LSLLEKTCQEMGVSLPKIIEKPDNYNQVQRIYELKPDLVITGMSHANPLEARGINTKWSV
EFTFAQIHGFGNARDILELVTRPLRRNNNLKDLGWDKLVREATTA
>EFA73533.1
MSRIGAIALFAPLLVINGWALSSIFNYFHSLIVILVGASVLAFLLNYPITWMQKQGARRE
QVAVLVFLIALSILLALGVTLFPLALTQARQLVNRLPEWIDSGRSQLMILNEKAEMMGLP
INLDAIVVQINDRVKSQLQAITAQVLNLAVVTVTSLLDFLLTMVLTFYLLQHGDELWQSL
VEWLPNKFREPFSRTVRLSFQNFFITQLILSTCMASALIPSFLWLKVPYGLLFGLTIGIM
ALIPFGGSVGIAITTLLVSLQDISMGVRVLAAAVIVQQILENILAPRILGNFTGLNPVWV
LISVLTGARVGGLLGVIVAVPCAVVIKTVIGVIRSPMVIDQTNHQLDETGEQDLAPAENN
LSINTLVNHPHIQPKN
>EFA73536.1
MTLPANPASLNSNAMKTAQTPTDIVRTYLREIGKVPLLTHEQEIYYGKQVQKSTALYETK
DNLAQELNRQPSLEEWAKAAQLDPQELNRLLAQGEIAKRKMVEANLRLLVSVAKKYIKRN
VDLLDLIQEGSIGMQRGVEKFDPSKGYRFSTYAYWWIRQAITRAIAEKARTIRLPIHITE
KLNKIKKAQRDLSQTLGRSPSIAELAQELEITPKQVRDCLEKARLPLSLDLRLGDNYDTE
LGEMLEDPGASPEDYVIQSSLSSELERMMTHLTTQQREVIKLRFGLVDGQSMTLARIGEV
LNISRERVRQIEREALTRLRRCKTSMSEYLVS
>EFA73546.1
MKEFNTAGICLPKKHYMCDITAKFNRCRHLIESGKYFAINFPRQYGKTTMQHLLEKSFQG
VEEYLVISTSFEGIGDLPFQSEEQMASVVPAVLAKGLFFNNPELADWLEVKSKQINSFKQ
LSRFVSDWVRESKLKAGSG
>EFA73547.1
MDVDIDFTFSQEEIEPMLADYASEHGFQMDCPVLARLLFYYTSGNPFLVSKMCQVIDEMH
QNQRPWSVSDIEDAYKHLVDVSYSNTNFNDVYKNIENNPDFSQLVRAIAIDGEDLVFDRG
NPLIDLGATYGIIKSNSVGRCDIANKIYEFRIISYFISKRETAHQHLDRYRDSGFISNGK
LQISLILERFQWFMREHHSSRDNDFLEKNGRMLLMSFFRRIINGNGYMFKENVVAENRRM
DLVVTYKNQRYVIELKIWYGEKRLQDGIEQLCSYLDSYGLNEGHILIFNFNSNKTYDIRE
INHQGKHLTAFFV
>EFA73586.1

MYKWLSIIGIGEDGIAGLSKTAILLVNQAEILVGGDRHLAMLPPDDGRKKISWNSPFGNS
IQQVIQYRNQNKLVCVLSSGDPMCYGVGASLIRHIPKEEITIIPTNSAFSLACSRLGWNY
SEVETVSVCGRPVSLLACYIYPNAKLLILSEGKHTPAIVANILTTRGYGGSKITVLEKMG
GVKERILTGLAQSWQETEIADLNTMAVECIAQIGISGLSRLPGLPDDAFHHDGQLTKREV
RSLTLSTLAPLPGELLWDIGSGCGSIAIEWMRTNMRCAAVAIEQNSRRSHYIADNASALG
TPNLQIISGQAPQVLRDLPTPNAIFIGGGISTPGLLEECWNFLCPGGRIVANVVTIEGEK
TLFTWYEQVGGSFTRISIQRAQPLGKFFGLEGNVSGYPMGGT
>EFA73598.1
MNAHLGSAVLNSASLTMHPVTTGVTDNQRLRLFSGSANIQLAQEVARYLGLDLGPMIRKR
FADGELYIQIQESIRGCDVYLIQPTCKPVNDHLMELLIMIDACRRASARQITAVVPYYGY
ARADRKTAGRESITAKLVANLITQAGANRVLAMDLHAAQIQGYFDIPFDHVYGSPVLLEY
LQNKALQDIVVVSPDVGGVARARAFAKKLNDAPLAIIDKRRQAHNVAEVLNVIGDVKGKT
AVLVDDMIDTGGTITQGAKLLREEGASQVYACATHAVFSPPAIERLSSGLFEEVIVTNTI
PIPQSDQFPQLVVLSVANLLGEAIWRIHEDTSLSSLFR
>EFA73608.1
MGSILQVSHGILEIEMGANLGAGFLMVGEGKIGANACIGAGTTVFNDSVAAQQVIPAGSI
LGDGSRQANSQESGESVDEGDTSSQSGEQVVSKTQFTATFVNFQTQSTSVPPLSPTPKSQ
SPPETESSTESQEISDGKPRSRDPTEPYPLGTQIYGQGSINRLLSTLFPHRQSLSDQDAN
NGAE
>EFA73609.1
MEAYSQPTLSTIQSPRRHNNLKDTALGLVSTLSFPAIVGTADMMLKSAGVHLVGYEKIGS
GHCTAIVRGNIADVRLAVEAGVQTAEQFGQLVSSLVIPRPYPNLDIVLPINRLTQIMADG
SYSRLSNQAIGLVETRGFPAMVGACDAMLKSAEVHLASYEKIGAGLCTAIIRGTVANVAV
AVEAGMYEAERIGELNAVMVIPRPLDELEQTLPIASCWVEEHQPLDISLHVKEKIVDAQA
VELPDLAKLPVKIREELLVEE
>EFA73612.1
MLDSILPRTSVIVMTSTLATLIVACSGHKQTNQGINRATDVGNLAAVQPPKRLPHSQEVS
VLELALDKGVSAVRISQWAQSAEDWQLVESNFNRAIALLKQIRPNSPNFAFARKKIIEYE
GQLQLARQQGNPSKGSSLSPDSPVVTRSNPGRKILYSPPIPVRETPTITDFQSPSPDIAT
KKIPTEKTNDQVYIVPIKRRIGGTPIVEVTFNGKSRFEMIVDTGASGSVITQEMARSLGV
VPVGVARANTVSSQGIEFPIAYVDSMEVGGVMAKRVPVAIAGLALTTGLLGHDFFGNYDI
TIKRNVVEFRPQNTASTSVKIPRAVPTFPKDYQLPEFP
>EFA73618.1
MSQVFKNWIVRISSILLSTLLWSALILPTYAQTQIDLKLEQQVLEIIRRNPITIIEAVQA
YQEEQQQRVITRRQEFIKNFSQNPQDVIANSPTIGSSKLQTVLLEFSDFECPYCSEAHKT
LKNLLNKYPNRFTLVYKHFPLFQIHSQALPAARAAWAAHQQGKFWQYHDTLFTKQNQLGE
SLYIETAKSLKLDLGKFNQDRQLADKAIQKDLDLVNNLNLSGTPLLLSLVQILPDLSSYQ
SWKHFWQLLEIK
>EFA73620.1
MVESNLFSKLENLLRSQNFREADTETLIDNVQFLRKLRNSIAHSNVNIDKDTGYIRFKNF
NKNRDCDFEVTNNIPGLAKFVEEISGFFIDENNII
>EFA73621.1
MLTNNNYQNVFPWELSGLGADAVNLLKKYQLKYLRTYQKLIDIYEGNPRYLQIVGHLIQD
LFAGDTNKFLQLQQPSIDIDLMTFLRQTLDGLSSLETEILKFIASQSGIPGSNFTGNTGS
LSN
>EFA73630.1
MEPTPPTPPTETLSTQETVEGKLEPTPPTPPTESLSTPETVEGKSEPTPPTPPTESLSTP
ETLT
>EFA73633.1
MIGCQQILINGDKDTLAIIEYLCNESNDLYNCAVYYARQIWFKTKRIVTGYELTAQMKGN
KHFNAGYASSMQQTCLTVGEAFKSFKGLLREAKKGNIDQKPKALNYRKPNGLFTVSYPKR
WLKLVDGLIRFPLGNQVKAWFGISEFYLPMPTNLRL

>EFA73643.1
MNNYLYQYNQLYCGKLLKRYKRFFVDVELESGEIVTAHCANTGPMTGVSQIGSAVQLSKT
DNPQRKLAYTLELIQVDNSGPTWVGVNTNLPNNIIKLALEKQLFPQLGEYKQIKGEVVYG
KEKRSRVDFYLTGNEMPIYLEIKNTTWSQGSLALFPDTETTRGQKHLRELMEVLPKNRAV
MLYFINRGDCVKFAPGDSADPLYGQLLRQAIKVGLEVLPCRFDVSPQGVSYLGLAELKI
>EFA73644.1
MICCLNPDCQNPQNHDSSKFCQSCDTPLVPLLRNRFRIIHVLSDEGGFGRTYLAEDTDKL
NDSCVVKQLAPKFQGTWSQKKAMELFSQEAKRLQELGEHPQIPTLLAYFEQHNCLYLVQQ
FIDGNNLLQELRLRKHYKDSDIQSLLLDLLPILKFIHSRGVIHRDIKPENIIRRKRDGRL
ILIDFGSAKRLTAAVQKKYGTSIGSHGYSAIEQIRDGKAYPASDLFSLGATCFHLLTGLS
PFQLWIENGYGWVKNWQDCLENSRSVELIIIILDKLLQLDLKNRYQSADEVIKDLSKKNIY
GSEKSGIYPKNGKHKKSKDTILPNIFLTLVTISVVGGLGYRNLGQIQTAIFSQFKPLLMP
SNKSSTSPKYGQISRRNSSIRANKVSLFKTITQVDKSLAVVAITPDGNTIVSAGHKEIKL
WNSKTGKQIISLPGHTQNINALAISPDGNNLVSAGDDKTIKVWNLQTKKLTFNLVGHQDS
VQALAISQDSKTLVSAGDDKTIKVWSLLTGKFLKTLLDHNYWVRSLALSPDGFTLASGSF
DKTIKIWNINQTSGQKPTTLLDTTSQTVTSLTFSPDSSKLVSTSRDRQIKFWDIKNKEMI
FASKKQNVTSVIFSPDGKTLISGAKSCLDCSDKISSMIKLWDVSKKEEIYALPVTTKIVT
SLVLSADGKTLVGGTKDNKINIWEISP
>EFA73645.1
MTSESNKLRFYTQEEVQEILNLAIARQSKDLSQEFSYQQILEIAKELQIEPESVLQAKTE
WLAQQSETQQRKAFDTYRQNKLKEPYRNYIIINTFFVLTNLIGSASLTWSLYILMFSGLV
ILVEVWKTFRNQGEEYEAAFQKWNRQHQIKETVNTVMSAVINKWFKRRG
>EFA73656.1
MRLGLKVQILAGLITFLSVLSVNQYQFIHAQLTNKPLHTQQRVEQHQSQESSVLYSVIDE
WQKYKYSVNGKSILEPMKLPNTKVSFNANDLLFVLTNTRKYFQDYSQKDQNISRNGLLVN
QGVKVEDVLKTLDFMISTLREDVGKNRPPRLQDPNFINANFRVIKWKAYNPDKPEQKQLR
ITKYAVFTHPGSHTKTNTYNIPIYSLKDNSATDKFYTKYTKQDVLSGIYEPGGKEFGKVE
TLAYLTRQGLEEALMQGTILINFTDGSKGFFNVDRNNGISYIRGVKDTLQKRYWYFRPVD
QIKGYGYKIDAKISIRPGVTFAGDVLNIGLGRVVLIEYNQGKNKRLQMGVVADTGGAFLP
NLHQLDYLAGVFKNQTEFRQYIRQLPEYASAYILVKK
>EFA73671.1
MKIFPRLLYIDGLGEPGSDGETYQKMMDANTRSIVEGLGVDI
>EFA73672.1
MNRLLKCNFCNICLPIGLPIILAILGCNHRTPNNNYTQDQPGQQNQKISQNLPQVVVTTT
ILCDITNQIARESINLICLVPPGKEPPRYQPTLGDIKAIKQADLILYHGYNFEPRLIKSF
KNSRKNIAKISVGQRAVKQPQKLQQNGKIINEPHIWHDVRNAIKMVEVVVNFQLGKLSAEN
RQKYNSNTRQLTEELKKLNQWIKSTLSTIPDKNRKLLTTHRAMIYYVKAYGLDYKGTLPD
ISNEDKLTSKKAKSLAEYIKTTQAPTIFADRAVNLMLLAPIAKKNKSENFSSTTLY
>EFA73677.1
MVKLSELKIEPVTLEHLTDLLELDQACFDGLWTIEGYRQEIESISSHFLGLFSLIPSHDL
LGIGCFWSVLEEAHITILAVHPEYQGQGLGQALLYSLIKDAVDMGLERATLEVRVSNTPA
ISLYKKFGWKTAGIRPRYYQDNQEDGLILWISQLQHPHFLQTLDKWHVLVQERLGQFSWL
LIQEEN
>EFA73680.1
MTIQQTELLYLPPDLKPELLPKHVAVIMDGNGRWAKGQGLPRIMGHKRGVDALKNLLRCC
KDWGIQALTAYAFSTENWNRPHEEVEFLMTLFQGVLRQELREMVEENVEIQFVGNLQALP
VALQREISHSMAETRGNQSIKFTVATNYGGRQEIVQACQAIAQKVKEGLLNPEEISEELF
AGHLYTAGIADPDLLIRTSGEMRLSNFLLWQMAYAEIYITDTLWPDFGRREFHLALLAYQ
QRERRFGVV
>EFA73687.1
MQNYILALAKYRQTQLAEDRLQQGDRLGAATMLQAAAKTALQMGDANAGTVLQNSATRLQ
AGEDLSASDRSKTKIVSKTVLQDPTLT

>EFA73688.1
MKVNLQSGVNNTNVDASKSSSQRLIAISVSAIGETIDSRVPLNLCLILDHSGSMKGQPVE
NVKRAAWLLVDKLRDQDRLSIVVFNHRAEVLLSNQNVVDRDHIKQQINRLSANGGTSIDE
GLRLGIEELAKGRRDTISQAFLLTDGENEHGDNNRCLKFAQLAADYNLTVNTLGFGNNWN
QHILEKISDAGLGSLSHIEHPDQAVDKFNSLLMRMQTVGLTNAYLLFSLAPNIRLAEFKP
VAQVSPDTIELPVQVEPDGRFGVKLGDLMKDVERVILTNIYLEQLPEGQQAIANVQVRYN
DPSVDKTGLYSQIYQSMSMWSALTKKILVQKCKITF
>EFA73694.1
MSIYVGNLSYDVTLEDLKSAFSKYGNVSKVQLPTDRETGRPRGFGFVEMSNEAEENAAID
ALNGKEFKGRQLKVNKARPREERESSRGGWGGGRSGSAGHDHRY
>EFA73697.1
MGRCSPPSSCLEMKFMQAEPKPGTLYIVGTPIGNLEDMTFRAVRILQAVDMIAAEDTRHT
GKLLQHFQVHTPQISYHEHNRTGRIPEILTYLHYGKAIALVSDAGMPGVSDPGHELITAC
VAAGIDVVPIPGATAVITALSVSGLATSKFVFDGFLPAKRQHRREYLETLLMETRTLVFY
ESPHRLRETLEDLGEILGGSRTIVMGRELTKLYEEIWRGDIKDAIAYYQGKDPQGEYTLV
LGGASPSQPEITEAQLRAELLEIIKQGVSRSQASRQLAQETSISRRYLYQLALSIDQEGV
>EFA73699.1
MKQSFFNRQLTQFVGILLGMGVALFILRGLRIITFLPSGIIGLLFLGAIAIGVLSYLQTK
WWRF
>EFA73700.1
MLLSQDKHKSLIEGEILVQTRPHNAWGGAVTASIYLPLVRSYVWEQITEYPRWVNYFPDL
TKSELISPGNSSQGNVKFLYQRAQKAFLFFTAQVEIYLTVIEVLGKQIQFRMERGTFEDF
YANLQFQDMGDGTLLIYTVEATPNIPIPSMLIEQGMSRGLPDNLRKMRQFLCSRA
>EFA73707.1
MILGGGIGSEAKAFGKAKGYADRATITQNRSH
>EFA73709.1
MQTVTLGDRGPSVSALGMGTWAWGDTLFWAYGKSFGESEVAAAFQASLAAGITFFDTAEI
YGFGESERLLGQFCQQTQQPVQIATKYFPLPWRWNRVAIVDALTASLDRLRMSRICLYQI
HWPLEFLLKTQDFMEVLAAEVKKGRIQAVGVSNYGAKQMTLAHEYLAAKGIPLATNQVPY
SLLTRQIESNGILDQARQLGVTILAYSPLAQGLLTGKYTPETAPSLQGARRLDPRFSPQG
LKKLAPLISALQQIGEKYDKTPAQVSLNWLIAQGNVIPIPGAKNANQAKQNAGALGWLLS
PEEVESLGTIV
>EFA73710.1
MFRFPKSLISQKAIEPCCIDNCISLSFVGDWAILDWEFSQEEGFDYWIKGEGILSELIGL
RQEILQQDYRGLYLAWLKAIALSEEYIDIDNTQLEPPLPPGLNQLSPSQKAFVEIFDLDE
NLLNVACASSGQPTTISEQVWQQAITKLSASECQDFLLRLLKEESNLSAKFKRRLSQLIP
PSPASNQPRRTIQELLEASSEEGKKAEKRQQKRSRSQFRKSSP
>EFA73713.1
MSSAYLLVSHGSKDSRPNLATEELARLLFEKLTAGEDPEKHLVGVGTLEFNSQPLSGQIK
DFAQRAVVCDCKSIKVIPLFLLPGVHLMSDLPMEVKLAQDSLGNDRPIKIKPYLGSHAMM
GKLLALGMAQISAEAFILLAHGSRRANSHHPVETIANQLGAFSAYWSVPPSLEARVIDLV
SAGYQHVGILPYFLFSGGITDEIAIMVERLTLKFPEIKLELASPLGVTSQLVDIIWDLAT
QEE
>EFA73720.1
MIEPSISEVSPESGAQLTPDQAIANLQSSDLSLRYYAAWWLGKFRINRTEAVEALIEALT
DEADRTELGGYPLRRNAARSLGKLGSSQAIEGLISSLNCEDFYVREAAAESLAMLNAKTA
VPELIKMLDGGLAQAVQVPGRPHLVQPYEAVLEALGSLGATEAISLVEPFLEHSVPRVKC
AAARAMYQLTQDSQYGEYLVEMLGSSDLKLQRVILGDLGAIGYMGAAEAIFQAKAENSFK
LMALKGLLEHHLKDVSQSISESAILVMNLMDSLL
>EFA73722.1
MSIPLLEYSPSSQNQRVEGYEVANEDTPIVYKLNYATSDQEIDEIIWASYRQIFSEHLIL
TSYRQKFLESQLRNRAINVRDFIRGLGKSEVFRTQIAEVNSNYRLVDIILQRFLGRKAYN

REEEIAWSIVIGTKGVHGFIDALLESEEYINNFGDDIVPYQRRRFGSRPFNLVNPRYSSY
WRDAQSMRVLDYRSFYSARTYGTATSEDIRKAIPANFFAMAGKIITPERNYQRTIASVTT
QIGSLEIPDTSREVSTSEVTIKPVAVSLPYSYIPPISKN
>EFA73725.1
MVKITGIPPYLLPSPSRIIQTLISDWFILFPALLITIKITVVAFFYAAVLGLLISMIMAQ
SKWIEKSLYPYAVILQTMPIAAIAPLIIIWLRNNTFAALVLCAWIVAFFPIISNTTFGLN
SIDPNLRDLFRLYKANPWQTMVYLRLPSALPYFLSALRISGGLSLIGAVVAEFVAGSGGT
NSGLAYQMLMASYNLQIPRMFAALIMISALGILIFLILTAFSNLLLGKWHESSLKRTP
>EFA73736.1
MSYSSQELSEFLHKIPGFERLSATEINHLLSKQQPLRYRLGQKIVGKEKLPERIVIVYQG
KVRLLAYHLQSQLPITLQMLEPGAIIGEISYLRQIACETAIASTEVVCLTWNPVDYLSVF
SQNSDFAKERQEKNHIIEVFDVISHHVAQKAYGNLNLKDITRDILPESKIQYLPPGKTPL
DQLGSDRTWFVSSGQITNFPVNSQIVSSNNREVLQVKGKIPARLLGISCEALLLLEQQEN
REPEKIEDPRKKTDVDVIDIPFAITTEFSPQENRKAKSSKNRQKYPFFAGWGELNSTLAC
FQMLAKHLQIPLRKEVVRRILSDNLKRQGNVSFQLCAYVGKLLG
>EFA73737.1
MNPVLQFRNFSGRESYHTLTAPEVLKLLNKYQLIPMFLQEVIMEQAIAQISCTPEEERLA
YEQLTQEYQGQKEQGISQEQLQNMATRQLKLERFKEVTWAKDIDSYFYQRKPQLDRVIYS
LITTNDRGVAQEIYFRVQEGEQSFAQLAQEYSQGPEAKTSGLVGPVELQSLHPLLVRILS
TSQPQQLSLPTAIEDWIVIVRLEKMLPAQLDSGMRQRLINERFQSWLKAQVSPQNWQIKE
SEN
>EFA73738.1
MVQPEDFLAVVDREWQMLSSDGLPLSLIFCAIDNFDPNQETTKNTSNLRQIALVIRKCLY
PPSCFAYYKQGNFGVLLPNMSLDAAVRLAHDLHETVVSTVEISFSLGITGTFPRSSSTVE
KLLNTANQALTGAQRHGGNTFCLYPG
>EFA73739.1
MKILVVDDDTSLCQLVKTSLVAHRYVVDVANDGEMGLEMGYQFNYDLILLDILMPKLDGL
SLCHTLRNKGYQGQIIMVTAKTTQEDIIIGLDAGADDYLIKPYYIHELLARIRACSRRIG
SQLTLNTLVYNSLSLDPELIEVKYDEASINLSRREFQLLELFLRYPQKIFTRGQIIDKLW
SIDDSPTDGAVTNLVKDLRHKLKKAGMQEELIETVHGLGYRLTSKRELEREVVETIPTQA
SSPITDSSVSEGLVLMEQIKLEFEMSLPKKIADIRAGLVKLETEITNGQQQQYLMRMTHS
LLGSLGTFGYPQCSQVAAKMENILVKESLTNVDIQAIDSLIHALEWEVNPKKRKNRSQFQ
HFF
>EFA73750.1
MVALIGASGSGKSTLLRHINGLQTADAGEITIYGTTLQSQGKLHSKVRLLRSRIGCIFQQ
FNLVNRLTVIENVLVGNLARVSPLRSTLHLFTKEEKTQALAALEQVGIIEHAYKRASTIS
GGQQQRVAIARCLVQRAKIILADEPIASLDPESARKVMELLVQLNRDNGISIVASLHQIQ
MVRNYFSRAIALKDGEVKFDGPTVELDDRKLNQIYGAAVEELVMRGHSEVLL
>EFA73755.1
MQGTLAGISRLGKPTPYVFYFGKESGDTVIYFFETQSPVGKKITRVCKQNTLCRGKAQLQ
VVKSIPKSIPESTSGTYKIISVSQVNGKR
>EFA73762.1
MTEQPQKQKSSRSFAGIAGIVAAATLISKIFGLIRQQAIAAAFGVGAAATAYSYAYIIPG
FLLILLGGVNGPLHSALVSVLAKRKQEEAAPIVETVTTLVSGLLLIVTVAQIFLAEPLID
LVGYGLDVKTREIAVRQLQIMSPMALFSGLIGLGFGTLNVANQYWLLSISPLLSSITVVI
GIGILALEYGKQIIQPEFAFIGGMVLAWGTLIGAILQWSVQLIFQWRLGLGKLKLRFDFK
SPAVQEVIKIMIPATISSGMMPINVATDLFFASPIKGAAAAFNYANLLVQTPLGIISNII
FTSLLPMFAKLAEPQSWPDLKLRIRQGIILTAVTMLPLGGLMIGLSTPIVQVVYQRGAFN
QQATELVASLLIAYGIGMFAYLGRDVLVRVFYALGDGQTPFKISAFNIFLNVVLDFVLVK
PFGAPGLVLATVGVNCSSMLMLLWLLNRKLNGLPLREWTWPILGFNFWQCGCWC
>EFA73764.1
MIRFIKREVLPIITDLKLAIVLLLVIAIFSVTGTVIEQGETPAFIRPIIPNIQLYFGFLS

WKVIQVVGLDHVYRTWWFLSLLVLFGLSLTACTFTRQLPALKTAQKWQYYDEPRKFNKLA
LSAELNNVNLNSITDLLRKSQYKIFQQEETSHLLYARKGIIGKIGPIIVHIGIVMILLGG
IWGAITGFMAQEMITSGETFQVKNIIDAGAWSSQDVLKNWSVKVNRFWIDYTPKGGIDQF
YSDLSVLNDQGAEVNHEKIYVNKPLRYHGVVFYQTDWGIAGVKVKINNSPIFQLPMALLN
TNGQGRLWGTWIPTKPDLSAGVSLLAKDLQGMVLIYDNEGKLVNTVRAGMSIPVNGVNLQ
ILDVVGSTGLQIKYDPGIPIVYIGFALLMLGVVMSYFSHSQIWALQNGDVLYVGGKTNRA
QVTFEREVLGILDQLSQTAKK
>EFA73765.1
MLENLQTQIYHLEQFANILVAQQLNHLTITSIAIIFLAGLLTSLTPCMLSMLPITIGYIG
GYEAKTRLQAAAQSTWFALGLATTLAGLGILAGLVGKVYGQVGMGLPIIVSILAIIMGLN
LLEALPLQFPSWGETNWISNDMPSGVRSYFIGLTFGLVASPCSTPVLASLLGWVANSQDL
LLGAFLLLAYTAGYVTPLILAGTFTATIKKLLEFRRWSGWINPVSGALLVGFGVFSLLSR
LPIY
>EFA73766.1
MAKLFAAIDGNEAVARVAYKLNEVIAIYPITPSSNMGGWRKINLIYGGIIPQVTQMQSEG
GAAAAVHGALQTGSLSTTFTASQGLLLMIPNLFKIAGELTSFVLHVSARSLATHALSIFG
DHSDVMAARTTGFDLLCSASVQESHDFDLIAQVATLQARVPFLHFFDGFRTSHNDKGGET
PTPYLIS
>EFA73767.1
MPVIEKKRTRDLPQINERIRFPKIRVIDNEGAQLGIMAPLEALRLAEEKELDLVLLSDKA
DPPVCRIMDYGKYKFEQEKKAREARKKQHTADVKEVKMRYKIEEHDYNVRVKQAERFLKD
GDKVKATVMFRGREIQHSDLAETLLNRMATDLEPFGEIQQAPKKEGRNMMMLISPKK
>EFA73770.1
MNQLPPMPLPESLWGEQWCFASVSAGDILEEFGSRSIPFKKIPDSFLPVKLGLAVTVSIP
GVIIYGGKQSLRLARWLSENNPVSLNYIAGAPDGLILQSSSTNRWIVATFTDTDVTAAAK
VYQQRKKVSEGVHFLLVQPDNSGMTFSWFLVIKRHLVVNG
>EFA73772.1
MAARGIDRQLPFLICFIQGKLEIVRSIFNHSAYPPV
>EFA73773.1
MPQVVLINPQIPPNTGNIARTCAATATELHLVGPLGFEITDRYLKRAGLDYWPHVKLNYH
ESYHTFLKVHQQRGGRLLGFSVRGNINYIQHEFHPDDWLLFGSETTGIPKNILDICDSTL
YIPMDQPGVRSLNLSVSVAVALFESRRQLTYLK
>EFA73775.1
MKIKNPNLSVFAARQFRYSLYQNSLELTIKESRPFNVLEEFIIRAAIEFKPAPTPDELAA
ILALDPVFVHSTISILQSLQTLAFQTPITVTQEGRLFYEKGTVPQPPYNVHIYANTDILE
GKFTLTTEAFNHDVPRHLPDLSLLLNLEDGTLDVSVLTLTDIQEIIQSSHLSFHLPETGK
IVTGFRILPPSQMIWKLVSLFVVFDVTEHKFHIQIRSGKRILPAASTRVGYFLDQGRISL
SELCDLPQATINFLLDDKNR
>EFA73777.1
MQTLEKKKDRANELIFECVGKQKCLFYIRRQGNVRDTEEEILDLTIATDESEFTLPKRLK
ELDKTLGLRAALQKNGNGGLKILSTYLLPIARGKKDAYTVPLNLQLVPNHYEPVHIPPKT
LAQIVKMPICGNHVPTEEQLRAWKAFLKVEERVAKARQFCVNFLNSYYDLKQREIVLKIN
INSATLDGDEENYINEDNFWERVRQAKNQEIKFSDVIPTEKSVAPVVNWEQLKR
>EFA73778.1
MATAATHGQLLAQESVPIQIPTPQPVQEKKSGFTLYIWQPNGTISPVIARISLKAKHGQK
YLQERFLGDYQYRIKQKAKFENGFKWGDRIVVRLYNTQNQLVGYTEFACLPNHTTVNLIL
PANSPPNPLVRVFYGVDGDEDGIVDPDTTMSYDYFTEIRNQQVTFLSSPPDIDLSPYQAA
GFAKVAQPSTYPGSFIQGPFALVGKPISINDSQLAKAFLYPPGEIVQVTKLSESAYQLSN
LLSKYREVGVGKGIRVRFSDLPQNYWAKDYIEELAAMEIIDGYPDGTFRPNAPITRAQLA
TLLPKIFFKDKIRGEVAFRDIPKNHWAHNAIQKTYQMGILTPSANRRFRPDQKLTRLDVL
TTIAKALNYKFTGSTRDILSIYQDASTIRSEHRGLIAAITENGVVVNYPNIRLLNTRKLV
TRAEVCALLYRAMVSKGDVPDFSSVYTVRSNGSNIK

>EFA73782.1
MNTRKSPIQIFGVYALLLAIALLTLFPLLWLISTALKSPTENLLETPPKLLPLEPTLDNF
LRVWESLPFGQYLYNSFLVAILTVALNLLFCSLAAYPLARLSFPGRNTIFIAIVSTIMIP
FQIVMIPLYIITVQLGLTNSYLGMIFPSLASAFGIFLLRQAFMGVPKEIEEAARIDGSSE
LGLWWFIMLPAIKPALITLAIFVFIGAWSDFLWPLIVIQDESLYTLPLGVAKLAGTFSLD
WRLVAAGSIISVAPVLLLFLFLQKFIVPTDTGSGIKG
>EFA73792.1
MFEHFTSEAIRVIMLAQEEARRLGHNFVGTEQILLGLIGEGTGVAAKVLTDLGVTLKDAR
REVEKLLVGVLVSSHPKSLSPRKSRVYLSSLLEKLTVLVTTT
>EFA73793.1
MLGLTDAGEGVAAKVLKSLGIELQTVRSRVMSILGEDNRVVAGRQDNPRRNQNLSIEEFG
RNLTKLAQQGRLDPVVGRQTEIERTVQILGRRTKNNPVLIGEPGVGKTAIAEGLAQRIVN
QDVPEILLNKQVISLDMGLLVAGTRFRGDFEERLKKVMEDIRSVGNIILVIDEIHTIVGA
GGTEGGLDAANILKPALARGELQCIGATTLDEYRKYIERDAALERRFQPILVGEPSVAET
IEILRGLRSAYEQHHKVNISDDAVIAAAELSDRYISDRFLPDKAIDLIDEAGSRVRLRHS
RIIDNKELKQQLKNTSQEKAEAVRVQDFGKASKLRQEELDLQTQLAIAQNLPRITIPQVD
EEDIAEIVSSWTGVPVNKLTESESELLLHLEDTLHKRLIGQEQAVTAVSRSLRRARVGLK
SPNRPIASFIFSGPTGVGKTELAKALAAYFFGAEDSMIRLDMSEYMESHNVSKLIGSPPG
YVGYDEGGQLTEAVRRKPYTLLLFDEIEKAHPDVFNMLLQILDDGHLTDAKGRKVDFKNT
LIILTSNIGSKLIEKGGMSLGFEFDNQANASYNRIRNLVNEELKSYFRPEFLNRVDEIIV
FSQLNKDEVKEISQIMLEEVAKRLQEKGIKLEVTEAFKDLVVTEGYDPSYGARPLRRAIM
RLLEDSLAEAILSGEIGEDDQAIVDVDDDGLVKVKKAETRDLVLVSAPA
>EFA73798.1
MKIIPDKNNINFDPHGSNPYLWKFWGVLPLYPYNQRRTVRQEVVKDTIWTFDQIQGVFYV
VVPIRMTVIRLAQGGLLVYAPVAPTQECIKLVNELVALHGDVKYIILSTVSGLEHKVFVG
PFAREFSQARVFVAPGQWSFPLNLPLSWLGLPGNRTQILPEDPHETPFSDEFDYAILGPI
NLGLGKFGEVALLHKQSRTLMVTDTIVSVSVDSPAIIQLDPFPLLYHAQEKSGDTVPDNL
QNRRKGWRRITLFAFYFRPSAVDVPPWKDIWQDAKNVSQRNLRNYFGFFPFHWKPDWEES
YELLTRGGEIFVAPVLRTLILNRAPQVTINWADRVAKWDFAWVIPCHFDAPISVTPQQFR
RAFSFLEKGSTDYLPHNTKVEEDLQVLRDIDQGLNNWNIVPPALPNPNSRVR
>EFA73799.1
MTPQLLFNSSTMKNLPSLFTLFISVVLATWILAIALISVQNTASISLRFLVFQSIQVPFG
LMLAFWVVVGLITVSFGQLLGNMGGSGNSADEEGDFFVDEDFR
>EFA73800.1
MSNPSNSGKIKFGTDGWRGIIADDFTFSNVRKVTRAIAAYLETAYDKSRPVLIAYDTRFL
ADEFARTSGAVLADLGWNVKITVRDCPTPVIAYNAKHLNSAGALMFTASHNPAPYCGIKY
IPDYAGPATPEITDTIVANIETASEELPGSNPSGTVSTFDPKPDYLDFIYKLLDVRKIRS
ANLKVKYDALYSTSRGYLDEVLQYCGTQLESFHTWRDVLFGGGMPEPKGDQLVELVTAVK
NDKADLGLATDGDSDRFGIVDELGNVLTPNTVLLVLARHLIKNKGKSGAIVRTVATTHLL
DNFAAKYGLPIYETAVGFKYIGEKMRETTVLIGGEESGGLSVIGHIPEKDGVLADMLVAE
AIAYEGKPLSQLVQEAIAEADGPLYNNRLDLHLTEAHKNAVIQSYTQNPPSQVAGIRVKE
VGRKDGIKLYLAEGSWILLRPSGTEPLVRVYIETNSPEKLGEIAKTMEAEIAQLA
>EFA73803.1
MYLQSLELRNFRNYQEQKVEFTAPKTILVGNNAQGKSNLLESVELLATLRSHRLGKDSDF
IQEGQDMARINAILDRTTGISNLTLNLRRNSRRTVAINGETVRRQMDFLGILNAVEFSSL
DLELVRGSPAIRRSWLDTLLVQLEPVYAHILHQYNQVLRQRNAFLKTIQQKGIKNHDSEL
AIWDAQLVTTGTKVMRRRNRAIQRLGPIATHWHSSISGKMEKLEINYMPNVPILIDEQQE
LLQFFLDRVQEHSAIESYRGTTLVGPHRDEIELVVNGTPARQYASQGQQRTLVLALKLAE
LQLIEEVVNDTPLLLLDDVLAELDLSRQNQLLDAIQDRFQTLITTTHLGAFDAQWLNSSQ
ILFVKCGSISQ
>EFA73805.1
MLRLNEDSHIRDNTQQDHISTLSEDVSYLVEQYRSRHERHIYPTINLPSELQPDTTHGSQ

FEFVIHNGRNHGKSRDTIYKIIQEASEFLLVSSYIIEDEELTELICQKSLELPKGVWILT
DLNNELLDRIDEQILEHISIPERYRISDERKKSCLKMLLNSNVSIRSGAFHLKTYISDRS
AYLGSSNLTRGSLDFNKEAGIIFSNNFEHQNLINFFQTFWYTRSKYQVFPEPNGCGFRLR
SVMYSPQKRYGAEFSVESNFLTPAQYQRDVVENWRNLPRDMEVRIYSRDFKPSQEMAVYL
NSNTSVFINSSVSENNRRFYIERFNLENFHAVDNLHAKITILGNRVAYIGGINFSFNSYY
NSNSNSTLYDLMYKTTDVNKINQLINQLYLLR
>EFA73806.1
MKIQIELSGKFARSLLNATEVIPRTLNHLIHEVQAIGTRYHRRTPQLLRQSFGLDFIPEP
VLINLCKNSRGVTKESDIYQVPPLKLLKPLKSQLDNLDIYQYAYNFICEQGNLNLTPRDP
QLPVFSSDVCEQMGSYIITISEPNYPDLNLDFIQIIWNNSWQYWQKLSDIEKIDYALKSH
ELS
>EFA73807.1
MSPAYVYKLREQFRLHPKIAETVYPVICDHWVYTHYPNIDYNVSNLSQIARGVTWKNMSE
RLTVSQSVGEKIYELVENLTQTLSSEIISEIGIITFSETQRDNLREVLTANHPQFSPIFI
GTAKEWSGAERRIIIVNCMTACSGGPNNIALEDINIALTRAKDQLFLVGDHVLWQESRSP
IRSLLYQSSLSIETQVVI
>EFA73808.1
MTNRITRQNLRQQLQNLIYESDEISPISQAQSEKLVEIYNYIYNSPSLPTNLIIETLCNQ
GYYSQKRASLNFPSIKYKTFLSASQQIALEMALSNSPITLISGTAATGKTAIAKNLVQAA
IDCSYKVLILAHNLASLNPYYNLTTYPFLLSQQENYSQRMVNQLRDHILHNLARTRMDYL
PLYLLPDGELAKLRTPAILETLLPEIVDKTNEQIMEILRSEFTEFAHLAEPRLQLLAHRL
QQLLPFLQEQLRLMQIYNNLSEVEIEELANQLVANPQVTIMGTVSDFMQLENKPLWDSDF
DLIIVEEANLLNWRELIFLSGLCQKLVLFGEDIPRRYFSCYSHIQQHQENHNSRRNSFFR
>EFA73809.1
MGDWMLTLEIHQLDSMELNSNKLQFPIWVFWYLPSRDVINQMGYYYLHNLRNQELENNGL
FAGAIVLYLQDHTQNYSLDDSPSYEELFPNLRLVKLPENFTEQGFSQTVEIIFNTLIEVV
GGFTL
>EFA73810.1
MADAIYIYLIVGLLRLQGVNIDPTVGVSCKLIPDQGRYLSPDQNIKVRVFYKPGFVYLG
>EFA73811.1
MRIYPSLFAYLNQHQSPLISDIFALEASHRPILVLELDEDELLSLPSWFIPISQGIYEVG
FPYLQPTDLHYDHDHHASLRYVLSLAIPGQGYNLSITDVLDLETIKEEVEIPEWEHRLGN
RLDRTASELVRLFIGMFTHISSGTTRINSEANILLSGLSQKLDSVNIEDANLPIVISLDN
KYQLHQKLELIGSRLRHQLTRKAELIPVGKIQEMDSYCLRDYIRRPGSTPEEKAGSKQEL
MGIKRYQDFNTPENKFLVYFARIFHLNCVQYEISNANQFRSQINKIRLVIDLFQQLPTVK
TIQNRGYQFTKPNYVLQQNTIYKSFYQAYLEYIRKKHEKENLWGLEIICWQMLSIFI
>EFA73812.1
MEQRAAELELLRFTYDAILSQKQDYEAKVSQLNPEIDRLELEKQRILQTIEENQNNYQKL
IELREQYRSLTLTIRDQESRLRELENEGNNLQTRNHNLQTETDNLQQTKLEIETTIANLR
GEIREIENSGRVALEPLREKLWTNLPRQVRQLEGETQFIQNFITSLRSEGLSFSSRTIKA
FHTSLKVQDISALVILAGISGTGKSELPQRYANYIGAQLLTLAVQPRWDSPQDLQGFYNY
LEKKFKPTQLMRGLYQYKDPEMSNRIVIVLLDEMNLARVEYYFSEFLSKLESRRNHATYL
EIDVGSLPIAESERRLEIPKAFLFVGTMNEDETTQTLSDKVLDKANVLTFGRPQNLKLRQ
RSGQQITVNQHSDNLVNNRDSSYVTYSDFQNWVRTPVPESDVVKEVESYLNEANKVMEKM
GHPFAHRVYQAIAQYVVNYPEVNGIDSESFKFALADQFGQKLLPKLRGVMVDEAHQQLQE
MKEIIDKINDQPLIKAFDKAKEGRYGQFSWQGLVYEDEEA
>EFA73815.1
MAVVKLLEVIGEAVKKIPNERRELYPDIPWKSIAGMKDMLVHEYWQVDVAVVWATVQHSL
PSLKAIVVMELEKMQG
>EFA73817.1
MHKLRSMAEFTQRFPDVKEVIVNGTEPPVQRPQNRERQKEYYSCKKKQHTCKQITVSTRK
KRVIILTKTRAGKVHDKRLLQESEIVQYIPDEVAIEGDLGFQGL

>EFA73818.1
MFKSLYFLSSVLLTSIVISSMPHSTSYAQVGRSGFRSISRGSYGSIRGSLRLPQQIKVSR
YGYASFYFNLGNDCGAGISYGKKGGMDGQWYTFMNCGNNRGGSGRPIGLQPINRIELSYD
GISQVTLLVNGRRVQSAMTTLSPSSVSMVHAYYDEPGGTEKTYYDSAAFEEVECNYSRSS
CNFSSPEFFGTARRLFYRSTPNTLNTYMR
>EFA73825.1
MPAVIVMKEPVPDEFAREFLERFLIHFVKECNFLSVAVEKAAKDILYKEDQEFPGATWLP
ITYYSNAKSEEFTWSKKNQIYKNRIDIKIHNYLYQRQEENSLCPYQGLFHFGPEEAEYFF
GRQIFIEQLYEATQNRKFIPILGASGSGKSSVVLAGLVPKLKNQGNWEFTYFRPGKEPFP
ALATALIPLLYTSELDTIEKMAQGRILATHLQTGTIPLSDVFTQIQQKHPTEKVLIIADQ
FEEIYTMCPEPETRQQFLDCLIASLSIPNSPIVLVTTMRADFLSDALSYRPFGDTLQNAD
LKILAMNREELTEVIIKPAEKLGVSFEQGLAARILNDLEQQPGNLPLLEFALTQLWNQHH
EWQLTHNAYEKIGQVQGALARYADEKYSNLTKTEQETVEVLHEALIRDWGERIEYHSQAY
YKEPKTSTLRK
>EFA73826.1
MNITQSTFINKISIGWTSLLIFTLTSVPTSAQTIDRIRDILEKTTVQINSNANPGGSGVI
IKKEGNTYTVLTANHVVCENLGVETIRCRTDFTYSIRTYDGREYPIKKRQILQTKLQDPD
LAIVTFESQQNYKIASLGNSSQVKIQSDIFVAGFPTIFGFTGRDRTFSITNGKVVAFIPQ
QKAPKGYGLIYDATTRIGNSGGPVFDSNGNVIGIHGLADADALENNNTNQSETRNNLRPN
LSTIRKLINPQTESGVSFQVKTGFNAGIPIDILYGVLPQTYFNTRSITSPSDSMNSGESG
TSNENIDDSIKSQSIAYFDKAAEQYNGGDKQGAIANYNQAIKLDPDYTDAYINRGLVRSE
LGDRQGAIADYNQAIKLDPNYALAYYNRGIVHSELGDRQGAIADYNQAIKLDSNYTDAYY
NRGIVRSELGDKQGAIVDYNQAIKLNPNYTNAYINRGLVRSELGDNQVAIADYNQAIKLN
PNYALAYYNRGIVHSELGDKRGAIADYNQAIRLDHNYTDAYYNRGILRSELGDKQGAIVD
YNQAIKLNPNYTNAYINRGLVRSELGDNQVAIADYNQAIKLNPNYALAYYNRGTVHSELG
DKRGAIADYNQAIRLDPNYTDAYYNRGILRSELGDKQGAIDDYNQAIKLDPNYANAYYNR
GIIRSELGYNQGAIADFQKAVNIYQQQGKENNYRDALDRIREIQQR
>EFA73834.1
MTLVFQFALVSLVLFSFVLVVGVPVAYATPQNWVDSKKLLWLGSGIWIALVLLVGVLNFF
VV
>EFA73837.1
MNFSLALPTFVITLREGVEAALVVGIVLALLNKSKQSQLNVWVYAGVVVGVIVSGLIGIL
FTGLIKFLGSVNPEYSSTVEPILEGIFSILAIIMLSWMLIWMTQQAKFLKLQVEGAVRQA
LTKNSHAGLGIFSLVLIAVVREGFETVLFVAANFQQGLLPTLGAIGGLATAAGIGVLLFK
LGVRINIGRFFQVMGILLVLIVAGLLVSGLGHFDDAIASLAMTSRASENLCFYYEHFTKI
HSCILGPLVWNSSSILPDEKFPGIIFKSLFGYRDKLYLVQGIGYLLLLTTVGGLYFRSLN
NRNNPPKKNSPNHSRIAIITVYLL
>EFA73842.1
MTSLTSYSSLTNFFPETLVLEVRNLMYAYTNKYTILKNISFKLKTGDRVALIGATGSGKS
TLLENLIGLKYPQSGTITINGIPVEPNTVAKIRKQIGFVFQDANDQLFMPTVLEDITFGP
LNYGVAPAVAKENARQLLADFGLEKYAHRSHHELSGGQRRLAAIASVLALEPEILILDEP
TTGLDPILFG
>EFA73843.1
MSLVNFQVQTACLNPQQIFLALSQVIPSQTITKAIESTCSSQRRLRILPTYIIVTLVIAM
SFWSSDSIVDVFKNLIHGLSSLHIPSGLRLQTPSASSITEARQRTGAAVMRRLFELVAKP
LATILTPGAFLGELRIMAVDGTVFDVPDTSTNARVFGYPGSPKGTYPGFPKVRLVFLVEA
GTHLIIDAFCYPYRMGERRGALKLLRSINSSMLLMWDRGLHSFKMVHTVIKQQGNFLGRV
PGNVKFQVVKTLADGSYLSWIAPDGQSRKKGSKKNGGSY
>EFA73844.1
MTDQLGRILDKMTAPVCERYQSVGEVIKELNLAESNHSETESDVNPVNYWQKLVSRAVDK
GKQSVQQWFSINDGQVAEILATVSSQLPTTEALLIGKPQTGKSSIVRGFTGISPEIIGQG
FRPHTQNTQRYIYPNNDLPLIIFTDTVGLGDTDKDTEVIIQEIIKDLNTGNKRARVFILT

VKINDFATDTLKNIAQKLRQQYTHIPCLLAVTCLHEIYPPDMENHPDYPPNFPEINRAFD
EIKANFSGLYDRVTLVDFTLEEDGYSPVFYGLESFRDSLTSLLPEAEAKTVYQLLDEQMG
DKLGNIYRDTARRYILPFSIMATTLAAVPLPFTTMPVLTALQVSMVGILGKLYGQTLTPS
QAGGIVSTIAGWFLAQAIGRELVKFIPGFGTVIAASWAGAYTWSLGEAACVYFGDLMGGK
KPDLQTIQNVMQQTFQSKENTQKEE
>EFA73851.1
MKMKFVYILAGIALFLKMLFLSDGTSNLSGMSTIKDTMSTIVPAGISITEKVVAESGVVN
AVSGIIFRNRLYDTIFEVIVFTIAILGCNFLLASENPSCAIYQFKDRASITLARLGATIA
ALVGIELAIRGHLSPGGGFAAGVAGGTAIGLIAITSSYQWMQDIYQRYHAAVWEKISVLI
FYCVITGHFIGT
>EFA73855.1
MNTITTVWVGIPFFLGFMVFLVPQLNRHLALLGTLVSAAYGIELLIKQPEIQLNLLDSFG
VTLVADQLSGYFILTNALVTIAVVLYCWRSNKNAFFYAQTLLVHGSLNAAFICADFISLY
VALEVSGIAAFLLIAYSRSDRAIWVGLRYLFVSNISMLFYLVGAVLIYEKTLSFSFVGLK
DAPPEAIALIFLGLLVKAGIFVSGLWLPLTHSESETPVSALLSGIVVKASVLPLLRCAEI
SENIHQIVVIFGVGTALMGVCYAVLEKDTKRMLAFSTISQLGFILAAPAVGGFYALTHGL
VKSSLFLIVGSLPTRNFKELKSQRISTGIWIPLVIASLSICGFPLLAGFSAKVLTLKNIT
SWQFIAMNLAAVGTVIYYARFIFLPHKWDDVQDDKPAVKPELWIAIIVLITSLFIANIGY
LSAYNTDDIIKAIITIAIGWLAYYVVFQKLAPLLPKISLYIPRLVEKFEHLVGVMSLTFN
FVILDGIIMIGHLILRLTIWFLLTSDFSTVNIIIGVAIAFLLPRGYSSRAKFTEWLRVFG
QVLVAIVVAFKEAFEIILFPHHREEIIRENVKHKHSYLLIFMDIFLITFTPKTIVFNHNE
QGFYEVHQIKPGGKE
>EFA73858.1
MYYDEDANLDFLAGKTVAIIGYGSQGHAHALNLKDSGVNVIVGLYPGSKSIAKAEAAGLT
VKNVADAAKAADLIMILLPDEVQKTIYTNEILPNLEAGNILAFAHGFNIHFGQIVPPGDV
DVIMVAPKGPGHLVRRTYEQGQGVPALFAVYQDATGKARDRAMAYAKGIGGTRAGVLETT
FREETETDLFGEQAVLCGGLSALIKAGFETLIEAGYQPELAYFECLHEVKLIVDLVVEGG
LATMRDSISNTAEYGDYTRGPRVVTAQTKAEMKKILGEIQSGQFAREFVLENQAGKPGFT
AMRRQEAEHPIEEVGKDLRAMFSWLKKA
>EFA73862.1
MSSIWEENQVKSKFFRTFFIFLSLVGLGGCIYFSLFDQQPWLIIMSVVLMISMLITSQLI
SQHNRQFIPILFGGMYLVLFFLMISKSWVWELNEKFPVQPVAALIRQHVPPGTKIYTSFP
DARPSLDFYSDCQIIPTSLTDLSSRFYHQSYLLINNKQLVKINLKNSKLLGEYQEFTLIA
PI
>EFA73863.1
MYRHLTHLMTQLDGDAELILVDDGSQDRSLTMIRELHDHDGRIRYLSLARNFGHQVAVTA
GLNFVQGQAIIVMDADLQDPPELILSMIDKWHEGYQVVYAQRISRQKESLFKRLTAYLFY
RLLQRLSKVKMPVDTGDFCLMDKQVVDILNAMPERNRYIRGLRAWVGFKQTSVLFERGPR
YAGKVKYTFSKSFSLAIDGIISFSTVPLRLATYLGIISATIALIMILLVLYWRIFAPVSQ
LIGYSLITIAIFFLGSVQLICIGILGEYIGRIYEEVKARPLYTLKETGGFDPLNI
>EFA73864.1
MSLQEFTLLLFSVLISVTGQFLLKTGALKLGKVDAGNFITHILNMITVPELLLGLACYGV
GAIAYILLLTRVNLSIAGPAVSVGYIFQS
>EFA73870.1
MYQGIYNHPKKPNSALRKVARVRLTSGFEVTAYIPGIGHNLQEHSVVMIRGGRVKDLPGV
RYHIIRGTLDTAGVKDRKQGRSKYGTKRPKQTKK
>EFA73871.1
MIMRRVMRHGKKSLAARIVYDAFKTIEDRTGGSPLEVFERAVRNATPLVEVKARRVGGAT
YQVPMEVRADRGTALALRWLVQFSRSRPGRTMASRLANELMDASNETGSAIRKREETHRM
AEANKAFAHYRY
>EFA73876.1
MMNTILESDRRFGVLMVNPINGAIANVGCCAEIIHYQRLEDGRMEILTLGQQRFRVLEYV

REKPYRVGLVEWMEENPPALDLRPLAGEVEQLLRDVVRLSSKLTDRDIELPEDLPDLPRE
LSYWVASNLYGVADEQQALLELQDTQARLNREAEILTSTRNHLAARSVLKDTFDDIK
>EFA73888.1
MPKAGIIYNDVKPIAVRVAIELKDQLTAAGWDVCMTASIGGILGYSNPDSPVRHTPISGL
TPPGFDSDMGFAVVLGGDGTVLAASRQVAPSGVPLLTVNTGHMGFLTETYLNQLPTAMEQ
VIEGHYEIEDRAMLNVQVWRGDSVLWEALCLNEMVLHREPLTSMCHFEIEIGRHAAVDIA
ADGVIVSTPTGSTAYSLSAGGPVIAPGVPVMQLVPICPHSLASRALVFPDHEPVSIYPVN
IPRLVMVVDGNGGCFVLSEDRVYLRRSEYKARFIRLQPPEFFRILREKLGWGLPHIAKPS
SVELP
>EFA73889.1
MPSHGSRVLVIEPDETLASQLGFDLQEAGYEAIIATDANSGLHKCRDLQPALIVVDRMLP
GESGLSFCKNLRATGNTKPVLILMARDAMDDRVACLEAGADDYILKPYRSEEFSKLISLY
LKPDIDSSEQLRFVDLVLDIGTRRAIYNNKAIDLTMKEFELLKFFMEHPREVLTREQILE
NVWGYDFVGESNVIEVYIRYLRLKIEENGQKRLIQTVRGVGYVLREH
>EFA73891.1
MHHRNCLEVHNLQVEFSGDGAQVQAINGISFDLPQGQTLGIVGESGSGKSVTALTIMGLL
PYPGQVTGGQILFRNQKNAQPLDLLALPSREIQLYRGGDMAMIFQEPMTSLNPVYNIGFQ
LTEAIERHQNVTLAEAKRIAIARLQEVKLLPKDENIKQQYLENWHQTHLKTPLPSDYQLA
QLVKEYKMAMLERYPHELSGGQLQRVMIAMAICCNPSLLIADEPTTALDVTVQATIMELM
RELQKSRNMAMIFISHDLGLVAEIADQVAVMYKGKIVECGLASQIFTHPQHPYTKGLVAC
RPTLNQRPQKLLTVSDYMIVQRYPNGELIIKSKEPNIPREITPAEIETRIDDLTRKSPLL
QIHNLKVGFPVRGLLGGNKRYHIAVNGVSFDVYPGETLGLVGESGCGKTTLGRTLIRLIQ
PMAGKIIFEGENITNFSGTQLQTLRKQMQIIFQNPFSSLDPRIKIGAAIMEPLLIHSVGK
TKQQRQEIVVQLLERVGLSADDMNRYPHQFSGGQRQRICIARSLALNPKFIICDESVSAL
DVSVQAQVLNLLKELQQEFQLTYIFISHDLSVVKFMSDRILVMNEGRIVEVGTSENIYRH
PKEEYTRKLIAAIPRGNTNHN
>EFA73893.1
MDWIFYCWFSRILNGVRRGDYYSPLLTSVFGVDIRYAIGASLVSVIATSLGSASTYIKKG
FTNIRLGMFLEVATTTGAIIGALMATFISVKFLTIILAFVLIYSAYLSQSPKSEYQEVAT
PDTLAEYLELNGTYPISDGVIPYYVNCLPAGFSIMLLAGILSGLLGIGSGAFKVLAMDQV
MRLPFKVSTTTSNFMIGVTAATSAGVYLSRGYIDPGLSMPVMLGVLPGAFLGARILIGAK
TQTIRIIFSIILIVMALKMVYNGLTGGV
>EFA73898.1
MPMAVGVIETLGFPAVLAAADAMVKSAAVTIVYYGIAESGRLLVAVRGQVAEVKTAVAAG
IASEETVYGGQVITHYIVPNPPENVETILPIHFTSESEPFRYFLTCTGNFIYTGD
>EFA73905.1
MLYTHRRKFISSVDNPQFLPPVSRWTSLAGIFLVGTVATAIALASWVKYNVTVKADAVVR
PMGEIRVVQPEIEGTIKSILVKPNQTVKIGDVIAYLNTDDLLIQKKSVTRKYPTG
>EFA73906.1
MICSYKKSQLQENIQQGNLQILQIHAQVRILDRQILAETQVAQNAINSARVDLLRNQREY
QQQQVSTQGEFLSAQANWQKAKASLDKARADLNFAKMDKERYQELSQIGAVGRRELEQKA
LTVQQTQSILDTERKSLEMAKIKVQSAQVAINPTPAAVMIAQERIAQETARGVSNIASLN
KEKQALIERRVQLETQIKQSIKELEQVENQMRKSMILATSNGIILKLNLRNPGQVIRPSE
SVAEIVPDSSELLIRALIPTEEIQKVNIGQQVKLRVGACPYPDYGTLHGVVQTISPDVIT
NQPNNPGTTTNRMGYFEAMVKPASLQFGQGRHQCYLQSGMEVKADIISKQETALEFMFRK
ARLITDL
>EFA73931.1
MTDKGEISLHQKVLGSRRLSNYSWATIVTLGASGFSLASISSYLKVNLLIVTDATELIFV
PQGLVMGIYGIAGMLLALYLWLVILWDVGGGYNEFNQETGKFKIFRWGFPGKDRQIAIES
SIGDIQSVRISIKEGLNPQRALYLKVKGRRDIPLTRVGQPLSLSELETQGAQLARFLGVP
LEGL
>EFA73932.1

MPRLLPLPGYLGDGNLMILNLSHYGKFWVENFVFDIGAIGGT
>EFA73934.1
MKNPSSYLILVMTLLLGNPALLIGTGTSAIAQFSDCQKAVTQAQLNQCAAINAKTADQKL
NDAYNKVLAIYKGKR
>EFA73942.1
MKLWVDLAQAAKTDAIKDTVDYRSVISLVQNLLTTSKFDLLERLAGAIADAILQESSLTT
QVQVTLIKPAAPIPDFDGNIRIELTRSKSNL
>EFA73955.1
MNIFQENYQKPDLNYSLNYSFLFPWQFPQNNGQDNCCLEVKAEEGAYSFYLNRTILEKIA
QLKLQGISLDIPKGLLLNLWYYSIFDSAVDRDENYDKSADIYPHPQLSVLIYFFLVKTWK
ILTSQNRNKTIKFQCGFTFNSYYQSTENIMMQSTILFDGDIIQKISQNLISNGDASKIVN
SHYWLTEQVTKCLRSNLNSVYWFVSAIFPAAVITWKITSGMSLWLSILISTLGWIIIFLV
LGTLGLLIISQLKKLLVRIKNKSVDKFIEPVISWLVWVGTNLVISINVFSRDSVLLIANS
LFMLLLPNLLKYFLPKASKYLFKLFMK
>EFA73956.1
MLAKDSNSLESQPNQGTAGRAKDLLGKFVDTVGVIVRDITSLEVNTIVVNNISGSKFNPW
QSYYNIYAISEPNYFDSKNIVSELEERYINIFLQLEREYIYTFLTKELESPSPKAQVVQG
YRSRLKYIDENRLNSDGTKASQIVLPSPFDQESQVENYQKIAELVTDDKFVLTLRKVSEM
KAALDGGDVTSENVDTIYAQTIIQLDGDIITRYHRELFGLKESDKDLIMKVHNDGVVSGE
KQWREVIDFLINFIKGIAS
>EFA73965.1
MTITVKDVSTCKWDFFVWNADIMTQPNYGWQWKIGTEEFAIVFHMFCFAFNQQNDGTSPS
GNVEWFVGGV
>EFA73969.1
MNGLPRRYHITTFGCQMNKADSERMAGILEDMGFEWSENPQNANLIVYNTCTIRDNAEQK
VYSYLGRQAKRKHQEPDLTLVVAGCVAQQEGQALLRRVPELDLVMGPQYANRLQDLLDSV
FAGNQVVATEEVHILEDITQPRRDSRVTAWVNVIYGCNERCTYCVVPNVRGVEQSRTPEA
IRGEMEILGQQGYREITLLGQNIDAYGRDLPGSTTEGRNLHTFTDLLYYVHDVPGIERIR
FATSHPRYFTERLIKACAELPKICEHFHIPFQSGDNEILKAMSRGYTQEKYRRIINTIRS
YMPDASISADAIVGFPGETEIQFQNTLQLVDDIGFDHLNTAAYSPRPGTPAALWDNQLSE
EIKSDRLQRLNHLVNVKAAQRSQRYLGRMEEVLVEDQNPKDQSQVMGRTRGNRLAFFQGD
INQLRGRMVRVKIQEVRAFSLSGELVE
>EFA73975.1
MGFNLYETDFYEWTLKQSRLLKQGDFPQLDIINLVEEIESLGKQERRELENRLAILMGHL
LKWEYQAEKRSKSWRATIREQRRIVQKLLSQNPSLKPYLFELIADGYESGKDLVVRETPL
DYLDLPEKCPYTPEQILDREYPRE
>EFA73976.1
MKTLVNLPTKPRAEDSFAITLAPLLPEEIYTAAQDLGNGAVVLMSGVVRNQTDGKPVVAL
EYQAYQPMALQVFYQIAAHIRHQWSNVNRVVIHHRIGKLRVGEVSVLVGIGSPHRSEAFA
ACQYAIDTLKHQAPIWKKEYWQDGSSSWISIGLCQQEC
>EFA73981.1
MKFLNALIEAEGIQLDGAPGANGRGGRSASYRVSVQSNNNLLIGSAYTKQMNLKPGDEFV
ISLGKKHIRLRQVEPEERDDDDQLEEVTA
>EFA73984.1
MNLPVVLDIALGLVFTYLILSLLASEIQELLATVLQWRAVHLKKSIEIFLAGDIKNSDAK
DVLDLVNRIYNNPIIRSINQETKGFLSTLPRKMTWKVADIVSSVKKLVSRSSRENKIFAN
QNSGPSYISSDSFVSGLLEELKLPKIIHSLVEVRLENFKIQHLRAIKLILTRSLRQIASN
ELSTNVTHDINDDFVNLDSEYRQIVDDFKNQKFDVDTSMNRMQDSLNKYINNFQANIENN
HPALMETHKRLQTLQNHIFPSLEEAITVAGLKPSISEIIQLMETGKAGYNEIQTTSQLRD
GERYVTIRDLIDSLPPGMKQNIATMAKRAQYKAKTTEEGIRVLRQEIENSFDSSMQRAGG
VYKRNAKGVAILIGIVIAFGANADTFYIIDRLSKDTALREAIVYKAQQTIDQQVLDPNLK
NIDTNQILEDISLPIGWSEKNLNRATGY

>EFA73985.1
MIMGWLVSGFAIAMGAPFWFDLLGKVMNVRNTGKGNKGTGN
>EFA73988.1
MTTVTAKRRSSNNGSENFILAFINKQMPEILPPLIAIVAFLVVWQVFSLTGGTLPGPIQV
VQDTWELIIYPFYDRGGIDKGLFWQVFASLQRVAISYTLAAVVGIGLGILIGVNTTMSKA
LDPLFQLLRTVPPLAWVPISLAALRQNEPAALFVIFITSLWPILINTAVGVKEIPVDYNN
VAKVLQLSQKEYFFNVLIPAALPYIFTGLRISIGLAWLAIIAAEIVMSGIVGIGFFIWDS
YQANKVSEVILALVYIGVVGLLLDKFMAWLQTRILPEQK
>EFA73991.1
MLQRLAHQPLTLGDVLETTSQRGFILVIALLVLPFLFPMPPGLTGPLGSACLLLSLQMLL
GRRSPWLPKKIANYQFPRVFAQTILQNLSRVTRLLEKIARPRLTKLANHDITWRCNGFCI
SWLAILLISPVPLTNPIPTIGILLFAAASIESDGLLICICYVLTLLITLIFYLIVYGVLQ
FPGLIT
>EFA73997.1
MKYWRETLAIAQRILTELLRRRRSLIFWTIFPVSILILNGFVLSERGELAMEVALEKAAP
SSLVGAALFFSCLGGTVATVVAEREQQTLKRLFISPLSGTSYFLGIFLAHSCIGIGQTLL
VYIVAGFWGATFQGSILLGIVIIFLSIIGYVGLGFVLGTQLARRTEDVNALVATFGVPLL
ILGGTFFPSSLFPKTLLDIAKYNPIYHMNEALLGVSANGEKFSDVSLHLRFLFIFCYTHP
RLWLDIL
>EFA73999.1
MLSPNMATTYLPPMGERISMEEVISPTGRTITLIRS
>EFA74000.1
MFAASHQLLWSMIGLLLTIGGTFLEAYGTTLPWSWSQQGIKTFPLGASYQIAAVLFVGCL
GVKVLVPFPKLLI
>EFA74001.1
MFRGKSAGALSQIAYLVMGLTLLPVFAQGGGIGYVKLPNLVTYWGLFQEHGFADILPLKP
DLD
>EFA74003.1
MNGIYSLSDVTKKGLGFWGGCWELMVWDLVKLIYIKINWEFYA
>EFA74006.1
MSKETLFALSLFPYLGFLWFISRSPQMPRLALYGFYGTLVFVGVTIPAGIYAKIAYQEAL
ANIDWLHGSAELFLTLANILIVLGFSQAVQNLETKRK
>EFA74024.1
MTLHLIRGKIHRRNTVEQLAVVGPDSLFIALLTAIFVGAVFTIQVAREFINFGAGNLVGG
VLAVALTRELSPVLTAVILAGRVGSAFAAEIGTMRVTEQIDALLMLKTDPIDFFSHTSSF
GLFINATNFDLAIPGYWNVGRLIIATNLYNLSDTVFLDSARNFLDLRDIISAMIKAACFG
ILIAIIGCSWGLTTTGGAKGVGQSTTTAVVTALLVIFISNFFLSWLMFQGAGNPSIRP
>EFA74028.1
MSRCVNNWGNLFTLINGEYVNTPTIIDSLNPSDFSQVVGKIGLISVEQAEEAMQAARAAF
PHWQKTPVPQRADILRRAANLMEIRRAELAAWIVLEVGKPVKEADAEVSEAIDFCRYYAQ
EMERLDGGVVYDVAGETNRYIYQPKGIAVVISPWNFPLAIACGMTVAALVAGNCTLLKPA
ETSSVITAKFTQILLEAGIPKGVFQYVPGKGSQVGAYLVNHPQTHVIAFTGSQEVGCRIY
AEAAIVKPGQKHLKKVIAEMGGKNAIIVDESADLDQAVVGVVHSAFGYSGQKCSACSRVI
VLQPIYETFLSRLIEATKSLNIGAAELPSTQVGPVIDSQAKNRILEYIEMGKKEAKLVLQ
LESPSQGYFVGPVIFAEVPPQGAIAQQEIFGPVLAVIPAPDFHQAVEIANSTNYALTGGI
YSRTPSHIEQAKRELEVGNLYINRNITGAIVARQPFGGFKLSGVGSKAGGPDYLLQFLEP
RTITENIQRQGFAPIEGAE
>EFA74029.1
MALKTQNSNYEVKTQEIAQQILTHTQEGRSFFAALRDQMRWDDKLLGWTMDNPGLRVQLF
RFIDTLPALHSKVEIASHLQEYLGDDSVELPPALKSLLNFAYPDSMPGQVAATTVETAVQ
TLAHKYISGENIQQVIKTVEKLRKEKMAFTIDLLGEAVITEVEAQSYLEKYLELMQQLVE
ASKKWHHVPIIDEADGEIIPKVQVSVKLTAFYSQFDPLNAEGSEAKVSERIRILLRRSRE

LGAAVHFDMEQYAYKDITLKILKKLLLEEEFRQRTDIGITIQAYLRDSKEDVQGVIDWLK
QRGYPLTIRLVKGAYWDQETIKAAQKHWHQPVYNDKVATDANFEAITQLLLENHQYVYSA
LGSHNVRSQARAIAIAETLKVPRRCFEMQVLYGMGDKIAKALVDKGYRVRVYCPYGDLLP
GMAYLIRRLLENTANSSFLRQNLENRPVAELLAPPIVEETPSPENPTQDFRGAADIDYAE
EQKREDSRLAFEQVRQQLGKPIYPN
>EFA74031.1
MDYRDAGVDVEAGRAFVGQIRNLVHSTFRPEVLGGLGGFSGCFQLPTGYHEPVLVSGTDG
VGTKLKIAQILNCHHTVGIDLVAMCVNDVLTSGAEPLFFLDYVATGKLEGEQLTQVVSGI
AQGCKLAGAALLGGETAEMPGFYQVGEYDLAGFCVGIVEKSKMLDGSQVQIGDLVMGLAS
SGIHSNGLSLVRKIISDGGFAWDHTPELFHGKTLGSTFLTPTRIYVKSVLSALHQGLEIH
GMAHITGGGLPENLPRCLGSNQSVKVHPASWPIPPVFKWLADFGSVGAEAMYNTFNMGLG
FVLILPPTQAQQAITHFQSQHIPAFTIGEVVGGSGELIALFDS
>EFA74037.1
MGERLISNLNIIATYNSSEFNLQFKQKHKKRLIIITIL
>EFA74048.1
MVLGLIITLFFVLLAFMAPVWQNWGWLSDPKELLTNPIHQPPSGKYWFGTSRLGYDVFSR
TIFGAQAALQVVILATGLSMVVGVPLGMISGYLGGKLDKTLLFLMDSIYTLPGLLLSVTL
AFVVGRGILNAAIAISIAYIPQYYRVVRNHTVSVKTEVYIEAAQAMGASTWTVLSRYLFL
NVIQSVPVLFTLNAADAILVLGGLGFLGLGLPEEVPEWGYDLRQALEALPTGIWWTTLFP
GLAMTIMVVGLSLLGEGLNELIHPRMQKRF
>EFA74050.1
MEESFQGVNPEYFLKQLNTFPLLGKQIKLWLKLGFLNHQQFSLPSLLLGAALGELERRIN
WSLTSLIADSNQAGITTSSCDVIPHFVKHRGGFVLVHQELEVIILCEHIITQWLSEVGLE
LKGEGIRVSHSLYDYQGNVGCNFLGFYIRQFVAQDKSHDKSSQNSDLGSPHYNTSYNTLI
SISKESLEHHTQSLGAIIDQHRSAKQSVLISRLNPLIERWTKYYSPLISDRIFGKVDFEL
HSKLRAWSRRRHNQKGKRWISKKYWSICKEGWRFCHQNSEGKTYELMKHQYIHQDLKLRK
RERRSE
>EFA74051.1
MVVFAIYNQALTLLISMAMSPEWDAKFNLGLHEANSDISCYNAVRSIRREVVKNLSMF
>EFA74052.1
MAITEISSAKTSFCSGEKDLAPWFGGNTPFTAIHLADQDLINSFWSL
>EFA74053.1
MTKSTVENAENLVIIGSGPAGYTAAIYAGRANLKPIVFEGFATGGLPGGQLMSAIR
>EFA74054.1
MKTKLQVKLLQYILIRDKTAQGFTLLELLVVIVIIGILSAIALPSFLNQANKAKESEAKT
YISSFNKAQTLYRLENTGFATTLNQLSIIIPPSTEFYNYTIGGVALLPTLQQVLRTLTPS
KGLVVE
>EFA74055.1
MSATGQTQSVACQTEQVQQIHPVVVPILDSTQAKCDDTKNMTTMK
>EFA74067.1
MAELVAFCQINGIPGDSIGDFVAALGEYAGESLIRMVALICVDGILPLGPDFVQKSLSFL
SSMNPQELDQNSTFRSIKDAIPGNGTGGKIDFIGQSLDSVQGWMNGIVSANALTAQKVLN
NISGFLEFADDKLDYVAAFLDVSTNYYEHTGTQTLARRLIERALAEI
>EFA74068.1
MSKPIFELVDELPTSGLTVSLLNALDFVAPGEWQNTVGFVNTIKTVTGETDEDLIQAIGE
RAVYLFNDKSQGYQTALWLYQTVDGTDKALGAAALANKVGEPYPYLVS
>EFA74072.1
MLNEKYAQEIKGFHSINVPSEWELNNIGNLILLGGHKGSVSIQLMSPASGNNTI
>EFA74076.1
MNTNELKFMLKLLGYQTYRGNWSLFKDKEKTKVCQELERREYVDYSREIVSAQILPAGKA
LLQIETSQLPITAEELKVLEKIAKAGKKIAPSAIKITKLKVRSKRGNPKNPR
>EFA74077.1

MIKTELKIKKNQAEVWLTDRGLEFLRSEYIPSKTYNPAISLELLGNYIRFLRKNAIPADP
SGAKPVVDKITDRKITDEEILQTIKNLDRELGTENYLPIFHLREKLQPPLLRDEVDQALY
RLQKSDKIDFSSLQEVTAYTPEQIDAGIPQNIGGQLFFIMVN
>EFA74079.1
MYNNRGEAKTLLPRGIQMHISDGERFMISMVSDLARRMAIANPKIRKSIERQGDYFN
>EFA74082.1
MHYLTDVTEIYRQISQLTRFRTLWLDTEIANWNTPYPRLSLIQVLAYPSDLTGEFAYIFD
VLDKPDLTAYFIQHIMVNSNIQKVFHNADFDLKYLGKNQAQNVVCTFKLAKKINRKVLQT
TNLKLKNLSC
>EFA74083.1
MGTSDWGERPLSSRQLKYAAMDVIYLAAVHHRLLEISDPNAVSTIFSISFDSSFNGNFDN
SNDSNLNNWDNRVNLERENSPFSPTKLRLAFECPRLFYLNYKFNCKSIFIPADSTPGIGN
IFHQLADNLIDVLLTDPSFTNLFIPPSDQLDVEKLAAQIQRLFYQISFFPYLQTTISQEK
SKGVLLYKVWARLQELIKKITKLLVDNRRYCTAKALMANTFINEDRNLEYDFNLPDGTKE
LIRGKYDCLVFSFASQRLSVIEFKTYQPMDISGQLAQTAIYSYMLSQRKKMPVDSAVYCF
LPEFQEYTYTWEQLENTTHQIIHHKLLQMRQWSGWESPQPNPPPMTTQTHLCQICPEQQR
CQTFFAAEVTTPQSTQAIQSIQLPKINEIPEPKIVDANAIEVKLVNTLASFGIGVEYQGT
MVGPAFIRVKLKPHLGVKVNSFVEIV
>EFA74084.1
MLKLSKDLQVQLELENPPLIASQAGYVSVDLPRKDRQIARFEDYIQRNFYLQPLS
>EFA74085.1
MNIDGHLLEADLSDSNTCHFLVGGTTGSGKSEFLRSLLLSLLYRHSPQHLKIVLVDPKRV
TFPEFERIPWLYSPVVKDSDRAVEIMGELVAEMDSRYQKFELVKCPNITTYNQNSGKILP
RLVCIFDEYADFMAEKEIRSVLEQSIKRLGAMARAAGIHLIISTQRPEAGVVTPIIRSNL
PGRIALRTSSAADSQIYFRCQDITSSRFIG
>EFA74092.1
MLERGIYLAPSQFEAGFTSLAHTEEDINHTLEVVKEVLSNL
>EFA74093.1
MVDTTIQTTKSQEIFAAAQNLMPGGVSSPVRAFKSVGGQPIVFDKVKGAHIWDVDGNKYI
DYVGTWGPAICGHANPEVISALHQALEKGTSFGAPCVLENILAEMVIDAVPSIEMVRFVN
SGTEACMAVLRLMRAYTNREKIIKFEGCYHGHADMFLVKAGSGVATLGLPDSPGVPKSAT
KGTLTAPFNDLEAVKALFQQNPGEIAGVILEPVVGNAGFIAPDAGFLEGLREITQENGAL
LVFDEVMTGFRIAYGGAQAKFGITPDLTTLGKVIGGGLPVGAYGGRREIMSMIAPAGPVY
QAGTLSGNPLAMTAGIKTLELLQKPGTYDYLERITQKLVNGMLTIAKEAGHSVCGGSISA
MFGLFFTSGPVHNYEDAKKIRHSQIW
>EFA74094.1
MLSPQSLSLESSIPVEKIRYDQHGLVPAIIQDYLDGTVLMMAWMNQESLQRTLTTGETWF
WSRSRQEFWHKGATSGHIQKVKTARYDCDSDALLFSVEQIGDIACHTGERSCFHQLDNQV
GKIIPPPPDSLSQLFQVICDRRDYPAEGSYTCKLLAGGDNKILKKIGEESAEVVMAFKDD
DPDAIAGEVADLFYHTLVALAHHQVDLRSVYRKLEERRK
>EFA74096.1
MMRQAGRYMKAYRDLREKYPSFRERSEIPEVAIEVSLQPWRAFQPDGVILFSDIVTPLPG
MGIDMDIAEGKGPIIFSPIRTQKQVDGLHPLDPETALPFIRQILGALRQEVGSASTVLGF
VGAPWTLAAYAVEGKGSKTYSIIKNMAFSDSNILHQLLDKLAESIAVYIRYQIDCGAQVV
QMFDSWAGQLSPQDYDIFALPYQKKVFELVKRTHPQTPLILLVTGSAGLLERMATSGADI
LTIDWTVDMADARRRLGNNVKVQGNLDPGVLFGSKQFIRERILDTVRKAGNWGHILNLGH
GVLPETPEENVAFFFETAKNLHVSV
>EFA74101.1
MIEREKKRAGLVAKYAAKREALLEEFRTTESPLEKLEVHRKIQQLPRNSAPTRRQNRCWL
TGRPRGVYRDFGLSRNVLREWAHQGLLPGVVKSSW
>EFA74105.1
MDSLAERLREDRVKAIIQPMLSGSDLPDTPEDDRRFLLDLGLVKRSPLGGLTIANPIYQE

VIPRVLSQGSQDSLPQTYGREN
>EFA74106.1
DVKNLYEQHTQATGQIFTPDAINHAYYLTQGQPWLVNALARQATEFLNTDPSVPITVDLI
NEAKEILIQRQDTHLDSLAERLRENRVQAIIEPILSGEQLPDVPQDDIRFVLDLGLCRQD
TGRGLEIANPIYKEVLPRVLAYTPTMSLGVIEPEWLSPTGELVPEQLLAAFLEFWRQHGE
PLFKSTPYPEIAPHLVLMAFLHRVVNGGGTLEREYAIGSGRMDICLRYGQVVLAMELKVW
RKGSKDPLTAGLKQLDKYLSGLNLKTGWLVIFDRRPNLPPVSDRTTTEMAISPQGRSITV
IRG
>EFA74113.1
MLTTLIIVIFTGVLREQYGQNSLSDSETSQPVVVNPYPKQLLQAQSKTIQPKSIQIQNFS
QSLIERIKQKKVVKKYITTNNFSKYQPRLAAAQVHPSNYGERFSRDTKGMAVSNQPIIVL
HETTYSASSAINYFQNHNVDEDIQASYHAIIARDGTVIYLVPPDKRAFGAGNSVFKNTDG
IIETVQTNPKLAPSVNNFAYHVSLETPPDGWGKRDIREHSGYTHEQYNSLAWLIAQSQVP
DERITTHRAVDVANGKVDPLSFDFDRFFKKLHSFRKLYSIAQSHS
>EFA74118.1
MIVPTGAGAAMSAGRKILALTAAKATKNTKTTSRGAMY
>EFA74122.1
MENLRKTLCQLADGVLPLQLISNLLSQVQLQGQKELRLKQIQLQNQVARDILTTRDKRLL
SFLNQLNLELETITSIENFLVQDIDSLYVDLSTKLYDSEPVKANNYQECFLHADEETLTS
LDRLVYSLPTVQNNAKNLLLELRNCLELIVSLDRQIQAAAPPEAYIKLQKIRELAEKEWS
RANTNLEILNRQFINLTTTIDKTKKELNSYTNKNLKYRSNQHLIDSVHRVQENLKLFKEK
LTLRKLNKLEEEVKNCFLYLLHKSDLVYRVGIDSKSFQLSLYDLHGKLVPKHRLSAGEKQ
LLAIAFLWGLAKVSGKQLPVAIDTPLGRLDSSHRNNLLERYFPTASHQVILLSTDTEIAR
KEIAILREHQAIAREYVLEYDSGKRETSIKEGYFW
>EFA74123.1
MIFLELVLQNFGPYAGKQVINLDTRIDRNNIRPIILLGGMNGGGKTTLMDAIRLALYGAR
AQCSTRGNLSYGDFLTQCVNNKADPINKTRIELVFEHIEDDKPVRYRVVRTWEKNPKDGK
DSLGILGEDETWPQSLANIWDEYIENILPLGISNLFLFDGEQVKELAEQEVPTPIVVDAI
NGLLGLELVDKLSLDLEILVNRKKKENADDRDLAKLEELENRLHEQMEQKNSQKAQLQDL
EEQGKKFKV
>EFA74125.1
MAKIPQDNRNIFPQYPVGQPPQLPSQIMVQQTKMGNVTAYLGSVTLEWFAKKVKFASTLP
ILQNKYNPVTDNIEIDADSIEQIQERSLDWSRQSSLLQYLAIHRNHKFPPVLVVISQPWV
NDEKSDLWDGEGRAKKATTDFIPSDANSHIGLLNVAEEDTSIYVLDGQHRLMGVQGLLEL
LENGRIARYNRDKIPTDSYIDLSELVDQYRIDVDYLKQLPQEKLALNLFAQSIVEKPTQK
PNRESDQFLCM
>EFA74126.1
MNLMATPLTKGQLIQLNEDNGFAIVARKIAVSHPLLAHRENRKPRINWNSATVAANSTVL
TTLQALQDMSTKYLGYKFPHWQPQIKGLIPMRPDPDQLGEGMREFQQLFDHLATLPSYKI
LDHEDTPVLRRFSFEKGGGEGNILFRPVAQVALAEALGILVFKNGLLLENIFKKLQKFDL
QGGFSQMEFPNSVWYGVLYDPNKKRVQVSKKELAVKLLIYLLGGIKDRMEIAQLRKALAS
ARTREDRAMDFDGNWVSDQEVGLPSI
>EFA74143.1
MAAGGEAVQVFSDLNELIEVSEQWPAQKRLRAGERGGGKVAGFELGNSPLDCTAELVEGR
RLFISTTNGTRALKRIEDAPIVLAAALINRSAVVEFLLEKQPQTIWIVGSGWEGSFSLED
TVCAGAIAHSVWQKTGLELDEIAGNDEVTSAIALYEQWQDNLIGLFHQASHGKRLLRLEC
VEDLKYCSQVDILDILPLQKQPGILTSHNAH
>EFA74145.1
MIKNLDKKTWAHAISQPAKEFPLSQLPIISGKIPQGLRGTLYRNGPGRLQRGNICVGHWF
DGDGAILAVHFRDEGARAVYRYVETRGYQEETQADKFLYGNYGMTAPGMIWHKWRRPVKN
AANTSVLALPDQLLALWEGDHPHALDLENLETKGLTNLGGLTEGKAYSAHPKVDYQTGEI
FNFGVNIGTNFGIGSPGSGILNVYKSDFTGRIIKHAQFDLTQFSLIHDFVLAGKYLVFFI

PPVQLNIWSVVLGISSYSQALTWQPHIGTQIIVIDRENLSLVSRGETESWFQWHFSNGYV
DNRGIVIIDFAKYADFQTNEYLREVATGKVKTTAKTTFTRVELNPQTGRIKQLATLLNRT
CEFPSVPSKNVGKFSPATYMSIFKEGTETQGEILNSMAKFDHENGILTEANLGLNCYPSE
PIYVEDRTNSDRGWVLTVVYDGNSHSSKICIFDSHGMNEEPICQLALPSVIPHSFHGTWK
PDK
>EFA74146.1
MLKMQDVKTEKAGWSLTHRDPQFIAKMMPLLAILYNCYFRVETSGWENIPEGKILVVGSH
NGGLASPDTSMMMYDWLRRFGTERLIYGLMHPKVWEVFPPAAEIAMKGGAIRAHPKMAYA
AFRAGASVLVYPGGAEDVFRPHQMRDKIYFAQRRGFIKLALRENVPIVPAISWGSHDTLI
VLTDLYQIMARFHQWGMPWLFDVDPIVFPLYLGLPWGLALGPLPNIPLPAKIHTRVCPPI
IFERYGKDAVSDVSYVNECYELVSNQMQRELNQLIKQAS
>EFA74148.1
MYFIASSIASSSKLPVSAPYSFSLKLPFVRENGGDYNVNSFLPGCPKIPLHVELRGYQKQ
AITSWFANNGRGTLKMATGSGKTITALAIAYELYKQIGLQVLLVVCPHRHLVTQWGRECE
KFNLKPILAYENIRNWHSQMSVELYNLGSGYQKFLTIITTNLTLIGEGFQSQLKYFPPKT
LIIGDEAHNLGAPRLEESLPRSVGLRLALSATPERYFDEEGTESLLEYFGPVLQPEFSLK
DAIDQGALVHYLYYPVFVELTELESINYLRLTKKIGRCLQNREIKETVSFEDMEGIKSLL
IKRARLISSAENKLVALRHLMLNRLNTSHTLFYCSDGSQEGGNHSYQLEEVTKILGVELG
YKISTYTARTSLPEREVLRKQFESGDLQGLVAIRCLDEGVDIPAIRTAVILSSSSNPRQF
IQRRGRVLRPHPGKERATIFDMIVSPPDLDREAMEVERNLLRKELKRFVEFSNLADNAGE
ARMKLFNLQKRYSLLDI
>EFA74150.1
MLFSKASTRTRVSFTVAMYQLGGQVIDLHPNVTQVSRGEPVQDTARVLDRYLDVLAIRTF
AQQELVTFAHYAKIPVINALTDLEHPCQILADLLTIREVFSDLTGLTLTYVGDGNNVANS
LMLGCALAGMNVRIAFPQGYAPNQDIVEKSRVIAGDKTEVMLTHDPVIASKNAHILYTDV
WASMGQESEADNRLPIFQPYQISQDLLSLADPQAIVLHCLPAHRGEEITDEVIEGSQSKV
WDQAENRLHAQKALLASILGAS
>EFA74156.1
MTDQDISGGFSPVETTKPEQNPRLSPDSEVQVNQSSGRNAIQHSDRNKIWELGMWKKSWI
WWFLVLAFIPSTMGIISVAILLKLPSAPNCPRIFWPLASASMRVHCANLAASKQTVNDLL
QAIALVKDLPQDHPLRGQINDLLQEWSRDIINLAEKSFQSGNLEEAIATDQKNSRKFGRS
SVCGRENTQMAIYLVNS
>EFA74166.1
MNKDISLLLVEDGQFVEASTEVVKDIFCQTSGVVEITQKNDILREIVIKPGELLMVDDPE
AVLGRDNTFIHPQEELLGTTVTEMRYIQYVESPEGPALLSRPVVEFQVSSHPNLPSTTSV
SQQTGRSIQLRAVQRLPYKDGERVKAVDGVELLRTQLVLEIEQEGEQDHASPLAADIELV
PDSENSEIQRLQLVILESLSLRRDMAADATQGSTHTTLEVEDGITIPPGSVVARTQILSK
EGGIIRGVRPGAEAVRRCLILRDTDMIVVKTNLPPTVKKGDLLVQGTEIAPGITAPVSGQ
LVDISHQLPPNNNQQEQLVEAAYSLKIRTGRPYRVSPGAVLQIEDGGLVQRGDNLVLLVF
ERAKTGDIIQGLPRIEELLEARKPKEACILSRRSGEVKVVYGEGDENLVSREAYSVKVVE
SDGVVIDYPLGPGQNLIVPDGAMVEAGQPLTDGPSNPHEILEIFFSLGSEDGLYACASHA
LQKVQSFLVNEVQMVYQSQGIDIADKHIEVIVRQMTNKVRIDDGGDTIMLPGELVELKQV
EQVNEAMSITGGARAQYTPVLLGITKASLNTDSFISAASFQETTRVLTEAAIEGKSDWLR
GLKENVIIGRLIPAGTGYNTYEEVGAIEDYGTDVATGVLDEVDDPLDIVLDDRTAKLYSL
DNSGLDGTYDDSYDHDNTYGSNHSTVLEDEDEDLITDEVITGVDEDEDEDDYEDEDEDDF
DIDVDL
>EFA74176.1
MWSQHPAPSLEAIVPWTRLFCDRRDQFQNDQDATKDFQQKLAEFLKALFITTEPKELDNE
IQNWLKLAAFVKRKRDIKVGGDN
>EFA74178.1
MFLYVISYDIPNDKRRKKIADLLEGYGQRVQYSVFECQLNTKKYNDLRCRLRKIVKLEED
NVRFYPLSRHTLTQVEIWGMGVPVIEPPGSIIV

>EFA74179.1
MGFEGTGAAQYFSVFSECLTNPDFVFSGRSRRPPGNPVNAMLSFGYQVLWNHLLALIEVQ
GLDPYYACLHQANDGHAALASDLIEEFRAPLVDSLVMWLINRNIMDVKGDFQFKNGGCYL
NDSGRKKFLRGFLQRMTEEIQTNDGKQPKWDLLTQQVRNYKQFVYNPSHHYKPYQID
>EFA74180.1
MQTLYISEQNCYVCLQKETLIVKQGDTVHVEVQLPLLEQILIFGRSQITTQVIRACLWRD
IPIAYLSRMGYCYGRILPISRGYRQLSRYQQELSPVDRLITARAIVKGKLKIVEFY
>EFA74182.1
MYISLTKLFLQCQKNLSHPYTEPLAFERLLLLIATLLKYPGVGSPDFLEFSNNKNHDAIG
VVKIYLQQLAKNLNIKLPDNYPAVDTLRKDLKTLRRYGILDQRMYRWGYYLGTGALSITE
LKVAFNTLASQAQYQGDAQTRRIYETLSKRLRSLDRELKGEFFYPIRQHLNRAIIHTDPE
EMAAKGENRDTLFHQLPLLEQAISQGQAIEISRSKSFYGSGNIGRFQVFPLQLIYHDIAW
YLLYELCEGGHLVMGRINRFNSYCSLVKIPARGIEKQRESLAKAYQLLENGWGLNLGEPE
PQQLELAGNLKFIEVEVRFYPPISNFIVEGERRHPNQKIVVGEPDKNTGEYKFIEYSIKL
PPRSLNEFIQWVYRYMDKAQILSPPELIKQHHQLAQNLYNRYQKV
>EFA74197.1
MSFANNKKTNEYKELKEKPHWQGTTVTPATKTTTRSRDTKSQEPVQGADMSDDLNRGRVA
IFIDGLNLFHAALQIGIEIDYVKLLCRLTQTSRLLRAFFYTGVDTSKEKQQGFLLWMRRN
GYRVVTKDIIALTENGKKPKSQCGNCCGYDYLSSLL
>EFA74198.1
MVKNPNLNVEIAVDMITLAPYYDTAVLVSGDGDLAYAVNAVTSLGSRVEVIGLQTMTSDS
LIDVADYFIDFDSIKQYIQKDSQFGYNYRSSPTSHL
>EFA74206.1
MEGWSWEDAAYMTVITLATVGYGETHPLGSHGRLFTIALILMGVINIGYIVNRFTEAIIG
GYFQQGIKVRQQRRLMESLIDHYIICGFSRTGKQIAKEFQSESVSFVVIDSKIESVQQAE
SQGYIVYQGDATLDDTLSKVGIQRAVCIVAALPSDAENLYTVLSAKTLNPQIRAIARAST
EESLQKLQRGGADTVISPYITGGKRMAAAALRPQVLDFVDGILSGTDRQLYMEEFLLDGD
CPFLGQTLQKAKLRSQTGALVLAIRRSDGNLIGGPTGDTILISGDTLICMGTAEQLRGLN
KVLAPINSRQLRRPKHI
>EFA74210.1
MLVKGVNQNVNALGYFGYAYYKKNQDKLKLVAVDNKKGPILPSPETVGKSKYQPLSRPLF
IYVNLWSGKHRADLYKFVDFYLKKAPAIVNSVGSVPLPKEAYNIGYVHLHNGKAGTVFGG
KSEFNLTIGELLRKQKEF
>EFA74211.1
MKPTAVRLLITVGMLTLAASCTADVNKNSSVEATKDVNSQTASTIRIDGSSTVYPITQAI
VKEFSKDGKNPTSVQVSFSGTGGGFKKFCRNETEINNASRPISAKEMAECNKNGVRYIEI
PVAFDALTVVVHPQNNWVKDITVDELRKIWEPPIPGENFPVESSTCFLARPPIKIIRTR
>EFA74219.1
MFKRLKDHQKKVLINGRWVVGDASCKYNAHSEIIRCAIKPDGPCNSCGFKE
>EFA74228.1
MNQPIQLSLEQQFNVYSFASQVKEMSHEQAQEFLVKLYEQMVVREATYKELLKHQWGLDL
GSMA
>EFA74229.1
MGGHAGGEEASRIAIQQIQAYLTNQWDSPENTQSLLRLAISQANNSILQDQLSHPERSDM
GTTVVVVVFRSPEPPICAHLGDSRLYLLTNSQLCQITQDHTWIAKAMEIGEISPEEARSH
PYRHVLSRCLGRADLSDPEVQSFKLNPGDRLLLCSDGLTEELLDEEIFQLYQNLDLGQTA
SSLVQAAKAKGGKDNITVILVAAEKQE
>EFA74233.1
MGPVLRVLFSIPIALVYLRWGNRASWMAAITSGLLLSVLTGPVRSLLFIMPFAFLGVLLG
ASWHRRVPWFVSITLGTILCTLGVFFRLWLLSILSGEDLWIYLTNQVTEILQWLFLNLRI
LTPPSTLGVKLAALLLIVINNLIYMFIVHLAAWLLLERLGNSIPNPPHWIQTLMNYED
>EFA74238.1

MAFSLAFIWGFGYFVPSAIALTQQQKLVAEAWRIVNRSYIDATFNNQNWERVRQRAFKQP
LGNDQAAYKVVRDMLKSLDDPFTRFLDPDQYRSLQVNTSGELTGVGLQIALNSETGILEV
ITPIQGSPAQRAGLKPRDRILQIEGLSTENITVDEAAARMRGPIGTVLTLLIGREGQPNQ
EVVLVRDRIELNPVLADLRLSPEGIAIGYICLSQFNANAALELANAINSLEERGATAYIL
DLRNNPGGLLQAGIEVARQWLDSGMVVYTVNRQGIQGSFEAFGPALTQDPLVILVNQGTA
SASEILAGALQDNGRAQLVGETTFGKGLIQSLFELSDGSGLAVTIAKYETPNHRDINKLG
IKPDVEVKQSVISREEVGSLDDQQYQVALKILNEKLLIAESLKKSN
>EFA74239.1
MASVYDWFEERLELEAIAEDVTSKYVPPHVNIFYCLGGITLVCFLIQFATGFAMTFYYKP
TVAEAFSSVQYIMNEVNFGWLIRSIHRWSASMMVLMMILHVFRVYLTGGFKNQRELTWVS
GVILAVITVSFGVTGYSLPWDQVGYWAVKIVSGVPEAIPVVGVLISDLLRGGSSVGQATL
TRYYSAHTFVLPWLIAVFMLFHFLMIRKQGISGPL
>EFA74243.1
MIIRNATELDLPAIVAIYNAAVPTRMATADLEPVTLESRMTWFQERVPSRSPLWVVEVND
NIGGWLSFQSFYGRPAYHATAEISIYISPSFHRRGLGKTLLLKAIHESPNLGIKTLLSFY
FCP
>EFA74245.1
MRAANPLRKAHTRRPLTTMEAKCLVIITSYMAKRLTVVIRQLLMIYQQLSEKQIPLSHNL
RLANYLERFRTHFKSRMNPRRSVLLTPNNSDEKLDQLAIDLLAKLLFCTGTAGMQRFWIS
LFDGEVE
>EFA74249.1
MVSSLAVIITLIWWWNRRSSVSDMPNIASSRETVEAINLQTAKTEIVSSQAITELSQGNL
ERGLVAVEKLLDRGDFKLADTALSSIPSKHWKNPAVNFLRGRLAWQSAQMRDKKYSIDDA
RRYWEVAVKSQPSSIMYNNALGFAYYAEGKLNYANDFWFRSVNLSLKSNKVSLDSLTAHG
GLALGLYKSANDQPPNRRQKYIDEAIKLRQMIIEKEPEQFTVERLTRNWLWTEQAISDWQ
SLLEENPKR
>EFA74250.1
MTQEFHISVTPVGQSDYLVRTEQVAPGVPLAEELVTWPVAEWLAAAEHLMNDPLKSVLQG
EGLTQPAAGTARSSVNLVALGQKLYNALFQGTLRDSWITAQGVAQNQQQLLRLRLGLKDN
KLARLPWEVMHAGDRPVATGPYIAFSRYQNGVLSVSSSPTRLPTRQEENLIKVLMVISCPV
DQAKLDLLKQEAFNLKSELNRNDWVQKESNKDLPEIELTVLEQPGREELTRSLEQGRYHV
LHYSGHSNVGSQGGEIYLVSRRTGLTESLTGDDLAGLLVNNNIQMAVFNSCLGAYRAKSD
SEGDTGEQNLTESLVKRGISSVLAMSERIPDEVALTLTQLFYRNLRQGYPLDLCVSRVRQ
GLISAYGSHQVYWALPTLYLHREFEGFVRPQLGPSGSSEFFGDYQSTISGFESEVPEHKI
DDLEANPGLEEIMDYGLTKETSDLDWLEDSQWDLMGGVNHDQENDDQDAAIVADLLRRID
NKPVASEQLIVKAEPGILIKGQESTGGENEFDGWNDSKTAKSSNQLHRDPRRRVSPDVPL
PPITKVAIDRKPKLIWECWWSVV
>EFA74253.1
MSINIPITPLSGKTILVTRAVGQSSELSHGITNLGARVIELPALEIGPPSSWRSLDQSIL
ELSTFDWLVFTSTNGVESFFARMALQYKNQQALIRRAINRTKIAAVGEKTAQSLKMHQIE
PDFIPPDFIADSLVNNFPENLAGKKILFPRVESGGRDILVKELTQKGAQVVEVPAYESLC
PQSIPTTAQQALINHSVDVITFASSKTVKFFCVLLENNLPQKAEQYLNKSAIASIGPQTS
KTCIDLLGRVDITAQKHTIDGLIKAIVEWSKSLN
>EFA74256.1
MSTKIYQQIQEFYDASSGLWEQIWGEHMHHGYYGADGRERKERRQAQIDLIEAVLNWSGV
KHADDILDVGCGIGGSSLYLAQKFHAMSTGITLSPVQCARAKERALEANLQSRSSFLVAN
AQEMPFDDNSFDLVWSLESGEHMPDKTKFLQECYRVLKPGGTLIMVTWCHRPTDVLTLSG
DEQKHLQDIYRAYCLPYVISLPEYDTIASQLGLKNIRTADWSTGVAPFWNVVIDSAFTPQ
AFLGLLFSGWTTIQAALSLSLMRRGYERGLIKFGLLCGVK
>EFA74257.1
MAVILCGTILLMMPFSTSNGTWNDPIVALFTSTSAVCVTGLSVVDPGTYFSFWGQLLILL
LVQIGGLGYMTTTTFLILLIGKRFDLRQKVAIQQALDRPGMSGSSQIIRSIIATTIIFEI

TGIFLLLPAFVPDHGWSYGMWLAIFHSINSFNNAGFSLFKDNLIGYQTSLLVVFTVTGLI
IFGGIGYQVILDMYLWLRDRLKRKTTFMAFSLDFKVAVSTTLLLLVVGTVAFFLIEIRNP
ETFGKFRFSDQLLLAWFQSVTPRTAGFNTIDIGKMSDAGLFITIALMFIGASPGGTGGGI
KTTTLRVLTSCTKAILQGKEEVWLYERKIAINLILKAIGVVFGSLATVLSATVLISLTDP
KLEFIQILFEVVSAFGTVGLSTGITGSISTAAKIVIIVTMYIGRVGVLLSMSAILGDPRP
SRVHYPEGNLLVG
>EFA74259.1
MQNSDLDISLSGYDYELPPELIAQNPVVPRDNSRLMVINPPAGTLTPPLDHIFRDLPELL
KPGDLLVMNNTRVIPARLYGRKSTGARVEVLLLEEKQHNCWLALVKPGKYFNIGTEIIFD
DQGLSSVNKLIPQLTATVIERDENTGGRLLRFDLPINVSLLHFLHKLGKIPLPPYINNSH
ALEEQYQTVYAKHDGAVAAPTAGLHFTPELLVKLRESGIQQEFLTLHVGVGTFRPVEVED
VTTHQMHEEWIEVSGDLVEKIYATKNAGGRIIAVGTTVVRALEGAAQSGSLQPYTGKVNL
FYLSRLSMACRGRSYY
>EFA74260.1
MSQKNVLCFDGKWNHVVLPPDNSDYSQGITVEAWVWYGSFGQNWSRIVDFGNGQGRNNIV
LAHAGTSNSLAFHTFTSTGGYAVEVPNALEIGKWAHVAATIDKSGEAKLYKNGKLIQTKP
FRLPDNVERKLNYIGKSNWPNNDGFFQGKMAEVRLWNVARTPEEIEQNMNRRLSGNEAGL
VAYYPLNGDANDKTKNARHGIIIGATWQQEELPIQEPNVNQNQTTTQTTTFITPDRLLPK
YDAVIVGAGIAGAIVAKTLSQQGKTVLILEAGEAKELTLSGFQHYLDTFYGSTEKHPNSP
YPENHYVQSPMDDNGYFVEKGPMTLSGSYTRVLGGTTMHWEAKTPRMLPNDFKTKSLYGQ
GLDWPISYDDLMPYYQQAEYEIGVSGDVEEQKSLGLKFEDGYVFPMEKMPPSFLDQKVIE
KVDGTKVQVCGETVELKFSTFPQGRNGIPNPKYNQGNLFVPQGVSSVTPVQYGERCQGNA
NCVPICPAQAKYDARRTLARALETGRVHILPKAVAFHINYNRENGRIQSIEYKYYGNEKT
GSVDSPLKVEGTLFVLGANAVENARLMLSCNLPNTSGMIGRHLMDHPFVLAWALMPEVTG
TMRGPLVTSGIGTLRDGTFRAKQSGFAMDIHNDGWGWATGSPDTELFDAVDHKNKYGAEL
RETLINRISRQLLLAFMCEMPPDISNRVTIDPKYKDKLGNYRPVINYNLP
>EFA74261.1
MSIIIYSHQAIAQTRNRDSKDNIYNPILLKLEGEITDKLTIKDIPTGQGGFARDYQINLN
KGDNLVIDASSENFDTIITLLGPNGSTVGENDDGPDGTSNSLLFVRITETGKYIIRVRSF
GETGVGSYKLKVTKLLPAK
>EFA74263.1
MSKVLVSDPIDQAGIDILSQVATVDVKTGLKPAELVAIIGEYDALMIRSGTRVTEEIIEA
GTQLKIIGRAGVGVDNVDVPTATRKGIVVVNSPEGNTIAAAEHTLAMMLSLSRHIPDANT
SLKKGEWDRKTFVGAEIYKKILGVVGLGKIGSHVAHVAKAMGMKLLAYDPFISTERAEQI
GCQLVDLDLLFQQADYITLHIPKHRKTTNLINAKTLGKMKPTTRIINCARGGIIDELALA
DAIKNGKIAGAALDVFQSEPLGDSPLRSLGKEIILTPHLGASTTEAQVNVSIDVAEQIRD
VLLGLPARSAVNIPGLGPDIIEELKPYMQLAETLGNLVGQLAGGRIETLNVKLQGDLATN
KSQPLVVAALKGLLYQALRERVNYVNATIEAKERGIRVIETRDASARDYAGSLHLEATGT
LGTHSVTGALLGEKEIHLTDVDGFPINVPPSKYMLFTLHRDMPGIIGKLGSLLGSFNVNI
ASMQVGRKIVRGDAVMALSIDDPLPDGILEEIKQVSGIRDAYTVTL
>EFA74264.1
MNISNDKNIMTNTWWEIQILCTPDLEDSIFWRLETLGCRGVAVEKKDRLLLIRAYLSTLQ
TQLSDLTNLSTLLHEDSTAIGLPTPELNWHQINEEDWSTSWKQYWQPQEIGNQFLINPAW
LPIPLSTSRLVIRLDPGVAFGTGNHATTQLCLESLEKYLTQNSDPQVIADIGCGSGILGI
GALLLGAKKVYGVDNDPLAVESTNSNCILNHLNPEKLTCALGSVHTLTEILTEPLDGIVC
NILADVIIELIPQMTDLVKPGSWGIFSGILVEQSPSVITILEKTVGY
>EFA74266.1
MHPYPTFEDNPQISNQFLTLEEIPKSPIGVFDSGVGGLTVLRQVYQQLPNESVIYFGDTA
HLPYGIRSQKEILGYVREILNWMEQQGVKMVIMACNTSSALTLETVRLEYKFPILGMISP
ATKFAVNVGKRIGVIATPATAKSNAYRQAIMELKPNVQVWQVSCPEFVPLIEQNRLHDPY
TLAVAKSYLEPLLAQEIDTLIYGCTHYPLLEPIIKTLIPNHIHLVDPAVHVVRVCQRELE
ILNIKNHLLPMPTRFVVSGSPQHFSQSGTPWLGYTPLVEQVQFCPTVPYSLR

>EFA74268.1
MPLINTIDVTIFTRFTIPLLGSSTNEPVDNTMVVAAVLLSLVVIYLASKVGGELSNKLGF
PPVLGELVGGVVIGTSVLHLLVFPEGGTDSSSSLIMSFLQITAGLTPEATPAVFAAQSEV
ISVLAELGVIILLFEIGLESNLKDLMEVGIQAFVVAVVGVAVPFAAGTAGLMIIFGIAPI
PAIFAGAALTATSIGITSRVLSEIGRLNSKEGQIILGAAVIDDILGIIVLAVVASLAKDG
AVDVGNVIYLIASATGFIIGAVILGNIFNKSFVAIADVLKTRGSGNSRFYFCVHHVLPGG
HY
>EFA74270.1
MCDRHFGIGADGVIFALPGQKGTDYTMRIFNSDGSEPEMCGNGIRCLAAFLTELEGISRS
KDYYLIHTLAGIIRPQLTDDGQVKVDMGSPRLLAQEIPTTLVPGDSKVINLPLEIGKQTW
HITCVSMGNPHCITFVEDVASIPVEIIGPQFEHHPVFPQRTNTEFIQVVNPSHLKMRVWE
RGAGITLACGTGACASLVAGVLNNLCDRMATIELPGGNLEIQWSETDQRIYMTGPAERVF
TGKI
>EFA74275.1
MEMTNKLPRWLTFALAFPIIILNGWLLIQVIKYFQPLVSVVVLAVLLSFVLNYPIKFFHS
LGVPRILAIVGVLLLTIVMLGAIGVILLPLIFQQLNELINILPAWISSGTQQLQAFLDWA
ATQQDLPVNIIGIATQLLEKISNQIQSFTGKILSFAFDTIGFLLNLILTIVLTIYLILNG
EKLWDGLYGWLPTYLGVKARALLKEDFQNYFIGQATLGAVLGVTVTLTFIALKIPPSLII
WNYHWFFSLFPFGTGIGIGIVSLLIALENFWQAVEVALIAVTIDQINSNIVAPRLLGNLT
GLNPVWVVISLLVGAKLGGVLGLLIAIPIASFIKDFADIWRQGQLQTDSMEGKITTPS
>EFA74282.1
MGKYHYVLASQKFLLEEEPLEEVLRERIRNYHEREKEIDFWLVKNPAFLEAPEMSDIKKK
CPQLPVAIISTDSQFITWLKLRLEHVIEGEFTTPLGTIHDPLASLTNV
>EFA74285.1
MREAVFNIWQGKIDQCRWLDLCAGSGSMGAEALCRGAKLVVGIEKSSHACTIIQENWQHL
ITEQQVFHILRGDVIQQLKKLSGQTFDRIYFDPPYTSDLYDQVLNAIAGFKLLHKDGEIA
VEHSSDFKVPIIPVWQVIRQRKYGNTSLTFYSCREECESDGALYYDTSPSLGTHN
>EFA74286.1
MPVIAVVDYDMGNLHSVCKALEKAGAIARVTACGKELARADAIVLPGVGAFDPAMQNLRS
RNLEQPIKDVVTSGKPFLGICLGLQILFESSAEGNEPGLGIIRGKVRRFRPEAGIAIPQM
GWNQLQLTQPNSILWEHLPSQPWVYFVHSYYVDPLEPEVQSATVTHGSQTVTAAIARENL
LAVQFHPEKSSNIGLQVLSNFVAQVREKIPA
>EFA74289.1
MNAILPQFLKSFYRKDPIISVLITMGIVDALIGGLDDSWSLFAFGLGTTGVALGLKLASK
LSRAQQRSPREEGRTFQYYLPPTSSSSSLPIIKATKNKSYY
>EFA74291.1
MQIGVPKETKDQEFRVGLTPASVRVITENNHSVFIETMAGYPAGFTDEDYLAAGAQIVAS
AESAWHQDLVVKVKEPLRSEYQFLRKDLVLFTYLHLAADRELTEHLIDSGTCAIAYETVE
QVGTNRLPLLTPMSIIAGRLAVQFGARFLESQQGGRGVLLGVFPELSPVKW
>EFA74292.1
MVILGGGVVGTEAAKIAVGMGAGVQILDVNVERLSYLETLFGSRVELLYSNSAQIEAVVR
EADLLIGAVLIPGKKAPILVPRKLVKQMRPGSVIVDVAVDQGGCVETLYPTSHSNPVYVD
EGIVHYGVPNMPGAVPWTSTQALNNSTLPYVLQLANLGLEALEVNSALAKGLNIKSHHLL
HPAVQEVFPDL
>EFA74304.1
MENWSPNTTPIDLTINGAFIQMKRSNYATGTYSTSINSQPQDFQYKIAVNFGRSSGHLPP
RRQWHYDVGLLSQPLDLFAQKIPIKSQKLSKDYFRQVGRDDEWIQTLLCAKQTSDATYAV
DPDQRFNCF
>EFA74310.1
MAGTDFKDYYSILGISKTASPEEIKQAFRKLARKYHPDVNPGNKQAEARFKEINEAYEVL
SDPDKRKKYDQYGQYWKQVGEGGFLVVRGVDMSGFDFGQYGSFNDFLNDLFGGVGPRSST
SSRSAGRPGGFGDFGFPDMGAQDSEFVITLSFAEAFGGVQKRFSLGNETIEVRIPAGAKT

GTRLRVRGKGAVNPKTGQRGDLYLKTELSPHSFFQLEGDQLICEVPIAPEEAVLGATIQV
PTPDGHASVKLPAGVRSGQSLRLRGKGWPLPKGGRSDQLVKVAIVPPKDLTPQEREYYEK
LRAIRSYDPRAHLQQIKF
>EFA74312.1
MTWGNVGDKTVICALSLKDEGGCDGNNTLFALKPENARNPNRVLETLTQISIRGSSAGVI
TETRGRRPNRASVNLGEVISQAAKRLPKQTLRTRPNPGVKPTVRPTRGGL
>EFA74313.1
MSQGNGKWATVGQRPGGQPIPVIIWTQSASKYFGSQFAPENRCQIVTSKFNTAVRESGGS
LQDVLLTSGNVNGQVVICVISDRDNGCQEKNTLFTLKPENAKNPDKVLSQLLEISTKGSD
GGVITETRSNRRRVTVKLSDVLSIAARRSSSSPVKRPKVQNNRRNQRGL
>EFA74316.1
MNLSITTKTPRKTTIKTARINLFLLASALVTAITSTTSLATDTISAQDIAQIAKKTSVQI
NTEGDITPGGSGVIIAQQGNRYSVLTANHVVCDIIDRPGKITCAKDITYSVRTNDGKEYP
IQSQDIIVLQSTKNDPDLALVSFVATEEYPVANLGDSDQMTEASDVFVGGFPAVFGKVGS
ARDFSFSKGIVLSRGRTSINGYSLIYDAKTLTGNSGGPVFDIKGRVVGIHGLADASNKSK
TETGELVSQKTGFNAGIPINTFLNFNNPLVKDLPIKRNTIATGEAPQERLNSPQSARDFY
ARGITKLEQFNYKESLADLDQAIKIDPKYAEAYFKRGYALSWLRRYEEALLDFNQVIALD
PNYLDGYLNRGWTYIWLQNDQAALEDFNRVIRINPNYAEAYAHQGMAYIKLGKYQAALES
SKQAIRLDPNKSYGYTIQGDVFNYLKDYPAAINVSTFAIKN
>EFA74317.1
MFRLLQLKIDPDDFNAYINRALAYTLTGNYSDALVDYQKSAEIFERRYTRKPSN
>EFA74323.1
MSTSQQTVSNPLKTPLLPPLLGASVDELTTWVQQQGQPGYRGKQLHNWIYHHGVHRISDI
SVFPKTWREQVTDVSIGRSSVNYQCSATDGTVKYLLNLADGEIIETVGIPSDKRLTVCVS
TQVGCPMACDFCATGKGGFKRNLNRGEIVDQVLTVQEDFQQRVSHVVFMGMGEPLLNTEN
VILALKCLNQDLGIGQRSLTVSTVGIRDRIRQLAEHHLQVTLAVSLHAPNQILREQIIPS
AKTYPIEQLLAECRQYVEITGRRVTFEYILLSGVNDLPEQALELSQRLRGFQSHVNLIPL
>EFA74331.1
MTLTFPKRAIQIPVTYRLARDFNVAANIIRAQVAPNQIGKLVVELSGDIDQLDAAIDWMR
SQHIAVSHNLGEIFIDDQLCVDCGLCTGVCPTEALSLNRETYKLTFTRSRCIVCEQCISS
CPLQAISTNL
>EFA74359.1
MGSDSDLPTMKEAIAICEEFSVSYEVAIVSAHRTPLRMVEYAQTAHIRGLKVIIAGAGGA
AHLPGMVASLTPLPVIGVPVPTRHLQGVDSLYSIVQMPAGIPVATVAIGGAKNAGLLAVQ
ILATYKPDLLVKVQAYRQSLSDMVMDKQNRLNS
>EFA74363.1
MGLIEELVLQVYRDDLKVFISELEYPEVKAAQKHIPVSIGILTGLKNRSVPMEQIATQVK
KTRDRNFAGVAFFFYETLWNMSEETVAKRQASFQKLFPRTVQRNT
>EFA74364.1
MAPADSLLAQARPEWITTRRDGTKIVKEGIHDRVWLNPFHPEVQKFMENLIVEIVRNYDI
DGIQFDDHFGLPSELGYDSYTVGLYKQEHQGKAPSENFQDPEWVKWRADKITNLMKRVFF
AIKANKKELSSFCCTQSPKIFLRILFSRLAKMGTDGINRRTGITSI
>EFA74365.1
MFPKEKRLGKMGISESRGVWLTNIDSDVLFARERLKRSLKTLGKLNFNTVYPTVWNWGYT
LYPSQIASRVIGKSLDPTPGLKTEIY
>EFA74366.1
MNIKLDKYTPSSLASLFILLMEGGITPNQIMSGIVLLAIQNYELEGTMFSANCLHFLMKA
IPVDTTATGVTEFILSLANESINIGMLLDAFAFACQKQGSRNIASLVSLTYQRLEADRVI
SQLINDQL
>EFA74367.1
MPLFSFVLTNTRSLTPEAADRTTREVCHNLKIALAQENIEDFLVVSRSDSTLRGHYPIET
DVIAEELGPFDAHFLIPAFFEGGRITRDAIHYLIIDGIPTPVHETEFARDSVFGYNYSYL

PKYVEEKTQGRIPESAVTKFTLEDIRQGSLNRLLTLHNNQCGVVDGESQEDLNMFARDIL
TAATGGKRFLFRSAASILTALANLPPQPIAPENMAKCVRGGKPGVVIVGSHVKKTTQQLE
SLLKIPQTVGIEVNVAKLLYESVNESGELLEILQQVEQVHHAGKIPVVYTSRQELTFPDV
KTRLDFGIRVSALLMDIVRGLPKDIGFLISKGGITSNDVLSTGLSLTSARLLGQILPGCS
MVTTPSDHPHFPLLPVVLFPGNVGNTDALATICQRLTISNPVK
>EFA74373.1
MGPKPPSSFKITGKYPQGWELISDEQGQWGKIE
>EFA74380.1
MECIINRRAQFSASHRYWLPELGEAQNVGKFGLCAKFPGHGHNYVLFISMSGELDEYGMV
LNLSDVKQVIKSEVTSELDFSYLNEVWEEFQATLPTTENIARVIWNRLTPHLPLVRVQLF
EHPQLWTEYEGKGEKISLTARSHFSAAHRLAPNLRAEKYGKCTRTHGHNYHLEVTVEGEM
DGRTGTIVDLGCLHETVEREILELFDHSCINEDIPYFSTSHIVPTTENIARYMSDLLQFP
ISELGVKLSRVKLFESDHLWVEYEGKDSEIFFSVATGFSAAHRLADPTLSLEKNQTIYGK
CSRINGHGHNYHLEVTVRGEIDSVTGMSVDLVGLNQIIQHYVIEPMDHSFLNQDLPYFTE
VVPTAENIAVYISDVVRSPIEELGAKLHKVKLIESPNNSCEIYAKDIEESRVDRVDRELA
VV
>EFA74386.1
MEVIGETYEQEDIELILPTHSSPLFDINFDINPI
>EFA74388.1
MNHAKFESDLAQLQDEVSNCERDVLNLIKGKKTMEYSQNYNGGNQSNQIAIIGMASLFPE
SRNLQEYWQVIMDKIDCITDVPASRWSVEDYYDPNPKAPDKTYCKRGGFIPDIDFNPMEF
GLPPNILEVTDISQLLGLVVAKAAMEDAGYSESQQFDRDRTGVILGVAIGRQLAVPLGSR
LQYPIWKKVFKNCGLSDDETEKVIEKLKSAYIQWEENAFPGMLANVISGRIANRLDLGGT
NCVVDAACASSLGALNMAISELLAHRADMMITGGVDTDNSIFAYMCFSKTPAVSPSENVR
PFDVNSDGMMLGEGVGMLVLKRLEDAVKDGDRIYAVIKGIGSSSDGKYKSIYAPHSQGQV
KAIRRAYENAGFAPQTVGLIEAHGTGTMVGDPTEFISINQVFGDNNSLKQHIALGTVKSQ
IGHTKAAAGAASLIKTALALHHKVLPPTINITQPHPKLNIENSPFYLNTETRPWISNQPR
RAGVSAFGFGGTNYHVVLEEYESEHHQSYRLHNCAKSIFLSAPTTPELLSQCQHLYQQLE
STDKEQHYQRIIAESEQLIIPVDHARVGFTTLSLSQAIAHLAIIIDLLKNQPSVDFWEHP
KGIYYRQQGMETTGKVVALFSGQGSQYLEMGRELVINFPCLRQTYSHLDDLFSREGLESL
SQVVFPTPVFSPQERQEQLEKLQKTEYAQPAIGVLSAGLYKILQQAGLKVDFVAGHSFGE
LTALWSAGVLTEEDYFFLVKARGKAMSTSPEVDAGGMLAVKGNISQVTEFIKDFPQVAIA
NYNSPQQIVLAGNKSEITQLQNVLQAQGFSCFLLGVSAAFHTPLVSHAQKPFAHAIAQVN
FQPPRIPVYSNVTGKLYPNKPGSMQKILQEHLLNQVLFQQQIENIYQAGGNCFIEFGPKN
ILTNLVKEILVDKPHIAVALNANYRQDSDLLLREAVTKLRVFGVPLKNLDPYQIPTKISS
ASQKNEQKTLNIRLNATNINDRSQKAFAQALATGSVIPITRVSEENYQTQPEKTLVEINP
EITSSSILNSKTNSEVKNYSMVEQKIEIPVENYDRILDSLEQSLAEFTRQQSEINHAHQQ
SLQNQIEYNKTFYELMQQQCLFLGKEETNEYQAQTQQLAISSTERSMMQLHDHQAETIRI
HEKYLNYQQEYTNNYFQLLEQHYSLLEVGSPNGYSHLPSSHVAQSDPPAQKLIYPLEPEN
NSQNNLPDITVDFPIATHIDKGKLRETLINIVSDKTGYPVEMLDLSMDIEADLGIDSIKR
VEILGGLLELYPDLPRPNPEELAQLATLEQIAEYINNLITQLGQNQPLEETLSEHHNHPQ
FLVLPHDDLHEDLSNRSTTITIPDNLSQILLTIVSDKTGYPVEMLDPSMDMEADLGIDSI
KRVEILGGLLELYPDLPRPNPEELAQLATLEQIADYMRNQAETVERSNLSTSEKPDTSTT
EVADKILRLPVQLKALPQPDSLDLTIPENHFVLITNDGSEVTHRLVAKLADKGCKTVVLT
FPYLESNLSEEIAQIRLNDWHEETLQEHLTELTSKFGCVGGFIHLHPYSNNNLGIDQAIV
QHVFLIAKHLKEDLNQLTKKERACFFAVVRLDGELGTAKTHNFSPISGGLFGLTKSLNQE
WPEVFCRTLDLSPDLDGDTTVKHILAELQDPNLLVTEVGYNKTDRFTLVAEPTKSSIIPD
SLNITKDQVFLVSGGAKGITAKCVIKLAEEYQCKFILLGRSSAEIEPVWSEGYEDENELK
RRIMEDFLSKGEKPTPIMVQKKYQTIASQREIHNTLKAINEAGGKAEYICVDITDGMMLK
EKLRPIIDQFGTITGIIHGAGNLADKRIEKKTVQDFETVYAAKVQGLENLLNIVETNQLE
YLILFSSVVGFYGNVGQTDYAIANEILNKSAHVIKHHKYPNCHVVSINWGPWDSGMVSPEL
QTAFAQRGIETIPQELGSSILVDQLRTSDSSMTQVVIGSPLVYIPSTLSSELKTHQITRQ

LKLNYNPFLQDHVIAGNPVLPATCGLSWISSSCEQLYPGFQIFHCPNFKVLKGIVFDQNS
PHEYILEIQEIAKIDNQEIHLVGKISSVTNHGKIRYHFSSNLILKRQVPPADNYELFNLT
QDGQFLASNSLLYQTGVGSLFHGNTFQGVKSVLNISPGKLTMKCELPEPTLHQQGQFRVQ
TFNPYIADVQVHSLWIWTQHFHQVGCLPSEIENFEQFAPVPFGETFYVTCEIKSKTESYV
VADVITHSQKGQVYNRMKGAKATILPNSY
>EFA74390.1
MVDKEKHLLVNFRSWLLISLATTTFISTLLISFQSISNFQTKSQSKVAIPINTPSPIITG
VAALGRLEPQGEIIRLSASNSLGGGIRIAKLLVKKGDKIRQGQLIAFLDNYSLNLAALEK
AKRQVEVAKANLEKVEAGAKQGDIYAQKATIGRLEAELRGETSAQKAIIARLQAELNNAQ
RENQRYEDLYENGAVSASNAESKRLRRDTLQQQINEAKAALNRTQQTLQKQLNEAQSRLN
SIVEIRPTDLQVAKADLASAKASITQVQAELDLSIIRSPIDGQVLKINAWPGEIISSQGI
AELGRTQQMYVVAEVYETDVKKVKLGQSVDITADAFPGKIQGTVTDIGLQVNQQNIFNNH
PGADTDNKIVDVKIRINNPKDNQRVADLTNLQVQVWINM
>EFA74395.1
MWQSQEKFAVLLLDVKNRFLGTKIITIGTATETLASPRDIFREVIRHGATRMIIAHNHPS
GNLDPSQADLDLTRQLLSGAQLLNIPILDHLILGNGNHQSLREITSLWDDFPQEN
>EFA74396.1
MTYCLRISDLPENERPRERLITHGAKVLATAELIAILLGTGQGPGKLSAVGLGQHILQEL
GRDQGDPLAALRDATPAQLTEIHGVGSAKATSILAAVELGKEFFISSCGGSDY
>EFA74400.1
MTTASAYSLNVPGHPTGQISKEELRSLLKEVESQLYHSHAYRAVVDKAQKLFDISHEQLT
NFNKLIQAISREAIALTFERFVTKQSATSTDSSKNDSADSTNSNEIKELSNQTVEVCEQV
LENRSQPNDPEPTAKKQQKSDRVKGENEPDPTKKTQKLAMEKSKTF
>EFA74401.1
MKTNQIQRKKLKSWPWKNQKPSKSRLAAKKAEEERLESLRQIGEQLKKARESQGLTLHKL
TMYTYISPNQMAAVENGDMEKLPEDVLLRGFIRIMGNALGLNGADLANSLPVRNECKSIL
PSWYKNQRPSRSLSIELSPTHLYLGYTALVAGAVSGLSMMYEQANNQGIKNFQSTTPCLS
SLCGSDEKATNVNDQSTMQHGRVGNSFGPQISPPEAL
>EFA74407.1
MSKPILEGFDAKLEEKNPVEGGSDAIEQPTFPLIL
>EFA74409.1
MLANKFPELTKEIIMEMLEIKTMDVTKSRFYQQIYTEGRQEGEVNLVLRLLTRKLANLSE
TMVKRVKKLTIPQLENLGEALLGFTQIGDLETFLSKLESKPEQSTLTQLDTEQPDQEN
>EFA74410.1
MTTKILVTGGAGYIGSHVVLQLAESGYDIVVYDNCSTGTPESVLHGELVVGDLADVDRLY
QIFSQHKFSAVLHFAASLVVPESVAHPLDYYANNTRNTLNLLRCCSVMGVNQFVFSALHN
KV
>EFA74412.1
MLNAYSLGVSRRTVVSLALQIANERVNKDRLVRRRTAQVQIAFDIYLRALYQNRPDISFF
FTNHVASSLHRYWPSLFPNDFKSLKYDQFWVERWSDEILFAMREAAYQIGQLMAFVERYP
GSSRWSQ
>EFA74416.1
MQCLFMTKNQNPSLSSHVNVLSTNLLDASPINQRLNVLQGLAQFSLCFLIVGCQQLGMQV
VCYPLVIVISLVFSKRIKLLSYKYTVAPLALISLAALSLNLRDFPSGDVLNLIRFFTGFL
LISFFFANSTVKLRYFSYSFIAWSLIELCFLIFLNDQPPYIKSYLSNSDIESFSRAEFTY
GNLRLLGPFINSSINGTYAGVLTLMLLLDPDLIFGKFELRSKKREILLLSILSGVTLLYS
GSATGFITTGFLLFLRLSPYLFILRSKYVKILLTLSLIAFASFSLGGIYFSKLDSNYLSF
IFDLKLNQFFDRVNNVYDLVLGSPYETGFIYGGDFLVLSMIDNFGIILAMLVFFTAWNQC
GRKYRPYFLALSFSSLHYGTIFSLTGQVLFASLVLSPNKSYDLK
>EFA74419.1
MSVLDREFLGMGEEVKLFEEELTAFLGRQVACVVNGTAALHLGLQACGIGQGDEVLVQSL
TYVASFQAISAVGATPIPCDIDPKTLTIDWKDAEKRITPRTRAIMPVHYSGGVGELDQIY

AFAQSHEIRVIEDAAHAFGTKYQNQLVGSFGDIACFSFDGIKNITSGEGGCIVSNSPDIM
SRVRDARLLGVEKDTEKRFQGQRSWDFDVRSQGWRYHMSNIMAAIGRVQLRRFPELSFRR
QQLAHCYDKRLSGISFITLLKHNYEEVVPHIYPIYLTEGVSRDYVRQRLLEHGIQTGLHY
QPNHHLSFFSHGLDNELPITDRTYSGLITLPLHPDLSEEQIDFICSILIQVINNIDNATL
N
>EFA74420.1
MKVIILAGGFGTRLAEYTDIIPKPMVSVGGLPVLLHIMQLFDNYGHKNFYIACGYKAEVI
KQYFLNYPTLNADFTIDLATGQVQTVKKKEIDWQVTLIQTGLKSMTGGRVKRLQSLIGNE
TCFLTYGDGLANINLDDLLSYHYNHGRMVTITAVRPSARFGELEICDGQVISFEEKPQLR
QGWINGGFFVIEPSFFDLIAGDDTVLEAEPLEKATALGELMAYQHDGFWQCMDTKRDLDL
LESLWKSGKPPWIKE
>EFA74421.1
MLDGVILTPIRRIPSDAGDVLHGIKASDPGYVKFGEAYFSTIHYGTIKPWKRHNQMTLNL
VVPCGCIRFVLYDDRPESTSYGRVESFILSPEDQDYRRLTIPPGIWMAFQGVSQNLNLVL
NIADIEHDPKESDRADIDFITFDWSLKP
>EFA74423.1
MYYVLLLIGRIFGKLSRIVFNLAKDFEPEDIQSKLVQKSTQYDMVTAPDEPYYFQQYWKF
ILPHIEKLLEPNQVLELGCSQGRFTLPLARLFPNTQVIACDFSATAISTAKKYASEALVT
NIDYRVQSISECLKSLKAGTCDIVFMTEVTFFYPKWKLDLPKIVEVLKPGGVVVMSFKTK
YFYALTIARHRFWDNVDMLITQTQGRIGVGSPVEFTWQTSTEIREILSKFGLVLLDLVGI
GVLSGMKGHDPHDGIVQPSLLDDSEREALMKLELNLASEVPDGGRYILAVARKT
>EFA74424.1
MKTWEKQLVGNRISLKEFIQIESIRQRPLILDWIRRQREANNDSIHWWMTDLAGRNNVSC
RFFDDVIHISALKSWITTNASYPDEILVVCENSYVLAAAKTNLESTVQLRCQIGWQITMA
FETSYLILRACVNFARQILQFWKHHRAAQISRPFNLQPPQGEVYLINQCLDDKAFNKDGP
LACRYFTVLPEWLEKQGKQVYRLPWLYNVSLPLEEVYQKLRDSSCFLPEDWLTWRDYVRA
FYDGIKTVSTIRKSIPYKDNFHLEFLLIRERFRQLTTGISVSRFLLYLPALRRWGNNLTK
LVSITHFESMQPERVQPYFCRFVLGNKSKSIGYYHSLVASDFLGYHIPLGESESKFFPDI
IVTNGGLGHSVLISRGLDEKKLLSGPGLRQNFVQTATENSGNCLGIPLPLDLQGAVEMLD
GIYAHFDWVRNYLKSSVILKPHPMMSRREINILLSHMGWTDLPQDWQWCEGEMTDLLKIS
RCCLVMSTASIIDVLLSGRVAISLGRELTSSWNYGDFLENEFPILRSVSPKNLCQRLEEV
FLTDTQWYDDEFRKIRQKFLAGLNQTSDQTLSVFL
>EFA74425.1
MKLEPQVCPLCNSSKPHQRIRSTDVYGGVSEQAFWQCNDCNAIYLYPVTSREDDKAFYEN
EFDKWMAKRSGDESWSDPESQFKSLSSREMPLRKPWLDKLCPAGSRVLEIGSSSGFMLKP
LQDSGCEVIGVELSHQYRDFANSKGVKTVASMDALNRDHTGYFDILLHYYVLEHVTDPLS
FLKSLLPFLKPGGKMMFEVPNGNDPLISFYKVPEFEKFYWWRAHHWYFTPESLKYVLAGL
DRPFEIFPGQRYDLSNHIHWLMTGKPGGFRKYSHIFSQETERAYAEDLKRSGWCDYLIAI
VS
>EFA74426.1
MKAIVTGGAGFIGSHLVVRLIDDGWDVIVVDNLSSGHERNIPGGAHFIWMDLTTEDSFSL
LPDNGVDAIFHLASHVGQELSFENPTYDLKANALSTIFLLKWALAKRVPRFIFASTMNIY
GDPLNLPVSEDSEIKPPSPYSVGKVASEYLCKIYQGFGIHTTCLRLFNVYGPLQDMKNMK
QGMVSIFMSYVAKNVPIHVKGSKDRFRDFIYVDDVVDAFVKSLDNRASGKIYNVSTGTKT
YVWELIDYILDAFGKKADEYPITFGDGTPKDQFGIYGDNSSLVGDLDWVPRTDLKSGLKV
MADWVRTLPSEMLPNL
>EFA74427.1
MYDFFLGSCPKNLDEEINYLISVKRLLPRWINSIPDSEFATICTIIHDLGNRATITGEKL
CLIETGVGASSIAIVFYAIKHHGLALTWDFNSKKGSEIRTACVETICTLFSANINSVWKL
IGYNSRSPYLGIGITSEWTDTVHFTMHDSEHVWENVEGELNLVDPFLKDGSVVAVDDAYY
TFLHTDTAYINLTRKKLGLKAINSIEGNVCQSHSIEAENFLKKRWGKVKSLAGDYQKICQ
DDVSIAYFNNELKVRSSLGMEQVQQLETRFGVWEVKSRLRQKEEK

>EFA74428.1
MANKVLNTTSRVAVTSRSFSRHFDLRQELLASYAQVTFNESGQVLAGNDLVEFLQGHDKA
ITALEKIDENLLAQLPQLKVISKYGVGLDTIDLEAMDRHGVQLGWKGGVNRRSVAEMVIA
AAISLLHRTSESHAEVRAGQWRQLQGRQLTGKTVGIVGCGHIGKEVAILLSGFGCRILAH
DIKNYAEFYIAKDIVPVSLEDLLSDADVVTIHLPLDISTRKMFSLERLKLMKKGACLINF
ARGGIVDESGLKQLLKDGYLAGAALDVFNEEPPLDLELLNLPNLIATGHIGGSTGEAVLA
MGRAAIIGLDSAKPARNYTEIF
>EFA74429.1
MCEALVIQSHRGSYTVRYAQNALHLLALESHEKRHFIIDANVVAIYHEVLNPIISGSSAL
VIEATESAKTLDRFTGYVEALVSQSMRRDHRLIAIGGGITQDITAFLATTLLRGVAWEFY
PTTLLAQADSCIGSKSSINVGPVKNILGTYCPPNQITIDPDILKTLKEVDFRSGIGEILK
VHAIAGPSHYDEIAAVQDQLKTDHNLLRYFINRSLEIKKTFIESDEFDTGPRNVMNYGHS
FGHAIEAATDFFVPHGIAVTIGMDLANHVAMQLGHVSDDHFNRMHPTLMKNSEGFHTVEV
PRDRFFAALSKDKKNIGTQLSLILLDAAGLPKRTLIDNSEEFCTICNNYFLDVLPYG
>EFA74430.1
MSFYPVDFADTHATESFVDKIRSSKFDILVNNAGINKVAPFANIAPKDFDLIHLVNVRAP
FRLCQAVIPYMLEQRWGRIVNVSSIFGIVSREHRASYSTSKFAIDGMTAALAAEVAQFGI
LANCVAPGFIDTELTRSILGTQGIADLADRIPARRLGQAEEVASLVCWLCSPENTYISGQ
RLVIDGGFVRV
>EFA74433.1
MKIVRYVTHNPPGGVHNGVVKDGNVFELEGDILSGNTHAGKMVGSIEDLRLVSPCQPAKI
ICVAINFPGILHFSPTMSEPLVFVKPPSCICGPGDTVENPFPGLSWWGEAELAVVIGKRL
RNISDCEAREGILGFTIANDTTVENVDHRDHHLARSKCADKFCAVGPWIDTEFDASDCVI
EAIQNGEVIRRGRSSDQFWQWQRIISWLSTWMTLNPWDLVLTGNPPDTVGMRFLGDDDIY
TARVSGLGELTNRFVRTLPIRRSF
>EFA74434.1
MYNYIQSYADSLKYALDQQAMQLIPNLGEALRNAWLQGKVVYLCGNGGSAGNAVHLANDL
IYGAGMQAGLGLRVEALAANSAILTCLANDLGYEQIYAQQLRVKAEIGDVLIVLSGSGNS
PNVVKALEVGNNLGMVTCAILGFSGGRCKDLAQIPIHFPVNDMQIAEDLQLIVGHICMQW
LCEVLGKDLEVKPIPKARLIA
>EFA74450.1
MGDKQGAISDFTQAINLNPNFAQPYYNRGATRNDLGDKQGAINDFSQFINFYPRNSLAYF
NRGIAWHELGDKQRAISDFTQVIKLNPNNVAAYYNRGASRSDLGDKHGAINDFTTVINLN
PNFAQPYYNRGLARHNLGDKQGAISDFTQSLRLDYRDPTAYNNRGIARYDMGDRQGAIND
FTQAIQISPNFAQAYNNRGLARHNMGDKQGAISDFTQAIQINPNFAQAYNNRGATRNDLG
DKEGAIRDFNKASQLFDSRR
>EFA74451.1
MYKILKNKIYVANLSLFVLISVSTSSTSAQTTTGIEDIARKTTVQINSNANPGGSGVIIK
KEGTIYTVLTANHVVCDNLGTIKIRCRTDSNYTVVTYDGKEYPMKYRQSLQINVQDPDLA
IIRFESRENYQIAPLGNSDNVKIQSDILVAGFPTIFGRVGKQRTFTITNGKVVTFIPNSD
RGYGLVYNATTFIGNSGGPVFDIYGRVIGIHGLADTDDGETNNNNQSETVNGVKPTQKTG
FNAGIPINIFFSLSNFNQQVNPTVP
>EFA74453.1
MTKLKTLIAAPGRTIYVTTQAVINKIHMPLWLRPFAMTFIITTGIGLTGAGLWSFGNFLV
KTLTSVSLPKVEIPQFPSVPSLPFGRSQRSIENVFTRLQQLEISRRFFIKTVDETFYTSR
PELKNRSLRDTAGDRALRKEWNNKADELLWKIEQANLSQSARKKMGSYSQQDLQRWNRLA
KAGKLGKYRSLEEVRKETYRKFDSLFPGQERGKLNRGTFLQIWCAIASDLVGNK
>EFA74455.1
MSRKGGDFQVDFNRYGGPDLVIIDEAHEGIPNYRNRIHQTCLQIQQSDRLYSIQDNQQEL
DLGILEYGVTSLVALMRWK
>EFA74456.1
MEVEKDLTIRKQLLDVIIIKRYKDSGFNLYPAGLENMSKHNLISYKSIHESFTVWSLMEL

IGHYVNYRKQTSKSLSNLLPEKDYKLYGLTTHEPTQLMGKLKWKSVSQGVYDIECVGMNV
RLIVLSKIPKSANNELWRLFSAKAEVVEEAISHYQESYRNSEYSVLMQQLYEFYLKEKLP
MTYTLKQFKKDFVISHLREIPTEEVLKQYSPEEVLKQYSLQDLLEGLSPETLERLAIILS
QLGVNQIKNQEQ
>EFA74460.1
MGTAIVIGYGNELFGDDGIGPLIAKVIQRWRLPCVQSLAVHQLTPELAEPIANSRLAIFV
DTCINSYIIRCKYNYYCHYH
>EFA74468.1
MLEPLFRCNLACSGCGKIQHPTEVLKQNLSPEQCFAAVEECGAPVVSIPGGEPLLHPQIN
QIVQGLVDRKKYVYLCTNGLLLEKSLHKFKPSPYLTFSVHLDGMKEWHDHCVDRKGVFDV
AVAAIRAAKSQGFRVTTNTTIFTGCNVEEMQGFFDFLETLKVDGMMISPGYSYEWAPDQD
HFLQREQTRALFREILAPYQSGKKNWNFNHNPLFLDFLIGEKDYECTPWGSPSYSVLGWQ
KPCYLLNEGYYQTFQELLDQTDWSKYGHASGNPKCADCMVHCGYEPTAAIDAMQPQNIAR
SLSTVFGR
>EFA74470.1
MDICLRYGKVVMGIELKVRREKLDPLTKGLTQLDKYLDGLGLDTGWLVIFDRRPGLPPMG
ERISTEEAISPGGRTIMVIRS
>EFA74471.1
MKPRSRPQNTDKYFKESVSWSKVTSSYFSLRFYPQGFIFADAGMSIFSNDKTIIKAILGV
MNSPVMNGTTGSLSPTLNFEVGQISNFPILNNTIDTAQIKITENIDQAINISRQDWDNLE
TSWDFLTHPLLRHNSPSISQCFTQWQNQSETAFRQLQLLEEENNRYWIQTYGLETELTPE
VPEDQITINRADQQRDIRSLLSYIVGCIMGRYSLDKPGIIHAGNPFDPSLHQQFPASNHA
IIPITDQTYFTDDIITRFEEFIQIAYSADTCSENLKFIADTLTLKNGESPRERIQRYFLQ
EFISDHIQTYKKRPIYWLFTSGKKRAFNALIYLHRYQEDALARMRTDYVLELQIKLEGEI
TKYQKQLEISTNNADKKIASKRLKELQDQQSELAEYQEKLQHLADARIKLDLDDGVAYNY
CRFKGLVYEGSDLKMADLEKASQWKK

-Protein sequences of the non-toxic group which were not included in the Veen diagram.

>1170769.6.peg.1
MCNGDLESTVKAVKRLFAGIPWRNFTNNDLANFEGYYASVLYAFLSSLNARIIPEDITNY
GQADITAILGDHIYVMEIKVIDGDKVKENLALKQIRKCNYAQKYRGEPGKTVHEVGLVFS
SSKRNLIQADWE
>1170769.6.peg.7
MSNQNDNSQNDNSRDKSQKPQSSGWFKIKRLRPPKGIGKPKTE
>1170769.6.peg.17
MKYWRETLAIAQRILTELLRRRRSLIFWTIFPVSILILNGFVLSERGELAMEVALEKAAP
SSLVGAALFFSCLGGTVATVVAEREQQTLKRLFISPLSGTSYFLGIFLAHSCIGIGQTLL
VYIVAGFWGATFQGSILLGIVIIFLSIIGYVGLGFVLGTQLARRTEDVNALVATFGVPLL
ILGGTFFPSSLFPKTLLDIAKYNPIYHMNEALLGVSANGEKFSDVSLHLRFLLFFATLTL
VCGWISYRRMLMVEKRL
>1170769.6.peg.23
MLLGRRSPWLPKKIANYQFPRVFAQTILHNLGRVTRLLEKIARPRLTKLANHDITWRCNG
FCISWLAILLISPVPLTNPIPTIGILLFAAASIESDGLLICICYVLTLLITLIFYLIVYG
VLQLPGLIT
>1170769.6.peg.24
MSPGGIGNKNGKTNKAMTRMNPRWEVVSSTSPRVRGWCARRCSKDFISSEKVKRIVQGLG
I
>1170769.6.peg.43
MESNNYREIMNSKNFTFSGLTLKSLILLSSLLICNGLTIRGLELSANAQLLSNAEKEELS

RLRTETKIQKQLQSYLSIWLTLLSLFAVGLIATLWFLRKAIIRDIVERAMRQIGNIENLQ
TELIAANQKTTGLIEYSQDLALELEQKVNHLKTTIEGEGGKLSVLLSDLPKSKQEFLTAL
EREVIAAQENISSLEFKLNTQLEQVTLAAQQQRVTIENIKKLESELFAQFSEIKLSIENH
RDTSVSDINKYRSELMEQFEILALETLESKTQVVQSITEHASQFTSNLSEFQTHAQEQMD
GFTSNLSGFQTHAQEQMDGFTSNLSEFQTHAQNQMDGFTSNLSEFQTHAQNQMDGFTSNL
SEFQTYAQEQMDGFTSNLSEFQTHAQNQIDSFTSSLSDLNQTSALQLIDLQTDTQKQKDH
IFKDLAKSQSEFSHYLLELRNTTESRQREIIENWQKSVDEVEQMLSNLHNDVEEQKTDLL
ENLQVLGLGFQKQIGDLQNSIEQRHRKFFQTSQNSVEQLISEFMSELSVMKSDIKTDVDH
QKATLIARLAKLEKLESQFVEQLKKLQLDAENRQYETLKELSQMTPQTPVKHISPLSSVE
TDTNTTNTIDVQTVNNLDNHLDHEIQEAEELLSQNRYEDALSIYEKITSIQPDHGEYWLK
RGFILNKLKRYKEAIGAYNQVIRINPAHYQGWFDIGITCGKLGKHQEAFNCFNKATEIKP
EESVAWLNRGLSLVELENYEEAISSFDKALEIQPSSFKIWDKRGYTLVRLGRDEEAITNF
NKALELNPDYGSALYHKSACYALQKNVELALVNLQQAIKHKPSYREDAAGDIDFDDINND
TRFQQLIHGE
>1170769.6.peg.57
MDIVKTVLADVIELQIETSVIDESSYNDNASSTKPENKMVTRINLVDGDIRNEIGTSFIG
SGPYTELREFHLSQVQEGREIIQKNIESVQKLGEILMSMVKQSQNSQSSQLAKLP
>1170769.6.peg.67
MGRSPAKNSSPYYTSSRAGVTPNLLLRENKGAKVLFSDIC
>1170769.6.peg.68
MPKLVIHEYQRTAQYFREDLGNDIQLDIDVDSWWHFCHGFTGRREG
>1170769.6.peg.71
MLGVAGVFGGSLFSAMHGSLVTSSLLDSKNTFNTVFYKFI
>1170769.6.peg.72
MYHLEKGRYKQVKANRRNHYAIPELGVELGILLDQERPPIPWLRWWDSRGNLLLTGNERA
EEESQRRELAEAIAIQERQQKELAEALAIQERQQKEMAEALAIQERGKKEKLAAYLRSLG
INPDEI
>1170769.6.peg.73
MQVTTTSTPIPPGELSPSNWPDHTQLPDSDDNFVKNFQEHPQSVILTTSIEPLLDKIHPD
KESGEVLGL
>1170769.6.peg.74
MGSSFIGLINSRLFNFSWFGDGRIRVVLFLLGVVILYERFKGC
>1170769.6.peg.76
MELIQFFDSLIGRSRIICGVIFLELTSNLSNLLLILD
>1170769.6.peg.78
MPGIRNAWNGKVWWKSGSLQMFTDAGNLLCVTLCMSG
>1170769.6.peg.79
MQYNPFSDFYSTLTNNPGYFPGPQGVGNFVAISAIAALLEWVRVGNAPSIPDIFGYLV
>1170769.6.peg.80
MNLSITTKTPRKTTIKTARINLFLLASALITAITSTTSLATDTISAQDIAQIAKKTSVQI
NTEGDITPGGSGVIIAQQGNRYSVLTANHVVCDIIDRPGKITCAKDITYSVRTNDGKEYP
IQSQDIIVLQSTKNDPDLALVSFVATEEYPAANLGDSDQMTEASDVFVGGFPAVFGKVGS
ARDFSFSKGIVLSRGRTSINGYSLIYDAKTLTGNSGGPVFDIKGRVVGIHGLADASNKSK
TETGELVSQKTGFNAGIPINTFLNFNNPLVKDLPIKRNTIATGEAPQERLNSPQSARDFY
ARGITKLEQFNYKESLADLDQAIKIDPKYAEAYFKRGYALSWLRRYEEALLDFNQVIALD
PNYLDGYLNRGWTYIWLQNDQAALEDFNRVIRINPNYAEAYAHQGMAYIKLGKYQAALES
SKQAIRLDPNKSYGYTIQGDVFNYLKDYPAAINVSTFAIKIDPDDFNAYINRALAYTLTG
NYSDALVDYQKSAEIFERRYTRKPSN
>1170769.6.peg.99
MVMERLKQDLKNDLIAGLLVVIPLATTIWLTITIANWVINFLTQIPKQLNPFDGLNPILV
NLLNFLVGLAVPLISILFIGLMARNIFGKWLLDFGERILHAIPLAGQVYKTLKQLLETIL
KDSNGKFRRVVLLEYPRRGIWSIGFVTGVIASDIQAKLSRPMLSIFIPTTPNPTTGWYAV

VPEDEAINLTMSIEDAFKIIVSGGIVAPSNGIVMSQLPLTTPALTTESKSHLVGVEPDF
>1170769.6.peg.105
MQIGVPKETKDQEFRVGLTPASVRVITENNHSVFIETMAGYPAGFTDEDYLAAGAQIVAS
AESAWHQDLVVKVKEPLRSEYQFLRKDLVLFTYLHLAADRELTEHLIDSGTCAIAYETVE
QVGTNRLPLLTPMSIIAGRLAVQFGARFLESQQGGRGVLLGGVPGVKPGKVVILGGGVVG
TEAAKIAVGMGAGVQILDVNVERLSYLETLFGSRVELLYSNSAQIEAVVREADLLIGAVL
IPGKKAPILVPRKLVKQMRPGSVIVDVAVDQGGCVETLYPTSHSNPVYVDEGIVHYGVPN
MPGAVPWTSTQALNNSTLPYVLQLANLGLEALEVNSALAKGLNIKSHHLLHPAVQEVFPD
L
>1170769.6.peg.107
MGIVDALIGGLDDSWSLFAFGLGTTGVALGLKLASKLSRAQQRSPREEGRTFQYYLPPTS
SSSSLPIIKATKNKSYY
>1170769.6.peg.110
MGNLHSVCKALEKAGAIARVTACGKELARADAIVLPGVGAFDPAMQNLRSRNLEQPIKDV
VTSGKPFLGICLGLQILFESSAEGNEPGLGIIRGKVRRFRPEAGIAIPQMGWNQLQLTQP
NSILWEHLPSQPWVYFVHSYYVDPLEPEVQSATVTHGSQTVTAAIARENLLAVQFHPEKS
SNIGLQVLSNFVAQVREKIPA
>1170769.6.peg.115
MLASQKFLLEEEPLEEVLRERIRNYHEREKEIDFWLVKNPAFLEAPEMSDIKKKCPQLPV
AIISTDSQFITWLKLRLEHVIEGEFTTPLGTIHDPLASLTNV
>1170769.6.peg.118
MKQNKPQVRPVALGCRKYRRCPAWYFGGVMELRAAVVC
>1170769.6.peg.121
MGANITPIAKIKPVNHPETIVYIQGRIEKHAPLIGEQAYQIADSTGKIWVVINQNSGQNQ
NNLQLGQEVVIKGKVKYKGITLHQQEYGEVYLEEE
>1170769.6.peg.128
MTIEFTKYHGLGNDFILIDNRCDLTPLITPEMAIKMCDRHFGIGADGVIFALPGQKGTDY
TMRIFNSDGSEPEMCGNGIRCLAAFLTELEGISRSKDYYLIHTLAGIIRPQLTDDGQVKV
DMGSPRLLAQEIPTTLVPGDSKVINLPLEIGKQTWHITCVSMGNPHCITFVEDVASIPVE
IIGPQFEHHPVFPQRTNTEFIQVVNPSHLKMRVWERGAGITLACGTGACASLVAGVLNNL
CDRMATIELPGGNLEIQWSETDQRIYMTGPAERVFTGKI
>1170769.6.peg.130
MVTSIVFMSGIGFINVYEFFVLTFLYHFPHIFYLWFQIRSLFQFYTG
>1170769.6.peg.131
MVVAAVLLSLVVIYLASKVGGELSNKLGFPPVLGELVGGVVIGTSVLHLLVFPEGGTDSS
SSLIMSFLQITAGLTPEATPAVFAAQSEVISVLAELGVIILLFEIGLESNLKDLMEVGIQ
AFVVAVVGVAVPFGAGTAGLMIIFGIAPVPAIFAGAALTATSIGITSRVLSEIGRLNSKE
GQIILGAAVIDDILGIIVLAVVASLAKDGAVDVGNVIYLIASATGFIIGAVILGNVFNKS
FVAIADVLKTRGGVVIPAFIFAFIMSYLADIINLEAILGAFAAGLVLEETEKRKELQKQV
IPIADMLVPIFFVAVGAKTDLGVLNPAIPTNREGLVMATFLITIAIIGKVITGLAVFGQP
GINRLAIGVGMIPRGEVGLVFAGVGAASGVLSKPLGAAIIMMVIITTFLAPPLLRVVFPQ
GESSVAIDSTSEV
>1170769.6.peg.136
MSKVLVSDPIDQAGIDILSQVATVDVKTGLKPAELVAIIGEYDALMIRSGTRVTEEIIEA
GTQLKIIGRAGVGVDNVDVPTATRKGIVVVNSPEGNTIAAAEHTLAMMLSLSRHIPDANT
SLKKGEWDRKTFVGAEIYKKILGVVGLGKIGSHVAHVAKAMGMKLLAYDPFISTERAEQI
GCQLVDLDLLFQQADYITLHIPKTPETTNLINAKTLGKMKPTTRIINCARGGIIDELALA
DAIKNGKIAGAALDVFQSEPLGDSPLRSLGKEIILTPHLGASTTEAQVNVSIDVAEQIRD
VLLGLPARSAVNIPGLGPDIIEELKPYMQLAETLGNLVGQLAGGRIETLNVKLQGDLATN
KSQPLVVAALKGLLYQALRERVNYVNATIEAKERGIRVIETRDASARDYAGSLHLEATGT
LGTHSVTGALLGEKEIHLTDVDGFPINVPPSKYMLFTLHRDMPGIIGKLGSLLGSFNVNI
ASMQVGRKIVRGDAVMALSIDDPLPDGILEEIKQVSGIRDAYTVTL

>1170769.6.peg.144
MVRALVGDSTITRVDMEISKKRIALKLNSQVEIILAESQG
>1170769.6.peg.145
MPSKSIFIFNLCLTYGGCAWRIKLGNSDSSLILPFTWVHDLVHNSIISSYRS
>1170769.6.peg.158
MTVHWGIFRLQYFTVLRWLAIAFLRLLSSNFSNILFYGIKNTKQ
>1170769.6.peg.168
MKANPVNLPNFRSAVDGDSQDSPVASSKTLVQLLSEEIESQVKASSRCIQAVVSRITKEV
ERICDKSSRIQTSGQIKSWQNNLAKHRLQKCLRYYQLGSRQGRIELHSSLGSIIYRHVTT
SGSELGFEARYNLIEDFLQAFYMESIKAFRRENELAQEYTPRTQLELAEYMAFTEQYAKR
RINLPSGVNQQLIVLRAQGFARRQPQETTVDIEMAVDSAKTEETESYQRNLAVQQIRSQM
VAKSTFDPSEDSERDRVITELMKYLQSQGQGDCMNYLSLKLQDLSAPEIDQILGLTSRQR
DYLQQRFKYHVEKFAKQHHWQLVHQWLGAGLEHKLGLSSQQWDLFWNQLTQQQQQIFQLK
TTMENDQAIAKAVQCTPKQLQKRWTQMLELAWAIRNGNVEVKTC
>1170769.6.peg.171
MRKGIVSRQQPIYTLCQYIPAREWVLVEYELEKCDFLLRDRIGDLIGREQWQND
>1170769.6.peg.191
MIADYGQLCQLYCILACQIWTLGSLKMTRKLTAKAQPA
>1170769.6.peg.196
MIVHVPGSRADDPTHPLKVVGWGNLAKDIHQNYHAGDRVILEGRLGMNTFDRPEGFKEKR
AELTVQKIHPVTKNTGTSQPAGQLPQETPNYQASRPTPTPVVEETARSVTTLDPSPQAVI
QPTPNPDEIPF
>1170769.6.peg.201
MSSLNARIIPEDITNYGQADITAILGDHIYVMEIKVVDGEKVKENLALKQIQECNYAQKY
RGEPGKTVHEVGLIFSRSKRNLIRADWE
>1170769.6.peg.203
MPTSIAELLPTISELSRADKLQLVQIVLQQLAAEEGIKKPKKSLRGTLKHYANPSLIEQE
QTAWLNAVGDDYEPS
>1170769.6.peg.204
MPQVFSKLNSLLQAQNFKQADRETRKIMLAIAKREEEGWLRIEDAEKFPCKELRSIDQLW
LKYSGGKFGISVQQQIYQSLGGTKEYNYDVAKHSPCDRS
>1170769.6.peg.217
MSDGGNVEVVELDGPIVKLRLQGACGSCPSSTMTLRMGIERRLKEMIPEIGEVEQVF
>1170769.6.peg.219
MNLQKPILVGGLGLSFSLWMLNTWHDSIIQMAELGLLGALAVGGGLWFLPKSQPSEQLHD
NPPDRSRVEAVISQAQSIIHQIANEVVNHPSLSILQEQLAKLTLELDRKEVQIAVTGGKS
VGKSTIIRLLKTVSNLSANPWELVETLPLFTRLSQDDDPAILLTLQKSDLVLFITNGDLT
ASQLDVLQKLRSTQQLVLLVFNKQDQYMTEERIIILQSLQQTFPGHVLATSASPLPIKVR
KHLADGSIQEGMEQPTPNIQQLVEQLTVILAQQIPQLVCATTWRKSLFLKTQARSCLNSI
RRDRSLPIVEQNQWIAGAAAFANPLPALDILATVAITGQMVMDLSNIYLQKISLDQAQKV
ARTLANLMFKLGLVELSTKAVTGVLKTNAVTFFAGGMVEGVSAAYLTRIAGLSLIEYFEQ
QDIALTSDRQLNMETLGGVLQRVFQENQTLLLLETFVKQGVKRLSRETKPSTTSVHF
>1170769.6.peg.229
MIERSGRNLIPWVICYGFLILCPLPLTIRLTDFQILE
>1170769.6.peg.234
MVEETNQKPVGKKEKPPAVEDKPFQEFMQEHYLPALQKAIAQEGISDVKLTFVKQKYAIV
GFNSSQECWQITGSWQNGAKQFNVYFPDQDIQGKKGFSCHEGKKPSTLESFLIDERKITL
DLLVSRLIYRLNGQKWLGRN
>1170769.6.peg.243
MMIIANMAGVVPDVYPFLSHALWNGCTPTDLVYPFFLFIVGVAMSFSLSKYSLESKLDKF
VYFNLCRRAVILFTLGLLLNGFWNQGVGSFDLQSLRVMGVLQRISLAYLVASLIVLKFPE
KTQWALAGILLIFYWLTMMYIPVPDYGAGMLTREGNFGAFIDRLIIAKPHLYAGDGFNFR

GDPEGLFSTIPAIVNVLFGYFAGQWMRKSTINSHTSMDLVLWGLCSLVVGMIWDGLFPIN
KKLWTSSYVLFSTGWGLVFLAACYDLIEVRKIKRWSKGFEIIGLNAIALFVASVFLIKVT
VKLKIGEGENAISVYNWIYRNLFASWVGNTNGSFLFALATLLLWYGLAFFMYRQRWFIKV
>1170769.6.peg.252
MTQSETTTAKSFPQLLAGLSLISLSLVISTWIGSRAILDFKKANDALIVTGSAKRPIRSD
YIVLRLSLVSQRPTIEEAFKDLKNQTIRVQAYLKENGVPDAAITSNPVETMTIPEITENG
RETGRILAHKLTQNLQIRSQDVDKYSQLSQKSTELINEGINLTVQPPEYLYTQLSKLRVE
MVAEATKDAQARAKAIADSAGGQVGSVRSAQTGVFQITARNSTDVSDSGLYDTSSIDKDI
TAVVSITFSMK
>1170769.6.peg.261
MVDQWQAINKMSKSVIDLLKQTTEGLFMPSESEYPFNVVHWEFFNLNETTIQEKTGIIGN
VRTVTVDDFFRGVTKQEDWYEEEEQNIAKRFESLVLVLKSNLDEAKVYEIGNREVHAYIL
GTKDGEIIGISTVVIRT
>1170769.6.peg.265
MYHLEQGRYKQVKANRRNHYPIPELGVELGMLLDQERPPIPWLRWWDNGGNLLLTGNERA
EQECQRRELAEAIAIQERFEKEQERQQKELAESLAIQEREKKEMAEALAIQERTEKEQER
QQKELAEALAIQEREKKEKLAAYLRSLGINPDEI
>1170769.6.peg.271
MPKPYSVDLRNRVIVVWATQEGSQRQLAERFKVSLSFVTNLVRRYRETGQVEPKQCGGYE
KPIIEGQYLNMIKAWIDEKNDLLLSELSDRLREKTGIKLMLVSQPCIEHYKSWVYIVKKM
VWESTICWCSI
>1170769.6.peg.272
MLTHNIQTKIKNSPLLFLLPLALAVRLYNINSPIIGIHSWRQADTAAIARNFYESRFNIF
YPQIDWGGNTPGYCETEFPIYSFIVAIFYKIFGVHEIFGRLTSVIFSLLTIYFLYKLIAK
FLDPKIAIWSCLFFAILPLTVYYSRTFQPEAILLTCSVAGIYYFSNWLESEKIHFLCISG
IFVSLACLIKVLPIIYVGLPLLYLAYCKFKHKIFTNIYLWIYTLFIGISFGMWYYHAHQL
YLEYGNTFGFWGSSANGRYQYNIIFTLKFWLDIIFRTVVRHFAVFMFPIFIAGLFIPRTN
KQEYLFDIWLISVIITWVLVPTVSLVHEYYQLPLMLPGVVFIGKFLAKYLDHNSLKINIK
KISITCICLSIITGSLIYTIDYMFKEKIHKSATFQLAQIVQKKTDAKSLMIFTTGGDPTL
MYLSHRKGWLIDPSDLTRQYLETKIKLGANYLISSFQFVESYNFNINENQKQNIIDTLVK
YPNILDSKELIIAFLNLRKN
>1170769.6.peg.273
METVWDAQAEEVILSGTTSQLGILTGHAPLLSALDTGVMRVRATRNSNWQAIALLQAIAL
LGGFAEVENNEVTILVNGAETGDKINLEEAHAAYNEAQQRLSQVPAGESQAQIKATQAFK
RARLQAAGDMV
>1170769.6.peg.274
MLLNNPHFHLPYKNQQSQGKNTLLSRGILSIIGRVRVRCRSYPKE
>1170769.6.peg.294
MSAGNHFPLAFTLVYVVGFVAAVTIGSIAWYNSKRPPGWESKDRPDFVPKIDKE
>1170769.6.peg.306
MTKITNQALNSALNQLWVFSLSSDFWEVFDAAFGTEYNRKNAEILRSQWQIGDFSQLPEI
EILDSSILGSANGAYSSSENRIYLSSNLMENGTSSKIREVLIEEIGHFVDSRINQIDTPG
DEGEYFAHLLTDQKLNKDEIDRLKAEDDRVVVTIDGKGVEVEQNNATLAIAPANASQTEG
NSGTKPFTFTVTRTGDTSGTSSANWAVTGSGINPANATDFGGILPSGTVNFAVGETSQTI
TINVSGDTTIENDEGFTVTLSDPIGAVLGTSSSGNTINESASGGFGVTEKYYNISSPGGT
FQLNYDMYGIPDRAEIFVNNVLQNQTTGGTNGDGFVSGSGSLNLNSIQLKAGDQIKVTIT
GNVQGTAWDYNVNYTGGLSSVNYIASGLITNDDNPLNSNVVTPKVNISTGLIFTAQSNGN
ITLDTNRGSATPDEVTSVQNGTESNFDHIIGLYEVLNSQGEIKDNQGNTLKPGDANYALH
ALTTARVKNFTVQAGGNDTPSTATQLGSGVSVLAGKLYAPFAIANGGTYFPGNQGIEDFV
AAEQGDINRFSAAPQYVRNLVDIEGKNGDIFNNAPRFVQEPVAYFSFGAANPDVSPHFRS
YGNGVYGFEDLPVSYTQYSNNDFNDGVFALTLSI
>1170769.6.peg.308

MDFSCSSIVYQLSAQITSIDVSRTISAKGSSRKSVKEVLAVWN
>1170769.6.peg.314
MWSGLHFVDVLESFSQSYHQNQGAGYDPASWRVIALLVGWELRWWLLAWLMRGSGIGSSF
IGLINSRLFNFSWFGDGRISVVLFLCSYIVPADILTPNIKLSNMQYLILKILLILYFTGL
FK
>1170769.6.peg.327
MSGKGIVLQEPSVVAIDQNEKIALAVGEDAKRMLGRTPGNIIAVRPLRDGVIADFDTAEL
MLKSFIQRVNEGKPLLLPRIVIGIPSGVTGVERRAVMDAATQAGAREVYLIDEPVAAAIG
AGLPVAEPTGNMIIDIGGGTTEVAVLSLQGTVVSESVRIAGDELTESIIQYMKKVHNLVI
GERTAEEIKIRIGSAYPSTEEEQGSMEVRGLHLLSGLPRTVTVQGQEVRESMLEPLAIII
EAVKRTLERTPPELAADIIDRGIMLAGGGALLKGLDTLISHETGIVTHIAADPLSCVVLG
TGRVLENFKQLERVFSGRSRNT
>1170769.6.peg.331
MNLSNSNLNSNLEKIYAFDYLRAISCIFVIALHSNIICFVKENQIIHDLIIFNLFDLAVP
LFFQISLILFFLKREKQPDYFFKKRFFKLLKMYIFWGLFYQLFSWGLHFKNIDFTNIQNL
IHIEANMKNIIIFIITDGKSPTFYFLFSLFFITSLAELFVYCLEKIKTRYQINPELISYT
ALCLTVWYNS
>1170769.6.peg.332
MSPITYSDFKNMSKIPKIIHQIFFLGAAAIPEKYKRYQQTVLQNHSHWEYQFWNEEKARQ
FMAENFSWFLPTFDAYPHDIQRRDAIRYFILYHYGGFYLDMDVESIKPLDNLLEEYELVL
SKLIVFSNAVMGSIPRHPLWLMVFEELKKRQHNLDNKTMPLYIGYSTGPIMLNHCVIAGK
FYEHSTVLVCPGYIFEPGAPMELNGKVFKSKRNLETYTIHHMTTSWLSRKDKILRFLFAI
VLEPYWFFQSLFNNRYGN
>1170769.6.peg.335
MNQLLPLSINQQEIYIDQRMWTNGSHLNIGAIVIVRGVFNREILNCAMSKIIDCHPGLRT
RVYEVDGKPLQTVTEYQHRDICFIDFSNYESSEAKAQEYISQEFAKPFDFNIDAPLADFK
LIKLSDDSHIIYAKYHHVITDGWGTAIFFREVIKTYTQIINDGKDSQIPRDWVIQEYIEE
EKKYLESSIFLRDRDYWQQRLQNVSPKIFSLIQPQGLEGKRHSIYIPREEYNRVNRLCQD
MQSNVFHFILSLISIYLTRRYLKDDVVVGLSLLNRSKKNFKDAIGLFVSTIPFRLTINRQ
QTIHQLLDNIRSLLRQDYRHQRFPLGEMKRFSGLQNKIKENLFEVFLSYERHDYSESFPD
TQTSCIPLYSGQQKIPLIVYVREYEETSDVKIDFDYNLSYLDEKTVEQIVTGFQNLFIQA
TDNLEIFIGDLEDSLADSRDISKNISVSQPEIPFVNYQETLISAFEEIATQYPGNPAVQF
NNKILGYAQLNAHANRLANYLIGQGIKPQSKVGICLERSDQMIIAILGIIKTGSSYVPLD
PNSPIARRQLILQDSGMTALITQSSLLTELNTENIIAFTLESIDSELNKEPNTSPQIAIS
TDFPAYVIYTSGSTGTPKGCIVTHRNAVRLMRATEAWFRFNETDIWTLFHSFAFDFSVWE
LWGALLYGGKVIIVPFGLSRNPEKFREFLTTEKVTVLNQTPSAFYQLIRADESAVGDLSL
RYIIFGGEALDLQSLQAWLEKYGDKKPRLINMYGITETTVHVTYRPITRQDLKTKGSFIG
REIPDLAIYLLDDQLIPVADGVSGEIYVSGAGVTNGYLNRPTLTAERFLPNPFGTGRIYR
TGDLARRLPNGDLEYLGRADQQVKIRGFRIELGEIQAALTSHGEVQEAVVVTDEWQEEKR
LVAYYVPGESSPTVNELRQFLKNTLPDYMIPAAYVSLKAFPLNVNGKIDIQALPAPDWNS
LRVEEDYIGPRNIDEEILCTIVAKILGLEKVGIDDNFFEIGGDSILALQVIAKAKKEGFA
ISARELYELTTVRNLATKKAAVATLEDIAETSILSLVSDTDKAVLPKDIEDAYPLSSLQG
GMLYHSELHTGSAIFHQIFTFNLEISYSELAWRQAIADVCLANPVLRTSFHWTGYSQPLQ
IVHRQVESPLSIVDLRSSPNANQHVQEWVELEKTRGFDIGNPPLFRFQIHRISNENLCFS
FSFHHVILDGWSVATLLTQLLRRYVQYLDGKNLPPLVFPETIYRQFIAQEQNAIANEEIR
EFWSQHLSNLQVTFLPRLNTTGIKITTTDYHNRQLKRLSLTISDELTDRLRKISQNIGVP
LKTCLLALHLRVVSFVTGQKEVVTGNVTNARSETSDSENALGLFVNTIPLRLELPSGSWI
DLIKAVFHAETAVLPYRMFPLAEIQRLLGKRPLFEVGFNYVHFHVYDSLLSLPQVQVGNV
DIFEETDFPFLAEFCLVPGSQTLQLNLIYDTQQFADAQVEQYGRYYQTAMVEMTTAPQMA
YHRRSLISISERQQLLQAANLDPQNFPSPDTLVSTFTQAVVKYTNKTALVYQQTTLSFAE
LETRANCLAHYLRTKKVNTEKLVGVCLERSEQLIITILGILKAGGCYVPIDPSYPSDRLE
FIFQDSQMMLLITEKSLISQLPECGGEIVTIEDITTEIDTRNCHPPVVQILPENPAYVIY

TSGSTGKPKGCIVTHSNVIRLLKATQSWFNFNSEDVWTLFHSYAFDFSVWEMWGALLYGG
QIVIVPYWTSRSPKDLLQLLTKNRVTVLNQTPSAFKQLIPAVQEQSEKLSLRYVIFGGEA
LEPATLQPWFDLYGDEKPKLINMYGITETTVHVTYKPITQTDIISNRGSVIGQPIPDLEL
YILDENLEPTPIGVTGEIYIGGAGVTRGYLQQPRLSAERFIPHPYSEKPGSRLYRSGDLA
KRLSDGEIQYLGRSDQQIKIRGFRIELGEISSVISSYPQVKQALVMVQKAPNGENRIIAY
VIFDSTAQAIDPLKEFLKTKLPDYMIPSALMAIETIPLTINGKIDYESLPIHDWHWKNKD
YVSPRNEREATICSLMASLLKLEQVGVQDDFFEIGGDSLLVTQLAIRLRQTYNIEFPLPQ
LFTHRTPEAIALLLPEVSQSSVETQIPKLRRTSRSVTLSDDGVLSKN
>1170769.6.peg.336
MYNNEQNDNIIYLVVVNHEEQYSIWPKWKRELPLGWRTVGKEGTKAECLAYIEEVWTDMR
PLSLRKAMEAVVE
>1170769.6.peg.337
MKLEETKNIVTGAASGIGRCIALELARSGAKVVGGDVDIDGLKSLELEAGELPGEILGMQ
LDVANESSVKEFISSASQKIGYPNTLVNNAGILRDGLLVTQDEDGWLRKLPTAQWKRVID
VNLTGAFFMAREFAAVAIEHSISPALIINISSVTRSGNPGQSNYSASKAGLDADTRTWAL
ELAPFGFRVGGVAPGLTNTPILSRVSSDALADMTANIPLGRIAQPYEIWQAVRFIIECDY
FTGSVVDVDGGARF
>1170769.6.peg.338
MFRSEILNTAQPPMFFDSKEFDFVSTLEKGWLDVKAELVKLQPKNFIDWPEKNIYNQGWE
VFGLYAFEQRIEENCKLCPKTVQLIEKIPGMLTAGFSSLAPGTYIGPHFGATNAVLRCHL
GVVVPDNDCGIRVDKETKSWQEGKCLIFDDTYEHEAWNRSDRTRIVLLIDFLRSNPNSTE
KDFTDKDFDQEYWLKILNQSEVRL
>1170769.6.peg.339
MEVYVLPLSLSQKQFWYREKLSSGNTAYNIPLALSLLGNLDQVVLETCFQKIINRHEILR
TTFALENGEPVQLVHDKKEFYLECQTLPLHLEKRPIEVSTIKEILERESRQPFDLINGPL
MRVKLYQISSEEHILLINLHHIISDGWSLGIFIQELTKLYATSGKINLPELPIQYGDYAQ
WEETYLQADKIQEQLTYWQDKLTLPLPILDLPLDKNRPILQTFNGAVLRQSLPGDVISSL
EILAAKEGVTFFMLTLAVYQVLLFRYSRQTDIIVGSPCANRTRSEVQNLIGCFINTLPIV
CSLEGEISFRQILQQISITSVEAFANQDVPLELIIDQLRIKRDPSRSQVFQTLFALQNAP
IGTIEMAGITVQPIHLDNGGAKFDISLMLEPDFEHGWTAALEYNTDLFTAETAQEILTRY
QQLLVAVVDNFDTRIDALPWLSKQEIKELLTLGSTEQTAEKIEPISLIDIFTQTVNNYKN
KVAVIDSSQKITYQELDHISNQLAASLIQKGVGRETRVGIFQERNIELIASILAVLKAGA
TCVPLDPQYPGERLNFIARDSGIELVLTTELLQSKIPVGIPEVLLVESGKFTEKLEQLNL
PAKIFPQQAAYIIYTSGSTGQPKGCVVTHQNVVRLMRNTQKWFEFNEKDVWTMFHSFAFD
FSVWEIWGALLYGGKLVIVPYFESRSPQAFRELLLAHEVTILNQTPSAFRQLIRADEEFS
HPLNNLRAIIFGGEALELQSLRPWIERYGDSHPRLVNMYGITETTVHVTYHQILAQDLLE
QRPSVIGIPIPDLCLYILNDSFEPVPYGVAGEIYVGGMGVSRGYLNLRSLTAQRFIPDPI
SQKPGARLYRTGDLARKLRNGDIEYLGRCDFQVKIRGFRIELGEIEAALIKLLQVSEAVV
TVHSFTEEEQRLVAYIVVSSVGNKGITTSELRTELKQKLPDYMIPAAFVFLDTMPLTSQG
KINRSALPAPDWNQSATKKDFTPPATDAEKILCHAWQLTLGVDNIGIEDNFFDLGGDSIL
ALRMVTQMRNQGWIVTPKQIFQEQTVKKLALVAQKQTNLQTLTVENHIARNEVPLSPIQQ
WFFDLTLSNPNHWNQSLLLQVHPSLEPKEVLAAMKVVCAHHDSFRLRFEKQAKGWRQFYA
SESQDGFSWEEKVDLDFKTEIEQNVIISQVRERVEKSLDLTHGPLCRLVWFNLGESLPPQ
LLIVIHHLIIDGVSWRILLQDLVEVISGAEISPTTGSWQNWCQFLQDSVKLQNIQDERQF
WQSTLAKETAKLPLDFSEGVKTNLECHVKTVSCQLTAQETQILLTTANKSYRTNPQELLL
AALGKTLANVTQSSDVRIMLEGHGREELSSGLDITRTLGWFTTLYPFTLKLPETDCHSEI
IKSVKEQLRAVPQKGFSYGILRYYNQETKTSLTTTVEVSFNYLGQVRNEGGNKHQLFSLL
NSQGSPARDPQGVRPHLIDINAIVLEGKLQVDWLYSSNLHRTTTIEQWVSDFKKNLLEIL
ELCLDLGVSEYTPTDFPLIKIPQSKLDIIQNQYPWIEDIYPLSPLQEGMLFHAIYEPEEG
IYFEQVIGKIIGKLDADNFAHAWQVVVDRHSILRTCFVWEEQEEPLQIVNKQTTFSVTCM
DWRNLSQELQLERVQEYLIADKQEGFSLSQNPLMRVTLVRLNDDTWQWLWSHHHIILDGW
SLPVIFKEVLTIYQWINQNSQRVTGERATNTLPPAVPYRHYIQWLRSRDQQKAQQFWRKY

LAGISTVTRLAWNTHDLETNPGLPAYQEIELKLTESEFALVQKMAQSWRVTINTITQGAW
AICLQKHGAGEDVVFGVTVSGRPPELPEMENMVGLFINTLPMRVKIDPTLSIANWLQNIQ
QHHLEMRDYEYSKLADIQKDCNLAGSALFESLLVFENYPVDQSLRGKLGDFQVEDIQFYE
RTNYPLTVGVIPNQGILLKLNYQTSFLSRGAAEKLISRFRNIIVNMAVEADETLDRIQTL
SVSEQKELITSSRGNIIKWQGFKSAHQLFENSADLYPDVTALVCGDRNISYGELEKRANN
LAFKLLAKGIRYESLVGLYFEPSIDYIVALLAVLKVGAAFLPLDRNYPDNRLEFIFLDSQ
IPLVLTNNSVVPSKLPEKVEVLDISQIDWRENHPRLNLQVSPENIAYVIYTSGSTGKPKG
VLVPHSGIENLVRAQIDSFGVNTESRVYQFASLNFDAAISEIFMALGSGAMLYMQPQAER
SPSSELWEKLTSWKITHLTLPPSLVAAISTTALPQLKTLIMAGEAVSGDLLRLWGGEQRK
CFNAYGPTEATVCSSLIDCTNLLGDASIGRTIPNVEIYLLDSFLDPVAPGVVGEIYIGGV
GLARGYLQRPGMTASVFIPHPFSEKPGQRLYKTGDRGAYDFHGNICFMGRYDDQVKVNGY
RIELSEIESALTKHKAVDSAVVILRQDLIGRNRILGYALVKPAEGKDELTGQELKDYLTN
ILPAYMVPGAIILMREWPLTPNGKIDRRLLPTPEFANAELIPKTYTEQILGQIWMELLGI
ETVNPQDNFFELGGDSIISLQVVARARTAGWEISPKDIFEAQTLSRVATRAKLISQQAEI
VEPLTGLIPLSPIQNWFFAQNLLHPHHWNQSVALTCREPLNTEALIVALDALVAYHDIFR
IGFSQDKGKWQQFYVSETKSPSLKIIDFASDSPETQLASLDTALEAEHASFQLDAPPLLR
VLYATNLTEYGDILFVFAHHLITDGVSWRILLEDLSKAYQQTIDQQTISLSLKTGSYRQW
TTYLQTLANTDKVIQDIPFWQNILDASVTRLPIDKSGKNSVDSTNAICTQLSLQETLLLV
KQATATYHASVQEIMLAALLSTLVNVYKSDHWLIDLEGHGREQIGDALDTSRTVGWFTCL
YPILLKLPANQDNHEVLLKEVKTQLRSIPHYGISFGLLRYLNQNQPLHRQGNSDISFNYL
GKIDNLSKKNGIFGLSNVPTGTGLFYLQERTHFLAINAKIQNEILQIEWSYSTNIHHDHT
IENIAKTYLQYLALYVTGCESPDSLFYTASDFHLADISESELGSILEDLE
>1170769.6.peg.340
MTGLKDKIEDIFPLTPLQKGLLFHTLYEPQSGVYFEQLHCQLQGEVSLVAVRQAWQTLVD
RHSILRTAIVTKGQTEPVQVVFRHLTFNIAEEDWRGLSDDAQKEYLNQFLEADKHKGFVL
NRPPLMRVTLIRLREDIWQLVWSHHHIILDGWSWPILLKEFLMLHKAAKENVAISLPRVR
PYTDFIAWLKQRNHQESKKFWQQYMLGFESATPLLMISKTRINSKFKSGEITTEFSPEKT
DLLDKLARNCSVTLNTVIQGAWAILLNRYSRSNDVVYGITVAGRPPEIPGVETMIGPFIN
TLPLRVLISGEETLDYWLQNLQSQVALMRQFEHTSLSDIQGWSDIPRGKQLFESLLAFEN
FPVDKSLKASDFGLNVPESYFSESTHYPITLVVIPGNVISLKLSYNENRFDASTMKLLLQ
QFSDLLLNMTGNPRAFLRDISLLGYQEKQYLMEKREPSELPPAQVTINEVFAQSVSIYPE
RIALSYEDKKFTYQQLDQSSNTIANYLHTLGIGSEKRVVICLERTPELIIAMLSVVKAGG
VYVPVDPTYPPDRIEFIIDDCNAGVIITTSNIDLEFPDRIIRINLDTLDINTTYVNIFTN
KLNTSLSIGTEPSVINLNPDSGAYIIYTSGSTGKPKGVLVTHNNVTRLFKSTEDWFKFNQ
EDVWTFFHSFAFDFSVWEIWGALLYGGRLVIVPYCVSRNPQAFVQLLREQEVTVLNQTPS
AFIQMLACQDLADGFCSLRYIIFGGEALNLGSLSPWFERYGENPTRLVNMYGITETTVHV
TYRPITKQDVDNSSGSLIGKPIPDLHISILDPDGNHLPKGVIGEMYVGGAGVARGYLNRP
ELTAERFIQDRLKPNSRLYRTGDLGRFLPDGDLEYLGRIDSQVKIRGFRIEIGEIENALA
QIPDVQENVVIVTTDDKTSEKRLVAYLVCTVEKQPTVKSIREHLQSYLPEYMIPSQIVYL
EEFPLTANGKVDRKSLPVPQITRENLGIEFVAPVTEIEQQLASIWHQVLEVEKIGRFDNY
FVLGGDSIRSIRVCSLAQSIGLNLKIEQIFSHPVLAELAAFLETVPAAKTQNCYEAPFTL
IAAEDREKLATIADDAYPLAQLQAGMLFHGEYSQTSTTYHDLFSFRVRISFSLEIWQQAY
TEMFDRHPVLRTAFFLGEFSQPLQAIVKDVSARIIFTDLRDLSASAQEDYIEAFIEQEQA
SRFAYNKAPLIRFHIHQLENDVVEATLVIHHAIMDGWSFANFLYELTGLYLQKMGRGIPA
LPSASGLEYSQFIALEKQALQDQKQKEFWQQQLLEIPFTKLPRLPIMTSWEFIPKVGKVD
ITLTQTTSQGLKDISHDLGVPLRTVLLALHLHILSIFSGEEEIVTGLVSNGRPDTTDSDR
VLGLFLNTLPLRMKLPHGSWVELVKKTWQAEQAIMPNRRFPLAELQRLNSNLPLYETSFN
FIHFHVYQGLLNWRDIELLQSKSFEETNIPFAVTWNQEVGTANISLNITYNHSEFTQEQV
DNIASCYRTCADLLTQNPLSDREFLLTPQEVITAEQLSTSQTITPVHEIITEQAALNPDV
IAVVHEGKSWTYQQLNERANQLARFLQHQGVSQEKPVGICLERSLDMVCAMMGVLKAGSC
YVPIDPYYPSVRIQSMLEDAKVHLLLTHSNLKIDCLKSTSQENTHKIIFLDTCSREIEAE
SLENLKVPVLANNIAYIIFTSGSTGRAKGVAIPHGALISHQNWFLENFGVNNRDVVLQKT

PFSFDASVWEFWTPLMVGAKLVMAKPGGHQDPLYLIRTIQQENITLLQLVPSFLEVILSE
AEFSQCTSLRLVFSGGEMLKTPAWQKFREKLSVPLVNLYGPTETTIDISFHHCRENENNH
QIPIGEPVSNSKLYILNSLMHPVPIGTPGNLFVSGCQLARGYWNMPAMTAERFLPDPFVP
GQRMYHTGDRARYLPDGKIEFLGRVDQQVKIRGFRIEINEIIAALEKQSWVHRAVVRAIS
GSQNSPRLVAYLELTTTSAPDNWQKILRLELAQILPEYMIPALFFKVDSWPLLANSKIDI
NSLPDPEFPTTTAYQDYIAPQTEAQKMLVTLWQQVLKVPRLSIHDNFFELGGDSIIGLQI
IAKARDLGFYFTPQDLFKYPQVADLAAQVRKLEDHSFNLPTIITGEIPFTPIQKWFFQQS
LAHPEYWNQAILLDIKPPFNMDNCQKSIKQIVNKHSVFQLRFRQTETGWIQELGHSSQRI
NWDFIDLTDTPEYELSGQLQTIATKFQGQLNLETGLLFRVVYFQTPVTTNDKILLIIHHL
IVDGVSWRVILQDFAENLAQDTPPISPKSKVSVGFPQWSYYLHQVSANMIADTNINWQSD
VDFWKNQIAANFALPLDFSENHNKENSADQIEFTLTEAETDKLLLEIPQTRKVRIQEVLL
TALAVTVLEWTGQSEMTIALETHGRESTAEVDISDSVGWFTSIFPYKLTTQSQSNDILRH
LVGIKQQLSRLPNNGLSYGIISQKPEYVDVLPPIPPGIIFNYLGQFDEQFPPNAPFALAK
EDTGISRHPENQRAFQLEITGLVVNGKLQMRFGFSKNLHHEATIRHVADIYQRCLQNLLT
ICEQEFTSWVPEDFPLVNLNQEQLNIVVSGVTDLQDVYPLAPVQEGILFHTNYEVEKDIY
LQQVTGNIIGNLNIELFKNAWEKCISRHSSLRTSFIWRSLPRPLARVHSRVKLPFIYEDW
SSIDHWEQKWADFLTRDRQEGLSTEIAPLMRLSLFRTDHEKWRFCWTHHHVLLDGWSLPL
VFEDVIAFYQAQQNQRDLNLPKLPNYRSFISWLNGKPIEEAKVFWRGQLMGLESATSLGL
TTQKLAPTEDYQILQATLEPKIYSQLKTYANKHQITVSTLVKAAWAILLNKYSRGEEVIF
GVTVSGRPAELSGFERMVGLFINTLPLRLSLQADLPMYQWLKSVSDRILSINEYSSSSLV
DIQGWSGINRGDPLFESIVIYENYPLSENLDPGDLLINSVESLEKNHYPVSVYALPGKED
LTLKVAFQKSTGTREERHQILQYIVDILTTFATKSPEFLGEICLPTSSSIPIIPQILPVV
TLLDLFNQVVLESPDSPAVLHGEKYLTYRELNNQANQVAQALMQLGVKRETLVAVCLERH
GGLAVALLGIMKAGATYLPMDPNLPTERWKWIIDDSQATIILTESSIVSQLPESSATILL
LDAIKTIPQQQIVISPHPLDLAYIIYTSGSTGRPKGVQIQHQSLLNFLLSFREKLQLTPT
DTLVAVTTISFDIAGLEILLPLISGAKLVVANRETTQDGFKLADLLQRSQATIMQATPTT
WQLLLTTDWQPKNPFSAICGGEAIPQELATSLLTLGINLWNVYGPTETTIWSSIKKLEKP
QDAVSIGKEIANTCLYILDSCLNPVPQGIIGELYIGGIGLARGYQNNPKLTSEKFVPNPF
SQEPGSRLYRTGDLARWLSDGEIEFLGRIDYQVKIRGHRIELGEIETVLSSHPAINQAIV
QAIGDTAADKKLVAYLVAKNQPPTIEELRLYLSAKLSNYMIPSAWVFIDKVPLTPNNKIN
RRALPIPDYTEEQSEYLAPRNPIEEALAYIWQELLKLEKVGVKDNFFYLGGHSLLAGQFH
GYIKKVFAIDLGLRELFDAVTIERIAILLIEKESKLGNTEKIAKAFLRIKAMTPEEKAKL
VQKSKLEGQN
>1170769.6.peg.341
MKKLLVTGSSGLIGSEVCAYFAQKGWSIHGIDNNQRAVFFGQQGDTRWNQKRLESQIKGF
VHHEVDIRNRKSILELIERIIPDAIVHTAAQPSHDLAAKIPFDDFDINAVGTLNLLEATR
QFAASIPFVYLSTNKVYGDAPNEIPLIELETRWDYNVTKYQNGIAESFRIDQSKHSLFGA
SKLAADIMVQEYGRYFGLNTCCLRGGCLTGPNHSGVELHGFLSYLVKCNLEGRTYKVFGY
KGKQVRDNIHSYDVARFIEEFIAAPRTGEVYNLGGGKENTCSILEAFKIVTELTNKPMVH
EYIATNREGDHICYYSNLQKIREHYPNWSITKPLNSIFVELVAAWSKKMYTT
>1170769.6.peg.346
MLSETANRAPANPDSIAIPPIPMTNNNQRPDFDFLGFLPLDLLLTGLLPAADVL
>1170769.6.peg.351
MKFDITNPGMQFSEYLPWNETLGLAYQLGVDGLSILMLILNSLLTWIAIYSSSENTERPK
LFYSLVLLVSGGVAGAFLAQNLLLFFLFYEMELIPFYLLISIWGGNKRAYAGMKFLIYTA
VSGALILATFLGIVWLSGSHSFALDAVNTQNLSTTMQIVLLAGIILGFGIKIPLIPFHTW
LPDAYVEASAPIAILLGGILAKLGTYGLLRFGFGLFPQAWNVVAPTLAIWGAISAIYGAV
VAISQKDIKRMVAYSSIGHMGYILLATASGTKLALVGAVAQMFSHGLILAILFHLVGIIE
GKVGTRELDKLNGLMSPIRGLPIVSALLVLGGMASAGIPGLTGFIAEFIVFQGSFSTFPI
PTLLCVASSGLTAVYFVILLNRTCFGKLNNDLAYYPKVVWAEKIPALVLAGLIIFLGVQP
TWLVRWNETTTSAMVGAISTAGTIIISQVETNQHQQ
>1170769.6.peg.371

MVILLSTFKYLKVFYRKIDPPDIPSDIKVAGKLFDGGWEVLVGLRCNHL
>1170769.6.peg.379
MLKLKLHVQQLTNGKTGIFLDILGIPNFVKLGLFASYSNSNTK
>1170769.6.peg.395
MKLEDRIDNAIDSIDKYLTIVMIWTKSPRFACQISKQQMPTL
>1170769.6.peg.396
MVKYLSMLSIALSILSSSFNPQLNKLTSFLKSQTTEVLVSKTLPAFPGAEGFGANVTGGR
GGKVIYVTTTANSGPGSLQWAIDQPGPKYILFKVSGLINTRIHLRNGDVTIAGHTSPGGI
TIRGFVTDETPFQDQAIRAPSDYAENWILQHVRIRPGLNGPSDDGLRLRYTRRAMVDHVS
VGNATDEAVEISYSNNITIQNSILAETLGGHSFYGGMLINYSNPTHGFGLDNLSIHHNLF
VRIEGRLPEASRESRAAARSTMNLEISNNLYWDPRFFIALGANTGQLVDGNSRPYPIYYR
LNAVGNYFRTGSTFPYGMWDDQILRETSASGNEVYVQDNKISLYPTRSDYELFYCCNDYA
QEKNPDKTSRQAKKRTSRHNFPTITYTPATSLPIMLPKQVGAWPRDGMDKRLVQPIQNNR
IDTANINTNPARDALIPAYLGSPPAPPTDRDNDGMPDAWEIARGLNPNLSNHNGYNLSSR
GYTNLEVYLHELSTKILGVGFPRNNL
>1170769.6.peg.397
MFLRCLSTFCILSPIESPGLFILIYSQSALMRLRLLRLKTNPTSIGNDRE
>1170769.6.peg.428
MSNQERMDNMENQLIDIRLAVSALLETSVIYQRNFEMMQRNFDSVVIEIREMQSEIREMR
SDIREMQSEIREIQSEVREIQLDVRGLQTENRRILDVLQNVPPDKYE
>1170769.6.peg.432
MVIFRRLITSGLIGFLASSCANAAQSSTEYTKQIVQENNVARLMAQVQDSQKVSNLLSQG
NGFLDSGRYEEALQLYNRAIEIEKDSVPSWVNRGNALLSLKRHQEALESYNQAIALRPNK
NEAWYNRGNALSALGRYEEAIRSYNESIVIDPNKFEAWINKGIALTKLQRYQEGLASYNQ
AISINPNFPTAYYNKACNYALQKQVNLAVESLAKAIKIDGQKYTQLARVDKDFAKISDNR
DFQELLK
>1170769.6.peg.434
MEWNNTQTDYPHQCIHQLFEQQVERTPNAIAVQWENQQVTYRELNNRANQLAHYLQFLGV
SAQTLVGIYLERSPKIIIAMLGILKAGGAYLPLDPTYPSDRLAFMLQDAKAFLILTEQQL
GGKLTDVEQICLDRDWSVIAQKNQQNLNCDTTPDNLAYVIYTSGSTGQPKGVMVPHCGVV
RLVVNTDYITLQPTDVIAQVSNNSFDVATCEIWGALLNGAKVAIFDRETILSPRDFATSL
QNEGITILMITTALFNQMVQQVPMAFRHLHYLLFGGEAVDTQWVRHLLAVDGPQKLLHAY
GPTENTTISTCYYIQQIEPKAKTVPIGRAIANTQVYILDRHLQPVSIGVSGELYLGGTGL
AIGYLNRPELTSERFLPNPFVANDRLYKTGDLARFLPDGNIEFIGRVDHQVKIRGFRIEL
GEIETVLTQHPQVKQVVVIVREDNPGNKYLTAYIVSESLTLSSSELRQFLKERLPEYMIP
LAFVILNAFPLNPNGKIDRRALPIPIVDNPQTVFVAPRNPTEETIAHIIANVLGLEKVGI
YDNFFELGGHSLLATSVISRIRESLSLELPLRSLFKAPTIVQLSQVINVHQLEVEQQSAG
SMTFDTLPPLLPQVRNTYIPLSFAQESIWHSQQLAPDNYAYNSFVTLRFTGSLSATVLES
SFNEIIRRHEILRTAFTLIEGQPVQVITPLLTIPLEIIDLQNLPNTKRTSEAERLAALEY
EHHFDLGSLPLIKTKLLQVNQKEYWLTINMHHIITDGWSLGLLLEELGILYTAFSNGLSS
PLPELPVQYADFTLWQHQCFNEKVIEKQLAYWVEKLTNTSPISHNVSNIPPQVSSSASVY
SIVLPVSMVRSIQAFSLEQKVTIFVIILTAVNILLFNYSGKDDILVITTVGNRSSVKTET
MLGCFINDVILHSHFSSEETGLTLLQQVQQTLMEAINNKEIACQTVIDTVTSKQPLNIST
SLTMLPPQNWHNRMLDFEFVSIKRDRSLWDEEIPLEIYVSSPSVNNPTMEIKVFYSRELF
TDNAIEFMFSYYQEILQKLVQHPNSPIG
>1170769.6.peg.438
MELFLGTWQGTKEGRTGYWLRWWRETGNLLPWALELIEQERQQVEQERQRAEREHQRAEQ
EHQRAEQERQRAEQERQEKEMLIAYLRSQGIDPNNLPNHAE
>1170769.6.peg.442
MDHRQILTMAKHFNTAGPCQSDIHYMLPPTARLPDLRALIHGRNYLEGDVPAVVMEFLSD
KDGGEYSFKRTYPPGKWFFYEQILQVPVYIIFDPDGGLLEYYELKNERYELKQPDENGRH
WIESMELFLGTWQGAKEGRTGYWLRWWEEAGNLLPWALELIEQERQQVEQERQEKEMLIA

YLRSQGIDPNNLPNHTE
>1170769.6.peg.445
MKSGFLLGSLVAVCVVGFPLALHHHKAIRPVLVELGLVLQ
>1170769.6.peg.446
MSGNTPTTSTTATCARNLSKPSSTSWSTGVLRKPTSPDPRYSVRQKGRSSGPFHFKNHGC
MTQPPLNSGPVNRPPALMPFLANQPWFISSTNRTGPDEQWESVVWGFMSARLTMVPSSLT
KAAVRGSSVFIIQKHCTDGCSKTKIMPSCWGISLRNMRPVLRCSGVDATCASIWCIPAES
LTRGRSICGDWAPTKGPVRARRAAKAYWTSGFMGRHFARSP
>1170769.6.peg.448
MQVPVYIIFDPDGGLLEYYELKNERYELKQPDENGRHWIESMELFLGTWQGAKEGRTGYW
LRWWEETGNLLPWALELIEQERQRAEQEHQRAEQEHQRAEQERQLAEQERQLAEQERQLA
EQERQRAEREHQLAEQERQEKERLIAYLRSQGIDPNNLPNHAE
>1170769.6.peg.449
MQVPVYIIFDPDGGLLEYYELKNERYELKQPDENGRHWIESMELFLGTWQGTKEGRTGYW
LRWWGETGNLLPWALELIEQERQQVEQERQRAEREHQRAEQERQEKEREHQRAEQERQRA
EQERQEKEMLIAYLRSQGIDPNNLPNHTE
>1170769.6.peg.450
MFEDELELAYAVMDGVNARGKRNKHSTERIKFNNSCLSQPFVT
>1170769.6.peg.451
MQRFPLSGYHVQRSIVNPPGRKFGGAVIGDKVNSDRFLVKGENISNVK
>1170769.6.peg.454
MTIVSQQSDRYLPYLLLQHRRILRVEPRYEHDVPMKRSLFTLSPNYSTAGFLEIEY
>1170769.6.peg.456
MHHDSLPVLIAEIEEGQKVSKDLEKLYNTYKPFFTDESKRDIRDAVLLADIFCNTYTCLE
TIFLRISREFENHLDDSQWHKELLRKMRIEIPGIRQALLSHHSYELKGLR
>1170769.6.peg.463
MQTLPKERRYETLSYLPPLSDVQIEKQVQYILSQGYIPAVEFNETSEPTEFFWTLWKLPL
FNAKTTREVLGEVQSCRSQYSNCYIRVVGFDNIKQCQVLSFIVHKPTRY
>1170769.6.peg.473
MDVMAVFIFNIQVKNVTMNRELASVSEFSQVPNSSSQLSGISLQQQKLP
>1170769.6.peg.491
MQVKAVIITHSETSTGVINDLAAINSHVKAHGEALIIVDAVTSLGAYNVAVDELGLDVVA
SGSQKGYMIPPGLGFVSVSTKAWEAYKTAKLPKFYLDLGKYRKSTAKNTTPFTPPVNLIV
ALHTTLGMMKKEGLESIFTRHERQKNATRAAMKALNLPLFAADECASPAITAVSVPGMEA
DKIRSLMKKRFDIALAGGQDHLTNKIFRIGHLGFVSDRDILSCVSSLEVVLGELGYENFT
PGTGVAAGVKVFAAH
>1170769.6.peg.492
MSLSSDGGIILARQVEEKVKICQDMADCLTDNRDQTKVKHSLSQLISQRIYQIIAGYEDS
NDSNKLRQDPIFKLVCNQVPTVGENLLASQPTMSRLENQVTQKDIKQIRRLFVDKFLESY
PRESKEIVLDIDAWDALTHGHQQLSLFNGYHRHDIYFPVLINEASSGYPLVLQLRAGNSH
SGKGVAGILKWLFLRIKRALPEIRIVLRGDGGFSLPEIIEVCEKSGVGYVFGFSNNDVLK
RKINYLLDRARLEYCRTGEKVRLFDDVYYAARSWSEPRRVIMKAEWLEKGPNPRFIITSL
ETEAQDLYDKFYVQRGATSEHRIKELKLGIKSDRLSCEKFIVNQFRLFLSQAAYILMLGI
RQAAQGTKLAKAQVPRLRETIIKIAAKVTVSARRVLVELPYYCPFSSEINLIMERLASEF
EIIFS
>1170769.6.peg.493
MSRGDRILVGNNGKFGERWVEVGEAFGLTWIIHQSRIKIAERVILQ
>1170769.6.peg.501
MQLEQAQQDLEQKLDLDFEGFTVEHLVINQQQALAIENLPGYHLEGVYDLKVKLPTRQIA
QSHNHFNIYLQIQKEGKSWRLLIPEKSGEKQPLVWRGYLII
>1170769.6.peg.507
MISNLLKFSLLPILTAVSLIFTLALTSKANGQNPAITSTEITNYAQTVMIMEPKRQQAFK

EIKTLINTRSIPIIICNDVNSIKLLPRDAQKIAINYCNEYEETVSENNLTVDRFNQITVE
VNNNSVLRERVQKLMMEKMGL
>1170769.6.peg.508
MEYVVQVLLSTIPSLTQPQAVTIMMEAHSNGLALVISCALEHAEFYCESLNNHGLTSTIE
PDD
>1170769.6.peg.509
MFRPSQIALWISQRPAPQRLGCFVLCLLVLWLPFVIPIYLLVEDGNLESIFSMVLLYGEF
IVLVRLWGKKIYQRDKILTDYGLELSSLNGVDFCQGLSIGVLSIILLFSIQGLLGWILWQ
PPKAFIVQIIWEGLLVASGVGFAEELLFRGWLLDELNRDYGSRLSTAINAILFAVAHFIR
PISAIISTLPQFPALVLLGLTQVWGKHKKRGRLGLPMGLHSGLVWGYYIINVGGLVQPSG
IVPDWVTGVNNNPFQGIVGMLGMALLAYQMRVK
>1170769.6.peg.510
MTSIHLLLGNPSNATSSLDNPDNYLMIKPQYALSYNRSHGSANWVTWQLDKSWLGDAKRQ
DDFRPDDTLPNGWTRVKPSVYNSSGYDRGHIARSADRTQSVEDNSATFLMTNIIPQTPDN
NRNTWGNLEDYSMKLAGEGKQLYIIAGGFGDKGKLKNLVTIPQYTWKIIVVLDRPGLGLK
DVNVNTRVIAVNIPNDEQLDNNWRVFRTSVDKLEELTGYDFLSTVSPDIQKVIESQVDNL
>1170769.6.peg.512
MNIETLKSEKTKQLPGANLEDQDLSEFDLTAANLAGANLMGAHLVSVNLEGSHLEGANLM
GASLQGADLRANLLGANLMQADLTGADLRGSNLRGANLMGATVAGASLTAAFLSGANLMS
VNLQGVDLRDADLRGANLTGANLKGADLSRADLQGALLNQANLEESDLRGANLAGANLAG
ANLLCAELEAASLNGANLYQACLLGTILETYHD
>1170769.6.peg.518
MIGYGNALNPISGFCPRKFMTESTTFFLSPLIRITLLTLYIALTLPLPFLAEVTQAPVTP
LVLWLGIIIGLVALYGVLTQRVIVNEQEIQVTYPIWVPKLLRQDWSLPWSEIKSLKCRTT
GQGGLVYYFLTDEGKAYLLPMRVAGFNRLVKFVQLKTGMDTTDVRPLSQPWMYLILLICT
IFLLLVDAWTINIANSMI
>1170769.6.peg.523
MVITSCFYSYLVSQLLVSYLYLQRRQFVFGGSPKTMPTASI
>1170769.6.peg.554
MRGGKSFWENFAPNHLTGKKNLVIICGVGENNENLLGL
>1170769.6.peg.558
MFSFFMVWHVYTWFSIGVIMPPTYILLSLGTTCLLTNLGAIVWRVNFRHGTSSPRSKLID
EINSSF
>1170769.6.peg.567
MADLILKLLPTNKKAKEAFVYYRDGMSAQAEGEYAEALEYYEEALSLEEDTNDKSFIVYN
MGLIYASNGDHDRALDFYHKAIDLNPRLPQALNNIAVIYHYKGEKAKEEGDNDGGEALFD
QAADYWIRAIRLAPNNYIEAQNWLKTTGRSQIDVFF
>1170769.6.peg.577
MSFYAVGDRHFHLFSKRILEEVSNLYDLWYNGICSQDLCIS
>1170769.6.peg.578
MPPRSFSWQTFIYLSVFSWGISSLATGIIKDIIAFTGWIFLFAGTAWYTTDSPVRIPGTF
MPVGALLTGFIFSVFVFGHGENTITVRTIVLWPTIAAIITALPQFFTGNGISPKATLPKL
EVRQKIILLLSWSMLISCWLQFNFVTDKWLKEYPSLSAQSFQRSNFVIKFEPKANKPEPG
NVILNRIEPLILQQITNRPWSEAERWLLEANQQVDNLGKIIINKNLQKFEEKGLWKIEPR
VVNIKSGYRLDLLSIWQGPTDSKKGFYLRKSCYIEPIASEYTTDNSNVKAKIKCDPKTKF
FRGSPPAQQ
>1170769.6.peg.584
MGDCYPLPPVHTGGTVLPTIIDGYGGGWGFPVSPTYDYLIFYYTI
>1170769.6.peg.604
MNVWLLSLLFHYFQHPITILSIAAILAFLLNYPVQLLEKARITRTYSVIIVLVITLTLLV
ILGFTLVPMLVEQTTQLLRNIPDWVTSSQENLSKLQVLARQKRLHIDFSVVSSQINASVQ
NILQQIASGAVGFAGTLLSALLNIVLVVVLAFYMLIYGDRLWSGLINQLPSYIGLPLSKS

LQLNFHNFFLSQLLLALFMVIALTPIFLFLRVPFALLFAIIIGISELVPVIGATLGIGLV
TLLVSLQTWWLAFPVAMVAIIIQQIRDNILAPKLLGNFTGLNPLWIFIAILMGFEIGGLL
GTLVAVPIAGTIKSTIDAIKNNKSGIT
>1170769.6.peg.606
MKAMILAAGKGTRVRPITYTIPKPMIPILQKPVMEFLLELLRKHGFDQIMVNVSHLAEEI
ENYFRDGQRFGVQIGYSFEGKIDDQGKLVGEAIGSAGGMRRIQDFSPFFDDTFVVLCGDA
LIDLDLTAAVKWHRSRGAMATIITKSVPQEEVSSYGVVVTDNENRVRAFQEKPSVEEALS
TNINTGIYIFEPEVFKYIPSGVQYDIGSQLFPHLVEINAPFYAIPMDFEWVDIGKVPDYW
RAIRGVLLGEIKNVQIPGYQVAPSIYTGLNVAVNWDRVDITGPVYIGAMTRIEDGAKIVG
PSMIGPNCWICGGVTVDNSVIFEWSRLGPGVRLIDKLVFGRYCVDKMGTAIDVQAAALDW
LITDARQTPPAEIPPEHQAIAEFLGGITV
>1170769.6.peg.609
MSAKVKLATFPFQEFGLVDAEVLQISPITRRFNEAFSVR
>1170769.6.peg.618
MHHFSEQYAKRTGTYFCSDPSVTAVVIEGLAKHKDDLGAPLCPCRHYEDKEAEVSAAYWN
CPCVPMRERKECHCMLFLTPDNEFAGKNLQEIPVETIKEVRDSMG
>1170769.6.peg.626
MEDLMAIIHCFSCRLYGLRNYSKEIKDNLKNAIDKPA
>1170769.6.peg.630
MEPFGGAWIQFNIRYYMFALVFVVFDVETVFLYPWAVAFHRLGLLAFIEALIFIAILVIA
LVYAWRKGALEWS
>1170769.6.peg.636
MVYVNLFEMKMRVRMSMRSVITSAYCQGLPGLYQTYENSDRSQ
>1170769.6.peg.640
MLIVLVLLYLIRAIAAIALQKTINKYKQIFATGVNYFSYK
>1170769.6.peg.660
MGIKPWSTKELLVSFIGFRTLEVITVATFRGMEGGLSKPDLYLGFYNPKDSKNFMNQNSS
FSHI
>1170769.6.peg.663
MTPTEVKDLAARLELDNYSNAFDGLNDWHLLRAIAFQRPELVEPYVYLLDLEPYDEG
>1170769.6.peg.684
MRVLRYFSNQEVLDLRENLGRGGEAFIYTVSSDENLVAKIYHRPSSDHIKKLQAMIANPP
ANPAASFGHISIAWPQELLTAVDGSDTIIGFLMPRIRDMVPIIDFYNPGNRRQNCPLFNY
QYLLRTARNLAAAVAALHASNYCIGDVNESNILVSNTALVSLVDTDSFQVPDLSQSRVYR
CLVGKPEYTPPELQNKTFADYNRETYHDLFGLGVLIFQLLMEGNHPFSGVFQGLGDPPSY
EYRILAGHFTYSQKQKVPYLPTPITPSWQTLHPAVRDLFVSCFEDGHHSPHLRPSAQTWL
SVLSTAEASLVSCAVNPQHVYHFDLNTCPWCERTVKLGGRDPFPSLQTISAREYHQPRPK
SRKRYRYASHVRKSATPVLVTYTQSSLKSSSAIYKPIQISNRSKFYALMFGFLGLGVLGY
LDIMIKFTRIFISPSPYTKQSLLSSRSENVHSPLSLSFHDYYQRGNQAYQQQDYQQAIED
FTQGIKQNTNFSKLYMHRGNARYNLNDYQGALSDYSIALKINPQEVKAFINSGNAYFKLA
DYSNDPDYEYKKAIDSFNNAININKQDDDAYVRRGVVRSQIAKYSHNSQQEYKKSISDFT
QAIKLNPFKAEAYFQRGLSRYQFGQYSSNYAEIYKQAIADFDQALNINPQMAEVFLKRGT
IYYELAQYGERTTKNNQQKALEDLEKSAQLYLNKKDFNNYQQAMSNICVIAEKKCDYFLQ
NSSIIYSVNP
>1170769.6.peg.693
MLLLLVANKVTSELIITLGTASEEIFRGDRLPPLKFPQESCAEDRITS
>1170769.6.peg.694
MVFSQGLKRNSFVRTQSTPYIIASVISNVTSDSKTTNPQNKSCLLRFPIPLYYK
>1170769.6.peg.695
MEGFPSSFVKKSWIHHIEQFFFDIFVTFVAIEMSQDSLDG
>1170769.6.peg.706
MTITLEEIRKKLKQVWGYENFRPPQEEIVSSLLSQKDALIIMPTGAGKSICFQLPALLSN

GLTLVVSPLIALIENQVEELKQRNQKADLLHSELPASQRYKVLESISKQQLRLLYLSPET
LLSSAVWEKISHPHIGITSLILDEAHCLVQWGETFRPVYRRLAAVRPSLLNTKPPGTKIS
VAAFTATADPSTQNIIKDVLQLQQPDIYRLNPYRQNLQPTVKTVWTPKARKQQLLKFLQL
HPHQTGLIYVRTRKDSEELAQWLMNLGYDTASYHGGLSGEERRAIEKSWLHGKKSFVVCT
CAFGMGINKADVRWIVHFHAPYLLSEYVQEIGRAGRDGIVAEVLTFISEPTGFFDGEDQR
RKLFFQQQILQQYKKAQELIKKLPLQGEVKSVVKEFQHGATALAILHSSGRLLWNDPFHY
QILGKDIHQSRTYFNPAQQMVEYLRTKNCRWQFLLQSFGFNKDRENWRCGHCDNCRFKSG
GFQGIS
>1170769.6.peg.710
MTTPQEVLKMIQDQNIQMIDLKFIDAPGTWQHLTVYHNQIDESSFTSGVPFDGSSIRGWK
GIEESDMTMVLDASTAWIDPFMKEPTLSIICSIKEPRTGEWYNRCPRVIAQKAIDYLGTT
GIGDTAFFGPEAEFFIFDDVRYDQTANEGYYHVDSVEGRWNTGRKGKNGEADGPNLGYKT
RFKEGYFPVPPTDTFHDMRTEMLLTMAKCGVPVEKQHHEVATGGQCELGFRFGKLIEAAD
WLMTYKYVIKNVARKYGKTVTFMPKPIFGDNGSGMHCHQSIWKGGQPLFAGDKYANMSDM
GLYYIGGILKHAPALLAITNPTTNSYKRLVPGYEAPVNLAYSQGNRSASVRIPLSGDNPK
AKRLEFRCPDATSNPYLAFAAMLCAGIDGIKNKIHPGEPLDRNIYELSPEELAKIPSTPG
SLELALEALENDHAFLTETGVFSEDFIQNWIDYKLANEVKQLQLRPHPYEFFLYYDC
>1170769.6.peg.711
MRDAVTNLINSYDLAGKYLDRNALDSLKSYFDSGTSRVQAATAINANAAAIVKQAGSKLF
EELPELIRPGGNAYTTRRYAACLRDMDYYLRYATYALIAANMNVLDERVLQGLKETYNSL
DVPIGSTVRGIQIMKDLAKEQAIAAGVANAAFVDEPFDYITRELSEQNI
>1170769.6.peg.718
MIYGTAALMAYVTGVDSQLALYVAIAYLTARVLFSVFYILNIPILRSLMFGVGSACIISL
FVLSILKGT
>1170769.6.peg.720
MSIFNTAILYDIENLTKGYSFSKDFIKELSLKQIYRQILEVDIVNKICLQRAYANWSDHR
LSLLRGEINELGIDPIQIFGFARYHKKNAADIQLVVDTMDITIRFPHIEVYVIVSGDGGF
ASLAKKLHEYGKQVIGCAYENAANDIFKSVCDYFIKLELPEEYSPEDINTDPKNTTFGNN
KGLGIGITHPLVVRMANNIQPIYQADKKTIFSHGQKIISWFGQDPESRKQMYGHGIPLST
VREAFKYAIPEFKPEMVGFMRFAEFLQFICANTEFCVGTLPPSNTLLVFRNSIPNGVVIL
LDILNQDLHTPERYQSLLASGKPRITIEDKYSLETFVDTLMSKRDILMNISEILDIFSQE
LPDFESNKLNNLCLSLIHCNILKGYPEDENISEQKFHISQDFKDTAQILEHVKQTSLNKL
ISILVDDFKSDVFKEVIPF
>1170769.6.peg.728
MERKKQRSNLHRDRNLGLPGGIMIPQKYFNKFLPFTNSHAELL
>1170769.6.peg.732
MHPDFEIMVFRDGELRNHRYVDSLYAGFINVGNQYAPS
>1170769.6.peg.735
MVFPFTAIVGQEEMKLALLLNVIDPKIGGVMIMGDRGTGKSTTIRALADLLPEIPVVAND
PFNSDPEDPDLMSDEVRQQVAQGLEIAIGHKKVQMVDLPLGATEDRVCGTIDIEKALSEG
VKAFEPGLLAKANRGILYVDEVNLLDDHLVDVLLDSAASGWNTVEREGISIRHPARFVLV
GSGNPEEGELRPQLLDRFGMHAEIHTVKEPALRVQIVEQRSEFDQNPAQFLENYQFQQES
LQQQIVSSQELLPKVNIDYDMRVKISEVCSQLDVDGLRGDIVTNRAAKALTAFEGRTEVT
VEDIRRVITLCLRHRLRKDPLESIDSGYKVEKAFARVFGVELAEDVAGKNGTGMRSGVR
>1170769.6.peg.745
MGYNPLHYIPQRTYSRIAKENNPVVTTAIPIPQEALETINSHS
>1170769.6.peg.751
MAIARRGMCLKEKMHDSITAYLSVKDGKHVWSQWNQLNKTLKSRTDVSGSVSF
>1170769.6.peg.759
MFKTQAESEEFLRGFLPELEETPSIREVVLCPPFTNLNVMSKYLHGSRVGLGAQNVHWAE
NGAYTGEIAAPMLLEIGVRYVIIGHSERRQYFGETDETVNLRLKAAQNYGLTPILCVGET
KQQRDAGETEKLISYQLEHDLVDIDQTKLVIAYEPIWAIGTGDTCESAEANRVIGLIRSQ

LTNDKVPIQYGGSVKPNNIDEIMAQSEIDGVLVGGASLEPDSFARIVNYR
>1170769.6.peg.762
MGDKQFFPRRFAIPIEKYGDVASLNQLRSLVQPFKQSEPIMKHLQNIYRDVTQKACLLAM
NMG
>1170769.6.peg.774
MTLISLIAAISQDRILADSKNEHIRGGIPWDIPSDGRYFKEITWRHPVIMGRKTYATFNH
PLPNRTNLIVTKNTDYQAPGCVVFHSLEEAIKWSKMCETEEIFIAGGEQIYTQTMELAHK
LYLTIVEGSFEGDIYFPEFSNFGKLTKEEKLEENGFKFKFVEIERQ
>1170769.6.peg.775
MLGYVRGGETNYVSTLKSIRNLFELSDKLPVEYYRIGSPMQKITSSEYYNYGVTTTFYDG
SSNQFPEVSSPIDAAIISPEKEQKKMTVIITDLQQNSGDVTKLNKLINNTYYNIDNRDYA
VGIWAIKSEFDGKIYLEGNNPRSFDYNTGQESAKFRPFYVLFVGPYGDIKYYFSQLKKYN
TNQALLNSDNSKFMIFHPDHILDKISVLDATPMSLPQGITEPFSLVNGGVVVSKSNQEML
KLSSSLKQSSTINYGVSFLHSEYSLLLDPSTIKAQVKGEKFDRFKRTFVRVDSNSEIISS
IELKDWQILPEENQAKFAAVIQPDKLSEPGIYKLQFDLINSSLAVPNWWKEWDWQTRTGE
EDGSKTYNLQEFFTALKVRTETMQSEIAKSPQHSGWFIGTLCYAIQKD
>1170769.6.peg.776
MVTIGIIPNNHQSNLHRAVAAPSLKRLFSANAHQSNVETRQAKNMARAALV
>1170769.6.peg.777
MVTIAIAVFYINRSSEAHFSLMAFFLLDAILLYWLPTATSSPEAVKYVPPLAFWLRHKLM
GD
>1170769.6.peg.788
MIGKIDGRTRFLIDLQQKLPSFKTISTVLKPNTKSINQLKNLLFL
>1170769.6.peg.810
MGFIAYGLDNATLNLVGFFYGFPLLLGGLALKANELQPIPFSEPTTESVLELRKQQATIT
QNKIRKDITRFCYGQQAHLDRALDYLGLNPTDEERPVVVGLRETSLDGSYCLILEFDSPL
IPLDTWLEKQEKMTKYFAPNVHVKITQPDEDKIELELITTNHSN
>1170769.6.peg.815
MSSIVKLGMWGKEKGDRLLRTFADKEIGAIAMETYRSP
>1170769.6.peg.819
MSYIIPVFGRSLPLVNRLVMVTSILFILIVGGIFPFRWWGEHLRESRVVGFLGNS
>1170769.6.peg.821
MINNRQSKVYKINLSVLLKRFIFLVLLPTSLLIITHKLVINQLINVQGDPSKKQGTKPKP
YNINGDNMDARNVKPKLETQPPISTANTLNSSTITSGISEVKESPTSSLMRQDSVRLIQP
EDYDNLSGNKTNKIKTLSYNTKPPHFKHGDNLDQIVNMIVAQVDAKGLPTHKFSISLLDI
SNSKHICAGGYLSNKPRFPGSIVKLFWMVYLYGNYNNNIEHKHLKKMIQDSDNESSSLIV
DKITKTESGESLSDKELNSWIYKRLAMNSFFQKAGYQNLNISQKVFPTSYQKNDAPSGRD
LQIRDNEINPIRNYVTSYDVARLLYEIYTNKSISKNYSLKMKKLIERDLAPTAWQNKPFN
SIEGFLGEGLPENVKFYSKMGWNSRTRNDAAIIISPDKKHKYILVVLGDDPSFFQDKKLF
PEISRVVYKAMTINTSSSMSDSDRSSARVKVY
>1170769.6.peg.822
MKILVLNAGSSSQKSCLYEIGEFLPDSPIPPIWEGSIDWGTTQAKITIKVSGHIKTALLP
SSNRFHALKNLILTVIQGEHSVVESLKEINRVGHRVVHGGRRYQQATLITPEVEAEIERL
IPLAPNHNPCHLEGIAAIRQILEDVPQIAVFDTAFHAQIPQSIAAYPIPYKWYEQGIRRY
GFHGISHQYCAKRAAHLLGCELDKLKIITCHLGNGASLAAIRNGISINTTMGFTPLEGLM
MGTRCGSIDPSILIYLLKNQGLSTDELNHILNRESGLKGIFGKSGDMRDVLASWTAGDEQ
AVLALDMYIERLKSAIGAMTATLGGVDCLVFTAGIGENSAVVRQLTCNGLGFLGISVDHN
LNEGVSSDIDIATPDSRVRIFVIHTQEDWEIAAECWWLTLHHGNSYN
>1170769.6.peg.823
MIYLRANPLLREPLEEAHIKHRLLGHWGASPALSFSYIHLNRLINKYDLNVLFMAGPGHG
APGVIGPVYLEGTYSEVYPDKSEDIEGMEKLFRWFSFPGGIGSHCTPELPGSIHEGGELG
YSLAHAYGAAYDHPDLLVACVVGDGEAETGPLATAWHSNKFLNPIRDGAVLPILNLNGYK

IANPSILARISHSELESLFRGYGYNPYWVEGDDPEIMHQTMATTLERCFQEIRHYQKEAR
TTGVVSRPHWPMIILKSPKGWTGPKTVDGHITEGFWRSHQVPMGAMHSNPQHLKMLEDWL
GSYRPNELFDENGTLFPEFKAIAPQGHRRMSANPIANGGIIRKELRMPDFRDFAVSFTKP
GTIEVENTYILGNFLREVMRKNMTNFRIFSPDETASNRFQSVYEVTEKAWVGDYLPEDKD
GGHLAVDGRVMEMLSEHTLQGWLEGYLLSGRHGFFHTYEAFAHVVDSMFNQHAKWLDICK
TKVPWRSPISSLNILLSSLVWRQDHNGFSHQDPGYIDLVMNKSPDVIRVYFPPDANCLLS
VADHCLRSRDYVNVIVSDKQMHLQYLTMDEAIAHCTKGLGIWKWASNDDCGTAPDEPDVV
MACCGDIPTMESLAATAILREEFPDLKVRFINVVDLLTLNDEREHPHGLSHRDFDTLFTP
DKPIIFNFHGYPWLIHKLVYRRSNQERIHVRGYKEKGNINTPLDLAIKNQIDRFNLVIDV
IDRVTQLGSRAAYVKERMKNRIIECVHYAYTEGIDEPDITNWKWPY
>1170769.6.peg.825
MSIFDLIQNLMSKPKNPELDKVRQQHDNLPTSKYFDDTNISDEVFLHDYKESVERGHELA
LVFVATIQEHYTRDEIEKVTEDIVRCVSYLIGNCRMFPDVDYKDIWYTDYYDLIQLAQDM
RLGDYSLPHWPEEQNISRRYLELTVSDLVQIFLAFLEESIIEEYSFEDNTEEFTNYLLRE
TLKTRRRVRRQKDRESQTIPFIERIPTEL
>1170769.6.peg.826
MYQPVNVAVVDCGIEYQEPSKFNRIIILDKFGISMTIPANYRAMAKDNGTVEILDGGTYD
AFVCHAQNPGATGGSGYYSIEIYKSKASYLYENVWDKVPGKENMYIVWEKTYAGQELNYH
YIKLRIKTKKGLVEIDAGSEHSTQTEDDVKAALSTHAGLTQSALLYSAQTSPNSPG
>1170769.6.peg.827
MNPKKLTQLITIGILVTFITIFHPFIAIALTSKKIGAIATENDLIRNKNNRFTLLPSSSL
LRFLPNVL
>1170769.6.peg.828
MGQFFGVHPLFLVDDKKVPAIAILENEKALLFKGVRGNR
>1170769.6.peg.834
MELLGSPQPPLKRGANCFMFILGEVGAAYIIVNGVNAREE
>1170769.6.peg.837
MSTSSTSAQTTTGIEDIARKTTVQINSNANPGGSGVIIKKEGTIYTVLTANHVVCDNLGT
IKIRCRTDSNYTVVTYDGKEYPMKYRQSLQINVQDPDLAIIRFESRENYQIAPLGNSDNV
KIQSDILVAGFPTIFGRVGKQRTFTITNGKVVTFIPNSDRGYGLVYNATTFIGNSGGPVF
DIYGRVIGIHGLADTDDGETNNNNQSETVNGVKPTQKTGFNAGIPINIFFSLSNFNQQVN
PTVSINRQPNPINPNNVNLSNGAIAYNDRGVNRYQLGDKQGAISDFTQAINLNPNFAQPY
YNRGATRNDLGDKQGAINDFSQFINFYPRNSLAYFNRGIARHELGDKQRAISDFTQVIKL
NPNNVAAYYNRGASRSDLGDKQGAINDFSTVINLNPNFAQAYNNRGLARHNLGDKQGAIS
DFTQSLRLDYRDPTAYNNRGIARHDMGDRQGAINDFTQAIQISPNFAQAYNNRGLARHNM
GDKQGAISDFTQAIQINPNFAQAYNNRGATRNDLGDKEGAIRDFNKASQLFDSRR
>1170769.6.peg.838
MYIGGDINPDLPASNYSYGSLSGGFGSQSDVEDITGNRKVGDIRFNSAFSFNAAIGRQLE
QFRVEIEFGNQFLSAKEFKFNGDLIPPTTSLSGNINASTILLNGYYDIPTGSKFRPYVGG
GLGVARISGKVTDNEGFYDDDDVSLNGTSFAYQLKGGVQYEVTKKGNVFGEVKYSSISSY
KAEDYTNVDFGPYNSFSFAVGYRQGF
>1170769.6.peg.870
MQRIVARFRSKSDMDGYIQHLRQLVPNDSFKMFFDSQILLSAI
>1170769.6.peg.873
MFLQEVIMEQAIAQISCTPEEERLAYEQLTQEYQGQKEQGISQEQLQNMATRQLKLERFK
EVTWAKDIDSYFYQRKPQLDRVIYSLITTNDRGVAQEIYFRVQEGEQSFAQLAQEYSQGP
EAKTSGLVGPVELQSLHPLLVRILSTSQPQQLSLPTAIEDWIVIVRLEKMLPAQLDSGMR
QRLINERFQSWLKAQVSPQNWQIKESEN
>1170769.6.peg.883
MGLLATGCVSTVKESAEAQSQSSPGKKSAKPISVDVAIARTGSLNEELIYTGSTVPRKII
SVRSQVEGRLIGLDLEIGDKVSKGQRVGRLDDILLKTGLEQQEAELANRESEVERVRAQV
KNIEAEVEKVRLELMQAKSDSDRQQKLLQEGAISQQAAQQALTRVRTYQQILKATIEKQR

TEKKAVAAAQNRVLAQRAVVKAARERLSYSDLISPITGVVTEKITEPGNLLQTGNEVIKI
ADLSQIKVVVKVSELELGKVEIGKSVGVNLDAFPDEKIMGRIERISPVADSTARVVPVEI
VIPNSQGKIRSGMLARVNFSRQESSRVVIFKTAINNQEQETSLTNNNSTIFVIERKEERV
KVKEQPVVLGKEADGKVEIISGIKPGDSYVFRSSKPLEDGQIVKLSALSELPN
>1170769.6.peg.897
MDYLPDVDDRFDQFMMWYILFQLTAKHCKATTLEDAIAIVYLLLGMS
>1170769.6.peg.911
MPMAVGVIETLGFPAVLAAADAMVKSAAVTIVYYGIAESGRLLVAVRGQVAEVKTAVAAG
IASEETVYGGQVITHYIVPNPPENVETILPIHFTSESEPFRIF
>1170769.6.peg.920
MNLTSCLPILFLVPTLMLSSSPQSLAKTTLKQESSCELEREGEFYSPGQLKTIAQRITVR
VIADNSGGSGTLIAREGNSYLVLTTNDVISGTTPTALRITTHDGRTHQGRMLYNYKLDDK
QKLDKANLAILEFTSSRKYCLTKQIVNTESRQDTAVMASGYSINSSKIIFSPGRIKRIVS
QPTFSQGYEIGYDSTIQQGMGGGPIINSTGDLIGIHGKSAFPILNNGYVYADGKKPPLSE
IAEFRKLSWGIPVSSVLAQLKPEILTRYGLPIPRINRSVPEIPKLPEWLGNIESKVRQFT
VRIDGGGNNGSGVIIAREGNTYTVLTSAHVVCKRPQKISRSKEDKNKCIEENYTVIAASG
QKYPLDNSSIKLGGGGGRFGHSKI
>1170769.6.peg.921
MATVKFNSGENYPVATLADYPVANHQYIFTVGYPKLGQTPSWRFTIGQIFSRENGLLALT
STGQDLKSIDNTIQDANLGKEYELVYTSITLGGMSGGPVVDSQGRVIGIHGKAEGQVLME
DTTRVQLGYSLGIPISTFLRIAPELNTRVNRVENTPAPQLKSWEIESIRKAILSVNVSGG
NASAIEWIERGNQLWLLGRYQEAATAFENAIEQRPAFIHLAYYGKGLSLWWNGNDTAAVE
AFKQVVQAKPDFVPALYFLSLTNQNLGNLDQALFNVRQAIFHQNQAKYQPISPNLYALKG
NLLSDLKRYKEAIEAIDQAILVDPRAIFYVIRGDIHNNLGDKQEAMNDYTQALDLNPNVA
YTYTARGSVRKELGDNKGAIDDYTQALRLNSYGVVIWDIRNGK
>1170769.6.peg.922
MFSSSPQSLAKTTPKREPSCELQREGEFYSSGQLKTIAQRITVRVIGDNSGGSGTLIARE
GGSYLVLTSSDVIRGITPSALRIQTHDGRIHQGRTLYNYKLADQQQLDKINLVVLEFTSN
QKYCLTKQIVNTETRQDTAVLSSGYSVNSSKIVFSPGRIKRIVSQPTFAQGYEIGYDSTI
QQGMGGGPIINSTGELIGIHGRSAFPILNNGYVYADGKKPLVSEIKEFRQLSWGIPVRSI
LVQLRPEILARYRLPVPRRNRSVPETPILPEWLGNIESKVRQFTVRIDGGVDNGSGVIIT
REGNTYTVLTSAHLLCKISQKISSNRQNQYKCGNKNYTLVTASGKKYPLDNIKLGEGVDI
ATVKFNSGENYPVAKLANYPVENNQYVFTIGDPRVGQTPGFTVGQIFSRENGLLALTITG
QDLNGIDYTRIENANSGKEYELVYTSITPGGMSGAPVVDSQGRVIAIHGKSEGQVVMEER
TEDEGISIDNRVQLGYGTGIPISRFLSIVPQLNTQVDEVENTAAPALKLGEIESIRKIIV
LVNASMGKASAIEWIERGNQLWLLGRYQEAAMAFENAIERKPAFIHLAYYGKGLSLESNG
NDTEAIGAFEQAVKAKFDFSVAWNRLTALNIKFGRLRMALAATNQAIKLKPRDTSLYSQK
FYILISLTMYQQAVEVMDQAILLNPHQDFYINRGFARRELGDYKEAIDDYTQAIRVSPEF
ASAYYERANVRRELRDYKGAVDDYTQAIRISPEFALAYYERANVHRELRDYKKAVDDYTQ
AIRISPEFALAYYERANVHRELRDYKGAIDDYTQVIKIDPESVPSVYYERANVRRQLGDY
KGAIDDYTQVIRISPEFASAYYERANVRRELGDHQGAGADFQKASDLQVSSPKIPPKD
>1170769.6.peg.925
MTASIGGILGYSNPDSPVRHTPISGLTPPGFDSDMGFAVVLGGDGTVLAASRQVAPSGVP
LLTVNTGHMGFLTETYLNQLPTAMEQVIEGHYEIEDRAMLNVQVWRRDSVLWEALCLNEM
VLHREPLTSMCHFEIEIGRHAAVDIAADGVIVSTPTGSTAYSLSAGGPVIAPGVPVLQLV
PICPHSLASRALVFPDHEPVSIYPVNIPRLVMVVDGNGGCFVLSEDRVYLRRSEYKARFI
RLQPPEFFRILREKLGWGLPHIAKPSSVELP
>1170769.6.peg.933
MRRLFALILVIGLWFNFAPQAHALGANLVPCKDSPAFQDLALNARNTTADPESGKKRFER
YSQALCGPEGYPHLIVDGRLDRAGDFLIPSILFLYIAGWIGWVGRAYLQAIKKEADSEQK
EIQIDLGLALPIITTGFAWPAAAIKEFLSGELTAKDSEITVSPR
>1170769.6.peg.935

MAQAANKSKNLPNGPRNQQTVRAAGGNAQDGNLETPINSSPLVKWFVGNLPAYRPGLTPF
RRGLEVGMAHGYLLFGPFDKLGPLRDSANANLAGLLGSVGLVIILTACLSLYANSNPAPA
LASVTVTKVPGDAFNSKESWNNFTSAFLIGGIGGAVVAFFLTLNSGIIQGLIG
>1170769.6.peg.940
MVKIHLCSFFFPQALNLKFTEPGKLKMATLQQQKIRIRLQAFDRRLLDTSCEKIVDTANR
TNATAIGPIPLPTKRKIYCVLRSPHVDKDSREHFETRTHRRIIDIHQPSSKTIDALMKLD
LPSGVDIEVKL
>1170769.6.peg.943
MRRVMRHGKKSLAARIVYDAFKTIEDRTGGSPLEVFERAVRNATPLVEVKARRVGGATYQ
VPMEVRADRGTALALRWLVQFSRSRPGRTMASRLANELMDASNETGSAIRKREETHRMAE
ANKAFAHYRY
>1170769.6.peg.948
MANSLTKHISSYIYSPILIHTCINSYLVFVGGWGQRVPFLLKDKTWAGIWFYLTLGYGFY
RLIVNPY
>1170769.6.peg.975
MTKITNQALNSALNQLWVFSLSSDFWAVFDAVFGTEYNRKNAEILRSQWQIGDFSQLPEI
EILDSSILGSANGAYSSSENRIYLSSNLMENGTSSKIREVLIEEIGHFVDSRINQIDTPG
DEGEYFAGLVTDKKLNKDEIDRLKAEDDSNWISVDGERLLIEQSSPGTVTRTPIAPASPG
RTRYEVGNYNAFAALKSNGSVVTWGDSSYGGDSSSVASQLTSGVTQIFSTYYAFAALKSD
GSVVTWGDSSGGGDSSSVASQLTSGITQIFSNGSAFAALKSNGSVVTWGDSSGGGDSSSV
ASQLTSGVTQIFSTGFAFAALKSDGSVVTWGSGSGGDSSSVASQLTSGVTQIFSNWFAFA
ALKSDGSVVTWGSDWSGGDSSSVASQLTSGVTQIFSNGLAFAALKSDGSVVTWGSDWSGG
DSSIVTYNYNTGSSSYVSVASQLTSGVTQIFSNWRAFAALKSDGSVVTWGFSDQGGNSSI
ATYNYNTNSYSYVSVASQLTSGVTQIFSTPYAFAALKSDGSVVTWGDSSYGGDSSSVASQ
LTSGVTQIFSTGWAVPFKNASNEGISISGEGAFAALKSDGSVVTWGSGSGGDSSSVATQL
TSGVTQIFSNGSAFAALKSDGSVVTWGSYNYGGDSSSVASQLTSGVVSFADPFNDDRLVP
GSSVTLAVSPSTVAEDGTSNLIYTFTRTGVISNELTVNYTAGGTATNGTDYSNIGTSVTF
AANSATATVTVDPTADTTVESDETVSLTLASGTGYTIGTTSAVTGTITNDDNPLNSNVDT
PKVNISTGLIFTAQSNGNITLDTNRGSATPDEVTSVQNGTKSNFNHIIGLYEVLNSQGEI
KDNQGNTLKPGDANYALHALTTARVKNFAVRAGGNDTPSTATQLGSGVSVLAGKLYAPFA
IANGGTYFPGNQGIEDFVAAEQGDINRFSSAPQYVRNLVDIEGKNGDVFNNAPRFVQEPV
AYFSFGAANPDGSPHFRSHGNGVYGFEDLPVSYTQYSNNDFNDGVFALTLSI
>1170769.6.peg.977
MSAFAKHSPDGIINKTLIVNPNLVFGKGDLFNNETLLYHMSWR
>1170769.6.peg.979
MQVTTTSTPPGELSPSSWPDHTQLPDSDDNFVKNFQEHPQSVILTTSIEPLLDKIHPEKD
YCIGQDSGIYWRFTEPVEKGVEAPDWFYVPGVPSRLKGKLRRSYVMWKEKVPPLIVIEFV
SGDGKEEKDNSPPPERDEVDPKTKKVKKAGKFWVYEQAVKIPYYAIFDGFEGTLEMYHLE
KGRYEQVKANTRNHYPIPELGVELGMLLDQEKPPVPWLRWWDNRGNLLLTGNERAEEECQ
RRELAEAIAIQEREKKEKLAAYLRSIGINPDEI
>1170769.6.peg.980
MVGWGLRWWLLAWLMRGSGIGSSFIGLINSRLFNFPWFGDGRIRVVLFLLGVVILYQWIF
>1170769.6.peg.987
MEVFLRDRLWVNPRTTIPDFTRLQPFSSQQFFNTSHN
>1170769.6.peg.1022
MSATNSRRIIIGDVHGHYQGLMLLMEKIAPNSGDQVYFLGDLIDRGPQSAQVVKFVKENN
YPCLLGNHEEMIMNVMVHHYTSSKAVQSWLYSGGQATMASYRSARIPQEDLDWFGSLPTY
LDLGDILLAHAGVNPNKSLSEQTEGDLCWIREEFHSMKTPYFTNKLIVTGHTITFTLPGV
KPGDLAQGQGWLDIDTGAYHPRSGWLTGLDITNSLVYQVNVFKNSTRCLPLEKALSKINP
QEIKLSGRYKQVS
>1170769.6.peg.1025
MSFPFHVHHKVQSFNLDTTYYFFLPNVDYGMGNPDSDHQTV

>1170769.6.peg.1027
MLLSIKTKLKLNKTQETLMAVQPLIASAKAHSKAIHSKRTQL
>1170769.6.peg.1034
MTTKSDPNRILRLLPLVVGSLGTVLLLVNRVLTPQLTESQARGDVLGVILSAVLILTGLI
WQQVQPKSPDTVELIGKEGFILASDLPETIKTELAWASRLLLTNTVTRSLVVYYKGQVLL
RRGILGSKAEVTPGPILERVLGTQKPIYLVALRVYPGKIEFDYLPDNTQGVICQPIGKEG
VLILGANAPRSYTKQDENWIEGIADKLAVTLENRTE
>1170769.6.peg.1036
MRTIDPGTTPPPSTLSNSEYPDSHRAIWRSLIWSKVSDALGLEGLVDLADGWALCQPPTP
KELAFCSTRES
>1170769.6.peg.1037
MVLIGGDPGIGKSTLLLQVSNELSQRYRILYVTGEESGQQVKLRASRLGMSKPLQVVSME
DSNTSSESSHPLPETDPGNNVGDAVEDNHRVTVEHHTISSDLYVLPETDLEEILREIDSL
KPNLAVIDSIQTVFFPALTSAPGSVAQVRECTAALMKVAKHEDITMLIVGHVTKEGTIAG
PRVLEHLVDTVLYFEGDRFASHRLLRTVKNRFGATHEIGIFEMVSNGLREVPNPSELFLG
NRDEPSPGTAIVVACEGTRPIVVELQALVSPTSYPSPRRAGTGVDFNRLVQILAVLEKRV
GVPMSKLDSYVASAGGLSVEEPAVDLGIAVAIVASFRDRIVDPQTVLIGEVGLGGQVRSV
SQMELRLKEAAKLGFKRAIIPKGQKFPDLKIEILEVSKVIDAIIAAIPHQSLENGDLDLE
EEDED
>1170769.6.peg.1047
MPSLSFGNPILVKSYSTLGRLQKAKHRTYKFQEKSDKL
>1170769.6.peg.1050
MIPVKKNLLVIYILNLRFPIFWTITGQGEKNQSSLDSLTNS
>1170769.6.peg.1061
MQESQYTETKTKETPIPDLSTQTGSITKLQSPPKSQEQWLKYGQEVSNFLGTLPEYLVGL
FDKYKQPLLTLGLIVTAGVTVKVILAVLDSLNDIPLVAPTFELIGIGYSGWFVYRYLLKA
STREELTSEIDTLKSQVFGQD
>1170769.6.peg.1063
MKTLVSVHESYKLCQELTAKYAKTFYLGTLLMSPTKRQSVWSIYAWCRRTDELVDGPTAT
KTTPETLAQWENQLDSIFAGCPLDDYDVALVDTLQHFPLDIQPFRDMIAGQRMDLYRSRY
ETFDDLYLYCYRVAGTVGLMSTTIMGVDTSVYSAPWYRDIQPYFPVEEAIALGIANQLTN
ILRDVGEDARRGRIYIPLEDLKKFSYSPEELLQGVLDDRWRSLMRFQIKRAREFYTKADR
GISYLAQDARWPVWAASMLYGKILDVIERNDYQVFSQRAYVSQLQKISTLPLAWMRSQVL
>1170769.6.peg.1068
MLHDLCCPQTVLITHPIKLMKLIAIQNVEGFDITGVLS
>1170769.6.peg.1081
MANCATPVREFQQFSFLKLGLKIRKTTSFSYCLCYHLE
>1170769.6.peg.1087
MGAGVFGFVGFFAAGGFLLSGFLVASGFTSVVFCTSGFLVASGFTSVVFCPLLIVGPACA
ENGATPKRSIMLASKDPATL
>1170769.6.peg.1124
MTTPQEKLFGPDYPNQDLRDNQESRVGYWQILTISLC
>1170769.6.peg.1128
MAENKPNLWGIIPQVTQMQSEGGAAAAVHGALQTGSLSTTFTASQGLLLMIPNLFKIAGE
LTSFVLHVSARSLATHALSIFGDHSDVMAARTTGFDLLCSASVQESHDFDLIAQVATLQA
RVPFLHFFDGFRTSHNDKGGKPPPLILFLRRNEHHHISPFFLRSLLNLTKGFQVCSHTI
>1170769.6.peg.1129
MAKLFAAIDGNEAVARVAYKLNEVIAIYPITPSSNMGGWRKINLIYGG
>1170769.6.peg.1161
MIDELTILPFIAAGAIAMHQFVSPARIWFGQKSDSQRIFGYHRSGYVWIGAALFTSISFV
ALQVVWQLGTSLQNTGWGLQTYSWSALLALTFGMYGLALSASSTPFTALLVDITDEDDRP
KLIAVVWSMLMMGIVIGASISSRLLERPEICGTALLAYDPSQMNKLVDISKLQTTINPVF

IILPGAVFVLTLLATLGIEKKYSRYGIRGNMVEREDQITLGKALKILTANRQTGIFFGFL
MVLTLSIFMQDSILEPYGGEVFGMCISQTTRLNIPFGIGTLLGIGSTGFLLIPRLGKKQT
TKTGCIGAAMSFCLMIMAGVSQNSGLLMGSLFFFGLASGVITTGATNLMLDLTATETAGT
FVGAWGLSQAMARGMATVLGGTVLNIGKLVFTSPTLAYGMVFALQAMGMLLAIWLLRRVN
VVEFQQNSKQVLASVLESDLD
>1170769.6.peg.1162
MQDKALANVFRQMATGAFPPVVETFERNKTIFFPGDPAERVYFLLRGAVKLSRVYEAGEE
ITVALLRENSVFGVLSLLTGNKSDRFYHAVAFTSVELLSSPIEQVEQSLKENPELSMLML
RGLSSRILQTEMMIETLAHRDMGSRLVSFLLILCRDFGVPCADGVTVDLKLSHQAIAEAI
GSTRVTVTRLLGDLREKKIISIYKKKITVHKPVALSKQFT
>1170769.6.peg.1178
MTPAIEKVALRALLYLRSGFPVHLRGPAGTGKTTLALHLAHCLDRPVMLLFGDDEFKSSD
LIGSESGYTHKKLLDNYIHSVVKIEDEFRQNWMDSRLTLACREGFTLVYDEFNRSRPEVN
NVLLSALEEKILSLPPSSNQPEYLHVNPQFRVIFTSNPEEYCGVHSTQDALMDRLVTISM
PEPEHLTQTEILAQKTNIDKESAGFIVDLVRSFRLATHAEKTSGLRAGLMIAKVCADNDI
LVAPEDPTFREIAMDILFNRSSLPVAECTDIFMDLLNLDEPELPIEKEVNGHINEEENNH
TAIPIEEIEGLVTAKVVPFEKEVYNYLLQKRSESVNGIKKFLNSEYHTALNALRSLEQKG
LVSKNKRIYTI
>1170769.6.peg.1183
MIAQTNKQSSRIYPNSMIFGSQVQKGSFFTNWLNRLGISIFILNKCSFL
>1170769.6.peg.1184
MSIENNPASVTKQALAALASAIEAKGHKREYAEAMAASIIFQADLDLRNAQLANLLGWLK
QEHEKIYESALSVIESTRQEFENRVSRG
>1170769.6.peg.1191
MIDQEADIAVDSRNITGGGTSDWLGLATKRVLPFILPPLLV
>1170769.6.peg.1197
MGIGSAGMVQASKLPFTHGITPAVVWGFSQTVGTVAVTRLSVTGTFPNNPVLIAIAAMIL
GNLFGYLSEAWGKAMTTSNVATVGE
>1170769.6.peg.1210
MAKKSMIEREKKRAGLVAKYAAKREALLEEFRTTESPLEKLEVHRKIQQLPRNSAPTRRQ
NRCWLTGRPRGVYRDFGLSRNVLREWAHQGLLPGVVKSSW
>1170769.6.peg.1214
MVTTLKPVSTLPTQDEILRQLRSGFPHSVGLIGMRDVRDYKVKSGGSERLNTSSPFNIKA
ESLTLSNFSLPEVEELYLQHTQATGQVFTTEAIQRAYYLTDGQLWLVNALARQATQVLVK
DVTQPITTEVINRAKEILIQRQDTHLDSLAERLREDRVKAIIQPMLSGSDLPDTPEDDRR
FLLDLGLVKRSPLGGLTIANPIYQEVIPRVLSHGSQDSLPQTYGREN
>1170769.6.peg.1215
MVREERSQGVQCDRREEEIKMNCPNCDFNNIRKNGQRRGKQSFASLFS
>1170769.6.peg.1219
MLKHSDLFEELPEKFHDSAFLDRIHFYIPGWEVDIIRGEMFSDNYGFVVDYLAEILRAMR
NHDYSDRYKQYFSLSPDISTRDRDGIHKTFSGLMKILFPHGGATEREVEELLQMAMEGRK
RVKDQLLRIDSTYAKVNFAYENKEGISKTVVTLEEEEYPKYYHQTLTEEREGEEVVKLEG
ASAPESKSGVHPGEAHLTFQENQRGVSFDSLFGPYLKGAKKITITDPYIRVFYQARNLMD
LIETVIKLKPQEDEVKINLITTLDDFKGEQQVEYLGRIQESCATVGIDFTWEFDGTCTIH
GRHIVTDTGWKILLDRGLDIFQHYDIKDAFSIANRLQEFRGCKAFEVTFLRRETVEGN
>1170769.6.peg.1220
MKFLRELLEKVRVFEHVRHCVLGVSYEYHRCFRPQGFYSPGKGLVGHVVFHNVHKRLVHG
LLFASKLIEGYNIPVAHQADLTSRVIDKELRHSYLAARYQNAMGRELREDMGFPSALGTK
FDQVVIALAERYEANELEQLAPSTEHLRIEADTLNKQVNPLLSG
>1170769.6.peg.1223
MISGFVDTYLDLSPLEEIEFEEEIRKFSQPIQEGALHLHGMMYNKGKNF
>1170769.6.peg.1238

MELIQGLDSLIGNPRIISGVILLELTNNLSILLLILD
>1170769.6.peg.1239
MRKYKEIDDYNQDIKINPNDPEYYYNRGNTRRELGDKQGAIDDYTQAITLDHNFAYAYIP
QGNVRTASGDNQDAIDDYIQVITLVNPDPKNRIIFIEQQ
>1170769.6.peg.1243
MLHKLFTEIKEYELAAYFYQISQSKISSKILKLLVNSNNITPEIILGLPIKESRPWINSI
SKFFNKDIDEQEKIFKKRREKYLELLRKYKEIDDYNQDIKINPNDPEYYYNRGNTRSDLG
DKQGAIADYNQAITLDPNYANSYNNRGNARRDLGDKQGAIADYNQAITLNPNYGNAYYNR
GLARYELGDKQGAIADFQKAADLYQKQGRDEWYRDALDRIREIQQR
>1170769.6.peg.1244
MNNNIIAGLGKAKQFAFWGAKLAVHTKIKFGAVKGEIR
>1170769.6.peg.1247
MPVGLPEFVDNPENRCPVILLLDTSASMSGKPIEQLNEALIDFKQDITRDDQACLSVEIA
LVTFGPVKLVHDFVTVENFIPPSLKADGMTPMGEAIEYALDLLEQRKQNYKTHGIVYYRP
WIFLITDGAPTDDWQTAAHRIRIAEEEHRLLFFTVAVQGADIHTLKQIAPLERPPVTLRG
LDFRSLFLWLSTSMKRISGSKIGQAVSLPPMGWGQINT
>1170769.6.peg.1248
MGWKAITRSVIGTSHKENNTPCQDYGACKIFDNIIVGAVADGAGSAQYSHIGAKKAVDTI
INSFQNIINKPTQKLNPNLELSQEVVKKVFIKITDNIIKELEKEADKHKYEIKDLACTLI
AFIATPQWLAAMQIGDGFILIDTPSQKCRMLFKPDKGEFFNETTFITSTHALEEMQIEVV
FDKIEFICVSTDGIEKVAIHLATWQPHPPFFSPLRQYLEETNNPEKEDSYIQNFLESEKL
NARTDDDKTLMLCLQI
>1170769.6.peg.1251
MIRKSICLSTWRNPRSEALIWVEVWIPISERIEQFIEPDKNLLLVWNY
>1170769.6.peg.1256
MVLTLYEKPRKCLVVGGAGYIGSHVVLTLNEAGYHVTVFDNLSTGLESSVLSPAKLIVGD
LNNLDHLEEVIRNGEFDAILHFAASIVVSESITDPLSYYTNNVVNTINLLTLAHRYRIPR
FIFSSSAAVYGITEQVPVPETAGLFPISPYGRTKLVTEWAIQDLARSAPWFSYGILRYFN
VAGCQFTSGLGGNNSRATHVIKLACQTALGKRPVFQIYGSDYPTADGTGVRDFIHVTDLS
QAHLSVLNYLEDSAESSIFNCGYGQGYSVLDVIKTVQEVSGVKFPIEIVPRRIGDPPEVV
ADVSSILQRTSWQPQHNDLKLIIQSAWNWEKGLMNNDSVS
>1170769.6.peg.1257
MPRRIFAEAMDCYLAMGFVPGNLCILQGYNKLPPAHALSLDAA
>1170769.6.peg.1258
MCGIIGLASTTSQRDRAWLSIARDTLIHRGPDDAGEWWSEDQRVEMAHRRLSILDLSPAG
HQPMELPSHGLTIVFNGEIYNYRELKQQLSTIGFTFHSTSDT
>1170769.6.peg.1259
MSINHSKLILGFDGWTLGSHHYQRLVQSLKDSGYRIKLIHFGSYGHDIRQQTSEFLGPLE
VCDISHYKGKSVREILLEEQPKAVVFLSTQSFLHQAVNRYCQALKIPTLHLFHGFVTVQA
VETSQPNKYRFWPQSKLVSQRAFKNLTKIFPIYLKSLIETKADILSWLYFFRDVVSKIFG
LFQKVASPNCSTTFCAVYGLSDVTYAHNTYRIPIHNIKVVGNPDFIKFRLSDELILSCVS
PCSSKTKIIYIDDGSPTCGLTFASQNDFLNFLVKTKLKLAEQGYELLVKLHPSQAQFDTA
QELIRLGVLLSTDASFTSDLLDCRAAITGPSSAAVIPASLGLTLLLAQYDQFEGQKYGIV
YRDYPRSLYLRNLMELKRLLNQETKPDVNRMKMWVQQYLYPLPAEDMPKRVVEIIDQMVH
KHERPCAE
>1170769.6.peg.1260
MVLKLFTPILDRYRLVLKRVHDLEIVQNTLLFDETWVNDPTKFLNGQLQRQSIVLQLRQA
FDFSIIIETGSFIGNTTGWFAELWGGEKIHSCEIDPRFHALSQIRCANMNISFFMGDSRS
FLENLRNSDISQIAFFYLDAHWGNDLPLKQELMIIKEKWPCSVVMIDDFEVPGDIGYSYD
NYGPGKALTFQEFSPFFLENKFYVYSPTVKSQDESGFRRGMVLLTLDPAISKVIDDIPSL
YRHYHH
>1170769.6.peg.1261

MQRNKIFIAHSNSGQQLLDWHARRENCARNLGYKIETMAMMDYHPYTIFPYLDKKWKKRD
TVLMRFYEKLGEKIADSDIFIHYNGALIHPKFLQQFTQLKIYHCADDPEASDVISRPVVH
AYDIHAISNPTCLDLYRSWGCKHVFFWPLGAFHYDFAVENRSQTAVRDISLSFVGTKYGV
TRWRYVHRIPILKYWSGLYTKKAFFDRLEQAFPSMVAYGGGWRMGRIEDSDIPDLYRRTL
VGINVHNNMGVHNGRLFDLAAYGVCQICDNKQHLHHVFVPGKEIIGYESTNEAIDLIRYY
LAHPNEANAIGIAGRERYYRDYTMDAIWNKFFEDLGRLSITADLSGV
>1170769.6.peg.1262
MGTYFLDALIFMPRLHAITHKTSNIDLFTTPYLIIGNALMVRVHEVAKQGRSALRQLFEQ
ATNIAASSALILAVVWILLDPISRWLFPDTENVEKFFAPLLFLIPAIITNALYVPLSDYY
GGLTKRNLFLSVMAIIQLGVLWGCSRWGDGYSAVILVVVIYLITVRGYAMIASRVLLGDF
KPPIPKEAKAFILINLMSLATSSFISETIRQSSNLHGLPPFILHLAIFFVMTCAMTGSRS
DLRRSFLQLGILRL
>1170769.6.peg.1263
MRSWLLSESVRMLKSRTLTPGFGVFFDPSKTTSGQRFFAGLCQELNQSAVPFEQRPRVVL
FNISVPWREVVKAKFRGQKIVVRVDGLYFDRLSPDFLASFLPPLRWLFGLGVRYPQFHNL
LAHLANLLDQNYKAFFRILLADYVIYQSEFSHRVHETYFPNKPSCVIVNGARYVNGSNEP
ARTTKACIRLVSIYDAWKPAKRVYDVLRFVCWLNERQQPATLTVLGYTGTVPEGSPQEMR
QMLEDSSFVSTLPRFSTFEGCFADALVGSDCYITFSYRDPCPNAVVEAMAHGVPVLALTS
GGIPDIVSDAGRLIPTDDFAQGFFSDHRFGSDFPPIDFEAVSTALNDILVNLPMYRQRVA
RRFAEQLDTTVTAKKYLQVLNYLSNS
>1170769.6.peg.1264
MQCPICKSTNYSIDPVASRLIGLCEPFKVMTCSGCGLGQLMPHLDLKEMKNLYDQAYFDS
PNADKAGLDNISLVDDYANAVESRIPKFNNTLNDLTNRFPESRTFLDIGAATGEMVMMAR
RAGYQAEGVEFSDFAVNKAREKWGIILKQTLLSEMESESFDIVHLNHVFEHFTDPVAELK
NVHRILTAGGGLYIEIPYQFHVVERLKHRFASRSVPFSLHSIHHPFFYTPKTIQRLLRDH
GFHILKLNVFAADRYPALTPSQQVKRLFWRAASWVSVGNYIEIMAIKRVSPNAKK
>1170769.6.peg.1265
MCGLTGFIETKDFSSDEARNVIVRMAQALVHRGPDDWGVWLDDEHGLALGHRRLAIVDLS
DAGHQPMVSGSGRFVLVFNGEIYNHRELRQCLPDRSWRGHSDTETLLAGIEYWGLERTLK
AAVGMFALALWDKEENTLSLARDRMGEKPLYYGWMHGTFLFASELKALRRHPAFTGEING
QALAHYVRCGDVPAPFSIFQGISKLPPGTIAILRNADRLARQEPVLTNYWSLRTVVAQRA
QSTFQGCAGEAVEQLEDLLTQSVAGQCLADVPVGAFLSGGIDSSSVVALLQSVSKKAVRT
FSIGFDESGYDESQHARNVAAHLKTEHTEFRVTAADALKIIPELPVIYDEPFADASQIPT
LLVSRLARQYVTVAITGDGGDELFCGYGRYPHTRDRWQRLARLPSVMRGVGSRVLPASPL
QECLRANSLDEFYHFTNRQWKGFPDLVCGSQEAPHALKIPDELTAANERMMFADALDYLP
NDILVKVDRAAMSCSLETRVPLLDHRIVEFAWSLPDAIKYHQGIGKWPLKQLLYRHVPRS
LVERTKMGFGVPIDHWLRGKLRDWAEDLLNEKRLQREGFFNPAPIRQEWNRHLSGKYDRH
YGLWTILIFQCWLRDWKETS
>1170769.6.peg.1266
MDGVAYLRDDLRFSPKLSRVSSYFLPLSSQLLYQTVRQSAVISNWYEIQRNLFTLVVREH
IFANLNNDRMI
>1170769.6.peg.1267
MLAELMRPTGCLPPVVENNKQSSNLVDVKVVIRVDSSQNMGSGHLVRCRTLAEALQHRGA
EVKFICRQHPGNLIHLLTHANFSVTVLPPPTSLQPTVEDYTLWLGVNLETDAAETIEALK
GKMPDWLIVDHYGLDNFWEEKLRSYVGKILVIDDLANRPHNCDALLNQNYHFPQTNRYDG
WRCIFAG
>1170769.6.peg.1271
MRPELIALGTAQFGLSYGVANQKGQVPAREAYQILKYAASVGINTLDTAIAYGNSEECLG
SIGVKDWQVISKIPEFPLETYDIQGWVRNSVQGSLERLKTPQLYGLLLHNPQQLLEPQGV
ELYDALNLLKTEGLVKKIGISVYSPEELTLLFNHFSFDLVQAPFNVVDRSLDQSGWLNRL
SGLGVEVHVRSIFLQGLLLMQAEQRPAYFQSWAALWKAWEEWLLVTGITPLQACLAFVLH
YPGINRVVVGVDSLEQLQEILSATRIKSVTLPDYLYSNDTDLINPAQWRLT

>1170769.6.peg.1272
MSWWVGLTNSSSVARKPSICFAFQKDFLLIHNLCSGLELN
>1170769.6.peg.1273
MSKQKNIFLESEGDAWFERNHYAIQNREFGDQDQIIHALLRCQLSVARGVNCWKLVAGKV
SDWNGFRKALI
>1170769.6.peg.1274
MGFREGDFPVAEAYYGRAISLPMYPELTDADQLRVVEILGHVLGFGNYEQAKKHISRK
>1170769.6.peg.1275
MDRSKVAIIVPAFNEAQSIAGVISELLPYGIVIVIDDASTDGTSSTALESSAVVVKHPYN
QGYDAALNSGFSKAADLGCDYAITFDADGQHDASLIPKFIDLFSHGVDMVIGVRPQPARL
GEYVFSLYARWKFGVRDPLCGMKGYRMSIYQARGWFDSYQSIGTELMLFGLRKQYTYEQM
EVPIAARQGSGPRFGNRIKANWRILRAITMSLWHYPVG
>1170769.6.peg.1276
MKAITIQDRKVGLHHPTYFVADIAANHDGDIGRSKDLIYLCAEAGANAAKFQHFSAKTIV
SDYGFKSLGTQISHQAEWKKSVFEVYEDASLNIDWTSILKETCDDAGIAFFTSPYSLELV
DAVDPFVPAYKVGSGDITWLEIIQYMATKKKPLLLATGAATIDEVDRAVATALTRTDDVV
VMQCNTNYTGSVDNFYHINLNVLKLYREMYPDLVLGLSDHTPGPTTVLGAVALGARVIEK
HFTDDANREGPDHGFSMTPSTWREMVDRTRELEAALGKAIKKVETNEQETVILQRRGIRA
KETLTPGVTLTRDLLEVLRPCPSDALPPYRLDEILGKTVVNQIQEGDHLRWTDLK
>1170769.6.peg.1277
MKVLITGASGLLGTALVDHLADDFEVVGVSRKPGFCPKHVSWVLADLLDLSETSKLLQRI
QPQAVIHCAALVNVDLCEKDGYVADQLHRRTTEVIVKTLGKWNGRLIYISTDSVFNGRKD
DPYTEKDLPDPPNSYARTKLGGELAALSYSESVVLRTNIFGWSRAEKLSFAEWVLKGLVL
GIPLTMFTDVAYTPIHVSHLANIILQVLQCISLKGVYHATGSQVLTKYDFAMTMASLFNL
ENDHIKPISVDDLNLVADRPKNMALLNQALASSLECTIPGAQSGIELMKYQYDTGWVSRI
KNRPMKTGYQFWETL
>1170769.6.peg.1278
MCSANHGVAVNSATSALHIACLALGLQPGCRLWTVPNTFVASANCGRYCGAEVDFVDIDP
QTYNLSIPALEKKLAIAREGGNLPQVLIPVHFAGQSCEMAKIYALSQEYGFRIIADASHA
VGAEYQGEPVGNGRYSDITVLLLCSSIAFSISCFNCF
>1170769.6.peg.1279
MKITPESSILITGGTGSFGKKFVEMLLQRFPDIHRLVIYSRDELKQYEMSLQFPDRQYRG
LRYFLGDVRDEQRLAHACEGIDIIVHAAALKQVPTAEYNPMECIKTNVLGAQNVITAALD
TGVKQVVALSTDKAAAPINLYGATKLCSDKLFIAANNIKGKRDISFSVVRYGNVMGSRGS
VIPFFLQQRDQGVLPITDAQMTRFNITLEEGVKMVLWTIDHGKGSEIFVPKIPSYRITDV
ARAIAPQAEQRVVGIRPGEKIHEEMITSSDSYTTLDLGCYYAILPSQVSDGLQDYYSETG
AVKVAPGFSYNSGSNNTFLTVSQLRELIRQHVDPEFRVELSV
>1170769.6.peg.1280
MATALRILQSRPHLRLVLLEKECTLAKHQTGNNSGVIHSGLYYRPGSLKALNCIAGYRQL
LAFCQDEGIPHEICGKVVVATKEKELPQLEMLYHRGIANDLDGIHY
>1170769.6.peg.1281
MGKVGVKGDRLVLYKNVNRHSYPLCTAQPLISELRGRN
>1170769.6.peg.1282
MHTTTNNTKLGKMQHLILKILLVLSLLFKSTHTWRLSMGEDRE
>1170769.6.peg.1284
METHFEEKKEYLITLKHQIREQERANKEMEKKLDQRFAAASARAEAEALKKLEKLPSYEA
LHICGMI
>1170769.6.peg.1285
MCWIFPYLSVLKGNLSRSLSAIALGLLRKCSGYYHDLPILKGNL
>1170769.6.peg.1286
MVKRFWRSQFWRMKTPCLKRGINAFCLVYEKLGLLGSPQPSLKRGTNCFVFSL
>1170769.6.peg.1289

MRSQFPPIPPYIDYKLLNAIAQFWLATCNNVQFPAVH
>1170769.6.peg.1290
MAINLQKGQRISLTKEAPGLKKIICGLGWDVVEQTGGGLLGMFTNKKNYDLDASVICLDN
NGKISDVGNIVYFGNLTHKSGAITHLGDNLTGEGEGDDEQIIVDLPLVPSQISKLIFTVN
IYECVAREQDFCQVRNAFVRLVDMANNKELARYNLSGIEYKGMTGMIMAEIYRYKEEWKL
AAIGNGIKINGLREIAHYYM
>1170769.6.peg.1294
MGTTANTTTSHPPPKVSIQLMSPASGNWEDSQEEMGKHAVAFPFN
>1170769.6.peg.1295
MNGNAFTPWRRFSLLSLGSHSLGTLIEWKLIIPYPESLLVWVSFNVPTRWGH
>1170769.6.peg.1297
MGKGRSRKCECFANARSEYFANALDIRLPPLSQGKFIKVIGCDRT
>1170769.6.peg.1299
MWFWDIGCDRMAVTPEHLPSTPSAIALYPFIISLALTPSIII
>1170769.6.peg.1301
MSAIARRECFAIALGFLSSQGNGGDLAWRVLRKRNLLGLLKGKFEDGKQAIAILENENVW
RCDRL
>1170769.6.peg.1314
MWVCLMAWDPPQPPLKRGAFRLNVLCDNMLSRIGFPPQPPLKRGAFRLNVFYNNMLSGMG
SPPNPP
>1170769.6.peg.1319
METILQQEKEQLIYQGSSHSLGTLIEWKLVVKATGLISSGSSHSLGTLIEWKQELTRTYQ
RLQRVLQFPLAGDIN
>1170769.6.peg.1335
MVFPTAKKWEVALIIETTLLKDQQVTESDIKSGGVLHTLT
>1170769.6.peg.1336
MFLKTKISYYGAIVQRCKTEKGLTQITATVTKDLPKSTQH
>1170769.6.peg.1337
MMEHEAQESEKLGRIRVIVQVTYQLSILLTLFNPKLISRFGT
>1170769.6.peg.1340
MRPARQHVGFGAREFGGRAAAAIERLSDRHRRRDRRARALVIDRLLVARTVGAVDGGGRI
VEVARLAGIARQRDRRAPLRQRLRHGFVGRAQSGTLGEQFGIAAIGVRERLVDRLGAGHR
RQRHHGRQGRARHRRLQCHPSRRHRITLFQISNAPCPDRPARPIDRLRPPS
>1170769.6.peg.1341
MEPMVFARLVQALALEPGQRVLIVGDFTGYAAAVLKDMGVTLASDADDSAVDAVLFAGAI
GELLDTYTRRLNEGGRIVGVLTAPGEPGRATLWRKFAGDVTSITMFDAATPVLPGFEKQP
GFVF
>1170769.6.peg.1342
MHQPREHHRLHEILSAAQRQILVHVSDASELGGHEFFARDRGHRRDQALVDHLVGAQLAF
HHVAAAFGEIHVFGLPWCRAIRLADLYMVRRHKEQLKARKCR
>1170769.6.peg.1343
MAEQPERARRRRAYGRRRRGRDRFRSPPSRASCRQRRRPFLFRRLERTGIAVDLDAPDER
DRDDRCVRTARFGAKPRPCGNDRCGRHLGRHLSGRRRARRTICSRRRLHDRGRRRPDPER
RLRQFLEEIRHRGRLAAGGGDRRRRWQGAHRQRSPRPRAVLRAARRRRRQFRRRYEADAA
HARSARNVRRRVRADRREIGHGLRRTS
>1170769.6.peg.1344
MKRDVPMPARVEQRAGEVGLARLFQRRGGQPGGPVGVQELAGFFAIARPENAVFRRAGLV
VL
>1170769.6.peg.1345
MFDASRHWNVSLHMNKGLAGAPDAVRAQAAQTAINPAVNTAFALAICGAAGPPAYPGIAG
HEPDLARARANADKVAAAMQALETRVPSTGTYLAESDYFDANWRSGYWGGANAARLEAIK
RHYDPDNLFRVHHGIGA

>1170769.6.peg.1346
MRAIIGLTGLLLASTAEAEPKQERCLPPAVFAPRVLYMPCRDQRAEEPKSNERGPFDRRH
CLEPPWRRDKSC
>1170769.6.peg.1347
MAPRQIMLILRPLLALAALLALSACGPPMRWEHPALSDAQAQAEMGDCRQQAWSEAQSRA
FYNRFAYGPSYVRGRDGRLYMADPWMRPGFNNTWFEEQRLRDFCLRNKGFRLVPGE
>1170769.6.peg.1348
MGPQSYAEQTQKEYHKLIGARAPMGWNNQLKNEPGIALFYERKWRRMRTPLPDFPLFELD
MTPHVGASVGNVFTYGAAGATFRIGRDLGVDYGPPRIRPGLAGSLHIDPPLDRYAYYAFF
GFEGRAVARDITLDGNTFARSHSVNRRPLVGDLQMGFAVVVERIRGTFSYVMRTREFEDQ
KNPDRFGALSISYRF
>1170769.6.peg.1349
MVLLLRLLGVALRAHDAELQFQRVEPAAILPLAQIGGQRRREIRPARIGPVVGDQVAPRN
ILGRVHVLPEAEPRARLARGVEHRDPAREFAQGAPAQAGLRHPVEPNPVGEIAVGLLGLI
EGIELVVFDQHRIGAAPRPFRRILGGDGTGESCKNDGNKGAIHRNALNRTRRLRLVCRS
>1170769.6.peg.1356
MVREIAPKTQLGVIKSKVNQGGETIAPEIVYPESDGKPMADNTKQFAWIVKIKENLEILF
KSNPDVFVAGDLFWYPVKGSNRIKLAPDTMVVFGRPKGQRGSYRQWEEDNIPPQVVFEIL
SPCNSKGEMTRKKLFYLKHGVEEYYVYDPDEISLEVSIRENNSFKEVEDFATWTSPRLNI
RFDMTGDELVIYYPDGSRFLSPVELSNYAEQERFLKEQANQRAEQERLLKEQANQRAEQE
RFLKEQANQRAEQERFLKEQSNQRAEQERLLKEQERFLKEQANQRAEQERFLKEQEQLKY
QTLLSQLKANGINITGLE
>1170769.6.peg.1364
MDEFLIAGLAEINGAFNLSPSWYVEALKYIKANHGLGGQAANEANTYIDYAINALS
>1170769.6.peg.1365
MSHIIHPILLCGGSGTRLWPLSRKSYPKQFARIVGNESLFQASARRLSGADFAAPTIITG
SDYRFIVVEQLAGIEIAADAILIEPSARNTAAVAAAAVALQAQSPGALMLVAPSDHVIP
DAAAFRAAVKAAVPVAKAGGLVTFGIRPDRAETGYGWLELSADPGADFAPVPQPLRRFVE
KPNAATAADMLAKGTFLWNAGIFLFSTDGILAAFRAHAPTLLEQVEAAVAAAKRDLGFTR
LDAAAWGKIEDISIDYAVMEKASNLSVVPYAGVWSDLGGWEAVWRDGAQDGTGVVTSGPA
TALDCHDTLLRSESSAQQIVGIGLTDIVAVAMPDAVLIAHKDRAQDVKLAVAAMKKAGVS
QAETLPRDYRPWGWYESLVIGSRFQVKRIVVHPGAALSLQSHHHRAEHWIVVEGTAKVTI
DAEVRIISENQSVYIPLGAVHRMENPGKIPMVLIEVQTGSYLGEDDIIRYEDVYARS
>1170769.6.peg.1366
MARYYIAGHRGMVGGAILRRLDQRRAAGEAVEIVTRTHAELDLTDQAQVRAFMQAERPDV
VILAAAKVGGIHANNTYPADFIYENLMIECNVIHQAFAAGVTRLLQLGSSCIYPRAVAQP
MAESALLTGVLEPTNEPYAVAKIAGIKLCESYNRQHGVDYRSVMPTNLYGPGDNFHPQNS
HVLPALIRRFHEAALAGAEVVTIWGTGTPMREFLHVDDMAEASLFVLDLPKAIYDANTEP
MLSHINVGSGSDVSILNLARMVAEVTGFTGRIETDPTKPDGTKRKLMDVGRLAAMGWRAK
IGLRDGIADAYRWFLAHQADLRV
>1170769.6.peg.1367
MKKALITGITGQDGSYLAEFLLAKGYEVHGIKRRASLFNTQRIDHIYEDPHSNHQRLKLH
YGDLTDTSNLTRILQEVQPDEVYNLGAQSHVAVSFEAPEYTADVDGVGTLRLLEAIRFLG
LEKKSRFYQASTSELYGLVQEIPQRETTPFHPRSPYAVAKMYAYWITVNYREAYGIYACN
GILFNHESPRRGETFVTRKITRGLSNIAQGLEPCLYMGNIDSLRDWGHAKDYVRMQWMML
QQDAPDDFVIATGVQYSVREFISWSAAELGITLDFRGEGVEEQAIVADVTGDKAPAVKPG
DVIVRIDPRYFRPAEVETLLGDPSKAKQKLGWVPEITAQEMCAEMVAEDLKTARRHALLK
QHGYGLPVSLEG
>1170769.6.peg.1369
MVRFSIDLDPRAVHGRAGGMRAVYGLPERFFYLPNQFWSHKNHATVVEALGLLAQAGRLD
ALPPVVMTGRTEDARDPGLFGQVMARAKALGVQDHFRHLGLIPYADVFALNAAAHRLINP
SLFEGWSTTVEEAKALGTPMILSDIPLHREQAPEATFFARSSAQALAEALVAAAAAGPRP

AVDLEALDQAQTARRNAHADAFLAAVAAARTGGRT
>1170769.6.peg.1370
MREIPTEEVLKQYSLEEVLKQYSSEEVLKQYSPEEVLKQYSPEEVLKQYSSEEVLKQYSS
EEVLKQYSPEEVLKQYSPEEVLKQYSPEEVLKQYSPEEIKQYSPKEVLKQYSPQEFLEGL
SPETLEHLAIILSQLGVNQIKNQEQ
>1170769.6.peg.1408
MVKRVQLVLTKDVSKLGKSGDLVEVAPGYARNYLIPQSLATQVTPGILKQVERRREIERQ
RQLELKQQAEEQKSALEKLIKVAIAKQVGENEAIFGTVTTQDVVDAIQAATGQIIDRRGI
TIPDINHLGTYKADIKLHSEVTAKIDIEVVAS
>1170769.6.peg.1415
MTPQRETILHIFQELPQGEHLSAEDLHHRLENGGESISLSTVYRTLKLMARLGILRELEL
GEGHKHYELNQPYPHHHHHLICVKCNATIEFKNESILKIGTKTAQKEGYQLLDCQLTIHA
VCPRCQRALMPL
>1170769.6.peg.1453
MAQIDEKFLEEAALLGSYRKNIGWTELQRNTSNQDSTYLIIN
>1170769.6.peg.1469
MPGMEKARNSKPSSKSGNLQVFTDAGNPYVLHLKSMEGKLSVIKIVWYVLILSEKNIRRG
K
>1170769.6.peg.1470
MGEFYLVQEYIEGKNLSQVGQIKPEQATVILSSLLNTL
>1170769.6.peg.1474
MVREIAPKTQLGVIKSNVNQGGETIAPEIVYPESDGKPMADNTKQFTWIVKIKENLEILF
KFNPEVFVAGDLFWYPVKGSNRIKLAPDTMVVFGRPKGQRGSYRQWEEDNIPPQVVFEIL
SPCNSKGEMTRKKLFYLKHGVEEYYVYDPDEISLEVSIREDNSFREVEDFATWTSPRLNI
RFDMTGDELVIYYPDGSRFLSPVELSNYAEQERFLKEQANQRAEQERFLKEQANQRAEQE
RLLKEQERFLKEQANQRAEQERLLKEQSNQRAEQERLLKEQERFLKEQEQLKYQTLLSQL
KANGIDVTGLE
>1170769.6.peg.1476
MVREIAPKTQLGVIKSNVNQGGETIAPDIVYPESDGKPMADNTKQFAWIVKIKENLEILF
KSNPEVFVAGDLFWYPVKGSNRIKLAPDTMVVFGRPKGQRGSYRQWEEDNIPPQVVFEIL
SPCNSKGEMTRKKLFYLKHGVEEYYVYDPDEISLEVSIREDNSFKEVEDFATWTSPRLNI
RFDMTGDELVIYYPDGSRFISPVELSNYAEQERLLKEQANQRAEQERFLKEQANQRAEQE
RLLKEQANQRAEQERFLKEQSNQRAEQERLLKEQERFLKEQEQLKYQTLLSQLKAKGIDV
TGLE
>1170769.6.peg.1481
MDLSRGNYFIKYSPREYQRRSHLLETLPNVRHFWQLFQS
>1170769.6.peg.1485
MTLIIDRSYCPLDQWQTQVVIPYEDLYRKYEDKQLKIESITLFSDLGQESSEKIPTPQEI
AKLNTYGKLSSNRQKSLEKLSQTSKILGCQ
>1170769.6.peg.1496
MFIIGEGNFSEMLEGVGHEGKLSAIAQIIKFVLFITVMIYHWVNR
>1170769.6.peg.1497
MELMETNLKFQLQGRTLEEVRFLLGSGINGNITPSIISV
>1170769.6.peg.1498
METAVLLERVPFIILMVRFLLGSGINGNTLDLSNQETQVDTSAFF
>1170769.6.peg.1500
MTVPKLRFPEFRDVREWEEKKLADVTQNIGDGIHSTPIYNENGEYYFINGNNLSKLD
>1170769.6.peg.1501
MSEIEREKSYLSLVDEIAARVTEGRKKAVTQLNTTLLETNWHIGRHIVEFEQQGAERAEY
GTQLLTNLARDITARYGKGFSRSSLFLCRQLYLRIPKIQTLSGKLSWSHYCEILKADDDL
EVNFYMKQCEAENWSVRELKRQMKSMLFHRLALSSDKAKVLELAEKGQVVSTPLDIVKDP
YVLEFLNLPQRQVYLESDLEEALISNLQSFLLELGKGFAFIGRQYRINIGGRQFKVDLVF

YHRILKCFVLIDLKQGEIEHGDIGQMNLYLNYFKQEESVDDDNEPIGIVLGAYKDKILME
YATQNISNQLFVSRYQLYLPDRKQLEQELQRLLESRESQDGGEL
>1170769.6.peg.1502
MTEQIIEQELIEKLGNLKYIYREDIRDRITLEKNFREKFETLNRVHLTDAEFTRLLDQII
TADIFAAARHLRDRNSFERDDGTPLFYTLVNIKDWCKNTFEVINQLWINTTNSYHRYDVI
LLINGIPVVQIELKTLTISPRRAIQQIIDYKNDLGNGYTKTLLCFLQLFIVSNRSDTWYF
ANNNNRHFKFDAEERFLPIYQFAAEDNKKITHLDSFAEKFLTKCTLGQMISRYMVLVASE
QKLMIMRPYQIYAVKSIVECIHQNCGNGYIWHTTGSGKTLTSFKASTLLKEVQRLKTQLD
QYTDLTTENINEIGEVISQDQLQGFRGVYLETAKRLKAEQGKDKPIREVEQLDFEFVLFA
STVIDYDYIMGLIARYTQEESGRQEMTREQLIGLIQSDAKFMDEDITAYIDTLPTGEGLN
ETAIREGYERFKSEKNARELATIAEKHGLEAAALQGFVDGIVRRMIFDGEQLSNLLSPLE
LGWKARARAESALMADLIPFLHRLAQGREITGLKAYE
>1170769.6.peg.1503
MKIDRHSKAKILSQEEIQRLFTTGLTTARDRTLCAVMLYTGCRVNEAVTLKISDVYDKKG
RIRTELILRKGNTKGHLATRTIPVLEDLKHFLEQYQPPATKDGFLFPGRWGRGHLHSDSA
SIIFREGCKQVDIEGASTHSFRRTALTLMSNAGIPLRVIQEISVHRNLEQLQRYLEVESS
QVRGAIASLSMLTPVAPSPEIDDITITKITVKTDS
>1170769.6.peg.1504
MNGEDFKKFVEEELLPKLWKGAVVVMDNLKAHKMKGIIEMIESVGARVVYLSPYSPEFNP
IEHLWWQLKAFIRKFSPKNSLTVVQLLSVGVLLCSGQQLQNYFSHCCYCTS
>1170769.6.peg.1505
MFQDKLEFVYAVMDGVKGRGKRNKHSAKRIKFNNSCFA
>1170769.6.peg.1507
MNSDRLVGILWSHPVFNPQKSRGAVMGGKVNSDRSLVKGGKY
>1170769.6.peg.1512
MSIRKKTAQIPIVRVPCPTVNSQPPRKKFRGAVIGDKVNSDRLVGILWSHPVFNPQKSRG
AVIRR
>1170769.6.peg.1514
MSISVRVYLLKCQLRTSSDYSTTGFLGVEPRCDQISQSSDRYLPYLLLQRHGIFFEGG
>1170769.6.peg.1518
MVTLYEQDFALWSEKMADLIVRKCFDELDITNLVEEIRDLSKRERDRLFSSMRLILHHLL
KWDYQPELRSRRWLLTIQRERSNIEDYLAGSPSLKKYMTDEYLYKTYQKARLDAIAETGL
EMPISCSYTLNDIISRSLTLT
>1170769.6.peg.1520
MAGDRKALRAIARRRYCDRLHNCSLAIEYKRIYGYDEVRKYSWEGMISQCTYEASDLTFG
LNSNSGNKKRIKTLIEEARSRYFSDCY
>1170769.6.peg.1522
MEEWITQELERTELGDKRRTKRLIKIVSNLSASPGASVPQASGTWSQTKATYDFWDSPYI
KPSYRVTRIEGLRQLYF
>1170769.6.peg.1529
MYQSEAPPLKTIATDLPSQDIDEELNSTIHPRPPWETLPTMYDLPSENPEEPGLPDEFHN
FQPQLLRETCQSSVYPRAEREKQRAEQERLAKEQAEAMTIQERQQRELAEAIVIQEREKK
EKLAARLRSLGINPDDI
>1170769.6.peg.1534
MKVNLQPGVNNTNVDASKSSSQRLIAISVSAIGETIDSRVPLNLCLILDHSGSMKGQPVE
NVKRAAWLLVDKLRDQDRLSIVVFNHRAEVLLSNQNVVDRDHIKQQINRLSANGGTSIDE
GLRLGIEELAKGRRDTISQAFLLTDGENEHGDNNRCLKFAQLAADYNLTVNTLGFGNNWN
QHILEKISDAGLGSLSHIEHPDQAVDKFNSLLMRMQTVGLTNAYLLFSLAPNIRLAEFKP
VAQVSPDTIELPVQVEPDGRFGVKLGDLMKDVERVILTNIYLEQLPEGQQAIANVQVRYD
DPSVDKTGLYSPNLPVYVNVERTYQENISPKVQNYILALAKYRQTQLAEDRLQQGDRLGA
VTMLQAAAKTALQMGDANAGTVLQNSATRLQAGEDLSASDRSKTKIVSKTVLQDPTLT
>1170769.6.peg.1535

MPQRRGRGGRGQPVQRGQCPRDRQRVGRQPGRPAFDGLDQQRAQRQAGRGRERIEQARGV
AAGGGALEAGALVGQVVHGRQAHLALLGHTGQPGRASQPHMAVRQPRVGQRLQRRRRQRG
VGGAQRPAQGVGQVGNGLGHGGGRNDRSEGRCNNAPRSNDHRANHSGHCREHPQIAVAAG
LGRCAGAVLGARPRSLPEPGTTQGQPGCLHRALPAPPRPGGRGLRRPVRAGHRALAARGH
AHHPGRRCDLRPVLGHGDRVVRLQHRRHAGLSGGALPVARRGAGALRPAAGRHPARPRPR
RRVLPVHAAADPGRALLPHQFAHGPHAHEGAHLLRGEPVGHARGHRGVRERGHTTRAARF
AARHPEPGTDRLVRPAGRVPAAGALAGERRAAPAGVRALARPAAAPLRPQPDRDRRRRGR
SGHRLHRRRGESARDAGRGAQDGRRLPELRLRAEQGADPQRQAGAADAPGLAAGAAGHPA
HVPLQGRDAARARRDPRHRAARQRRALHRPGRGGAARPCAAGEPVDGVDRAGRRRHADPH
RAQHRARHRGAALRAAAARPRRGPRADQRHGVGPPGRARTRARAAGGARRWPHRLRTGAG
LRASGLAGDADRNGTAPAGARRRRRERLRPRLARGRWRAGVHRAQGPARGTRGRAAMAGG
RTRWA
>1170769.6.peg.1536
MAEHGGREERFAFDELLCAVGRVARLDGFGLQELGIPTGRVIETNAWLQTRYPHIYAVGD
AAGPHQFTHVAAHQAWYAAVNALFGEWRRFRVDYRVIPWTTFIDPEVARVGLNERDAREQ
GVPFEVTRFEIGELDRAIADGETRGFVKVLTVPGKDRILGVTIAGAHAGELIAEYVLAMK
HGLGLNRLLGTIHAYPTLAEANKYAAGVWKRAHAPEGLLRWVQRYHDWKR
>1170769.6.peg.1537
MARVPHVADGGGGQGLAGGAARQPQHLALDEGHARQRVVEHCADLERGVVAGLVQQGGAV
GGQVQRAVQPPTPAQRRGRGVAQGKRQRAVRPVGDDLHAAHAVALARQEARGRDLAVGRG
VAHHAPGRGDGAVGGGQRRQGLGAGDGGDQRGQGQAGHADHRGHTGEGEGESGGHHRFQS
W
>1170769.6.peg.1538
MTALAQRQAAVLQPRGRVGADGEHRHVAAQQRAVAQVHAAVVDGGAGFAQHLDAGAPQAP
QQPLAHRGRVARTHLRPAGDQGEGGAGHTVAAQPFGGRKHGFDAGRTTADHGHRQGACAG
QRDHVGPEPHQAFDGPQRERVLARAGQFGVEPLGPQHRAGVDRQQVVVLGVQVRAVGRHP
LHARVGGVDGGAAPGVPAHPPGLHQRGQRNLHLGLAVAPGQQARHHAAVGLGRVRRDQRD
LPVGGVVLGEAGQQVQVRMARAQQKESTDAAVHGRRWYVGLQRAHCGSATQPSLPLAVLS
ASAPMTICVSLGGGSPAGCSSANRRRKSAPGSTRSLSQRPVGSPWPRCTTKRTRRLWGLA
PSVPVGWRASHT
>1170769.6.peg.1539
MVGGHVYVMEIKVVEGNQVQGNAALDQILQRNYAEKYRGEPGKSVHEIGLIFSRNQRNLI
QADWR
>1170769.6.peg.1553
MWGGAAIAACVIGSLSGILPGNTSRLQLAQQKIDSVQTQAPISPLMVALNNPIVEIPKTA
VVRPAKIEEIDYIQEEMEFEGN
>1170769.6.peg.1562
MNIFGIGLPEMIVIGVVALLIFGPKKLPEIGRSLAKTIRSFQQASSEFQNEFKKEVQQLE
ETIKTTAEIEPKQIESSKEQKHS
>1170769.6.peg.1571
MIAEATAVTGELAEGAEHSGIGLNTNIFETNLINLAIIITVLFVFGRKVLGNTLKTRREN
IETAIKSAEERAANAAKQLKVAEEKLTQAQVEANRIKADAETSAKAAGEAILVQAAADVE
KMQAAGAADLNAELERVISQLRQKVVAKALQKAEAELKAGIAEDAQIRIIDRSIAQLGG
>1170769.6.peg.1572
MTHWITLLAVEEVAKKGGLFDLDATLPLMAIQFLVLALILNATLYKPLGQAIDGRNDYIR
NNQLDAQQRLSQAEKLAAQYEQELAGARRQAQAVIAQAQAEAQKVAAQKIAAAQQEAQAQ
REKAASEIEQQKQQALASLEAQVDALSRQILEKLLGADLISQR
>1170769.6.peg.1577
MHQLLLAVQQRCYEHHHQQIVSISLAIDFIDPLFVLDKLGQRNTLSFYFENKSKGEAIVA
IDAIKKLDINGQNRFNKTEDFIKDCLKNIINFGNLNEPFTGSHFFCSFSFFEQHQNSNYP
FAAATIFLPKLQVAVKNSSCTLVINKVVDADVDVNHILQDIQNKIRTLQSLTNGLPIAVT
TCKGSQTSQINNPDVFKNSVLSALQKIEAKQLRKIVLADTLDVYSYSSFNLLKSLNNLRN

LHPNCYVFCISNGRGQNFLGASPERLISIQDHQLITDALAGSAPRGKTPKEDAVNAHRLV
NSSKEKHEHNLVIDFISHRLSQLGLFPQPLTPRLRQLANIQHLWTPITAVVPNNVHPIKI
VAQLHPTPAVAGATREIACAEIRHYEKFDRGLYAAPLGWLDSRGNCEFIVGIRSALINNN
CARLYAGAGIVAGSDPEREFAEVQLKLQALLKALV
>1170769.6.peg.1592
MMLKAKIGLPLAYIPAERPVFGREAYKKLVPWREHCTLMKSVVRTGVRKILN
>1170769.6.peg.1609
MNSIYWWNGEVQMEKEAILIMKSRQDLFAQVKDKIDSVHSYDTPCIVAMPIDYISETYLS
WLIKETETLN
>1170769.6.peg.1613
MAGFFGLFQGRTIFTPSGDIPQELTLEIVALCPIKPGCQN
>1170769.6.peg.1623
MSPELKNYLRLYAIEPFVLKCSFGFIGRFLIEQTDVSLLNQLAYI
>1170769.6.peg.1625
MVVKNRQICYIIAPNILKDGINLRYNLDLFNHIEQTKNSQLYCF
>1170769.6.peg.1632
MRKYWIERSPAVIKVYFLPEKVREKNFLELVDIPEIIG
>1170769.6.peg.1638
MKYLTPEQVYKQFGYHPKTTVEWADLGKIECIQNVSVG
>1170769.6.peg.1643
MIKAAGQIYHTYSQVHPEIIGQVSGVAISRTTYRGKVIFNQQPILLPQECFIPLQQIESC
VY
>1170769.6.peg.1644
MGWTLKLLRSGCERYTGTLWLLYRWSYIWFWKPLANNNAKFFLLDYLNL
>1170769.6.peg.1687
MVRIKSGFNADDQHPIQATSADIILRQQLEHSISRYFYHGCDRNIQNLLSYCRWYMKTDT
KALTLVIECPDQVSNWRILQKIVPMATLLYSVVSSAKIRVCPPEPRAIPFEMRVDELYVY
RDWA
>1170769.6.peg.1688
MIPTLLAATTCFIIAFIAAPPVDIDGIREPVAGSLIYGNNIISGAVVPSSNAIGLHFYPI
WEAASLDEWLYNGGPYQLVIFHFLIGCACYLGRQWELSYRLGMRPWICVAYSAPLASATA
VFLIYPIGQGSFSDGMPLGISGTFNFMIVFQAEHNILMHPFHMLGVAGVFGGSLFSAMHG
SLVTSSLVRETTETESQNYGYKFGQEEETYNIVAAHGYFGRLIFQYASFNNSRSLHFFLA
AWPVVGIWFTALGVSTMAFNLNGFNFNQSIIDSQGRVIGTWADVINRANLGMEVMHERNA
HNFPLDLAAVEVSPVALTAPAING
>1170769.6.peg.1719
MRLNDVKPQKGSKKRRKRVGRGISAGQGASAGLGMRGQKSRSGSSTRPGFEGGQQPLYRR
IPKLKGFPVVNRRVYTTINVEKLASLPANSEVNLASLKEAGILTSAKGPLKVLGNGELGV
PLKVQAAAFTGQARSKIEAAGGSCEVLS
>1170769.6.peg.1740
MLRLEHISKIYPTGEVLKDINWEVKPGDRIGLVGVNGAGKSTQLKIISGEIEPTAGQIIR
PASLHIAYLNQEFEVDPTRTVQEEFWTVFKEANAVQLALYEVQRDMETATVEQLDKLINK
LDKLQRQFESLDGYNLDARIGKILPEMGFQVEDGDRLVSAFSGGWQMRMSLGKILLQKPD
LLLLDEPTNHLDLETIEWLENYLRSLTTPMVIISHDREFLDRLCTQIVETERGVSTTYLG
NYSAYLQQKAENEASQLSAFERQQKEIEKQQAFVDRFRASATRSTQAKSREKQLEKVERI
EAPTTGVRTLNFRFPPAPRSGREVVEIKDLTHTYGDKILFLGANLLIERGDRIAFLGPNG
AGKSTLLKMITGMEIPTEGTVKLGDHNVIPGYFEQNQAEALDLNKTVMETIHDEVPDWKN
EEVRTLLGRFLFAGDTVFKQVGDLSGGEKARLALAKMLLCPVNLIILDEPTNHLDIPAKE
MLEEALQNYDGTAILVSHDRYFISQVANKIVEIRDGEFRVYLGDYHYYLTKIEEEKEQAK
LAAKKATKTAKAAAKKK
>1170769.6.peg.1749
MYPSNVAKSLDIQPFVFRGSDGHDILADIINQAHSQNLLAIPWFEFGFMTPNTGELALNK

PEWLTKMRDGSTVSMSAAGEVSWLNPFHPQVQKFIIDLLVELTNNYDIDGIQFDDHTSLP
HQFGYDDYTVNLYKQETGKNPPANSQDSEWVAWRANKITEFMVRLNHTVKQIKPKVIFSV
SPNYYDHAYKFQLQDWLNWVRLNIVDELVMQVYRDDLESFTSKIARNEIQEVRQIIPTGI
GIMAGLRTSPVPMQQITKQVRTVQREELGIVFFYYETMWNRSPETLEQRIQGFKNFFPYP
AVRVAAE
>1170769.6.peg.1752
MAENTPLYERKLLSEPPEYVKLAWEWTSGKLPTCDVRGIEVHGEFLSWQDILLKLLNTPS
IASKSWVYRQYDHQVQNNTVLLPGGADAAVLRLRPLSGSTVTPWESGVAATVDCNSRYVY
LDPYEGAKAVVAEAARNLSCVGAQPLAVTDNLNFGSPEKEIGYWQLAYACKGISEGCKEL
GTPVTGGNVSLYNETFDAQGNPQAIYPTPVVGMVGLIEDLQKICGQGWQNVGDGIYLLGL
PVSVKLELGGSEYLAVIHHTVAGKPPKIDFALERDVQQVCRYGISHKWISSAHDSAEGGL
VVALAECCLSGNLGASINLGISSNGECRFDEVLFGEGGARILVSIPGTYQQVWESYLQAH
LGNNWQKLGSVVNLSSGLTVSTLDDYEVMRMDIPQMGNVYHQAIAQRLAFYE
>1170769.6.peg.1755
MPFLLLGVLFSSLLLFFVEERKLVNIMPKNPLLGALFGSMIGFLFPVCECGNVPVARRLL
MQGVPTSVAIGFLLAAPTINPIVIWATWTAFRDQPEIVVLRVVLSLLIATIIGFVFSFQK
DITPYLQPQIARYLKFNPPAQTEPKTPTRQLQEQVITPSPLLQSGTYILGGKAGISTRLS
GNSSQTVAKTPNKTLMVKLGLVLDNAIQELRELGAIMVLGSAMAAAIQVLAPRDVILSLG
AGPISSILTMLLLATVVSICSTVDSFFALSFASTFTSGSLLAFLVFGPMIDIKGVGLMLS
VFKPKALFYLFALAGQLTLLSTLFLNLHIM
>1170769.6.peg.1762
MGNCHDLMFFMAPSTVPCKERNFSNRTSLTAWVTRALLTIWKLPWAFWACLANCTRAPKP
ELSIKSICDRSRIVLFGPSPILASSTLRNVGSEKASNNPVRRKSWQLSRTSRVPLRLTVK
FSTSVFSAITPSCSY
>1170769.6.peg.1774
MKKILKNLIVVRLTYSKPRFVLSLFKKEKLEPDDLDE
>1170769.6.peg.1791
MKNTTQSPDFLEMPMTTSRQPKQPQSTLVFECHLLHDDSKELSPHIHLVGRKPKDVEILF
MR
>1170769.6.peg.1795
MNSFNQVSYPVDFRHLLFLEDINRIVGSEDFHPLIFP
>1170769.6.peg.1809
MVIPFPHSVLSLVYRLVYGVIGQNGLRLRNIFGYPVTRK
>1170769.6.peg.1815
MCNPITQLQKAMGFLVCEAVSVRKIFITSSSPRATSGARGIK
>1170769.6.peg.1832
MLASKIAATYGFRIDGFVPAFLGSIVLSIASTLINYFMRLIV
>1170769.6.peg.1840
MSVELVESFAQSVPDVEIIKLNNVGHYPQEHYHEVILQDLLAFVRLTST
>1170769.6.peg.1841
MPEKRGIYNSVQLAQKLEAERSVKLSPDRLIRVTSKNSVAPVNTIANFSKYV
>1170769.6.peg.1842
MGARLRVFLTSEEDKTLFNLRSADVPQKVKDRAEVIRLNAHGWYVEKIAAHFNWTSQTVR
EVLHKWEKFGLEGLWEKSGRGGKPKYKDSDIEFLEECLKKEAYTTVFN
>1170769.6.peg.1843
MKYNYKVEEQTLEKYYQQAVQLIETQALDQIQKEIKESLDIFIERIETDKSLTQVIITTL
LKKIIKPQQDIRLHMAKFPNGYSARVLDTKVTTPFFKRKFLKYANKETAFLTKATRAEII
WNFEEGIKLPLKSKILVEPFLKLIDKIENQTIDIEQCLVYILSQLYFLCQSHKVVFSETL
KVANSVNIININRVVKMVEKHFEEPYSSRLPVIVIFAIYKQLFKTVRRFENKILLPLNVH
TSADKHGYGDIEIRDNDNNPFEILEIKHNIPIDRNIILDIVKKSTDTKIQRYYILTTYKE
CFISKDDEEYINELILNIKRESGLEIIANGIVNTLKYYLRFVEDYSEFINTYTEELVRES
LTSTEVRDSHIRTWQRILQE

>1170769.6.peg.1845
MEKICSNLIEDRVNSNSSFEKVREYQILAMLETSVGYGSPL
>1170769.6.peg.1846
MNAVRSRKFDCEWGCQGLIFGKSAFWVIWAEIIKVLFLTKALSK
>1170769.6.peg.1853
MHLFHMLGVAGVFGGSLFSAMHGSLVTSSLVRETTETDPWI
>1170769.6.peg.1860
MNYLTYSYLLTPLHTGASTQAGNLLGIAREAQTELPYIPSSSLRGKLRSSLESMAEIRSE
AGSFFGERIKDGQQPTEGEVWFADATLLFFPVGSFSHQFLWITCPLWLSRWGRWLRNDQL
NKLIEQWQSDLLSNGKKAITSASGKQIYLQGAILNEADIKTINNIDWNLFKDIPDGNGIL
DLKNKLVILSNEDCGALVEIGLQREVRIALDENEKIVAGGSFRSEEAIPSETIMFFPWGM
KLEKEANKTHKVRESLINILNDRLQFGGLEGLGRGWTENKTIAVNKKEE
>1170769.6.peg.1861
MQKLDTRDFSRHAYNGLIEIKKNVRSVDHKKASGIVQGLSAYISTWGLHRLSGDGLKYIN
SRSDDTKYKGQIYQEFLKTLQKLSKVPFAYDDASSLINLELEKYTGLNRLAIELAKEWSF
WAVPILGEAEQS
>1170769.6.peg.1862
MRNQKSEADIWQDFVQTEIKSKSKLGKILESSQYGGYEKRELTIYFPDENFRKGAQGQIE
AIKKKLREPYGLLCDRITVKTGSVSANVMNTSVTRGNSKSVSGTPKNHNPLQALYWVEPN
LPENDASQRMSILKETVAAEQGCNQIYTKLRQRTLQLVNGEENTVSVSFNWRIRIGGTRG
FRELLLPVLHPIFGIPYIPASTLKGAARAWARKNDAPARVQELLGMLNGKDAKAAKIEFL
DAFPTKHCLSIDVATPQWVWKDKKVMYEPVPHPLLSLEQPQFLIGLRPTSRQNSDCQDDL
KTVKSWLENALNSGIGSRVSSGYGKALGTIPITNTRKSYDFELWTQGMYGSNPPSKENNY
QGTPEFRPSAVRGILRYWFRAVALRLYDVPTCQKLEEQIFGNLGKQGKISLSTKINPSTK
KDPYFYDGKIYLEATEIKYLNLAEKLLFLALQLGGVGRGSRRPLHLLNGRMRGCHWQIVG
KELTLDYDSEQWREFFTEIEQAFQAIEATKATIGSYIVSPGKRGARQQDVLDKNAQIWLL
KSPSQIHPAKITNWQTDGSSSKVRGTALDLLYGDNRFKGQSKGQGNANVGGALETPSFVW
IKSIFTDSLPYQVITIFGSDDQDRKEFAKELKNQGAILVFPEMSPSKSTTSSPIKRK
>1170769.6.peg.1863
MESVLIATIGTRDLMFQIASGEWFNIGNDRVQNGEIISEQLEVISDLGLKDNTTFRDLTK
YLLDHIQKHIDHIQPPIIGKMFIEQASNIEKVYLIATDQEKNVKQREKDTLYSAELIKQW
LMHKFSHLHNHNVHIILLGQDGTNPSIFEDMFNWWRKIWKNTITVNKSQSVWVCLKGGVG
QTSEAARISGLSFYGDRIQFFEFKENTPANRNGIPSDYSGPFLGTNYLWDRTQKQALKLL
ESYDYTEAYELLEPYFQQPSANFGAIPNLLKAGKLWNQGQFERFLSLARSSTQISGVQGR
LWMAYEQAYLGVIRLEQMNTTEAMLHSYRAIEGLLYWWAADSFPDHIEERKNQYPSIKDS
ILQKYPSLKNYFNRPEPKREVNLQGYLLEDLLNLAIPETANSIDFKAFWGRSKDTRNYFS
HRLGGLAEQDIFTAWGEDITDSPQWQKRILNCINLVTGKSFKTLSQASIFSQIHTQVLEA
IKKQEIINYDNNK
>1170769.6.peg.1864
MVLITNFIRFIFVSLINGYQKYLSPYKGYSCAHHILHQGESCSQYVKRSLLQQDLQTAIK
LSQQRFVDCGKAAQVLSHQRSPYSPINKSSNQPRFYRPISRRIFILVILPSLFTFGLISP
ALAGRIPNRGFQKAGQCFGEAGMDEDRRDGDYGDPNMFYGLCCLSLIGAGILTEER
>1170769.6.peg.1874
MFATVSSQKNYQLICTHTTRNTRIARNLQVLQFHRKILGKILQLLTKVL
>1170769.6.peg.1878
MGLNTGEDSSIIIIYYIIVNADFHLKIGDLNGSYRSY
>1170769.6.peg.1904
MMSGLIPLDKGEIRIHGKPIHRTRIGYVFQNYRDSLFPWMSAYDNIAYPLRVKGISEREC
RHSVEHLIETFNICLDLKRYPYSFSGGQQQLVSILRALVAQPEVLFLDEPFSALDFETTL
FVRDKLQEIFMASTIPMLMVVHNLEEAIFLADKILLLSKRPTQVVEMVSFDAPRPRTPET
LTSRNFVEVSRYCLDIFRQEMQK
>1170769.6.peg.1927

MDICLRYGQVVLAMELKVWRKGSKDPLTAGLKQLDKYLSGLNLKTGWLVIFDRRPNLPPV
SDRTTTEMAISPQGRSITVIRG
>1170769.6.peg.1951
MDWLARFSPTYKERIIYHEAGHFLVAHLLGITVTGYTLSAWEAWKVGQPGQGGIILGDDE
IAKQLERGKIGVSMVEKYCNIWMAGIAAELLVFNSAEGGGDDKAKLNQFLTVLGFQETLF
EQKQRFHLLQAKNLLEQNWHTYQHLVQAMRNRLDVEECKKLIA
>1170769.6.peg.1952
MVPPLPSNRKLSKVAIPKNPNMVSAGMVGDRWISGPNRVDRVIKKTQMAIRLMAV
>1170769.6.peg.1959
METGDREFLKMIQVYNEDDCYATRRVKDWLVSFLVKKNLIKNEDADSESASTQ
>1170769.6.peg.1964
MALTCKDPLATEIFYTKNFGFRRVRVAKLPDGDQIVFIKMADSAFYFELFKAKEELPIPR
PTLDGPQYPGLRHLAFKVDNVDAKLAEIGSDAVITLGPINFDDYIPGWRTVWIADPDGRI
VEISQGFQDEIDVPPLKFI
>1170769.6.peg.1965
MKNVPDSPLGKLYRDHIELIMNKDIEAILDQYTDDALLISSFMKTPKYYQGRDQLREHMQ
GILGIVDLETEINFWAETENPQTLMITEIIHMKVGGEKLTMRFADSWVLQDGKIAIHFAG
MVQHPDGTLA
>1170769.6.peg.1975
MAQYFLPRPNRKFLFSHLLLILPILGGNIHQQELFSP
>1170769.6.peg.1981
MGGDRQTIIAQRQYSREVSAPKILAKNFFEKKVEKVVDILKEVRYIE
>1170769.6.peg.1982
MRDIPTEEVLKQYSPEEIKQYSPKEVLKQYSLQDLLEGLSPETLERLAIILSQLGVNQIK
NQEQ
>1170769.6.peg.1991
MLSGLLKFVLGFLLAIAVLLGSGMTIAIYFINRTAITPQKPMFPNDNPDPKPNLPRVTRK
KVVKVKPKPIATPDLPRESPTPLPSGSYTAVVTWSQGLTMRDKPAFEGQAIGGVAGNQKV
IILETSQDGKWEKIRIPDTNQEGWVKSGNTEKSN
>1170769.6.peg.2001
MCGGNLNQLATPIVHSLFYLKVVFSQPHIHSTHGDFLLQWRGLV
>1170769.6.peg.2007
MVIFENLPILVHQLRIYHQFFNLCWIYLPNWLINKQGMVYNG
>1170769.6.peg.2027
MTGSVSAFLGFSMGSSALKGVTSPDGRPTSKLISSGNNNLQSVPISFLKEEDIINQVKKR
IEQNKTKTNRTKKVEEEEQTVSTKDKSQQKAQELPEEPPQPGFPVVAESEGVNMSVQSAS
YSGGQLILKVKMHNQSNESVRFLYSFLDVTDNRGRVLTATTEGLPAELPGNGSVFMGNIS
IPTALLNDVSSLTLSLTDYPAQKLKLQLSDIPVEK
>1170769.6.peg.2028
MRARRTVTIANSAATKKALIVIKISIDNNFNPISVQINWGKVITSQARVE
>1170769.6.peg.2029
MVTVRRARIHQLVQSGDAPAIAVEETKFPPGCVLLIEITTNSAKSSEST
>1170769.6.peg.2039
MDFIRVSLPSLFQEAAKILQEKEEIILSAEEYAQQVIEAAQVKRSQILADNDIIHQVERE
TVQLRREAQQKCDAIMQDTLAEIERKRLDCDQEMEETRQNAIAHAREIENGADEYADRVL
ENIEEDLQKMLRIVTNGRLQLGGETRKQRGSSDIPKE
>1170769.6.peg.2044
MLVLTVGLFSLSLVPLSRTVIPGASRYTLVYDNGSNQAVIATSPDITPTQLEATLRQAAS
NLYSYGRMGSGRNNSLTIRARTVLHPESGISTPVYLGKVERTLVTREDPQMLVEVFLDKF
PQLPPS
>1170769.6.peg.2046
MQLIAMKVPIINSSRGFLYKSQILSYSIGYQQLQFYV

>1170769.6.peg.2047
MLAANLKQIQQFFVHPHSSLQLPWNSLLLGLLILPINPFFGAIAIGWASYKTWRKKYSSI
QRKTLNHLLVILSFWFLITTGFTVFARDQPDTLLGLFNFLPYFVVFAGLTPLITTVSQLR
QLTWIIVWSSLPVVIIGLGQLFLGWHARWQFLSVVVNLTIDPGGEPLAIYLGGEPLVRMS
SVFMNPNTLAAYLITVLILGLGLWLENYHKIRKKANPLGFIFLSVVIIANFLALILTGSR
NGWGIAVMACIAYALYQGWRLIIAGVIGLTTSIILASFATERIASVFRSFVPRFIWARLN
SDTPLALMRKTQWQFAWDLTLQQPLSGWGLRSFPHLYEQKMGVSVNHPHNLFLMLSAETG
LVTTCLFLVFLAWILIAAIKFWYKCSLPKENRLVFLSYLLAFIGWIMFNMADVTTFDLIL
SALFWVILAALYGAAHRYEGSHH
>1170769.6.peg.2055
MLRVENDTFISKLPGDKTRNYPTFLAFLGCIGVKLSKP
>1170769.6.peg.2065
MQRSEDLINAASNRYRITVQVANRAKRRRYEDFENNEDSIMKPVLRAIIEMSDELTQPEI
IGELPSDWL
>1170769.6.peg.2090
MKVLVIGGDGYCGWATALYLSNRGHEVGILDSLVRRHWDNTLGVETLTPIAPIQQRLQRW
QDLTGKSIDLFIGDITDYGFLHKALHEFEPEAIVHFGEQRSAPFSMIDREHAVLTQVNNV
VGTLNLLYIMREDFPDCHLVKLGTMGEYGTPNIDIEEGYITIEHNGRKDTLPYPKQPGSM
YHLSKVHDSHNIHFACRIWGLRATDLNQGIVYGVITEETGLDELLINRLDYDGVFGTALN
RFCIQAAVGHPLTVYGKGGQTRGFLDIRDTVRCIELAIVNPAQPGEFRVFNQFTELFSVG
DLALMVKKASYAMGLNVEIDHIDNPRIEKEEHYFNARNTKLLDLGLQPHYLSDSLLDSLL
NFAVKYKGRVDNNQILPKVSWHRK
>1170769.6.peg.2092
MAISDFPQPGTPKINIPFGCGNPYSLALSVKALDLCFSHFFNSSNPPTASIVSSSSMYSK
MPLRRISCFFSDKTISTSSSVSLPLVTWAFRYTFSASSLVSPKAALSSFSLASVVKRRPL
FCSWWAVRTLLNSVMSGSWKSITTTSFSNSIGICCIGDIKIMFFCIPLKLAIASRRDLVV
AGASIKGCKSLKINIPGSFCLIAHSIAASGDTVLCWFVLLVCPYSTMPWVTVHT
>1170769.6.peg.2093
MLILGVPGCGKSLIAKTTSRLWGLPILRLDMGRVYDGSMVGRSEANLRNALKTAESISPT
ILFIDELDKSFAGSTGSSDSDGGTSSRIFGSFLTWMQEKKSPVFVMATANRVERLPGEFL
RKGRFDEIFFVDLPTPEERQDIFRIHLTKRREEIARFDLEQLAKMSDGFSGAEIEQAIIA
AMYEAFAQDREFTQLDIIAALKSTLPLSRTMQEQVTALRDWARQRARPAASSVAEYQRLE
F
>1170769.6.peg.2124
MILKSPGQLKFTVITFTLAMLGLLGGCLGLTVSFDSQPSDSMSQAPEKSPFLENHTATPS
PQLQRVEPKITSNPTLEKSKFGNLRISNKTYQPIRLALLLRHSPSSSSGKKGLIPAHWDF
APQEGSQGGLILSLPEGSLKLEKGDILVAFAQDGSRRYWGPYIVGETSGPLWDPKTGEWQ
LTLVP
>1170769.6.peg.2127
MILVILSLYPALEKFLHSLFTHYYNLINTLISLAPDSFISVFTSNLFVKNISGLELPNTY
LLSYIKEKRYLGLDQKEGYTLVLACENSHTNQQFQDMTDGERQEILKKLKLDYGKILLNY
FSVDENLKTKIDQFISTLFCANIPVPQVIEIHMELIDEFSKQLKLEGRSDETLLDYRLTL
IDVLANLCEVYRCSTSKIN
>1170769.6.peg.2130
MKGQQLFHSFLPGVTAAVLTTQSAWAGTFKANDLKLVSSPVVSTATNPKVSVVENNWHLV
ANTVDHALGFDYQLDFGQSILPELPSSSSRTNVPSPTKFKTVLSLPPVNPKVRSGKTYNQ
VTQITLPTDKSSETEPVKPNSQSVNPTSDSPQMILERLKPNPNLLDVPQDSQGVKVQTTE
AISLEQALDLAKQNNNDLQVAVLQLQRSQSSLKEAQAALLPSLNLVGGVTRSRSSSATLR
ARQENAPENPEATSVFDSQAELRYDLYTSGRRTAAIKEAEEQVRLQQFEVEKQSEEIRLN
VATEYYSLQQSDESVRIARSAVENSQASLRDAQALERAGVGTKFDVLRSQVNLANSQQEL
TDALSQQAIARRRLALRLNLPQSVSITASDPVQLAGLWKSSLEDSIVLAYQNRAELQQKL
AERNIREQQRKQALATLGPQISFIARYDLLDRFNDGVAINDGYSVGLRASMNLYDGGASQ

ARAARAKTEIAIAEAEFAERRNQVRFQVEEAYSSHLANLENVQTAATALDQAKESLRLAR
LRFQAGVGTQTDVINAQSELTRSEANRVRAILNYNLALTRLQRYVTSRAVQKS
>1170769.6.peg.2145
MRNYHPCPKNPLTIHTSNGQIKNSSLCDFCWNEYCFCWSNYCIDEWLELQNKYMQQRLQQ
KTSNIKT
>1170769.6.peg.2150
MFKTLAISKQQLYNVQVVVVEQAPPAKYTNSQNGQNHRVAVT
>1170769.6.peg.2152
MARDYYEILGVSRDADKEQIKQAYRRQARKYHPDVNKEPGAEEKFKEINRAYEVLSEAET
RERYNRFGEAGVSGAAGFQDMGDAGGFADIFESIFSGFAGGMGGPTQQQRRRGGPVRGDD
LRLDLKLEFREAVFGGEKEIRIAHQETCEVCSGTGAKPGTRPRTCSTCSGSGQVRRVTRT
PFGSFTQVSTCPTCNGTGSVIEDKCESCDGKGMKQVTKKLKVTIPPGVDNGTRLRISQEG
DSGQRGGPAGDLYVYLFVNDDEEFQRDGINILSELKISYLQAILGCRIDVNTVDGPVELT
IPPGTQPNTVIKLENRGVPRLGNAVSRGDHLLTVLIDIPTKVTLEERELLEKLAKIKGDR
TGKGGLEGFLGNLFKA
>1170769.6.peg.2158
MPKRINNAGPLYLLYDHDLRIGLLTGCKSVNWQLGRSSLVGNLLPGNPCYIIAKSLIGKF
IKFFVETPLQN
>1170769.6.peg.2160
MGQSGLIKQCLIAFLAGGHIILEGVPGTGKTLLVKVLAQLIQGEFKRIQLTPDVLPSDIT
GTNIFDLNTRNFYLRKGPIFTEILLADEINRTPPKTQAALLEAMEELQVTLDGESLPLPD
LFWVVATQNPLEFEGTYPLPEAQLDRFLFKLVVGYPDQTAEKQMLFNRQSGFTGRRIDIS
NLNPVTTVDNILQARQAVKQVNVAEAIVDYILEIVSKTRKHPDLALGASPRAAGAWLQTS
QACAWLAGRDFVTPDDVKAVAAPLLRHRLILNPEAMLDGSKIDSVITTVINQVPVPR
>1170769.6.peg.2170
MKGRVGLLRLGMPWAKLSGDTAIEEIRRPVETNISAFTGVY
>1170769.6.peg.2174
MLIWNEPDEQISYGQLSAMIDLALLIFIFAGAENGQKI
>1170769.6.peg.2178
MLLIGEKTQKNAIALDLPSCDRPLITFNISPEKNEGNGDYRSSRFLKTLMKDFN
>1170769.6.peg.2180
MQFGITQVSIAQVATAQVGTVQVSTAQVGMRQVGTAQVSIA
>1170769.6.peg.2181
MEVRSHLGFNLLKEMGAIALSLIFSREILRAVKKAIAILENENVGRCDRA
>1170769.6.peg.2182
METYPNFTLPPTPIHRSHSLGTLIEWKRARASGLGAYQEIPKGVPTRWGH
>1170769.6.peg.2183
MYSSHSLGTLIEWKQEIRELNADLKELIGSHSLGTLIEWKHIPKITI
>1170769.6.peg.2184
MSAAVAPVKIVSIQLMSPASGNPKPNLQSRAWGCFHSINVPSEWEPKK
>1170769.6.peg.2186
MICSKISKIIRDRTSPVLLKGKTEGGKQAIAWRLFPNPTSAILLKGNFERGEKAIAWQLI
QCDRL
>1170769.6.peg.2187
MNDLPRQKLREIIVQHGRALCDDPKRCEAFLRDYCGQYGREIFILISALKKGVVKDILNS
SNIPVELLLGRLTKQMQNDLGLTEEAARYAVESWAMVLDKMTSQQIQQPIIKPPTTISRN
QQSILKSTYPKVTPRIQEPIIKPSNTISRNQQPIVKSIYLVPTEIFQIDYTNLERLLQSQ
NFRAADTETGKIILAVVKRGKEGWLRIEDVKNFPCKELRSIDKLWLKYSGGKFGISVQQQ
IYQSLGGTKKFNYDLWKYMGERVGWRLDGDWISYSDYDFSQTAPFGHLPAVGPYRAVSAG
PSLLLRYAECNT
>1170769.6.peg.2191
MFGLLGALGCLFGWLLGEWFLSSTMLSQPQQQASTPAIIFNPELSKRLEREGAKTGDVQL

SLSWNNYNDLDIHCIDPLGEEIYFSRKRSQSGGELDVDMNAGGQQSKEPVENIYWPAGGA
PSGKYQVFVNYYGQHDSADSTSFTVGVKEKSKTQEFTGSISRGQAKRLIYEFTVDGKVQE
VKAQPLWYTVLIIGLWTALLAIGLSLALVIGQNLYLHRPFLSIHQGAILLAGGLAAGLVS
GGSAQALLSLVSQSEFLAKTGWIGGWTLLGGLLGWGMGFFISNLQAKRAIMAGGVGGFFG
ALAFLLVSDSAGDASGRLVGAIILGFFIGLMVVFVEVAFRTAWLEIQYNPNEIRTVNLGI
DPVSIGSDVKSCTIYVSNSPPVATRYRLTQGKILCEDVTKGVTQTLQPGDTQRISNITVI
VRSSDILEQSEHQNVSSYAQSQTEFSLHIKRQVISLTNGTCLTGVEIPGLEAKGSDGVVA
RVNTNPNDPRILGLQNCSHRVWNVTLADGEQKQIESGRSIKLATGTKIKFGSIEGEVI
>1170769.6.peg.2195
MEINHRISRTNRYKLTMIYSGISTITDNLLNVGFLLSLNLSKKGK
>1170769.6.peg.2198
MIKQNFNYTSVALIALSILGLAGGFYVTQQMLQAQERAAELEREKKEAEEKKEIAEKERL
IAQEKAMEAENLRQTAEQQRLEIQQRQAQEERRRLAAESRQARLERQRLENFSSQHDISR
QDALHLVQKWYAAKPQIFAPPFNTGLVDQLATGKLHTFTTRSNGPVEWLRQNDAYYEYNY
SEIKRVLDFSTSGRYPYIKIRVSEELYLHGKNGIDKNNSGASTNNLIYFFEKENGIWKIY
DYRKVR
>1170769.6.peg.2199
MNDIPRQKLKEIIIQHGHALCDDPKRCEAFLRDYCGEYAREIFILISALKQGIVKDLLNS
NNIPIELLLGRLTKQMQNNLGLTEEAARYGVESWAVVLDKMTPQQIQQPIIKPPTTISRN
QQPIVESTHPKPTEISQARQIDYTNLESLLQAQNFRAADEETCQVMLAVANREREGWLRT
EDVEKFPCKELSSIDQLWVKYSKGKFGISVQQQIYQSLKMDKNNRDVWRSMGERVGWGWR
GGWLGRTNWLDYNNFNFSQTAPSGHLPVARGGWWGPGNWERERVFVVSLLSRHAECNT
>1170769.6.peg.2200
MKKNRWGSKLLAGMKPDKKGKKPPPGQLRLFWDEVDMGNEEIIGVATKFVKANCYSPNPK
SF
>1170769.6.peg.2201
MVKKGDRIASKVRDRPFISKNQLKASSFFSFRHKFGGVCNQI
>1170769.6.peg.2202
MGARLRVFLTSEEDKTLFNLISADVPQKVKDRAEVIRLNAHSDPLC
>1170769.6.peg.2205
MRKIKRCLRSADVPQKVKDRQYGVPEDVVRTVTENCQTLKFNNQAFEQE
>1170769.6.peg.2206
MRLIGSIIVVYLHENCCNMISLVSRSASLRMLLFLAPWLFI
>1170769.6.peg.2214
MELSNYAEQEKLLKEQAHQRAEQERLLKEQANQRAEQERLLREQERFLKEQAHQRAEQER
LLKEQAHQRAEQERLLKEQANQRAEQERLLKEQEQLKYQTLLSQLKAKGIDITTLE
>1170769.6.peg.2217
MVRQIAPKNQLGLIKSNVNQGGETIGPEIVYPESDGKPMADNTKQFT
>1170769.6.peg.2220
MIENYIQQLIRKQRPYYLIQGTPIKGVNNQYWVVFKHRDSHNLLHKVITFLGSGKKQATH
KLFRIFSAKYLLSCAESIYNEVVQLKQQLSGEIEQPETLENITITSEKISKIQRRFSKMD
SLPNCLNKASVNSTLKKLNSWKETKLVQKLSKDYTDGDKAKLDDEQFAIQLVEDTDKLNL
VLEEGIKSTSLEISLAALLLRA
>1170769.6.peg.2235
MFTYHINGVGKIKKKLKKLGESKNQPGIAPWIVRTEQGA
>1170769.6.peg.2238
MFATLVNWAWFLVTKCMKPIAIVACSVGKPECPPGLLSIKAVYCL
>1170769.6.peg.2263
MFTSAATGLAVGIAFIKGLTGRKLGNFYIDLIRAITRILLPISIIGAIALVALGVPETLE
GTLVVKTLEGRTQYITRGPVASFEMIKMIGQNGGGFFAANSAHPFENPNSVSNLIETIAM
IIIPASLIHSYGVFANNLKQSWLLFWMVFLIFVVFIWVTVAGEMQGNYLANQIIGIEIPN
LEGKEMRFGVGETALWAVITATTMTGAVNGMLDSFMPQGIFCNLSSLFLQMVWGGQGTGT

ANLFIYLILTVFITGLMVGKTPEFLGRKIEQREIFLASVILLIHPIIVLVPSAIALAYPN
SLSGISNPGFHGISQVVYEYASASANNGSGLEGLQDNTLWWNLSTSLTILLGRYVPIIAM
LLLANSMSSKSTVPQTRSTLRTDSVMFITITACLTMILTLLTFFPVLVLGPVAEGLNLVS
GN
>1170769.6.peg.2267
MNQPIQLSLEQQFNVYSFASQVKEMSREQAQEFLVKLYEQMVVREATYKELLKHQWGLDL
GSMA
>1170769.6.peg.2284
MNSYASTTTFDPPTASSTPIILENLPDPPLVNGVCPRRTATDIDLILLAIESIELGGSEA
ILTFAQELELNQVIRNRVNLWRMRAANPLRKAHTRRPLTTMEAKCLVIITSYMAKRLTVV
IRQLLMIYQQLSEKQIPLSHNLRLANYLERFRTHFKSRMNPRRSVLLTPNNSDEKLDQLA
IDLLAKLLFCTGTAGMQRFWISLFDGEVE
>1170769.6.peg.2294
MMPFSTSNGTWNDPIVALFTSTSAVCVTGLSVVDPGTYFSFWGQLLILLLVQIGGLGYMT
TTTFLILLIGKRFDLRQKVAIQQALDRPGMSGSSQIIRSIIATTIIFEITGIFLLLPAFV
PDHGWSYGMWLAIFHSINSFNNAGFSLFKDNLIGYQTSLLVVFTVTGLIIFGGIGYQVIL
DMYLWLRDRLKRKTTFMAFSLDFKVAVSTTLLLLVVGTVAFFLIEIRNPETFGKFRFSDQ
LLLAWFQSVTPRTAGFNTIDIGKMSDAGLFITIALMFIGASPGGTGGGIKTTTLRVLTSC
TKAILQGKEEVWLYERKIAINLILKAIGVVFGSLATVLSATVLISLTDPKLEFIQILFEV
VSAFGTVGLSTGITGSISTAAKIVIIVTMYIGRVGVLLSMSAILGDPRPSRVHYPEGNLL
VG
>1170769.6.peg.2297
MVFTYRSLNSGISVMTEFDVLITGGSGFIGSAIARYLVSVGKSVVCMDAVDQGRLDDIDQ
SQVNRIHKISGNVLDAEFVDQWISRCGRVIHLAAVVGVDEYVTRPHDVLDVNILGTRNVL
MACLQHNRPVLIASSSETYGLNNGILEEDSDRIYGTSRNHRWSYAISKTAGEHYAYALGR
LGLTVTSVRYFNVYGPQLDAPGQGRVISKFLGRIRDSLPLMLVDGGHAVRTLCYIDDAAE
ATARLALELSPDCGYNHSAVNIGRPEPTTMRELADIMIRLSGHKAGTQEISGKEFFGEGF
EEIPVRVPDVSKLERVINFKARIDLEEGLRRTLDYWGLLNPETNQDTSLSSSPAAVVPMV
RPHFAPNGVLLQTLHRSLATGQVTNGGLHLRSFEEELAEYLGVPDVVVLSNGADALTLGL
QVLGRKGKAILPSYTFIATLNSVESAGLEPIFCDIDPETFTMSPTALAQILDQERDVAAV
IPVNVFGVTPDLPAIADLCRQGGVEIIYDNCHGFGTETHGRRVPQEARLQMFSFHATKVL
PAVEGGALVGADEELLDLVRKLRNHGIDSHNPSASGLGMNAKMDELRAATGRHVLRQFPE
QLEQRRYYAQQLRTFFQESCHGALIPQRIPDGVVSNFQNLGVLIPSASQFGLKAAITALH
DRGVECRSYFNPALHTLTRARSYARYPLPVTDQVWNSLLCFPIHSQMDPQDIDQVQLAAR
SVVDALVMQQV
>1170769.6.peg.2299
MTIEPLPITPWSSMQWYNPPAVWQNRGDRLWVKTGLKTDFWRTTHYGFIRDSGHCYYREV
LGNFRARVCFSGQYQDLYDQAGLMVRVSKTHWLKCGIEYVDGVQYASTVVTRGSSDWAVA
PLAGQPDRLWFEVRRRNEAIAVLYSVDGEKFVLLRLTDFPEDPMVWVGPMCASPEREGFE
VTFESFTVEPSTAPVLNHV
>1170769.6.peg.2300
MTTETPDQSPDLDRSPLAFLWLILGLIALSSTAIFIKLSIQEISAEATVFNRLWIATLAF
TGLNWIRPVNTQSSETEPDQSGEVDKSRKGPLGLYWSWEIIGLLLTLGFVHLIGRYLWTW
SLTSTTAANGAMLANMPPLFTALGGWLFLGQRFDRRFLLGLAIAVVGAITLALGDWIQPK
EVLFGTGALLGDGAALLSSVFYAASFLLVEKLRRNLSTSTILVWRCALGLMLATPLVWLI
DDTIFPISTLGWVAVFGLGLISELTGHGLIVYSLKYFSSAFVTIVLLLEPAPVAVVAWLW
FGEFLDPLNIAGFCLITVGIYLAKTGSGSTQDSPNRNILKLEPSDISSDIPSES
>1170769.6.peg.2308
MIQFDTCHNFLLENLFLLVNMGAFEICSTDTPIFMGENLKNQGFELC
>1170769.6.peg.2316
MNIQAQAPTANDYFNSGLDKQNQGDNSGAIADYTEAISIDPLHTFSYYNRGLARYDLGDK
QGAIEDYNQAIDVDPEYVNAYINRGLARSDLGDKQGAIEDYNQAIKISPNNDLAYYNRGL

ANYELGNKQEAIDDYKESIRINPNSVVAYYNMGLARYDLGDKQGAIEDYDKAIDVDPGYV
NAYINRGLARYDLGDKQGAIKDYNQAIELNPSNFLAYYDRGLARYDLGNIIGAIEDYNQS
IKFKSDYPNSYINRGLARYDLGDKQGAIRDYNQAIELNPNDVLPHYNRGIARHDLGDKQG
AIEDYNQAIELNPNLAQAHYNRGVIRSEIGDTYGALRDLKNAAQIYREQGKTQDYQETLD
RISRLEKL
>1170769.6.peg.2322
MLDLTKLAGQMQGLSQHLCSEVAESNRKLELAKENLKKACQCQDEIVKRQEKWRDRITFA
NATPLEPLDTCIQISTPPKVHTIIATDGSQIAPNHHEIAYCYLLNIGRVVLYYGQNRHPL
LDSLPEIFYREEDLYIARQWGLKTEEWMSHCRTASEITVLADLACSVKNDHPSLAMVDGS
LIYWFLEQLPLEARDQILPPILEAWGKLRQAGIPIVGYLSAARNNEAKNLLRLLNCPYPV
PDCINYCPDQLDYVPCKKFEGLRDTTLWATQLPQGQRSALWRSNSRILQLYDDQVIYFCY
LHVGTEIARIEFPAWVVNDPQMIDQALGLVLAQIQKGYGYPVAIAEAHNQAVVRSGDRNQ
FFALLEREMIKAGVKNVSISYKEARKRGSIA
>1170769.6.peg.2324
MNKIESDQKQATAKKLLVITEFFPPDYAATEQLIEELVRQLNQEKLKVEVFTGQPGYVYS
VDKAPSREYLEGVHIISTERGLQHKRK
>1170769.6.peg.2326
MIKSKLAAKVTLCQVLSVGVDLGAALATSMGHTKSGRADKTLCDRSLREWIKKKVINPLF
SRVLYISLILSLML
>1170769.6.peg.2332
MTQNLLKIQQRMSYILNLEKNCGCRENLLISTRLLPKSSINWY
>1170769.6.peg.2343
MGNLGYGIDPEVVKGIAQELAEVVATGVQVAIVVGGGNIFRGVKAASAGMDRATADYIGM
IATVMNAMTLQDSLERIGVQTRVQTAIAMQEVAEPYIRRRAIRHLEKGRVVIFGAGSGNP
FFTTDTTAALRAAEIEANVIFKATKVDGIYNADPKVHSNAKRYKTLTYGHVLAEDLRVMD
STAIALCKENNIPILVFDLTVRGNIRRAVMGESIGTLVGGSCEIS
>1170769.6.peg.2348
MSGLLAGCFGYPRILNYPLDSGGRGLNSLASEFNPHIGGRYIVFVTDRRGSQDVYLFDTI
TRSLVDLPELNTFDSMADHPAVSQDGRYIVFAASRQGKSGIFLYDRETRQSRNLTTNLAA
QFRNPTISADGNRIAFEFTNSGQWDILVYDQFGQKLNVP
>1170769.6.peg.2362
MSKKSAEIPYVTTVEIIEDFKPFLPHLQTSVREYSQYINDKDLVWSYTGIANYYNGQGLY
AIAETYYQDCLTVNRIRLGDDHPHVASSLNNLAVLYESQGRYTEAEPLYLQALDLRKRLL
GDDHPDVANSLNNLAVLYAAQGRYKEAEPLYLQALDLRKRLLGDDHPHLATSLNNLALLY
QSQGRYTEAEPLYLQALDLTKRLLGDDHPHVATSLNNLALLYQSQGRYTEAEPLYLQALD
LTKRLLGDDHPHVASSLNNLGGFYQYQGRYTEAEPLYLQALDLRKRLLGDDHPDVATSLN
SLAVLYESQGRYTEAEHLFIQALDLRKRLLGDDHPDVATFLNNLGELYAAQGRYTEAEPL
YLQVLDLTKRFLGDDHPHVAISLNKIGGLYESQGRYTEAEPLYLQALDLRKRLLGDDHPH
VAISLNKIGGLYESQGRYTEAEPLYLQALDLRKRLLGDDHPDVAISLNSLGGLYESQGRY
TEAEPLYLQALDLTKRLLGDDHLHVATFLNNLGGLYESQRRYTEAEPLYLQALDLTKRLL
GDDHPHVATFLNNLGELYRSQGRYTEAEPLYLQALDLRKRLLGDNHPHVASSLNNLALLY
AAQGRYKEAEPLFIQALDLTKRLLGDNHPNTQTVYHNYLSMLSQYPEA
>1170769.6.peg.2363
MFNLKAPFLRGLGGILKMLNRTGHFWEKRYHSTGFLTTETMMAFTVLC
>1170769.6.peg.2365
MELFLGTWQGAKEGRTGYWLRRWWEETGNLLPWALELIEQERQQVEQERQRAEQERQRAEQ
ERQEKERLIAYLRSQGIDPNNLPNHAE
>1170769.6.peg.2382
MYEPQSQPGRFFAWAIPQSLGGGWNPQNRCQAIASRLELYRPDGLQELQIARQNNENIIC
VTTEVVSTCRIVFTVPRTRDPYSVRSSIFSSLTAADQGQQTVGVNTYISSPRRSGNNPHS
RRGINLKPFLSMEDGGTGTNLNNGLLIRSRTPGKTILNPRLFR
>1170769.6.peg.2393

MYGNISCYFFFRQIRQFIPTWSLICIIHEAKSWQICQ
>1170769.6.peg.2394
MSEFVLEQQNEAVENHPQELIRRLVWKICIATLILMAIGSATRVMNAGLACPDWPLCYGE
LVPTKQMNLQVFLEWFHRLDAALIGLSAIALTGLCWWHRRVLPLWLPGAATFALLLIVFQ
GILGGLTVTELLRFDIVTAHLATALLFFTTLLVIGTFLIPYKGTGAVGKLPWVSLTAAVL
VYLQSLLGALVGSRWALHQCLAGEELCGVMYSHIFGLLPPTVATLSMVFISWRTPALHPV
LRKLANLAGGLLISQLLLGLATFRLHLQVEPLTITHQAVGATLLGTLVVFTVLALRDRSI
SNPSYLNN
>1170769.6.peg.2395
MIETNVSRHHDSFLQVIQSYYQLTKPRIIPLLLITTSGSMWIAAKGQVDPWLLLVTLLGG
TLAAASAQTINCIYDRDIDYEMERTRHRPMPSGKVQFRDALIFAIALAIASFTLLTVFAN
LLAALLAFSGIVFYVLVYTHWLKRHSTQNIVIGGAAGAIPALVGWAAVTDTLSWAAWLLF
AIVFLWTPPHFWALAMMIRDDYAKVGIPMLPVVAGNQATVRQIWFYTVITVTSTVLLFYP
LHASGIVYVVIAMTLGGIFLHKSWRLLQNPEDKTIAKELFLYSISYMMLLCLGMVIDSLP
FTHYLVNTILHSFHLVG
>1170769.6.peg.2397
MEDPNILLTIGLPNSANFANTISRTNGDENFDQLVARSGAAVVVNGTFAYTNPQKTVMGN
LVAGGRSLKYSPWENFGTTLGLGVGNKPEMITARVEGRPEWNKHWFSITSGPRLLRNGEV
SVNPRLEGFKDPAVLGTSLRTAIGFSEDGKKLFLANFDEKLYLEEEAEAMKAIGCYEAMN
LDGGPSRALASDNVILVPPARKLTNVILVYDGKNPPPEELKLSWERFQTRWRPDR
>1170769.6.peg.2398
MHYARQGVITEEIHHVAKRENLPPELIREEVARGRMIIPANINHTNLEPMAIGIASRCKV
NANIGASPTSSNVEEELDKLRLSIKYGADTVMDLSTGGGNLDQIRTAIINASSVPIGTVP
VYQALESVHGKMENFTADDFLHVIEKHAQQGVDYQTIHAGLLIEHLPLVKTRLTGIVSRG
GGILAKWMLLHRKQNPLYTRFQDIIEIFKKYDVSFSLGDSLRPGCTHDASDAAQLAELKT
LGQLTRKAWEHDVQVMVEGPGHVPMDQIEFNVRKQMEECSEAPFYVLGPLVTDIAPGYDH
ITSAIGAAMAGWYGTAMLCYVTPKEHLGLPNAEDVRNGLIAYKIAAHAADIGRHRPGARD
RDDELSQARYNFDWNRQFELALDPDRAREYHDETLPEEVYKQAEFCSMCGPKFCPMQTKV
DADAIAELEQFLAKEPVGQV
>1170769.6.peg.2418
MYFFWDLRFHSYKQSPPLRYDRGQRYQFIGINTEGKTLWLELQSMVSGALAETLPVAG
>1170769.6.peg.2438
MKLELHLKTFNFDGYWGVHTVQTTPSSQIRKFWEIDHKVE
>1170769.6.peg.2450
MGFYVWISKYKEILVEYLYRYYKDDTVGWEMIIFGSKCM
>1170769.6.peg.2451
MTLEQAEKILLSLKGQGICEILILTGEVHPLSSRRQEWLELIYDLCLLALSMGFLPHTNA
GILSFSEMQKLKSVNASMGLMLEQLTPKLLHTVHRHAPSKIPELRLQHLQWAGELQIPFT
TGLLLGIGENEDDRRQTLMAIADLHLKYQNIQEVILQPHSPGSQQTFDEVAFDPYQLPGV
IAQAREILPSDITIQIPPNLVPEENWLLTCLDAGARDLGGIGPKDEVNPDYPHREVNRLR
EVLLSGGWTLLPRLPVYQRTHSVLGTMVFS
>1170769.6.peg.2462
MQLQPEFTSYFQGLISTFESQNPGIKVKWVDVPWAAMESKILTAVSAKTPPDVVNLNPDF
AAQLAGRNAWLDLDTKVSPEVRSSYLPNIWQASTLNGKSFGIPWYLTTRLTIYNTDLLKQ
ASMSKPPATYQELAQAARQIHDKTGKYAFFTTFVPQDSGEVLESLVQMGVNLVDKQEKAA
FNTPEGRAGFQYWVDLYQQGLLPKECLTQGHRHAIDLYQSGETAFLASGAEFLKTIANNA
PQIAKSSTIAPQITGNTGKKNVAVMNVVIPRSSKNPDAALKFALFVTNDDNQLAFAKAAN
VLPSTVRALSNSYFREVPANASTVEKARIISAQEMQKAQILTPKMKDFKLLQRAIYENLQ
AAMLGQKTVDQAVSDAAKQWDSR
>1170769.6.peg.2467
MCIRHGAFEADPKTEELVHLQSGRHWRKWGNDWYRQHTHPDGIRFEIHEALDKLYTQGYR
ATRVIIARRYQELMSGYLERSSSWRSEQTETGNARLYGLPVEFGPDPLDEPCWDVINFDL

DKEPGAPVRYPYFRLFE
>1170769.6.peg.2470
MEGGIEGWGEASPFSVGSYRETTDVIFQSLQEMSAVLESYSPWQRDDISNILTQHQIPSA
AKTAIDMALHDWMGKNVGLPLWQLWGLNINTIVPTSVTIGINSPQGAATRAREWLDYMDV
QLLKVKLGAKEGIDADKKMILAVKDAAPKVDLFVDANGGWSLADAIAMSHWLADLGVKYL
EQPLPKGEEEKLASLKKQSPLPIFVDESCFTSTDIPPLADYVDGINIKLMKSGGLREAWR
MLNTAKAHNLQVMFGCYSDSSLANTAASHIAPLADYLDLDSHLNLINDPFVGASVTEGRI
IPNSLPGLGVQYSALTT
>1170769.6.peg.2474
MIICPNCNHPNPDAAVQCEACYTPLPNTTTCPSCGANVQADAAFCGQCGYNLVSTPIEVP
PLVTPDPLEISTQSPCSVPPTLVSTAPDPGPPVKVLKTQLQQVVGRLFHLQGNQEVELPQ
NLSVIHIGKPNDRIPPDIDVAGFSNSEIVSRIHADIRVEGDAYYIEDVGSSNGTYINNLP
LLPGNRHRLRPGDRISLGKGDLVTFLFQLS
>1170769.6.peg.2498
MVDFSLQDLTFPLVSEIEANKNRWLFSKDNLPIGFIDL
>1170769.6.peg.2507
MYAYTNKYTILKNISFKLKTGDRVALIGATGSGKSTLLENLIGLKYPQSGTITINGIPVE
PNTVAKIRKQIGFVFQDANDQLFMPTVLEDITFGPLNYGVAPAVAKENARQLLADFGLEK
YAHRSHHELSGGQRRLAAIASVLALEPEILILDEPTTGLDPAWRRHLAQVLLKLPVQVLL
IASHDLNWLGKVTQRALVLTDGKIQIDHPIQPLLADGKTLDNLGLPLGW
>1170769.6.peg.2523
MNITQSTFINKISIGWTSLLIFTLISVPTSAQTIDRIRDILEKTTVQINSNANPGGSGVI
IKKEGNTYTVLTANHVVCENLGVETIRCRTDFTYSIRTYDGREYPIKKRQILQTKLQDPD
LAIVTFESQQNYKIASLTPPSPSISTPQTSGIPKPTPSFNSATGP
>1170769.6.peg.2525
MQRVYEVLATGDREALAMRDRPFYHSPKNPWGEECFFCGSV
>1170769.6.peg.2526
MFFLWECLIALDPPQPPLKRGAFRLNMMYKDIYIVNSVNV
>1170769.6.peg.2531
MVTAQEREYFRELVRQDQKLQSKRTTTNHAFRRFKLSLKQQK
>1170769.6.peg.2533
MVSGKDGLFSQQQKMTKTAMIEKTTFKNSPDEKHQYFLT
>1170769.6.peg.2537
MRSPFLPFSKKSLGRRVFFLWECLIALDPPQPPFLSYPL
>1170769.6.peg.2539
MRKYKEIDDYNQDIKINPNDPEYYYNRGNTRRELGDKQGAIDDYTQVIKLNPNSAYAYIL
RGNVRSALGDKQGATDDYIQTIKLDHNFAYAYIPQGNARTASGDNQDAIDDYIQVITLVN
PDPKNRIIFIEQVLNCKYHGTEFLLEVLKKDDSWEVKDAAYSLLSSKDSELAKSSGYIHT
TNIPQIFLKLDSLLKAQDFRAADEETQKVMLAVANREREGWLRIEDAEKFPCKKLRSIDQ
LWLKYSGGKFGISVQQQIYQSLGGTKQYNQDVWSSMGDCVGWRQRGKWLSYNDLSFSQTA
PSGHLPRFVGLVGVGVISLLSRYAECNT
>1170769.6.peg.2542
MATNADQVWELLAQLVESQAQLTESQKETDLQIKELGKQIGGLGNKFGSFTEGLALPSMQ
TILREQFGMEIISPSVRVKNRQMVL
>1170769.6.peg.2545
MGARLRVFLTSEKDKTLFNLRSADVPQKVKDRAEVIRLNAHGWYVEKIAAHFNWTSQTVR
EVLHKWDGY
>1170769.6.peg.2547
MNAVRSHSLGTLIEWKPIVLGSKPKALFCSHSLGTLIEWKLLVLGYTSH
>1170769.6.peg.2548
METKMPKYAGQLLALSGSHSLGTLIEWKLGVQVIAVVTDTDLFPLAGDIN
>1170769.6.peg.2553

MKVKSKASKTQQGKGCTMSNCTSGGMELELPLFAIAELVPQNNEMTLIGKLPTCNPSIQE
QRSSRMSGQDLTLKGKVCSPYWTDFCAEINSSLLLPVGIDSQDSDLISYNTWSSRMVDKS
WFSIKLYTAHNQNLPRIYSQFFTSFPTEYRDSGNTLKKSKKIYLKMTSAKKQKIKYWFGV
SRFVYNETIGYLQNMVEP
>1170769.6.peg.2587
MLILVEVGKICSLPLYCKKHTWQKWQSFFDLTIAFFKILVYS
>1170769.6.peg.2589
MLLLVGCQSRDKIKDSIVQARVVRVVSGQTVEVVKIGEPTSLVSSVRLIGLEAPDLRQYP
WGEDARKLLEKLIEDANSDNTSNNNTNSAQIKLEFDLQTQDKFGRNLAYVWKDQVLVNEQ
IIKQGYALFAGRSPNHKYDLRLENAQHWARLMGEGIWNPENPLRLAPGQFRRING
>1170769.6.peg.2598
MFPPNHTKKTLQGKYYNNYHYHAIGNGKSRKREWRRSNILMGAMDTTTIKWEINQGLLT
>1170769.6.peg.2633
MASSLLISCEQVCKTWGFQDIYLHVLEKNDQARKLYLKLGYEIYRVESSWQDFFFPSRQF
FLHKRLR
>1170769.6.peg.2643
MYQPMHEDKFLTHDSNPGYTGKTVNLDLRVLNFLNTTLVLGIRLRKKYASSLAIVGTSWQ
VDN
>1170769.6.peg.2644
MFSEKELSNELKLEQKSLDSVGKLPRVTALVSLFTALVALSFAAIFIRLSELEIGSNATI
FNRLWIATLFFGLWNSIKQIQAKDPKNLSSSAFKLNTRHFSFFNYLEANPKPDLKRPNNI
ATSLVAEGLHEQRMNYEDLILSLLSDRLDYLLTYHQQIIQHIIEMID
>1170769.6.peg.2655
MFLAKCNYAQKYREEPRKTIHEVGLVFSSSKRNLIQADWE
>1170769.6.peg.2656
MGEVGFGVTTDGGVSSINGDVRELVDPAKKADFGKFGHSGHEDELQVGILLLEDGV
>1170769.6.peg.2661
MDHRQILTMAKHFNTAGPCQSDIHYMLPPTARLPDLRALIHGRNYLEGDVPAVVMEFLSD
KDGGEYSFKRTYPPGKWFFYEQILQVPVYIIFDPDGGLLEYYELKNERYELKQPDENGRH
WIESMELFLGTWQGAKEGRTGYWLRWWEEAGNLLPWALELIEQERQQVEQERQEKEMLIA
YLRSQGIDPNNLPNHTE
>1170769.6.peg.2664
MELFLGTWQGAKEGRTGYWLRWWEETGNLLPWALELIEQERQRAEQEHQRAEQERQLAEQ
ERQEKEREHQRAEQERQLAEREHQLAEQERQEKERLIAYLRSQGIDPNNLPNHTE
>1170769.6.peg.2665
MTGDELVIYYPDGSRFLSPVELSNYAEQERFLKEQANQRAEQERLLKEQERFLKEQANER
AEQERFLKEQANQRAEQERLLKEQERFLKEQEQLKYQTLLAQLKANGIDVTGLE
>1170769.6.peg.2666
MLLDLNKMLPASAYLGRGFLKTLLLVESALNRLSQFFKSINFK
>1170769.6.peg.2668
MRTLTWDEEGTAITLGYWGPGDITGHVLSKVTPYQIQCLTSVEATIIPPDLWHEDLKALL
SHIQQTEQILHILHCKPTSLRLWHFLLWLGNKFGRDLERGKLIDLNLTHQDISEVLNTTR
VTITRLLQKFETQGKISRHKRSIILRLTQDLQQD
>1170769.6.peg.2673
MWKLAKFGCTNSQKLGTPVLLLIQGFLDYISITIFPIWK
>1170769.6.peg.2680
MFYHRPVNLHVHINLQLTKVEGQPDLTHNQDSSINNVPKTVKLGTKLEWFGQIKQSFYPS
VHRCTGENENTKINSLSYC
>1170769.6.peg.2684
MRDRAGVKQLSTALTLARFRFEDINAKPVAKPKRKMEGQGKFMSSPKIADYILTIIYIY
>1170769.6.peg.2685
MPATILQMNPLINNGQVIPGTQLQIPPFDGMVVQVPNGQGWQQIAKKYGVRPDTLFELNG

CQQNPRVVFVPSSSKIKPIYGSAPSITTIMGSPVSNSTGVSFPYGWQIHPITNQVFFHSG
IDLLAEVGTPVRATASGVVVFAKEQGSYGNLVIINHQGGMQTRYAQLESIKVKLGQQVKV
NQVLGTVGATGEPSSREPHLHFEVRAREDLGWTAKDPVEYLK
>1170769.6.peg.2691
MLIIVGWGYAAVAADPPKLEDLAKQSADLKVGIDTMWVMFAGMLVFFMNAGFGMLETGLC
RQKNAVNVLAKNLIVFALSTVAFWAIGFGLMFSDGNPFIGLGGLFLQGADNSPAMGDAYK
GIFSSLNWTGVPLGAKFFFQLVFAGTAATIVSGAVAERVKFLSFLVFSLLLVGLAYPITG
HWIWGGGWLYKLGFWDFAGCTVVHSVGGWSALVGAFLLGPRIGRYNPDGSANAMPGHNMS
IATLGCLILWLGWFGFNPGSTMSVSDGTAIAHIALTTNTAGAFGGIAATIAAWAFLGKPD
LSMIINGILAGLVGVTASCAYISVPSSAIVGAIAGVLVVKAVPFFDKLKIDDPVGAVSVH
LCCGVWGTLAVGLFSQGNVYYQGGPTGLFFGGGIGQLWTQFVGVLTVGLFTVLISGIFFL
ALKYTMGIRVEESEELEGLDVGEHGMEAYPGFVKEASSPDLLGFGNR
>1170769.6.peg.2708
MALASIREWIITQLNRWFNFAERFLYTSNTEFEKTRTARESQNAFYASIMSILPFLVFGA
LSYWSLEISLGSSWGISTGILTTVGASIYELGRINGKNSD
>1170769.6.peg.2716
MASILEKIAQKYGLTTWNEASYNAYLSCPYFWIFLNQ
>1170769.6.peg.2717
MFADYGEVKRVVLPTDRETGKMRGFAFVEMIEDAQEDAAISELDGAEWMGRQLRVNKAKP
KEENRPRKGHSKG
>1170769.6.peg.2720
MLHLDPYLYLYPKINILGKGKHILFWTGLCKMSIFLGILFSALGKTREN
>1170769.6.peg.2722
MHGFMGNIYEFDNVIKLLNNNFSYLTVDLPGHGKTEVLGGNDYYQMENTAQAIINLLDEL
KIEKCFLVGYSMGGRIALYLTINFPERFIKVVLESSSPGLSTDSQRIMRIKSDAGIIQKL
TRISTRNEFGVFLKNWYSQPIFGQIKNHPAYPKMIETRLENSPLKISKSLQFMGTGYQPS
LWHKLEYSQIPLLLLVGEYDQKFIDINTVIYNLIPGSKLVTINRAAHNTHLENPLMFVEQ
IMEFFKP
>1170769.6.peg.2728
MSTKIIALAISTLAMSGCGLGNNSLGEGQKPPAIAQFTDPVVPAKINSVVNPVIESNKNQ
VIPSASQNRSVRSLVKARRDPFAQIIRVPQTIPTLPSVVKSNNTSRLNSLKKPLAIRQAD
LEIRGKNPIKPSPPKPPDTKLAQSILVSGVILVNQQAQAIIKLPDDSNSRYVHAGETLTN
GILVKRIEVNQCDNPVVVLEQFGVEVRRVISGQEDGIC
>1170769.6.peg.2740
MAENVDRGWDEELNTIIDDNYFVFNAKLAQKIKFSVA
>1170769.6.peg.2762
MIKEVQWLENQLGIQQVTALEHDGKMQQLMSVETQPVSNPLPGLGTEVKAEHVNSQRTVI
KSMGEIPYQLFEKLGLSMPGWLLWVLTFAISITLSGLLMSAVALWTPLWSNLEQAEDDGY
TPTNRENLKVSDGLWNKLSLYQLSKPMNILVMGVETIKGTLDGSPESFAGSSDTMLLVRL
NPSDKSIRVLSIPKGTMVSLPEDGLSKISEANAKGGPVLAARVISRTFSDAPIDRYIRIS
TSGLRELVDQLGGVDIFVPQSMTSQDQSGRTPINLVTGWQTLNGEQAELFARFRESSVGD
MARVQRQQALIGGLVQRLNNPVVLPRLPQLTRMMRKYFDTNLRMEEMMALANFAVNVERD
KFEMTMLPGTFSKFSKDPESYWLNLTGQQSLLKNYVGVDIYQVRSDSRSVFQLKIAIQNA
SSQPQVTAKVINHLKSKGFANIYTVPDWAENQRQTQIFVRRGTRHPGVELRKILGVGQIE
VSAQGDLDADLTIRIGEDWK
>1170769.6.peg.2764
MTIGNGKKIDLEVAKTPEQLMMGLMYRPALPDNRGMLFVFSSPQPVGFWMKNVPVSLDMV
FINRGVVKYIKTAPPCENEPCPTYGPRVLIDQVVELRAERARELGLKIGDRVKIEVFKSL
R
>1170769.6.peg.2767
MELVIFLHLPEQTLFVASPGQHHYRWWEFLSKEALSFSSDISQCFCDVVVNVPKLT
>1170769.6.peg.2768

MHWMSPRANAGLNIFEASKEPDAPPAPTMVCSSSMNKITSGDFSNSFITAFIRSSNWPRY
LVPATSEARSSVTTRLPNNTRDTFFCTILRAKPSAIAVLPTPGSPMRTGLFFLRRLSTWA
TRSISFSRPTIGSNLPNSAIFVRSRPKLSNTGVRDFSALLGSFPCCEPPLNILRSSSSSV
SGPAWGLVPDD
>1170769.6.peg.2769
MQDSPSSKNLSILSGSPCTAQYATVVYAWFFAGIIHGAIA
>1170769.6.peg.2771
MELPIGTTIGQIFAITFLPIAIGMSIRQFVPKLSTKLEKVTSISATILLAVIILLLIIKE
WSRLPNFIVQVGIGVLLLNTLSMGAGFYLSKLFNLNYKQQICISIEVGMQNGTLAIAITA
GLLNNPDMAIPGAIYSLLMYLTGCMVIIYGRNLSASRV
>1170769.6.peg.2772
MGIVNTVIKLLKAVSVTLKATSPIAKNVTKFEETPPGHKAKIIKPVAIAGCMGKSFAIAN
PMRGSMSNWLINPIATAFGYLPTR
>1170769.6.peg.2773
MLQGQFLKTLINGIQYLYIPDITYRLRSNDAWYGFIPCARRLSASG
>1170769.6.peg.2776
MFLKTSSQFVGLFILSYLDGLVFECDRIFLSGEPSHLCS
>1170769.6.peg.2784
MGFFIGFQPYIWKIGKGINYGMYYLIAIVLIAIVIRLN
>1170769.6.peg.2785
MFWFFPWSLVLTVIYQYCCQNSKTVLINTFAKKEYEKVGTM
>1170769.6.peg.2787
MKNDQGHGSVFLISQDRLVITNISNAYVNKLNFFNYCGFFT
>1170769.6.peg.2788
MGVSSVNTGTESSNKQDVMVKSGFISGSFLLSGFIERVIRT
>1170769.6.peg.2789
MKPDFTITSCLLLLSVPVLTLLTPTPSFPKSTTPRSCQLQSENKEGYSPEQLKTIAERIT
VRIRGNNTAASGTLIAKQGNSYFLVLTNQHVTRRIAPGNIKIQTVDGKIHQGRVLDKFNL
LNQQEFAKYDLAILEFTTSENYCLPTRVFNNQVPDQIEVLASGYSVQTGRITFAEGQIKK
IVTEPALARGYEIGYDSRVEQGMSGGPIISSKGELLGINGKSAFPILNGGYIYADGTKPT
STQIQELRKLSWGIPITSILAQMKPEFLTAYELPVPNIAPKVPVQTLTLPQWIGKIEEKS
KQFTVRIDDSKGENGSGVIIAKQENTTASNWLETGNQLWRLGRYPEAITAFDNAIKQKPE
FIHLAYYGKGLALSRSGKVMEAVIALEEAVKAKSDSVLAWTILSLANTKLGRSDQALLAI
NQAIKLQPNNPNLYNEKLVVLSNLKRYQEAIDAIDQAIKLSPHADFYYNRGAVRSRLGDN
QGAIADLQKAADLLQKQGLSAE
>1170769.6.peg.2796
MDQLIWLRKNLFNNWYNGVLTIVCVIVLFSLGKGILFWIFNQAKWEVVTANIHLFLVGRF
PQALYWRIWLALAINSIIGLITWYGLMQKCHLPENQLLQKAYQLIVPLLAPIWFLTFIIT
IWLLGGGLGLQSVPTNLWNGLLLTLLMAFVSIVLSFPLGVLLALGRNSELPVIRWFSILY
IELVRGLPLIGILFIAQVMLPLFLPDNWRLDRLVRGVAGLVLFSAAYMAENVRGGLQSIP
RGQFEAARALGLNTFLLLILVILPQALRAVIPTIVGQFIGLFKDTSLLSLVGLVELTGIA
RSILAQPQFLGRYMEVYIFIGVIYWVFCYSMSLVARQLEI
>1170769.6.peg.2804
MILVHLKQFATFTNDNLVLYKPLYLHLLDTMALNLNL
>1170769.6.peg.2824
MDLPEPLGPEMTVHPGSKGTEVVPPKDLKLVSSTRLICTII
>1170769.6.peg.2855
MAIRFNLSDNLANLPNLTSFSWNNNKDIWINNHIFGLYLLKLKNSHFCLELK
>1170769.6.peg.2862
MDVPDMGRRQFMNLLTFGTVTGVAAGVLYPVVNYFIPPASGGAGGGSVAKDELGNDVSVS
KFLSSHNVGDRTLVQGLKGDPTYIVVESKEAIADYGINAICTHLGCVVPWNVAENKFKCP
CHGSQYDATGKVVRGPAPKSLALAHANVNDDTIVLTPWTETDFRTNENPWWA

>1170769.6.peg.2869
MNQICCLNPTYECDNPQVPDNNSYCPTCSIPLVILKNRYQPVKRLGGGGFAKTYLALDTH
KLNEPCVIKQLAPSLGNQTTQALIKATELFLQEAQQLQKLAEHTQIPSLFAYFEENRQLY
LVEQFVDGKNLLEELQTEGVFNEAKIREFLQDLLLILQEVHKQGIIHRDIKPENIMRRHK
DGKLVLIDFGASKELQGGATSGTRIGTDGYAPWEQRVDGVASTAGDLYSLGVTCFYLLTS
KNPYELWLKDGYNWVANWRNYLNQPLSQKLQQILDKLLVASSDNRYGLAEKVLEELRQPY
SIIPSNSPKTIISHTEKPQRFSYILALISVFILGIGYLVITKSPQFQPRTEPNVIPETDR
GL
>1170769.6.peg.2872
MGTWNNEPDYQWDEVSIQLMSPASGNEGRRYVSVARVDPNLGVQSFHSINVPSEWEQSLR
C
>1170769.6.peg.2873
MNGNSKILIFLTPKNCSHSLGTLIEWKPEGCGVAVALTDGSTGSHSLGTLIEWKLFVESF
SFIIVMFPLAGDIN
>1170769.6.peg.2877
MCLSSYTIYGDWRLTRIIPLRRIAQVRLGSTRHARSPPFAWEDKTPNAIALQSVWEIVLF
YHLQIFP
>1170769.6.peg.2878
MVSIQLMSPASGNIEVQPATIFNGSFHSINVPSEWELTLV
>1170769.6.peg.2879
MNKTELLNQLLGFHSINVPSEWEQERGGLREDKKEEFPFN
>1170769.6.peg.2881
MIRKRAGYSLTSPFSREIYQGHWVRSPWEYFVNALDITMTSPFSREICENGK
>1170769.6.peg.2883
MFVDESGINLGMSRLFARSQDGQRAIGSVPGNKGKNISLIGALNMDGILAAMTVEGSTNT
EVFVTYVNQVLVPQLWKGAIVVMDNLKVHYAERVRLSIESVGAKVKFLPPYSPDLSPIEL
CWSKLKQFLRSREARTLEALNEAVTSAVNYITAEDALNWFNHCGLFT
>1170769.6.peg.2884
MQQVKKAPDAVAIIFENKQLTYGELNCKANQLAHYLQSIGVGPEVFVGLCVTRSIEIVIG
IMGILKAGGAYVPLDPAYPQERLAFMLEDASPKVVLTESQWLEALPVINATVLCLDAEWE
KIEQQSQDSPSCSVTSENLAYLIYTSGSTGKPKGVQMPHASIVNYLQGITNIIPVDNQDI
YLHIASFSFTASVRQLFLPLSQGAAIVIATREQTRDPLRLFELIETQEITICDGVPSVWR
YGLMALESLDKKYTRAIGESKLKYLIFGGELLPYQLIKKLRNLFQTPPQFFNILGQTESI
GNAFYPIPENCDTEQGYVPVGNPLQDMQQVYVLNSQLEPVKNGESGELHIAGGTLARGYL
NRAQANAEKFINNPFNPQQKLFKTGDIARHSQDGNLEILGRIDFQANIRGMRVELEEIEA
ILKLHPSVKEGAISLREDIPGDQRLVAYIVPNTQTLDLAEIRNFIEHKLPDYMIPNAFVL
IEKLPVLPNGKLDRNNLPAPNLSATIGNFVAPRNPQEELIANIWAEVLGLEKVGIYDNFL
ELGGHSLLASLVISRLREALSLELSISILFEAPTIASLSEKVTTFRDDFHPTNSLSVLQP
VSRTAESPLSLIQQRFWIVDQMEGANAAYNITRALRLVGSLNLMALQQAVQSIIQRHETL
RTSFGISEGKPVQFIAETLPFTLPLVDLQTLAEVAREAELQRLITTEYTEAFDLSQAPLL
RVKLIRLESNSHILLVTMHHIISDAWSVAIFFQELSSLYANSPLANLPVQYADYAYWQRQ
GLQNDVINTQISYWKQQLADAPPITELPTDYPRSTIETFHGSIHRFRLGNHLTNKLKILS
QKSGTSLFMTLQAAFVTFLYRYTGQEDIIIGSPITNRNRQALESLIGFFVNTLVLRTRLE
NNPTFKQLLSQVRQVSLDGYVHQELPFDILVEALQPKRQKNLSPLFQVMFVLQNSPREKF
NLPGLNVTQIELNRPTAGATFDLTLSMQEANLELIGAFEYNANLFDATTIARMVDTAVFM
>1170769.6.peg.2885
MRIYHNSNTLAKKGYQKAGMTYLTLQTNSSFISVPNK
>1170769.6.peg.2886
MAVVIIITPTSNSTGWVRYTVNDVITSAEWCTFKMQRQKYTQLARVDKDFAKISDNREFQ
ELLIYSD
>1170769.6.peg.2902
MSITQNYKLNIIQWYPGHIAKAEKNLKEQLKRVDVVLEVRDARIPLSTHHPQVKEWIVNK

SRVLVINRLDMILPQVKSIWSEWLKKQGEVPYFANAQQGQGITAIAKAAQIAGTELNERR
KQRGMLPRPVRAVVIGFPNVGKSALINRLLGKRVVESAARPGVTRQLRWVKISEHLELLD
APGIIPSRLEDQQAAVKLAICDDIGEASYDNQLIAAAFVDMVNQFQETSPHLLPPHPLLS
RYGVDSIIHTGEAYLEVLAASRYQGDVERTARTIISDFRKGLLGAIVLEVPKINNS
>1170769.6.peg.2903
MQLLESPPIKFIPSIYVEWQVDHLRLGIFRPQKDHESEFVVHGKNPLSLCIEVSNLENAI
SDLKSLGYLPTGEISITSHGQEIYAQDPDGNRLILYSSSNIDKQCP
>1170769.6.peg.2908
MFYTSIPLPHLPDLKFQRVALFAPAIGLLIGAILGLLDMLFDYLGISALTQSVLIVIIWI
GITGGLHLDGAMDTADGLAVTDPQRRLEVMTDSATGAFGVMAALAILLIKMAALTDISQN
RFFLLMVACGWGRWGQQLAIFQYPYLKSTGKGAFHKQAIRHQMDLFPSWVLLLGFTLLIW
AFNQGNFVLVIFTLIIGNIISFIVPAWFNHKLGGHTGDTYGAVVEWTEALFLCCMSSLT
>1170769.6.peg.2934
MKPTPKRSGKMTGSNPKVKTLRVPAHPKKYAHPSRKARLKPAANIILYALRLLIVGVGLG
AIVGTLLSVLDPANRITTNSINPPVTPSSSPQSPINSSGLVISREITPLKTTIENLSAAN
PNLIPGVFIVDIDSGAYVDVSGNKNFPAASTIKIPVLVAFLEDVDRGKIRLDEILTMEQE
MVAGGSGNLRTMPVGTKLKSIEVATKMMTISDNTATNILISKLGGKELLNARFRSWGLVN
TAIQSPLPDLEGTNTTSPKELASLIAKVNQGELISMRSRDLMLDIMRRTQRDDLLPAGLG
EGATAYHKTGDIGTMLADAGLIDVPTGKRYIASIMVKRPHNEPAAAKLINSISQATYSYL
SQSNFPPDGSTNNQPSSNQQLNNSSTPVPQLQPFTQPFLQPQGGNSNIRNATINNAPLGN
YQSPLNNPPYYPPQSHPN
>1170769.6.peg.2941
MAIIHCFSCRLYGLRNYSKEIKDNLKNAIDKPARDMSELRDINAAIMLSRLAKACKSTEG
>1170769.6.peg.2955
MITSGLQIYNANPVFGGRGGLHIPPIFTLGAWLAGGRHWHFAAMWLFSVNLFSYGIYILL
TRRWQHRFVGNNDIKALQKTDNIKRLTYSWHRIVYTAIIPILLLAICTGIGMYKPAQFPW
LVDIFGNWQGLRIVHFASVPLIIIFVVIHWQLGKRAGGDKLLESMFW
>1170769.6.peg.2967
MIVVFLTSFSSASESLGLTGKSWTWANYLESWERGRFLVVFANSTLVAIAVYK
>1170769.6.peg.2968
MFDLTIPDSDATTLFMTQKSLVFPSITMEIRVGGDRVLSLKFQSQYNIIE
>1170769.6.peg.2977
MLSFTPQPTILCFGWEILYPQPVPCSLLVYTQRKIPVIMGILVIFT
>1170769.6.peg.2984
MLRNIKRVRRRILGKIHGDCYSQSYISRDIWGIGSSEPNPIWASNS
>1170769.6.peg.2989
MALVEASWLRSVFSVSLVSKLHPSSLATASKRKWMSLSLS
>1170769.6.peg.2990
MTTAIQQRQSANLWDRFCEFITSTNNRLYIGWFGVLMIPTLLAATTCFIIAFIAAPPVDI
DGIREPVAGSLMYGNNIISGAVVPSSNAIGLHFYPIWEAASLDEWLYNGGPYQLVIFHFL
IGVACYLGREWELSFRLGMRPWICVAFSAPLAAATAVFLIYPIGQGSFSDGMPLGISGTF
NFMIVFQAEHNILMHPFHMLGVAGVFGGSLFSAMHGSLVTSSLVRETTETESQNYGYKFG
QEEETYNIVAAHGYFGRLIFQYASFNNSRSLHFFLAAWPVVGIWFTALGVSTMAFNLNGF
NFNQSIIDSQGRVIGTWADVINRANLGMEVMHERNAHNFPLDLAAGEVAPVALTAPAING
>1170769.6.peg.2992
MYHLEKGRYKQVKANKRKHYPIPELGVELGMLLERERPPIPWLRWWDNRGGCFMISAIMG
FTRDWGMME
>1170769.6.peg.2993
MGDDGITTWHPAFLALGTSLEECAIKYKGFCQKYRVQEKPVKKNRWG
>1170769.6.peg.2994
MVNERSHLVIPVLLFSREIYKHGKCAIALGVLCKCAGYSLTSPFSREIYQGH
>1170769.6.peg.2995

MVGHFPTVPETAFSGRSHSLGTLIEWKLAKSKLASINSV
>1170769.6.peg.2996
MSKKFEYKNIRFDFKGRGITQEINLLDIDGKRVKGWFANTEEVPTLPELLSAAGADGWDL
VSHSVNQDNQANGVTFHYLYFKRELV
>1170769.6.peg.2999
MLGIGCFWSVLEEAHITILAVHPEYQGQGLGQALLYSLIKDAVDMGLERATLEVRVSNTP
AISLYKKFGWKTAGIRPRYYQDNQEDGLILWISQLQHPHFLQTLDKWHVLVQERLGQFSW
LLIQEEN
>1170769.6.peg.3001
MGNMTLPSSRFNWAEDIPRHLRKAWDFYQERFGQVPRGLWPSEQSVSPEILPYVIKQGFK
WICSDEAVLGWTTRHFFHRDGAGNVQEPELLYRPYRLQTPEGEVSIVFRDHRLSDLIGFT
YSSMQPRQAVANLVGHLEAISRQQKERSTEQPWLVTIALDGENCWEFYPEDGKPFLETLY
QTLSQEPNIQLVTVSEFLDKYPPTATINGDRLHSGSWVDGSFTTWIGDPVKNRAWDYLVQ
ARQTLARHPEATEENNPAAWEALYAAEGSDWFWWFGEGHSSNQDAIFDQLFREHLYGIYR
ALNEPIPAYLNSPLEVHEVKADRRPESFIHPVIDGKGDEQDWDKAGRVEVGGARGTMHQS
SLIQRLWYGVDHLNFYLRVDFKNGLTPGKELPPELNLLWYYPDRPMVNSSIPLAEVPDMA
PVNYLFHHHLEINLISQAVQFREAVENYQWLPRASRAQVALNTCLEVAVPWVDLQVPPDY
PLRLILVLADDGCFHSYLPENALIPIEVP
>1170769.6.peg.3007
MTTASAYSLNVPGHPTGQISKEELRSLLKEVESQLYHSHAYRAVVDKAQKLFDISHEQLT
NFNKLIQAISREAIALTFERFVTKQSATSTDSSKNDSADSTNSNEIKELSNQTVEVCEQV
LENRSQPNDPEPTAKKQQKSDRVKGENEPDPRKKLKSWPWKNQKPSKSRLAAKKAEEERL
ESLRQIGEQLKKARESQGLTLHKLTMYTYISPNQMAAVENGDMEKLPEDVLLRGFIRIMG
NALGLNGADLANSLPVRNECKSILPSWYKNQRPSRSLSIELSPTHLYLGYTALVAGAVSG
LSMMYEQANNQGIKNFQSTTPCLSSLCGSDEKATNVNDQSTMQHGRVGNSFGPQISPPEA
L
>1170769.6.peg.3008
MLLIFDENSNFIYFAYSEIYCYIRIFIHKFIIGTINTLGGI
>1170769.6.peg.3040
MEGGITPNQIMSGIVLLAIQNYELEGTMFSANCLHFLMKAIPVDTTATGVTEFILSLANE
SINIGMLLDAFAFACQKQGSRNIASLVSLTYQRLEADRVISQLINDQL
>1170769.6.peg.3050
MNQICCLNPTYECDNPPIPDNNSYCPTCSRRLVRLKTRYQPVKRLGGGGFAKTYLALDTH
KLNELCVIKQLAPSLGNQTTQALIKATELFLQEAQQLQKLAEHTQIPSLFAYFEEDRQLY
LVEQFVDGKNLLEELQTEGVFNEAKIREFLQDLY
>1170769.6.peg.3054
MIPGEIDQKAEELLCVEGSSDFYRKIIFIMFLYFCHISI
>1170769.6.peg.3066
MGRSGVTPESGGWDFHPYPTESSTFIYVLLIVEVGEVITD
>1170769.6.peg.3069
MDVLQWYLTFRLLPLQKLLLILSKKGHSPHYIWKGGAIKAP
>1170769.6.peg.3077
MKKRVTLTFPKRAIQIPVTYRLARDFNVAANIIRAQVAPNQIGKLVVELSGDIDQLDAAI
DWMRSQHIAVSHNLGEIFIDDQLCVDCGLCTGVCPTEALSLNRETYKLTFTRSRCIVCEQ
CISSCPLQAISTNL
>1170769.6.peg.3081
MLSIPGGRQYCAHPFGYSENYDDVFAEPGNILIRYYVPTRMGIKMIFQGLFFQLNNPQNW
SLTFPKTISKE
>1170769.6.peg.3091
MVNTSSSSSVAGTYLVIDDVLPGFQSSNDLLVNITGYSGVLPSFGTIPVTSFFV
>1170769.6.peg.3092
MRSPEPFYHQCLGLFLILINPKLRKKAAIAVWKRVFKKSFCNLPPTLKTPCLGS

>1170769.6.peg.3095
MSHRPSLFKKISLVKKLFHLLVNCPRWQKSLILYFADSIVFFLATYLALIISSQTLLSFS
IVQKHTVYIIPFICAKLFLFTSLGLYRGIVKYTEFGFLYTASKSIFLGQGLLIFISFILP
IKITNLLPILPISVQVIDGLITLILVIKIRLIARWLLYSYKSPLKLTQPDQTNIIYQPKS
RQTVIIYGAGQAGFQLYQALHQDNLFQVVAFTDDNEQLWKHLINGIQVYSPQKLEYLINR
YQVTLVLLAIPSTTPQRKQQILTQLQNLGLEVKTVPTLAEIISGKVSIAQVRKIDICDIL
GREEVLPDKDLLALNITGKSVLVTGAGGSIGAELCRQIAQQQPRHLVLYELNEFALYSIE
IELRETYPDLSCVACLGSVSDYERIKQVINKYEVETIYHAAAYKHVPLVESNPVQGVINN
IYGTLVTVKTANECKVDTFVLISTDKAVRPTNIMGATKRVAELILQGLSKEKDTHTRLMM
VRFGNVLNSTGSVVPRFQKQIANRQPITITHPDIIRYFMSIPEAARLVIQAGALGKGGEV
FLLDMGEPIRIYDLAVQMVELSGLVIGKDIEIQITGLRPGEKLYEELLIEDGNAQKTIHP
KIYAAKEAMIPWNKLESYLDQLFLAANSQDIDSLLSILKIIVPEYLGVRVVIADRRPGDP
ACVTADAQKIKQVLNWQPKHEDLDEIIASTIDWEKSRPT
>1170769.6.peg.3097
MILDILHHQGRLIKAFVDDNPPEMLHSIHGTPIIHSQVLSQITVDSSLWIVGIGNNRNRK
TIVEKLKNQGHSFTSAIHPSAQIGLGVEIGVGTVVMANAVVNIDTVLGDHVIINTGATID
HDCNIGDYCHVAPGSNVSGHVKLGNSVLLGVGTHVSSCVEIGDNTTCGAGSVVIRSIDGN
CLAYGCPAKVVEVLPP
>1170769.6.peg.3098
MTQIFTENHTKNFLDWFVAILFLTLTSPLLLVITILVRIKLGTPVLFSQQRPGLKGRPFT
ILKFRTMIDKRDAHGNLLPDSARLTPFGRWLRSTSLDELPELLNVIRGEMSFVGPRPLLM
KYLNLYTPEQMRRHNMKPGITGWAQVNGRNALEWEEKFKLDLWYIDHWSLMLDLRILFIT
VVKVIKRQGISSSNHATMGEFTGANLKSTR
>1170769.6.peg.3099
MLIRIAVVASYAPSLVNFRGSLIVTLEKMGAKLLLIAPELTSKTIEPLELERAETIDLPM
NRNGVNPFEDLSTCYFLWKSLRKFSPDIVLTYTAKPVIYGTLASYFARVPKSYALVTGLG
STFTDNSDISKYTAFLTRYLYKIAMRCVTASIFQNPDDEETFREKKILPASVTSYVVNGS
GVDLKYFSPSPLPESASFLMMARLLVDKGVREYVAAARQIKLQHPQTRFLLAGPLDNNPT
SISADELQSWIDDSTVEYLGFLNDVRPAISRCSVYVLPSYREGTPRSVLEAMSMGRAIIT
TNTPGCRETVQHGVNGYLIPVRDVNALVSSMKELFDPALRCQLGLASRSIAEEKYDVHKV
NQQMLTAMGL
>1170769.6.peg.3102
MRNESSFTPLISIIIPALKADIELRRCIDSVRVACPDHRKCEVIVVLPVSEIDKASLLLP
EEYVITEGNPGIYSAMNNGVGASSGRYLYFLGKDDIVLPRFSKALDLIESNSPDSLFFDV
YWGVIGLVKGNPSRWRILRGNICHQGIIYSREVLLIHGPYLTKMHVRADHLLNIRLLWDR
VHSFQVEYINLPLVWYSGTGFSSVNTDNTFWHLYPLILQKYVGKWAACLLIAYRKLRILV
PLLPDR
>1170769.6.peg.3103
MNCSNCDSNNIRKNGQRRGKQNYQCKNCGCQFIESYSPRGYSQEVKEACLTMYVNGNGFR
AIETMTKVNHNTVIRWVKKLGYQLPDTNNNSQTPEVCQLDELETFIDKKNKIWVWTVVDN
KRSGIIEYIVGDRSAKTFAKLWKKIERNPIFGLRMAIKFTQNLFPMETKLLVKHQ
>1170769.6.peg.3106
MLTLYENLGASSRLRFFQYVPWLERCGIQVTISSLLSNEYIRHLQDGGKNKLEVLKGYFS
RLKIIPSLEKYDILWIEKECYRWLPAWFERILVLNRIPYVLDYDDAVFHNYDQHPNFLIR
YLLANKHPQLMRSSALVIAGNYYLADFARKAGAKNIETIPTAIDLNRYQSYPSPSVKSQL
PCIGWIGQRSTASYLHPFAPLFKRLLAEGKSQFLAIGINTQQLGIPMESVPWSEETEVEQ
ISRINIGIMPLDNSPFIHGKCGYKLIQYMACGLPVVASPVGVNTKIIEHGVNGFLAGTLM
EWETALERLLSDPDLSFRMGQAGRQKVEREYCVQVTAPRLIESLRNVMYSRHDSDK
>1170769.6.peg.3108
MKLGIKLVKKYCGSYLCKQAIAFNLPLRKTIKHNLNPSPSAIARH
>1170769.6.peg.3111
MIERLNSEGFTLWAIQKGFTDPRDGRTLQVDAIFFRLD

>1170769.6.peg.3112
MSPNNWKVRIANLKKHYTVREAIRKVFFYPPPYLPYMEHISNSLQSFFATTHRILNFRKE
KYYLDLPLAVYDLRFSPASYDLAYFLYEADCYFRGNGYEKFNLVIVPEIAESTPSGINKD
WKQVISEDSRRQRIFNMLLPMASMYESCSSVSLINDVELIVHICRSHFCVFPQNYDGVFI
RTHMSYLNVYDFERKKTYFSGFSSFSSDLDKVKSWISSQGINLPFITFTLRHYVHQPERN
SNIADYLKFANYLCEVGISSVFIPDTDDLSYISELSEYPVFFTGAFNLYQRQAIYELALT
NIFACSGCHSLCALNKRCSYIMSGIINENYNMENLYKPRGLKYGDQPFCDNRGVWEWGGE
TFDSLKNSFDLLLKLKSKQLYIQHFV
>1170769.6.peg.3115
MVNEFSSCPVLIVGSSGTIGRSLHRFFAQREVPVWLTTRNVDQANKQFDQRTLLLDLSQP
ASIWKLDDLSVCVAIICAAVTSQKACQEDYAATYAINVTATVELAKRLMDAGVFVVFLST
NLVFDGSIPHVPPDQPVNPKTAYGQQKAESEQMLLTMGCDSVAIVRLTKVVDQGFPLFHH
WVDSLGSGQSIHPFEDLYFAPVSLDFTTQLLFEISQRRINGIIQVSATKDISYADAAYYL
CQKLQLNINLIRPVSSRTAGISHSPRFTTLDTKRLESFGMFPPEPWQFIDGIFASIILQN
SNNPDTTDNN
>1170769.6.peg.3116
MQTKEPQYNLIFDVIEKHGITQLGLMINESWNQDPKRTLFTLARYKFVAKILADQNRVLE
IGCADAFGTRLVQQTVQHITAVDFDPVFVQDVQNRLDPNWPLDCFVHDLLECPVPGEFDA
VYSLDVLEHIPPEREAEFLKNALASLKETGVMILGMPSLESQIYASPQSKAGHINCKSGN
DFRVLMRQYFHQVFLFSMNDEVVHTGFYPMAHYLMALCCGKR
>1170769.6.peg.3117
MTRLVFLVPVWGRSYVQVMIEGLLPSLLTPRNFPLLCQHRRSTLKIISNSEGEMLARDIE
VIEKCREFLDVDFVVDDNTIDSNKYASMSRMYLHGLSTLNKENRLSKEDNFIVYLTPDMF
CSDGTIETLLECSKSSFKKVMVLGIRVIKDKFLSGIKNYSPGDSSNSLDFLIQLMLSTLH
PISEVLNVNAGTFNNQWPSHLYWIEKNILIAHGFHWHPLMQSHSEIWIKTLLEQYSTSTP
PTIDDHRFMRESDGKFSYFEHCCVNTDPKRIMLVELSDLPKQRQNNRKTLVSIPSILLWC
HSNVDEFQWYLFTHEVIFSLSSSILNEKVINESRVFTEKILAFNKKMPRFFIKLIIKFRH
LIGFVKLETKRLLRKITQFVT
>1170769.6.peg.3118
MENEYFESNCHICGGNLSHIASFSNFLQVTSDCRPWKTGGSLVLCQSCGTVQKPVTEIYL
KEAEIIYAQYEIYSQSGGVEQSAFDSITGAAKSRSTKLVEWLTTNWNLPSTGKLLDIGCG
NGAFLRAFGSTYPKWLMTGLELNDRNREVVEAISGVQSLHVGSIESLQDKFNVISLIHAL
EHIPDPSSFLHGLKEKLLPGGILLIEVPNLRTSPFDILIADHCTHFTSTILSKVVDRANF
SVIRLDQDYIPKELTVAIEVKNGSGFSLEPQSDDNQEYTQNTHTLLHQHINYLQSLLDLA
KSVPGNVGILGTSIAGTWLAESLHNKVKFFLDEDPSRIGRYHLKRPIISPDAVEDGYPVL
VPLPHAIAVKVAKRLSHLNCNFLTVR
>1170769.6.peg.3119
MSPESLLKLTSQYLRKERIIIVKGWFKDTVPNIPESKKFALLHIDGDLYESAIDVLDSLF
SRNMISKGACLFFDDWNCNAADPKFGERRAWQEMVEKYNVKFSDLGSYGIVSHRFIVHEY
AREY
>1170769.6.peg.3120
MLISKLNTLNKIYKSRGVTGLLDATLTRIFRRTYNFNRVAQEVTNHLSNPTSPEVYNRLS
EGVYYVFGMGVEGDIAEFGTMSGRTAVALAASLNYCNNTLSDSDLLHGFKEPRKLWLFDS
FEGLPQARTNVDQSSLHVASGIWGG
>1170769.6.peg.3122
MALVDFMSVLHKSTTRDYLARVNDPEYPKDKAAKLAKQWDYDYWDGDRRINYGGYRYIEG
RWEKVARAMVDHYDLPRRPRILDIGCGKGFLLFDFLKVLPDAEVYGLDVSNYAISHSKEE
IRDQLTVGSATQLPWTDNYFDLVISINTFHNLHNYDLDVALREMERVGKTHKYLCVESYR
TEEEKTNLLYWQVTCEAFCTPKEWEWWFQQTGYTGDYSFIYFE
>1170769.6.peg.3123
MNVLTTGVEDFLLGFGLINPQLERIRAGRNSRVWRVKCYDEVYILKEYFRHPSDPRDRLS
TEYNFLTFLGSQGITHVPQPLNRDPDRGIALYSCLPGVSINTIRQDYIQQAAKFIHEINQ

KRNVIVAQSLPQASEACFSLLEHIERVKFRMELLQAALVETTNQWQRKAAQLVYKRLCPT
YNKVESEIRSYYSFAQLSKKLNRDHWILSPSDFGFHNMLESRGELYFLDFEYAGWDDPVK
LICDFACQPQVPVSSLQSDMFREHLNLLLELDQEQVHLLLPLYRLKWCCILLNEFRSQDL
ERRQHAGDGHGDILEQQLQKAQNYFYQHLGDG
>1170769.6.peg.3124
MLLGLDFDNTLISYDTLFHLVAVERGLIPVDLPAQKNCVRNYLRQQNQEDEWTSMQGEVY
GNRITEAVSFPGVLPTLQKLTELGVPMCLVSHKTRTPYQGPSYDLHQAARNWLTQQGFFS
PVGLNWGTGQVFFELTKQEKVNRIVELGCSHYVDDLPEILEMLPKTVQPILFAPTGEVST
PTDWYQFQSWMDLPQLIA
>1170769.6.peg.3125
MRNAFAKAVTELADRNPNLVLLAGDIGNRMFDSFKEKYPTRFYNCGVAEAGMTGIAAGLA
ASGLQPITYTITPFNTLRCLEQIRDDICYPDLPVILVGTGSGLSYASLGATHHSMDDIAA
LRILPNMHVICPGDPVEVELAVGAALDLGRPTYMRIGKKGEPVVHHQPPVFRLGKGITLQ
DGTDIALVSVGNVLPLALECSQVLNDKGFSTGVVSLHTVKPLDDSLLADLFNRYRLIAVL
EEHGLAGGAGSAILEWGCTQKVDLRKLRCFAGPDRFLSACGNQDQARAAIGLDVATIVQK
LVQESMP
>1170769.6.peg.3126
MTTTPNFFTVSAQLRSQIVQMSHQAKAAHLASALSCVDILTVLYGQVLNLDPSNPRHADR
DRFILSKGHAAAALYATLAWKGIISPEQLPTYGKQHSLLEEHPSPKLPGVEAATGSLGHG
LPIANGMTLAARIQNRSYRVFVLMSDGECNEGSVWEAAMFASAQKLDNLTAIVDFNKWQA
TGRSQEVLQLDPLPDKWASFGWEVTELDGHDHGQLLAALTQRPTGRPHAIIAHTIKGKGI
SFMEDDNNWHYRVPTAQEVLDAKLELGVI
>1170769.6.peg.3127
MGKFKILRISQLAYPKALIVFESKHPEVQILPYEEQKRLFFSEKLVYSDSFSRAMRQLGH
EADEIVSDVDWIQKTWAKENSVNYSDSSWQEEILLSQISKIRPEILYFQHNPPLPYGVWK
NLKHTCPSIKKILVHRAFPGNFNTLGAADLLMVGTRRLVSQYADHDIKAKLLYHYFDEAV
PDLLDRPQIKYPLTFLGSSGFSYGISHATRYWLLRTVLEQTQAKMWLEEPLSISPNANPT
QQVKKLLSSIIKGFVNFLPRTSQKSLGNAAWLPLKARNLIQSEYEQIELRPTGLKVPEKR
LLDLFPQRCHSPVYGLDYYEIIANSLISFNCHTDAAVDQVGNMRMFQATGMGSCLLTDTG
DNMSDLFEEDKEVVTYSSKEEALEKIDYLLQNETKCREIASAGQNRTLRDHTALKRCEQI
DEWLQEIMMC
>1170769.6.peg.3128
MSFKPVPMVDLHAQYLSIKTEIDEAIASVIAESAFIRGKYVDNFESAFAQLLGVNHCVSC
ANGTDALYIAMKSLGVKPGDEVITTAHSWISTSETITQAGGKVVFCDTDSSTFTIDPSQI
TDKITARTVGIIPVHLYGQPADMDYIMNIAQKYGLWVIEDCAQSHLARYKGQLVGTIGQF
GTFSFYPGKNLGAMGDAGCLVTQDAKLADWCSLYARHGGKGNHQMEGVNSRMDGLQAAIL
LAKLPHLPNWTEKRQRVAELYNRKFNNFPFVMTPFVAPGRDHVYHLYVICVSNRDRVREY
LSMQGILTGINYPKLLPFYPAYGYLNHQPSDFPHAVSNQERILSLPMYPEMTENQVEYVV
SSIQKFFFQ
>1170769.6.peg.3129
MPVTICDDVVIGAGSVVTRNITSPGIYAGSPARLLRKIDNTIEKV
>1170769.6.peg.3130
MGLNSRTIANLYTSLNFNKWSDKYIVTKDASVDIYGFDQYNPFAKHRI
>1170769.6.peg.3131
MALSLEDADSMIKACDYYGINLFVVKQNRFNLPILKLREAVQSQRFGKMVLGTVRVRWCR
TQSYYDQDSWRGTWSMDGGVISNQASHHIDMLLWMMGDVDTVYALGATQLVNIEAEDTAV
VSLRFRNGALGVIEATTATRPQDQEGSISILGEEGMVEVGGFAMNVIKTWQFSHSTHEDQ
EVATKYSVNPPNVYGFGHQEYYAHVVRSILSEEPNMIDGLAGRKSLELIIAIYESMEKEQ
PIKLLLSPNHTRLGKSYP
>1170769.6.peg.3132
MCGISGWWSFNRPLGKEFNIVGLTSGLSHRGPDGKSIISLDNDMLQLGHTRLSILDLSDS
GKQPMSYAQGKFYITFNGEIYNFLELRQELVSLGHQFRSDSDTEVILASYVEWGEDCLFK

FNGMWAFALWDSCKQRLFLSRDRFGVKPLFYLFDGQNFIFASELKAFMALHKEVKPDLDP
EIISLFSNLESTSLTLLKGVKNLNASHSLILARYGQPHLKQWWRTSEHLIEVPSNYADQR
EQYRELFFDACKVRMRSDVPIGTALSGGMDSSSVICAMAEIQNSNGDLFHSRQASNWRKA
FVLDYTGSTHSERHYAQEVINYVQAEPNFRELGLEKIEPEDLNRSIFALEAIQEPGLGPW
LIYQQMRSQGIVVSLDGHGGDEQLAGYHFYCQAAFQDALWRWRGIGSFGDIQNVFNGLYD
VADLPEGMSSQLPPSQLKVLFDFFRQGLREKVFSKPTLYQLLRQIKYLLNNRAFSFSKIA
TARTLPVGNRQSASIATTFLKKPKIKGYDYLNQVLYDDFHGGTLPVILRNFDRLSMSHGV
EIRAPFLDYRLVTYAFSLPSSTKLGNGFTKRILRDAMHGFMPESIRTRKSKIGFASPMIK
WIEAPLKDFVLDQVHSQIFLDCPIWDGKQVRERVVQAYRNFQPENVYKDWKYIQAHTLMQ
AFYQAY
>1170769.6.peg.3139
MIIFTSGSTGKSKAAIHDFILMQEKFQIIRYAQRTLLFLLFDHIGGINTLLHVLANGGCA
VIPSDHSSETVASAIAQYGVQVLPTSPTFLTLLLLSGVYQRYDLSSLEIITYGTEVMPES
VLTSLNQVFPHIRFHQTYGLTELGIMRSKSQSSDSLWFKVGGEGYETRIVNGMLEIKAKS
AMLGYLNAPAPFTTYGWYMTGDVVEIDGEWLKILGRKSEIINVGGQKVYPGEVENVLQTM
PGVAEVSVKGEANPITGNIVTAKVRLKTDESLRDFRVRLRAFCQDKLESYKVPVRVTLVQ
ERLHSDRFKKSR
>1170769.6.peg.3141
MSRVVFFNVSAPGHIIPTLGLVKELIDRGEEVIYYEVPRFEEEILSFGAEFRPYPPLNPE
TAPGYVTHLYTPK
>1170769.6.peg.3143
MIIAHICRALLTLVLLFIVTTTGIQLWYIYLLNIFVSIIGAFNNPAYKAAITSLVPQKDL
PQASGMVQLSFSLQQIISPLIAGLLLELIHLQGIVIIDFLGLLIALTILILVKFGEKIDH
NQEDNWELNQSPSLWQEIIGGWTYLRERPGLINFVVFLTVYQFLIGFVNVLFYPLVLTIT
TPPELGKIIFLSGIGMLLGSIFMSNWKYSWENLITTVLVAMSLSGIWIAIGGSRPSLIQV
YIGTILFFITHTFVNGLVQLIIQTKVNESFQGRVFALTGTLSASATPLASIIAAPLSDFV
FEPLMAFDGPWSKLLVGQLIGSGPGRGTGFLFVIVGCFILITAIIASQYPAFRKLEGKAQ
>1170769.6.peg.3153
MVFLGLLALSGVYSISYLRGQNQVKSSFNPPSVQVAKVVTNRPSSIKVARPVVKINKQKA
VKAVWNLRQVQRKAKEIKTLSQGAINVGVVVSSYPKISQPFYTVKVLENHPDSTTSPVYW
FRVSSSNGAIQPLDLVSNKYTTLANWNPDGI
>1170769.6.peg.3188
MLKRTIHKLLLLExIGSxLLIxPVDMGFCTGASEKIALESRSSFLRAGLVEDGESSPYQI
GEESGLNQELFVETADLGNADIIVMNAKFEPEQVAC
>1170769.6.peg.3202
MAIDPVTVTMKLGQVPSKLEGLNLVKVAIQLDYDSLALEA
>1170769.6.peg.3206
MIDEWQKYKYSVNGKSILEPMKLPNTKVSFNANDLLFVLTNTRKYFQDYSQKDQNISRNG
LLVSQGVKVEDVLKTLDFMISTLREDVGKNRPPRLQDPNFINANFRVIKWKAYNPDKPEQ
KQLRITKYAVFTHPGSHTKTNTYNIPIYSLKDNSATDKFYTKYTKQDVLSGIYEPGGKEF
GKVETLAYLTRQGLEEALMQGTILINFTDGSKGFFNVDRNNGISYIRGVKDTLQKRYWYF
RPVDQIKGYGYKIDAKISIRPGVTFAGDVLNIGLGRVVLIEYNQGKNKRLQMGVVADTGG
AFLPNLHQLDYLAGVFKNQTEFRQYIRQLPEYASAYILVKK
>1170769.6.peg.3208
MGGCALIWKYIPKPRQVEFSANSLSHSRSDQVDTKGW
>1170769.6.peg.3226
MKRSYTEFLQDILDAITEIGLFVNGVSYEAFESNREKTLAVVKLLEVIVEAVKKISNERR
EQYPDIPWKSIAGMNDMLVHEY
>1170769.6.peg.3227
MLVELRDYLSQLFGLKVDIVTKNGLKPLIRERFLAEAIYI
>1170769.6.peg.3249
MQWLGRWFGVLEFLNCFPNPWQHRFRQFVAERGRKLLGIIQVSPFNRTRSTWHVDQVILD

PCADKLGIGSQLLRHCFESILEARMWILEVNINDVNALALYRQNGFQRLAETTYWEIKPE
LLQELAQAEPDLPNLLPVSNADAPLLYQLDTASMPPLVRQVFDRHTHDFKTSLFDIIGDA
IKQWVEQTEVVSAYVFEPQRKAAIGYFELQLDRKGTSPHVATLTVHPAYTWLYPELLSQL
ARISQDFPQQSLKLASSDYQPEREQYLENIGAQRIEHTLIMSRSVWHKIRESKFVSLEGI
QWTEVLQGLQPSRKPIPGGMSWVPKSPPVPEVTMGKSEMVALTIEPSEPPQENN
>1170769.6.peg.3262
MQFCISVVKDSVIIIIIIKTPDLFGGQFQKFILAKVWRSNWPLWQ
>1170769.6.peg.3288
MPSDNGELQVNLFTVNRPQEEAKYTVAYIDYPAQYIQLLRSKNLVEQAIEQGKSTALQRV
RGTIVSEEKKTLGDNVGIEVNYTTADAKVVKQRVFLVGNRFYQITAETTQKRQRFLTRSM
QGFCDSFKLLP
>1170769.6.peg.3292
MSVELVLPRDEINHHKSPHKSPHKSPSLPTIYLYEFRNR
>1170769.6.peg.3298
MRRLGELPGLTRKSARRAYAPGQHGQNRKKRSEYAIRLEEKQKLRLNYGLTEKQMLRYVR
KARRVTGSTGQVLLQLLEMRLDNTVFRLGMAPTISAARQLVSHGHVTINGRVVNIASYQC
RPGEEIGVKDKEASRKLVENNLQYPGLANLPNHLEFDKNKLTGKVNGVIEREWVALQVNE
LLVVEYYSRQA
>1170769.6.peg.3299
MDQIMIYLAFSAMRTSGHRHGAFLDAAATAAKCAIYMTYLEQGQNLRMTGHLHHLEPKRV
KIIVEEIREALTEGKLLKMLGSQEPRYLIQLPHVWMEKYSWQPGKSRIPSSNLTTEEKKQ
IERKLPANLPDAQLVTSFEFLELIEFLHKRSQEELPHHHQMPLSEALAEHIKRRLIYSGT
VTRIDSPWGMPFYVLTRQFYAPADDQERTYTMIEDTARYFRMMKNWAERKSHAMRAVEEL
DIAPEKIQAAMDELDEIIRVWADRYHQEGGKPVVLQMAFGEQDD
>1170769.6.peg.3300
MEVSQDQPIHFEAPLQLLLFVDGRPKSRQQVQRICAYLQDLEVDYSFDLQIIDVREEPYL
AEHFRLVATPALVKIHPKPQQTLAGSNIIAQLQNWWPRWQTVIDTSLALQKDLHELPEPD
ISMTHPPSTIHSVALSAERIKLSDQIFYLNQEKAKLQEQLQFKERIIAMLAHDLRNPLTA
AAIAIDTLQSDYNPDLGQFQRLKPNMTENLLKQARHQTRIIDRMIADLLEIGRENDNDFN
IAPQRLELGKLSFEVLEELRDRYVGKSQTVETDLPSDLPCVYADPERIRQVLINLLDNAI
KYTPKEGKISLAGLHRTTQKVQFSVGDTGPGIPHENRDHIFENHFRLERDQAADGYGIGL
SLCQRIIRAHYGQIWVDSTPNGGAWFHFTLPVYPS
>1170769.6.peg.3302
MIITPSALALTSIKLFDLSYQDCPSDLAQGAVVSGSSRFANCFIITGKAENGTYKTVYDA
DVYGRIYDANNDPILQNRSRLGSIAQVPPGISNFELRISVPANQPTPLKLKQFKASGFST
MIRK
>1170769.6.peg.3303
MLIVRVLLTWFPQINWYNQPFAALSQVSDPYLNLFRNIIPSLGGIDISPILAFLVLNIVS
SLLENLSRVTSLGGF
>1170769.6.peg.3317
MRGSGIGSSFIRLINSRLFINFPWFGDGRVSRRKVVFEVCHKFN
>1170769.6.peg.3327
MRARRDRRRHARHRRRDRQRRAARPARHCDGVPILRPLSAYECRAEHGLRAALGRYAARR
NRQARGRGRRNPAPHAVARPQAAPIVGRSASARGDRPRYRARAAHFPVRRAAVQSRRLAA
RRYARGNRGAQTASRHDDDLRDPRSDGSDDARRQDRGDERGPPRTSRPAARSLSQTGQPF
RRALHRLAAHESAAGRFVGRSRGRDRPAPGGFEAG
>1170769.6.peg.3328
MIHQWRVYSFPASKRVSARPRCCTASISKSATANSPCSSVRRVAANPRCCA
>1170769.6.peg.3329
MRDDLRCAVPSSCFLGEGPVWSAADKRLLFVDILAPSVILADPESGQFVSKPMPELIGAA
VPRARGGFLAATQNGFKTFDWSSDTLTPIAHPEAGKPGNRFNDGKCDRRGRFWAGTLAIT
TAPGEGALYRLDPDGSVATMGKGFHISNGLGWSPDDRRFYFTDSGARRIYVYDFDLDGGE

IANRRVFVQLPENAGIPDGLTVDAEGFVWSAHWDGWCVTRYDPDGTVDRVVTLPVPRPTS
CCFGGPDLSTLYITTARIRMSQRQLAEAPLSGGVFALRAGVRGQADTPFAG
>1170769.6.peg.3330
MEYLECDPVVFCPHPDGKYFLAHKSLEKTCAEIARYNPKDAKKYAEFTQYWQRVINAMMP
IFNAPPKSIIDIFGNYNLEKFQDLFSVVGSTQKSLDFIRTMLTSAEDILNEWFDEEFLKA
PLSRLASELGAPPSQKNLAIGVMMMSMRHHPGMSRPRGGTGALIKALVNLVTAKQGKILT
DQLVEKVLIDNGEAVGVRVAGGREYRAKYGVISNIDAQRLFLQLVDPTEVDDADPQLRER
LARRIVNNNETILKIDLALDEPLRFPYHEHKDEYLVGSILIADSMHHVEQAHSKCTLGEI
PDSNPSMYVVVPSFLDPSLAPPGKHTVWIEFFAPYQIAGAEGRGLKGTGWTDELKNRVAD
KVVEKLATYAPNVKTATIARRVESPAELGERLGAYKGNYYHIDMTLDQMIFFRPLPELAN
YKTPINNLFLTGAGTHPGGSISGMPGRNCAKVFLQSKHPITQTLKDAGNSIKSTMGSVFG
IS
>1170769.6.peg.3332
MFIILRFSRFLKTLIKDFDSINVPSKWEPDLIIFCHL
>1170769.6.peg.3333
MNDLPRQKLREIIVQHGRALCDDPKRCEAFLRDYCGEYGREIFILISALKQGIVKDLLNS
NNIPIELLLGRLTKQMQNNLGLTEEAARYGVESWAVVLDKMTPQQIQQPIIKPPTTISRN
QQPIVQSTHPKATEISQTRQIDYTNLESLLQQQNFRAADEETYKVMLAVAKREIKGDLDV
KSIDNFPCRDLCTIDKLWVKYSTGKFGFSVQKRIYQGLGGTRNYDRKIWYAWADKVVWKT
ESSWLYTKRFYPIMNFTYDMKAPEGHLPTPRVYQHWASTFWFEEAAKGLSLFSRLETCKL
>1170769.6.peg.3334
MGNSRHAIACFPPSVYPLRRTGEVRSRIILLILERIIISSYILILEIL
>1170769.6.peg.3337
MGARLRVFLTSEEDKTLFNLRSADVPQKVKDRAEVIRLNAHGWYVEKIAAHFNWTSQTVR
EVLHKWEKFGLEGLWEKSGRGGKPKYYGSYS
>1170769.6.peg.3338
MENRRSLLVYEFEEGDRCCGRGSKLLAVRLFWDEVDMDNEEIIGVATKFVKANCYSPNPK
SF
>1170769.6.peg.3340
MCGGAITRSEGSPINFPASSQGKIMEWSTGLSPIVTAVRSHFP
>1170769.6.peg.3341
MILPLFFLNRTVLTGDRTSPRFHSPELRSPFNPLQIFP
>1170769.6.peg.3343
MLLDPLSIDALLPAITGESNKISPYRSGPDLISWFNPYGFQDEYLSGLPENKSRSKYALA
RVQEINNDDEKMRRFIESIVDPRRFIDSELDVAKTVEFVNSIIKMDGLELRESLRGYRLY
PLRDSSEPAGITPVFEEIQSQILDSIEAAEFCIWVAVAWFTDPVLYNALAKKRREGISVR
VILIDDEINRNRSNFKEFPVKWISPEGTHNNLMHCKFCVIDLKKVIHGSYNWTNKARFNN
EQITVIEDKICAEDFAKEFVKLAAD
>1170769.6.peg.3344
MNCPNCDSNNIRKNGQRRGKQNYQCKNCGRQFIESYSPRGYSQEVKEACLTMYVNGNGFR
AIERMTKVNHNTVIRWVKKLGRDAMLFN
>1170769.6.peg.3347
MYCNFELGSREQDGRSRVGSTVRSPKLINNTLAKKGYEKVGPNLQKA
>1170769.6.peg.3352
MEMLEIKTMDVTKSRFYQQIYTEGRQEEAANLVLRQLTRKLANLSETMVKRVKKLTIPQL
ENLGEALLEFTQIVDLERFLSKLESKPEESTLIQPDQEN
>1170769.6.peg.3353
MDRALSLVAALHGDRRDERARAACGDARQLPSVVGSRALRRGGGHRAHLRASAPSVELVI
TLRSTLDRAQAPAHQSIPRPPVSATCDHSFEVRPVVSTVGERVTLFSRMGKAKCPTAPPT
RFPACTPKAGAAESSSHEKGRTATGTEAMPTAVAASPPYGVPSLAKEMDALLPPSEKKPV
PST
>1170769.6.peg.3354

MVAWIGRVVRTGLERLCGVGGGYVPQHEPCGHEKPDQRRRPRGARGARGARGARGARGAR
AEKARGPWRAGVVRTRGRQGVFGHESRLKEGAVAAMTTA
>1170769.6.peg.3355
MLRVRKGDTEEPAVVPLGQSRILDHAVSGSERVAVGEILEGHHRRDVDRAAGDESAEELG
TPERIRREQGENRRRRKRVVLEQHGAADQEAHHERGHRRGPRRTISGAPKLTFDQREGGE
HHRRFERVAREHEGVPEEVRTETERCGGAQTNTCIPHVRAQPSLEKHEEGEAGKLHGQET
QPPGGVTEPPGSRLGWICRRRATVSEELGALLEGQVSGHAEEAVGRSLHRDMLVGAVLEG
HLGRVVGPAEGGDLVVHREGAVIDQRHGHVRAHVLVRVVHRGEVREHEKQRAHHEGEEEP
RDRGRFPGGRPPPGANVAVHLEGHGLSTPRRVSPPLPTSPTRPTLLIFRDFLPRLGDASG
RGAHVSHDARENGLLPESGDVARGVRVYCSSMGILVDGKWHTDMVGATSKTGAFERKPVT
FRERIEPKSEANQAARFVAEAGRYHLYVSLACPWAHRTLVLRALKGLEEVVSVSVVDPLM
LDQGWVFSDGPGCVPDSLFGSSALHEIYTRAMPDYSGRVTVPVLWDKRHGTIVNNESSEI
VRMFNTAFDALATKELPDLYPEALRAEIDALNARIYDTLNNGVYRSGFATTQSAYETAVT
ELFATLDMLEARLEKGPYLFGERMTEADWRLFPTLVRFDAVYHGHFKCNLRKLVEYPNLW
AYTKRLYHVPGVAPTVSIDHIKRHYYGSHLHLNPKGIVPVGPALTFD
>1170769.6.peg.3356
MSTAVLTRVRDAEGVHGRGGEACIPTESEIEPSQEPRAHEADGPVEHVPNDDRGGRVRER
HGERDGRAHHTLRVVPEWQTATLVRVPQGELVVAPKAYFARVEHLLEVLAVATSRVRIEK
DRGRPRDGGEHEPGDGDHPQLAARGGGREQTLELLFGADDSAHGQTAPGGTAR
>1170769.6.peg.3357
MYAFGVSHACQYGGGHTPLAPFIFLPLCLYLWRKAETSYEHAVGLGLVYALTFYNGGVYP
LGFVSLVVACETLTRMWPVPRAGRVVRAGVVAGLVFVTVSAARLVPVVDQLAHHKRALEP
EIDFISWQTLKDMYLDRTHAWHPVGQTYVWPEYSTYTGILGVSLALLGILHLRRKEAWLF
AVAAVTFLLMLGHFASWAPWSFLKANVFPYKSMRVPSRFRLLESAFVAFFVGYAIDRAPA
TFAKILRKPTALRQARLAVSCLALIGIGDIFGTASSVVASKFDGPPETRVVASTRLYVDN
ESANFEDQPRQNRARLACWDEWNFTMGAPLWYGDVPQARAVDPGAVVEVANRTQNSFTLD
VDVKREGAEVLVNGAWDRGWRTSVGRIFERNKQIVLSLPQGRHRVRVHYWPVGLTAGLVT
TGLSLVAVALFFARGRLFGKPVAEEAAGASASTASTTPEDTSDESASNDEPSEATPREAS
SDAKVEAHEPSTETSSEPLEIPKPEPSKPRSPSTLADSPDAKKRES
>1170769.6.peg.3358
MRIVIAYVHAFRHHLRDQDTKAGLDEVLTKDELARVSLQENVPLAILALLGDRLASARRR
GWLHVMHAPVLEASLVSLTDVQGACERIKTTPIPYSYTVLMHRIVGGYCILLPFGIGEAM
GWVSVPVVVVVSYALFGLDAVGDELEQPFGTDTHDLALFAISRTIEINLLRRIGATDVPA
PAKPHQGVLA
>1170769.6.peg.3359
MEAPGHRRSERRDHGHAPPDVRQGPSPHDHVVKPGIFRFDPHRTAELNSLRHRRAARVSR
SERSLLDRSTPGSNPGNPGESKETREKEAM
>1170769.6.peg.3360
MVELEPAPLEAIDLEVGRGPGVARERDPGLVALHGAVGVVRARGEDRVLSSNRGARTARR
HVVGLELLAREQREALHVEGAERLAIDHVRSRGDARERLFGRLLRGSVGRLGRAPGERAG
REHRDGEEGEGTSESEHAPP
>1170769.6.peg.3361
MKKDPSFKPVPTVTKTLQCTGPRVDARLTLDKSENRRGTLKITRKSGATDDDPKSATIGV
SITLDEATRDWVHYEGTNGEQDFFFGIVKADLDRATGAAKVNLSWAEFGQEFRVNVDCSF
VR
>1170769.6.peg.3362
MLGGAEREVHVDLDLARIDALKLSPLAILQQLKAQNLNVPAGHFEEGTKEISVRTVGELK
TVEAIRDTIVATTKDGSSVRLSDVATVEDGHEELRTRIRTNGQPAVSFDVVKQSGKNTVA
IADAVKAKLATIEATFPKGYKTSVIVDQSKFIRENAHEVEIAIVFGGAMAILVILVFMLD
LRSTLISSVALPTSVVSTFFVMYALNFTLNMMTLLALSLAIGLLIDDAVVVRENISKHLE
RGVDPKTAALEGTKEISLSVLATTLTIVAVFVPVAFMSGIVGQFFRQFGLTITAAVLVSL
FVAFTLDPMLSSRFSKAHVKGAVDRFMIVKRPFLAVFEGIEALYRRVLGFALNHKILVGV

VAFLALFSIGPVAGLMGNEFVNQEDRGQFVLEAELAAGTKLDETSRLSLPAEQKVLEDKR
FLTILSTLGPSGEVNKVRWRVVTVPKSERDVPLSELKERARTIALAALPGAKVTVTDPAF
VEGAATEAPIMLQVRGASYEELAPLAREFEQAMKAIPGIADLQVKYSPGQPELRVGVDRD
KAARAQVPVAQIALALRAAVEGDEAGKMRQGKDEVPIKVRLRQGDRSTVDDVLRMTVQTP
QGPMALADLATVERGEGPSVIEREDRERQIVVWASTKGRSLGEVVPEMTAAFAKIKMPPG
ATYHFDGQIRQMNETNGSMGAAMILGIIFIYLILASQFESFIHPLTIMLTLPLGFVGAFY
ALFMAERTMAMGAMIGIILLMGLVTKNAILLLDRALVRVREHGETPLQAILEAGPERLRP
ILMTSAAMILGMLPTATSNGEGSEFRSPMAIAVIGGVISSTILSLVVVPVVYLTIENAKG
FLGRLFGITPKTPEPTAPPPAPAE
>1170769.6.peg.3363
MHLAFGAAEHVHRRYAVDLFEARFDDVTGEFRSLRERASSALEGVGDGRSRAHVDAGDDG
LFGLGGQLVAGLRHALAHLGGGLLDVDVELEEHDDLRGALAGVRLDAVDAVERDDRVFER
LRDQLLHLARRRARIRHGDGDDREGHVREEVGAEVLVAHDPEDHEGRRDHHGEDRALDRD
L
>1170769.6.peg.3364
MDLDSNRPYQGPVDAPPPATKAGGKAVIVVGAVIFLGFLGVMGSRVGKALDKRKATAVER
ENAVVELAKKLPVEVASPSAMRWKPRIELTGTLRPWREADIGFELSGRLSKLNVQTGDKV
KSGSLLAVLDASRAGAQVNQAVAQSKAAEANLALAEDNLKRTEALVLTKSIPEAQAEQAR
QQVALAKAQLDGARASTSLAQQGAGMHAIVAPFEGVITKAPTAIGAVVNPGVPLIRVEDV
SKFRLSASVGEDDAALVKVDSPVTITYQGRTVTGKVIAVIPSLDQATRRAPVEVEVPNDP
KSPLLGYGFVRAHIDGKNEVDALRVPAAARRPGSQNEVVRLVGGKAQRTRVSHTVDTDGS
WIVTDGLTAGDTLVLNPSDDVKDGDALEVAAPKADKGQAPAPAEKK
>OHY31526.1
MILLQNPCFNYEQETKKPGVKITVNKFNQLLIGGTAMVSMLGWNINMAQAQSLPAYCTGS
AIGALLGLAIGANPRAAANAIPAECTTQNQNSPSSSPKLIAAYNSTKSDSDVGIKQDCIS
FFYDSGDFLEKCSSFINSDYTRPRTRITNIEGIWKRTGDLVYVKVRSSAGLSGERKYQII
PDGLLNLDTGTRLTKE
>OHY31530.1
MTMNKFNQLLMGTTTVVLTAVMLTSCGGGTSSNTGGGTTQGGGGTTQGGGSVTPVQPTPT
TPQTLTVTVPSLGYVQTIDKYTTLADITTAARDGAAECINRFGLSSASNTCLGEVRAAQS
QAVNFIN
>OHY31549.1
MKWQLLTHNKQVLGKIFTILVFTGLTGILCVSCNRNQDLLVTEIGVNPPKRPTRKTSGAG
EFYLQGQNQHSRGNFQAAIAAYSKSISLNSDYAPAFKARGLAYFDLNNKERAINDYNQSL
QINPNDPETYNYRGNARASLGDQKGAIEDYNEAIRLSPNYAEAFNNRGNSHAAQGNKNAA
LEDYTQAIRIDQNYSVAYNNRGNAYSSLGNTSKAIADYNQAIRLNPQFAPAYNNRGNAFA
SSGDKRRALQDLQKAATIFDQEGNRGLYQQTMKNIEELGN
>OHY31590.1
MAITTAASRLGTEPFSEARRVELRPSASREEVELVIRTAYRQVLGNDYILASDRLVSAES
LLRDGNLTVREFVRAIAKSELYKVKFFYNSFQTRLIELNYKHLLGRAPLDESEVVYHLDL
YDNKGYDAEIDSYIDSVEYQNNFGENIVPYYRGFDFQPGQTSVGFTRMFRLYRGYANSDT
AQVEGSKSRLARELAGKKASSIVGPSGSNDNWSFRPSADNAPKQNLGNAVGQSDRVYRIE
VAGIRSPGYPSVRRSSTAFIVPYERLSDKIQQIHKQGGKIVSITAT
>OHY31928.1
MAERLREERVKAIIQPMLAGLDLPDTPEDDRRFSLDLGLVKRIPLGRMTIANPIYQEVIP
RVLSQGSQDSLTQIQPTWLNTDK
>OHY31994.1
MNDLLIAAKNGNVQQVRQLLGSGFPVDTGDRHGTTALMFAANFGYTEIVRCLLDFGADID
LPRKLHGLTALMLAAAHNQVDVVKLLTSQGANTNAVNEDGSTALMIAVEKGYIETVQNLL
DFGADPKIVDQHNEDAFKLAIRQNNRVILNILVKNSQIKGETESLLIMGADNGNLEIVKT
SLLYGVNPNLENSDGTTALLAAAAGGHTEIIQVLLDRGAEINHQDQEGETAMHFSVVENH
LETVQTLVNRGANLEIRNNLGDTPLILAAFQGYQEIVKVLLDAGADGGKKNLGEFPLTLA

AFQGHTETVKVLLESGVNIDVIAEDGKSALVKAIIGNHPEIFQLLLTKGANVNLQDPVGV
TPLMYATAQGYTQAVDMLIQAGANVNIKNQGGYTALMIAKSNNYTKTSNLLIQAGAKE
>OHY32218.1
MGLFDESTLKCKAEVWRSLINRWRDIRGGYNKCEEYRPLAQLFERFKEYDLAGYFYQVSD
GVVKKDLFYEIPKAQNRPSPVIYGLGIEREVTLEDIIIGLADGVVKFFNQDVDMKLVFVI
PVSLLLILGSGWFIGSKTWQYVYANEAEKFLCQKSGGGENCPVIVLDGKNHYSFDEIKQVI
PKVVNQVIDQQTKAPTAEPTQSGSNTSGQNYGQQDIKKKVIENLIQILGDKNLKYQDLNS
TDKIEEAVKTQWVIAVYNYQLKSKIDQKVTTQKSNKEECLLQIFFCLKKNIKTEETYSLE
AKLTNDINKKINPPKKNQPGQNSPVK
>OHY32222.1
MTTALSWQKRVGNQRDWVWRGWQIRYTFIRPVNHHQTATPLILLHGFGASIGHWRHNLEV
LGKHHTVYALDMLGFGGSEKVPANYSVNLWVEQLYDFWQTFIHHPVILIGNSLGSLVTLV
AAAVHPDMVQGMVMMSLPDPNLEQEVLPPFLHPLVRGIKGIFASPLLLKPLFNFIRQPAV
LRRWAGLAYAHPQAITDELIDILAGPPQDRGSTRAFIALFKA
>OHY32223.1
MGNIIANDADGNTLTYSISGGADQSLFTINANTGVLSFVTAPNFEAPGDVGADNRYNLQI
QVTDGNNRVTQVLIIDVTNLNEAPTDLTLSATTIEENQASGTVVGNFSTTDPDAGNTFTY
SLVTGAGSTDNSFFTIDGGKLKTAAAFDFETKNSYSIRVRSTDQGGLFFEKQLTIGVKGV
NEPPVFSAVSFSVRENSKSIGRISVQDPEGDNITFALAGVDAKLLSIDPTTGELTFNQAP
DFEKPEDADNNKIYQVQVTVRDGNTPVTRNIDIKVEDVNEAPAAIGDFLAIVGDTSGSIE
PLRNDTDPDSGDKLKIIGVTDGKQGKVEIIGDQLKYTLLDAAYTGDDVFSYTISDQGNLT
ATANVKVNVTGTKVVVNSGVITDVQPGDPLIPSEAGSLSGIVNNVSFNFRAGYNPTQARD
ILQRTLVRTDAAFNNLFGLYEIDDATGTVNGVAPGQPGYARAALNRAVSSFAVRAGGSGN
GITGNVVVGGDKFYAPFVIANGGNLFGSMQDAINTFFQLNADNSRATAENYTSFPVAYFS
FGAANPDGAAHIKSFGNNIFGFEDLPAGVGVNDYDFNDTVFSFG
>OHY32224.1
MSPASGSVAGAMVPTVVLFSAVEKVAVALAKLGASLIPVTLTVTVWVAFKPLTSVAVMVR
L
>OHY32225.1
MGNITATDADGNILTYSISGGADRSLFTINANANTGVLSFVTAPNFEVPTDVGTNNVYNL
QIQVTDGNNPVTQDLIINVTNVNETPTDLTLSATTIAENQAIGTVVGNLSTIDPDAGNTF
TYSLVTGAGATDNSSFTIDGVQLKNRCCF
>OHY32226.1
MSTPKLVITPKIQSVSPRSGKINLDYRLENFTDQAVSSASIEVYFSNELQIDVNAITIGD
NTGTVPLGGRITGADTNNADGDGTTGNFVVLTFQNLTTSATNKSFINIPFVTTSTFDGQA
AVNFIARSTNSNLTVDTIAPVAINNQVAAILEVGPDQTYKTIQAAIDAASNGDVVRVLSG
VYNENVTINKSVTLEGPNKGIRPTTPDINLTGGININQGYRTNPEAWIKGTVTVTADNVT
IDGFRLRNENGPLQWTGTPDNFKLLNNYVTGYNANKGPRFGDANSNNPTNVVTGWQIDAN
YIGGLLGGGGTGGSMYLAGLSNSSINNNTFWRPRAAHLYLASLTNVTIDGNKFYHGLHAG
GADFDGFGKFFSGTGYGYGGYGGYGGSYGGGYGRNYWLELKGTNNTVNIKNNSGEYNSGG
IQLYGEVNDPFLFNKVTIENNTFPANNFINAYTQASNNNLSGLIPAVMATARVVNGGPSG
SDLVIRDNKITMDLAQVKYDKDHKSSLEVRGNFNGVTIENNTLTPTGTNGGVNLITGLNL
YGSLPGQVSVKNNEFFGEGGTRQNASYYGIDVNPTFTGYGTYNSNLNIQNNTIRNWEVGV
VLRDAVQITANSINIAGNNFSNNSSNVFDGINPTITASQVLSYPENQQQGATLGTVSASD
NLPNTDNVGIIQYSISSGNESGFFSINSSSGQITLTSAGISAAANNFESLPNTFTLGITV
TDGGGLTATNAVTLSVTNVNEAPSFANATATFSRAENSTTVGTISAAIDPDAGDTLTYTL
SGADVAKFNIDNTTRSLTFKTAPDFEAPGSAAGTNTYSVTVIATDGGGLTATQAVTVNVT
DVDDTPPDAPQIINFIDNVAPVTGTFGNGTTTDDLTPTLNIKAEAGSSVQVFRNSLTYGD
ATAANTPGDYTFTTANLAPGNTYSFTARATDAAGNVSPLSNPFTLTVGLPGYQRYNFTYR
YGNGDSYSGYVYAPVGTYTQGQNIPVSNTNETGQTGSYTIDSFGEITTDSSFNNLVYLTS
YNDADTGFGTTTNIWPPQGTVSGSSGLGSEYGFAYDANFFSSDPYFSNFFEADIRSNNVF
FEFTYYYGEDTNNDYYKGYGYASRDYINAPGRYLAINSKPNDTGKTGYYQVTSVQNSFDF

GLRNINTYIWVNEYFDIQTDQDGIGTGGYGKANYIWSYGGNRGLGSEEGYAYNLGFEGGD
NQFNHINSADIATTKTFLSIRNPGNAWLQTRFEGNQGTSTNYTFEVVRQGNLNSALSVNW
NTQSFFFPNADANDFVGSTFPSGTVNFTPGQSTAPLTISVQGDIDIEFPEWFQAVIDNPD
PTSIVLAQNYAWSLILNDDGWFWGWGFGDPHLVTLDGLAYDFMAVGEFVLVETTPGSANP
FQVQVRYEPYPGSEVVSVTTRMAVKLGERRIELQLGPDPLLVDGSIVSIAPTEAGVDING
DGTLDVERNGNVYIITLNDLGEQVRVEIYDAFMDVNVLIVESPSGVNRGFRGLLGNRNND
RTDDLTGRDGTLYSQPVSFENLYGAFANSWRLDAVGTNNGKASLFSYGVGERFGGFDRSN
FPQGVIDLDQVPADLLTAARTAAAGITDPILKDAAIYDYLLTGERSFIAAAEVFPDKPKD
DTDPTLARVITSVGVAATPLSITEGNSASQDVTFRVWRTNPSGNLTVDYRLEGSINADDL
SPGTPFSGLINFADGETEKLVKVTVLGDTLIETDEQLVMRIETPNIGSVMVAAGQAATTI
ISDDLPPVTIGVIAGNDILNEAEKAAGAVITGTATGLAQVQVTIAGQRKTVNVIDGNWTA
NFTPQELPGDGSYTVEAIGIAQSGSQTIPASRTLLLDTIPPNAPVINPVTGDDIINPAER
SSGITITGTAEANSRVRLTFGNVTRTVTAINGQWSVNISASELPSEGILSLLATATDTAG
NTSAAIAREVRFNRAPSFANATATFSTAENSTRVEIITAATDPDAGDTLIYTLSGADGDK
FNIDSSTRLLSFKTAPNFEAPGSAAGTNAYSVTVTATDRGGLTATQAVTVNVTDVVEIGN
PPVITSGSTFSIAENSTTVATIIATDVESSTLNYSISGGVDQNLFAIDPTTGVLRFVTAP
NFEAPTDVGADNRYNLQIQVKDSDNNTVTKDLIITVTDVNEAPSFNTPTATFSTGENTIL
VGSVVATDPDRGDTLTYTLSGADAGKFDIDSTTQFLTFKTAPNFEAPGSAGGNNTYNVTV
TARDGAGLTTTQAVTISVTNVNEAPSFANPTATFSTAENTTTVEIITATDPDGGDTLTYS
LSGADEGKFNIDSSTRLLSFKTPPDFEAPGSAAGTNTYRVTVTAKDAAGLTATQEVTVNV
TGVVENGNPPLITSPSTFSIAENSTAVETIIATDADSNTLIYSISGGVDRSLFTINANTG
ALSFVNAPNFEAPGTDNIYNVQIQVTDGNNPVTQDLIINVTNVNEAPSFTNTTATFPVAE
NSTTVGTIAPATDPDAGDTLTYTLSGADAGKFNIDSSTRLLSFKTPPDFEAKGSAAGSNT
YSVTVTATDGGGLTTTQASYC
>OHY32227.1
MSFAAGETTQTITVNVSGDSTVEPDEGFTVTLSNPTNATITTATATGTITNDDTTPVVNA
NQTFSYAERQVANFQVGTVTATGAVGVTSFAIASGNNSGFFAINNSGVITLTAAGAAASA
ASNDFETTPNTFTLGITASDAAGNTSTSTNITINVTDVDDTAPVVNGNQTFSYAERQVAN
FQVGTVTATDAVGVTSFAIASGNNSGFFAISNIGVITLTAAGAAASAASNDFETTPNTFT
LGITGSDAANNTSSPVNVTINVTDIPNDVPTVTIAATDPYAAEIQTPRVNNGKFTFTLSE
AAPVGGIAVNYTVSGTAIGREDYTLLPGTVTIAGGQTTAVVDVLPINDAVVEGNQSVILS
LTDGVTYDLGATTGATVTIADGAIGDIDGNGVFTGSDAFLINQFLAERNNPNRNFILETT
FARFPSETVGSTNTTGATLANGIEAQLSLFDIDGNSTTSPGDIFLMNQYLLLGSNPNRNQ
ILQLVASAFGSEFSGPNNTGTELNQALSNLIGTNI
>OHY32617.1
MGKLAEVSYFGCNMINSLKPAVYIVGAGPGDPDLLTVKAQRLLAAADLVLFADSLVPQQI
LDICRPDAQVIGTATKTLEEIVGTIIAAVQSNKFVVRLHSGDVSLYSAIHEQIELLNASN
IPFEIVPGVSAFQAAAAKLKVELTVPDLVQSIILTRVSGRTKVPDQEQLASLAAHQASLC
LYLSARHVASAQNQLLQHYPPQTPVAICFRVGWPDEKIFIVPLEKMAESTDEQKLLRTTL
YIISPALSTVSGRSHLYDAKYNHLFRVSTQ
>OHY32795.1
MCGPLAVAFSFSYPEKTQSWQRQLQFHILLNLGRVFSYALVGTAIGTLGSALVQGGQLGG
VGSDLRRWIAIITGMMLIWLGLAQVKPHLIPKIPIFHPLLQNSLHNRLSAVMVNLSLHTK
WWTPALLGMTWGLMPCGFLYAAQIKAAATGNGWHGAITMLAFGLGTLPTMLGVSISTSLM
SKDQRSQLFRMGGWVSMIIGIITISRTGETMVDYSGYAALICLILALIARPTSVLLPALI
RYRRGLGVGAFLLSLVHTVHKLEHFLAWNVSAIWFLPVEFQWGMGAGILALIFMTPAAFT
SFDFMQKSLGPNWRRIHLLTVPALILTTIHAVLIGSTYLGALKLTIFNQMATLLLVFIIL
AVLMMRSPLVWSIFKLKEFYTPLKQKE
>OHY32901.1
MGIELKVRKEKLDPLTQGLIQLDKYLDGLGLDTGWLVIFDRRPGLPPMGERISTEEAISP
GGRTITVIRS
>OHY33029.1

MKAINPSISIVTPSYNQAEFLEATIDSVLSQNYPSLEYIIIDGGSTDGSVDIIKKHEKHL
TFWCSEPDSGQYDAINKGFRYSTGEIMAWINSDDMYLPWALKTVADIMSSLPTVEWLTTL
YPGHWDYCGFCKGFGNTPGFSLDAFLDGYYLPTNGVGYWIQQESTFWRRSLWVRSGSCLN
NNLKLAGDFELWCRFYLHTNLYGTASPLGGFRFQYCQKSKNIQEYISEATLALLAMRQHL
NWNYYIKTGKNRVFHQIRQIPKLTSSIISRWGYNGKKIVRVDSDHPDGYWKIEDYRF
>OHY33030.1
MQRPELIVIIVKLILKRLGLPKIPKTSLTKDLSLQSALSRITTKDIKIKTVIDIGASDGQ
WTKVVKAYFPWAFYYLIEANPIHCSISK
>OHY33031.1
MSIKQAQEPEKDNLEFPLVLVGSDQGNVSYVKGKVKELNLSKQVYFLGFVPQKDMASLYI
NAFALTFVSFFGPDNLPPLEAMALNCPVIASKVSGSEEQLGNSALLVNPKEPQEIANAIK
SLWHDSTLRQNLIRKGKDRAFQWTARDYVQSLFCLLDQFEPIRRCWQ
>OHY33032.1
MIPIQKILLRSLPRYNTPAYRTITTTMNRNRSLVVFTFVSNLLSATLETATLGIIFLALG
VLQDNQLPQLPDTIKSALPWLADRWKGENQEVFLLLIGLAVLSQVVRSLMTYISLVSSGD
LTARVQAQMTEKVFARIMSFTFSCASRYKIGDLSTYVGQAGSTVDMQMRLWSQFLTGIMM
FFAYSITVLTISLPLSAVALLLFVLLIWLQRYLIPRIQSTARELSQAQVDVAKDMVENIQ
GLRVVHTFGYQHSTINRVVYLQKQVLVFLQRQARLLSITSPLNNALTILVIAALLLTGSF
LLQRGQGNVLPALATFILALNRLSMQVQSLAGTMNGLAERSGMMDRLDAILGGEGQELSR
VGGEIFEGLKSAITFNHVSLKYEGTSLPALSDICFKLPRNRVVALIGSSGAGKSSVADLL
IGLYAPTTGEILVDGLNLQSYSWESWRSKLGVVSQDTFIFNQSILENIRYGMTNATDEQV
LEAARVAQADQFIQLLPRGYETVVGERGYRLSGGQRQRVALARAILKQPEILILDEATSA
LDSESERLVQQALGQFQAERTVLVIAHRLSTIVNADEILVMEQGCIVERGTHQELLELGA
KYANYWQMQSAH
>OHY33051.1
MEETRKIVAETNKNMGSITSRWGEFVENLVRPAAVRLFKEQGINVHYTSLQVKADDYAGS
IEIDIWAENDGEIVAIEVKSHLKVRDIKRFIKVLDRFKDIFPKYKNYRLYGAVAGIKVDE
>OHY33097.1
MRSHFSAAHRLAPNLSAEKYGKCTRTHGHNYHLEVTVEGEMDGRTGMIVDLGCLHEIVER
EILELFDHSCINEDIPYFSTSHIVPTTENIARYMSDLLQFPISQLGVKLSRVKLFESDHL
WVEYEGKDSEIFFSVATGFSAAHRLADPTLSLEKNQTIYGKCSRINGHGHNYYLEVTVQG
EIDSVTGMSVDLVGLNQIIQHYVIEPMDHSFLNQDLPYFTEVVPTAENIAVYITNVVRSP
IEELGAKLHKVKLIESPNNSCEIYARDIEESKVDRIYRELAAV
>OHY33260.1
MWGFTGDGGAMYTIQSLWTAARHNVDAKFVICNNSSYRLLQLNIQAYWNERNIPLHDFPL
SFDLSKPSIQFAQLAQAMGVEAIRVEDPNQIEPAIQKALEHSGPFLIDLVLEGDVHPELV
GVRCG
>OHY33415.1
MIGANLDAQSFFSQSVYLSANIQYLNWDLDVLLNGTEGFINVHALVQFVGGPDFFLSHAF
SLLGASVGLWLLTQSWLLLFPQGKKYLGWVILLYTLYPSILTFQSYILREVWQNVCILGL
GWLALKIKAKGWSGGRILGLVTLTIAGSLLHKAMPLVMPLLLIISIMLANKVSLINWLYS
PIRLVKLLIVLLVLSSLILPVISQSSYFSALTEGELIEQTDQYSEIALKDARTQYGKLFF
ANQPWTIIPTFLAYELMPLPFQIRNPADLVAFIQNLFRVWLVWVYWRHRQYLDKNTLQSV
NMLLLMWLVVDLVYAAGTINWGTAARHHIKSLALLLLSGLLVWARFREYHTLSIPEQSHK
KTIRSTKKQSRLAS
>OHY33416.1
MQKNNIKNILIQALKPNRFSVMIGKIFKRFNDKQGMHSSNENLSWIESHSCEFQQLAMKL
DMELWQEAECFSQKLQINSEEKLKNINYPLGGGGLYPLLYFITRYVKPSSILETGVAAGW
SSCAFLEAIKINGKGKLYSSDFPYFRLPNPERYIGILVDESLKSNWDLYIDGDEKNLPMI
LNKLDAIDMFHYDSDKSYSGREMVMFGIETKLSQNSIILMDDIQDNSFFYDYIERNNIQE
WYVFEFQGKYVGMIGSLH
>OHY33418.1

MKILVTGAAGFIGFHLSQRLLSRGDMVVGLDNLNNYYDASLKQDRLSQLESQRTFTFAKL
DLEDQEGVNSLFKKHNFDTVVNLAAQAGVRYSLENPHAYINSNILGFTNILEACRYKQVK
HLVFASSSSVYGANTKTPFSVHDNVDHPISLYAATKKANELMAHTYSHLYGIPSTGLRFF
TVYGPWGRPDMALFLFTKAILSGKPIDVFNFGKMKRDFTYIDDIVEGLVRVIDRIPQGNS
NWSGYNPDPGTSKAPYKIYNIGNNNPVELLHFINVIEECLGIKAQKNMLPLQLGDVTMTY
ADVDDLVADVGFKPSTSIELGVQRFIEWYKKYYIS
>OHY33430.1
MNQAHKAVFLDRDGVINRSLVKQGKPYPPATIDELEILPGVDEALISLKKEGFLLIVVTN
QPDVARGKTKKEFVNAINSRLASSLPIDDFFTCFHDDSDNCDCRKPKPGSLFSAATRHDI
CLPSSFMVGDRWRDIEAGYGAGCRTIFIDYGYDEKQPDHFDFRVSSLFEAARIILKTPEK
FDEKD
>OHY33431.1
MTYAQQHLEEATRIIEKIDFDTVEQVADILACVKAEEGRIFFLGVGGSAGNCSHAVNDFR
KIVGIESYAPTDNVSELTARVNDEGWATIFVEWLKTSKLNSKDCVFVFSVGGGNLEKNIS
PNLVEALKYTKTVSAKITGVVGRDGGYTAKVADACVIIPTVNPDTITPHSEAFQAVIWHL
LVSHPKLKANQTKWESTVK
>OHY33432.1
MLPVAILAGGLATRLRPITEKIPKSLVPVAGKPFICHQLNYLREQGLEKVVLCIGYLGEM
IQEVVGNGENFGLSVNYSLDGSVLLGTGGALKQALPLLGDEFFVLYGDSFLPIDFSAVEN
FFLSCNKPALMTILRNANQWDKSNVIFRNGTLEEYNKSIYRTDMEFIDYGLGILSRSVLD
KYPIAQPFDLADVYHSLSTEGNLLGYQVHKRFYEIGSTVGLQETETYFLRKL
>OHY33433.1
MIITRSPLRITLGGGGTDLPSYYRDHEGFLIAAAIDKYVYVTVMRPFTEGIFLKYSQLEH
VNEIAEVKHPIIRECLHILDLKTPQVEITTLADIPAGTGLGSSGSFTTALLKALYTHRRC
HLHQEELAELACHIEIDRLGEPIGKQDQYAAAIGGITCFTFHKDDQVTANPLAISMDTMF
DLEDNLLLFFTGFSRSASGILKDQKERTQKSDGDMLANLHYVKDLGYRSKAALESGDTYL
FGQLMHEHWEHKKKRSGGMSNPQIDEWYQLAMNNDAIGGKLVGAGGGGFLMFMASDRNKL
RHAMTNAGLEEVRFGFDFEGTKVVLTS
>OHY33434.1
MIFNLLYKSAYILEKLSAFAQGKGYGSRSIRQEIAVAKKLMQSLQPELLMIDIGGNIGDY
TYQLRKGFKQAEVHIFEPSIVNVNKLSQRFKGDPLVILNPVGVSNCEGSFLLYSNEQGSG
IASLSKRRLDHFDISFDFSEQIQTICFENYWINQLNRKRINLVKLDIEGHELDALRGFGS
AIWATELIQFEFGGSNLDTHTTFQDFFYFFKEHNYEIYSRKNK
>OHY33436.1
MLLYQFQGQSKKYSFYTKNNAGYKLMRRLGIKLSKGYPKTTMEKLFYSHIFKASEVAFFR
RRFDWAIK
>OHY33437.1
MNSKVALITGASRGLGKVLAHRFWESNYSLYLIARSYEELQKVRSSLPPRPSQNCDIYGC
DLGISESIERLRSEIYNNLSRLNVLINNAGTHGPIGQSWINNTSDWQKTIQVNLFAPVAL
CQIAVPLMEQTGGGVIINLSGGGATGPRPNFSAYATAKAALVRFSETLAEETRGISIRVN
CIAPGAMKTALLAEILEKGTQLSGEREFDLASKVLVEGGASMDRVADLALFLASEDSKGI
TGKLISAVWDRWEDWPLYLDELSKTDVYTLRRIVGRDRGMTWGDK
>OHY33438.1
MKKVFITGCAGFIGSNLTDRLLSLGTKVTGYDNFSTGQERFLALASKNANFNLVRGDLLD
QTVLTNAMEGCEMVFHLAANADVRFGTNHPRRDLEQNTIATYNVLEAMRQNGIQHIAFSS
TGSVYGEAPVIPTPEDAPFPIQTSLYGASKLAGEGLIAAYCEGFGFQSWIFRFVSILGER
YTHGHVFDFYKQLKADPTRLAVLGNGTQRKSYLYIQDCLDAILLALERASNRVNIFNLGV
DDYCQVNNSIGWICQELGVNPQLEYSGGDRGWIGDNPFIHLDVSKIQALGWEPKLTIREG
VIKTVQYLRANEWVFEVRK
>OHY33563.1
MKVREGDLEIDFTDAIEALIFDQMKDKTLPDYHGVAEMYRVDFVVEFETRIVFVEIKDPG
NPKAQVKGLEKFWEELKNGSLSRTFANKFVDSFLYRWAEEKIHKPVDYLNIITLDDAVLL

DNFSDEIRKVIPPMGKTVPRWRRQLVENCQVFNLETWNETFPGWPVTRLNQSTERREN
>OHY33719.1
MTSQAIKRGNFAFFLTQPKQLENQTRLFQLAPQDIALINPNTLTCPVFRTSKDAELTKKI
YQNVPVLENEKTGINPWNFSFMRMFDMANDSGLFHTQDPGLIEEVPDLKVPLYEAKMFHQ
FDHRWATYTDNGDTRDLTDDEKSDLSFTIKPRYWVDKKEVENKLSGRWDKNWLLGFRDIT
NSTNERTAIFSLLPKVGIGNNAPVILTNIKEAKLISCLLANFCSLTFDFVTRHKVGGTHM
NFFIVKQLPVIPPERYTEKDIEYIAPRVLELVYTSWDMQPFALDMGYEGEPFIWNPNRRA
LIRAELDAYYAKLYGLTRDELRYILDPADVYGADFPSETFRVLKNNEIKQYGEYRTQRLV
LEAWDRIIRNS
>OHY33721.1
MCYGTKQINAALSTTLNPKVMTSFRKFAQNCQVGDVNPCFDKYSDLDSGAAAFVNNEVDA
FFGYSERLNYILKNSSNSDVQLSSLPLSEGSNPLLFADALVLRKDCDQTCENAANTFAAY
LDNPDTQEWILSSKDAGENAVPRYLIPATYSAFTTNSLAKDSYYQTLEKVVKNADAYPNS
GFAEIRKTLKKAILQELQSSS
>OHY33730.1
MKKLQNLLFSFLIFTLVFLGITAFFIPNIAYAQTTSKTILKVALFPYIPDSAEDQYQTLL
NRIESEFETQNPNIDLVLKPLNPEEEGFYDIDTLKQWLTNPSKQDGYDLVEVDTLLLGDL
VKANVVKTWNKPENIKDWYPAGLQGVTVNGDIYGVPHLLCGHFIISRNDKVAKTKSVEKL
LNILTAITPDTPNLAGDLTGSWNLPALYLDGWADTYGTRSVEC
>OHY33748.1
MNDLPRQKLKEIIIQHGRSLCDDPHRCEAFLRDYCGQYKREIFLLISALKQGVVKDLLNS
NNTPIEVLLGRLINQMQNDLGLTKEAAQYAVESWAQALDKMPQIVTLQGQNKVGKTENLK
LQGEITALLETRRDKQKQETTSLVPIPSSYKELETLLKSQKFKRADEETGNVMLAVANRQ
NEGWLRVEDAENFPCEELRAIDNLWLKYSQGRFGISVQQEIYKNLGGTKQYNKNVWRSFG
DHVGWRSAGSWLNYSDLNFSLSAPKGQLPTVRRSMTMVWGGWVLGGFVVSLLSRHVECNP
YHLIDYLYP
>OHY33754.1
MNDLPRQKLKEIIIQHGRSLCDNPQRCEAFLRDYCGGYRREIFILISALKQDAAKDLLNS
NNVPLELLVSRLIKKMQNELGLTEEAAHYAVESWAQALDKMPQQQIQQPRFDVINKANKK
LNHPVSSQQTTVVSPLFTTNQQQQKLVLGRGLLKKAGVSVGLFILVVIVQQIFTANSTEP
EINGYPTTEITELPTPEITRSSTPKVTRSLRRKITEPTEPEITEPTEPEITEPTEPEITE
PTEPEITEPTEPEITESTEVEVDESSKPENAEFLEREIREVNESPTPEVTESPTLEVTES
PTPEVTESPTPEKTNPI
>OHY33755.1
MNDLPRQKLKEIIIQHGRSLCDNPQRCEAFLRDYSGEYKREIFLLISALKQGVAKDLLNS
SNIPIELLLGRLTKQMQNDLGLTEEAARYAVESWAQVLDKMPQQQIQQPRVQRTNKVDKP
TIASHQNAVKLPLSPNNKQQKLDLRRGILRKAGFAVGLFTLAIVALVVLLYRPKDPPTVI
PTLEETPITTPSRYTKLETLLKAQDFREADLETDRVMLAVANRQSEGRLRIEDAENFPCK
ELRTIDNLWLKYSQGKFGISVQQEIYKNLGGTKQFDSEIWESFGERVGWRKQGSWIFRKQ
GSWISYSDLNFSLSAPTGHLPQGIRVWIWVVGGTWFTWLDLGRVSLLSRHVECNP
>OHY33899.1
MLIKDNQMADALDLASAILGSGVAITDANFTGNEQSAAVFSAADPSTGVGITSGIVLSTG
KVADIYGPNDSDTTSTDFFRAGDTDLNVLVTPSDTKDAAVLEFDFVPTNSFLTFNYVFAS
EEYNEWVSTQFNDVFGFFVTDSLGVTTNIAVIPGTTTPIATNNVNSSTNPAYYLNNDTSD
FGLSTTPYNTEFDGFTTVLTAAIQVIPDKKYHFKLAIADNSDGIYDSAVFITKGSFASLP
LVPVTDSFLVSSYSSTPLDVLANDLVLEGKQPFIKAFDSSSINGGTVYLNDNQTPDNLLD
DNLLYTPQSGFSGVDSFNYTLGDGSGNAVTGTVYIQVNMVGGVSTLSVGDVSVVENGSYE
VWIPVMLTGIADQTFTVDYSLSPDTATENQDYTPVTGTLTFNPGDSIQYIVVPLQDDDSV
EGNETFFVNLSNVSGGAVLGQTTGTVTITENEKYTFTYFYGNGDSYSGYGFAQLGTHGVG
KLPYNYDNETGTQKGYYFIDSVEDSETGTDGYVYITSYTDADTGFGETTNIWYGGGYSGL
GSEYGYAYNSDGNTFDPYFNSYYEADIIGQEYTFTYFYGNGDNYSGYGFAPLGTYTVGQL
PDYYDNETGTQKGYYVINSVEDGATGTNGYVYITSYTDADTGFGETTNIWYGGGYSGLGS

EYGYAYNSDGNTFDPYFNSYYEADIIGQEYTFTYFYGNGDNYSGYGFAPLGTYTVGQLPD
YYDNETGTQKGYYVINSVEDGATNTKDYVQVTSYTDADTGFGETTSIYSGSGYYGLGSEG
GQAFNANPWTGDTYFSRYYEADLPAALQVQVNLLADDGGKPGEVLADNRVGLNHSFFVQI
QAGDFRPNAAGVVGLNLDFAWNGSILESINFNPSLDITSNFPYQKGGTLASDGLIDDLSG
GSLPEFGTGKAIGVNQLETFALLHFSTQNYGNWWGNYFTTTVNDVSLADDNPYYSLNVET
DQPVYVVTPYAKYNFTYTYANGDSYTGYVYAVEGTYTEGQIIAGTTNETGFAGSYTIGST
VETTLDVWYNNRVYVTSYTDADTGFGETTNISKYWWGGNGDYGLGSENGYAYDSNGLSSD
PFFGADGSGIHKEADILNQKYTFTYTYGNGDSYSGYGYALAGTYTEGQIVGYYPNETESS
GTPGHYTINSVEAGTTSSSVNNHVYITSYTDNDTFYGETTNVWKHWWGGNGDSGLGSEYG
YAYDSNGSAWDGGYFNKYYEADLVKATLSISDNSGNASDSSIKFTTQFPKRINYTESDFV
RPNFADTTKYIEIANNGTGILQVVSLQVSDDVLGVTTNFEDLISSEVDYLLINPGASQQI
KLTYDPSVAGENFSVANGLLLVTNDPYNPQHAIALQGKSTYDADINYDGKVSFGDLGPLN
NAYKNFKEGIYDSTADINGDGDVGLGDLGLLTAQWGSVL
>OHY33900.1
MALQLKLQLFTNNNGLIGDPINEVPFENSFFLQILAGDFRSDAEGLIGFVTDLQWQPGQI
QALDDPFGPKTLVTSSFPLFVGGTLDKTLGLINDLTGGSLPEFEIGEAIGIKKWEPFATL
LFQAIGTKIINVTDFTLTPDLSNLSFADGYINNAPIISDPGIVLENSNPTILTVSDPNPS
DILIFKITGGADQQWFTLNTNGELLFNLGENKSPNYEDPLDSDQNNSYQVEITAYDNFGE
LKFGDLEKTVLGVTTVRMLIIEVINVNETPTNISLNATTVDENIPTNTVIGTFSTTDPDA
GNTFTYSLVGGDTDNSVFSIVDNQLQINNSPDFETKSSYSIRVKTRDQGGLGFEKTLTIT
VNNLNEAPTFPTDTATFSSVENSTSVGTITGATDPDGHTLTYSIEGADANKFNFDTSNRV
LSFKTAPDFEASGSAVGTNVYTVTVTATDGAGLTATQAVTVNVTDVVEVGNPPLITSSST
FFVAENSTVVEAITATDANLEDILTYIISGGLDQSLFTIDANTGVLGFVTAPDFEAPGDT
GTDNFYNLQIQVTDSKNPVTQDLIVAVNNLNEAPTDIILININLYENVPINTVIGAFSTT
DPDAGNTFTYSLVGGGADNPVFSIVGNQLQINNSPDFETKSSYSIRVKTTDQDGLEFEKT
LGITVNNLNEAPTDIILDNFSIDENVPINTVIGAFSTTDPDAGNTFTYSLVGGGADNPVF
SIVGNQLQINNSPDFETKSSYSIRVKTTDQDGLEFEKTLGITVNNLNEVPSRVISGTDQA
DNINATTGQTTVMPGKGDDIIRVNSASVVIIELPNEGNDTVFSSINYNLASLPQIENLTL
TGTGDINGIGNRKDNVITGNSGQNVLTGLQGNDTFVFNFGDSVVGKLDRIGDFQFGKDKI
KVNAVSPSVLTRASNSGASTLSSLVDSVFIDANGAESGNQGLGTNSAALVVSIAQGIAGT
YLIVNDGVGEFNPTTDLVINLTGYSGTLPGVGNIGIANLFV
>OHY33901.1
MIEILLLRLSSLTIFSRSRFTCSVVAIKVEAGNFANSVLKTFSICSKNERAEQAINNRPS
LLSIFGGMP
>OHY34129.1
MGEGGGNIISGGAGADQFWILTDDPTRLQTPNRIVDYTVGTDVIGITNQFASSVGDLTFS
GSDISLNGVLIATLNGVNAAGATFVFANPPANLP
>OHY34199.1
MYWQNFINKSTNRKIFGAALVVAAMTALVYLSRTANELIIAWKFGTGDDIEAFLIALLIP
SLLITVIGGSFNAAFIPTYIQVKQLQGSPASQKLLSGVNGWSSVLLVLATIIMLLTAPVY
LRFVAGGFGEEKLHLTFQLLCIISTKVVFSGTLTIWRAVLNAGERFAYAALTPTITPILS
IFLLLALPSWGIYAVAGGLVGGSILELTAMGIALKRQRISLIPKFNSFDANLRQVAAQYV
PMVAGAFLMSSTTLVDQSMAAMLSPGSVAALNYGNKLVGLPMVLATTALSTAVTPYFSQM
VAAQDWSSVRNTLKKYLFCIFLISIPLTGIFILLSHPITAIFLQRGSFTSEDTNLVSQIQ
SCYALQIPFYIGGIFVVRLISALKCNNILMLAAAINLLLNISFNFILMNIIGAAGIALST
SLVYMFSFLFVLFSLHQKIQCFDS
>OHY34201.1
MQQEKIWQYYQNEGLSSFKNNWGRLEFLARQIRKKLSGKPRGLNIGVGNGSLEKIAISLG
LDIYSLDPDNCAIERLIQELGMDGKAKVGYLQNLPFPDNFFDFIVVSEVLEHLSDDILDQ
SLHEFNRVLKSKGMIIGTVPARENLQDKIIICPSCGEKFHRWGHVQSFDSVRLFNLLSKH
FQVEKIKEKWFFTIPNWKAKIVDCIKITIKITMEKCGISVPGKTIYFLISKK
>OHY34232.1

MAILAVGAGQTYTTIQAAINAANNDDIIVVRPGIYQEDLTINKSVTLIGPYGTFEGIDGF
ENRLGVKPLDPDINAALGVGGLPTANEDFRRYQDDNGTIDNAFGNTQEAWIKGTITVTED
NVTIDGFRLRNENGPLKWNDTPDNFKLLNNYLTGYTANNSPSFGDASINNPTGVVTGWQI
AGNYIGGLLGGGGTGGSIYLAGLQDSNIDDNTFWRPRAAHLYLASLTNVTIEDNKFYHGL
HTGGANFDGFGEFFSGSGYGYGGYGDGYGGYGDGFFGRNYWLELKGDNDQVLIKNNEGEY
NSGGIQLFGETDSPFAFDNITIEGNTFPDNNFINAYSEAPSNGKSGLIPAVMATARLSGP
SGSNLVIRDNDITMDLAQVKFITDHKSSLEVRGNFNGVTVEGNTLTPKNINGGVDIITGL
SLYGSLPGETLIRGNQLLGQDGDPLEASYYGIDLIPTFADYGTYTGDLTVEDNTINSWQV
GVNLRNTNEITGDININGNTFENNAYGVVLDATATTNSINIAGNTFSNNFSNVFDGIDPV
ITMDQVLSYEENQDLGSVLGTVSATDNLPNTDNVGVTQYFISSGNDDGFFTINSSGEITL
TEAGLAAANDFETSLSSFNLGIIVTDGGGLQDTETITLSIINVNEGLGQLPPITTEGAGF
TVGATLIAAIPFDDPDGIPTDISYQWQRLIEGVWTNIPDATEQNYVATEDDNNNQLRVEV
TYIAGGFEKVIYSNNVSISLVLVSGTFDSITGDNNLDISEKEAGVTLTGSVSEIGTTVTI
LFGGQTRVAQVDGLSWSYVLKPNDYNFFAAGSNLFTAIFTRTDGGETGSFTTFQTLTIPD
GILPPNTSNAFDPTQPKGLKSEVIDAAQTLEIDGVSILEISKTVGILGEGESFNDPNIAV
LPIGTRDIFDQGANAGAANYAAFKTEPGTSIQSIYIVPAVEEEGVKKLQVVLADGTVVEA
EIPPDLISPIGDPLAVTISGVQPGGTTTFVLYLSQNVINQLPEDLNLARYVKFNYESQQF
ELYDDFNYDYIFNDVDGDGVRDFGEVYLTVNLTDGDIWDGDGLANGIIVDPGQLGIATDS
GTDNNPPIAIELRGIVAENDPGAFIGSLTVTDDPGDSHTFTVNDSRFEVINVDGNNILKL
REGESLDYEAASNIVLTITAIDNGGLEITQDFTITVTDVNEAPVAIELNQITVIENDPGA
IIGTLTVSDPDGNDGHTLKVNNDTRFEIVDFDGNQTLKLKAGESLDYEAGSVKLSITATD
NGGLEVTQDLTVSITDVNEPPVVSFSFFVPESTTLVSNLTVEDPENDPITLSLAGVDASL
FSISPTGELTFNTAPDFEEPLNADKNNLYKLQVVARDEQNNKSIQDISILVTNVNEAPIA
IDDVLAIIPGSSFGTLNPLDNDSDPDLNDPLTIINKTDGNYGRVEIRDNELIYTLLDATY
IGDDVFSYTIIDEQGLAATANVNVTITGTDIITYPVEILDPEDSLIPDEAGPLSDIVNDI
SFNFLTDYDKVQAKLALQEALSKTEASFTNLFGLYEVDNALTGSVNGVLPEDKSAYAKAA
LSRVVPNFVVRAGGSGDGVNGDVIVSEGKIYAPFVIAHGGNFSGSVQDAVNAFFQVNPDN
SPATAQNYTTLPVAYFSFGSANPDGAAHIKSFGNNVFGFEDLPAGVGVSDYDFNDTVFSF
G
>OHY34268.1
MAFNLNGFNFNQSIIDSQGRVIGTWADVINRANLGMEVMHERNAHNFPLDLAAGEVAPVA
LTAPAING
>OHY34437.1
MYCISALDSKNCQLLISVNLLSIHKIYARAAKVKFLPPYSPDLSPIELCWSKLKQFLRSR
EARTLEALNEAMTSAVNYITAEDALNWFNHCGLFT
>OHY34438.1
MSNTKVDVSFILGDIFAAGDQNIHIDLNAYEIGKNHPVDIGLVADPKLTLARMAEILRES
MTPAQQEAAELRVKMLKNAKQNTIRDQRVKDREDWDNRPLHIVQFAEALGRLLPDNVVVF
DEALTNSPPLSRYLRVTEPGSYFLTRGGFKLFGNFYP
>OHY34530.1
MLQATTIQISCKNNLHNILPDIKELLDTCYPRPPRNVFYLLIEKYCVGFPVYIAIDNFSR
IVGFTYLAINSKGGTLESLAVHPDFRNQNLGSQLVNTLLKENKGVIQITTRIPKFFEKLG
FEYVKTLPDQSHYMININFS
>OHY34532.1
MIILIPTYYKNEKYCNLLLDLLEVNWPNHPELYFLTDGGNINYPNVIKVDNKNWLIVLYK
GLKYLINKYPDLDYIYLVLEDLIPLWSLSVEELTKIENVVINNKLKCVCFPTYPAYWGQE
NEVKLDGITLYKTPEEFDFYSQLQPSIWQVGHLMKICEHALENNLLDAWSFEWIKSEEQH
YVSSYQWSTVFNGFLVRGRVNLAAINKIKLPEGKKLKNQLLKSFIFDLPSLIKYRINRKL
KLIG
>OHY34602.1
MGDNTGGSGTLIAQEGGSYLVLTSGDVISGITPAALRIQTHDGRVHQGRALYNYKLADQQ
QLDKINLVILEFTPNRKYCLTKQILNTAIKQDTAVMASGYSVNSSKIIFSPGTIKQIVSQ

PTFVQGYEIGYDSASATQQGMSGGPIINSTGDLIGIHGKSAFPILNNGYVYADGKKPLVS
EIKEFRKLSWGIPVGSILAQLKPEILARYGLPVPRRNRSVPEIPILPEWLNNIESKVREF
TVRIDGDGNNGSGVIIAREGNTYTVLTSAHVLCKIPHKTSNNHCATKNHTLVTASGQKYP
LDNSSIKLVQGVDIATVKFNSGENYPVATLANYAVENHQYVFTVGEPKLGQTPRLTVGQI
FSKENGLLALKSAGQELKDIDYTTIEDANLGKEYELVYTSVSQRGMSGGPVVDSQGRVIG
IHGKSEGQVVMEETTEDGGVDDRVQLGYNLGIPISTFLRIAPQLNTRPERVENTPAPQLK
SWEIESIRKTIVLVNASMGKASAIEWIERGNQLWLLGRYQEAVTAFENAIERKPAFIHLA
YYGKGLSLESNGNDTEATGAFEQAVKAKFDFSVAWNRLAALNIKFGRLSMALAATNQAIK
LQSMDTSLYSQKFYILLSLTMYQEAIQVMDQAILLNPHHGFYINRGAARRELGDYKGAID
DYTQAIEISPELASVYYERGGARRELGDYKGAIDDYTQAIKISPELASVYYERGGARREL
GDYKGAIDDYTQAIEISPEFASAYYDRGGVRRKLRDYKGAIDDYTQTIKTDPESAFLAYY
ERGGARRELGDYKGAIDDYTQAIEISPEFASAYYERGFARRELGDENGAGFDFQRASDLQ
VSRPRILPKD
>OHY34603.1
MNLTSCLPILFLVPTLVLSSSPQSLAKTTPKQESSCELEREGEFYSPEQLKTIAQRITVR
VIADNSGGSGTLIAQEGNSYLVLTSNDVISGTTPSALRIQTHDGRIHQGRMLYNYNNAEQ
QQLDKINLVILEFTSNRKYCLTKQIVNTEIRQDTAVLASGYSVNSSKIIFSPGTIKQIVS
QPTFAQGYEIGYDSTIQQGMGGGPIINSTGDLIGIHGKSAFPILNNGYVYADGKTPPLSE
IEEFRKLSWGIPVSSILAQLKPEVLSRYGLPIPRRNRSVPEIPILPEWLGNIESKVRQFT
VRIDGGGNNGSGVIIAREGNTYTVLTSAHVVCKIPQKISRSKEDKNKCVEENYTVIAASG
REYPLDNGSIKLGKGVDLATVKFNSGENYPVATLADYPVANHQYIFTVGYPKLGRTPSWR
FTIGQIFSRENGLLALTSTRQNLKSIDYTIQDANVGKEYELVYTSITLGGMSGGAVVDSQ
GRVIGIHGKAEGQVVMEETTEDEAVSIGNRVQLGYSLGIPISTFLRIAPELNTRVDRVEN
TPAPQLKSWEVESIRKAILSVNVSRGNASAIEWVERGNQLWLLGRYQEAATAFENAIERR
PAFIHLAYYGKALSLWWNGNDTETGRGILLSRTIKNHSSKNYSQSYGRQHWWFRNSNCPG
RWLLFGADKW
>OHY34663.1
MNIETLKSEKTKQLPGANLEDQDLSEFDLTAVNLAGANLMGAHLVSANLEGSHLEGANLM
GASLQGADLRANLLGANLMQADLTGADLRGSNLRGANLMGATVAGASLTAAFLSGANLMS
VNFQGVDLRGADLRGANLIGANLKGADLSRADLQGALLNQANLEESDLRGANLAGANLAG
ANLLCAELEAASLNGANLYQACLLGTILETYHD
>OHY34729.1
KSTSQSSFFDCDNIPKFGEKPEGWKASGKRVSRGVYKTSDGFKINADCSSAANILKKVAV
MLGIDLSGISRGCLSQPQKVRLWALQKSPCLPEKLNKNRVLTSFSVKSEKKLI
>OHY34808.1
MNRTTIKNFAIWARNHLKEQVSTRATQLTITEKTITEKTITDQRTFAGGLLSGEQTLNSE
EAKQYQKLHSHIEYLLKQQASKNLDKKFNSNNRRKQLSRVVEKNKRGSDFDGGFNIHTVI
SLGDQIDQRLPQHQPRECFAIALKFL
>OHY34821.1
MNPKNLPLGINTLSMLRENNCVYVDKTEIAHGLIRIPGRFFLSRPRRFGKSLFIDTLKEI
FEGNQKLFEGLYIHDQWDWSRKFPVIKIDFAGGVLKNRQELDLRILDILHENAEYLGVSY
ESTDIPGKLGTLIRKAMAKYGERAVVLVDEYDKPILDNIDNPPIAAEMREGLKNLLPGDK
VVKQLVS
>OHY34844.1
MIPACDGVSFDLYPGQVLGIVGESGSGKSTLLKAIAHYITVDEGSIIYRNRQEQYLKIQE
LAEYQRRWLMRTEWGFVQQNPRDGLRMQVSAGANIGERLLDIGMRNYGQIRQEAIRWLQE
VEIDPDRLDDLPTTFSGGMQQRLQLARVLVTRPRLILMDEPTGGLDVSVQARLLDLLRSL
VRNFNLSVILVTHDIGVVRLLAHRLLVMQQGKVVESGLTDQVLDDPQHPYTQLLVSAALT
P
>OHY35003.1
MKDLIIVSGLSAGVGIGVILTGIANGQASIGVLSSTAGAIMGASVVRKLEDDRNRVTLVK
LEELKNRELTRQESTNLIQQIETLRPTVKQLQQDREREEENLVVLRGKLGNQQAELEFIR

REWEQNQSRLEDITQTVANLTAQKQELENRISVINQENPRLLELEELRRAIEQSRIEKSA
LEGKISGLTSQLESLDSRRQETVSIELELRAKEIDLEKLTTQVENLKNQSQELEQRAADL
ELLRFTYDNIIKQNQDYEVKVNQLRPEIQRLELEKQQILHSIQENQNEYQKVQELREKLH
SLRLSIADESSRLREVETETQTWQGKKAALEESNGHLQEQKQELERTISRYKQEIVEIEN
SAELALQPLKEKLWNNLPQIIKPRKQESEFIADFITRIRSQGLTFSTRTVNAFHTSLKVQ
DISALVILAGISGTGKSELPQRYANYLGAQLLTLAVQPRWDSPQDLQGFYNYVEKKFKPT
DLMRGLYQYGQPDMQDRIVIVLLDEMNLARVEYYFSEFLSKLESRRNHATYLELDVGSLR
IRENERRLQIPNSFLFVGTMNEDETTQTLSDKVLDRANVLTFGRPQNLKLRQENQGAAVQ
HHDSSYLPYSHFQSWVKLPNPNSEIVRSVKYFLNQANEVMEKMGHPFAHRVYQAITQYVV
NYPQVESIESPAFKFALADQFGQKLLPKLRGVMVDEAHEQLEEMGRIIDDINDQPLITAF
DKARKGRYGQFTWQGLVYEDEEV
>OHY35020.1
MYQSEAPPLKTIATNLPSEDIDEELNSTIHPRPPWETLPTMYDLPSENPEEPGLPDEFHD
FQPQLLRETCQSSVYPREKMFIGTDLNLYYDVHHFSWYKRPDWFLVLGAAASETQQDMRL
SYVIWQEGFAPFLIVELLSPGTEAEDLGKTLRSANKPPTKWQTYEQYLRSPYYIIFDRYE
NQLRVFQLSGIKYQAVELTEPKFWFPELKLGVGVWLGKYQGTEGLWLRWYNEDGDWIATL
AETAEQEKQRAEQEKQRTEQEKQRAEQEKQRAEQEKQRAEQEKLRAEQEKLRAEQEKLRA
DKLAEKLAALGVNLDE
>OHY35093.1
MPKTNSFCIEVLVEEPSAEEALKTLLPKLLKGRSRHKIINFGSKSKLLKLLSQRLSAYGD
RISKGENVRIIVLVDQDNDDCKKLKNQLEAIAQQTGLSTKSKPDSEGRFVVLNRIVIKEL
ESWFIGDTEALRKAFSSLPSISPKSGIFRSPDNGGSWEALHRFLKKHGIYKNGYPKIEAA
RRISLHMNPDQNYSPSFNCFKAGIEAFLSQ
>OHY35094.1
MKRLLTVALLTTGTITGSFFLNVYAEPSLTAFQSDSNSYFSIPSAEAANRKGSRYVGGTS
SDGTSRSASGRCNSPSGRAASVRPGGR
>OHY35099.1
MMNQNKTPPRIQYLKIENFRALRKVELKNLTPLTVLLGPNGSGKSTLFDVFAFLSECFEL
GLRRAWDKRGRAKELKTRGSDEAIVIEIQYKEPDYPTITYHLAVDERNGAPFVKEEWLKW
RRGKHGQPFNFLDYKEGNGKAISGELPDEKDKRIEIPLKSPDLLAVNALGQFAEHPRVAA
LRDFITGWHVSYLSADSIRTQPEAGPQEHLNRSGDNLANVIQYLNEQHRDRLEAIFTTLR
RRIPRIERVLTESMPDGRLLLQIKDTPFTHPIIARFASDGSLKMLAYLVLLQDPAPPSFI
GIEEPENYLHPRLLPELAEECQQATARTQMLVTTHSPFFINQLKPKQVRIIYRGTDGYTR
TQLIEDIPGIKEFIQHGANLGDLWMEGHFEVGDPLNQEGI
>OHY35592.1
MGIELGILYDNQKPPTPWLRWWDEKGSLLLTGNERAEQAEAIAIRERLAKEQEQEAKEQA
EAIAIRERLAKEQAEAVAIRERLAKEQAEAIAIRERLAKEQEQEAKEQAEAIATRERQQK
EKLVAYLRSLGIDPEKI
>OHY35701.1
MALIVQKYGGTSVGSVERIQAVARRIHGTAQVGNSVVVVVSAMGKTTDGLVKLAHEISPS
PTRREMDMLLSTGEQVTIALLSMALQEIGQPAISLTGAQVGIVTEAEHTRARILHIETER
LISHLNAGQVVVVAGFQGISNTSAMEITTLGRGGSDTSAVALAAALKADFCEIYTDVPGI
LTTDPRLVPEAQLMKEITCDEMLELASLGAKVLHPRAVEIAKNYGVPLVVRSSWTDQPGT
WVTSNKVQERAMVNLELARPVDAVEFDVDQAKVSLLRVPDQPGVAARLFNEIADQQVDVD
LIIQSIYEGNSNDIAFTVNTLILKRAEAVASAIAPALRNNDGSDEVEVLVERNTAKVSIS
GAGMIGRPGVAAKMFATLAKAGVNIQMISTSEVKVSCLVAAVDCDRAILSLCQEFEVNAS
TGNVSSSNSIYSTVCGVALDMNQSRLAIRHVPDQPGIAGKLFGLLAESNISVDMIIQSQR
CRVIDGVACRDIAFTTNRTDGENAQAKINQVAAQLGWGEVILDDAIAKVSVVGSGMVGQP
GVAAKMFTALAQNQINIQMITTSEIKISCVVSEKEGVKALQIIHTAFGLAGTHKFVVPA
>OHY35704.1
MVSKNFWKPIIHSSTALILLTTLNTAWPLVSLAQSKPQPKVNSASSSLFTDYLLGGGDRI
RVNVFEAPEYTGEYQIPPGGEINMPLIGSIPVSGLTTQQAADEIARRYARFLKRPLISVN

LLAPRPINVFVAGEVTRPGSYSLSLQGTGGNNPGVQYPTVLAALTTAEGVTLAADITKVQ
LRRQVGRSGEQVISLDLKQITQTGRIPIDITLRDGDTIFVPTATDFNVAEARNLFAASYA
ASRTAPRRVAITGQVYRPGSYLVAAGGGNDSGGLPTVMRAIQLSGGITSQADVRNIKVRR
PTRTDKEQTLNINLWELLQSGDLNQDVVVQDGDTIIVPTATQVNTAEVTQLATTTLSPAT
IKVGVVGEVKRPGVTELQPNSSLNQALLAAGGFNDARASSSSVDLVRLNPNGTVTKRAVK
VDLSKGINEETNPILRNNDVIVVNRSVLARTGDTLGAVTAPLAPVFSIISLFRLLGF
>OHY35767.1
MKFGIDIGHNCPPDTGAAGIRSEDKLTMEVGNKVISKLRGLGHQVIPCKPDSASSVSQSL
GRRCDIANRNRVDVFASIHFNAFNGKANGTEVLAGSDAGRKIAQSIVNEIVSLGFFNRGV
KNGSHLYVLRNTNMTSVLIECCFVDSAKDMQLYDGEAMANAIVKGLTGKLPTAPVKPVED
VTGDKDTKDTSILRLQKALNQLKITDRNNRPLTEDNFTGPATSSAVEKFQRVVGIIPTGM
ATQTTWDAINQILAKRTVQGNQTSGPIMRYLQYRVGTTPDGIYGRQTEAAIKRFQQQNGL
TADGIIGPATWGKLLG
>OHY35795.1
MWFQYVFKNWITLFPTPRIFPIRVTSIDPGMVETEFSQVRFHGDREKAKKVYEGIKPLTA
QM
>OHY35854.1
MNERLPLSINQKEIYVDQIMSPDSCHMHIGATVTVRGIFDRQILNYAMSKTIDCHPGLKT
RIYELDGQPFQTIASDTSNHIPFIDFSGHDNSDEQAENYINQEFIKPLTFGENVPLADFQ
LIKVCDDKHIVYAKYHHVITDGWGAAIFFREVIKTYNQILQEGREGQETRDWVITEYIEE
ERKYLASDIFMRDQHYWQQRLNNVSPMIFSPIKQPQELDGKRHSIYIPRHQYDQVDELCK
NVKSNVFHFILSLIAIYVSKHYLKNDVVLGLSLLNRSKNIFKDAIGMFVSTIPFRLEVEQ
EDTIHQLLDKIRYTLRQDYRHQKFPVAEMKQLSGLKATSKQHLFEVFLSYERHDYADNFL
GTKTTCVPLYSQQQKVPLIIYVREYEKTDDVKIDFDYNLSYLDGEAVGEMVRSFETLFTQ
AATNLEISIGDLAICDSETINISQPDSPSTKFFADDTETLVSAFEKVVSQYPQNLAVQFD
GELYKKFLSYTELNDQANRLANYLISQGVKPGSRVGICLERSEQIIVAILAIIKTGSAYV
PIDPHAPSVRRQFIVQDSGMTTLITETSLIAELVTENISTLTIESINLALAKQANTLPRI
SIKPDFPAYIIYTSGSTGTPKGCVVTHKNAIRLMRATEPWFGFNEKDIWTLFHSFAFDFS
VWELWGALLYGGKVIVVPFWLSRNPEKFREFLSTEKVTVLNQTPSAFYQLIHADQSSVQD
IYLRYIMFGGEALNIQSLQPWLERYGDKKPYLINMYGITETTVHVTYRPITRQDLKVRGS
FIGKEIADLHIYLLDEKLSPVADGIPGEIYVGGAGVTNGYLNRPALTAERFLPNPFGSGR
MYRSGDLAKRLPNGDLEYLGRIDQQVKIRGFRIELGEIQAALISHYQVREAVVITDEWEE
EKRLVAYYVPDESSPTAHELRQYLKNKLPDYMIPAAYVKLEVLPLNVNGKVDIKALPMPD
WNLLRVEEDYIAPRNLDEEILCTIVAEILGLQKVGIDDNFFEIGGDSILALQVVARAKNA
GFLISAGELYELATVRYLATKKAIITSGDGVKKLPNIGSALVSDADKLLLPQDVGDAYPL
SSLQSGMLYHSELDPDSAIFHQIFTFDLQIGYSELAWKQAIADICSANPVLRTSFHWTGY
SQPLQMVHEQVELPLSVVDLRGCDNANEQIREWIELEKNHNFDITQAPLFRLQIHRVSDL
KLSFSFSFHHVILDGWSVATLLTQLLRRYVEYLATENLPPLPVTQISYKDFIAQEQNVIT
NHTVREFWLQHLRNLQVTFLPRLSTNTAKTTAASHQKRQLKRMSVLVDTKLAEKLRQITK
NLGVPLKTSLLALHLRVLSFITGQKQVVTGNVINARPETSGSENLLGLFVNTIPFRLELP
QGNWLDLVRAVFRLETEILPHRTFPLAEIQRVLDQRPLFDVGFNYVHFHVYEGLLNLPQI
QVENVDIFEETDFPFLTEFCLVPGSAALQLNLIYDTQQFADTQVDQYANYYQAAMFDMVT
NPQTPYHRRSLISSQERQHLIQSANQNLKDFVSSQTLVSAFNQTVAKHANKTALVYQQTS
LSFGELEIQANRLAHYLQAKGIGPETLVGVCLERSEQLIISILAVLKAGGAYVPIDPAYP
SDRLEFLFRDSGIMLLITQRSVISQLPECGAEIIILEDIAKEIEGNNSQTPAVNILPENA
AYVIYTSGSTGKPKGCIVTHANVIRLFNSTTTWFNFHSEDIWTLFHSYAFDFSVWEMWGG
LLYGGQVVVVPHWTVRSPKEFLQMLATHRVTVLNQTPSAFKQLISVVRQKPEKLSLRYVI
FGGEALELADLQPWIDLYGHTQPELINMYGITETTVHVTYRPITQKDIINNLNHRPSVIG
QSIPDLELYILDENLDPTPTGVTGEIYIGGAGVTRGYLQQPGLTAQRFIPHAHRPGSRLY
RSGDLARYLPDGEIQYLGRADQQIKIRGFRIELEEIQLVITSHPDVKQALVVCQKSPTGE
NRIVAYVIFSGVAQPQNDLGKFLKTKLPDYMVPSVFVPIETIPLTINGKVDYAALPVHNW
NSIKKDYIAPRNDREATICSLMASLLKLERVGVDDDFFEIGGDSLLVTQLAISLRQTYDT

EFPLPELFTHRTPGEIALLVGDESPALPEIEIPKASRTRRSVTLTDDGILSKY
>OHY35864.1
MTNFSQADDIDLLGLLLEEEGIELEIKEVVPRVGSTEAPTSFQQRRLWFLYELEPTSSAY
NICSIFDLKGTLNITALRVAFKQLQQRHESLRTTFMDVDGEPWQKIHANSATELRLEDWS
NDRSEDKIPEIIAEIARHESDHQFNLQTGPLIRAQLFKIESKQHILSINLHHIIADAWSV
GIILEEIAMLYQAEISKTPMALPELKFQYTDYALWEKGNFQNSNILEKSLTYWEKQLAQL
PTLQFPLDFPRPRLQTFRGGLIKFEISKETTNRIRNFIVKEGATLFMFLMAVFQTLLSRY
TGQEDIAVGTSIANRPSDSENLIGFFVNMLVIRTNLADEPNFNSLLKTVKKTILSAFEHK
EIPFETLVEKLNLPRDTSRNPLFQIAFTLLNAPKPQFGTGDLEVCILATQEAARFDLELF
ITETEDTLNGAVSYNIDLLKRETVERVARHFCQLLDSVLAQPEIPVSRLPFLLSEEIAVL
APSQPTQTFPVHFCLHDIFTQQAKLRPQQTALIFGQERLTYLEVNERANQLAHYLMHVGV
KPEARVGLWLSRSLDLVIGIIAILKAGGVYVPFDPNYPKDRITYMLEDSQIRVLLTHSEF
EAQIPFEFTNNTNNVSNTNNNILTIFIDKYKSEFTQVVTTEPEVLILPDNAAYIIYTSGS
TGKPKGVVVTHRHVVRLMLSTEKWFKFNAKDVWTLFHSCAFDFSVWEIWGALFYGGVLVI
VPYLISRSPEEFYNLLCEEKVTVLNQTPSAFQQLIQAESTLCREGELELRYVIFGGEALD
LASLEPWFERHDDQFPLLVNMYGITETTVHVTYLPLTFKDVKKGSGSLIGKQIPDLSLYI
LDRHLQPVPIGVVGEMYVGGAGVTRGYFHRPQLTAERMIPNPFATNDLTSVRLYKTGDLA
RFLDNGNIEYIGRNDHQVKIRGFRIELGEIEALIKSHPEVRDALVIAREESKEDVRLDAY
IIPINQIANTETLTQEQTQEWQYTFNDTYNITSGETEEDFNIIGWNSSYSNQPIPGVEMR
QWLNNTLLRIQSLKPRKVLEIGCGTGMILLNIAPQVESYWGTDFSQAAINRLDTIVKNRS
LKNVNLLTREAIDFSEIPTGYFDTVVINSVAQYFPSIEYLQQVIKSVWQLLKTGGSLFIG
DNRNLSLSNYFYASVAYFQANDNTDCETFKTQVRRIAKKENELILAPHFFTDLRKSFPDL
TAVEIQIKSENNENELTKYRYDVILHKLGISTEQPPEIIWRDWETDNLQLTDLKQQVIEM
RSIGWHSIPNGRLSKDAAIYQWMLKNSHENEQKTIGELRTVLNNIHEPKGFNPADFYAIA
EEIGLEVSISYSPGKVDCFDVCFYPAGSGKSMAPSMPIVNDLLGRDNHPSWIDPLKNRLT
KLLISQLKQRLEEKLPEYMCPSSFMILENFPLTPSGKLDRRALPIPDRDLIINQQSLVPP
KTPTEYKLSQLWMDVLGIDKIGVTEDFFHLGGHSLLATKLVSRIREEFNVALPLRSIFEY
STIARLGDEIDCLIDVNTTKTGPEDIIPVSNRENLPLSFSQSRLWFLDLLEKENAAYNIS
VAFRLEGDLNVDALQESWQNIIQRHEVLRTTFDNVQGSPIQIVHDWSELKLTIRNLSCLD
FQTQQETLRKSIQEVVITPFNLNQLPLLRIHLYQLSADVSVLLLVIHHIIADGWSLGVMV
KELSLFYTAICQRNIPSIPPLSIQYGDFANWQREVFQKTQLPIQLAYWKQKLTGANQILE
LPTDYPRSPIPSYQGSAVNFAINPQTTQEFKKLCESQGATLFMGLLAVFSILLMRYSGQE
DLLIGTPIANRNRKQTEDLIGFFVNTLVIRNNLSGNPNFINLLSITKEETLQAYAHQDVP
FEKIVEEINPQRNLSQHPLFQVMFVWQNAPMNKLELPNLQLSPWRLEQRLAKFDLTLLMT
ETEQGIDGTWEYRTDLFAPETINRMIGHFETLLKGIIAEPQKPITHLPILTSHEKNQLLF
QWNQTQFEYPLYQQNKCLHQLFELQVEKTPNNVAVVFKNQSLTYFQLNQRANQLAHYLQS
RGVRPDVLVGICMERSLEMVIGLLGILKAGGAYVPMDSNYPRERLDFMLVDAGISLLLTQ
ENQVTTLDILPPHQIICLDKEWQVIAQEDTHNPSTNLVVENLAYLIYTSGSTGQPKGVMI
SHSAICNHMLWMQKTFSFGEREKVLQKTPFSFDASVWEFYAPLLTGGQLIIAEKDGHKDV
SYLLKLICEQQVTVLQMVPSLLQMFLEYGEIENCHSLTHIFCGGEALPVAMVENLLSKLN
VNFHNLYGPTEACIDATFLSFTKENNHYIKQNMLPIGRPIANTQTYVLDAHLQPVPIGVP
GELYIGGMGLARGYCQLPQLTRDKFIAHPFSDNPDSRLYKTGDLVRYLPDGNIEFIGRID
HQVKIRGFRIELGEIEAVLTQHPNVLNAVVVISGDSSATNSLIAYCLSTEKQFTSSGVLR
DFLKEKLPDYMIPNSFIVLDHLPMTPNGKIDRKLLAGLNINRNFDAHQHVSPRTLLEYKL
VEIWEEILQVSPISVTENFFDLGGHSLLAIRLIAAIEQKLKCNLPVVSLFREGTIEKIAL
LLDQDHQKASNHSDILIPLQTQGDLLPLFLVHQAGGYGLSYSVLAEKLAVGMGKKLPIYA
IQSPGLDGKQSPLESIEEMANTYIHTIREIQPHGPYLLGGHSLGGLIAFAMASQLEAMGE
QIERVLIIDTHPPMPTDETIASLEDNAGIICFMVEQIALFFNKNVTINYQTISSLDQDSQ
LDYVAQTLEQHNLIPPNSGNSLIARLIKVYKANLRASVVYQPPVNRSNITLFITPSLAAK
FPNDPTVGWQKLTTQKVQVCRVMGEHQTMLKEPEVENLVTEIMATLVNTP
>OHY37328.1
MTTISFPSGAVCIEQNNSNNYGYTITAHCSAATVSGESLSLKEFRGQLVSVPGSGGDKCE

GQIELTLRDESNGVDVKYDVQLAENFFMRLITSNGSYIKSGAVEINRSSKEGGSFGRPRV
FTMDMTINLSNLSFADAEDM
>OHY37808.1
MSKDVDVDVKSKEAEEKPAYELLEKIQQEYFQVRNRVERTTNQPEKYELQSDLIRKLLSI
LGYEYKIDFKLLDDDRIVPIVAEFKKNSGEPYMWILSAFNLGIETSDILSLEINQEQINE
INSTYLLNVQKGKKSSREPQVVQGNLEELLNDAIFAQNAPPKWVILINMEQIVLIDRYKW
NASRLIRFDLAKLLEERDKKSLMVTANLLHRESIVLKDGTCLLDIIEEKSYRHSYSISEN
LKFALREAIEILGNEAIYYWQKSGKRVFDNQKQKEQGVVEIDPQQLKKECLRWIYRLLFL
FYIEARPELGYIPMGSDVYRQGYSLEALRDVELAKLTEEDEENYYLDWSIRLLFELLWSG
YPLDKQKQLTTENPSLADTLIHKTFRLPSLQSHLFDPKNTKMLNEVKFRNVALQKIIRLM
SLSTEEGKRGRISYAQLGVNQLGEVYEGLLSLSAFLAQEDLYEVQPSNKKESGDESEEQE
EEDLETNERENVTRGKTVKGKENLKQDLEVAYFIPEHRMDEFKQEELVIDWEKGEFRKHA
KGKFLFRLAGRDREKSASYYTPQSLTKCLVKYALKELLEGKQADDILELTICEPAMGSAA
FLNEVIDQLAETYLERKQKEKGELISHEQVMREQQKIKMLLADRNVFGIDKNPIAMELAE
VSLWLNCIYASEADKDNIFIPWFGFQLQCGNSLIGARRQIYYLNNITEKKKSKSSKNTKW
YEQEPEQLSLGKNLPKGGIFHFLLGDSGMSDYTDKIIKKMSGENLKKIDNWRKEFCQELT
DEQGSYAHTLSERIDKLWHHYAQELKRIRKRTTDSLNIWGQEEEKKQEIALEKKDKIHDQ
EKLAEGIENATSYRRLKLVMDYWCALWFWPIEKAEELPDREQFFCDLAIILGETEMLLDS
NRQLSLFPETQTPHEGEEFINKWGFVNLNKLKQKNIRLQIVDEVVNNQRFFHWELEFADI
FWERGGFDLILGNPPWIKIEWEEKGILGDYDPRIAIRNLSASELAKKREELFDRHPGLRA
AYIQEYEEISGTKNFLNAEQNYPLLKGSPTNLFKCFLPQGWRYTRAGGVSAFLHPEGVYD
DPKGGRLRRAIYQRLRYHFQFQNQYILFPIGHRVKYSLNVYGSEKLPEFTTIANLFTSKT
VDECLEHDGKGEVKGIKDESNKWNVQGHKERIITVNRERLNLFAQLYDDEGTPGEEARLP
TLHSQQLMGVLEKFAAQERRLGNLQGEYFATEMWHETNSQKDQTIKRNTQFAQGAEQLIL
SGPHFFVGNPLNKTPRKVCKTKSDYDVIDLTQIPPDYLPRTNYLPDCAPRDYLDRTPRVP
WGEQKPVSDFYRVINREMLSQSGERTFITALIPKGIAHINTCISIAFRETTKAIDFLGMG
LSLPVDFFIKTTGMGHANQNLLRQLPFIGDDFSLRYSLHIRTLALNCLTKYYGDLWEENW
HNEYTQESWSKPQDPRLNNNFFGQLTPFWQRNNALRTDYERRQALIEIDVLAAMSLGMTL
DELNTIYRVQFPVMQQYEKDTYYDMNGRIVFTINKGLVGVGLPRKGNPKTKSQGWEDIKD
MTTGPVEVPIVNQTLSNNPANQSIIYQAPFVKCDRIEDYRKAWDYFRCLD
>OHY37831.1
MLRLLIYSVTSGFLVLLNTGIVDLANAQETSGRYVKTFVGNQSVTIDCIDETYDKKGDGS
VWISVYDYHQRYSTLRKIIQSEYVRLCGRMLNSDSIRAGLNQERQRKAWERRFQKERQRE
REFQREQQRQQKREQRFQKEQQRERRRKRKF
>OHY37848.1
MDICLRYGVVVMGMELKMWKPGKKDPLPQGLQQLDKYLAGLGLDTGWLVIFDRRPDLPPI
EERTTTEEVVSPGGRAIIVIRG
>OHY38788.1
MVQELKRPRQIASFPETAPAANPVFFRTYSRRTQTGLRESWSNVCDRTLKGLVELGKLNL
EETALLEKMQLQMKALPSGRWLWVGGV
>OHY38887.1
MKIISVAAIQKREYWIEEIRKLSGNFGDDSERLEKELSYEIQNEGLETLLNHLRLSGDIP
ESYGHDTSEEKQYSKYTDALLSELYKALGLKSFVLKERADAADVEVVAKDYSFVADAKAF
RLSRTAKNQKDFKVQAMDGWKRGKPFAMVVCPIYQLPARSSQIYEQASTRNVCIFTYSHL
AMLVSYSMIEGKSKAEELIHEVFKVIPALNPSKEASAYWLAVNKTMLGFSPQIEKLWKIE
KMAAIESIAAAKEEALKFLANEREKIMRMSHDEAIKELINVHKIESRIRTIKTVSDNGIL
GIR
>OHY38888.1
MEDYINKILNEDSIKGIKKLSDNSIHLILSDIPYGIGVEDWDVLHDNTNSAYLGTSPGQE
KAGAVFKKRGKPINGWSEADREIPKQYYEWCSSWASEWLRVMKPGGSVFIFAGRRYAHRC
ISALEDAGFSFKDMFAWMRQRAPHRAQRISVVYDRRGDTDSSQIWEGWRVGNLRPTFEPV
LWFTKPYKIGTTIADNVLSHGVGAFNESAFMKYEKSPDNILTSGFMSGETGYHPTQKPVR

LMQSLIELATQEEQIVLDPFCGSGSTLIAAKLLNRKFIGYELNKEYYSIAEERLKEKKEV
QLSLL
>OHY39335.1
MQRGATSFGTSTGSVTFATGSSVVILPIDPSSDVVSDGNETVALTLAAGTGYAVGSSGAV
TGIILDNDVAPGTVVRGSVAIRLVCWVSCLNPTYDYLRQN
>OHY39387.1
MICCLNPDCQNPQNPHGSKFCQSCDTPLVPLLRNRFRIIRVLSDEGGFGRTYLAEDTDKL
NDNCVVKQLAPKFQGTWAQKKAMELFSQEAKRLQELGEHPQIPTLLAYFEQDKCLYLVQQ
FIDGNNLLQELQLRKHYKDSDIQSLLLDLLPILKFLHSRGIIHRDIKPENIIRRKRDGRL
ILIDFGSAKQLTVAVQKKYGTSIGSHGYSAIEQIRDGKAYPASDLFSLGATCFHLLTGLS
PFQLWIEHGYSWVKNWQDCLNNSRSAELIIIILDKLLQLDLKNRYQSADEVIKDLSKKNVY
GSKKFGIYLKKGKHEKHKHTILRNIFLILVTISVVGGLGYRNLGQIQTAIFSQFNPLLIP
SNKSSTSPEYEQISGRTSSIRTNKVSLLKTITQVDKSLAVVAITPDGNIIVSAGHKEIKL
WNSKTGKQIISLPGHTQNINALAISPDGNNLVSAGDDKTIKVWNLQTKKLTFNLVGHQDS
IQALAISQDSKILVSAGDDKTIKVWSLLTGKFLKTLLGHNYWVRSLALSPDGFTLASGSF
DKTIKIWNINQTSGQKPTTLLDTTSQTVTSLAFSPDTSTLVSTSRDRQIKFWDIKNKEII
FASKKQNVTSVIFSPDGKTLISKAKSCPDCSEKISSVIKLWDVSTKEEIYALPGNTKIVT
SLVLSADGKTLVGGTEDNKINIWEISP
>OHY39512.1
MIKIKTKKTVINEEIAKVKKKRWNAYYFVGNLAALWCKGFSHYFEGSGGEKQRNFADLRK
VGLDTLTG
>OHY39803.1
MVALLLDLLIRDRHQNTVSLNDVMVEMWRKFGTQETGYTTQDLKEVIEQVAQIDLSDFFK
QYIDGLEHLPFLKYLEPFGLALVEQSESAPYLGIRVEPENGQETIKFVEAHSPASTAGLD
VGDELLAIDGIKVGINQLGHRLQDYQPKDTIEITVFHQDELRNHKVTLGKPRPHKYQLRP
VENPSTTQKNNFEGWLEVPITTIQ
>OHY39892.1
MDAYQHPEALEALQKEQLVQIARHIGLETSLRGKAKTAPALKAEIRNYLNYCEQLTIPEV
MPLSG
>OHY40959.1
MGLFDESTLKCKAEVWRSLINRWRDIRGGYNKCEEYRPLAQLFERFKEYDLAVYFYQVSD
GVVKKDLFYEIPPRSRNLRLSEKDVLGLKIKTKLNLVDNFGLRIGDMINSFINFSRYDMK
IPQVMIVSLVFFVSGLSLGNIFPIRNSLNEQTTVNQSKPSTNPTEEPDVENVNNDWNKTT
KAFQEIISDLSNNIKKENTPKIIKDLYPNLHTHIFPLISPAQPNKQLKEELIFSLILNEM
NITQYQKFSYAKLGNITDEQKIKIYQAIKKFQTSNQSKVKDGYFDFENPKDTSLNKLKER
VRQKI
>OHY41055.1
MVGGLETLNNQTLSLIVIEGVSHAVHDKLTQAGFIKVFYNPYTREILSNPVASLKSGNNI
YIRNQKFVSDKLAQSPAYFIHNMGLRI
>OHY41057.1
MTINKIIDDWYLENLICPQDNTKLNLVGNDLVSQSGNTYPIVNGIPIMLIDDVPQTIDLA
NTSLADSKLKNDSDPYFINTLGISEYEKNQVKKYTENLQVQERIIGIINTSVDPVIEFLI
GATNGILYSQNIGNLKRLPIPDINLPVATRKQFNL
>OHY41059.1
MSNSTDNTHGILIIGFTTHPHDVGSALVLAAQSLNLPLAVANISDYESSLNHLWGKVFYR
IAQRRTLEWFDFQRKILDLIGKFQPQLVIVTGIIPLGQDIFHTIHNYQGKIVNYLTDNPW
NPALGSPIFRDNLRLYDCVFSTKTAIIPQLLRVGVRNLFSHF
>OHY41062.1
MKTVVIVAPHFPPSNLTAGHRCRYFATHLPKFGWNVKVISIQPQYYEEKLEPQLTELLPP
ELEVIRTTALPTRPLRLIGDVGIRAFWWHYQALCKLIESGTINPKTDLIYIPIPSNYSSL
LGYLIYKRYGIAYGIDYIDPWVNTWPGCEVWLSKAWFSYNLGKILEPIALRHVRIITAVA
PGYYEGVLKSYPWINPDCCFAMPYGVEPEDFKYIESHPRATYLFDPLDGDYHIIYAGAML

PKAYSTLEALFTAINQIKSVNPQLGKRLKFHFVGTGQNPTDPESYSIKPYAEKYQLLDTV
TEHPARIPYLDVLNHLQQAQGVLIVGSPERHYTPSKVFQSVLSRRPVIALLHSESTAVSI
LNQVNTGYLVTFDERKPAHVCIDDMATAIEKAVTNPDNTEQINWDAFYTYSTVAMTEKLA
QAFDLTLSANVR
>OHY41063.1
MRILITVDPEIPVPPQLYGGIERIVDILVRGLRKRGHDVGLVANGKSTTSASELFPWWGR
RSQNQLDTVKNTILLWSIVQRFRPDIIHSFSRIFYLLPWLGSPLPKIMSYQRDPSYRTTS
WAVKLSGNSLKFTGCSRYICDLGKRSGGNWYPIHNCVELEKYTFNPTVSQDSPLVFLSRV
EKIKGAHTAIQIALKTGHPLIIAGNHNPDDPYWQREILPHINNNGQIQYIGPVNDQQKNH
LLGQAKAMIVPIEWEEPFGIVFAEALACGTPVISCPRGALPEIIRPGVDGYLINSIEEGC
QAVQKLNHIHRVNCRQRVEEYFASDVIVEQYEQLYKVHLS
>OHY41064.1
MLTDTIYQSLKSVPSLQRQFKRVVLKLPHRQRLVNHFQCKLWVDPAELHGFYLYYEREYD
DYIFEFLLTQVRNLQSKYHRAIDIGANIGIYTTFLAQISNHVDAFEPEKQVLARLRKNLS
LNGINNVAIHEKCVGQFSGNVGFTSPDKHNQGVGSISLESGIGQVPCITLDDFLGGVLSE
SCLIKMDIEGGEWLALQGAREALTQRKAPVSILLEVHPEEIERLGGTVKELKQLLESMRL
EVSALTPQGLKPLPENGNRAQVLNPAIKLYNLCVLPR
>OHY41065.1
MPSLKQKVGAWLIPKLPINRHVFDHIRLELNALRVRGLHSFHPGIRRKVKQLQSQNNLLV
NIGCGPFGEDGWVNLDLFTHPNLTLVADTRRRLPLADSSCVGIHVEHFFEHLNPEDECPA
FLSECRRCLQPDGVLRIIVPDAELYIKAYLSPGWDMFNAIGCGGDQPETAFNSKMQALNH
VFIQGWEHYGGYDTQSLTLMLQQAGFTKINRCGWRSGDFPVTPIDREQHRPYSLYLEAIL
>OHY41066.1
MVLYLLNRHVNPITAALLGLSFLLDPIFVASSRLRVDCWAIALCLGSCYLLRVSLKLIQN
NQRCFVNIGVAGSLSAAAFFVWPSVLIIYPLILLELWYVLVAVIQGKKIWKDACQLLLVF
GTSFLLICLLLIIPIIGNLDMIISDFSRATRAVSKIGIGEFINFATSFIRGRSLVLPILA
SFALVYYVTEKSLAITTLLALSIVISTGVTPDRCLYAFPYLILLVSNLYSQPTKSIKSRDL
NPRIKAAFLLVLVGYAVTISLIIRPVIGLSNSSGRNTNLLVNAGKTHIGEGEYKIWDSTW
QFYETGRLLKWKMYQPFWAFWGIEDNDNSHRFIDNLDHVILQHKSEYKIIEKRGFRLQKT
IRLDSGNQSNEGVFLNHLTKSLNSQTAYGPYDLYSR
>OHY41067.1
MLLTNKLNQLRHNFWLIWFLILLLVALIHSLTLTVSPPIMGDEVQIIEYGRAFLSPNTDW
SMNWDVANNRPFTSLFYLGCLLQEIAFSVANFSIFGSRFMGLKAIRIKYSSNHIGIILIK
SPC
>OHY41069.1
MQLLLSSPGVGPFIQQTAKALYEASILHSYATTFVSYPESTWQKSLCGMAKIFKFNLERE
LQRRAITEIPLTYVHNYPWREILRTISSKLDQDGRLTDKLWEWSTKGFDHWVANHHLGTV
GGVYGYETACLATFRAAKKQGLATIYELPAPEHDFVANILEQELTLYPQLRTSYYQYTQQ
LQQQRTEHGRQEWELADVIIVNSQFTKNSYAAAGLDMDKVRLIPLGAPPVREKLPNNSIN
SDKTMQFLWAGTFSIRKGAHYLVSAWQKLQPQEARLKVFGAMGLPENLLINLPKSIEFFP
TVPRTELVKIYQVCDVLVFPTLCDGFGMVITEALAQGLPVITTNCAGASDLIQDGVNGLI
IPPRDTEALAAAINWCLTHPSQVKEMSTAALKTAAQWQWSDYRQSLVANIQAGLKSAGYQ
>OHY41070.1
MTDPDKIQSDYYTQTASSYDDMHGDPEHDVALSYISSLITGLNISNILDVGCGTGRGIKY
FLSKHKNLTIKGVEPVEALIEIAVNKNHISHQLISKGNGENLPFTDQSFDAVFELAMLHH
VPHPNLVVSEMIRVARKAIFISDSNRFGQGSYLARWVKLILYKLGLWKWADLIRTQGKGY
TITPGDGLAYSYSVFDSYDLLAQWADRIILIPTVPFSSKTWFHPLLTSGHILMCAIRE
>OHY41610.1
MSVKASGGGSVARPQLYQTLPVATISQAEQQDRFLGRGELTELESYFASGAKRLEIAQVL
TENSETIVSRAANRIFVGGSPMAFLEKPQEPEMALVGAGTMNVQEGMKLGTITYVESRGG
FFESLRSIFNTSAGGPTPPGFRPINIARYGPSNMSKSLRDLSWFLRYATYAIVAGDPNII
SVNTRGLREIIENACSGEATIVALQEIKVASLSFFRKDPVATEIVTQYMDVLLTEFQAPT

PSTKVRQRPSADQQGLQLPQIYFNAAERRPKYVMKPGLSATEKTEVVKAAYRQIFERDIT
RAYSLSISDLESKVKNGSISMKEFVRRLAKSPLYQKQFYQPFINSRVIELAFRHILGRGP
SSREEVQKYFAIISNGGLPALVDTLVDSREYSDYFGEETVPYIRGLGQEAQECRNWGPQQ
DLFKYSAPFRKVPQFITTFAAYEQPLPDQHPYGSGNDPLEIQFGAIFPKETRNPSSSPAP
FGKDTRRILIHQGPGINNQLSNPKARGLAPSSLGAKVFKLDQLPGTIGKKAAKGASVKFS
ESSTQAVIKACYLQVFGRDVYEGQRLKVAEIKLENGEITVREFIRILAKSDLFRKMYWTS
LYVCKAIEYIHRRLLGRPTYGREENNKYFDIASKKGFYAVVDAILDTVEYSESFGEDTVP
YERYLTPAGLSLRQLRVGTIREDINKVEKEETPRFVELGAVKELRTQPSVDFRINQGVSK
QREQTKVFTLFATDKVAVQTLIGAAYRQVFERDIPPYIIQNEFTDLESKLGNGEITVREF
IQSLGGSGLYIKEFYTPYPNTKVIELGTKHFLGRAPLDQAEIRKYNQILATQGIKAFINA
MVNTAEYAESFGEDTVPYRRFPTLPAANFPNTEKLYNQLTKQNDDLVVPSFETLQPRIKS
ENTPLLGNAIADLAIKAKQMDKSKPLFIELGRSFNDGRGQSVEVGVGTSRRKPARIYRMT
VGTNKPEINQVINAIYVQVMDVFSGQIPDYIRRTDLDSRLRNGEITVREFVIELASSEIY
RKRFYTPYPNTKVIEFLFRHILGRAPATQSEIRQYNKILADSGLRTAVETIVNTGEYSRY
FGEDVVPYNRFPSLPAGNYLGSVKAEADLVKQSWSSLSPSVLTGRGTNR
>OHY41613.1
MSESLPLRDRYLALIDEIVSNTLKGKISSVYQIYQMLLNGISLDTGEVFELALSDRTRDI
QLQVDNEKDETKKGKANRSLRAIKTVQSQWQRWEEQNQATEVITSAVREIISASPEENLT
EFLRYLDPNQKHALNLSQLQQIAKSLDQFSTVNRDIKEICDWC
>OHY41660.1
MGLDLNLSGKTAIVTGGSAGIGLATAKGLYKEGVNVVIAARSPSTLEEAIQEIKNAPSPN
SQNEVISINADLTKVEDIEKVVLGTSEKFGRIDILINNAGSARAGSFLDLEDEVFLDAWH
LKLLGYIRFVRLVVPYFKQQGDGRIVNIIGGAGRTPRPNFLPGGTTNAALLNFTRGISKE
LAQNNIRINAISPGLTDTRRAKTLAQQNAQSLGISVEEYNLQAVKGIPLGKIVQPDEIAA
LALFLVSDLASSITGTEIQVDGGATPGV
>OHY42057.1
MINLEQTLNEQQNLSELCSDTPPKLLNTCEVFAPNAYYGNDLIYKLYADLPVKYPLKAVL
PHAPDFYVNSRDKVWESELVNSLPEIWCYGNRSTQIYSQALKNIKIDKKVVPSASPFLYL
LKLIQPDSIIPERRGTIFFPTHSTHHIIDNTSFEILASKLDCLGEEYRPISVCIYWRDFN
LGRHLPFEKRGMKIVSAGHMYDPEFLFRFYHLCSLHKYSCANDYGTAILYSIKSGCSYFH
LDADDLYSNTVKKINSNVCLPDDPASYVSTEVTSLEEKINEVKTFRDLFAVPRQELISNQ
IEFVNELLGNQSLKTPTELRDMIIAAEIKYVATAEIAIRDNNLLPRLLRRLRGYPLYKLL
RKN
>OHY42059.1
MKTRIHIISASFNAGNCIGKLIQSLESQTDKDFVWILVDGGSNDDTLGKAEKIQGIAKKS
CWKIRDNNQADK
>OHY42232.1
MQYRNLGDSDLLVSELCLGTMNYGKQNTLEEAKNQLSYAFDRGINFIDTAEMYPAPTCSE
TQGKTEEYIGKWLVTQPRDQVIIATKIAGPSGGQTLPITWIREGKNRIDRKNIQEAVEGS
LRRLQTDYIDLYQIHWPDRYVPLFGAPDYDPNYERETVAIAEQLEAFAELVKAGKIRYLG
VSNETPWGVCEFCHLAQQLGLPKIVSIQNAFNLTNRVFQINLAETCRFHNVGLIAYSTLG
FGHLTGKYLSQTPPRSRIDLFPKFDRRYRKPHFQEAVQAYVDLADKNGLTPVQLALGFVR
SRWFVTSTIIAASTLEQLQENISCVDVVLTPEILTEIDQIHARYPNPVP
>OHY42509.1
MNDLENIPNHHYEPIKTILRVIDFSQGQFSLIFLHCNDAELREQVAAQLRERSPNKIEEI
TLPHSAISLYDNISLTLDRHPEVLMVFGLETVNNLDSILQFSNQIREEFRKNFTFPLLIW
IDDQILRKILRVAPDLETWGSIIGTPNYYNGENQVKSLAQIHRFLAEAALENQQWSEAKK
QAQYAQEILTSVDHIILPDDGLYHLLIARSQIGLGETSTAIKSLEIAKTHSAPPQDPQLY
IEILKTLASLYFDHGNYLEAFYVRQEQLQVEQQYGLRAFLGSSYLNPQRPIMNGTNGSNG
RSMAFGREEDIKRLWQRISDNEHPLVVIHGQFAVGKTSIIQGGLVPILEQELIDDRQLLP
IILREYTNWLEELWEQLLNKLENKLQSTIAIETFNGLSPQERIIKLLNISGDKNLLTVLI
FDQFEEFFFVANNLEQKRTFYQLLRSCLDIPFVKIILTLREEYLHYLLEIDRLVDLRVTN

NNILDKTIRYYLGNFSKAVAKRIIQTLMVKDQFELQMELTDQLVEELGDNLGEVRPLELQ
IVGVQLEKEGIDTLEQYEQFGGKQKLLEKFLQDVIKSCGPENESTAQLVLYLLTGENGTK
PLKTQAEIITQLSVESDKLDLVLKIFVGSGLVWLVRESLRDRYQLVHDCLVQFIRYQYAR
SYYTQLSAQLERIQAELRQEREAEEQAQLVTKLEEDALIALDQFQTDPLLSLVTAVGNAN
LLKSIVQNNPLDKYPTVRPIYTLNTILDTISDRNIIKGHEGGITSVCFSPDGQSIGTGSW
DKTIRLWNLRGENIQQFRGHEGGITSVCFSPDGQSIGTGSEDGTARLWNLQGKNIQQFRG
HEGGITSVCFSPDGQSIGTGSEDGTARLWNLQGKNIQQFRSHEGGVTSICFSPDGQSIGT
GSEDGTARLWNLQGENIQQFHGHEDWVTSVSFSPDGQILATTSVDKTVRLWNLQGETIQQ
FHGHENWVTSVSFSPDGKTLATTSVDKTARLWGLHRQKIQEIRGHEDWVTSVSFSPDGQN
IATGSRDNTARLWNWEGRLIQEFKGHQSRVTSVNFSPDGQTIGTGSADKTARLWNLQGDI
LGEFQGHEDWVTSVSFSPNGQILATGSRDKIARLWSLQGDLLGEFPGHEDWVTSVSFSPN
GQTLATGSADKIARLWNLQGDLLGKFPGHEGGVTSVSFSPDGQTLVTGSVDKIARLWNLN
GYLIREFKGHDSGITNVSFSPDGQTLATASVDKTVRLWDLKGQLIQEFKGYDDTVTSVSF
SPDGQTLATGSLDKIARLWPVRYLDRALKDGNTWLIDTNF
>OHY42533.1
MATDITKILIGETTALIPQAGASTAPEHLLDGGSGDTDFPYGKFKAMATVGEIDPKTGHV
LTGWPDGQAAWLLDEDTIRVAYQSESYATFVKETYGWKMDSGVSFTGSHVHVIDYNRAAF
ADFLNNNSPASKMFEGAGHLFNTVYNVFGEIVDGKNADPKDLSAKWGNQTGADGTLYEFD
ERYRLTQGDWFFHSFCGAYYEEANKYGNGIGFADDVWLMGEEWNIGQMYSSRGGDKFFTD
NTMGLASMVVDIANKTAYTVPVLGQSGYEKILPINSGHKDYVVLVMSGYNLEVEPAPLKI
YIGKKNVDAAGKAMNYNTASARDAFLGRNGLLFGQLYGMAATNDTYADLGIANVDADTEM
LNAYTADADAPDTFKVRYYPTKYRWDGFDTPENAGKTEVYRWLQDGDTVGGVKEANEQPE
GYTFFNGDSKVEHPAVDPDITQSRYVINLTDARSILGIDFNNIVTDLTNDADGNGLPDYL
SADVTRVLAGVDGALVLETNGKGAAPTGPNNPASSLTHAIHVEQGKAYADQPDGLQWVKT
SDGDYLILDEDSGNDYGERKYVLPIDSETLQLTDPGTGYFLASAGGSLNPRAKAKVAAIP
GTFSRATGAEFSGTWNVTHLVAKKEDGSFYTQEELDGTGAQRIIGSLPLEEQTFIGVVQQ
GGESGGILAERKADQGGQIFMFNITEPLEFVKPLITGTPNADTIEAGVGEFTGVNSLVFT
GAGKDEVDIPIGGAKLYLGSNSIFTGSGADTISVTDKDRAFGGSGDDKFDATEATGYRIS
GGVGNDIFYLGVNGRAIGGDGDDRFFVGEGGGNIISGGAGADQFWILTDDPTKLKASNTI
VDYTIGTDVIGIANQVADSVDDLTLSGSNISLNGVLIATLNGVNVASATFVFGNPLAS
>OHY42534.1
MGEGGGNIISGGAGADQFWILTDDPTKLKASNTIVDYTISTDVIGIANQVADSVDDLTLS
GSNISLKGVLIATLNGVNAASATFVFGSPLAS
>OHY42587.1
MEPNWLNPDGTLNPQILLDSFLEFWRQHGEPLLRSAPYHEIAPHLVLMAFLHRVVNGGGT
LEREYAIGSGRMDICLRYGQVVMGIELKVRREKLDPLTKGLTQLDKYLDGLGLDTGWLVI
FDRRPGLPPMGERISTEEAISPGGRTITVIRS
>OHY42712.1
MEKFLATYECAGCDLSGARLFSAYLSGARLSRANLSRADLGDADLSRANLSGANLSGANL
EAADLRNARLSYANLSGANLYYAKLSGADLSGANLEGADLSELDPFD
>OHY42751.1
MRLVSTLLANIALAAQTHCDVILFNIDSKADANNKNDKETITKVFQKVFDDHLGYFGTTP
EIARFERQLENKGKYQPFKEAFYRQTNQSWEETREAWAFYQDDIVAALTVSTGMTGEQAN
RLLDFNEKYSLSPEEFAKTVKEHLHTKGPKHHLVFMVDEVGQYIGEDTKLMLNLQTVVED
LGIHCQGRAWVLVTSQEAMDEITKNKIKGFKLFGNFYP
>OHY43326.1
MNMVWSRNLRVFRPEKLNLEGTVVPTPQEAVRIEVDKGIAMVERERLKAIQAQQSVEQER
LRALQAEQDAKQSKAELRELQDKVKTLGISID
>OHY43438.1
MNSKFSAAFLVGTLALSGILGLGHGQLKAQSSDDAFFDPSDPSQYNGTKFTCVAQGSDWS
TVGQRPGGRPIPIIMWTKKGAQSFGENYNPKSRCEIVTANLNQAVEDSAGKLYDVVLTWG
KLDDGKTVICALSLKDEGGCDGNNTLFALKPENAKDPNKVLETLTQISIKGSSAGTVRET

TSRLSVNLGKVVSQAAKRLPQETPKTRTTPVNRPTKGTL
>OHY43451.1
MPIIVWTKQGAQSFGENYNPKSRCEIVTANLNQAVEDSAGNLYDVVLTWGKLDDGKTVIC
ALSLKDEGGCDGSNTLFALKPENAKNPNRVLETLTQISIKGSSAGVVSETKPRGRLSVNL
GRVVSQAAKRLPRETPKTRKTPRSTKGSV
