# Supplementary material for: Genomic and Genotypic Characterization of Cylindrospermopsis raciborskii: Toward an Intraspecific Phylogenetic Evaluation by Comparative Genomics
Source: Front Microbiol. 2018 Feb 26;9:306. doi: 10.3389/fmicb.2018.00306 (PMC5834425; doi:10.3389/fmicb.2018.00306)
Supplement: Supplementary file 3 [file Table_1.pdf]

Supplementary Table S1. Details regarding the *Cylindrospermopsis raciborskii* strains included in this study.

Cyanobacteria strain	Locality	Collecting date	Genome reference
Cylindrospermopsis raciborskii CS-505	Australia: Solomon Dam, Palm Island, Queensland, 18°43’27.1”S 146°35’38.4”E	01.02.1996	Fuentes-Valdés et al., 2016a
Cylindrospermopsis raciborskii CS-508	Australia: Aquaculture pond,Townsville, North Qld, Queensland, 19°15’57.6”S, 146°48’57.6”E	01.08.1997	Fuentes-Valdés et al., 2016b
Cylindrospermopsis racoborskii CR12	Singapore: tropical freshwater lake, Singapore city	2015	Mohamed Nor et al., 2016
Cylindrospermopsis raciborski MVCC14	Uruguay: Laguna Blanca, Maldonado, 34°53'59.3"S 54°50'10.8"W	Unknown	Fuentes-Valdés et al., 2016b
Cylindrospermopsis raciborskii ITEP-A1	Brazil: Arcoverde reservoir, Pedra, PE, Brazil, 8°33’32.5”S, 36°59’07.5”W	09.11.1999	Lorenzi et al., 2016
Cylindrospermopsis raciborskii CENA302	Brazil: Riacho Grande branch of the Billings reservoir, São Bernardo do Campo, SP, 23°46’33.9”S, 46°31’54.4”W	26.05.2008	This study
Cylindrospermopsis raciborskii CENA303	Brazil: Theobaldo Dick lake, Lajeado, RS, 29°27’54.47"S, 51°58’15.51"W	03.07.2009	This study
Raphidiopsis brookii D9	Brazil: Taquacetuba branch of the Billings reservoir, São Paulo, SP, 23°48’04.51”S, 46°37’35.41”W	07.05.1997	Stucken et al., 2010

Fuentes-Valdés, J. J., Plominsky, A. M., Allen, E. E., Tamames, J., and Vásquez, M. (2016a). Complete genome sequence of a cylindrospermopsin-producing cyanobacterium, *Cylindrospermopsis raciborskii* CS505, containing a circular chromosome and a single extrachromosomal element. *Genome Announc.* 4(4), e00823-16.

Fuentes-Valdes, J. J., Soto, K., Belmar, L., Pantoja, D., Tamames, J., Pedros, C., Garrido, D., and Vasquez, M. (2016b). National Center for Biotechnology Information (NCBI) project accessions NZ_MBQX00000000 and NZ_MBQY00000000.

Lorenzi, A. S., Silva, G. G. Z., Lopes, F. A. C., Chia, M. A., Edwards, R. A., Bittencourt-Oliveira, M. C. (2016). Draft genome sequence of *Cylindrospermopsis raciborskii* (Cyanobacteria) strain ITEP-A1 isolated from a Brazilian semiarid freshwater body: evidence of saxitoxin and cylindrospermopsin synthetase genes. *Genome Announc,* 4(3), e00228-16.

Mohamed Nor, N. H., Tan, B. F., Te, S. H., Thompson, J. R., and Gin, K.Y.-H. (2016). Draft genome sequence of *Cylindrospermopsis* sp. strain CR12 extracted from the minimetagenome of a nonaxenic unialgal culture from a tropical freshwater lake. *Genome Announc.* 4(1), e01726-15.

Stucken, K., John, U., Cembella, A., Murillo, A. A., Soto-Liebe K, et al. (2010). The smallest known genomes of multicellular and toxic cyanobacteria: comparison, minimal gene sets for linked traits and the evolutionary implications. *PLoS ONE* 5(2), e9235.
